# A computational method for predicting regulation of human microRNAs on the influenza virus genome

**DOI:** 10.1186/1752-0509-7-S2-S3

**Published:** 2013-10-14

**Authors:** Hao Zhang, Zhi Li, Yanpu Li, Yuanning Liu, Junxin Liu, Xin Li, Tingjie Shen, Yunna Duan, Minggang Hu, Dong Xu

**Affiliations:** 1 Symbol Computation and Knowledge Engineering of Ministry of Education, College of Computer Science and Technology, Jilin University, Changchun, China; 2 College of Applied Technique, Changchun University of Science & Technology, Changchun, China; 3 Department of Computer Science, Christopher S. Bond Life Sciences Center, University of Missouri, USA

## Abstract

**Background:**

While it has been suggested that host microRNAs (miRNAs) may downregulate viral gene expression as an antiviral defense mechanism, such a mechanism has not been explored in the influenza virus for human flu studies. As it is difficult to conduct related experiments on humans, computational studies can provide some insight. Although many computational tools have been designed for miRNA target prediction, there is a need for cross-species prediction, especially for predicting viral targets of human miRNAs. However, finding putative human miRNAs targeting influenza virus genome is still challenging.

**Results:**

We developed machine-learning features and conducted comprehensive data training for predicting interactions between H1N1 genome segments and host miRNA. We defined our seed region as the first ten nucleotides from the 5' end of the miRNA to the 3' end of the miRNA and integrated various features including the number of consecutive matching bases in the seed region of 10 bases, a triplet feature in seed regions, thermodynamic energy, penalty of bulges and wobbles at binding sites, and the secondary structure of viral RNA for the prediction.

**Conclusions:**

Compared to general predictive models, our model fully takes into account the conservation patterns and features of viral RNA secondary structures, and greatly improves the prediction accuracy. Our model identified some key miRNAs including hsa-miR-489, hsa-miR-325, hsa-miR-876-3p and hsa-miR-2117, which target HA, PB2, MP and NS of H1N1, respectively. Our study provided an interesting hypothesis concerning the miRNA-based antiviral defense mechanism against influenza virus in human, i.e., the binding between human miRNA and viral RNAs may not result in gene silencing but rather may block the viral RNA replication.

## Background

Influenza is an infectious disease caused by RNA influenza viruses in the family orthomyxoviridae. Influenza viruses are classified into three types: A, B and C. Influenza A infects a wide variety of avian and mammalian species including humans, which can be subdivided into different serotypes based on the antibody response to these viruses [1]. Influenza B virus almost exclusively infects humans, and it has only one known subtype and is less common than influenza A. Influenza C virus can cause a mild upper respiratory disease [2,3], but it is rare. Influenza A and B genomes each contain eight segments of single-stranded RNA, and C contains seven segments of single-stranded RNA. Each RNA segment encodes one or two proteins [4]. Take influenza A for example; the eight RNA segments are HA (hemagglutinin), NA (neuraminidase), NP (nucleoprotein), M (matrix protein), NS (nonstructural protein), PA (polymerase A), PB1 (polymerase B1), and PB2 (polymerase B2), altogether coding 11 proteins. Influenza virus genome is prone to gene reassortment. The novel influenza, A/H1N1, is a mixed strain [5] first reported in Mexico and the United States (March 2009), and soon spread over several other nations in 2009.

Influenza viruses only replicate within living cells, and they deliver their genes and accessory proteins into the host cells [6]. Host cells do not passively accept viral infection, but trigger resistance and neutralization actively. Studying the interaction between viruses and host cells is important for understanding the mechanism of pathogenicity so as to search for an appropriate anti-virus method. Recent studies demonstrate miRNAs encoded by viruses or humans may exert an important influence on the interaction between virus and host [7,8].

**Figure 1:**
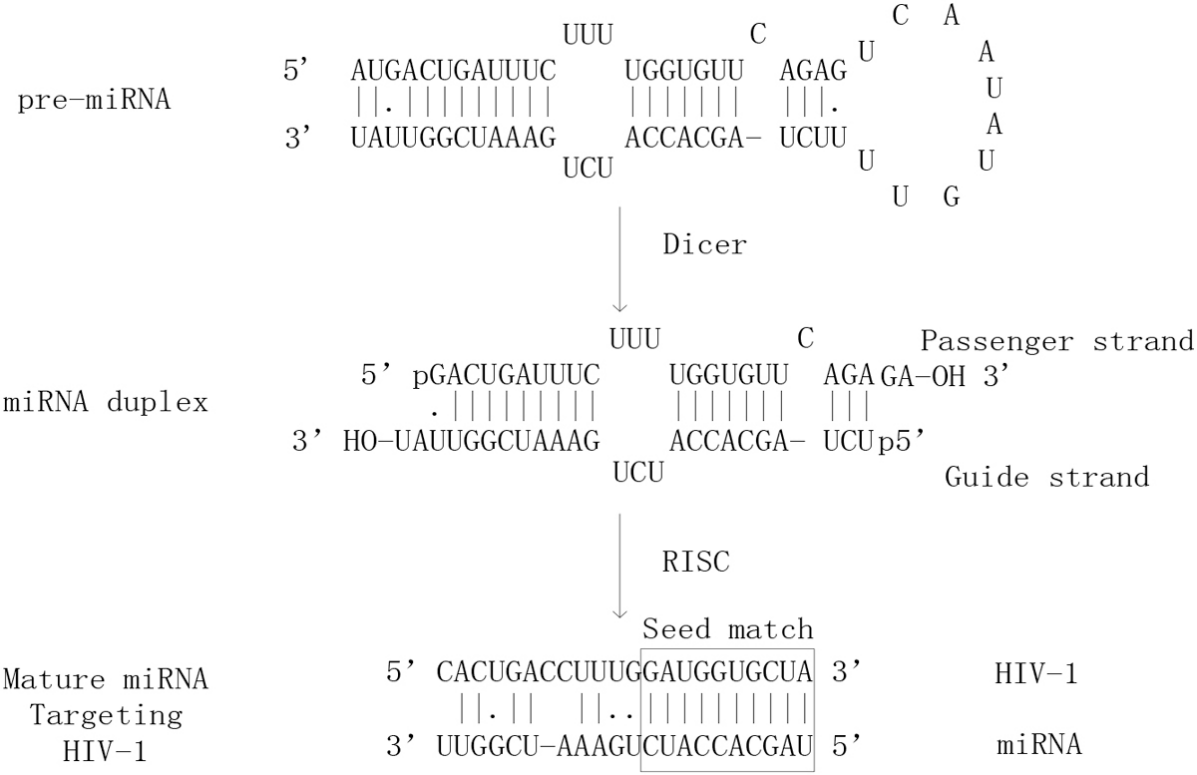
**Process of has2miR229a targeting HIV21 Sequence**.

MiRNAs are small noncoding and endogenous RNAs, ~22 nucleotides in length, and have been discovered in a variety of organisms. MiRNAs play important roles in many biological processes such as development and apoptosis. They establish cell lineage by targeting message RNA (mRNAs) that can direct the RNA-induced silencing complex (RISC) to downregulate gene expression by either mRNA cleavage or translational repression [9]. If complementarities between 3' untranslated region (UTR) of the mRNA and the miRNA (especially between nucleotides 2-7, the so-called seed region) [10] is sufficient, the miRNA can result in mRNA cleavage; however, if complementarities are insufficient, it may repress translation. Regulatory effects of miRNAs on virus replication and pathogenicity have been studied. Among the effects, some studies showed the binding mode between human-encoded miRNAs and viruses. For example, a transcript in human foamy virus (PFV) can be used as the target site of human-encoded miR-32 [11]; hepatitis C virus (HCV) replication is regulated by miR-199a* that may serve as a novel antiviral therapy [12]; and human-encoded miRNAs can target crucial HIV-1 genes [13]. Human miR-326 is physiologically functional in moderating HIV-1 replication in human cells [14]. Heiss et al. (1986) noted in the Journal of Virology that "In the developing CNS of highly permissive suckling mice, the miRNA-targeted viruses can revert to a neurovirulent phenotype by accumulating deletions or mutations within the miRNA target sequence" [15]. Figure 1 shows the process of host miRNA 'hsa-miR-229a' targeting an HIV-1 transcript. Scaria et al. (2006) also found that human-encoded miRNAs could target critical genes involved in the pathogenesis and tropism of influenza virus A/H5N1, and the target regions in the respective genes were found to be conserved across different viral strains [16]. Cellular miRNAs expression and its relation to virulence in influenza expression were reported in [17]. However, no detailed analysis has been conducted for the interactions between human miRNA and influenza virus genome. The direct role of miRNAs in influenza and related mechanisms has not been well established. Hence, it is of great significance to study host-virus interactions mediated by miRNAs in flu.

Unlike many plant miRNA targets, which are almost completely complementary in open reading frames (ORFs) [18], the binding between animal miRNAs and their target sites has incomplete complementarity in base-pairing, and binding sites can be found in 3' UTRs, 5' UTRs and coding regions of target genes [19-21]. Experimental miRNA-recognition methods are laborious and time-consuming; hence these methods cannot achieve high throughput currently. Therefore, numerous miRNA target prediction methods have been proposed such as miRanda [22], TargetScan(S) [23], RNA22 [24], Diana-MicroT [25], PicTar [26], RNAhybrid [27], and miTarget [28], based on seed complementarity, thermodynamics, conservation, Bayesian statistics, SVM, HMM, artificial neural networks, etc. However, available methods suffer from the lack of gold standards of negative examples to build an effective classifier and can hardly make a good balance between high sensitivity and high specificity, which leads to high false positive and false negative rates. Current prediction algorithms lack consistency when compared to each other, and none of the existing prediction tools have been able to incorporate comprehensive features efficiently. While computational analysis of miRNA-mediated antiviral defense has been conducted [29], no available software tool to predict cross-species miRNA-mediation mechanism has been available until now. It is likely that some special characteristics exist for binding between human miRNA and viral RNA. In particular, influenza viruses mostly have negative-sense single-strand RNAs. In this study, we focused on interaction between human miRNA and viral negative-sense RNAs, which may prevent the viral RNAs from replication and possibly lead to RNA degradation. Such a binding may have different features from interaction between human miRNA and human mRNA, which results in gene silencing through translational repression or target degradation.

In this study, we developed an influenza virus-based multi-level scoring neural network model to predict human miRNAs that may target influenza RNAs. Our model combines viral genome characteristics, RNA secondary structure characteristics, genetic conservative characteristics, and interaction features at seed regions, which work together to greatly improve prediction accuracy and search speed. A hypothesis is proposed for the interaction between the human miRNA and viral negative-sense RNA. Our study may help find a new approach for the prevention and control of the influenza virus.

## Results

In this section we conducted seed region feature analyses and compared our method's performance with the other five prevailing prediction algorithms, using a completely independent test data set.

### 10-nt sequence base-pairing value in seed region of the binding site

**Figure 2:**
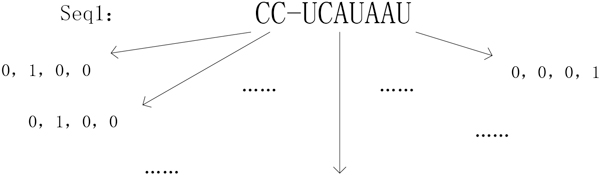
**Base-bit representation of one sequence**. Each base of a miRNA sequence is represented by a 4-dimentioanl vector, indicating the presence of A, C, G, and U, respectively. The miRNA seed position shown in the figure is represented by (0,1,0,0,0,1,0,0,0,0,0,0,0,0,0,1,0,1,0,0,1,0,0,0,0,0,0,1,1,0,0,0,1,0,0,0,0,0,0,1).

MiRNA targets commonly have at least one region that has Watson-Crick pairing to the 5' part of miRNA. Normally miRNA seed is defined as the consecutive 7 to 8-nt sequence starting from either the first or second base at the 5' end of an miRNA. Seed region has the most important features for target recognition. In order to get better results for predicting H1N1 RNA targets from human microRNAs, we conducted base-pairing comparative studies between four segments of H1N1-2009 from NCBI and human miRNAs from miRBase. For H1N1 genomes, results showed that the consecutive 10-nt sequence pattern is better than the 7, 8 or 9-nt sequence pattern that is frequently observed in the binding between human mRNA and human miRNA. So our prediction model improves traditional statistical seed region features at the binding site and defines the viral RNA seed region with the consecutive 10-nt sequence (seed sequence of miRNA also corresponds to the consecutive 10-nt sequence accordingly). Typically, the region sequence we refer to is 10-nt from the 5' end of the miRNA. Each base position is a vector of four according to the order of A, C, G, U for the Boolean type representation. For example, if the base symbol bit is G, then the corresponding bit in the base of the third vector element is set to 1, and the rest is marked as 0. Hence, the 10-nt seed region of the miRNA sequence is encoded into a 40-dimensional vector. An example is shown in Figure 2.

### N3 statistical information in seed region

The complexity of miRNA-RNA interactions may lead to an inefficient search for miRNA-RNA sequence matching in the miRNA target recognition, as the current search is often based on minimum free energy (MFE) of miRNA:target duplexes. To further reduce the search time, we developed a statistical energy formula for constructing the triplet (N3) feature to represent local MFE concept at the seed region. N3 is produced by consecutive three-base pairings between miRNA and RNA in the seed region. This is a novel feature of miRNA-target base pairing, in contrast to traditional thermodynamic parameters. There are 216 types of triplet base-pairings according to their MFEs. First, based on the statistical energy formula and experimental data, we calculated their MFEs and mapped the results into discrete consecutive integers from 1 to 27 using the formula Mapping Function: SCORE(x)=[MFE(x)*(-1)/6.7*27+1], where x∈[1,216] and if MFE(x)≥0, MFE(x)=0. The MFE of a triplet is calculated based on dimer thermodynamic parameters. The SCORE(x) discretizes triplet base-pairing scores, as shown in Additional File 1. As an example, with regard to the seed region instance in Figure 2, the triplet feature value is (18, 13, 12, 11, 11, 0, 0, 0) with 8 dimensions.

### Gap penalty function calculation of binding sites

Because of the limited complementarity between miRNAs and their targets, mismatch and gaps between sequences should be considered. Here, mismatches are known as wobbles and bugles in the seed region, based on its upstream or downstream activity. A wobble can be considered as a special bugle. Our method extracts information including the number of bulges and size of every bulge. We developed an empirical gap penalty score as shown in Table 1. The basic idea is to impose more penalty for large gaps and gaps close to the center of miRNA.

**Table 1:** Gap penalty of bulge in relationship with gap size and starting position

		Starting position of bulge (from 5' end of miRNA)					
		
		1	2	3	4	5	>5
gap size	1	-1	-1	-2	-2	-5	-20
	
	2	-1	-1	-2	-2	-5	-20
	
	3	-2	-2	-3	-3	-8	-25
	
	4	-2	-2	-3	-3	-8	-25
	
	>4	-5	-5	-6	-6	-10	-40

### Feature analysis

**Figure 3:**
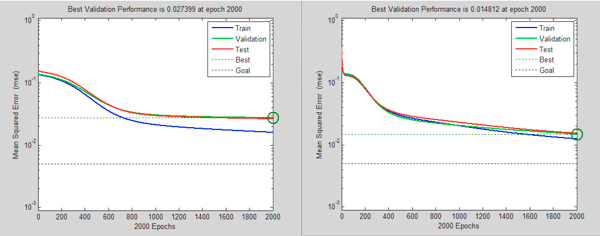
**Comparison of the MSE curve trend of control model (left) and new model (right)**. In both graphs, the horizontal axis indicates the number of iterations during training; the vertical axis shows the model error (MSE) values. Blue curve represents the MSE of the training model in the neural network building process, green curve indicates the MSE trend under data cross-validation; red curve represents the test data (sampled from the neural network training set by removing the extraordinarily good or poor models as outliers).

**Figure 4:**
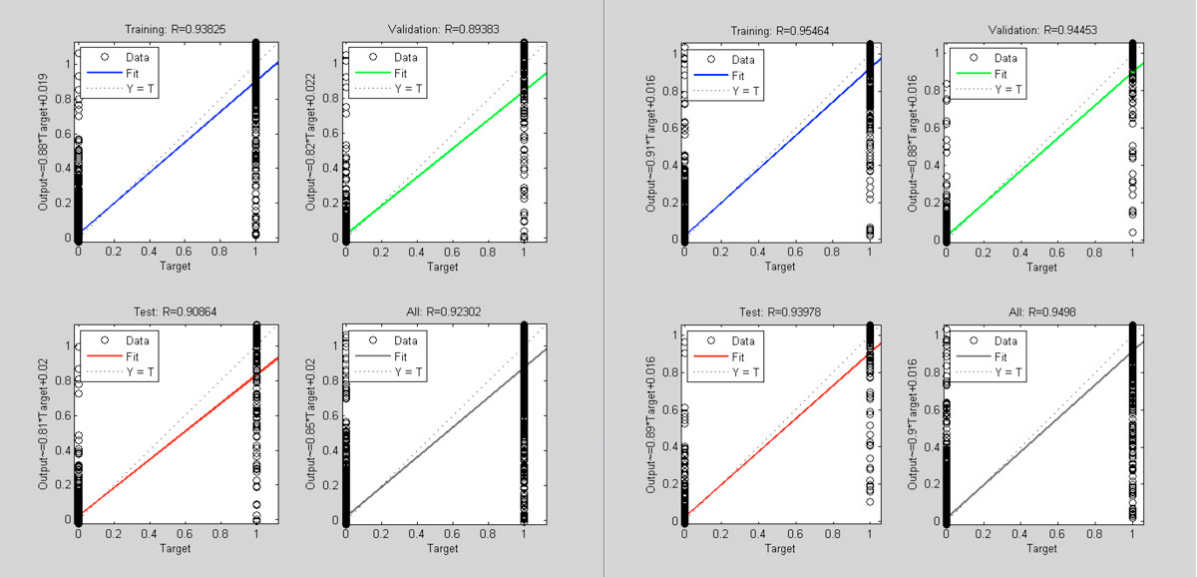
**Comparison of data dependence analysis for control model (left) and new model (right)**. In both graphs, the horizontal axis indicates the objective output value of the model training; the vertical axis shows the model output values. Blue curve represents the current training model data correlation in the process of neural network building; green curve represents the data correlation under cross-validation data, red curve is the corresponding model correlation with test data (sampled from the neural network training set by removing the extraordinarily good or poor models as outliers).

**Figure 5:**
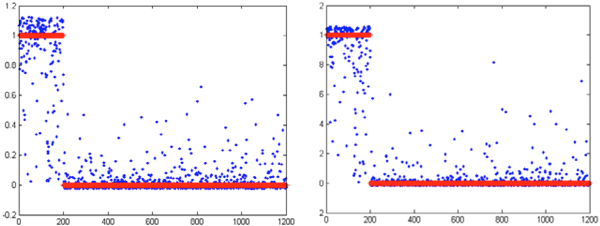
**Comparison of classification between the control model (left) and the new model (right)**. In both graphs, the horizontal axis indicates the data sample numbers, and the vertical axis is classification value. Blue indicates the actual output value of the model, red indicates the objective (target) value.

**Figure 6:**
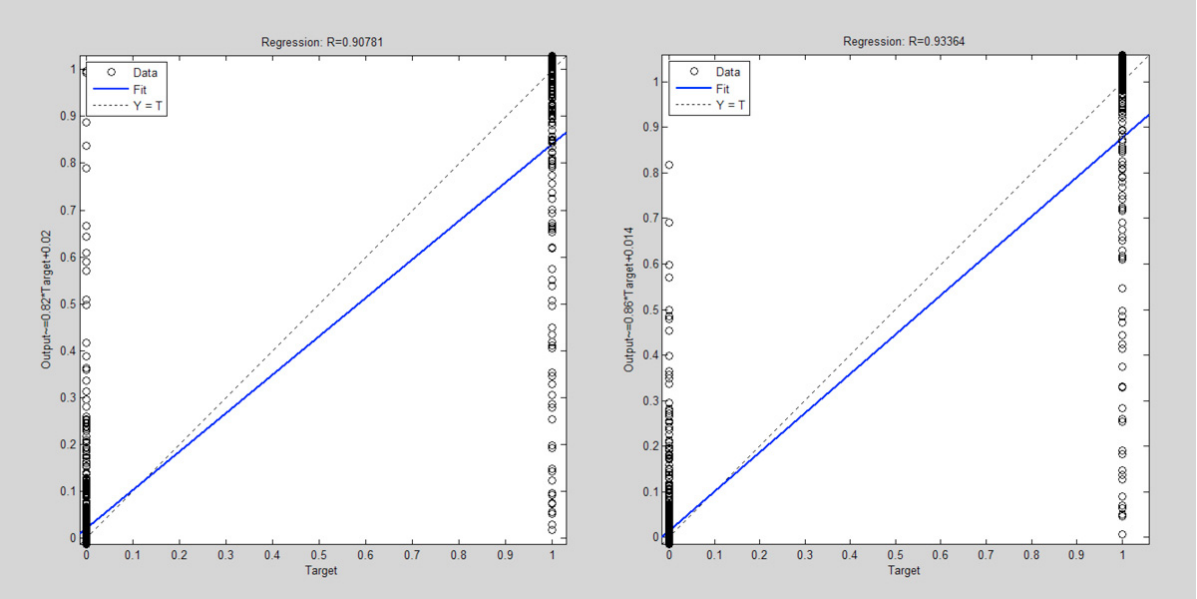
**Comparison of test set correlation analysis of control model (left) and new model (right)**. In both graphs, the horizontal axis indicates the output objective (target) values, and the vertical axis is the output value of model.

We compared the traditional feature selection method (defined as the control model) and our improved feature selection method (defined as new model) by a 10-fold cross-validation. Figures 3-6 illustrate one of 10 test cases in the 10-fold cross-validation tests. The criteria for comparing between the control model and the new model include: MSE (Mean Squared Error, which refers to the expected difference square between the estimated value and the true value) and correlation analysis. The figures demonstrate that the new feature extraction method is a significant improvement over the traditional feature selection method.

In Figure 3, the model MSE trend shows that the new model converged slower, but reached a lower MSE value than the control model. This indicates that the introduction of new features affects convergence of the model. More features used in the new model may result in longer convergence iterations, but better results. Figure 4 shows after adding new features, the correlation between the objective values and the model output values improves significantly in both test and validation sets over the control models.

It is worth mentioning that the new target genes feature an extraction method, which can be easily integrated into the traditional target gene model without significant additional computing time.

### Performance on completely independent test data

In order to evaluate the performance of the classifier, three objective functions including sensitivity $SN=TP/\left(TP+FN\right)$, specificity $SP=TN/\left(TN+FP\right)$, Matthew's correlation coefficient $MCC=\frac{TP^{*}TN-FP^{*}FN}{\sqrt{\left(TP+FN\right)\left(TP+FP\right)\left(TN+FP\right)\left(TN+FN\right)}}$ and average class-wise accuracy ACA=(TP+FN)/(TP+FN)/(FP+TP+TN+FN) are computed. Here, SN and SP control false negatives and false positives, respectively, and MCC measures the balance of the classification results. We chose two independent test data sets for our performance assessment. First we selected 200 groups' positive target genes and 1000 groups' negative target genes using TarBase as our test data set. All the chosen data had no more than 50% sequence identity from any of our training data sets. Then we chose a subset which consisted of 137 positive and 67 negative examples to test our model again. Both tests showed better results for our method than others, as shown in Table 2. In the first test, our model provided MCC (0.645) and ACA (0.8), and in the second test, MCC (0.6553) and ACA (0.8479).

**Table 2:** A comparison among different methods in their ACA values

Methods	MCC	ACA
PITA	0.4023	0.5710

miRANDA	0.2427	0.6242

Diana MicroT 3.0	-0.0089	0.5236

TargetScan	0.3769	0.6430

Our Algorithm	0.6553	0.8479

### Predicted human miRNAs that regulate the influenza virus genome

**Figure 7:**
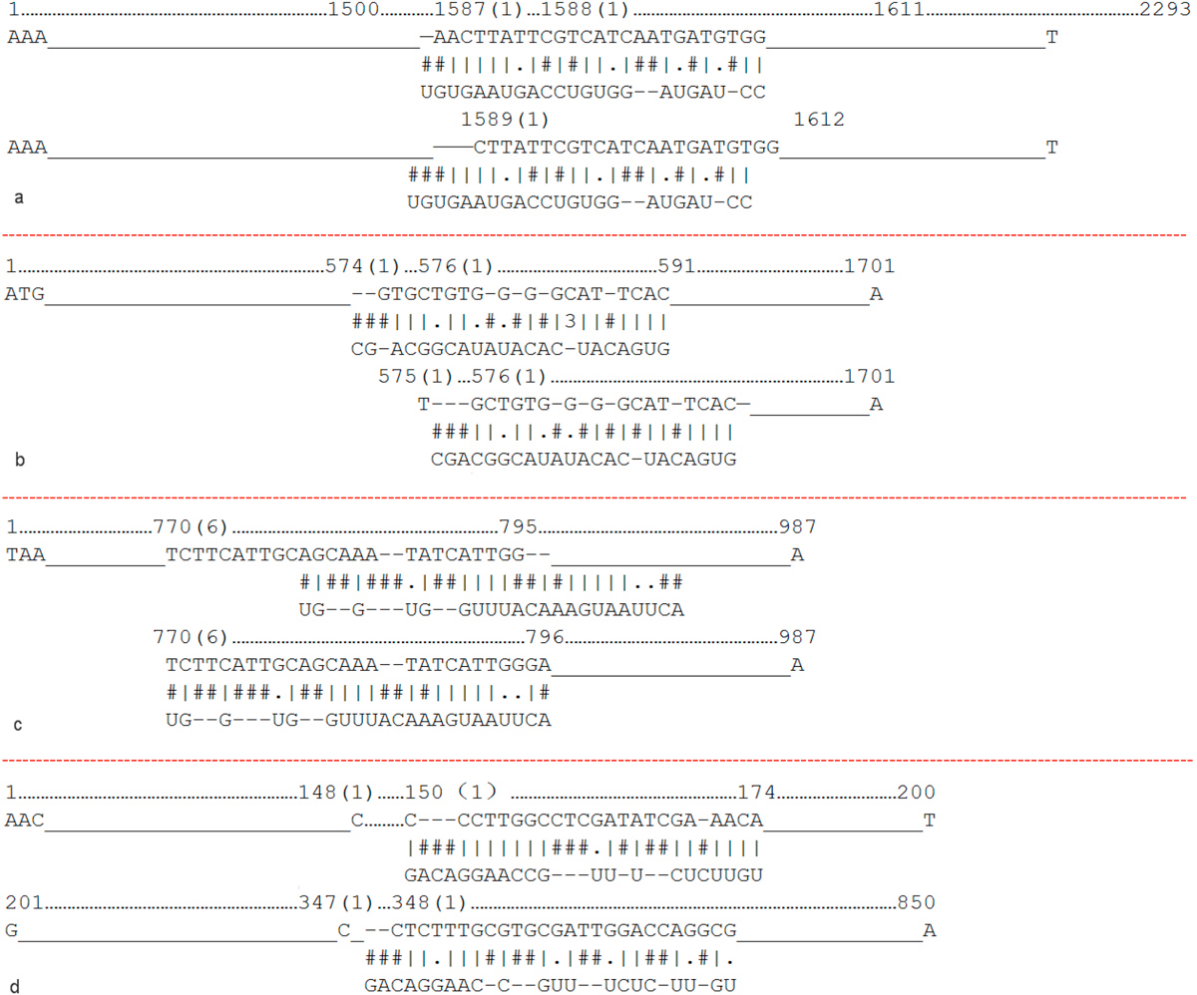
**The binding modes of predicted miRNA-RNA pairs**. hsa-miR-489, hsa-miR-325, hsa-miR-876-3p and hsa-miR-2117 are predicted to target HA, PB2, MP and NS of influenza A, respectively.

**Figure 8:**
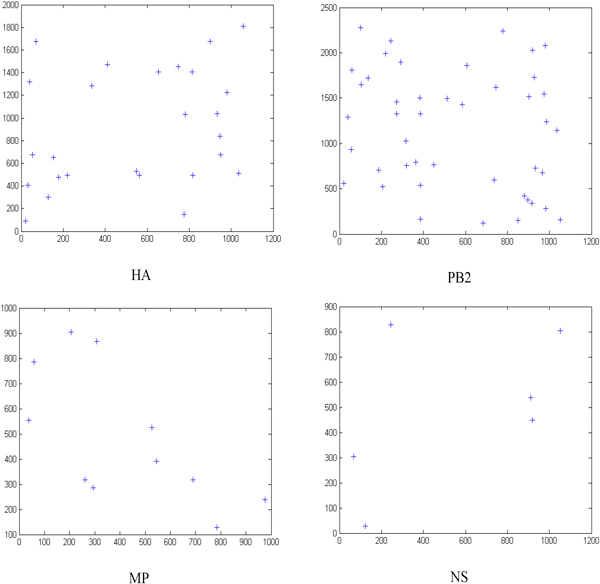
**H1N1 genome complementary site profile**. The horizontal axis indicates the order number of human encoded miRNA that is predicted to bind the segment. The vertical axis represents the nucleotide sequence order of a segment.

**Figure 9:**
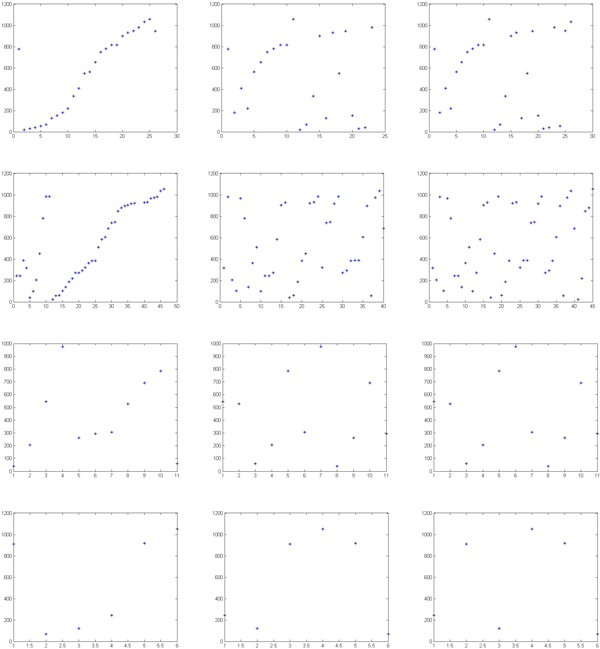
**Profile of different scores**. The four rows of 3-set graphs correspond to HA, PB2, MP and NS, respectively. The three rows show results with three methods. The first row represents the results using traditional features. The second row represents the results with new features only. The third row shows the results with the combination of traditional features and the new features. The horizontal axis indicates the order number of miRNAs and the vertical axis is the position of binding sites. Here, we reordered the miRNA candidates according to their binding energies.

Using our model, hsa-miR-489, hsa-miR-325, hsa-miR-876-3p and hsa-miR-2117 are predicted to target HA, PB2, MP and NS of influenza A, respectively. Table 3 and Figure 7 provide start positions and binding energies, as well as the modes of binding pairs. The number of binding sites of hsa-miR-489 is the least, and only the seed region was paired. Because the binding energy of hsa-miR-325 and PB2 was the lowest, their binding was the most stable. Figure 8 shows the distribution of complementary sites. Figure 9 shows the impact of new features. The miRNAs predicted with new features and were significantly different from those predicted by traditional features. Using new features only had a similar result to the prediction from combined features, but predicted fewer miRNAs. This showed the new features to be dominant in the combined prediction, but the inclusion of traditional features helped obtain more putative miRNA-target candidates. The most complementary sites were in the PB2 segment and the least were in the NS segment. This means PB2 may be the most important segment for the binding of human encoded miRNAs and H1N1 segments.

**Table 3:** Start position of binding and binding energy

MiRNA	Start Position of Binding	Energy (kcal/mol)
hsa-miR-489	151	-11.19

hsa-miR-325	1029	-13.72

hsa-miR-876-3p	526	-13.23

hsa-miR-2117	827	-11.74

## Discussion

In this section we discuss parameter selection rules and seed region features. We also discuss our key finding and its potential biological implication.

### H1N1 segments and genome sequences selection

Given the differences in quantities, regions, acquisition methods and data completeness of H1N1 genome sequences in NCBI (http://www.ncbi.nlm.nih.gov/), we could not choose all possible H1N1 genome sequences. It would have led to biases in the result. For H1N1 genome sequences, only a certain number of sequences of each segment per year from the past 13 years (2000-2012) were selected as non-redundant representatives. In order to identify what number of sequences was the most suitable, we chose 10, 20, 30, 40, 50, 70, 90, 100, and 200 sequences per year to perform comparative analyses. The result indicated that 50 sequences were the best [final data sets selected from this process are in Additional Files 2-10.

It has not been proven that human miRNAs could play any direct role in any of the segments of influenza genome. To simplify our research, we chose HA, PB2, MP and NS segments of H1N1 from 2000 to 2012 for our study, since these four influenza virus proteins play essential roles in influenza virus's pathogenicity and infectiousness [30-34]. For example, the strength of the virulence is directly linked to whether HA can be cleaved to HA1 and HA2. NS plays a regulatory role in viral transcription and replication process. PB2 generates the primer required for viral RNA transcription. MP contains matrix proteins and coding proteins (m1, m2, and m3).

### H1N1 segments secondary structure and MFE calculation simplification

In our model we used the different scores for the test to determine which step had significant effect on the result. The test confirmed that RNA secondary structure is an important factor for discovering human encoded miRNAs that regulate the influenza virus genome. This is consistent with our previous study [35], which showed that local RNA structure had a much stronger effect than a global one on the miRNA-RNA binding.

### Cross-hybridized binding

In this study, cross-hybridized binding was considered. We assumed that when an miRNA targets many segments, it loses specificity and its biological effect to inhibit viral RNA will be substantially reduced. Hence, for one miRNA targeting multiple RNAs, we lowered the miRNA's score through dividing by the number of targets. For multiple miRNAs targeting one RNA, we assumed they added more effectiveness for inhibiting the viral RNA. This might be a mechanism of human endogenous miRNAs to improve strengths in targeting influenza virus. So we took multiple miRNAs targeting one RNA into account in our study.

### Biological implication

Our result showed that the predicted binding mode between human miRNA and viral negative-sense single-strand RNAs are significantly different from the observed binding mode between human miRNA and human mRNA. In particular, the former has a consecutive 10-nt fully complementary sequence pattern while the latter has 7, 8 or 9-nt complementary sequence pattern. The A:U and G:C ratio in seed, up-stream and down-stream regions, are also different between the two cases. This indicates that the binding between human miRNA and viral RNAs may be much tighter than that between human miRNA and human mRNA. This also suggests that the binding between human miRNA and viral RNAs may not result in gene silencing through translational repression or target degradation, but rather may prevent the viral RNA replication by forming double-strand RNAs between human miRNA and viral negative-sense single-strand RNAs.

## Conclusion

In conclusion, we developed a novel model for cross-species miRNA target prediction based on machine learning approach. Compared to general predictive models, our model fully takes into account the conservation patterns and features of viral RNA secondary structures, and greatly improves the prediction accuracy. Using our model, we discovered human encoded miRNAs hsa-miR-489, hsa-miR-325, hsa-miR-876-3p and hsa-miR-2117 targeting HA, PB2, MP and NS of influenza A, respectively. This number of candidates was very small, and thus the results can be used as a basis for biological reverse genetics test experiments for verification. Moreover, next-generation sequencing can also be used to test the effectiveness of our method and our biological hypothesis. In future work, we will extend our study from the four segments to all eight segments. Different score's weight will be considered by adding more H1N1 segment characteristics.

## Materials and methods

**Figure 10:**
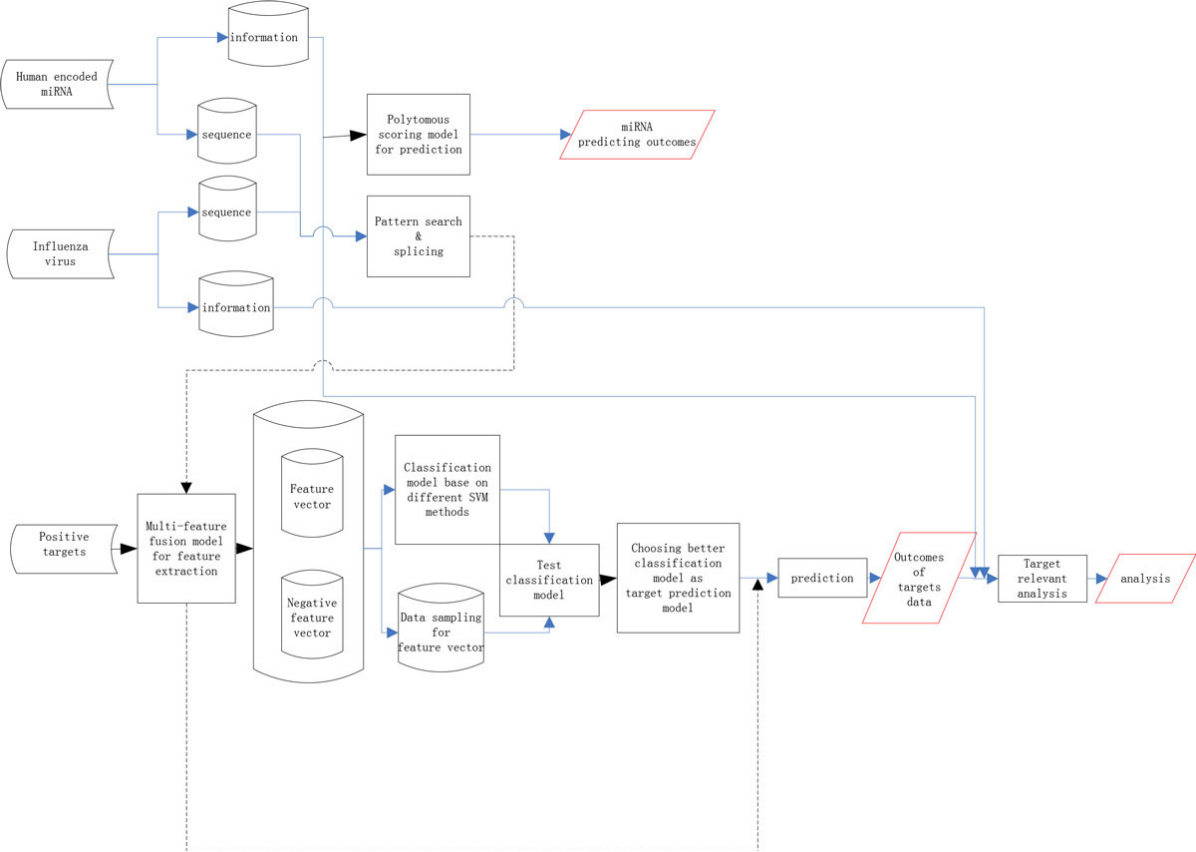
**Workflow of our system**.

Figure 10 shows the workflow of our model in studying the interactions between human miRNAs and HA, PB2, MP or NS segments of H1N1. We included novel target binding site features and conducted comprehensive data trainings in our target prediction model.

### Data preparation

The human miRNA sequences were downloaded from miRBase [36-38]. The data of this database include the predicted hairpin portion of miRNA transcript, information on the genomic location and mature miRNA sequences. Mature sequences were used in this study. The number of mature sequences is 1100 [Additional File 11]. Each sequence is represented in the FASTA format, as shown in Table 4 as examples. The sequences of human encoded miRNAs are numbered. The RNA segments of Influenza A genome, HA, PB2, MP and NS were chosen for this study. We chose these four segments since their data are relatively complete, covering a significant range of time and geography. 50 sequences of each segment from the past thirteenyears (2000-2012) different from our preliminary work's data set (2000-2009) were randomly sampled from the NCBI collection.

**Table 4:** Three of human encoded miRNA sequences used in this study as examples

miRNA	Sequence
>hsa-miR-576-3p MIMAT0004796	AAGAUGUGGAAAAAUUGGAAUC

>hsa-miR-194* MIMAT0004671	CCAGUGGGGCUGCUGUUAUCUG

>hsa-miR-140-5p MIMAT0000431	CAGUGGUUUUACCCUAUGGUAG

We obtained a consensus secondary structure using RNAalifold of the Vienna RNA package (http://rna.tbi.univie.ac.at/cgi-bin/RNAalifold.cgi) with the default parameters (new RNAalifold with RIBOSUM scoring; fold algorithms and basic options: minimum free energy (MFE) and partition function, and avoiding isolated base pairs). We then aligned all the 50 influenza sequences using ClustalW2 with the default parameters (ALINMENT: full; SCORE TYPE: percent; NO END GAPS: yes; ITERATION: none; NUMITER: 1; OUTPUT FORMAT: aln w/numbers; OUTPUT ORDER: aligned; TREE TYPE: none; CORRECT DIST: off; IGNORE GAPS: off; CLUSTERING: NJ).

### Artificial neural network design

An artificial neural network was used for the miRNA target prediction, with a suitable selection and representation of the binding site features used as input features. In this study, in addition to the traditional binding site features, we proposed three novel features which have been described.

MiRNA target gene prediction models all employ 3-layer neural network structure with a single hidden layer input layer nodes with the number (N) representing characteristics input of the target gene data to be predicted; the number of hidden layer is 2*N+1, the output layer is single node; the transfer function from input layer to the middle hidden layer is TANSIG, the transfer function from middle layer to the output hidden layer is LOGSIG (TANSIG and LOGSIG both are in the MATLAB transfer function package). The building and testing of comparison model based on the classification model framework can be accomplished using the following steps:

1) Sample positive and negative target genes data as a training model of the data set, from positive target genes (788 groups) and negative target genes (4000 groups) randomly, with total of 4788 target genes. The remaining 1200 groups (including 200 positive target genes and 1000 negative target genes) form a model test data set.

2) Perform feature selection for model training and extract selected features accordingly in the form of vectors, which are applied to the model training.

3) In order to achieve good convergence of the training, the maximum number of iterations of the neural network training parameters is set to 2000; the MSE difference threshold between two iterations is set at 0.005, and other parameters adopt the default settings.

4) Test the current training model by the classification accuracy, correlation test, etc.

5) Repeat steps 1) to 4) 10 times and compare results. Filter out the outlier models that are extraordinarily good (to avoid over-training) and those that are particularly poor. The remaining models should be used to measure the test performance.

The training data sets including positive and negative data can be found in Additional Files 12-17.

### N3 statistical information in seed region

**Figure 11:**
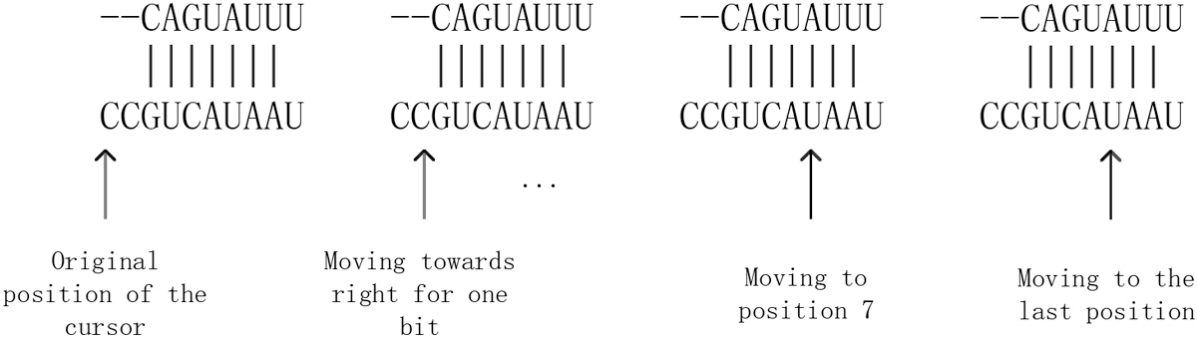
**Schematic diagram for cursor in seed region**.

Before constructing N3 statistical information table, we had to set up a sequence cursor to indicate the triple consecutive elements, as shown in Figure 11. During the N3 information extraction process in a given sequence, the sequence cursor moves to the next element position after processing the current three elements. In order to move through the whole sequence, the sequence cursor can be moved up to n-(k-1) times, where n  is the string length of the sequence, and k  represents the k  mer. As for 10 -bit seed region for a triplet, n=10,k=3, and hence sequence cursor can be moved up to 8 positions. Hence, we can get eight-dimension information by the triplet representation of a 10-nt seed region. If sequence alignment generates a gap ("-" represents gap in this paper), then when the cursor points to a position, it is treated as a new type of nucleotide element.

**Figure 12:**
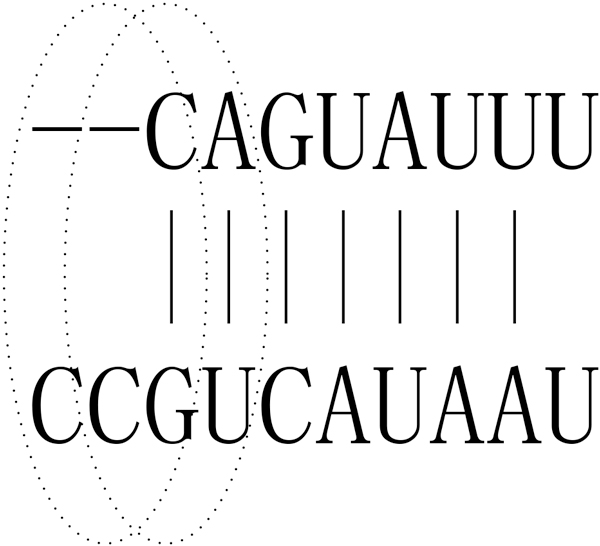
**3-mer instance containing gap in seed region**.

Figure 12 shows the basic idea of N3 statistical information. Each dotted ellipse includes a triplet, which may contain gap. We calculated all 216 triplet base-pairings' MFEs according to Turner (2004) (http://rna.urmc.rochester.edu/NNDB/ and http://rna.urmc.rochester.edu/NNDB/turner04/stack.txt).

### Build a target gene prediction model based on multi-feature fusion machine learning

This paper presents an efficient miRNA target prediction approach based on artificial neural networks trained on both positive and negative data as described above. We ranked all the features including 3-mer matching, penalty assessment of binding sites and alignment feature function at binding sites. Through the feature-ranking test, our new features turned out to be non-redundant with higher scores than features used in the traditional methods. Our method can distinguish all six known types of miRNA-target interactions (7mer1A, 7mer-m8, 8mer, 6mer canonical sites, 3'-supplementary sites, and 3'-compensatory sites).

### Discover the candidates using our model

The method proposed in this paper was based on scoring, and the secondary structure of RNAs were also considered as an important factor. Viral RNA structure has been demonstrated to be crucial for the adaptability of viruses [39]. For this reason, scoring based on secondary structure was considered in the method. We focused on the bind sites in the stems of RNA secondary structure. Because the stem is stable, we believe that if the bind sites are in the stem region, miRNA will perturb the RNAs more strongly. The secondary structure of any single sequence may not be representative; as a result, the consensus nucleotide sequence was used.

There are three factors to build the score to find the human miRNA sequences that can regulate influenza virus genome:

(1) Based on the complementary sites: A sliding window method is applied to search for complete complementary fragments.

(2) Based on the secondary structure of the complementary sites: If the nucleotide was in the stem region, additional reward score is given.

(3) Based on sequence conservation: If nucleotides at the bind site are conserved or nearly conserved across virus strands in different years, additional reward score is given.

The weights of the above three factors were trained empirically. By combining all score components, we ranked the composite scores in ascending order. The miRNA with the highest composite score and the target subsequence were used. We then used RNAfold of Vienna RNA package to get its binding energy. If more than one miRNA had the same score, the one with the lowest binding energy was used for the final result.

## Competing interests

The authors declare that they have no competing interests.

## Authors' contributions

HZ, YL, ZL and JL conceived the initial study and prepared relevant data and their pre-processing. HZ, XL, YD and TS designed the prediction model. YL, XL and TS implemented the prediction algorithm. HZ and DX performed the data analyses. All wrote the manuscript.

## Supplementary Material

Additional File 1 N3 energy values Click here for file

Additional File 2 **Description of selection of H1N1 genomic fragments**. Click here for file

Additional File 3 **The coding sequence of the HA fragment from year 2000 to 2012 recorded in Genbank**. Click here for file

Additional File 4 **The coding sequence of the gene fragment of MP from 2000 to 2012 recorded in Genbank**. Click here for file

Additional File 5 **The coding sequence of the gene fragment of NA from 2000 to 2012 recorded in Genbank**. Click here for file

Additional File 6 **The coding sequence of the gene fragment of NP from 2000 to 2012 recorded in Genbank**. Click here for file

Additional File 7 **The coding sequence of the gene fragment of NS from 2000 to 2012 recorded in Genbank**. Click here for file

Additional File 8 **The coding sequence of the gene fragment of PA from 2000 to 2012 recorded in Genbank**. Click here for file

Additional File 9 **The coding sequence of the gene fragment of PB1 from 2000 to 2012 recorded in Genbank**. Click here for file

Additional File 10 **The coding sequence of the gene fragment of PB2 from 2000 to 2012 recorded in Genbank**. Click here for file

Additional File 11 1100 miRNAs from miRBase Click here for file

Additional File 12 **58 miRNAs that were used as the test dataset, including positive samples and negative samples**. Click here for file

Additional File 13 **A list of test datasets including positive samples and negative samples**. Click here for file

Additional File 14 **The coding sequence of the gene fragments which were used as test datasets including positive samples and negative samples**. Click here for file

Additional File 15 **204 miRNAs that were used as the training datasets including positive samples and negative samples**. Click here for file

Additional File 16 **The list of training datasets including positive samples and negative samples**. Click here for file

Additional File 17 **The coding sequence of the gene fragments which were used as training datasets including positive samples and negative samples**. Click here for file

## Declarations

The publication of this work was supported by the National Natural Science Foundation of China (60673099,60873146, and 60971089) and Jilin University Natural Science Foundation (2010).

This article has been published as part of *BMC Systems Biology *Volume 7 Supplement 2, 2013: Selected articles from The 6th International Conference of Computational Biology. The full contents of the supplement are available online at http://www.biomedcentral.com/bmcsystbiol/supplements/7/S2.
