# Supplementary figures and images for: A computational method for predicting regulation of human microRNAs on the influenza virus genome
Source: BMC Syst Biol. 2013 Oct 14;7(Suppl 2):S3. doi: 10.1186/1752-0509-7-S2-S3 (PMC3851852; doi:10.1186/1752-0509-7-S2-S3)

| No. | Triplet Base-pairing Type | MFE Value | Score |
| --- | --- | --- | --- |
| 1-8 | 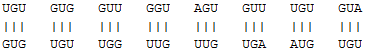 | + | 1 |
| 9-14 | 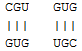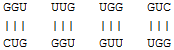 | -0.1/-0.2 | 2 |
| 15-18 | 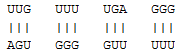 | -1 | 4 |
| 19-22 | 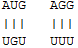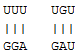 | -1.1 | 5 |
| 23-27 | 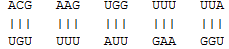 | -1.4/-1.5 | 6 |
| 28-35 | 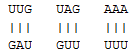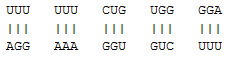 | -1.6/-1.8 | 7 |
| 36-53 | 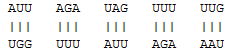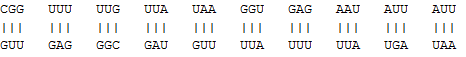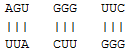 | -1.9/-2.0 | 8 |
| 54-65 | 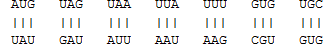  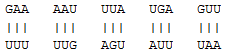 | -2.1/-2.2/-2.3 | 9 |
| 66-73 | 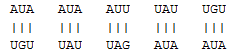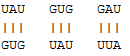 | -2.4 | 10 |
| 74-89 | 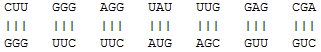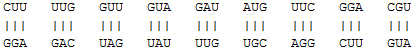 | -2.6/-2.7/-2.8 | 11 |
| 90-101 | 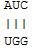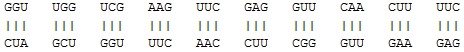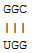 | -2.9/-3 | 12 |
| 102-113 | 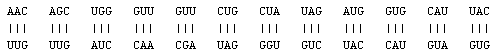 | -3.1/-3.2 | 13 |
| 114-131 | 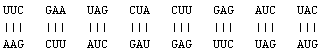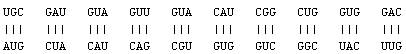 | -3.3/-3.4/-3.5 | 14 |
| 132-140 | 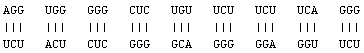 | -3.6 | 15 |
| 141-152 | 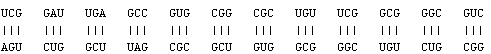 | -3.8/-3.9/4 | 16 |
| 153-154 | 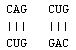 | -4.2 | 17 |
| 155-170 | 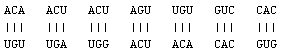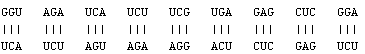 | -4.3/-4.5 | 18 |
| 171-182 | 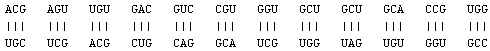 | -4.6/-4.7 | 19 |
| 183-190 | 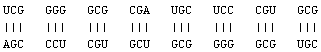 | -4.8/-4.9 | 20 |
| 191-204 | 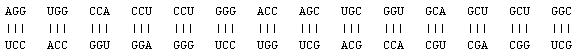 | -5.4/-5.5 | 22 |
| 205-208 | 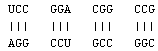 | -5.7 | 23 |
| 209-212 | 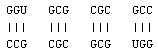 | -5.8 | 24 |
| 213-214 | 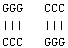 | -6.6 | 26 |
| 215-216 | 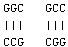 | -6.7 | 27 |

Supplement: Additional File 1 — N3 energy values [file 1752-0509-7-S2-S3-S1.docx]
