# Supplementary material for: A computational method for predicting regulation of human microRNAs on the influenza virus genome
Source: BMC Syst Biol. 2013 Oct 14;7(Suppl 2):S3. doi: 10.1186/1752-0509-7-S2-S3 (PMC3851852; doi:10.1186/1752-0509-7-S2-S3)
Supplement: Additional File 2 — Description of selection of H1N1 genomic fragments. [file 1752-0509-7-S2-S3-S2.PDF]

Genomic Fragments

The H1N1 data are totally from NCBI. Incomplete statistics, H1N1 has 8 genomic fragments, they are HA，PB2，NS，MP，PA，PB1，NP，NA. We selected the H1N1 fragments data from the year 2000 to 2012, and these fragments have the complete coding sequence of the gene(complete CDs) fragments. And during the selection, we try to select the data which are from different regions.

For each of the genomic fragments, from the year 2000 to 2009 we select 5 data for every year. And from the year 2010 to 2012 we select 10 data for every year because that this time is closer. But as the data in 2012 are not enough, so most of the data of the year 2012 are less than 10.

After calculation, we select 583 data totally. The following tables are data list from the year 2010 to 2012 given as an example, and the detailed data message are all in the additional files(HA.pdf, PB2.pdf, NS.pdf, MP.pdf, PA.pdf, PB1.pdf, NP.pdf, NA.pdf).

HA：

District	Continent	2010	GenBank	2011	GenBank	2012	GenBank
Totorri	Asia	2	AB745263.1<br>AB745278.1				
HangZhou	Asia	1	JQ365014.1				
Thailand	Asia	1	CY096809.1				
Singapore	Asia	1	JX309997.1	1	CY091720.1		
Pernambuco	South America	1	CY103938.1				
Shenzhen	Asia	1	JQ065243.1				
Finland	Europe	1	JN601105.1				
Karaj	Asia	1	JF500446.1				
Sydney	Oceania	1	CY092393.1	1	CY092864.1		
Mexico	North America			1	CY089854.2		
Tula	Europe			1	JN185117.1		
St.Petersburg	North America			1	JN185105.1		
Guangdong	Asia			1	JF929779.1		
Moscow	Europe			1	JN714526.1		
Budapest	Europe			1	CY097988.1		
Brazil	South America			1	CY120755.1		
Kenya	Africa			1	JQ396242.1		
Vladivostok	Europe					1	JX473010.1

PB2：

District	Continent	2010	GenBank	2011	GenBank	2012	GenBank
Singapore	Asia	1	JX309805.1				
Tochigi	Asia	1	AB704483.1				
Bangkok	Asia	1	CY098169.1				
Chile	South America	1	CY098097.1				
Mexico City	North America	1	CY097961.1				
Amman	Asia	1	CY097953.1				
District of Columbia	North America	1	CY097842.1				
Dakar	Africa	1	CY097786.1				
Sydney	Oceania	1	CY093156.1	1	CY092863.1		
Netherlands	Europe	1	JF906180.1				
Pennsylvania	North America			1	JN655532.1		
Moscow	Europe			1	CY098057.1		
Prague	Europe			1	CY098001.1		
Totorri	Asia			1	JN185090.1		
Taiwan	Asia			1	JN187181.1		
Brazil	Latin America			1	CY120762.1		
Mexico	North America			1	CY120019.1		
Illinois	North America			1	CY092879.1		
Denmark	Europe			1	CY090829.1		
Vladivostok	Europe					1	JX473007.1
Tomsk	Europe					1	JQ768350.1

NS:

District	Continent	2010	GenBank	2011	GenBank	2012	GenBank
Bangkok	Asia	1	CY098557.1				
Chile	South America	1	CY098174.1				
Cambridge	Europe	1	CY096606.1				
District of Columbia	North America	1	CY096590.1				
Westmead	Oceania	1	CY096573.1				
Khon Kaen	Asia	1	CY096549.1				
Melbourne	Oceania	1	CY096254.1				
Managua	North America	1	CY089375.1				

Athens	Europe	1	CY083814.1				
Finland	Europe	1	JF327347.1				
Iowa	North America			1	JQ290182.1		
Pennsylvania	North America			1	JN655538.1		
Georgia	Asia			1	CY092908.1		
Sydney	Oceania			1	CY092860.1		
Indiana	North America			1	JN655553.1		
St. Petersburg	Europe			1	CY121820.1		
Brazil	Latin America			1	CY120759.1		
Boston	North America			1	CY111258.1	2	CY125779.1<br>CY125787.1
California	North America			1	CY092884.1		
Illinois	North America			1	CY092876.1		

MP:

District	Continent	2010	GenBank	2011	GenBank	2012	GenBank
Bangkok	Asia	1	CY098564.1				
Chile	South America	1	CY093206.1				
Khon Kaen	Asia	1	CY098099.1				
Sydney	Oceania	1	CY093150.1	1	CY092865.1		
Netherlands	Europe	1	CY090862.1				
Managua	North America	1	CY090838.1				
Los Angeles	North America	1	CY083818.1				
Cambridge	Europe	1	CY096603.1				
District of Columbia	North America	1	CY096595.1				
North Fitzroy	Oceania	1	CY098546.1				
Pennsylvania	North America			1	JN655534.1		
Indiana	North America			1	JQ070787.1		
Georgia	Asia			1	CY092905.1		
Brazil	Latin America			1	CY120748.1		
South Carolina	North America			1	CY092897.1		
California	North America			1	CY092881.1		
Illinois	North America			1	CY092873.1		
Boston	North America			1	CY111561.1	2	CY125776.1<br>CY125784.1

PA：

District	Continent	2010	GenBank	2011	GenBank	2012	GenBank
Singapore	Asia	1	JX309983.1				
Tochigi	Asia	1	AB704477.1				

Chile	South America	1	CY098095.1				
Mexico City	North America	1	CY097963.1				
Amman	Asia	1	CY097955.1				
District of Columbia	North America	1	CY097844.1				
Dakar	Africa	1	CY097788.1				
Sydney	Oceania	1	CY092747.1	1	CY092861.1		
Melbourne	Oceania	1	CY096255.1				
Thailand	Asia	1	CY089436.1				
Iowa	North America			1	JQ290184.1		
Volgograd	Europe			1	JN714507.1		
Moscow	Europe			1	CY098027.1	1	JX046925.1
Taiwan	Asia			1	JN187269.1		
Illinois	North America			1	CY092877.1		
Budapest	Europe			1	CY097995.1		
Prague	Europe			1	CY098003.1		
Ulaanbaatar	Asia			1	CY081057.1		
Brazil	South America			1	CY120752.1		
Vladivostok	Europe					1	JX473009.1
Tomsk	Europe					1	JQ768352.1

PB1：

District	Continent	2010	GenBank	2011	GenBank	2012	GenBank
Singapore	Asia	1	JX309982.1				
Tochigi	Asia	1	AB704484.1				
Chile	South America	1	CY098096.1				
Mexico	North America	1	CY097962.1				
Amman	Asia	1	CY097954.1				
District of Columbia	North America	1	CY097843.1				
Dakar	Africa	1	CY097787.1				
Managua	North America	1	CY090843.1				
Melbourne	Oceania	1	CY096280.1				
Chile	South America	1	CY093195.1				
Novosibirsk	Europe			1	JQ041360.1		
Moscow	Europe			1	CY098058.1		
Iowa	North America			1	JQ290186.1		
Prague	Europe			1	CY098002.1		
Budapest	Europe			1	CY097994.1		
Taiwan	Asia			1	JN187239.1		
Tula	Europe			1	JN185091.1		
Pennsylvania	North America			1	JN655539.1		

Sydney	Oceania			1	CY092870.1		
Ulaanbaatar	Asia			1	CY080572.1		
Boston	North America					2	CY125781.1<br>CY125789.1
Tomsk	Europe					1	JQ768351.1

NP：

District	Continent	2010	GenBank	2011	GenBank	2012	GenBank
Singapore	Asia	1	JX309984.1				
Tochigi	Asia	1	AB704487.1				
Bangkok	Asia	1	CY098556.1				
Sydney	Oceania	1	CY098190.1				
Chile	South America	1	CY098173.1				
Cambridge	Europe	1	CY096605.1				
District of Columbia	North America	1	CY096597.1				
Melbourne	Oceania	1	CY096253.1				
Netherlands	Europe	1	JF906184.1				
Managua	North America	1	CY089382.1				
Novosibirsk	Europe			1	JQ041357.1		
Moscow	Europe			1	CY098061.1		
District of Columbia	North America			1	CY097869.1		
Taiwan	Asia			1	JN187297.1		
California	North America			1	CY092883.1		
Sydney	Oceania			1	CY092867.1		
Thailand	Asia			1	CY089464.1		
Ulaanbaatar	Asia			1	CY080573.1		
Prague	Europe			1	CY098005.1		
Budapest	Europe			1	CY097997.1		
Moscow	Europe					1	JX046927.1
Tomsk	Europe					1	JQ768354.1

NA：

District	Continent	2010	GenBank	2011	GenBank	2012	GenBank
Taiwan	Asia	1	JN381442.1	1	JN381480.1		
Singapore	Asia	1	JX309996.1	1	CY091725.1		
Ecuador	South America	1	CY090053.1				
Ontario	North America	1	JN632581.1				
Sydney	Oceania	1	CY098180.1	1	CY092866.1		
Chile	South America	1	CY098172.1				
Amman	Asia	1	CY097958.1				

District of Columbia	North America	1	CY097847.1	1	CY097870.1		
Dakar	Africa	1	CY097791.1				
Netherlands	Europe	1	CY090863.1				
Moscow	Europe			1	JN714539.1	1	JX046928.1
Prague	Europe			1	CY098006.1		
Budapest	Europe			1	CY097998.1		
California	North America			1	CY092882.1		
Thailand	Asia			1	CY089465.1		
Brazil	South America			1	CY120757.1		
Boston	North America					2	CY125777.1<br>CY125785.1
Vladivostok	Europe					1	JX473012.1
Mexico	North America					2	CY120057.1<br>CY120059.1
Ulaanbaatar	Asia					1	CY116605.1
Tomsk	Europe					1	JQ768355.1
