# Supplementary material for: A computational method for predicting regulation of human microRNAs on the influenza virus genome
Source: BMC Syst Biol. 2013 Oct 14;7(Suppl 2):S3. doi: 10.1186/1752-0509-7-S2-S3 (PMC3851852; doi:10.1186/1752-0509-7-S2-S3)
Supplement: Additional File 3 — The coding sequence of the HA fragment from year 2000 to 2012 recorded in Genbank. [file 1752-0509-7-S2-S3-S3.PDF]

miRNA of Human

The sequences of miRNA of Human are totally from miRTarBase, and this additional file gives 1100 miRNA of Human in FASTA format.

>hsa-miR-576-3p MIMAT0004796
AAGAUGUGGAAAAAUUGGAAUC
>hsa-miR-140-5p MIMAT0000431
CAGUGGUUUUACCCUAUGGUAG
>hsa-miR-124* MIMAT0004591
CGUGUUCACAGCGGACCUUGAU
>hsa-miR-132* MIMAT0004594
ACCGUGGCUUUCGAUUGUUACU
>hsa-miR-376b MIMAT0002172
AUCAUAGAGGAAAAUCCAUGUU
>hsa-miR-517a MIMAT0002852
AUCGUGCAUCCCUUUAGAGUGU
>hsa-miR-557 MIMAT0003221
GUUUGCACGGGUGGGCCUUGUCU
>hsa-miR-665 MIMAT0004952
ACCAGGAGGCUGAGGCCCCU
>hsa-miR-518d-5p MIMAT0005456
CUCUAGAGGGAAGCACUUUCUG
>hsa-miR-637 MIMAT0003307
ACUGGGGGCUUUCGGGCUCUGCGU
>hsa-miR-200b MIMAT0000318
UAAUACUGCCUGGUAAUGAUGA
>hsa-miR-520b MIMAT0002843
AAAGUGCUUCCUUUUAGAGGG
>hsa-miR-105* MIMAT0004516
ACGGAUGUUUGAGCAUGUGCUA
>hsa-miR-518a-3p MIMAT0002863
GAAAGCGCUUCCCUUUGCUGGA
>hsa-miR-645 MIMAT0003315
UCUAGGCUGGUACUGCUGA
>hsa-miR-16 MIMAT0000069
UAGCAGCACGUAAAUAUUGGCG
>hsa-miR-3157 MIMAT0015031
UUCAGCCAGGCUAGUGCAGUCU
>hsa-miR-448 MIMAT0001532
UUGCAUAUGUAGGAUGUCCCAU
>hsa-miR-2909 MIMAT0013863
GUUAGGGCCAACAUCUCUUGG
>hsa-miR-1306 MIMAT0005950

ACGUUGGCUCUGGUGGUG
>hsa-miR-548s MIMAT0014987
AUGGCCAAAACUGCAGUUAUUUU
>hsa-miR-20b* MIMAT0004752
ACUGUAGUAUGGGCACUUCCAG
>hsa-miR-515-5p MIMAT0002826
UUCUCCAAAAGAAAGCACUUUCUG
>hsa-miR-27b* MIMAT0004588
AGAGCUUAGCUGAUUGGUGAAC
>hsa-miR-1915 MIMAT0007892
CCCCAGGGCGACGCGGCGGG
>hsa-miR-625 MIMAT0003294
AGGGGGAAAGUUCUAUAGUCC
>hsa-miR-138 MIMAT0000430
AGCUGGUGUUGUGAAUCAGGCCG
>hsa-miR-130b* MIMAT0004680
ACUCUUUCCCUGUUGCACUAC
>hsa-miR-1228* MIMAT0005582
GUGGGCGGGGGCAGGUGUGUG
>hsa-miR-648 MIMAT0003318
AAGUGUGCAGGGCACUGGU
>hsa-miR-562 MIMAT0003226
AAAGUAGCUGUACCAUUUGC
>hsa-miR-4328 MIMAT0016926
CCAGUUUUCCCAGGAUU
>hsa-miR-4314 MIMAT0016868
CUCUGGGAAAUGGGACAG
>hsa-miR-3136 MIMAT0015003
CUGACUGAAUAGGUAGGGUCAUU
>hsa-miR-1276 MIMAT0005930
UAAAGAGCCCUGUGGAGACA
>hsa-miR-520g MIMAT0002858
ACAAAGUGCUUCCCUUUAGAGUGU
>hsa-miR-616 MIMAT0004805
AGUCAUUGGAGGGUUUGAGCAG
>hsa-miR-615-5p MIMAT0004804
GGGGGUCCCCGGUGCUCGGAUC
>hsa-miR-550* MIMAT0003257
UGUCUUACUCCCUCAGGCACAU
>hsa-miR-101* MIMAT0004513
CAGUUAUCACAGUGCUGAUGCU
>hsa-miR-3155 MIMAT0015029
CCAGGCUCUGCAGUGGGAACU
>hsa-miR-181a MIMAT0000256

AACAUUCAACGCUGUCGGUGAGU
>hsa-miR-551b* MIMAT0004794
GAAAUCAAGCGUGGGUGAGACC
>hsa-miR-1911* MIMAT0007886
CACCAGGCAUUGUGGUCUCC
>hsa-miR-519b-3p MIMAT0002837
AAAGUGCAUCCUUUUAGAGGUU
>hsa-miR-513b MIMAT0005788
UUCACAAGGAGGUGUCAUUUAU
>hsa-miR-195* MIMAT0004615
CCAAUAUUGGCUGUGCUGCUCC
>hsa-miR-302c* MIMAT0000716
UUUAACAUGGGGGUACCUGCUG
>hsa-miR-2110 MIMAT0010133
UUGGGGAAACGGCCGCUGAGUG
>hsa-miR-487a MIMAT0002178
AAUCAUACAGGGACAUCCAGUU
>hsa-miR-483-3p MIMAT0002173
UCACUCCUCUCCUCCCGUCUU
>hsa-miR-3195 MIMAT0015079
CGCGCCGGGCCCGGGUU
>hsa-miR-204 MIMAT0000265
UUCCCUUUGUCAUCCUAUGCCU
>hsa-miR-193a-5p MIMAT0004614
UGGGUCUUUGCGGGCGAGAUGA
>hsa-miR-96 MIMAT0000095
UUUGGCACUAGCACAUUUUUGCU
>hsa-miR-1289 MIMAT0005879
UGGAGUCCAGGAAUCUGCAUUUU
>hsa-miR-3140 MIMAT0015008
AGCUUUUGGGAAUUCAGGUAGU
>hsa-miR-563 MIMAT0003227
AGGUUGACAUACGUUUCCC
>hsa-miR-2278 MIMAT0011778
GAGAGCAGUGUGUGUUGCCUGG
>hsa-miR-1179 MIMAT0005824
AAGCAUUCUUUCAUUGGUUGG
>hsa-miR-214 MIMAT0000271
ACAGCAGGCACAGACAGGCAGU
>hsa-miR-532-3p MIMAT0004780
CCUCCCACACCCAAGGCUUGCA
>hsa-miR-377 MIMAT0000730
AUCACACAAAGGCAACUUUUGU
>hsa-miR-30c-1* MIMAT0004674

CUGGGAGAGGGUUGUUUACUCC
>hsa-miR-218-1* MIMAT0004565
AUGGUUCCGUCAAGCACCAUGG
>hsa-miR-1304 MIMAT0005892
UUUGAGGCUACAGUGAGAUGUG
>hsa-miR-3122 MIMAT0014984
GUUGGGACAAGAGGACGGUCUU
>hsa-miR-4274 MIMAT0016906
CAGCAGUCCCUCCCCCUG
>hsa-miR-4300 MIMAT0016853
UGGGAGCUGGACUACUUC
>hsa-miR-27a MIMAT0000084
UUCACAGUGGCUAAGUUCCGC
>hsa-miR-1267 MIMAT0005921
CCUGUUGAAGUGUAAUCCCCA
>hsa-miR-1248 MIMAT0005900
ACCUUCUUGUAUAAGCACUGUGCUAAA
>hsa-miR-335 MIMAT0000765
UCAAGAGCAAUAACGAAAAAUGU
>hsa-miR-920 MIMAT0004970
GGGGAGCUGUGGAAGCAGUA
>hsa-miR-92a-2* MIMAT0004508
GGGUGGGGAUUUGUUGCAUUAC
>hsa-miR-363* MIMAT0003385
CGGGUGGAUCACGAUGCAAUUU
>hsa-miR-548b-5p MIMAT0004798
AAAAGUAAUUGUGGUUUUGGCC
>hsa-miR-516a-3p MIMAT0006778
UGCUUCCUUUCAGAGGGU
>hsa-miR-3121 MIMAT0014983
UAAAUAGAGUAGGCAAAGGACA
>hsa-miR-452 MIMAT0001635
AACUGUUUGCAGAGGAAACUGA
>hsa-miR-16-2* MIMAT0004518
CCAAUAUUACUGUGCUGCUUUA
>hsa-miR-575 MIMAT0003240
GAGCCAGUUGGACAGGAGC
>hsa-miR-496 MIMAT0002818
UGAGUAUUACAUGGCCAAUCUC
>hsa-miR-320c MIMAT0005793
AAAAGCUGGGUUGAGAGGGU
>hsa-miR-511 MIMAT0002808
GUGUCUUUUGCUCUGCAGUCA
>hsa-miR-661 MIMAT0003324

UGCCUGGGUCUCUGGCCUGCGCGU
>hsa-miR-375 MIMAT0000728
UUUGUUCGUUCGGCUCGCGUGA
>hsa-miR-31 MIMAT0000089
AGGCAAGAUGCUGGCAUAGCU
>hsa-miR-513a-3p MIMAT0004777
UAAAUUUCACCUUUCUGAGAAGG
>hsa-miR-99a MIMAT0000097
AACCCGUAGAUCCGAUCUUGUG
>hsa-miR-148a* MIMAT0004549
AAAGUUCUGAGACACUCCGACU
>hsa-miR-4302 MIMAT0016855
CCAGUGUGGCUCAGCGAG
>hsa-miR-26a-1* MIMAT0004499
CCUAUUCUUGGUUACUUGCACG
>hsa-miR-142-5p MIMAT0000433
CAUAAAGUAGAAAGCACUACU
>hsa-miR-379* MIMAT0004690
UAUGUAACAUGGUCCACUAACU
>hsa-miR-3162 MIMAT0015036
UUAGGGAGUAGAAGGGUGGGGAG
>hsa-miR-486-3p MIMAT0004762
CGGGGCAGCUCAGUACAGGAU
>hsa-miR-507 MIMAT0002879
UUUUGCACCUUUUGGAGUGAA
>hsa-miR-449a MIMAT0001541
UGGCAGUGUAUUGUUAGCUGGU
>hsa-miR-3171 MIMAT0015046
AGAUGUAUGGAAUCUGUAUAUAUC
>hsa-miR-29b-1* MIMAT0004514
GCUGGUUUCAUAUGGUGGUUUAGA
>hsa-miR-183* MIMAT0004560
GUGAAUUACCGAAGGGCCAUAA
>hsa-miR-330-3p MIMAT0000751
GCAAAGCACACGGCCUGCAGAGA
>hsa-miR-4257 MIMAT0016878
CCAGAGGUGGGGACUGAG
>hsa-miR-4264 MIMAT0016899
ACUCAGUCAUGGUCAUU
>hsa-miR-302a* MIMAT0000683
ACUUAAACGUGGAUGUACUUGCU
>hsa-miR-191 MIMAT0000440
CAACGGAAUCCCAAAAGCAGCUG
>hsa-miR-19b MIMAT0000074

UGUGCAAAUCCAUGCAAAACUGA
>hsa-miR-3202 MIMAT0015089
UGGAAGGGAGAAGAGCUUUAAU
>hsa-miR-943 MIMAT0004986
CUGACUGUUGCCGUCCUCCAG
>hsa-miR-194 MIMAT0000460
UGUAACAGCAACUCCAUGUGGA
>hsa-miR-3149 MIMAT0015022
UUUGUAUGGAUAUGUGUGUGUAU
>hsa-miR-146b-3p MIMAT0004766
UGCCCUGUGGACUCAGUUCUGG
>hsa-miR-1908 MIMAT0007881
CGGCGGGGACGGCGAUUGGUC
>hsa-miR-885-5p MIMAT0004947
UCCAUUACACUACCCUGCCUCU
>hsa-miR-302e MIMAT0005931
UAAGUGCUUCCAUGCUU
>hsa-miR-1282 MIMAT0005940
UCGUUUGCCUUUUUCUGCUU
>hsa-miR-93* MIMAT0004509
ACUGCUGAGCUAGCACUUCCCG
>hsa-let-7c* MIMAT0004483
UAGAGUUACACCCUGGGAGUUA
>hsa-miR-3186-3p MIMAT0015068
UCACGCGGAGAGAUGGCUUUG
>hsa-miR-769-5p MIMAT0003886
UGAGACCUCUGGGUUCUGAGCU
>hsa-miR-544b MIMAT0015004
ACCUGAGGUUGUGCAUUUCUAA
>hsa-miR-603 MIMAT0003271
CACACACUGCAAUUACUUUUGC
>hsa-miR-19a* MIMAT0004490
AGUUUUGCAUAGUUGCACUACA
>hsa-miR-938 MIMAT0004981
UGCCCUUAAAGGUGAACCCAGU
>hsa-miR-574-3p MIMAT0003239
CACGCUCAUGCACACACCCACA
>hsa-miR-128 MIMAT0000424
UCACAGUGAACCGGUCUCUUU
>hsa-miR-134 MIMAT0000447
UGUGACUGGUUGACCAGAGGGG
>hsa-miR-342-3p MIMAT0000753
UCUCACACAGAAAUCGCACCCGU
>hsa-miR-298 MIMAT0004901

AGCAGAAGCAGGGAGGUUCUCCCA
>hsa-miR-1308 MIMAT0005947
GCAUGGGUGGUUCAGUGG
>hsa-miR-4326 MIMAT0016888
UGUUCCUCUGUCUCCCAGAC
>hsa-miR-92b MIMAT0003218
UAUUGCACUCGUCCCGGCCUCC
>hsa-miR-130b MIMAT0000691
CAGUGCAAUGAUGAAAGGGCAU
>hsa-miR-542-5p MIMAT0003340
UCGGGGAUCAUCAUGUCACGAGA
>hsa-miR-1231 MIMAT0005586
GUGUCUGGGCGGACAGCUGC
>hsa-miR-663 MIMAT0003326
AGGCGGGGCGCCGCGGGACCGC
>hsa-miR-4255 MIMAT0016885
CAGUGUUCAGAGAUGGA
>hsa-miR-370 MIMAT0000722
GCCUGCUGGGGUGGAACCUGGU
>hsa-miR-199a-5p MIMAT0000231
CCCAGUGUUCAGACUACCUGUUC
>hsa-miR-937 MIMAT0004980
AUCCGCGCUCUGACUCUCUGCC
>hsa-miR-1280 MIMAT0005946
UCCCACCGCUGCCACCC
>hsa-miR-1207-5p MIMAT0005871
UGGCAGGGAGGCUGGGAGGGG
>hsa-miR-1287 MIMAT0005878
UGCUGGAUCAGUGGUUCGAGUC
>hsa-miR-623 MIMAT0003292
AUCCCUUGCAGGGGCUGUUGGGU
>hsa-miR-875-3p MIMAT0004923
CCUGGAAACACUGAGGUUGUG
>hsa-miR-657 MIMAT0003335
GGCAGGUUCUCACCCUCUCUAGG
>hsa-miR-424* MIMAT0004749
CAAAACGUGAGGCGCUGCUAU
>hsa-miR-181c* MIMAT0004559
AACCAUCGACCGUUGAGUGGAC
>hsa-miR-339-3p MIMAT0004702
UGAGCGCCUCGACGACAGAGCCG
>hsa-let-7d* MIMAT0004484
CUAUACGACCUGCUGCCUUUCU
>hsa-miR-1252 MIMAT0005944

AGAAGGAAAUUGAAUUCAUUUA
>hsa-miR-651 MIMAT0003321
UUUAGGAUAAGCUUGACUUUUG
>hsa-miR-630 MIMAT0003299
AGUAUUCUGUACCAGGGAAGGU
>hsa-miR-580 MIMAT0003245
UUGAGAAUGAUGAAUCAUUAGG
>hsa-miR-129* MIMAT0004548
AAGCCCUUACCCCAAAAAGUAU
>hsa-miR-148a MIMAT0000243
UCAGUGCACUACAGAACUUUGU
>hsa-miR-1246 MIMAT0005898
AAUGGAUUUUUGGAGCAGG
>hsa-miR-379 MIMAT0000733
UGGUAGACUAUGGAACGUAGG
>hsa-miR-600 MIMAT0003268
ACUUACAGACAAGAGCCUUGCUC
>hsa-miR-576-5p MIMAT0003241
AUUCUAAUUUCUCCACGUCUUU
>hsa-miR-4268 MIMAT0016896
GGCUCCUCCUCUCAGGAUGUG
>hsa-miR-586 MIMAT0003252
UAUGCAUUGUAUUUUUAGGUCC
>hsa-miR-876-5p MIMAT0004924
UGGAUUUCUUUGUGAAUCACCA
>hsa-miR-621 MIMAT0003290
GGCUAGCAACAGCGCUUACCU
>hsa-miR-3173 MIMAT0015048
AAAGGAGGAAAUAGGCAGGCCA
>hsa-miR-582-5p MIMAT0003247
UUACAGUUGUUCAACCAGUUACU
>hsa-miR-338-5p MIMAT0004701
AACAAUAUCCUGGUGCUGAGUG
>hsa-miR-1296 MIMAT0005794
UUAGGGCCCUGGCUCCAUCUCC
>hsa-miR-30b MIMAT0000420
UGUAAACAUCCUACACUCAGCU
>hsa-miR-1470 MIMAT0007348
GCCCUCCGCCCGUGCACCCCG
>hsa-miR-543 MIMAT0004954
AAACAUUCGCGGUGCACUUCUU
>hsa-miR-4291 MIMAT0016922
UUCAGCAGGAACAGCU
>hsa-miR-374a* MIMAT0004688

CUUAUCAGAUUGUAUUGUAAUU
>hsa-miR-208a MIMAT0000241
AUAAGACGAGCAAAAAGCUUGU
>hsa-miR-186 MIMAT0000456
CAAAGAAUUCUCCUUUUGGGCU
>hsa-miR-301b MIMAT0004958
CAGUGCAAUGAUAUUGUCAAAGC
>hsa-miR-1236 MIMAT0005591
CCUCUUCCCCUUGUCUCUCCAG
>hsa-miR-618 MIMAT0003287
AAACUCUACUUGUCCUUCUGAGU
>hsa-miR-4282 MIMAT0016912
UAAAAUUUGCAUCCAGGA
>hsa-miR-33a* MIMAT0004506
CAAUGUUUCCACAGUGCAUCAC
>hsa-miR-491-5p MIMAT0002807
AGUGGGGAACCCUUCCAUGAGG
>hsa-miR-2114* MIMAT0011157
CGAGCCUCAAGCAAGGGACUU
>hsa-miR-4286 MIMAT0016916
ACCCCACUCCUGGUACC
>hsa-miR-3177 MIMAT0015054
UGCACGGCACUGGGGACACGU
>hsa-miR-218-2* MIMAT0004566
CAUGGUUCUGUCAAGCACCGCG
>hsa-miR-410 MIMAT0002171
AAUAUAACACAGAUGGCCUGU
>hsa-miR-641 MIMAT0003311
AAAGACAUAGGAUAGAGUCACCUC
>hsa-miR-320e MIMAT0015072
AAAGCUGGGUUGAGAAGG
>hsa-miR-1193 MIMAT0015049
GGGAUGGUAGACCGGUGACGUGC
>hsa-miR-3153 MIMAT0015026
GGGGAAAGCGAGUAGGGACAUUU
>hsa-miR-92b* MIMAT0004792
AGGGACGGGACGCGGUGCAGUG
>hsa-miR-29c* MIMAT0004673
UGACCGAUUUCUCCUGGUGUUC
>hsa-miR-548d-3p MIMAT0003323
CAAAAACCACAGUUUCUUUUGC
>hsa-miR-4312 MIMAT0016864
GGCCUUGUUCCUGUCCCCA
>hsa-miR-516b* MIMAT0002860

UGCUUCCUUUCAGAGGGU
>hsa-miR-1912 MIMAT0007887
UACCCAGAGCAUGCAGUGUGAA
>hsa-miR-4316 MIMAT0016867
GGUGAGGCUAGCUGGUG
>hsa-miR-146a* MIMAT0004608
CCUCUGAAAUUCAGUUCUUCAG
>hsa-miR-525-5p MIMAT0002838
CUCCAGAGGGAUGCACUUUCU
>hsa-miR-494 MIMAT0002816
UGAAACAUACACGGGAAACCUC
>hsa-miR-30a* MIMAT0000088
CUUUCAGUCGGAUGUUUGCAGC
>hsa-miR-3161 MIMAT0015035
CUGAUAAGAACAGAGGCCCAGAU
>hsa-miR-29a* MIMAT0004503
ACUGAUUUCUUUUGGUGUUCAG
>hsa-miR-3124 MIMAT0014986
UUCGCGGGCGAAGGCAAAGUC
>hsa-miR-4297 MIMAT0016846
UGCCUUCCUGUCUGUG
>hsa-miR-3065-5p MIMAT0015066
UCAACAAAAUCACUGAUGCUGGA
>hsa-miR-548o MIMAT0005919
CCAAAACUGCAGUUACUUUUGC
>hsa-miR-1 MIMAT0000416
UGGAAUGUAAAGAAGUAUGUAU
>hsa-miR-1227 MIMAT0005580
CGUGCCACCCUUUUCCCCAG
>hsa-miR-373 MIMAT0000726
GAAGUGCUUCGAUUUUGGGGUGU
>hsa-miR-616* MIMAT0003284
ACUCAAAACCCUUCAGUGACUU
>hsa-miR-874 MIMAT0004911
CUGCCCUGGCCCGAGGGACCGA
>hsa-miR-323b-3p MIMAT0015050
CCCAAUACACGGUCGACCUCUU
>hsa-miR-1273c MIMAT0015017
GGCGACAAAACGAGACCCUGUC
>hsa-miR-373* MIMAT0000725
ACUCAAAAUGGGGGCGCUUUCC
>hsa-miR-2276 MIMAT0011775
UCUGCAAGUGUCAGAGGCGAGG
>hsa-miR-548f MIMAT0005895

AAAAACUGUAAUUACUUUU
>hsa-miR-3119 MIMAT0014981
UGGCUUUUAACUUUGAUGGC
>hsa-miR-26b MIMAT0000083
UUCAAGUAAUUCAGGAUAGGU
>hsa-miR-569 MIMAT0003234
AGUUAAUGAAUCCUGGAAAGU
>hsa-miR-99a* MIMAT0004511
CAAGCUCGCUUCUAUGGGUCUG
>hsa-miR-147b MIMAT0004928
GUGUGCGGAAAUGCUUCUGCUA
>hsa-miR-518b MIMAT0002844
CAAAGCGCUCCCCUUUAGAGGU
>hsa-miR-3126-5p MIMAT0014989
UGAGGGACAGAUGCCAGAAGCA
>hsa-miR-2116* MIMAT0011161
CCUCCCAUGCCAAGAACUCCC
>hsa-let-7f MIMAT0000067
UGAGGUAGUAGAUUGUAUAGUU
>hsa-miR-573 MIMAT0003238
CUGAAGUGAUGUGUAACUGAUCAG
>hsa-miR-4276 MIMAT0016904
CUCAGUGACUCAUGUGC
>hsa-miR-32* MIMAT0004505
CAAUUUAGUGUGUGUGAUAUUU
>hsa-miR-1269 MIMAT0005923
CUGGACUGAGCCGUGCUACUGG
>hsa-miR-302d MIMAT0000718
UAAGUGCUUCCAUGUUUGAGUGU
>hsa-miR-1202 MIMAT0005865
GUGCCAGCUGCAGUGGGGGAG
>hsa-miR-450a MIMAT0001545
UUUUGCGAUGUGUUCCUAAUAU
>hsa-miR-765 MIMAT0003945
UGGAGGAGAAGGAAGGUGAUG
>hsa-miR-628-3p MIMAT0003297
UCUAGUAAGAGUGGCAGUCGA
>hsa-miR-4304 MIMAT0016854
CCGGCAUGUCCAGGGCA
>hsa-miR-3131 MIMAT0014996
UCGAGGACUGGUGGAAGGGCCUU
>hsa-miR-532-5p MIMAT0002888
CAUGCCUUGAGUGUAGGACCGU
>hsa-miR-296-5p MIMAT0000690

AGGGCCCCCCCUCAAUCCUGU
>hsa-miR-15b MIMAT0000417
UAGCAGCACAUCAUGGUUUACA
>hsa-miR-188-3p MIMAT0004613
CUCCCACAUGCAGGGUUUGCA
>hsa-miR-196b* MIMAT0009201
UCGACAGCACGACACUGCCUUC
>hsa-miR-96* MIMAT0004510
AAUCAUGUGCAGUGCCAAUAUG
>hsa-miR-2117 MIMAT0011162
UGUUCUCUUUGCCAAGGACAG
>hsa-miR-599 MIMAT0003267
GUUGUGUCAGUUUAUCAAAC
>hsa-miR-590-3p MIMAT0004801
UAAUUUUAUGUAUAAGCUAGU
>hsa-miR-548u MIMAT0015013
CAAAGACUGCAAUUACUUUUGCG
>hsa-miR-3200 MIMAT0015085
CACCUUGCGCUACUCAGGUCUG
>hsa-miR-4263 MIMAT0016898
AUUCUAAGUGCCUUGGCC
>hsa-miR-548v MIMAT0015020
AGCUACAGUUACUUUUGCACCA
>hsa-miR-29c MIMAT0000681
UAGCACCAUUUGAAAUCGGUUA
>hsa-miR-132 MIMAT0000426
UAACAGUCUACAGCCAUGGUCG
>hsa-miR-7-1* MIMAT0004553
CAACAAAUCACAGUCUGCCAUA
>hsa-miR-589 MIMAT0004799
UGAGAACCACGUCUGCUCUGAG
>hsa-miR-939 MIMAT0004982
UGGGGAGCUGAGGCUCUGGGGGUG
>hsa-miR-635 MIMAT0003305
ACUUGGGCACUGAAACAAUGUCC
>hsa-miR-1915* MIMAT0007891
ACCUUGCCUUGCUGCCCGGGCC
>hsa-miR-589* MIMAT0003256
UCAGAACAAAUGCCGGUUCCCAGA
>hsa-miR-1291 MIMAT0005881
UGGCCCUGACUGAAGACCAGCAGU
>hsa-miR-190b MIMAT0004929
UGAUAUGUUUGAUAUUGGGUU
>hsa-miR-1200 MIMAT0005863

CUCCUGAGCCAUUCUGAGCCUC
>hsa-miR-885-3p MIMAT0004948
AGGCAGCGGGGUGUAGUGGAUA
>hsa-miR-3199 MIMAT0015084
AGGGACUGCCUUAGGAGAAAGUU
>hsa-miR-643 MIMAT0003313
ACUUGUAUGCUAGCUCAGGUAG
>hsa-miR-3169 MIMAT0015044
UAGGACUGUGCUUGGCACAUAG
>hsa-miR-135a MIMAT0000428
UAUGGCUUUUUAUUCCUAUGUGA
>hsa-miR-579 MIMAT0003244
UUCAUUUGGUAUAAACCGCGAUU
>hsa-miR-411* MIMAT0004813
UAUGUAACACGGUCCACUAACC
>hsa-miR-520f MIMAT0002830
AAGUGCUUCCUUUUAGAGGGUU
>hsa-miR-26a-2* MIMAT0004681
CCUAUUCUUGAUUACUUGUUUC
>hsa-miR-361-5p MIMAT0000703
UUAUCAGAAUCUCCAGGGGUAC
>hsa-miR-146a MIMAT0000449
UGAGAACUGAAUUCCAUGGGUU
>hsa-miR-605 MIMAT0003273
UAAAUCCCAUGGUGCCUUCUCCU
>hsa-miR-103-as MIMAT0007402
UCAUAGCCCUGUACAAUGCUGCU
>hsa-miR-200c* MIMAT0004657
CGUCUUACCCAGCAGUGUUUGG
>hsa-miR-323b-5p MIMAT0001630
AGGUUGUCCGUGGUGAGUUCGCA
>hsa-miR-3191 MIMAT0015075
UGGGGACGUAGCUGGCCAGACAG
>hsa-miR-1233 MIMAT0005588
UGAGCCCUGUCCUCCCGCAG
>hsa-miR-520c-3p MIMAT0002846
AAAGUGCUUCCUUUUAGAGGGU
>hsa-miR-4310 MIMAT0016862
GCAGCAUUCAUGUCCC
>hsa-miR-518a-5p MIMAT0005457
CUGCAAAGGGAAGCCCUUUC
>hsa-miR-608 MIMAT0003276
AGGGGUGGUGUUGGGACAGCUCCGU
>hsa-miR-770-5p MIMAT0003948

UCCAGUACCACGUGUCAGGGCCA

>hsa-miR-18b MIMAT0001412

UAAGGUGCAUCUAGUGCAGUUAG

>hsa-miR-4277 MIMAT0016908

GCAGUUCUGAGCACAGUACAC

>hsa-miR-509-3p MIMAT0002881

UGAUUGGUACGUCUGUGGGUAG

>hsa-miR-185* MIMAT0004611

AGGGGCUGGCUUUCCUCUGGUC

>hsa-miR-2115 MIMAT0011158

AGCUUCCAUGACUCCUGAUGGA

>hsa-miR-1323 MIMAT0005795

UCAAAACUGAGGGGCAUUUUCU

>hsa-miR-214* MIMAT0004564

UGCCUGUCUACACUUGCUGUGC

>hsa-miR-886-5p MIMAT0004905

CGGGUCGGAGUUAGCUCAAGCGG

>hsa-miR-323-5p MIMAT0004696

AGGUGGUCCGUGGCGCGUUCGC

>hsa-miR-4292 MIMAT0016919

CCCCUGGGCCGGCCUUGG

>hsa-miR-384 MIMAT0001075

AUUCCUAGAAAUUGUUCAUA

>hsa-miR-653 MIMAT0003328

GUGUUGAAACAAUCUCUACUG

>hsa-miR-220b MIMAT0004908

CCACCACCGUGUCUGACACUU

>hsa-miR-302a MIMAT0000684

UAAGUGCUUCCAUGUUUUGGUGA

>hsa-miR-675* MIMAT0006790

CUGUAUGCCCUCACCGCUCA

>hsa-miR-4253 MIMAT0016882

AGGGCAUGUCCAGGGGGU

>hsa-miR-127-3p MIMAT0000446

UCGGAUCCGUCUGAGCUUGGCU

>hsa-miR-654-3p MIMAT0004814

UAUGUCUGCUGACCAUCACCUU

>hsa-miR-30e* MIMAT0000693

CUUUCAGUCGGAUGUUUACAGC

>hsa-miR-122* MIMAT0004590

AACGCCAUUAUCACACUAAAUA

>hsa-miR-126 MIMAT0000445

UCGUACCGUGAGUAAUAAUGCG

>hsa-miR-429 MIMAT0001536

UAAUACUGUCUGGUAAAACCGU
>hsa-miR-503 MIMAT0002874
UAGCAGCGGGAACAGUUCUGCAG
>hsa-miR-624* MIMAT0003293
UAGUACCAGUACCUUGUGUUCA
>hsa-miR-488 MIMAT0004763
UUGAAAGGCUAUUUCUUGGUC
>hsa-miR-508-3p MIMAT0002880
UGAUUGUAGCCUUUUGGAGUAGA
>hsa-let-7g MIMAT0000414
UGAGGUAGUAGUUUGUACAGUU
>hsa-miR-718 MIMAT0012735
CUUCCGCCCCGCCGGGCGUCG
>hsa-miR-517* MIMAT0002851
CCUCUAGAUGGAAGCACUGUCU
>hsa-miR-548d-5p MIMAT0004812
AAAAGUAAUUGUGGUUUUUGCC
>hsa-miR-500b MIMAT0016925
AAUCCUUGCUACCUGGGU
>hsa-miR-325 MIMAT0000771
CCUAGUAGGUGUCCAGUAAGUGU
>hsa-miR-324-5p MIMAT0000761
CGCAUCCCCUAGGGCAUUGGUGU
>hsa-miR-4280 MIMAT0016911
GAGUGUAGUUCUGAGCAGAGC
>hsa-miR-3158 MIMAT0015032
AAGGGCUUCCUCUCUGCAGGAC
>hsa-miR-1294 MIMAT0005884
UGUGAGGUUGGCAUUGUUGUCU
>hsa-miR-432 MIMAT0002814
UCUUGGAGUAGGUCAUUGGGUGG
>hsa-miR-210 MIMAT0000267
CUGUGCGUGUGACAGCGGCUGA
>hsa-miR-1270 MIMAT0005924
CUGGAGAUAUGGAAGAGCUGUGU
>hsa-miR-3168 MIMAT0015043
GAGUUCUACAGUCAGAC
>hsa-miR-769-3p MIMAT0003887
CUGGGAUCUCCGGGGUCUUGGUU
>hsa-miR-593 MIMAT0004802
UGUCUCUGCUGGGGUUUCU
>hsa-miR-136* MIMAT0004606
CAUCAUCGUCUCAAAUGAGUCU
>hsa-miR-4323 MIMAT0016875

CAGCCCCACAGCCUCAGA
>hsa-miR-34b* MIMAT0000685
UAGGCAGUGUCAUUAGCUGAUUG
>hsa-miR-148b* MIMAT0004699
AAGUUCUGUUAUACACUCAGGC
>hsa-miR-4299 MIMAT0016851
GCUGGUGACAUGAGAGGC
>hsa-miR-512-3p MIMAT0002823
AAGUGCUGUCAUAGCUGAGGUC
>hsa-miR-18b* MIMAT0004751
UGCCCUAAAUGCCCCUUCUGGC
>hsa-miR-195 MIMAT0000461
UAGCAGCACAGAAAUAUUGGC
>hsa-miR-423-3p MIMAT0001340
AGCUCGGUCUGAGGCCCCUCAGU
>hsa-miR-1910 MIMAT0007884
CCAGUCCUGUGCCUGCCGCCU
>hsa-miR-331-5p MIMAT0004700
CUAGGUAUGGUCCCAGGGAUCC
>hsa-miR-487b MIMAT0003180
AAUCGUACAGGGUCAUCCACUU
>hsa-miR-4303 MIMAT0016856
UUCUGAGCUGAGGACAG
>hsa-miR-3132 MIMAT0014997
UGGGUAGAGAAGGAGCUCAGAGGA
>hsa-miR-610 MIMAT0003278
UGAGCUAAAUGUGUGCUGGGA
>hsa-miR-519d MIMAT0002853
CAAAGUGCCUCCCUUUAGAGUG
>hsa-miR-198 MIMAT0000228
GGUCCAGAGGGGAGAUAGGUUC
>hsa-miR-144* MIMAT0004600
GGAUAUCAUCAUAUACUGUAAG
>hsa-miR-545 MIMAT0003165
UCAGCAAACAUUUAUUGUGUGC
>hsa-miR-452* MIMAT0001636
CUCAUCUGCAAAGAAGUAAGUG
>hsa-miR-181d MIMAT0002821
AACAUUCAUUGUUGUCGGUGGGU
>hsa-miR-504 MIMAT0002875
AGACCCUGGUCUGCACUCUAUC
>hsa-miR-935 MIMAT0004978
CCAGUUACCGCUUCCGCUACCGC
>hsa-miR-612 MIMAT0003280

GCUGGGCAGGGCUUCUGAGCUCCUU
>hsa-miR-762 MIMAT0010313
GGGGCUGGGGCCGGGGCCGAGC
>hsa-miR-3130-5p MIMAT0014995
UACCCAGUCUCCGGUGCAGCC
>hsa-miR-323-3p MIMAT0000755
CACAUUACACGGUCGACCUCU
>hsa-miR-1307 MIMAT0005951
ACUCGGCGUGGCGUCGGUCGUG
>hsa-miR-1258 MIMAT0005909
AGUUAGGAUUAGGUCGUGGAA
>hsa-miR-200a MIMAT0000682
UAACACUGUCUGGUAACGAUGU
>hsa-miR-4315 MIMAT0016866
CCGCUUUCUGAGCUGGAC
>hsa-miR-3138 MIMAT0015006
UGUGGACAGUGAGGUAGAGGGAGU
>hsa-miR-30d MIMAT0000245
UGUAAACAUCCCCGACUGGAAG
>hsa-miR-3142 MIMAT0015011
AAGGCCUUUCUGAACCUUCAGA
>hsa-miR-10b* MIMAT0004556
ACAGAUUCGAUUCUAGGGGAAU
>hsa-miR-93 MIMAT0000093
CAAAGUGCUGUUCGUGCAGGUAG
>hsa-miR-3181 MIMAT0015061
AUCGGGCCCUCGGCGCCGG
>hsa-miR-29b-2* MIMAT0004515
CUGGUUUCACAUGGUGGCUUAG
>hsa-miR-3074 MIMAT0015027
GAUAUCAGCUCAGUAGGCACCG
>hsa-miR-4259 MIMAT0016880
CAGUUGGGUCUAGGGGUCAGGA
>hsa-miR-566 MIMAT0003230
GGGCGCCUGUGAUCCCAAC
>hsa-miR-139-3p MIMAT0004552
GGAGACGCGGCCCUGUUGGAGU
>hsa-miR-554 MIMAT0003217
GCUAGUCCUGACUCAGCCAGU
>hsa-miR-523 MIMAT0002840
GAACGCGCUUCCCUAUAGAGGGU
>hsa-miR-491-3p MIMAT0004765
CUUAUGCAAGAUUCCCUUCUAC
>hsa-miR-1260 MIMAT0005911

AUCCCACCUCUGCCACCA
>hsa-miR-942 MIMAT0004985
UCUUCUCUGUUUUGGCCAUGUG
>hsa-miR-152 MIMAT0000438
UCAGUGCAUGACAGAACUUGG
>hsa-miR-422a MIMAT0001339
ACUGGACUUAGGGUCAGAAGGC
>hsa-miR-4261 MIMAT0016890
AGGAAACAGGGACCCA
>hsa-miR-101 MIMAT0000099
UACAGUACUGUGAUAACUGAA
>hsa-miR-23a* MIMAT0004496
GGGGUUCCUGGGGAUGGGAUUU
>hsa-miR-362-3p MIMAT0004683
AACACACCUAUUCAAGGAUUCA
>hsa-miR-1263 MIMAT0005915
AUGGUACCCUGGCAUACUGAGU
>hsa-miR-539 MIMAT0003163
GGAGAAAUUAUCCUUGGUGUGU
>hsa-miR-1257 MIMAT0005908
AGUGAAUGAUGGGUUCUGACC
>hsa-miR-548x MIMAT0015081
UAAAAACUGCAAUUACUUUCA
>hsa-miR-1229 MIMAT0005584
CUCUCACCACUGCCCUCCCACAG
>hsa-miR-146b-5p MIMAT0002809
UGAGAACUGAAUUCCAUAGGCU
>hsa-miR-412 MIMAT0002170
ACUUCACCUGGUCCACUAGCCGU
>hsa-miR-634 MIMAT0003304
AACCAGCACCCCAACUUUGGAC
>hsa-miR-133b MIMAT0000770
UUUGGUCCCCUUCAACCAGCUA
>hsa-miR-567 MIMAT0003231
AGUAUGUUCUUCCAGGACAGAAC
>hsa-miR-149* MIMAT0004609
AGGGAGGGACGGGGGCUGUGC
>hsa-miR-572 MIMAT0003237
GUCCGCUCGGCGGUGGCCCA
>hsa-miR-548q MIMAT0011163
GCUGGUGCAAAAGUAAUGGCGG
>hsa-miR-2052 MIMAT0009977
UGUUUUGAUAACAGUAAUGU
>hsa-miR-4307 MIMAT0016860

AAUGUUUUUUCCUGUUUCC
>hsa-miR-136 MIMAT0000448
ACUCCAUUUGUUUUGAUGAUGGA
>hsa-miR-328 MIMAT0000752
CUGGCCCUCUCUGCCCUUCCGU
>hsa-miR-330-5p MIMAT0004693
UCUCUGGGCCUGUGUCUUAGGC
>hsa-miR-3197 MIMAT0015082
GGAGGCGCAGGCUCGGAAAGGCG
>hsa-miR-139-5p MIMAT0000250
UCUACAGUGCACGUGUCUCCAG
>hsa-miR-941 MIMAT0004984
CACCCGGCUGUGUGCACAUGUGC
>hsa-miR-141 MIMAT0000432
UAACACUGUCUGGUAAAGAUGG
>hsa-miR-223 MIMAT0000280
UGUCAGUUUGUCAAAUACCCCA
>hsa-let-7a MIMAT0000062
UGAGGUAGUAGGUUGUAUAGUU
>hsa-miR-889 MIMAT0004921
UUAAUAUCGGACAACCAUUGU
>hsa-miR-518c MIMAT0002848
CAAAGCGCUUCUCUUUAGAGUGU
>hsa-miR-449b* MIMAT0009203
CAGCCACAACUACCCUGCCACU
>hsa-miR-98 MIMAT0000096
UGAGGUAGUAAGUUGUAUUGUU
>hsa-miR-571 MIMAT0003236
UGAGUUGGCCAUCUGAGUGAG
>hsa-miR-548h MIMAT0005928
AAAAGUAAUCGCGGUUUUUGUC
>hsa-miR-492 MIMAT0002812
AGGACCUGCGGGACAAGAUUCUU
>hsa-miR-625* MIMAT0004808
GACUAUAGAACUUUCCCCCUCA
>hsa-miR-381 MIMAT0000736
UAUACAAGGGCAAGCUCUCUGU
>hsa-miR-3175 MIMAT0015052
CGGGGAGAGAACGCAGUGACGU
>hsa-miR-708* MIMAT0004927
CAACUAGACUGUGAGCUUCUAG
>hsa-miR-597 MIMAT0003265
UGUGUCACUCGAUGACCACUGU
>hsa-miR-1185 MIMAT0005798

AGAGGAUACCCUUUGUAUGUU
>hsa-miR-744* MIMAT0004946
CUGUUGCCACUAACCUCAACCU
>hsa-miR-519e* MIMAT0002828
UUCUCCAAAAGGGAGCACUUUC
>hsa-miR-3151 MIMAT0015024
GGUGGGGCAAUGGGAUCAGGU
>hsa-miR-1238 MIMAT0005593
CUUCCUCGUCUGUCUGCCCC
>hsa-miR-150 MIMAT0000451
UCUCCCAACCCUUGUACCAGUG
>hsa-miR-3128 MIMAT0014991
UCUGGCAAGUAAAAAACUCUCAU
>hsa-miR-526a MIMAT0002845
CUCUAGAGGGAAGCACUUUCUG
>hsa-miR-181a-2* MIMAT0004558
ACCACUGACCGUUGACUGUACC
>hsa-miR-451 MIMAT0001631
AAACCGUUACCAUUACUGAGUU
>hsa-miR-3125 MIMAT0014988
UAGAGGAAGCUGUGGAGAGA
>hsa-let-7e* MIMAT0004485
CUAUACGGCCUCCUAGCUUUCC
>hsa-miR-3117 MIMAT0014979
AUAGGACUCAUAUAGUGCCAG
>hsa-miR-501-5p MIMAT0002872
AAUCCUUUGUCCCUGGGUGAGA
>hsa-miR-199b-5p MIMAT0000263
CCCAGUGUUUAGACUAUCUGUUC
>hsa-miR-342-5p MIMAT0004694
AGGGGUGCUAUCUGUGAUUGA
>hsa-miR-493 MIMAT0003161
UGAAGGUCUACUGUGUGCCAGG
>hsa-miR-224 MIMAT0000281
CAAGUCACUAGUGGUUCCGUU
>hsa-miR-1284 MIMAT0005941
UCUAUACAGACCCUGGCUUUUC
>hsa-miR-221* MIMAT0004568
ACCUGGCAUACAAUGUAGAUUU
>hsa-miR-3185 MIMAT0015065
AGAAGAAGGCGGUCGGUCUGCGG
>hsa-miR-527 MIMAT0002862
CUGCAAAGGGAAGCCCUUUC
>hsa-miR-185 MIMAT0000455

UGGAGAGAAAGGCAGUUCCUGA
>hsa-miR-4270 MIMAT0016900
UCAGGGAGUCAGGGGAGGGC
>hsa-miR-17* MIMAT0000071
ACUGCAGUGAAGGCACUUGUAG
>hsa-miR-20b MIMAT0001413
CAAAGUGCUCAUAGUGCAGGUAG
>hsa-miR-556-5p MIMAT0003220
GAUGAGCUCAUUGUAAUAUGAG
>hsa-miR-1321 MIMAT0005952
CAGGGAGGUGAAUGUGAU
>hsa-miR-523* MIMAT0005449
CUCUAGAGGGAAGCGCUUUCUG
>hsa-miR-100* MIMAT0004512
CAAGCUUGUAUCUAUAGGUAUG
>hsa-miR-106a MIMAT0000103
AAAAGUGCUUACAGUGCAGGUAG
>hsa-miR-4305 MIMAT0016857
CCUAGACACCUCCAGUUC
>hsa-miR-759 MIMAT0010497
GCAGAGUGCAAACAAUUUUGAC
>hsa-miR-138-2* MIMAT0004596
GCUAUUUCACGACACCAGGGUU
>hsa-miR-877* MIMAT0004950
UCCUCUUCUCCCUCCUCCCAG
>hsa-miR-18a* MIMAT0002891
ACUGCCCUAAGUGCUCCUUCUGG
>hsa-miR-208b MIMAT0004960
AUAAGACGAACAAAAGGUUUGU
>hsa-miR-30b* MIMAT0004589
CUGGGAGGUGGAUGUUUACUUC
>hsa-miR-1539 MIMAT0007401
UCCUGCGCGUCCCAGAUGCCC
>hsa-miR-191* MIMAT0001618
GCUGCGCUUGGAUUUCGUCCCC
>hsa-miR-483-5p MIMAT0004761
AAGACGGGAGGAAAGAAGGGAG
>hsa-miR-550 MIMAT0004800
AGUGCCUGAGGGAGUAAGAGCCC
>hsa-miR-2115* MIMAT0011159
CAUCAGAAUUCAUGGAGGCUAG
>hsa-miR-758 MIMAT0003879
UUUGUGACCUGGUCCACUAACC
>hsa-miR-520h MIMAT0002867

ACAAAGUGCUUCCCUUUAGAGU
>hsa-miR-4311 MIMAT0016863
GAAAGAGAGCUGAGUGUG
>hsa-miR-10b MIMAT0000254
UACCCUGUAGAACCGAAUUUGUG
>hsa-miR-555 MIMAT0003219
AGGGUAAGCUGAACCUCUGAU
>hsa-let-7c MIMAT0000064
UGAGGUAGUAGGUUGUAUGGUU
>hsa-miR-339-5p MIMAT0000764
UCCCUGUCCUCCAGGAGCUCACG
>hsa-miR-378b MIMAT0014999
ACUGGACUUGGAGGCAGAA
>hsa-miR-486-5p MIMAT0002177
UCCUGUACUGAGCUGCCCCGAG
>hsa-miR-202* MIMAT0002810
UUCCUAUGCAUAUACUUCUUUG
>hsa-miR-4320 MIMAT0016871
GGGAUUCUGUAGCUUCCU
>hsa-miR-135a* MIMAT0004595
UAUAGGGAUUGGAGCCGUGGCG
>hsa-miR-17 MIMAT0000070
CAAAGUGCUUACAGUGCAGGUAG
>hsa-miR-196a* MIMAT0004562
CGGCAACAAGAAACUGCCUGAG
>hsa-miR-663b MIMAT0005867
GGUGGCCCGGCCGUGCCUGAGG
>hsa-miR-369-5p MIMAT0001621
AGAUCGACCGUGUUAUAUUCGC
>hsa-miR-500* MIMAT0002871
AUGCACCUGGGCAAGGAUUCUG
>hsa-miR-1279 MIMAT0005937
UCAUAUUGCUUCUUUCU
>hsa-miR-4321 MIMAT0016874
UUAGCGGUGGACCGCCCUGCG
>hsa-miR-656 MIMAT0003332
AAUAUUAUACAGUCAACCUCU
>hsa-miR-454 MIMAT0003885
UAGUGCAAUAUUGCUUAUAGGGU
>hsa-miR-3186-5p MIMAT0015067
CAGGCGUCUGUCUACGUGGCUU
>hsa-miR-2053 MIMAT0009978
GUGUUAAUUAAACCUCUAUUUAC
>hsa-miR-345 MIMAT0000772

GCUGACUCCUAGUCCAGGGCUC
>hsa-miR-548k MIMAT0005882
AAAAGUACUUGCGGAUUUUGCU
>hsa-miR-99b* MIMAT0004678
CAAGCUCGUGUCUGUGGGUCCG
>hsa-miR-1274b MIMAT0005938
UCCCUGUUCGGGCGCCA
>hsa-miR-764 MIMAT0010367
GCAGGUGCUCACUUGUCCUCCU
>hsa-miR-675 MIMAT0004284
UGGUGCGGAGAGGGCCCACAGUG
>hsa-miR-654-5p MIMAT0003330
UGGUGGGCCGCAGAACAUGUGC
>hsa-miR-4330 MIMAT0016924
CCUCAGAUCAGAGCCUUGC
>hsa-miR-506 MIMAT0002878
UAAGGCACCCUUCUGAGUAGA
>hsa-miR-626 MIMAT0003295
AGCUGUCUGAAAAUGUCUU
>hsa-miR-181b MIMAT0000257
AACAUUCAUUGCUGUCGGUGGGU
>hsa-miR-187* MIMAT0004561
GGCUACAACACAGGACCCGGGC
>hsa-miR-1251 MIMAT0005903
ACUCUAGCUGCCAAAGGCGCU
>hsa-miR-21 MIMAT0000076
UAGCUUAUCAGACUGAUGUUGA
>hsa-miR-670 MIMAT0010357
GUCCCUGAGUGUAUGUGGUG
>hsa-miR-485-5p MIMAT0002175
AGAGGCUGGCCGUGAUGAAUUC
>hsa-miR-4251 MIMAT0016883
CCUGAGAAAAGGGCCAA
>hsa-miR-614 MIMAT0003282
GAACGCCUGUUCUUGCCAGGUGG
>hsa-miR-3193 MIMAT0015077
UCCUGCGUAGGAUCUGAGGAGU
>hsa-miR-888* MIMAT0004917
GACUGACACCUCUUUGGGUGAA
>hsa-miR-892a MIMAT0004907
CACUGUGUCCUUUCUGCGUAG
>hsa-miR-206 MIMAT0000462
UGGAAUGUAAGGAAGUGUGUGG
>hsa-miR-125b-1* MIMAT0004592

ACGGGUUAGGCUCUUGGGAGCU

>hsa-miR-33a MIMAT0000091

GUGCAUUGUAGUUGCAUUGCA

>hsa-miR-196a MIMAT0000226

UAGGUAGUUUCAUGUUGUUGGG

>hsa-miR-4295 MIMAT0016844

CAGUGCAAUGUUUUCCUU

>hsa-miR-1979 MIMAT0009454

CUCCCACUGCUUCACUUGACUA

>hsa-miR-19a MIMAT0000073

UGUGCAAAUCUAUGCAAAACUGA

>hsa-miR-1972 MIMAT0009447

UCAGGCCAGGCACAGUGGCUCA

>hsa-miR-4272 MIMAT0016902

CAUUCAACUAGUGAUUGU

>hsa-miR-3183 MIMAT0015063

GCCUCUCUCGGAGUCGCUCGGA

>hsa-miR-485-3p MIMAT0002176

GUCAUACACGGCUCUCCUCUCU

>hsa-miR-632 MIMAT0003302

GUGUCUGCUUCCUGUGGGA

>hsa-miR-219-5p MIMAT0000276

UGAUUGUCCAAACGCAAUUCU

>hsa-miR-374a MIMAT0000727

UUAUAAUACAACCUGAUAAGUG

>hsa-miR-1244 MIMAT0005896

AAGUAGUUGGUUUGUAUGAGAUGGUU

>hsa-miR-556-3p MIMAT0004793

AUAUUACCAUUAGCUCAUCUUU

>hsa-miR-1537 MIMAT0007399

AAAACCGUCUAGUUACAGUUGU

>hsa-miR-564 MIMAT0003228

AGGCACGGUGUCAGCAGGC

>hsa-miR-4289 MIMAT0016920

GCAUUGUGCAGGGCUAUCA

>hsa-miR-1298 MIMAT0005800

UUCAUUCGGCUGUCCAGAUGUA

>hsa-miR-601 MIMAT0003269

UGGUCUAGGAUUGUUGGAGGAG

>hsa-miR-4327 MIMAT0016889

GGCUUGCAUGGGGGACUGG

>hsa-miR-425 MIMAT0003393

AAUGACACGAUCACUCCCGUUGA

>hsa-miR-183 MIMAT0000261

UAUGGCACUGGUAGAAUUCACU
>hsa-miR-876-3p MIMAT0004925
UGGUGGUUUACAAAGUAAUUCA
>hsa-miR-423-5p MIMAT0004748
UGAGGGGCAGAGAGCGAGACUUU
>hsa-miR-455-3p MIMAT0004784
GCAGUCCAUGGGCAUAUACAC
>hsa-miR-196b MIMAT0001080
UAGGUAGUUUCCUGUUGUUGGG
>hsa-miR-126* MIMAT0000444
CAUUAUUACUUUUGGUACGCG
>hsa-miR-363 MIMAT0000707
AAUUGCACGGUAUCCAUCUGUA
>hsa-miR-1272 MIMAT0005925
GAUGAUGAUGGCAGCAAAUUCUGAAA
>hsa-miR-338-3p MIMAT0000763
UCCAGCAUCAGUGAUUUUGUUG
>hsa-miR-509-5p MIMAT0004779
UACUGCAGACAGUGGCAAUCA
>hsa-miR-4313 MIMAT0016865
AGCCCCCUGGCCCCAAACCC
>hsa-miR-767-5p MIMAT0003882
UGCACCAUGGUUGUCUGAGCAUG
>hsa-miR-944 MIMAT0004987
AAAUUAUUGUACAUCGGAUGAG
>hsa-miR-1265 MIMAT0005918
CAGGAUGUGGUCAAGUGUUGUU
>hsa-miR-219-2-3p MIMAT0004675
AGAAUUGUGGCUGGACAUCUGU
>hsa-miR-515-3p MIMAT0002827
GAGUGCCUUCUUUUGGAGCGUU
>hsa-miR-140-3p MIMAT0004597
UACCACAGGGUAGAACCACGG
>hsa-miR-3166 MIMAT0015040
CGCAGACAAUGCCUACUGGCCUA
>hsa-miR-887 MIMAT0004951
GUGAACGGGCGCCAUCCCGAGG
>hsa-miR-497 MIMAT0002820
CAGCAGCACACUGUGGUUUGU
>hsa-miR-369-3p MIMAT0000721
AAUAAUACAUGGUUGAUCUUU
>hsa-let-7f-2* MIMAT0004487
CUAUACAGUCUACUGUCUUUCC
>hsa-miR-4317 MIMAT0016872

ACAUUGCCAGGGAGUUU
>hsa-miR-3164 MIMAT0015038
UGUGACUUUAAGGGAAAUGGCG
>hsa-miR-3190-5p MIMAT0015073
UGUGGAAGGUAGACGGCCAGAGA
>hsa-miR-517c MIMAT0002866
AUCGUGCAUCCUUUUAGAGUGU
>hsa-miR-639 MIMAT0003309
AUCGCUGCGGUUGCGAGCGCUGU
>hsa-miR-218 MIMAT0000275
UUGUGCUUGAUCUAACCAUGU
>hsa-miR-551b MIMAT0003233
GCGACCCAUACUUGGUUUCAG
>hsa-miR-1183 MIMAT0005828
CACUGUAGGUGAUGGUGAGAGUGGGCA
>hsa-miR-10a* MIMAT0004555
CAAAUUCGUAUCUAGGGGAAUA
>hsa-miR-497* MIMAT0004768
CAAACCACACUGUGGUGUUAGA
>hsa-miR-3144-5p MIMAT0015014
AGGGGACCAAAGAGAUAUAUAG
>hsa-miR-223* MIMAT0004570
CGUGUAUUUGACAAGCUGAGUU
>hsa-miR-595 MIMAT0003263
GAAGUGUGCCGUGGUGUGUCU
>hsa-miR-154 MIMAT0000452
UAGGUUAUCCGUGUUGCCUUCG
>hsa-miR-662 MIMAT0003325
UCCCACGUUGUGGCCCAGCAG
>hsa-miR-3187 MIMAT0015069
UUGGCCAUGGGGCUGCGCGG
>hsa-miR-26a MIMAT0000082
UUCAAGUAAUCCAGGAUAGGCU
>hsa-miR-1275 MIMAT0005929
GUGGGGGAGAGGCUGUC
>hsa-miR-1255b MIMAT0005945
CGGAUGAGCAAAGAAAGUGGUU
>hsa-miR-142-3p MIMAT0000434
UGUAGUGUUUCCUACUUUAUGGA
>hsa-miR-520d-5p MIMAT0002855
CUACAAAGGGAAGCCCUUUC
>hsa-miR-200a* MIMAT0001620
CAUCUUACCGGACAGUGCUGGA
>hsa-miR-1205 MIMAT0005869

UCUGCAGGGUUUGCUUUGAG
>hsa-miR-891b MIMAT0004913
UGCAACUUACCUGAGUCAUUGA
>hsa-miR-425* MIMAT0001343
AUCGGGAAUGUCGUGUCCGCCC
>hsa-miR-3147 MIMAT0015019
GGUUGGGCAGUGAGGAGGGUGUGA
>hsa-miR-95 MIMAT0000094
UUCAACGGGUAUUUAUUGAGCA
>hsa-miR-3179 MIMAT0015056
AGAAGGGGUGAAAUUUAAACGU
>hsa-miR-744 MIMAT0004945
UGCGGGGCUAGGGCUAACAGCA
>hsa-miR-154* MIMAT0000453
AAUCAUACACGGUUGACCUAUU
>hsa-miR-671-5p MIMAT0003880
AGGAAGCCCUGGAGGGGCUGGAG
>hsa-miR-1975 MIMAT0009450
CCCCCACAACCGCGCUUGACUAGCU
>hsa-miR-340* MIMAT0000750
UCCGUCUCAGUUACUUUAUAGC
>hsa-miR-194* MIMAT0004671
CCAGUGGGGCUGCUGUUAUCUG
>hsa-miR-4275 MIMAT0016905
CCAAUUACCACUUCUUU
>hsa-miR-502-3p MIMAT0004775
AAUGCACCUGGGCAAGGAUUCA
>hsa-miR-3170 MIMAT0015045
CUGGGGUUCUGAGACAGACAGU
>hsa-miR-510 MIMAT0002882
UACUCAGGAGAGUGGCAAUCAC
>hsa-miR-509-3-5p MIMAT0004975
UACUGCAGACGUGGCAAUCAUG
>hsa-miR-490-5p MIMAT0004764
CCAUGGAUCUCCAGGUGGGU
>hsa-miR-877 MIMAT0004949
GUAGAGGAGAUGGCGCAGGG
>hsa-miR-34c-5p MIMAT0000686
AGGCAGUGUAGUUAGCUGAUUGC
>hsa-miR-1266 MIMAT0005920
CCUCAGGGCUGUAGAACAGGGCU
>hsa-miR-4306 MIMAT0016858
UGGAGAGAAAGGCAGUA
>hsa-miR-32 MIMAT0000090

UAUUGCACAUUACUAAGUUGCA
>hsa-miR-224* MIMAT0009198
AAAAUGGUGCCCUAGUGACUACA
>hsa-miR-1254 MIMAT0005905
AGCCUGGAAGCUGGAGCCUGCAGU
>hsa-miR-642 MIMAT0003312
GUCCCUCUCCAAAUGUGUCUUG
>hsa-miR-380 MIMAT0000735
UAUGUAAUAUGGUCCACAUCUU
>hsa-miR-4296 MIMAT0016845
AUGUGGGCUCAGGCUCA
>hsa-miR-431* MIMAT0004757
CAGGUCGUCUUGCAGGGCUUCU
>hsa-miR-3180-5p MIMAT0015057
CUUCCAGACGCUCCGCCCCACGUCG
>hsa-miR-519a* MIMAT0005452
CUCUAGAGGGAAGCGCUUUCUG
>hsa-miR-500 MIMAT0004773
UAAUCCUUGCUACCUGGGUGAGA
>hsa-miR-518e MIMAT0002861
AAAGCGCUUCCCUUCAGAGUG
>hsa-miR-1180 MIMAT0005825
UUUCCGGCUCGCGUGGGUGUGU
>hsa-miR-380* MIMAT0000734
UGGUUGACCAUAGAACAUGCGC
>hsa-miR-141* MIMAT0004598
CAUCUUCCAGUACAGUGUUGGA
>hsa-miR-522* MIMAT0005451
CUCUAGAGGGAAGCGCUUUCUG
>hsa-miR-542-3p MIMAT0003389
UGUGACAGAUUGAUAACUGAAA
>hsa-miR-1468 MIMAT0006789
CUCCGUUUGCCUGUUUCGCUG
>hsa-miR-1909* MIMAT0007882
UGAGUGCCGGUGCCUGCCCUG
>hsa-miR-3123 MIMAT0014985
CAGAGAAUUGUUUAAUC
>hsa-miR-187 MIMAT0000262
UCGUGUCUUGUGUUGCAGCCGG
>hsa-miR-4283 MIMAT0016914
UGGGGCUCAGCGAGUUU
>hsa-miR-22 MIMAT0000077
AAGCUGCCAGUUGAAGAACUGU
>hsa-miR-922 MIMAT0004972

GCAGCAGAGAAUAGGACUACGUC
>hsa-miR-493* MIMAT0002813
UUGUACAUGGUAGGCUUUCAUU
>hsa-miR-4284 MIMAT0016915
GGGCUCACAUCACCCCAU
>hsa-miR-216a MIMAT0000273
UAAUCUCAGCUGGCAACUGUGA
>hsa-miR-219-1-3p MIMAT0004567
AGAGUUGAGUCUGGACGUCCCG
>hsa-miR-19b-2* MIMAT0004492
AGUUUUGCAGGUUUGCAUUUCA
>hsa-let-7d MIMAT0000065
AGAGGUAGUAGGUUGCAUAGUU
>hsa-miR-513a-5p MIMAT0002877
UUCACAGGGAGGUGUCAU
>hsa-miR-875-5p MIMAT0004922
UAUACCUCAGUUUUAUCAGGUG
>hsa-miR-4269 MIMAT0016897
GCAGGCACAGACAGCCCUGGC
>hsa-miR-192 MIMAT0000222
CUGACCUAUGAAUUGACAGCC
>hsa-miR-650 MIMAT0003320
AGGAGGCAGCGCUCUCAGGAC
>hsa-miR-766 MIMAT0003888
ACUCCAGCCCCACAGCCUCAGC
>hsa-miR-3172 MIMAT0015047
UGGGGUUUUGCAGUCCUUA
>hsa-miR-9 MIMAT0000441
UCUUUGGUUAUCUAGCUGUAUGA
>hsa-miR-1184 MIMAT0005829
CCUGCAGCGACUUGAUGGCUUCC
>hsa-miR-4256 MIMAT0016877
AUCUGACCUGAUGAAGGU
>hsa-miR-133a MIMAT0000427
UUUGGUCCCCUUCAACCAGCUG
>hsa-miR-125b-2* MIMAT0004603
UCACAAGUCAGGCUCUUGGGAC
>hsa-miR-3118 MIMAT0014980
UGUGACUGCAUUAUGAAAAUUCU
>hsa-miR-150* MIMAT0004610
CUGGUACAGGCCUGGGGGACAG
>hsa-miR-1290 MIMAT0005880
UGGAUUUUUGGAUCAGGGA
>hsa-miR-3163 MIMAT0015037

UAUAAAAUGAGGGCAGUAAGAC
>hsa-miR-222* MIMAT0004569
CUCAGUAGCCAGUGUAGAUCCU
>hsa-miR-374b MIMAT0004955
AUAUAAUACAACCUGCUAAGUG
>hsa-miR-1301 MIMAT0005797
UUGCAGCUGCCUGGGAGUGACUUC
>hsa-miR-454* MIMAT0003884
ACCCUAUCAAUAUUGUCUCUGC
>hsa-miR-182* MIMAT0000260
UGGUUCUAGACUUGCCAACUA
>hsa-miR-378c MIMAT0016847
ACUGGACUUGGAGUCAGAAGAGUGG
>hsa-miR-644 MIMAT0003314
AGUGUGGCUUUCUUAGAGC
>hsa-miR-125a-5p MIMAT0000443
UCCCUGAGACCCUUUAACCUGUGA
>hsa-miR-337-5p MIMAT0004695
GAACGGCUUCAUACAGGAGUU
>hsa-miR-221 MIMAT0000278
AGCUACAUUGUCUGCUGGGUUUC
>hsa-miR-548g MIMAT0005912
AAAACUGUAAUUACUUUUGUAC
>hsa-let-7g* MIMAT0004584
CUGUACAGGCCACUGCCUUGC
>hsa-miR-4278 MIMAT0016910
CUAGGGGGUUUGCCCUUG
>hsa-miR-604 MIMAT0003272
AGGCUGCGGAAUUCAGGAC
>hsa-miR-28-3p MIMAT0004502
CACUAGAUUGUGAGCUCCUGGA
>hsa-miR-668 MIMAT0003881
UGUCACUCGGCUCGGCCCACUAC
>hsa-miR-1909 MIMAT0007883
CGCAGGGGCCGGGUGCUCACCG
>hsa-miR-106b* MIMAT0004672
CCGCACUGUGGGUACUUGCUGC
>hsa-miR-514b-5p MIMAT0015087
UUCUCAAGAGGGAGGCAAUCAU
>hsa-miR-549 MIMAT0003333
UGACAACUAUGGAUGAGCUCU
>hsa-miR-638 MIMAT0003308
AGGGAUCGCGGGCGGGUGGCGGCCU
>hsa-miR-3146 MIMAT0015018

CAUGCUAGGAUAGAAAGAAUGG
>hsa-miR-4267 MIMAT0016893
UCCAGCUCGGUGGCAC
>hsa-miR-890 MIMAT0004912
UACUUGGAAAGGCAUCAGUUG
>hsa-miR-4325 MIMAT0016887
UUGCACUUGUCUCAGUGA
>hsa-miR-548a-5p MIMAT0004803
AAAAGUAAUUGCGAGUUUUACC
>hsa-miR-376c MIMAT0000720
AACAUAGAGGAAAUUCCACGU
>hsa-miR-1826 MIMAT0006766
AUUGAUCAUCGACACUUCGAACGCAAU
>hsa-miR-490-3p MIMAT0002806
CAACCUGGAGGACUCCAUGCUG
>hsa-miR-647 MIMAT0003317
GUGGCUGCACUCACUUCCUUC
>hsa-miR-559 MIMAT0003223
UAAAGUAAAUAUGCACCAAAA
>hsa-miR-3196 MIMAT0015080
CGGGGCGGCAGGGGCCUC
>hsa-miR-138-1* MIMAT0004607
GCUACUUCACAACACCAGGGCC
>hsa-miR-548t MIMAT0015009
CAAAAGUGAUCGUGGUUUUUG
>hsa-miR-127-5p MIMAT0004604
CUGAAGCUCAGAGGGCUCUGAU
>hsa-miR-3134 MIMAT0015000
UGAUGGAUAAAAGACUACAUAUU
>hsa-miR-4265 MIMAT0016891
CUGUGGGCUCAGCUCUGGG
>hsa-miR-520a-3p MIMAT0002834
AAAGUGCUUCCCUUUGGACUGU
>hsa-miR-3154 MIMAT0015028
CAGAAGGGGAGUUGGGAGCAGA
>hsa-miR-15b* MIMAT0004586
CGAAUCAUUAUUUGCUGCUCUA
>hsa-miR-31* MIMAT0004504
UGCUAUGCCAACAUAUUGCCAU
>hsa-miR-585 MIMAT0003250
UGGGCGUAUCUGUAUGCUA
>hsa-miR-1471 MIMAT0007349
GCCCGCGUGUGGAGCCAGGUGU
>hsa-miR-544 MIMAT0003164

AUUCUGCAUUUUUAGCAAGUUC
>hsa-miR-143 MIMAT0000435
UGAGAUGAAGCACUGUAGCUC
>hsa-miR-3156 MIMAT0015030
AAAGAUCUGGAAGUGGGAGACA
>hsa-miR-24-1* MIMAT0000079
UGCCUACUGAGCUGAUAUCAGU
>hsa-miR-220a MIMAT0000277
CCACACCGUAUCUGACACUUU
>hsa-miR-335* MIMAT0004703
UUUUUCAUUAUUGCUCCUGACC
>hsa-miR-3190-3p MIMAT0015074
UGGAAGGUAGACGGCCAGAGAG
>hsa-miR-548m MIMAT0005917
CAAAGGUAUUUGUGGUUUUUG
>hsa-miR-28-5p MIMAT0000085
AAGGAGCUCACAGUCUAUUGAG
>hsa-miR-378 MIMAT0000732
ACUGGACUUGGAGUCAGAAGG
>hsa-miR-1226* MIMAT0005576
GUGAGGGCAUGCAGGCCUGGAUGGGG
>hsa-miR-190 MIMAT0000458
UGAUAUGUUUGAUAUAUUAGGU
>hsa-miR-1237 MIMAT0005592
UCCUUCUGCUCCGUCCCCCAG
>hsa-miR-561 MIMAT0003225
CAAAGUUUAAGAUCCUUGAAGU
>hsa-miR-3065-3p MIMAT0015378
UCAGCACCAGGAUAUUGUUGGAG
>hsa-miR-519c-3p MIMAT0002832
AAAGUGCAUCUUUUUAGAGGAU
>hsa-miR-129-5p MIMAT0000242
CUUUUUGCGGUCUGGGCUUGC
>hsa-miR-541* MIMAT0004919
AAAGGAUUCUGCUGUCGGUCCCACU
>hsa-miR-548n MIMAT0005916
CAAAAGUAAUUGUGGAUUUUGU
>hsa-miR-217 MIMAT0000274
UACUGCAUCAGGAACUGAUUGGA
>hsa-miR-1178 MIMAT0005823
UUGCUCACUGUUCUUCCCUAG
>hsa-miR-892b MIMAT0004918
CACUGGCUCCUUUCUGGGUAGA
>hsa-miR-1288 MIMAT0005942

UGGACUGCCCUGAUCUGGAGA
>hsa-miR-658 MIMAT0003336
GGCGGAGGGAAGUAGGUCCGUUGGU
>hsa-miR-3180-3p MIMAT0015058
UGGGGCGGAGCUUCCGGAGGCC
>hsa-miR-3143 MIMAT0015012
AUAACAUUGUAAAGCGCUUCUUUCG
>hsa-miR-548c-5p MIMAT0004806
AAAAGUAAUUGCGGUUUUUGCC
>hsa-miR-3160 MIMAT0015034
AGAGCUGAGACUAGAAAGCCCA
>hsa-miR-1277 MIMAT0005933
UACGUAGAUAUAUAUGUAUUUU
>hsa-miR-886-3p MIMAT0004906
CGCGGGUGCUUACUGACCCUU
>hsa-miR-3189 MIMAT0015071
CCCUUGGGUCUGAUGGGGUAG
>hsa-miR-1825 MIMAT0006765
UCCAGUGCCCUCCUCUCC
>hsa-miR-1914* MIMAT0007890
GGAGGGGUCCCGCACUGGGAGG
>hsa-miR-519a MIMAT0002869
AAAGUGCAUCCUUUUAGAGUGU
>hsa-miR-409-5p MIMAT0001638
AGGUUACCCGAGCAACUUUGCAU
>hsa-miR-1268 MIMAT0005922
CGGGCGUGGUGGUGGGGG
>hsa-miR-508-5p MIMAT0004778
UACUCCAGAGGGCGUCACUCAUG
>hsa-miR-455-5p MIMAT0003150
UAUGUGCCUUUGGACUACAUCG
>hsa-miR-619 MIMAT0003288
GACCUGGACAUGUUUGUGCCCAGU
>hsa-let-7e MIMAT0000066
UGAGGUAGGAGGUUGUAUAGUU
>hsa-miR-587 MIMAT0003253
UUUCCAUAGGUGAUGAGUCAC
>hsa-miR-1303 MIMAT0005891
UUUAGAGACGGGGUCUUGCUCU
>hsa-miR-216b MIMAT0004959
AAAUCUCUGCAGGCAAAUGUGA
>hsa-miR-103 MIMAT0000101
AGCAGCAUUGUACAGGGCUAUGA
>hsa-miR-1182 MIMAT0005827

GAGGGUCUUGGGAGGGAUGUGAC
>hsa-miR-660 MIMAT0003338
UACCCAUUGCAUAUCGGAGUUG
>hsa-miR-299-5p MIMAT0002890
UGGUUUACCGUCCCACAUACAU
>hsa-miR-548p MIMAT0005934
UAGCAAAAACUGCAGUUACUUU
>hsa-miR-367 MIMAT0000719
AAUUGCACUUUAGCAAUGGUGA
>hsa-miR-1203 MIMAT0005866
CCCGGAGCCAGGAUGCAGCUC
>hsa-miR-3137 MIMAT0015005
UCUGUAGCCUGGGAGCAAUGGGGU
>hsa-miR-615-3p MIMAT0003283
UCCGAGCCUGGGUCUCCCUCUU
>hsa-miR-25* MIMAT0004498
AGGCGGAGACUUGGGCAAUUG
>hsa-miR-19b-1* MIMAT0004491
AGUUUUGCAGGUUUGCAUCCAGC
>hsa-miR-524-5p MIMAT0002849
CUACAAAGGGAAGCACUUUCUC
>hsa-miR-143* MIMAT0004599
GGUGCAGUGCUGCAUCUCUGGU
>hsa-miR-34a* MIMAT0004557
CAAUCAGCAAGUAUACUGCCCU
>hsa-miR-25 MIMAT0000081
CAUUGCACUUGUCUCGGUCUGA
>hsa-miR-516a-5p MIMAT0004770
UUCUCGAGGAAAGAAGCACUUUC
>hsa-miR-23a MIMAT0000078
AUCACAUUGCCAGGGAUUUCC
>hsa-miR-2116 MIMAT0011160
GGUUCUUAGCAUAGGAGGUCU
>hsa-miR-30c-2* MIMAT0004550
CUGGGAGAAGGCUGUUUACUCU
>hsa-miR-365 MIMAT0000710
UAAUGCCCCUAAAAAUCCUUAU
>hsa-miR-449b MIMAT0003327
AGGCAGUGUAUUGUUAGCUGGC
>hsa-miR-3129 MIMAT0014992
GCAGUAGUGUAGAGAUUGGUUU
>hsa-miR-3201 MIMAT0015086
GGGAUAUGAAGAAAAAU
>hsa-miR-10a MIMAT0000253

UACCCUGUAGAUCCGAAUUUGUG
>hsa-miR-337-3p MIMAT0000754
CUCCUAUAUGAUGCCUUUCUUC
>hsa-miR-1305 MIMAT0005893
UUUUCAACUCUAAUGGGAGAGA
>hsa-miR-598 MIMAT0003266
UACGUCAUCGUUGUCAUCGUCA
>hsa-miR-302c MIMAT0000717
UAAGUGCUUCCAUGUUUCAGUGG
>hsa-miR-302f MIMAT0005932
UAAUUGCUUCCAUGUUU
>hsa-miR-1201 MIMAT0005864
AGCCUGAUUAAACACAUGCUCUGA
>hsa-miR-548i MIMAT0005935
AAAAGUAAUUGCGGAUUUUGCC
>hsa-miR-202 MIMAT0002811
AGAGGUAUAGGGCAUGGGAA
>hsa-miR-520a-5p MIMAT0002833
CUCCAGAGGGAAGUACUUUCU
>hsa-miR-606 MIMAT0003274
AAACUACUGAAAAUCAAAGAU
>hsa-miR-421 MIMAT0003339
AUCAACAGACAUUAAUUGGGCGC
>hsa-miR-24-2* MIMAT0004497
UGCCUACUGAGCUGAAACACAG
>hsa-miR-212 MIMAT0000269
UAACAGUCUCCAGUCACGGCC
>hsa-miR-521 MIMAT0002854
AACGCACUUCCCUUUAGAGUGU
>hsa-miR-151-3p MIMAT0000757
CUAGACUGAAGCUCCUUGAGG
>hsa-let-7b* MIMAT0004482
CUAUACAACCUACUGCCUUCCC
>hsa-miR-329 MIMAT0001629
AACACACCUGGUUAACCUCUUU
>hsa-miR-646 MIMAT0003316
AAGCAGCUGCCUCUGAGGC
>hsa-miR-153 MIMAT0000439
UUGCAUAGUCACAAAAGUGAUC
>hsa-miR-708 MIMAT0004926
AAGGAGCUUACAAUCUAGCUGGG
>hsa-miR-145* MIMAT0004601
GGAUUCCUGGAAAUACUGUUCU
>hsa-miR-590-5p MIMAT0003258

GAGCUUAUUCAUAAAAGUGCAG
>hsa-miR-1281 MIMAT0005939
UCGCCUCCUCCUCUCCC
>hsa-miR-4262 MIMAT0016894
GACAUUCAGACUACCUG
>hsa-miR-582-3p MIMAT0004797
UAACUGGUUGAACAACUGAACC
>hsa-miR-411 MIMAT0003329
UAGUAGACCGUAUAGCGUACG
>hsa-miR-371-5p MIMAT0004687
ACUCAAACUGUGGGGGCACU
>hsa-miR-652 MIMAT0003322
AAUGGCGCCACUAGGGUUGUG
>hsa-miR-30a MIMAT0000087
UGUAAACAUCCUCGACUGGAAG
>hsa-miR-489 MIMAT0002805
GUGACAUCACAUAUACGGCAGC
>hsa-miR-3148 MIMAT0015021
UGGAAAAAACUGGUGUGUGCUU
>hsa-miR-2113 MIMAT0009206
AUUUGUGCUUGGCUCUGUCAC
>hsa-miR-4324 MIMAT0016876
CCCUGAGACCCUAACCUUAA
>hsa-miR-324-3p MIMAT0000762
ACUGCCCCAGGUGCUGCUGG
>hsa-miR-622 MIMAT0003291
ACAGUCUGCUGAGGUUGGAGC
>hsa-miR-376a MIMAT0000729
AUCAUAGAGGAAAAUCCACGU
>hsa-miR-200c MIMAT0000617
UAAUACUGCCGGGUAAUGAUGGA
>hsa-miR-581 MIMAT0003246
UCUUGUGUUCUCUAGAUCAGU
>hsa-miR-1207-3p MIMAT0005872
UCAGCUGGCCCUCAUUUC
>hsa-miR-145 MIMAT0000437
GUCCAGUUUUCCCAGGAAUCCCU
>hsa-miR-130a MIMAT0000425
CAGUGCAAUGUUAAAAGGGCAU
>hsa-miR-3126-3p MIMAT0015377
CAUCUGGCAUCCGUCACACAGA
>hsa-miR-552 MIMAT0003215
AACAGGUGACUGGUUAGACAA
>hsa-miR-2355 MIMAT0016895

AUCCCCAGAUACAAUGGACAA
>hsa-miR-148b MIMAT0000759
UCAGUGCAUCACAGAACUUUGU
>hsa-let-7i* MIMAT0004585
CUGCGCAAGCUACUGCCUUGCU
>hsa-miR-220c MIMAT0004915
ACACAGGGCUGUUGUGAAGACU
>hsa-miR-362-5p MIMAT0000705
AAUCCUUGGAACCUAGGUGUGAGU
>hsa-miR-1286 MIMAT0005877
UGCAGGACCAAGAUGAGCCCU
>hsa-miR-1914 MIMAT0007889
CCCUGUGCCCGGCCCACUUCUG
>hsa-miR-484 MIMAT0002174
UCAGGCUCAGUCCCCUCCCGAU
>hsa-miR-664 MIMAT0005949
UAUUCAUUUAUCCCCAGCCUACA
>hsa-miR-720 MIMAT0005954
UCUCGCUGGGGCCUCCA
>hsa-miR-3145 MIMAT0015016
AGAUAUUUUGAGUGUUUGGAAUUG
>hsa-miR-1247 MIMAT0005899
ACCCGUCCCGUUCGUCCCCGGA
>hsa-let-7a-2* MIMAT0010195
CUGUACAGCCUCCUAGCUUUCC
>hsa-miR-624 MIMAT0004807
CACAAGGUAUUGGUAUUACCU
>hsa-miR-1913 MIMAT0007888
UCUGCCCCCUCCGCUGCUGCCA
>hsa-miR-4309 MIMAT0016859
CUGGAGUCUAGGAUUCCA
>hsa-miR-320d MIMAT0006764
AAAAGCUGGGUUGAGAGGA
>hsa-miR-1324 MIMAT0005956
CCAGACAGAAUUCUAUGCACUUUC
>hsa-miR-4290 MIMAT0016921
UGCCCUCCUUUCUUCCCUC
>hsa-miR-617 MIMAT0003286
AGACUUCCCAUUUGAAGGUGGC
>hsa-miR-4285 MIMAT0016913
GCGGCGAGUCCGACUCAU
>hsa-miR-3176 MIMAT0015053
ACUGGCCUGGGACUACCGG
>hsa-miR-495 MIMAT0002817

AAACAAACAUGGUGCACUUCUU
>hsa-miR-502-5p MIMAT0002873
AUCCUUGCUAUCUGGGUGCUA
>hsa-miR-1271 MIMAT0005796
CUUGGCACCUAGCAAGCACUCA
>hsa-miR-513c MIMAT0005789
UUCUCAAGGAGGUGUCGUUUAU
>hsa-miR-1297 MIMAT0005886
UUCAAGUAAUUCAGGUG
>hsa-miR-591 MIMAT0003259
AGACCAUGGGUUCUCAUUGU
>hsa-miR-1208 MIMAT0005873
UCACUGUUCAGACAGGCGGA
>hsa-miR-215 MIMAT0000272
AUGACCUAUGAAUUGACAGAC
>hsa-miR-34c-3p MIMAT0004677
AAUCACUAACCACACGGCCAGG
>hsa-miR-767-3p MIMAT0003883
UCUGCUCAUACCCCAUGGUUUCU
>hsa-miR-26b* MIMAT0004500
CCUGUUCUCCAUUACUUGGCUC
>hsa-miR-1253 MIMAT0005904
AGAGAAGAAGAUCAGCCUGCA
>hsa-miR-34a MIMAT0000255
UGGCAGUGUCUUAGCUGGUUGU
>hsa-miR-936 MIMAT0004979
ACAGUAGAGGGAGGAAUCGCAG
>hsa-miR-921 MIMAT0004971
CUAGUGAGGGACAGAACCAGGAUUC
>hsa-miR-29b MIMAT0000100
UAGCACCAUUUGAAAUCAGUGUU
>hsa-miR-193a-3p MIMAT0000459
AACUGGCCUACAAAGUCCCAGU
>hsa-miR-2861 MIMAT0013802
GGGGCCUGGCGGUGGGCGG
>hsa-miR-1260b MIMAT0015041
AUCCCACCACUGCCACCAU
>hsa-miR-1273d MIMAT0015090
GAACCCAUGAGGUUGAGGCUGCAGU
>hsa-miR-1538 MIMAT0007400
CGGCCCGGGCUGCUGCUGUUCCU
>hsa-miR-376a* MIMAT0003386
GUAGAUUCUCCUUCUAUGAGUA
>hsa-miR-1243 MIMAT0005894

AACUGGAUCAAUUAUAGGAGUG
>hsa-miR-1259 MIMAT0005910
AUAUAUGAUGACUUAGCUUUU
>hsa-miR-3116 MIMAT0014978
UGCCUGGAACAUAGUAGGGACU
>hsa-miR-761 MIMAT0010364
GCAGCAGGGUGAAACUGACACA
>hsa-miR-374b* MIMAT0004956
CUUAGCAGGUUGUAUUAUCAUU
>hsa-miR-3198 MIMAT0015083
GUGGAGUCCUGGGGAAUGGAGA
>hsa-miR-186* MIMAT0004612
GCCCAAAGGUGAAUUUUUUGGG
>hsa-miR-147 MIMAT0000251
GUGUGUGGAAAUGCUUCUGC
>hsa-miR-3139 MIMAT0015007
UAGGAGCUCAACAGAUGCCUGUU
>hsa-miR-873 MIMAT0004953
GCAGGAACUUGUGAGUCUCCU
>hsa-miR-27a* MIMAT0004501
AGGGCUUAGCUGCUUGUGAGCA
>hsa-miR-498 MIMAT0002824
UUUCAAGCCAGGGGGCGUUUUUC
>hsa-miR-1226 MIMAT0005577
UCACCAGCCCUGUGUUCCCUAG
>hsa-miR-4318 MIMAT0016869
CACUGUGGGUACAUGCU
>hsa-miR-1469 MIMAT0007347
CUCGGCGCGGGGCGCGGGCUCC
>hsa-miR-4260 MIMAT0016881
CUUGGGGCAUGGAGUCCCA
>hsa-miR-517b MIMAT0002857
UCGUGCAUCCCUUUAGAGUGUU
>hsa-miR-1262 MIMAT0005914
AUGGGUGAAUUUGUAGAAGGAU
>hsa-miR-144 MIMAT0000436
UACAGUAUAGAUGAUGUACU
>hsa-miR-633 MIMAT0003303
CUAAUAGUAUCUACCACAAUAAA
>hsa-miR-125b MIMAT0000423
UCCCUGAGACCCUAACUUGUGA
>hsa-miR-548w MIMAT0015060
AAAAGUAACUGCGGUUUUUGCCU
>hsa-miR-505* MIMAT0004776

GGGAGCCAGGAAGUAUUGAUGU
>hsa-miR-888 MIMAT0004916
UACUCAAAAAGCUGUCAGUCA
>hsa-miR-1224-5p MIMAT0005458
GUGAGGACUCGGGAGGUGG
>hsa-miR-30d* MIMAT0004551
CUUUCAGUCAGAUGUUUGCUGC
>hsa-miR-553 MIMAT0003216
AAAACGGUGAGAUUUUGUUUU
>hsa-miR-568 MIMAT0003232
AUGUAUAAAUGUAUACACAC
>hsa-miR-802 MIMAT0004185
CAGUAACAAAGAUUCAUCCUUGU
>hsa-miR-592 MIMAT0003260
UUGUGUCAAUAUGCGAUGAUGU
>hsa-miR-3127 MIMAT0014990
AUCAGGGCUUGUGGAAUGGGAAG
>hsa-miR-21* MIMAT0004494
CAACACCAGUCGAUGGGCUGU
>hsa-miR-596 MIMAT0003264
AAGCCUGCCCGGCUCCUCGGG
>hsa-miR-29a MIMAT0000086
UAGCACCAUCUGAAAUCGGUUA
>hsa-miR-105 MIMAT0000102
UCAAAUGCUCAGACUCCUGUGGU
>hsa-miR-570 MIMAT0003235
CGAAAACAGCAAUUACCUUUGC
>hsa-miR-124 MIMAT0000422
UAAGGCACGCGGUGAAUGCC
>hsa-miR-155 MIMAT0000646
UUAAUGCUAAUCGUGAUAGGGGU
>hsa-miR-940 MIMAT0004983
AAGGCAGGGCCCCCGCUCCCC
>hsa-miR-34b MIMAT0004676
CAAUCACUAACUCCACUGCCAU
>hsa-miR-588 MIMAT0003255
UUGGCCACAAUGGGUUAGAAC
>hsa-miR-671-3p MIMAT0004819
UCCGGUUCUCAGGGCUCCACC
>hsa-miR-1261 MIMAT0005913
AUGGAUAAGGCUUUGGCUU
>hsa-miR-222 MIMAT0000279
AGCUACAUCUGGCUACUGGGU
>hsa-miR-184 MIMAT0000454

UGGACGGAGAACUGAUAAGGGU
>hsa-miR-518f* MIMAT0002841
CUCUAGAGGGAAGCACUUUCUC
>hsa-miR-125a-3p MIMAT0004602
ACAGGUGAGGUUCUUGGGAGCC
>hsa-miR-449c* MIMAT0013771
UUGCUAGUUGCACUCCUCUCUGU
>hsa-miR-450b-3p MIMAT0004910
UUGGGAUCAUUUUGCAUCCAUA
>hsa-miR-1285 MIMAT0005876
UCUGGGCAACAAAGUGAGACCU
>hsa-miR-4258 MIMAT0016879
CCCCGCCACCGCCUUGG
>hsa-miR-1181 MIMAT0005826
CCGUCGCCGCCACCCGAGCCG
>hsa-miR-520d-3p MIMAT0002856
AAAGUGCUUCUCUUUGGUGGGU
>hsa-miR-205* MIMAT0009197
GAUUUCAGUGGAGUGAAGUUC
>hsa-miR-450b-5p MIMAT0004909
UUUUGCAAUAUGUUCCUGAAUA
>hsa-miR-15a* MIMAT0004488
CAGGCCAUAUUGUGCUGCCUCA
>hsa-miR-3182 MIMAT0015062
GCUUCUGUAGUGUAGUC
>hsa-miR-4271 MIMAT0016901
GGGGGAAGAAAAGGUGGGG
>hsa-miR-2114 MIMAT0011156
UAGUCCCUUCCUUGAAGCGGUC
>hsa-miR-92a-1* MIMAT0004507
AGGUUGGGAUCGGUUGCAAUGCU
>hsa-miR-20a* MIMAT0004493
ACUGCAUUAUGAGCACUUAAAG
>hsa-miR-135b MIMAT0000758
UAUGGCUUUUCAUUCCUAUGUGA
>hsa-miR-1204 MIMAT0005868
UCGUGGCCUGGUCUCCAUUAU
>hsa-miR-514 MIMAT0002883
AUUGACACUUCUGUGAGUAGA
>hsa-miR-9* MIMAT0000442
AUAAAGCUAGAUAACCGAAAGU
>hsa-miR-299-3p MIMAT0000687
UAUGUGGGAUGGUAAACCGCUU
>hsa-miR-27b MIMAT0000419

UUCACAGUGGCUAAGUUCUGC

>hsa-miR-636 MIMAT0003306

UGUGCUUGCUCGUCCCGCCCGCA

>hsa-miR-664* MIMAT0005948

ACUGGCUAGGGAAAAUGAUUGGAU

>hsa-miR-548j MIMAT0005875

AAAAGUAAUUGCGGUCUUUGGU

>hsa-miR-520e MIMAT0002825

AAAGUGCUUCCUUUUUGAGGG

>hsa-miR-607 MIMAT0003275

GUUCAAAUCCAGAUCUAUAAC

>hsa-miR-4254 MIMAT0016884

GCCUGGAGCUACUCCACCAUCUC

>hsa-miR-520c-5p MIMAT0005455

CUCUAGAGGGAAGCACUUUCUG

>hsa-miR-578 MIMAT0003243

CUUCUUGUGCUCUAGGAUUGU

>hsa-miR-383 MIMAT0000738

AGAUCAGAAGGUGAUUGUGGCU

>hsa-miR-629 MIMAT0004810

UGGGUUUACGUUGGGAGAACU

>hsa-miR-629* MIMAT0003298

GUUCUCCCAACGUAAGCCCAGC

>hsa-miR-155* MIMAT0004658

CUCCUACAUAUUAGCAUUAACA

>hsa-miR-192* MIMAT0004543

CUGCCAAUUCCAUAGGUCACAG

>hsa-miR-3174 MIMAT0015051

UAGUGAGUUAGAGAUGCAGAGCC

>hsa-miR-193b MIMAT0002819

AACUGGCCCUCAAAGUCCCGCU

>hsa-miR-1206 MIMAT0005870

UGUUCAUGUAGAUGUUUAAGC

>hsa-miR-934 MIMAT0004977

UGUCUACUACUGGAGACACUGG

>hsa-miR-518c* MIMAT0002847

UCUCUGGAGGGAAGCACUUUCUG

>hsa-miR-200b* MIMAT0004571

CAUCUUACUGGGCAGCAUUGGA

>hsa-miR-548b-3p MIMAT0003254

CAAGAACCUCAGUUGCUUUUGU

>hsa-miR-3178 MIMAT0015055

GGGGCGCGGCCGGAUCG

>hsa-let-7i MIMAT0000415

UGAGGUAGUAGUUUGUGCUGUU
>hsa-miR-1292 MIMAT0005943
UGGGAACGGGUUCCGGCAGACGCUG
>hsa-miR-23b MIMAT0000418
AUCACAUUGCCAGGGAUUACC
>hsa-miR-1322 MIMAT0005953
GAUGAUGCUGCUGAUGCUG
>hsa-miR-3152 MIMAT0015025
UGUGUUAGAAUAGGGGCAAUAA
>hsa-miR-1274a MIMAT0005927
GUCCCUGUUCAGGCGCCA
>hsa-miR-545* MIMAT0004785
UCAGUAAAUGUUUAUUAGAUGA
>hsa-miR-609 MIMAT0003277
AGGGUGUUUCUCUCAUCUCU
>hsa-miR-1295 MIMAT0005885
UUAGGCCGCAGAUCUGGGUGA
>hsa-miR-548a-3p MIMAT0003251
CAAAACUGGCAAUUACUUUUGC
>hsa-miR-23b* MIMAT0004587
UGGGUUCCUGGCAUGCUGAUUU
>hsa-miR-1255a MIMAT0005906
AGGAUGAGCAAAGAAAGUAGAUU
>hsa-miR-33b MIMAT0003301
GUGCAUUGCUGUUGCAUUGC
>hsa-miR-516b MIMAT0002859
AUCUGGAGGUAAGAAGCACUUU
>hsa-miR-526b MIMAT0002835
CUCUUGAGGGAAGCACUUUCUGU
>hsa-miR-33b* MIMAT0004811
CAGUGCCUCGGCAGUGCAGCCC
>hsa-miR-4322 MIMAT0016873
CUGUGGGCUCAGCGCGUGGGG
>hsa-let-7b MIMAT0000063
UGAGGUAGUAGGUUGUGUGGUU
>hsa-miR-302d* MIMAT0004685
ACUUUAACAUGGAGGCACUUGC
>hsa-miR-620 MIMAT0003289
AUGGAGAUAGAUAUAGAAAU
>hsa-miR-4329 MIMAT0016923
CCUGAGACCCUAGUUCCAC
>hsa-miR-551a MIMAT0003214
GCGACCCACUCUUGGUUUCCA
>hsa-miR-301a MIMAT0000688

CAGUGCAAUAGUAUUGUCAAAGC
>hsa-miR-3167 MIMAT0015042
AGGAUUUCAGAAAUACUGGUGU
>hsa-miR-526b* MIMAT0002836
GAAAGUGCUUCCUUUUAGAGGC
>hsa-miR-22* MIMAT0004495
AGUUCUUCAGUGGCAAGCUUUA
>hsa-miR-203 MIMAT0000264
GUGAAAUGUUUAGGACCACUAG
>hsa-miR-92a MIMAT0000092
UAUUGCACUUGUCCCGGCCUGU
>hsa-miR-211 MIMAT0000268
UUCCCUUUGUCAUCCUUCGCCU
>hsa-miR-3144-3p MIMAT0015015
AUAUACCUGUUCGGUCUCUUUA
>hsa-miR-181a* MIMAT0000270
ACCAUCGACCGUUGAUUGUACC
>hsa-miR-361-3p MIMAT0004682
UCCCCCAGGUGUGAUUCUGAUUU
>hsa-miR-518e* MIMAT0005450
CUCUAGAGGGAAGCGCUUUCUG
>hsa-miR-4281 MIMAT0016907
GGGUCCCGGGGAGGGGGG
>hsa-let-7f-1* MIMAT0004486
CUAUACAAUCUAUUGCCUUCCC
>hsa-miR-593* MIMAT0003261
AGGCACCAGCCAGGCAUUGCUCAGC
>hsa-miR-432* MIMAT0002815
CUGGAUGGCUCCUCCAUGUCU
>hsa-miR-628-5p MIMAT0004809
AUGCUGACAUAUUUACUAGAGG
>hsa-miR-4301 MIMAT0016850
UCCCACUACUUCACUUGUGA
>hsa-miR-3192 MIMAT0015076
UCUGGGAGGUUGUAGCAGUGGAA
>hsa-miR-30c MIMAT0000244
UGUAAACAUCCUACACUCUCAGC
>hsa-miR-1273 MIMAT0005926
GGGCGACAAAGCAAGACUCUUUCUU
>hsa-miR-181c MIMAT0000258
AACAUUCAACCUGUCGGUGAGU
>hsa-miR-3133 MIMAT0014998
UAAAGAACUCUUAAAACCCAAU
>hsa-miR-583 MIMAT0003248

CAAAGAGGAAGGUCCCAUUAC
>hsa-miR-760 MIMAT0004957
CGGCUCUGGGUCUGUGGGGA
>hsa-miR-4298 MIMAT0016852
CUGGGACAGGAGGAGGAGGCAG
>hsa-miR-611 MIMAT0003279
GCGAGGACCCCUCGGGGUCUGAC
>hsa-miR-205 MIMAT0000266
UCCUUCAUUCCACCGGAGUCUG
>hsa-miR-18a MIMAT0000072
UAAGGUGCAUCUAGUGCAGAUAG
>hsa-miR-1911 MIMAT0007885
UGAGUACCGCCAUGUCUGUUGGG
>hsa-miR-151-5p MIMAT0004697
UCGAGGAGCUCACAGUCUAGU
>hsa-miR-4287 MIMAT0016917
UCUCCCUUGAGGGCACUUU
>hsa-miR-659 MIMAT0003337
CUUGGUUCAGGGAGGGUCCCCA
>hsa-miR-378* MIMAT0000731
CUCCUGACUCCAGGUCCUGUGU
>hsa-miR-1256 MIMAT0005907
AGGCAUUGACUUCUCACUAGCU
>hsa-miR-640 MIMAT0003310
AUGAUCCAGGAACCUGCCUCU
>hsa-miR-631 MIMAT0003300
AGACCUGGCCCAGACCUCAGC
>hsa-miR-199a-3p MIMAT0000232
ACAGUAGUCUGCACAUUGGUUA
>hsa-miR-1224-3p MIMAT0005459
CCCCACCUCCUCUCUCCUCAG
>hsa-miR-1245 MIMAT0005897
AAGUGAUCUAAAGGCCUACAU
>hsa-miR-149 MIMAT0000450
UCUGGCUCCGUGUCUUCACUCCC
>hsa-miR-4266 MIMAT0016892
CUAGGAGGCCUUGGCC
>hsa-miR-4273 MIMAT0016903
GUGUUCUCUGAUGGACAG
>hsa-miR-525-3p MIMAT0002839
GAAGGCGCUUCCCUUUAGAGCG
>hsa-miR-99b MIMAT0000689
CACCCGUAGAACCGACCUUGCG
>hsa-miR-130a* MIMAT0004593

UUCACAUUGUGCUACUGUCUGC
>hsa-miR-519e MIMAT0002829
AAGUGCCUCCUUUUAGAGUGUU
>hsa-miR-4279 MIMAT0016909
CUCUCCUCCCGGCUUC
>hsa-miR-466 MIMAT0015002
AUACACAUACACGCAACACACAU
>hsa-miR-711 MIMAT0012734
GGGACCCAGGGAGAGACGUAAG
>hsa-miR-320a MIMAT0000510
AAAAGCUGGGUUGAGAGGGCGA
>hsa-miR-1225-5p MIMAT0005572
GUGGGUACGGCCCAGUGGGGGG
>hsa-miR-4288 MIMAT0016918
UUGUCUGCUGAGUUUCC
>hsa-miR-522 MIMAT0002868
AAAAUGGUUCCCUUUAGAGUGU
>hsa-miR-3120 MIMAT0014982
CACAGCAAGUGUAGACAGGCA
>hsa-miR-431 MIMAT0001625
UGUCUUGCAGGCCGUCAUGCA
>hsa-miR-197 MIMAT0000227
UUCACCACCUUCUCCACCCAGC
>hsa-miR-1228 MIMAT0005583
UCACACCUGCCUCGCCCCCC
>hsa-miR-1249 MIMAT0005901
ACGCCCUUCCCCCCCUUCUUCA
>hsa-miR-129-3p MIMAT0004605
AAGCCCUUACCCCAAAAAGCAU
>hsa-miR-24 MIMAT0000080
UGGCUCAGUUCAGCAGGAACAG
>hsa-miR-1827 MIMAT0006767
UGAGGCAGUAGAUUGAAU
>hsa-miR-3165 MIMAT0015039
AGGUGGAUGCAAUGUGACCUCA
>hsa-miR-518f MIMAT0002842
GAAAGCGCUUCUCUUUAGAGG
>hsa-miR-100 MIMAT0000098
AACCCGUAGAUCCGAACUUGUG
>hsa-miR-548c-3p MIMAT0003285
CAAAAAUCUCAAUUACUUUUGC
>hsa-miR-1299 MIMAT0005887
UUCUGGAAUUCUGUGUGAGGGA
>hsa-miR-4308 MIMAT0016861

UCCCUGGAGUUUCUUCUU
>hsa-miR-3130-3p MIMAT0014994
GCUGCACCGGAGACUGGGUAA
>hsa-miR-7 MIMAT0000252
UGGAAGACUAGUGAUUUUGUUGU
>hsa-miR-106a* MIMAT0004517
CUGCAAUGUAAGCACUUCUUAC
>hsa-miR-382 MIMAT0000737
GAAGUUGUUCGUGGUGGAUUCG
>hsa-miR-320b MIMAT0005792
AAAAGCUGGGUUGAGAGGGCAA
>hsa-miR-182 MIMAT0000259
UUUGGCAAUGGUAGAACUCACACU
>hsa-miR-297 MIMAT0004450
AUGUAUGUGUGCAUGUGCAUG
>hsa-miR-1264 MIMAT0005791
CAAGUCUUAUUUGAGCACCUGUU
>hsa-miR-449c MIMAT0010251
UAGGCAGUGUAUUGCUAGCGGCUGU
>hsa-miR-3188 MIMAT0015070
AGAGGCUUUGUGCGGAUACGGGG
>hsa-miR-548l MIMAT0005889
AAAAGUAUUUGCGGGUUUUGUC
>hsa-miR-424 MIMAT0001341
CAGCAGCAAUUCAUGUUUUGAA
>hsa-miR-302b MIMAT0000715
UAAGUGCUUCCAUGUUUUAGUAG
>hsa-miR-1283 MIMAT0005799
UCUACAAAGGAAAGCGCUUUCU
>hsa-miR-548e MIMAT0005874
AAAAACUGAGACUACUUUUGCA
>hsa-miR-3150 MIMAT0015023
CUGGGGAGAUCCUCGAGGUUGG
>hsa-miR-655 MIMAT0003331
AUAAUACAUGGUUAACCUCUUU
>hsa-miR-518d-3p MIMAT0002864
CAAAGCGCUUCCCUUUGGAGC
>hsa-miR-4319 MIMAT0016870
UCCCUGAGCAAAGCCAC
>hsa-miR-891a MIMAT0004902
UGCAACGAACCUGAGCCACUGA
>hsa-miR-3135 MIMAT0015001
UGCCUAGGCUGAGACUGCAGUG
>hsa-miR-137 MIMAT0000429

UUAUUGCUUAAGAAUACGCGUAG
>hsa-miR-199b-3p MIMAT0004563
ACAGUAGUCUGCACAUUGGUUA
>hsa-miR-331-3p MIMAT0000760
GCCCCUGGGCCUAUCCUAGAA
>hsa-miR-1278 MIMAT0005936
UAGUACUGUGCAUAUCAUCUAU
>hsa-miR-924 MIMAT0004974
AGAGUCUUGUGAUGUCUUGC
>hsa-miR-488* MIMAT0002804
CCCAGAUAAUGGCACUCUCAA
>hsa-miR-577 MIMAT0003242
UAGAUAAAAUAUUGGUACCUG
>hsa-miR-3184 MIMAT0015064
UGAGGGGCCUCAGACCGAGCUUUU
>hsa-miR-122 MIMAT0000421
UGGAGUGUGACAAUGGUGUUUG
>hsa-miR-7-2* MIMAT0004554
CAACAAAUCCCAGUCUACCUAA
>hsa-miR-1973 MIMAT0009448
ACCGUGCAAAGGUAGCAUA
>hsa-miR-377* MIMAT0004689
AGAGGUUGCCCUUGGUGAAUUC
>hsa-miR-3115 MIMAT0014977
AUAUGGGUUUACUAGUUGGU
>hsa-miR-107 MIMAT0000104
AGCAGCAUUGUACAGGGCUAUCA
>hsa-miR-300 MIMAT0004903
UAUACAAGGGCAGACUCUCUCU
>hsa-miR-188-5p MIMAT0000457
CAUCCCUUGCAUGGUGGAGGG
>hsa-miR-15a MIMAT0000068
UAGCAGCACAUAAUGGUUUGUG
>hsa-miR-933 MIMAT0004976
UGUGCGCAGGGAGACCUCUCCC
>hsa-miR-4293 MIMAT0016848
CAGCCUGACAGGAACAG
>hsa-miR-20a MIMAT0000075
UAAAGUGCUUAUAGUGCAGGUAG
>hsa-miR-409-3p MIMAT0001639
GAAUGUUGCUCGGUGAACCCCU
>hsa-miR-627 MIMAT0003296
GUGAGUCUCUAAGAAAAGAGGA
>hsa-miR-519b-5p MIMAT0005454

CUCUAGAGGGAAGCGCUUUCUG
>hsa-let-7a* MIMAT0004481
CUAUACAAUCUACUGUCUUUC
>hsa-miR-2054 MIMAT0009979
CUGUAAUAUAAAUUUAAUUUAUU
>hsa-miR-103-2* MIMAT0009196
AGCUUCUUUACAGUGCUGCCUUG
>hsa-miR-2277 MIMAT0011777
UGACAGCGCCCUGCCUGGCUC
>hsa-miR-501-3p MIMAT0004774
AAUGCACCCGGGCAAGGAUUCU
>hsa-miR-340 MIMAT0004692
UUAUAAAGCAAUGAGACUGAUU
>hsa-miR-574-5p MIMAT0004795
UGAGUGUGUGUGUGUGAGUGUGU
>hsa-miR-499-5p MIMAT0002870
UUAAGACUUGCAGUGAUGUUU
>hsa-miR-193b* MIMAT0004767
CGGGGUUUUGAGGGCGAGAUGA
>hsa-miR-3194 MIMAT0015078
GGCCAGCCACCAGGAGGGCUG
>hsa-miR-371-3p MIMAT0000723
AAGUGCCGCCAUCUUUUGAGUGU
>hsa-miR-365* MIMAT0009199
AGGGACUUUCAGGGGCAGCUGU
>hsa-miR-106b MIMAT0000680
UAAAGUGCUGACAGUGCAGAU
>hsa-miR-296-3p MIMAT0004679
GAGGGUUGGGUGGAGGCUCUCC
>hsa-miR-524-3p MIMAT0002850
GAAGGCGCUUCCCUUUGGAGU
>hsa-miR-519c-5p MIMAT0002831
CUCUAGAGGGAAGCGCUUUCUG
>hsa-miR-1197 MIMAT0005955
UAGGACACAUGGUCUACUUCU
>hsa-miR-3141 MIMAT0015010
GAGGGCGGGUGGAGGAGGA
>hsa-miR-346 MIMAT0000773
UGUCUGCCCGCAUGCCUGCCUCU
>hsa-miR-16-1* MIMAT0004489
CCAGUAUUAACUGUGCUGCUGA
>hsa-miR-302b* MIMAT0000714
ACUUUAACAUGGAAGUGCUUUC
>hsa-miR-372 MIMAT0000724

AAAGUGCUGCGACAUUUGAGCGU
>hsa-miR-649 MIMAT0003319
AAACCUGUGUUGUUCAAGAGUC
>hsa-miR-367* MIMAT0004686
ACUGUUGCUAAUAUGCAACUCU
>hsa-miR-326 MIMAT0000756
CCUCUGGGCCCUUCCUCCAG
>hsa-miR-505 MIMAT0002876
CGUCAACACUUGCUGGUUUCCU
>hsa-miR-433 MIMAT0001627
AUCAUGAUGGGCUCCUCGGUGU
>hsa-miR-499-3p MIMAT0004772
AACAUCACAGCAAGUCUGUGCU
>hsa-miR-602 MIMAT0003270
GACACGGGCGACAGCUGCGGCCC
>hsa-miR-1234 MIMAT0005589
UCGGCCUGACCACCCACCCCAC
>hsa-miR-4252 MIMAT0016886
GGCCACUGAGUCAGCACCA
>hsa-miR-541 MIMAT0004920
UGGUGGGCACAGAAUCUGGACU
>hsa-miR-1250 MIMAT0005902
ACGGUGCUGGAUGUGGCCUUU
>hsa-miR-3159 MIMAT0015033
UAGGAUUACAAGUGUCGGCCAC
>hsa-miR-1302 MIMAT0005890
UUGGGACAUACUUAUGCUAAA
>hsa-miR-558 MIMAT0003222
UGAGCUGCUGUACCAAAAU
>hsa-miR-135b* MIMAT0004698
AUGUAGGGCUAAAAGCCAUGGG
>hsa-miR-514b-3p MIMAT0015088
AUUGACACCUCUGUGAGUGGA
>hsa-miR-613 MIMAT0003281
AGGAAUGUUCCUUCUUUGCC
>hsa-miR-1293 MIMAT0005883
UGGGUGGUCUGGAGAUUUGUGC
>hsa-miR-1225-3p MIMAT0005573
UGAGCCCCUGUGCCGCCCCCAG
>hsa-miR-4294 MIMAT0016849
GGGAGUCUACAGCAGGG
>hsa-miR-512-5p MIMAT0002822
CACUCAGCCUUGAGGGCACUUUC
>hsa-miR-1976 MIMAT0009451

CCUCCUGCCCUCCUUGCUGU
>hsa-miR-30e MIMAT0000692
UGUAAACAUCCUUGACUGGAAG
>hsa-miR-584 MIMAT0003249
UUAUGGUUUGCCUGGGACUGAG
