# Supplementary material for: A computational method for predicting regulation of human microRNAs on the influenza virus genome
Source: BMC Syst Biol. 2013 Oct 14;7(Suppl 2):S3. doi: 10.1186/1752-0509-7-S2-S3 (PMC3851852; doi:10.1186/1752-0509-7-S2-S3)
Supplement: Additional File 4 — The coding sequence of the gene fragment of MP from 2000 to 2012 recorded in Genbank. [file 1752-0509-7-S2-S3-S4.PDF]

HA

>gi|145278771|gb|CY021693.1| Influenza A virus (A/Memphis/15/2000(H1N1)) segment 4, complete sequence

GAAAATAGAAACAACCAAAATGAAAGCAAAACTAATGGTCCTGTTATGTACATTTACAGCTACATATGCA
GACACAATATGTATCGGCTACCATGCCAACAACTCAACCGACACTGTTGACACAGTACTTGAGAAGAATG
TGACAGTGACACACTCTGTCAACCTACTTGAGGACAGTCACAATGGAAAACTATGTCTACTAAAAGGAAT
AGCCCCACTACAATTGGGTAACTGCAGCGTTGCCGGATGGATCTTAGGAAACCCAGAATGCGAATTACTG
ATTTCCAAGGAATCATGGTCCTACATTGTAGAAACACCAAATCCTGAAAATGGAACATGTTACCCAGGGT
ATTTCGCCGACTATGAGGAACTGAGGGAGCAATTGAGTTCAGTATCTTCATTTGAGAGATTCGAAATATT
CCCCAAAGAAAGCTCATGGCCCAACCACACCGTAACCGGAGTATCAGCATCATGCTCCCATAATGGGAAA
AGCAGTTTTTACAGAAATTTGCTATGGCTGACGGGGAAGAATGGTTTGTACCCAAACCTGAGCAAGTCCT
ATGCAAACAACAAAGAGAAAGAAGTCCTTGTACTATGGGGTGTTCATCACCCGCCTAACATAGGGGACCA
AAGGGCCCTCTATCATACAGAAAATGCTTATGTCTCTGTAGTGTCTTCACATTATAGCAGAAGATTCACC
CCAGAAATAGCCAAAAGACCCAAAGTAAGAGATCAGGAAGGAAGAATCAACTACTACTGGACTCTGCTGG
AACCCGGGGATACAATAATATTTGAGGCAAATGGAAATCTAATAGCGCCATGGTATGCTTTCGCACTGAG
TAGAGGCTTTGGATCAGGAATCATCACCTCAAATGCACCAATGGGTGAATGTGATGCGAAGTGTCAAACA
CCTCAGGGAGCTATAAACAGCAGTCTTCCTTTCCAGAATGTACACCCAGTCACAATAGGAGAGTGTCCAA
AGTATGTCAGGAGTGCAAAATTAAGGATGGTTACAGGACTAAGGAACATCCCATCCATTCAATCCAGAGG
TTTGTTTGGAGCCATTGCCGGTTTCATTGAAGGGGGGTGGACTGGAATGGTAGATGGGTGGTATGGTTAT
CATCATCAGAATGAGCAAGGATCTGGCTATGCTGCAGATCAAAAAAGCACACAAAATGCCATTAACGGGA
TTACAAACAAGGTGAATTCTGTAATTGAGAAAATGAACACTCAATTCACAGCTGTGGGCAAAGAATTCAA
CAAATTGGAAAGAAGGATGGAAAACTTAAATAAAAAAGTTGATGATGGGTTTCTAGACATTTGGACATAT
AATGCAGAATTGTTGGTTCTACTGGAAAATGAAAGGACTTTGGATTTCCATGACTCCAATGTGAAGAATC
TGTATGAGAAAGTAAAAAGCCAATTAAAGAATAATGCCAAAGAAATAGGAAACGGGTGTTTTGAATTCTA
TCACAAGTGTAACAATGAATGCATGGAGAGTGTGAAAAATGGAACTTATGACTATCCAAAATATTCCGAA
GAATCAAAGTTAAACAGGGAGAAAATTGATGGAGTGAAATTGGAATCAATGGGAGTCTATCAGATTCTGG
CGATCTACTCAACTGTCGCCAGTTCCCTGGTTCTTTTGGTCTCCCTGGGGGCAATCAGCTTCTGGATGTG
TTCCAATGGGTCTTTGCAGTGTAGAATATGCATCTGAGACCAGAATT

>gi|70907639|gb|CY000449.2| Influenza A virus (A/New York/146/2000(H1N1)) segment 4, complete sequence

AGCAAAAGCAGGGGAAAATAAAAACAACCAAAATGAAAGCAAAACTACTAGTTCTGTTGTGTGCATTTAC
AGCTACATATGCAGACACAATATGTATAGGCTACCATGCGAACAACTCAACTGACACTGTTGACACAGTA
CTTGAGAAGAACGTGACAGTGACACACTCTGTCAACCTACTTGAGGACAGTCACAACGGAAAACTATGCC
GACTAAAAGGAACAGCCCCACTACAATTGGGTAATTGCAGCATTGCCGGATGGATCTTAGGAAATCCAGA
ATGCGAATCACTGTTTTCTAAGGAATCATGGTCTTACATTGCAGAAACACCAAACCCTAAAAATGGAACA
TGTTACCCAGGGTATTTCGCCGACTATGAGGAACTGAGGGAGCAATTGAGCTCAGTATCATCATTCGAGA
GATTTGAAATATTCCCCAAGGATAGCTCATGGCCCAACCACACTGTAACCAAAGGAGTGACGGCATCATG
CTCCCATAATGGGAAAAGCAGCTTTTACAAAAATTTGCTATGGCTGACGGAGAAGAATGGCTTGTACCCA
AATCTGAGCAAGTCCTATGTAAACAAAAAGGGAAAAGAAGTCCTTGTGCTATGGGGTGTTCATCACCCGT
CTAACATGGGGGACCAACGGGCCATCTATCATAAAGAAAATGCTTATGTTTCTGTGTTGTCTTCACATTA
TAGCAGAAGATTCACCCCAGAAATAGCAAAAAGACCAAAAGTAAGAGATCAAGAAGGGAGAATTAACTAC
TACTGGACTCTGCTGGAACCCGGGGACACAATAATATTTGAGGCAAATGGAAATCTAATAGCGCCGTGGT
ACGCTTTCGCACTGAGTAGAGGCTTTGGGTCAGGAATCATCATCTCAAACGCATCAATGGGTGAATGTGA

CGCTAAGTGTCAAACACCCCAAGGAGCTATAAACAGTAGTCTCCCCTTCCAGAATGTACACCCAGTCACA
ATAGGAGAGTGTCCAAAGTATGTCAGGAGTACAAAATTAAGGATGGTTACAGGACTAAGGAACGTCCCAT
CCATTCAATCCAGAGGTTTGTTTGGAGCCATTGCCGGTTTCATTGAAGGAGGGTGGACTGGAATGATAGA
TGGATGGTATGGTTATCATCATCAAAATGAACAAGGATCTGGCTATGCTGCGGACCAAAAAAGCACACAA
AATGCCATTAATGGGATTACAAACAAGGTGAATTCTATAATCGAGAAAATGAACACTCAATTCACAGCTG
TAGGCAAAGAATTCAACAAATTAGAAAAAAGGATGGAAAACTTAAATAAGAAAGTTGATGATGGATTTCT
GGACATTTGGACATATAATGCAGAATTGTTAGTTCTCCTGGAAAATGAAAGGACTTTGGATTTTCATGAC
TTAAATGTGAAGAACCTGTATGAGAAAGTGAAAAACCAATTGAAGAATAATGCCAAAGAAATAGGGAACG
GGTGTTTTGAATTCTATCACAAGTGTAACAATGAATGCATGGAAAGTGTGAAAAATGGAACTTATGACTA
TCCAAAATATTCCAAAGAATCAAAGTTAAACAGGGAAAAAATTGATGGAGTGAAATTGGAATCAATGGGA
GTCTATCAGATTCTGGCGATCTACTCAACTGTCGCCAGTTCGCTGGTGCTTTTGGTCTCCCTGGGGGCAA
TCAGCTTCTGGATGTGTTCTAATGGGTCTTTGCAGTGTAGAATATGCATCTGAGACCAGAATTTCAGAAA
TATAAGAAAAAACACCCTTGTTTCTACT
>gi|145278904|gb|CY021749.1| Influenza A virus (A/South Australia/44/2000(H1N1)) segment 4, complete sequence
ATGAAAGCAAAACTACTAGTTCTGTTGTGTGCATTTACAGCTACATATGCAGACACAATATGTATAGGCT
ACCATGCGAACAACTCAACTGACACTGTTGACACAGTACTTGAGAAGAACGTGACAGTGACACACTCTGT
CAACCTACTTGAGGACAGTCACAACGGAAAACTATGCCGACTAAAAGGAACAGCCCCACTACAATTGGGT
AATTGCAGCATTGCCGGATGGATCTTAGGAAATCCAGAATGCGAATCACTGTTTTCTAAGGAATCATGGT
CTTACATTGCAGAAACACCAAACCCTAAAAATGGAACATGTTACCCAGGGTATTTCGCCGACTATGAGGA
ACTGAGGGAGCAATTGAGCTCAGTATCATCATTCGAGAGATTTGAAATATTCCCCAAGGATAGCTCATGG
CCCAACCACACTGTAACCAAAGGAGTGACGGCATCATGCTCCCATAATGGGAAAAGCAGCTTTTACAAAA
ATTTGCTATGGCTGACGGAGAAGAATGGCTTGTACCCAAATCTGAGCAAGTCCTATGTAAACAAAAAGGG
AAAAGAAGTCCTTGTGCTATGGGGTGTTCATCACCCGTCTAACATGGGGGACCAACGGGCCATCTATCAT
AAAGAAAATGCTTATGTTTCTGTGTTGTCTTCACATTATAGCAGAAGATTCACCCCAGAAATAGCAAGAA
GACCAAAAGTAAGAGATCAAGAAGGGAGAATTAACTACTACTGGACTCTGCTGGAACCCGGGGACACAAT
AATATTTGAGGCAAATGGAAATCTAATAGCGCCGTGGTACGCTTTCGCACTGAGTAGAGGCTTTGGGTCA
GGAATCATCATCTCAAACGCATCAATGGGTGAATGTGACGCTAAGTGTCAAACACCCCAAGGAGCTATAA
ACAGTAGTCTCCCCTTCCAGAATGTACACCCAGTCACAATAGGAGAGTGTCCAAAGTATGTCAGGAGTAC
AAAATTAAGGATGGTTACAGGACTAAGGAACGTCCCATCCATTCAATCCAGAGGTTTGTTTGGAGCCATT
GCCGGTTTCATTGAAGGAGGGTGGACTGGAATGATAGATGGATGGTATGGTTATCATCATCAAAATGAAC
AAGGATCTGGCTATGCTGCGGACCAAAAAAGCACACAAAATGCCATTAATGGGATTACAAACAAGGTGAA
TTCTATAATCGAGAAAATGAACACTCAATTCACAGCTGTAGGCAAAGAATTCAACAAATTAGAAAGAAGG
ATGGAAAACTTAAATAAGAAAGTTGATGATGGATTTCTGGACATTTGGACATATAATGCAGAATTGTTAG
TTCTCCTGGAAAATGAAAGGACTTTGGATTTTCATGACTCAAATGTGAAGAACCTGTATGAGAAAGTGAA
AAACCAATTGAAGAATAATGCCAAAGAAATAGGGAACGGGTGTTTTGAATTCTATCACAAGTGTAACAAT
GAATGCATGGAAAGTGTGAAAAATGGAACTTATGACTATCCAAAATATTCCAAAGAATCAAAGTTAAACA
GGGAAAAAATTGATGGAGTGAAATTGGAATCAATGGGAGTCTATCAGATTCTGGCGATCTACTCAACTGT
CGCCAGTTCGCTGGTGCTTTTGGTCTCCCTGGGGGCAATCAGCTTCTGGATGTGTTCTAATGGGTCTTTG
CAGTGTAGAATATGCATCTGAGACCAGAATTTCAGAAATATAAGAAAAAA
>gi|157367765|gb|CY026155.1| Influenza A virus (A/Auckland/585/2000(H1N1)) segment 4, complete sequence
AATAAAACCAACCAAAATGAAAGTAAAACTACTGGTCCTGTTATGTACATTTACAGCTACATATGCAGAC
ACAATATGTATAGGGTACCATGCCAACAACTCAACCGACACTGTTGACACAGTACTTGAGAAGAATGTGA

CAGTGACACACTCTGTCAACCTACTTGAGGACAGTCACAATGGAAAACTATGTCTACTAAAAGGAATAGC
CCCACTACAATTGGGTAATTGCAGCGTTGCCGGATGGATCTTAGGAAACCCAGAATGCGAATTACTGATT
TCCAAGGAATCATGGTCCTACATTGTAGAAACACCAAATCCTGAGAATGGAACATGTTACCCAGGGTATT
TCGCCGACTATGAGGAACTGAGGGAGCAATTGAGTTCAGTATCTTCATTTGAGAGATTCGAAATATTCCC
CAAAGAAAGCTCATGGCCCAACCACACCGTAACCGGAGTATCAGCATCATGCTCCCATAATGGGAAAAGC
AGTTTTTACAGAAATTTGCTATGGCTGACGRGGAAGAATGGTTTGTACCCAAACCTGAGCAAGTCCTATG
CAAACAACAAAGAGAAAGAAGTCCTTGTACTATGGGGTGTTCATCACCCGCCTAACATAGGGGACCAAAG
GGCCCTCTATCATACAGAAAATGCTTATGTCTCTGTAGTGTCTTCACATTATAGCAGAAGATTCACCCCA
GAAATAGCCAAAAGACCCAAAGTAAGAGATCAGGAAGGAAGAATCAACTACTACTGGACTCTGCTGGAAC
CCGGGGATACAATAATATTTGAGGCAAATGGAAATCTAATAGCGCCACGGTATGCTTTCGCACTGAGTAG
AGGCTTTGGATCAGGAATCATCACCTCAAATGCACCAATGGATGAATGTGATGCGAAGTGTCAAACACCT
CAGGGAGCTATAAACAGCAGTCTTCCTTTCCAGAACGTACACCCAGTCACAATAGGAGAGTGTCCAAAGT
ATGTCAGGAGTGCAAAATTAAGGATGGCTACAGGACTAAGGAACATCCCATCCATTCAATCCAGAGGTTT
GTTTGGAGCCATTGCCGGTTTCATTGAAGGGGGGTGGACTGGAATGGTAGATGGGTGGTATGGTTATCAT
CATCAGAATGAGCAAGGATCTGGCTATGCCGCAGATCAAAAAAGCACACAAAATGCCATTAACGGGATTA
CAAACAAGGTGAATTCTGTAATTGAGAAAATGAACACTCAATTCACAGCTGTGGGCAAAGAATTCAACAA
ATTGGAAAGAAGGATGGAAAACTTAAATAAAAAAGTTGATGATGGGTTTCTAGACATTTGGACATATAAT
GCAGAATTGTTGGTTCTACTGGAAAATGAAAGGACTTTGGATTTCCATGACTCCAATGTGAAGAATCTGT
ATGAGAAAGTAAAAAGCCAATTAAAGAATAATGCCAAAGAAATAGGAAACGGGTGTTTTGAATTCTATCA
CAAGTGTAACAATGAATGCATGGAGAGTGTGAAAAATGGAACTTATGACTATCCAAAATATTCCGAAGAA
TCAAAGTTAAACAGGGAGAAAATTGATGGAGTGAAATTGGAATCAATGGGAGTCTATCAGATTCTGGCGA
TCTACTCAACTGTCGCCAGTTCCCTGGTTCTTTTGGTCTCCCTGGGGGCAATCAGCTTCTGGATGTGTTC
CAATGGGTCTTTGCAGTGTAGAATATGCATCTGAGACCAGAATTTCAGAAATATAAGAAAA
>gi|145278617|gb|CY021629.1| Influenza A virus (A/Wellington/4/2000(H1N1)) segment 4, complete sequence
ACCAACCAAAATGAAAGTAAAACTACTGGTCCTGTTATGTACATTTACAGCTACATATGCAGACACAATA
TGTATAGGGTACCATGCCAACAACTCAACCGACACTGTTGACACAGTACTTGAGAAGAATGTGACAGTGA
CACACTCTGTCAACCTACTTGAGGACAGTCACAATGGAAAACTATGTCTACTAAAAGGAATAGCCCCACT
ACAATTGGGTAATTGCAGCGTTGCCGGATGGATCTTAGGAAACCCAGAATGCGAATTACTGATTTCCAAG
GAATCATGGTCCTACATTGTAGAAACACCAAATCCTGAGAATGGAACATGTTACCCAGGGTATTTCGCCG
ACTATGAGGAACTGAGGGAGCAATTGAGTTCAGTATCTTCATTTGAGAGATTCGAAATATTCCCCAAAGA
AAGCTCATGGCCCAACCACACCGTAACCGGAGTATCAGCATCATGCTCCCATAATGGGAAAAGCAGTTTT
TACAGAAATTTGCTATGGCTGACGGGGAAGAATGGTTTGTACCCAAACCTGAGCAAGTCCTATGCAAACA
ACAAAGAGAAAGAAGTCCTTGTACTATGGGGTGTTCATCACCCGCCTAACATAGGGGACCAAAGGGCCCT
CTATCATACAGAAAATGCTTATGTCTCTGTAGTGTCTTCACATTATAGCAGAAGATTCACCCCAGAAATA
GCCAAAAGACCCAAAGTAAGAGATCAGGAAGGAAGAATCAACTACTACTGGACTCTGCTGGAACCCGGGG
ATACAATAATATTTGAGGCAAATGGAAATCTAATAGCGCCACGGTATGCTTTCGCACTGAGTAGAGGCTT
TGGATCAGGAATCATCACCTCAAATGCACCAATGGATGAATGTGATGCGAAGTGTCAAACACCTCAGGGA
GCTATAAACAGCAGTCTTCCTTTCCAGAACGTACACCCAGTCACAATAGGAGAGTGTCCAAAGTATGTCA
GGAGTGCAAAATTAAGGATGGTTACAGGACTAAGGAACATCCCATCCATTCAATCCAGAGGTTTGTTTGG
AGCCATTGCCGGTTTCATTGAAGGGGGGTGGACTGGAATGGTAGATGGGTGGTATGGTTATCATCATCAG
AATGAGCAAGGATCTGGCTATGCCGCAGATCAAAAAAGCACACAAAATGCCATTAACGGGATTACAAACA
AGGTGAATTCTGTAATTGAGAAAATGAACACTCAATTCACAGCTGTGGGCAAAGAATTCAACAAATTGGA
AAGAAGGATGGAAAACTTAAATAAAAAAGTTGATGATGGGTTTCTAGACATTTGGACATATAATGCAGAA

TTGTTGGTTCTACTGGAAAATGAAAGGACTTTGGATTTCCATGACTCCAATGTGAAGAATCTGTATGAGA
AAGTAAAAAGCCAATTAAAGAATAATGCCAAAGAAATAGGAAACGGGTGTTTTGAATTCTATCACAAGTG
TAACAATGAATGCATGGAGAGTGTGAAAAATGGAACTTATGACTATCCAAAATATTCCGAAGAATCAAAG
TTAAACAGGGAGAAAATTGATGGAGTGAAATTGGAATCAATGGGAGTCTATCAGATTCTGGCGATCTACT
CAACTGTCGCCAGTTCCCTGGTTCTTTTGGTCTCCCTGGGGGCAATCAGCTTCTGGATGTGTTCCAATGG
GTCTTTGCAGTGTAGAATATGCATCTGAGACCAGAATTTCAGAAATATAAGAAAAAA
>gi|131058466|gb|CY020149.1| Influenza A virus (A/Memphis/7/2001(H1N1)) segment 4, complete sequence
AACAACCAAAATGAAAGCAAAACTACTGGTCCTGTTATGTACATTTACAGCTACATACGCAGACACAATA
TGTATAGGCTACCATGCCAACAACTCAACCGACACTGTTGACACAGTACTTGAGAAGAATGTGACAGTGA
CACACTCTGTCAACCTACTTGAGGACAGTCACAATGGAAAACTATGTCTACTAAAAGGAATAGCCCCACT
ACAATTGGGTAATTGCAGCGTTGCCGGATGGATCTTAGGAAACCCAGAATGCGAATTACTGATTTCCAAG
GAATCATGGTCCTACATTGTAGAAACACCAAATCCTGAGAATGGAACATGTTACCCAGGGTATTTCGCTG
ACTATGAGGAACTGAGGGAGCAATTGAGTTCAGTATCTTCATTTGAGAGATTCGAAATATTCCCCAAAGA
AAGCTCATGGCCCAACCACACCGTAACCGGAGTATCAGCATCATGCTCCCATAATGGGAAAAGCAGTTTT
TACAGAAATTTGCTATGGCTGACGGGGAAGAATGGTTTGTACCCAAACCTGAGCAGGTCCTATGTAAACA
ACAAAGAGAAAGAAGTCCTTGTGCTATGGGGTGTTCATCACCCACCTAACATAGGGGACCAAAGGGCCCT
CTACCATACAGAAAATGCTTATGTCTCTGTAGTGTCTTCACATTATAGCAGAAGATTCACCCCAGAAATA
GCCAAAAGACCCAAAGTAAGAGATCAGGAAGGAAGAATCAACTACTACTGGACTCTGCTGGAACCCGGGG
ACACATTAATATTTGAGGCAAATGGAAATCTAATAGCGCCATGGTATGCTTTCGCACTGAGTAGAGGCTT
TGGATCAGGAATCATCACCTCAAATGCACCAATGGATGAATGTGATGCGAAGTGTCAAACACCTCAGGGA
GCTATAAACAGCAGTCTTCCTTTCCAGAATGTACACCCAGTTACAATAGGAGAGTGTCCAAAGTATGTCA
GGAGTACAAAATTAAGGATGGTTACAGGACTAAGGAACATCCCATCCATTCAATCCAGAGGTTTGTTTGG
AGCCATTGCCGGTTTCATTGAAGGGGGGTGGACTGGAATGGTAGATGGGTGGTATGGTTATCATCATCAG
AATGAGCAAGGATCTGGCTATGCTGCAGATCAAAAAAGCACACAAAATGCCATTAACGGGATTACAAACA
AGGTGAATTCTGTAATTGAGAAAATGAACACTCAATTCACAGCTGTGGGCAAAGAATTCAACAAATTAGA
AAGAAGGATGGAAAACTTAAATAAAAAAGTTGATGATGGGTTTCTAGACATTTGGACATATAATGCAGAA
TTGTTGGTTCTACTGGAAAATGAAAGGACTTTGGATTTCCATGACTCCAATGTGAAGAATCTGTATGAGA
AAGTAAAAAGCCAATTAAAGAATAATGCCAAAGAAATAGGAAACGGGTGTTTTGAATTCTATCACAAGTG
TAACAATGAATGCATGGAAAGTGTGAAAAATGGAACTTATGACTATCCAAAATATTCCGAAGAATCAAAG
TTAAACAGGGAGAAAATTGATGGAGTAAAATTGGAATCAATGGGAGTCTATCAGATTCTGGCGATCTACT
CAACTGTCGCCAGTTCCCTGGTTCTTTTGGTCTCCCTGGGGGCAATCAGCTTCTGGATGTGTTCCAATGG
GTCTTTGCAATGTAGAATATGCATCTGAGACCAGAATTTCAGAAAATATAA
>gi|73761473|gb|CY002528.1| Influenza A virus (A/New York/220/2002(H1N1)) segment 4, complete sequence
AAGCAACCAAAATGAAAGTAAAACTACTGGTTCTGTTATGTACATTTACAGCTACATATGCAGACACAAT
ATGTATAGGCTACCATGCCAACAACTCAACCGACACTGTTGACACAGTACTTGAGAAGAATGTGACAGTG
ACACACTCTGTCAACCTACTTGAGGACAGTCACAATGGAAAACTATGTCTACTAAAAGGAATAGCCCCAC
TACAATTGGGTAATTGCAGCGTTGCCGGATGGATCTTAGGAAACCCAGAATGCGAATTACTGATTTCCAA
GGAATCATGGTCCTACATTGTAGAAACACCAAATCCTGAAAATGGAACATGTTACCCAGGGTATTTCGCC
GACTATGAGGAACTGAGGGAGCAATTGAGTTCAGTATCTTCATTTGAGAGGTTCGAAATATTCCCCAAAG
AGAGCTCATGGCCCAACCACACCGTAACCGGAGTATCAGCATCATGCTCCCATAACGGGAAAAGCAGTTT
TTACAGAAATTTGCTATGGCTGACGGGGAAGAATGGTTTGTACCCAAACCTGAGCAAGTCCTATGCAAAC
AACAAAGAGAAAGAAGTCCTTGTACTATGGGGTGTTCATCACCCGCCTAACATAGGGGACCAAAGGGCCC

TCTATCATACAGAAAATGCTTATATCTCTGTAGTGTCTTCACATTATAGCAGAAGATTCACCCCAGAAAT
AGCCAAAAGACCCAAGGTAAGAGATCAGGAAGGAAGAATCAACTACTACTGGACTCTGCTGGAACCCGGG
GATACAATAATATTTGAGGCAAATGGAAATCTAATAGCGCCAAGGTATGCTTTCGCACTGAGTAGAGGCT
TTGGATCAGGAATCATCACCTCAAATGCACCAATGGATGAATGTGATGCGAAGTGTCAAACACCTCAGGG
AGCTATAAACAGCAGTCTTCCTTTCCAGAATGTACACCCAGTCACAATAGGAGAGTGTCCAAAGTATGTC
AGGAGTGCAAAATTAAGGATGGTTACAGGACTAAGGAACATCCCATCCATTCAATCCAGAGGTTTGTTTG
GAGCCATTGCCGGTTTCATTGAAGGGGGGTGGACTGGAATGGTAGATGGTTGGTATGGTTATCATCATCA
GAATGAGCAAGGATCTGGGTATGCTGCAGATCAAAAAAGCACACAAAATGCCATTAACGGGATTACAAAC
AAGGTGAATTCTGTAATTGAGAAAATGAACACTCAATTCACAGCTGTGGGCAAAGAATTCAACAAATTGG
AAAGAAGGATGGAAAACTTAAATAAAAAGGTTGATGATGGGTTTCTAGACATTTGGACATATAATGCAGA
ATTGTTGGTTCTACTGGAAAATGAAAGGACTTTGGATTTCCATGACTCCAACGTGAAGAATCTGTATGAG
AAAGTAAAAAGCCAATTAAAGAATAATGCCAAAGAAATAGGAAACGGGTGTTTTGAATTCTATCACAAGT
GTAACGATGAATGCATGGAGAGTGTGAAAAATGGAACTTATGACTATCCAAGATATTCCGAAGAATCAAA
GTTAAACAGGGAGAAAATTGATGGAGTGAAATTGGAATCAATGGGAGTCTATCAGATTCTGGCGATCTAC
TCAACAGTCGCCAGTTCCCTGGTTCTTTTGGTCTCCCTGGGGGCAATCAGCTTCTGGATGTGTTCCAATG
GGTCTTTGCAGTGTAGAATATGCATCTAAGACCAGAATTTCAGAAATATAAGGAAAAACACCCTTGTTTC
TACT
>gi|156536317|gb|CY025034.1| Influenza A virus (A/Auckland/597/2000(H1N1)) segment 4, complete sequence
ATGAAAGTAAAACTACTGGTCCTGTTATGTACATTTACAGCTACATATGCAGACACAATATGTATAGGGT
ACCATGCCAACAACTCAACCGACACTGTTGACACAGTACTTGAGAAGAATGTGACAGTGACACACTCTGT
CAACCTACTTGAGGACAGTCACAATGGAAAACTATGTCTACTAAAAGGAATAGCCCCACTACAATTGGGT
AATTGCAGCGTTGCCGGATGGATCTTAGGAAACCCAGAATGCGAATTACTGATTTCCAAGGAATCATGGT
CCTACATTGTAGAAACACCAAATCCTGAGAATGGAACATGTTACCCAGGGTATTTCGCCGACTATGAGGA
ACTGAGGGAGCAATTGAGTTCAGTATCTTCATTTGAGAGATTCGAAATATTCCCCAAAGAAAGCTCATGG
CCCAACCACACCGTAACCGGAGTATCAGCATCATGCTCCCATAATGGGAAAAGCAGTTTTTACAGAAATT
TGCTATGGCTGACGGGGAAGAATGGTTTGTACCCAAACCTGAGCAAGTCCTATGCAAACAACAAAGAGAA
AGAAGTCCTTGTACTATGGGGTGTTCATCACCCGCCTAACATAGGGAACCAAAGGGCCCTCTATCATACA
GAAAATGCTTATGTCTCTGTAGTGTCTTCACATTATAGCAGAAGATTCACCCCAGAAATAGCCAAAAGAC
CCAAAGTAAGAGATCAGGAAGGAAGAATCAACTACTACTGGACTCTGCTGGAACCCGGGGATACAATAAT
ATTTGAGGCAAATGGAAATCTAATAGCGCCACGGTATGCTTTCGCACTGAGTAGAGGCTTTGGATCAGGA
ATCATCACCTCAAATGCACCAATGGATGAATGTGATGCGAAGTGTCAAACACCTCAGGGAGCTATAAACA
GCAGTCTTCCTTTCCAGAACGTACACCCAGTCACAATAGGAGAGTGTCCAAAGTATGTCAGGAGTGCAAA
ATTAAGGATGGTTACAGGACTAAGGAACATCCCATCCATTCAATCCAGAGGTTTGTTTGGAGCCATTGCC
GGTTTCATTGAAGGGGGGTGGACTGGAATGGTAGATGGGTGGTATGGTTATCATCATCAGAATGAGCAAG
GATCTGGCTATGCCGCAGATCAAAAAAGCACACAAAATGCCATTAACGGGATTACAAACAAGGTGAATTC
TGTAATTGAGAAAATGAACACTCAATTCACAGCTGTGGGCAAAGAATTCAACAAATTGGAAAGAAGGATG
GAAAACTTAAATAAAAAAGTTGATGATGGGTTTCTAGACATTTGGACATATAATGCAGAATTGTTGGTTC
TACTGGAAAATGAAAGGACTTTGGATTTCCATGACTCCAATGTGAAGRATCTGTATGAGAAAGTAAAAAG
CCAATTAAAGAATAATGCCAAAGAAATAGGAAACGGGTGTTTTGAATTCTATCACAAGTGTAACAATGAA
TGCATGGAGAGTGTGAAAAATGGAACTTATGACTATCCAAAATATTCCGAAGAATCAAAGTTAAACAGGG
AAAAAATTGATGGAGTGAAATTGGAATCAATGGGAGTCTATCAGATTCTGGCGATCTACTCAACTGTCGC
CAGTTCCCTGGTTCTTTTGGTCTCCCTGGGGGCAATCAGCTTCTGGATGTGTTCCAATGGGTCTTTGCAG
TGTAGAATATGCATCTGAGACCAGAATTTCAGAAATATAAGAAAAAA

>gi|149780526|gb|CY022533.1| Influenza A virus (A/Auckland/605/2001(H1N1)) segment 4, complete sequence
AATAAAAACAACCAAAATGAAAGCAAAACTACTGGTCCTGTTATGTACATTTACAGCTACATACGCAGAC
ACAATATGTATAGGCTACCATGCCAACAACTCAACCGACACTGTTGACACAGTACTTGAGAAGAATGTGA
CAGTGACACACTCTGTCAACCTACTTGAGGACAGTCACAATGGAAAACTATGTCTACTAAAAGGAATAGC
CCCACTACAATTGGGTAATTGCAGCGTTGCCGGATGGATCTTAGGAAACCCAGAATGCGAATTACTGATT
TCCAAGGAATCATGGTCCTACATTGTAGAAACACCAAATCCCGAGAATGGAACATGTTACCCAGGGTATT
TCGCCGACTATGAGGAACTGAGGGAGCAATTGAGTTCAGTATCTTCATTTGAGAGATTCGAAATATTCCC
CAAAGGAAGCTCATGGCCCAACCACACCGTGACCGGAGTATCAGCATCATGCTCCCATAATGGGAAAAGC
AGTTTTTACAGAAATTTGCTATGGCTGACGGGGAAGAATGGTTTGTACCCAAACCTGAGCATGTCCTATG
TAAACAACAAAGAGAAAGAAGTCCTTGTGCTATGGGGTGTTCATCACCCACCTAACATAGGGAACCAAAG
GGCCCTCTACCATACAGAAAATGCTTATGTCTCTGTAGTGTCTTCACATTATAGCAGAAGATTCACCCCA
GAAATAGCCAAAAGACCCAAAGTAAGAGATCAGGAAGGAAGAATCAACTACTACTGGACTCTGCTGGAAC
CCGGGGACACAATAATATTTGAGGCAAATGGAAATCTAATAGCGCCATGGTATGCTTTCGCACTGAGTAG
AGGCTTTGGATCAGGAATCATCACCTCAAATGCACCAATGGGTGAATGTGATGCGAAGTGTCAAACACCT
CAGGGAGCTATAAACAGTAGTCTTCCTTTCCAGAATGTACACCCAGTTACAATAGGAGAGTGTCCAAAGT
ATGTCAGGAGTGCAAAATTAAGGATGGTTACAGGACTAAGGAACATCCCATCCATTCAATCCAGAGGTTT
GTTTGGAGCCATTGCCGGTTTCATTGAAGGGGGGTGGACTGGAATGGTAGATGGGTGGTATGGTTATCAT
CATCAGAATGAGCAAGGATCTGGCTATGCTGCAGATCAAAAAAGCACACAAAATGCCATTAACGGGATTA
CAAACAAGGTGAATTCTGTAATTGAGAAAATGAACACTCAATTCACAGCTGTGGGCAAAGAATTCAACAA
ATTAGAAAGAAGGATGGAAAACTTAAATAAAAAAGTTGATGATGGGTTTCTAGACATTTGGACATATAAT
GCAGAATTGTTGGTTCTACTGGAAAATGAAAGGACTTTGGATTTCCATGACTCCAATGTGAAGAATCTGT
ATGAGAAAGTAAAAAGCCAATTAAAGAATAATGCCAAAGAAATAGGAAACGGGTGTTTTGAATTCTATCA
CAAGTGTAACAATGAATGCATGGAAAGTGTGAAAAATGGAACTTATGACTATCCAAAATATTCCGAAGAA
TCAAAGTTAAACAGGGAGAAAATTGATGGAGTAAAATTGGAATCAATGGGAGTCTATCAGATTCTGGCGA
TCTACTCAACTGTCGCCAGTTCCCTGGTTCTTTTGGTCTCCCTGGGGGCAATCAGCTTCTGGATGTGTTC
CAATGGGTCTTTGCAGTGTAGAATATGCATCTGAGACCAGAATTTCAGAAATATAAGAAAAAA
>gi|237688862|gb|CY040074.1| Influenza A virus (A/Taiwan/567/2002(H1N1)) segment 4, complete sequence
ATGAAAGCAAAACTACTGGTCCTGTTATGTACATTTACAGCTACATACGCAGACACAATATGTATAGGCT
ACCATGCCAACAACTCAACCGACACTGTTGACACAGTACTTGAGAAGAATGTGACAGTGACACACTCTGT
CAACCTACTTGAGGACAGTCACAATGGAAAACTATGTCTACTAAAAGGAATAGCCCCACTACAATTGGGT
AATTGCAGCGTTGCCGGATGGATCTTAGGAAACCCAGAATGCGAATTACTGATTTCCAAGGAATCATGGT
CCTACATTGTAGAAACACCAAATCCCGAGAATGGAACATGTTACCCAGGGTATTTCGCCGACTATGAGGA
ACTGAGGGAGCAATTGAGTTCAGTATCTTCATTTGAGAGATTCGAAATATTCCCCAAAGGAAGCTCATGG
CCCAACCACACCGTGACCGGAGTATCAGCATCATGCTCCCATAATGGGAAAAGCAGTTTTTACAGAAATT
TGCTATGGCTGACGGGGAAAAATGGTTTGTACCCAAACCTGAGCATGTCCTATGTAAACAACAAAGAGAA
AGAAGTCCTTGTGCTATGGGGTGTTCATCACCCACCTAACATAGGGAACCAAAGGGCCCTCTACCATACA
GAAAATGCTTATGTCTCTGTAGTGTCTTCACATTATAGCAGAAGATTCACCCCAGAAATAGCCAAAAGAC
CCAAAGTAAGAGATCAGGAAGGAAGAATCAACTACTACTGGACTCTGCTGGAACCCGGGGACACAATAAT
ATTTGAGGCAAATGGAAATCTAATAGCGCCATGGTATGCTTTCGCACTGAGTAGAGGCTTTGGATCAGGA
ATCATCACCTCAAATGCACCAATGGGTGAATGTGATGCGAAGTGTCAAACACCTCAGGGAGCTATAAACA
GTAGTCTTCCTTTCCAGAATGTACACCCAGTTACAATAGGAGAGTGTCCAAAGTATGTCAGGAGTGCAAA
ATTAAGGATGGTTACAGGACTAAGGAACATCCCATCCATTCAATCCAGAGGTTTGTTTGGAGCCATTGCC

GGTTTCATTGAAGGAGGGTGGACTGGAATGGTAGATGGGTGGTATGGTTATCATCATCAGAATGAGCAAG
GATCTGGCTATGCTGCAGATCAAAAAAGCACACAAAATGCCATTAACGGGATTACAAACAAGGTGAATTC
TGTAATTGAGAAAATGAACACTCAATTCACAGCTGTGGGCAAAGAATTCAACAAATTAGAAAGAAGGATG
GAAAACTTAAATAAAAAAGTTGATGATGGGTTTCTAGACATTTGGACATATAATGCAGAATTGTTGGTTC
TACTGGAAAATGAAAGGACTTTGGATTTTCATGACTCCAATGTGAAGAATCTGTATGAGAAAGTAAAAAG
CCAATTAAAGAATAATGCCAAAGAAATAGGAAACGGGTGTTTTGAATTCTATCACAAGTGTAACAATGAA
TGCATGGAAAGTGTGAAAAATGGAACTTATGACTATCCAAAATATTCCGAAGAATCAAAGTTAAACAGGG
AGAAAATTGATGGAGTAAAATTGGAATCAATGGGAGTCTATCAGATTCTGGCGATCTACTCAACTGTCGC
CAGTTCCCTGGTTCTTTTGGTCTCCCTGGGGGCAATCAGCTTCTGGATGTGTTCCAATGGGTCTTTGCAG
TGTAGAATATGCATCTGAGACCAGA
>gi|122855944|gb|CY019341.1| Influenza A virus (A/Memphis/6/2003(H1N1)) segment 4, complete sequence
AGCAACCAAAATGAAAGTAAAACTACTGGTTCTGTTATGTACATTTACAGCTACATATGCAGACACAATA
TGTATAGGCTACCATGCCAACAACTCAACCGACACTGTTGACACAGTACTTGAGAAGAATGTGACAGTGA
CACACTCTGTCAACCTACTTGAGGACAGTCACAATGGAAAACTATGTCTACTAAAAGGAATAGCCCCACT
ACAATTGGGTAATTGCAGCGTTGCCGGATGGATCTTAGGAAACCCAGAATGCGAATTACTGATTTCCAAG
GAATCATGGTCCTACATTGTAGAAACACCAAATCCTGAGAATGGAACATGTTACCCAGGGTATTTCGCCG
ACTATGAGGAACTGAGGGAGCAATTGAGTTCAGTATCTTCATTTGAGAGGTTCGAAATATTCCCCAAAGA
GAGCTCATGGCCCAACCACACCGTAACCGGAGTATCAGCATCATGCTCCCATAACGGGAAAAGCAGTTTT
TACAGAAATTTGCTATGGCTGACGGGGAAGAATGGTTTGTACCCAAACCTGAGCAAGTCCTATGCAAACA
ACAAAGAGAAAGAAGTCCTTGTACTATGGGGTGTTCACCACCCGCCTAACATAGGGGACCAAAGGGCCCT
CTATCATACAGAAAATGCTTATGTCTCTGTAGTGTCTTCACATTATAGCAGAAGATTCACCCCAGAAATA
GCCAAAAGACCCAAGGTGAGAGATCAGGAAGGAAGAATCAACTACTACTGGACTCTGCTGGAACCCGGGG
ATACAATAATATTTGAGGCAAATGGAAATCTAATAGCGCCAAGGTATGCTTTCGCACTGAGTAGAGGCTT
TGGATCAGGAATCATCACCTCAAATGCACCAATGGATGAATGTGATGCGAAGTGTCAAACACCTCAGGGA
GCTATAAACAGCAGTCTTCCTTTCCAGAATGTACACCCAGTCACAATAGGAGAGTGTCCAAAGTATGTCA
GGAGTGCAAAATTAAGGATGGTTACAGGACTAAGGAACATCCCATCCATTCAATCCAGAGGTTTGTTTGG
AGCCATTGCCGGTTTCATTGAAGGGGGGTGGACTGGAATGGTGGATGGTTGGTATGGTTATCATCATCAG
AATGAGCAAGGATCTGGGTATGCTGCAGATCAAAAAAGCACACAAAATGCCATTAACGGGATTACAAACA
AGGTGAATTCTGTAATTGAGAAAATGAACACTCAATTCACAGCTGTGGGCAAAGAATTCAACAAATTGGA
AAGAAGGATGGAAAACTTAAATAAAAAAGTTGATGATGGGTTTCTAGACATTTGGACATATAATGCAGAA
TTGTTGGTTCTACTGGAAAATGAAAGGACTTTGGATTTCCATGACTCCAACGTGAAGAATCTGTATGAGA
AAGTAAAAAGCCAATTAAAGAATAATGCCAAAGAAATAGGAAACGGGTGTTTTGAATTCTATCACAAGTG
TAACGATGAATGCATGGAGAGTGTGAAAAATGGAACTTATGACTATCCAAAATATTCCGAAGAATCAAAG
TTAAACAGGGAGAAAATTGATGGAGTGAAATTGGAATCAATGGGAGTCTATCAGATTCTGGCGATCTACT
CAACAGTCGCCAGTTCCCTGGTTCTTTTGGTCTCCCTGGGGGCAATCAGCTTCTGGATGTGTTCCAATGG
GTCTTTGCAGTGTAGAATATGCATCTAAGACCAGAATTTCAGAAATATAAGGAAAA
>gi|82546774|gb|CY006675.1| Influenza A virus (A/New York/494/2002(H1N1)) segment 4, complete sequence
GGGAAAATAAAAACAACCAAAATGAAAGTAAAACTACTGGTCCTGTTATGTACATTTACAGCTACATATG
CAGACACAATATGTATAGGCTACCATGCCAACAACTCAACCGACACTGTTGACACAGTACTTGAGAAGAA
TGTGACAGTGACACACTCTGTCAACCTACTTGAGGACAGTCACAATGGAAAACTATGTCTACTAAAAGGA
ATAGCCCCACTACAATTGGGTAATTGCAGCGTTGCCGGATGGATCTTAGGAAACCCAGAATGCGAATTGC
TGATTTCCAAGGAATCATGGTCCTACATTGTAGAAACACCAAATCCTGAGAATGGAACATGTTACCCAGG

GTATTTCGCCGACTATGAGGAACTGAGGGAGCAATTGAGTTCAGTATCTTCATTTGAGAGGTTCGAAATA
TTCCCCAAAGAAAGCTCATGGCCCAACCACACCGTAACCGGAGTATCATCATCATGCTCCCATAATGGGA
AAAGCAGTTTTTACAGAAATTTGCTATGGCTGACGGGGAAGAATGGTTTGTACCCAAACCTGAGCAAGTC
CTATGTAAACAACAAAGAGAAAGAAGTCCTTGTACTATGGGGTGTTCATCACCCGCCTAACATAGGGGAC
CAAAGGGCCCTCTATCATACAGAAAATGCTTATGTCTCTGTAGTGTCTTCACATTATAGCAGAAGATTCA
CCCCAGAAATAGCCAAAAGACCCAAAGTAAGAGATCAGGAAGGAAGAATCAACTACTACTGGACTCTGCT
GGAACCCGGGGACACAATAATATTTGAGGCAAATGGAAATCTAATAGCGCCAAGGTATGCTTTCGCACTG
AGTAGGGGCTTTGGATCAGGAATCATCACCTCAAATGCACCAATGGATGAATGTGATGCGAAGTGTCAAA
CACCTCAGGGAGCTATAAACAGCAGTCTTCCTTTCCAGAATGTACACCCAGTCACAATAGGAGAGTGTCC
AAAGTATGTCAGGAGTGCAAAATTAAGGATGGTTACAGGACTAAGGAACATCCCATCCATTCAATCCAGA
GGTTTGTTTGGGGCCATTGCCGGTTTCATTGAAGGGGGGTGGACTGGAATGGTAGATGGTTGGTATGGTT
ATCATCATCAGAATGAGCAAGGATCTGGCTATGCTGCAGATCAAAAAAGCACACAAAATGCCATTAACGG
GATTACAAACAAGGTGAATTCTGTAATTGAGAAAATGAACACTCAATTCACAGCTGTGGGCAAAGAATTC
AATAAATTGGAAAGAAGGATGGAAAACTTAAATAAAAAAGTTGATGATGGGTTTCTAGACATTTGGACAT
ATAATGCAGAATTGTTGGTTCTACTGGAAAATGAAAGGACTTTGGATTTCCATGACTCCAATGTGAAGAA
TCTGTATGAGAAAGTAAAAAGCCAATTAAAGAATAATGCCAAAGAAATAGGAAACGGGTGTTTTGAATTC
TATCACAAGTGTAACGATGAATGCATGGAGAGTGTGAAAAATGGAACTTATGACTATCCAAAATATTCCG
AAGAATCAAAGTTAAACAGGGAGAAAATTGATGGAGTGAAATTGGAATCAATGGGAGTCTATCAGATTCT
GGCGATCTACTCAACAGTCGCCAGTTCCCTGGTTCTTTTGGTCTCCCTGGGGGCAATCAGCTTCTGGATG
TGTTCCAATGGGTCTTTGCAGTGTAGAATATGCATCTAAGACCAGAATTCAGAA
>gi|77543341|gb|CY003304.1| Influenza A virus (A/New York/291/2002(H1N1)) segment 4, complete sequence
GCCAGTGGGGAAAATAAATACAACCAAAATGAAAGTAAAACTACTGGTCCTGTTATGTACATTTACAGCT
ACATATGCAGACACAATATGTATAGGCTACCATGCCAACAACTCAACCGACACTGTTGACACAGTACTTG
AGAAGAATGTGACAGTGACACACTCTGTCAACCTACTTGAGGACAGTCACAATGGAAAACTATGTCTACT
AAAAGGAATAGCCCCACTACAATTGGGTAATTGCAGCGTTGCCGGATGGATCTTAGGAAACCCAGAATGC
GAATTACTGATTTCCAAGGAATCATGGTCCTACATTATAGAAACACCAAATCCTGAGAATGGAACATGTT
ACCCAGGGTATTTCGCCGACTATGAGGAACTGAGGGAGCAATTGAGTTCAGTATCTTCATTTGAGAGGTT
CGAAATATTCCCCAAAGAAAGCTCATGGCCCAACCACACCGTAACCGGAGTATCAGCATCATGCTCCCAT
AATGGGAAAAGCAGTTTTTACAGAAATTTGCTATGGCTGACGGGGAAGAATGGTTTGTACCCAAACCTGA
GCAAGTCCTATGCAAACAACAAAGAGAAAGAAGTCCTTGTACTATGGGGTGTTCATCACCCGCCTAACAT
GGGGGACCAAAGGGCCCTCTATCATACAGAAAATGCTTATGTCTCTGTAGTGTCTTCACATTATAGCAGA
AGATTCACCCCAGAAATAGCCAAAAGACCCAAAGTAAGAGATCAGGAAGGAAGAATCAACTACTACTGGA
CTCTGCTGGAACCCGGGGATACAATAATATTTGAGGCAAATGGAAATCTAATAGCGCCAAGGTATGCTTT
CGCACTGAGTAGAGGCTTTGGATCAGGAATCATCACCTCAAATGCACCAATGGATGAATGTGATGCGAAG
TGTCAAACACCTCAGGGAGCTATAAACAGCAGTCTTCCTTTCCAGAATGTACACCCAGTCACAATAGGAG
AGTGTCCAAAGTATGTCAGGAGTGCAAAATTAAGGATGGTTACAGGACTAAGGAACATCCCATCCATTCA
ATCCAGAGGTTTGTTTGGAGCCATTGCCGGTTTCATTGAAGGGGGGTGGACTGGAATGGTAGATGGTTGG
TATGGTTATCATCATCAGAATGAGCAAGGATCTGGCTATGCTGCAGATCAAAAAAGCACACAAAATGCCA
TTAACGGGATTACAAACAAGGTGAATTCTGTAATTGAGAAAATGAACACTCAATTCACAGCTGTGGGCAA
AGAATTCAACAAATTGGAAAGAAGGATGGAAAACTTGAATAAAAAAGTTGATGATGGGTTTCTAGACATT
TGGACATATAATGCAGAATTGTTGGTTCTACTGGAAAATGAAAGGACTTTGGATTTCCATGACTCCAATG
TGAAGAATCTGTATGAGAAAGTAAAAAGCCAATTAAAGAATAATGCCAAAGAAATAGGAAACGGGTGTTT
TGAATTCTATCACAAGTGTAACGATGAATGCATGGAGAGTGTGAAAAATGGAACTTATGACTATCCAAAA

TATTCCGAAGAATCAAAGTTAAACAGGGAGAAAATTGATGGAGTGAAATTGGAATCAATGGGAGTCTATC
AGATTCTGGCGATCTACTCAACAGTCGCCAGTTCCCTGGTTCTTTTGGTCTCCCTGGGGGCAATCAGCTT
CTGGATGTGTTCCAATGGGTCTTTGCAGTGTAGAATATGCATCTAAGACCAGAATTTCAGAAATATAAGG
AAAAA

>gi|237689035|gb|CY040146.1| Influenza A virus (A/Taiwan/52/2002(H1N1)) segment 4, complete sequence

ATGAAAGCAAAACTACTGGTCCTGTTATGTACATTTACAGCTACATACGCAGACACAATATGTATAGGCT
ACCATGCCAACAACTCAACCGACACTGTTGACACAGTACTTGAGAAGAATGTGACAGTGACACACTCTGT
CAACCTACTTGAGGACAGTCACAATGGAAAACTATGTCTACTAAAAGGAATAGCCCCACTACAATTGGGT
AATTGCAGCGTTGCCGGATGGATCTTAGGAAACCCAGAATGCGAATTACTGATTTCCAAGGAATCATGGT
CCTACATTGTAGAAACACCAAATCCCGAGAATGGAACATGTTACCCAGGGTATTTCGCCGACTATGAGGA
ACTGAGGGAGCAATTGAGTTCAGTATCTTCATTTGAGAGATTCGAAATATTCCCCAAAGGAAGCTCATGG
CCCAACCACACCGTGACCGGAGTATCAGCATCATGCTCCCATAATGGGAAAAGCAGTTTTTACAGAAATT
TGCTATGGCTGACGGGGAAAAATGGTTTGTACCCAAACCTGAGCATGTCCTATGTAAACAACAAAGAGAA
AGAAGTCCTTGTGCTATGGGGTGTTCATCACCCACCTAACATAAGGGACCAGAGGGCCCTCTACCATACA
GAAAATGCTTATGTCTCTGTAGTGTCTTCACATTATAGCAGAAGATTCACCCCAGAAATAGCCAAAAGAC
CCAAAGTAAGAGATCAGGAAGGAAGAATCAACTACTACTGGACTCTGCTGGAACCCGGGGACACAATAAT
ATTTGAGGCAAATGGAAATCTAATAGCGCCATGGTATGCTTTCGCACTGAGTAGAGGCTTTGGATCAGGA
ATCATCACCTCAAATGCACCAATGGGTGAATGTGATGCGAAGTGTCAAACACCTCAGGGAGCTATAAACA
GTAGTCTTCCTTTCCAGAATGTACACCCAGTTACAATAGGAGAGTGTCCAAAGTATGTCAGGAGTGCAAA
ATTAAGGATGGTTACAGGACTAAGGAACATCCCATCCATTCAATCCAGAGGTTTGTTTGGAGCCATTGCC
GGTTTCATTGAAGGAGGGTGGACTGGAATGGTAGATGGGTGGTATGGTTATCATCATCAGAATGAGCAAG
GATCTGGCTATGCTGCAGATCAAAAGAGCACACAAAATGCCATTAACGGGATTACAAACAAGGTGAATTC
TGTAATTGAAAAAATGAACACTCAATTCACAGCTGTGGGCAAAGAATTCAACAAATTAGAAAGAAGGATG
GAAAACTTAAATAAAAAAGTTGATGATGGGTTTCTAGACATTTGGACATATAATGCAGAATTGTTGGTTC
TACTGGAAAATGAAAGGACTTTGGATTTTCATGACTCCAATGTGAAGAATCTGTATGAGAAAGTAAAAAG
CCAATTAAAGAATAATGCCAAAGAAATAGGAAACGGGTGTTTTGAATTCTATCACAAGTGTAACAATGAA
TGCATGGAAAGTGTGAAAAATGGAACTTATGACTATCCAAAATATTCCGAAGAATCAAAGTTAAACAGGG
AGAAAATTGATGGAGTAAAATTGGAATCAATGGGAGTCTATCAGATTCTGGCGATCTACTCAACTGTCGC
CAGTTCCCTGGTTCTTTTGGTCTCCCTGGGGGCAATCAGCTTCTGGATGTGTTCCAATGGGTCTTTGCAG
TGTAGAATATGCATCTGAGACCAGAATTTCAGAAATATAAGAA

>gi|237689054|gb|CY040154.1| Influenza A virus (A/Taiwan/123/2002(H1N1)) segment 4, complete sequence

ATAAAAACAACCAAAATGAAAGCAAAACTACTGGTCCTGTTATGTACATTTACAGCTACATACGCAGACA
CAATATGTATAGGCTACCATGCCAACAACTCAACCGACACTGTTGACACAGTACTTGAGAAGAATGTGAC
AGTGACACACTCTGTCAACCTACTTGAGGACAGTCACAATGGAAAACTATGTCTACTAAAAGGAATAGCC
CCACTACAATTGGGTAATTGCAGCGTTGCCGGATGGATCTTAGGAAACCCAGAATGCGAATTACTGATTT
CCAAGGAATCATGGTCCTACATTGTAGAAACACCAAATCCCGAGAATGGAACATGTTACCCAGGGTATTT
CGCCGACTATGAGGAACTGAGGGAGCAATTGAGTTCAGTATCTTCATTTGAGAGATTCGAAATATTCCCC
AAAGGAAGCTCATGGCCCAACCACACCGTGACCGGAGTATCAGCATCATGCTCCCATAATGGGAAAAGCA
GTTTTTACAGAAATTTGCTATGGCTGACGGGGAAAAATGGTTTGTACCCAAACCTGAGCATGTCCTATGT
AAACAACAAAGAAAAAGAAGTCCTTGTGCTATGGGGTGTTCATCACCCACCTAACATAGGGAACCAAAGG
GCCCTCTACCATACAGAAAATGCTTATGTCTCTGTAGTGTCTTCACATTATAGCAGAAGATTCACCCCAG
AAATAGCCAAAAGACCCAAAGTAAGAGATCAGGAAGGAAGAATCAACTACTACTGGACTCTGCTGGAACC

CGGGGACACAATAATATTTGAGGCAAATGGAAATCTAATAGCGCCATGGTATGCTTTCGCACTGAGTAGA
GGCTTTGGATCAGGAATCATCACCTCAAATGCACCAATGGGTGAATGTGATGAGAAGTGTCAAACACCTC
AGGGAGCTATAAACAGTAGTCTTCCTTTCCAGAATGTACACCCAGTTACAATAGGAGAGTGTCCAAAGTA
TGTCAGGAGTGCAAAATTAAGGATGGTTACAGGACTAAGGAACATCCCATCCATTCAATCCAGAGGTTTG
TTTGGAGCCATTGCCGGTTTCATTGAAGGAGGGTGGACTGGAATGGTAGATGGGTGGTATGGTTATCATC
ATCAGAATGAGCAAGGATCTGGCTATGCTGCAGATCAAAAAAGCACACAAAATGCCATTAACGGGATTAC
AAACAAGGTGAATTCTGTAATTGAGAAAATGAACACTCAATTCACAGCTGTGGGCAAAGAATTCAACAAA
TTAGAAAGAAGGATGGAAAACTTAAATAAAAAAGTTGATGATGGGTTTCTAGACATTTGGACATATAATG
CAGAATTGTTGGTTCTACTGGAAAATGAAAGGACTTTGGATTTCCATGACTCCAATGTGAAGAATCTGTA
TGAGAAAGTAAAAAGCCAATTAAAGAATAATGCCAAAGAAATAGGAAACGGGTGTTTTGAATTCTATCAC
AAGTGTAACAATGAATGCATGGAAAGTGTGAAAAATGGAACTTATGACTATCCAAAATATTCCGAAGAAT
CAAAGTTAAACAGGGAGAAAATTGATGGAGTAAAATTGGAATCAATGGGAGTCTATCAGATTCTGGCGAT
CTACTCAACTGTCGCCAGTTCCCTGGTTCTTTTGGTCTCCCTGGGGGCAATCAGCTTCTGGATGTGTTCC
AATGGGTCTTTGCAGTGTAGAATATGCATCTGAGACCAGAATTTCAGAAATATAAGAAA
>gi|77747421|gb|CY003688.1| Influenza A virus (A/New York/486/2003(H1N1)) segment 4, complete sequence
AAATGAAAGTAAAACTACTGGTTCTGTTATGTACATTTACAGCTACATATGCAGACACAATATGTATAGG
GTACCATGCCAACAACTCAACCGACACTGTTGACACAGTACTTGAGAAGAATGTGACAGTGACACACTTT
GTCAACCTACTTGAGGACAGTCACAATGGAAAACTATGTCTACTAAAAGGAATAGCCCCACTACAATTGG
GTAATTGCAGCGTTGCCGGATGGATCTTAGGAAACCCAGAATGCGAATTACTGATTTCCAAGGAATCATG
GTCCTACATTGTAGAAACACCAAATCCTGAGAATGGAACATGTTACCCAGGGTATTTCGCCGACTATGAG
GAACTGAGGGAGCAATTGAGTTCAGTATCTTCATTTGAGAGGTTCGAAATATTCCCCAAAGAGAGCTCAT
GGCCCAACCACACCGTAACCGGAGTATCAGCATCATGCTCCCATAACGGGAAAAGCAGTTTTTACAGAAA
TTTGCTATGGCTGACGGGGAAGAATGGTTTGTACCCAAACCTGAGCAAGTCCTATGCAAACAACAAAGAG
AAAGAAGTCCTTGTACTATGGGGTGTTCATCACCCGCCTAACATAGGGGACCAAAGGGCCCTCTATCATA
CAGAAAATGCTTATGTCTCTGTAGTGTCTTCACATTATAGCAGAAGATTCACCCCAGAAATAGCCAAAAG
ACCCAAGGTGAGAGATCAGGAAGGAAGAATCAACTACTACTGGACTCTGCTGGAACCCGGGGATACAATA
ATATTTGAGGCAAATGGAAATCTAATAGCGCCAAGGTATGCTTTCGCACTGAGTAGAGGCTTTGGATCAG
GAATCATCACCTCAAATGCACCAATGGATGAATGTGATGCGAAGTGTCAAACACCTCAGGGAGCTATAAA
CAGCAGTCTTCCTTTCCAGAATGTACACCCAGTCACAATAGGAGAGTGTCCAAAGTATGTCAGGAGTGCA
AAATTAAGGATGGTTACAGGACTAAGGAACATCCCATCCATTCAATCCAGAGGTTTGTTTGGAGCCATTG
CCGGTTTCATTGAAGGGGGGTGGACTGGAATGGTAGATGGTTGGTATGGTTATCATCATCAGAATGAGCA
AGGATCTGGGTATGCTGCAGATCAAAAAAGCACACAAAATGCCATTAACGGGATTACAAACAAGGTGAAT
TCTGTAATTGAGAAAATGAACACTCAATTCACAGCTGTGGGCAAAGAATTCAACAAATTGGAAAGAAGGA
TGGAAAACTTAAATAAAAAAGTTGATGATGGGTTTCTAGACATTTGGACATATAATGCAGAATTGTTGGT
TCTACTGGAAAATGAAAGGACTTTGGATTTCCATGACTCCAACGTGAAGAATCTGTATGAGAAAGTAAAA
AGCCAATTAAAGAATAATGCCAAAGAAATAGGAAACGGGTGTTTTGAATTCTATCACAAGTGTAACGATG
AATGCATGGAGAGTGTGAAAAATGGAACTTATGACTATCCAAAATATTCCGAAGAATCAAAGTTAAACAG
AGAGAAAATTGATGGAGTGAAATTGGAATCAATGGGAGTCTATCAGATTCTGGCGATCTACTCAACAGTC
GCCAGTTCCCTGGTTCTTTTGGTCTCCCTGGGGGCAATCAGCTTCTGGATGTGTTCCAATGGGTCTTTGC
AGTGTAGAATATGCATCTAAGACCAGAATTTCAGAAATATAAGGAAAAA
>gi|125664175|gb|CY019883.1| Influenza A virus (A/Memphis/5/2003(H1N1)) segment 4, complete sequence
AGCAACCAAAATGAAAGTAAAACTACTGGTTCTGTTATGTACATTTACAGCTACATATGCAGACACAATA

TGTATAGGCTACCATGCCAACAACTCAACCGACACTGTTGACACAGTACTTGAGAAGAATGTGACAGTGA
CACACTCTGTCAACCTACTTGAGGACAGTCACAATGGAAAACTATGTCTACTAAAAGGAATAGCCCCACT
ACAATTGGGTAATTGCAGCGTTGCCGGATGGATCTTAGGAAACCCAGAATGCGAATTACTGATTTCCAAG
GAATCATGGTCCTACATTGTAGAAACACCAAATCCTGAGAATGGAACATGTTACCCAGGGTATTTCGCCG
ACTATGAGGAACTGAGGGAGCAATTGAGTTCAGTATCTTCATTTGAGAGGTTCGAAATATTCCCCAAAGA
GAGCTCATGGCCCAACCATACCGTAACCGGAGTATCAGCATCATGCTCCCATAACGGGAAAAGCAGTTTT
TACAGAAATTTGCTATGGCTGACGGGGAAGAATGGTTTGTACCCAAACCTGAGCAAGTCCTATGCAAACA
ACAAAGAGAAAGAAGTCCTTGTACTATGGGGTGTTCATCACCCGCCTAACATAGGGGACCAAAGGGCCCT
CTATCATACAGAAAATGCTTATGTCTCTGTAGTGTCTTCACATTATAGCAGAAGATTCACCCCAGAAATA
GCCAAAAGACCCAAGGTGAGAGATCAGGAAGGAAGAATCAACTACTACTGGACTCTGCTGGAACCCGGGG
ATACAATAATATTTGAGGCAAATGGAAATCTAATAGCGCCAAGATATGCTTTCGCACTGAGTAGAGGCTT
TGGATCAGGAATCATCACCTCAAATGCACCAATGGATGAATGTGATGCGAAGTGTCAAACACCTCAGGGA
GCTATAAACAGCAGTCTTCCTTTCCAGAATGTACACCCAGTCACAATAGGAGAGTGTCCAAAGTATGTCA
GGAGTGCAAAATTAAGGATGGTTACAGGACTAAGGAACATCCCATCCATTCAATCCAGAGGTTTGTTTGG
AGCCATTGCCGGTTTCATTGAGGGGGGGTGGACTGGAATGGTAGATGGTTGGTATGGTTATCATCATCAG
AATGAGCAAGGATCTGGGTATGCTGCAGATCAAAAAAGCACACAAAATGCCATTAACGGGATTACAAACA
AGGTGAATTCTGTAATTGAGAAAATGAACACTCAATTCACAGCTGTGGGCAAAGAATTCAACAAATTGGA
AAGAAGGATGGAAAACTTAAATAAAAAAGTTGATGATGGGTTTCTAGACATTTGGACATATAATGCAGAA
TTGTTGGTTCTACTGGAAAATGAAAGGACTTTGGATTTCCATGACTCCAACGTGAAGAATCTGTATGAGA
AAGTAAAAAGCCAATTAAAGAATAATGCCAAAGAAATAGGAAACGGGTGTTTTGAATTCTATCACAAGTG
TAACGATGAATGCATGGAGAGTGTGAAAAATGGAACTTATGACTATCCAAAATATTCCGAAGAATCAAAG
TTAAACAGGGAGAAAATTGATGGAGTGAAATTGGAATCAATGGGAGTCTATCAGATTCTGGCGATCTACT
CAACAGTCGCCAGTTCCCTGGTTCTTTTGGTCTCCCTGGGGGCAATCAGCTTCTGGATGTGTTCCAATGG
GTCTTTGCAGTGTAGAATATGCATCTAAGACCAGAATTTCAGAAATATAAG
>gi|73763194|gb|CY002536.1| Influenza A virus (A/New York/227/2003(H1N1)) segment 4, complete sequence
AAAGTAAAACTACTGGTTCTGTTATGTACATTTACAGCTACATATGCAGACACAATATGTATAGGCTACC
ATGCCAACAACTCAACCGACACTGTTGACACAGTACTTGAGAAGAATGTGACAGTGACACACTCTGTCAA
CCTACTTGAGGACAGTCACAATGGAAAACTATGTCTACTAAAAGGAATAGCCCCACTACAATTGGGTAAT
TGCAGCGTTGCCGGATGGATCTTAGGAAACCCAGAATGCGAATTACTGATTTCCAAGGAATCATGGTCCT
ACATTGTAGAAACACCAAATCCTGAGAATGGAACATGTTACCCAGGGTATTTCGCCGACTATGAGGAACT
GAGGGAGCAATTGAGTTCAGTATCTTCATTTGAGAGGTTCGAAATATTCCCCAAAGAGAGCTCATGGCCC
AACCACACCGTAACCGGAGTATCAGCATCATGCTCCCATAACGGGAAAAGCAGTTTTTACAGAAATTTGC
TATGGCTGACGGGGAAGAATGGTTTGTACCCAAACCTGAGCAAGTCCTATGCAAACAACAAAGAGAAAGA
AGTCCTTGTACTATGGGGTGTTCATCACCCGCCTAACATAGGGGACCAAAGGGCCCTCTATCATACAGAA
AATGCTTATGTCTCTGTAGTGTCTTCACATTATAGCAGAAGATTCACCCCAGAAATAGCCAAAAGACCCA
AGGTGAGAGATCAGGAAGGAAGAATCAACTACTACTGGACTCTGCTGGAACCCGGGGATACAATAATATT
TGAGGCAAATGGAAATCTAATAGCGCCAAGGTATGCTTTCGCACTGAGTAGAGGCTTTGGATCAGGAATC
ATCACCTCAAATGCACCAATGGATGAATGTGATGCGAAGTGTCAAACACCTCAGGGAGCTATAAACAGCA
GTCTTCCTTTCCAGAATGTACACCCAGTCACAATAGGAGAGTGTCCAAAGTATGTCAGGAGTGCAAAATT
AAGGATGGTTACAGGACTAAGGAACATCCCATCCATTCAATCCAGAGGTTTGTTTGGAGCCATTGCCGGT
TTCATTGAGGGGGGGTGGACTGGAATGGTAGATGGTTGGTATGGTTATCATCATCAGAATGAGCAAGGAT
CTGGGTATGCTGCAGATCAAAAAAGCACACAAAATGCCATTAACGGGATTACAAACAAGGTGAATTCTGT
AATTGAGAAAATGAACACTCAATTCACAGCTGTGGGCAAAGAATTCAACAAATTGGAAAGAAGGATGGAA

AACTTAAATAAAAAAGTTGATGATGGGTTTCTAGACATTTGGACATATAATGCAGAATTGTTGGTTCTAC
TGGAAAATGAAAGGACTTTGGATTTCCATGACTCCAACGTGAAGAATCTGTATGAGAAAGTAAAAAGCCA
ATTAAAGAATAATGCCAAAGAAATAGGAAACGGGTGTTTTGAATTCTATCACAAGTGTAACGATGAATGC
ATGGAGAGTGTGAAAAATGGAACTTATGACTATCCAAAATATTCCGAAGAATCAAAGTTAAACAGGGAGA
AAATTGATGGAGTGAAATTGGAATCAATGGGAGTTTATCAGATTCTGGCGATCTACTCAACAGTCGCCAG
TTCCCTGGTTCTTTTGGTCTCCCTGGGGGCAATCAGCTTCTGGATGTGTTCCAATGGGTCTTTGCAGTGT
AGAATATGCATCTAAGACCAGAATTTCAGAAATATAAGGAAAAACACCCTTGTTTCTACT
>gi|89112165|gb|CY008996.1| Influenza A virus (A/New York/484/2003(H1N1)) segment 4, complete sequence
ATGAAAGTAAAACTACTGGTTCTGTTATGTACATTTACAGCTACATATGCAGACACAATATGTATAGGCT
ACCATGCCAACAACTCAACCGACACTGTTGACACAGTACTTGAGAAGAATGTGACAGTGACACACTCTGT
CAACCTACTTGAGGACAGTCACAATGGAAAACTATGTCTACTAAAAGGAATAGCCCCACTACAATTGGGT
AATTGCAGCGTTGCCGGATGGATCTTAGGAAACCCAGAATGCGAATTACTGATTTCCAAGGAATCATGGT
CCTACATTGTAGAAACACCAAATCCTGAAAATGGAACATGTTACCCAGGGTATTTCGCCGACTATGAGGA
ACTGAGGGAGCAATTGAGTTCAGTATCTTCATTTGAGAGGTTCGAAATATTCCCCAAAGAGAGCTCATGG
CCCAACCACACCGTAACCGGAGTATCAGCATCATGCTCCCATAACGGGAAAAGCAGTTTTTACAGAAATT
TGCTATGGCTGACGGGGAAGAATGGTTTGTACCCAAACCTGAGCAAGTCCTATGCAAACAACAAAGAGAA
AGAAGTCCTTGTACTATGGGGTGTTCATCACCCGCCTAACATAGGGGACCAAAGGGCCCTCTATCATACA
GAAAATGCTTATATCTCTGTAGTGTCTTCACATTATAGCAGAAGATTCACCCCAGAAATAGCCAAAAGAC
CCAAGGTAAGAGATCAGGAAGGAAGAATCAACTACTACTGGACTCTGCTGGAACCCGGGGATACAATAAT
ATTTGAGGCAAATGGAAATCTAATAGCGCCAAGGTATGCTTTCGCACTGAGTAGAGGCTTTGGATCAGGA
ATCATCACCTCAAATGCACCAATGGATGAATGTGATGCGAAGTGTCAAACACCTCAGGGAGCTATAAACA
GCAGTCTTCCTTTCCAGAATGTACACCCAGTCACAATAGGAGAGTGTCCAAAGTATGTCAGGAGTGCAAA
ATTAAGGATGGTTACAGGACTAAGGAACATCCCATCCATTCAATCCAGAGGTTTGTTTGGAGCCATTGCC
GGTTTCATTGAAGGGGGGTGGACTGGAATGGTAGATGGTTGGTATGGTTATCATCATCAGAATGAGCAAG
GATCTGGGTATGCTGCAGATCAAAAAAGCACACAAAATGCCATTAACGGGATTACAAACAAGGTGAATTC
TGTAATTGAGAAAATGAACACTCAATTCACAGCTGTGGGCAAAGAATTCAACAAATTGGAAAGAAGGATG
GAAAACTTAAATAAAAAGGTTGATGATGGGTTTCTAGACATTTGGACATATAATGCAGAATTGTTGGTTC
TACTGGAAAATGAAAGGACTTTGGATTTCCATGACTCCAACGTGAAGAATCTGTATGAGAAAGTAAAAAG
CCAATTAAAGAATAATGCCAAAGAAATAGGAAACGGGTGTTTTGAATTCTATCACAAGTGTAACGATGAA
TGCATGGAGAGTGTGAAAAATGGAACTTATGACTATCCAAGATATTCCGAAGAATCAAAGTTAAACAGGG
AGAAAATTGATGGAGTGAAATTGGAATCAATGGGAGTCTATCAGATTCTGGCGATCTACTCAACAGTCGC
CAGTTCCCTGGTTCTTTTGGTCTCCCTGGGGGCAATCAGCTTCTGGATGTGTTCCAATGGGTCTTTGCAG
TGTAGAATATGCATCTAAGACCAGAATTTCAGAAATATAAGGAAAAA
>gi|83727841|gb|CY006915.1| Influenza A virus (A/New York/488/2003(H1N1)) segment 4, complete sequence
GGGAAAATAAAAGCAACCAAAATGAAAGTAAAACTACTGGTTCTGTTATGTACATTTACAGCTACATATG
CAGACACAATATGTATAGGCTACCATGCCAACAACTCAACCGACACTGTTGACACAGTACTTGAGAAGAA
TGTGACAGTGACACACTCTGTCAACCTACTTGAGGACAGTCACAATGGAAAACTATGTCTACTAAAAGGA
ATAGCCCCACTACAATTGGGTAATTGCAGCGTTGCCGGATGGATCTTAGGAAACCCAGAATGCGAATTAC
TGATTTCCAAGGAATCATGGTCCTACATTGTAGAAACACCAAATCCTGAGAATGGAACATGTTATCCAGG
GTATTTCGCCGACTATGAGGAACTGAGGGAGCAATTGAGTTCAGTATCTTCATTTGAGAGGTTCGAAATA
TTCCCCAAAGAGAGCTCATGGCCCAACCACACCGTAACCGGAGTATCAGCATCATGCTCCCATAACGGGA
AAAGCAGTTTTTACAGAAATTTGCTATGGCTGACGGGGAAGAATGGTTTATACCCAAACCTGAGCAAGTC

CTATGCAAACAACAAAGAGAAAGAAGTCCTTGTACTATGGGGTGTTCATCACCCGCCTAACATAGGGGAC
CAAAGGGCCCTCTATCATACAGAAAATGCTTATGTCTCTGTAGTGTCTTCACATTATAGCAGAAGATTCA
CCCCAGAAATAGCCAAAAGACCCAAGGTAAGAGATCAGGAAGGAAGAATCAACTACTACTGGACTCTGCT
GGAACCCGGGGATACAATAATATTTGAGGCAAATGGAAATCTAATAGCGCCAAGGTATGCTTTCGCACTG
AGTAGAGGCTTTGGATCAGGAATCATCACCTCAAATGCACCAATGGATGAATGTGATGCGAAGTGTCAAA
CACCTCAGGGAGCTATAAACAGCAGTCTTCCTTTCCAGAATGTACACCCAGTCACAATAGGAGAGTGTCC
AAAGTATGTCAGGAGTGCAAAATTAAGGATGGTTACAGGACTAAGGAACATCCCATCCATTCAATCCAGA
GGTTTGTTTGGAGCCATTGCCGGTTTCATTGAAGGGGGGTGGACTGGAATGGTAGATGGTTGGTATGGTT
ATCATCATCAGAATGAGCAAGGATCTGGGTATGCTGCAGATCAAAAAAGCACACAAAATGCCATTAACGG
GATTACAAACAAGGTGAATTCTGTAATTGAGAAAATGAACACTCAATTCACAGCTGTGGGCAAAGAATTC
AACAAATTGGAAAGAAGGATGGAAAACTTAAATAAAAAAGTTGATGATGGATTTCTAGACATTTGGACAT
ATAATGCAGAATTGTTGGTTCTACTGGAAAATGAAAGGACTTTGGATTTCCATGACTCCAACGTGAAGAA
TCTGTATGAGAAAGTAAAAAGCCAATTAAAGAATAATGCCAAAGAAATAGGAAACGGGTGTTTTGAATTC
TATCACAAGTGTAACGATGAATGCATGGAGAGTGTGAAAAATGGAACTTATGACTATCCAAAATATTCCG
AAGAATCAAAGTTAAACAGGGAGAAAATTGATGGAGTGAAATTGGAATCAATGGGAGTCTATCAGATTCT
GGCGATCTACTCAACAGTCGCCAGTTCCCTGGTTCTTTTGGTCTCCCTGGGGGCAATCAGCTTCTGGATG
TGTTCCAATGGGTCTTTGCAGTGTAGAATATGCATCTAAGACCAGAATTTCAGAAATATAAGGAAAAA
>gi|157281257|gb|CY025213.1| Influenza A virus (A/Texas/UR06-0012/2006(H1N1)) segment 4, complete sequence
ATAAAAGCAACAAAAATGAAAGTGAAACTACTGGTTCTGTTATGTACATTTACAGCTACATATGCAGACA
CAATATGTATAGGCTACCATGCCAACAACTCGACCGACACTGTTGACACAGTACTTGAGAAGAATGTGAC
AGTGACACACTCTGTCAACCTACTTGAGGACCGTCACAATGGAAAACTATGTCTACTAAAAGGAATAGCC
CCACTACAATTGGGTAATTGCAGCGTTGCCGGATGGATCTTAGGAAACCCAGAATGCGAATTACTGATTT
CCAAGGAATCATGGTCTTACATTGTAGAAACACCAAATCCTGAGAATGGAACATGTTACCCAGGGTATTT
CGCCGACTATGAGGAGCTGAGGGAGCAATTGAGTTCAGTATCTTCATTTGAGAGGTTCGAAATATTCCCC
AAAGAGAGCTCATGGCCCAACCACACCGTAACCGGAGTATCAGCATCATGCTCCCATAACGGGAAAAGCA
GTTTTTACAGAAATTTGCTATGGCTGACGGGAAAGAATGGTTTGTACCCAAACCTGAGCAGATCCTATGC
AAACAACAAAGAGAAAGAAGTCCTTGTACTGTGGGGTGTTCATCACCCGCCTAACATAGGGGACCAAAGG
GCCCTCTATCATACAGAAAATGCTTATGTCTCTGTAGTGTCTTCACATTATAGCAGAAGATTCACCCCAG
AAATAGCCAAAAGACCCAAGGTGAGAGATCAGGAAGGAAGAATCAATTACTACTGGACTCTACTGGAACC
CGGGGATACAATAATATTTGAGGCAAATGGGAATCTAATAGCGCCAAGGTTTGCTTTCGCACTGAGTAGA
GGCTTTGGATCAGGAATCATCACCTCAAATGCACCAATGGATGAATGTGATGCGAAGTGTCAAACACCTC
AGGGAGCTATAAACAGCAGTCTTCCTTTCCAGAATGTACACCCAGTCACAATAGGAGAGTGTCCAAAGTA
TGTCAGGAGTGCAAAATTAAGAATGGTTACAGGACTAAGGAACATCCCATCCATTCAATCCAGAGGTTTG
TTTGGAGCCATTGCCGGTTTCATTGAAGGGGGGTGGACTGGAATGGTAGATGGTTGGTATGGTTATCACC
ACCAGAATGAGCAAGGATCTGGGTATGCTGCAGATCAAAAAAGCACACAAAATGCCATTAACGGGATTAC
AAACAAGGTGAATTCTGTGATTGAGAAAATGAACACTCAATTCACAGCTGTGGGCAAAGAATTCAACAAA
TTGGAAAGAAGAATGGAAAACTTAAATAAAAAAGTTGATGATGGGTTTCTAGACATTTGGACGTATAACG
CAGAGTTGTTGGTTCTACTGGAAAATGAAAGGACTTTGGACTTCCATGACTCCAACGTGAAGAATCTGTA
TGAGAAAGTAAAAAGCCAATTAAAGAATAATGCCAAAGAAATAGGAAACGGGTGTTTTGAATTCTATCAC
AAGTGTAACGATGAATGCATGGAGAGTGTGAAAAATGGAACTTATGACTATCCAAAATATTCCGAAGAAT
CAAAGTTAAACAGGGAGAAAATTGATGGAGTGAAATTGGAATCAATGGGAGTCTATCAGATTCTGGCGAT
CTACTCAACAGTCGCCAGTTCCCTGGTTCTTTTGGTCTCCCTGGGGGCAATCAGCTTCTGGATGTGTTCC
AATGGGTCTTTGCAGTGTAGAATATGCATCTAAGACCAGAATTTCAGAAATATAAGGAAAAA

>gi|94959532|gb|CY010764.1| Influenza A virus (A/Canterbury/20/2001(H1N1)) segment 4, complete sequence
GAAATTAAAAACAACCAAAATGAAAGCAAAACTACTGGTCCTGTTATGTACATTTACAGCTACATACGCA
GACACAATATGTATAGGCTACCATGCCAACAACTCAACCGACACTGTTGACACAGTACTTGAGAAGAATG
TGACAGTGACACACTCTGTCAACCTACTTGAGGACAGTCACAATGGAAAACTATGTCTACTAAAAGGAAT
AGCCCCACTACAATTGGGTAATTGCAGCGTTGCCGGATGGATCTTAGGAAACCCAGAATGCGAATTACTG
ATTTCCAAGGAATCATGGTCCTACATTGTAGAAACACCAAATCCCGAGAATGGAACATGTTACCCAGGGT
ATTTCGCCGACTATGAGGAACTGAGGGAGCAATTGAGTTCAGTATCTTCATTTGAGAGATTCGAAATATT
CCCCAAAGGAAGCTCATGGCCCAACCACACCGTGACCGGAGTATCAGCATCATGCTCCCATAATGGGAAA
AGCAGTTTTTACAGAAATTTGCTATGGCTGACGGGGAAGAATGGTTTGTACCCAAAACTGAGCATGTCCT
ATGTAAACAACAAAGAGAAAGAAGTCCTTGTGCTATGGGGTGTTCATCACCCACCTAACATAGGGGACCA
AAGGGCCCTCTACCATACAGAAAATGCTTATGTCTCTGTAGTGTCTTCACATTATAGCAGAAGATTCACC
CCAGAAATAGCCAAAAGACCCAAAGTAAGAGATCAGGAAGGAAGAATCAACTACTACTGGACTCTGCTGG
AACCCGGGGACACAATAATATTTGAGGCAAATGGAAATCTAATAGCGCCATGGTATGCTTTCGCACTGAG
TAGAGGCTTTGGATCAGGAATCATCACCTCAAATGCACCAATGGGTGAATGTGATGCGAAGTGTCAAACA
CCTCAGGGAGCTATAAACAGTAGTCTTCCTTTCCAGAATGTACACCCAGTTACAATAGGAGAGTGTCCAA
AGTATGTCAGGAGTGCAAAATTAAGGATGGTTACAGGACTAAGGAACATCCCATCCATTCAATCCAGAGG
TTTGTTTGGAGCCATTGCCGGTTTCATTGAAGGGGGGTGGACTGGAATGGTAGATGGGTGGTATGGTTAT
CATCATCAGAATGAGCAAGGATCTGGCTATGCTGCAGATCAAAAAAGCACACAAAATGCCATTAACGGGA
TTACAAACAAGGTGAATTCTGTAATTGAGAAAATGAACACTCAATTCACAGCTGTGGGCAAAGAATTCAA
CAAATTAGAAAGAAGGATGGAAAACTTAAATAAAAAAGTTGATGATGGGTTTCTAGACATTTGGACATAT
AATGCAGAATTGTTGGTTCTACTGGAAAATGAAAGGACTTTGGATTTCCATGACTCCAATGTGAAGAATC
TGTATGAGAAAGTAAAAAGCCAATTAAAGAATAATGCCAAAGAAATAGGAAACGGGTGTTTTGAATTCTA
TCACAAGTGTAACAATGAATGCATGGAAAGTGTGAAAAATGGAACTTATGACTATCCAAAATATTCCGAA
GAATCAAAGTTAAACAGGGAGAAAATTGATGGAGTAAAATTGGAATCAATGGGAGTCTATCAGATTCTGG
CGATCTACTCAACTGTCGCCAGTTCCCTGGTTCTTTTGGTCTCCCTGGGGGCAATCAGCTTCTGGATGTG
TTCCAATGGGTCTTTGCAGTGTAGAATATGCATCTGAGACCAGAATTTCAGAAATATAAGAAAAAA
>gi|91119026|gb|CY010404.1| Influenza A virus (A/West Coast/33/2001(H1N1)) segment 4, complete sequence
GAAAATAAAAACAACCAAAATGAAAGCAAAACTACTGGTCCTGTTATGTACATTTACAGCTACATACGCA
GACACAATATGTATAGGCTACCATGCCAACAACTCAACCGACACTGTTGACACAGTACTTGAGAAGAATG
TGACAGTGACACACTCTGTCAACCTACTTGAGGACAGTCACAATGGAAAACTATGTCTACTAAAAGGAAT
AGCCCCACTACAATTGGGTAATTGCAGCGTTGCCGGATGGATCTTAGGAAACCCAGAATGCGAATTACTG
ATTTCCAAGGAATCATGGTCCTACATTGTAGAAACACCAAATCCCGAGAATGGAACATGTTACCCAGGGT
ATTTCGCCGACTATGAGGAACTGAGGGAGCAATTGAGTTCAGTATCTTCATTTGAGAGATTCGAAATATT
CCCCAAAGGAAGCTCATGGCCCAACCACACCGTGACCGGAGTATCAGCATCATGCTCCCATAATGGGAAA
AGCAGTTTTTACAGAAATTTGCTATGGCTGACGGGGAAGAATGGTTTGTACCCAAACCTGAGCATGTCCT
ATGTAAACAACAAAGAGAAAGAAGTCCTTGTGCTATGGGGTGTTCATCACCCACCTAACATAGGGAACCA
AAGGGCCCTCTACCATACAGAAAATGCTTATGTCTCTGTAGTGTCTTCACATTATAGCAGAAGATTCACC
CCAGAAATAGCCAAAAGACCCAAAGTAAGAGATCAGGAAGGAAGAATCAACTACTACTGGACTCTGCTGG
AACCCGGGGACACAATAATATTTGAGGCAAATGGAAATCTAATAGCGCCATGGTATGCTTTCGCACTGAG
TAGAGGCTTTGGATCAGGAATCATCACCTCAAATGCACCAATGGGTGAATGTGATGCGAAGTGTCAAACA
CCTCAGGGAGCTATAAACAGTAGTCTTCCTTTCCAGAATGTACACCCAGTTACAATAGGAGAGTGTCCAA
AGTATGTCAGGAGTGCAAAATTAAGGATGGTTACAGGACTAAGGAACATCCCATCCATTCAATCCAGAGG

TTTGTTTGGAGCCATTGCCGGTTTCATTGAAGGGGGGTGGACTGGAATGGTAGATGGGTGGTATGGTTAT
CATCATCAGAATGAGCAAGGATCTGGCTATGCTGCAGATCAAAAAAGCACACAAAATGCCATTAACGGGA
TTACAAACAAGGTGAATTCTGTAATTGAGAAAATGAACACTCAATTCACAGCTGTGGGCAAAGAATTCAA
CAAATTAGAAAGAAGGATGGAAAACTTAAATAAAAAAGTTGATGATGGGTTTCTAGACATTTGGACATAT
AATGCAGAATTGTTGGTTCTACTGGAAAATGAAAGGACTTTGGATTTCCATGACTCCAATGTGAAGAATC
TGTATGAGAAAGTAAAAAGCCAATTAAAGAATAATGCCAAAGAAATAGGAAACGGGTGTTTTGAATTCTA
TCACAAGTGTAACAATGAATGCATGGAAAGTGTGAAAAATGGAACTTATGACTATCCAAAATATTCCGAA
GAATCAAAGTTAAACAGGGAGAAAATTGATGGAGTAAAATTGGAATCAATGGGAGTCTATCAGATTCTGG
CGATCTACTCAACTGTCGCCAGTTCCCTGGTTCTTTTGGTCTCCCTGGGGGCAATCAGCTTCTGGATGTG
TTCCAATGGGTCTTTGCAGTGTAGAATATGCATCTGAGACCAGAATTTCAGAAATATAAGAAAAAA
>gi|131052777|gb|CY019997.1| Influenza A virus (A/Waikato/17/2005(H1N1)) segment 4, complete sequence
AGCAACCAAAATGAAAGTAAAACTACTGGTTCTGTTATGTACATTTACAGCTACATATGCAGACACAATA
TGTATAGGCTACCATGCCAACAACTCAACCGACACTGTTGACACAGTACTTGAGAAGAATGTGACAGTGA
CACACTCTGTCAACCTACTTGAGGACAGTCACAATGGAAAACTATGTCTACTAAAAGGAATAGCCCCATT
ACAATTGGGTAATTGCAGCGTTGCCGGATGGATCTTAGGAAACCCAGAATGCGAATTACTGATTTCCAAG
GAATCATGGTCCTACATTGTAGAAACACCAAATCCTGAGAATGGAACATGTTACCCAGGGTATTTCGCCG
ACTATGAGGAACTGAGGGAGCAATTGAGTTCAGTATCTTCATTTGAGAGGTTCGAAATATTCCCCAAAGA
GAGCTCATGGCCCAACCACACCGTAACCGGAGTATCAGCATCATGCTCCCATAACGGGAAAAGCAGTTTT
TACAGAAATTTGCTATGGCTGACGGGGAAGAATGGTTTGTACCCAAACCTGAGCAAGTCTTATGCAAACA
ACAAAGAAAAAGAAGTCCTTGTACTATGGGGTGTTCATCACCCGCCTAACATAGGGGACCAAAGGGCTCT
CTATCATACAGAAAATGCTTATGTCTCTGTAGTGTCTTCACATTATAGCAGAAGATTCACCCCAGAAATA
GCCAAAAGACCCAAGGTGAGAGATCAGGAAGGAAGAATCAACTACTACTGGACACTGCTGGAACCCGGG
G
ATACAATAATATTTGAGGCAAATGGAAATCTAATAGCGCCAAGGTTTGCTTTCGCACTGAGTAGAGGCTT
TGGATCAGGAATCATCACCTCAAATGCACCAATGGATGAATGTGATGCAAAGTGTCAAACACCTCAAGGA
GCTATAAACAGCAGTCTTCCTTTCCAGAATGTACACCCAGTCACAATAGGGGAGTGTCCAAAGTATGTCA
GGAGTGCAAAATTAAGGATGGCTACAGGACTAAGGAACATCCCATCCATTCAATCCAGAGGTTTGTTTGG
AGCCATTGCCGGTTTCATTGAAGGGGGGTGGACTGGAATGGTAGATGGTTGGTATGGTTATCATCATCAG
AATGAGCAAGGATCTGGGTATGCTGCAGATCAAAAAAGCACACAAAATGCCATTAACGGGATTACAAACA
AGGTGAATTCTGTAATTGAAAAAATGAACACTCAATTCACAGCTGTGGGCAAAGAATTCAGCAAATTGGA
GAGAAGGATGGAAAACTTACATAAAAAAGTTGATGATGGGTTTCTAGACATTTGGACATATAATGCAGAA
TTGTTGGTTCTACTGGAAAATGAAAGGACTTTGGATTTCCACGACTCCAACGTGAAGAATCTGTATGAGA
AAGTGAAAAGCCAATTAAAGAATAATGCCAAAGAAATAGGAAACGGGTGTTTTGAATTCTATCACAAATG
TAACGATGAATGCATGGAGAGTGTGAAAAATGGAACTTATGACTATCCAAAATATTCCGAAGAATCAAAG
TTAAACAGGGAGAAAATTGATGGAGTGAAATTGGAATCAATGGGAGTCTATCAGATTCTGGCGATCTACT
CAACAGTCGCCAGTTCCCTGGTTCTTTTGGTCTCCCTGGGGGCAATCAGCTTCTGGATGTGTTCCAATGG
GTCTTTGCAGTGTAGAATATGCATCTAAGACCAGAATTTCAGAAATATAAGGAAAAA
>gi|83744834|gb|CY007467.1| Influenza A virus (A/Canterbury/106/2004(H1N1)) segment 4, complete sequence
GGGAAAATAAAAACAACCAAAATGAAAGTAAAACTACTGGTCCTGTTATGCACATTTACAGCTACATATG
CAGACACAATATGTATAGGCTACCATGCCAACAACTCAACCGACACTGTTGACACAGTACTTGAAAAGAA
TGTGACAGTGACACACTCTGTCAACCTGCTTGAGGACAGTCACAATGGAAAACTATGTCTATTAAAAGGA
ATAGCCCCACTACAATTGGGTAATTGCAGCGTTGCCGGGTGGATCTTAGGAAACCCAGAATGCGAATTAC

TGATTTCCAAGGAATCATGGTCCTACATTGTAGAAAAACCAAATCCTGAGAATGGAACATGTTACCCAGG
GCATTTCGCTGACTATGAGGAACTGAGGGAGCAATTGAGTTCAGTATCTTCATTTGAGAGGTTCGAAATA
TTCCCCAAAGAAAGCTCATGGCCCAACCACACCGTAACCGGAGTATCAGCATCATGCTCCCATAATGGGA
AAAGCAGTTTTTACAAAAATTTGCTATGGCTGACGGGGAAGAATGGTTTGTACCCAAACCTGAGCAAGTC
CTATGCAAACAACAAAGAGAAAGAAGTCCTTGTACTATGGGGTGTTCATCACCCGCCAAACATAGGTGAC
CAAAAGGCCCTCTATCATACAGAAAATGCTTATGTCTCTGTAGTGTCTTCACATTATAGCAGAAAATTCA
CCCCAGAAATAGCCAAAAGACCCAAAGTAAGAGATCAAGAAGGAAGAATCAACTACTACTGGACTCTGCT
TGAACCCGGGGATACAATAATATTTGAGGCAAATGGAAATCTAATAGCGCCAAGATATGCTTTCGCACTG
AGTAGAGGCTTTGGATCAGGAATCATCAACTCAAATGCACCAATGGATGAATGTGATGCGAAGTGCCAAA
CACCTCAGGGAGCTATAAACAGCAGTCTTCCTTTCCAGAATGTACACCCAGTCACAATAGGAGAGTGTCC
AAAGTATGTCAGGAGTGCAAAATTAAGGATGGTTACAGGACTAAGGAACATCCCATCCATTCAATCCAGA
GGTTTGTTTGGAGCCATTGCCGGTTTCATTGAAGGGGGGTGGACTGGAATGGTAGATGGTTGGTATGGTT
ATCATCATCAGAATGAGCAAGGATCTGGCTATGCTGCAGATCAAAAAAGCACACAAAATGCCATTAATGG
GATTACAAACAAGGTGAATTCTGTAATTGAGAAAATGAACACTCAATTCACAGCTGTGGGCAAAGAATTC
AACAAATTGGAAAGAAGGATGGAAAACTTAAATAAAAAAGTTGATGATGGGTTTATAGACATTTGGACAT
ATAATGCAGAACTGTTGGTTCTACTGGAAAATGAAAGGACTTTGGATTTCCATGACTCCAATGTGAAGAA
TCTGTATGAGAAAGTAAAAAGCCAATTAAAGAATAATGCCAAAGAAATAGGAAATGGGTGTTTTGAATTC
TATCACAAGTGTAACGATGAATGCATGGAGAGTGTAAAAAATGGAACTTATGACTATCCAAAATATTCCG
AAGAATCAAAGTTAAACAGGGAGAAAATTGATGGAGTGAAATTGGAATCAATGGGAGTCTATCAGATTCT
GGCGATCTACTCAACAGTCGCCAGTTCTCTGGTTCTTTTGGTCTCCCTGGGGGCAATCAGCTTCTGGATG
TGTTCCAATGGGTCTTTGCAGTGTAGAATATGCATCTAAGACCAGAATTTCAGAAATATAAGGAAAAA
>gi|115607821|gb|CY016699.1| Influenza A virus (A/South Australia/58/2005(H1N1)) segment 4, complete sequence
CCAAAATGAAAGTAAAACTACTGGTTCTGTTATGTACATTTACAGCTACATATGCAGACACAATATGTAT
AGGCTACCATGCCAACAACTCAACCGACACTGTTGACACAGTACTTGAGAAGAATGTGACAGTGACACAC
TCTGTCAACCTACTTGAGGACAGTCACAATGGAAAACTATGTCTACTAAAAGGAATAGCCCCATTACAAT
TGGGTAATTGCAGCGTTGCCGGATGGATCTTAGGAAACCCAGAATGCGAATTACTGATTTCCAAGGAATC
ATGGTCCTACATTGTAGAAACACCAAATCCTGAGAATGGAACATGTTACCCAGGGTATTTCGCCGACTAT
GAGGAACTGAGGGAGCAATTGAGTTCAGTATCTTCATTTGAGAGGTTCGAAATATTCCCCAAAGAGAGCT
CATGGCCCAACCACACCGTAACCGGAGTATCAGCATCATGCTCCCATAACGGGAAAAGCAGTTTTTACAG
AAATTTGCTATGGCTGACGGGGAAGAATGGTTTGTACCCAAACCTGAGCAAGTCCTATGCAAACAACAAA
GAGAAAGAAGTCCTTGTACTATGGGGTGTTCATCACCCGCCTAACATAGGGGACCAAAGGGCCCTCTATC
ATACAGAAAATGCTTATGTCTCTGTAGTGTCTTCACATTATAGCAGAAGATTCACCCCAGAAATAGCCAA
AAGACCCAAGGTGAGAGATCAGGAAGGAAGAATCAACTACTACTGGACTCTGCTGGAACCCGGGGATACA
ATAATATTTGAGGCAAATGGAAATCTAATAGCGCCAAGGTTTGCTTTCGCACTGAGTAGAGGCTTTGGAT
CAGGAATCATCACCTCAAATGCACCAATGGATGAATGTGATGCGAAGTGTCAAACACCTCAAGGAGCTAT
AAACAGCAGTCTTCCTTTCCAGAATGTACACCCAGTCACAATAGGGGAGTGTCCAAAGTATGTCAGGAGT
GCAAAATTAAGGATGGCTACAGGACTAAGGAACATCCCATCCATTCAATCCAGAGGTTTGTTTGGAGCCA
TTGCCGGTTTCATTGAAGGGGGGTGGACTGGAATGGTAGATGGTTGGTATGGTTATCATCATCAGAATGA
GCAAGGATCTGGGTATGCTGCAGATCAAAAAAGCACACAAAATGCCATTAACGGGATTACAAACAAGGTG
AATTCTGTAATTGAGAAAATGAACACTCAATTCACAGCTGTGGGCAAAGAATTCAGCAAATTGGAGAGAA
GGATGGAAAACTTAAATAAAAAAGTTGATGATGGGTTTCTAGACATTTGGACATATAATGCAGAATTGTT
GGTTCTACTGGAAAATGAAAGAACTTTGGATTTCCATGACTCCAACGTGAAGAATCTGTATGAGAAAGTG
AAAAGCCAATTAAAGAATAATGCCAAAGAAATAGGAAACGGGTGTTTTGAATTCTATCACAAATGTAACG

ATGAATGCATGGAGAGTGTGAAAAATGGAACTTATGACTATCCAAAATATTCCGAAGAATCAAAGTTAAA
CAGGGAGAAAATTGATGGAGTGAAATTGGAATCAATGGGAGTCTATCAGATTCTGGCGATCTACTCAACA
GTCGCCAGTTCCCTGGTTCTTTTGGTCTCCCTGGGGGCAATCAGCTTCTGGATGTGTTCCAATGGGTCTT
TGCAGTGTAGAATATGCATCTAA
>gi|113170881|gb|CY014007.1| Influenza A virus (A/Wellington/11/2005(H1N1)) segment 4, complete sequence
ATGAAAGTAAAACTACTGGTTCTGTTATGTACATTTACAGCTACATATGCAGACACAATATGTATAGGCT
ACCATGCCAACAACTCGACCGACACTGTTGACACAGTACTTGAGAAGAATGTGACAGTGACACACTCTGT
CAACCTACTTGAGGACAGTCACAATGGAAAACTATGTCTACTAAAAGGAATAGCCCCACTACAATTGGGT
AATTGCAGCGTTGCCGGATGGATCTTAGGAAACCCAGAATGCGAATTACTGATTTCCAAGGAATCATGGT
CCTACATTGTAGAAACACCAAATCCTGAGAATGGAACATGTTACCCAGGGTATTTCGCCGACTATGAGGA
GCTGAGGGAGCAATTGAGTTCAGTATCTTCATTTGAGAGGTTCGAAATATTCCCCAAAGAGAGCTCATGG
CCCAACCACACCGTAACCGGAGTATCAGCATCATGCTCCCATAACGGGAAAAGCAGTTTTTACAGGAATT
TGCTATGGCTGACGGGGAAGAATGGTTTGTATCCAAACCTGAGCAAGTCCTATGCAAACAACAAAGAGAA
AGAAGTCCTTGTACTGTGGGGTGTTCATCACCCGCCTAACATAGGGGACCAAAGGGCCCTCTATCATACA
GAAAATGCTTATGTCTCTGTAGTGTCTTCACATTATAGCAGAAGATTCACCCCAGAAATAGCCAAAAGAC
CCAAGGTGAGAGATCAGGAAGGAAGAATCAACTACTACTGGACTCTGCTGGAACCCGGGGATACAATAAT
ATTTGAGGCAAATGGAAATCTAATAGCGCCAAGGTTTGCTTTCGCACTGAGTAGAGGCTTTGGATCAGGA
ATCATCACCTCAAATGCACCAATGGATGAATGTGATGCGAAGTGTCAAACACCTCAGGGAGCTATAAACA
GCAGTCTTCCTTTCCAGAATGTACACCCAGTCACAATAGGAGAGTGTCCAAAGTATGTCAGGAGTGCAAA
ATTAAGAATGGTTACAGGACTAAGGAACATCCCATCCATTCAATCCAGAGGTTTGTTTGGAGCCATTGCC
GGTTTCATTGAAGGGGGGTGGACTGGAATGGTAGATGGTTGGTATGGTTATCACCATCAGAATGAGCAAG
GATCTGGGTATGCTGCAGATCAAAAAAGCACACAAAATGCCATTAACGGGATTACAAACAAGGTGAATTC
TGTAATTGAGAAAATGAACACTCAATTCACAGCTGTGGGCAAAGAATTCAACAAATTGGAAAGAAGGATG
GAAAACTTAAATAAAAAAGTTGATGATGGGTTTCTAGACATTTGGACATATAACGCAGAATTGTTGGTTC
TACTGGAAAATGAAAGGACTTTGGACTTCCATGACTCCAACGTGAAGAATCTGTATGAGAAAGTAAAAAG
CCAATTAAAGAATAATGCCAAAGAAATAGGAAACGGGTGTTTTGAATTCTATCACAAGTGTAACGATGAA
TGCATGGAGAGTGTGAAAAATGGAACTTATGACTATCCAAAATATTCCGAAGAATCAAAGTTGAACAGGG
AGAGAATTGATGGAGTGAAATTGGAATCAATGGGAGTCTATCAGATTCTGGCGATCTACTCAACAGTCGC
CAGTTCCCTGGTTCTTTTGGTCTCCCTGGGGGCAATCAGCTTCTGGATGTGTTCCAATGGGTCTTTGCAG
TGTAGAATATGCATCTAAGACCAGAGTTTCAGAAATATAAG
>gi|145278923|gb|CY021757.1| Influenza A virus (A/South Australia/51/2005(H1N1)) segment 4, complete sequence
GAAAAATAAAAGCAACCAAAATGAAAGTAAAACTACTGGTTCTGTTATGTACATTTACAGCTACATATGC
AGACACAATATGTATAGGCTACCATGCCAACAACTCGACCGACACTGTTGACACAGTACTTGAGAAGAAT
GTGACAGTGACACACTCTGTCAACCTACTTGAGGACAGTCACAATGGAAAACTATGTCTACTAAAAGGAA
TAGCCCCACTACAATTGGGTAATTGCAGCGTTGCCGGATGGATCTTAGGAAACCCAGAATGCGAATTACT
GATTTCCAAGGAATCATGGTCCTACATTGTAGAAACACCAAATCCTGAGAATGGAACATGTTACCCAGGG
TATTTCGCCGACTATGAGGAGCTGAGGGAGCAATTGAGTTCAGTATCTTCATTTGAGAGGTTCGAAATAT
TCCCCAAAGAGAGCTCATGGCCCAACCACACCGTAACCGGAGTATCAGCATCATGCTCCCATAACGGGAA
AAGCAGTTTTTACAGAAATTTGCTATGGCTGACGGGGAAGAATGGTTTGTATCCAAACCTGAGCAAGTCC
TATGCAAACAACAAAGAGAAAGAAGTCCTTGTACTGTGGGGTATTCATCACCCGCCTAACATAGGGGACC
AAAGGGCCCTCTATCATACAGAAAATGCTTATGTCTCTGTAGTGTCTTCACATTACAGCAGAAGATTCAC
CCCAGAAATAGCCAAAAGACCCAAGGTGAGAGATCAGGAAGGAAGAATCAACTACTACTGGACTCTGCTG

GAACCCGGGGATACAATAATATTTGAGGCAAATGGAAATCTAATAGCGCCAAGGTTTGCTTTCGCACTGA
GTAGAGGCTTTGGATCAGGAATCATCACCTCAAATGCACCAATGGATGAATGTGATGCGAAGTGTCAAAC
ACCTCAGGGAGCTATAAACAGCAGTCTTCCTTTCCAGAATGTACACCCAGTCACAATAGGAGAGTGTCCA
AAGTATGTCAGGAGTGCAAAATTAAGAATGGTTACAGGACTAAGGAACATCCCATCCATTCAATCCAGAG
GTTTGTTTGGAGCCATTGCCGGTTTCATTGAAGGGGGGTGGACTGGAATGGTAGATGGTTGGTATGGTTA
TCACCATCAGAATGAGCAAGGATCTGGGTATGCTGCAGATCAAAAAAGCACACAAAATGCCATTAACGGG
ATTACAAACAAGGTGAATTCTGTAATTGAGAAAATGAACACTCAATTCACAGCTGTGGGCAAAGAATTCA
ACAAATTGGAAAGAAGGATGGAAAACTTAAATAAAAAAGTTGATGATGGGTTTCTAGACATTTGGACATA
TAACGCAGAATTGTTGGTTCTACTGGAAAATGAAAGGACTTTGGACTTCCATGACTCCAACGTGAAGAAT
CTGTATGAGAAAGTAAAAAGCCAATTAAAGAATAATGCCAAAGAAATAGGAAACGGGTGTTTTGAATTCT
ATCACAAGTGTAACGATGAATGCATGGAGAGTGTGAAAAATGGAACTTATGACTATCCAAAATATTCCGA
AGAATCAAAGTTGAACAGGGAGAGAATTGATGGAGTGAAATTGGAATCAATGGGAGTCTATCAGATTCTG
GCGATCTACTCAACAGTCGCCAGTTCCCTGGTTCTTTTGGTCTCCCTGGGGGCAATCAGCTTCTGGATGT
GTTCCAATGGGTCTTTGCAGTGTAGAATATGCATCTAAGACCAGAGTTTCAGAAATATAAGGAAAAA
>gi|149780692|gb|CY022581.1| Influenza A virus (A/Auckland/619/2005(H1N1)) segment 4, complete sequence
AATAAAAGCAACCAAAATGAAAGTAAAACTACTGGTTCTGTTATGTACATTTACAGCTACATATGCAGAC
ACAATATGTATAGGCTACCATGCCAACAACTCGACCGACACTGTTGACACAGTACTTGAGAAGAATGTGA
CAGTGACACACTCTGTCAACCTACTTGAGGACAGTCACAATGGAAAACTATGTCTACTAAAAGGAATAGC
CCCACTACAATTGGGTAATTGCAGCGTTGCCGGATGGATCTTAGGAAACCCAGAATGCGAATTACTGATT
TCCAAGGAATCATGGTCCTACATTGTAGAAACACCAAATCCTGAGAATGGAACATGTTACCCAGGGTATT
TCGCCGACTATGAGGAGCTGAGGGAGCAATTGAGTTCAGTATCTTCATTTGAGAGGTTCGAAATATTCCC
CAAAGAGAGCTCATGGCCCAACCACACCGTAACCGGAGTATCAGCATCATGCTCCCATAACGGGAAAAGC
AGTTTTTACAGAAATTTGCTATGGCTGACGGGGAAGAATGGTTTGTATCCAAACCTGAGCAAGTCCTATG
CAAACAACAAAGAGAAAGAAGTCCTTGTACTGTGGGGTGTTCATCACCCGCCTAACATAGGGGACCAAAG
GGCCCTCTATCATACAGAAAATGCTTATGTCTCTGTAGTGTCTTCACATTATAGCAGAAGATTCACTCCA
GAAATAGCCAAAAGACCCAAGGTGAGAGATCAGGAAGGAAGAATCAACTACTACTGGACTCTGCTGGAAC
CCGGGGATACTATAATATTTGAGGCAAATGGAAATCTAATAGCGCCAAGGTTTGCTTTCGCACTGAGTAG
AGGCTTTGGATCAGGAATCATCACCTCAAATGCACCAATGGATGAATGTGATGCGAAGTGTCAAACACCT
CAGGGAGCTATAAACAGCAGTCTTCCTTTCCAGAATGTACACCCAGTCACAATAGGAGAGTGTCCAAAGT
ATGTCAGGAGTGCAAAATTAAGAATGGTTACAGGACTAAGGAACATCCCATCCATTCAATCCAGAGGTTT
GTTTGGAGCCATTGCCGGTTTCATTGAAGGGGGGTGGACTGGAATGGTAGATGGTTGGTATGGTTATCAC
CATCAGAATGAGCAAGGATCTGGGTATGCTGCAGATCAAAAAAGCACACAAAATGCCATTAACGGGATTA
CAAACAAGGTGAATTCTGTAATTGAGAAAATGAACACTCAATTCACAGCTGTGGGCAAAGAATTCAACAA
ATTGGAAAGAAGGATGGAAAACTTAAATAAAAAAGTTGATGATGGGTTTCTAGACATTTGGACATATAAC
GCAGAATTGTTGGTTCTACTGGAAAATGAAAGGACTTTGGACTTCCATGACTCCAACGTGAAGAATCTGT
ATGAGAAAGTAAAAAGCCAATTAAAGAATAATGCCAAAGAAATAGGAAACGGGTGTTTTGAATTCTATCA
CAAGTGTAACGATGAATGCATGGAGAGTGTGAAAAATGGAACTTATGACTATCCAAAATATTCCGAAGAA
TCAAAGTTGAACAGGGAGAGAATTGATGGAGTGAAATTGGAATCAATGGGAGTCTATCAGATTCTGGCGA
TCTACTCAACAGTCGCCAGTTCCCTGGTTCTTTTGGTCTCCCTGGGGGCAATCAGCTTCTGGATGTGTTC
CAATGGGTCTTTGCAGTGTAGAATATGCATCTAAGACCAGAGTTTCAGAAATATAAGGAAAAA
>gi|117572938|gb|CY017315.1| Influenza A virus (A/Waikato/4/2005(H1N1)) segment 4, complete sequence
AAGCAACCAAAATGAAAGTAAAACTACTGGTTCTGTTATGTACATTTACAGCTACATATGCAGACACAAT

ATGTATAGGCTACCATGCCAACAACTCAACCGACACTGTTGACACAGTACTTGAGAAGAATGTGACAGTG
ACACACTCTGTCAACCTACTTGAGGACAGTCACAATGGAAAACTATGTCTACTAAAAGGAATAGCCCCAT
TACAATTGGGTAATTGCAGCGTTGCCGGATGGATCTTAGGAAACCCAGAATGCGAATTACTGATTTCCAA
GGAATCATGGTCCTACATTGTAGAAACACCAAATCCTGAGAATGGAACATGTTACCCAGGGTATTTCGCC
GACTATGAGGAACTGAGGGAGCAATTGAGTTCAGTATCTTCATTTGAGAGGTTCGAAATATTCCCCAAAG
AGAGCTCATGGCCCAACCACACCGTAACCGGAGTATCAGCATCATGCTCCCATAACGGGAAAAGCAGTTT
TTACAGAAATTTGCTATGGCTGACGGGGAAGAATGGTTTGTACCCAAACCTGAGCAAGTCTTATGCAAAC
AACAAAGAAAAAGAAGTCCTTGTACTATGGGGTGTTCATCACCCGCCTAACATAGGGAACCAAAGGGCTC
TCTATCATACAGAAAATGCTTATGTCTCTGTAGTGTCTTCACATTATAGCAGAAGATTCACCCCAGAAAT
AGCCAAAAGACCCAAGGTGAGAGATCAGGAAGGAAGAATCAACTACTACTGGACACTGCTGGAACCCGGG
GATACAATAATATTTGAGGCAAATGGAAATCTAATAGCGCCAAGGTTTGCTTTCGCACTGAGTAGAGGCT
TTGGATCAGGAATCATCACCTCAAATGCACCAATGGATGAATGTGATGCAAAGTGTCAAACACCTCAAGG
AGCTATAAACAGCAGTCTTCCTTTCCAGAATGTACACCCAGTCACAATAGGGGAGTGTCCAAAGTATGTC
AGGAGTGCAAAATTAAGGATGGCTACAGGACTAAGGAACATCCCATCCATTCAATCCAGAGGTTTGTTTG
GAGCCATTGCCGGTTTCATTGAAGGGGGGTGGACTGGAATGGTAGATGGTTGGTATGGTTATCATCATCA
GAATGAGCAAGGATCTGGGTATGCTGCAGATCAAAAAAGCACACAAAATGCCATTAACGGGATTACAAAC
AAGGTGAATTCTGTAATTGAAAAAATGAACACTCAATTCACAGCTGTGGGCAAAGAATTCAGCAAATTGG
AGAGAAGGATGGAAAACTTAAATAAAAAAGTTGATGATGGGTTTCTAGACATTTGGACATATAATGCAGA
ATTGTTGGTTCTACTGGAAAATGAAAGGACTTTGGATTTCCACGACTCCAACGTGAAGAATCTGTATGAG
AAAGTGAAAAGCCAATTAAAGAATAATGCCAAAGAAATAGGAAACGGGTGTTTTGAATTCTATCACAAAT
GTAACGATGAATGCATGGAGAGTGTGAAAAATGGAACTTATGACTATCCAAAATATTCCGAAGAATCAAA
GTTAAACAGGGAGAAAATTGATGGAGTGAAATTGGAATCAATGGGAGTCTATCAGATTCTGGCGATCTAC
TCAACAGTCGCCAGTTCCCTGGTTCTTTTGGTCTCCCTGGGGGCAATCAGCTTCTGGATGTGTTCCAATG
GGTCTTTGCAGTGTAGAATATGCATCTAAGACCAGAATTTCAGAAATATAAGGAAA
>gi|161139444|gb|CY028195.1| Influenza A virus (A/Kentucky/UR06-0007/2006(H1N1)) segment 4, complete sequence
GAAAATAAAAGCAACCAAAATGAAAGTAAAACTACTGATTCTGTTATGTACATTTACAGCTACATATGCA
GACACAATATGTATAGGCTACCATGCCAACAACTCGACCGACACTGTTGACACAGTACTTGAGAAGAATG
TGACAGTGACACACTCTGTCAACCTACTTGAGGACAGTCACAATGGAAAACTATGTCTACTAAAAGGAAT
AGCCCCACTACAATTGGGTAATTGCAGCGTTGCCGGATGGATCTTAGGAAACCCAGAATGCGAATTGCTG
ATTTCCAAGGAATCATGGTCCTACATTGTAGAGACACCAAATCCTGAGAATGGAACATGTTACCCAGGGT
ATTTCGCCGACTATGAGGAGCTGAGAGAGCAATTGAGTTCAGTATCTTCATTTGAGAGGTTCGAAATATT
CCCCAAAGAGAGCTCATGGCCCAACCACACCGTAACCGGAGTATCAGCATCATGCTCCCATAACGGGAAA
AGCAGTTTTTACAGAAATTTGCTATGGCTGACGGGGAAGAATGGTTTGTATCCAAACCTGAGCAAGTCCT
ATGCAAACAACAAAGAGAAAGAAGTCCTTGTACTGTGGGGTGTTCATCACCCGCCTAACATAGGGGACCA
AAGGGCCCTCTATCATACAGAAAATGCTTATGTCTCTGTAGTGTCTTCACATTATAGCAGAAGATTCACC
CCAGAAATAGCCAAAAGACCCAAGGTGAGAGATCAGGAAGGAAGAATCAACTACTACTGGACTCTGCTTG
AACCCGGGGATACAATAATATTTGAGGCAAATGGAAATCTAATAGCGCCAAGGTTTGCTTTCGCACTGAG
TAGAGGCTTTGGATCAGGAATCATCACCTCAAATGCACCAATGGATGAATGTGATGCGAAATGTCAAACA
CCTCAGGGAGCTATAAACAGCAGTCTTCCTTTCCAGAATGTACACCCAGTCACAATAGGAGAGTGTCCAA
AGTATGTCAGGAGTACAAAATTAAGAATGGTTACAGGACTAAGGAACATCCCATCCATTCAATCCAGAGG
TTTATTTGGAGCCATTGCCGGTTTCATTGAAGGGGGGTGGACTGGAATGGTAGATGGTTGGTATGGTTAT
CACCATCAGAATGAGCAAGGATCTGGGTATGCTGCAGATCAAAAAAGCACACAAAATGCCATTAACGGGA
TTACAAACAAGGTGAATTCTGTAATTGAGAAAATGAACACTCAATTCACAGCTGTGGGCAAAGAATTCAA

CAAATTGGAAAGAAGGATGGAAAACTTAAATAAAAAAGTTGATGATGGGTTTCTAGACATTTGGACATAT
AACGCAGAATTGTTGGTTCTACTGGAAAATGAAAGGACTTTGGACTTCCATGACTCCAACGTGAAGAATC
TGTATGAGAAAGTAAAAAGCCAATTAAAGAATAATGCCAAAGAAATAGGAAACGGGTGTTTTGAATTCTA
TCACAAGTGTAACGATGAATGCATGGAGAGTGTGAAAAATGGAACTTATGACTATCCAAAATATTCCGAA
GAATCAAAGTTGAACAGGGAGAAAATTGATGGAGTGAAATTGGAATCAATGGGAGTCTATCAGATTTTGG
CGATCTACTCAACAGTCGCCAGTTCCCTGGTTCTTTTGGTCTCCCTGGGGGCAATCAGCTTCTGGATGTG
TTCCAATGGGTCTTTGCAGTGTAGAATATGCATCTAAGACCAGAGTTTCAGAAATATAAGGAA
>gi|157281276|gb|CY025221.1| Influenza A virus (A/Michigan/UR06-0015/2006(H1N1)) segment 4, complete sequence
AATAAAAGCAACCAAAATGAAAGTAAAACTACTTGTTCTGTTATGTACATTTACAGCTACATATGCAGAC
ACAATATGTATAGGCTACCATGCCAACAACTCGACCGACACTGTTGACACAGTACTTGAGAAGAATGTGA
CAGTGACACACTCTGTCAACCTACTTGAGGACAGTCACAATGGAAAACTATGTCTACTAAAAGGAATAGC
CCCACTACAATTGGGTAATTGCAGCGTTGCCGGATGGATCTTAGGAAACCCAGAATGCGAATTACTGATT
TCCAAGGAATCATGGTCCTACATTGTAGAAACACCAAATCCTGAGAATGGAACATGTTACCCAGGATATT
TCGCCGACTATGAGGAGCTGAGGGAGCAATTGAGTTCAGTATCTTCATTTGAGAGGTTCGAAATATTCCC
CAAAGAGAGCTCATGGCCCAACCACACCGTAACCGGAGTATCAGCATCATGCTCCCATAGCGGGAAAAGC
AGTTTTTACAGAAATTTGCTATGGCTGACGGGGAAGAATGGTTTGTATCCAAACCTGAGCAAGTCCTATG
CAAACAACAAAGAGAAAGAAGTCCTTGTACTGTGGGGTGTTCATCACCCGCCTAACATAGGGGACCAAAG
GGCCCTCTATCATACAGAAAATGCTTATGTCTCTGTAGTGTCTTCACATTATAGCAGAAGATTCACCCCA
GAAATAGCCAAAAGACCCAAGGTGAGAGATCAGGAAGGAAGAATCAACTACTACTGGACTCTGCTGGAAC
CCGGGGATACAATAATATTTGAGGCAAATGGAAATCTAATAGCGCCAAGGTTTGCTTTCGCACTGAGTAG
AGGCTTTGGATCAGGAATCATCACCTCAAATGCACCAATGGGTGAATGTGATGCGAAGTGTCAAACACCT
CAGGGAGCTATAAACAGTAGTCTTCCTTTCCAGAATGTACATCCAGTCACAATAGGGGAGTGTCCAAAGT
ATGTCAGGAGTGCAAAATTAAGAATGGTTACAGGACTAAGGAACATCCCATCCATTCAATCCAGAGGTTT
GTTTGGAGCCATTGCCGGTTTCATTGAAGGGGGGTGGACTGGAATGGTAGATGGTTGGTATGGTTATCAC
CATCAGAATGAGCAAGGATCTGGGTATGCTGCAGATCAAAAAAGCACACAAAATGCCATTAACGGGATTA
CAAACAAGGTGAATTCTGTAATTGAGAAAATGAACACTCAATTCACAGCTGTGGGCAAAGAATTCAACAA
ATTGGAAAGAAGGATGGAAAACTTAAATAAAAAGGTTGATGATGGGTTTCTAGACATTTGGACATATAAC
GCAGAATTGTTGGTTCTACTGGAAAATGAAAGGACTTTGGACTTCCATGACTCCAACGTGAAGAATCTGT
ATGAGAAAGTAAAAAGCCAATTAAAGAATAATGCCAAAGAAATAGGAAACGGGTGTTTTGAATTCTATCA
CAAGTGTAACGATGAATGCATGGAGAGTGTGAAAAATGGAACTTATGACTATCCAAAATATTCCGAAGAA
TCAAAGTTGAACAGGGAGAAAATTGATGGAGTGAAATTGGAATCAATGGGAGTCTATCAGATTCTGGCGA
TCTACTCAACAGTCGCCAGTTCCCTGGTTCTTTTGGTCTCCCTGGGGGCAATCAGCTTCTGGATGTGTTC
CAATGGGTCTTTGCAGTGTAGAATATGCATCTAAGACCAGAGTTTCAGAAATATAAGGAAAAAA
>gi|218875168|gb|CY036919.1| Influenza A virus (A/NYMC X-163A(NYMC X-157-St. Petersburg/8/2006)(H1N1)) segment 4, complete sequence
AAACAACCAAAATGAAAGTAAAACTACTGGTCCTATTATGCACATTTACAGCTACATATGCAGACACAAT
ATGTATAGGCTACCATGCCAACAACTCAACCGACACTGTTGACACAGTACTTGAAAAGAATGTGACAGTG
ACACACTCTGTAAACCTGCTTGAGGACAGCCACAATGGAAAATTATGTCTATTAAAAGGAATAGCCCCAC
TACAATTGGGTAATTGCAGCGTTGCCGGTTGGATCTTAGGAAACCCAGAATGCGAATTACTGATTTCCAA
GGAGTCATGGTCCTACATTGTAGAAAAACCAAATCCTGAGAATGGAACATGTTACCCAGGGCATTTCGCC
GACTATGAGGAACTGAGGGAGCAATTGAGTTCAGTATCTTCATTTGAGAGGTTCGAAATATTCCCCAAAG
AAAGCTCATGGCCCAACCACACCGTAACCGGAGTATCAGCATCATGCTCCCATAATGGGAAAAGCAGTTT
TTACAAAAATTTGCTATGGCTGACGGGAAAGAATGGTTTGTACCCAAACCTGAGCAAGTCCTATGCAAAC

AACAAAGAGAAAGAAGTCCTTGTACTATGGGGTGTTCATCACCCGCCAAACATAGGTAACCAAAGGGCCC
TCTATCATACAGAAAATGCTTATGTCTCTGTAGTGTCTTCACATTATAGCAGAAAATTCACCCCAGAAAT
AGCCAAAAGACCCAAAGTAAGAGATCAAGAAGGAAGAATCAACTACTACTGGACTCTGCTTGAACCCGGG
GATACAATAATATTTGAGGCAAATGGAAATCTAATAGCGCCAAGATATGCTTTCGCACTGAGTAGAGGCT
TTGGATCAGGAATCATCAACTCAAATGCACCAATGGATGAATGTGATGCGAAGTGCCAAACACCTCAGGG
AGCTATAAACAGCAGTCTTCCTTTTCAGAATGTACACCCAGTCACAATAGGAGAATGTCCAAAGTATGTC
AGGAGTGCAAAATTAAGGATGGTTACAGGACTAAGGAACATCCCATCCATTCAATCCAGAGGTTTGTTTG
GAGCCATTGCCGGTTTCATTGAAGGGGGGTGGACTGGAATGGTAGATGGTTGGTATGGTTATCATCACCA
GAATGAGCAAGGATCTGGCTATGCTGCAGATCAAAAAAGCACACAAAATGCCATTAATGGGATTACAAAC
AAGGTGAATTCTGTAATTGAGAAGATGAACACTCAGTTCACAGCTGTGGGCAAAGAATTCAACAAATTGG
AAAGAAGGATGGAAAACTTAAATAAAAAAGTTGATGACGGGTTTATAGACGTTTGGACATATAATGCAGA
ACTGTTGGTTCTACTGGAAAATGAAAGGACTTTGGATTTCCATGACTCCAATGTGAAGAATTTGTATGAG
AAAGTAAAAAGCCAATTAAAGAATAATGCCAAAGAAATAGGAAATGGGTGTTTTGAATTTTATCACAAGT
GTAACGATGAATGCATGGAGAGTGTAAAAAATGGAACTTATGACTATCCAAAATATTCCGAAGAATCAAA
GTTAAGCAGGGAGAAAATTGATGGAGTGAAATTGGAATCAATGGGAGTCTATCAGATTCTGGCGATCTAC
TCAACAGTCGCCAGTTCTCTGGTTCTTTTGGTCTCCCTGGGGGCAATCAGCTTCTGGATGTGTTCCAATG
GGTCTTTGCAGTGTAGAATATGCATCTAAGACCAGAATTTCAGAAATATAAGGAAAA
>gi|208344083|gb|CY035126.1| Influenza A virus (A/St. Petersburg/8/2006(H1N1)) segment 4, complete sequence
CAACCAAAATGAAAGTAAAACTACTGGTCCTATTATGCACATTTACAGCTACATATGCAGACACAATATG
TATAGGCTACCATGCCAACAACTCAACCGACACTGTTGACACAGTACTTGAAAAGAATGTGACAGTGACA
CACTCTGTAAACCTGCTTGAGGACAGCCACAATGGAAAATTATGTCTATTAAAAGGAATAGCCCCACTAC
AATTGGGTAATTGCAGCGTTGCCGGTTGGATCTTAGGAAACCCAGAATGCGAATTACTGATTTCCAAGGA
GTCATGGTCCTACATTGTAGAAAAACCAAATCCTGAGAATGGAACATGTTACCCAGGGCATTTCGCCGAC
TATGAGGAACTGAGGGAGCAATTGAGTTCAGTATCTTCATTTGAGAGGTTCGAAATATTCCCCAAAGAAA
GCTCATGGCCCAACCACACCGTAACCGGAGTATCAGCATCATGCTCCCATAATGGGAAAAGCAGTTTTTA
CAAAAATTTGCTATGGCTGACGGGAAAGAATGGTTTGTACCCAAACCTGAGCAAGTCCTATGCAAACAAC
AAAGAGAAAGAAGTCCTTGTACTATGGGGTGTTCATCACCCGCCAAACATAGGTAACCAAAGGGCCCTCT
ATCATACAGAAAATGCTTATGTCTCTGTAGTGTCTTCACATTATAGCAGAAAATTCACCCCAGAAATAGC
CAAAAGACCCAAAGTAAGAGATCAAGAAGGAAGAATCAACTACTACTGGACTCTGCTTGAACCCGGGGAT
ACAATAATATTTGAGGCAAATGGAAATCTAATAGCGCCAAGATATGCTTTCGCACTGAGTAGAGGCTTTG
GATCAGGAATCATCAACTCAAATGCACCAATGGATGAATGTGATGCGAAGTGCCAAACACCTCAGGGAGC
TATAAACAGCAGTCTTCCTTTTCAGAATGTACACCCAGTCACAATAGGAGAATGTCCAAAGTATGTCAGG
AGTGCAAAATTAAGGATGGTTACAGGACTAAGGAACATCCCATCCATTCAATCCAGAGGTTTGTTTGGAG
CCATTGCCGGTTTCATTGAAGGGGGGTGGACTGGAATGGTAGATGGTTGGTATGGTTATCATCACCAGAA
TGAGCAAGGATCTGGCTATGCTGCAGATCAAAAAAGCACACAAAATGCCATTAATGGGATTACAAACAAG
GTGAATTCTGTAATTGAGAAGATGAACACTCAGTTCACAGCTGTGGGCAAAGAATTCAACAAATTGGAAA
GAAGGATGGAAAACTTAAATAAAAAAGTTGATGACGGGTTTATAGACGTTTGGACATATAATGCAGAACT
GTTGGTTCTACTGGAAAATGAAAGGACTTTGGATTTCCATGACTCCAATGTGAAGAATTTGTATGAGAAA
GTAAAAAGCCAATTAAAGAATAATGCCAAAGAAATAGGAAATGGGTGTTTTGAATTTTATCACAAGTGTA
ACGATGAATGCATGGAGAGTGTAAAAAATGGAACTTATGACTATCCAAAATATTCCGAAGAATCAAAGTT
AAGCAGGGAGAAAATTGATGGAGTGAAATTGGAATCAATGGGAGTCTATCAGATTCTGGCGATCTACTCA
ACAGTCGCCAGTTCTCTGGTTCTTTTGGTCTCCCTGGGGGCAATCAGCTTCTGGATGTGTTCCAATGGGT
CTTTGCAGTGTAGAATATGCATCTAAGACCAGAATTTCAGAAATATAAGGAA

>gi|226954746|gb|CY038879.1| Influenza A virus (A/Taiwan/2645/2006(H1N1)) segment 4, complete sequence
ATGAAAGTAAAACTACTGGTCCTGTTATGCACATTTACAGCTACATATGCAGACACAATATGTATAGGCT
ACCATGCCAACAACTCAACCGACACTGTTGACACAGTACTTGAGAAGAATGTGACAGTGACACACTCTGT
CAACCTGCTTGAGGACAGTCACAATGGAAAATTATGTCTATTAAAAGGAATAGCCCCACTACAATTGGGT
AATTGCAGCGTTGCCGGATGGATCTTAGGAAACCCAGAATGCGAATTACTGATTTCCAGGGAATCATGGT
CCTACATTGTAGAAAAACCAAATCCTGAGAATGGAACATGTTACCCAGGGCATTTCGCCGACTATGAGGA
ACTGAGGGAGCAATTGAGTTCAGTATCTTCATTTGAGAGATTCGAAATATTCCCCAAAGAAAGCTCATGG
CCCAACCACACCACAACCGGAGTATCAGCATCATGCTCCCATAATGGGGAAAGCAGTTTTTACAAAAATT
TGCTATGGCTGACGGGGAAGAATGGTTTGTACCCAAACCTGAGCAAGTCCTATGCAAACAACAAAGAGAA
AGAAGTCCTTGTACTATGGGGTGTTCATCACCCGCCTAACATAGGTAACCAAAGGGCTCTCTATCATACA
GAAAATGCTTATGTCTCTGTAGTGTCTTCACATTATAGCAGAAAATTCACCCCAGAAATAGCCAAAAGAC
CCAAAGTAAGAGATCAAGAAGGAAGAATCAACTACTACTGGACTCTACTTGAACCCGGGGATACAATAAT
ATTTGAGGCAAATGGAAATCTAATAGCGCCAAGATATGCTTTCGCACTGAGTAGAGGCTTTGGATCAGGA
ATCATCAACTCAAATGCACCAATGGATGGATGTGATGCGAAGTGCCAAACACCTCAGGGAGCTATAAACA
GCAGTCTTCCTTTCCAGAATGTACACCCTGTCACAATAGGAGAGTGTCCAAAGTATGTCAGGAGTGCAAA
ATTAAGGATGGTTACAGGACTAAGGAACATCCCATCCATTCAATCCAGAGGTTTGTTTGGAGCCATTGCC
GGTTTCATTGAAGGGGGGTGGACTGGAATGGTAGATGGTTGGTATGGTTATCATCATCAGAATGAGCAAG
GATCTGGCTATGCTGCAGATCAAAAAAGCACACAAAATGCCATTAATGGGATTACAAACAAGGTGAATTC
TGTAATTGAGAAAATGAACACTCAATTCACAGCTGTGGGCAAAGAATTCAACAAATTGGAAAGAAGGATG
GAAAACTTAAATAAAAAAGTTGATGATGGGTTTATAGACATTTGGACATATAATGCAGAATTGTTGGTTC
TACTGGAAAATGAAAGGACTTTGGATTTCCATGACTCCTATGTGAAGAATCTGTATGAGAAAGTAAAAAG
CCAATTAAAGAATAATGCCAAAGAAATAGGAAATGGGTGTTTTGAATTCTATCACAAGTGTAACGATGAA
TGCATGGAGAGTGTAAAAAATGGAACTTATGACTATCCAAAATATTCCGAAGAATCAAAGTTAAACAGGG
AGAAAATTGATGGAGTGAAATTGGAATCAATGGGAGTCTATCAGATTCTGGCGATCTACTCAACAGTCGC
CAGTTCTCTGGTTCTTTTGGTCTCCCTGGGGGCAATCAGCTTCTGGATGTGTTCCAATGGGTCTTTGCAG
TGTAGAATATGCATCTAAGACCAGAATTTCAGAAATATAAG
>gi|256385511|gb|CY044349.1| Influenza A virus (A/South Korea/AF10/2008(H1N1)) segment 4, complete sequence
ATGAAAGTAAAACTACTGGTCCTATTATGCACATTTACAGCTACATATGCGGACACAATATGTATCGGCT
ACCATGCCAACAACTCAACCGACACTGTTGACACAGTACTTGAAAAGAATGTGACAGTGACACACTCTGT
CAACCTGCTTGAGGACAACCACAATGGAAAACTATGTCTATTAAAAGGAAAAGCCCCATTACAATTGGGT
AACTGCAGCGTTGCCGGGTGGATCTTAGGAAACCCAGAATGCGGATTACTGATTTCCAAGGAGTCATGGT
CCTACATTGTAGAAAGACCAAATCCTGAGAATGGAACATGTTACCCAGGGCATTTCGCCGACTATGAGGA
ACTGAGGGAGCAATTGAGTTCAGTATCTTCATTTGAGAGGTTCGAAATATTCCCCAAAGAAAGCTCATGG
CCCAACCACACCGTAACCGGAGTATCAGCATCATGCTCCCATAACGGGGAAAGCAGCTTTTACAAAAATT
TGCTATGGCTGACGGGAAAGAATGGTTTGTACCCAAACCTGAGCAAGTCCTATGCAAACAACAAAGAGAA
AGAAGTCCTCGTACTATGGGGTGTTCATCACCCGCCAAACATAGGTGACCAAATGACCCTCTATCATAAA
GAAAATGCTTATGTCTCTGTAGTGTCTTCACATTATAGCAGAAAATTCACCCCAGAAATAGCCAAAAGAC
CCAAAGTAAGAGATCAAGAAGGAAGAATCAACTACTACTGGACTCTGCTTGAACCCGGGGATACAATAAT
ATTTGAGGCAAATGGGAATCTAATAGCGCCAAGATATGCTTTCGCACTGAGTAGAGGCTTTGGATCAGGA
ATCATCAACTCAAATGCACCAATGGATGAATGTGATGCGAAGTGCCAAACACCTCAGGGAGCTATAAACA
GCAGTCTTCCTTTCCAGAATGTACACCCAGTCACAATAGGAGAATGTCCAAAGTATGTCAGGAGTGCAAA
ATTAAGGATGGTTACAGGACTAAGGAACATCCCATCCATTCAATCCAGAGGTTTGTTTGGAGCCATTGCC

GGTTTCATTGAAGGGGGGTGGACTGGAATGGTAGATGGTTGGTATGGTTATCATCACCAGAATGAGCAAG
GATCTGGCTATGCTGCAGATCAAAAAAGCACACAAAATGCCATTAATGGGATTACAAACAAGGTGAACTC
TGTAATTGAGAAGATGAACACTCAATTCACAGCTGTAGGCAAAGAATTCAACAAATTGGAAAGAAGGATG
GAAAACTTAAATAAAAAAGTTGATGACGGGTTTATAGACGTTTGGACATATAATGCAGAACTGTTGGTTC
TACTAGAAAATGAAAGGACTTTGGATTTCCATGACTCCAATGTGAAGAATTTGTATGAGAAAGTAAAAAA
CCAATTAAAAAATAATGCCAAAGAAATAGGAAATGGGTGTTTTGAATTTTATCACAAGTGTAACGATGAA
TGCATGGAGAGTGTAAAAAATGGAACTTATGATTATCCAAAATATTCCGAAGAATCAAAGTTAAGCAGGG
AGAAAATTGATGGAGTGAAATTGGAATCAATGGGAGTCTATCAGATTCTGGCGATCTACTCAACAGTCGC
CAGTTCTCTGGTTCTTTTGGTCTCCCTGGGGGCAATCAGCTTCTGGATGTGTTCCAATGGGTCTTTGCAG
TGTAGAATATGCATCTAAGACCAGAAT
>gi|163964716|gb|CY028459.1| Influenza A virus (A/California/UR06-0442/2007(H1N1)) segment 4, complete sequence
AAAGCAACCAAAATGAAAGTAAAACTACTTGTTCTGTTATGTACATTTACAGCTACATATGCAGACACAA
TATGTATAGGCTACCATGCCAACAACTCGACCGACACTGTTGACACAGTACTTGAGAAGAATGTGACAGT
GACACACTCTGTCAACCTACTTGAGGACAGTCACAATGGAAAACTATGTCTACTAAAAGGAATAGCCCCA
CTACAATTGGGTAATTGCAGCGTTGCCGGATGGATCTTAGGAAACCCAGAATGCGAATTACTGATTTCCA
AGGAATCATGGTCCTACATTGTAGAAACACCAAATCCTGAGAATGGAACATGTTACCCAGGATATTTCGC
CGACTATGAGGAGCTGAGGGAGCAATTGAGTTCAGTATCTTCATTTGAGAGGTTCGAAATATTCCCCAAA
GAGAGCTCATGGCCCAACCACACCGTAACCGGAGTATCAGCATCATGCTCCCATAACGGGAAAAGCAGTT
TCTACAGAAATTTGCTATGGCTGACGGGGAAGAATGGTTTGTATCCAAACCTGAGCAAGTCCTATGCAAA
CAACAAAGAGAAAGAAGTCCTTGTACTGTGGGGTGTTCATCACCCGCCTAACATAGGGGACCAAAGGGCC
CTCTATCATACAGAAAATGCTTATGTCTCTGTAGTGTCTTCACATTATAGCAGAAGATTCACCCCAGAAA
TAGCCAAAAGACCCAAGGTGAGAGATCAGGAAGGAAGAATCAACTACTACTGGACTCTGCTGGAACCCGG
GGATACAATAATATTTGAGGCAAATGGAAATCTAATAGCGCCAAGGTTTGCTTTCGCACTGAGTAGAGGC
TTTGGATCAGGAATCATCACCTCAAATGCACCAATGGGTGAATGTGATGCGAAGTGTCAAACACCTCAGG
GAGCTATAAACAGTAGTCTTCCTTTCCAGAATGTACATCCAGTCACAATAGGAGAGTGTCCAAAGTATGT
CAGGAGTGCAAAATTAAGAATGGTTACAGGACTAAGGAACATCCCATCCATTCAATCCAGAGGTTTGTTT
GGAGCCATTGCCGGTTTCATTGAAGGGGGGTGGACTGGAATGGTAGATGGTTGGTATGGTTATCACCATC
AGAATGAGCAAGGATCTGGGTATGCTGCAGATCAAAAAAGCACACAAAATGCCATTAACGGGATTACAAA
CAAGGTGAATTCTGTAATTGAGAAAATGAACACTCAATTCACAGCTGTGGGCAAAGAATTCAACAAATTG
GAAAGAAGGATGGAAAACTTAAATAAAAAGGTTGATGATGGGTTTCTAGACATTTGGACATATAACGCAG
AATTGTTGGTTCTACTGGAAAATGAAAGGACTTTGGACTTCCATGACTCCAACGTGAAGAATCTGTATGA
GAAAGTAAAAAGCCAATTAAAGAATAATGCCAAAGAAATAGGAAACGGGTGTTTTGAATTCTATCACAAG
TGTAACGATGAATGCATGGAGAGTGTGAAAAATGGAACTTATGACTATCCAAAATATTCCGAAGAATCAA
AGTTGAACAGGGAGAAAATTGATGGAGTGAAATTGGAATCAATGGGAGTCTATCAGATTCTGGCGATCTA
CTCAACAGTCGCCAGTTCCCTGGTTCTTTTGGTCTCCCTGGGGGCAATCAGCTTCTGGATGTGTTCCAAT
GGGTCTTTGCAGTGTAGAATATGCATCTAAGACCAGAGTTTCAGAAATATAA
>gi|157281600|gb|CY025357.1| Influenza A virus (A/Kentucky/UR06-0363/2007(H1N1)) segment 4, complete sequence
AAATAAAAGCAACCAAAATGAAAGTAAAACTACTTGTTCTGTTATGTACATTTACAGCTACATATGCAGA
CACAATATGTATAGGCTACCATGCCAACAACTCGACTGACACTGTTGACACAGTACTTGAGAAGAATGTG
ACAGTGACACACTCTGTCAACCTACTTGAGGACAGTCACAATGGAAAACTATGTCTACTAAAAGGAATAG
CCCCACTACAATTGGGTAATTGCAGCGTTGCCGGATGGATCTTAGGAAACCCAGAATGCGAATTACTGAT
TTCCAAGGAATCATGGTCCTACATTGTAGAAACACCAAATCCTGAGAATGGAACATGTTACCCAGGATAT

TTCGCCGACTATGAGGAGCTGAGGGAGCAATTGAGTTCAGTATCTTCATTTGAGAGGTTCGAAATATTCC
CCAAAGAGAGCTCATGGCCCAACCACACCGTAACCGGAGTATCAGCATCATGCTCCCATAACGGGGAAAG
CAGTTTTTACAGAAATTTGTTATGGCTGACGGGGAAGAATGGTTTGTATCCAAACCTGAGCAAGTCCTAT
GCAAACAACAAAGAGAAAGAAGTCCTTGTACTGTGGGGTGTTCATCACCCGCCTAACATAGGAGACCAAA
GGGCCCTCTATCATACAGAAAATGCTTATGTCTCTGTAGTGTCTTCACATTATAGCAGAAGATTCACCCC
AGAAATAGCCAAAAGACCCAAGGTGAGAGATCAGGAAGGAAGAATCAACTACTACTGGACTCTGCTGGAA
CCCGGGGATACAATAATATTTGAGGCAAATGGAAATCTAATAGCGCCAAGGTTTGCTTTCGCACTGAGTA
GAGGCTTTGGATCAGGAATCATCACCTCAAATGCACCAATGGATAAATGTGATGCGAAGTGTCAAACACC
TCAGGGAGCTATAAACAGCAGTCTTCCTTTCCAGAATGTACACCCAGTCACAATAGGAGAGTGTCCAAAG
TATGTCAGGAGTACAAAATTAAGAATGGTTACAGGACTAAGGAACATCCCATCCATTCAATCCAGAGGTT
TGTTTGGAGCCATTGCCGGTTTCATTGAAGGGGGGTGGACTGGAATGGTAGATGGTTGGTATGGTTATCA
CCATCAGAATGAGCAAGGATCTGGGTATGCTGCAGATCAAAAAAGCACACAAAATGCCATTAACGGGATT
ACAAACAAGGTGAATTCTGTAATTGAGAAAATGAACACTCAATTCACAGCTGTGGGCAAAGAATTCAACA
AATTGGAAAGAAGGATGGAAAATTTAAATAAAAAGGTTGATGATGGGTTTCTAGACATTTGGACATATAA
TGCAGAATTGTTGGTTCTACTGGAAAATGAAAGGACTTTGGACTTCCATGACTCCAACGTGAAGAATCTG
TATGAGAAAGTAAAAAGCCAATTAAAGAATAATGCCAAAGAAATAGGAAACGGGTGTTTTGAATTCTATC
ACAAGTGTGACGATGAATGCATGGAGAGTGTGAAAAATGGAACTTATGACTATCCAAAATATTCCGAAGA
ATCAAAGTTGAACAGGGAGAAAATTGATGGAGTGAAATTGGAATCAATGGGAGTCTATCAGATTCTGGCG
ATCTACTCAACAGTCGCCAGTTCCTTGGTTCTTTTGGTCTCCCTGGGGGCAATCAGCTTCTGGATGTGTT
CCAATGGGTCTTTGCAGTGTAGAATATGCATCTAAGGCCAGAGTTTCAGAAATATAAGGAAAAA
>gi|158957789|gb|CY027411.1| Influenza A virus (A/Alabama/UR06-0536/2007(H1N1)) segment 4, complete sequence
ATGAAAGTAAAACTACTGGTTCTGTTATGTACATTTACAGCTACATATGCAGACACAATATGTATAGGCT
ACCATGCCAACAACTCGACCGACACTGTTGACACAGTACTTGAGAAGAATGTGACAGTGACACACTCTGT
CAACCTACTTGAGGACAGTCACAATGGAAAACTATGTCTACTAAAAGGAATAGCCCCACTACAATTGGGT
AATTGCAGCGTTGCCGGATGGATCTTAGGAAACCCAGAATGCGAATTACTGATTTCCAAGGAATCATGGT
CCTACATTGTAGAGACACCAAATCCTGAGAATGGAACATGTTACCCAGGGTATTTCGCCGACTATGAGGA
GCTGAGAGAGCAATTGAGTTCAGTATCTTCATTTGAGAGGTTCGAAATATTCCCCAAAGAGAGCTCATGG
CCCAACCACACCGTAACCGGAGTATCAGCATCATGCTCCCATAACGGGAAAAGCAGTTTTTACAGAAATT
TGCTATGGCTGACGGGGAAGAATGGTTTGTATCCAAACCTGAGCAAGTCCTATGCAAACAACAAAGAGAA
AGAAGTCCTTGTACTGTGGGGTGTTCATCACCCGCCTAACATAGGGGACCAAAGGGCCCTCTATCATACA
GAAAATGCTTATGTCTCTGTAGTGTCTTCACATTATAGCAGAAGATTCACCCCAGAAATAGCCAAAAGAC
CCAAGGTGAGAGATCAGGAAGGAAGAATCAACTACTACTGGACTCTGCTTGAACCCGGGGATACAATAAT
ATTTGAGGCAAATGGAAATCTAATAGCGCCAAGGTTTGCTTTCGCACTGAGTAGAGGCTTTGGATCAGGA
ATCATCACCTCAAATGCACCAATGGATGAATGTGATGCGAAATGTCAAACACCTCAGGGAGCTATAAACA
GCAGTCTTCCTTTCCAGAATGTACACCCAGTCACAATAGGAGAGTGTCCAAAGTATGTCAGGAGTACAAA
ATTAAGAATGGTTACAGGACTAAGGAACATCCCATCCATTCAATCCAGAGGTTTGTTTGGAGCCATTGCC
GGTTTCATTGAAGGGGGGTGGACTGGAATGGTAGATGGATGGTATGGTTACCACCATCAGAATGAGCAAG
GATCTGGGTATGCTGCTGATCAAAAAAGCACACAAAATGCCATTAACGGGATTACAAACAAGGTGAATTC
TGTAATTGAGAAAATGAACACTCAATTCACAGCTGTGGGCAAAGAATTCAACAAATTGGAAAGAAGGATG
GAAAACTTAAATAAAAAAGTTGATGATGGGTTTCTAGACATTTGGACATATAACGCAGAATTGTTGGTTC
TACTGGAAAATGAAAGGACTTTGGACTTCCATGACTCCAACGTGAAGAATCTGTATGAGAAAGTAAAAAG
CCAATTAAAGAATAATGCCAAAGAAATAGGAAACGGGTGTTTTGAATTCTATCACAAGTGTAACGATGAA
TGCATGGAGAGTGTGAAAAATGGAACTTATGACTATCCAAAATATTCCGAAGAATCAAAGTTGAACAGGG

AGAAAAATTGATGGAGTGAAATTGGAATCAATGGGAGTCTATCAGATTTTGGCGATCTACTCAACAGTCGC
CAGTTCCCTGGTTCTTTTGGTCTCCCTGGGGGCAATCAGCTTCTGGATGTGTTCCAATGGGTCTTTGCAG
TGTAGAATATGCATCTAAGACCAGAGTTTCAGAAATATAAGGAAA
>gi|237688824|gb|CY040058.1| Influenza A virus (A/Taiwan/71720/2007(H1N1)) segment 4, complete sequence
GAAAATAAAAACAACCAGAATGAAAGTAAAACTACTGGTCCTGTTATGCACATTTACAGCTACATATGCA
GACACAATATGTATAGGCTACCATGCTAACAACTCGACCGACACTGTTGACACAGTACTTGAAAAGAATG
TGACAGTGACACACTCTGTCAACCTGCTTGAGAACAGTCATAATGGAAAACTATGTCTATTAAAAGGAAT
AGCCCCACTACAATTGGGTAATTGCAGCGTTGCCGGGTGGATCTTAGGAAACCCAGAATGCGAATTACTG
ATTTCCAAGGAGTCATGGTCCTACATTGTAGAAAAACCAAATCCTGAGAATGGAACATGTTACCCAGGGC
ATTTCGCTGACTATGAGGAACTGAGGGAGCAATTGAGTTCAGTATCTTCATTTGAGAGGTTCGAAATATT
CCCCAAAGAAAGCTCATGGCCCAACCACACCGTAACCGGAGTGTCAGCATCATGCTCCCATAATGGGGAA
AGCAGTTTTTACAGAAATTTGCTATGGCTGACGGGGAAGAATGGTTTGTACCCAAACCTGAGCAAGTCCT
ATGCAAACAACAAAGAAAAAGAAGTCCTTGTACTATGGGGTGTTCATCACCCGCCAAACATAGGTAACCA
AAAGGCCCTCTATCATACAGAAAATGCTTATGTCTCTGTAGTGTCTTCACATTATAGCAGAAAATTCACC
CCAGAAATAGCCAAAAGACCCAAAGTAAGAGATCAAGAAGGAAGAATCAACTACTACTGGACTCTGCTTG
AACCCGGGGATACAATAATATTTGAGGCAAATGGAAATCTAATAGCGCCAAGATATGCTTTCGCACTGAG
TAGAGGCCTTGGATCAGGAATCATCAACTCAAATGCACCAATGGATAAATGTGATGCGAAGTGCCAAACA
CCTCAGGGAGCTATAAACAGCAGTCTTCCTTTCCAGAACGTACACCCAGTCACAATAGGAGAGTGTCCAA
AGTATGTCAGGAGTGCAAAATTAAGGATGGTTACAGGACTAAGGAACATCCCATCCATTCAATCCAGAGG
TTTGTTTGGAGCCATTGCCGGTTTCATTGAAGGGGGGTGGACTGGAATGGTAGATGGTTGGTATGGTTAT
CATCATCAGAATGAGCAAGGATCTGGCTATGCTGCAGATCAAAAAAGCACACAAAATGCCATTAATGGGA
TTACAAACAAGGTGAATTCTGTAATTGAGAAAATGAACACTCAATTCACAGCAGTGGGCAAAGAATTCAA
CAAATTGGAAAGAAGGATGGAAAACTTGAATAAAAAAGTTGATGATGGTTTTATAGACATTTGGACATAT
AATGCAGAACTGTTGGTTTTACTGGAAAATGAAAGGACTTTGGATTTCCATGACTCCAATGTGAAGAATC
TGTATGAGAAAGTAAAAAGCCAGTTAAAGAATAATGCTAAAGAAATAGGAAATAGGTGTTTTGAATTCTA
TCACAAGTGTAACGATGAATGCATGGAGAGTGTAAAGAATGGAACTTATGACTATCCAAAATATTCCGAA
GAATCAAAGTTAAACAGGGAGAAAATTGATGGAGTGAAATTGGAATCAATGGGAGTCTATCAGATTCTGG
CGATCTACTCAACAGTCGCCAGTTCTCTGGTTCTTTTGGTCTCCCTGGGGGCAATCAGCTTCTGGATGTG
TTCCAATGGGTCTTTGCAGTGTAGAATATGCATCTAAGACCAGAA
>gi|237689282|gb|CY040250.1| Influenza A virus (A/Managua/3153.01/2008(H1N1)) segment 4, complete sequence
AACCAGAATGAAAGTAAAACTACTGGTCCTGTTATGCACATTTACAGCTACATATGCAGACACAATATGT
ATAGGCTACCATGCTAACAACTCGACCGACACTGTTGACACAGTACTTGAAAAGAATGTGACAGTGACAC
ACTCTGTCAACCTGCTTGAGAACAGTCACAATGGAAAACTATGTCTATTAAAAGGAATAGCCCCACTACA
ATTGGGTAATTGCAGCGTTGCCGGGTGGATCTTAGGAAACCCAGAATGCGAATTACTGATTTCCAAGGAG
TCATGGTCCTACATTGTAGAAAACCCAAATCCTGAGAATGGAACATGTTACCCAGGGCATTTCGCTGACT
ATGAGGAACTGAGGGAGCAATTGAGTTCAGTATCTTCATTTGAGAGGTTCGAAATATTCCCCAAAGAAAG
CTCATGGCCCAACCACACCGTAACCGGAGTGTCAGCATCATGCTCCCACAATGGGGAAAGCAGTTTTTAC
AGAAATTTGCTATGGCTGACGGGGAAGAATGGTTTGTACCCAAACCTGAGCAAGTCCTATGCAAACAACA
AAGAAAAAGAAGTCCTTGTACTATGGGGTGTTCATCACCCGCCAAACATAGGTGACCAAAAGGCCCTCTA
CCATACAGAAAATGCTTATGTTTCTGTAGTGTCTTCACATTATAGCAGAAAATTCACCCCAGAAATAGCC
AAAAGACCCAAAGTAAGAGATCAAGAAGGAAGAATCAACTACTACTGGACTCTGCTTGAACCCGGGGATA
CAATAATATTTGAGGCAAATGGAAATCTAATAGCGCCAAGATATGCTTTCGCACTGAGTAGAGGCTTTGG

ATCAGGAATCATCAACTCAAATGCACCAATGGATAAATGTGATGCGAAGTGCCAAACACCTCAGGGAGCT
ATAAACAGCAGTCTTCCTTTCCAGAACGTACACCCAGTCACAATAGGAGAGTGTCCAAAGTATGTCAGGA
GTGCAAAATTAAGGATGGTTACAGGACTAAGAAACATCCCATCTATTCAATCCAGAGGTTTGTTTGGAGC
CATTGCCGGTTTCATTGAAGGAGGGTGGACTGGAATGGTAGATGGTTGGTATGGTTATCATCATCAGAAT
GAGCAAGGATCTGGCTATGCTGCAGATCAAAAAAGCACACAAAATGCCATTAATGGGATTACAAACAAGG
TGAATTCTGTAATTGAGAAAATGAACACTCAATTCACAGCAGTGGGCAAAGAATTCAACAAATTGGAAAG
AAGGATGGAAAACTTGAATAAAAAAGTTGATGATGGGTTTATAGACATTTGGACATATAATGCAGAACTG
TTGGTTCTACTGGAAAATGAAAGGACTTTGGATTTCCATGACTCCAATGTGAAGAATCTGTATGAGAAAG
TAAAAAGCCAGTTAAAGAATAATGCTAAAGAAATAGGAAATGGGTGTTTTGAATTCTATCACAAGTGTAA
CGATGAATGCATGGAGAGTGTAAAGAATGGAACTTATGACTATCCAAAATATTCCGAAGAATCAAAGTTA
AACAGGGAGAAAATTGATGGAGTGAAATTGGAATCAATGGGAGTCTATCAGATTCTGGCGATCTACTCAA
CAGTCGCCAGTTCTCTGGTTCTTCTGGTCTCCCTGGGGGCAATCAGCTTCTGGATGTGTTCCAATGGGTC
TTTGCAGTGTAGAATATGCATCTAAGACCAGAATTTCAGAAATATAAGAAA
>gi|224020929|gb|CY037327.1| Influenza A virus (A/Washington/AF06/2007(H1N1)) segment 4, complete sequence
TAAAAACAACCAGAATGAAAGTAAAACTACTGGTCCTGTTATGCACATTTACAGCTACATATGCAGACAC
AATATGTATAGGCTACCATGCTAACAACTCGACCGACACTGTTGACACAGTACTTGAAAAGAATGTGACA
GTGACACACTCTGTCAACCTGCTTGAGAACAGTCACAATGGAAAACTGTGTCTATTAAAAGGAATAGCCC
CACTACAATTGGGTAATTGCAGCGTTGCCGGGTGGATCTTAGGAAACCCAGAATGCGAATTACTGATTTC
CAAGGAGTCATGGTCCTACATTGTAGAAAAACCAAATCCTGAGAATGGAACATGTTACCCAGGGCATTTC
GCTGACTATGAGGAACTGAGGGAGCAATTGAGTTCAGTATCTTCGTTTGAGAGGTTCGAAATATTCCCCA
AAGAAAGCTCATGGCCCAACCACACCGTAACCGGAGTGTCAGCATCATGCTCCCATAATGGGGAAAGCAG
TTTTTACAGAAATTTGCTATGGCTGACGGGGAAGAATGGTTTGTACCCAAACCTGAGCAAGTCCTATGCA
AACAACAAAGAAAAAGAAGTCCTTGTACTATGGGGTGTTCATCACCCGCCAAACATAGGTGACCAAAAGG
CCCTCTATCATACAGAAAATGCTTATGTCTCTGTAGTGTCTTCACATTATAGCAGAAAATTCACCCCAGA
AATAGCCAAAAGACCCAAAGTAAGAGATCAAGAAGGAAGAATCAACTACTACTGGACTCTGCTTGAACCC
GGGGATACTATAATATTTGAGGCAAATGGAAATCTAATAGCGCCAAGATATGCTTTCGCACTGAGTAGAG
GCTTTGGATCAGGAATCATCAACTCAAATGCTCCAATGGATAAATGTGATGCGAAGTGCCAAACACCTCA
GGGAGCTATAAACAGCAGTCTTCCTTTCCAGAACGTACACCCAGTCACAATAGGAGAGTGTCCAAAGTAT
GTCAGGAGTGCAAAATTAAGGATGGTTACAGGACTAAGGAACATCCCATCCATTCAATCCAGAGGTTTGT
TTGGAGCCATTGCCGGTTTCATTGAAGGGGGGTGGACTGGAATGGTAGATGGTTGGTATGGTTATCATCA
TCAGAATGAGCAAGGATCTGGCTATGCTGCAGATCAAAAAAGCACACAAAATGCCATCAATGGGATTACA
AACAAGGTGAATTCTGTAATTGAGAAAATGAACACTCAATTCACAGCAGTGGGCAAAGAATTCAACAAAT
TGGAAAGAAGGATGGAAAACTTGAATAAAAAAGTTGATGATGGGTTTATAGACATTTGGACATATAATGC
AGAACTGTTGGTTCTACTGGAAAATGAAAGGACGTTGGATTTCCATGACTCCAATGTGAAGAATCTGTAT
GAGAAAGTAAAAAGCCAGTTAAAGAATAATGCTAAAGAAATAGGAAATGGGTGTTTTGAATTCTATCACA
AGTGTAACGATGAATGCATGGAGAGTGTAAAGAATGGAACTTATGACTATCCAAAATATTCCGAAGAATC
AAAGTTAAACAGGGAGAAAATTGATGGAGTGAAATTGGAATCAATGGGAGTCTATCAGATTCTAGCGATC
TACTCAACAGTCGCCAGTTCTCTGGTTCTTTTGGTCTCCCTGGGGGCAATCAGCTTCTGGATGTGTTCCA
ATGGGTCTTTGCAGTGTAGAATATGCATCTAAGACCAGAATTTCAGAAATATAAGGAAAAA
>gi|224021234|gb|CY037335.1| Influenza A virus (A/Japan/AF07/2008(H1N1)) segment 4, complete sequence
AATAAAAACAACCAGAATGAAAGCAAAACTACTGGTCCTGTTATGCACATTTACAGCTACATATGCAGAC
ACAATATGTATAGGCTACCATGCTAACAACTCGACCGACACTGTTGACACAGTACTTGAAAAGAATGTGA

CAGTGACGCACTCTGTCAACCTGCTTGAGAACAGTCACAATGGAAAACTATGTCTATTAAAAGGAATAGC
CCCACTACAATTGGGTAATTGCAGCGTTGCCGGTTGGATCTTAGGAAACCCAGAATGCGAATTACTGATT
TCCAAGGAGTCATGGTCCTACATTGTAGAAAAACCAAATCCTGAGAATGGAACATGTTACCCAGGGCATT
TCGCTGACTATGAGGAACTGAGGGAGCAATTGAGTTCAGTATCTTCATTTGAGAGGTTCGAAATATTCCC
CAAAGAAAGCTCATGGCCCAACCACACCGTAACCGGAGTGTCAGCATCATGCTCCCATAATGGGGAAAGC
AGTTTTTACAGAAATTTGCTATGGCTGACGGGGAAGAATGGTTTGTACCCAAACCTGAGCAAGTCCTATG
CAAACAACAAAGAAAAAGAAGTCCTTGTACTATGGGGTGTTCATCACCCGCCAAACATAGGTGACCAAAA
GGCCCTCTATCATACAGAAAATGCTTATGTCTCTGTAGTGTCTTCACATTATAGCAGAAAATTCACCCCA
GAAATAGCCAAAAGACCCAAAGTAAGAGATCAAGAAGGAAGAATCAACTACTACTGGACTCTGCTTGAAC
CCGGGGATACAATAATATTTGAGGCAAATGGAAATCTAATAGCGCCAAGATATGCTTTCGCACTGAGTAG
AGGCTTAGGATCAGGAATCATCAACTCAAATGCACCAATGGATAAATGTGATGCGAAGTGCCAAACACCT
CAGGGAGCTATAAACAGCAGTCTTCCTTTCCAGAACGTACACCCAGTCACAATAGGAGAGTGTCCAAAGT
ATGTCAGGAGTGCAAAATTAAGGATGGTTACAGGACTAAGGAACATCCCATCCATTCAATCCAGAGGTTT
GTTTGGAGCCATTGCCGGTTTCATTGAAGGGGGGTGGACTGGAATGGTAGATGGTTGGTATGGTTATCAT
CATCAGAATGAGCAAGGATCTGGCTATGCTGCAGATCAAAAAAGCACACAAAATGCCATTAATGGGATTA
CAAACAAGGTGAATTCTGTAATTGAGAAAATGAACACTCAATTCACAGCAGTGGGCAAAGAATTCAACAA
ATTGGAAAGAAGGATGGAAAACTTGAATAAAAAAGTTGATGATGGGTTTATAGACATTTGGACATATAAT
GCAGAACTGTTGGTTCTACTGGAAAATGAAAGGACTTTGGATTTCCATGACTCCAATGTGAAGAATCTGT
ATGAGAAAGTAAAAAGCCAGTTAAAGAATAATGCTAAAGAAATAGGAAATGGGTGTTTTGAATTCTATCA
CAAGTGTAACGATGAATGCATGGAGAGTGTAAAGAATGGAACTTATGACTATCCAAAATATTCCGAAGAA
TCAAAGTTAAACAGGGAGAAAATTGATGGAGTGAAATTGGAATCAATGGGAGTCTATCAGATTCTGGCGA
TCTACTCAACAGTCGCCAGTTCTCTGGTTCTTTTGGTCTCCCTGGGGGCAATCAGCTTCTGGATGTGTTC
CAATGGGTCTTTGCAGTGTAGAATATGCATCTAAGACCAGAA
>gi|212381594|gb|FJ445028.1| Influenza A virus (A/England/26/2008(H1N1)) segment 4 hemagglutinin (HA) gene, complete cds
ATGAAAGTAAAACTACTGGTCCTGTTATGCACATTTACAGCTACATATGCAGACACAATATGTATAGGCT
ACCATGCTAACAACTCGACCGACACTGTTGACACAGTACTTGAAAAGAATGTGACAGTGACACACTCTGT
CAACCTGCTTGAGAACAGTCACAACGGAAAACTATGTCTATTAAAAGGAATAGCCCCACTACAATTGGGT
AACTGCAGCGTTGCCGGGTGGATCTTAGGAAACCCAGAATGCGAATTACTGATTTCCAAGGAGTCATGGT
CCTACATTGTAGAAAAACCAAATCCTGAGAATGGAACATGTTACCCAGGGCATTTCGCTGACTATGAGGA
ACTGAGGGAGCAATTGAGTTCAGTATCTTCATTTGAGAGGTTCGAAATATTCCCCAAAGAAAGCTCATGG
CCCAACCACACCGTAACCGGAGTGTCAGCATCATGCTCCCATAATGGGGAAAGCAGTTTTTACAGAAATT
TGCTATGGCTGACGGGGAAGAATGGTTTGTACCCAAACCTGAGCAAGTCCTATGCAAACAACAAAGAAAA
AGAAGTCCTTGTACTATGGGGTGTTCATCACCCGCCAAACATAGGTGACCAAAAGGCCCTCTATCATACA
GAAAATGCTTATGTTTCTGTAGTGTCTTCACATTATAGCAGAAAATTCACCCCAGAAATAGCCAAAAGAC
CCAAAGTAAGAGATCAAGAAGGAAGAATCAACTACTACTGGACTCTGCTTGAACCCGGGGATACAATAAT
ATTTGAGGCAAATGGAAATCTAATAGCGCCAAGATATGCTTTCGCACTGAGTAGAGGCTTTGGATCAGGA
ATCATCAACTCAAATGCACCAGTGGATAAATGTGATGCGAAGTGCCAAACACCTCAGGGAGCTATAAACA
GCAGTCTTCCTTTCCAGAACGTACACCCAGTCACAATAGGAGAGTGTCCAAAGTATGTCAGGAGTGCAAA
ATTAAGGATGGTTACAGGACTAAGGAACATCCCATCCATTCAATCCAGAGGTTTGTTTGGAGCCATTGCC
GGTTTCATTGAAGGGGGGTGGACTGGAATGGTAGATGGTTGGTATGGTTATCATCATCAGAATGAGCAAG
GATCTGGCTATGCTGCAGATCAAAAAAGCACACAAAATGCCATTAATGGGATTACAAACAAGGTGAATTC
TGTAATTGAGAAAATGAACACTCAATTCACAGCAGTGGGCAAAGAATTCAACAAATTGGAAAGAAGGATG
GAAAACTTGAATAAAAAAGTTGATGATGGGTTTATAGACATTTGGACATATAATGCAGAACTGTTGGTTC

TACTGGAAAATGAAAGGACTTTGGATTTCCATGACTCCAATGTGAAGAATCTGTATGAGAAAGTAAAAAG
CCAGTTAAAGAATAATGCTAAAGAAATAGGAAATGGGTGTTTTGAATTCTATCACAAGTGTAACGATGAA
TGCATGGAGAGTGTAAAGAATGGAACTTATGACTATCCAAAATATTCCGAAGAATCAAAGTTAAACAGGG
AGAAAATTGATGGAGTGAAATTGGAATCAATGGGAGTCTATCAGATTCTGGCGATCTACTCAACAGTCGC
CAGTTCTCTGGTTCTTTTGGTCTCCCTGGGGGCAATCAGCTTCTGGATGTGTTCCAATGGGTCTTTGCAG
TGTAGAATATGCATCTAA
>gi|224027204|gb|CY037679.1| Influenza A virus (A/Florida/UR07-0022/2008(H1N1)) segment 4, complete sequence
GAAAATAAAAACAACCAGAATGAAAGTAAAACTACTGGTCCTGTTATGCACATTTACAGCTACATATGCA
GACACAATATGTATAGGCTACCATGCTAACAACTCGACCGACACTGTTGACACAGTACTTGAAAAGAATG
TGACAGTGACACACTCTGTCAACCTGCTTGAGAACAGTCACAATGGAAAACTATGTCTATTAAAAGGAAT
AGCCCCACTACAATTGGGTAATTGCAGCGTTGCCGGGTGGATCTTAGGAAACCCAGAATGCGAATTACTG
ATTTCCAAGGAGTCATGGTCCTACATTGTAGAAAAACCAAATCCTGAGAATGGAACATGTTACCCAGGGC
ATTTCGCTGACTATGAGGAACTGAGGGAGCAATTGAGTTCAGTATCTTCATTTGAGAGGTTCGAAATATT
CCCCAAAGAAAGCTCATGGCCCAACCACACCGTAACCGGAGTGTCAGCATCATGCTCCCATAATGGGGAA
AGCAGTTTTTACAGAAATTTGCTATGGCTGACGGGGAAGAATGGTTTGTACCCAAACCTGAGCAAGTCCT
ATGCAAACAACAAAGAAAAAGAAGTCCTTGTACTATGGGGTGTTCATCACCCGCCAAACATAGGTGACCA
AAAGGCCCTCTATCATACAGAAAATGCTTATGTTTCTGTAGTGTCTTCACATTATAGCAGAAAATTCACC
CCAGAAATAGCCAAAAGACCCAAAGTAAGAGATCAAGAAGGAAGAATCAACTACTACTGGACTCTGCTTG
AACCCGGGGATACAATAATATTTGAGGCAAATGGAAATCTAATAGCGCCAAGATATGCTTTCGCACTGAG
TAGAGGCTTTGGATCAGGAATCATCAACTCAAATGCACCAATGGATAAATGTGATGCGAAGTGCCAAACA
CCTCAGGGAGCTATAAACAGCAGTCTTCCTTTCCAGAACGTACACCCAGTCACAATAGGAGAGTGTCCAA
AGTATGTCAGAAGTGCAAAATTAAGGATGGTTACAGGACTAAGGAACATCCCATCCATTCAATCCAGAGG
TTTGTTTGGAGCCATTGCCGGTTTCATTGAAGGGGGGTGGACTGGAATGGTAGATGGTTGGTATGGTTAT
CATCATCAGAATGAGCAAGGATCAGGCTATGCTGCAGATCAAAAAAGCACACAAAATGCCATTAATGGGA
TTACAAACAAGGTGAATTCTGTAATTGAGAAAATGAACACTCAATTCACAGCAGTGGGCAAAGAATTCAA
CAAATTGGAAAGAAGGATGGAAAACTTGAATAAAAAAGTTGATGATGGGTTTATAGACATTTGGACATAT
AATGCAGAACTGTTGGTTCTACTGGAAAATGAAAGGACTTTGGATTTCCATGACTCCAATGTGAAGAATC
TGTATGAGAAAGTAAAAAGCCAGTTAAAGAACAATGCTAAAGAAATAGGAAATGGGTGTTTTGAATTCTA
TCACAAGTGTAACGATGAATGCATGGAGAGTGTAAAGAATGGAACTTATGACTATCCAAAATATTCCGAA
GAATCAAAGTTAAACAGGGAGAAAATTGATGGAGTGAAATTGGAATCAATGGGAGTCTATCAGATTCTGG
CGATCTACTCAACAGTCGCCAGTTCTCTGGTTCTTTTGGTCTCCCTGGGGGCAATCAGCTTCTGGATGTG
TTCCAATGGGTCTTTGCAGTGTAGAATATGCATCTAAGACCAGAATTTCAGAAATATAAGGAA

>gi|296240579|gb|CY063606.1| Influenza A virus (A/Aalborg/INS133/2009(H1N1)) segment 4, complete sequence
AAAAGCAACAAAAATGAAGGCAATACTAGTAGTTCTGCTATATACATTTGCAACCGCAAATGCAGACACA
TTATGTATAGGTTATCATGCGAACAATTCAACAGACACTGTAGACACAGTACTAGAAAAGAATGTAACAG
TAACACACTCTGTTAACCTTCTAGAAGACAAGCATAACGGGAAACTATGCAAACTAAGAGGGGTAGCCCC
ATTGCATTTGGGTAAATGTAACATTGCTGGCTGGATCCTGGGAAATCCAGAGTGTGAATCACTCTCCACA
GCAAGCTCATGGTCCTACATTGTGGAAACATCTAGTTCAGACAATGGAACGTGTTACCCAGGAGATTTCA
TCGATTATGAGGAGCTAAGAGAGCAATTGAGCTCAGTGTCATCATTTGAAAGGTTTGAGATATTCCCCAA
GACAAGTTCATGGCCCAATCATGACTCGAACAAAGGTGTAACGGCAGCATGTCCTCATGCTGGAGCAAAA
AGCTTCTACAAAAATTTAATATGGCTAGTTAAAAAAGGAAATTCATACCCAAAGCTCAGCAAATCCTACA

TTAATGATAAAGGGAAAGAAGTCCTCGTGCTATGGGGCATTCACCATCCATCTACTAGTGCTGACCAACA
AAGTCTCTATCAGAATGCAGATGCATATGTTTTTGTGGGGACATCAAGATACAGCAAGAAGTTCAAGCCG
GAAATAGCAATAAGACCCAAAGTGAGGGATCAAGAAGGGAGAATGAACTATTACTGGACACTAGTAGAGC
CGGGAGACAAAATAACATTCGAAGCAACTGGAAATCTAGTGGTACCGAGATATGCATTCGCAATGGAAAG
AAATGCTGGATCTGGTATTATCATTTCAGATACACCAGTCCACGATTGCAATACAACTTGTCAGACACCC
AAGGGTGCTATAAACACCAGCCTCCCATTTCAGAATATACATCCGATCACAATTGGAAATTGTCCAAAAT
ATGTAAAAAGCACAAAATTGAGACTGGCCACAGGATTGAGGAATGTCCCGTCTATTCAATCTAGAGGCCT
ATTTGGGGCCATTGCCGGTTTCATTGAAGGGGGGTGGACAGGGATGGTAGATGGATGGTACGGTTATCAC
CATCAAAATGAGCAGGGGTCAGGATATGCAGCCGACCTGAAGAGCACACAGAATGCCATTGACAAGATTA
CTAACAAAGTAAATTCTGTTATTGAAAAGATGAATACACAGTTCACAGCAGTAGGTAAAGAGTTCAACCA
CCTGGAAAAAAGAATAGAGAATTTAAATAAAAAAGTTGATGATGGTTTCCTGGACATTTGGACTTACAAT
GCCGAACTGTTGGTTCTATTGGAAAATGAAAGAACTTTGGACTACCACGATTCAAATGTGAAGAACTTAT
ATGAAAAGGTAAGAAGCCAGTTAAAAAACAATGCCAAGGAAATTGGAAACGGCTGCTTTGAATTTTACCA
TAAATGCGATAACACGTGCATGGAAAGTGTCAAAAATGGGACTTATGACTACCCAAAATACTCAGAGGAA
GCAAAATTAAACAGAGAAGAAATAGATGGGGTAAAGCTGGAATCAACAAGGATTTACCAGATTTTGGCGA
TCTATTCAACTGTCGCCAGTTCATTGGTACTGATAGTCTCCCTGGGGGCAATCAGTTTCTGGATGTGCTC
TAATGGGTCTCTACAGTGTAGAATATGTATTTAACATTAGGATTTCAGAAGCAT
>gi|296240561|gb|CY063598.1| Influenza A virus (A/Bonn/INS128/2009(H1N1)) segment 4, complete sequence
AAAAGCAACAAAAATGAAGGCAATACTAGTAGTTCTGCTATATACATTTGCAACCGCAAATGCAGACACA
TTATGTATAGGTTATCATGCGAACAATTCAACAGACACTGTAGACACAGTACTAGAAAAGAATGTAACAG
TAACACACTCTGTTAACCTTCTAGAAGACAAGCATAACGGGAAACTATGCAAACTAAGAGGGGTAGCCCC
ATTGCATTTGGGTAAATGTAACATTGCTGGCTGGATCCTGGGAAATCCAGAGTGTGAATCACTCTCCACA
GCAAGCTCATGGTCCTACATTGTGGAAACATCTAGTTCAGACAATGGAACGTGTTACCCAGGAGATTTCA
TCGATTATGAGGAGCTAAGAGAGCAATTGAGCTCAGTGTCATCATTTGAAAGGTTTGAGATATTCCCCAA
GACAAGTTCATGGCCCAATCATGACTCGAACAAAGGTGTAACGGCAGCATGTCCTCATGCTGGAGCAAAA
AGCTTCTACAAAAATTTAATATGGCTAGTTAAAAAAGGAAATTCATACCCAAAGCTCAGCAAATCCTACA
TTAATGATAAAGGGAAAGAAGTCCTCGTGCTATGGGGCATTCACCATCCATCTACTAGTGCTGACCAACA
AAGTCTCTATCAGAATGCAGATGCATATGTTTTTGTGGGGACATCAAGATACAGCAAGAAGTTCAAGCCG
GAAATAGCAATAAGACCCAAAGTGAGGGATCAAGAAGGGAGAATGAACTATTACTGGACACTAGTAGAGC
CGGGAGACAAAATAACATTCGAAGCAACTGGAAATCTAGTGGTACCGAGATATGCATTCGCAATGGAAAG
AAATGCTGGATCTGGTATTATCATTTCAGATACACCAGTCCACGATTGCAATACAACTTGTCAGACACCC
AAGGGTGCTATAAACACCAGCCTCCCATTTCAGAATATACATCCGATCACAATTGGAAAATGTCCAAAAT
ATGTAAAAAGCACAAAATTGAGACTGGCCACAGGATTGAGGAATGTCCCGTCTATTCAATCTAGAGGCCT
ATTTGGGGCCATTGCCGGTTTCATTGAAGGGGGGTGGACAGGGATGGTAGATGGATGGTACGGTTATCAC
CATCAAAATGAGCAGGGGTCAGGATATGCAGCCGACCTGAAGAGCACACAGAATGCCATTGACAAGATTA
CTAACAAAGTAAATTCTGTTATTGAAAAGATGAATACACAGTTCACAGCAGTAGGTAAAGAGTTCAACCA
CCTGGAAAAAAGAATAGAGAATTTAAATAAAAAAGTTGATGATGGTTTCCTGGACATTTGGACTTACAAT
GCAGAACTGTTGGTTCTATTGGAAAATGAAAGAACTTTGGACTACCACGATTCAAATGTGAAGAACTTAT
ATGAAAAGGTAAGAAGCCAGTTAAAAAACAATGCCAAGGAAATTGGAAACGGCTGCTTTGAATTTTACCA
CAAATGCGATAACACGTGCATGGAAAGTGTCAAAAATGGGACTTATGACTACCCAAAATACTCAGAGGAA
GCAAAATTAAACAGAGAAGAAATAGATGGGGTAAAGCTGGAATCAACAAGGATTTACCAGATTTTGGCGA
TCTATTCAACTGTCGCCAGTTCATTGGTACTGGTAGTCTCCCTGGGGGCAATCAGTTTCTGGATGTGCTC
TAATGGGTCTCTACAGTGTAGAATATGTATTTAACATTAGGATTTCAGAAGCAT

>gi|296240309|gb|CY063486.1| Influenza A virus (A/Boston/110/2009(H1N1)) segment 4, complete sequence
AAAAGCAACAAAAATGGAGGCAATACTAGTAGTTCTGCTATATACATTTGCAACCGCAAATGCAGACACA
TTATGTATAGGTTATCATGCGAACAATTCAACAGACACTGTAGACACAGTACTAGAAAAGAATGTAACAG
TAACACACTCTGTTAACCTTCTAGAAGACAAGCATAATGGGAAACTATGCAAACTAAGAGGGGTAGCCCC
ATTGCATTTGGGTAAATGTAACATTGCTGGCTGGATCCTGGGAAATCCAGAGTGTGAATCACTCTCCACA
GCAAGCTCATGGTCCTACATTGTGGAAACATCTAGTTCAGACAATGGAACGTGTTACCCAGGAGATTTCA
TCGATTATGAGGAGCTAAGAGAGCAATTGAGCTCAGTGTCATCATTTGAAAGGTTTGAGATATTCCCCAA
GACAAGTTCATGGCCCAATCATGACTCGAACAAAGGTGTAACGGCAGCATGTCCTCATGCTGGAGCAAAA
AGCTTCTACAAAAATTTAATATGGCTAGTTAAAAAAGGAAATTCATACCCAAAGCTCAGCAAATCCTACA
TTAATGATAAAGGGAAAGAAGTCCTCGTGCTATGGGGCATTCACCATCCATCTACTAGTGCTGACCAACA
AAGTCTCTATCAGAATGCAGATGCATATGTTTTTGTGGGGTCATCAAGATACAGCAAGAAGTTCAAGCCG
GAAATAGCAATAAGACCCAAAGTGAGGGATCAGGAAGGGAGAATGAACTATTACTGGACACTAGTAGAGC
CGGGAGACAAAATAACATTCGAAGCAACTGGAAATCTAGTGGTACCGAGATATGCATTCGCAATGGAAAG
AAATGCTGGATCTGGTATTATCATTTCAGATACACCAGTCCACGATTGCAATACAACTTGTCAGACACCC
AAGGGTGCTATAAACACCAGCCTCCCATTTCATAATATACATCCGATCACAATTGGAAAATGTCCAAAAT
ATGTAAAAAGCACAAAATTGAGACTGGCCACAGGATTGAGGAATGTCCCGTCTATTCAATCTAGAGGCCT
ATTTGGGGCCATTGCCGGTTTCATTGAAGGGGGGTGGACAGGGATGGTAGATGGATGGTACGGTTATCAC
CATCAAAATGAGCAGGGGTCAGGATATGCAGCCGACCTGAAGAGCACACAGAATGCCATTGACGAGATTA
CTAACAAGGTAAATTCTGTTATTGAAAAGATGAATACACAGTTCACAGCAGTAGGTAAAGAGTTCAACCA
CCTGGAAAAAAGAATAGAGAATTTAAATAAAAAAGTTGATGATGGTTTCCTGGACATTTGGACTTACAAT
GCCGAACTGTTGGTTCTATTGGAAAATGAAAGAACTTTGGACTACCACGATTCAAATGTGAAGAACTTAT
ATGAAAAGGTAAGAAGCCAGTTAAAAAACAATGCCAAGGAAATTGGAAACGGCTGCTTTGAATTTTACCA
CAAATGCGATAACACGTGCATGGAAAGTGTCAAAAATGGGACTTATGACTACCCAAAATACTCAGAGGAA
GCAAAATTAAACAGAGAAGAAATAGATGGGGTAAAGCTGGAATCAACAAGGATTTACCAGATTTTGGCGA
TCTATTCAACTGTCGCCAGTTCATTGGTACTGGTAGTCTCCCTGGGGGCAATCAGTTTCTGGATGTGCTC
TAATGGGTCTCTACAGTGTAGAATATGTATTTAACATTAGGATTTCAGAAGCAT
>gi|296240615|gb|CY063622.1| Influenza A virus (A/New York/INS150/2009(H1N1)) segment 4, complete sequence
ATGAAGGCAATACTAGTAGTTCTGCTATATACATTTGCAACCGCAAATGCAGACACATTATGTATAGGTT
ATCATGCGAACAATTCAACAGACACTGTAGACACAGTACTAGAAAAGAATGTAACAGTAACACACTCTGT
TAACCTTCTAGAAGACAAGCATAACGGGAAATTATGCAAACTAAGAGGGGTAGCCCCATTGCATTTGGGT
AAATGTAACATTGCTGGCTGGATCTTGGGAAATCCAGAGTGTGAATCACTCTCCACAGCAAGCTCATGGT
CCTACATTGTGGAAACATCTAGTTCAGACAATGGAACGTGTTACCCAGGAGATTTCATCGATTATGAGGA
GCTAAGAGAGCAATTGAGCTCAGTGTCATCATTTGAAAGGTTTGAGATATTCCCCAAGACAAGTTCATGG
CCCAATCATGACTCGAACAAGGGTGTAACGGCAGCATGTCCTCATGCTGGAGCAAAAAGCTTCTACAAAA
ATTTAATATGGCTAGTTAAAAAAGGAAATTCATACCCAAAGCTCAGCAAATCCTACATTAATGATAAAGG
GAAAGAAGTCCTCGTGCTATGGGGCATTCACCATCCATCTACTAGTGCTGACCAACAAAGTCTCTATCAG
AATGCAGATGCATATGTTTTTGTAGGGACATCAAGATACAGCAAGAAGTTCAAGCCGGAAATAGCAATAA
GACCCAAAGTGAGGGATCAAGAAGGGAGAATGAACTATTACTGGACACTAGTGGAGCCGGGAGACAAAAT
AACATTCGAAGCAACTGGAAATCTAGTGGTACCGAGATATGCATTCGCAATGGAAAGAAATGCTGGATCT
GGTATTATCATTTCAGATACACCAGTCCACGATTGCAATACAACTTGTCAGACACCCAAGGGTGCTATAA
ACACCAGCCTCCCATTTCAGAATATACATCCGATCACAATTGGAAAATGTCCAAAATATGTAAAAAGCAC
AAAATTGAGACTGGCCACAGGATTGAGGAATGTCCCGTCTATTCAATCTAGAGGCCTATTTGGGGCCATT

GCCGGTTTCATTGAAGGGGGGTGGACAGGGATGGTAGATGGATGGTACGGTTATCACCATCAAAATGAGC
AGGGGTCAGGATATGCAGCCGACCTGAAGAGCACACAGAATGCCATTGACAAGATTACTAACAAAGTAAA
TTCTGTTATTGAAAAGATGAATACACAGTTCACAGCAGTAGGTAAAGAGTTCAACCACCTGGAAAAAAGA
ATAGAGAATTTAAATAAAAAAGTTGATGATGGTTTCCTGGACATTTGGACTTACAATGCCGAACTGTTGG
TTCTATTGGAAAATGAAAGAACTTTGGACTACCACGATTCAAATGTGAAGAACTTATATGAAAAGGTAAG
AAGCCAGTTAAAAAACAATGCCAAGGAAATTGGAAACGGCTGCTTTGAATTTTACCACAAATGCGATAAC
ACGTGCATGGAAAGTGTCAAAAATGGGACTTATGACTACCCAAAATACTCAGAGGAAGCAAAATTAAACA
GAGAAGAAATAGATGGGGTAAAGCTGGAATCAACAAGGATTTACCAGATTTTGGCGATCTATTCAACTGT
CGCCAGTTCATTGGTACTGGTAGTCTCCCTGGGGGCAATCAGTTTCTGGATGTGCTCTAATGGGTCTCTA
CAGTGTAGAATATGTATTTAACATTAGGATTTCAGAAGCAT

>gi|296240543|gb|CY063590.1| Influenza A virus (A/Athens/INS122/2009(H1N1)) segment 4, complete sequence

AAAAGCAACAAAAATGAAGGCAATACTAGTAGTTCTGCTATATACATTTGCAACCGCAAATGCAGACACA
TTATGTATAGGTTATCATGCGAACAATTCAACAGACACTGTAGACACAGTACTAGAAAAGAATGTAACAG
TAACACACTCTGTTAACCTTCTAGAAGACAAGCATAACGGGAAACTATGCAAACTAAGAGGGGTAGCCCC
ATTGCATTTGGGTAAATGTAACATTGCTGGCTGGATCCTGGGAAATCCAGAGTGTGAATCACTCTCCACA
GCAAGCTCATGGTCCTACATTGTGGAAACATCTAGTTCAGACAATGGAACGTGTTACCCAGGAGATTTCA
TCGATTATGAGGAGCTAAGAGAGCAATTGAGCTCAGTGTCATCATTTGAAAGGTTTGAGATATTCCCCAA
GACAAGTTCATGGCCCAATCATGACTCGAACAAAGGTGTAACGGCAGCATGTCCTCATGCTGGAGCAAAA
AGCTTCTACAAAAATTTAATATGGCTAGTTAAAAAAGGAAATTCATACCCAAAGCTCAGCAAATCCTACA
TTAATGATAAAGGGAAAGAAGTCCTCGTGCTATGGGGCATTCACCATCCATCTACTAGTGCTGACCAACA
AAGTCTCTATCAGAATGCAGATGCATATGTTTTTGTGGGGACATCAAGATACAGCAAGAAGTTCAAGCCG
GAAATAGCAATAAGACCCAAAGTGAGGGATCAAGAAGGGAGAATGAACTATTACTGGACACTAGTAGAGC
CGGGAGACAAAATAACATTCGAAGCAACTGGAAATCTAGTGGTACCGAGATATGCATTCGCAATGGAAAG
AAATGCTGGATCTGGTATTATCATTTCAGATACACCAGTTCACGATTGCAATACAACTTGTCAGACACCC
AAGGGTGCTATAAACACCAGCCTCCCATTTCAGAATATACATCCGATCACAATTGGAAAATGTCCAAAAT
ATGTAAAAAGCACAAAATTGAGACTGGCCACAGGATTGAGGAATGTCCCGTCTATTCAATCTAGAGGCCT
ATTTGGGGCCATTGCCGGTTTCATTGAAGGGGGGTGGACAGGGATGGTAGATGGATGGTACGGTTATCAC
CATCAAAATGAGCAGGGGTCAGGATATGCAGCCGACCTGAAGAGCACACAGAATGCCATTGACGAGATTA
CTAATAAAGTAAATTCTGTTATTGAAAAGATGAATACACAGTTCACAGCAGTAGGTAAAGAGTTCAACCA
CCTGGAAAAAAGAATAGAGAATTTAAATAAAAAAGTTGATGATGGTTTCCTGGACATTTGGACTTACAAT
GCCGAACTGTTGGTTCTATTGGAAAATGAAAGAACTTTGGACTACCACGATTCAAATGTGAAGAACTTAT
ATAAAAAGGTAAGAAGCCAGTTAAAAAACAATGCCAAGGAAATTGGAAACGGCTGCTTTGAATTTTACCA
CAAGTGCGATAACACGTGCATGGAAAGTGTCAAAAATGGGACTTATGACTACCCAAAATACTCAGAGGAA
GCAAAATTAAACAGAGAAGAAATAGATGGGGTAAAGCTGGAATCAACAAGGATTTACCAGATTTTGGCGA
TCTATTCAACTGTCGCCAGTTCATTGGTACTGGTAGTCTCCCTGGGGGCAATCAGTTTCTGGATGTGCTC
TAATGGGTCTCTACAGTGTAGAATATGTATTTAATATTAGGATTTCAGAAGCAT

>gi|402784380|dbj|AB745263.1| Influenza A virus (A/Tottori/ST505/2010(H1N1)) HA gene for hemagglutinin, complete cds

AAAACAAAAGCAACAAAAATGAAGGCAATACTAGTAGTTCTGCTATATACATTTGCAACCGCAAATGCAG
ACACATTATGTATAGGTTATCATGCGAACAATTCAACAGACACTGTAGACACAATACTAGAAAAGAATGT
AACAGTAACACACTCTGTTAACCTTCTAGAAGACAAGCATAACGGGAAACTATGCAAACTAAGAGGGGTA
GCCCCATTGCATTTGGGTAAATGTAACATTGCTGGCTGGATCCTGGGAAATCCAGAGTGTGAATCACTCT
CCACAGCAAGCTCATGGTCCTACATTGTGGAAACATCTAGTTCAGACAATGGAACGTGTTACCCAGGAGA

TTTCATCGATTATGAGGAGCTAAGAGAGCAATTGAGCTCAGTGTCATCATTTGAAAGGTTTGAGATATTC
CCCAAGACAAGTTCATGGCCCAATCATGACTCGAACAAAGGTGTAACGGCAGCCTGTCCTCATGCTGGAG
CAAAAAGCTTCTACAAAAATTTAATATGGCTAGTTAAAAAAGGAAATTCATACCCAAAGCTCAGCAAATC
CTACATTAATGATAAAGGGAAAGAAGTCCTCGTGCTATGGGGCATTCACCATCCATCTACTAGTGCTGAC
CAACAAAGTCTCTATCAGAATGCAGATGCATATGTTTTTGTGGGGACATCAAGATACAGCAAGAAGTTCA
AGCCGGAAATAGCAATAAGACCCAAAGTGAGGGATCAAGAAGGGAGAATGAACTATTACTGGACACTAGT
AGAGCCGGGAGACAAAATAACATTCGAAGCAACTGGAAATCTAGTGGTACCGAGATATGCATTCGCAATG
GAAAGAAATGCTGGATCTGGTATTATCATTTCAGATACACCAGTCCACGATTGCAATACAACTTGTCAGA
CACCCAAGGGTGCTATAAACACCAGCCTCCCATTTCAGAATATACATCCGATCACAATTGGAAAATGTCC
AAAATATGTAAAAAGCACAAAATTGAGACTGGCCACAGGATTGAGGAATGTCCCGTCTATTCAATCTAGA
GGCCTATTTGGGGCCATTGCCGGTTTCATTGAAGGGGGGTGGACAGGGATGGTAGATGGATGGTACGGTT
ATCACCATCAAAATGAGCAGGGGTCAGGATATGCAGCCGACCTGAAGAGCACACAGAATGCCATTGACGA
GATTACTAACAAAGTAAATTCTGTTATTGAAAAGATGAATACACAGTTCACAGCAGTAGGTAAAGAGTTC
AACCACCTAGAAAAAAGAATAGAGAATTTAAATAAAAAGGTTGATGATGGTTTCCTTGACATTTGGACTT
ACAATGCCGAACTGTTGGTTCTATTGGAAAATGAAAGAACTTTGGACTACCACGATTCAAATGTGAAGAA
CTTATATGAAAAGGTAAGAAGCCAGTTAAAAAACAATGCCAAGGAAATTGGAAACGGCTGCTTTGAATTT
TACCACAAATGCGATAACACGTGCATGGAAAGTGTCAAAAATGGGACTTATGACTACCCAAAATACTCAG
AGGAAGCAAAATTAAACAGAGAAGAAATAGATGGGGTAAAGCTGGAATCAACAAGGATTTACCAGATTTT
GGCGATCTATTCAACTGTCGCCAGTTCATTGGTACTGGTAGTCTCCCTGGGGGCAATCAGTTTCTGGATG
TGCTCTAATGGGTCTCTACAGTGTAGAATATGTATTTAACATTAGGATTTCAGAAGCATGAGAA
>gi|402783820|dbj|AB745278.1| Influenza A virus (A/Tottori/ST511/2010(H1N1)) HA gene for hemagglutinin, complete cds
AAAACAAAAGCAACAAAAATGAAGGCAATACTAGTAGTTCTGCTATATACATTTGCAACCGCAAATGCAG
ACACATTATGTATAGGTTATCATGCGAACAATTCAACAGACACTGTAGACACAGTACTAGAAAAGAATGT
AACAGTAACACACTCTGTTAATCTTCTAGAAGACAAGCATAACGGAAAACTATGCAAACTAAGAGGGGTA
GCCCCATTGCATTTGGGTAAATGTAACATTGCTGGCTGGATCCTGGGAAATCCAGAGTGTGAATCACTCT
CCACAGCAAGCTCATGGTCCTACATTGTGGAAACATCTAGTTCAGACAATGGAACGTGTTACCCAGGAGA
TTTCATCGATTATGAGGAGCTAAGAGAGCAATTGAGCTCAGTGTCATCATTTGAAAGGTTTGAGATATTC
CCCAAGACAAGTTCATGGCCCAATCATGACTCGAACAAAGGTGTAACGGCAGCATGTCCTCATGCTGGAG
CAAAAAGCTTCTACAAAAATTTAATATGGCTAGTTAAAAAAGGAAATTCATACCCAAAGCTCAGCAAATC
CTACATTAATGATAAAGGGAAAGAAGTCCTCGTGCTATGGGGCATTCACCATCCATCTACTACTGCTGAC
CAACAAAGTCTCTATCAGAATGCAGATACATATGTTTTTGTGGGGACATCAAGATACAGCAAGAAGTTCA
AGCCGGAAATAGCAATAAGACCCAAAGTGAGGGATCAAGAAGGGAGAATGAACTATTACTGGACACTAGT
AGAGCCGGGAGACAAAATAACATTCGAAGCAACTGGAAATCTAGTGGTACCGAGATATGCATTCGCAATG
GAAAGAAATGCTGGATCTGGTATTATCATTTCAGATACACCAGTCCACGATTGCAATACAACTTGTCAGA
CACCCAAGGGTGCTATAAACACCAGCCTCCCATTTCAAGATATACATCCGATCACAATTGGAAAATGTCC
AAAATATGTAAAAAGCACAAAATTGAGACTGGCCACAGGATTGAGGAATGTCCCGTCTATTCAATCTAGA
GGCCTATTTGGGGCCATTGCCGGCTTCATTGAAGGGGGGTGGACAGGGATGGTAGATGGATGGTACGGTT
ATCACCATCAAAATGAGCAGGGGTCAGGATATGCAGCCGACCTGAAGAGCACACAGAATGCCATTGACAA
GATTACTAACAAAGTAAATTCTGTTATTGAAAAGATGAATACACAGTTCACAGCAGTAGGTAAAGAGTTC
AACCACCTGGAAAAAAGAATAGAGAATTTAAATAAAAAAGTTGATGATGGTTTCCTGGACATTTGGACTT
ACAATGCCGAACTGTTGGTTCTATTGGAAAATGAAAGAACTTTGGACTACCACGATTCAAATGTGAAGAA
CTTATATGAAAAAGTAAGAAACCAGTTAAAAAACAATGCCAAGGAAATTGGAAATGGCTGCTTTGAATTT
TACCACAAATGCGATAACACGTGCATGGAAAGTGTCAAAAATGGGACTTATGACTACCCAAAATACTCAG

AGGAAGCAAAATTAAACAGAGAAAAAATAGATGGGGTAAAGCTGGAATCAACAAGGATTTACCAGATTTT
GGCGATCTATTCAACTGTCGCCAGTTCATTGGTACTGGTAGTCTCCCTGGGGGCAATCAGTTTTTGGATG
TGCTCTAATGGGTCTCTACAGTGTAGAATATGTATTTAACATTAGGATTTCAGAAGCATGAGAA
>gi|374256412|gb|JQ365014.1| Influenza A virus (A/Hangzhou/178/2010(H1N1)) segment 4 hemagglutinin (HA) gene, complete cds
ATGAAGGCAATACTAGTAGTTCTGCTATATACATTTGCAACCGCAAATGCAGACACATTATGTATAGGTT
ATCATGCGAACAATTCAACAGACACTGTAGACACAGTACTAGAAAAGAATGTAACAGTAACACACTCTGT
TAACCTTCTAGAAGACAAGCATAACGGGAAATTATGCAAACTAAGAGGGGTAGCCCCATTGCATTTGGGT
AAATGTAACATTGCTGGCTGGATCCTGGGAAATCCAGAGTGTGAATCACTCTCCACAGCAAGCTCATGGT
CCTACATTGTGGAAACATCTAGTTCAGACAATGGAACGTGTTACCCAGGAGATTTCATCGATTATGAGGA
GCTAAGAGAGCAATTGAGCTCAGTGTCATCATTTGAAAGGTTTGAGATATTCCCCAAGACAAGTTCATGG
CCCAATCATGACTCGAACAAAGGTGTAACGGCAGCATGTCCTCATGCTGGAGCAAAAAGCTTCTACAAAA
ATTTAATATGGCTAGTTAAAAAAGGAAATTCATACCCAAAGCTCAGCAAATCCTACATTAATGATAAAGG
GAAAGAAGTCCTCGTGCTATGGGGCATTCACCATCCATCTACTAGTGCTGACCAACAAAGTCTCTATCAG
AATGCAGATGCATATGTTTTTGTGGGGACATCAAGATACAGCAAGAAGTTCAAGCCGGAAATAGCAATAA
GACCCAAAGTGAGGGATCAAGAAGGGAGAATGAACTATTACTGGACACTAGTAGAGCCGGGAGACAAAAT
AACATTCGAAGCAACTGGAAATCTAGTGGTACCGAGATATGCATTCTCAATGGAAAGAAATGCTGGATCT
GGTATTATCATTTCAGATACACCAGTCCACGATTGCAATACAACTTGTCAGACACCCAAGGGTGCTATAA
ACACCAGCCTCCCATTTCAGAATATACATCCGATCACAATTGGAAAATGTCCAAAATATGTAAAAAGCAC
AAAATTGAGACTGGCCACAGGATTGAGGAATGTCCCGTCTATTCAATCTAGAGGCCTATTTGGGGCCATT
GCCGGTTTTATTGAAGGGGGGTGGACAGGGATGGTAGATGGATGGTACGGTTATCACCATCAAAATGAGC
AGGGGTCAGGATATGCAGCCGACCTGAAGAGCACACAGAATGCCATTGACGAGATTACTAACAAAGTAAA
TTCTGTTATTGAAAAGATGAATACACAGTTCACAGCAGTAGGTAAAGAGTTCAACCACCTGGAAAAAAGA
ATAGAGAATTTAAATAAAAAGGTTGATGATGGTTTCCTGGACATTTGGACTTACAATGCCGAACTGTTGG
TTCTATTGGAAAATGAAAGAACTTTGGACTACCACGATTCAAATGTGAAGAACTTATATGAAAAGGTAAG
AAGCCAGTTAAAAAACAATGCCAAGGAAATTGGAAACGGCTGCTTTGAATTTTACCACAAATGCGATAAC
ACGTGCATGGAAAGTGTCAAAAATGGGACTTATGACTACCCAAAATACTCAGAGGAAGCAAAATTAAACA
GAGAAGAAATAGATGGGGTAAAGCTGGAATCAACAAGGATTTACCAGATTTTGGCGATCTATTCAACTGT
CGCCAGTTCATTGGTACTGGTAGTCTCCCTGGGGGCAATCAGTTTCTGGATGTGCTCTAATGGGTCTCTA
CAGTGTAGAATATGTATTTAA
>gi|377520207|gb|CY096809.1| Influenza A virus (A/Thailand/ICRC-NMA1/2010(H1N1)) hemagglutinin (HA) gene, complete cds
ATGAAGGCAATACTAGTAGTTCTGCTATATACATTTGCAACCGCAAATGCAGACACATTATGTATAGGTT
ATCATGCGAACAATTCAACAGACACTGTAGACACAGTACTAGAAAAGAATGTAACAGTAACACACTCTGT
TAACCTTCTAGAAGACAAGCATAACGGGAAACTATGCAAACTAAGAGGGGTAGCCCCATTGCATTTGGGT
AAATGTAACATTGCTGGCTGGATCCTGGGAAATCCAGAGTGTGAATCACTCTCCACAGCAAGCTCATGGT
CCTACATTGTGGAAACATCTAGTTCAGACAATGGAACGTGTTACCCAGGAGATTTCATCGATTATGAGGA
GCTAAGAGAGCAATTGAGCTCAGTGTCATCATTTGAAAGGTTTGAGATATTCCCCAAGACAAGTTCATGG
CCCAATCATGACTCGAACAAAGGTGTAACGGCAGCATGTCCTCATGCTGGAGCAAAAAGCTTCTACAAAA
ATTTAATATGGCTAGTTAAAAAAGGAAATTCATACCCAAAGCTCAGCAAATCCTACATTAATGATAAAGG
GAAAGAAGTCCTCGTGCTATGGGGCATTCACCATCCATCTACTAGTGCTGACCAACAAAGTCTCTATCAG
AATGCAGATGCATATGTTTTTGTGGGGACATCAAGATACAGCAAGAAGTTCAAGCCGGAAATAGCAATAA
GACCCAAAGTGAGGGATCAAGAAGGGAGAATGAACTATTACTGGACACTAGTAGAGCCGGGAGACAAAAT
AACATTCGAAGCAACTGGAAATCTAGTGGTACCGAGATATGCATTCGCAATGGAAAGAAATGCTGGATCT

GGTATTATCATTTCAGACACACCAGTCCACGATTGCAATACAACTTGTCAGACACCCAAGGGTGCTATAA
ACACCAGCCTCCCATTTCAGAATATACATCCGATCACAATTGGAAAATGTCCAAAATATGTAAAAAGCAC
AAAATTGAGACTGGCCACAGGATTGAGGAATGTCCCGTCTATTCAATCTAGAGGCCTATTTGGGGCCATT
GCCGGTTTCATTGAAGGGGGGTGGACAGGGATGGTAGATGGATGGTACGGTTATCACCATCAAAATGAGC
AGGGGTCAGGATATGCAGCCGACCTGAAGAGCACACAGAATGCCATTGACAAGATTACTAACAAAGTAAA
TTCTGTTATTGAAAAGATGAATACACAGTTCACAGCAGTAGGTAAAGAGTTCAACCACCTGGAAAAAAGA
ATAGAGAATTTAAATAAAAAAGTTGATGATGGTTTCCTGGACATTTGGACTTACAATGCCGAACTGTTGG
TTCTATTGGAAAATGAAAGAACTTTGGACTACCACGATTCAAATGTGAAGAACTTATATGAAAAGGTAAG
AAGCCAGTTAAAAAACAATGCCAAGGAAATTGGAAACGGCTGCTTTGAATTTTACCACAAATGCGATAAC
ACGTGCATGGAAAGTGTCAAAAATGGGACTTATGACTACCCAAAATACTCAGAGGAGGCAAAATTAAACA
GAGAAGAAATAGATGGGGTGAAGCTGGAATCAACAAGGATTTACCAGATTTTGGCGATCTATTCAACTGT
CGCCAGTTCATTGGTACTGGTAGTCTCCCTGGGGGCAATCAGTTTCTGGATGTGCTCTAATGGGTCTCTA
CAGTGTAGAATATGTATTTAA

>gi|399226457|gb|JX309997.1| Influenza A virus (A/Singapore/TT495/2010(H1N1)) segment 4 hemagglutinin (HA) gene, complete cds

AGCAAAAGCAGGGGAAAACAAAAGCAACAAAAATGAAGGCAATACTAGTAGTTCTGCTATATACATTTGC
AACCGCAAATGCAGACACATTATGTATAGGTTACCATGCGAACAATTCAACAGACACTGTAGACACAGTA
CTAGAAAAGAATGTAACAGTAACACACTCTGTCAATCTTCTAGAAGACAAGCATAACGGGAAACTATGCA
AACTAAGAGGGGTAGCCCCATTGCATTTGGGTAAATGTAACATTGCTGGCTGGATCCTGGGAAATCCAGA
GTGTGAATCACTCTCCACAGCAAGCTCATGGTCCTACATTGTGGAAACATCTAGTTCAGACAATGGAACG
TGTTACCCAGGAGATTTCATTGATTATGAGGAGCTAAGAGAGCAATTGAGCTCAGTGTCATCATTTGAAA
GATTTGAGATATTCCCCAAGACAAGTTCATGGCCCAATCATGACTCGAACAAAGGTGTAACGGCAGCATG
TCCTCATGCTGGAGCAAAAGGCTTCTACAAAAATTTAATATGGCTAGTTAAAAAAGGAAATTCATACCCA
AAGCTCAGCAAATCCTACATTAATGATAAAGGGAAAGAAGTCCTCGTGCTATGGGGCATTCACCATCCAT
CTACTACTGCTGACCAACAAAGTCTCTATCAGAATGCAGATACATATGTTTTTGTGGGGACATCAAGATA
CAGCAAGAAGTTCAAGCCGGAAATAGCAATAAGACCCAAAGTGAGGGATCAAGAAGGGAGAATGAACTAT
TACTGGACAATAGTAGAGCCGGGAGACAAAATAACATTCGAAGCAACTGGAAATCTAGTGGTACCGAGAT
ATGCATTCGCAATGGAAAGAAATGCTGGATCTGGTATTATCATTTCAGATACACCAGTCCACGATTGCAA
TACAACTTGTCAGACACCCAAGGGTGCTATAAACACCAGCCTCCCATTTCAGAATATACATCCGATCACA
ATTGGGAAATGTCCAAAATATGTAAAAAGCACCAAATTGAGACTGGCCACAGGATTGAGGAATGTCCCGT
CTATTCAATCTAGAGGCCTATTTGGGGCCATTGCCGGCTTCATTGAAGGGGGGTGGACAGGGATGGTAGA
TGGATGGTACGGTTATCACCATCAAAATGAGCAGGGGTCAGGATATGCAGCCGACCTGAAGAGCACACAG
AATGCCATTGACAAGATTACTAACAAAGTAAATTCTGTTATTGAAAAGATGAATACACAGTTCACAGCAG
TAGGTAAAGAGTTCAACCACCTGGAAAAAAGAATAGAGAATTTAAATAAAAAAGTTGATGATGGTTTCCT
GGACATTTGGACTTACAATGCCGAACTGTTGGTTCTATTGGAAAATGAAAGAACTTTGGACTACCACGAT
TCAAATGTGAAGAACTTATATGAAAAGGTAAGAAACCAGTTAAAAAACAATGCCAAGGAAATTGGAAATG
GCTGCTTTGAATTTTACCACAAATGCGATAACACGTGCATGGAAAGTGTCAAAAATGGGACTTATGACTA
CCCAAAATACTCAGAGGAAGCAAAATTAAACAGAGAAGAAATAGATGGGGTAAAGCTGGAATCAACAAGG
ATTTACCAGATTTTGGCGATCTATTCAACTGTCGCCAGTTCATTGGTACTGGTAGTCTCCCTGGGGGCAA
TCAGTTTTTGGATGTGCTCTAATGGGTCTCTACAGTGTAGAATATGTATTTAACATTAGGATTTCAGAAG
CATGAGAAAAACACCCTTGTTTCTACT

>gi|370076915|gb|CY103938.1| Influenza A virus (A/Pernambuco/732/2010(H1N1)) hemagglutinin (HA) gene, complete cds

ATGAAGGCAATACTAGTAGTTCTGCTATATACATTTGCAACCGCAAATGCAGACACATTATGTATAGGTT

ATCATGCGAACAATTCAACAGACACTGTAGACACAGTACTAGAAAAGAATGTAACAGTAACACACTCTGT
TAACCTTCTAGAAGACAAGCATAACGGGAAACTATGCAAACTAAGAGGGGTAGCCCCATTGCATTTGGGT
AAATGTAACATTGCTGGCTGGATCCTGGGAAATCCAGAGTGTGAGTCACTCTCCACAGCAAGCTCATGGT
CCTACATTGTGGAAACATCTAGTTCAGACAATGGTACGTGTTACCCAGGAGATTTCATCGATTATGAGGA
GCTAAGAGAGCAATTGAGCTCAGTGTCATCATTTGAAAGGTTTGAGATATTCCCTAAGACAAGTTCATGG
CCCAATCATGACTCGAACAAAGGTGTAACGGCAGCATGTCCTCATGCTGGAGCAAAAAGCTTCTACAAAA
ATTTAATATGGCTAGTTAAAAAAGGAAATTCATACCCAAAGCTCAGCAAATCCTACATTAATGATAAAGG
GAAAGAAGTCCTCGTGCTATGGGGCATTCACCATCCATCTWYTAGTGCTGACCAACAAAGTCTTGWTCAG
AAWGCRSATGYATATGTTTTTGTGGGGACATCAAGATACAGCAAGAAGTTCAAGCCGGAAATAGCAATAA
GACCCAAAGTGAGGGATCAAGAAGGGAGAATGAACTATTACTGGACACTAGTAGAGCCGGGAGACAAAAT
AACATTCGAAGCAACTGGAAATCTAGTGGTACCGAGATATGCATTCGCAATGGAAAGAAATGCTGGATCT
GGTATTATCATTTCAGATACACCAGTCCACGATTGCAATACAACTTGTCAGACACCCAAGGGTGCTATAA
ACACCAGCCTCCCATTTCAGAATATACATCCGATCACAATTGGAAAATGTCCAAAATATGTAGAAAGCAC
AAAATTGAGACTGGCCACAGGATTGAGGAATGTCCCGTCTATTCAATCTAGAGGCCTATTTGGGGCCATT
GCCGGTTTCATTGAAGGGGGGTGGACAGGGATGGTAGATGGATGGTACGGTTATCACCATCAAAATGAGC
AGGGGTCAGGATATGCAGCCGACCTGAAGAGCACACAGAATGCCATTGACGAGATTACTAACAAAGTAAA
TTCTGTTATTGAAAAGATGAATACACAGTTCACAGCAGTAGGTAAAGAGTTCAACCACCTGGAAAAAAGA
ATAGAGAATTTAAATAAAAAAGTTGATGATGGTTTCCTGGACATTTGGACTTACAATGCCGAACTGTTGG
TTCTATTGGAAAATGAAAGAACTTTGGACTACCACGATTCAAATGTGAAGAACTTATATGAAAAGGTAAG
AAGCCAGTTAAAAAACAATGCCAAGGAAATTGGAAACGGCTGCTTTGAATTTTACCACAAATGCGATAAC
ACGTGCATGGAAAGTGTCAAAAATGGGACTTATGACTACCCAAAATACTCAGAGGAAGCAAAATTAAACA
GAGAAGAAATAGATGGGGTAAAGCTGGAATCAACAAGGATTTACCAGATTTTGGCGATCTATTCAACTGT
CGCCAGTTCATTGGTACTGGTAGTCTCCCTGGGGGCAATCAGTTTCTGGATGTGCTCTAATGGGTCTCTA
CAGTGTAGAATATGTATTTAA
>gi|359340816|gb|JQ065243.1| Influenza A virus (A/Shenzhen/1089/2010(H1N1)) segment 4 hemagglutinin (HA) gene, complete cds
ATGAAGGCAATACTAGTAGTTCTGCTATATACATTTGCAACCGCAAATGCAGACACATTATGTATAGGTT
ATCATGCGAACAATTCAACAGACACTGTAGACACAGTACTAGAAAAGAATGTAACAGTAACACACTCTGT
TAACCTTCTAGAAGACAAGCATAACGGGAAACTATGCAAACTAAGAGGGGTAGCCCCATTGCATTTGGGT
AAATGTAACATAGCTGGCTGGATCCTGGGAAATCCAGAGTGTGAATCACTCTCCACAGCAAGCTCATGGT
CCTACATTGTGGAAACATCTAGTTCAGACAATGGAACGTGTTACCCAGGAGATTTCATCGATTATGAGGA
GCTAAGAGAGCAATTGAGCTCAGTGTCATCATTTGAAAGATTTGAGATATTCCCCAAGACAAGTTCATGG
CCCAATCATGACCCGAACAAAGGTGTAACGGCAGCATGTCCTCATGCTGGAGCAAAAAGCTTCTACAAAA
ATTTAATATGGCTAGTTAAAAAAGGAAATTCATACCCAAAGCTCAGCACATCCTACATTAATGATAAAGG
GAAAGAAGTCCTCGTGCTATGGGGCATTCACCATCCATCTACTAGTGCTGACCAACAAAGTCTCTATCAG
AATGCAGATGCATATGCTTTTGTGGGGACATCAAGATACAGCAAGAAGTTCAAGCCGGAAATAGCAATAA
GACCCAAAGTGAGGGATCAAGAAGGGAGAATGAACTATTACTGGACACTAGTAGAGCCGGGAGACAAAAT
AACATTCGAAGCAACTGGAAATCTAGTGGTACCGAGATATGCATTCGCAATGGAAAGAAATGCTGGATCT
GGTATTATCATTTCAGATACATCAGTCCACGATTGCAATACAACTTGTCAGACACCCAAGGGTGCTATAA
ACACCAGCCTCCCATTTCAGAATGTACATCCGATCACAATTGGAAAATGTCCAAAATATGTAAAAAGCAC
AAAATTGAGACTGGCCACAGGATTGAGAAATGTCCCGTCTATTCAATCTCGAGGCCTATTTGGGGCCATT
GCCGGTTTCATTGAAGGGGGGTGGACAGGGATGGTAGATGGATGGTACGGTTATCATCATCAAAATGAGC
AGGGGTCAGGATATGCAGCCGACCTGAAGAGCACACAGAATGCCTTTGACGAGATTACTAACAAAGTAAA
TTCTGTTATTGAAAAGATGAATACACAGTTCACAGCAGTAGGTAAAGAGTTCAACCACCTGGAAAAAAGA

ATAGAGAATTTAAATAAAAAAGTTGATGATGGTTTCCTGGACATTTGGACTTACAATGCCGAACTGTTGG
TTCTATTGGAAAATGAAAGAACTTTGGACTACCACGATTCAAATGTGAAGAACTTATATGAAAAGGTAAG
AAGCCAGTTAAAAAACAATGCCAAGGAAATTGGAAACGGCTGCTTTGAATTTTACCACAAATGCGATAAC
ACGTGCATGGAAAGTGTCAAAAATGGGACTTATGACTACCCAAAATACTCAGAGGAAGCAAAATTAAACA
GAGAAGAAATAGATGGGGTAAAGCTGGAATCAACAAGGATTTACCAGATTTTGGCGATCTATTCAACTGT
CGCCAGTTCATTGGTACTGGTAGTCTCCCTGGGGGCAATCAGTTTCTGGATGTGCTCTAATGGGTCTCTA
CAGTGTAGAATATGTATTTAA
>gi|345098364|gb|JN601105.1| Influenza A virus (A/Finland/44/2010(H1N1)) segment 4 hemagglutinin (HA) gene, complete cds
ATGAAGGCAATACTAGTAGTTCTGCTATATACATTTGCAACCGCAAATGCAGACACATTATGTATAGGTT
ATCATGCGAACAATTCAACAGACACTGTAGACACAGTACTAGAAAAGAATGTAACAGTAACACACTCTGT
TAACCTTCTAGAAGACAAGCATAACGGGAAACTATGCAAACTAAGAGGGGTAGCCCCATTGCATTTGGGT
AAATGTAACATTGCTGGCTGGATCCTGGGAAATCCAGAGTGTGAATCACTCTCCACAGCAAGCTCATGGT
CCTACATTGTGGAAACATCTAGTTCAGACAATGGAACGTGTTACCCAGGAGATTTCATCGATTATGAGGA
GCTAAGAGAGCAATTGAGCTCAGTGTCATCATTTGAAAGGTTTGAGATATTCCCCAAGACAAGTTCATGG
CCCAATCATGACTCGAACAAAGGTGTAACGACAGCATGTCCTCATGCTGGAGCAAAAAGCTTCTACAAAA
ATTTAATATGGCTAGTTAAAAAAGGAAATTCATACCCAAAGCTCAGCAAATCCTACATTAATGATAAAGG
GAAAGAAGTCCTCGTGCTATGGGGCATTCACCATCCACCTACTAGTGCTGACCAACAAAGTCTCTATCAG
AATGCAGATGCATATGTTTTTGTGGGGACATCAAGATACAGCAAGGAGTTCAAGCCGGAAATAGCAATAA
GACCCAAAGTGAGGGATCAAGAAGGGAGAATGAACTATTACTGGACACTAGTAGAGCCGGGAGACAAAAT
AACATTCGAAGCAACTGGAAATCTAGTGGTACCGAGATATGCATTCGCAATGGAAAGAAATGCTGGATCT
GGTATTATCATTTCAGATACACCAGTCCACGATTGCAATACAACTTGTCAGACACCCAAGGGTGCTATAA
ACACCAGCCTCCCATTTCAGAACATACATCCGATCACAATTGGAAAATGTCCAAAATATGTAAAAAGCAC
AAAATTGAGACTGGCCACAGGATTGAGGAATGTCCCGTCCATTCAATCTAGAGGCCTATTTGGGGCCATT
GCCGGTTTCATTGAAGGGGGGTGGACAGGGATGGTAGATGGATGGTACGGTTATCACCATCAAAATGAGC
AGGGGTCAGGATATGCAGCCGACCTGAAGAGCACACAGAATGCCATTGACGAGATTACTAACAAAGTAAA
CTCTGTTATTGAAAAAATGAATACACAGTTCACAGCAGTAGGTAAAGAGTTCAACCACCTGGAAAAAAGA
ATAGAGAATTTAAATAAAAAAGTTGATGATGGTTTCCTGGACATTTGGACTTACAATGCCGAACTGTTGG
TTCTATTGGAAAATGAAAGAACTTTGGACTACCACGATTCAAAGGTGAAGAACTTATATGAAAAGGTAAG
AAGCCAGTTAAAAAACAATGCCAAGGAAATTGGAAACGGCTGCTTTGAATTTTACCACAAATGTGATAAC
ACGTGCATGGAAAGTGTCAAAAATGGGACTTATGACTACCCAAAATACTCAGAGGAAGCAAAATTAAACA
GAGAAGAAATAGATGGGGTAAAGCTGGAATCAACAAGGATTTACCAGATTTTGGCGATCTATTCAACTGT
CGCCAGTTCATTGGTACTGGTAGTCTCCCTGGGGGCAATCAGCTTCTGGATGTGCTCTAATGGATCTCTA
CAGTGTAGAATATGTATTTAA
>gi|325587300|gb|JF500446.1| Influenza A virus (A/Karaj/5987/2010(H1N1)) segment 4 hemagglutinin (HA) gene, complete cds
ATGAAGGCAATACTAGTAGTTCTGCTATATACATTTGCAACCGCAAATGCAGACACATTATGTATAGGTT
ATCATGCGAACAATTCAACAGACACTGTAGACACAGTACTAGAAAAGAATGTAACAGTAACACACTCTGT
TAATCTTCTAGAAGACAAGCATAACGGGAAACTATGCAAACTAAGAGGGGTAGCCCCATTGCATTTGGGT
AAATGTAACATTGCTGGCTGGATCCTGGGAAATCCAGAGTGTGAATCACTCTCCACAGCAAGCTCATGGT
CCTACATTGTGGAAACATCTAGTTCAGACAATGGAACGTGTTACCCAGGAGATTTCATCGATTATGAGGA
GCTAAGAGAGCAATTGAGCTCAGTGTCATCATTTGAAAGGTTTGAGATATTCCCCAAGACAAGTTCATGG
CCCAATCATGACTCGAACAAAGGTGTAACGGCAGCATGTCCTCATGCTGGAGCAAAAGGCTTCTACAAAA
ATTTAATATGGCTAGTTAAAAAAGGAAATTCATACCCAAAGCTCAGCAAATCCTACATTAATGATAAAGG

GAAAGAAGTCCTCGTGCTATGGGGCATTCACCATCCATCTACTACTGCTGACCAACAAAGTCTCTATCAG
AATGCAGATACATATGTTTTTGTGGGGACATCAAGATACAGCAAGAAGTTCAAGCCGGAAATAGCAATAA
GACCCAAAGTGAGGGATCAAGAAGGGAGAATGAACTATTACTGGACACTAGTAGAGCCGGGAGACAAAAT
AACATTCGAAGCAACTGGAAATCTAGTGGTACCGAGATATGCATTCGCAATGGAAAGAAATGCTGGATCT
GGTATTATCATTTCAGATACACCAGTCCACGATTGCAATACAACTTGTCAGACACCCAAGGGTGCTATAA
ACACCAGCCTCCCATTTCAGAATATACATCCGATCACAATTGGAAAATGTCCAAAATATGTAAAAAGCAC
AAAATTGAGACTGGCCACAGGATTGAGGAATGTCCCGTCTATTCAATCTAGAGGCCTATTTGGGGCCATT
GCCGGCTTCATTGAAGGGGGGTGGACAGGGATGGTAGATGGATGGTACGGTTATCACCATCAAAATGAGC
AGGGGTCAGGATATGCAGCCGACCTGAAGAGCACACAGAATGCCATTGACAAGATTACTAACAAAGTAAA
TTCTGTTATTGAAAAGATGAATACACAGTTCACAGCAGTAGGTAAAGAGTTCAACCACCTGGAAAAAAGA
ATAGAGAATTTAAATAAAAAAGTTGATGATGGTTTCCTGGACATTTGGACTTACAATGCCGAACTGTTGG
TTCTATTGGAAAATGAAAGAACTTTGGACTACCACGATTCAAATGTGAAGAACTTATATGAAAAGGTAAG
AAACCAGTTAAAAAACAATGCCAAGGAAATTGGAAATGGCTGCTTTGAATTTTACCACAAATGCGATAAC
ACGTGCATGGAAAGTGTCAAAAATGGGACTTATGACTACCCAAAATACTCAGAGGAAGCAAAATTAAACA
GAGAAGAAATAGATGGGGTAAAGCTGGAATCAACAAGGATTTACCAGATTTTGGCGATCTATTCAACTGT
CGCCAGTTCATTGGTACTGGTAGTCTCCCTGGGGGCAATCAGTTTTTGGATGTGCTCTAATGGGTCTCTA
CAGTGTAGAATATGTATTTAA

>gi|336477973|gb|CY092393.1| Influenza A virus (A/Sydney/DD3-32/2010(H1N1)) hemagglutinin (HA) gene, complete cds

AAAAGCAACAAAAATGAAGGCAATACTAGTAGTTCTGCTATATACATTTGCAACCGCAAATGCAGACACA
TTATGTATAGGTTATCATGCGAACAATTCAACAGACACTGTAGACACAGTACTAGAAAAGAATGTAACAG
TAACACACTCTGTTAACCTTCTAGAAGACAAGCATAACGGGAAAYTATGCAAACTAAGAGGGGTAGCCCC
ATTGCATTTGGGTAAATGTAACATTGCTGGCTGGATCCTGGGAAATCCAGAGTGTGAATCACTCTCCACA
GCAAGCTCATGGTCCTACATTGTGGAAACATCTAGTTCAGACAATGGAACGTGTTACCCAGGAGATTTCA
TCAATTATGAGGAGCTAAGARARCAATTGAGCTCAGTGTCATCATTTGAAAGGTTTGAGATATTCCCCAA
GACAAGTTCATGGCCCAATCATGACTCGAACAAAGGTGTAACGGCAGCATGTCCTCATGCTGGAGCAAAA
AGCTTCTACAAAAATTTAATATGGCTAGTTAAAAAAGGAAATTCATACCCAAAGCTCAGCAAATCCTACA
TTAATGATAAAGGGAAAGAAGTCCTCGTGCTATGGGGCATTCACCATCCATCTACTACTGCTGACCAACA
AAGTCTCTATCAGAATGCAGATGCATATGTTTTTGTGGGGACATCAAGATACAGCAAGAAGTTCAAGCCG
GAAATAGCAATAAGACCCAAAGTGAGGGATCAAGAAGGGAGAATGAACTATTACTGGACACTAGTAGAGC
CGGGAGACAAAATAACATTCGAAGCAACTGGAAATCTAGTGGTACCGAGATATGCATTCGCAATGGAAAG
AAATGCTGGATCTGGTATTATCATTTCAGATACACCATTCCACGATTGCAATACAACTTGTCAGACACCC
AAGGGTGCTATAAACACCAGCCTCCCATTTCAGAATATACATCCGATCACAATTGGAAAATGTCCAAAAT
ATGTAAAAAGCACAAAATTGAGACTGGCCACAGGATTGAGGAATGTCCCGTCTATTCAATCTAGAGGCCT
ATTTGGGGCCATTGCCGGYTTCATTGAAGGGGGGTGGACAGGGATGGTAGATGGATGGTACGGTTATCAC
CATCAAAATGAGCAGGGGTCAGGATATGCAGCCGACCTGAAGAGCACACAGAATGCCATTGACAAGATTA
CTAACAAAGTAAATTCTGTTATTGAAAAGATGAATACACAGTTCACAGCAGTAGGTAAAGAGTTCAACCA
CCTGGAAAAAAGAATAGAGAATTTAAATAAAAAAGTTGATGATGGTTTCCTGGACATTTGGACTTACAAT
GCCGAACTGTTGGTTCTATTGGAAAATGAAAGAACTTTGGACTACCACGATTCAAATGTGAAGAACTTAT
ATGAAAAGGTAAGAAACCAGTTAAAAAACAATGCCAAGGAAATTGGAAACGGCTGCTTTGAATTTTACCA
CAAATGCGATAACACGTGCATGGAAAGTGTCAAAAATGGGACTTATGACTACCCAAAATACTCAGAGGAA
GCAAAATTAAACAGAGAAGAAATAGATGGGGTAAAGCTGGAATCAACAAGGATTTACCAGATTTTGGCGA
TCTATTCAACTGTCGCCAGTTCATTGGTACTGGTAGTCTCCCTGGGGGCAATCAGTTTCTGGATGTGCTC
TAATGGGTCTCTACAGTGTAGAATATGTATTTAACATTAGGATTTCAGAAGCAT

>gi|339730804|gb|CY089854.2| Influenza A virus (A/Mexico/InDRE2424/2011(H1N1)) hemagglutinin (HA) gene, complete cds
ATGAAGGCAATACTAGTAGTTCTGCTATATACATTTGCAACCGCAAATGCAGACACATTATGTATAGGTT
ATCATGCGAACAATTCAACAGACACTGTAGACACAGTACTAGAAAAGAATGTAACAGTAACACACTCTGT
TAACCTTCTAGAAGACAAGCATAACGGGAAACTATGCAAACTAAGAGGGGTAGCCCCATTGCATTTGGGT
AAATGTAACATAGCTGGCTGGATCCTGGGAAATCCAGAGTGTGAATCACTCTCCACAGCAAGCTCATGGT
CCTACATTGTGGAAACATCTAGTTCAGACAATGGAACGTGTTACCCAGGAGATTTCATCGATTATGAGGA
GCTAAGGGAGCAATTGAGCTCAGTGTCATCATTTGAAAGATTTGAGATATTCCCCAAGACAAGTTCATGG
CCCAATCATGACCCGAACAAAGGTGTAACGGCAGCATGTCCTCATGCTGGAGCAAAAAGCTTCTACAAAA
ATTTAATATGGCTAGTTAAAAAAGGAAATTCATACCCAAAGCTCAGCACATCCTATATTAATGATAAAGG
GAAAAAAGTACTCGTGCTATGGGGCATTCACCATCCATCTACTAGTGCTGACCAACAAAGTCTCTATCAG
AATGCAGATGCATATGCTTTTGTGGGGACATCAAGATACAGCAAGAAGTTCAAGCCGGAAATAGCAATAA
GACCCAAAGTGAGGGATCAAGAAGGGAGAATGAACTATTACTGGACACTAGTAGAGCCGGGAGACAAAAT
AACATTCGAAGCAACTGGAAATCTAGTGGTGCCGAGATATGCATTCGCAATGGAAAGAAATGAGGGATCT
GGTATTATCATTTCAGATACATCAGTCCACGATTGCAATACAACTTGTCAAACACCCAAGGGTGCTATAA
ACACCAGCCTCCCATTTCAGAATGTACATCCGATCACAATTGGAAAATGTCCAAAATATGTAAAAAGCAC
AAAATTGAGACTGGCCACAGGATTGAGGAATGTCCCGTCTATTCAATCTCGAGGCCTATTTGGGGCCATT
GCCGGTTTCATTGAAGGGGGGTGGACAGGGATGGTAGATGGATGGTACGGTTACCATCATCAAAATGAGC
AGGGGTCAGGATATGCAGCCGACCTGAAGAGCACACAGAATGCCTTTGACAAGATTACTAACAAAGTAAA
TTCTGTTATTGAAAAGATGAATACACAGTTCACAGCAGTAGGTAAAGAGTTCAACCACCTGGAAAAAAGA
ATAGAGAATTTAAATAAAAAAGTTGATGATGGTTTCCTGGACATTTGGACTTACAATGCCGAACTGTTGG
TTCTATTGGAAAATGAAAGAACTTTGGACTACCACGATTCAAATGTGAAGAACTTATATGAAAAGGTAAG
AAGCCAGTTAAAAAACAATGCCAAGGAAATTGGAAACGGCTGCTTTGAATTTTACCACAAATGCGATAAC
ACGTGCATGGAAAGTGTCAAAAATGGGACTTATGACTACCCAAAATACTCAGAGGAAGCAAAATTAAACA
GAGAAGAAATAGATGGGGTAAAGCTGGAATCAACAAGGATTTACCAGATTTTGGCGATCTATTCAACTGT
CGCCAGTTCATTGGTACTGGTAGTCTCCCTGGGGGCAATCAGTTTCTGGATGTGCTCTAATGGGTCTCTA
CAGTGTAGAATATGTATTTAACATTAGGATTTCAGAAG
>gi|339518916|gb|JN185117.1| Influenza A virus (A/Tula/CRIE-LMV/2011(H1N1)) segment 4 hemagglutinin (HA) gene, complete cds
ATGAAGGCAATACTAGTAGTTCTGCTATATACATTTGCAACCGCAAATGCAGACACATTATGTATAGGTT
ATCATGCGAACAATTCAACAGACACTGTAGACACAGTACTAGAAAAGAATGTAACAGTAACACACTCTGT
TAACCTTCTAGAAGACAAGCATAACGGGAAACTATGCAAACTAAGAGGGGTAGCCCCATTGCATTTGGGT
AAATGTAACATTGCTGGCTGGATCCTGGGAAATCCAGAGTGTGAATCATTCTCCACAGCAAGCTCATGGT
CCTACATTGTGGAAACATCTAGTTCAGACAATGGAACGTGTTACCCAGGAGATTTCATCAATTATGAGGA
GCTAAGAGAGCAATTGAGCTCAGTGTCATCATTTGAAAGGTTTGAGATATTCCCCAAGACAAGTTCATGG
CCCAATCATGACTCGAACAAAGGTGTAACGGCAGCATGTCCTCATGCTGGAGCAAAAAGCTTCTACAAAA
ATTTAATATGGCTAGTTAAAAAAGGAAATTCATACCCAAAGCTCAGCAAATCCTACATTAATGATAAAGG
GAAAGAAGTCCTCGTGCTGTGGGGCATTCACCATCCATCTACTACTGCTGACCAACAAAGTCTCTATCAG
AATGCAGATGCATATGTTTTTGTGGGGACATCAAGATACAGCAAGAAGTTCAAGCCGGAAATAGCAATAA
GACCCAAAGTGAGGGATCAAGAAGGGAGAATGAACTATTACTGGACACTAGTAGAGCCGGGAGACAAAAT
AACATTCGAAGCAACTGGAAATCTAGTGGTACCGAGATATGCATTCGCAATGGAAAGAAATGCTGGATCT
GGTATTATCATTTCAGATACACCAGTCCACGATTGCAATACAACTTGTCAGACACCCAAGGGTGCTATAA
ACACCAGCCTCCCATTTCAGAATATACATCCGATCACAATTGGAAAATGTCCAAAATATGTAAAAAGCAC
AAAATTGAGACTGGCCACAGGATTGAGGAATGTCCCGTCTATTCAATCTAGAGGCCTACTTGGGGCCATT

GCCGGCTTCATTGAAGGGGGGTGGACAGGGATGGTAGATGGATGGTACGGTTATCACCATCAAAATGAGC
AGGGGTCAGGATATGCAGCCGACCTGAAGAGCACACAGAATGCCATTGACAAGATTACTAACAAAGTAAA
TTCTGTTATTGAAAAGATGAATACACAGTTCACAGCAGTAGGTAAAGAGTTCAACCACCTGGAAAAAAGA
ATAGAGAATTTAAATAAAAAAGTTGATGATGGTTTCCTGGACATTTGGACTTACAATGCCGAACTGTTGG
TTCTATTGGAAAATGAAAGAACTTTGGACTACCACGATTCAAATGTGAAGAACTTGTATGAAAAGGTAAG
AAACCAGTTAAAAAACAATGCCAAGGAAATTGGAAACGGCTGCTTTGAATTTTACCACAAATGCGATAAC
ACGTGTATGGAAAGTGTCAAAAATGGGACTTATGACTACCCAAAATACTCAGAGGAAGCAAAATTAAACA
GAGAAGAAATAGATGGGGTAAAGCTGGAATCAACAAGGATTTACCAGATTTTGGCGATCTATTCAACTGT
CGCCAGTTCATTGGTACTGGTAGTCTCCCTGGGGGCAATCAGTTTCTGGATGTGCTCCAATGGGTCTCTA
CAGTGTAGAATATGTATTTAA
>gi|339518892|gb|JN185105.1| Influenza A virus (A/St.Petersburg/CRIE-RME/2011(H1N1)) segment 4 hemagglutinin (HA) gene, complete cds
ATGAAGGCAATACTAGTAGTTCTGCTATATACATTTGCAACCGCAAATGCAGACACATTATGTATAGGTT
ATCATGCGAACAATTCAACAGACACTGTAGACACAGTACTAGAAAAGAATGTAACAGTAACACACTCTGT
TAACCTTCTAGAAGACAAGCATAACGGGAAACTATGCAAACTAAGAGGGGTAGCCCCATTGCATTTGGGT
AAATGTAACATCGCTGGCTGGATCCTGGGAAATCCAGAGTGTGAATCACTCTCCACAGCAAGCTCATGGT
CCTACATTGTGGAAACATCTAGTTCAGACAATGGAACGTGTTACCCAGGAGATTTCATCGATTATGAGGA
GCTAAGAGAGCAATTGAGCTCAGTGTCATCATTTGAAAGGTTTGAGATATTCCCCAAGACAAGTTCATGG
CCCGATCATGACTCGAACAAAGGTGTAACGGCAGCATGTCCTCATGCTGGAGCAAAAAGCTTCTACAAAA
ATTTAATATGGCTAGTTAAAAAAGGAAATTCATACCCAAAGCTCAGCAAATCCTACATTAATGATAAAGG
GAAAGAAGTCCTCGTGCTATGGGGCATTCACCATCCATCTACTAGTGCTGACCAACAAAGTCTCTATCAG
AATGCAGATGCATATGTTTTTGTGGGGACATCAAGATACAGCAAGAAGTTCAAGCCGGAAATAGCAATAA
GACCCAAAGTGAGGRRTCAAGAAGGGAGAATGAACTATTACTGGACACTAGTAGAGCCGGGAGACAAAAT
AACATTCGAAGCAACTGGAAATCTAGTGGTACCGAGATATGCATTCGCAATGGAAAGAAATGCTGGATCT
GGTATTATCATTTCAGATACACCAGTCCACGATTGCAATACAACTTGTCAGACACCCAAGGGTGCTATAA
ACACCAGCCTCCCATTTCAGAATATACATCCGATCACAATTGGAAAATGTCCAAAATATGTAAAAAGCAC
AAAATTGAGACTGGCCACAGGATTGAGGAATGTCCCGTCTATTCAATCTAGAGGCCTATTTGGGGCCATT
GCCGGTTTCATTGAAGGGGGGTGGACAGGGATGGTAGATGGATGGTACGGTTATCACCATCAAAATGAGC
AGGGGTCAGGATATGCAGCCGACCTGAAGAGCACACAGAATGCCATTGACAAGATTACTAACAAAGTAAA
TTCTGTTATTGAAAAGATGAATACACAGTTCACAGCAGTAGGTAAAGAGTTCAACCACCTGGAAAAAAGA
ATAGAGAATTTAAATAAAAAAGTTGATGATGGTTTCCTGGACATTTGGACTTACAATGCCGAACTGTTGG
TTCTATTGGAAAATGAAAGAACTTTGGACTACCACGATTCAAATGTGAAGAACTTATATGAAAAGGTAAG
AAGCCAGTTAAAAAACAATGCCAAGGAAATTGGAAACGGCTGCTTTGAGTTTTACCACAAATGCGATAAC
ACGTGCATGGAAAGTGTCAAAAATGGGACTTATGACTACCCAAAATACTCAGAGGAAGCAAAATTAAACA
GAGAAGAAATAGATGGGGTAAAGCTGGAATCAACAAGGATTTACCAGATTTTGGCGATCTATTCAACTGT
CGCCAGTTCATTGGTACTGGTAGTCTCCCTGGGGGCAATCAGTTTCTGGATGTGCTCCAATGGGTCTCTA
CAGTGTAGAATATGTATTTAA
>gi|338826617|gb|CY092864.1| Influenza A virus (A/Sydney/DD3-59/2011(H1N1)) hemagglutinin (HA) gene, complete cds
AAAAGCAACAAAAATGAAGGCAATACTAGTAGTTCTGCTATATACATTTGCAACCGCAAATGCAGACACA
TTATGTATAGGTTATCATGCGAACAATTCAACAGACACTGTAGACACAGTACTAGAAAAGAATGTAACAG
TAACACACTCTGTTAACCTTCTAGAAGACAAGCATAACGGGAAACTATGCAAACTAAGAGGGGTAGCCCC
ATTGCATTTGGGTAAATGTAGCATTGCTGGCTGGATCCTGGGAAATCCAGAATGTGAATCACTCTCCACA
GCAAGCTCATGGTCCTACATTGTGGAAACATCTAGTTCAGACAATGGAACGTGTTACCCAGGAGATTTCA

TCAATTATGAGGAGCTAAGAGAGCAATTGAGCTCAGTGTCATCATTTGAAAGGTTTGAGATATTCCCCAA
GACAAGTTCATGGCCCAATCATGACTCGAACAAAGGTGTAACGGCAGCATGTCCTCATGCTGGAGCAAAA
AGCTTCTACAAAAATTTAATATGGCTAGTTAAAAAAGGAAATTCATACCCAAAGCTCAGCAAATCCTACA
TTAATGATAAAGGGAAAGAAGTCCTCGTGCTATGGGGCATTCACCATCCATCTACTACTGCTGACCAACA
AAGTCTCTATCAGAATGCAGATGCATATGTTTTTGTGGGGACATCAAGATACAGCAAGAAGTTCAAGCCG
GAAATAGCAATAAGACCCAAAGTGAGGGATCAAGAAGGGAGAATGAACTATTACTGGACACTAGTAGAGC
CGGGAGACAAAATAACATTCGAAGCAACTGGAAATCTAGTGGTACCGAGATATGCATTCGCAATGGAAAG
AAATGCTGGATCTGGTATTATCATTTCAGATACACCAGTCCACGATTGCAATACAACTTGTCAGACACCC
AAGGGTGCTATAAACACCAGCCTCCCATTTCAGAATATACATCCGATCACAATTGGGAAATGTCCAAAAT
ATGTAAAAAGCACAAAATTGAGACTGGCCACAGGATTGAGGAATGTCCCGTCTATTCAATCTAGAGGCCT
ATTTGGGGCCATTGCCGGCTTCATTGAAGGGGGGTGGACAGGGATGGTAGATGGATGGTACGGTTATCAC
CATCAAAATGAGCAGGGGTCAGGATATGCAGCCGACCTGAAGAGCACACAGAATGCCATTGACAAGATTA
CTAACAAAGTAAATTCTGTTATTGAAAAGATGAATACACAGTTCACAGCAGTAGGTAAAGAGTTCAACCA
CCTGGAAAAAAGAATAGAGAATTTAAATAAAAAAGTTGATGATGGTTTCCTGGACATTTGGACTTACAAT
GCCGAACTGTTGGTTCTATTGGAAAATGAAAGAACTTTGGACTACCACGATTCAAATGTGAAGAACTTAT
ATGAAAAGGTAAGAAACCAGTTAAAAAACAATGCCAAGGAAATTGGAAACGGCTGCTTTGAATTTTACCA
CAAATGCGATAACACGTGCATGGAAAGTGTCAAAAATGGGACTTATGACTACCCAAAATACTCAGAGGAA
GCAAAATTAAACAGAGAAGAAATAGATGGGGTAAAGCTGGAATCAACAAGGATTTACCAGATTTTGGCGA
TCTATTCAACTGTCGCCAGTTCATTGGTACTGGTAGTCTCCCTGGGGGCAATCAGCTTCTGGATGTGCTC
TAATGGGTCTCTACAGTGTAGAATATGTATTTAACATTAGGATTTCAGAAGCAT
>gi|335883166|gb|CY091720.1| Influenza A virus (A/Singapore/TT5/2011(H1N1)) hemagglutinin (HA) gene, complete cds
AGCAAAAGCAGGGGAAAACAAAAGCAACAAAAATGAAGGCAATACTAGTAGTTCTGCTATATACATTTGC
AACCGCAAATGCAGACACATTATGTATAGGTTACCATGCGAACAATTCAACAGACACTGTAGACACAGTA
CTAGAAAAGAATGTAACAGTAACACACTCTGTCAATCTTCTAGAAGACAAGCATAACGGGAAACTATGCA
AACTAAGAGGGGTAGCCCCATTGCATTTGGGTAAATGTAACATTGCTGGCTGGATCCTGGGAAATCCAGA
GTGTGAATCACTCTCCACAGCAAGCTCATGGTCCTACATTGTGGAAACATCTAGTTCAGACAATGGAACG
TGTTACCCAGGAGATTTCATTGATTATGAGGAGCTAAGAGAGCAATTGAGCTCAGTGTCATCATTTGAAA
GGTTTGAGATATTCCCCAAGACAAGTTCATGGCCCAATCATGACTCGAACAAAGGTGTAACGGCAGCATG
TCCTCATGCTGGAGCAAAAGGCTTCTACAAAAATTTAATATGGCTAGTTAAAAAAGGAAATTCATACCCA
AAGCTCAGCAAATCCTACATTAATGATAAAGGGAAAGAAGTCCTCGTGCTATGGGGCATTCACCATCCAT
CTACTACTGCTGACCAACAAAGTCTCTATCAGAATGCAGATACATATGTTTTTGTGGGGACATCAAGATA
CAGCAAGAAGTTCAAGCCGGAAATAGCAATAAGACCCAAAGTGAGGGATCAAGAAGGGAGAATGAACTAT
TACTGGACACTAGTAGAGCCGGGAGACAAAATAACATTCGAAGCAACTGGAAATCTAGTGGTACCGAGAT
ATGCATTCGCAATGGACAGAAATGCTGGATCTGGTATTATCATTTCAGATACACCAGTCCACGATTGCAA
TACAACTTGTCAGACACCCAAGGGTGCTATAAACACCAGCCTCCCATTTCAGAATATACATCCGATCACA
ATTGGAAAATGTCCAAAATATGTAAAAAGCACAAAATTGAGACTGGCCACAGGATTGAGGAATGTCCCGT
CTATTCAATCTAGAGGCCTATTTGGGGCCATTGCCGGCTTCATTGAAGGGGGGTGGACAGGGATGGTAGA
TGGATGGTACGGTTATCACCATCAAAATGAGCAGGGGTCAGGATATGCAGCCGACCTGAAGAGCACACAG
AATGCCATTGACAAGATTACTAACAAAGTAAATTCTGTTATTGAAAAGATGAATACACAGTTCACAGCAG
TAGGTAAAGAGTTCAACCACCTGGAAAAAAGAATAGAGAATTTAAATAAAAAAGTTGATGATGGTTTCCT
GGACATTTGGACTTACAATGCCGAACTGTTGGTTCTATTGGAAAATGAAAGAACTTTGGACTACCACGAT
TCAAATGTGAAGAACTTATATGAAAAGGTAAGAAACCAGTTGAAAAACAATGCCAAGGAAATTGGAAATG
GCTGCTTTGAATTTTACCACAAATGCGATAACACGTGCATGGAAAGTGTCAAAAATGGGACTTATGACTA

CCCAAAATACTCAGAGGAAGCAAAATTAAACAGAGAAGAAATAGATGGGGTAAAGCTGGAATCAACAAGG
ATTTACCAGATTTTGGCGATCTATTCAACTGTCGCCAGTTCATTGGTACTGGTAGTCTCCCTGGGGGCAA
TCAGTTTTTGGATGTGCTCTAATGGGTCTCTACAGTGTAGAATATGTATTTAACATTAGGATTTCAGAAG
CATGAGAAAAACACCCTTGTTTCTACT
>gi|333123620|gb|JF929779.1| Influenza A virus (A/Guangdong/067/2011(H1N1)) segment 4 hemagglutinin (HA) gene, complete cds
ATGAAGGCAATACTAGTAGTTCTGCTATATACATTTGCAACCGCAAATGCAGACACATTATGTATAGGTT
ATCATGCGAACAATTCAACAGACACTGTAGACACAGTACTAGAAAAGAATGTAACAGTAACACACTCTGT
TAACCTTCTAGAAGACAAGCATAACGGGAAACTATGCAAACTAAGAGGGGTAGCCCCATTGCATTTGGGT
AAATGTAACATAGCTGGCTGGATCCTGGGAAATCCAGAGTGTGAATCACTCTCCACAGCAAGCTCATGGT
CCTACATTGTGGAAACATCTAGTTCAGACAATGGAACGTGTTACCCAGGAGATTTCATCGATTATGAGGA
GCTAAGAGAGCAATTGAGCTCAGTGTCATCATTTGAAAGATTTGAGATATTCCCCAAGACAAGTTCATGG
CCCAATCATGACCCGAACAAAGGTGTAACGGCAGCATGTCCTCATGCTGGAGCAAAAAGCTTCTACAAAA
ATTTAATATGGCTAGTTAAAAAAGGAAATTCCTACCCAAAACTCAGCAAATCGGTCATACTTGATAAAGG
GAAAGAAGTCCTCGTGCTATGGGGCATTCACCATCCATCTACTAGTGCTGACCAACAAAGTCTCTATCAG
AATGCAGATGCATATGCTTTTGTGGGGACATCAAGATACAGCAAGAAGTTCAAGCCGGAAATAGCAATAA
GACCCAAAGTGAGGGGTCAAGAAGGGAGAATGAACTATTACTGGACACTAGTAGAGCCGGGAGACAAAAT
AACATTCGAAGCAACTGGAAATCTAGTGGTGCCGAGATATGCATTCGCAATGGAAAGAAATGCGGGATCT
GGTATTATCATTTCAGATACATCAGTCCACGATTGCAATACAACTTGTCAGACACCCAAGGGTGCTATAA
ACACCAGCCTCCCATTTCAGAATGTACATCCGATCACAATTGGAAAATGTCCAAAATATGTAAAAAGCAC
AAAATTGAGACTGGCCACAGGATTGAGGAATGTCCCGTCTATTCAATCTCGAGGCCTATTTGGGGCCATT
GCCGGTTTCATTGAAGGGGGGTGGACAGGGATGGTAGATGGATGGTACGGTTATCATCATCAAAATGAGC
AGGGGTCAGGATATGCAGCCGACCTGAAGAGCACACAGAATGCCTTTGACAAGATTACTAACAAAGTAAA
TTCTGTTATTGAAAAGATGAATACACAGTTCACAGCAGTAGGTAAAGAGTTCAACCACCTGGAAAAAAGA
ATAGAGAATTTAAATAAAAAAGTTGATGATGGTTTCCTGGACATTTGGACTTACAATGCCGAACTGTTGG
TTCTATTGGAAAATGAAAGAACTTTGGACTACCACGATTCAAATGTGAAGAACTTATATGAAAAGGTAAG
AAGCCAGTTAAAAAACAATGCCAAGGAAATTGGAAACGGCTGCTTTGAATTTTACCACAAATGCGATAAC
ACGTGCATGGAAAGTGTCAAAAATGGGACTTATGACTACCCAAAATACTCAGAGGAAGCAAAATTAAACA
GAGAAGAAATAGATGGGGTAAAGCTGGAATCAACAAGGATTTACCAGATTTTGGCGATCTATTCAACTGT
CGCCAGTTCATTGGTACTGGTAGTCTCCCTGGGGGCAATCAGTTTCTGGATGTGCTCTAATGGGTCTCTA
CAGTGTAGAATATGTATTTAA
>gi|350612129|gb|JN714526.1| Influenza A virus (A/Moscow oblast/CRIE-HDV/2011(H1N1)) segment 4 hemagglutinin (HA) gene, complete cds
ATGAAGGCAATACTAGTAGTTCTGCTATATACATTTGCAACCGCAAATGCAGACACATTATGTATAGGTT
ATCATGCGAACAATTCAACAGACACTGTAGACACAGTACTAGAAAAGAATGTAACAGTAACACACTCTGT
TAACCTTCTAGAAGACAAGCATAACGGGAAACTATGCAAACTAAGAGGGGTAGCCCCATTGCATTTGGGT
AAATGTAACATTGCTGGCTGGATCCTGGGAAATCCAGAGTGTGAATCATTCTCCACAGCAAGCTCATGGT
CCTACATTGTGGAAACATCTAGTTCAGACAATGGAACGTGTTACCCAGGAGATTTCATCAATTATGAGGA
GCTAAGAGAGCAATTGAGCTCAGTGTCATCATTTGAAAGGTTTGAGATATTCCCCAAGACAAATTCATGG
CCCAATCATGACTCGAACAAAGGTGTAACGGCAGCATGTCCTCATGCTGGAGCAAAAAGCTTCTACAAAA
ATTTAATATGGCTAGTTAAAAAAGGAAATTCATACCCAAAGCTCAGCAAATCCTACATTAATGATAAAGG
GAAAGAAGTCCTCGTGCTGTGGGGCATTCACCATCCATCTACTACTGCTGACCAACAAAGTCTCTATCAG
AATGCAGATGCATATGTTTTTGTGGGGACATCAAGATACAGCAAGAAGTTCAAGCCGGAAATAGCAATAA
GACCCAAAGTGAGGGATCAAGAAGGGAGAATGAACTATTACTGGACACTAGTAGAGCCGGGAGACAAAAT

AACATTCGAAGCAACTGGAAATCTAGTGGTACCGAGATATGCATTCGCAATGGAAAGAAATGCTGGATCT
GGTATTATCATTTCAGATACACCAGTCCACGATTGCAATACAACTTGTCAGACACCCAAGGGTGCTATAA
ACACCAGCCTCCCATTTCAGAATATACATCCGATCACAATTGGAAAATGTCCAAAATATGTAAAAAGCAC
AAAATTGAGACTGGCCACAGGATTGAGGAATGTCCCGTCTATTCAATCTAGAGGCCTATTTGGGGCCATT
GCCGGCTTCATTGAAGGGGGGTGGACAGGGATGGTAGATGGATGGTACGGTTATCACCATCAAAATGAGC
AGGGGTCAGGATATGCAGCCGACCTGAAGAGCACACAGAATGCCATTGACAAGATTACTAACAAAGTAAA
TTCTGTTATTGAAAAGATGAATACACAGTTCACAGCAGTAGGTAAAGAGTTCAACCACCTGGAAAAAAGA
ATAGAGAATTTAAATAAAAAAGTTGATGATGGTTTCCTGGACATTTGGACTTACAATGCCGAACTGTTGG
TTCTATTGGAAAATGAAAGAACTTTGGACTACCACGATTCAAATGTGAAGAACTTGTATGAAAAGGTAAG
AAACCAGTTAAAAAACAATGCCAAGGAAATTGGAAACGGCTGCTTTGAATTTTACCACAAATGCGATAAC
ACGTGTATGGAAAGTGTCAAAAATGGGACTTATGACTACCCAAAATACTCAGAGGAAGCAAAATTAAACA
GAGAAGAAATAGATGGGGTAAAGCTGGAATCAACAAGGATTTACCAGATTTTGGCGATCTATTCAACTGT
CGCCAGTTCATTGGTACTGGTAGTCTCCCTGGGGGCAATCAGTTTCTGGATGTGCTCCAATGGGTCTCTA
CAGTGTAGAATATGTATTTAA
>gi|344166342|gb|CY097988.1| Influenza A virus (A/Budapest/WRAIR3794N/2011(H1N1)) hemagglutinin (HA) gene, complete cds
ATGAAGGCAATACTAGTAGTTCTGCTATATACATTTGCAACCGCAAATGCAGACACATTATGTATAGGTT
ATCATGCGAACAATTCAACAGACACTGTAGACACAGTACTAGAAAAGAATGTAACAGTAACACACTCTGT
TAACCTTCTAGAAGACAAGCATAACGGGAAACTATGCAAACTAAGAGGGGTAGCCCCATTGCATTTGGGT
AAATGTAGCATTGCTGGCTGGATCCTGGGAAATCCAGAATGTGAATCACTCTCCACAGCAAGCTCATGGT
CCTACATTGTGGAAACATCTAGTTCAGACAATGGAACGTGTTACCCAGGAGATTTCATCAATTATGAGGA
GCTAAGAGAGCAATTGAGCTCAGTGTCATCATTTGAAAGGTTTGAGATATTCCCCAAGACAAGTTCATGG
CCCAATCATGACTCGAACAAAGGTGTAACGGCAGCATGTCCTCATGCTGGAGCAAAAAGCTTCTACAAAA
ATTTAATATGGCTAGTTAAAAAAGGAAATTCATACCCAAAGCTCAGCAAATCCTACATTAATGATAAAGG
GAAAGAAGTCCTCGTGCTATGGGGCATTCACCATCCATCTACTACTGCTGACCAACAAAGTCTCTATCAG
AATGCAGATGCATATGTTTTTGTGGGGACATCAAGATACAGCAAGAAGTTCAAGCCGGAAATAGCAATAA
GACCCAAAGTGAGGGATCAAGAAGGGAGAATGAACTATTACTGGACACTAGTAGAGCCGGGAGACAAAAT
AACATTCGAAGCAACTGGAAATCTAGTGGTACCGAGATATGCATTCGCAATGGAAAGAAATGCTGGATCT
GGTATTATCATTTCAGATACACCAGTCCACGATTGCAATACAACTTGTCAGACACCCAAGGGTGCTATAA
ACACCAGCCTCCCATTTCAGAATATACATCCGATCACAATTGGAAAATGTCCAAAATATGTAAAAAGCAC
AAAATTGAGACTGGCCACAGGATTGAGGAATGTCCCGTCTATTCAATCTAGAGGCCTATTTGGGGCCATT
GCCGGCTTCATTGAAGGGGGGTGGACAGGGATGGTAGATGGATGGTACGGTTATCACCATCAAAATGAGC
AGGGGTCAGGATATGCAGCCGACCTGAAGAGCACACAGAATGCCATTGACAAGATTACTAACAAAGTAAA
TTCTGTTATTGAAAAGATGAATACACAGTTCACAGCAGTAGGTAAAGAGTTCAACCACCTGGAAAAAAGA
ATAGAGAATTTAAATAAAAAAGTTGATGATGGTTTCCTGGACATTTGGACTTACAATGCCGAACTGTTGG
TTCTATTGGAAAATGAAAGAACTTTGGACTACCACGATTCAAATGTGAAGAACTTATATGAAAAGGTAAG
AAACCAGTTAAAAAACAATGCCAAGGAAATTGGAAACGGCTGCTTTGAATTTTACCACAAATGCGATAAC
ACGTGCATGGAAAGTGTCAAAAATGGGACTTATGACTACCCAAAATACTCAGAGGAAGCAAAATTAAACA
GAGAAGAAATAGATGGGGTAAAGCTGGAATCAACAAGGATTTACCAGATTTTGGCGATCTATTCAACTGT
CGCCAGTTCATTGGTACTGGTAGTCTCCCTGGGGGCAATCAGTTTCTGGATGTGCTCTAATGGGTCTCTA
CAGTGTAGAATATGTATTTAA
>gi|388774706|gb|CY120755.1| Influenza A virus (A/Brazil/AVS11/2011(H1N1)) hemagglutinin (HA) gene, complete cds
GGAAAACAAAAGCAACAAAAATGAAGGCAATACTAGTAGTTCTGCTATATACATTTGCAACCGCAAATGC

AGACACATTATGTATAGGTTATCATGCGAACAATTCAACAGACACTGTAGACACAGTACTAGAAAAGAAT
GTAACAGTAACACACTCTGTTAACCTTCTAGAAGACAAGCATAACGGGAAACTATGCAAACTGAGAGGGG
TAGCCCCATTGCATTTGGGTAAATGTAACATTGCTGGCTGGATCCTGGGAAATCCAGAGTGTGAATCACT
CTCCACAGCAAGCTCATGGTCCTACATTGTGGAAACATCTAGTTCAGACAATGGAACGTGTTACCCAGGA
GATTTCATCAATTATGAAGAGCTAAGAGAGCAATTGAGCTCAGTGTCATCATTTGAAAGGTTTGAGATAT
TCCCAAAGACAAGTTCATGGCCCAATCATGACTCGAACAAAGGTGTAACGGCAGCATGTCCTCATGCTGG
AGCAAAAAGCTTCTACAAAAATTTAATATGGCTAGTTAAAAAAGGAAATTCATACCCAAAGCTCAGCAAA
TCCTATATTAACGATAAAGGGAAAGAAGTCCTAGTGCTATGGGGCATTCACCATCCATCTACTAGTGCTG
ACCAACAAAGTCTCTATCAGAATGCAGATGCATATGTTTTTGTGGGGACATCAAAATACAGCAAGAAGTT
CAAGCCGGAAATAGCAGTAAGACCTAAAGTGAGGGATCAAGAAGGGAGAATGAACTATTACTGGACACTA
GTAGAGCCGGGAGACAAAATAACATTCGAAGCAACTGGAAATCTATTGGTACCGAGATATGCATTCGCAA
TGGAAAGAAATGCTGGATCTGGTATTATCATTTCAGATACACCAGTCCACGATTGCAATACAACTTGTCA
GACACCCAAGGGTGCTATAAACACCAGCCTCCCATTTCAGAATATACATCCGATCACAATTGGAAAATGT
CCAAAATATGTAAAAAGCACAAAATTGAGACTGGCCACGGGATTGAGGAATGTCCCGTCTATTCAATCTA
GAGGCCTATTTGGGGCCATTGCCGGTTTCATTGAAGGGGGGTGGACAGGGATGGTAGATGGATGGTACGG
TTATCACCATCAAAATGAGCAGGGGTCAGGATATGCAGCCGACCTGAAGAGCACACAGAATGCCATTGAC
AAGATTACTAACAAAGTAAATTCTGTTATTGAAAAGATGAATACACAGTTCACAGCAGTAGGTAAAGAGT
TCAACCACCTGGAAAAAAGAATAGAGAATTTAAATAAAAAAGTTGATGATGGTTTCCTGGACATTTGGAC
TTACAATGCCGAACTGTTGGTTCTATTGGAAAATGAAAGAACTTTGGACTACCACGATTCAAATGTGAAA
AACTTATATGAAAAGGTAAGAAGCCAGTTAAAAAACAATGCCAAGGAAATTGGAAACGGCTGCTTTGAAT
TTTACCACAAATGCGATAACACGTGCATGGAAAGTGTCAAAAATGGGACTTATGACTACCCAAAATACTC
AGAGGAAGCAAAATTAAACAGAGAAGAAATAGATGGGGTAAAGCTGGAATCAACAAGGATTTACCAGATT
TTGGCGATCTATTCAACTGTCGCCAGTTCATTGGTACTGGTAGTCTCCCTGGGGGCAATCAGTTTCTGGA
TGTGCTCTAATGGGTCTCTACAGTGTAGAATATGTATTTAACATTAGGATTTCAGAAGCATGAGAAAAAC
AC

>gi|377824117|gb|JQ396242.1| Influenza A virus (A/Kenya/149/2011(H1N1)) segment 4 hemagglutinin (HA) gene, complete cds

AGCAAAAGCAGGGGAAAACAAAAGCAACAAAAATGAAGGCAATACTAGTAGTTCTGCTATATACATTTGC
AACCGCAAATGCAGACACATTGTGTATAGGTTATCATGCGAACAATTCAACAGACACTGTAGACACAGTA
CTAGAAAAGAATGTAACAGTAACACACTCTGTTAACCTTCTAGAAGACAAGCATAACGGGAAACTATGCA
AACTAAGAGGGGTAGCCCCATTACATTTGGGTAAATGTAACATTGCTGGCTGGATCCTGGGAAATCCAGA
GTGTGAATCACTCTCCACAGCAAGCTCATGGTCCTACATTGTGGAAACATCTAGTTCAGACAATGGAACG
TGTTACCCAGGAGATTTCATCAATTATGAGGAGCTAAGAGAGCAATTGAGCTCAGTGTCATCATTTGAAA
GGTTTGAGATATTCCCCAAGACAAGTTCATGGCCCAATCATGACTCGAACAAAGGTGTAACGGCAGCATG
TCCTCATGCTGGAGCAAAAAGCTTCTACAAAAATTTAATATGGCTAGTTAAAAAAGGAAATTCATACCCA
AAGCTCAGCAAATCCTACATTAATGATAAAGGGAAAGAAGTCCTCGTGCTGTGGGGCATTCACCATCCAT
CTACTACTGCTGACCAACAAAGTCTCTATCAGAATGCAGATGCATATGTTTTTGTGGGGACATCAAGATA
CAGCAAGAAGTTCAAGCCGGAAATAGCAATAAGACCCAAAGTGAGGGATCAAGAAGGGAGAATGAACTAT
TACTGGACACTAGTAGAGCCGGGAGACAAAATAACATTCGAAGCAACTGGAAATCTAGTGGTACCGAGAT
ATGCATTCGCAATGGAAAGAAATGCTGGATCTGGTATTATCATTTCAGATACACCAGTCCACGATTGCAA
TACAACTTGTCAGACACCCAAGGGGGCTATAAACACCAGCCTCCCATTTCAGAATATACATCCGATCACA
ATTGGAAAATGTCCAAAATATGTAAAAAGCACAAAATTGAGACTGGCCACAGGATTGAGGAATGTCCCGT
CTATTCAATCTAGAGGCCTATTTGGGGCCATTGCCGGCTTCATTGAAGGGGGGTGGACAGGGATGGTAGA
TGGATGGTACGGTTATCACCATCAAAATGAGCAGGGGTCAGGATATGCAGCCGACCTGAAGAGCACACAG

AATGCCATTGACAAGATTACTAACAAAGTAAATTCTGTTATTGAAAAGATGAATACACAGTTCACAGCAG
TAGGTAAAGAGTTCAACCACCTGGAAAAAAGAATAGAGAATTTAAATAAAAAAGTTGATGATGGTTTCCT
GGACATTTGGACTTACAATGCCGAACTGTTGGTTCTATTGGAAAATGAAAGAACTTTGGACTACCACGAT
TCAAATGTGAAGAACTTGTATGAAAAGGTAAGAAACCAGTTAAAAAACAATGCTAAGGAAATTGGAAACG
GCTGCTTTGAATTTTACCACAAATGCGATAACATGTGCATGGAAAGTGTCAAAAATGGGACTTATGACTA
CCCAAAATACTCAGAGGAAGCAAAATTAAACAGAGAAGAAATAGATGGGGTAAAGCTGGAATCAACAAGG
ATTTACCAGATTTTGGCGATCTATTCAACTGTTGCCAGTTCATTGGTACTGGTAGTCTCCCTGGGGGCAA
TCAGTTTCTGGATGTGCTCTAATGGGTCTCTACAGTGTAGAATATGTATTTAACATTAGGATTCAGAAGC
ATGATAAAAACACCCTTGTTTCTACT

>gi|401716662|gb|JX473010.1| Influenza A virus (A/Vladivostok/28/2012(H1N1)) segment 4 hemagglutinin (HA) gene, complete cds

ATGAAGGCAATACTAGTAGTTCTGCTATATACATTTGCAACCGCAAATGCAGACACATTATGTATAGGTT
ATCATGCGAACAATTCAACAGACACTGTAGACACAGTACTAGAAAAGAATGTAACAGTAACTCACTCTGT
TAACCTTCTAGAAGACAAGCATAACGGGAAACTATGCAAACTAAGAGGGGTAGCCCCATTGCATTTGGGT
AAATGTAACATTGCGGGCTGGATCCTGGGAAATCCAGAGTGTGAATCACTCTCCACAGCAAGCTCATGGT
CCTACATTGTGGAAACATCTAGTTCAGACAATGGAACGTGTTACCCAGGAGATTTCATCAATTATGAGGA
GCTAAGAGAGCAATTGAGCTCAGTGTCATCATTTGAAAGGTTTGAAATATTCCCCAAGACAAGTTCATGG
CCCAATCATGACTCGAACAAAGGTGTAACGGCAGCATGTCCTCGTGCTGGAGCAAAAAGCTTCTACAAAA
ATTTAATATGGCTAGTTAAAAAAGGAAATTCATACCCAAAGCTCAGCAAATCCTACATTAATGATAAAGG
GAAAGAAGTCCTCGTGCTGTGGGGCATTCACCATCCATCTACTACTGCTGACCAACAAAGTCTCTATCAG
AATGCAGATGCATATGTTTTTGTGGGGACATCAAGATACAGCAAGAAGTTCAAGCCGGAAATAGCAATAA
GACCCAAAGTGAGGGATCAAGAAGGGAGAATGAACTATTACTGGACACTAGTAGAGCCGGGAGACAAAAT
AACATTCGAAGCAACTGGAAATCTATTGGTACCGAGATATGCATTCGCAATGGAAAGAAATGCTGGATCT
GGTATTATCATTTCAGATACACCAGTCCACGATTGCAATACAACTTGTCAGACACCCAAGGGTGCTATAA
ACACCAGCCTCCCATTTCAGAATATACATCCGATCACAATTGGAAAATGTCCAAAATATGTAAAAAGCAC
AAAATTGAGACTGGCCACAGGATTGAGGAATGTCCCGTCTATTCAATCTAGAGGCCTATTTGGAGCCATT
GCCGGCTTCATTGAAGGGGGGTGGACAGGGATGGTAGATGGATGGTACGGTTATCACCATCAAAATGAGC
AGGGGTCAGGATATGCAGCCGACCTGAAGAGCACACAGAATGCCATTGACAAGATTACTAACAAAGTAAA
TTCTGTTATTGAAAAGATGAATACACAGTTCACAGCAGTAGGTAAAGAGTTCAACCACCTGGAAAAAAGA
ATAGAGAATTTAAATAAAAAAGTTGATGATGGTTTCCTGGACATTTGGACTTACAATGCCGAACTGTTGG
TTCTATTGGAAAATGAAAGAACTTTGGACTACCACGATTCAAATGTGAAGAACTTGTATGAAAAGGTAAG
AAACCAGTTAAAAAACAATGCCAAGGAAATTGGAAACGGCTGCTTTGAATTTTACCACAAATGCGATAAC
ACGTGCATGGAAAGTGTCAAAAATGGGACTTATGACTACCCAAAATACTCAGAGGAAGCAAAATTAAACA
GAGAAGAAATAGATGGGGTAAAGCTGGAATCAACAAGGATTTACCAGATTTTGGCGATCTATTCAACTGT
CGCCAGTTCATTGGTACTGGTAGTCTCCCTGGGGGCAATCAGTTTCTGGATGTGCTCTAATGGGTCTCTA
CAGTGTAGAATATGTATTTAA
