# Supplementary material for: A computational method for predicting regulation of human microRNAs on the influenza virus genome
Source: BMC Syst Biol. 2013 Oct 14;7(Suppl 2):S3. doi: 10.1186/1752-0509-7-S2-S3 (PMC3851852; doi:10.1186/1752-0509-7-S2-S3)
Supplement: Additional File 6 — The coding sequence of the gene fragment of NP from 2000 to 2012 recorded in Genbank. [file 1752-0509-7-S2-S3-S6.PDF]

NA

>gi|145278776|gb|CY021695.1| Influenza A virus (A/Memphis/15/2000(H1N1)) segment 6, complete sequence
TTTAAAATGAATCCAAATCAAAAAATAATAACCATTGGATCAATCAGTATAGCAATCGGAATAATTAGTC
TAATGTTGCAAATAGGAAATATTATTTCAATATGGGCTAGTCACTCAATCCAAACTGGAAGTCAAAACCA
CACTGGAATATGCAACCAAAGAATCATCACATATGAAAACAGCACCTGGGTGAATCACACATATGTTAAT
ATTAACAACACTAATGTTGTTGCTGGAAAGGACAAAACTTCAGTGACATTGGCCGGCAATTCATCTCTTT
GTTCTATCAGTGGATGGGCTATATACACAAAAGACAACAGCATAAGAATTGGCTCCAAAGGAGATGTTTT
TGTCATAAGAGAACCTTTCATATCATGTTCTCACTTGGAATGCAGAACCTTTTTTCTGACCCAAGGTGCT
CTATTAAATGACAAACATTCAAATGGGACCGTTAAGGACAGAAGTCCTTATAGGGCCTTAATGAGCTGTC
CTCTAGGTGAAGCTCCGTCCCCATACAATTCAAAGTTTGAATCAGTTGCATGGTCAGCAAGCGCATGCCA
TGATGGCATGGGCTGGTTAACAATCGGAATTTCTGGTCCAGACAATGGAGCTGTGGCTGTACTAAAATAC
AACGGCATAATAACTGAAACCATAAAAAGTTGGAAAAAGCAAATATTAAGAACACAAGAGTCTGAATGTG
TCTGTGTGAACGGGTCATGTTTCACCATAATGACCGATGGCCCGAGTAATGGGGCTGCCTCGTATAAAAT
CTTCAAGATCGAAAAAGGGAAAGTTACTAAATCAATAGAGTTGAATGCACCCAATTTTCATTATGAGGAA
TGTTCCTGTTACCCAGACACTGGCACAGTGATGTGTGTGTGCAGGGACAACTGGCATGGTTCAAATCGAC
CTTGGGTGTCTTTTAATCAAAACCTGGATTATCAAATAGGATACATCTGCAGTGGGGTGTTCGGTGACAA
TCCGCGTCCCAAAGATGGAGAGGGCAGCTGTAATCCAGTGACTGTTGATGGGGCAGACGGAGTAAAGGGG
TTTTCATACAAATATGGTAATGGTGTTTGGATAGGAAGGACTAAAAGTAACAGACTTAGAAAGGGGTTTG
AGATGATTTGGGATCCTAATGGATGGACAGATACCGACAGTGATTTCTCAGTGAAACAGGATGTTGTGGC
AATAACTGATTGGTCAGGGTACAGCGGAAGTTTCGTTCAACATCCTGAGTTAACAGGATTGGACTGTATA
AGACCTTGCTTCTGGGTTGAGTTAGTCAGAGGACTGCCTAGAGAAAATACAACAATCTGGACTAGTGGGA
GCAGCATTTCTTTTTGTGGCGTAAATAGTGATACTGCAAACTGGTCTTGGCCAGACGGTGCTGAGTTGCC
GTTCACCATTGACAAGTAGTTCGTTGAAAAAAA
>gi|70907644|gb|CY000451.2| Influenza A virus (A/New York/146/2000(H1N1)) segment 6, complete sequence
AGCAAAAGCAGGAGTTTAAAATGAATCCAAATCAAAAAATAATAACCATTGGATCAATCAGTGTGGCAAT
CGGAATAATTAGTCTAATATTGCAAATAGGAAATATTATTTCAATATGGGCTAGCCACTCAATCCAAACT
GGAAGTCAAAACTACACTGGAATATGCAACCAAAGAATCATTACATATGAAAATAGAACCTGGGTAAATC
AAACATATGTTAATATTAACAACACTAATGTTGTTGCTGGAAAGGACAAAACCTCGATGACATTGGCCGG
CAATTCATCTCTTTGCCCTATCCGTGGATGGGCAATATACACAAAAGACAACAGCATAAGAATTGGTTCC
AAAGGAGATGTTTTTGTCATAAGAGAGCCTTTTATATCATGTTCTCACTTGGAATGCAGAACCTTTTTTC
TGACCCAAGGTGCTCTATTAAATGACAAGCATTCAAATGGGACCATTAAGGACAGAAGCCCTTTTAGGGC
CTTAATGAGCTGTCCTCTGGGTGAAGCTCCGTCTCCATACAATTCAAGATTTGAATCAGTTGCTTGGTCA
GCAAGCGCATGCCATGATGGCCTGGGCTGGCTAACAATCGGAATTTCTGGTCCAGATAATGGAGCAGTGG
CTGTACTAAAATACAACGGCATAATAACTGAAACCATAAAAAGTTGGAAAAAGCAAATCCTAAGAACACA
AGAGTCTGAATGTGTCTGTGTGAACGGTTCATGTTTTACCATAATGACCGATGGCCCGAGTAACAGGGCC
GCATCGTACAGAATCTTCAAAATTGAGAAGGGGAGAGTTACTAAATCAATAGAGTTGGATGCACCCAATT
ATCATTACGAGGAATGTTCCTGTTATCCAGACACCGGCACAGTAATGTGTGTGTGCAGGGACAATTGGCA
CGGTTCAAATCGACCTTGGGTGTCCTTTAATCAAAACCTGGATTATCAAATAGGATACATCTGCAGTGGG
GTGTTCGGTGACAATCCGCGTCCCAAAGATGGAGAAGGCAGCTGTAATCCAGTGACTGTTGATGGAGCAG
ACGGAGTAAAGGGGTTTTCATACAGATATGGTAATGGTGTTTGGATAGGAAGGACTAAAAGTAACAGACT
AAGAAAGGGATTTGAGATGATTTGGGATCCTAATGGATGGACAGATACCGACAGTGATTTCTCAATGAAA

CAGGATGTCGTGGCAATGAATGATTGGTCAGGGTACAGCGGAAGTTTTGTTCAACATCCTGAGCTAACAG
GATTGGACTGTATGAGACCTTGCTTCTGGGTTGAATTAGTCAGAGGGCTACCTAGAGAAAATACAACAAT
CTGGACTAGTGGGAGCAGCATTTCTTTTTGTGGCGTGAATAGCGAGACTGCAAACTGGTCTTGGCCAGAC
GGTGCTGAGTTGCCATTCACCATTGACAAGTAGTCCGTTGAAAAAAAACTCCTTGTTTCTACT
>gi|145278909|gb|CY021751.1| Influenza A virus (A/South Australia/44/2000(H1N1)) segment 6, complete sequence
ATGAATCCAAATCAAAAAATAATAACCATTGGATCAATCAGTGTGGCAATCGGAATAATTAATCTAACAT
TGCAAATAGGAAATATTATTTCAATATGGGCTAGCCACTCAATCCAAACTGGAAGTCAAAACTACACTGG
AATATGCAACCAAAGAATCATTACATATGAAAATAGAACCTGGGTAAATCAAACATATGTTAATATTAAC
AACACTAATGTTGTTGCTGGAAAGGACAAAACCTCGATGACATTGGCCGGCAATTCATCTCTTTGCCCTA
TCCGTGGATGGGCAATATACACAAAAGACAACAGCATAAGAATTGGTTCCAAAGGAGATGTTTTTGTCAT
AAGAGAGCCTTTTATATCATGTTCTCACTTGGAATGCAGAACCTTTTTTCTGACCCAAGGTGCTCTATTA
AATGACAAGCATTCAAATGGGACCATTAAGGACAGAAGCCCTTTTAGGGCCTTAATGAGCTGTCCTCTGG
GTGAAGCTCCGTCTCCATACAATTCAAGATTTGAATCAGTTGCTTGGTCAGCAAGCGCATGCCATGATGG
CCTGGGCTGGCTAACAATCGGAATTTCTGGTCCAGATAATGGAGCAGTGGCTGTACTAAAATACAACGGC
ATAATAACTGAAACCATAAAAAGTTGGAAAAAGCAAATCCTAAGAACACAAGAGTCTGAATGTGTCTGTG
TGAACGGTTCATGTTTTACCATAATGACCGATGGCCCGAGTAACAGGGCCGCATCGTACAGAATCTTCAA
AATTGAGAAGGGGAGAGTTACTAAATCAATAGAGTTGGATGCACCCAATTATCATTACGAGGAATGTTCC
TGTTATCCAGACACCGGCACAGTAATGTGTGTGTGCAGGGACAATTGGCACGGTTCAAATCGACCTTGGG
TGTCCTTTAATCAAAACCTGGATTATCAAATAGGATACATCTGCAGTGGGGTGTTCGGTGACAATCCGCG
TCCCAAAGATGGAGAAGGCAGCTGTAATCCAGTGACTGTTGATGGAGCAGACGGAGTAAAGGGGTTTTCA
TACAGATATGGTAATGGTGTTTGGATAGGAAGGACTAAAAGTAACAGACTAAGAAAGGGATTTGAGATGA
TTTGGGATCCTAATGGATGGACAGATACCGACAGTGATTTCTCAATGAAACAGGATGTCGTGGCAATGAC
TGATTGGTCAGGGTACAGCGGAAGTTTTGTTCAACATCCTGAGCTAACAGGATTGGACTGTATGAGACCT
TGCTTCTGGGTTGAATTAGTCAGAGGGCTACCTAGAGAAAATACAACAATCTGGACTAGTGGGAGCAGCA
TTTCTTTTTGTGGCGTGAATAGCGAGACTGCAAACTGGTCTTGGCCAGACGGTGCTGAGTTGCCATTCAC
CATTGACAAGTAGTCCGTTGAAAAAAAA
>gi|157367770|gb|CY026157.1| Influenza A virus (A/Auckland/585/2000(H1N1)) segment 6, complete sequence
ATGAATCCAAATCAAAAAATAATAACCATTGGATCAATCAGTATAGCAATCGGAATAATTAGTCTAATGT
TGCAAATAGGAAATATTATTTCAATATGGGCTAGTCACTCAATCCAAACTGGAAGTCAAAACCACACTGG
AATATGCAACCAAAGAATCATCACATATGAAAACAGCACCTGGGTGAATCACACATATGTTAATATTAAC
AACACTAATGTTGTTGCTGGAAAGGACAAAACTTCAGTGACATTGGCCGGCAATTCATCTCTTTGTTCCA
TCAGTGGATGGGCTATATACACAAAAGACAACAGCATAAGAATTGGATCCAAAGGAGATGTTTTTGTCAT
AAGAGAACCTTTCATATCATGTTCTCACTTGGAATGCAGAACCTTTTTTCTGACCCAAGGTGCTCTATTA
AATGACAAACATTCAAATGGGACCGTTAAGGACAGAAGTCCTTATAGGGCCTTAATGAGCTGCCCTCTAG
GTGAAGCTCCGTCCCCATACAATTCAAAGTTTGAATCAGTTGCATGGTCAGCAAGCGCATGCCATGATGG
CATGGGCTGGTTAACAATCGGAATTTCTGGTCCAGACAATGGAGCTGTGGCTGTACTAAAATACAACGGC
ATAATAACTGAAACCATAAAAAGTTGGAAAAAGCGAATATTAAGAACACAAGAGTCTGAATGTGTCTGTG
TGAACGGGTCATGTTTCACCATAATGACCGATGGCCCGAATAATGGGGCCGCCTCGTACAAAATCTTCAA
GATCGAAAAGGGGAAGGTTACTAAATCAATAGAGTTGAATGCACCCAATTTTCATTATGAGGAATGTTCC
TGTTACCCAGACACTGGCACAGTGATGTGTGTATGCAGGGACAACTGGCATGGTTCAAATCGACCTTGGG
TGTCTTTTAATCAAAACCTGGATTATCAAATAGGATACATCTGCAGTGGGGTGTTCGGTGACAATCCGCG
TCCCAAAGATGGAGAGGGCAGCTGTAATCCAGTGACTGTTGATGGAGCAGACGGAGTAAAGGGGTTTTCA

TACAAATATGGTAATGGTGTTTGGATAGGAAGGACTAAAAGTAACAGACTTAGAAAGGGGTTTGAGATGA
TTTGGGATCCTAATGGATGGACAGATACCGACAGTAATTTCTCAGTGAAACAGGATGTTGTGGCAATAAC
TGATTGGTCAGGGTACAGCGGAAGTTTCGTTCAACATCCTGAGTTAACAGGATTGGACTGTATAAGACCT
TGCTTCTGGGTTGAGTTAGTCAGAGGACTGCCTAGAGAAAATACAACAATCTGGACTAGTGGGAGCAGCA
TTTCTTTTTGTGGCGTAAATAGTGATACTGCAAACTGGTCTTGGCCAGACGGTGCTGAGTTGCCGTTCAC
CATTGACAAGTAGTTCGTTGA

>gi|145278622|gb|CY021631.1| Influenza A virus (A/Wellington/4/2000(H1N1)) segment 6, complete sequence

ATGAATCCAAATCAAAAAATAATAACCATTGGATCAATCAGTATAGCAATCGGAATAATTAGTCTAATGT
TGCAAATAGGAAATATTATTTCAATATGGGCTAGTCACTCAATCCAAACTGGAAGTCAAAACCACACTGG
AATATGCAACCAAAGAATCATCACATATGAAAACAGCACCTGGGTGAATCACACATATGTTAATATTAAC
AACACTAATGTTGTTGCTGGAAAGGACAAAACTTCAGTGACATTGGCCGGCAATTCATCTCTTTGTTCTA
TCAGTGGATGGGCTATATACACAAAAGACAACAGCATAAGAATTGGATCCAAAGGAGATGTTTTTGTCAT
AAGAGAACCTTTCATATCATGTTCTCACTTGGAATGCAGAACCTTTTTTCTGACCCAAGGTGCTCTATTA
AATGACAAACATTCAAATGGGACCGTTAAGGACAGAAGTCCTTATAGGGCCTTAATGAGCTGCCCTCTAG
GTGAAGCTCCGTCCCCATACAATTCAAAGTTTGAATCAGTTGCATGGTCAGCAAGCGCATGCCATGATGG
CATGGGCTGGTTAACAATCGGAATTTCTGGTCCAGACAATGGAGCTGTGGCTGTACTAAAATACAACGGC
ATAATAACTGAAACCATAAAAAGTTGGAAAAAGCGAATATTAAGAACACAAGAGTCTGAATGTGTCTGTG
TGAACGGGTCATGTTTCACCATAATGACCGATGGCCCGAGTAATGGGGCCGCCTCGTACAAAATCTTCAA
GATCGAAAAGGGGAAGGTTACTAAATCAATAGAGTTGAATGCACCCAATTTTCATTATGAGGAATGTTCC
TGTTACCCAGACACTGGCACAGTGATGTGTGTATGCAGGGACAACTGGCATGGTTCAAATCGACCTTGGG
TGTCTTTTAATCAAAACCTGGATTATCAAATAGGATACATCTGCAGTGGGGTGTTCGGTGACAATCCGCG
TCCCAAAGATGGAGAGGGCAGCTGTAATCCAGTGACTGTTGATGGAGCAGACGGAGTAAAGGGGTTTTCA
TACAAATATGGTAATGGTGTTTGGATAGGAAGGACTAAAAGTAACAGACTTAGAAAGGGGTTTGAGATGA
TTTGGGATCCTAATGGATGGACAGATACCGACAGTAATTTCTCAGTGAAACAGGATGTTGTGGCAATAAC
TGATTGGTCAGGGTACAGCGGAAGTTTCGTTCAACATCCTGAGTTAACAGGATTGGACTGTATAAGACCT
TGCTTCTGGGTTGAGTTAGTCAGAGGACTGCCTAGAGAAAATACAACAATCTGGACTAGTGGGAGCAGCA
TTTCTTTTTGTGGCGTAAATAGTGATACTGCAAACTGGTCTTGGCCAGACGGTGCTGAGTTGCCGTTCAC
CATTGACAAGTAGTTCGTTGAAAAAAAA

>gi|131058493|gb|CY020151.1| Influenza A virus (A/Memphis/7/2001(H1N1)) segment 6, complete sequence

ATGAATCCAAATCAAAAAATAATAACCATTGGATCAATCAGTATAGCAATCGGAATAATTAGTCTAATGT
TGCAAATAGGAAATATTATTTCAATATGGGCTAGTCACTCAATCCAAACTGGAAGTAAAAACCACACTGG
AGTATGCAACCAAAGAATCATCACATATGAAAACAGCACCTGGGTGAATCACACATATGTTAATATTAAC
AACACTAATGTTGTTGCTGGAAAGGACAAAACTTCAGTGACATTGGCCGGCAATTCATCTCTTTGTTCTA
TCAGTGGATGGGCTATATACACAAAAGACAACAGCATAAGAATTGGTTCCAAAGGAGATGTGTTTGTCAT
AAGAGAGCCTTTCATATCATGTTCTCACTTGGAATGCAGAACCTTTTTTCTGACCCAAGGTGCTCTATTA
AATGACAAACATTCAAATGGGACCGTTAAGGACAGAAGTCCCTATAGGGCTTTAATGAGCTGTCCTCTAG
GTGAAGCTCCGTCCCCATACAATTCAAAGTTTGAATCAGTTGCTTGGTCAGCAAACGCATGCCATGATGG
CATGGGCTGGTTAACAATCGGAATTTCTGGTCCAGACAATGGAGCAGTGGCTGTACTAAAATACAACGGC
ATAATAACTGAAACCATAAAAAGTTGGAAAAAGCAAATATTAAGAACACAAGAGTCTGAATGTGTCTGTG
TGAACGGGTCATGTTTCACCATAATGACCGATGGCCCGAGTAATGGGGCCGCCTCGTACAAAATCTTCAA
GATAAAAAAGGGAAAGGTTACTAAATCAATAGAGTTGAATGCACCCAATTTTCATTATGAGGAATGTTCC
TGTTACCCAGACACTGGCACAGTGATGTGTGTATGCAGGGACAACTGGCATGGTTCAAATCGACCTTGGG

TGTCTTTTAATCAAAACCTGGATTATCAAATAGGATACATCTGCAGTGGGGTGTTCGGTGACAATCCGCG
TCCCAAAGATGGAGAGGGCAGCTGTAATCCAGTGACTGTTGATGGAGCAAACGGAGTAAAGGGGTTTTCA
TACAAATATGGTAATGGTGTTTGGATAGGAAGGACTAAAAGTAACAGACTTAGAAAGGGGTTTGAGATGA
TTTGGGATCCTAATGGATGGACAGATACCGACAGTGATTTCTCAGTGAGACAGGATGTTGTGGCAATAAC
TGATTGGTCAGGATACAGCGGAAGTTTCGTTCAACATCCTGAGTTAACAGGATTGGACTGTATAAGACCT
TGCTTCTGGGTTGAGTTAGTCAGAGGACTGCCTAGAGAGAATACAACAATCTGGACTAGTGGGAGCAGCA
TTTCTTTTTGTGGCGTAAATAGTGATACTGCAAACTGGTCTTGGCCAGACGGTGCTGAGTTGCCGTTCAC
CATTGACAAGTAG
>gi|73761478|gb|CY002530.1| Influenza A virus (A/New York/220/2002(H1N1)) segment 6, complete sequence
AGCAAAAGCAGGAGTTTAAAATGAATCCAAATCAAAAAATAATAACCATTGGATCAATCAGTATAGCAAT
CGGAATAATTAGTCTAATGTTGCAAATAGGAAATATTATTTCAATATGGGCTAGTCACTCAATCCAAACT
GGAAGTCAAAACCACACTGGAATATGCAACCAAAGAATCATCACATATGAAAACAGCACCTGGGTGAATC
ACACATATGTTAATATTAACAACACTAATGTTGTTGCTGGAAAGGACAAAACTTCAGTGACATTGGCCGG
CAATTCATCTCTTTGTTCTATCAGTGGATGGGCTATATACACAAAAGACAACAGCATAAGAATTGGCTCC
AAAGGAGATGTTTTTGTCATAAGAGAACCTTTCATATCATGTTCTCACTTGGAATGCAGAACCTTTTTTC
TGACCCAAGGTGCTCTATTAAATGACAAACATTCAAATGGAACCGTTAAGGACAGAAGTCCTTATAGGGC
CTTAATGAGCTGTCCTCTAGGTGAAGCTCCGTCCCCATACAATTCAAAGTTTGAATCAGTTGCATGGTCA
GCAAGCGCATGCCATGATGGCATGGGCTGGTTAACAATCGGAATTTCTGGTCCAGACAATGGAGCTGTGG
CTGTACTAAAATACAACGGAATAATAACTGAAACCATAAAAAGTTGGAAAAAGCGAATATTGAGAACACA
AGAGTCTGAATGTGTCTGTGTGAACGGGTCATGTTTCACCATAATGACCGATGGCCCGAGTAATGGGGCC
GCCTCGTACAAAATCTTCAAGATCGAAAAGGGGAAGGTTACTAAATCAATAGAGTTGAATGCACCCAATT
TCCATTATGAGGAATGTTCCTGTTACCCAGACACTGGCACAGTGATGTGTGTATGCAGGGACAACTGGCA
TGGTTCAAATCGACCTTGGGTGTCTTTTAATCAAAACTTGGATTATCAAATAGGATACATCTGCAGTGGA
GTGTTCGGTGACAATCCGCGTCCCAAAGATGGAAAGGGCAGCTGTAATCCAGTGACTGTTGATGGAGCAG
ACGGAGTTAAGGGGTTTTCATACAAATATGGTAATGGTGTTTGGATAGGAAGGACTAAAAGTAACAGACT
TAGAAAGGGGTTTGAGATGATTTGGGATCCTAATGGATGGACAGATACCGACAGTGATTTCTCAGTGAAA
CAGGATGTTGTGGCAATAACTGATTGGTCAGGGTACAGCGGAAGTTTCGTTCAACATCCTGAGTTAACAG
GATTGGACTGTATAAGACCTTGCTTCTGGGTTGAGTTAGTCAGAGGACTGCCTAGAGAAAATACAACAAT
CTGGACTAGTGGGAGCAGCATTTCTTTTTGTGGCATTGATAGTGATACTGCAAACTGGTCTTGGCCAGAC
GGTGCTGAGTTGCCGTTCACCATTGACAAGTAGTTCGTTGAAAAAACTCCTTGTTTCTACT
>gi|156536322|gb|CY025036.1| Influenza A virus (A/Auckland/597/2000(H1N1)) segment 6, complete sequence
ATGAATACAAATCAAAAAATAATAACCATTGGATCAATCAGTATAGCAATCGGAATAATTAGTCTAATGT
TGCAAATAGGAAATATTATTTCAATATGGGCTAGTCACTCAATCCAAACTGGAAGTCAAAACCACACTGG
AATATGCAACCAAAGAATCATCACATATGAAAACAGCACCTGGGTGAATCACACATATGTTAATATTAAC
AACACTAATGTTGTTGCTGGAAAGGACAAAACTTCAGTGACATTGGCCGGCAATTCATCTCTTTGTTCCA
TCAGTGGATGGGCTATATACACAAAAGACAACAGCATAAGAATTGGATCCAAAGGAGATGTTTTTGTCAT
GAGAGAACCTTTCATATCATGTTCTCACTTGGAATGCAGAACCTTTTTTCTGACCCAAGGTGCTCTATTA
AATGACAAACATTCAAATGGGACCGTTAAGGACAGAAGTCCTTATAGGGCCTTAATGAGCTGCCCTCTAG
GTGAAGCTCCGTCCCCATACAATTCAAAGTTTGAATCAGTTGCATGGTCAGCAAGCGCATGCCATGATGG
CATGGGCTGGTTAACAATCGGAATTTCTGGTCCAGACAATGGAGCTGTGGCTGTACTAAAATACAACGGC
ATAATAACTGAAACCATAAAAAGTTGGAAAAAGCGAATATTAAGAACACAAGAGTCTGAATGTGTCTGTG
TGAACGGGTCATGTTTCACCATAATGACCGATGGCCCGAGTAATGGGGCCGCCTCGTACAAAATCTTCAA

GATCGAAAAGGGGAAGGTTACTAAATCAATAGAGTTGAATGCACCCAATTTTCATTATGAGGAATGTTCC
TGTTACCCAGACACTGGCACAGTGATGTGTGTATGCAGGGACAACTGGCATGGTTCAAATCGACCTTGGG
TGTCTTTTAATCAAAACCTGGATTATCAAATAGGATACATCTGCAGTGGGGTGTTCGGTGACAATCCGCG
TCCCAAAGATGGAGAGGGCAGCTGTAATCCAGTGACTGTTGATGGAGCAGACGGAGTAAAGGGGTTTTCA
TACAAATATGGTAATGGTGTTTGGATAGGAAGGACTAAAAGTAACAGACTTAGAAAGGGGTTTGAGATGA
TTTGGGATCCTAATGGATGGACAGATACCGACAGTAATTTCTCAGTGAAACAGGATGTTGTGGCAATAAC
TGATTGGTCAGGGTACAGCGGAAGTTTCGTTCAACATCCTGAGTTAACAGGATTGGACTGTATAAGACCT
TGCTTCTGGGTTGAGTTAGTCAGAGGACTGCCTAGAGAAAATACAACAATCTGGACTAGTGGGAGCAGCA
TTTCTTTTTGTGGCGTAAATAGTGATACTGCAAACTGGTCTTGGCCAGACGGTGCTGAGTTGCCGTTCAC
CATTGACAAGTAGTTCGTTGAAAAAAAA
>gi|149780531|gb|CY022535.1| Influenza A virus (A/Auckland/605/2001(H1N1)) segment 6, complete sequence
ATGAATCCAAATCAAAAAATAATAACCATTGGATCAATCAGTATAGCAATCGGAATAATTAGCCTAATGT
TGCAAATAGGAAACATTATTTCAATATGGGCTAGTCACTCAATCCAAACTGGAAGTCAAAACCACACTGG
AGTATGCAACCAAAGAATCATCACATATGAAAACAGCACCTGGGTGAATCACACATATGTTAATATTAAC
AACACCAATGTTATTGCTGGAAAGGACAAAACTTCAGTGACATTGGCCGGCAATTCATCCCTTTGTTCTA
TCAGTGGATGGGCTATATACACAAAAGACAACAGCATAAGAATTGGTTCCAAAGGAGATGTTTTTGTCAT
AAGAGAGCCTTTCATATCATGTTCTCACTTGGAATGCAGAACCTTTTTTCTGACCCAAGGTGCTCTATTA
AATGACAAACATTCAAATGGGACCGTTAAGGACAGAAGCCCCTATAGGGCTTTAATGAGCTGTCCTCTAG
GTGAAGCTCCGTCCCCATACAATTCAAAGTTTGAATCAGTTGCTTGGTCAGCAAGCGCATGCCATGATGG
CATGGGCTGGTTAACAATCGGAATTTCTGGTCCAGACAATGGAGCAGTGGCTGTACTAAAATACAACGGC
ATAATAACTGAAACCATAAAAAGTTGGAAAAAGCAAATATTAAGAACACAAGAGTCTGAATGTGTCTGTG
TGAACGGGTCATGTTTCACCATAATGACCGATGGTCCGAGTAATGGGGCCGCCTCGTACAAAATCTTCAA
GATCGAAAAGGGAAAGGTTACTAAATCAATAGAGTTGAATGCACCCAATTTTCATTATGAGGAATGTTCC
TGTTACCCAGACACTGGCACAGTGATGTGTGTATGCAGGGACAACTGGCATGGTTCAAATCGACCCTGGG
TGTCTTTTAATCAAAACCTGGATTATCAAATAGGATACATCTGCAGTGGGGTGTTCGGTGACAATCCGCG
TCCCAAAGATGGAGAGGGCAGCTGTAATCCAGTGACTGTTGATGGAGCAAACGGAGTAAAGGGGTTTTCA
TACAAATATGGTAATGGTGTTTGGATAGGAAGGACTAAAAGTAACAGACTTAGAAAGGGGTTTGAGATGA
TTTGGGATCCTAATGGATGGACAGATACCGACAGTGATTTCTCAGTGAAACAGGATGTTGTGGCAATAAC
TGATTGGTCTGGGTACAGCGGAAGTTTCGTTCAACATCCTGAGTTAACAGGATTGGACTGTATAAGACCT
TGCTTCTGGGTTGAGTTAGTCAGAGGACTGCCAAAAGAAAATACAACAATCTGGACTAGTGGGAGCAGCA
TTTCTTTTTGTGGCGTAAATAGTGATACTGCAAACTGGTCTTGGCCAGACGGTGCTGAGTTGCCGTTTAC
CATTGACAAGTAGTTCGTTGAAAAAAAA
>gi|237688867|gb|CY040076.1| Influenza A virus (A/Taiwan/567/2002(H1N1)) segment 6, complete sequence
ATGAATCCAAATCAAAAAATAATAACCATTGGATCAATCAGTATAGCAATCGGAATAATTAGCCTAATGT
TGCAAATAGGAAACATTATTTCAATATGGGCTAGTCACTCAATCCAAACTGGAAGTCAAAATCACACTGG
AGTATGCAACCAAAGAATCATCACATATGAAAACAGCACCTGGGTGAATCACACATATGTTAATATTAAC
AACACCAATGTTATTGCTGGAAAGGACAAAACTTCAGTGACATTGGCCGGCAATTCATCTCTTTGTTCTA
TCAGTGGATGGGCTATATACACAAAAGACAACAGCATAAGAATTGGTTCCAAAGGAGATGTTTTTGTCAT
AAGAGAGCCTTTCATATCATGTTCTCACTTGGAATGCAGAACCTTTTTTCTGACCCAAGGTGCTCTATTA
AATGACAAACATTCAAATGGGACCGTTAAGGACAGAAGCCCCTATAGGGCTTTAATGAGCTGTCCTCTAG
GTGAAGCTCCGTCCCCATACAATTCAAAGTTTGAATCAGTTGCTTGGTCAGCAAGCGCATGCCATGATGG
CATGGGCTGGTTAACAATCGGAATTTCTGGTCCAGACAATGGAGCAGTGGCTGTACTAAAATACAACGGC

ATAATAACTGAAACCATAAAAAGTTGGAAAAAGCAAATATTAAGAACACAAGAGTCTGAATGTGTCTGTG
TGAACGGGTCGTGTTTCACCATAATGACCGATGGCCCGAGTAATGGGGCCGCCTCGTACAAAATCTTCAA
GATCGAAAAGGGAAAGGTTACTAAATCAATAGAGTTGAATGCACCCAATTTTCATTATGAGGAATGTTCC
TGTTACCCAGACACTGGCACAGTGATGTGTGTATGCAGGGACAACTGGCATGGTTCAAATCGACCCTGGG
TGTCCTTTAATCAAAACCTGGATTATCAAATAGGATACATCTGCAGTGGGGTGTTCGGTGACAATCCGCG
TCCCAAAGATGGAGAGGGCAGCTGTAATCCAGTGRCTGTTGATGGAGCAAACGGAGTAAAGGGGTTTTCA
TACAAATATGGTAATGGTGTTTGGATAGGAAGGACTAAAAGTAACAGACTTAGAAAGGGGTTTGAGATGA
TTTGGGATCCTAATGGATGGACAGATACCGACAGTGATTTCTCAGTGAAACAGGATGTTGTGGCAATAAC
TGATTGGTCTGGGTACAGCGGAAGTTTCGTTCAACATCCTGAGTTAACAGGATTGGACTGTATAAGACCT
TGCTTCTGGGTTGAGTTAGTCAGAGGACTGCCAAAAGAAAATACAACAATCTGGACTAGTGGGAGCAGCA
TTTCTTTTTGTGGCGTAAATAGTGATACTGCAAACTGGTCTTGGCCAGACGGTGCTGAGTTGCCGTTTAC
CATTGACAAGTAATTC

>gi|122855950|gb|CY019343.1| Influenza A virus (A/Memphis/6/2003(H1N1)) segment 6, complete sequence

ATGAATCCAAATCAAAAAATAATAACCATTGGATCAATCAGTATAGCAATCGGAACAATTAGTCTAATGT
TGCAAATAGGAAATATGATTTCAATATGGGCTAGTCACTCAATCCAAACTGGAAGTCAAAACCACACTGG
AATATGCAACCAAAGAATCATCACATATGAAAACAGCACCTGGGTGAATCACACATATGTTAATATTAAC
AACACTAATGTTGTTGCTGGAAAGGACAAAACTTCAGTGACATTGGCCGGCAATTCATCTCTTTGTTCTA
TCAGTGGATGGGCTATATACACAAAAGACAACAGCATAAGAATTGGCTCCAAAGGAGATGTTTTTGTCAT
AAGAGAACCTTTCATATCATGTTCTCACTTGGAATGCAGAACCTTTTTTCTGACCCAAGGTGCTCTATTA
AATGACAAACATTCAAATGGAACCGTTAAGGACAGAAGTCCTTATAGGGCCTTAATGAGCTGTCCTCTAG
GTGAAGCTCCGTCCCCATACAATTCAAAGTTTGAATCAGTTGCATGGTCAGCAAGCGCATGCCATGATGG
CATGGGCTGGTTAACAATCGGAATTTCTGGTCCAGACAATGGAGCTGTGGCTGTACTAAAATACAACGGA
ATAATAACTGAAACCATAAAAAGTTGGAAAAAGCGAATATTGAGAACACAAGAGTCTGAATGTGTCTGTG
TGAACGGGTCATGTTTCACCATAATGACCGATGGCCCGAGTAATGGGGCCGCCTCGTACAAAATCTTCAA
GATCGAAAAGGGGAAGGTTACTAAATCAATAGAGTTGAATGCACCCAATTTTCATTATGAGGAATGTTCC
TGTTACCCAGACACTGGCACAGTGATGTGTGTATGCAGGGACAATTGGCATGGCTCAAATCGACCTTGGG
TGTCTTTTAATCAAAACTTGGATTATCAAATAGGATACATCTGCAGTGGAGTGTTCGGTGACAATCCGCG
TCCCAAAGATGGAAAGGGCAGCTGTAATCCAGTGACTGTTGATGGAGCAGACGGAGTTAAGGGGTTTTCA
TACAAATATGGTAATGGTGTTTGGATAGGAAGGACTAAAAGTAACAGACTTAGAAAGGGGTTTGAGATGA
TTTGGGATCCTAATGGATGGACAGATACCGACAGTGATTTCTCAGTGAAACAGGATGTTGTGGCAATAAC
TGATTGGTCAGGGTACAGCGGAAGTTTCGTTCAACATCCTGAGTTAACAGGATTGGACTGTATAAGACCT
TGCTTCTGGGTTGAGTTAGTCAGAGGACTGCCTAGAGAAAATACAACAATCTGGACTAGTGGGAGCAGCA
TTTCTTTTTGTGGCGTTGATAGTGATACTGCAAACTGGTCTTGGCCAGACGGTGCTGAGTTGCCGTTCAC
CATTGACAAGTAG

>gi|82546779|gb|CY006677.1| Influenza A virus (A/New York/494/2002(H1N1)) segment 6, complete sequence

GAGTTTAAAATGAATCCAAATCAAAAAATAATAACCATTGGATCAATCAGTATAGCAATCGGAATAATTA
GTCTAATGTTGCAAATAGGAAATATTATTTCAATATGGGCTAGTCACTCAATCCAAACTGGAAGTCAAAA
CCACACTGGAATATGCAACCAAAGAATCATCACATATGAAAACAGCACCTGGGTGAATCACACATATGTT
AATATTAACAACACTAATGTTATTGCTGAAAAGGACAAAACTTCAGTGACATTGGCCGGCAATTCATCTC
TTTGTTCCATCAGTGGATGGGCTATATACACAAAAGACAACAGCATAAGAATTGGCTCCAAAGGAGATGT
TTTTGTCATAAGAGAACCTTTCATATCATGTTCTAACTTGGAATGCAGAACCTTTTTTCTGACCCAAGGT
GCTCTATTAAATGACAAACATTCAAATGGGACCGTTAAGGACAGAAGTCCTTATAGGGCCTTAATGAGCT

GTCCTCTAGGTGAAGCTCCGTCCCCATACAATTCAAAGTTTGAATCAGTTGCATGGTCAGCAAGCGCATG
CCATGATGGCATGGGCTGGTTAACAATCGGAATTTCTGGTCCAGACAATGGAGCTGTGGCTGTACTAAAA
TACAACGGAATAATAACTGAAACCATAAAAAGTTGGAAAAAGCGAATATTAAGAACACAAGAGTCTGAAT
GTGTCTGTGTGAACGGGTCATGTTTCACCATAATGACCGATGGCCCGAGTAATGGGGCCGCCTCGTACAA
AATCTTCAAGATCGAAAAGGGGAAGGTTACTAAATCAATAGAGTTGAATGCACCCAATTTTCATTATGAG
GAATGTTCCTGTTACCCAGACACTGGCACAGTGATGTGTGTATGCAGGGACAACTGGCATGGTTCAAATC
GACCTTGGGTGTCTTTTAATCAAAACTTGGATTATCAAATAGGATACATCTGCAGTGGAGTGTTCGGTGA
CAATCCTCGTCCCAAAGATGGAGAGGGCAGCTGTAATCCAGTGACTGTTGATGGAGCAGACGGAGTAAAG
GGGTTTTCATACAAATATGGTAATGGTGTTTGGATAGGAAGGACTAAAAGTAACAGACTTAGAAAGGGGT
TTGAGATGATTTGGGATCCTAATGGATGGACAGATACCGACAGTGATTTCTCAGTGAAACAGGATGTTGT
GGCAATAACTGATTGGTCAGGGTACAGCGGAAGTTTTGTTCAACATCCTGAGTTAACAGGATTGGACTGT
ATAAGACCTTGCTTCTGGGTTGAGTTAGTCAGAGGACTGCCTAGAGAAAATACAACAATCTGGACTAGTG
GGAGCAGCATTTCTTTTTGTGGCGTTAATAGTGATGCTGCAGACTGGTCTTGGCCAGACGGTGCTGAGTT
GCCGTTCACCATTGACAAGTAGTTCGTTGAAAAAA
>gi|77543346|gb|CY003306.1| Influenza A virus (A/New York/291/2002(H1N1)) segment 6, complete sequence
GTTTAAAATGAATCCAAATCAAAAAATAATAACCATTGGATCAATCAGTATAGCAATCGGAATAATTAGT
CTAATGTTGCAAATAGGAAATATTATTTCAATATGGGCTAGTCACTCAATCCAAACTGGAAGTCAAAACC
ACACTGGAATATGCAACCAAAAAATCATCACATATAAAAACAGCACCTGGGTGAATCACACATATGTTAA
TATTAACAACACTAATGTTGTTGCTGGAAAGGACAAAACTTCAGTGACATTGGCCGGCAATTCATCTCTT
TGTTCTATCAGTGGATGGGCTATATACACAAAAGATAACAGCATAAGAATTGGCTCCAAAGGAGATGTTT
TTGTCATAAGAGAACCTTTCATATCATGTTCTCACTTGGAATGCAGAACCTTTTTTCTGACCCAAGGTGC
TCTATTAAATGACAAACATTCAAATGGGACCGTTAAGGACAGAAGTCCTTATAGGGCCTTAATGAGCTGT
CCTCTAGGTGAAGCTCCGTCTCCATACAATTCAAAGTTTGAATCAGTTGCATGGTCAGCAAGCGCATGCC
ATGATGGCATGGGCTGGTTAACAATCGGAATTTCTGGTCCAGACAATGGAGCTGTGGCTGTACTAAAATA
CAACGGAATAATAACTGAAACCATAAAAAGTTGGAAAAAGCGAATATTAAGAACACAAGAGTCTGAATGT
GTCTGTGTGAACGGGTCATGTTTCACCATAATGACCGATGGCCCGAGTAATGGGGCCGCCTCGTACAAAA
TCTTCAAGATCGAAAAGGGGAAGGTTACTAAATCAATAGAGTTGAATGCACCCAATTTTCATTATGAGGA
ATGTTCCTGTTACCCAGACACTGGCACAGTGATGTGTGTATGCAGGGACAACTGGCATGGTTCAAATCGA
CCTTGGGTGTCTTTTAATCAAAACTTGGATTATCAAATAGGATACATCTGCAGTGGAGTGTTCGGTGACA
ATCCGCGTCCCAAAGATGGAGAGGGCAGCTGTAATCCAGTGACTGTTGATGGAGCAGACGGAGTAAAGGG
GTTTTCATACAAATATGGTAATGGTGTTTGGATAGGAAGGACTAAAAGTAACAGACTTAGAAAGGGGTTT
GAGATGATTTGGGATCCTAATGGATGGACAGATACCGACAGTGTTTTCTCAGTGAAACAGGATGTTGTGG
CAATAACTGATTGGTCAGGGTACAGCGGAAGTTTCGTTCAACATCCTGAGTTAACAGGATTGGACTGTAT
AAGACCTTGTTTCTGGGTTGAGTTAGTCAGAGGACTGCCTAGAGAAAATACAACAATCTGGACTAGTGGG
AGCAGCATTTCTTTTTGTGGCGTTAATAGTGATACTGCAAACTGGTCTTGGCCAGACGGTGCTGAGTTGC
CGTTCACCATTGACAAGTAGTTCGTTGAAAAAAAC
>gi|237689040|gb|CY040148.1| Influenza A virus (A/Taiwan/52/2002(H1N1)) segment 6, complete sequence
AATGAATCCAAATCAAAAAATAATAACCATTGGATCAATCAGTATAGCAATCGGAATAATTAGCCTAATG
TTGCAAATAGGAAACATTATTTCAATATGGGCTAGTCACTCAATCCAAACTGGAAGTCAAAACCACACTG
GAGTATGCAACCAAAGAATCATCACATATGAAAACAGCACCTGGGTGAATCACACATATGTTAATATTAA
CAACACCAATGTTATTGCTGGAAAGGACAAAACTTCAGTGACATTGGCCGGCAATTCATCTCTTTGTTCT
ATCAGTGGATGGGCTATATACACAAAAGACAACAGCATAAGAATTGGTTCCAAAGGAGATGTTTTTGTCA

TAAGAGAGCCTTTCATATCATGTTCTCACTTGGAATGCAGAACCTTTTTCTGACTCAAGGTGCTCTATT
AAATGACAAACATTCAAATGGGACCGTTAAGGACAGAAGCCCCTATAGGGCTTTAATGAGCTGTCCTCTA
GGTGAAGCTCCGTCCCCATACAATTCAAAGTTTGAATCAGTTGCTTGGTCAGCAAGCGCATGCCATGATG
GCATGGGCTGGTTAACAATCGGAATTTCTGGTCCAGACAATGGAGCAGTGGCTGTACTAAAATACAACGG
CATAATAACTGAAACCATAAAAAGTTGGAAAAGGCAAATATTAAGAACACAAGAGTCTGAATGTGTCTGT
GTGAACGGGTCATGTTTCACCATAATGACCGATGGCCCGAGTAATGGGGCCGCCTCGTACAAAATCTTCA
AGATCGAAAAGGGAAAGGTTACTAAATCAATAGAGTTGAATGCACCCAATTTTCATTATGAGGAATGTTC
CTGTTACCCAGACACTGGCACAGTGATGTGTGTATGCAGGGACAACTGGCATGGTTCAAATCGACCCTGG
GTGTCCTTTAATCAAAACCTGGATTATCAAATAGGATACATCTGCAGTGGGGTGTTCGGTGACAATCCGC
GTCCCAAAGATGGAGAGGGCAGCTGTAATCCAGTGACTGTTGATGGAGCAAACGGAGTAAAGGGGTTTTC
ATACAAATATGGTAATGGTGTTTGGATAGGAAGGACTAAAAGTAACAGACTTAGAAAGGGGTTTGAGATG
ATTTGGGATCCTAATGGATGGACAGATACCGACAGTGATTTCTCAGTGAAACAGGATGTTGTGGCAATAA
CTGATTGGTCTGGGTACAGCGGAAGTTTCGTTCAACATCCTGAGTTAACAGGATTGGACTGTATAAGACC
TTGCTTCTGGGTTGAGTTAGTCAGAGGACTGCCAAAAGAAAATACAACAATCTGGACTAGTGGGAGCAGC
ATTTCTTTTTGTGGCGTAAATAGTGATACTGCAAACTGGTCTTGGCCAGACGGTGCTGAGTTGCCGTTTA
CCATTGACAAGTAATTCGTTGAAA
>gi|237689059|gb|CY040156.1| Influenza A virus (A/Taiwan/123/2002(H1N1)) segment 6, complete sequence
AATGAATCCAAATCAAAAAATAATAACCATCGGATCAATCAGTATAGCAATCGGAATAATTAGCCTAATG
TTGCAAATAGGAAACATTATTTCAATATGGGCTAGTCACTCAATCCAAACTAGAAGTCAAAACCACACTG
GAGTATGCAACCAAAGAATCATCACATATGAAAACAGCACCTGGGTGAATCACACATATGTTAATATTAA
CAACACCAATGTTATTGCTGGAAAGGACAAAACTTCAGTGACATTGGCCGGCAATTCATCTCTTTGTTCT
ATCAGTGGATGGGCTATATACACAAAAGACAACAGCATAAGAATTGGTTCCAAAGGAGATGTTTTTGTCA
TAAGAGAGCCTTTCATATCATGTTCTCACTTGGAATGCAGAACCTTTTTCTGACCCAAGGTGCTCTATT
AAATGACAAACATTCAAATGGGACCGTTAAGGACAGAAGCCCCTATAGGGCTTTAATGAGCTGTCCTCTA
GGTGAAGCTCCGTCCCCATACAATTCAAAGTTTGAATCAGTTGCTTGGTCGGCAAGCGCATGCCATGATG
GCATGGGCTGGTTAACAATCGGAATTTCTGGTCCAGACAATGGAGCAGTGGCTGTACTAAAATACAACGG
CATAATAACTGAAACCATAAAAAGTTGGAAAAAGCAAATATTAAGAACACAGGAGTCTGAATGTGTCTGT
GTGAACGGGTCATGTTTCACCATAATGACCGATGGCCCGAGTAATGGGGCCGCCTCGTACAAAATCTTCA
AGATCGAAAAGGGAAAGGTTACTAAATCAATAGAGTTGAATGCACCCAATTTTCATTATGAGGAATGTTC
CTGTTACCCAGACACTGGCACAGTGATGTGTGTATGCAGGGACAACTGGCATGGTTCAAATCGACCCTGG
GTGTCTTTTAATCAAAACCTGGATTATCAAATAGGATACATCTGCAGTGGGGTGTTCGGTGACAATCCGC
GTCCCAAAGATGGAGAGGGCAGCTGTAATCCAGTGACTGTTGATGGAGCGAACGGAGTAAAGGGGTTTTC
ATACAAATATGGTAATGGTGTTTGGATAGGAAGGACTAAAAGTAACAGACTTAGAAAGGGGTTTGAGATG
ATTTGGGATCCTAATGGATGGACAGATACCGACAGTGATTTCTCAGTGAAACAGGATGTTGTGGCAATAA
CTGATTGGTCTGGGTACAGCGGAAGTTTCGTTCAACATCCTGAGTTAACAGGATTGGACTGTATAAGACC
TTGCTTCTGGGTTGAGTTAGTCAGAGGACTGCCAAAAGAAAATACAACAATCTGGACTAGTGGGAGCAGC
ATTTCTTTTTGTGGTGTAAATAGTGATACTGCAAACTGGTCTTGGCCAGACGGTGCTGAGTTGCCGTTTA
CCATTGACAAGTAATTCGTTGAAA
>gi|77747426|gb|CY003690.1| Influenza A virus (A/New York/486/2003(H1N1)) segment 6, complete sequence
CAAAAGCAGGAGTTTAAAATGAATCCAAATCAAAAAATAATAACCATTGGATCAATCAGTATAGCAATCG
GAATAATTAGTCTAATGTTGCAAATAGGAAATATTATTTCAATATGGGCTAGTCACTCAATCCAAACTGG
AAGTCAAAACCACACTGGAATATGCAACCAAAGAATCATCACATATGAAAACAGCACCTGGGTGAATCAC

ACATATGTTAATATTAACAACACTAATGTTGTTGCTGGAAAGGACAAAACTTCAGTGACATTGGCCGGCA
ATTCATCTCTTTGTTCTATCAGTGGATGGGCTATATACACAAAAGACAACAGCATAAGAATTGGCTCCAA
AGGAGATGTTTTTGTCATAAGAGAACCTTTCATATCATGTTCTCACTTGGAATGCAGAACCTTTTTTCTG
ACCCAAGGTGCTCTATTAAATGACAAACATTCAAATGGAACCGTTAAGGACAGAAGTCCTTATAGGGCCT
TAATGAGCTGTCCTCTAGGTGAAGCTCCGTCCCCATACAATTCAAAGTTTGAATCAGTTGCATGGTCAGC
AAGCGCATGCCATGATGGCATGGGCTGGTTAACAATCGGAATTTCTGGTCCAGACAATGGAGCTGTGGCT
GTACTAAAATACAACGGAATAATAACTGAAACCATAAAAAGTTGGAAAAAGCGAATATTGAGAACACAAG
AGTCTGAATGTGTCTGTGTGAACGGGTCATGTTTCACCATAATGACCGATGGCCCGAGTAATGGGGCCGC
CTCGTACAAAATCTTTAAGATCGAAAAGGGGAAGGTTACTAAATCAATAGAGTTGAATGCACCCAATTTT
CATTATGAGGAATGTTCCTGTTACCCAGACACTGGCACAGTGATGTGTGTATGCAGGGACAACTGGCATG
GTTCAAATCGACCTTGGGTGTCTTTTAATCAAAACTTGGATTATCAAATAGGATACATCTGCAGTGGAGT
GTTCGGTGACAATCCGCGTCCCAAAGATGGAAAGGGCAGCTGTAATCCAGTGACTGTTGATGGAGCAGAC
GGAGTTAAGGGGTTTTCATACAAATATGGTAATGGTGTTTGGATAGGAAGGACTAAAAGTAACAGACTTA
GAAAGGGGTTTGAGATGATTTGGGATCCTAATGGATGGACAGATACCGACAGTGATTTCTCAGTGAAACA
GGATGTTGTGGCAATAACTGATTGGTCAGGGTACAGCGGAAGTTTCGTTCAACATCCTGAGTTAACAGGA
TTGGACTGTATAAGACCTTGCTTCTGGGTTGAGTTAGTCAGAGGACTGCCTAGAGAAAATACAACAATCT
GGACTAGTGGGAGCAGCATTTCTTTTTGTGGCGTTGATAGTGATACTGCAAACTGGTCTTGGCCAGACGG
TGCTGAGTTGCCGTTCACCATTGACAAGTAGTTCGTTGAAAAAAAACTCCTTGTTTCTACT
>gi|125664180|gb|CY019885.1| Influenza A virus (A/Memphis/5/2003(H1N1)) segment 6, complete sequence
ATGAATCCAAATCAAAAAATAATAACCATTGGATCAATCAGTATAGCAATCGGAATAATTAGTCTAATGT
TGCAAATAGGAAATATTATTTCAATATGGGCTAGTCACTCAATCCAAACTGGAAGTCAAAACCACACTGG
AATATGCAACCAAAGAATCATCACATATGAAAACAGCACCTGGGTGAATCACACATATGTTAATATTAAC
AACACTAATGTTGTTGCTGGAAAGGACAAAACTTCAGTGACATTGGCCGGCAATTCATCTCTTTGTTCTA
TCAGTGGATGGGCTATATACACAAAAGACAACAGCATAAGAATTGGCTCCAAAGGAGATGTTTTTGTCAT
AAGAGAACCTTTCATATCATGTTCTCACTTGGAATGCAGAACCTTTTTTCTGACCCAAGGTGCTCTATTA
AATGACAAACATTCAAATGGAACCGTTAAGGACAGAAGTCCTTATAGGGCCTTAATGAGCTGTCCTCTAG
GTGAAGCCCCGTCCCCATACAATTCAAAGTTTGAATCAGTTGCATGGTCAGCAAGCGCATGCCATGATGG
CATGGGCTGGTTAACAATCGGAATTTCTGGTCCAGACAATGGAGCTGTGGCTGTACTAAAATACAACGGA
ATAATAACTGAAACCATAAAAAGTTGGAAAAAGCGAATATTGAGAACACAAGAGTCTGAATGTGTCTGTG
TGAACGGGTCATGTTTCACCATAATGACCGATGGCCCGAGTAATGGGGCCGCCTCGTACAAAATCTTCAA
GATCGAAAAGGGGAAGGTTACTAAATCAATAGAGTTGAATGCACCCAATTTTCATTATGAGGAATGTTCC
TGTTACCCAGACACTGGCACAGTGATGTGTGTATGCAGGGACAACTGGCATGGTTCAAATCGACCTTGGG
TGTCTTTTAATCAAAACTTGGATTATCAAATAGGATACATCTGCAGTGGAGTGTTCGGTGACAATCCGCG
TCCCAAAGATGGGAAGGGCAGCTGTAATCCAGTGACTGTTGATGGAGCAGACGGAGTTAAGGGGTTTTCA
TACAAATATGGTAATGGTGTTTGGATAGGAAGGACTAAAAGTAACAGACTTAGAAAGGGGTTTGAGATGA
TTTGGGATCCTAATGGATGGACAGATACCGACAGTGATTTCTCAGTGAAACAGGATGTTGTGGCAATAAC
TGATTGGTCAGGGTACAGCGGAAGTTTCGTTCAACATCCTGAGTTAACAGGATTGGACTGTATAAGACCT
TGCTTCTGGGTTGAGTTAGTCAGAGGACTGCCTAGAGAAAATACAACAATCTGGACTAGTGGGAGCAGCA
TTTCTTTTTGTGGCGTTGATAGTGATACTGCAAACTGGTCTTGGCCAGACGGTGCTGAGTTGCCGTTCAC
CATTGACAAGTAG
>gi|73763199|gb|CY002538.1| Influenza A virus (A/New York/227/2003(H1N1)) segment 6, complete sequence
AGCAAAAGCAGGAGTTTAAAATGAATCCAAATCAAAAAATAATAACCATTGGATCAATCAGTATAGCAAT

CGGAATAATTAGTCTAATGTTGCAAATAGGAAATATTATTTCAATATGGGCTAGTCACTCAATCCAAACT
GGAAGTCAAAACCACACTGGAATATGCAACCAAAGAATCATCACATATGAAAACAGCACCTGGGTGAATC
ACACATATGTTAATATTAACAACACTAATGTTGTTGCTGGAAAGGACAAAACTTCAGTGACATTGGCCGG
CAATTCATCTCTTTGTTCTATCAGTGGATGGGCTATATACACAAAAGACAACAGCATAAGAATTGGCTCC
AAAGGAGATGTTTTTGTCATAAGAGAACCTTTCATATCATGTTCTCACTTGGAATGCAGAACCTTTTTTC
TGACCCAAGGTGCTCTATTAAATGACAAACATTCAAATGGAACCGTTAAGGACAGAAGTCCTTATAGGGC
CTTAATGAGCTGTCCTCTAGGTGAAGCTCCGTCCCCATACAATTCAAAGTTTGAATCAGTTGCATGGTCA
GCAAGCGCATGCCATGATGGCATGGGCTGGTTAACAATCGGAATTTCTGGTCCAGACAATGGAGCTGTGG
CTGTACTAAAATACAACGGAATAATAACTGAAACCATAAAAAGTTGGAAAAAGCGAATATTGAGAACACA
AGAGTCTGAATGTGTCTGTGTGAACGGGTCATGTTTCACCATAATGACCGATGGCCCGAGTAATGGGGCC
GCCTCGTACAAAATCTTCAAGATCGAAAAGGGGAAGGTTACTAAATCAATAGAGTTGAATGCACCCAATT
TTCATTATGAGGAATGTTCCTGTTACCCAGACACTGGCACAGTGATGTGTGTATGCAGGGACAACTGGCA
TGGTTCAAATCGACCTTGGGTGTCTTTTAATCAAAACTTGGATTATCAAATAGGATACATCTGCAGTGGA
GTGTTCGGTGACAATCCGCGTCCCAAAGATGGAAAGGGCAGCTGTAATCCAGTGACTGTTGATGGAGCAG
ACGGAGTTAAGGGGTTTTCATACAAATATGGTAATGGTGTTTGGATAGGAAGGACTAAAAGTAACAGACT
TAGAAAGGGGTTTGAGATGATTTGGGATCCTAATGGATGGACAGATACCGACAGTGATTTCTCAGTGAAA
CAGGATGTTGTGGCAATAACTGATTGGTCAGGGTACAGCGGAAGTTTCGTTCAACATCCTGAGTTAACAG
GATTGGACTGTATAAGACCTTGCTTCTGGGTTGAGTTAGTCAGAGGACTGCCTAGAGAAAATACAACAAT
CTGGACTAGTGGGAGCAGCATTTCTTTTTGTGGCGTTGATAGTGATACTGCAAACTGGTCTTGGCCAGAC
GGTGCTGAGTTGCCGTTCACCATTGACAAGTAGTTCGTTGAAAAAAAACTCCTTGTTTCTACT
>gi|89112170|gb|CY008998.1| Influenza A virus (A/New York/484/2003(H1N1)) segment 6, complete sequence
GTTTAAAATGAATCCAAATCAAAAAATAATAACCATTGGATCAATCAGTATAGCAATCGGAATAATTAGT
CTAATGTTGCAAATAGGAAATATTATTTCAATATGGGCTAGTCACTCAATCCAAACTGGAAGTCAAAACC
ACACTGGAATATGCAACCAAAGAATCATCACATATGAAAACAGCACCTGGGTGAATCACACATATGTTAA
TATTAACAACACTAATGTTGTTGCTGGAAAGGACAAAACTTCAGTGACATTGGCCGGCAATTCATCTCTT
TGTTCTATCAGTGGATGGGCTATATACACAAAAGACAACAGCATAAGAATTGGCTCCAAAGGAGATGTTT
TTGTCATAAGAGAACCTTTCATATCATGTTCTCACTTGGAATGCAGAACCTTTTTTCTGACCCAAGGTGC
TCTATTAAATGACAAACATTCAAATGGAACCGTTAAGGACAGAAGTCCTTATAGGGCCTTAATGAGCTGT
CCTCTAGGTGAAGCTCCGTCCCCATACAATTCAAAGTTTGAATCAGTTGCATGGTCAGCAAGCGCATGCC
ATGATGGCATGGGCTGGTTAACAATCGGAATTTCTGGTCCAGACAATGGAGCTGTGGCTGTACTAAAATA
CAACGGAATAATAACTGAAACCATAAAAAGTTGGAAAAAGCGAATATTGAGAACACAAGAGTCTGAATGT
GTCTGTGTGAACGGGTCATGTTTCACCATAATGACCGATGGCCCGAGTAATGGGGCCGCCTCGTACAAAA
TCTTCAAGATCGAAAAGGGGAAGGTTACTAAATCAATAGAGTTGAATGCACCCAATTTCCATTATGAGGA
ATGTTCCTGTTACCCAGACACTGGCACAGTGATGTGTGTATGCAGGGACAACTGGCATGGTTCAAATCGA
CCTTGGGTGTCTTTTAATCAAAACTTGGATTATCAAATAGGATACATCTGCAGTGGAGTGTTCGGTGACA
ATCCGCGTCCCAAAGATGGAAAGGGCAGCTGTAATCCAGTGACTGTTGATGGAGCAGACGGAGTTAAGGG
GTTTTCATACAAATATGGTAATGGTGTTTGGATAGGAAGGACTAAAAGTAACAGACTTAGAAAGGGGTTT
GAGATGATTTGGGATCCTAATGGATGGACAGATACCGACAGTGATTTCTCAGTGAAACAGGATGTTGTGG
CAATAACTGATTGGTCAGGGTACAGCGGAAGTTTCGTTCAACATCCTGAGTTAACAGGATTGGACTGTAT
AAGACCTTGCTTCTGGGTTGAGTTAGTCAGAGGACTGCCTAGAGAAAATACAACAATCTGGACTAGTGGG
AGCAGCATTTCTTTTTGTGGCGTTGATAGTGATACTGCAAACTGGTCTTGGCCAGACGGTGCTGAGTTGC
CGTTCACCATTGACAAGTAGTTCGTTGAAAAAA
>gi|83727846|gb|CY006917.1| Influenza A virus (A/New York/488/2003(H1N1)) segment 6,

complete sequence
GAGTTTAAAATGAATCCAAATCAAAAAATAATAACCATTGGATCAATCAGTATAGCAATCGGAATAATTA
GTCTAATGTTGCAAATAGGAAATATTATTTCAATATGGGCTAGTCACTCAATCCAAACTGGAAGTCAAAA
TCACACTGGAATATGCAACCAAAGAATCATCACATATGAAAACAGCACCTGGGTGAATCACACATATGTT
AATATTAACAACACTAATGTTGTTGCTGGAAAGGACAAAACTTCAGTGACATTGGCCGGCAATTCATCTC
TTTGTTCTATCAGTGGATGGGCTATATACACAAAAGACAACAGCATAAGAATTGGCTCCAAAGGAGATGT
TTTTGTCATAAGAGAACCTTTCATATCATGTTCTCACTTGGAATGCAGAACCTTTTTTCTGACCCAAGGT
GCTCTATTAAATGACAAACATTCAAATGGAACCGTTAAGGACAGAAGTCCTTATAGGGCCTTAATGAGCT
GTCCTCTAGGTGAAGCTCCGTCCCCATACAATTCAAAGTTTGAATCAGTTGCATGGTCAGCAAGCGCATG
CCATGATGGCATGGGCTGGTTAACAATCGGAATTTCTGGTCCAGACAATGGAGCTGTGGCTGTACTAAAA
TACAACGGAATAATAACTGAAACCATAAAAAGTTGGAAAAAGCGAATATTGAGAACACAAGAGTCTGAAT
GTGTCTGTGTGAACGGGTCATGTTTCACCATAATGACCGATGGCCCGAGTAATGGGGCCGCCTCGTACAA
AATCTTCAAGATCGAAAAGGGGAAGGTTACTAAATCAATAGAGTTGAATGCACCCAATTTTCATTATGAG
GAATGTTCCTGTTACCCAGACACTGGCACAGTGATGTGTGTATGCAGGGACAACTGGCATGGTTCAAATC
GACCTTGGGTGTCTTTTAATCAAAACTTGGATTATCAAATAGGATACATCTGCAGTGGAGTGTTCGGTGA
CAATCCGCGTCCCAAAGATGGAAAGGGCAGCTGTAATCCAGTGACTGTTGATGGAGCAGACGGAGTTAAG
GGGTTTTCATACAAATATGGTAATGGTGTTTGGATAGGAAGGACTAAAAGTAACAGACTTAGAAAGGGGT
TTGAGATGATTTGGGATCCTAATGGATGGACAGATACCGACAGTGATTTCTCAGTGAAACAGGATGTTGT
GGCAGTAACTGATTGGTCAGGGTACAGCGGAAGTTTCGTTCAACATCCTGAGTTAACAGGATTGGACTGT
ATAAGACCTTGCTTCTGGGTTGAGTTAGTCAGAGGACTGCCTAGAGAAAATACAACAATCTGGACTAGTG
GGAGCAGCATTTCTTTTTGTGGCGTTGATAGTGATACTGCAAACTGGTCTTGGCCAGACGGTGCTGAATT
GCCGTTCACCATTGACAAGTAGTTCGTTGAAAAAAA
>gi|157281262|gb|CY025215.1| Influenza A virus (A/Texas/UR06-0012/2006(H1N1)) segment 6, complete sequence
TTTAAAATGAATCCAAATCAAAAAATAATAACCATTGGATCAATCAGTATAGCAATCGGAATAATTAGTC
TAATGTTGCAAATAGGAAATATTATTTCAATATGGGCTAGTCACTCAATCCAAACTGGAAGTCAAAACCA
CACTGGAATATGCAACCAAAGAATCATCACATATGAAAACAGCACCTGGGTGAATCACACATATGTTAAT
ATTAACAACACTAATGTTGTTGCTGGAAAGGACAAAACTTCAGTGACATTGGCCGGCAATTCATCTCTTT
GTTCTATCAGTGGATGGGCTATATACACAAAAGACAACAGCATAAGAATTGGCTCCAAGGGAGATGTTTT
TGTCATAAGAGAACCTTTCATATCATGTTCTCATTTGGAATGCAGGACCTTTTTTCTGACCCAAGGTGCT
CTATTAAATGACAAACATTCAAATGGAACCGTTAAGGACAGAAGTCCTTATAGGGCCTTAATGAGCTGTC
CTCTAGGTGAAGCTCCGTCCCCATACAATTCAAAGTTTGAATCAGTTGCATGGTCAGCAAGCGCATGCCA
TGATGGCATGGGCTGGTTAACAATCGGAATTTCTGGTCCAGACAATGGAGCTGTGGCTGTACTAAAATAC
AACGGAATAATAACTGAAACCATAAAAAGTTGGAAAAAGCGAATATTGAGAACACAAGAGTCTGAATGTG
TCTGTGTGAACGGGTCTTGTTTCACCATAATGACCGATGGCCCGAGTAATGGGGCCGCCTCGTACAAAAT
CTTCAAGATCGAAAAGGGAAAGGTTACTAAATCAATAGAGTTAAATGCACCCAATTTTCATTATGAGGAA
TGTTCCTGTTACCCAGACACTGGCACAGTGATGTGTGTATGCAGAGACAACTGGCATGGTTCAAATCGAC
CTTGGGTGTCTTTTAATCAAAACTTGGATTATCAAATAGGATACATCTGCAGTGGAGTGTTCGGTGACAA
TCCGCGTCCCAAAGATGGAAAGGGCAGCTGTAATCCAGTGACTGTTGATGGAGCAGACGGAATTAAGGGG
TTTTCATACAAATATGGTAATGGTGTTTGGATAGGAAGAACTAAAAGTAACAGACTTAGAAAGGGGTTTG
AGATGATTTGGGATCCTAATGGATGGACAAATACCGACAGTGATTTCTCAGTGAAACAGGATGTTGTGGC
AATAACTGATTGGTCAGGGTACAGTGGAAGTTTCGTTCAACATCCTGAGTTAACAGGATTGGACTGTATA
AGACCTTGCTTCTGGGTTGAATTAGTCAGAGGACTGCCTAGAGAAAATACAACAATCTGGACTAGTGGGA
GCAGCATTTCTTTTTGTGGCGTTGATGGTGATACTGCAAACTGGTCTTGGCCAGACGGTGCTGAGTTGCC

GTTCACCATTGACAAATAGTTCGTTGAAAAAAA
>gi|94959537|gb|CY010766.1| Influenza A virus (A/Canterbury/20/2001(H1N1)) segment 6, complete sequence
GAGTTTAAAATGAATCCAAATCAAAAAATAATAACCATTGGATCAATCAGTATAGCAATCGGAATAATTA
GCCTAATGTTGCAAATAGGAAACATTATTTCAATATGGGCTAGTCACTCAATCCAAACTGGAAGTCAAAA
CCACACTGGAGTATGCAACCAAAGAATCATCACATATGAAAACAGCACCTGGGTGAATCACACATATGTT
AATATTAACAACACCAATGTTATTGCTGGAAAGGACAAAACTTCAGTGACATTGGCCGGCAATTCATCTC
TTTGTTCTATCAGTGGATGGGCTATATACACAAAAGACAACAGCATAAGAATTGGTTCCAAAGGAGATGT
TTTTGTCATAAGAGAGCCTTTCATATCATGTTCTCACTTGGAATGCAGAACCTTTTTTCTGACCCAAGGT
GCTCTATTAAATGACAAACATTCAAATGGGACCGTTAAGGACAGAAGCCCCTATAGGGCTTTAATGAGCT
GTCCTCTAGGTGAAGCTCCGTCCCCATACAATTCAAAGTTTGAATCAGTTGCTTGGTCAGCAAGCGCATG
CCATGATGGCATGGGCTGGTTAACAATCGGAATTTCTGGTCCAGACAATGGAGCAGTGGCTGTACTAAAA
TACAACGGCATAATAACTGAAACCATAAAAAGTTGGAAAAAGCAAATATTAAGAACACAAGAGTCTGAAT
GTGTCTGTGTGAACGGGTCATGTTTCACCATAATGACCGATGGCCCGAGTAATGGGGCCGCCTCGTACAA
AATCTTCAAGATCGAAAAGGGAAAGGTTACTAAATCAATAGAGTTGAATGCACCCAATTTTCATTATGAG
GAATGTTCCTGTTACCCAGACACTGGCACAGTGATGTGTGTATGCAGGGACAACTGGCATGGTTCAAATC
GACCCTGGGTGTCTTTTAATCAAAACCTGGATTATCAAATAGGATACATCTGCAGTGGGGTGTTCGGTGA
CAATCCGCGTCCCAAAGATGGAGAGGGCAGCTGTAATCCAGTGACTGTTGATGGAGCAAACGGAGTAAAG
GGGTTTTCATACAAATATGGTAATGGTGTTTGGATAGGAAGGACTAAAAGTAACAGACTTAGAAAGGGGT
TTGAGATGATTTGGGATCCTAATGGATGGACAGATACCGACAGTGATTTCTCAGTGAAACAGGATGTTGT
GGCAATAACTGATTGGTCTGGGTACAGCGGAAGTTTCGTTCAACATCCTGAGTTAACAGGATTGGACTGT
ATAAGACCTTGCTTCTGGGTTGAGTTAGTCAGAGGACTGCCAAAAGAAAATACAACAATCTGGACTAGTG
GGAGCAGCATTTCTTTTTGTGGCGTAAATAGTGATACTGCAAACTGGTCTTGGCCAGACGGTGCTGAGTT
GCCGTTTACCATTGACAAGTAGTTCGTTGAAAAAAACTT
>gi|91119031|gb|CY010406.1| Influenza A virus (A/West Coast/33/2001(H1N1)) segment 6, complete sequence
GAGTTTAAAATGAATCCAAATCAAAAAATAATAACCATTGGATCAATCAGTATAGCAATCGGAATAATTA
GCCTAATGTTGCAAATAGGAAACATTATTTCAATATGGGCTAGTCACTCAATCCAAACTGGAAGTCAAAA
CCACACTGGAGTATGCAACCAAAGAATCATCACATATGAAAACAGCACCTGGGTGAATCACACATATGTT
AATATTAACAACACCAATGTTATTGCTGGAAAGGACAAAACTTCAGTGACATTGGCCGGCAATTCATCTC
TTTGTTCTATCAGTGGATGGGCTATATACACAAAAGACAACAGCATAAGAATTGGTTCCAAAGGAGATGT
TTTTGTCATAAGAGAGCCTTTCATATCATGTTCTCACTTGGAATGCAGAACCTTTTTTCTGACCCAAGGT
GCTCTATTAAATGACAAACATTCAAATGGGACCGTTAAGGACAGAAGCCCCTATAGGGCTTTAATGAGCT
GTCCTCTAGGTGAAGCTCCGTCCCCATACAATTCAAAGTTTGAATCAGTTGCTTGGTCAGCAAGCGCATG
CCATGATGGCATGGGCTGGTTAACAATCGGAATTTCTGGTCCAGACAATGGAGCAGTGGCTGTACTAAAA
TACAACGGCATAATAACTGAAACCATAAAAAGTTGGAAAAAGCAAATATTAAGAACACAAGAGTCTGAAT
GTGTCTGTGTGAACGGGTCATGTTTCACCATAATGACCGATGGCCCGAGTAATGGGGCCGCCTCGTACAA
AATCTTCAAGATCGAAAAGGGAAAGGTTACTAAATCAATAGAGTTGAATGCACCCAATTTTCATTATGAG
GAATGTTCCTGTTACCCAGACACTGGCACAGTGATGTGTGTATGCAGGGACAACTGGCATGGTTCAAATC
GACCCTGGGTGTCTTTTAATCAAAACCTGGATTATCAAATAGGATACATCTGCAGTGGGGTGTTCGGTGA
CAATCCGCGTCCCAAAGATGGAGAGGGCAGCTGTAATCCAGTGACTGTTGATGGAGCAAACGGAGTAAAG
GGGTTTTCATACAAATATGGTAATGGTGTTTGGATAGGAAGGACTAAAAGTAACAGACTTAGAAAGGGGT
TTGAGATGATTTGGGATCCTAATGGATGGACAGATACCGACAGTGATTTCTCAGTGAAACAGGATGTTGT
GGCAATAACTGATTGGTCTGGGTACAGCGGAAGTTTCGTTCAACATCCTGAGTTAACAGGATTGGACTGT

ATAAGACCTTGCTTCTGGGTTGAGTTAGTCAGAGGACTGCCAAAAGAAAATACAACAATCTGGACTAGTG
GGAGCAGCATTTCTTTTTGTGGCGTAAATAGTGATACTGCAAACTGGTCTTGGCCAGACGGTGCTGAGTT
GCCGTTTACCATTGACAAGTAGTTCGTTGAAAAAAAA

>gi|131052804|gb|CY019999.1| Influenza A virus (A/Waikato/17/2005(H1N1)) segment 6, complete sequence

ATGAATCCAAATCAAAAAATAATAACCATTGGATCAATCAGTATAGCAATCGGAATAATTAGTCTAATGT
TGCAAATAGGAAATATTATTTCAATATGGGCTAGTCACTCAATCCAAACTGGAAGTCAAAACCACACTGG
AATATGCAACCAAAGAATCATCACATATGAAAACAGCACCTGGGTGAATCACACATATGTTAATATTAAC
AACACTAATGTTGTTGCTGGAAAGGACAAAACTTCAGTGACATTGGCCGGCAATTCATCTCTTTGTTCTA
TCAGTGGATGGGCTATATACACAAAAGACAACAGCATAAGAATTGGCTCCAAGGGAGATGTTTTTGTCAT
AAGAGAACCTTTCATATCATGTTCTCACTTGGAATGCAGAACCTTTTTTCTGACCCAAGGTGCTCTATTA
AATGACAAACATTCAAATGGAACCGTTAAGGACAGAAGTCCTTATAGGGCCTTAATGAGCTGTCCTCTAG
GTGAAGCTCCGTCCCCATACAATTCAAAGTTTGAATCAGTTGCATGGTCAGCAAGCGCATGCCATGATGG
CATGGGCTGGTTAACAATCGGAATTTCTGGTCCAGACAATGGAGCTGTGGCTGTACTAAAATACAACGGA
ATAATAACTGAAACCATAAAAAGTTGGGAAAAGCGAATATTGAGAACACAAGAGTCTGAATGTGTCTGTG
TGAACGGGTCATGTTTCACCATAATGACCGATGGCCCAAGTAATGGGGCCGCCTCGTACAAAATCTTCAA
GATCGAAAAGGGAAAGGTTACTAAATCAGTAGAGTTAAATGCACCCAATTTTCATTATGAGGAATGTTCC
TGTTACCCAGACACTGGCACAGTAATGTGTGTATGCAGGGACAACTGGCATGGTTCAAATCGACCTTGGG
TGTCTTTTAATCAAAACTTGGATTATCAAATAGGATACATCTGCAGTGGAGTGTTCGGTGACAATCCGCG
CCCCAAAGATGGAAAGGGCAGCTGTAATCCAGTGACTGTTGATGGAGCAGACGGAGTTAAGGGGTTTTCA
TACAAATATGGTAATGGTGTTTGGATAGGAAGGACTAAAAGTAACAGACTTAGAAAGGGTTTTGAGATGA
TTTGGGATCCTAATGGATGGACAGATACCGACAGTGATTTCTCAGTGAAACAGGATGTTGTGGCAATAAC
TGATTGGTCAGGGTACAGCGGAAGTTTCGTTCAACATCCTGAGTTGACAGGATTGGACTGTATAAGACCT
TGCTTCTGGGTTGAGTTAGTCAGAGGACTGCCTAGAGAAAATACAACAATCTGGACTAGTGGGAGCAGCA
TTTCTTTTTGTGGCGTTGATAGTGATACTGCAAACTGGTCTTGGCCAGACGGTGCTGAGTTGCCGTTCAC
CATTGACAAGTAGTTCGTTGAAAAAAA

>gi|83744839|gb|CY007469.1| Influenza A virus (A/Canterbury/106/2004(H1N1)) segment 6, complete sequence

GAGTTTAAAATGAACCCAAATCAAAAGATAATAACCATTGGATCAATCAGTATAGCAATCGGAATAATTA
GTCTAATGTTGCAAATAGGAAATATTATTTCAATATGGGCTAGTCACTCAATCCAAACTGGAAGTCAAAA
CCACACTGGAATATGCAACCAAAGAATCATCACATATGAAAACAGCACCTGGGTGAATCACACATATGTT
AATATTAACAACACTAATGTTGTTGCTGGAAAGGACAAAACTTCAGTGACATTGGCCGGCAATTCATCTC
TTTGTTCTATCAGTGGATGGGCTATATACACAAAAGACAACAGCATAAGAATTGGCTCCAAAGGAGATGT
TTTTGTCATAAGAGAACCTTTCATATCATGTTCTCACTTGGAATGCAGAACCTTTTTTCTGACCCAAGGC
GCTCTATTAAATGACAAACATTCAAATGGGACCGTAAAGGACAGAAGTCCTTATAGGGCCTTAATGAGCT
GTCCTCTAGGTGAAGCTCCGTCCCCATACAATTCAAAGTTCGAATCAGTTGCATGGTCAGCAAGCGCATG
CCATGATGGCATGGGCTGGTTAACAATCGGAATTTCTGGTCCAGACAATGGAGCTGTGGCTGTACTAAAA
TACAACGGAATAATAACTGGAACCATAAAAAGTTGGAAAAAGCAAATATTAAGAACACAAGAGTCTGAAT
GTGTCTGTATGAACGGGTCATGTTTCACCATAATGACCGATGGCCCGAGTAATGGGGCCGCCTCGTACAA
AATTTTCAAGATCGAAAAGGGGAAGGTTACTAAATCAATAGAGTTGAATGCACCCAATTTTCATTATGAG
GAATGTTCCTGTTACCCAGACACTGGCACAGTGATGTGTGTATGCAGGGACAACTGGCATGGTTCAAATC
GACCTTGGGTGTCTTTTAATCAAAACTTGGATTATCAAATAGGATACATCTGCAGTGGAGTGTTCGGTGA
CAATCCGCGTCCCAAAGATGGAGAGGGCAGCTGCAATCCAGTGACTGTTGATGGAGCAGACGGAGTAAAA
GGGTTTTCATACAAATATGGTAATGGTGTTTGGATAGGAAGGACCAAAAGTAACAGACTTAGAAAGGGGT

TTGAGATGATTTGGGATCCTAATGGATGGACAAATACCGACAGTGATTTCTCAGTGAAACAGGATGTTGT
AGCAATAACTGATTGGTCAGGGTACAGCGGAAGTTTCGTCCAACATCCTGAGTTAACAGGATTGGACTGT
ATAAGACCTTGCTTCTGGGTTGAGTTAGTCAGAGGGCTGCCTAGAGAAAATACAACAATCTGGACTAGTG
GGAGCAGCATTTCTTTTTGTGGCGTTAATAGTGATACTGCAAACTGGTCTTGGCCAGACGGTGCTGAGTT
GCCGTTCACCATTGACAAGTAGTTCGTTGAAAAAAA
>gi|115607826|gb|CY016701.1| Influenza A virus (A/South Australia/58/2005(H1N1)) segment 6, complete sequence
ATGAATCCAAATCAAAAAATAATAACCATTGGATCAATCAGTATAGCAATCGGAATAATTAGTCTAATGT
TGCAAATAGGAAATATTATTTCAATATGGGCTAGTCACTCAATCCAAACTGGAAGTCAAAACCACACTGG
AATATGCAACCAAAGAATCATCACATATGAAAACAGCACCTGGGTGAATCACACATATGTTAATATTAAC
AACACTAATGTTGTTGCTGGAAAGGACAAAACTTCAGTGACATTGGCCGGCAATTCATCTCTTTGTTCTA
TCAGTGGATGGGCTATATACACAAAAGACAACAGCATAAGAATTGGCTCCAAAGGAGATGTTTTTGTCAT
AAGAGAACCTTTCATATCATGTTCTCACTTGGAATGCAGAACCTTTTTTCTGACCCAAGGTGCTCTATTA
AATGACAAACATTCAAATGGAACCGTTAAGGACAGAAGTCCTTATAGGGCCTTAATGAGCTGTCCTCTAG
GTGAAGCTCCGTCCCCATACAATTCAAAGTTTGAATCAGTTGCATGGTCAGCAAGCGCATGCCATGATGG
CATGGGCTGGTTAACAATCGGAATTTCTGGTCCAGACAATGGAGCTGTGGCTGTACTAAAATACAACGGA
ATAATAACTGAAACCATAAAAAGTTGGAAAAAGCGAATATTGAGAACACAAGAGTCTGAATGTGTCTGTG
TGAACGGGTCATGTTTCACCATAATGACCGATGGCCCAAGTAATGGGGCCGCCTCGTACAAAATCTTCAA
GATCGAAAAGGGAAAGGTTACTAAATCAATAGAGTTAAATGCACCCAATTTTCATTATGAGGAATGTTCC
TGTTACCCAGACACTGGCACAGTGATGTGTGTATGCAGGGACAACTGGCATGGTTCAAATCGACCTTGGG
TGTCTTTTAATCAAAACTTGGATTATCAAATAGGATACATCTGCAGTGGAGTGTTCGGTGACAATCCGCG
TCCCAAAGATGGAAAGGGCAGCTGTAATCCAGTGACTGTTGATGGAGCAGACGGAGTTAAGGGGTTTTCA
TACAAATATGGTAATGGTGTTTGGATAGGAAGGACTAAAAGTAACAGACTTAGAAAGGGTTTTGAGATGA
TTTGGGATCCTAATGGATGGACAGATACCGACAGTGATTTCTCAGTGAAACAGGATGTTGTGGCAATAAC
TGATTGGTCAGGGTACAGCGGAAGTTTCGTTCAACATCCTGAGTTAACAGGATTGGACTGTATAAGACCT
TGCTTCTGGGTTGAGTTAGTCAGAGGACTGCCTAGAGAAAATACAACAATCTGGACTAGTGGGAGCAGCA
TTTCTTTTTGTGGCGTTGATAGTGATACTGCAAACTGGTCTTGGCCAGACGGTGCTGAGTTGCCGTTCAC
CATTGACAAGTAG
>gi|113170886|gb|CY014009.1| Influenza A virus (A/Wellington/11/2005(H1N1)) segment 6, complete sequence
AATCAAAAAATAATAACCATTGGATCAATCAGTATAGCAATCGGAATAATTAGTCTAATGTTGCAAATAG
GAAATATTATTTCAATATGGGCTAGTCACTCAATCCAAACTGGAAGTCAAAACCACACTGGAATATGCAA
CCAAAGAATCATCACATATGAAAACAGCACCTGGGTGAATCACACATATGTTAATATTAACAACACTAAT
GTTGTTGCTGGAAAGGACAAAACTTCAGTGACATTGGCCGGCAATTCATCTCTTTGTTCTATCAGTGGAT
GGGCTATATACACAAAAGACAACAGCATAAGAATTGGCTCCAAAGGAGATGTTTTTGTCATAAGAGAACC
TTTCATATCATGTTCTCATTTGGAATGCAGGACCTTTTTTCTGACCCAAGGTGCTCTATTAAATGACAAA
CATTCAAATGGAACCGTTAAGGACAGAAGTCCTTATAGGGCCTTAATGAGCTGTCCTCTAGGTGAAGCTC
CGTCCCCATACAATTCAAAGTTTGAATCAGTTGCATGGTCAGCAAGCGCATGCCATGATGGCATGGGCTG
GTTAACAATCGGAATTTCTGGTCCAGACAATGGAGCTGTGGCTGTACTAAAATACAACGGAATAATAACT
GAAACCATAAAAAGTTGGAAAAAGCGGATATTGAGAACACAAGAGTCTGAATGTGTCTGTGTGAACGGGT
CATGTTTCACCATAATGACCGATGGCCCGAGTAATGGGGCCGCCTCGTACAAAATCTTCAAGATCGAAAA
GGGGAAGGTTACTAAATCAATAGAGTTAAATGCACCCAATTTTCATTATGAGGAATGTTCCTGTTACCCA
GACACTGGCACAGTGATGTGTGTATGCAGGGACAACTGGCATGGTTCAAATCGACCTTGGGTGTCTTTTA
ATCAAAACTTGGATTATCAAATAGGATACATCTGCAGTGGAGTGTTCGGTGACAATCCGCGTCCCAAAGA

TGGAAAGGGCAGCTGTAATCCAATGACTGTTGATGGAGCAGACGGAGTTAAGGGGTTTTCATACAAATAT
GGTAATGGTGTTTGGATAGGAAGGACTAAAAGTAACAGACTTAGAAAGGGGTTTGAGATGATTTGGGATC
CTAATGGATGGACAGATACCGACAGTGATTTCTCAGTGAAACAGGATGTTGTGGCAATAACTGATTGGTC
AGGGTACAGTGGAAGTTTCGTTCAACATCCTGAGTTAACAGGATTGGACTGTATAAGACCTTGCTTCTGG
GTTGAGTTAGTCAGAGGACTGCCTAGAGAAAATACAACAATCTGGACTAGTGGGAGCAGCATTTCCTTTT
GTGGCGTTGATAGTGATACTGCAAACTGGTCTTGGCCAGACGGTGCTGAGTTGCCGTTCACCATTGACAA
ATAGTTCGT
>gi|145278928|gb|CY021759.1| Influenza A virus (A/South Australia/51/2005(H1N1)) segment 6, complete sequence
ATGAATCCAAATCAAAAAATAATAACCATTGGATCAATCAGTATAGCAATCGGAATAATTAGTCTAATGT
TGCAAATAGGAAATATTATTTCAATATGGGCTAGTCACTCAATCCAAACTGGAAGTCAAAACCACACTGG
AATATGCAACCAAAGAATCATCACATATGAAAACAGCACCTGGGTGAATCACACATATGTTAATATTAAC
AACACTAATGTTGTTGCTGGAAAGGACAAAACTTCAGTGACATTGGCCGGCAATTCATCTCTTTGTTCTA
TCAGTGGATGGGCTATATACACAAAAGACAACAGCATAAGAATTGGCTCCAAAGGAGATGTTTTTGTCAT
AAGAGAACCTTTCATATCATGTTCTCATTTGGAATGCAGGACCTTTTTTCTGACCCAAGGTGCTCTATTA
AATGACAAACATTCAAATGGAACCGTTAAGGACAGAAGTCCTTATAGGGCCTTAATGAGCTGTCCTCTAG
GTGAAGCTCCGTCCCCATACAATTCAAAGTTTGAATCAGTTGCATGGTCAGCAAGCGCATGCCATGATGG
CATGGGCTGGTTAACAATCGGAATTTCTGGTCCAGACAATGGAGCTGTGGCTGTACTAAAATACAACGGA
ATAATAACTGAAACCATAAAAAGTTGGAAAAAGCGAATATTGAGAACACAAGAGTCTGAATGTGTCTGTG
TGAACGGGTCATGTTTCACCATAATGACCGATGGCCCGAGTAATGGGGCCGCCTCGTACAAAATCTTCAA
GATCGAAAAGGGGAAGGTTACTAAATCAATAGAGTTAAATGCACCCAATTTTCATTATGAGGAATGTTCC
TGTTACCCAGACACTGGCACAGTGATGTGTGTATGCAGGGACAACTGGCATGGTTCAAATCGACCTTGGG
TGTCTTTTAATCAAAACTTGGATTATCAAATAGGATACATCTGCAGTGGAGTGTTCGGTGACAATCCGCG
TCCCAAAGATGGAAAGGGCAGCTGTAATCCAGTGACTGTTGATGGAGCAGACGGAGTTAAGGGGTTTTCA
TACAAATATGGTAATGGTGTTTGGATAGGAAGGACTAAAAGTAACAGACTTAGAAAGGGGTTTGAGATGA
TTTGGGATCCTAATGGATGGACAGATACCGACAGTGATTTCTCAGTGAAACAGGATGTTGTGGCAATAAC
TGATTGGTCAGGGTACAGTGGAAGTTTCGTTCAACATCCTGAGTTAACAGGATTGGACTGTATAAGACCT
TGCTTCTGGGTTGAGTTAGTCAGAGGACTGCCTAGAGAAAATACAACAATCTGGACTAGTGGGAGCAGCA
TTTCTTTTTGTGGCGTCGATAGTGATACTGCAAACTGGTCTTGGCCAGACGGTGCTGAGTTGCCGTTCAC
CATTGACAAATAGTTCGTTGAAAAAA
>gi|149780698|gb|CY022583.1| Influenza A virus (A/Auckland/619/2005(H1N1)) segment 6, complete sequence
ATGAATCCAAATCAAAAAATAATAACCATTGGATCAATCAGTATAGCAATCGGAATAATTAGTCTAATGT
TGCAAATAGGAAATATTATTTCAATATGGGCTAGTCACTCAATCCAAACTGGAAGTCAAAACCACACTGG
AATATGCAACCAAAGAATCATCACATATGAAAACAGCACCTGGGTGAATCACACATATGTTAATATTAAC
AACACTAATGTTGTTGCTGGAAAGGACAAAACTTCAGTGACATTGGCCGGCAATTCATCTCTTTGTTCTA
TCAGTGGATGGGCTATATACACAAAAGACAACAGCATAAGAATTGGCTCTAAAGGAGATGTTTTTGTCAT
AAGAGAACCTTTCATATCATGTTCTCATTTGGAATGCAGGACCTTTTTTCTGACCCAAGGTGCTCTATTA
AATGACAAACATTCAAATGGAACCGTTAAGGACAGAAGTCCTTATAGGGCCTTAATGAGCTGTCCTCTAG
GTGAAGCTCCGTCCCCATACAATTCAAAGTTTGAATCAGTTGCATGGTCAGCAAGCGCATGCCATGATGG
CATGGGCTGGTTAACAATCGGAATTTCTGGTCCAGACAATGGAGCTGTGGCTGTACTAAAATACAACGGA
ATAATAACTGAAACCATAAAAAGTTGGAAAAAGCGGATATTGAGAACACAAGAGTCTGAATGTGTCTGTG
TGAACGGGTCATGTTTCACCATAATGACCGATGGCCCGAGTAATGGGGCCGCCTCGTACAAAATCTTCAA
GATCGAAAAGGGGAAGGTTACTAAATCAATAGAGTTAAATGCACCCAATTTTCATTATGAGGAATGTTCC

TGTTACCCAGACACTGGCACAGTGATGTGTGTATGCAGGGACAACTGGCATGGTTCAAATCGACCTTGGG
TGTCTTTTAATCAAAACTTGGATTATCAAATAGGATACATCTGCAGTGGAGTGTTCGGTGACAATCCGCG
TCCCAAAGATGGAAAGGGCAGCTGTAATCCAGTGACTGTTGATGGAGCAGACGGAGTTAAGGGGTTTTCA
TACAAATATGGTAATGGTGTTTGGATAGGAAGGACTAAAAGTAACAGACTTAGAAAGGGGTTTGAGATGA
TTTGGGATCCTAATGGATGGACAGATACCGACAGTGATTTCTCAGTGAAACAGGATGTTGTGGCAATAAC
TGATTGGTCAGGGTACAGTGGAAGTTTCGTTCAACATCCTGAGTTAACAGGATTGGACTGTATAAGACCT
TGCTTCTGGGTTGAGTTAGTCAGAGGACTGCCTAGAGAAAATACAACAATCTGGACTAGTGGGAGCAGCA
TTTCTTTTTGTGGCGTTGATAGTGATACTGCAAACTGGTCTTGGCCAGACGGTGCTGAGTTGCCGTTCAC
CATTGACAAGTAGTTCGTTGAAAAA
>gi|117572943|gb|CY017317.1| Influenza A virus (A/Waikato/4/2005(H1N1)) segment 6, complete sequence
ATGAATCCAAATCAAAAAATAATAACCATTGGATCAATCAGTATAGCAATCGGAATAATTAGTCTAATGT
TGCAAATAGGAAATATTATTTCAATATGGGCTAGTCACTCAATCCAAACTGGAAGTCAAAACCACACTGG
AATATGCAACCAAAGAATCATCACATATGAAAACAGCACCTGGGTGAATCACACATATGTTAATATTAAC
AACACTAATGTTGTTGCTGGAAAGGACAAAACTTCAGTGACATTGGCCGGCAATTCATCTCTTTGTTCTA
TCAGTGGATGGGCTATATACACAAAAGACAACAGCATAAGAATTGGCTCCAAGGGAGATGTTTTTGTCAT
AAGAGAACCTTTCATATCATGTTCTCACTTGGAATGCAGAACCTTTTTTCTGACCCAAGGTGCTCTATTA
AATGACAAACATTCAAATGGAACCGTTAAGGACAGAAGTCCTTATAGGGCCTTAATGAGCTGTCCTCTAG
GTGAAGCTCCGTCCCCATACAATTCAAAGTTTGAATCAGTTGCATGGTCAGCAAGCGCATGCCATGATGG
CATGGGCTGGTTAACAATCGGAATTTCTGGTCCAGACAATGGAGCTGTGGCTGTACTAAAATACAACGGA
ATAATAACTGAAACCATAAAAAGTTGGGAAAAGCGAATATTGAGAACACAAGAGTCTGAATGTGTCTGTG
TGAACGGGTCATGTTTCACCATAATGACCGATGGCCCAAGTAATGGGGCCGCCTCGTACAAAATCTTCAA
GATCGAAAAGGGAAAGGTTACTAAATCAGTAGAGTTAAATGCACCCAATTTTCATTATGAGGAATGTTCC
TGTTACCCAGACACTGGCACAGTAATGTGTGTATGCAGGGACAACTGGCATGGTTCAAATCGACCTTGGG
TGTCTTTTAATCAAAACTTGGATTATCAAATAGGATACATCTGCAGTGGAGTGTTCGGTGACAATCCGCG
CCCCAAAGATGGAAAGGGCAGCTGTAATCCAGTGACTGTTGATGGAGCAGACGGAGTTAAGGGGTTTTCA
TACAAATATGGTAATGGTGTTTGGATAGGAAGGACTAAAAGTAACAGACTTAGAAAGGGTTTTGAGATGA
TTTGGGATCCTAATGGATGGACAGATACCGACAGTGATTTCTCAGTGAAACAGGATGTTGTGGCAATAAC
TGATTGGTCAGGGTACAGCGGAAGTTTCGTTCAACATCCTGAGTTGACAGGATTGGACTGTATAAGACCT
TGCTTCTGGGTTGAGTTAGTCAGAGGACTGCCTAGAGAAAATACAACAATCTGGACTAGTGGGAGCAGCA
TTTCTTTTTGTGGCGTTGATAGTGATACTGCAAACTGGTCTTGGCCAGACGGTGCTGAGTTGCCGTTCAC
CATTGACAAGTAGTTCGTTGAAAA
>gi|161139449|gb|CY028197.1| Influenza A virus (A/Kentucky/UR06-0007/2006(H1N1)) segment 6, complete sequence
GAGTTTAAAATGAATCCAAATCAAAAAATAATAACCATTGGATCAATCAGTATAGCAATCGGGATAATTA
GTCTAATGTTGCAAATAGGAAATATTATTTCAATATGGGCTAGTCACTCAATCCAAACTGGAAGTCAAAA
CCACACTGGAATATGCAACCAAAGAATCATCACATATGAAAACAGCACCTGGGTGAATCACACATATGTT
AGTATTAACAACACTAATGTTGTTGCTGGAAAGGACAAAACTTCAGTGACATTGGCCGGCAATTCATCTC
TTTGTTCTATCAGTGGATGGGCTATATACACAAAAGACAACAGCATAAGAATTGGCTCCAAAGGAGATGT
TTTTGTCATAAGAGAACCTTTCATATCATGTTCTCATTTGGAATGCAGGACCTTTTTTCTGACCCAAGGT
GCTCTATTAAATGACAAACATTCAAATGGAACCGTTAAGGACCGAAGTCCTTATAGGGCCTTAATGAGCT
GTCCTCTAGGTGAAGCTCCGTCCCCATACAATTCAAAGTTTGAATCAGTTGCATGGTCAGCAAGCGCATG
CCATGATGGCATGGGCTGGTTAACAATCGGAATTTCTGGTCCAGACAATGGAGCTGTGGCTGTACTAAAA
TACAACGGAATAATAACTGAAACCATAAAAAGTTGGAAAAAGCGAATATTGAGAACACAAGAGTCTGAAT

GTGTCTGTGTGAACGGGTCATGTTTCACCATAATGACCGATGGCCCGAGTAATGGGGCCGCCTCGTACAA
AATCTTCAAGATCGAAAAGGGGAAGGTTACTAAATCAATAGAGTTAAATGCACCCAATTTTCATTATGAG
GAATGTTCCTGTTACCCAGACACTGGCACAATGATGTGTGTATGCAGGGACAACTGGCATGGTTCAAATC
GACCTTGGGTGTCTTTTAATCAAAACTTGGATTATCAAATAGGATACATCTGCAGTGGAGTGTTCGGTGA
CAATCCGCGTCCCAAAGATGGAAAGGGCAGCTGTAATCCAGTGACTGTTGATGGAGCAGACGGGGTTAAG
GGGTTTTCATACAAATATGGTAATGGTGTTTGGATAGGAAGAACTAAAAGTAACAGACTTAGAAAGGGGT
TTGAGATGATTTGGGATCCTAATGGATGGACAGATACCGACAGTGATTTCTCAGTGAAACAGGATGTTGT
GGCAATAACTGATTGGTCAGGGTACAGTGGAAGTTTCGTTCAACATCCTGAGTTAACAGGATTGGACTGT
ATAAGACCTTGCTTCTGGGTTGAGTTAGTCAGAGGACTGCCTAGAGAAAATACAACAATCTGGACTAGTG
GGAGCAGCATTTCTTTTTGTGGCGTTGATAGTGATACTGCAAACTGGTCTTGGCCAGACGGTGCTGAGTT
GCCGTTCACCATTGACAAGTAGTTCGTTGA
>gi|157281281|gb|CY025223.1| Influenza A virus (A/Michigan/UR06-0015/2006(H1N1)) segment 6, complete sequence
ATGAATCCAAATCAAAAAATAATAACCATTGGATCAATCAGTATAGCAATTGGAATAATTAGTCTAATGT
TGCAAATAGGAAATATTATTTCAATATGGGCTAGTCACTCAATCCAAACTGGAAGTCAAAACCACACTGG
AATATGCAACCAAAGAATCATCACATATGAAAACAGCACCTGGGTGAATCACACATATGTTAATATTAAC
AACACTAATGTTGTTGCTGGAAAGGACAAAACTTCAGTGACATTGGCCGGCAATTCATCTCTTTGTTCTA
TCAGTGGATGGGCTATATACACAAAAGACAACAGCATAAGAATTGGCTCCAAAGGAGATGTTTTTGTCAT
AAGAGAACCTTTCATATCATGTTCTCATTTGGAATGCAGGACCTTTTTTCTGACCCAAGGTGCTCTATTA
AATGACAAACATTCAAATGGAACCGTTAAGGACAGAAGTCCTTATAGGGCCTTAATGAGCTGTCCTCTAG
GTGAAGCTCCGTCCCCATACAATTCAAAGTTTGAATCAGTTGCATGGTCAGCAAGCGCATGCCATGATGG
CATGGGCTGGTTAACAATCGGGATTTCTGGTCCAGACAATGGAGCTGTGGCTGTACTAAAATACAACGGA
ATAATAACTGAAACCATAAAAAGTTGGAAAAAGCGAATATTGAGAACACAGGAGTCTGAATGTGTCTGTG
TGAACGGGTCATGTTTCACCATAATGACCGATGGCCCGAGTAATGGGGCCGCCTCGTACAAAATCTTCAA
GATCGAAAAGGGGAAGGTTACTAAATCAATAGAGTTAAATGCACCCAATTTTCATTATGAGGAGTGTTCC
TGTTACCCAGACACTGGCACAGTGATGTGTGTATGCAGGGATAACTGGCATGGTTCAAATCGACCTTGGG
TGTCTTTTAATCAAAACTTGGATTATCAAGTAGGATACATCTGCAGTGGAGTGTTCGGTGACAATCCGCG
TCCCAAAGATGGAAAGGGCAGCTGTAATCCAATGACTGTTGATGGAGCAGACGGAGTTAAGGGGTTTTCA
TACAAATATGGTAATGGTGTTTGGATAGGAAGGACTAAAAGTAACAAACTTAGAAAGGGGTTTGAGATGA
TTTGGGATCCTAATGGATGGACAGATACCGACAGTGATTTCTCAGTGAAACAGGATGTTGTGGCAATAAC
CGATTGGTCAGGGTACAGTGGAAGTTTCGTTCAACATCCTGAGTTAACAGGATTGGACTGTATAAGACCT
TGCTTCTGGGTTGAGTTAGTCAGAGGACTGCCTAGAGAAAATGCAACAATCTGGACTAGTGGGAGCAGCA
TTTCTTTTTGTGGCGTTGATAGTGATACTGCAAACTGGTCTTGGCCAGACGGTGCTGAGTTGCCGTTCAC
CATTGACAAATAGTTCGTTGA
>gi|218875173|gb|CY036921.1| Influenza A virus (A/NYMC X-163A(NYMC X-157-St. Petersburg/8/2006)(H1N1)) segment 6, complete sequence
ATGAACCCAAATCAAAAAATAATAACCATTGGATCAATCAGTATAGCAATCGGAATAATTAGTCTAATGT
TGCAAATAGGAAATATTATTTCAATATGGGCTAGTCACTCAATCCAAACTGGAAGTCAAAACCACACTGG
AATATGCAACCAAAGAATCATCACATATGAAAACAGCACCTGGGTGAATCACACATATGTTAATATTAAC
AACACTAATGTTGTTGCTGGAAAGGACAAAACTTCAGTGACATTGGCCGGCAACTCATCTCTTTGTTCTA
TCAGTGGATGGGCTATATACACAAAAGACAACAGCATAAGAATTGGCTCCAAAGGAGATGTTTTTGTCAT
AAGAGAACCTTTCATATCATGTTCTCACTTGGAATGCAAAACCTTTTTTCTGACCCAAGGCGCTCTATTA
AATGACAAACATTCAAATGGGACCGTAAAGGACAGAAGTCCTTATAGGGCCTTAATGAGCTGTCCTCTAG
GTGAAGCTCCGTCCCCATACAATTCAAAGTTCGAATCAGTTGCATGGTCAGCAAGCGCATGCCATGATGG

CATGGGCTGGTTAACAATCGGAATTTCTGGTCCAGACAATGGAGCTGTGGCTGTACTAAAATACAACGGA
ATAATAACTGGAACCATAAAAAGTTGGAAAAAGCAAATATTAAGAACACAAGAGTCTGAATGTGTCTGTA
TGAACGGGTCATGTTTCACCATAATGACCGATGGTCCGAGTAATGGGGCCGCCTCGTACAAAATTTTCAA
GATCGAAAAGGGGAAGGTTACTAAATCAATAGAGTTGAATGCACCCAATTTTCATTATGAGGAATGTTCC
TGTTACCCAGACACTGGCACAGTGATGTGTGTATGCAGGGACAACTGGCATGGTTCAAATCGACCTTGGG
TGTCTTTTAATCAAAACTTGGATTATCAAATAGGATACATCTGCAGTGGAGTGTTCGGTGACAATCCGCG
TCCCAAAGATAGAGAGGGCAGCTGCAATCCAGTGACTATTGATGGAGCAGACGGAGTAAAAGGGTTTTCA
TACAAATATGGTAATGGTGTTTGGATAGGAAGGACCAAAAGTAACAGACTTAGAAAGGGGTTTGAAATGA
TTTGGGATCCTAATGGATGGACAAATACCGACAGTGATTTCTCAGTGAAACAGGATGTTGTAGCAATAAC
TGATTGGTCAGGGTACAGCGGAAGTTTCGTCCAACATCCTGAGTTAACAGGATTGGACTGTATAAGACCT
TGCTTCTGGGTTGAGTTAGTCAGAGGGCTGCCTAGAGAAAATACAACAATCTGGACTAGTGGGAGCAGCA
TTTCTTTTTGTGGCGTTAATAGTGATACTGCAAACTGGTCTTGGCCAGACGGTGCTGAGTTGCCGTTCAC
CATTGACAAGTAGTTCGTTGAA
>gi|208344088|gb|CY035128.1| Influenza A virus (A/St. Petersburg/8/2006(H1N1)) segment 6, complete sequence
TTTAAAATGAACCCAAATCAAAAAATAATAACCATTGGATCAATCAGTATAGCAATCGGAATAATTAGTC
TAATGTTGCAAATAGGAAATATTATTTCAATATGGGCTAGTCACTCAATCCAAACTGGAAGTCAAAACCA
CACTGGAATATGCAACCAAAGAATCATCACATATGAAAACAGCACCTGGGTGAATCACACATATGTTAAT
ATTAACAACACTAATGTTGTTGCTGGAAAGGACAAAACTTCAGTGACATTGGCCGGCAACTCATCTCTTT
GTTCTATCAGTGGATGGGCTATATACACAAAAGACAACAGCATAAGAATTGGCTCCAAAGGAGATGTTTT
TGTCATAAGAGAACCTTTCATATCATGTTCTCACTTGGAATGCAAAACCTTTTTTCTGACCCAAGGCGCT
CTATTAAATGACAAACATTCAAATGGGACCGTAAAGGACAGAAGTCCTTATAGGGCCTTAATGAGCTGTC
CTCTAGGTGAAGCTCCGTCCCCATACAATTCAAAGTTCGAATCAGTTGCATGGTCAGCAAGCGCATGCCA
TGATGGCATGGGCTGGTTAACAATCGGAATTTCTGGTCCAGACAATGGAGCTGTGGCTGTACTAAAATAC
AACGGAATAATAACTGGAACCATAAAAAGTTGGAAAAAGCAAATATTAAGAACACAAGAGTCTGAATGTG
TCTGTATGAACGGGTCATGTTTCACCATAATGACCGATGGTCCGAGTAATGGGGCCGCCTCGTACAAAAT
TTTCAAGATCGAAAAGGGGAAGGTTACTAAATCAATAGAGTTGAATGCACCCAATTTTCATTATGAGGAA
TGTTCCTGTTACCCAGACACTGGCACAGTGATGTGTGTATGCAGGGACAACTGGCATGGTTCAAATCGAC
CTTGGGTGTCTTTTAATCAAAACTTGGATTATCAAATAGGATACATCTGCAGTGGAGTGTTCGGTGACAA
TCCGCGTCCCAAAGATAGAGAGGGCAGCTGCAATCCAGTGACTATTGATGGAGCAGACGGAGTAAAAGGG
TTTTCATACAAATATGGTAATGGTGTTTGGATAGGAAGGACCAAAAGTAACAGACTTAGAAAGGGGTTTG
AAATGATTTGGGATCCTAATGGATGGACAAATACCGACAGTGATTTCTCAGTGAAACAGGATGTTGTAGC
AATAACTGATTGGTCAGGGTACAGCGGAAGTTTCGTCCAACATCCTGAGTTAACAGGATTGGACTGTATA
AGACCTTGCTTCTGGGTTGAGTTAGTCAGAGGGCTGCCTAGAGAAAATACAACAATCTGGACTAGTGGGA
GCAGCATTTCTTTTTGTGGCGTTAATAGTGATACTGCAAACTGGTCTTGGCCAGACGGTGCTGAGTTGCC
GTTCACCATTGACAAGTAGTTCGTTGAA
>gi|226954751|gb|CY038881.1| Influenza A virus (A/Taiwan/2645/2006(H1N1)) segment 6, complete sequence
AAATGAACCCAAATCAAAAAATAATAACCATTGGATCAATCAGTATAGCAATCGGAATAATTAGTCTAAT
ATTGCAAATAGGAAATATTATTTCAATATGGGCTAGTCACTCAATCCAAACTGGAAGTCAAAACCACACT
GGAATATGCAACCAAAGAATCATTACATATGAAAACAGCACCTGGGTGAATAACACATATGTTAATATTA
ACAACACCAATGTTGTTGCTGAAAAGGACAAAACTTCAGTGACATTGGCCGGCAATTCATCTCTTTGTTC
TATCAGTGGATGGGCTATATACACAAAAGACAACAGCATAAGAATTGGCTCCAAAGGAGATGTTTTTGTC
ATAAGAGAACCTTTCATATCATGTTCTCACTTGGAATGCAGAACCTTTTTTCTGACCCAAGGTGCTCTAT

TAAATGACAAACATTCAAATGGGACCGTAAAGGACAGAAGTCCTTATAGGGCCTTAATGAGCTGTCCTCT
AGGTGAAGCTCCGTCCCCATACAATTCAAGGTTCGAATCAGTTGCATGGTCAGCAAGCGCATGCCATGAT
GGCATGGGCTGGTTAACAATCGGAATTTCTGGTCCAGACAATGGAGCTGTGGCTGTACTAAAATACAACG
GAATAATAACTGAAACCATAAAAAGTTGGAAAAAGCGAATATTAAGAACACAAGAGTCTGAATGTGTCTG
TATGAACGGGTCATGTTTCACCATAATGACCGATGGCCCGAGTAATGGGACCGCCTCGTACAAAATTTTC
AAGATCGAAAAGGGGAAGGTTACCAAAACAATAGAGTTGAATGCACCCAATTTTCATTATGAGGAATGTT
CCTGTTACCCAGACACTGGCACAGTGATGTGTGTATGCAGAGACAACTGGCATGGTTCAAATCGACCTTG
GGTGTCTTTTAATCAAAACTTGGATTATCAAATAGGATACATCTGCAGTGGAGTGTTCGGTGACAATCCG
CGTCCCAAAGATGGGGAGGGCAGCTGCAATCCAGTGACTGTTGATGGAGCAGACGGAGTAAAAGGGTTTT
CATACAAATATGGTAATGGTGTTTGGATAGGAAGGACCAAAAGTAACAGACTTAGAAAGGGGTTTGAGAT
GATTTGGGATCCTAATGGATGGACAAATACCGACAGTGATTTCTCAGTTAAACAGGATGTTGTAGCAATA
ACTGATTGGTCAGGGTACAGCGGAAGTTTTGTTCAACATCCTGAGTTAACAGGATTGGACTGTATAAGAC
CTTGCTTCTGGGTTGAGTTAGTCAGAGGGCTGCCTAGGGAAAATACAACAATCTGGACTAGTGGGAGCAG
CATTTCTTTTTGTGGCGTTAATAGTGGTACTGCAAACTGGTCTTGGCCAGACGGTGCTGAGTTGCCGTTC
ACCATTGACAAGTAGTTCGTTGAA

>gi|256385516|gb|CY044351.1| Influenza A virus (A/South Korea/AF10/2008(H1N1)) segment 6, complete sequence

ATGAACCCAAATCAAAAAATAATAACCATTGGATCAATCAGTATAGCAATCGGAATAATTAGTTTAATGT
TGCAAATAGGAAATATTATTTCGATATGGGCTAGTCACTCAATCCAAACTGGAAGTCAAAACCACACTGG
AATATGCAACCAAAGAATCATTACATATGAAAACAGCACCTGGGTGAATCACACATATGTTAATATTAAC
AACACTAATGTTGTTGCTGGAAAGGACAAAACTCCAGTTACATTGGCCGGCAACTCATCTCTTTGTTCTA
TCAGTGGATGGGCTATACACACAAAAGACAACAGCATAAGAATTGGCTCCAAAGGAGATGTTTTTGTCAT
AAGAGAACCTTTCATATCATGTTCTCACTTGGAATGCAAAACCTTTTTTCTTACCCAAGGCGCTCTATTA
AATGACAAACATTCAAATGGGACCGTAAAGGACAGAAGTCCTTATAGGGCCTTAATGAGCTGTCCTCTAG
GTGAAGCTCCGTCCCCATACAATTCAAAGTTCGAATCAGTTGCATGGTCAGCAAGCGCATGCCATGATGG
CATAGGCTGGTTAACAATCGGAATTTCTGGTCCAGACAATGGAGCTGTGGCTGTACTAAAATACAACGGA
ATAATAACTGGAACCATAAAAAGTTGGAAAAAGCAAATATTAAGAACACAAGAGTCTGAATGTGTCTGTA
TGAACGGGTCATGTTTCACCATAATGACTGATGGTCCGAGTAATGGGGCCGCCTCGTACAAAATTTTCAA
GATCGAAAAGGGGAAGGTTACTAAATCAATGGAGTTAAATGCACCCAATTTTCATTATGAGGAATGTTCT
TGTTACCCAGACACTGGCACAGTGATGTGTGTATGCAGGGACAACTGGCATGGTTCAAATCGACCTTGGG
TGTCTTTTAATCAAAACTTGGATTATCAAATAGGATACATCTGCAGTGGAGTGTTCGGTGACAATCCGCG
TCCCGAAGATGGAGAGGGCAGCTGCAATCCAGTGACTGTTGATGGAGCAAACGGAGTAAAAGGGTTTTCA
TACAAATATGGTAATGGTGTTTGGATAGGAAGGACCAAAAGTAACAGAATTAGAAAGGGGTTTGAGATGA
TTTGGGATCCTAATGGATGGACAAATACCGACAGTGATTTCTCAGTGAAACAGGATATTGTAGCAATAAC
TGATTGGTCAGGGTACAGCGGAAGTTTCGTCCAACATCCTGAGTTAACAGGATTGGACTGTATAAGACCT
TGCTTCTGGGTTGAGTTAGTCAGAGGGCTGCCTAGAGAAAATACGACAATCTGGACTAGTGGAAGCAGCA
TTTCCTTTTGTGGCGTTAATAGTGATATTGCAAACTGGTCTTGGCCAGACGGTGCTGAGCTGCCGTTCAC
CATTGACAAGTAGTTCGTT

>gi|163964721|gb|CY028461.1| Influenza A virus (A/California/UR06-0442/2007(H1N1)) segment 6, complete sequence

GAGTTTAAAATGAATCCAAATCAAAAAATAATAACCATTGGATCAATCAGTATAGCAATTGGAATAATTA
GTCTAATGTTGCAAATAGGAAATATTATTTCAATATGGGCTAGTCACTCAATCCAAACTGGAAGTCAAAA
CCACACTGGAATATGCAACCAAAGAATCATCACATATGAAAACAGCACCTGGGTGAATCACACATATGTT
AATATTAACAACACTAATGTTGTTGCTGGAAAGGACAAAACTTCAGTGACATTGGCCGGCAATTCATCTC

TTTGTTCTATCAGTGGATGGGCTATATACACAAAAGACAACAGCATAAGAATTGGCTCCAAAGGAGATGT
TTTTGTCATAAGAGAACCTTTCATATCATGTTCTCATTTGGAATGCAGGACCTTTTTTCTGACCCAAGGT
GCTCTATTAAATGACAAACATTCAAATGGAACCGTTAAGGACAGAAGTCCTTATAGGGCCTTAATGAGCT
GTCCTCTAGGTGAAGCTCCGTCCCCATACAATTCAAAGTTTGAATCAGTTGCATGGTCAGCAAGCGCATG
CCATGATGGCATGGGCTGGTTAACAATCGGGATTTCTGGTCCAGACAATGGAGCTGTGGCTGTACTAAAA
TACAACGGAATAATAACTGAAACCATAAAAAGTTGGAAAAAGCGAATATTGAGAACACAGGAGTCTGAAT
GTGTCTGTGTGAACGGGTCATGTTTCACCATAATGACCGATGGCCCGAGTAATGGGGCCGCCTCGTACAA
AATCTTCAAGATCGAAAAGGGGAAGGTTACTAAATCAATAGAGTTAAATGCACCCAATTTTCATTATGAG
GAATGTTCCTGTTACCCAGACACTGGCACAGTGATGTGTGTATGCAGGGATAACTGGCATGGTTCAAATC
GACCTTGGGTGTCTTTTAATCAAAACTTGGATTATCAAGTAGGATACATCTGCAGTGGAGTGTTCGGTGA
CAATCCGCGTCCCAAAGATGGAAAGGGCAGCTGTAATCCAATGACTGTTGATGGAGCAGACGGAGTTAAG
GGGTTTTCATACAAATATGGTAATGGTGTTTGGATAGGAAGGACTAAAAGTAACAAACTTAGAAAGGGGT
TTGAGATGATTTGGGATCCTAATGGATGGACAGATACCGACAGTGATTTCTCAGTGAAACAGGATGTTGT
GGCAATAACCGATTGGTCAGGGTACAGTGGAAGTTTCGTTCAACATCCTGAGTTAACAGGATTGGACTGT
ATAAGACCTTGCTTCTGGGTTGAGTTAGTTAGAGGACTGCCTAGAGAAAATGCAACAATCTGGACTAGTG
GGAGCAGCATTTCTTTTTGTGGCGTTGATAGTGATACTGCAAACTGGTCTTGGCCAGACGGTGCTGAGTT
GCCGTTCACCATTGACAAATAGTTCGTTGAA
>gi|157281605|gb|CY025359.1| Influenza A virus (A/Kentucky/UR06-0363/2007(H1N1)) segment 6, complete sequence
ATGAATCCAAATCAAAAAATAATAACCATTGGATCAATCAGTATAGCAATCGGAATAATTAGTCTAATGT
TGCAAATAGGAAATATTATTTCAATATGGGCTAGTCACTCAATCCAAACTGGAAGTCAAAACCACACTGG
AATATGCAACCAAAGAATCATCACATATGAAAACAGCACCTGGGTGAATCACACATATGTTAATATTAAC
AACACTAATGTTGTTACTGGAAAGGACAAAACTTCAGTGACATTGGCCGGCAATTCATCTCTTTGTTCTA
TCAGTGGATGGGCTATATACACAAAAGACAACAGCATAAGAATTGGCTCCAAAGGAGATGTTTTTGTCAT
AAGAGAACCTTTCATATCATGTTCTCATTTGGAATGCAGGACCTTTTTTCTGACCCAAGGTGCTCTATTA
AATGACAAACATTCAAATGGAACCGTTAAGGACAGAAGTCCTTATAGGGCCTTAATGAGCTGTCCTCTAG
GTGAAGCTCCGTCCCCATACAATTCAAAGTTTGAATCAGTTGCATGGTCAGCAAGCGCATGCCATGATGG
CATGGGCTGGTTAACAATCGGAATTTCTGGTCCAGACAATGGAGCTGTGGCTGTACTAAAATACAACGGA
ATAATAACTGAAACCATAAAAAGTTGGAAAAAGCGAATATTGAGAACACAAGAGTCTGAATGTGTCTGTG
TGAACGGGTCATGTTTCACTATAATGACCGATGGCCCGAGTGATGGGGCCGCCTCGTACAAAATCTTCAA
GATCGAAAAGGGGAAGGTTACTAAATCAATAGAGTTAAATGCACCCAATTTTCATTATGAGGAATGTTCC
TGTTACCCAGACACTGGCACAGTGATGTGTGTATGCAGGGACAACTGGCATGGTTCAAATCGACCTTGGG
TGTCTTTTAATCAAAACTTGGATTATCAAATAGGATACATCTGCAGTGGAGTGTTCGGTGACAATCCGCG
TCCCAAAGATGGAAAGGGCAGCTGTAATCCAGTGACTGTTGATGGAGCAGACGGAGTTAAGGGGTTTTCA
TACAAATATGGTAATGGTGTTTGGATAGGAAGGACTAAAAGTAACAAACTTAGAAAAGGGTTTGAGATGA
TTTGGGATCCTAATGGATGGACAGATACCGACAATGATTTCTCAGTGAAACAGGATGTTGTGGCAATAAC
TGATTGGTCAGGGTACAGTGGAAGTTTCGTTCAACATCCTGAGTTAACAGGATTGGACTGTATAAGACCT
TGCTTCTGGGTTGAGTTAGTCAGAGGACTGCCTAGAGAAAATACAACAATCTGGACTAGTGGGAGCAGCA
TTTCTTTTTGTGGCGTTGATAGTGATACTGCAAACTGGTCTTGGCCAGACGGTGCTGAGTTGCCGTTCAC
CATTGACAAATAGTTCGTTGAAAAA
>gi|158957794|gb|CY027413.1| Influenza A virus (A/Alabama/UR06-0536/2007(H1N1)) segment 6, complete sequence
ATGAATCCAAATCAAAAAATAATAACCATTGGATCAATCAGTATAGCAATCGGGATAATTAGTCTAATGT
TGCAAATAGGAAATATTATTTCAATATGGGCTAGTCACTCAATCCAAACTGGAAGTCAAAACCACACTGG

AATATGCAACCAAAGAATCATCACATATGAAAACAGCACCTGGGTGAATCACACATATGTTAGTATTAAC
AACACTAATGTTGTTGCTGGAAAGGACAAAACTTCAGTGACATTGGCCGGCAATTCATCTCTTTGTTCTA
TCAGTGGATGGGCTATATACACAAAAGACAACAGCATAAGAATTGGCTCCAAAGGAGATGTTTTTGTCAT
AAGAGAACCTTTCATATCATGTTCTCATTTGGAATGCAGGACCTTTTTTCTGACCCAAGGTGCTCTATTA
AATGACAAACATTCAAATGGAACCGTTAAGGACCGAAGTCCTTATAGGGCCTTAATGAGCTGTCCTCTAG
GTGAAGCTCCGTCCCCATACAATTCAAAGTTTGAATCAGTTGCATGGTCAGCAAGCGCATGCCATGATGG
CATGGGCTGGTTAACAATCGGAATTTCTGGTCCAGACAATGGAGCTGTGGCTGTACTAAAATACAACGGA
ATAATAACTGAAACCATAAAAAGTTGGAAAAAGCGAATATTGAGAACACAGGAGTCTGAATGTGTCTGTG
TGAATGGGTCATGTTTCACCATAATGACCGATGGCCCGAGTAATGGGGCCGCCTCGTACAAAATCTTCAA
AATCGAAAAGGGGAAGGTTACTAAATCAATAGAGTTAAATGCACCCAATTTTCATTATGAGGAATGTTCC
TGTTACCCAGACACTGGCACAGTGATGTGTGTATGCAGGGACAACTGGCATGGTTCAAATCGACCTTGGG
TGTCTTTTAATCAAAACTTGGATTATCAAATAGGATACATCTGCAGTGGAGTGTTCGGTGACAATCCGCG
TCCCAAAGATGGAAAGGGCAGCTGTAATCCAGTGACTGTTGATGGAGCAGACGGGGTTAAGGGGTTTTCA
TACAAATATGGTAATGGTGTTTGGATAGGAAGAACTAAAAGTAACAGACTTAGAAAGGGGTTTGAGATGA
TTTGGGATCCTAATGGATGGACAGATACCGACAGTGATTTCTCAGTGAAACAGGATGTTGTGGCAATAAC
TGATTGGTCAGGGTACAGTGGAAGTTTCGTTCAACATCCTGAGTTAACAGGATTGGACTGTATAAGACCT
TGCTTCTGGGTTGAGTTAGTCAGAGGACTGCCTAGAGAAAATACAACAATCTGGACTAGTGGGAGCAGCA
TTTCTTTTTGTGGCGTTGATAGTGATACTGCAAACTGGTCTTGGCCAGACGGTGCTGAGTTGCCGTTCAC
CATTGACAAGTAGTTCGTTGA
>gi|237688829|gb|CY040060.1| Influenza A virus (A/Taiwan/71720/2007(H1N1)) segment 6, complete sequence
GGAGTTTAAAATGAACCCAAATCAAAAGATAATAACCATTGGATCAATCAGTATAGCAATCGGAATAATT
AGTTTAATGTTGCAAATAGGAAATATTATTTCAATATGGGCTAGTCACTCAATCCAAACTGGAAGTCAAA
ACAACACTGGAATATGCAACCAAAGAATCATCACATATGAAAACAGCACCTGGGTGAATCACACATATGT
TAATATTAACAACACTAATGTTGTTGCTGGAGAGGACAAAACTTCAGTGACATTGGCCGGCAATTCATCT
CTCTGTTCTATCAGTGGATGGGCTATATACACAAAAGACAACAGCATAAGAATTGGCTCCAAAGGAGATG
TTTTTGTCATAAGAGAACCTTTCATATCATGTTCTCACTTGGAATGCAGAACCTTTTTTCTGACCCAAGG
CGCTCTATTAAATGACAAACATTCAAATGGGACCGTAAAGGACAGAAGTCCTTATAGGGCCTTAATGAGC
TGTCCTCTAGGTGAAGCTCCGTCCCCATACAATTCAAAGTTCGAATCAGTTGCATGGTCAGCAAGCGCAT
GCCATGATGGCATGGGCTGGTTAACAATCGGAATTTCTGGTCCAGACAATGGAGCCGTGGCTGTACTAAA
ATACAACGGAATAATAACTGGAACCATAAAAAGTTGGAAAAAGCAAATATTAAGAACACAAGAGTCTGAA
TGTGTCTGTATGAACGGGTCATGTTTCACCATAATGACCGATGGCCCGAGTAATAAGGCCGCCTCGTACA
AAATTTTCAAAATCGAAAAGGGGAAGGTTACTAAATCAATAGAGTTGAATGCACCCAATTTTCATTATGA
GGAATGTTCCTGTTACCCAGACACTGGCATAGTGATGTGTGTATGCAGGGACAACTGGCATGGTTCAAAT
CGACCTTGGGTGTCTTTTAATCAAAACTTGGATTATCAAATAGGATACATCTGCAGTGGAGTGTTCGGTG
ACAATCCGCGTCCCGAAGATGGAGAGGGCAGCTGCAATCCAGTGACTGTTGATGGAGCAAACGGAGTAAA
AGGGTTTTCATACAAATATGATAATGGTGTTTGGATAGGAAGGACCAAAAGTAACAGACTTAGAAAGGGG
TTTGAGATGATTTGGGATCCTAATGGATGGACAAATACCGACAGTGATTTCTCAGTGAAACAGGATGTTG
TAGCAATAACTGATTGGTCAGGGTACAGCGGAAGTTTCGTCCAACATCCTGAGTTAACAGGATTGGACTG
TATAAGACCTTGCTTCTGGGTTGAGTTAGTCAGAGGGCTGCCTAGAGAAAATACAACAATCTGGACTAGT
GGGAGCAGCATTTCTTTTTGTGGCGTTAATAGTGATACTGCAAACTGGTCTTGGCCAGACGGTGCTGAGT
TGCCGTTCACCATTGACAAGTAGTTCG
>gi|237689287|gb|CY040252.1| Influenza A virus (A/Managua/3153.01/2008(H1N1)) segment 6, complete sequence

AAATGAACCCAAATCAAAAGATAATAACCATTGGATCAATCAGTATAGCAATCGGAATAATTAGTCTAAT
GTTGCAAATAGGAAATATTATTTCAATATGGGCTAGTCACTCAATCCAAACTGGAAGTCAAAACAACACT
GGAATATGCAACCAAAGAATCATCACATATGAAAACAGCACCTGGGTGAATCACACATATGTTAATATTA
ACAACACTAATGTTGTTGCTGGAGAGGATAAAACTTCAGTGACATTGGCCGGCAATTCATCTCTTTGTTC
TATCAGTGGATGGGCTATATACACAAAAGACAACAGCATAAGAATTGGCTCCAAAGGAGATGTTTTTGTC
ATAAGAGAACCTTTCATATCATGTTCTCACTTGGAATGCAGAACCTTTTTTCTGACCCAAGGCGCTCTAT
TAAATGACAAACATTCAAATGGGACCGTAAAGGACAGAAGTCCTTATAGGGCCTTAATGAGCTGTCCTCT
AGGTGAAGCTCCGTCCCCATACAATTCAAAGTTCGAATCAGTTGCATGGTCAGCAAGCGCATGCCATGAT
GGCATGGGCTGGTTAACAATCGGAATTTCTGGTCCAGACAATGGAGCTGTGGCTGTACTAAAATACAACG
GAATAATAACTGGAACCATAAAAAGTTGGAAAAAGCAAATATTAAGAACACAAGAGTCTGAATGTGTCTG
TATGAACGGGTCATGTTTCACTATAATGACCGACGGCCCGAGTAATAAGGCCGCCTCGTACAAAATTTTC
AAGATCGAAAAGGGGAAGGTTACTAAATCAATAGAGTTGAATGCACCCAATTTTTATTATGAGGAATGTT
CCTGTTACCCAGACACTGGCATAGTGATGTGTGTATGCAGGGACAACTGGCATGGTTCAAATCGACCTTG
GGTGTCTTTTAATCAAAACTTGGATTATCAAATAGGATACATCTGCAGTGGAGTGTTTGGTGACAATCCG
CGTCCCGAAGATGGAGAGGGCAGCTGCAATCCAGTGACTGTTGATGGAGCAAACGGAGTAAAAGGGTTTT
CATACAAATATGATAATGGTGTTTGGATAGGAAGGACCAAAAGTAACAGACTTAGAAAGGGGTTTGAGAT
GATTTGGGATCCTAATGGATGGACAAATACCGACAGTGATTTCTCAGTGAAACAGGATGTTGTAGCAATA
ACTGATTGGTCAGGGTACAGTGGAAGTTTCGTCCAACATCCTGAGTTAACAGGATTGGACTGTATAAGAC
CTTGCTTCTGGGTTGAGTTAGTCAGAGGGCTGCCTAGAGAAAATACAACAATCTGGACTAGTGGGAGCAG
CATTTCTTTTTGTGGCGTTAATAGTGATACTGCAAACTGGTCTTGGCCAGACGGTGCTGAGTTGCCGTTC
ACCATTGACAAGTAGTTCGTTGA
>gi|224020934|gb|CY037329.1| Influenza A virus (A/Washington/AF06/2007(H1N1)) segment 6, complete sequence
AATCAAAAGATAATAACCATTGGATCAATCAGTATAGCAATCGGAATAATTAGTCTAATGTTGCAAATAG
GAAATATTATTTCAATATGGGCTAGTCACTCAATCCAAACTGGAAGTCAAAACAACACTGGAATATGCAA
CCAAAGAATCATCACATATGAAAACAGCACCTGGGTGAATCACACATATGTTAATATTAACAACACTAAT
GTTGTTGCTGGAGAGGACAAAACTTCAGTGACATTGGCCGGCAATTCATCTCTTTGTTCTATCAGTGGAT
GGGCTATATACACAAAAGACAACAGCATAAGAATTGGCTCCAAAGGAGATGTTTTTGTCATAAGAGAACC
TTTCATATCATGTTCTCACTTGGAATGCAGAACCTTTTTTCTGACCCAAGGCGCTCTATTAAATGACAAA
CATTCAAATGGGACCGTAAAGGATAGAAGTCCTTATAGGGCCTTAATGAGCTGTCCCTTAGGTGAAGCTC
CGTCCCCATACAATTCAAAGTTCGAATCAGTTGCATGGTCAGCAAGCGCATGCCATGATGGCATGGGCTG
GTTAACAATCGGAATTTCTGGTCCAGACAATGGAGCTGTGGCTGTACTAAAATACAACGGAATAATAACT
GGAACCATAAAAAGTTGGAAAAAGCAAATATTAAGAACACAAGAGTCTGAATGTGTCTGTATGAACGGGT
CATGTTTCACCATAATGACCGATGGCCCGAGTAATAAGGCCGCCTCGTACAAAATTTTCAAGATCGAAAA
GGGGAAGGTTACTAAATCAATAGAGTTGAATGCACCCAATTTTCATTATGAGGAATGTTCCTGTTACCCA
GACACTGGCATAGTGATGTGTGTATGCAGGGACAACTGGCATGGTTCAAATCGACCTTGGGTGTCTTTTA
ATCAAAACTTGGATTATCAAATAGGATACATCTGCAGTGGAGTGTTCGGTGACAATCCGCGTCCCGAAGA
TGGAGAGGGCAGCTGCAATCCAGTGACTGTTGATGGAGCAAACGGAGTAAAAGGGTTTTCATACAAATAT
GATAATGGTGTTTGGATAGGAAGGACCAAAAGTAACAGACTTAGAAAGGGGTTTGAGATGATCTGGGATC
CTAATGGATGGACAAATACCGACAGTGATTTCTCAGTGAAACAGGATGTTGTAGCAATAACTGATTGGTC
AGGATACAGCGGAAGTTTCGTCCAACATCCTGAGTTAACAGGATTGGACTGTATAAGACCTTGCTTCTGG
GTTGAGTTAGTCAGAGGACTGCCTAGAGAAAATACAACAATCTGGACTAGTGGGAGCAGCATTTCTTTTT
GTGGCGTTAATAGTGATACTGCAAACTGGTCTTGGCCAGACGGTGCTGAGTTGCCGTTCACCATTGACAA
GTAGTTCGTTGA

>gi|224021239|gb|CY037337.1| Influenza A virus (A/Japan/AF07/2008(H1N1)) segment 6, complete sequence
AACCCAAATCAAAAGATAATAACCATTGGATCAATCAGTATAGCAATCGGAATAATTAGTCTAATGTTGC
AAATAGGAAATATTATTTCAATATGGGCTAGTCACTCAATCCAAACTGGAAGTCAAAACAATACTGGAAT
ATGCAACCAAAGAATCATCACATATGAAAACAGCACCTGGGTGAATCACACATATGTTAATATTAACAAC
ACTAATGTTGTTGCTGGAGAGGACAAAACTTCAGTGACATTGGCCGGCAATTCATCTCTTTGTTCTATCA
GTGGATGGGCTATATACACAAAAGACAACAGCATAAGAATTGGCTCCAAAGGAGATGTTTTTGTCATAAG
AGAACCTTTCATATCATGTTCTCACTTGGAATGCAGAACCTTTTTTCTGACCCAAGGCGCTCTATTGAAT
GACAAACATTCAAATGGGACCGTAAAGGACAGAAGTCCTTATAGGGCCTTAATGAGCTGTCCTCTAGGTG
AAGCTCCGTCCCCATACAATTCAAAGTTCGAATCAGTTGCATGGTCAGCAAGCGCATGCCATGATGGCAT
GGGCTGGTTAACAATTGGAATTTCTGGTCCAGACAATGGAGCTGTGGCTGTACTAAAATACAACGGAATA
ATAACTGGAACCATAAAAAGTTGGAAAAAGCAAATATTAAGAACACAAGAGTCTGAATGTGTCTGTATGA
ACGGGTCATGTTTTACCATAATGACCGATGGCCCGAGTAATAAGGCCGCCTCGTACAAAATTTTCAAGAT
CGAAAAGGGGAAGGTTACTAAATCAATAGAGTTGAATGCACCCAATTTTCATTATGAGGAATGTTCCTGT
TACCCAGACACTGGCATAGTGATGTGTGTATGCAGGGACAACTGGCATGGTTCAAATCGACCTTGGGTGT
CTTTTAATCAAAACTTGGATTATCAAATAGGATACATCTGCAGTGGAGTGTTCGGTGACAATCCGCGTCC
CGAAGATGGAGAGGGCAGCTGCAATCCAGTGACTGTTGATGGAGCAAACGGAGTAAAAGGGTTTTCATAC
AAATATGATAATGGTGTTTGGATAGGAAGGACCAAAAGTAACAGACTTAGAAAGGGGTTTGAGATGATCT
GGGATCCTAATGGATGGACAAATACCGACAGTGATTTCTCAGTGAAACAGGATGTTGTAGCAATAACTGA
TTGGTCAGGGTACAGCGGAAGTTTCGTCCAACATCCTGAGTTAACAGGATTGGACTGTATAAGACCTTGC
TTCTGGGTTGAGTTAGTCAGAGGGCTGCCTAGAGAAAATACAACAATCTGGACTAGTGGGAGCAGCATTT
CTTTTTGTGGCGTTAATAGTGATACTGCAAACTGGTCTTGGCCAGACGGTGCTGAGTTGCCGTTCACCAT
TGACAAGTAGTTCGTTGA
>gi|212381596|gb|FJ445029.1| Influenza A virus (A/England/26/2008(H1N1)) segment 6 neuraminidase (NA) gene, complete cds
ATGAACCCAAATCAAAAGATAATAACCATTGGATCAATCAGTATAGCAATCGGAATAATTAGTCTAATGT
TGCAAATAGGAAATATTATTTCAATATGGGCTAGTCACTCAATCCAAACTGGAAGTCAAAACAACACTGG
AATATGCAACCAAAGAATCATCACATATGAAAACAGCACCTGGGTGAATCACACATATGTTAATATTAAC
AACACTAATGTTGTTGCTGGAGAAGACAAAACTTCAGTGACATTGGCCGGCAATTCATCTCTTTGTTCTA
TCAGTGGATGGGCTATATACACAAAAGACAACAGCATAAGAATTGGCTCCAAAGGAGATGTTTTTGTCAT
AAGAGAACCTTTCATATCATGTTCTCACTTGGAATGCAGAACCTTTTTTCTGACCCAAGGCGCTCTATTA
AATGACAAACATTCAAATGGGACCGTAAAGGACAGAAGTCCTTATAGGGCCTTAATGAGCTGTCCTCTAG
GTGAAGCTCCGTCCCCATACAATTCAAAGTTCGAATCAGTTGCATGGTCAGCAAGCGCATGCCATGATGG
CATGGGCTGGTTAACAATCGGAATTTCTGGTCCAGACAATGGAGCTGTGGCTGTACTAAAATACAACGGA
ATAATAACTGGAACCATAAAAAGTTGGAAAAAGCAAATATTAAGAACACAAGAGTCTGAATGTGTCTGTA
TGAACGGGTCATGTTTCACCATAATGACCGATGGCCCGAGTAATAAGGCCGCCTCGTACAAAATTTTCAA
GATCGAAAAGGGGAAGGTTACTAAATCAATAGAGTTGAATGCACCCAATTTTTATTATGAGGAATGCTCC
TGTTACCCAGACACTGGCATAGTGATGTGTGTATGCAGGGACAACTGGCATGGTTCAAATCGACCTTGGG
TGTCTTTTAATCAAAACTTGGATTATCAAATAGGATACATCTGCAGTGGAGTGTTTGGTGACAATCCGCG
TCCCGAAGATGGAGAGGGCAGCTGCAATCCAGTGACTGTTGATGGAGCAAACGGAGTAAAAGGGTTTTCA
TACAAATATGGTAATGGTGTTTGGATAGGAAGGACCAAAAGTAACAGACTTAGAAAGGGGTTTGAGATGA
TTTGGGATCCTAATGGATGGACAAATACCGACAGTGATTTCTCAGTGAAACAGGATGTTGTAGCAATAAC
TGATTGGTCAGGGTACAGCGGAAGTTTCGTCCAACATCCTGAGTTAACAGGATTGGACTGTATAAGACCT
TGCTTCTGGGTTGAGTTAGTCAGAGGGCTGCCTAGAGAAAATACAACAATCTGGACTAGTGGGAGCAGCA

TTTCTTTTTGTGGCGTTAATAGTGATACTGCAAACTGGTCTTGGCCAGACGGTGCTGAGTTGCCGTTCAC
CATTGACAAGTAG
>gi|224027209|gb|CY037681.1| Influenza A virus (A/Florida/UR07-0022/2008(H1N1)) segment 6, complete sequence
AAATGAACCCAAATCAAAAGATAATAACCATTGGATCAATCAGTATAGCAATCGGAATAATTAGTCTAAT
GTTGCAAATAGGAAATATTATTTCAATATGGGCTAGTCACTCAATCCAAACTGGAAGTCAAAACAACACT
GGAATATGCAACCAAAGAATCATCACATATGAAAACAGCACCTGGGTGAATCACACATATGTTAATATTA
ACAACACTAATGTTGTTGCTGGAGAGGACAAAACTTCAGTGACATTGGCCGGCAGTTCATCTCTTTGTTC
TATCAGTGGATGGGCTATATACACAAAAGACAACAGCATAAGAATTGGCTCCAAAGGAGATGTTTTTGTC
ATAAGAGAACCTTTCATATCATGTTCTCACTTGGAATGCAGAACCTTTTTTCTGACCCAAGGTGCCCTAT
TAAATGACAAACATTCAAATGGGACCGTAAAGGACAGAAGTCCTTATAGGGCCTTAATGAGCTGTCCTCT
AGGTGAAGCTCCGTCCCCATACAATTCAAAGTTCGAATCAGTTGCATGGTCAGCAAGCGCATGCCATGAT
GGCATGGGCTGGTTAACAATCGGAATTTCTGGTCCAGACAATGGAGCTGTGGCTGTACTAAAATACAACG
GAATAATAACTGGAACCATAAAAAGTTGGAAAAAGCAAATATTAAGAACACAAGAGTCTGAATGTGTCTG
TATGAACGGGTCATGTTTCACCATAATGACCGATGGCCCGAGTAATAAGGCCGCCTCGTACAAAATTTTC
AAGATCGAAAAGGGGAAGGTTACTAAATCAATAGAGTTGAATGCACCCAATTTTCATTATGAGGAATGTT
CCTGTTACCCAGACACTGGCATAGTGATGTGTGTATGCAGGGACAACTGGCATGGTTCAAATCGACCTTG
GGTGTCTTTTAATCAAAACTTGGATTATCAAATAGGATACATCTGCAGTGGAGTGTTTGGTGACAATCCG
CGTCCCGAAGATGGAGAGGGCAGCTGCAATCCAGTGACTGTTGATGGAGCAAACGGGGTAAAAGGGTTTT
CATACAAATATGATAATGGTGTTTGGATAGGAAGGACCAAAAGTAACAGACTTAGAAAGGGGTTTGAGAT
GATTTGGGATCCTAATGGATGGACAAATACCGACAGTGATTTCTCAGTGAAACAGGATGTTGTAGCAATA
ACTGATTGGTCAGGGTACAGCGGAAGTTTCGTCCAACATCCTGAGTTAACAGGATTGGACTGTATAAGAC
CTTGCTTCTGGGTTGAGTTAGTCAGAGGGCTGCCTAGAGAAAATACAACAATCTGGACTAGTGGGAGCAG
CATTTCTTTTTGTGGCGTTAATAGTGATACTGCAAACTGGTCTTGGCCAGACGGTGCTGAGTTGCCGTTC
ACCATTGACAAGTAGTTCGTTGA
>gi|296240584|gb|CY063608.1| Influenza A virus (A/Aalborg/INS133/2009(H1N1)) segment 6, complete sequence
AAATGAATCCAAACCAAAAGATAATAACCATTGGTTCGGTCTGTATGACAATTGGAATGGCTAACTTAAT
ATTACAAATTGGAAACATAATCTCAATATGGATTAGCCACTCAATTCAACTTGGGAATCAAAATCAGATT
GAAACATGCAATCAAAGCGTCATTACTTATGAAAACAACACTTGGGTAAATCAGACATATGTTAACATCA
GCAACACCAACTTTGCTGCTGGACAGTCAGTGGTTTCCGTGAAATTAGCGGGCAATTCCTCTCTCTGCCC
TGTTAGTGGATGGGCTATATACAGTAAAGACAACAGTATAAGAATCGGTTCCAAGGGGGATGTGTTTGTC
ATAAGGGAACCATTCATATCATGCTCCCCCTTGGAATGCAGAACCTTCTTCTTGACTCAAGGGGCCTTGC
TAAATGACAAACATTCCAATGGAACCATTAAAGACAGGAGCCCATATCGAACCCTAATGAGCTGTCCTAT
TGGTGAAGTTCCCTCTCCATACAACTCAAGATTTGAGTCAGTCGCTTGGTCAGCAAGTGCTTGTCATGAT
GGCATCAATTGGCTAACAATTGGAATTTCTGGCCCAGACAATGGGGCAGTGGCTGTGTTAAAGTACAACG
GCATAATAACAGACACTATCAAGAGTTGGAGAAACAATATATTGAGAACACAAGAGTCTGAATGTGCATG
TGTAAATGGTTCTTGCTTTACTGTAATGACCGATGGACCAAGTGATGGACAGGCCTCATACAAGATCTTC
AGAATAGAAAAGGGAAAGATAGTCAAATCAGTCGAAATGAATGCCCCTAATTATCACTATGAGGAATGCT
CCTGTTATCCTGATTCTAGTGAAATCACATGTGTGTGCAGGGATAACTGGCATGGCTCGAATCGACCGTG
GGTGTCTTTCAACCAGAATCTGGAATATCAGATAGGATACATATGCAGTGGGATTTTCGGAGACAATCCA
CGCCCTAATGATAAGACAGGCAGTTGTGGTCCAGTATCGTCTAATGGAGCAAATGGAGTAAAAGGATTTT
CATTCAAATACGGCAATGGTGTTTGGATAGGGAGAACTAAAAGCATTAGTTCAAGAAACGGTTTTGAGAT
GATTTGGGATCCGAACGGATGGACTGGGACAGACAATAACTTCTCAATAAAGCAAGATATCGTAGGAATA

AATGAATGGTCAGGATATAGCGGGAGTTTTGTTCAGCATCCAGAACTAACAGGGCTGGATTGTATAAGAC
CTTGCTTCTGGGTTGAACTAATCAGAGGGCGACCCAAAGAGAACACAATCTGGACTAGCGGGAGCAGCAT
ATCCTTTTGTGGTGTAAACAGTGACACTGTGGGTTGGTCTTGGCCAGACGGTGCTGAGTTGCCATTTACC
ATTGACAAGTAATTTGTTCA
>gi|296240566|gb|CY063600.1| Influenza A virus (A/Bonn/INS128/2009(H1N1)) segment 6, complete sequence
AAATGAATCCAAACCAAAAGATAATAACCATTGGTTCGGTCTGTATGACAATTGGAATGGCTAACTTAAT
ATTACAAATTGGAAACATAATCTCAATATGGATTAGCCACTCAATTCAACTTGGGAATCAAAATCAGATT
GAAACATGCAATCAAAGCGTCATTACTTATGAAAACAACACTTGGGTAAATCAGACATATGTTAACATCA
GCAACACCAACTTTGCTGCTGGACAGTCAGTGGTTTCCGTGAAATTAGCGGGCAATTCCTCTCTCTGCCC
TGTTAGTGGATGGGCTATATACAGTAAAGACAACAGTATAAGAATCGGTTCCAAGGGGGATGTGTTTGTC
ATAAGGGAACCATTCATATCATGCTCCCCCTTGGAATGCAGAACCTTCTTCTTGACTCAAGGGGCCTTGC
TAAATGACAAACATTCCAATGGAACCATTAAAGACAGGAGCCCATATCGAACCCTAATGAGCTGTCCTAT
TGGTGAAGTTCCCTCTCCATACAACTCAAGATTTGAGTCAGTCGCTTGGTCAGCAAGTGCTTGTCATGAT
GGCATCAATTGGCTAACAATTGGAATTTCTGGCCCAGACAATGGGGCAGTGGCTGTGTTAAAGTACAACG
GCATAATAACAGACACTATCAAGAGTTGGAGAAACAATATATTGAGAACACAAGAGTCTGAATGTGCATG
TGTAAATGGTTCTTGCTTTACTGTAATGACCGATGGACCAAGTGATGGACAGGCCTCATACAAGATCTTC
AGAATAGAAAAGGGAAAGATAGTCAAATCAGTCGAAATGAATGCCCCTAATTATCACTATGAGGAATGCT
CCTGTTATCCTGATTCTAGTGAAATCACATGTGTGTGCAGGGATAACTGGCATGGCTCGAATCGACCGTG
GGTGTCTTTCAACCAGAATCTGGAATATCAGATAGGATACATATGCAGTGGGATTTTCGGAGACAATCCA
CGCCCTAATGATAAGACAGGCAGTTGTGGTCCAGTATCGTCTAATGGAGCAAATGGAGTAAAAGGATTTT
CATTCAAATACGGCAATGGTGTTTGGATAGGGAGAACTAAAAGCATTAGTTCAAGAAACGGTTTTGAGAT
GATTTGGGATCCGAACGGATGGACTGGGACAGACAATAACTTCTCAATAAAGCAAGATATCGTAGGAATA
AATGAATGGTCAGGATATAGCGGGAGTTTTGTTCAGCATCCAGAACTAACAGGGCTGGATTGTATAAGAC
CTTGCTTCTGGGTTGAACTAATCAGAGGGCGACCCAAAGAGAACACAATCTGGACTAGCGGGAGCAGCAT
ATCCTTTTGTGGTGTAAACAGTGACACTGTGGGTTGGTCTTGGCCAGACGGTGCTGAGTTGCCATTTACC
ATTGACAAGTAATTTGTTCA
>gi|296240314|gb|CY063488.1| Influenza A virus (A/Boston/110/2009(H1N1)) segment 6, complete sequence
AAATGAATCCAAACCAAAAGATAATAACCATTGGTTCGGTCTGTATGACAATTGGAATGGCTAACTTAAT
ATTACAAATTGGAAACATAATCTCAATATGGATTAGCCACTCAATTCAACTTGGGAATCAAAATCAGATT
GAAACATGCAATCAAAGCGTCATTACTTATGAAAACAACACTTGGGTAAATCAGACATATGTTAACATCA
GCAACACCAACTTTGCTGCTGGACAGTCAGTGGTTTCCGTGAAATTAGCGGGCAATTCCTCTCTCTGCCC
TGTTAGTGGATGGGCTATATACAGTAAAGACAACAGTATAAGAATCGGTTCCAAGGGGGATGTGTTTGTC
ATAAGGGAACCATTCATATCATGCTCCCCCTTGGAATGCAGAACCTTCTTCTTGACTCAAGGGGCCTTGC
TAAATGACAAACATTCCAATGGAACCATTAAAGACAGGAGCCCATATCGAACCCTAATGAGCTGTCCTAT
TGGTGAAGTTCCCTCTCCATACAACTCAAGATTTGAGTCAGTCGCTTGGTCAGCAAGTGCTTGTCATGAT
GGCATCAATTGGCTAACAATTGGAATTTCTGGCCCAGACAATGGGGCAGTGGCTGTGTTAAAGTACAACG
GCATAATAACAGACACTATCAAGAGTTGGAGAAACAATATATTGAGAACACAAGAGTCTGAATGTGCATG
TGTAAATGGTTCTTGCTTTACTGTAATGACCGATGGACCAAGTGATGGACAGGCCTCATACAAGATCTTC
AGAATAGAAAAGGGAAAGATAGTCAAATCAGTCGAAATGAATGCCCCTAATTATCACTATGAGGAATGCT
CCTGTTATCCTGATTCTAGTGAAATCACATGTGTGTGCAGGGATAACTGGCATGGCTCGAATCGACCGTG
GGTGTCTTTCAACCAGAATCTGGAATATCAGATAGGATACATATGCAGTGGGATTTTCGGAGACAATCCA
CGCCCTAATGATAAGACAGGCAGTTGTGGTCCAGTATCGTCTAATGGAGCAAATGGAGTAAAAGGATTTT

CATTCAAATACGGCAATGGTGTTTGGATAGGGAGAACTAAAAGCATTAGTTCAAGAAACGGTTTTGAGAT
GATTTGGGATCCGAACGGATGGACTGGGACAGACAATAACTTCTCAATAAAGCAAGATATCGTAGGAATA
AATGAGTGGTCAGGATATAGCGGGAGTTTTGTTCAGCATCCAGAACTAACAGGGCTGGATTGTATAAGAC
CTTGCTTCTGGGTTGAACTAATCAGAGGGCGACCCAAAGAGAACACAATCTGGACTAGCGGGAGCAGCAT
ATCCTTTTGTGGTGTAAACAGTGACACTGTGGGTTGGTCTTGGCCAGACGGTGCTGAGTTGCCATTTACC
ATTGACAAGTAATTTGTTCA

>gi|296240620|gb|CY063624.1| Influenza A virus (A/New York/INS150/2009(H1N1)) segment 6, complete sequence

ATGAATCCAAACCAAAAGATAATAACCATTGGTTCGGCCTGTATGACAATTGGAATGGCTAACTTAATAT
TACAAATTGGAAACATAATCTCAATATGGATTAGCCACTCAATTCAACTTGGGAATCAAAATCAGACTGA
AACATGCAATCAAAGCGTCATTACTTATGAAAACAACACTTGGGTAAATCAGACATATGTTAACATCAGC
AACACCAACTTTGCTGCTGGACAGTCAGTGGTTTCCGTGAAATTAGCGGGCAATTCCTCTCTCTGCCCTG
TTAGTGGATGGGCTATATACAGCAAAGACAACAGTATAAGAATCGGTTCCAAGGGGGATGTGTTTGTCAT
AAGGGAACCATTCATATCATGCTCCCCCTTGGAATGCAGAACCTTCTTCTTGACTCAAGGGGCCTTGCTA
AATGACAAACATTCCAATGGAACCATTAAAGACAGGAGCCCATATCGAACCTTAATGAGCTGTCCTATTG
GTGAAGTTCCCTCTCCATACAACTCAAGATTTGAGTCAGTCGCTTGGTCAGCAAGTGCTTGTCATGATGG
CATCAATTGGCTAACAATTGGAATTTCTGGCCCAGACAATGGGGCAGTGGCTGTGTTAAAGTACAATGGC
ATAATAACAGACACTATCAAGAGTTGGAGAAACAATATATTGAGAACACAAGAGTCTGAATGTGCATGTG
TAAATGGTTCTTGCTTTACTGTAATGACCGATGGACCAAGTGATGGACAGGCCTCATACAAGATCTTCAG
AATAGAAAAGGGAAAGATAGTCAAATCAGTCGAAATGAATGCCCCTAATTATCACTATGAGGAATGCTCC
TGTTATCCTGATTCTAGTGAAATCACATGTGTGTGCAGGGATAACTGGCATGGCTCGAATCGACCGTGGG
TGTCTTTCAACCAGAATCTGGAATATCAGATAGGATACATATGCAGTGGGATTTTCGGAGACAACCCACG
CCCTAATGATAAGACAGGCAGTTGTGGTCCAGTATCGTCTAATGGAGCAAATGGAGTAAAAGGATTTTCA
TTCAAATACGGCAATGGTGTTTGGATAGGGAGAACTAAAAGCATTAGTTCAAGAAACGGTTTTGAGATGA
TTTGGGATCCGAACGGATGGACTGGGACAGACAATAACTTCTCAATAAAGCAAGATATCGTAGGAATAAA
TGAGTGGTCAGGATATAGCGGGAGTTTTGTTCAGCATCCAGAACTAACAGGGCTGGATTGTATAAGACCT
TGCTTCTGGGTTGAACTAATCAGAGGGCGACCCAAAGAGAACACAATCTGGACTAGCGGGAGCAGCATAT
CCTTTTGTGGTGTAAACAGTGACACTGTGGGTTGGTCTTGGCCAGACGGTGCTGAGTTGCCATTTACCAT
TGACAAGTAATTTGTTCA

>gi|296240548|gb|CY063592.1| Influenza A virus (A/Athens/INS122/2009(H1N1)) segment 6, complete sequence

AAATGAATCCAAACCAAAAGATAATAACCATTGGTTCGGTCTGTATGACAATTGGAATGGCTAACTTAAT
ATTACAAATTGGAAACATAATCTCAATATGGATTAGCCACTCAATTCAACTTGGGAATCAAAATCAGATT
GAAACATGCAATCAAAGCGTCATTACTTATGAAAACAACACTTGGGTAAATCAGACATATGTTAACATCA
GCAACACCAACTTTGCTGCTGAACAGTCAGTGGTTTCCGTGAAATTAGCGGGCAATTCCTCTCTCTGCCC
TGTTAGTGGATGGGCTATATACAGTAAAGACAACAGTATAAGAATCGGTTCCAAGGGGGATGTGTTTGTC
ATAAGGGAACCATTCATATCATGCTCCCCCTTGGAATGCAGAACCTTCTTCTTGACTCAAGGGGCCTTGC
TAAATGACAAACATTCCAATGGAACCATTAAAGACAGGAGCCCATATCGAACCCTAATGAGCTGTCCTAT
TGGTGAAGTTCCCTCTCCATACAACTCAAGATTTGAGTCAGTCGCTTGGTCAGCAAGTGCTTGTCATGAT
GGCATCAATTGGCTAACAATTGGAATTTCTGGCCCAGACAATGGGGCAGTGGCTGTGTTAAAGTACAACG
GCATAATAACAGACACTATCAAGAGTTGGAGAAACAATATATTGAGAACACAAGAGTCTGAATGTGCATG
TGTAAATGGTTCTTGCTTTACTGTAATGACCGATGGACCAAGTGATGGACAGGCCTCATACAAGATCTTC
AGAATAGAAAAGGGAAAGATAGTCAAATCAGTCGAAATGAATGCCCCTAATTATCACTATGAGGAATGCT
CCTGTTATCCTGATTCTAGTGAAATCACATGTGTGTGCAGGGATAACTGGCATGGCTCGAATCGACCGTG

GGTGTCTTTCAACCAGAATCTGGAATATCAGATAGGATACATATGCAGTGGGATTTTCGGAGACAATCCA
CGCCCTAATGATAAGACAGGCAGTTGTGGTCCAGTATCGTCTAATGGAGCAAATGGAGTAAAAGGATTTT
CATTCAAATACGGCAATGGTGTTTGGATAGGGAGAACTAAAAGCATTAGTTCAAGAAACGGTTTTGAGAT
GATTTGGGATCCGAACGGATGGACTGGGACAGACAATAACTTCTCAATAAAGCAAGATATCGTAGGAATA
AATGAGTGGTCAGGATATAGCGGGAGTTTTGTTCAGCATCCAGAACTAACAGGGCTGGATTGTATAAGAC
CTTGCTTCTGGGTTGAACTGATCAGAGGGCGACCCAAAGAGAACACAATCTGGACTAGCGGGAGCAGCAT
ATCCTTTTGTGGTGTAAACAGTGACACTGTGGGTTGGTCTTGGCCAGACGGTGCTGAGTTGCCATTTACC
ATTGACAAGTAATTTGTTCA

>gi|341612315|gb|JN381442.1| Influenza A virus (A/Taiwan/66259/2010(H1N1)) segment 6 neuraminidase (NA) gene, complete cds

ATGAATCCAAACCAAAAGATAATAACCATTGGTTCGGTCTGTATGACAATTGGAATGGCTAACTTAATAT
TACAAATTGGAAACATAATCTCAATATGGATTAGCCACTCAATTCAACTTGGAAATCAAAATCAGATTGA
AACATGCAATCAAAGCGTCATTACTTATGAAAACAACACTTGGGTAAATCAGACATATGTTAACATCAGC
AACACCAACTTTGCTGTTGGACAGTCAGTGGTTTCCGTGAAATTAGCGGGCAATTCATCTCTCTGCCCTG
TTAGTGGATGGGCTATATACAGTAAAGACAACAGTATAAGAATCGGTTCCAAGGGGGATGTGTTTGTCAT
AAGGGAACCATTCATATCATGCTCCCCCTTGGAATGCAGAACCTTCTTCTTGACTCAAGGGGCCTTGCTA
AATGACATACATTCCAATGGAACCATTAAAGACAGGAGCCCATATCGAACCCTAATGAGCTGTCCTATTG
GTGAAGTTCCCTCTCCATACAACTCAAGATTTGAGTCAGTCGCTTGGTCAGCAAGTGCTTGTCATGATGG
CATCAATTGGCTAACAATTGGAATTTCTGGCCCAGACAATGGGGCAGTGGCTGTGTTAAAGTACAACGGC
ATAATAACAGACACTATCAAGAGTTGGAGAAACAATATATTGAGAACACAAGAGTCTGAATGTGCATGTG
TAAATGGTTCTTGCTTTACCATAATGACCGATGGACCAAATGATGGACAGGCCTCATACAAGATCTTCAG
AATAGAAAAGGGAAAGATAGTCAAATCAGTCGAAATGAATGCCCCTAATTATCACTATGAGGAATGCTCC
TGTTATCCTGATTCTAGTGAAATCACATGTGTGTGCAGGGATAACTGGCATGGCTCGAATCGACCGTGGG
TGTCTTTCAACCAGAATCTGGAATATCAGATAGGATACATATGCAGTGGGATTTTCGGAGACAATCCACG
CCCTAATGATAAGACAGGCAGTTGTGGTCCAGTATCGTCTAATGGAGCAAATGGAGTAAAAGGATTTTCA
TTCAAATACGGCAATGGTGTTTGGATAGGGAGAACTAAAAGCATTAGTTCAAGAAAAGGTTTTGAGATGA
TTTGGGATCCAAACGGATGGACTGGGACAGACAATAACTTCTCAATAAAGCAAGATATCGTAGGAATAAA
TGAGTGGTCAGGATATAGCGGGAGTTTTGTTCAGCATCCAGAACTAACAGGGCTGGATTGTATAAGACCT
TGCTTCTGGGTTGAACTAATCAGAGGGCGACCCAAAGAGAACACAATCTGGACTAGCGGGAGCAGCATAT
CCTTTTGTGGTGTAAACAGTGACACTGTGGGTTGGTCTTGGCCAGACGGTGCTGAGTTGCCATTTACCAT
TGACAAGTAA

>gi|399226455|gb|JX309996.1| Influenza A virus (A/Singapore/TT491/2010(H1N1)) segment 6 neuraminidase (NA) gene, complete cds

AGCAAAAGCAGGAGTTTAAAATGAATCCAAACCAAAAGATAATAACCATTGGTTCGGTCTGTATGACAAT
TGGAATGGCTAACTTAATATTACAAATTGGAAACATAATCTCAATATGGATTAGCCACTCAATTCAACTT
GGGAATCAAAGTCAGATTGAAACATGCAATCAAAGCGTCATTACTTATGAAAACAACACTTGGGTAAATC
AGACATATGTTAACATCAGCAACACCAACTTTGCTGCTGGACAGTCAGTGGTTTCCGTGAAATTAGCGGG
CAATTCCTCTCTCTGCCCTGTTAGTGGATGGGCTATATACAGTAAAGACAACAGTATAAGAATCGGTTCC
AAGGGGGATGTGTTTGTCATAAGGGAACCATTCATATCATGCTCCCCCTTGGAATGCAGAACCTTCTTCT
TGACTCAAGGGGCCTTGCTAAATGACAAACATTTCAATGGAACCATTAAAGACAGGAGCCCATATCGAAC
CCTAATGAGCTGTCCTATTGGTGAAGTTCCCTCTCCATACAACTCAAGATTTGAGTCAGTCGCTTGGTCA
GCAAGTGCTTGTCATGATGGCATCAATTGGCTAACAATTGGAATTTCTGGCCCAGACAATGGGGCAGTGG
CTGTGTTAAAGTACAACGGCATAATAACAGACACTATCAAGAGTTGGAGAAACAATATATTGAGAACACA
AGAGTCTGAATGTGCATGTGTAAATGGTTCTTGCTTTACCATAATGACCGATGGACCAAGTGATGGACAG

GCCTCATACAAGATCTTCAGAATAGAAAAGGGAAAGATAGTCAAATCAGTCGAAATGAATGCCCCTAATT
ATCACTATGAGGAATGCTCCTGTTATCCAGATTCTAGTGAAATCACATGTGTGTGCAGGGATAACTGGCA
TGGCTCGAATCGACCGTGGGTGTCTTTCAACCAGAATCTGGAATATCAGATAGGATACATATGCAGTGGG
ATTTTCGGAGACAATCCACGCCCTAATGATAAGACAGGCAGTTGTGGTCCAGTATCGTCTAATGGAGCAA
ATGGAGTAAAAGGATTTTCATTCAAATACGGCAATGGCGTTTGGATAGGGAGAACTAAAAGCATTAGTTC
AAGAAAAGGTTTTGAGATGATTTGGGATCCAAACGGATGGACTGGGACAGACAATAACTTCTCAATAAAG
CAAGATATCGTAGGAATAAATGAGTGGTCAGGATATAGCGGGAGTTTTGTTCAGCATCCAGAACTAACAG
GGCTGGATTGTATAAGACCTTGCTTCTGGGTTGAACTAATCAGAGGGCGACCCAAAGAGAACACAATCTG
GACTAGCGGGAGCAGCATATCCTTTTGTGGTGTAAACAGTGACACTGTGGGTTGGTCTTGGCCAGACGGT
GCTGAGTTGCCATTTACCATTGACAAGTAATTTGTTCAAAAAACTCCTTGTTTCTACT
>gi|355467686|gb|CY090053.1| Influenza A virus (A/Ecuador/IEC00027/2010(H1N1)) neuraminidase (NA) gene, complete cds
CAGGAGTGAAAATGAATCCAAACCAAAAGATAATAACCATTGGTTCGGTCTGTATGACAATTGGAATGGC
CAACTTAATATTACAAATTGGAAACATAATCTCAATATGGATTAGCCACTCAATTCAACTTGGGAATCAA
AATCAGATTGAAACATGCAATCAAAGCGTCATTACTTATGAAAACAACACTTGGGTAAATCAGACATATG
TTAACATCAGCAACACCAACTTTGCTGCTGGACAGTCAGTGGTTTCCGTGAAATTAGCGGGCAATTCCTC
TCTCTGCCCTGTTAGTGGATGGGCTATATACAGTAAAGACAACAGTATAAGAATCGGTTCCAAGGGGGAT
GTGTTTGTCATAAGGGAACCATTCATATCATGCTCCCCCTTGGAATGCAGAACCTTCTTCTTGACTCAAG
GGGCCTTACTAAATGACAAACATTCCAATGGAACCATTAAAGACAGGAGCCCATATCGAACCCTAATGAG
CTGTCCTATTGGTGAAGTTCCCTCTCCATACAACTCAAGATTTGAGTCAGTCGCTTGGTCAGCAAGTGCT
TGTCATGATGGCATCAATTGGCTAACAATTGGAATTTCTGGCCCAGACAATGGGGCAGTGGCTGTGTTAA
AGTACAACGGCATAATAACAGACACTATCAAGAGTTGGAGAAACAATATATTGAGAACACAAGAGTCTGA
ATGTGCATGTGTAAATGGTTCTTGCTTTACTGTAATGACCGATGGACCAAGTGATGGACAGGCCTCATAC
AAGATCTTCAGAATAGAAAAGGGAAAGATAGTCAAATCAGTCGAAATGAATGCCCCTAATTATCACTATG
AGGAATGCTCCTGTTATCCTGATTCTAGTGAAATCACATGTGTGTGCAGGGATAACTGGCATGGCTCGAA
CCGACCGTGGGTGTCTTTCAACCAGAATCTGGAATATCAGATAGGATACATATGCAGTGGGATTTTCGGA
GACAATCCACGTCCTAATGACAAGACAGGCAGTTGTGGTCCAGTATCGTCTAATGGAGCAAATGGAGTAA
AAGGATTTTCATTCAAATACGGCAATGGTGTTTGGATAGGGAGAACTAAAAGCATTAGTTCAAGAAACGG
TTTTGAGATGATTTGGGATCCGAACGGATGGACTGGGACAGACAATAACTTCTCAATAAAGCAAGATATC
GTAGGAATAAATGAGTGGTCAGGATATAGCGGGAGTTTTGTTCAGCATCCAGAACTAACAGGGCTGGATT
GTATAAGACCTTGCTTCTGGGTTGAACTAATCAGAGGGCGACCCAAAGAGAACACAATCTGGACTAGCGG
GAGCAGCATATCCTTTTGTGGTGTAAACAGTGACACTGTTGGTTGGTCTTGGCCAGACGGTGCTGAGTTG
CCATTTACCATTGACAAGTAATTTGTTCAAAAAACTCCTTG
>gi|345846646|gb|JN632581.1| Influenza A virus (A/Ontario/842001/2010(H1N1)) segment 6 neuraminidase (NA) gene, complete cds
ATGAATCCAAACCAAAAGATAATAACCATTGGTTCGGTCTGTATGACAATTGGAATGGCTAACTTAATAT
TACAAATTGGAAACATAATCTCAATATGGATTAGCCACTCAATTCAACTTGGGAATCAAAATCAGATTGA
AACATGCAATCAAAGCGTCATTACTTATGAAAACAACACTTGGGTAAATCAGACATATGTTAACATCAGC
AACACCAACTTTGCTGCTGGACAGTCAGTGGTTTCCGTGAAATTAGCGGGCAATTCCTCTCTCTGCCCTG
TTAGTGGATGGGCTATATACAGTAAAGACAACAGTATAAGAATCGGTTCCAAGGGGGATGTGTTTGTCAT
AAGGGAACCATTCATATCATGCTCCCCCTTGGAATGCAGAACCTTCTTCTTGACTCAAGGGGCCTTGCTA
AATGACAAACATTCCAATGGAACCATTAAAGACAGGAGCCCATATCGAACCCTAATGAGCTGTCCTATTG
GTGAAGTTCCCTCTCCATACAACTCAAGATTTGAGTCAGTCGCTTGGTCAGCAAGTGCTTGTCATGATGG
CATCAATTGGCTAACAATTGGAATTTCTGGCCCAGACAATGGGGCAGTGGCTGTGTTAAAGTACAACGGC

ATAATAACAGACACTATCAAGAGTTGGAGAAACAATAGATTGAGAACACAAGAGTCTGAATGTGCATGTG
TAAATGGTTCTTGCTTTACCATAATGACCGATGGACCAAGTGATGGACAGGCCTCATACAAGATCTTCAG
AATAGAAAAGGGAAAGATAGTCAAATCAGTCGAAATGAATGCCCCTAATTATCACTATGAGGAATGCTCC
TGTTATCCTGATTCTAGTGAAATCACATGTGTGTGCAGGGATAACTGGCATGGCTCGAATCGACCGTGGG
TGTCTTTCAACCAGAATCTGGAATATCAGATAGGATACATATGCAGTGGGATTTTCGGAGACAATCCACG
CCCTAATGATAAGACAGGCAGTTGTGGTCCAGTATCGTCTAATGGAGCAAATGGAGTAAAAGGATTTTCA
TTCAAATACGGCAATGGTGTTTGGATAGGGAGAACTAAAAGCATTAGTTCAAGAAAAGGTTTTGAGATGA
TTTGGGATCCAAACGGATGGACTGGGACAGACAATAACTTCTCAATAAAGCAAGATATCGTAGGAATAAA
TGAGTGGTCAGGATATAGCGGGAGTTTTGTTCAGCATCCAGAACTAACAGGGCTGGACTGTATAAGACCT
TGCTTCTGGGTTGAACTAATCAGAGGGCGACCCAAAGAGAACACAATCTGGACTAGCGGGAGCAGCATAT
CCTTTTGTGGTGTAAACAGTGACACTGTGGGTTGGTCTTGGCCAGACGGTGCTGAGTTGCCATTTACCAT
TGACAAGTAATTTGTTCAAAAAACTCCTTGTTTCTACT
>gi|345101142|gb|CY098180.1| Influenza A virus (A/Sydney/DD3-21/2010(mixed)) neuraminidase (NA) gene, complete cds
AAATGAATCCAAACCAAAAGATAATAACCATTGGTTCGGTCTGTATAACAATTGGAATGGCTAACTTAAT
ATTACAAATTGGAAACATAATCTCAATATGGATTAGCCACTCAATTCAACTTGGGAATCAAAATCAGATT
GAAACATGCAATCAAAGCGTCATTACTTATGAAAACAACACTTGGGTAAATCAGACATATGTTAACATCA
GCAACACCAACTTTGCTGCTGGACAGTCAGTGGTTTCCGTGAAATTAGCGGGCAATTCCTCTCTCTGCCC
TGTTAGTGGATGGGCTATATACAGTAAAGACAACAGTATAAGAATCGGTTCCAAGGGGGATGTGTTTGTC
ATAAGGGAACCATTCATATCATGCTCCCCCTTGGAATGCAGAACCTTCTTCTTGACTCAAGGGGCCTTGC
TAAATGACAAACATTCCAATGGAACCATTAAAGACAGGAGCCCATATCGAACCCTAATGAGCTGTCCTAT
TGGTGAAGTTCCCTCTCCATACAACTCAAGATTTGAGTCAGTCGCTTGGTCAGCAAGTGCTTGTCATGAT
GGCATCAGTTGGCTAACAATTGGAATTTCTGGCCCAGACAATGGGGCAGTGGCTGTGTTAAAGTACAACG
GCATAATAACAGACACTATCAAGAGTTGGAGAAACAATATATTGAGAACACAAGAGTCTGAATGTGCATG
TGTAAATGGTTCTTGTTTTACTGTAATGACCGATGGACCAAGTGATGGACAGGCCTCATACAAGATCTTC
AGAATAGAAAAGGGAAAGATAGTCAAATCAGTCGAAATGAATGCCCCTAATTATCACTATGAGGAATGCT
CCTGTTATCCTGATTCTAGTGAAATCACATGTGTGTGCAGGGATAACTGGCATGGCTCGAATCGACCGTG
GGTGTCTTTCAACCATAATCTGGAATATCAGATAGGATACATATGCAGTGGGATTTTCGGAGACAATCCA
CGCCCTAATGATAAGACAGGCAGTTGTGGTCCAGTATCGTCTAATGGAGCAAATGGAGTAAAAGGATTTT
CATTCAAATACGGCAATGGTGTTTGGATAGGGAGAACTAAAAGCATTAGTTCAAGAAACGGTTTTGAGAT
GATTTGGGATCCGAACGGATGGACTGGGACAGACAATAACTTCTCAATAAAGCAAGATATCGTAGGAATA
AATGAGTGGTCAGGATATAGCGGGAGTTTTGTTCAGCATCCAGAACTAACAGGGCTGGATTGTATAAGAC
CTTGCTTCTGGGTTGAACTAATCAGAGGGCGACCCAAAGAGAACACAATCTGGACTAGCGGGAGCAGCAT
ATCCTTTTGTGGTGTAAACAGTGACACTGTGGGTTGGTCTTGGCCAGACGGTGCTGAGTTGCCATTTACC
ATTGACAAGTAATTTGTTCA
>gi|345101125|gb|CY098172.1| Influenza A virus (A/Chile/70/2010(H1N1)) neuraminidase (NA) gene, complete cds
AAATGAATCCAAACCAAAAGATAATAACCATTGGTTCGGTCTGTATAACAATTGGAATGGCTAACTTAAT
ATTACAAATTGGAAACATAATCTCAATATGGATTAGCCACTCAATTCAACTTGGGAATCAAAATCAGATT
GAAACATGCAATCAAAGCGTCATTACTTATGAAAACAACACTTGGGTAAATCAGACATATGTTAACATCA
GCAACACCAACTTTGCTGCTGGACAGTCAGTGGTTTCCGTGAAATTAGCGGGCAATTCCTCTCTCTGCCC
TGTTAGTGGATGGGCTATATACAGTAAAGACAACAGTATAAGAATCGGTTCCAAGGGGGATGTGTTTGTC
ATAAGGGAACCATTCATATCATGCTCCCCCTTGGAATGCAGAACCTTCTTCTTGACTCAAGGGGCCTTGC
TAAATGACAAACATTCCAATGGAACCATTAAAGACAGGAGCCCATATCGAACCCTAATGAGCTGTCCTAT

TGGTGAAGTTCCCTCCCCATACAACTCAAGATTTGAGTCAGTCGCTTGGTCAGCAAGTGCTTGTCATGAT
GGCATCAGTTGGCTAACAATTGGAATTTCTGGCCCAGACAATGGGGCAGTGGCTGTGTTAAAGTACAACG
GCATAATAACAGACACTATCAAGAGTTGGAGAAACAATATATTGAGAACACAAGAGTCTGAATGTGCATG
TGTAAATGGTTCTTGTTTTACTGTAATGACCGATGGACCAAGTGATGGACAGGCCTCATACAAGATCTTC
AGAATAGAAAAGGGAAAGATAGTCAAATCAGTCGAAATGAATGCCCCTAATTATCACTATGAGGAATGCT
CCTGTTATCCTGATTCTAGTGAAATCACATGTGTGTGCAGGGATAACTGGCATGGCTCGAATCGACCGTG
GGTGTCTTTCAACCAGAATCTGGAATATCAGATAGGATACATATGCAGTGGGATTTTCGGAGACAATCCA
CGCCCTAATGATAAGACAGGCAGTTGTGGTCCAGTATCGTCTAATGGAGCAAATGGAGTAAAAGGATTTT
CATTCAAATACGGCAATGGTGTTTGGATAGGGAGAACTAAAAGCATTAGTTCAAGAAACGGTTTTGAGAT
GATTTGGGATCCGAACGGATGGACTGGGACAGACAATAACTTCTCAATAAAGCAAGATATCGTAGGAATA
AATGAGTGGTCAGGATATAGCGGGAGTTTTGTTCAGCATCCAGAACTAACAGGGCTGGATTGTATAAGAC
CTTGCTTCTGGGTTGAACTAATCAGAGGGCGACCCAAAGAGAACACAATCTGGACTAGCGGGAGCAGCAT
ATCCTTTTGTGGTGTAAACAGTGACACTGTGGGTTGGTCTTGGCCAGACGGTGCTGAGTTGCCATTTACC
ATTGACAAGTAATTTGTTCA
>gi|344166274|gb|CY097958.1| Influenza A virus (A/Amman/WRAIR3448T/2010(H1N1)) neuraminidase (NA) gene, complete cds
ATGAATCCAAACCAAAAGATAATAACCATTGGTTCGGTCTGTATGACAATTGGAATGGCTAACTTAATGT
TACAAATTGGAAACATAATCTCAATATGGATTAGCCACTCAATTCAACTTGGGAATCAAAATCAGATTGA
AACATGCAATCAAAGCGTCATTACTTATGAAAACAACACTTGGGTAAATCAGACATATGTTAACATCAGC
AACACCAACTTTGCTGCTGGACAGTCAGTGGTTTCCGTGAAATTAGCGGGCAATTCCTCTCTCTGCCCTG
TTAGTGGATGGGCTATATACAGTAAAGACAACAGTATAAGAATCGGTTCCAAGGGGGATGTGTTTGTCAT
AAGGGAACCATTCATATCATGCTCCCCCTTGGAATGCAGAACCTTCTTCTTGACTCAAGGGGCCCTGCTA
AATGACAAACATTCCAATGGAACCATTAAAGACAGGAGCCCATATCGAACCCTAATGAGCTGTCCTATTG
GTGAAGTTCCCTCTCCATACAACTCAAGATTTGAGTCAGTCGCTTGGTCAGCAAGTGCTTGTCATGATGG
CATCAATTGGCTAACAATTGGAATTTCTGGCCCAGACAATGGGGCAGTGGCTGTGTTAAAGTACAACGGC
ATAATAACAGACACTATCAAGAGTTGGAGAAACAATATATTGAGAACACAAGAGTCTGAATGTGCATGTG
TAAATGGGTCTTGCTTTACTGTAATGACCGATGGACCAAGTGATGGACAGGCCTCATACAAGATCTTCAG
AATAGAAAAGGGAAAGATAGTCAAATCAGTCGAAATGAATGCCCCTAATTACCACTATGAGGAATGCTCC
TGTTATCCTGATTCTAGTGAAATCACATGTGTGTGCAGGGATAACTGGCATGGCTCGAATCGACCGTGGG
TGTCTTTCAACCAGAATCTGGAATATCAGATAGGATACATATGCAGTGGGATTTTCGGAGACAATCCACG
CCCTAATGATAAGACAGGCAGTTGTGGTCCAGTATCGTCTAATGGAGCAAATGGAGTAAAAGGATTTTCA
TTCAAATACGGCAATGGTGTTTGGATAGGGAGAACTAAAAGCATTAGTTCAAGAAACGGTTTTGAAATGA
TTTGGGATCCGAACGGATGGACTGGGACAGACAATAACTTCTCAATAAAGCAAGATATCATAGGAATAAA
TGAGTGGTCAGGATATAGCGGGAGTTTTGTTCAGCATCCAGAACTAACAGGGCTGGATTGTATAAGACCT
TGCTTCTGGGTTGAACTAATCAGAGGGCGACCCAAAGAGAACACAATCTGGACTAGCGGGAGCAGCATAT
CCTTTTGTGGTGTAAACAGTGACACTGTGGGTTGGTCTTGGCCAGACGGTGCTGAGTTGCCATTTACCAT
TGACAAGTAA
>gi|344165964|gb|CY097847.1| Influenza A virus (A/District of Columbia/WRAIR0310/2010(H1N1)) neuraminidase (NA) gene, complete cds
ATGAATCCAAACCAAAAGATAATAACCATTGGTTCGGTCTGTATGACAATTGGAATGGCTAACTTAATAT
TACAAATTGGAAACATAATCTCAATATGGATTAGCCACTCAATTCAACTTGGGAATCAAAATCAGATTGA
AACATGCAATCAAAGCGTCATTACTTATGAAAACAACACTTGGGTAAATCAGACATATGTTAACATCAGC
AACACCAACTTTGCTGCTGGACAGTCAGTGGTTTCCGTAAAATTAGCGGGCAATTCCTCTCTCTGCCCTG
TTAGTGGATGGGCTATATACAGTAAAGACAACAGTATAAGAATCGGTTCCAAGGGGGATGTGTTTGTCAT

AAGGGAACCATTCATATCATGCTCCCCCTTGGAATGCAGAACCTTCTTCTTGACTCAAGGGGCCTTGCTA
AATGACAAACATTCCAATGGAACCATTAAAGACAGGAGCCCATATCGAACCCTAATGAGCTGTCCTATTG
GTGAAGTTCCCTCTCCATACAACTCAAGATTTGAGTCAGTCGCTTGGTCAGCAAGTGCTTGTCATGATGG
CATCAATTGGCTAACAATTGGAATTTCTGGCCCAGACAATGGGGCAGTGGCTGTGTTAAAGTACAACGGC
ATAATAACAGACACTATCAAGAGTTGGAGAAACAATATATTGAGAACACAAGAGTCTGAATGTGCATGTG
TAAATGGTTCTTGCTTTACCATAATGACCGATGGACCAAGTGATGGACAGGCCTCATACAAGATCTTCAG
AATAGAAAAGGGAAAGATAGTCAAATCAGTCGAAATGAATGCCCCTAATTATCACTATGAGGAATGCTCC
TGTTATCCTGATTCTAGTGAAATCACATGTGTGTGCAGGGATAACTGGCATGGCTCGAATCGACCGTGGG
TGTCTTTCAACCAGAATCTGGAATATCAGATAGGATACATATGCAGTGGGATTTTCGGAGACAATCCACG
CCCCAATGATAAGACAGGCAGTTGTGGTCCAGTATCGTCTAATGGAGCAAATGGAGTAAAAGGATTTTCA
TTCAAATACGGCAATGGTGTTTGGATAGGGAGAACTAAAAGCATTAGTTCAAGAAAAGGTTTTGAGATGA
TTTGGGATCCAAACGGATGGACTAGGACAGACAATAACTTCTCAATAAAGCAAGATATCGTAGGAATAAA
TGAGTGGTCAGGATATAGCGGGAGTTTTGTTCAGCATCCAGAACTAACAGGGCTGGATTGTATAAGACCT
TGCTTCTGGGTTGAACTAATCAGAGGGCGACCCAAAGAGAACACAATCTGGACTAGCGGGAGCAGCATAT
CCTTTTGTGGTGTAAACAGTGACACTGTGGGTTGGTCTTGGCCAGACGGTGCTGAGTTGCCATTTACCAT
TGACAAGTAA

>gi|344165838|gb|CY097791.1| Influenza A virus (A/Dakar/WRAIR0020T/2010(H1N1)) neuraminidase (NA) gene, complete cds

ATGAATCCAAACCAAAAGATAATAACCATTGGTTCGGTCTGTATGACAATTGGAATGGCTAACTTAATAT
TACAAATTGGAAACATAATCTCAATATGGATTAGCCACTCAATTCAACTTGGGAATCAAAATCAGATTGA
AACATGCAATCAAAGCGTCATTACTTATGAAAACAACACTTGGGTAAATCAGACATATGTTAACATCAGC
AACACCAACTTTGCTGCTGGACAGTCAGTGGTTTCCGTGAAATTAGCGGGCAATTCCTCTCTCTGCCCTG
TTAGTGGATGGGCTATATACAGTAAAGACAACAGTATAAGAATCGGTTCCAAGGGGGATGTGTTTGTCAT
AAGGGAACCATTCATATCATGCTCCCCCTTGGAATGCAGAACCTTCTTCTTGACTCAAGGGGCCTTGCTA
AATGACAAACATTCCAATGGAACCATTAAAGACAGGAGCCCATATCGAACCCTAATGAGCTGTCCTATTG
GTGAAGTTCCCTCTCCATACAACTCAAGATTTGAGTCAGTCGCTTGGTCAGCAAGTGCTTGTCATGATGG
CATCAATTGGCTAACAATTGGAATTTCTGGCCCAGACAATGGGGCAGTGGCTGTGTTAAAGTACAACGGC
ATAATAACAGACACTATCAAGAGTTGGAGAAACAATATATTGAGAACACAAGAGTCTGAATGTGCATGTG
TAAATGGTTCTTGCTTTACTGTAATGACCGATGGACCAAGTGATGGACAGGCCTCATACAAGATCTTCAG
AATAGAAAAGGGAAAGATAGTCAAATCAGTCGAAATGAATGCCCCTAATTATCACTATGAGGAATGCTCC
TGTTATCCTGATTCTAGTGAAATCACATGTGTGTGCAGGGATAACTGGCATGGCTCGAATCGACCGTGGG
TTTCTTTCAACCAGAATCTGGAATATCAGATAGGATACATATGCAGTGGGATTTTCGGAGACAATCCACG
CCCTAATGATAAGACAGGCAGTTGTGGTCCAGTATCGTCTAATGGAGCAAATGGAGTAAAAGGATTTTCA
TTCAAATACGGCAATGGTGTTTGGATAGGGAGAACTAAAAGCATTAGTTCAAGAAACGGTTTTGAGATGA
TTTGGGATCCGAACGGATGGACTGGGACAGACAATAACTTCTCAATAAAGCAAGATATCGTAGGAATAAA
TGAGTGGTCAGGATATAGCGGGAGTTTTGTTCAGCATCCAGAACTAACAGGGCTGGATTGTATAAGACCT
TGCTTCTGGGTTGAACTAATCAGAGGGCGACCCAAAGAGAACACAATCTGGACTAGCGGGAGCAGCATAT
CCTTTTGTGGTGTAAACAGTGACACTGTGGGTTGGTCTTGGCCAGACGGTGCTGAGTTGCCATTTACCAT
TGACAAGTAA

>gi|334134089|gb|CY090863.1| Influenza A virus (A/Netherlands/2631c/2010(H1N1)) neuraminidase (NA) gene, complete cds

AGTTTAAAATGAATCCAAACCAAAAGATAATAACCATTGGTTCGGTCTGTATGACAATTGGAATGGCTAA
CTTAATATTACAAATTGGAAACATAATCTCAATATGGATTAGCCACTCAATTCAACTTGGGAATCAAAAT
CAGATTGAAACATGCAATCAAAGCGTCATTACTTATGAAAACAACACTTGGGTAAATCAGACATATGTTA

ACATCAGCAACACCAACTTTGCTGCTGGACAGTCAGTGGTTTCCGTGAAATTAGCGGGCAATTCCTCTCT
CTGCCCTGTTAGTGGATGGGCTATATACAGTAAAGACAACAGTATAAGAATCGGTTCCAAGGGGGATGTG
TTTGTCATAAGGGAACCATTCATATCATGCTCCCCCTTGGAATGCAGAACCTTCTTCTTGACTCAAGGGG
CCTTGCTAAATGACAAACATTCCAATGGAACCATTAAAGACAGGAGCCCATATCGAACCCTAATGAGCTG
TCCTATTGGTGAAGTTCCCTCTCCATACAACTCAAGATTTGAGTCAGTCGCTTGGTCAGCAAGTGCTTGT
CATGATGGCATCAATTGGCTAACAATTGGAATTTCTGGCCCAGACAATGGGGCAGTGGCTGTGTTAAAGT
ACAACGGCATAATAACAGACACTATCAAGAGTTGGAGAAACAATAGATTGAGAACACAAGAGTCTGAATG
TGCATGTGTAAATGGTTCTTGCTTTACTGTAATGACCGATGGACCAAGTGATGGACAGGCCTCATACAAG
ATCTTCAGAATAGAAAAGGGAAAGATAGTCAAATCAGTCGAAATGAATGCCCCTAATTATCACTATGAGG
AATGCTCCTGTTATCCTGATTCTAGTGAAATCACATGTGTGTGCAGGGATAACTGGCATGGCTCGAATCG
ACCGTGGGTGTCTTTCAACCAGAATCTGGAATATCAGATAGGATACATATGCAGTGGGATTTTCGGAGAC
AATCCACGCCCTAATGATAAGACAGGCAGTTGTGGTCCAGTATCGTCTAATGGAGCAAATGGAGTAAAAG
GATTTTCATTCAAATACGGCAATGGTGTTTGGATAGGGAGAACTAAAAGCATTAGTTCAAGAAACGGTTT
TGAGATGATTTGGGACCCGAACGGATGGACTGGGACAGACAATGACTTCTCAATAAAGCAAGATATCGTA
GGAATAAATGAGTGGTCAGGATATAGCGGGAGTTTTGTTCAGCATCCAGAACTAACAGGGCTGGATTGTA
TAAGACCTTGCTTCTGGGTTGAATTAATCAGAGGGCGACCCAAAGAGAACACAATCTGGACTAGCGGGAG
CAGCATATCCTTTTGTGGTGTAAACAGTGACACTGTGGGTTGGTCTTGGCCAGACGGTGCTGAGTTGCCA
TTCACCATTGACAAGTAATTTGTTCAAAAAAC
>gi|341612391|gb|JN381480.1| Influenza A virus (A/Taiwan/65569/2011(H1N1)) segment 6 neuraminidase (NA) gene, complete cds
ATGAATCCAAACCAAAAGATAATAACCATTGGTTCGGTCTGTATGACAATTGGAATGGCTAACTTAATAT
TACAAATTGGAAACATAATCTCAATATGGATTAGCCACTCAATTCAACTTGGGAATCAAAATCAGATTGA
AACATGCAATCAAAGCGTCATTACTTATGAAAACAACACTTGGGTAAATCAGACATATGTTAACATCAGC
AACACCAACTTTGCTGCTGGACAGTCAGTGGTTTCCGTGAAATTAGCGGGCAATTCCTCTCTCTGCCCTG
TTAGTGGATGGGCTATATACAGTAAAGACAACAGTATAAGAATCGGTTCCAAGGGGGATGTGTTTGTCAT
AAGGGAACCATTCATATCATGCTCCCCCTTGGAATGCAGAACCTTCTTCTTGACTCAAGGGGCCTTGCTA
AATGACAAACATTCCAATGGAACCATTAAAGACAGGAGCCCATATCGAACCCTAATGAGCTGTCCTATTG
GTGAAGTTCCCTCCCCATACAACTCAAGATTTGAGTCAGTCGCTTGGTCAGCAAGTGCTTGTCATGATGG
CATCAATTGGCTAACAATTGGAATTTCTGGCCCAGACAATGGGGCAGTGGCTGTGTTAAAGTACAACGGC
ATAATAACAGACACTATCAAGAGTTGGAGAAACAATATATTGAGAACACAAGAGTCTGAATGTGCATGTG
TAAATGGTTCTTGCTTTACCATAATGACCGATGGACCAAGTGATGGACAGGCCTCATACAAGATCTTCAG
AATAGAAAAGGGAAAGATAGTCAAATCAGTCGAAATGAATGCCCCTAATTATCACTATGAGGAATGCTCC
TGTTATCCTGATTCTAGTGAAATCACATGTGTGTGCAGGGATAACTGGCATGGCTCGAATCGACCGTGGG
TGTCTTTCAACCAGAATCTGGAATATCAGATAGGATACATATGCAGTGGGATTTTCGGAGACAATCCACG
CCCTAATGATAAGACAGGCAGTTGTGGTCCAGTATCGTCTAATGGAGCAAATGGAGTAAAAGGATTTTCA
TTCAAATACGGCAATGGTGTTTGGATAGGAAGAACTAAAAGTATTAGTTCAAGAAAAGGTTTTGAGATGA
TTTGGGATCCAAACGGATGGACTGGGACAGACAATAACTTCTCAATAAAGCAAGATATCGTAGGAATAAA
TGAGTGGTCAGGATATAGCGGGAGTTTTGTTCAGCATCCAGAACTAACAGGGCTGGACTGTATAAGACCT
TGCTTCTGGGTTGAACTAATCAGAGGGCGACCCAAAGAGAACACAATCTGGACTAGCGGGAGCAGCATAT
CCTTTTGTGGTGTAAACAGTGACACTGTGGGTTGGTCTTGGCCAGACGGTGCTGAGTTGCCATTTACCAT
TGACAAGTAA
>gi|350612127|gb|JN714539.1| Influenza A virus (A/Moscow oblast/CRIE-HDV/2011(H1N1)) segment 6 neuraminidase (NA) gene, complete cds
ATGAATCCAAACCAAAAGATAATAACCATTGGTTCGGTCTGTATGACAATTGGAATGGCTAACTTAATAT

TACAAATTGGAAACATAATCTCAATATGGATTAGCCACTCAATTCAACTTGGGAATCAAAATCAGATTGA
AACATGCAATCAAAGCGTCATTACTTATGAAAACAACACTTGGGTAAATCAGACATATGTTAACATCAGC
AACACCAACTTTGCTGCTGGACAGTCAGTGGTTTCCGTGAAATTAGCGGGCAATTCCTCTCTCTGCCCTG
TTAGTGGATGGGCTATATACAGTAAAGACAACAGTATAAGAATCGGTTCCAAGGGGGATGTGTTTGTCAT
AAGGGAACCATTCATATCATGCTCCCCCTTGGAATGCAGAACCTTCTTCTTGACTCAAGGGGCCTTGCTA
AATGACAAACATTCCAATGGAACCATTAAAGACAGGAGCCCATATCGAACCCTAATGAGCTGTCCTATTG
GTGAAGTTCCCTCTCCATACAACTCAAGATTTGAGTCAGTCGCTTGGTCAGCAAGTGCTTGTCATGATGG
CATCAATTGGCTAACAATTGGAATTTCTGGCCCAGACAATGGGGCAGTGGCTGTGTTAAAGTACAACGGC
ATAATAACAGACACTATCAAGAGTTGGAGAAACAATATATTGAGAACACAAGAGTCTGAATGTGCATGTG
TAAATGGTTCTTGCTTTACCATAATGACCGATGGACCAAGTGATGGACAGGCCTCATACAAGATCTTCAG
AATAGAAAAGGGAAAGATAGTCAAATCAGTCGAAATGAATGCCCCTAATTATCACTATGAGGAATGCTCC
TGTTATCCTGATTCTAGTGAAATCACATGTGTGTGCAGGGATAACTGGCATGGCTCGAATCGACCGTGGG
TGTCTTTCAACCAGAATCTGGAATATCAGATAGGATACATATGCAGTGGGATTTTCGGAGACAATCCACG
CCCTAATGATAAGACAGGCAGTTGTGGTCCAGTATCGTCTAATGGAGCAAATGGAGTAAAAGGATTTTCA
TTCAAAYACGGCAATGGTGTTTGGATAGGGAGAACTAAAAGCATTAGTTCAAGAAAAGGTTTTGAGATGA
TTTGGGATCCAAACGGATGGACTGGGACAGACAATAACTTCTCAATAAAGCAAGATATCGTAGGAATAAA
TGAGTGGTCAGGATATAGCGGGAGTTTTGTTCAGCATCCAGAACTAACAGGGCTGGACTGTATAAGACCT
TGCTTCTGGGTTGAACTAATCAGAGGGCGACCCAAAGAGAACACAATCTGGACTAGCGGGAGCAGCATAT
CCTTTTGTGGTGTAAACAGTGACACTGTGGGTTGGTCTTGGCCAGACGGTGCTGAGTTGCCATTTACCAT
TGACAAGTAA
>gi|344166382|gb|CY098006.1| Influenza A virus (A/Prague/WRAIR4146N/2011(H1N1)) neuraminidase (NA) gene, complete cds
ATGAATCCAAACCAAAAGATAATAACCATTGGTTCGGTCTGTATGACAATTGGAATGGCTAACTTAATAT
TACAAATTGGAAACATAATCTCAATATGGATTAGCCACTCAATTCAACTTGGGAATCAAAATCAGATTGA
AACATGCAATCAAAGCGTCATTACTTATGAAAACAACACTTGGGTAAATCAGACATATGTTAACATCAGC
AACACCAACTTTGCTGCTGGACAGTCAGTGGTTTCCGTGAAATTAGCGGGCAATTCCTCTCTCTGCCCTG
TTAGTGGATGGGCTATATACAGTAAAGACAACAGTATAAGAATCGGTTCCAAGGGGGATGTGTTTGTCAT
AAGGGAACCATTCATATCATGCTCCCCCTTGGAATGCAGAACCTTCTTCTTGACTCAAGGGGCCTTGCTA
AATGACAAACATTCCAATGGAACCATTAAAGACAGGAGCCCATATCGAACCCTAATGAGCTGTCCTATTG
GTGAAGTTCCCTCTCCATACAACTCAAGATTTGAGTCAGTCGCTTGGTCAGCAAGTGCTTGTCATGATGG
CATCAATTGGCTAACAATTGGAATTTCTGGCCCAGACAATGGGGCAGTGGCTGTGTTAAAGTACAACGGC
ATAATAACAGACACTATCAAGAGTTGGAGAAACAATATATTGAGAACACAAGAGTCTGAATGTGCATGTG
TAAATGGTTCTTGCTTTACCATAATGACCGATGGACCAAGTAATGGACAGGCCTCATACAAGATCTTCAG
AATAGAAAAGGGAAAGATAGTCAAATCAGTCGAAATGAATGCCCCTAATTATCACTATGAGGAATGCTCC
TGTTATCCTGATTCTAGTGAAATCACATGTGTGTGCAGGGATAACTGGCATGGCTCGAATCGACCGTGGG
TGTCTTTCAACCAGAATCTGGAATATCAGATAGGATACATATGCAGTGGGATTTTCGGAGACAATCCACG
CCCTAATGATAAGACAGGCAGTTGTGGTCCAGTATCGTCTAATGGAGCAAATGGAGTAAAAGGATTTTCA
TTCAAATACGGCAATGGTGTTTGGATAGGGAGAACTAAAAGCATTAGTTCAAGAAAAGGTTTTGAGATGA
TTTGGGATCCAAACGGATGGACTGGGACAGACAATAACTTCTCAATAAAGCAAGATATCGTAGGAATAAA
TGAGTGGTCAGGATATAGCGGGAGTTTTGTTCAGCATCCAGAACTAACAGGGCTGGACTGTATAAGACCT
TGCTTCTGGGTTGAACTAATCAGAGGGCGACCCAAAGAGAACACAATCTGGACTAGCGGGAGCAGCATAT
CCTTTTGTGGTGTAAACAGTGACACTGTGGGTTGGTCTTGGCCAGACGGTGCTGAGTTGCCATTTACCAT
TGACAAGTAA
>gi|344166364|gb|CY097998.1| Influenza A virus (A/Budapest/WRAIR3794T/2011(H1N1))

neuraminidase (NA) gene, complete cds
ATGAATCCAAACCAAAAGATAATAACCATTGGTTCGGTCTGTATGACAATTGGAATGGCTAACTTAATAT
TACAAATTGGAAACATAATCTCAATATGGATTAGCCACTCAATTCAACTTGGGAATCAAAATCAGATTGA
AACATGCAATCAAAGCGTCATTACTTATGAAAACAACACTTGGGTAAATCAGACATATGTTAACATCAGC
AACACCAACTTTGCTGCTGGACAGTCAGTGGTTTCCGTGAAATTAGCGGGCAATTCCTCTCTCTGCCCTG
TTAGTGGATGGGCTATATACAGTAAAGACAACAGTATAAGAATCGGTTCCAAGGGGAATGTGTTTGTCAT
AAGGGAACCATTCATATCATGCTCCCCCTTGGAATGCAGAACCTTCTTCTTGACTCAAGGGGCCTTGCTA
AATGATAAACATTCCAATGGAACCATTAAAGACAGGAGCCCATATCGAACCCTAATGAGCTGTCCTATTG
GTGAAGTTCCCTCTCCATACAACTCAAGATTTGAGTCAGTCGCTTGGTCAGCAAGTGCTTGTCATGATGG
CATCAATTGGCTAACAATTGGAATTTCTGGCCCAGACAATGGGGCAGTGGCTGTGTTAAAGTACAACGGC
ATAATAACAGACACTATCAAGAGTTGGAGAAACAATATATTGAGAACACAAGAGTCTGAATGTGCATGTG
TAAATGGTTCTTGCTTTACCATAATGACCGATGGACCAAGTGATGGACAGGCCTCATACAAGATCTTCAG
AATAGAAAAGGGAAAGATAGTCAAATCAGTCGAAATGAATGCCCCTAATTATCACTATGAGGAATGCTCC
TGTTATCCTGATTCTAGTGAAATCACATGTGTGTGCAGGGATAACTGGCATGGCTCGAATCGACCGTGGG
TGTCTTTCAACCAGAATCTGGAATATCAGATAGGATACATATGCAGTGGGATTTTCGGAGACAATCCACG
CCCTAATGATAAGACAGGCAGTTGTGGTCCAGTATCGTCTAATGGAGCAAATGGAGTAAAAGGATTTTCA
TTCAAATACGGCAATGGTGTTTGGATAGGGAGAACTAAAAGCATTAGTTCAAGAAAAGGTTTTGAGATGA
TTTGGGATCCAAACGGATGGACTGGGACAGACAATAACTTCTCAATAAAGCAAGATATCGTAGGAATAAA
TGAGTGGTCAGGATATAGCGGGAGTTTTGTTCAGCATCCAGAACTAACAGGGCTGGACTGTATAAGACCT
TGCTTCTGGGTTGAACTAATCAGAGGGCGACCCAAAGAGAACACAATCTGGACTAGCGGGAGCAGCATAT
CCTTTTGTGGTGTAAACAGTGACACTGTGGGTTGGTCTTGGCCAGACGGTGCTGAGTTGCCATTTACCAT
TGACAAGTAA
>gi|344166016|gb|CY097870.1| Influenza A virus (A/District of Columbia/WRAIR0313/2011(H1N1)) neuraminidase (NA) gene, complete cds
ATGAATCCAAACCAAAAGATAATAACCATTGGTTCGGTCTGTATGACAATTGGAATGGCTAACTTAATAT
TACAAATTGGAAACATAATCTCAATATGGATTAGCCACTCAATTCAACTTGGGAATCAAAATCAGATTGA
AACATGCAATCAAAGCGTCATTACTTATGAAAACAACACTTGGGTAAATCAGACATATGTTAACATCAGC
AACACCAACTTTGCTGCTGGACAGTCAGTGGTGTCCGTGAAATTAGCGGGCAATTCCTCTCTCTGCCCTG
TTAGTGGATGGGCTATATACAGTAAAGACAACAGTATAAGAATCGGTTCCAAGGGGGATGTGTTTGTCAT
AAGGGAACCATTCATATCATGCTCCCCCTTGGAATGCAGAACCTTCTTCTTGACTCAAGGGGCCTTGCTA
AATGACAAACATTCCAATGGAACCATTAAAGACAGGAGCCCATATCGAACCCTAATGAGCTGTCCTATTG
GTGAAGTTCCCTCTCCATACAACTCAAGATTTGAGTCAGTCGCTTGGTCAGCAAGTGCTTGTCATGATGG
CACCAATTGGCTAACAATTGGAATTTCTGGCCCAGACAATGGGGCAGTGGCTGTGTTAAAGTACAACGGC
ATAATAACAGACACTATCAAGAGTTGGAGAAACAATATATTGAGAACACAAGAGTCTGAATGTGCATGTG
TAAATGGTTCTTGCTTTACTGTAATGACCGATGGACCAAGTGATGGACAGGCCTCATACAAGATCTTCAG
AATAGAAAAGGGAAAGATAGTCAAATCAGTCGAAATGAATGCCCCTAATTATCACTATGAGGAATGCTCC
TGTTATCCTGATTCTAGTGAAATCACATGTGTGTGCAGGGATAACTGGCATGGCTCGAATCGACCGTGGG
TGTCTTTCAACCAGAATCTGGAATATCAGATAGGATACATATGCAGTGGGATTTTCGGAGACAATCCACG
CCCTAATGACAAGACAGGCAGTTGTGGTCCAGTATTGTCTAATGGAGCAAATGGAGTAAAAGGATTTTCA
TTCAAATACGGCAATGGTGTTTGGATAGGGAGAACTAAAAGCATTAGTTCAAGAAACGGGTTTGAGATGA
TTTGGGATCCGAACGGATGGACTGGGACAGACAATAACTTCTCAATAAAGCAAGATATCGTAGGAATAAA
TGAGTGGTCAGGATATAGCGGGAGTTTTGTTCAGCATCCAGAACTAACAGGGCTGGATTGTATAAGACCT
TGCTTCTGGGTTGAACTAATCAGAGGGCGACCCAAAGAGAACACAATCTGGACTAGCGGGAGCAGCATAT
CCTTTTGTGGTGTAAACAGTGACACTGTGGGTTGGTCTTGGCCAGACGGTGCTGAGTTGCCATTTACCAT

TGACAAGTAA
>gi|338826658|gb|CY092882.1| Influenza A virus (A/California/NHRC0001/2011(H1N1)) neuraminidase (NA) gene, complete cds
AAATGAATCCAAACCAAAAGATAATAACCATTGGTTCGGTCTGTATGACAATTGGAATGGCTAACTTAAT
ATTACAAATTGGAAACATAATCTCAATATGGATTAGCCACTCAATTCAACTTGGGAATCAAAATCAGATT
GAAACATGCAATCAAAGCGTCATTACTTATGAAAACAACACTTGGGTAAATCAGACATATGTTAACATCA
GCAACACCAACTTTGCTGCTGGACAGTCAGTGGTTTCCGTGAAATTAGCGGGCAATTCTTCTCTCTGCCC
TGTTAGTGGATGGGCTATATACAGTAAAGACAACAGTATAAGAATCGGTTCCAAGGGGGATGTGTTTGTC
ATAAGGGAACCATTCATATCATGCTCCCCTTTGGAATGCAGAACCTTCTTCTTGACTCAAGGGGCCTTGC
TAAATGACAAACATTCCAATGGAACCATTAAAGACAGGAGCCCATATCGAACCCTAATGAGCTGTCCTAT
TGGTGAAGTTCCCTCTCCATACAACTCAAGATTTGAGTCAGTCGCTTGGTCAGCAAGTGCTTGTCATGAT
GGCATCAATTGGCTAACAATTGGAATTTCTGGCCCAGACAATGGGGCAGTGGCTGTGTTAAAGTACAACG
GCATAATAACAGACACTATCAAGAGTTGGAGAAACAATATATTGAGAACACAAGAGTCCGAATGTGCATG
TGTAAATGGCTCTTGCTTTACTGTGATGACCGATGGACCAAGTGATGGACAGGCCTCATACAAGATCTTC
AGAATAGAAAAGGGAAAGATAGTCAAATCAGTCGAAATGAATGCCCCTAATTATCACTATGAGGAATGCT
CCTGTTATCCTGATTCTAGTGAAATCACATGTGTGTGCAGGGATAACTGGCATGGCGCGAATCGACCGTG
GGTGTCTTTCAACCAGAATCTGGAATATCAGATAGGATACATATGCAGTGGGATTTTCGGAGACAATCCA
CGCCCTAATGATAAGACAGGCAGTTGTGGTCCAGTATCGTCTAATGGAGCAAATGGAGTAAAAGGATTTT
CATTCAAATACGGCAATGGTGTTTGGATAGGGAGAACTAAAAGCATTAGTTCAAGAAACGGTTTTGAGAT
GATTTGGGATCCGAACGGATGGACTGGGACAGACAATAACTTCTCAATAAAGCAAGATATCGTAGGAATA
AATGAGTGGTCAGGATATAGCGGGAGTTTTGTTCAGCATCCAGAACTAACAGGGCTGGATTGTATAAGAC
CTTGCTTCTGGGTTGAACTAATCAGAGGGCGACCCAAAGAGAACACAATCTGGACTAGCGGGAGCAGCAT
ATCCTTTTGTGGTGTAAACAGTGACACTGTGGGTTGGTCTTGGCCAGACGGTGCTGAGTTGCCATTTACC
ATTGACAAGTAATTTGTTCA
>gi|338826622|gb|CY092866.1| Influenza A virus (A/Sydney/DD3-59/2011(H1N1)) neuraminidase (NA) gene, complete cds
AAATGAATCCAAACCAAAAGATAATAACCATTGGTTCGGTCTGTATGACAATTGGAATGGCTAACTTAAT
ATTACAAATTGGAAACATAATCTCAATATGGATTAGCCACTCAATTCAACTTGGGAATCAAAATCAGATT
GAAACATGCAATCAAAGCGTCATTACTTATGAAAACAACACTTGGGTAAATCAGACATATGTTAACATCA
GCAACACCAACTTTGCTGCTGGACAGTCAGTGGTTTCCGTGAAATTAGCGGGCAATTCCTCTCTCTGCCC
TGTTAGTGGATGGGCTATATACAGTAAAGACAACAGTATAAGAATCGGTTCCAAGGGGGATGTGTTTGTC
ATAAGGGAACCATTCATATCATGCTCCCCCTTGGAATGCAGAACCTTCTTCTTGACTCAAGGGGCCTTGC
TAAATGACAAACATTCCAATGGAACCATTAAAGACAGGAGCCCATATCGAACCCTAATGAGCTGTCCTAT
TGGTGAAGTTCCCTCTCCATACAACTCAAGATTTGAGTCAGTCGCTTGGTCAGCAAGTGCTTGTCATGAT
GGCATCAATTGGCTAACAATTGGAATTTCTGGCCCAGACAATGGGGCAGTGGCTGTGTTAAAGTACAACG
GCATAATAACAGACACTATCAAGAGTTGGAGAAACAATATATTGAGAACACAAGAGTCTGAATGTGCATG
TGTAAATGGTTCTTGCTTTACCATAATGACCGATGGACCAAGTGATGGACAGGCCTCATACAAGATCTTC
AGAATAGAAAAGGGAAAGATAGTCAAATCAGTCGAAATGAATGCCCCTAATTATCACTATGAGGAATGCT
CCTGTTATCCTGATTCTAGTGAAATCACATGTGTGTGCAGGGATAACTGGCATGGCTCGAATCGACCGTG
GGTGTCTTTCAACCAGAATCTGGAATATCAGATAGGATACATATGCAGTGGGATTTTCGGAGACAATCCA
CGCCCTAATGATAAGACAGGCAGTTGTGGTCCAGTATCGTCTAATGGAGCAAATGGAGTAAAAGGATTTT
CATTCAAATACGGCAATGGTGTTTGGATAGGGAGAACTAAAAGCATTAGTTCAAGAAAAGGTTTTGAGAT
GATTTGGGATCCAAACGGATGGACTGGGACAGACAATAACTTCTCAATAAAGCAAGATATCGTAGGAATA
AATGAGTGGTCAGGATATAGCGGGAGTTTTGTTCAGCATCCAGAACTAACAGGGCTGGACTGTATAAGAC

CTTGCTTCTGGGTTGAACTAATCAGAGGGCGACCCAAAGAGAACACAATCTGGACTAGCGGGAGCAGCAT
ATCCTTTTGTGGTGTAAACAGTGACACTGTGGGTTGGTCTTGGCCAGACGGTGCTGAGTTGCCATTTACC
ATTGACAAGTAATTTGTTCA
>gi|335883176|gb|CY091725.1| Influenza A virus (A/Singapore/TT89/2011(H1N1)) neuraminidase (NA) gene, complete cds
AGCAAAAGCAGGAGTTTAAAATGAATCCAAACCAAAAGATAATAACCATTGGTTCGGTCTGTATGACAAT
TGGAATGGCTAACTTAATATTACAAATTGGAAACATAATCTCAATATGGATTAGCCACTCAATTCAACTT
GGGAATCAAAATCAGATTGAAACATGCAATCAAAGCGTCATTACTTATGAAAACAACACTTGGGTAAATC
AGACATATGTTAACATCAGCAACACCAACTTTGCTGCTGGACAGTCAGTGGTTTCCGTGAAATTAGCGGG
CAATTCTTCTCTCTGCCCTGTTAGTGGATGGGCTATATACAGTAAAGACAACAGTATAAGAATCGGTTCC
AAGGGGGATGTGTTTGTCATAAGGGAACCATTCATATCATGCTCCCCTTTGGAATGCAGAACCTTCTTCT
TGACTCAAGGGGCCTTGCTAAATGACAAACATTCCAATGGAACCATTAAAGACAGGAGCCCATATCGAAC
CCTAATGAGCTGTCCTATTGGTGAAGTTCCCTCTCCATACAACTCAAGATTTGAGTCAGTCGCTTGGTCA
GCAAGTGCTTGTCATGATGGCATCAATTGGCTAACAATTGGAATTTCTGGCCCAGACAATGGGGCAGTGG
CTGTGTTAAAGTACAACGGCATAATAACAGACACTATCAAGAGTTGGAGAAACAATATATTGAGAACACA
AGAGTCTGAATGTGCATGTGTAAATGGTTCTTGCTTTACCATAATGACCGATGGACCAAGTGATGGACAG
GCCTCATACAAGATCTTCAGAATAGAAAAGGGAAAGATAGTCAAATCAGTCGAAATGAATGCCCCTAATT
ATCACTATGAGGAATGCTCCTGTTATCCTGATTCTAGTGAAATCACATGTGTGTGCAGGGATAACTGGCA
TGGCTCGAATCGACCGTGGGTGTCTTTCAACCAGAATCTGGAATATCAGATAGGATACATATGCAGTGGG
ATTTTCGGAGACAATCCACGCCCTAATGATAAGACAGGCAGTTGTGGTCCAGTATCGTCTAATGGAGCAA
ATGGAGTAAAAGGATTTTCATTCAAATACGGCAATGGTGTTTGGATAGGGAGAACTAAAAGCATTAGTTC
AAGAAAAGGTTTTGAGATGATTTGGGATCCAAATGGATGGACTGGGACAGACAATAACTTCTCAATAAAG
CAAGATATCGTAGGAATAAATGAGTGGTCAGGATATAGCGGGAGTTTTGTTCAGCATCCAGAACTAACAG
GGCTGGATTGTATAAGACCTTGCTTCTGGGTTGAACTAATCAGAGGGCGACCCAAAGAGAACACAATCTG
GACTAGCGGGAGCAGCATATCCTTTTGTGGTGTAAACAGTGACACTGTGGGTTGGTCTTGGCCAGACGGT
GCTGAGTTGCCATTTACCATTGACAAGTAATTTGTTCAAAAAAACTCCTTGTTTCTAC
>gi|328496422|gb|CY089465.1| Influenza A virus (A/Thailand/CU-H2911/2011(H1N1)) neuraminidase (NA) gene, complete cds
AAAGCCAGGAGTTCAAAATGAATCCAAACCAAAAGATAATAACCATTGGTTCGGTCTGTATGACAATTGG
AATGGCTAACTTAATATTACAAATTGGAAACATAATCTCAATATGGATTAGCCACTCAATTCAACTTGGG
AATCAAAGTCAGATTGAAACATGCAATCAAAGCGTCATTACTTATGAAAACAACACTTGGGTAAATCAGA
CATATGTTAACATCAGCAACACCAACTTTGCTGCTGGACAGTCAGTGGTTTCCGTGAAATTAGCGGGCAA
TTCCTCTCTCTGCCCTGTTAGTGGATGGGCTATATACAGTAAAGACAACAGTATAAGAATCGGTTCCAAG
GGGGATGTGTTTGTCATAAGGGAACCATTCATATCATGCTCCCCCTTGGAATGCAGAACCTTCTTCTTGA
CTCAAGGGGCCTTGCTAAATGACAAACATTCCAATGGAACCATTAAAGACAGGAGCCCATATCGAACCCT
AATGAGCTGTCCTATTGGTGAAGTTCCCTCTCCATACAACTCAAGATTTGAGTCAGTCGCTTGGTCAGCA
AGTGCTTGTCATGATGGCATCAATTGGCTAACAATTGGAATTTCTGGCCCAGACAATGGGGCAGTGGCTG
TGTTAAAGTACAACGGCATAATAACAGACACTATCAAGAGTTGGAGAAACAATATATTGAGAACACAAGA
GTCTGAATGTGCATGTGTAAATGGTTCTTGCTTTACCATAATGACCGATGGACCAAGTGATGGACAGGCC
TCATACAAGATCTTCAGAATAGAAAAGGGAAAGATAGTCAAATCAGTCGAAATGAATGCCCCTAATTATC
ACTATGAGGAATGCTCCTGTTATCCTGATTCTAGTGAAATCACATGTGTGTGCAGGGATAACTGGCATGG
CTCGAATCGACCGTGGGTGTCTTTCAACCAGAATCTGGAATATCAGATAGGATACATATGCAGTGGGATT
TTCGGAGACAATCCACGCCCTAATGATAAGACAGGCAGTTGTGGTCCAGTATCGTCTAATGGAGCAAATG
GAGTAAAAGGATTTTCATTCAAATACGGCAATGGTGTTTGGATAGGGAGAACTAAAAGCATTAGTTCAAG

AAAAGGTTTTGAGATGATTTGGGATCCAAATGGATGGACTGGGACAGACAATAACTTCTCAATAAAGCAA
GATATCGTAGGAATAAATGAGTGGTCAGGATATAGCGGGAGTTTTGTTCAGCATCCAGAACTAACAGGGC
TGGATTGTATAAGACCTTGCTTCTGGGTTGAACTAATCAGAGGGCGACCCAAAGAGAACACAATCTGGAC
TAGCGGGAGCAGCATATCCTTTTGTGGTGTAAACAGTGACACTGTGGGTTGGTCTTGGCCAGACGGTGCT
GAGTTGCCATTTACCATTGACAAGTAATTTGTTCAAAAAACTCCTTGTTTCTAC
>gi|388774723|gb|CY120757.1| Influenza A virus (A/Brazil/AVS11/2011(H1N1)) neuraminidase (NA) gene, complete cds
AGTTTAAAATGAATCCAAACCAAAAGATAATAACCATTGGTTCGGTCTGTATGACAATTGGAATGGCTAA
CTTAATATTACAAATTGGAAACATAATCTCAATATGGATTAGCCACTCAATTCAACTTGGGAATCAAAAT
CAGATTGAAACATGCAATCAAAGCGTCATTACTTATGAAAACAACACTTGGGTAAATCAGACATATGTTA
ACATCAGCAACACCAACTTTGCTGCTGGACAGTCAGTGGCTTCCGTGAAATTAGCGGGCAATTCCTCTCT
CTGCCCTGTTAGTGGATGGGCTATATACAGTAAAGACAACAGTATAAGAATCGGTTCCAAGGGGGATGTG
TTTGTCATAAGGGAACCATTCATATCATGCTCCCCCTTGGAATGCAGAACCTTCTTCTTGACTCAAGGGG
CTTTGCTAAATGACAAACATTCCAATGGAACCATTAAAGACAGGAGCCCATATCGAGCCCTAATGAGCTG
TCCTATTGGTGAAGTTCCCTCTCCATACAACTCAAGATTTGAGTCAGTCGCTTGGTCAGCAAGTGCTTGT
CATGATGGCATCAATTGGCTAACAATTGGAATTTCTGGCCCAGACAATGGGGCAGTGGCTGTGTTAAAGT
ACAACGGCATAATAACAGACACTATCAAGAGTTGGAGAAACAATATATTGAGAACACAAGAGTCTGAATG
TGCATGTGTAAATGGTTCTTGCTTTACCATAATGACCGATGGACCAAGTGATGGACAGGCCTCATACAAG
ATCTTCAGAATAGAAAAGGGAAAGATAGTCAAATCAGTCGAAATGAATGCCCCTAATTATCACTATGAGG
AATGCTCCTGTTATCCTGATTCTAGTGAAATCACATGTGTGTGCAGGGATAACTGGCATGGCTCGAATCG
ACCGTGGGTGTCTTTCAACCAGAATCTGGAATATCAGATAGGATACATATGCAGTGGGATTTTCGGAGAC
AACCCACGCCCTAATGATAAGACAGGCAGTTGTGGTCCAGTATCGTCTAATGGAGCAAATGGAGTAAAAG
GATTTTCATTCAAATACGGCAATGGTGTTTGGATAGGGAGAACTAAAAGCATTAGTTCAAGAAAAGGTTT
TGAGATGATTTGGGATCCAAACGGATGGACTGGGACAGACAATAACTTCTCAATAAAGCAAGATATCGTA
GGAATGAATGAGTGGTCAGGATATAGCGGGAGTTTTGTTCAGCATCCAGAACTAACAGGACTGGATTGTA
TAAGACCTTGCTTCTGGGTTGAACTAATCAGAGGGCGACCCAAAGAGAACACAATCTGGACTAGCGGGAG
CAGCATATCCTTTTGTGGTGTAAACAGTGACACTGTGGGTTGGTCTTGGCCAGACGGTGCTGAGTTGCCA
TTTACCATTGACAAGTAATTTGTTCAAAAAAC
>gi|404425398|gb|CY125777.1| Influenza A virus (A/Boston/DOA90/2012(H1N1)) neuraminidase (NA) gene, complete cds
GAGTTTAAAATGAATCCAAACCAAAAGATAATAACCATTGGTTCGGTCTGTATGACAATTGGAATGGCTA
ACTTAATATTACAAATTGGAAACATAATCTCAATATGGATTAGCCACTCAATTCAACTTAGGAATCAAAG
TCAGATTGAAACATGCAATCAAAGCGTCATTACTTATGAAAACAACACTTGGGTAAATCAGACATATGTT
AACATCAGCAACACCAACTTTGCTGCTGGACAGTCAGTGGTTTCCGTGAAATTAGCGGGCAATTCCTCTC
TCTGCCCTGTTAGTGGATGGGCTATATACAGTAAAGACAACAGTATAAGAATCGGTTCCAAGGGGGATGT
GTTTGTCATAAGGGAACCATTCATATCATGCTCCCCCTTGGAATGCAGAACCTTCTTCTTGACCCAAGGG
GCCTTGCTAAATGACAAACATTCCAATGGAACCATTAAAGACAGGAGCCCATATCGAACCCTAATGAGCT
GTCCTATTGGTGAAGTTCCCTCTCCATACAACTCAAGATTTGAGTCAGTCGCTTGGTCAGCAAGTGCTTG
TCATGATGGCATCAATTGGCTAACAATTGGAATTTCTGGCCCAGACAATGGGGCAGTGGCTGTGTTAAAG
TACAATGGCATAATAACAGACACTATCAAGAGTTGGAGAAATAATATATTGAGAACACAAGAATCTGAAT
GTGCATGTGTAAATGGTTCTTGCTTTACCATAATGACCGATGGACCAAGTGATGGACAGGCCTCATACAA
GATCTTCAGAATAGAAAAGGGAAAGATAGTCAAATCAGTCGAAATGAATGCCCCTAATTATCACTATGAG
GAATGCTCCTGTTATCCTGATTCTAGTGAAATCACATGTGTGTGCAGGGATAACTGGCATGGCTCGAATC
GACCGTGGGTTTCTTTCAACCAGAATCTGGAATATCAGATAGGATACATATGCAGTGGGATTTTCGGAGA

CAATCCACGCCCTAATGATAAGACAGGCAGTTGTGGTCCAGTATCGTCTAATGGAGCAAATGGAGTAAAA
GGATTTTCATTCAAATACGGCAATGGTGTTTGGATAGGGAGAACTAAAAGCATTAGTTCAAGAAAAGGTT
TTGAGATGATTTGGGATCCAAACGGATGGACTGGGACAGACAATAACTTCTCAATAAAGCAAGATATCGT
AGGAATAAATGAGTGGTCAGGATACAGCGGGAGTTTTGTTCAGCATCCAGAACTAACAGGGCTGGATTGT
ATAAGACCTTGCTTCTGGGTTGAACTAATCAGAGGACGACCCAAAGAGAACACAATCTGGACTAGCGGGA
GCAGCATATCCTTTTGTGGTGTAAACAGTGACACTGTGGGTTGGTCTTGGCCAGACGGTGCTGAGTTGCC
ATTTACCATTGACAAGTAATTTGTTCAAAAAACT
>gi|404425416|gb|CY125785.1| Influenza A virus (A/Boston/DOA93/2012(H1N1)) neuraminidase (NA) gene, complete cds
GAGTTTAAAATGAATCCAAACCAAAAGATAATAACCATTGGTTCGGTCTGTATGACAATTGGAATGGCTA
ACTTAATATTACAAATTGGAAACATAATCTCAATATGGATTAGCCACTCAATTCAACTTAGGAATCAAAG
TCAGATTGAAACATGCAATCAAAGCGTCATTACTTATGAAAACAACACTTGGGTAAATCAGACATATGTT
AACATCAGCAACACCAACTTTGCTGCTGGACAGTCAGTGGTTTCCGTGAAATTAGCGGGCAATTCCTCTC
TCTGCCCTGTTAGTGGATGGGCTATATACAGTAAAGACAACAGTATAAGAATCGGTTCCAAGGGGGATGT
GTTTGTCATAAGGGAACCATTCATATCATGCTCCCCCTTGGAATGCAGAACCTTCTTCTTGACCCAAGGG
GCCTTGCTAAATGACAAACATTCCAATGGAACCATTAAAGACAGGAGCCCATATCGAACCCTAATGAGCT
GTCCTATTGGTGAAGTTCCCTCTCCATACAACTCAAGATTTGAGTCAGTCGCTTGGTCAGCAAGTGCTTG
TCATGATGGCATCAATTGGCTAACAATTGGAATTTCTGGCCCAGACAATGGGGCAGTGGCTGTGTTAAAG
TACAACGGCATAATAACAGACACTATCAAGAGTTGGAGAAATAATATATTGAGAACACAAGAATCTGAAT
GTGCATGTGTAAATGGTTCTTGCTTTACCATAATGACCGATGGACCAAGTGATGGACAGGCCTCATACAA
GATCTTCAGAATAGAAAAGGGAAAGATAGTCAAATCAGTCGAAATGAATGCCCCTAATTATCACTATGAG
GAATGCTCCTGTTATCCTGATTCTAGTGAAATCACATGTGTGTGCAGGGATAACTGGCATGGCTCGAATC
GACCGTGGGTGTCTTTCAACCAGAATCTGGAATATCAGATAGGATACATATGCAGTGGGATTTTCGGAGA
CAATCCACGCCCTAATGATAAGACAGGCAGTTGTGGTCCAGTATCGTCTAATGGAGCAAATGGAGTAAAA
GGATTTTCATTCAAATACGGCAATGGTGTTTGGATAGGGAGAACTAAAAGCATTAGTTCAAGAAAAGGTT
TTGAGATGATTTGGGATCCAAACGGATGGACTGGGACAGACAATAACTTCTCAATAAAGCAAGATATCGT
AGGAATAAATGAGTGGTCAGGATACAGCGGGAGTTTTGTTCAGCATCCAGAACTAACAGGGCTGGATTGT
ATAAGACCTTGCTTCTGGGTTGAACTAATCAGAGGACGACCCAAAGAGAACACAATCTGGACTAGCGGGA
GCAGCATATCCTTTTGTGGTGTAAACAGTGACACTGTGGGTTGGTCTTGGCCAGACGGTGCTGAGTTGCC
ATTTACCATTGACAAGTAATTTGTTCAAAAAACT
>gi|401716666|gb|JX473012.1| Influenza A virus (A/Vladivostok/28/2012(H1N1)) segment 6 neuraminidase (NA) gene, complete cds
ATGAATCCAAACCAAAAGATAATAACCATTGGTTCGGTCTGTATGACAATTGGAATAGCTAACTTAATAT
TACAAATTGGAAACATAATCTCAATATGGATTAGCCACTCAATTCAACTTGGGAATCAAAATCAGATTGA
AACATGCAATCAAAGCGTCATTACTTATGAAAACAACACTTGGGTAAATCAGACATATGTTAACATCAGC
AACACCAACTTTGCTGCTGGACAGTCAGTGGTTTCCGTGAAATTAGCGGGCAATTCCTCTCTCTGCCCTG
TTAGTGGATGGGCTATATACAGTAAAGACAACAGTATAAGAATCGGTTCCAAGGGGGATGTGTTTGTCAT
AAGGGAACCATTCATATCATGCTCCCCCTTGGAATGCAGAACCTTCTTCTTGACTCAAGGGGCATTGCTA
AATGACAAACATTCCAATGGAACCATTAAAGACAGGAGCCCATATCGAACCTTAATGAGCTGTCCTATTG
GTGAAGTTCCCTCTCCATACAACTCAAGATTTGAGTCAGTCGCTTGGTCAGCAAGTGCTTGTCATGATGG
CATCAATTGGCTAACAATTGGAATTTCTGGCCCAGACAATGGGGCAGTGGCTGTGTTAAAGTACAACGGC
ATAATAACAGACACTATCAAGAGTTGGAGAAACAATATATTGAGAACACAAGAGTCTGAATGTGCATGTG
TAAATGGTTCTTGCTTTACCATAATGACCGATGGACCAAGTGATGGACAGGCCTCATACAAGATCTTCAG
AATAGAAAAGGGAAAGATAGTCAAATCAGTCGAAATGAATGCCCCTAATTATCACTATGAGGAATGCTCC

TGTTATCCTGATTCTAGTGAAATCACATGTGTGTGCAGGGATAACTGGCATGGCTCGAATCGACCGTGGG
TGTCTTTCAACCAGAATCTGGAATATCAGATAGGATACATATGCAGTGGGATTTTCGGAGACAATCCACG
CCCTAATGATAAGACAGGCAGTTGTGGTCCAGTATCGTCTAATGGAGCAAATGGAGTAAAAGGATTTTCA
TTCAAATACGGCAATGGTGTTTGGATAGGGAGAACTAAAAGCATTAGTTCACGAAAAGGTTTTGAGATGA
TTTGGGATCCAAACGGATGGACTGGGACAGACAATAGCTTCTCAATAAAGCAAGATATCGTAGGAATAAA
TGAGTGGTCAGGATATAGCGGGAGTTTTGTTCAGCATCCAGAACTAACAGGGCTGGACTGTATAAGACCT
TGCTTCTGGGTTGAACTAATCAGAGGGCGACCCAAAGAGAACACAATCTGGACTAGCGGGAGCAGCATAT
CCTTTTGTGGTGTAAACAGTGGCACTGTGGGTTGGTCTTGGCCAGACGGTGCTGAGTTGCCATTTACCAT
TGACAAGTAA
>gi|390135407|gb|JX046928.1| Influenza A virus (A/Moscow/IIV-45/2012(H1N1)) segment 6 neuraminidase (NA) gene, complete cds
ATGAATCCAAACCAAAAGATAATAACCATTGGTTCGGTCTGTATGACAATTGGAATAGCTAACTTAATAT
TACAAATTGGAAACATAATCTCAATATGGATTAGCCACTCAATTCAACTTGGGAATCAAAATCAGATTGA
AACATGCAATCAAAGCGTCATTACTTATGAAAACAACACTTGGGTAAATCAGACATATGTTAACATCAGC
AACACCAACTTTGCTGCTGGACAGTCAGTGGTTTCCGTGAAATTAGCGGGCAATTCCTCTCTCTGCCCTG
TTAGTGGATGGGCTATATACAGTAAAGACAACAGTATAAGAATCGGTTCCAAGGGGGATGTGTTTGTCAT
AAGGGAACCATTCATATCATGCTCCCCCTTGGAATGCAGAACCTTCTTCTTGACTCAAGGGGCATTGCTA
AATGACAAACATTCCAATGGAACCATTAAAGACAGGAGCCCATATCGAACCTTAATGAGCTGTCCTATTG
GTGAAGTTCCCTCTCCATACAACTCAAGATTTGAGTCAGTCGCTTGGTCAGCAAGTGCTTGTCATGATGG
CATCAATTGGCTAACAATTGGAATTTCTGGCCCAGACAATGGGGCAGTGGCTGTGTTAAAGTACAACGGC
ATAATAACAGACACTATCAAGAGTTGGAGAAACAATATATTGAGAACACAAGAGTCTGAATGTGCATGTG
TAAATGGTTCTTGCTTTACCATAATGACCGATGGACCAAGTGATGGACAGGCCTCATACAAGATCTTCAG
AATAGAAAAGGGAAAGATAGTCAAATCAGTCGAAATGAATGCCCCTAATTATCACTATGAGGAATGCTCC
TGTTATCCTGATTCTAGTGAAATCACATGTGTGTGCAGGGATAACTGGCATGGCTCGAATCGACCGTGGG
TGTCTTTCAACCAGAATCTGGAATATCAGATAGGATACATATGCAGTGGGATTTTCGGAGACAATCCACG
CCCTAATGATAAGACAGGCAGTTGTGGTCCAGTATCGTCTAATGGAGCAAATGGAGTAAAAGGATTTTCA
TTCAAATACGGCAATGGTGTTTGGATAGGGAGAACTAAAAGCATTAGTTCAAGAAAAGGTTTTGAGATGA
TTTGGGATCCAAACGGATGGACTGGGACAGACAATAGCTTCTCAATAAAGCAAGATATCGTAGGAATAAA
TGAGTGGTCAGGATATAGCGGGAGTTTTGTTCAGCATCCAGAACTAACAGGGCTGGACTGTATAAGACCT
TGCTTCTGGGTTGAACTAATCAGAGGGCGACCCAAAGAGAACACAATCTGGACTAGCGGGAGCAGCATAT
CCTTTTGTGGTGTAAACAGTGGCACTGTGGGTTGGTCTTGGCCAGACGGTGCTGAGTTGCCATTTACCAT
TGACAAGTAA
>gi|388260779|gb|CY120057.1| Influenza A virus (A/Mexico/InDRE5726/2012(H1N1)) neuraminidase (NA) gene, complete cds
ATGAATCCAAACCAAAAGATAATAACCATTGGTTCGGTCTGTATGACAATTGGAATGGCTAACTTAATAT
TACAAATTGGAAACATAATCTCAATATGGATTAGCCACTCAATTCAACTTAGGAATCAAAGTCAGATTGA
AACATGCAATCAAAGCGTCATTACTTATGAAAACAACACTTGGGTAAATCAGACATATGTTAACATCAGC
AACACCAACTTTGCTGCTGGACAGTCAGTGGTTTCCGTGAAATTAGCGGGCAATTCCTCTCTCTGCCCTG
TTAGTGGATGGGCTATATACAGTAAAGACAACAGTATAAGAATCGGTTCCAAGGGGGATGTGTTTGTCAT
AAGGGAACCATTCATATCATGCTCCCCCTTGGAATGCAGAACCTTCTTCTTGACCCAAGGGGCCTTGCTA
AATGACAAACATTCCAATGGAACCATTAAAGACAGGAGCCCATATCGAACCCTAATGAGCTGTCCTATTG
GTGAAGTTCCCTCTCCATACAACTCAAGATTTGAGTCAGTCGCTTGGTCAGCAAGTGCTTGTCATGATGG
CATCAATTGGCTAACAATTGGAATTTCTGGCCCAGACAATGGGGCAGTGGCTGTGTTAAAGTACAACGGC
ATAATAACAGACACTATCAAGAGTTGGAGAAATAATATATTGAGAACACAAGAATCTGAATGTGCATGTG

TAAATGGTTCTTGCTTTACCATAATGACCGATGGACCAAGTGATGGACAGGCCTCATACAAGATCTTCAG
AATAGAAAAGGGAAAGATAGTCAAATCAGTCGAAATGAATGCCCCTAATTATTACTATGAGGAATGCTCC
TGTTATCCTGATTCTAGTGAAATCACATGTGTGTGCAGGGATAACTGGCATGGCTCGAATCGACCGTGGG
TGTCTTTCAACCAGAATCTGGAATATCAGATAGGATACATATGCAGTGGGATTTTCGGAGACAATCCACG
CCCTAATGATAAGACAGGCAGTTGTGGTCCAGTATCGTCTAATGGAGCAAATGGAGTAAAAGGATTTTCA
TTCAAATACGGCAATGGTGTTTGGATAGGGAGAACTAAAAGCATTAGTTCAAGAAAAGGTTTTGAGATGA
TTTGGGATCCAAACGGATGGACTGGGACAGACAATAACTTCTCAATAAAGCAAGATATCGTAGGAATAAA
TGAGTGGTCAGGATACAGCGGGAGTTTTGTTCAGCATCCAGAACTAACAGGGCTGGATTGTATAAGACCT
TGCTTCTGGGTTGAACTAATCAGAGGACGACCCAAAGAGAACACAATCTGGACTAGCGGGAGCAGCATAT
CCTTTTGTGGTGTAAACAGTGACACTGTGGGTTGGTCTTGGCCAGACGGTGCTGAGTTGCCATTTACCAT
TGACAAGTAATTTGTTCAAAAA

>gi|388260783|gb|CY120059.1| Influenza A virus (A/Mexico/InDRE6023/2012(H1N1)) neuraminidase (NA) gene, complete cds

ATGAATCCAAACCAAAAGATAATAACCATTGGTTCGGTCTGTATGACAATTGGAATGGCTAACTTAATAT
TACAAATTGGAAACATAATCTCAATATGGATTAGCCACTCAATTCAACTTAGGAATCAAAGTCAGATTGA
AACATGCAATCAAAGCGTCATTACTTATGAAAACAACACTTGGGTAAATCAGACATATGTTAACATCAGC
AACACCAACTTTGCTGCTGGACAGTCAGTGGTTTCCGTGAAATTAGCGGGCAATTCCTCTCTCTGCCCTG
TTAGTGGATGGGCTATATACAGTAAAGACAACAGTATAAGAATCGGTTCCAAGGGGGATGTGTTTGTCAT
AAGGGAACCATTCATATCATGCTCCCCCTTGGAATGCAGAACCTTCTTCTTGACCCAAGGGGCCTTGCTA
AATGACAAACATTCCAATGGAACCATTAAAGACAGGAGCCCATATCGAACCCTAATGAGCTGTCCTATTG
GTGAAGTTCCCTCTCCATACAACTCAAGATTTGAGTCAGTCGCTTGGTCAGCAAGTGCTTGTCATGATGG
CATCAATTGGCTAACAATTGGAATTTCTGGCCCAGACAATGGGGCAGTGGCTGTGTTAAAGTACAACGGC
ATAATAACAGACACTATCAAGAGTTGGAGAAATAATATATTGAGAACACAAGAATCTGAATGTGCATGTG
TAAATGGTTCTTGCTTTACCATAATGACCGATGGACCAAGTGATGGACAGGCCTCATACAAGATCTTCAG
AATAGAAAAGGGAAAGATAGTCAAATCAGTCGAAATGAATGCCCCTAATTATTACTATGAGGAATGCTCC
TGTTATCCTGATTCTAGTGAAATCACATGTGTGTGCAGGGATAACTGGCATGGCTCGAATCGACCGTGGG
TGTCTTTCAACCAGAATCTGGAATATCAGATAGGATACATATGCAGTGGGATTTTCGGAGACAATCCACG
CCCTAATGATAAGACAGGCAGTTGTGGTCCAGTATCATCTAATGGAGCAAATGGAGTAAAAGGATTTTCA
TTCAAATACGGCAATGGTGTTTGGATAGGGAGAACTAAAAGCATTAGTTCAAGAAAAGGTTTTGAGATGA
TTTGGGATCCAAACGGATGGACTGGGACAGACAATAACTTCTCAATAAAGCAAGATATCGTAGGAATAAA
TGAGTGGTCAGGATACAGCGGGAGTTTTGTTCAGCATCCAGAACTAACAGGGCTGGATTGTATAAGACCT
TGCTTCTGGGTTGAACTAATCAGAGGACGACCCAAAGAGAACACAATCTGGACTAGCGGGAGCAGCATAT
CCTTTTGTGGTGTAAACAGTGACACTGTGGGTTGGTCTTGGCCAGACGGTGCTGAGTTGCCATTTACCAT
TGACAAGTAATTTGTTCAAAAAACTCCTTGTTTCTACT

>gi|385268626|gb|CY116605.1| Influenza A virus (A/Ulaanbaatar/1687/2012(H1N1)) neuraminidase (NA) gene, complete cds

AGCAAAAGCAGGAGTTTAAAATGAATCCAAACCAAAAGATAATAACCATTGGTTCGGTCTGTATGACAAT
TGGAATGGCTAACTTAATATTACAAATTGGAAACATAATCTCAATATGGATTAGTCACTCAATTCAACTT
GGGAATCAAAATCAGATTGAAACATGCAATCAAAGCGTCATTACTTATGAAAACAACACTTGGGTAAATC
AGACATATGTTAACATCAGCAACACCAACTTTGCTGCTGGACAGTCAGTGGTTTCCGTGAAATTAGCGGG
CAATTCCTCTCTCTGCCCTGTTAGTGGATGGGCTATATACAGTAAAGACAACAGTATAAGAATCGGTTCC
AAGGGGGAATGTGTTTGTCATAAGGGAACCATTCATATCATGCTCCCCCTTGGAATGCAGGACCTTCTTCT
TGACTCAAGGGGCCTTGCTAAATGACAAACATTCCAATGGAACCATTAAAGACAGGAGCCCATATCGAAC
CTTAATGAGCTGTCCTATTGGTGAAGTTCCCTCTCCATACAACTCAAGATTTGAGTCAGTCGCTTGGTCA

GCAAGTGCTTGTCATGATGGCATCAATTGGCTAACAATTGGAATTTCTGGCCCAGACAATGGGGCAGTGG
CTGTGTTAAAGTACAACGGCATAATAACAGACACTATCAAGAGTTGGAGAAACAATATATTGAGAACACA
AGAGTCTGAATGTGCATGTGTAAATGGTTCTTGCTTTACCATAATGACCGATGGACCAAGTGATGGACAG
GCCTCATACAAGATCTTCAGAATAGAAAAGGGAAAGATAGTCAAATCAGTCGAAATGAATGCCCCTAATT
ATCACTATGAGGAATGCTCCTGTTATCCTGATTCTAGTGAAATCACATGTGTGTGCAGGGATAACTGGCA
TGGCTCGAATCGACCGTGGGTGTCTTTCAACCAGAATCTGGAATATCAGATAGGATACATATGCAGTGGG
ATTTTCGGAGACAATCCACGCCCTAATGATAAGACAGGCAGTTGTGGTCCAGTATCGTCTAATGGAGCAA
ATGGAGTAAAAGGATTTTCATTCAAATACGGCAATGGTGTTTGGATAGGGAGAACTAAAAGCATTAGTTC
AAGAAAAGGTTTTGAGATGATTTGGGATCCAAACGGATGGACTGGGACAGACAATAGCTTCTCAATAAAG
CAAGATATCGTAGGAATAAATGAGTGGTCAGGATATAGCGGGAGTTTTGTTCAGCATCCAGAACTAACAG
GGCTGGACTGTATAAGACCTTGCTTCTGGGTTGAACTAATCAGAGGGCGACCCAAAGAGAACACAATCTG
GACTAGCGGGAGCAGCATATCCTTTTGTGGTGTAAACAGTGGCACTGTGGGTTGGTCTTGGCCAGACGGT
GCTGAGTTGCCATTTACCATTGACAAGTAATTTGTTCAAAAAACTCCTTGTTTCTACT

>gi|383513272|gb|JQ768355.1| Influenza A virus (A/Tomsk/IIV-19/2012(H1N1)) segment 6 neuraminidase (NA) gene, complete cds

ATGAATCCAAACCAAAAGATAATAACCATTGGTTCGGTCTGTATGACAATTGGAATAGCTAACTTAATAT
TACAAATTGGAAACATAATCTCAATATGGATTAGCCACTCAATTCAACTTGGGAATCAAAATCAGATTGA
AACATGCAATCAAAGCGTCATTACTTATGAAAACAACACTTGGGTAAATCAGACATATGTTAACATCAGC
AACACCAACTTTGCTGCTGGACAGTCAGTGGTTTCCGTGAAATTAGCGGGCAATTCCTCTCTCTGCCCTG
TTAGTGGATGGGCTATATACAGTAAAGACAACAGTATAAGAATCGGTTCCAAGGGGGATGTGTTTGTCAT
AAGGGAACCATTCATATCATGCTCCCCCTTGGAATGCAGAACCTTCTTCTTGACTCAAGGGGCCTTGCTA
AATGACAAACATTCCAATGGAACCATTAAAGACAGGAGCCCATATCGAACCTTAATGAGCTGTCCTATTG
GTGAAGTTCCCTCTCCATACAACTCAAGATTTGAGTCAGTCGCTTGGTCAGCAAGTGCTTGTCATGATGG
CATCAATTGGCTAACAATTGGAATTTCTGGCCCAGACAATGGGGCAGTGGCTGTGTTAAAGTACAACGGC
ATAATAACAGACACTATCAAGAGTTGGAGAAACAATATATTGAGAACACAAGAGTCTGAATGTGCATGTG
TAAATGGTTCTTGCTTTACCATAATGACCGATGGACCAAGTGATGGACAGGCCTCATACAAGATCTTCAG
AATAGAAAAGGGAAAGATAGTCAAATCAGTCGAAATGAATGCCCCTAATTATCACTATGAGGAATGCTCC
TGTTATCCTGATTCTAGTGAAATCACATGTGTGTGCAGGGATAACTGGCATGGCTCGAATCGACCGTGGG
TGTCTTTCAACCAGAATCTGGAATATCAGATAGGATACATATGCAGTGGGATTTTCGGAGACAATCCACG
CCCTAATGATAAGACAGGCAGTTGTGGTCCAGTATCGTCTAATGGAGCAAATGGAGTAAAAGGATTTTCA
TTCAAATACGGCAATGGTGTTTGGATAGGGAGAACTAAAAGCATTAGTTCAAGAAAAGGTTTTGAGATGA
TTTGGGATCCAAACGGATGGACTGGGACAGACAATAGCTTCTCAATAAAGCAAGATATCGTAGGAATAAA
TGAGTGGTCAGGATATAGCGGGAGTTTTGTTCAGCATCCAGAACTAACAGGGCTGGACTGTATAAGACCT
TGCTTCTGGGTTGAACTAATCAGAGGGCGACCCAAAGAGAACACAATCTGGACTAGCGGGAGCAGCATAT
CCTTTTGTGGTGTAAACAGTGGCACTGTGGGTTGGTCTTGGCCAGACGGTGCTGAGTTGCCATTTACCAT
TGACAAGTAA
