# Supplementary material for: A computational method for predicting regulation of human microRNAs on the influenza virus genome
Source: BMC Syst Biol. 2013 Oct 14;7(Suppl 2):S3. doi: 10.1186/1752-0509-7-S2-S3 (PMC3851852; doi:10.1186/1752-0509-7-S2-S3)
Supplement: Additional File 7 — The coding sequence of the gene fragment of NS from 2000 to 2012 recorded in Genbank. [file 1752-0509-7-S2-S3-S7.PDF]

NP

>gi|145278778|gb|CY021696.1| Influenza A virus (A/Memphis/15/2000(H1N1)) segment 5, complete sequence

ACTCACTGAGTGACATCAAAGTTATGGCGTCCCAAGGCACCAAACGGTCTTACGAACAGATGGAGACTGA
TGGGGAACGCCAGAATGCAACTGAAATCAGAGCATCCGTCGGAAGAATGATTGGTGGAATTGGGCGATTC
TACATCCAAATGTGCACCGAGCTTAAACTCAATGATTATGAGGGACGACTGATCCAGAACAGCTTAACAA
TAGAGAGAATGGTGCTCTCTGCTTTTGATGAGAGGAGGAATAAATATCTGGAAGAACATCCCAGTGCGGG
GAAAGATCCTAAGAAAACTGGAGGACCCATATACAAGAGAGTAGATGGAAAGTGGGTGAGGGAACTCGTC
CTTTATGACAAAGAAGAAATAAGGCGGATTTGGCGCCAAGCCAACAATGGTGATGATGCAACGGCTGGTT
TGACTCACATTATGATCTGGCATTCTAATTTGAATGATACAACTTACCAGAGGACAAGAGCTCTTGTCCG
CACCGGAATGGATCCCAGGATGTGCTCTTTGATGCAAGGTTCAACTCTCCCTAGAAGATCTGGAGCAGCA
GGCGCTGCAGTCAAAGGAGTTGGGACAATGGTGTTGGAGTTAATCAGGATGATCAAACGTGGGATCAATG
ACCGAAACTTCTGGAGGGGTGAGAATGGAAGAAAAACAAGGATTGCTTATGAGAGAATGTGCAACATTCT
CAAAGGAAAATTTCAAACAGCTGCACAAAAAGCAATGATGGATCAAGTGAGAGAAAGCCGGAACCCAGGA
AATGCTGAGATCGAAGATCTCACTTTTCTGGCACGGTCTGCACTTATATTAAGAGGGTCAGTTGCTCACA
AGTCTTGCCTGCCTGCCTGTGTGTATGGACCAGCCGTAGCCAGTGGGTACGACTTCGAAAAAGAGGGATA
CTCTTTGGTAGGGGTAGACCCTTTCAAACTGCTTCAAACCAGTCAGGTATACAGCCTAATCAGACCAAAC
GAGAATCCCGCACACAAGAGTCAGTTGGTGTGGATGGCATGCAATTCTGCTGCATTTGAAGATCTAAGAG
TGTCAAGCTTCATCAGAGGGACAAGAGTACTTCCAAGGGGGAAGCTCTCCACTAGAGGAGTACAAATTGC
TTCAAATGAAAACATGGATGCTATTGTATCAAGTACTCTTGAACTGAGAAGCAGATACTGGGCCATAAGA
ACCAGAAGTGGAGGGAACACTAATCAACAAAGGGCCTCTGCGGGCCAAATCAGCACACAACCTACGTTTT
CTGTGCAGAGAAACCTCCCATTTGACAAAACAACCATCATGGCAGCATTCACCGGGAATACGGAGGGAAG
AACATCAGACATGAGGGCAGAAATCATAAAGATGATGGAAAGTGCAAGACCAGAAGAAGTGTCCTTCCAG
GGGCGGGGAGTCTTTGAGCTCTCGGACGAAAGGGCAACGAACCCGATCGTGCCCTCCTTTGACATGAGTA
ATGAAGGATCTTATTTCTTCGGAGACAATGCAGAGGAGTACGACAATTAATGAAAAAT

>gi|70907646|gb|CY000452.2| Influenza A virus (A/New York/146/2000(H1N1)) segment 5, complete sequence

CAAAAGCAGGGTAGATAATCACTCACTGAGTGACATCAAAGTCATGGCGTCCCAAGGCACCAAACGGTCT
TACGAACAGATGGAGACTGATGGGGAACGCCAGAATGCAACTGAAATCAGAGCATCCGTTGGAAAAATGA
TTGGTGGAATTGGGCGATTCTACATCCAAATGTGCACCGAGCTTAAACTCAACGATCATGAGGGAAGACT
GATCCAGAACAGCTTAACAATAGAGAGAATGGTGCTCTCTGCTTTTGACGAGAGGAGGAATAAATATCTG
GAAGAACATCCCAGCGCAGGGAAAGATCCTAAGAAAACTGGAGGACCCATCTACAGGAGAGTAGATGGAA
AGTGGGTGAGGGAACTCGTCCTTTATGACAAAGAAGAAGTAAGGCGAATTTGGCGCCAAGCCAACAATGG
TGATGATGCAACGGCTGGTTTGACTCACATTATGATCTGGCATTCCAATTTGAATGATACAACTTATCAG
AGGACAAGAGCTCTTGTCCGCACCGGGTTGGATCCCAGGATGTGCTCTTTGATGCAGGGTTCAACCCTTC
CTAGGAGATCTGGAGCAGCAGGCGCTGCAGTCAAAGGAGTTGGAACAATGGTGCTGGAGTTGATCAGGAT
GATCAAACGTGGGATCAATGATCGAAATTTCTGGAGGGGTGAGAATGGAAGAAAAACCAGGATTGCTTAT
GAGAGAATGTGCAATATCCTCAAAGGGAAATTTCAAACAGCTGCACAAAGAGCTATGATGGATCAAGTGA
GAGAAAGCCGAAACCCAGGGAATGCTGAGATCGAAGATCTCACCTTTCTGGCACGGTCTGCACTCATATT
GAGAGGATCAGTTGCTCACAAGTCTTGTCTGCCTGCCTGTGTGTATGGACCTGCCGTAGCCAGTGGGTAC
GACTTCGAAAAAGAGGGATACTCTTTGGTAGGGATAGACCCTTTCAAACTGCTTCAAACCAGCCAAGTAT
ATAGCCTAATCAGACCAAACGAGAATCCCGCACACAAGAGTCAGTTGGTGTGGATGGCATGCAATTCTGC
TGCATTTGAAGACCTAAGAGTGTCGAGCTTCATCAGAGGGACAAGAGTGCTTCCAAGGGGGAAGCTCTCT

ACTAGAGGAGTACAAATTGCTTCAAATGAAAACATGGATTCTATTGTATCAAGTACTCTTGAACTGAGAA
GCAGGTACTGGGCCATAAGAACCAGAAGTGGAGGAAACACTAATCAACAAAAAGCCTCTGCGGGCCAAAT
CAGTATACAACCTACGTTTTCTGTGCAGAGAAACCTCCCATTTGACAAAACAACCATCATGGCAGCATTC
ACTGGGAATGCGGAGGGAAGAACATCAGACATGAGGGCAGAAATCATAAAAATGATGGAAAGTGCAAGAC
CAGAAGAAGTGTCCTTCCAGGGGAGGGGAGTCTTCGAGCTCTCGGACGAAAGGGCAACGAACCCGATCG
T
GCCCTCCTTTGACATGAGTAATGAAGGATCTTATTTCTTCGGAGACAATGCAGAGGAGTACGACAATTAA
TGAAAAATACCCTTGTTTCTACT
>gi|145278911|gb|CY021752.1| Influenza A virus (A/South Australia/44/2000(H1N1)) segment 5, complete sequence
ACTCACTGAGTGACATCAAAGTCATGGCGTCCCAAGGCACCAAACGGTCTTACGAACAGATGGAGACTGA
TGGGGAACGCCAGAATGCAACTGAAATCAGAGCATCCGTTGGAAAAATGATTGGTGGAATTGGGCGATTC
TACATCCAAATGTGCACCGAGCTTAAACTCAACGATCATGAGGGAAGACTGATCCAGAACAGCTTAACAA
TAGAGAGAATGGTGCTCTCTGCTTTTGACGAGAGGAGGAATAAATATCTGGAAGAACATCCCAGCGCAGG
GAAAGATCCTAAGAAAACTGGAGGACCCATCTACAGGAGAGTAGATGGAAAGTGGGTGAGGGAACTCGTC
CTTTATGACAAAGAAGAAGTAAGGCGAATTTGGCGCCAAGCCAACAATGGTGATGATGCAACGGCTGGTT
TGACTCACATTATGATCTGGCATTCCAATTTGAATGATACAACTTATCAGAGGACAAGAGCTCTTGTCCG
CACCGGGTTGGATCCCAGGATGTGCTCTTTGATGCAGGGTTCAACCCTTCCTAGGAGATCTGGAGCAGCA
GGCGCTGCAGTCAAAGGAGTTGGAACAATGGTGCTGGAGTTGATCAGGATGATCAAACGTGGGATCAATG
ATCGAAATTTCTGGAGGGGTGAGAATGGAAGAAAAACCAGGATTGCTTATGAGAGAATGTGCAATATCCT
CAAAGGGAAATTTCAAACAGCTGCACAAAGAGCTATGATGGATCAAGTGAGAGAAAGCCGAAACCCAGGG
AATGCTGAGATCGAAGATCTCACCTTTCTGGCACGGTCTGCACTCATATTGAGAGGATCAGTTGCTCACA
AGTCTTGTCTGCCTGCCTGTGTGTATGGACCTGCTGTAGCCAGTGGGTACGACTTCGAAAAAGAGGGATA
CTCTTTGGTAGGGATAGACCCTTTCAAACTGCTTCAAACCAGCCAAGTATATAGCCTAATCAGACCAAAC
GAGAATCCCGCACACAAGAGTCAGTTGGTGTGGATGGCATGCAATTCTGCTGCATTTGAAGACCTAAGAG
TGTCGAGCTTCATCAGAGGGACAAAAGTGCTTCCAAGGGGGAAGCTCTCTACTAGAGGAGTACAAATTGC
TTCAAATGAAAACATGGATTCTATTGTATCAAGTACTCTTGAACTGAGAAGCAGGTACTGGGCCATAAGA
ACCAGAAGTGGAGGAAACACTAATCAACAAAAAGCCTCTGCGGGCCAAATCAGTATACAACCTACGTTTT
CTGTGCAGAGAAACCTCCCATTTGACAAAACAACCATCATGGCAGCATTCACTGGGAATGCGGAGGGAAG
AACATCAGACATGAGGGCAGAAATCATAAAAATGATGGAAAGTGCAAGACCAGAAGAAGTGTCCTTCCAG
GGGAGGGGAGTCTTCGAGCTCTCGGACGAAAGGGCAACGAACCCGATCGTGCCCTCCTTTGACATGAGTA
ATGAAGGATCTTATTTCTTCGGAGACAATGCAGAGGAGTACGACAATTAA
>gi|157367772|gb|CY026158.1| Influenza A virus (A/Auckland/585/2000(H1N1)) segment 5, complete sequence
ACTCACTGAGTGACATCAAAGTCATGGCGTCCCAAGGCACCAAACGGTCTTACGAACAGATGGAGACTGA
TGGGGAACGCCAGAATGCAACTGAAATCAGAGCATCCGTCGGAAGAATGATTGGTGGAATTGGGCGATTC
TACATCCAAATGTGCACCGAGCTTAAACTCAATGATTATGAGGGACGACTGATCCAGAACAGCTTAACAA
TAGAGAGAATGGTGCTCTCTGCTTTTGATGAGAGGAGAAATAAATATCTGGAAGAACATCCCAGCGCGGG
GAAAGATCCTAAGAAAACTGGAGGACCCATATACAAGAGAGTAGATGGAAAGTGGGTGAGGGAACTCGTC
CTTTATGACAAAGAAGAAATAAGGCGGATTTGGCGCCAAGCCAACAATGGTGATGATGCAACGGCTGGTT
TGACTCACATTATGATCTGGCATTCTAATTTGAATGATACAACTTACCAGAGGACAAGAGCTCTTGTCCG
CACCGGAATGGATCCCAGGATGTGCTCTTTGATGCAAGGTTCAACTCTCCCTAGAAGATCTGGAGCAGCA
GGCGCTGCAGTCAAAGGAGTTGGGACAATGGTATTGGAGTTAATCAGGATGATCAAACGTGGGATCAATG
ACCGAAACTTCTGGAGGGGTGAGAATGGAAGAAAAACAAGGATTGCTTATGAGAGAATGTGCAACATTCT

CAAAGGAAAATTTCAAACAGCTGCACAAAAAGCAATGATGGATCAAGTGAGAGAAAGCCGGAACCCAGGA
AATGCTGAAATCGAAGATCTCACTTTTCTGGCACGGTCTGCACTCATATTAAGAGGGTCAGTTGCTCACA
AGTCTTGCCTGCCTGCCTGTGTGTATGGACCAGCCGTAGCCAGTGGGTACGACTTCGAAAAAGAGGGATA
CTCTTTGGTAGGGGTAGACCCTTTCAAACTGCTTCAAACCAGTCAGGTATACAGCCTAATCAGACCAAAC
GAGAATCCCGCACACAAGAGCCAGTTGGTGTGGATGGCATGCAATTCTGCTGCATTTGAAGATCTAAGAG
TGTCAAGCTTCATCAGAGGGACAAGAGTACTTCCAAGGGGGAAGCTCTCCACTAGAGGAGTACAAATTGC
TTCAAATGAAAACATGGATGCTATTGTATCAAGTACTCTTGAACTGAGAAGCAGATACTGGGCCATAAGG
ACCAGAAGTGGAGGGAACACTAATCAACAAAGGGCCTCTGCGGGCCAAATCAGCACACAACCTACGTTTT
CTGTGCAGAGAAACCTCCCATTTGACAAAACAACCATCATGGCAGCATTCACTGGGAATACGGAGGGAAG
AACATCAGACATGAGGGCAGAAATCATAAGGATGATGGAAAGTGCAAGACCAGAAGAAGTGTCCTTCCAG
GGGCGGGGAGTCTTTGAGCTCTCGGACGAAAGGGCAACGAACCCGATCGTGCCCTCCTTTGACATGAGTA
ATGAAGGATCTTATTTCTTCGGAGACAATGCAGAGGAGTACGACAATTAA
>gi|145278624|gb|CY021632.1| Influenza A virus (A/Wellington/4/2000(H1N1)) segment 5, complete sequence
ACTCACTGAGTGACATCAAAGTCATGGCGTCCCAAGGCACCAAACGGTCTTACGAACAGATGGAGACTGA
TGGGGAACGCCAGAATGCAACTGAAATCAGAGCATCCGTCGGAAGAATGATTGGTGGAATTGGGCGATTC
TACATCCAAATGTGCACCGAGCTTAAACTCAATGATTATGAGGGACGACTGATCCAGAACAGCTTAACAA
TAGAGAGAATGGTGCTCTCTGCTTTTGATGAGAGGAGAAATAAATATCTGGAAGAACATCCCAGCGCGGG
GAAAGATCCTAAGAAAACTGGAGGACCCATATACAAGAGAGTAGATGGAAAGTGGGTGAGGGAACTCGTC
CTTTATGACAAAGAAGAAATAAGGCGGATTTGGCGCCAAGCCAACAATGGTGATGATGCAACGGCTGGTT
TGACTCACATTATGATCTGGCATTCTAATTTGAATGATACAACTTACCAGAGGACAAGAGCTCTTGTCCG
CACCGGAATGGATCCCAGGATGTGCTCTTTGATGCAAGGTTCAACTCTCCCTAGAAGATCTGGAGCAGCA
GGCGCTGCAGTCAAAGGAGTTGGGACAATGGTATTGGAGTTAATCAGGATGATCAAACGTGGGATCAATG
ACCGAAACTTCTGGAGGGGTGAGAATGGAAGAAAAACAAGGATTGCTTATGAGAGAATGTGCAACATTCT
CAAAGGAAAATTTCAAACAGCTGCACAAAAAGCAATGATGGATCAAGTGAGAGAAAGCCGGAACCCAGGA
AATGCTGAGATCGAAGATCTCACTTTTCTGGCACGGTCTGCACTCATATTAAGAGGGTCAGTTGCTCACA
AGTCTTGCCTGCCTGCCTGTGTGTATGGACCAGCCGTAGCCAGTGGGTACGACTTCGAAAAAGAGGGATA
CTCTTTGGTAGGGGTAGACCCTTTCAAACTGCTTCAAACCAGTCAGGTATACAGCCTAATCAGACCAAAC
GAGAATCCCGCACACAAGAGCCAGTTGGTGTGGATGGCATGCAATTCTGCTGCATTTGAAGATCTAAGAG
TGTCAAGCTTCATCAGAGGGACAAGAGTACTTCCAAGGGGGAAGCTCTCCACTAGAGGAGTACAAATTGC
TTCAAATGAAAACATGGATGCTATTGTATCAAGTACTCTTGAACTGAGAAGCAGATACTGGGCCATAAGG
ACCAGAAGTGGAGGGAACACTAATCAACAAAGGGCCTCTGCGGGCCAAATCAGCACACAACCTACGTTTT
CTGTGCAGAGAAACCTCCCATTTGACAAAACAACCATCATGGCAGCATTCACTGGGAATACGGAGGGAAG
AACATCAGACATGAGGGCAGAAATCATAAGGATGATGGAAAGTGCAAGACCAGAAGAAGTGTCCTTCCAG
GGGCGGGGAGTCTTTGAGCTCTCGGACGAAAGGGCAACGAACCCGATCGTGCCCTCCTTTGACATGAGTA
ATGAAGGATCTTATTTCTTCGGAGACAATGCAGAGGAGTACGACAATTAATGAAAAAT
>gi|131058505|gb|CY020152.1| Influenza A virus (A/Memphis/7/2001(H1N1)) segment 5, complete sequence
CTCACTGAGTGACATCAAAGTCATGGCGTCCCAAGGCACCAAACGGTCTTACGAACAGATGGAGACTGAT
GGGGAACGCCAGAATGCAACTGAAATCAGAGCATCCGTCGGAAGAATGGTTGGTGGAATTGGGCGATTCT
ACATCCAAATGTGCACCGAGCTTAAACTCAATGATTATGAGGGACGACTGATCCAGAACAGCTTAACAAT
AGAGAGAATGGTGCTCTCTGCTTTTGATGAGAGGAGGAATAAATATCTGGAAGAACATCCCAGCACGGGG
AAAGATCCTAAGAAAACTGGAGGACCCATATACAAGAGGGTAGATGGAAAGTGGGTGAGGGAACTCGTCC
TTTATGACAAAGAAGAAATAAGGCGAATTTGGCGCCAAGCCAACAATGGTGATGATGCAACGGCTGGTTT

GACTCACATTATGATCTGGCATTCTAATTTGAATGATACAACTTACCAGAGGACAAGAGCTCTTGTCCGC
ACAGGAATGGATCCCAGGATGTGCTCTTTGATGCAAGGTTCAACTCTCCCTAGAAGATCTGGAGCAGCAG
GCGCTGCAGTCAAAGGAGTTGGGACAATGGTGTTGGAGTTAATCAGGATGATCAAACGTGGGATCAATGA
CCGAAACTTCTGGAGGGGTGAGAATGGAAGGAAAACAAGGATTGCTTATGAGAGAATGTGCAACATTCTC
AAAGGAAAATTTCAAACAGCTGCACAAAAAGCAATGATGGATCAAGTGAGAGAAAGCCGGAACCCAGGA
A
ATGCTGAGATCGAAGATCTCACTTTTCTGGCACGGTCTGCACTCATATTAAGAGGGTCAGTTGCTCACAA
ATCTTGCCTGCCTGCCTGTGTGTATGGACCAGCCGTAGCCAGTGGGTACGACTTCGAAAAAGAGGGATAC
TCTTTGGTAGGGGTAGACCCTTTTAAACTGCTTCAAACCAGTCAGGTATACAGCCTAATCAGACCAAACG
AGAATCCCGCACACAAGAGTCAGTTGGTGTGGATGGCATGCAACTCTGCTGCATTTGAAGATCTAAGAGT
GTCAAGCTTCATCAGAGGGACAAGAGTACTTCCAAGGGGGAAGCTCTCCACTAGAGGAGTACAAATTGCT
TCAAATGAAAACATGGATGCTATTGTATCAAGTACTCTTGAACTGAGAAGCAGATACTGGGCCATAAGAA
CCAGAAGTGGAGGAAACACTAACCAACAAAGGGCCTCTGCGGGCCAAATCAGCACACAACCTACGTTTTC
TGTGCAGAGAAACCTCCCATTTGACAAAACAACCATCATGGCAGCATTCACTGGGAATACGGAGGGAAGA
ACATCAGACATGAGGGCAGAAATCATAAAGATGATGGAAAGTGCAAGACCAGAAGAAGTGTCCTTCCAGG
GGCGGGGAGTCTTTGAGCTCTCGGACGAAAGGGCAACGAACCCGATCGTGCCCTCCTTTGACATGAGTAA
TGAAGGATCTTATTTCTTCGGAGACAATGCAGAGGAGTACGACAATTAATGAAAAAT
>gi|73761480|gb|CY002531.1| Influenza A virus (A/New York/220/2002(H1N1)) segment 5, complete sequence
GCAAAAGCAGGGTAGATAATCACTCACTGAGTGACATCAAAGTCATGGCGTCCCAAGGCACCAAACGGTC
TTACGAACAGATGGAGACTGATGGGGAACGCCAGAATGCAACTGAAATCAGAGCATCCGTCGGAAGAATG
ATTGGTGGAATTGGGCGATTCTACATCCAAATGTGCACCGAGCTCAAGCTCAATGATTATGAGGGACGAC
TGATCCAGAACAGCTTAACAATAGAGAGAATGGTGCTCTCTGCTTTTGATGAGAGGAGAAATAAATATCT
GGAAGAACATCCCAGCGCGGGGAAAGATCCTAAGAAAACTGGAGGACCCATATACAAGAGAGTAGATGGA
AAGTGGATGAGGGAACTCGTCCTTTATGACAAAGAAGAAATAAGGCGGATTTGGCGCCAAGCCAACAATG
GTGATGATGCAACGGCTGGTTTGACTCACATTATGATCTGGCATTCTAATTTGAATGATACAACTTACCA
GAGGACAAGGGCTCTTGTCCGCACCGGAATGGATCCCAGGATGTGCTCTTTGATGCAAGGTTCAACTCTC
CCTAGAAGATCTGGAGCAGCAGGCGCTGCAGTCAAAGGAGTTGGGACAATGGTATTGGAGTTAATCAGGA
TGATCAAACGTGGGATCAATGACCGAAACTTCTGGAGGGGTGAGAATGGAAGAAAAACAAGGATTGCTTA
TGAGAGAATGTGCAACATTCTCAAAGGAAAATTTCAAACAGCTGCACAAAAAGCAATGATGGATCAAGTG
AGAGAAAGCCGGAACCCAGGAAATGCTGAGATCGAAGATCTCACTTTTCTGGCACGGTCTGCACTCATAT
TGAGAGGATCAGTTGCTCACAAGTCTTGCCTGCCTGCCTGTGTGTATGGACCAGCCGTAGCCAGTGGGTA
CGACTTCGAAAAAGAGGGATACTCTTTGGTAGGAGTAGACCCTTTCAAACTGCTTCAAACCAGTCAGGTA
TACAGCCTAATCAGACCAAACGAGAATCCCGCACACAAGAGCCAGTTGGTGTGGATGGCATGCAATTCTG
CTGCATTTGAAGATCTAAGAGTGTCAAGCTTCATCAGAGGGACAAGAGTACTTCCAAGGGGGAAGCTCTC
CACTAGAGGAGTACAAATTGCTTCAAATGAAAACATGGATGCTATTGTATCAAGTACTCTTGAACTGAGA
AGCAGATACTGGGCCATAAGAACCAGAAGTGGAGGGAACACTAATCAACAAAGGGCCTCTGCGGGCCAAA
TCAGCACACAACCTACGTTTTCTGTGCAGAGAAACCTCCCATTTGACAAAACAACCATCATGGCAGCATT
CACTGGGAATACAGAGGGAAGAACATCAGACATGAGGGCAGAAATCATAAAGATGATGGAAAGTGCAAGA
CCAGAAGAAGTGTCCTTCCAGGGGCGGGGAGTCTTTGAGCTCTCGGACGAAAGGGCAACGAACCCGATC
G
TGCCCTCCTTTGACATGAGTAATGAAGGATCTTATTTCTTCGGAGACAATGCAGAGGAGTACGACAATTA
ATGAAAAATACCCTTGTTTCTACT
>gi|156536324|gb|CY025037.1| Influenza A virus (A/Auckland/597/2000(H1N1)) segment 5,

complete sequence
ACTCACTGAGTGACATCAAAGTCATGGCGTCCCAAGGCACCAAACGGTCTTACGAACAGATGGAGACTGA
TGGGGAACGCCAGAATGCAAATGAAATCAGAGCATCCGTCGGAAGAATGATTGGTGGAATTGGGCGATTC
TACATCCAAATGTGCACCGAGCTTAAACTCAATGATTATGAGGGACGACTGATCCAGAACAGCTTAACAA
TAGAGAGAATGGTGCTCTCTGCTTTTGATGAGAGGAGAAATAAATATCTGGAAGAACATCCCAGCGCGGG
GAAAGATCCTAAGAAAACTGGAGGACCCATATACAAGAGAGTAGATGGAAAGTGGGTGAGGGAACTCGTC
CTTTATGACAAAGAAGAAATAAGGCGGATTTGGCGCCAAGCCAACAATGGTGATGATGCAACGGCTGGTT
TGACTCACATTATGATCTGGCATTCTAATTTGAATGATACAACTTACCAGAGGACAAGAGCTCTTGTCCG
CACCGGAATGGATCCCAGGATGTGCTCTTTGATGCAAGGTTCAACTCTCCCTAGAAGATCTGGAGCAGCA
GGCGCTGCAGTCAAAGGAGTTGGGACAATGGTATTGGAGTTAATCAGGATGATCAAACGTGGGATCAATG
ACCGAAACTTCTGGAGGGGTGAGAATGGAAGAAAAACAAGGATTGCTTATGAGAGAATGTGCAACATTCT
CAAAGGAAAATTTCAAACAGCTGCACAAAAAGCAATGATGGATCAAGTGAGAGAAAGCCGGAACCCAGGA
AATGCTGAAATCGAAGATCTCACTTTTCTGGCACGGTCTGCACTCATATTAAGAGGGTCAGTTGCTCACA
AGTCTTGCCTGCCTGCCTGTGTGTATGGACCAGCCGTAGCCAGTGGGTACGACTTCGAAAAAGAGGGATA
CTCTTTGGTAGGGGTAGACCCTTTCAAACTGCTTCAAACCAGTCAGGTATACAGCCTAATCAGACCAAAC
GAGAATCCCGCACACAAGAGCCAGTTGGTGTGGATGGCATGCAATTCTGCTGCATTTGAAGATCTAAGAG
TGTCAAGCTTCATCAGAGGGACAAGAGTACTTCCAAGGGGGAAGCTCTCCACTAGAGGAGTACAAATTGC
TTCAAATGAAAACATGGATGCTATTGTATCAAGTACTCTTGAACTGAGAAGCAGATACTGGGCCATAAGG
ACCAGAAGTGGAGGGAACACTAATCAACAAAGGGCCTCTGCGGGCCAAATCAGCACACAACCTACGTTTT
CTGTGCAGAGAAACCTCCCATTTGACAAAACAACCATCATGGCAGCATTCACTGGGAATACGGAGGGAAG
AACATCAGACATGAGGGCAGAAATCATAAGGATGATGGAAAGTGCAAGACCAGAAGAAGTGTCCTTCCAG
GGGCGGGGAGTCTTTGAGCTCTCGGACGAAAGGGCAACGAACCCGATCGTGCCCTCCTTTGACATGAGTA
ATGAAGGATCTTATTTCTTCGGAGACAATGCAGAGGAGTACGACAATTAATGAA
>gi|149780533|gb|CY022536.1| Influenza A virus (A/Auckland/605/2001(H1N1)) segment 5, complete sequence
ATCACTCACTGAGTGACATCAAAGTCATGGCGTCCCAAGGCACCAAACGGTCTTACGAACAGATGGAGAC
TGATGGGGAACGCCAGAATGCAACTGAAATCAGAGCATCCGTCGGAAGAATGATTGGTGGAATTGGGCGA
TTCTACATCCAAATGTGCACCGAGCTTAAACTCAATGATTATGAGGGACGACTGATCCAGAACAGCTTAA
CAATAGAGAGAATGGTGCTCTCTGCTTTTGATGAGAGGAGAAATAAATATCTGGAAGAACATCCCAGCGC
GGGGAAAGATCCTAAGAAAACTGGAGGACCCATATACAAGAGAGTAGATGGAAAGTGGGTGAGGGAACT
C
GTCCTTTATGACAAAGAAGAAATAAGGCGGATTTGGCGCCAAGCCAACAATGGTGATGATGCAACGGCTG
GTTTGACTCACATTATGATCTGGCATTCTAATTTGAATGATACAACTTACCAGAGGACAAGAGCTCTTGT
CCGCACCGGGATGGATCCCAGGATGTGCTCTTTGATGCAAGGTTCAACTCTCCCTAGAAGATCTGGAGCA
GCAGGCGCTGCAGTAAAGGGAGTTGGGACAATGGTATTGGAGTTAATTAGGATGATCAAACGTGGGATCA
ATGACCGAAACTTCTGGAGGGGTGAGAATGGAAGAAAAACAAGAATTGCTTATGAGAGAATGTGCAATAT
TCTCAAAGGAAAATTTCAAACAGCTGCACAAAAAGCAATGATGGATCAAGTGAGAGAAAGCCGGAACCCA
GGAAATGCTGAGATCGAAGATCTCACTTTTCTGGCAAGGTCTGCACTCATATTAAGAGGGTCAGTTGCTC
ACAAGTCTTGCCTGCCTGCCTGTGTGTATGGACCAGCCGTAGCCAGTGGGTACGACTTCGAAAAAGAGGG
ATACTCTTTGGTAGGGGTAGACCCTTTCAAACTGCTTCAAACCAGTCAGGTATACAGCCTAATCAGACCA
AACGAGAATCCCGCACACAAGAGCCAGTTGGTGTGGATGGCATGCAATTCTGCTGCATTTGAAGATCTAA
GGGTGTCAAGCTTCATCAGAGGGACAAGAGTACTTCCAAGGGGGAAGCTCTCCACTAGAGGAGTACAAAT
TGCTTCAAATGAAAACATGGATGCTATTGTATCAAGTACTCTTGAACTGAGAAGCAGATACTGGGCCATA
AGAACCAGAAGTGGAGGGAACACTAATCAACAAAGGGCCTCTGCGGGCCAAATCAGCACACAACCTACGT

TTTCTGTGCAGAGAAACCTCCCATTTGACAAAACAACCATCATGGCAGCATTCACTGGGAATACGGAGGG
AAGAACATCAGACATGAGGGCAGAAATCATAAAGATGATGGAAAGTGCAAGACCAGAAGAAGTGTCCTTC
CAGGGGCGGGGAGTCTTTGAGCTCTCGGACGAAAGGGCAACGAACCCGATCGTGCCCTCCTTTGACATGA
GTAATGAAGGATCTTATTTCTTCGGAGACAATGCAGAGGAGTACGACAATTAATGA

>gi|237688869|gb|CY040077.1| Influenza A virus (A/Taiwan/567/2002(H1N1)) segment 5, complete sequence

ATGGCGTCCCAAGGCACCAAACGGTCTTACGAACAGATGGAGACTGATGGGGAACGCCAGAATGCAACTG
AAATCAGAGCATCCGTCGGAAGAATGATTGGTGGAATTGGGCGATTCTACATCCAAATGTGCACCGAGCT
TAAACTCAATGATTATGAGGGACGACTGATCCAGAACAGCTTAACAATAGAGAGAATGGTGCTCTCTGCT
TTTGATGAGAGGAGAAATAAATATCTGGAAGAACATCCCAGCGCGGGGAAAGATCCTAAGAAAACTGGAG
GACCCATATACAAGAGAGTAGATGGAAAGTGGGTGAGGGAACTCGTCCTTTATGACAAAGAAGAAATAAG
GCGGATTTGGCGCCAAGCCAACAATGGTGATGATGCAACGGCTGGTTTGACTCACATTATGATCTGGCAT
TCTAATTTGAATGATACAACTTACCAGAGGACAAGAGCTCTTGTCCGCACCGGGATGGATCCCAGGATGT
GCTCTTTGATGCAAGGTTCAACTCTCCCTAGAAGATCTGGAGCAGCAGGCGCTGCCGTAAAAGGAGTTGG
GACAATGGTATTGGAGTTAATTAGGATGATCAAACGTGGGATCAATGACCGAAACTTCTGGAGGGGTGAG
AATGGAAGAAAAACAAGAATTGCTTATGAGAGAATGTGCAATATTCTCAAAGGAAAATTTCAAACAGCTG
CACAAAAAGCAATGATGGATCAAGTGAGAGAAAGCCGGAACCCAGGAAATGCTGAGATCGAAGATCTCAC
TTTTCTGGCACGGTCTGCACTCATATTAAGAGGGTCAGTTGCTCACAAGTCTTGCCTGCCTGCCTGTGTG
TATGGACCAGCCGTAGCCAGTGGGTACGACTTCGAAAAAGAGGGATACTCTTTGGTAGGGGTAGACCCTT
TCAAACTGCTTCAAACCAGTCAGGTATACAGCCTAATCAGACCAAACGAGAATCCCGCACACAAGAGTCA
GTTGGTGTGGATGGCATGCAATTCTGCTGCATTTGAAGATCTAAGGGTGTTAAGCTTCATCAGAGGGACA
AGAGTACTTCCAAGGGGGAAGCTCTCCACTAGAGGAGTACAAATTGCTTCAAATGAAAACATGGATGCTA
TTGTATCAAGTACTCTTGAACTGAGAAGCAGATACTGGGCCATAAGAACCAGAAGTGGAGGGAACACTAA
TCAACAAAGGGCCTCTGCGGGCCAAATCAGCACACAACCTACGTTTTCTGTGCAGAGAAACCTCCCATTT
GACAAAACAACCATCATGGCAGCATTCACTGGGAATACGGAGGGAAGAACATCAGACATGAGGGCAGAAA
TCATAAAGATGATGGAAAGTGCAAGACCAGAAGAAGTGTCCTTCCAGGGGCGGGGAGTCTTTGAGCTCTC
GGACGAAAGGGCAACGAACCCGATCGTGCCCTCCTTTGACATGAGTAATGAAGGATCTTATTTCTTCGGA
GACAATGCAGAGGAGTACGACAATTAACGA

>gi|122855952|gb|CY019344.1| Influenza A virus (A/Memphis/6/2003(H1N1)) segment 5, complete sequence

CTCACTGAGTGACATCAAAGTCATGGCGTCCCAAGGCACCAAACGGTCTTACGAACAGATGGAGACTGAT
GGGGAACGCCAGAATGCAACTGAAATCAGAGCATCCGTCGGAAGAATGATTGGTGGAATTGGGCGATTCT
ACATCCAAATGTGCACCGAGCTTAAGCTCAATGATTATGAGGGACGACTGATCCAGAACAGCTTAACAAT
AGAGAGAATGGTGCTCTCTGCTTTTGATGAGAGGAGAAATAAATATCTGGAAGAACATCCCAGCACGGGG
AAAGATCCTAAGAAAACTGGAGGACCCATATACAAGAGAGTAGATGGAAAATGGGTGAGGGAACTCGTCC
TTTATGACAAAGAAGAAATAAGGCGGATTTGGCGCCAAGCCAACAATGGTGATGATGCAACGGCTGGTTT
GACTCACATTATGATCTGGCATTCCAATTTGAATGATACAACTTACCAGAGGACAAGAGCTCTTGTCCGC
ACCGGAATGGATCCCAGGATGTGCTCTTTGATGCAAGGTTCAACTCTCCCTAGAAGATCTGGAGCAGCAG
GCGCTGCAGTCAAAGGAGTTGGGACAATAGTATTGGAGTTAATCAGGATGATCAAACGTGGGATCAATGA
CCGAAACTTCTGGAGGGGTGAGAATGGAAGAAAAACAAGGATTGCTTATGAGAGAATGTGCAACATTCTC
AAAGGAAAATTTCAAACAGCTGCACAAAAAGCAATGATGGATCAAGTGAGAGAAAGCCGGAACCCAGGA
A
ATGCTGAGATCGAAGATCTCACTTTTCTGGCACGGTCTGCACTCATATTGAGAGGATCAGTTGCTCACAA
GTCTTGCCTGCCTGCCTGTGTGTATGGACCAGCCGTAGCCAGTGGGTACGACTTCGAAAAAGAGGGATAC

TCTTTGGTAGGAGTAGACCCTTTCAAACTGCTTCAAACCAGTCAGGTATACAGCCTAATCAGACCAAACG
AGAATCCCGCACACAAGAGCCAGTTGGTGTGGATGGCATGCAATTCTGCTGCATTTGAAGATCTAAGAGT
GTCAAGCTTCATCAGAGGGACAAGAGTACTTCCAAGGGGGAAGCTCTCCACTAGAGGAGTACAAATTGCT
TCAAATGAAAACATGGATGCTATTGTATCAAGTACTCTTGAACTGAGAAGCAGATACTGGGCCATAAGAA
CCAGAAGTGGAGGGAACACTAATCAACAAAGGGCCTCTGCGGGCCAAATCAGCACACAACCTACGTTTTC
TGTGCAGAGAAACCTCCCATTTGACAAAACAACCATCATGGCAGCATTCACTGGGAATACAGAGGGAAGA
ACATCAGACATGAGGGCAGAAATCATAAAGATGATGGAAAGTGCAAGACCAGAAGAAGTGTCCTTCCAGG
GGCGGGGAGTCTTTGAGCTCTCGGACGAAAGGGCAACGAACCCGATCGTGCCCTCCTTTGACATGAGTAA
TGAAGGATCTTATTTCTTCGGAGACAATGCAGAGGAGTACGACAATTAATGAAAAAT
>gi|82546781|gb|CY006678.1| Influenza A virus (A/New York/494/2002(H1N1)) segment 5, complete sequence
CAAAAGCAGGGTAGATAATCACTCACTGAGTGACATCAAAGTCATGGCGTCCCAAGGCACCAAACGGTCT
TACGAACAGATGGAGACTGATGGGGAACGCCAGAATGCAACTGAAATCAGAGCATCCGTCGGAAGAATGA
TTGGTGGAATTGGGCGATTCTACATCCAAATGTGCACCGAGCTTAAGCTCAATGATTATGAGGGACGACT
GATCCAGAACAGCTTAACAATAGAGAGAATGGTGCTCTCTGCTTTTGATGAGAGGAGAAATAAATATCTG
GAAGAACATCCCAGCGCGGGGAAAGATCCTAAGAAAACTGGAGGACCCATATACAAGAGAGTAGATGGAA
AGTGGGTGAGGGAACTCGTCCTTTATGACAAAGAAGAAATAAGGCGGATTTGGCGCCAAGCCAACAATGG
TGATGATGCAACGGCTGGTTTGACTCACATTATGATCTGGCATTCTAATTTGAATGATACAACTTACCAG
AGGACAAGAGCTCTTGTCCGCACCGGAATGGATCCCAGGATGTGCTCTTTGATGCAAGGTTCAACTCTCC
CTAGAAGATCTGGAGCAGCAGGCGCTGCAGTCAAAGGAGTTGGGACAATGGTATTGGAGTTAATCAGGAT
GATCAAACGTGGGATCAATGACCGAAACTTCTGGAGGGGTGAGAATGGAAGAAAAACAAGGATTGCTTAT
GAGAGAATGTGCAACATTCTCAAAGGAAAATTTCAAACAGCTGCACAAAAAGCAATGATGGATCAAGTGA
GAGAAAGCCGGAACCCAGGAAATGCTGAGATCGAAGATCTCACTTTTCTGGCACGGTCTGCACTCATATT
GAGAGGGTCCGTTGCTCACAAGTCTTGCCTGCCTGCCTGTGTGTATGGACCAGCCGTAGCCAGTGGGTAC
GACTTCGAAAAAGAGGGATACTCTTTGGTAGGAGTAGACCCTTTCAAACTGCTTCAAACCAGTCAGGTAT
ACAGCCTAATCAGACCAAACGAGAATCCCGCACACAAGAGCCAGTTGGTGTGGATGGCATGCAATTCTGC
TGCTTTTGAAGATCTAAGAGTGTCTAGCTTCATCAGAGGGACAAGAGTACTTCCAAGGGGGAAGCTCTCC
ACTAGAGGAGTACAAATTGCTTCAAATGAAAACATGGATGCTATTGTGTCAAGTACTCTTGAACTGAGAA
GCAGATACTGGGCCATAAGAACCAGAAGTGGAGGGAACACTAATCAACAAAGGGCCTCTGCGGGCCAAAT
CAGCACACAACCTACGTTTTCTGTGCAGAGAAACCTCCCATTTGACAAAACAACCATCATGGCAGCATTT
ACTGGGAATACAGAGGGTAGAACATCAGACATGAGGGCAGAAATCATAAAGATGATGGAAAGTGCAAGAC
CAGAAGAAGTGTCCTTCCAGGGGCGGGGAGTCTTTGAGCTCTCGGACGAAAGGGCAACGAACCCGATCGT
GCCCTCCTTTGACATGAGTAATGAAGGATCTTATTTCTTCGGAGACAATGCAGAGGAGTACGACAATTAA
TGAAAAAT
>gi|77543348|gb|CY003307.1| Influenza A virus (A/New York/291/2002(H1N1)) segment 5, complete sequence
ACTGAGTGACATCAAAGTCATGGCGTCCCAAGGCACCAAACGGTCTTACGAACAGATGGAGACTGATGGG
GAACGCCAGAATGCAACTGAAATCAGAGCATCCGTCGGAAGAATGATTGGTGGAATTGGGCGATTCTACA
TCCAAATGTGCACCGAGCTTAAACTCAATGATTATGAGGGACGACTGATCCAGAACAGCTTAACAATAGA
GAGAATGGTGCTCTCTGCTTTTGATGAGAGGAGAAATAAATATCTGGAAGAACATCCCAGCGCGGGGAAA
GATCCTAAGAAAACTGGAGGACCCATATACAAGAGAGTAGATGGAAAGTGGGTGAGGGAACTCGTCCTTT
ATGACAAAGAAGAAATAAGGCGGATTTGGCGCCAAGCCAACAATGGTGATGATGCAACGGCTGGTTTGAC
TCACATTATGATCTGGCATTCTAATTTGAATGATACAACTTACCAGAGGACAAGAGCTCTTGTCCGCACC
GGAATGGATCCCAGGATGTGCTCTTTGATGCAAGGTTCAACTCTCCCTAGAAGATCTGGAGCAGCAGGCG

CTGCAGTCAAAGGAGTTGGGACAATGGTATTGGAGTTAATCAGGATGATCAAACGTGGGATCAATGACCG
AAACTTCTGGAGGGGTGAGAATGGAAGAAAAACAAGGATTGCTTATGAGAGAATGTGCAACATTCTCAAA
GGGAAATTTCAAACAGCTGCACAAAAAGCAATGATGGATCAAGTGAGAGAAAGCCGGAACCCAGGAAATG
CTGAGATCGAAGATCTCACTTTTCTGGCACGGTCTGCACTCATATTGAGAGGGTCAGTTGCTCACAAGTC
TTGCCTGCCTGCCTGTGTGTATGGACCAGCCGTAGCCAGTGGGTACGACTTCGAAAAAGAGGGATACTCT
TTGGTAGGAGTAGACCCTTTCAAACTGCTTCAAACCAGTCAGGTATACAGCCTAATCAGACCAAACGAGA
ATCCCGCACACAAGAGCCAGTTAGTGTGGATGGCATGTAATTCTGCTGCATTTGAAGATCTAAGAGTGTC
AAGCTTCATCAGAGGGACAAGAGTACTTCCAAGGGGGAAGCTCTCCACTAGAGGAGTACAAATTGCTTCA
AATGAAAACATGGATGCTATTATATCAAGTACTCTTGAATTGAGAAGCAGATACTGGGCCATAAGAACCA
GAAGTGGAGGGAACACTAATCAACAAAGGGCCTCTGCGGGCCAAATCAGCACACAACCTACGTTTTCTGT
GCAGAGAAACCTCCCATTTGACAAAACAACCATCATGGCAGCATTCACTGGGAATACAGAGGGAAGAACA
TCAGACATGAGGGCAGAAATCATAAAGATGATGGAAAGTGCAAGACCAGAAGAAGTGTCCTTCCAGGGGC
GGGGAGTCTTTGAGCTCTCGGACGAAAGGGCAACGAACCCGATCGTGCCCTCCTTTGACATGAGTAATGA
AGGATCTTATTTCTTCGGAGACAATGCAGAGGAGTACGACAATTAATGAAAAAT
>gi|237689042|gb|CY040149.1| Influenza A virus (A/Taiwan/52/2002(H1N1)) segment 5, complete sequence
CTCACTGAGTGACATCAAAGTCATGGCGTCCCAAGGCACCAAACGGTCTTACGAACAGATGGAGACTGAT
GGGGAACGCCAGAATGCAACTGAAATCAGAGCATCCGTCGGAAGAATGATTGGTGGAATTGGGCGATTCT
ACATCCAAATGTGCACCGAGCTTAAACTCAATGATTATGAGGGACGACTGATCCAGAACAGCTTAACAAT
AGAGAGAATGGTGCTCTCTGCTTTTGATGAGAGGAGAAATAAATATCTGGAAGAACATCCCAGCGCGGGG
AAAGATCCTAAGAAAACTGGAGGACCCATATACAAGAGAGTAGATGGAAAGTGGGTGAGGGAACTCGTCC
TTTATGACAAAGAAGAAATAAGGCGGATTTGGCGCCAAGCCAACAATGGTGATGATGCAACAGCTGGTTT
GACTCACATTATGATCTGGCATTCTAATTTGAATGATACAACTTACCAGAGGACAAGAGCTCTTGTCCGC
ACCGGGATGGATCCCAGGATGTGCTCTTTGATGCAAGGTTCAACTCTCCCTAGAAGATCTGGAGCAGCAG
GCGCTGCCGTAAAAGGAGTTGGGACAATGGTATTGGAGTTAATTAGGATGATCAAACGTGGGATCAATGA
CCGAAACTTCTGGAGGGGTGAGAATGGAAGAAAAACAAGAATTGCTTATGAGAGAATGTGCAATATTCTC
AAAGGAAAATTTCAAACAGCTGCACAAAAAGCAATGATGGATCAAGTGAGAGAAAGCCGGAACCCAGGA
A
ATGCTGAGATCGAAGATCTCACTTTTCTGGCACGGTCTGCACTCATATTAAGAGGGTCAGTTGCTCACAA
GTCTTGCCTGCCTGCCTGTGTGTATGGACCAGCCGTAGCCAGTGGGTACGACTTCGAAAAAGAGGGATAC
TCTTTGGTAGGGGTAGACCCTTTCAAACTGCTTCAAACCAGTCAGGTATACAGCCTAATCAGACCAAACG
AGAATCCCGCACACAAGAGCCAGTTGGTGTGGATGGCATGCAATTCTGCTGCATTTGAAGATCTAAGGGT
GTTAAGCTTCATCAGAGGGACAAGAGTACTTCCAAGGGGGAAGCTCTCCACTAGAGGAGTACAAATTGCT
TCAAATGAAAACATGGATGCTATTGTATCAAGTACTCTTGAACTGAGAAGCAGATACTGGGCCATAAGAA
CCAGAAGTGGAGGGAACACTAATCAACAAAGGGCCTCTGCGGGCCAAATCAGCACACAACCTACGTTTTC
TGTGCAGAGAAACCTCCCATTTGACAAAACAACCATCATGGCAGCATTCACTGGGAATACGGAGGGAAGA
ACATCAGACATGAGGGCAGAAATCATAAAGATGATGGAAAGTGCAAGACCAGAAGAAGTGTCCTTCCAGG
GGCGGGGAGTCTTTGAGCTCTCGGACGAAAGGGCAACGAACCCGATCGTGCCCTCCTTTGACATGAGTAA
TGAAGGATCTTATTTCTTCGGAGACAATGCAGAGGAGTACGACAATTAATGAA
>gi|237689061|gb|CY040157.1| Influenza A virus (A/Taiwan/123/2002(H1N1)) segment 5, complete sequence
CTCACTGAGTGACATCAAAGTCATGGCGTCCCAAGGCACCAAACGGTCTTACGAACAGATGGAGACTGAT
GGGGAACGCCAGAATGCAACTGAAATCAGAGCATCCGTCGGAAGAATGATTGGTGGAATTGGGCGATTCT
ACATCCAAATGTGCACCGAGCTTAAACTCAATGATTATGAGGGACGACTGATCCAGAACAGCTTAACAAT

AGAGAGAATGGTGCTCTCTGCTTTTGATGAGAGGAGAAATAAATATCTGGAAGAACATCCCAGCGCGGGG
AAAGATCCTAAGAAAACTGGAGGACCCATATACAAGAGAGTAGATGGAAAGTGGGTGAGGGAACTCGTCC
TTTATGACAAAGAAGAAATAAGGCGGATTTGGCGCCAAGCCAACAATGGTGATGATGCAACGGCTGGTTT
GACTCACATTATGATCTGGCATTCTAATTTGAATGACACAACTTACCAGAGGACAAGAGCTCTTGTCCGC
ACCGGGATGGATCCCAGGATGTGCTCTTTGATGCAAGGTTCAACTCTCCCTAGAAGATCTGGAGCAGCAG
GCGCTGCCGTAAAAGGAGTTGGGACAATGGTATTGGAGTTAATTAGGATGATTAAACGTGGGATCAATGA
CCGAAACTTCTGGAGGGGTGAGAATGGAAGAAAAACAAGAATTGCTTATGAGAGAATGTGCAATATTCTC
AAAGGAAAATTTCAAACAGCTGCACAAAAAGCAATGATGGATCAAGTGAGAGAAAGCCGGAACCCAGGA
A
ATGCTGAGATCGAAGATCTCACTTTTCTGGCACGGTCTGCACTCATATTAAGAGGGTCAGTTGCCCACAA
GTCTTGCCTGCCTGCCTGTGTGTATGGACCAGCCGTAGCCAGTGGGTACGACTTCGAAAAAGAGGGATAC
TCTTTGGTAGGGGTAGACCCTTTCAAACTGCTTCAAACCAGTCAGGTATACAGCCTAATCAGACCAAACG
AGAATCCCGCACACAAGAGCCAGTTGGTGTGGATGGCATGCAATTCTGCTGCATTTGAAGATCTAAAGGT
GTTAAGCTTCATCAGAGGGACAAGAGTACTTCCAAGGGGGAAGCTCTCCACTAGAGGAGTACAAATTGCT
TCAAATGAAAACATGGATGCTATTGTATCAAGTACTCTTGAACTGAGAAGCAGATACTGGGCCATAAGAA
CCAGAAGTGGAGGGAACACTAATCAACAAAGGGCCTCTGCGGGCCAAATCAGCACACAACCTACGTTTTC
TGTGCAGAGAAACCTCCCATTTGACAAAACAACCATCATGGCAGCATTCACTGGGAATACGGAGGGAAGA
ACATCAGACATGAGGGCAGAAATCATAAAGATGATGGAAAGTGCAAGACCAGAAGAAGTGTCCTTCCAGG
GGCGGGGAGTCTTTGAGCTCTCGGACGAAAGGGCAACGAACCCGATCGTGCCCTCCTTTGACATGAGTAA
TGAAGGATCTTATTTCTTCGGAGACAATGCAGAGGAGTACGACAATTAATGAA
>gi|77747428|gb|CY003691.1| Influenza A virus (A/New York/486/2003(H1N1)) segment 5, complete sequence
AGCAAAAGCAGGGTAGATAATCACTCACTGAGTGACATCAAAGTCATGGCGTCCCAAGGCACCAAACGGT
CTTACGAACAGATGGAGACTGATGGGGAACGCCAGAATGCAACTGAAATCAGAGCATCCGTCGGAAGAAT
GATTGGTGGAATTGGGCGATTTTACATCCAAATGTGCACCGAGCTTAAGCTCAATGATTATGAGGGACGA
CTGATCCAGAACAGCTTAACAATAGAGAGAATGGTGCTCTCTGCTTTTGATGAGAGGAGAAATAAATATC
TGGAAGAACATCCCAGCGCGGGGAAAGATCCTAAGAAAACTGGAGGACCCATATACAAGAGAGTAGATGG
AAAGTGGGTGAGGGAACTCGTCCTTTATGACAAAGAAGAAATAAGGCGGATTTGGCGCCAAGCCAACAAT
GGTGATGATGCAACGGCTGGTTTGACTCACATTATGATCTGGCATTCTAATTTGAATGATACAACTTACC
AGAGGACAAGAGCTCTTGTCCGCACCGGAATGGATCCCAGGATGTGCTCTTTGATGCAAGGTTCAACTCT
CCCTAGAAGATCTGGAGCAGCAGGAGCTGCAGTCAAAGGAGTTGGGACAATGGTATTGGAGTTAATCAGG
ATGATCAAACGTGGGATCAATGACCGAAACTTCTGGAGGGGTGAGAATGGAAGAAAAACAAGGATTGCTT
ATGAGAGAATGTGCAACATTCTCAAAGGAAAATTTCAAACAGCTGCACAAAAAGCAATGATGGATCAAGT
GAGAGAAAGCCGGAACCCAGGAAATGCTGAGATCGAAGATCTCACTTTTCTGGCACGGTCTGCACTCATA
TTGAGAGGATCAGTTGCTCACAAGTCTTGCCTGCCTGCCTGTGTGTATGGACCAGCCGTAGCCAGTGGGT
ACGACTTCGAAAAAGAGGGATACTCTTTGGTAGGAGTAGACCCTTTCAAACTGCTTCAAACCAGTCAGGT
ATACAGCCTAATCAGACCAAACGAGAATCCCGCACACAAGAGCCAGTTGGTGTGGATGGCATGCAATTCT
GCTGCATTTGAAGATCTAAGAGTGTCAAGCTTCATCAGAGGGACAAGAGTACTTCCAAGGGGGAAGCTCT
CCACTAGAGGAGTACAAATTGCTTCAAATGAAAACATGGATGCTATTGTATCAAGTACTCTTGAACTGAG
AAGCAGATACTGGGCCATAAGAACCAGAAGTGGAGGGAACACTAATCAACAAAGGGCCTCTGCGGGCCAA
ATCAGCACACAACCTACGTTTTCTGTGCAGAGAAACCTCCCATTTGACAAAACAACCATCATGGCAGCAT
TCACTGGGAATACAGAGGGAAGAACATCAGACATGAGGGCAGAAATCATAAAGATGATGGAAAGTGCAAG
ACCAGAAGAAGTGTCCTTCCAGGGGCGGGGAGTCTTTGAGCTCTCGGACGAAAGGGCAACGAACCCGATC
GTGCCCTCCTTTGACATGAGTAATGAAGGATCTTATTTCTTCGGAGACAATGCAGAGGAGTACGACAATT

AATGAAAAAT

>gi|125664182|gb|CY019886.1| Influenza A virus (A/Memphis/5/2003(H1N1)) segment 5, complete sequence

AGTCATGGCGTCCCAAGGCACCAAACGGTCTTACGAACAGATGGAGACTGATGGGGAACGCCAGAATGCA
ACTGAAATCAGAGCATCCGTCGGAAGAATGATTGGTGGAATTGGGCGATTCTACATCCAAATGTGCACCG
AGCTTAAGCTCAATGATTATGAGGGACGACTGATCCAGAACAGCTTAACAATAGAGAGAATGGTGCTCTC
TGCTTTTGATGAGAGGAGAAATAAATATCTGGAAGAACATCCCAGCGCGGGGAAAGATCCTAAGAAAACT
GGAGGACCCATATACAAGAGAGTAGATGGAAAGTGGGTGAGGGAACTCGTCCTTTATGACAAAGAAGAAA
TAAGGCGGATTTGGCGCCAAGCCAACAATGGTGATGATGCAACGGCTGGTTTGACTCACATTATGATCTG
GCATTCTAATTTGAATGATACAACTTACCAGAGGACAAGAGCTCTTGTCCGCACCGGAATGGATCCCAGG
ATGTGCTCTTTGATGCAAGGTTCAACTCTCCCTAGAAGATCTGGAGCAGCAGGCGCTGCAGTCAAAGGAG
TTGGGACAATGGTATTGGAGTTAATCAGGATGATCAAACGTGGGATCAATGACCGAAACTTCTGGAGGGG
TGAGAATGGAAGAAAAACAAGGATTGCTTATGAGAGAATGTGCAACATTCTCAAAGGAAAATTTCAAACA
GCTGCACAAAAAGCAATGATGGATCAAGTGAGAGAAAGCCGGAACCCAGGAAATGCTGAGATCGAAGATC
TCACTTTTCTGGCACGGTCTGCACTCATATTGAGAGGATCAGTTGCTCACAAGTCTTGCCTGCCTGCCTG
TGTGTATGGACCAGCCGTAGCCAGTGGGTACGACTTCGAAAAAGAGGGATACTCTTTGGTAGGAGTAGAC
CCTTTCAAACTGCTTCAAACCAGTCAGGTATACAGCCTAATTAGACCAAACGAGAATCCCGCACACAAGA
GCCAGTTGGTGTGGATGGCATGCAATTCTGCTGCATTTGAAGATCTAAGAGTGTCAAGCTTCATCAGAGG
GACAAGAGTACTTCCAAGGGGGAAGCTCTCCACTAGAGGAGTACAAATTGCTTCAAATGAAAACATGGAT
GCTATTGTATCAAGTACTCTTGAACTGAGAAGCAGATACTGGGCCATAAGAACCAGAAGTGGAGGGAACA
CTAATCAACAAAGGGCCTCTGCGGGCCAAATCAGCACACAACCTACATTTTCTGTGCAGAGAAACCTCCC
ATTCGACAAAACAACCATCATGGCAGCATTCACTGGGAATACAGAGGGAAGAACATCAGACATGAGGGCA
GAAATCATAAAGATGATGGAAAGTGCAAGACCAGAAGAAGTGTCCTTCCAGGGGCGGGGAGTCTTTGAGC
TCTCGGACGAAAGGGCAACGAACCCGATCGTGCCCTCCTTTGACATGAGTAATGAAGGATCTTATTTCTT
CGGAGACAATGCAGAGGAGTACGACAATTAATGAAAAAT

>gi|73763201|gb|CY002539.1| Influenza A virus (A/New York/227/2003(H1N1)) segment 5, complete sequence

GTAGATAATCACTCACTGAGTGACATCAAAGTCATGGCGTCCCAAGGCACCAAACGGTCTTACGAACAGA
TGGAGACTGATGGGGAACGCCAGAATGCAACTGAAATCAGAGCATCCGTCGGAAGAATGATTGGTGGAAT
TGGGCGATTCTACATCCAAATGTGCACCGAGCTTAAGCTCAATGATTATGAGGGACGACTGATCCAGAAC
AGCTTAACAATAGAGAGAATGGTGCTCTCTGCTTTTGATGAGAGGAGAAATAAATATCTGGAAGAACATC
CCAGCGCGGGGAAAGATCCTAAGAAAACTGGAGGACCCATATACAAGAGAGTAGATGGAAAGTGGGTGA
G
GGAACTCGTCCTTTATGACAAAGAAGAAATAAGGCGGATTTGGCGCCAAGCCAACAATGGTGATGATGCA
ACGGCTGGTTTGACTCACATTATGATCTGGCATTCTAATTTGAATGATACAACTTACCAGAGGACAAGAG
CTCTTGTCCGCACCGGAATGGATCCCAGGATGTGCTCTTTGATGCAAGGTTCAACTCTCCCTAGAAGATC
TGGAGCAGCAGGCGCTTCAGTCAAAGGAGTTGGGACAATGGTATTGGAGTTAATCAGGATGATCAAACGT
GGGATCAATGACCGAAACTTCTGGAGGGGTGAGAATGGAAGAAAAACAAGGATTGCTTATGAGAGAATGT
GCAACATTCTCAAAGGAAAATTTCAAACAGCTGCACAAAAAGCAATGATGGATCAAGTGAGAGAAAGCCG
GAACCCAGGAAATGCTGAGATCGAAGATCTCACTTTTCTGGCACGGTCTGCACTCATATTGAGAGGATCA
GTTGCTCACAAGTCTTGCCTGCCTGCCTGTGTGTATGGACCAGCCGTAGCCAGTGGGTACGACTTCGAAA
AAGAGGGATACTCTTTGGTAGGAGTAGACCCTTTCAAACTGCTTCAAACCAGTCAGGTATACAGCCTAAT
TAGACCAAACGAGAATCCCGCACACAAGAGCCAGTTGGTGTGGATGGCATGCAATTCTGCTGCATTTGAA
GATCTAAGAGTGTCAAGCTTCATCAGAGGGACAAGAGTACTTCCAAGGGGGAAGCTCTCCACTAGAGGAG

TACAAATTGCTTCAAATGAAAACATGGATGCTATTGTATCAAGTACTCTTGAACTGAGAAGCAGATACTG
GGCCATAAGAACCAGAAGTGGAGGGAACACTAATCAACAAAGGGCCTCTGCGGGCCAAATCAGCACACAA
CCTACATTTTCTGTGCAGAGAAACCTCCCATTTGACAAAACAACCATCATGGCAGCATTCACTGGGAATA
CAGAGGGAAGAACATCAGACATGAGGGCAGAAATCATAAAGATGATGGAAAGTGCAAGACCAGAAGAAG
T
GTCCTTCCAGGGGCGGGGAGTCTTTGAGCTCTCGGACGAAAGGGCAACGAACCCGATCGTGCCCTCCTTT
GACATGAGTAATGAAGGATCTTATTTCTTCGGAGACAATGCAGAGGAGTACGACAATTAATGAAAAATAC
>gi|89112172|gb|CY008999.1| Influenza A virus (A/New York/484/2003(H1N1)) segment 5, complete sequence
GGTAGATAATCACTCACTGAGTGACATCAAAGTCATGGCGTCCCAAGGCACCAAACGGTCTTACGAACAG
ATGGAGACTGATGGGGAACGCCAGAATGCAACTGAAATCAGAGCATCCGTCGGAAGAATGATTGGTGGAA
TTGGGCGATTCTACATCCAAATGTGCACCGAGCTTAAGCTCAATGATTATGAGGGACGACTGATCCAGAA
CAGCTTAACAATAGAGAGAATGGTGCTCTCTGCTTTTGATGAGAGGAGAAATAAATATCTGGAAGAACAT
CCCAGCGCGGGGAAAGATCCTAAGAAAACTGGAGGACCCATATACAAGAGAGTAGATGGAAAGTGGGTGA
GGGAACTCGTCCTTTATGACAAAGAAGAAATAAGGCGGATTTGGCGCCAAGCCAACAATGGTGATGATGC
AACGGCTGGTTTGACTCACATTATGATCTGGCATTCTAATTTGAATGATACAACTTACCAGAGGACAAGA
GCTCTTGTCCGCACCGGAATGGATCCCAGGATGTGCTCTTTGATGCAAGGTTCAACTCTCCCTAGAAGAT
CTGGAGCAGCAGGCGCTGCAGTCAAAGGAGTTGGGACAATGGTATTGGAGTTAATCAGGATGATCAAACG
TGGGATCAATGACCGAAACTTCTGGAGGGGTGAGAATGGAAGAAAAACAAGGATTGCTTATGAGAGAATG
TGCAACATTCTCAAAGGAAAATTTCAAACAGCTGCACAAAAAGCAATGATGGATCAAGTGAGAGAAAGCC
GGAACCCAGGAAATGCTGAGATCGAAGATCTCACTTTTCTGGCACGGTCTGCACTCATATTGAGAGGATC
AGTTGCTCACAAGTCTTGCCTGCCTGCCTGTGTGTATGGACCAGCCGTAGCCAGTGGGTACGACTTCGAA
AAAGAGGGATACTCTTTGGTAGGAGTAGACCCTTTCAAACTGCTTCAAACCAGTCAGGTATACAGCCTAA
TCAGACCAAACGAGAATCCCGCACACAAGAGCCAGTTGGTGTGGATGGCATGCAATTCTGCTGCATTTGA
AGATCTAAGAGTGTCAAGCTTCATCAGAGGGACAAGAGTACTTCCAAGGGGGAAGCTCTCCACTAGAGGA
GTACAAATTGCTTCAAATGAAAACATGGATGCTATTGTATCAAGTACTCTTGAACTGAGAAGCAGATACT
GGGCCATAAGAACCAGAAGTGGAGGGAACACTAATCAACAAAGGGCCTCTGCGGGCCAAATCAGCACACA
ACCTACGTTTTCTGTGCAGAGAAACCTCCCATTTGACAAAACAACCATCATGGCAGCATTCACTGGGAAT
ACAGAGGGAAGAACATCAGACATGAGGGCAGAAATCATAAAGATGATGGAAAGTGCAAGACCAGAAGAA
G
TGTCCTTCCAGGGGCGGGGAGTCTTTGAGCTCTCGGACGAAAGGGCAACGAACCCGATCGTGCCCTCCTT
TGACATGAGTAATGAAGGATCTTATTTCTTCGGAGACAATGCAGAGGAGTACGACAATTAATGAAAAAT
>gi|83727848|gb|CY006918.1| Influenza A virus (A/New York/488/2003(H1N1)) segment 5, complete sequence
GGTAGATAATCACTCACTGAGTGACATCAAAGTCATGGCGTCCCAAGGCACCAAACGGTCTTACGAACAG
ATGGAGACTGATGGGGAACGCCAGAATGCAACTGAAATCAGAGCATCCGTCGGAAGAATGATTGGTGGAA
TTGGGCGATTCTACATCCAAATGTGCACCGAGCTTAAGCTCAATGATTATGAGGGACGACTGATCCAGAA
CAGCTTAACAATAGAGAGAATGGTGCTCTCTGCTTTTGATGAGAGGAGAAATAAATATCTGGAAGAACAT
CCCAGCGCGGGGAAAGATCCTAAGAAAACTGGAGGACCCATATACAAGAGAGTAGATGGAAAGTGGGTGA
GGGAACTCGTCCTTTATGACAAAGAAGAAATAAGGCGGATTTGGCGCCAAGCCAACAATGGTGATGATGC
AACGGCTGGTTTGACTCACATTATGATCTGGCATTCTAATTTGAATGATACAACTTACCAGAGGACAAGA
GCTCTTGTCCGCACCGGAATGGATCCCAGGATGTGCTCTTTGATGCAAGGTTCAACTCTCCCTAGAAGAT
CTGGAGCAGCAGGCGCTGCAGTCAAAGGAGTTGGGACAATGGTATTGGAGTTAATCAGGATGATCAAACG
TGGGATCAATGACCGAAACTTCTGGAGGGGTGAGAATGGAAGAAAAACAAGGATTGCTTATGAGAGAATG

TGCAACATTCTCAAAGGAAAATTTCAAACAGCTGCACAAAAAGCAATGATGGATCAAGTGAGAGAAAGCC
GGAACCCAGGAAATGCTGAGATCGAAGATCTCACTTTTCTGGCACGGTCTGCACTCATATTGAGAGGATC
AGTTGCTCACAAGTCTTGCCTGCCTGCCTGTGTGTATGGACCAGCCGTAGCCAGTGGGTACGACTTCGAA
AAAGAGGGATACTCTTTGGTAGGAGTAGACCCTTTCAAACTGCTTCAAACCAGTCAGGTATACAGCCTAA
TCAGACCAAACGAGAATCCCGCACACAAGAGCCAGTTGGTGTGGATGGCATGCAATTCTGCTGCATTTGA
AGATCTAAGAGTGTCAAGCTTCATCAGAGGGACAAGAGTACTTCCAAGGGGGAAGCTCTCCACTAGAGGA
GTACAAATTGCTTCAAATGAAAACATGGATGCTATTGTATCAAGTACTCTTGAACTGAGAAGCAGATACT
GGGCCATAAGAACCAGAAGTGGAGGGAACACTAATCAACAAAGGGCCTCTGCGGGCCAAATCAGCACACA
ACCTACGTTTTCTGTGCAGAGAAACCTCCCATTTGACAAAACAACCATCATGGCAGCATTCACTGGGAAT
ACAGAGGGAAGAACATCAGACATGAGGGCAGAAATCATAAAGATGATGGAAAGTGCAAGACCAGAAGAA
G
TGTCATTCCAGGGGCGGGGAGTCTTTGAGCTCTCGGACGAAAGGGCAACGAACCCAATCGTGCCCTCCTT
TGACATGAGTAATGAAGGATCTTATTTCTTCGGAGACAATGCAGAGGAGTACGACAATTAATGAAAAAT
>gi|157281264|gb|CY025216.1| Influenza A virus (A/Texas/UR06-0012/2006(H1N1)) segment 5, complete sequence
ACTCACTGAGTGACATTAAAGTCATGGCGTCCCAAGGCACCAAACGGTCTTACGAACAGATGGAGACTGA
TGGGGAACGCCAGAATGCAACTGAAATCAGAGCATCCGTCGGAAGAATGATTGGTGGAATTGGGCGATTC
TACATCCAAATGTGCACTGAGCTTAAGCTCAATGATTATGAGGGACGGCTGATCCAGAACAGCTTAACAA
TAGAGAGAATGGTGCTCTCTGCTTTTGATGAGAGGAGAAATAAATATCTGGAAGAACATCCCAGCGCGGG
GAAAGATCCTAAGAAAACTGGAGGACCCATATACAAGAGAGTAGATGGAAAGTGGGTGAGGGAACTCGTC
CTTTATGACAAAGAAGAAATAAGGCGGATTTGGCGCCAAGCCAACAATGGTGATGATGCAACGGCTGGTT
TGACTCACATTATGATCTGGCATTCTAATTTGAATGATACAACTTACCAGAGGACAAGAGCTCTTGTCCG
CACCGGAATGGATCCCAGGATGTGCTCTTTGATGCAAGGTTCAACTCTCCCTAGAAGATCTGGAGCAGCA
GGCGCTGCAGTCAAAGGAGTTGGGACAATGGTATTGGAGTTAATCAGGATGATCAAACGTGGGATCAATG
ACCGAAACTTCTGGAGGGGTGAGAATGGAAGAAAAACAAGGATTGCTTATGAGAGAATGTGCAACATTCT
CAAAGGAAAATTTCAAACAGCTGCACAAAAAGCAATGATGGATCAAGTGAGAGAAAGCCGGAACCCAGGA
AATGCTGAGATCGAAGATCTCACTTTTCTGGCACGGTCTGCACTCATATTGAGAGGATCAGTTGCTCACA
AGTCTTGCCTGCCTGCCTGTGTGTATGGACCAGCCGTAGCCAGTGGGTATGACTTCGAAAAAGAGGGTTA
CTCTTTGGTAGGAGTAGACCCTTTCAAACTGCTTCAAACCAGTCAGGTATACAGTCTAATTAGACCAAAC
GAGAATCCCGCACACAAGAGCCAGTTGGTGTGGATGGCATGCAATTCTGCTGCATTTGAAGATCTAAGAG
TGTCAAGCTTCATCAGAGGGACAAGAGTACTTCCAAGGGGGAAGCTCTCCACTAGAGGAGTACAAATTGC
TTCAAATGAAAACATGGATGCTATTGTATCAAGTACTCTTGAACTGAGAAGCAGATACTGGGCCATAAGA
ACCAGAAGTGGAGGGAACACTAATCAACAAAGGGCCTCTGCGGGCCAAATCAGCACACAACCTACGTTTT
CTGTGCAGAGAAACCTCCCATTTGACAAAGCAACCATCATGGCAGCATTCACTGGGAATACAGAGGGAAG
AACATCAGACATGAGGGCAGAAATCATAAAGATGATGGAAAGTGCAAGACCAGAAGAAGTGTCCTTCCAG
GGGCGGGGAGTCTTTGAGCTCTCGGACGAAAGGGCAACGAACCCGATCGTGCCCTCCTTTGACATGAGTA
ATGAAGGATCTTATTTCTTCGGAGACAATGCAGAGGAGTACGACAATTAATGAAAAAT
>gi|94959539|gb|CY010767.1| Influenza A virus (A/Canterbury/20/2001(H1N1)) segment 5, complete sequence
TAGATAATCACTCACTGAGTGACATCAAAGTCATGGCGTCCCAAGGCACCAAACGGTCTTACGAACAGAT
GGAGACTGATGGGGAACGCCAGAATGCAACTGAAATCAGAGCATCCGTCGGAAGAATGATTGGTGGAATT
GGGCGATTCTACATCCAAATGTGCACCGAGCTTAAACTCAATGATTATGAGGGACGACTGATCCAGAACA
GCTTAACAATAGAGAGAATGGTGCTCTCTGCTTTTGATGAGAGGAGAAATAAATATCTGGAAGAACATCC
CAGCGCGGGGAAAGATCCTAAGAAAACTGGAGGACCCATATACAAGAGAGTAGATGGAAAGTGGGTGAG

G
GAACTCGTCCTTTATGACAAAGAAGAAATAAGGCGGATTTGGCGCCAAGCCAACAATGGTGATGATGCAA
CGGCTGGTTTGACTCACATTATGATCTGGCATTCTAATTTGAATGATACAACTTACCAGAGGACAAGAGC
TCTTGTCCGCACCGGGATGGATCCCAGGATGTGCTCTTTGATGCAAGGTTCAACTCTCCCTAGAAGATCT
GGAGCAGCAGGCGCTGCAGTAAAAGGAGTTGGGACAATGGTATTGGAGTTAATTAGGATGATCAAACGTG
GGATCAATGACCGAAACTTCTGGAGGGGTGAGAATGGAAGAAAAACAAGAATTGCTTATGAGAGAATGTG
CAATATTCTCAAAGGAAAATTTCAAACAGCTGCACAAAAAGCAATGATGGATCAAGTGAGAGAAAGCCGG
AACCCAGGAAATGCTGAGATCGAAGATCTCACTTTTCTGGCACGGTCTGCACTCATATTAAGAGGGTCAG
TTGCTCACAAGTCTTGCCTGCCTGCCTGTGTGTATGGACCAGCCGTAGCCAGTGGGTACGACTTCGAAAA
AGAGGGATACTCTTTGGTAGGGGTAGACCCTTTCAAACTGCTTCAAACCAGTCAGGTATACAGCCTAATC
AGACCAAACGAGAATCCCGCACACAAGAGCCAGTTGGTGTGGATGGCATGCAATTCTGCTGCATTTGAAG
ATCTAAGGGTGTCAAGCTTCATCAGAGGGACAAGAGTACTTCCAAGGGGGAAGCTCTCCACTAGAGGAGT
ACAAATTGCTTCAAATGAAAACATGGATGCTATTGTATCAAGTACTCTTGAACTGAGAAGCAGATACTGG
GCCATAAGAACCAGAAGTGGAGGGAACACTAATCAACAAAGGGCCTCTGCGGGCCAAATCAGCACACAAC
CTACGTTTTCTGTGCAGAGAAACCTCCCATTTGACAAAACAACCATCATGGCAGCATTCACTGGGAATAC
GGAGGGAAGAACATCAGACATGAGGGCAGAAATCATAAAGATGATGGAAAGTGCAAGACCAGAAGAAGT
G
TCCTTCCAGGGGCGGGGAGTCTTTGAGCTCTCGGACGAAAGGGCAACGAACCCGATCGTGCCCTCCTTTG
ACATGAGTAATGAAGGATCTTATTTCTTCGGAGACAATGCAGAGGAGTACGACAATTAATGAAAAAT
>gi|91119033|gb|CY010407.1| Influenza A virus (A/West Coast/33/2001(H1N1)) segment 5, complete sequence
TAGATAATCACTCACTGAGTGACATCAAAGTCATGGCGTCCCAAGGCACCAAACGGTCTTACGAACAGAT
GGAGACTGATGGGGAACGCCAGAATGCAACTGAAATCAGAGCATCCGTCGGAAGAATGATTGGTGGAATT
GGGCGATTCTACATCCAAATGTGCACCGAGCTTAAACTCAATGATTATGAGGGACGACTGATCCAGAACA
GCTTAACAATAGAGAGAATGGTGCTCTCTGCTTTTGATGAGAGGAGAAATAAATATCTGGAAGAACATCC
CAGCGCGGGGAAAGATCCTAAGAAAACTGGAGGACCCATATACAAGAGAGTAGATGGAAAGTGGGTGAG
G
GAACTCGTCCTTTATGACAAAGAAGAAATAAGGCGGATTTGGCGCCAAGCCAACAATGGTGATGATGCAA
CGGCTGGTTTGACTCACATTATGATCTGGCATTCTAATTTGAATGATACAACTTACCAGAGGACAAGAGC
TCTTGTCCGCACCGGGATGGATCCCAGGATGTGCTCTTTGATGCAAGGTTCAACTCTCCCTAGAAGATCT
GGAGCAGCAGGCGCTGCAGTAAAAGGAGTTGGGACAATGGTATTGGAGTTAATTAGGATGATCAAACGTG
GGATCAATGACCGAAACTTCTGGAGGGGTGAGAATGGAAGAAAAACAAGAATTGCTTATGAGAGAATGTG
CAATATTCTCAAAGGAAAATTTCAAACAGCTGCACAAAAAGCAATGATGGATCAAGTGAGAGAAAGCCGG
AACCCAGGAAATGCTGAGATCGAAGATCTCACTTTTCTGGCAAGGTCTGCACTCATATTAAGAGGGTCAG
TTGCTCACAAGTCTTGCCTGCCTGCCTGTGTGTATGGACCAGCCGTAGCCAGTGGGTACGACTTCGAAAA
AGAGGGATACTCTTTGGTAGGGGTCGACCCTTTCAAACTGCTTCAAACCAGTCAGGTATACAGCCTAATC
AGACCAAACGAGAATCCCGCACACAAGAGCCAGTTGGTGTGGATGGCATGCAATTCTGCTGCATTTGAAG
ATCTAAGGGTGTCAAGCTTCATCAGAGGGACAAGAGTACTTCCAAGGGGGAAGCTCTCCACTAGAGGAGT
ACAAATTGCTTCAAATGAAAACATGGATGCTATTGTATCAAGTACTCTTGAACTGAGAAGCAGATACTGG
GCCATAAGAACCAGAAGTGGAGGGAACACTAATCAACAAAGGGCCTCTGCGGGCCAAATCAGCACACAAC
CTACGTTTTCTGTGCAGAGAAACCTCCCATTTGACAAAATAACCATCATGGCAGCATTCACTGGGAATAC
GGAGGGAAGAACATCAGACATGAGGGCAGAAATCATAAAGATGATGGAAAGTGCAAGACCAGAAGAAGT
G
TCCTTCCAGGGGCGGGGAGTCTTTGAGCTCTCGGACGAAAGGGCAACGAACCCGATCGTGCCCTCCTTTG

ACATGAGTAATGAAGGATCTTATTTCTTCGGAGACAATGCAGAGGAGTACGACAATTAATGAAAAAT
>gi|131052818|gb|CY020000.1| Influenza A virus (A/Waikato/17/2005(H1N1)) segment 5, complete sequence
ACTCACTGAGTGACATCGAAGTCATGGCGTCCCAAGGCACCAAACGGTCTTACGAACAGATGGAGACTGA
TGGGGAACGCCAGAATGCAACTGAAATCAGAGCATCCGTCGGAAGAATGATTGGTGGAATTGGGCGATTC
TACATCCAAATGTGCACCGAGCTTAAGCTCAATGATTATGAGGGACGACTGATCCAGAACAGCTTAACAA
TAGAGAGAATGGTGCTCTCTGCTTTTGATGAGAGGAGAAATAAATATCTGGAAGAACATCCCAGCGCGGG
GAAAGATCCTAAGAAAACTGGAGGACCCATATACAAGAGAGTAGATGGAAAGTGGGTGAGGGAACTCGTC
CTTTATGACAAAGAAGAAATAAGGCGGATTTGGCGCCAAGCCAACAATGGTGATGATGCAACGGCTGGTT
TGACTCACATTATGATCTGGCATTCTAATTTGAATGATACAACTTACCAGAGGACAAGAGCTCTTGTCCG
CACCGGAATGGATCCCAGGATGTGCTCTTTAATGCAAGGTTCAACTCTCCCTAGAAGATCTGGAGCAGCA
GGCGCTGCAGTCAAAGGAGTTGGGACAATGGTATTGGAGTTAATCAGGATGATCAAACGTGGGATCAATG
ACCGAAACTTCTGGAGGGGTGAGAATGGAAGAAAAACAAGGATTGCTTATGAGAGAATGTGCAACATTCT
CAAAGGAAAATTTCAAACAGCTGCACAAAAAGCAATGATGGATCAAGTGAGAGAAAGCCGGAACCCAGGA
AATGCTGAGATCGAAGATCTCACTTTTCTGGCGCGGTCTGCACTCATATTGAGAGGATCAGTTGCTCACA
AGTCTTGCCTGCCTGCCTGTGTGTATGGACCAGCCGTAGCCAGTGGGTATGACTTCGAAAAAGAGGGATA
CTCTTTGGTAGGAGTAGACCCTTTCAAACTGCTTCAAACCAGTCAGGTATACAGCCTAATTAGACCTAAC
GAGAATCCCGCACACAAGAGCCAGTTGGTGTGGATGGCATGCAATTCTGCTGCATTTGAAGATCTAAGAG
TGTCAAGCTTCATCAGAGGGACAAGAGTACTTCCAAGGGGGAAGCTCTCCACTAGAGGAGTACAAATTGC
TTCAAATGAAAACATGGATGCTATTGTATCAAGTACTCTTGAACTGAGAAGCAGATACTGGGCCATAAGA
ACCAGAAGTGGAGGGAACACTAATCAACAAAGGGCCTCTGCGGGCCAAATCAGCACACAACCTACGTTTT
CTGTGCAGAGAAACCTCCCATTTGACAAAACAACCATCATGGCAGCATTCACTGGGAATACAGAGGGAAG
AACATCAGACATGAGGGCAGAAATCATAAAGATGATGGAAAGTGCAAGACCAGAAGAAGTGTCCTTCCAG
GGGCGGGGAGTCTTTGAGCTCTCGGACGAAAGGGCAACGAACCCGATCGTGCCCTCCTTTGACATGAGTA
ATGAAGGATCTTATTTCTTCGGAGACAATGCAGAGGAGTACGACAATTAATGAAAAAT
>gi|83744841|gb|CY007470.1| Influenza A virus (A/Canterbury/106/2004(H1N1)) segment 5, complete sequence
GGTAGATAATCACTCACTGAGTGACATCAAAGTCATGGCGTCCCAAGGCACCAAACGGTCTTATGAACAG
ATGGAGACTGATGGGGAACGCCAGAATGCAACTGAAATCAGAGCATCCGTCGGAAGAATGATTGGTGGAA
TTGGGCGATTCTACATCCAAATGTGCACCGAGCTTAAGCTCAATGATTATGAGGGACGGCTGATCCAGAA
CAGCTTAACAATAGAGAGAATGGTGCTCTCTGCTTTTGATGAGAGGAGAAATAAATATCTGGAAGAACAT
CCCAGCGCGGGGAAAGATCCTAAGAAAACTGGAGGACCCATATACAAGAGAGTAGATGGAAAGTGGGTGA
GGGAACTCGTCCTTTATGACAAAGAAGAAATAAGGCGGATTTGGCGCCAAGCCAACAATGGTGATGATGC
AACGGCTGGCTTGACTCACATTATGATCTGGCATTCTAATTTGAATGATACAACTTACCAGAGGACAAGA
GCTCTTGTTCGCACCGGAATGGATCCCAGGATGTGCTCTTTGATGCAAGGTTCAACTCTCCCTAGAAGAT
CTGGAGCAGCAGGAGCTGCAGTCAAAGGAGTTGGGACAATGGTATTGGAGTTAATCAGGATGATCAAACG
TGGGATCAATGACCGAAACTTCTGGAGGGGTGAGAATGGAAGAAAAACAAGGATTGCTTATGAGAGAATG
TGCAACATTCTCAAAGGAAAATTTCAAACAGCTGCACAAAAAGCAATGATGGATCAAGTGAGAGAAAGCC
GGAACCCAGGAAATGCTGAGATCGAAGATCTCACTTTTCTGGCACGGTCTGCACTCATATTGAGAGGGTC
AGTTGCTCACAAGTCTTGCCTGCCTGCCTGTGTGTACGGACCAGCCGTAGCCAGTGGGTACGACTTCGAA
AAAGAGGGATACTCTTTGGTAGGAGTAGACCCTTTCAAACTACTCCAAACCAGTCAGGTATACAGCCTAA
TCAGACCAAACGAGAATCCCGCACACAAGAGCCAGTTGGTGTGGATGGCTTGCAATTCTGCTGCATTTGA
AGATCTAAGAGTGTCAAGCTTCATCAGAGGGACAAGAGTACTTCCAAGGGGGAAGCTCTCCACTAGAGGA
GTACAAATTGCTTCAAATGAAAACATGGATGCTATTGTATCAAGTACTCTTGAACTGAGAAGCAGATACT

GGGCCATAAGAACCAGAAGTGGAGGGAACACTAATCAACAAAGGGCCTCTGCGGGCCAAATCAGCACACA
ACCTACGTTTTCTGTGCAGAGAAACCTCCCATTTGACAAAACAACCATCATGGCAGCATTCACTGGGAAT
ACAGAGGGAAGAACATCAGACATGAGGGCAGAAATCATAAAGATGATGGAAAGTGCAAGACCAGAAGAA
G
TGTCCTTCCAGGGGCGGGGAGTCTTTGAGCTCTCGGACGAAAGGGCAACGAACCCGATCGTGCCCTCCTT
TGACATGAGTAATGAAGGATCTTATTTCTTCGGAGACAATGCAGAGGAGTACGACAATTAATGAAAAAT
>gi|115607828|gb|CY016702.1| Influenza A virus (A/South Australia/58/2005(H1N1)) segment 5, complete sequence
ATCGAAGTCATGGCGTCCCAAGGCACCAAACGGTCTTACGAACAAATGGAGACTGATGGGGAACGCCAGA
ATGCAACTGAAATCAGAGCATCCGTCGGAAGAATGATTGGTGGAATTGGGCGATTCTACATCCAAATGTG
CACCGAGCTTAAGCTCAATGATTATGAGGGACGACTGATCCAGAACAGCTTAACAATAGAGAGAATGGTG
CTCTCTGCTTTTGATGAGAGGAGAAATAAATATCTGGAAGAACATCCCAGCGCGGGGAAAGATCCTAAGA
AAACTGGAGGACCCATATACAAGAGAGTAGATGGAAAGTGGGTGAGGGAACTCGTCCTTTATGACAAAGA
AGAAATAAGGCGGATTTGGCGCCAAGCCAACAATGGTGATGATGCAACGGCTGGTTTGACTCACATTATG
ATATGGCATTCTAATTTGAATGATACAACTTACCAGAGGACAAGAGCTCTTGTCCGCACCGGAATGGATC
CCAGGATGTGCTCTTTAATGCAAGGTTCAACTCTCCCTAGAAGATCTGGAGCAGCAGGCGCTGCAGTCAA
AGGAGTTGGGACAATGGTATTGGAGTTAATCAGGATGATTAAACGTGGGATCAATGACCGAAACTTCTGG
AGGGGTGAGAATGGAAGAAAAACAAGGATTGCTTATGAGAGAATGTGCAACATTCTCAAAGGAAAATTTC
AAACAGCTGCACAAAAAGCAATGATGGATCAAGTGAGAGAAAGCCGGAACCCAGGAAATGCTGAGATCGA
AGATCTCACTTTTCTGGCACGGTCTGCACTCATATTGAGAGGATCAGTTGCTCACAAGTCTTGCCTGCCT
GCCTGTGTGTATGGACCAGCCGTAGCCAGTGGGTATGACTTCGAAAAAGAGGGATACTCTTTGGTAGGAG
TAGACCCTTTCAAACTGCTTCAAACCAGTCAGGTATACAGCCTAATTAGACCTAACGAGAATCCCGCACA
CAAGAGCCAGTTGGTGTGGATGGCATGCAATTCTGCTGCATTTGAAGATCTAAGAGTGTCAAGCTTCATC
AGAGGGACAAGAGTACTTCCAAGGGGGAAGCTCTCCACTAGAGGAGTACAAATTGCTTCAAATGAAAACA
TGGATGCTATTGTATCAAGTACTCTTGAACTGAGAAGCAGATACTGGGCCATAAGAACCAGAAGTGGAGG
GAACACTAATCAACAAAGGGCCTCTGCGGGCCAAATCAGCACACAACCTACGTTTTCTGTGCAGAGAAAC
CTCCCATTTGACAAAACAACCATCATGGCAGCATTCACTGGGAATACAGAGGGAAGAACATCAGACATGA
GGGCAGAAATCATAAAGATGATGGAAAGTGCAAGACCAGAAGAAGTGTCCTTCCAGGGGCGGGGAGTCTT
TGAGCTCTCGGACGAAAGGGCAACGAACCCGATCGTGCCCTCCTTTGACATGAGTAATGAAGGATCTTAT
TTCTTCGGAGACAATGCAGAGGAGTACGACAATTAATGAAAAAT
>gi|113170888|gb|CY014010.1| Influenza A virus (A/Wellington/11/2005(H1N1)) segment 5, complete sequence
ATCAAAGTCATGGCGTCCCAAGGCACCAAACGGTCTTACGAACAGATGGAGACTGATGGGGAACGCCAGA
ATGCAACTGAAATCAGAGCATCCGTCGGAAGAATGATTGGTGGAATTGGGCGATTCTACATCCAAATGTG
CACCGAGCTTAAGCTCAATGATTATGAGGGACGGCTGATCCAGAACAGCTTAACAATAGAGAGAATGGTG
CTCTCTGCTTTTGATGAGAGGAGAAATAAATATCTGGAAGAACATCCCAGCGCGGGGAAAGATCCTAAGA
AAACTGGAGGACCCATATACAAGAGAGTAGATGGAAAGTGGGTGAGGGAACTCGTCCTTTATGACAAAGA
AGAAATAAGGCGGATTTGGCGCCAAGCCAACAATGGTGATGATGCAACGGCTGGTTTGACTCACATTATG
ATCTGGCATTCTAATTTGAATGATACAACTTACCAGAGGACAAGAGCTCTTGTCCGCACCGGAATGGATC
CCAGGATGTGCTCTTTGATGCAAGGTTCAACTCTCCCTAGAAGATCTGGAGCAGCAGGCGCTGCAGTCAA
AGGAGTTGGGACAATGGTATTGGAGTTAATCAGGATGATCAAACGTGGGATCAATGACCGAAACTTCTGG
AGGGGTGAGAATGGAAGAAAAACAAGGATTGCCTATGAGAGAATGTGCAACATTCTCAAAGGAAAATTTC
AAACAGCTGCACAAAAAGCAATGATGGATCAAGTGAGAGAAAGCCGGAACCCAGGAAATGCTGAGATCGA
AGATCTCACTTTTCTGGCACGGTCTGCACTCATATTGAGAGGATCAGTTGCTCACAAGTCTTGCCTGCCT

GCCTGTGTGTATGGACCAGCCGTAGCCAGTGGGTATGACTTCGAAAAAGAGGGTTACTCTTTGGTAGGAG
TAGACCCTTTCAAACTGCTTCAAACCAGTCAGGTATACAGTCTAATTAGACCAAACGAGAATCCTGCACA
CAAGAGCCAGTTGGTGTGGATGGCATGCAATTCTGCTGCATTTGAAGATCTAAGAGTGTCAAGCTTCATC
AGAGGGACAAGAGTACTTCCAAGGGGGAAGCTCTCCACTAGAGGAGTACAAATTGCTTCAAATGAAAACA
TGGATGCTATTGTATCAAGTACTCTTGAACTGAGAAGCAGATACTGGGCCATAAGAACCAGAAGTGGAGG
GAACACTAATCAACAAAGGGCATCTGCGGGCCAAATCAGCACACAACCTACGTTTTCTGTGCAGAGAAAC
CTCCCATTTGACAAAGCAACCATCATGGCAGCATTCACTGGGAATACAGAGGGAAGAACATCAGACATGA
GGGCAGAAATCATAAAGATGATGGAAAGTGCAAGACCAGAAGAAGTGTCCTTCCAGGGGCGGGGAGTCTT
TGAGCTCTCGGACGAAAGGGCAACGAACCCGATCGTGCCCTCCTTTGACATGAGTAATGAAGGATCTTAT
TTCTTCGGAGACAATGCAGAGGAGTACGACAATTAATGAAAAAT
>gi|145278930|gb|CY021760.1| Influenza A virus (A/South Australia/51/2005(H1N1)) segment 5, complete sequence
ACTCACTGAGTGACATCAAAGTCATGGCGTCCCAAGGCACCAAACGGTCTTACGAACAGATGGAGACTGA
TGGGGAACGCCAGAATGCAACTGAAATCAGAGCATCCGTCGGAAGAATGATTGGTGGAATTGGGCGATTC
TACATCCAAATGTGCACCGAGCTTAAGCTCAATGATTATGAGGGACGGCTGATCCAGAACAGCTTAACAA
TAGAGAGAATGGTGCTCTCTGCTTTTGATGAGAGGAGAAATAAATATCTGGAAGAACATCCCAGCGCGGG
GAAAGATCCTAAGAAAACTGGAGGACCCATATACAAGAGAGTAGATGGAAAGTGGGTAAGGGAACTCGTC
CTTTATGACAAAGAAGAAATAAGGCGGATTTGGCGCCAAGCCAACAATGGTGATGATGCAACGGCTGGTT
TGACTCACATTATGATCTGGCATTCTAATTTGAATGATACAACTTACCAGAGGACAAGAGCTCTTGTCCG
CACCGGAATGGATCCCAGGATGTGCTCTTTGATGCAAGGTTCAACTCTCCCTAGAAGATCTGGAGCAGCA
GGCGCTGCAGTCAAAGGAGTTGGGACAATGGTATTGGAGTTAATCAGGATGATCAAACGTGGGATCAATG
ACCGAAACTTCTGGAGGGGTGAGAATGGAAGAAAAACAAGGATTGCTTATGAGAGAATGTGCAACATTCT
CAAAGGAAAATTTCAAACAGCTGCACAAAAAGCAATGATGGATCAAGTGAGAGAAAGCCGGAACCCAGGA
AATGCTGAGATCGAAGATCTCACTTTTCTGGCACGGTCTGCACTCATATTGAGAGGATCAGTTGCTCACA
AGTCTTGCCTGCCTGCCTGTGTGTATGGACCAGCCGTAGCCAGTGGGTATGACTTCGAAAAAGAGGGTTA
CTCTTTGGTAGGAGTAGACCCTTTCAAACTGCTTCAAACCAGTCAGGTATACAGTCTAATTAGACCAAAC
GAGAATCCCGCACACAAGAGCCAGTTGGTGTGGATGGCATGCAATTCTGCTGCATTTGAAGATCTAAGAG
TGTCAAGCTTCATCAGAGGGACAAGAGTACTTCCAAGGGGGAAGCTCTCCACTAGAGGAGTACAAATTGC
TTCAAATGAAAACATGGATGCTATTGTATCAAGTACTCTTGAACTGAGAAGCAGATACTGGGCCATAAGA
ACCAGAAGTGGAGGGAACACTAATCAACAAAGGGCCTCTGCGGGCCAAATCAGCACACAACCTACGTTTT
CTGTGCAGAGAAACCTCCCATTTGACAAAGCAACCATCATGGCAGCATTCACTGGGAATACAGAGGGAAG
AACATCAGACATGAGGGCAGAAATCATAAAGATGATGGAAAGTGCAAGACCAGAAGAAGTGTCCTTCCAG
GGGCGGGGAGTCTTTGAGCTCTCGGACGAAAGGGCAACGAACCCGATCGTGCCCTCCTTTGACATGAGTA
ATGAAGGATCTTATTTCTTCGGAGACAATGCAGAGGAGTACGACAATTAA
>gi|149780700|gb|CY022584.1| Influenza A virus (A/Auckland/619/2005(H1N1)) segment 5, complete sequence
ATCACTCACTGAGTGACATCAAAGTCATGGCGTCCCAAGGCACCAAACGGTCTTACGAACAGATGGAGAC
TGATGGGGAACGCCAGAATGCAACTGAAATCAGAGCATCCGTCGGAAGAATGATTGGTGGAATTGGGCGA
TTCTACATCCAAATGTGCACCGAGCTTAAGCTCAATGATTATGAGGGACGGCTGATCCAGAACAGCTTAA
CAATAGAGAGAATGGTGCTCTCTGCTTTTGATGAGAGGAGAAATAAATATCTGGAAGAACATCCCAGCGC
GGGGAAAGATCCTAAGAAAACTGGAGGACCCATATACAAGAGAGTAGATGGAAAGTGGGTGAGGGAACT
C
GTCCTTTATGACAAAGAAGAAATAAGGCGGATTTGGCGCCAAGCCAACAATGGTGATGATGCAACGGCTG
GTTTGACTCACATTATGATCTGGCATTCTAATTTGAATGATACAACTTACCAGAGGACAAGAGCTCTTGT

CCGCACCGGAATGGATCCCAGGATGTGCTCTTTGATGCAAGGTTCAACTCTCCCTAGAAGATCTGGAGCA
GCAGGCGCTGCAGTCAAAGGAGTTGGGACAATGGTATTGGAGTTAATCAGGATGATCAAACGTGGGATCA
ATGACCGAAACTTCTGGAGGGGTGAGAATGGAAGAAAAACAAGGATTGCTTATGAGAGAATGTGCAACAT
TCTCAAAGGAAAATTTCAAACAGCTGCACAAAAAGCAATGATGGATCAAGTGAGAGAAAGCCGGAACCCA
GGAAATGCTGAGATCGAAGATCTCACTTTTCTGGCACGGTCTGCACTCATATTGAGAGGATCAGTTGCTC
ACAAGTCTTGCCTGCCTGCCTGTGTGTATGGACCAGCCGTAGCCAGTGGGTATGACTTCGAAAAAGAGGG
TTACTCTTTGGTAGGAGTAGACCCTTTCAAACTGCTTCAAACCAGTCAGGTATACAGTCTAATTAGACCA
AACGAGAATCCCGCACACAAGAGCCAGTTGGTGTGGATGGCATGCAATTCTGCTGCATTTGAAGATCTAA
GAGTGTCAAGCTTCATCAGAGGGACAAGAGTACTTCCAAGGGGGAAGCTCTCCACTAGAGGAGTACAAAT
TGCTTCAAATGAAAACATGGATGCTATTGTATCAAGTACTCTTGAACTGAGAAGCAGATACTGGGCCATA
AGAACCAGAAGTGGAGGGAACACTAATCAACAAAGGGCCTCTGCGGGCCAAATCAGCACACAACCTACGT
TTTCTGTGCAGAGAAACCTCCCATTTGACAAAGCAACCATCATGGCAGCATTCACTGGGAATACAGAGGG
AAGAACATCAGACATGAGGGCAGAAATCATAAAGATGATGGAAAGTGCAAGACCAGAAGAAGTGTCCTTC
CAGGGGCGGGGAGTCTTTGAGCTCTCGGACGAAAGGGCAACGAACCCGATCGTGCCCTCCTTTGACATGA
GTAATGAAGGATCTTATTTCTTCGGAGACAATGCAGAGGAGTACGACAATTAATGAAAAA
>gi|117572945|gb|CY017318.1| Influenza A virus (A/Waikato/4/2005(H1N1)) segment 5, complete sequence
ATCGAAGTCATGGCGTCCCAAGGCACCAAACGGTCTTACGAACAGATGGAGACTGATGGGGAACGCCAGA
ATGCAACTGAAATCAGAGCATCCGTCGGAAGAATGATTGGTGGAATTGGGCGATTCTACATCCAAATGTG
CACCGAGCTTAAGCTCAATGATTATGAGGGACGACTGATCCAGAACAGCTTAACAATAGAGAGAATGGTG
CTCTCTGCTTTTGATGAGAGGAGAAATAAATATCTGGAAGAACATCCCAGCGCGGGGAAAGATCCTAAGA
AAACTGGAGGACCCATATACAAGAGAGTAGATGGAAAGTGGGTGAGGGAACTCGTCCTTTATGACAAAGA
AGAAATAAGGCGGATTTGGCGCCAAGCCAACAATGGTGATGATGCAACGGCTGGTTTGACTCACATTATG
ATCTGGCATTCTAATTTGAATGATACAACTTACCAGAGGACAAGAGCTCTTGTCCGCACCGGAATGGATC
CCAGGATGTGCTCTTTAATGCAAGGTTCAACTCTCCCTAGAAGATCTGGAGCAGCAGGCGCTGCAGTCAA
AGGAGTTGGGACAATGGTATTGGAGTTAATCAGGATGATCAAACGTGGGATCAATGACCGAAACTTCTGG
AGGGGTGAGAATGGAAGAAAAACAAGGATTGCTTATGAGAGAATGTGCAACATTCTCAAAGGAAAATTTC
AAACAGCTGCACAAAAAGCAATGATGGATCAAGTGAGAGAAAGCCGGAACCCAGGAAATGCTGAGATCGA
AGATCTCACTTTTCTGGCGCGGTCTGCACTCATATTGAGAGGATCAGTTGCTCACAAGTCTTGCCTGCCT
GCCTGTGTGTATGGACCAGCCGTAGCCAGTGGGTATGACTTCGAAAAAGAGGGATACTCTTTGGTAGGAG
TAGACCCTTTCAAACTGCTTCAAACCAGTCAGGTATACAGCCTAATTAGACCTAACGAGAATCCCGCACA
CAAGAGCCAGTTGGTGTGGATGGCATGCAATTCTGCTGCATTTGAAGATCTAAGAGTGTCAAGCTTCATC
AGAGGGACAAGAGTACTTCCAAGGGGGAAGCTCTCCACTAGAGGAGTACAAATTGCTTCAAATGAAAACA
TGGATGCTATTGTATCAAGTACTCTTGAACTGAGAAGCAGATACTGGGCCATAAGAACCAGAAGTGGAGG
GAACACTAATCAACAAAGGGCCTCTGCGGGCCAAATCAGCACACAACCTACGTTTTCTGTGCAGAGAAAC
CTCCCATTTGACAAAACAACCATCATGGCAGCATTCACTGGGAATACAGAGGGAAGAACATCAGACATGA
GGGCAGAAATCATAAAGATGATGGAAAGTGCAAGACCAGAAGAAGTGTCCTTCCAGGGGCGGGGAGTCTT
TGAGCTCTCGGACGAAAGGGCAACGAACCCGATCGTGCCCTCCTTTGACATGAGTAATGAAGGATCTTAT
TTCTTCGGAGACAATGCAGAGGAGTACGACAATTAATGA
>gi|161139451|gb|CY028198.1| Influenza A virus (A/Kentucky/UR06-0007/2006(H1N1)) segment 5, complete sequence
ATAATCACTCACTGAGTGACATCAAAGTCATGGCGTCCCAAGGCACCAAACGGTCTTACGAACAAATGGA
GACTGATGGGGAACGCCAGAATGCAACTGAAATCAGAGCATCCGTCGGAAGAATGATTGGTGGAATTGGG
CGATTCTACATCCAAATGTGCACCGAGCTTAAGCTCAATGATTATGAGGGACGGCTGATCCAGAACAGCT

TAACAATAGAGAGAATGGTGCTCTCTGCTTTTGATGAGAGGAGAAATAAATATCTGGAAGAACATCCCAG
CGCGGGGAAAGATCCTAAGAAAACTGGAGGACCCATATACAAGAGAGTAGATGGAAAGTGGGTGAGGGA
A
CTCGTCCTTTATGACAAAGAAGAAATAAGGCGGATTTGGCGCCAAGCCAACAATGGTGATGATGCAACGG
CTGGTTTGACTCACATTATGATCTGGCATTCTAATTTGAATGATACAACTTACCAGAGGACAAGAGCTCT
TGTCCGCACCGGAATGGATCCCAGGATGTGCTCTTTGATGCAAGGTTCAACTCTCCCCAGAAGATCTGGA
GCAGCAGGCGCTGCAGTCAAAGGAGTTGGGACAATGGTATTGGAGTTAATCAGGATGATCAAACGTGGGA
TCAATGACCGAAACTTCTGGAGGGGTGAGAATGGAAGAAAAACAAGGATTGCTTATGAGAGAATGTGCAA
CATTCTCAAAGGAAAATTTCAAACAGCTGCACAAAAAGCAATGATGGATCAAGTGAGAGAAAGCCGGAAC
CCAGGAAATGCTGAGATCGAAGATCTCACTTTTCTGGCACGGTCTGCACTCATATTGAGAGGATCAGTTG
CTCACAAGTCTTGCCTGCCTGCCTGTGTGTATGGACCAGCCGTAGCCAGTGGGTATGACTTCGAAAAAGA
GGGATACTCTTTGGTAGGAGTAGACCCTTTCAAACTGCTTCAAACCAGTCAGGTATACAGTCTAATTAGA
CCAAACGAGAATCCCGCACACAAGAGCCAGTTAGTGTGGATGGCATGCAATTCTGCTGCATTTGAAGATC
TAAGAGTGTCAAGCTTCATCAGAGGGACAAGAGTACTTCCAAGGGGGAAACTCTCCACTAGAGGAGTACA
AATTGCTTCAAATGAAAACATGGATGCTATTGTATCAAGTACTCTTGAACTGAGAAGCAGATACTGGGCC
ATAAGAACCAGAAGTGGAGGGAACACTAATCAACAAAGGGCCTCTGCGGGCCAAATCAGCACACAGCCTA
CGTTTTCTGTGCAGAGAAACCTCCCATTTGACAAAGCAACCATCATGGCAGCATTCACTGGGAATACAGA
GGGAAGAACATCAGACATGAGGGCAGAAATCATAAAGATGATGGAAAGTGCAAGACCAGAAGAAGTGTCC
TTCCAGGGGCGGGGAGTCTTTGAGCTCTCGGACGAAAGGGCAACGAACCCGATCGTGCCCTCCTTTGACA
TGAGTAATGAAGGATCTTATTTCTTCGGAGACAATGCAGAGGAGTACGACAATTAATGAA
>gi|157281283|gb|CY025224.1| Influenza A virus (A/Michigan/UR06-0015/2006(H1N1)) segment 5, complete sequence
ACTCACTGAGTGACATCAAAGTCATGGCGTCCCAAGGCACCAAACGGTCCTACGAACAGATGGAGACTGA
TGGGGAACGCCAGAATGCAACTGAAATCAGAGCATCTGTCGGAAGAATGATTGGTGGAATTGGGCGATTC
TACATCCAAATGTGCACCGAGCTTAAGCTCAATGATTATGAGGGACGGCTGATCCAGAACAGCTTAACAA
TAGAGAGAATGGTGCTCTCTGCTTTTGATGAGAGGAGAAATAAATATCTGGAAGAACATCCCAGCGCGGG
GAAAGATCCTAAGAAAACTGGAGGACCCATATACAAAAGAGTAGATGGAAAGTGGGTGAGGGAACTCGTC
CTTTATGACAAAGAAGAAATAAGGCGGATTTGGCGCCAAGCCAACAATGGTGATGATGCAACGGCTGGTT
TGACTCACATTATGATCTGGCATTCTAATTTGAATGATACAACTTACCAGAGGACAAGAGCTCTTGTCCG
CACCGGAATGGATCCCAGGATGTGCTCTTTGATGCAAGGTTCAACCCTCCCTAGAAGATCTGGAGCAGCA
GGCGCTGCAGTCAAAGGAGTTGGGACAATGGTATTGGAGTTAATCAGGATGATCAAACGTGGGATCAACG
ACCGAAACTTCTGGAGGGGTGAGAATGGAAGAAAAACAAGGATTGCTTATGAGAGAATGTGCAACATTCT
CAAAGGAAAATTTCAAACAGCTGCACAAAAAGCAATGATGGATCAAGTGAGAGAAAGCCGGAACCCAGGA
AATGCTGAGATCGAAGATCTCACTTTTCTGGCACGGTCTGCACTCATATTGAGAGGATCAGTTGCTCACA
AGTCTTGCCTGCCTGCCTGTGTGTATGGACCAGCCGTAGCCAGTGGGTATGACTTCGAAAAAGAGGGTTA
CTCTTTGGTAGGAGTAGACCCTTTCAAACTGCTTCAAACCAGTCAGGTATACAGTCTAATTAGACCAAAC
GAGAATCCCGCACACAAGAGCCAGTTGGTGTGGATGGCATGCAATTCTGCTGCATTTGAAGATCTAAGAG
TGTCAAGCTTCATCAGAGGGACAAGAGTACTTCCAAGGGGGAAGCTCTCCACTAGAGGAGTACAAATTGC
TTCAAATGAAAACATGGATGCTATTGTATCAAGTACTCTTGAACTGAGAAGCAGATACTGGGCCATAAGA
ACCAGAAGTGGAGGGAACACTAATCAACAAAGGGCCTCTGCGGGCCAAATCAGCACACAACCTACGTTTT
CTGTGCAGAGAAACCTCCCATTTGACAAAGCAACCATCATGGCAGCATTCACTGGGAATACAGAGGGAAG
AACATCAGACATGAGGGCAGAAATCATAAAGATGATGGAAAGTGCAAGACCAGAGGAAGTGTCCTTCCAG
GGGCGGGGAGTCTTTGAGCTCTCGGACGAAAGGGCAACGAACCCGATCGTGCCCTCCTTTGACATGAGTA
ACGAAGGATCTTATTTCTTCGGAGACAATGCAGAGGAGTACGACAATTAATGA

>gi|218875175|gb|CY036922.1| Influenza A virus (A/NYMC X-163A(NYMC X-157-St. Petersburg/8/2006)(H1N1)) segment 5, complete sequence
CTCACTGAGTGACATCAAAATCATGGCGTCTCAAGGCACCAAACGATCTTACGAACAGATGGAGACTGAT
GGAGAACGCCAGAATGCCACTGAAATCAGAGCATCCGTCGGAAAAATGATTGGTGGAATTGGACGATTCT
ACATCCAAATGTGCACCGAACTCAAACTCAGTGATTATGAGGGACGGTTGATCCAAAACAGCTTAACAAT
AGAGAGAATGGTGCTCTCTGCTTTTGACGAAAGGAGAAATAAATACCTTGAAGAACATCCCAGTGCGGGG
AAAGATCCTAAGAAAACTGGAGGACCTATATACAGGAGAGTAAACGGAAAGTGGATGAGAGAACTCATCC
TTTATGACAAAGAAGAAATGAGGCGAATCTGGCGCCAAGCTAATAATGGTGACGATGCAACGGCTGGTCT
GACTCACATGATGATCTGGCATTCCAATTTGAATGATGCAACTTATCAGAGGACAAGAGCTCTTGTTCGC
ACCGGAATGGATCCCAGGATGTGCTCTCTGATGCAAGGTTCAACTCTCCCTAGGAGGTCTGGAGCCGCAG
GTGCTGCAGTCAAAGGAGTTGGAACAATGGTGATGGAATTGGTCAGAATGATCAAACGTGGGATCAATGA
TCGGAACTTCTGGAGGGGTGAGAATGGACGAAAAACAAGAATTGCTTATGAAAGAATGTGCAACATTCTC
AAAGGGAAATTTCAAACTGCTGCACAAAAAGCAATGATGGATCAAGTGAGAGAGAGCCGGAACCCAGGG
A
ATGCTGAGTTCGAAGATCTCACTTTTCTAGCACGGTCTGCACTCATATTGAGAGGGTCGGTTGCTCACAA
GTCCTGCCTGCCTGCCTGTGTGTATGGACCTGCCGTAGCCAGTGGGTACGACTTTGAAAGGGAGGGATAC
TCTCTAGTCGGAATAGACCCTTTCAGACTGCTTCAAAACAGCCAAGTGTACAGCCTAATCAGACCAAATG
AGAATCCAGCACACAAGAGTCAACTGGTGTGGATGGCATGCCATTCTGCCGCATTTGAAGATCTAAGAGT
ATTAAGCTTCATCAAAGGGACGAAGGTGCTCCCAAGAGGGAAGCTTTCCACTAGAGGAGTTCAAATTGCT
TCCAATGAAAATATGGAGACTATGGAATCAAGTACACTTGAACTGAGAAGCAGGTACTGGGCCATAAGGA
CCAGAAGTGGAGGAAACACCAATCAACAGAGGGCATCTGCGGGCCAAATCAGCATACAACCTACGTTCTC
AGTACAGAGAAATCTCCCTTTTGACAGAACAACCATTATGGCAGCATTCAATGGGAATACAGAGGGGAGA
ACATCTGACATGAGGACCGAAATCATAAGGATGATGGAAAGTGCAAGACCAGAAGATGTGTCTTTCCAGG
GGCGGGGAGTCTTCGAGCTCTCGGACGAAAAGGCAGCGAGCCCGATCGTGCCTTCCTTTGACATGAGTAA
TGAAGGATCTTATTTCTTCGGAGACAATGCAGAGGAGTACGACAATTAAAGAA
>gi|208344090|gb|CY035129.1| Influenza A virus (A/St. Petersburg/8/2006(H1N1)) segment 5, complete sequence
ACTCACTGAGTGACATCAAAGTCATGGCGTCCCAAGGCACCAAACGGTCTTATGAACAGATGGAGACTGA
TGGGGAACGCCAGAATGCAACTGAAATCAGAGCATCCGTCGGAAGAATGATTGGTGGAATTGGGCGATTC
TACATCCAAATGTGCACCGAGCTTAAGCTCAATGATTATGAGGGACGGCTGATCCAGAATAGCTTAACAA
TAGAGAGAATGGTGCTCTCTGCTTTTGATGAGAGGAGAAATAAATATCTGGAAGAACATCCCAGCACGGG
GAAAGATCCTAAGAAAACTGGAGGACCCATATACAAGAGAGTAGATAGAAAGTGGGTGAGGGAACTCGTC
CTTTATGACAAAGAAGAAATAAGGCGGATTTGGCGCCAAGCCAACAATGGTGATGATGCAACGGCTGGCT
TGACTCACATTATGATCTGGCATTCTAATTTGAATGATACAACTTACCAGAGGACAAGAGCTCTTGTTCG
CACCGGAATGGATCCCAGGATGTGCTCTTTGATGCAAGGTTCAACTCTCCCTAGAAGATCTGGAGCAGCA
GGAGCTGCAGTCAAAGGAGTTGGGACAATGGTATTGGAATTAATCAGGATGATTAAACGTGGGATCAATG
ACCGAAACTTCTGGAGGGGTGAGAATGGAAGAAAAACAAGGATTGCTTATGAGAGAATGTGCAACATTCT
CAAAGGAAAATTTCAAACAGCTGCACAAAAAGCAATGACGGATCAAGTGAGAGAAAGCCGAAACCCAGG
G
AATGCTGAGATCGAAGATCTCACTTTTCTGGCACGATCTGCACTCATATTGAGAGGGTCAGTTGCTCACA
AGTCTTGCCTGCCCGCCTGTGTGTACGGACCAGCCGTAGCCAGTGGGTACGACTTCGAAAAAGAGGGATA
CTCTTTGGTAGGAGTAGACCCTTTCAAACTACTCCAAACCAGTCAGGTATACAGCCTAATCAGACCAAAC
GAGAATCCCGCACACAAGAGCCAGTTGGTGTGGATGGCTTGCAATTCTGCTGCATTTGAAGATCTAAGAG
TGTCAAGCTTCATCAGAGGGACAAGAGTACTTCCAAGGGGGAAGCTCTCCACTAGAGGAGTACAAATTGC

TTCAAATGAAAACATGGATGCTATTGTATCAAGTACTCTTGAACTGAGAAGCAGATATTGGGCCATAAGA
ACCAGAAGTGGAGGGAACACTAATCAACAAAGGGCCTCTGCGGGCCAAATCAGCACACAACCTACGTTTT
CTGTGCAGAGAAACCTCCCATTTGACAAAACAACCATCATGGCAGCATTCACTGGGAATACAGAGGGAAG
AACATCAGACATGAGGGCAGAAATCATAAAGATGATGGAAAGTGCAAGACCAGAAGAAGTGTCCTTCCAG
GGGCGGGGAGTCTTTGAGCTCTCGGACGAAAGGGCAACGAACCCGATCGTGCCCTCCTTTGACATGAGTA
ATGAAGGATCTTATTTCTTCGGAGACAATGCAGAGGAGTACGACAATTAATGAA
>gi|226954753|gb|CY038882.1| Influenza A virus (A/Taiwan/2645/2006(H1N1)) segment 5, complete sequence
CTCACTGAGTGACATCAAAGTCATGGCGTCCCAAGGCACCAAACGGTCTTATGAACAGATGGAGACAGAT
GGGGAACGCCAGAATGCAACTGAAATCAGAGCATCCGTCGGAAGAATGATTGGTGGAATTGGGCGATTCT
ACATCCAAATGTGCACCGAGCTTAAGCTCAATGATTATGAGGGACGGCTGATCCAGAACAGCTTAACAAT
AGAGAGAATGGTGCTCTCTGCTTTTGATGAGAGGAGAAATAAATATCTGGAAGAACATCCCAGCGCGGGG
AAAGATCCTAAGAAAACTGGAGGACCCATATACAAGAGAGTAGATGGAAAGTGGGTGAGGGAACTCGTCC
TTTATGACAAAGAAGAAATAAGGCGGATTTGGCGCCAAGCCAACAATGGTGATGATGCAACGGCTGGCTT
GACTCACATTATGATCTGGCATTCTAATTTGAATGATACAACTTACCAGAGGACAAGAGCTCTTGTTCGC
ACCGGAATGGATCCCAGGATGTGCTCTTTGATGCAAGGTTCAACTCTCCCTAGAAGATCTGGAGCAGCAG
GAGCTGCAGTCAAAGGAGTTGGGACAATGGTATTGGAGTTAATCAGGATGATCAAACGTGGGATCAATGA
CCGAAACTTCTGGAGGGGTGAGAATGGAAGAAAAACAAGGATTGCTTATGAGAGAATGTGCAACATTCTC
AAAGGAAAATTTCAAACAGCTGCACAAAAAGCAATGATGGATCAAGTGAGAGAAAGCCGGAACCCAGGA
A
ATGCTGAGATCGAAGATCTCACTTTTCTGGCACGGTCTGCACTCATATTGAGAGGGTCAGTTGCTCACAA
GTCTTGCCTGCCTGCCTGTGTGTACGGACCAGCCGTAGCCAGTGGGTACGACTTCGAAAAAGAGGGATAC
TCTTTGGTAGGAGTAGACCCTTTCAAACTGCTCCAAACCAGTCAGGTATACAGCCTAATCAGACCAAACG
AGAATCCCGCACACAAGAGTCAGTTGGTGTGGATGGCTTGCAATTCTGCTGCATTTGAAGATCTAAGAGT
GTCAAGCTTCATCAGAGGGACAAGAGTACTTCCAAGGGGGAAGCTCTCCACTAGAGGAGTACAAATTGCT
TCAAATGAAAACATGGATGCTATTGTATCAAGTACTCTTGAACTGAGAAGCAGATACTGGGCCATAAGAA
CCAGAAGTGGAGGGAACACTAATCAACAAAGGGCCTCTGCGGGCCAAATCAGCACACAACCTACGTTTTC
TGTGCAGAGAAACCTCCCGTTTGACAAAACAACCATCATGGCAGCATTCACTGGGAATACAGAGGGAAGA
ACATCAGACATGAGGGCAGAAATCATAAAGATGATGGAAAGTGCAAGACCAGAAGACGTGTCCTTCCAGG
GGCGGGGAGTCTTTGAGCTCTCGGACGAAAGGGCAACGAACCCAATCGTGCCCTCCTTTGACATGAGTAA
TGAAGGATCTTATTTCTTCGGAGACAATGCAGAGGAGTACGACAGTTAATGAA
>gi|256385518|gb|CY044352.1| Influenza A virus (A/South Korea/AF10/2008(H1N1)) segment 5, complete sequence
ATGGCGTCCCAAGGCACCAAACGGTCTTATGAACAGATGGAGACTGATGGGGAACGCCAGAATGCAACTG
AAATCAGAGCATCCGTCGGAAGAATGATTGGTGGAATTGGGCGATTCTACATCCAAATGTGCACTGAGCT
TAAGCTCAATGATTATGAGGGACGGCTGATCCAGAACAGCTTAACAATAGAGAGAATGGTGCTCTCTGCT
TTTGATGAGAGGAGAAATAAATATCTGGAAGAACATCCCAGCGCGGGGAAAGATCCTAAGAAAACTGGAG
GACCCATATACAAGAGAGTAGATGGAAAGTGGGTGAGGGAACTCGTCCTTTATGACAAAGAAGAAATAAG
GCGGATTTGGCGCCAAGCCAACAATGGTGATGATGCAACGGCTGGCTTGACTCACATTATGATCTGGCAT
TCTAATTTGAATGATACAACTTACCAGAGGACAAGAGCTCTTGTTCGCACCGGAATGGATCCCAGGATGT
GCTCTTTGATGCAAGGTTCAACTCTCCCTAGAAGATCTGGAGCAGCAGGAGCTGCAGTCAAAGGAGTTGG
GACAATGGTATTGGAGTTAATCAGGATGATTAAACGTGGGATCAATGACCGAAACTTCTGGAGGGGTGAG
AATGGAAGAAAAACAAGGATTGCTTATGAGAGAATGTGCAACATTCTCAAAGGAAAATTTCAAACAGCTG
CACAAAAAGCAATGATGGATCAAGTGAGAGAAAGCCGAAACCCAGGAAATGCTGAGATCGAAGATCTCAC

CTTTCTGGCACGGTCTGCACTCATATTAAGAGGGTCAGTTGCTCACAAGTCTTGCCTGCCTGCCTGTGTG
TACGGACCGGCCGTAGCCAGTGGGTACGACTTCGAGAAAGAGGGATACTCTTTGGTAGGAGTAGACCCTT
TCAAATTACTCCAAACCAGTCAGGTATACAGCCTAATCAGACCAAACGAGAATCCCGCACACAAGAGCCA
GTTGGTGTGGATGGCTTGCAATTCTGCTGCATTTGAAGATCTAAGAGTGTCAAGCTTCATCAGAGGGACA
AGAGTACTTCCAAGAGGGAAGCTCTCCACTAGAGGAGTACAAATTGCTTCAAATGAAAACATGGATGCTA
TTGTATCAAGTACTCTTGAACTGAGAAGCAGATATTGGGCCATAAGAACCAGAAGTGGAGGGAACACTAA
CCAACAAAGGGCCTCTGCGGGCCAAATCAGCACACAACCTACGTTTTCTGTGCAGAGAAACCTCCCATTT
GACAAAACAACCATCATGGCAGCATTCACTGGGAATACAGAGGGAAGAACATCAGACATGAGGGCAGAAA
TCATAAAGATGATGGAAAGTGCAAGACCAGAAGAAGTGTCCTTCCAGGGGCGGGGAGTCTTTGAGCTCTC
GGACGAAAGGGCAACGAACCCAATCGTGCCCTCCTTTGACATGAGTAATGAAGGATCTTATTTCTTCGGA
GACAATGCAGAGGAGTACGACAATTAA
>gi|163964723|gb|CY028462.1| Influenza A virus (A/California/UR06-0442/2007(H1N1)) segment 5, complete sequence
TCACTCACTGAGTGACATCAAAGTCATGGCGTCCCAAGGCACCAAACGGTCCTACGAACAGATGGAGACT
GATGGGGAACGCCAGAATGCAACTGAAATCAGAGCATCTGTCGGAAGAATGATTGGTGGAATTGGGCGAT
TCTACATCCAAATGTGCACCGAGCTTAAGCTCAATGATTATGAGGGACGGCTGATCCAGAACAGCTTAAC
AATAGAGAGAATGGTGCTCTCTGCTTTTGATGAGAGGAGAAATAAATATCTGGAAGAACATCCCAGCGCG
GGGAAAGATCCTAAGAAAACTGGAGGACCCATATACAAAAGAGTAGATGGAAAGTGGGTGAGGGAACTCG
TCCTTTATGACAAAGAAGAAATAAGGCGGATTTGGCGCCAAGCCAACAATGGTGATGATGCAACAGCTGG
TTTGACTCACATTATGATCTGGCATTCTAATTTGAATGATACAACTTACCAGAGGACAAGAGCTCTTGTC
CGCACCGGAATGGATCCCAGGATGTGCTCTTTGATGCAAGGTTCAACCCTCCCTAGAAGATCTGGAGCAG
CAGGCGCTGCAGTCAAAGGAGTTGGGACAATGGTATTGGAGTTAATCAGGATGATCAAACGTGGGATCAA
CGACCGAAACTTCTGGAGGGGTGAGAATGGAAGAAAAACAAGGATTGCTTATGAGAGAATGTGCAACATT
CTCAAAGGAAAATTTCAAACAGCTGCACAAAAAGCAATGATGGATCAAGTGAGAGAAAGCCGGAACCCAG
GAAATGCTGAGATCGAAGATCTCACTTTTCTGGCACGGTCTGCACTCATATTGAGAGGATCAGTTGCTCA
CAAGTCTTGCCTGCCTGCCTGTGTGTATGGACCAGCCGTAGCCAGTGGGTATGACTTCGAAAAAGAGGGT
TACTCTTTGGTAGGAGTAGACCCTTTCAAACTGCTTCAAACCAGTCAGGTATACAGTCTAATTAGACCAA
ACGAGAATCCCGCACACAAGAGCCAGTTGGTGTGGATGGCATGCAATTCTGCTGCATTTGAAGATCTAAG
AGTGTCAAGCTTCATCAGAGGGACAAGAGTACTTCCAAGGGGGAAGCTCTCCACTAGAGGAGTACAAATT
GCTTCAAATGAAAACATGGATGCTATTGTATCAAGTACTCTTGAACTGAGAAGCAGATACTGGGCCATAA
GAACCAGAAGTGGAGGGAACACTAATCAACAAAGGGCCTCTGCGGGCCAAATCAGCACACAACCTACGTT
TTCTGTGCAGAGAAACCTCCCATTTGACAAAGCAACCATCATGGCAGCATTCACTGGGAATACAGAGGGA
AGAACATCAGACATGAGGGCAGAAATCATAAAGATGATGGAAAGTGCAAGACCAGAGGAAGTGTCCTTCC
AGGGGCGGGGAGTCTTTGAGCTCTCGGACGAAAGGGCAACGAACCCGATCGTGCCCTCCTTTGACATGAG
TAACGAAGGATCTTATTTCTTCGGAGACAATGCAGAGGAGTACGACAATTAATGAA
>gi|157281607|gb|CY025360.1| Influenza A virus (A/Kentucky/UR06-0363/2007(H1N1)) segment 5, complete sequence
ACTCACTGAGTGACATCAAAGTCATGGCGTCCCAAGGCACCAAACGGTCTTACGAACAGATGGAGACTGA
TGGGGAACGCCAGAATGCAACTGAAATCAGAGCATCTGTCGGAAGAATGATTGGTGGAATTGGGCGATTC
TACATCCAAATGTGCACCGAGCTTAAGCTCAATGATTATGAGGGACGGCTGATCCAGAACAGCTTAACAA
TAGAGAGAATGGTGCTCTCTGCTTTTGATGAGAGGAGAAATAAATATCTGGAAGAACATCCCAGCGCGGG
GAAAGATCCTAAGAAAACTGGAGGACCCATATACAAGAGAGTAGATGGAAAATGGGTGAGGGAACTCGTC
CTTTATGACAAAGAAGAAATAAGGCGGATTTGGCGCCAAGCCAACAATGGTGATGATGCAACGGCTGGTT
TGACTCACATTATGATCTGGCATTCTAATTTGAATGATACAACTTACCAGAGGACAAGAGCTCTTGTCCG

CACCGGAATGGATCCCAGGATGTGCTCTTTGATGCAAGGTTCAACTCTCCCTAGAAGATCTGGAGCAGCA
GGCGCTGCAGTCAAAGGAGTTGGGACAATGGTATTGGAGTTAATCAGGATGATCAAACGTGGGATCAATG
ACCGAAACTTCTGGAGGGGTGAGAATGGAAGAAAAACAAGGATTGCTTATGAGAGAATGTGCAACATTCT
CAAAGGAAAATTTCAAACAGCTGCACAAAAAGCAATGATGGATCAAGTGAGAGAAAGCCGGAACCCAGGA
AATGCTGAGATCGAAGATCTCACTTTTCTGGCACGGTCTGCACTCATATTGAGAGGATCAGTTGCTCACA
AGTCTTGCCTGCCTGCCTGTGTGTATGGACCAGCCGTAGCCAGTGGGTATGACTTCGAAAAAGAGGGTTA
CTCTTTGGTAGGAGTAGACCCTTTCAAACTGCTTCAAACCAGTCAGGTATACAGTCTAATTAGACCAAAC
GAGAATCCCGCACACAAGAGCCAGTTGGTGTGGATGGCATGCAATTCTGCTGCATTTGAAGATCTAAGAG
TGTCAAGCTTCATCAGAGGGACAAGAGTACTTCCAAGGGGGAAGCTCTCCACTAGAGGAGTACAAATTGC
TTCAAATGAAAACATGGATGCTATTGTATCAAGTACTCTTGAACTGAGAAGCAGATACTGGGCCATAAGA
ACCAGAAGTGGAGGGAACACTAATCAACAAAGGGCCTCTGCGGGCCAAATCAGCACACAACCTACGTTTT
CTGTGCAGAGAAACCTCCCATTTGACAAAGCAACCATCATGGCAGCATTCACTGGGAATACAGAGGGAAG
AACATCAGACATGAGGGCAGAAATCATAAAGATGATGGAAAGTGCAAGACCAGAGGAAGTATCCTTCCAG
GGGCGGGGAGTCTTTGAGCTCTCGGACGAAAGGGCAACGAACCCGATCGTGCCCTCCTTTGACATGAGTA
ATGAAGGATCTTATTTCTTCGGAGACAATGCAGAGGAGTACGACAATTAATGAAAA
>gi|158957796|gb|CY027414.1| Influenza A virus (A/Alabama/UR06-0536/2007(H1N1)) segment 5, complete sequence
CACTGAGTGACATCAAAGTCATGGCGTCCCAAGGCACCAAACGGTCTTACGAACAAATGGAGACTGATGG
AGAACGCCAGAATGCAACTGAAATCAGAGCATCCGTCGGAAGAATGATTGGTGGAATTGGGCGATTCTAC
ATCCAAATGTGCACCGAGCTTAAGCTCAATGATTATGAGGGACGGCTGATCCAGAACAGCTTAACAATAG
AGAGAATGGTGCTCTCTGCTTTTGATGAGAGGAGAAATAAATATCTGGAAGAACATCCCAGCGCGGGGAA
AGATCCTAAGAAAACTGGAGGACCCATATACAAGAGAGTAGATGGAAAGTGGGTGAGGGAACTCGTCCTT
TATGACAAAGAAGAAATAAGGCGGATTTGGCGCCAAGCCAACAATGGTGATGATGCAACGGCTGGTTTGA
CTCACATTATGATCTGGCATTCTAATTTGAATGATACAACTTACCAGAGGACAAGAGCTCTTGTCCGCAC
CGGAATGGATCCCAGGATGTGCTCTTTGATGCAAGGTTCAACTCTCCCCAGAAGATCTGGAGCAGCAGGC
GCTGCAGTCAAAGGAGTTGGGACAATGGTATTGGAGTTAATCAGGATGATCAAACGTGGGATCAATGACC
GAAACTTCTGGAGGGGTGAGAATGGAAGAAAAACAAGGATTGCTTATGAGAGAATGTGCAACATTCTCAA
AGGAAAATTTCAAACAGCTGCACAAAAAGCAATGATGGATCAAGTGAGAGAAAGCCGGAACCCAGGAAAT
GCTGAGATCGAAGATCTCACTTTTCTGGCACGGTCTGCACTCATATTGAGAGGATCAGTTGCTCACAAGT
CTTGCCTGCCTGCCTGTGTGTATGGACCAGCCGTAGCCAGTGGGTATGACTTCGAAAAAGAGGGATACTC
TTTGGTAGGAGTAGACCCTTTCAAACTGCTTCAAACCAGTCAGGTATACAGTCTAATTAGACCAAACGAG
AATCCCGCACACAAGAGCCAGTTAGTGTGGATGGCATGCAATTCTGCTGCATTTGAAGATCTAAGAGTGT
CAAGCTTCATCAGAGGGACAAGAGTACTTCCAAGGGGGAAACTCTCCACTAGAGGAGTACAAATTGCTTC
AAATGAAAACATGGATGCTATTGTATCAAGTACTCTTGAACTGAGAAGCAGATACTGGGCCATAAGAACC
AGAAGTGGAGGGAACACTAATCAACAAAGGGCCTCTGCGGGCCAAATCAGCACACAACCTACGTTTTCTG
TGCAGAGAAACCTCCCATTTGACAAAGCAACCATCATGGCAGCATTCACCGGGAATACAGAGGGAAGAAC
ATCAGACATGAGGGCAGAAATCATAAAGATGATGGAAAGTGCAAGACCAGAAGAAGTGTCCTTCCAGGGG
CGGGGAGTCTTTGAGCTCTCGGACGAAAGGGCAACGAACCCGATCGTGCCCTCCTTTGACATGAGTAATG
AAGGATCTTATTTCTTCGGAGACAATGCAGAGGAGTACGACAATTAATGA
>gi|237688831|gb|CY040061.1| Influenza A virus (A/Taiwan/71720/2007(H1N1)) segment 5, complete sequence
TGAGTGACATCAAAGTCATGGCGTCCCAAGGCACCAAACGGTCTTACGAACAGATGGAGACTGATGGGGA
ACGCCAGAATGCAACTGAAATCAGAGCATCCGTCGGAAGAATGATTGGTGGAATTGGGCGATTCTACATC
CAAATGTGCACCGAGCTTAAGCTCAATGATTATGAGGGACGGCTGATCCAGAACAGCTTAACAATAGAGA

GAATGGTGCTCTCTGCTTTTGATGAGAGGAGAAATAAATATCTAGAGGAACATCCCAGCGCGGGGAAAGA
TCCTAAGAAAACTGGAGGACCCATATACAAGAGAGTAGATGGAAAGTGGGTGAGGGAACTCGTCCTTTAT
GACAAAGAAGAAATAAGGCGGATTTGGCGCCAAGCCAACAATGGTGATGATGCAACGGCTGGTTTGACTC
ACATTATGATCTGGCATTCTAATTTGAATGATACAACTTACCAGAGGACAAGAGCTCTTGTCCGCACCGG
AATGGATCCCAGGATGTGCTCTTTGATGCAAGGTTCAACTCTCCCTAGAAGATCTGGAGCAGCAGGCGCC
GCAGTCAAAGGAGTTGGGACAATGGTATTGGAGTTAATCAGGATGATCAAACGTGGGATCAATGACCGAA
ACTTCTGGAGGGGTGAGAATGGAAGAAAAACAAGGATTGCTTATGAGAGAATGTGCAACATTCTCAAAGG
AAAATTTCAAACAGCTGCACAAAAAGCAATGATGGACCAAGTGAGAGAAAGCCGGAACCCAGGAAATGCT
GAGATCGAAGATCTCACTTTTCTGGCACGGTCTGCACTCATATTGAGAGGATCAGTTGCTCACAAATCTT
GCCTGCCTGCCTGTGTGTATGGACCAGCCGTAGCCAGTGGGTATGACTTCGAAAAAGAGGGTTACTCTTT
GGTAGGAGTAGACCCTTTCAAACTGCTTCAAACCAGTCAGGTATACAGTCTAATTAGACCAAACGAGAAT
CCCGCACACAAGAGCCAGTTGGTGTGGATGGCATGCAATTCTGCTGCATTTGAAGATCTAAGAGTGTCAA
GCTTCATCAGAGGAACAAGAGTACTTCCAAGGGGGAAGCTCTCCACTAGAGGAGTACAAATTGCTTCAAA
TGAAAACATGGATGCTATTGTGTCAAGTACTCTTGAACTGAGAAGCAGATACTGGGCCATAAGAACCAGA
AGTGGAGGGAACACTAATCAACAAAGGGCCTCTGCGGGCCAAATCAGCACACAACCTACGTTTTCTGTGC
AGAGAAACCTCCCATTTGACAAAGCAACCATCATGGCAGCATTCTCTGGGAATACAGAGGGAAGAACATC
AGACATGAGGGCAGAAATCATAAAGATGATGGAAAGTGCAAGACCAGAAGAAGTGTCCTTCCAGGGGCGG
GGAGTCTTTGAGCTCTCGGACGAAAGGGCAACGAACCCGATCGTGCCCTCCTTTGACATGAGTAATGAAG
GATCTTATTTCTTCGGAGACAATGCAGAGGAGTACGACAATTAATGAA
>gi|237689289|gb|CY040253.1| Influenza A virus (A/Managua/3153.01/2008(H1N1)) segment 5, complete sequence
ATAATCACTCACTGAGTGACATCAAAGTCATGGCGTCCCAAGGCACCAAACGGTCTTACGAACAGATGGA
GACTGATGGGGAACGCCAGAATGCAACTGAAATCAGAGCATCAGTCGGAAGAATGATTGGTGGAATTGGG
CGATTCTACATCCAAATGTGCACCGAGCTTAAGCTCAATGATTATGAGGGACGGCTGATCCAGAACAGCT
TAACAATAGAGAGAATGGTGCTCTCTGCTTTTGATGAGAGGAGAAATAAATATCTAGAGGAACATCCCAG
CGCGGGGAAAGATCCTAAGAAAACTGGAGGACCCATATACAAGAGAGTAGATGGAAAGTGGGTGAGGGA
A
CTCGTCCTTTATGACAAAGAAGAAATAAGGCGGATTTGGCGCCAAGCCAACAATGGTGATGATGCAACGG
CTGGTTTGACTCACATTATGATCTGGCATTCTAATTTGAATGATACAACTTACCAGAGGACAAGAGCTCT
TGTTCGCACCGGAATGGATCCCAGGATGTGCTCTTTGATGCAAGGTTCAACTCTCCCTAGAAGATCTGGA
GCAGCAGGCGCTGCAGTCAAAGGAGTTGGGACAATGGTATTGGAATTAATCAGGATGATCAAACGTGGGA
TCAATGACCGAAACTTTTGGAGGGGTGAGAATGGAAGAAAAACAAGGATTGCTTATGAGAGAATGTGCAA
CATTCTCAAAGGAAAATTTCAAACAGCTGCACAAAAAGCAATGATGGACCAAGTGAGAGAAAGCCGGAAC
CCAGGAAATGCTGAGATTGAAGATCTCACTTTTCTGGCACGGTCTGCACTCATATTGAGAGGATCAGTTG
CTCACAAGTCTTGCCTGCCTGCCTGTGTGTATGGACCAGCCGTAGCCAGTGGGTATGACTTCGAAAAAGA
GGGTTACTCTTTGGTAGGAGTAGACCCTTTCAAACTGCTTCAAACCAGTCAGGTATACAGTCTAATTAGA
CCAAACGAGAATCCCGCACACAAGAGCCAGTTGGTGTGGATGGCATGCAATTCTGCTGCATTTGAAGATC
TAAGAGTGTCAAGCTTCATCAGAGGAACAAGAGTACTTCCAAGGGGGAAGCTCTCCACTAGAGGAGTACA
AATTGCTTCAAATGAAAACATGGATGCTATTGTGTCAAGTACTCTTGAACTGAGGAGCAGATACTGGGCC
ATAAGAACCAGAAGTGGAGGGAACACTAATCAACAAAGGGCCTCTGCGGGCCAAATCAGCACACAACCTA
CGTTTTCTGTGCAGAGAAACCTCCCATTTGACAAAGCAACCATCATGGCAGCATTCTCTGGGAACACAGA
GGGAAGAACATCAGACATGAGGGCAGAAATCATAAAGATGATGGAAAGTGCAAGACCAGAAGAAGTGTCC
TTCCAGGGGCGGGGAGTCTTTGAGCTCTCGGACGAAAGGGCAACGAACCCGATCGTGCCCTCCTTTGACA
TGAATAATGAAGGATCTTATTTCTTCGGAGACAATGCAGAGGAGTACGACAATTAATGAA

>gi|224020936|gb|CY037330.1| Influenza A virus (A/Washington/AF06/2007(H1N1)) segment 5, complete sequence
ACTCACTGAGTGACATCAAAGTCATGGCGTCCCAAGGCACCAAACGGTCTTACGAACAGATGGAGACTGA
TGGGGAACGCCAGAATGCAACTGAAATCAGAGCATCCGTCGGAAGAATGATTGGTGGAATTGGGCGATTC
TACATCCAAATGTGCACCGAGCTTAAGCTCAATGATTATGAGGGACGGCTGATCCAGAACAGCTTAACAG
TAGAGAGAATGGTGCTCTCTGCTTTTGATGAGAGGAGAAATAAATATCTAGAGGAACATCCCAGCGCGGG
GAAAGATCCTAAGAAAACTGGAGGACCCATATACAAGAGAGTAGATGGAAAGTGGGTGAGGGAACTCGTC
CTTTATGACAAAGAAGAAATAAGGCGGATTTGGCGCCAAGCCAATAATGGTGATGATGCAACGGCTGGTT
TGACTCACATTATGATCTGGCATTCTAATTTGAATGATACAACTTACCAGAGGACAAGAGCTCTTGTCCG
CACCGGAATGGATCCCAGGATGTGCTCTTTGATGCAAGGTTCAACTCTCCCTAGAAGATCTGGAGCAGCA
GGCGCCGCAGTCAAAGGAGTTGGGACAATGGTATTGGAGTTAATCAGGATGATCAAACGTGGGATCAATG
ACCGAAACTTCTGGAGGGGTGAGAATGGAAGAAAAACAAGGATTGCTTATGAGAGAATGTGCAACATTCT
CAAAGGAAAATTTCAAACAGCTGCACAAAAAGCAATGATGGACCAAGTGAGAGAAAGCCGGAACCCAGG
A
AATGCTGAGATCGAAGATCTCACTTTTCTGGCACGGTCTGCACTCATATTGAGAGGATCAGTTGCTCACA
AGTCTTGCCTGCCTGCCTGTGTGTATGGACCAGCCGTAGCCAGTGGGTATGACTTCGAAAAAGAGGGTTA
CTCTTTGGTAGGAGTAGACCCTTTCAAACTGCTTCAAACCAGTCAGGTATACAGTCTAATTAGACCAAAC
GAGAATCCCGCACACAAGAGCCAGTTGGTGTGGATGGCATGCAATTCTGCTGCATTTGAAGATCTAAGAG
TGTCAAGCTTCATCAGAGGAACAAGAGTACTTCCAAGGGGGAAGCTCTCCACTAGAGGAGTACAAATTGC
TTCAAATGAAAACATGGATGCTATTGTGTCAAGTACTCTTGAACTGAGAAGCAGATACTGGGCCATAAGA
ACCAGAAGTGGAGGGAACACTAATCAACAAAGGGCCTCTGCGGGCCAAATCAGCACACAACCTACGTTTT
CTGTGCAGAGAAACCTCCCATTTGACAAAGCAACCATCATGGCAGCATTCTCTGGGAATACAGAGGGAAG
AACATCAGACATGAGGGCAGAAATCATAAAGATGATGGAAAGTGCAAGACCAGAAGAAGTGTCCTTCCAG
GGGCGGGGAGTCTTTGAGCTCTCGGACGAAAGGGCAACGAACCCGATCGTGCCCTCCTTTGACATGAGTA
ATGAAGGATCTTATTTCTTCGGAGACAATGCAGAGGAGTACGACAATTAATGAA
>gi|224021241|gb|CY037338.1| Influenza A virus (A/Japan/AF07/2008(H1N1)) segment 5, complete sequence
ATAATCACTCACTGAGTGACATCAAAGTCATGGCGTCCCAAGGCACCAAACGGTCTTACGAACAGATGGA
GACTGATGGGGAACGCCAGAATGCAACTGAAATCAGAGCATCCGTCGGAAGAATGATTGGTGGAATTGGG
CGATTCTACATCCAAATGTGCACCGAGCTTAAGCTCAATGATTATGAGGGACGGCTGATCCAGAACAGCT
TAACAATAGAGAGAATGGTGCTCTCTGCTTTTGATGAGAGGAGAAATAAATATCTAGAGGAACATCCCAG
CGCGGGGAAAGATCCTAAGAAAACTGGAGGACCCATATACAAGAGAGTAGATGGAAAGTGGGTGAGGGA
A
CTCGTCCTTTATGACAAAGAAGAAATAAGGCGGATTTGGCGCCAAGCCAACAATGGTGATGATGCAATGG
CTGGTTTGACTCACATTATGATCTGGCATTCTAATTTGAATGATACAACTTACCAGAGGACAAGAGCTCT
TGTCCGCACCGGAATGGATCCCAGGATGTGCTCTTTGATGCAAGGTTCAACTCTCCCTAGAAGATCTGGA
GCAGCAGGCGCCGCAGTCAAAGGAGTTGGGACAATGGTATTGGAGTTAATCAGGATGATCAAACGTGGGA
TCAATGACCGAAACTTCTGGAGGGGTGAGAATGGAAGAAAAACAAGGATTGCTTATGAGAGAATGTGCAA
CATTCTCAAAGGAAAATTTCAAACAGCTGCACAAAAAGCAATGATGGACCAAGTGAGAGAAAGCCGGAAC
CCAGGAAATGCTGAGATCGAAGATCTCACTTTTCTGGCACGGTCTGCACTCATATTGAGAGGATCAGTTG
CTCACAAGTCTTGCCTGCCTGCCTGTGTGTATGGACCAGCCGTAGCCAGTGGGTATGACTTCGAAAAAGA
GGGTTACTCTTTGGTAGGAGTAGACCCTTTCAAACTGCTTCAAACCAGTCAGGTATACAGTCTAATTAGA
CCAAACGAGAATCCCGCACACAAGAGCCAGTTGGTGTGGATGGCATGCAATTCTGCTGCATTTGAAGATC
TAAGAGTGTCAAGCTTCATCAGAGGAACAAGAGTACTTCCAAGGGGGAAGCTCTCCACTAGAGGAGTACA

AATTGCTTCAAATGAAAACATGGATGCTATTGTGTCAAGTACTCTTGAACTGAGAAGCAGATACTGGGCC
ATAAGAACCAGAAGTGGAGGGAACACTAATCAACAAAGGGCCTCTGCGGGCCAAATCAGCACACAACCTA
CATTTTCTGTGCAGAGAAACCTCCCATTTGACAAAGCAACCATCATGGCAGCATTCTCTGGGAATACAGA
GGGAAGAACATCAGACATGAGGGCAGAAATCATAAAGATGATGGAAAGTGCAAGACCAGAAGAAGTGTCC
TTCCAGGGGCGGGGAGTCTTTGAGCTCTCGGACGAAAGGGCAACGAACCCGATCGTGCCCTCCTTTGACA
TGAGTAATGAAGGATCTTATTTCTTCGGAGACAATGCAGAGGAGTACGACAATTAATGAA
>gi|212381598|gb|FJ445060.1| Influenza A virus (A/England/26/2008(H1N1)) segment 5 nucleocapsid protein (NP) gene, complete cds
ATGGCGTCCCAAGGCACCAAACGGTCTTACGAACAGATGGAGACTGATGGGGAACGCCAGAATGCAACTG
AAATCAGAGCATCCGTCGGAAGAATGATTGGTGGAATTGGGCGATTCTACATCCAAATGTGCACCGAGCT
TAAGCTCAATGATTATGAGGGACGGCTGATCCAGAACAGCTTAACAATAGAGAGAATGGTGCTCTCTGCT
TTTGATGAGAGGAGAAATAAATATCTAGAGGAACATCCCAGCGCGGGGAAAGATCCTAAGAAAACTGGAG
GACCCATATACAAGAGAGTAGATGGAAAGTGGGTGAGGGAACTCGTCCTTTATGACAAAGAAGAAATAAG
GCGGATTTGGCGCCAAGCCAACAATGGTGATGATGCAACGGCTGGTTTGACTCACATTATGATCTGGCAT
TCTAATTTGAATGATACAACTTACCAGAGGACAAGAGCTCTTGTCCGCACCGGAATGGATCCCAGGATGT
GCTCTTTGATGCAAGGTTCAACTCTCCCTAGAAGATCTGGAGCAGCAGGCGCCGCAGTCAAAGGAGTTGG
GACAATGGTATTGGAATTAATCAGGATGATCAAACGTGGGATCAATGACCGAAACTTCTGGAGGGGTGAG
AATGGAAGAAAAACAAGGATTGCTTATGAGAGAATGTGCAACATTCTAAAAGGAAAATTTCAAACAGCTG
CACAAAAAGCAATGATGGACCAAGTGAGAGAAAGCCGGAACCCAGGAAATGCTGAGATCGAAGATCTCAC
TTTTCTGGCACGGTCTGCACTCATATTGAGAGGATCAGTTGCTCACAAGTCTTGCCTGCCTGCCTGTGTG
TATGGACCAGCCGTAGCCAGTGGGTATGACTTCGAAAAAGAGGGTTACTCTTTGGTAGGAGTAGACCCTT
TCAAACTGCTTCAAACCAGTCAGGTATACAGTCTAATTAGACCAAACGAGAATCCCGCACACAAGAGCCA
GTTGGTGTGGATGGCATGCAATTCTGCTGCATTTGAAGATCTAAGAGTGTCAAGCTTCATCAGAGGAACA
AGAGTACTTCCAAGGGGGAAGCTCTCCACTAGAGGAGTACAAATTGCTTCAAATGAAAACATGGATGCTA
TTGTGTCAAGTACTCTTGAACTGAGAAGCAGATACTGGGCCATAAGAACCAGAAGTGGAGGGAACACTAA
TCAACAAAGGGCCTCTGCGGGCCAAATCAGCACACAACCTACGTTTTCTGTGCAGAGAAACCTCCCATTT
GACAAAGCAACCATCATGGCAGCATTCTCTGGGAATACAGAGGGAAGAACATCAGACATGAGGGCAGAAA
TCATAAAGATGATGGAAAGTGCAAGACCAGAAGAAGTGTCCTTCCAGGGGCGGGGAGTCTTTGAGCTCTC
GGACGAAAGGGCAACGAACCCGATCGTGCCCTCCTTTGACATGAGTAATGAAGGATCTTATTTCTTCGGA
GACAATGCAGAGGAGTACGACAATTAA
>gi|224027211|gb|CY037682.1| Influenza A virus (A/Florida/UR07-0022/2008(H1N1)) segment 5, complete sequence
ACATCAAAGTCATGGCGTCCCAAGGCACCAAACGGTCTTACGAACAGATGGAGACTGATGGGGAACGCCA
GAATGCAACTGAAATCAGAGCATCCGTCGGAAGAATGATTGGTGGAATTGGGCGATTCTACATCCAAATG
TGCACCGAGCTTAAGCTCAATGATTATGAGGGACGGCTGATCCAGAACAGCTTAACAATAGAGAGAATGG
TGCTCTCTGCTTTTGATGAGAGGAGAAATAAATATCTAGAGGAACATCCCAGCGCGGGGAAAGATCCTAA
GAAAACTGGAGGACCCATATACAAGAGAGTAGATGGAAAGTGGGTGAGGGAACTCGTCCTTTATGACAAA
GAAGAAATAAGGCGGATTTGGCGCCAAGCCAACAATGGTGATGATGCAACGGCTGGTTTGACTCACATTA
TGATCTGGCATTCTAATTTGAATGATACAACTTACCAGAGGACAAGAGCTCTTGTCCGCACCGGAATGGA
TCCCAGGATGTGCTCTTTGATGCAAGGTTCAACTCTCCCTAGAAGATCTGGAGCAGCAGGCGCCGCAGTC
AAAGGAGTTGGGACAATGGTATTGGAATTAATCAGGATGATCAAACGTGGGATCAATGACCGAAACTTCT
GGAGGGGTGAGAATGGAAGAAAAACAAGGATTGCTTATGAGAGAATGTGCAACATTCTCAAAGGAAAATT
TCAAACAGCTGCACAAAAAGCAATGATGGACCAAGTGAGAGAAAGCCGGAACCCAGGAAATGCTGAGATC
GAAGATCTCACTTTTCTGGCACGGTCTGCACTCATATTGAGAGGATCAGTTGCTCACAAGTCTTGCCTGC

CTGCCTGTGTGTATGGACCAGCCGTAGCCAGTGGGTATGACTTCGAAAAAGAGGGTTACTCTTTGGTAGG
AGTAGACCCTTTCAAACTGCTTCAAACCAGTCAGGTATACAGTCTAATTAGACCAAACGAGAATCCCGCA
CACAAGAGCCAGTTGGTGTGGATGGCATGCAATTCTGCTGCATTTGAAGATCTAAGAGTGTCAAGCTTCA
TCAGAGGAACAAGAGTACTTCCAAGGGGGAAGCTCTCCACTAGAGGAGTACAAATTGCTTCAAATGAAAA
CATGGATGCTATTGTATCAAGTACTCTTGAACTAAGAAGCAGATACTGGGCCATAAGAACCAGAAGTGGA
GGGAACACTAATCAACAAAGGGCCTCTGCGGGCCAAATCAGCACACAACCTACGTTTTCTGTGCAGAGAA
ACCTCCCATTTGACAAAGCAACCATCATGGCAGCATTCTCTGGGAATACAGAGGGAAGAACATCAGATAT
GAGGGCAGAAATCATAAAGATGATGGAAAGTGCAAGACCAGAAGAAGTGTCCTTCCAGGGGCGGGGAGT
C
TTCGAGCTCTCGGACGAAAGGGCAACGAACCCGATCGTGCCCTCCTTTGACATGAGTAATGAAGGATCTT
ATTTCTTCGGAGACAATGCAGAGGAGTACGACAATTAATGAA
>gi|296240586|gb|CY063609.1| Influenza A virus (A/Aalborg/INS133/2009(H1N1)) segment 5, complete sequence
TCACTCAATGAGTGACATCGAAACCATGGCGTCTCAAGGCACCAAACGATCATATGAACAAATGGAGACT
GGGGGGGAGCGCCAGGATGCCACAGAAATCAGAGCATCTGTCGGAAGAATGATTGGTGGAATCGGGAGA
T
TCTACATCCAAATGTGCACTGAACTCAAACTCAGTGATTATGATGGACGACTAATCCAGAATAGCATAAC
AATAGAGAGGATGGTGCTTTCTGCTTTTGATGAGAGAAGAAATAAATACCTAGAAGAGCATCCCAGTGCT
GGGAAGGACCCTAAGAAAACAGGAGGACCCATATATAGAAGAATAGACGGAAAGTGGATGAGAGAACTCA
TCCTTTATGACAAAGAAGAAATAAGGAGAGTTTGGCGCCAAGCAAATAATGGCGAAGATGCAACAGCAGG
TCTTACTCATATCATGATTTGGCATTCCAACCTGAATGATGCCACATATCAGAGAACAAGAGCGCTTGTT
CGCACCGGAATGGATCCCAGAATGTGCTCTCTAATGCAAGGTTCAACACTTCCCAGAAGGTCTGGTGCCG
CAGGTGCTGCGGTGAAAGGAGTTGGAACAATAGCAATGGAGTTAATCAGAATGATCAAACGTGGAATCAA
TGACCGAAATTTCTGGAGGGGTGAAAATGGACGAAGGACAAGGGTTGCTTATGAAAGAATGTGCAATATC
CTCAAAGGAAAATTTCAAACAGCTGCCCAGAGGGCAATGATGGATCAAGTAAGAGAAAGTCGAAACCCAG
GAAACGCTGAGATTGAAGACCTCATTTTCCTGGCACGGTCAGCCCTCATTCTGAGGGGATCAGTTGCACA
TAAATCCTGCCTGCCTGCTTGTGTGTATGGGCTTGCAGTAGCAAGTGGGCATGACTTTGAAAGGGAAGGG
TACTCACTGGTCGGGATAGACCCATTCAAATTACTCCAAAACAGCCAAGTGGTCAGCCTGATGAGACCAA
ATGAAAACCCAGCTCACAAGAGTCAATTGGTGTGGATGGCATGCCACTCTGCTGCATTTGAAGATTTAAG
AGTATCAAGTTTCATAAGAGGAAAGAAAGTGATTCCAAGAGGAAAGCTTTCCACAAGAGGGGTCCAGATT
GCTTCAAATGAGAATGTGGAAACCATGGACTCCAATACCCTGGAACTAAGAAGCAGATACTGGGCCATAA
GGACCAGGAGTGGAGGAAATACCAATCAACAAAAGGCATCCGCAGGCCAGATCAGTGTGCAGCCTACATT
CTCAGTGCAGCGAAATCTCCCTTTTGAAAGAGCAACCGTTATGGCAGCATTCAGCGGGAACAATGAAGGA
CGGACATCCGACATGCGAACAGAAGTTATAAGAATGATGGAAAGTGCAAAGCCAGAAGATTTGTCCTTTC
AGGGGCGGGGAGTCTTCGAGCTCTCGGACGAAAAGGCAACGAACCCGATCGTGCCTTCCTTTGACATGAG
TAATGAAGGGTCTTATTTCTTCGGAGACAATGCAGAGGAGTATGACAGTTGAG
>gi|296240568|gb|CY063601.1| Influenza A virus (A/Bonn/INS128/2009(H1N1)) segment 5, complete sequence
TCACTCAATGAGTGACATCGAAACCATGGCGTCTCAAGGCACCAAACGATCATATGAACAAATGGAGACT
GGGGGGGAGCGCCAGGATGCCACAGAAATCAGAGCATCTGTCGGAAGAATGATTGGTGGAATCGGGAGA
T
TCTACATCCAAATGTGCACTGAACTCAAACTCAGTGATTATGATGGACGACTAATCCAGAATAGCATAAC
AATAGAGAGGATGGTGCTTTCTGCTTTTGATGAGAGAAGAAATAAATACCTAGAAGAGCATCCCAGTGCT
GGGAAGGACCCTAAGAAAACAGGAGGACCCATATATAGAAGAATAGACGGAAAGTGGATGAGAGAACTCA

TCCTTTATGACAAAGAAGAAATAAGGAGAGTTTGGCGCCAAGCAAATAATGGCGAAGATGCAACAGCAGG
TCTTACTCATATCATGATTTGGCATTCCAACCTGAATGATGCCACATATCAGAGAACAAGAGCGCTTGTT
CGCACCGGAATGGATCCCAGAATGTGCTCTCTAATGCAAGGTTCAACACTTCCCAGAAGGTCTGGTGCCG
CAGGTGCTGCGGTGAAAGGAGTTGGAACAATAGCAATGGAGTTAATCAGAATGATCAAACGTGGAATCAA
TGACCGAAATTTCTGGAGGGGTGAAAATGGACGAAGGACAAGGGTTGCTTATGAAAGAATGTGCAATATC
CTCAAAGGAAAATTTCAAACAGCTGCCCAGAGGGCAATGATGGATCAAGTAAGAGAAAGTCGAAACCCAG
GAAACGCTGAGATTGAAGACCTCATTTTCCTGGCACGGTCAGCCCTCATTCTGAGGGGATCAGTTGCACA
TAAATCCTGCCTGCCTGCTTGTGTGTATGGGCTTGCAGTAGCAAGTGGGCATGACTTTGAAAGGGAAGGG
TACTCACTGGTCGGGATAGACCCATTCAAATTACTCCAAAACAGCCAAGTGGTCAGCCTGATGAGACCAA
ATGAAAACCCAGCTCACAAGAGTCAATTGGTGTGGATGGCATGCCACTCTGCTGCATTTGAAGATTTAAG
AGTATCAAGTTTCATAAGAGGAAAGAAAGTGATTCCAAGAGGAAAGCTTTCCACAAGAGGGGTCCAGATT
GCTTCAAATGAGAATGTGGAAACCATGGACTCCAATACCCTGGAACTAAGAAGCAGATACTGGGCCATAA
GGACCAGGAGTGGAGGAAATACCAATCAACAAAAGGCATCCGCAGGCCAGATCAGTGTGCAGCCTACATT
CTCAGTGCAGCGAAATCTCCCTTTTGAAAGAGCAACCGTTATGGCAGCATTCAGCGGGAACAATGAAGGA
CGGACATCCGACATGCGAACAGAAGTTATAAGAATGATGGAAAGTGCAAAGCCAGAAGATTTGTCCTTTC
AGGGGCGGGGAGTCTTCGAGCTCTCGGACGAAAAGGCAACGAACCCGATCGTGCCTTCCTTTGACATGAG
TAATGAAGGGTCTTATTTCTTCGGAGACAATGCAGAGGAGTATGACAGTTGAG
>gi|296240316|gb|CY063489.1| Influenza A virus (A/Boston/110/2009(H1N1)) segment 5, complete sequence
TCACTCAATGAGTGACATCGAAGCCATGGCGTCTCAAGGCACCAAACGATCATATGAACAAATGGAGACT
GGTGGGGAGCGCCAGGATGCCACAGAAATCAGAGCATCTGTCGGAAGAATGATTGGTGGAATCGGGAGAT
TCTACATCCAAATGTGCACTGAACTCAAACTCAGCGATTATGATGGACGACTAATCCAGAATAGCATAAC
AATAGAGAGGATGGTGCTTTCTGCTTTTGATGAGAGAAGAAATAAATACCTAGAAGAGCATCCCAGTGCT
GGGAAGGACCCTAAGAAAACAGGAGGACCCATATATAGAAGAATAGACGGAAAGTGGATGAGAGAACTCA
TCCTTTATGACAAAGAAGAAATAAGGAGAGTTTGGCGCCAAGCAAACAATGGCGAAGATGCAACAGCAGG
TCTTACTCATATCATGATTTGGCATTCCAACCTGAATGATGCCACATATCAGAGAACAAGAGCGCTTGTT
CGCACCGGAATGGATCCCAGAATGTGCTCTCTAATGCAAGGTTCAACACTTCCCAGAAGGTCTGGTGCCG
CAGGTGCTGCGGTGAAAGGAGTTGGAACAATAGCAATGGAGTTAATCAGAATGATCAAACGTGGAATCAA
TGACCGAAATTTCTGGAGGGGTGAAAATGGACGAAGGACAAGGGTTGCTTATGAAAGAATGTGCAATATC
CTCAAAGGAAAATTTCAAACAGCTGCCCAGAGGGCAATGATGGATCAAGTAAGAGAAAGTCGAAACCCAG
GAAACGCTGAGATTGAAGACCTCATTTTCCTGGCACGGTCAGCACTCATTCTGAGGGGATCAGTTGCACA
TAAATCCTGCCTGCCTGCTTGTGTGTATGGGCTTGCAGTAGCAAGTGGGCATGACTTTGAAAGGGAAGGG
TACTCACTGGTCGGGATAGACCCATTCAAATTACTCCAAAACAGCCAAGTGGTCAGCCTGATGAGACCAA
ATGAAAACCCAGCTCACAAGAGTCAATTGGTGTGGATGGCATGCCACTCTGCTGCATTTGAAGATTTAAG
AGTATCAAGTTTCATAAGAGGAAAGAAAGTGATTCCAAGAGGAAAGCTTTCCACAAGAGGGGTCCAGATT
GCTTCAAATGAGAATGTGGAAACCATGGACTCCAATACCCTGGAACTAAGAAGCAGATACTGGGCCATAA
GGACCAGGAGTGGAGGAAATACCAATCAACAAAAGGCATCCGCAGGCCAGATCAGTGTGCAGCCTACATT
CTCAGTGCAGCGGAATCTCCCTTTTGAAAGAGCAACCGTTATGGCAGCATTCAGCGGGAACAATGAAGGA
CGGACATCCGACATGCGAACAGAAGTTATAAGAATGATGGAAAGTGCAAAGCCAGAAGATTTGTCCTTCC
AGGGGCGGGGAGTCTTCGAGCTCTCGGACGAAAAGGCAACGAACCCGATCGTGCCTTCCTTTGACATGAG
TAATGAAGGGTCTTATTTCTTCGGAGACAATGCAGAGGAGTATGACAGTTGAG
>gi|296240622|gb|CY063625.1| Influenza A virus (A/New York/INS150/2009(H1N1)) segment 5, complete sequence
ATGAGTGACATCGAAGCCATGGCGTCTCAAGGCACCAAACGATCATATGAACAAATGGAGACTGGTGGGG

AGCGCCAGGATGCCACAGAAATCAGAGCATCTGTCGGAAGAATGATTGGTGGAATCGGGAGATTCTACAT
CCAAATGTGCACTGAACTCAAACTCAGTGATTATGATGGACGACTAATCCAGAATAGCATAACAATAGAG
AGGATGGTGCTTTCTGCTTTTGATGAGAGAAGAAATAAATACCTAGAAGAGCATCCCAGTGCTGGGAAGG
ACCCTAAGAAAACAGGAGGACCCATATATAGAAGAATAGACGGAAAGTGGATGAGAGAACTCATCCTTTA
TGACAAAGAAGAAATAAGGAGAGTTTGGCGCCAAGCAAACAATGGCGAAGATGCAACAGCAGGTCTTACT
CATATCATGATTTGGCATTCCAACCTGAATGATGCCACATATCAGAGAACAAGAGCGCTTGTTCGCACCG
GAATGGATCCCAGAATGTGCTCTCTAATGCAAGGTTCAACACTTCCCAGAAGGTCTGGTGCCGCAGGTGC
TGCGGTGAAAGGAGTTGGAACAATAGCAATGGAGTTAATCAGAATGATCAAACGTGGAATCAATGACCGA
AATTTCTGGAGGGGTGAAAATGGACGAAGGACAAGGGTTGCTTATGAAAGAATGTGCAATATCCTCAAAG
GAAAATTTCAAACAGCTGCCCAGAGGGCAATGATGGATCAAGTAAGAGAAAGTCGAAACCCAGGAAACGC
TGAGATTGAAGACCTCATTTTCCTGGCACGGTCAGCACTCATTCTGAGGGGATCAGTTGCACATAAATCC
TGCCTGCCTGCTTGTGTGTATGGGCTTGCAGTAGCAAGTGGGCATGACTTTGAAAGGGAAGGGTACTCAC
TGGTCGGGATAGACCCATTCAAATTACTCCAAAACAGCCAAGTGGTCAGCCTGATGAGACCAAATGAAAA
CCCAGCTCACAAGAGTCAATTGGTGTGGATGGCATGCCACTCTGCTGCATTTGAAGATTTAAGAGTATCA
AGTTTCATAAGAGGAAAGAAAGTGATTCCAAGAGGAAAGCTTTCCACAAGAGGGGTCCAGATTGCTTCAA
ATGAGAATGTGGAAACCATGGACTCCAATACCCTGGAACTAAGAAGCAGATACTGGGCAATAAGGACCAG
GAGTGGAGGAAATACCAATCAACAAAAGGCATCCGCAGGCCAGATCAGTGTGCAGCCTACATTCTCAGTG
CAGCGAAATCTCCCTTTTGAAAGAGCAACCGTCATGGCAGCATTCAGCGGGAACAATGAAGGACGGACAT
CCGACATGCGAACAGAAGTTATAAGAATGATGGAAAGTGCAAAGCCAGAAGATTTGTCCTTCCAGGGGCG
GGGAGTCTTCGAGCTCTCGGACGAAAAGGCAACGAACCCGATCGTGCCTTCCTTTGACATGAGTAATGAA
GGGTCTTATTTCTTCGGAGACAATGCAGAGGAGTATGACAGTTGAG
>gi|296240550|gb|CY063593.1| Influenza A virus (A/Athens/INS122/2009(H1N1)) segment 5, complete sequence
TCACTCAATGAGTGACATCGAAGCCATGGCGTCTCAAGGCACCAAACGATCATATGAACAAATGGAGACT
GGTGGGGAGCGCCAGGATGCCACAGAAATCAGAGCATCTGTCGGAAGAATGATTGGTGGAATCGGGAGAT
TCTACATCCAAATGTGCACTGAACTCAAACTCAGTGATTATGATGGACGACTAATCCAGAATAGCATAAC
AATAGAGAGGATGGTGCTTTCTGCTTTTGATGAGAGAAGAAATAAATACCTAGAAGAGCATCCCAGTGCT
GGGAAGGACCCTAAGAAAACAGGAGGACCCATATATAGAAGAATAGACGGAAAGTGGATGAGAGAACTCA
TCCTTTATGACAAAGAAGAAATAAGGAGAGTTTGGCGCCAAGCAAACAATGGCGAAGATGCAACAGCAGG
TCTTACTCATATCATGATTTGGCATTCCAACCTGAATGATGCCACATATCAGAGAACAAGAGCGCTTGTT
CGCACCGGAATGGATCCCAGAATGTGCTCTCTAATGCAAGGTTCAACACTTCCCAGAAGGTCTGGTGCCG
CAGGTGCTGCGGTGAAAGGAGTTGGAACAATAGCAATGGAGTTAATCAGAATGATCAAACGTGGAATCAA
TGACCGAAATTTCTGGAGGGGTGAAAATGGACGAAGGACAAGGGTTGCTTATGAAAGAATGTGCAATATC
CTCAAAGGAAAATTTCAAACAGCTGCCCAGAGGGCAATGATGGATCAAGTAAGAGAAAGTCGAAACCCAG
GAAACGCTGAGATTGAAGACCTCATTTTCCTGGCACGGTCAGCACTCATTCTGAGGGGATCAGTTGCACA
TAAATCCTGCCTGCCTGCTTGTGTGTATGGGCTTGCAGTAGCAAGTGGGCATGACTTTGAAAGGGAAGGG
TACTCACTGGTCGGGATAGACCCATTCAAATTACTCCAAAACAGCCAAGTGGTCAGCCTGATGAGACCAA
ATGAAAACCCAGCTCACAAGAGTCAATTGGTGTGGATGGCATGCCACTCTGCTGCATTTGAAGATTTAAG
AGTATCAAGTTTCATAAGAGGAAAGAAAGTGATTCCAAGAGGAAAGCTTTCCACAAGAGGGGTCCAGATT
GCTTCAAATGAGAATGTGGAAACCATGGACTCCAATACCCTGGAACTAAGAAGCAGATACTGGGCCATCA
GGACCAGGAGTGGAGGAAATACCAATCAACAAAAGGCATCCGCAGGCCAGATCAGTGTGCAGCCTACATT
CTCAGTGCAGCGAAATCTCCCTTTTGAAAGAGCAACCGTTATGGCAGCATTCAGCGGGAACAATGAAGGA
CGGACATCCGACATGCGAACAGAAGTTATAAGAATGATGGAAAGTGCAAAGCCAGAAGATTTGTCCTTCC
AGGGGCGGGGAGTCTTCGAGCTCTCGGACGAAAAGGCAACGAACCCGATCGTGCCTTCTTTTGACATGAG

TAATGAAGGGTCTTATTTCTTCGGAGACAATGCAGAGGAGTATGACAGTTGAG
>gi|399226429|gb|JX309984.1| Influenza A virus (A/Singapore/KK734/2010(H1N1)) segment 5 nucleocapsid protein (NP) gene, complete cds
CAGTCAGGGTAGATAATCACTCACTGAGTGACATCGAAGCCATGGCGTCTCAAGGCACCAAACGATCATA
TGAACAAATGGAGACTGGTGGGGAGCGCCAGGATGCCACAGAAATCAGAGCATCTGTCGGAAGAATGATT
GGTGGAATCGGGAGATTCTACATCCAAATGTGCACTGAACTCAAACTCAGTGATTATGATGGACGACTAA
TCCAGAATAGCATAACAATAGAGAGGATGGTGCTTTCTGCTTTTGATGAGAGAAGAAATAAATACCTAGA
AGAGCATCCCAGTGCTGGGAAGGACCCTAAGAAAACAGGAGGACCCATATATAGAAGAATAGACGGAAAG
TGGATGAGAGAACTCATCCTTTATGACAAAGAAGAAATAAGGAGAGTTTGGCGCCAAGCAAACAATGGCG
AAGATGCAACAGCAGGTCTTACTCATATCATGATTTGGCATTCCAACCTGAATGATGCCACATATCAGAG
AACAAGAGCGCTTGTTCGCACCGGAATGGATCCCAGAATGTGCTCTCTAATGCAAGGTTCAACACTTCCC
AGAAGGTCTGGTGCCGCAGGTGCTGCGGTGAAAGGAGTTGGAACAATAGCAATGGAGTTAATCAGAATGA
TCAAACGTGGAATCAATGACCGAAATTTCTGGAGGGGTGAAAATGGACGAAGGACAAGGGTTGCTTATGA
AAGAATGTGCAATATCCTCAAAGGAAAATTTCAAACAGCTGCCCAGAGGGCAATGATGGATCAAGTAAGA
GAAAGTCGAAACCCAGGAAACGCTGAGATTGAAGACCTCATTTTCCTGGCACGTTCAGCACTCATTCTGA
GGGGATCAGTTGCACATAAATCCTGCCTGCCTGCTTGTGTGTATGGGCTTGCAGTAGCAAGTGGGCATGA
CTTTGAAAGGGAAGGGTACTCACTGGTCGGGATAGACCCATTCAAATTACTCCAAAACAGCCAAGTGGTC
AGCCTGATGAGACCAAATGAAAACCCAGCTCACAAGAGTCAATTGGTGTGGATGGCATGCCACTCTGCTG
CATTTGAAGATTTAAGAGTATCAAGTTTCATAAGAGGAAAGAAAGTGATCCCAAGAGGAAAGCTTTCCAC
AAGAGGGGTCCAGATTGCTTCAAATGAGAATGTGGAAACCATGGACTCCAATACCCTGGAACTAAGAAGC
AGATACTGGGCCATAAGGACCAGGAGTGGAGGAAATACCAATCAACAAAAGGCATCCGCAGGCCAAATCA
GTGTGCAGCCTACATTCTCAGTGCAGCGAAATCTCCCTTTTGAAAGAGCAACCGTTATGGCAGCATTCAG
CGGGAACAATGAAGGACGGACATCCGACATGAGAACAGAAGTTATAAGAATGATGGAAAGTGCAAAGCCA
GAGGATTTGTCCTTCCAGGGGCGGGGAGTCTTCGAGCTCTCGGACGAAAAGGCAACGAACCCGATCGTGC
CTTCCTTTGACATGAGTAATGAAGGGTCTTATTTCTTCGGAGACAATGCAGAGGAGTATGACAGTTGAGG
AAAAATACCCTTGTTTCTACT
>gi|396940869|dbj|AB704487.1| Influenza A virus (A/Tochigi/10/2010(H1N1)) NP gene for nucleocapsid protein, complete cds
ATGGCGTCTCAAGGCACCAAACGATCATATGAACAAATGGAGACTGGTGGGGAGCGCCAGGATGCCACAG
AGATCAGAGCATCTGTCGGAAGAATGATTGGTGGAATCGGGAGATTCTACATCCAAATGTGCACTGAACT
CAAACTCAGTGATTATGATGGACGACTAATCCAGAATAGCATAACAATAGAGAGGATGGTGCTTTCTGCT
TTTGATGAGAGAAGAAATAAATACCTAGAAGAGCATCCCAGTGCTGGGAAGGACCCTAAGAAAACAGGAG
GACCCATATATAGAAGAATAGACGGAAAGTGGATGAGAGAACTCATCCTTTATGACAAAGAAGAAATAAG
GAGAGTTTGGCGCCAAGCAAACAATGGCGAAGATGCAACAGCAGGTCTTACTCATATCATGATTTGGCAT
TCCAACCTGAATGATGCCACATATCAGAGAACAAGAGCGCTTGTTCGCACCGGAATGGATCCCAGAATGT
GCTCTCTAATGCAAGGTTCAACACTTCCCAGAAGGTCTGGTGCCGCAGGTGCTGCGGTGAAAGGAGTTGG
AACAATAGCAATGGAGTTAATCAGAATGATCAAACGTGGAATCAATGACCGAAATTTCTGGAGGGGTGAA
AATGGACGAAGGACAAGGGTTGCTTATGAAAGAATGTGCAATATCCTCAAAGGAAAATTTCAAACAGCTG
CCCAGAGGGCAATGATGGATCAAGTAAGAGAAAGTCGAAACCCAGGAAACGCTGAGATTGAAGACCTCAT
TTTCCTGGCACGGTCAGCACTCATTCTGAGGGGATCAGTTGCACATAAATCCTGCCTGCCTGCTTGTGTG
TATGGGCTTGCAGTAGCAAGTGGGCATGACTTTGAAAGGGAAGGGTACTCACTGGTCGGGATAGACCCAT
TCAAATTACTCCAAAACAGCCAAGTGGTCAGCCTGATGAGACCAAATGAAAACCCAGCTCACAAGAGTCA
ATTGGTGTGGATGGCATGCCACTCTGCTGCATTTGAAGATTTAAGAGTATCAAGTTTCATAAGAGGAAAG
AAAGTGATTCCAAGAGGAAAGCTTTCCACAAGAGGGGTCCAGATTGCTTCAAATGAGAATGTGGAAACCA

TGGACTCCAATACCCTGGAACTAAGAAGCAGATACTGGGCCATAAGGACCAGGAGTGGAGGAAATACCAA
TCAACAAAAGGCATCCGCAGGCCAGATCAGTGTGCAGCCTACATTCTCAGTGCAGCGAAATCTCCCTTTT
GAAAGAGCAACCGTTATGGCAGCATTCAGCGGGAACAATGAAGGACGGACATCCGACATGCGAACAGAAG
TTATAAGAATGATGGAAAGTGCAAAGCCAGAAGATTTGTCCTTCCAGGGGCGGGGAGTCTTCGAGCTCTC
GGACGAAAAGGCAACGAACCCGATCGTGCCTTCCTTTGACATGAGTAATGAAGGGTCTTATTTCTTCGGA
GACAATGCAGAGGAGTATGACAGTTGA
>gi|346642631|gb|CY098556.1| Influenza A virus (A/Bangkok/INS516/2010(H1N1)) nucleocapsid protein (NP) gene, complete cds
TCACTCAATGAGTGACATCGAAGCCATGGCGTCTCAAGGCACCAAACGATCATATGAACAAATGGAGACT
GGTGGGGAGCGCCAGGATGCCACAGAAATCAGAGCATCTGTCGGAAGAATGATTGGTGGAATCGGGAGAT
TCTACATCCAAATGTGCACTGAACTCAAACTCAGTGATTATGATGGACGACTAATCCAGAATAGCATAAC
AATAGAGAGGATGGTGCTTTCTGCTTTTGATGAGAGAAGAAATAAATACCTAGAAGAGCATCCCAGTGCT
GGGAAGGACCCTAAGAAAACAGGAGGACCCATATATAGAAGAATAGACGGAAAGTGGATGAGAGAACTCA
TCCTTTATGACAAAGAAGAAATAAGGAGAGTTTGGCGCCAAGCAAACAATGGCGAAGATGCAACAGCAGG
TCTTACTCATATCATGATTTGGCATTCCAACCTGAATGATGCCACATATCAGAGAACAAGAGCGCTTGTT
CGCACCGGAATGGATCCCAGAATGTGCTCTCTAATGCAAGGTTCAACACTTCCCAGAAGGTCTGGTGCCG
CAGGTGCTGCGGTGAAAGGAGTTGGAACAATAGCAATGGAGTTAATCAGAATGATCAAACGTGGAATCAA
TGACCGAAATTTCTGGAGGGGTGAAAATGGACGAAGGACAAGGGTTGCTTATGAAAGAATGTGTAATATC
CTCAAAGGAAAATTTCAAACAGCTGCCCAGAGGGCAATGATGGATCAAGTAAGAGAAAGTCGAAACCCAG
GAAACGCTGAGATTGAAGACCTCATTTTCCTGGCACGGTCAGCACTCATTCTGAGGGGATCAGTTGCACA
TAAATCCTGCCTGCCTGCTTGTGTGTATGGGCTTGCAGTAGCAAGTGGGCATGACTTTGAAAGGGAAGGG
TATTCACTGGTCGGGATAGACCCATTCAAATTACTCCAAAACAGCCAAGTGGTCAGTCTGATGAGACCAA
ATGAAAACCCAGCTCACAAGAGTCAATTGGTGTGGATGGCATGCCACTCTGCTGCATTTGAAGATTTAAG
AGTATCAAGTTTCATAAGAGGAAAGAAAGTGATTCCAAGAGGAAAGCTTTCCACAAGAGGGGTCCAGATT
GCTTCAAATGAGAATGTGGAAACCATGGACTCCAATACCCTGGAACTAAGAAGCAGATACTGGGCCATAA
GGACCAGGAGTGGAGGAAATACCAATCAACAAAAGGCATCCGCAGGCCAGATCAGTGTGCAGCCTACATT
CTCAGTGCAGCGAAATCTCCCTTTTGAAAGAGCAACCGTTATGGCAGCATTCAGCGGGAACAATGAAGGA
CGGACATCCGACATGCGAACAGAAGTTATAAGAATGATGGAAAGTGCAAAGCCAGAAGATTTGTCCTTCC
AGGGGCGGGGAGTCTTCGAGCTCTCGGACGAAAAGGCAACGAACCCGATCGTGCCTTCCTTTGACATGAG
TAATGAAGGGTCTTATTTCTTCGGAGACAATGCAGAGGAGTATGACAGTTGAG
>gi|345101183|gb|CY098190.1| Influenza A virus (A/Sydney/DD3-33/2010(mixed)) nucleocapsid protein (NP) gene, complete cds
TCACTCAATGAGTGACATCGAAGCCATGGCGTCTCAAGGCACCAAACGATCATATGAACAAATGGAGACT
GGTGGGGAGCACCAGGATGCCACAGAAATCAGAGCATCTGTCGGAAGAATGATTGGTGGAATCGGGAGAT
TCTACATCCAAATGTGCACTGAACTCAAACTCAGTGATTATGATGGACGACTAATCCAGAATAGCATAAC
AATAGAGAGGATGGTGCTTTCTGCTTTTGATGAGAGAAGAAATAAATACCTAGAAGAGCATCCCAGTGCT
GGGAAGGACCCTAAGAAAACAGGAGGACCCATATATAGAAGAATAGACGGAAAGTGGATGAGAGAACTCA
TCCTTTATGACAAAGAAGAAATAAGGAGAGTTTGGCGCCAAGCAAACAATGGCGAAGATGCAACAGCAGG
TCTTACTCATATCATGATTTGGCATTCCAACCTGAATGATGCCACATATCAGAGAACAAGAGCGCTTGTT
CGCACTGGAATGGATCCCAGAATGTGCTCTCTAATGCAAGGTTCAACACTTCCCAGAAGGTCTGGTGCCG
CAGGTGCTGCGGTGAAAGGAGTTGGAACAATAGCAATGGAGTTAATCAGAATGATCAAACGTGGAATCAA
TGACCGAAATTTCTGGAGGGGTGAAAATGGACGAAGGACAAGGGTTGCTTATGAAAGAATGTGCAATATC
CTCAAAGGAAAATTTCAAACAGCTGCCCAGAGGGCAATGATGGATCAAGTAAGAGAAAGTCGAAACCCAG
GAAACGCTGAGATTGAAGACCTCATTTTCCTGGCACGGTCAGCACTCATTCTGAGGGGATCAGTTGCACA

TAAATCCTGCCTGCCTGCTTGTGTGTATGGGCTTGCAGTAGCAAGTGGGCATGACTTTGAAAGGGAAGGG
TACTCACTGGTCGGGATAGACCCATTCAAACTACTCCAAAACAGCCAAGTGGTCAGCCTTATGAGACCAA
ATGAAAACCCAGCTCACAAGAGTCAATTGGTGTGGATGGCATGCCACTCTGCTGCATTTGAAGATTTAAG
AGTATCAAGTTTCATAAGAGGAAAGAAAGTGATTCCAAGAGGAAAGCTTTCCACAAGAGGGGTCCAAATT
GCTTCAAATGAGAATGTGGAAACCATGGACTCCAATACCCTGGAACTAAGAAGCAGATACTGGGCCATAA
GGACCAGGAGTGGAGGAAATACCAATCAACAAAAGGCATCCGCAGGCCAGATCAGTGTGCAGCCTACATT
CTCAGTGCAGCGAAATCTCCCTTTTGAAAGAGCAACCGTTATGGCAGCATTCAGCGGGAACAATGAAGGA
CGGACATCCGACATGCGAACAGAAGTTATAAGAATGATGGAAAGTGCAAAGCCAGAAGATTTGTCCTTCC
AGGGGCGGGGAGTCTTCGAGCTCTCGGACGAAAAGGCAACGAACCCGATCGTGCCTTCCTTTGACATGAG
TAATGAAGGGTCTTATTTCTTCGGAGACAATGCAGAGGAGTATGACAGTTGAG

>gi|345101127|gb|CY098173.1| Influenza A virus (A/Chile/70/2010(H1N1)) nucleocapsid protein (NP) gene, complete cds

TCACTCAATGAGTGACATCGAAGCCATGGCGTCTCAAGGCACCAAACGATCATATGAACAAATGGAGACT
GGTGGGGAGCGCCAGGATGCCACAGAAATCAGAGCATCTGTCGGAAGAATGATTGGTGGAATCGGGAGAT
TCTACATCCAAATGTGCACTGAACTCAAACTCAGTGATTATGATGGACGACTAATCCAGAATAGCATAAC
AATAGAGAGGATGGTGCTTTCTGCTTTTGATGAGAGGAGAAATAAATACCTAGAAGAGCATCCCAGTGCT
GGGAAGGACCCTAAGAAAACAGGAGGACCCATATATAGAAGAATAGACGGAAAGTGGATGAGAGAACTCA
TCCTTTATGACAAAGAAGAAATAAGGAGAGTTTGGCGCCAAGCAAACAATGGCGAAGATGCAACAGCAGG
TCTTACTCATATCATGATTTGGCATTCCAACCTGAATGATGCCACATATCAGAGAACAAGAGCGCTTGTT
CGCACCGGAATGGATCCCAGAATGTGCTCTCTAATGCAAGGTTCAACACTTCCCAGAAGGTCTGGTGCCG
CAGGTGCTGCGGTGAAAGGAGTTGGAACAATAGCAATGGAGTTAATCAGAATGATCAAACGTGGAATCAA
TGACCGAAATTTCTGGAGGGGTGAAAATGGACGAAGGACAAGGGTTGCTTATGAAAGAATGTGCAATATC
CTCAAAGGAAAATTTCAAACAGCTGCCCAGAGGGCAATGATGGATCAAGTAAGAGAAAGTCGAAACCCAG
GAAACGCTGAGATTGAAGACCTCATTTTCCTGGCACGGTCAGCACTCATTCTGAGAGGATCAGTTGCACA
TAAATCCTGCCTGCCTGCTTGTGTGTATGGGCTTGCAGTAGCAAGTGGGCATGACTTTGAAAGGGAAGGG
TACTCACTGGTCGGGATAGACCCATTCAAATTACTCCAAAATAGCCAAGTGGTCAGCCTGATGAGACCAA
ATGAAAATCCAGCTCACAAGAGTCAATTGGTGTGGATGGCATGCCACTCTGCTGCATTTGAAGATTTAAG
AGTATCAAGTTTCATAAGAGGAAAGAAAGTGATTCCAAGAGGAAAGCTTTCCACAAGAGGGGTCCAGATT
GCTTCAAATGAGAATGTGGAAACCATGGACTCCAATACTCTGGAACTAAGAAGCAGATACTGGGCCATAA
GGACCAGGAGTGGAGGAAATACCAATCAACAAAAGGCATCCGCAGGCCAGATCAGTGTGCAGCCTACATT
CTCAGTGCAGCGAAATCTCCCTTTTGAAAGAGCAACCGTTATGGCAGCATTCAGCGGGAACAATGAAGGA
CGGACATCCGACATGCGAACAGAAGTTATAAGAATGATGGAAAGTGCAAGGCCAGAAGATTTGTCCTTCC
AGGGGCGGGGAGTCTTCGAGCTCTCGGACGAAAAGGCAACGAACCCGATCGTGCCTTCCTTTGACATGAG
TAATGAAGGGTCTTATTTCTTCGGAGACAATGCAGAGGAGTATGACAGTTGAG

>gi|343174021|gb|CY096605.1| Influenza A virus (A/Cambridge/INS528/2010(H1N1)) nucleocapsid protein (NP) gene, complete cds

TCACTCAATGAGTGACATCGAAGCCATGGCGTCTCAAGGCACCAAACGATCATATGAACAAATGGAGACT
GGTGGGGAGCGCCAGGATGCCACAGAAATCAGAGCATCTGTCGGAAGAATGATTGGTGGAATCGGGAGAT
TCTACATCCAAATGTGCACTGAACTCAAACTCAGTGATTATGATGGACGACTAATTCAGAATAGCATAAC
AATAGAGAGGATGGTGCTTTCTGCTTTTGATGAGAGAAGAAATAAATACCTAGAAGAGCATCCCAGTGCT
GGGAAGGACCCTAAGAAAACAGGAGGACCCATATATAGAAGAATAGACGGGAAGTGGATGAGAGAACTCA
TCCTTTATGACAAAGAAGAAATAAGGAGAGTTTGGCGCCAAGCAAACAATGGCGAAGATGCAACAGCAGG
TCTTACTCATATCATGATTTGGCATTCCAACCTGAATGATGCCACATATCAGAGAACAAGAGCGCTTGTT
CGCACCGGAATGGATCCCAGAATGTGCTCTCTAATGCAAGGTTCAACACTTCCCAGAAGGTCTGGTGCCG

CAGGTGCTGCGGTGAAAGGAGTTGGAACAATAGCAATGGAGTTAATCAGAATGATCAAACGTGGAATCAA
TGACCGAAATTTCTGGAGGGGTGAAAATGGACGAAGGACAAGGGTTGCTTATGAAAGAATGTGCAATATC
CTCAAAGGAAAATTTCAAACAGCTGCCCAGAGGGCAATGATGGATCAAGTAAGAGAAAGTCGAAACCCAG
GAAACGCTGAGATTGAAGACCTCATTTTCCTGGCACGGTCAGCACTCATTCTGAGGGGATCAGTTGCACA
TAAATCCTGCCTGCCTGCTTGTGTGTATGGGCTTGCAGTAGCAAGTGGGCATGACTTTGAAAGGGAAGGG
TACTCACTGGTCGGGATAGACCCATTCAAATTACTCCAAAACAGCCAAGTGGTCAGCCTGATGAGACCAA
ATGAAAACCCAGCTCACAAGAGTCAATTGGTGTGGATGGCATGCCACTCTGCTGCATTTGAAGATTTAAG
AGTATCAAGTTTCATAAGAGGAAAGAAAGTGATTCCAAGAGGAAAGCTTTCCACAAGAGGGGTCCAGATT
GCTTCAAATGAGAATGTGGAAACCATGGACTCCAATACCCTGGAACTAAGAAGCAGATACTGGGCCATAA
GGACCAGGAGTGGAGGAAATACCAATCAACAAAAGGCATCCGCAGGCCAGATCAGTGTGCAGCCTACATT
CTCAGTGCAGCGAAATCTCCCTTTTGAAAGAGCAACCGTTATGGCAGCATTCAGCGGGAACAATGAAGGA
CGGACATCCGACATGCGAACAGAAGTTATAAGAATGATGGAAAGTGCAAAGCCAGAAGATTTGTCCTTCC
AGGGGCGGGGAGTCTTCGAGCTCTCGGACGAAAAGGCAACGAACCCGATCGTGCCTTCTTTTGACATGAG
TAATGAAGGGTCTTATTTCTTCGGAGACAATGCAGAGGAGTATGACAGTTGAG
>gi|343174003|gb|CY096597.1| Influenza A virus (A/District of Columbia/INS527/2010(H1N1)) nucleocapsid protein (NP) gene, complete cds
TCACTCAATGAGTGACATCGAAGCCATGGCGTCTCAAGGCACCAAACGATCATATGAACAAATGGAGACT
GGTGGGGAGCGCCAGGATGCCACAGAAATCAGAGCATCTGTCGGAAGAATGATTGGTGGAATCGGGAGAT
TCTACATCCAAATGTGCACTGAACTCAAACTCAGTGATTATGATGGACGACTAATCCAGAATAGCATAAC
AATAGAGAGGATGGTGCTTTCTGCTTTTGATGAGAGAAGAAATAAATACCTAGAAGAGCATCCCAGTGCT
GGGAAGGACCCTAAGAAAACAGGAGGACCCATATATAGAAGAATAGACGGAAAGTGGATGAGAGAACTCA
TTCTTTATGACAAAGAAGAAATAAGGAGAGTTTGGCGCCAAGCAAACAATGGCGAAGATGCAACAGCAGG
TCTTACTCATATCATGATTTGGCATTCCAACCTGAATGATGCCACATATCAGAGAACAAGAGCTCTTGTT
CGCACCGGAATGGATCCCAGAATGTGCTCTCTAATGCAAGGTTCAACACTTCCCAGAAGGTCTGGTGCCG
CAGGTGCTGCGGTGAAAGGAGTTGGAACAATAGCAATGGAATTAATCAGAATGATCAAACGTGGAATCAA
TGACCGAAATTTCTGGAGAGGTGAAAATGGACGAAGGACAAGGGTTGCTTATGAAAGAATGTGCAATATC
CTCAAAGGAAAATTTCAAACAGCTGCCCAGAGGGCAATGATGGATCAAGTAAGAGAAAGTCGAAACCCAG
GAAACGCTGAGATTGAAGACCTCATTTTCCTGGCACGGTCAGCACTCATTCTGAGGGGATCAGTTGCACA
TAAATCCTGCCTGCCTGCTTGTGTGTATGGGCTTGCAGTAGCAAGTGGGCATGACTTTGAAAGGGAAGGG
TACTCACTGGTCGGGATAGACCCATTCAAATTACTCCAAAACAGCCAAGTGGTCAGCCTGATGAGACCAA
ATGAAAACCCAGCTCACAAGAGTCAATTGGTGTGGATGGCATGCCACTCTGCTGCATTTGAAGATTTAAG
AGTATCAAGTTTCATAAGAGGAAAGAAAGTGATTCCAAGAGGAAAGCTTTCCACAAGAGGGGTCCAGATT
GCTTCAAATGAGAATGTAGAAACCATGGACTCCAATACCCTGGAACTAAGAAGCAGATACTGGGCCATAA
GGACCAGGAGTGGAGGAAATACCAATCAACAAAAGGCATCCGCAGGCCAGATCAGTGTGCAGCCTACATT
CTCAGTGCAGCGAAATCTCCCTTTTGAAAGAGCAACCGTTATGGCAGCATTCAGCGGGAACAATGAAGGA
CGGACATCCGACATGCGAACAGAAGTTATAAGAATGATGGAAAGTGCAAAGCCAGAAGATTTGTCCTTCC
AGGGGCGGGGAGTCTTCGAGCTCTCGGACGAAAAGGCAACGAACCCGATCGTGCCTTCCTTTGACATGAG
TAATGAAGGGTCTTATTTCTTCGGAGACAATGCAGAGGAGTATGACAGTTGAG
>gi|343131998|gb|CY096253.1| Influenza A virus (A/Melbourne/INS472/2010(H1N1)) nucleocapsid protein (NP) gene, complete cds
TCACTCAATGAGTGACATCGAAGCCATGGCGTCTCAAGGCACCAAACGATCATATGAACAAATGGAGACT
GGTGGGGAGCGCCAGGATGCCACAGAAATCAGAGCATCTGTCGGAAGAATGATTGGTGGAATCGGGAGAT
TCTACATCCAAATGTGCACTGAACTCAAACTCAGTGATTATGATGGACGACTAATCCAGAATAGCATAAC
AATAGAGAGGATGGTGCTTTCTGCTTTTGATGAGAGAAGAAATAAATACCTAGAAGAGCATCCCAGTGCT

GGGAAGGACCCTAAGAAAACAGGAGGACCCATATATAGAAGAATAGACGGAAAGTGGATGAGAGAACTCA
TCCTTTATGACAAAGAAGAAATAAGGAGAGTTTGGCGCCAAGCAAACAATGGCGAAGATGCAACAGCAGG
TCTTACTCATATCATGATTTGGCATTCCAACCTGAATGATGCCACATATCAGAGAACAAGAGCGCTTGTT
CGCACCGGAATGGATCCCAGAATGTGCTCTCTAATGCAAGGTTCAACACTTCCCAGAAGGTCTGGTGCCG
CAGGTGCTGCGGTGAAAGGAGTTGGAACAATAGCAATGGAGTTAATCAGAATGATCAAACGTGGAATCAA
TGACCGAAATTTCTGGAGGGGTGAAAATGGACGAAGGACAAGGGTTGCTTATGAAAGAATGTGCAATATC
CTCAAAGGAAAATTTCAAACAGCTGCCCAGAGGGCAATGATGGATCAAGTAAGAGAAAGTCGAAACCCAG
GAAACGCTGAGATTGAAGACCTCATTTTCCTGGCACGGTCAGCACTCATTCTGAGAGGATCAGTTGCACA
TAAATCCTGCCTGCCTGCTTGTGTGTATGGGCTTGCAGTAGCAAGTGGGCATGACTTTGAAAGGGAAGGG
TACTCACTGGTCGGGATAGACCCATTCAAATTACTCCAAAATAGCCAAGTGGTCAGCCTGATGAGACCAA
ATGAAAACCCAGCTCACAAGAGTCAATTGGTGTGGATGGCATGCCACTCTGCTGCATTTGAAGATTTAAG
AGTATCAAGTTTCATAAGAGGAAAGAAAGTGATTCCAAGAGGAAAGCTTTCCACAAGAGGGGTCCAGATT
GCTTCAAATGAGAATGTGGAAACCATGGACTCCAATACCCTGGAACTAAGAAGCAGATACTGGGCCATAA
GGACCAGGAGTGGAGGAAATACCAATCAACAAAAGGCATCCGCAGGCCAGATCAGTGTGCAGCCTACATT
CTCAGTGCAGCGAAATCTCCCTTTTGAAAGAGCAACCGTTATGGCAGCATTCAGCGGGAACAATGAAGGA
CGGACATCCGACATGCGAACAGAAGTTATAAGAATGATGGAAAGTGCAAGGCCAGAAGATTTGTCCTTCC
AGGGGCGGGGAGTCTTCGAGCTCTCGGACGAAAAGGCAACGAACCCGATCGTGCCTTCCTTTGACATGAG
TAATGAAGGGTCTTATTTCTTCGGAGACAATGCAGAGGAGTATGACAGTTGAG
>gi|332384142|gb|JF906184.1| Influenza A virus (A/Netherlands/2631_1202/2010(H1N1)) segment 5 nucleocapsid protein (NP) gene, complete cds
ATGGCGTCTCAAGGCACCAAACGATCATATGAACAAATGGAGACTGGTGGGGAGCGCCAGGATGCCACAG
AAATCAGAGCATCTGTCGGAAGAATGATTGGTGGAATCGGGAGATTCTACATCCAAATGTGCACTGAACT
CAAACTCAGTGATTATGATGGACGACTAATCCAGAATAGCATAACAATAGAGAGGATGGTGCTTTCTGCT
TTTGATGAGAGAAGAAATAAATACCTAGAAGAGCATCCCAGTGCTGGGAAGGACCCTAAGAAAACAGGAG
GACCCATATATAGAAGAATAGACGGAAAGTGGATGAGAGAACTCATCCTTTATGACAAAGAAGAAATAAG
GAGAGTTTGGCGCCAAGCAAACAATGGCGAAGATGCAACAGCAGGTCTTACTCATATCATGATTTGGCAT
TCCAACCTGAATGATGCCACATATCAGAGAACAAGAGCGCTTGTTCGCACCGGAATGGATCCCAGAATGT
GCTCTCTAATGCAAGGTTCAACACTTCCCAGAAGGTCTGGTGCCGCAGGTGCTGCGGTGAAAGGAGTTGG
AACAATAGCAATGGAGTTAATCAGAATGATCAAACGTGGAATCAATGACCGAAATTTCTGGAGGGGTGAA
AATGGACGAAGGACAAGGGTTGCTTATGAAAGAATGTGCAATATCCTCAAAGGAAAATTTCAAACAGCTG
CCCAGAGGGCAATGATGGATCAAGTAAGAGAAAGTCGAAACCCAGGAAACGCTGAGATTGAAGACCTCAT
TTTCCTGGCACGGTCAGCACTCATTCTGAGGGGATCAGTTGCACATAAATCCTGCCTGCCTGCTTGTGTG
TATGGGCTTGCAGTAGCAAGTGGGCATGACTTTGAAAGGGAAGGGTACTCACTGGTCGGGATAGACCCAT
TCAAATTACTCCAAAACAGCCAAGTGGTCAGCCTGATGAGACCAAATGAAAACCCAGCTCACAAGAGTCA
ATTGGTGTGGATGGCATGCCACTCTGCTGCATTTGAAGATTTAAGAGTATCAAGTTTCATAAGAGGAAAG
AAAGTGATTCCAAGAGGAAAGCTTTCCACAAGAGGGGTCCAGATTGCTTCAAATGAGAGTGTGGAAACCA
TGGACTCCAATACCCTGGAACTAAGAAGCAGATACTGGGCCATAAGGACCAGGAGTGGAGGAAATACCAA
TCAACAAAAGGCATCCGCAGGCCAGATCAGTGTGCAGCCTACATTCTCAGTGCAGCGAAATCTCCCTTTT
GAAAGAGCAACCGTTATGGCAGCATTCAGCGGGAACAATGAAGGACGGACATCCGACATGCGAACAGAAG
TTATAAGAATGATGGAAAGTGCAAAGCCAGAAGATTTGTCCTTCCAGGGGCGGGGAGTCTTCGAGCTCTC
GGACGAAAAGGCAACGAACCCGATCGTGCCTTCCTTTGACATGAGTAATGAAGGGTCTTATTTCTTCGGA
GACAATGCAGAGGAGTATGACAGTTGA
>gi|327409537|gb|CY089382.1| Influenza A virus (A/Managua/3246.01/2010(H1N1)) nucleocapsid protein (NP) gene, complete cds

TCACTCAATGAGTGACATCGAAGCCATGGCGTCTCAAGGCACCAAACGATCATATGAACAAATGGAGACT
GGTGGGGAGCGCCAGGATGCCACAGAAATCAGAGCATCTGTCGGAAGAATGATTGGTGGAATCGGGAGAT
TCTACATCCAAATGTGCACTGAACTCAAACTCAGTGATTATGATGGACGACTAATCCAGAATAGCATAAC
AATAGAGAGGATGGTGCTTTCTGCTTTTGATGAGAGAAGAAATAAATACCTAGAAGAGCATCCCAGTGCT
GGGAAGGACCCTAAGAAAACAGGAGGACCCATATATAGAAGAATAGACGGAAAGTGGATGAGAGAACTCA
TCCTTTATGACAAAGAAGAAATAAGGAGAGTTTGGCGCCAAGCAAACAATGGCGAAGATGCAACAGCAGG
TCTTACTCATATCATGATTTGGCATTCCAACCTGAATGATGCCACATATCAGAGAACAAGAGCGCTTGTT
CGCACCGGAATGGATCCCAGAATGTGCTCTCTAATGCAAGGTTCAACACTTCCCAGAAGGTCTGGTGCCG
CAGGTGCTGCGGTGAAAGGAGTTGGAACAATAGCAATGGAGTTAATCAGAATGATCAAACGTGGAATCAA
TGACCGAAATTTCTGGAGGGGTGAAAATGGACGAAGGACAAGGGTTGCTTATGAAAGAATGTGCAATATC
CTCAAAGGAAAATTTCAAACAGCTGCCCAGAGGGCAATGATGGATCAAGTAAGAGAAAGTCGAAACCCAG
GAAACGCTGAGATTGAAGACCTCATTTTCCTGGCACGGTCAGCACTCATTCTGAGGGGATCAGTTGCACA
TAAATCCTGCCTGCCTGCTTGTGTGTATGGGCTTGCAGTAGCAAGTGGGCATGACTTTGAAAGGGAAGGG
TACTCACTGGTCGGGATAGACCCATTCAAATTACTCCAAAACAGCCAAGTGGTCAGCCTGATGAGACCAA
ATGAAAACCCAGCTCACAAGAGTCAATTGGTGTGGATGGCATGCCACTCTGCTGCATTTGAAGATTTAAG
AGTATCAAGTTTCATAAGAGGAAAGAAAGTGATTCCAAGAGGAAAGCTTTCCACAAGAGGGGTCCAGATT
GCTTCAAATGAGAATGTGGAAACCATGGACTCCAATACCCTGGAACTAAGAAGCAGATACTGGGCCATAA
GGACCAGGAGTGGAGGAAATACCAATCAACAAAAGGCATCCGCAGGCCAGATCAGTGTGCAGCCTACATT
CTCAGTGCAGCGAAATCTCCCTTTTGAAAGAGCAACCGTTATGGCAGCATTCAGCGGGAACAATGAAGGA
CGGACATCCGACATGCGAACAGAAGTTATAAGAATGATGGAAAGTGCAAAGCCAGAAGATTTGTCCTTCC
AGGGGCGGGGAGTCTTCGAGCTCTCGGACGAAAAGGCAACGAACCCGATCGTGCCTTCCTTTGACATGAG
TAATGAAGGGTCTTATTTCTTCGGAGACAATGCAGAGGAGTATGACAGTTGAG
>gi|359828251|gb|JQ041357.1| Influenza A virus (A/Novosibirsk/KSH/2011(H1N1)) segment 5 nucleocapsid protein (NP) gene, complete cds
TGAGTGACATCGAAGCCATGGCGTCTCAAGGCACCAAACGATCATATGAACAAATGGAGACCGGTGGGGA
GCGCCAGGATGCCACAGAAATCAGAGCATCTGTCGGAAGAATGATTGGTGGAATCGGGAGATTCTACATC
CAAATGTGCACTGAACTCAAACTCAGTGATTATGATGGACGACTAATCCAGAATAGCATAACAATAGAGA
GGATGGTGCTTTCTGCTTTTGATGAGAGAAGAAATAAATACCTAGAAGAGCATCCCAGTGCTGGGAAGGA
CCCTAAGAAAACAGGAGGACCCATATATAGAAGAATAGACGGAAAGTGGATGAGAGAACTCATCCTTTAT
GACAAAGAAGAAATAAGGAGAGTTTGGCGCCAAGCAAACAATGGCGAAGATGCAACAGCAGGTCTTACTC
ATATCATGATTTGGCATTCCAACCTGAATGATGCCACATATCAGAGAACAAGAGCGCTTGTTCGCACCGG
AATGGATCCCAGAATGTGCTCTCTAATGCAAGGTTCAACACTTCCCAGAAGGTCTGGTGCCGCAGGTGCT
GCGGTGAAAGGAGTTGGAACAATAGCAATGGAGTTAATCAGAATGATCAAACGTGGAATCAATGACCGAA
ATTTCTGGAGGGGTGAAAATGGAAGAAGGACAAGGGTTGCTTTTGAAAGAATGTGCAATATCCTCAAAGG
AAAATTTCAAACAGCTGCCCAGAGGGCAATGATGGATCAAGTAAGAGAAAGTCGAAACCCAGGAAACGCT
GAGATTGAAGACCTCATTTTCCTGGCACGGTCAGCACTCATTCTGAGGGGATCAGTTGCACATAAATCCT
GCCTGCCTGCTTGTGTGTATGGGCTTGCAGTAGCAAGTGGGCATGACTTTGAAAGGGAAGGGTACTCACT
GGTCGGGATAGACTCATTCAAATTACTCCAAAACAGTCAAGTGGTCAGCCTGATGAGACCAAATGAAAAT
CCAGCTCACAAGAGTCAATTGGTATGGATGGCATGCCACTCTGCTGCATTTGAAGATTTAAGAGTATCAA
GTTTCATAAGAGGAAAGAAAGTGATCCCAAGAGGAAAGCTTTCCACAAGAGGGGTCCAGATTGCTTCAAA
TGAGAATGTGGAAACCATGGACTCCAATACCCTGGAACTAAGAAGCAGATACTGGGCCATAAGGACCAGG
AGTGGAGGAAATACCAATCAACAAAAGGCATCCGCAGGCCAGATCAGTGTGCAGCCTACATTCTCAGTGC
AGCGAAATCTCCCTTTTGAAAGAGCAACCGTTATGGCAGCATTCAGCGGGAACAATGAAGGACGGACATC
CGACATGCGAACAGAGGTTATAAGAATGATGGAAAGTGCAAAGCCAGAAGATTTGTCCTTCCAGGGGCGG

GGAGTCTTCGAGCTCTCGGACGAAAAGGCAACGAACCCGATCGTGCCTTCCTTTGACATGAGTAATGAAG
GGTCTTATTTCTTCGGAGACAATGCAGAGGAGTATGACAGTTGAGGAAAAATAC
>gi|344166506|gb|CY098061.1| Influenza A virus (A/Moscow/WRAIR4316T/2011(H1N1)) nucleocapsid protein (NP) gene, complete cds
ATGGCGTCTCAAGGCACCAAACGATCATATGAACAAATGGAGACTGGTGGGGAGCGCCAGGATGCCACAG
AAATCAGAGCATCTGTCGGAAGAATGATTGGTGGAATCGGGAGATTCTACATCCAAATGTGCACTGAACT
CAAACTCAGTGATTATGATGGACGACTAATCCAGAATAGCATAACAATAGAGAGGATGGTGCTTTCTGCT
TTTGATGAGAGAAGAAATAAATACCTAGAAGAGCATCCCAGTGCTGGGAAGGACCCTAAGAAAACAGGAG
GACCCATATATAGAAGAGTAGACGGAAAGTGGATGAGAGAACTCATCCTTTATGACAAAGAAGAAATAAG
GAGAGTTTGGCGCCAAGCAAACAATGGCGAAGATGCAACAGCAGGTCTTACTCATATCATGATTTGGCAT
TCCAACCTGAATGATGCCACATATCAGAGAACAAGAGCGCTTGTTCGCACCGGAATGGATCCCAGAATGT
GCTCTCTAATGCAAGGTTCAACACTTCCCAGAAGGTCTGGTGCCGCAGGTGCTGCGGTGAAAGGAGTTGG
AACAATAGCAATGGAGTTAATCAGAATGATCAAACGTGGAATCAATGACCGAAATTTCTGGAGGGGTGAA
AATGGACGAAGGACAAGGGTTGCTTATGAAAGAATGTGCAATATCCTCAAAGGAAAATTTCAGACAGCTG
CCCAGAGGGCAATGATGGATCAAGTAAGAGAAAGTCGAAACCCAGGAAACGCTGAGATTGAAGACCTCAT
TTTCCTGGCACGGTCAGCACTCATTCTGAGGGGATCAGTTGCACATAAATCCTGCCTGCCTGCTTGTGTG
TATGGGCTTGCAGTAGCAAGTGGGCATGACTTTGAAAGGGAAGGGTACTCACTGGTCGGGATAGACCCAT
TCAAATTACTCCAAAACAGCCAAGTGGTCAGCCTGATGAGACCAAATGAAAACCCAGCTCACAAGAGTCA
ATTGGTGTGGATGGCATGCCACTCTGCTGCATTTGAAGATTTAAGAGTATCAAGTTTCATAAGAGGAAAG
AAAGTGATTCCAAGAGGAAAGCTTTCCACAAGAGGGGTCCAGATTGCTTCAAATGAGAATGTGGAAACCA
TGGACTCCAATACCCTGGAACTGAGAAGCAGGTACTGGGCCATAAGGACCAGGAGTGGAGGAAATACCAA
TCAACAAAAGGCATCCGCAGGCCAGATCAGTGTGCAGCCTACATTCTCAGTGCAGCGGAATCTCCCTTTT
GAAAGAGCAACCGTTATGGCAGCATTCAGCGGGAACAATGAAGGACGGACATCCGACATGCGAACAGAAG
TTATAAGAATGATGGAAAGTGCAAAGCCAGAAGATTTGTCCTTCCAAGGGCGGGGAGTCTTCGAGCTCTC
GGACGAAAAGGCAACGAACCCGATCGTGCCTTCCTTTGACATGAGTAATGAAGGGTCTTATTTCTTCGGA
GACAATGCAGAGGAGTATGACAGTTGA
>gi|344166014|gb|CY097869.1| Influenza A virus (A/District of Columbia/WRAIR0313/2011(H1N1)) nucleocapsid protein (NP) gene, complete cds
ATGGCGTCTCAAGGCACCAAACGATCATATGAACAAATGGAGACTGGTGGGGAGCGCCAGGATGCCACAG
AAATCAGAGCATCTGTCGGAAGAATGATTGGTGGAATCGGGAGATTCTACATCCAAATGTGCACTGAACT
CAAACTCAGTGATTATGATGGACGACTAATCCAGAATAGCATAACAATAGAGAGGATGGTGCTTTCTGCT
TTTGATGAGAGAAGAAATAAATACCTAGAAGAGCATCCCAGTGCTGGGAAGGACCCTAAGAAAACAGGAG
GACCCATATATAGAAGAATAGACGGAAAGTGGATGAGAGAACTCATCCTTTATGACAAAGAAGAAATAAG
GAGAGTTTGGCGCCAAGCAAACAATGGCGAAGATGCAACAGCAGGTCTTACTCATATCATGATTTGGCAT
TCCAACCTGAATGATGCCACATATCAGAGAACAAGAGCGCTTGTTCGCACTGGAATGGATCCCAGAATGT
GCTCTCTAATGCAAGGTTCAACACTTCCCAGAAGGTCTGGTGCCGCAGGTGCTGCGGTGAAAGGGGTTGG
AACAATAGCAATGGAGTTAATCAGAATGATCAAACGTGGAATCAATGACCGAAATTTCTGGAGGGGTGAA
AATGGACGAAGGACAAGGGTTGCTTATGAAAGAATGTGCAATATCCTCAAAGGAAAATTTCAAACAGCTG
CCCAGAGGGCAATGATGGATCAAGTAAGGGAAAGTCGAAACCCAGGAAACGCTGAGATTGAAGACCTCAT
TTTCCTGGCACGGTCAGCACTCATTCTGAGGGGATCAGTTGCACATAAATCCTGCCTGCCTGCTTGTGTG
TATGGGCTTGCAGTAGCAAGTGGGCATGACTTTGAAAGGGAAGGGTACTCACTGGTCGGGATAGACCCAT
TCAAATTACTCCAAAACAGCCAAGTGGTCAGCCTGATGAGACCAAATGAAAACCCAGCTCACAAGAGTCA
ATTGGTGTGGATGGCATGCCACTCTGCTGCATTTGAAGATTTAAGAGTATCAAGTTTCATAAGAGGAAAG
AAAGTGATTCCAAGAGGAAAGCTTTCCACAAGAGGGGTCCAGATTGCTTCAAATGAGAATGTGGAAACCA

TGGACTCCAATACCCTGGAACTAAGAAGCAGATACTGGGCCATAAGGACCAGGAGTGGAGGAAATACCAA
TCAACAAAAGGCATCCGCAGGCCAGATCAGTGTGCAGCCTACATTCTCAGTGCAGCGAAATCTCCCTTTT
GAAAGAGCAACCGTTATGGCAGCATTCAGCGGGAACAATGAAGGACGGACATCCGACATGCGAACAGAAG
TTATAAGAATGATGGAAAGTGCAAAGCCAGAAGATTTGTCCTTCCAGGGGCGGGGAGTCTTCGAGCTCTC
GGACGAAAAGGCAACGAACCCGATCGTGCCTTCCTTTGACATGAGTAATGAAGGGTCTTATTTCTTCGGA
GACAATGCAGAGGAGTATGACAGTTGA
>gi|339517261|gb|JN187297.1| Influenza A virus (A/Taiwan/3697/2011(H1N1)) segment 5 nucleocapsid protein (NP) gene, complete cds
ATGGCGTCTCAAGGCACCAAACGATCATATGAACAAATGGAGACTGGTGGGGAGCGCCAGGATGCCACAG
AAATCAGAGCATCTGTCGGAAGAATGATTGGTGGAATCGGGAGATTCTACATCCAAATGTGCACTGAACT
CAAACTCAGTGATTATGATGGACGACTAATCCAGAATAGCATAACAATAGAGAGGATGGTGCTTTCTGCT
TTTGATGAGAGAAGAAATAAATACCTAGAAGAGCATCCCAGTGCTGGGAAGGACCCTAAGAAAACAGGAG
GACCCATATATAGAAGAATAGACGGAAAGTGGATGAGAGAACTCATCCTTTATGACAAAGAAGAAATAAG
GAGAGTTTGGCGCCAAGCAAACAATGGCGAAGATGCAACAGCAGGTCTTACTCATATCATGATTTGGCAT
TCCAACCTGAATGATGCCACATATCAGAGAACAAGAGCGCTTGTTCGCACCGGAATGGATCCCAGAATGT
GCTCTCTAATGCAAGGTTCAACACTTCCCAGAAGGTCTGGTGCCGCAGGTGCTGCGGTGAAAGGAGTTGG
AACAATAGCAATGGAGTTAATCAGAATGATCAAACGTGGAATCAATGACCGAAATTTCTGGAGGGGTGAA
AATGGACGAAGGACAAGGGTTGCTTATGAAAGAATGTGCAATATCCTCAAAGGAAAATTTCAAACAGCTG
CCCAGAGGGCAATGATGGATCAAGTAAGAGAAAGTCGAAGCCCAGGAAACGCTGAGATTGAAGACCTCAT
TTTCCTGGCACGGTCAGCACTCATTCTGAGGGGATCAGTTGCACATAAATCCTGCCTGCCTGCTTGTGTG
TATGGGCTTGCAGTAGCAAGTGGGCATGACTTTGAAAGGGAAGGGTACTCACTGGTCGGGATAGACCCAT
TCAAATTACTCCAAAACAGTCAAGTGGTCAGCCTGATGAGACCAAATGAAAATCCAGCTCACAAGAGTCA
ATTGGTATGGATGGCATGCCACTCTGCTGCATTTGAAGATTTAAGAGTATCAAGTTTCATAAGAGGAAAG
AAAGTGATCCCAAGAGGAAAGCTTTCCACAAGAGGGGTCCAGATTGCTTCAAATGAGAATGTGGAAACCA
TGGACTCCAATACCCTGGAACTAAGAAGCAGATACTGGGCCATAAGGACCAGGAGTGGAGGAAATACCAA
TCAACAAAAGGCATCCGCAGGCCAGATCAGTGTGCAGCCTACATTCTCAGTGCAGCGAAATCTCCCTTTT
GAAAGAGCAACCGTTATGGCAGCATTCAGCGGGAACAATGAAGGACGGACATCCGACATGCGAACAGAGG
TTATAAGAATGATGGAAAGTGCAAAGCCAGAAGATTTGTCCTTCCAGGGGCGGGGAGTCTTCGAGCTCTC
GGACGAAAAGGCAACGAACCCGATCGTGCCTTCCTTTGACATGAGTAATGAAGGGTCTTATTTCTTCGGA
GACAATGCAGAGGAGTATGACAGTTGA
>gi|338826660|gb|CY092883.1| Influenza A virus (A/California/NHRC0001/2011(H1N1)) nucleocapsid protein (NP) gene, complete cds
TCACTCAATGAGTGACATCGAAGCCATGGCGTCTCAAGGCACCAAACGATCATATGAACAAATGGAGACT
GGTGGGGAGCGCCAGGATGCCACAGAAATCAGAGCATCTGTCGGAAGAATGATTGGTGGAATCGGGAGAT
TCTACATCCAAATGTGCACTGAACTCAAACTCAGTGATTATGATGGACGACTAATCCAGAATAGCATAAC
AATAGAGAGGATGGTGCTTTCTGCTTTTGATGAGAGAAGAAATAAATACCTAGAAGAGCATCCCAGTGCT
GGGAAGGACCCTAAGAAAACAGGAGGACCCATATATAGAAGAATAGACGGAAAGTGGATGAGAGAACTCA
TCCTTTATGACAAAGAAGAAATAAGGAGAGTTTGGCGCCAAGCAAACAATGGCGAAGATGCAACAGCAGG
TCTTACTCATATCATGATTTGGCATTCCAACCTGAATGATGCCACATATCAGAGAACAAGAGCGCTTGTT
CGCACCGGAATGGATCCCAGAATGTGCTCTCTAATGCAAGGTTCAACACTTCCCAGAAGGTCTGGTGCCG
CAGGTGCTGCGGTGAAAGGAGTTGGAACAATAGCAATGGAGTTAATCAGAATGATCAAACGTGGAATCAA
TGACCGAAATTTCTGGAGGGGTGAAAATGGACGAAGGACAAGGGTTGCTTATGAAAGAATGTGCAATATC
CTCAAAGGAAAATTTCAAACAGCTGCCCAGAGGGCAATGATGGATCAAGTAAGAGAAAGTCGAAACCCAG
GAAACGCTGAGATTGAAGACCTCATTTTCCTGGCACGTTCAGCACTCATTCTGAGGGGATCAGTTGCACA

TAAATCCTGCCTGCCTGCTTGTGTGTATGGGCTTGCAGTAGCAAGTGGGCATGACTTTGAAAGGGAAGGG
TACTCACTGGTCGGGATAGACCCATTCAAATTACTCCAAAACAGCCAAGTGGTCAGCCTGATGAGACCAA
ATGAAAACCCAGCTCACAAGAGTCAATTGGTGTGGATGGCATGCCACTCTGCTGCATTTGAAGATTTAAG
AGTATCAAGTTTCATAAGAGGAAAGAAAGTGATCCCAAGAGGAAAGCTTTCCACAAGAGGGGTCCAGATT
GCTTCAAATGAGAATGTGGAAACCATGGACTCCAATACCCTGGAACTAAGAAGCAGATACTGGGCCATAA
GGACCAGGAGTGGAGGAAATACCAATCAACAAAAGGCATCCGCAGGCCAAATCAGTGTGCAGCCTACATT
CTCAGTGCAGCGAAATCTCCCTTTTGAAAGAGCAACCGTTATGGCAGCATTCAGCGGGAACAATGAAGGA
CGGACATCCGACATGAGAACAGAAGTTATAAGAATGATGGAAAGTGCAAAGCCAGAGGATTTGTCCTTCC
AGGGGCGGGGAGTCTTCGAGCTCTCGGACGAAAAGGCAACGAACCCGATCGTGCCTTCCTTTGACATGAG
TAATGAAGGGTCTTATTTCTTCGGAGACAATGCAGAGGAGTATGACAGTTGAG

>gi|338826624|gb|CY092867.1| Influenza A virus (A/Sydney/DD3-59/2011(H1N1)) nucleocapsid protein (NP) gene, complete cds

TCACTCAATGAGTGACATCGAAGCCATGGCGTCTCAAGGCACCAAACGATCATATGAACAAATGGAGACT
GGTGGGGAGCGCCAGGATGCCACAGAAATCAGAGCATCTGTCGGAAGAATGATTGGTGGAATCGGGAGAT
TCTACATCCAAATGTGCACTGAACTCAAACTCAGTGATTATGATGGACGACTAATCCAGAATAGCATAAC
AATAGAGAGGATGGTGCTTTCTGCTTTTGATGAGAGAAGAAATAAATACCTAGAAGAGCATCCCAGTGCT
GGGAAGGACCCTAAGAAAACAGGAGGACCCATATATAGAAGAATAGACGGAAAGTGGATGAGAGAACTCA
TCCTTTATGACAAAGAAGAAATAAGGAGAGTTTGGCGCCAAGCAAACAATGGCGAAGATGCAACAGCAGG
CCTTACTCATATCATGATTTGGCATTCCAACCTGAATGATGCCACATATCAGAGAACAAGAGCGCTTGTT
CGCACCGGAATGGATCCCAGAATGTGCTCTCTAATGCAAGGTTCAACACTTCCCAGAAGATCTGGTGCCG
CAGGTGCTGCGGTGAAAGGAGTTGGAACAATAGCAATGGAGTTAATCAGAATGATCAAACGTGGAATCAA
TGACCGAAATTTCTGGAGGGGTGAAAATGGACGAAGGACAAGGGTTGCTTATGAAAGAATGTGCAATATC
CTCAAAGGAAAATTTCAAACAGCTGCCCAGAGGGCAATGATGGATCAAGTAAGAGAAAGTCGAAACCCAG
GAAACGCTGAGATTGAAGACCTCATCTTCCTGGCACGGTCAGCACTTATTCTGAGGGGATCAGTTGCACA
TAAATCCTGCCTGCCTGCTTGTGTGTATGGGCTTGCAGTAGCAAGTGGGCATGACTTTGAAAGGGAAGGG
TACTCACTGGTCGGGATAGACCCATTCAAATTACTCCAAAACAGCCAAGTGGTCAGCCTGATGAGACCAA
ATGAAAACCCAGCTCACAAGAGTCAATTGGTGTGGATGGCATGCCACTCTGCTGCATTTGAAGATTTAAG
AGTATCAAGTTTCATAAGAGGAAAGAAAGTGATTCCAAGAGGAAAGCTTTCCACAAGAGGGGTCCAGATT
GCTTCAAATGAGAATGTGGAAACCATGGACTCCAATACCCTGGAACTAAGAAGCAGATACTGGGCCATAA
GGACCAGGAGTGGAGGAAATACCAATCAACAAAAGGCATCCGCAGGCCAGATCAGTGTGCAGCCTACATT
CTCAGTGCAGCGAAATCTCCCTTTTGAAAGAGCAACCGTTATGGCAGCATTCAGCGGGAACAATGAAGGA
CGGACATCCGACATGCGAACAGAGGTTATAAGAATGATGGAAAGTGCAAAGCCAGAAGATTTGTCCTTCC
AGGGGCGGGGAGTCTTCGAGCTCTCGGACGAAAAGGCAACGAACCCGATCGTGCCTTCCTTTGACATGAG
TAATGAAGGGTCTTATTTCTTCGGAGACAATGCAGAGGAGTATGACAGTTGAG

>gi|328496420|gb|CY089464.1| Influenza A virus (A/Thailand/CU-H2911/2011(H1N1)) nucleocapsid protein (NP) gene, complete cds

ATGAGTGACATCGAAGCCATGGCGTCTCAAGGCACCAAACGATCATATGAACAAATGGAGACTGGTGGGG
AGCGCCAGGATGCCACAGAAATCAGAGCATCTGTCGGAAGAATGATTGGTGGAATCGGGAGATTCTACAT
CCAAATGTGCACTGAACTCAAACTCAGTGATTATGATGGACGACTAATCCAGAATAGCATAACAATAGAG
AGGATGGTGCTTTCTGCTTTTGATGAGAGAAGAAATAAATACCTAGAAGAGCATCCCAGTGCTGGGAAGG
ACCCTAAGAAAACAGGAGGACCCATATATAGAAGAATAGACGGAAAGTGGATGAGAGAACTCATCCTTTA
TGACAAAGAAGAAATAAGGAGAGTTTGGCGCCAAGCAAACAATGGCGAAGATGCAACAGCAGGTCTTACT
CATATCATGATTTGGCATTCCAACCTGAATGATGCCACATATCAGAGAACAAGAGCGCTTGTTCGCACCG
GAATGGATCCCAGAATGTGCTCTCTAATGCAAGGTTCAACACTTCCCAGAAGGTCTGGTGCCGCAGGTGC

TGCGGTGAAAGGAGTTGGAACAATAGCAATGGAGTTAATCAGAATGATCAAACGTGGAATCAATGACCGA
AATTTCTGGAGGGGTGAAAATGGACGAAGGACAAGGGTTGCTTATGAAAGAATGTGCAATATCCTCAAAG
GAAAATTTCAAACAGCTGCCCAGAGGGCAATGATGGATCAAGTAAGAGAAAGTCGAAACCCAGGAAACGC
TGAGATTGAAGACCTCATTTTCCTGGCACGGTCAGCACTCATTCTGAGGGGATCAGTTGCACATAAATCC
TGCCTGCCTGCTTGTGTGTATGGGCTTGCAGTAGCAAGTGGGCATGACTTTGAAAGGGAAGGGTACTCAC
TGGTCGGGATAGACCCATTCAAATTACTCCAAAACAGTCAAGTGGTCAGCCTGATGAGACCAAATGAAAA
TCCAGCTCACAAGAGTCAATTGGTATGGATGGCATGCCACTCTGCTGCATTTGAAGATTTAAGAGTATCA
AGTTTCATAAGAGGAAAGAAAGTGATCCCAAGAGGAAAGCTTTCCACAAGAGGGGTCCAGATTGCTTCAA
ATGAGAATGTGGAAACCATGGACTCCAATACCCTGGAACTAAGAAGCAGATACTGGGCCATAAGGACCAG
GAGTGGAGGAAATACCAATCAACAAAAGGCATCCGCAGGCCAGATCAGTGTGCAGCCTACATTCTCAGTG
CAGCGAAATCTCCCTTTTGAAAGAGCAACCGTTATGGCAGCATTCAGCGGGAACAATGAAGGACGGACAT
CCGACATGCGAACAGAAGTTATAAGAATGATGGAAAGTGCAAAGCCAGAAGATTTGTCCTTCCAGGGGCG
GGGAGTCTTCGAGCTCTCGGACGAAAAGGCAACGAACCCGATCGTGCCTTCCTTTGACATGAGTAATGAA
GGGTCTTATTTCTTCGGAGACAATGCAGAGGAGTATGACAGTTGAG
>gi|319918320|gb|CY080573.1| Influenza A virus (A/Ulaanbaatar/190/2011(H1N1)) nucleocapsid protein (NP) gene, complete cds
ATGGCGTCTCAAGGCACCAAACGATCATATGAACAAATGGAGACTGGTGGGGAGCGCCAGGATGCCACAG
AAATCAGAGCATCTGTCGGAAGAATGATTGGTGGAATCGGGAGATTCTACATCCAAATGTGCACTGAACT
CAAACTCAGTGATTATGATGGACGACTAATCCAGAATAGCATAACAATAGAGAGGATGGTGCTTTCTGCT
TTTGATGAGAGAAGAAATAAATACCTAGAAGAGCATCCCAGTGCTGGGAAGGACCCTAAGAAAACAGGAG
GACCCATATATAGAAGAATAGACGGAAAGTGGATGAGAGAACTCATCCTTTATGACAAAGAAGAAATAAG
GAGAGTTTGGCGCCAAGCAAACAATGGCGAAGATGCAACAGCAGGTCTTACTCATATCATGATTTGGCAT
TCCAACCTGAATGATGCCACATATCAGAGAACAAGAGCGCTTGTTCGCACCGGAATGGATCCCAGAATGT
GCTCTCTAATGCAAGGTTCAACACTTCCCAGAAGGTCTGGTGCCGCAGGTGCTGCGGTGAAAGGAGTTGG
AACAATAGCAATGGAGTTAATCAGAATGATCAAACGTGGAATCAATGACCGAAATTTCTGGAGGGGTGAA
AATGGACGAAGGACAAGGGTTGCTTATGAAAGAATGTGCAATATCCTCAAAGGAAAATTTCAAACAGCTG
CCCAGAGGGCAATGATGGATCAAGTAAGAGAAAGTCGAAACCCAGGAAACGCTGAGATTGAAGACCTCAT
TTTCCTGGCACGGTCAGCACTCATTCTGAGGGGATCAGTTGCACATAAATCCTGCCTGCCTGCTTGTGTG
TATGGGCTTGCAGTAGCAAGTGGGCATGACTTTGAAAGGGAAGGGTACTCACTGGTCGGGATAGACCCAT
TCAAATTACTCCAAAACAGTCAAGTGGTCAGCCTGATGAGACCAAATGAAAATCCAGCTCACAAGAGTCA
ATTGGTATGGATGGCATGCCACTCTGCTGCATTTGAAGATTTAAGAGTATCAAGTTTCATAAGAGGAAAG
AAAGTGATCCCAAGAGGAAAGCTTTCAACAAGAGGGGTCCAGATTGCTTCAAATGAGAATGTGGAAACCA
TGGACTCCAATACCCTGGAACTAAGAAGCAGATACTGGGCCATAAGGACCAGGAGTGGAGGAAATACCAA
TCAACAAAAGGCATCCGCAGGCCAGATCAGTGTGCAGCCTACATTCTCAGTGCAGCGAAATCTCCCTTTT
GAAAGAGCAACCGTTATGGCAGCATTCAGCGGGAACAATGAAGGACGGACATCCGACATGCGAACAGAGG
TTATAAGAATGATGGAAAGTGCAAAGCCAGAAGATTTGTCCTTCCAGGGGCGGGGAGTCTTCGAGCTCTC
GGACGAAAAGGCAACGAACCCGATCGTGCCTTCCTTTGACATGAGTAATGAAGGGTCTTATTTCTTCGGA
GACAATGCAGAGGAGTATGACAGTTGA
>gi|344166380|gb|CY098005.1| Influenza A virus (A/Prague/WRAIR4146N/2011(H1N1)) nucleocapsid protein (NP) gene, complete cds
ATGGCGTCTCAAGGCACCAAACGATCATATGAACAAATGGAAACTGGTGGGGAGCGCCAGGATGCCACAG
AAATCAGAGCATCTGTCGGAAGAATGATTGGTGGAATCGGGAGATTCTACATCCAAATGTGCACTGAACT
CAAACTCAGTGATTATGATGGACGACTAATCCAGAATAGCATAACAATAGAGAGGATGGTGCTTTCTGCT
TTTGATGAGAGAAGAAATAAATACCTAGAAGAGCATCCCAGTGCTGGGAAGGACCCTAAGAAAACAGGAG

GACCCATATATAGAAGAATAGACGGAAAGTGGATGAGAGAACTCATCCTTTATGACAAAGAAGAAATAAG
GAGAGTTTGGCGCCAAGCAAACAATGGCGAAGATGCAACAGCAGGCCTTACTCATATCATGATTTGGCAT
TCCAACCTGAATGATGCCACATATCAGAGAACAAGAGCGCTTGTTCGCACCGGAATGGATCCCAGAATGT
GCTCTCTAATGCAAGGTTCAACACTTCCCAGAAGATCTGGTGCCGCAGGTGCTGCGGTGAAAGGAGTTGG
AACAATAGCAATGGAGTTAATCAGAATGATCAAACGTGGAATCAATGACCGAAATTTCTGGAGGGGTGAA
AATGGACGAAGGACAAGGGTTGCTTATGAAAGAATGTGCAATATCCTCAAAGGAAAATTTCAAACAGCTG
CCCAGAGGGCAATGATGGATCAAGTAAGAGAAAGTCGAAACCCAGGAAACGCTGAGATTGAAGACCTCAT
CTTCCTGGCACGGTCAGCACTTATTCTGAGAGGATCAGTTGCACATAAATCCTGCCTGCCTGCTTGTGTG
TATGGGCTTGCAGTAGCAAGTGGGCATGACTTTGAAAGGGAAGGGTACTCACTGGTCGGGATAGACCCAT
TCAAATTACTCCAAAACAGCCAAGTGGTCAGCCTGATGAGACCAAATGAAAACCCAGCTCACAAGAGTCA
ATTGGTGTGGATGGCATGCCACTCTGCTGCATTTGAAGATTTAAGAGTATCAAGTTTCATAAGAGGAAAG
AAAGTGATTCCAAGAGGAAAGCTTTCCACAAGAGGGGTCCAGATTGCTTCAAATGAGAATGTGGAAACCA
TGGACTCCAATACCCTGGAACTAAGAAGCAGATACTGGGCCATAAGGACCAGGAGTGGAGGAAATACCAA
TCAACAAAAGGCATCCGCAGGCCAGATCAGTGTGCAGCCTACATTCTCAGTGCAGCGAAATCTCCCTTTT
GAAAGAGCAACCGTTATGGCAGCATTCAGCGGGAACAATGAAGGACGGACATCCGACATGCGAACAGAGG
TTATAAGAATGATGGAAAGTGCAAAGCCAGAAGATTTGTCCTTCCAGGGGCGGGGAGTCTTCGAGCTCTC
GGACGAAAAGGCAACGAACCCGATCGTGCCTTCCTTTGACATGAGTAATGAAGGGTCTTATTTCTTCGGA
GACAATGCAGAGGAGTATGACAGTTGA
>gi|344166362|gb|CY097997.1| Influenza A virus (A/Budapest/WRAIR3794T/2011(H1N1)) nucleocapsid protein (NP) gene, complete cds
ATGGCGTCTCAAGGCACCAAACGATCATATGAACAAATGGAGACTGGTGGGGAGCGCCAGGATGCCACAG
AAATCAGAGCATCTGTCGGAAGAATGATTGGTGGAATCGGGAGATTCTACATCCAAATGTGCACTGAACT
CAAACTCAGTGATTATGATGGACGACTAATCCAGAATAGCATAACAATAGAGAGGATGGTGCTTTCTGCT
TTTGATGAGAGAAGAAATAAATACCTAGAAGAGCATCCCAGTGCTGGGAAGGACCCTAAGAAAACAGGAG
GACCCATATATAGAAGAATAGACGGAAAGTGGATGAGAGAACTCATCCTTTATGACAAAGAAGAAATAAG
GAGAGTTTGGCGCCAAGCAAACAATGGCGAAGATGCAACAGCAGGCCTTACTCATATCATGATTTGGCAT
TCCAACCTGAATGATGCCACATATCAGAGAACAAGAGCGCTTGTTCGCACCGGAATGGATCCCAGAATGT
GCTCTCTAATGCAAGGTTCAACACTTCCCAGAAGATCTGGTGCCGCAGGTGCTGCGGTGAAAGGAGTTGG
AACAATAGCAATGGAGTTAATCAGAATGATCAAACGTGGAATCAATGACCGAAATTTCTGGAGGGGTGAA
AATGGACGAAGGACAAGGGTTGCTTATGAAAGAATGTGCAATATCCTCAAAGGAAAATTTCAAACAGCTG
CCCAGAGGGCAATGATGGATCAAGTAAGAGAAAGTCGAAACCCAGGAAACGCTGAGATTGAAGACCTCAT
CTTCCTGGCACGGTCAGCACTTATTCTGAGGGGATCAGTTGCACATAAATCCTGCCTGCCTGCTTGTGTG
TATGGGCTTGCAGTAGCAAGTGGGCATGACTTTGAAAGGGAAGGGTACTCACTGGTCGGGATAGACCCAT
TCAAATTACTCCAAAACAGCCAAGTGGTCAGCCTGATGAGACCAAATGAAAACCCAGCTCACAAGAGTCA
ATTGGTGTGGATGGCATGCCACTCTGCTGCATTTGAAGATTTAAGAGTATCAAGTTTCATAAGAGGAAAG
AAAGTGATTCCAAGAGGAAAGCTTTCCACAAGAGGGGTCCAGATTGCTTCAAATGAGAATGTGGAAACCA
TGGACTCCAATACCCTGGAACTAAGAAGCAGATACTGGGCCATAAGGACCAGGAGTGGAGGAAATACCAA
TCAACAAAAGGCATCCGCAGGCCAGATCAGTGTGCAGCCTACATTCTCAGTGCAGCGAAATCTCCCTTTT
GAAAGAGCAACCGTTATGGCAGCATTCAGCGGGAACAATGAAGGACGGACATCCGACATGCGAACAGAGG
TTATAAGAATGATGGAAAGTGCAAAGCCAGAAGATTTGTCCTTCCAGGGGCGGGGAGTCTTCGAGCTCTC
GGACGAAAAGGCAACGAACCCGATCGTGCCTTCCTTTGACATGAGTAATGAAGGGTCTTATTTCTTCGGA
GACAATGCAGAGGAGTATGACAGTTGA
>gi|390135405|gb|JX046927.1| Influenza A virus (A/Moscow/IIV-45/2012(H1N1)) segment 5 nucleocapsid protein (NP) gene, complete cds

ATGGCGTCTCAAGGCACCAAACGATCATATGAACAAATGGAGACTGGTGGGGAGCGCCAGGATGCCACAG
AAATCAGAGCATCCGTCGGAAGAATGATTGGTGGAATCGGGAGATTCTACATCCAAATGTGCACTGAACT
CAAACTCAGTGATTATGATGGACGACTAATCCAGAATAGCATAACAATAGAGAGGATGGTGCTTTCTGCT
TTTGATGAGAGAAGAAATAAATACCTAGAAGAGCATCCCAGTGCTGGGAAGGACCCTAAGAAAACAGGAG
GACCCATATATAGAAGAATAGACGGAAAGTGGATGAGAGAACTCATCCTTTATGACAAAGAAGAAATAAG
GAGAGTTTGGCGCCAAGCAAACAATGGCGAAGATGCAACAGCAGGTCTTACTCATATCATGATTTGGCAT
TCCAACCTGAATGATGCCACATATCAGAGAACAAGAGCGCTTGTTCGCACCGGAATGGATCCCAGAATGT
GCTCTCTAATGCAAGGTTCAACACTTCCCAGAAGGTCTGGTGCCGCAGGTGCTGCGGTGAAAGGAGTTGG
AACAATAGCAATGGAGTTAATCAGAATGATCAAACGTGGAATCAATGACCGAAATTTCTGGAGGGGTGAA
AATGGACGAAGGACAAGGGTTGCTTATGAAAGAATGTGCAATATCCTCAAAGGAAAATTTCAAACAGCTG
CCCAGAGGGCAATGATGGATCAAGTAAGAGAAAGTCGAAACCCAGGAAACGCTGAGATTGAAGACCTCAT
TTTCCTGGCACGGTCAGCACTCATTCTGAGGGGATCAGTTGCACATAAATCCTGCCTGCCTGCTTGTGTG
TATGGGCTTGCAGTAGCAAGTGGGCATGACTTTGAAAGGGAAGGGTACTCACTGGTCGGGATAGACCCAT
TCAAATTACTCCAAAACAGCCAAGTGGTCAGCCTGATGAGACCAAATGAAAACCCAGCTCACAAGAGTCA
ATTGGTGTGGATGGCATGCCACTCTGCTGCATTTGAAGATTTAAGAGTATCAAGTTTCATAAGAGGAAAG
AAAGTGATTCCAAGAGGAAAGCTTTCCACAAGAGGGGTCCAGATTGCTTCAAATGAGAATGTGGAAACCA
TGGACTCCAATACCCTGGAACTAAGAAGCAGATACTGGGCCATAAGGACCAGGAGTGGCGGAAATACCAA
TCAACAAAAGGCATCCGCAGGCCAGATCAGTGTGCAGCCTACATTCTCAGTGCAGCGAAATCTCCCCTTT
GAAAGAGCAACCGTTATGGCAGCATTCAGCGGAAACAATGAAGGACGGACATCCGACATGCGAACAGAGG
TTATAAGAATGATGGAAAGTGCAAAGCCAGAAGATTTGTCCTTCCAGGGGCGGGGAGTCTTCGAGCTCTC
GGACGAAAAGGCAACGAACCCGATCGTGCCTTCCTTTGACATGAGTAATGAAGGGTCTTATTTCTTCGGA
GACAATGCAGAGGAGTATGACAGTTGA
>gi|383513270|gb|JQ768354.1| Influenza A virus (A/Tomsk/IIV-19/2012(H1N1)) segment 5 nucleocapsid protein (NP) gene, complete cds
ATGGCGTCTCAAGGCACCAAACGATCATATGAACAAATGGAGACTGGTGGGGAGCGCCAGGATGCCACAG
AAATCAGAGCATCCGTCGGAAGAATGATTGGTGGAATCGGGAGATTCTACATCCAAATGTGCACTGAACT
CAAACTCAGTGATTATGATGGACGACTAATCCAGAATAGCATAACAATAGAGAGGATGGTGCTTTCTGCT
TTTGATGAGAGAAGAAATAAATACCTAGAAGAGCATCCCAGTGCTGGGAAGGACCCTAAGAAAACAGGAG
GACCCATATATAGAAGAATAGACGGAAAGTGGATGAGAGAACTCATCCTTTATGACAAAGAAGAAATAAG
GAGAGTTTGGCGCCAAGCAAACAATGGCGAAGATGCAACAGCAGGTCTTACTCATATCATGATTTGGCAT
TCCAACCTGAATGATGCCACATATCAGAGAACAAGAGCGCTTGTTCGCACCGGAATGGATCCCAGAATGT
GCTCTCTAATGCAAGGTTCAACACTTCCCAGAAGGTCTGGTGCCGCAGGTGCTGCGGTGAAAGGAGTTGG
AACAATAGCAATGGAGTTAATCAGAATGATCAAACGTGGAATCAATGACCGAAATTTCTGGAGGGGTGAA
AATGGACGAAGGACAAGGGTTGCTTATGAAAGAATGTGCAATATCCTCAAAGGAAAATTTCAAACAGCTG
CCCAGAGGGCAATGATGGATCAAGTAAGAGAAAGTCGAAACCCAGGAAACGCTGAGATTGAAGACCTCAT
TTTCCTGGCACGGTCAGCACTCATTCTGAGGGGATCAGTTGCACATAAATCCTGCCTGCCTGCTTGTGTG
TATGGGCTTGCAGTAGCAAGTGGGCATGACTTTGAAAGGGAAGGGTACTCACTGGTCGGGATAGACCCAT
TCAAATTACTCCAAAACAGCCAAGTGGTCAGCCTGATGAGACCAAATGAAAACCCAGCTCACAAGAGTCA
ATTGGTGTGGATGGCATGCCACTCTGCTGCATTTGAAGATTTAAGAGTATCAAGTTTCATAAGAGGAAAG
AAAGTGATTCCAAGAGGAAAGCTTTCCACAAGAGGGGTCCAGATTGCTTCAAATGAGAATGTGGAAACCA
TGGACTCCAATACCCTGGAACTAAGAAGCAGATACTGGGCCATAAGGACCAGGAGTGGCGGAAATACCAA
TCAACAAAAGGCATCCGCAGGCCAGATCAGTGTGCAGCCTACATTCTCAGTGCAGCGAAATCTCCCTTTT
GAAAGAGCAACCGTTATGGCAGCATTCAGCGGAAACAATGAAGGACGGACATCCGACATGCGAACAGAGG
TTATAAGAATGATGGAAAGTGCAAAGCCAGAAGATTTGTCCTTCCAGGGGCGGGGAGTCTTCGAGCTCTC

GGACGAAAAGGCAACGAACCCGATCGTGCCTTCCTTTGACATGAGTAATGAAGGGTCTTATTTCTTCGGA
GACAATGCAGAGGAGTATGACAGTTGA
