# Supplementary material for: A computational method for predicting regulation of human microRNAs on the influenza virus genome
Source: BMC Syst Biol. 2013 Oct 14;7(Suppl 2):S3. doi: 10.1186/1752-0509-7-S2-S3 (PMC3851852; doi:10.1186/1752-0509-7-S2-S3)
Supplement: Additional File 8 — The coding sequence of the gene fragment of PA from 2000 to 2012 recorded in Genbank. [file 1752-0509-7-S2-S3-S8.PDF]

NS

>gi|145278780|gb|CY021697.1| Influenza A virus (A/Memphis/15/2000(H1N1)) segment 8, complete sequence
GACATAATGGATTCCCACACTGTGTCAAGCTTTCAGGTAGATTGCTTCCTTTGGCATGTCCGCAAACAAG
TTGCAGACCAAGGTCTAGGCGATGCCCCATTCCTTGATCGACTTCGCCGAGATCAGAAGTCTCTAAAGGG
AAGAGGCAGCACTCTCGGTCTGAACATCGAAACAGCCACTTGTGTTGGAAAGCAAATAGTAGAGAGGATT
CTGAAAGAAGAATCCGATGAGGCATTTAAAATGACCATGGCCTCCGCACTTGCTTCGCGGTACCTAACTG
ACATGACTATTGAAGAAATGTCAAGGGACTGGTTCATGCTCATGCCCAAGCAGAAAGTGGCTGGCCCTCT
TTGTGTCAGAATGGACCAGGCAATAATGGATAAGAACATCATACTGAAAGCGAATTTCAGTGTGATTTTT
GACCGGTTGGAGAATCTGACATTACTAAGGGCTTTCACCGAAGAGGGAGCAATTGTTGGCGAAATTTCAC
CACTGCCTTCTCTTCCAGGACATACTAATGAGGATGTCAAAAATGCAATTGGGGTCCTCATCGGGGGACT
TGAATGGAATGATAACACAGTTCGAATCTCTGAAACTCTACAGAGATTCGCTTGGAGAAGCAGTAATGAG
ACTGGGGGACCTCCATTCACTCCAACACAGAAACGGAAAATGGCGGGAACAATTAGGTCAGAAGTTTGAA
GAAATAAGATGGCTGATTGAAGAAGTGAGGCATAAATTGAAGACAACAGAGAATAGTTTTGAGCAAATAA
CATTTATGCAAGCATTACAGCTATTGTTTGAAGTGGAACAAGAGATTAGAACGTTTTCGTTTCAGCTTAT
TTAATGATAAAAAA
>gi|70907648|gb|CY000453.2| Influenza A virus (A/New York/146/2000(H1N1)) segment 8, complete sequence
AGCAAAAGCAGGGTGGCAAAGACATAATGGATTCCAACACTTTGTCAAGCTTTCAGGTAGATTGCTTCCT
TTGGCATGTCCGCAAACGAGTTGCAGACCAAGAGCTAGGTGATGCCCCATTCCTTGATCGGCTTCGCCGA
GATCAGAAGTCCCTAAAGGGAAGAGGCAGCACTCTCGGTCTGAACATCGAAACAGCCACCTGTGTTGGAA
AGCAAATAGTAGAGAGGATTCTGAAGGAAGAATCCGATGAGGCATTTAAAATGACCATGGCCTCCGCACT
TGCTTCGCGATACCTAACTGACATGACTATTGAAGAGATGTCAAGGGGCTGGTTCATGCTTATGCCCAAG
CAGAAAGTGTCAGGCCCTCTTTGTGTCAGAATGGACCAGGCGATAACGGATAAGAACATCATACTGAAAG
CGAATTTCAGTGTGATTTTTGACCGGTTGGAAACTCTGACATTACTAAGGGCTTTCACCGAAGAGGGAGC
AATTGTTGGCGAAATTTCACCATTGCCTTCTCTTCCAGGACATACTAATGAGGATGTCAAAAATGCAATT
GGGATCCTCATCGGGGGACTTGAATGGAATGATAACACAGTTCGAGTCTCTGAAATTCTACAGAGATTCG
CTTGGAGAAGCAGTAATGAGAATGGGGGACCTCCACTCACTCCAACACAGAAACGGAAAATGGCGGGAAC
AATTAGGTCAGAAGTTTGAAGAAATAAGATGGCTGATTGAAGAAGTGAGACACAAATTGAAGACAACAGA
GAATAGTTTTGAGCAAATAACATTCATGCAAGCCTTACAGCTATTGTTTGAAGTGGAACAAGAGATCAGA
ACTTTTTCGTTTCAGCTTATTTAATGATAAAAAACACCCTTGTTTCTACT
>gi|145278913|gb|CY021753.1| Influenza A virus (A/South Australia/44/2000(H1N1)) segment 8, complete sequence
ATAATGGATTCCAACACTTTGTCAAGCTTTCAGGTAGATTGCTTCCTTTGGCATGTCCGCAAACGAGTTG
CAGACCAAGAGCTAGGTGATGCCCCATTCCTTGATCGGCTTCGCCGAGATCAGAAGTCCCTAAAGGGAAG
AGGCAGCACTCTCGGTCTGAACATCGAAACAGCCACCTGTGTTGGAAAGCAAATAGTAGAGAGGATTCTG
AAGGAAGAATCCGATGAGGCATTTAAAATGACCATGGCCTCCGCACTTGCTTCGCGATACCTAACTGACA
TGACTATTGAAGAGATGTCAAGGGGCTGGTTCATGCTTATGCCCAAGCAGAAAGTGTCAGGCCCTCTTTG
TGTCAGAATGGACCAGGCGATAACGGATAAGAACATCATACTGAAAGCGAATTTCAGTGTGATTTTTGAC
CGGTTGGAAACTCTGACATTACTAAGGGCTTTCACCGAAGAGGGAGCAATTGTTGGCGAAATTTCACCAT
TGCCTTCTCTTCCAGGACATACTAATGAGGATGTCAAAAATGCAATTGGGATCCTCATCGGGGGACTTGA
ATGGAATGATAACACAGTTCGAGTCTCTGAAATTCTACAGAGATTCGCTTGGAGAAGCAGTAATGAGAAT
GGGGGACCTCCACTCACTCCAACACAGAAACGGAAAATGGCGGGAACAATTAGGTCAGAAGTTTGAAGAA

ATAAGATGGCTGATTGAAGAAGTAAGACACAAATTGAAGACAACAGAGAATAGTTTTGAGCAAATAACAT
TCATGCAAGCCTTACAGCTATTGTTTGAAGTGGAACAAGAGATCAGAACTTTTTCGTTTCAGCTTATTTA
A
>gi|157367774|gb|CY026159.1| Influenza A virus (A/Auckland/585/2000(H1N1)) segment 8, complete sequence
TAATGGATTCCCACACTGTGTCAAGCTTTCAGGTAGATTGCTTCCTTTGGCATGTCCGCAAACAAGTTGC
AGACCAAGATCTAGGCGATGCCCCCTTCCTTGATCGGCTTCGCCGAGATCAGAAGTCTCTAAAGGGAAGA
GGCAGCACTCTCGGTCTGAACATCGAAACAGCCACTTGTGTTGGAAAGCAAATAGTAGAGAGGATTCTGA
AAGAAGAATCCGATGAGGCATTTAAAATGACCATGGCCTCCGCACTTGCTTCGCGGTACCTAACTGACAT
GACTATTGAAGAAATGTCAAGGGACTGGTTCATGCTCATGCCCAAGCAGAAAGTGGCTGGCCCTCTTTGT
GTCAGAATGGACCAGGCGATAATGGATAAGAACATCATACTGAAAGCGAATTTCAGTGTGATTTTTGACC
GGTTGGAGAATCTGACATTACTAAGGGCTTTCACCGAAGAGGGAGCAATTGTTGGCGAAATTTCACCATT
GCCTTCTCTTCCAGGACATACTAATGAGGATGTCAAAAATGCAATTGGGGTCCTCATCGGGGGACTTGAA
TGGAATGATAACACAGTTCGAGTCTCTGAAACTCTACAGAGATTCACTTGGAGAAGCAGTAATGAGACTG
GGGGACCTCCATTCACTCCAACACAGAAACGGAAAATGGCGGGAACAATTAGGTCAGAAGTTTGAAGAAA
TAAGATGGCTGATTGAAGAAGTGAGGCATAAATTGAAGACGACAGAGAATAGTTTTGAGCAAATAACATT
TATGCAAGCATTACAGCTATTGTTTGAAGTGGAACAAGAGATTAGAACGTTTTCGTTTCAGCTTATTTAA
>gi|145278626|gb|CY021633.1| Influenza A virus (A/Wellington/4/2000(H1N1)) segment 8, complete sequence
ATAATGGATTCCCACACTGTGTCAAGCTTTCAGGTAGATTGCTTCCTTTGGCATGTCCGCAAACAAGTTG
CAGACCAAGATCTAGGCGATGCCCCCTTCCTTGATCGGCTTCGCCGAGATCAGAAGTCTCTAAAGGGAAG
AGGCAGCACTCTCGGTCTGAACATCGAAACAGCCACTTGTGTTGGAAAGCAAATAGTAGAGAGGATTCTG
AAAGAAGAATCCGATGAGGCATTTAAAATGACCATGGCCTCCGCACTTGCTTCGCGGTACCTAACTGACA
TGACTATTGAAGAAATGTCAAGGGACTGGTTCATGCTCATGCCCAAGCAGAAAGTGGCTGGCCCTCTTTG
TGTCAGAATGGACCAGGCGATAATGGATAAGAACATCATACTGAAAGCGAATTTCAGTGTGATTTTTGAC
CGGTTGGAGAATCTGACATTACTAAGGGCTTTCACCGAAGAGGGAGCAATTGTTGGCGAAATTTCACCAT
TGCCTTCTCTTCCAGGACATACTAATGAGGATGTCAAAAATGCAATTGGGGTCCTCATCGGGGGACTTGA
ATGGAATGATAACACAGTTCGAGTCTCTGAAACTCTACAGAGATTCGCTTGGAGAAGCAGTAATGAGACT
GGGGGACCTCCATTCACTCCAACACAGAAACGGAAAATGGCGGGAACAATTAGGTCAGAAGTTTGAAGAA
ATAAGATGGCTGATTGAAGAAGTGAGGCATAAATTGAAGACGACAGAGAATAGTTTTGAGCAAATAACAT
TTATGCAAGCATTACAGCTATTGTTTGAAGTGGAACAAGAGATTAGAACGTTTTCGTTTCAGCTTATTTA
ATGATAAAA
>gi|131058517|gb|CY020153.1| Influenza A virus (A/Memphis/7/2001(H1N1)) segment 8, complete sequence
ATGGATTCCCACACTGTGTCAAGCTTTCAGGTAGATTGCTTCCTTTGGCATGTCCGCAAACAAGTTGCAG
ACCAGGATCTAGGCGATGCCCCATTCCTTGATCGGCTTCGCCGAGATCAGAAGTCTCTAAAGGGAAGAGG
CAGCACTCTCGGTCTGAACATCGAAACAGCCACCTGTGTTGGAAAGCAAATAGTAGAGAGGATTCTGAAA
GAAGAATCCGATGAGGCATTTAAAATGACCATGGCCTCCGCACTTGCTTCACGGTACCTAACTGACATGA
CCATTGAAGAAATGTCAAGGGACTGGTTCATGCTCATGCCCAAGCAGAAAGTGGCTGGCCCTCTTTGTGT
CAGAATGGACCAGGCGATAATGGATAAGAACATCATACTGAAAGCGAATTTCAGTGTGATTTTTGACCGG
TTGGAGACTCTGACATTACTAAGGGCTTTCACCGAAGAGGGAGCAATTGTTGGCGAAATTTCACCATTGC
CTTCTCTTCCAGGACATACTAATGAGGATGTCAAAAATGCAATTGGGGTCCTCATCGGGGGACTTGAATG
GAATGATAACACAGTTCGAGTCTCTGAAACTCTACAGAGATTCGCTTGGAGAAGCAGTAATGAGACTGGG
GGACCTCCATTCACTCCAACACAGAAACGGAAAATGGCGGGAACAATTAGGTCAGAAGTTTGAAGAAATA

AGATGGCTGATTGAAGAAGTGAGACATAAATTGAAGACGACAGAGAATAGTTTTGAACAAATAACATTTA
TGCAAGCATTACAGCTATTGTTTGAAGTGGAACAAGAGATTAGAACGTTCTCGTTTCAGCTTATTTAA
>gi|73761482|gb|CY002532.1| Influenza A virus (A/New York/220/2002(H1N1)) segment 8, complete sequence
AGCAAAAGCAGGGTGGCAAAGACATAATGGATTCCCACACTGTGTCAAGCTTTCAGGTAGATTGCTTCCT
TTGGCATGTCCGCAAACAAGTTGCAGACCAAAATCTAGGCGATGCCCCCTTCCTTGATCGGCTTCGCCGA
GATCAGAAGTCTCTAAAGGGAAGAGGCAGCACTCTCGGTCTGAACATCGAAACAGCCACTTGTGTTGGAA
AGCAAATAGTAGAGAGGATTCTGAAAGAAGAATCCGATGAGGCATTTAAAATGACCATGGCCTCCGCACT
TGCTTCGCGGTACCTAACTGACATGACTGTTGAAGAAATGTCAAGGGACTGGTTCATGCTCATGCCCAAG
CAGAAAGTGGCTGGCCCTCTTTGTGTCAGAATGGACCAGGCGATAATGGATAAGAACATCATACTGAAAG
CGAATTTCAGTGTGATTTTTGACCGGTTGGAGAATCTGACATTACTAAGGGCTTTCACCGAAGAGGGAGC
AATTGTTGGCGAAATTTCACCATTGCCTTCTTTTCCAGGACATACTAATGAGGATGTCAAAAATGCAATT
GGGGTCCTCATCGGGGGACTTGAATGGAATGATAACACAGTTCGAGTCTCTGAAACTCTACAGAGATTCG
CTTGGAGAAGCAGTAATGAGACTGGGGGACCTCCATTCACTACAACACAGAAACGGAAAATGGCGGGAAC
AATTAGGTCAGAAGTTTGAAGAAATAAGATGGCTGATTGAAGAAGTGAGGCATAAATTGAAGACGACAGA
GAATAGTTTTGAGCAAATAACATTTATGCAAGCATTACAGCTATTATTTGAAGTGGAACAAGAGATTAGA
ACGTTTTCGTTTCAGCTTATTTAATGATAAAAAACACCCTTGTTTCTACT
>gi|156536326|gb|CY025038.1| Influenza A virus (A/Auckland/597/2000(H1N1)) segment 8, complete sequence
ATAATGGATTCCCACACTGTGTCAAGCTTTCAGGTAGATTGCTTCCTTTGGCATGTCCGCAAACAAGTTG
CAGACCAAGATCTAGGCGATGCCCCCTTCCTTGATCGGCTTCGCCGAGATCAGAAGTCTCTAAAGGGAAG
AGGCAGCACTCTCGGTCTGAACATCGAAACAGCCACTTGTGTTGGAAAGCAAATAGTAGAGAGGATTCTG
AAAGAAGAATCCGATGAGGCATTTAAAATGACCATGGCCTCCGCACTTGCTTCGCGGTACCTAACTGACA
TGACTATTGAAGAAATGTCAAGGGACTGGTTCATGCTCATGCCCAAGCAGAAAGTGGCTGGCCCTCTTTG
TGTCAGAATGGACCAGGCGATAATGGATAAGAACATCATACTGAAAGCGAATTTCAGTGTGATTTTTGAC
CGGTTGGAGAATCTGACATTACTAAGGGCTTTCACCGAAGAGGGAGCAATTGTTGGCGAAATTTCACCAT
TGCCTTCTCTTCCAGGACATACTAATGAGGATGTCAAAAATGCAATTGGGGTCCTCATCGGGGGACTTGA
ATGGAATGATAACACAGTTCGAGTCTCTGAAACTCTACAGAGATTCACTTGGAGAAGCAGTAATGAGACT
GGGGGACCTCCATTCACTCCAACACAGAAACGGAAAATGGCGGGAACAATTAGGTCAGAAGTTTGAAGAA
ATAAGATGGCTGATTGAAGAAGTGAGGCATAAATTGAAGACGACAGAGAATAGTTTTGAGCAAATAACAT
TTATGCAAGCATTACAGCTATTGTTTGAAGTGGAACAAGAGATTAGAACGTTTTCGTTTCAGCTTATTTA
ATGATAAAAAA
>gi|149780536|gb|CY022537.1| Influenza A virus (A/Auckland/605/2001(H1N1)) segment 8, complete sequence
ATAATGGATTCCCACACTGTGTCAAGCTTTCAGGTAGACTGCTTCCTTTGGCATGTCCGCAAACAAGTTG
CAGACCAAGGTCTAGGCGATGCCCCCTTCCTTGATCGGCTTCGCCGAGATCAGAAGTCTCTAAAGGGAAG
AGGCAGCACTCTCGGTCTGAACATCGAAACAGCCACTTGTGTTGGAAAGCAAATAGTAGAGAGGATTCTG
AAAGAAGAATCCGATGAGGCATTTAAAATGACAATGGCCTCCGCACTTGCTTCGCGGTACCTAATTGACA
TGACTATTGAAGAAATGTCAAGGGACTGGTTCATGCTCATGCCCAAGCAGAAAGTGGCTGGCCCTCTTTG
TGTCAAAATGGACCAGGCGATAATGGATAAGAACATCATACTGAAAGCGAATTTCAGTGTGATTTTTGAT
CGGTTGGAGAATCTGACATTACTAAGGGCTTTCACCGAAGAGGGAGCAATTGTTGGCGAAATTTCACCAT
TGCCTTCTCTTCCAGGACATACTAATGAGGATGTCAAAAATGCAATTGGGGTCCTCATCGGGGGACTTGA
ATGGAATGATAACACAGTTCGAGTCTCTGAAACTCTACAGAGATTCGCTTGGAGAAGCAGTAATGAGACT
GGGGGACCTCCATTCACTCCAACACAGAAACGGAAAATGGCGGGAACAATTAGGTCAGAAGTTTGAAGAA

ATAAGATGGCTGATTGAAGAAGTGAGGCATAAATTGAAGACGACAGAGAATAGTTTTGAGCAAATAACAT
TTATGCAAGCATTACAGCTATTGTTTGAAGTGGAACAAGAGATTAGAACGTTTTCGTTTCAGCTTATTTA
ATGATAAAAAA
>gi|237688871|gb|CY040078.1| Influenza A virus (A/Taiwan/567/2002(H1N1)) segment 8, complete sequence
ATGGATTCCAACACTGTGTCAAGCTTTCAGGTAGACTGCTTCCTTTGGCATGTCCGCAAACAAGTTGCAG
ACCAAGGTCTAGGCGATGCCCCCTTCCTTGATCGGCTTCGCCGAGATCAGAAGTCTCTAAAGGGAAGAGG
CAGCACTCTCGGTCTGAACATCGAAACAGCCACTTGTGTTGGAAAGCAAATAGTAGAGAGGATTCTGAAA
GAAGAATCCGATGAGGCATTTAAAATGACAATGGCCTCCGCACTTGCTTCGCGGTACCTAACTGACATGA
CTATTGAAGAAATGTCAAGGGACTGGTTCATGCTCATGCCCAAGCAGAAAGTGGCTGGCCCTCTTTGTGT
CAAAATGGACCAGGCGATAATGGATAAGAACATCATACTGAAAGCGAATTTCAGTGTGATTTTTGATCGG
TTGGAGAATCTGACATTACTAAGGGCTTTCACCGAAGAGGGAGCAATTGTTGGCGAAATTTCACCATTGC
CTTCTCTTCCAGGACATACTAATGAGGATGTCAAAAATGCAATTGGGGTCCTCATCGGGGGACTTGAATG
GAATGATAACACAGTTCGAGTCTCTGAAACTCTACAGAGATTCGCTTGGAGAAGCAGTAATGAGACTGGG
GGACCTCCATTCACTCCAACACAGAAACGGAAAATGGCGGGAACAATTAGGTCAGAAGTTTGAAGAAATA
AGATGGCTGATTGAAGAAGTGAGGCATAAATTGAAGACGACAGAGAATAGTTTTGAGCAAATAACATTTA
TGCAAGCATTACAGCTATTGTTTGAAGTGGAACAAGAGATTAGAACGTTTTCGTTTCAGCTTATTTAATG
ATAAAAA
>gi|122855956|gb|CY019345.1| Influenza A virus (A/Memphis/6/2003(H1N1)) segment 8, complete sequence
ATAATGGATTCCCACACTGTGTCAAGCTTTCAGGTAGATTGCTTCCTTTGGCATGTCCGCAAACAAGTTG
CAGACCAAGATCTAGGCGATGCCCCCTTCCTTGATCGGCTTCGCCGAGATCAGAAGTCTCTAAAGGGAAG
AGGCAGCACTCTCGGTCTGAACATCGAAACAGCCACTTGTGTTGGAAAGCAAATAGTAGAGAGGATTCTG
AAAGAAGAATCCGATGAGGCATTTAAAATGACCATGGCCTCCGCACTTGCTTCGCGGTACCTAACTGACA
TGACTGTTGAAGAAATGTCAAGGGACTGGTTCATGCTCATGCCCAAGCAGAAAGTGGCTGGCCCTCTTTG
TGTCAGAATGGACCAGGCGATAATGGATAAGAACATCATACTGAAAGCGAATTTCAGTGTGATTTTTGAC
CGGTTGGAGAATCTGACATTACTAAGGGCTTTCACCGAAGAGGGAGCAATTGTTGGCGAAATTTCACCAT
TGCCTTCTTTTCCAGGACATACTAATGAGGATGTCAAAAATGCAATTGGGGTCCTCATCGGGGGACTTGA
ATGGAATGATAACACAGTTCGAGTCTCTGAAACTCTACAGAGATTCGCTTGGAGAAGCAGTAATGAGACT
GGGGGACCTCCATTCACTACAACACAGAAACGGAAAATGGCGGGAACAATTAGGTCAGAAGTTTGAAGAA
ATAAGATGGCTGATTGAAGAAGTGAGGCATAAATTGAAGACGACAGAGAATAGTTTTGAGCAAATAACAT
TTATGCAAGCATTACAGCTATTATTTGAAGTGGAACAAGAGATTAGAACGTTTTCGTTTCAGCTTATTTA
ATGATAA
>gi|82546783|gb|CY006679.1| Influenza A virus (A/New York/494/2002(H1N1)) segment 8, complete sequence
AGCAAAAGCAGGGTGGCAAAGACATAATGGATTCTCACACTGTGTCAAGCTTTCAGGTAGATTGCTTCCT
TTGGCATGTCCGCAAACAAGTTGCAGACCAAGATCTAGGCGATGCCCCCTTCCTTGATCGGCTTCGCCGA
GATCAGAAGTCTCTAAAGGGAAGAGGCAGCACTCTCGGCCTGAACATCGAAACAGCCACTTGTGTTGGAA
AGCAAATAGTAGAGAGGATTCTGAAAGAAGAATCCGATGAGGCATTTAAAATGACCATGGCCTCCGCACT
TGCTTCGCGGTACCTAACTGACATGACTGTTGAAGAAATGTCAAGGGACTGGTTCATGCTCATGCCCAAG
CAGAAAGTGGCTGGCCCTCTTTGTGTCAGAATGGACCAGGCGATAATGGATAAGAACATCATACTGAAAG
CGAATTTCAGTGTGATTTTTGACCGGTTGGAGAATCTGACATTACTAAGGGCTTTCACCGAAGAGGGAGC
AATTGTTGGCGAAATTTCACCATTGCCTTCTTTTCCAGGACATACTAATGAGGATGTCAAAAATGCAATT
GGGGTCCTCATCGGGGGACTTGAATGGAATGATAACACAGTTCGAGTCTCTGAAACTCTACAGAGATTCG

CTTGGAGAAGCAGTAATGAGACTGGGGGACCTCCATTCACTACAACACAGAAACGGAAAATGGCGGGAAC
AATTAGGTCAGAAGTTTGAAGAAATAAGATGGCTGATTGAAGAAGTGAGGCATAAATTGAAGACAACAGA
AAATAGTTTTGAGCAAATAACATTTATGCAAGCATTACAGCTATTGTTTGAAGTGGAACAAGAGATTAGA
ACATTTTCGTTTCAGCTTATTTAATGATAAAAAAC
>gi|77543350|gb|CY003308.1| Influenza A virus (A/New York/291/2002(H1N1)) segment 8, complete sequence
ATTCCCACACTGTGTCAAGCTTTCAGGTAGATTGCTTCCTTTGGCATGTCCGCAAACAAGTTGCAGACCA
AGATCTAGGCGATGCCCCCTTCCTTGATCGGCTTCGCCGAGATCAGAAGTCTCTAAAGGGAAGAGGCAGC
ACTCTTGGTCTGAACATCGAAACTGCCACTTGTGTTGGAAAGCAAATAGTAGAGAGGATTCTGAAAGAAG
AATCCGATGAGGCATTTAAAATGACCATGGCCTCCGCACTTGCTTCGCGGTACCTAACTGACATGACTGT
TGAAGAAATGTCAAGGGACTGGTTCATGCTCATGCCCAAGCAGAAAGTGGCTGGCCCTCTTTGTGTCAGA
ATGGACCAGGCGATAATGGATAAGAACATCATACTGAAAGCGAATTTCAGTGTGATTTTTGACCGGTTGG
AGAATCTGACATTACTAAGGGCTTTCACCGAAGAGGGAGCAATTGTTGGCGAAATTTCACCATTGCCTTC
TTTTCCAGGACATACTAATGAGGATGTCAAAAATGCAATTGGGGTCCTCATCGGGGGACTTGAATGGAAT
GATAACACAGTTCGAGTCTCTGAAACTCTACAGAGATTCGCTTGGAGAAGCAGTAATGAGACTGGGGGAC
CTCCATTCACTACAACACAGAAACGGAAAATGGCGGGAACAATTAGGTCAGAAGTTTGAAGAAATAAGAT
GGCTGATTGAAGAAGTGAGGCATAAATTGAAAACGACAGAGAATAGTTTTGAGCAAATAACATTTATGCA
AGCATTACAGCTATTGTTTGAAGTGGAACAAGAGATTAGAACGTTTTCGTTTCAGCTTATTTAATGATAA
AAAA
>gi|237689044|gb|CY040150.1| Influenza A virus (A/Taiwan/52/2002(H1N1)) segment 8, complete sequence
ATGGATTCCCACACTGTGTCAAGCTTTCAGGTAGACTGCTTCCTTTGGCATGTCCGCAAACAAGTTGCAG
ACCAAGGTCTAGGCGATGCCCCCTTCCTTGATCGGCTTCGCCGAGATCAGAAGTCTCTAAAGGGAAGAGG
CAGCACTCTCGGTCTGAACATCGAAACAGCCACTTGTGTTGGAAAGCAAATAGTAGAGAGGATTCTGAAA
GAAGAATCCGATGAGGCATTTAAAATGACAATGGCCTCCGCACTTGCTTCGCGGTACCTAACTGACATGA
CTATTGAAGAAATGTCAAGGGACTGGTTCATGCTCATGCCCAAGCAGAAAGTGGCTGGCCCTCTTTGTGT
CAAAATGGACCAGGCGATAATGGATAAGAACATCATACTGAAAGCGAATTTCAGTGTGATTTTTGATCGG
TTGGAGAATCTGACATTACTAAGGGCTTTCACCGAAGAGGGAGCAATTGTTGGCGAAATTTCACCATTGC
CTTCTCTTCCAGGACATACTAATGAGGATGTCAAAAATGCAATTGGGGTCCTCATCGGGGGACTTGAATG
GAATGATAACACAGTTCGAGTCTCTGAAACTCTACAGAGATTCGATTGGAGAAGCAGTAATGAGACTGGG
GAACCTCCATTCACTCCAACACAGAAACGGAAAATGGCGGGAACAATTAGGTCAGAAGTTTGAAGAAATA
AGATGGCTGATTGAAGAAGTGAGGCATAAATTGAAGACGACAGAGAATAGTTTTGAGCAAATAACATTTA
TGCAAGCATTACAGCTATTGTTTGAAGTGGAACAAGAGATTAGAACGTTTTCGTTTCAGCTTATTTAA
>gi|237689063|gb|CY040158.1| Influenza A virus (A/Taiwan/123/2002(H1N1)) segment 8, complete sequence
TAATGGATTCCCACACTGTGTCAAGCTTTCAGGTAGACTGCTTCCTTTGGCATGTCCGCAAACAAGTTGC
AGACCAAGGTCTAGGCGATGCCCCCTTTCTTGATCGGCTTCGCCGAGATCAGAAGTCTCTAAAGGGAAGA
GGCAGCACTCTCGGTCTGAACATCGAAACAGCCACTTGTGTTGGAAAGCAAATAGTAGAGAGGGTTCTGA
AAGAAGAATCCGATGAGGCATTTAAAATGACAATGGCCTCCGCACTTGCTTCGCGGTACCTAACTGACAT
GACTATTGAAGAAATGTCAAGGGACTGGTTCATGCTCATGCCCAAGCAGAAAGTGGCTGGCCCTCTTTGT
GTCAAAATGGACCAGGCGATAATGGATAAGAACATCATACTGAAAGCGAATTTCAGTGTGATCTTTGATC
GGTTGGAGAATCTGACATTACTAAGGGCTTTCACCGAAGAGGGAGCAATTGTTGGCGAAATTTCACCATT
GCCTTCTCTTCCAGGACATACTAATGAGGATGTCAAAAATGCAATTGGGGTCCTCATCGGGGGACTTGAA
TGGAATGATAACACAGTTCGAGTCTCTGAAACTCTACAGAGATTCGCTTGGAGAAGCAGTAATGAGACTG

GGGGACCTCCATTCACTCCAACACAGAAACGGAAAATGGCGGGAACAATTAGGTCAGAAGTTTGAAGAAA
TAAGATGGCTGATTGAAGAAGTGAGGCATAAATTGAAGACGACAGAGAATAGTTTTGAGCAAATAACATT
TATGCAAGCATTACAGCTATTGTTTGAAGTGGAACAAGAGATCAGAACGTTTTCGTTTCAGCTTATTTAA
>gi|77747430|gb|CY003692.1| Influenza A virus (A/New York/486/2003(H1N1)) segment 8, complete sequence
GCAAAAGCAGGGTGACAAAGACATAATGGATTCCCACACTGTGTCAAGCTTTCAGGTAGATTGCTTCCTT
TGGCATGTCCGCAAACAAGTTGCAGACCAAGATCTAGGCGATGCCCCCTTCCTTGATCGGCTTCGCCGAG
ATCAGAAGTCTCTAAAGGGAAGAGGCAGCACTCTCGGTCTGAACATCGAAACAGCCACTTGTGTTGGAAA
GCAAATAGTAGAGAGGATTCTGAAAGAAGAATCCGATGAGGCATTTAAAATGACCATGGCCTCCGCACTT
GCTTCGCGGTACCTAACTGACATGACTGTTGAAGAAATGTCAAGGGACTGGTTCATGCTCATGCCCAAGC
AGAAAGTGGCTGGCCCTCTTTGTGTCAGAATGGACCAGGCGATAATGGATAAGAACATCATACTGAAAGC
GAATTTCAGTGTGATTTTTGACCGGTTGGAGAATCTGACATTACTAAGGGCTTTCACCGAAGAGGGAGCA
ATTGTTGGCGAAATTTCACCATTGCCTTCTTTTCCAGGACATACTAATGAGGATGTCAAAAATGCAATTG
GGGTCCTCATCGGGGGACTTGAATGGAATGATAACACAGTTCGAGTCTCTGAAACTCTACAGAGATTCGC
TTGGAGAAGCAGTAATGAGACTGGGGGACCTCCATTCACTACAACACAGAAACGGAAAATGGCGGGAACA
ATTAGGTCAGAAGTTTGAAGAAATAAGATGGCTGATTGAAGAAGTGAGGCATAAATTGAAGACGACAGAG
AATAGTTTTGAGCAAATAACATTTATGCAAGCATTACAGCTATTATTTGAAGTGGAACAAGAGATTAGAA
CGTTTTCGTTTCAGCTTATTTAATGATAAAAAACACCCTTGTTTCTACT
>gi|125664184|gb|CY019887.1| Influenza A virus (A/Memphis/5/2003(H1N1)) segment 8, complete sequence
ATAATGGATTCCCACACTGTGTCAAGCTTTCAGGTAGATTGCTTCCTTTGGCATGTCCGCAAACAAGTTG
CAGACCAAGATCTAGGCGATGCCCCCTTCCTTGATCGGCTTCGCCGAGATCAGAAGTCTCTAAAGGGAAG
AGGCAGCACTCTCGGTCTGAACATCGAGACAGCCACTTGTGTTGGAAAGCAAATAGTAGAGAGGATTCTG
AAAGAAGAATCCGATGAGGCATTTAAAATGACCATGGCCTCCGCACTTGCTTCGCGGTACCTAACTGACA
TGACTGTTGAAGAAATGTCAAGGGACTGGTTCATGCTCATGCCCAAGCAGAAAGTGGCTGGCCCTCTTTG
TGTCAGAATGGACCAGGCGATAATGGATAAGAACATCATACTGAAAGCGAATTTCAGTGTGATTTTTGAC
CGGTTGGAGAATCTGACATTACTAAGGGCTTTCACCGAAGAGGGAGCAATTGTTGGCGAAATTTCACCAT
TGCCTTCTTTTCCAGGACATACTAATGAGGATGTCAAAAATGCAATTGGGGTCCTCATCGGGGGACTTGA
ATGGAATGATAACACAGTTCGAGTCTCTGAAACTCTACAGAGATTCGCTTGGAGAAGCAGTAATGAGACT
GGGGGACCTCCATTCACTACAACACAGAAACGGAAAATGGCGGGAACAATTAGGTCAGAAGTTTGAAGAA
ATAAGATGGCTGATTGAAGAAGTGAGGCATAAATTGAAGACGACAGAGAATAGTTTTGAGCAAATAACAT
TTATGCAAGCATTACAGCTATTATTTGAAGTGGAACAAGAGATTAGAACGTTTTCGTTTCAGCTTATTTA
A
>gi|73763203|gb|CY002540.1| Influenza A virus (A/New York/227/2003(H1N1)) segment 8, complete sequence
AGCAAAAGCAGGGTGGCAAAGACATAATGGATTCCCACACTGTGTCAAGCTTTCAGGTAGATTGCTTCCT
TTGGCATGTCCGCAAACAAGTTGCAGACCAAGATCTAGGCGATGCCCCCTTCCTTGATCGGCTTCGCCGA
GATCAGAAGTCTCTAAAGGGAAGAGGCAGCACTCTCGGTCTGAACATCGAAACAGCCACTTATGTTGGAA
AGCAAATAGTAGAGAGGATTCTGAAAGAAGAATCCGATGAGGCATTTAAAATGACCATGGCCTCCGCACT
TGCTTCGCGGTACCTAACTGACATGACTGTTGAAGAAATGTCAAGGGACTGGTTCATGCTCATGCCCAAG
CAGAAAGTGGCTGGCCCTCTTTGTGTCAGAATGGACCAGGCGATAATGGATAAGAACATCATACTGAAAG
CGAATTTCAGTGTGATTTTTGACCGGTTGGAGAATCTGACATTACTAAGGGCTTTCACCGAAGAGGGAGC
AATTGTTGGCGAAATTTCACCATTGCCTTCTTTTCCAGGACATACTAATGAGGATGTCAAAAATGCAATT
GGGGTCCTCATCGGGGGACTTGAATGGAATGATAACACAGTTCGAGTCTCTGAAACTCTACAGAGATTCG

CTTGGAGAAGCAGTAATGAGACTGGGGGACCTCCATTCACTACAACACAGAAACGGAAAATGGCGGGAAC
AATTAGGTCAGAAGTTTGAAGAAATAAGATGGCTGATTGAAGAAGTGAGGCATAAATTGAAGACGACAGA
GAATAGTTTTGAGCAAATAACATTTATGCAAGCATTACAGCTATTATTTGAAGTGGAACAAGAGATTAGA
ACGTTTTCGTTTCAGCTTATTTAATGATAAAAAACACCCTTGTTTCTACT
>gi|89112174|gb|CY009000.1| Influenza A virus (A/New York/484/2003(H1N1)) segment 8, complete sequence
CAAAAGCAGGGTGGCAAAGACATAATGGATTCCCACACTGTGTCAAGCTTTCAGGTAGATTGCTTCCTTT
GGCATGTCCGCAAACAAGTTGCAGACCAAAATCTAGGCGATGCCCCCTTCCTTGATCGGCTTCGCCGAGA
TCAGAAGTCTCTAAAGGGAAGAGGCAGCACTCTCGGTCTGAACATCGAAACAGCCACTTGTGTTGGAAAG
CAAATAGTAGAGAGGATTCTGAAAGAAGAATCCGATGAGGCATTTAAAATGACCATGGCCTCCGCACTTG
CTTCGCGGTACCTAACTGACATGACTGTTGAAGAAATGTCAAGGGACTGGTTCATGCTCATGCCCAAGCA
GAAAGTGGCTGGCCCTCTTTGTGTCAGAATGGACCAGGCGATAATGGATAAGAACATCATACTGAAAGCG
AATTTCAGTGTGATTTTTGACCGGTTGGAGAATCTGACATTACTAAGGGCTTTCACCGAAGAGGGAGCAA
TTGTTGGCGAAATTTCACCATTGCCTTCTTTTCCAGGACATACTAATGAGGATGTCAAAAATGCAATTGG
GGTCCTCATCGGGGGACTTGAATGGAATGATAACACAGTTCGAGTCTCTGAAACTCTACAGAGATTCGCT
TGGAGAAGCAGTAATGAGACTGGGGGACCTCCATTCACTACAACACAGAAACGGAAAATGGCGGGAACAA
TTAGGTCAGAAGTTTGAAGAAATAAGATGGCTGATTGAAGAAGTGAGGCATAAATTGAAGACGACAGAGA
ATAGTTTTGAGCAAATAACATTTATGCAAGCATTACAGCTATTATTTGAAGTGGAACAAGAGATTAGAAC
GTTTTCGTTTCAGCTTATTTAATGATAAAAAA
>gi|83727850|gb|CY006919.1| Influenza A virus (A/New York/488/2003(H1N1)) segment 8, complete sequence
GGTGGCAAAGACATAATGGATTCCCACACTGTGTCAAGCTTTCAGGTAGATTGCTTCCTTTGGCATGTCC
GCAAACAAGTTGCAGACCAAGATCTAGGCGATGCCCCCTTCCTTGATCGGCTTCGCCGAGATCAGAAGTC
TCTAAAGGGAAGAGGCAGCACTCTCGGTCTGAACATCGAAACAGCCACTTGTGTTGGAAAGCAAATAGTA
GAGAGGATTCTGAAAGAAGAATCCGATGAGGCATTTAAAATGACCATGGCCTCCGCACTTGCTTCGCGGT
ACCTAACTGACATGACTGTTGAAGAAATGTCAAGGGACTGGTTCATGCTCATGCCCAAGCAGAAAGTGGC
TGGCCCTCTTTGTGTCAGAATGGACCAGGCGATAATGGATAAGAACATCATACTGAAAGCGAATTTCAGT
GTGATTTTTGACCGGTTGGAGAATCTGACATTACTAAGGGCTTTCACCGAAGAGGGAGCAATTGTTGGCG
AAATTTCACCATTGCCTTCTTTTCCAGGACATACTAATGAGGATGTCAAAAATGCAATTGGGGTCCTCAT
CGGGGGACTTGAATGGAATGATAACACAGTTCGAGTCTCTGAAACTCTACAGAGATTCGCTTGGAGAAGC
AGTAATGAGACTGGGGGACCTCCATTCACTACAACACAGAAACGGAAAATGGCGGGAACAATTAGGTCAG
AAGTTTGAAGAAATAAGATGGCTGATTGAAGAAGTGAGGCATAAATTGAAGACGACAGAGAATAGTTTTG
AGCAAATAACATTTATGCAAGCATTACAGCTATTATTTGAAGTGGAACAAGAGATTAGAACGTTTTCGTT
TCAGCTTATTTAATGATAAAAAA
>gi|157281266|gb|CY025217.1| Influenza A virus (A/Texas/UR06-0012/2006(H1N1)) segment 8, complete sequence
TAATGGATTCCCACACTGTGTCAAGCTTTCAGGTAGATTGCTTCCTTTGGCATGTCCGCAAACAAGTTGC
AGACCAAAATCTAGGCGATGCCCCCTTCCTTGATCGGCTTCGCCGAGATCAGAAGTCTCTAAAGGGAAGA
GGCAGCACTCTCGGTCTGAACATCGAAACAGCTACTTGTGTTGGAAAGCAAATAGTAGAGAGGATTCTGA
AAGAAGAATCCGATGAGGCACTTAAAATGACCATTGCCTCCGCACTTGCTTCGCGGTACCTAACTGACAT
GACTGTTGAAGAAATGTCAAGGGACTGGTTCATGCTCATGCCCAAGCAAAAAGTGGCTGGCCCTCTTTGT
GTCAGAATGGACCAGGCAATAATGGATAAGAACATCATACTGAAAGCGAATTTCAGTGTGATTTTTGACC
GGTTGGAGAATCTGACATTACTAAGGGCTTTCACCGAAGAGGGAGCAATTGTTGGCGAAATTTCACCATT
GCCTTCTTTTCCAGGACATACTAATGAGGATGTCAAAAATGCAATTGGGGTCCTCATCGGGGGACTTGAA

TGGAATGATAACACAGTTCGAGTCTCTGAAACTCTACAGAGATTCGCTTGGAGAAGCAGTAATGAGACTG
GGGGACCTCCATACACTACAACACAGAAACGGAAAATGGCGGGAACAACTAGGTCAGAAGTTTGAAGAAA
TAAGATGGCTGATTGAAGAAGTGAGGCATAAATTGAAGACGACAGAGAATAGTTTTGAGCAAATAACATT
TATGCAAGCATTACAGCTATTATTTGAAGTGGAACAAGAGATTAGAACGTTTTCGTTTCAGCTTATTTAG
TGATAAAAA
>gi|94959541|gb|CY010768.1| Influenza A virus (A/Canterbury/20/2001(H1N1)) segment 8, complete sequence
TGGCAAAGACATAATGGATTCCCACACTGTGTCAAGCTTTCAGGTAGACTGCTTCCTTTGGCATGTCCGC
AAACAAGTTGCAGACCAAGGTCTAGGCGATGCCCCCTTCCTTGATCGGCTTCGCCGAGATCAGAAGTCTC
TAAAGGGAAGAGGCAGCACTCTCGGTCTGAACATCGAAACAGCCACTTGTGTTGGAAAGCAAATAGTAGA
GAGGATTCTGAAAGAAGAATCCGATGAGGCATTTAAAATGACAATGGCCTCCGCACTTGCTTCGCGGTAC
CTAACTGACATGACTATTGAAGAAATGTCAAGGGACTGGTTCATGCTCATGCCCAAGCAGAAAGTGGCTG
GCCCTCTTTGTGTCAAAATGGACCAGGCGATAATGGATAAGAACATCATACTGAAAGCGAATTTCAGTGT
GATTTTTGATCGGTTGGAGAATCTGACATTACTAAGGGCTTTCACCGAAGAGGGAGCAATTGTTGGCGAA
ATTTCACCATTGCCTTCTCTTCCAGGACATACTAATGAGGATGTCAAAAATGCAATTGGGGTCCTCATCG
GGGGACTTGAATGGAATGATAACACAGTTCGAGTCTCTGAAACTCTACAGAGATTCGCTTGGAGAAGCAG
TAATGAGACTGGGGGACCTCCATTCACTCCAACACAGGAACGGAAAATGGCGGGAACAATTAGGTCAGAA
GTTTGAAGAAATAAGATGGCTGATTGAAGAAGTGAGGCATAAATTGAAGACGACAGAGAATAGTTTTGAG
CAAATAACATTTATGCAAGCATTACAGCTATTGTTTGAAGTGGAACAAGAGATTAGAACGTTTTCGTTTC
AGCTTATTTAATGATAAAAAA
>gi|91119035|gb|CY010408.1| Influenza A virus (A/West Coast/33/2001(H1N1)) segment 8, complete sequence
TGGCAAAGACATAATGGATTCCCACACTGTGTCAAGCTTTCAGGTAGACTGCTTCCTTTGGCATGTCCGC
AAACAAGTTGCAGACCAAGGTCTAGGCGATGCCCCCTTCCTTGATCGGCTTCGCCGAGATCAGAAGTCTC
TAAAGGGAAGAGGCAGCACTCTCGGTCTGAACATCGAAACAGCCACTTGTGTTGGAAAGCAAATAGTAGA
GAGGATTCTGAAAGAAGAATCCGATGAGGCATTTAAAATGACAATGGCCTCCGCACTTGCTTCGCGGTAC
CTAACTGACATGACTATTGAAGAAATGTCAAGGGACTGGTTCATGCTCATGCCCAAGCAGAAAGTGGCTG
GCCCTCTTTGTGTCAAAATGGACCAGGCGGTAATGGATAAGAACATCATACTGAAAGCGAATTTCAGTGT
GATTTTTGATCGGTTGGAGAATCTGACATTACTAAGGGCTTTCACCGAAGAGGGAGCAATTGTTGGCGAA
ATTTCACCATTGCCTTCTCTTCCAGGACATACTAATGAGGATGTCAAAAATGCAATTGGGGTCCTCATCG
GGGGACTTGAATGGAATGATAACACAGTTCGAGTCTCTGAAACTCTACAGAGATTCGCTTGGAGAAGCAG
TAATGAGACTGGGGGACCTCCATTCACTCCAACACAGAAACGGAAAATGGCGGGAACAATTAGGTCAGAA
GTTTGAAGAAATAAGATGGCTGATTGAAGAAGTGAGGCATAAATTGAAGACGACAGAGAATAGTTTTGAG
CAAATAACATTTATGCAAGCATTACAGCTATTGTTTGAAGTGGAACAAGAGATTAGAACGTTTTCGTTTC
AGCTTATTTAATGATAAAAAA
>gi|131052834|gb|CY020001.1| Influenza A virus (A/Waikato/17/2005(H1N1)) segment 8, complete sequence
TAATGGATTCCCACACTGTGTCAAGCTTTCAGGTAGATTGCTTCCTTTGGCATGTCCGCAAACAAGTTGC
AGACCAAGATCTAGGAGATGCTCCCTTCCTTGATCGGCTTCGCCGAGATCAGAAGTCTCTAAAGGGAAGA
GGCAGCACTCTCGGTCTGAATATCGAAACAGCTACTTGTGTTGGAAAGCAAATAGTAGAGAGGATTCTGA
AAGAAGAATCCGATGAGGCATTTAAAATGACCATGGCCTCCGCACTTGCTTCGCGGTACCTAACCGACAT
GACTGTTGAAGAAATGTCAAGGGACTGGTTCATGCTCATGCCCAAGCAGAAAGTGGCTGGCCCTCTTTGT
GTCAGAATGGACCAGGCGATAATGGATAAGAACATCATACTGAAAGCTAATTTCAGTGTGATTTTTGACC
GGTTGGAAAATCTGACATTACTAAGGGCTTTCACCGAAGAGGGAGCAATTGTTGGCGAAATTTCACCATT

GCCTTCTTTTCCAGGACATACTAATGAGGATGTCAAAAATGCAATTGGGGTCCTCATCGGGGGACTTGAA
TGGAATGATAACACAGTTCGAGTCTCTGAAACTCTACAGAGATTCGCTTGGAGAAGCAGTAATGAGACTG
GGGGACCTCCATTCACTACAACACAGAAACGGAAAATGGAGGGAACAATTAGGTCAGAAGTTTGAAGAAA
TAAGATGGCTGATTGAAGAAGTGAGGCATAAATTGAAGACGACAGAGAATAGTTTTGAGCAAATAACATT
TATGCAAGCATTACAACTATTATTTGAAGTGGAACAAGAGATTAGAACGTTTTCGTTTCAGCTTATTTAG
TGATAAAAAA
>gi|83744843|gb|CY007471.1| Influenza A virus (A/Canterbury/106/2004(H1N1)) segment 8, complete sequence
GGTGACAAAGACATAATGGATTCCCACACTGTGTCAAGCTTTCAGGTAGATTGCTTCCTTTGGCATGTCC
GCAAACAAGTTGCAGACAAAGATCTAGGCGATGCCCCCTTCCTTGATCGGCTTCGCCGAGATCAGAAGTC
TCTAAAGGGAAGAGGCAGCACTCTCGGTTTGAACATCGAAACAGCCACTTGTGTTGGAAAGCAAATAGTA
GAGAGGATTCTGAAAGAAGAATCCGATGAGGCATTTAAAATGACCATGGCTTCCGCACTTGCTTCGCGGT
ACCTAACTGACATGACTGTTGAAGAAATGTCAAGGGACTGGTTCATGCTCATGCCCAAGCAGAAAGTGGC
TGGCCCTCTTTGTGTCAGAATGGACCAGGCGATAATGGATAAGAACATCATACTGAAAGCTAATTTCAGT
GTGATTTTTGACCGGTTGGAGAATCTGACATTACTAAGGGCTTTCACCGAAGAGGGAGCAATTGTTGGCG
AAATTTCACCATTGCCTTCTTTTCCAGGACATACTAATGAGGATGTCAAAAATGCAATTGGGGTCCTCAT
CGGGGGACTTGAATGGAATGATAACACAGTTCGAGTCTCTGAAACTCTACAGAGATTCGCTTGGAGAAGC
AGTAATGAGACTGGGGGACCTCCATTCACTACAACACAGAAACGGAAAATGGCGGGAACAATTAGGTCAG
AAGTTTGAAGAAATAAGATGGCTGATTGAAGAAGTGAGGCATAAATTGAAGACGACAGAGAATAGTTTTG
AGCAAATAACATTTATGCAAGCATTACAGCTATTGTTTGAAGTGGAACAAGAGATTAGAACGTTCTCGTT
TCAGCTTATTTAATGATAAAAAA
>gi|115607830|gb|CY016703.1| Influenza A virus (A/South Australia/58/2005(H1N1)) segment 8, complete sequence
GACATAATGGATTCCCACACTGTGTCAAGCTTTCAGGTAGATTGCTTCCTTTGGCATGTCCGCAAACAAG
TTGCAGACCAAGATCTAGGCGATGCCCCCTTCCTTGATCGGCTTCGCCGAGATCAGAAGTCTCTAAAGGG
AAGAGGCAGCACTCTCGGTCTGAACATCGAAACAGCTACTTGTGTTGGAAAGCAAATAGTAGAGAGGATT
CTGAAAGAAGAATCCGATGAGGCATTTAAAATGACCATGGCCTCCGCACTTGCTTCGCGGTACCTAACCG
ACATGACTGTTGAAGAAATGTCAAGGGACTGGTTCATGCTCATGCCCAAGCAGAAAGTGGCTGGCCCTCT
TTGTGTCAGAATGGACCAGGCGATAATGGATAAGAACATCATACTGAAAGCTAATTTCAGTGTGATTTTT
GACCGGTTGGAGAATCTGACATTACTAAGGGCTTTCACCGAAGAGGGAGCAATTGTTGGCGAAATTTCAC
CATTGCCTTCTTTTCCAGGACATACTAATGAGGATGTCAAAAATGCAATTGGGGTCCTCATCGGGGGACT
TGAATGGAATGATAACACAGTTCGAGTCTCTGAAACTCTACAGAGATTCGCTTGGAGAAGCAGTAATGAG
ACTGGGGGACCTCCATTCACTACAACACAGAAACGGAAAATGGCGGGAACAATTAGGTCAGAAGTTTGAA
GAAATAAGATGGCTGATTGAAGAAGTGAGGCATAAATTGAAGACGACAGAGAATAGTTTTGAGCAAATAA
CATTTATGCAAGCATTACAACTATTATTTGAAGTGGAACAAGAGATTAGAACGTTTTCGTTTCAGCTTAT
TTAGTGATAAAAAA
>gi|113170890|gb|CY014011.1| Influenza A virus (A/Wellington/11/2005(H1N1)) segment 8, complete sequence
GACATAATGGATTCCCACACTGTGTCAAGCTTTCAGGTAGATTGCTTCCTTTGGCATGTCCGCAAACAAG
TTGCAGACCAAGATTTAGGCGATGCCCCCTTCCTTGATCGGCTTCGCCGAGATCAGAAGTCTCTAAAGGG
AAGAGGCAGCACTCTCGGTCTGAACATCGAAACAGCTACTTGTGTTGGAAAGCAAATAGTAGAGAGGATT
CTGAAAGAAGAATCCGATGAGGCACTTAAAATGACCATGGCCTCCGCACTTGCTTCGCGGTACCTAACTG
ACATGACTGTTGAAGAAATGTCAAGGGACTGGTTCATGCTCATGCCCAAGCAAAAAGTGGCTGGCCCTCT
TTGTATCAGAATGGACCAGGCAATAATGGATAAGAACATCATACTGAAAGCGAATTTCAGTGTGATTTTT

GACCGGTTGGAGAATCTGACATTACTAAGGGCTTTCACCGAAGAGGGAGCAATTGTTGGCGAAATTTCAC
CATTGCCTTCTTTTCCAGGACATACTAATGAGGATGTCAAAAATGCAATTGGGGTCCTCATCGGGGGACT
TGAATGGAATGATAACACAGTTCGAGTCTCTGAAACTCTACAGAGATTCGCTTGGAGAAGCAGTAATGAG
ACTGGGGGACCTCCATTCACTACAACACAGAAACGGAAAATGGCGGGAACAACTAGGTCAGAAGTTTGAA
GAAATAAGATGGCTGATTGAAGAAATGAGGCATAAATTGAAGACGACAGAGAATAGTTTTGAGCAAATAA
CATTTATGCAAGCATTACAGCTATTATTTGAAGTGGAACAAGAGATTAGAACGTTTTCGTTTCAGCTTAT
TTAGTAATAAAAA
>gi|145278932|gb|CY021761.1| Influenza A virus (A/South Australia/51/2005(H1N1)) segment 8, complete sequence
ATAATGGATTCCCACACTGTGTCAAGCTTTCAGGTAGATTGCTTCCTTTGGCATGTCCGCAAACAAGTTG
CAGACCAAGATCTAGGCGATGCCCCCTTCCTTGATCGGCTTCGCCGAGATCAGAAGTCTCTAAAGGGAAG
AGGCAGCACTCTCGGTCTGAACATCGAAACAGCTACTTGTGTTGGAAAGCAAATAGTAGAGAGGATTCTG
AAAGAAGAATCCGATGAGGCACTTAAAATGACCATGGCCTCCGCACTTGCTTCGCGGTACCTAACTGACA
TGACTGTTGAAGAAATGTCAAGGGACTGGTTCATGCTCATGCCCAAGCAAAAAGTGGCTGGCCCTCTTTG
TGTCAGAATGGACCAGGCAATAATGGATAAGAACATCATACTGAAAGCGAATTTCAGTGTGATTTTTGAC
CGGTTGGAGAATCTGACATTACTAAGGGCTTTCACCGAAGAGGGAGCAATTGTTGGCGAAATTTCACCAT
TGCCTTCTTTTCCAGGACATACTAATGAGGATGTCAAAAATGCAATTGGGGTCCTCATCGGGGGACTTGA
ATGGAATGATAACACAGTTCGAGTCTCTGAAACTCTACAGAGATTCGCTTGGAGAAGCAGTAATGAGACT
GGGGGACCTCCATTCACTACAACACAGAAACGGAAAATGGCGGGAACAACTAGGTCAGAAGTTTGAAGAA
ATAAGATGGCTGATTGAAGAAATGAGGCATAAATTGAAGACGACAGAGAATAGTTTTGAGCAAATAACAT
TTATGCAAGCATTACAGCTATTATTTGAAGTGGAACAAGAGATTAGAACGTTTTCGTTTCAGCTTATTTA
GTAATAAAAAA
>gi|149780702|gb|CY022585.1| Influenza A virus (A/Auckland/619/2005(H1N1)) segment 8, complete sequence
TGGCAAAGACATAATGGATTCCCACACTGTGTCAAGCTTTCAGGTAGATTGCTTCCTTTGGCATGTCCGC
AAACAAGTTGCAGACCAAGATCTAGGCGATGCCCCCTTCCTTGATCGGCTTCGCCGAGATCAGAAGTCTC
TAAAGGGAAGAGGCAGCACTCTCGGTCTGAACATCGAAACAGCTACTTGTGTTGGAAAGCAAATAGTAGA
GAGGATTCTGAAAGAAGAATCCGATGAGGCACTTAAAATGACCATGGCCTCCGCACTTGCTTCGCGGTAC
CTAACTGACATGACTGTTGAAGAAATGTCAAGGGACTGGTTCATGCTCATGCCCAAGCAAAAAGTGGCTG
GCCCTCTTTGTGTCAGAATGGACCAGGCAATAATGGATAAGAACATCATACTGAAAGCGAATTTCAGTGT
GATTTTTGACCGGTTGGAGAATCTGACATTACTAAGGGCTTTCACCGAAGAGGGAGCAATTGTTGGCGAA
ATTTCACCATTGCCTTCTTTTCCAGGACATACTAATGAGGATGTCAAAAATGCAATTGGGGTCCTCATCG
GAGGACTTGAATGGAATGATAACACAGTTCGAGTCTCTGAAACTCTACAGAGATTCGCTTGGAGAAGCAG
TAATGAGACTGGGGGACCTCCATTCACTACAACACAGAAACGGAAAATGGCGGGAACAACTAGGTCAGAA
GTTTGAAGAAATAAGATGGCTGATTGAAGAAATGAGGCATAAATTGAAGACGACAGAGAATAGTTTTGAG
CAAATAACATTTATGCAAGCATTACAGCTATTATTTGAAGTGGAACAAGAGATTAGAACGTTTTCGTTTC
AGCTTATTTAGTAATAA
>gi|117572947|gb|CY017319.1| Influenza A virus (A/Waikato/4/2005(H1N1)) segment 8, complete sequence
ATAATGGATTCCCACACTGTGTCAAGCTTTCAGGTAGATTGCTTCCTTTGGCATGTCCGCAAACAAGTTG
CAGACCAAGATCTAGGAGATGCTCCCTTCCTTGATCGGCTTCGCCGAGATCAGAAGTCTCTAAAGGGAAG
AGGCAGCACTCTCGGTCTGAATATCGAAACAGCTACTTGTGTTGGAAAGCAAATAGTAGAGAGGATTCTG
AAAGAAGAATCCGATGAGGCATTTAAAATGACCATGGCCTCCGCACTTGCTTCGCGGTACCTAACCGACA
TGACTGTTGAAGAAATGTCAAGGGACTGGTTCATGCTCATGCCCAAGCAGAAAGTGGCTGGCCCTCTTTG

TGTCAGAATGGACCAGGCGATAATGGATAAGAACATCATACTGAAAGCTAATTTCAGTGTGATTTTTGAC
CGGTTGGAAAATCTGACATTACTAAGGGCTTTCACCGAAGAGGGAGCAATTGTTGGCGAAATTTCACCAT
TGCCTTCTTTTCCAGGACATACTAATGAGGATGTCAAAAATGCAATTGGGGTCCTCATCGGGGGACTTGA
ATGGAATGATAACACAGTTCGAGTCTCTGAAACTCTACAGAGATTCGCTTGGAGAAGCAGTAATGAGACT
GGGGGACCTCCATTCACTACAACACAGAAACGGAAAATGGAGGGAACAATTAGGTCAGAAGTTTGAAGAA
ATAAGATGGCTGATTGAAGAAGTGAGGCATAAATTGAAGACGACAGAGAATAGTTTTGAGCAAATAACAT
TTATGCAAGCATTACAACTATTATTTGAAGTGGAACAAGAGATTAGAACGTTTTCGTTTCAGCTTATTTA
GTGATAAAAAA
>gi|161139453|gb|CY028199.1| Influenza A virus (A/Kentucky/UR06-0007/2006(H1N1)) segment 8, complete sequence
ATAATGGATTCCCACACTGTGTCAAGCTTTCAGGTAGATTGCTTCCTTTGGCATGTCCGCAAACAAGTTG
CAGACCAAGATCTAGGCGATGCCCCCTTCCTTGATCGGCTTCGCCGGGATCAGAAGTCTCTAAAGGGAAG
AGGCAGCACTCTCGGTCTGAACATCGAAACAGCTACTTGTGTTGGAAAGCAAATAGTAGAGAGGATTCTG
AAGGAAGAATCCGATGAGGCACTTAAAATGACCATGGCCTCCGCACTTGCTTCGCGGTACCTAACTGACA
TGACTGTTGAAGAAATGTCAAGGGACTGGTTCATGCTCATGCCCAAGCAAAAAGTGGCTGGCCCTCTTTG
TGTCAGAATGGACCAGGCAATAATGGATAAGAACATCATACTGAAAGCGAATTTCAGTGTGATTTTTGAC
CGGTTGGAGAATCTGACATTACTAAGGGCTTTCACCGAAGAGGGAGCAATTGTTGGCGAAATTTCACCAT
TGCCTTCTTTTCCAGGACATACTAATGAGGATGTCAAAAATGCAATTGGGGTCCTCATCGGGGGACTTGA
ATGGAATGATAACACAGTTCGAGTCTCTGAAACTCTACAGAGATTCGCTTGGAGAAGCAGTAATGAGACT
GGGGGACCTCCATTCACTACAACACAGAAACGGCAAATGGCGGGAACAACTAGGTCAGAAGTTTGAAGAA
ATAAGATGGCTGATTGAAGAAGTGAGGCATAAATTGAAGACGACAGAGAATAGTTTTGAGCAAATAACAT
TTATGCAAGCATTACAGCTGTTATTTGAAGTGGAACAAGAGATTAGAACGTTTTCGTTTCAGCTTATTTA
GT
>gi|157281285|gb|CY025225.1| Influenza A virus (A/Michigan/UR06-0015/2006(H1N1)) segment 8, complete sequence
ATAATGGATTCCCACACTGTGTCAAGCTTTCAGGTAGATTGCTTCCTTTGGCATGTCCGCAAACAAGTTG
CAGACCAAGATCTAGGCGATGCCCCCTTCCTAGATCGGCTTCGCCGAGATCAGAAGTCTCTAAAGGGAAG
AGGCAGCACTCTCGGTCTGAACATCGAAACAGCTACTTGTGTTGGAAAGCAAATAGTAGAGAGGATTCTG
AAAGAAGAATCCGATGAGGCACTTAAAATGACCATGGCCTCCGCACTTGCTTCGCGGTACCTAACTGACA
TGACTGTTGAAGAAATGTCAAGGGACTGGTTCATGCTCATGCCCAAGCAAAAAGTGGCTGGCCCTCTTTG
TGTCAGAATGGACCAGGCAATAATGGATAAGAACATCATACTGAAAGCGAATTTCAGTGTGATTTTTGAC
CGGTTGGAGAATCTGACATTACTAAGGGCTTTCACCGAAGAGGGAGCAATTGTTGGCGAAATTTCACCAT
TGCCTTCTTTTCCAGGACATACTAATGAGGATGTCAAAAATGCAATTGGGGTCCTCATCGGGGGACTTGA
ATGGAATGATAACACAGTTCGAGTCTCTGAAACTCTACAGAGATTCGCTTGGAGAATCAGTAATGAGACT
GGGGGACCTCCATTCACTACAACACAGAAACGGAAAATGGCGGGAACAACTAGGTCAGAAATTTGAAGAA
ATAAGATGGCTGATTGAAGAAGTGAGGCATAAATTGAAGACGACAGAGAATAGTTTTGAGCAAATAACAT
TTATGCAAGCATTACAGCTATTATTTGAAGTGGAACAAGAGATTAGAACGTTTTCGTTTCAGCTTATTTA
GTGATAAAAAA
>gi|218875177|gb|CY036923.1| Influenza A virus (A/NYMC X-163A(NYMC X-157-St. Petersburg/8/2006)(H1N1)) segment 8, complete sequence
ATGGATCCAAACACTGTGTCAAGCTTTCAGGTAGATTGCTTTCTTTGGCATGTCCGCAAACGAGTTGCAG
ACCAAGAACTAGGTGATGCCCCATTCCTTGATCGGCTTCGCCGAGATCAGAAATCCCTAAGAGGAAGGGG
CAGCACCCTCGGTCTGGACATCGAGACAGCCACACGTGCTGGAAAGCAGATAGTGGAGCGGATTCTGAAA
GAAGAATCCGATGAGGCACTTAAAATGACCATGGCCTCTGTACCTGCGTCGCGTTACCTAACTGACATGA

CTCTTGAGGAAATGTCAAGGGACTGGTCCATGCTCATACCCAAGCAGAAAGTGGCAGGCCCTCTTTGTAT
CAGAATGGACCAGGCGATCATGGATAAGAACATCATACTGAAAGCGAACTTCAGTGTGATTTTTGACCGG
CTGGAGACTCTAATATTGCTAAGGGCTTTCACCGAAGAGGGAGCAATTGTTGGCGAAATTTCACCATTGC
CTTCTCTTCCAGGACATACTGCTGAGGATGTCAAAAATGCAGTTGGAGTCCTCATCGGGGGACTTGAATG
GAATGATAACACAGTTCGAGTCTCTGAAACTCTACAGAGATTCGCTTGGAGAAGCAGTAATGAGAATGGG
AGACCTCCACTCACTCCAAAACAGAAACGAGAAATGGCGGGAACAATTAGGTCAGAAGTTTGAAGAAATA
AGATGGTTGATTGAAGAAGTGAGACACAAACTGAAGATAACAGAGAATAGTTTTGAGCAAATAACATTTA
TGCAAGCCTTACATCTATTGCTTGAAGTGGAGCAAGAGATAAGAACTTTCTCGTTTCAGCTTATTTAGTA
ATAAAAAA
>gi|208344092|gb|CY035130.1| Influenza A virus (A/St. Petersburg/8/2006(H1N1)) segment 8, complete sequence
ATGGATTCCCACACTGTGTCAAGCTTTCAGGTAGATTGCTTCCTTTGGCATGTCCGCAAACAAGTTGCAG
ACAAAGATCTAGGCGATGCCCCCTTCCTTGATCGGCTTCGCCGAGATCAGAAGTCTCTAAAGGGAAGAGG
CAGCACTCTCGGTTTGAACATCGAAACAGCCACTTGTGTTGGAAAGCAAATAGTAGAAAGGATTCTGAAA
GAAGAATCCGATGAGGCATTTAAAATGACCATGGCTTCCGCACTTGCTTCGCGGTACCTAACTGACATGA
CTGTTGAAGAAATGTCAAGGGACTGGTTCATGCTCATGCCCAAGCAGAAAGTGGCTGGTCCTCTTTGTGT
CAGAATGGACCAGGCGATAATGGATAAGAACATCATACTGAAAGCGAATTTCAGTGTGATTTTTGACCGG
TTGGAGAATTTGACATTACTAAGGGCTTTCACCGAAGAGGGAGCAATTGTTGGCGAAATTTCACCATTGC
CTTCTTTTCCAGGACATACTAATGAGGATGTCAAAAATGCAATTGGGGTCCTCATCGGGGGACTTGAATG
GAATGATAACACAGTTCGAGTCTCTGAAACTCTACAGAGATTCGCTTGGAGAAGCAGTAATGAGACTGGG
GGACCTCCATTCACTACAACACAGAAACGGAAAATGGCGGGAACAATTAGGTCAGAAGTTTGAAGAAATA
AGATGGCTGATTGAAGAAGTGAGGCATAAATTGAAGACGACAGAGAATAGTTTTGAGCAAATAACATTTA
TGCAAGCATTACAGCTATTGTTTGAAGTGGAACAAGAGATTAGAACGTTCTCGTTTCAGCTTATTTAATG
ATAAAAAA
>gi|226954755|gb|CY038883.1| Influenza A virus (A/Taiwan/2645/2006(H1N1)) segment 8, complete sequence
TAATGGATTCCCACACTGTGTCAAGCTTTCAGGTAGATTGCTTCCTTTGGCATGTCCGCAAACAAGTTGC
AGACAAAGATCTAGGCGATGCCCCCTTCCTTGATCGGCTTCGCCGAGATCAGAAGTCTCTAAAGGGAAGA
GGCAGCACTCTCGGTTTGAACATCGAAACAGCCACTTGTGTTGGAAAGCAAATAGTAGAGAGGATTCTGA
AAGAAGAATCCGATGAGGCATTTAAAATGACCATGGCCTCCGCACTTGCTTCGCGGTACCTAACTGACAT
GACTGTTGAAGAAATGTCAAGGGACTGGTTCATGCTCATGCCCAAGCAGAAAGTGGCTGGCCCTCTTTGT
GTCAGAATGGACCAGGCGATAATGGATAAGAACATCATACTGAAAGCGAATTTCAGTGTGATTTTTGACC
GGTTGGAGAATCTGACATTACTAAGGGCTTTCACCGAAGAGGGAGCAATTGTTGGCGAAATTTCACCATT
GCCTTCTTTTCCAGGACATACTAATGAGGATGTCAAAAATGCAATTGGGGTCCTCATCGGGGGACTTGAA
TGGAATGATAACACAGTTCGAGTCTCTGAAACTCTACAGAGATTCGCTTGGAGAAGCAGTAATGAGACTG
GGGGACCTCCATTCACTACAACACAGAAACGGAAAATGGCGGGAACAATTAGGTCAGAAGTTTGAAGAAA
TAAGATGGCTAATTGAAGAAGTGAGGCATAAATTGAAGACGACAGAGAATAGTTTTGAGCAAATAACATT
TATGCAAGCGTTACAGCTATTGTTTGAAGTGGAACAAGAGATTAGAACGTTCTCGTTTCAGCTTATTTAA
>gi|256385520|gb|CY044353.1| Influenza A virus (A/South Korea/AF10/2008(H1N1)) segment 8, complete sequence
ATGGATTCCCACACTGTGTCAAGCTTTCAGGTAGATTGCTTCCTTTGGCATGTCCGCAAACAAGTTGCAG
ACAAAGATCTAGGCGATGCCCCCTTCCTTGATCGGCTTCGCCGAGATCAGAAGTCTCTAAAGGGAAGAGG
CAGCACTCTCGGTTTGAACATCGAAACAGCCACTTGTGTTGGAAAGCAAATAGTAGAAAGGATTCTGAAA
GAAGAATCCGATGAGGCATTTAAAATGACCATGGCTTCCGCACTTGCTTCGCGGTACCTAACTGACATGA

CTGTTGAAGAAATGTCAAGGGACTGGTTCATGCTCATGCCCAAGCAGAAAGTGGCTGGCCCTCTTTGCGT
CAGAATGGATCAGGCGATAATGGATAAGGACATCATACTGAAAGCAAATTTCAGTGTGATTTTTGACCGG
TTGGAGAATTTGACATTACTAAGGGCTTTCACCGAAGAGGGAGCAATTGTTGGCGAAATTTCACCATTGC
CTTCTTTTCCAGGACATACTAATGAGGATGTCAAAAATGCAATTGGGGTCCTCATCGGGGGACTTGAATG
GAATGATAACACAGTTCGAGTCTCTGAAACTCTACAGAGATTCGCTTGGAGAAGCAGTAATGAGACTGGG
GGACCTCCATTCACTACAACACAGAAACGGAAAATGGCGGGAACAATTAGGTCAGAAGTTTGAAGAAATA
AGATGGCTGATTGAAGAAGTGAGGCATAAATTGAAGACGACAGAGAATAGTTTTGAGCAAATAACATTTA
TGCAAGCATTACAGCTATTGTTTGAAGTGGAACAAGAGATTAGAACGTTCTCGTTTCAGCTTATTTAATG
ATAAAAA
>gi|163964725|gb|CY028463.1| Influenza A virus (A/California/UR06-0442/2007(H1N1)) segment 8, complete sequence
GACATAATGGATTCCCACACTGTGTCAAGCTTTCAGGTAGATTGCTTCCTTTGGCATGTCCGCAAACAAG
TTGCAGACCAAGATCTAGGCGATGCCCCCTTCCTAGATCGGCTTCGCCGAGATCAGAAGTCTCTAAAGGG
AAGAGGCAGCACTCTCGGTCTGAACATCGAAACAGCTACTTGTGTTGGAAAGCAAATAGTAGAGAGGATT
CTGAAAGAAGAATCCGATGAGGCACTTAAAATGACCATGGCCTCCGCACTTGCTTCGCGGTACCTAACTG
ACATGACTGTTGAAGAAATGTCAAGGGACTGGTTCATGCTCATGCCCAAGCAAAAAGTGGCTGGCCCTCT
TTGTGTCAGAATGGACCAGGCAATAATGGATAAGAACATCATACTGAAAGCGAATTTCAGTGTGATTTTT
GACCGGTTGGAGAATCTGACATTACTAAGGGCTTTCACCGAAGAGGGAGCAATTGTTGGCGAAATTTCAC
CATTGCCTTCTTTTCCAGGACATACTAATGAGGATGTCAAAAATGCAATTGGGGTCCTCATCGGGGGACT
TGAATGGAATGATAACACAGTTCGAGTCTCTGAAACTATACAGAGATTCGCTTGGAGAATCAGTAATGAG
ACTGGGGAACCTCCATTCACTACAACACAGAAACGGAAAATGGCGGGAACAACTAGGTCAGAAATTTGAA
GAAATAAGATGGCTGATTGAAGAAGTGAGGCATAAATTGAAGACGACAGAGAATAGTTTTGAGCAAATAA
CATTCATGCAAGCATTACAGCTATTATTTGAAGTGGAACAAGAGATTAGAACGTTTTCGTTTCAGCTTAT
TTAG
>gi|157281609|gb|CY025361.1| Influenza A virus (A/Kentucky/UR06-0363/2007(H1N1)) segment 8, complete sequence
ATAATGGATTCCCACACTGTGTCAAGCTTTCAGGTAGATTGCTTCCTTTGGCATGTCCGCAAACAAGTTG
CAGACCAAGATCTAGGCGATGCCCCCTTCCTTGATCGGCTTCGCCGAGATCAGAAGTCTCTAAAGGGAAG
AGGCAGCACTCTCGGTCTGAACATCGAAACAGCTACTTGTGTTGGAAAGCAACTAGTAGAGAGGATTCTG
AAAGAAGAATCCGATGAGGCACTTAAAATGACCATGGCCTCCGCACTTGCTTCGCGGTACCTAACTGACA
TGACTGTTGAAGAAATGTCAAGGGACTGGTTCATGCTCATGCCCAAGCAAAAAGTTGCTGGCCCTCTTTG
TGTCAGAATGGACCAGGCGATAATGGATAAGAACATCATACTGAAAGCAAATTTCAGTGTGATTTTTGAC
CGGTTGGAGAATCTGACATTACTAAGGGCTTTCACCGAAGAGGGAGCAATTGTTGGCGAAATTTCACCAT
TGCCTTCTTTTCCAGGACATACTAATGAGGATGTCAAAAATGCAATTGGGGTCCTCATCGGGGGACTTGA
ATGGAATGATAACACAGTTCGAGTCTCTGAAACTCTACAGAGATTCGCTTGGAGAAGCAGTAATGAGACT
GGGGGACCTCCATTCACTACAACACAGAAACGGAAAATGGCGGGAACAACTAGGTCTGAAATTTGAAGAA
ATAAGATGGCTGATTGAAGAAGTGAGGCATAAATTGAAGACGACAGAGAATAGTTTTGAGCAAATAACAT
TTATGCAAGCATTACAGCTATTATTTGAAGTGGAACAAGAGATTAGAACGTTTTCGTTTCAGCTTATTTA
GTGATAAAAA
>gi|158957798|gb|CY027415.1| Influenza A virus (A/Alabama/UR06-0536/2007(H1N1)) segment 8, complete sequence
ATAATGGATTCCCACACTGTGTCAAGCTTTCAGGTAGATTGCTTCCTTTGGCATGTCCGCAAACAAGTTG
CAGACCAAGATCTAGGCGATGCCCCCTTCCTTGATCGGCTTCGCCGGGATCAGAAGTCTCTAAAGGGAAG
AGGCAGCACTCTCGGTCTGAATATCGAAACAGCTACTTGCGTTGGAAAGCAAATAGTAGAGAGGATTCTG

AAGGAAGAATCCGATGAGGCACTTAAAATGACCATGGCCTCCGCACTTGCTTCGCGGTACCTAACTGACA
TGACTGTTGAAGAAATGTCAAGGGACTGGTTCATGCTCATGCCCAAGCAAAAAGTGGCTGGCCCTCTTTG
TGTCAGAATGGACCAGGCAATAATGGATAAGAACATCATACTGAAAGCGAATTTCAGTGTGATTTTTGAC
CGGTTGGAGAATCTGACATTACTAAGGGCTTTCACCGAAGAGGGAGCAATTGTTGGCGAAATTTCACCAT
TGCCTTCTTTTCCAGGACATACTAATGAGGATGTCAAAAATGCAATTGGGGTCCTCATCGGGGGACTTGA
ATGGAATGATAACACAGTTCGAGTCTCTGAAACTCTACAGAGATTCGCTTGGAGAAGCAGTAATGAGACT
GGGGGACCTCCATTCACTACAACACAGAAACGGAAAATGGCGGGAACAACTAGGTCAGAAGTTTGAAGAA
ATAAGATGGCTGATTGAAGAAGTGAGGCATAAATTGAAGACGACAGAGAATAGTTTTGAGCAAATAACAT
TTATGCAAGCATTACAGCTATTATTTGAAGTGGAACAAGAGATTAGAACGTTTTCGTTTCAGCTTATTTA
GTGATAAAAA
>gi|237688833|gb|CY040062.1| Influenza A virus (A/Taiwan/71720/2007(H1N1)) segment 8, complete sequence
TAATGGATTCCCACACTGTGTCAAGCTTTCAGGTAGATTGCTTCCTTTGGCATGTCCGCAAACAAGTTGC
AGACCAAGATCTAGGCGATGCCCCCTTCCTTGATCGGCTTCGCCGAGATCAGAAGTCTCTAAAGGGAAGA
GGCAGCACTCTCGGTCTGAACATCGAAACAGCTACTTGTGTTGGAAAGCAAATAGTAGAGAGGATTCTGA
AAGAAGAATCCGATGATGCACTTAAAATGACCATGGCCTCCGCACTTGCTTCGCGGTACCTAACTGACAT
GACTGTTGAAGAAATGTCAAGGGACTGGTTCATGCTCATGCCCAAGCAAAAAGTGGCTGGCCCTCTTTGT
GTCAGAATGGACCAGGCAATAATGGATAAGAACATCATACTGAAAGCGAATTTCAGTGTGATTTTTGACC
GGTTGGAGAATCTGACATTACTAAGGGCTTTCACCGAAGAGGGAGCAATTGTTGGCGAAATTTCACCATT
GCCTTCTTTTCCAGGACATACTAATGAGGATGTCAAAAATGCAATTGGGGTCCTCATCGGGGGACTTGAA
TGGAATGATAACACAGTTCGAGTCTCTGAAACTCTACAGAGATTCGCTTGGAGAAGCAGTAATGAGACTG
GGGGACCTCCATTCACTACAACACAGAAACGGAAAATGGCGGGAACAACTAGGTCAGAAGTTTGAAGAAG
TAAGATGGCTGATTGAAGAAGTGAGGCATAAATTGAAGACGACAGAGAATAGTTTTGAGCAAATAACATT
TATGCAAGCATTACAGCTATTATTTGAAGTGGAACAAGAGATTAGAACGTTTTCGTTTCAGCTTATTTAG
TGATAAAAAA
>gi|237689291|gb|CY040254.1| Influenza A virus (A/Managua/3153.01/2008(H1N1)) segment 8, complete sequence
ATGGATTCCCACACTGTGTCAAGCTTTCAGGTAGATTGCTTCCTTTGGCATGTTCGCAAACAAGTTGCAG
ACCAAGATCTAGGCGATGCCCCCTTCCTTGATCGGCTCCGCCGAGATCAGAAGTCTCTAAAGGGAAGAGG
CAGCACTCTCGGTCTGAACATCGAAACAGCTACTTGTGTTGGAAAGCAAATAGTAGAGAGGATTCTGAAA
AAAGAATCCGATGAGGCACTTAAAATGACCATGGCCTCCGCACTTGCTTCGCGGTACCTAACTGACATGA
CTGTTGAAGAAATGTCAAGGGACTGGTTCATGCTCATGCCCAAGCAAAAAGTGGCTGGCCCTCTTTGTGT
CAGAATGGACCAGGCAATAATGGATAAGAACATCATACTGAAAGCGAATTTCAGTGTGATTTTTGACCGG
TTGGAGAATCTGACATTATTAAGGGCTTTCACCGAAGAGGGAGCAATTGTTGGCGAAATTTCACCATTGC
CTTCTTTTCCAGGACATACTAATGAGGATGTCAAAAATGCAATTGGGGTCCTCATCGGGGGACTTGAATG
GAATGATAACACAGTTCGAGTCTCTGAAACTCTACAGAGATTCGCTTGGAGAAGCAGTAATGAGACTGGG
GGACCTCCATTCACTACAACACAGAAACGGAAAATGGCGGGAACAACTAGGTCAGAAGTTTGAAGAAGTA
AGATGGCTGATTGAGGAAGTGAGGCATAAATTGAAGACGACAGAGAATAGTTTTGAGCAAATAACATTTA
TGCAAGCATTACAGCTTTTATTTGAAGTGGAACAAGAGATTAGAACGTTTTCGTTTCAGCTTATTTAGTG
ATAAAA
>gi|224020938|gb|CY037331.1| Influenza A virus (A/Washington/AF06/2007(H1N1)) segment 8, complete sequence
ATGGATTCCCACACTGTGTCAAGCTTTCAGGTAGATTGCTTCCTTTGGCATGTCCGCAAACAAGTTGCAG
ACCAAGATCTAGGCGATGCCCCCTTCCTTGATCGGCTTCGCCGAGATCAGAAGTCTCTAAAGGGAAGAGG

CAGCACTCTCGGTCTGAACATCGAAACAGCTACTTGTGTTGGAAAGCAAATAGTAGAGAGGATTCTGAAA
GAAGAATCCGATGAGGCACTTAAAATGACCATGGCCTCCGCACTTGCTTCGCGGTACTTAACTGACATGA
CTGTTGAAGAAATGTCAAGGGACTGGTTCATGCTCATGCCCAAGCAAAAAGTGGCTGGCCCTCTTTGTGT
CAGAATGGACCAGGCAATAATGGATAAGAACATCATACTGAAAGCGAATTTCAGTGTGATTTTTGACCGG
TTGGAGAATCTGACATTACTAAGGGCTTTCACCGAAGAGGGAGCAATTGTTGGCGAAATTTCACCATTGC
CTTCTTTTCCAGGACATACTAATGAGGATGTCAAAAATGCAATTGGGGTCCTCATCGGGGGACTTGAATG
GAATGATAACACAGTTCGAGTCTCTGAAACTCTACAGAGATTCGCTTGGAGAAGCAGTAATGAGGCTGGG
GGACCTCCATTCACTACAACACAGAAACGGAAAATGGCGGGAACAACTAGGTCAGAAGTTTGAAGAAGTA
AGATGGCTGATTGAAGAAGTGAGGCATAAATTGAAGACGACAGAAAATAGTTTTGAGCAAATAACATTTA
TGCAAGCATTACAGCTATTATTTGAAGTGGAACAAGAGATTAGAACGTTTTCGTTTCAGCTTATTTAGTG
A
>gi|224021243|gb|CY037339.1| Influenza A virus (A/Japan/AF07/2008(H1N1)) segment 8, complete sequence
ATAATGGATTCCCACACTGTGTCAAGCTTTCAGGTAGATTGCTTCCTTTGGCATGTCCGCAAACAAGTTG
CAGACCAAGATCTAGGCGATGCCCCCTTCCTTGATCGGCTTCGCCGAGATCAGAAGTCTCTAAAGGGAAG
AGGCAGCACTCTCGGTCTGAACATCGAAACAGCTACTTGTGTTGGAAAGCAAATAGTAGAGAGGATTCTG
AAAGAAGAATCCGATGAGGCACTTAAAATGACCATGGCCTCCGCACTTGCTTCGCGGTACCTAACTGACA
TGACTGTTGAAGAAATGTCAAGGGACTGGTTCATGCTCATGCCCAAGCAAAAAGTGGCTGGCCCTCTTTG
TGTCAGAATGGACCAGGCAATAATGGATAAGAACATCATACTGAAAGCGAATTTCAGTGTGATTTTTGAC
CGGTTGGAGAATCTGACATTACTAAGGGCTTTCACCGAAGAGGGAGCAATTGTTGGCGAAATCTCACCAT
TGCCTTCTTTTCCAGGACATACTAATGAGGATGTCAAAAATGCAATTGGGGTCCTCATCGGGGGACTTGA
ATGGAATGATAACACAGTTCGAGTCTCTGAAACTCTACAGAGATTCGCTTGGAGAAGCAGTAATGAGACT
GGGGGACCTCCATTCACTACAACACAGAAACGGAAAATGGCGGGAACAACTAGGTCAGAAGTTTGAAGAA
GTAAGATGGCTGATTGAAGAAGTGAGGCATAAATTGAAGACGACAGAGAATAGTTTTGAGCAAATAACAT
TTATGCAAGCATTACAGCTATTATTTGAAGTGGAACAAGAGATTAGAACGTTTTCGTTTCAGCTTATTTA
GTGATAAAAAA
>gi|212381600|gb|FJ445062.1| Influenza A virus (A/England/26/2008(H1N1)) segment 8 nuclear export protein (NEP) and nonstructural protein 1 (NS1) genes, complete cds
ATGGATTCCCACACTGTGTCAAGCTTTCAGGTAGATTGCTTCCTTTGGCATGTCCGCAAACAAGTTGCAG
ACCAAGATCTAGGCGATGCCCCCTTCCTTGATCGGCTTCGCCGAGATCAGAAGTCTCTAAAGGGAAGAGG
CAGCACTCTCGGTCTGAACATCGAAACAGCTACTTGTGTTGGAAAGCAAATAGTAGAGAGGATTCTGAAA
GAAGAATCCGATGAGGCACTTAAAATGACCATGGCCTCCGCACTTGCTTCGCGGTACCTAACTGACATGA
CTGTTGAAGAAATGTCAAGGGACTGGTTCATGCTCATGCCCAAGCAAAAAGTGGCTGGCCCTCTCTGTGT
CAGAATGGACCAGGCAATAATGGATAAGAACATCATACTGAAAGCGAATTTCAGTGTGATTTTTGACCGG
TTGGAGAATCTGACATTATTAAGGGCTTTCACCGAAGAGGGAGCAATTGTTGGCGAAATTTCACCATTGC
CTTCTTTTCCAGGACATACTAATGAGGATGTCAAAAATGCAATTGGGGTCCTCATCGGGGGACTTGAATG
GAATGATAACACAGTTCGAGTCTCTGAAACTCTACAGAGATTCGCTTGGAGAAGCAGTAATGAGACTGGG
GGACCTCCATTCACTACAACACAGAAACGGAAAATGGCGGGAACAACTAGGTCAGAAGTTTGAAGAAGTA
AGATGGCTGATTGAAGAAGTGAGGCATAAATTGAAGACGACAGAGAATAGTTTTGAGCAAATAACATTTA
TGCAAGCATTACAGCTATTATTTGAAGTGGAACAAGAGATTAGAACGTTTTCGTTTCAGCTTATTTAGTG
ATAAAA
>gi|224027213|gb|CY037683.1| Influenza A virus (A/Florida/UR07-0022/2008(H1N1)) segment 8, complete sequence
ATAATGGATTCCCACACTGTGTCAAGCTTTCAGGTAGATTGCTTTCTTTGGCATGTCCGCAAGCAAGTTG

CAGACCAAGATCTAGGCGATGCCCCCTTCCTTGATCGGCTTCGCCGAGATCAGAAGTCTCTAAAGGGAAG
AGGCAGCACTCTCGGTCTGAACATCGAAACAGCTACTTGTGTTGGAAAGCAAATAGTAGAGAGGATTCTG
AAAGAAGAATCCGATGAGGCACTTAAAATGACCATGGCCTCCGCACTTGCTTCGCGGTACCTAACTGACA
TGACTGTTGAAGAAATGTCAAGGGACTGGTTCATGCTCATGCCCAAGCAAAAAGTGGCTGGCCCTCTTTG
TGTCAGAATGGACCAGGCAATAATGGATAAGAACATCATACTGAAAGCGAATTTCAGTGTGATTTTTGAC
CGGTTGGAGAATCTGACATTATTAAGGGCTTTCACCGAAGAGGGAGCAATTGTTGGCGAAATTTCACCAT
TGCCTTCTTTTCCAGGACATACTAATGAGGATGTCAAAAATGCAATTGGGGTCCTCATCGGGGGACTTGA
ATGGAATGATAACACAGTTCGAGTCTCTGAAACTCTACAGAGATTCGCTTGGAGAAGCAGTAATGAGACT
GGGGGACCTCCATTCACTACAACACAGAAACGGAAAATGGCGGGAACAACTAGGTCAGAAGTTTGAAGAA
GTAAGATGGCTGATTGAAGAAGTGAGGCATAAATTGAAGACGACAGAGAATAGTTTTGAGCAAATAACAT
TTATGCAAGCATTACAGCTATTATTTGAAGTGGAACAAGAGATTAGAACGTTTTCGTTTCAGCTTATTTA
GTGATAAAAAA
>gi|296240588|gb|CY063610.1| Influenza A virus (A/Aalborg/INS133/2009(H1N1)) segment 8, complete sequence
AACATAATGGACTCCAACACCATGTCAAGCTTTCAGGTAGACTGTTTCCTTTGGCATATCCGCAAGCGAT
TTGCAGACAATGGATTGGGTGATGCCCCATTCCTTGATCGGCTCCGCCGAGATCAAAAGTCCTTAAAAGG
AAGAGGCAACACCCTTGGCCTCGATATCGAAACAGCCACTCTTGTTGGGAAACAAATCGTGGAATGGATC
TTGAAAGAGGAATCCAGCGAGACACTTAGAATGACAATTGCATCTGTACCTACTTCGCGCTACCTTTCTG
ACATGACCCTCGAGGAAATGTCACGAGACTGGTTCATGCTCATGCCTAGGCAAAAGAAAATAGGCCCTCT
TTGCGTGCGATTGGACCAGGCGGTCATGGAAAAGAACATAGTACTGAAAGCGAACTTCAGTGTAATCTTT
AACCGATTAGAGACCTTGATACTACTAAGGGCTTTCACTGAGGAGGGAGCAATAGTTGGAGAAATTTCAC
CATTACCTTCTCTTCCAGGACATACTTATGAGGATGTCAAAAATGCAGTTGGGGTCCTCATCGGAGGACT
TGAATGGAATGGTAACACGGTTCGAGTCTCTGAAAATATACAGAGATTCGCTTGGAGAAACTGTGATGAG
AATGGGAGACCTTCACTACCTCCAGAGCAGAAATGAAAAGTGGCGAGAGCAATTGGGACAGAAATTTGAG
GAAATAAGGTGGTTAATTGAAGAAATGCGGCACAGATTGAAAGCGACAGAGAATAGTTTCGAACAAATAA
CATTTATGCAAGCCTTACAACTACTGCTTGAAGTAGAACAAGAGATAAGAGCTTTCTCGTTTCAGCTTAT
TTAATGATAA
>gi|296240570|gb|CY063602.1| Influenza A virus (A/Bonn/INS128/2009(H1N1)) segment 8, complete sequence
AACATAATGGACTCCAACACCATGTCAAGCTTTCAGGTAGACTGTTTCCTTTGGCATATCCGCAAGCGAT
TTGCAGACAATGGATTGGGTGATGCCCCATTCCTTGATCGGCTCCGCCGAGATCAAAAGTCCTTAAAAGG
AAGAGGCAACACCCTTGGCCTCGATATCGAAACAGCCACTCTTGTTGGGAAACAAATCGTGGAATGGATC
TTGAAAGAGGAATCCAGCGAGACACTTAGAATGACAATTGCATCTGTACCTACTTCGCGCTACCTTTCTG
ACATGACCCTCGAGGAAATGTCACGAGACTGGTTCATGCTCATGCCTAGGCAAAAGATAATAGGCCCTCT
TTGCGTGCGATTGGACCAGGCGGTCATGGAAAAGAACATAGTACTGAAAGCGAACTTCAGTGTAATCTTT
AACCGATTAGAGACCTTGATACTACTAAGGGCTTTCACTGAGGAGGGAGCAATAGTTGGAGAAATTTCAC
CATTACCTTCTCTTCCAGGACATACTTATGAGGATGTCAAAAATGCAGTTGGGGTCCTCATCGGAGGACT
TGAATGGAATGGTAACACGGTTCGAGTCTCTGAAAATATACAGAGATTCGCTTGGAGAAACTGTGATGAG
AATGGGAGACCTTCACTACCTCCAGAGCAGAAATGAAAAGTGGCGAGAGCAATTGGGACAGAAATTTGAG
GAAATAAGGTGGTTAATTGAAGAAATGCGGCACAGATTGAAAGCGACAGAGAATAGTTTCGAACAAATAA
CATTTATGCAAGCCTTACAACTACTGCTTGAAGTAGAACAAGAGATAAGAGCTTTCTCGTTTCAGCTTAT
TTAATGATAA
>gi|296240318|gb|CY063490.1| Influenza A virus (A/Boston/110/2009(H1N1)) segment 8, complete sequence

AACATAATGGACTCCAACACCATGTCAAGCTTTCAGGTAGACTGTTTCCTTTGGCATATCCGCAAGCGAT
TTGCAGACAATGGATTGGGTGATGCCCCATTCCTTGATCGGCTCCGCCGAGATCAAAAGTCCTTAAAAGG
AAGAGGCAACACCCTTGGCCTCGATATCGAAACAGCCACTCTTGTTGGGAAACAAATCGTGGAATGGATC
TTGAAAGAGGAATCCAGCGAGACACTTAGAATGACAATTGCATCTGTACCTACTTCGCGCTACCTTTCTG
ACATGACCCTCGAGGAAATGTCACGAGACTGGTTCATGCTCATGCCTAGGCAAAAGATAATAGGCCCTCT
TTGCGTGCGATTGGACCAGGCGATCATGGAAAAGAACATAGTACTGAAAGCGAACTTCAGTGTAATCTTT
AACCGATTAGAGACCTTGATACTACTAAGGGCTTTCACTGAGGAGGGAGCAATAGTTGGAGAAATTTCAC
CATTACCTTCTCTTCCAGGACATACTTATGAGGATGTCAAAAATGCAGTTGGGGTCCTCATCGGAGGACT
TGAATGGAATGGTAACACGGTTCGAGTCTCTGAAAATATACAGAGATTCGCTTGGAGAAACTGTGATGAG
AATGGGAGACCTTCACTACCTCCAGAGCAGAAATGAAAAGTGGCGAGAGCAATTGGGACAGAAATTTGAG
GAAATAAGGTGGTTAATTGAAGAAATGCGGCACAGATTGAAAGCGACAGAGAATAGTTTCGAACAAATAA
CATTTATGCAAGCCTTACAACTACTGCTTGAAGTAGAACAAGAGATAAGAGCTTTCTCGTTTCAGCTTAT
TTAATGATAA
>gi|296240624|gb|CY063626.1| Influenza A virus (A/New York/INS150/2009(H1N1)) segment 8, complete sequence
ATGGACTCCAACACCATGTCAAGCTTTCAGGTAGACTGTTTCCTTTGGCATATCCGCAAGCGATTTGCAG
ACAATGGATTGGGTGATGCCCCATTCCTTGATCGGCTCCGCCGAGATCAAAAGTCCTTAAAAGGAAGAGG
CAACACCCTTGGCCTCGATATCGAAACAGCCACTCTTGTTGGGAAACAAATCGTGGAATGGATCTTGAAA
GAGGAATCCAGCGAGACACTTAGAATGACAATTGCATCTGTACCTACTTCGCGCTACCTTTCTGACATGA
CCCTCGAGGAAATGTCACGAGACTGGTTCATGCTCATGCCTAGGCAAAAGATAATAGGCCCTCTTTGCGT
GCGATTGGACCAGGCGGTCATGGAAAAGAACATAGTACTGAAAGCGAACTTCAGTGTAATCTTTAACCGA
TTAGAGACCTTGATACTACTAAGGGCTTTCACTGAGGAGGGAGCAATAGTTGGAGAAATTTCACCATTAC
CTTCTCTTCCAGGACATACTTATGAGGATGTCAAAAATGCAGTTGGGGTCCTCATCGGAGGACTTGAATG
GAATGGTAACACGGTTCGAGTCTCTGAAAATATACAGAGATTCGCTTGGAGAAACTGTGATGAGAATGGG
AGACCTTCACTACCTCCAGAGCAGAAATGAAAAGTGGCGAGAGCAATTGGGACAGAAATTTGAGGAAATA
AGGTGGTTAATTGAAGAAATGCGGCACAGATTGAAAGCGACAGAGAATAGTTTCGAACAAATAACATTTA
TGCAAGCCTTACAACTGCTGCTTGAAGTAGAACAAGAGATAAGAGCTTTCTCGTTTCAGCTTATTTAATG
ATAA
>gi|296240552|gb|CY063594.1| Influenza A virus (A/Athens/INS122/2009(H1N1)) segment 8, complete sequence
AACATAATGGACTCCAACACCATGTCAAGCTTTCAGGTAGACTGTTTCCTTTGGCATATCCGCAAGCGAT
TTGCGGACAATGGATTGGGTGATGCCCCATTCCTTGATCGGCTCCGCCGAGATCAAAAGTCCTTAAAAGG
AAGAGGCAACACCCTTGGCCTCGATATCGAAACAGCCACTCTTGTTGGGAAACAAATCGTGGAATGGATC
TTGAAAGAGGAATCCAGCGAGAAACTTAGAATGACAATTGCATCTGTACCTACTTCGCGCTACCTTTCTG
ACATGACCCTCGAGGAAATGTCACGAGACTGGTTCATGCTCATGCCTAGGCAAAAGATAATAGGCCCTCT
TTGCGTGCGATTGGACCAGGCGGTAATGGAAAAGAACATAGTACTGAAAGCGAACTTCAGTGTAATCTTT
AACCGATTAGAGACCTTGATACTACTAAGGGCTTTCACTGAGGAGGGAGCAATAGTTGGAGAAATTTCAC
CATTACCTTCTCTTCCAGGACATACTTATGAGGATGTCAAAAATGCAGTTGGGGTCCTCATCGGAGGACT
TGAATGGAATGGTAACACGGTTCGAGTCTCTGAAAATATACAGAGATTCGCTTGGAGAAACTGTGATGAG
AATGGGAGACCTTCACTACCTCCAGAGCAGAAATGAAAAGTGGCGAGAGCAATTGGGACAGAAATTTGAG
GAAATAAGGTGGTTAATTGAAGAAATGCGACACAGATTGAAAGCGACAGAGAATAGTTTCGAACAAATAA
CATTTATGCAAGCCTTACAACTACTGCTTGAAGTAGAACAAGAGATAAGAGCTTTCTCGTTTCAGCTTAT
TTAATGATAA>gi|346642633|gb|CY098557.1| Influenza A virus (A/Bangkok/INS516/2010(H1N1)) nuclear export protein (NEP) and nonstructural protein 1 (NS1) genes, complete cds

AACATAATGGACTCCAACACCATGTCAAGCTTTCAGGTAGACTGTTTCCTTTGGCATATCCGCAAGCGAT
TTGCAGACAATGGATTGGGTGATGCCCCATTCCTTGATCGGCTCCGCCGAGATCAAAAGTCCTTAAAAGG
AAGAGGCAACACCCTTGGCCTCGATATCCAAACAGCCACTCTTGTTGGGAAACAAATCGTGGAATGGATC
TTGAAAGAGGAATCCAGCGAGACACTTAGAATGACAATTGCATCTGTACCTACTTCGCGCTACCTTTCTG
ACATGACCCTCGAGGAAATGTCACGAGACTGGTTCATGCTCATGCCTAGGCAAAAGATAATAGGCCCTCT
TTGCGTGCGATTGGACCAGGCGGCCATGGAAAAGAACATAGTACTGAAAGCGAACTTCAGTGTAATCTTT
AACCGATTAGAGACCTTGATACTACTAAGGGCTTTCACTGAGGAGGGAGCAATAGTTGGAGAAATTTCAC
CATTACCTTCTCTTCCAGGACATACTTATGAGGATGTCAAAAATGCAGTTGGGGTCCTCATCGGAGGACT
TGAATGGAATGGTAACACGGTTCGAGTCTCTGAAAATATACAGAGATTCGCTTGGAGAAACTGTGATGAG
AATGGGAGACCTTCACTACCTCCAGAGCAGAAATGAAAAGTGGCGAGAGCAATTGGGACAGAAATTTGAG
GAAATAAGGTGGTTAATTGAAGAAATGCGGCACAGATTGAAAGCGACAGAGAATAGTTTCGAACAAATAA
CATTTATGCAAGCCTTACAACTACTGCTTGAAGTAGAACAAGAGATAAGAGCTTTCTCGTTTCAGCTTAT
TTGATGATAA
>gi|345101129|gb|CY098174.1| Influenza A virus (A/Chile/70/2010(H1N1)) nuclear export protein (NEP) and nonstructural protein 1 (NS1) genes, complete cds
AACATAATGGACTCCAACACCATGTCAAGCTTTCAGGTAGACTGTTTCCTTTGGCATATCCGCAAGCGAT
TTGCAGACAATGGATTGGGTGATGCCCCATTCCTTGATCGGCTCCGCCGAGATCAAAAGTCCTTAAAAGG
AAGAGGCAACACCCTTGGCCTCGATATCGAAACAGCCACTCTTGTTGGGAAACAAATCGTGGAATGGATC
TTGAAAGAGGAATCCAGCGAGACACTTAGAATGACAATTGCATCTGTACCTACTTCGCGCTACCTTTCTG
ACATAACCCTCGAGGAAATGTCACGAGACTGGTTCATGCTCATGCCTAGGCAAAAGATAATAGGCCCTCT
TTGCGTGCGATTGGACCAGGCGGTCATGGAAAAGAACATAGTACTGAAAGCGAACTTCAGTGTAATCTTT
AACCGATTAGAGACCTTGATACTACTAAGGGCTTTCACTGAGGAGGGAGCAATAGTTGGAGAAATTTCAC
CATTACCTTCTCTTCCAGGACATACTTATGAGGATGTCAAAAATGCAGTTGGGGTCCTCATCGGAGGACT
TGAATGGAATGGTAACACGGTTCGAGTCTCTGAAAATATACAGAGATTCGCTTGGAGAAACTGTGATGAG
AATGGGAGACCTTCACTACCTCCAGAGCAGAAATGAAAAGTGGCGAGAGCAATTGGGACAGAAATTTGAG
GAAATAAGGTGGTTAATTGAAGAAATGCGGCACAGATTGAAAGCGACAGAGAATAGTTTCGAACAAATAA
CATTTATGCAAGCCTTACAACTACTGCTTGAAGTAGAACAAGAGATAAGAGCTTTCTCGTTTCAGCTTAT
TTAATGATAA
>gi|343174023|gb|CY096606.1| Influenza A virus (A/Cambridge/INS528/2010(H1N1)) nuclear export protein (NEP) and nonstructural protein 1 (NS1) genes, complete cds
AACATAATGGACTCCAACACCATGTCAAGCTTTCAGGTAGACTGTTTCCTTTGGCATATCCGCAAGCGAT
TTGCAGACAATGGATTGGGTGATGCCCCATTCCTTGATCGGCTCCGCCGAGATCAAAAGTCCTTAAAAGG
AAGAGGCAACACCCTTGGCCTCGATATCGAAACAGCCACTCTTGTTGGGAAACAAATCGTGGAATGGATC
TTGAAAGAGGAATCCAGCGAGACACTTAGAATGACAATTGCATCTGTACCTACTTCGCGCTACCTTTCTG
ACATGACCCTCGAGGAAATGTCACGAGACTGGTTCATGCTCATGCCTAGGCAAAAGATAATAGGCCCTCT
TTGCGTGCGATTGGACCAGGCGGTCATGGAAAAGAACATAGTACTGAAAGCGAACTTCAGTGTAATCTTT
AACCGATTAGAGACCTTGATACTACTAAGGGCTTTCACTGAGGAGGGAGCAATAGTTGGAGAAATTTCAC
CATTACCTTCTCTTCCAGGACATACTTATGAGGATGTCAAAAATGCAGTTGGGGTCCTCATCGGAGGACT
TGAATGGAATGGTAACACGGTTCGAGTCTCTGAAAATATACAGAGATTCGCTTGGAGAAACTGTGATGAG
AATGGGAGACCTTCACTACCTCCAGAGCAGAAATGAAAAGTGGCGAGAGCAATTGGGACAGAAATTTGAG
GAAATAAGGTGGTTAATTGAAGAAATGCGACACAGATTGAAAGCGACAGAGAATAGTTTCGAACAAATAA
CATTTATGCAAGCCTTACAACTACTGCTTGAAGTAGAACAAGAGATAAGAGCTTTCTCGTTTCAGCTTAT
TTAATGATAA
>gi|343170673|gb|CY096590.1| Influenza A virus (A/District of Columbia/INS525/2010(H1N1))

nuclear export protein (NEP) and nonstructural protein 1 (NS1) genes, complete cds
AACATAATGGACTCCAACACCATGTCAAGCTTTCAGGTAGACTGTTTCCTTTGGCATATCCGCAAGCGAT
TTGCAGACAATGGATTGGGTGATGCCCCATTCCTTGATCGGCTCCGCCGAGATCAAAAGTCCTTAAAAGG
AAGAGGCAACACCCTTGGCCTCGATATCGAAACAGCCACTCTTGTTGGGAAACAAATCGTGGAATGGATC
TTGAAAGAGGAATCCAGCGAGACACTTAGAATAACAATTGCATCTGTACCTACTTCGCGCTACCTTTCCG
ACATGACCCTCGAGGAAATGTCACGAGACTGGTTCATGCTCATGCCTAAGCAAAAGATAATAGGCCCTCT
TTGCGTGCGATTGGACCAGGCGGTCATGGAAAAGAACATAGTACTGAAAGCGAACTTCAGTGTAATCTTT
AACCGATTAGAGACCTTGATACTACTAAGGGCTTTCACTGAGGAGGGAGCAATAGTTGGAGAAATTTCAC
CATTACCTTCTCTTCCAGGACATACTTATGAGGATGTCAAAAATGCAGTTGGGGTCCTCATCGGAGGACT
TGAATGGAATGGTAACACGGTTCGAGTCTCTGAAAATATACAGAGATTCGCTTGGAGAAACTGTGATGAG
AATGGGAGACCTTCACTACCTCCAGAGCAGAAATGAAAAGTGGCGAGAGCAATTGGGACAGAAATTTGAG
GAAATAAGGTGGTTAATTGAAGAAATGCGGCACAGATTGAAAGCGACAGAGAATAGTTTCGAACAAATAA
CATTTATGCAAGCCTTACAACTACTGCTTGAAGTAGAACAAGAGATAAGAGCTTTCTCGTTTCAGCTTAT
TTAATGATAA
>gi|343143579|gb|CY096573.1| Influenza A virus (A/Westmead/INS524/2010(H1N1)) nuclear export protein (NEP) and nonstructural protein 1 (NS1) genes, complete cds
AACATAATGGACTCCAACACCATGTCAAGCTTTCAGGTAGACTGTTTCCTTTGGCATATCCGCAAGCGAT
TTGCAGACAATGGATTGGGTGATGCCCCATTCCTTGATCGGCTCCGCCGAGATCAAAAGTCCTTAAAAGG
AAGAGGCAACACCCTTGGCCTCGATATCGAAACAGCCACTCTTGTTGGGAAACAAATCGTGGAATGGATC
TTGAAAGAGGAATCCAGCGAGACACTTAAAATAACAATTGCATCTGTACCTACTTCGCGCTACCTTTCTG
ACATAACCCTCGAGGAAATGTCACGAGACTGGTTCATGCTCATGCCTAGGCAAAAGATAATAGGCCCTCT
TTGCGTGCGATTGGACCAGGCGGTCATGGAAAAGAACATAGTACTGAAAGCGAACTTCAGTGTAATCTTT
AACCGATTAGAGACCTTGATACTACTAAGGGCTTTCACTGAGGAGGGAGCAATAGTTGGAGAAATTTCAC
CATTACCTTCTCTTCCAGGACATACTTATGAGGATGTCAAAAATGCAGTTGGGGTCCTCATCGGAGGACT
TGAATGGAATGGTAACACGGTTCGAGTCTCTGAAAATATACAGAGATTCGCTTGGAGAAACTGTGATGAG
AATGGGAGACCTTCACTACCTCCAGAGCAGAAATGAAAAGTGGCGAGAGCAATTGGGACAGAAATTTGAG
GAAATAAGGTGGTTAATTGAAGAAATGCGGCACAGATTGAAAGCGACAGAGAATAGTTTCGAACAAATAA
CATTTATGCAAGCCTTACAACTACTGCTTGAAGTAGAACAAGAGATAAGAGCTTTCTCGTTTCAGCTTAT
TTAATGATAA
>gi|343142257|gb|CY096549.1| Influenza A virus (A/Khon Kaen/INS521/2010(H1N1)) nuclear export protein (NEP) and nonstructural protein 1 (NS1) genes, complete cds
AACATAATGGACTCCAACACCATGTCAAGCTTTCAGGTAGACTGTTTCCTTTGGCATATCCGCAAGCGAT
TTGCAGACAATGGATTGGGTGATGCCCCATTCCTTGATCGGCTTCGCCGAGATCAAAAGTCCTTAAAAGG
AAGAGGCAACACCCTTGGCCTCGATATCGAAACAGCCACTCTTGTTGGGAAACAAATCGTGGAATGGATC
TTGAAAGAGGAATCCAGCGAGACACTTAGAATGACAATTGCATCTGTACCTACTTCGCGTTACATTTCTG
ACATGACCCTCGAGGAAATGTCACGAGACTGGTTCATGCTCATGCCTAGGCAAAAGATAATAGGCCCTCT
TTGCGTGCGATTGGACCAGGCGGTCATGGAAAAGAACATAGTACTGAAAGCGAACTTCAGTGTAATCTTC
AACCGATTAGAGACCTTGATACTACTAAGGGCTTTCACTGAGGAGGGAGCAATAGTTGGAGAAATTTCAC
CATTACCTTCTCTTCCAGGACATACTTATGAGGATGTCAAAAATGCAGTTGGGGTCCTCATCGGAGGACT
TGAATGGAATGGTAACACGGTTCGAGTCTCTGAAAATATACAGAGATTCGCTTGGAGAAACTGTGATGAG
AATGGGAGACCTTCACTACCTCCAGAGCAGAAATGAAAAGTGGCGAGAGCAATTGGGACAGAAATTTGAG
GAAATAAGGTGGTTAATTGAAGAAATGCGGCACAGATTGAAAGCGACAGAGAATAGTTTCGAACAAATAA
CATTTATGCAAGCCTTACAACTACTGCTTGAAGTAGAACAAGAGATAAGAGCTTTCTCGTTTCAGCTTAT
TTAATGATAA

>gi|343132000|gb|CY096254.1| Influenza A virus (A/Melbourne/INS472/2010(H1N1)) nuclear export protein (NEP) and nonstructural protein 1 (NS1) genes, complete cds
AACATAATGGACTCCAACACCATGTCAAGCTTTCAGGTAGACTGTTTCCTTTGGCATATCCGCAAGCGAT
TTGCAGACAATGGATTGGGTGATGCCCCATTCCTTGATCGGCTCCGCCGAGATCAAAAGTCCTTAAAAGG
AAGAGGCAACACCCTTGGCCTCGATATCGAAACAGCCACTCTTGTTGGGAAACAAATCGTGGAATGGATC
TTGAAAGAGGAATCCAGCGAGACACTTAGAATGACAATTGCATCTGTACCTACTTCGCGCTACCTTTCTG
ACATAACCCTCGAGGAAATGTCACGAGACTGGTTCATGCTCATGCCTAGGCAAAAGATAATAGGCCCTCT
TTGCGTGCGTTTGGACCAGGCGGTCATGGAAAAGAACATAGTACTGAAAGCGAACTTCAGTGTAATCTTT
AACCGATTAGAGACCTTGATACTACTAAGGGCTTTCACTGAGGAGGGAGCAATAGTTGGAGAAATTTCAC
CATTACCTTCTCTTCCAGGACATACTTATGAGGATGTCAAAAATGCAGTTGGGGTCCTCATCGGAGGACT
TGAATGGAATGGTAACACGGTTCGAGTCTCTGAAAATATACAGAGATTCGCTTGGAGAAACTGTGATGAG
AATGGGAGACCTTCACTACCTCCAGAGCAGAAATGAAAAGTGGCGAGAGCAATTGGGACAGAAATTTGAG
GAAATAAGGTGGTTAATTGAAGAAATGCGGCACAGATTGAAAGCGACAGAGAATAGTTTCGAACAAATAA
CATTTATGCAAGCCTTACAACTACTGCTTGAAGTAGAACAAGAGATAAGAGCTTTCTCGTTTCAGCTTAT
TTAATGATAA
>gi|327409521|gb|CY089375.1| Influenza A virus (A/Managua/5708.01/2010(H1N1)) nuclear export protein (NEP) and nonstructural protein 1 (NS1) genes, complete cds
AACATAATGGACTCCAACACCATGTCAAGCTTTCAGGTAGACTGTTTCCTTTGGCATATCCGCAAGCGAT
TTGCAGACAATGGATTGGGTGATGCCCCATTCCTTGATCGGCTCCGCCGAGATCAAAAGTCCTTAAAAGG
AAGAGGCAACACCCTTGGCCTCAATATCGAAACAGCCACTCTTGTTGGGAAGCAGATCGTGGAATGGATC
TTGAAAGAGGAATCCAGCGAGACACTTAGAATGACAATTGCATCTGTACCTACTTCGCGCTACCTTTCTG
ACATGACCCTCGAGGAAATGTCAAGAGACTGGTTCATGCTCATGCCTAGGCAAAAGATAATGGGCCCTCT
TTGCGTGCGATTGGACCAGGCGGTCATGGAAAAGAACATAGTACTGAAAGCGAACTTCAGTGTAATCTTT
AACCGATTAGAGACCTTGATACTACTAAGGGCTTTCACTGAGGAGGGAGCAATAGTTGGAGAAATTTCAC
CATTACCTTCTCTTCCAGGACATACTTATGAGGATGTCAAAAATGCAGTTGGGGTCCTCATCGGAGGACT
TGAATGGAATGGTAACACGGTTCGAGTCTCTGAAAATATACAGAGATTCGCTTGGAGAAACTGTGATGAG
AATGGGAGACCTTCACTACCTCCAGAGCAGAAATGAAAAGTGGCGAGAGCAATTGGGACAGAAATTTGAG
GAAATAAGGTGGTTAATTGAAGAAATGCGGCACAGATTGAAAGCGACAGAGAATAGTTTCGAACAAATAA
CATTTATGCAAGCCTTACAACTACTGCTTGAAGTAGAACAAGAGATAAGAGCTTTCTCGTTTCAGCTTAT
TTAATGATAA
>gi|323668711|gb|CY083814.1| Influenza A virus (A/Athens/INS387/2010) nuclear export protein (NEP) and nonstructural protein 1 (NS1) genes, complete cds
GTGACAAAAACATAATGGACTCCAACACCATGTCAAGCTTTCAGGTAGACTGTTTCCTTTGGCATATCCG
CAAGCGATTTGCAGACAATGGATTGGGTGATGCCCCATTCCTTGATCGGCTCCGCCGAGATCAAAAGTCC
TTAAAAGGAAGAGGCAACACCCTTGGCCTCGGTATCGAAACAGCCACTCTTGTTGGGAAACAAATCGTGG
AATGGATCTTGAAAGAGGAATCCAGCGAGACACTTAGAATGACAATTGCATCTGTACCTACTTCGCGCTA
CCTTTCTGACATGACCCTCGAGGAAATGTCACGAGACTGGTTCATGCTCATGCCTAGGCAAAAGATAATA
GGCCCTCTTTGCGTGCGATTAGACCAGGCGGTCATGGAAAAGAACATAGTACTGAAAGCGAACTTCAGTG
TAATCTTTAACCGATTGGAGACCTTGATACTACTAAGGGCTTTCACTGAGGAGGGAGCAATAGTTGGAGA
AATTTCACCATTACCTTCTCTTCCAGGACATACTTATGAGGATGTCAAAAATGCAGTTGGGGTCCTCATC
GGAGGACTTGAATGGAATGGTAACACGGTTCGAGTCTCTGAAAATATACAGAGACTCGCTTGGAGAAACT
GTGATGAGAATGGGAGACCTTCACTACCTCCAGAGCAGAAATGAAAAGTGGCGAGAGCAATTGGGACAGA
AATTTGAGGAAATAAGGTGGTTAATTGAAGAAATGCGGCACAGATTGAAAGCGACAGAGAATAGTTTCGA
ACAAATAACATTTATGCAAGCCTTACAACTACTGCTTGAAGTAGAACAAGAGATAAGAGCTTTCTCGTTT

CAGCTTATTTAATGATAAAAAAC
>gi|324033803|gb|JF327347.1| Influenza A virus (A/Finland/4/2010(H1N1)) segment 8 nuclear export protein (NEP) and nonstructural protein 1 (NS1) genes, complete cds
ATGGACTCCAACACCATGTCAAGCTTTCAGGTAGACTGTTTCCTTTGGCATATCCGCAAGCGATTTGCAG
ACAATGGATTGGGTGATGCCCCATTCCTTGATCGGCTCCGCCGAGATCAAAAGTCCTTAAAAGGAAGAGG
CAACACCCTTGGCCTCGATATCGAAACAGCCACTCTTGTTGGGAAACAAATCGTGGAATGGATCTTGAAA
GAGGAATCCAGCGAGACACTTAGAATGACAATTGCATCTGTACCTACTTCGCGCTACCTTTCTGACATAA
CCCTCGAGGAAATGTCACGAGACTGGTTCATGCTCATGCCTAGGCAAAAGATAATAGGCCCTCTTTGCGT
GCGATTGGACCAGGCGGTCATGGAAAAGAACATAGCACTGAAAGCGAACTTCAGTGTAATCTTTAACCGA
TTAGAGACCTTGATACTACTAAGGGCTTTCACTGAGGAGGGAGCAATAGTTGGAGAAATTTCACCATTAC
CTTCTCTTCCAGGACATACTTATGAGGATGTCAAAAATGCAGTTGGGGTCCTCATCGGAGGACTTGAATG
GAATGGTAACACGGTTCGAGTCTCTGAAAATATACAGAGATTCGCTTGGAGAAACTGTGATGAGAATGGG
AGACCTTCACTACCTCCAGAGCAGAAATGAAAAGTGGCGAGAGCAATTGGGACAGAAATTTGAGGAAATA
AGGTGGTTAATTGAAGAAATGCGGCACAGATTGAAAGCGACAGAGAATAGTTTCGAACAAATAACATTTA
TGCAAGCCTTACAACTACTGCTTGAAGTAGAACAAGAGATAAGAGCTTTCTCGTTTCAGCTTATTTAGTG
>gi|363805059|gb|JQ290182.1| Influenza A virus (A/Iowa/09/2011(H3N2)) segment 8 nuclear export protein (NEP) and nonstructural protein 1 (NS1) genes, complete cds
ATGGACTCCAATACTGTGTCAAGTTTTCAGGTAGACTGTTTCCTTTGGCACATCCGCAAACGGTTTGCAG
ACAATGGATTGGGTGATGCCCCATTCCTTGATCGGCTCCGCCGAGATCAAAAGTCCCTAAAAGGAAGAGG
CAACACCCTTGGCCTTGATATCGAAACTGCCACTCTTGTTGGGAAGCAAATTGTGGAGTGGATTTTGAGA
GAGGAATCCAGCGAGACACTTAAGATGACCATTGCGTCTGTACCTACTTCGCGCTACATAGCTGACATGA
CCCTCGAGGAAATGTCACGAGACTGGTTCATGCTCATGCCTAGGCAAAAGATAATAGGCCCTCTTTGTGT
GCGAATGGACCAGGCGATCATGGAAAAGAACATTATACTGAAAGCGAACTTCAGTGTGATCTTTAACCGA
TTAGAGACTTTGATACTACTAAGGGCTTTCACTGAGGAGGGAGCAATCGTTGGAGAAATTTCACCATTAC
CTTCTCTTCCAGGACATACTAACGAGGATGTCAAAAATGCAGTTGGGGTCCTCATCGGAGGACTTGAATG
GAATGGTAACACGGTTCGAGGCTCTGAAAATCTACAGAGATTCGCTTGGAGAAACCGTAATGAGGATGGG
AGACCTTCACTACCTCCAGAGCAGAAATGAAAAGTGGCGAGAGCAATTGGGACAGAAATTTGAGGAAATA
AGGTGGTTAATTGAAGAAGTACGACACAGATTGAAGGCAACAGAGAATAGTTTCGAACAAATAACATTTA
TGCAAGCCTTACAACTACTGCTTGAAGTAGAGCAAGAGATAAGGACTTTCTCGTTTCAGCTTATTTAATG
ATAA
>gi|345722626|gb|JN655538.1| Influenza A virus (A/Pennsylvania/09/2011(H3N2)) segment 8 nuclear export protein (NEP) and nonstructural protein 1 (NS1) genes, complete cds
ATGGACTCCAATACTGTGTCAAGTTTTCAGGTAGACTGTTTCCTTTGGCACATCCGCAAACGGTTTGCAG
ACAATGGATTGGGTGATGCCCCATTCCTTGATCGGCTCCGCCGAGATCAAAAGTCCCTAAAAGGAAGAGG
CAACACCCTTGGCCTCGATATCGAAACTGCCACTCTTGTTGGGAAGCAAATTGTGGAGTGGATTTTGAGA
GAGGAATCCAGCGAGACACTTAAGATGACCATTGCATCTGTACCTACTTCGCGCTACATAGCTGACATGA
CCCTCGAGGAAATGTCACGAGACTGGTTCATGCTCATGCCTAGGCAAAAGATAATAGGCCCTCTTTGTGT
GCGAATGGACCAGGCGATCATGGAAAAGAACATTATACTGAAAGCGAACTTCAGTGTGATCTTTAACCGA
TTAGAGACTTTGATACTACTAAGGGCTTTCACTGAGGAGGGAGCAATCGTTGGAGAAATTTCACCATTAC
CTTCTCTTCCAGGACATACTAACGAGGATGTCAAAAATGCAGTTGGGGTCCTCATCGGAGGACTTGAATG
GAATGGTAACACGGTTCGAGGCTCTGAAAATCTACAGAGATTCGCTTGGAGAAACCGTAATGAGGATGGG
AGACCTTCACTACCTCCAGAGCAGAAATGAAAAGTGGCGAGAGCAATTGGGACAGAAATTTGAGGAAATA
AGGTGGTTAATTGAAGAAGTACGACACAGATTGAAGGCAACAGAGAATAGTTTCGAACAAATAACATTTA
TGCAAGCCTTACAACTACTGCTTGAAGTAGAGCAAGAGATAAGGACTTTCTCGTTTCAGCTTATTTAATG

ATAA
>gi|338826716|gb|CY092908.1| Influenza A virus (A/Georgia/NHRC0001/2011(H1N1)) nuclear export protein (NEP) and nonstructural protein 1 (NS1) genes, complete cds
AACATAATGGACTCCAACACCATGTCAAGCTTTCAGGTAGACTGTTTCCTTTGGCATATCCGCAAGCGAT
TTGCAGACAATGGATTGGGTGATGCCCCATTCCTTGATCGGCTCCGCCGAGATCAAAAGTCCTTAAAAGG
AAGAGGCAACACCCTTGGCCTCGATATCGAAACAGCCACTCTTGTTGGGAAACAAATCGTGGAATGGATC
TTGAAAGAGGAATCCAGCGAGACACTTAGAATGACAATTGCATCTGTACCTACTTCGCGTTACCTTTCTG
ACATGACCCTCGAGGAAATGTCACGAGACTGGTTCATGCTCATGCCTAGGCAAAAGATAATAGGCCCTCT
TTGCGTGCGATTGGACCAGGCGGTCATGGAAAAGAACATAGTACTGAAAGCGAACTTCAGTGTAATCTTC
AACCGATTAGAGACCTTGATACTACTAAGGGCTTTCACTGAGGAGGGAGCAATAGTTGGAGAAATTTCAC
CATTACCTTCTCTTCCAGGACATACTTATGAGGATGTCAAAAATGCAGTTGGGGTCCTCATCGGAGGACT
TGAATGGAATGGTAACACGGTTCGAGTCTCTGAAAATATACAGAGATTCGCTTGGAGAAACTGTGATGAG
AATGGGAGACCTTCACTACCTCCAGAGCAGAAATGAAAAGTGGCGAGAGCAATTGGGACAGAAATTTGAG
GAAATAAGGTGGTTAATTGAAGAAATGCGGCACAGATTGAAAGCGACAGAGAATAGTTTCGAACAAATAA
CATTTATGCAAGCCTTACAACTACTGCTTGAAGTAGAACAAGAGATAAGAGCTTTCTCGTTTCAGCTTAT
TTAATGATAA
>gi|338826608|gb|CY092860.1| Influenza A virus (A/Sydney/DD3-58/2011(H1N1)) nuclear export protein (NEP) and nonstructural protein 1 (NS1) genes, complete cds
AACATAATGGACTCCAACACCATGTCAAGCTTTCAGGTAGACTGTTTCCTTTGGCATATCCGCAAGCGAT
TTGCAGACAATGGATTGGGTGATGCCCCATTCCTTGATCGGCTCCGCCGAGATCAAAAGTCCTTAAAAGG
AAGAGGCAACACCCTTGGCCTCGATATCGAAACAGCCACTCTTGTTGGGAAACAAATCGTGGAATGGATC
TTGAAAGAGGAATCCAGCGAGACACTTAGAATGACAATTGCATCTGTACCTACTTCGCGTTACATTTCTG
ACATGACCCTCGAGGAAATGTCACGAGACTGGTTCATGCTTATGCCTAGGCAAAAGATAATAGGCCCTCT
TTGCGTGCGATTGGACCAGGCGGTCATGGAAAAGAACATAGTACTGAAAGCGAACTTCAGTGTAATCTTC
AACCGATTAGAGACCTTGATACTACTAAGGGCTTTCACTGAGGAGGGAGCAATAGTTGGAGAAATTTCAC
CATTACCTTCTCTTCCAGGACATACTTATGAGGATGTCAAAAATGCAGTTGGGGTCCTCATCGGAGGACT
TGAATGGAATGGTAACACGGTTCGAGTCTCTGAAAATATACAGAGATTCGCTTGGAGAAACTGTGATGAG
AATGGGAGACCTTCACTACCTCCAGAGCAGAAATGAAAAGTGGCGAGAGCAATTGGGACAGAAATTTGAG
GAAATAAGGTGGTTAATTGAAGAAATGCGGCACAGATTGAAAGCGACAGAGAATAGTTTCGAACAAATAA
CATTTATGCAAGCCTTACAACTACTGCTTGAAGTAGAACAAGAGATAAGAGCTTTCTCGTTTCAGCTTAT
TTAATGATAA
>gi|345722470|gb|JN655553.1| Influenza A virus (A/Indiana/08/2011(H3N2)) segment 8 nuclear export protein (NEP) and nonstructural protein 1 (NS1) genes, complete cds
ATGGACTCCAATACTGTGTCAAGTTTTCAGGTAGACTGTTTCCTTTGGCACATCCGCAAACGGTTTGCAG
ACAATGGATTGGGTGATGCCCCATTCCTTGATCGGCTCCGCCGAGATCAAAAGTCCCTAAAAGGAAGAGG
CAACACCCTTGGCCTCGATATCGAAACTGCCACTCTTGTTGGGAAGCAAATTGTGGAGTGGATTTTGAGA
GAGGAATCCAGCGAGACACTTAAGATGACCATTGCATCTGTACCTACTTCGCGCTACATAGCTGACATGA
CCCTCGAGGAAATGTCACGAGACTGGTTCATGCTCATGCCTAGGCAAAAGATAATAGGCCCTCTTTGTGT
GCGAATGGACCAGGCGATCATGGAAAAGAACATTATACTGAAAGCGAACTTCAGTGTGATCTTTAGCCGA
TTAGAGACTTTGATACTACTAAGGGCTTTCACTGAGGAGGGAGCAATCGTTGGAGAAATTTCACCATTAC
CTTCTCTTCCAGGACATACTAACGAGGATGTCAAAAATGCAGTTGGGGTCCTCATCGGAGGACTTGAATG
GAATGGTAACACGGTTCGAGGCTCTGAAAATCTACAGAGATTCGCTTGGAGAAACCGTAATGAGGATGGG
AGACCTTCACTACCTCCAGAGCAGAAATGAAAAGTGGCGAGAGCAATTGGGACAGAAATTTGAGGAAATA
AGGTGGTTAATTGAAGAAGTACGACACAGATTGAAGGCAACAGAGAATAGTTTCGAACAAATAACATTTA

TGCAAGCCTTACAACTACTGCTTGAAGTAGAGCAAGAGATAAGGACTTTCTCGTTTCAGCTTATTTAATGATA
A
>gi|392357391|gb|CY121820.1| Influenza A virus (A/St. Petersburg/100/2011(H1N1)) nuclear export protein (NEP) and nonstructural protein 1 (NS1) genes, complete cds
GTGACAAAAACATAATGGACTCCAACACCATGTCAAGCTTTCAGGTAGACTGTTTCCTTTGGCATATCCG
CAAGCGATTTGCAGACAATGGATTGGGTGATGCCCCATTCCTTGATCGGCTCCGCCGAGATCAAAAGTCC
TTAAAAGGAAGAGGCAACACCCTTGGCCTCGATATCGAAACAGCCACTCTTGTTGGGAAACAAATCGTGG
AATGGATCTTGAAAGAGGAATCCAGCGAGACACTTAGAATGACAATTGCATCTGTACCTACTTCGCGTTA
CATTTCTGACATGACCCTCGAGGAAATGTCACGAGACTGGTTCATGCTCATGCCTAGGCAAAAGATAATA
GGCCCTCTTTGCGTGCGTTTGGACCAGGCGGTCATGGAAAAGAACATAGTACTGAAAGCGAACTTCAGTG
TAATCTTCAACCGATTAGAGACCTTGATACTACTAAGGGCTTTCACTGAGGAGGGAGCAATAGTTGGAGA
AATTTCACCATTACCTTCTCTTCCAGGACATACTTATGAGGATGTCAAAAATGCAGTTGGGGTCCTCATC
GGAGGACTTGAATGGAATGGTAACACGGTTCGAGTCTCTGAAAATATACAGAGATTCGCTTGGAGAAACT
GTGATGAGAATGGGAGACCTTCACTACCTCCAGAGCAGAAATGAAAAGTGGCGAGAGCAATTGGGACAGA
AATTTGAGGAAATAAGGTGGTTAATTGAAGAAATGCGGCACAGATTGAAAGCGACAGAGAATAGTTTCGA
ACAAATAACATTTATGCAAGCCTTACAACTACTGCTTGAAGTAGAACAAGAGATAAGAGCTTTCTCGTTT
CAGCTTATTTAATGATAAAAAACCC
>gi|388774753|gb|CY120759.1| Influenza A virus (A/Brazil/AVS11/2011(H1N1)) nuclear export protein (NEP) and nonstructural protein 1 (NS1) genes, complete cds
GTGACAAAAACATAATGGACTCCAACACCATGTCAAGCTTTCAGGTAGACTGTTTCCTTTGGCATATCCG
CAAGCGATTTGCAGACAATGGATTGGGTGATGCCCCATTCCTTGATCGGCTCCGCAGAGATCAAAAGTCC
TTAAAAGGAAGAGGCAACACCCTTGGCCTCGATATCGAAACAGCCACTCTTGTTGGGAAACAAATCGTGG
AATGGATCTTGAAAGAGGAATCCAGCGAGACACTTAGAATGACAATTGCATCTGTACCTACTTCGCGCTA
CCTTTCTGACATGACCCTCGAGGAAATGTCACGAGACTGGTTCATGCTCATGCCTAGGCAAAAGATAATA
GGCCCTCTTTGCGTGCGATTGGACCAGGTGGTCATGGAAAAGAACATAGTACTGAAAGCGAACTTCAGTG
TAATCTTTAACCGATTAGAGACCTTGATACTACTAAGGGCTTTCACTGAGGAGGGAGCAATAGTTGGAGA
AATTTCACCATTACCTTCTCTTCCAGGACATACTTATGAGGATGTCAAAAATGCAGTTGGGGTCCTCATC
GGAGGACTTGAATGGAATGGTAACACGGTTCGAGTCTCTGAAAATATACAGAGATTCGCTTGGAGAAACT
GTGATGAGAATGGGAGACCTTCACTACCTCCAGAGCAGAAATGAAAAGTGGCGAGAGCAATTGGGACAGA
AATTTGAGGAAATAAGGTGGTTAATTGAAGAAATGCGGCACAGATTGAAAGCGACAGAGAATAGTTTCGA
ACAAATAACATTTATGCAAGCCTTACAACTACTGCTTGAAGTAGAACAAGAGATAAGAGCTTTCTCGTTT
CAGCTTATTTAATGATAAAAAACCC
>gi|380709128|gb|CY111258.1| Influenza A virus (A/Boston/DOA21/2011(H1N1)) nuclear export protein (NEP) and nonstructural protein 1 (NS1) genes, complete cds
AACATAATGGACTCCAACACCATGTCAAGCTTTCAGGTAGACTGTTTCCTTTGGCATATCCGCAAGCGAT
TTGCAGACAATGGATTGGGTGATGCCCCATTCCTTGATCGGCTCCGCCGAGATCAAAAGTCCTTAAAAGG
AAGAGGCAACACCCTTGGCCTCGATATCGAAACAGCCACTCTTGTTGGGAAACAAATCGTGGAATGGATC
TTGAAAGAGGAATCCAGCGAGACACTTAGAATGACAATTGCATCTGTACCTACTTCGCGTTACCTTTCTG
ACATGACCCTCGAGGAAATGTCACAAGACTGGTTCATGCTCATGCCTAGGCAAAAGATAATAGGCCCTCT
TTGCGTGCGATTGGACCAGGCGGTCATGGAAAAGAACATAGTACTGAAAGCGAACTTCAGTGTAATCTTC
AACCGATTAGAGACCTTGATACTACTAAGGGCTTTCACTGAGGAGGGAGCAATAGTTGGAGAAATTTCAC
CATTACCTTCTCTTCCAGGACATACTTATGAGGATGTCAAAAATGCAGTTGGGGTCCTCATCGGAGGACT
TGAATGGAATGGTAACACGGTTCGAGTCTCTGAAAATATACAGAGATTCGCTTGGAGAAACTGTGATGAG
AATGGGAGACCTTCACTACCTCCAGAGCAGAAATGAAAAGTGGCGAGAGCAATTGGGACAGAAATTTGAG

GAAATAAGGTGGTTAATTGAAGAAATGCGGCACAGATTGAAAGCGACAGAGAATAGTTTCGAACAAATAA
CATTTATGCAAGCCTTACAACTACTGCTTGAAGTAGAACAAGAGATAAGAGCTTTCTCGTTTCAGCTTAT
TTAATGATAA
>gi|338826662|gb|CY092884.1| Influenza A virus (A/California/NHRC0001/2011(H1N1)) nuclear export protein (NEP) and nonstructural protein 1 (NS1) genes, complete cds
AACATAATGGACTCCAACACCATGTCAAGCTTTCAGGTAGACTGTTTCCTTTGGCATATCCGCAAGCGAT
TTGCAGACAATGGATTGGGTGATGCCCCATTCCTTGATCGGCTCCGCAGAGATCAAAAGTCCTTAAAAGG
AAGAGGCAACACCCTTGGCCTCGATATCGAAACAGCCACTCTTGTTGGGAAACAAATCGTGGAATGGATC
TTGAAAGAGGAATCCAGCGAGACACTTAGAATGACAATTGCATCTGTACCTACTTCGCGCTACCTTTCTG
ACATGACCCTCGAGGAAATGTCACGAGACTGGTTCATGCTCATGCCTAGGCAAAAGATAATAGGCCCTCT
TTGCGTGCGATTGGACCAGGCGGTCATGGAAAAGAACATAGTACTGAAAGCGAACTTCAGTGTAATCTTT
AACCGATTAGAGACCTTGATACTACTAAGGGCTTTCACTGAGGAGGGAGCAATAGTTGGAGAAATTTCAC
CATTACCTTCTCTTCCAGGACATACTTATGAGGATGTCAAAAATGCAGTTGGGGTCCTCATCGGAGGACT
TGAATGGAATGGTAACACGGTTCGAGTCTCTGAAAATATACAGAGATTCGCTTGGAGAAACTGTGATGAG
AATGGGAGACCTTCACTACCTCCAGAGCAGAAATGAAAAGTGGCGAGAGCAATTGGGACAGAAATTTGAG
GAGATAAGGTGGTTAATTGAAGAAATGCGGCACAGATTGAAAGCGACAGAGAATAGTTTCGAACAAATAA
CATTTATGCAAGCCTTACAACTACTGCTTGAAGTAGAACAAGAGATAAGAGCTTTCTCGTTTCAGCTTAT
TTAATGATAA
>gi|338826644|gb|CY092876.1| Influenza A virus (A/Illinois/NHRC0001/2011(H1N1)) nuclear export protein (NEP) and nonstructural protein 1 (NS1) genes, complete cds
AACATAATGGACTCCAACACCATATCAAGCTTTCAGGTAGACTGTTTCCTTTGGCATATCCGCAAGCGAT
TTGCAGACAATGGATTGGGTGATGCCCCATTCCTTGATCGGCTCCGCCGAGATCAAAAGTCCTTAAAAGG
AAGAGGCAACACCCTTGGCCTCGATATCGAAACAGCCACTCTTGTTGGGAAACAAATAGTGGAATGGATC
TTGAAAGAGGAATCCAGCGAGACACTTAGAATGACAATTGCATCTGTACTTACTTCGCGCTACCTTTCTG
ACATGACCCTCGAGGAAATGTCACGAGAATGGTTCATGCTCATGCCTAGGCAAAAGATAATAGGCCCTCT
TTGCGTGCGATTGGACCAGGCGGTCATGGAAAAGAACATAGTACTGAAAGCGAACTTCAGTGTAATCTTT
AACCGATTAGAGACCTTGATACTACTAAGGGCTTTCACTGAGGAGGGAGCAATAGTTGGAGAAATTTCAC
CATTACATTCTCTTCCAGGACATACTTATGAGGATGTCAAAAATGCAGTTGGGGTCCTCATCGGAGGACT
TGAATGGAATGGTAACACGGTTCGAGTCTCTGAAAATATACAGAGATTCGCTTGGAGAAACTGTGATGAG
AATGGGAGACCTTCACTACCTCCAGAGCAGAAATGAAAAGTGGCGAGAGCAATTGGGACAGAAATTTGAG
GAAATAAGGTGGTTAATTGAAGAAATGCGGCACAGATTGAAAGCGACAGAGAATAGTTTCGAACAAATAA
CATTTATGCAAGCCTTACAACTACTGCTTGAAGTAGAACAAGAGATAAGAGCTTTCTCGTTTCAGCTTAT
TTAATGATAA
>gi|404425402|gb|CY125779.1| Influenza A virus (A/Boston/DOA90/2012(H1N1)) nuclear export protein (NEP) and nonstructural protein 1 (NS1) genes, complete cds
ATGGACTCCAACACCATGTCAAGCTTTCAGGTAGACTGTTTCCTTTGGCATATCCGCAAGCGATTTGCAG
ACAATGGATTGGGTGATGCCCCATTCCTTGATCGGCTCCGCCGAGATCAAAAGTCCTTAAAAGGAAGAGG
CAACACCCTTGGCCTCGATATCGAAACAGCCACTCTTGTTGGGAAACAAATCGTGGAATGGATCTTGAAA
GAGGAATCCAGCGAGACACTTAGAATGACAATTGCATCTGTACCTACTTCGCGTTACATTTCTGACATGA
CCCTCGAGGAAATGTCACGAGACTGGTTCATGCTCATGCCTAGGCAAAAGATAATAGGCCCTCTTTGCGT
GCGATTGGACCAGGCGGTCATGGAAAAAAACATAGTACTGAAAGCGAACTTCAGTGTAATCTTCAACCGA
TTAGAGACCTTGATACTACTAAGGGCTTTCACTGAGGAGGGAGCAATAGTTGGAGAAATTTCACCATTAC
CTTCTCTTCCAGGACATACTTATGAGGATGTCAAAAATGCAGTTGGGGTCCTCATCGGAGGACTTGAATG
GAATGGTAACACGGTTCGAGTCTCTGAAAATATACAGAGATTCGCTTGGAGAAACTGTGATGAGAATGGG

AGACCTTCACTACCTCCAGAGCAGAAATGAAAAGTGGCGAGAGCAATTGGGACAGAAATTTGAGGAAATA
AGGTGGTTAATTGAAGAAATGCGGCACAGATTGAAAGCGACAGAGAATAGTTTCGAACAAATAACATTTA
TGCAAGCCTTACAACTACTGCTTGAAGTAGAACAAGAGATAAGAGCTTTCTCGTTTCAGCTTATTTAATG
ATAAAAAACAC
>gi|404425420|gb|CY125787.1| Influenza A virus (A/Boston/DOA93/2012(H1N1)) nuclear export protein (NEP) and nonstructural protein 1 (NS1) genes, complete cds
GTGACAAAAACATAATGGACTCCAACACCATGTCAAGCTTTCAGGTAGACTGTTTCCTTTGGCATATCCG
CAAGCGATTTGCAGACAATGGATTGGGTGATGCCCCATTCCTTGATCGGCTCCGCCGAGATCAAAAGTCC
TTAAAAGGAAGAGGCAACACCCTTGGCCTCGATATCGAAACAGCCACTCTTGTTGGGAAACAAATCGTGG
AATGGATCTTGAAAGAGGAATCCAGCGAGACACTTAGAATGACAATTGCATCTGTACCTACTTCGCGTTA
CATTTCTGGCATGACCCTCGAGGAAATGTCACGAGACTGGTTCATGCTCATGCCTAGGCAAAAGATAATA
GGCCCTCTTTGCGTGCGATTGGACCAGGCGGTCATGGAAAAAAACATAGTACTGAAAGCGAACTTCAGTG
TAATCTTCAACCGATTAGAGACCTTGATACTACTAAGGGCTTTCACTGAGGAGGGAGCAATAGTTGGAGA
AATTTCACCATTACCTTCTCTTCCAGGACATACTTATGAGGATGTCAAAAATGCAGTTGGGGTCCTCATC
GGAGGACTTGAATGGAATGGTAACACGGTTCGAGTCTCTGAAAATATACAGAGATTCGCTTGGAGAAACT
GTGATGAGAATGGGAGACCTTCACTACCTCCAGAGCAGAAATGAAAAGTGGCGAGAGCAATTGGGACAGA
AATTTGAGGAAATAAGGTGGTTAATTGAAGAAATGCGACACAGATTGAAAGCGACAGAGAATAGTTTCGA
ACAAATAACATTTATGCAAGCCTTACAACTACTGCTTGAAGTAGAACAAGAGATAAGAGCTTTCTCGTTT
CAGCTTATTTAATGATAAAAAACAC
