# Supplementary material for: A computational method for predicting regulation of human microRNAs on the influenza virus genome
Source: BMC Syst Biol. 2013 Oct 14;7(Suppl 2):S3. doi: 10.1186/1752-0509-7-S2-S3 (PMC3851852; doi:10.1186/1752-0509-7-S2-S3)
Supplement: Additional File 9 — The coding sequence of the gene fragment of PB1 from 2000 to 2012 recorded in Genbank. [file 1752-0509-7-S2-S3-S9.PDF]

PA

>gi|145278783|gb|CY021698.1| Influenza A virus (A/Memphis/15/2000(H1N1)) segment 3, complete sequence
TTCGAAATGGAAGATTTTGTGCGACAATGCTTCAATCCGATGATTGTCGAGCTTGCGGAAAAGGCAATGA
AAGAGTATGGAGAGGACCTGAAAATCGAAACAAACAAATTTGCAGCAATATGCACTCACTTGGAAGTATG
CTTCATGTATTCAGATTTTCATTTCATCAATGAGCAAGGCGAATCAATAATAGTAGAGCCTGAGGACCCA
AATGCACTTTTAAAGCACAGATTTGAGATAATAGAGGGACGAGATCGTACAATGGCATGGACAGTTGTAA
ACAGTATTTGCAACACCACAGGAGCTGAGAAACCAAAATTTCTGCCAGACCTGTATGATTACAAAGAGAA
TAGATTCATCGAGATTGGAGTGACAAGGAGGGAAGTTCACATATACTATCTGGAAAAGGCCAACAAAATT
AAATCTGAAAAGACACACATTCACATTTTCTCATTCACTGGCGAAGAAATGGCCACAAAGGCCGATTACA
CTCTCGATGAAGAAAGCAGGGCTAGGATTAAAACCAGACTATTCACCATAAGACAAGAAATGGCAAGCAG
AGGTCTTTGGGACTCCTTTCGTCAGTCCGAAAGAGGCGAAGAAACAATTGAAGAAAGATTTGAAATCACA
GGGACAATGCGCAGGCTCGCTGACCAAAGCCTTCCGCCGAACTTCTCCTGCATTGAGAATTTTAGAGCCT
ATGTGGATGGATTTGAACCGAACGGCTACATTGAGGGCAAGCTTTCTCAAATGTCCAAAGAAGTAAATGC
TAGAATTGAGCCTTTTTTGAAAACAACACCACGATCAATTAGACTTCCGGATGGGCCTCCTTGTTTTCAG
CGGTCAAAATTCCTGCTGATGGATTCTTTAAAATTAAGCATTGAGGATCCAAATCATGAAGGAGAGGGAA
TACCACTATATGATGCAATCAAGTGTATGAAAACATTCTTTGGATGGAAAGAACCCTCTGTTGTCAAGCC
ACACGGGAAGGGAATAAATCCGAATTATCTGCTGTCATGGAAGCAGGTATTGGAAGAGCTGCAGGACATT
GAGAGTGAGGAGAAGATTCCAAGAACAAAAAACATGAAAAAAACGAGTCAGCTAAAGTGGGCACTTGGT
G
AGAACATGGCACCAGAGAAGGTGGATTTTGATGACTGTAAAGATATAAGCGATTTGAAGCAATATGATAG
TGACGAACCTGAATTAAGGTCATTTTCAAGTTGGATCCAGAATGAGTTCAACAAGGCATGCGAGCTGACC
GATTCAATCTGGATAGAGCTCGATGAGATTGGAGAAGATGTGGCCCCGATTGAACACATTGCAAGCATGA
GAAGAAATTACTTCACAGCTGAGGTGTCCCATTGCAGAGCCACAGAATATATAATGAAGGGGGTATACAT
TAATACTGCTTTGCTTAATGCATCCTGTGCAGCAATGGATGATTTCCAACTAATTCCCATGATAAGCAAA
TGTAGAACCAAAGAGGGAAGGAGAAAAACCAATTTGTACGGCTTCATCGTAAAAGGAAGATCTCACTTAA
GGAATGACACCGATGTGGTAAACTTTGTGAGCATGGAGTTTTCCCTCACTGACCCAAGACTTGAGCCACA
CAAATGGGAGAAGTACTGTGTTCTTGAGATAGGAGATATGCTTCTAAGGAGTGCAATAGGCCAGGTGTCA
AGGCCCATGTTCTTGTACGTAAGGACAAATGGAACCTCAAAAATTAAAATGAAATGGGGAATGGAGATGA
GGCGTTGCCTCCTCCAATCCCTTCAACAAATAGAGAGCATGATTGAGGCTGAGTCCTCTGTCAAGGAGAA
AGACATGACAAAAGAGTTTTTTGAGAATAGATCAGAAACATGGCCCATTGGAGAGTCACCAAAAGGAGTG
GAAGAAGGTTCCATTGGGAAAGTATGCAGGACACTATTGGCTAAGTCAGTATTCAATAGTCTGTATGCAT
CTCCACAATTAGAAGGATTTTCAGCTGAGTCAAGAAAGTTGCTCCTCATTGTTCAGGCTCTTAGGGACAA
TCTGGAACCTGGGACCTTTGATCTTGGGGGGCTATATGAAGCAATTGAGGAGTGCCTGATTAATGATCCC
TGGGTTTTGCTTAATGCTTCTTGGTTCAACTCCTTCCTAACACATGCATTGAGATAACTGGGGCAATGCT
ACTATTTACTATCCATAC
>gi|70907651|gb|CY000454.2| Influenza A virus (A/New York/146/2000(H1N1)) segment 3, complete sequence
AAAGCAGGTACTGATTCGAAATGGAAGATTTTGTGCGACAATGCTTCAATCCGATGATTGTCGAGCTTGC
GGAAAAGGCAATGAAAGAGTATGGAGAGGACCTGAAGATCGAAACAAACAAATTTGCAGCAATATGCACT
CACTTGGAAGTATGCTTCATGTATTCAGATTTTCATTTCATCAATGAGCAAGGCGAGTCAATAATAGTAG
AGCCTGAAGACCCAAACGCACTTTTAAAACACAGATTTGAAATAATAGAGGGACGAGATCGTACAATGGC
ATGGACAGTTGTAAACAGTATTTGCAACACCACAGGAGTTGAGAAACCAAAGTTCCTGCCAGATTTGTAT

GATTACAAGGAGAATAGATTCATCGAGATTGGAGTGACAAGGAGGGAAGTTCACATATACTATCTGGAAA
AGGCAAACAAAATTAAGTCTGAGAAGACACACATACACATTTTCTCATTCACTGGTGAAGAAATGGCCAC
AAAGGCCGATTACACTCTCGATGAAGAAAGCAGGGCTAGGATTAAAACCAGACTATTCACCATAAGACAG
GAAATGGCAAGCAGAGGCCTTTGGGATTCCTTTCGTCAGTCCGAGAGAGGCGAAGAAACAATTGAAGAAA
GATTTGAAATTACAGGAACAATGCGCAGGCTCGCTGACCAAAGCCTCCCGCCGAACTTCTCCTGCCTTGA
GAATTTTAGAGCCTATGTGGATGGATTCGAACCGAACGGCTACATTGAGGGCAAGCTTTCTCAAATGTCC
AAAGAAGTAAATGCTAGAATTGAGCCTTTTTTGAAAACAACACCACGACCAATTAGGCTGCCGGATGGGC
CTCCTTGTTTTCAGCGGTCAAAATTCCTGCTGATGGATTCTTTAAAATTAAGCATTGAGGATCCAAGTCA
TGAAGGAGAGGGAATACCACTACATGATGCGATCAAGTGTATGAGAACATTCTTTGGATGGAAAGAACCC
TCTGTTGTCAAACCACACGAGAAGGGAATAAATCCGAATTATCTGCTGTCATGGAAGCAGGTACTGGCAG
AACTGCAGGACATTGAGAGTGAGGAGAAGATTCCAAGAATCAAAAACATGAAAAAGACGAGTCAGCTTAA
GTGGGCGCTTGGTGAGAACATGGCACCAGAGAAGGTAGATTTTGACGACTGTAAAGATATAGGCGACTTG
AAGCAATATGATAGTGATGAGCCTGAGTTAAGGTCACTTTCAAGTTGGATCCAGAATGAGTTCAACAAGG
CATGCGAGCTGACTGATTCAATCTGGATAGAGCTTGATGAGATTGGAGAAGATGTGGCTCCGATTGAACA
CATTGCAAGCATGCGAAGAAATTACTTCACAGCAGAGGTGTCTCATTGCAGAGCCACAGAATATATAATG
AAGGGGGTATACATTAATACCGCTTTGCTTAATGCATCCTGTGCAGCAATGGATGATTTCCAACTAATCC
CCATGATAAGCAAATGTAGGACTAAAGAGGGAAGGCGAAAGACCAATTTGTATGGCTTCATCATAAAAGG
AAGATCTCACTTAAGGAATGACACCGATGTGGTAAACTTTGTGAGCATGGAGTTTTCCCTCACTGACCCG
AGACTTGAGCCACACAAATGGGAGAAGTACTGTGTTCTTGAGATAGGGGATATGCTTCTAAGAAGTGCAA
TAGGCCAAGTGTCAAGGCCCATGTTCTTGTATGTGAGGACAAATGGAACCTCCAAAATTAAAATGAAATG
GGGGATGGAGATGAGGCGTTGCCTCCTCCAATCCCTCCAACAAATAGAGAGTATGATTGAAGCTGAGTCC
TCTGTCAAGGAGAAAGACATGACAAAAGAGTTTTTTGAGAATAGATCAGAAACATGGCCCATTGGGGAGT
CACCCAAAGGAGTGGAAGAAGGTTCCATTGGGAAAGTATGCAGAACTTTATTGGCTAAGTCAGTATTCAA
TAGTTTGTATGCATCTCCACAATTAGAAGGATTTTCAGCTGAATCAAGAAAGTTGCTCCTTATTGTTCAG
GCTCTTCGGGACAATCTGGAACCTGGGACCTTTGATCTTGGGGGGCTATATGAAGCAATTGAGGAGTGCC
TGATTAATGATCCCTGGGTTTTGCTTAATGCTTCTTGGTTCAACTCCTTCCTAACACATGCATTGAGATA
GCTGTGGCAATGCTACTATTTGCTATCCATACTGTCCAAAAAAGTACCTTGTTTCTACT
>gi|145278916|gb|CY021754.1| Influenza A virus (A/South Australia/44/2000(H1N1)) segment 3, complete sequence
ATTCGAAATGGAAGATTTTGTGCGACAATGCTTCAATCCGATGATTGTCGAGCTTGCGGAAAAGGCAATG
AAAGAGTATGGAGAGGACCTGAAGATCGAAACAAACAAATTTGCAGCAATATGCACTCACTTGGAAGTAT
GCTTCATGTATTCAGATTTTCATTTCATCAATGAGCAAGGCGAGTCAATAATAGTAGAGCCTGAAGACCC
AAACGCACTTTTAAAACACAGATTTGAAATAATAGAGGGACGAGATCGTACAATGGCATGGACAGTTGTA
AACAGTATTTGCAACACCACAGGAGTTGAGAAACCAAAGTTCCTGCCAGATTTGTATGATTACAAGGAGA
ATAGATTCATCGAGATTGGAGTGACAAGGAGGGAAGTTCACATATACTATCTGGAAAAGGCAAACAAAAT
TAAGTCTGAGAAGACACACATACACATTTTCTCATTCACTGGTGAAGAAATGGCCACAAAGGCCGATTAC
ACTCTCGATGAAGAAAGCAGGGCTAGGATTAAAACCAGACTATTCACCATAAGACAGGAAATGGCAAGCA
GAGGCCTTTGGGATTCCTTTCGTCAGTCCGAGAGAGGCGAAGAAACAATTGAAGAAAGATTTGAAATTAC
AGGAACAATGCGCAGGCTCGCTGACCAAAGCCTCCCGCCGAACTTCTCCTGCCTTGAGAATTTTAGAGCC
TATGTGGATGGATTCGAACCGAACGGCTACATTGAGGGCAAGCTTTCTCAAATGTCCAAAGAAGTAAATG
CTAGAATTGAGCCTTTTTTGAAAACAACACCACGACCAATTAGGCTGCCGGATGGGCCTCCTTGTTTTCA
GCGGTCAAAATTCCTGCTGATGGATTCTTTAAAATTAAGCATTGAGGATCCAAGTCATGAAGGAGAGGGA
ATACCACTATATGATGCGATCAAGTGTATGAGAACATTCTTTGGATGGAAAGAACCCTCTGTTGTCAAAC
CACACGAGAAGGGAATAAATCCGAATTATCTGATGTCATGGAAGCAGGTACTGGCAGAACTGCAGGACAT

TGAGAGTGAGGAGAAGATTCCAAGAATCAAAAACATGAAAAAGACGAGTCAGCTTAAGTGGGCACTTGGT
GAGAACATGGCACCAGAGAAGGTAGATTTTGACGACTGTAAAGATATAGGCGACTTGAAGCAATATGATA
GTGATGAGCCTGAGTTAAGGTCACTTTCAAGTTGGATCCAGAATGAGTTCAACAAGGCATGCGAGCTGAC
TGATTCAATCTGGATAGAGCTTGATGAGATTGGAGAAGATGTGGCTCCGATTGAACACATTGCAAGCATG
CGAAGAAATTACTTCACAGCAGAGGTGTCTCATTGCAGAGCCACAGAATATATAATGAAGGGGGTATACA
TTAATACCGCTTTGCTTAATGCATCCTGTGCAGCAATGGATGATTTCCAACTAATCCCCATGATAAGCAA
ATGTAGGACTAAAGAGGGAAGGCGAAAGACCAATTTGTATGGCTTCATCATAAAAGGAAGATCTCACTTA
AGGAATGACACCGATGTGGTAAACTTTGTGAGCATGGAGTTTTCCCTCACTGACCCGAGACTTGAGCCAC
ACAAATGGGAGAAGTACTGTGTTCTTGAGATAGGGGATATGCTTCTAAGAAGTGCAATAGGCCAAGTGTC
AAGGCCCATGTTCTTGTATGTGAGGACAAATGGAACCTCCAAAATTAAAATGAAATGGGGGATGGAGATG
AGGCGTTGCCTCCTCCAATCCCTCCAACAAATAGAGAGTATGATTGAAGCTGAGTCCTCTGTCAAGGAGA
AAGACATGACAAAAGAGTTTTTTGAGAATAGATCAGAAACATGGCCCATTGGGGAGTCACCCAAAGGAGT
GGAAGAAGGTTCCATTGGGAAAGTATGCAGAACTTTATTGGCTAAGTCAGTATTCAATAGTTTGTATGCA
TCTCCACAATTAGAAGGATTTTCAGCTGAATCAAGAAAGTTGCTCCTTATTGTTCAGGCTCTTCGGGACA
ATCTGGAACCTGGGACCTTTGATCTTGGGGGGCTATATGAAGCAATTGAGGAGTGCCTGATTAATGATCC
CTGGGTTTTGCTTAATGCTTCTTGGTTCAACTCCTTCCTAACACATGCATTGAGATAGCTGTGGCAATGC
TACTATTTGCTATCCATACTGTCCAAAAAA

>gi|157367777|gb|CY026160.1| Influenza A virus (A/Auckland/585/2000(H1N1)) segment 3, complete sequence

ATTCGAAATGGAAGATTTTGTGCGACAATGCTTCAATCCGATGATTGTCGAGCTTGCGGAAAAGGCAATG
AAAGAGTATGGAGAGGACCTGAAAATCGAAACAAACAAATTTGCAGCAATATGCACTCACTTGGAAGTAT
GCTTCATGTATTCAGATTTTCATTTCATCAATGAGCAAGGCGAATCAATAATAGTAGAGCCTGAGGACCC
AAATGCACTTTTAAAGCACAGATTTGAGATAATAGAGGGACGAGATCGTACAATGGCATGGACAGTTGTA
AACAGTATTTGCAACACCACAGGAGCTGAGAAACCAAAGTTTCTGCCAGATCTGTACGATTACAAAGAGA
ATAGATTCATCGAGATTGGAGTGACAAGGAGGGAAGTTCACATATACTATCTGGAAAAGGCCAACAAAAT
TAAATCTGAGAAGACACACATTCACATTTTCTCATTCACTGGCGAAGAAATGGCCACAAAGGCCGATTAC
ACTCTCGATGAAGAAAGCAGGGCTAGGATTAAAACCAGACTATTCACCATAAGACAAGAAATGGCAAGCA
GAGGTCTTTGGGACTCCTTTCGTCAGTCCGAAAGAGGCGAAGAAACAATTGAAGAAAGATTTGAAATCAC
AGGGACAATGCGCAGGCTCGCTGACCAAAGCCTTCCGCCGAACTTCTCCTGCATTGAGAATTTTAGAGCC
TATGTGGATGGATTTGAACCGAACGGCTACATTGAGGGCAAGCTTTCTCAAATGTCCAAAGAAGTAAATG
CTAGAATTGAGCCTTTTTTGAAAACAACACCACGACCAATTAGACTTCCGGATGGGCCTCCTTGTTTTCA
GCGGTCAAAATTCCTGCTGATGGATTCTTTAAAATTAAGCATTGAGGATCCAAATCATGAAGGGGAGGGA
ATACCACTATATGATGCAATCAAGTGTATGAGAACATTCTTTGGATGGAAAGAACCCTCTGTTGTCAAGC
CACACGGGAAGGGAATAAATCCGAATTATCTGCTGTCATGGAAGCAGGTATTGGAAGAGCTGCAGGACAT
TGAGAGTGAGGAGAAGATTCCAAGAACAAAAAACATGAAAAAAACGAGTCAGCTAAAGTGGGCACTTGG
T
GAGAACATGGCACCAGAGAAGGTGGATTTTGATGACTGTAAAGATATAAGCGATTTGAAGCAATATGATA
GTGACGAACCTGAATTAAGGTCATTTTCAAGTTGGATCCAGAATGAGTTCAACAAAGCATGCGAGCTGAC
CGATTCAATCTGGATAGAGCTCGATGAGATTGGAGAAGATGTGGCCCCGATTGAACACATTGCAAGCATG
AGAAGAAATTACTTCACAGCTGAGGTGTCCCATTGCAGAGCCACAGAATATATAATGAAGGGGGTATACA
TTAATACTGCTTTGCTTAATGCATCCTGTGCAGCAATGGATGATTTCCAACTAATTCCCATGATAAGCAA
ATGTAGAACTAAAGAGGGAAGGAGAAAGACCAATTTGTACGGCTTCATCGTAAAAGGAAGATCTCACTTA
AGGAATGACACCGATGTGGTAAACTTTGTGAGCATGGAGTTCTCCCTCACTGACCCAAGACTTGAGCCAC
ACAAATGGGAGAAGTACTGTGTTCTTGAGATAGGAGATATGCTTCTAAGGAGTGCAATAGGCCAAGTGTC

AAGGCCCATGTTCTTGTATGTAAGGACAAATGGAACCTCAAAAATTAAAATGAAATGGGGAATGGAGATG
AGGCGTTGCCTCCTCCAATCCCTTCAACAAATAGAGAGCATGATTGAAGCTGAGTCTTCTGTCAAGGAGA
AAGACATGACAAAAGAGTTTTTTGAGAATAGATCAGAAACATGGCCCATTGGAGAGTCACCAAAAGGAGT
GGAAGAAGGTTCCATTGGGAAAGTATGCAGGACACTATTGGCTAAGTCAGTATTCAATAGTCTGTATGCA
TCTCCACAATTAGAAGGATTTTCAGCTGAGTCAAGAAAGTTGCTCCTCATTGTTCAGGCTCTTAGGGACA
ATCTGGAACCTGGGACCTTTGATCTTGGGGGGCTATATGAAGCAATTGAGGAGTGCCTGATTAATGATCC
CTGGGTTTTGCTTAATGCTTCTTGGTTCAACTCCTTCCTAACACATGCATTGAGATAGCTGGGGCAATGC
TACTATTTACTATCCATA
>gi|145278629|gb|CY021634.1| Influenza A virus (A/Wellington/4/2000(H1N1)) segment 3, complete sequence
ATGGAAGATTTTGTGCGACAATGCTTCAATCCGATGATTGTCGAGCTTGCGGAAAAGGCAATGAAAGAGT
ATGGAGAGGACCTGAAAATCGAAACAAACAAATTTGCAGCAATATGCACTCACTTGGAAGTATGCTTCAT
GTATTCAGATTTTCATTTCATCAATGAGCAAGGCGAATCAATAATAGTAGAGCCTGAGGACCCAAATGCA
CTTTTAAAGCACAGATTTGAGATAATAGAGGGACGAGATCGTACAATGGCATGGACAGTTGTAAACAGTA
TTTGCAACACCACAGGAGCTGAGAAACCAAAGTTTCTGCCAGATCTGTATGATTACAAAGAGAATAGATT
CATCGAGATTGGAGTGACAAGGAGGGAAGTTCACATATACTATCTGGAAAAGGCCAACAAAATTAAATCT
GAGAAGACACACATTCACATTTTCTCATTCACTGGCGAAGAAATGGCCACAAAGGCCGATTACACTCTCG
ATGAAGAAAGCAGGGCTAGGATTAAAACCAGACTATTCACCATAAGACAAGAAATGGCAAGCAGAGGTCT
TTGGGACTCCTTTCGTCAGTCCGAAAGAGGCGAAGAAACAATTGAAGAAAGATTTGAAATCACGGGGACA
ATGCGCAGGCTCGCTGACCAAAGCCTTCCGCCGAACTTCTCCTGCATTGAGAATTTTAGAGCCTATGTGG
ATGGATTTGAACCGAACGGCTACATTGAGGGCAAGCTTTCTCAAATGTCCAAAGAAGTAAATGCTAGAAT
TGAGCCTTTTTTGAAAACAACACCACGACCAATTAGACTTCCGGATGGGCCTCCTTGTTTTCAGCGGTCA
AAATTCCTGCTGATGGATTCTTTAAAATTAAGCATTGAGGATCCAAATCATGAAGGGGAGGGAATACCAC
TATATGATGCAATCAAGTGTATGAGAACATTCTTTGGATGGAAAGAACCCTCTGTTGTCAAGCCACACGG
GAAGGGAATAAATCCGAATTATCTGCTGTCATGGAAGCAGGTATTGGAAGAGCTGCAGGACATTGAGAGT
GAGGAGAAGATTCCAAGAACAAAAAACATGAAAAAAACGAGTCAGCTAAAGTGGGCACTTGGTGAGAAC
A
TGGCACCAGAGAAGGTGGATTTTGATGACTGTAAAGATATAAGCGATTTGAAGCAATATGATAGTGACGA
ACCTGAATTAAGGTCATTTTCAAGTTGGATCCAGAATGAGTTCAACAAAGCATGCGAGCTGACCGATTCA
ATCTGGATAGAGCTCGATGAGATTGGAGAAGATGTGGCCCCGATTGAACACATTGCAAGCATGAGAAGAA
ATTACTTCACAGCTGAGGTGTCCCATTGCAGAGCCACAGAATATATAATGAAGGGGGTATACATTAATAC
TGCTTTGCTTAATGCATCCTGTGCAGCAATGGATGATTTCCAACTAATTCCCATGATAAGCAAATGTAGA
ACTAAAGAGGGAAGGAGAAAGACCAATTTGTACGGCTTCATCGTAAAAGGAAGATCTCACTTAAGGAATG
ACACCGATGTGGTAAACTTTGTGAGCATGGAGTTCTCCCTCACTGACCCAAGACTTGAGCCACACAAATG
GGAGAAGTACTGTGTTCTTGAGATAGGAGATATGCTTCTAAGGAGTGCAATAGGCCAAGTGTCAAGGCCC
ATGTTCTTGTATGTAAGGACAAATGGAACCTCAAAAATTAAAATGAAATGGGGAATGGAGATGAGGCGTT
GCCTCCTCCAATCCCTTCAACAAATAGAGAGCATGATTGAAGCTGAGTCTTCTGTCAAGGAGAAAGACAT
GACAAAAGAGTTTTTTGAGAATAGATCAGAAACATGGCCCATTGGAGAGTCACCAAAAGGAGTGGAAGAA
GGTTCCATTGGGAAAGTATGCAGGACACTATTGGCTAAGTCAGTATTCAATAGTCTGTATGCATCTCCAC
AATTAGAAGGATTTTCAGCTGAGTCAAGAAAGTTGCTCCTCATTGTTCAGGCTCTTAGGGACAATCTGGA
ACCTGGGACCTTTGATCTTGGGGGGCTATATGAAGCAATTGAGGAGTGCCTGATTAATGATCCCTGGGTT
TTGCTTAATGCTTCTTGGTTCAACTCCTTCCTAACACATGCATTGAGATAGCTGGGGCAATGCTACTATT
TACTATCCATAC
>gi|131058530|gb|CY020154.1| Influenza A virus (A/Memphis/7/2001(H1N1)) segment 3,

complete sequence
TTCGAAATGGAAGATTTTGTGCGACAATGCTTCAATCCGATGATTGTCGAGCTTGCGGAGAAGGCAATGA
AAGAGTATGGAGAGGACCTAAAAATCGAAACAAACAAATTTGCAGCAATATGCACTCACTTGGAAGTATG
CTTCATGTATTCAGATTTCCATTTCATCAATGAGCAAGGCGAATCAATAATAGTAGAGCCTGAGGACCCA
AATGCACTTTTAAAACACAGATTTGAGATAATAGAGGGACGAGATCGTACAATGGCATGGACAGTTGTAA
ACAGTATTTGCAACACCACAGGAGCTGAGAAACCAAAGTTTCTGCCAGATCTGTATGATTACAAAGAGAA
TAGATTCATCGAGATTGGAGTGACAAGGAGGGAAGTTCACATATACTATCTGGAAAAGGCCAACAAAATT
AAATCTGAAAAGACACACATTCACATTTTCTCATTCACTGGCGAAGAAATGGCCACAAAGGCCGATTACA
CTCTCGATGAAGAAAGCAGGGCTAGGATTAAAACCAGACTATTCACCATAAGACAAGAAATGGCAAGCAG
AGGTCTTTGGGACTCCTTTCGTCAGTCCGAAAGAGGCGAAGAAACAATTGAAGAAAGATTTGAAATCACA
GGGACAATGCGCAGGCTCGCTGACCAAAGCCTTCCGCCGAACTTCTCCTGCATTGAGAATTTTAGAGCCT
ATGTGGATGGATTTGAACCGAACGGCTACATTGAGGGCAAGCTTTCTCAAATGTCCAAAGAAGTAAATGC
TAGAATTGAGCCTTTTTTGAAAACAACACCACGACCAATTAGACTTCCGGATGGGCCTCCTTGTTTTCAG
CGGTCAAAATTCCTGCTGATGGATTCTTTAAAATTAAGCATTGAGGATCCAAATCATGAAGGAGAGGGAA
TACCACTATATGATGCAATCAAGTGTATGAGAACATTCTTTGGATGGAAAGAACCCTCTGTTGTCAAGCC
ACACGGGAAGGGAATAAATCCGAATTATCTGCTGTCATGGAAGCAGGTATTGGAAGAGCTGCAGGACATT
GAGAGTGAAGAGAAGATTCCAAGAACAAAAAACATGAAAAAAACGAGTCAGCTAAAGTGGGCACTTGGT
G
AGAACATGGCACCAGAGAAAGTAGATTTTGATGACTGTAAAGATATAAGCGATTTGAAGCAATATGATAG
TGACGAACCTGAATTAAGGTCATTTTCAAGTTGGATCCAGAATGAATTCAACAAGGCATGTGAGCTGACC
GATTCAATCTGGATAGAGCTCGATGAGATTGGAGAAGATGTGGCCCCGATTGAACACATTGCAAGCATGA
GGAGAAATTACTTCACAGCTGAGGTGTCCCATTGCAGAGCCACAGAATATATAATGAAGGGAGTATACAT
TAATACTGCTTTGCTTAATGCATCCTGTGCAGCAATGGATGATTTCCAACTAATTCCCATGATAAGCAAA
TGTAGAACTAAAGAGGGAAGGAGAAAGACCAATTTGTACGGCTTCATCATAAAAGGAAGATCTCACTTAA
GGAATGACACCGATGTGGTAAACTTTGTGAGCATGGAGTTTTCCCTCACTGACCCAAGACTTGAGCCACA
CAAATGGGAGAAGTACTGTGTTCTCGAGATAGGAGATATGCTTCTAAGGAGTGCAATAGGCCAAGTGTCA
AGGCCCATGTTCTTGTATGTAAGGACAAATGGAACCTCAAAAATTAAAATGAAATGGGGAATGGAGATGA
GGCGTTGCCTCCTCCAATCCCTTCAACAAATAGAGAGCATGATTGAAGCTGAGTCCTCTGTCAAGGAGAA
AGACATGACAAAAGAGTTTTTTGAGAATAGATCAGAAACATGGCCCATTGGAGAGTCACCAAAAGGAGTG
GAAGAAGGTTCCATTGGGAAAGTATGCAGGACACTATTGGCTAAGTCAGTATTCAATAGTCTGTATGCAT
CACCACAATTAGAAGGATTCTCAGCTGAGTCAAGAAAGTTGCTCCTCATTGTTCAGGCTCTTAGGGACAA
TCTGGAGCCTGGGACCTTTGATCTTGGGGGGCTATATGAAGCAATTGAGGAGTGCCTGATTAATGATCCC
TGGGTTTTGCTTAATGCTTCTTGGTTCAACTCCTTCCTAACACATGCATTGAGATAGTTGGGGCAATGCT
ACTATT
>gi|73761485|gb|CY002533.1| Influenza A virus (A/New York/220/2002(H1N1)) segment 3, complete sequence
AAAGCAGGTACTGATTCGAAATGGAAGATTTTGTGCGACAATGCTTCAATCCGATGATTGTCGAGCTTGC
GGAAAAGGCAATGAAAGAGTATGGAGAGGACCTGAAAATCGAAACAAACAAATTTGCAGCAATATGCACC
CACTTGGAAGTATGCTTCATGTATTCAGATTTTCATTTCATCAATGAGCAAGGCGAATCAATAATAGTAG
AGCCTGAGGACCCAAATGCACTTTTAAAACACAGATTTGAGATAATAGAGGGACGAGATCGTACAATGGC
ATGGACAGTTGTAAACAGTATTTGCAACACCACAGGAGCTGAGAAACCAAAGTTTCTGCCAGATCTGTAT
GATTACAAAGAGAATAGATTCATCGAGATTGGAGTGACAAGGAGAGAAGTTCACATATACTATCTGGAAA
AGGCCAACAAAATTAAATCTGAGAAGACACACATTCACATTTTCTCATTCACTGGCGAAGAAATGGCCAC
AAAGGCCGATTACACTCTCGATGAAGAAAGCAGGGCTAGGATTAAAACCAGACTATTCACCATAAGACAA

GAAATGGCAAGCAGAGGTCTTTGGGACTCCTTTCGTCAGTCCGAAAGAGGCGAAGAAACAATTGAAGAAA
GATTTGAAATCACAGGGACAATGCGCAGGCTCGCTGATCAAAGCCTTCCGCCGAACTTCTCCTGCATTGA
GAATTTTAGAGCCTATGTGGATGGATTTGAACCGAACGGCTACATTGAGGGCAAGCTTTCTCAAATGTCC
AAAGAAGTAAATGCTAGAATTGAGCCTTTTTTGAAAACAACACCTCGACCAATTAGACTTCCGAATGGGC
CTCCTTGTTTTCAGCGGTCAAAATTCCTGCTGATGGATTCTTTAAAATTAAGCATTGAGGATCCAAATCA
TGAAGGGGAGGGAATACCACTATATGATGCAATCAAGTGTATGAGAACATTCTTTGGATGGAAAGAACCC
ACTGTTGTCAAGCCACACGAGAAGGGAATAAATCCGAATTATCTGCTGTCGTGGAAGCAGGTATTGGAAG
AGCTGCAGGACATTGAGAGTGAGGAGAAGATTCCAAGAACAAAAAACATGAAAAAAACGAGTCAGCTAA
A
GTGGGCACTTGGTGAGAACATGGCACCAGAGAAGGTGGATTTTGATGACTGTAAAGGTATAAGCGATTTG
AAGCAATATGATAGTGACGAACCTGAATTAAGGTCATTTTCAAGTTGGATCCAGAATGAGTTCAACAAGG
CATGCGAGCTGACCGATTCAATCTGGATAGAGCTCGATGAGATTGGAGAAGATGTGGCCCCGATTGAACA
CATTGCAAGCATGAGAAGAAATTACTTCACAGCTGAGGTGTCCCATTGCAGAGCCACTGAATATATAATG
AAGGGGGTATACATTAATACTGCTTTGCTTAATGCATCCTGTGCAGCAATGGATGATTTCCAACTAATTC
CCATGATAAGCAAATGTAGAACTAAAGAGGGAAGGAGAAAGACCAATTTGTACGGCTTCATCATAAAAGG
AAGATCTCACTTAAGGAATGACACCGATGTGGTAAACTTTGTGAGCATGGAGTTTTCCCTCACTGACCCA
AGACTTGAGCCACACAAATGGGAGAAGTACTGTGTTCTTGAGATAGGAGATATGCTTCTAAGGAGTGCAA
TAGGCCAAGTGTCAAGGCCCATGTTCTTGTATGTAAGAACAAATGGAACCTCAAAAATTAAAATGAAATG
GGGAATGGAGATGAGGCGTTGCCTCCTCCAATCCCTCCAACAAATAGAGAGCATGATTGAAGCTGAGTCC
TCTGTCAAGGAGAAAGACATGACAAAAGAGTTTTTTGAGAATAGATCAGAAACATGGCCCATTGGAGAGT
CACCAAAAGGAGTGGAAGAAGGTTCCATTGGGAAAGTATGCAGGACACTATTGGCTAAGTCAGTATTCAA
TAGTCTGTATGCATCTCCACAATTAGAAGGATTTTCAGCTGAGTCAAGAAAGTTGCTCCTCATTGTTCAG
GCTCTTAGGGACAACCTGGAACCTGGGACCTTTGATCTTGGGGGACTATATGAAGCAATTGAGGAGTGCC
TGATTAATGATCCCTGGGTTTTGCTTAATGCTTCTTGGTTCAACTCCTTCCTAACACATGCATTGAGATA
GCTGAGGCAATGCTACTATTTGTTATCCATACTGTCCAAAAAAGTACCTTGTTTCTACT
>gi|156536329|gb|CY025039.1| Influenza A virus (A/Auckland/597/2000(H1N1)) segment 3, complete sequence
ATTCGAAATGGAAGATTTTGTGCGACAATGCTTCAATCCGATGATTGTCGAGCTTGCGGAAAAGGCAATG
AAAGAGTATGGAGAGGACCTGAAAATCGAAACAAACAAATTTGCAGCAATATGCACTCACTTGGAAGTAT
GCTTCATGTATTCAGATTTTCATTTCATCAATGAGCAAGGCGAATCAATAATAGTAGAGCCTGAGGACCC
AAATGCACTTTTAAAGCACAGATTTGAGATAATAGAGGGACGAGATCGTACAATGGCATGGACAGTTGTA
AACAGTATTTGCAACACCACAGGAGCTGAGAAACCAAAGTTTCTGCCAGATCTGTACGATTACAAAGAGA
ATAGATTCATCGAGATTGGAGTGACAAGGAGGGAAGTTCACATATACTATCTGGAAAAGGCCAACAAAAT
TAAATCTGAGAAGACACACATTCACATTTTCTCATTCACTGGCGAAGAAATGGCCACAAAGGCCGATTAC
ACTCTCGATGAAGAAAGCAGGGCTAGGATTAAAACCAGACTATTCACCATAAGACAAGAAATGGCAAGCA
GAGGTCTTTGGGACTCCTTTCGTCAGTCCGAAAGAGGCGAAGAAACAATTGAAGAAAGATTTGAAATCAC
AGGGACAATGCGCAGGCTCGCTGACCAAAGCCTTCCGCCGAACTTCTCCTGCATTGAGAATTTTAGAGCC
TATGTGGATGGATTTGAACCGAACGGCTACATTGAGGGCAAGCTTTCTCAAATGTCCAAAGAAGTAAATG
CTAGAATTGAGCCTTTTTTGAAAACAACACCACGACCAATTAGACTTCCGGATGGGCCTCCTTGTTTTCA
GCGGTCAAAATTCCTGCTGATGGATTCTTTAAAATTAAGCATTGAGGATCCAAATCATGAAGGGGAGGGA
ATACCACTATATGATGCAATCAAGTGTATGAGAACATTCTTTGGATGGAAAGAACCCTCTGTTGTCAAGC
CACACGGGAAGGGAATAAATCCGAATTATCTGCTGTCATGGAAGCAGGTATTGGAAGAGCTGCAGGACAT
TGAGAGTGAGGAGAAGATTCCAAGAACAAAAAACATGAAAAAAACGAGTCAGCTAAAGTGGGCACTTGG
T

GAGAACATGGCACCAGAGAAGGTGGATTTTGATGACTGTAAAGATATAAGCGATTTGAAGCAATATGATA
GTGACGAACCTGAATTAAGGTCATTTTCAAGTTGGATCCAGAATGAGTTCAACAAAGCATGCGAGCTGAC
CGATTCAATCTGGATAGAGCTCGATGAGATTGGAGAAGATGTGGCCCCGATTGAACACATTGCAAGCATG
AGAAGAAATTACTTCACAGCTGAGGTGTCCCATTGCAGAGCCACAGAATATATAATGAAGGGGGTATACA
TTAATACTGCTTTGCTTAATGCATCCTGTGCAGCAATGGATGATTTCCAACTAATTCCCATGATAAGCAA
ATGTAGAACTAAAGAGGGAAGGAGAAAGACCAATTTGTACGGCTTCATCGTAAAAGGAAGATCTCACTTA
AGGAATGACACCGATGTGGTAAACTTTGTGAGCATGGAGTTCTCCCTCACTGACCCAAGACTTGAGCCAC
ACAAATGGGAGAAGTACTGTGTTCTTGAGATAGGAGATATGCTTCTAAGGAGTGCAATAGGCCAAGTGTC
AAGGCCCATGTTCTTGTATGTAAGGACAAATGGAACCTCAAAAATTAAAATGAAATGGGGAATGGAGATG
AGGCGTTGCCTCCTCCAATCCCTTCAACAAATAGAGAGCATGATTGAAGCTGAGTCTTCTGTCAAGGAGA
AAGACATGACAAAAGAGTTTTTTGAGAATAGATCAGAAACATGGCCCATTGGAGAGTCACCAAAAGGAGT
GGAAGAAGGTTCCATTGGGAAAGTATGCAGGACACTATTGGCTAAGTCAGTATTCAATAGTCTGTATGCA
TCTCCACAATTAGAAGGATTTTCAGCTGAGTCAAGAAAGTTGCTCCTCATTGTTCAGGCTCTTAGGGACA
ATCTGGAACCTGGGACCTTTGATCTTGGGGGGCTATATGAAGCAATTGAGGAGTGCCTGATTAATGATCC
CTGGGTTTTGCTTAATGCTTCTTGGTTCAACTCCTTCCTAACACATGCATTGAGATAGCTGGGGCAATGC
TACTATTTACTATCCATACTGTCCAAAAAA

>gi|149780539|gb|CY022538.1| Influenza A virus (A/Auckland/605/2001(H1N1)) segment 3, complete sequence

ATTCGAAATGGAAGATTTTGTGCGACAATGCTTCAATCCGATGATTGTCGAGCTTGCGGAAAAGGCAATG
AAAGAGTATGGAGAGGACCTGAAAATCGAAACAAACAAATTTGCAGCAATATGCACTCACTTGGAAGTAT
GCTTCATGTATTCAGATTTTCATTTCATCAATGAGCAAGGCGAATCAATAATAGTAGAGCCTGAGGACCC
AAATGCACTTTTAAAACACAGATTTGAGATAATAGAGGGACGAGATCGTACAATGGCATGGACAGTTGTA
AACAGTATTTGCAACACCACAGGAGCTGAGAAACCAAAGTTTCTGCCAGATCTGTATGATTACAAGGAGA
ATAGATTCATCGAGATTGGAGTGACAAGGAGGGAAGTTCACATATACTATCTGGAAAAGGCCAACAAAAT
TAAATCTGAAAAGACACACATTCACATTTTCTCATTCACTGGCGAAGAAATGGCCACAAAGGCCGATTAC
ACTCTCGATGAAGAAAGCAGGGCTAGGATTAAAACCAGACTATTCACCATAAGACAAGAAATGGCAAGCA
GAGGTCTTTGGGACTCCTTTCGTCAGTCCGAAAGAGGCGAAGAAACAATTGAAGAAAGATTTGAAATCAC
AGGGACAATGCGCAGGCTTGCTGACCAAAGCCTTCCGCCGAACTTCTCCTGCATTGAGAATTTTAGAGCC
TATGTGGATGGATTTGAACCGAACGGCTACATTGAGGGCAAGCTTTCTCAAATGTCCAAAGAAGTAAATG
CTAGAATTGAGCCTTTTTTGAAAACAACACCACGACCAATTAGACTTCCGGATGGGCCTCCTTGTTTTCA
GCGGTCAAAATTCCTGCTGATGGATTCTTTAAAATTAAGCATTGAGGATCCAAATCACGAAGGAGAGGGA
ATACCACTATATGATGCAATCAAGTGTATGAGAACATTCTTTGGATGGAAAGAACCCTCTGTTGTCAAGC
CACACGAGAAGGGAATAAATCCGAATTATCTGCTGTCATGGAAGCAGGTATTGGAAGAGCTGCAGGACAT
TGAGAGTGAGGAGAAGATTCCAAGAACAAAGAACATGAAAAAAACGAGTCAGCTAAAGTGGGCACTTGG
T
GAGAACATGGCACCAGAGAAGGTTGATTTTGATGACTGTAAAGATATAAGCGATTTGAAGCAATATGATA
GTGACGAACCTGAATTAAGGTCATTTTCAAGTTGGATCCAGAATGAATTCAACAAGGCATGCGAGCTGAC
CGATTCAATCTGGATAGAGCTTGATGAGATTGGAGAAGATGTGGCTCCGATTGAACACATTGCAAGCATG
AGAAGAAATTACTTCACAGCTGAGGTGTCCCATTGCAGAGCCACAGAATATATAATGAAGGGGGTATACA
TTAATACTGCTTTGCTTAATGCATCCTGTGCAGCAATGGATGATTTCCAACTAATTCCCATGATAAGCAA
ATGTAGAACTAAAGAGGGAAGGAGAAAGACCAATTTGTACGGCTTCATCATAAAAGGAAGATCTCACTTA
AGGAATGACACCGATGTGGTAAACTTTGTGAGCATGGAATTTTCCCTCACTGACCCAAGACTTGAGCCAC
ACAAATGGGAGAAGTACTGTGTTCTCGAGATAGGAGATATGCTTCTAAGGAGTGCAATAGGCCAAGTGTC
AAGGCCCATGTTCTTGTATGTAAGGACAAATGGAACCTCAAAAATTAAAATGAAATGGGGAATGGAGATG

AGGCGTTGCCTCCTCCAATCCCTTCAACAAATAGAGAGCATGATTGAAGCTGAATCCTCTGTCAAGGAGA
AAGACATGACAAAAGAGTTTTTTGAGAATAGATCAGAAACATGGCCCATTGGAGAGTCACCAAAAGGAGT
GGAAGAAGGTTCCATTGGAAAAGTATGCAGGACACTATTGGCCAAGTCAGTATTCAATAGTCTGTATGCA
TCGCCACAATTAGAAGGATTTTCAGCTGAGTCAAGAAAGTTGCTCCTCATTGTTCAGGCTCTTAGGGACA
ATCTGGAGCCTGGGACCTTTGATCTTGGGGGGCTATATGAAGCAATTGAGGAGTGCCTGATTAATGATCC
CTGGGTTTTGCTTAATGCTTCTTGGTTCAACTCCTTCCTAACACATGCATTGAGATAGTTGGGGCAATGC
TACTATTTACTATCCATACTGTCCAAAAAA
>gi|237688874|gb|CY040079.1| Influenza A virus (A/Taiwan/567/2002(H1N1)) segment 3, complete sequence
ATGGAAGATTTTGTGCGACAATGCTTCAATCCGATGATTGTCGAGCTTGCGGAAAAGGCAATGAAAGAGT
ATGGAGAGGACCTGAAAATCGAAACAAACAAATTTGCAGCAATATGCACTCACTTGGAAGTATGCTTCAT
GTATTCAGATTTTCATTTCATCAATGAGCAAGGCGAATCAATAATAGTAGAGCCTGAGGACCCAAATGCA
CTTTTAAAACACAGATTTGAGATAATAGAGGGACGAGATCGTACAATGGCATGGACAGTTGTAAACAGTA
TTTGCAACACCACAGGAGCTGAGAAACCAAAGTTTCTGCCAGATCTGTATGATTACAAGGAGAATAGATT
CATCGAGATTGGAGTGACAAGGAGGGAAGTTCACATATACTATCTGGAAAAGGCCAACAAAATTAAATCT
GAAAAGACACACATTCACATTTTCTCATTCACTGGCGAAGAAATGGCCACAAAGGCCGATTACACTCTCG
ATGAAGAAAGCAGGGCTAGGATTAAAACCAGACTATTCACCATAAGACAAGAAATGGCAAGCAGAGGTCT
TTGGGACTCCTTTCGTCAGTCCGAAAGAGGCGAAGAAACAATTGAAGAAAGATTTGAAATCACAGGGACA
ATGCGCAGGCTTGCTGACCAAAGCCTTCCGCCGAACTTCTCCTGCATTGAGAATTTTAGAGCCTATGTGG
ATGGATTTGAACCGAACGGCTACATTGAGGGCAAGCTTTCTCAAATGTCCAAAGAAGTAAATGCTAGAAT
TGAGCCTTTTTTGAAAACAACACCACGACCAATTAGACTTCCGGATGGGCCTCCTTGTTTTCAGCGGTCA
AAATTCCTGCTGATGGATTCTTTAAAATTAAGCATTGAGGATCCAAATCACGAAGGAGAGGGAATACCAC
TATATGATGCAATCAAGTGTATGAGAACATTCTTTGGATGGAAAGAACCCTCTGTTGTCAAGCCACACGA
GAAGGGAATAAATCCGAATTATCTGCTGTCATGGAAGCAGGTATTGGAAGAGCTGCAGGACATTGAGAGT
GAGGAGAAGATTCCAAGAACAAAGAACATGAAAAAAACGAGTCAGCTAAAGTGGGCACTTGGTGAGAAC
A
TGGCACCAGAGAAGGTTGATTTTGATGACTGTAAAGATATAAGCGATTTGAAGCAATATGATAGTGACGA
ACCTGAATTAAGGTCATTTTCAAGTTGGATCCAGAATGAATTCAACAAGGCATGCGAGCTGACCGATTCA
ATCTGGATAGAGCTTGATGAGATTGGAGAAGATGTGGCTCCGATTGAACACATTGCAAGCATGAGAAGAA
ATTACTTCACAGCTGAGGTGTCCCATTGCAGAGCCACAGAATATATAATGAAGGGGGTATACATTAATAC
TGCTTTGCTTAATGCATCCTGTGCAGCAATGGATGATTTCCAACTAATTCCCATGATAAGCAAATGTAGA
ACTAAAGAGGGAAGGAGAAAGACCAATTTGTACGGCTTCATCATAAAAGGAAGATCTCACTTAAGGAATG
ACACCGATGTGGTAAACTTTGTGAGCATGGAATTTTCCCTCACTGACCCAAGACTTGAGCCACACAAATG
GGAGAAGTACTGTGTTCTCGAGATAGGAGATATGCTTCTAAGGAGTGCAATAGGCCAAGTGTCAAGGCCC
ATGTTCTTGTATGTAAGGACAAATGGAACCTCAAAAATTAAAATGAAATGGGGAATGGAGATGAGGCGTT
GCCTCCTCCAATCCCTTCAACAAATAGAGAGCATGATTGAAGCTGAATCCTCTGTCAAGGAGAAAGACAT
GACAAAAGAGTTTTTTGAGAATAGATCAGAAACATGGCCCATTGGAGAGTCACCAAAAGGAGTGGAAGAA
GGTTCCATTGGGAAAGTATGCAGGACACTATTGGCCAAGTCAGTATTCAATAGTCTGTATGCATCGCCAC
AATTAGAAGGATTTTCAGCTGAGTCAAGAAAGTTGCTTCTCATTGTTCAGGCTCTTAGGGACAATCTGGA
GCCTGGGACCTTTGATCTTGGGGGGCTATATGAAGCAATTGAGGAGTGCCTGATTAATGATCCCTGGGTT
TTGCTTAATGCTTCTTGGTTCAACTCCTTCCTAACGCATGCATTGAGATAGTTGGGGCAATGCTACT
>gi|122855959|gb|CY019346.1| Influenza A virus (A/Memphis/6/2003(H1N1)) segment 3, complete sequence
ATGGAAGATTTTGTGCGACAATGCTTCAATCCGATGATTGTCGAGCTTGCGGAAAAGGCAATGAAAGAGT

ATGGAGAGGACCTGAAAATCGAAACAAACAAATTTGCAGCAATATGCACCCACTTGGAAGTATGCTTCAT
GTATTCAGATTTTCATTTCATCAATGAGCAAGGCGAATCAATAATAGTAGAGCCTGAGGACCCAAATGCA
CTTTTAAAACACAGATTTGAGATAATAGAGGGACGAGATCGTACAATGGCATGGACAGTTGTAAACAGTA
TTTGCAACACCACAGGAGCTGAGAAACCAAAGTTTCTGCCAGATCTGTATGATTACAAAGAGAATAGATT
CATCGAGATTGGAGTGACAAGGAGAGAAGTTCACATATACTATCTGGAAAAGGCCAACAAAATTAAATCT
GAGAAGACACACATTCACATTTTCTCATTCACTGGCGAAGAAATGGCCACAAAGGCCGATTACACTCTCG
ATGAAGAAAGCAGGGCTAGGATTAAAACCAGACTATTCACCATAAGACAAGAAATGGCAAGCAGAGGTCT
TTGGGACTCCTTTCGTCAGTCCGAAAGAGGCGAAGAAACAATTGAAGAAAGATTTGAAATCACAGGGACA
ATGCGCAGGCTCGCTGATCAAAGCCTTCCGCCGAACTTCTCCTGCATTGAGAATTTTAGAGCCTATGTGG
ATGGATTTGAACCGAACGGCTACATTGAGGGCAAGCTTTCTCAAATGTCCAAAGAAGTAAATGCTAGAAT
TGAGCCTTTTTTGAAAACAACACCTCGACCAATTAGACTTCCGAATGGGCCTCCTTGTTTTCAGCGGTCA
AAATTCCTGCTGATGGATTCTTTAAAATTAAGCATTGAGGATCCAAATCATGAAGGGGAGGGAATACCAC
TATATGATGCAATCAAGTGTATGAGAACATTCTTTGGATGGAAAGAACCCACTGTTGTCAAACCACACGA
GAAGGGAATAAATCCGAATTATCTGCTGTCGTGGAAGCAGGTATTGGAAGAGCTGCAGGACATTGAGAGT
GAGGAGAAGATTCCAAGAACAAAAAACATGAAAAAAACGAGTCAGCTAAAGTGGGCACTTGGTGAGAAC
A
TGGCACCAGAGAAGGTGGATTTTGATGACTGTAAAGATATAAGCGATTTGAAGCAATATGATAGTGACGA
ACCTGAATTAAGGTCATTTTCAAGTTGGATCCAGAATGAGTTCAACAAGGCATGCGAGCTGACCGATTCA
ATCTGGATAGAGCTCGATGAGATTGGAGAAGATGTGGCTCCGATTGAACACATTGCAAGCATGAGAAGAA
ATTACTTCACAGCTGAGGTGTCCCATTGCAGAGCCACTGAATATATAATGAAGGGGGTATACATTAATAC
TGCTTTGCTTAATGCATCCTGTGCAGCAATGGATGATTTCCAACTAATTCCCATGATAAGCAAATGTAGA
ACTAAAGAGGGAAGGAGAAAGACCAATTTGTACGGCTTCATCATAAAAGGAAGATCTCACTTAAGGAATG
ACACCGATGTGGTAAACTTTGTGAGCATGGAGTTTTCCCTCACTGACCCAAGACTTGAGCCACACAAATG
GGAGAAGTACTGTGTTCTTGAGATAGGAGATATGCTTCTAAGGAGTGCAATAGGCCAAGTGTCAAGGCCC
ATGTTCTTGTATGTAAGAACAAATGGAACCTCAAAAATTAAAATGAAATGGGGAATGGAGATGAGGCGTT
GCCTCCTCCAATCCCTCCAACAAATAGAGAGCATGATTGAAGCTGAGTCCTCTGTCAAGGAGAAAGACAT
GACAAAAGAGTTTTTTGAGAATAGATCAGAAACATGGCCCATTGGAGAGTCACCAAAAGGAGTGGAAGAA
GGTTCCATTGGGAAAGTATGCAGGACACTATTGGCTAAGTCAGTATTCAATAGTCTGTATGCATCTCCAC
AATTAGAAGGATTTTCAGCTGAGTCAAGAAAGTTGCTCCTCATTGTCCAGGCTCTTAGGGACAATCTGGA
ACCTGGGACCTTTGATCTTGGGGGACTATATGAAGCAATTGAGGAGTGCCTGATTAATGATCCCTGGGTT
TTGCTTAATGCTTCTTGGTTCAACTCCTTCCTAACACATGCATTGAGATAGCTGAGGCAATGCTACTA
>gi|82546786|gb|CY006680.1| Influenza A virus (A/New York/494/2002(H1N1)) segment 3, complete sequence
GTACTGATTCGAAATGGAAGATTTTGTGCGACAATGCTTCAATCCGATGATTGTCGAGCTTGCGGAAAAG
GCAATGAAAGAGTATGGAGAGGACCTGAAAATCGAAACAAACAAATTTGCAGCAATATGCACCCACTTGG
AAGTATGCTTCATGTATTCAGATTTTCATTTCATCAATGAGCAAGGCGAATCAATAATAGTAGAGCCTGA
GGACCCAAATGCACTTTTAAAGCACAGATTTGAGATAATAGAGGGACGAGATCGTACAATGGCATGGACA
GTTGTAAACAGTATTTGCAACACCACAGGAGCTGAGAAACCAAAGTTTCTGCCAGATCTGTATGATTACA
AAGAGAATAGATTCATCGAGATTGGAGTGACAAGGAGGGAAGTTCACATATACTATCTGGAAAAGGCCAA
CAAAATTAAGTCTGAGAAGACACACATTCACATTTTCTCGTTCACTGGCGAAGAAATGGCCACAAAGGCC
GATTACACTCTCGATGAAGAAAGCAGGGCTAGGATTAAAACCAGACTATTCACCATAAGACAAGAAATGG
CAAGCAGAGGTCTTTGGGACTCCTTTCGTCAGTCCGAAAGAGGCGAAGAAACAATTGAAGAAAGATTTGA
AATCACAGGGACAATGCGCAGGCTCGCTGATCAAAGCCTTCCGCCGAACTTCTCCTGTATTGAGAATTTT
AGAGCCTATGTGGATGGATTTGAACCGAACGGCTACATTGAGGGCAAGCTTTCTCAAATGTCCAAAGAAG

TAAATGCCAGAATTGAGCCTTTTTTGAAAACAACACCTCGACCAATTAGACTTCCGAATGGGCCTCCTTG
TTTTCAGCGGTCAAAATTCCTGCTGATGGATTCTTTAAAATTAAGCATTGAGGATCCAAATCATGAAGGG
GAGGGAATACCACTATATGATGCAATCAAGTGTATGAGAACATTCTTTGGATGGAAAGAACCCACTGTTG
TCAAGCCACACGAGAAGGGAATAAATCCGAATTATCTGCTGTCGTGGAAGCAGGTATTGGAAGAGCTGCA
GGACATTGAGAGTGAGGAGAAGATTCCAAGAACAAAAAACATGAAAAAAACGAGTCAGCTAAAGTGGGC
A
CTTGGTGAGAACATGGCACCAGAGAAGGTGGATTTTGATGACTGTAAAGATATAAGCGATTTGAAGCAAT
ATGATAGTGACGAACCTGAATTAAGGTCATTTTCAAGTTGGATCCAGAATGAGTTCAACAAGGCATGCGA
GCTGACCGATTCAATCTGGATAGAGCTCGATGAGATTGGAGAAGATGTGGCCCCGATTGAACACATTGCA
AGCATGAGAAGAAATTACTTCACAGCTGAGGTGTCCCATTGCAGAGCCACTGAATATATAATGAAGGGGG
TATACATTAATACTGCTTTGCTTAATGCATCCTGTGCAGCAATGGATGATTTCCAACTAATTCCCATGAT
AAGCAAATGTAGAACTAAAGAGGGAAGGAGAAAGACCAATTTGTACGGCTTCATCGTAAAAGGAAGATCT
CACTTAAGGAATGACACCGATGTGGTAAACTTTGTGAGCATGGAGTTTTCCCTCACTGACCCAAGACTTG
AGCCACACAAATGGGAGAAGTACTGTGTTCTTGAGATAGGAGATATGCTTCTAAGGAGTGCAATAGGCCA
AGTGTCAAGGCCCATGTTCTTGTATGTAAGGACAAATGGAACCTCAAAAATTAAAATGAAATGGGGAATG
GAGATGAGGCGTTGCCTCCTCCAATCCCTCCAACAAATAGAGAGCATGATTGAAGCTGAGTCCTCTGTCA
AGGAGAAAGACATGACAAAAGAGTTTTTTGAGAATAGATCAGAAACATGGCCCATTGGAGAGTCACCAAA
AGGAGTGGAAGAAGGTTCCATTGGGAAAGTATGCAGGACACTATTGGCTAAGTCAGTATTCAATAGTCTG
TATGCATCTCCACAATTAGAAGGATTTTCAGCTGAGTCAAGAAAGTTGCTCCTCATTGTTCAGGCTCTTA
GGGACAATCTGGAACCTGGGACCTTTGATCTTGGGGGGCTATATGAAGCAATTGAGGAGTGCCTGATTAA
TGATCCCTGGGTTTTGCTTAATGCTTCTTGGTTCAACTCCTTCCTAACACATGCATTGAGATAGCTGGGG
CAATGCTACTATTTGTTATCCATACTGTCCAAAAAA
>gi|77543353|gb|CY003309.1| Influenza A virus (A/New York/291/2002(H1N1)) segment 3, complete sequence
GCAAAAGCAGGTACTGATTCGAAATGGAAGATTTTGTGCGACAATGCTTCAATCCGATGATTGTCGAGCT
TGCGGAAAAGGCAATGAAAGAGTATGGAGAGGACCTGAAAATCGAAACAAACAAATTTGCAGCAATATGC
ACCCACTTGGAAGTATGCTTCATGTATTCAGATTTTCATTTCATCAATGAGCAAGGCGAATCAATAATAG
TAGAGCCTGAGGACCCAAATGCACTTTTAAAACACAGATTTGAGATAATAGAGGGACGAGATCGTACAAT
GGCATGGACAGTTGTAAACAGTATTTGCAACACCACAGGAGCTGAGAAACCAAAGTTTCTGCCAGATCTG
TATGATTACAAAGAGAATAGATTCATCGAGATTGGAGTGACAAGGAGGGAAGTTCACATATACTATCTGG
AAAAGGCCAACAAAATTAAATCTGAGAAGACACACATTCACATTTTCTCATTCACTGGCGAAGAAATGGC
CACAAAGGCCGATTACACTCTCGATGAAGAAAGCAGGGCTAGGATTAAAACCAGACTATTCACCATAAGA
CAAGAGATGGCAAGCAGAGGTCTTTGGGACTCCTTTCGTCAGTCCGAAAGAGGCGAAGAAACAATTGAAG
AAAGATTTGAAATCACAGGGACAATGCGCAGGCTCGCTGATCAAAGCCTTCCGCCGAACTTCTCCTGCAT
TGAGAATTTTAGAGCCTATGTGGATGGATTTGAACCGAACGGCTACATTGAGGGCAAGCTTTCTCAAATG
TCCAAAGAAGTAAATGCTAGAATTGAACCTTTTTTGAAAACAACACCTCGACCAATTAGACTTCCGAATG
GGCCTCCTTGTTTTCAGCGGTCAAAATTTCTGCTGATGGATTCTTTAAAATTAAGCATTGAGGATCCAAA
TCATGAAGGGGAGGGAATACCACTATATGATGCAATCAAGTGTATGAGAACATTCTTTGGATGGAAAGAA
CCCACTGTTGTCAAGCCACACGAGAAGGGAATAAATCCGAATTATCTGCTGTCGTGGAAGCAGGTATTGG
AAGAGCTGCAGGACATTGAGAGTGAGGAGAAGATTCCAAGAACAAAAAACATGAAAAAAACGAGTCAGC
T
AAAGTGGGCACTTGGTGAGAACATGGCACCAGAGAAGGTGGATTTTGATGACTGTAAAGATATAAGCGAT
TTGAAGCAATATGATAGTGACGAACCTGAATTAAGGTCATTTTCAAGTTGGATCCAGAATGAGTTCAACA
AGGCATGCGAGCTGACCGATTCAATCTGGATAGAGCTCGATGAGATTGGAGAAGATGTGGCCCCGATTGA

ACACATTGCAAGCATGAGAAGAAATTACTTCACAGCTGAGGTGTCCCATTGCAGAGCCACTGAATATATA
ATGAAGGGGGTATACATTAATACTGCTTTGCTTAATGCATCCTGTGCAGCAATGGATGATTTCCAACTAA
TTCCCATGATAAGCAAATGTAGAACTAAAGAGGGAAGGAGAAAGACCAATTTGTACGGCTTCATCGTAAA
AGGAAGATCTCACTTAAGGAATGACACCGATGTGGTAAACTTTGTGAGCATGGAGTTTTCCCTCACTGAC
CCAAGACTTGAGCCACACAAATGGGAGAAGTACTGTGTTCTTGAGATAGGAGATATGCTTCTAAGGAGTG
CAATAGGCCAAGTGTCAAGGCCCATGTTCTTGTATGTAAGGACAAATGGAACCTCAAAAATTAAAATGAA
ATGGGGAATGGAGATGAGGCGTTGCCTCCTCCAATCCCTCCAACAAATAGAGAGCATGATTGAAGCTGAG
TCCTCTGTCAAGGAGAAAGACATGACAAAAGAGTTTTTTGAGAATAGATCAGAAACATGGCCCATTGGAG
AGTCACCAAAAGGAGTGGAAGAAGGTTCCATTGGGAAAGTATGCAGGACACTATTGGCTAAGTCAGTATT
CAATAGTCTGTATGCATCTCCACAACTAGAAGGATTTTCAGCTGAGTCAAGAAAGTTGCTCCTCATTGTT
CAGGCTCTTAGGGACAATCTGGAACCTGGGACCTTTGATCTTGGGGGGCTATATGAAGCAATTGAGGAGT
GCCTGATTAATGATCCCTGGGTTTTGCTTAATGCTTCTTGGTTCAACTCCTTCCTAACACATGCATTAAG
ATAGCTGGGGCAATGCTACTATTTGTTATCCATACTGTCCAAAAAAGTACCTTGTTTCTACT
>gi|237689047|gb|CY040151.1| Influenza A virus (A/Taiwan/52/2002(H1N1)) segment 3, complete sequence
CTGATTCGAAATGGAAGATTTTGTGCGACAATGCTTCAATCCGATGATTGTCGAGCTTGCGGAAAAGGCA
ATGAAAGAGTATGGAGAGGACCTGAAAATCGAAACAAACAAATTTGCAGCAATATGCACTCACTTGGAAG
TATGCTTCATGTATTCAGATTTTCATTTCATCAATGAGCAAGGCGAATCAATAATAGTAGAGCCTGAGGA
CCCAAATGCACTTTTAAAACACAGATTTGAGATAATAGAGGGACGAGATCGTACAATGGCATGGACAGTT
GTAAACAGTATTTGCAACACCACAGGAGCTGAGAAACCAAAGTTTCTGCCAGATCTGTATGATTACAAGG
AGAATAGATTCATCGAGATTGGAGTGACAAGGAGGGAAGTTCACATATACTATCTGGAAAAGGCCAACAA
AATTAAATCTGAAAAGACACACATTCACATTTTCTCATTCACTGGCGAAGAAATGGCCACAAAGGCCGAT
TACACTCTCGATGAAGAAAGCAGGGCTAGGATTAAAACCAGACTATTCACCATAAGACAAGAAATGGCAA
GCAGAGGTCTTTGGGACTCCTTTCGTCAGTCCGAAAGAGGCGAAGAAACAATTGAAGAAAGATTTGAAAT
CACAGGGACAATGCGCAGGCTTGCTGACCAAAGCCTTCCGCCGAACTTCTCCTGCATTGAGAATTTTAGA
GCCTATGTGGATGGATTTGAACCGAACGGCTACATTGAGGGCAAGCTTTCTCAAATGTCCAAAGAAGTAA
ATGCTAGAATTGAGCCTTTTTTGAAAACAACACCACGACCAATTAGACTTCCGGATGGGCCTCCTTGTTT
TCAGCGGTCAAAATTCCTGCTGATGGATTCTTTAAAATTAAGCATTGAGGATCCAAATCACGAAGGAGAG
GGAATACCACTATATGATGCAATCAAGTGTATGAGAACATTCTTTGGATGGAAAGAACCCTCTGTTGTCA
AGCCACACGAGAAGGGAATAAATCCGAATTATCTGCTGTCATGGAAGCAGGTATTGGAAGAGCTGCAGGA
CATTGAGAGTGAGGAGAAGATTCCAAGAACAAAGAACATGAAAAAAACGAGTCAGCTAAAGTGGGCACTT
GGTGAGAACATGGCACCAGAGAAGGTTGATTTTGATGACTGTAAAGATATAAGCGATTTGAAGCAATATG
ATAGTGACGAACCTGAATTAAGGTCATTTTCAAGTTGGATCCAGAATGAATTCAACAAGGCATGCGAGCT
GACCGATTCAATCTGGATAGAGCTTGATGAGATTGGAGAAGATGTGGCTCCGATTGAACACATTGCAAGC
ATGAGAAGAAATTACTTCACAGCTGAGGTGTCCCATTGCAGAGCCACAGAATATATAATGAAGGGGGTAT
ACATTAATACTGCTTTGCTTAATGCATCCTGTGCAGCAATGGATGATTTCCAACTAATTCCCATGATAAG
CAAATGTAGAACTAAAGAGGGAAGGAGAAAGACCAATTTGTACGGCTTCATCATAAAAGGAAGATCTCAC
TTAAGGAATGACACCGATGTGGTAAACTTTGTGAGCATGGAATTTTCCCTCACTGACCCAAGACTTGAGC
CACACAAATGGGAGAAGTACTGTGTTCTCGAGATAGGAGATATGCTTCTAAGGAGTGCAATAGGCCAAGT
GTCAAGGCCCATGTTCTTGTATGTAAGGACAAATGGAACCTCAAAAATTAAAATGAAATGGGGAATGGAG
ATGAGGCGTTGCCTCCTCCAATCCCTTCAACAAATAGAGAGCATGATTGAAGCTGAATCCTCTGTCAAGG
AGAAAGACATGACAAAAGAGTTTTTTGAGAATAGATCAGAAACATGGCCCATTGGAGAGTCACCAAAAGG
AGTGGAAGAAGGTTCCATTGGGAAAGTATGCAGGACACTATTGGCCAAGTCAGTATTCAATAGTCTGTAT
GCATCGCCACAATTAGAAGGATTTTCAGCTGAGTCAAGAAAGTTGCTTCTCATTGTTCAGGCTCTTAGGG

ACAATCTGGAGCCTGGGACCTTTGATCTTGGGGGGCTATATGAAGCAATTGAGGAGTGCCTGATTAATGA
TCCCTGGGTTTTGCTTAATGCTTCTTGGTTCAACTCCTTCCTAACACATGCATTGAGATAGTTGGGGCAA
TGCTACTATTTACTAT
>gi|237689066|gb|CY040159.1| Influenza A virus (A/Taiwan/123/2002(H1N1)) segment 3, complete sequence
GTACTGATTCGAAATGGAAGATTTTGTGCGACAATGCTTCAATCCGATGATTGTCGAGCTTGCGGAAAAG
GCAATGAAAGAGTATGGAGAGGACCTGAAAATCGAAACAAACAAATTTGCAGCAATATGCACTCACTTGG
AAGTATGCTTCATGTATTCAGATTTTCATTTCATCAATGAGCAAGGCGAATCAATAATAGTAGAGCCTGA
GGACCCAAATGCACTTTTAAAACACAGATTTGAGATAATAGAGGGACGAGATCGTACAATGGCATGGACA
GTTGTAAACAGTATTTGCAACACCACAGGAGCTGAGAAACCAAAGTTTCTGCCAGATCTGTATGATTACA
AGGAGAATAGATTCATCGAGATTGGAGTGACAAGGAGGGAAGTTCACATATACTATCTGGAAAAGGCCAA
CAAAATTAAATCTGAAAAGACACACATTCACATTTTCTCATTCACTGGCGAAGAAATGGCCACAAAGGCC
GATTACACTCTCGATGAAGAAAGCAGGGCTAGGATTAAAACCAGACTATTCACCATAAGACAAGAAATGG
CAAGCAGAGGTCTTTGGGACTCCTTTCGTCAGTCCGAAAGAGGCGAAGAAACAATTGAAGAAAGATTTGA
AATCACAGGGACAATGCGCAGGCTTGCTGACCAAAGCCTTCCGCCGAACTTCTCCTGCATTGAGAATTTT
AGAGCCTATGTGGATGGATTTGAACCGAACGGCTACATTGAGGGCAAGCTTTCTCAAATGTCCAAAGAAG
TAAATGCTAGAATTGAGCCTTTTTTGAAAACAACACCACGACCAATTAGACTTCCGGATGGGCCTCCTTG
TTTTCAGCGGTCAAAATTCCTGCTGATGGATTCTTTAAAATTAAGCATTGAGGATCCAAATCACGAAGGA
GAGGGAATACCACTATATGATGCAATCAAGTGTATGAGAACATTCTTTGGATGGAAAGAACCCTCTGTTG
TCAAGCCACACGAGAAGGGAATAAATCCGAATTATCTGCTGTCATGGAAGCAGGTATTGGAAGAGCTGCA
GGACATTGAGAGTGAGGAGAAGATTCCAAGAACAAAGAACATGAAAAAAACGAGTCAGCTAAAGTGGGC
A
CTTGGTGAGAACATGGCACCAGAGAAGGTTGATTTTGATGACTGTAAAGATATAAGCGATTTGAAGCAAT
ATGATAGTGACGAACCTGAATTAAGGTCATTTTCAAGTTGGATCCAGAATGAATTCAACAAGGCATGCGA
GCTGACCGATTCAATCTGGATAGAGCTTGATGAGATTGGAGAAGATGTGGCTCCGATTGAACACATTGCA
AGCATGAGAAGAAATTACTTCACAGCTGAGGTGTCCCATTGCAGAGCCACAGAATATATAATGAAGGGGG
TATACATTAATACTGCTTTGCTTAATGCATCCTGTGCAGCAATGGATGATTTCCAACTAATTCCCATGAT
AAGCAAATGTAGAACTAAAGAGGGAAGGAGAAAGACCAATTTGTACGGCTTCATCATAAAAGGAAGATCT
CACTTAAGGAATGACACCGATGTGGTAAACTTTGTGAGCATGGAATTTTCCCTCACTGACCCAAGACTTG
AGCCACACAAATGGGAGAAGTACTGTGTTCTCGAGATAGGAGATATGCTTCTAAGGAGTGCAATAGGCCA
AGTGTCAAGGCCCATGTTCTTGTATGTAAGGACAAATGGAACCTCAAAAATTAAAATGAAATGGGGAATG
GAGATGAGGCGTTGCCTCCTCCAATCCCTTCAACAAATAGAGAGCATGATTGAAGCTGAATCCTCTGTCA
AGGAGAAAGACATGACAAAAGAGTTTTTTGAGAATAGATCAGAAACATGGCCCATTGGAGAGTCACCAAA
AGGAGTGGAAGAAGGTTCCATTGGGAAAGTATGCAGGACACTATTGGCCAAGTCAGTATTCAATAGTCTG
TATGCATCGCCACAATTAGAAGGATTTTCAGCTGAGTCAAGAAAGTTGCTTCTCATTGTTCAGGCTCTTA
GGGACAATCTGGAGCCAGGGACCTTTGATCTTGGGGGGCTATATGAAGCAATTGAGGAGTGCCTGATTAA
TGATCCCTGGGTTTTGCTTAATGCTTCTTGGTTCAACTCCTTCCTAACACATGCATTGAGATAGTTGGGG
CAATGCTACTATTAACTAT
>gi|77747433|gb|CY003693.1| Influenza A virus (A/New York/486/2003(H1N1)) segment 3, complete sequence
AAAGCAGGTACTGATTCGAAATGGAAGATTTTGTGCGACAATGCTTCAATCCGATGATTGTCGAGCTTGC
GGAAAAGGCAATGAAAGAGTATGGAGAGGACCTGAAAATCGAAACAAACAAATTTGCAGCAATATGCACC
CACTTGGAAGTATGCTTCATGTATTCAGATTTTCATTTCATCAATGAGCAAGGCGAATCAATAATAGTAG
AGCCTGAGGACCCAAATGCACTTTTAAAACACAGATTTGAGATAATAGAGGGACGAGATCGTACAATGGC

ATGGACAGTTGTAAACAGTATTTGCAACACCACAGGAGCTGAGAAACCAAAGTTTCTGCCAGATCTGTAT
GATTACAAAGAGAATAGATTCATCGAGATTGGAGTGACAAGGAGAGAAGTTCACATATACTATCTGGAAA
AGGCCAACAAAATTAAATCTGAGAAGACACACATTCACATTTTCTCATTCACTGGCGAAGAAATGGCCAC
AAAGGCCGATTACACTCTCGATGAAGAAAGCAGGGCTAGGATTAAAACCAGACTATTCACCATAAGACAA
GAAATGGCAAGCAGAGGTCTTTGGGACTCCTTTCGTCAGTCCGAAAGAGGCGAAGAAACAATTGAAGAAA
GATTTGAAATCACAGGGACAATGCGCAGGCTCGCTGATCAAAGCCTTCCGCCGAACTTCTCCTGCATTGA
GAATTTTAGAGCCTATGTGGATGGATTTGAACCGAACGGCTACATTGAGGGCAAGCTTTCTCAAATGTCC
AAAGAAGTAAATGCTAGAATTGAGCCTTTTTTGAAAACAACACCTCGACCAATTAGACTTCCGAATGGGC
CTCCTTGTTTTCAGCGGTCAAAATTCCTGCTGATGGATTCTTTAAAATTAAGCATTGAGGATCCAAATCA
TGAAGGGGAGGGAATACCACTATATGATGCAATCAAGTGTATGAGAACATTCTTTGGATGGAAAGAACCC
ACTGTTGTCAAGCCACACGAGAAGGGAATAAATCCGAATTATCTGCTGTCGTGGAAGCAGGTATTGGAAG
AGCTGCAGGACATTGAGAGTGAGGAGAAGATTCCAAGAACAAAAAACATGAAAAAAACGAGTCAGCTAA
A
GTGGGCACTTGGTGAGAACATGGCACCAGAGAAGGTGGATTTTGATGACTGTAAAGATATAAGCGATTTG
AAGCAATATGATAGTGACGAACCTGAATTAAGGTCATTTTCAAGTTGGATCCAGAATGAGTTCAACAAGG
CATGCGAGCTGACCGATTCAATCTGGATAGAGCTCGATGAGATTGGAGAAGATGTGGCCCCGATTGAACA
CATTGCAAGCATGAGAAGAAATTACTTCACAGCTGAGGTGTCCCATTGCAGAGCCACTGAATATATAATG
AAGGGGGTATACATTAATACTGCTTTGCTTAATGCATCCTGTGCAGCAATGGATGATTTCCAACTAATTC
CCATGATAAGCAAATGTAGAACTAAAGAGGGAAGGAGAAAGACCAATTTGTACGGCTTCATCATAAAAGG
AAGATCTCACTTAAGGAATGACACCGATGTGGTAAACTTTGTGAGCATGGAGTTTTCCCTCACTGACCCA
AGACTTGAGCCACACAAATGGGAGAAGTACTGTGTTCTTGAGATAGGAGATATGCTTTTAAGGAGTGCAA
TAGGCCAAGTGTCAAGGCCCATGTTCTTGTATGTAAGAACAAATGGAACCTCAAAAATTAAAATGAAATG
GGGAATGGAGATGAGGCGTTGCCTCCTCCAATCCCTCCAACAAATAGAGAGCATGATTGAAGCTGAGTCC
TCTGTCAAGGAGAAAGACATGACAAAAGAGTTTTTTGAGAATAGATCAGAAACATGGCCCATTGGAGAGT
CACCAAAAGGAGTGGAAGAAGGTTCCATTGGGAAAGTATGCAGGACACTATTGGCTAAGTCAGTATTCAA
TAGTCTGTATGCATCTCCACAATTAGAAGGATTTTCAGCTGAGTCAAGAAAGTTGCTCCTCATTGTTCAG
GCTCTTAGGGACAATCTGGAACCTGGGACCTTTGATCTTGGGGGACTATATGAAGCAATTGAGGAGTGCC
TGATTAATGATCCCTGGGTTTTGCTTAATGCTTCTTGGTTCAACTCCTTCCTAACACATGCATTGAGATA
GCTGAGGCAATGCTACTATTTGTTATCCATACTGTCCAAAAAAGTACCTTGTTTCTACT
>gi|125664187|gb|CY019888.1| Influenza A virus (A/Memphis/5/2003(H1N1)) segment 3, complete sequence
ATGGAAGATTTTGTGCGACAATGCTTCAATCCGATGATTGTCGAGCTTGCGGAAAAGGCAATGAAAGAGT
ATGGAGAGGACCTGAAAATCGAAACAAACAAATTTGCAGCAATATGCACCCACTTGGAAGTATGCTTCAT
GTATTCAGATTTTCATTTCATCAATGAGCAAGGCGAATCAATAATAGTAGAGCCTGAGGACCCAAATGCA
CTTTTAAAACACAGATTTGAGATAATAGAGGGACGAGATCGTACAATGGCATGGACAGTTGTAAACAGTA
TTTGCAACACCACAGGAGCTGAGAAACCAAAGTTTCTGCCAGATCTGTATGATTACAAAGAGAATAGATT
CATCGAGATTGGAGTGACAAGGAGAGAAGTTCACATATACTATCTGGAAAAGGCCAACAAAATTAAATCT
GAGAAGACACACATTCACATTTTCTCATTCACTGGCGAAGAAATGGCCACAAAGGCCGATTACACTCTCG
ATGAAGAAAGCAGGGCTAGGATTAAAACCAGACTATTCACCATAAGACAAGAAATGGCAAGCAGAGGTCT
TTGGGACTCCTTTCGTCAGTCCGAAAGAGGCGAAGAAACAATTGAAGAAAGATTTGAAATCACAGGGACA
ATGCGCAGGCTCGCTGATCAAAGCCTTCCGCCGAACTTCTCCTGCATTGAGAATTTTAGAGCCTATGTGG
ATGGATTTGAACCGAACGGCTACATTGAGGGCAAGCTTTCTCAAATGTCCAAAGAAGTAAATGCTAGAAT
TGAGCCTTTTTTGAAAACAACACCTCGACCAATTAGACTTCCGAATGGGCCTCCTTGTTTTCAGCGGTCA
AAATTCCTGCTGATGGATTCTTTAAAATTAAGCATTGAGGATCCAAATCATGAAGGGGAGGGAATACCAC

TATATGATGCAATCAAGTGTATGAGAACATTCTTTGGATGGAAAGAACCCACTGTTGTCAAACCACACGA
GAAGGGAATAAATCCGAATTATCTGCTGTCGTGGAAGCAGGTATTGGAAGAGCTGCAGGACATTGAGAGT
GAGGAGAAGATTCCAAGAACAAAAAACATGAAAAAAACGAGTCAGCTAAAGTGGGCACTTGGTGAGAAC
A
TGGCACCAGAGAAGGTGGATTTTGATGACTGTAAAGATATAAGCGATTTGAAGCAATATGATAGTGACGA
ACCTGAATTAAGGTCATTTTCAAGTTGGATCCAGAATGAGTTCAACAAGGCATGCGAGCTGACCGATTCA
ATCTGGATAGAGCTCGATGAGATTGGAGAAGATGTGGCCCCGATTGAACACATTGCAAGCATGAGAAGAA
ATTACTTCACAGCTGAGGTGTCCCATTGCAGAGCCACTGAATATATAATGAAGGGGGTATACATTAATAC
TGCTTTGCTTAATGCATCCTGTGCAGCAATGGATGATTTCCAACTAATTCCCATGATAAGCAAATGTAGA
ACTAAAGAGGGAAGGAGAAAGACCAATTTGTACGGCTTCATCATAAAAGGAAGATCTCACTTAAGGAATG
ATACCGATGTGGTAAACTTTGTGAGCATGGAGTTTTCCCTCACTGACCCAAGACTTGAGCCACACAAATG
GGAGAAGTACTGTGTTCTTGAGATAGGAGATATGCTTCTAAGGAGTGCAATAGGCCAAGTGTCAAGGCCC
ATGTTCTTGTATGTAAGAACAAATGGAACCTCAAAAATTAAAATGAAATGGGGAATGGAGATGAGGCGTT
GCCTCCTCCAATCCCTCCAACAAATAGAGAGCATGATTGAAGCTGAGTCCTCTGTCAAGGAGAAAGACAT
GACAAAAGAGTTTTTTGAGAATAGATCAGAAACATGGCCCATTGGAGAGTCACCAAAAGGAGTGGAAGAA
GGTTCCATTGGGAAAGTATGCAGGACACTATTGGCTAAGTCAGTATTCAATAGTCTGTATGCATCTCCAC
AATTAGAAGGATTTTCAGCTGAGTCAAGAAAGTTGCTCCTCATTGTTCAGGCTCTTAGGGACAATCTGGA
ACCTGGGACCTTTGATCTTGGGGGACTATATGAAGCAATTGAGGAGTGCCTGATTAATGATCCCTGGGTT
TTGCTTAATGCTTCTTGGTTCAACTCCTTCCTAACACATGCATTGAGATAGCTGAGGCAATGCTACTATT
TGTTA
>gi|73763206|gb|CY002541.1| Influenza A virus (A/New York/227/2003(H1N1)) segment 3, complete sequence
AGCAAAAGCAGGTACTGATTCGAAATGGAAGATTTTGTGCGACAATGCTTCAATCCGATGATTGTCGAGC
TTGCGGAAAAGGCAATGAAAGAGTATGGAGAGGACCTGAAAATCGAAACAAACAAATTTGCAGCAATATG
CACCCACTTGGAAGTATGCTTCATGTATTCAGATTTTCATTTCATCAATGAGCAAGGCGAATCAATAATA
GTAGAGCCTGAGGACCCAAATGCACTTTTAAAACACAGATTTGAGATAATAGAGGGACGAGATCGTACAA
TGGCATGGACAGTTGTAAACAGTATTTGCAACACCACAGGAGCTGAGAAACCAAAGTTTCTGCCAGATCT
GTATGATTACAAAGAGAATAGATTCATCGAGATTGGAGTGACAAGGAGAGAAGTTCACATATACTATCTG
GAAAAGGCCAACAAAATTAAATCTGAGAAGACACACATTCACATTTTCTCATTCACTGGCGAAGAAATGG
CCACAAAGGCCGATTACACTCTCGATGAAGAAAGCAGGGCTAGGATTAAAACCAGGCTATTCACCATAAG
ACAAGAAATGGCAAGCAGAGGTCTTTGGGACTCCTTTCGTCAGTCCGAAAGAGGCGAAGAAACAATTGAA
GAAAGGTTTGAAATCACAGGGACAATGCGCAGGCTCGCTGATCAAAGCCTTCCGCCGAACTTCTCCTGCA
TTGAGAATTTTAGAGCCTATGTGGATGGATTTGAACCGAACGGCTACATTGAGGGCAAGCTTTCTCAAAT
GTCCAAAGAAGTAAATGCTAGGATTGAGCCTTTTTTGAAAACAACACCTCGACCAATTAGACTTCCGAAT
GGGCCTCCTTGTTTTCAGCGGTCAAAATTCCTGCTGATGGATTCTTTAAAATTAAGCATTGAGGATCCAA
ATCATGAAGGGGAGGGAATACCACTATATGATGCAATCAAGTGTATGAGAACATTCTTTGGATGGAAAGA
ACCCACTGTTGTCAAGCCACACGAGAAGGGAATAAATCCGAATTATCTGCTGTCGTGGAAGCAGGTATTG
GAAGAGCTGCAGGACATTGAGAGTGAGGAGAAGATTCCAAGAACAAAAAACATGAAAAAAACGAGTCAG
C
TAAAGTGGGCACTTGGTGAGAACATGGCACCAGAGAAGGTGGATTTTGATGACTGTAAAGATATAAGCGA
TTTGAAGCAATATGATAGTGACGAACCTGAATTAAGGTCATTTTCAAGTTGGATCCAGAATGAGTTCAAC
AAGGCATGCGAGCTGACCGATTCAATCTGGATAGAGCTCGATGAGATTGGAGAAGATGTGGCCCCGATTG
AACACATTGCAAGCATGAGAAGAAATTACTTCACAGCTGAGGTGTCCCATTGCAGAGCCACTGAATATAT
AATGAAGGGGGTATACATTAATACTGCTTTGCTTAATGCATCCTGTGCAGCAATGGATGATTTCCAACTA

ATTCCCATGATAAGCAAATGTAGAACTAAAGAGGGAAGGAGAAAGACCAATTTGTACGGCTTCATCATAA
AAGGAAGATCTCACTTAAGGAATGACACCGATGTGGTAAACTTTGTGAGCATGGAGTTTTCCCTCACTGA
CCCAAGACTTGAGCCACACAAATGGGAGAAGTACTGTGTTCTTGAGATAGGAGATATGCTTCTAAGGAGT
GCAATAGGCCAAGTGTCAAGGCCCATGTTCTTGTATGTAAGAACAAATGGAACCTCAAAAATTAAAATGA
AATGGGGAATGGAGATGAGGCGTTGCCTCCTCCAATCCCTCCAACAAATAGAGAGCATGATTGAAGCTGA
GTCCTCTGTCAAGGAGAAAGACATGACAAAAGAGTTTTTTGAGAATAGATCAGAAACATGGCCCATTGGA
GAGTCACCAAAAGGAGTGGAAGAAGGTTCCATTGGGAAAGTATGCAGGACACTATTGGCTAAGTCAGTAT
TCAATAGTCTGTATGCATCTCCACAATTAGAAGGATTTTCAGCTGAGTCAAGAAAGTTGCTCCTCATTGT
TCAGGCCCTTAGGGACAATCTGGAACCTGGGACCTTTGATCTTGGGGGACTATATGAAGCAATTGAGGAG
TGCCTGATTAATGATCCCTGGGTTTTGCTTAATGCTTCTTGGTTCAACTCCTTCCTAACACATGCATTGA
GATAGCTGAGGCAATGCTACTATTTGTTATCCATACTGTCCAAAAAAGTACCTTGTTTCTACT
>gi|89112177|gb|CY009001.1| Influenza A virus (A/New York/484/2003(H1N1)) segment 3, complete sequence
AAAGCAGGTACTGATTCGAAATGGAAGATTTTGTGCGACAATGCTTCAATCCGATGATTGTCGAGCTTGC
GGAAAAGGCAATGAAAGAGTATGGAGAGGACCTGAAAATCGAAACAAACAAATTTGCAGCAATATGCACC
CACTTGGAAGTATGCTTCATGTATTCAGATTTTCATTTCATCAATGAGCAAGGCGAATCAATAATAGTAG
AGCCTGAGGACCCAAATGCACTTTTAAAACACAGATTTGAGATAATAGAGGGACGAGATCGTACAATGGC
ATGGACAGTTGTAAACAGTATTTGCAACACCACAGGAGCTGAGAAACCAAAGTTTCTGCCAGATCTGTAT
GATTACAAAGAGAATAGATTCATCGAGATTGGAGTGACAAGGAGAGAAGTTCACATATACTATCTGGAAA
AGGCCAACAAAATTAAATCTGAGAAGACACACATTCACATTTTCTCATTCACTGGCGAAGAAATGGCCAC
AAAGGCCGATTACACTCTCGATGAAGAAAGCAGGGCTAGGATTAAAACCAGACTATTCACCATAAGACAA
GAAATGGCAAGCAGAGGTCTTTGGGACTCCTTTCGTCAGTCCGAAAGAGGCGAAGAAACAATTGAAGAAA
GATTTGAAATCACAGGGACAATGCGCAGGCTCGCTGATCAAAGCCTTCCGCCGAACTTCTCCTGCATTGA
GAATTTTAGAGCCTATGTGGATGGATTTGAACCGAACGGCTACATTGAGGGCAAGCTTTCTCAAATGTCC
AAAGAAGTAAATGCTAGAATTGAGCCTTTTTTGAAAACAACACCTCGACCAATTAGACTTCCGAATGGGC
CTCCTTGTTTTCAGCGATCAAAATTCCTGCTGATGGATTCTTTAAAATTAAGCATTGAGGATCCAAATCA
TGAAGGGGAGGGAATACCACTATATGATGCAATCAAGTGTATGAGAACATTCTTTGGATGGAAAGAACCC
ACTGTTGTCAAGCCACACGAGAAGGGAATAAATCCGAATTATCTGCTGTCGTGGAAGCAGGTATTGGAAG
AGCTGCAGGACATTGAGAGTGAGGAGAAGATTCCAAGAACAAAAAACATGAAAAAAACGAGTCAGCTAA
A
GTGGGCACTTGGTGAGAACATGGCACCAGAGAAGGTGGATTTTGATGACTGTAAAGGTATAAGCGATTTG
AAGCAATATGATAGTGACGAACCTGAATTAAGGTCATTTTCAAGTTGGATCCAGAATGAGTTCAACAAGG
CATGCGAGCTGACCGATTCAATCTGGATAGAGCTCGATGAGATTGGAGAAGATGTGGCCCCGATTGAACA
CATTGCAAGCATGAGAAGAAATTACTTCACAGCTGAGGTGTCCCATTGCAGAGCCACTGAATATATAATG
AAGGGGGTATACATTAATACTGCTTTGCTTAATGCATCCTGTGCAGCAATGGATGATTTCCAACTAATTC
CCATGATAAGCAAATGTAGAACTAAAGAGGGAAGGAGAAAGACCAATTTGTACGGCTTCATCATAAAAGG
AAGATCTCACTTAAGGAATGACACCGATGTGGTAAACTTTGTGAGCATGGAGTTTTCCCTCACTGACCCA
AGACTTGAGCCACACAAATGGGAGAAGTACTGTGTTCTTGAGATAGGAGATATGCTTCTAAGGAGTGCAA
TAGGCCAAGTGTCAAGGCCCATGTTCTTGTATGTAAGAACAAATGGAACCTCAAAAATTAAAATGAAATG
GGGAATGGAGATGAGGCGTTGCCTCCTCCAATCCCTCCAACAAATAGAGAGCATGATTGAAGCTGAGTCC
TCTGTCAAGGAGAAAGACATGACAAAAGAGTTTTTTGAGAATAGATCAGAAACATGGCCCATTGGAGAGT
CACCAAAAGGAGTGGAAGAAGGTTCCATTGGGAAAGTATGCAGGACACTATTGGCTAAGTCAGTATTCAA
TAGTCTGTATGCATCTCCACAATTAGAAGGATTTTCAGCTGAGTCAAGAAAGTTGCTCCTCATTGTTCAG
GCTCTTAGGGACAATCTGGAACCTGGGACCTTTGATCTTGGGGGACTATATGAAGCAATTGAGGAGTGCC

TGATTAATGATCCCTGGGTTTTGCTTAATGCTTCTTGGTTCAACTCCTTCCTAACACATGCATTGAGATA
GCTGAGGCAATGCTACTATTTGTTATCCATACTGTCCAAAAAAGTACCTTGTTTCTACT
>gi|83727853|gb|CY006920.1| Influenza A virus (A/New York/488/2003(H1N1)) segment 3, complete sequence
AAAGCAGGTACTGATTCAAAATGGAAGATTTTGTGCGACAATGCTTCAATCCAATGATTGTCGAGCTTGC
GGAAAAGGCAATGAAAGAGTATGGAGAGGACCTGAAAATCGAAACAAACAAATTTGCAGCAATATGCACC
CACTTGGAAGTATGCTTCATGTATTCAGATTTTCATTTCATCAATGAGCAAGGCGAATCAATAATAGTAG
AGCCTGAGGACCCAAATGCACTTTTAAAACACAGATTTGAGATAATAGAGGGACGAGATCGTACAATGGC
ATGGACAGTTGTAAACAGTATTTGCAACACCACAGGAGCTGAGAAACCAAAGTTTCTGCCAGATCTGTAT
GATTACAAAGAGAATAGATTCATCGAGATTGGAGTGACAAGGAGAGAAGTTCACATATACTATCTGGAAA
AGGCCAACAAAATTAAATCTGAAAAGACACACATTCACATTTTCTCATTCACTGGCGAAGAAATGGCCAC
AAAGGCCGATTACACTCTCGATGAAGAAAGCAGGGCTAGGATTAAAACCAGACTATTCACCATAAGACAA
GAAATGGCAAGCAGAGGTCTTTGGGACTCCTTTCGTCAGTCCGAAAGAGGCGAAGAAACAATTGAAGAAA
GATTTGAAATCACAGGGACAATGCGCAGGCTCGCTGATCAAAGCCTTCCGCCGAACTTCTCCTGCATTGA
GAATTTTAGAGCCTATGTGGATGGATTTGAACCGAACGGCTACATTGAGGGCAAGCTTTCTCAAATGTCC
AAAGAAGTAAACGCTAGAATTGAGCCTTTTTTGAAAACAACACCTCGACCAATTAGACTTCCGAATGGGC
CTCCTTGTTTTCAGCGGTCAAAATTCCTGCTGATGGATTCTTTAAAATTAAGCATTGAGGATCCAAATCA
TGAAGGGGAGGGAATACCACTATATGATGCAATCAAGTGTATGAGAACATTCTTTGGATGGAAAGAACCC
ACTGTTGTCAAGCCACACGAGAAGGGAATAAATCCGAATTATCTGCTATCGTGGAAGCAGGTATTGGAAG
AGCTGCAGGACATTGAGAGTGAGGAGAAGATTCCAAGAACAAAAAACATGAAAAAAACGAGTCAGCTAA
A
GTGGGCACTTGGTGAGAACATGGCACCAGAGAAGGTGGATTTTGATGACTGTAAAGATATAAGCGATTTG
AAGCAATATGATAGTGACGAACCTGAATTAAGGTCATTTTCAAGTTGGATCCAGAATGAGTTCAACAAGG
CATGCGAGCTGACCGATTCAATCTGGATAGAGCTCGATGAGATTGGAGAAGATGTGGCCCCGATTGAACA
CATTGCAAGCATGAGAAGAAATTACTTCACAGCTGAGGTGTCCCATTGCAGAGCCACTGAATATATAATG
AAGGGGGTATACATTAATACTGCTTTGCTTAATGCGTCCTGTGCAGCAATGGATGATTTCCAACTAATTC
CCATGATAAGCAAATGTAGAACTAAAGAGGGAAGGAGAAAGACCAATTTGTACGGCTTCATCATAAAAGG
AAGATCTCACTTAAGGAATGACACCGATGTGGTAAACTTTGTGAGCATGGAGTTTTCCCTCACTGACCCA
AGACTTGAGCCACACAAATGGGAGAAGTACTGTGTTCTTGAGATAGGAGATATGCTTCTAAGGAGTGCAA
TAGGCCAAGTGTCAAGGCCTATGTTCTTGTATGTAAGAACAAATGGAACCTCAAAAATTAAAATGAAATG
GGGAATGGAGATGAGGCGTTGCCTCCTCCAATCCCTCCAACAAATAGAGAGCATGATTGAAGCTGAGTCC
TCTGTCAAGGAGAAAGACATGACAAAAGAGTTTTTTGAGAATAGATCAGAAACATGGCCCATTGGAGAGT
CACCAAAAGGAGTGGAAGAAGGTTCCATTGGGAAAGTATGCAGGACACTATTGGCTAAGTCAGTATTCAA
TAGTCTGTATGCATCTCCACAATTAGAAGGATTTTCAGCTGAGTCAAGAAAGTTGCTCCTCATTGTTCAG
GCTCTTAGGGACAATCTGGAACCTGGGACCTTTGATCTTGGGGGACTATATGAAGCAATTGAGGAGTGCC
TGATTAATGATCCCTGGGTTTTGCTTAATGCTTCTTGGTTCAACTCCTTCCTAACACATGCATTGAGATA
GCTGAGGCAATGCTACTATTTGTTATCCATACTGTCCAAAAAA
>gi|157281269|gb|CY025218.1| Influenza A virus (A/Texas/UR06-0012/2006(H1N1)) segment 3, complete sequence
TGATTCGAAATGGAAGATTTTGTGCGACAATGCTTCAATCCGATGATTGTCGAGCTTGCGGAAAAGGCAA
TGAAAGAGTATGGAGAGGACCTGAAAATCGAAACAAACAAATTTGCAGCAATATGCACCCACTTGGAAGT
ATGCTTCATGTATTCAGATTTTCATTTCATCAATGAGCAAGGCGAATCAATAATAGTAGAGCCTGAGGAC
CCAAATGCACTTTTAAAACACAGATTTGAGATAATAGAGGGACGAGATCGTACAATGGCATGGACAGTTG
TAAACAGTATTTGCAACACCACAGGAGCTGAGAAACCAAAGTTTCTGCCAGATCTGTATGATTACAAAGA

GAATAGATTCATCGAGATTGGAGTGACAAGGAGAGAAGTTCACATATACTATCTGGAAAAGGCCAACAAA
ATTAAATCTGAGAAGACACACATTCACATTTTCTCATTCACTGGCGAAGAAATGGCCACAAAGGCCGATT
ATACTCTCGATGAAGAAAGCAGGGCTAGGATTAAAACCAGACTATTCACCATAAGACAAGAAATGGCAAG
CAGAGGTCTTTGGGACTCCTTTCGTCAGTCCGAAAGAGGCGAAGAAACAATTGAAGAAAGATTTGAAATC
ACAGGGACAATGCGCAGGCTCGCTGATCAAAGCCTTCCGCCGAACTTCTCCTGCATTGAGAATTTTAGAG
CCTATGTGGATGGATTCGAACCGAACGGATACATTGAGGGCAAGCTTTCTCAAATGTCCAAAGAAGTAAA
TGCTAAAATTGAGCCTTTTTTGAAAACAACACCTCGACCAATTAGACTTCCGAATGGGCCTCCTTGTTTT
CAGCGGTCAAAATTCCTGCTGATGGATTCTTTAAAATTAAGCATTGAGGATCCAAATCATGAAGGGGAGG
GAATACCACTATATGATGCAATCAAGTGTATGAGAACATTCTTTGGATGGAAAGAACCCACTGTTGTCAA
ACCACATGAGAAGGGAATAAATCCGAATTATCTGCTGTCATGGAAGCAGGTATTGGAAGAGCTGCAGGAC
ATTGAGAATGAGGAGAAAATTCCAAGAACAAAAAACATGAAAAAAACGAGTCAGCTAAAGTGGGCACTTG
GTGAGAACATGGCACCAGAGAAGGTGGATTTTGATGACTGTAAAGATATAAGCGATTTGAAGCAATATGA
TAGTGACGAACCTGAATTAAGGTCATTTTCAAGTTGGATCCAGAATGAGTTCAACAAGGCATGCGAGCTG
ACCGATTCAATCTGGATAGAGCTCGATGAGATTGGAGAAGATGTGGCCCCAATTGAACACATTGCAAGCA
TGAGAAGAAATTACTTCACAGCTGAGGTGTCCCATTGCAGAGCCACTGAATATATAATGAAGGGGGTATA
CATTAATACTGCTTTGCTTAATGCATCCTGTGCAGCAATGGATGATTTCCAACTAATTCCCATGATAAGC
AAATGTAGAACTAAAGAGGGAAGGAGAAAGACCAATTTGTACGGCTTCATTATAAAAGGAAGATCTCACT
TAAGGAATGACACCGATGTGGTAAACTTTGTGAGCATGGAGTTTTCCCTCACTGACCCAAGACTTGAGCC
ACACAAATGGGAGAAGTACTGTGTTCTTGAGATAGGAGATATGCTTCTAAGGAGTGCAATAGGCCAAGTG
TCAAGGCCCATGTTCTTGTATGTAAGAACAAATGGAACCTCAAAAATTAAAATGAAATGGGGAATGGAGA
TGAGGCGTTGCCTCCTCCAATCCCTCCAACAAATAGAGAGCATGATTGAAGCTGAGTCCTCTGTCAAGGA
GAAAGACATGACAAAAGAGTTTTTTGAGAATAGATCAGAAACATGGCCCATTGGAGAGTCACCAGAAGGA
GTGGAAGAAGGTTCCATTGGGAAAGTATGCAGGACACTATTGGCTAAGTCAGTATTCAATAGTCTGTATG
CATCTCCACAATTAGAAGGATTTTCAGCTGAGTCAAGAAAGTTGCTCCTCATTGTTCAGGCTCTTAGGGA
CAATCTGGAACCTGGGACCTTTGATCTTGGGGGACTATATGAAGCAATTGAGGAGTGCCTGATTAATGAT
CCCTGGGTTTTGCTTAATGCTTCTTGGTTCAACTCCTTCCTAACACATGCATTGAGATAGCTGAGGCAAT
GCTACTATTTGTTATCCATACTGTCCAAAA
>gi|94959544|gb|CY010769.1| Influenza A virus (A/Canterbury/20/2001(H1N1)) segment 3, complete sequence
GTACTGATTCGAAATGGAAGATTTTGTGCGACAATGCTTCAATCCGATGATTGTCGAGCTTGCGGAAAAG
GCAATGAAAGAGTATGGAGAGGACCTGAAAATCGAAACAAACAAATTTGCAGCAATATGCACTCACTTGG
AAGTATGCTTCATGTATTCAGATTTTCATTTCATCAATGAGCAAGGCGAATCAATAATAGTAGAGCCTGA
GGACCCAAATGCACTTTTAAAACACAGATTTGAGATAATAGAGGGACGAGATCGTACAATGGCATGGACA
GTTGTAAACAGCATTTGCAACACCACAGGAGCTGAGAAACCAAAGTTTCTGCCAGATCTGTATGATTACA
AGGAGAATAGATTCATCGAGATTGGAGTGACAAGGAGGGAAGTTCACATATACTATCTGGAAAAGGCCAA
CAAAATTAAATCTGAAAAGACACACATTCACATTTTCTCATTCACTGGCGAAGAAATGGCCACAAAGGCC
GATTACACTCTCGATGAAGAAAGCAGGGCTAGGATTAAAACCAGACTATTCACCATAAGACAAGAAATGG
CAAGCAGAGGTCTTTGGGACTCCTTTCGTCAGTCCGAAAGAGGCGAAGAAACAATTGAAGAAAGATTTGA
AATCACAGGGACAATGCGCAGGCTTGCTGACCAAAGCCTTCCGCCGAACTTCTCCTGCATTGAGAATTTT
AGAGCCTATGTGGATGGATTTGAACCGAACGGCTACATTGAGGGCAAGCTTTCTCAAATGTCCAAAGAAG
TAAATGCTAGAATTGAGCCTTTTTTGAAAACAACACCACGACCAATTAGACTTCCGGATGGGCCTCCTTG
TTTTCAGCGGTCAAAATTCCTGCTGATGGATTCTTTAAAATTAAGCATTGAGGATCCAAATCACGAAGGA
GAGGGAATACCACTATATGATGCAATCAAGTGTATGAGAACATTCTTTGGATGGAAAGAACCCTCTGTTG
TCAAGCCACACGAGAAGGGAATAAATCCGAATTATCTGCTGTCATGGAAGCAGGTATTGGAAGAGCTGCA

GGACATTGAGAGTGAGGAGAAGATTCCAAGAACAAAGAACATGAAAAAAACGAGTCAGCTAAAGTGGGC
A
CTTGGTGAGAACATGGCACCAGAGAAGGTTGATTTTGATGACTGTAAAGATATAAGCGATTTGAAGCAAT
ATGATAGTGACGAACCTGAATTAAGGTCATTTTCAAGTTGGATCCAGAATGAATTCAACAAGGCATGCGA
GCTGACCGATTCAATCTGGATAGAGCTTGATGAGATTGGAGAAGATGTGGCTCCGATTGAACACATTGCA
AGCATGAGAAGAAATTACTTCACAGCTGAGGTGTCCCATTGCAGAGCCACAGAATATATAATGAAGGGGG
TATACATTAATACTGCTTTGCTTAATGCATCCTGTGCAGCAATGGATGATTTCCAACTAATTCCCATGAT
AAGCAAATGTAGAACTAAAGAGGGAAGGAGAAAGACCAATTTGTACGGCTTCATCATAAAAGGAAGATCT
CACTTAAGGAATGACACCGATGTGGTAAACTTTGTGAGCATGGAATTTTCCCTCACTGACCCAAGACTTG
AGCCACACAAATGGGAGAAGTACTGTGTTCTCGAGATAGGAGATATGCTTCTAAGGAGTGCAATAGGCCA
AGTGTCAAGGCCCATGTTCTTGTATGTAAGGACAAATGGAACCTCAAAAATTAAAATGAAATGGGGAATG
GAGATGAGGCGTTGCCTCCTCCAATCCCTTCAACAAATAGAGAGCATGATTGAAGCTGAATCCTCTGTCA
AGGAGAAAGACATGACAAAAGAGTTTTTTGAGAATAGATCAGAAACATGGCCCATTGGAGAGTCACCAAA
AGGAGTGGAAGAAGGTTCCATTGGAAAAGTATGCAGGACACTATTGGCCAAGTCAGTATTCAATAGTCTG
TATGCATCGCCACAATTAGAAGGATTTTCAGCTGAGTCAAGAAAGTTGCTCCTCATTGTTCAGGCTCTTA
GGGACAATCTGGAGCCTGGGACCTTTGATCTTGGGGGGCTATATGAAGCAATTGAGGAGTGCCTGATTAA
TGATCCCTGGGTTTTGCTTAATGCTTCTTGGTTCAACTCCTTCCTAACACATGCATTGAGATAGTTGGGG
CAATGCTACTATTTACTATCCATACTGTCCAAAAAA
>gi|91119038|gb|CY010409.1| Influenza A virus (A/West Coast/33/2001(H1N1)) segment 3, complete sequence
GTACTGATTCGAAATGGAAGATTTTGTGCGACAATGCTTCAATCCGATGATTGTCGAGCTTGCGGAAAAG
GCAATGAAAGAGTATGGAGAGGACCTGAAAATCGAAACAAACAAATTTGCAGCAATATGCACTCACTTGG
AAGTATGCTTCATGTATTCAGATTTTCATTTCATCAATGAGCAAGGCGAATCAATAATAGTAGAGCCTGA
GGACCCAAATGCACTTTTAAAACACAGATTTGAGATAATAGAGGGACGAGATCGTACAATGGCATGGACA
GTTGTAAACAGTATTTGCAACACCACAGGAGCTGAGAAACCAAAGTTTCTGCCAGATCTGTATGATTACA
AGGAGAATAGATTCATCGAGATTGGAGTGACAAGGAGGGAAGTTCACATATACTATCTGGAAAAGGCCAA
CAAAATTAAATCTGAAAAGACACACATTCACATTTTCTCATTCACTGGCGAAGAAATGGCCACAAAGGCC
GATTACACTCTCGATGAAGAAAGCAGGGCTAGGATTAAAACCAGACTATTCACCATAAGACAAGAAATGG
CAAGCAGAGGTCTTTGGGACTCCTTTCGTCAGTCCGAAAGAGGCGAAGAAACAATTGAAGAAAGATTTGA
AATCACAGGGACAATGCGCAGGCTTGCTGACCAAAGCCTTCCGCCGAACTTCTCCTGCATTGAGAATTTT
AGAGCCTATGTGGATGGATTTGAACCGAACGGCTACATTGAGGGCAAGCTTTCTCAAATGTCCAAAGAAG
TAAATGCTAGAATTGAGCCTTTTTTGAAAACAACACCACGACCAATTAGACTTCCGGATGGGCCTCCTTG
TTTTCAGCGGTCAAAATTCCTGCTGATGGATTCTTTAAAATTAAGCATTGAGGATCCAAATCACGAAGGA
GAGGGAATACCACTATATGATGCAATCAAGTGTATGAGAACATTCTTTGGATGGAAAGAACCCTCTGTTG
TCAAGCCACACGAGAAGGGAATAAATCCGAATTATCTGCTGTCATGGAAGCAGGTATTGGAAGAGCTGCA
GGACATTGAGAGTGAGGAGAAGATTCCAAGAACAAAGAACATGAAAAAAACGAGTCAGCTAAAGTGGGC
A
CTTGGTGAGAACATGGCACCAGAGAAGGTTGATTTTGATGACTGTAAAGATATAAGCGATTTGAAGCAAT
ATGATAGTGACGAACCTGAATTAAGGTCATTTTCAAGTTGGATCCAGAATGAATTCAACAAGGCATGCGA
GCTGACCGATTCAATCTGGATAGAGCTTGATGAGATTGGAGAAGATGTGGCTCCGATTGAACACATTGCA
AGCATGAGAAGAAATTACTTCACAGCTGAGGTGTCCCATTGCAGAGCCACAGAATATATAATGAAGGGGG
TATACATTAATACTGCTTTGCTTAATGCATCCTGTGCAGCAATGGATGATTTCCAACTAATTCCCATGAT
AAGCAAATGTAGAACTAAAGAGGGAAGGAGAAAGACCAATTTGTACGGCTTCATCATAAAAGGAAGATCT
CACTTAAGGAATGACACCGATGTGGTAAACTTTGTGAGCATGGAATTTTCCCTCACTGACCCAAGACTTG

AGCCACACAAATGGGAGAAGTACTGTGTTCTCGAGATAGGAGATATGCTTCTAAGGAGTGCAATAGGCCA
AGTGTCAAGGCCCATGTTCTTGTATGTAAGGACAAATGGAACCTCAAAAATTAAAATGAAATGGGGAATG
GAGATGAGGCGTTGCCTCCTCCAATCCCTTCAACAAATAGAGAGCATGATTGAAGCTGAATCCTCTGTCA
AGGAGAAAGACATGACAAAAGAGTTTTTTGAGAATAGATCAGAAACATGGCCCATTGGAGAGTCACCAAA
AGGAGTGGAAGAAGGTTCCATTGGAAAAGTATGCAGGACACTATTGGCCAAGTCAGTATTCAATAGTCTG
TATGCATCGCCACAATTAGAAGGATTTTCAGCTGAGTCAAGAAAGTTGCTCCTCATTGTTCAGGCTCTTA
GGGACAATCTGGAGCCTGGGACCTTTGATCTTGGGGGGCTATATGAAGCAATTGAGGAGTGCCTGATTAA
TGATCCCTGGGTTTTGCTTAATGCTTCTTGGTTCAACTCCTTCCTAACACATGCATTGAGATAGTTGGGG
CAATGCTACTATTTACTATCCATACTGTCCAAAAAA

>gi|131052848|gb|CY020002.1| Influenza A virus (A/Waikato/17/2005(H1N1)) segment 3, complete sequence

ATTCGAAATGGAAGATTTTGTGCGACAATGCTTCAACCCGATGATTGTCGAGCTTGCGGAAAAGGCAATG
AAAGAGTATGGAGAGGACCTTAAAATCGAAACAAACAAATTTGCAGCAATATGCACCCACTTGGAAGTAT
GCTTCATGTATTCAGATTTTCATTTCATCAATGAGCAAGGCGAATCAATAATAGTAGAGCCTGAGGACCC
AAATGCACTTTTAAAACACAGATTTGAGATAATAGAGGGACGAGATCGTACAATGGCATGGACAGTTGTA
AACAGTATTTGCAACACCACAGGAGCTGAGAAACCAAAGTTTCTGCCAGATCTGTATGATTACAAAGAGA
ATAGGTTCATCGAGATTGGAGTGACAAGGAGAGAAGTTCACATATACTATCTGGAAAAGGCCAACAAAAT
CAAATCTGAGAAGACACACATCCACATTTTCTCATTCACTGGCGAAGAAATGGCCACAAAGGCCGATTAC
ACTCTCGATGAAGAAAGCAGGGCTAGGATTAAAACCAGACTATTCACCATAAGACAAGAAATGGCAAGCA
GAGGTCTTTGGGACTCCTTTCGTCAGTCCGAAAGAGGCGAAGAAACAATTGAAGAAAGATTTGAAATCAC
AGGGACAATGCGCAGGCTCGCTGATCAAAGCCTTCCGCCGAACTTCTCCTGCATTGAGAATTTTAGAGCC
TATGTGGATGGATTTGAACCAAACGGCTACATTGAGGGCAAGCTTTCTCAAATGTCCAAAGAAGTAAATG
CTAGAATTGAGCCCTTTTTGAAAACAACACCTCGACCAATTAGACTTCCGAATGGGCCTCCTTGTTTTCA
GCGGTCAAAATTCCTGCTGATGGATTCTTTAAAATTAAGCATTGAGGATCCAAATCATGAAGGGGAGGGA
ATACCACTATATGATGCAATCAAATGTATGAGAACATTCTTTGGATGGAAAGAACCCACTGTTGTCAAGC
CACACGAGAAGGGAATAAATCCGAATTATCTGCTGTCGTGGAAGCAGGTATTGGAAGAGCTGCAGGACAT
TGAGAGTGAGGAGAAGATTCCAAGAACAAAAAACATGAAAAAAACGAGTCAGCTAAAGTGGGCACTTGG
T
GAGAACATGGCACCAGAGAAGGTGGATTTTGATGACTGTAAAGATATAAGCGATTTAAAGCAATATGATA
GTGACGAACCTGAATTAAGGTCATTTTCAAGTTGGATCCAGAATGAGTTCAACAAGGCATGCGAGCTGAC
CGATTCAATCTGGATAGAGCTCGATGAGATTGGAGAAGATGTGGCCCCGATTGAACACATTGCAAGCATG
AGAAGAAATTACTTCACAGCTGAGGTGTCCCATTGCAGAGCCACTGAATATATAATGAAGGGGGTATACA
TTAATACTGCTTTGCTTAATGCATCCTGTGCAGCAATGGATGATTTCCAACTAATTCCCATGATAAGCAA
ATGTAGAACTAAAGAGGGAAGGAGAAAGACCAATTTGTACGGCTTCATCATAAAAGGAAGATCTCACTTA
AGGAATGACACCGATGTGGTAAACTTTGTGAGCATGGAGTTTTCCCTCACTGACCCAAGACTTGAGCCAC
ACAAATGGGAGAAGTACTGTGTTCTTGAGATAGGAGATATGCTTCTAAGGAGTGCAATAGGCCAAGTGTC
AAGGCCCATGTTCTTGTATGTAAGAACAAATGGAACCTCAAAAATTAAAATGAAATGGGGAATGGAGATG
AGGCGTTGTCTCCTCCAATCCCTCCAACAAATAGAGAGCATGATTGAAGCTGAGTCCTCTGTCAAGGAGA
AAGACATGACAAAAGAGTTTTTTGAGAATAGATCAGAAACATGGCCCATTGGAGAGTCACCAAAAGGAGT
GGAAGAAGGTTCCATTGGAAAAGTATGCAGGACACTACTGGCTAAGTCAGTATTCAATAGTCTGTATGCA
TCTCCACAATTAGAAGGGTTTTCAGCTGAGTCAAGGAAGTTGCTACTCATTGTTCAGGCTCTTAGGGACA
ATCTGGAACCTGGGACCTTTGATCTTGGGGGACTATATGAAGCAATTGAGGAGTGCCTGATTAATGATCC
CTGGGTTTTGCTTAATGCTTCTTGGTTCAACTCCTTCCTAACACATGCATTGAGATAGCTGAGGCAATGC
TACTATTTGTTATCCATACTGTCCAAAAAA

>gi|83744846|gb|CY007472.1| Influenza A virus (A/Canterbury/106/2004(H1N1)) segment 3, complete sequence
GTACTGATTCGAAATGGAAGATTTTGTGCGACAATGCTTCAATCCGATGATTGTCGAGCTTGCGGAAAAG
GCAATGAAAGAGTATGGAGAGGACCTGAAAATCGAAACAAACAAATTTGCAGCAATATGCACCCACTTGG
AAGTGTGCTTCATGTATTCAGATTTTCATTTCATCAATGAGCAAGGCGAATCAATAATAGTAGAACCTGA
GGACCCAAATGCACTTTTAAAGCACAGATTTGAGATAATAGAGGGACGAGATCGTACAATGGCATGGACA
GTTGTAAACAGTATTTGCAACACCACAGGAGCTGAGAAACCAAAGTTTCTGCCAGATCTGTATGATTACA
AAGAGAATAGATTCATCGAGATTGGAGTGACAAGGAGGGAAGTTCACATATACTATCTGGAAAAGGCCAA
CAAAATTAAATCTGAGAAGACACACATTCACATTTTCTCATTCACTGGCGAAGAAATGGCCACAAAGGCC
GATTACACTCTTGATGAAGAAAGCAGAGCTAGGATTAAAACCAGACTATTCACCATAAGACAAGAAATGG
CAAGCAGAGGTCTTTGGGACTCCTTTCGTCAGTCCGAAAGAGGCGAAGAAACAATTGAAGAAAGGTTTGA
AATCACAGGGACAATGCGCAGGCTCGCTGACCAAAGCCTCCCGCCGAACTTCTCCTGCATTGAGAATTTT
AGAGCCTATGTGGATGGATTTGAACCGAACGGCTACATTGAGGGCAAGCTTTCTCAAATGTCCAAAGAAG
TAAATGCTAGAATTGAGCCTTTTTTGAAAACAACACCTCGGCCAATTAGACTTCCGAATGGGCCTCCTTG
TTTTCAGCGGTCAAAATTCCTGCTGATGGATTCTTTAAAATTAAGCATTGAGGATCCAAATCATGAAGGT
GAGGGAATACCACTATATGATGCAATCAAGTGTATGAGAACATTCTTTGGATGGAAAGAACCCACTGTTG
TCAAGCCACACGAGAAGGGAATAAATCCGAATTATCTGCTGTCGTGGAAGCAAGTATTGGAAGAGCTGCA
GGACATTGAGAGTGAGGAGAAGATTCCAAGAACAAAAAACATGAAAAAAACGAGTCAGCTAAAGTGGGC
A
CTTGGTGAGAACATGGCACCAGAGAAGGTGGATTTTGATGACTGTAAAGATATAAGCGATTTGAAGCAAT
ATGACAGTGACGAACCTGAATTAAGGTCATTTTCAAGTTGGATCCAGAATGAGTTCAACAAGGCATGCGA
GCTGACCGATTCAATCTGGATAGAGCTTGATGAGATTGGAGAAGATGTGGCCCCGATTGAACACATTGCA
AGCATGAGAAGGAATTACTTCACAGCTGAGGTGTCCCATTGCAGAGCCACAGAATATATAATGAAGGGGG
TATACATTAATACTGCTTTGCTCAATGCATCCTGTGCAGCAATGGATGATTTCCAACTAATTCCCATGAT
AAGCAAATGTAGAACTAAAGAGGGAAGGAGAAAGACCAATTTGTACGGCTTCATCGTAAAAGGAAGATCT
CACTTAAGGAATGACACCGATGTGGTAAACTTTGTGAGCATGGAGTTTTCCCTCACTGACCCAAGACTTG
AGCCACACAAATGGGAGAAGTACTGTGTTCTTGAGATAGGGGATATGCTTCTAAGGAGTGCAATAGGCCA
AGTGTCAAGGCCCATGTTCTTGTATGTAAGGACAAATGGAACCTCAAAAATTAAAATGAAATGGGGAATG
GAGATGAGGCGTTGCCTCCTCCAATCCCTCCAACAAATAGAGAGCATGATTGAAGCTGAGTCCTCTGTCA
AAGAGAAAGACATGACAAAAGAGTTTTTTGAGAATAGATCAGAAACATGGCCCATTGGAGAGTCACCAAA
AGGAGTGGAAGAAGGTTCCATTGGGAAAGTATGCAGGACACTATTGGCTAAGTCAGTATTCAATAGTCTG
TATGCATCTCCACAATTAGAAGGATTTTCAGCTGAGTCAAGGAAGTTGCTCCTCATTGTTCAGGCTCTTA
GGGACAATCTGGAACCTGGGACCTTTGATCTTGGGGGGCTATATGAAGCAATTGAGGAGTGCCTGATTAA
TGATCCCTGGGTTTTGCTTAATGCTTCTTGGTTCAACTCCTTCCTAACACATGCATTGAGATAGCTGGGG
CAATGCTACTATTTGTTATCCATACTGTCCAAAAAA
>gi|115607833|gb|CY016704.1| Influenza A virus (A/South Australia/58/2005(H1N1)) segment 3, complete sequence
ATTCGAAATGGAAGATTTTGTGCGACAATGCTTCAACCCGATGATTGTCGAGCTTGCGGAAAAGGCAATG
AAAGAGTATGGAGAGGACCTGAAAATCGAAACAAACAAATTTGCAGCAATATGCACCCACCTGGAAGTAT
GCTTCATGTATTCAGATTTTCATTTCATCAATGAGCAAGGCGAATCAATAATAGTAGAGCCTGAGGACCC
AAATGCACTTTTAAAACACAGATTTGAGATAATAGAGGGACGAGATCGTACAATGGCATGGACAGTTGTA
AACAGTATTTGCAACACCACAGGAGCTGAGAAACCAAAGTTTCTGCCAGATCTGTATGATTACAAAGAGA
ATAGGTTCATCGAGATTGGAGTGACAAGGAGAGAAGTTCACATATACTATCTGGAAAAGGCCAACAAAAT
CAAATCTGAGAAGACACACATCCACATTTTCTCATTCACTGGCGAAGAAATGGCCACAAAGGCCGATTAC

ACTCTCGATGAAGAAAGCAGGGCTAGGATAAAAACCAGACTATTCACCATAAGACAAGAAATGGCAAGCA
GAGGTCTTTGGGACTCCTTTCGTCAGTCCGAAAGAGGCGAAGAAACAATTGAAGAAAGATTTGAAATCAC
AGGGACAATGCGCAGGCTCGCTGATCAAAGCCTTCCGCCGAACTTCTCCTGCATTGAGAATTTTAGAGCC
TATGTGGATGGATTTGAACCAAACGGCTACATTGAGGGCAAGCTTTCTCAAATGTCCAAAGAAGTAAATG
CTAGAATTGAGCCCTTTTTGAAAACAACACCTCGACCAATTAGACTTCCGAATGGGCCTCCTTGTTTTCA
GCGGTCAAAATTCCTGCTGATGGATTCTTTAAAATTAAGCATTGAGGATCCAAATCATGAAGGGGAGGGA
ATACCACTATATGATGCAATCAAATGTATGAGAACATTCTTTGGATGGAAAGAACCCACTGTTGTCAAGC
CACACGAGAAGGGAATAAATCCGAATTATCTGCTGTCGTGGAAGCAGGTATTGGAAGAGCTGCAGGACAT
TGAGAGTGAGGAGAAGATTCCAAGAACAAAAAACATGAAAAAAACGAGTCAGCTAAAGTGGGCACTTGG
T
GAGAACATGGCACCAGAGAAGGTGGATTTTGATGACTGTAAAGATATAAGCGATTTGAAGCAATATGATA
GTGACGAACCTGAATTAAGGTCATTTTCAAGTTGGATCCAGAATGAGTTCAACAAGGCATGCGAGCTGAC
CGATTCAATCTGGATAGAGCTCGATGAGATTGGAGAAGATGTGGCCCCGATTGAACACATTGCAAGCATG
AGAAGAAATTACTTCACAGCTGAGGTGTCCCATTGCAGAGCCACTGAATATATAATGAAGGGGGTATACA
TTAATACTGCTTTGCTTAATGCATCCTGTGCAGCAATGGATGATTTCCAACTAATTCCCATGATAAGCAA
ATGTAGAACTAAAGAGGGAAGGAGAAAGACCAATTTGTACGGCTTCATCATAAAAGGAAGATCTCACTTA
AGGAATGACACCGATGTGGTAAACTTTGTGAGCATGGAGTTTTCCCTCACTGACCCAAGACTTGAGCCAC
ACAAATGGGAGAAGTACTGTGTTCTTGAGATAGGAGATATGCTTCTAAGGAGTGCAATAGGCCAAGTGTC
AAGGCCCATGTTCTTGTATGTAAGAACAAATGGAACCTCAAAAATTAAAATGAAATGGGGAATGGAGATG
AGGCGTTGTCTCCTCCAATCCCTCCAACAAATAGAGAGCATGATTGAAGCTGAGTCCTCTGTCAAGGAGA
AAGACATGACAAAAGAGTTTTTTGAGAATAGATCAGAAACATGGCCCATTGGAGAGTCACCAAAAGGAGT
GGAAGAAGGTTCCATTGGGAAAGTATGCAGGACACTACTGGCTAAGTCAGTATTCAATAGTCTGTATGCA
TCTCCACAATTAGAAGGGTTTTCAGCTGAGTCAAGGAAGTTGCTCCTCATTGTTCAGGCTCTTAGGGACA
ATCTGGAACCTGGGACCTTTGATCTTGGGGGACTATATGAAGCAATTGAGGAGTGCCTGATTAATGATCC
CTGGGTTTTGCTTAATGCTTCTTGGTTCAACTCCTTCCTAACACATGCATTGAGATAGCTGAGGCAATGC
TACTATTTGTCATCCATACTGTCCAAAA
>gi|113170893|gb|CY014012.1| Influenza A virus (A/Wellington/11/2005(H1N1)) segment 3, complete sequence
ATTCGAAATGGAAGATTTTGTGCGACAATGCTTCAATCCGATGATTGTCGAGCTTGCGGAAAAGGCAATG
AAAGAGTATGGAGAGGACCTGAAAATCGAAACAAACAAATTTGCAGCAATATGCACCCACTTGGAAGTAT
GCTTCATGTATTCAGATTTTCATTTCATCAATGAGCGAGGCGAATCAATAATAGTAGAGCCTGAGGACCC
AAATGCACTTTTAAAACACAGATTTGAGATAATAGAGGGACGAGATCGTACAATGGCATGGACAGTTGTA
AACAGTATTTGCAACACCACAGGAGCTGAGAAACCGAAGTTTCTGCCAGATCTGTATGATTACAAAGAGA
ATAGATTCATCGAGATTGGAGTGACAAGGAGAGAAGTTCACATATACTATCTGGAAAAGGCCAACAAAAT
TAAATCTGAGAAGACACACATTCACATTTTCTCATTCACTGGCGAAGAAATGGCCACAAAGGCCGATTAT
ACCCTCGATGAAGAAAGCAGGGCTAGGATTAAAACCAGACTATTCACCATAAGACAAGAAATGGCAAGCA
GAGGTCTTTGGGACTCCTTTCGTCAGTCCGAAAGAGGCGAAGAAACAATTGAAGAAAGATTTGAAATCAC
AGGGACAATGCGCAGGCTCGCTGATCAAAGCCTTCCGCCGAACTTCTCCTGCATTGAGAATTTTAGAGCC
TATGTGGATGGATTCGAACCGAACGGCTACATTGAGGGCAAGCTTTCTCAAATGTCCAAAGAAGTAAATG
CTAGAATTGAGCCTTTTTTGAAAACAACACCTCGACCAATTAGACTTCCGAATGGGCCTCCTTGTTTTCA
GCGGTCAAAATTCCTGCTGATGGATTCTTTAAAATTAAGCATTGAGGATCCAAATCATGAAGGGGAGGGA
ATACCACTATATGATGCAATCAAGTGTATGAGAACATTCTTTGGATGGAAAGAACCCACTGTTGTCAAGC
CACACGAGAAGGGAATAAATCCGAATTATCTGCTGTCATGGAAGCAGGTATTGGAAGAGCTGCAGGACAT
TGAGAGTGAGGAGAAGATTCCAAGAACAAAAAACATGAAAAAAACGAGTCAGCTAAAGTGGGCACTTGG

T
GAGAACATGGCACCAGAGAAGGTGGATTTTGATGACTGTAAAGATGTAAGCGATTTGAAGCAATATGATA
GTGACGAACCTGAATTAAGGTCATTTTCAAGTTGGATCCAGAATGAGTTCAACAAGGCATGCGAGCTGAC
CGATTCAATCTGGATAGAGCTCGATGAGATTGGAGAAGATGTGGCCCCGATTGAACACATTGCAAGCATG
AGAAGAAATTACTTCACAGCTGAGGTGTCCCATTGCAGAGCCACTGAATATATAATGAAGGGGGTATACA
TTAATACTGCTTTGCTTAATGCATCCTGTGCAGCAATGGATGATTTCCAACTAATTCCCATGATAAGCAA
ATGTAGAACTAAAGAGGGAAGGAGAAAGACCAATTTGTACGGCTTCATTATAAAAGGAAGATCTCACTTA
AGGAATGACACCGATGTGGTAAACTTTGTGAGCATGGAGTTTTCCCTCACTGACCCAAGACTTGAGCCAC
ACAAATGGGAGAAGTACTGTGTTCTTGAGATAGGAGATATGCTTCTAAGGAGTGCAATAGGCCAAGTGTC
AAGGCCCATGTTCTTGTATGTAAGAACAAATGGAACCTCAAAAATTAAAATGAAATGGGGAATGGAGATG
AGGCGTTGCCTCCTCCAATCCCTCCAACAAATAGAGAGCATGATTGAAGCTGAGTCCTCTGTCAAGGAGA
AAGACATGACAAAAGAGTTTTTTGAGAATAGATCAGAAACATGGCCCATTGGAGAGTCACCAGAAGGAGT
GGAGGAAGGTTCCATTGGGAAAGTATGCAGGACACTATTGGCTAAGTCAGTATTCAATAGTCTGTATGCA
TCTCCACAATTAGAAGGATTTTCAGCTGAGTCAAGAAAGTTGCTCCTCATTGTTCAGGCTCTTAGGGACA
ATCTGGAACCTGGGACCTTTGATCTTGGGGGACTATATGAAGCAATTGAGGAGTGCCTGATTAATGATCC
CTGGGTTTTGCTTAATGCTTCTTGGTTCAACTCCTTCCTAACACATGCATTGAGATAGCTGAGGCAATGC
TACTATTTGTTATCCATACTGTCCAAAAA
>gi|145278935|gb|CY021762.1| Influenza A virus (A/South Australia/51/2005(H1N1)) segment 3, complete sequence
ATTCGAAATGGAAGATTTTGTGCGACAATGCTTCAATCCGATGATTGTCGAGCTTGCGGAAAAGGCAATG
AAAGAGTATGGAGAGGACCTGAAAATCGAAACAAACAAATTTGCAGCAATATGCACCCACTTGGAAGTAT
GCTTCATGTATTCAGATTTTCATTTCATCAATGAGCAAGGCGAATCAATAATAGTAGAGCCTGAGGACCC
AAATGCACTTTTAAAACACAGATTTGAGATAATAGAGGGACGAGATCGTACAATGGCATGGACAGTTGTA
AACAGTATTTGCAACACCACAGGAGCTGAGAAACCGAAGTTTCTGCCAGATCTGTATGATTACAAAGAGA
ATAGATTCATCGAGATTGGAGTGACAAGGAGAGAAGTTCACATATACTATCTGGAAAAGGCCAACAAAAT
TAAATCTGAGAAGACACACATTCACATTTTCTCATTCACTGGCGAAGAAATGGCCACAAAGGCCGATTAT
ACCCTCGATGAAGAAAGCAGGGCTAGGATTAAAACCAGACTATTCACCATAAGACAAGAAATGGCAAGCA
GAGGTCTTTGGGACTCCTTTCGTCAGTCCGAAAGAGGCGAAGAAACAATTGAAGAAAGATTTGAAATCAC
AGGGACAATGCGCAGGCTCGCTGATCAAAGCCTTCCGCCGAACTTCTCCTGCATTGAGAATTTTAGAGCC
TATGTGGATGGATTCGAACCGAACGGCTACATTGAGGGCAAGCTTTCTCAAATGTCCAAAGAAGTAAATG
CTAGAATTGAGCCTTTTTTGAAAACAACACCTCGACCAATTAGACTTCCGAATGGGCCTCCTTGTTTTCA
GCGGTCAAAATTCCTGCTGATGGATTCTTTAAAATTAAGCATTGAGGATCCAAATCATGAAGGGGAGGGA
ATACCACTATATGATGCAATCAAGTGTATGAGAACATTCTTTGGATGGAAAGAACCCACTGTTGTCAAGC
CACACGAGAAGGGAATAAATCCGAATTATCTGCTGTCATGGAAGCAGGTATTGGAAGAGCTGCAGGACAT
TGAGAGTGAGGAGAAGATTCCAAGAACAAAAAACATGAAAAAAACGAGTCAGCTAAAGTGGGCACTTGG
T
GAGAACATGGCACCAGAGAAGGTGGATTTTGATGACTGTAAAGATGTAAGCGATTTGAAGCAATATGATA
GTGACGAACCTGAATTAAGGTCAGTTTCAAGTTGGATCCAGAATGAGTTCAACAAGGCATGCGAGCTGAC
CGATTCAATCTGGATAGAGCTCGATGAGATTGGAGAAGATGTGGCCCCGATTGAACACATTGCAAGCATG
AGAAGAAATTACTTCACAGCTGAGGTGTCCCATTGCAGAGCCACTGAATATATAATGAAGGGGGTATACA
TTAATACTGCTTTGCTTAATGCATCCTGTGCAGCAATGGATGATTTCCAACTAATTCCCATGATAAGCAA
ATGTAGAACTAAAGAGGGAAGGAGAAAGACCAATTTGTACGGCTTCATTATAAAAGGAAGATCTCACTTA
AGGAATGACACCGATGTGGTAAACTTTGTGAGCATGGAGTTTTCCCTCACTGACCCAAGACTTGAGCCAC
ACAAATGGGAGAAGTACTGTGTTCTTGAGATAGGAGATATGCTTCTAAGGAGTGCAATAGGCCAAGTGTC

AAGGCCCATGTTCTTGTATGTAAGAACAAATGGAACCTCAAAAATTAAAATGAAATGGGGAATGGAGATG
AGGCGTTGCCTCCTCCAATCCCTCCAACAAATAGAGAGCATGATTGAAGCTGAGTCCTCTGTCAAGGAGA
AAGACATGACAAAAGAGTTTTTTGAGAATAGATCAGAAACATGGCCCATTGGAGAGTCACCAGAAGGAGT
GGAAGAAGGTTCCATTGGGAAAGTATGCAGGACACTATTGGCTAAGTCAGTATTCAATAGTCTGTATGCA
TCTCCACAATTAGAAGGATTTTCAGCTGAGTCAAGAAAGTTGCTCCTCATTGTTCAGGCTCTTAGGGACA
ATCTGGAACCTGGGACCTTTGATCTTGGGGGACTATATGAAGCAATTGAGGAGTGCCTGATTAATGATCC
CTGGGTTTTGCTTAATGCTTCTTGGTTCAACTCCTTCCTAACACATGCATTGAGATAGCTGAGGCAATGC
TACTATTTGTTATCCATAC
>gi|149780705|gb|CY022586.1| Influenza A virus (A/Auckland/619/2005(H1N1)) segment 3, complete sequence
ATTCGAAATGGAAGATTTTGTGCGACAATGCTTCAATCCGATGATTGTCGAGCTTGCGGAAAAGGCAATG
AAAGAGTATGGAGAGGACCTGAAAATCGAAACAAACAAATTTGCAGCAATATGCACCCACTTGGAAGTAT
GCTTCATGTATTCAGATTTTCATTTCATCAATGAGCAAGGCGAATCAATAATAGTAGAGCCTGAGGACCC
AAATGCACTTTTAAAACACAGATTTGAGATAATAGAGGGACGAGATCGTACAATGGCATGGACAGTTGTA
AACAGTATTTGCAACACCACAGGAGCTGAGAAACCGAAGTTTCTGCCAGATCTGTATGATTACAAAGAGA
ATAGATTCATCGAGATTGGAGTGACAAGGAGAGAAGTTCACATATACTATCTGGAAAAGGCCAACAAAAT
TAAATCTGAGAAGACACACATTCACATTTTCTCATTCACTGGCGAAGAAATGGCCACAAAGGCCGATTAT
ACCCTCGATGAAGAAAGCAGGGCTAGGATTAAAACCAGACTATTCACCATAAGACAAGAAATGGCAAGCA
GAGGTCTTTGGGACTCCTTTCGTCAGTCCGAAAGAGGCGAAGAAACAATTGAAGAAAGATTTGAAATCAC
AGGGACAATGCGCAGGCTCGCTGATCAAAGCCTTCCGCCGAACTTCTCCTGCATTGAGAATTTTAGAGCC
TATGTGGATGGATTCGAACCGAACGGCTACATTGAGGGCAAGCTTTCTCAAATGTCCAAAGAAGTAAATG
CTAGAATTGAGCCTTTTTTGAAAACAACACCTCGACCAATTAGACTTCCGAATGGGCCTCCTTGTTTTCA
GCGGTCAAAATTCCTGCTGATGGATTCTTTAAAATTAAGCATTGAGGATCCAAATCATGAAGGGGAGGGA
ATACCACTATATGATGCAATCAAGTGTATGAGAACATTCTTTGGATGGAAAGAACCCACTGTTGTCAAGC
CACACGAGAAGGGAATAAATCCGAATTATCTGCTGTCATGGAAGCAGGTATTGGAAGAGCTGCAGGACAT
TGAGAGTGAGGAGAAGATTCCAAGAACAAAAAACATGAAAAAAACGAGTCAGCTAAAGTGGGCACTTGG
T
GAGAACATGGCACCAGAGAAGGTGGATTTTGATGACTGTAAAGATGTAAGCGATTTGAAGCAATATGATA
GTGACGAACCTGAATTAAGGTCATTTTCAAGTTGGATCCAGAATGAGTTCAACAAGGCATGCGAGCTGAC
CGATTCAATCTGGATAGAGCTCGATGAGATTGGAGAAGATGTGGCCCCGATTGAACACATTGCAAGCATG
AGAAGAAATTACTTCACAGCTGAGGTGTCCCATTGCAGAGCCACTGAATATATAATGAAGGGGGTATACA
TTAATACTGCTTTGCTTAATGCATCCTGTGCAGCAATGGATGATTTCCAACTAATTCCCATGATAAGCAA
ATGTAGAACTAAAGAGGGAAGGAGAAAGACCAATTTGTACGGCTTCATTATAAAAGGAAGATCTCACTTA
AGGAATGACACCGATGTGGTAAACTTTGTGAGCATGGAGTTTTCCCTCACTGACCCAAGACTTGAGCCAC
ACAAATGGGAGAAGTACTGTGTTCTTGAGATAGGAGATATGCTTCTAAGGAGTGCAATAGGCCAAGTGTC
AAGGCCCATGTTCTTGTATGTAAGAACAAATGGAACCTCAAAAATTAAAATGAAATGGGGAATGGAGATG
AGGCGTTGCCTCCTCCAATCCCTCCAACAAATAGAGAGCATGATTGAAGCTGAGTCCTCTGTCAAGGAGA
AAGACATGACAAAAGAGTTTTTTGAGAATAGATCAGAAACATGGCCCATTGGAGAGTCACCAGAAGGAGT
GGAAGAAGGTTCCATTGGGAAAGTATGCAGGACACTATTGGCTAAGTCAGTATTCAATAGTCTGTATGCA
TCTCCACAATTAGAAGGATTTTCAGCTGAGTCAAGAAAGTTGCTCCTCATTGTTCAGGCTCTTAGGGACA
ATCTGGAACCTGGGACCTTTGATCTTGGGGGACTATATGAAGCAATTGAGGAGTGCCTGATTAATGATCC
CTGGGTTTTGCTTAATGCTTCTTGGTTCAACTCCTTCCTAACACATGCATTGAGATAGCTGAGGCAATGC
TACTATTTGTTATCCATACTGTCCAAAA
>gi|117572950|gb|CY017320.1| Influenza A virus (A/Waikato/4/2005(H1N1)) segment 3,

complete sequence
ATTCGAAATGGAAGATTTTGTGCGACAATGCTTCAACCCGATGATTGTCGAGCTTGCGGAAAAGGCAATG
AAAGAGTATGGAGAGGACCTTAAAATCGAAACAAACAAATTTGCAGCAATATGCACCCACTTGGAAGTAT
GCTTCATGTATTCAGATTTTCATTTCATCAATGAGCAAGGCGAATCAATAATAGTAGAGCCTGAGGACCC
AAATGCACTTTTAAAACACAGATTTGAGATAATAGAGGGACGAGATCGTACAATGGCATGGACAGTTGTA
AACAGTATTTGCAACACCACAGGAGCTGAGAAACCAAAGTTTCTGCCAGATCTGTATGATTACAAAGAGA
ATAGGTTCATCGAGATTGGAGTGACAAGGAGAGAAGTTCACATATACTATCTGGAAAAGGCCAACAAAAT
CAAATCTGAGAAGACACACATCCACATTTTCTCATTCACTGGCGAAGAAATGGCCACAAAGGCCGATTAC
ACTCTCGATGAAGAAAGCAGGGCTAGGATTAAAACCAGACTATTCACCATAAGACAAGAAATGGCAAGCA
GAGGTCTTTGGGACTCCTTTCGTCAGTCCGAAAGAGGCGAAGAAACAATTGAAGAAAGATTTGAAATCAC
AGGGACAATGCGCAGGCTCGCTGATCAAAGCCTTCCGCCGAACTTCTCCTGCATTGAGAATTTTAGAGCC
TATGTGGATGGATTTGAACCAAACGGCTACATTGAGGGCAAGCTTTCTCAAATGTCCAAAGAAGTAAATG
CTAGAATTGAGCCCTTTTTGAAAACAACACCTCGACCAATTAGACTTCCGAATGGGCCTCCTTGTTTTCA
GCGGTCAAAATTCCTGCTGATGGATTCTTTAAAATTAAGCATTGAGGATCCAAATCATGAAGGGGAGGGA
ATACCACTATATGATGCAATCAAATGTATGAGAACATTCTTTGGATGGAAAGAACCCACTGTTGTCAAGC
CACACGAGAAGGGAATAAATCCGAATTATCTGCTGTCGTGGAAGCAGGTATTGGAAGAGCTGCAGGACAT
TGAGAGTGAGGAGAAGATTCCAAGAACAAAAAACATGAAAAAAACGAGTCAGCTAAAGTGGGCACTTGG
T
GAGAACATGGCACCAGAGAAGGTGGATTTTGATGACTGTAAAGATATAAGCGATTTAAAGCAATATGATA
GTGACGAACCTGAATTAAGGTCATTTTCAAGTTGGATCCAGAATGAGTTCAACAAGGCATGCGAGCTGAC
CGATTCAATCTGGATAGAGCTCGATGAGATTGGAGAAGATGTGGCCCCGATTGAACACATTGCAAGCATG
AGAAGAAATTACTTCACAGCTGAGGTGTCCCATTGCAGAGCCACTGAATATATAATGAAGGGGGTATACA
TTAATACTGCTTTGCTTAATGCATCCTGTGCAGCAATGGATGATTTCCAACTAATTCCCATGATAAGCAA
ATGTAGAACTAAAGAGGGAAGGAGAAAGACCAATTTGTACGGCTTCATCATAAAAGGAAGATCTCACTTA
AGGAATGACACCGATGTGGTAAACTTTGTGAGCATGGAGTTTTCCCTCACTGACCCAAGACTTGAGCCAC
ACAAATGGGAGAAGTACTGTGTTCTTGAGATAGGAGATATGCTTCTAAGGAGTGCAATAGGCCAAGTGTC
AAGGCCCATGTTCTTGTATGTAAGAACAAATGGAACCTCAAAAATTAAAATGAAATGGGGAATGGAGATG
AGGCGTTGTCTCCTCCAATCCCTCCAACAAATAGAGAGCATGATTGAAGCTGAGTCCTCTGTCAAGGAGA
AAGACATGACAAAAGAGTTTTTTGAGAATAGATCAGAAACATGGCCCATTGGAGAGTCACCAAAAGGAGT
GGAAGAAGGTTCCATTGGAAAAGTATGCAGGACACTACTGGCTAAGTCAGTATTCAATAGTCTGTATGCA
TCTCCACAATTAGAAGGGTTTTCAGCTGAGTCAAGGAAGTTGCTACTCATTGTTCAGGCTCTTAGGGACA
ATCTGGAACCTGGGACCTTTGATCTTGGGGGACTATATGAAGCAATTGAGGAGTGCCTGATTAATGATCC
CTGGGTTTTGCTTAATGCTTCTTGGTTCAACTCCTTCCTAACACATGCATTGAGATAGCTGAGGCAATGC
TACTATTTGTTATCCATAC
>gi|161139456|gb|CY028200.1| Influenza A virus (A/Kentucky/UR06-0007/2006(H1N1)) segment 3, complete sequence
GTACTGATTCAAAATGGAAGATTTTGTGCGACAATGCTTCAATCCGATGATTGTCGAGCTTGCGGAAAAG
GCAATGAAAGAGTATGGAGAGGACCTGAAAATCGAAACAAACAAATTTGCAGCAATATGCACCCACTTGG
AAGTATGCTTCATGTATTCAGATTTTCATTTCATCAATGAGCAAGGCGAATCAATAATAGTAGAGCCTGA
GGACCCAAATGCACTTTTAAAACACAGATTTGAGATAATAGAGGGACGAGATCGTACAATGGCATGGACA
GTTGTAAACAGTATTTGCAACACCACAGGAGCTGAGAAACCGAAGTTTCTGCCAGATCTGTATGATTACA
AAGAGAATAGATTCATCGAGATTGGAGTGACAAGGAGAGAAGTTCACATATACTATCTGGAAAAGGCCAA
CAAAATTAAATCTGAGAAGACACACATTCACATTTTCTCATTCACTGGCGAAGAAATGGCCACAAAGGCA
GATTATACTCTCGATGAAGAAAGCAGGGCTAGGATTAAAACCAGACTATTCACCATAAGACAAGAAATGG

CAAGCAGAGGTCTTTGGGACTCCTTTCGTCAGTCCGAAAGAGGCGAAGAAACAATTGAAGAAAGATTTGA
AATCACAGGGACAATGCGCAGGCTCGCTGATCAAAGCCTTCCGCCGAACTTCTCCTGCATTGAGAATTTT
AGAGCCTATGTGGATGGATTCGAACCGAACGGCTACATTGAGGGCAAGCTTTCTCAAATGTCCAAAGAAG
TAAATGCTAGAATTGAGCCTTTTTTGAAAACAACACCTCGACCAATTAGACTTCCGAATGGGCCTCCTTG
TTTTCAGCGGTCAAAATTCCTGCTGATGGATTCTTTAAAATTAAGCATTGAGGATCCAGATCATGAAGGG
GAGGGAATACCACTATATGATGCAATCAAGTGTATGAGAACATTCTTTGGATGGAAAGAACCCACTGTTG
TCAAGCCACACGAGAAGGGAATAAATCCTAATTATCTGCTGTCATGGAAGCAGGTATTGGAAGAGCTGCA
GGACATTGAGAGTGAGGAGAAGATTCCAAGAACAAAAAACATGAAAAAAACAAGTCAGCTAAAGTGGGC
A
CTTGGTGAGAACATGGCACCAGAGAAGGTGGATTTTGATGACTGTAAAGATACAAGCGATTTGAAGCAAT
ATGATAGTGACGAACCTGAATTAAGGTCGTTTTCAAGTTGGATCCAGAATGAGTTCAACAAGGCATGCGA
GCTGACCGATTCAATCTGGATAGAGCTCGATGAGATTGGAGAAGATGTGGCCCCGATTGAACACATTGCA
AGTATGAGAAGAAATTACTTCACAGCTGAGGTGTCCCATTGCAGAGCCACTGAATATATAATGAAGGGGG
TATACATTAATACTGCTTTGCTTAATGCATCCTGTGCAGCAATGGATGATTTCCAACTAATTCCCATGAT
AAGCAAATGTAGAACTAAAGAGGGAAGGAGAAAGACCAATTTGTACGGCTTCATTATAAAAGGAAGATCT
CACTTAAGGAATGACACCGATGTGGTAAACTTTGTGAGCATGGAGTTTTCCCTCACTGACCCAAGACTTG
AGCCACACAAATGGGAGAAGTACTGTGTTCTTGAAATAGGAGATATGCTTCTAAGGAGTGCAATAGGCCA
AGTGTCAAGGCCCATGTTCTTGTATGTAAGAACAAATGGAACCTCAAAAATTAAAATGAAATGGGGAATG
GAGATGAGGCGTTGCCTCCTCCAATCCCTCCAACAAATAGAGAGCATGATTGAAGCTGAGTCCTCTGTCA
AGGAGAAAGACATGACAAAAGAATTTTTTGAGAATAGATCAGAAACATGGCCCATTGGAGAGTCACCAGA
AGGAGTGGAAGAAGGTTCCATTGGGAAAGTATGCAGGACACTATTGGCTAAGTCAGTATTCAATAGTCTG
TATGCATCTCCACAATTAGAAGGATTTTCAGCTGAGTCAAGAAAGTTGCTCCTCATTGTTCAGGCTCTTA
GGGACAATCTGGAACCTGGGACCTTTGATCTTGGGGGACTATATGAAGCAATTGAGGAGTGCCTGATTAA
TGATCCCTGGGTTTTGCTTAATGCTTCTTGGTTCAACTCCTTCCTAACACATGCATTGAGATAGCTGAGG
CAATGCTACTATTTGTTATCCATACTGTCCAAAAAA
>gi|157281288|gb|CY025226.1| Influenza A virus (A/Michigan/UR06-0015/2006(H1N1)) segment 3, complete sequence
ATTCGAAATGGAAGATTTTGTGCGACAATGCTTCAATCCGATGATTGTCGAGCTTGCGGAAAAGGCAATG
AAAGAGTATGGAGAGGACCTGAAAATCGAAACAAACAAATTTGCAGCAATATGCACCCACTTGGAAGTAT
GCTTCATGTATTCAGATTTTCATTTCATCAATGAGCAAGGCGAATCAATAATAGTAGAGCCTGAGGACCC
AAATGCACTTTTAAAACACAGATTTGAGATAATAGAGGGACGAGATCGTACAATGGCATGGACAGTTGTA
AACAGTATTTGCAACACCACAGGAGCTGAGAAACCGAAGTTTCTGCCAGATCTGTATGATTACAAAGAGA
ATAGATTCATCGAGATTGGAGTGACAAGGAGAGAAGTTCACATATACTATCTGGAAAAGGCCAACAAAAT
TAAATCTGAGAAGACACACATTCACATTTTCTCATTCACTGGCGAAGAAATGGCCACAAAGGCCGATTAT
ACTCTCGATGAAGAAAGCAGGGCTAGGATTAAAACCAGACTATTCACCATAAGACAAGAAATGGCAAGCA
GAGGTCTTTGGGACTCCTTTCGTCAGTCCGAAAGAGGCGAAGAAACAATTGAAGAAAGATTTGAAATCAC
AGGGACAATGCGCAGGCTCGCTGATCAAAGCCTTCCGCCGAACTTCTCCTGCATTGAGAATTTTAGAGCC
TATGTGGATGGATTCGAACCGAACGGCTACATTGAGGGCAAGCTTTCTCAAATGTCCAAAGAAGTAAATG
CTAGAATTGAGCCTTTTTTGAAAACAACACCTCGTCCAATTAGACTTCCGAATGGGCCTCCTTGTTTTCA
GCGGTCAAAATTCCTGCTGATGGATTCTTTAAAATTAAGCATTGAGGATCCAAATCATGAAGGGGAGGGA
ATACCACTATATGATGCAATCAAGTGTATGAGAACATTCTTTGGATGGAAAGAACCCACTGTTGTCAAGC
CACACGAGAAAGGAATAAATCCGAATTATCTGCTGTCATGGAAGCAGGTATTGGAAGAGCTGCAGGACAT
TGAGAGTGAGGAGAAGATTCCAAGAACAAAAAACATGAAAAAAACGAGTCAGCTAAAGTGGGCACTTGG
T

GAGAACATGGCACCAGAGAAGGTGGATTTTGATGACTGTAAAGATGTAAGCGATTTGAAGCAATATGATA
GTGACGAACCTGAATTAAGGTCATTTTCAAGTTGGATCCAGAATGAGTTCAACAAGGCATGCGAGCTAAC
CGATTCAATCTGGATAGAGCTCGATGAGATTGGAGAAGATGTGGCCCCGATTGAACACATTGCAAGCATG
AGAAGAAATTACTTCACAGCTGAGGTGTCCCATTGCAGAGCCACTGAATATATAATGAAGGGGGTATACA
TTAATACTGCTTTGCTTAATGCATCCTGTGCAGCAATGGATGATTTTCAACTAATTCCCATGATAAGCAA
ATGTAGAACTAAAGAGGGAAGGAGAAAGACCAATTTGTACGGCTTCATTATAAAAGGAAGATCTCACTTA
AGGAATGACACCGATGTGGTAAACTTTGTGAGCATGGAGTTTTCCCTCACTGACCCAAGACTTGAGCCAC
ACAAATGGGAGAAGTACTGTGTTCTTGAGATAGGAGATATGCTTCTAAGGAGTGCAATAGGCCAAGTATC
AAGGCCCATGTTCTTGTATGTAAGAACAAATGGAACCTCAAAAATTAAAATGAAATGGGGAATGGAGATG
AGGCGTTGCCTCCTCCAATCCCTCCAACAAATAGAGAGCATGATTGAAGCTGAGTCCTCTGTCAAGGAGA
AAGACATGACAAAAGAGTTTTTTGAAAATAGATCAGAAACATGGCCCATTGGAGAGTCACCAAAAGGAGT
GGAAGAAGGTTCCATTGGGAAAGTATGCAGGACACTATTGGCTAAGTCAGTATTCAATAGCATGTATGCA
TCTCCACAATTAGAAGGATTTTCAGCTGAGTCAAGAAAGTTGCTCCTCATTGTTCAGGCTCTTAGGGACA
ATCTGGAACCTGGGACCTTTGATCTTGGGGGACTATATGAAGCAATTGAGGAGTGCCTGATTAATGATCC
CTGGGTTTTGCTTAATGCTTCTTGGTTCAACTCCTTCATAACACATGCATTGAGATAGCTGAGGCAATGC
TACTATTTGTTATCCATACTGTCCAAAAAA
>gi|218875180|gb|CY036924.1| Influenza A virus (A/NYMC X-163A(NYMC X-157-St. Petersburg/8/2006)(H1N1)) segment 3, complete sequence
ATCCAAAATGGAAGATTTTGTGCGACAATGCTTCAATCCGATGATTGTCGAGCTTGCGGAAAAAACAATG
AAAGAGTATGGGGAGGACCTGAAAATCGAAACAAACAAATTTGCAGCAATATGCACTCACTTGGAAGTAT
GCTTCATGTATTCAGATTTTCACTTCATCAATGAGCAAGGCGAGTCAATAATCGTAGAACTTGGTGATCC
AAATGCACTTTTGAAGCACAGATTTGAAATAATCGAGGGAAGAGATCGCACAATGGCCTGGACAGTAGTA
AACAGTATTTGCAACACTACAGGGGCTGAGAAACCAAAGTTTCTACCAGATTTGTATGATTACAAGGAGA
ATAGATTCATCGAAATTGGAGTAACAAGGAGAGAAGTTCACATATACTATCTGGAAAAGGCCAATAAAAT
TAAATCTGAGAAAACACACATCCACATTTTCTCGTTCACTGGGGAAGAAATGGCCACAAAGGCAGACTAC
ACTCTCGATGAAGAAAGCAGGGCTAGGATCAAAACCAGACTATTCACCATAAGACAAGAAATGGCCAGCA
GAGGCCTCTGGGATTCCTTTCGTCAGTCCGAGAGAGGAGAAGAGACAATTGAAGAAAGGTTTGAAATCAC
AGGAACAATGCGCAAGCTTGCCGACCAAAGTCTCCCGCCGAACTTCTCCAGCCTTGAAAATTTTAGAGCC
TATGTGGATGGATTCGAACCGAACGGCTACATTGAGGGCAAGCTGTCTCAAATGTCCAAAGAAGTAAATG
CTAGAATTGAACCTTTTTTGAAAACAACACCACGACCACTTAGACTTCCGAATGGGCCTCCCTGTTCTCA
GCGGTCCAAATTCCTGCTGATGGATGCCTTAAAATTAAGCATTGAGGACCCAAGTCATGAAGGAGAGGGA
ATACCGCTATATGATGCAATCAAATGCATGAGAACATTCTTTGGATGGAAGGAACCCAATGTTGTTAAAC
CACACGAAAAGGGAATAAATCCAAATTATCTTCTGTCATGGAAGCAAGTACTGGCAGAACTGCAGGACAT
TGAGAATGAGGAGAAAATTCCAAAGACTAAAAATATGAAGAAAACAAGTCAGCTAAAGTGGGCACTTGGT
GAGAACATGGCACCAGAAAAGGTAGACTTTGACGACTGTAAAGATGTAGGTGATTTGAAGCAATATGATA
GTGATGAACCAGAATTGAGGTCGCTTGCAAGTTGGATTCAGAATGAGTTTAACAAGGCATGCGAACTGAC
AGATTCAAGCTGGATAGAGCTCGATGAGATTGGAGAAGATGTGGCTCCAATTGAACACATTGCAAGCATG
AGAAGGAATTATTTCACATCAGAGGTGTCTCACTGCAGAGCCACAGAATACATAATGAAGGGAGTGTACA
TCAATACTGCCTTGCTTAATGCATCTTGTGCAGCAATGGATGATTTCCAATTAATTCCAATGATAAGCAA
GTGTAGAACTAAGGAGGGAAGGCGAAAGACCAACTTGTATGGTTTCATCATAAAAGGAAGATCCCACTTA
AGGAATGACACCGACGTGGTAAACTTTGTGAGCATGGAGTTTTCTCTCACTGACCCAAGACTTGAACCAC
ATAAATGGGAGAAGTACTGTGTTCTTGAGATAGGAGATATGCTTATAAGAAGTGCCATAGGCCAGGTTTC
AAGGCCCATGTTCTTGTATGTGAGAACAAATGGAACCTCAAAAATTAAAATGAAATGGGGAATGGAGATG
AGGCGTTGCCTCCTCCAGTCACTTCAACAAATTGAGAGTATGATTGAAGCTGAGTCCTCTGTCAAAGAGA

AAGACATGACCAAAGAGTTCTTTGAGAACAAATCAGAAACATGGCCCATTGGAGAGTCCCCCAAAGGAGT
GGAGGAAAGTTCCATTGGGAAGGTCTGCAGGACTTTATTAGCAAAGTCGGTATTCAACAGCTTGTATGCA
TCTCCACAACTAGAAGGATTTTCAGCTGAATCAAGAAAACTGCTTCTTATCGTTCAGGCTCTTAGGGACA
ACCTGGAACCTGGGACCTTTGATCTTGGGGGGCTATATGAAGCAATTGAGGAGTGCCTGATTAATGATCC
CTGGGTTTTGCTTAATGCTTCTTGGTTCAACTCCTTCCTTACACATGCATTGAGTTAGTTGTGGCAGTGC
TACTATTTGCTATCCATACTGTCCAAAAAA
>gi|208344095|gb|CY035131.1| Influenza A virus (A/St. Petersburg/8/2006(H1N1)) segment 3, complete sequence
ATTCGAAATGGAAGATTTTGTGCGACAATGCTTCAATCCGATGATTGTCGAGCTTGCGGAAAAGGCAATG
AAAGAGTATGGAGAGGACCTGAAAATCGAAACAAACAAATTTGCAGCAATATGCACCCACTTGGAAGTGT
GCTTCATGTATTCAGACTTTCATTTCATCAATGAGCAAGGCGAATCAATAATAGTAGAACCTGAGGATCC
AAATGCACTTTTAAAGCACAGATTTGAGATAATAGAGGGACGAGATCGTACAATGGCATGGACAGTTGTA
AACAGTATTTGCAACACCACAGGAGCTGAGAAACCAAAGTTTCTGCCAGATCTGTATGATTACAAAGAGA
ATAGATTCATCGAGATTGGAGTGACAAGGAGGGAAGTTCACATATACTATCTGGAAAAGGCCAACAAAAT
TAAATCTGAGAAGACACACATTCACATTTTCTCATTCACTGGCGAAGAAATGGCCACAAAGGCCGATTAC
ACTCTTGATGAAGAAAGCAGGGCTAGGATTAAAACCAGACTATTCACCATAAGACAAGAAATGGCAAGTA
GAGGTCTTTGGGACTCCTTTCGTCAGTCCGAAAGAGGCGAAGAAACAATTGAAGAAAGATTTGAAATCAC
AGGGACAATGCGCAGGCTCGCTGACCAAAGCCTCCCGCCGAACTTCTCCTGCATTGAGAATTTTAGAGCC
TATGTGGATGGATTTGAACCGAACGGCTACATTGAGGGCAAGCTTTCTCAAATGTCCAAAGAAGTAAATG
CTAGAATTGAGCCTTTTTTGAAAACAACACCTCGACCAATTAGACTTCCGAATGGGCCTCCTTGTTTTCA
GCGGTCAAAATTCCTGCTGATGGATTCTTTAAAATTAAGCATTGAGGATCCAAATCATGAAGGTGAGGGA
ATACCACTATATGATGCAATCAAGTGTATGAAAACATTCTTTGGATGGAAAGAACCCACTGTTGTCAAGC
CACACGAGAAGGGAATAAATCCGAATTATCTGCTGTCGTGGAAGCAAGTATTGGAAGAGCTGCAGGACAT
TGAGAGTGAGGAGAAGATTCCAAGAACAAAAAATATGAAAAAAACGAGTCAGCTAAAGTGGGCACTTGGT
GAGAACATGGCACCAGAGAAAGTGGATTTTGATGACTGTAAAGATATAAGCGATTTGAAGCAATATGACA
GTGACGAACCTGAATTAAGGTCATTTTCAAGTTGGATCCAGAATGAGTTCAACAAGGCATGCGAGCTGAC
CGATTCAATCTGGATAGAGCTTGATGAGATTGGAGAAGATGTGGCCCCGATTGAACACATTGCAAGCATG
AGAAGGAATTACTTCACAGCTGAGGTGTCCCATTGCAGAGCCACAGAATATATAATGAAAGGGGTATACA
TTAATACTGCTTTGCTCAATGCATCCTGTGCAGCAATGGATGATTTCCAACTAATTCCCATGATAAGCAA
ATGTAGAACTAAAGAGGGAAGGAGAAAGACAAATTTGTACGGCTTCATCGTAAAAGGAAGATCTCACTTA
AGGAATGACACCGATGTGGTAAACTTTGTGAGCATGGAGTTTTCCCTCACTGACCCAAGACTTGAGCCAC
ACAAATGGGAGAAGTACTGTGTTCTTGAGATAGGGGATATGCTTCTAAGGAGTGCAATAGGCCAAGTGTC
AAGGCCCATGTTCTTGTATGTAAGGACAAATGGAACCTCAAAAATTAAAATGAAATGGGGAATGGAGATG
AGGCGTTGCCTCCTCCAATCCCTCCAACAAATAGAGAGCATGATTGAAGCTGAGTCCTCTGTCAAAGAGA
AAGACATGACAAAAGAGTTTTTTGAGAATAGATCAGAAACATGGCCCATTGGAGAATCACCAAAAGGAGT
GGAAGAAGGTTCCATTGGGAAAGTATGCAGGACACTATTGGCTAAGTCAGTATTCAATAGTCTGTATGCA
TCTCCACAATTAGAAGGATTTTCAGCTGAGTCAAGGAAGTTGCTCCTCATTGTTCAGGCTCTTAGGGACA
ATCTGGAACCTGGGACCTTTGATCTTGGGGGGCTATATGAAGCAATTGAGGAGTGCCTGATTAATGATCC
CTGGGTTTTGCTTAATGCTTCTTGGTTCAACTCCTTCCTAACACATGCATTGAGATAGCTGGGGCAATGC
TACTATTTGTTATCCATACTGTCCAAAAAAA
>gi|226954758|gb|CY038884.1| Influenza A virus (A/Taiwan/2645/2006(H1N1)) segment 3, complete sequence
GGAGATTTTGTGCGACAATGCTTCAATCCGATGATTGTCGAGCTTGCGGAAAAGGCAATGAAAGAGTATG
GAGAGGACCTGAAAATCGAAACAAACAAATTTGCAGCAATATGCACCCACTTGGAAGTGTGCTTCATGTA

TTCAGATTTTCATTTCATCAATGAGCAAGGCGAATCAATAATAGTAGAACCTGAGGACCCAAATGCACTT
TTAAAGCACAGATTTGAGATAATAGAGGGGCGAGATCGTACAATGGCATGGACAGTTGTAAACAGTATTT
GCAACACCACAGGAGCTGAGAAACCAAAGTTTCTGCCAGATCTGTATGATTACAAAGAGAATAGATTCAT
CGAGATTGGAGTGACAAGGAGGGAAGTTCACATATACTATCTGGAAAAAGCCAACAAAATTAAATCTGAG
AAGACACACATTCACATTTTCTCATTCACCGGCGAAGAAATGGCTACAAAGGCCGATTACACTCTTGATG
AAGAAAGCAGAGCTAGGATTAAAACCAGACTATTCACCATAAGACAAGAAATGGCAAGCAGAGGTCTTTG
GGACTCCTTTCGTCAGTCCGAAAGAGGCGAAGAAACAATTGAAGAAAGATTTGAAATCACAGGGACAATG
CGCAGGCTCGCTGATCAAAGCCTCCCGCCGAACTTCTCCTGCATTGAGAATTTTAGAGCCTATGTGGATG
GATTTGAACCGAACGGCTACATTGAGGGCAAGCTTTCTCAAATGTCCAAAGAAGTAAATGCTAGAATTGA
GCCTTTTTTGAAAACAACACCTCGGCCAATTAGACTTCCGAATGGGCCTCCTTGTTTTCAGCGGTCAAAA
TTCCTGCTGATGGATTCTTTAAAATTAAGCATTGAGGATCCAAATCATGAAGGTGAAGGAATACCACTAT
ATGATGCAATCAAATGTATGAGAACATTTTTTGGATGGAAAGAACCCACTGTTGTCAAGCCACACGAGAA
GGGAATAAATCCGAATTATCTGCTGTCGTGGAAGCAAGTATTGGAAGAACTGCAGGACATTGAGAGTGAG
GAGAAGATTCCAAGAACAAAAAACATGAAGAAAACGAGTCAGCTAAAGTGGGCACTTGGTGAGAACATGG
CACCAGAGAAGGTGGATTTTGATGACTGTAAAGATATAAGCGATTTGAAGCAATATGACAGTGACGAACC
TGAATTAAGGTCATTTTCAAGTTGGATCCAAAATGAGTTCAACAAGGCATGCGAGCTGACCGATTCAATC
TGGATAGAGCTTGATGAGATTGGAGAAGATGTGGCCCCGATTGAACACATTGCAAGCATGAGAAGGAATT
ACTTCACAGCTGAGGTGTCCCATTGCAGAGCCACGGAATATATAATGAAGGGAGTATACATTAATACTGC
TTTGCTCAATGCATCCTGTGCAGCAATGGATGATTTCCAACTAATTCCCATGATAAGCAAATGTAGAACT
AAAGAGGGAAGGAGAAAGACCAATTTGTACGGCTTCATCGTAAAAGGAAGATCTCACTTGAGGAATGACA
CCGATGTGGTAAACTTTGTGAGCATGGAGTTTTCCCTCACTGACCCAAGACTTGAGCCACACAAATGGGA
GAAGTACTGTGTTCTTGAGATAGGAGATATGATTCTAAGGAGTGCAATAGGCCAAGTGTCAAGGCCCATG
TTCTTGTATGTAAGGACAAATGGAACCTCAAAAATTAAAATGAAATGGGGAATGGAGATGAGGCGTTGCC
TCCTCCAATCCCTCCAACAAATAGAGAGCATGATTGAAGCTGAGTCCTCTGTCAAAGAGAAAGACATGAC
AAAAGAGTTTTTTGAGAATAGATCAGAAACATGGCCCATTGGAGAGTCACCAAAAGGAGTGGAAGAAGGT
TCCATTGGGAAAGTATGCAGGACACTATTGGCTAAGTCAGTATTCAATAGTCTGTACGCATCTCCACAAT
TAGAAGGATTTTCAGCTGAGTCAAGGAAGTTGCTCCTCATTGTTCAGGCTCTTAGGGACAATCTGGAACC
TGGGACCTTTGATCTTGGGGGGCTATATGAAGCAATTGAGGAGTGCCTGATTAATGATCCCTGGGTTTTG
CTTAATGCTTCTTGGTTCAACTCCTTCCTAACACATGCATTGAGATAGCTGAGGCAATGCTACTATTTGT
TATCCATACTGTCCAAAAA
>gi|256385523|gb|CY044354.1| Influenza A virus (A/South Korea/AF10/2008(H1N1)) segment 3, complete sequence
ATGGAAGATTTTGTGCGACAATGCTTCAATCCGATGATTGTCGAGCTTGCGGAAAAGGCAATGAAAGAGT
ATGGAGAGGACCTGAAAATCGAAACAAACAAATTTGCAGCAATATGCACCCACTTGGAAGTGTGCTTCAT
GTATTCAGATTTCCATTTCATCAATGAGCAAGGCGAATCAATAATAGTAGAACCTGAGGACCCAAATGCA
CTTTTAAAGCACAGATTTGAGATAATAGAGGGACGAGATCGTACAATGGCATGGACAGTTGTAAACAGTA
TTTGCAACACCACAGGAGCTGAGAAACCAAAGTTTTTGCCAGATCTGTATGATTACAAAGAGAATAGATT
TATCGAGATTGGAGTGACAAGGAGGGAAGTTCACCTATACTATCTGGAAAAGGCCAACAAAATTAAATCT
GAGAAGACACACATTCACATTTTCTCATTCACTGGCGAAGAAATGGCCACAAAGGCCGATTACACTCTTG
ATGAAGAAAGCAGGGCTAGAATTAAAACCAGACTATTCACCATAAGACAAGAAATGGCAAGTAGAGGTCT
TTGGGACTCCTTTCGTCAGTCCGAAAGAGGCGAAGAAACAATTGAAGAGAGATTTGAAATCACAGGGACA
ATGCGCAGGCTCGCTGACCAAAGCCTCCCGCCGAACTTCTCCTGCATTGAGAATTTTAGAGCCTATGTGG
ATGGATTTGAACCGAACGGCTACATTGAGGGCAAGCTTTCTCAAATGTCCAAAGAAGTAAATGCTAGAAT
TGAGCCTTTTTTGAAAACAACACCTCGACCAATTAGACTTCCGAATGGGCCTCCTTGCTTTCAGCGGTCA

AAATTCCTACTGATGGATTCTTTAAAGTTAAGCATTGAGGATCCAAATCATGAAGGTGAGGGAATACCAC
TATATGATGCAATCAAGTGTATGAAAACATTCTTTGGATGGAAAGAACCCACTGTTGTCAAGCCACACGA
GAAGGGAATAAATCCGAATTATCTGCTGTCGTGGAAGCAAGTATTAGAAGAGCTGCAGGACATTGAGAGT
GAGGAGAAGATTCCAAGAACAAAAAACATGAAAAAAACGAGTCAGCTAAAGTGGGCACTTGGTGAGAAC
A
TGGCACCAGAGAAAGTGGATTTTGATGACTGTAAAGATGTAGGCGATTTGAAGCAATATGACAGTGACGA
ACCTGAATTAAGGTCATTTTCAAGTTGGATCCAGAATGAGTTCAACAAGGCATGCGAGCTGACCGATTCA
ATCTGGATAGAGCTTGATGAGATTGGAGAAGATGTGGCCCCGATTGAACACATTGCAAGCATGAGAAGGA
ATTACTTCACAGCTGAGGTGTCCCATTGCAGAGCCACAGAATATATAATGAAGGGGGTATACATTAATAC
TGCTTTGCTCAATGCATCCTGTGCTGCAATGGATGATTTCCAACTAATTCCCATGATAAGCAAATGTAGA
ACTAAAGAGGGAAGGAGAAATACCAATTTGTACGGCTTCATCGTAAAAGGAAGATCTCACTTAAGGAATG
ACACCGATGTGGTAAACTTTGTTAGCATGGAGTTTTCCCTCACTGACCCAAGACTTGAGCCACACAAATG
GGAGAAGTACTGTGTTCTTGAGATAGGGGATATGCTTCTAAGGAGCGCAATAGGCCAAGTGTCAAGGCCC
ATGTTCTTGTACGTAAGGACAAATGGAACCTCAAAAATTAAAATGAAATGGGGAATGGAGATGAGGCGTT
GCCTCCTCCAATCCCTCCAACAAATAGAGAGCATGATTGAAGCTGAGTCCTCTGTCAAAGAGAAAGACAT
GACAAAAGAGTTTTTTGAGAATAGATCAGAAACATGGCCCATTGGAGAATCACCAAAAGGAGTGGAAGAA
GGTTCCATTGGGAAAATATGCAGGACACTATTAGCTAAGTCAGTATTCAATAGTCTGTATGCATCTCCAC
AGTTAGAAGGATTTTCAGCTGAGTCAAGGAAGTTGCTCCTCATTGTTCAGGCTCTTAGGGACAATCTGGA
ACCTGGGACCTTTGATCTTGAGGGGCTATATGAAGCAATTGAGGAGTGCCTGATTAATGATCCCTGGGTT
TTGCTTAATGCTTCTTGGTTCAACTCCTTCCTAACACATGCATTGAGATAGCTGGGGCAATGCTACTATT
TGTTAT
>gi|163964728|gb|CY028464.1| Influenza A virus (A/California/UR06-0442/2007(H1N1)) segment 3, complete sequence
CTGATTCGAAATGGAAGATTTTGTGCGACAATGCTTCAATCCGATGATTGTCGAGCTTGCGGAAAAGGCA
ATGAAAGAGTATGGAGAGGACCTGAAAATCGAAACAAACAAATTTGCAGCAATATGCACCCACTTGGAAG
TATGCTTCATGTATTCAGATTTTCATTTCATCAATGAGCAAGGCGAATCAATAATAGTAGAGCCTGAGGA
CCCAAATGCACTTTTAAAACACAGATTTGAGATAATAGAGGGACGAGATCGTACAATGGCATGGACAGTT
GTAAACAGTATTTGCAACACCACAGGAGCTGAGAAACCGAAGTTTCTGCCAGATCTGTATGATTACAAAG
AGAATAGATTCATCGAGATTGGAGTGACAAGGAGAGAAGTTCACATATACTATCTGGAAAAGGCCAACAA
AATTAAATCTGAGAAGACACACATTCACATTTTCTCATTCACTGGCGAAGAAATGGCCACAAAGGCCGAT
TATACTCTCGATGAAGAAAGCAGGGCTAGGATTAAAACCAGACTATTCACCATAAGACAAGAAATGGCAA
GCAGAGGTCTTTGGGACTCCTTTCGTCAGTCCGAAAGAGGCGAAGAAACAATTGAAGAAAGATTTGAAAT
CACAGGGACAATGCGCAGGCTCGCTGATCAAAGCCTTCCGCCGAACTTCTCCTGCATTGAGAATTTTAGA
GCCTATGTGGATGGATTCGAACCGAACGGCTACATTGAGGGCAAGCTTTCTCAAATGTCCAAAGAAGTAA
ATGCTAGAATTGAGCCTTTTTTGAAAACAACACCTCGTCCAATCAGACTTCCGAATGGGCCTCCTTGTTT
TCAGCGGTCAAAATTCCTGCTGATGGATTCTTTAAAATTAAGCATTGAGGATCCAAATCATGAAGGGGAG
GGAATACCACTATATGATGCAATCAAATGTATGAGAACATTCTTTGGATGGAAAGAACCCACTGTTGTCA
AGCCACACGAGAAAGGAATAAATCCGAATTATCTGCTGTCATGGAAGCAGGTATTGGAAGAGCTGCAGGA
CATTGAGAGTGAGGAGAAGATTCCAAGAACAAAAAACATGAAAAAAACGAGTCAGCTAAAGTGGGCACTT
GGTGAGAACATGGCACCAGAGAAGGTGGATTTTGATGACTGTAAAGATGTAAGCGATTTGAAGCAATATG
ATAGTGACGAACCTGAATTAAGGTCATTTTCAAGTTGGATCCAGAATGAGTTCAACAAGGCATGCGAGCT
GACCGATTCAATCTGGATAGAGCTCGATGAGATTGGAGAAGATGTGGCCCCGATTGAACACATTGCAAGC
ATGAGAAGAAATTACTTCACAGCTGAGGTGTCCCATTGCAGAGCCACTGAATATATAATGAAGGGGGTAT
ACATTAATACTGCTTTGCTTAATGCATCCTGTGCAGCAATGGATGATTTTCAACTAATTCCCATGATAAG

CAAATGTAGAACTAAAGAGGGAAGGAGAAAGACCAATTTGTACGGCTTCATTATAAAAGGAAGATCTCAC
TTAAGGAATGACACCGATGTGGTAAACTTCGTGAGCATGGAGTTTTCCCTCACTGACCCAAGACTTGAGC
CACACAAATGGGAGAAGTACTGTGTTCTTGAGATAGGAGATATGCTTCTAAGGAGTGCAATAGGCCAAGT
ATCAAGGCCCATGTTCTTGTATGTAAGAACAAATGGAACCTCAAAAATTAAAATGAAATGGGGAATGGAG
ATGAGGCGTTGCCTCCTCCAATCCCTCCAACAAATAGAGAGCATGATTGAAGCTGAGTCCTCTGTCAAGG
AGAAAGACATGACAAAAGAGTTTTTTGAAAATAGATCAGAAACATGGCCCATTGGAGAGTCACCAAAAGG
AGTGGAAGAAGGTTCCATTGGGAAAGTATGCAGGACACTATTGGCTAAGTCAGTATTCAATAGCCTGTAT
GCATCTCCACAATTAGAAGGATTTTCAGCTGAGTCAAGAAAGTTGCTCCTCATTGTTCAGGCTCTTAGGG
ACAATCTGGAACCTGGGACCTTTGATCTTGGGGGACTATATGAAGCAATTGAGGAGTGCCTGATTAATGA
TCCCTGGGTTTTGCTTAATGCTTCTTGGTTCAACTCCTTCCTAACACATGCATTGAGATAGCTGAGGCAA
TGCTACTATTTGTTATCCATACTGTCCAAAAAA
>gi|157281612|gb|CY025362.1| Influenza A virus (A/Kentucky/UR06-0363/2007(H1N1)) segment 3, complete sequence
CTGATTCGAAATGGAAGATTTTGTGCGACAATGCTTCAATCCGATGATTGTCGAGCTTGCGGAAAAGGCA
ATGAAAGAGTATGGAGAGGACCTGAAAATCGAAACAAACAAATTTGCAGCAATATGCACCCACTTGGAAG
TATGCTTCATGTATTCAGATTTTCATTTCATCAATGAGCAAGGCGAATCAATAATAGTAGAGCCTGAGGA
CCCAAATGCACTTTTAAAACACAGATTTGAGATAATAGAGGGACGAGATCGTACAATGGCATGGACAGTT
GTAAACAGTATTTGCAACACCACAGGAGCTGAGAAACCGAAGTTTCTGCCAGATCTGTATGATTACAAAG
AGAATAGATTCATCGAGATTGGAGTGACAAGGAGAGAAGTTCACATATACTATCTGGAAAAGGCCAACAA
AATTAAATCTGAGAAGACACACATTCACATTTTCTCATTCACTGGCGAAGAAATGGCCACAAAGGCCGAT
TATACTCTCGATGAAGAAAGCAGGGCTAGGATTAAAACCAGACTATTCACCATAAGACAAGAAATGGCAA
GCAGAGGTCTTTGGGACTCCTTTCGTCAGTCCGAAAGAGGCGAAGAAACAATTGAAGAAAGGTTTGAAAT
CACAGGGACAATGCGCAGGCTCGCTGATCAAAGCCTTCCGCCGAACTTCTCCTGCATTGAGAATTTTAGA
GCCTATGTGGATGGATTCGAACCGAACGGCTACATTGAGGGCAAGCTTTCTCAAATGTCCAAAGAAGTAA
ATGCTAGAATTGAGCCTTTTTTGAAAACAACACCTCGACCAATTAGACTTCCGAATGGGCCTCCTTGTTT
TCAGCGGTCAAAATTCCTGCTGATGGATTCTTTAAAATTAAGCATTGAGGATCCAAATCATGAAGGGGAG
GGAATACCACTATATGATGCAATCAAGTGTATGAGAACATTCTTTGGATGGAAAGAACCCACTGTTGTCA
AGCCACACGAGAAAGGAATAAATCCGAATTATCTGCTGTCATGGAAGCAGGTATTGGAAGAGCTGCAGGA
CATTGAGAGTGAGGAGAAGATTCCAAGAACAAAAAACATGAAAAAAACGAGTCAGCTAAAGTGGGCACTT
GGTGAGAACATGGCACCAGAGAAGGTGGATTTTGATGACTGTAAAGATGTAAGCGATTTGAAGCAATATG
ATAGTGACGAACCTGAATTAAGGTCATTTTCAAGTTGGATCCAGAATGAGTTCAACAAGGCATGCGAGCT
GACCGATTCAATCTGGATAGAGCTCGATGAGATTGGAGAAGATGTGGCCCCGATTGAACACATTGCAAGC
ATGAGAAGAAATTACTTCACAGCTGAGGTGTCCCATTGCAGAGCCACTGAATATATAATGAAGGGGGTAT
ACATTAATACTGCTTTGCTTAATGCATCCTGTGCAGCAATGGATGATTTTCAACTAATTCCCATGATAAG
CAAATGTAGAACTAAAGAGGGAAGGAGAAAGACCAATTTGTACGGCTTCATTATAAAAGGAAGATCTCAC
TTAAGGAATGACACCGATGTGGTAAACTTTGTGAGCATGGAGTTTTCCCTCACTGACCCAAGACTTGAGC
CACACAAATGGGAGAGATACTGTGTTCTTGAGATAGGAGATATGCTTCTAAGGAGTGCAATAGGCCAAGT
GTCAAGGCCTATGTTCTTGTATGTAAGAACAAATGGAACCTCAAAAATTAAAATGAAATGGGGAATGGAG
ATGAGGCGTTGCCTCCTCCAATCCCTCCAACAAATAGAGAGCATGATTGAAGCTGAGTCCTCTGTCAAGG
AGAAAGACATGACAAAAGAGTTTTTTGAGAATAGATCAGAAACATGGCCCATTGGAGAGTCACCAGAAGG
AGTGGAAGAAGGTTCCATTGGGAAAGTATGCAGGACACTACTGGCTAAGTCAGTATTCAATAGTCTGTAT
GCATCTCCACAATTAGAAGGATTTTCAGCTGAGTCAAGAAAGTTGCTCCTCATTGTTCAGGCTCTTAGGG
ACAATCTGGAACCTGGGACCTTTGATCTTGGGGGACTATATGAAGCAATTGAGGAGTGCCTGATTAATGA
TCCCTGGGTTTTGCTTAATGCTTCTTGGTTCAACTCCTTCCTAACACATGCATTGAGATAGCTGAGGCAA

TGCTACTATTTGTTATCCATACTGTCCAAAAA
>gi|158957801|gb|CY027416.1| Influenza A virus (A/Alabama/UR06-0536/2007(H1N1)) segment 3, complete sequence
ATGGAAGATTTTGTACGACAATGCTTCAATCCGATGATTGTCGAGCTTGCGGAAAAGGCAATGAAAGAGT
ATGGAGAGGACCTGAAAATCGAAACAAACAAATTTGCAGCAATATGCACCCACTTGGAAGTATGCTTCAT
GTATTCAGATTTTCATTTCATCAATGAGCAAGGCGAATCAATAATAGTAGAGCCTGAGGACCCAAATGCA
CTTTTAAAACACAGATTTGAGATAATAGAGGGACGAGATCGTACAATGGCATGGACAGTTGTAAACAGTA
TTTGCAACACCACAAGAGCTGAGAAACCGAAGTTTCTGCCAGATCTGTATGATTACAAAGAGAATAGATT
CATCGAGATTGGAGTGACAAGGAGAGAAGTTCACATATACTATCTGGAAAAGGCCAACAAAATTAAATCT
GAGAAGACACACATTCACATTTTCTCATTCACTGGCGAAGAAATGGCCACAAAGGCAGATTATACTCTCG
ATGAAGAAAGCAGGGCTAGGATTAAAACCAGACTATTCACCATAAGACAAGAAATGGCAAGCAGAGGTCT
TTGGGACTCCTTTCGTCAGTCCGAAAGAGGCGAAGAAACAATTGAAGAAAGATTTGAAATCACAGGGACA
ATGCGCAGGCTCGCTGATCAAAGCCTTCCGCCGAACTTCTCCTGCATTGAGAATTTTAGAGCCTATGTGG
ATGGATTCGAACCGAACGGCTACATTGAGGGCAAGCTTTCTCAAATGTCCAAAGAAGTAAATGCTAGAAT
TGAGCCTTTTTTGAAAACAACACCTCGACCAATTAGACTTCCGAATGGGCCTCCTTGTTTTCAGCGGTCA
AAATTCCTGCTGATGGATTCTTTGAAATTAAGCATTGAGGATCCAGATCATGAAGGGGAGGGAATACCAC
TATATGATGCAATCAAGTGTATGAGAACATTCTTTGGATGGAAAGAACCCACTGTTGTCAAGCCACACGA
GAAGGGAATAAATCCTAATTATCTGCTTTCATGGAAGCAGGTATTGGAAGAGCTGCAGGACATTGAGAGT
GAGGAGAAGATTCCAAGAACAAAAAACATGAAAAAAACAAGTCAGCTAAAGTGGGCACTTGGTGAAAAC
A
TGGCACCAGAGAAGGTGGATTTTGATGACTGTAAAGATACAAGCGATTTGAAGCAATATGATAGTGACGA
ACCTGAATTAAGGTCGTTTTCAAGTTGGATCCAGAATGAGTTCAACAAGGCATGCGAGCTGACCGATTCA
ATCTGGATAGAGCTCGATGAGATTGGAGAAGATGTGGCCCCGATTGAACACATTGCAAGTATGAGAAGAA
ATTACTTCACAGCTGAGGTGTCCCATTGCAGAGCCACTGAATATATAATGAAGGGGGTATACATTAATAC
TGCTTTGCTTAATGCATCCTGTGCAGCAATGGATGATTTCCAACTAATTCCCATGATAAGCAAATGTAGA
ACTAAAGAGGGAAGGAGAAAGACCAATTTGTACGGCTTCATTATAAAAGGAAGATCTCACTTAAGGAATG
ACACCGATGTGGTAAACTTTGTGAGCATGGAGTTTTCCCTCACTGACCCAAGACTTGAGCCACACAAATG
GGAGAAGTACTGTGTTCTTGAGATAGGAGATATGCTTCTAAGGAGTGCAATAGGCCAAGTGTCAAGGCCC
ATGTTCTTGTATGTAAGAACAAATGGAACCTCAAAAATTAAAATGAAATGGGGAATGGAGATGAGGCGTT
GCCTCCTCCAATCCCTCCAACAAATAGAGAGCATGATTGAAGCTGAGTCCTCTGTCAAGGAGAAAGACAT
GACAAAAGAATTTTTTGAGAATAGATCAGAAACATGGCCCATTGGAGAGTCACCAGAAGGAGTGGAAGAA
GGTTCCATTGGGAAAGTATGCAGGACACTATTGGCTAAGTCAGTATTCAATAGTCTGTATGCATCTCCAC
AATTAGAAGGATTTTCAGCTGAGTCAAGAAAGTTGCTCCTCATTGTTCAGGCTCTTAGGGACAATCTGGA
ACCTGGGACCTTTGATCTTGGGGGACTATATGAAGCAATTGAGGAGTGCCTGATTAATGATCCCTGGGTT
TTGCTTAATGCTTCTTGGTTCAACTCCTTCCTAACACATGCATTGAGATAGCTGAGGCAATGCTACTATT
TGTTATCCATACTGTCCAAAA
>gi|237688836|gb|CY040063.1| Influenza A virus (A/Taiwan/71720/2007(H1N1)) segment 3, complete sequence
GTACTGATTCAAAATGGAAGATTTTGTACGACAATGCTTCAATCCGATGATTGTCGAGCTTGCAGAAAAA
GCGATGAAAGAGTATGGAGAGGACCTGAAAATCGAAACAAACAAATTTGCAGCAATATGCACCCACTTGG
AAGTGTGCTTCATGTATTCAGATTTTCATTTCATCAATGAGCAAGGCGAATCAATAATAGTAGAGCCTGA
GGACCCAAATGCACTTTTAAAGCACAGATTTGAGATAATAGAGGGACGAGATCGTACAATGGCATGGACA
GTTGTAAACAGTATTTGCAACACCACAGGAGCTGAGAAACCAAAGTTTCTGCCAGATCTGTATGATTACA
AAGAGAATAGATTCATCGAGATTGGAGTGACAAGGAGGGAAGTTCACATATACTATCTGGAAAAGGCCAA

CAAAATTAAATCTGAGAAGACACACATTCACATTTTCTCATTCACCGGCGAAGAAATGGCCACAAAGGCC
GATTACACTCTCGATGAAGAAAGCAGAGCTAGGATTAAAACCAGATTGTTCAYCATAAGACAAGAAATGG
CAAGCAGAGGTCTTTGGGACTCCTTTCGTCAGTCCGAAAGAGGCGAAGAAACAATTGAAGAAAGATTTGA
AATCACAGGGACAATGCGCAGGCTCGCTGACCAAAGCCTTCCGCCGAACTTCTCCTGCATTGAGAATTTT
AGAGCCTATGTGGATGGATTTGAACCGAACGGCTACATTGAGGGCAAGCTTTCTCAAATGTCCAAAGAAG
TAAATGCTAGAATTGAGCCTTTTTTGAAAACAACACCTCGACCAATTAGACTTCCGAATGGGCCTCCTTG
TTTTCAGCGGTCAAAATTCCTGCTGATGGATTCTTTAAAATTAAGCATTGAGGATCCAAATCATGAAGGT
GAGGGGATACCACTATATGATGCAATCAAGTGTATGAGAACATTCTTTGGATGGAAAGAACCCACTGTTG
TCAAGCCACACGAGAAGGGAATAAATCCGAATTATCTGTTGTCGTGGAAGCAAGTATTGGAAGAGCTGCA
GGACATTGAGAGTGAGGAGAAGATTCCAAGAACAAAAAACATGAAAAAAACTAGTCAGCTAAAGTGGGCA
CTTGGTGAGAACATGGCACCAGAGAAGGTGGATTTTGATGACTGTAAAGATATAAGCGATTTGAAGCAAT
ATGACAGTGACGAACCTGAATTAAGATCATTTTCAAGTTGGATCCAGAATGAGTTCAACAAGGCATGCGA
GCTGACCGATTCAATCTGGATAGAGCTTGATGAGATTGGAGAAGATGTGGCTCCGATTGAACACATTGCA
AGCATGAGAAGGAATTACTTCACAGCTGAGGTGTCCCATTGCAGAGCCACAGAATATATAATGAAGGGGG
TATACATTAATACTGCTTTGCTCAATGCATCCTGTGCAGCAATGGATGATTTCCAACTAATTCCCATGAT
AAGCAAATGTAGAACTAAAGAGGGAAGGAGAAAGACCAATTTGTACGGCTTCATCGTAAAAGGAAGATCT
CACTTAAGGAATGACACCGATGTGGTAAACTTTGTGAGCATGGAGTTTTCCCTCACTGACCCAAGACTTG
AGCCACACAAATGGGAGAAGTACTGCGTTCTTGAGATAGGAGATATGCTTCTAAGGAGTGCAATAGGCCA
AGTGTCAAGGCCCATGTTCTTGTATGTAAGGACAAATGGAACCTCAAAAATTAAAATGAAATGGGGAATG
GAGATGAGGCGTTGCCTCCTCCAATCCCTCCAACAAATAGAGAGCATGATTGAAGCTGAGTCCTCTGTCA
AAGAGAAAGACATGACAAAAGAGTTTTTTGAGAATAAATCAGAAACATGGCCCATTGGAGAGTCACCAAA
AGGAGTGGAAGAAGGTTCCATTGGGAAAGTATGCAGGACACTGTTGGCTAAGTCAGTATTCAATAGCCTG
TATGCATCTCCACAATTAGAAGGATTTTCAGCTGAGTCAAGAAAGTTGCTCCTCATTGTTCAGGCTCTTA
GGGACAATCTGGAACCTGGGACCTTTGATCTTGGGGGGCTATATGAAGCAATTGAGGAGTGCCTGATTAA
TGATCCCTGGGTTTTGCTTAATGCTTCTTGGTTCAACTCCTTCCTAACACATGCATTGAGATAGCTGGGG
CAATGCTACTATTTGTTATCCATAC
>gi|237689294|gb|CY040255.1| Influenza A virus (A/Managua/3153.01/2008(H1N1)) segment 3, complete sequence
ATGGAGGATTTTGTACGACAATGCTTCAATCCGATGATTGTCGAGCTTGCAGAAAAAGCAATGAAAGAGT
ATGGAGAGGACCTGAAAATCGAAACAAACAAATTTGCAGCAATATGCACCCACTTGGAAGTGTGCTTCAT
GTATTCAGATTTTCATTTCATCAATGAGCAAGGCGAATCAATAATAGTAGAGCCTGAGGACCCAAATGCA
CTTTTAAAGCACAGATTTGAGATAATAGAGGGACGAGATCGTACAATGGCATGGACAGTTGTAAACAGTA
TTTGCAACACCACAGGAGCTGAGAAACCAAAGTTTCTGCCAGATCTGTATGATTACAAAGAGAATAGATT
CATCGAGATTGGGGTGACAAGGAGGGAAGTTCACATATACTATCTGGAAAAGGCCAACAAAATTAAATCT
GAGAAGACACACATTCACATTTTCTCATTCACCGGCGAAGAAATGGCCACAAAGGCTGATTACACTCTCG
ATGAAGAAAGCAGAGCTAGGATTAAAACCAGATTGTTCACCATAAGACAAGAAATGGCAAGCAGAGGTCT
TTGGGACTCCTTTCGTCAGTCCGAAAGAGGCGAAGAAACAATTGAAGAAAGATTTGAAATCACAGGGACA
ATGCGCAGGCTCGCTGACCAAAGCCTTCCGCCGAACTTCTCCTGCATTGAGAATTTTAGAGCCTATGTGG
ATGGATTTGAACCGAACGGCTACATTGAGGGCAAGCTTTCTCAAATGTCCAAAGAAGTAAATGCTAGAAT
TGAGCCTTTTTTGAAAACAACACCTCGACCAATTAGACTTCCGAATGGGCCTCCTTGTTTTCAGCGGTCA
AAGTTCCTGCTGATGGATTCTTTAAAATTAAGCATTGAGGATCCAAATCATGAAGGTGAGGGGATACCAC
TATATGATGCAATCAAGTGTATGAGAACATTCTTTGGATGGAAAGAACCCACTGTTGTCAAGCCACACGA
GAAGGGAATAAATCCAAATTATCTGTTGTCGTGGAAGCAAGTATTGGAAGAGCTGCAGGACATTGAGAGT
GAGGAGAAGATTCCAAGAACAAAAAACATGAAAAAAACTAGTCAGCTAAAGTGGGCACTTGGTGAGAACA

TGGCACCAGAGAAGGTGGATTTTGATGACTGTAAAGATATAAGCGATTTGAAGCAATATGACAGTGACGA
ACCTGAATTAAGATCATTTTCAAGTTGGATCCAGAATGAGTTCAACAAGGCATGCGAGCTGACCGATTCA
ATCTGGATAGAGCTTGATGAGATTGGAGAAGATGTGGCTCCGATTGAACACATTGCAAGCATGAGAAGGA
ATTACTTCACAGCTGAGGTGTCCCACTGCAGAGCCACAGAATATATAATGAAGGGGGTATACATTAATAC
TGCTTTGCTCAATGCATCCTGTGCAGCAATGGATGATTTCCAACTAATTCCCATGATAAGCAAATGTAGA
ACTAAAGAGGGAAGGAGAAAGACCAATTTGTACGGCTTCATCATAAAAGGAAGATCTCACTTAAGGAATG
ACACCGATGTGGTAAACTTTGTGAGCATGGAGTTTTCCCTCACTGACCCAAGACTTGAGCCACACAAATG
GGAGAAGTACTGCGTTCTTGAGATAGGAGATATGCTTTTAAGGAGTGCAATAGGCCAAGTGTCAAGGCCC
ATGTTCTTGTATGTAAGGACAAATGGAACCTCAAAAATTAAAATGAAATGGGGAATGGAGATGAGGCGTT
GCCTCCTCCAATCCCTCCAACAAATAGAGAGCATGATTGAAGCTGAGTCCTCTGTCAAAGAGAAAGACAT
GACAAAAGAGTTTTTTGAGAATAAATCAGAAACATGGCCCATTGGAGAGTCACCAAAAGGAGTGGAAGAA
GGTTCCATTGGGAAAGTATGCAGGACACTGTTGGCTAAGTCAGTATTCAATAGCCTGTATGCATCTCCAC
AATTAGAAGGATTTTCAGCTGAGTCAAGAAAGTTGCTCCTCATTGTTCAGGCTCTTAGGGACAATCTGGA
ACCTGGGACCTTTGATCTTGGGGGGCTATATGAAGCAATTGAGGAGTGCCTGATTAATGATCCCTGGGTT
TTGCTTAATGCTTCTTGGTTCAACTCCTTCCTAACACATGCATTGAGATAGCTGGGGCAATGCTACTATT
TGTTAT
>gi|224020941|gb|CY037332.1| Influenza A virus (A/Washington/AF06/2007(H1N1)) segment 3, complete sequence
TTCAAAATGGAAGATTTTGTAAGACAATGCTTCAATCCGATGATTGTCGAGCTTGCAGAAAAAACGATGA
AAGAGTATGGAGAGGACCTGAAAATCGAAACAAACAAATTTGCAGCAATATGCACCCACTTGGAAGTGTG
CTTCATGTATTCAGATTTTCATTTCATCAATGAGCAAGGCGAATCAATAATAGTAGAGCCTGAGGACCCA
AATGCACTTTTAAAGCACAGATTTGAGATAATAGAGGGACGAGATCGTACAATGGCATGGACAGTTGTAA
ACAGTATTTGCAACACCACAGGAGCTGAGAAACCAAAGTTTCTGCCAGATCTGTATGATTACAAAGAGAA
TAGATTCATCGAGATTGGAGTGACAAGGAGGGAAGTTCACATATACTATCTGGAAAAGGCCAACAAAATT
AAATCTGAGAAGACACACATTCACATTTTCTCATTCACCGGCGAAGAAATGGCCACAAAGGCCGATTACA
CTCTCGATGAAGAAAGCAGAGCTAGGATTAAAACCAGATTGTTCACCATAAGACAAGAAATGGCAAGCAG
AGGTCTTTGGGACTCCTTTCGTCAGTCCGAAAGAGGCGAAGAAACAATTGAAGAAAGATTTGAAATCACA
GGGACAATGCGCAGGCTCGCTGACCAAAGCCTTCCGCCGAACTTCTCCTGCATTGAGAATTTTAGAGCCT
ATGTGGATGGATTTGAACCGAACGGCTACATTGAGGGCAAGCTTTCTCAAATGTCCAAAGAAGTAAATGC
TAGAATTGAGCCTTTTTTGAAAACAACACCTCGACCAATTAGACTTCCGAATGGGCCTCCTTGTTTTCAG
CGGTCAAAATTCCTGCTGATGGATTCTTTAAAATTAAGCATTGAGGATCCAAATCATGAAGGTGAGGGGA
TACCACTATATGATGCAATCAAGTGTATGAGAACATTCTTTGGATGGAAAGAACCCACTGTTGTCAAGCC
ACACGAGAAGGGAATAAATCCGAACTATCTGTTGTCGTGGAAGCAAGTATTGGAAGAACTGCAGGACATT
GAGAGTGAGGAGAAGATTCCAAGAACAAAAAACATGAAAAAAACTAGTCAGCTAAAGTGGGCACTTGGTG
AGAACATGGCACCAGAGAAGGTGGATTTTGATGACTGTAAAGATATAAGCGATTTGAAGCAATATAACAG
TGACGAACCTGAATTAAGATCATTTTCAAGTTGGATCCAGAATGAGTTCAACAAGGCATGCGAGCTGACC
GATTCAATCTGGATAGAGCTTGATGAGATTGGAGAAGATGTGGCTCCGATTGAACACATTGCAAGCATGA
GAAGGAATTACTTCACAGCTGAGGTGTCCCATTGCAGAGCCACAGAATATATAATGAAGGGGGTATACAT
TAATACTGCTTTGCTCAATGCATCCTGTGCAGCAATGGATGATTTCCAACTAATTCCCATGATAAGCAAA
TGTAGAACTAAAGAGGGAAGGAGAAAGACCAATTTGTACGGCTTCATCGTAAAAGGAAGATCTCACTTAA
GGAATGACACCGATGTGGTAAACTTTGTGAGCATGGAGTTTTCCCTCACTGACCCAAGACTTGAGCCACA
CAAATGGGAGAAGTACTGCGTTCTTGAGATAGGAGATATGCTTCTAAGGAGTGCAATAGGCCAAGTGTCA
AGGCCCATGTTCTTGTATGTAAGGACAAATGGAACCTCAAAAATTAAAATGAAATGGGGAATGGAGATGA
GGCGTTGCCTCCTCCAATCCCTCCAACAAATAGAGAGCATGATTGAAGCTGAGTCCTCTGTCAAAGAGAA

AGACATGACAAAAGAGTTTTTTGAGAATAAATCAGAAACATGGCCCATTGGAGAGTCACCAAAAGGAGTG
GAAGAAGGTTCCATTGGGAAAGTATGCAGGACACTGTTGGCTAAGTCAGTATTCAATAGCCTGTATGCAT
CTCCACAATTAGAGGGATTTTCAGCTGAGTCAAGAAAGTTGCTCCTCATTGTTCAGGCTCTTAGGGACAA
TCTGGAACCTGGGACCTTTGATCTTGGGGGGCTATATGAAGCAATTGAGGAGTGCCTGATTAATGATCCC
TGGGTTTTGCTTAATGCTTCTTGGTTCAACTCCTTCCTAACACATGCATTGAGATAGCTGGGGCAATGCT
ACAATTTGTTATCCATACTGTCCAAAAAA
>gi|224021246|gb|CY037340.1| Influenza A virus (A/Japan/AF07/2008(H1N1)) segment 3, complete sequence
GTACTGATTCAAAATGGAAGATTTTGTACGACAATGCTTCAATCCGATGATTGTCGAGCTTGCAGAAAAA
GCGATGAAAGAGTATGGAGAGGACCTGAAAATCGAAACAAACAAATTTGCAGCAATATGCACCCACTTGG
AAGTGTGCTTCATGTATTCAGATTTTCATTTCATCAATGAGCAAGGCGAATCAATAATAGTAGAGCCTGA
GGACCCAAATGCACTTTTAAAGCACAGATTTGAGATAATAGAGGGACGAGATCGTACAATGGCATGGACA
GTTGTAAACAGTATTTGCAACACCACAGGAGCTGAGAAACCAAAGTTTCTGCCAGATCTGTATGATTACA
AAGAGAATAGATTCATCGAGATTGGAGTGACAAGGAGGGAAGTTCACATATACTATCTGGAAAAGGCCAA
CAAAATTAAATCTGAGAAGACACACATTCACATTTTCTCATTCACCGGCGAAGAAATGGCCACAAAGGCC
GATTACACTCTCGATGAAGAAAGCAGAGCTAGGATTAAAACCAGATTGTTCACCATAAGACAAGAAATGG
CAAGCAGAGGTCTTTGGGACTCCTTTCGTCAGTCCGAAAGAGGCGAAGAAACAATTGAAGAAAGATTTGA
AATCACAGGGACAATGCGCAGGCTCGCTGACCAAAGCCTTCCGCCGAACTTCTCCTGCATTGAGAATTTT
AGAGCCTATGTGGATGGATTTGAACCGAACGGCTACATTGAGGGCAAGCTTTCTCAAATGTCCAAAGAAG
TAAATGCTAGAATTGAGCCTTTTTTGAAAACAACACCTCGACCAATTAGACTTCCGAATGGGCCTCCTTG
TTTTCAGCGGTCAAAATTCCTGCTGATGGATTCTTTAAAATTAAGCATTGAGGATCCAAATCATGAAGGT
GAGGGGATACCACTATATGATGCAATCAAGTGTATGAGAACATTCTTTGGATGGAAAGAACCCACTGTTG
TCAAGCCACACGAGAAGGGAATAAATCCGAATTATCTGTTGTCGTGGAAGCAAGTATTGGAAGAGCTGCA
GGACATTGAGAGTGAGGAGAAGATTCCAAGAACAAAAAACATGAAAAAAACTAGTCAGCTAAAGTGGGCA
CTTGGTGAGAACATGGCACCAGAGAAGGTGGATTTTGATGACTGTAAAGATATAAGCGATTTGAAGCAAT
ATGACAGTGACGAACCTGAATTAAGATCATTTTCAAGTTGGATCCAGAATGAGTTCAACAAGGCATGCGA
GCTGACCGATTCAATCTGGATAGAGCTTGATGAGATTGGAGAAGATGTGGCTCCAATTGAACACATTGCA
AGCATGAGAAGGAATTACTTCACAGCTGAGGTGTCCCATTGCAGAGCCACAGAATATATAATGAAGGGGG
TATACATTAATACTGCTTTGCTCAATGCATCCTGTGCAGCAATGGATGATTTCCAACTAATTCCCATGAT
AAGCAAATGTAGAACTAAAGAGGGAAGGAGAAAGACCAATTTGTACGGCTTCATCGTAAAAGGAAGATCT
CACTTAAGGAATGACACCGATGTGGTAAACTTTGTGAGCATGGAGTTTTCCCTCACTGACCCAAGACTTG
AGCCACACAAATGGGAGAAGTACTGCGTTCTTGAGATAGGAGATATGCTTCTAAGGAGTGCAATAGGCCA
AGTGTCAAGGCCCATGTTCTTGTATGTAAGGACAAATGGAACCTCAAAAATTAAAATGAAATGGGGAATG
GAGATGAGGCGTTGCCTCCTCCAATCCCTCCAACAAATAGAGAGCATGATTGAAGCTGAGTCCTCTGTCA
AAGAGAAAGACATGACAAAAGAGTTTTTTGAGAATAAATCAGAAACATGGCCCATTGGAGAGTCACCAAA
AGGAGTGGAAGAAGGTTCCATTGGGAAAGTATGCAGGACACTGTTGGCTAAGTCAGTATTCAATAGCCTG
TATGCATCTCCACAATTAGAAGGATTTTCAGCTGAGTCAAGGAAGTTGCTCCTCATTGTTCAGGCTCTTA
>gi|212381608|gb|FJ445076.1| Influenza A virus (A/England/26/2008(H1N1)) segment 3 polymerase PA (PA) gene, complete cds
ATGGAAGATTTTGTACGACAATGCTTCAATCCGATGATTGTCGAGCTTGCAGAAAAAGCAATGAAAGAGT
ATGGAGAGGACCTGAAAATCGAAACAAACAAATTTGCAGCAATATGCACCCACTTGGAAGTGTGCTTCAT
GTATTCAGATTTTCATTTCATCAATGAGCAAGGCGAATCAATAATAGTAGAGCCTGAGGACCCAAATGCA
CTTTTAAAGCACAGATTTGAGATAATAGAGGGACGAGATCGTACAATGGCATGGACAGTTGTAAACAGTA
TTTGCAACACCACAGGAGCTGAGAAACCAAAGTTTCTGCCAGATCTGTATGATTACAAAGAGAATAGATT

CATCGAGATTGGAGTGACAAGGAGGGAAGTTCACATATACTATCTGGAAAAGGCCAACAAAATTAAATCT
GAGAAGACACACATTCACATTTTCTCATTCACCGGCGAAGAAATGGCCACAAAGGCTGATTACACTCTCG
ATGAAGAAAGCAGAGCTAGGATTAAAACCAGATTGTTCACCATAAGACAAGAAATGGCAAGCAGAGGTCT
TTGGGACTCCTTTCGTCAGTCCGAAAGAGGCGAAGAAACAATTGAAGAAAGATTTGAAATCACAGGGACA
ATGCGCAGGCTCGCTGACCAAAGCCTTCCGCCGAACTTCTCCTGCATTGAGAATTTTAGAGCCTATGTGG
ATGGATTTGAACCGAACGGCTACATTGAGGGCAAGCTTTCTCAAATGTCCAAAGAAGTAAATGCTAGAAT
TGAGCCTTTTTTGAAAACAACACCTCGACCAATTAGACTTCCGAATGGGCCTCCTTGTTTTCAGCGGTCA
AAATTCCTGCTGATGGATTCTTTAAAATTAAGCATTGAGGATCCAAATCATGAAGGTGAGGGGATACCAC
TATATGATGCAATCAAGTGTATGAGGACATTCTTTGGATGGAAAGAACCCACTGTTGTCAAGCCACACGA
GAAGGGAATAAATCCAAATTATCTGTTGTCGTGGAAGCAAGTATTGGAAGAGCTGCAGGACATTGAGAGT
GAGGAGAAGATTCCAAGAACAAAAAACATGAAAAAAACTAGTCAGCTAAAGTGGGCACTTGGTGAGAACA
TGGCACCAGAGAAAGTGGATTTTGATGACTGTAAAGATATAAGCGATTTGAAGCAATATGACAGTGACGA
ACCTGAATTAAGATCATTTTCAAGTTGGATCCAGAATGAGTTCAACAAGGCATGCGAGCTGACCGACTCA
ATCTGGATAGAGCTTGATGAGATTGGAGAAGATGTGGCTCCGATTGAACACATTGCAAGCATGAGAAGGA
ATTACTTCACAGCTGAGGTATCCCACTGCAGAGCCACAGAATATATAATGAAGGGGGTATACATTAATAC
TGCTTTGCTCAATGCATCCTGTGCAGCAATGGATGATTTCCAACTAATTCCCATGATAAGCAAATGTAGA
ACTAAAGAGGGAAGGAGAAAGACCAATTTGTACGGCTTCATCGTAAAAGGAAGATCTCACTTAAGGAATG
ACACCGATGTGGTAAACTTTGTGAGCATGGAGTTTTCCCTCACTGACCCAAGACTTGAGCCACACAAATG
GGAGAAGTACTGCGTTCTTGAGATAGGAGATATGCTTCTAAGGAGTGCAATAGGCCAAGTGTCAAGGCCC
ATGTTCTTGTATGTAAGGACAAATGGAACCTCAAAAATTAAAATGAAATGGGGAATGGAGATGAGGCGTT
GCCTCCTCCAATCCCTCCAACAAATAGAGAGCATGATTGAAGCTGAGTCCTCTGTCAAAGAGAAAGACAT
GACAAAAGAGTTTTTTGAGAATAAATCAGAAACATGGCCCATTGGAGAGTCACCAAAAGGAGTGGAAGAA
GGTTCCATTGGGAAAGTATGCAGGACACTGTTGGCTAAGTCAGTATTCAATAGCCTGTATGCATCTCCAC
AATTAGAAGGATTTTCAGCTGAGTCAAGAAAGTTGCTCCTCATTGTTCAGGCTCTTAGGGACAATCTGGA
ACCTGGGACCTTTGATCTTGGGGGGCTATATGAAGCAATTGAGGAGTGCCTGATTAATGATCCCTGGGTT
TTGCTTAATGCTTCTTGGTTCAACTCCTTCCTAACACATGCATTGAGATAG
>gi|224027216|gb|CY037684.1| Influenza A virus (A/Florida/UR07-0022/2008(H1N1)) segment 3, complete sequence
AATGGAAGATTTTGTACGACAATGCTTCAATCCGATGATTGTCGAGCTTGCAGAAAAAGCAATGAAAGAG
TATGGAGAGGACCTGAAAATCGAAACAAACAAATTTGCAGCAATATGCACCCACTTGGAAGTGTGCTTCA
TGTATTCAGATTTTCATTTCATCAATGAGCAAGGCGAATCAATAATAGTAGAGCCTGAGGACCCAAATGC
ACTTTTAAAGCACAGATTTGAGATAATAGAGGGACGAGATCGTACAATGGCATGGACAGTTGTAAACAGT
ATTTGCAACACCACAGGAGCTGAGAAACCAAAGTTTCTGCCAGATCTGTATGATTACAAAGAGAATAGAT
TCATCGAGATTGGAGTGACAAGGAGGGAAGTTCACATATACTATCTGGAAAAGGCCAACAAAATTAAATC
TGAGAAGACACACATTCACATTTTCTCATTCACCGGCGAAGAAATGGCCACAAAGGCTGATTACACTCTC
GATGAAGAAAGCAGAGCTAGGATTAAAACCAGATTGTTCACCATAAGACAAGAAATGGCAAGCAGAGGTC
TTTGGGACTCCTTTCGTCAGTCCGAAAGAGGCGAAGAAACAATTGAAGAAAGATTTGAAATCACAGGGAC
AATGCGCAGGCTCGCTGACCAAAGCCTTCCGCCGAACTTCTCCTGCATTGAGAATTTTAGAGCCTATGTG
GATGGATTTGAACCGAACGGCTACATTGAGGGCAAGCTTTCTCAAATGTCCAAAGAAGTAAATGCTAGAA
TTGAGCCTTTTTTGAAAACAACACCTCGACCAATTAGACTTCCGAATGGGCCTCCTTGTTTTCAGCGGTC
AAAATTCCTGCTGATGGATTCTTTAAAATTAAGCATTGAGGATCCAAATCATGAAGGTGAGGGGATACCA
CTATATGATGCAATCAAGTGTATGAGAACATTCTTTGGATGGAAAGAACCCACTGTTGTCAAGCCACACG
AGAAGGGAATAAATCCAAATTATCTGTTGTCGTGGAAGCAAGTATTGGAAGAGCTGCAAGACATTGAGAG
TGAGGAGAAGATTCCAAGAACAAAAAACATGAAAAAAACTAGTCAGCTAAAGTGGGCACTTGGTGAGAAC

ATGGCACCAGAGAAGGTGGATTTTGATGACTGTAAAGATATAAGCGATTTGAAGCAATATGACAGTGACG
AACCTGAATTAAGATCATTTTCAAGTTGGATCCAGAATGAGTTCAACAAGGCATGCGAGCTGACCGATTC
AATCTGGATAGAGCTTGATGAGATTGGAGAAGATGTGGCTCCGATTGAACACATTGCAAGCATGAGAAGG
AATTACTTCACAGCTGAGGTGTCCCACTGCAGAGCCACAGAATATATAATGAAGGGGGTATACATTAATA
CTGCTTTGCTCAATGCATCCTGTGCAGCAATGGATGATTTCCAACTAATTCCCATGATAAGCAAATGTAG
AACTAAAGAGGGAAGGAGAAAGACCAATTTGTACGGCTTCATCGTAAAAGGAAGATCTCACTTAAGGAAT
GACACCGATGTGGTAAACTTTGTGAGCATGGAGTTTTCCCTCACTGACCCAAGACTTGAGCCACACAAAT
GGGAGAAGTACTGCGTTCTTGAGATAGGAGATATGCTTCTAAGGAGTGCAATAGGCCAAGTGTCAAGGCC
CATGTTCTTGTATGTAAGGACAAATGGAACCTCAAAAATTAAAATGAAATGGGGAATGGAGATGAGGCGT
TGCCTCCTCCAATCCCTCCAACAAATAGAGAGCATGATTGAAGCTGAGTCCTCTGTCAAAGAGAAAGACA
TGACAAAAGAGTTTTTTGAGAATAAATCAGAAACATGGCCCATTGGAGAGTCACCAAAAGGAGTGGAAGA
AGGTTCCATTGGGAAAGTATGCAGAACACTGTTGGCTAAGTCAGTATTCAATAGCCTGTATGCATCTCCA
CAATTAGAAGGATTTTCAGCTGAGTCAAGAAAGTTGCTCCTCATTGTTCAGGCTCTTAGGGACAATCTGG
AACCTGGGACCTTTGATCTTGGGGGGCTATATGAAGCAATTGAGGAGTGCCTGATTAATGATCCCTGGGT
TTTGCTTAATGCTTCTTGGTTCAACTCCTTCCTAACACATGCATTGAGATAGCTGGGGCAATGCTACTAT
TTGTTATCCATACTGTCCAAAAAA
>gi|296240591|gb|CY063611.1| Influenza A virus (A/Aalborg/INS133/2009(H1N1)) segment 3, complete sequence
TCCAAAATGGAAGACTTTGTGCGACAATGCTTCAATCCAATGATCATCGAGCTTGCGGAAAAGGCAATGA
AAGAATATGGGGAAGATCCGAAAATCGAAACTAACAAGTTTGCTGCAATATGCACACATTTGGAAGTTTG
TTTCATGTATTCGGATTTCCATTTCATCGACGAACGGGGTGAATCAATAATTGTAGAATCTGGTGACCCG
AATGCACTATTGAAGCACCGATTTGAGATAATTGAAGGAAGAGACCGAATCATGGCCTGGACAGTGGTGA
ACAGTATATGTAACACAACAGGGGTAGAGAAGCCTAAATTTCTTCCTGATTTGTATGATTACAAAGAGAA
CCGGTTCATTGAAATTGGAGTAACACGGAGGGAAGTCCACATATATTACCTAGAGAAAGCCAACAAAATA
AAATCTGAGAAGACACACATTCACATCTTTTCATTCACTGGAGAGGAGATGGCCACCAAAGCGGACTACA
CCCTTGACGAAGAGAGCAGGGCAAGAATCAAAACTAGGCTTTTCACTATAAGACAAGAAATGGCCAGTAG
GAGTCTATGGGATTCCTTTCGTCAGTCCGAAAGAGGCGAAGAGACAATTGAAGAAAAATTTGAGATTACA
GGAACTATGCGCAAGCTTGCCGACCAAAGTCTCCCACCGAACTTCTCCAGCCTTGAAAACTTTAGAGCCT
ATGTAGATGGATTCGAGCCGAACGGCTGCATTGAGGGCAAGCTTTCCCAAATGTCAAAAGAAGTGAACGC
CAAAATTGAACCATTCTTGAGGACGACACCACGCCCCCTCAGATTGCCTGATGGGCCTCTTTGCCATCAG
CGGTCAAAGTTCCTGCTGATGGATGCTCTGAAATTAAGTATTGAAGACCCGAGTCACGAGGGGGAGGGAA
TACCACTATATGATGCAATCAAATGCATGAAGACATTCTTTGGCTGGAAAGAGCCTAACATAGTCAAACC
ACATGAGAAAGGCATAAATCCCAATTACCTCATGGCTTGGAAGCAGGTGCTAGCAGAGCTACAGGACATT
GAAAATGAAGAGAAGATCCCAAGGACAAAGAACATGAAGAGAACAAGCCAATTGAAGTGGGCACTCGGT
G
AAAATATGGCACCAGAAAAAGTAGACTTTGATGACTGCAAAGATGTTGGAGACCTTAAACAGTATGACAG
TGATGAGCCAGAGCCCAGATCTCTAGCAAGCTGGGTCCAAAATGAATTCAATAAGGCATGTGAATTGACT
GATTCAAGCTGGATAGAACTTGATGAAATAGGAGAAGATGTTGCCCCGATTGAACATATCGCAAGCATGA
GGAGGAACTATTTTACAGCAGAAGTGTCCCACTGCAGGGCTACTGAATACATAATGAAGGGAGTGTACAT
AAATACGGCCTTGCTCAATGCATCCTGTGCAGCCATGGATGACTTTCAGCTGATCCCAATGATAAGCAAA
TGTAGGACCAAAGAAGGAAGACGGAAAACAAACCTGTATGGGTTCATTATAAAAGGAAGGTCTCATTTGA
GAAATGATACTGATGTGGTGAACTTTGTAAGTATGGAGTTCTCACTCACTGACCCGAGACTGGAGCCACA
CAAATGGGAAAAATACTGTGTTCTTGAAATAGGAGACATGCTCTTGAGGACTGCGATAGGCCAAGTGTCG
AGGCCCATGTTCCTATATGTGAGAACCAATGGAACCTCCAAGATCAAGATGAAATGGGGCATGGAAATGA

GGCGCTGCCTTCTTCAGTCTCTTCAGCAGATTGAGAGCATGATTGAGGCCGAGTCTTCTGTCAAAGAGAA
AGACATGACCAAGGAATTCTTTGAAAACAAATCGGAAACATGGCCAATCGGAGAGTCACCCAGGGGAGTG
GAGGAAGGCTCTATTGGGAAAGTGTGCAGGACCTTACTGGCAAAATCTGTATTCAACAGTCTATATGCGT
CTCCACAACTTGAGGGGTTTTCGGCTGAATCGAGAAAATTGCTTCTCATTGTTCAGGCACTTAGGGACAA
CCTGGAACCTGGAACCTTCGATCTTGGGGGGCTATATGAAGCAATCGAGGAGTGCCTGATTAATGATCCC
TGGGTTTTGCTTAATGCATCTTGGTTCAACTCCTTCCTCACACATGCACTGCAGTAGTTGTGGCAATGCT
ACTAT
>gi|296240573|gb|CY063603.1| Influenza A virus (A/Bonn/INS128/2009(H1N1)) segment 3, complete sequence
TCCAAAATGGAAGACTTTGTGCGACAATGCTTCAATCCAATGATCATCGAGCTTGCGGAAAAGGCAATGA
AAGAATATGGGGAAGATCCGAAAATCGAAACTAACAAGTTTGCTGCAATATGCACACATTTGGAAGTTTG
TTTCATGTATTCGGATTTCCATTTCATCGACGAACGGGGTGAATCAATAATTGTAGAATCTGGTGACCCG
AATGCACTATTGAAGCACCGATTTGAGATAATTGAAGGAAGAGACCGAATCATGGCCTGGACAGTGGTGA
ACAGTATATGTAACACAACAGGGGTAGAGAAGCCTAAATTTCTTCCTGATTTGTATGATTACAAAGAGAA
CCGGTTCATTGAAATTGGAGTAACACGGAGGGAAGTCCACATATATTACCTAGAGAAAGCCAACAAAATA
AAATCTGAGAAGACACACATTCACATCTTTTCATTCACTGGAGAGGAGATGGCCACCAAAGCGGACTACA
CCCTTGACGAAGAGAGCAGGGCAAGAATCAAAACTAGGCTTTTCACTATAAGACAAGAAATGGCCAGTAG
GAGTCTATGGGATTCCTTTCGTCAGTCCGAAAGAGGCGAAGAGACAATTGAAGAAAAATTTGAGATTACA
GGAACTATGCGCAAGCTTGCCGACCAAAGTCTCCCACCGAACTTCTCCAGCCTTGAAAACTTTAGAGCCT
ATGTAGATGGATTCGAGCCGAACGGCTGCATTGAGGGCAAGCTTTCCCAAATGTCAAAAGAAGTGAACGC
CAAAATTGAACCATTCTTGAGGACGACACCACGCCCCCTCAGATTGCCTGATGGGCCTCTTTGCCATCAG
CGGTCAAAGTTCCTGCTGATGGATGCTCTGAAATTAAGTATTGAAGACCCGAGTCACGAGGGGGAGGGAA
TACCACTATATGATGCAATCAAATGCATGAAGACATTCTTTGGCTGGAAAGAGCCTAACATAGTCAAACC
ACATGAGAAAGGCATAAATCCCAATTACCTCATGGCTTGGAAGCAGGTGCTAGCAGAGCTACAGGACATT
GAAAATGAAGAGAAGATCCCAAGGACAAAGAACATGAAGAGAACAAGCCAATTGAAGTGGGCACTCGGT
G
AAAATATGGCACCAGAAAAAGTAGACTTTGATGACTGCAAAGATGTTGGAGACCTTAAACAGTATGACAG
TGATGAGCCAGAGCCCAGATCTCTAGCAAGCTGGGTCCAAAATGAATTCAATAAGGCATGTGAATTGACT
GATTCAAGCTGGATAGAGCTTGATGAAATAGGAGAAGATGTTGCCCCGATTGAACATATCGCAAGCATGA
GGAGGAACTATTTTACAGCAGAAGTGTCCCACTGCAGGGCTACTGAATACATAATGAAGGGAGTGTACAT
AAATACGGCCTTGCTCAATGCATCCTGTGCAGCCATGGATGACTTTCAGCTGATCCCAATGATAAGCAAA
TGTAGGACCAAAGAAGGAAGACGGAAAACAAACCTGTATGGGTTCATTATAAAAGGAAGGTCTCATTTGA
GAAATGATACTGATGTGGTGAACTTTGTAAGTATGGAGTTCTCACTCACTGACCCGAGACTGGAGCCACA
CAAATGGGAAAAATACTGTGTTCTTGAAATAGGAGACATGCTCTTGAGGACTGCGATAGGCCAAGTGTCG
AGGCCCATGTTCCTATATGTGAGAACCAATGGAACCTCCAAGATCAAGATGAAATGGGGCATGGAAATGA
GGCGCTGCCTTCTTCAGTCTCTTCAGCAGATTGAGAGCATGATTGAGGCCGAGTCTTCTGTCAAAGAGAA
AGACATGACCAAGGAATTCTTTGAAAACAAATCGGAAACATGGCCAATCGGAGAGTCACCCAGGGGAGTG
GAGGAAGGCTCTATTGGGAAAGTGTGCAGGACCTTACTGGCAAAATCTGTATTCAACAGTCTATATGCGT
CTCCACAACTTGAGGGGTTTTCGGCTGAATCGAGAAAATTGCTTCTCATTGTTCAGGCACTTAGGGACAA
CCTGGAACCTGGAACCTTCGATCTTGGGGGGCTATATGAAGCAATCGAGGAGTGCCTGATTAATGATCCC
TGGGTTTTGCTTAATGCATCTTGGTTCAACTCCTTCCTCACACATGTACTGCAGTAGTTGTGGCAATGCT
ACTAT
>gi|296240321|gb|CY063491.1| Influenza A virus (A/Boston/110/2009(H1N1)) segment 3, complete sequence

TCCAAAATGGAAGACTTTGTGCGACAATGCTTCAATCCAATGATCGTCGAGCTTGCGGAAAAGGCAATGA
AAGAATATGGGGAAGATCCGAAAATCGAAACTAACAAGTTTGCTGCAATATGCACACATTTGGAAGTTTG
TTTCATGTATTCGGATTTCCATTTCATCGACGAACGGGGTGAATCAATAATTGTAGAATCTGGTGACCCG
AATGCACTATTGAAGCACCGATTTGAGATAATTGAAGGAAGAGACCGAATCATGGCCTGGACAGTGGTGA
ACAGTATATGTAACACAACAGGGGTAGAGAAGCCTAAATTTCTTCCTGATTTGTATGATTACAAAGAGAA
CCGGTTCATTGAAATTGGAGTAACACGGAGGGAAGTCCACATATATTACCTAGAGAAAGCCAACAAAATA
AAATCTGAGAAGACACACATTCACATCTTTTCATTCACTGGAGAGGAGATGGCCACCAAAGCGGACTACA
CCCTTGACGAAGAGAGCAGGGCAAGAATCAAAACTAGGCTTTTCACTATAAGACAAGAAATGGCCAGTAG
GAGTCTATGGGATTCCTTTCGTCAGTCCGAAAGAGGCGAAGAGACAATTGAAGAAAAAATTTGAGATTACA
GGAACTATGCGCAAGCTTGCCGACCAAAGTCTCCCACCGAACTTCTCCAGCCTTGAAAACTTTAGAGCCT
ATGTAGATGGATTCGAGCCGAACGGCTGCATTGAGGGCAAGCTTTCCCAAATGTCAAAAGAAGTGAACGC
CAAAATTGAACCATTCTTGAGGACGACACCACGCCCCCTCAGATTGCCTGATGGGCCTCTTTGCCATCAG
CGGTCAAAGTTCCTGCTGATGGATGCTCTGAAATTAAGTATTGAAGACCCGAGTCACGAGGGGGAGGGAA
TACCACTATATGATGCAATCAAATGCATGAAGACATTCTTTGGCTGGAAAGAGCCTAACATAGTCAAACC
ACATGAGAAAGGCATAAATCCCAATTACCTCATGGCTTGGAAGCAGGTGCTAGCAGAGCTACAGGACATT
GAAAATGAAGAGAAGATCCCAAGGACAAAGAACATGAAGAGAACAAGCCAATTGAAGTGGGCACTCGGT
G
AAAATATGGCACCAGAAAAAGTAGACTTTGATGACTGCAAAGATGTTGGAGACCTTAAACAGTATGACAG
TGATGAGCCAGAGCCCAGATCTCTAGCAAGCTGGGTCCAAAATGAATTCAATAAGGCATGTGAATTGACT
GATTCAAGCTGGATAGAACTTGATGAAATAGGAGAAGATGTTGCCCCGATTGAACATATCGCAAGCATGA
GGAGGAACTATTTTACAGCAGAAGTGTCCCACTGCAGGGCTACTGAATACATAATGAAGGGAGTGTACAT
AAATACGGCCTTGCTCAATGCATCCTGTGCAGCCATGGATGACTTTCAGCTGATCCCAATGATAAGCAAA
TGTAGGACCAAAGAAGGAAGACGGAAAACAAACCTGTATGGGTTCATTATAAAAGGAAGGTCTCATTTGA
GAAATGATACTGATGTGGTGAACTTTGTAAGTATGGAGTTCTCACTCACTGACCCGAGACTGGAGCCACA
CAAATGGGAAAAATACTGTGTTCTTGAAATAGGAGACATGCTCTTGAGGACTGCGATAGGCCAAGTGTCG
AGGCCCATGTTCCTATATGTGAGAACCAATGGAACCTCCAAGATCAAGATGAAATGGGGCATGGAAATGA
GGCGCTGCCTTCTTCAGTCTCTTCAGCAGATTGAGAGCATGATTGAGGCCGAGTCTTCTGTCAAAGAGAA
AGACATGACCAAGGAATTCTTTGAAAACAAATCGGAAACATGGCCAATCGGAGAGTCACCCAGGGGAGTG
GAGGAAGGCTCTATTGGGAAAGTGTGCAGGACCTTACTGGCAAAATCTGTATTCAACAGTCTATATGCGT
CTCCACAACTTGAGGGGTTTTCGGCTGAATCGAGAAAATTGCTTCTCATTGTTCAGGCACTTAGGGACAA
CCTGGAACCTGGAACCTTCGATCTTGGGGGGCTATATGAAGCAATCGAGGAGTGCCTGATTAATGATCCC
TGGGTTTTGCTTAATGCATCTTGGTTCAACTCCTTCCTCACACATGCACTGAAGTAGTTGTGGCAATGCT
ACTAT
>gi|296240627|gb|CY063627.1| Influenza A virus (A/New York/INS150/2009(H1N1)) segment 3, complete sequence
ATGGAAGACTTTGTGCGACAATGCTTCAATCCAATGATCGTCGAGCTTGCGGAAAAGGCAATGAAAGAAT
ATGGAGAAGATCCGAAAATCGAAACTAACAAGTTTGCTGCAATATGCACACATTTGGAAGTTTGTTTCAT
GTATTCGGATTTCCATTTCATCGACGAACGGGGTGAATCAATAATTGTAGAATCTGGTGACCCGAATGCA
CTATTGAAGCACCGATTTGAGATAATTGAAGGAAGAGACCGAATCATGGCCTGGACAGTGGTGAACAGTA
TATGTAACACAACAGGGGTAGAGAAGCCTAAATTTCTTCCTGATTTGTATGATTACAAAGAGAACCGGTT
CATTGAAATTGGAGTAACACGGAGGGAAGTCCACATATATTACCTAGAGAAAGCCAACAAAATAAAATCT
GAGAAGACACACATTCACATCTTTTCATTCACTGGAGAGGAGATGGCCACCAAAGCGGACTACACCCTTG
ACGAAGAGAGCAGGGCAAGAATCAAAACTAGGCTTTTCACTATAAGACAAGAAATGGCCAGTAGGAGTCT
ATGGGATTCCTTTCGTCAGTCCGAAAGAGGCGAAGAGACAATTGAAGAAAAAATTTGAGATTACAGGAACT

ATGCGCAAGCTTGCCGACCAAAGTCTCCCACCGAACTTCTCCAGCCTTGAAAACTTTAGAGCCTATGTAG
ATGGATTCGAGCCGAACGGCTGCATTGAGGGCAAGCTTTCCCAAATGTCAAAAGAAGTGAACGCCAAAAT
TGAACCATTCTTGAGGACGACACCACGCCCCCTCAGATTGCCTGATGGGCCTCTTTGCCATCAGCGGTCA
AAGTTCCTGCTGATGGATGCTCTGAAATTAAGTATTGAAGACCCGAGTCACGAGGGGGAGGGAATACCAC
TATATGATGCAATCAAATGCATGAAGACATTCTTTGGCTGGAAAGAGCCTAACATAGTCAAACCACATGA
GAAAGGCATAAATCCCAATTACCTCATGGCTTGGAAGCAGGTGCTAGCAGAGCTACAGGACATTGAAAAT
GAAGAGAAGATCCCAAGGACAAAGAACATGAAGAGAACAAGCCAATTGAAGTGGGCACTCGGTGAAAAT
A
TGGCACCAGAAAAAGTAGACTTTGATGACTGCAAAGATGTTGGAGACCTTAAACAGTATGACAGTGATGA
GCCAGAGCCCAGATCTCTAGCAAGCTGGGTCCAAAGTGAATTCAATAAGGCATGTGAATTGACTGATTCA
AGCTGGATAGAACTTGATGAAATAGGAGAAGATGTTGCCCCGATTGAACATATCGCAAGCATGAGGAGGA
ACTATTTTACAGCAGAAGTGTCCCACTGTAGGGCTACTGAATACATAATGAAGGGAGTGTACATAAATAC
GGCCTTGCTCAATGCATCCTGTGCAGCCATGGATGACTTTCAGCTGATCCCAATGATAAGCAAATGTAGG
ACCAAAGAAGGAAGACGGAAAACAAACCTGTATGGGTTCATTATAAAAGGAAGGTCTCATTTGAGAAATG
ATACTGATGTGGTGAACTTTGTAAGTATGGAGTTCTCACTCACTGACCCGAGACTGGAGCCACACAAATG
GGAAAAATACTGTGTTCTTGAAATAGGAGACATGCTCTTGAGGACTGCGATAGGCCAAGTGTCGAGGCCC
ATGTTCCTATATGTAAGAACCAATGGAACCTCCAAGATCAAGATGAAATGGGGCATGGAAATGAGGCGCT
GCCTTCTTCAGTCTCTTCAGCAGATTGAGAGCATGATTGAGGCCGAGTCTTCTGTCAAAGAGAAAGACAT
GACCAAGGAATTCTTTGAAAACAAATCGGAAACATGGCCAATCGGAGAGTCACCCAGGGGAGTGGAAGAA
GGCTCTATTGGGAAAGTGTGCAGGACCTTACTGGCAAAATCTGTATTCAACAGTCTATATGCGTCTCCAC
AACTTGAGGGGTTTTCGGCTGAATCGAGAAAATTGCTTCTCATTGTTCAGGCACTTAGGGACAACCTGGA
ACCTGGAACCTTCGATCTTGGGGGGCTATATGAAGCAATCGAGGAGTGCCTGATTAATGATCCCTGGGTT
TTGCTTAATGCATCTTGGTTCAACTCCTTCCTCACACATGCACTGAAGTAGTTGTGGCAATGCTACTAT
>gi|296240555|gb|CY063595.1| Influenza A virus (A/Athens/INS122/2009(H1N1)) segment 3, complete sequence
TCCAAAATGGAAGACTTTGTGCGACAATGCTTCAATCCAATGATCGTCGAGCTTGCGGAAAAGGCAATGA
AAGAATATGGGGAAGATCCGAAAATCGAAACTAACAAGTTTGCTGCAATATGCACACATTTGGAAGTTTG
TTTCATGTATTCGGATTTCCATTTCATCGACGAACGGGGTGAATCAATAATTGTAGAATCTGGTGACCCG
AATGCACTATTGAAGCACCGATTTGAGATAATTGAAGGAAGAGACCGAATCATGGCCTGGACAGTGGTGA
ACAGTATATGTAACACAACAGGGGTAGAGAAGCCTAAATTTCTTCCTGATTTGTATGATTACAAAGAGAA
CCGGTTCATTGAAATTGGAGTAACACGGAGGGAAGTCCACATATATTACCTAGAGAAAGCCAACAAAATA
AAATCTGAGAAGACACACATTCACATCTTTTCATTCACTGGAGAGGAGATGGCCACCAAAGCGGACTACA
CCCTTGACGAAGAGAGCAGGGCAAGAATCAAAACTAGGCTTTTCACTATAAGACAAGAAATGGCCAGTAG
GAGTCTATGGGATTCCTTTCGTCAGTCCGAAAGAGGCGAAGAGACAATTGAAGAAAAATTTGAGATTACA
GGAACTATGCGCAAGCTTGCCGACCAAAGTCTCCCACCGAACTTCTCCAGCCTTGAAAACTTTAGAGCCT
ATGTAGATGGATTCGAGCCGAACGGCTGCATTGAGGGCAAGCTTTCCCAAATGTCAAAAGAAGTGAACGC
CAAAATTGAACCATTCTTGAGGACGACACCACGCCCCCTCAGATTGCCTGATGGGCCTCTTTGCCATCAG
CGGTCAAAGTTCCTGCTGATGGATGCTCTGAAATTAAGTATTGAAGACCCGAGTCACGAGGGGGAGGGAA
TACCACTATATGATGCAATCAAATGCATGAAGACATTCTTTGGCTGGAAAGAGCCTAACATAGTCAAACC
ACATGAGAAAGGCATAAATCCCAATTACCTCATGGCTTGGAAGCAGGTGCTAGCAGAGCTACAGGACATT
GAAAATGAAGAGAAGATCCCAAGGACAAAGAACATGAAGAGAACAAGCCAATTGAAGTGGGCACTCGGT
G
AAAATATGGCACCAGAAAAAGTAGACTTTGATGACTGCAAAGATGTTGGAGACCTTAAACAGTATGACAG
TGATGAGCCAGAGCCCAGATCTCTAGCAAGCTGGGTCCAAAATGAATTCAATAAGGCATGTGAATTGACT

GATTCAAGCTGGATAGAACTTGATGAAATAGGAGAAGATGTTGCCCCGATCGAACATATCGCAAGCATGA
GGAGGAACTATTTTACAGCAGAAGTGTCCCACTGCAGGGCTACTGAATACATAATGAAGGGAGTGTACAT
AAATACGGCCTTGCTCAATGCATCCTGTGCAGCCATGGATGACTTTCAGCTGATCCCAATGATAAGCAAA
TGTAGGACCAAAGAAGGAAGACGGAAAACAAACCTGTATGGGTTCATTATAAAAGGAAGGTCTCATTTGA
GAAATGATACTGATGTGGTGAACTTTGTAAGTATGGAGTTCTCACTCACTGACCCGAGACTGGAGCCACA
CAAATGGGAAAAATACTGTGTTCTTGAAATAGGAGACATGCTCTTGAGGACTGCGATAGGCCAAGTGTCG
AGGCCCATGTTCCTATATGTGAGAACCAATGGAACCTCCAAGATCAAGATGAAATGGGGCATGGAAATGA
GGCGCTGCCTTCTTCAGTCTCTTCAGCAGATTGAGAGCATGATTGAGGCCGAGTCTTCTGTCAAAGAGAA
AGACATGACCAAGGAATTCTTTGAAAACAAATCGGAAACATGGCCAATCGGAGAGTCACCCAGGGGAGTG
GAGGAAGGCTCTATTGGGAAAGTGTGCAGGACCTTACTGGCAAAATCTGTATTCAACAGTCTATATGCGT
CTCCACAACTTGAGGGGTTTTCGGCTGAATCGAGAAAATTGCTTCTCATTGTTCAGGCACTTAGGGACAA
CCTGGAACCTGGAACCTTCGATCTTGGGGGGCTATATGAAGCAATCGAGGAGTGCCTGATTAATGATCCC
TGGGTTTTGCTTAATGCATCTTGGTTCAACTCCTTCCTCACACATGCACTGAAGTAGTTGTGGCAATGCT
ACTAT>gi|399226427|gb|JX309983.1| Influenza A virus (A/Singapore/KK734/2010(H1N1)) segment 3 polymerase PA (PA) gene, complete cds
AGCAAAAGCAGGTACTGATCCAAAATGGAAGACTTTGTGCGACAATGCTTCAATCCAATGATCATCGAGC
TTGCGGAAAAGGCAATGAAAGAATATGGGGAAGATCCGAAAATCGAAACTAACAAGTTTGCTGCAATATG
CACACATTTGGAAGTTTGTTTCATGTATTCGGATTTCCATTTCATCGACGAACGGGGTGAATCAATAATT
GTAGAATCTGGTGACCCGAATGCACTATTGAAGCACCGATTTGAGATAATTGAAGGAAGAGACCGAATCA
TGGCCTGGACAGTGGTGAACAGTATATGTAACACAACAGGGGTAGAGAAGCCTAAATTTCTTCCTGATTT
GTATGATTACAAAGAGAACCGGTTCATTGAAATTGGAGTAACACGGAGGGAAGTCCACATATATTACCTA
GAGAAAGCCAACAAAATAAAATCTGAGAAGACACACATTCACATCTTCTCATTCACTGGAGAGGAGATGG
CCACCAAAGCGGACTACACCCTTGACGAAGAGAGCAGGGCAAGAATCAAAACTAGGCTTTTCACTATAAG
ACAAGAAATGGCCAGTAGGAGTCTATGGGATTCCTTTCGTCAGTCCGAAAGAGGCGAAGAGACAATTGAA
GAAAAATTTGAGATTACAGGAACTATGCGCAAGCTTGCCGACCAAAGTCTCCCACCGAACTTCTCCAGCC
TTGAAAACTTTAGAGCCTATGTAGATGGATTCGAGCCGAACGGCTGCATTGAGGGCAAGCTTTCCCAAAT
GTCAAAAGAAGTGAACGCCAAAATTGAACCATTCTTGAGAACGACACCACGCCCCCTCAGATTGCCTGAT
GGGCCTCTTTGCCATCAGCGGTCAAAGTTCCTGCTGATGGATGCTCTGAAATTAAGTATTGAAGACCCGA
GTCACGAGGGGGAGGGAATACCACTATATGATGCAATCAAATGCATGAAGACATTCTTTGGCTGGAAGGA
GCCTAACATAGTCAAACCACATGAGAAAGGCATAAATCCCAATTACCTCATGGCTTGGAAGCAGGTGCTA
GCAGAGCTACAGGACATTGAAAATGAAGAGAAGATCCCAAGGACAAAGAACATGAAGAGAACAAGCCAAT
TGAAGTGGGCACTCGGTGAAAATATGGCACCAGAAAAAGTAGACTTTGATGATTGCAAAGATGTTGGAGA
CCTTAAACAGTATGACAGTGATGAGCCGGAGCCCAGAGCTCTAGCAAGCTGGGTCCAAAATGAATTCAAT
AAGGCATGTGAATTGACTGATTCAAGCTGGATAGAACTTGATGAAATAGGAGAAGATGTTGCCCCGATTG
AACATATCGCAAGCATGAGGAGGAACTATTTTACAGCAGAAGTGTCCCACTGCAGGGCTACTGAATACAT
AATGAAGGGAGTGTACATAAATACGGCCTTGCTCAATGCATCCTGTGCAGCCATGGATGACTTTCAGCTG
ATCCCAATGATAAGCAAATGTAGGACCAAAGAAGGAAGACGGAAAACAAACCTGTATGGGTTCATTATAA
AAGGAAGGTCTCATTTGAGAAATGATACTGATGTGGTGAACTTTGTAAGTATGGAGTTCTCACTCACTGA
CCCGAGACTGGAGCCACACAAATGGGAAAAATACTGTGTTCTTGAAATAGGAGACATGCTCTTGAGGACT
GCGATAGGCCAAGTGTCGAGGCCCATGTTCCTATATGTGAGAACCAATGGAACCTCCAAGATCAAGATGA
AATGGGGCATGGAAATGAGGCGCTGCCTTCTTCAGTCTCTTCAGCAAATTGAGAGCATGATTGAGGCCGA
GTCTTCTGTCAAAGAGAAAGACATGACCAAGGAATTCTTTGAAAACAAATCGGAAACATGGCCAATCGGA
GAGTCACCCAGGGGAGTGGAGGAAGGCTCTATTGGGAAAGTGTGCAGGACCTTACTGGCAAAATCTGTAT
TCAACAATCTATATGCGTCTCCACAACTTGAGGGGTTTTCGGCTGAATCGAGAAAATTGCTTCTCATTGT

TCAGGCACTTAGGGACAACCTGGAACCTGGAACCTTTGATCTTGGGGGGCTATATGAAGCAATCGAGGAG
TGCCTGATTAATGATCCCTGGGTTTTGCTTAATGCATCTTGGTTCAACTCCTTCCTCACACATGCACTGA
AATAGTTGTGGCAATGCTACTATTTGCTATCCATACTGTCCAAAAAGGTACCTTGTTTCTACT
>gi|396940847|dbj|AB704477.1| Influenza A virus (A/Tochigi/2/2010(H1N1)) PA gene for polymerase PA, complete cds
ATGAAAGACTTTGTGCGACAATGCTTCAATCCAATGATCGTCGAGCTTGCGGAAAAGGCAATGAAAGAAT
ATGGGGAAGATCCGAAAATCGAAACTAACAAGTTTGCTGCAATATGCACACATTTGGAAGTTTGTTTCAT
GTATTCGGATTTCCATTTCATCGACGAACGGGGTGAATCAATAATTGTAGAATCTGGTGACCCGAATGCA
CTATTGAAACACCGATTTGAGATAATTGAAGGAAGAGACCGAATCATGGCCTGGACAGTGGTGAACAGTA
TATGTAACACAACAGGGGTAGAGAAGCCTAAATTTCTTCCTGATTTGTATGATTACAAAGAGAACCGGTT
CATTGAAATTGGAGTAACACGGAGGGAAGTCCACATATATTACCTAGAGAAAGCCAACAAAATAAAATCT
GAGAAGACACACATTCACATCTTTTCATTCACTGGAGAGGAGATGGCCACCAAAGCAGACTACACCCTTG
ACGAAGAGAGCAGGGCAAGAATCAAAACTAGGCTTTTCACTATAAGACAAGAAATGGCCAGTAGGAGTCT
ATGGGATTCCTTTCGTCAGTCCGAAAGAGGCGAAGAGACAATTGAAGAAAAATTTGAGATTACAGGAACT
ATGCGCAAGCTTGCCGACCAAAGTCTCCCACCGAACTTCTCCAGCCTTGAAAACTTTAGAGCCTATGTAG
ATGGATTCGAGCCGAACGGCTGCATTGAGGGCAAGCTTTCCCAAATGTCAAAAGAAGTGAACGCCAAAAT
TGAACCATTCTTGAGGACGACACCACGCCCCCTCAGATTGCCTGATGGGCCTCTTTGCCATCAGCGGTCA
AAGTTCCTGCTGATGGATGCTCTGAAATTAAGTATTGAAGACCCGAGTCACGAGGGGGAGGGAATACCAC
TATATGATGCAATCAAATGCATGAAGACATTCTTTGGCTGGAAAGAGCCTAACATAGTCAAACCACATGA
GAAAGGCATAAATCCCAATTACCTCATGGCTTGGAAGCAGGTGCTAGCAGAGCTACAGGACATTGAAAAT
GAAGAGAAGATCCCAAGGACAAAGAACATGAAGAGAACAAGCCAATTGAAGTGGGCACTCGGTGAAAAT
A
TGGCACCAGAAAAAGTAGACTTTGATGACTGCAAAGATGTTGGAGACCTTAAACAGTATGACAGTGATGA
GCCAGAGCCCAGATCTCTAGCAAGCTGGGTCCAAAATGAATTCAATAAGGCATGTGAATTGACTGATTCA
AGCTGGATAGAACTTGATGAAATAGGAGAAGATTTTGCCCCGATTGAACATATCGCAAGCATGAGGAGGA
ACTATTTTACAGCAGAAGTGTCCCACTGCAGGGCTACTGAATACATAATGAAGGGAGTGTACATAAATAC
GGCCTTGCTCAATGCATCCTGTGCAGCCATGGATGACTTTCAGCTGATCCCAATGATAAGCAAATGTAGG
ACCAAAGAAGGAAGACGGAAAACAAACCTGTATGGGTTCATTATAAAAGGAAGGTCTCATTTGAGAAATG
ATACTGATGTGGTGAACTTTGTAAGTATGGAGTTCTCACTCACTGATCCGAGACTGGAGCCACACAAATG
GGAAAAATACTGTGTTCTTGAAATAGGAGACATGCTCTTGAGGACTGCGATAGGCCAAGTGTCGAGGCCC
ATGTTCCTATATGTGAGAACCAATGGAACCTCCAAGATCAAGATGAAATGGGGCATGGAAATGAGGCGCT
GCCTTCTTCAGTCTCTTCAGCAGATTGAGAGCATGATTGAGGCCGAGTCTTCTGTCAAAGAGAAAGACAT
GACCAAGGAATTCTTTGAAAACAAATCGGAAACATGGCCAATCGGAGAGTCACCCAGGGGAGTGGAGGA
A
GGATCTATTGGGAAAGTGTGCAGGACCTTACTGGCAAAATCTGTATTCAACAGTCTATATGCGTCTCCAC
AACTTGAGGGGTTTTCGGCTGAATCGAGAAAATTGCTTCTCATTGTTCAGGCACTTAGGGACAACCTGGA
ACCTGGAACCTTCGATCTTGGGGGGCTATATGAAGCAATCGAGGAGTGCCTGATTAATGATCCCTGGGTT
TTGCTTAATGCATCTTGGTTCAACTCCTTCCTCACACATGCACTGAAGTAG
>gi|344995021|gb|CY098095.1| Influenza A virus (A/Chile/89/2010(H1N1)) polymerase PA (PA) gene, complete cds
TCCAAAATGGAAGACTTTGTGCGACAATGCTTCAATCCAATGATCGTCGAGCTTGCGGAAAAGGCAATGA
AAGAATATGGGGAAGATCCGAAAATCGAAACTAACAAGTTTGCTGCAATATGCACACATTTGGAAGTTTG
TTTCATGTATTCGGATTTCCATTTCATCGACGAACGGGGTGAATCAATAATTGTAGAATCTGGTGACCCG
AATGCACTATTGAAGCACCGATTTGAGATAATTGAAGGAAGAGACCGAATCATGGCCTGGACAGTGGTGA

ACAGTATATGTAACACAACAGGGGTAGAGAAGCCTAAATTTCTTCCTGATTTGTATGACTACAAAGAGAA
CCGGTTCATTGAAATTGGAGTAACACGGAGGGAAGTCCACATATATTACCTAGAGAAAGCCAACAAAATA
AAATCTGAGAAGACACACATTCACATCTTTTCATTCACTGGAGAGGAGATGGCCACCAAAGCAGACTACA
CCCTTGACGAAGAGAGCAGGGCAAGAATCAAAACTAGGCTTTTCACTATAAGACAAGAAATGGCCAGTAG
GAGTCTATGGGATTCCTTTCGTCAGTCCGAAAGAGGCGAAGAGACAATTGAAGAAAAATTTGAGATTACA
GGAACTATGCGCAAGCTTGCCGACCAAAGTCTCCCACCGAACTTCTCCAGCCTTGAAAACTTTAGAGCCT
ATGTAGATGGATTCGAGCCGAACGGCTGCATTGAGGGCAAGCTTTCCCAAATGTCAAAAGAAGTGAACGC
CAAAATTGAACCATTCTTGAGGACGACACCACGCCCCCTCAGATTGCCTGATGGGCCTCTTTGCCATCAG
CGGTCAAAGTTCCTGCTGATGGATGCTCTGAAATTAAGTATTGAAGACCCGAGTCACGAGGGGGAGGGAA
TACCACTATATGATGCAATCAAATGCATGAAGACATTCTTTGGCTGGAAAGAGCCTAACATAGTCAAACC
ACATGAGAAAGGCATAAATCCCAATTACCTCATGGCTTGGAAGCAGGTGCTAGCAGAGCTACAGGACATT
GAAAATGAAGAGAAGATCCCAAGGACAAAGAACATGAAGAGAACAAGCCAATTGAAGTGGGCACTCGGT
G
AAAATATGGCACCAGAAAAAGTAGACTTTGATGACTGCAAAGATGTTGGAGACCTTAAACAGTATGACAG
TGATGAGCCAGAGCCCAGATCTCTAGCAAGCTGGGTCCAAAATGAATTCAATAAGGCATGTGAATTGACT
GATTCAAGCTGGATAGAACTTGATGAAATAGGAGAAGATGTTGCCCCGATTGAACATATCGCAAGCATGA
GGAGGAACTATTTTACAGCAGAAGTGTCCCACTGCAGGGCTACTGAATACATAATGAAGGGAGTGTACAC
AAATACGGCCTTGCTCAATGCATCCTGTGCAGCCATGGATGACTTTCAGCTGATCCCAATGATAAGCAAA
TGTAGGACCAAAGAAGGAAGACGGAAAACAAACCTGTATGGGTTCATTATAAAAGGAAGGTCTCATTTGA
GAAATGATACTGATGTGGTGAACTTTGTAAGTATGGAGTTCTCACTCACTGATCCGAGACTGGAGCCACA
CAAATGGGAAAAATACTGTGTTCTTGAAATAGGAGACATGCTCTTGAGGACTGCGATAGGCCAAGTGTCG
AGGCCCATGTTCCTATATGTGAGAACCAATGGAACCTCCAAGATCAAAATGAAATGGGGCATGGAAATGA
GGCGCTGCCTTCTTCAGTCTCTTCAGCAGATTGAGAGCATGATTGAGGCCGAGTCTTCTGTCAAAGAGAA
AGACATGACCAAGGAATTCTTTGAAAACAAATCGGAAACATGGCCAATCGGAGAGTCACCCAGGGGAGTG
GAGGAAGGCTCTATTGGGAAAGTGTGCAGGACCTTACTGGCAAAATCTGTATTCAACAGTCTATATGCGT
CTCCACAACTTGAGGGGTTTTCGGCTGAATCGAGAAAATTGCTTCTCATTGTTCAGGCACTTAGGGACAA
CCTGGAACCTGGAACCTTCGATCTTGGGGGGCTATATGAAGCAATCGAGGAGTGCCTGATTAATGATCCC
TGGGTTTTGCTTAATGCATCTTGGTTCAACTCCTTCCTCACACATGCACTAAAGTAGTTGTGGCAATGCT
ACTAT
>gi|344166286|gb|CY097963.1| Influenza A virus (A/Mexico City/WRAIR3569N/2010(H1N1)) polymerase PA (PA) gene, complete cds
ATGGAAGACTTTGTGCGACAATGCTTCAATCCAATGATCGTCGAGCTTGCGGAAAAGGCAATGAAAGAAT
ATGGGGAAGATCCGAAAATCGAAACTAACAAGTTTGCTGCAATATGCACACATTTGGAAGTTTGTTTCAT
GTATTCGGATTTCCATTTCATCGACGAACGGGGTGAATCAATAATTGTAGAATCTGGTGACCCGAATGCA
CTATTGAAACACCGATTTGAGATAATTGAAGGAAGAGACCGAATCATGGCCTGGACAGTGGTGAACAGTA
TATGTAACACAACAGGGGTAGAGAAGCCTAAATTTCTTCCTGATTTGTATGATTACAAAGAGAACCGGTT
CATTGAAATTGGAGTAACACGGAGGGAAGTCCACATATATTACCTAGAGAAAGCCAACAAAATAAAATCT
GAGAAGACACACATTCACATCTTTTCATTCACTGGAGAGGAGATGGCCACCAAAGCGGACTACACCCTTG
ACGAAGAGAGCAGAGCAAGAATCAAAACTAGGCTTTTCACTATAAGACGAGAAATGGCCAGTAGGAGTCT
ATGGGATTCCTTTCGTCAGTCCGAAAGAGGCGAAGAGACAATTGAAGAAAAATTTGAGATTACAGGAACT
ATGCGCAAGCTTGCCGACCAAAGTCTCCCACCAAACTTCTCCAGCCTTGAAAACTTTAGAGCCTATGTAG
ATGGATTCGAGCCGAACGGCTGCATTGAGGGCAAGCTTTCCCAAATGTCAAAAGAAGTGAACGCCAAAAT
TGAACCATTCTTGAGGACGACACCACGCCCCCTCAGATTGCCTGATGGGCCTCTTTGCCATCAGCGGTCA
AAGTTCCTGCTGATGGATGCTCTGAAATTAAGCATTGAAGACCCGAGTCACGAGGGGGAGGGAATACCAC

TATATGATGCAATCAAATGCATGAAGACATTCTTTGGATGGAAAGAGCCTAAAATAGTCAAACCACATGA
GAAAGGCATAAATCCCAATTACCTCATGACTTGGAAGCAGGTGCTAGCAGAGCTACAGGACATTGAAAAT
GAAGAGAAGATCCCAAGGACAAAGAACATGAAGAGAACAAGCCAATTGAAGTGGGCACTCGGTGAAAAT
A
TGGCACCAGAAAAAGTAGGCTTTGATGACTGCAAAGATGTTGGAGACCTTAAACAGTATGACAGTGATGA
GCCAGAGCCCAGATCTCTAGCAAGCTGGATCCAAAATGAATTCAATAAGGCATGTGAATTGACTGATTCA
AGCTGGATAGAACTTGATGAAATAGGAGAAGATGTTGCCCCGATTGAACATATCGCAAGCATGAGGAGGA
ACTATTTTACAGCAGAAGTGTCCCACTGCAGGGCTACTGAATACATAATGAAGGGAGTGTACATAAATAC
GGCCCTGCTCAATGCATCCTGTGCAGCCATGGATGACTTTCAGCTGATCCCAATGATAAGCAAATGTAGG
ACCAAAGAAGGAAGACGGAAAACAAACCTGTATGGGTTCATTATAAAAGGAAGGTCTCATTTGAGAAATG
ATACTGATGTGGTGAACTTTGTAAGTATGGAGTTCTCACTCACTGACCCGAGACTGGAGCCACACAAATG
GGAAAAATACTGTGTTCTTGAAATAGGAGACATGCTCTTGAGGACTGCAATAGGCCAAGTGTCGAGGCCC
ATGTTCCTATATGTGAGAACCAATGGAACCTCCAAGATCAAGATGAAATGGGGCATGGAAATGAGGCGCT
GTCTTCTTCAGTCTCTTCAGCAGATTGAGAGCATGATTGAAGCCGAGTCTTCTGTCAAAGAGAAAGACAT
GACCAAGGAATTCTTTGAAAACAAATCGGAAACATGGCCAATCGGAGAGTCACCCAGGGGAGTGGAGGA
A
GGCTCTATTGGGAAAGTGTGCAGGACCTTACTGGCAAAATCTGTATTCAACAGTCTATATGCGTCTCCAC
AACTTGAGGGGTTTTCGGCTGAATCAAGAAAATTGCTTCTCATTGTTCAGGCACTTAGGGACAACCTGGA
ACCTGGAACCTTCGATCTTGGGGGGCTATATGAAGCAATCGAGGAGTGCCTGATTAATGATCCCTGGGTT
TTGCTTAATGCATCTTGGTTCAACTCCTTCCTCACTCATGCACTGAAGTAG
>gi|344166268|gb|CY097955.1| Influenza A virus (A/Amman/WRAIR3448T/2010(H1N1)) polymerase PA (PA) gene, complete cds
ATGGAAGACTTTGTGCGACAATGCTTCAATCCAATGATCGTCGAGCTTGCGGAAAAGGCAATGAAAGAAT
ATGGGGAAGATCCGAAAATCGAAACTAACAAGTTTGCTGCAATATGCACACATTTGGAAGTTTGTTTCAT
GTATTCGGATTTCCATTTCATCGACGAACGGGGTGAATCAATAATTGTAGAATCTGGTGACCCGAATGCA
CTATTGAAACACCGATTTGAGATAATTGAAGGAAGAGACCGAATCATGGCCTGGACAGTGGTGAACAGTA
TATGTAACACAACAGGGGTAGAGAAGCCTAAATTTCTTCCTGATTTGTATGATTACAAAGAGAACCGGTT
CATTGAAATTGGAGTAACACGGAGGGAAGTCCACATATATTACCTAGAGAAAGCCAACAAAATAAAATCT
GAGAAGACACACATTCACATCTTTTCATTCACTGGAGAGGAGATGGCCACCAAAGCGGACTACACCCTTG
ACGAAGAGAGCAGAGCAAGAATCAAAACTAGGCTTTTCACTATAAGACAAGAAATGGCCAGTAGGAGTCT
ATGGGATTCCTTTCGTCAGTCCGAAAGAGGCGAAGAGACAATTGAAGAAAAATTTGAGATTACAGGAACT
ATGCGCAAGCTTGCCGACCAAAGTCTCCCACCGAACTTCTCCAACCTTGAAAACTTTAGAGCCTATGTAG
ATGGATTCGAGCCGAACGGCTGCATTGAGGGCAAGCTTTCCCAAATGTCAAAAGAAGTGAATGCCAAAAT
TGAACCATTCTTGAGGACGACACCACGCCCTCTCAGATTGCCTGATGGACCTCTTTGCCATCAGCGGTCA
AAGTTCCTGCTGATGGATGCTCTGAAATTAAGTATTGAAGACCCGAGTCACGAGGGGGAGGGAATACCAC
TATATGATGCAATCAAATGCATGAAGACATTCTTTGGCTGGAAAGAGCCTAACATAGTCAAACCACATGA
GAAAGGCATAAATCCCAATTACCTCATGGCTTGGAAGCAGGTGCTAGCAGAGCTACAGGACATTGAAAAT
GAAGAGAAGATCCCAAGGACAAAGAACATGAAGAGAACAAGCCAATTGAAGTGGGCACTCGGTGAAAAT
A
TGGCACCAGAAAAAGTAGACTTTGATGACTGCAAAGATGTTGGAGACCTTAAACAGTATGACAGTGATGA
GCCAGAGCCCAAATCTCTAGCAAGCTGGGTCCAAAATGAATTCAATAAGGCATGTGAATTGACTGATTCA
AGCTGGATAGAACTTGATGAAATAGGAGAAGATGTTGCCCCGATTGAACATATCGCAAGCATGAGGAGGA
ACTATTTTACAGCAGAAGTGTCCCACTGCAGGGCTACTGAATACATAATGAAGGGAGTGTACATAAATAC
GGCCTTGCTCAATGCATCCTGTGCAGCCATGGATGAATTTCAGCTGATCCCAATGATAAGCAAATGTAGG

ACCAAAGAAGGAAGACGGAAAACAAACCTGTATGGGTTCATTATAAAAGGAAGGTCTCATTTGAGAAATG
ATACTGATGTGGTGAACTTTGTAAGTATGGAGTTCTCACTCACTGACCCGAGACTGGAGCCACACAAATG
GGAAAAATACTGTGTTCTTGAAATAGGAGACATGCTCTTGAGGACTGCGATAGGCCAAGTGTCGAGGCCC
ATGTTCCTATATGTGAGAACCAATGGAACCTCCAAGATCAAGATGAAATGGGGCATGGAAATGAGGCGCT
GCCTTCTTCAGTCTCTTCAGCAGATTGAGAGCATGATTGAGGCCGAGTCTTCTGTCAAAGAGAAAGACAT
GACCAAGGAATTCTTTGAAAACAAATCGGAAACATGGCCAATCGGAGAGTCACCCAGGGGAGTGGAGGA
A
GGCTCTATTGGGAAAGTGTGCAGGACCTTGCTGGCAAAATCTGTATTCAACAGTCTATATGCGTCTCCAC
AACTTGAGGGGTTTTCGGCTGAATCGAGAAAGTTGCTTCTCATTGTTCAGGCACTTAGGGACAACCTGGA
ACCTGGAACCTTCGATCTTGGGGGGCTATATGAAGCAATCGAGGAGTGCCTGATTAATGATCCCTGGGTT
TTGCTTAATGCATCTTGGTTCAACTCCTTCCTCACACATGCACTGAAGTAG
>gi|344165958|gb|CY097844.1| Influenza A virus (A/District of Columbia/WRAIR0310/2010(H1N1)) polymerase PA (PA) gene, complete cds
ATGGAAGACTTTGTGCGACAATGCTTCAATCCGATGATCGTCGAGCTTGCGGAAAAGGCAATGAAAGAAT
ATGGGGAAGATCCGAAAATCGAAACTAATAAGTTTGCTGCAATATGCACACATTTGGAAGTTTGTTTCAT
GTATTCGGATTTCCATTTCATCGATGAACGGGGTGAATCAATAATTGTAGAATCTGGTGACCCGAATGCA
CTATTGAAGCACCGATTTGAGATAATTGAAGGAAGAGACCGAATCATGGCCTGGACAGTGGTGAACAGTA
TATGTAACACAACAGGGGTAGAGAAGCCTAAATTTCTTCCTGATTTGTATGATTACAAAGAGAACCGGTT
CATTGAAATTGGAGTAACACGGAGGGAAGTCCACATATATTACCTAGAGAAAGCCAACAAAATAAAATCT
GAGAAGACACACATTCACATCTTTTCATTCACTGGAGAGGAGATGGCCACCAAAGCGGACTACACCCTTG
ACGAAGAGAGCAGGGCAAGAATCAAAACTAGGCTTTTCACTATAAGACAAGAAATGGCCAGTAGGAGTCT
ATGGGATTCCTTTCGTCAGTCCGAAAGAGGCGAAGAGACAATTGAAGAAAAATTTGAGATTACAGGAACT
ATGCGCAAGCTTGCCGACCAAAGTCTCCCACCAAACTTCTCCAGCCTTGAAAACTTTAGAGCCTATGTAG
ATGGGTTCGAGCCGAACGGCTGCATTGAGGGCAAGCTTTCCCAAATGTCAAAAGAAGTGAACGCCAAAAT
TGAACCATTCTTGAGGACGACACCACGCCCCCTCAGATTGCCTGATGGGCCTCTTTGCCATCAGCGGTCA
AAGTTCCTTCTGATGGATGCTCTGAAATTAAGCATTGAAGACCCGAGTCACGAGGGGGAGGGAATACCAC
TATATGATGCAATCAAATGCATGAAGACATTCTTTGGCTGGAAAGAGCCTAAAATAGTCAAACCACATGA
GAAAGGCATAAATCCCAATTACCTCATGACTTGGAAGCAGGTGCTAGCAGAGCTACAGGACATTGAAAAT
GAAGAGAAGATCCCAAGGACAAAGAACATGAAGAGAACAAGCCAATTGAAGTGGGCACTCGGTGAAAAT
A
TGGCACCAGAAAAAGTAGACTTTGATGACTGCAAAGATGTTGGAGACCTTAAACAGTATGACAGTGATGA
GCCAGAGCCCAGATCTCTAGCAAGCTGGATCCAAAATGAATTCAATAAGGCATGTGAATTGACTGATTCA
AGCTGGATAGAACTTGATGAAATAGGAGAAGATGTTGCCCCGATTGAACATATCGCAAGCATGAGGAGGA
ACTATTTTACAGCAGAAGTGTCCCACTGCAGGGCTACTGAATACATAATGAAGGGAGTGTACATAAATAC
GGCCTTGCTCAATGCATCCTGTGCAGCCATGGATGACTTTCAGCTGATCCCAATGATAAGCAAATGTAGG
ACCAAAGAAGGAAGACGGAAAACAAACCTGTATGGGTTCATTATAAAAGGAAGGTCTCATTTGAGAAATG
ATACTGATGTGGTGAACTTTGTAAGTATGGAGTTCTCACTCACTGACCCGAGACTGGAGCCACACAAATG
GGAAAAATACTGTGTTCTTGAAATAGGAGACATGCTCTTGAGGACTGCAATAGGCCAAGTGTCGAGGCCC
ATGTTCCTATATGTGAGAACCAATGGAACCTCCAAGATCAAGATGAAATGGGGCATGGAAATGAGGCGCT
GTCTTCTTCAGTCTCTTCAGCAGATTGAGAGCATGATTGAAGCCGAGTCTTCTGTCAAAGAGAAAGACAT
GACCAAGGAATTCTTTGAAAACAAATCGGAAACATGGCCAATCGGAGAGTCACCCAGGGGAGTGGAGGA
A
GGCTCTATTGGGAAAGTGTGCAGGACCTTACTGGCAAAATCTGTATTCAACAGTCTATATGCGTCTCCAC
AACTTGAGGGGTTTTCGGCTGAATCAAGAAAATTGCTTCTCATTGTTCAGGCACTTAGGGACAACCTGGA

ACCTGGAACCTTCGAAATTGGGGGGCTGTATGAAGCAATCGAGGAGTGCCTGATTAATGATCCCTGGGTT
TTGCTTAATGCATCTTGGTTCAACTCCTTCCTCACACATGCACTGAAGTAG
>gi|344165832|gb|CY097788.1| Influenza A virus (A/Dakar/WRAIR0020T/2010(H1N1)) polymerase PA (PA) gene, complete cds
ATGGAAGAATTTGTGCGACAATGCTTCAATCCAATGATCGTCGAGCTTGCGGAAAAGGCAATGAAAGAAT
ATGGGGAAGATCCGAAAATCGAAACTAACAAGTTTGCTGCAATATGCACACATTTGGAAGTTTGTTTCAT
GTATTCGGATTTCCATTTCATCGACGAACGGGGTGAATCAATAATTGTAGAATCTGGTGACCCGAATGCA
CTATTGAAGCACCGATTTGAGATAATTGAAGGAAGAGACCGAATCATGGCCTGGACAGTGGTGAACAGTA
TATGTAACACAACAGGGGTAGAGAAGCCTAAATTTCTTCCTGATTTGTATGATTACAAAGAGAACCGGTT
CATTGAAATTGGAGTAACACGGAGGGAAGTCCACATATATTACCTAGAGAAAGCCAACAAAATAAAATCT
GAGAAGACACACATACACATCTTTTCATTCACTGGAGAGGAGATGGCCACCAAAGCGGACTACACCCTTG
ACGAAGAGAGCAGGGCAAGAATCAAAACTAGGCTTTTCACTATAAGACAAGAAATGGCCAGTAGGAGTCT
ATGGGATTCCTTTCGTCAGTCCGAAAGAGGCGAAGAGACAATTGAAGAAAAATTTGAGATTACAGGAACT
ATGCGCAAGCTTGCCGACCAAAGTCTCCCACCGAACTTCTCCAGCCTTGAAAACTTTAGAGCCTATGTAG
ATGGATTCGAGCCGAACGGCTGCATTGAGGGCAAGCTTTCCCAAATGTCAAAAGAAGTGAACGCCAAAAT
TGAACCATTCTTGAGGACGACACCACGCCCCCTCAGATTGCCTGATGGGCCTCTTTGCCATCAGCGGTCA
AAGTTCCTGCTGATGGATGCTCTGAAATTAAGTATTGAAGACCCGAGTCACGAGGGGGAGGGAATACCAC
TATATGATGCAATCAAATGCATGAAGACATTCTTTGGCTGGAAAGAGCCTAACATAGTCAAACCACATGA
GAAAGGCATAAATCCCAATTACCTCATGGCTTGGAAGCAGGTGCTAGCAGAGCTACAGGACATTGAAAAT
GAAGAGAAGATCCCAAGGACAAAGAACATGAAGAGAACAAGCCAATTGAAGTGGGCACTCGGTGAAAAT
A
TGGCACCCGAAAAAGTAGACTTTGATGACTGCAAAGATGTTGGAGACCTTAAACAGTATGACAGTGATGA
GCCAGAGCCCAGATCTCTAGCAAGCTGGGTCCAAAATGAATTCAATAAGGCATGTGAATTGACTGATTCA
AGCTGGATAGAACTTGATGAAATAGGAGAAGATGTTGCCCCGATTGAACATATCGCAAGCATGAGGAGGA
ACTATTTTACAGCAGAAGTGTCCCACTGCAGGGCTACTGAATACATAATGAAGGGAGTGTACATAAATAC
GGCCTTGCTCAATGCATCCTGTGCAGCCATGGATGACTTTCAGCTGATCCCAATGATAAGCAAATGTAGG
ACCAAAGAAGGAAGACGGAAAACAAACCTGTATGGGTTCATTATAAAAGGAAGGTCTCATTTGAGAAATG
ATACTGATGTGGTGAACTTTGTAAGTATGGAGTTCTCACTCACTGACCCGAGACTGGAGCCACACAAATG
GGAAAAATACTGTGTTCTTGAAATAGGAGACATGCTCTTGAGGACTGCGATAGGCCAAGTGTCGAGGCCC
ATGTTCCTATATGTGAGAACCAATGGAACCTCCAAGATCAAGATGAAATGGGGCATGGAAATGAGGCGCT
GCCTTCTTCAGTCTCTTCAACAGATTGAGAGCATGATTGAGGCCGAGTCTTCTGTCAAAGAGAAAGACAT
GACCAAGGAATTCTTTGAAAACAAATCGGAAACATGGCCAATCGGAGAGTCACCCAGGGGAGTGGAGGA
A
GGCTCTATTGGGAAAGTGTGCAGGACCTTACTGGCAAAATCTGTATTCAACAGTCTATATGCGTCTCCAC
AACTTGAGGGGTTTTCGGCTGAATCGAGAAAATTGCTTCTCATTGTTCAGGCACTTAGGGACAACCTGGA
ACCTGGAACCTTCGATCTTGGGGGGCTATATGAAGCAATCGAGGAGTGCCTGATTAATGATCCCTGGGTT
TTGCTTAATGCATCTTGGTTCAACTCCTTCCTCACACATGCACTGAAGTAG
>gi|338221022|gb|CY092747.1| Influenza A virus (A/Sydney/DD3-56/2010(H1N1)) polymerase PA (PA) gene, complete cds
TCCAAAATGGAAGACTTTGTGCGACAATGCTTCAATCCAATGATCGTCGAGCTTGCGGAAAAGGCAATGA
AAGAATATGGGGAAGATCCGAAAACCGAAACTAACAAGTTTGCTGCAATATGCACACATTTGGAAGTTTG
TTTCATGTATTCGGATTTCCATTTCATCGACGAACGGGGTGAATCAATAATTGTAGAATCTGGTGACCCG
AATGCACTATTGAAGCACCGATTTGAGATAATTGAAGGAAGAGACCGAATCATGGCCTGGACAGTGGTGA
ACAGTATATGTAACACAACAGGGGTAGAGAAGCCTAAATTTCTTCCTGATTTGTATGACTACAAAGAGAA

CCGGTTCATTGAAATTGGAGTAACACGGAGGGAAGTCCACATATATTACCTAGAGAAAGCCAACAAAATA
AAATCTGAGAAGACACACATTCACATCTTTTCATTCACTGGAGAGGAGATGGCCACCAAAGCAGACTACA
CCCTTGACGAAGAGAGCAGGGCAAGAATCAAAACTAGGCTTTTCACTATAAGACAAGAAATGGCCAGTAG
GAGTCTATGGGATTCCTTTCGTCAGTCCGAAAGAGGCGAAGAGACAATTGAAGAAAAATTTGAGATTACA
GGAACTATGCGCAAGCTTGCCGACCAAAGTCTCCCACCGAACTTCTCCAGCCTTGAAAACTTTAGAGCCT
ATGTAGATGGATTCGAGCCGAACGGCTGCATTGAGGGCAAGCTTTCCCAAATGTCAAAAGAAGTGAACGC
CAAAATTGAACCATTCTTGAGGACGACACCACGCCCCCTCAGATTGCCTGATGGGCCTCTTTGCCATCAG
CGGTCAAAGTTCCTGCTGATGGATGCTCTGAAATTAAGTATTGAAGACCCGAGTCACGAGGGGGAGGGAA
TACCACTATATGATGCAATCAAATGCATGAAGACATTCTTTGGCTGGAAAGAGCCTAACATAGTCAAACC
ACATGAGAAAGGCATAAATCCCAATTACCTCATGGCTTGGAAGCAGGTGCTAGCAGAGCTACAGGACATT
GAAAATGAAGAGAAGATCCCAAGGACTAAGAACATGAAGAGAACAAGCCAATTGAAGTGGGCACTCGGT
G
AAAATATGGCACCAGAAAAAGTAGACTTTGATGACTGCAAAGATGTTGGAGACCTTAAACAGTATGACAG
TGATGAGCCAGAGCCCAGATCTCTAGCAAGCTGGGTCCAAAATGAATTCAATAAGGCATGTGAATTGACT
GATTCAAGCTGGATAGAACTTGATGAAATAGGAGAAGATGTTGCCCCGATTGAACATATCGCAAGCATGA
GGAGGAACTATTTTACAGCAGAAGTGTCCCACTGCAGGGCTACTGAATACATAATGAAGGGAGTGTACAT
AAATACGGCCTTGCTCAATGCATCCTGTGCAGCCATGGATGACTTTCAGCTGATCCCAATGATAAGCAAA
TGTAGGACCAAAGAAGGAAGACGGAAAACAAACCTGTATGGGTTCATTATAAAAGGAAGGTCTCATTTGA
GAAATGATACTGATGTGGTGAACTTTGTAAGTATGGAGTTCTCACTCACTGATCCGAGACTGGAGCCACA
CAAATGGGAAAAATACTGTGTTCTTGAAATAGGAGACATGCTCTTGAGGACTGCGATAGGCCAAGTGTCG
AGGCCCATGTTCCTATATGTGAGAACCAATGGAACCTCCAAGATCAAGATGAAATGGGGCATGGAAATGA
GGCGCTGCCTTCTTCAGTCTCTTCAGCAGATTGAGAGCATGATTGAGGCCGAGTCTTCTGTCAAAGAGAA
AGACATGACCAAGGAATTCTTTGAAAACAAATCGGAAACATGGCCAATCGGAGAGTCACCCAGGGGAGTG
GAGGAAGGCTCTATTGGGAAAGTGTGCAGGACCTTACTGGCAAAATCTGTATTCAACAGTCTATATGCGT
CTCCACAACTTGAGGGGTTTTCGGCTGAATCGAGAAAATTGCTTCTCATTGTTCAGGCACTTAGGGACAA
CCTGGAACCTGGAACCTTCGATCTTGGGGGGCTATATGAAGCAATCGAGGAGTGCCTGATTAATGATCCC
TGGGTTTTGCTTAATGCATCTTGGTTCAACTCCTTCCTCACACATGCACTAAAGTAGTTGTGGCAATGCT
ACTAT
>gi|343132003|gb|CY096255.1| Influenza A virus (A/Melbourne/INS472/2010(H1N1)) polymerase PA (PA) gene, complete cds
TCCAAAATGGAAGACTTTATGCGACAATGCTTCAATCCAATGATCGTCGAGCTTGCGGAAAAGGCAATGA
AAGAATATGGGGAAGATCCGAAAATCGAAACTAACAAGTTTGCTGCAATATGCACACATTTGGAAGTTTG
TTTCATGTATTCGGATTTCCATTTCATCGACGAAAGGGGTGAATCAATAATTGTAGAATCTGGTGACCCG
AATGCACTATTGAAGCACCGATTTGAGATAATTGAAGGAAGAGACCGAATCATGGCCTGGACAGTGGTGA
ACAGTATATGTAACACAACAGGGGTAGAGAAGCCTAAATTTCTTCCTGATTTGTATGATTACAAAGAGAA
CCGGTTCATTGAAATTGGAGTAACACGGAGGGAAGTCCACATATATTACCTAGAGAAAGCCAACAAAATA
AAATCTGAGAAGACACACATTCACATCTTTTCATTCACTGGAGAGGAGATGGCCACCAAAGCAGACTACA
CCCTTGACGAAGAGAGCAGGGCAAGAATCAAAACTAGGCTTTTCACTATAAGACAAGAAATGGCCAGTAG
GAGTCTATGGGATTCCTTTCGTCAGTCCGAAAGAGGCGAAGAGACAATTGAAGAAAAATTTGAGATTACA
GGAACTATGCGCAAGCTTGCCGACCAAAGTCTCCCACCGAACTTCTCCAGCCTTGAAAACTTTAGAGCCT
ATGTAGATGGATTCGAGCCGAACGGCTGCATTGAGGGCAAGCTTTCCCAAATGTCAAAAGAAGTGAACGC
CAAAATTGAACCATTCTTGAGGACGACACCACGCCCCCTCAGATTGCCTGATGGGCCTCTTTGCCATCAG
CGGTCAAAGTTCCTACTGATGGATGCTCTGAAATTAAGTATTGAAGACCCGAGTCACGAGGGGGAGGGAA
TACCACTATATGATGCAATCAAATGCATGAAGACATTCTTTGGCTGGAAAGAGCCTAACATAGTCAAACC

ACATGAGAAAGGCATAAATCCCAATTACCTCATGGCTTGGAAGCAGGTGCTAGCAGAGCTACAGGACATT
GAAAATGAAGAGAAGATCCCAAGGACAAAGAACATGAAGAGAACAAGCCAATTGAAGTGGGCACTCGGT
G
AAAATATGGCACCAGAAAAAGTAGACTTTGATGACTGCAAAGATGTTGGAGACCTTAAACAGTATGACAG
TGATGAGCCAGAGCCCAGATCTCTAGCAAGCTGGGTCCAAAATGAATTCAATAAGGCATGTGAATTGACT
GATTCAAGCTGGATAGAACTTGATGAAATAGGAGAAGATGTTGCCCCGATTGAACATATCGCAAGCATGA
GGAGGAACTATTTTACAGCAGAAGTGTCCCACTGCAGGGCTACTGAATACATAATGAAGGGAGTGTACAT
AAATACGGCCTTGCTCAATGCATCCTGTGCAGCCATGGATGACTTTCAGCTGATCCCAATGATAAGCAAA
TGTAGGACCAAAGAAGGAAGACGGAAAACAAACCTGTATGGGTTCATTATAAAAGGAAGGTCTCATTTGA
GAAATGATACTGATGTGGTGAACTTTGTAAGTATGGAGTTCTCACTCACTGATCCGAGACTGGAGCCACA
CAAATGGGAAAAATACTGTGTTCTTGAAATAGGAGACATGCTCTTGAGGACTGCGATAGGCCAAGTGTCG
AGGCCCATGTTCCTATATGTGAGGACCAATGGAACCTCCAAGATCAAGATGAAATGGGGCATGGAAATGA
GGCGCTGCCTTCTTCAGTCTCTTCAGCAGATTGAGAATATGATTGAGGCCGAGTCTTCTGTCAAAGAGAA
AGACATGACCAAGGAATTCTTTGAAAACAAATCGGAAACATGGCCAATCGGAGAGTCACCCAGGGGAGTG
GAGGAAGGCTCTATTGGGAAAGTGTGCAGGACCTTACTGGCAAAATCTGTATTCAACAGTCTATATGCGT
CTCCACAACTTGAGGGGTTTTCGGCTGAATCGAGAAAATTGCTTCTCATTGTTCAGGCACTTAGGGACAA
CCTGGAACCTGGAACCTTCGATCTTGGGGGGCTATATGAAGCAATTGAGGAGTGCCTGATTAATGATCCC
TGGGTTTTGCTTAATGCATCTTGGTTCAACTCCTTCCTCACACATGCACTAAAGTAGTTGTGGCAATGCT
ACTAT
>gi|328496344|gb|CY089436.1| Influenza A virus (A/Thailand/CU-B4339/2010(H1N1)) polymerase PA (PA) gene, complete cds
ATGGAAGACTTTGTGCGACAATGCTTCAATCCAATGATCGTCGAGCTTGCGGAAAAGGCAATGAAAGAAT
ATGGGGAAGATCCGAAAATCGAAACTAATAAGTTTGCTGCAATATGCACACATTTGGAAGTTTGTTTCAT
GTATTCGGATTTCCATTTCATCGACGAACGGGGTGAATCAATAATTGTAGAATCTGGTGACCCGAATGCA
CTATTGAAGCACCGATTTGAGATAATTGAAGGAAGAGACCGAATCATGGCCTGGACAGTGGTGAACAGTA
TATGTAACACAACAGGGGTAGAGAAGCCTAAATTTCTTCCTGATTTGTATGATTATAAAGAGAACCGGTT
CATTGAAATTGGAGTAACACGGAGGGAAGTCCACATATATTACCTAGAGAAAGCCAACAAAATAAAATCT
GAGAAGACACACATTCACATCTTTTCATTCACTGGAGAGGAAATGGCCACCAAAGCGGACTACACCCTTG
ACGAAGAGAGCAGGGCGAGAATCAAAACTAGGCTTTTCACTATAAGACAAGAAATGGCCAGTAGAAGTCT
ATGGGATTCCTTTCGTCAGTCCGAAAGAGGCGAAGAGACAATTGAAGAAAAATTTGAGATTACAGGAACT
ATGCGCAAGCTTGCCGACCAAAGTCTCCCACCAAACTTCTCCAGCCTTGAAAACTTTAGAGCCTATGTAG
ATGGATTCGAGCCGAACGGCTGCATTGAGGGCAAGCTTTCCCAAATGTCAAAAGAAGTGAACGCCAAAAT
TGAACCATTCTTGAGGACGACACCACGCCACCTCAGATTGCCTGATGGGCCTCTTTGCCATCAGCGGTCA
AAGTTCCTGCTGATGGATGCTCTGAAATTAAGCATTGAAGACCCGAGTCACGAGGGGGAGGGAATACCAC
TATATGATGCAATCAAATGCATGAAGACATTCTTTGGCTGGAAAGAGCCTAAAATAGTCAAACCACATGA
GAAAGGCATAAATCCCAATTACCTCATGGCTTGGAAGCAGGTGCTAACAGAGCTACAGGACATTGAAAAT
GAAGAGAAGATCCCAAGGACAAAGAACATGAAGAGAACAAGCCAATTGAAGTGGGCACTCGGTGAAAAT
A
TGGCACCAGAAAAAGTAGACTTTGATGACTGCAAAGATGTTGGAGACCTTAAACAGTATGACAGTGATGA
GCCAGAGCCCAGATCTCTAGCAAGCTGGATCCAAAATGAATTCAATAAGGCATGTGAATTGACTGATTCA
AGCTGGATAGAACTTGATGAAATAGGAGAAGATGTTGCCCCGATTGAACATATCGCAAGCATGAGGAGGA
ACTATTTTACAGCAGAAGTGTCCCACTGCAGGGCTACTGAATACATAATGAAGGGAGTGTACATAAATAC
GGCCTTGCTCAATGCATCCTGTGCAGCTATGGATGACTTTCAGCTGATCCCAATGATAAGCAAATGTAGG
ACCAAAGAAGGAAGACGGAAAACAAACCTGTATGGGTTCATTATAAAAGGAAGGTCTCATTTGAGAAATG

ATACTGATGTGGTGAACTTTGTAAGTATGGAGTTCTCACTCACTGACCCGAGACTGGAGCCACACAAATG
GGAAAAATACTGTGTTCTTGAAATAGGAGACATGCTCTTGAGGACTGCGATAGGCCAAGTGTCGAGGCCC
ATGTTCCTATATGTGAGAACCAATGGAACCTCCAAGATCAAGATGAAATGGGGCATGGAAATGAGGCGCT
GCCTTCTTCAGTCTCTTCAGCAGATCGAGAGCATGATTGAGGCCGAGTCTTCTGTCAAAGAGAAAGACAT
GACCAAGGAATTCTTTGAAACCAAATCGGAAACATGGCCAATCGGAGAGTCACCCAGGGGAGTGGAGGAA
GGCTCTATTGGGAAAGTATGCAGGACCTTACTGGCAAAATCTGTATTCAACAGTCTATATGCGTCCCCAC
AACTTGAGGGGTTTTCGGCTGAATCAAGAAAATTGCTTCTCATTGTTCAGGCACTTAGGGACAACCTGGA
ACCTGGAACCTTCGATCTTGGGGGGCTATATGAAGCAATCGAGGAGTGCCTGATTAATGATCCCTGGGTT
TTGCTTAATGCATCCTGGTTCAACTCCTTCCTCACACATGCACTGAAGTAGTTGTGGCAATGCTACTATT
TGCTATCC
>gi|363805065|gb|JQ290184.1| Influenza A virus (A/Iowa/09/2011(H3N2)) segment 3 polymerase PA (PA) gene, complete cds
ATGGAAGACTTTGTGCGACAATGCTTCAATCCAATGATCGTCGAGCTTGCGGAAAAGGCAATGAAAGAAT
ATGGGGAAGATTCGAAAATCGAAACCAACAAGTTCGCTGCAATATGCACGCACTTGGAAGTCTGTTTCAT
GTATTCGGACTTCCATTTCATTGACGAACAGGGTGAATCAATCATTGTAGAATCTGGTGATCCAAATGCA
TTGCTGAAGCACCGATTTGAGATAATTGAAGGAAGAGACAGGACTATGGCTTGGACAGTAGTGAACAGTA
TCTGCAACACCACAGGGGTAGAGAAGCCTAAATTTCTTCCGGATTTATACGACTACAAAGAAAATCGATT
CGTTGAAATTGGAGTGACACGAAGGGAGGTCCACATATACTACCTAGAGAAAGCCAACAAAATAAAATCC
GAGAAGACACACATTCATATTTTTTCATTCACTGGAGAGGAGATGGCCACCAAAGCGGACTACACCCTTG
ACGAAGAGAGCAGGGCAAGAATCAAGACCAGGCTCTTCACCATAAGACAAGAGATGGCCAGTAGGGGTCT
ATGGGATTCCTTTCGTCAGTCCGAGAGAGGCGAAGAGACAATTGAAGAAAGATTTGAAATTACAGGAACC
ATGCGCAGGCTTGCCGACCAAAGTCTCCCACCGAACTTCTCCAGCCTTGAAAACTTTAGAGCCTATGTAG
ATGGATTCGAACCAAACGGCTGCATTGAGGGCAAGCTTTCTCAAATGTCAAAAAAAGTGAGTGCCCAAAT
TGAACCATTCTTGAAGACAACACCACGCCCTCTCAGATTGCCTGATGGGCCCCCTTGCTCTCAGCGGTCA
AAGTTCTTGCTGATGGATGCTCTGAAACTAAGTATTGAAGACCCGAGTCATGAAGGGGAAGGAATACCAC
TATATGATGCAATCAAGTGCATGAAGACATTTTTTGGCTGGAAAGAGCCTAACATAATCAAACCTCATGA
GAAAGGCATAAACCCCAATTATCTACTGGCTTGGAAGCAAGTACTAGCAGAGCTCCAGGACATTGAAAAT
GAAGAGAAGATCCCAAAGACAAAGAACATGAAGAGAACAAGCCAATTGAAGTGGGCACTTGGTGAGAAT
A
TGGCACCAGAGAAAGTAGATTTTGATGACTGCAAAGATGTCGGTGATCTCAAACAGTATGACAGCGATGA
GCCAGAGCCTAGATCTCTAGCAAGCTGGGTCCAAAATGAGTTCAACAAAGCATGTGAATTAACCGATTCA
AGCTGGATAGAACTTGATGAGATAGGAGAAGATGTTGCCCCGATTGAACACATCGCAAGCATGAGGAGGA
ACTATTTTACAGCAGAAGTGTCCCATTGCAGGGCTACTGAATACATAATGAAGGGAGTGTACATAAATAC
GGCTCTCCTTAATGCATCTTGTGCAGCCATGGATGACTTTCAGCTGATCCCAATGATAAGCAAATGTAGG
ACCAAAGAAGGAAGACGAAGAACAAATCTGTACGGCTTCATTATAAAGGGAAGGTCCCATCTGAGAAATG
ATACTGACGTGGTGAACTTTGTAAGCATGGAGTTCTCCCTCACTGACCCGAGGCTGGAGCCACACAAATG
GGAAAAGTACTGTGTTCTTGAAATAGGAGACATGCTCCTGAGGACTGCAATAGGCCAAGTGTCGAGGCCC
ATGTTCCTATATGTGAGAACCAATGGAACCTCCAAGATCAAGATGAAATGGGGCATGGAAATGAGGCGCT
GCCTTCTCCAGTCTCTTCAGCAGATTGAGAGTATGATTGAGGCTGAGTCTTCTGTCAAAGAGAAAGACAT
GACCAAGGAATTCTTTGAAAACAAATCGGAAACATGGCCAATCGGAGAATCACCCAAAGGAGTGGAGGAA
GGCTCCATTGGGAAAGTGTGCAGGACCTTACTGGCAAAATCTGTATTCAACAGTCTATACGCGTCTCCAC
AGCTTGAGGGGTTTTCGGCTGAATCGAGAAAGTTGCTCCTCATTGTTCAGGCACTTAGGGACAACTTGGA
ACCTGGGACCTTTGATCTTGGGGGGCTATATGAAGCAATCGAGGAGTGCCTGATTAATGATCCCTGGGTT
TTGCTTAATGCATCTTGGTTCAACTCCTTCCTCACACATGCACTGAAATAG

>gi|350612171|gb|JN714507.1| Influenza A virus (A/Volgograd/CRIE-DMV/2011(H1N1)) segment 3 polymerase PA (PA) gene, complete cds
ATGGAAGACTTTGTGCGACAATGCTTCAATCCAATGATCGTCGAGCTTGCGGAAAAGGCAATGAAAGAAT
ATGGGGAAGATCCGAAAATCGAAACTAACAAGTTTGCTGCAATATGCACACATTTGGAAGTTTGTTTCAT
GTATTCGGATTTCCATTTCATCGACGAACGGGGTGAATCAATAATTGTAGAATCTGGTGACCCGAATGCA
CTATTGAAGCACCGATTTGAGATAATTGAAGGAAGAGACCGAATCATGGCCTGGACAGTGGTGAACAGTA
TATGTAACACAACAGGGGTAGAGAAGCCTAAATTTCTTCCTGATTTGTATGATTACAAAGAGAACCGGTT
CATTGAAATTGGAGTAACACGGAGGGAAGTCCACATATATTACCTAGAGAAAGCCAACAAAATAAAATCT
GAGAAGACACACATTCACATCTTTTCATTCACTGGAGAGGAGATGGCCACCAAAGCAGACTACACCCTTG
ACGAAGAGAGCAGGGCAAGAATCAAAACTAGGCTTTTCACTATAAGACAAGAAATGGCCAGTAGGAGTCT
ATGGGATTCCTTTCGTCAGTCCGAAAGAGGCGAAGAGACAATTGAAGAAAAATTTGAGATTACAGGAACT
ATGCGCAAGCTTGCCGACCAAAGTCTCCCACCGAACTTCTCCAGCCTTGAAAACTTTAGAGCCTATGTAG
ATGGATTCGAGCCGAACGGCTGCATTGAGGGCAAGCTTTCCCAAATGTCAAAAGAAGTGAACGCCAAAAT
TGAACCATTCTTGAGGACGACACCACGCCCCCTCAGATTGCCTGATGGGCCTCTTTGCCATCAGCGGTCA
AAGTTCCTGCTGATGGATGCTCTGAAATTAAGTATTGAAGACCCGAGTCACGAGGGGGAGGGAATACCAC
TATATGATGCAATCAAATGCATGAAGACATTCTTTGGCTGGAAAGAGCCTAACATAGTCAAACCACATGA
GAAAGGCATAAATCCCAATTACCTCATGGCTTGGAAGCAGGTGCTAGCAGAGCTACAGGACATTGAAAAT
GAAGAGAAGATCCCAAGGACAAAGAACATGAAGAGAACAAGCCAATTGAAGTGGGCACTCGGTGAAAAT
A
TGGCACCAGAAAAAGTAGACTTTGATGACTGCAAAGATGTTGGAGACCTTAAACAGTATGACAGTGATGA
GCCAGAGCCCAGATCTCTAGCAAGCTGGGTCCAAAATGAATTCAATAAGGCATGTGAATTGACTGATTCG
AGCTGGATAGAACTTGATGAAATAGGAGAAGATGTTGCCCCGATTGAACATATCGCAAGCATGAGGAGGA
ACTATTTTACAGCAGAAGTGTCCCACTGCAGGGCTACTGAATACATAATGAAGGGAGTGTACATAAATAC
GGCCTTGCTCAATGCATCCTGTGCAGCCATGGATGACTTTCAGCTGATCCCAATGATAAGCAAGTGTAGG
ACCAAAGAAGGAAGACGGAAAACAAACCTGTATGGGTTCATTATAAAAGGAAGGTCTCATTTGAGAAATG
ATACTGATGTGGTGAACTTTGTAAGTATGGAGTTCTCACTCACTGATCCGAGACTGGAGCCACACAAATG
GGAAAAATACTGTGTTCTTGAAATAGGAGACATGCTCTTGAGGACTGCGATAGGCCAAGTGTCGAGGCCC
ATGTTCCTATATGTGAGAACCAATGGAACCTCCAAGATCAAGATGAAATGGGGCATGGAAATGAGGCGCT
GCCTTCTTCAGTCTATTCAGCAGATTGAGAGCATGATTGAGGCCGAGTCTTCTGTCAAAGAGAAAGACAT
GACCAAGGAATTCTTTGAAAACAAATCGGAAACATGGCCAATCGGAGAGTCACCCAGGGGAGTGGAGGA
A
GGCTCTATTGGGAAAGTGTGCAGGACCTTACTGGCAAAATCTGTATTCAACAGTCTATATGCGTCTCCAC
AACTTGAAGGGTTTTCGGCTGAATCGAGAAAATTGCTTCTCATTGTTCAGGCACTTAGGGACAACCTGGA
ACCTGGAACCTTCGATCTTGGGGGGCTATATGAAGCAATTGAGGAGTGCCTGATTAATGATCCCTGGGTT
TTGCTTAATGCATCTTGGTTCAACTCCTTCCTCACACATGCACTAAAGTAGTTGTGGCAATGCTACTATT
TGCTATCCATACTGTCCAAAAAAGTACCT
>gi|344166430|gb|CY098027.1| Influenza A virus (A/Moscow/WRAIR4308T/2011(H1N1)) polymerase PA (PA) gene, complete cds
ATGGAAGACTTTGTGCGACAATGCTTCAATCCAATGATCGTCGAGCTTGCGGAAAAGGCAATGAAAGAAT
ATGGGGAAGATCCGAAAATCGAAACTAACAAGTTTGCTGCAATATGCACACATTTGGAAGTTTGTTTCAT
GTATTCGGATTTCCATTTCATCGACGAACGGGGTGAATCAATAATTGTAGAATCTGGTGACCCGAATGCA
CTATTGAAGCACCGATTTGAGATAATTGAAGGAAGAGACCGAATCATGGCCTGGACAGTGGTGAACAGTA
TATGTAACACAACAGGGGTAGAGAAGCCTAAATTTCTTCCTGATTTGTATGATTACAAAGAGAACCGGTT
CATTGAAATTGGAGTAACACGGAGGGAAGTCCACATATATTACCTAGAGAAAGCCAACAAAATAAAATCT

GAGAAGACACACATTCACATCTTTTCATTCACTGGAGAGGAGATGGCCACCAAAGCGGACTACACCCTTG
ACGAAGAGAGCAGAGCAAGAATCAAAACTAGGCTTTTCACTATAAGACAGGAAATGGCCAGTAGGAGTCT
ATGGGATTCCTTTCGTCAGTCCGAAAGAGGCGAAGAGACAATTGAAGAAAAATTTGAGATTACAGGAACT
ATGCGCAAGCTTGCCGACCAAAGTCTCCCACCGAACTTCTCCAGCCTTGAAAACTTTAGAGCCTATGTAG
ATGGATTCGAGCCGAACGGCTGCATTGAGGGCAAGCTTTCCCAAATGTCAAAAGAAGTGAACGCCAAAAT
TGAACCATTCTTGAGGACGACACCACGCCCCCTCAGATTGCCTGATGGGCCTCTTTGCCATCAGCGGTCA
AAGTTCCTGCTGATGGATGCTCTGAAATTAAGCATTGAAGACCCGAGTCACGAGGGGGAGGGGATACCAC
TATATGATGCAATCAAATGCATGAAGACATTCTTTGGCTGGAAAGAGCCTAACATAGTCAAACCACATGA
GAAAGGCATAAATCCCAATTACCTCATGGCTTGGAAGCAGGTGCTAGCAGAGCTACAGGACATTGAAAAT
GAAGAGAAGATCCCAAGGACAAAGAACATGAAGAGAACAAGCCAATTGAAGTGGGCACTCGGTGAAAAT
A
TGGCACCAGAAAAAGTAGACTTTGATGACTGCAAAGATGTTGGAGACCTTAAACAGTATGACAGTGATGA
GCCAGAGCCCAGATCTCTAGCAAGCTGGGTCCAAAATGAATTCAATAAGGCATGTGAATTGACTGATTCA
AGCTGGATAGAACTTGATGAAATAGGAGAAGATGTTGCCCCGATTGAACATATCGCAAGCATGAGGAGGA
ACTATTTTACAGCAGAAGTGTCCCACTGCAGGGCTACTGAATACATAATGAAGGGAGTGTACATAAATAC
GGCCTTACTCAATGCATCCTGTGCAGCCATGGATGACTTTCAGCTGATCCCAATGATAAGCAAATGTAGG
ACCAAAGAAGGAAGACGGAAAACAAACCTGTATGGGTTCATTATAAAAGGAAGGTCTCATTTGAGAAATG
ATACTGATGTGGTGAACTTTGTAAGTATGGAGTTCTCACTCACTGACCCGAGACTGGAGCCACACAAATG
GGAAAAATACTGTGTTATTGAAATAGGAGACATGCTCTTGAGGACTGCGATAGGCCAAGTGTCGAGGCCC
ATGTTCCTATATGTGAGAACCAATGGGACCTCCAAGATCAAGATGAAATGGGGCATGGAAATGAGGCGCT
GCCTTCTTCAGTCTCTTCAGCAGATTGAGAGCATGATTGAGGCCGAGTCTTCTGTCAAAGAGAAAGACAT
GACCAAGGAATTCTTTGAAAACAAATCGGAAACATGGCCAATCGGAGAGTCACCCAGGGGAGTGGAGGA
A
GGCTCTATTGGGAAAGTGTGCAGGACCTTACTGGCAAAATCTGTATTCAACAGTCTATATGCGTCTCCAC
AACTTGAGGGGTTTTCGGCTGAATCGAGAAAATTGCTTCTCATTGTTCAGGCACTTAGGGACAACCTGGA
ACCTGGAACCTTCGATCTTGGGGGGCTATATGAAGCAATCGAGGAGTGCCTGATTAATGATCCCTGGGTT
TTGCTTAATGCATCTTGGTTCAACTCCTTCCTCACACATGCACTGAAGTAG
>gi|339517205|gb|JN187269.1| Influenza A virus (A/Taiwan/4611/2011(H1N1)) segment 3 polymerase PA (PA) gene, complete cds
ATGGAAGACTTTGTGCGACAATGCTTCAATCCAATGATCGTCGAGCTTGCGGAAAAGGCAATGAAAGAAT
ATGGGGAAGATCCGAAAATCGAAACTAATAAGTTTGCTGCAATATGCACACATTTGGAAGTTTGTTTCAT
GTATTCGGATTTCCATTTCATCGACGAACGGGGTGAATCAATAATTGTAGAATCTGGTGACCCGAATGCA
CTATTGAAGCACCGATTTGAGATAATTGAAGGAAGAGACCGAATCATGGCCTGGACAGTGGTGAACAGTA
TATGTAACACAACAGGGGTAGAGAAGCCTAAATTTCTTCCTGATTTGTATGATTATAAAGAGAACCGGTT
CATTGAAATTGGAGTAACACGGAGGGAAGTCCACATATATTACCTAGAGAAAGCCAACAAAATAAAATCT
GAGAAGACACACATTCACATCTTTTCATTCACTGGAGAGGAGATGGCCACCAAAGCGGACTACACCCTTG
ACGAAGAGAGCAGGGCGAGAATCAAAACTAGGCTTTTCACTATAAGACAAGAAATGGCCAGTAGGAGTCT
ATGGGATTCCTTTCGTCAGTCCGAAAGAGGCGAAGAGACAATTGAAGAAAAATTTGAGATTACAGGAACT
ATGCGCAAGCTTGCCGACCAAAGTCTCCCACCAAACTTCTCCAGCCTTGAAAACTTTAGAGCCTATGTAG
ATGGATTCGAGCCGAACGGCTGCATTGAGGGCAAGCTTTCCCAAATGTCAAAAGAAGTGAACGCCAAAAT
TGAACCATTCTTGAGGACGACACCACGCCCCCTCAGATTGCCTGATGGGCCTCTTTGCCATCAGCGGTCA
AAGTTCCTGCTGATGGATGCTCTGAAATTAAGCATTGAAGACCCGAGTCACGAGGGGGAGGGAATACCAC
TATATGATGCAATCAAATGCATGAAGACATTCTTTGGCTGGAAAGAGCCTAAAATAGTCAAACCACATGA
GAAAGGCATAAATCCCAATTACCTCATGGCTTGGAAGCAGGTGCTAACAGAGCTACAGGACATTGAAAAT

GAAGAGAAGATCCCAAGGACCAAGAACATGAAGAGAACAAGCCAATTGAAATGGGCACTCGGTGAAAATA
TGGCACCAGAAAAAGTAGACTTTGATGACTGCAAAGATGTTGGAGACCTTAAACAGTATGACAGTGATGA
GCCAGAGCCCAGATCTCTAGCAAGCTGGGTCCAAAATGAATTCAATAAGGCTTGTGAATTGACTGATTCA
AGCTGGATAGAACTTGATGAAATAGGAGAAGATGTTGCCCCGATTGAACATATCGCAAGCATGAGGAGGA
ACTATTTTACAGCAGAAGTGTCCCACTGCAGGGCTACTGAATACATAATGAAGGGAGTGTACATAAATAC
GGCCTTGCTCAATGCATCCTGTGCAGCTATGGATGACTTTCAGCTGATCCCAATGATAAGCAAATGTAGG
ACCAAAGAAGGAAGACGGAAAACAAACCTGTATGGGTTCATTATAAAAGGAAGGTCTCATTTGAGAAATG
ATACTGATGTGGTGAACTTTGTAAGTATGGAGTTCTCACTCACTGACCCGAGACTGGAGCCACACAAATG
GGAAAAATACTGTGTTCTTGAAATAGGAGACATGCTCTTGAGGACTGCGATAGGCCAAGTGTCGAGGCCC
ATGTTCCTATATGTGAGAACCAATGGAACCTCCAAGATCAAGATGAAATGGGGCATGGAAATGAGGCGCT
GCCTTCTTCAGTCTCTTCAGCAGATCGAGAGCATGATTGAGGCCGAGTCTTCTGTCAAAGAGAAAGACAT
GACCAAGGAATTCTTTGAAAACAAATCGGAAACATGGCCAATCGGAGAGTCACCCAGGGGAGTGGAGGA
A
GGCTCTATTGGGAAAGTGTGCAGGACCTTACTGGCAAAATCTGTATTCAACAGTCTATATGCGTCTCCAC
AACTTGAGGGGTTTTCGGCTGAATCAAGAAAATTGCTTCTCATTGTTCAGGCACTTAGGGACAACCTGGA
ACCTGGAACCTTCGATCTTGGGGGGCTATATGAAGCAATCGAGGAGTGCCTGATTAATGATCCCTGGGTT
TTGCTTAATGCATCCTGGTTCAACTCCTTCCTCACACATGCACTGAAGTAG
>gi|338826611|gb|CY092861.1| Influenza A virus (A/Sydney/DD3-58/2011(H1N1)) polymerase PA (PA) gene, complete cds
TCCAAAATGGAAGACTTTGTGCGACAATGCTTCAATCCAATGATCGTCGAGCTTGCGGAAAAGGCAATGA
AAGAATATGGGGAAGATCCGAAAATCGAAACTAATAAGTTTGCTGCAATATGCACACATTTGGAAGTTTG
TTTCATGTATTCGGATTTCCATTTCATCGACGAACGGGGTGAATCAATAATTGTAGAATCTGGTGACCCG
AATGCACTATTGAAGCACCGATTTGAGATAATTGAAGGAAGAGACCGAATCATGGCCTGGACAGTGGTGA
ACAGTATATGTAACACAACAGGGGTAGAGAAGCCTAAATTTCTTCCTGATTTGTATGATTATAAAGAGAA
CCGGTTCATTGAAATTGGAGTAACACGGAGGGAAGTCCACATATATTACCTAGAGAAAGCCAACAAAATA
AAATCTGAGAAGACACACATTCACATCTTTTCATTCACTGGAGAAGAAATGGCCACCAAAGCGGACTACA
CCCTTGACGAAGAGAGCAGGGCGAGAATCAAAACTAGGCTTTTCACTATAAGACAAGAAATGGCCAGTAG
GAGTCTATGGGATTCCTTTCGTCAGTCCGAAAGAGGCGAAGAGACAATTGAAGAAAAATTTGAGATTACT
GGAACTATGCGCAAGCTTGCCGACCAAAGTCTCCCACCAAACTTCTCCAGCCTTGAAAACTTTAGAGCCT
ATGTAGATGGATTCGAGCCGAACGGCTGCATTGAGGGCAAGCTTTCCCAAATGTCAAAAGAAGTGAACGC
CAAAATTGAACCATTCTTGAGGGCGACACCACGCCCCCTCAGATTGCCTGATGGGCCTCTTTGCCATCAG
CGGTCAAAGTTCCTGCTGATGGATGCTCTGAAATTAAGCATTGAAGACCCGAGTCACGAGGGGGAGGGAA
TACCACTATATGATGCAATCAAATGCATGAAGACATTCTTTGGCTGGAAAGAGCCTAAAATAGTCAAACC
ACATGAGAAAGGCATAAATCCCAATTACCTCATGGCTTGGAAGCAGGTGCTAACAGAGCTACAGGACATT
GAAAATGAAGAGAAGATCCCAAGGACAAAAAACATGAAGAGAACAAGCCAATTGAAGTGGGCACTCGGT
G
AAAATATGGCACCAGAAAAAGTAGACTTTGATGACTGCAAAGATGTTGGAGACCTTAAACAGTATGACAG
TGATGAGCCAGAGCCCAGATCTCTAGCAAGCTGGGTCCAAAATGAATTCAATAAGGCATGTGAATTGACT
GATTCAAGCTGGATAGAACTTGATGAAATAGGAGAAGATGTTGCCCCGATTGAACATATCGCAAGCATGA
GGAGGAACTATTTTACAGCAGAAGTGTCCCACTGCAGGGCTACTGAATACATAATGAAGGGAGTGTACAT
AAATACGGCCTTGCTCAATGCATCCTGTGCAGCTATGGATGACTTTCAGCTGATCCCAATGATAAGCAAA
TGTAGGACCAAAGAAGGAAGACGGAAAACAAACCTGTATGGGTTCATTATAAAAGGAAGGTCTCATTTGA
GAAATGATACTGATGTGGTGAACTTTGTAAGTATGGAGTTCTCACTCACTGACCCGAGACTGGAGCCACA
CAAATGGGAAAAATACTGTGTTCTTGAAATAGGAGACATGCTCTTGAGGACTGCGATAGGCCAAGTGTCG

AGGCCCATGTTCCTATATGTGAGAACCAATGGAACCTCCAAGATCAAGATGAAATGGGGCATGGAAATGA
GGCGCTGCCTTCTTCAGTCTCTTCAGCAGATCGAGAGCATGATTGAGGCCGAGTCTTCTGTCAAAGAGAA
AGACATGACTAAGGAATTCTTTGAAAACAAATCGGAAACATGGCCAATCGGAGAGTCACCCAGGGGAGTG
GAGGAAGGCTCTATTGGGAAAGTGTGCAGGACCTTACTGGCAAAATCTGTATTCAACAGTCTATATGCGT
CTCCACAACTTGAGGGGTTTTCGGCTGAATCAAGAAAATTGCTTCTCATTGTTCAGGCTCTTAGGGACAA
CCTGGAACCTGGAACCTTCGATCTTGGGGGGCTATATGAAGCAATCGAGGAGTGCCTGATTAATGATCCC
TGGGTTTTGCTTAATGCATCCTGGTTCAACTCCTTCCTCACACATGCACTGAAGTAGTTGTGGCAATGCT
ACTAT
>gi|338826647|gb|CY092877.1| Influenza A virus (A/Illinois/NHRC0001/2011(H1N1)) polymerase PA (PA) gene, complete cds
TCCAAAATGGAAGACTTTGTGCGACAATGCTTCAATCCAATGATCGTCGAGCTTGCGGAAAAGGCAATGA
AAGAATATGGGGAAGATCCGAAAATCGAAACTAACAAGCTTGCTGCAATATGCACACATTTGGAAGTTTG
TTTCATGTATTCGGATTTCCATTTCATCGACGAACGGGGTGAATCAATAATTGTAGAATCTGGTGACCCG
AATGCACTATTGAAGCACCGATTTGAGATAATTGAAGGAAGAGACCGAATCATGGCCTGGACAGTGGTGA
ACAGTATATGTAACACAACAGGGGTAGAGAAGCCTAAATTTCTTCCTGATTTGTATGATTACAAAGAGAA
CCGGTTCATTGAAATTGGAGTAACACGGAGGGAAGTCCACATATATTACCTAGAGAAAGCCAACAAAATA
AAATCTGAGAAGACACACATTCACATCTTTTCATTCACTGGAGAGGAAATGGCCACCAAAGCGGACTACA
CCCTTGACGAAGAGAGCAGGGCAAGAATCAAAACTAGGCTTTTCACTATAAGACAAGAAATGGCCAGTAG
GAGTCTATGGGATTCCTTTCGTCAGTCCGAAAGAGGCGAAGAGACAATTGAAGAAAAATTTGAGATTACA
GGAACTATGCGCAAGCTTGCCGACCAAAGTCTCCCACCGAACTTCTCCAGCCTTGAAAACTTCAGAGCCT
ATGTAGATGGATTCGAGCCAAACGGCTGCATTGAGGGCAAGCTTTCCCAAATGTCAAAAGAAGTGAACGC
CAAAATTGAACCATTCTTGAGGACGACACCACGCCCCCTCAGATTGCCTGATGGACCTCTTTGCCATCAG
CGGTCAAAGTTCCTGCTGATGGATGCTTTGAAATTAAGTATTGAAGACCCGAGTCACGAGGGGGAGGGAA
TACCACTATATGATGCAATCAAATGCATGAAGACATTCTTTGGCTGGAAAGAGCCTAATATAGTCAAACC
ACATGAGAAAGGCATAAATCCCAATTACCTCATGGCTTGGAAGCAGGTGCTAGCAGAGCTACAGGACATT
GAAAATGAAGAGAAGATCCCAAGGACAAAGAACATGAAGAGAACAAGCCAATTGAAGTGGGCACTCGGT
G
AAAATATGGCACCAGAAAAAGTAGACTTTGATGACTGCAAAGATGTTGGAGACCTTAAACAGTATGACAG
TGATGAGCCAGAGCCCAGATCTCTAGCAAGCTGGGTCCAAAATGAATTCAATAAGGCATGTGAATTGACT
GATTCAAGCTGGATAGAACTTGATGAAATAGGAGAAGATGTTGCCCCGATTGAACATATCGCAAGCATGA
GGAGGAACTATTTTACAGCAGAAGTGTCCCACTGCAGGGCTACTGAATACATAATGAAGGGAGTGTACAT
AAATACGGCCTTGCTCAATGCATCCTGTGCAGCCATGGATGACTTTCAGCTGATCCCAATGATAAGCAAA
TGTAGGACCAAAGAAGGAAGACGGAAAACAAACCTGTATGGGTTCATTATAAAGGGAAGGTCTCATTTGA
GAAATGATACTGATGTGGTGAACTTTGTAAGTATGGAGTTCTCACTCACTGACCCGAGACTGGAGCCACA
CAAATGGGAAAAATACTGTGTTCTTGAAATAGGAGACATGCTCTTGAGGACTGCGGTAGGCCAAGTGTCG
AGGCCCATGTTCCTATATGTGAGAACCAATGGAACCTCCAAGATCAAGATGAAATGGGGCATGGAAATGA
GGCGCTGCCTTCTTCAGTCTCTTCAGCAGATTGAGAGCATGATTGAGGCCGAGTCTTCTGTCAAAGAGAA
AGACATGACCAAGGAATTCTTTGAAAACAAATCGGAAACATGGCCAATCGGAGAGTCACCCAGGGGAGTG
GAGGAAGGCTCTATTGGGAAAGTATGCAGGACCTTACTGGCAAAATCTGTATTCAACAGTCTATATGCGT
CTCCACAACTTGAGGGGTTTTCGGCTGAATCGAGAAAATTGCTTCTCATTGTTCAGGCACTTAGGGACAA
CCTGGAACCTGGAACCTTCGATCTTGGGGGGCTATATGAAGCAATCGAGGAGTGCCTTATTAATGATCCC
TGGGTTTTGCTTAATGCATCTTGGTTCAACTCCTTCCTCACACGTGCATTGAAGTAGTTGTGGCAATGCTACTA
T
>gi|344166358|gb|CY097995.1| Influenza A virus (A/Budapest/WRAIR3794T/2011(H1N1))

polymerase PA (PA) gene, complete cds
ATGGAAGACTTTGTGCGACAATGCTTCAATCCAATGATCGTCGAGCTTGCGGAAAAGGCAATGAAAGAAT
ATGGAGAAGATCCGAAAATCGAAACTAATAAGTTTGCTGCAATATGCACACATTTGGAAGTTTGTTTCAT
GTATTCGGATTTCCATTTCATCGACGAACGGGGTGAATCAATAATTGTAGAATCTGGTGACCCGAATGCA
CTATTGAAGCACCGATTTGAGATAATTGAAGGAAGAGACCGAATCATGGCCTGGACAGTGGTGAACAGTA
TATGTAACACAACAGGGGTAGAGAAGCCTAAATTTCTTCCTGATTTGTATGATTACAAAGAGAACCGGTT
CATTGAAATTGGAGTAACACGGAGGGAAGTCCACATATATTACCTGGAGAAAGCCAACAAAATAAAATCT
GAGAAGACACACATTCACATCTTTTCATTCACTGGAGAGGAGATGGCCACCAAAGCGGACTACACCCTTG
ACGAAGAGAGCAGAGCAAGAATCAAAACTAGGCTTTTCACTATAAGACAGGAAATGGCCAGTAGGAGTCT
ATGGGATTCCTTTCGTCAGTCCGAAAGAGGCGAAGAGACAATTGAAGAAAAATTTGAGATTACAGGAACT
ATGCGCAAGCTTGCCGACCAAAGTCTCCCACCAAACTTCTCCAGCCTTGAAAACTTTAGAGCCTATGTAG
ATGGATTCGAGCCGAACGGCTGCATTGAGGGTAAGCTTTCCCAAATGTCAAAAGAAGTGAACGCCAAAAT
TGAACCATTCTTGAGGACGACACCACGCCCCCTCAGATTGCCTGATGGGCCTCTTTGCCATCAGCGGTCA
AAGTTCCTGCTGATGGATGCTCTGAAATTAAGCATTGAAGACCCGAGTCACGAGGGGGAGGGAATACCAC
TATATGATGCAATCAAATGCATGAAGACATTCTTTGGCTGGAAAGAGCCTAAAATAGTCAAACCACATGA
GAAAGGCATAAATCCCAATTACCTCATGGCTTGGAAGCAGGTGCTAGCAGAGCTACAGGACATTGAAAAT
GAAGAGAAGATCCCAAGGACAAAGAACATGAAGAGAACAAGCCAATTGAAGTGGGCACTCGGTGAAAAT
A
TGGCACCAGAAAAAGTAGACTTTGATGACTGCAAAGATGTTGGAGACCTTAAACAGTATGACAGTGATGA
GCCAGAGCCCAGATCTCTAGCAAGCTGGGTCCAAAATGAATTCAATAAGGCATGTGAATTGACTGATTCA
AGCTGGATAGAACTTGATGAAATAGGAGAAGATGTTGCCCCGATTGAACATATCGCAAGCATGAGGAGGA
ACTATTTTACAGCAGAAGTGTCCCACTGCAGGGCTACTGAATACATAATGAAGGGAGTGTACATAAATAC
GGCCTTGCTCAATGCATCCTGTGCAGCCATGGATGACTTTCAGCTGATCCCAATGATAAGCAAATGTAGG
ACCAAAGAAGGAAGACGGAAAACAAACCTGTATGGGTTCATTATAAAAGGAAGGTCTCATTTGAGAAATG
ATACTGATGTGGTGAACTTTGTAAGTATGGAGTTCTCACTCACTGACCCGAGACTGGAGCCACACAAATG
GGAAAAATACTGTGTTCTTGAAATAGGAGACATGCTCTTGAGGACTGCGATAGGCCAAGTGTCAAGGCCC
ATGTTCCTATATGTGAGAACCAATGGAACCTCCAAGATCAAGATGAAATGGGGCATCGAAATGAGGCGCT
GCCTTCTTCAGTCTCTTCAGCAGATTGAGAGTATGATTGAGGCCGAGTCTTCTGTCAAAGAGAAAGACAT
GACCAAGGAATTCTTTGAAAACAAATCGGAAACATGGCCAATCGGAGAGTCACCCAGGGGAGTGGAGGA
A
GGCTCTATTGGGAAAGTGTGCAGGACCTTACTGGCAAAATCTGTATTCAACAGTCTATATGCGTCTCCAC
AACTTGAGGGGTTTTCGGCTGAATCAAGAAAATTGCTTCTCATTGTTCAGGCACTTAGGGACAACCTGGA
ACCTGGAACCTTCGAAATTGGGGGGCTGTATGAAGCAATCGAGGAGTGCCTGATTAATGATCCCTGGGTT
TTGCTTAATGCATCTTGGTTCAACTCCTTCCTCACACATGCACTGAAGTAG
>gi|344166376|gb|CY098003.1| Influenza A virus (A/Prague/WRAIR4146N/2011(H1N1)) polymerase PA (PA) gene, complete cds
ATGGAAGACTTTGTGCGACAATGCTTCAATCCAATGATCGTCGAGCTTGCGGAAAAGGCAATGAAAGAAT
ATGGGGAAGATCCGAAAATCGAAACTAATAAGTTTGCTGCAATATGCACACATTTGGAAGTTTGTTTCAT
GTATTCGGATTTCCATTTCATCGACGAACGGGGTGAATCAATAATTGTAGAATCTGGTGACCCGAATGCA
CTATTGAAGCACCGATTTGAGATAATTGAAGGAAGAGACCGAATCATGGCCTGGACAGTGGTGAACAGTA
TATGTAACACAACAGGGGTAGAGAAGCCTAAATTTCTTCCTGATTTGTATGATTACAAAGAGAACCGGTT
CATTGAAATTGGAGTAACACGGAGGGAAGTCCACATATATTACCTGGAGAAAGCCAACAAAATAAAATCT
GAGAAGACACACATTCACATCTTTTCATTCACTGGAGAGGAGATGGCCACCAAAGCGGACTACACCCTTG
ACGAAGAGAGCAGAGCAAGAATCAAAACTAGGCTTTTCACTATAAGACAAGAAATGGCCAGTAGGAGTCT

ATGGGATTCCTTTCGTCAGTCCGAAAGAGGCGAAGAGACAATTGAAGAAAAATTTGAGATTACAGGAACT
ATGCGCAAGCTTGCCGACCAAAGTCTCCCACCAAACTTCTCCAGCCTTGAAAACTTTAGAGCCTATGTAG
ATGGATTCGAGCCGAACGGCTGCATTGAGGGCAAGCTTTCCCAAATGTCAAAAGAAGTGAACGCCAAAAT
TGAACCATTCTTGAGGACGACACCACGCCCCCTCAGATTGCCTGATGGGCCTCTTTGCCATCAGCGGTCA
AAGTTCCTGCTGATGGATGCTCTGAAATTAAGCATTGAAGACCCGAGTCACGAGGGGGAGGGAATACCAC
TATATGATGCAATCAAATGCATGAAGACATTCTTTGGCTGGAAAGAGCCTAAAATAGTCAAACCACATGA
GAAAGGCATAAATCCCAATTACCTCATGGCTTGGAAGCAGGTGCTAGCAGAGCTACAGGACATTGAAAAT
GAAGAGAAGATCCCAAGGACAAAGAACATGAAGAGAACAAGCCAATTGAAGTGGGCACTCGGTGAAAAT
A
TGGCACCAGAAAAAGTAGACTTTGATGACTGCAAAGATGTTGGAGACCTTAAACAGTATGACAGTGATGA
GCCAGAGCCCAGATCTCTAGCAAGCTGGGTCCAAAATGAATTCAATAAGGCATGTGAATTGACTGATTCA
AGCTGGATAGAACTTGATGAAATAGGAGAAGATGTTGCCCCGATTGAACATATCGCAAGCATGAGGAGGA
ACTATTTTACAGCAGAAGTGTCCCACTGCAGGGCTACTGAATACATAATGAAGGGAGTGTACATAAATAC
GGCCTTGCTCAATGCATCCTGTGCAGCCATGGATGACTTTCAGCTGATCCCAATGATAAGCAAATGTAGG
ACCAAAGAAGGAAGACGGAAAACAAACCTGTATGGGTTCATTATAAAAGGAAGGTCTCATTTGAGAAATG
ATACTGATGTGGTGAACTTTGTAAGTATGGAGTTCTCACTCACTGACCCGAGACTGGAGCCACACAAATG
GGAAAAATACTGTGTTCTTGAAATAGGAGACATGCTCTTGAGGACTGCGATAGGCCAAGTGTCGAGGCCC
ATGTTCCTATATGTGAGAACCAATGGAACCTCCAAGATCAAGATGAAATGGGGCATGGAAATGAGGCGCT
GCCTTCTTCAGTCTCTTCAGCAGATTGAGAGCATGATTGAGGCCGAGTCTTCTGTCAAAGAGAAAGACAT
GACCAAGGAATTCTTTGAAAACAAATCGGAAACATGGCCAATCGGAGAGTCACCCAGGGGAGTGGAGGA
A
GGCTCTATTGGGAAAGTGTGCAGGACCTTACTGGCAAAATCTGTATTCAACAGTCTATATGCGTCTCCAC
AACTTGAGGGGTTTTCGGCTGAATCAAGAAAATTGCTTCTCATTGTTCAGGCACTTAGGGACAACCTGGA
ACCTGGAACCTTCGATCTTGGGGGGCTGTATGAAGCAATCGAGGAGTGCCTGATTAATGATCCCTGGGTT
TTGCTTAATGCATCTTGGTTCAACTCCTTCCTCACACATGCACTGAAGTAG
>gi|320202781|gb|CY081057.1| Influenza A virus (A/Ulaanbaatar/190/2011(H1N1)) polymerase PA (PA) gene, complete cds
ATGGAAGACTTTGTGCGACAATGCTTCAATCCAATGATCGTCGAGCTTGCGGAAAAGGCAATGAAAGAAT
ATGGGGAAGATCCGAAAATCGAAACTAATAAGTTTGCTGCAATATGCACACATTTGGAAGTTTGTTTCAT
GTATTCGGATTTCCATTTCATCGACGAACGGGGTGAATCAATAATTGTAGAATCTGGTGACCCGAATGCA
CTATTGAAGCACCGATTTGAGATAATTGAAGGAAGAGACCGAATCATGGCCTGGACAGTGGTGAACAGTA
TATGTAACACAACAGGGGTAGAGAAGCCTAAATTTCTTCCTGATTTGTATGATTATAAAGAGAACCGGTT
CATTGAAATTGGAGTAACACGGAGGGAAGTCCACATATATTACCTAGAGAAAGCCAACAAAATAAAATCT
GAGAAGACACACATTCACATCTTTTCATTCACTGGAGAGGAAATGGCCACCAAAGCGGACTACACCCTTG
ACGAAGAGAGCAGGGCGAGAATCAAAACTAGGCTTTTCACTATAAGACAAGAAATGGCCAGTAGGAGTCT
ATGGGATTCCTTTCGTCAGTCCGAAAGAGGCGAAGAGACAATTGAAGAAAAATTTGAGATTACAGGAACT
ATGCGCAAGCTTGCCGACCAAAGTCTCCCACCAAACTTCTCCAGCCTTGAAAACTTTAGAGCCTATGTAG
ATGGATTCGAGCCGAACGGCTGCATTGAGGGCAAGCTTTCCCAAATGTCAAAAGAAGTGAACGCCAAAAT
TGAATCATTCTTGAGGACGACACCACGCCCCCTCAGATTGCCTGATGGGCCTCTTTGCCATCAGCGGTCA
AAGTTCCTGCTGATGGATGCTCTGAAATTAAGCATTGAAGACCCGAGTCACGAGGGGGAGGGAATACCAC
TATATGATGCAATCAAATGCATGAAGACATTCTTTGGCTGGAAAGAGCCTAAAATAGTCAAACCACATGA
GAAAGGCATAAATCCCAATTACCTCATGGCTTGGAAGCAGGTGCTAACCGAGCTACAGGACATTGAAAAT
GAAGAGAAGATCCCAAGGACAAAGAACATGAAGAGAACAAGCCAATTGAAGTGGGCACTCGGTGAAAAT
A

TGGCACCAGAAAAAGTAGACTTTGATGACTGCAAAGATGTTGGAGACCTTAAACAGTATGACAGTGATGA
GCCAGAGCCCAGATCTCTAGCAAGCTGGGTCCAAAATGAATTCAATAAGGCATGTGAATTGACTGATTCA
AGCTGGATAGAACTTGATGAAATAGGAGAAGATGTTGCCCCGATTGAACATATCGCAAGCATGAGGAGGA
ACTATTTTACAGCAGAAGTGTCCCACTGCAGGGCTACTGAATACATAATGAAGGGAGTGTACATAAATAC
GGCCTTGCTCAATGCATCCTGTGCAGCTATGGATGACTTTCAGCTGATCCCAATGATAAGCAAATGTAGG
ACCAAAGAAGGAAGACGGAAAACAAACCTGTATGGGTTCATTATAAAAGGAAGGTCTCATTTGAGAAATG
ATACTGATGTGGTGAACTTTGTAAGTATGGAGTTCTCACTCACTGACCCGAGACTGGAGCCACACAAATG
GGAAAAATACTGTGTTCTTGAAATAGGAGACATGCTCTTGAGGACTGCGATAGGCCAAGTGTCGAGGCCC
ATGTTCCTATATGTGAGAACCAATGGAACCTCCAAGATCAAGATGAAATGGGGCATGGAAATGAGGCGCT
GCCTTCTTCAGTCTCTTCAGCAGATCGAGAGCATGATTGAGGCCGAGTCTTCTGTCAAAGAGAAAGACAT
GACCAAGGAATTCTTTGAAAACAAATCGGAAACATGGCCAATCGGAGAGTCACCCAGGGGAGTGGAGGA
A
GGCTCTATTGGGAAAGTGTGCAGGACCTTACTGGCAAAATCTGTATTCAACAGTCTATATGCGTCTCCAC
AACTTGAAGGGTTTTCGGCTGAATCAAGAAAATTGCTTCTCATTGTTCAGGCACTTAGGGACAACCTGGA
ACCTGGAACCTTCGATCTTGGGGGGCTATATGAAGCAATCGAGGAGTGCCTGATTAATGATCCCTGGGTT
TTGCTTAATGCATCCTGGTTCAACTCCTTCCTCACACATGCACTGAAGTAG

>gi|388770783|gb|CY120752.1| Influenza A virus (A/Brazil/AVS08/2011(H1N1)) polymerase PA (PA) gene, complete cds

AAAATGGAAGACTTTGTGCGACAATGCTTCAATCCAATGATCATCGAGCTTGCGGAAAAGGCAATGAAAG
AATATGGGGAAGATCCGAAAATCGAAACTAACAAGTTTGCTGCAATATGCACACATTTGGAAGTTTGTTT
CATGTATTCGGATTTCCATTTCATCGACGAACGGGGTGAATCAATAATTGTAGAATCTGGTGACCCGAAT
GCACTATTGAAGCACCGATTTGAGATAATTGAAGGAAGAGACCGAATCATGGCCTGGACAGTGGTGAACA
GTATATGTAACACAACAGGGGTAGAGAAGCCTAAATTTCTTCCTGATTTGTATGATTACAAAGAGAACCG
GTTCATTGAAATTGGAGTAACACGGAGGGAAGTCCACATATATTACCTAGAGAAAGCCAACAAAATAAAA
TCTGAGAAGACACACATTCACATCTTTTCATTCACTGGAGAGGAGATGGCCACCAAAGCGGACTACACCC
TTGACGAAGAGAGCAGGGCAAGAATCAAAACTAGGCTTTTCACTATAAGACAAGAAATGGCCAGTAGGAG
TCTATGGGATTCCTTTCGTCAGTCCGAAAGAGGCGAAGAGACAATTGAAGAAAAATTTGAGATTACAGGA
ACTATGCGCAAGCTTGCCGACCAAAGTCTCCCACCGAACTTCTCCGGCCTTGAAAACTTTAGAGCCTATG
TAGATGGATTCGAGCCGAACGGCTGCATTGAGGGCAAGCTTTCCCAAATGTCAAAAGAAGTGAACGCCAA
AATTGAACCATTCTTGAGGACGACACCACGCCCCCTCAGATTGCCTGATGGGCCTCTTTGCCATCAGCGG
TCAAAGTTCTTGCTGATGGATTCTCTGAAATTAAGTATTGAAGACCCGAGTCACGAGGGGGAGGGAATAC
CACTATATGATGCAATCAAATGCATGAAGACATTCTTTGGCTGGAAGGAGCCTAACATAGTCAAACCACA
TGAGAAAGGCATAAATCCCAATTACCTCATGGCTTGGAAGCAGGTGCTAGCAGAGCTACAGGACATTGAA
AATGAAGAGAAGATCCCAAGGACAAAGAACATGAAGAAAACAAGCCAATTGAAGTGGGCACTCGGTGAA
A
ATATGGCACCAGAAAAAGTAGACTTTGATGACTGCAAAGATGTTGGAGACCTTAAACAGTATAACAGTGA
TGAGCCGGAGCCCAGATCTCTAGCAAGCTGGGTCCAAAATGAATTCAATAAGGCATGTGAATTGACTGAT
TCAAGCTGGATAGAACTTGATGAAATAGGAGAAGATGTTGCCCCGATTGAACATATCGCAAGCATGAGGA
GGAACTATTTTACAGCAGAAGTGTCCCACTGCAGGGCTACTGAATACATAATGAAGGGAGTGTACATAAA
TACGGCCTTGCTCAATGCATCCTGTGCAGCCATGGATGACTTTCAGCTGATCCCAATGATAAGCAAATGT
AGGACCAAAGAAGGAAGACGGAAAACAAACCTGTATGGGTTCATTATAAAAGGAAGGTCTCATTTGAGAA
ATGATACTGATGTGGTGAACTTTGTAAGTATGGAGTTCTCACTCACTGACCCGAGACTGGAGCCACACAA
ATGGGGAAAAATACTGTGTTCTTGAAATAGGAGACATGCTCTTGAGGACTGCGATAGGCCAAGTGTCGAGG
CCCATGTTCCTATATGTGAGAACCAATGGAACCTCCAAGATCAAGATGAAATGGGGCATGGAAATGAGGC

GCTGCCTTCTTCAGTCTCTTCAGCAGATTGAGAGCATGATTGAGGCCGAGTCTTCTGTCAAAGAGAAAGA
CATGACCAAGGAATTCTTTGAAAACAAATCGGAAACATGGCCAATCGGAGAGTCACCCAGGGGAGTGGAG
GAAGGCTCTATTGGGAAAGTGTGCAGGACCTTACTGGCAAAATCTGTATTCAACAGTCTATATGCGTCTC
CACAACTTGAGGGGTTTTCGGCTGAATCGAGAAAATTGCTTCTCATTGTTCAGGCACTTAGGGACAACCT
GGAACCTGGAACCTTTGATCTTGGGGGGCTATATGAAGCAATCGAGGAGTGCCTGATTAATGATCCCTGG
GTTTTGCTTAATGCATCTTGGTTCAACTCCTTCCTCACACATGCACTGAAATAGTTGTGGCAATGCTACT
ATTTGCTATC
>gi|401716660|gb|JX473009.1| Influenza A virus (A/Vladivostok/28/2012(H1N1)) segment 3 polymerase PA (PA) gene, complete cds
ATGGAAGACTTTGTGCGACAATGCTTCAATCCAATGATCGTCGAGCTTGCGGAAAAGGCAATGAAAGAAT
ATGGGGAAGATCCGAAAATCGAAACTAATAAGTTTGCTGCAATATGCACACATTTGGAAGTTTGTTTCAT
GTATTCGGATTTCCATTTCATCGACGAACGGGGTGAATCAATAATTGTAGAATCTGGTGACCCAAATGCA
CTATTGAAGCACCGATTTGAGATAATTGAAGGAAGAGACCGAATCATGGCCTGGACAGTGGTGAACAGTA
TATGTAACACAACAGGGGTAGAGAAGCCTAAATTTCTTCCTGATTTGTATGATTACAAAGAGAACCGGTT
CATTGAAATTGGAGTAACACGGAGGGAAGTCCACATATATTACCTAGAGAAAGCCAACAAAATAAAATCT
GAGAAGACACACATTCACATCTTTTCATTCACTGGAGAGGAGATGGCCACCAAAGCGGACTACACCCTTG
ACGAAGAGAGCAGGGCAAGAATCAAAACTAGGCTTTTCACTATAAGACAAGAAATGGCCAGTAGGAGTCT
ATGGGATTCCTTTCGTCAGTCCGAAAGAGGCGAAGAGACAATTGAAGAAAAATTTGAGATTACAGGAACT
ATGCGCAAGCTTGCCGACCAAAGTCTCCCACCAAACTTCTCCAGCCTTGAAAACTTCAGAGCCTATGTAG
ATGGATTCGAGCCGAACGGCTGCATTGAGGGCAAGCTTTCCCAAATGTCAAAAGAAGTGAACGCCAAAAT
TGAACCATTCTTGAGGACGACACCACGCCCCCTCAGATTGCCTGATGGACCTCTTTGCCATCAGCGGTCA
AAGTTCCTGCTGATGGATGCTCTGAAATTAAGCATTGAAGACCCGAATCACGAGGGGGAGGGAATACCAC
TATATGATGCAATCAAATGCATGAAGACATTCTTTGGCTGGAAAGAGCCCAAAATAGTCAAACCACATGA
GAAAGGCGTAAATCCCAATTACCTCATGGCTTGGAAGCAGGTGCTAGCAGAGCTACAGGACATTGAAAAT
GAAGATAAGATCCCAAGGACAAAGAACATGAAGAGAACAAGCCAATTGAAGTGGGCACTCGGTGAAAATA
TGGCACCAGAAAAAGTAGACTTTGATGACTGCAAAGATGTTGGAGACCTTAAACAGTATGACAGTGATGA
GCCAGAGCCCAAATCTCTAGCAAGCTGGGTCCAAAATGAATTCAATAAGGCATGTGAATTGACTGATTCA
AGCTGGATAGAACTTGATGAAATAGGAGAAGATGTTGCCCCGATTGAACATATCGCAAGCATGAGGAGGA
ACTATTTTACAGCAGAAGTGTCCCACTGCAGGGCTACTGAATACATAATGAAGGGAGTGTACATAAATAC
GGCCTTGCTCAATGCATCCTGTGCAGCCATGGATGACTTTCAGCTGATCCCAATGATAAGCAAATGTAGG
ACCAAAGAAGGAAGACGGAAAACAAACCTGTATGGGTTCATTATAAAAGGAAGGTCTCATTTGAGAAATG
ATACTGATGTGGTGAACTTTGTAAGTATGGAGTTCTCACTCACTGACCCGAGACTGGAGCCACACAAATG
GGAAAAATACTGTGTTCTTGAAATAGGAGACATGCTCTTGAGGACTGCGATAGGCCAAGTGTCGAGGCCC
ATGTTCCTATATGTGAGAACCAATGGAACCTCCAAGATCAAGATGAAATGGGGCATGGAAATGAGGCGCT
GCCTTCTTCAGTCTCTTCAGCAGATTGAGAGCATGATTGAGGCCGAGTCTTCTGTCAAAGAGAAAGACAT
GACCAAGGAATTCTTTGAAAACAAATCGGAAACATGGCCAATCGGAGAGTCACCCAGGGGAGTGGAGGA
A
GGCTCTATTGGGAAAGTGTGCAGGACCTTACTGGCAAAATCAGTATTCAACAGTCTATATGCGTCTCCAC
AACTTGAGGGGTTTTCGGCTGAATCAAGAAAATTGCTTCTCATTGTTCAGGCACTTAGGGACAACCTGGA
ACCTGGAACCTTCGATCTTGGGGGGCTATATGAAGCAATCGAGGAGTGCCTGATTAATGATCCCTGGGTT
TTGCTTAATGCATCTTGGTTCAACTCCTTCCTCACACATGCACTGAAGTAGTTGTGGCAATGCTACTATT
TGCTATCCATAC
>gi|390135401|gb|JX046925.1| Influenza A virus (A/Moscow/IIV-45/2012(H1N1)) segment 3 polymerase PA (PA) gene, complete cds

ATGGAAGACTTTGTGCGACAATGCTTCAATCCAATGATCGTCGAGCTTGCGGAAAAGGCAATGAAAGAAT
ATGGGGAAGATCCGAAAATCGAAACTAATAAGTTTGCTGCAATATGCACACATTTGGAAGTTTGTTTCAT
GTATTCGGATTTCCATTTCATCGACGAACGGGGTGAATCAATAATTGTAGAATCTGGTGACCCAAATGCA
CTATTGAAGCACCGATTTGAGATAATTGAAGGAAGAGACCGAATCATGGCCTGGACAGTGGTGAACAGTA
TATGTAACACAACAGGGGTAGAGAAGCCTAAATTTCTTCCTGATTTGTATGATTACAAAGAGAACCGGTT
CATTGAAATTGGAGTAACACGGAGGGAAGTCCACATATATTACCTAGAGAAAGCCAACAAAATAAAATCT
GAGAAGACACACATTCACATCTTTTCATTCACTGGAGAGGAGATGGCCACCAAAGCGGACTACACCCTTG
ACGAAGAGAGCAGGGCAAGAATCAAAACTAGGCTTTTCACTATAAGACAAGAAATGGCCAGTAGGAGTCT
ATGGGATTCCTTTCGTCAGTCCGAAAGAGGCGAAGAGACAATTGAAGAAAAATTTGAGATTACAGGAACT
ATGCGCAAGCTTGCCGACCAAAGTCTCCCACCAAACTTCTCCAGCCTTGAAAACTTCAGAGCCTATGTAG
ATGGATTCGAGCCGAACGGCTGCATTGAGGGCAAGCTTTCCCAAATGTCAAAAGAAGTGAACGCCAAAAT
TGAACCATTCTTGAGGACGACACCACGCCCCCTCAGATTGCCTGATGGACCTCTTTGCCACCAGCGGTCA
AAGTTCCTGCTGATGGATGCTCTGAAATTAAGCATTGAAGACCCGAATCACGAGGGGGAGGGAATACCAC
TATATGATGCAATCAAATGCATGAAGACATTCTTTGGCTGGAAAGAGCCCAAAATAGTCAAACCACATGA
GAAAGGCGTAAATCCCAATTACCTCATGGCTTGGAAGCAGGTGCTAGCAGAGCTACAGGACATTGAAAAT
GAAGATAAGATCCCAAGGACAAAGAACATGAAGAGAACAAGCCAATTGAAGTGGGCACTCGGTGAAAATA
TGGCACCAGAAAAAGTAGACTTTGATGACTGCAAAGATGTTGGAGACCTTAAACAGTATGACAGTGATGA
GCCAGAGCCCAAATCTCTAGCAAGCTGGGTCCAAAATGAATTCAATAAGGCATGTGAATTGACTGATTCA
AGCTGGATAGAACTTGATGAAATAGGAGAAGATGTTGCCCCGATTGAACATATCGCAAGCATGAGGAGGA
ACTATTTTACAGCAGAAGTGTCCCACTGCAGGGCTACTGAATACATAATGAAGGGAGTGTACATAAATAC
GGCCTTGCTCAATGCATCCTGTGCAGCCATGGATGACTTTCAGCTGATCCCAATGATAAGCAAATGTAGG
ACCAAAGAAGGAAGACGGAAAACAAACCTGTATGGGTTCATTATAAAAGGAAGGTCTCATTTGAGAAATG
ATACTGATGTGGTGAACTTTGTAAGTATGGAGTTCTCACTCACTGACCCGAGACTGGAGCCACACAAATG
GGAAAAATACTGTGTTCTTGAAATAGGAGACATGCTCTTGAGGACTGCGATAGGCCAAGTGTCGAGGCCC
ATGTTCCTATATGTGAGAACCAATGGAACCTCCAAGATCAAGATGAAATGGGGCATGGAAATGAGGCGCT
GCCTTCTTCAGTCTCTTCAGCAGATTGAGAGCATGATTGAGGCCGAGTCTTCTGTCAAAGAGAAAGACAT
GACCAAGGAATTCTTTGAAAACAAATCGGAAACATGGCCAATCGGAGAGTCACCCAGGGGAGTGGAGGA
A
GGCTCTATTGGGAAAGTGTGCAGGACCTTACTGGCAAAATCTGTATTCAACAGTCTATATGCGTCTCCAC
AACTTGAGGGGTTTTCGGCTGAATCAAGAAAATTGCTTCTCATTGTTCAGGCACTTAGGGACAACCTGGA
ACCTGGAACCTTCGATCTTGGGGGGCTATATGAAGCAATCGAGGAGTGCCTGATTAATGATCCCTGGGTT
TTGCTTAATGCATCTTGGTTCAACTCCTTCCTCACACATGCACTGAAGTAG
>gi|383513266|gb|JQ768352.1| Influenza A virus (A/Tomsk/IIV-19/2012(H1N1)) segment 3 polymerase PA (PA) gene, complete cds
ATGGAAGACTTTGTGCGACAATGCTTCAATCCAATGATCGTCGAGCTTGCGGAAAAGGCAATGAAAGAAT
ATGGGGAAGATCCGAAAATCGAAACTAATAAGTTTGCTGCAATATGCACACATTTGGAAGTTTGTTTCAT
GTATTCGGATTTCCATTTCATCGACGAACGGGGTGAATCAATAATTGTAGAATCTGGTGACCCAAATGCA
CTATTGAAGCACCGATTTGAGATAATTGAAGGAAGAGACCGAATCATGGCCTGGACAGTGGTGAACAGTA
TATGTAACACAACAGGGGTAGAGAAGCCTAAATTTCTTCCTGATTTGTATGATTACAAAGAGAACCGGTT
CATTGAAATTGGAGTAACACGGAGGGAAGTCCACATATATTACCTAGAGAAAGCCAACAAAATAAAATCT
GAGAAGACACACATTCACATCTTTTCATTCACTGGAGAGGAGATGGCCACCAAAGCGGACTACACCCTTG
ACGAAGAGAGCAGGGCAAGAATCAAAACTAGGCTTTTCACTATAAGACAAGAAATGGCCAGTAGGAGTCT
ATGGGATTCCTTTCGTCAGTCCGAAAGAGGCGAAGAGACAATTGAAGAAAAATTTGAGATTACAGGAACT
ATGCGCAAGCTTGCCGACCAAAGTCTCCCACCAAACTTCTCCAGCCTTGAAAACTTCAGAGCCTATGTAG

ATGGATTCGAGCCGAACGGCTGCATTGAAGGCAAGCTTTCCCAAATGTCAAAAGAAGTGAACGCCAAAAT
TGAACCATTCTTGAGGACGACACCACGCCCCCTCAGATTGCCTGATGGACCTCTTTGCCATCAGCGGTCA
AAGTTCCTGCTGATGGATGCTCTGAAATTAAGCATTGAAGACCCGAATCACGAGGGGGAGGGAATACCAC
TATATGATGCAATCAAATGCATGAAGACATTCTTTGGCTGGAAAGAGCCCAAAATAGTCAAACCACATGA
GAAAGGCGTAAATCCCAATTACCTCATGGCTTGGAAGCAGGTGCTAGCAGAGCTACAGGACATTGAAAAT
GAAGATAAGATCCCAAGGACAAAGAACATGAAGAGAACAAGCCAATTGAAGTGGGCACTCGGTGAAAATA
TGGCACCAGAAAAAGTAGACTTTGATGACTGCAAAGATGTTGGAGACCTTAAACAGTATGACAGTGATGA
GCCAGAGCCCAAATCTCTAGCAAGCTGGGTCCAAAATGAATTCAATAAGGCATGTGAATTGACTGATTCA
AGCTGGATAGAACTTGATGAAATAGGAGAAGATGTTGCCCCGATTGAACATATCGCAAGCATGAGGAGGA
ACTATTTTACAGCAGAAGTGTCCCACTGCAGGGCTACTGAATACATAATGAAGGGAGTGTACATAAATAC
GGCCTTGCTCAATGCATCCTGTGCAGCCATGGATGACTTTCAGCTGATCCCAATGATAAGCAAATGTAGG
ACCAAAGAAGGAAGACGGAAAACAAACCTGTATGGGTTCATTATAAAAGGAAGGTCTCATTTGAGAAATG
ATACTGATGTGGTGAACTTTGTAAGTATGGAGTTCTCACTCACTGACCCGAGACTGGAGCCACACAAATG
GGAAAAATACTGTGTTCTTGAAATAGGAGACATGCTCTTGAGGACTGCGATAGGCCAAGTGTCGAGGCCC
ATGTTCCTATATGTGAGAACCAATGGAACCTCCAAGATCAAGATGAAATGGGGCATGGAAATGAGGCGCT
GCCTTCTTCAGTCTCTTCAGCAGATTGAGAGCATGATTGAGGCCGAGTCTTCTGTCAAAGAGAAAGACAT
GACCAAAGAATTCTTTGAAAACAAATCGGAAACATGGCCAATCGGAGAGTCACCCAGGGGAGTGGAGGAA
GGCTCTATTGGGAAAGTGTGCAGGACCTTACTGGCAAAATCTGTATTCAACAGTCTATATGCGTCTCCAC
AACTTGAGGGGTTTTCGGCTGAATCAAGAAAATTGCTTCTCATTGTTCAGGCACTTAGGGACAACCTGGA
ACCTGGAACCTTCGATCTTGGGGGGCTATATGAAGCAATCGAGGAGTGCCTGATTAATGATCCCTGGGTT
TTGCTTAATGCATCTTGGTTCAACTCCTTCCTCACACATGCACTGAAGTAG
