# Supplementary material for: A computational method for predicting regulation of human microRNAs on the influenza virus genome
Source: BMC Syst Biol. 2013 Oct 14;7(Suppl 2):S3. doi: 10.1186/1752-0509-7-S2-S3 (PMC3851852; doi:10.1186/1752-0509-7-S2-S3)
Supplement: Additional File 10 — The coding sequence of the gene fragment of PB2 from 2000 to 2012 recorded in Genbank. [file 1752-0509-7-S2-S3-S10.PDF]

PB1

>gi|145278785|gb|CY021699.1| Influenza A virus (A/Memphis/15/2000(H1N1)) segment 2, complete sequence

ATGGATGTCAATCCGACATTACTTTTCTTAAAAGTGCCAGCACAAAATGCTATAAGCACAACTTTTCCTT
ATACTGGTGACCCTCCTTACAGCCATGGGACAGGAACAGGGTACACCATGGATACAGTCAACAGGACACA
TCAGTACTCAGAAAGAGGAAGGTGGACAAAAAATACCGAAACTGGAGCACCGCAACTCAACCCAATTGAT
GGGCCACTACCAAAAGACAATGAACCAAGTGGCTATGCCCAAACAGATTGTGTATTAGAAGCAATGGCTT
TCCTTGAGGAATCCCATCCTGGTATTTTTGAAAACTCTTGTATTGAAACAATGGAGGTTGTTCAGCAAAC
AAGGGTGGACAAACTGACACAAGGCAGACAGACCTATGACTGGACTCTAAATAGGAACCAGCCTGCTGCC
ACAGCATTGGCCAACACTATAGAAGTGTTCAGATCAAATGGCCTCATAGCAAATGAATCTGGGAGGCTAA
TAGACTTCCTTAAAGATGTAATGGAGTCGATGGACAGAGACGAAGTAGAGATCACAACTCATTTTCAGAG
AAAGAGGAGAGTAAGAGACAATGTAACTAAAAAAATGGTGACCCAAAGAACAATAGGCAAAAAGAAACAT
AAATTAGACAAAAGAAGTTACCTAATTAGGGCATTAACCCTGAACACAATGACCAAAGATGCTGAGAGGG
GGAAACTAAAACGCAGAGCAATTGCAACCCCAGGAATGCAAATAAGGGGGTTTGTATACTTTGTTGAGAC
ACTGGCAAGAAGCATATGTGAAAAGCTTGAACAATCAGGGTTGCCAGTTGGAGGAAATGAAAAGAAAGCA
AAGTTAGCAAATGTTGTAAGGAAGATGATGACCAACTCCCAGGACACTGAAATTTCTTTCACAATCACTG
GAGATAACACAAAATGGAACGAAAATCAAAACCCTAGAATGTTCTTGGCCATGATCACATATATAACCAA
AAATCAGCCTGAATGGTTCAGAAATATTTTAAGTATTGCTCCAATAATGTTTTCAAACAAAATGGCGAGA
CTAGGTAAGGGGTACATGTTTGAAAGCAAGAGTATGAAACTGAGAACTCAAATACCTGCAGAGATGCTAG
CCAACATAGATTTGAAATATTTCAATGATTCAACTAAAAAGAAAATTGAAAAAATCCGGCCATTATTAAT
AGATGGAACTGCATCATTGAGTCCTGGAATGATGATGGGCATGTTCAATATGTTAAGCACCGTCTTGGGC
GTCTCCATTCTGAATCTTGGGCAAAAGAGATACACCAAGACTACTTACTGGTGGGATGGTCTTCAATCGT
CTGATGATTTTGCTCTGATTGTGAATGCACCCAACCATGCAGGCATTCAAGCTGGAGTTGACAGGTTTTA
TCGAACCTGTAAGCTGCTCGGAATTAATATGAGCAAAAAGAAGTCTTACATAAACAGAACAGGTACCTTT
GAATTCACGAGCTTCTTCTATCGTTATGGGTTTGTTGCCAATTTCAGCATGGAGCTTCCTAGTTTTGGGG
TGTCTGGGGTCAATGAATCTGCAGACATGAGTATTGGAGTCACTGTCATCAAAAACAATATGATAAACAA
TGACCTTGGCCCAGCAACTGCTCAAATGGCCCTTCAGTTATTTATAAAAGATTACAGGTACACGTATCGA
TGCCACAGAGGTGACACACAAATACAAACCCGGAGATCATTTGAAATAAAGAAACTATGGGACCAAACCC
GCTCCAAAGCTGGGCTGTTGGTCTCTGATGGAGGCCCAAATTTATATAACATTAGAAATCTCCATATTCC
TGAAGTCTGCTTGAAATGGGAGTTGATGGATGAGGATTACCAGGGGCGTTTATGCAACCCATTGAACCCG
TTTGTCAGTCATAAAGAGATTGAATCAGTGAACAATGCAGTGATGATGCCGGCACATGGTCCAGCCAAAA
ATATGGAGTATGACGCTGTTGCAACAACACACTCCTGGGTCCCCAAAAGGAATCGATCCATTTTGAATAC
GAGCCAAAGGGGGATACTTGAGGATGAGCAAATGTATCAGAGGTGCTGCAATTTATTTGAAAAATTCTTC
CCAAGTAGCTCATACAGAAGACCAGTTGGAATATCCAGTATGGTAGAGGCTATGGTTTCCAGAGCCCGAA
TTGATGCACGGATTGATTTCGAATCTGGAAGGATAAAGAAAGAGGAATTCGCTGAGATCATGAAGACCTG
TTCCACCATTGAAGACCTCAAACGGCAAAAATAGAGAATTTGGCTTGT

>gi|70907653|gb|CY000456.2| Influenza A virus (A/New York/146/2000(H1N1)) segment 2, complete sequence

AGCGAAAGCAGGCAAACCATTTGAATGGATGTCAATCCGACTTTACTTTTCTTAAAAGTGCCAGCACAAA
ATGCTATAAGCACAACTTTCCCCTATACTGGAGACCCCCCTTACAGCCATGGGACAGGGACAGGGTACAC
CATGGATACAGTTAACAGGACACATCAGTACTCAGAAAGAGGAAGATGGACAAAAAATACCGAAACTGGA
GCACCGCAACTCAACCCGATTGATGGGCCATTACCAAAAGACAATGAACCAAGTGGCTATGCCCAAACAG
ATTGTGTATTAGAAGCAATGGCTTTCCTTGAGGAATCCCATCCTGGTATTTTTGAAAACTCATGTATTGA

AACGATGGAGGCTGTTCAGCAAACAAGAGTGGACAAACTGACACAAGGAAGACAGACCTATGACTGGACT
CTAAATAGAAACCAGCCTGCTGCCACAGCATTGGCCAACACTATAGAAGTGTTCAGATCAAACGGCCTCA
TAGCAAATGAGTCTGGGAGGCTAATAGACTTCCTTAAAGATGTAATGGAGTCAATGGACAGAGAAGAAGT
AGAGATCACAACTCACTTTCAGAGAAAGAGAAGAGTGAGAGACAATGTAACTAAAAAAATGGTGACACAA
AGAACAATAGGCAAAAAGAAACATAAATTGGACAAAAGAAGTTATCTAATTAGGGCATTAACCCTGAACA
CAATGACCAAAGATGCTGAGAGGGGGAAACTAAAACGTAGAGCTATTGCAACCCCAGGAATGCAGATAAG
GGGGTTTGTATACTTTGTTGAGACACTGGCAAGGAGCATATGTGAGAAACTTGAGCAATCAGGGTTACCA
GTTGGAGGAAATGAGAAGAAAGCAAAGTTAGCAAATGTTGTAAGGAAGATGATGACCAACTCCCAGGACA
CTGAAATTTCTTTCACCATCACTGGAGATAACACAAAATGGAACGAAAATCAGAACCCTAGAATGTTTTT
GGCTATGATCACATATATAACCAGAAATCAGCCTGAATGGTTCAGAAACATTCTAAGTATTGCTCCAATA
ATGTTTTCAAACAAAATGGCGAGACTAGGTAAGGGGTACATGTTTGAAAGCAAGAGTATGAAGCTGAGAA
CTCAAATACCTGCAGAAATGCTGGCCAACATAGATTTGAAATATTTCAATGATTCAACAAAAAAGAAAAT
TGAAAAAATCCGGCCATTATTAATAGATGGAACTGCATCACTGAGTCCTGGAATGATGATGGGCATGTTC
AATATGTTGAGCACTGTCTTGGGCGTCTCCATCCTGAATCTTGGACAAAAGAGATACACCAAGACCACTT
ACTGGTGGGATGGTCTTCAATCGTCTGATGATTTTGCTCTGATTGTGAATGCACCCAACTATGCAGGAAT
TCAAGCTGGAGTTGACAGGTTTTATCGAACCTGTAAGCTGCTCGGAATTAATATGAGCAAAAAGAAGTCT
TACATAAACAGGACAGGTACTTTTGAATTTACAAGCTTTTTCTATCGTTATGGGTTTGTTGCCAATTTTA
GCATGGAACTTCCTAGTTTTGGGGTGTCTGGGGTTAATGAGTCTGCAGACATGAGTATTGGAGTCACTGT
CATCAAAAATAATATGATAAACAATGATCTTGGTCCAGCAACTGCCCAAATGGCCCTTCAATTATTCATC
AAAGATTACAGGTATACTTATCGATGCCACAGAGGTGACACACAAATACAAACCCGGAGATCATTTGAGA
TAAAAAAACTGTGGGACCAAACCCGTTCCAAAGCTGGGCTGTTGGTCTCTGATGGAGGCCCCAATTTATA
TAATATTAGAAATCTCCACATTCCTGAAGTCTGCTTGAAATGGGAGTTGATGGATGAGGATTACCAAGGG
CGTTTATGCAATCCATTGAACCCTTTTGTCAGTCATAAAGAAATTGAATCAGTGAACAATGCAGTGATGA
TGCCGGCACATGGTCCAGCCAAAAATGTGGAATATGACGCTGTTGCAACAACGCACTCCTGGGTCCCCAA
AAGGAATCGATCCATTTTGAATACGAGCCAAAGAGGAATACTTGAGGATGAGCAAATGTATCAAAGGTGC
TGCAATTTGTTTGAAAAGTTCTTCCCAAGCAGCTCATATAGAAGACCAGTTGGAATATCCAGTATGGTAG
AGGCCATGGTCTCCAGAGCCCGAATCGATGCACGGATTGATTTCGAATCTGGAAGGATAAAGAAAGAAGA
GTTCGCTGAGATCATGAAGACCTGTTCCACCATTGAAGACCTCAGACGGCAAAAATAGGGAATTTGGCTT
GTCCTTCGTGAAAAAATGCCTTGTTTCTACT
>gi|145278918|gb|CY021755.1| Influenza A virus (A/South Australia/44/2000(H1N1)) segment 2, complete sequence
CATTTGAATGGATGTCAATCCGACTTTACTTTTCTTAAAAGTGCCAGCACAAAATGCTATAAGCACAACT
TTCCCCTATACTGGAGACCCCCCTTACAGCCATGGGACAGGGACAGGGTACACCATGGATACAGTTAACA
GGACACATCAGTACTCAGAAAGAGGAAGATGGACAAAAAATACCGAAACTGGAGCACCGCAACTCAACCC
GATTGATGGGCCATTACCAAAAGACAATGAGCCAAGTGGCTATGCCCAAACAGATTGTGTATTAGAAGCA
ATGGCTTTCCTTGAGGAATCCCATCCTGGTATTTTTGAAAACTCATGTATTGAAACGATGGAGGCTGTTC
AGCAAACAAGAGTGGACAAACTGACACAAGGAAGACAGACCTATGACTGGACTCTAAATAGAAACCAGCC
TGCTGCCACAGCATTGGCCAACACTATAGAAGTGTTCAGATCAAACGGCCTCATAGCAAATGAGTCTGGG
AGGCTAATAGACTTCCTTAAAGATGTAATGGAGTCAATGGACAGAGAAGAAGTAGAGATCACAACTCACT
TTCAGAGAAAGAGAAGAGTGAGAGACAATGTAACTAAAAAAATGGTGACACAAAGAACAATAGGCAAAAA
GAAACATAAATTGGACAAAAGAAGTTATCTAATTAGGGCATTAACCCTGAACACAATGACCAAAGATGCT
GAGAGGGGGAAACTAAAACGTAGAGCTATTGCAACCCCGGGAATGCAGATAAGGGGGTTTGTATACTTTG
TTGAGACACTGGCAAGGAGCATATGTGAGAAACTTGAGCAATCAGGGTTACCAGTTGGAGGAAATGAGAA
GAAAGCAAAGTTAGCAAATGTTGTAAGGAAGATGATGACCAACTCCCAGGACACTGAAATTTCTTTCACC

ATCACTGGAGATAACACAAAATGGAACGAAAATCAGAACCCTAGAATGTTTTTGGCTATGATCACATATA
TAACCAGAAATCAGCCTGAATGGTTCAGAAACATTCTAAGTATTGCTCCAATAATGTTTTCAAACAAAAT
GGCGAGACTAGGTAAGGGGTACATGTTTGAAAGCAAGAGTATGAAGCTGAGAACTCAAATACCTGCAGAA
ATACTGGCCAACATAGATTTGAAATATTTCAATGATTCAACAAAAAAGAAAATTGAAAAAATCCGGCCAT
TATTAATAGATGGAACTGCATCACTGAGTCCTGGAATGATGATGGGCATGTTCAATATGTTGAGCACTGT
CTTGGGCGTCTCCATCCTGAATCTTGGACAAAAGAGATACACCAAGACCACTTACTGGTGGGATGGTCTT
CAATCGTCTGATGATTTTGCTCTGATTGTGAATGCGCCCAACTATGCAGGAATTCAAGCTGGAGTTGACA
GGTTTTATCGAACCTGTAAGCTGCTCGGAATTAATATGAGCAAAAAGAAGTCTTACATAAACAGGACAGG
TACTTTTGAATTTACAAGCTTTTTCTATCGTTATGGGTTTGTTGCCAATTTTAGCATGGAACTTCCTAGT
TTTGGGGTGTCTGGGGTTAATGAGTCTGCAGACATGAGTATTGGAATCACTGTCATCAAAAATAATATGA
TAAACAATGATCTTGGTCCAGCAACTGCCCAAATGGCCCTTCAGTTATTCATCAAAGATTACAGGTATAC
GTATCGATGCCACAGAGGTGACACACAAATACAAACCCGGAGATCATTTGAGATAAAAAAACTGTGGGAC
CAAACCCGTTCCAAAGCTGGGCTGTTGGTCTCTGATGGAGGCCCCAATTTATATAATATTAGAAATCTCC
ACATTCCTGAAGTCTGCTTGAAATGGGAGTTGATGGATGAGGATTACCAAGGGCGTTTATGCAATCCATT
GAACCCTTTTGTCAGTCATAAAGAAATTGAATCAGTGAACAATGCAGTGATGATGCCGGCACATGGTCCA
GCCAAAAATGTGGAATATGACGCTGTTGCAACAACGCACTCCTGGGTCCCCAAAAGGAATCGATCCATTT
TGAATACGAGCCAAAGAGGAATACTTGAGGATGAGCAAATGTATCAAAGGTGCTGCAATTTGTTTGAAAA
GTTCTTCCCAAGCAGCTCATATAGAAGACCAGTTGGAATATCCAGTATGGTAGAGGCCATGGTTTCCAGA
GCCCGAATCGATGCACGGATTGATTTCGAATCTGGAAGGATAAAGAAAGAAGAGTTCGCTGAGATCATGA
AGACCTGTTCCACCATTGAAGACCTCAGACGGCAAAAATAGGGAATTTGGCTTGT
>gi|157367779|gb|CY026161.1| Influenza A virus (A/Auckland/585/2000(H1N1)) segment 2, complete sequence
ATTTGAATGGATGTCAATCCGACATTACTTTTCTTAAAAGTGCCAGCACAAAATGCTATAAGCACAACTT
TTCCTTATACTGGTGACCCTCCTTACAGCCATGGGACAGGAACAGGGTACACCATGGATACAGTCAACAG
GACACATCAGTACTCAGAAAGAGGAAGATGGACAAAAAATACCGAAACTGGAGCACCGCAACTCAACCCA
ATTGATGGGCCACTACCAAAAGACAATGAACCAAGTGGCTATGCCCAAACAGATTGTGTATTAGAAGCAA
TGGCTTTCCTTGAGGAATCCCATCCTGGTATTTTTGAAAACTCTTGTATTGAAACAATGGAGGTTGTTCA
GCAAACAAGGGTGGACAAACTGACACAAGGCAGACAGACCTATGACTGGACTCTAAATAGGAACCAGCCT
GCTGCCACAGCATTGGCCAACACTATAGAAGTGTTCAGATCAAACGGCCTCATAGCAAATGAATCTGGGA
GGCTAATAGACTTCCTTAAAGATGTAATGGAGTCGATGGACAGAGACGAAATAGAGGTCACAACTCATTT
TCAGAGAAAGAGGAGAGTGAGAGACAATGTAACTAAAAAAATGGTGACCCAAAGAACAATAGGCAAAAA
G
AAACATAAATTAGATAAAAGAAGTTACCTAATTAGGGCATTAACCCTGAACACAATGACCAAAGATGCTG
AGAGGGGGAAACTAAAACGCAGAGCAATTGCAACCCCAGGAATGCAAATAAGGGGGTTTGTATACTTTGT
TGAGACACTGGCAAGAAGCATATGTGAAAAGCTTGAACAATCAGGGTTGCCAGTTGGAGGAAATGAAAAG
AAAGCAAAGTTAGCAAATGTTGTAAGGAAGATGATGACCAACTCCCAGGACACTGAAATTTCTTTCACCA
TCACTGGAGATAACACAAAATGGAACGAAAATCAAAACCCTAGAATGTTCTTGGCCATGATCACATATAT
AACCAAAAATCAGCCTGAATGGTTCAGAAATATTCTAAGTATTGCTCCAATAATGTTTTCAAACAAAATG
GCGAGACTAGGTAAGGGGTACATGTTTGAAAGCAAGAGTATGAAACTGAGAACTCAAATACCTGCAGAGA
TGCTAGCCAACATAGATTTGAAATATTTCAATGATTCAACTAAAAAGAAAATTGAAAAAATCCGGCCATT
ATTAATAGATGGAACTGCATCATTGAGTCCTGGAATGATGATGGGCATGTTCAATATGTTAAGCACCGTC
TTGGGCGTCTCCATTCTGAATCTTGGGCAAAAGAGATACACCAAGACTACTTACTGGTGGGATGGTCTTC
AATCGTCTGATGATTTTGCTCTGATTGTGAATGCACCCAACTATGCAGGAATTCAAGCTGGAGTTGACAG
GTTTTATCGAACCTGTAAGCTGCTCGGAATTAATATGAGCAAAAAGAAGTCTTACATAAACAGAACAGGT

ACCTTTGAATTCACGAGCTTTTTCTATCGTTATGGGTTTGTTGCCAATTTCAGCATGGAGCTTCCTAGTT
TTGGGGTGTCTGGGGTCAATGAATCTGCAGACATGAGTATTGGAGTCACTGTCATCAAAAACAATATGAT
AAACAATGACCTTGGCCCAGCAACTGCTCAAATGGCCCTTCAGTTATTTATAAAAGATTACAGGTACACG
TATCGATGCCACAGAGGTGACACACAAATACAAACCCGGAGATCATTTGAGATAAAGAAACTATGGGACC
AAACCCGCTCCAAAGCTGGGCTGTTGGTCTCTGATGGAGGCCCCAATTTATATAACATTAGAAATCTCCA
TATTCCTGAAGTCTGCTTGAAATGGGAGTTGATGGATGAGGATTACCAAGGGCGTTTATGCAACCCATTG
AACCCGTTTGTCAGTCATAAAGAGATTGAATCAGTGAACAATGCAGTGATGATGCCGGCACATGGTCCAG
CCAAAAATATGGAGTATGACGCTGTTGCAACAACACACTCCTGGGTTCCCAAAAGGAATCGATCCATTTT
GAATACGAGCCAAAGGGGGATACTTGAGGATGAGCAAATGTATCAGAGGTGCTGCAATTTATTTGAAAAA
TTCTTCCCAAGTAGCTCATACAGAAGACCAGTTGGAATATCCAGTATGGTAGAGGCTATGGTTTCCAGAG
CCCGAATTGATGCACGGATTGATTTCGAATCTGGAAGGATAAAGAAAGAGGAATTCGCTGAGATCATGAA
GACCTGTTCCACCATTGAAGACCTCAGACGGCAAAAATAGGGAATTTGGCTTGTCCTTCATGA
>gi|145278631|gb|CY021635.1| Influenza A virus (A/Wellington/4/2000(H1N1)) segment 2, complete sequence
AATGGATGTCAATCCGACATTACTTTTCTTAAAAGTGCCAGCACAAAATGCTATAAGCACAACTTTTCCT
TATACTGGTGACCCTCCTTACAGCCATGGGACAGGAACAGGGTACACCATGGATACAGTCAACAGGACAC
ATCAGTACTCAGAAAGAGGAAGATGGACAAAAAATACCGAAACTGGAGCACCGCAACTCAACCCAATTGA
TGGGCCACTACCAAAAGACAATGAACCAAGTGGCTATGCCCAAACAGATTGTGTATTAGAAGCAATGGCT
TTCCTTGAGGAATCCCATCCTGGTATTTTTGAAAACTCTTGTATTGAAACAATGGAGGTTGTTCAGCAAA
CAAGGGTGGACAAACTGACACAAGGCAGACAGACCTATGACTGGACTCTAAATAGGAACCAGCCTGCTGC
CACAGCATTGGCCAACACTATAGAAGTGTTCAGATCAAACGGCCTCATAGCAAATGAATCTGGGAGGCTA
ATAGACTTCCTTAAAGATGTAATGGAGTCGATGGACAGAGACGAAATAGAGGTCACAACTCATTTTCAGA
GAAAGAGGAGAGTGAGAGACAATGTAACTAAAAAAATGGTGACCCAAAGAACAATAGGCAAAAAGAAAC
A
TAAATTAGATAAAAGAAGTTACCTAATTAGGGCATTAACCCTGAACACAATGACCAAAGATGCTGAGAGG
GGGAAACTAAAACGCAGAGCAATTGCAACCCCAGGAATGCAAATAAGGGGGTTTGTATACTTTGTTGAGA
CACTGGCAAGAAGCATATGTGAAAAGCTTGAACAATCAGGGTTGCCAGTTGGAGGAAATGAAAAGAAAGC
AAAGTTAGCAAATGTTGTAAGGAAGATGATGACCAACTCCCAGGACACTGAAATTTCTTTCACCATCACT
GGAGATAACACAAAATGGAACGAAAATCAAAACCCTAGAATGTTCTTAGCCATGATCACATATATAACCA
AAAATCAGCCTGAATGGTTCAGAAATATTCTAAGTATTGCTCCAATAATGTTTTCAAACAAAATGGCGAG
ACTAGGTAAGGGGTACATGTTTGAAAGCAAGAGTATGAAACTGAGAACTCAAATACCTGCAGAGATGCTA
GCCAACATAGATTTGAAATATTTCAATGATTCAACTAAAAGGAAAATTGAAAAAATCCGGCCATTATTAA
TAGATGGAACTGCATCATTGAGTCCTGGAATGATGATGGGCATGTTCAATATGTTAAGCACCGTCTTGGG
CGTCTCCATTTTGAATCTTGGGCAAAAGAGATACACCAAGACTACTTACTGGTGGGATGGTCTTCAATCG
TCTGATGATTTTGCTCTGATTGTGAATGCACCCAACTATGCAGGAATTCAAGCTGGAGTTGACAGGTTTT
ATCGAACCTGTAAGCTGCTCGGAATTAATATGAGCAAAAAGAAGTCTTACATAAACAGAACAGGTACCTT
TGAATTCACGAGCTTTTTCTATCGTTATGGGTTTGTTGCCAATTTCAGCATGGAGCTTCCTAGTTTTGGG
GTGTCTGGGGTCAATGAATCTGCAGACATGAGTATTGGAGTCACTGTCATCAAAAACAATATGATAAACA
ATGACCTTGGCCCAGCAACTGCTCAAATGGCCCTTCAGTTATTTATAAAAGATTACAGGTACACGTATCG
ATGCCACAGAGGTGACACACAAATACAAACCCGGAGATCATTTGAGATAAAGAAACTATGGGACCAAACC
CGCTCCAAAGCTGGGCTGTTGGTCTCTGATGGAGGCCCCAATTTATATAACATTAGAAATCTCCATATTC
CTGAAGTCTGCTTGAAATGGGAGTTGATGGATGAGGATTACCAAGGGCGTTTATGCAACCCATTGAACCC
GTTTGTCAGTCATAAAGAGATTGAATCAGTGAACAATGCAGTGATGATGCCGGCACATGGTCCAGCCAAA
AATATGGAGTATGACGCTGTTGCAACAACACACTCCTGGGTTCCCAAAAGGAATCGATCCATTTTGAATA

CGAGCCAAAGGGGGATACTTGAGGATGAGCAAATGTATCAGAGGTGCTGCAATTTATTTGAAAAATTCTT
CCCAAGTAGCTCATACAGAAGACCAGTTGGAATATCCAGTATGGTAGAGGCTATGGTTTCCAGAGCCCGA
ATTGATGCACGGATTGATTTCGAATCTGGAAGGATAAAGAAAGAGGAATTCGCTGAGATCATGAAGACCT
GTTCCACCATTGAAGACCTCAGACGGCAAAAATAGGGAATTTGGCTTGTCCTTCATGAAAA
>gi|131058545|gb|CY020155.1| Influenza A virus (A/Memphis/7/2001(H1N1)) segment 2, complete sequence
AATGGATGTCAATCCGACCTTACTTTTCTTAAAAGTGCCAGCACAAAATGCTATAAGCACAACTTTCCCT
TATACTGGTGACCCTCCTTACAGCCATGGGACAGGAACAGGGTACACCATGGATACAGTCAACAGGACAC
ATCAGTACTCAGAAAGAGGAAGGTGGACAAAGAATACCGAAACTGGAGCACCGCAACTCAACCCAATTGA
TGGGCCACTACCAAAAGACAATGAACCGAGTGGCTATGCCCAAACAGATTGTGTATTAGAAGCAATGGCT
TTCCTTGAGGAATCCCATCCTGGTATTTTTGAAAACTCTTGTATTGAAACAATGGAGGTTGTTCAGCAAA
CAAGGGTGGACAAACTGACACAAGGCAGACAGACCTATGACTGGACTCTAAATAGAAACCAGCCTGCTGC
CACAGCATTGGCCAACACTATAGAAGTGTTCAGATCAAACGGCCTCATAGCAAATGAATCTGGGAGGCTA
ATAGACTTCCTTAAAGATGTAATGGAGTCGATGGACAGAGACGAAGTAGAGATCACAACTCATTTTCAGA
GAAAGAGGAGAGTGAGAGACAATGTAACTAAAAAAATGGTGACCCAAAGAACAATAGGCAAAAAGAAAC
A
TAAATTAGACAAAAGAAGTTACCTAATTAGGGCATTAACCCTGAACACAATGACCAAAGATGCTGAGAGG
GGGAAACTAAAACGTAGAGCAATTGCAACCCCAGGAATGCAAATAAGGGGGTTTGTATACTTTGTTGAGA
CACTGGCAAGAAGCATATGTGAAAAGCTTGAACAATCAGGGTTGCCAGTTGGGGGAAATGAAAAGAAAGC
AAAGTTAGCAAATGTTGTAAGGAAGATGATGACCAACTCCCAGGACACTGAAATTTCTTTCACCATCACT
GGAGATAACACAAAATGGAACGAAAATCAAAACCCTAGAATGTTTTTGGCCATGATCACATATATAACCA
AAAATCAGCCTGAATGGTTCAGAAATATTCTAAGTATTGCTCCAATAATGTTTTCAAACAAAATGGCGAG
ACTAGGTAAGGGGTACATGTTTGAAAGCAAGAGTATGAAACTGAGAACTCAAATACCTGCAGAGATGCTA
GCCAACATAGATTTGAAATATTTCAATGATTCAACTAAAAAGAAAATTGAAAAAATCCGGCCATTATTAA
TAGATGGAACTGCATCATTGAGTCCTGGAATGATGATGGGCATGTTCAATATGCTAAGCACCGTCTTGGG
CGTCTCCATTCTGAATCTTGGGCAAAAGAGATACACCAAGACTACTTACTGGTGGGATGGTCTTCAATCG
TCTGATGATTTTGCTCTTATTGTGAATGCACCCAACTATGCAGGAATTCAAGCTGGAGTTGACAGGTTTT
ATCGAACCTGCAAGCTGCTCGGAATTAATATGAGCAAAAAGAAGTCTTACATAAACAGAACAGGTACCTT
TGAATTCACGAGCTTTTTCTATCGTTATGGGTTTGTTGCCAATTTCAGCATGGAGCTTCCTAGTTTTGGG
GTGTCTGGGGTCAATGAGTCTGCAGACATGAGTATTGGAGTCACTGTCATCAAAAACAATATGATAAACA
ATGACCTTGGCCCAGCAACTGCTCAAATGGCCCTCCAGTTATTTATCAAAGATTACAGGTACACGTATCG
ATGCCACAGAGGTGACACACAAATACAAACCCGGAGATCATTTGAGATAAAGAAATTATGGGACCAAACC
CGCTCCAAAGCTGGGCTGTTGGTCTCTGATGGAGGCCCCAATTTATATAACATTAGAAATCTCCATATTC
CTGAAGTCTGCTTGAAATGGGAGTTGATGGATGAGGATTACCAGGGGCGTTTATGCAACCCATTGAACCC
GTTTGTCAGTCATAAAGAGATTGAATCAGTGAACAATGCAGTGATGATGCCGGCACATGGTCCAGCCAAA
AATATGGAGTATGACGCTGTTGCAACAACACACTCCTGGGTTCCCAAAAGGAATCGATCCATTTTGAATA
CGAGCCAAAGGGGGATACTTGAGGATGAGCAAATGTATCAGAGGTGCTGCAATTTATTTGAAAAATTCTT
CCCAAGTAGCTCATACAGAAGACCAGTTGGAATATCCAGTATGGTAGAGGCTATGGTTTCCAGAGCCCGA
ATTGATGCACGGATTGATTTCGAATCTGGAAGGATAAAGAAAGAGGAATTCGCTGAGATCATGAAGACCT
GTTCCACCATTGAAGACCTCAGACGGCAAAAATAGGGAATTTGGC
>gi|73761487|gb|CY002534.1| Influenza A virus (A/New York/220/2002(H1N1)) segment 2, complete sequence
AGCGAAAGCAGGCAAACCATTTGAATGGATGTCAATCCGACATTACTTTTCTTAAAAGTGCCAGCACAAA
ATGCTATAAGCACAACTTTTCCTTATACTGGTGACCCTCCTTACAGCCATGGGACAGGAACAGGGTACAC

CATGGATACAGTCAACAGGACACATCAGTACTCAGAAAGAGGAAGATGGACAAAAAATACCGAAACTGGA
GCACCGCAACTCAACCCAATTGATGGGCCACTACCAGAAGACAATGAACCAAGTGGCTATGCCCAAACAG
ATTGTGTATTAGAAGCAATGGCTTTCCTTGAAGAATCCCATCCTGGTATTTTTGAAAACTCTTGTATTGA
AACAATGGAGGTTGTTCAGCAAACAAGGGTGGACAAACTGACACAAGGCAGACAGACCTATGACTGGACT
CTAAATAGGAACCAGCCTGCTGCCACAGCATTGGCAAACACTATAGAAGTATTCAGATCAAACGGCCTCA
TAGCAAATGAATCTGGGAGGCTAATAGACTTCCTTAAAGATGTAATGGAGTCGATGGACAGAGACGAAGT
AGAGGTCACAACTCATTTTCAAAGAAAGAGGAGAGTGAGAGACAATGTAACTAAAAAAATGGTGACCCAA
AGAACAATAGGCAAAAAGAAACATAAATTAGACAAAAGAAGTTACCTAATTAGGGCATTAACCCTGAACA
CAATGACCAAAGATGCTGAGAGGGGGAAACTAAAACGCAGAGCAATTGCAACCCCAGGAATGCAAATAAG
GGGGTTTGTATACTTTGTTGAGACACTGGCAAGAAGCATATGTGAAAAGCTTGAACAATCAGGGTTGCCA
GTTGGAGGAAATGAGAAGAAAGCAAAGTTAGCAAATGTTGTAAGGAAGATGATGACCAACTCCCAGGACA
CTGAAATTTCTTTCACCATCACTGGAGATAACACAAAATGGAACGAAAATCAAAACCCTAGAATGTTCTT
GGCCATGATCACATATATAACCAAAAATCAGCCTGAATGGTTCAGAAATATTCTAAGTATTGCTCCAATA
ATGTTTTCAAACAAGATGGCGAGACTAGGTAAGGGGTACATGTTTGAAAGCAAGAGTATGAAACTGAGAA
CTCAAATACCTGCAGAGATGCTAGCCAACATAGATTTGAAATATTTCAATGATTCAACTAAAAAGAAAAT
TGAAAAAATCCGACCATTATTAATAGATGGAACTGCATCATTGAGTCCTGGAATGATGATGGGCATGTTC
AATATGTTAAGCACCGTCTTGGGCGTCTCCATTCTGAATCTTGGGCAAAAGAGATACACCAAGACTACTT
ACTGGTGGGATGGTCTTCAATCGTCTGATGATTTTGCTTTGATTGTGAATGCACCCAACTATGCAGGAAT
TCAAGCTGGAGTCGACAGGTTTTATCGAACCTGTAAGCTGCTCGGAATTAATATGAGCAAAAAGAAGTCT
TACATAAACAGAACAGGTACCTTTGAATTCACGAGCTTTTTCTATCGTTATGGGTTTGTTGCCAATTTCA
GCATGGAGCTTCCTAGTTTTGGGGTGTCTGGGGTCAATGAATCTGCAGACATGAGTATTGGAGTCACTGT
CATCAAAAACAATATGATAAACAATGACCTTGGCCCAGCAACTGCTCAAATGGCCCTTCAGTTATTTATA
AAAGATTACAGGTACACTTATCGATGCCACAGAGGTGACACACAAATACAAACCCGGAGATCATTTGAGA
TAAAAAAACTATGGGACCAAACTCGCTCCAAAGCTGGGCTGTTGGTCTCTGATGGAGGCCCCAATTTATA
TAATATTAGAAATCTCCATATTCCTGAAGTCTGCTTGAAATGGGAGTTGATGGATGAGGATTACCAGGGG
CGTTTATGCAACCCATTGAACCCGTTTGTCAGTCATAAAGAGATTGAATCAGTGAACAATGCAGTGATAA
TGCCGGCACATGGTCCAGCCAAAAATATGGAGTATGACGCTGTTGCAACAACACACTCCTGGGTCCCCAA
AAGGAATCGATCCATTTTGAACACGAGCCAAAGGGGGATACTTGAAGATGAGCAAATGTATCAGAGGTGC
TGCAATTTATTTGAAAAATTCTTCCCAAGTAGCTCATACAGAAGACCAGTTGGAATATCCAGTATGGTAG
AGGCTATGGTTTCAAGAGCCCGAATTGATGCACGGATTGATTTCGAATCTGGAAGGATAAAGAAAGAGGA
ATTCGCTGAGATCATGAAGACCTGTTCCACCATTGAAGACCTCAGACGGCAAAAATAGGGAATTTGGCTT
GTCCTTCATGAAAAAATGCCTTGTTTCTACT
>gi|156536331|gb|CY025040.1| Influenza A virus (A/Auckland/597/2000(H1N1)) segment 2, complete sequence
CATTTGAATGGATGTCAATCCGACATTACTTTTCTTAAAAGTGCCAGCACAAAATGCTATAAGCACAACT
TTTCCTTATACTGGTGACCCTCCTTACAGCCATGGGACAGGAACAGGGTACACCATGGATACAGTCAACA
GGACACATCAGTACTCAGAAAGAGGAAGATGGACAAAAAATACCGAAACTGGAGCACCGCAACTCAACCC
AATTGATGGGCCACTACCAAAAGACAATGAACCAAGTGGCTATGCCCAAACAGATTGTGTATTAGAAGCA
ATGGCTTTCCTTGAGGAATCCCATCCTGGTATTTTTGAAAACTCTTGTATTGAAACAATGGAGGTTGTTC
AGCAAACAAGGGTGGACAAACTGACACAAGGCAGACAGACCTATGACTGGACTCTAAATAGGAACCAGCC
TGCTGCCACAGCATTGGCCAACACTATAGAAGTGTTCAGATCAAACGGCCTCATAGCAAATGAATCTGGG
AGGCTAATAGACTTCCTTAAAGATGTAATGGAGTCGATGGACAGAGACGAAATAGAGGTCACAACTCATT
TTCAGAGAAAGAGGAGAGTGAGAGACAATGTAACTAAAAAAATGGTGACCCAAAGAACAATAGGCAAAAA
GAAACATAAATTAGATAAAAGAAGTTACCTAATTAGGGCATTAACCCTGAACACAATGACCAAAGATGCT

GAGAGGGGGAAACTAAAACGCAGAGCAATTGCAACCCCAGGAATGCAAATAAGGGGGTTTGTATACTTTG
TTGAGACACTGGCAAGAAGCATATGTGAAAAGCTTGAACAATCAGGGTTGCCAGTTGGAGGAAATGAAAA
GAAAGCAAAGTTAGCAAATGTTGTAAGGAAGATGATGACCAACTCCCAGGACACTGAAATTTCTTTCACC
ATCACTGGAGATAACACAAAATGGAACGAAAATCAAAACCCTAGAATGTTCTTGGCCATGATCACATATA
TAACCAAAAATCAGCCTGAATGGTTCAGAAATATTCTAAGTATTGCTCCAATAATGTTTTCAAACAAAAT
GGCGAGACTAGGTAAGGGGTACATGTTTGAAAGCAAGAGTATGAAACTGAGAACTCAAATACCTGCAGAG
ATGCTAGCCAACATAGATTTGAAATATTTCAATGATTCAACTAAAAAGAAAATTGAAAAAATCCGGCCAT
TATTAATAGATGGAACTGCATCATTGAGTCCTGGAATGATGATGGGCATGTTCAATATGTTAAGCACCGT
CTTGGGCGTCTCCATTCTGAATCTTGGGCAAAAGAGATACACCAAGACTACTTACTGGTGGGATGGTCTT
CAATCGTCTGATGATTTTGCTCTGATTGTGAATGCACCCAACTATGCAGGAATTCAAGCTGGAGTTGACA
GGTTTTATCGAACCTGTAAGCTGCTCGGAATTAATATGAGCAAAAAGAAGTCTTACATAAACAGAACAGG
TACCTTTGAATTCACGAGCTTTTTCTATCGTTATGGGTTTGTTGCCAATTTCAGCATGGAGCTTCCTAGT
TTTGGGGTGTCTGGGGTCAATGAATCTGCAGACATGAGTATTGGAGTCACTGTCATCAAAAACAATATGA
TAAACAATGACCTTGGCCCAGCAACTGCTCAAATGGCCCTTCAGTTATTTATAAAAGATTACAGGTACAC
GTATCGATGCCACAGAGGTGACACACAAATACAAACCCGGAGATCATTTGAGATAAAGAAACTATGGGAC
CAAACCCGCTCCAAAGCTGGGCTGTTGGTCTCTGATGGAGGCCCCAATTTATATAACATTAGAAATCTCC
ATATTCCTGAAGTCTGCTTGAAATGGGAGTTGATGGATGAGGATTACCAAGGGCGTTTATGCAACCCATT
GAACCCGTTTGTCAGTCATAAAGAGATTGAATCAGTGAACAATGCAGTGATGATGCCGGCACATGGTCCA
GCCAAAAATATGGAGTATGACGCTGTTGCAACAACACACTCCTGGGTTCCCAAAAGGAATCGATCCATTT
TGAATACGAGCCAAAGGGGGATACTTGAGGATGAGCAAATGTATCAGAGGTGCTGCAATTTATTTGAAAA
ATTCTTCCCAAGTAGCTCATACAGAAGACCAGTTGGAATATCCAGTATGGTAGAGGCTATGGTTTCCAGA
GCCCGAATTGATGCACGGATTGATTTCGAATCTGGAAGGATAAAGAAAGAGGAATTCGCTGAGATCATGA
AGACCTGTTCCACCATTGAAGACCTCAGACGGCAAAAATAGGGAATTTGGCTTGTCCTTCATGAAAA
>gi|149780541|gb|CY022539.1| Influenza A virus (A/Auckland/605/2001(H1N1)) segment 2, complete sequence
CATTTGAATGGATGTCAATCCGACCTTACTTTTCTTAAAAGTGCCAGCACAAAATGCTATAAGCACAACT
TTCCCTTATACTGGTGATCCTCCTTACAGCCATGGGACAGGAACAGGGTACACCATGGATACAGTCAACA
GGACACATCAGTACTCAGAAAGAGGAAGATGGACAAAAAATACCGAAACTGGAGCACCGCAACTCAACCC
AATTGATGGACCACTACCAAAAGACAATGAACCAAGTGGCTATGCCCAAACAGATTGTGTATTAGAAGCA
ATGGCTTTCCTTGAGGAATCCCATCCTGGTATTTTTGAAAACTCTTGTATTGAAACAATGGAGGTTGTTC
AGCAAACAAGGGTGGACAAACTGACACAAGGCAGACAGACCTATGACTGGACTCTAAATAGAAACCAGCC
TGCTGCCACAGCATTGGCCAACACTATAGAAGTGTTCAGATCAAACGGCCTCATAGCAAATGAATCTGGG
AGGCTAATAGACTTCCTTAAAGATGTAATGGAGTCGATGGACAGAGACGAAGTAGAGATCACAACACATT
TTCAGAGAAAGAGGAGGGTGAGAGACAATGTAACTAAAAAAATGGTGACCCAAAGAACAATAGGCAAAA
A
GAAACATAAATTAGACAAAAGAAGTTACCTAATTAGGGCATTAACCCTGAACACAATGACCAAAGATGCT
GAGAGGGGGAAACTAAAACGTAGAGCAATTGCAACCCCAGGAATGCAAATAAGGGGGTTTGTATACTTTG
TTGAGACACTGGCAAGAAGCATATGTGAAAAGCTTGAACAATCAGGGTTGCCAGTTGGGGGAAATGAAAA
GAAAGCAAAGTTAGCAAATGTTGTAAGGAAGATGATGACCAACTCCCAGGACACTGAAATTTCTTTCACC
ATCACTGGAGATAACACAAAATGGAACGAAAATCAAAACCCTAGAATGTTTTTGGCCATGATCACATATA
TAACCAAAAATCAGCCTGAATGGTTCAGAAATATTCTAAGTATTGCTCCAATAATGTTTTCAAACAAAAT
GGCGAGACTAGGTAAGGGGTACATGTTTGAAAGCAAGAGTATGAAACTGAGAACTCAAATACCTGCAGAG
ATGCTAGCCAACATAGATTTGAAATATTTCAATGATTCAACTAAAAAGAAAATTGAAAAAATCCGGCCAT
TATTAATAGATGGAACTGCATCATTGAGTCCTGGAATGATGATGGGCATGTTCAATATGTTAAGCACTGT

CTTGGGCGTCTCCATTCTGAATCTTGGGCAAAAGAGATACACCAAGACTACTTACTGGTGGGATGGTCTT
CAATCGTCTGATGATTTTGCTCTGATTGTGAATGCATCCAACTATGCAGGAATTCAAGCTGGAGTTGACA
GGTTTTATCGAACCTGTAAGCTGCTCGGAATTAATATGAGCAAAAAGAAGTCTTACATAAACAGAACAGG
TACCTTTGAATTCACGAGCTTTTTCTATCGTTATGGGTTTGTTGCCAATTTCAGCATGGAGCTTCCTAGT
TTTGGGGTGTCTGGGGTCAATGAGTCTGCAGACATGAGTATTGGAGTCACTGTCATCAAAAACAATATGA
TAAACAATGACCTTGGCCCAGCAACTGCTCAAATGGCCCTTCAGTTATTTATCAAAGATTACAGGTACAC
GTATCGATGCCACAGAGGTGACACACAAATACAAACCCGGAGATCATTTGAGATAAAGAAACTATGGGAC
CAAACCCGCTCCAAAGCTGGGCTGTTGGTCTCTGATGGAGGCCCCAATTTATATAACATTAGAAATCTCC
ATATTCCTGAAGTCTGCTTGAAATGGGAGTTGATGGATGAGGATTACCAGGGGCGTTTATGCAACCCATT
GAACCCATTTGTCAGTCATAAAGAGATTGAGTCAGTGAACAATGCAGTGATGATGCCGGCACATGGTCCA
GCCAAAAATATGGAGTATGACGCTGTTGCAACAACACACTCCTGGGTTCCCAAAAGGAATCGATCCATTT
TGAATACGAGCCAAAGGGGGATACTTGAGGATGAGCAAATGTATCAGAGGTGCTGCAATTTATTTGAAAA
ATTCTTCCCAAGTAGTTCATACAGAAGACCAGTTGGAATATCCAGTATGGTAGAGGCTATGGTTTCCAGA
GCCCGAATTGATGCAAGAATTGATTTCGAATCTGGAAGGATAAAGAAAGAGGAATTCGCTGAGATCATGA
AGACCTGTTCCACCATTGAAGACCTCAGACGGCAAAAATAGGGAATTTGGCTTGTCCTTCATGA
>gi|237688876|gb|CY040080.1| Influenza A virus (A/Taiwan/567/2002(H1N1)) segment 2, complete sequence
ATGGATGTCAATCCGACCTTACTTTTCTTAAAAGTGCCAGCACAAAATGCTATAAGCACAACTTTCCCTT
ATACTGGTGATCCTCCTTACAGCCATGGGACAGGAACAGGGTACACCATGGATACAGTCAACAGGACACA
TCAGTACTCAGAAAGAGGAAGATGGACAAAAAATACCGAAACTGGAGCACCGCAACTCAACCCAATTGAT
GGACCACTACCAAAAGACAATGAACCAAGTGGCTATGCCCAAACAGATTGTGTATTAGAAGCAATGGCTT
TCCTTGAGGAATCCCATCCTGGTATTTTTGAAAACTCTTGTATTGAAACAATGGAGGTYGTTCAGCAAAC
AAGAGTGGACAAACTGACACAAGGCAGACAGACCTATGACTGGACTCTAAATAGAAACCAGCCTGCTGCC
ACAGCATTGGCCAACACTATAGAAGTGTTCAGATCAAACGGCCTCATGGCAAATGAATCTGGGAGGCTAA
TAGACTTCCTTAAAGATGTAATGGAGTCGATGGACAGAGACGAAGTAGAGATCACAACACATTTTCAGAG
AAAGAGGAGGGTGAGAGACAATGTAACTAAAAAAATGGTGACCCAAAGAACAATAGGCAAAAAGAAACAT
AAATTAGACAAAAGAAGTTACCTAATTAGGGCATTAACCCTAAACACAATGACCAAAGATGCTGAGAGGG
GGAAACTAAAACGTAGAGCAATTGCAACCCCAGGAATGCAAATAAGGGGGTTTGTATACTTTGTTGAGAC
ACTGGCAAGAAGCATATGTGAAAAGCTTGAACAATCAGGGTTGCCAGTTGGGGGGAATGAAAAGAAAGCA
AAGTTAGCAAATGTTGTAAGGAAGATGATGACCAACTCCCAGGACACTGAAATTTCTTTCACCATCACTG
GAGATAACACAAAATGGAACGAAAATCAAAACCCTAGAATGTTTTTGGCCATGATCACATATATAACCAA
AAATCAGCCTGAATGGTTCAGAAATATTCTAAGTATTGCTCCAATAATGTTTTCAAACAAAATGGCGAGA
CTAGGTAAGGGGTACATGTTTGAAAGCAAGAGTATGAAACTGAGAACTCAAATACCTGCAGAGATGCTAG
CCAACATAGATTTGAAATATTTCAATGATTCAACTAAAAAGAAAATTGAAAAAATCCGGCCATTATTAAT
AGATGGAACTGCATCATTGAGTCCTGGAATGATGATGGGCATGTTCAATATGTTAAGCACTGTCTTGGGC
GTCTCCATTCTGAATCTTGGGCAAAAGAGATACACCAAGACTACTTACTGGTGGGATGGTCTTCAATCGT
CTGATGATTTTGCTCTGATTGTGAATGCACCCAACTATGCAGGAATTCAAGCTGGAGTTGACAGGTTTTA
TCGAACCTGTAAGCTGCTCGGAATTAATATGAGCAAAAAGAAGTCTTACATAAACAGAACAGGTACCTTT
GAATTCACGAGCTTTTTCTATCGTTATGGGTTTGTTGCCAATTTCAGCATGGAGCTTCCTAGTTTTGGGG
TGTCTGGGGTCAATGAGTCTGCAGACATGAGTATTGGAGTCACTGTCATCAAAAACAATATGATAAACAA
TGACCTTGGCCCAGCAACTGCTCAAATGGCCCTTCAGTTATTTATCAAAGATTACAGGTACACGTATCGA
TGCCACAGAGGTGACACACAAATACAAACCCGGAGATCATTTGAGATAAAGAAACTATGGGACCAAACCC
GCTCCAAAGCTGGGCTGTTGGTCTCTGATGGAGGCCCCAATTTGTATAACATTAGAAATCTCCATATTCC
TGAAGTCTGCTTGAAATGGGAGTTGATGGATGAGGATTACCAGGGGCGTTTATGCAACCCATTGAACCCA

TTTGTCAGTCATAAAGAGATTGAGTCAGTGAACAATGCAGTGATGATGCCGGCACATGGTCCAGCCAAAA
ATATGGAGTATGACGCTGTTGCAACAACACACTCCTGGGTTCCCAAAAGGAATCGATCCATTTTGAATAC
GAGCCAAAGGGGGATACTTGAGGATGAGCAAATGTATCAGAGGTGCTGCAATTTATTTGAAAAATTCTTC
CCAAGTAGTTCATACAGAAGACCAGTTGGAATATCCAGTATGGTAGAGGCTATGGTTTCCAGAGCCCGAA
TTGATGCAAGAATTGATTTCGAATCTGGAAGGATAAAGAAAGAGGAATTCGCTGAGATCATGAAGACCTG
TTCCACCATTGAAGACCTCAGACGGCAAAAATAGGGAATTTGGCTTGTC
>gi|122855963|gb|CY019347.1| Influenza A virus (A/Memphis/6/2003(H1N1)) segment 2, complete sequence
AATGGATGTCAATCCGACATTACTTTTCTTAAAAGTGCCAGCACAAAATGCTATAAGCACAACTTTTCCT
TATACTGGTGACCCTCCTTACAGCCATGGAACAGGAACAGGGTACACCATGGATACAGTCAACAGGACAC
ATCAGTACTCAGAAAGAGGAAGATGGACAAAAAATACCGAAACTGGAGCACCGCAACTCAACCCAATTGA
TGGGCCACTACCAGAAGACAATGAACCAAGTGGCTATGCCCAAACAGATTGTGTATTAGAAGCAATGGCT
TTCCTTGAAGAATCCCATCCTGGTATTTTTGAAAACTCTTGTATTGAAACAATGGAGGTTGTTCAGCAAA
CAAGGGTGGACAAACTGACACAAGGCAGACAGACCTATGACTGGACTCTAAATAGGAACCAGCCTGCTGC
CACAGCATTGGCAAACACTATAGAAGTATTCAGATCAAACGGCCTCATAGCAAATGAATCTGGGAGGCTA
ATAGACTTCCTTAAAGATGTAATGGAGTCGATGGACAGAGACGAAGTAGAGGTCACAACTCATTTTCAAA
GAAAGAGGAGAGTGAGAGACAATGTAACTAAAAAAATGGTGACCCAAAGAACAATAGGCAAAAAGAAAC
A
TAAATTAGACAAAAGAAGTTACCTAATTAGGGCATTAACCCTGAACACAATGACCAAAGATGCTGAGAGG
GGGAAACTAAAACGCAGAGCAATTGCAACCCCAGGAATGCAAATAAGGGGGTTTGTATACTTTGTTGAGA
CACTGGCAAGAAGCATATGTGAAAAGCTTGAACAATCAGGGTTGCCAGTTGGAGGAAATGAGAAGAAAGC
AAAGTTAGCAAATGTTGTAAGGAAGATGATGACCAACTCCCAGGACACTGAAATTTCTTTCACCATCACT
GGAGATAACACAAAATGGAACGAAAATCAAAACCCTAGAATGTTCTTGGCCATGATCACATATATAACCA
AAAATCAGCCTGAATGGTTCAGAAATATTCTAAGTATTGCTCCAATAATGTTTTCAAACAAGATGGCGAG
ACTAGGTAAGGGGTACATGTTTGAAAGCAAGAGTATGAAACTGAGAACTCAAATACCTGCAGAGATGCTA
GCCAACATAGATTTGAAATATTTCAATGATTCAACTAAAAAGAAAATTGAAAAAATCCGACCGTTATTAA
TAGATGGAACTGCATCATTGAGTCCTGGAATGATGATGGGCATGTTCAATATGTTAAGCACCGTCTTGGG
CGTCTCCATTCTGAATCTTGGGCAAAAGAGATACACCAAGACTACTTACTGGTGGGATGGTCTTCAATCG
TCTGATGATTTTGCTTTGATTGTGAATGCACCCAACTATGCAGGAATTCAAGCTGGAGTTGACAGGTTTT
ATCGAACCTGTAAGCTGCTCGGAATTAATATGAGCAAAAAGAAGTCTTACATAAACAGAACAGGTACCTT
TGAATTCACGAGCTTTTTCTATCGTTATGGGTTTGTTGCCAATTTCAGCATGGAGCTTCCTAGTTTTGGG
GTGTCTGGGGTCAATGAATCTGCAGACATGAGTATTGGAGTCACTGTCATCAAAAACAATATGATAAACA
ATGACCTTGGCCCAGCAACTGCTCAAATGGCCCTTCAGTTATTTATAAAAGATTACAGGTACACTTATCG
ATGCCACAGAGGTGACACACAAATACAAACCCGGAGATCATTTGAGATAAAGAAACTATGGGACCAAACC
CGTTCCAAAGCTGGGCTGTTGGTCTCTGATGGAGGCCCCAATTTATATAACATTAGAAATCTCCATATTC
CTGAAGTCTGCTTGAAATGGGAGTTGATGGATGAGGATTACCAGGGGCGTTTATGCAACCCATTGAACCC
GTTTGTCAGTCATAAAGAGATTGAATCAGTGAACAATGCAGTGATAATGCCGGCACATGGTCCAGCCAAA
AATATGGAGTATGACGCTGTTGCAACAACACACTCCTGGGTCCCCAAAAGGAATCGATCCATTTTGAACA
CGAGCCAAAGGGGGATACTTGAAGATGAGCAAATGTATCAGAGGTGCTGCAATTTATTTGAAAAATTCTT
CCCAAGTAGCTCATACAGAAGACCAGTTGGAATATCCAGTATGGTAGAGGCTATGGTTTCAAGAGCCCGA
ATTGATGCACGGATTGATTTCGAATCTGGAAGGATAAAGAAAGAGGAATTCGCTGAGATCATGAAGACCT
GTTCCACCATTGAAGACCTCAGACGGCAAAAATAGGGAATTTGGCTTGT
>gi|82546788|gb|CY006681.1| Influenza A virus (A/New York/494/2002(H1N1)) segment 2, complete sequence

ATGGATGTCAATCCGACATTACTTTTCTTAAAAGTGCCAGCACAAAATGCTATAAGCACAACTTTTCCTT
ATACTGGTGACCCTCCTTACAGCCATGGGACAGGAACAGGGTACACCATGGATACAGTCAACAGGACACA
TCAGTACTCAGAAAGAGGAAGATGGACAAAAAATACCGAAACTGGAGCACCGCAACTCAACCCAATTGAT
GGACCACTACCAGAAGACAATGAACCAAGTGGCTATGCCCAAACAGATTGTGTATTAGAAGCAATGGCTT
TCCTTGAAGAATCCCATCCTGGTATTTTTGAAAATTCTTGTATTGAAACAATGGAGGTTGTTCAGCAAAC
AAGGGTGGACAAACTGACACAAGGCAGACAGACCTATGACTGGACTCTAAATAGGAACCAGCCTGCTGCC
ACAGCATTGGCCAACACTATAGAAGTATTCAGATCAAACGGCCTCATAGCAAATGAATCTGGGAGGCTAA
TAGACTTCCTTAAAGATGTAATGGAGTCGATGGACAGAGACGAAGTAGAGGTCACAACTCATTTTCAGAG
AAAGAGGAGAGTGAGAGACAATGTAACTAAAAAAATGGTGACCCAAAGAACAATAGGCAAAAAGAAACAT
AAATTAGACAAAAGAAGTTACCTAATTAGGGCATTAACCCTGAACACAATGACCAAAGATGCTGAGAGGG
GGAAACTAAAACGCAGAGCAATTGCAACCCCAGGAATGCAAATAAGGGGGTTTGTATACTTTGTTGAGAC
ACTGGCAAGAAGCATATGTGAAAAGCTTGAACAATCAGGGTTGCCAGTTGGAGGAAATGAGAAGAAAGCA
AAGTTAGCAAATGTTGTAAGGAAGATGATGACCAACTCCCAGGACACTGAAATTTCTTTCACCATCACTG
GAGATAACACAAAATGGAACGAAAATCAAAACCCTAGAATGTTCTTGGCCATGATCACATATATAACCAA
AAATCAGCCTGAATGGTTCAGAAATATTCTAAGTATTGCTCCAATAATGTTTTCAAACAAGATGGCGAGA
CTAGGTAAGGGGTACATGTTTGAAAGCAAGAGTATGAAACTGAGAACTCAAATACCTGCAGAGATGTTAG
CCAACATAGATTTGAAATATTTCAATGATTCAACTAAAAAGAAAATTGAAAAAATCCGACCATTATTAAT
AGATGGAACTGCATCATTGAGTCCTGGAATGATGATGGGCATGTTCAATATGTTAAGCACCGTCTTGGGC
GTCTCCATTCTGAATCTTGGGCAAAAGAGATACACCAAGACTACTTACTGGTGGGATGGTCTTCAATCGT
CTGATGATTTTGCTCTGATTGTGAATGCACCCAACTATGCAGGAATTCAAGCTGGAGTTGACAGGTTTTA
TCGAACCTGTAAGCTGCTCGGAATTAATATGAGCAAAAAGAAATCTTACATAAACAGAACAGGTACCTTT
GAATTCACGAGCTTTTTCTATCGTTATGGGTTTGTTGCCAATTTCAGCATGGAGCTTCCTAGTTTTGGGG
TGTCTGGGGTCAATGAATCTGCAGACATGAGTATTGGAGTCACTGTCATCAAAAACAATATGATAAACAA
TGACCTTGGCCCAGCAACTGCTCAAATGGCCCTTCAGTTATTTATAAAAGATTACAGGTACACTTATCGA
TGCCACAGAGGTGACACACAAATACAAACCCGGAGATCATTTGAGATAAAGAAACTATGGGACCAAACCC
GCTCCAAAGCTGGACTGTTGGTCTCTGATGGAGGCCCCAATTTATATAACATTAGAAATCTCCATATTCC
TGAAGTCTGCTTGAAATGGGAGTTGATGGATGAGGATTACCAGGGGCGTTTATGCAACCCATTGAACCCA
TTTGTCAGTCATAAAGAGATTGAATCAGTGAACAATGCAGTGATGATGCCGGCACATGGTCCAGCCAAAA
ATATGGAGTATGACGCTGTTGCAACAACACACTCCTGGGTTCCCAAAAGGAATCGATCCATTTTGAACAC
GAGCCAAAGGGGGATACTTGAAGATGAGCAAATGTATCAGAGGTGCTGCAATTTATTTGAAAAATTCTTC
CCAAGTAGCTCATACAGAAGACCAGTTGGAATATCCAGTATGGTAGAGGCTATGGTTTCAAGAGCCCGAA
TTGATGCACGGATTGATTTCGAATCTGGAAGGATAAAGAAAGAGGAATTCGCTGAGATCATGAAGACCTG
TTCCACCATTGAAGACCTCAGACGGCAAAAATAGGGAATTTGGCTTGTCCTTCATGAAAA
>gi|77543355|gb|CY003310.1| Influenza A virus (A/New York/291/2002(H1N1)) segment 2, complete sequence
GCAAACCATTTGAATGGATGTCAATCCGACATTACTTTTCTTAAAAGTGCCAGCACAAAATGCTATAAGC
ACAACTTTTCCTTATACTGGTGACCCTCCTTACAGCCATGGGACAGGAACAGGATACACCATGGATACAG
TCAACAGGACACATCAGTACTCAGAAAGAGGAAGATGGACAAAAAATACCGAAACTGGAGCACCGCAACT
CAACCCAATTGATGGGCCACTACCAGAAGACAATGAACCAAGTGGCTATGCCCAAACAGATTGTGTATTA
GAAGCAATGGCTTTCCTTGAAGAATCCCATCCTGGTATTTTTGAAAGCTCTTGTATTGAAACAATGGAGG
TTGTTCAGCAAACAAGGGTGGACAAACTGACACAAGGCAGACAGACCTATGACTGGACTCTAAATAGGAA
CCAGCCTGCTGCCACAGCATTGGCCAACACTATAGAAGTATTCAGATCAAACGGCCTCATAGCAAATGAA
TCTGGGAGGCTAATAGACTTCCTTAAAGATGTAATGGAGTCGATGGACAGAGACGAAGTAGAGGTCACAA
CTCATTTTCAGAGAAAGAGGAGAGTGAGAGACAATGTAACTAAAAAAATGGTGACCCAAAGAACAATAGG

CAAAAAGAAACATAAATTAGACAAAAGAAGTTACCTAATTAGGGCATTAACCCTGAACACAATGACCAAA
GATGCTGAGAGGGGGAAACTAAAACGCAGAGCAATTGCAACCCCAGGAATGCAAATAAGGGGGTTTGTAT
ACTTTGTTGAGACACTGGCAAGATGCATATGTGAAAAGCTTGAACAATCAGGGTTGCCAGTTGGAGGAAA
TGAGAAGAAAGCAAAGTTAGCAAATGTTGTAAGGAAGATGATGACCAACTCCCAGGACACTGAAATTTCT
TTCACCATCACTGGAGATAACACAAAATGGAACGAAAATCAAAACCCTAGAATGTTCTTGGCCATGATCA
CATATATAACCAAAAATCAGCCTGAATGGTTCAGAAATATTCTAAGTATTGCTCCAATAATGTTTTCAAA
CAAGATGGCGAGACTAGGTAAGGGGTACATGTTTGAAAGCAAGAGTATGAAACTGAGAACTCAAATACCT
GCAGAGATGCTAGCCAACATAGATTTGAAATATTTCAATGATTCAACTAAAAAGAAAATTGAAAAAATCC
GACCATTATTAATAGATGGAACTGCATCATTGAGTCCTGGAATGATGATGGGCATGTTCAATATGTTAAG
CACCGTCTTGGGCGTCTCCATTCTGAATCTTGGGCAAAAGAGATACACCAAGACTACTTACTGGTGGGAT
GGTCTTCAATCGTCTGATGATTTTGCTCTGATTGTGAACGCACCCAACTATGCAGGAATTCAAGCTGGAG
TTGACAGGTTTTATCGAACCTGTAAGCTGCTCGGAATTAATATGAGCAAAAAGAAGTCTTACATAAACAG
AACAGGTACCTTTGAATTCACGAGCTTTTTCTATCGTTATGGGTTTGTTGCCAATTTCAGCATGGAGCTT
CCTAGTTTTGGGGTGTCTGGGGTCAATGAATCTGCAGACATGAGTATTGGAGTCACTGTCATCAAAAACA
ATATGATAAACAATGACCTTGGCCCAGCAACTGCTCAAATGGCCCTTCAGTTATTTATAAAAGATTACAG
GTACACTTATCGATGCCACAGAGGTGACACACAAATACAAACCCGGAGATCATTTGAGATAAAGAAACTA
TGGGACCAAACCCGCTCCAAAGCTGGGCTGTTGGTCTCTGATGGAGGCCCCAATTTATATAACATTAGAA
ATCTCCATATTCCTGAAGTCTGCTTGAAATGGGAGTTGATGGATGAGGATTACCAGGGGCGTTTATGCAA
CCCATTGAACCCGTTTGTCAGTCATAAAGAGATTGAATCAGTGAACAATGCAGTGATGATGCCGGCACAT
GGTCCAGCCAAAAATATGGAGTATGACGCTGTTGCAACAACACACTCCTGGGTTCCCAAAAGGAATCGAT
CCATTTTGAACACGAGCCAAAGGGGGATACTTGAAGATGAGCAAATGTATCAGAGGTGCTGCAATTTATT
TGAAAAATTCTTCCCAAGTAGCTCATACAGAAGACCAGTTGGAATATCCAGTATGGTAGAGGCTATGGTT
TCAAGAGCCCGAATTGATGCACGGATTGATTTCGAATCTGGAAGGATAAAGAAAGAGGAATTCGCTGAGA
TCATGAAGACCTGTTCCACCATTGAAGACCTCAGACGGCAAAAATAGGGAATTTGGCTTGTCCTTTATGA
AAA
>gi|237689049|gb|CY040152.1| Influenza A virus (A/Taiwan/52/2002(H1N1)) segment 2, complete sequence
CATTTGAATGGATGTCAATCCGACCTTACTTTTCTTAAAAGTGCCAGCACAAAATGCTATAAGCACAACT
TTCCCTTATACTGGTGATCCTCCTTACAGCCATGGGACAGGAACAGGGTACACCATGGATACAGTCAACA
GGACACATCAGTACTCAGAAAGAGGAAGATGGACAAAAAATACCGAAACTGGAGCACCGCAACTCAACCC
AATTGATGGACCACTACCAAAAGACAATGAACCAAGTGGCTATGCCCAAACAGATTGTGTATTAGAAGCA
ATGGCTTTCCTTGAGGAATCCCATCCTGGTATTTTTGAAAACTCTTGTATTGAAACAATGGAGGTTGTTC
AGCAAACAAGGGTGGACAAACTGACACAAGGCAGACAGACCTATGACTGGACTCTAAATAGAAACCAGCC
TGCTGCCACAGCATTGGCCAACACTATAGAAGTGTTCAGATCAAACGGCCTCATGGCAAATGAATCTGGG
AGGCTAATAGACTTCCTTAAAGATGTAATGGAGTCGATGGACAGAGACGAAGTAGAGATCACAACACATT
TTCAGAGAAAGAGGAGGGTGAGAGACAATGTAACTAAAAAAATGGTGACCCAAAGAACAATAGGCAAAA
A
GAAACATAAATTAGACAAAAGAAGTTACCTAATTAGGGCATTAACCCTGAACACAATGACCAAAGATGCT
GAGAGGGGGAAACTAAAACGTAGAGCAATTGCAACCCCAGGAATGCAAATAAGGGGGTTTGTATACTTTG
TTGAGACACTGGCAAGAAGCATATGTGAAAAGCTTGAACAATCAGGGTTGCCAGTTGGGGGGAATGAAAA
GAAAGCAAAGTTAGCAAATGTTGTAAGGAAGATGATGACCAACTCCCAGGACACTGAAATTTCTTTCACC
ATTACTGGAGATAACACAAAATGGAACGAAAATCAAAACCCTAGAATGTTTTTGGCCATGATCACATATA
TAACCAAAAATCAGCCTGAATGGTTCAGAAATATTCTAAGTATTGCTCCAATAATGTTTTCAAACAAAAT
GGCGAGACTAGGTAAGGGGTACATGTTTGAAAGCAAGAGTATGAAACTGAGAACTCAAATACCTGCAGAG

ATGCTAGCCAACATAGATTTGAAATATTTCAATGATTCAACTAAAAAGAAAATTGAAAAAATCCGGCCAT
TATTAATAGATGGAACTGCATCATTGAGTCCTGGAATGATGATGGGCATGTTCAATATGTTAAGCACTGT
CTTGGGCGTCTCCATTCTGAATCTTGGGCAAAAGAGATACACCAAGACTACTTACTGGTGGGATGGTCTT
CAATCGTCTGATGATTTTGCTCTGATTGTGAATGCACCCAACTATGCAGGAATTCAAGCTGGAGTTGACA
GGTTTTATCGAACCTGTAAGCTGCTCGGAATTAATATGAGCAAAAAGAAGTCTTACATAAACAGAACAGG
TACCTTTGAATTCACGAGCTTTTTCTATCGTTATGGGTTTGTTGCCAATTTCAGCATGGAGCTTCCTAGT
TTTGGGGTGTCTGGGGTCAATGAGTCTGCAGACATGAGTATTGGAGTCACTGTCATCAAAAACAATATGA
TAAACAATGACCTTGGCCCAGCAACTGCTCAAATGGCCCTTCAGTTATTTATCAAAGATTACAGGTACAC
GTATCGATGCCACAGAGGTGACACACAAATACAAACCCGGAGATCATTTGAGATAAAGAAACTATGGGAC
CAAACCCGCTCCAAAGCTGGGCTGTTGGTCTCTGATGGAGGCCCCAATTTGTATAACATTAGAAATCTCC
ATATTCCTGAAGTCTGCTTGAAATGGGAGTTGATGGATGAGGATTACCAGGGGCGTTTATGCAACCCATT
GAACCCATTTGTCAGTCATAAAGAGATTGAGTCAGTGAACAATGCAGTGATGATGCCGGCACATGGTCCA
GCCAAAAATATGGAGTATGACGCTGTTGCAACAACACACTCCTGGGTTCCCAAAAGGAATCGATCCATTT
TGAATACGAGCCAAAGGGGGATACTTGAGGATGAGCAAATGTATCAGAGGTGCTGCAATTTATTTGAAAA
ATTCTTCCCAAGTAGTTCATACAGAAGACCAGTTGGAATATCCAGTATGGTAGAGGCTATGGTTTCCAGA
GCCCGAATTGATGCAAGAATTGATTTCGAATCTGGAAGGATAAAGAAAGAGGAATTCGCTGAGATCATGA
AGACCTGTTCCACCATTGAAGACCTCAGACGGCAAAAATAGGGAATTTGGCTTGTCCTTCATGAAAA
>gi|237689068|gb|CY040160.1| Influenza A virus (A/Taiwan/123/2002(H1N1)) segment 2, complete sequence
AATGGATGTCAATCCGACCTTACTTTTCTTAAAAGTGCCAGCACAAAATGCTATAAGCACAACTTTCCCT
TATACTGGTGATCCTCCTTACAGCCATGGGACAGGAACAGGGTACACCATGGATACAGTCAACAGGACAC
ATCAGTACTCAGAAAGAGGAAGATGGACAAAAAATACCGAAACTGGAGCACCGCAACTCAACCCAATTGA
TGGACCACTACCAAAAGACAATGAACCAAGTGGCTATGCCCAAACAGATTGTGTATTAGAAGCAATGGCT
TTCCTTGAGGAATCCCATCCTGGTATTTTTGAAAACTCTTGTATTGAAACAATGGAGGTTGTTCAGCAAA
CAAGGGTGGACAAACTGACACAAGGCAGACAGACCTATGACTGGACTCTAAATAGAAACCAGCCTGCTGC
CACAGCATTGGCCAACACTATAGAAGTGTTCAGATCAAACGGCCTCATAGCAAATGAATCTGGGAGGCTA
ATAGACTTCCTTAAAGATGTAATGGAGTCGATGGACAGAGACGAAGTAGAGATCACAACACATTTTCAGA
GAAAGAGGAGGGTGAGAGACAATGTAACTAAAAAAATGGTGACCCAAAGAACAATAGGCAAAAAGAAAC
A
TAAATTAGACAAAAGAAGTTACCTAATTAGGGCATTAACCCTGAACACAATGACCAAAGATGCTGAGAGG
GGGAAACTAAAACGTAGAGCAATTGCAACCCCAGGAATGCAAATAAGGGGGTTTGTATACTTTGTTGAGA
CACTGGCAAGAAGCATATGTGAAAAGCTTGAACAATCAGGGTTGCCAGTCGGGGGAAATGAAAAGAAAGC
AAAGTTAGCAAATGTTGTAAGGAAGATGATGACCAACTCCCAGGACACTGAAATTTCTTTCACCATCACT
GGAGATAACACAAAATGGAACGAAAATCAAAACCCTAGAATGTTTTTGGCCATGATCACATATATAACCA
AAAATCAGCCTGAATGGTTCAGAAATATTCTAAGTATTGCTCCAATAATGTTTTCAAACAAAATGGCGAG
ACTAGGTAAGGGGTACATGTTTGAAAGCAAGAGTATGAAACTGAGAACTCAAATACCTGCAGAGATGCTA
GCCAACATAGATTTGAAATATTTCAATGATTCAACTAAAAAGAAAATTGAAAAAATCCGGCCATTATTAA
TAGATGGAACTGCATCATTGAGCCCTGGAATGATGATGGGCATGTTCAATATGTTAAGCACTGTCTTGGG
CGTCTCCATTCTGAATCTTGGGCAAAAGAGATACACCAAGACTACTTACTGGTGGGATGGTCTTCAATCG
TCTGATGATTTTGCTCTGATTGTGAATGCACCCAACTATGCAGGAATTCAAGCTGGAGTTGACAGGTTTT
ATCGAACCTGTAAGCTGCTCGGAATTAATATGAGCAAAAAGAAGTCTTACATAAACAGAACAGGTACCTT
TGAATTCACGAGCTTTTTCTATCGTTATGGGTTTGTTGCCAATTTCAGCATGGAGCTTCCTAGTTTTGGG
GTGTCTGGGGTCAATGAGTCTGCAGACATGAGTATTGGAGTCACTGTCATCAAAAACAATATGATAAACA
ATGACCTTGGCCCAGCAACTGCTCAAATGGCCCTTCAGTTATTTATCAAAGATTACAGGTACACGTATCG

ATGCCACAGAGGTGACACACAAATACAAACCCGGAGATCATTTGAGATAAAGAAACTATGGGACCAAACC
CGCTCCAAAGCTGGGCTGTTGGTCTCTGATGGAGGCCCCAATTTGTATAACATTAGAAATCTCCATATTC
CTGAAGTCTGCTTGAAATGGGAGTTGATGGATGAGGATTACCAGGGGCGTTTATGCAACCCATTGAACCC
ATTTGTCAGTCATAAAGAGATTGAGTCAGTGAACAATGCAGTGGTGATGCCGGCACATGGTCCAGCCAAA
AATATGGAGTATGACGCTGTTGCAACAACACACTCCTGGGTTCCCAAAAGGAATCGATCCATTTTGAATA
CGAGCCAAAGGGGGATACTTGAGGATGAGCAAATGTATCAGAGGTGCTGCAATTTATTTGAAAAATTCTT
CCCAAGTAGTTCATACAGAAGACCAGTTGGAATATCCAGTATGGTAGAGGCTATGGTTTCCAGAGCCCGA
ATTGATGCAAGAATTGATTTCGAATCTGGAAGGATAAAGAAAGAGGAATTCGCTGAGATCATGAAGACCT
GTTCCACCATTGAAGACCTCAGACGGCAAAAATAGGGAATTTGGCTTGTCCTTCATGAAAA
>gi|77747435|gb|CY003694.1| Influenza A virus (A/New York/486/2003(H1N1)) segment 2, complete sequence
GAAAGCAGGCAAACCATTTGAATGGATGTCAATCCGACATTACTTTTCTTAAAAGTGCCAGCACAAAATG
CTATAAGCACAACTTTTCCTTATACTGGTGACCCTCCTTACAGCCATGGGACAGGAACAGGGTACACCAT
GGATACAGTCAACAGGACACACCAGTACTCAGAAAGAGGAAGATGGACAAAAAATACCGAAACTGGAGCA
CCGCAACTCAACCCAATTGATGGGCCACTACCAGAAGACAATGAACCAAGTGGCTATGCCCAAACAGATT
GTGTATTAGAAGCAATGGCTTTCCTTGAAGAATCCCATCCTGGTATTTTTGAAAACTCTTGTATTGAAAC
AATGGAGGTTGTTCAGCAAACAAGGGTGGACAAACTGACACAAGGCAGACAGACCTATGACTGGACTCTA
AATAGGAACCAGCCTGCTGCCACAGCATTGGCAAACACTATAGAAGTATTCAGATCAAACGGCCTCATAG
CAAATGAATCTGGGAGGCTAATAGACTTCCTTAAAGATGTAATGGAGTCGATGGACAGAGACGAAGTAGA
GGTCACAACTCATTTTCAAAGAAAGAGGAGAGTGAGAGACAATGTAACTAAAAAAATGGTGACCCAAAGA
ACAATAGGCAAAAAGAAACATAAATTAGACAAAAGAAGTTACCTAATTAGGGCATTAACCCTGAACACAA
TGACCAAAGATGCTGAGAGGGGGAAACTAAAACGCAGAGCAATTGCAACCCCAGGAATGCAAATAAGGG
G
GTTTGTATACTTTGTTGAGACACTGGCAAGAAGCATATGTGAAAAGCTTGAACAATCAGGGTTGCCAGTT
GGAGGAAATGAGAAGAAAGCAAAGTTAGCAAATGTTGTAAGGAAGATGATGACCAACTCCCAGGACACTG
AAATTTCTTTCACCATCACTGGAGATAACACAAAATGGAACGAAAATCAAAACCCTAGAATGTTCTTGGC
CATGATCACATATATAACCAAAAACCAGCCTGAATGGTTCAGAAATATTCTAAGTATTGCTCCTATAATG
TTTTCAAACAAGATGGCGAGACTAGGTAAGGGGTACATGTTTGAAAGCAAGAGTATGAAACTGAGAACTC
AAATACCTGCAGAGATGCTAGCCAACATAGATTTGAAATATTTCAATGATTCAACTAAAAAGAAAATTGA
AAAAATCCGACCATTATTAATAGATGGAACTGCATCATTGAGTCCTGGAATGATGATGGGCATGTTCAAT
ATGTTAAGCACCGTCTTGGGCGTCTCCATTCTGAATCTTGGGCAAAAGAGATACACCAAGACTACTTACT
GGTGGGATGGTCTTCAATCGTCTGATGATTTTGCTTTGATTGTGAATGCACCCAACTATGCAGGAATTCA
AGCTGGAGTTGACAGGTTTTATCGAACCTGTAAGCTGCTCGGAATTAATATGAGCAAAAAGAAGTCTTAC
ATAAACAGAACAGGTACCTTTGAATTCACGAGCTTTTTCTATCGTTATGGGTTTGTTGCCAATTTCAGCA
TGGAGCTTCCTAGTTTTGGGGTGTCTGGGGTCAATGAATCTGCAGACATGAGTATTGGAGTCACTGTCAT
CAAAAACAATATGATAAACAATGACCTTGGCCCAGCAACTGCTCAAATGGCCCTTCAGTTATTTATAAAA
GATTACAGGTACACTTATCGATGCCACAGAGGTGACACACAAATACAAACCCGGAGATCATTTGAGATAA
AGAAACTATGGGACCAAACCCGCTCCAAAGCTGGGCTGTTGGTCTCTGATGGAGGCCCCAATTTATATAA
CATTAGAAATCTCCATATTCCTGAAGTCTGCTTGAAATGGGAGTTGATGGATGAGGATTACCAGGGGCGT
TTATGCAACCCATTGAACCCGTTTGTCAGTCATAAAGAGATTGAATCAGTGAACAATGCAGTGATAATGC
CGGCACATGGTCCAGCCAAAAATATGGAGTATGACGCTGTTGCAACAACACATTCCTGGGTCCCCAAAAG
GAATCGATCCATTTTGAACACGAGCCAAAGGGGGATACTTGAAGATGAGCAAATGTATCAGAGGTGCTGC
AATTTATTTGAAAAATTCTTCCCAAGTAGCTCATACAGAAGACCAGTTGGAATATCCAGTATGGTAGAGG
CTATGGTTTCAAGAGCCCGAATTGATGCACGGATTGATTTCGAATCTGGAAGGATAAAGAAAGAGGAATT

CGCTGAGATCATGAAGACCTGTTCCACCATTGAAGACCTCAGACGGCAAAAATAGGGAATTTGGCTTGTC
CTTCATGAAAA
>gi|125664189|gb|CY019889.1| Influenza A virus (A/Memphis/5/2003(H1N1)) segment 2, complete sequence
AATGGATGTCAATCCGACATTACTTTTCTTAAAAGTGCCAGCACAAAATGCTATAAGCACAACTTTTCCT
TATACTGGTGACCCTCCTTACAGCCATGGGACAGGAACAGGGTACACCATGGATACAGTCAACAGGACAC
ATCAGTACTCAGAAAGAGGAAGATGGACAAAAAATACCGAAACTGGAGCACCGCAACTCAACCCAATTGA
TGGGCCACTACCAGAAGACAATGAACCAAGTGGCTATGCCCAAACAGATTGTGTATTAGAAGCAATGGCT
TTCCTTGAAGAATCCCATCCTGGTATTTTTGAAAACTCTTGTATTGAAACAATGGAGGTTGTTCAGCAAA
CAAGGGTGGACAAACTGACACAAGGCAGACAGACCTATGACTGGACTCTAAATAGGAACCAGCCTGCTGC
CACAGCATTGGCAAACACTATAGAAGTATTCAGATCAAACGGCCTCATAGCAAATGAATCTGGGAGGCTA
ATAGACTTCCTTAAAGATGTAATGGAGTCGATGGACAGAGACGAAGTAGAGGTCACAACTCATTTTCAAA
GAAAGAGGAGAGTGAGAGACAATGTAACTAAAAAAATGGTGACCCAAAGAACAATAGGCAAAAAGAAAC
A
TAAATTAGACAAAAGAAGTTACCTAATTAGGGCATTAACCCTGAACACAATGACCAAAGATGCTGAGAGG
GGGAAACTAAAACGCAGAGCAATTGCAACCCCAGGAATGCAAATAAGGGGGTTTGTATACTTTGTTGAGA
CACTGGCAAGAAGCATATGTGAAAAGCTTGAACAATCAGGGTTGCCAGTTGGAGGAAATGAGAAGAAAGC
AAAGTTAGCAAATGTTGTAAGGAAGATGATGACCAACTCCCAGGACACTGAAATTTCTTTCACCATCACT
GGAGATAACACAAAATGGAACGAAAATCAAAACCCTAGAATGTTCTTGGCCATGATCACATATATAACCA
AAAATCAGCCTGAATGGTTCAGAAATATTCTAAGTATTGCTCCAATAATGTTTTCAAACAAGATGGCGAG
ACTAGGTAAGGGGTACATGTTTGAAAGCAAGAGTATGAAACTGAGAACTCAAATACCTGCAGAGATGCTA
GCCAACATAGATTTGAAATATTTCAATGATTCAACTAAAAAGAAAATTGAAAAAATCCGACCATTATTAA
TAGATGGAACTGCATCATTGAGTCCTGGAATGATGATGGGCATGTTCAATATGTTAAGCACCGTCTTGGG
CGTCTCCATTCTGAATCTTGGGCAAAAGAGATACACCAAGACTACTTACTGGTGGGATGGTCTTCAATCG
TCTGATGATTTTGCTTTGATTGTGAATGCACCCAACTATGCAGGAATTCAAGCTGGAGTTGACAGGTTTT
ATCGAACCTGTAAGCTGCTCGGAATTAATATGAGCAAAAAGAAGTCTTACATCAACAGAACAGGTACCTT
TGAATTCACGAGCTTTTTCTATCGTTATGGGTTTGTTGCCAATTTCAGCATGGAGCTTCCTAGTTTTGGG
GTGTCTGGGGTCAATGAATCTGCAGACATGAGTATTGGAGTCACTGTCATCAAAAACAATATGATAAACA
ATGACCTTGGCCCAGCAACTGCTCAAATGGCCCTTCAGTTATTTATAAAAGATTACAGGTACACTTATCG
ATGCCACAGAGGTGACACACAAATACAAACCCGGAGATCATTTGAGATAAAGAAACTATGGGACCAAACC
CGCTCCAAAGCTGGGCTGTTGGTCTCTGATGGAGGCCCCAATTTATATAACATTAGAAATCTCCATATTC
CTGAAGTCTGCTTGAAATGGGAGTTGATGGATGAGGATTACCAGGGGCGTTTATGCAACCCATTGAACCC
GTTTGTCAGCCATAAAGAGATTGAATCAGTGAACAATGCAGTGATAATGCCGGCACATGGTCCAGCCAAA
AATATGGAGTATGACGCTGTTGCAACAACACACTCCTGGGTCCCCAAAAGAAATCGATCCATTTTGAACA
CGAGCCAAAGGGGGATACTTGAAGATGAGCAAATGTATCAGAGGTGCTGCAATTTATTTGAAAAATTCTT
CCCAAGTAGCTCATACAGAAGACCAGTTGGAATATCCAGTATGGTAGAGGCTATGGTTTCAAGAGCCCGA
ATTGATGCACGGATTGATTTCGAATCTGGAAGGATAAAGAAAGAGGAATTCGCTGAGATCATGAAGACCT
GTTCCACCATTGAAGACCTCAGACGGCAAAAATAGGGAATTTGGC
>gi|73763208|gb|CY002542.1| Influenza A virus (A/New York/227/2003(H1N1)) segment 2, complete sequence
AGCGAAAGCAGGCAAACCATTTGAATGGATGTCAATCCGACATTACTTTTCTTAAAAGTGCCAGCACAAA
ATGCTATAAGCACAACTTTTCCTTATACTGGTGACCCTCCTTACAGCCATGGGACAGGAACAGGGTACAC
CATGGACACAGTCAACAGGACACATCAGTACTCAGAAAGAGGAAGATGGACAAAAAATACCGAAACTGGA
GCACCGCAACTCAACCCAATTGATGGGCCACTACCAGAAGACAATGAACCAAGTGGCTATGCCCAAACAG

ATTGTGTATTAGAAGCAATGGCTTTCCTTGAAGAATCCCATCCTGGTATTTTTGAAAACTCTTGTATTGA
AACAATGGAGGTTGTTCAGCAAACAAGGGTGGACAAACTGACACAAGGCAGACAGACCTATGACTGGACT
CTAAATAGGAACCAGCCTGCTGCCACAGCATTGGCAAACACTATAGAAGTATTCAGATCAAACGGCCTCA
TAGCAAATGAATCTGGGAGGCTAATAGACTTCCTTAAAGATGTAATGGAGTCGATGGACAGAGACGAAGT
AGAGGTCACAACTCATTTTCAAAGAAAGAGGAGAGTGAGAGACAATGTAACTAAAAAAATGGTGACCCAA
AGAACAATAGGCAAAAAGAAACATAAATTAAACAAAAGAAGTTACCTAATTAGGGCATTAACCCTGAACA
CAATGACCAAAGATGCTGAGAGGGGGAAACTAAAACGCAGAGCAATTGCAACCCCAGGAATGCAAATAAG
GGGGTTTGTATACTTTGTTGAGACACTGGCAAGAAGCATATGTGAAAAGCTTGAACAATCAGGGTTGCCA
GTTGGAGGAAATGAGAAGAAAGCAAAGTTAGCAAATGTTGTAAGGAAGATGATGACCAACTCCCAGGACA
CTGAAATTTCTTTCACCATCACTGGAGATAACACAAAATGGAACGAAAATCAAAACCCTAGAATGTTCTT
GGCCATGATCACATATATAACCAAAAATCAGCCTGAATGGTTCAGAAATATTCTAAGTATTGCTCCAATA
ATGTTTTCAAACAAGATGGCGAGACTAGGTAAGGGGTACATGTTTGAAAGCAAGAGTATGAAACTGAGAA
CTCAAATACCTGCAGAGATGCTAGCCAACATAGATTTGAAATATTTCAATGATTCAACTAAAAAGAAAAT
TGAAAAAATCCGACCATTATTAATAGATGGAACTGCATCATTGAGTCCTGGAATGATGATGGGCATGTTC
AATATGTTAAGCACCGTCTTGGGCGTCTCCATTCTGAATCTTGGGCAAAAGAGATACACCAAGACTACTT
ACTGGTGGGATGGTCTTCAATCGTCTGATGATTTTGCTTTGATTGTGAATGCACCCAACTATGCAGGAAT
TCAAGCTGGAGTTGACAGGTTTTATCGAACCTGTAAGCTGCTCGGAATTAATATGAGCAAAAAGAAGTCT
TACATCAACAGAACAGGTACCTTTGAATTCACGAGCTTTTTCTATCGTTATGGGTTTGTTGCCAATTTCA
GCATGGAGCTTCCTAGTTTTGGGGTGTCTGGGGTCAATGAATCTGCAGACATGAGTATTGGAGTCACTGT
CATCAAAAACAATATGATAAACAATGACCTTGGCCCAGCAACTGCTCAAATGGCCCTTCAGTTATTTATA
AAAGATTACAGGTACACTTATCGATGCCACAGAGGTGACACACAAATACAAACCCGGAGATCATTTGAGA
TAAAGAAACTATGGGACCAAACCCGCTCCAAAGCTGGGCTGTTGGTCTCTGATGGAGGCCCCAATTTATA
TAACATTAGAAATCTCCATATTCCTGAAGTCTGCTTGAAATGGGAGTTGATGGATGAGGATTACCAGGGG
CGTTTATGCAACCCATTGAACCCGTTTGTCAGCCATAAAGAGATTGAATCAGTGAACAATGCAGTGATAA
TGCCGGCACATGGTCCAGCCAAAAATATGGAGTATGACGCTGTTGCAACAACACACTCCTGGGTCCCCAA
AAGAAATCGATCCATTTTGAACACGAGCCAAAGGGGGATACTTGAAGATGAGCAAATGTATCAGAGGTGC
TGCAATTTATTTGAAAAATTCTTCCCAAGTAGCTCATACAGAAGACCAGTTGGAATATCCAGTATGGTAG
AGGCTATGGTTTCAAGAGCCCGAATTGATGCACGGATTGATTTCGAATCTGGAAGGATAAAGAAAGAGGA
ATTCGCTGAGATCATGAAGACCTGTTCCACCATTGAAGACCTCAGACGGCAAAAATAGGGAATTTGGCTT
GTCCTTCATGAAAAAATGCCTTGTTTCTACT
>gi|89112179|gb|CY009002.1| Influenza A virus (A/New York/484/2003(H1N1)) segment 2, complete sequence
CATTTGAATGGATGTCAATCCGACATTACTTTTCTTAAAAGTGCCAGCACAAAATGCTATAAGCACAACT
TTTCCTTATACTGGTGACCCTCCTTACAGCCATGGGACAGGAACAGGGTACACCATGGATACAGTCAACA
GGACACATCAGTACTCAGAAAGAGGAAGATGGACAAAAAATACCGAAACTGGAGCACCGCAACTCAACCC
AATTGATGGGCCACTACCAGAAGACAATGAACCAAGTGGCTATGCCCAAACAGATTGTGTATTAGAAGCA
ATGGCTTTCCTTGAAGAATCCCATCCTGGTATTTTTGAAAACTCTTGTATTGAAACAATGGAGGTTGTTC
AGCAAACAAGGGTGGACAAACTGACACAAGGCAGACAGACCTATGACTGGACTCTAAATAGGAACCAGCC
TGCTGCCACAGCATTGGCAAACACTATAGAAGTATTCAGATCAAACGGCCTCATAGCAAATGAATCTGGG
AGGCTAATAGACTTCCTTAAAGATGTAATGGAGTCGATGGACAGAGACGAAGTAGAGGTCACAACTCATT
TTCAAAGAAAGAGGAGAGTGAGAGACAATGTAACTAAAAAAATGGTGACCCAAAGAACAATAGGCAAAAA
GAAACATAAATTAGACAAAAGAAGTTACCTAATTAGGGCATTAACCCTGAACACAATGACCAAAGATGCT
GAGAGGGGGAAACTAAAACGCAGAGCAATTGCAACCCCAGGAATGCAAATAAGGGGGTTTGTATACTTTG
TTGAGACACTGGCAAGAAGCATATGTGAAAAGCTTGAACAATCAGGGTTGCCAGTTGGAGGAAATGAGAA

GAAAGCAAAGTTAGCAAATGTTGTAAGGAAGATGATGACCAACTCCCAGGACACTGAAATTTCTTTCACC
ATCACTGGAGATAACACAAAATGGAACGAAAATCAAAACCCTAGAATGTTCTTGGCCATGATCACATATA
TAACCAAAAATCAGCCTGAATGGTTCAGAAATATTCTAAGTATTGCTCCAATAATGTTTTCAAACAAGAT
GGCGAGACTAGGTAAGGGGTACATGTTTGAAAGCAAGAGTATGAAACTGAGAACTCAAATACCTGCAGAG
ATGCTAGCCAACATAGATTTGAAATATTTCAATGATTCAACTAAAAAGAAAATTGAAAAAATCCGACCAT
TATTAATAGATGGAACTGCATCATTGAGTCCTGGAATGATGATGGGCATGTTCAATATGTTAAGCACCGT
CTTGGGCGTCTCCATTCTGAATCTTGGGCAAAAGAGATACACCAAGACTACTTACTGGTGGGATGGTCTT
CAATCGTCTGATGATTTTGCTTTGATTGTGAATGCACCCAACTATGCAGGAATTCAAGCTGGAGTCGACA
GGTTTTATCGAACCTGTAAGCTGCTCGGAATTAATATGAGCAAAAAGAAGTCTTACATAAACAGAACAGG
TACCTTTGAATTCACGAGCTTTTTCTATCGTTATGGGTTTGTTGCCAATTTCAGCATGGAGCTTCCTAGT
TTTGGGGTGTCTGGGGTCAATGAATCTGCAGACATGAGTATTGGAGTCACTGTCATCAAAAACAATATGA
TAAACAATGACCTTGGCCCAGCAACTGCTCAAATGGCCCTTCAGTTATTTATAAAAGATTACAGGTACAC
TTATCGATGCCACAGAGGTGACACACAAATACAAACCCGGAGATCATTTGAGATAAAGAAACTATGGGAC
CAAACTCGCTCCAAAGCTGGGCTGTTGGTATCTGATGGAGGCCCCAATTTATATAACATTAGAAATCTCC
ATATTCCTGAAGTCTGCTTGAAATGGGAGTTGATGGATGAGGATTACCAGGGGCGTTTATGCAACCCATT
GAACCCGTTTGTCAGTCATAAAGAGATTGAATCAGTGAACAATGCAGTGATAATGCCGGCACATGGTCCA
GCCAAAAATATGGAGTATGACGCTGTTGCAACAACACACTCCTGGGTCCCCAAAAGGAATCGATCCATTT
TGAACACGAGCCAAAGGGGGATACTTGAAGATGAGCAAATGTATCAGAGGTGCTGCAATTTATTTGAAAA
ATTCTTCCCAAGTAGCTCATACAGAAGACCAGTTGGAATATCCAGTATGGTAGAGGCTATGGTTTCAAGA
GCCCGAATTGATGCACGGATTGATTTTGAATCTGGAAGGATAAAGAAAGAGGAATTCGCTGAGATCATGA
AGACCTGTTCCACCATTGAAGACCTCAGACGGCAAAAATAGGGAATTTGGCTTGTCCTTCATGAAAA
>gi|83727855|gb|CY006921.1| Influenza A virus (A/New York/488/2003(H1N1)) segment 2, complete sequence
GCAAACCATTTGAATGGATGTCAATCCGACATTACTTTTCTTAAAAGTGCCAGCACAAAATGCTATAAGC
ACAACTTTTCCTTATACTGGTGACCCTCCTTACAGCCATGGGACAGGAACAGGGTACACCATGGATACAG
TCAACAGGACACATCAGTACTCAGAAAGAGGAAGATGGACAAAAAATACCGAAACTGGAGCACCGCAACT
CAACCCAATTGATGGGCCACTACCAGAAGACAATGAACCAAGTGGCTATGCCCAAACAGATTGTGTATTA
GAAGCAATGGCTTTCCTTGAAGAATCCCATCCTGGTATTTTTGAAAACTCTTGTATTGAAACAATGGAGG
TTGTTCAGCAAACAAGGGTGGACAAACTGACACAAGGCAGACAGACCTATGACTGGACTCTAAATAGGAA
CCAGCCTGCTGCTACAGCATTGGCAAACACTATAGAAGTATTCAGGTCAAACGGCCTCATAGCAAATGAA
TCTGGGAGGCTAATAGACTTCCTTAAAGATGTAATGGAGTCAATGGACAGAGACGAAGTAGAGGTCACAA
CTCATTTTCAAAGAAAGAGGAGAGTGAGAGACAATGTAACTAAAAAAATGGTGACCCAAAGAACAATAGG
CAAAAAGAAACATAAATTAGACAAAAAAAGTTACCTAATTAGGGCATTAACCCTGAACACGATGACCAAA
GATGCTGAGAGGGGAAAACTAAAACGCAGAGCAATTGCAACCCCAGGAATGCAAATAAGGGGGTTTGTAT
ACTTTGTTGAGACACTGGCAAGAAGCATATGTGAAAAGCTTGAACAATCAGGGTTGCCAGTTGGAGGAAA
TGAGAAGAAAGCAAAGTTAGCAAATGTTGTAAGGAAGATGATGACCAACTCCCAGGACACTGAAATTTCT
TTCACCATCACTGGAGATAACACAAAATGGAACGAAAATCAAAACCCTAGAATGTTCTTGGCCATGATCA
CATATATAACCAAAAATCAGCCTGAATGGTTCAGAAATATTCTGAGTATTGCTCCAATAATGTTTTCAAA
CAAGATGGCGAGACTAGGTAAGGGGTACATGTTTGAAAGCAAGAGTATGAAACTGAGAACTCAAATACCT
GCAGAGATGCTAGCCAACATAGATTTGAAATATTTCAATGATTCAACTAAAAAGAAAATTGAAAAAATCC
GACCATTATTAATAGATGGAACTGCATCATTGAGTCCTGGAATGATGATGGGCATGTTCAATATGTTAAG
CACCGTCTTGGGCGTCTCCATTCTGAATCTTGGGCAAAAGAGATACACCAAGACTACTTACTGGTGGGAT
GGTCTTCAATCGTCTGATGATTTTGCTTTGATTGTGAATGCACCCAACTATGCAGGAATTCAAGCTGGAG
TTGACAGGTTCTATCGAACCTGTAAGCTGCTCGGAATTAATATGAGCAAAAAGAAGTCTTACATAAACAG

AACAGGTACCTTTGAATTCACGAGCTTTTTCTATCGTTATGGGTTTGTTGCCAATTTCAGCATGGAGCTT
CCTAGTTTTGGGGTGTCTGGGGTCAATGAATCTGCAGACATGAGCATTGGAGTCACTGTCATCAAAAACA
ATATGATAAACAATGACCTTGGCCCAGCAACTGCTCAAATGGCCCTTCAGTTATTTATAAAAGATTACAG
GTACACTTATCGATGCCACAGAGGTGACACACAAATACAAACCCGGAGATCATTTGAGATAAAGAAACTA
TGGGACCAAACCCGCTCCAAAGCTGGGCTGTTGGTCTCTGATGGAGGCCCCAATTTATATAACATTAGAA
ATCTCCATATTCCTGAAGTCTGCTTGAAATGGGAGTTGATGGATGAGGATTACCAGGGGCGTTTATGCAA
CCCATTGAACCCGTTTGTCAGTCATAAAGAGATTGAATCAGTGAACAATGCAGTGATAATGCCGGCACAT
GGTCCAGCCAAAAATATGGAGTATGACGCTGTTGCAACAACACACTCCTGGGTCCCCAAAAGGAATCGAT
CCATTTTGAACACGAGCCAAAGGGGGATACTTGAAGATGAGCAAATGTATCAGAGGTGCTGCAATTTATT
TGAAAAATTCTTCCCAAGTAGCTCATACAGAAGACCAGTTGGAATATCCAGTATGGTAGAGGCTATGGTT
TCAAGAGCCCGAATTGATGCACGGATTGATTTCGAATCTGGAAGGATAAAGAAAGAGGAATTCGCTGAGA
TCATGAAGACCTGTTCCACCATTGAAGACCTCAGACGGCAAAAATAGGGAATTTGGCTTGTCCTTCATGA
AAA
>gi|157281271|gb|CY025219.1| Influenza A virus (A/Texas/UR06-0012/2006(H1N1)) segment 2, complete sequence
CATTTGAATGGATGTCAATCCGACATTACTTTTCTTAAAAGTGCCAGCACAAAATGCTATAAGCACAACT
TTTCCTTATACTGGTGACCCTCCTTACAGCCATGGGACAGGAACAGGGTACACCATGGATACAGTCAACA
GGACACACCAGTACTCAGAAAGAGGAAGATGGACAAAAAATACCGAAACTGGAGCACCGCAACTCAACCC
AATTGATGGGCCACTACCAGAAGACAATGAACCAAGTGGCTATGCCCAAACAGATTGTGTATTAGAAGCA
ATGGCTTTCCTTGAAGAATCCCATCCTGGTATCTTTGAAAACTCTTGTATTGAAACAATGGAGGTTGTTC
AGCAAACAAGGGTGGACAAACTGACACAAGGCAGACAGACCTATGACTGGACTCTAAATAGGAACCAGCC
TGCTGCCACAGCATTGGCAAACACTATAGAAGTATTCAGATCAAACGGCCTCATAGCAAATGAATCTGGG
AGGCTAATAGACTTCCTTAAAGATGTAATGGAGTCAATGGACAGAGACGAAGTAGAGATCACAACTCATT
TTCAAAGAAAGAGGAGAGTGAGAGACAATGTAACTAAAAAAATGGTGACCCAAAGAACAATAGGCAAAAA
GAAACATAAATTAGACAAAAGAAGTTACCTAATTAGGGCATTAACCCTGAACACAATGACCAAAGATGCT
GAAAGGGGGAAACTAAAACGCAGAGCAATTGCAACCCCAGGAATGCAAATAAGGGGGTTTGTGTACTTTG
TTGAAACACTGGCAAGAAGCATATGTGAAAAGCTTGAACAATCAGGATTGCCAGTTGGAGGAAATGAGAA
GAAAGCAAAGTTAGCAAATGTTGTAAGGAAGATGATGACCAACTCCCAGGACACTGAAATTTCTTTCACC
ATTACTGGAGATAACACAAAATGGAACGAAAATCAAAACCCTAGAATGTTCTTGGCCATGATCACATATA
TAACCAAAAATCAGCCTGAATGGTTCAGAAATATTCTAAGTATTGCTCCAATAATGTTTTCAAACAAGAT
GGCGAGACTAGGTAAAGGGTACATGTTTGAAAGCAAGAGTATGAAACTGAGAACTCAAATACCTGCAGAA
ATGCTAGCCAACATAGATTTGAAATATTTCAATGATTCAACTAAAAAGAAAATTGAAAAAATCCGACCAT
TATTAATAGATGGAACTGCATCATTGAGTCCTGGAATGATGATGGGCATGTTCAATATGTTGAGCACCGT
CTTGGGCGTCTCCATTCTGAATCTTGGGCAAAAGAGATACACCAAGACTACTTACTGGTGGGATGGTCTT
CAATCGTCTGATGATTTTGCTTTGATTGTGAATGCACCCAACTATGCAGGAATTCAAGCTGGAGTTGACA
GGTTTTATCGAACCTGTAAGCTGCTCGGAATTAATATGAGCAAAAAGAAGTCTTACATAAACAGAACAGG
TACCTTTGAATTCACGAGCTTTTTCTATCGTTATGGGTTTGTTGCCAATTTCAGCATGGAGCTTCCTAGT
TTTGGGGTGTCTGGGGTCAATGAATCTGCAGACATGAGTATTGGAGTCACTGTCATCAAAAACAATATGA
TAAACAATGACCTTGGCCCAGCAACTGCTCAAATGGCCCTTCAGTTATTTATAAAAGATTACAGGTACAC
TTATCGATGCCACAGAGGTGACACACAAATACAAACCCGGAGATCATTTGAGATAAAGAAACTATGGGAC
CAAACCCACTCCAAAGCTGGGCTGTTGGTCTCTGATGGAGGCCCCAATTTATATAACATTAGAAATCTCC
ATATTCCTGAAGTTTGCTTGAAATGGGAGTTGATGGATGAGGATTACCAGGGGCGTTTATGCAACCCATT
AAACCCGTTTGTCAGCCATAAAGAGATTGAATCAGTGAACAATGCAGTGATAATGCCCGCACATGGTCCA
GCCAAGAATATGGAGTATGACGCTGTTGCAACAACACACTCCTGGGTCCCCAAAAGAAATCGATCCATTT

TGAACACGAGCCAAAGGGGGATACTTGAAGATGAGCAAATGTATCAGAGGTGCTGCAATTTATTTGAAAA
ATTCTTCCCAAGTAGCTCATACAGAAGACCAGTTGGAATATCCAGTATGGTAGAGGCTATGGTTTCAAGA
GCCAGAATTGATGCACGGATTGATTTCGAATCTGGAAGGATAAAGAAAGAGGAATTTGCTGAGATCATGA
AGATCTGTTCCACCATTGAAGACCTCAGACGGCAAAAATAGGGAATTTGGCTTGTCCTTCATGAAAA
>gi|94959546|gb|CY010770.1| Influenza A virus (A/Canterbury/20/2001(H1N1)) segment 2, complete sequence
GCAAACCATTTGAATGGATGTCAATCCGACCTTACTTTTCTTAAAAGTGCCAGCACAAAATGCTATAAGC
ACAACTTTCCCTTATACTGGTGATCCTCCTTACAGCCATGGGACAGGAACAGGGTACACCATGGATACAG
TCAACAGGACACATCAGTACTCAGAAAGAGGAAGATGGACAAAAAATACCGAAACTGGAGCACCGCAACT
CAACCCAATTGATGGACCACTACCAAAAGACAATGAACCAAGTGGCTATGCCCAAACAGATTGTGTATTA
GAAGCAATGGCTTTCCTTGAGGAATCCCATCCTGGTATTTTTGAAAACTCTTGTATTGAAACAATGGAGG
TTGTTCAGCAAACAAGGGTGGACAAACTGACACAAGGCAGACAGACCTATGACTGGACTCTAAATAGAAA
CCAGCCTGCTGCCACAGCATTGGCCAACACTATAGAAGTGTTCAGATCAAACGGCCTCATAGCAAATGAA
TCTGGGAGGCTAATAGACTTCCTTAAAGATGTAATGGAGTCGATGGACAGAGACGAAGTAGAGATCACAA
CACATTTTCAGAGAAAGAGGAGGGTGAGAGACAATGTAACTAAAAAAATGGTGACCCAAAGAACAATAGG
CAAAAAGAAACATAAATTAGACAAAAGAAGTTACCTAATTAGGGCATTAACCCTGAACACAATGACCAAA
GATGCTGAGAGGGGGAAACTAAAACGTAGAGCAATTGCAACCCCAGGAATGCAAATAAGGGGGTTTGTAT
ACTTTGTTGAGACACTGGCAAGAAGCATATGTGAAAAGCTTGAACAATCAGGGTTGCCAGTTGGGGGAAA
TGAAAAGAAAGCAAAGTTAGCAAATGTTGTAAGGAAGATGATGACCAACTCCCAGGACACTGAAATTTCT
TTCACCATCACTGGAGATAACACAAAATGGAACGAAAATCAAAACCCTAGAATGTTTTTGGCCATGATCA
CATATATAACCAAAAATCAGCCTGAATGGTTCAGAAATATTCTAAGTATTGCTCCAATAATGTTTTCAAA
CAAAATGGCGAGACTAGGTAAGGGGTACATGTTTGAAAGCAAGAGTATGAAACTGAGAACTCAAATACCT
GCAGAGATGCTAGCCAACATAGATTTGAAATATTTCAATGATTCAACTAAAAAGAAAATTGAAAAAATCC
GGCCATTATTAATAGATGGAACTGCATCATTGAGTCCTGGAATGATGATGGGCATGTTCAATATGTTAAG
CACTGTCTTGGGCGTCTCCATTCTGAATCTTGGGCAAAAGAGATACACCAAGACTACTTACTGGTGGGAT
GGTCTTCAATCGTCTGATGATTTTGCTCTGATTGTGAATGCATCCAACTATGCAGGAATTCAAGCTGGAG
TTGACAGGTTTTATCGAACCTGTAAGCTGCTCGGAATTAATATGAGCAAAAAGAAGTCTTACATAAACAG
AACAGGTACCTTTGAATTCACGAGCTTTTTCTATCGTTATGGGTTTGTTGCCAATTTCAGCATGGAGCTT
CCTAGTTTTGGGGTGTCTGGGGTCAATGAGTCTGCAGACATGAGTATTGGAGTCACTGTCATCAAAAACA
ATATGATAAACAATGACCTTGGCCCAGCAACTGCTCAAATGGCCCTTCAGTTATTTATCAAAGATTACAG
GTACACGTATCGATGTCACAGAGGTGACACACAAATACAAACCCGGAGATCATTTGAGATAAAGAAACTA
TGGGACCAAACCCGCTCCAAAGCTGGGCTGTTGGTCTCTGATGGAGGCCCCAATTTATATAACATTAGAA
ATCTCCATATTCCTGAAGTCTGCTTGAAATGGGAGTTGATGGATGAGGATTACCAGGGGCGTTTATGCAA
CCCATTGAACCCATTTGTCAGTCATAAAGAGATTGAGTCAGTGAACAATGCAGTGATGATGCCGGCACAT
GGTCCAGCCAAAAATATGGAGTATGACGCTGTTGCAACAACACACTCCTGGGTTCCCAAAAGGAATCGAT
CCATTTTGAATACGAGCCAAAGGGGGATACTTGAGGATGAGCAAATGTATCAGAGGTGCTGCAATTTATT
TGAAAAATTCTTCCCAAGTAGTTCATACAGAAGACCAGTTGGAATATCCAGTATGGTAGAGGCTATGGTT
TCCAGAGCCCGAATTGATGCAAGAATTGATTTCGAATCTGGAAGGATAAAGAAAGAGGAATTCGCTGAGA
TCATGAAGACCTGTTCCACCATTGAAGACCTCAGACGGCAAAAATAGGGAATTTGGCTTGTCCTTCATGA
AAA
>gi|91119040|gb|CY010410.1| Influenza A virus (A/West Coast/33/2001(H1N1)) segment 2, complete sequence
GCAAACCATTTGAATGGATGTCAATCCGACCTTACTTTTCTTAAAAGTGCCAGCACAAAATGCTATAAGC
ACAACTTTCCCTTATACTGGTGATCCTCCTTACAGCCATGGGACAGGAACAGGGTACACCATGGATACAG

TCAACAGGACACATCAGTACTCAGAAAGAGGAAGATGGACAAAAAATACCGAAACTGGAGCACCGCAACT
CAACCCAATTGATGGACCACTACCAAAAGACAATGAACCAAGTGGCTATGCCCAAACAGATTGTGTATTA
GAAGCAATGGCTTTCCTTGAGGAATCCCATCCTGGTATTTTTGAAAACTCTTGTATTGAAACAATGGAGG
TTGTTCAGCAAACAAGGGTGGACAAACTGACACAAGGCAGACAGACCTATGACTGGACTCTAAATAGAAA
CCAGCCTGCTGCCACAGCATTGGCCAACACTATAGAAGTGTTCAGATCAAACGGCCTCATAGCAAATGAA
TCTGGGAGGCTAATAGACTTCCTTAAAGATGTAATGGAGTCGATGGACAGAGACGAAGTAGAGATCACAA
CACATTTTCAGAGAAAGAGGAGGGTGAGAGACAATGTAACTAAAAAAATGGTGACCCAAAGAACAATAGG
CAAAAAGAAACATAAATTAGACAAAAGAAGTTACCTAATTAGGGCATTAACCCTGAACACAATGACCAAA
GATGCTGAGAGGGGGAAACTAAAACGTAGAGCAATTGCAACCCCAGGAATGCAAATAAGGGGGTTTGTAT
ACTTTGTTGAGACACTGGCAAGAAGCATATGTGAAAAGCTTGAACAATCAGGGTTGCCAGTTGGGGGAAA
TGAAAAGAAAGCAAAGTTAGCAAATGTTGTAAGGAAGATGATGACCAACTCCCAGGACACTGAAATTTCT
TTCACCATCACTGGAGATAACACAAAATGGAACGAAAATCAAAACCCTAGAATGTTTTTGGCCATGATCA
CATATATAACCAAAAATCAGCCTGAATGGTTCAGAAATATTCTAAGTATTGCTCCAATAATGTTTTCAAA
CAAAATGGCGAGACTAGGTAAGGGGTACATGTTTGAAAGCAAGAGTATGAAACTGAGAACTCAAATACCT
GCAGAGATGCTAGCCAACATAGATTTGAAATATTTCAATGATTCAACTAAAAAGAAAATTGAAAAAATCC
GGCCATTATTAATAGATGGAACTGCATCATTGAGTCCTGGAATGATGATGGGCATGTTCAATATGTTAAG
CACTGTCTTGGGCGTCTCCATTCTGAATCTTGGGCAAAAGAGATACACCAAGACTACTTACTGGTGGGAT
GGTCTTCAATCGTCTGATGATTTTGCTCTGATTGTGAATGCATCCAACTATGCAGGAATTCAAGCTGGAG
TTGACAGGTTTTATCGAACCTGTAAGCTGCTCGGAATTAATATGAGCAAAAAGAAGTCTTACATAAACAG
AACAGGTACCTTTGAATTCACGAGCTTTTTCTATCGTTATGGGTTTGTTGCCAATTTCAGCATGGAGCTT
CCTAGTTTTGGGGTGTCTGGGGTCAATGAGTCTGCAGACATGAGTATTGGAGTCACTGTCATCAAAAACA
ATATGATAAACAATGACCTTGGCCCAGCAACTGCTCAAATGGCCCTTCAGTTATTTATCAAAGATTACAG
GTACACGTATCGATGCCACAGAGGTGACACACAAATACAAACCCGGAGATCATTTGAGATAAAGAAACTA
TGGGACCAAACCCGCTCCAAAGCTGGGCTGTTGGTCTCTGATGGAGGCCCCAATTTATATAACATTAGAA
ATCTCCATATTCCTGAAGTCTGCTTGAAATGGGAGTTGATGGATGAGGATTACCAGGGGCGTTTATGCAA
CCCATTGAACCCATTTGTCAGTCATAAAGAGATTGAGTCAGTGAACAATGCAGTGATGATGCCGGCACAT
GGTCCAGCCAAAAATATGGAGTATGACGCTGTTGCAACAACACACTCCTGGGTTCCCAAAAGGAATCGAT
CCATTTTGAATACGAGCCAAAGGGGGATACTTGAGGATGAGCAAATGTATCAGAGGTGCTGCAATTTATT
TGAAAAATTCTTCCCAAGTAGTTCATACAGAAGACCAGTTGGAATATCCAGTATGGTAGAGGCTATGGTT
TCCAGAGCCCGAATTGATGCAAGAATTGATTTCGAATCTGGAAGGATAAAGAAAGAGGAATTCGCTGAGA
TCATGAAGACCTGTTCCACCATTGAAGACCTCAGACGGCAAAAATAGGGAATTTGGCTTGTCCTTCATGA
AAA

>gi|131052866|gb|CY020003.1| Influenza A virus (A/Waikato/17/2005(H1N1)) segment 2, complete sequence

AATGGATGTCAATCCGACATTACTTTTCTTAAAAGTGCCAGCACAAAATGCTATAAGCACAACTTTTCCT
TATACTGGTGACCCTCCTTACAGCCATGGGACAGGAACAGGGTACACCATGGATACAGTCAACAGGACAC
ATCAGTACTCAGAAAGAGGAAAATGGACAAAAAATACCGAAACTGGAGCACCGCAACTCAACCCGATTGA
TGGGCCACTACCAGAAGACAATGAACCAAGTGGCTATGCCCAAACAGATTGTGTATTAGAAGCAATGGCT
TTCCTTGAAGAATCCCATCCTGGTATTTTTGAAAACTCTTGTATTGAAACAATGGAGGTTGTTCAGCAAA
CAAGGGTGGACAAACTGACACAAGGCAGACAGACCTATGACTGGACTTTAAATAGGAACCAGCCTGCTGC
CACAGCTTTGGCAAACACTATAGAAGTATTCAGATCAAACGGCCTCATAGCAAATGAATCTGGGAGGCTA
ATAGACTTCCTTAAAGATGTAATGGAGTCGATGGACAGAGACGAAGTAGAGGTCACAACTCATTTTCAAA
GAAAGAGGAGAGTGAGAGACAATGTAACTAAAAAAATGGTGACCCAAAGAACAATAGGCAAAAAGAAAA
A

TAAATTAGACAAAAGAAGTTACCTAATTAGGGCATTAACCCTGAACACAATGACCAAAGATGCTGAGAGG
GGGAAACTAAAACGCAGAGCAATTGCAACCCCAGGAATGCAAATAAGGGGGTTCGTATACTTTGTTGAGA
CACTGGCAAGAAGCATATGTGAAAAGCTTGAACAATCAGGGTTGCCAGTTGGAGGAAATGAGAAGAAAGC
AAAGTTAGCAAATGTTGTAAGAAAGATGATGACCAACTCCCAGGACACTGAAATTTCTTTCACCATCACT
GGAGATAACACAAAATGGAACGAAAATCAAAACCCTAGAATGTTCTTGGCCATGATCACATATATAACCA
AAAATCAACCTGAATGGTTCAGAAATATTCTAAGTATTGCTCCAATAATGTTTTCAAACAAGATGGCGAG
ACTAGGTAAGGGGTACATGTTTGAAAGCAAGAGTATGAAACTGAGAACTCAAGTACCTGCAGAGATGCTA
GCCAACATAGATTTGAAATATTTCAATGATTCAACTAAAAAGAAAATTGAAAAAATCCGACCATTATTAA
TAGATGGAACTGCATCATTGAGTCCTGGAATGATGATGGGCATGTTCAATATGTTAAGCACCGTCTTGGG
CGTCTCCATTCTGAATCTTGGGCAAAAGAGATACACCAAGACTACTTACTGGTGGGATGGTCTTCAATCG
TCTGATGATTTTGCTTTGATTGTGAATGCACCCAACTATGCAGGAATTCAAGCTGGAGTTGACAGATTTT
ATCGAACCTGTAAGCTGCTCGGAATTAATATGAGCAAAAAGAAGTCTTACATAAACAGAACAGGTACCTT
TGAATTCACGAGCTTTTTCTATCGTTATGGGTTTGTTGCCAATTTCAGCATGGAGCTTCCTAGTTTTGGG
GTGTCTGGGGTCAATGAATCTGCAGACATGAGTATTGGAGTCACTGTTATCAAAAACAATATGATAAACA
ATGACCTTGGCCCAGCAACTGCTCAAATGGCCCTTCAGTTATTTATAAAAGATTACAGGTACACTTATCG
ATGCCACAGAGGTGACACACAAATACAAACCCGGAGATCATTTGAGATAAAGAAACTATGGGACCAAACC
CGCTCCAAAGCTGGGCTGTTGGTCTCTGATGGAGGGCCCAATTTATATAACATTAGAAATCTCCATATTC
CTGAAGTCTGCTTGAAGTGGGAGTTGATGGATGAGGATTACCAGGGGCGTTTATGCAACCCATTGAACCC
ATTTGTCAGCCATAAAGAGATTGAATCAGTGAACAATGCAGTGATAATGCCGGCACATGGTCCAGCCAAA
AATATGGAGTATGACGCTGTTGCAACAACACACTCCTGGGTCCCCAAAAGAAACCGATCCATTTTGAACA
CGAGCCAAAGGGGGATACTTGAAGATGAGCAAATGTATCAGAGGTGCTGCAATTTATTTGAAAAATTCTT
CCCAAGTAGCTCATACAGAAGACCAGTTGGAATATCCAGTATGGTAGAGGCTATGGTTTCAAGAGCCCGA
ATTGATGCACGGATTGATTTCGAATCTGGAAGGATAAAGAAAGAGGAATTCGCTGAGATCATGAAGACCT
GTTCCACCATTGAAGACCTCAGACGGCAAAAATAGGGAA
>gi|83744848|gb|CY007473.1| Influenza A virus (A/Canterbury/106/2004(H1N1)) segment 2, complete sequence
GCAAACCATTTGAATGGATGTCAATCCGACATTACTTTTCTTAAAAGTGCCAGCACAAAATGCTATAAGC
ACAACTTTTCCTTATACTGGTGACCCTCCTTACAGCCATGGGACAGGAACAGGGTACACCATGGATACAG
TCAACAGGACACATCAGTACTCAGAAAGAGGAAGATGGACAAAAAATACCGAAACTGGAGCACCGCAACT
CAACCCAATTGATGGGCCACTACCAGAAGACAATGAACCAAGTGGCTATGCCCAAACAGATTGTGTATTA
GAAGCAATGGCTTTCCTTGAAGAATCCCATCCTGGTATTTTTGAAAACTCTTGTATTGAAACAATGGAGG
TTGTTCAGCAAACAAGGGTGGACAAACTGACACAAGGCAGACAGACCTATGACTGGACTCTAAATAGGAA
CCAGCCTGCTGCCACAGCATTGGCCAACACTATAGAAGTATTCAGATCAAACGGCCTCATAGCAAATGAA
TCTGGGAGGCTAATAGACTTCCTTAAAGATGTAATGGAGTCGATGGACAGAGAAGAAGTAGAGGTCACAA
CTCATTTTCAGAGAAAGAGGAGAGTGAGAGACAATGTAACTAAAAAAATGGTGACCCAAAGAACAATAGG
CAAAAAGAAACATAAATTAGACAAAAGGAGTTACCTAATTAGAGCATTAACCCTGAACACAATGACCAAA
GATGCTGAGAGGGGGAAACTAAAACGCAGAGCAATTGCAACCCCAGGAATGCAAATAAGGGGGTTTGTAT
ACTTTGTTGAAACACTGGCAAGAAGCATATGTGAAAAGCTTGAACAATCAGGGTTGCCAGTTGGAGGAAA
TGAGAAAAAAGCAAAGTTAGCAAATGTTGTAAGGAAGATGATGACCAACTCCCAGGACACTGAAATTTCT
TTCACCATCACTGGAGATAACACAAAATGGAACGAAAATCAAAACCCTAGAATGTTTTTGGCCATGATCA
CATATATAACCAGAAATCAGCCTGAATGGTTCAGAAATATTCTAAGTATTGCTCCAATAATGTTTTCAAA
CAAAATGGCGAGACTAGGTAAGGGGTACATGTTTGAAAGCAAGAGTATGAAACTGAGAACTCAAATACCT
GCAGAGATACTAGCCGACATAGATTTGAAGTATTTCAATGAGTCAACTAAAAAGAAAATTGAAAAAATCC
GACCATTATTAATAGATGGAACTGCATCATTGAGTCCTGGAATGATGATGGGCATGTTCAATATGTTAAG

CACTGTCTTGGGCGTCTCCATTCTGAATCTTGGGCAAAAGAGATACACCAAGACTACTTACTGGTGGGAT
GGTCTTCAATCGTCTGATGATTTTGCTCTGATAGTGAATGCGCCCAACTATGCAGGAATTCAAGCTGGAG
TTGACAGGTTTTATCGAACCTGTAAGCTGCTCGGAATTAATATGAGCAAAAAGAAGTCTTACATAAACAG
GACAGGTACCTTTGAATTCACGAGCTTTTTCTATCGTTATGGGTTTGTTGCCAATTTCAGCATGGAGCTT
CCTAGTTTTGGGGTGTCTGGGGTCAATGAATCTGCAGACATGAGTATTGGAGTCACTGTTATCAAAAACA
ATATGATAAACAATGACCTTGGCCCAGCAACTGCTCAAATGGCCCTTCAGTTATTTATAAAAGATTACAG
ATACACGTATCGATGCCACAGAGGTGACACACAAATACAAACCCGGAGATCATTTGAGATAAAGAAATTA
TGGGACCAAACCCGCTCCAAAGCTGGGCTGTTGGTTTCTGATGGAGGCCCCAATTTATATAACATTAGAA
ATCTCCATATTCCTGAAGTCTGCTTGAAATGGGAGTTGATGGATGAGGATTACCAGGGGCGTTTATGCAA
CCCATTGAACCCGTTTGTCAGTCATAAAGAGATTGAATCAGTGAACAATGCAGTGATGATGCCGGCACAT
GGTCCAGCCAAAAATATGGAGTATGACGCTGTTGCAACAACACATTCCTGGGTTCCCAAAAGGAATCGAT
CCATTTTGAACACGAGCCAAAGGGGGATACTTGAGGATGAGCAAATGTATCAGAGGTGCTGCAATTTATT
TGAAAAATTCTTCCCAAGTAGCTCATACAGAAGACCAGTTGGGATATCCAGTATGGTAGAGGCTATGGTT
TCCAGAGCCCGAATTGATGCACGGATTGATTTCGAATCTGGAAGGATAAAGAAAGAGGAATTCGCTGAGA
TCATGAAGACCTGTTCCACCATTGAAGACCTCAGACGGCAAAAATAGGGAGTTTGGCTTGTCCTTCATGA
AAA
>gi|115607835|gb|CY016705.1| Influenza A virus (A/South Australia/58/2005(H1N1)) segment 2, complete sequence
ATTTGAATGGATGTCAATCCGACATTACTTTTCTTAAAAGTGCCAGCACAAAATGCTATAAGCACAACTT
TTCCTTATACTGGTGACCCTCCTTACAGCCATGGGACAGGAACAGGGTACACCATGGATACAGTCAACAG
GACACATCAGTACTCAGAAAGAGGAAGATGGACAAAAAATACCGAAACTGGAGCACCGCAACTCAACCCA
ATTGATGGGCCACTACCAGAAGACAATGAACCAAGTGGCTATGCCCAAACAGATTGTGTATTAGAAGCAA
TGGCTTTCCTTGAAGAATCCCATCCTGGTATTTTTGAAAACTCTTGTATTGAAACAATGGAGGTTGTTCA
GCAAACAAGGGTGGACAAACTGACACAAGGCAGACAGACCTATGACTGGACTTTAAATAGGAACCAGCCT
GCTGCCACAGCTTTGGCAAACACTATAGAAGTATTCAGATCAAACGGCCTCATAGCAAATGAATCTGGGA
GGCTAATAGACTTCCTTAAAGATGTAATGGAGTCGATGGACAGAGACGAAGTAGAGGTCACAACTCATTT
TCAAAGAAAGAGGAGAGTGAGAGACAATGTAACTAAAAAAATGGTGACCCAAAGAACAATAGGCAAAAA
G
AAACATAAATTAGACAAAAGAAGTTACCTAATTAGGGCATTAACCCTGAACACAATGACCAAAGATGCTG
AGAGGGGGAAACTAAAACGCAGAGCAATTGCAACCCCAGGAATGCAAATAAGGGGGTTCGTATACTTTGT
TGAGACACTGGCAAGAAGCATATGTGAAAAGCTTGAACAATCAGGGTTGCCAGTTGGAGGAAATGAGAAG
AAAGCAAAGTTAGCAAATGTTGTAAGAAAGATGATGACCAACTCCCAGGACACTGAAATTTCTTTCACCA
TCACTGGAGATAACACAAAATGGAACGAAAATCAAAACCCTAGAATGTTCTTGGCCATGATCACATATGT
AACCAAAAATCAACCTGAATGGTTCAGAAATATTCTAAGTATTGCTCCAATAATGTTTTCAAACAAGATG
GCGAGACTAGGTAAGGGGTACATGTTTGGAAGCAAGAGTATGAAACTGAGAACTCAAATACCTGCAGAGA
TGCTAGCCAACATAGATTTGAAATATTTCAATGATTCAACTAAAAAGAAAATTGAAAAAATCCGACCATT
ATTAATAGATGGAACTGCATCATTGAGTCCTGGAATGATGATGGGCATGTTCAATATGTTAAGCACCGTC
TTGGGCGTCTCCATTCTGAATCTTGGGCAAAAGAGATACACCAAGACTACTTACTGGTGGGATGGTCTTC
AATCGTCTGATGATTTTGCTTTGATTGTGAATGCACCCAACTATGCAGGAATTCAAGCTGGAGTTGACAG
ATTTTATCGAACCTGTAAGCTGCTCGGAATTAATATGAGCAAAAAGAAGTCTTACATAAACAGAACAGGT
ACCTTTGAATTCACGAGCTTTTTCTATCGTTATGGGTTTGTTGCCAATTTCAGCATGGAGCTTCCTAGTT
TTGGGGTGTCTGGGGTCAATGAATCTGCAGACATGAGTATTGGAGTCACTGTTATCAAAAACAATATGAT
AAACAATGACCTTGGCCCAGCAACTGCTCAAATGGCCCTTCAGTTATTTATAAAAGATTACAGGTACACT
TATCGATGCCACAGAGGTGACACACAAATACAAACCCGGAGATCATTTGAGATAAAGAAACTATGGGACC

AAACCCGCTCCAAAGCTGGGCTGTTGGTCTCTGATGGAGGCCCCAATTTATATAACATTAGAAATCTCCA
TATTCCTGAAGTCTGCTTGAAGTGGGAGTTGATGGATGAGGATTACCAGGGGCGTTTATGCAACCCATTG
AACCCGTTTGTCAGCCATAAAGAGATTGAATCAGTGAACAATGCAGTGATAATGCCGGCACATGGTCCAG
CCAAAAATATGGAGTATGACGCTGTTGCAACAACACACTCCTGGGTCCCCAAAAGAAACCGATCCATTTT
GAACACGAGCCAAAGGGGGATACTTGAAGATGAGCAAATGTATCAGAGGTGCTGCAATTTATTTGAAAAA
TTCTTCCCAAGTAGCTCATACAGAAGACCAGTTGGAATATCCAGTATGGTAGAGGCTATGGTTTCAAGAG
CCCGAATTGATGCACGGATTGATTTCGAATCTGGAAGGATAAAGAAAGAGGAATTCGCTGAGATCATGAA
GACCTGTTCCACCATTGAAGACCTCAGACGGCAAAAATAGGGAATTTGGCTTGTCCTTCATGAAAA
>gi|113170895|gb|CY014013.1| Influenza A virus (A/Wellington/11/2005(H1N1)) segment 2, complete sequence
ACCATTTGAATGGATGTCAATCCGACATTACTTTTCTTAAAAGTGCCAGCACAAAATGCTATAAGCACAA
CTTTTCCTTATACTGGTGACCCTCCTTACAGCCATGGGACAGGAACAGGGTACACCATGGATACAGTCAA
CAGGACACACCAGTACTCAGAAAGAGGAAGATGGACAAAAAATACCGAAACTGGAGCACCGCAACTCAAC
CCAATTGATGGGCCACTACCAGAAGACAATGAACCAAGTGGCTATGCCCAAACAGATTGTGTATTAGAAG
CAATGGCTTTCCTTGAAGAATCCCATCCTGGTATCTTTGAAAACTCTTGTATTGAAACAATGGAGGTTGT
TCAGCAAACAAGGGTGGACAAACTGACACAAGGCAGACAGACCTATGACTGGACTCTAAATAGGAACCAG
CCTGCTGCCACAGCATTGGCAAACACTATAGAAGTATTCAGATCAAACGGCCTCATAGCAAATGAATCTG
GGAGGCTAATAGACTTCCTTAAAGATGTAATGGAGTCGATGGACAGAGACGAAGTAGAGGTCACAACTCA
TTTTCAAAGAAAGAGGAGAGTGAGAGACAATGTAACTAAAAAAATGGTGACCCAAAGAACAATAGGCAAA
AAGAAACATAAATTAGACAAAAGAAGTTACCTAATTAGGGCATTAACCCTGAACACAATGACCAAAGATG
CTGAGAGGGGGAAACTAAAACGCAGAGCAATTGCAACCCCAGGAATGCAAATAAGGGGGTTTGTATACTT
TGTTGAGACACTGGCAAGAAGCATATGTGAAAAGCTTGAACAATCAGGATTGCCAGTTGGAGGGAATGAG
AAGAAAGCAAAGTTAGCAAATGTTGTAAGGAAGATGATGACCAACTCCCAGGACACTGAAATTTCTTTCA
CCATCACTGGAGATAACACAAAATGGAACGAAAATCAAAACCCTAGAATGTTCTTGGCCATGATCACATA
TATAACCAAAAATCAGCCTGAATGGTTCAGAAATATTCTAAGTATTGCTCCAATAATGTTTTCAAACAAG
ATGGCGAGACTAGGTAAGGGGTACATGTTTGAAAGCAAGAGTATGAAACTGAGAACTCAAATACCTGCAG
AGATGCTAGCCAACATAGATTTGAAATATTTCAATGATTCAACTAAAAAGAAAATTGAAAAAATCCGACC
ATTATTAATAGATGGAACTGCATCATTGAGTCCTGGAATGATGATGGGCATGTTCAATATGTTGAGCACC
GTCTTGGGCGTCTCCATTCTGAATCTTGGGCAAAAGAGATACACCAAGACTACATACTGGTGGGATGGTC
TTCAATCGTCTGATGATTTTGCTTTGATTGTGAATGCACCCAACTATGCAGGAATTCAAGCTGGAGTTGA
CAGGTTTTATCGAACCTGTAAGCTGCTCGGAATTAATATGAGCAAAAAGAAGTCTTACATAAACAGAACA
GGTACCTTCGAATTCACGAGCTTTTTCTATCGTTATGGGTTTGTTGCCAATTTCAGCATGGAGCTTCCTA
GTTTTGGGGTGTCTGGGGTCAATGAATCTGCAGACATGAGTATTGGAGTCACTGTCATCAAAAACAATAT
GATAAACAATGACCTTGGCCCAGCAACTGCTCAAATGGCCCTTCAGTTATTTATAAAAGATTACAGGTAC
ACTTATCGATGCCACAGAGGTGACACACAAATACAAACCCGGAGATCATTTGAGATAAAGAAACTATGGG
ACCAAACCCGCTCCAAAGCTGGGCTGTTGGTCTCTGATGGAGGCCCCAATTTATATAACATTAGAAATCT
CCATATTCCTGAAGTTTGCTTGAAATGGGAGTTGATGGATGAGGATTACCAGGGGCGTTTATGCAACCCA
TTAAACCCGTTTGTCAGCCATAAAGAGATTGAATCAGTGAACAATGCAGTGATAATGCCGGCACATGGTC
CAGCCAAAAATATGGAGTATGACGCTGTTGCAACAACACACTCCTGGGTCCCCAAAAGAAATCGATCCAT
TTTGAACACGAGCCAAAGGGGGATACTTGAAGATGAGCAAATGTATCAGAGGTGCTGCAATTTATTTGAA
AAATTCTTCCCAAGTAGCTCATACAGAAGACCAGTTGGAATATCCAGTATGGTAGAGGCTATGGTTTCAA
GAGCCCGAATTGATGCACGGATTGATTTCGAATCTGGAAGGATAAAGAAAGAGGAATTTGCTGAGATCAT
GAAGATCTGTTCCACCATTGAAGACCTCAGACGGCAAAAATAGGGAATTTGGCTTGT
>gi|145278937|gb|CY021763.1| Influenza A virus (A/South Australia/51/2005(H1N1)) segment 2,

complete sequence
CATTTGAATGGATGTCAATCCGACATTACTTTTCTTAAAAGTGCCAGCACAAAATGCTATAAGCACAACT
TTTCCTTATACTGGTGACCCTCCTTACAGCCATGGGACAGGAACAGGGTACACCATGGATACAGTCAACA
GGACACACCAGTACTCAGAAAGAGGAAGATGGACAAAAAATACCGAAACTGGAGCACCGCAACTCAACCC
AATTGATGGGCCACTACCAGAAGACAATGAACCAAGTGGCTATGCCCAAACAGATTGTGTATTAGAAGCA
ATGGCTTTCCTTGAAGAATCCCATCCTGGTATCTTTGAAAACTCTTGTATTGAAACAATGGAGGTTGTTC
AGCAAACAAGGGTGGACAAACTGACACAAGGCAGACAGACCTATGACTGGACTCTAAATAGGAACCAGCC
TGCTGCCACAGCATTGGCAAACACTATAGAAGTATTCAGATCAAACGGCCTCATAGCAAATGAATCTGGG
AGGCTAATAGACTTCCTTAAAGATGTAATGGAGTCGATGGACAGAGACGAAGTAGAGGTCACAACTCATT
TTCAAAGAAAGAGGAGAGTGAGAGACAATGTAACTAAAAAAATGGTGACCCAAAGAACAATAGGCAAAAA
GAAACATAAATTAGACAAAAGAAGTTACCTAATTAGGGCATTAACCCTGAACACAATGACCAAAGATGCT
GAGAGGGGGAAACTAAAACGCAGAGCAATTGCAACCCCAGGAATGCAAATAAGGGGGTTTGTATACTTTG
TTGAGACACTGGCAAGAAGCATATGTGAAAAGCTTGAACAATCAGGATTGCCAGTTGGAGGAAATGAGAA
GAAAGCAAAGTTAGCAAATGTTGTAAGGAAGATGATGACCAACTCCCAGGACACTGAAATTTCTTTCACC
ATCACTGGAGATAACACAAAATGGAACGAAAATCAAAACCCTAGAATGTTCTTGGCCATGATCACATATA
TAACCAAAAATCAGCCTGAATGGTTCAGGAATATTCTAAGTATTGCTCCAATAATGTTTTCAAACAAGAT
GGCGAGACTAGGTAAGGGGTACATGTTTGAAAGCAAGAGTATGAAACTGAGAACTCAAATACCTGCAGAG
ATGCTAGCCAACATAGATTTGAAATATTTCAATGATTCAACTAAAAAGAAAATTGAAAAAATCCGACCAT
TATTAATAGATGGAACTGCATCATTGAGTCCTGGAATGATGATGGGCATGTTCAATATGTTGAGCACCGT
CTTGGGCGTCTCCATTTTGAATCTTGGGCAAAAGAGATACACCAAGACTACATACTGGTGGGATGGTCTT
CAATCGTCTGATGATTTTGCTTTGATTGTGAATGCACCCAACTATGCAGGAATTCAAGCTGGAGTTGACA
GGTTTTATCGAACCTGTAAGCTGCTCGGAATTAATATGAGCAAAAAGAAGTCTTACATAAACAGAACAGG
TACCTTCGAATTCACGAGCTTTTTCTACCGTTATGGGTTTGTTGCCAATTTCAGCATGGAGCTTCCTAGT
TTTGGGGTGTCTGGGGTCAATGAATCTGCAGACATGAGTATTGGAGTCACTGTCATCAAAAACAATATGA
TAAACAATGACCTTGGCCCAGCAACTGCTCAAATGGCCCTTCAGTTATTTATAAAAGATTACAGGTACAC
TTATCGATGCCACAGAGGTGACACACAAATACAAACCCGGAGATCATTTGAGATAAAGAAACTATGGGAC
CAAACCCGCTCCAAAGCTGGGCTGTTGGTCTCTGATGGAGGCCCCAATTTATATAACATTAGAAATCTCC
ATATTCCTGAAGTTTGCTTGAAATGGGAGTTGATGGATGAGGATTACCAGGGGCGTTTATGCAACCCATT
AAACCCGTTTGTCAGCCATAAAGAGATTGAATCAGTGAACAATGCAGTGATAATGCCGGCACATGGTCCA
GCCAAAAATATGGAGTATGACGCTGTTGCAACAACACACTCCTGGGTCCCCAAAAGAAATCGATCCATTT
TGAACACGAGCCAAAGGGGGATACTTGAAGATGAGCAAATGTATCAGAGGTGCTGCAATTTATTTGAAAA
ATTCTTCCCAAGTAGCTCATACAGAAGACCAGTTGGAATATCCAGTATGGTAGAGGCTATGGTTTCAAGA
GCCCGAATTGATGCACGGATTGATTTCGAATCTGGAAGGATAAAGAAAGAGGAATTTGCTGAGATCATGA
AGATCTGTTCCACCATTGAAGACCTCAGACGGCAAAAATAGGGAATTTGGCTTGT
>gi|149780708|gb|CY022587.1| Influenza A virus (A/Auckland/619/2005(H1N1)) segment 2, complete sequence
CATTTGAATGGATGTCAATCCGACATTACTTTTCTTAAAAGTGCCAGCACAAAATGCTATAAGCACAACT
TTTCCTTATACTGGTGACCCTCCTTACAGCCATGGGACAGGAACAGGGTACACCATGGATACAGTCAACA
GGACACACCAGTACTCAGAAAGAGGAAGATGGACAAAAAATACCGAAACTGGAGCACCGCAACTCAACCC
AATTGATGGGCCACTACCAGAAGACAATGAACCAAGTGGCTATGCCCAAACAGATTGTGTATTAGAAGCA
ATGGCTTTCCTTGAAGAATCCCATCCTGGTATCTTTGAAAACTCTTGTATTGAAACAATGGAGGTTGTTC
AGCAAACAAGGGTGGACAAACTGACACAAGGCAGACAGACCTATGACTGGACTCTAAATAGGAACCAGCC
TGCTGCCACAGCATTGGCAAACACTATAGAAGTATTCAGATCAAACGGCCTCATAGCAAATGAATCTGGG
AGGCTAATAGACTTCCTTAAAGATGTAATGGAGTCGATGGACAGAGACGAAGTAGAGGTCACAACTCATT

TTCAAAGAAAGAGGAGAGTGAGAGACAATGTAACTAAAAAAATGGTGACCCAAAGAACAATAGGCAAAAA
GAAACATAAATTAGACAAAAGAAGTTACCTAATTAGGGCATTAACCCTGAACACAATGACCAAAGATGCT
GAGAGGGGGAAACTAAAACGCAGAGCAATTGCAACCCCAGGAATGCAAATAAGGGGGTTTGTATACTTTG
TTGAGACACTGGCAAGAAGCATATGTGAAAAGCTTGAACAATCAGGATTGCCAGTTGGAGGAAATGAGAA
GAAAGCAAAGTTAGCAAATGTTGTAAGGAAGATGATGACCAACTCCCAGGACACTGAAATTTCTTTCACC
ATCACTGGAGATAACACAAAATGGAACGAAAATCAAAACCCTAGAATGTTCTTGGCCATGATCACATATA
TAACCAAAAATCAGCCTGAATGGTTCAGAAATATTCTAAGTATTGCTCCAATAATGTTTTCAAACAAGAT
GGCGAGACTAGGTAAGGGGTACATGTTTGAAAGCAAGAGTATGAAACTGAGAACTCAAATACCTGCAGAG
ATGCTAGCCAACATAGATTTGAAATATTTCAATGATTCAACTAAAAAGAAAATTGAAAAAATCCGACCAT
TATTAATAGATGGAACTGCATCATTGAGTCCTGGAATGATGATGGGCATGTTCAATATGTTGAGCACCGT
CTTGGGCGTCTCCATTCTGAATCTTGGGCAAAAGAGATACACCAAGACTACATACTGGTGGGATGGTCTT
CAATCGTCTGATGATTTTGCTTTGATTGTGAATGCACCCAACTATGCAGGAATTCAAGCTGGAGTTGACA
GGTTTTATCGAACCTGTAAGCTGCTCGGAATTAATATGAGCAAAAAGAAGTCTTACATAAACAGAACAGG
TACCTTCGAATTCACGAGCTTTTTCTATCGTTATGGGTTTGTTGCCAATTTCAGCATGGAGCTTCCTAGT
TTTGGGGTGTCTGGGGTCAATGAATCTGCAGACATGAGTATTGGAGTCACTGTCATCAAAAACAATATGA
TAAACAATGACCTTGGCCCAGCAACTGCTCAAATGGCCCTTCAGTTATTCATAAAAGATTACAGGTACAC
TTATCGATGCCACAGAGGTGACACACAAATACAAACCCGGAGATCATTTGAGATAAAGAAACTATGGGAC
CAAACCCGCTCCAAAGCTGGGCTGTTGGTCTCTGATGGAGGCCCCAATTTATATAACATTAGAAATCTCC
ATATTCCTGAAGTTTGCTTGAAATGGGAGTTGATGGATGAGGATTACCAGGGGCGTTTATGCAACCCATT
AAACCCGTTTGTCAGCCATAAAGAGATTGAATCAGTGAACAATGCAGTGATAATGCCGGCACATGGTCCA
GCCAAAAATATGGAGTATGACGCTGTTGCAACAACACACTCCTGGGTCCCCAAAAGAAATCGATCCATTT
TGAACACGAGCCAAAGGGGGATACTTGAAGATGAGCAAATGTATCAGAGGTGCTGCAATTTATTTGAAAA
ATTCTTCCCAAGTAGCTCATACAGAAGACCAGTTGGAATATCCAGTATGGTAGAGGCTATGGTTTCAAGA
GCCCGAATTGATGCACGGATTGATTTCGAATCTGGAAGGATAAAGAAAGAGGAATTTGCTGAGATCATGA
AGATCTGTTCCACCATTGAAGACCTCAGACGGCAAAAATAGGGAATTTGGCTTGTCCTTCATGA
>gi|117572952|gb|CY017321.1| Influenza A virus (A/Waikato/4/2005(H1N1)) segment 2, complete sequence
AATGGATGTCAATCCGACATTACTTTTCTTAAAAGTGCCAGCACAAAATGCTATAAGCACAACTTTTCCT
TATACTGGTGACCCTCCTTACAGCCATGGGACAGGAACAGGGTACACCATGGATACAGTCAACAGGACAC
ATCAGTACTCAGAAAGAGGAAAATGGACAAAAAATACCGAAACTGGAGCACCGCAACTCAACCCGATTGA
TGGGCCACTACCAGAAGACAATGAACCAAGTGGCTATGCCCAAACAGATTGTGTATTAGAAGCAATGGCT
TTCCTTGAAGAATCCCATCCTGGTATTTTTGAAAACTCTTGTATTGAAACAATGGAGGTTGTTCAGCAAA
CAAGGGTGGACAAACTGACACAAGGCAGACAGACCTATGACTGGACTTTAAATAGGAACCAGCCTGCTGC
CACAGCTTTGGCAAACACTATAGAAGTATTCAGATCAAACGGCCTCATAGCAAATGAATCTGGGAGGCTA
ATAGACTTCCTTAAAGATGTAATGGAGTCGATGGACAGAGACGAAGTAGAGGTCACAACTCATTTTCAAA
GAAAGAGGAGAGTGAGAGACAATGTAACTAAAAAAATGGTGACCCAAAGAACAATAGGCAAAAAGAAAA
A
TAAATTAGACAAAAGAAGTTACCTAATTAGGGCATTAACCCTGAACACAATGACCAAAGATGCTGAGAGG
GGGAAACTAAAACGCAGAGCAATTGCAACCCCAGGAATGCAAATAAGGGGGTTCGTATACTTTGTTGAGA
CACTGGCAAGAAGCATATGTGAAAAGCTTGAACAATCAGGGTTGCCAGTTGGAGGAAATGAGAAGAAAGC
AAAGTTAGCAAATGTTGTAAGAAAGATGATGACCAACTCCCAGGACACTGAAATTTCTTTCACCATCACT
GGAGATAACACAAAATGGAACGAAAATCAAAACCCTAGAATGTTCTTGGCCATGATCACATATATAACCA
AAAATCAACCTGAATGGTTCAGAAATATTCTAAGTATTGCTCCAATAATGTTTTCAAACAAGATGGCGAG
ACTAGGTAAGGGGTACATGTTTGAAAGCAAGAGTATGAAACTGAGAACTCAAGTACCTGCAGAGATGCTA

GCCAACATAGATTTGAAATATTTCAATGATTCAACTAAAAAGAAAATTGAAAAAATCCGACCATTATTAA
TAGATGGAACTGCATCATTGAGTCCTGGAATGATGATGGGCATGTTCAATATGTTAAGCACCGTCTTGGG
CGTCTCCATTCTGAATCTTGGGCAAAAGAGATACACCAAGACTACTTACTGGTGGGATGGTCTTCAATCG
TCTGATGATTTTGCTTTGATTGTGAATGCACCCAACTATGCAGGAATTCAAGCTGGAGTTGACAGATTTT
ATCGAACCTGTAAGCTGCTCGGAATTAATATGAGCAAAAAGAAGTCTTACATAAACAGAACAGGTACCTT
TGAATTCACGAGCTTTTTCTATCGTTATGGGTTTGTTGCCAATTTCAGCATGGAGCTTCCTAGTTTTGGG
GTGTCTGGGGTCAATGAATCTGCAGACATGAGTATTGGAGTCACTGTTATCAAAAACAATATGATAAACA
ATGACCTTGGCCCAGCAACTGCTCAAATGGCCCTTCAGTTATTTATAAAAGATTACAGGTACACTTATCG
ATGCCACAGAGGTGACACACAAATACAAACCCGGAGATCATTTGAGATAAAGAAACTATGGGACCAAACC
CGCTCCAAAGCTGGGCTGTTGGTCTCTGATGGAGGGCCCAATTTATATAACATTAGAAATCTCCATATTC
CTGAAGTCTGCTTGAAGTGGGAGTTGATGGATGAGGATTACCAGGGGCGTTTATGCAACCCATTGAACCC
ATTTGTCAGCCATAAAGAGATTGAATCAGTGAACAATGCAGTGATAATGCCGGCACATGGTCCAGCCAAA
AATATGGAGTATGACGCTGTTGCAACAACACACTCCTGGGTCCCCAAAAGAAACCGATCCATTTTGAACA
CGAGCCAAAGGGGGATACTTGAAGATGAGCAAATGTATCAGAGGTGCTGCAATTTATTTGAAAAATTCTT
CCCAAGTAGCTCATACAGAAGACCAGTTGGAATATCCAGTATGGTAGAGGCTATGGTTTCAAGAGCCCGA
ATTGATGCACGGATTGATTTCGAATCTGGAAGGATAAAGAAAGAGGAATTCGCTGAGATCATGAAGACCT
GTTCCACCATTGAAGACCTCAGACGGCAAAAATAGGGAATTTGGCTTGT

>gi|161139458|gb|CY028201.1| Influenza A virus (A/Kentucky/UR06-0007/2006(H1N1)) segment 2, complete sequence

GCAAACCATTTGAATGGATGTCAATCCGACATTACTTTTCTTAAAAGTGCCAGCACAAAATGCTATAAGC
ACAACTTTTCCTTATACTGGTGACCCTCCTTACAGCCATGGGACAGGAACAGGGTACACCATGGATACAG
TCAACAGGACACACCAGTACTCAGAAAGAGGAAGATGGACAAAAAATACCGAAACTGGAGCACCGCAACT
CAACCCAATTGATGGGCCACTACCAGAAGACAATGAACCAAGTGGCTATGCCCAAACAGATTGTGTATTA
GAAGCAATGGCTTTCCTTGAAGAATCCCATCCTGGTATCTTTGAAAACTCTTGTATTGAAACAATGGAGG
TTGTTCAGCAAACAAGGGTGGACAAACTGACACAAGGCAGACAGACCTATGACTGGACTCTAAATAGGAA
CCAGCCTGCTGCAACAGCATTGGCAAACACTATAGAAGTATTCAGATCAAACGGCCTCATAGCAAATGAA
TCTGGAAGGCTAATAGACTTCCTTAAAGATGTAATGGAGTCGATGGACAGAGACGAAGTAGAGGTCACAA
CTCATTTTCAAAGAAAGAGGAGAGTAAGAGACAATGTAACTAAAAAAATGGTGACTCAAAGAACAATAGG
CAAAAAGAAACATAAATTAGACAAAAGAAGTTACCTAATTAGGGCATTAACCCTGAACACAATGACCAAA
GACGCTGAGAGGGGGAAACTAAAACGCAGAGCAATTGCAACCCCAGGAATGCAAATAAGAGGGTTTGTAT
ACTTTGTTGAGACACTGGCAAGAAGCATATGTGAAAAGCTTGAACAATCAGGATTGCCAGTTGGAGGAAA
TGAGAAGAAAGCAAAGTTAGCAAATGTTGTAAGGAAGATGATGACCAACTCCCAGGACACTGAAATTTCT
TTCACCATCACTGGAGATAACACAAAATGGAACGAAAATCAAAACCCTAGAATGTTCTTGGCCATGATCA
CATATATAACCAAAAATCAGCCTGAATGGTTCAGAAATATTCTAAGTATTGCTCCAATAATGTTTTCAAA
CAAGATGGCGAGACTAGGTAAGGGGTACATGTTTGAAAGCAAGAGTATGAAACTGAGAACTCAAATACCT
GCAGAGATGCTAGCCAACATAGATTTGAAATATTTCAATGATTCAACTAAAAAGAAAATTGAAAAAATCC
GACCATTATTAATAGATGGAACTGCATCATTGAGTCCTGGAATGATGATGGGCATGTTCAATATGTTGAG
CACCGTCTTGGGCGTCTCCATTCTGAATCTTGGGCAAAAGAGATACACCAAGACTACTTACTGGTGGGAT
GGTCTTCAATCGTCTGATGATTTTGCTTTGATTGTGAATGCACCCAACTATGCAGGAATTCAAGCTGGAG
TTGACAGGTTTTATCGAACCTGTAAGCTGCTCGGAATTAATATGAGCAAAAAGAAGTCTTACATAAACAG
AACAGGTACCTTTGAATTCACGAGCTTTTTCTATCGTTATGGGTTTGTTGCCAATTTCAGCATGGAGCTT
CCTAGTTTTGGGGTGTCTGGGGTCAATGAATCTGCAGACATGAGTATTGGAGTCACTGTCATCAAAAACA
ATATGATAAACAATGACCTTGGCCCAGCAACTGCTCAAATGGCCCTTCAGTTATTTATAAAAGATTACAG
GTACACTTATCGATGCCACAGAGGTGACACACAAATACAAACCCGGAGATCATTTGAGATAAAGAAACTA

TGGGACCAAACCCGCTCCAAAGCTGGGCTGTTGGTCTCTGATGGAGGCCCCAATTTATATAACATTAGAA
ATCTCCATATTCCTGAAGTTTGCTTGAAATGGGAGTTGATGGATGAGGATTACCAAGGGCGTTTATGCAA
CCCATTAAACCCGTTTGTCAGCCATAAAGAGATTGAATCAGTGAACAATGCAGTGATAATGCCGGCACAT
GGCCCAGCCAAAAATATGGAGTATGACGCTGTTGCAACAACACACTCCTGGGTCCCCAAAAGAAATCGAT
CCATTTTGAACACGAGCCAAAGGGGGATACTTGAAGATGAGCAAATGTATCAGAGGTGCTGCAATTTATT
TGAAAAATTCTTCCCAAGTAGCTCATACAGAAGACCAGTTGGAATATCCAGTATGGTAGAGGCTATGGTT
TCAAGAGCCCGAATTGATGCACGGATTGATTTCGAATCTGGAAGGATAAAGAGGGAGGAATTTGCTGAGA
TCATGAAGATCTGTTCCACCATTGAAGACCTCAGACGGCAAAAATAGGGAATTTGGCTTGTCCTTCATGA
AAA
>gi|157281290|gb|CY025227.1| Influenza A virus (A/Michigan/UR06-0015/2006(H1N1)) segment 2, complete sequence
TTGAATGGATGTCAATCCGACATTACTTTTCTTAAAAGTGCCAGCACAAAATGCTATAAGCACAACTTTT
CCTTATACTGGTGACCCTCCCTACAGCCATGGGACAGGAACAGGGTACACCATGGATACAGTCAACAGGA
CACACCAGTACTCAGAAAGAGGAAGATGGACAAAAAATACCGAAACTGGAGCACCGCAACTCAACCCAAT
TGATGGGCCACTACCAGAAGGCAATGAACCGAGTGGCTATGCCCAAACAGATTGTGTATTAGAAGCAATG
GCTTTCCTTGAAGAATCCCATCCTGGTATCTTTGAAAACTCTTGTATTGAGACAATGGAGGTTGTTCAGC
AAACAAGGGTGGACAAACTGACACAGGGCAGACAGACCTATGACTGGACTCTAAATAGGAACCAGCCTGC
TGCCACAGCATTGGCAAACACTATAGAAGTATTCAGATCAAACGGCCTCATAGCAAATGAATCTGGGAGG
CTAATAGACTTCCTTAAAGATGTAATGGAGTCGATGGACATAGACGAAGTAGAGGTCACAACTCATTTTC
AAAGAAAGAGGAGAGTGAGAGACAATGTAACTAAAAAAATGGTGACCCAAAGAACAATAGGCAAAAAGA
A
ACATAAATTAGACAAAAGAAGTTACCTAATTAGGGCATTAACCCTGAACACAATGACCAAAGATGCTGAG
AGAGGGAAACTAAAACGCAGAGCAATTGCAACCCCAGGAATGCAAATAAGGGGGTTTGTATACTTTGTTG
AGACACTGGCAAGAAGCATATGTGAAAAGCTTGAACAATCAGGATTGCCAGTTGGAGGAAATGAGAAGAA
AGCAAAGTTAGCAAATGTTGTAAGGAAGATGATGACCAACTCCCAGGACACTGAAATTTCTTTCACCATC
ACTGGAGATAACACAAAATGGAACGAAAATCAAAACCCTAGAATGTTCTTGGCCATGATCACATATATAA
CCAAAAATCAGCCTGAATGGTTCAGAAATATTCTAAGTATTGCCCCAATAATGTTTTCAAACAAGATGGC
GAGACTAGGTAAGGGGTACATGTTTGAAAGCAAGAGTATGAAACTGAGAACTCAAATACCTGCAGAGATG
CTAGCCAACATAGATTTGAAATATTTCAATGATTCAACTAAAAAGAAAATTGAAAAAATCCGACCATTAT
TAATAGATGGAACTGCATCATTGAGTCCTGGAATGATGATGGGCATGTTCAATATGTTGAGCACCGTCTT
GGGCGTCTCCATTCTGAATCTTGGGCAAAAGAGATACACCAAGACTACTTACTGGTGGGATGGTCTTCAA
TCGTCTGATGATTTTGCTTTGATTGTGAATGCACCCAACTATGCAGGAATTCAAGCTGGAGTTGACAGGT
TTTATCGAACCTGTAAGCTGCTCGGAATTAATATGAGCAAAAAGAAGTCTTACATAAACAGAACAGGTAC
CTTTGAATTCACGAGCTTTTTCTATCGTTATGGGTTTGTTGCCAATTTCAGCATGGAGCTTCCTAGTTTT
GGGGTGTCTGGGGTCAATGAATCTGCAGACATGAGTATTGGAGTCACTGTCATCAAAAACAATATGATAA
ACAATGACCTTGGCCCAGCAACTGCTCAAATGGCCCTTCAGTTATTTATAAAAGATTACAGGTACACTTA
TCGATGCCACAGAGGTGACACACAAATACAAACCCGGAGATCATTTGAGATAAAGAAACTATGGGACCAA
ACCCGCTCTAAAGCTGGGCTGTTGGTCTCTGATGGAGGCCCCAATTTATATAACATTAGAAATCTCCATA
TTCCTGAAGTTTGCTTGAAATGGGAGTTGATGGATGAGGATTATCAGGGGCGTTTATGCAACCCATTAAA
CCCGTTTGTCAGCCATAAAGAGATTGAATCAGTAAACAATGCAGTGATAATGCCGGCACATGGTCCAGCC
AAAAATATGGAGTATGACGCTGTTGCAACAACACACTCCTGGGTCCCCAAAAGAAATCGATCCATTTTGA
ACACGAGCCAACGGGGGATACTTGAAGATGAGCAAATGTATCAGAGGTGCTGCAATTTATTTGAAAAATT
CTTCCCAAGTAGCTCATACAGAAGACCAGTTGGAATATCCAGTATGGTAGAGGCTATGGTTTCAAGAGCC
CGAATTGATGCACGGATTGATTTCGAATCTGGAAGGATAAAGAAAGAGGAATTTGCTGAGATCATGAAGA

TCTGTTCCACCATTGAAGACCTCAGACGGCAAAAATAGGGAATTTGGCTTGTCCTTCATGAAAA

>gi|218875182|gb|CY036925.1| Influenza A virus (A/NYMC X-163A(NYMC X-157-St. Petersburg/8/2006)(H1N1)) segment 2, complete sequence

ATGGATGTCAATCCGACCTTACTTTTCTTAAAAGTGCCAGCACAAAATGCTATAAGCACAACTTTCCCTT
ATACTGGAGACCCTCCTTACAGCCATGGGACAGGAACAGGATACACCATGGATACTGTCAACAGGACACA
TCAGTACTCAGAAAAGGGAAGATGGACAACAAACACCGAAACTGGAGCACCGCAACTCAACCCGATTGAT
GGGCCACTTCCAGAAGACAATGAACCAAGTGGTTATGCCCAAACAGATTGTGTATTGGAGGCGATGGCTT
TCCTTGAGGAATCCCATCCTGGTATTTTTGAAAACTCGTGTATTGAAACGATGGAGGTTGTTCAGCAAAC
ACGAGTAGACAAGCTGACACAAGGCCGACAGACCTATGACTGGACTCTAAATAGAAACCAACCTGCTGCA
ACAGCATTGGCCAACACAATAGAAGTGTTCAGATCAAATGGCCTCACGGCCAATGAGTCTGGAAGGCTCA
TAGACTTCCTTAAGGATGTAATGGAGTCAATGAACAAAGAAGAAATGGGGATCACAACTCATTTTCAGAG
AAAGAGACGGGTGAGAGACAATATGACTAAGAAAATGATAACACAGAGAACAATGGGTAAAAAGAAGCAG
AGATTGAACAAAAGGAGTTATCTAATTAGAGCATTGACCCTGAACACAATGACCAAAGATGCTGAGAGAG
GGAAGCTAAAACGGAGAGCAATTGCAACCCCAGGGATGCAAATAAGGGGGTTTGTATACTTTGTTGAGAC
ACTGGCAAGGAGTATATGTGAGAAACTTGAACAATCAGGGTTGCCAGTTGGAGGCAATGAGAAGAAAGCA
AAGTTGGCAAATGTTGTAAGGAAGATGATGACCAATTCTCAGGACACCGAACTTTCTTTCACCATCACTG
GAGATAACACCAAATGGAACGAAAATCAGAATCCTCGGATGTTTTTGGCCATGATCACATATATGACCAG
AAATCAGCCCGAATGGTTCAGAAATGTTCTAAGTATTGCTCCAATAATGTTCTCAAACAAAATGGCGAGA
CTGGGAAAAGGGTATATGTTTGAGAGCAAGAGTATGAAACTTAGAACTCAAATACCTGCAGAAATGCTAG
CAAGCATCGATTTGAAATATTTCAATGATTCAACAAGAAAGAAGATTGAAAAAATCCGACCGCTCTTAAT
AGAGGGGACTGCATCATTGAGCCCTGGAATGATGATGGGCATGTTCAATATGTTAAGCACTGTATTAGGC
GTCTCCATCCTGAATCTTGGACAAAAGAGATACACCAAGACTACTTACTGGTGGGATGGTCTTCAATCCT
CTGACGATTTTGCTCTGATTGTGAATGCACCCAATCATGAAGGGATTCAAGCCGGAGTCGACAGGTTTTA
TCGAACCTGTAAGCTACTTGGAATCAATATGAGCAAGAAAAAGTCTTACATAAACAGAACAGGTACATTT
GAATTCACAAGTTTTTTCTATCGTTATGGGTTTGTTGCCAATTTCAGCATGGAGCTTCCCAGTTTTGGGG
TGTCTGGGATCAACGAGTCAGCGGACATGAGTATTGGAGTTACTGTCATCAAAAACAATATGATAAACAA
TGATCTTGGTCCAGCAACAGCTCAAATGGCCCTTCAGTTGTTCATCAAAGATTACAGGTACACGTACCGA
TGCCATAGAGGTGACACACAAATACAAACCCGAAGATCATTTGAAATAAAGAAACTGTGGGAGCAAACCC
GTTCCAAAGCTGGACTGCTGGTCTCCGACGGAGGCCCAAATTTATACAACATTAGAAATCTCCACATTCC
TGAAGTCTGCCTAAAATGGGAATTGATGGATGAGGATTACCAGGGGCGTTTATGCAACCCACTGAACCCA
TTTGTCAGCCATAAAGAAATTGAATCAATGAACAATGCAGTGATGATGCCAGCACATGGTCCAGCCAAAA
ACATGGAGTATGATGCTGTTGCAACAACACACTCCTGGATCCCCAAAAGAAATCGATCCATCTTGAATAC
AAGTCAAAGAGGAGTACTTGAGGATGAACAAATGTACCAAAGGTGCTGCAATTTATTTGAAAAATTCTTC
CCCAGCAGTTCATACAGAAGACCAGTCGGGATATCCAGTATGGTGGAGGCTATGGTTTCCAGAGCCCGAA
TTGATGCACGGATTGATTTCGAATCTGGAAGGATAAAGAAAGAAGAGTTCACTGAGATCATGAAGATCTG
TTCCACCATTGAAGAGCTCAGACGGCAAAAATAGTGAATTTAGCTTGTCCTTCATGAAAA

>gi|208344097|gb|CY035132.1| Influenza A virus (A/St. Petersburg/8/2006(H1N1)) segment 2, complete sequence

TTTGAATGGATGTCAATCCGACATTACTTTTCTTAAAAGTGCCAGCACAAAATGCTATAAGCACAACTTT
TCCTTATACTGGTGACCCTCCTTACAGCCATGGGACAGGAACAGGGTACACCATGGATACAGTCAACAGG
ACACATCAGTACTCAGAAAGAGGAAGATGGACAAAAAATACCGAAACTGGAGCACCGCAACTCAACCCAA
TTGATGGGCCACTACCAGAAGACAATGAACCAAGTGGCTATGCCCAAACAGATTGTGTATTAGAAGCAAT
GGCTTTCCTTGAAGAATCCCATCCTGGTATTTTTGAAAACTCTTGTATTGAAACAATGGAGGTTGTTCAG
CAAACAAGGGTGGACAAACTGACACAGGGCAGACAGACCTATGACTGGACTCTAAATAGGAACCAGCCTG

CTGCCACAGCATTGGCCAACACTATAGAAGTATTCAGATCAAACGGCCTCATAGCAAATGAATCTGGGAG
GCTAATAGACTTCCTTAAAGATGTAATGGAGTCGATGGACAGAGAAGAAGTAGAGGTCACAACTCATTTT
CAGAGAAAGAGGAGAGTGAGAGACAATGTAACTAAAAAAATGGTGACCCAAAGAACAATAGGCAAAAAG
A
AACATAAATTAGACAAAAGGAGTTACCTAATTAGAGCATTAACCCTGAACACAATGACCAAAGATGCTGA
GAGGGGGAAACTAAAACGCAGAGCAATTGCAACCCCAGGAATGCAAATAAGGGGATTTGTATACTTTGTT
GAAACACTGGCAAGAAGCATATGTGAAAAGCTTGAACAATCAGGGTTGCCAGTTGGAGGAAATGAGAAGA
AAGCAAAGTTAGCAAATGTTGTAAGGAAGATGATGATCAACTCCCAGGACACTGAAATTTCTTTCACCAT
CACTGGAGATAACACAAAATGGAACGAAAATCAAAACCCTAGAATGTTTTTGGCCATGATCACATATATA
ACCAGAAATCAGCCTGAATGGTTCAGAAATATTCTAAGTGTTGCTCCAATAATGTTTTCAAACAAAATGG
CGAGACTAGGTAAGGGGTACATGTTTGAAAGCAAGAGTATGAAACTGAGAACTCAAATACCTGCAGAGAT
ACTAGCCGACATAGATTTGAAGTATTTCAATGAGTCAACTAAAAAGAAGATTGAAAAAATCCGACCATTA
TTAATAGATGGAACTGCATCATTGAGTCCTGGAATGATGATGGGCATGTTCAATATGTTAAGCACTGTCT
TGGGCGTCTCCATTCTGAATCTTGGGCAAAAGAGATACACCAAGACTACTTACTGGTGGGATGGTCTTCA
ATCGTCTGATGATTTTGCTCTGATAGTGAATGCGCCCAACTATGCAGGAATTCAAGCTGGAGTTGACAGG
TTTTATCGAACCTGTAAGCTGCTCGGAATTAATATGAGCAAAAAGAAGTCTTACATAAACAGGACAGGTA
CCTTTGAATTCACAAGCTTTTTCTATCGTTATGGGTTTGTTGCCAATTTCAGCATGGAGCTTCCTAGTTT
TGGGGTGTCTGGGGTCAATGAATCTGCAGACATGAGTATTGGAGTCACTGTTATCAGAAACAATATGATA
AACAATGACCTTGGCCCAGCAACTGCTCAAATGGCCCTTCAGTTATTTATAAAAGATTACAGATACACGT
ATCGATGCCACAGAGGTGACACACAAATACAAACCCGGAGATCATTTGAGATAAAGAAATTATGGACCA
AACCCGCTCCAAAGCTGGGCTGTTGGTTTCTGATGGAGGCCCCAATTTATATAACATTAGAAATCTCCAT
ATTCCTGAAGTCTGCTTGAAATGGGAGTTGATGGATGAGGATTACCAGGGGCGTTTATGCAACCCATTGA
ACCCATTTGTCAGTCATAAAGAGATTGAATCAGTGAACAATGCAGTGATGATGCCGGCACATGGTCCAGC
CAAAAATATGGAGTATGACGCTGTTGCAACAACACATTCCTGGGTTCCCAAAAGGAATCGATCCATTTTG
AACACGAGCCAAAGGGGGATACTTGAGGATGAGCAAATGTATCAGAGGTGCTGCAATTTATTTGAAAAAT
TCTTCCCAAGTAGCTCATACAGAAGACCAGTTGGGATATCCAGTATGGTAGAGGCTATGGTTTCCAGAGC
CCGAATTGATGCACGGATTGATTTCGAATCTGGAAGGATAAAGAAAGAGGAATTCGCTGAGATCATGAAG
ACCTGTTCCACCATTGAAGACCTCAGACGGCAAAAATAGGGAGTTTGGCTTGTCCTTCATGAAA
>gi|226954760|gb|CY038885.1| Influenza A virus (A/Taiwan/2645/2006(H1N1)) segment 2, complete sequence
GATGGATGTCAATCCGACATTACTTTTCTTAAAAGTGCCAGCACAAAATGCTATAAGCACAACTTTTCCT
TATACTGGTGACCCTCCTTACAGCCATGGGACAGGAACAGGGTACACAATGGATACAGTCAACAGGACAC
ATCAGTACTCAGAAAGAGGAAGATGGACAAAGAATACCGAAACTGGAGCACCGCAACTCAACCCAATTGA
TGGGCCACTACCAGAAGACAATGAACCAAGTGGCTATGCCCAAACAGATTGTGTATTAGAAGCAATGGCT
TTCCTTGAAGAATCCCATCCTGGCATTTTTGAAAACTCTTGTATTGAAACAATGGAGGTTGTTCAGCAAA
CAAGGGTGGACAAACTGACACAAGGCAGACAGACCTATGACTGGACTCTAAATAGGAACCAGCCTGCTGC
CACAGCATTGGCCAACACTATAGAAGTATTCAGATCAAACGGCCTCATAGCAAATGAATCAGGGAGGCTA
ATAGACTTCCTTAAAGATGTAATGGAGTCGATGGACAGAGACGAAGTAGAGGTCACAACTCATTTTCAGA
GAAAGAGGAGAGTGAGAGACAATGTAACTAAAAAAATGGTGACCCAAAGAACAATAGGCAAAAAGAAAC
A
TAAATTAGACAAAAGGAGTTACCTAATTAGAGCATTAACCCTGAACACAATGACCAAAGATGCTGAGAGG
GGGAAACTAAAACGCAGAGCAATTGCAACCCCAGGAATGCAAATAAGGGGGTTTGTATACTTTGTTGAGA
CACTGGCAAGAAGCATATGTGAAAAGCTTGAACAATCAGGGTTGCCCGTTGGAGGGAACGAGAAGAAAGC
AAAGTTAGCAAATGTTGTAAGGAAGATGATGACCAACTCCCAGGACACTGAAATTTCTTTCACCATCACT

GGAGATAACACAAAATGGAACGAAAATCAAAACCCTAGAATGTTTTTGGCCATGATCACATATATAACCA
GAAATCAGCCCGAATGGTTCAGAAATATTCTAAGTATTGCTCCAATAATGTTTTCAAACAAAATGGCGAG
ACTAGGTAAGGGGTACATGTTTGAAAGCAAGAGTATGAAACTGAGAACTCAAATACCTGCAGAGATACTA
GCCGACATAGATTTGAAATATTTCAATGAGTCAACTAAAAAGAAAATTGAAAAAATCCGACCATTATTAA
TAGATGGAACTGCATCATTGAGTCCTGGAATGATGATGGGAATGTTCAATATGTTAAGCACTGTCTTGGG
CGTCTCCATTCTGAATCTTGGGCAAAAGAGATACACCAAGACTACTTACTGGTGGGATGGTCTTCAATCG
TCTGATGATTTTGCTCTGATAGTGAATGCGCCCAACTATGCAGGAATTCAAGCAGGAGTTGACAGGTTTT
ATCGAACCTGTAAGCTGCTCGGAATTAATATGAGCAAAAAGAAGTCTTACATAAATAGGACAGGTACCTT
TGAATTCACGAGCTTTTTCTATCGTTATGGGTTTGTTGCCAATTTCAGCATGGAGCTTCCTAGTTTTGGG
GTGTCTGGAGTCAATGAATCTGCAGACATGAGTATTGGAGTCACTGTTATCAAAAACAATATGATAAACA
ATGACCTTGGCCCAGCAACTGCTCAAATGGCCCTTCAGTTATTTATAAAAGATTACAGATACACGTATCG
ATGCCACAGAGGTGACACACAAATACAAACCCGGAGATCATTTGAGATAAAGAAATTATGGGACCAAACC
CGCTCCAAAGCTGGGCTGTTGGTCTCTGATGGAGGGCCCAATCTATATAACATTAGAAATCTCCATATTC
CTGAAGTCTGCTTGAAATGGGAGTTGATGGATGAGGATTACCAGGGGCGTTTATGCAACCCATTGAACCC
GTTTGTCAGTCATAAAGAGATTGAATCAGTGAACAATGCAGTGATGATGCCAGCACATGGTCCAGCCAAA
AATATGGAGTATGACGCTGTTGCAACAACACATTCCTGGGTTCCCAAAAGGAATCGATCCATTTTGAACA
CGAGCCAAAGGGGAATACTTGAGGATGAGCAAATGTATCAGAGGTGCTGCAATTTATTTGAAAAATTCTT
CCCAAGTAGCTCATACAGAAGACCAGTTGGGATATCCAGTATGGTAGAGGCTATGATTTCCAGAGCCCGA
ATTGATGCACGGATTGATTTCGAATCTGGAAGGATAAAGAAAGAGGAATTCGCTGAGATCATGAAGACCT
GTTCCACCATTGAAGACCTCAGACGGCAAAAATAGGGAATTTGGCTTGTCCTTCATGAAAA
>gi|256385525|gb|CY044355.1| Influenza A virus (A/South Korea/AF10/2008(H1N1)) segment 2, complete sequence
ATGGATGTCAATCCGACATTACTTTTCTTAAAAGTGCCAGCACAAAATGCTATAAGCACAACTTTTCCCT
ATACTGGTGACCCTCCTTACAGCCATGGGACAGGAACAGGGTACACCATGGATACAGTCAACAGGACACA
TCAGTACTCAGAAAGAGGAAGATGGACAAAAAATACCGAAACTGGAGCACCGCAACTCAACCCAATTGAT
GGGCCACTACCAGAAGACAATGAACCAAGTGGCTATGCCCAAACAGATTGTGTATTAGAAGCAATGGCTT
TCCTTGAAGAATCCCATCCTGGTATTTTTGAAAACTCTTGTATTGAAACAATGGAGATTGTTCAGCAAAC
AAGGGTGGACAAACTGACACAAGGCAGACAGACCTATGACTGGACTCTAAATAGGAACCAGCCTGCTGCC
ACAGCATTGGCCAACACTATAGAAGTATTCAGATCAAACGGCCTCATAGCAAATGAATCTGGGAGGCTAA
TAGACTTCCTTAAAGATGTAATGGAGTCGATGGACAGAGAAGAAGTAGAGGTCACAACTCATTTTCAGAG
AAAGAGGAGAGTGAGAGACAATGTAACTAAAAAAATGGTGACCCAAAGAACAATAGGCAAAAAGAAACAT
AAATTAGACAAAAGGAGTTACCTAATTAGAGCATTAACCCTGAACACAATGACCAAAGATGCTGAGAGGG
GGAAACTAAAACGCAGAGCAATTGCAACCCCAGGAATGCAAATAAGGGGGTTTGTATACTTTGTTGAAAC
ACTGGCAAGAAGCATATGTGAAAAGCTTGAACAATCAGGGTTGCCAGTTGGGGGAAATGAGAAGAAAGCA
AAGTTAGCAAATGTTGTAAGGAAGATGATGACCAACTCCCAGGACACTGAAATTTCTTTCACCATCACTG
GAGATAACACAAAATGGAACGAAAATCAAAACCCTAGAATGTTTTTGGCCATGATCACATATATAACCAG
AAATCAGCCTGAATGGTTCAGAAATATTCTAAGTGTTGCTCCAATAATGTTTTCAAACAAAATGGCGAGA
CTAGGTAAGGGGTACATGTTTGAAAGCAAGAGTATGAAACTGAGAACTCAAATACCTGCAGAGATACTAG
CCGACATAGATTTGAAGTATTTCAATGAGTCAACTAAAAAGAAAATTGAAAAAATCCGACCATTATTAAT
AGATGGAACTGCATCATTGAGTCCTGGAATGATGATGGGCATGTTCAATATGCTAAGCACTGTCTTGGGC
GTCTCCATTCTGAATCTTGGGCAAAAGAGATACACCAAGACTACTTACTGGTGGGATGGTCTCCAATCGT
CTGATGATTTTGCTCTGATAGTGAATGCGCCCAACTATGCAGGAATTCAAGCTGGAGTTGACAGGTTTTA
TCGAACCTGTAAGCTGCTCGGAATTAATATGAGCAAAAAGAAGTCTTACATAAACAGGACGGGTACCTTT
GAATTCACAAGTTTTTTCTATCGTTATGGGTTTGTTGCCAATTTCAGCATGGAGCTTCCTAGTTTTGGAG

TGTCAGGGGTCAATGAATCTGCAGACATGAGTATTGGAGTCACTGTTATCAGAAACAATATGATAAACAA
TGACCTTGGCCCAGCAACTGCTCAAATGGCCCTTCAGTTATTTATAAAAGATTACAGATACACGTATCGA
TGCCACAGAGGTGACACACAAATACAAACCCGGAGATCATTTGAGATAAAGAAATTATGGGACCAAACCC
GCTCCAAAGCTGGGCTGTTGGTTTCTGATGGAGGCCCCAATTTATATAACATTAGAAATCTCCATATTCC
TGAAGTCTGCTTGAAATGGGAGTTGATGGATGAGGATTACCAGGGGCGTTTATGCAACCCATTGAACCCA
TTTGTCAGTCATAAAGAGATTGAATCAGTGAACAATGCAGTGATGATGCCGGCACATGGTCCAGCCAAAA
ATATGGAGTATGACGCTGTTGCAACAACACATTCCTGGGTTCCCAAAAGGAATCGATCCATTTTAAACAC
GAGCCAAAGGGGGATACTTGAGGATGAGCAAATGTATCAGAGGTGCTGCAATTTATTTGAAAAATTCTTT
CCAAGTAGCTCATACAGACGACCAGTTGGGATATCCAGTATGGTAGAGGCTATGGTTTCCAGAGCCCGAA
TTGATGCACGGATTGATTTCGAATCTGGAAGGATAAAGAAAGAGGAATTCGCTGAGATCATGAAGACCTG
TTCCACCATTGAAGACCTCAGACGGCAAAAATAGGGAGTTTGG
>gi|163964730|gb|CY028465.1| Influenza A virus (A/California/UR06-0442/2007(H1N1)) segment 2, complete sequence
AATGGATGTCAATCCGACATTACTTTTCTTAAAAGTGCCAGCACAAAATGCTATAAGCACAACTTTTCCT
TATACTGGTGACCCTCCTTACAGCCATGGGACAGGAACAGGGTACACCATGGATACAGTCAACAGGACAC
ACCAGTACTCAGAAAGAGGAAGATGGACAAAAAATACCGAAACTGGAGCACCGCAACTCAACCCAATTGA
TGGGCCACTACCAGAAGACAATGAACCGAGTGGCTATGCCCAAACAGATTGTGTATTAGAAGCAATGGCT
TTCCTTGAAGAATCCCATCCTGGTATCTTTGAAAACTCTTGTATTGAGACAATGGAGGTTGTTCAGCAAA
CAAGGGTGGACAAACTGACACAGGGCAGACAGACCTATGACTGGACTCTAAATAGGAACCAGCCTGCTGC
CACAGCATTGGCAAACACTATAGAAGTATTCAGATCAAACGGCCTCATAGCAAATGAATCTGGGAGGCTA
ATAGACTTCCTTAAAGATGTAATGGAGTCGATGGACATAGACGAAGTAGAGGTCACAACTCATTTTCAAA
GGAAGAGGAGAGTGAGAGACAGTGTAACTAAAAAAATGGTGACCCAAAGAACAATAGGCAAAAAGAAAC
A
TAAATTAGACAAAAGAAGTTACCTAATTAGGGCATTAACCCTGAACACAATGACCAAAGATGCTGAGAGA
GGGAAACTAAAACGCAGAGCAATTGCAACCCCAGGAATGCAAATAAGGGGGTTTGTATACTTTGTTGAGA
CACTGGCAAGAAGCATATGTGAAAAGCTTGAACAATCAGGATTGCCAGTTGGAGGAAATGAGAAGAAAGC
AAAGTTAGCAAATGTTGTAAGGAAGATGATGACCAACTCCCAGGACACTGAAATTTCTTTCACCATCACT
GGAGATAACACAAAATGGAACGAAAATCAAAACCCTAGAATGTTCTTGGCCATGATCACATATATAACCA
AAAATCAGCCTGAATGGTTCAGAAATATTCTAAGTATTGCCCCAATAATGTTTTCAAACAAGATGGCGAG
ACTAGGTAAGGGGTACATGTTTGAAAGCAAGAGTATGAAACTGAGAACTCAAATACCTGCAGAGATGCTA
GCCAACATAGATTTGAAATATTTCAATGATTCAACTAAAAAGAAAATTGAAAAAATCCGACCATTATTAA
TAGATGGAACTGCATCATTGAGTCCTGGAATGATGATGGGCATGTTCAATATGTTGAGCACCGTCTTGGG
CGTCTCCATTCTGAATCTTGGGCAAAAGAGATACACCAAGACTACTTACTGGTGGGATGGTCTTCAATCG
TCTGATGATTTTGCTTTGATTGTGAATGCACCCAACTATGCAGGAATTCAAGCTGGAGTTGACAGGTTTT
ATCGAACCTGTAAGCTGCTCGGAATTAATATGAGCAAAAAGAAGTCTTACATAAACAGAACAGGTACCTT
TGAATTCACGAGCTTTTTCTATCGTTATGGGTTTGTTGCCAATTTCAGCATGGAGCTTCCTAGTTTTGGG
GTGTCTGGGGTCAATGAATCTGCAGACATGAGTATTGGAGTCACTGTCATCAAAAACAATATGATAAACA
ATGACCTTGGCCCAGCAACTGCTCAAATGGCCCTTCAGTTATTTATAAAAGATTACAGGTACACTTATCG
ATGCCACAGAGGTGACACACAAATACAAACCCGGAGATCATTTGAGATAAAGAAACTATGGGACCAAACC
CGCTCTAAAGCTGGGCTGTTGGTCTCTGATGGAGGCCCCAATTTATATAACATTAGAAATCTCCATATTC
CTGAAGTTTGCTTGAAATGGGAGTTGATGGATGAGGATTATCAGGGGCGTTTATGCAACCCATTAAACCC
GTTTGTCAGCCATAAAGAGATTGAATCAGTAAACAATGCAGTGATAATGCCGGCACATGGTCCAGCCAAA
AATATGGAGTATGACGCTGTTGCAACAACACACTCCTGGGTCCCCAAAAGAAATCGATCCATTTTGAACA
CGAGCCAACGGGGGATACTTGAAGATGAGCAAATGTATCAGAGGTGCTGCAATTTATTTGAAAAATTCTT

CCCAAGTAGCTCATACAGAAGACCAGTTGGAATATCCAGTATGGTAGAGGCTATGGTTTCAAGAGCCCGA
ATTGATGCACGGATTGATTTCGAATCTGGAAGGATAAAGAAAGAGGAATTTGCTGAGATCATGAAGATCT
GTTCCACCATTGAAGACCTCAGACGGCAAAAATAGGGAATTTGGCTTGTCCTTCATGAAAA
>gi|157281614|gb|CY025363.1| Influenza A virus (A/Kentucky/UR06-0363/2007(H1N1)) segment 2, complete sequence
CATTTGAATGGATGTCAATCCGACATTACTTTTCTTAAAAGTGCCAGCACAAAATGCTATAAGCACAACT
TTTCCTTATACTGGTGACCCTCCTTACAGCCATGGGACAGGAACAGGGTACACCATGGATACAGTCAACA
GGACACACCAGTACTCAGAAAGAGGAAGATGGACAAAAAATACCGAAACTGGAGCACCGCAACTCAACCC
AATTGATGGGCCACTACCAGAAGACAATGAACCAAGTGGCTATGCCCAAACAGATTGTGTATTAGAAGCA
ATGGCTTTCCTTGAAGAATCCCATCCTGGTATCTTTGAAAACTCTTGTATTGAGACAATGGAGGTTGTTC
AGCAAACAAGGGTGGACAAACTGACACAAGGCAGACAGACCTATGACTGGACTCTAAATAGGAACCAGCC
TGCTGCCACAGCATTGGCAAACACTATAGAAGTATTCAGATCAAACGGCCTCATAGCAAATGAATCTGGG
AGGCTAATAGACTTCCTTAAAGATGTAATGGAGTCGATGGACAGAGACGAAGTAGAGGTCACAACTCATT
TTCAAAGAAAGAGGAGAGTGAGAGACAATGTAACTAAAAAAATGGTGACCCAAAGAACAATAGGCAAAAA
GAAACATAAATTAGACAAAAGAAGTTACCTAATTAGGGCATTAACCCTGAACACAATGACCAAAGATGCT
GAGAGGGGGAAACTAAAACGCAGAGCAATTGCAACCCCAGGAATGCAAATAAGGGGGTTTGTATACTTTG
TTGAGACACTGGCAAGAAGCATATGTGAAAAGCTTGAACAATCAGGATTGCCAGTTGGAGGAAATGAGAA
GAAAGCAAAGTTAGCAAATGTTGTAAGGAAGATGATGACCAACTCCCAGGACACTGAAATTTCTTTCACC
ATCACTGGAGATAACACAAAATGGAACGAAAATCAAAATCCTAGAATGTTCTTGGCCATGATCACATATA
TAACCAAAAATCAGCCTGAATGGTTCAGAAATATTCTAAGTATTGCCCCAATAATGTTTTCAAACAAGAT
GGCGAGACTAGGTAAGGGGTACATGTTTGAAAGCAAGAGTATGAAACTGAGAACTCAAATACCTGCAGAG
ATGCTAGCCAACATAGATTTGAAATATTTCAATGATTCAACTAAAAAGAAAATTGAAAAAATCCGACCAT
TATTAATAGATGGAACTGCATCATTGAGTCCTGGAATGATGATGGGCATGTTCAATATGTTGAGCACCGT
CTTGGGCGTCTCCATTCTGAATCTTGGGCAAAAGAGATACACCAAGACTACTTACTGGTGGGATGGTCTT
CAATCGTCTGATGATTTTGCTTTGATTGTGAATGCACCCAACTATGCAGGAATTCAAGCTGGAGTTGACA
GGTTTTATCGAACCTGTAAGCTGCTCGGAATTAATATGAGCAAAAAGAAGTCTTACATAAACAGAACAGG
TACCTTTGAATTCACGAGCTTTTTCTATCGTTATGGGTTTGTTGCCAATTTCAGCATGGAGCTTCCTAGT
TTTGGGGTGTCTGGGGTCAATGAATCTGCAGACATGAGTATTGGAGTCACTGTCATCAAAAACAATATGA
TAAACAATGACCTTGGCCCAGCAACAGCTCAAATGGCCCTTCAGTTATTTATAAAAGATTACAGGTACAC
TTATCGATGCCACAGAGGTGACACACAAATACAAACCCGGAGATCATTTGAGATAAAGAAACTATGGGAC
CAAACCCGCTCCAAAGCTGGGCTGTTGGTCTCTGATGGAGGCCCCAATTTATATAACATTAGAAATCTCC
ATATTCCTGAAGTTTGCTTGAAATGGGAGTTGATGGATGAGGATTACCAGGGGCGTTTATGCAACCCATT
AAACCCGTTTGTCAGCCATAAAGAGATTGAGTCAGTGAACAATGCAGTGATAATGCCGGCACATGGTCCA
GCCAAAAATATGGAGTATGACGCTGTTGCAACAACACACTCCTGGGTCCCCAAAAGAAATCGATCCATTT
TGAACACGAGCCAAAGGGGGATACTTGAAGATGAGCAAATGTATCAGAGATGCTGTAATTTATTTGAAAA
ATTCTTCCCAAGTAGCTCATACAGAAGACCAGTTGGAATATCCAGTATGGTAGAGGCTATGGTTTCAAGA
GCCCGAATTGATGCACGGATTGATTTCGAATCTGGAAGGATAAAGAAAGAGGAATTTGCTGAGATCATGA
AGATCTGTTCCACCATTGAAGACCTCAGACGGCAAAAATAGGGAATTTGGCTTGTCCTTCATGAAAA
>gi|158957803|gb|CY027417.1| Influenza A virus (A/Alabama/UR06-0536/2007(H1N1)) segment 2, complete sequence
ATGGATGTCAATCCGACATTACTTTTCTTAAAAGTGCCAGCACAAAATGCTATAAGCACAACTTTTCCTT
ATACTGGTGACCCTCCTTACAGCCATGGGACAGGAACAGGGTACACCATGGATACAGTCAACAGGACACA
CCAGTACTCAGAAAGAGGAAGATGGACTAAAAATACCGAAACTGGAGCACCGCAACTCAACCCAATTGAT
GGGCCACTACCAGAAGACAATGAACCAAGTGGCTATGCCCAAACAGATTGTGTATTAGAAGCAATGGCTT

TCCTTGAAGAATCCCATCCTGGTATCTTTGAAAACTCTTGTATTGAAACAATGGAGGTTGTTCAGCAAAC
AAGGGTGGACAAACTGACACAAGGCAGACAGACCTATGACTGGACTCTAAATAGGAACCAGCCTGCTGCA
ACAGCATTGGCAAACACTATAGAAGTATTCAGATCAAACGGCCTCATAGCAAATGAATCTGGAAGGCTAA
TAGACTTCCTTAAAGATGTAATGGAGTCGATGGACAGAGACGAAGTAGAGGTCACAACTCATTTTCAAAG
AAAGAGGAGAGTGAGAGACAATGTAACTAAAAAAATGGTGACTCAAAGAACAATAGGCAAAAAGAAACAT
AAATTAGACAAAAGAAGTTACCTAATTAGGGCATTAACCCTGAACACAATGACCAAAGATGCTGAGAGGG
GGAAACTAAAACGCAGAGCAATTGCAACCCCAGGAATGCAAATAAGAGGGTTTGTATACTTTGTTGAGAC
ACTGGCAAGAAGCATATGTGAAAAGCTTGAACAATCAGGTTTGCCAGTTGGAGGAAATGAGAAGAAAGCA
AAATTAGCAAATGTTGTAAGGAAGATGATGACCAACTCCCAGGACACTGAAATTTCCTTCACCATCACTG
GAGATAACACAAAATGGAACGAAAATCAAAACCCTAGAATGTTCTTGGCCATGATCACATATATAACCAA
AAATCAGCCTGAATGGTTCAGAAATATTCTAAGTATTGCTCCAATAATGTTTTCAAACAAGATGGCGAGA
CTAGGTAAGGGGTACATGTTTGAAAGCAAGAGTATGAAACTGAGAACTCAAATACCTGCAGAGATGCTAG
CCAACATAGATTTGAAATATTTCAATGATTCAACTAAAAAGAAAATTGAAAAAATCCGACCATTATTAAT
AGATGGAACTGCATCATTGAGTCCTGGAATGATGATGGGCATGTTCAATATGTTGAGCACCGTCTTGGGC
GTCTCCATTCTGAATCTTGGGCAAAAGAGATACACCAAGACTACTTACTGGTGGGATGGTCTTCAATCGT
CTGATGATTTTGCTTTGATTGTGAATGCACCCAACTATGCAGGAATTCAAGCTGGAGTTGACAGGTTTTA
TCGAACCTGTAAGCTGCTCGGAATTAATATGAGCAAAAAGAAGTCTTACATAAACAGAACAGGTACCTTT
GAATTCACGAGCTTTTTCTATCGTTATGGGTTTGTTGCCAATTTCAGCATGGAGCTTCCTAGTTTTGGGG
TGTCTGGGGTCAATGAATCTGCAGACATGAGTATTGGAGTCACTGTCATCAAAAACAATATGATAAACAA
TGACCTTGGCCCAGCAACTGCTCAAATGGCCCTTCAGTTATTTATAAAAGATTACAGGTACACTTATCGA
TGCCACAGAGGTGACACACAAATACAAACCCGGAGATCATTTGAGATAAAGAAACTATGGGACCAAACCC
GCTCCAAAGCTGGGCTGTTGGTCTCTGATGGAGGCCCCAATTTATATAACATTAGAAATCTCCATATTCC
TGAAGTTTGCTTGAAATGGGAGTTGATGGATGAGGATTACCAGGGGCGTTTATGCAACCCATTAAACCCG
TTTGTCAGCCATAAAGAGATTGAATCAGTGAACAATGCAGTGATAATGCCGGCACATGGCCCAGCCAAAA
ATATGGAGTATGACGCTGTTGCAACAACACACTCCTGGGTCCCCAAAAGAAATCGATCCATTTTGAACAC
GAGCCAAAGGGGGATACTTGAAGATGAGCAAATGTATCAGAGGTGCTGCAATTTATTTGAAAAATTCTTC
CCAAGTAGCTCATACAGAAGACCAGTTGGAATATCCAGTATGGTAGAGGCTATGGTTTCAAGAGCCCGAA
TTGATGCACGGATTGATTTCGAATCTGGAAGGATAAAGAGGGAGGAATTTGCTGAGATCATGAAGATCTG
TTCCACCATTGAAGACCTCAGACGGCAAAAATAGGGAATTTGGCTTGTCCTTCATGA
>gi|237688838|gb|CY040064.1| Influenza A virus (A/Taiwan/71720/2007(H1N1)) segment 2, complete sequence
GCAAACCATTTGAATGGATGTCAATCCGACATTACTTTTCTTAAAAGTGCCAGCACAAAATGCTATAAGC
ACAACTTTTCCTTATACTGGTGACCCTCCTTACAGCCATGGGACAGGAACAGGGTACACCATGGATACAG
TCAACAGGACACACCAGTACTCAGAAAGAGGAAGATGGACAAAAAATACCGAAACGGGAGCACCGCAACT
TAACCCAATTGATGGTCCACTACCGGAAGACAATGAACCAAGTGGCTATGCCCAAACAGATTGTGTATTA
GAAGCAATGGCTTTCCTTGAAGAATCCCATCCCGGTATCTTTGAAAACTCTTGTATTGAAACAATGGAGG
TTGTTCAGCAAACAAGGGTGGACAAACTGACACAAGGCAGACAGACCTATGACTGGACTCTAAATAGGAA
CCAGCCTGCTGCCACAGCATTGGCAAACACTATAGAAGTATTCAAATCAAACGGCCTCATAGCAAATGAA
TCTGGGAGGCTAATAGACTTCCTTAAAGATGTAATGGAGTCGATGGACAGAGGCGAAGTAGAGGTCACAA
CTCATTTTCAAAGAAAGAGGAGAGTGAGAGACAATGTAACTAAAAAAATGGTGACCCTAAGAACAATAGG
CAAAAAGAAACATAAATTAGACAAAAGAAGTTACCTAATTAGGGCATTAACCCTGAACACAATGACCAAA
GATGCTGAGAGGGGGAAACTAAAACGCAGAGCAATTGCAACCCCAGGAATGCAAATAAGGGGGTTTGTAT
ACTTTGTTGAGACACTGGCAAGAAGCATATGTGAAAAGCTTGAACAATCAGGATTGCCAGTTGGAGGAAA
TGAGAAGAAAGCAAAGTTAGCAAATGTTGTAAGGAAGATGATGACCAACTCCCAGGACACTGAAATTTCT

TTCACCATAACCGGAGATAACACAAAATGGAACGAAAATCAAAACCCTAGAATGTTCTTGGCCATGATCA
CATATATAACCAAAAATCAGCCTGAATGGTTCAGAAATATTCTAAGTATTGCTCCAATAATGTTTTCAAA
CAAGATGGCGAGACTAGGTAAGGGGTACATGTTTGAAAGCAAGAGTATGAAACTGAGAACTCAAATACCT
GCAGAGATGCTAGCCAACATAGATTTGAAATATTTCAATGATTCAACTAAAAAGAAAATTGAAAAAATCC
GACCATTATTAATAGATGGAACTGCATCATTGAGTCCTGGAATGATGATGGGCATGTTCAATATGTTGAG
CACCGTCTTGGGCGTCTCCATTCTGAATCTTGGGCAAAAGAGATACACCAAGACTACTTACTGGTGGGAT
GGTCTTCAATCGTCTGATGATTTTGCTTTGATTGTGAATGCACCCAACTATGCAGGAATTCAAGCTGGAG
TTGACAGGTTTTATCGAACCTGTAAGCTGCTCGGAATTAATATGAGCAAAAAGAAGTCTTACATAAACAG
AACAGGTACCTTTGAATTCACGAGCTTTTTCTATCGTTATGGGTTTGTTGCCAATTTCAGCATGGAGCTT
CCTAGTTTTGGGGTGTCTGGGGTCAATGAATCTGCAGACATGAGTATTGGAGTCACTGTCATCAAAAACA
ATATGATAAACAATGACCTTGGCCCAGCAACTGCTCAAATGGCCCTCCAGTTATTTATAAAAGATTACAG
GTACACTTATCGATGCCACCGAGGTGACACACAAATACAAACCCGGAGATCATTTGAGATAAAGAAACTA
TGGGACCAAACCCGCTCAAAAGCTGGGCTGTTGGTCTCTGATGGAGGCCCCAATTTATATAACATTAGAA
ATCTCCATATTCCTGAAGTTTGCTTGAAATGGGAGTTGATGGATGAGGATTACCAGGGGCGTTTATGCAA
CCCATTAAACCCGTTTGTCAGCCATAAAGAGATTGAATCAGTGAACAATGCAGTGATAATGCCGGCACAT
GGTCCAGCCAAAAATATGGAGTATGACGCTGTTGCAACAACACACTCCTGGGTCCCCAAAAGAAATCGAT
CCATTTTGAACACGAGCCAAAGGGGGATACTTGAAGATGAGCAAATGTATCAGAGGTGCTGCAATTTATT
TGAAAAATTCTTCCCAAGTAGCTCATACAGAAGACCAGTTGGAATATCCAGTATGGTAGAGGCTATGGTC
TCAAGAGCCCGAATTGATGCACGGATTGATTTCGAATCTGGAAGGATAAAGAAAGAGGAATTTGCTGAGA
TCATGAAGATCTGTTCCACCATTGAAGACCTCAGACGGCAAAAATGAGGAATTTGGCTTGTCCTTCATGA
AAA
>gi|237689296|gb|CY040256.1| Influenza A virus (A/Managua/3153.01/2008(H1N1)) segment 2, complete sequence
ATGGATGTCAATCCGACATTACTTTTCTTAAAAGTGCCAGCACAAAATGCTATAAGCACAACTTTTCCTT
ATACTGGTGACCCTCCTTACAGCCATGGGACAGGAACAGGGTACACCATGGATACAGTCAACAGGACACA
CCAGTACTCAGAAAGAGGAAGATGGACAAAAAATACCGAAACTGGAGCACCGCAACTTAACCCAATTGAT
GGTCCACTACCGGAAGACAATGAACCAAGTGGCTATGCCCAAACAGATTGTGTATTAGAAGCAATGGCTT
TCCTTGAAGAATCCCATCCCGGTATCTTTGAAAACTCTTGTATTGAAACAATGGAGGTTGTTCAGCAAAC
AAGGGTGGACAAACTGACACAAGGCAGACAGACCTATGACTGGACTCTAAATAGGAACCAGCCTGCTGCC
ACAGCATTGGCAAACACTATAGAAGTGTTCAGATCAAACGGCCTCATAGCAAATGAATCTGGAAGGCTAA
TAGACTTCCTTAAAGATGTAATGGAGTCGATGGACAGAGGCGAAGTAGAGGTCACAACTCATTTTCAAAG
AAAGAGGAGAGTGAGAGACAATGTAACTAAAAAAATGGTGACCCAAAGAACAATAGGCAAAAAGAAACAT
AAATTAGACAAAAGAAGTTACCTAATTAGGGCATTAACCCTGAACACAATGACCAAAGATGCTGAGAGGG
GGAAACTAAAACGCAGAGCAATTGCAACCCCAGGAATGCAAATAAGGGGGTTTGTATACTTTGTTGAGAC
ACTGGCAAGAAGCATATGTGAAAAGCTTGAACAATCAGGATTGCCAGTTGGAGGAAATGAGAAGAAAGCA
AAGTTAGCAAATGTTGTAAGGAAGATGATGACCAACTCCCAGGACACTGAAATTTCTTTCACCATAACCG
GAGATAACACAAAATGGAACGAAAATCAAAACCCTAGAATGTTCTTGGCCATGATCACATATATAACCAA
AAATCAGCCTGAATGGTTCAGAAATATTCTAAGTATTGCTCCAATAATGTTTTCAAACAAGATGGCGAGA
CTAGGTAAGGGGTACATGTTTGAAAGCAAGAGTATGAAACTGAGAACTCAAATACCTGCAGAGATGCTAG
CCAACATAGATTTGAAATATTTCAATGATTCAACTAAAAAGAAAATTGAAAAAATCCGACCATTATTAAT
AGATGGAACTGCATCATTGAGTCCTGGAATGATGATGGGCATGTTCAATATGTTGAGCACCGTCTTGGGC
GTCTCCATTCTGAATCTTGGGCAAAAGAGATACACCAAGACTACTTACTGGTGGGATGGTCTTCAATCGT
CTGATGATTTTGCTTTGATTGTGAATGCACCCAATTATGCAGGAATTCAAGCTGGAGTTGACAGGTTTTA
TCGAACCTGTAAGCTGCTCGGAATTAATATGAGCAAAAAGAAGTCTTACATAAACAGAACAGGTACCTTT

GAATTCACGAGCTTTTTCTATCGTTATGGGTTTGTTGCCAATTTCAGCATGGAGCTCCCTAGTTTTGGGG
TGTCTGGGGTCAATGAATCTGCAGACATGAGTATTGGAGTCACTGTCATCAAAAACAATATGATAAACAA
TGACCTTGGCCCAGCAACTGCTCAAATGGCCCTTCAGTTATTTATAAAAGATTACAGGTACACTTATCGA
TGCCACCGAGGTGACACACAAATACAAACCCGGAGATCATTTGAGATAAAGAAACTATGGGACCAAACCC
GCTCAAAAGCTGGGCTGTTGGTCTCTGATGGAGGCCCAAATTTATATAACATTAGAAATCTCCATATTCC
TGAAGTTTGCTTGAAATGGGAGTTGATGGATGAGGATTACCAGGGGCGTTTATGCAACCCATTAAACCCG
TTTGTCAGCCATAAAGAGATTGAATCAGTGAACAATGCAGTGATAATGCCGGCACATGGTCCAGCCAAAA
ATATGGAGTATGACGCTGTTGCAACAACACACTCCTGGGTCCCCAAGAGAAATCGATCCATTTTGAACAC
GAGCCAAAGGGGGATACTTGAAGATGAGCAAATGTATCAGAGGTGCTGCAATTTATTTGAAAAATTCTTC
CCAAGTAGCTCATACAGAAGACCAGTTGGAATATCCAGTATGGTAGAGGCTATGGTCTCAAGAGCCCGAA
TTGATGCACGGATTGATTTCGAATCTGGAAGGATAAAGAAAGAGGAATTTGCTGAGATCATGAAGATCTG
TTCCACCATTGAAGACCTCAGACGGCAAAAATGAGGAATTTGGCTTGTCCTTCATGAAAA
>gi|224020943|gb|CY037333.1| Influenza A virus (A/Washington/AF06/2007(H1N1)) segment 2,
complete sequence
ATGGATGTCAATCCGACATTACTTTTCTTAAAAGTGCCAGCACAAAATGCTATAAGCACAACTTTTCCTT
ATACTGGTGACCCTCCTTACAGCCATGGGACAGGAACAGGGTACACCATGGATACAGTCAACAGGACACA
CCAGTACTCAGAAAGAGGAAGATGGACAAAAAATACCGAAACAGGAGCACCGCAACTTAACCCAATTGAT
GGTCCCCTACCGGAAGACAATGAACCAAGTGGCTATGCCCAAACAGATTGTGTATTAGAAGCAATGGCTT
TCCTTGAAGAATCCCATCCCGGTATCTTTGAAAACTCTTGTATTGAAACAATGGAGGTTGTTCAGCAAAC
AAGGGTGGACAAACTGACACAAGGCAGACAGACCTATGACTGGACTCTAAATAGGAACCAGCCTGCTGCC
ACAGCATTGGCAAACACTATAGAAGTATTCAGATCAAACGGCCTCATAGCAAATGAATCTGGGAGGCTAA
TAGACTTCCTTAAAGATGTAATGGAGTCGATGGACAGAGGCGAAGTAGAGGTCACAACTCATTTTCAAAG
AAAGAGGAGAGTGAGAGACAATGCAACTAAAAAAATGGTGACCCAAAGAACAATAGGCAAAAAGAAACA
T
AAATTAGACAAAAGAAGTTACCTAATTAGGGCATTAACCCTGAACACAATGACCAAAGATGCTGAGAGGG
GGAAACTAAAACGCAGAGCAATTGCAACCCCAGGAATGCAAATAAGGGGGTTTGTATACTTTGTTGAGAC
ACTGGCAAGAAGCATATGTGAAAAGCTTGAACAATCAGGATTGCCAGTTGGAGGAAATGAGAAGAAAGCA
AAGTTAGCAAATGTTGTAAGGAAGATGATGACCAACTCCCAGGACACTGAAATTTCTTTCACCATAACCG
GAGATAACACAAAATGGAACGAAAATCAAAACCCTAGAATGTTCTTGGCCATGATCACATATATAACCAA
AAATCAGCCTGAATGGTTCAGAAATATTCTAAGTATTGCTCCAATAATGTTTTCAAACAAGATGGCGAGG
CTAGGTAAGGGGTACATGTTTGAAAGCAAGAGTATGAAACTGAGAACTCAAATACCTGCAGAGATGCTAG
CCAACATAGATTTGAAATATTTCAATGATTCAACTAAAAAGAAAATTGAAAAAATCCGACCATTATTAAT
AGATGGAACTGCATCATTGAGTCCTGGAATGATGATGGGCATGTTCAATATGTTGAGCACCGTCTTGGGC
GTCTCCATTCTGAATCTTGGGCAAAAGAGATACACCAAGACTACTTACTGGTGGGATGGTCTTCAATCGT
CTGATGATTTTGCTTTGATTGTGAATGCACCCAACTATGCAGGAATTCAAGCTGGAGTTGACAGGTTTTA
TCGAACCTGTAAGCTGCTCGGAATTAATATGAGCAAAAAGAAGTCTTACATAAACAGAACAGGTACCTTT
GAATTCACGAGCTTTTTCTATCGTTATGGGTTTGTTGCCAATTTCAGCATGGAGCTTCCTAGTTTTGGGG
TGTCTGGGGTCAATGAATCTGCAGACATGAGTATTGGAGTCACTGTCATCAAAAACAATATGATAAACAA
TGACCTTGGCCCAGCAACTGCTCAAATGGCCCTTCAGTTATTTATAAAAGATTACAGGTACACTTATCGA
TGCCACCGAGGTGACACACAAATACAAACCCGGAGATCATTTGAGATAAAGAAACTATGGGACCAAACCC
GCTCAAAAGCTGGGCTGTTGGTCTCTGATGGAGGCCCCAATTTATATAACATTAGAAATCTCCATATTCC
TGAAGTTTGCTTGAAATGGGAGTTGATGGATGAGGATTACCAGGGGCGTTTATGCAACCCATTAAACCCG
TTTGTCAGCCATAAAGAGATTGAATCAGTGAACAATGCAGTGATAATGCCGGCACATGGTCCAGCCAAAA
ATATGGAGTATGACGCTGTTGCAACAACACACTCCTGGGTCCCCAAAAGAAATCGATCCATTTTGAACAC

GAGCCAAAGGGGGATACTTGAAGATGAGCAAATGTATCAGAGGTGCTGCAATTTATTTGAAAAATTCTTC
CCAAGTAGCTCATACAGAAGACCAGTTGGAATATCCAGTATGGTAGAGGCTATGGTCTCAAGAGCCCGAA
TTGATGCACGGATTGATTTCGAATCTGGAAGGATAAAGAAAGAGGAATTTGCTGAGATCATGAAGATCTG
TTCCACCATTGAAGACCTCAGACGGCAAAAATGAGGAATTT
>gi|224021248|gb|CY037341.1| Influenza A virus (A/Japan/AF07/2008(H1N1)) segment 2, complete sequence
ATGGATGTCAATCCGACATTACTTTTCTTAAAAGTGCCAGCACAAAATGCTATAAGCACAACTTTTCCTT
ATACTGGTGACCCTCCTTACAGCCATGGGACAGGAACAGGGTACACCATGGATACAGTCAACAGGACACA
CCAGTACTCAGAAAGAGGAAGATGGACAAAAAATACCGAAACGGGAGCACCGCAACTTAACCCAATTGAT
GGTCCCTTACCGGAAGACAATGAACCAAGTGGCTATGCCCAAACAGATTGTGTATTAGAAGCAATGGCTT
TCCTTGAAGAATCCCATCCCGGTATCTTTGAAAACTCTTGTATTGAAACAATGGAGGTTGTTCAGCAAAC
AAGGGTGGACAAACTGACACAAGGCAGACAGACCTATGACTGGACTCTAAATAGGAACCAGCCTGCTGCC
ACAGCATTGGCAAACACTATAGAAGTATTCAGATCAAATGGCCTCATAGCAAATGAATCTGGGAGGCTAA
TAGACTTCCTTAAAGATGTAATGGAGTCGATGGACAGAGGCGAAGTAGAGGTCACAACTCATTTTCAAAG
AAAGAGGAGAGTGAGAGACAATGTAACTAAAAAAATGGTGACCCAAAGAACAATAGGCAAAAAGAAACAT
AAATTAGACAAAAGAAGTTACCTAATTAGGGCATTAACCCTGAACACAATGACCAAAGATGCTGAGAGGG
GGAAACTAAAACGCAGAGCAATTGCAACCCCAGGAATGCAAATAAGGGGGTTTGTATACTTTGTTGAGAC
ACTGGCAAGAAGCATATGTGAAAAGCTTGAACAATCAGGATTGCCAGTTGGAGGAAATGAGAAGAAAGCA
AAGTTAGCAAATGTTGTAAGGAAGATGATGACCAACTCCCAGGACACTGAAATTTCTTTCACCATAACCG
GAGATAACACAAAATGGAACGAAAATCAAAACCCTAGAATGTTCTTGGCCATGATCACATATATAACCAA
AAATCAGCCTGAATGGTTCAGAAATATTCTAAGTATTGCTCCAATAATGTTTTCAAACAAGATGGCGAGA
CTAGGTAAGGGGTACATGTTTGAAAGCAAGAGTATGAAACTGAGAACTCAAATACCTGCAGAGATGCTAG
CCAACATAGATTTGAAATATTTCAATGATTCAACTAAAAAGAAAATTGAAAAAATCCGACCATTATTAAT
AGATGGAACTGCATCATTGAGTCCTGGAATGATGATGGGCATGTTCAATATGTTGAGCACCGTCTTGGGC
GTCTCCATTCTGAATCTTGGGCAAAAGAGATACACCAAGACTACTTACTGGTGGGATGGTCTTCAATCGT
CTGATGATTTTGCTTTGATTGTGAATGCACCCAACTATGCAGGAATTCAAGCTGGAGTTGACAGGTTTTA
TCGAACCTGTAAGCTGCTCGGAATTAATATGAGCAAAAAGAAGTCTTACATAAACAGAACAGGTACCTTT
GAATTCACGAGCTTTTTCTATCGTTATGGGTTTGTTGCCAATTTCAGCATGGAGCTTCCTAGTTTTGGGG
TGTCTGGGGTCAATGAATCTGCAGACATGAGTATTGGAGTCACTGTCATCAAAAACAATATGATAAACAA
TGACCTTGGCCCAGCAACTGCTCAAATGGCCCTTCAGTTATTTATAAAAGATTACAGATACACTTATCGA
TGCCACCGAGGTGACACACAAATACAAACCCGGAGATCATTTGAGATAAAGAAACTATGGGACCAAACCC
GCTCAAAAGCTGGGCTGTTGGTCTCTGATGGAGGCCCCAATTTATATAACATTAGAAATCTCCATGTTCC
TGAAGTTTGCTTGAAATGGGAGTTGATGGATGAGGATTACCAGGGGCGTTTATGCAACCCATTAAACCCG
TTTGTCAGCCATAAAGAGATTGAATCAGTGAACAATGCAGTGATAATGCCGGCACATGGTCCAGCCAAAA
ATATGGAGTATGACGCTGTTGCAACAACACACTCCTGGGTCCCCAAAAGAAATCGATCCATTTTGAACAC
GAGCCAAAGGGGAATACTTGAAGATGAGCAAATGTATCAGAGGTGCTGCAATTTATTTGAAAAATTCTTC
CCAAGTAGCTCATACAGAAGACCAGTTGGAATATCCAGTATGGTAGAGGCTATGGTCTCAAGAGCCCGAA
TTGATGCACGGATTGATTTCGAATCTGGAAGGATAAAGAAAGAGGAATTTGCTGAGATCATGAAGATCTG
TTCCACCATTGAAGACCTCAGACGGCAAAAATGAGGAATTTGGCTTGTCCTTCATGAAA
>gi|212381610|gb|FJ445084.1| Influenza A virus (A/England/26/2008(H1N1)) segment 2 polymerase PB1 (PB1) and PB1-F2 protein (PB1-F2) genes, complete cds
ATGGATGTCAATCCGACATTACTTTTCTTAAAAGTGCCAGCACAAAATGCTATAAGCACAACTTTTCCTT
ATACTGGTGACCCTCCTTACAGCCATGGGACAGGAACAGGGTACACCATGGATACAGTCAACAGGACACA
CCAGTACTCAGAAAGAGGAAGATGGACAAAAAATACCGAAACGGGAGCACCGCAACTTAACCCAATTGAT

GGTCCCTTACCGGAAGACAATGAACCAAGTGGCTATGCCCAAACAGATTGTGTATTAGAAGCAATGGCTT
TCCTTGAAGAATCCCATCCCGGTATCTTTGAAAACTCTTGTATTGAAACAATGGAGGTTGTTCAACAAAC
AAGGGTGGACAAACTGACACAAGGCAGACAGACCTATGACTGGACTCTAAATAGGAACCAGCCTGCTGCC
ACAGCATTGGCAAACACTATAGAAGTATTCAGATCAAACGGCCTCATAGCAAATGAATCTGGGAGGCTAA
TAGACTTCCTTAAAGATGTAATGGAGTCGATGGACAGAGGCGAAGTAGAGGTCACAACTCATTTTCAAAG
AAAGAGGAGAGTGAGAGACAATGTAACTAAAAAAATGGTGACCCAAAGAACAATAGGCAAAAAGAAACAT
AAATTAGACAAAAGAAGTTACCTAATTAGGGCATTAACCCTGAACACAATGACCAAAGATGCTGAGAGGG
GGAAACTAAAACGCAGAGCAATTGCAACCCCAGGAATGCAAATAAGGGGGTTTGTATACTTTGTTGAGAC
ACTGGCAAGAAGCATATGTGAAAAGCTTGAACAATCAGGATTGCCAGTTGGAGGAAATGAGAAGAAAGCA
AAGTTGGCAAATGTTGTAAGGAAGATGATGACCAACTCCCAGGACACTGAAATTTCTTTCACCATAACCG
GAGATAACACAAAATGGAACGAAAATCAAAACCCTAGAATGTTCTTGGCCATGATCACATATATAACCAA
AAATCAGCCTGAATGGTTCAGAAATATTCTAAGTATTGCTCCAATAATGTTTTCAAACAAGATGGCGAGA
CTAGGTAAGGGGTACATGTTTGAAAGCAAGAGTATGAAACTGAGAACTCAAATACCTGCAGAGATGCTAG
CCAACATAGACTTGAAATATTTCAATGATTCAACTAAAAAGAAAATTGAAAAAATCCGACCATTATTAAT
AGATGGAACTGCATCATTGAGTCCTGGAATGATGATGGGCATGTTCAATATGTTGAGCACCGTCTTGGGC
GTCTCCATTCTGAATCTTGGGCAAAAGAGATACACCAAGACTACTTACTGGTGGGATGGTCTTCAATCGT
CTGATGATTTTGCTTTGATTGTGAACGCACCCAACTATGCAGGAATTCAAGCTGGAGTTGACAGGTTTTA
TCGAACCTGTAAGCTGCTCGGAATTAATATGAGCAAAAAGAAGTCTTACATAAACAGAACAGGTACCTTT
GAATTCACGAGCTTTTTCTATCGTTATGGGTTTGTTGCCAATTTCAGCATGGAGCTTCCTAGTTTTGGGG
TGTCTGGGGTCAATGAATCTGCAGACATGAGTATTGGAGTCACTGTCATCAAAAACAATATGATAAACAA
TGACCTTGGCCCAGCAACTGCTCAAATGGCCCTTCAGTTATTTATAAAAGATTACAGGTACACTTATCGA
TGCCACCGAGGTGACACACAAATACAAACCCGGAGATCATTTGAGATAAAGAAACTATGGGACCAAACCC
GCTCAAAAGCTGGGCTGTTGGTCTCTGATGGAGGCCCCAATTTGTATAACATTAGAAATCTCCATATTCC
TGAAGTTTGCTTGAAATGGGAGTTGATGGATGAGGATTACCAGGGGCGTTTATGCAACCCATTAAACCCG
TTTGTCAGCCATAAAGAGATTGAATCAGTGAACAATGCAGTGATAATGCCGGCACATGGCCCAGCCAAAA
ATATGGAGTATGACGCTGTTGCAACAACACACTCCTGGGTCCCCAAAAGAAATCGATCCATTTTGAACAC
GAGCCAAAGGGGGATACTTGAAGATGAGCAAATGTATCAGAGGTGCTGCAATTTATTTGAAAAATTCTTC
CCAAGTAGCTCATACAGAAGACCAGTTGGAATATCCAGTATGGTAGAGGCTATGGTCTCAAGAGCCCGAA
TTGATGCACGGATTGATTTCGAATCTGGAAGGATAAAGAAAGAGGAATTTGCTGAGATCATGAAGATCTG
TTCCACCATTGAAGACCTCAGACGGCAAAAATGA
>gi|224027218|gb|CY037685.1| Influenza A virus (A/Florida/UR07-0022/2008(H1N1)) segment 2, complete sequence
ATGGATGTCAATCCGACATTACTTTTCTTAAAAGTGCCAGCACAAAATGCCATAAGCACAACTTTTCCTT
ATACTGGTGACCCTCCTTACAGCCATGGGACAGGAACAGGGTACACCATGGATACAGTCAACAGGACACA
CCAGTACTCAGAAAGAGGAAGATGGACAAAAAATACCGAAACTGGAGCACCGCAACTTAACCCAATTGAT
GGTCCACTACCGGAAGACAATGAACCAAGTGGCTATGCCCAAACAGATTGTGTATTAGAAGCAATGGCTT
TCCTTGAAGAAACCCATCCCGGTATCTTTGAAAACTCTTGTATTGAAACAATGGAGGTTGTTCAGCAAAC
AAGGGTGGACAAACTGACACAAGGCAGACAGACCTATGACTGGACTCTAAATAGGAACCAGCCTGCTGCC
ACAGCATTGGCAAACACTATAGAAGTATTCAGATCAAACGGCCTCATAGCAAATGAATCTGGGAGGCTAA
TAGACTTCCTTAAAGATGTAATGGAGTCGATGGACAGAGGCGAAGTAGAGGTCACAACTCATTTTCAAAG
AAAGAGGAGAGTGAGAGACAATGTAACTAAAAAAATGGTGACCCAAAGAACAATAGGCAAAAAGAAACAT
AAATTAGACAAAAGAAGTTACCTAATTAGGGCATTAACCCTGAACACAATGACCAAAGATGCTGAGAGGG
GGAAACTAAAACGCAGAGCAATTGCAACCCCAGGAATGCAAATAAGGGGGTTTGTATACTTTGTTGAGAC
ACTGGCAAGAAGCATATGTGAAAAGCTTGAACAATCAGGATTGCCAGTTGGAGGAAATGAGAAGAAAGCA

AAGTTAGCAAATGTTGTAAGGAAGATGATGACCAACTCCCAGGACACTGAAATTTCTTTCACCATAACCG
GAGATAACACAAAATGGAACGAAAATCAAAACCCTAGAATGTTCTTGGCCATGATCACATATATAACCAA
AAATCAGCCTGAATGGTTCAGAAATATTCTAAGTATTGCTCCAATAATGTTTTCAAACAAGATGGCGAGA
CTAGGTAAGGGGTACATGTTTGAAAGCAAGAGTATGAAACTGAGAACTCAAATACCTGCAGAGATGCTAG
CCAACATAGATTTGAAATATTTCAATGATTCAACTAAAAAGAAAATTGAAAAAATCCGACCATTATTAAT
AGATGGAACTGCATCATTGAGTCCTGGAATGATGATGGGCATGTTCAATATGTTGAGCACCGTCTTGGGC
GTCTCCATTCTGAATCTTGGGCAAAAGAGATACACCAAGACTACTTACTGGTGGGATGGTCTTCAATCGT
CTGATGATTTTGCTTTGATTGTGAATGCACCCAACTATGCAGGAATTCAAGCTGGAGTTGACAGGTTTTA
TCGAACCTGTAAGCTGCTCGGAATTAATATGAGCAAAAAGAAGTCTTACATAAACAGAACAGGTACCTTT
GAATTCACGAGCTTTTTCTATCGTTATGGGTTTGTTGCTAATTTCAGCATGGAGCTTCCTAGTTTTGGGG
TGTCTGGGGTCAATGAATCTGCAGACATGAGTATTGGAGTCACTGTCATCAAAAACAATATGATAAACAA
TGACCTTGGCCCAGCAACTGCTCAAATGGCCCTTCAGTTATTTATAAAAGATTACAGGTACACTTATCGA
TGCCACCGAGGTGACACACAAATACAAACCCGGAGATCATTTGAGATAAAGAAACTATGGGACCAAACCC
GCTCAAAAGCTGGGCTGTTGGTCTCTGATGGAGGCCCCAATTTATATAACATTAGAAATCTCCATATTCC
TGAAGTTTGCTTGAAATGGGAGTTGATGGATGAGGATTACCAGGGGCGTTTATGCAACCCATTAAACCCG
TTTGTCAGCCATAAAGAGATTGAATCAGTGAACAATGCAGTGATAATGCCGGCACATGGTCCAGCCAAAA
ATATGGAGTATGACGCTGTTGCAACAACACACTCCTGGGTCCCCAAAAGAAATCGATCCATTTTGAACAC
GAGCCAAAGGGGGATACTTGAAGATGAGCAAATGTATCAGAGGTGCTGCAATTTATTTGAAAAATTCTTC
CCAAGTAGCTCATACAGAAGACCAGTTGGAATATCCAGTATGGTAGAGGCTATGGTCTCAAGAGCCCGAA
TTGATGCACGGATTGATTTCGAATCTGGAAGGATAAAGAAAGAGGAATTTGCTGAGATCATGAAGATCTG
TTCCACCATTGAAGACCTCAGACGGCAAAAATGAGGAATTTGGCTTGTCCTTCATGAAAAAA
>gi|296240593|gb|CY063612.1| Influenza A virus (A/Aalborg/INS133/2009(H1N1)) segment 2, complete sequence
TTGAATGGATGTCAATCCGACTCTACTTTTCCTAAAAATTCCAGCGCAAAATGCCATAAGCACCACATTC
CCTTATACTGGAGATCCTCCATACAGCCATGGAACAGGAACAGGATACACCATGGACACAGTAAACAGAA
CACACCAATACTCAGAAAAGGGAAAGTGGACGACAAACACAGAGACTGGTGCACCCCAGCTCAACCCGAT
TGATGGACCACTACCTGAGGATAATGAACCAAGTGGGTATGCACAAACAGACTGTGTTCTAGAGGCTATG
GCTTTCCTTGAAGAATCCCACCCAGGAATATTTGAGAATTCATGCCTTGAAACAATGGAAGTTGTTCAAC
AAACAAGGGTAGATAAACTAACTCAAGGTCGCCAGACTTATGATTGGACATTAAACAGAAATCAACCGGC
AGCAACTGCGTTGGCCAACACCATAGAAGTCTTTAGATCGAATGGCCTAACAGCTAATGAGTCAGGAAGG
CTAATAGATTTCTTAAAGGATGTAATGGAATCAATGAACAAAGAGGAAATAGAGATAACAACCCACTTTC
AAAGAAAAAGGAGAGTAAGAGACAACATGACCAAGAAGATGGTCACGCAAAGAACAATAGGGAAGAAAA
A
ACAAAGACTGAATAAGAGAGGCTATCTAATAAGAGCACTGACATTAAATACGATGACCAAAGATGCAGAG
AGAGGCAAGTTAAAAAGAAGGGCTATCGCAACACCTGGGATGCAGATTAGAGGTTTCGTATACTTTGTTG
AAACTTTAGCTAGGAGCATTTGCGAAAAGCTTGAACAGTCTGGGCTCCCAGTAGGGGGCAATGAAAAGAA
GGCCAAACTGGCAAATGTTGTGAGAAAGATGATGACTAATTCACAAGACACAGAGATTTCTTTCACAATC
ACTGGGGACAACACTAAGTGGAATGAAAATCAAAATCCTCGAATGTTCCTGGCGATGATTACATATATCA
CCAGAAATCAACCCGAGTGGTTCAGAAACATCCTGAGCATGGCACCCATAATGTTCTCAAACAAAATGGC
AAGACTAGGGAAAGGGTACATGTTCGAGAGTAAAAGAATGAAGATTCGAACACAAATACCAGCAGAAATG
CTAGCAAGCATTGACCTGAAGTACTTCAATGAATCAACAAAGAAGAAAATTGAGAAAATAAGGCCTCTTC
TAATAGATGGCACAGCATCACTGAGTCCTGGGATGATGATGGGCATGTTCAACATGCTAAGTACGGTCTT
GGGAGTCTCGATACTGAATCTTGGACAAAAGAAATACACCAAGACAATATACTGGTGGGATGGGCTCCAA
TCATCCGACGATTTTGCTCTCATAGTGAATGCACCAAACCATGAGGGAATACAAGCAGGAGTGGACAGAT

TCTACAGGACCTGCAAGTTAGTGGGAATCAACATGAGCAAAAAGAAGTCCTATATAAATAAGACAGGGAC
ATTTGAATTCACAAGCTTTTTTTATCGCTATGGATTTGTGGCTAATTTTAGCATGGAGCTACCCAGCTTT
GGAGTGTCTGGAGTAAATGAATCAGCTGACATGAGTATTGGAGTAACAGTGATAAAGAACAACATGATAA
ACAATGACCTTGGACCTGCAACGGCCCAGATGGCTCTTCAACTGTTCATCAAAGACTACAGATACACATA
TAGGTGCCATAGGGGAGACACACAAATTCAGACGAGAAGATCATTTGAGTTAAAGAAGCTGTGGGATCAA
ACCCAATCAAAGGTAGGGCTATTAGTATCAGATGGAGGACCAAACTTATACAATATACGGAATCTTCACA
TTCCTGAAGTCTGCTTAAAATGGGAGCTAATGGATGATGATTATCGGGGAAGACTTTGTAATCCCCTGAA
TCCCTTTGTCAGTCATAAAGAGATTGATTCTGTAAACAATGCTGTGGTAATGCCAGCCCATGGTCCAGCC
AAAAGCATGGAATATGATGCCGTTGCAACTACACATTCCTGGATTCCCAAGAGGAATCGTTCCATTCTCA
ACACAAGCCAAAGGGGAATTCTTGAGGATGAACAGATGTACCAGAAGTGCTGCAATCTATTCGAGAAATT
TTTCCCTAGCAGTTCATATAGGAGACCAGTTGGAATTTCTAGCATGGTGGAGGCCATGGTGTCTAGGGCC
CGGATTGATGCCAGGGTCGACTTCGAGTCTGGACGGATCGGGAAGGAAGAGTTCTCTGAGATCATGAAGA
TCTGTTCCACCATTGAAGAACTCAGACGGCAAAAATAATGAATTTAACTTGTCCTTCATGAAA
>gi|296240575|gb|CY063604.1| Influenza A virus (A/Bonn/INS128/2009(H1N1)) segment 2, complete sequence
TTGAATGGATGTCAATCCGACTCTACTTTTCCTAAAAATTCCAGCGCAAAATGCCATAAGCACCACATTC
CCTTATACTGGAGATCCTCCATACAGCCATGGAACAGGAACAGGATACACCATGGACACAGTAAACAGAA
CACACCAATACTCAGAAAAGGGAAAGTGGACGACAAACACAGAGACTGGTGCACCCCAGCTCAACCCGAT
TGATGGACCACTACCTGAGGATAATGAACCAAGTGGGTATGCACAAACAGACTGTGTTCTAGAGGCTATG
GCTTTCCTTGAAGAATCCCACCCAGGAATATTTGAGAATTCATGCCTTGAAACAATGGAAGTTGTTCAAC
AAACAAGGGTAGATAAACTAACTCAAGGTCGCCAGACTTATGATTGGACATTAAACAGAAATCAACCGGC
AGCAACTGCATTGGCCAACACCATAGAAGTCTTTAGATCGAATGGCCTAACAGCTAATGAGTCAGGAAGG
CTAATAGATTTCTTAAAGGATGTAATGGAATCAATGAACAAAGAGGAAATAGAGATAACAACCCACTTTC
AAAGAAAAAGGAGAGTAAGAGACAACATGACCAAGAAGATGGTCACGCAAAGAACAATAGGGAAGAAAA
A
ACAAAGACTGAATAAGAGAGGCTATCTAATAAGAGCACTGACATTAAATACGATGACCAAAGATGCAGAG
AGAGGCAAGTTAAAAAGAAGGGCTATCGCAACACCTGGGATGCAGATTAGAGGTTTCGTATACTTTGTTG
AAACTTTAGCTAGGAGCATTTGCGAAAAGCTTGAACAGTCTGGGCTCCCAGTAGGGGGCAATGAAAAGAA
GGCCAAACTGGCAAATGTTGTGAGAAAGATGATGACTAATTCACAAGACACAGAGATTTCTTTCACAATC
ACTGGGGACAACACTAAGTGGAATGAAAATCAAAATCCTCGAATGTTCCTGGCGATGATTACATATATCA
CCAGAAAYCAACCCGAGTGGTTCAGAAACATCCTGAGCATGGCACCCATAATGTTCTCAAACAAAATGGC
AAGACTAGGGAAAGGGTACATGTTCGAGAGTAAAAGAATGAAGATTCGAACACAAATACCAGCAGAAATG
CTAGCAAGCATTGACCTGAAGTACTTCAATGAATCAACAAAGAAGAAAATTGAGAAAATAAGGCCTCTTC
TAATAGATGGCACAGCATCACTGAGTCCTGGGATGATGATGGGCATGTTCAACATGCTAAGTACGGTCTT
GGGAGTCTCGATACTGAATCTTGGACAAAAGAAATACACCAAGACAATATACTGGTGGGATGGGCTCCAA
TCATCCGACGATTTTGCTCTCATAGTGAATGCACCAAACCATGAGGGAATACAAGCAGGAGTGGACAGAT
TCTACAGGACCTGCAAGTTAGTGGGAATCAACATGAGCAAAAAGAAGTCCTATATAAATAAGACAGGGAC
ATTTGAATTCACAAGCTTTTTTTATCGCTATGGATTTGTGGCTAATTTTAGCATGGAGCTACCCAGCTTT
GGAGTGTCTGGAGTAAATGAATCAGCTGACATGAGTATTGGAGTAACAGTGATAAAGAACAACATGATAA
ACAATGACCTTGGACCTGCAACGGCCCAGATGGCTCTTCAATTGTTCATCAAAGACTACAGATACACATA
TAGGTGCCATAGGGGAGACACACAAATTCAGACGAGAAGATCATTTGAGTTAAAGAAGCTGTGGGATCAA
ACCCAATCAAAGGTAGGGCTATTAGTATCAGATGGAGGACCAAACTTATACAATATACGGAATCTTCACA
TTCCTGAAGTCTGCTTAAAATGGGAGCTAATGGATGATGATTATCGGGGAAGACTTTGTAATCCCCTGAA
TCCCTTTGTCAGTCATAAAGAGATTGATTCTGTAAACAATGCTGTGGTAATGCCAGCCCATGGTCCAGCC

AAAAGCATGGAATATGATGCCGTTGCAACTACACATTCCTGGATTCCCAAGAGGAATCGTTCTATTCTCA
ACACAAGCCAAAGGGGAATTCTTGAGGATGAACAGATGTACCAGAAGTGCTGCAATCTATTCGAGAAATT
TTTCCCTAGCAGTTCATATAGGAGACCGGTTGGAATTTCTAGCATGGTGGAGGCCATGGTGTCTAGGGCC
CGGATTGATGCCAGGGTCGACTTCGAGTCTGGACGGATCAAGAAAGAAGAGTTCTCTGAGATCATGAAGA
TCTGTTCCACCATTGAAGAACTCAGACGGCAAAAATAATGAATTTAACTTGTCCTTCATGAAA
>gi|296240323|gb|CY063492.1| Influenza A virus (A/Boston/110/2009(H1N1)) segment 2, complete sequence
TTGAATGGATGTCAATCCGACTCTACTTTTCCTAAAAATTCCAGCGCAAAATGCCATAAGCACCACATTC
CCTTATACTGGAGATCCTCCATACAGCCATGGAACAGGAACAGGATACACCATGGACACAGTAAACAGAA
CACACCAATACTCAGAAAAGGGAAAGTGGACGACAAACACAGAGACTGGTGCACCCCAGCTCAACCCGAT
TGATGGACCACTACCTGAGGATAATGAACCAAGTGGGTATGCACAAACAGACTGTGTTCTAGAGGCTATG
GCTTTCCTTGAAGAATCCCACCCAGGAATATTTGAGAATTCATGCCTTGAAACAATGGAAGTTGTTCAAC
AAACAAGGGTAGATAAACTAACTCAAGGTCGCCAGACTTATGATTGGACATTAAACAGAAATCAACCGGC
AGCAACTGCATTGGCCAACACCATAGAAGTCTTTAGATCGAATGGCCTAACAGCTAATGAGTCAGGAAGG
CTAATAGATTTCTTAAAGGATGTAATGGAATCAATGAACAAAGAGGAAATAGAGATAACAACCCACTTTC
AAAGAAAAAGGAGAGTAAGAGACAACATGACCAAGAAGATGGTCACACAAAGAACAATAGGGAAGAAAA
A
ACAAAGACTGAATAAGAGAGGCTATCTAATAAGAGCACTGACATTAAATACGATGACCAAAGATGCAGAG
AGAGGCAAGTTAAAAAGAAGGGCTATCGCAACACCTGGGATGCAGATTAGAGGTTTCGTATACTTTGTTG
AAACTTTAGCTAGGAGCATTTGCGAAAAGCTTGAACAGTCTGGGCTCCCAGTAGGGGGCAATGAAAAGAA
GGCCAAACTGGCAAATGTTGTGAGAAAGATGATGACTAATTCACAAGACACAGAGATTTCTTTCACAATC
ACTGGGGACAACACTAAGTGGAATGAAAATCAAAATCCTCGAATGTTCCTGGCGATGATTACATATATCA
CCAGAAATCAACCCGAGTGGTTCAGAAACATCCTGAGCATGGCACCCATAATGTTCTCAAACAAAATGGC
AAGACTAGGGAAAGGGTACATGTTCGAGAGTAAAAGAATGAAGATTCGAACACAAATACCAGCAGAAATG
CTAGCAAGCATTGACCTGAAGTACTTCAATGAATCAACAAAGAAGAAAATTGAGAAAATAAGGCCTCTTC
TAATAGATGGCACAGCATCACTGAGTCCTGGGATGATGATGGGCATGTTCAACATGCTAAGTACGGTCTT
GGGAGTCTCGATACTGAATCTTGGACAAAAGAAATACACCAAGACAATATACTGGTGGGATGGGCTCCAA
TCATCCGACGATTTTGCTCTCATAGTGAATGCACCAAACCATGAGGGAATACAAGCAGGAGTGGACAGAT
TCTACAGGACCTGCAAGTTAGTGGGAATCAACATGAGCAAAAAGAAGTCCTATATAAATAAGACAGGGAC
ATTTGAATTCACAAGCTTTTTTTATCGCTATGGATTTGTGGCTAATTTTAGCATGGAGCTACCCAGCTTT
GGAGTGTCTGGAGTAAATGAATCAGCTGACATGAGTATTGGAGTAACAGTGATAAAGAACAACATGATAA
ACAATGACCTTGGACCTGCAACGGCCCAGATGGCTCTTCAATTGTTCATCAAAGACTACAGATACACATA
TAGGTGCCATAGGGGAGACACACAAATTCAGACGAGAAGATCATTTGAGTTAAAGAAGCTGTGGGATCAA
ACCCAATCAAAGGTAGGGCTATTAGTATCAGATGGAGGACCAAACTTATACAATATACGGAATCTTCACA
TTCCTGAAGTCTGCTTAAAATGGGAGCTAATGGATGATGATTATCGGGGAAGACTTTGTAATCCCCTGAA
TCCCTTTGTCAGTCATAAAGAGATTGATTCTGTAAACAATGCTGTGGTAATGCCAGCCCATGGTCCAGCC
AAAAGCATGGAATATGATGCCGTTGCAACTACACATTCCTGGATTCCCAAGAGGAATCGTTCTATTCTCA
ACACAAGCCAAAGGGGAATTCTTGAGGATGAACAGATGTACCAGAAGTGTTGCAATCTATTCGAGAAATT
TTTCCCTAGCAGTTCATATAGGAGACCGGTTGGAATTTCTAGCATGGTGGAGGCCATGGTGTCTAGGGCC
CGGATTGATGCCAGGGTCGACTTCGAGTCTGGACGGATCAAGAAAGAAGAGTTCTCTGAGATCATGAAGA
TCTGTTCCACCATTGAAGAACTCAGACGGCAAAAATAATGAATTTAACTTGTCCTTCATGAAA
>gi|296240629|gb|CY063628.1| Influenza A virus (A/New York/INS150/2009(H1N1)) segment 2, complete sequence
ATGGATGTCAATCCGACTCTACTTTTCCTAAAAATTCCAGCGCAAAATGCCATAAGCACCACATTCCCTT

ACACTGGAGATCCTCCATACAGCCATGGAACAGGAACAGGATACACCATGGACACAGTAAACAGAACACA
CCAATACTCAGAAAAGGGAAAGTGGACGACAAACACAGAGACTGGTGCACCCCAGCTCAACCCGATTGAT
GGACCACTACCTGAGGATAATGAACCAAGTGGGTATGCACAAACAGACTGTGTTCTAGAGGCTATGGCCT
TCCTTGAAGAATCCCACCCAGGAATATTTGAGAATTCATGCCTTGAAACAATGGAAGTTGTTCAACAAAC
AAGGGTAGATAAACTAACTCAAGGTCGCCAGACTTATGATTGGACATTAAACAGAAATCAACCGGCAGCA
ACTGCATTGGCCAACACCATAGAAGTCTTTAGATCGAATGGCCTAACAGCCAATGAGTCAGGAAGGCTAA
TAGATTTCTTAAAGGATGTAATGGAATCAATGAACAAAGAGGAAATAGAGATAACAACCCACTTTCAAAG
AAAAAGGAGAGTAAGAGACAACATGACCAAGAAGATGGTCACGCAAAGAACAATAGGGAAGAAAAAACA
A
AGGCTGAATAAGAGAGGCTATCTAATAAGAGCACTGACATTAAATACGATGACCAAAGATGCAGAGAGAG
GCAAGTTAAAAAGAAGGGCTATCGCAACACCTGGGATGCAGATTAGAGGTTTCGTATACTTTGTTGAAAC
TTTAGCTAGGAGCATTTGCGAAAAGCTTGAACAGTCTGGGCTCCCAGTAGGGGGCAATGAAAAGAAGGCC
AAACTGGCAAATGTTGTGAGAAAGATGATGACTAATTCACAAGACACAGAGATTTCTTTCACAATCACTG
GGGACAACACTAAGTGGAATGAAAATCAAAATCCTCGAATGTTCCTGGCGATGATTACATATATCACCAG
AAATCAACCCGAGTGGTTCAGAAACATCCTGAGCATGGCACCCATAATGTTCTCAAACAAAATGGCAAGA
CTAGGGAAAGGGTACATGTTCGAGAGTAAAAGAATGAAGATTCGAACACAAATACCAGCAGAAATGCTAG
CAAGCATTGACCTGAAGTACTTCAATGAATCAACAAAGAAGAAAATTGAGAAAATAAGGCCTCTTCTAAT
AGATGGCACAGCATCACTGAGTCCTGGGATGATGATGGGCATGTTCAACATGCTAAGTACGGTCTTGGGA
GTCTCGATACTGAATCTTGGACAAAAGAAATACACCAAGACAATATACTGGTGGGATGGGCTCCAATCAT
CCGACGATTTTGCTCTCATAGTGAATGCACCAAACCATGAGGGAATACAAGCAGGAGTGGACAGATTCTA
CAGGACCTGCAAGTTAGTGGGAATCAACATGAGCAAAAAGAAGTCCTATATAAATAAGACAGGGACATTT
GAATTCACAAGCTTTTTTTATCGCTATGGATTTGTGGCTAATTTTAGCATGGAGCTACCCAGCTTTGGAG
TGTCTGGAGTAAATGAATCAGCTGACATGAGTATTGGAGTAACAGTGATAAAGAACAACATGATAAACAA
TGACCTTGGACCTGCAACGGCCCAGATGGCTCTTCAATTGTTCATCAAAGACTACAGATACACATATAGG
TGTCATAGGGGAGACACACAAATTCAGACGAGAAGATCATTTGAGTTAAAGAAGCTGTGGGATCAAACCC
AATCAAAGGCAGGACTATTAGTATCAGATGGAGGACCAAACTTATACAATATACGGAATCTTCACATTCC
TGAAGTCTGCTTAAAATGGGAGCTAATGGATGATGATTATCGGGGAAGACTTTGTAATCCCCTGAATCCC
TTTGTCAGTCATAAAGAGATTGATTCTGTAAACAATGCTGTGGTAATGCCAGCCCATGGTCCAGCCAAAA
GCATGGAATATGATGCCGTTGCAACTACACATTCCTGGATTCCCAAGAGGAATCGTTCTATTCTCAACAC
AAGCCAAAGGGGAATTCTTGAGGATGAACAGATGTACCAGAAGTGCTGCAATCTATTCGAGAAATTTTTC
CCTAGCAGTTCATATAGGAGACCGGTTGGAATTTCTAGCATGGTGGAGGCCATGGTGTCTAGGGCCCGGA
TTGATGCCAGGGTCGACTTCGAGTCTGGACGGATCAAGAAAGAAGAGTTCTCTGAGATCATGAAGATCTG
TTCCACCATTGAAGAACTCAGACGGCAAAAATAATGAATTTAACTTGTCCTTCATGAAA
>gi|296240557|gb|CY063596.1| Influenza A virus (A/Athens/INS122/2009(H1N1)) segment 2, complete sequence
TTGAATGGATGTCAATCCGACTCTACTTTTCCTAAAAATTCCAGCGCAAAATGCCATAAGCACCACATTC
CCTTATACTGGAGATCCTCCATACAGCCATGGAACAGGAACAGGATACACCATGGACACAGTAAACAGAA
CACACCAATACTCAGAAAAAGGAAAGTGGACGACAAACACAGAGACTGGTGCACCCCAGCTCAACCCGAT
TGATGGACCACTACCTGAGGATAATGAACCAAGTGGGTATGCACAAACAGACTGTGTTCTAGAGGCTATG
GCTTTCCTTGAAGAATCCCACCCAGGAATATTTGAGAATTCATGCCTTGAAACAATGGAAGTTGTTCAAC
AAACAAGGGTAGATAAACTAACTCAAGGTCGCCAGACTTATGATTGGACATTAAACAGAAATCAACCGGC
AGCAACTGCATTGGCCAACACCATAGAAGTCTTTAGATCGAATGGCCTAACAGCTAATGAGTCAGGAAGG
CTAATAGATTTCTTAAAGGATGTAATGGAATCAATGAACAAAGAGGAAATAGAGATAACAACTCACTTTC
AAAGAAAAAGGAGAGTAAGAGACAACATGACCAAGAAGATGGTCACGCAAAGAACAATAGGGAAGAAAA

A
ACAAAGACTGAATAAGAGAGGCTATCTAATAAGAGCACTGACATTAAATACGATGACCAAAGATGCAGAG
AGAGGCAAGTTAAAAAGAAGGGCTATCGCAACACCTGGGATGCAGATTAGAGGTTTCGTATACTTTGTTG
AAACTTTAGCTAGAAGCATTTGCGAAAAGCTTGAACAGTCTGGGCTCCCAGTAGGGGGCAATGAAAAGAA
GGCCAAACTGGCAAATGTTGTGAGAAAGATGATGACTAATTCACAAGACACAGAGATTTCTTTCACAATC
ACTGGGGACAACACTAAGTGGAATGAAAATCAAAATCCTCGAATGTTCCTGGCGATGATTACATATATCA
CCAGAAATCAACCCGAGTGGTTCAGAAACATCCTGAGCATGGCACCCATAATGTTCTCAAACAAAATGGC
AAGACTAGGGAAAGGGTACATGTTCGAGAGTAAAAGAATGAAGATTCGAACACAAATACCAGCAGAAATG
CTAGCAAGCATTGACCTGAAGTACTTCAATGAATCAACAAAGAAGAAAATTGAGAAAATAAGGCCTCTTC
TAATAGATGGCACAGCATCACTGAGTCCTGGGATGATGATGGGCATGTTCAACATGCTAAGTACGGTCTT
GGGAGTCTCGATACTGAATCTTGGACAAAAGAAATACACCAAGACAATATACTGGTGGGATGGGCTCCAA
TCATCCGACGATTTTGCTCTCATAGTGAATGCACCAAACCATGAGGGAATACAAGCAGGAGTGGACAGAT
TCTACAGGACCTGCAAGTTAGTGGGAATCAACATGAGCAAAAAGAAGTCCTATATAAATAAGACAGGGAC
ATTTGAATTCACAAGCTTTTTTTATCGCTATGGATTYGTGGCTAATTTTAGCATGGAGCTACCCAGCTTT
GGAGTGTCTGGAGTAAATGAATCAGCTGACATGAGTATTGGAGTAACAGTGATAAAGAACAACATGATAA
ACAATGACCTTGGACCTGCAACGGCCCAGATGGCTCTTCAATTGTTCATCAAAGACTACAGATACACATA
TAGGTGCCATAGGGGAGACACACAAATTCAGACGAGAAGATCATTTGAGTTAAAGAAGCTGTGGGATCAA
ACCCAATCAAAGGTAGGGCTATTAGTATCAGATGGAGGACCAAACTTATACAATATACGGAATCTTCACA
TTCCTGAAGTCTGCTTAAAATGGGAGCTAATGGATGATGATTATCGGGGAAGACTTTGTAATCCCCTGAA
TCCCTTTGTCAGTCATAAAGAGATTGATTCTGTAAACAATGCTGTGGTAATGCCAGCCCATGGTCCAGYC
AAAAGCATGGAATATGATGCCGTTGCAACTACACATTCCTGGATTCCCAAGAGGAATCGTTCTATTCTCA
ACACAAGCCAAAGGGGAATTCTTGAGGATGAACAGATGTACCAGAAGTGCTGCAATCTATTCGAGAAATT
TTTCCCTAGCAGTTCATATAGGAGACCGGTTGGAATTTCTAGCATGGTGGAGGCCATGGTGTCTAGGGCC
CGGATTGATGCCAGGGTCGACTTCGAGTCTGGACGGATCAAGAAAGAAGAGTTCTCTGAGATCATGAAGA
TCTGCTCCACCATTGAAGAACTCAGACGGCAAAAATAATGAATTTAACTTGTCCTTCATGAAA>gi|3992264
25|gb|JX309982.1| Influenza A virus (A/Singapore/KK734/2010(H1N1)) segment 2 polymerase
PB1 (PB1) gene, complete cds; and PB1-F2 gene, complete sequence
AGCAAAAGCAGGCAAACCATTTGAATGGATGTCAATCCGACTCTACTTTTCCTAAAAATTCCAGCGCAAA
ATGCCATAAGCACCACATTCCCTTATACTGGAGATCCTCCATACAGCCATGGAACAGGAACAGGATACAC
CATGGACACAGTAAACAGAACACACCAATACTCAGAAAAGGGAAGGTGGACGACAAACACAGAGACTGGT
GCACCCCAGCTCAACCCGATTGATGGACCACTACCTGAGGATAATGAACCAAGTGGGTATGCACAAACAG
ACTGTGTTCTAGAGGCTATGGCTTTCCTTGAAGAATCCCACCCAGGAATATTTGAGAATTCATGCCTTGA
AACAATGGAAGCTGTTCAACAAACAAGGGTAGATAAACTAACTCAAGGTCGCCAGACTTATGATTGGACA
TTGAACAGAAATCAACCGGCAGCAACTGCATTGGCCAACACCATAGAAGTCTTTAGATCGAATGGCCTAA
CAGCTAATGAGTCAGGAAGGCTAATAGATTTCTTAAAGGATGTAATGGAATCAATGAACAAAGAGGAAAT
AGAGATAACAACCCACTTTCAAAGAAAAAGGAGAGTAAGAGACAACATGACCAAGAAGATGGTCACGCAA
AGAACAATAGGAAAGAAAAAGCAAAGACTGAATAAGAGAGGCTATCTAATAAGAGCACTGACATTAAATA
CGATGACCAAAGATGCAGAGAGAGGCAAGTTAAAAAGAAGGGCTATCGCAACACCTGGGATGCAGATTAG
AGGTTTCGTATACTTTGTTGAAACTTTAGCTAGGAGCATTTGCGAAAAGCTTGAACAGTCTGGGCTCCCA
GTAGGGGGCAATGAAAAGAAGGCCAAACTGGCAAATGTTGTGAGAAAGATGATGACTAATTCACAAGACA
CAGAGATTTCTTTCACAATCACTGGGGACAACACTAAGTGGAATGAAAATCAAAATCCTCGAATGTTCCT
GGCGATGATTACATATATCACCAGAAATCAACCCGAGTGGTTCAGAAACATCCTGAGCATGGCACCCATA
ATGTTCTCAAACAAAATGGCAAGACTAGGGAAAGGGTACATGTTCGAGAGTAAAAGAATGAAGATTCGAA
CACAAATTCCAGCAGAAATGCTAGCAAGCATTGACCTGAAGTACTTCAATGAATCAACAAAGAGGAAAAT

TGAGAAAATAAGGCCTCTTCTAATAGATGGCACAGCATCACTGAGTCCTGGGATGATGATGGGCATGTTC
AACATGCTAAGTACGGTCTTGGGAGTCTCGATACTGAATCTTGGACAAAAGAAATACACCAAGACAACAT
ACTGGTGGGATGGGCTCCAATCATCCGACGATTTTGCTCTCATAGTGAATGCACCAAACCATGAGGGAAT
ACAAGCAGGAGTGGACAGATTCTACAGGACCTGCAAGTTAGTGGGAATCAACATGAGCAAAAAGAAGTCA
TATATAAATAAAACAGGGACATTTGAATTCACAAGCTTTTTTTATCGCTATGGATTTGTGGCTAATTTTA
GCATGGAGCTACCCAGCTTTGGAGTGTCTGGAGTAAATGAATCAGCTGACATGAGTATTGGAGTAACAGT
GATAAAGAACAACATGATAAACAATGACCTTGGACCTGCAACGGCCCAGATGGCTCTTCAATTGTTCATC
AAAGACTACAGATACACATATAGGTGCCATAGGGGAGACACACAAATTCAGACGAGAAGATCATTTGAGT
TAAAAAAGCTGTGGGATCAAACCCAATCAAAGGTAGGGCTATTAGTATCAGATGGAGGACCAAACTTATA
CAATATACGGAATCTTCACATTCCTGAAGTCTGCTTAAAATGGGAGCTAATGGATGATGATTATCGGGGA
AGACTTTGCAATCCCCTGAATCCCTTTGTCAGTCATAAAGAGATTGATTCTGTAAACAATGCTGTGGTAA
TGCCAGCCCATGGTCCAGCCAAAAGCATGGAATATGATGCCGTTGCAACTACACATTCCTGGATTCCCAA
GAGGAATCGTTCTATTCTCAACACAAGCCAAAGGGGAATTCTTGAGGATGAACAGATGTACCAGAAGTGC
TGCAATCTATTCGAGAAATTTTTCCCTAGCAGTTCATATAGGAGACCGGTTGGAATTTCTAGCATGGTGG
AGGCCATGGTGTCTAGGGCCCGGATTGATGCCAGGGTCGACTTCGAGTCTGGACGGATCAAGAAAGAAGA
GTTCTCTGAGATCATGAAGATCTGTTCCACCATTGAAGAACTCAGACGGCAAAAATAATGAATTTAACTT
GTCCTTCATGAAAAAATGCCTTGTTTCTACT
>gi|396940863|dbj|AB704484.1| Influenza A virus (A/Tochigi/10/2010(H1N1)) PB1 gene for polymerase PB1, complete cds
ATGGATGTCAATCCGACTCTACTTTTCCTAAAAATTCCAGCACAAAATGCCATAAGCACCACATTCCCTT
ATACTGGAGATCCTCCATACAGCCATGGAACAGGAACAGGATACACCATGGACACAGTAAACAGAACACA
CCAATACTCAGAAAAGGGAAAGTGGATGACAAACACAGAGACTGGTGCACCCCAGCTCAACCCGATTGAT
GGACCACTACCTGAGGATAATGAACCAAGTGGGTATGCACAAACAGACTGTGTTCTAGAGGCTATGGCTT
TCCTTGAAGAATCCCACCCAGGAATATTTGAGAATTCATGCCTTGAAACAATGGAAGTTGTTCAACAAAC
AAGGGTAGATAAACTAACTCAAGGTCGCCAGACTTATGATTGGACATTAAACAGAAATCAACCGGCAGCA
ACTGCATTGGCCAACACCATAGAAGTCTTTAGATCGAATGGCCTAACAGCTAATGAGTCAGGAAGGCTAA
TAGATTTCTTAAAGGATGTAATGGAATCAATGAACAAAGAGGAAATAGAGATAACAACCCACTTTCAAAG
AAAAAGGAGAGTAAGAGACAACATGACCAAGAAGATGGTCACGCAAAGAACAATAGGGAAGAAAAAACA
A
AGACTGAATAAGAGAGGCTATCTAATAAGAGCACTGACATTAAATACGATGACCAAAGATGCAGAGAGAG
GCAAGTTAAAAAGAAGGGCTATCGCAACACCTGGGATGCAGATTAGAGGTTTCGTATACTTTGTTGAAAC
TTTAGCTAGGAGCATTTGCGAAAAGCTTGAACAGTCTGGGCTCCCAGTAGGGGGCAATGAAAAGAAGGCC
AAACTGGCAAATGTTGTGAGAAAGATGATGACTAATTCACAAGACACAGAGATTTCTTTCACAATCACTG
GGGACAACACTAAGTGGAATGAAAATCAAAATCCTCGAATGTTCCTGGCGATGATTACATATATCACCAG
AAATCAACCCGAGTGGTTCAGAAACATCCTGAGCATGGCACCCATAATGTTCTCAAACAAAATGGCAAGA
CTAGGGAAAGGGTACATGTTCGAGAGTAAAAGAATGAAGATTCGAACACAAATACCAGCAGAAATGCTAG
CAAGCATTGACCTGAAGTACTTCAATGAATCAACAAAGAAGAAAATTGAGAAAATAAGGCCTCTTCTAAT
AGATGGCACAGCATCACTGAGTCCTGGGATGATGATGGGCATGTTCAACATGCTAAGTACGGTCTTGGGA
GTCTCGATACTGAATCTTGGACAAAAGAAATACACCAAGACAATATACTGGTGGGATGGGCTCCAATCAT
CCGACGATTTTGCTCTCATAGTGAATGCACCAAACCATGAGGGAATACAAGCAGGAGTGGACAGATTCTA
CAGGACCTGCAAGTTAGTGGGAATCAACATGAGCAAAAAGAAGTCCTATATAAATAAGACAGGGACATTT
GAATTCACAAGCTTTTTTTATCGCTATGGATTTGTGGCTAATTTTAGCATGGAGCTACCCAGCTTTGGAG
TGTCTGGAGTAAATGAATCAGCTGACATGAGTATTGGAGTAACAGTGATAAAGAACAACATGATAAACAA
TGACCTTGGACCTGCAACGGCCCAGATGGCTCTTCAATTGTTCATCAAAGACTACAGATACACATATAGG

TGCCATAGGGGAGACACACAAATTCAGACGAGAAGATCATTTGAGTTAAAGAAGCTGTGGGATCAAACCC
AATCAAAGGTAGGGCTATTAGTATCAGATGGAGGACCAAACTTATACAATATACGGAATCTTCACATTCC
TGAAGTCTGCTTAAAATGGGAGCTAATGGATGATGATTATCGGGGAAGACTTTGTAATCCCCTGAATCCC
TTTGTCAGTCATAAAGAGATTGATTCTGTAAACAATGCTGTGGTAATGCCAGCCCATGGTCCAGCCAAAA
GCATGGAATATGATGCCGTTGCAACTACACATTCCTGGATTCCCAAGAGGAATCGTTCTATTCTCAACAC
AAGCCAAAGGGGAATTCTTGAGGATGAACAGATGTACCAGAAGTGCTGCAATCTATTCGAGAAATTTTTC
CCTAGCAGTTCATATAGGAGACCGGTTGGAATTTCTAGCATGGTGGAGGCCATGGTGTCTAGGGCCCGGA
TTGATGCCAGGGTCGACTTCGAGTCTGGACGGATCAAGAAAGAAGAGTTCTCTGAGATCATGAAGATCTG
TTCCACCATTGAAGAACTCAGACGGCAAAAATAA
>gi|344995023|gb|CY098096.1| Influenza A virus (A/Chile/89/2010(H1N1)) polymerase PB1 (PB1) gene, complete cds; and PB1-F2 gene, complete sequence
TTGAATGGATGTCAATCCGACTCTACTTTTCCTAAAAATTCCAGCGCAAAATGCCATAAGCACCACATTC
CCTTATACTGGAGATCCTCCATACAGCCATGGAACAGGAACAGGATACACCATGGACACAGTAAACAGAA
CACACCAATACTCAGAAAAGGGAAAGTGGACGACAAACACAGAGACTGGTGCACCCCAGCTCAACCCGAT
TGATGGACCACTACCTGAGGATAATGAACCAAGTGGGTATGCACAAACAGACTGTGTTCTAGAGGCTATG
GCTTTCCTTGAAGAATCCCACCCAGGAATATTTGAGAATTCATGCCTTGAAACAATGGAAGTTGTTCAAC
AAACAAGGGTAGATAAACTAACTCAAGGTCGCCAGACTTATGATTGGACATTAAACAGAAATCAACCGGC
AGCAACTGCATTGGCCAACACCATAGAAGTCTTTAGATCGAATGGCCTAACAGCTAATGAGTCAGGAAGG
CTAATAGATTTCTTAAAGGATGTAATGGAATCAATGAATAAAGAGGAAATAGAGATAACAACCCACTTTC
AAAGAAAAAGGAGAGTAAGAGACAACATGACCAAGAAGATGGTCACGCAAAGAACAATAGGGAAGAAAA
A
ACAAAGACTGAATAAGAGAGGCTATCTAATAAGAGCACTGACATTAAATACGATGACCAAAGATGCAGAG
AGAGGCAAGTTAAAAAGAAGGGCTATCGCAACACCTGGGATGCAGATTAGAGGTTTCGTATACTTTGTTG
AAGCTTTAGCTAGGAGCATTTGCGAAAAGCTTGAACAGTCTGGGCTCCCAGTAGGGGGCAATGAAAAGAA
GGCCAAACTGGCAAATGTTGTGAGAAAGATGATGACTAATTCACAAGACACAGAGATTTCTTTCACAATC
ACTGGGGACAACACTAAGTGGAATGAAAATCAAAATCCTCGAATGTTCCTGGCAATGATTACATATATCA
CCAGAAATCAACCCGAGTGGTTCAGAAACATCCTGAGCATGGCACCCATAATGTTCTCAAACAAAATGGC
AAGACTAGGGAAAGGGTACATGTTCGAGAGTAAAAGAATGAAGATTCGAACACAAATACCAGCAGAAATG
CTAGCAAGCATTGACCTGAAGTACTTCAATGAATCAACGAAGAAGAAAATTGAGAAAATAAGGCCTCTTC
TAATAGATGGCACAGCATCACTGAGTCCTGGGATGATGATGGGCATGTTCAACATGCTAAGTACGGTCTT
GGGAGTCTCGATACTGAATCTTGGACAAAAGAAATACACCAAGACAATATACTGGTGGGATGGACTCCAA
TCATCCGACGATTTTGCTCTCATAGTGAATGCACCAAACCATGAGGGAATACAAGCAGGAGTGGACAGAT
TCTACAGGACCTGCAAGTTAGTGGGAATCAACATGAGCAAAAAGAAGTCCTATATAAATAAGACAGGGAC
ATTTGAATTCACAAGCTTTTTTTATCGCTATGGATTTGTGGCTAATTTTAGCATGGAGCTACCCAGCTTT
GGAGTGTCTGGAGTAAATGAATCAGCTGACATGAGTATTGGAGTAACAGTGATAAAGAACAACATGATAA
ACAATGACCTTGGACCTGCAACGGCCCAGATGGCTCTTCAATTGTTCATCAAAGACTACAGATACACATA
TAGGTGCCATAGGGGAGACACACAAATTCAGACGAGAAGATCATTTGAGTTAAAGAAGCTGTGGGATCAA
ACCCAATCAAAGGTAGGGCTATTAGTATCAGATGGAGGACCAAACTTATACAATATACGGAATCTTCACA
TTCCTGAAGTCTGCTTAAAATGGGAGCTAATGGATGATGATTATCGGGGAAGACTTTGTAATCCCCTGAA
TCCCTTTGTCAGTCATAAAGAGATTGATTCTGTAAACAATGCTGTGGTAATGCCAGCCCATGGTCCAGTC
AAAAGCATGGAATATGATGCCGTTGCAACTACACATTCCTGGATTCCCAAGAGGAATCGTTCTATTCTCA
ACACAAGCCAAAGGGGAATTCTTGAGGATGAACAGATGTACCAGAAGTGCTGCAATCTATTCGAGAAATT
TTTCCCTAGCAGTTCATATAGGAGACCGGTTGGAATTTCTAGCATGGTGGAGGCCATGGTGTCTAGGGCC
CGGATTGATGCCAGGGTCGACTTCGAGTCTGGACGGATCAAGAAAGAAGAGTTCTCTGAGATCATGAAGA

TCTGTTCCACCATTGAAGAACTCAGACGGCAAAAATAATGAATTTAACTTGTCCTTCATGAAA
>gi|344166284|gb|CY097962.1| Influenza A virus (A/Mexico City/WRAIR3569N/2010(H1N1)) polymerase PB1 (PB1) gene, complete cds; and PB1-F2 gene, complete sequence
ATGGATGTCAATCCGACTCTACTTTTCCTAAAAATTCCAGCGCAAAATGCCATAAGCACCACATTCCCTT
ATACTGGAGATCCTCCATACAGCCATGGAACAGGAACAGGATACACCATGGACACAGTAAACAGAACACA
CCAATACTCAGAAAAGGGAAAGTGGACGACAAACACAGAGACTGGTGCACCCCAGCTCAACCCGATTGAT
GGACCACTACCTGAGGATAATGAACCAAGTGGGTATGCACAAACAGACTGTGTTCTAGAGGCTATGGCTT
TCCTTGAAGAATCCCACCCAGGAATATTTGAGAATTCATGCCTTGAAACAATGGAAGTTGTTCAACAAAC
AAGGGTAGATAAACTAACTCAAGGTCGCCAGACTTATGATTGGACATTAAACAGAAATCAACCGGCAGCA
ACTGCATTGGCCAACACCATAGAAGTCTTTAGATCGAATGGCCTAACAGCTAATGAGTCAGGAAGGCTAA
TAGATTTCTTAAAGGATGTAATGGAATCAATGAACAAAGAGGAAATAGAGATAACAACCCACTTTCAAAG
AAAAAGGAGAGTAAGAGACAACATGACCAAGAAGATGGTCACGCAAAGAACAATAGGGAAGAAAAAACA
A
AGACTGAATAAGAGAGGCTATCTAATAAGAGCACTGACATTAAATACGATGACCAAAGATGCAGAGAGAG
GCAAGTTAAAAAGAAGGGCTATCGCAACACCTGGGATGCAGATTAGAGGTTTCGTATACTTTGTTGAAAC
TTTAGCTAGGAGCATTTGCGAAAAGCTTGAACAGTCTGGGCTCCCAGTAGGGGGCAATGAAAAGAAGGCC
AAACTGGCAAATGTTGTGAGAAAGATGATGACTAATTCACAAGACACAGAGATTTCTTTCACAATCACTG
GAGACAACACTAAGTGGAATGAAAATCAAAATCCTCGAATGTTCCTGGCGATGATTACATATATCACCAG
AAATCAACCCGAGTGGTTCAGAAACATCCTGAGCATGGCACCCATAATGTTCTCAAACAAAATGGCAAGA
CTAGGGAAAGGGTACATGTTCGAGAGTAAAAGAATGAAGATTCGAACACAAATACCAGCAGAAATGCTAG
CAAGCATTGACCTGAAGTACTTCAATGAATCAACAAAGAAGAAAATTGAGAAAATAAGGCCTCTTTTAAT
AGATGGCACAGCATCACTGAGTCCTGGGATGATGATGGGCATGTTCAACATGCTAAGTACGGTCTTGGGA
GTCTCGATACTGAATCTTGGACAAAAGAAATACACCAAGACAATATACTGGTGGGATGGGCTCCAATCAT
CCGACGATTTTGCTCTCATAGTGAATGCACCAAACCATGAGGGAATACAAGCAGGAGTGGACAGATTCTA
CAGGACCTGCAAGTTAGTGGGAATCAACATGAGCAAAAAGAAGTCCTATATAAATAAGACAGGGACATTT
GAATTCACAAGCTTTTTTTATCGCTATGGATTTGTGGCTAATTTTAGCATGGAGCTGCCCAGTTTTGGAG
TGTCTGGAGTAAATGAATCAGCTGACATGAGTATTGGAGTAACAGTAATAAAGAACAACATGATAAACAA
TGACCTTGGGCCTGCAACGGCCCAGATGGCTCTTCAATTGTTCATCAAAGACTACAGATACACATATAGG
TGCCATAGGGGAGACACACAAATTCAGACGAGAAGATCATTTGAGTTAAAGAAGCTGTGGGATCAAACCC
AATCAAAGGTAGGGCTATTAGTATCAGATGGAGGACCAAATTTATACAATATACGGAATCTTCACATTCC
TGAAGTCTGCTTAAAATGGGAGCTAATGGATGATGATTATCGGGGAAGACTTTGTAATCCCCTGAATCCC
TTTGTAAGTCATAAAGAGATTGATTCTGTAAACAATGCTGTGGTAATGCCAGCCCATGGTCCAGCCAAAA
GCATGGAGTATGATGCCGTTGCAACTACACATTCCTGGATTCCCAAGAGGAATCGTTCTATTCTCAACAC
AAGCCAAAGGGGAATTCTTGAGGATGAACAGATGTACCAGAAGTGCTGCAATCTATTCGAGAAATTTTTC
CCTAGCAGTTCATATAGGAGACCGGTTGGAATTTCTAGCATGGTGGAGGCCATGGTGTCTAGGGCCCGGA
TTGATGCCAGGGTCGACTTTGAGTCTGGACGGATCAAGAAAGAAGAGTTCTCTGAGATCATGAAGATCTG
TTCCACCATTGAAGAACTCAGACGGCAAAAATAA
>gi|344166266|gb|CY097954.1| Influenza A virus (A/Amman/WRAIR3448T/2010(H1N1)) polymerase PB1 (PB1) gene, complete cds; and PB1-F2 gene, complete sequence
ATGGATGTCAATCCGACTCTACTTTTCCTGAAAATTCCAGCGCAAAATGCCATAAGCACCACATTCCCTT
ATACTGGAGATCCTCCATACAGCCATGGAACAGGAACAGGATACACCATGGACACAGTAAACAGAACACA
CCAATACTCAGAAAAGGGAAAGTGGACGACAAACACAGAGACTGGTGCACCCCAGCTCAACCCGATTGAT
GGACCACTACCTGAGGATAATGAACCAAGTGGGTATGCACAAACAGACTGTGTTCTAGAGGCTATGGCTT
TCCTTGAAGAATCCCACCCAGGAATATTTGGGAATTCATGCCTTGAAACAATGGAAGTTGTTCAACAAAC

AAGGGTAGATAAACTAACTCAAGGTCGCCAGACTTATGATTGGACATTAAACAGAAATCAACCGGCAGCA
ACTGCATTGGCCAACACCATAGAAGTCTTTAGATCGAATGGCCTAACAGCTAATGAGTCAGGAAGGCTAA
TAGATTTCTTAAAGGATGTAATGGAATCAATGAACAAAGAGGAAATAGAGATAACAACCCACTTTCAAAG
AAAAAGGAGAGTAAGAGACAACATGACCAAGAAGATGGTCACGCAAAGAACAATAGGGAAGAAAAAACA
A
AAACTGAATAAGAGAGGCTATCTAATAAGAGCACTGACGTTAAATACGATGACCAAAGATGCAGAGAGAG
GCAAGTTAAAAAGAAGGGCTATCGCAACACCTGGGATGCAGATTAGAGGTTTCGTATACTTTGTTGAAAC
TTTAGCTAGGAGCATTTGCGAAAAGCTTGAACAGTCTGGGCTCCCAGTAGGGGGCAATGAAAAGAAGGCC
AAACTGGCAAATGTTGTGAGAAAGATGATGACTAATTCACAAGACACAGAGATTTCTTTCACAATCACTG
GGGACAACACTAAGTGGAATGAAAATCAAAATCCTCGAATGTTCCTGGCGATGATTACATATATCACCAG
AAATCAACCCGAGTGGTTCAGAAACATCCTGAGCATGGCACCCATAATGTTCTCAAACAAAATGGCAAGG
CTAGGGAAAGGGTACATGTTCGAGAGTAAAAGAATGAAGATTCGAACACAAATACCAGCAGAAATGCTAG
CAAGCATTGACCTGAAGTACTTCAATGAATCAACAAAGAAGAAAATTGAGAAAATAAGGCCTCTTCTAAT
AGATGGCACAGCATCACTGAGTCCTGGGATGATGATGGGCATGTTCAACATGCTAAGTACGGTCTTGGGA
GTCTCGATACTGAATCTTGGACAAAAGAAATACACCAAGACAATATACTGGTGGGATGGGCTCCAATCAT
CCGACGATTTTGCTCTCATAGTGAATGCACCAAACCATGAGGGAATACAAGCAGGAGTGGACAGATTCTA
CAGGACCTGCAAGTTAGTGGGAATCAACATGAGCAAAAAGAAGTCCTATATAAATAAGACAGGGACATTT
GAATTCACAAGCTTTTTTTATCGCTATGGATTTGTGGCTAATTTTAGCATGGAGCTACCCAGCTTTGGAG
TGTCTGGAGTAAATGAATCAGCTGACATGAGTATTGGAGTAACAGTGATAAAGAACAACATGATAAACAA
TGACCTTGGACCTGCAACGGCCCAGATGGCTCTTCAATTGTTCATCAAAGACTACAGATACACATATAGG
TGCCATAGGGGAGACACACAAATTCAGACGAGAAGATCATTTGAGTTAAAGAAGCTGTGGGATCAAACCC
AATCAAAGGTAGGGCTATTAGTATCAGATGGAGGACCAAACTTATACAATATACGGAATCTTCACATTCC
TGAAGTCTGCTTAAAATGGGAGCTGATGGATGATGATTATCGGGGAAGACTTTGTAATCCCCTGAATCCC
TTTGTCAGTCATAAAGAGATTGATTCTGTAAACAATGCTGTGGTAATGCCAGCCCATGGTCCAGTCAAAA
GCATGGAATATGATGCCGTTGCAACTACACATTCCTGGATTCCCAAGAGGAATCGTTCTATTCTCAACAC
AAGCCAAAGGGGAATTCTTGAGGATGAACAGATGTATCAGAAGTGCTGCAATCTATTCGAGAAATTTTTC
CCTAGCAGTTCATATAGGAGACCGGTTGGAATTTCTAGCATGGTGGAGGCCATGGTGTCTAGGGCCCGGA
TTGATGCCAGGGTCGACTTCGAGTCTGGACGGATCAAGAAAGAAGAGTTCTCTGAGATCATGAAGATCTG
TTCCACCATTGAAGAACTCAGACGGCAAAAATAA
>gi|344165956|gb|CY097843.1| Influenza A virus (A/District of Columbia/WRAIR0310/2010(H1N1)) polymerase PB1 (PB1) gene, complete cds; and PB1-F2 gene, complete sequence
ATGGATGTCAATCCGACTCTACTTTTCCTAAAAATTCCAGCGCAAAATGCCATAAGCACCACATTCCCTT
ATACTGGAGATCCTCCATACAGCCATGGAACGGGAACAGGATACACCATGGACACAGTAAACAGAACACA
CCAATACTCAGAAAAGGGAAAGTGGACGACAAACACAGAGACTGGTGCACCCCAGCTCAACCCGATTGAT
GGACCACTACCTGAGGATAATGAACCAAGTGGGTATGCACAAACAGACTGTGTTCTAGAGGCTATGGCTT
TCCTTGAAGAATCCCACCCAGGAATATTTGAGAATTCATGCCTTGAAACAATGGAAGTTGTTCAACAAAC
AAGGGTAGATAAACTAACTCAAGGTCGCCAGACTTATGATTGGACATTAAACAGAAATCAACCGGCAGCA
ACTGCATTGGCCAACACCATAGAAGTCTTTAGATCGAATGGCCTAACAGCTAATGAATCAGGAAGACTAA
TAGATTTCTCAAAGGATGTGATGGAATCAATGGATAAAGAGGAAATGGAGATAACAACACACTTTCAAAG
AAAAAGGAAAGTAAGGGACAACATGACCAAGAAAATGGTCACACAAAGAACAATAGGGAAGAAAAAACA
A
AGAGTGAATAAAAGAGGCTACCTAATAAGAGCTTTGACATTGAACACGATGACCAAAGATGCAGAGAGAG
GCAAATTAAAAAGAAGGGCTATTGCAACACCCGGGATGCAAATTAGAGGTTTCGTATACTTTGTTGAAAC

TTTAGCTAGGAGCATTTGCGAAAAGCTTGAACAGTCTGGGCTCCCAGTAGGGGGCAATGAAAAGAAGGCC
AAACTGGCAAATGTTGTGAGAAAAATGATGACTAATTCACAAGACACAGAGCTTTCTTTCACAATCACTG
GGGACAACACTAAGTGGGATGAAAATCAAAACCCTCGAATGTTTCTGGCGATGATTACATACATCACAAA
GAATCAACCTGAGTGGTTCAGAAACATCCTGAGCATCGCACCAATAATGTTCTCAAACAAAATGGCAAGA
CTGGGAAAAGGATACATGTTCGAGAGTAAGAAAATGAAGCTCCGGACACAAATACCTGCAGAAATGCTAG
CAAGCATTGACCTGAAGTACTTCAATGAATCAACAAAGAAGAAAATTGAGAAAATAAGGCCTCTTTTAAT
AGATGGCACAGCATCACTGAGTCCTGGGATGATGATGGGCATGTTCAACATGCTAAGTACGGTCTTGGGA
GTCTCGATACTGAATCTTGGACAAAAGAAATACACCAAGACAATATACTGGTGGGATGGGCTCCAATCAT
CCGACGATTTTGCTCTCATAGTGAATGCACCAAACCATGAGGGAATACAAGCAGGAGTGGACAGATTCTA
CAGGACCTGCAAGTTAGTGGGAATCAACATGAGCAAAAAGAAGTCCTATATAAATAAGACAGGGACATTT
GAATTCACAAGCTTTTTTTATCGCTATGGATTTGTGGCTAATTTTAGCATGGAGCTACCCAGCTTTGGAG
TGTCTGGAGTAAATGAATCAGCTGACATGAGTATTGGAGTAACAGTAATAAAGAACAACATGATAAACAA
TGACCTTGGGCCTGCAACGGCCCAGATGGCTCTTCAATTGTTCATCAAAGACTACAGATACACATATAGG
TGCCATAGGGGAGACACACAAATTCAGACGAGAAGATCATTTGAGTTAAAGAAGCTGTGGGATCAAACCC
AATCAAAGGTAGGGCTATTAGTATCAGATGGAGGACCAAATTTATACAATATACGGAATCTTCACATTCC
TGAAGTCTGCTTAAAATGGGAGCTAATGGATGATGATTATCGGGTAAGACTTTGTAATCCCCTGAATCCC
TTTGTAAGTCATAAAGAGATTGATTCTGTAAACAATGCTGTGGTAATGCCAGCCCATGGTCCAGCCAAAA
GCATGGAGTATGATGCCGTTGCAACTACACATTCCTGGATTCCCAAGAGGAATCGTTCTATTCTCAACAC
AAGCCAAAGGGGAATTCTTGAGGATGAACAGATGTACCAGAAGTGCTGCAATCTATTCGAGAAATTTTTC
CCTAGCAGTTCATATAGGAGACCGGTTGGAATTTCTAGCATGGTGGAGGCCATGGTGTCTAGGGCCCGGA
TTGATGCCAGGGTCGACTTTGAGTCTGGACGGATCAAGAAAGAAGAGTTCTCTGAGATCATGAAGATCTG
TTCCACCATTGAAGAACTCAGACGGCAAAAATAA
>gi|344165830|gb|CY097787.1| Influenza A virus (A/Dakar/WRAIR0020T/2010(H1N1)) polymerase PB1 (PB1) gene, complete cds; and PB1-F2 gene, complete sequence
ATGGATGTCAATCCGACTCTACTTTTCCTAAAAATTCCAGCGCAAAATGCCATAAGCACCACATTCCCTT
ATACTGGAGATCCTCCATACAGCCATGGAACAGGAACAGGATACACCATGGACACAGTAAACAGAACACA
CCAATACTCAGAAAAGGGAAAGTGGACGACAAACACAGAGACTGGTGCACCCCAGCTCAACCCGATTGAT
GGACCACTACCTGAGGATAATGAACCAAGTGGGTATGCACAAACAGACTGTGTTCTAGAGGCTATGGCTT
TCCTTGAAGAATCCCACCCGGGAATATTTGAGAATTCATGCCTTGAAACAATGGAAGTTGTTCAACAAAC
AAGGGTAGATAAACTAACTCAAGGTCGCCAGACTTATGATTGGACATTAAACAGAAATCAACCGGCAGCA
ACTGCATTGGCCAACACCATAGAAGTCTTTAGATCGAATGGCCTAACAGCTAATGAGTCAGGAAGGCTAA
TAGATTTCTTAAAGGATGTAATGGAATCAATGAACAAAGAGGAAATAGAGATAACAACCCACTTTCAAAG
AAAAAGGAGAGTAAGAGACAACATGACCAAGAAGATGGTCACGCAAAGAACAATAGGGAAGAAAAAACA
A
AGACTGAATAAGAGAGGCTATCTAATAAGAGCACTGACATTAAATACGATGACCAAAGATGCAGAGAGAG
GCAAGTTAAAAAGAAGGGCTATCGCAACACCTGGGATGCAGATTAGAGGTTTCGTATACTTTGTTGAAAC
TTTAGCTAGGAGCATTTGCGAAAAGCTTGAACAGTCTGGGCTCCCAGTAGGGGGCAATGAAAAGAAAGCC
AAATTGGCAAATGTTGTGAGAAAGATGATGACTAATTCACAAGACACAGAGATTTCTTTCACAATCACTG
GGGACAACACTAAGTGGAATGAAAATCAAAATCCTCGAATGTTCCTGGCGATGATTACATATATCACCAG
AAATCAACCCGAGTGGTTCAGAAACATCCTGAGCATGGCACCCATAATGTTCTCAAACAAAATGGCAAGA
ATAGGGAGAGGGTACATGTTCGAGAGTAAAAGAATGAAGATTCGAACACAAATACCAGCAGAAATGCTAG
CAAGCATTGACCTGAAGTACTTCAATGAATCAACAAAGAAGAAAATTGAGAAAATAAGGCCTCTTCTAAT
AGATGGCACAGCATCACTGAGTCCTGGGATGATGATGGGCATGTTCAACATGCTAAGTACGGTCTTGGGA
GTCTCGATACTGAATCTTGGACAAAAGAAATACACCAAGACAATATACTGGTGGGATGGGCTCCAATCAT

CCGACGATTTTGCTCTCATAGTGAATGCACCAAACCATGAGGGAATACAAGCAGGAGTGGACAGATTCTA
CAGGACCTGCAAGTTAGTGGGAATCAACATGAGCAAAAAGAAGTCCTATATAAATAAGACAGGGACATTT
GAATTCACAAGCTTTTTTTATCGCTATGGATTTGTGGCTAATTTTAGCATGGAGCTACCCAGCTTTGGAG
TATCTGGAGTAAATGAATCAGCTGACATGAGTATTGGAGTAACAGTGATAAAGAACAACATGATAAACAA
TGACCTTGGACCTGCAACGGCCCAGATGGCTCTTCAATTGTTCATCAAAGACTACAGATACACATATAGG
TGCCATAGGGGAGACACACAAATTCAGACGAGAAGATCATTTGAGTTAAAGAAGCTGTGGGATCAAACCC
AATCAAAGGCAGGGCTATTAGTATCAGATGGAGGACCAAACTTATACAATATACGGAATCTTCACATTCC
TGAAGTCTGCTTAAAATGGGAGCTAATGGATGATGATTATCGGGGAAGACTTTGTAATCCCCTGAATCCC
TTTGTCAGTCATAAAGAGATTGATTCTGCAAACAATGCTGTGGTAATGCCAGCCCATGGTCCAGCCAAAA
GCATGGAATATGATGCCGTTGCAACTACACATTCCTGGATTCCCAAGAGGAATCGTTCTATTCTCAACAC
AAGCCAAAGGGGAATTCTTGAGGATGAACAGATGTACCAGAAGTGCTGCAATCTATTCGAGAAATTTTTC
CCTAGCAGTTCATATAGGAGACCGGTTGGAATTTCAAGCATGGTGGAGGCCATGGTGTCTAGGGCCCGGA
TTGATGCCAGGGTCGACTTCGAGTCTGGACGGATCAAGAAAGAAGAGTTCTCTGAGATCACGAAGATCTG
TTCCACCATTGAAGAACTCAGACGGCAAAAATAA
>gi|334127602|gb|CY090843.1| Influenza A virus (A/Managua/0305_10/2010(H1N1)) polymerase PB1 (PB1) gene, complete cds; and PB1-F2 gene, complete sequence
TTGAATGGATGTCAATCCGACTCTACTTTTCCTAAAAATTCCAGCGCAAAATGCCATAAGCACCACATTC
CCTTATACTGGAGATCCTCCATACAGCCATGGAACAGGAACAGGATACACCATGGACACAGTAAACAGAA
CACACCAATACTCAGAAAAGGGAAAGTGGACGACAAACACAGAGACTGGTGCACCCCAGCTCAACCCGAT
TGATGGACCACTACCTGAGGATAATGAACCAAGTGGGTATGCACAAACAGACTGTGTTCTAGAGGCTATG
GCTTTCCTTGAAGAATCCCACCCAGGAATATTTGAGAATTCATGCCTTGAAACAATGGAAGTTGTTCAAC
AAACAAGGGTAGATAAACTAACTCAAGGTCGCCAGACTTATGATTGGACATTAAACAGAAATCAACCGGC
AGCAACTGCATTGGCCAACACCATAGAAGTCTTTAGATCGAATGGCCTAACAGCTAATGAGTCAGGAAGG
CTAATAGATTTCTTAAAGGATGTAATGGAGTCAATGAACAAAGAGGAAATAGAGATAACAACCCACTTTC
AAAGAAAAAGGAGAGTAAGAGACAACATGACCAAGAAGATGGTCACGCAAAGAACAATAGGGAAGAAAA
A
ACAAAGACTGAATAAGAGAGGCTATCTAATAAGAGCACTGACATTAAATACGATGACCAAAGATGCAGAG
AGAGGCAAGTTAAAAAGAAGGGCTATCGCAACACCTGGGATGCAGATTAGAGGTTTCGTATACTTTGTTG
AAACTTTAGCTAGGAGCATTTGCGAAAAGCTTGAACAGTCTGGGCTCCCAGTAGGGGGCAATGAAAAGAA
GGCCAAACTGGCAAATGTTGTGAGAAAGATGATGACTAATTCACAAGACACAGAGATTTCTTTCACAATC
ACTGGGGACAACACTAAGTGGAATGAAAATCAAAATCCTCGAATGTTCCTGGCGATGATTACATATATCA
CCAGAAATCAACCCGAGTGGTTCAGAAACATCCTGAGCATGGCACCCATAATGTTCTCAAACAAAATGGC
AAGACTAGGGAAAGGGTACATGTTCGAGAGTAAAAGAATGAAGATTCGAACACAAATACCAGCAGAAATG
CTAGCAAGCATTGACCTGAAATACTTCAATGAATCAACAAAGAAGAAAATTGAGAAAATAAGGCCTCTTC
TAATAGATGGCACAGCATCACTGAGTCCTGGGATGATGATGGGCATGTTCAACATGCTAAGTACGGTCTT
GGGAGTCTCGATATTGAATCTTGGACAAAAGAAATACACCAAGACAATATACTGGTGGGATGGGCTCCAA
TCATCCGACGATTTTGCTCTCATAGTGAATGCACCAAACCATGAGGGAATACAAGCAGGAGTGGACAGAT
TCTACAGGACCTGCAAGTTAGTGGGAATCAACATGAGCAAAAAGAAGTCCTATATAAATAAGACAGGGAC
ATTTGAATTCACAAGCTTTTTTTATCGCTATGGATTTGTGGCTAATTTTAGCATGGAGCTACCCAGCTTT
GGAGTGTCTGGAGTAAATGAATCAGCTGACATGAGTATTGGAGTAACAGTGATAAAGAACAACATGATAA
ACAATGACCTTGGACCTGCAACGGCCCAGATGGCTCTTCAATTGTTCATCAAAGACTACAGATACACATA
TAGGTGCCATAGGGGAGACACACAAATTCAGACGAGAAGATCATTTGAGTTAAAAAAGCTGTGGGATCAA
ACCCAATCAAAGGCAGGGCTATTAGTATCAGATGGAGGACCAAACGTATACAATATACGGAATCTTCACA
TTCCTGAAGTCTGCTTAAAATGGGAGCTAATGGATGATGATTATCGGGGAAGACTTTGTAATCCCCTGAA

CCCCTTTGTCAGTCATAAAGAGATTGATTCTGTAAACAATGCTGTGGTAATGCCAGCCCATGGTCCAGTC
AAAAGCATGGAATATGATGCCGTTGCAACTACACATTCCTGGATTCCCAAGAGGAATCGTTCTATTCTCA
ACACAAGCCAAAGGGGAATTCTTGAGGATGAACAGATGTACCAGAAGTGCTGCAATCTATTCGAGAAATT
TTTCCCTAGCAGTTCATATAGGAGACCGGTTGGAATTTCTAGCATGGTGGAGGCCATGGTGTCTAGGGCC
CGGATTGATGCCAGGGTCGACTTCGAGTCTGGACGGATCAAGAAAGAAGAGTTCTCTGAGATCATGAAGA
TCTGTTCCACCATTGAAGAACTCAGACGGCAAAAATAATGAATTTAACTTGTCCTTCATGAAAAAATGC
>gi|343132059|gb|CY096280.1| Influenza A virus (A/Melbourne/INS475/2010(H1N1)) polymerase PB1 (PB1) gene, complete cds; and PB1-F2 gene, complete sequence
TTGAATGGATGTCAATCCGACTCTACTTTTCCTAAAAATTCCAGCGCAAAATGCCATAAGCACCACATTC
CCTTATACTGGAGATCCTCCATACAGCCATGGAACAGGAACAGGATACACCATGGACACAGTAAACAGAA
CACACCAATACTCAGAAAAGGGAAAGTGGACGACAAACACAGAGACTGGTGCACCCCAGCTCAACCCGAT
TGATGGACCACTACCTGAGGATAATGAACCAAGTGGGTATGCACAAACAGACTGTGTTCTAGAGGCTATG
GCTTTCCTTGAAGAATCCCACCCAGGAATATTTGAGAATTCATGCCTTGAAACAATGGAAGTTGTTCAAC
AAACAAGGGTAGATAAACTAACTCAAGGTCGCCAGACTTATGATTGGACATTAAACAGAAATCAACCGGC
AGCAACTGCATTGGCCAACACCATAGAAGTCTTTAGATCGAATGGCCTAACAGCTAATGAGTCAGGAAGG
CTAATAGATTTCTTAAAGGATGTAATGGAATCAATGAACAAAGAGGAAATAGAGATAACAACCCACTTTC
AAAGAAAAAGGAGAGTAAGAGACAACATGACCAAGAAGATGGTCACGCAAAGAACAATAGGGAAGAAAA
A
ACAAAGACTGAATAAGAGAGGCTATCTAATAAGAGCACTGACATTAAATACGATGACCAAAGATGCAGAG
AGAGGCAAGTTAAAAAGAAGGGCTATCGCAACACCTGGGATGCAGATTAGAGGTTTCGTATACTTTGTTG
AAGCTTTAGCTAGGAGCATTTGCGAAAAGCTTGAACAGTCTGGGCTCCCAGTAGGGGGCAATGAAAAGAA
GGCCAAACTGGCAAATGTTGTGAGAAAGATGATGACTAATTCACAAGACACAGAGATTTCTTTCACAATC
ACTGGGGACAACACTAAGTGGAATGAAAATCAAAATCCTCGAATGTTCCTGGCAATGATTACATATATCA
CCAGAAATCAACCCGAGTGGTTCAGAAATATCCTGAGCATGGCACCCATAATGTTCTCAAACAAAATGGC
AAGACTAGGGAAAGGGTACATGTTCGAGAGTAAAAGAATGAAGATTCGAACACAAATACCAGCAGAAATG
CTAGCAAGCATTGACCTGAAGTACTTCAATGAATCAACGAAGAAGAAAATTGAGAAAATAAGGCCTCTTC
TAATAGATGGCACAGCATCACTGAGTCCTGGGATGATGATGGGCATGTTCAACATGCTAAGTACGGTCTT
GGGAGTCTCGATACTGAATCTTGGACAAAAGAAATACACCAAGACAATATACTGGTGGGATGGGCTCCAA
TCATCCGACGATTTTGCTCTCATAGTGAATGCACCAAACCATGAGGGAATACAAGCAGGAGTGGACAGAT
TCTACAGGACCTGCAAGTTAGTGGGAATCAACATGAGCAAAAAGAAGTCCTATATAAATAAGACAGGGAC
ATTTGAATTCACAAGCTTTTTTTATCGCTATGGATTTGTGGCTAATTTTAGCATGGAGCTACCCAGCTTT
GGAGTGTCTGGAGTAAATGAATCAGCTGACATGAGTATTGGAGTAACAGTGATAAAGAACAACATGATAA
ACAATGACCTTGGACCTGCAACGGCCCAGATGGCTCTTCAATTGTTCATCAAAGACTACAGATACACATA
TAGGTGCCATAGGGGAGACACACAAATTCAGACGAGAAGATCATTTGAGTTAAAGAAGCTGTGGGATCAA
ACCCAATCAAAGGTAGGGCTATTAGTATCAGATGGAGGACCAAACTTATACAATATACGGAATCTTCACA
TTCCTGAAGTCTGCTTAAAATGGGAGCTAATGGATGATGATTATCGGGGAAGACTTTGTAATCCCCTGAA
TCCCTTTGTCAGTCATAAAGAGATTGATTCTGTAAATAATGCTGTGGTAATGCCAGCCCATGGTCCAGTC
AAAAGCATGGAATATGATGCCGTTGCAACTACACATTCCTGGATTCCCAAGAGGAATCGTTCTATTCTCA
ACACAAGCCAAAGGGGAATTCTTGAGGATGAACAGATGTACCAGAAGTGCTGCAATCTATTCGAGAAATT
TTTCCCTAGCAGTTCATATAGGAGACCGGTTGGAATTTCTAGCATGGTGGAGGCCATGGTGTCTAGGGCC
CGGATTGATGCCAGGGTCGACTTCGAGTCTGGACGGATCAAGAAAGAAGAGTTCTCCGAGATCATGAAGA
TCTGTTCCACCATTGAAGAACTCAGACGGCAAAAATAATGAATTTAACTTGTCCTTCATGAAA
>gi|339851070|gb|CY093195.1| Influenza A virus (A/Chile/106/2010(H1N1)) polymerase PB1 (PB1) gene, complete cds; and PB1-F2 gene, complete sequence

TTGAATGGATGTCAATCCGACTCTACTTTTCCTAAAAATTCCAGCGCAAAATGCCATAAGCACCACATTC
CCTTATACTGGAGATCCTCCATACAGCCATGGAACAGGAACAGGATACACCATGGACACAGTAAACAGAA
CACACCAATACTCAGAAAAGGGAAAGTGGACGACAAACACAGAGACTGGTGCACCCCAGCTCAACCCGAT
TGATGGACCACTACCTGAGGATAATGAACCAAGTGGGTATGCACAAACAGACTGTGTTCTAGAGGCTATG
GCTTTCCTTGAAGAATCCCACCCAGGAATATTTGAGAATTCATGCCTTGAAACAATGGAAGTTGTTCAAC
AAACAAGGGTAGATAAACTAACTCAAGGTCGCCAGACTTATGATTGGACATTAAACAGAAATCAACCGGC
AGCAACTGCATTGGCCAACACCATAGAAGTCTTTAGATCGAATGGCCTAACAGCTAATGAGTCAGGAAGG
CTAATAGATTTCTTAAAGGATGTAATGGAATCAATGAACAAAGAGGAAATAGAGATAACAACCCACTTTC
AAAGAAAAAGGAGAGTAAGAGACAACATGACCAAGAAGATGGTCACGCAAAGAACAATAGGGAAGAAAA
A
ACAAAGGCTGAATAAGAGAGGCTATCTAATAAGAGCACTGACATTAAATACGATGACCAAAGATGCAGAG
AGAGGCAAGTTAAAAAGAAGGGCTATCGCAACACCTGGGATGCAGATTAGAGGTTTCGTATACTTTGTTG
AAGCTTTAGCTAGGAGCATTTGCGAAAAGCTTGAACAGTCTGGGCTCCCAGTAGGGGGCAATGAAAAGAA
GGCCAAACTGGCAAATGTTGTGAGAAAGATGATGACTAATTCACAAGACACAGAGATTTCTTTCACAATC
ACTGGGGACAACACTAAGTGGAATGAAAATCAAAATCCTCGAATGTTCCTGGCAATGATTACATATATCA
CCAGAAATCAACCCGAGTGGTTCAGAAACATCCTGAGCATGGCACCCATAATGTTCTCAAACAAAATGGC
AAGACTAGGGAAAGGGTACATGTTCGAGAGTAAAAGAATGAAGATTCGAACACAAATACCAGCAGAAATG
CTAGCAAGCATTGACCTGAAGTACTTCAATGAATCAACGAAGAAGAAAATTGAGAAAATAAGGCCTCTTC
TAATAGATGGCACAGCATCACTGAGTCCTGGGATGATGATGGGCATGTTCAACATGCTAAGTACGGTCTT
GGGAGTCTCGATACTGAATCTTGGACAAAAGAAATACACCAAGACAATATACTGGTGGGATGGACTCCAA
TCATCCGACGATTTTGCTCTCATAGTGAATGCACCAAACCATGAGGGAATACAAGCAGGAGTGGACAGAT
TCTACAGGACCTGCAAGTTAGTGGGAATCAACATGAGCAAAAAGAAGTCCTATATAAATAAGACAGGGAC
ATTTGAATTCACAAGCTTTTTTTATCGCTATGGATTTGTGGCTAATTTTAGCATGGAGCTACCCAGCTTT
GGAGTGTCTGGAGTAAATGAATCAGCTGACATGAGTATTGGAGTAACAGTGATAAAGAACAACATGATAA
ACAATGACCTTGGACCTGCAACGGCCCAGATGGCTCTTCAATTGTTCATCAAAGACTACAGATACACATA
TAGGTGCCATAGGGGAGACACACAAATTCAGACGAGAAGATCATTTGAGTTAAAGAAGCTGTGGGATCAA
ACCCAATCAAAGGTAGGGCTATTAGTATCAGATGGAGGACCAAACTTATACAATATACGGAATCTTCACA
TTCCTGAAGTCTGCTTAAAATGGGAGCTAATGGATGATGATTATCGGGGAAGACTTTGTAATCCCCTGAA
TCCCTTTGTCAGTCATAAAGAGATTGATTCTGTAAACAATGCTGTGGTAATGCCAGCCCATGGTCCAGTC
AAAAGCATGGAATATGATGCCGTTGCAACTACACATTCCTGGATTCCCAAGAGGAATCGTTCTATTCTCA
ACACAAGCCAAAGGGGAATTCTTGAGGATGAACAGATGTACCAGAAGTGCTGCAATCTATTCGAGAAATT
TTTCCCTAGCAGTTCATATAGGAGACCGGTTGGAATTTCTAGCATGGTGGAGGCCATGGTGTCTAGGGCC
CGGATTGATGCCAGGGTCGACTTCGAGTCTGGACGGATCAAGAAAGAAGAGTTCTCTGAGATCATGAAGA
TCTGTTCCACCATTGAAGAACTCAGACGGCAAAAATAATGAATTTAACTTGTCCTTCATGAAA
>gi|359828258|gb|JQ041360.1| Influenza A virus (A/Novosibirsk/KSH/2011(H1N1)) segment 2 polymerase PB1 (PB1) gene, complete cds; and nonfunctional PB1-F2 protein (PB1-F2) gene, complete sequence
CAAACCATTTGAATGGATGTCAATCCGACTCTACTTTTCCTAAAAATTCCAGCGCAAAATGCCATAAGCA
CCACATTCCCTTATACTGGAGATCCTCCATACAGCCATGGAACAGGAACAGGATACACCATGGACACAGT
AAACAGAACACACCAATACTCAGAAAAGGGAAAGTGGACGACAAACACAGAGACTGGTGCACCCCAGCTC
AACCCGATTGATGGACCACTACCTGAGGATAATGAACCAAGTGGGTATGCACAAACAGACTGTGTTCTAG
AGGCTATGGCTTTCCTTGAAGAATCCCACCCAGGAATATTTGAGAATTCATGCCTTGAAACAATGGAAGT
TGTTCAACAAACAAGGGTAGATAAACTGACTCAAGGTCGCCAGACTTATGATTGGACATTAAACAGAAAT
CAACCGGCAGCAACTGCATTGGCCAACACCATAGAAGTCTTTAGATCGAATGGCCTAACAGCTAATGAGT

CAGGAAGGCTAATAGATTTCTTAAAGGATGTAATGGAATCAATGAACAAAGAGGAAATAGAGATAACAAC
CCACTTTCAAAGAAAAAGGAGAGTAAGAGACAACATGACCAAGAAGATGGTCACGCAAAGAACAATAGGG
AAGAAAAAACAGAGACTGAATAAGAGAGGCTATCTAATAAGAGCACTGACATTAAATACGATGACCAAAG
ATGGGGAGAGAGGCAAGTTAAAAAGAAGGGCTATCGCAACACCTGGGATGCAGATTAGAGGTTTCGTATA
CTTTGTTGAAACTTTAGCTAGGAGCATTTGCGAAAAGCTTGAACAGTCTGGGCTCCCAGTAGGGGGCAAT
GAAAAGAAGGCCAAACTGGCAAATGTTGTGAGAAAGATGATGACTAATTCACAAGACACAGAGATTTCTT
TCACAATCACTGGGGACAACACTAAGTGGAATGAAAATCAAAATCCTCGAATGTTCCTGGCGATGATTAC
ATATATCACCAGAAATCAACCCGAGTGGTTTAGAAACATCCTGAGCATGGCACCCATAATGTTCTCAAAC
AAAATGGCAAGACTAGGGAAAGGGTACATGTTCGAGAGTAAAAGAATGAAGATTCGAACACAAATACCAG
CAGAAATGCTAGCAAGCATTGACCTGAAGTACTTCAATGAATCAACAAAGAAGAAAATTGAGAAAATAAG
GCCTCTTTTAATGGATGGCACAGCATCACTGAGTCCTGGGATGATGATGGGCATGTTCAACATGCTAAGT
ACGGTCTTGGGAGTCTCGATACTGAATCTTGGACAAAAGAAATACACCAAGACAACATACTGGTGGGATG
GGCTCCAATCATCCGACGATTTTGCTCTCATAGTGAATGCACCAAACCATGAGGGAATACAAGCAGGAGT
GGACAGATTCTACAGGACCTGCAAGTTAGTGGGAATCAACATGAGCAAAAAGAAGTCCTATATAAATAAG
ACAGGGACATTTGAATTCACAAGCTTTTTTTATCGCTATGGATTTGTGGCTAATTTTAGCATGGAGCTAC
CCAGCTTTGGAGTGTCTGGAGTAAATGAATCAGCTGACATGAGTATTGGAGTAACAGTGATAAAGAACAA
CATGATAAACAATGACCTTGGACCTGCAACGGCCCAGATGGCTCTTCAATTGTTCATCAAAGACTACAGA
TACACATATAGGTGCCATAGGGGAGACACACAAATTCAGACGAGAAGATCATTTGAGTTAAAGAAGCTGT
GGGATCAAACCCAATCAAAGGTAGGGCTATTAGTATCAGATGGAGGACCAAACTTATACAATATACGGAA
TCTTCACATTCCTGAAGTCTGCTTAAAATGGGAGCTAATGGATGATGATTATCGGGGAAGACTTTGTAAT
CCCCTGAATCCCTTTGTAAGTCATAAGGAGATTGATTCTGTAAACAATGCTGTGGTAATGCCAGCCCATG
GTCCAGCCAAAAGCATGGAATATGATGCCGTTGCAACTACACATTCCTGGATTCCCAAGAGGAATCGTTC
TATTCTCAACACAAGCCAAAGGGGAATTCTTGAGGATGAACAGATGTACCAGAAGTGCTGCAATCTATTC
GAGAAATTTTTCCCTAGCAGTTCATATAGGAGACCGGTTGGAATTTCTAGCATGGTGGAGGCCATGGTGT
CTAGGGCCCGGATTGATGCCAGGGTCGACTTTGAGTCTGGACGGATCAAGAAAGAAGAGTTCTCTGAGAT
CATGAAGATCTGTTCCACCATTGAAGAACTCAGACGGCAAAAATAATGAATTTAACTTGTCCTTCATGAA
AAAATG
>gi|344166500|gb|CY098058.1| Influenza A virus (A/Moscow/WRAIR4316T/2011(H1N1)) polymerase PB1 (PB1) gene, complete cds; and PB1-F2 gene, complete sequence
ATGGATGTCAATCCGACTCTACTTTTCCTAAAAATTCCAGCGCAAAATGCCATAAGCACCACATTCCCTT
ATACTGGAGATCCTCCATACAGCCATGGAACAGGAACAGGATACACCATGGACACAGTAAACAGAACACA
CCAATACTCAGAAAAGGGAAAGTGGACGACAAACACAGAGACTGGTGCACCCCAGCTCAACCCGATTGAT
GGACCACTACCTGAGGATAATGAACCAAGTGGGTATGCACAAACAGACTGTGTTCTAGAGGCTATGGCTT
TCCTTGAAGAATCCCACCCAGGAATATTTGAGAATTCATGCCTTGAAACAATGGAAGCTGTTCAACAAAC
AAGGGTAGATAAACTAACTCAAGGTCGCCAGACTTATGATTGGACATTAAACAGAAATCAACCGGCAGCA
ACTGCATTGGCCAACACCATAGAAGTCTTTAGATCGAATGGCCTAACAGCTAATGAGTCAGGAAGGCTAA
TAGATTTCTTAAAGGATGTAATGGAATCAATGAACAAAGAGGAAATAGAGATAACAACCCACTTTCAAAG
AAAAAGGAGAGTAAGAGACAACATGACCAAGAAGATGGTCACGCAAAGAACAATAGGAAAGAAAAAACA
A
AGACTGAATAAGAGAGGCTATATAATAAGAGCACTGACATTAAATACGATGACCAAAGATGCAGAGAGAG
GCAAGTTAAAAAGAAGGGCTATCGCAACACCTGGGATGCAGATTAGAGGTTTCGTATACTTTGTTGAAAC
TTTAGCTAGGAGCATTTGCGAAAAGCTTGAACAGTCTGGGCTCCCAGTAGGGGGCAATGAAAAGAAGGCC
AAACTGGCAAATGTTGTGAGAAAGATGATGACTAATTCACAAGACACAGAGATTTCTTTCACAATCACTG
GGGACAACACTAAGTGGAATGAAAATCAAAATCCTCGAATGTTCCTGGCGATGATTACATATATCACCAG

AAATCAACCCGAGTGGTTCAGAAACATCCTGAGCATGGCACCCATAATGTTCTCAAACAAAATGGCAAGA
CTAGGGAGAGGGTACATGTTCGAGAGTAAAAGAATGAAGATTCGAACACAAATACCAGCAGAAATGCTAG
CAAGCATTGACCTGAAGTACTTCAATGAATCAACAAAGAAGAAAATTGAGAAAATAAGGCCTCTTCTAAT
AGATGGCACAGCATCACTGAGTCCTGGGATGATGATGGGCATGTTCAACATGCTAAGTACAGTCTTGGGA
GTCTCGATACTGAACCTTGGACAAAAGAAATACACCAAGACAACATACTGGTGGGATGGGCTCCAATCAT
CCGACGATTTTGCTCTCATAGTGAATGCACCAAACCATGAGGGGATACAAGCAGGAGTGGACAGATTCTA
CAGGACCTGCAAGTTAGTGGGAATCAACATGAGCAAAAAGAAGTCCTATATAAATAAAACAGGGACATTT
GAATTCACAAGCTTTTTTTATCGCTATGGATTTGTGGCTAATTTTAGCATGGAGCTACCCAGCTTTGGAG
TGTCTGGAGTAAATGAATCAGCTGACATGAGTATTGGAGTAACAGTGATAAAGAACAACATGATAAACAA
TGACCTTGGACCTGCAACGGCCCAGATGGCTCTTCAATTGTTCATCAAAGACTACAGATACACATACAGG
TGCCATAGGGGAGACACACAAATTCAGACGAGAAGATCATTTGAGTTAAAGAAGCTGTGGGACCAAACCC
AATCAAAGGTAGGGCTATTAGTATCAGATGGAGGACCAAACTTATACAATATACGGAATCTTCACATTCC
TGAAGTCTGCTTAAAATGGGAGCTAATGGATGATGATTATCGGGGAAGACTTTGTAATCCCCTGAATCCC
TTTGTCAGTCATAAAGAGATTGATTCTGTAAACAATGCTGTGGTAATGCCAGCCCATGGTCCAGCCAAAA
GCATGGAATATGATGCCGTTGCAACTACACATTCCTGGATTCCCAAGAGGAATCGTTCTATTCTCAACAC
AAGCCAAAGGGGAATTCTTGAGGATGAACAGATGTACCAGAAGTGCTGCAATCTATTCGAGAAATTTTTC
CCTAGCAGTTCATATAGGAGACCGGTTGGAATTTCTAGCATGGTGGAGGCCATGGTGTCTAGGGCCCGGA
TTGATGCCAGGGTCGACTTCGAGTCTGGACGGATCAAGAAAGAAGAGTTCTCTGAGATCATGAAGATCTG
TTCCACCATTGAAGAACTCAGACGGCAGAAATAA
>gi|363805069|gb|JQ290186.1| Influenza A virus (A/Iowa/09/2011(H3N2)) segment 2 polymerase PB1 (PB1) and PB1-F2 protein (PB1-F2) genes, complete cds
ATGGATGTCAATCCGACTCTACTTTTCCTAAAGGTTCCAGCGCAGAATGCCATAAGCACCACATTCCCTT
ATACTGGAGATCCTCCATACAGCCATGGAACAGGAACAGGATACACCATGGACACAGTCAACAGAACACA
CCAATATTCAGAAAAAGGGAAATGGACGACAAATACAGAGACTGGGGCACCCCAGCTCAATCCGATTGAT
GGACCACTACCTGAAAATAATGAACCAAGTGGATATGCACAAACAGACTGTGTCCTGGAGGCCATGGCTT
TCCTTGAAGAATCCCACCCAGGGATATTTGAGAATTCATGCCTTGAAACAATGGAAATTGTCCAACAAAC
AAGGGTGGATAAACTAACCCAGGGTCGCCAGACATATGATTGGACATTAAATAGAAACCAACCGGCAGCA
ACTGCATTGGCCAACACCATAGAAGTTTTTAGATCAAATGGTCTAACAGCTAATGAGTCAGGAAGGCTGA
TAGATTTCTTAAAAGATGTAATGGAATCAATGGATAAAGAGGAAATAGAGATAACAACACACTTTCAAAG
AAAAAGGAGAGTGAGGGATAACATGACCAAGAAGATGGTCACACAAAGAACAATAGGGAAGAAAAAACA
A
AGAGTGAATAAGAGGAGTTATTTAATAAGAGCACTAACATTGAATACGATGACCAAAGATGCAGAGAGAG
GCAAATTGAAAAGAAGGGCTATTGCAACACCTGGGATGCAAATTAGAGGGTTCGTGTACTTTGTTGAGAC
TCTAGCTAGAAGCATTTGCGAGAAGCTTGAACAATCCGGACTCCCAGTAGGGGGCAATGAAAAGAAAGCC
AAATTGGCAAACGTTGTGAGAAAGATGATGACTAATTCACAAGACACAGAGCTTTCCTTCACAATTACTG
GGGACAACACTAAATGGAATGAAAACCAAAATCCTCGAATGTTCCTGGCGATGATTACATACATCACCAG
AAACCAACCCGAGTGGTTCAGAAACATCCTGAGCATGGCACCCATAATGTTCTCAAACAAAATGGCAAGA
CTAGGAAAAGGGTACATGTTCGAGAGTAAAAGGATGAAGCTCCGAACACAGATACCAGCAGAAATGCTAT
CAAGCATTGACCTGAAGTATTTCAATGAATCAACAAGGAAGAAAATTGAGAAAATAAGGCCTCTCCTAAT
AGATGGCACAGCATCACTGAGCCCTGGGATGATGATGGGCATGTTCAACATGCTAAGTACGGTTTTGGGA
GTCTCAATACTGAATCTTGGACAAAAGAAATACACCAGGACAACATACTGGTGGGACGGACTCCAATCCT
CCGACGATTTTGCCCTCATAGTAAACGCACCAAATCATGAGGGAATCCAAGCAGGAGTGGATAGATTCTA
CAGGACCTGCAAGTTAGTAGGGATCAACATGAGCAAAAAGAAGTCCTATATAAATAAGACAGGGACATTT
GAATTCACAAGCTTTTTTTATCGCTATGGGTTTGTAGCTAATTTTAGCATGGAGCTGCCCAGTTTTGGAG

TGTCTGGAATAAACGAATCAGCTGATATGAGTATTGGGGTAACAGTGATAAAGAACAACATGATAAACAA
TGATCTTGGACCTGCAACAGCTCAGATGGCCCTTCAGTTGTTCATCAAAGACTACAGATACACCTATAGA
TGCCATAGAGGGGACACACAAATTCAAACGAGAAGATCATTCGAGCTAAAGAAGCTGTGGGATCAAACCC
AATCAAAGGCAGGATTACTAGTATCTGATGGAGGACCAAATTTATACAATATCCGGAATCTTCACATTCC
TGAAGTCTGCTTAAAATGGGAGCTAATGGATGAGGATTATCGAGGAAGACTTTGCAATCCTCTGAATCCC
TTTGTCAGCCATAAAGAGATTGATTCTGTAAACAGTGCTGTGGTGATGCCAGCCCATGGTCCAGCCAAAA
GCATGGAATATGATGCCGTTGCAACTACACACTCCTGGATCCCCAAGAGGAACCGTTCTATTCTCAACAC
AAGCCAAAGGGGAATTCTTGAGGATGAACAGATGTACCAGAAGTGCTGCAATCTGTTCGAGAAATTTTTC
CCTAGTAGTTCGTACAGGAGACCGGTTGGAATTTCTAGCATGGTAGAGGCCATGGTGTCTAGGGCCCGAA
TTGATGCCAGGATTGACTTCGAGTCTGGACGGATTAAGAAAGAAGAGTTCTCTGAGATCATGAAGATCTG
TTCCACCATTGAAGAACTCAGACGGCAAAAGTAA
>gi|344166374|gb|CY098002.1| Influenza A virus (A/Prague/WRAIR4146N/2011(H1N1)) polymerase PB1 (PB1) gene, complete cds; and PB1-F2 gene, complete sequence
ATGGATGTCAATCCGACTCTACTTTTCCTAAAAATTCCAGCGCAAAATGCCATAAGCACCACATTCCCTT
ATACTGGAGATCCTCCATACAGCCATGGAACAGGAACAGGATACACCATGGACACAGTGAACAGAACACA
CCAATACTCAGAAAAGGGAAAGTGGACGACAAACACAGAGACTGGTGCACCCCAGCTCAACCCGATTGAT
GGACCACTACCTGAGGATAATGAACCAAGTGGGTATGCACAAACAGACTGTGTTCTAGAGGCTATGGCTT
TCCTTGAAGAATCCCACCCAGGAATATTTGAGAATTCATGCCTTGAAACAATGGAAGTTGTTCAACAAAC
AAGGGTAGATAAACTAACTCAAGGTCGCCAGACTTATGATTGGACATTAAACAGAAATCAACCGGCAGCA
ACTGCATTGGCCAACACCATAGAAGTCTTTAGATCGAATGGCCTAACAGCTAATGAGTCAGGCAGGCTAA
TAGATTTCTTAAAGGATGTAATGGAATCAATGAACAAAGAGGAAATAGAGATAACAACCCACTTTCAAAG
AAAAAGGAGAGTAAGAGACAACATGACCAAGAAGATGGTCACGCAAAGAACAATAGGGAAGAAAAAACA
A
AGACTGAATAAGAGAGGCTATCTAATAAGAGCACTGACATTAAATACGATGACCAAAGATGCAGAGAGAG
GCAAGTTAAAAAGAAGGGCTATCGCAACACCTGGGATGCAGATTAGAGGTTTCGTATACTTTGTTGAAAC
TTTAGCTAGGAGCATTTGCGAAAAGCTTGAACAGTCTGGGCTCCCAGTAGGGGGCAATGAAAAGAAAGCC
AAATTGGCAAATGTTGTGAGAAAGATGATGACTAATTCACAAGACACAGAGATTTCTTTCACAATCACTG
GGGACAACACTAAGTGGAATGAAAATCAAAATCCTCGAATGTTCCTGGCGATGATTACATATATCACCAG
AAATCAACCCGAGTGGTTCAGAAACATCCTGAGCATGGCACCCATAATGTTCTCAAACAAAATGGCAAGA
CTAGGGAAAGGGTACATGTTCGAGAGTAAAAGAATGAAGATTCGAACACAAATACCAGCAGAAATGCTAG
CAAGCATTGACCTGAAGTACTTCAATGAATCAACAAAGAAGAAAATTGAGAAAATAAGGCCTCTTTTAAT
AGATGGCACAGCATCACTGAGTCCTGGGATGATGATGGGCATGTTCAACATGCTAAGTACGGTCTTGGGA
GTCTCGATACTGAATCTTGGACAAAAGAAATACACCAAGACAATATACTGGTGGGATGGGCTCCAATCAT
CCGACGATTTTGCTCTCATAGTGAATGCACCAAACCATGAGGGAATACAAGCAGGAGTGGACAGATTCTA
CAGGACCTGCAAGTTAGTGGGAATCAACATGAGCAAAAAGAAGTCCTATATAAATAAGACAGGGACATTT
GAATTCACAAGCTTTTTTTATCGCTATGGATTTGTGGCTAATTTTAGCATGGAGCTACCCAGCTTTGGAG
TGTCTGGAGTAAATGAATCAGCTGACATGAGTATTGGAGTAACAGTGATAAAGAACAACATGATAAACAA
TGACCTTGGACCTGCAACGGCCCAGATGGCTCTTCAATTGTTCATCAAAGACTACAGATACACATATAGG
TGCCATAGGGGAGACACACAAATTCAGACGAGAAGATCATTTGAGTTAAAGAAGCTGTGGGATCAAACCC
AATCAAAGGTAGGGCTATTAGTATCAGATGGAGGACCAAACTTATACAATATACGGAATCTTCACATTCC
TGAAGTCTGCTTAAAATGGGAGCTAATGGATGATGATTATCGGGGAAGACTTTGTAATCCCCTGAATCCC
TTTGTAAGTCATAAAGAGATTGATTCTGTAAACAATGCTGTGGTAATGCCAGCCCATGGTCCAGCCAAAA
GCATGGAATATGATGCCGTTGCAACTACACATTCCTGGATTCCCAAGAGGAATCGTTCTATTCTCAACAC
AAGCCAAAGGGGAATTCTTGAGGATGAACAGATGTACCAGAAGTGCTGCAATCTATTCGAGAAATTTTTC

CCTAGCAGTTCATATAGGAGACCGGTTGGAATTTCTAGCATGGTGGAGGCCATGGTGTCTAGGGCCCGGA
TTGATGCCAGGGTCGACTTTGAGTCTGGACGGATCAAGAAAGAAGAGTTCTCTGAGATCATGAAGATCTG
TTCCACCATTGAAGAACTCAGACGGCAAAAATAA

>gi|344166356|gb|CY097994.1| Influenza A virus (A/Budapest/WRAIR3794T/2011(H1N1)) polymerase PB1 (PB1) gene, complete cds; and PB1-F2 gene, complete sequence

ATGGATGTCAATCCGACTCTACTTTTCCTAAAAACTCCAGCGCAAAATGCCATAAGCACCACATTCCCTT
ATACTGGAGATCCTCCATACAGCCATGGAACAGGAACAGGATACACCATGGACACAGTGAACAGAACACA
CCAATACTCAGAAAAGGGAAAGTGGACGACAAACACAGAGACTGGTGCACCCCAGCTCAACCCGATTGAT
GGACCACTACCTGAGGATAATGAACCAAGTGGGTATGCACAAACAGACTGTGTTCTAGAGGCTATGGCTT
TCCTTGAAGAATCCCACCCAGGGATATTTGAGAATTCATGCCTTGAAACAATGGAAGTTGTTCAACAAAC
AAGGGTAGATAAACTAACTCAAGGTCGCCAGACTTATGATTGGACATTAAACAGAAATCAACCGGCAGCA
ACTGCATTGGCCAACACCATAGAAGTCTTTAGATCGAATGGCCTAACAGCTAATGAGTCAGGCAGGCTAA
TAGATTTCTTAAAGGATGTAATGGAATCAATGAACAAAGAGGAAATAGAGATAACAACCCACTTTCAAAG
AAAAAGGAGAGTAAGAGACAACATGACCAAGAAGATGGTCACGCAAAGAACAATAGGGAAGAAAAAACA
A
AGACTGAATAAGAGAGGCTATCTAATAAGAGCACTGACATTAAATACGATGACCAAAGATGCAGAGAGAG
GCAAGTTAAAAAGAAGGGCTATCGCAACACCTGGGATGCAGATTAGAGGTTTCGTATACTTTGTTGAAAC
TTTAGCTAGGAGCATTTGCGAAAAGCTTGAACAGTCTGGGCTCCCAGTAGGGGGCAATGAAAAGAAGGCC
AAACTGGCAAATGTTGTGAGAAAGATGATGACTAATTCACAAGACACAGAGATTTCTTTCACAATCACTG
GGGACAACACTAAGTGGAATGAAAATCAAAATCCTCGAATGTTCCTGGCGATGATTACATATATCACCAG
AAATCAACCCGAGTGGTTCAGAAACATCCTGAGCATGGCACCCATAATGTTCTCAAACAAAATGGCAAGA
CTAGGGAAAGGGTACATGTTCGAGAGTAAAAGAATGAAGATTCGAACACAAATACCAGCAGAAATGCTAG
CAAGCATTGACCTGAAGTACTTCAATGAATCAACAAAGAAGAAAATTGAGAAAATAAGGCCTCTTTTAAT
AGATGGCACAGCATCACTGAGTCCTGGGATGATGATGGGCATGTTCAACATGCTAAGTACGGTCTTGGGA
GTCTCGATACTGAATCTTGGACAAAAGAAATACACCAAGACAATATACTGGTGGGATGGGCTCCAATCAT
CCGACGATTTTGCTCTCATAGTGAATGCACCAAACCATGAGGGAATACAAGCAGGAGTGGACAGATTCTA
CAGGACCTGCAAGTTAGTGGGAATCAACATGAGCAAAAAGAAGTCCTATATAAATAAGACAGGGACATTT
GAATTCACAAGCTTTTTTTATCGCTATGGATTTGTGGCTAATTTTAGCATGGAGCTACCCAGCTTTGGAG
TGTCTGGAGTAAATGAATCAGCTGACATGAGTATTGGAGTAACAGTGATAAAGAACAACATGATAAACAA
TGACCTTGGACCTGCAACGGCCCAGATGGCTCTTCAATTGTTCATCAAAGACTACAGATACACATATAGG
TGCCATAGGGGAGACACACAAATTCAGACGAGAAGATCATTTGAGTTAAAGAAGCTGTGGGATCAAACCC
AATCAAAGGTAGGGCTATTAGTATCAGATGGAGGACCAAACTTATACAATATACGGAATCTTCACATTCC
TGAAGTCTGCTTAAAATGGGAGCTAATGGATGATGATTATCGGGGAAGACTTTGTAATCCCCTGAATCCC
TTTGTAAGTCATAAAGAGATTGATTCTGTAAACAATGCTGTGGTAATGCCAGCCCATGGTCCAGCCAAAA
GCATGGAATATGATGCCGTTGCAACTACACATTCCTGGATTCCCAAGAGGAATCGTTCTATTCTCAACAC
AAGCCAAAGGGGAATTCTTGAGGATGAACAGATGTACCAGAAGTGCTGCAATCTATTCGAGAAATTTTTC
CCTAGCAGTTCATATAGGAGACCGGTTGGAATTTCTAGCATGGTGGAGGCCATGGTGTCTAGGGCCCGGA
TTGATGCCAGGGTCGACTTTGAGTCTGGACGGGTCAAGAAAGAAGAGTTCTCTGAGATCATGAAGATCTG
TTCCACCATTGAAGAACTCAGACGGCAAAAATAA

>gi|339517145|gb|JN187239.1| Influenza A virus (A/Taiwan/3697/2011(H1N1)) segment 2 polymerase PB1 (PB1) gene, complete cds; and nonfunctional PB1-F2 protein (PB1-F2) gene, complete sequence

ATGGATGTCAATCCGACTCTACTTTTCCTAAAAATTCCAGCGCAAAATGCCATAAGCACCACATTCCCTT
ATACTGGAGATCCTCCATACAGCCATGGAACAGGAACAGGATACACCATGGACACAGTAAACAGAACACA

CCAATACTCAGAAAAGGGAAAGTGGACGACAAACACAGAGACTGGTGCACCCCAGCTCAACCCGATTGAT
GGACCACTACCTGAGGATAATGAACCAAGTGGGTATGCACAAACAGACTGTGTTCTAGAGGCTATGGCTT
TCCTTGAAGAATCCCACCCAGGAATATTTGAGAATTCATGCCTTGAAACAATGGAAGTTGTTCAACAAAC
AAGGGTAGATAAACTGACTCAAGGTCGCCAGACTTATGATTGGACATTAAACAGAAATCAACCGGCAGCA
ACTGCATTGGCCAACACCATAGAAGTCTTTAGATCGAATGGCCTAACAGCTAATGAGTCAGGAAGGCTAA
TAGATTTCTTAAAGGATGTAATGGAATCAATGAACAAAGAGGAAATAGAGATAACAACCCACTTTCAAAG
AAAAAGGAGAGTAAGAGACAACATGACCAAGAAGATGGTCACGCAAAGAACAATAGGGAAGAAAAAACA
G
AGACTGAATAAGAGAGGCTATCTAATAAGAGCACTGACATTAAATACGATGACCAAAGATGCAGAGAGAG
GCAAGTTAAAAAGAAGGGCTATCGCAACACCTGGGATGCAGATTAGAGGTTTCGTATACTTTGTTGAAAC
TTTAGCTAGGAGCATTTGCGAAAAGCTTGAACAGTCTGGGCTCCCAGTAGGGGGCAATGAAAAGAAGGCC
AAACTGGCAAATGTTGTGAGAAAGATGATGACTAATTCACAAGACACAGAGATTTCTTTCACAATCACTG
GGGACAACACTAAGTGGAATGAAAATCAAAATCCTCGAATGTTCCTGGCGATGATTACATATATCACCAG
AAATCAACCCGAGTGGTTTAGAAACATCCTGAGCATGGCACCCATAATGTTCTCAAACAAAATGGCAAGA
CTAGGGAAAGGGTACATGTTCGAGAGTAAAAGAATGAAGATTCGAACACAAATACCAGCAGAAATGCTAG
CAAGCATTGACCTGAAGTACTTCAATGAATCAACAAAGAAGAAAATTGAGAAAATAAGGCCTCTTTTAAT
GGATGGCACAGCATCACTGAGTCCTGGGATGATGATGGGCATGTTCAACATGCTAAGTACGGTCTTGGGA
GTCTCGATACTGAATCTTGGACAAAAGAAATACACCAAGACAACATACTGGTGGGATGGGCTCCAATCAT
CCGACGATTTTGCTCTCATAGTGAATGCACCAAACCATGAGGGAATACAAGCAGGAGTGGACAGATTCTA
CAGGACCTGCAAGTTAGTGGGAATCAACATGAGCAAAAAGAAGTCCTATATAAATAAGACAGGGACATTT
GAATTCACAAGCTTTTTTTATCGCTATGGATTTGTGGCTAATTTTAGCATGGAGCTACCCAGCTTTGGAG
TGTCTGGAGTAAATGAATCAGCTGACATGAGTATTGGAGTAACAGTGATAAAGAACAACATGATAAACAA
TGACCTTGGACCTGCAACGGCCCAGATGGCTCTTCAATTGTTCATCAAAGACTACAGATACACATATAGG
TGCCATAGGGGAGACACACAAATTCAGACGAGAAGATCATTTGAGTTAAAGAAGCTGTGGGATCAAACCC
AATCAAAGGTAGGGCTATTAGTATCAGATGGAGGACCAAACTTATACAATATACGGAATCTTCACATTCC
TGAAGTCTGCTTAAAATGGGAGCTAATGGATGATGATTATCGGGGAAGACTTTGTAATCCCCTGAATCCC
TTTGTAAGTCATAAGGAGATTGATTCTGTAAACAATGCTGTGGTAATGCCAGCCCATGGTCCAGCCAAAA
GCATGGAATATGATGCCGTTGCAACTACACATTCCTGGATTCCCAAGAGGAATCGTTCTATTCTCAACAC
AAGCCAAAGGGGAATTCTTGAGGATGAACAGATGTACCAGAAGTGCTGCAATCTATTCGAGAAATTTTTC
CCTAGCAGTTCATATAGGAGACCGGTTGGAATTTCTAGCATGGTGGAGGCCATGGTGTCTAGGGCCCGGA
TTGATGCCAGGGTCGACTTTGAGTCTGGACGGATCAAGAAAGAAGAGTTCTCTGAGATCATGAAGATCTG
TTCCACCATTGAAGAACTCAGACGGCAAAAATAA
>gi|339518922|gb|JN185091.1| Influenza A virus (A/Tula/CRIE-SIA/2011(H1N1)) segment 2 polymerase PB1 (PB1) gene, complete cds; and nonfunctional PB1-F2 protein (PB1-F2) gene, complete sequence
ATGGATGTCAATCCGACTCTACTTTTCCTAAAAATTCCAGCGCAAAATGCTATAAGCACCACATTCCCTT
ATACTGGAGATCCTCCATACAGCCATGGAACAGGAACAGGATACACCATGGACACAGTAAACAGAACACA
CCAATACTCAGAAAAGGGAAAGTGGACGACAAACACAGAGACTGGTGCACCCCAGCTCAACCCGATTGAT
GGACCACTACCTGAGGATAATGAACCAAGTGGGTATGCACAAACAGACTGTGTTCTAGAGGCTATGGCTT
TCCTTGAAGAATCCCACCCAGGAATATTTGAGAATTCATGCCTTGAAACAATGGAAGTTGTTCAACAAAC
AAGGGTAGATAAACTAACTCAAGGTCGCCAGACTTATGATTGGACATTAAACAGAAATCAACCGGCAGCA
ACTGCATTGGCCAACACCATAGAAGTCTTTAGATCGAATGGCCTAACAGCTAATGAGTCAGGAAGGCTAA
TAGATTTTTTAAAGGATGTAATGGAATCAATGAACAAAGAGGAAATAGAGATAACAACCCACTTTCAAAG
AAAAAGGAGAGTAAGAGACAACATGACCAAGAAGATGGTCACGCAAAGAACAATAGGGAAGAAAAAACA

A
AGACTGAATAAGAGAGGCTATCTAATAAGAGCACTGACATTAAATACGATGACCAAAGATGCAGAGAGAG
GCAAGTTAAAAAGAAGGGCTATCGCAACACCTGGGATGCAGATTAGAGGTTTCGTATACTTTGTTGAAGC
TTTAGCTAGGAGCATTTGCGAAAAGCTTGAACAGTCTGGGCTCCCAGTAGGGGGCAATGAAAAAAAGGCC
AAACTGGCAAATGTTGTGAGAAAGATGATGACTAATTCACAAGACACAGAGATTTCTTTCACAATCACTG
GGGACAACACTAAGTGGAATGAAAATCAAAATCCTCGAATGTTCCTGGCAATGATTACATATATCACCAG
AAATCAACCCGAGTGGTTCAGAAACATCCTGAGCATGGCACCCATAATGTTCTCAAACAAAATGGCAAGA
CTAGGGAAAGGGTACATGTTCGAGAGTAAAAGAATGAAGATTCGAACACAAATACCAGCAGAAATGCTAG
CAAGCATTGACCTGAAGTACTTCAATGAATCAACGAAGAAGAAAATTGAGAAAATAAGGCCTCTTCTAAT
AGATGGCACAGCATCACTGAGTCCTGGGATGATGATGGGCATGTTCAACATGCTAAGTACGGTCTTGGGA
GTCTCGATACTGAATCTTGGACAAAAGAAATACACCAAGACAATATACTGGTGGGATGGGCTCCAATCGT
CCGACGATTTTGCTCTCATAGTGAATGCACCAAACCATGAGGGAATACAAGCAGGAGTGGACAGATTCTA
CAGGACCTGCAAGTTAGTGGGAATCAACATGAGCAAAAAGAAGTCCTATATAAATAAGACAGGGACATTT
GAATTCACAAGCTTTTTTTATCGCTATGGATTTGTGGCTAATTTTAGCATGGAGCTACCCAGCTTTGGAG
TGTCTGGAGTAAATGAATCAGCTGACATGAGTATTGGAGTAACAGTGATAAAGAACAACATGATAAACAA
TGACCTTGGACCTGCAACGGCCCAGATGGCTCTTCAATTGTTCATCAAAGACTACAGATACACATATAGG
TGCCATAGGGGAGACACACAAATTCAGACGAGGAGATCATTTGAGTTAAAGAAGCTGTGGGATCAAACCC
AATCAAAGGTAGGGCTATTAGTATCAGATGGAGGACCAAACTTATACAATATACGGAATCTTCACATTCC
TGAAGTCTGCTTAAAATGGGAGCTAATGGATGATGATTATCGGGGAAGACTTTGTAATCCCCTGAATCCC
TTTGTCAGTCATAAAGAGATTGATTCTGTAAACAATGCTGTGGTAATGCCAGCCCATGGTCCAGTCAAAA
GCATGGAATATGATGCCGTTGCAACTACACATTCCTGGATTCCCAAGAGGAATCGTTCTATTCTCAACAC
AAGCCAAAGGGGAATTCTTGAGGATGAACAGATGTACCAGAAGTGCTGCAATCTATTCGAGAAATTTTTC
CCTAGCAGTTCATATAGGAGACCGGTTGGAATTTCTAGCATGGTGGAGGCCATGGTGTCTAGGGCCCGGA
TTGATGCCAGGGTCGACTTCGAGTCTGGACGGATAAAGAAAGAAGAGTTCTCTGAGATCATGAAGATCTG
TTCCACCATTGAAGAACTCAGACGGCAAAAATAA
>gi|345722629|gb|JN655539.1| Influenza A virus (A/Pennsylvania/09/2011(H3N2)) segment 2 polymerase PB1 (PB1) and PB1-F2 protein (PB1-F2) genes, complete cds
ATGGATGTCAATCCGACTCTACTTTTCCTAAAGGTTCCAGCGCAAAATGCCATAAGCACCACATTCCCTT
ATACTGGAGATCCTCCATACAGCCATGGAACAGGAACAGGATACACCATGGACACAGTCAACAGAACACA
CCAATATTCAGAAAAAGGGAAATGGACGACAAATACAGAGACTGGGGCACCCCAGCTCAATCCGATTGAT
GGACCACTACCTGAAGATAATGAACCAAGTGGATATGCACAAACAGACTGTGTCCTGGAGGCCATGGCTT
TCCTTGAAGAATCCCACCCAGGGATATTTGAGAATTCATGCCTTGAAACAATAGAAATTGTCCAACAAAC
AAGGGTGGATAAACTAACCCAGGGTCGCCAGACATATGATTGGACATTAAATAGAAATCAACCGGCAGCA
ACTGCATTGGCCAACACCATAGAAGTTTTTAGATCAAATGGTCTAACAGCTAATGAGTCAGGAAGGCTGA
TAGATTTCTTAAAAGATGTAATGGAATCAATGGATAAAGAGGAAATAGAGATAACAACACACTTTCAAAG
AAAAAGGAGAGTAAGGGATAACATGACCAAGAAGATGGTCACACAAAGAACAATAGGGAAGAAAAAACA
A
AGAGTGAATAAGAGGAGTTATTTAATAAGAGCACTAACATTGAATACGATGACCAAAGATGCAGAGAGAG
GCAAATTAAAAAGAAGGGCTATTGCAACACCTGGAATGCAAATTAGAGGGTTCGTGTACTTTGTTGAGAC
TCTAGCTAGAAGCATTTGCGAGAAGCTTGAACAATCCGGACTCCCAGTAGGGGGCAATGAAAAAAAGCC
AAATTGGCAAACGTTGTGAGAAAGATGATGACTAATTCACAAGACACAGAGCTTTCCTTCACAATTACTG
GGGACAACACTAAATGGAATGAAAACCAAAATCCTCGAATGTTCCTGGCGATGATTACATACATCACCAG
AAACCAACCCGAGTGGTTCAGAAACATCCTGAGCATGGCACCCATAATGTTCTCAAACAAAATGGCAAGA
CTAGGAAAAGGGTACATGTTCGAGAGTAAAAGGATGAAGCTCCGAACACAGATACCAGCAGAAATGCTAT

CAAGCATTGACCTGAAGTATTTCAATGAATCAACAAGGAAGAAAATTGAGAAAATAAGGCCCCTCCTAAT
AGATGGCACAGCATCACTGAGCCCTGGGATGATGATGGGCATGTTCAACATGCTAAGTACGGTTTTGGGA
GTCTCGATACTGAATCTTGGACAAAAGAAATACACCAGGACAACATACTGGTGGGACGGACTCCAATCCT
CCGACGATTTTGCCCTCATAGTAAACGCACCAAATCATGAGGGAATCCAAGCAGGAGTGGATAGATTCTA
CAGGACCTGCAAGTTAGTAGGAATCAACATGAGCAAAAAGAAGTCCTATATAAATAAGACTGGGACATTT
GAATTCACAAGCTTTTTTTATCGCTATGGGTTTGTAGCTAATTTTAGCATGGAGCTGCCCAGTTTTGGAG
TGTCTGGAATAAACGAATCAGCTGATATGAGTATTGGGGTAACAGTGATAAAGAACAACATGATAAACAA
TGATCTTGGACCTGCAACAGCTCAGATGGCCCTTCAGTTGTTCATCAAAGACTACAGATACACCTATAGA
TGCCATAGAGGGGACACACAGATTCAAACGAGAAGATCATTCGAGCTAAAGAAGCTGTGGGATCAAACCC
AATCAAAGGCAGGATTACTAGTATCTGATGGAGGACCAAATTTATACAATATCCGGAATCTTCACATTCC
TGAAGTCTGCTTAAAATGGGAGCTAATGGATGAGGATTATCGGGGAAGACTTTGCAATCCCCTGAATCCC
TTTGTCAGCCATAAAGAGATTGATTCTGTAAACAGTGCTGTGGTGATGCCAGCCCATGGTCCAGCCAAAA
GCATGGAATATGATGCCGTTGCAACTACACACTCCTGGATCCCCAAGAGGAACCGCTCTATTCTCAACAC
AAGCCAAAGGGGAATTCTTGAGGATGAACAGATGTACCAGAAGTGCTGCAATCTGTTCGAGAAATTTTTC
CCTAGTAGTTCGTACAGGAGACCGGTTGGAATTTCTAGTATGGTAGAGGCCATGGTGTCTAGGGCCCGAA
TTGATGCCAGGATTGACTTCGAGTCTGGACGGATTAAGAAAGAAGAGTTCTCTGAGATCATGAAGATCTG
TTCCACCATTGAAGAACTCAGACGGCAAAAGTAA
>gi|338826631|gb|CY092870.1| Influenza A virus (A/Sydney/DD3-59/2011(H1N1)) polymerase PB1 (PB1) gene, complete cds; and PB1-F2 gene, complete sequence
TTGAATGGATGTCAATCCGACTCTACTTTTCCTAAAAACTCCAGCGCAAAATGCCATAAGCACCACATTC
CCTTATACTGGAGATCCTCCATACAGCCATGGAACAGGAACAGGATACACCATGGACACAGTGAACAGAA
CACACCAATACTCAGAAAAGGGAAAGTGGACGACAAACACAGAGACTGGTGCACCCCAGCTCAACCCGAT
TGATGGACCACTACCTGAGGATAATGAACCAAGTGGGTATGCACAAACAGACTGTGTTCTAGAGGCTATG
GCTTTCCTTGAAGAATCCCACCCAGGGATATTTGAGAATTCATGCCTTGAAACAATGGAAGTTGTTCAAC
AAACAAGGGTAGATAAACTAACTCAAGGTCGCCAGACTTATGATTGGACATTAAACAGAAATCAACCGGC
AGCAACTGCATTGGCCAACACCATAGAAGTCTTTAGATCGAATGGCCTAACAGCTAATGAGTCAGGCAGG
CTAATAGATTTCTTAAAGGATGTAATGGAATCAATGAACAAAGAGGAAATAGAGATAACAACCCACTTTC
AAAGAAAAAGGAGAGTAAGAGACAACATGACCAAGAAGATGGTCACGCAAAGAACAATAGGGAAGAAAA
A
ACAAAGACTGAATAAGAGAGGCTATCTAATAAGAGCACTGACATTAAATACGATGACCAAAGATGCAGAG
AGAGGCAAGTTAAAAAGAAGGGCTATCGCAACACCTGGGATGCAGATTAGAGGTTTCGTATACTTTGTTG
AAACTTTAGCTAGGAGCATTTGCGAAAAGCTTGAACAGTCTGGGCTCCCAGTAGGGGGCAATGAAAAGAA
GGCCAAACTGGCAAATGTTGTGAGAAAGATGATGACTAATTCACAAGACACAGAGATTTCTTTCACAATC
ACTGGGGACAACACTAAGTGGAATGAAAATCAAAATCCTCGAATGTTCCTGGCGATGATTACATATATCA
CCAGAAATCAACCCGAGTGGTTCAGAAACATCCTGAGCATGGCACCCATAATGTTCTCAAACAAAATGGC
AAGACTAGGGAAAGGGTACATGTTCGAGAGTAAAAGAATGAAGATTCGAACACAAATACCAGCAGAAATG
CTAGCAAGCATTGACCTGAAGTACTTCAATGAATCAACAAAGAAGAAAATTGAGAAAATAAGGCCTCTTT
TAATAGATGGCACAGCATCACTGAGTCCTGGGATGATGATGGGCATGTTCAACATGCTAAGTACGGTCTT
GGGAGTCTCGATACTGAATCTTGGACAAAAGAAATACACCAAGACAATATACTGGTGGGATGGGCTCCAA
TCATCCGACGATTTTGCTCTCATAGTGAATGCACCAAACCATGAGGGAATACAAGCAGGAGTGGACAGAT
TCTACAGGACCTGCAAGTTAGTGGGAATCAACATGAGCAAAAAGAAGTCCTATATAAATAAGACAGGGAC
ATTTGAATTCACAAGCTTTTTTTATCGCTATGGATTTGTGGCTAATTTTAGCATGGAGCTACCCAGCTTT
GGAGTGTCTGGAGTAAATGAATCAGCTGACATGAGTATTGGAGTAACAGTGATAAAGAACAACATGATAA
ACAATGACCTTGGACCTGCAACGGCCCAGATGGCTCTTCAATTGTTCATCAAAGACTACAGATACACATA

TAGGTGCCATAGGGGAGACACACAAATTCAGACGAGAAGATCATTTGAGTTAAAGAAGCTGTGGGATCAA
ACCCAATCAAAGGTAGGGCTATTAGTATCAGATGGAGGACCAAACTTATACAATATACGGAATCTTCACA
TTCCTGAAGTCTGCTTAAAATGGGAGCTAATGGATGATGATTATCGGGGAAGACTTTGTAATCCCCTGAA
TCCCTTTGTAAGTCATAAAGAGATTGATTCTGTAAACAATGCTGTGGTAATGCCAGCCCATGGTCCAGCC
AAAAGCATGGAATATGATGCCGTTGCAACTACACATTCCTGGATTCCCAAGAGGAATCGTTCTATTCTCA
ACACAAGCCAAAGGGGAATTCTTGAGGATGAACAGATGTACCAGAAGTGCTGCAATCTATTCGAGAAATT
TTTCCCTAGCAGTTCATATAGGAGACCGGTTGGAATTTCTAGCATGGTGGAGGCCATGGTGTCTAGGGCC
CGGATTGATGCCAGGGTCGACTTTGAGTCTGGACGGATCAAGAAAGAAGAGTTCTCTGAGATCATGAAGA
TCTGTTCCACCATTGAAGAACTCAGACGGCAAAAATAATGAATTTAACTTGTCCTTCATGAAA
>gi|319918318|gb|CY080572.1| Influenza A virus (A/Ulaanbaatar/190/2011(H1N1)) polymerase PB1 (PB1) gene, complete cds; and PB1-F2 gene, complete sequence
ATGGATGTCAATCCGACTCTACTTTTCCTAAAAATTCCAGCGCAAAATGCCATAAGCACCACATTCCCTT
ATACTGGAGATCCTCCATACAGCCATGGAACAGGAACAGGATACACCATGGACACAGTAAACAGGACACA
CCAATACTCAGAAAAGGGAAAGTGGACGACAAACACAGAGACTGGTGCACCCCAGCTCAACCCGATTGAT
GGACCACTACCTGAGGATAATGAACCAAGTGGGTATGCACAAACAGACTGTGTTCTAGAGGCTATGGCTT
TCCTTGAAGAATCCCACCCAGGAATATTTGAGAATTCATGCCTTGAAACAATGGAAGTTGTTCAACAAAC
AAGGGTAGATAAACTGACTCAAGGTCGCCAGACTTATGATTGGACATTAAACAGAAATCAACCGGCAGCA
ACTGCATTGGCCAACACCATAGAAGTCTTTAGATCGAATGGCCTAACAGCTAATGAGTCAGGAAGGCTAA
TAGATTTCTTAAAGGATGTAATGGAATCAATGAACAAAGAGGAAATAGAGATAACAACCCACTTTCAAAG
AAAAAGGAGAGTAAGAGACAACATGACCAAGAAGATGGTCACGCAAAGAACAATAGGGAAGAAAAAACA
G
AGACTGAATAAGAGAGGCTATCTAATAAGAGCACTGACATTAAATACGATGACCAAAGATGCAGAGAGAG
GCAAGTTAAAAAGAAGGGCTATCGCAACACCTGGGATGCAGATTAGAGGTTTCGTATACTTTGTTGAAAC
TTTAGCTAGGAGCATTTGCGAAAAGCTTGAACAGTCTGGGCTCCCAGTGGGGGGCAATGAAAAGAAGGCC
AAACTGGCAAATGTTGTGAGAAAGATGATGACTAATTCACAAGACACAGAGATTTCTTTCACAATCACTG
GGGACAACACTAAGTGGAATGAAAATCAAAATCCTCGAATGTTCCTGGCGATGATTACATATATCACCAG
AAATCAACCCGAGTGGTTTAGAAACATCCTGAGCATGGCACCCATAATGTTCTCAAACAAAATGGCAAGA
CTAGGGAAAGGGTACATGTTCGAGAGTAAAAGAATGAAGATTCGAACACAAATACCAGCAGAAATGCTAG
CAAGCATTGACCTGAAGTACTTCAATGAATCAACAAAGAAGAAAATTGAGAAAATAAGGCCTCTTTTAAT
GGATGGCACAGCATCACTGAGTCCTGGGATGATGATGGGCATGTTCAACATGCTAAGTACGGTCTTGGGA
GTCTCGATACTGAATCTTGGACAAAAGAAATACACCAAGACAACATACTGGTGGGATGGGCTCCAATCAT
CCGACGATTTTGCTCTCATAGTGAATGCACCAAACCATGAGGGAATACAAGCAGGAGTGGACAGATTCTA
CAGGACCTGCAAGTTAGTGGGAATCAACATGAGCAAAAAGAAGTCTTATATAAATAAGACAGGGACATTT
GAATTCACAAGCTTTTTTTATCGCTATGGATTTGTGGCTAATTTTAGCATGGAGCTACCCAGCTTTGGAG
TGTCTGGAGTAAATGAATCAGCTGACATGAGTATTGGAGTAACAGTGATAAAGAACAACATGATAAACAA
TGACCTTGGACCTGCAACGGCCCAGATGGCTCTTCAATTGTTCATCAAAGACTACAGGTACACATATAGG
TGCCATAGGGGAGACACACAAATTCAGACGAGAAGATCATTTGAGTTAAAGAAGCTGTGGGATCAAACCC
AATCAAAGGTAGGGCTATTAGTATCAGATGGAGGACCAAACTTATACAATATACGGAATCTTCACATTCC
TGAAGTCTGCTTAAAATGGGAGCTAATGGATGATGATTATCGGGGAAGACTTTGTAATCCCCTGAATCCC
TTTGTAAGTCATAAGGAGATTGATTCTGTAAACAATGCTGTGGTAATGCCAGCCCATGGTCCAGCCAAAA
GCATGGAATATGATGCCGTTGCAACTACACATTCCTGGATTCCCAAGAGGAATCGTTCTATTCTCAACAC
AAGCCAAAGGGGAATTCTTGAGGATGAACAGATGTACCAGAAGTGCTGCAATCTATTCGAGAAATTTTTC
CCTAGCAGTTCATATAGGAGACCGGTTGGAATTTCTAGCATGGTGGAGGCCATGGTGTCTAGGGCCCGGA
TTGATGCCAGGGTCGACTTTGAGTCTGGACGGATCAAGAAAGAAGAGTTCTCTGAGATCATGAAGATCTG

TTCCACCATTGAAGAACTCAGACGGCAAAAATAA
>gi|404425407|gb|CY125781.1| Influenza A virus (A/Boston/DOA90/2012(H1N1)) polymerase PB1 (PB1) gene, complete cds; and PB1-F2 gene, complete sequence
ATGGATGTCAATCCGACTCTACTTTTCCTAAAAATTCCAGCGCAAAATGCCATAAGCATCACATTCCCTT
ATACTGGAGATCCTCCATACAGCCATGGAACAGGAACAGGATACACCATGGACACAGTAAACAGAACACA
CCAATACTCAGAAAAGGGAAAGTGGACGACAAACACAGAGACTGGTGCACCCCAGCTCAACCCGATTGAT
GGACCACTACCTGAGGATAACGAACCAAGTGGGTATGCACAAACAGACTGTGTCCTAGAGGCTATGGCTT
TCCTTGAAGAATCCCACCCAGGAATATTTGAGAATTCATGCCTTGAAACAATGGAAGTTGTTCAACAAAC
AAGAGTAGATAAACTGACTCAAGGTCGCCAGACTTATGATTGGACATTAAACAGAAATCAACCGGCAGCA
ACTGCATTGGCCAACACCATAGAAGTCTTTAGATCGAATGGCCTAACAGCTAATGAGTCAGGAAGGCTAG
TAGATTTCTTAAAGGATGTAATGGAATCAATGAACAAAGAGGAAATAGAGATAACAACCCACTTTCAAAG
AAAAAGGAGAGTAAGAGACAACATGACCAAGAAGATGGTCACGCAAAGAACAATAGGGAAGAAAAAACA
G
AGACTGAATAAGAGAGGCTATCTAATAAGAGCACTGACATTAAATACGATGACCAAAGATGCAGAGAGAG
GCAAGTTAAAAAGAAGGGCTATCGCAACACCTGGGATGCAGATTAGAGGTTTCGTATACTTTGTTGAAAC
TTTAGCTAGGAGCATTTGCGAAAAGCTTGAACAGTCTGGGCTCCCAGTAGGGGGCAATGAAAAGAAGGCC
AAACTGGCAAATGTTGTGAGAAAGATGATGACTAATTCACAAGACACAGAGATTTCTTTCACAATCACTG
GGGACAACACTAAGTGGAATGAAAACCAAAATCCTCGAATGTTCCTGGCGATGATTACATATATCACCAA
AAATCAACCCGAGTGGTTTAGAAACATCCTGAGCATGGCACCCATAATGTTCTCAAACAAAATGGCAAGA
CTAGGGAAAGGGTACATGTTCGAGAGTAAAAGAATGAAGATTCGAACACAAATACCAGCAGAAATGCTAG
CAAGCATTGACCTGAAGTACTTCAATGAATCAACAAAGAAGAAAATTGAGAAAATAAGGCCTCTTTTAAT
GGATGGCACAGCATCACTGAGTCCTGGGATGATGATGGGCATGTTCAACATGCTAAGTACGGTCTTGGGG
GTCTCGATACTGAATCTTGGACAAAAGAAATACACCAAGACAACATACTGGTGGGATGGGCTCCAATCAT
CCGACGATTTTGCTCTCATAGTGAATGCACCAAACCATGAGGGAATACAAGCAGGAGTGGACAGATTCTA
CAGGACCTGCAAGTTAGTGGGAATCAACATGAGCAAAAAGAAGTCCTATATAAATAAGACAGGGACATTT
GAATTCACAAGCTTTTTTTATCGCTATGGATTTGTGGCTAATTTTAGCATGGAGCTACCCAGCTTTGGAG
TGTCTGGAGTAAATGAATCAGCTGACATGAGTATTGGAGTAACAGTGATAAAGAACAACATGATAAACAA
TGACCTTGGACCTGCAACAGCCCAGATGGCTCTTCAATTGTTCATCAAAGACTACAGATACACATATAGG
TGCCATAGGGGAGACACACAAATTCAGACGAGAAGATCATTTGAGTTAAAGAAGCTGTGGGATCAAACCC
AATCAAAGGTAGGGCTATTAGTATCAGATGGAGGACCAAACTTATACAATATACGGAATCTTCACATTCC
TGAAGTCTGCTTAAAATGGGAGCTAATGGATGATGATTATCGGGGAAGACTTTGTAATCCCCTGAATCCC
TTTGTAAGTCATAAGGAGATTGATTCTGTAAACAATGCTGTGGTAATGCCAGCCCATGGTCCAGCCAAAA
GCATGGAATATGATGCCGTTGCAACTACACATTCCTGGATTCCCAAGAGGAATCGTTCTATTCTCAACAC
AAGCCAAAGGGGAATTCTTGAGGATGAACAGATGTACCAGAAGTGCTGCAATCTATTCGAGAAATTTTTC
CCTAGCAGTTCATATAGGAGACCGGTTGGAATTTCTAGCATGGTGGAGGCCATGGTGTCTAGGGCCCGGA
TTGATGCCAGGGTCGACTTTGAGTCTGGACGGATCAAGAAAGAAGAGTTCTCTGAGATCATGAAGATCTG
TTCCACCATTGAAGAACTCAGACGGCAAAAATAA
>gi|404425425|gb|CY125789.1| Influenza A virus (A/Boston/DOA93/2012(H1N1)) polymerase PB1 (PB1) gene, complete cds; and PB1-F2 gene, complete sequence
ATGGATGTCAATCCGACTCTACTTTTCCTAAAAATTCCAGCGCAAAATGCCATAAGCATCACATTCCCTT
ATACTGGAGATCCTCCATACAGCCATGGAACAGGAACAGGATACACCATGGACACAGTAAACAGAACACA
CCAATACTCAGAAAAGGGAAAGTGGACGACAAACACAGAGACTGGTGCACCCCAGCTCAACCCGATTGAT
GGACCACTACCTGAGGATAACGAACCAAGTGGGTATGCACAAACAGACTGTGTCCTAGAGGCTATGGCTT
TCCTTGAAGAATCCCACCCAGGAATATTTGAGAATTCATGCCTTGAAACAATGGAAGTTGTTCAACAAAC

AAGAGTAGATAAACTGACTCAAGGTCGCCAGACTTATGATTGGACATTAAACAGAAATCAACCGGCAGCA
ACTGCATTGGCCAACACCATAGAAGTCTTTAGATCGAATGGCCTAACAGCTAATGAGTCAGGAAGGCTAG
TAGATTTCTTAAAGGATGTAATGGAATCAATGAACAAAGAGGAAATAGAGATAACAACCCACTTTCAAAG
AAAAAGGAGAGTAAGAGACAACATGACCAAGAAGATGGTCACGCAAAGAACAATAGGGAAGAAAAAACA
G
AGACTGAATAAGAGAGGCTATCTAATAAGAGCACTGACATTAAATACGATGACCAAAGATGCAGAGAGAG
GCAAGTTAAAAAGAAGGGCTATCGCAACACCTGGGATGCAGATTAGAGGTTTCGTATACTTTGTTGAAAC
TTTAGCTAGGAGCATTTGCGAAAAGCTTGAACAGTCTGGGCTCCCAGTAGGGGGCAATGAAAAGAAGGCC
AAACTGGCAAATGTTGTGAGAAAGATGATGACTAATTCACAAGACACAGAGATTTCTTTCACAATCACTG
GGGACAACACTAAGTGGAATGAAAATCAAAATCCTCGAATGTTCCTGGCGATGATTACATATATCACCAA
AAATCAACCCGAGTGGTTTAGAAACATCCTGAGCATGGCACCCATAATGTTCTCAAACAAAATGGCAAGA
CTAGGGAAAGGGTACATGTTCGAGAGTAAAAGAATGAAGATTCGAACACAAATACCAGCAGAAATGCTAG
CAAGCATTGACCTGAAGTACTTCAATGAATCAACAAAGAAGAAAATTGAGAAAATAAGGCCTCTTTTAAT
GGATGGCACAGCATCACTGAGTCCTGGGATGATGATGGGCATGTTCAACATGCTAAGTACGGTCTTGGGA
GTCTCGATACTGAATCTTGGACAAAAGAAATACACCAAGACAACATACTGGTGGGATGGGCTCCAATCAT
CCGACGATTTTGCTCTCATAGTGAATGCACCAAACCATGAGGGAATACAAGCAGGAGTGGACAGATTCTA
CAGGACCTGCAAGTTAGTGGGAATCAACATGAGCAAAAAGAAGTCCTATATAAATAAGACAGGAACATTT
GAATTCACAAGCTTTTTTTATCGCTATGGATTTGTGGCTAATTTTAGCATGGAGCTACCCAGCTTTGGAG
TGTCTGGAGTAAATGAATCAGCTGACATGAGTATTGGAGTAACAGTGATAAAGAACAACATGATAAACAA
TGACCTTGGACCTGCAACAGCCCAGATGGCTCTTCAATTGTTCATCAAAGACTACAGATACACATATAGG
TGCCATAGGGGAGACACACAAATTCAGACGAGAAGATCATTTGAGTTAAAGAAGCTGTGGGATCAAACCC
AATCAAAGGTAGGGCTATTAGTATCAGATGGAGGACCAAACTTATACAATATACGGAATCTTCACATTCC
TGAAGTCTGCTTAAAATGGGAGCTAATGGATGATGATTATCGGGGAAGACTTTGTAATCCCCTGAATCCC
TTTGTAAGTCATAAGGAAATTGATTCTGTAAACAATGCTGTGGTAATGCCAGCCCATGGTCCAGCCAAAA
GCATGGAATATGATGCCGTTGCAACTACACATTCCTGGATTCCCAAGAGGAATCGTTCTATTCTCAACAC
AAGCCAAAGGGGAATTCTTGAGGATGAACAGATGTACCAGAAGTGCTGCAATCTATTCGAGAAATTTTTC
CCTAGCAGTTCATATAGGAGACCGGTTGGAATTTCTAGCATGGTGGAGGCCATGGTGTCTAGGGCCCGGA
TTGATGCCAGGGTCGACTTTGAGTCTGGACGGATCAAGAAAGAAGAGTTCTCTGAGATCATGAAGATCTG
TTCCACCATTGAAGAACTCAGACGGCAAAAATAA

>gi|383513264|gb|JQ768351.1| Influenza A virus (A/Tomsk/IIV-19/2012(H1N1)) segment 2 polymerase PB1 (PB1) gene, complete cds; and nonfunctional PB1-F2 protein (PB1-F2) gene, complete sequence

ATGGATGTCAATCCGACTCTACTTTTCCTAAAAATTCCAGCGCAAAATGCCATAAGCACCACATTCCCTT
ATACTGGAGATCCTCCATACAGCCATGGAACAGGAACAGGATACACCATGGACACAGTAAACAGAACACA
CCAATACTCAGAAAAGGGAAAGTGGACGACAAACACAGAGACTGGTGCACCCCAGCTCAACCCGATTGAT
GGACCACTACCTGAGGATAATGAACCAAGTGGGTATGCACAAACAGACTGTGTTCTAGAGGCTATGGCTT
TCCTTGAAGAATCCCACCCAGGAATATTTGAGAATTCATGCCTTGAAACAATGGAAGTTGTTCAACAAAC
AAGGGTAGACAAACTAACTCAAGGTCGCCAGACTTATGATTGGACATTAAACAGAAATCAACCGGCAGCA
ACTGCATTGGCCAACACCATAGAAGTCTTTAGATCGAATGGCCTAACAGCTAATGAGTCAGGAAGGCTAA
TAGATTTCTTAAAGGATGTAATGGAATCAATGAACAAAGAGGAAATAGAGATAATAACCCACTTTCAAAG
AAAAAGGAGAGTAAGAGACAACATGACCAAGAAGATGGTCACGCAAAGAACAATAGGGAAGAAAAAACA
A
AGACTGAATAAGAGAGGCTATCTAATAAGAGCACTGACATTAAATACGATGACCAAAGATGCAGAGAGAG
GCAAGTTAAAAAGAAGGGCTATCGCAACACCTGGAATGCAGATTAGAGGTTTCGTATACTTTGTTGAAAC

TTTAGCTAGGAGCATTTGCGAAAAGCTTGAACAGTCTGGGCTCCCAGTAGGGGGCAATGAAAAGAAGGCC
AAACTGGCAAATGTTGTGAGAAAGATGATGACTAATTCACAAGACACAGAGATCTCTTTCACAATCACTG
GGGACAACACTAAGTGGAATGAAAACCAAAATCCTCGAATGTTCCTGGCGATGATTACATATATCACCAG
AAATCAACCCGAATGGTTCAGAAACATCCTGAGCATGGCACCCATAATGTTCTCAAACAAAATGGCAAGA
CTAGGGAAAGGGTACATGTTCGAGAGTAAAAGAATGAAGATTCGAACGCAAATACCAGCAGAAATGCTAG
CAAGCATTGACCTGAAGTACTTCAATGAATCAACAAAGAAGAAAATTGAGAAAATAAGGCCTCTTTTAAT
AGATGGCACAGCATCACTGAGTCCTGGGATGATGATGGGCATGTTCAACATGCTAAGTACGGTCTTGGGA
GTCTCGATACTGAATCTTGGACAAAAGAAATACACCAAGACAATATACTGGTGGGATGGGCTCCAATCAT
CCGACGATTTTGCTCTCATAGTGAATGCACCAAACCATGAGGGAATACAAGCAGGAGTGGACAGATTTTA
CAGGACCTGCAAGTTAGTGGGAATCAACATGAGCAAAAAGAAGTCCTATATAAATAAGACAGGGACATTT
GAATTCACAAGCTTTTTTTATCGCTATGGATTTGTGGCTAATTTTAGCATGGAGCTACCCAGCTTTGGAG
TGTCCGGAGTAAATGAATCAGCTGACATGAGTATTGGAGTAACAGTGATAAAGAACAACATGATAAACAA
TGACCTTGGACCTGCAACGGCCCAGATGGCTCTTCAATTGTTCATCAAAGACTACAGATACACATATAGG
TGCCATAGGGGAGACACACAAATTCAGACGAGAAGATCATTTGAGTTAAAGAAGCTGTGGGATCAAACCC
AATCAAAGGTAGGGCTATTAGTATCAGATGGAGGACCAAACTTATACAATATACGGAATCTTCACATTCC
TGAAGTCTGCTTAAAATGGGAGCTAATGGATGATGATTATCGGGGAAGACTTTGTAATCCCCTGAATCCC
TTTGTAAGTCATAAAGAGATTGATTCTGTAAACAATGCTGTGGTAATGCCAGCCCATGGTCCAGCCAAAA
GCATGGAATATGATGCCGTTGCAACTACACATTCCTGGATTCCCAAGAGGAATCGTTCTATTCTCAACAC
AAGCCAAAGGGGAATTCTTGAGGATGAACAGATGTACCAGAAGTGCTGCAATCTATTCGAGAAATTTTTC
CCTAGCAGTTCATATAGGAGACCGGTTGGAATTTCTAGCATGGTGGAGGCCATGGTGTCTAGGGCCCGGA
TTGATGCCAGGGTCGACTTTGAGTCTGGACGGATCAAGAAAGAAGAGTTCTCTGAGATCATGAAGATCTG
TTCCACCATTGAAGAACTCAGACGGCAAAAATAA
