# Supplementary material for: A computational method for predicting regulation of human microRNAs on the influenza virus genome
Source: BMC Syst Biol. 2013 Oct 14;7(Suppl 2):S3. doi: 10.1186/1752-0509-7-S2-S3 (PMC3851852; doi:10.1186/1752-0509-7-S2-S3)
Supplement: Additional File 11 — 1100 miRNAs from miRBase [file 1752-0509-7-S2-S3-S11.PDF]

PB2

>gi|145278788|gb|CY021700.1| Influenza A virus (A/Memphis/15/2000(H1N1)) segment 1, complete sequence

TTCAATATGGAAAGAATAAAAGAGCTAAGGAATCTGATGTCACAATCTCGCACTCGCGAGATACTTACAA
AAACTACTGTAGACCACATGGCCATAATCAAGAAATACACATCAGGAAGACAGGAGAAAAACCCATCACT
TAGGATGAAATGGATGATGGCAATGAAATACCCAATTACAGCAGATAAAAGGATAACGGAAATGATTCCT
GAAAGAAATGAGCAAGGACAGACATTATGGAGTAAAGTGAATGATGCCGGATCAGACCGAGTGATGATAT
CACCCCTGGCTGTGACATGGTGGAACAGAAATGGACCAGTGGCAAGTACTATTCACTATCCAAAAATCTA
CAAAACTTACTTTGAAAAGGTTGAAAGGTTAAAACATGGAACCTTTGGCCCTGTACACTTTAGAAACCAA
GTCAAAATACGCCGAAGAGTCGACATAAATCCTGGTCATGCAGACCTCAGCGCCAAGGAGGCACAGGATG
TAATTATGGAAGTTGTTTTCCCTAATGAAGTGGGAGCCAGAATACTAACATCAGAATCGCAATTAACGAT
AACCAAGGAGAAAAAAGAAGAACTCCAGAATTGCAAAATTTCCCCTTTGATGGTTGCATACATGTTAGAG
AGGGAGCTTGTCCGCAAAACGAGATTTCTCCCGGTTGCTGGTGGAACAAGCAGTGTGTACATTGAAGTTT
TGCATTTAACACAGGGGACATGCTGGGAGCAGATGTACACTCCAGGTGGGGAGGTGAGGAATGATGATGT
TGATCAAAGCCTAATTATTGCTGCTAGGAACATAGTGAGAAGAGCTGCAGTATCAGCAGATCCACTAGCA
TCTTTATTAGAAATGTGCCATAGCACACAGATTGGTGGAACAAGGATGGTGGATATTCTCAGGCAAAATC
CAACAGAAGAACAAGCTGTGGATATATGCAAAGCAGCAATGGGGCTGAGGATCAGTTCATCCTTCAGTTT
TGGCGGATTCACATTTAAGAGAACAAGTGGATCATCAGTCAAAAAGGAGGAAGAAGTGCTCACGGGCAAT
CTGCAAACATTGAAGTTAACTGTGCATGAGGGATATGAGGAGTTCACAATGGTTGGGAAAAGGGCAACAG
CTATACTCAGAAAAGCAACCAGGAGATTGATTCAACTAATAGTGAGTGGAAGAGACGAACAGTCAATAGT
CGAAGCAATAGTTGTAGCAATGGTATTCTCACAAGAAGATTGCATGGTAAAAGCAGTTAGAGGTGATCTG
AATTTCGTTAATAGAGCGAACCAGCGGTTGAATCCCATGCATCAACTTTTGAGACATTTTCAGAAAGATG
CTAAAGTACTTTTCTTAAATTGGGGAATTGAACCTATCGACAATGTGATGGGAATGATTGGGATATTACC
TGATATGACTCCAAGTACCGAGATGTCAATGAGAGGAGTGAGAGTCAGCAAAATGGGTGTAGATGAATAC
TCCAATGCTGAAAGGGTAGTGGTGAGCATTGACCGTTTTTTGAGAGTCCGGGACCAAAGAGGAAATGTAC
TACTGTCTCCAGAGGAAGTCAGTGAAACACAGGGAACAGAGAAACTGACAATAACTTACTCTTCATCAAT
GATGTGGGAGATTAATGGCCCTGAGTCAGTGTTGATCAATACCTATCAGTGGATCATCAGAAACTGGGAG
ACTGTTAAAATTCAGTGGTCTCAGAACCCTACAATGCTATACAATAAAATGGAATTCGAGCCATTTCAGT
CTCTAGTCCCTAAGGCCATTAGAGGCCAATACAGTGGGTTTGTTAGAACTCTATTTCAACAAATGAGGGA
TGTGCTTGGGACCTTTGACACAACTCAGATAATAAAACTTCTTCCCTTTGCAGCCGCTCCACCAAAGCAA
AGTAGAATGCAATTCTCATCATTGACTGTGAATGTGAGGGGATCAGGAATGAGAATACTTGTAAGGGGTA
ATTCTCCAGTATTCAACTACAACAAGACCACTAAGAGACTCACAGTCCTCGGAAAGGATGCTGGCACTTT
AACTGAAGACCCAGATGAAGGCACAGCTGGAGTGGAATCTGCTGTTCTAAGGGGATTCCTCATTCTAGGC
AAAGAAGATAGAAGATATGGGCCAGCATTAAGCATCAATGAATTGAGCAACCTTGCGAAAGGGGAAAAAG
CTAATGTGCTAATTGGGCAAGGGGACGTAGTGTTGGTAATGAAACGAAAACGGGACTCTAGCATACTTAC
TGACAGCCAGACAGCGACCAAAAGAATTCGGATGGCCATCAATTAAT

>gi|70907656|gb|CY000455.2| Influenza A virus (A/New York/146/2000(H1N1)) segment 1, complete sequence

AAAGCAGGTCAATTATATTCAATATGGAAAGAATAAAAGAGCTAAGGGACCTGATGTCGCAATCCCGCAC
GCGCGAGATACTCACAAAAACCACTGTAGACCACATGGCCATAATCAAGAAGTACACATCAGGAAGACAG
GAGAAAAACCCGTCACTTAGGATGAAATGGATGATGGCAATGAAATACCCAATTACAGCAGACAAAAAAA
TAACGGAAATGATTCCTGAAAGGAATGAGCAAGGGCAAACATTATGGAGTAAAGTGAATGATGCCGGATC
AGACCGAGTGATGATATCACCCCTGGCTGTGACATGGTGGAACAGAAATGGACCAGTGGCAAGTACTATT

CACTATCCAAAAATCTACAAAACTTACTTTGAAAAAGTCGAAAGGTTAAAACATGGAACCTTTGGCCCTG
TACACTTTAGAAATCAAGTCAAAATACGCAGAAGGGTCGACATAAATCCTGGTCATGCAGACCTCAGCGC
CAAGGAGGCACAGGATGTGATTATGGAAGTTGTTTTCCCTAATGAAGTGGGAGCCAGAATACTAACATCG
GAATCGCAATTAACGATAACCAAAGAGAAAAAAGAAGAACTCCAGGATTGCAAAATTTCCCCTTTGATGG
TTGCATACATGTTAGAGAGGGAACTTGTCCGCAAAACGAGATTTCTCCCGGTTGCTGGTGGAACAAGCAG
TGTGTACATTGAAGTTTTGCATTTAACACAGGGAACATGCTGGGAACAGATGTACACTCCAGGTGGGGAG
GTGAAGAATGATGATGTTGATCAAAGTCTAATTATTGCTGCTAGGAATATAGTGAGAAGGGCTGCGGTAT
CAGCAGATCCACTAGCATCTTTATTAGAAATGTGCCACAGCACACAGATTGGTGGAACAAGAATGGTGGA
CATTCTCAGGCAAAATCCAACAGAAGAACAAGCAGTAGATATATGCAAAGCAGCAATGGGACTGAGAATC
AGTTCATCCTTCAGTTTTGGTGGATTCACATTTAAAAGAACAAGTGGATCATCAGTCAAAAGGGAGGAAG
AAGTGCTCACGGGCAATCTTCAAACATTGAAGCTAAATGTGCATGAGGGATATGAAGAGTTCACAATGGT
TGGGAAAAGGGCAACAGCTATACTCAGAAAAGCAACCAGGAGATTGATTCAACTGATAGTGAGTGGAAGA
GACGAACAATCGATAGTCGAAGCAATGGTTGTAGCAATGGTATTCTCACAAGAAGATTGCATGATAAAAG
CAGTCAGAGGTGATCTTAATTTCGTTAATAGAGCGAATCAGCGGTTGAATCCCATGCATCAACTTTTGAG
ACATTTTCAGAAGGATGCTAAAGTGCTTTTCCAAAATTGGGGAATTGAACCTATCGACAATGTGATGGGA
ATGATTGGGATATTACCTGACATGACTCCAAGTACCGAGATGTCAATGAGAGGAGTGAGAGTCAGCAAGA
TGGGTGTAGATGAATACTCCAATGCGGAAAGAGTAGTGGTGAGCATTGACCGTTTTTTGAGAGTCCGGGA
CCAAAGAGGAAATGTACTACTGTCTCCGGAGGAGGTCAGTGAGACACAGGGAACAGAAAAACTGACAATA
ACTTACTCTTCATCAATGATGTGGGAGATTAATGGCCCTGAGTCAGTGCTGATCAATACATATCAGTGGA
TCATCAGAAACTGGGAGACTGTTAAAATTCAGTGGTCTCAGAACCCTACACTGCTATACAATAAAATGGA
ATTCGAGCCATTCCAGTCTTTAGTCCCTAAGGCCATTAGAGGCCAATACAGCGGGTTTGTTAGAACTCTA
TTCCAACAAATGAGGGATGTGCTTGGGACCTTTGACACCATTCAGATAATAAAACTTCTTCCCTTTGCAG
CCGCTCCACCAAAGCAAAGTAGAATGCAGTTCTCCTCATTGACTGTGAATGTGAGGGGGTCAGGAATGAG
AATACTTGTAAGGGGTAATTCTCCAGTATTTAACTACAACAAGACCACCAAGAGACTCACTGTCCTCGGA
AAGGACGCTGGCACTTTAACTGAAGACCCAGATGAAGGCACAGCTGGAGTGGAATCTGCTGTTCTAAGGG
GATTCCTCATTCTAGGCAAAGAGGATAGGAGATATGGGCCAGCATTGAGCATCAATGAACTGAGCAACCT
TGCGAAAGGGGAAAAAGCCAATGTGCTAATTGGACAAGGGGACATAGTGTTGGTAATGAAACGAAAACGG
GACTCTAGCATACTTACTGACAGCCAGACAGCGACCAAAAGAATTCGAATGGCCATCAATTAATTTC
>gi|145278921|gb|CY021756.1| Influenza A virus (A/South Australia/44/2000(H1N1)) segment 1, complete sequence
AATATGGAAAGAATAAAAGAGCTAAGGGATCTGATGTCGCAATCCCGCACGCGCGAGATACTCACAAAAA
CCACTGTAGACCACATGGCCATAATCAAGAAGTACACATCAGGAAGACAGGAGAAAAACCCGTCACTTAG
GATGAAATGGATGATGGCAATGAAATACCCAATTACAGCAGACAAAAAAATAACGGAAATGATTCCTGAA
AGGAATGAGCAAGGGCAAACATTATGGAGTAAAGTGAATGATGCCGGATCAGACCGAGTGATGATATCAC
CCCTGGCTGTGACATGGTGGAACAGAAATGGACCAGTGGCAAGTACTATTCACTATCCAAAAATCTACAA
AACTTACTTTGAAAAAGTCGAAAGGTTAAAACATGGAACCTTTGGCCCTGTACACTTTAGAAATCAAGTC
AAAATACGCAGAAGGGTCGACATAAATCCTGGTCATGCAGACCTCAGCGCCAAGGAGGCACAGGATGTGA
TTATGGAAGTTGTTTTCCCTAATGAAGTGGGAGCCAGAATACTAACATCGGAATCGCAATTAACGATAAC
CAAAGAGAAAAAAGAAGAACTCCAGGATTGCAAAATTTCCCCTTTGATGGTTGCATACATGTTAGAGAGG
GAACTTGTCCGCAAAACGAGATTTCTCCCGGTTGCTGGTGGAACAAGCAGTGTGTACATTGAAGTTTTGC
ATTTAACACAGGGAACATGCTGGGAACAGATGTACACTCCAGGTGGGGAGGTGAAGAATGATGATGTTGA
TCAAAGTCTAATTATTGCTGCTAGGAATATAGTGAGAAGGGCTGCGGTATCAGCAGATCCACTAGCATCT
TTATTAGAAATGTGCCACAGCACACAGATTGGTGGAACAAGAATGGTGGACATTCTCAGGCAAAATCCAA
CAGAAGAACAAGCAGTAGATATATGCAAAGCAGCAATGGGACTGAGAATCAGTTCATCCTTCAGTTTTGG

TGGATTCACATTTAAGAGAACAAGTGGATCATCAGTCAAAAGGGAGGAAGAAGTGCTCACGGGCAATCTT
CAAACATTGAAGCTAAATGTGCATGAGGGATATGAAGAGTTCACAATGGTTGGGAAAAGGGCAACAGCTA
TACTCAGAAAAGCAACCAGGAGATTGATTCAACTGATAGTGAGTGGAAGAGACGAACAATCGATAGTCGA
AGCAATGGTTGTAGCAATGGTATTCTCACAAGAAGATTGCATGATAAAAGCAGTTAGAGGTGATCTTAAT
TTCGTTAATAGAGCGAATCAGCGGTTGAATCCCATGCATCAACTTTTGAGACATTTTCAGAAGGATGCTA
AAGTGCTTTTCCAAAATTGGGGAATTGAACCTATCGACAATGTGATGGGAATGATTGGGATATTACCTGA
CATGACTCCAAGTACCGAGATGTCAATGAGAGGAGTGAGAGTCAGCAAGATGGGTGTAGATGAATACTCC
AATGCGGAAAGAGTAGTGGTGAGCATTGACCGTTTTTTGAGAGTCCGGGACCAAAGAGGAAATGTACTAC
TGTCTCCGGAGGAGGTCAGTGAGACACAGGGAACAGAAAAACTGACAATAACTTACTCTTCATCAATGAT
GTGGGAGATTAATGGCCCTGAGTCAGTGCTGATCAATACATATCAGTGGATCATCAGAAACTGGGAGACT
GTTAAAATTCAGTGGTCTCAGAACCCTACACTGCTATACAATAAAATGGAATTCGAGCCATTCCAGTCTT
TAGTCCCTAAGGCCATTAGAGGCCAATACAGCGGGTTTGTTAGAACTCTATTCCAACAAATGAGGGATGT
GCTTGGGACCTTTGACACCATTCAGATAATAAAACTTCTTCCCTTTGCAGCCGCTCCACCAAAGCAAAGT
AGAATGCAGTTCTCCTCATTGACTGTGAATGTGAGGGGGTCAGGAATGAGAATACTTGTAAGGGGTAATT
CTCCAGTATTTAACTACAACAAGACCACCAAGAGACTCACTGTCCTCGGAAAGGACGCTGGCACTTTAAC
TGAAGACCCAGATGAAGGCACAGCTGGAGTGGAATCTGCTGTTCTAAGGGGATTCCTCATTCTAGGCAAA
GAGGATAGGAGATATGGGCCAGCATTGAGCATCAATGAACTGAGCAACCTTGCGAAAGGGGAAAAAGCCA
ATGTGCTAATTGGACAAGGGGACATAGTGTTGGTAATGAAACGAAAACGGGACTCTAGCATACTTACTGA
CAGCCAGACAGCGACCAAAAGAATTCGAATGGCCATCAATTAATTTCGAATAATTTA

>gi|157367782|gb|CY026162.1| Influenza A virus (A/Auckland/585/2000(H1N1)) segment 1, complete sequence

ATATGGAAAGAATAAAAGAGCTAAGGAATCTGATGTCACAATCTCGCACTCGCGAGATACTTACAAAAAC
TACTGTAGACCACATGGCCATAATCAAGAAATACACATCAGGAAGACAGGAGAAAAACCCATCACTTAGG
ATGAAATGGATGATGGCAATGAAATACCCAATTACAGCTGATAAAAGGATAACGGAAATGATTCCTGAAA
GAAATGAGCAAGGACAGACATTATGGAGTAAAGTGAATGATGCCGGATCAGACCGAGTGATGATATCACC
CCTGGCTGTGACATGGTGGAACAGAAATGGACCAGTGGCAAGTACTATTCACTATCCAAAAATCTACAAA
ACTTACTTTGAAAAGGTTGAAAGGTTAAAACATGGAACCTTTGGCCCTGTACACTTTAGAAACCAAGTCA
AAATACGTCGAAGAGTCGACATAAATCCTGGTCATGCAGACCTCAGCGCCAAGGAGGCACAGGATGTAAT
TATGGAAGTTGTTTTCCCTAATGAAGTGGGAGCCAGAATACTAACATCAGAATCGCAATTAACGATAACC
AAGGAGAAAAAAGAAGAACTCCAGAATTGCAAAATTTCCCCTTTGATGGTTGCATACATGTTAGAGAGGG
AACTTGTCCGCAAAACAAGATTTCTCCCGGTTGCTGGTGGAACAAGCAGTGTGTACATTGAAGTTTTGCA
TTTAACACAGGGGACATGCTGGGAGCAGATGTACACTCCAGGTGGGGAGGTGAAGAATGATGATGTTGAT
CAAAGCCTAATTATTGCTGCTAGGAACATAGTGAGAAGAGCTGCAGTATCAGCAGATCCACTAGCATCTT
TATTAGAAATGTGCCATAGCACACAGATTGGTGGAACAAGGATGGTGGATATTCTCAGGCAAAATCCAAC
AGAAGAACAAGCTGTGGATATATGCAAAGCAGCAATGGGGCTGAGAATCAGTTCATCCTTCAGTTTTGGC
GGATTCACATTTAAGAGAACAAGTGGATCATCAGTCAAAAGGGAGGAAGAAGTGCTAACGGGCAATCTGC
AAACATTGAAGCTAACTGTGCATGAGGGATATGAAGAGTTCACAATGGTTGGGAAAAGGGCAACAGCTAT
ACTCAGAAAAGCAACCAGGAGATTGATTCAACTAATAGTGAGTGGAAGAGACGAACAGTCAATAGTCGAA
GCAATAGTTGTAGCAATGGTATTCTCACAAGAAGATTGCATGATAAAAGCAGTTAGAGGTGATCTGAATT
TCGTTAATAGAGCGAATCAGCGGTTGAATCCCATGCATCAACTTTTGAGACATTTTCAGAAGGATGCTAA
AGTACTTTTCTTAAATTGGGGAATTGAACCTATCGACAATGTGATGGGAATGATTGGGATATTACCTGAT
ATGACTCCAAGTACTGAGATGTCAATGAGAGGAGTGAGAGTCAGCAAAATGGGTGTAGATGAATACTCCA
ATGCTGAAAGGGTAGTGGTGAGCATTGACCGTTTTTTGAGAGTCCGGGACCAAAGAGGAAATGTACTACT
GTCTCCAGAGGAAGTCAGTGAAACACAGGGAACAGAGAAACTGACAATAACTTACTCTTCATCAATGATG

TGGGAGATTAATGGCCCTGAGTCAGTGTTGATCAATACCTATCAGTGGATCATCAGAAACTGGGAGACTG
TTAAAATTCAGTGGTCTCAGAACCCTACGATGCTATACAATAAAATGGAATTTGAACCATTTCAGTCTCT
AGTCCCTAAGGCCATTAGAGGCCAATACAGTGGGTTTGTTAGAACTCTATTTCAACAAATGAGGGATGTG
CTTGGGACCTTTGACACAACTCAGATAATAAAACTTCTTCCCTTTGCAGCCGCTCCACCAAAGCAAAGTA
GAATGCAATTCTCGTCATTAACTGTGAATGTGAGGGGATCAGGAATGAGAATACTTGTAAGGGGTAATTC
TCCAGTATTCAACTACAACAAGACCACCAAGAGACTCACAGTCCTCGGAAAGGATGCTGGCACTTTAACT
GAAGACCTAGATGAAGGCACAGCTGGAGTGGAATCTGCTGTTCTAAGGGGATTCCTCATTCTAGGCAAAG
AAGATAGAAGATATGGGCCAGCATTAAGCATCAATGAATTGAGCAACCTTGCGAAAGGGGAAAAAGCTAA
TGTGCTAATTGGACAAGGGGACGTAGTGTTGGTAATGAAACGAAAACGGGACTCTAGCATACTTACTGAC
AGCCAGACAGCGACCAAAAGAATTCGGATGGCCATCAATTAATTTCGAATAA
>gi|145278634|gb|CY021636.1| Influenza A virus (A/Wellington/4/2000(H1N1)) segment 1, complete sequence
AATATGGAAAGAATAAAAGAGCTAAGGAATCTGATGTCACAATCTCGCACTCGCGAGATACTTACAAAAA
CTACTGTAGACCACATGGCCATAATCAAGAAATACACATCAGGAAGACAGGAGAAAAACCCATCACTTAG
GATGAAATGGATGATGGCAATGAAATACCCAATTACAGCTGATAAAAGGATAACGGAAATGATTCCTGAA
AGAAATGAGCAAGGACAGACATTATGGAGTAAAGTGAATGATGCCGGATCAGACCGAGTGATGATATCAC
CCCTGGCTGTGACATGGTGGAACAGAAATGGACCAGTGGCAAGTACTATTCACTATCCAAAAATCTACAA
AACTTACTTTGAAAAGGTTGAAAGGTTAAAACATGGAACCTTTGGCCCTGTACACTTTAGAAACCAAGTC
AAAATACGCCGAAGAGTCGACATAAATCCTGGTCATGCAGACCTCAGCGCCAAGGAGGCACAGGATGTAA
TTATGGAAGTTGTTTTCCCTAATGAAGTGGGAGCCAGAATACTAACATCAGAATCGCAATTAACGATAAC
CAAGGAGAAAAAAGAAGAACTCCAGAATTGCAAAATTTCCCCTTTGATGGTTGCATACATGTTAGAGAGG
GAACTTGTCCGCAAAACAAGATTTCTCCCGGTTGCTGGTGGAACAAGCAGTGTGTACATTGAAGTTTTGC
ATTTAACACAGGGGACATGCTGGGAGCAGATGTACACTCCAGGTGGGGAGGTGAAGAATGATGATGTTGA
TCAAAGCCTAATTATTGCTGCTAGGAACATAGTGAGAAGAGCTGCAGTATCAGCAGATCCACTAGCATCT
TTATTAGAAATGTGCCATAGCACACAGATTGGTGGAACAAGGATGGTGGATATTCTCAGGCAAAATCCAA
CAGAAGAACAAGCTGTGGATATATGCAAAGCAGCAATGGGGCTGAGAATCAGTTCATCCTTCAGTTTTGG
CGGATTCACATTTAAGAGAACAAGTGGATCATCAGTCAAAAGGGAGGAAGAAGTGCTCACGGGCAATCTG
CAAACATTGAAGCTAACTGTGCATGAGGGATATGAAGAGTTCACAATGGTTGGGAAAAGGGCAACAGCTA
TACTCAGAAAAGCAACCAGGAGATTGATTCAACTAATAGTGAGTGGAAGAGACGAACAGTCAATAGTCGA
AGCAATAGTTGTAGCAATGGTATTCTCACAAGAAGATTGCATGATAAAAGCAGTTAGAGGTGATCTGAAT
TTCGTTAATAGAGCGAATCAGCGGTTGAATCCCATGCATCAACTTTTGAGACATTTTCAGAAGGATGCTA
AAGTACTTTTCTTAAATTGGGGAATTGAACCTATCGACAATGTGATGGGAATGATTGGGATATTACCTGA
TATGACTCCAAGTACCGAGATGTCAATGAGAGGAGTGAGAGTCAGCAAAATGGGTGTAGATGAATACTCC
AATGCTGAAAGGGTAGTGGTGAGCATTGACCGTTTTTTGAGAGTCCGGGACCAAAGAGGAAATGTACTAC
TGTCTCCAGAGGAAGTCAGTGAAACACAGGGAACAGAGAAACTGACAATAACTTACTCTTCATCAATGAT
GTGGGAGATTAATGGCCCTGAGTCAGTGTTGATCAATACCTATCAGTGGATCATCAGAAACTGGGAGACT
GTTAAAATTCAGTGGTCTCAGAACCCTACGATGCTATACAATAAAATGGAATTTGAACCATTTCAGTCTC
TAGTCCCTAAGGCCATTAGAGGCCAATACAGTGGGTTTGTTAGAACTCTATTTCAACAAATGAGGGATGT
GCTTGGGACCTTTGACACAACTCAGATAATAAAACTTCTTCCCTTTGCAGCCGCTCCACCAAAGCAAAGT
AGAATGCAATTTTCGTCATTAACTGTGAATGTGAGGGGATCAGGAATGAGAATACTTGTAAGGGGTAATT
CTCCAGTATTCAACTACAACAAGACCACTAAGAGACTCACAGTCCTCGGAAAGGATGCTGGCACTTTAAC
TGAAGACCCAGATGAAGGCACAGCTGGAGTGGAATCTGCTGTTCTAAGGGGATTTCTCATTCTAGGCAAA
GAAGATAGAAGATATGGGCCAGCATTAAGCATCAATGAATTGAGCAACCTTGCGAAAGGGGAAAAAGCTA
ATGTGCTAATTGGACAAGGGGACGTAGTGTTGGTAATGAAACGAAAACGGGACTCTAGCATACTTACTGA

CAGCCAGACAGCGACCAAAAGAATTCGGATGGCCATCAATTAATTTCGAA
>gi|73761490|gb|CY002535.1| Influenza A virus (A/New York/220/2002(H1N1)) segment 1, complete sequence
AAAGCAGGTCAATTATATTCAATATGGAAAGAATAAAAGAGCTAAGGAATCTGATGTCACAATCTCGCAC
TCGCGAGATACTTACCAAAACTACTGTAGACCACATGGCCATAATCAAGAAATACACATCAGGAAGACAG
GAGAAAAACCCATCACTTAGGATGAAATGGATGATGGCAATGAAATACCCAATTACAGCTGATAAAAGGA
TAACGGAAATGATTCCTGAAAGAAATGAGCAAGGACAGACACTATGGAGTAAAGTGAATGATGCCGGATC
AGACCGAGTGATGATATCACCCCTGGCTGTGACATGGTGGAACAGAAATGGACCAGTGGCAAATACTATT
CACTATCCAAAAATCTACAAAACTTACTTTGAAAAGGTTGAAAGGTTAAAACATGGAACCTTTGGCCCTG
TACACTTTAGAAACCAAGTCAAAATACGCCGAAGAGTCGACATAAATCCTGGTCATGCAGACCTCAGCGC
CAAGGAGGCACAGGATGTAATTATGGAAGTTGTTTTCCCTAATGAAGTGGGAGCCAGAATACTAACATCA
GAATCGCAATTAACGATAACCAAGGAGAAAAAAGAAGAACTCCAGAATTGCAAAATTTCCCCTTTGATGG
TTGCATACATGTTAGAGAGGGAACTTGTCCGCAAAACAAGATTTCTCCCGGTTGCAGGTGGAACAAGCAG
TGTGTACATTGAAGTTTTGCATTTAACACAGGGGACATGCTGGGAGCAGATGTACACTCCAGGTGGGGAG
GTGAGGAATGATGATGTTGATCAAAGCCTAATTATTGCTGCTAGGAACATAGTGAGAAGAGCTGCAGTAT
CAGCAGATCCACTAGCATCTTTATTAGAAATGTGCCATAGCACACAGATTGGTGGAACAAGGATGGTGGA
TATTCTCAGGCAAAATCCAACAGAAGAACAAGCTGTGGACATATGCAAAGCAGCAATGGGGCTGAGAATC
AGTTCATCCTTCAGTTTTGGCGGATTCACATTTAAGAGAACAAGTGGATCATCAGTCAAAAGGGAGGAAG
AAGTGCTCACGGGCAATCTGCAAACATTGAAGCTAACTGTGCATGAGGGATATGAAGAATTCACAATGGT
TGGGAAAAGGGCAACAGCTATACTCAGAAAAGCAACCAGGAGATTGATTCAACTAATAGTGAGTGGAAGA
GACGAACAGTCAATAGTCGAAGCAATAGTTGTAGCAATGGTATTCTCACAAGAAGATTGCATGGTAAAAG
CAGTTAGAGGTGATCTGAATTTCGTTAATAGAGCGAATCAGCGGTTGAATCCCATGCATCAACTTTTGAG
ACATTTTCAGAAGGATGCTAAAGTACTTTTCTTAAATTGGGGAATTGAACATATTGACAATGTGATGGGA
ATGATTGGGATATTACCTGATATGACTCCAAGTACCGAGATGTCAATGAGAGGAGTGAGAGTCAGCAAAA
TGGGTGTAGATGAATACTCCAATGCTGAAAGGGTGGTGGTGAGCATTGACCGTTTTTTGAGAGTCCGGGA
CCAAAGAGGAAATGTACTACTGTCTCCAGAGGAAGTCAGTGAAACACAAGGAACAGAGAAACTGACAATA
ACTTACTCTTCATCAATGATGTGGGAGATTAATGGCCCTGAGTCAGTGTTGATCAATACCTATCAGTGGA
TCATCAGAAACTGGGAGACTGTTAAGATTCAGTGGTCTCAGAACCCTACGATGCTATACAATAAAATGGA
ATTTGAACCATTTCAATCTCTAGTCCCCAAGGCCATTAGAGGCCAATACAGTGGGTTTGTTAGAACTCTA
TTTCAACAAATGAGGGATGTGCTCGGGACCTTTGACACAACTCAGATAATAAAACTTCTTCCCTTTGCAG
CCGCTCCACCAAAGCAAAGTAGAATGCAATTCTCGTCATTAACTGTGAATGTGAGGGGATCAGGAATGAG
AATACTTGTAAGGGGTAATTCTCCAGTATTCAACTACAACAAGACCACTAAGAGACTCACAATCCTCGGA
AAGGATGCTGGCACTTTAACTGAAGACCCAGATGAAGGCACAGCTGGAGTGGAATCTGCTGTTTTAAGGG
GATTCCTCATTCTAGGCAAAGAAGATAGAAGATATGGGCCAGCATTAAGCATCAATGAATTGAGCAACCT
TGCGAAAGGGGAAAAAGCTAATGTGCTAATTGGGCAAGGGGATGTAGTGTTGGTAATGAAACGAAAACGG
GACTCTAGCATACTTACTGACAGCCAGACAGCGACCAAAAGAATTCGGATGGCCATCAATTAATTTCGAA
TAATTTAAAAACGACCTTGTTTCTACT
>gi|131058562|gb|CY020156.1| Influenza A virus (A/Memphis/7/2001(H1N1)) segment 1, complete sequence
ATATGGAAAGAATAAAAGAGCTAAGGAATCTGATGTCACAATCTCGAACTCGCGAGATACTTACAAAAAC
TACTGTAGACCACATGGCCATAATCAAAAAATACACATCAGGAAGACAGGAGAAAAACCCATCACTTAGG
ATGAAATGGATGATGGCAATGAAATACCCAATTACAGCAGACAAAAGGATAACAGAAATGATTCCTGAAA
GAAATGAGCAAGGACAGACATTATGGAGTAAAGTGAATGATGCCGGGTCGGACCGAGTGATGATATCACC
CCTGGCTGTGACATGGTGGAACAGAAATGGACCAGTGGCAAGTACTATTCACTATCCAAAAATCTACAAA

ACTTACTTTGAAAGGGTTGAAAGGTTAAAACATGGAACCTTTGGCCCTGTACACTTTAGAAACCAAGTCA
AAATACGCCGAAGAGTCGACATAAATCCTGGTCATGCAGACCTCAGCGCCAAGGAGGCACAGGATGTAAT
TATGGAAGTTGTTTTCCCTAATGAAGTGGGAGCCAGAATACTAACATCAGAATCGCAATTAACGATAACC
AAAGAGAAAAAAGAAGAACTCCAGAATTGCAAAATTTCCCCTTTGATGGTTGCATACATGTTAGAGAGGG
AACTTGTCCGCAAAACGAGATTTCTCCCGGTTGCTGGTGGAACAAGCAGTGTGTACATTGAAGTTTTACA
TTTAACACAGGGGACATGCTGGGAACAGATGTACACTCCAGGTGGGGAGGTGAGGAATGATGATGTTGAT
CAAAGCTTAATTATTGCTGCTAGGAACATAGTGAGAAGAGCTGCAGTATCAGCAGATCCACTAGCATCTT
TATTAGAAATGTGCCATAGCACACAGATTGGTGGAACAAGGATGGTGGATATTCTCCGGCAAAACCCAAC
AGAAGAACAAGCTGTGGATATATGCAAAGCAGCAATGGGGCTGAGAATCAGTTCATCCTTCAGTTTTGGC
GGATTCACATTTAAGAGAACAAGTGGATCATCAGTCAAAAGGGAGGAAGAAGTGCTCACGGGCAATCTGC
AAACATTGAAGCTAACTGTGCATGAGGGATATGAAGAGTTCACAATGGTTGGGAAAAGGGCAACAGCTAT
ACTCAGAAAAGCAACCAGGAGATTGATTCAACTAATAGTGAGTGGACGAGACGAACAGTCGATAGTCGAA
GCAATAGTTGTAGCAATGGTATTCTCACAAGAGGATTGCATGGTAAAAGCAGTTAGAGGTGATCTGAATT
TCGTCAATAGAGCGAATCAGCGGTTGAATCCCATGCATCAACTTTTGAGACATTTTCAGAAGGATGCTAA
AGTACTTTTCTTAAATTGGGGAATTGAACCTATCGACAATGTGATGGGAATGATTGGGATATTACCTGAT
ATGACTCCAAGTACCGAGATGTCAATGAGAGGAGTGAGAGTCAGCAAAATGGGTGTAGATGAATACTCCA
ATGCTGAAAGGGTAGTGGTGAGCATTGACCGTTTTTTGAGAGTCCGGGACCAAAGAGGAAATGTACTACT
GTCTCCAGAGGAAGTCAGTGAAACACAGGGAACAGAGAAACTGACAATAACTTACTCTTCATCAATGATG
TGGGAGATTAATGGCCCTGAGTCAGTGTTGATCAATACCTATCAGTGGATCATCAGAAACTGGGAGACTG
TTAAAATTCAGTGGTCTCAGAACCCTACAATGCTATACAATAAAATGGAATTCGAGCCATTTCAGTCTCT
AGTCCCTAAGGCCATTAGAGGCCAATACAGTGGGTTTGTTAGAACTCTATTCCAACAAATGAGGGATGTG
CTTGGGACCTTTGACACAACTCAGATAATAAAACTTCTTCCCTTTGCAGCCGCTCCACCAAAGCAAAGTA
GAATGCAATTCTCATCATTGACTGTGAATGTGAGGGGATCAGGAATGAGAATACTTGTAAGGGGTAATTC
TCCAGTATTCAACTACAACAAGACCACTAAGAGACTCACAGTCCTCGGAAAGGATGCTGGCACTTTAACT
GAAGACCCAGATGAAGGCACAGCTGGAGTGGAATCTGCTGTTTTAAGGGGATTCCTCATTCTAGGCAAAG
AAGATAGAAGATATGGGCCAGCATTAAGCATCAATGAATTGAGCAACCTTGCGAAAGGGGAAAAAGCTAA
TGTGCTAATTGGGCAAGGGGACGTAGTGTTGGTAATGAAACGAAAACGGGACTCTAGCATACTTACTGAC
AGCCAGACAGCGACCAAAAGAATTCGGATGGCCATCAATTAA
>gi|156536334|gb|CY025041.1| Influenza A virus (A/Auckland/597/2000(H1N1)) segment 1, complete sequence
AATATGGAAAGAATAAAAGAGCTAAGGAATCTGATGTCACAATCTCGCACTCGCGAGATACTTACAAAAA
CTACTGTAGACCACATGGCCATAATCAAGAAATACACATCAGGAAGACAGGAGAAAAACCCATCACTTAG
GATGAAATGGATGATGGCAATGAAATACCCAATTACAGCTGATAAAAGGATAACGGAAATGATTCCTGAA
AGAAATGAGCAAGGACAGACATTATGGAGTAAAGTGAATGATGCCGGATCAGACCGAGTGATGATATCAC
CCCTGGCTGTGACATGGTGGAACAGAAATGGACCAGTAGCAAGTACTATTCACTATCCAAAAATCTACAA
AACTTACTTTGAAAAGGTTGAAAGGTTAAAACATGGAACCTTTGGCCCTGTACACTTTAGAAACCAAGTC
AAAATACGTCGAAGAGTCGACATAAATCCTGGTCATGCAGACCTCAGCGCCAAGGAGGCACAGGATGTAA
TTATGGAAGTTGTTTTCCCTAATGAAGTGGGAGCCAGAATACTAACATCAGAATCGCAATTAACGATAAC
CAAGGAGAAAAAAGAAGAACTCCAGAATTGCAAAATTTCCCCTTTGATGGTTGCATACATGTTAGAGAGG
GAACTTGTCCGCAAAACAAGATTTCTCCCGGTTGCTGGTGGAACAAGCAGTGTGTACATTGAAGTTTTGC
ATTTAACACAGGGGACATGCTGGGAGCAGATGTACACTCCAGGTGGGGAGGTGAAGAATGATGATGTTGA
TCAAAGCCTAATTATTGCTGCTAGGAACATAGTGAGAAGAGCTGCAGTATCAGCAGATCCACTAGCATCT
TTATTAGAAATGTGCCATAGCACACAGATTGGTGGAACAAGGATGGTGGATATTCTCAGGCAAAATCCAA
CAGAAGAACAAGCTGTGGATATATGCAAAGCAGCAATGGGGCTGAGAATCAGTTCATCCTTCAGTTTTGG

CGGATTCACATTTAAGAGAACAAGTGGATCATCAGTCAAAAGGGAGGAAGAAGTGCTCACGGGCAATCTG
CAAACATTGAAGCTAACTGTGCATGAGGGATATGAAGAGTTCACAATGGTTGGGAAAAGGGCAACAGCTA
TACTCAGAAAAGCAACCAGGAGATTGATTCAACTAATAGTGAGTGGAAGAGACGAACAGTCAATAGTCGA
AGCAATAGTTGTAGCAATGGTATTCTCACAAGAAGATTGCATGATAAAAGCAGTTAGAGGTGATCTGAAT
TTCGTTAATAGAGCGAATCAGCGGTTGAATCCCATGCATCAACTTTTGAGACATTTTCAGAAGGATGCTA
AAGTACTTTTCTTAAATTGGGGAATTGAACCTATCGACAATGTGATGGGAATGATTGGGATATTACCTGA
TATGACTCCAAGTACCGAGATGTCAATGAGAGGAGTGAGAGTCAGCAAAATGGGTGTAGATGAATACTCC
AATGCTGAAAGGGTAGTGGTGAGCATTGACCGTTTTTTGAGAGTCCGGGACCAAAGGGGAAATGTACTAC
TGTCTCCAGAGGAAGTCAGTGAAACACAGGGAACAGAGAAACTGACAATAACTTACTCTTCATCAATGAT
GTGGGAGATTAATGGCCCTGAGTCAGTGTTGATCAATACCTATCAGTGGATCATCAGAAACTGGGAGACT
GTTAAAATTCAGTGGTCTCAGAACCCTACGATGCTATACAATAAAATGGAATTTGAACCATTTCAGTCTC
TAGTCCCTAAGGCCATTAGAGGCCAATACAGTGGGTTTGTTAGAACTCTATTTCAACAAATGAGGGATGT
GCTTGGGACCTTTGACACAACTCAGATAATAAAACTTCTTCCCTTTGCAGCCGCTCCACCAAAGCAAAGT
AGAATGCAATTCTCGTCATTAACTGTGAATGTGAGGGGATCAGGAATGAGAATACTTGTAAGGGGTAATT
CTCCAGTATTCAACTACAACAAGACCACCAAGAGACTCACAGTCCTCGGAAAGGATGCTGGCACTTTAAC
TGAAGACCCAGATGAAGGCACAGCTGGAGTGGAATCTGCTGTTCTAAGGGGATTCCTCATTCTAGGCAAA
GAAGATAGAAGATATGGGCCAGCATTAAGCATCAATGAATTGAGCAACCTTGCGAAAGGGGAAAAAGCTA
ATGTGCTAATTGGACAAGGGGACGTAGTGTTGGTAATGAAACGAAAACGGGACTCTAGCATACTTACTGA
CAGCCAGACAGCGACCAAAAGAATTCGGATGGCCATCAATTAATTTCGAATAATTTAA
>gi|149780544|gb|CY022540.1| Influenza A virus (A/Auckland/605/2001(H1N1)) segment 1, complete sequence
AATATGGAAAGAATAAAAGAGCTAAGGAATCTGATGTCACAATCTCGCACTCGCGAGATACTCACAAAAA
CTACTGTAGACCACATGGCCATAATCAAAAAATACACATCCGGAAGACAGGAGAAAAACCCATCACTTAG
GATGAAATGGATGATGGCAATGAAATACCCAATTACAGCAGACAAAAGGATAACGGAAATGATTCCTGAA
AGAAATGAGCAAGGACAGACATTATGGAGTAAAGTGAATGATGCCGGTTCAGACCGAGTGATGATATCAC
CCCTGGCTGTGACATGGTGGAATAGAAATGGACCAGTGGCAAGTACTATTCACTATCCAAAAATCTACAA
AACTTACTTTGAAAAGGTTGAAAGGTTAAAACATGGAACCTTTGGCCCTGTACAATTTAGAAACCAAGTC
AAAATACGCCGAAGAGTCGACATAAATCCTGGTCATGCAGACCTCAGCGCCAAGGAGGCACAGGATGTAA
TTATGGAAGTTGTTTTCCCTAATGAAGTGGGAGCCAGAATACTAACATCAGAATCGCAATTAACGATAAC
CAAAGAGAAAAAAGAAGAACTCCAGAATTGCAAAATTTCCCCTTTGATGGTTGCATACATGTTAGAGAGG
GAACTTGTCCGCAAAACGAGATTTCTCCCGGTTGCTGGTGGAACAAGCAGTGTGTACATTGAAGTTTTGC
ATTTAACACAGGGGACATGCTGGGAACAGATGTACACTCCAGGTGGAGAGGTGAGGAATGATGATGTTGA
TCAAAGCCTAATTATTGCTGCTAGGAACATAGTGAGAAGAGCTGCAGTATCAGCAGATCCACTAGCATCT
TTATTAGAAATGTGCCACAGCACACAGATTGGTGGAACAAGGATGGTGGATATTCTCAGGCAAAACCCAA
CAGAAGAACAAGCTGTGGATATATGCAAAGCAGCAATGGGGCTGAGAATCAGTTCATCCTTCAGTTTTGG
CGGATTCACATTCAAGAGAACAAGTGGATCATCAGTCAAAAGGGAGGAAGAAATGCTCACGGGCAATCTG
CAAACATTGAAGCTAACTGTGCATGAGGGATATGAAGAGTTCACAATGGTTGGGAAAAGGGCAACAGCTA
TACTCAGAAAAGCAACCAGGAGATTGATTCAACTAATAGTGAGTGGACGAGACGAACAGTCGATAGTCGA
AGCAATAGTTGTAGCAATGGTATTCTCACAAGAAGATTGCATGGTAAAAGCAGTTAGAGGTGATCTGAAT
TTCGTCAATAGAGCGAATCAGCGGTTGAATCCCATGCATCAACTTTTGAGACATTTTCAGAAGGATGCTA
AAGTACTTTTCTTAAATTGGGGAATTGAACCTATCGACAATGTGATGGGAATGATTGGGATACTACCTGA
TATGACTCCAAGCACCGAGATGTCAATGAGAGGAGTGAGAATCAGCAAAATGGGTGTAGATGAATACTCC
AATGCTGAAAAGGTAGTGGTGAGTATTGACCGTTTTTTGAGAGTCCGGGACCAAAGAGGAAATGTACTAC
TGTCTCCAGAGGAAGTCAGTGAAACACAGGGAACAGAGAAACTGACAATAACTTACTCTTCATCAATGAT

GTGGGAGATTAATGGCCCTGAGTCAGTGTTGATCAATACCTATCAGTGGATCATCAGAAACTGGGAGACT
GTTAAAATTCAGTGGTCTCAGAACCCTACAATGCTATACAATAAAATGGAATTCGAGCCATTTCAGTCTC
TAGTCCCTAAGGCCATTAGAGGCCAATACAGTGGGTTTGTTAGAACTCTATTCCAACAAATGAGGGATGT
GCTTGGGACCTTTGACACAACTCAGATAATAAAACTTCTTCCCTTTGCAGCCGCTCCACCAAAGCAAAGT
AGAATGCAATTCTCATCATTGACTGTGAATGTGAGAGGATCAGGAATGAGAATACTTGTAAGGGGTAATT
CTCCAGTATTCAACTACAACAAGACCACTAAGAGACTCACAGTCCTCGGAAAGGATGCTGGCACTTTAAC
TGAAGACCCAGATGAAGGCACAGCTGGAGTGGAATCTGCTGTTTTAAGGGGATTCCTCATTTTAGGCAAA
GAAGATAGAAGATATGGGCCAGCATTAAGCATCAATGAATTGAGCAACCTTGCGAAAGGAGAAAAAGCTA
ATGTGCTAATTGGGCAAGGGGACGTAGTGTTGGTGATGAAACGAAAACGGGACTCTAGCATACTTACTGA
CAGCCAGACAGCGACCAAAAGAATTCGGATGGCCATCAATTAATTTCGAATAATTTA
>gi|237688879|gb|CY040081.1| Influenza A virus (A/Taiwan/567/2002(H1N1)) segment 1, complete sequence
ATGGAAAGAATAAAAGAGCTAAGGAATCTGATGTCACAATCTCGCACTCGCGAGATACTTACAAAAACTA
CTGTAGACCACATGGCCATAATCAAAAAATACACATCCGGAAGACAGGAGAAAAACCCATCACTTAGGAT
GAAATGGATGATGGCAATGAAATACCCAATTACAGCAGACAAAAGGATAACGGAAATGATTCCTGAAAGA
AATGAGCAAGGACAGACATTATGGAGTAAAGTGAATGATGCAGGTTCAGACCGAGTGATGATATCACCCC
TGGCTGTGACATGGTGGAATAGAAATGGACCAGTGGCAAGTACTATTCACTATCCAAAAATCTACAAAAC
TTACTTTGAAAAGGTTGAAAGGTTAAAACATGGAACCTTTGGCCCTGTACACTTTAGAAACCAAGTCAAA
ATACGCCGAAGAGTCGACATAAATCCTGGTCATGCAGACCTCAGCGCCAAGGAGGCACAGGATGTAATTA
TGGAAGTTGTTTTCCCTAATGAAGTGGGAGCCAGAATACTAACATCAGAATCACAATTAACGATAACCAA
AGAGAAAAAAGAAGAACTCCAGAATTGCAAAATTTCCCCTTTGATGGTTGCATACATGTTAGAGAGGGAA
CTTGTCCGCAAAACGAGATTTCTCCCGGTTGCTGGTGGAACAAGCAGTGTGTACATTGAAGTTTTGCATT
TAACACAGGGGACATGCTGGGAACAGATGTACACTCCAGGTGGAGAGGTGAGGAATGATGATGTTGATCA
AAGCCTAATTATTGCTGCTAGGAACATAGTGAGAAGAGCTGCAGTATCAGCAGATCCACTAGCATCTTTA
TTAGAAATGTGCCACAGCACACAGATTGGTGGAACAAGGATGGTGGATATTCTCAGGCAAAACCCAACAG
AAGAACAAGCTGTGGATATATGCAAAGCAGCAATGGGGCTGAGAATCAGTTCATCCTTCAGTTTTGGCGG
ATTCACATTCAAGAGAACAAGTGGATCATCAGTCAAAAGGGAGGAAGAAATGCTCACGGGCAATCTGCAA
ACATTGAAGCTAACTGTGCATGAGGGATATGAAGAGTTCACAATGGTTGGGAAAAGGGCAACAGCTATAC
TCAGAAAAGCAACCAGGAGATTGATTCAACTAATAGTGAGTGGACGAGACGAACAGTCGATAGTCGAAGC
AATAGTTGTAGCAATGGTATTCTCACAAGAAGATTGCATGGTAAAAGCAGTTAGAGGTGATCTGAATTTC
GTCAATAGAGCGAATCAGCGGTTGAATCCCATGCATCAACTTTTGAGACATTTTCAGAAGGATGCTAAAG
TACTTTTCTTAAATTGGGGAATTGAACCTATCGACAATGTGATGGGAATGATTGGGATATTACCTGATAT
GACTCCAAGTACCGAGATGTCAATGAGAGGAGTGAGAGTCAGCAAAATGGGTGTAGATGAGTACTCCAAT
GCTGAAAAGGTAGTGGTGAGCATTGACCGTTTTTTGAGAGTCCGGGACCAAAGAGGAAATGTACTACTGT
CTCCAGAGGAAGTCAGTGAAACACAGGGAACAGAGAAACTGACAATAACTTACTCTTCATCAATGATGTG
GGAGATTAATGGCCCTGAGTCAGTGTTGATCAATACCTATCAGTGGATCATCAGAAACTGGGAGACTGTT
AAAATTCAGTGGTCTCAGAACCCTACAATGCTATACAATAAAATGGAATTCGAGCCATTTCAGTCTCTAG
TCCCTAAGGCCATTAGAGGCCAATACAGTGGGTTTGTTAGAACTCTATTCCAACAAATGAGGGATGTGCT
TGGGACCTTTGACACAACTCAGATAATAAAACTTCTTCCCTTTGCAGCCGCTCCACCAAAGCAAAGTAGA
ATGCAATTCTCATCATTGACTGTGAATGTGAGAGGATCAGGAATGAGAATACTTGTAAGGGGTAATTCTC
CAGTATTCAACTACAACAAGACCACTAAGAGACTCACAGTCCTCGGAAAGGATGCTGGCACTTTAACTGA
AGACCCAGATGAAGGCACAGCTGGAGTGGAATCTGCTGTTTTAAGGGGATTCCTCATTCTAGGCAAAGAA
GATAGAAGATATGGGCCAGCATTAAGCATCAATGAATTGAGCAACCTTGCGAAAGGAGAAAAAGCTAATG
TGCTAATTGGGCAAGGGGACGTAGTGTTGGTGATGAAACGAAAACGGGACTCTAGCATACTTACTGACAG

CCAGACAGCGACCAAAAGAATTCGGATGGCCATCAATTAATTTCGAATAATT
>gi|122855966|gb|CY019348.1| Influenza A virus (A/Memphis/6/2003(H1N1)) segment 1, complete sequence
ATATGGAAAGAATAAAAGAGCTAAGGAATCTGATGTCACAATCTCGCACTCGCGAGATACTTACCAAAAC
TACTGTAGACCACATGGCCATAATCAAGAAATACACATCAGGAAGACAGGAGAAAAACCCATCACTTAGG
ATGAAATGGATGATGGCAATGAAATACCCAATTACAGCTGATAAAAGGATAACGGAAATGATTCCTGAAA
GAAATGAGCAAGGACAGACACTATGGAGTAAAGTGAATGATGCCGGATCAGACCGAGTGATGATATCACC
CCTGGCTGTGACATGGTGGAACAGAAATGGACCAGTGGCAAATACTATTCACTATCCAAAAATCTACAAA
ACTTACTTTGAAAAGGTTGAAAGGTTAAAACATGGAACCTTTGGCCCTGTACACTTTAGAAACCAAGTCA
AAATACGCCGAAGAGTCGACATAAATCCTGGTCATGCAGACCTCAGCGCCAAGGAGGCACAGGATGTAAT
TATGGAAGTTGTTTTCCCTAATGAAGTGGGAGCCAGAATACTAACATCAGAATCGCAATTAACGATAACC
AAGGAGAAAAAAGAAGAACTCCAGAATTGCAAAATTTCCCCTTTGATGGTTGCATACATGTTAGAGAGGG
AACTTGTCCGCAAAACAAGATTTCTCCCGGTTGCAGGTGGAACAAGCAGTGTGTACATTGAAGTTTTGCA
TTTAACACAGGGGACATGCTGGGAGCAGATGTACACTCCAGGTGGGGAGGTGAGGAATGATGATGTTGAT
CAAAGCCTAATTATTGCTGCTAGGAACATAGTGAGAAGAGCTGCAGTATCAGCAGATCCACTAGCATCTT
TATTAGAAATGTGCCATAGCACACAGATTGGTGGAACAAGAATGGTGGATATTCTCAGGCAAAATCCAAC
AGAAGAACAAGCTGTGGACATATGCAAAGCAGCAATGGGGCTGAGAATCAGTTCATCCTTCAGTTTTGGC
GGATTCACATTTAAGAGAACAAGTGGATCATCAGTCAAAAGGGAGGAAGAAGTGCTCACGGGCAATCTGC
AAACATTGAAGCTAACTGTGCATGAGGGATATGAAGAGTTCACAATGGTTGGGAAAAGGGCAACAGCTAT
ACTCAGAAAAGCAACCAGGAGATTGATTCAACTAATAGTGAGTGGAAGAGACGAACAGTCAATAGTCGAA
GCAATAGTTGTAGCAATGGTATTCTCACAAGAAGATTGCATGGTAAAAGCAGTTAGAGGTGATCTGAATT
TCGTTAATAGAGCGAATCAGCGGTTGAATCCCATGCATCAACTTTTGAGACATTTTCAGAAGGATGCTAA
AGTACTTTTCTTAAATTGGGGAATTGAACATATTGACAATGTGATGGGAATGATTGGGATATTACCTGAT
ATGACTCCAAGTACCGAGATGTCAATGAGAGGAGTGAGAGTCAGCAAAATGGGTGTAGATGAATACTCCA
ATGCTGAAAGGGTAGTGGTGAGCATTGACCGTTTTTTGAGAGTCCGGGATCAAAGAGGAAATGTACTACT
GTCTCCAGAGGAAGTCAGTGAAACACAAGGAACAGAGAAACTGACAATAACTTACTCTTCATCAATGATG
TGGGAGATTAATGGCCCTGAGTCAGTGTTGATCAATACCTATCAGTGGATCATCAGAAACTGGGAGACTG
TTAAAATTCAGTGGTCTCAGAACCCTACGATGCTATACAATAAAATGGAATTTGAACCATTTCAATCTCT
AGTCCCCAAGGCCATTAGAGGCCAATACAGTGGGTTTGTTAGAACTCTATTTCAACAAATGAGGGATGTG
CTCGGGACCTTTGACACAACTCAGATAATAAAACTTCTTCCCTTTGCAGCCGCTCCACCAAAGCAAAGTA
GAATGCAATTCTCGTCATTAACTGTGAATGTGAGGGGATCAGGAATGAGAATACTTGTAAGGGGCAATTC
TCCAGTATTCAACTACAACAAGACCACTAAGAGACTCACAATCCTCGGAAAGGATGCTGGCACTTTAACT
GAAGACCCAGATGAAGGCACAGCTGGAGTGGAATCTGCTGTTTTAAGGGGATTCCTCATTCTAGGCAAAG
AAGATAGAAGATATGGGCCAGCATTAAGCATCAATGAATTGAGCAACCTTGCGAAAGGGGAAAAAGCTAA
TGTGCTAATTGGGCAAGGGGATGTAGTGTTGGTAATGAAACGAAAACGGGACTCTAGCATACTTACTGAC
AGCCAGACAGCGACCAAAAGAATTCGGATGGCCATCAATTAA
>gi|82546791|gb|CY006682.1| Influenza A virus (A/New York/494/2002(H1N1)) segment 1, complete sequence
TCAATTATATTCAATATGGAAAGAATAAAAGAGCTAAGGAATCTGATGTCACAATCTCGCACTCGCGAGA
TACTTACCAAAACTACTGTAGACCACATGGCCATAATCAAGAAATACACATCAGGAAGACAGGAGAAAAA
CCCATCACTTAGGATGAAATGGATGATGGCAATGAAATACCCAATTACAGCTGATAAAAGGATAACGGAA
ATGATTCCTGAAAGAAATGAGCAAGGACAGACACTATGGAGTAAAGTGAATGATGCCGGATCAGACCGAG
TGATGATATCACCCCTGGCTGTGACATGGTGGAACAGAAATGGACCAGTGGCAAGTACTATTCACTATCC
AAAAATCTACAAAACTTACTTTGAAAAGGTTGAAAGGTTAAAACATGGAACCTTTGGCCCTGTACACTTT

AGAAACCAAGTCAAAATACGCCGAAGAGTCGACATAAATCCTGGTCATGCAGACCTCAGCGCCAAGGAGG
CACAGGATGTAATTATGGAAGTTGTTTTCCCTAATGAAGTGGGAGCCAGAATACTAACATCAGAATCGCA
ATTAACGATAACCAAGGAGAAAAAAGAAGAACTCCAGAATTGCAAAATTTCCCCTTTGATGGTTGCATAC
ATGTTAGAGAGGGAACTTGTCCGCAAAACAAGATTTCTCCCGGTTGCAGGTGGAACAAGCAGTGTGTACA
TTGAAGTTTTGCATTTAACACAGGGGACATGCTGGGAGCAGATGTACACTCCAGGTGGGGAGGTGAGGAA
TGATGATGTTGATCAAAGCCTAATTATTGCTGCTAGGAACATAGTGAGAAGAGCTGCAGTATCAGCAGAT
CCACTAGCATCTTTATTAGAAATGTGCCATAGCACACAGATTGGTGGAACAAGGATGGTGGATATTCTCA
GGCAAAATCCAACAGAAGAACAAGCTGTGGACATATGCAAAGCAGCAATGGGGCTGAGAATCAGTTCATC
CTTCAGTTTTGGCGGATTCACATTTAAGAGAACAAGTGGATCATCAGTCAAAAGGGAGGAAGAAGTGCTC
ACGGGCAATCTGCAAACATTGAAGCTAACTGTGCATGAGGGATATGAAGAATTCACAATGGTTGGGAAAA
GGGCAACAGCTATACTCAGAAAAGCAACCAGGAGATTGATTCAACTAATAGTGAGTGGAAGAGACGAACA
GTCAATAGTCGAAGCAATAGTTGTAGCAATGGTATTCTCACAAGAAGATTGCATGGTAAAAGCAGTTAGA
GGTGATCTGAATTTCGTTAATAGAGCGAATCAGCGATTGAATCCCATGCATCAACTTTTGAGACATTTTC
AGAAGGATGCTAAAGTACTTTTCTTAAATTGGGGAATTGAACCTATTGACAATGTGATGGGAATGATTGG
GATATTACCTGATATGACTCCAAGTACCGAGATGTCAATGAGAGGAGTGAGAGTCAGCAAAATGGGTGTA
GATGAATATTCCAATGCTGAAAGGGTAGTGGTGAGTATTGACCGTTTTTTGAGAGTCCGGGACCAAAGAG
GAAATGTACTACTGTCTCCAGAGGAAGTCAGTGAAACACAAGGAACAGAGAAACTGACAATAACTTACTC
TTCATCAATGATGTGGGAGATTAATGGCCCTGAGTCAGTGTTGATCAATACCTATCAGTGGATCATCAGA
AACTGGGAGACTGTTAAAATTCAGTGGTCTCAGAACCCTACGATGCTATACAATAAAATGGAATTTGAAC
CATTTCAGTCTCTAGTCCCCAAGGCCATTAGAGGCCAATACAGTGGGTTTGTTAGAACTCTATTTCAACA
AATGAGGGATGTGCTTGGGACCTTTGACACAACTCAGATAATAAAACTTCTTCCCTTTGCAGCCGCTCCA
CCAAAGCAGAGTAGAATGCAATTCTCGTCATTAACTGTGAATGTGAGGGGATCAGGAATGAGAATACTTG
TAAGGGGTAATTCTCCAGTATTCAACTACAACAAGACCACTAAGAGACTCACAGTCCTCGGAAAGGATGC
TGGCACTTTAACTGAAGACCCAGATGAAGGCACAGCTGGAGTGGAATCTGCTGTTCTAAGGGGATTCCTC
ATTCTAGGCAAAGAAGATAGAAGATATGGGCCAGCATTAAGCATCAATGAATTGAGCAACCTTGCGAAAG
GGGAAAAAGCTAATGTGCTAATTGGGCAAGGGGATGTAGTGTTGGTAATGAAACGAAAACGGGACTCTAG
CATACTTACTGACAGCCAGACAGCGACCAAAAGAATTCGGATGGCCATCAATTAA
>gi|77543358|gb|CY003311.1| Influenza A virus (A/New York/291/2002(H1N1)) segment 1, complete sequence
CAATTATATTCAATATGGAAAGAATAAAAGAGCTAAGGAATCTGATGTCACAATCTCGCACTCGCGAGAT
ACTTACCAAAACTACTGTAGACCACATGGCCATAATCAAGAAATACACATCAGGAAGACAGGAGAAAAAC
CCATCACTTAGGATGAAATGGATGATGGCAATGAAATACCCAATTACAGCTGATAAAAGGATAACGGAAA
TGATTCCTGAAAGAAATGAGCAAGGACAGACACTATGGAGTAAAGTGAATGATGCCGGATCAGACCGAGT
GATGATATCACCCCTGGCTGTGACATGGTGGAACAGAAATGGACCAGTGGCAAGTACTATTCACTATCCA
AAAATCTACAAAACTTACTTTGAAAAGGTTGAAAGGTTAAAACATGGAACCTTTGGCCCTGTACACTTTA
GAAACCAAGTCAAAATACGCCGAAGAGTCGACATAAACCCTGGTCATGCAGACCTCAGCGCCAAGGAGGC
ACAGGATGTAATTATGGAAGTTGTTTTCCCTAATGAAGTGGGAGCCAGAATACTAACATCAGAATCGCAA
TTAACGATAACCAAGGAGAAAAAAGAAGAACTCCAGAATTGCAAAATTTCCCCTTTGATGGTTGCATACA
TGTTAGAGAGGGAACTTGTCCGCAAAACAAGATTTCTCCCGGTTGCAGGTGGAACAAGCAGTGTGTACAT
TGAAGTTTTGCATTTAACACAGGGGACATGCTGGGAGCAGATGTACACTCCAGGTGGGGAGGTGAGGAAT
GATGATGTTGATCAAAGCCTAATTATTGCTGCTAGGAACATAGTGAGAAGAGCTGCAGTATCAGCAGATC
CACTAGCATCTTTATTAGAAATGTGCCATAGCACACAGATTGGTGGAACAAGGATGGTGGATATTCTCAG
GCAAAATCCAACAGAAGAACAAGCTGTGGACATATGCAAAGCAGCAATGGGGCTGAGAATCAGTTCATCC
TTCAGTTTTGGCGGATTCACATTTAAGAGAACAAGTGGATCATCAGTCAAAAGGGAGGAAGAAGTGCTCA

CGGGCAATCTGCAAACATTGAAGCTAACTGTGCATGAGGGATATGAAGAATTCACAATGGTTGGGAAAAG
GGCAACAGCTATACTCAGAAAAGCAACCAGGAGATTGATTCAACTAATAGTGAGTGGAAGAGACGAACAG
TCAATAGTCGAAGCAATAGTTGTAGCAATGGTATTCTCACAAGAAGATTGCATGGTAAAAGCAGTTAGAG
GTGATCTGAATTTCGTTAATAGAGCGAATCAGCGGTTGAATCCCATGCATCAACTTTTGAGACATTTTCA
GAAGGATGCTAAAGTACTTTTCTTAAATTGGGGAATTGAACCTATTGACAATGTGATGGGAATGATTGGG
ATATTACCTGATATGACTCCAAGTACCGAGATGTCAATGAGAGGAGTGAGAGTCAGCAAAATGGGTGTAG
ATGAATACTCCAATGCTGAAAAGGTAGTGGTGAGCATTGACCGGTTTTTGAGAGTCCGGGACCAAAGAGG
AAATGTACTACTGTCTCCAGAGGAAGTCAGTGAAACACAAGGAACAGAGAAACTGACAATAACTTACTCT
TCATCAATGATGTGGGAGATTAATGGCCCTGAGTCAGTGTTGATCAATACCTATCAGTGGATCATCAGAA
ACTGGGAGACTGTTAAAATTCAGTGGTCTCAGAACCCTACGATGCTATACAATAAAATGGAATTTGAACC
ATTTCAGTCTCTAGTCCCCAAGGCCATTAGAGGCCAATACAGTGGGTTTGTTAGAACTCTATTTCAACAA
ATGAGGGATGTGCTTGGGACCTTTGACACAACTCAGATAATAAAACTTCTTCCCTTTGCAGCCGCTCCAC
CAAAGCAAAGTAGAATGCAATTCTCGTCATTAACTGTGAATGTGAGGGGATCAGGAATGAGAATACTTGT
AAGGGGTAATTCTCCAGTATTCAACTACAACAAGACCACTAAGAGACTCACAGTCCTCGGAAAGGATGCT
GGCACTTTAACTGAAGACCCAGATGAAGGCACAGCTGGAGTGGAATCTGCTGTTCTAAGGGGATTCCTCA
TTCTAGGCAAAGAAGATAGAAGATATGGGCCAGCATTAAGCATCAATGAATTGAGCAACCTTGCGAAAGG
GGAAAAAGCTAATGTGCTAATTGGGCAAGGGGATGTAGTGTTGGTAATGAAACGAAAACGGGACTCTAAC
ATACTTACTGACAGCCAGACAGCGACCAAAAGAATTCGGATGGCCATCAATTAATTTCAAATAATTTAAA
AA
>gi|237689052|gb|CY040153.1| Influenza A virus (A/Taiwan/52/2002(H1N1)) segment 1, complete sequence
ATATGGAAAGAATAAAAGAGCTAAGGAATCTGATGTCACAATCTCGCACTCGCGAGATACTTACAAAAAC
TACTGTAGACCACATGGCCATAATCAAAAAATACACATCCGGAAGACAGGAGAAAAACCCATCACTTAGG
ATGAAATGGATGATGGCAATGAAATACCCAATTACAGCAGACAAAAGGATAACGGAAATGATTCCTGAAA
GAAATGAGCAAGGACAGACATTATGGAGTAAAGTGAATGATGCAGGTTCAGACCGAGTGATGATATCACC
CCTGGCTGTGACATGGTGGAACAGAAATGGACCAGTGGCAAGTACTATTCACTATCCAAAAATCTACAAA
ACTTACTTTGAAAAGGTTGAAAGGTTAAAACATGGAACCTTTGGCCCTGTACACTTTAGAAACCAAGTCA
AAATACGCCGAAGAGTCGACATAAATCCTGGTCATGCAGACCTCAGCGCCAAGGAGGCACAGGATGTAAT
TATGGAAGTTGTTTTCCCTAATGAAGTGGGAGCCAGAATACTAACATCAGAATCGCAATTAACGATAACC
AAAGAGAAAAAAGAAGAACTCCAGAATTGCAAAATTTCCCCTTTGATGGTTGCATACATGTTAGAGAGGG
AACTTGTCCGCAAAACGAGATTTCTCCCGGTTGCTGGTGGAACAAGCAGTGTGTACATTGAAGTTTTGCA
TTTAACACAGGGGACATGCTGGGAACAGATGTACACTCCAGGTGGAGAGGTGAGGAATGATGATGTTGAT
CAAAGCCTAATTATTGCTGCTAGGAACATAGTGAGAAGAGCTGCAGTATCAGCAGATCCACTAGCATCTT
TATTAGAAATGTGCCACAGCACACAGATTGGTGGAACAAGGATGGTGGATATTCTCAGGCAAAACCCAAC
AGAAGAACAAGCTGTGGATATATGCAAAGCAGCAATGGGGCTGAGAATCAGTTCATCCTTCAGTTTTGGC
GGATTCACATTCAAGAGAACAAGTGGATCATCAGTCAAAAGGGAGGAAGAAATGCTCACGGGCAATCTGC
AAACATTGAAGCTAACTGTGCATGAGGGATATGAAGAGTTCACAATGGTTGGGAAAAGGGCAACAGCTAT
ACTCAGAAAAGCAACCAGGAGATTGATTCAACTAATAGTGAGTGGACGAGACGAACAGTCGATAGTCGAA
GCAATAGTTGTAGCAATGGTATTCTCACAAGAAGATTGCATGGTAAAAGCAGTTAGAGGTGATCTGAATT
TCGTCAATAGAGCGAATCAGCGGTTGAATCCCATGCATCAACTTTTGAGACATTTTCAGAAGGATGCTAA
AGTACTTTTCTTAAATTGGGGAATTGAACCTATCGACAATGTGATGGGAATGATTGGGATATTACCTGAT
ATGACTCCAAGTACCGAGATGTCAATGAGAGGAGTGAGAGTCAGCAAAATGGGTGTAGATGAATACTCCA
ATGCTGAAAAGGTAGTGGTGAGCATTGACCGTTTTTTGAGAGTCCGGGACCAAAGAGGAAATGTACTACT
GTCTCCAGAGGAAGTCAGTGAAACACAGGGAACAGAGAAACTGACAATAACTTACTCTTCATCAATGATG

TGGGAGATTAATGGCCCTGAGTCAGTGTTGATCAATACCTATCAGTGGATCATCAGAAACTGGGAGACTG
TTAAAATTCAGTGGTCTCAGAACCCTACAATGCTATACAATAAAATGGAATTCGAGCCATTTCAGTCTCT
AGTCCCTAAGGCCATTAGAGGCCAATACAGTGGGTTTGTTAGAACTCTATTCCAACAAATGAGGGATGTG
CTTGGGACCTTTGACACAACTCAGATAATAAAACTTCTTCCCTTTGCAGCCGCTCCACCAAAGCAAAGTA
GAATGCAATTCTCATCATTGACTGTGAATGTGAGAGGATCAGGAATGAGAATACTTGTAAGGGGTAATTC
TCCAGTATTCAACTACAACAAGACCACTAAGAGACTCACAGTCCTCGGAAAGGATGCTGGCACTTTAACT
GAAGACCCAGATGAAGGCACAGCTGGAGTGGAATCTGCTGTTTTAAGGGGATTCCTCATTCTAGGCAAAG
AAGATAGAAGATATGGGCCAGCATTAAGCATCAATGAATTGAGCAACCTTGCGAAAGGAGAAAAAGCTAA
TGTGCTAATTGGGCAAGGGGACGTAGTGTTGGTGATGAAACGAAAACGGGACTCTAGCATACTTACTGAC
AGCCAGACAGCGACCAAAAGAATTCGGATGGCCATCAATTAATTTCGAATAA
>gi|237689071|gb|CY040161.1| Influenza A virus (A/Taiwan/123/2002(H1N1)) segment 1, complete sequence
TATGGAAAGAATAAAAGAGCTAAGGAATCTGATGTCACAATCTCGCACTCGCGAGATACTTACAAAAACT
ACTGTAGACCACATGGCCATAATCAAAAAATACACATCAGGAAGACAGGAGAAAAACCCATCACTTAGGA
TGAAATGGATGATGGCAATGAAATACCCAATTACAGCAGACAAAAGGATAACGGAAATGATTCCTGAAAG
AAATGAGCAAGGACAGACATTATGGAGTAAAGTGAATGATGCCGGTTCAGATCGAGTGATGATATCACCC
CTGGCTGTGACATGGTGGAATAGAAATGGACCAGTGGCAAGTACTATTCACTATCCAAAAATCTACAAAA
CTTACTTTGAAAAGGTTGAAAGGTTAAAACATGGAACCTTTGGCCCTGTACACTTTAGAAACCAAGTCAA
AATACGCCGAAGAGTCGACATAAATCCTGGTCATGCAGACCTCAGCGCCAAGGAGGCACAGGATGTAATT
ATGGAAGTTGTTTTCCCTAATGAAGTGGGAGCCAGAATACTAACATCAGAATCGCAATTAACGATAACCA
AAGAGAAAAAAGAAGAACTCCAGAATTGCAAAATTTCCCCTTTGATGGTTGCATACATGTTAGAGAGGGA
ACTTGTCCGCAAAACGAGATTTCTCCCGGTTGCTGGTGGAACAAGCAGTGTGTACATTGAAGTTTTGCAT
TTAACACAGGGGACATGCTGGGAACAGATGTACACTCCAGGTGGAGAGGTGAGGAATGATGATGTTGATC
AAAGCCTAATTATTGCTGCTAGGAACATAGTGAGAAGAGCTGCAGTATCAGCAGATCCACTAGCATCTTT
ATTAGAAATGTGCCACAGCACACAGATTGGTGGAACAAGGATGGTGGATATTCTCAGGCAAAACCCAACA
GAAGAACAAGCTGTGGATATATGCAAAGCAGCAATGGGGCTGAGAATCAGTTCATCCTTCAGTTTTGGCG
GATTCACATTCAAGAGAACAAGTGGATCATCAGTCAAAAGGGAGGAAGAAATGCTCACGGGCAATCTGCA
AACATTGAAGCTAACTGTGCATGAGGGATATGAAGAGTTCACAATGGTCGGGAAAAGGGCAACAGCTATA
CTCAGAAAAGCAACCAGGAGATTGATTCAACTAATAGTGAGTGGACGAGACGAACAGTCGATAGTCGAAG
CAATAGTTGTAGCAATGGTATTCTCACAAGAAGATTGCATGGTAAAAGCAGTTAGAGGTGATCTGAATTT
CGTCAATAGAGCGAATCAGCGGTTGAATCCCATGCATCAACTTTTGAGACATTTTCAGAAGGATGCTAAA
GTACTTTTCTTAAATTGGGGAATTGAACCTATCGACAATGTGATGGGGATGATTGGGATATTACCTGATA
TGACTCCAAGTACCGAGATGTCAATGAGAGGAGTGAGAGTCAGCAAAATGGGTGTAGATGAATACTCCAA
TGCTGAAAAGGTAGTGGTGAGCATTGACCGTTTTTTGAGAGTCCGGGACCAAAGAGGAAATGTACTACTG
TCTCCAGAGGAAGTCAGTGAAACACAGGGAACAGAGAAACTGACAATAACTTACTCTTCATCAATGATGT
GGGAGATTAATGGCCCTGAGTCAGTGTTGATCAATACCTATCAGTGGATCATCAGAAACTGGGAGACTGT
TAAAATTCAGTGGTCTCAGAACCCTACAATGCTATACAACAAAATGGAATTCGAGCCATTTCAGTCTCTA
GTCCCTAAGGCCATTAGAGGCCAATACAGTGGGTTTGTTAGAACTCTATTCCAACAAATGAGGGATGTGC
TTGGGACCTTTGACACAACTCAGATAATAAAACTTCTTCCCTTTGCAGCCGCTCCACCAAAGCAAAGTAG
AATGCAATTCTCATCATTGACTGTGAATGTGAGAGGATCAGGAATGAGAATACTTGTAAGGGGTAATTCT
CCAGTATTCAACTACAACAAGACCACTAAGAGACTCACAGTCCTCGGAAAGGATGCTGGCACTTTAACTG
AAGACCCAGATGAAGGCACAGCTGGAGTGGAATCTGCTGTTTTAAGGGGATTCCTCATTCTAGGCAAAGA
AGATAGAAGATATGGGCCAGCATTAAGCATCAATGAATTGAGCAACCTTGCGAAAGGAGAAAAAGCTAAT
GTGCTAATTGGGCAAGGGGACGTAGTGTTGGTGATGAAACGAAAACGGGACTCTAGCATACTTACTGACA

GCCAGACAGCGACCAAAAGAATTCGGATGGCCATCAATTAATTTCGAATAATTTAA
>gi|125664192|gb|CY019890.1| Influenza A virus (A/Memphis/5/2003(H1N1)) segment 1, complete sequence
AATATGGAAAGAATAAAAGAGCTAAGGAATCTGATGTCACAATCTCGCACTCGCGAGATACTTACCAAAA
CTACTGTAGACCACATGGCCATAATCAAGAAATACACATCAGGAAGACAGGAGAAAAACCCATCACTTAG
GATGAAATGGATGATGGCAATGAAATACCCAATTACAGCTGATAAAAGGATAACGGAAATGATTCCTGAA
AGAAATGAGCAAGGACAGACACTATGGAGTAAAGTGAATGATGCCGGATCAGACCGAGTGATGATATCAC
CCCTGGCTGTGACATGGTGGAACAGAAATGGACCAGTGGCAAATACTATTCACTATCCAAAAATCTACAA
AACTTACTTTGAAAAGGTTGAAAGGTTAAAACATGGAACCTTTGGCCCTGTACACTTTAGAAACCAAGTC
AAAATACGCCGAAGAGTCGACATAAATCCTGGTCATGCAGACCTCAGCGCCAAGGAGGCACAGGATGTAA
TTATGGAAGTTGTTTTCCCTAATGAAGTGGGAGCCAGAATACTAACATCAGAATCGCAATTAACGATAAC
CAAGGAGAAAAAAGAGGAACTCCAGAATTGCAAAATTTCCCCTTTGATGGTTGCATACATGTTAGAGAGG
GAACTTGTCCGCAAAACAAGATTTCTCCCGGTTGCAGGTGGAACAAGCAGTGTGTACATTGAAGTTTTGC
ATTTAACACAGGGGACATGCTGGGAGCAGATGTACACTCCAGGTGGGGAGGTGAGGAATGATGATGTTGA
TCAAAGCCTAATTATTGCTGCTAGGAACATAGTGAGAAGAGCTGCAGTATCAGCAGATCCACTAGCATCT
TTATTAGAAATGTGCCATAGCACACAGATTGGTGGAACAAGGATGGTGGATATTCTCAGGCAAAATCCAA
CAGAAGAACAAGCTGTGGACATATGCAAAGCAGCAATGGGGCTGAGAATCAGTTCATCCTTCAGTTTTGG
AGGATTCACATTTAAGAGAACAAGTGGATCATCAGTCAAAAGGGAGGAAGAAGTGCTCACGGGCAATCTG
CAAACATTGAAGCTAACTGTGCATGAGGGATATGAAGAATTCACAATGGTTGGGAAAAGGGCAACAGCTA
TACTCAGAAAAGCAACCAGGAGATTGATTCAACTAATAGTGAGTGGAAGAGACGAACAGTCAATAGTCGA
AGCAATAGTTGTAGCAATGGTATTCTCACAAGAAGATTGCATGGTAAAAGCAGTTAGAGGTGATCTGAAT
TTCGTTAATAGAGCGAATCAGCGGTTGAATCCCATGCATCAACTTTTGAGACATTTTCAGAAGGATGCTA
AAGTACTTTTCTTAAATTGGGGAATTGAACATATTGACAATGTGATGGGAATGATTGGGATATTACCTGA
TATGACTCCAAGTACCGAGATGTCAATGAGAGGAGTGAGAGTCAGCAAAATGGGTGTAGATGAATACTCC
AATGCTGAAAGGGTAGTGGTGAGCATTGACCGTTTTTTGAGAGTCCGGGACCAAAGAGGAAATGTACTAC
TGTCTCCAGAGGAAGTCAGTGAAACACAAGGAACAGAGAAACTGACAATAACTTACTCTTCATCATTGAT
GTGGGAGATTAATGGCCCTGAGTCAGTGTTGATCAATACCTATCAGTGGATCATCAGAAACTGGGAGACT
GTTAAAATTCAGTGGTCTCAGAACCCTACGATGCTATACAATAAAATGGAATTTGAACCATTTCAATCTC
TAGTCCCCAAGGCCATTAGAGGCCAATACAGTGGGTTTGTTAGAACCCTATTTCAACAAATGAGGGATGT
GCTCGGGACCTTTGACACAACTCAGATAATAAAACTTCTTCCCTTTGCAGCCGCTCCACCAAAGCAAAGT
AGAATGCAATTCTCGTCATTAACTGTGAATGTGAGGGGATCAGGAATGAGAATACTTGTAAGGGGTAATT
CTCCAGTATTCAACTACAACAAGACCACTAAGAGACTCACAATCCTCGGAAAGGATGCTGGCACTTTAAC
TGAAGACCCAGATGAAGGCACAGCTGGAGTGGAATCTGCTGTTTTAAGGGGATTCCTCATTCTAGGCAAA
GAAGATAGAAGATATGGGCCAGCATTAAGCATCAATGAATTGAGCAACCTTGCGAAAGGGGAAAAAGCTA
ATGTGCTAATTGGGCAAGGGGATGTAGTGTTGGTAATGAAACGAAAACGGGACTCTAGCATACTTACTGA
CAGCCAGACAGCGACCAAAAGAATTCGGATGGCCATCAATTAA
>gi|73763211|gb|CY002543.1| Influenza A virus (A/New York/227/2003(H1N1)) segment 1, complete sequence
AGCAAAAGCAGGTCAATTATATTCAATATGGAAAGAATAAAAGAGCTAAGGAATCTGATGTCACAATCTC
GCACTCGCGAGATACTTACCAAAACTACTGTAGACCACATGGCCATAATCAAGAAATACACATCAGGAAG
ACAGGAGAAAAACCCATCACTTAGGATGAAATGGATGATGGCAATGAAATACCCAATTACAGCTGATAAA
AGGATAACGGAAATGATTCCTGAAAGAAATGAGCAAGGACAGACACTATGGAGTAAAGTGAATGATGCCG
GATCAGACCGAGTGATGATATCACCCCTGGCTGTGACATGGTGGAACAGAAATGGACCAGTGGCAAATAC
TATTCACTATCCAAAAATCTACAAAACTTACTTTGAAAAGGTTGAAAGGTTAAAACATGGAACCTTTGGC

CCTGTACACTTTAGAAACCAAGTCAAAATACGCCGAAGAGTCGACATAAATCCTGGTCATGCAGACCTCA
GCGCCAAGGAGGCACAGGATGTAATTATGGAAGTTGTTTTCCCTAATGAAGTGGGAGCCAGAATACTAAC
ATCAGAATCGCAATTAACGATAACCAAGGAGAAAAAAGAGGAACTCCAGAATTGCAAAATCTCCCCTTTG
ATGGTTGCATACATGTTAGAGAGGGAACTTGTCCGCAAAACAAGATTTCTCCCGGTTGCAGGTGGAACAA
GCAGTGTGTACATTGAAGTTTTGCATTTAACACAGGGGACATGCTGGGAGCAGATGTACACTCCAGGTGG
GGAGGTGAGGAATGATGATGTTGATCAAAGCCTAATTATTGCTGCTAGGAACATAGTGAGAAGAGCTGCA
GTATCAGCAGATCCACTAGCATCTTTATTAGAAATGTGCCATAGCACACAGATTGGTGGAACAAGGATGG
TGGATATTCTCAGGCAAAATCCAACAGAAGAACAAGCTGTGGACATATGCAAAGCAGCAATGGGGCTGAG
AATCAGTTCATCCTTCAGTTTTGGCGGATTCACATTTAAGAGAACAAGTGGATCATCAGTCAAAAGGGAG
GAAGAAGTGCTCACGGGCAATCTGCAGACATTGAAGCTAACTGTGCATGAGGGATATGAAGAATTCACAA
TGGTTGGGAAAAGGGCAACAGCTATACTCAGAAAAGCAACCAGGAGATTGATTCAACTAATAGTGAGTGG
AAGAGACGAACAGTCAATAGTCGAAGCAATAGTTGTAGCAATGGTATTCTCACAAGAAGATTGCATGGTA
AAAGCAGTTAGAGGTGATCTGAATTTCGTTAATAGAGCGAATCAGCGGTTGAATCCCATGCATCAACTTT
TGAGACATTTTCAGAAGGATGCTAAAGTACTTTTCTTAAATTGGGGAATTGAACATATTGACAATGTGAT
GGGAATGATTGGGATATTACCTGATATGACTCCAAGTACCGAGATGTCAATGAGAGGAGTGAGAGTCAGC
AAAATGGGTGTAGATGAATACTCCAATGCTGAAAGGGTAGTGGTGAGCATTGACCGTTTTTTGAGAGTCC
GGGACCAAAGAGGAAATGTACTACTGTCTCCAGAGGAAGTCAGTGAAACACAAGGAACAGAGAAACTGAC
AATAACTTACTCTTCATCATTGATGTGGGAGATTAATGGCCCTGAGTCAGTGTTGATCAATACCTATCAG
TGGATCATCAGAAACTGGGAGACTGTTAAAATTCAGTGGTCTCAGAACCCTACGATGCTATACAATAAAA
TGGAATTTGAACCATTCCAATCTCTAGTCCCCAAGGCCATTAGAGGCCAATACAGTGGGTTTGTTAGAAC
CCTATTTCAACAAATGAGGGATGTGCTCGGGACCTTTGACACAACTCAGATAATAAAACTTCTTCCCTTT
GCAGCCGCTCCACCAAAGCAAAGTAGAATGCAATTCTCGTCATTAACTGTGAATGTGAGGGGATCAGGAA
TGAGAATACTTGTAAGGGGTAATTCTCCAGTATTCAACTACAACAAGACCACTAAGAGACTCACAATCCT
CGGAAAGGATGCTGGCACTTTAACTGAAGACCCAGATGAAGGCACAGCTGGAGTGGAATCTGCTGTTTTA
AGGGGATTCCTCATTCTAGGCAAAGAAGATAGAAGATATGGGCCAGCATTAAGCATCAATGAATTGAGCA
ACCTTGCGAAAGGGGAAAAAGCTAATGTGCTAATTGGGCAAGGGGATGTAGTGTTGGTAATGAAACGAAA
ACGGGACTCTAGCATACTTACTGACAGCCAGACAGCGACCAAAAGAATTCGGATGGCCATCAATTAATTT
C
>gi|89112182|gb|CY009003.1| Influenza A virus (A/New York/484/2003(H1N1)) segment 1, complete sequence
ATATTCAATATGGAAAGAATAAAAGAGCTAAGGAATCTGATGTCACAATCTCGCACTCGCGAGATACTTA
CCAAAACTACTGTAGACCACATGGCCATAATCAAGAAATACACATCAGGAAGACAGGAGAAAAACCCATC
ACTTAGGATGAAATGGATGATGGCAATGAAATACCCAATTACAGCTGATAAAAGGATAACGGAAATGATT
CCTGAAAGAAATGAGCAAGGACAGACACTATGGAGTAAAGTGAATGATGCCGGATCAGACCGAGTGATGA
TATCACCCCTGGCTGTGACATGGTGGAACAGAAATGGACCAGTGGCAAATACTATTCACTATCCAAAAAT
CTACAAAACTTACTTTGAAAAGGTTGAAAGGTTAAAACATGGAACCTTTGGCCCTGTACACTTTAGAAAC
CAAGTCAAAATACGCCGAAGAGTCGACATAAATCCTGGTCATGCAGACCTCAGCGCCAAGGAGGCACAGG
ATGTAATTATGGAAGTTGTTTTCCCTAATGAAGTGGGAGCCAGAATACTAACATCAGAATCGCAATTAAC
GATAACCAAGGAGAAAAAAGAAGAACTCCAGAATTGCAAAATTTCCCCTTTGATGGTTGCATACATGTTA
GAGAGGGAACTTGTCCGCAAAACAAGATTTCTCCCGGTTGCAGGTGGAACAAGCAGTGTGTACATTGAAG
TTTTGCATTTAACACAGGGGACATGCTGGGAGCAGATGTACACTCCAGGTGGGGAGGTGAGGAATGATGA
TGTTGATCAAAGCCTAATTATTGCTGCTAGGAACATAGTGAGAAGAGCTGCAGTATCAGCAGATCCACTA
GCATCTTTATTAGAAATGTGCCATAGCACACAGATTGGTGGAACAAGGATGGTGGATATTCTCAGGCAAA
ATCCAACAGAAGAACAAGCTGTGGACATATGCAAAGCAGCAATGGGGCTGAGAATCAGTTCATCCTTCAG

TTTTGGCGGATTCACATTTAAGAGAACAAGTGGATCATCAGTCAAAAGGGAGGAAGAAGTGCTCACGGGC
AATCTGCAAACATTGAAGCTAACTGTGCATGAGGGATATGAAGAATTCACAATGGTTGGGAAAAGGGCAA
CAGCTATACTCAGAAAAGCAACCAGGAGATTGATTCAACTAATAGTGAGTGGAAGAGACGAACAGTCAAT
AGTCGAAGCAATAGTTGTAGCAATGGTATTCTCACAAGAAGATTGCATGGTAAAAGCAGTTAGAGGTGAT
CTGAATTTCGTTAATAGAGCGAATCAGCGGTTGAATCCCATGCATCAACTTTTGAGACATTTTCAGAAGG
ATGCTAAAGTACTTTTCTTAAATTGGGGAATTGAACATATTGACAATGTGATGGGAATGATTGGGATATT
ACCTGATATGACTCCAAGTACCGAGATGTCAATGAGAGGAGTGAGAGTCAGCAAAATGGGTGTAGATGAA
TACTCCAATGCTGAAAGGGTGGTGGTGAGCATTGACCGTTTTTTGAGAGTCCGGGACCAAAGAGGAAATG
TACTACTGTCTCCAGAGGAAGTCAGTGAAACACAAGGAACAGAGAAACTGACAATAACTTACTCTTCATC
AATGATGTGGGAGATTAATGGCCCTGAGTCAGTGTTGATCAATACCTATCAGTGGATCATCAGAAACTGG
GAGACTGTTAAGATTCAGTGGTCTCAGAACCCTACGATGCTATACAATAAAATGGAATTTGAACCATTTC
AATCTCTAGTCCCCAAGGCCATTAGAGGCCAATACAGTGGGTTTGTTAGAACTCTATTTCAACAAATGAG
GGATGTGCTCGGGACCTTTGACACAACTCAGATAATAAAACTTCTTCCCTTTGCAGCCGCTCCACCAAAG
CAAAGTAGAATGCAATTCTCGTCATTAACTGTGAATGTGAGGGGATCAGGAATGAGAATACTTGTAAGGG
GTAATTCTCCAGTATTCAACTACAACAAGACCACTAAGAGACTCACAATCCTCGGAAAGGATGCTGGCAC
TTTAACTGAAGACCCAGATGAAGGCACAGCTGGAGTGGAATCTGCTGTTTTAAGGGGATTCCTCATTCTA
GGCAAAGAAGATAGAAGATATGGGCCAGCATTAAGCATCAATGAATTGAGCAACCTTGCGAAAGGGGAAA
AAGCTAATGTGCTAATTGGGCAAGGGGATGTAGTGTTGGTAATGAAACGAAAACGGGACTCTAGCATACT
TACTGACAGCCAGACAGCGACCAAAAGAATTCGGATGGCCATCAATTAATTTCGAATAATTTAAAAA
>gi|77747438|gb|CY003695.1| Influenza A virus (A/New York/486/2003(H1N1)) segment 1, complete sequence
AAAGCAGGTCAATTATATTCAATATGGAAAGAATAAAAGAGCTAAGGAATCTGATGTCACAATCTCGCAC
TCGCGAGATACTTACCAAAACTACTGTAGACCACATGGCCATAATCAAGAGATACACATCAGGAAGACAG
GAGAAAAACCCATCACTTAGGATGAAATGGATGATGGCAATGAAATACCCAATTACAGCTGATAAAAGGA
TAACGGAAATGATTCCTGAAAGAAATGAGCAAGGACAGACACTATGGAGTAAAGTGAATGATGCCGGATC
AGACCGAGTGATGATATCACCCCTGGCTGTGACATGGTGGAACAGAAATGGACCAGTGGCAAATACTATT
CACTATCCAAAAATCTACAAAACTTACTTTGAAAAGGTTGAAAGGTTAAAACATGGAACCTTTGGTCCTG
TACACTTTAGAAACCAAGTCAAAATACGCCGAAGAGTCGACATAAATCCTGGTCATGCAGACCTCAGCGC
CAAGGAGGCACAGGATGTAATTATGGAAGTTGTTTTCCCTAATGAAGTGGGAGCCAGAATACTAACATCA
GAATCGCAATTAACGATAACCAAGGAGAAAAAAGAAGAACTCCAAAATTGCAAAATTTCCCCTTTGATGG
TTGCATACATGTTAGAGAGGGAACTTGTCCGCAAAACAAGATTTCTCCCGGTTGCAGGTGGAACAAGCAG
TGTGTACATTGAAGTTTTGCATTTAACACAGGGGACATGCTGGGAGCAGATGTACACTCCAGGTGGGGAG
GTGAGGAATGATGATGTTGATCAAAGCCTAATTATTGCTGCTAGGAACATAGTGAGAAGAGCTGCAGTAT
CAGCAGATCCACTAGCATCTTTATTAGAAATGTGCCATAGCACACAGATTGGTGGAACAAGGATGGTGGA
TATTCTCAGGCAAAATCCAACAGAAGAACAAGCTGTGGACATATGCAAAGCAGCAATGGGGCTGAGAATC
AGTTCATCCTTCAGTTTTGGCGGATTCACATTTAAGAGAACAAGTGGATCATCAGTCAAAAGGGAGGAAG
AAGTGCTCACGGGCAATCTGCAAACATTGAAGCTAACTGTGCATGAGGGATATGAAGAATTCACAATGGT
TGGGAAAAGGGCAACAGCTATACTCAGAAAAGCAACCAGGAGATTGATTCAACTAATAGTGAGTGGAAGA
GACGAACAGTCAATAGTCGAAGCAATAGTTGTAGCAATGGTATTCTCACAAGAAGATTGCATGGTAAAAG
CAGTTAGAGGTGATCTGAATTTCGTTAATAGAGCGAATCAGCGGTTGAATCCCATGCATCAACTTTTGAG
ACATTTTCAGAAGGATGCTAAAGTACTTTTCTTAAATTGGGGAATTGAACATATTGACAATGTGATGGGA
ATGATTGGGATATTACCTGATATGACTCCAAGTACCGAGATGTCAATGAGAGGAGTGAGAGTCAGCAAAA
TGGGTGTAGATGAATACTCCAATGCTGAAAGGGTAGTGGTGAGCATTGACCGTTTTTTGAGAGTCCGGGA
CCAAAGAGGAAATGTACTACTGTCTCCAGAGGAAGTCAGTGAAACACAAGGAACAGAGAAACTGACAATA

ACTTACTCTTCATCAATGATGTGGGAGATTAATGGCCCTGAGTCAGTGTTGATCAATACATATCAGTGGA
TCATCAGAAACTGGGAGACTGTTAAAATTCAGTGGTCTCAGAACCCTACGACGCTATACAATAAAATGGA
ATTTGAACCATTTCAATCTCTAGTCCCCAAGGCCATTAGAGGCCAATACAGTGGGTTTGTTAGAACTCTA
TTTCAACAAATGAGGGATGTGCTCGGGACCTTTGACACAACTCAGATAATAAAACTTCTTCCCTTTGCAG
CCGCTCCACCAAAGCAAAGTAGAATGCAATTCTCGTCATTAACTGTGAATGTGAGGGGATCAGGAATGAG
AATACTTGTAAGGGGTAATTCTCCAGTATTCAACTACAACAAGACCACTAAGAGACTCACAATCCTCGGA
AAGGATGCTGGCACTTTAACTGAAGACCCAGATGAAGGCACAGCTGGAGTGGAATCTGCTGTTTTAAGGG
GATTCCTCATTCTAGGCAAAGAAGATAGAAGATATGGGCCAGCATTAAGCATCAATGAATTGAGCAACCT
TGCGAAAGGGGAAAAAGCTAATGTGCTAATTGGGCAAGGGGATGTAGTGTTGGTAATGAAACGAAAACGG
GACTCTAGCATACTTACTGACAGCCAGACAGCGACCAAAAGAATTCGGATGGCCATCAATTAA
>gi|83727858|gb|CY006922.1| Influenza A virus (A/New York/488/2003(H1N1)) segment 1, complete sequence
AATAAAAGAGCTAAGGAATCTGATGTCACAATCTCGCACTCGCGAGATACTTACCAAAACTACTGTAGAC
CACATGGCCATAATCAAGAAATACACATCAGGAAGACAGGAGAAAAACCCATCACTTAGGATGAAATGGA
TGATGGCGATGAAATACCCAATTACAGCTGATAAAAGGATAACGGAAATGATTCCTGAAAGAAATGAGCA
AGGACAGACACTATGGAGTAAAGTGAATGATGCCGGATCAGACCGAGTGATGATATCACCCCTGGCTGTG
ACATGGTGGAACAGAAATGGACCAGTGGCAAATACTATTCACTATCCAAAAATCTACAAAACTTACTTTG
AAAAGGTTGAAAGGTTAAAACATGGAACCTTTGGCCCTGTACACTTTAGAAACCAAGTCAAAATACGCCG
AAGAGTCGACATAAATCCTGGTCATGCAGACCTCAGCGCCAAGGAGGCACAGGATGTAATTATGGAAGTT
GTTTTCCCTAATGAAGTGGGAGCCAGAATACTAACATCAGAATCGCAATTAACGATAACCAAGGAGAAAA
AAGAAGAACTCCAGAATTGCAAAATTTCCCCTTTGATGGTTGCATACATGTTAGAGAGGGAACTTGTCCG
CAAAACAAGATTTCTCCCGGTTGCAGGTGGAACAAGCAGTGTGTACATTGAAGTTTTGCATTTAACACAG
GGGACATGCTGGGAGCAGATGTACACTCCAGGTGGGGAGGTGAGGAATGATGATGTTGATCAAAGCCTAA
TTATTGCTGCTAGGAACATAGTGAGAAGAGCTGCAGTATCAGCAGATCCACTAGCATCTTTATTAGAAAT
GTGCCATAGCACACAGATTGGTGGAACAAGGATGGTGGATATTCTCAGGCAAAATCCAACAGAAGAACAA
GCTGTGGACATATGCAAAGCAGCAATGGGGCTGAGAATCAGTTCATCCTTCAGTTTTGGCGGATTCACAT
TTAAGAGAACAAGTGGATCATCAGTCAAAAGGGAGGAAGAAATGCTCACGGGCAATCTGCAAACATTGAA
GTTAACTGTACATGAGGGATATGAAGAATTCACAATGGTTGGGAAAAGGGCAACAGCTATACTCAGAAAA
GCAACCAGGAGATTGATTCAACTAATAGTGAGTGGAAGAGACGAACAGTCAATAGTCGAAGCAATAGTTG
TAGCAATGGTATTCTCACAAGAAGATTGCATGGTAAAAGCAGTTAGAGGTGATCTGAATTTCGTTAATAG
AGCGAATCAGCGGTTGAATCCCATGCATCAACTTTTGAGACATTTTCAGAAGGATGCTAAAGTACTTTTC
TTAAATTGGGGAATTGAACATATTGACAATGTGATGGGAATGATTGGGATATTACCTGATATGACTCCAA
GTACCGAGATGTCAATGAGAGGAGTGAGAGTCAGCAAAATGGGTGTAGATGAATACTCCAATGCTGAAAG
GGTAGTGGTGAGCATTGACCGTTTTTTGAGAGTCCGGGACCAAAGAGGAAATGTACTACTGTCTCCAGAG
GAAGTCAGTGAAACACAAGGAACAGAGAAACTGACAATAACTTACTCTTCATCAATGATGTGGGAGATTA
ATGGCCCTGAGTCAGTGTTGATCAATACCTATCAGTGGATCATCAGAAACTGGGAGACTGTTAAAATTCA
GTGGTCTCAGAACCCTACGATGCTATACAATAAAATGGAATTTGAACCATTTCAATCTCTAGTCCCCAAG
GCCATTAGAGGCCAATACAGTGGGTTTGTTAGAACTCTATTTCAACAAATGAGGGATGTGCTCGGGACCT
TTGACACAACTCAGATAATAAAACTTCTTCCCTTTGCAGCCGCTCCACCAAAGCAAAGTAGAATGCAATT
CTCGTCATTAACTGTGAATGTGAGGGGATCAGGAATGAGAATACTTGTAAGGGGTAATTCTCCAGTATTC
AACTACAACAAGACCACTAAGAGACTCACAATCCTCGGAAAGGATGCTGGCACTTTAACTGAAGACCCAG
ATGAAGGCACAGCTGGAGTGGAATCTGCTGTTTTAAGGGGATTCCTCATTCTAGGCAAAGAAGATAGAAG
ATATGGGCCAGCATTAAGCATCAATGAATTGAGCAACCTTGCGAAAGGGGAAAAAGCTAATGTGCTAATT
GGGCAAGGGGATGTAGTGTTGGTAATGAAACGAAAACGGGACTCTAGCATACTTACTGACAGCCAGACAG

CGACCAAAAGAATTCGGATGGCCATCAATTAATTTCGAATAATTTAAAAA
>gi|157281274|gb|CY025220.1| Influenza A virus (A/Texas/UR06-0012/2006(H1N1)) segment 1, complete sequence
AATATGGAAAGAATAAAAGAGCTAAGGAATCTGATGTCACAATCTCGCACTCGCGAGATACTTACCAGAA
CTACTGTAGACCACATGGCCATAATCAAGAAATACACATCAGGAAGACAGGAGAAAAACCCATCACTTAG
GATGAAATGGATGATGGCAATGAAATACCCAATTACAGCTGATAAAAGGATAACGGAAATGATTCCTGAA
AGAAATGAGCAAGGACAAACACTATGGAGTAAAGTGAACGATGCCGGATCAGACCGAGTGATGATATCAC
CCCTGGCTGTGACATGGTGGAACAGAAATGGACCAGTGGCAAATACTATTCACTATCCAAAAATCTACAA
AACTTACTTTGAAAAGATTGAAAGGTTAAAACATGGAACCTTTGGCCCTGTACACTTTAGAAACCAAGTC
AAAATACGCCGAAGAGTCGACATAAATCCTGGTCATGCAGACCTCAGCGCCAAGGAGGCACAGGATGTAA
TTATGGAAGTTGTTTTCCCTAATGAAGTGGGAGCCAGAATACTGACATCGGAATCGCAATTAACGATAAC
CAAGGAGAAAAAAGAAGAACTCCAGAATTGCAAAATTTCCCCTTTGATGGTTGCATACATGTTAGAGAGG
GAACTTGTCCGCAAAACAAGATTTCTCCCGGTTGCAGGTGGAACAAGCAGTGTGTACATTGAAGTTTTGC
ATTTAACACAGGGGACATGCTGGGAGCAGATGTACACTCCAGGTGGGGAGGTGAGGAATGATGATGTTGA
TCAAAGCCTAATTATTGCTGCTAGGAACATAGTGAGAAGAGCTGCAGTATCAGCAGATCCACTAGCATCT
TTATTAGAAATGTGCCATAGCACACAGATTGGTGGAACAAGGATGGTGGATATTCTCAGGCAAAATCCAA
CAGAAGAACAAGCTGTGGGCATATGCAAAGCAGCAATGGGGCTGAGAATCAGTTCATCCTTCAGTTTTGG
CGGATTCACATTTAAGAGAACAAGTGGATCATCAGTCAAAAGGGAGGAAGAAGTGCTCACGGGCAATCTG
CAAACATTGAAGCTAACTGTGCATGAGGGATATGAAGAATTCACAATGGTTGGGAAAAGGGCAACAGCTA
TACTCAAAAAAGCAACCAGGAGATTGATTCAACTAATAGTAAGTGGAAGAGACGAACAGTCAATAGTCGA
AGCAATAGTTGTAGCAATGGTATTCTCACAAGAAGATTGCATGGTAAAAGCAGTTAGAGGTGATCTGAAT
TTCGTTAATAGAGCAAATCAGCGGTTGAATCCCATGCATCAACTTTTGAGACATTTTCAGAAGGATGCTA
AAGTACTTTTCTTAAATTGGGGAATTGAACATATTGACAATGTTATGGGAATGATTGGGATATTACCTGA
TATGACTCCAAGTACCGAGATGTCAATGAGAGGAGTGAGAGTCAGCAAAATGGGTGTAGATGAATACTCC
AATGCTGAAAGGGTAGTGGTGAGCATTGACCGTTTTTTGAGAGTCCGGGACCAAAGAGGAAATGTACTAC
TGTCTCCAGAGGAAGTCAGTGAAACACAAGGAACAGAGAAACTGACAATAACTTACTCTTCATCATTGAT
GTGGGAGATTAATGGCCCTGAGTCAGTGTTGATCAATACCTATCAGTGGATCATCAGAAACTGGGAGACT
GTTAAAATTCAGTGGTCTCAGAACCCTACGATGCTATACAATAAAATGGAATTTGAACCATTTCAATCTC
TAGTCCCCAAGGCCATTAGAGGTCAATACAGTGGGTTTGTTAGAACTCTATTTCAACAAATGAGGGATGT
GCTCGGGACCTTTGACACAACTCAGATAATAAAACTTCTTCCCTTTGCAGCCGCTCCACCAAAGCAAAGT
AGAATGCAATTCTCGTCATTAACTGTGAATGTGAGGGGATCTGGAATGAGAATACTTGTGAGGGGTAATT
CTCCAGTATTCAATTACAACAAGACCACTAAGAGACTCACAATCCTTGGAAAGGATGCTGGCACTTTAAC
TGAAGACCCAGATGAAGGCACAGCTGGAGTGGAATCTGCTGTTTTAAGGGGATTCCTCATTCTAGGCAAA
GAAGATAGAAGATATGGGCCAGCATTAAGCATCAATGAATTGAGCAACCTTGCGAAAGGGGAAAAAGCTA
ATGTGCTAATTGGGCAAGGGGATGTAGTGTTGGTAATGAAACGAAAACGGGACTCTAGCATACTTACTGA
CAGCCAGACAGCGACCAAAAGAATTCGGATGGCCATCAATTAATTTCGAATAATTTAA
>gi|94959549|gb|CY010771.1| Influenza A virus (A/Canterbury/20/2001(H1N1)) segment 1, complete sequence
TCAATTATATTCAATATGGAAAGAATAAAAGAGCTAAGGAATCTGATGTCACAATCTCGCACTCGCGAGA
TACTTACAAAAACTACTGTAGACCACATGGCCATAATCAAAAAATACACATCCGGAAGACAGGAGAAAAA
CCCATCACTTAGGATGAAATGGATGATGGCAATGAAATACCCAATTACAGCAGACAAAAGGATAACGGAA
ATGATTCCTGAAAGAAATGAGCAAGGACAGACATTATGGAGTAAAGTGAATGATGCCGGTTCAGACCGAG
TGATGATATCACCCCTGGCTGTGACATGGTGGAATAGAAATGGACCAGTGGCAAGTACTATTCACTATCC
AAAAATCTACAAAACTTACTTTGAAAAGGTTGAAAGGTTAAAACATGGAACCTTTGGCCCTGTACACTTT

AGAAACCAAGTCAAAATACGCCGAAGAGTCGACATAAATCCTGGTCATGCAGACCTCAGCGCCAAGGAGG
CACAGGATGTAATTATGGAAGTTGTTTTCCCTAATGAAGTGGGAGCCAGAATACTAACATCAGAATCGCA
ATTAACGATAACCAAAGAGAAAAAAGAAGAACTCCAGAATTGCAAAATTTCCCCTTTGATGGTTGCATAC
ATGTTAGAGAGGGAACTTGTCCGCAAAACGAGATTTCTCCCGGTTGCTGGTGGAACAAGCAGTGTGTACA
TTGAAGTTTTGCATTTAACACAGGGGACATGCTGGGAACAGATGTACACTCCAGGTGGAGAGGTGAGGAA
TGATGATGTTGATCAAAGCCTAATTATTGCTGCTAGGAACATAGTGAGAAGAGCTGCAGTATCAGCAGAT
CCACTAGCATCTTTATTAGAAATGTGCCACAGCACACAGATTGGTGGAACAAGGATGGTGGATATTCTCA
GGCAAAACCCAACAGAAGAACAAGCTGTGGATATATGCAAAGCAGCAATGGGGCTGAGAATCAGTTCATC
CTTCAGTTTTGGCGGATTCACATTCAAGAGAACAAGTGGATCATCAGTCAAAAGGGAGGAAGAAATGCTC
ACGGGCAATCTGCAAACATTGAAGCTAACTGTGCATGAGGGATATGAAGAGTTCACAATGGTTGGGAAAA
GGGCAACAGCTATACTCAGAAAAGCAACCAGGAGATTGATTCAACTAATAGTGAGTGGACGAGACGAACA
GTCGATAGTCGAAGCAATAGTTGTAGCAATGGTATTCTCACAAGAAGATTGCATGGTAAAAGCAGTTAGA
GGTGATCTGAATTTCGTCAATAGAGCGAATCAGCGGTTGAATCCCATGCATCAACTTTTGAGACATTTTC
AGAAGGATGCTAAAGTACTTTTCTTAAATTGGGGAATTGAACCTATCGACAATGTGATGGGAATGATTGG
GATACTACCTGATATGACTCCAAGTACCGAGATGTCAATGAGAGGAGTGAGAGTCAGCAAAATGGGTGTA
GATGAATACTCCAATGCTGAAAAGGTAGTGGTGAGCATTGACCGTTTTTTGAGAGTCCGGGACCAAAGAG
GAAATGTACTACTGTCTCCAGAGGAAGTCAGTGAAACACAGGGAACAGAGAAACTGACAATAACTTACTC
TTCATCAATGATGTGGGAGATTAATGGCCCTGAGTCAGTGTTGATCAATACCTATCAGTGGATCATCAGA
AACTGGGAGACTGTTAAAATTCAGTGGTCTCAGAACCCTACAATGCTATACAATAAAATGGAATTCGAGC
CATTTCAGTCTCTAGTCCCTAAGGCCATTAGAGGCCAATACAGTGGGTTTGTTAGAACTCTATTCCAACA
AATGAGGGATGTGCTTGGGACCTTTGACACAACTCAGATAATAAAACTTCTTCCCTTTGCAGCCGCTCCA
CCAAAGCAAAGTAGAATGCAATTCTCATCATTGACTGTGAATGTGAGAGGATCAGGAATGAGAATACCTG
TAAGGGGTAATTCTCCAGTATTCAACTACAACAAGACCACTAAGAGACTCACAGTCCTCGGAAAGGATGC
TGGCACTTTAACTGAAGACCCAGATGAAGGCACAGCTGGAGTGGAATCTGCTGTTTTAAGGGGATTCCTC
ATTTTAGGCAAAGAAGATAGAAGATATGGGCCAGCATTAAGCATCAATGAATTGAGCAACCTTGCGAAAG
GAGAAAAAGCTAATGTGCTAATTGGGCAAGGGGACGTAGTGTTGGTGATGAAACGAAAACGGGACTCTAG
CATACTTACTGACAGCCAGACAGCGACCAAAAGAATTCGGATGGCCATCAATTAATTTCGAATAATTTAA
AAA
>gi|91119043|gb|CY010411.1| Influenza A virus (A/West Coast/33/2001(H1N1)) segment 1, complete sequence
TCAATTATATTCAATATGGAAAGAATAAAAGAGCTAAGGAATCTGATGTCACAATCTCGCACTCGCGAGA
TACTTACAAAAACTACTGTAGACCACATGGCCATAATCAAAAAATACACATCCGGAAGACAGGAGAAAAA
CCCATCACTTAGGATGAAATGGATGATGGCAATGAAATACCCAATTACAGCAGACAAAAGGATAACGGAA
ATGATTCCTGAAAGAAATGAGCAAGGACAGACATTATGGAGTAAAGTGAATGATGCCGGTTCAGACCGAG
TGATGATATCACCCCTGGCTGTGACATGGTGGAATAGAAATGGACCAGTGGCAAGTACTATTCACTATCC
AAAAATCTACAAAACTTACTTTGAAAAGGTTGAAAGGTTAAAACATGGAACCTTTGGCCCTGTACACTTT
AGAAACCAAGTCAAAATACGCCGAAGAGTCGACATAAATCCTGGTCATGCAGACCTCAGCGCCAAGGAGG
CACAGGATGTAATTATGGAAGTTGTTTTCCCTAATGAAGTGGGAGCCAGAATACTAACATCAGAATCGCA
ATTAACGATAACCAAAGAGAAAAAAGAAGAACTCCAGAATTGCAAAATTTCCCCTTTGATGGTTGCATAC
ATGTTAGAGAGGGAACTTGTCCGCAAAACGAGATTTCTCCCGGTTGCTGGTGGAACAAGCAGTGTGTACA
TTGAAGTTTTGCATTTAACACAGGGGACATGCTGGGAACAGATGTACACTCCAGGTGGAGAGGTGAGGAA
TGATGATGTTGATCAAAGCCTAATTATTGCTGCTAGGAACATAGTGAGAAGAGCTGCAGTATCAGCAGAT
CCACTAGCATCTTTATTAGAAATGTGCCACAGCACACAGATTGGTGGAACAAGGATGGTGGATATTCTCA
GGCAAAACCCAACAGAAGAACAAGCTGTGGATATATGCAAAGCAGCAATGGGGCTGAGAATCAGTTCATC

CTTCAGTTTTGGCGGATTCACATTCAAGAGAACAAGTGGATCATCAGTCAAAAGGGAGGAAGAAATGCTC
ACGGGCAATCTGCAAACATTGAAGCTAACTGTGCATGAGGGATATGAAGAGTTCACAATGGTTGGGAAAA
GGGCAACAGCTATACTCAGAAAAGCAACCAGGAGATTGATTCAACTAATAGTGAGTGGACGAGACGAACA
GTCGATAGTCGAAGCAATAGTTGTAGCAATGGTATTCTCACAAGAAGATTGCATGGTAAAAGCAGTTAGA
GGTGATCTGAATTTCGTCAATAGAGCGAATCAGCGGTTGAATCCCATGCATCAACTTTTGAGACATTTTC
AGAAGGATGCTAAAGTACTTTTCTTAAATTGGGGAATTGAACCTATCGACAATGTGATGGGAATGATTGG
GATACTACCTGATATGACTCCAAGTACCGAGATGTCAATGAGAGGAGTGAGAGTCAGCAAAATGGGTGTA
GATGAATACTCCAATGCTGAAAAGGTAGTGGTGAGCATTGACCGTTTTTTGAGAGTCCGGGACCAAAGAG
GAAATGTACTACTGTCTCCAGAGGAAGTCAGTGAAACACAGGGAACAGAGAAACTGACAATAACTTACTC
TTCATCAATGATGTGGGAGATTAATGGCCCTGAGTCAGTGTTGATCAATACCTATCAGTGGATCATCAGA
AACTGGGAGACTGTTAAAATTCAGTGGTCTCAGAACCCTACAATGCTATACAATAAAATGGAATTCGAGC
CATTTCAGTCTCTAGTCCCTAAGGCCATTAGAGGCCAATACAGTGGGTTTGTTAGAACTCTATTCCAACA
AATGAGGGATGTGCTTGGGACCTTTGACACAACTCAGATAATAAAACTTCTTCCCTTTGCAGCCGCTCCA
CCAAAGCAAAGTAGAATGCAATTCTCATCATTGACTGTGAATGTGAGAGGATCAGGAATGAGAATACTTG
TAAGGGGTAATTCTCCAGTATTCAACTACAACAAGACCACTAAGAGACTCACAGTCCTCGGAAAGGATGC
TGGCACTTTAACTGAAGACCCAGATGAAGGCACAGCTGGAGTGGAATCTGCTGTTTTAAGGGGATTCCTC
ATTTTAGGCAAAGAAGATAGAAGATATGGGCCAGCATTAAGCATCAATGAATTGAGCAACCTTGCGAAAG
GAGAAAAAGCTAATGTGCTAATTGGGCAAGGGGACGTAGTGTTGGTGATGAAACGAAAACGGGACTCTAG
CATACTTACTGACAGCCAGACAGCGACCAAAAGAATTCGGATGGCCATCAATTAA
>gi|131052882|gb|CY020004.1| Influenza A virus (A/Waikato/17/2005(H1N1)) segment 1, complete sequence
ATGGAAAGAATAAAAGAGCTAAGGAATTTGATGTCACAATCTCGCACTCGCGAGATACTTACCAAAACTA
CTGTAGACCACATGGCCATAATCAAGAAATACACATCAGGAAGACAGGAGAAAAACCCATCACTTAGGAT
GAAATGGATGATGGCAATGAAATACCCAATTACAGCTGATAAAAGGATAACGGAAATGATTCCTGAAAGA
AATGAGCAAGGACAGACACTATGGAGTAAAGTGAATGATGCTGGATCAGACCGAGTGATGATATCACCCC
TGGCTGTGACATGGTGGAACAGAAATGGACCAGTGGCAAATACTATTCACTATCCAAAAATCTACAAAAC
TTACTTTGAAAAGGTTGAAAGGTTAAAACATGGAACCTTTGGCCCTGTACACTTTAGAAACCAAGTCAAA
ATACGCCGAAGAGTCGACATAAATCCTGGTCATGCAGACCTCAGCGCCAAGGAGGCACAGGATGTAATTA
TGGAAGTTGTTTTCCCTAATGAAGTGGGAGCCAGAATACTAACATCAGAATCTCAATTAACGATAACCAA
GGAGAAAAAAGAAGAACTCCAGAATTGCAAAATTTCCCCTTTGATGGTTGCATACATGTTAGAGAGGGAA
CTTGTCCGCAAAACAAGATTTCTCCCGGTTGCAGGTGGAACAAGCAGTGTGTACATTGAAGTTTTGCATT
TAACACAGGGGACATGCTGGGAGCAGATGTACACTCCAGGTGGGGAGGTGAGGAATGATGATGTTGATCA
AAGCCTAATTATTGCTGCTAGGAACATAGTGAGAAGAGCTGCAGTATCAGCAGATCCACTAGCATCTTTA
TTAGAAATGTGCCATAGCACACAGATTGGTGGAACAAGGATGGTGGATATTCTCAGGCAAAATCCAACAG
AAGAACAAGCTGTTGACATATGCAAAGCAGCAATGGGGCTGAGAATCAGTTCATCCTTCAGTTTTGGCGG
ATTCACATTTAAGAGAACAAGTGGATCATCAGTCAAAAGGGAGGAAGAAGTGCTCACGGGCAATCTGCAA
ACATTGAAGCTAACTGTGCATGAGGGATATGAAGAATTCACAATGGTTGGGAAAAGGGCAACAGCTATAC
TCAGAAAAGCAACCAGGAGATTGATTCAACTAATAGTGAGTGGAAGAGACGAACAGTCAATAGTCGAAGC
AATAGTTGTAGCAATGGTATTCTCACAAGAAGATTGCATGGTAAAAGCAGTTAGAGGTGATCTGAATTTC
GTTAATAGAGCAAATCAGCGGTTGAATCCCATGCATCAACTTTTGAGACATTTTCAGAAGGATGCTAAAG
TACTTTTCTTAAATTGGGGAATTGAACATATTGACAATGTGATGGGAATGATTGGGATATTGCCTGATAT
GACTCCAAGTACCGAGATGTCAATGAGAGGAGTGAGAGTCAGCAAAATGGGTGTAGATGAATACTCCAAT
GCTGAAAGGGTAGTGGTGAGCATTGACCGTTTTTTGAGAGTCCGGGACCAAAGAGGAAATGTACTACTGT
CTCCAGAGGAAGTCAGTGAAACACAAGGAACAGAGAAACTGACAATAACTTACTCTTCATCATTGATGTG

GGAGATTAATGGCCCTGAGTCAGTGTTGATCAATACCTATCAGTGGATCATCAGAAACTGGGAGACTGTT
AAAATTCAGTGGTCTCAGAACCCTACGATGCTATACAATAAAATGGAATTTGAACCATTTCAATCTCTAG
TCCCCAAGGCCATTAGAGGCCAATACAGTGGGTTTGTTAGAACTCTATTTCAACAAATGAGGGATGTGCT
CGGGACCTTTGACACAACTCAGATAATAAAACTTCTTCCCTTTGCAGCCGCTCCACCAAAGCAAAGTAGA
ATGCAATTCTCGTCATTAACTGTGAATGTGAGGGGATCAGGAATGAGAATACTTGTAAGGGGTAATTCTC
CAGTATTCAACTACAACAAAACCACTAAGAGACTCACAATACTAGGAAAGGATGCTGGCACTTTAACTGA
AGACCCAGATGAAGGCACAGCTGGAGTGGAATCTGCTGTTTTAAGGGGATTCCTCATTTTAGGCAAAGAA
GATAGAAGATATGGGCCAGCATTAAGCATCAATGAATTGAGCAACCTTGCAAAAGGGGAAAAAGCTAATG
TGCTAATTGGGCAAGGGGATGTAGTGTTGGTAATGAAACGAAAACGGGACTCTAGCATACTTACTGACAG
CCAGACAGCGACCAAAAGAATTCGGATGGCCATCAATTAATTTCGAATAATTTAAAAA
>gi|83744851|gb|CY007474.1| Influenza A virus (A/Canterbury/106/2004(H1N1)) segment 1, complete sequence
TCAATTATATTCAATATGGAAAGAATAAAAGAGCTAAGGAATCTGATGTCACAATCTCGCACTCGCGAGA
TACTTACCAAAACTACTGTAGACCACATGGCCATAATCAAGAAATACACATCAGGAAGACAGGAGAAAAA
CCCATCACTTAGGATGAAATGGATGATGGCAATGAAATACCCAATTACAGCTGATAAAAGGATAACGGAA
ATGATTCCTGAAAGAAATGAGCAAGGACAGACATTATGGAGTAAGGTGAATGATGCCGGATCAGACCGAG
TGATGATATCACCCCTGGCTGTGACATGGTGGAACAGAAATGGACCAGTGGCAAGTACTATTCACTATCC
AAAAATCTACAAAACTTACTTTGAAAAGGTTGAAAGGTTAAAACATGGAACCTTTGGCCCTGTACACTTT
AGAAACCAAGTCAAAATACGCCGAAGAGTCGACATAAATCCTGGTCATGCAGACCTCAGCGCCAAGGAGG
CACAGGATGTAATTATGGAAGTTGTTTTCCCTAATGAAGTAGGAGCCAGAATACTAACATCAGAATCGCA
ATTAACGATAACCAAGGAGAAAAAAGAAGAACTCCAGAATTGCAAAATTTCCCCTTTGATGGTTGCATAC
ATGTTAGAGAGGGAACTTGTCCGCAAAACAAGATTTCTCCCGATTGCAGGTGGAACAAGCAGTGTGTACA
TTGAAGTTTTGCATTTAACACAGGGGACATGCTGGGAGCAGATGTACACTCCAGGTGGGGAGGTGAGGAA
TGATGATGTTGATCAAAGCCTAATTATTGCTGCTAGGAACATAGTGAGAAGAGCTGCAGTATCAGCAGAT
CCACTAGCATCTTTATTAGAAATGTGCCATAGCACACAGATTGGTGGAACAAGGATGGTGGATATTCTCA
GGCAAAATCCAACAGAAGAACAAGCTGTGGACATATGCAAAGCAGCAATGGGGCTGAGAATCAGTTCATC
CTTCAGTTTTGGCGGATTCACATTTAAGAGAACAAGTGGATCATCAGTCAAAAGGGAGGAAGAAGTGCTC
ACGGGCAATCTGCAAACATTGAAGCTAACTGTGCATGAGGGATATGAAGAGTTCACAATGGTTGGGAAAA
GGGCAACAGCTATATTCAGAAAAGCAACCAGGAGATTGATTCAACTGATAGTGAGTGGAAGAGACGAACA
GTCAATAGTCGAAGCAATAGTTGTAGCAATGGTATTCTCACAAGAAGATTGCATGGTAAAAGCAGTTAGA
GGTGATCTGAATTTCGTTAATAGAGCGAATCAGCGGTTGAATCCCATGCATCAACTTTTGAGACATTTTC
AGAAGGATGCTAGAGTACTTTTCTTAAATTGGGGAATTGAACCTATTGACAATGTGATGGGAATGATTGG
GATATTACCTGATATGACTCCAAGCACCGAGATGTCAATGAGAGGAGTGAGAGTCAGCAAAATGGGTGTA
GATGAATACTCCAATGCTGAAAGAGTAGTGGTGAGCATTGACCGTTTTTTGAGAGTCCGGGACCAAAGAG
GAAATGTACTACTGTCTCCAGAGGAAGTCAGTGAAACACAAGGGACAGAGAAACTGACAATAACATACTC
TTCATCAATGATGTGGGAGATTAATGGCCCTGAGTCAGTCTTGATCAATACCTATCAGTGGATCATCAGA
AATTGGGAGACTGTTAAAATTCAGTGGTCTCAGAATCCTACGATGCTATACAATAAAATGGAATTTGAAC
CATTTCAGTCTCTGGTCCCCAAGGCCATTAGAGGCCAATACAGTGGGTTTGTTAGAACTCTATTTCAACA
AATGAGGGATGTGCTTGGGACCTTTGACACAACTCAGATAATAAAACTTCTTCCCTTTGCAGCCGCTCCT
CCAAAGCAAAGCAGAATGCAATTCTCTTCATTAACTGTGAATGTGAGGGGATCAGGAATGAGAATACTTG
TAAGGGGTAATTCTCCAGTATTCAACTACAACAAGACTACTAAGAGACTCACAGTCCTCGGAAAGGATGC
TGGCACTTTAACTGAAGACCCAGATGAAGGCACAGCTGGAGTGGAATCTGCGGTTCTAAGGGGATTCCTC
ATTCTAGGCAAAGAAGATAGAAGATATGGGCCAGCATTAAGCATCAATGAATTGAGCAACCTTGCGAAAG
GGGAAAAAGCTAATGTGCTAATTGGGCAAGGGGATGTAGTGTTGGTAATGAAACGAAAACGGGACTCTAG

CATACTTACTGACAGCCAGACAGCGACCAAAAGAATTCGGATGGCCATCAATTAATTTCGAATAATTTAA
AAA
>gi|115607838|gb|CY016706.1| Influenza A virus (A/South Australia/58/2005(H1N1)) segment 1, complete sequence
AATATGGAAAGAATAAAAGAGCTAAGGAATCTGATGTCACAATCTCGCACTCGCGAGATACTTACCAAAA
CTACTGTAGACCACATGGCCATAATCAAGAAATACACATCAGGAAGACAGGAGAAAAACCCATCAATTAG
GATGAAATGGATGATGGCAATGAAATACCCAATTACAGCTGATAAAAAGATAACGGAAATGATTCCTGAA
AGAAATGAGCAAGGACAGACACTATGGAGTAAAGTGAATGATGCCGGATCAGACCGAGTGATGATATCAC
CCCTGGCTGTGACATGGTGGAACAGAAATGGACCAGTGGCAAATACTATTCACTATCCAAAAATCTACAA
AACTTACTTTGAAAAGGTTGAAAGGTTAAAACATGGAACCTTTGGCCCTGTACACTTTAGAAACCAAGTC
AAAATACGCCGAAGAGTCGACATAAATCCTGGTCATGCAGACCTCAGCGCCAAGGAGGCACAGGATGTAA
TTATGGAAGTTGTTTTCCCTAATGAAGTGGGAGCCAGAATACTAACATCAGAATCGCAATTAACGATAAC
CAAGGAGAAAAAAGAAGAACTCCAGAATTGCAAAATTTCCCCTTTGATGGTTGCATACATGTTAGAGAGG
GAACTTGTCCGCAAAACAAGATTTCTCCCGGTTGCAGGTGGAACAAGCAGTGTGTACATTGAAGTTTTGC
ATTTAACACAGGGGACATGCTGGGAGCAGATGTACACTCCAGGTGGGGAGGTGAGGAATGATGATGTTGA
TCAAAGCCTAATTATTGCTGCTAGGAACATAGTGAGAAGAGCTGCAGTATCAGCAGATCCACTAGCATCT
TTATTAGAAATGTGCCATAGCACACAGATTGGTGGAACAAGGATGGTGGATATTCTCAGGCAAAATCCAA
CAGAAGAACAAGCTGTTGACATATGCAAAGCAGCAATGGGGCTGAGAATCAGTTCATCCTTCAGTTTTGG
CGGATTCACATTTAAGAGAACAAGTGGATCATCAGTCAAAAGGGAGGAAGAAGTGCTCACGGGCAATCTG
CAAACATTGAAGCTAACTGTGCATGAGGGATATGAAGAATTCACAATGGTTGGGAAAAGGGCAACAGCTA
TACTCAGAAAAGCAACCAGGAGATTGATTCAACTAATAGTGAGTGGAAGAGACGAACAGTCAATAGTTGA
AGCAATAGTTGTAGCAATGGTATTCTCACAAGAAGATTGCATGGTAAAAGCAGTTAGAGGTGATCTGAAT
TTCGTTAATAGAGCAAATCAGCGGTTGAATCCCATGCATCAACTTTTGAGACATTTTCAGAAGGATGCTA
AAGTACTTTTCTTAAATTGGGGAATTGAACATATTGACAATGTGATGGGAATGATTGGGATATTGCCTGA
TATGACTCCAAGTACCGAGATGTCAATGAGAGGAGTGAGAGTCAGCAAAATGGGTGTAGATGAATACTCC
AATGCTGAAAGGGTAGTGGTGAGCATTGACCGTTTTTTGAGAGTCCGGGACCAAAGAGGAAATGTACTAC
TGTCTCCAGAGGAAGTCAGTGAAACACAAGGAACAGAGAAACTGACAATAACTTACTCTTCATCATTGAT
GTGGGAGATTAATGGCCCTGAGTCAGTGTTGATCAATACCTATCAGTGGATCATCAGAAACTGGGAGTCT
GTTAAAATTCAGTGGTCTCAGAACCCTACGATGCTATACAATAAAATGGAATTTGAACCATTTCAATCTC
TAGTCCCCAAGGCCATTAGAGGCCAATACAGTGGGTTTGTTAGAACTCTATTTCAACAAATGAGGGATGT
GCTCGGGACCTTTGACACAACTCAGATAATAAAACTTCTTCCCTTTGCAGCCGCTCCACCAAAGCAAAGT
AGAATGCAATTCTCGTCATTAACTGTGAATGTGAGGGGATCAGGAATGAGAATACTTGTAAGGGGTAATT
CTCCAGTATTCAACTACAACAAAACCACTAAGAGACTCACAATACTAGGAAAGGATGCTGGTACTTTAAC
TGAAGACCCAGATGAAGGCACAGCTGGAGTGGAATCTGCTGTTTTAAGGGGATTCCTCATTCTAGGCAAA
GAAGATAGAAGATATGGGCCAGCATTAAGCATCAATGAATTGAGCAACCTTGCGAAAGGGGAAAAAGCTA
ATGTGCTAATTGGGCAAGGGGATGTAGTGTTGGTAATGAAACGAAAACGGGACTCTAGCATACTTACTGA
CAGCCAGACAGCGACCAAAAGAATTCGGATGGCCATCAATTAATTTCGAATAA
>gi|113170898|gb|CY014014.1| Influenza A virus (A/Wellington/11/2005(H1N1)) segment 1, complete sequence
ATATGGAAAGAATAAAAGAGCTAAGGAATCTGATGTCACAATCTCGCACTCGCGAGATACTTACCAAAAC
TACTGTAGACCACATGGCTATAATCAAGAAATACACATCAGGAAGACAGGAGAAAAACCCATCACTTAGG
ATGAAATGGATGATGGCAATGAAATACCCAATTACAGCTGATAAAAGGATAACGGAAATGATTCCTGAAA
GAAATGAACAAGGACAAACACTATGGAGTAAAGTGAACGATGCCGGATCAGACCGAGTGATGATATCACC
CCTGGCTGTGACATGGTGGAACAGAAATGGACCAGTGGCAAATACTATTCACTATCCAAAAATATACAAA

ACTTACTTTGAAAAGGTTGAAAGGTTAAAACATGGAACCTTTGGCCCTGTACACTTTAGAAACCAAGTCA
AAATACGCCGAAGAGTCGACATAAATCCTGGTCATGCAGACCTCAGCGCCAAGGAGGCACAGGATGTAAT
TATGGAAGTTGTTTTCCCTAATGAAGTGGGAGCCAGAATACTGACATCGGAATCGCAATTAACGATAACC
AAGGAGAAAAAAGAAGAACTCCAGAATTGCAAAATTTCCCCTTTGATGGTTGCATACATGTTAGAGAGGG
AACTTGTCCGCAAAACAAGATTTCTCCCGGTTGCAGGTGGAACAAGCAGTGTGTACATTGAAGTTTTGCA
TTTAACACAGGGAACATGCTGGGAGCAGATGTACACTCCAGGTGGGGAGGTGAGGAATGATGATGTTGAT
CAAAGCCTAATTATTGCTGCTAGGAACATAGTGAGAAGAGCTGCAGTATCAGCAGATCCACTAGCATCTT
TATTAGAAATGTGCCATAGCACACAGATTGGTGGAACAAGGATGGTGGATATTCTCAGGCAAAATCCAAC
AGAAGAACAAGCTGTGGGCATATGCAAAGCAGCAATGGGGCTGAGAATCAGTTCATCCTTCAGTTTTGGC
GGATTCACATTTAAGAGGACAAGTGGATCATCAGTCAAAAGGGAGGAAGAAGTGCTCACGGGCAATCTGC
AAACATTGAAGCTAACTGTGCATGAGGGATATGAAGAATTCACAATGGTTGGGAAAAGGGCAACAGCTAT
ACTCAAAAAAGCAACCAGGAGATTGATTCAACTAATAGTGAGTGGAAGAGACGAACAGTCAATAGTCGAA
GCAATAGTTGTAGCAATGGTATTCTCACAAGAAGATTGCATGGTAAAAGCAGTTAGAGGTGATCTGAATT
TCGTTAATAGAGCAAATCAGCGGTTGAATCCCATGCATCAACTTTTGAGACATTTTCAGAAGGATGCTAA
AGTACTTTTCTTAAATTGGGGAATTGAACATATTGACAATGTGATGGGAATGATTGGGATATTACCTGAT
ATGACTCCAAGTACCGAGATGTCAATGAGAGGAGTGAGAGTCAGCAAAATGGGTGTAGATGAATACTCCA
ATGCTGAAAGGGTAGTGGTGAGCATTGACCGTTTTTTGAGAGTCCGGGACCAAAGAGGAAATGTACTACT
GTCTCCAGAGGAAGTCAGTGAAACACAAGGAACAGAGAAACTGACAATAACTTACTCTTCATCATTGATG
TGGGAGATTAATGGCCCTGAGTCAGTGTTGATCAATACCTATCAGTGGATCATCAGAAACTGGGAGACTG
TTAAAATTCAGTGGTCTCAGAACCCTACGATGCTATACAATAAAATGGAATTTGAACCATTTCAATCTCT
AGTCCCCAAGGCCATTAGAGGCCAATACAGTGGGTTTGTTAGAACTCTATTTCAACAAATGAGGGATGTG
CTCGGGACCTTTGACACAACTCAGATAATAAAACTTCTTCCCTTTGCAGCCGCTCCACCAAAGCAAAGTA
GAATGCAATTCTCGTCATTAACTGTGAATGTGAGGGGATCAGGAATGAGAATACTTGTGAGGGGTAATTC
TCCAGTATTCAATTACAACAAGACCACTAAGAGACTCACAATCCTTGGAAAGGATGCTGGCACTTTAACT
GAAGACCCAGATGAAGGCACAGCTGGAGTGGAATCTGCTGTTTTAAGGGGATTCCTCATTCTAGGCAAAG
AAGATAGAAGATATGGGCCAGCATTAAGCATCAATGAATTGAGCAACCTTGCGAAAGGGGAAAAAGCTAA
TGTGCTAATTGGGCAAGGGGATGTAGTGTTGGTAATGAAACGAAAACGGGACTCTAGCATACTTACTGAC
AGCCAGACAGCGACCAAAAGAATTCGGATGGCCATCAATTAA
>gi|145278940|gb|CY021764.1| Influenza A virus (A/South Australia/51/2005(H1N1)) segment 1, complete sequence
AATATGGAAAGAATAAAAGAGCTAAGGAATCTGATGTCACAATCTCGCACTCGCGAGATACTTACCAAAA
CTACTGTAGACCACATGGCCATAATCAAGAAATACACATCAGGAAGACAGGAGAAAAACCCATCACTTAG
GATGAAATGGATGATGGCAATGAAATACCCAATTACAGCTGATAAAAGGATAACGGAAATGATTCCTGAA
AGAAATGAGCAAGGACAAACACTATGGAGTAAAGTGAACGATGCCGGATCAGACCGAGTGATGATATCAC
CCCTGGCTGTGACATGGTGGAACAGAAATGGACCAGTGGCAAATACTATTCACTATCCAAAAATATACAA
AACTTACTTTGAAAAGGTTGAAAGGTTAAAACATGGAACCTTTGGCCCTGTACACTTTAGAAACCAAGTC
AAAATACGCCGAAGAGTCGACATAAATCCTGGTCATGCAGACCTCAGCGCCAAGGAGGCACAGGATGTAA
TTATGGAAGTTGTTTTCCCTAATGAAGTGGGAGCCAGAATACTGACATCGGAATCGCAATTAACGATAAC
CAAGGAGAAAAAAGAAGAACTCCAGAATTGCAAAATTTCCCCTTTGATGGTTGCATACATGTTAGAGAGG
GAACTTGTCCGCAAAACAAGATTTCTCCCGGTTGCAGGTGGAACAAGCAGTGTGTACATTGAAGTTTTGC
ATTTAACACAGGGGACATGCTGGGAGCAGATGTACACTCCAGGTGGGGAGGTGAGGAATGATGATGTTGA
TCAAAGCCTAATTATTGCTGCTAGGAACATAGTGAGAAGAGCTGCAGTATCAGCAGATCCACTAGCATCT
TTATTAGAAATGTGCCATAGCACACAGATTGGTGGAACAAGGATGGTGGATATTCTCAGGCAAAATCCAA
CAGAAGAACAAGCTGTGGGCATATGCAAAGCAGCAATGGGGCTGAGAATCAGTTCATCCTTCAGTTTTGG

CGGATTCACATTTAAGAGGACAAGTGGATCATCAGTCAAAAGGGAGGAAGAAGTGCTCACGGGCAATCTG
CAAACATTGAAGCTAACTGTGCATGAGGGATATGAAGAATTCACAATGGTTGGGAAAAGGGCAACAGCTA
TACTCAAAAAAGCAACCAGGAGATTGATTCAACTAATAGTGAGTGGAAGAGACGAACAGTCAATAGTCGA
AGCAATAGTTGTAGCAATGGTATTCTCACAAGAAGATTGCATGGTAAAAGCAGTTAGAGGTGATCTGAAT
TTCGTTAATAGAGCAAATCAGCGGTTGAATCCCATGCATCAACTTTTGAGACATTTTCAGAAGGATGCTA
AAGTACTTTTCTTAAATTGGGGAATTGAACATATTGACAATGTGATGGGAATGATTGGGATATTACCTGA
TATGACTCCAAGTACCGAGATGTCAATGAGAGGAGTGAGAGTCAGCAAAATGGGTGTAGATGAATACTCC
AATGCTGAAAGGGTAGTGGTGAGCATTGACCGTTTTTTGAGAGTCCGGGACCAAAGAGGAAATGTACTAC
TGTCTCCAGAGGAAGTCAGTGAAACACAAGGAACAGAGAAACTGACAATAACTTACTCTTCATCATTGAT
GTGGGAGATTAATGGCCCTGAGTCAGTGTTGATCAATACCTATCAGTGGATCATCAGAAACTGGGAGACT
GTTAAAATTCAGTGGTCTCAGAACCCTACGATGCTATACAATAAAATGGAATTTGAACCATTTCAATCTC
TAGTCCCCAAGGCCATTAGAGGCCAATACAGTGGGTTTGTTAGAACTCTATTTCAACAAATGAGGGATGT
GCTCGGGACCTTTGACACAACTCAGATAATAAAACTTCTTCCCTTTGCAGCCGCTCCACCAAAGCAAAGT
AGAATGCAATTCTCGTCATTAACTGTGAATGTGAGGGGATCAGGAATGAGAATACTTGTGAGGGGTAATT
CTCCAGTATTCAATTACAACAAGACCACTAAGAGACTCACAATCCTTGGAAAGGATGCTGGCACTTTAAC
TGAAGACCCAGATGAAGGCACAGCTGGAGTGGAATCTGCTGTTTTAAGGGGATTCCTCATTCTAGGCAAA
GAAGATAGAAGATATGGGCCAGCATTAAGCATCAATGAATTGAGCAACCTTGCGAAAGGGGAAAAAGCTA
ATGTGCTAATTGGGCAAGGGGATGTAGTGTTGGTAATGAAACGAAAACGGGACTCTAGCATACTTACTGA
CAGCCAGACAGCGACCAAAAGAATTCGGATGGCCATCAATTAATTTCGAATAATTTA
>gi|149780711|gb|CY022588.1| Influenza A virus (A/Auckland/619/2005(H1N1)) segment 1, complete sequence
ATTATATTCAATATGGAAAGAATAAAAGAGCTAAGGAATCTGATGTCACAATCTCGCACTCGCGAGATAC
TTACCAAAACTACTGTAGACCACATGGCCATAATCAAGAAATACACATCAGGAAGACAGGAGAAAAACCC
ATCACTTAGGATGAAATGGATGATGGCAATGAAATACCCAATTACAGCTGATAAAAGGATAACGGAAATG
ATTCCTGAAAGAAATGAACAAGGACAAACACTATGGAGTAAAGTGAACGATGCCGGATCAGACCGAGTGA
TGATATCACCCCTGGCTGTGACATGGTGGAACAGAAATGGACCTGTGGCAAATACTATTCACTATCCAAA
AATATACAAAACTTACTTTGAAAAGGTTGAAAGGTTAAAACATGGAACCTTTGGCCCTGTACACTTTAGA
AACCAAGTCAAAATACGCCGAAGAGTCGACATAAATCCTGGTCATGCAGACCTCAGCGCCAAGGAGGCAC
AGGATGTAATTATGGAAGTTGTTTTCCCTAATGAAGTGGGAGCCAGAATACTGACATCGGAATCGCAATT
AACGATAACCAAGGAGAAAAAAGAAGAACTCCAGAATTGCAAAATTTCCCCTTTGATGGTTGCATACATG
TTAGAGAGGGAACTTGTCCGCAAAACAAGATTTCTCCCGGTTGCAGGTGGAACAAGCAGTGTGTACATTG
AAGTTTTGCATTTAACACAGGGGACATGCTGGGAGCAGATGTACACTCCAGGTGGGGAAGTGAGGAATGA
TGATGTTGATCAAAGCCTAATTATTGCTGCTAGGAACATAGTGAGAAGAGCTGCAGTATCAGCAGATCCA
CTAGCATCTTTATTAGAAATGTGCCATAGCACACAGATTGGTGGAACAAGGATGGTGGATATTCTCAGGC
AAAATCCAACAGAAGAACAAGCTGTGGGCATATGCAAAGCAGCAATGGGGCTGAGAATCAGTTCATCCTT
CAGTTTTGGCGGATTCACATTTAAGAGGACAAGTGGATCATCAGTCAAAAGGGAGGAAGAAGTGCTCACG
GGCAATCTGCAAACATTGAAGCTAACTGTGCATGAGGGATATGAAGAATTCACAATGGTTGGGAAAAGGG
CAACAGCTATACTCAAAAAAGCAACCAGGAGATTGATTCAACTAATAGTGAGTGGAAGAGACGAACAGTC
AATAGTCGAAGCAATAGTTGTAGCAATGGTATTCTCACAAGAAGATTGCATGGTAAAAGCAGTTAGAGGT
GATCTGAATTTCGTTAATAGAGCAAATCAGCGGTTGAATCCCATGCATCAACTTTTGAGACATTTTCAGA
AGGATGCTAAAGTACTTTTCTTAAATTGGGGAATTGAACATATTGACAATGTGATGGGAATGATTGGGAT
ATTACCTGATATGACTCCAAGTACCGAGATGTCAATGAGAGGAGTGAGAGTCAGCAAAATGGGTGTAGAT
GAATACTCCAATGCTGAAAGGGTAGTGGTGAGCATTGACCGTTTTTTGAGAGTCCGGGACCAAAGAGGAA
ATGTACTACTGTCTCCAGAGGAAGTCAGTGAAACACAAGGAACAGAGAAACTGACAATAACTTACTCTTC

ATCATTGATGTGGGAGATTAATGGCCCTGAGTCAGTGTTGATCAATACCTATCAGTGGATCATCAGAAAC
TGGGAGACTGTTAAAATTCAGTGGTCTCAGAACCCTACGATGCTATACAATAAAATGGAATTTGAACCAT
TTCAATCTCTAGTCCCCAAGGCCATTAGAGGCCAATACAGTGGGTTTGTTAGAACTCTATTTCAACAAAT
GAGGGATGTGCTCGGGACCTTTGACACAACTCAGATAATAAAACTTCTTCCCTTTGCAGCCGCTCCACCA
AAGCAAAGTAGAATGCAATTCTCGTCATTAACTGTGAATGTGAGGGGATCAGGAATGAGAATACTTGTGA
GGGGTAATTCTCCAGTATTCAATTACAACAAGACCACTAAGAGACTCACAATCCTTGGAAAGGATGCTGG
CACTTTAACTGAAGACCCAGATGAAGGCACAGCTGGAGTGGAATCTGCTGTTTTAAGGGGATTCCTCATT
CTAGGCAAAGAAGATAGAAGATATGGGCCAGCATTAAGCATCAATGAATTGAGCAACCTTGCGAAAGGGG
AAAAAGCTAATGTGCTAATTGGGCAAGGGGATGTAGTGTTGGTAATGAAACGAAAACGGGACTCTAGCAT
ACTTACTGACAGCCAGACAGCGACCAAAAGAATTCGGATGGCCATCAATTAATTTCGAATAATTTAA
>gi|117572955|gb|CY017322.1| Influenza A virus (A/Waikato/4/2005(H1N1)) segment 1, complete sequence
AATATGGAAAGAATAAAAGAGCTAAGGAATTTGATGTCACAATCTCGCACTCGCGAGATACTTACCAAAA
CTACTGTAGACCACATGGCCATAATCAAGAAATACACATCAGGAAGACAGGAGAAAAACCCATCACTTAG
GATGAAATGGATGATGGCAATGAAATACCCAATTACAGCTGATAAAAGGATAACGGAAATGATTCCTGAA
AGAAATGAGCAAGGACAGACACTATGGAGTAAAGTGAATGATGCTGGATCAGACCGAGTGATGATATCAC
CCCTGGCTGTGACATGGTGGAACAGAAATGGACCAGTGGCAAATACTATTCACTATCCAAAAATCTACAA
AACTTACTTTGAAAAGGTTGAAAGGTTAAAACATGGAACCTTTGGCCCTGTACACTTTAGAAACCAAGTC
AAAATACGCCGAAGAGTCGACATAAATCCTGGTCATGCAGACCTCAGCGCCAAGGAGGCACAGGATGTAA
TTATGGAAGTTGTTTTCCCTAATGAAGTGGGAGCCAGAATACTAACATCAGAATCGCAATTAACGATAAC
CAAGGAGAAAAAAGAAGAACTCCAGAATTGCAAAATTTCCCCTTTGATGGTTGCATACATGTTAGAGAGG
GAACTTGTCCGCAAAACAAGATTTCTCCCGGTTGCAGGTGGAACAAGCAGTGTGTACATTGAAGTTTTGC
ATTTAACACAGGGGACATGCTGGGAGCAGATGTACACTCCAGGTGGGGAGGTGAGGAATGATGATGTTGA
TCAAAGCCTAATTATTGCTGCTAGGAACATAGTGAGAAGAGCTGCAGTATCAGCAGATCCACTAGCATCT
TTATTAGAAATGTGCCATAGCACACAGATTGGTGGAACAAGGATGGTGGATATTCTCAGGCAAAATCCAA
CAGAAGAACAAGCTGTTGACATATGCAAAGCAGCAATGGGGCTGAGAATCAGTTCATCCTTCAGTTTTGG
CGGATTCACATTTAAGAGAACAAGTGGATCATCAGTCAAAAGGGAGGAAGAAGTGCTCACGGGCAATCTG
CAAACATTGAAGCTAACTGTGCATGAGGGATATGAAGAATTCACAATGGTTGGGAAAAGGGCAACAGCTA
TACTCAGAAAAGCAACCAGGAGATTGATTCAACTAATAGTGAGTGGAAGAGACGAACAGTCAATAGTCGA
AGCAATAGTTGTAGCAATGGTATTCTCACAAGAAGATTGCATGGTAAAAGCAGTTAGAGGTGATCTGAAT
TTCGTTAATAGAGCAAATCAGCGGTTGAATCCCATGCATCAACTTTTGAGACATTTTCAGAAGGATGCTA
AAGTACTTTTCTTAAATTGGGGAATTGAACATATTGACAATGTGATGGGAATGATTGGGATATTGCCTGA
TATGACTCCAAGTACCGAGATGTCAATGAGAGGAGTGAGAGTCAGCAAAATGGGTGTAGATGAATACTCC
AATGCTGAAAGGGTAGTGGTGAGCATTGACCGTTTTTTGAGAGTCCGGGACCAAAGAGGAAATGTACTAC
TGTCTCCAGAGGAAGTCAGTGAAACACAAGGAACAGAGAAACTGACAATAACTTACTCTTCATCATTGAT
GTGGGAGATTAATGGCCCTGAGTCAGTGTTGATCAATACCTATCAGTGGATCATCAGAAACTGGGAGACT
GTTAAAATTCAGTGGTCTCAGAACCCTACGATGCTATACAATAAAATGGAATTTGAACCATTTCAATCTC
TAGTCCCCAAGGCCATTAGAGGCCAATACAGTGGGTTTGTTAGAACTCTATTTCAACAAATGAGGGATGT
GCTCGGGACCTTTGACACAACTCAGATAATAAAACTTCTTCCCTTTGCAGCCGCTCCACCAAAGCAAAGT
AGAATGCAATTCTCGTCATTAACTGTGAATGTGAGGGGATCAGGAATGAGAATACTTGTAAGGGGTAATT
CTCCAGTATTCAACTACAACAAAACCACTAAGAGACTCACAATACTAGGAAAGGATGCTGGCACTTTAAC
TGAAGACCCAGATGAAGGCACAGCTGGAGTGGAATCTGCTGTTTTAAGGGGATTCCTCATTTTAGGCAAA
GAAGATAGAAGATATGGGCCAGCATTAAGCATCAATGAATTGAGCAACCTTGCAAAAGGGGAAAAAGCTA
ATGTGCTAATTGGGCAAGGGGATGTAGTGTTGGTAATGAAACGAAAACGGGACTCTAGCATACTTACTGA

CAGCCAGACAGCGACCAAAAGAATTCGGATGGCCATCAATTAATTTCGAATAA
>gi|161139461|gb|CY028202.1| Influenza A virus (A/Kentucky/UR06-0007/2006(H1N1)) segment 1, complete sequence
TCAATTATATTCAATATGGAAAGAATAAAAGAGCTAAGAAATCTGATGTCACAATCTCGCACTCGCGAGA
TACTTACCAAAACTACTGTAGACCACATGGCCATAATCAAGAAATACACATCAGGAAGACAGGAGAAAAA
CCCATCACTTAGGATGAAATGGATGATGGCAATGAAATACCCAATTACAGCTGATAAAAGGATAACGGAA
ATGATTCCTGAAAGAAATGAGCAAGGACAAACACTATGGAGTAAAATGAACGATGCAGGATCAGACCGAG
TGATGATATCACCCCTGGCTGTGACATGGTGGAACAGAAATGGACCAGTGGCAAATACTATTCACTATCC
AAAAATCTACAAAACTTACTTTGAAAAAGTTGAAAGGTTAAAACATGGAACCTTTGGCCCTGTACACTTT
AGAAACCAAGTCAAAATACGCCGAAGAGTCGACATAAATCCTGGTCATGCAGACCTCAGCGCCAAGGAGG
CACAGGATGTAATTATGGAAGTTGTTTTCCCTAATGAAGTGGGAGCCAGAATACTGACATCAGAATCGCA
ATTAACGATAACCAAGGAGAAAAAAGAAGAACTCCAGGATTGCAAAATTTCCCCTTTGATGGTTGCATAC
ATGTTAGAGAGGGAACTTGTCCGCAAAACAAGATTTCTCCCGGTTGCAGGTGGAACAAGCAGTGTGTACA
TTGAAGTTTTGCATTTAACACAGGGGACATGCTGGGAGCAGATGTACACTCCAGGTGGGGAAGTGAGGAA
TGATGATGTTGATCAAAGCCTAATTATTGCTGCTAGGAACATAGTGAGAAGAGCTGCAGTATCAGCAGAT
CCACTAGCATCTTTATTAGAAATGTGCCATAGCACCCAGATTGGTGGAACAAGGATGGTGGATATTCTCA
GGCAAAATCCAACAGAAGAACAAGCTGTGGGCATATGCAAAGCAGCAATGGGGCTGAGAATCAGTTCATC
CTTCAGTTTTGGCGGATTCACATTTAAGAGAACAAGTGGATCATCAGTCAAAAGGGAGGAAGAAGTGCTC
ACGGGCAATCTGCAAACATTGAAGCTAACTGTGCATGAGGGATATGAAGAATTCACAATGGTTGGGAAAA
GGGCAACAGCTATACTCAAAAAAGCAACCAGGAGATTGATTCAACTAATAGTGAGTGGAAGAGACGAACA
GTCAATAGTCGAAGCAATAGTTGTAGCAATGGTATTCTCACAAGAAGATTGCATGATAAAAGCAGTTAGA
GGTGATCTGAATTTCGTTAATAGAGCAAATCAGCGGTTGAATCCCATGCATCAACTTTTGAGACATTTTC
AGAAGGATGCTAAAGTACTTTTCTTAAATTGGGGAATTGAACATATTGACAATGTGATGGGAATGATTGG
GATATTACCTGATATGACTCCAAGTACCGAGATGTCAATGAGAGGAGTGAGAGTCAGCAAAATGGGTGTA
GATGAATACTCCAATGCTGAAAGGGTAGTGGTGAGCATTGACCGTTTTTTGAGAGTCCGGGACCAAAGAG
GAAATGTACTACTGTCTCCAGAGGAAGTCAGTGAAACACAAGGAACAGAGAAACTGACAATAACTTACTC
TTCATCATTGATGTGGGAGATCAATGGCCCTGAGTCAGTTTTGATCAATACCTATCAGTGGATCATCAGA
AACTGGGAGACTGTTAAAATTCAGTGGTCTCAGAACCCCACGATGCTATACAATAAAATGGAATTTGAAC
CATTTCAATCTCTAGTCCCCAAGGCCATTAGAGGCCAATACAGTGGGTTTGTTAGAACTCTATTTCAACA
AATGAGGGATGTGCTCGGGACCTTTGATACAACTCAGATAATAAAACTTCTTCCCTTTGCAGCCGCTCCA
CCAAAGCAAAGTAGAATGCAATTCTCGTCATTAACTGTGAATGTGAGGGGATCAGGAATGAGAATACTTG
TGAGGGGTAATTCTCCAGTATTCAATTACAACAAGACCACTAAGAGACTCACAATCCTTGGAAAGGATGC
TGGCACTTTAACTGAAGACCCAGATGAAGGCACAGCTGGAGTGGAATCTGCTGTTTTAAGGGGATTCCTC
ATTCTAGGCAAAGAAGATAGAAGATATGGACCAGCATTAAGCATCAATGAATTGAGCAACCTTGCGAAAG
GGGAAAAAGCTAATGTGCTAATTGGGCAAGGGGATGTAGTGTTGGTAATGAAACGAAAACGGGACTCTAG
CATACTTACTGACAGCCAGACAGCGACCAAAAGAATTCGGATGGCCATCAATTAATTTCGAATAATTTAA
AAACA
>gi|157281293|gb|CY025228.1| Influenza A virus (A/Michigan/UR06-0015/2006(H1N1)) segment 1, complete sequence
ATGGAAAGAATAAAAGAGCTAAGGAATCTGATGTCACAATCTCGCACTCGCGAGATACTTACCAAAACTA
CTGTAGACCACATGGCCATAATCAAGAAATACACATCAGGAAGACAGGAGAAAAACCCATCACTCAGGAT
GAAATGGATGATGGCAATGAAATACCCAATTACAGCTGATAAAAGGATAACGGAAATGATTCCTGAAAGA
AATGAGCAAGGACAAACACTATGGAGTAAAGTGAACGATGCCGGATCAGACCGAGTGATGATATCACCCC
TGGCTGTGACATGGTGGAACAGAAATGGACCAGTGGCAAATACTATTCACTATCCAAAAATCTACAAAAC

TTACTTTGAAAAGGTTGAAAGGTTAAAACATGGAACCTTTGGTCCTGTACACTTTAGAAACCAAGTCAAA
ATACGCCGAAGAGTCGACATAAATCCTGGTCATGCAGACCTCAGCGCCAAGGAGGCACAGGATGTAATCA
TGGAAGTTGTTTTCCCTAATGAAGTGGGAGCCAGAATACTGACATCGGAATCGCAATTAACGATAACCAA
GGAGAAAAAAGAAGAACTCCAGAATTGCAAAATTTCCCCTTTGATGGTTGCATACATGTTAGAGAGGGAA
CTTGTCCGCAAAACAAGATTTCTCCCGGTTGCAGGTGGAACAAGCAGTGTGTACATTGAAGTTTTGCATT
TAACACAGGGGACATGCTGGGAGCAGATGTACACTCCAGGTGGGGAGGTGAGGAATGATGATGTTGATCA
AAGCCTAATTATTGCTGCTAGGAACATAGTGAGAAGAGCTGCAGTATCAGCAGATCCACTAGCATCTTTA
TTAGAAATGTGCCATAGCACACAGATTGGTGGAACAAGGATGGTGGATATTCTCAGGCAAAATCCAACAG
AAGAACAAGCTGTGGGCATATGCAAAGCAGCAATGGGGCTGAGAATCAGTTCATCCTTCAGTTTTGGCGG
ATTCACATTTAAGAGAACAAGTGGATCATCAGTCAAAAGGGAGGAAGAAGTGCTCACGGGCAATCTGCAA
ACATTGAAGCTAACTGTGCATGAGGGATATGAAGAATTCACAATGGTTGGGAAAAGGGCAACAGCTATAC
TCAAAAAAGCAACCAGGAGATTGATTCAACTAATAGTGAGTGGAAGAGACGAACAGTCAATAGTCGAAGC
AATAGTTGTAGCAATGGTATTCTCACAAGAAGATTGCATGGTAAAAGCAGTTAGAGGTGATCTGAATTTC
GTTAATAGAGCAAATCAGCGGTTGAATCCCATGCATCAACTTTTGAGACATTTTCAGAAGGATGCTAAAG
TACTTTTCTTAAATTGGGGAATTGAACATATTGACAATGTGATGGGAATGATTGGGATATTACCTGATAT
GACTCCAAGTGCCGAGATGTCAATGAGAGGAGTGAGAGTCAGCAAAATGGGTGTAGATGAATACTCCAAT
GCTGAAAGGGTAGTGGTGAGCATTGACCGTTTTTTGAGAGTCCGGGACCAAAGAGGAAATGTACTACTGT
CTCCAGAGGAAGTCAGTGAAACACAAGGAACAGAGAAACTGACAATAACTTACTCTTCCTCATTGATGTG
GGAGATTAATGGCCCTGAGTCAGTGTTGATCAATACCTATCAGTGGATCATCAGAAACTGGGAGACTGTT
AAAATTCAGTGGTCTCAGAACCCTACGATGCTATACAATAAAATGGAATTTGAACCATTTCAATCTCTAG
TCCCCAAGGCCATTAGAGGCCAATACAGTGGGTTTGTTAGAACTCTATTTCAACAAATGAGGGATGTGCT
CGGGACCTTTGACACAACTCAGATAATAAAACTTCTTCCCTTTGCAGCCGCTCCACCAAAGCAAAGTAGA
ATGCAATTCTCGTCGTTAACTGTGAATGTGAGGGGATCAGGAATGAGAATACTTGTGAGGGGTAATTCTC
CAGTATTCAATTACAACAAGACCACTAAGAGACTCACAATCCTTGGAAAGGATGCTGGCACTTTAACTGA
AGACCCAGATGAAGGCACAGCTGGAGTGGAATCTGCTGTTTTAAGGGGATTCCTCATTCTAGGTAAAGAA
GATAGAAGATATGGGCCAGCATTAAGCATCAATGAATTGAGCAACCTTGCGAAAGGGGAAAAAGCTAATG
TGCTAATTGGGCAAGGGGATGTAGTGTTGGTAATGAAACGAAAACGGGACTCTAGCATACTTACTGACAG
CCAGACAGCGACCAAAAGAATTCGGATGGCCATCAATTAATTTCGAATAATTTA

>gi|218875185|gb|CY036926.1| Influenza A virus (A/NYMC X-163A(NYMC X-157-St. Petersburg/8/2006)(H1N1)) segment 1, complete sequence

ATATGGAAAGAATAAAAGAACTACGAAATCTAATGTCGCAGTCTCGCACCCGCGAGATACTCACAAAAAC
CACCGTGGACCATATGGCCATAATCAAGAAGTACACATCAGGAAGACAGGAGAAGAACCCAGCACTTAGG
ATGAAATGGATGATGGCAATGAAATATCCAATTACAGCAGACAAGAGGATAACGGAAATGATTCCTGAGA
GAAATGAGCAAGGACAAACTTTATGGAGTAAAATGAATGATGCCGGATCAGACCGAGTGATGGTATCACC
TCTGGCTGTGACATGGTGGAATAGGAATGGACCAATAACAAATACAGTTCATTATCCAAAAATCTACAAA
ACTTATTTTGAAAGAGTCGAAAGGCTAAAGCATGGAACCTTTGGCCCTGTCCATTTTAGAAACCAAGTCA
AAATACGTCGGAGAGTTGACATAAATCCTGGTCATGCAGATCTCAGTGCCAAGGAGGCACAGGATGTAAT
CATGGAAGTTGTTTTCCCTAACGAAGTGGGAGCCAGGATACTAACATCGGAATCGCAACTAACGATAACC
AAAGAGAAGAAAGAAGAACTCCAGGATTGCAAAATTTCTCCTTTGATGGTTGCATACATGTTGGAGAGAG
AACTGGTCCGCAAAACGAGATTCCTCCCAGTGGCTGGTGGAACAAGCAGTGTGTACATTGAAGTGTTGCA
TTTGACTCAAGGAACATGCTGGGAACAGATGTATACTCCAGGAGGGGAAGTGAGGAATGATGATGTTGAT
CAAAGCTTGATTATTGCTGCTAGGAACATAGTGAGAAGAGCTGCAGTATCAGCAGATCCACTAGCATCTT
TATTGGAGATGTGCCACAGCACACAGATTGGTGGAATTAGGATGGTAGACATCCTTAGGCAGAACCCAAC
AGAAGAGCAAGCCGTGGATATATGCAAGGCTGCAATGGGACTGAGAATTAGCTCATCCTTCAGTTTTGGT

GGATTCACATTTAAGAGAACAAGCGGATCATCAGTCAAGAGAGAGGAAGAGGTGCTTACGGGCAATCTTC
AAACATTGAAGATAAGAGTGCATGAGGGATATGAAGAGTTCACAATGGTTGGGAGAAGAGCAACAGCCAT
ACTCAGAAAAGCAACCAGGAGATTGATTCAGCTGATAGTGAGTGGGAGAGACGAACAGTCGATTGCCGAA
GCAATAATTGTGGCCATGGTATTTTCACAAGAGGATTGTATGATAAAAGCAGTCAGAGGTGATCTGAATT
TCGTCAATAGGGCGAATCAGCGATTGAATCCTATGCATCAACTTTTAAGACATTTTCAGAAGGATGCGAA
AGTGCTTTTTCAAAATTGGGGAGTTGAACCTATCGACAATGTGATGGGAATGATTGGGATATTGCCCGAC
ATGACTCCAAGCATCGAGATGTCAATGAGAGGAGTGAGAATCAGCAAAATGGGTGTAGATGAGTACTCCA
GCACGGAGAGGGTAGTGGTGAGCATTGACCGTTTTTTGAGAATCCGGGACCAACGAGGAAATGTACTACT
GTCTCCCGAGGAGGTCAGTGAAACACAGGGAACAGAGAAACTGACAATAACTTACTCATCGTCAATGATG
TGGGAGATTAATGGTCCTGAATCAGTGTTGGTCAATACCTATCAATGGATCATCAGAAACTGGGAAACTG
TTAAAATTCAGTGGTCCCAGAACCCTACAATGCTATACAATAAAATGGAATTTGAACCATTTCAGTCTTT
AGTACCTAAGGCCATTAGAGGCCAATACAGTGGGTTTGTAAGAACTCTGTTCCAACAAATGAGGGATGTG
CTTGGGACATTTGATACCGCACAGATAATAAAACTTCTTCCCTTCGCAGCCGCTCCACCAAAGCAAAGTA
GAATGCAGTTCTCCTCATTTACTGTGAATGTGAGGGGATCAGGAATGAGAATACTTGTAAGGGGCAATTC
TCCTGTATTCAACTATAACAAGGCCACGAAGAGACTCACAGTTCTCGGAAAGGATGCTGGCACTTTAACT
GAAGACCCAGATGAAGGCACAGCTGGAGTGGAGTCCGCTGTTCTGAGGGGATTCCTCATTCTGGGCAAAG
AAGACAAGAGATATGGGCCAGCACTAAGCATCAATGAACTGAGCAACCTTGCGAAAGGAGAGAAGGCTAA
TGTGCTAATTGGGCAAGGAGACGTGGTGTTGGTAATGAAACGGAAACGGGACTCTAGCATACTTACTGAC
AGCCAGACAGCGACCAAAAGAATTCGGATGGCCATCAATTAGTGTCGAATAG
>gi|208344100|gb|CY035133.1| Influenza A virus (A/St. Petersburg/8/2006(H1N1)) segment 1, complete sequence
TTATATTCAATATGGAAAGAATAAAAGAGCTAAGGAATCTGATGTCACAATCTCGCACTCGCGAGATACT
TACCAAAACTACTGTAGACCACATGGCCATAATCAAGAAATACACATCAGGAAGACAGGAGAAAAACCCA
TCACTTAGGATGAAATGGATGATGGCAATGAAATACCCAATTACAGCTGATAAAAGGATAACGGAAATGA
TTCCTGAAAGAAATGAGCAAGGACAGACATTATGGAGTAAGGTGAATGATGCCGGATCAGACCGAGTGAT
GATATCACCCCTGGCTGTGACATGGTGGAACAGAAATGGACCAGTGGCAAGTACTATTCACTATCCAAAA
ATCTACAAAACTTACTTTGAAAAGGTTGAAAGGTTAAAACATGGAACCTTTGGCCCTGTACACTTTAGAA
ACCAAGTCAAAATACGCCGAAGAGTCGACATAAATCCTGGTCATGCAGACCTCAGCGCCAAGGAGGCACA
GGATGTAATTATGGAAGTTGTTTTCCCTAACGAAGTAGGAGCCAGAATACTAACATCAGAATCGCAATTA
ACGATAACCAAGGAGAAAAAAGAAGAACTCCAGAATTGCAAAATTTCCCCTTTGATGGTTGCATACATGT
TAGAGAGGGAACTTGTCCGCAAAACAAGATTTCTCCCGGTTGCAGGTGGAACAAGCAGTGTGTACATTGA
AGTTTTGCATTTAACACAAGGGACATGCTGGGAGCAGATGTACACTCCAGGTGGGGAGGTGAGAAATGAT
GATGTTGATCAAAGTCTGATTATTGCTGCTAGGAACATAGTGAGAAGAGCTGCAGTATCAGCAGATCCAC
TAGCATCTTTATTAGAAATGTGCCATAGCACACAGATTGGTGGAACAAGGATGGTGGATATTCTCAGGCA
AAATCCAACAGAAGAACAAGCTGTGGACATATGCAAAGCAGCAATGGGGCTGAGGATCAGTTCATCCTTC
AGTTTTGGCGGATTCACATTTAAGAGAACAAGTGGATCATCAGTCAAAAGGGAGGAAGAAGTGCTCACGG
GCAATTTGCAAACATTGAAGCTAACTGTGCATGAGGGATATGAAGAGTTCACAATGGTTGGGAAAAGGGC
AACAGCTATATTCAGAAAAGCAACCAGGAGATTGATTCAACTGATAGTGAGTGGAAGAGACGAACAGTCA
ATAGTCGAAGCAATAGTTGTAGCAATGGTATTCTCACAAGAAGATTGCATGGTAAAAGCAGTTAGAGGTG
ATCTGAATTTCGTTAATAGAGCGAATCAGCGGTTGAATCCCATGCATCAACTTTTGAGACATTTTCAGAA
GGATGCTAGAGTACTTTTCTTAAATTGGGGAATTGAACCTATTGACAATGTGATGGGAATGATTGGGATA
TTACCTGATATGACTCCAAGCACCGAGATGTCAATGAGAGGAGTGAGAGTCAGCAAAATGGGTGTAGATG
AATACTCCAATGCTGAAAGAGTAGTGGTGAGCATTGACCGTTTTTTGAGAGTCCGGGACCAAAGAGGGAA
TGTACTACTGTCTCCAGAGGAAGTCAGTGAAACACAAGGGACAGAGAAACTGACAATAACATACTCTTCA

TCAATGATGTGGGAGATTAATGGCCCTGAGTCAGTCTTGATCAATACCTATCAGTGGATCATCAGAAATT
GGGAGACTGTTAAAATTCAGTGGTCTCAGAATCCTACGATGCTATATAATAAAATGGAATTTGAACCATT
TCAGTCTCTAGTCCCCAAGGCCATTAGAGGCCAATACAGTGGGTTTGTCAGAACTCTATTTCAACAAATG
AGAGATGTGCTTGGGACCTTTGACACAATTCAGATAATAAAACTTCTTCCCTTTGCAGCCGCTCCTCCAA
AGCAAAGCAGAATGCAATTCTCTTCATTAACTGTGAATGTGAGGGGATCAGGAATGAGAATACTTGTAAG
GGGTAATTCTCCAGTATTTAACTACAACAAGACTACTAAGAGACTCACAGTCCTCGGAAAGGATGCTGGC
ACTTTAACTGAAGACCCAGATGAAGGCACAGCTGGAGTGGAATCTGCGGTTCTAAGGGGATTCCTCATTC
TAGGCAAAGAAGATAGAAGATATGGGCCAGCATTAAGCATCAATGAATTGAGCAACCTTGCGAAAGGGGA
AAAAGCTAATGTGCTAATTGGGCAAGGGGATGTAGTGTTGGTAATGAAACGAAAACGGGACTCTAGCATA
CTTACTGACAGCCAGACAGCGACCAAAAGAATTCGGATGGCCATCAATTAATTTCGAATAATTTAA
>gi|226954763|gb|CY038886.1| Influenza A virus (A/Taiwan/2645/2006(H1N1)) segment 1, complete sequence
TTCAATATGGAAAGAATAAAAGAGCTAAGGAATCTGATGTCACAATCTCGCACTCGCGAGATACTTACCA
AAACTACTGTAGACCACATGGCCATAATCAAGAAATACACATCAGGAAGACAGGAGAAAAACCCATCACT
TAGGATGAAATGGATGATGGCAATGAAATACCCAATTACAGCTGATAAAAGGATAACGGAAATGATTCCT
GAAAGAAATGAGCAAGGACAGACATTATGGAGTAAGGTGAATGATGCCGGATCAGACCGAGTGATGATAT
CACCCCTGGCTGTGACATGGTGGAACAGAAATGGACCAGTGGCAAGTACTATTCACTATCCAAAAATCTA
CAAAACTTACTTTGAAAAGGTTGAAAGGTTAAAACATGGAACCTTTGGCCCTGTACACTTTAGAAACCAA
GTCAAAATACGCCGAAGAGTCGACATAAATCCTGGTCATGCAGACCTCAGCGCCAAGGAGGCACAGGATG
TAATTATGGAAGTTGTTTTCCCTAATGAAGTAGGAGCCAGAATACTAACATCAGAATCGCAATTAACGAT
AACCAAGGAGAAAAAAGAAGAACTCCAGAATTGCAAAATTTCCCCTTTGATGGTTGCATACATGTTAGAG
AGGGAACTTGTCCGCAAAACAAGATTTCTCCCGGTTGCAGGTGGAACAAGCAGTGTGTACATTGAAGTTT
TGCATTTAACACAGGGGACATGTTGGGAGCAGATGTACACTCCAGGTGGGGAGGTGAGGAATGATGATGT
TGATCAAAGCCTAATTATTGCTGCTAGGAACATAGTGAGGAGAGCTGCAGTATCAGCAGATCCACTAGCA
TCTTTATTAGAAATGTGCCATAGCACACAGATTGGTGGAACAAGGATGGTGGATATTCTCAGGCAAAATC
CAACAGAAGAACAAGCTGTGGACATATGCAAAGCAGCAATGGGGCTGAGAATCAGTTCATCCTTCAGTTT
TGGCGGATTCACATTTAAGAGAACGAGTGGATCATCAGTCAAAAGGGAGGAAGAAGTGCTCACGGGCAAT
CTGCAAACATTGAAGCTAACTGTGCATGAGGGATATGAAGAGTTCACAATGGTTGGGAAAAGGGCAACAG
CTATACTCAGAAAAGCAACCAGGAGATTGATTCAACTAATAGTGAGTGGAAGAGACGAACAGTCAATAGT
CGAAGCAATAGTCGTAGCAATGGTATTCTCACAAGAAGATTGCATGGTAAAAGCAGTTAGAGGTGATCTG
AATTTCGTTAATAGAGCGAATCAGCGGTTGAATCCCATGCATCAACTTTTGAGACATTTTCAGAAGGATG
CTAGAGTACTTTTCTTAAATTGGGGAATTGAACCTATTGACAATGTGATGGGAATGATTGGGATATTACC
TGATATGACTCCAAGCACCGAGATGTCAATGAGAGGAGTGAGAGTCAGCAAAATGGGTGTAGATGAATAC
TCCAACGCTGAAAGAGTAGTGGTGAGCATTGATCGTTTTTTGAGAGTTCGGGACCAAAGAGGAAATGTAC
TACTGTCTCCAGAGGAAGTCAGTGAAACACAAGGGACAGAGAAACTGACAATAACATACTCTTCATCAAT
GATGTGGGAGATTAATGGCCCTGAGTCAGTCTTGATCAATACCTATCAGTGGATCATCAGAAACTGGGAG
ACTGTTAAAATTCAGTGGTCTCAGAATCCTACGATGCTATACAATAAAATGGAATTTGAACCATTTCAGT
CTCTAGTCCCCAAGGCCATTAGAGGCCAATACAGTGGGTTTGTTAGAACTCTATTTCAACAAATGAGGGA
TGTGCTTGGGACCTTTGACACAACTCAGATAATAAAACTTCTTCCCTTTGCAGCCGCTCCTCCAAAGCAA
AGCAGAATGCAATTCTCGTCATTAACTGTGAATGTGAGGGGATCAGGAATGAGAATACTTGTAAGGGGTA
ATTCTCCAGTTTTCAACTACAACAAGACTACTAAGAGACTCACAGTCCTCGGAAAGGATGCTGGCACTTT
AACTGAAGACCCAGATGAGGGCACAGCTGGAGTGGAATCTGCGGTTCTAAGGGGATTCCTCATTCTAGGC
AAAGAAGATAGAAGATATGGGCCAGCATTAAGCATCAATGAATTGAGCAACCTTGCGAAAGGGGAAAAAG
CTAATGTGCTAATTGGGCAAGGGGATGTAGTGTTGGTAATGAAACGAAAACGGGACTCTAGCATACTTAC

TGACAGCCAGACAGCGACCAAAAGAATTCGGATGGCCATCAATTAATTTCGAATAATTTAAA
>gi|256385528|gb|CY044356.1| Influenza A virus (A/South Korea/AF10/2008(H1N1)) segment 1, complete sequence
ATGGAAAGAATAAAAGAGCTAAGGAATCTGATGTCACAATCTCGCACTCGCGAGATACTTACCAAAACTA
CTGTAGACCACATGGCCATAATCAAGAAATACACATCAGGAAGACAGGAGAAAAACCCATCACTTAGGAT
GAAATGGATGATGGCAATGAAATACCCAATTACAGCTGATAAAAGGATAACGGAAATGATTCCTGAAAGA
AATGAGCAAGGACAGACATTATGGAGTAAGGTGAATGATGCCGGATCAGACCGAGTGATGATATCACCCC
TGGCTGTGACATGGTGGAACAGAAATGGACCAGTGGCAAGTACTATTCACTATCCAAAAATCTACAAAAC
TTACTTTGAAAAGGTTGAAAGGTTAAAACATGGAACCTTTGGCCCTGTACACTTTAGAAACCAAGTCAAA
ATACGCCGAAGAGTCGACATAAATCCTGGTCATGCAGACCTCAGCGCCAAGGAGGCACAGGATGTGATTA
TGGAAGTTGTTTTCCCTAATGAAGTAGGAGCCAGAATACTAACATCAGAATCGCAATTAACGATAACTAA
GGAGAAAAAAGAAGAACTCCAGAATTGCAAAATTTCCCCTTTGATGGTTGCATACATGTTAGAGAGGGAA
CTTGTCCGCAAAACAAGATTTCTCCCGGTTGCAGGTGGAACAAGCAGTGTGTACATTGAAGTTTTGCATT
TAACACAAGGGACATGCTGGGAGCAGATGTACACTCCGGGTGGGGAGGTGAGGAATGATGATGTTGATCA
AAGCTTAATTATTGCTGCTAGGAACATAGTGAGAAGAGCTGCAGTATCAGCAGATCCACTAGCATCTTTA
TTAGAAATGTGCCATAGCACACAGATTGGTGGAACAAGGATGGTGGATATTCTCAGGCAAAATCCAACAG
AAGAACAAGCTGTGGACATATGCAAAGCAGCAATGGGGCTGAGAATCAGTTCATCCTTCAGTTTTGGCGG
ATTCACATTTAAGAGAACAAGTGGATCATCAGTCAAAAGGGAAGAAGAAGTGCTCACAGGCAATTTGCAA
ACATTGAAGCTAACTGTGCATGAGGGATATGAAGAGTTCACAATGGTTGGGAAAAGGGCAACAGCTATAT
TCAGAAAAGCAACCAGAAGATTGATTCAACTGATAGTGAGTGGAAGAGACGAACAGTCAATAGTCGAAGC
AATAGTTGTAGCAATGGTATTCTCACAAGAAGATTGCATGGTAAAAGCAGTTAGAGGTGATCTGAATTTC
GTTAATAGAGCGAATCAGCGGTTGAATCCCATGCATCAACTTTTGAGACATTTTCAGAAGGATGCTAGAG
TACTTTTCTTAAATTGGGGAATTGAACCTATTGATAATGTGATGGGAATGATTGGGATATTACCTGATAT
GACTCCAAGCACTGAGATGTCAATGAGAGGAGTGAGAGTCAGCAAAATGGGTGTAGATGAATACTCCAAT
GCTGAAAGAGTAGTGGTGAGCATTGACCGTTTTTTGAGAGTCCGGGACCAAAGAGGAAATGTACTACTGT
CTCCAGAGGAAGTCAGTGAAACACAAGGGACAGAGAAACTGACAATAACATACTCTTCATCAATGATGTG
GGAGATTAATGGCCCTGAGTCAGTCTTGATCAATACCTATCAGTGGATCATCAGAAATTGGGAAACTGTT
AAAATTCAGTGGTCTCAGAATCCTACGATGCTATATAATAAAATGGAATTTGAACCATTTCAGTCTCTAG
TCCCCAAGGCCATTAGAGGCCAATACAGTGGGTTTGTTAGAACTCTATTTCAACAAATGAGGGATGTGCT
TGGGACCTTTGACACAATTCAGATAATAAAACTTCTTCCCTTTGCAGCCGCTCCTCCAAAGCAAAGCAGA
ATGCAATTCTCTTCATTAACTGTGAATGTGAGGGGATCAGGAATGAGAATACTTGTAAGGGGTAATTCTC
CAGTATTTAACTACAACAAGACTACTAAGAGACTCACAGTCCTCGGAAAGGATGCTGGCACTTTAACTGA
AGACCCAGATGAAGGCACAGCTGGAGTGGAATCTGCGGTTCTAAGAGGATTCCTCATTCTAGGCAAAGAA
GATAGAAGATATGGGCCAGCATTAAGCATCAATGAATTGAGCAACCTTGCGAAAGGGGAAAAAGCTAATG
TGCTAATTGGGCAAGGGGATGTAGTGTTGGTAATGAAACGAAAACGGGACTCTAGCATACTTACTGACAG
CCAGACAGCGACCAAAAGAATTCGGATGGCCATCAATTAA
>gi|163964733|gb|CY028466.1| Influenza A virus (A/California/UR06-0442/2007(H1N1)) segment 1, complete sequence
TATGGAAAGAATAAAAGAGCTAAGGAATCTGATGTCACAATCTCGCACTCGCGAGATACTTACCAAAACT
ACTGTAGACCACATGGCCATAATCAAGAAATACACATCAGGAAGACAGGAGAAAAACCCATCACTCAGGA
TGAAATGGATGATGGCAATGAAATACCCAATTACAGCTGATAAAAGGATAACGGAAATGATTCCTGAAAG
AAATGAGCAAGGACAAACACTATGGAGTAAAGTGAACGATGCCGGATCAGACCGAGTGATGATATCACCC
CTGGCTGTGACATGGTGGAACAGAAATGGACCAGTGGCAAATACTATTCACTATCCAAAAATCTACAAAA
CTTACTTTGAAAAGGTTGAAAGGTTAAAACATGGAACCTTTGGTCCTGTACACTTTAGAAACCAAGTCAA

AATACGCCGAAGAGTCGACATAAATCCTGGTCATGCAGACCTCAGCGCCAAGGAGGCACAGGATGTAATC
ATGGAAGTTGTTTTCCCTAATGAAGTGGGAGCCAGAATACTGACATCGGAATCGCAATTAACGATAACCA
AGGAGAAAAAAGAAGAACTCCAGAATTGCAAAATTTCCCCTTTGATGGTTGCATACATGTTAGAGAGGGA
ACTTGTCCGCAAAACAAGATTTCTCCCGGTTGCAGGTGGAACAAGCAGTGTGTACATTGAAGTTTTGCAT
TTAACACAGGGGACATGCTGGGAGCAGATGTACACTCCAGGTGGGGAGGTGAGGAATGATGATGTTGATC
AAAGCCTAATTATTGCTGCTAGGAACATAGTGAGAAGAGCTGCAGTATCAGCAGATCCACTAGCATCTTT
ATTAGAAATGTGCCATAGCACACAGATTGGTGGAACAAGGATGGTGGATATTCTCAGGCAAAATCCAACA
GAAGAACAAGCTGTGGGCATATGCAAAGCAGCAATGGGGCTGAGAATCAGTTCATCCTTCAGTTTTGGCG
GATTCACATTTAAGAGAACAAGTGGATCATCAGTCAAAAGGGAGGAAGAAGTGCTCACGGGCAATCTGCA
AACATTGAAGCTAACTGTGCATGAGGGATATGAAGAATTCACAATGGTTGGGAAAAGGGCAACAGCTATA
CTCAAAAAAGCAACCAGGAGATTGATTCAACTAATAGTGAGTGGAAGAGACGAACAGTCAATAGTCGAAG
CAATAGTTGTAGCAATGGTATTCTCACAAGAAGATTGCATGGTAAAAGCAGTTAGAGGTGATCTGAATTT
CGTTAATAGAGCAAATCAGCGGTTGAATCCCATGCATCAACTTTTGAGACATTTTCAGAAGGATGCTAAA
GTACTTTTCTTAAATTGGGGAATTGAACATATTGACAATGTGATGGGAATGATTGGGATATTACCTGATA
TGACTCCAAGTACCGAGATGTCAATGAGAGGAGTGAGAGTCAGCAAAATGGGTGTAGATGAATACTCCAA
TGCTGAAAGGGTAGTGGTGAGCATTGACCGTTTTTTGAGAGTCCGGGACCAAAGAGGAAATGTACTACTG
TCTCCAGAGGAAGTCAGTGAAACACAAGGAACAGAGAAACTGACAATAACTTACTCTTCCTCATTGATGT
GGGAGATTAATGGCCCTGAGTCAGTGTTGATCAATACCTATCAGTGGATCATCAGAAACTGGGAGACTGT
TAAAATTCAGTGGTCTCAGAACCCTACGATGCTATACAATAAAATGGAATTTGAACCATTTCAATCTCTA
GTCCCCAAGGCCATTAGAGGCCAATACAGTGGGTTTGTTAGAACTCTATTTCAACAAATGAGGGATGTGC
TCGGGACCTTTGACACAACTCAGATAATAAAACTTCTTCCCTTTGCAGCCGCTCCACCAAAGCAAAGTAG
AATGCAATTCTCGTCGTTAACTGTGAATGTGAGGGGATCAGGAATGAGAATACTTGTGAGGGGTAATTCT
CCAGTATTCAATTACAACAAGACCACTAAGAGACTCACAATCCTTGGAAAGGATGCTGGCACTTTAACTG
AAGACCCAGATGAAGGCACAGCTGGAGTGGAATCTGCTGTTTTAAGGGGATTCCTCATTCTAGGTAAAGA
AGATAGAAGATATGGACCAGCATTAAGCATCAATGAATTGAGCAACCTTGCGAAAGGGGAAAAAGCTAAT
GTGCTAATTGGGCAAGGGGATGTAGTGTTGGTAATGAAACGAAAACGGGACTCTAGCATACTTACTGACA
GCCAGACAGCGACCAAAAGAATTCGGATGGCCATCAATTAATTTCGAATAATTTAAAAA
>gi|157281617|gb|CY025364.1| Influenza A virus (A/Kentucky/UR06-0363/2007(H1N1)) segment 1, complete sequence
AATATGGAAAGAATAAAAGAGCTAAGGAATCTGATGTCACAATCTCGCACTCGCGAGATACTTACCAGAA
CTACTGTAGACCACATGGCCATAATCAAGAAATACACATCAGGAAGACAGGAGAAAAACCCATCACTTAG
GATGAAATGGATGATGGCAATGAAATACCCAATTACAGCTGATAAAAGGATAACGGAAATGATTCCTGAA
AGAAATGAGCAAGGACAAACACTATGGAGTAAAGTGAACGATGCCGGATCAGACCGAGTGATGATATCAC
CCCTGGCTGTGACATGGTGGAACAGAAATGGACCAGTGGCAAATACTATTCACTACCCAAAAATCTACAA
AACTTACTTTGAAAAGGTTGAAAGGTTAAAACATGGAACCTTTGGCCCTGTACACTTTAGAAACCAAGTC
AAAATACGCCGAAGAGTCGACATAAATCCTGGTCATGCAGACCTCAGCGCCAAGGAGGCACAGGATGTAA
TTATGGAAGTTGTTTTCCCTAATGAAGTGGGAGCCAGAATACTGACATCGGAATCGCAATTAACGATAAC
CAAGGAGAAAAAAGAAGAACTCCAGAATTGCAAAATTTCCCCTTTGATGGTTGCATACATGTTAGAGAGG
GAACTTGTCCGCAAAACAAGATTTCTCCCGGTTGCAGGTGGAACAAGCAGTGTGTACATTGAAGTTTTGC
ATTTAACACAGGGAACATGCTGGGAGCAGATGTACACTCCAGGTGGGGAGGTGAGGAATGATGATGTTGA
TCAAAGCCTAATTATTGCTGCTAGGAACATAGTGAGAAGAGCTGCAGTATCAGCAGATCCACTAGCATCT
TTATTAGAAATGTGCCATAGCACACAGATTGGTGGAACAAGGATGGTGGATATTCTCAGGCAAAATCCAA
CAGAAGAACAAGCTGTGGGCATATGCAAAGCAGCAATGGGGCTGAGAATCAGTTCATCCTTCAGTTTTGG
CGGATTCACATTTAAGAGAACAAGTGGATCATCAGTCAAAAGGGAGGAAGAAGTGCTCACGGGCAATCTG

CAAGCATTGAAGCTAACTGTGCATGAGGGATATGAAGAATTCACAATGGTTGGGAAAAGGGCAACAGCTA
TACTCAAAAAAGCAACCAGGAGATTGATTCAACTAATAGTGAGTGGAAGAGACGAACAGTCAATAGTCGA
AGCAATAGTTGTAGCAATGGTATTCTCACAAGAAGATTGCATGGTAAAAGCAGTTAGAGGTGATCTGAAT
TTCGTTAATAGAGCAAATCAGCGGTTGAATCCCATGCACCAACTTTTGAGACATTTTCAGAAGGATGCTA
AAGTACTTTTCTTAAATTGGGGAATTGAACATATTGACAATGTGATGGGAATGATTGGGATATTACCTGA
TATGACTCCAAGTACCGAGATGTCAATGAGAGGAGTGAGAGTCAGCAAAATGGGTGTAGATGAATACTCC
AATGCTGAAAGGGTAGTGGTGAGCATTGACCGTTTTCTGAGAGTCCGGGACCAAAGAGGAAATGTACTAC
TGTCTCCAGAGGAAGTCAGTGAAACACAAGGAACAGAGAAACTGACAATAACTTACTCTTCATCATTGAT
GTGGGAGATTAATGGCCCTGAGTCAGTGTTGATCAATACCTATCAGTGGATCATCAGAAACTGGGAGACT
GTTAAAATTCAGTGGTCTCAGAACCCTACGATGCTATACAATAAAATGGAATTTGAACCATTTCAATCTC
TAGTCCCCAAGGCCATTAGAGGCCAATACAGTGGGTTTGTTAGAACTCTATTTCAACAAATGAGGGATGT
GCTCGGGACCTTTGACACAACTCAGATAATAAAACTTCTTCCCTTTGCAGCCGCTCCACCAAAGCAAAGT
AGAATGCAATTCTCGTCGTTAACTGTGAATGTGAGGGGATCAGGAATGAGAATACTTGTGAGGGGTAATT
CTCCAGTATTCAATTACAACAAGACCACTAAGAGACTCACAATCCTTGGAAAGGATGCTGGCACTTTAAC
TGAAGACCCAGATGAAGGCACAGCTGGAGTGGAATCTGCTGTTTTAAGGGGATTCCTCATTTTAGGCAAA
GAAGATAGAAGATATGGGCCAGCATTAAGCATCAATGAATTGAGCAACCTTGCGAAAGGGGAAAAAGCTA
ATGTGCTAATTGGGCAAGGGGATGTAGTGTTGGTAATGAAACGAAAACGGGACTCTAGCATACTTACTGA
CAGCCAGACAGCGACCAAAAGAATTCGGATGGCCATCAATTAATTTCGAATAATTTAA
>gi|158957806|gb|CY027418.1| Influenza A virus (A/Alabama/UR06-0536/2007(H1N1)) segment 1, complete sequence
TTCAATATGGAAAGAATAAAAGAGCTAAGAAACCTGATGTCACAATCTCGCACTCGCGAGATACTTACCA
AAACTACTGTAGACCACATGGCCATAATCAAGAAATACACATCAGGAAGACAGGAGAAAAACCCATCACT
TAGGATGAAATGGATGATGGCAATGAAATACCCAATTACAGCTGATAAAAGGATAACGGAAATGATTCCT
GAAAGAAATGAGCAAGGACAAACACTATGGAGTAAAATGAACGATGCCGGATCAGACCGAGTGATGATAT
CACCCCTGGCTGTGACATGGTGGAACAGAAATGGACCAGTGGCAAATACTATTCACTATCCAAAAATCTA
CAAAACTTACTTTGAAAAGGTTGAAAGGTTAAAACATGGAACCTTTGGCCCTGTACACTTTAGAAACCAA
GTCAAAATACGCCGAAGAGTCGACATAAATCCTGGTCATGCAGACCTCAGCGCCAAGGAGGCACAGGATG
TAATTATGGAAGTTGTTTTCCCTAATGAAGTGGGAGCCAGAATACTGACATCAGAATCGCAATTAACGAT
AACCAAGGAGAAAAAAGAAGAACTCCAGGATTGCAAAATTTCCCCATTGATGGTTGCATACATGTTAGAG
AGGGAACTTGTCCGCAAAACAAGATTTCTCCCGGTTGCAGGTGGAACAAGCAGTGTGTACATTGAAGTTT
TGCATTTAACACAGGGGACATGCTGGGAGCAGATGTACACTCCAGGTGGGGAAGTGAGGAATGATGATGT
TGATCAAAGCCTAATTATTGCTGCTAGGAACATAGTGAGAAGAGCTGCAGTATCAGCAGATCCACTAGCA
TCTTTATTAGAAATGTGCCATAGCACACAGATTGGTGGAACAAGGATGGTGGATATTCTCAGGCAAAATC
CAACAGAAGAACAAGCTGTGGGCATATGCAAAGCAGCAATGGGGCTGAGAATCAGTTCATCCTTCAGTTT
TGGCGGATTCACATTTAAGAGAACAAGTGGATCATCAGTCAAAAGGGAGGAAGAAGTGCTCACGGGCAAT
CTGCAAACATTGAAGCTAACTGTGCATGAGGGATATGAAGAATTCACAATGGTTGGGAAAAGGGCAACAG
CTATACTCAAAAAAGCAACCAGGAGATTGATTCAACTAATAGTGAGTGGAAGAGACGAACAGTCAATAGT
CGAAGCAATAGTTGTAGCAATGGTATTCTCACAAGAAGATTGCATGATAAAAGCAGTTAGAGGTGATCTG
AATTTCGTTAATAGAGCAAATCAGCGGTTGAATCCCATGCATCAACTTTTGAGACATTTTCAGAAGGATG
CTAAAGTACTTTTCTTAAATTGGGGAATTGAACATATTGACAATGTGATGGGAATGATTGGGATATTACC
TGATATGACTCCAAGTACCGAGATGTCAATGAGAGGAGTGAGAGTCAGCAAAATGGGTGTAGATGAATAC
TCCAATGCTGAAAGGGTAGTGGTGAGCATTGACCGTTTTTTGAGAGTCCGGGACCAAAGAGGAAATGTAC
TACTGTCTCCAGAGGAAGTCAGTGAAACACAAGGAACAGAGAAACTGACAATAACTTACTCTTCATCATT
GATGTGGGAGATCAATGGCCCTGAGTCAGTTTTGATCAATACCTATCAGTGGATCATCAGAAACTGGGAG

ACTGTTAAAATTCAGTGGTCTCAGAACCCCACGATGCTATACAATAAAATGGAATTTGAACCATTTCAAT
CTCTAGTCCCCAAGGCCATTAGAGGCCAATACAGTGGGTTTGTTAGAACTCTATTTCAACAAATGAGGGA
TGTGCTCGGGACCTTTGATACAACTCAGATAATAAAACTTCTTCCCTTTGCAGCCGCTCCACCAAAGCAA
AGTAGAATGCAATTCTCGTCATTAACTGTGAATGTGAGGGGATCAGGAATGAGAATACTTGTGAGGGGTA
ATTCTCCAGTATTCAATTACAACAAGACCACTAAGAGACTCACAATCCTTGGAAAGGATGCTGGCACTTT
AACTGAAGACCCAGATGAAGGCACAGCTGGAGTGGAATCTGCCGTTTTAAGGGGATTCCTCATTCTAGGC
AAAGAAGATAGAAAATATGGACCAGCATTAAGCATCAATGAATTGAGCAACCTTGCGAAAGGGGAAAAAG
CTAATGTGCTAATTGGGCAAGGGGATGTAGTGTTGGTAATGAAACGAAAACGGGACTCTAGCATACTTAC
TGACAGCCAGACAGCGACCAAAAGAATTCGGATGGCCATCAATTAATTTCGAATAAT
>gi|237688841|gb|CY040065.1| Influenza A virus (A/Taiwan/71720/2007(H1N1)) segment 1, complete sequence
TTATATTCAATATGGAAAGAATAAAAGAGCTAAGGAATTTGATGTCACAATCTCGCACTCGCGAGATACT
TACCAAAACTACTGTAGACCACATGGCCATAATCAAGAAATACACATCAGGAAGACAGGAGAAAAACCCA
TCACTTAGGATGAAATGGATGATGGCAATGAAATACCCAATTACAGCTGATAAAAGGATAACGGAAATGA
TTCCTGAAAGAAATGAGCATGGACAGACATTATGGAGTAAGGTGAATGATGCCGGATCAGACCGAGTGAT
GGTATCACCCCTGGCTGTGACATGGTGGAACAGAAATGGACCAGTGGCAAGTACTATTCACTATCCAAAA
ATCTACAAAACTTACTTTGAAAAGGTTGAAAGGTTAAAACAAGGAACCTTTGGCCCTGTACACTTTAGAA
ACCAAGTCAAAATACGCCGAAGAGTCGACATAAATCCTGGTCATGCAGATCTCAGCGCCAAGGAGGCACA
GGATGTAATTATGGAAGTTGTTTTCCCTAATGAAGTAGGAGCCAGAATACTAACATCAGAATCGCAATTA
ACGATAACCAAGGAGAAAAAAGAAGAACTCCAGAATTGCAAAATTTCCCCTTTGATGGTTGCATACATGT
TAGAGAGGGAACTTGTCCGCAAAACAAGATTTCTCCCGGTTGCAGGTGGAACAAGCAGTGTGTACATTGA
AGTTTTGCATTTAACACAGGGGACATGCTGGGAGCAGATGTACACTCCAGGTGGGGAGGTGAGGAATGAT
GATGTTGATCAAAGCCTAATTATTGCTGCTAGGAACATAGTGAGAAGAGCTGCAGTATCAGCAGATCCAC
TAGCATCTTTATTAGAAATGTGCCATAGCACACAGATTGGTGGAACAAGGATGGTGGATATTCTCAGGCA
AAATCCAACAGAAGAACAAGCTGTGGACATATGCAAAGCAGCAATGGGGCTGAGAATCAGTTCATCCTTC
AGCTTTGGCGGATTCACATTTAAGAGAACAAGTGGATCATCAGTCAAAAGGGAGGAAGAAGTGCTCACGG
GCAATCTGCAAACATTGAAACTAACTGTGCATGAGGGATATGAAGAGTTCACAATGGTTGGGAAAAGGGC
AACAGCTATACTCAGAAAAGCAACCAGGAGATTGATTCAACTAATAGTGAGTGGAAGAGACGAACAATCA
ATAGTCGAAGCAATAGTTGTAGCAATGGTATTCTCACAAGAAGATTGCATGGTAAAAGCAGTTAGAGGTG
ATCTGAATTTCGTTAATAGAGCGAATCAGCGGTTGAATCCCATGCATCAACTATTGAGACATTTTCAGAA
GGATGCTAAAGTACTTTTCTTAAATTGGGGAGTTGAACCTATTGACAATGTGATGGGAATGATTGGGATA
TTACCTGATATGACTCCAAGTACCGAGATGTCAATGAGAGGAGTGAGAGTCAGCAAAATGGGTGTAGATG
AATACTCCAATGCTGAAAGGGTAGTGGTAAGCATTGACCGTTTTTTGAGAGTCCGGGACCAAAGAGGAAA
TGTACTACTGTCTCCAGAGGAAGTAAGTGAAACACAAGGGACAGAGAAACTGACAATAACTTACTCTTCA
TCAATGATGTGGGAGATTAATGGCCCTGAGTCAGTCTTGATCAATACCTATCAGTGGATCATCAGAAACT
GGGAGACTGTTAAAATTCAGTGGTCTCAGAATCCTACGATGCTGTACAATAAAATGGAATTTGAACCATT
TCAGTCTCTAGTCCCCAAGGCCATTAGAGGCCAATACAGTGGGTTTGTTAGAACTCTATTCCAACAAATG
AGGGATGTGCTTGGGACTTTTGACACAACTCAGATAATAAAACTTCTTCCCTTTGCAGCCGCTCCTCCAA
AGCAAAGCAGAATGCAATTCTCGTCATTAACTGTGAATGTGAGGGGATCAGGAATGAGAATACTTGTGAG
GGGTAATTCTCCAGTATTCAACTACAACAAGACTACCAAGAGACTCACAGTCCTCGGAAAGGATGCTGGC
ACTTTAACTGAAGACCCAGATGAAGGCACAGCTGGAGTGGAATCTGCGGTTCTAAGGGGATTCCTCATTT
TAGGCAAAGAAGATAGAAGATATGGGCCAGCATTAAGCATCAATGAATTGAGCAACCTTGCGAAAGGGGA
AAAAGCTAATGTGCTAATTGGGCAAGGGGATGTAGTGTTGGTAATGAAACGAAAACGGGACTCTAGCATA
CTTACTGACAGCCAGACAGCGACCAAAAGAATTCGGATGGCCATCAATTAATTTCGAATAATTTAAAAAC

A
>gi|237689299|gb|CY040257.1| Influenza A virus (A/Managua/3153.01/2008(H1N1)) segment 1, complete sequence
TTATATTCAATATGGAAAGAATAAAAGAGCTAAGGAATTTGATGTCACAATCTCGCACTCGCGAGATACT
TACCAAAACTACTGTAGACCACATGGCCATAATCAAGAAATACACATCAGGAAGACAGGAGAAAAACCCA
TCACTTAGGATGAAATGGATGATGGCAATGAAATACCCAATTACAGCTGATAAAAGGATAACGGAAATGA
TTCCTGAAAGAAATGAACATGGACAGACATTATGGAGTAAGGTGAATGATGCCGGATCAGACCGAGTGAT
GGTATCACCCCTGGCTGTGACATGGTGGAACAGAAATGGACCAGTGGCAAGTACTATTCACTATCCAAAA
ATCTACAAAACTTACTTTGAAAAGGTTGAAAGGTTAAAACAAGGAACCTTTGGCCCTGTACACTTTAGAA
ACCAAGTCAAAATACGCCGAAGAGTCGACATAAATCCTGGTCATGCAGACCTCAGCGCCATGGAGGCACA
GGATGTAATTATGGAAGTTGTTTTCCCTAATGAAGTAGGAGCCAGAATACTAACATCAGAATCGCAATTA
ACGATAACCAAGGAGAAAAAAGAAGAACTCCAGAATTGCAAAATTTCCCCTTTGATGGTTGCATACATGT
TAGAGAGGGAACTTGTCCGCAAAACAAGATTTCTCCCGGTTGCAGGTGGAACAAGCAGTGTGTACATTGA
AGTTTTGCATTTAACACAGGGGACATGCTGGGAGCAGATGTACACTCCAGGTGGAGAGGTGAGGAATGAT
GATGTTGATCAAAGCCTAATTATTGCTGCTAGGAACATAGTGAGAAGAGCTGCAGTATCAGCAGATCCAC
TAGCATCTTTATTAGAAATGTGCCATAGCACACAGATTGGTGGAACAAGGATGGTGGATATTCTCAGGCA
AAATCCAACAGAAGAACAAGCTGTGGACATATGCAAAGCAGCAATGGGGCTGAGAATCAGTTCATCCTTC
AGTTTTGGCGGATTCACATTTAAGAGAACAAGTGGATCATCAGTCAAAAGGGAGGAAGAAGTGCTCACGG
GCAATCTGCAAACATTGAAACTAACCGTGCATGAGGGATATGAAGAGTTCACAATGGTTGGGAAAAGGGC
AACAGCTATACTCAGAAAAGCAACCAGGAGATTGATTCAACTAATAGTGAGTGGAAGAGACGAACAATCA
ATAGTCGAAGCAATAGTTGTAGCAATGGTATTTTCACAAGAAGATTGCATGGTAAAAGCAGTTAGAGGTG
ATCTGAATTTCGTTAATAGAGCGAATCAGCGGTTGAATCCCATGCATCAACTATTGAGACATTTTCAGAA
GGATGCTAAAGTACTTTTCTTAAATTGGGGAGTTGAACCTATTGACAATGTGATGGGAATGATTGGGATA
TTACCTGATATGACTCCAAGTACCGAGATGTCAATGAGAGGAGTGAGAGTCAGCAAAATGGGTGTAGATG
AATACTCCAATGCTGAAAGGGTAGTGGTAAGCATTGACCGTTTTTTGAGAGTCCGGGACCAAAGAGGAAA
TGTACTACTGTCTCCAGAGGAAGTAAGTGAAACACAAGGGACAGAGAAACTGACAATAACTTATTCTTCA
TCAATGATGTGGGAGATTAATGGCCCTGAGTCAGTCTTGATCAATACTTATCAGTGGATCATCAGAAACT
GGGAGACTGTTAAGATTCAGTGGTCTCAGAATCCTACGATGCTGTACAATAAAATGGAATTTGAACCATT
TCAGTCTCTAGTCCCCAAGGCCATTAGAGGCCAATACAGTGGGTTTGTTAGAACTCTATTCCAACAAATG
AGGGATGTGCTTGGGACTTTTGACACAACTCAGATAATAAAACTTCTTCCCTTTGCAGCCGCTCCTCCAA
AGCAAAGCAGAATGCAATTCTCGTCATTAACTGTGAATGTGAGGGGATCAGGAATGAGAATACTTGTGAG
GGGTAATTCTCCAGTATTCAACTACAACAAGATTACCAAGAGACTCACAGTCCTCGGAAAGGATGCTGGC
ACTTTAACTGAAGACCCAGATGAAGGCACAGCTGGAGTGGAATCTGCGGTTCTAAGGGGATTCCTCATTT
TAGGCAAAGAAGATAGAAGATATGGGCCAGCGTTAAGCATCAATGAATTGAGCAACCTTGCGAAAGGGGA
AAAAGCTAATGTGCTAATTGGGCAAGGGGATGTAGTGTTGGTAATGAAACGAAAACGGGACTCTAGCATA
CTTACTGACAGCCAGACAGCGACCAAAAGAATTCGGATGGCCATCAATTAATTTCGAATAATTTAAAAAC
A
>gi|224020946|gb|CY037334.1| Influenza A virus (A/Washington/AF06/2007(H1N1)) segment 1, complete sequence
AATATGGAAAGAATAAAAGAGCTAAGGAATTTGATGTCACAATCTCGCACTCGCGAGATACTTACCAAAA
CTACTGTAGACCACATGGCCATAATCAAGAAATACACATCAGGAAGACAGGAGAAAAACCCATCACTTAG
GATGAAATGGATGATGGCAATGAAATACCCAATTACAGCTGATAAAAGGATAACGGAAATGATTCCTGAA
AGAAATGAGCATGGACAGACATTATGGAGTAAGGTGAATGATGCCGGATCAGACCGAGTGATGGTATCAC
CCCTGGCTGTGACATGGTGGAACAGAAATGGACCAGTGGCAAGTACTATTCACTATCCAAAAATCTACAA

AACTTACTTTGAAAAGGTTGAAAGGTTAAAACAAGGAACCTTTGGCCCTGTACACTTTAGAAACCAAGTC
AAAATACGCCGAAGAGTCGACATAAATCCTGGTCATGCAGACCTCAGCGCCAAGGAGGCACAAGATGTAA
TTATGGAAGTTGTTTTCCCTAATGAAGTAGGAGCCAGAATACTAACATCAGAATCGCAATTAACGATAAC
CAAGGAGAAAAAAGAAGAACTCCAGAATTGCAAAATTTCCCCTTTGATGGTTGCATACATGTTAGAGAGG
GAACTTGTCCGCAAAACAAGATTTCTCCCGGTTGCAGGTGGAACAAGCAGTGTGTACATTGAAGTTTTGC
ATTTAACACAGGGGACATGCTGGGAGCAGATGTACACTCCAGGTGGGGAGGTGAGGAATGATGATGTTGA
TCAAAGCCTAATTATTGCTGCTAGGAACATAGTGAGAAGAGCTGCAGTATCAGCAGATCCACTAGCATCT
TTATTAGAAATGTGCCATAGCACACAGATTGGTGGAACAAGGATGGTGGATATTCTCAGGCAAAATCCAA
CAGAAGAACAAGCTGTGGACATATGCAAAGCAGCAATGGGGCTGAGAATCAGTTCATCCTTCAGTTTTGG
CGGATTCACATTTAAGAGAACAAGTGGATCATCAGTCAAAAGGGAGGAAGAAGTGCTCACGGGCAATCTG
CAAACATTGAAACTAACTGTGCATGAGGGATATGAAGAGTTCACAATGGTTGGGAAAAGGGCAACAGCTA
TACTCAGAAAAGCAACCAGGAGATTGATTCAACTAATAGTGAGTGGAAGAGACGAACAATCAATAGTCGA
AGCAATAGTTGTAGCAATGGTATTCTCACAAGAAGATTGCATGGTAAAAGCAGTTAGAGGTGATCTGAAT
TTCGTTAATAGAGCGAATCAGCGGTTGAATCCCATGCATCAACTATTGAGACATTTTCAGAAGGATGCTA
AAGTACTTTTCTTAAATTGGGGGGTTGAACCTATTGACAATGTGATGGGAATGATTGGGATATTACCTGA
TATGACTCCAAGTACCGAGATGTCAATGAGAGGAGTGAGAGTCAGCAAAATGGGTGTAGATGAATACTCC
AATGCTGAAAGGGTAGTGGTAAGCATTGACCGTTTTTTGAGAGTCCGGGACCAAAGAGGAAATGTACTAC
TGTCTCCAGAGGAAGTAAGTGAAACACAAGGGACAGAGAAACTTACAATAACTTACTCTTCATCAATGAT
GTGGGAAATTAATGGCCCTGAGTCAGTCTTGATCAATACCTATCAGTGGATCATCAGAAACTGGGAGACT
GTTAAAATTCAGTGGTCTCAGAATCCTACGATGCTGTACAATAAAATGGAATTTGAACCATTTCAGTCTC
TAGTCCCCAAGGCCATTAGAGGCCAATACAGTGGGTTTGTTAGAACTCTATTCCAACAAATGAGGGATGT
GCTTGGGACTTTTGACACAACTCAGATAATAAAACTTCTTCCCTTTGCAGCCGCTCCTCCAAAGCAAAGC
AGAATGCAATTCTCGTCATTAACTGTGAATGTGAGGGGATCAGGAATGAGAATACTTGTGAGGGGTAATT
CTCCAGTATTCAACTACAACAAGACCACCAAGAGACTCACAGTCCTCGGAAAGGATGCTGGCACTTTAAC
TGAAGACCCAGATGAAGGCACAGCTGGAGTGGAATCTGCGGTTCTAAGGGGATTCCTCATTTTAGGCAAA
GAAGATAGAAGATATGGGCCAGCATTAAGCATCAATGAATTGAGCAACCTTGCGAAAGGGGAAAAAGCTA
ATGTGCTAATTGGGCAAGGGGATGTAGTGTTGGTAATGAAACGAAAACGGGACTCTAGCATACTTACTGA
CAGCCAGACAGCGACCAAAAGAATTCGGATGGCCATCAATTAATTTCGAATAATTTA
>gi|224021251|gb|CY037342.1| Influenza A virus (A/Japan/AF07/2008(H1N1)) segment 1, complete sequence
ATAAAAGAGCTAAGGAATTTGATGTCACAATCTCGCACTCGCGAGATACTTACCAAAACTACTGTAGACC
ACATGGCCATAATCAAGAAATACACATCAGGAAGACAGGAGAAAAACCCATCACTTAGGATGAAATGGAT
GATGGCAATGAAATACCCAATTACAGCTGATAAAAGGATAACGGAAATGATTCCTGAAAGAAATGAGCAT
GGACAGACATTATGGAGTAAGGTGAATGATGCCGGATCAGACCGAGTGATGGTATCACCCCTGGCTGTGA
CATGGTGGAACAGAAATGGACCAGTGGCAAGTACTATTCACTATCCAAAAATCTACAAAACTTACTTTGA
AAAGGTTGAAAGGTTAAAACAAGGAACCTTTGGCCCTGTACACTTTAGAAACCAAGTCAAAATACGCCGA
AGAGTCGACATAAATCCTGGTCATGCAGACCTCAGCGCCAAGGAGGCACAAGATGTAATTATGGAAGTTG
TTTTCCCTAATGAAGTAGGAGCCAGAATACTAACATCAGAATCGCAATTAACGATAACCAAGGAGAAAAA
AGAAGAACTCCAGAATTGCAAAATTTCCCCTTTGATGGTTGCATACATGTTAGAGAGGGAACTTGTCCGC
AAAACAAGATTTCTCCCGGTTGCAGGTGGAACAAGCAGTGTGTACATTGAAGTTTTGCATTTAACACAGG
GGACATGCTGGGAGCAGATGTACACTCCAGGTGGGGAGGTGAGGAATGATGATGTTGATCAAAGCCTAAT
TATTGCTGCTAGGAACATAGTGAGAAGAGCTGCAGTATCAGCAGATCCACTAGCATCTTTATTAGAAATG
TGCCATAGCACACAGATTGGTGGAACAAGGATGGTGGATATTCTCAGGCAAAATCCAACAGAAGAACAAG
CTGTGGACATATGCAAAGCAGCAATGGGGCTGAGAATCAGTTCATCTTTCAGTTTTGGCGGATTCACATT

TAAGAGAACAAGTGGATCATCAGTCAAAAGGGAGGAAGAAGTGCTCACAGGCAATCTGCAAACATTGAAA
CTAACTGTGCATGAGGGATATGAAGAGTTCACAATGGTTGGGAAAAGGGCAACAGCTATACTCAGAAAAG
CAACCAGGAGATTGATTCAACTAATAGTGAGTGGAAGAGACGAACAATCAATAGTCGAAGCAATAGTTGT
AGCAATGGTATTCTCACAAGAAGATTGCATGGTAAAAGCAGTTAGAGGTGATCTGAATTTCGTTAATAGA
GCGAATCAGCGGTTGAATCCCATGCATCAACTATTGAGACATTTTCAGAAGGATGCTAAAGTACTTTTCT
TAAATTGGGGAGTTGAACCTATTGACAATGTGATGGGAATGATTGGGATATTACCTGATATGACTCCAAG
TACCGAGATGTCAATGAGAGGAGTGAGAGTCAGCAAAATGGGTGTAGATGAATACTCCAATGCTGAAAGG
GTAGTGGTAAGCATTGACCGTTTTTTGAGAGTCCGGGACCAAAGAGGAAATGTACTACTGTCTCCAGAGG
AAGTAAGTGAAACACAAGGGACAGAGAAACTGACAATAACTTACTCTTCATCAATGATGTGGGAGATTAA
TGGCCCTGAGTCAGTCTTGATCAATACCTATCAGTGGATCATCAGAAACTGGGAGACTGTTAAAATTCAG
TGGTCTCAGAATCCTACGATGCTGTACAATAAAATGGAATTTGAACCATTTCAGTCTCTAGTCCCCAAGG
CCATTAGAGGCCAATACAGTGGGTTTGTTAGAACTCTATTCCAACAAATGAGGGATGTGCTTGGGACTTT
TGACACAACTCAGATAATAAAACTTCTTCCCTTTGCAGCCGCTCCTCCAAAGCAAAGCAGAATGCAATTC
TCGTCATTAACTGTGAATGTGAGGGGATCAGGAATGAGAATACTTGTGAGGGGTAATTCTCCAGTATTCA
ACTACAACAAAACTACCAAGAGACTCACAGTCCTCGGAAAGGATGCTGGCACTTTAACTGAAGACCCAGA
TGAAGGCACAGCTGGAGTGGAATCTGCGGTTCTAAGGGGATTCCTCATTTTAGGCAAAGAAGATAGAAGA
TATGGGCCAGCATTAAGCATCAATGAATTGAGCAATCTTGCGAAAGGGGAAAAAGCTAATGTGCTAATTG
GGCAAGGGGATGTAGTGTTGGTAATGAAACGAAAACGGGACTCTAGCATACTTACTGACAGCCAGACAGC
GACCAAAAGAATTCGGATGGCCATCAATTAATTTCGAATAATTTA
>gi|212381603|gb|FJ445065.1| Influenza A virus (A/England/26/2008(H1N1)) segment 1 polymerase PB2 (PB2) gene, complete cds
ATGGAAAGAATAAAAGAGCTAAGGAATTTGATGTCACAATCTCGCACTCGCGAGATACTTACCAAAACTA
CTGTAGACCACATGGCCATAATCAAGAAATACACATCAGGAAGACAGGAGAAAAACCCATCACTTAGGAT
GAAATGGATGATGGCAATGAAATACCCAATTACTGCTGATAAAAGGATAACGGAAATGATTCCTGAAAGA
AATGAGCATGGACAGACATTATGGAGTAAGGTGAATGATGCCGGATCAGACCGAGTGATGGTATCACCCC
TGGCTGTGACATGGTGGAACAGAAATGGACCAGTGGCAAGTACTATTCACTATCCAAAAATCTACAAAAC
TTACTTTGAAAAGGTTGAAAGGTTAAAACAAGGAACCTTTGGCCCTGTACACTTTAGAAACCAAGTCAAA
ATACGCCGAAGAGTCGACATAAATCCTGGTCATGCAGACCTCAGCGCCAAGGAGGCACAGGATGTAATTA
TGGAAGTTGTTTTCCCTAATGAAGTAGGAGCCAGAATACTAACATCAGAATCGCAATTAACGATAACCAA
GGAGAAAAAAGAAGAACTCCAGAATTGCAAAATTTCCCCTTTGATGGTTGCATACATGTTAGAGAGGGAA
CTTGTCCGCAAAACAAGATTTCTCCCGGTTGCAGGTGGAACAAGCAGTGTGTACATTGAAGTTTTGCATT
TAACACAGGGGACATGCTGGGAGCAGATGTACACTCCAGGTGGGGAGGTGAGGAATGATGATGTTGATCA
AAGCCTAATTATTGCTGCTAGGAACATAGTGAGAAGAGCTGCAGTATCAGCAGATCCACTAGCATCTTTA
TTAGAAATGTGCCATAGCACACAGATTGGTGGAACAAGGATGGTGGATATTCTCAGGCAAAATCCAACAG
AAGAACAAGCTGTGGACATATGCAAAGCAGCAATGGGGCTGAGAATCAGTTCATCCTTCAGTTTTGGCGG
ATTCACATTTAAGAGAACAAGTGGATCATCAGTCAAAAGGGAGGAAGAAGTGCTCACGGGCAATCTGCAA
ACATTGAAACTAACTGTGCATGAGGGATATGAAGAGTTCACAATGGTTGGGAAAAGGGCAACAGCTATAC
TCAGAAAAGCAACCAGGAGATTGATCCAACTAATAGTGAGTGGAAGAGACGAACAATCAATAGTCGAAGC
AATAGTTGTAGCAATGGTATTCTCACAAGAAGATTGCATGATAAAAGCAGTTAGAGGTGATCTGAATTTC
GTTAATAGAGCGAATCAGCGGTTGAATCCCATGCATCAACTATTGAGACATTTTCAGAAGGATGCTAAAG
TACTTTTCTTAAATTGGGGAGTTGAATCTATTGACAATGTGATGGGAATGATTGGGATATTACCTGATAT
GACTCCAAGTACCGAGATGTCAATGAGAGGAGTGAGAGTCAGCAAAATGGGTGTAGATGAATACTCCAAT
GCTGAAAGGGTAGTGGTAAGCATTGACCGTTTTTTGAGAGTCCGGGACCAAAGAGGAAATGTACTACTGT
CTCCAGAGGAAGTAAGTGAAACACAAGGGACAGAGAAACTGACAATAACTTATTCTTCATCAATGATGTG

GGAGATTAATGGCCCTGAGTCAGTCTTGATCAATACCTATCAGTGGATCATCAGAAACTGGGAGACTGTT
AAAATTCAGTGGTCTCAGAATCCTACGATGCTGTACAATAAAATGGAATTTGAACCATTTCAGTCTCTAG
TCCCCAAGGCCATTAGAGGCCAATACAGTGGGTTTGTTAGAACTCTATTCCAACAAATGAGGGATGTGCT
TGGGACTTTTGACACAACTCAGATAATAAAACTTCTTCCCTTTGCAGCCGCTCCTCCAAAGCAAAGCAGA
ATGCAATTCTCGTCATTAACTGTGAATGTGAGGGGATCAGGAATGAGAATACTTGTGAGGGGTAATTCTC
CAGTATTCAACTACAACAAGACTACCAAGAGACTCACAGTCCTCGGAAAGGATGCTGGCACTTTAACTGA
AGACCCAGATGAAGGCACAGCTGGAGTGGAATCTGCGGTTCTAAGGGGGTTCCTCATTTTAGGCAAAGAA
GATAGAAGATATGGGCCAGCATTAAGCATCAATGAATTGAGCAACCTTGCGAAAGGGGAAAAAGCTAATG
TGCTAATTGGGCAAGGGGATGTAGTGTTGGTAATGAAACGAAAACGGGACTCTAGCATACTTACTGACAG
CCAGACAGCGACCAAAAGAATTCGGATGGCCATCAATTAA
>gi|224027221|gb|CY037686.1| Influenza A virus (A/Florida/UR07-0022/2008(H1N1)) segment 1, complete sequence
TCAATTATATTCAATATGGAAAGAATAAAAGAGCTAAGGAATTTGATGTCACAATCTCGCACTCGCGAGA
TACTTACCAAAACTACTGTAGACCACATGGCCATAATCAAGAAATACACATCAGGAAGACAGGAGAAAAA
CCCATCACTTAGGATGAAATGGATGATGGCAATGAAATACCCAATTACAGCTGATAAAAGGATAACGGAA
ATGATTCCTGAAAGAAATGAGCATGGACAGACATTATGGAGTAAGGTGAATGATGCCGGATCAGACCGAG
TGATGGTATCACCCCTGGCTGTGACATGGTGGAACAGAAATGGACCAGTGGCAAGTACTATTCACTATCC
AAAAATCTACAAAACTTACTTTGAAAAGGTTGAAAGGTTAAAACAAGGAACCTTTGGCCCTGTACACTTT
AGAAACCAAGTCAAAATACGCCGAAGAGTCGACATAAATCCTGGTCATGCAGACCTCAGCGCCAAGGAGG
CACAGGATGTAATTATGGAAGTTGTTTTCCCTAATGAAGTAGGAGCCAGAATACTAACATCAGAATCGCA
ATTAACAATAACCAAGGAGAAAAAAGAAGAACTCCAAAATTGCAAAATTTCCCCTTTGATGGTTGCATAC
ATGTTAGAGAGGGAACTTGTCCGCAAAACAAGATTTCTCCCGGTTGCAGGTGGAACAAGCAGTGTGTACA
TTGAAGTTTTGCATTTAACACAGGGGACATGCTGGGAGCAGATGTACACTCCAGGTGGGGAGGTGAGGAA
TGATGATGTTGATCAAAGCCTAATTATTGCTGCTAGGAACATAGTGAGAAGAGCTGCAGTATCAGCAGAT
CCACTAGCATCTTTATTAGAAATGTGCCATAGCACACAGATTGGTGGAACAAGGATGGTGGATATTCTCA
GGCAAAATCCAACAGAAGAACAAGCTGTGGACATATGCAAAGCAGCAATGGGGCTGAGAATCAGTTCATC
CTTCAGTTTTGGCGGATTCACATTTAAGAGAACAAGTGGATCATCAGTCAAAAGGGAGGAAGAAGTGCTC
ACGGGCAATCTGCAAACATTGAAACTAACCGTGCATGAGGGATATGAAGAGTTCACAATGGTTGGGAAAA
GGGCAACAGCTATACTCAGAAAAGCAACCAGGAGATTGATTCAACTAATAGTGAGTGGAAGAGACGAACA
ATCAATAGTCGAAGCAATAGTTGTAGCAATGGTATTCTCACAAGAAGATTGCATGGTAAAAGCAGTTAGA
GGTGATCTGAATTTCGTTAATAGAGCGAATCAGCGGTTGAATCCCATGCATCAACTATTGAGACATTTTC
AGAAGGATGCTAAAGTACTTTTCTTAAATTGGGGAGTTGAACCTATTGACAATGTGATGGGAATGATTGG
GATATTACCTGATATGACTCCAAGTACCGAGATGTCAATGAGAGGAGTGAGAGTCAGCAAAATGGGTGTA
GATGAATACTCCAATGCTGAAAGGGTAGTGGTAAGCATTGACCGTTTTTTGAGAGTCCGGGACCAAAGAG
GAAATGTACTACTGTCTCCAGAGGAAGTAAGTGAAACACAAGGGACAGAGAAACTGACAATAACTTATTC
TTCATCAATGATGTGGGAGATTAATGGCCCTGAGTCAGTCTTGATCAATACCTATCAGTGGATCATCAGA
AACTGGGAGACTGTTAAAATTCAGTGGTCTCAGAATCCTACGATGCTGTACAATAAAATGGAATTTGAAC
CATTTCAGTCTCTAGTCCCCAAGGCCATTAGAGGCCAATACAGTGGGTTTGTTAGAACTCTATTCCAACA
AATGAGGGATGTGCTTGGGACTTTTGACACAACTCAGATAATAAAACTTCTTCCCTTTACAGCCGCTCCT
CCAAAGCAAAGCAGAATGCAATTCTCGTCATTAACTGTGAATGTGAGGGGATCAGGAATGAGAATACTTG
TGAGGGGTAATTCTCCAGTATTCAACTACAACAAGACTACCAAGAGACTCACAGTCCTCGGAAAGGATGC
TGGCACTTTAACTGAAGACCCAGATGAAGGCACAGCTGGAGTGGAATCTGCGGTTCTAAGGGGATTCCTC
ATTTTAGGCAAAGAAGATAGAAGATATGGGCCAGCATTAAGCATCAATGAATTGAGCAACCTTGCGAAAG
GGGAAAAAGCTAATGTGCTAATTGGGCAAGGGGATGTAGTGTTGGTAATGAAACGAAAACGGGACTCTAG

CATACTTACTGACAGCCAGACAGCGACCAAAAGAATTCGGATGGCCATCAATTAATTTCGAATAATTTAA
A
>gi|296240595|gb|CY063613.1| Influenza A virus (A/Aalborg/INS133/2009(H1N1)) segment 1, complete sequence
TATGGAGAGAATAAAAGAACTGAGAGATCTAATGTCGCAGTCCCGCACTCGCGAGATACTCACTAAGACC
ACTGTGGACCATATGGCCATAATCAAAAAGTACACATCAGGAAGGCAAGAGAAGAACCCCGCGCTCAGAA
TGAAGTGGATGATGGCAATGAGATACCCAATTACAGCAGACAAGAGAATAATGGACATGATTCCAGAGAG
GAATGAACAAGGACAAACCCTCTGGAGCAAAACAAACGATGCTGGATCAGACCGAGTGATGGTATCACCT
CTGGCCGTAACATGGTGGAATAGGAATGGCCCAACAACAAGTACAGTTCATTACCCTAAGGTATATAAAA
CTTATTTCGAAAAGGTCGAAAGGTTGAAACATGGTACCTTCGGCCCTGTCCACTTCAGAAATCAAGTTAA
AATAAGGAGGAGAGTTGATACAAACCCTGGCCATGCAGATCTCAGTGCCAAGGAGGCACAGGATGTGATT
ATGGAAGTTGTTTTCCCAAATGAAGTGGGGGCAAGAATACTGACATCAGAGTCACAGCTGGCAATAACAA
AAGAGAAGAAAGAAGAGCTCCAGGATTGTAAAATTGCTCCCTTGATGGTGGCGTACATGCTAGAAAGAGA
ATTGGTCCGTAAAACAAGGTTTCTCCCAGTAGCCGGCGGAACAGGCAGTGTTTATATTGAAGTGTTGCAC
TTAACCCAAGGGACGTGCTGGGAGCAGATGTACACTCCAGGAGGAGAAGTGAGAAATGATGATGTTGACC
AAAGTTTGATTATCGCTGCTAGAAACATAGTAAGAAGAGCAGCAGTGTCAGCAGACCCATTAGCATCTCT
CTTGGAAATGTGCCACAGCACGCAGATTGGAGGAGTAAGGATGGTGGACATCCTTAGACAGAATCCAACT
GAGGAACAAGCCGTAGACATATGCAAGGCAGCAATAGGGTTGAGGATTAGCTCATCTTTCAGTTTTGGTG
GGTTCACTTTCAAAAGGACAAGCGGATCATCAGTCAAGAAAGAAGAAGAAGTGCTAACAGGCAACCTCCA
AACTCTGAAAATAAGAGTACATGAAGGGTATGAAGAATTCACAATGGTTGGGAGAAGAGCAACAGCTATT
CTCAGAAAGGCAACCAGGAGATTGATCCAGTTGATAGTAAGCGGGAGAGACGAGCAGTCAATTGCTGAGG
CAATAATTGTGGCCATGGTATTCTCACAAGAGGATTGCATGATCAAGGCAGTTAGGGGCGATCTGAACTT
TGTCAATAGGGCAAACCAGCGACTAAACCCCATGCACCAACTCTTGAGGCATTTCCAAAAAGATGCAAAA
GTGCTTTTCCAGAACTGGGGAATTGAATCCATCGACAATGTGATGGGAATGATCGGAATACTGCCCGACA
TGACCCCAAGCACGGAGATGTCGCTGAGAGGGATAAGAGTCAGCAAAATGGGAGTAGATGAATACTCCAG
CACGGAGAGAGTGGTAGTGAGTATTGACCGATTTTTAAGGGTTAGAGATCAAAGAGGGAACGTACTATTG
TCTCCCGAAGAAGTCAGTGAAACGCAAGGAACTGAGAAGTTGACAATAACTTATTCGTCATCAATGATGT
GGGAGATCAATGGCCCTGAGTCAGTGCTAGTCAACACTTATCAATGGATAATCAGGAACTGGGAAATTGT
GAAAATTCAATGGTCACAAGATCCCACAATGTTATACAACAAAATGGAATTTGAACCATTTCAGTCTCTT
GTCCCTAAGGCAACCAGAAGCCGGTACAGTGGATTCGTAAGGACACTGTTCCAGCAAATGCGGGATGTGC
TTGGGACATTTGACACTGTCCAAATAATAAAACTTCTCCCCTTTGCTGCTGCTCCACCAGAACAGAGTAG
GATGCAATTTTCCTCATTGACTGTGAATGTGAGAGGATCAGGGTTGAGGATACTGGTAAGAGGCAATTCT
CCAGTATTCAATTACAACAAGGCAACCAAACGACTTACAGTTCTTGGAAAGGATGCAGGTGCATTGACAG
AAGATCCAGATGAAGGCACATCTGGGGTGGAGTCTGCTGTCCTGAGAGGATTTCTCATTTTGGGCAAAGA
AGACAAGAGATATGGCCCAGCATTAAGCATCAATGAACTGAGCAATCTTGCAAAAGGAGAGAAAGCTAAT
GTGCTAATTGGGCAAGGGGACGTAGTGTTGGTAATGAAACGAAAACGGGACTCTAGCATACTTACTGACA
GCCAGACAGCGACCAAAAGAATTCGGATGGCCATCAATTAGTGTCGAATTGTT
>gi|296240577|gb|CY063605.1| Influenza A virus (A/Bonn/INS128/2009(H1N1)) segment 1, complete sequence
TATGGAGAGAATAAAAGAACTGAGAGATCTAATGTCGCAGTCCCGCACTCGCGAGATACTCACTAAGACC
ACTGTGGACCATATGGCCATAATCAAAAAGTACACATCAGGAAGGCAAGAGAAGAACCCCGCACTCAGAA
TGAAGTGGATGATGGCAATGAGATACCCAATTACAGCAGACAAGAGAATAATGGACATGATTCCAGAGAG
GAATGAACAAGGACAAACCCTCTGGAGCAAAACAAACGATGCTGGATCAGACCGAGTGATGGTATCACCT
CTGGCCGTAACATGGTGGAATAGGAATGGCCCAACAACAAGTACAGTTCATTACCCTAAGGTATATAAAA

CTTATTTCGAAAAGGTCGAAAGGTTGAAACATGGTACCTTCGGCCCTGTCCACTTCAGAAATCAAGTTAA
AATAAGGAGGAGAGTTGATACAAACCCTGGCCATGCAGATCTCAGTGCCAAGGAGGCACAGGATGTGATT
ATGGAAGTTGTTTTCCCAAATGAAGTGGGGGCAAGAATACTGACATCAGAGTCACAGCTGGCAATAACAA
AAGAGAAGAAAGAAGAGCTCCAGGATTGTAAAATTGCTCCCTTGATGGTGGCGTACATGCTAGAAAGAGA
ATTGGTCCGTAAAACAAGGTTTCTCCCAGTAGCCGGCGGAACAGGCAGTGTTTATATTGAAGTGTTGCAC
TTAACCCAAGGGACGTGCTGGGAGCAGATGTACACTCCAGGAGGAGAAGTGAGAAATGATGATGTTGACC
AAAGTTTGATTATCGCTGCTAGAAACATAGTAAGAAGAGCAGCAGTGTCAGCAGACCCATTAGCATCTCT
CTTGGAAATGTGCCACAGCACGCAGATTGGAGGAGTAAGGATGGTGGACATCCTTAGACAGAATCCAACT
GAGGAACAAGCCGTAGACATATGCAAGGCAGCAATAGGGTTGAGGATTAGCTCATCTTTCAGTTTTGGTG
GGTTCACTTTCAAAAGGACAAGCGGATCATCAGTCAAGAAAGAAGAAGTGCTAACAGGCAACCTCCA
AACTCTGAAAATAAGAGTACATGAAGGGTATGAAGAATTCACAATGGTTGGGAGAAGAGCAACAGCTATT
CTCAGAAAGGCAACCAGGAGATTGATCCAGTTGATAGTAAGCGGGAGAGACGAGCAGTCAATTGCTGAGG
CAATAATTGTGGCCATGGTATTCTCACAAGAGGATTGCATGATCAAGGCAGTTAGGGGCGATCTGAACTT
TGTCAATAGGGCAAACCAGCGACTAAACCCCATGCACCAACTCTTGAGGCATTTCCAAAAAGATGCAAAA
GTGCTTTTCCAGAACTGGGGAATTGAATCCATCGACAATGTGATGGGAATGATCGGAATACTGCCCGACA
TGACCCCAAGCACGGAGATGTCGCTGAGAGGGATAAGAGTCAGCAAAATGGGAGTAGATGAATACTCCAG
CACGGAGAGAGTGGTAGTGAGTATTGACCGATTTTTAAGGGTTAGAGATCAAAGAGGGAACGTACTATTG
TCTCCCGAAGAAGTCAGTGAAACGCAAGGAACTGAGAAGTTGACAATAACTTATTCGTCATCAATGATGT
GGGAGATCAATGGCCCTGAGTCAGTGCTAGTCAACACTTATCAATGGATAATCAGGAACTGGGAAATTGT
GAAAATTCAATGGTCACAAGATCCCACAATGTTATACAACAAAATGGAATTTGAACCATTTCAGTCTCTT
GTCCCTAAGGCAACCAGAAGCCGGTACAGTGGATTCGTAAGGACACTGTTCCAGCAAATGCGGGATGTGC
TTGGGACATTTGACACTGTCCAAATAATAAAACTTCTCCCCTTTGCTGCTGCTCCACCAGAACAGAGTAG
GATGCAATTTTCCTCATTGACTGTGAATGTGAGAGGATCAGGGTTGAGGATACTGGTAAGAGGCAATTCT
CCAGTATTCAATTACAACAAGGCAACCAAACGACTTACAGTTCTTGGAAAGGATGCAGGTGCATTGACTG
AAGATCCAGATGAAGGCACATCTGGGGTGGAGTCTGCTGTCCTGAGAGGATTTCTCATTTTGGGCAAAGA
AGACAAGAGATATGGCCCAGCATTAAGCATCAATGAACTGAGCAATCTTGCAAAAGGAGAGAAAGCTAAT
GTGCTAATTGGGCAAGGGGACGTAGTGTTGGTAATGAAACGAAAACGGGACTCTAGCATACTTACTGACA
GCCAGACAGCGACCAAAAGAATTCGGATGGCCATCAATTAGTGTCGAATTGTT
>gi|296240325|gb|CY063493.1| Influenza A virus (A/Boston/110/2009(H1N1)) segment 1, complete sequence
TATGGAGAGAATAAAAGAACTGAGAGATCTAATGTCGCAGTCCCGCACTCGCGAGATACTCACTAAGACC
ACTGTGGACCATATGGCCATAATCAAAAAGTACACATCAGGAAGGCAAGAGAAGAACCCCGCACTCAGAA
TGAAGTGGATGATGGCAATGAGATACCCAATTACAGCAGACAAGAGAATAATGGACATGATTCCAGAGAG
GAATGAACAAGGACAAACCCTCTGGAGCAAAACAAACGATGCTGGATCAGACCGAGTGATGGTATCACCT
CTGGCCGTAACATGGTGGAATAGGAATGGCCCAACAACAAGTACAGTTCATTACCCTAAGGTATATAAAA
CTTATTTCGAAAAGGTCGAAAGGTTGAAACATGGTACCTTCGGCCCTGTCCACTTCAGAAATCAAGTTAA
AATAAGGAGGAGAGTTGATACAAACCCTGGCCATGCAGATCTCAGTGCCAAGGAGGCACAGGATGTGATT
ATGGAAGTTGTTTTCCCAAATGAAGTGGGGGCAAGAATACTGACATCAGAGTCACAGCTGGCAATAACAA
AAGAGAAGAAAGAAGAGCTCCAGGATTGTAAAATTGCTCCCTTGATGGTGGCGTACATGCTAGAAAGAGA
ATTGGTCCGTAAAACAAGGTTTCTCCCAGTAGCCGGCGGAACAGGCAGTGTTTATATTGAAGTGTTGCAC
TTAACCCAAGGGACGTGCTGGGAGCAGATGTACACTCCAGGAGGAGAAGTGAGAAATGATGATGTTGACC
AAAGTTTGATTATCGCTGCTAGAAACATAGTAAGAAGAGCAGCAGTGTCAGCAGACCCATTAGCATCTCT
CTTGGAAATGTGCCACAGCACACAGATTGGAGGAGTAAGGATGGTGGATATCCTTAGACAGAATCCAACT
GAGGAACAAGCCGTAGACATATGCAAGGCAGCAATAGGGTTGAGGATTAGCTCATCTTTCAGTTTTGGTG

GGTTCACTTTCAAAAGGACAAGCGGATCATCAGTCAAGAAAGAAGAAGAAGTGCTAACGGGCAACCTCCA
AACACTGAAAATAAGAGTACATGAAGGGTATGAAGAATTCACAATGGTTGGGAGAAGAGCAACAGCTATT
CTCAGAAAGGCAACCAGGAGATTGATCCAGTTGATAGTAAGCGGGAGAGACGAGCAGTCAATTGCTGAGG
CAATAATTGTGGCCATGGTATTCTCACAAGAGGATTGCATGATCAAGGCAGTTAGGGGCGATCTGAACTT
TGTCAATAGGGCAAACCAGCGACTGAACCCCATGCACCAACTCTTGAGGCATTTCCAAAAAGATGCAAAA
GTGCTTTTCCAGAACTGGGGAATTGAATCCATCGACAATGTGATGGGAATGATCGGAATACTGCCCGACA
TGACCCCAAGCACGGAGATGTCGCTGAGAGGGATAAGAGTCAGCAAAATGGGAGTAGATGAATACTCCAG
CACGGAGAGAGTGGTAGTGAGTATTGACCGATTTTTAAGGGTTAGAGATCAAAGAGGGAACGTACTATTG
TCTCCCGAAGAAGTCAGTGAAACGCAAGGAACTGAGAAGTTGACAATAACTTATTCGTCATCAATGATGT
GGGAGATCAATGGCCCTGAGTCAGTGCTAGTCAACACTTATCAATGGATAATCAGGAACTGGGAAATTGT
GAAAATTCAATGGTCACAAGATCCCACAATGTTATACAACAAAATGGAATTTGAACCATTTCAGTCTCTT
GTCCCTAAGGCAACCAGAAGCCGGTACAGTGGATTCGTAAGGACACTGTTCCAGCAAATGCGGGATGTGC
TTGGGACATTTGACACTGTCCAAATAATAAAACTTCTCCCCTTTGCTGCTGCTCCACCAGAACAGAGTAG
GATGCAATTTTCCTCATTGACTGTGAATGTGAGAGGATCAGGGTTGAGGATACTGGTAAGAGGCAATTCT
CCAGTATTCAATTACAACAAGGCAACCAAACGACTTACAGTTCTTGGAAAGGATGCAGGTGCATTGACTG
AAGATCCAGATGAAGGCACATCTGGGGTGGAGTCTGCTGTCCTGAGAGGATTTCTCATTTTGGGCAAAGA
AGACAAGAGATATGGCCCAGCATTAAGCATCAATGAACTGAGCAATCTTGCAAAAGGAGAGAAGGCTAAT
GTGCTAATTGGGCAAGGTGACGTAGTGTTGGTAATGAAACGAAAACGGGACTCTAGCATACTTACTGACA
GCCAGACAGCGACCAAAAGAATTCGGATGGCCATCAATTAGTGTCGAATTGTT
>gi|296240631|gb|CY063629.1| Influenza A virus (A/New York/INS150/2009(H1N1)) segment 1, complete sequence
ATGGAGAGAATAAAAGAACTGAGAGATCTAATGTCGCAGTCCCGCACTCGCGAGATATTCACTAAGACCA
CTGTGGACCATATGGCCATAATCAAAAAGTACACATCAGGAAGGCAAGAGAAGAACCCCGCACTCAGAAT
GAAGTGGATGATGGCAATGAGATACCCAATTACAGCAGACAAGAGAATAATGGACATGATTCCAGAGAGG
AATGAACAAGGACAAACCCTCTGGAGCAAAACAAACGATGCTGGATCAGACCGAGTGATGGTATCACCTC
TGGCCGTAACATGGTGGAATAGGAATGGCCCAACAACAAGTACAGTTCATTACCCTAAGGTATATAAAAC
TTATTTCGAAAAGGTCGAAAGGTTGAAACATGGTACCTTCGGCCCTGTCCACTTCAGAAATCAAGTTAAA
ATAAGGAGGAGAGTTGATACAAACCCTGGCCATGCAGATCTCAGTGCCAAGGAGGCACAGGATGTGATTA
TGGAAGTTGTTTTCCCAAATGAAGTGGGGGCAAGAATACTGACATCAGAGTCACAGCTGGCAATAACAAA
AGAGAAGAAAGAAGAGCTCCAGGATTGTAAAATTGCTCCCTTGATGGTGGCGTACATGCTAGAAAGAGAA
TTGGTTCGTAAAACAAGGTTTCTCCCAGTAGCCGGCGGAACAGGCAGTGTTTATATTGAAGTGTTGCACT
TAACCCAAGGGACGTGCTGGGAGCAGATGTACACTCCAGGAGGAGAAGTGAGAAATGATGATGTTGACCA
AAGTTTGATTATCGCTGCTAGAAACATAGTAAGAAGAGCAGCAGTGTCAGCAGACCCATTAGCATCTCTC
TTGGAAATGTGCCACAGCACACAGATTGGAGGAGTAAGGATGGTGGACATCCTTAGACAGAATCCAACTG
AGGAACAAGCCGTAGACATATGCAAGGCAGCAATAGGGTTGAGGATTAGCTCATCTTTCAGTTTTGGTGG
GTTCACTTTCAAAAGGACAAGCGGATCATCAGTCAAGAAAGAAGAAGAAGTGCTAACGGGCAGCCTCCAA
ACACTGAAAATAAGAGTACATGAAGGGTATGAAGAATTCACAATGGTTGGGAGAAGAGCAACAGCTATTC
TCAGAAAGGCAACCAGGAGATTGATCCAGTTGATAGTAAGCGGGAGAGACGAGCAGTCAATTGCTGAGGC
AATAATTGTGGCCATGGTATTCTCACAAGAGGATTGCATGATCAAGGCAGTTAGGGGCGATCTGAACTTT
GTCAATAGGGCAAACCAGCGACTGAACCCCATGCACCAACTCTTGAGGCATTTCCAAAAAGATGCAAAAG
TGCTTTTCCAGAACTGGGGAATTGAATCCATCGACAATGTGATGGGAATGATCGGAATACTGCCCGACAT
GACCCCAAGCACGGAGATGTCGCTGAGAGGGATAAGAGTCAGCAAAATGGGAGTAGATGAATACTCCAGC
ACGGAGAGAGTGGTAGTGAGTATTGACCGATTTTTAAGGGTTAGAGATCAAAGAGGGAACGTACTATTGT
CTCCCGAAGAAGTCAGTGAAACGCAAGGAACTGAGAAGTTGACAATAACTTATTCGTCATCAATGATGTG

GGAGATCAATGGCCCTGAGTCAGTGCTAGTCAACACTTATCAATGGATAATCAGGAACTGGGAAATTGTG
AAAATTCAATGGTCACAAGATCCCACAATGTTATACAACAAAATGGAATTTGAACCATTTCAGTCTCTTG
TCCCTAAGGCAACCAGAAGCCGGTACAGTGGATTCGTAAGGACACTGTTCCAGCAGATGCGGGATGTGCT
TGGGACATTTGACACTGTCCAAATAATAAAACTTCTACCCTTTGCTGCTGCTCCACCAGAACAGAGTAGG
ATGCAATTTTCCTCATTGACTGTGAATGTGAGAGGATCAGGGTTGAGGATACTGGTAAGAGGCAATTCTC
CAGTATTCAATTACAACAAGGCAACCAAACGACTTACAGTTCTTGGAAAGGATGCAGGTGCATTGACTGA
AGATCCAGATGAAGGCACATCTGGGGTGGAGTCTGCTGTCCTGAGAGGATTTCTCATTTTGGGCAAAGAA
GACAAGAGATATGGCCCAGCATTAAGCATCAATGAACTGAGCAATCTTGCAAAAGGAGAGAAAGCTAATG
TGCTAATTGGGCAAGGGGACGTAGTGTTGGTAATGAAACGAAAACGGGACTCTAGCATACTTACTGACAG
CCAGACAGCGACCAAAAGAATTCGGATGGCCATCAATTAGTGTCGAATTGTT
>gi|296240559|gb|CY063597.1| Influenza A virus (A/Athens/INS122/2009(H1N1)) segment 1, complete sequence
TATGGAGAGAATAAAAGAACTGAGAGATCTAATGTCGCAGTCCCGCACTCGCGAGATACTCACTAAGACC
ACTGTGGACCATATGGCCATAATCAAAAAGTACACATCAGGAAGGCAAGAGAAGAACCCCGCACTCAGAA
TGAAGTGGATGATGGCAATGAGGTACCCAATTACAGCAGACAAGAGAATAATGGACATGATTCCAGAGAG
GAATGAACAAGGACAAACCCTCTGGAGCAAAACAAACGATGCTGGATCAGACCGAGTGATGGTATCACCT
CTGGCCGTAACATGGTGGAATAGGAATGGCCCAACAACAAGTACAGTTCATTACCCTAAGGTATATAAAA
CTTATTTCGAAAAGGTCGAAAGGTTGAAACATGGTACCTTCGGCCCTGTCCACTTCAGAAATCAAGTTAA
AATAAGGAGGAGAGTTGATACAAACCCTGGCCATGCAGATCTCAGTGCCAAGGAGGCACAGGATGTGATT
ATGGAAGTTGTTTTCCCAAATGAAGTGGGGGCAAGAATACTGACATCAGAGTCACAGCTGGCAATAACAA
AAGAGAAGAAAGAAGAGCTCCAGGATTGTAAAATTGCTCCCTTGATGGTGGCGTACATGCTAGAAAGAGA
ATTGGTCCGTAAAACAAGGTTTCTCCCAGTAGCCGGCGGAACAGGCAGTGTTTATATTGAAGTGTTGCAC
TTAACCCAAGGGACGTGCTGGGAGCAGATGTACACTCCAGGAGGAGAAGTGAGAAATGATGATGTTGACC
AAAGTTTGATTATCGCTGCTAGAAACATAGTAAGAAGAGCAGCAGTGTCAGCAGACCCATTAGCATCTCT
CTTGGAAATGTGCCACAGCACACAGATTGGAGGAGTAAGGATGGTGGACATCCTTAGACAGAATCCAACT
GAGGAACAAGCCGTAGACATATGCAAGGCAGCAATAGGGTTGAGGATTAGCTCATCTTTCAGTTTTGGTG
GGTTCACTTTCAAAAGGACAAGCGGATCATCAGTCAAGAAAGAAGAAGTGCTAACGGGCAACCTCCA
AACACTGAAAATAAGAGTACATGAAGGGTATGAAGAATTCACAATGGTTGGGAGAAGAGCAACAGCTATT
CTCAGAAAGGCAACCAGGAGATTGATCCAGTTGATAGTAAGCGGGAGAGACGAGCAGTCAATTGCTGAGG
CAATAATTGTGGCCATGGTATTCTCACAAGAGGATTGCATGATCAAGGCAGTTAGGGGCGATCTGAACTT
TGTCAATAGGGCAAACCAGCGACTGAACCCCATGCACCAACTCTTGAGGCATTTCCAAAAAGATGCAAAA
GTGCTTTTCCAGAACTGGGGAATTGAATCCATCGACAATGTGATGGGAATGGTCGGAATACTGCCCGACA
TGACCCCAAGCACGGAGATGTCGCTGAGAGGGATAAGAGTCAGCAAAATGGGAGTAGATGAATACTCCAG
CACGGAGAGAGTGGTAGTGAGTATTGACCGATTTTTAAGGGTTAGAGATCAAAGAGGGAACGTACTATTG
TCTCCCGAAGAAGTCAGTGAAACGCAAGGAACTGAGAAGTTGACAATAACTTATTCGTCATCAATGATGT
GGGAGATCAATGGCCCTGAGTCAGTGCTAGTCAACACTTATCAATGGATAATCAGGAACTGGGAGATTGT
GAAAATTCAATGGTCACAAGATCCCACAATGTTATACAACAAAATGGAATTTGAACCATTTCAGTCTCTT
GTCCCTAAGGCAACCAGAAGCCGGTACAGTGGATTCGTAAGGACACTGTTCCAGCAAATGCGGGATGTGC
TTGGGACATTTGACACTGTCCAAATAATAAAACTTCTCCCCTTTGCTGCTGCTCCACCAGAACAGAGTAG
GATGCAATTTTCCTCATTGACTGTGAATGTGAGAGGATCAGGGTTGAGGATACTGGTAAGAGGCAATTCT
CCAGTATTCAATTACAACAAGGCAACCAAACGACTTACAGTTCTTGGAAAGGATGCAGGTGCATTGACTG
AAGATCCAGATGAAGGCACATCTGGGGTGGAGTCTGCTGTCCTGAGAGGATTTCTCATTTTGGGCAAAGA
AGACAAGAGATATGGCCCAGCATTAAGCATCAATGAACTGAGCAATCTTGCAAAAGGAGAGAAAGCTAAT
GTGCTAATTGGGCAAGGGGACGTAGTGTTGGTAATGAAACGAAAACGGGACTCTAGCATACTTACTGACA

GCCAGACAGCGACCAAAAGAATTCGAATGGCCATCAATTAGTGTCGAATTGTT
>gi|399226023|gb|JX309805.1| Influenza A virus (A/Singapore/TT450/2010(H1N1)) segment 1 polymerase PB2 (PB2) gene, complete cds
AGCAAAAGCAGGTCAAATATATTCAATATGGAGAGAATAAAAGAACTGAGAGATCTAATGTCGCAGTCCC
GCACTCGCGAGATACTCACTAAGACCACTGTGGACCATATGGCCATAATCAAAAAGTACACATCAGGAAG
GCAAGAGAAGAACCCCGCACTCAGAATGAAGTGGATGATGGCAATGAGATACCCAATTACAGCAGACAAG
AGAATAATGGACATGATTCCAGAGAGGAATGAACAAGGACAAACCCTCTGGAGCAAAACAAACGATGCTG
GATCAGACCGAGTGATGGTATCACCTCTGGCCGTAACATGGTGGAATAGGAATGGCCCAACAACAAGTAC
AGTTCATTACCCTAAGGTATATAAAACTTATTTCGAAAAGGTCGAAAGGTTGAAACATGGTACCTTCGGC
CCTGTCCACTTCAGAAATCAAGTTAAAATAAGGAGGAGAGTTGATACAAACCCTGGCCATGCAGATCTCA
GTGCCAAGGAGGCACAGGATGTGATTATGGAAGTTGTTTTCCCAAATGAAGTGGGGGCAAGAATACTGAC
ATCAGAGTCACAGCTGGCAATAACAAAAGAGAAGAAAGAAGAGCTCCAGGATTGTAAAATTGCTCCCTTG
ATGGTGGCGTACATGCTAGAAAGAGAATTGGTCCGTAAAACAAGGTTTCTCCCAGTAGCCGGTGGAACAG
GCAGTGTTTATATTGAAGTGTTGCACTTAACCCAAGGGACGTGCTGGGAGCAGATGTACACTCCAGGAGG
AGAAGTGAGAAATGATGATGTTGACCAAAGTTTGATTATCGCTGCTAGAAACATAGTAAGAAGAGCAGCA
GTGTCAGCAGACCCATTAGCATCTCTCTTGGAAATGTGCCACAGCACACAGATTGGAGGAGTAAGGATGG
TGGACATCCTTAGACAGAATCCAACTGAGGAACAAGCCGTAGACATATGCAAGGCAGCAATAGGGTTGAG
GATCAGCTCATCTTTCAGTTTTGGTGGGTTCACTTTCAAAAGGACAAGCGGATCATCAGTCAAGAAAGAA
GAAGAAATGCTAACGGGCAATCTCCAAACACTGAAATTAAGAGTACATGAAGGGTATGAAGAATTCACAA
TGGTTGGGAGAAGAGCAACAGCTATTCTCAGAAAGGCAACCAGGAGATTGATCCAATTGATAGTAAGCGG
GAGAGACGAGCAGTCAATTGCTGAGGCAATAATTGTGGCCATGGTATTCTCACAAGAGGATTGCATGATC
AAGGCAGTTAGGGGCGATCTGAACTTTGTCAATAGGGCAAACCAGCGACTGAACCCCATGCACCAACTCT
TGAGGCATTTCCAAAAAGATGCAAAAGTGCTTTTCCAGAACTGGGGAATTGAATCCATCGACAATGTGAT
GGGAATGATCGGAATACTGCCCGACATGACCCCAAGCACGGAGATGTCGCTGAGAGGGATAAGAGTCAGC
AAGATGGGAGTAGATGAATACTCCAGCACGGAGAGAGTGGTAGTGAGTATTGACCGATTTTTAAGGGTTA
GAGATCAAAGAGGGAACGTACTATTGTCTCCCGAAGAAGTCAGTGAAACGCAAGGAACTGAGAAGTTGAC
AATAACTTATTCGTCATCAATGATGTGGGAGATCAATGGCCCTGAGTCAGTGCTAGTCAACACTTATCAA
TGGATAATCAGGAACTGGGAAATTGTGAAAATTCAATGGTCACAAGATCCCACAATGTTATACAACAAAA
TGGAATTTGAACCATTTCAGTCTCTTGTCCCTAAGGCAACCAGAAGCCGGTACAGTGGATTCGTAAGGAC
ACTGTTCCAGCAAATGCGGGATGTGCTTGGGACATTTGACACTGTCCAAATAATAAAACTTCTCCCCTTT
GCTGCTGCTCCACCAGAACAGAGTAGGATGCAATTTTCCTCATTGACTGTGAATGTGAGAGGATCAGGGT
TGAGGATACTGGTAAGAGGCAATTCTCCAGTATTCAATTACAACAAGGCAACCAAACGACTTACAGTTCT
TGGAAAGGATGCAGGTGCATTGACTGAAGATCCAGATGAAGGCACATCTGGGGTGGAGTCTGCTGTCCTG
AGAGGATTTCTCATTTTAGGCAAAGAAGACAAGAGATATGGCCCAGCATTAAGCATCAATGAACTGAGCA
ATCTTGCAAAAGGAGAGAAAGCTAATGTGCTAATTGGGCAAGGGGACGTAGTGTTGGTAATGAAACGAAA
ACGGGACTCTAGCATACTTACTGACAGCCAGACAGCGACCAAAAGAATTCGGATGGCCATCAATTAGTGT
CGAATTGTTTAAAAACGACCTTGTTTCTACT
>gi|396940861|dbj|AB704483.1| Influenza A virus (A/Tochigi/10/2010(H1N1)) PB2 gene for polymerase PB2, complete cds
ATGGAGAGAATAAAAGAACTGAGAGATCTAATGTCGCAGTCCCGCACTCGCGAGATACTCACTAAGACCA
CTGTGGACCATATGGCCATAATCAAAAAGTACACATCAGGAAGGCAAGAGAAGAACCCCGCACTCAGAAT
GAAGTGGATGATGGCAATGAGATACCCAATTACAGCAGACAAGAGAATAATGGACATGATTCCAGAGAGG
AATGAACAAGGACAAACCCTCTGGAGCAAAACAAACGATGCTGGATCAGACCGAGTGATGGTATCACCTC
TGGCCGTAACATGGTGGAATAGGAATGGCCCAACAACAAGTACAGTTCATTACCCTAAGGTATATAAAAC

TTATTTCGAAAAGGTCGAAAGGTTGAAACATGGTACCTTCGGCCCTGTCCACTTCAGAAATCAAGTTAAA
ATAAGGAGGAGAGTTGATACAAACCCTGGCCATGCAGATCTCAGTGCCAAGGAGGCACAGGATGTGATTA
TGGAAGTTGTTTTCCCAAATGAAGTGGGAGCAAGAATACTGACATCAGAGTCACAGCTGGCAATAACAAA
AGAGAAGAAAGAAGAGCTCCAGGATTGTAAAATTGCTCCCTTAATGGTGGCGTACATGCTAGAAAGAGAA
TTGGTCCGTAAAACAAGGTTTCTCCCAGTAGCCGGCGGAACAGGCAGTGTTTATATTGAAGTGTTGCACT
TAACCCAAGGGACGTGCTGGGAGCAGATGTACACTCCAGGAGGAGAAGTGAGAAATGATGATGTTGACCA
AAGTTTGATTATCGCTGCTAGAAACATAGTAAGAAGAGCAGCAGTGTCAGCAGACCCATTAGCATCTCTC
TTGGAAATGTGCCACAGCACACAGATTGGAGGAGTAAGGATGGTGGATATCCTTAGACAGAATCCAACTG
AGGAACAAGCCGTAGACATATGCAAGGCAGCAATAGGGTTGAGGATTAGCTCATCTTTCAGTTTTGGTGG
GTTCACTTTCAAAAGGACAAGCGGATCATCAGTCAAGAAAGAAGAAGAAGTGCTAACGGGCAACCTCCAA
ACACTGAAAATAAGAGTACATGAAGGGTATGAAGAATTCACAATGGTTGGGAGAAGAGCAACAGCTATTC
TCAGAAAGGCAACCAGGAGATTGATCCAGTTGATAGTAAGCGGGAGAGACGAGCAGTCAATTGCTGAGGC
AATAATTGTGGCCATGGTGTTCTCACAAGAGGATTGCATGATCAAGGCAGTTAGGGGCGATCTAAACTTT
GTCAATAGGGCAAACCAGCGACTGAATCCCATGCACCAACTCTTGAGGCATTTCCAAAAAGATGCAAAAG
TGCTTTTCCAGAACTGGGGAATTGAATCCATCGACAATGTGATGGGAATGATCGGAATACTGCCCGACAT
GACCCCAAGCACGGAGATGTCGCTGAGAGGGATAAGAGTCAGCAAAATGGGAGTAGATGAATACTCCAGC
ACGGAGAGAGTGGTAGTGAGTATTGACCGATTTTTAAGGGTTAGAGATCAAAGAGGGAACGTACTATTGT
CTCCCGAAGAAGTCAGTGAAACGCAAGGAACTGAGAAGTTGACAATAACTTATTCGTCATCAATGATGTG
GGAGATCAATGGCCCTGAGTCAGTGCTAGTCAACACTTATCAATGGATAATCAGGAACTGGGAAATTGTG
AAAATTCAATGGTCACAAGATCCCACAATGTTATACAACAAAATGGAATTTGAACCATTTCAGTCTCTTG
TCCCTAAGGCAACCAGAAGCCGGTACAGTGGATTCGTAAGGACACTGTTCCAGCAAATGCGGGATGTGCT
TGGGACATTTGACACTGTCCAAATAATAAAACTTCTCCCCTTTGCTGCTGCTCCACCAGAACAGAGTAGG
ATGCAATTCTCCTCATTGACTGTGAATGTGAGAGGATCAGGGTTGAGGATACTGGTAAGAGGCAATTCTC
CAGTATTCAATTACAACAGGGCAACCAAACGACTTACAGTTCTTGGAAAGGATGCAGGTGCATTGACAGA
AGATCCAGATGAAGGCACATCTGGGGTGGAGTCTGCTGTCCTGAGAGGATTTCTCATTTTGGGCAAAGAA
GACAAAAGATATGGCCCAGCATTAAGCATCAATGAACTGAGCAATCTTGCAAAAGGAGAGAAAGCTAATG
TGCTAATTGGGCAAGGGGACGTAGTGTTGGTAATGAAACGAAAACGGGACTCTAGCATACTTACTGACAG
CCAGACAGCGACCAAAAGAATTCGGATGGCCATCAATTAG
>gi|344995323|gb|CY098169.1| Influenza A virus (A/Bangkok/INS509/2010(H1N1)) polymerase PB2 (PB2) gene, complete cds
TATGGAGAGAATAAAAGAACTGAGAGATCTAATGTCGCAGTCCCGCACTCGCGAGATACTCACTAAGACC
ACTGTGGACCATATGGCCATAATCAAAAAGTACACATCAGGAAGGCAAGAGAAGAACCCCGCACTCAGAA
TGAAGTGGATGATGGCAATGAGATACCCAATTACAGCAGACAAGAGAATAATGGACATGATTCCAGAGAG
GAATGAACAAGGACAAACCCTCTGGAGCAAAACAAACGATGCTGGATCAGACCGAGTGATGGTATCACCT
CTGGCCGTAACATGGTGGAATAGGAATGGCCCAACAACAAGTACAGTTCATTACCCTAAGGTATATAAAA
CTTATTTCGAAAAGGTCGAAAGGTTGAAACATGGTACCTTCGGCCCTGTCCACTTCAGAAATCAAGTTAA
AATAAGGAGGAGAGTTGATACAAACCCTGGCCATGCAGATCTCAGTGCCAAGGAGGCACAGGATGTGATT
ATGGAAGTTGTTTTCCCAAATGAAGTGGGGGCAAGAATACTGACATCAGAGTCACAGCTGGCAATAACAA
AAGAGAAGAAAGAAGAGCTCCAGGATTGTAAAATTGCTCCCTTGATGGTGGCGTACATGCTAGAAAGAGA
ATTGGTCCGTAAAACAAGGTTTCTCCCAGTAGCCGGTGGAACAGGCAGTGTTTATATTGAAGTGTTGCAC
TTAACCCAAGGGACGTGCTGGGAGCAGATGTACACTCCAGGAGGAGAAGTGAGAAATGATGATGTTGACC
AAAGTTTGATTATCGCTGCTAGAAACATAGTAAGAAGAGCAGCAGTGTCAGCAGACCCATTAGCATCTCT
CTTGGAAATGTGCCACAGCACACAGATTGGAGGAGTAAGGATGGTGGACATCCTTAGACAGAATCCAACT
GAGGAACAAGCCGTAGACATATGCAAGGCAGCAATAGGGTTGAGGATCAGCTCATCTTTCAGTTTTGGTG

GGTTCACTTTCAAAAGGACAAGCGGATCATCAGTCAAGAAAGAAGAAGAAATGCTAACGGGCAATCTCCA
AACACTGAAATTAAGAGTACATGAAGGGTATGAAGAATTCACAATGGTTGGGAGAAGAGCAACAGCTATT
CTCAGAAAGGCAACCAGGAGATTGATCCAATTGATAGTAAGCGGGAGAGACGAGCAGTCAATTGCTGAGG
CAATAATTGTGGCCATGGTATTCTCACAAGAGGATTGCATGATCAAGGCAGTTAGGGGCGATCTGAACTT
TGTCAATAGGGCAAACCAGCGACTGAACCCCATGCACCAACTCTTGAGGCATTTCCAAAAAGATGCAAAA
GTGCTTTTCCAGAACTGGGGAATTGAATCCATCGACAATGTGATGGGAATGATCGGAATACTGCCCGACA
TGACCCCAAGCACGGAGATGTCGCTGAGAGGGATAAGAGTCAGCAAGATGGGAGTAGATGAATACTCCAG
CACGGAGAGAGTGGTAGTGAGTATTGACCGATTTTTAAGGGTTAGAGATCAAAGAGGGAACGTACTATTG
TCTCCCGAAGAAGTCAGTGAAACGCAAGGAACTGAGAAGTTGACAATAACTTATTCGTCATCAATGATGT
GGGAGATCAATGGCCCTGAGTCAGTGCTAGTCAACACTTATCAATGGATAATCAGAAACTGGGAAATTGT
GAAAATTCAATGGTCACAAGATCCCACAATGTTATACAACAAAATGGAATTTGAACCATTTCAGTCTCTT
GTCCCTAAGGCAACCAGAAGCCGGTACAGTGGATTCGTAAGGACACTGTTCCAGCAAATGCGGGATGTGC
TTGGGACATTTGACACTGTCCAAATAATAAAACTTCTCCCCTTTGCTGCTGCTCCACCAGAACAGAGTAG
GATGCAATTTTCCTCATTGACTGTGAATGTGAGAGGATCAGGGTTGAGGATACTGGTAAGAGGCAATTCT
CCAGTATTCAATTACAACAAGGCAACCAAACGACTTACAGTTCTTGGAAAGGATGCAGGTGCATTGACTG
AAGATCCAGATGAAGGCACATCTGGGGTGGAGTCTGCTGTCCTGAGAGGATTTCTCATTTTAGGCAAAGA
AGACAAGAGATATGGCCCAGCATTAAGCATCAATGAACTGAGCAATCTTGCAAAAGGAGAGAAAGCTAAT
GTGCTAATTGGGCAAGGGGACGTAGTGTTGGTAATGAAACGAAAACGGGACTCTAGCATACTTACTGACA
GCCAGACAGCGACCAAAAGAATTCGGATGGCCATCAATTAGTGTCGAATTGTT
>gi|344995025|gb|CY098097.1| Influenza A virus (A/Chile/89/2010(H1N1)) polymerase PB2 (PB2) gene, complete cds
TATGGAGAGAATAAAAGAACTGAGAGATCTAATGTCGCAGTCCCGCACTCGCGAGATACTCACTAAGACC
ACTGTGGACCATATGGCCATAATCAAAAAGTACACATCAGGAAGGCAAGAGAAGAACCCCGCGCTCAGAA
TGAAGTGGATGATGGCAATGAGATACCCAATTACAGCAGACAAGAGAATAATGGACATGATTCCAGAGAG
GAATGAACAAGGACAAACCCTCTGGAGCAAAACAAACGATGCTGGATCAGACCGAGTGATGGTATCACCT
CTGGCCGTAACATGGTGGAATAGGAATGGCCCAACAACAAGTACAGTTCATTACCCTAAGGTATATAAAA
CTTATTTCGAAAAGGTCGAAAGGTTGAAACATGGTACCTTCGGCCCTGTCCACTTCAGAAATCAAGTTAA
AATAAGGAGGAGAGTTGATACAAACCCTGGCCATGCAGATCTCAGTGCCAAGGAGGCACAGGATGTGATT
ATGGAAGTTGTTTTCCCAAATGAAGTGGGGGCAAGAATACTGACATCAGAGTCACAGCTGGCAATAACAA
AAGAGAAGAAAGAAGAGCTCCAGGATTGTAAAATTGCTCCCTTGATGGTGGCGTACATGCTAGAAAGAGA
ATTGGTCCGTAAAACAAGGTTTCTCCCAGTAGCCGGCGGAACAGGCAGTGTTTATATTGAAGTGTTGCAC
TTAACCCAAGGGACGTGCTGGGAGCAGATGTACACTCCAGGAGGAGAAGTGAGAAATGATGATGTTGACC
AAAGTTTGATTATCGCTGCTAGAAACATAGTAAGAAGAGCAGCAGTGTCAGCAGACCCATTAGCATCTCT
CTTGGAAATGTGCCACAGCACACAGATTGGAGGAGTAAGGATGGTGGACATCCTTAGACAGAATCCAACT
GAGGAACAAGCCGTAGACATATGCAAGGCAGCAATAGGGTTGAGGATTAGCTCATCTTTCAGTTTTGGTG
GGTTCACTTTCAAAAGGACAAGCGGATCATCAGTCAAGAAAGAAGAAGTGCTAACGGGCAACCTCCA
AACACTGAAAATAAGAGTACATGAAGGGTATGAAGAATTCACAATGGTTGGGAGAAGAGCAACAGCTATT
CTCAGAAAGGCAACCAGGAGATTGATCCAGTTGATAGTAAGCGGGAGAGACGAGCAGTCAATTGCTGAGG
CAATAATTGTGGCCATGGTATTCTCACAAGAGGATTGCATGATCAAGGCAGTTAGGGGCGATCTGAACTT
TGTCAATAGGGCAAACCAGCGACTGAACCCCATGCACCAACTCTTGAGGCATTTCCAAAAAGATGCAAAA
GTGCTTTTCCAGAACTGGGGAATTGAATCCATCGACAATGTGATGGGAATGATCGGAATACTGCCCGACA
TGACCCCAAGCACGGAGATGTCGCTGAGAGGGATAAGAGTCAGCAAAATGGGAGTAGATGAATACTCCAG
CACGGAGAGAGTGGTAGTGAGTATTGACCGATTTTTAAGGGTTAGAGATCAAAGAGGGAACGTACTATTG
TCTCCCGAAGAAGTCAGTGAAACGCAAGGAACTGAGAAGTTGACAATAACTTATTCGTCATCAATGATGT

GGGAGATCAATGGCCCTGAGTCAGTGCTAGTTAACACTTATCAATGGATAATCAGGAACTGGGAAATTGT
GAAAATTCAATGGTCACAAGATCCCACAATGTTATACAACAAAATGGAATTTGAACCATTTCAGTCTCTT
GTCCCTAAGGCAACCAGAAGCCGGTACAGTGGATTCGTAAGGACACTGTTCCAGCAAATGCGGGATGTGC
TTGGGACATTTGACACTGTCCAAATAATAAAACTTCTCCCCTTTGCTGCTGCTCCACCAGAACAGAGTAG
GATGCAATTCTCCTCATTGACTGTGAATGTGAGAGGATCAGGGTTGAGGATACTGGTAAGAGGCAATTCT
CCAGTATTCAATTACAACAGGGCAACCAAACGACTTACAGTTCTTGGAAAGGATGCAGGTGCATTGACTG
AAGATCCAGATGAAGGCACATCTGGGGTGGAGTCTGCTGTCCTGAGAGGATTTCTCATTTTGGGCAAAGA
AGACAAGAGATACGGTCCAGCATTAAGCATCAATGAACTGAGCAATCTTGCAAAAGGAGAGAAAGCTAAT
GTGCTAATTGGGCAAGGGGACGTAGTGTTGGTAATGAAACGAAAACGGGACTCTAGCATACTTACTGACA
GCCAGACAGCGACCAAAAGAATTCGGATGGCCATCAATTAGTGTCGAATTGTT
>gi|344166282|gb|CY097961.1| Influenza A virus (A/Mexico City/WRAIR3569N/2010(H1N1)) polymerase PB2 (PB2) gene, complete cds
ATGGAGAGAATAAAAGAACTGAGAGATCTAATGTCGCAGTCCCGCACTCGCGAGATACTCACTAAGACCA
CTGTGGACCATATGGCCATAATCAAAAAGTACACATCAGGAAGGCAAGAGAAGAACCCCGCACTCAGAAT
GAAGTGGATGATGGCAATGAGATACCCAATTACAGCAGACAAGAGAATAATGGACATGATTCCAGAGAGG
AATGAACAAGGACAAACCCTCTGGAGCAAAACAAACGATGCTGGATCAGACCGAGTGATGGTATCACCTC
TGGCCGTAACATGGTGGAATAGGAATGGCCCAACAACAAGTACAGTTCATTACCCTAAGGTATATAAAAC
TTATTTCGAAAAGGTCGAAAGGTTGAAACATGGTACCTTCGGCCCTGTCCACTTCAGAAATCAAGTTAAA
ATAAGGAGGAGAGTTGATACAAACCCTGGCCATGCAGATCTCAGTGCCAAGGAGGCACAGGATGTGATTA
TGGAAGTTGTTTTCCCAAATGAAGTGGGGGCAAGAATACTGACATCAGAGTCACAGCTGGCAATAACAAA
AGAGAAGAAAGAAGAGCTCCAGGATTGTAAAATTGCTCCCTTGATGGTGGCGTACATGCTAGAAAGAGAA
TTGGTCCGTAAAACAAGGTTTCTCCCAGTAGCCGGTGGAACAGGCAGTGTTTATATTGAAGTGTTGCACT
TAACCCAAGGGACGTGCTGGGAGCAGATGTACACTCCAGGAGGAGAAGTGAGAAATGATGATATTGACCA
AAGTTTGATTATCGCTGCTAGAAACATAGTAAGAAGAGCAGCAGTGTCAGCAGACCCCTTAGCATCTCTC
TTGGAAATGTGCCACAGCACACAGATTGGAGGAGTAAGGATGGTGGACATCCTTAGACAGAATCCAACTG
AGGAACAAGCCGTAGACATATGCAAGGCAGCAATAGGGTTGAGGATCAGCTCATCTTTCAGTTTTGGTGG
GTTCACTTTCAAAAGGACAAGCGGATCATCAGTCAAGAAAGAAGAAGAAATGCTAACGGGCAACCTCCAA
ACACTGAAATTAAGAGTACATGAAGGGTATGAAGAATTCACAATGGTTGGGAGAAGAGCAACAGCTATTC
TCAGAAAGGCAACCAGGAGATTGATCCAATTGATAGTAAGCGGGAGAGACGAGCAATCAATTGCTGAGGC
AATAATTGTGGCCATGGTATTCTCACAAGAGGATTGCATGATCAAGGCAGTTAGGGGCGATCTGAACTTT
GTCAATAGGGCAAACCAGCGACTGAACCCCATGCACCAACTCTTGAGGCATTTCCAAAAAGATGCAAAAG
TGCTTTTCCAGAACTGGGGAATTGAATCCATCGACAATGTGATGGGAATGATCGGAATACTGCCCGACAT
GACCCCAAGCACGGAGATGTCGCTGAGAGGGATAAGAGTCAGCAAGATGGGAGTAGATGAATACTCCAGC
ACGGAGAGAGTGGTAGTGAGTATTGACCGATTTTTAAGGGTTAGAGATCAAAGAGGGAACGTACTATTGT
CTCCCGAAGAAGTCAGTGAAACGCAAGGAACTGAGAAGTTGACAATAACTTATTCGTCATCAATGATGTG
GGAGATCAATGGCCCTGAGTCAGTGCTAGTCAACACTTATCAATGGATAATCAGGAACTGGGAAATTGTG
AAAATTCAATGGTCACAAGATCCCACAATGTTATACAGCAAAATGGAATTTGAACCATTTCAGTCTCTTG
TCCCTAAGGCAATCAGAAGCCGGTACAGTGGATTCGTAAGGACACTGTTCCAGCAAATGCGGGATGTGCT
TGGGACATTTGACACTGTCCAAATAATAAAACTTCTCCCCTTTGCTGCTGCTCCACCAGAACAGAGTAGG
ATGCAATTTTCCTCATTGACTGTGAATGTGAGAGGATCAGGGTTGAGGATACTGGTAAGAGGCAATTCTC
CAGTATTCAATTACAACAAGGCAACCAAACGACTTACAGTTCTTGGAAAGGATGCAGGTGCATTGACTGA
AGATCCAGATGAAGGCACATCTGGGGTGGAGTCTGCTGTCCTGAGAGGATTTCTCATTTTGGGCAAAGAA
GACAAGAGATATGGCCCAGCATTAAGCATCAATGAACTGAGCAATCTTGCAAAAGGAGAGAAAGCTAATG
TGCTAATTGGGCAAGGGGACGTAGTGTTGGTAATGAAACGAAAACGGGACTCTAGCATACTTACTGACAG

CCAGACAGCGACCAAAAGAATTCGGATGGCCATCAATTAG
>gi|344166264|gb|CY097953.1| Influenza A virus (A/Amman/WRAIR3448T/2010(H1N1)) polymerase PB2 (PB2) gene, complete cds
ATGGAGAGAATAAAAGAACTGAGAGATCTAATGTCGCAGTCCCGCACTCGCGAGATACTCACTAAGACCA
CTGTGGACCATATGGCCATAATCAAAAAGTACACATCAGGAAGGCAAGAGAAGAACCCCGCACTCAGAAT
GAAGTGGATGATGGCAATGAGATACCCAATTACAGCAGACAAGAGAATAATGGACATGATTCCAGAGAGG
AATGAACAAGGACAAACCCTCTGGAGCAAAACAAACGATGCTGGATCAGACCGAGTGGTGGTATCACCTC
TGGCCGTAACATGGTGGAATAGGAATGGCCCAACAACAAGTACAGTTCATTACCCTAAGGTATATAAAAC
TTATTTCGAAAAGGTCGAAAGGTTGAAACATGGTACCTTCGGCCCTGTCCACTTCAGAAATCAAGTTAAA
ATAAGGAGGAGAGTTGATACAAACCCTGGCCATGCAGATCTCAGTGCCAAGGAGGCACAGGATGTTATTA
TGGAAGTTGTTTTCCCAAATGAAGTGGGGGCAAGAATACTGACATCAGAGTCACAACTGGCAATAACAAA
AGAGAAGAAAGAAGAGCTCCAGGATTGTAAAATTGCTCCCTTGATGGTGGCGTACATGCTAGAAAGAGAA
TTGGTCCGTAGAACAAGGTTTCTCCCAGTAGCCGGCGGAACAGGCAGTGTTTATATTGAAGTGTTGCACT
TAACCCAAGGGACGTGCTGGGAGCAGATGTACACTCCAGGAGGAGAAGTGAGAAATGATGATATTGACCA
AAGTTTGATTATCGCTGCTAGAAACATAGTAAGAAGAGCAGCAGTGTCAGCAGACCCATTAGCATCTCTC
TTGGAAATGTGCCACAGCACACAGATTGGAGGAGTAAGGATGGTGGACATCCTTAGACAGAATCCAACTG
AGGAACAAGCCGTAGACATATGCAAGGCAGCAATAGGGTTGAGGATTAGCTCATCTTTCAGTTTTGGTGG
GTTCACTTTCAAAAGGACAAGCGGATCATCAGTCAAGAAAGAAGAAGAAGTGCTAACGGGCAACCTCCAA
ACACTGAAAATAAGAGTACATGAAGGGTATGAAGAATTCACAATGGTTGGGAGAAGAGCAACAGCTATTC
TCAGAAAGGCAACCAGGAGATTGATCCAGTTGATAGTAAGCGGGAGAGACGAGCAGTCAATTGCTGAGGC
AATAATTGTGGCCATGGTATTCTCACAAGAGGATTGCATGATCAAGGCAGTTAGGGGCGATCTGAACTTT
GTCAATAGGGCAAACCAGCGACTGAACCCCATGCACCAACTCTTGAGGCATTTCCAAAAAGATGCAAAAG
TGCTTTTCCAGAACTGGGGAATTGAATCCATCGACAATGTGATGGGAATGATCGGAATACTGCCCGACAT
GACCCCAAGCACGGAGATGTCGCTGAGAGGGATAAGAGTCAGCAAAATGGGAGTGGATGAATACTCCAGC
ACGGAGAGAGTGGTAGTGAGTATTGACCGATTTTTAAGGGTTAGAGATCAAAGAGGGAACATACTATTGT
CTCCCGAAGAAGTTAGTGAAACGCAAGGAACTGAGAAGTTGACAATAACTTATTCGTCATCAATGATGTG
GGAGATCAATGGCCCTGAGTCAGTGCTAGTCAACACTTATCAATGGATAATCAGGAACTGGGAAATTGTG
AAAATTCAATGGTCACAAGATCCCACAATGTTATACAACAAAATGGAATTTGAACCATTTCAGTCTCTTG
TCCCTAAGGCAATCAGAAGCCGGTACAGTGGATTCGTAAGGACACTGTTCCAGCAAATGCGGGATGTGCT
TGGGACATTTGACACTGTCCAAATAATAAAACTTCTCCCCTTTGCTGCTGCTCCACCAGAACAGAGTAGG
ATGCAATTTTCCTCATTGACTGTGAATGTGAGAGGATCAGGGTTGAGGATACTGGTAAGAGGCAATTCTC
CAGTATTTAATTACAACAAGGCAACCAAACGACTTACAGTTCTTGGAAAGGATGCAGGTGCATTGACTGA
AGATCCAGATGAAGGCACATCTGGGGTGGAGTCTGCTGTCCTGAGAGGATTTCTCATTTTGGGCAAAGAA
GACAAGAGATATGGCCCAGCATTAAGCATCAATGAACTGAGCAATCTTGCAAGAGGAGAGAAAGCTAATG
TGCTAATTGGGCAAGGGGACGTAGTGTTGGTAATGAAACGAAAACGGGACTCTAGCATACTTACTGACAG
CCAGACAGCGACCAAAAGAATTCGGATGGCCATCAATTAG
>gi|344165954|gb|CY097842.1| Influenza A virus (A/District of Columbia/WRAIR0310/2010(H1N1)) polymerase PB2 (PB2) gene, complete cds
ATGGAGAGAATAAAAGAACTGAGAGATCTAATGTCGCAGTCCCGCACTCGCGAGATACTCACTAAGACCA
CTGTGGACCATATGGCCATAATCAAAAAGTACACATCAGGAAGGCAAGAGAAGAACCCCGCACTCAGAAT
GAAGTGGATGATGGCAATGAGATACCCAATTACAGCAGACAAGAGAATAATGGACATGATTCCAGAGAGG
AATGAACAAGGACAAACCCTCTGGAGCAAAACAAACGATGCTGGATCAGACCGAGTGATGGTATCACCTC
TGGCCGTAACATGGTGGAATAGGAATGGCCCAACAACAAGTACAGTTCATTACCCTAAGGTATATAAAAC
TTATTTCGAAAAGGTCGAAAGGTTGAAACATGGTACCTTCGGCCCTGTCCACTTCAGAAATCAAGTTAAA

ATAAGGAGGAGAGTTGATACAAACCCTGGCCATGCAGATCTCAGTGCCAAGGAGGCACAGGATGTGATTA
TGGAAGTTGTTTTCCCAAATGAAGTGGGGGCAAGAATACTGACATCAGAGTCACAGCTGGCAATAACAAA
AGAGAAGAAAGAAGAGCTCCAGGATTGTAAAATTGCTCCCTTGATGGTGGCGTACATGCTAGAAAGAGAA
TTGGTCCGTAAAACAAGGTTTCTCCCAGTAGCCGGTGGAACAGGCAGTGTTTATATTGAAGTGTTGCACT
TAACCCAAGGGACGTGCTGGGAGCAGATGTACACTCCAGGAGGAGAAGTGAGAAATGATGATATTGACCA
AAGTTTGATTATCGCTGCTAGAAACATAGTAAGAAGAGCAGCAGTGTCAGCAGACCCCTTAGCATCTCTC
TTGGAAATGTGCCACAGCACACAGATTGGAGGAGTAAGGATGGTGGACATCCTTAGACAGAATCCAACTG
AGGAACAAGCCGTAGACATATGCAAGGCAGCAATAGGGTTGAGGATCAGCTCATCTTTCAGTTTTGGTGG
GTTCACTTTCAAAAGGACAAGCGGATCATCAGTCAAGAAAGAAGAAGAAATGCTAACGGGCAACCTCCAA
ACACTGAAATTAAGAGTACATGAAGGGTATGAAGAATTCACAATGGTTGGGAGAAGAGCAACAGCTATTC
TCAGAAAGGCAACCAGGAGATTGATCCAATTGATAGTAAGCGGGAGAGACGAGCAATCAATTGCTGAGGC
AATAATTGTGGCCATGGTATTCTCACAAGAGGATTGCATGATCAAGGCAGTTAGGGGCGATCTGAACTTT
GTCAATAGGGCAAACCAGCGACTGAACCCCATGCACCAACTCTTGAGGCATTTCCAAAAAGATGCAAAAG
TGCTTTTCCAGAACTGGGGAATTGAATCCATCGACAATGTGATGGGAATGATCGGAATACTGCCCGACAT
GACCCCAAGCACGGAGATGTCGCTGAGAGGGATAAGAGTCAGCAAGATGGGAGTAGATGAATACTCCAGC
ACGGAGAGAGTGGTAGTGAGTATTGACCGATTTTTAAGGGTTAGAGATCAAAGAGGGAACGTACTATTGT
CTCCCGAAGAAGTCAGTGAAACGCAAGGAACTGAGAAGTTGACAATAACTTATTCGTCATCAATGATGTG
GGAGATCAATGGCCCTGAGTCAGTGCTAGTCAACACTTATCAATGGATAATCAGGAACTGGGAAATTGTG
AAAATTCAATGGTCACAAGATCCCACAATGTTATACAACAAAATGGAATTTGAACCATTTCAGTCTCTTG
TCCCTAAGGCAACCAGAAGCCGGTACAGTGGATTCGTAAGGACACTGTTCCAGCAAATGCGGGATGTGCT
TGGGACATTTGACACTGTCCAAATAATAAAACTTCTCCCCTTTGCTGCTGCTCCACCAGAACAGAGTAGG
ATGCAATTTTCCTCATTGACTGTGAATGTGAGAGGATCAGGGTTGAGGATACTGGTAAGAGGCAATTCTC
CAGTATTCAATTACAACAAGGCAACCAAACGACTTACAGTTCTTGGAAAGGATGCAGGTGCATTGACTGA
AGATCCAGATGAAGGCACATCTGGGGTGGAGTCTGCTGTCCTGAGAGGATTTCTCATTTTAGGCAAAGAA
GACAAGAGATATGGCCCAGCATTAAGCATCAATGAACTGAGCAATCTTGCAAAAGGAGAGAAAGCTAATG
TGCTAATTGGGCAAGGGGACGTAGTGTTGGTAATGAAACGAAAACGGGACTCTAGCATACTTACTGACAG
CCAGACAGCGACCAAAAGAATTCGGATGGCCATCAATTAG
>gi|344165828|gb|CY097786.1| Influenza A virus (A/Dakar/WRAIR0020T/2010(H1N1)) polymerase PB2 (PB2) gene, complete cds
ATGGAGAGAATAAAAGAACTGAGAGATCTAATGTCGCAGTCCCGCACTCGCGAGATACTCACTAAGACCA
CTGTGGACCATATGGCCATAATCAAAAAGTACACATCAGGAAGGCAAGAGAAGAACCCCGCACTCAGAAT
GAAGTGGATGATGGCAATGAGATACCCAATTACAGCAGACAAGAGAATAATGGACATGATTCCAGAGAGG
AATGAACAAGGACAAACCCTCTGGAGCAAAACAAACGATGCTGGATCAGACCGAGTGATGGTATCACCTC
TGGCCGTAACATGGTGGAATAGGAATGGCCCAACAACAAGTACAGTTCATTACCCTAAGGTATATAAAAC
TTATTTCGAAAAGGTCGAAAGGTTGAAACATGGTACCTTCGGCCCTGTCCACTTCAGAAATCAAGTTAAA
ATAAGGAGGAGAGTTGATACAAACCCTGGCCATGCAGATCTCAGTGCCAAGGAGGCACAGGATGTGATTA
TGGAAGTTGTTTTCCCAAATGAAGTGGGGGCAAGAATACTGACATCAGAGTCACAACTGGCAATAACAAA
AGAGAAGAAAGAAGAGCTCCAGGATTGTAAAATTGCTCCCTTGATGGTGGCGTACATGCTAGAAAGAGAA
TTGGTCCGTAAGACAAGGTTTCTCCCAGTAGCCGGAGGAACAGGCAGTGTTTATATTGAAGTGTTGCACT
TAACCCAAGGGACGTGCTGGGAGCAGATGTACACTCCAGGAGGAGAAGTGAGAAATGATGATGTTGACCA
AAGTTTGATTATCGCTGCTAGAAACATAGTAAGAAGAGCAGCAGTGTCAGCAGACCCATTAGCATCTCTC
TTGGAAATGTGCCACAGCACACAGATTGGAGGAGTAAGGATGGTGGACATCCTTAGACAGAATCCAACTG
AGGAACAAGCCGTAGACATATGCAAGGCAGCAATAGGGTTGAGGATTAGCTCATCTTTCAGTTTTGGTGG
GTTCACCTTCAAAAGGACAAGCGGATCATCAGTCAAGAAAGAAGAAGAAGTGCTAACGGGCAACCTCCAA

ACACTGAAAATAAGAGTACATGAAGGGTATGAAGAATTCACAATGGTTGGGAGAAGAGCAACAGCTATTC
TCAGAAAGGCAACCAGGAGATTGATCCAGTTGATAGTAAGCGGGAGAGACGAGCAGTCAATTGCTGAGGC
AATAATTGTGGCCATGGTATTCTCACAAGAGGATTGCATGATCAAGGCAGTTAGGGGCGATCTGAACTTT
GTCAATAGGGCAAACCAGCGACTGAACCCCATGCACCAACTCTTGAGGCATTTCCAAAAAGATGCAAAAG
TGCTTTTCCAGAACTGGGGAATTGAATCCATCGACAATGTGATGGGAATGATCGGAATACTGCCCGACAT
GACCCCAAGCACGGAGATGTCGCTGAGAGGGATAAGAGTCAGCAAAATGGGAGTAGATGAATACTCCAGC
ACGGAGAGAGTGGTAGTGAGTATTGACCGATTTTTAAGGGTTAGAGATCAAAGAGGGAACGTACTATTGT
CTCCCGAAGAAGTCAGTGAAACGCAAGGAACTGAGAAGTTGACAATAACTTATTCGTCATCAATGATGTG
GGAGATCAATGGCCCTGAGTCAGTGCTAGTCAACACTTATCAATGGATAATCAGGAACTGGGAAATTGTG
AAAATTCAATGGTCACAAGATCCCACAATGTTATACAACAAAATGGAATTTGAACCATTTCAGTCTCTTG
TCCCTAAGGCAACCAGAAGCCGGTACAGTGGATTCGTAAGGACACTGTTCCAGCAAATGCGGGATGTGCT
TGGGACATTTGACACTGTCCAAATAATAAAACTTCTCCCCTTTGCTGCTGCTCCACCAGAACAGAGTAGG
ATGCAAATTTCCTCATTGACTGTGAATGTGAGAGGATCAGGGTTAAGGATACTGGTAAGAGGCAATTCTC
CAGTATTCAATTACAACAAGGCAACCAAACGACTTACAGTTCTTGGAAAGGATGCAGGTGCATTGACTGA
AGATCCAGATGAAGGCACATCTGGGGTGGAGTCTGCTGTCCTGAGAGGATTTCTCATTTTGGGCAAAGAA
GACAAGAGATATGGCCCAGCATTAAGCATCAATGAACTGAGCAATCTTGCAAAAGGAGAGAAGGCTAATG
TGCTAATTGGGCAAGGGGACGTAGTGTTGGTAATGAAACGAAAACGGGACTCTAGCATACTTACTGACAG
CCAGACAGCGACCAAAAGAATTCGGATGGCCATCAATTAG
>gi|339840982|gb|CY093156.1| Influenza A virus (A/Sydney/DD3-23/2010(H1N1)) polymerase PB2 (PB2) gene, complete cds
TATGGAGAGAATAAAAGAACTGAGAGATCTAATGTCGCAGTCCCGCACTCGCGAGATACTCACTAAGACC
ACTGTGGACCATATGGCCATAATCAAAAAGTACACATCAGGAAGGCAAGAGAAGAACCCCGCACTCAGAA
TGAAATGGATGATGGCAATGAGATACCCAATTACAGCAGACAAGAGAATAATGGACATGATTCCAGAGAG
GAATGAACAAGGACAAACCCTCTGGAGCAAAACAAATGATGCTGGATCAGACCGAGTGATGGTATCACCT
CTGGCCGTAACATGGTGGAATAGGAATGGCCCAACAACAAGTACAGTTCATTACCCTAAGGTATATAAAA
CTTATTTCGAAAAGGTCGAAAGGTTGAAACATGGTACCTTCGGCCCTGTCCACTTCAGAAATCAAGTTAA
AATAAGGAGGAGAGTTGATACAAACCCTGGCCATGCAGATCTCAGTGCCAAGGAGGCACAGGATGTGATT
ATGGAAGTTGTTTTCCCAAATGAAGTGGGGGCAAGAATACTGACATCAGAGTCACAGCTGGCAATAACAA
AAGAGAAGAAAGAAGAGCTCCAGGATTGTAAAATTGCTCCCTTGATGGTGGCGTACATGCTAGAAAGAGA
ATTGGTCCGTAAAACAAGGTTTCTCCCAGTAGCCGGTGGAACAGGCAGTGTTTATATTGAAGTGTTGCAC
TTAACCCAAGGGACGTGCTGGGAGCAGATGTACACTCCAGGAGGAGAAGTGAGAAATGATGATGTTGACC
AAAGTTTGATTATCGCTGCTAGAAACATAGTAAGAAGAGCAGCAGTGTCAGCAGACCCATTAGCATCTCT
CTTGGAAATGTGCCACAGCACACAGATTGGAGGAGTAAGGATGGTGGACATCCTTAGACAGAATCCAACT
GAGGAACAAGCCGTAGACATATGCAAGGCAGCAATAGGGTTGAGGATCAGCTCATCTTTCAGTTTTGGTG
GGTTCACTTTCAAAAGGACAAGCGGGTCATCAGTCAAGAAAGAAGAAGAAATGCTAACGGGCAACCTCCA
AACACTGAAATTAAGAGTACATGAAGGGTATGAAGAATTCACAATGGTTGGGAGAAGAGCAACAGCTATT
CTCAGAAAGGCAACCAGGAGATTGATCCAATTGATAGTAAGCGGGAGAGACGAGCAGTCAATTGCTGAGG
CAATAATTGTGGCCATGGTATTCTCACAAGAGGATTGCATGATCAAGGCAGTTAGGGGCGATCTGAACTT
TGTCAATAGGGCAAACCAGCGACTGAACCCCATGCACCAACTCTTGAGGCATTTCCAAAAAGATGCAAAA
GTGCTTTTCCAGAACTGGGGAATTGAATCCATCGACAATGTGATGGGAATGATCGGAATACTGCCCGACA
TGACCCCAAGCACGGAGATGTCGCTGAGAGGGATAAGAGTCAGCAAGATGGGAGTAGATGAATACTCCAG
CACGGAGAGAGTGGTAGTGAGTATTGACCGATTTTTAAGGGTTAGAGATCAAAGAGGGAACGTACTATTG
TCTCCCGAAGAAGTCAGTGAAACGCAAGGAACTGAGAAGTTGACAATAACTTATTCGTCATCAATGATGT
GGGAGATCAATGGCCCTGAGTCAGTGCTAGTCAACACTTATCAATGGATAATCAGGAACTGGGAAATTGT

GAAAATTCAATGGTCACAAGATCCCACAATGTTATACAACAAAATGGAATTTGAACCATTCCAGTCTCTT
GTCCCTAAGGCAACCAGAAGCCGGTACAGTGGATTCGTAAGGACACTGTTCCAGCAAATGCGGGATGTGC
TTGGGACATTTGACACTGTCCAAATAATAAAACTTCTCCCCTTTGCTGCTGCTCCACCAGAACAGAGTAG
GATGCAATTTTCCTCATTGACTGTGAATGTGAGAGGATCAGGGTTGAGGATACTGGTAAGAGGCAATTCT
CCAGTATTCAATTACAACAAGGCAACCAAACGACTTACAGTTCTTGGAAAGGATGCAGGTGCATTGACTG
AAGATCCAGATGAAGGCACATCTGGGGTGGAGTCTGCTGTCCTGAGAGGATTTCTCATTTTAGGCAAAGA
AGACAAGAGATATGGCCCAGCATTAAGCATCAATGAACTGAGCAATCTTGCAAAAGGAGAGAAAGCTAAT
GTGCTAATTGGGCAAGGGGACGTAGTGTTGGTAATGAAACGAAAACGGGACTCTAGCATACTTACTGACA
GCCAGACAGCGACCAAAAGAATTCGGATGGCCATCAATTAGTGTCGAATTGTT
>gi|332384134|gb|JF906180.1| Influenza A virus (A/Netherlands/2631_1202/2010(H1N1)) segment 1 polymerase PB2 (PB2) gene, complete cds
ATGGAGAGAATAAAAGAACTGAGAGATCTAATGTCGCAGTCCCGCACTCGCGAGATACTCACTAAGACCA
CTGTGGACCATATGGCCATAATCAAAAAGTACACATCAGGAAGGCAAGAGAAGAACCCCGCACTCAGAAT
GAAGTGGATGATGGCAATGAGATACCCAATTACAGCAGACAAGAGAATAATGGACATGATTCCAGAGAGG
AATGAACAAGGACAAACCCTCTGGAGCAAAACAAACGATGCTGGATCAGACCGAGTGATGGTATCACCTC
TGGCCGTAACATGGTGGAATAGGAATGGCCCAACAACAAATACAGTTCATTACCCTAAGGTATATAAAAC
TTATTTCGAAAAGGTCGAAAGGTTGAAACATGGTACCTTCGGCCCTGTCCACTTCAGAAATCAAGTTAAA
ATAAGGAGGAGAGTTGATACAAACCCTGGCCATGCAGATCTCAGTGCCAAGGAGGCACAGGATGTGATTA
TGGAAGTTGTTTTCCCAAATGAAGTGGGGGCAAGAGTACTGACATCAGAGTCACAGCTGGCAATAACAAA
AGAGAAGAAAGAAGAGCTCCAGGATTGTAAAATTGCTCCCTTGATGGTGGCGTACATGCTAGAAAGAGAA
TTGGTCCGTAAAACAAGGTTTCTCCCAGTAGCCGGCGGAACAGGCAGTATTTATATTGAAGTGTTGCACT
TAACCCAAGGGACGTGCTGGGAGCAGATGTACACTCCAGGAGGAGAAGTGAGAAATGATGATGTTGACCA
AAGTTTGATTATCGCTGCTAGAAACATAGTAAGAAGAGCAGCAGTGTCAGCAGACCCATTAGCATCTCTC
TTGGAAATGTGCCACAGCACACAGATTGGAGGAGTAAGGATGGTGGACATCCTTAGACAGAATCCAACTG
AGGAACAAGCCGTAGACATATGCAAGGCAGCAATAGGGTTGAGGATTAGCTCATCTTTCAGTTTTGGTGG
GTTCACTTTCAAAAGGACAAGCGGATCATCAGTCAAGAAAGAAGAAGAAGTGCTAACGGGCAACCTCCAA
ACACTGAAAATAAGAGTACATGAAGGGTATGAAGAATTCACAATGGTTGGGAGAAGAGCAACAGCTATTC
TCAGAAAGGCAACCAGGAGATTGATCCAGTTGATAGTAAGTGGGAGAGACGAGCAGTCAATTGCTGAGGC
AATAATTGTGGCCATGGTATTCTCACAAGAGGATTGCATGATCAAGGCAGTTAGGGGCGATCTGAACTTT
GTCAATAGGGCAAACCAGCGACTGAACCCTATGCACCAACTCTTGAGGCATTTCCAAAAAGATGCAAAAG
TGCTTTTCCAGAACTGGGGAATTGAATCCATCGACAATGTGATGGGAATGATCGGAATACTGCCCGACAT
GACCCCAAGCACGGAGATGTCGCTGAGAGGGATAAGAGTCAGCAAAATGGGAGTAGATGAATACTCCAGC
ACGGAGAGAGTGGTAGTGAGTATTGACCGATTTTTAAGGGTTAGAGATCAAAGAGGGAACGTACTATTGT
CTCCCGAAGAAGTCAGTGAAACGCAAGGAACTGAGAAGTTGACAATAACTTATTCGTCATCAATGATGTG
GGAGATCAATGGCCCTGAGTCAGTGCTAGTCAACACTTATCAATGGATAATCAGGAACTGGGAAATTGTG
AAAATTCAATGGTCACAAGATCCCACAATGTTATACAACAAAATGGAATTTGAACCATTTCAGTCTCTTG
TCCCTAAGGCAACCAGAAGCCGGTACAGTGGATTCGTAAGGACACTGTTCCAGCAAATGCGGGATGTGCT
TGGGACATTTGACACTGTCCAAATAATAAAACTTCTCCCCTTTGCTGCTGCTCCACCAGAACAGAGTAGG
ATGCAATTTTCCTCATTGACTGTGAATGTGAGAGGATCAGGGTTGAGGATACTGGTAAGAGGCAATTCTC
CAGTATTCAATTACAACAAGGCAACCAAACGACTTACAGTTCTTGGAAAGGATGCAGGTGCATTGACTGA
AGATCCAGATGAAGGCACATCTGGGGTGGAGTCTGCTGTCCTGAGAGGATTTCTCATTTTGGGCAAAGAA
GACAAGAGATATGGCCCAGCATTAAGCATCAATGAACTGAGCAATCTTGCAAAAGGAGAGAAAGCTAATG
TGCTAATTGGACAAGGGGACGTAGTGTTGGTAATGAAACGAAAACGGGACTCTAGCATACTTACTGACAG
CCAGACAGCGACCAAAAGAATTCGGATGGCCATCAATTAG

>gi|338826615|gb|CY092863.1| Influenza A virus (A/Sydney/DD3-58/2011(H1N1)) polymerase PB2 (PB2) gene, complete cds
TATGGAGAGAATAAAAGAACTGAGAGATCTAATGTCGCAGTCCCGCACTCGCGAGATACTCACTAAGACC
ACTGTGGACCATATGGCCATAATCAAAAAGTACACATCAGGAAGGCAAGAGAAGAACCCCGCACTCAGAA
TGAAGTGGATGATGGCAATGAGATACCCAATTACAGCAGACAAGAGAATAATGGACATGATTCCAGAGAG
GAATGAACAAGGACAAACCCTCTGGAGCAAAACAAACGATGCTGGATCAGACCGAGTGATGGTATCACCT
CTGGCCGTAACATGGTGGAATAGGAATGGCCCAACAACAAGTACAGTTCATTACCCTAAGGTATATAAAA
CTTATTTCGAAAAGGTCGAAAGGTTGAAACATGGTACCTTCGGCCCTGTCCACTTCAGAAATCAAGTTAA
AATAAGGAGGAGAGTTGATACAAACCCTGGCCATGCAGATCTCAGTGCCAAGGAGGCACAGGATGTGATT
ATGGAAGTTGTTTTCCCAAATGAAGTGGGGGCAAGAATACTGACATCAGAGTCACAGCTGGCAATAACAA
AAGAGAAGAAAGAAGAGCTCCAGAATTGTAAAATTGCTCCCTTGATGGTGGCGTACATGCTAGAAAGAGA
ATTGGTCCGTAAAACAAGGTTTCTCCCAGTAGCCGGTGGAACAGGCAGTGTTTATATTGAAGTGTTGCAC
TTAACCCAAGGGACGTGCTGGGAGCAGATGTACACTCCAGGAGGAGAAGTGAGAAATGATGATGTTGACC
AAAGTTTGATTATCGCTGCTAGAAACATAGTAAGAAGAGCAGCAGTGTCAGCAGACCCATTAGCATCTCT
CTTGGAAATGTGCCACAGCACACAGATTGGAGGAGTGAAGATGGTGGACATCCTTAGACAGAATCCAACT
GAGGAACAAGCCGTAGACATATGCAAGGCAGCAATAGGGTTGAGGATCAGCTCATCTTTCAGTTTTGGTG
GGTTCACTTTCAAAAGGACAAGCGGATCATCAGTCAAGAAAGAAGAAGAAATGCTAACGGGCAATCTCCA
AACACTGAAATTAAGAGTACATGAAGGGTATGAAGAATTCACAATGGTTGGGAGAAGAGCAACAGCTATT
CTCAGAAAGGCAACCAGGAGATTGATCCAATTGATAGTAAGCGGGAGAGACGAGCAGTCAATTGCTGAGG
CAATAATTGTGGCCATGGTATTCTCACAAGAGGATTGCATGATCAAGGCAGTTAGGGGCGATCTGAACTT
TGTCAATAGGGCAAACCAGCGACTGAACCCCATGCACCAACTCTTGAGGCATTTCCAAAAAGATGCAAAA
GTGCTTTTCCAGAACTGGGGAATTGAATCCATCGACAATGTGATGGGAATGATCGGAATACTGCCCGACA
TGACCCCAAGCACGGAGATGTCGCTGAGAGGGATAAGAGTCAGCAAGATGGGAGTAGATGAATACTCCAG
CACGGAGAGAGTGGTAGTGAGTATTGACCGATTTTTAAGGGTTAGAGATCAAAGAGGGAACGTACTATTG
TCTCCCGAAGAAGTCAGTGAAACGCAAGGAACTGAGAAGTTGACAATAACTTATTCGTCATCAATGATGT
GGGAGATCAATGGCCCTGAGTCAGTGCTAGTCAACACTTATCAATGGATAATCAGAAACTGGGAAATTGT
GAAAATTCAATGGTCACAAGATCCCACAATGTTATACAATAAAATGGAATTTGAACCATTTCAATCTCTT
GTCCCTAAGGCAACCAGAAGCCGGTACAGTGGATTCGTAAGGACACTGTTCCAGCAAATGCGGGATGTGC
TTGGGACATTTGACACTGTCCAAATAATAAAACTTCTCCCCTTTGCTGCTGCTCCACCAGAACAGAGTAG
GATGCAATTTTCCTCATTGACTGTGAATGTGAGAGGATCAGGGTTGAGGATACTGGTAAGAGGCAATTCT
CCAGTATTCAATTACAACAAGGCAACCAAACGACTTACAGTTCTTGGAAAGGATGCAGGTGCATTGACTG
AAGATCCAGATGAAGGCACATCTGGGGTGGAGTCTGCTGTCCTGAGAGGATTTCTCATTTTAGGCAAAGA
AGACAAGAGATATGGCCCAGCATTAAGCATCAATGAACTGAGCAATCTTGCAAAAGGAGAGAAAGCTAAT
GTGCTAATTGGGCAAGGGGACATAGTGTTGGTAATGAAACGAAAACGGGACTCTAGCATACTTACTGACA
GCCAGACAGCGACCAAAAGAATTCGGATGGCCATCAATTAGTGTCGAATTGTT
>gi|345722613|gb|JN655532.1| Influenza A virus (A/Pennsylvania/09/2011(H3N2)) segment 1 polymerase PB2 (PB2) gene, complete cds
ATGGAGAGAATAAAAGAACTAAGAGATCTAATGTCGCAGTCTCGCACTCGCGAGATACTCACTAAGACCA
CTGTGGACCATATGGCCATAATCAAAAAGTACACGTCAGGAAGGCAGGAGAAGAACCCCGCACTCAGAAT
GAAATGGATGATGGCAATGAAATACCCAATTACAGCAGACAGGAGAATAATGGACATGATTCCAGAGAGG
AATGAACAGGGACAAACCCTCTGGAGCAAAACAACCGATGCTGGATCGGACCGTGTGATGGTATCACCCC
TGGCCGTAACATGGTGGAATAGGAATGGCCCAACAACAAGCACAGTTCACTACCCTAAGGTATACAAAAC
TTATTTCGAAAAAGTCGAAAGGTTAAAACATGGTACCTTTGGCCCTGTCCACTTCAGAAATCAAGTTAAA
ATAAGAAGGAGGGTTGACACAAACCCCGGTCATGCAGATCTCAGTGCCAAGGAGGCACARGATGTGATCA

TGGAAGTTGTTTTCCCAAACGAAGTGGGGGCAAGAATACTGACATCAGAGTCACAGCTGACAATAACAAA
AGAAAAGAAAGAAGAGCTCCAGGATTGTAAAATTGCTCCCTTGATGGTGGCATACATGCTAGAAAGAGAA
TTGGTTCGTAAGACGAGGTTTCTTCCGGTGGCTGGTGGAACAAGCAGTGTTTATATTGAAGTGCTGCACT
TAACTCAGGGAACATGTTGGGAACAAATGTACACTCCAGGAGGAGAAGTGAGAAATGATGATGTTGACCA
AAGTTTGATTATCGCCGCTAGAAACATAGTAAGAAGAGCAGCAGTGTCAGCAGACCCATTAGCATCTCTC
TTGGAAATGTGCCACAGCACACAAATTGGAGGAATAAGGATGATGGACATCCTTAGACAGAACCCAACGG
AGGAACAAGCCGTAGACATATGCAAGGCAGCAATGGGGCTGAGGATTAGCTCCTCTTTCAGCTTTGGTGG
GTTCACCTTCAAAAGGACAAGCGGATCATCTGTTAAGAAAGAAGAAGAAGTGCTCACGGGCAACCTCCAA
ACACTGAAAATAAGAGTACATGAAGGATATGAGGAATTCACAATGGTCGGGAGAAGAGCAACAGCTATTC
TCAGAAAAGCAACCAGGAGATTGATCCAGTTAATAGTAAGTGGAAGAGACGATCAATCAATTGCTGAGGC
AATAATTGTGGCCATGGTATTTTCACAAGAGGATTGCATGATCAAAGCAGTTAGGGGCGATCTGAACTTT
GTCAATAGGGCAAACCAGCGGCTGAATCCCATGCACCAACTCTTGAGGCATTTCCAAAAGGATGCAAAAG
TGCTTTTCCAGAACTGGGGGATTGAGCCCATCGACAGTGTAATGGGAATGATCGGAATATTGCCTGATAT
GACCCCAAGCACGGAAATGTCACTGAGAGGTATAAGAGTCAGCAAAATGGGAGTAGATGAATATTCAAGT
ACGGAGAGAGTGGTAGTGAGCATTGACCGATTTTTGAGAGTTCGGGATCAACGAGGGAACGTACTATTGT
CCCCCGAAGAGGTCAGCGAGACACAGGGAACTGAGAAATTGACCATAACTTATTCGTCATCAATGATGTG
GGAGATCAATGGTCCTGAGTCAGTGCTGGTCAACACTTATCAATGGATCATAAGGAACTGGGAAAGCTTG
AAAATTCAATGGTCACAGGATCCCACGATGTTATACAACAAAATGGAATTTGAACCATTCCAGTCTCTTG
TCCCTAAGGCAACCAGAAGTCGTTACAGTGGATTCGTGAGGACACTGTTCCAGCAAATGCGGGATGTGCT
TGGAACATTTGATACTGTCCAAATAATAAAGCTTCTCCCCTTTGCTGCTGCTCCACCGGAACAGAGTAGG
ATGCAGTTCTCCTCGCTGACTGTGAATGTAAGAGGATCAGGGCTGAGGATACTGGTAAGAGGCAATTCTC
CAGTGTTCAATTACAACAAAGCAACCAAAAGGCTTACAATTCTTGGAAAAGATGCAGGTGCATTGACTGA
AGATCCAGATGAAGGCACAGCTGGAGTGGAGTCTGCTGTCCTGAGGGGATTCCTCATTTTGGGTAAAGAA
GACAAGAGATATGGCCCAGCATTAAGCATCAATGAACTGAGCAATCTTGCAAAAGGAGAGAAGGCTAATG
TGCTAATTGGGCAAGGAGACGTGGTGTTGGTAATGAAACGGAAACGGGACTCTAGCATACTTACTGACAG
CCAGACAGCGACCAAAAGGATTCGGATGGCCATCAATTAG
>gi|344166498|gb|CY098057.1| Influenza A virus (A/Moscow/WRAIR4316T/2011(H1N1)) polymerase PB2 (PB2) gene, complete cds
ATGGAGAGAATAAAAGAACTGAGAGATCTAATGTCGCAGTCCCGCACTCGCGAGATACTCACTAAGACCA
CTGTGGACCATATGGCCATAATCAAAAAGTACACATCAGGAAGGCAAGAGAAGAACCCCGCACTCAGAAT
GAAGTGGATGATGGCAATGAGATACCCAATTACAGCAGACAAGAGAATAATGGACATGATTCCAGAGAGG
AATGAACAAGGACAAACCCTCTGGAGCAAAACAAACGATGCTGGATCAGACCGAGTGATGGTGTCACCTC
TGGCCGTAACATGGTGGAATAGGAATGGCCCAACAACAAGTACAGTTCATTACCCTAAGGTATATAAAAC
TTATTTCGAAAAGGTCGAAAGATTGAAACATGGTACCTTCGGCCCTGTCCACTTCAGAAATCAAGTTAAA
ATAAGGAGGAGAGTTGATACAAACCCTGGCCATGCAGATCTCAGTGCCAAGGAGGCACAGGATGTGATTA
TGGAAGTTGTTTTCCCAAATGAAGTGGGGGCAAGAATACTGACATCAGAGTCACAACTGGCAATAACAAA
AGAGAAGAAAGAAGAACTCCAGGATTGTAAAATTGCTCCCTTAATGGTGGCGTACATGCTAGAAAGAGAA
TTGGTCCGTAAGACAAGGTTTCTCCCAGTAGCCGGAGGAACAGGCAGTGTTTATATTGAAGTGTTGCACT
TAACCCAAGGGACGTGCTGGGAGCAGATGTACACTCCAGGAGGAGAAGTGAGAAATGATGATGTTGACCA
AAGTTTGATTATCGCTGCTAGAAACATAGTAAGAAGAGCAGCAGTGTCAGCAGACCCATTAGCATCTCTC
TTGGAAATGTGCCACAGCACACAGATTGGAGGAGTAAGGATGGTGGACATCCTTAGACAGAATCCAACTG
AGGAACAAGCCGTAGACATATGCAAGGCAGCAATAGGGTTGAGGATTAGCTCATCTTTCAGTTTTGGTGG
GTTCACCTTCAAAAGGACAAGCGGATCATCAGTCAAGAAAGAAGAAGAAGTGCTAACGGGCAACCTCCAA
ACACTGAAAATAAGAGTACATGAAGGGTATGAAGAATTCACAATGGTTGGGAGAAGAGCAACAGCTGTTC

TCAGAAAGGCAACCAGGAGATTGATCCAGTTGATAGTAAGCGGGAGAGACGAGCAGTCAATTGCTGAGGC
AATAATTGTGGCCATGGTATTCTCACAGGAGGATTGCATGATCAAGGCAGTTAGGGGCGATCTGAACTTT
GTCAATAGGGCAAACCAGCGACTGAACCCTATGCACCAACTCTTGAGACATTTCCAAAAAGATGCAAAAG
TGCTTTTCCAGAACTGGGGAATTGAATCCATCGACAATGTGATGGGAATGATCGGAATACTGCCCGACAT
GACCCCAAGCACGGAGATGTCACTGAGAGGGATAAGAGTCAGCAAAATGGGAGTAGATGAATACTCCAGC
ACGGAGAGAGTGGTAGTGAGCATTGACCGATTTTTAAGGGTTAGAGATCAAAGAGGGAACGTACTATTGT
CTCCCGAAGAAGTCAGTGAAACGCAAGGAACTGAGAAGTTGACAATAACTTATTCGTCATCAATGATGTG
GGAGATCAATGGCCCTGAGTCAGTGCTAGTCAACACTTATCAATGGATAATCAGGAACTGGGAAATTGTG
AAAATTCAATGGTCACAAGATCCCACAATGTTATACAACAAAATGGAATTTGAACCATTTCAGTCTCTTG
TCCCTAAGGCAACCAGAAGCCGGTACAGTGGATTCGTAAGGACACTGTTCCAGCAAATGCGGGATGTGCT
TGGGACATTTGACACTGTCCAAATAATAAAACTCCTCCCCTTTGCTGCTGCTCCACCAGAACAGAGTAGG
ATGCAATTTTCCTCATTGACTGTGAATGTGAGAGGATCAGGGTTGAGGATACTGGTAAGAGGCAATTCTC
CAGTATTCAATTACAACAAGGCAACCAAACGACTTACAGTTCTTGGAAAGGATGCAGGTGCATTGACTGA
AGATCCAGATGAAGGCACATCTGGGGTGGAGTCTGCTGTCCTGAGAGGATTTCTCATTTTGGGCAAAGAA
GACAAGAGATATGGCCCAGCATTAAGCATCAATGAACTGAGCAATCTTGCAAAAGGAGAGAAGGCTAATG
TGCTAATTGGGCAAGGGGACGTAGTGTTGGTAATGAAACGAAAACGGGACTCTAGCATACTTACTGACAG
CCAGACAGCGACCAAAAGAATTCGGATGGCCATCAATTAG
>gi|344166372|gb|CY098001.1| Influenza A virus (A/Prague/WRAIR4146N/2011(H1N1)) polymerase PB2 (PB2) gene, complete cds
ATGGAGAGAATAAAAGAACTGAGAGATCTAATGTCGCAGTCCCGCACTCGCGAGATACTCACTAAGACCA
CTGTGGACCATATGGCCATAATCAAAAAGTACACATCAGGAAGGCAAGAGAAGAACCCCGCACTCAGAAT
GAAGTGGATGATGGCAATGAGATACCCAATTACAGCAGACAAGAGAATAATGGACATGATTCCAGAGAGG
AATGAACAAGGACAAACCCTCTGGAGCAAAACAAACGATGCTGGATCAGACCGAGTGATGGTATCACCTC
TGGCCGTAACATGGTGGAATAGGAATGGCCCAACAACAAGTACAGTTCATTACCCTAAGGTATATAAAAC
TTATTTCGAAAAGGTCGAAAGGTTGAAACATGGTACCTTCGGCCCTGTCCACTTCAGAAATCAAGTTAAA
ATAAGGAGGAGAGTTGATACAAACCCTGGCCATGCAGATCTCAGTGCCAAGGAGGCACAGGATGTGATTA
TGGAAGTTGTTTTCCCAAATGAAGTGGGGGCAAGAATACTGACATCAGAGTCACAGCTGGCAATAACAAA
AGAGAAGAAAGAAGAGCTCCAGGATTGTAAAATTGCTCCCTTGATGGTGGCGTACATGCTAGAAAGAGAA
TTGGTCCGTAAAACAAGGTTTCTCCCAGTGGCCGGTGGAACAGGCAGTGTTTATATTGAAGTGTTGCACT
TAACCCAAGGGACGTGCTGGGAGCAGATGTACACTCCAGGAGGAGAAGTGAGAAATGATGATGTTGACCA
AAGTTTGATTATCGCTGCTAGAAATATAGTAAGAAGAGCAGCAGTGTCAGCAGACCCATTAGCATCTCTC
TTGGAACTGTGCCACAGCACACAGATTGGAGGAGTAAGGATGGTGGACATCCTTAGACAGAATCCAACTG
AGGAACAAGCCGTAGACATATGCAAGGCAGCAATAGGGTTGAGGATCAGCTCATCTTTCAGTTTTGGTGG
GTTCACTTTCAAAAGGACAAGCGGATCATCAGTCAAGAAAGAAGAAGAAATGCTAACGGGCAACCTCCAA
ACACTGAAATTAAGAGTACATGAAGGGTATGAAGAATTCACAATGGTTGGGAGAAGAGCAACAGCTATTC
TCAGAAAGGCAACCAGGAGATTGATCCAATTGATAGTAAGCGGGAGAGACGAGCAGTCAATTGCTGAGGC
AATAATTGTGGCCATGGTATTCTCACAAGAGGATTGCATGATCAAGGCAGTTAGGGGCGATCTGAACTTT
GTCAATAGGGCAAACCAGCGACTGAACCCCATGCACCAACTCTTGAGGCATTTCCAAAAAGATGCAAAAG
TGCTTTTCCAGAACTGGGGAATTGAATCCATCGACAATGTGATGGGAATGATCGGAATACTGCCCGACAT
GACCCCAAGCACGGAGATGTCGCTGAGAGGGATAAGAGTCAGCAAGATGGGAGTAGATGAATACTCCAGC
ACGGAGAGAGTGGTAGTGAGTATTGACCGATTTTTAAGGGTTAGAGATCAAAGAGGGAACGTACTATTGT
CTCCCGAAGAAGTCAGTGAAACGCAAGGAACTGAGAAGTTGACAATAACTTATTCGTCATCAATGATGTG
GGAGATCAATGGCCCTGAGTCAGTGCTAGTCAACACTTATCAATGGATAATCAGGAACTGGGAAATTGTG
AAAATTCAATGGTCACAAGATCCCACAATGTTATACAACAAAATGGAATTTGAACCATTTCAGTCTCTTG

TCCCTAAGGCAACCAGAAGCCGGTACAGTGGATTCGTAAGGACACTGTTCCAGCAAATGCGGGATGTGCT
TGGGACATTTGACACTGTCCAAATAATAAAACTACTCCCCTTTGCTGCTGCTCCACCAGAACAGAGTAGG
ATGCAATTTTCCTCATTGACTGTGAATGTGAGAGGATCAGGGTTGAGGATACTGGTAAGAGGCAATTCTC
CAGTATTCAATTACAACAAGGCAACCAAACGACTTACAGTTCTTGGAAAGGATGCAGGTGCATTGACTGA
AGATCCAGACGAAGGCACATCTGGGGTGGAGTCTGCTGTCCTGAGAGGATTTCTCATTTTAGGCAAAGAA
GACAAGAGATATGGCCCAGCATTAAGCATCAATGAACTGAGCAATCTTGCAAAAGGAGAGAAAGCTAATG
TGCTAATTGGGCAAGGGGACGTAGTGTTGGTAATGAAACGAAAACGGGACTCTAGCATACTTACTGACAG
CCAGACAGCGACCAAAAGAATTCGGATGGCCATCAATTAG
>gi|339518920|gb|JN185090.1| Influenza A virus (A/Tula/CRIE-SIA/2011(H1N1)) segment 1 polymerase PB2 (PB2) gene, complete cds
ATGGAGAGAATAAAAGAACTGAGAGATCTAATGTCGCAGTCCCGCACTCGCGAGATACTCACTAAGACCA
CTGTGGACCATATGGCCATAATCAAAAAGTACACATCAGGAAGGCAAGAGAAGAACCCCGCACTCAGAAT
GAAGTGGATGATGGCAATGAGATACCCAATTACAGCAGACAAGAGAATAATGGACATGATTCCAGAGAGG
AATGAACAAGGACAAACCCTCTGGAGCAAAACAAACGATGCTGGATCAGACCGAGTGATGGTATCACCTC
TGGCCGTAACATGGTGGAATAGGAATGGCCCAACAACAAGTACAGTTCATTACCCTAAGGTATATAAAAC
TTATTTCGAAAAGGTCGAAAGGTTGAAACATGGTACCTTCGGCCCTGTCCACTTCAGAAATCAAGTTAAA
ATAAGGAGGAGAGTTGATACAAACCCTGGCCATGCAGATCTCAGTGCCAAGGAGGCACAGGATGTGATTA
TGGAAGTTGTTTTCCCAAATGAAGTGGGGGCAAGAATACTGACATCAGAGTCACAGCTAGCAATAACAAA
AGAGAAGAAAGAAGAGCTCCAGGATTGTAAAATTGCTCCCTTGATGGTGGCGTACATGCTAGAAAGAGAA
TTGGTCCGTAAAACAAGGTTTCTCCCAGTAGCCGGCGGAACAGGCAGTGTTTATATTGAAGTGTTGCACT
TAACCCAAGGGACGTGCTGGGAGCAGATGTACACCCCAGGAGGAGAAGTGAGAAATGATGATGTTGACCA
AAGTTTGATTATCGCTGCTAGAAACATAGTAAGAAGAGCAGCAGTGTCAGCAGACCCATTAGCATCTCTC
TTGGAAATGTGCCACAGCACACAAATTGGAGGAGTAAGGATGGTGGACATCCTTAGACAGAATCCAACTG
AGGAACAAGCCGTAGACATATGCAAGGCAGCAATAGGGTTGAGGATTAGCTCATCTTTCAGTTTTGGTGG
GTTCACTTTCAAAAGGACAAGCGGATCATCAGTCAAGAAAGAAGAAGAAGTGCTAACGGGCAACCTCCAA
ACACTGAAAATAAGAGTACATGAAGGGTATGAAGAATTCACAATGGTTGGGAGAAGAGCAACAGCTATTC
TCAGAAAGGCAACCAGGAGATTGATCCAGTTGATAGTAAGCGGGAGAGACGAGCAGTCAATTGCTGAGGC
AATAATTGTGGCCATGGTATTCTCACAAGAGGATTGCATGATCAAGGCAGTTAGGGGCGATCTGAACTTT
GTTAATAGGGCAAACCAGCGACTGAACCCCATGCACCAACTCTTGAGGCATTTCCAAAAAGATGCAAAAG
TACTTTTCCAGAACTGGGGAATTGAATCCATCGACAATGTGATGGGAATGATCGGAATACTGCCTGACAT
GACTCCAAGCACGGAGATGTCGCTGAGAGGGATAAGAGTCAGCAAAATGGGAGTAGATGAATACTCCAGC
ACGGAGAGAGTGGTAGTGAGTATTGACCGATTTTTAAGGGTTAGAGATCAAAGAGGGAACGTACTATTGT
CTCCCGAAGAAGTCAGTGAAACGCAAGGAACTGAGAAGTTGACAATAACTTATTCGTCATCAATGATGTG
GGAGATCAATGGCCCTGAGTCAGTGCTAGTTAACACTTATCAATGGATAATCAGGAACTGGGAAATTGTG
AAAATTCAATGGTCACAAGATCCCACAATGTTATACAACAAAATGGAATTTGAACCATTTCAGTCTCTTG
TCCCTAAGGCAACCAGAAGCCGGTACAGTGGATTCGTAAGGACGCTGTTCCAGCAAATGCGGGATGTGCT
TGGGACATTTGACACTGTCCAAATAATAAAACTTCTCCCCTTTGCTGCTGCTCCACCAGAACAGAGTAGG
ATGCAATTCTCCTCATTGACTGTGAATGTGAGAGGATCAGGGTTGAGGATACTGGTAAGAGGCAATTCTC
CAGTATTCAATTACAACAGGGCAACCAAACGACTTACAGTTCTTGGAAAGGATGCAGGTGCATTGACTGA
AGATCCAGATGAAGGCACATCTGGAGTGGAGTCTGCTGTCCTGAGAGGATTTCTCATTTTGGGCAAAGAA
GACAAGAGATACGGCCCCGCATTAAGCATCAATGAACTGAGCAATCTTGCAAAAGGAGAGAAAGCTAATG
TGCTAATTGGGCAAGGGGACGTAGTGTTGGTAATGAAACGAAAACGGGACTCTAGCATACTTACTGACAG
CCAGACAGCGACCAAAAGAATTCGGATGGCCATCAATTAG
>gi|339517029|gb|JN187181.1| Influenza A virus (A/Taiwan/3697/2011(H1N1)) segment 1

polymerase PB2 (PB2) gene, complete cds
ATGGAGAGAATAAAAGAACTGAGAGATCTAATGTCGCAGTCCCGCACTCGCGAGATACTCACTAAGACCA
CTGTGGACCATATGGCCATAATCAAAAAGTACACATCAGGAAGGCAAGAGAAGAACCCCGCACTCAGAAT
GAAGTGGATGATGGCAATGAGATACCCAATTACAGCAGACAAGAGAATAATGGACATGATTCCAGAGAGG
AATGAACAAGGACAAACCCTCTGGAGCAAAACAAACGATGCTGGATCAGACCGAGTGATGGTATCACCTC
TGGCCGTAACATGGTGGAATAGGAATGGCCCAACAACAAGTACAGTTCATTACCCTAAGGTATATAAAAC
TTATTTCGAAAAGGTCGAAAGGTTGAAACATGGTACCTTCGGCCCTGTCCACTTCAGAAATCAAGTTAAA
ATAAGGAGGAGAGTTGATACAAACCCTGGCCATGCAGATCTCAGTGCCAAGGAGGCACAGGATGTGATTA
TGGAAGTTGTTTTCCCAAATGAAGTGGGGGCAAGAATACTGACATCAGAGTCACAGCTGGCAATAACAAA
AGAGAAGAAAGAAGAGCTCCAGGATTGTAAAATTGCTCCCTTGATGGTGGCGTACATGCTAGAAAGAGAA
TTGGTCCGTAAAACAAGGTTTCTCCCAGTAGCCGGTGGAACAGGCAGTGTTTATATTGAAGTGTTGCACT
TAACCCAAGGGACGTGCTGGGAGCAGATGTACACTCCAGGAGGAGAAGTGAGAAATGATGATGTTGACCA
AAGTTTGATTATCGCTGCTAGAAACATAGTAAGAAGAGCAGCAGTGTCAGCAGACCCATTAGCATCTCTC
TTGGAAATGTGCCACAGCACACAGATTGGAGGAGTAAGGATGGTGGACATCCTTAGACAGAATCCAACTG
AGGAACAAGCCGTAGACATATGCAAGGCAGCAATAGGGTTGAGAATCAGCTCATCTTTCAGTTTTGGTGG
GTTCACTTTCAAAAGGACAAGCGGATCATCAGTCAAGAAAGAAGAAGAAATGCTAACGGGCAATCTCCAA
ACACTGAAATTAAGAGTACATGAAGGGTATGAAGAATTCACAATGGTTGGGAGAAGAGCAACAGCTATTC
TCAGAAAGGCAACCAGGAGATTGATCCAATTGATAGTAAGCGGGAGAGACGAGCAATCAATTGCTGAGGC
AATAATTGTGGCCATGGTATTCTCACAAGAGGATTGCATGATCAAGGCAGTTAGAGGCGATCTGAACTTT
GTCAATAGGGCAAACCAGCGACTGAACCCCATGCACCAACTCTTGAGGCATTTCCAAAAAGATGCAAAAG
TGCTTTTCCAGAACTGGGGAATTGAATCCATCGACAATGTGATGGGAATGATCGGAATACTGCCCGACAT
GACCCCAAGCACGGAGATGTCGCTGAGAGGGATAAGAGTCAGCAAGATGGGAGTAGATGAATACTCCAGC
ACGGAGAGAGTGGTAGTGAGTATTGACCGATTTTTAAGGGTTAGAGATCAAAGAGGGAACGTACTATTGT
CTCCCGAAGAAGTCAGTGAAACACAAGGAACTGAGAAGTTGACAATAACTTATTCGTCATCAATGATGTG
GGAGATCAATGGCCCTGAGTCAGTGCTAGTCAACACTTATCAATGGATAATCAGAAACTGGGAAATTGTG
AAAATTCAATGGTCACAAGATCCCACAATGTTATACAACAAAATGGAATTTGAACCATTTCAGTCTCTTG
TCCCTAAGGCAACCAGAAGCCGGTACAGTGGATTCGTAAGGACACTGTTCCAGCAAATGCGGGATGTGCT
TGGGACATTTGACACTGTCCAAATAATAAAACTTCTCCCCTTTGCTGCTGCTCCACCAGAACAGAGTAGG
ATGCAATTTTCCTCATTGACTGTGAATGTGAGAGGATCAGGGTTGAGGATACTGGTAAGAGGCAATTCTC
CAGTATTCAATTACAACAAGGCAACCAAACGACTTACAGTTCTTGGAAAGGATGCAGGTGCATTGACTGA
AGATCCAGATGAAGGCACATCTGGGGTGGAGTCTGCTGTCCTGAGAGGATTTCTCATTTTAGGCAAAGAA
GACAAGAGATATGGCCCAACATTAAGCATCAATGAACTGAGCAATCTTGCAAAAGGAGAGAAAGCTAATG
TGCTAATTGGGCAAGGGGACGTAGTGTTGGTAATGAAACGAAAACGGGACTCTAGCATACTTACTGACAG
CCAGACAGCGACCAAAAGAATTCGGATGGCCATCAATTAG
>gi|388774802|gb|CY120762.1| Influenza A virus (A/Brazil/AVS11/2011(H1N1)) polymerase PB2 (PB2) gene, complete cds
TATGGAGAGAATAAAAGAACTGAGAGATCTAATGTCGCAGTCCCGCACTCGCGAGATACTCACTAAGACC
ACTGTGGACCATATGGCCATAATCAAAAAGTACACATCAGGAAGGCAAGAGAAGAACCCCGCACTCAGAA
TGAAGTGGATGATGGCAATGAGATACCCAATTACAGCAGACAAGAGAATAATGGACATGATTCCAGAGAG
GAATGAACAAGGACAAACCCTCTGGAGCAAAACAAGCGATGCTGGATCAGACCGAGTGATGGTATCACCT
CTGGCCGTAACATGGTGGAATAGGAATGGCCCAACAACAAGTACAGTTCATTACCCTAAGGTATATAAAA
CTTATTTCGAAAAGGTCGAAAGGTTGAAACATGGTACATTCGGCCCTGTCCACTTCAGAAATCAAGTTAA
AATAAGGAGGAGAGTTGATACAAACCCTGGCCATGCAGATCTCAGTGCCAAGGAGGCACAGGATGTGATT
ATGGAAGTTGTTTTCCCAAATGAAGTGGGGGCAAGAATATTGACATCAGAGTCACAGCTGGCAATAACAA

AAGAGAAGAAAGAAGAGCTCCAGGATTGTAAAATTGCTCCCTTGATGGTGGCGTACATGCTAGAAAGAGA
ATTGGTCCGTAAAACAAGGTTTCTCCCAGTAGCCGGTGGAACAGGCAGTGTTTATATTGAAGTGTTGCAC
TTAACCCAAGGGACGTGCTGGGAGCAGATGTACACTCCAGGAGGAGAAGTGAGAAATGATGATGTTGACC
AAAGTTTGATTATCGCTGCTAGAAACATAGTAAGAAGAGCAGCAGTGTCAGCAGACCCATTAGCATCTCT
CTTGGAAATGTGCCACAGCACACAGATTGGAGGAGTAAGGATGGTGGACATACTTAGACAGAATCCAACT
GAGGAACAAGCCGTAGACATATGCAAGGCAGCAATAGGGTTGAGGATCAGCTCATCTTTCAGTTTTGGTG
GGTTCACTTTCAAAAGGACAAGCGGGTCATCAGTCAAGAAAGAAGAAGAAATGCTAACGGGCAACCTTCA
AACACTGAAAATGAGAGTACATGAAGGGTATGAAGAATTCACAATGGTTGGGAGAAGAGCAACAGCTATT
CTCAGAAAGGCAACCAGGAGATTGATCCAATTGATAGTAAGCGGGAGAGACGAGCAGTCAATTGCTGAGG
CAATAATTGTGGCCATGGTATTCTCACAAGAGGATTGCATGATCAAGGCAGTTAGGGGCGATCTGAACTT
TGTCAATAGGGCAAACCAGCGGCTGAACCCCATGCACCAACTCTTGAGGCATTTCCAAAAAGATGCAAAA
GTGCTTTTCCAGAACTGGGGAATTGAATCCATCGACAATGTGATGGGAATGATCGGAATACTGCCCGACA
TGACCCCAAGCACGGAGATGTCGCTGAGAGGGATAAGAGTCAGCAAGATGGGAGTAGATGAATACTCCAG
CACGGAGAGAGTGGTAGTGAGTATTGACCGATTTTTAAGGGTTAGAGATCAAAGAGGGAACGTACTATTG
TCTCCCGAAGAAGTCAGTGAAACGCAAGGAACTGAGAAGTTGACAATAACTTATTCGTCATCAATGATGT
GGGAGATCAATGGCCCTGAGTCAGTGCTAGTCAACACTTATCAATGGATAATCAGGAACTGGGAAATTGT
GAAAATTCAATGGTCACAAGATCCCACAATGTTATACAACAAAATGGAATTTGAACCATTTCAGTCTCTT
GTCCCTAAGGCAACCAGAAGCCGGTACAGTGGATTCGTAAGGACACTGTTCCAGCAAATGCGGGATGTGC
TTGGGACATTTGACACTGTCCAAATAATAAAACTTCTCCCCTTTGCTGCTGCTCCACCAGAACAGAGTAG
GATGCAATTTTCCTCATTGACTGTGAATGTGAGAGGATCAGGGTTGAGGATACTGGTAAGAGGCAATTCT
CCAGTATTCAATTACAACAAGGCAACCAAACGACTTACAGTTCTTGGAAAGGATGCAGGTGCATTGACTG
AAGATCCAGATGAAGGCACATCTGGGGTGGAGTCTGCTGTCCTGAGAGGATTTCTCATTTTAGGCAAAGA
AGACAAGAGATATGGCCCAGCATTAAGCATCAATGAACTGAGCAATCTTGCAAAAGGAGAGAAAGCTAAT
GTGCTAATTGGGCAAGGGGACGTAGTGTTGGTAATGAAACGAAAACGGGACTCTAGCATACTTACTGACA
GCCAGACAGCGACCAAAAGAATTCGGATGGCCATCAATTAGTGTCGAATTGTT
>gi|387604495|gb|CY120019.1| Influenza A virus (A/Mexico/InDRE3740/2011(H1N1)) polymerase PB2 (PB2) gene, complete cds
ATGGAGAGAATAAAAGAACTGAGAGATCTAATGTCGCAGTCCCGCACTCGCGAGATACTCACTAAGACCA
CTGTGGACCATATGGCCATAATCAAAAAGTACACATCAGGAAGGCAAGAGAAGAACCCCGCACTCAGAAT
GAAGTGGATGATGGCAATGAGATACCCAATTACAGCAGACAAGAGAATAATGGACATGATTCCAGAGAGG
AATGAACAAGGACAAACCCTCTGGAGCAAAACAAATGATGCTGGATCAGACCGAGTGATGGTATCACCTC
TGGCCGTAACATGGTGGAATAGAAATGGCCCAACAACAAGTACAGTTCATTACCCTAAGGTATATAAAAC
TTATTTCGAAAAGGTCGAAAGGTTGAAACATGGTACCTTCGGCCCTGTCCACTTCAGAAATCAAGTTAAA
ATAAGGAGGAGAGTTGATACAAACCCTGGTCATGCAGACCTCAGTGCCAAGGAGGCACAGGATGTGATTA
TGGAAGTTGTTTTCCCAAATGAAGTAGGGGCAAGAATACTGACATCAGAGTCACAGCTGGCAATAACAAA
AGAGAAGAAAGAAGAGCTCCAGGATTGTAAAATTGCTCCCTTGATGGTGGCGTACATGCTAGAAAGAGAA
TTGGTCCGTAAAACAAGGTTTCTCCCAGTAGCCGGTGGAACAGGCAGTGTTTATATTGAAGTGTTGCACT
TAACCCAAGGGACATGCTGGGAGCAGATGTACACTCCAGGAGGAGAAGTGAGAAATGATGATGTTGACCA
AAGTTTGATTATCGCTGCTAGAAACATAGTAAGAAGAGCAGCAGTGTCAGCAGACCCATTAGCATCTCTC
TTGGAAATGTGCCACAGCACACAGATTGGAGGAGTAAGGATGGTGGACATCCTTAGACAGAATCCAACTG
AAGAACAAGCTGTAGACATATGCAAAGCAGCAATAGGGTTGAGGATCAGCTCATCTTTCAGTTTTGGTGG
GTTCACTTTCAAAAGGACAAGCGGATCATCAGTCAAGAAAGAAGAAGAAATGCTAACGGGCAATCTCCAA
ACACTGAAATTAAGAGTACATGAAGGGTATGAGGAATTCACAATGGTTGGGAGAAGAGCAACAGCTATTC
TCAGAAAGGCAACCAGGAGATTGATCCAATTGATAGTAAGCGGGAGAGACGAGCAGTCAATTGCTGAGGC

AATAATTGTGGCCATGGTATTCTCACAAGAGGATTGCATGATCAAGGCAGTTAGGGGCGATCTGAACTTT
GTCAATAGGGCAAACCAGCGACTGAACCCCATGCACCAACTCTTGAGGCATTTCCAAAAAGATGCAAAAG
TGCTTTTCCAGAACTGGGGGATTGAATCCATCGACAATGTGATGGGAATGATCGGAATACTGCCCGACAT
GACCCCAAGCACGGAGATGTCGCTGAGAGGGATAAGAGTCAGCAAGATGGGAGTAGATGAATACTCCAGC
ACGGAGAGAGTGGTAGTGAGTATTGACCGATTTTTAAGGGTTAGAGATCAAAGAGGGAACGTACTATTGT
CTCCCGAAGAAGTCAGTGAAACGCAAGGAACTGAGAAGTTGACAATAACTTATTCGTCATCAATGATGTG
GGAGATCAATGGCCCTGAATCAGTGCTAGTCAACACTTATCAATGGATAATCAGAAACTGGGAAATTGTC
AAAATTCAATGGTCACAAGATCCCACAATGTTATACAACAAAATGGAATTTGAACCATTTCAGTCTCTTG
TCCCTAAGGCAACCAGAAGCCGGTACAGTGGATTCGTAAGGACACTGTTCCAGCAAATGCGGGATGTGCT
TGGGACATTTGACACTGTCCAAATAATAAAACTTCTCCCCTTTGCTGCTGCTCCACCAGAGCAGAGTAGG
ATGCAATTTTCCTCATTGACTGTGAATGTGAGAGGATCAGGGTTGAGGATACTGGTAAGAGGCAATTCTC
CAGTATTCAATTACAACAAGGCAACCAAACGACTTACAGTTCTTGGAAAGGATGCAGGTGCATTGACTGA
AGATCCAGATGAAGGCACATCTGGGGTGGAGTCTGCTGTCCTGAGAGGATTTCTCATTTTAGGCAAAGAA
GACAAGAGATATGGCCCAGCATTAAGCATCAATGAACTGAGCAATCTTGCAAAAGGAGAGAAAGCTAATG
TGCTAATTGGGCAAGGGGACGTAGTGTTGGTAATGAAACGAAAACGGGACTCTAGCATACTTACTGACAG
CCAGACAGCGACCAAAAGAATTCGGATGGCCATCAATTAG
>gi|338826651|gb|CY092879.1| Influenza A virus (A/Illinois/NHRC0001/2011(H1N1)) polymerase PB2 (PB2) gene, complete cds
TATGGAGAGAATAAAAGAACTGAGAGATCTAATGTCGCAGTCCCGCACTCGCGAGATACTCACTAATACC
ACTGTGGACCATATGGCCATAATCAAAAAGTACACATCAGGAAGGCAAGAGAAGAACCCCGCACTCAGAA
TGAAGTGGATGATGGCAATGAAATACCCAATTACAGCAGACAAGAGAATAATGGACATGATTCCAGAGAG
GAATGAACAAGGACAAACCCTCTGGAACAAAACAAACGATGCTGGATCAGACCGAGTGATGGTATCACCT
CTGGCCGTAACATGGTGGAATAGGAATGGCCCAACAACAAGTACAGTTCATTACCCTAAGGTATATAAAA
CTTATTTCGAAAAGGTCGAAAGGTTGAAACATGGTACCTTTGGCCCTGTCCACTTCAGAAATCAAGTTAA
AATAAGGAGGAGAGTTGATACAAACCCTGGCCATGCAGATCTCAGTGCCAAGGAGGCACAGGATGTGATT
ATGGAAGTTGTTTTCCCAAATGAAGTGGGGGCAAGAATACTGACATCAGAATCACAGCTGGCAATAACAA
AAGAGAAGAAAGAAGAGCTCCAGGATTGTAAAATTGCTCCCTTGATGGTGGCGTACATGCTAGAAAGAGA
ATTGGTCCGTAAAACAAGGTTTCTCCCAGTAGCCGGCGGAACAGGCAGTGTTTATATTGAAGTGTTGCAC
TTAACCCAAGGGACGTGCTGGGAGCAGATGTACACTCCAGGAGGAGAAGTGAGAAATGATGATGTTGACC
AAAGTTTGATTATCGCTGCTAGAAACATAGTAAGAAGAGCAGCAGTGTCAGCAGACCCATTAGCATCTCT
CTTGGAAATGTGCCACAGCACACAGATTGGAGGAGTAAGGATGGTGGACATCCTTAGACAGAATCCAACT
GAGGAACAAGCCGTAGACATATGCAAGGCAGCAATAGGGTTGAGGATTAGCTCATCTTTCAGTTTTGGTG
GGTTCACTTTCAAAAGGACAAGCGGATCATCAGTCAAGAAAGAAGAAGTGCTAACGGGCAACCTCCA
AACACTGAAAATAAGAGTACATGAAGGATATGAAGAATTCACAATGGTTGGGAGAAGAGCAACAGCTATT
CTCAGAAAGGCAACCAGGAGATTGATCCAGTTGATAGTAAGCGGGAGAGACGAGCAGTCAATTGCTGAGG
CAATAATTGTGGCCATGGTATTCTCACAAGAGGATTGCATGATCAAGGCAGTTAGGGGCGATCTGAACTT
TGTCAATAGGGCAAACCAGCGACTGAACCCCATGCATCAACTCTTGAGGCATTTCCAAAAAGATGCAAAA
GTGCTTTTCCAGAACTGGGGAATTGAATCCATCGACAATGTGATGGGGATGATCGGAATACTGCCCGACA
TGACCCCAAGCACGGAGATGTCGCTGAGAGGGATAAGAGTCAGCAAAATGGGAGTAGATGAATACTCCAG
CACGGAGAGAGTGGTAGTGAGTATTGACCGATTTTTAAGGGTTAGAGATCAAAGAGGGAACGTAATATTG
TCTCCCGAAGAAGTCAGTGAAACGCAAGGAACTGAGAAGTTGACAATAACTTATTCGTCATCAATGATGT
GGGAGATCAATGGCCCTGAGTCAGTGCTAGTCAACACTTATCAATGGATAATCAGGAACTGGGAAATTGT
GAAAATTCAATGGTCACAAGATCCCACAATGTTATACAACAAAATGGAATTTGAACCATTTCAGTCTCTT
GTCCCTAAGGCAACCAGAAGCCGGTACAGTGGATTCGTAAGGACACTGTTCCAGCAAATGAGGGATGTGC

TTGGGACATTTGACACTGTCCAAATAATAAAACTTCTCCCCTTTGCTGCTGCTCCACCAGAACAGAGTAG
GATGCAATTTTCCTCATTGACTGTGAATGTGAGAGGTTCAGGGTTGAGGATACTGGTAAGAGGCAATTCT
CCAGTATTCAATTACAACAAGGCAACCAAACGACTTACAGTTCTTGGAAAGGATGCAGGTGCATTGACTG
AAGATCCAGATGAAGGAACATCTGGGGTGGAGTCTGCTGTCTTGAGAGGATTTCTCATTTTGGGCAAAGA
AGACAAGAGATATGGCCCAGCATTAAGCATCAATGAACTGAGCAATCTTGCAAAAGGAGAGAAAGCTAAT
GTGCTAATTGGGCAAGGGGACGTAGTGTTGGTAATGAAACGAAAACGGGACTCTAGCATACTTACTGACA
GCCAGACAGCGACCAAAAGAATTCGGATGGCCATCAATTAGTGTCGAATTGTT
>gi|333595991|gb|CY090829.1| Influenza A virus (A/Denmark/36/2011(H1N1)) polymerase PB2 (PB2) gene, complete cds
ATGGAGAGAATAAAAGAACTGAGAGATCTAATGTCGCAGTCCCGCACTCGCGAGATACTCACTAAGACCA
CTGTGGACCATATGGCCATAATCAAAAAGTACACATCAGGAAGGCAAGAGAAGAACCCCGCACTCAGAAT
GAAGTGGATGATGGCAATGAGATACCCAATTACAGCAGACAAGAGAATAATGGACATGATTCCAGAGAGG
AATGAACAAGGACAAACCCTCTGGAGCAAAACAAACGATGCTGGATCAGACCGAGTGATGGTATCACCTC
TGGCCGTAACATGGTGGAATAGGAATGGCCCAACAACAAGTACAGTTCATTACCCTAAGGTATATAAAAC
TTATTTCGAAAAGGTCGAAAGGTTGAAACATGGTACCTTCGGCCCTGTCCACTTCAGAAATCAAGTTAAA
ATAAGGAGGAGAGTTGATACAAACCCTGGCCATGCAGATCTAAGTGCCAAGGAGGCACAGGATGTGATTA
TGGAAGTTGTTTTCCCAAATGAAGTGGGGGCAAGAATACTGACATCAGAGTCACAGCTGGCAATAACAAA
AGAGAAGAAAGAAGAGCTCCAGGATTGTAAAATTGCTCCCTTGATGGTGGCGTACATGCTAGAAAGAGAA
TTGGTCCGTAAAACAAGGTTTCTCCCAGTAGCCGGTGGAACAGGCAGTGTTTATATTGAAGTGTTGCACT
TAACCCAAGGGACGTGCTGGGAGCAGATGTACACTCCAGGAGGAGAAGTGAGAAATGATGATGTTGACCA
AAGTTTGATTATCGCTGCTAGAAATATAGTAAGAAGAGCAGCAGTGTCAGCAGACCCATTAGCATCTCTC
TTGGAAATGTGCCACAGCACACAGATTGGAGGAGTAAGGATGGTGGACATCCTTAAACAGAATCCAACTG
AGGAACAAGCCGTAGACATATGCAAGGCAGCAATAGGGTTGAGGATCAGCTCATCTTTCAGTTTTGGTGG
GTTCACTTTCAAAAGGACAAGCGGATCATCAGTCAAGAAAGAAGAAGAAATGCTAACGGGCAACCTCCAA
ACACTGAAATTAAGAGTACATGAAGGGTATGAAGAATTCACAATGGTTGGGAGAAGAGCAACAGCTATTC
TCAGAAAGGCAACCAGGAGATTGATCCAATTGATAGTAAGCGGGAGAGACGAGCAGTCAATTGCTGAGGC
AATAATTGTGGCCATGGTATTCTCACAAGAGGATTGCATGATCAAGGCAGTTAGGGGCGATCTGAACTTT
GTCAACAGGGCAAACCAGCGATTGAACCCCATGCACCAACTCTTGAGGCATTTCCAAAAAGATGCAAAAG
TGCTTTTCCAGAACTGGGGAATTGAATCCATCGACAGTGTGATGGGAATGATCGGAATACTGCCCGACAT
GACCCCAAGCACGGAGATGTCGCTGAGAGGGATAAGAGTCAGCAAGATGGGAGTAGATGAATACTCCAGC
ACGGAGAGAGTGGTAGTGAGTATTGACCGATTTTTAAGGGTTAGAGATCAAAGAGGGAACGTACTATTGT
CTCCCGAAGAAGTCAGTGAAACGCAAGGAACTGAGAAGTTGACAATAACTTATTCGTCATCAATGATGTG
GGAGATCAATGGCCCTGAGTCAGTGCTAGTCAACACTTATCAATGGATAATCAGGAACTGGGAAATTGTG
AAAATTCAATGGTCACAAGATCCCACAATGTTATACAACAAAATGGAATTTGAACCATTTCAGTCTCTTG
TCCCTAAGGCAACCAGAAGCCGGTACAGTGGATTCGTAAGGACACTATTCCAGCAAATGCGGGATGTGCT
TGGGACATTTGACACTGTCCAAATAATAAAACTTCTCCCCTTTGCTGCTGCTCCACCAGAACAGAGTAGG
ATGCAATTTTCCTCATTGACTGTGAATGTGAGAGGATCAGGGTTGAGGATACTGGTAAGAGGCAATTCTC
CAGTATTCAATTACAACAAGGCAACCAAACGACTTACAGTTCTTGGAAAGGATGCAGGTGCATTGACTGA
AGATCCAGATGAAGGCACATCTGGGGTGGAGTCTGCTGTCCTGAGAGGATTTCTCATTTTAGGCAAAGAA
GACAAGAGATATGGCCCAGCATTAAGCATCAATGAACTGAGCAATCTTGCAAAAGGAGAGAAAGCTAATG
TGCTAATTGGGCAAGGGGACGTAGTGTTGGTAATGAAACGAAAACGGGACTCTAGCATACTTACTGACAG
CCAGACAGCGACCAAAAGAATTCGGATGGCCATCAATTAG
>gi|401716656|gb|JX473007.1| Influenza A virus (A/Vladivostok/28/2012(H1N1)) segment 1 polymerase PB2 (PB2) gene, complete cds

ATGGAGAGAATAAAAGAACTGAGAGATCTAATGTCGCAGTCCCGCACTCGCGAGATACTCACTAAGACCA
CTGTGGACCATATGGCCATAATCAAAAAGTACACATCAGGAAGGCAAGAGAAGAACCCCGCACTCAGAAT
GAAGTGGATGATGGCAATGAGATACCCAATTACAGCAGACAAGAGAATAATGGACATGATTCCAGAGAGG
AATGAACAAGGACAAACCCTCTGGAGCAAAACAAACGATGCTGGATCAGACCGAGTGATGGTATCACCTC
TGGCCGTAACATGGTGGAATAGGAATGGCCCAACAACAAGTACAGTTCATTACCCTAAGGTATATAAAAC
TTATTTCGAAAAGGTCGAAAGGTTGAAACATGGTACCTTCGGCCCTGTCCACTTCAGAAATCAAGTTAAA
ATAAGGAGGAGAGTTGATACAAACCCTGGCCATGCAGATCTCAGTGCCAAGGAGGCACAGGATGTGATTA
TGGAAGTTGTTTTCCCAAATGAAGTGGGGGCAAGAATACTGACATCAGAGTCACAGCTGGCAATAACAAA
AGAGAAGAAAGAAGAGCTCCAGGATTGTAAAATTGCTCCCTTGATGGTGGCGTACATGCTAGAAAGAGAA
TTGGTCCGTAAAACAAGGTTTCTCCCAGTAGCTGGTGGAACAGGCAGTGTTTATATTGAAGTGTTGCACT
TAACCCAAGGGACGTGCTGGGAGCAGATGTACACTCCAGGAGGAGAAGTGAGAAATGATGATGTTGACCA
AAGTTTGATCATCGCTGCTAGAAATATAGTAAGAAGAGCAGCAGTGTCAGCAGACCCATTAGCATCTCTC
TTGGAAATGTGCCACAGCACACAGATTGGAGGAGTAAGGATGGTGGACATCCTTAAACAGAATCCAACTG
AGGAACAAGCCGTAGACATATGCAAGGCAGCAATAGGGTTGAGGATCAGCTCATCTTTCAGTTTTGGTGG
GTTCACTTTCAAAAGGACAAGCGGATCATCAGTCAAGAAAGAAGAAGAAATGCTAACGGGCAACCTCCAA
ACACTGAAATTAAGAGTACATGAAGGGTATGAAGAATTCACAATGGTTGGGAGAAGAGCAACAGCTATTC
TCAGAAAGGCAACCAGGAGATTGATCCAATTGATAGTAAGCGGGAGAGACGAGCAGTCAATTGCTGAGGC
AATAATTGTGGCCATGGTATTCTCACAAGAGGATTGCATGATCAAGGCAGTTAGGGGCGAYCTGAACTTT
GTCAATAGGGCAAACCAGCGACTGAACCCCATGCACCAACTCTTGAGGCATTTCCAAAAAGATGCAAAAG
TGCTTTTCCAGAACTGGGGAATTGAATCCATCGACAGTGTGATGGGAATGATCGGAATACTGCCCGACAT
GACCCCAAGCACGGAGATGTCGCTGAGAGGGATAAGAGTCAGCAAGATGGGGGTGGATGAATACTCCAGC
ACGGAGAGAGTGGTAGTGAGTATTGACCGATTTTTAAGGGTTAGAGATCAAAGAGGGAACGTACTATTGT
CTCCCGAAGAAGTCAGTGAAACGCAAGGAACTGAGAAGTTGACAATAACTTATTCGTCATCAATGATGTG
GGAGATCAATGGCCCTGAGTCAGTGCTAGTCAACACTTATCAATGGATAATCAGGAACTGGGAAATTGTG
AAAATTCAATGGTCACAAGATCCCACAATGTTATACAACAAAATGGAATTTGAACCATTTCAGTCTCTTG
TCCCTAAGGCAACCAGAAGCCGGTACAGTGGATTCGTAAGGACACTATTCCAGCAAATGCGGGATGTGCT
TGGGACATTTGACACTGTCCAAATAATAAAACTTCTCCCCTTTGCTGCTGCTCCACCAGAACAGAGTAGG
ATGCAATTTTCCTCATTGACTGTGAATGTGAGAGGATCAGGGTTGAGGATACTGGTAAGAGGCAATTCTC
CAGTATTCAATTACAACAAGGCAACCAAACGACTTACAGTTCTTGGAAAGGATGCAGGTGCATTGACTGA
AGATCCAGATGAAGGCACATCTGGGGTGGAGTCTGCTGTCCTGAGAGGATTTCTCATTTTAGGCAAAGAA
GACAAGAGATATGGCCCAGCATTAAGCATCAATGAACTGAGCAATCTTGCAAAAGGAGAGAAAGCTAATG
TGCTAATTGGGCAAGGGGACGTAGTGTTGGTAATGAAACGAAAACGGGACTCTAGCATACTTACTGACAG
CCAGACAGCGACCAAAAGAATTCGGATGGCCATCAATTAG
>gi|383513262|gb|JQ768350.1| Influenza A virus (A/Tomsk/IIV-19/2012(H1N1)) segment 1 polymerase PB2 (PB2) gene, complete cds
ATGGAGAGAATAAAAGAACTGAGAGATCTAATGTCGCAGTCCCGCACTCGCGAGATACTCACTAAGACCA
CTGTGGACCATATGGCCATAATCAAAAAGTACACATCAGGAAGGCAAGAGAAGAACCCCGCACTCAGAAT
GAAGTGGATGATGGCAATGAGATACCCAATTACAGCAGACAAGAGAATAATGGACATGATTCCAGAGAGG
AATGAACAAGGACAAACCCTCTGGAGCAAAACAAACGATGCTGGATCAGACCGAGTGATGGTATCACCTC
TGGCCGTAACATGGTGGAATAGGAATGGCCCAACAACAAGTACAGTTCATTACCCTAAGGTATATAAAAC
TTATTTCGAAAAGGTCGAAAGGTTGAAACATGGTACCTTCGGCCCTGTCCACTTCAGAAATCAAGTTAAA
ATAAGGAGGAGAGTTGATACAAACCCTGGCCATGCAGATCTCAGTGCCAAGGAGGCACAGGATGTGATTA
TGGAAGTTGTTTTCCCAAATGAAGTGGGGGCAAGAATACTGACATCAGAGTCACAGCTGGCAATAACAAA
AGAGAAGAAAGAAGAGCTCCAGGATTGTAAAATTGCTCCCTTGATGGTGGCGTACATGCTAGAAAGAGAA

TTGGTCCGTAAAACAAGGTTTCTCCCAGTAGCTGGTGGAACAGGCAGTGTTTATATTGAAGTGTTGCACT
TAACCCAAGGGACGTGCTGGGAGCAGATGTACACTCCAGGAGGAGAAGTGAGAAATGATGATGTTGACCA
AAGTTTGATCATCGCTGCTAGAAATATAGTAAGAAGAGCAGCAGTGTCAGCAGACCCATTAGCATCTCTC
TTGGAAATGTGCCACAGCACACAGATTGGAGGAGTAAGGATGGTGGACATCCTTAAACAGAATCCAACTG
AGGAACAAGCCGTAGACATATGCAAGGCAGCAATAGGGTTGAGGATCAGCTCATCTTTCAGTTTTGGTGG
GTTCACTTTCAAAAGGACAAGCGGATCATCAGTCAAGAAAGAAGAAGAAATGCTAACGGGCAACCTCCAA
ACACTGAAATTACGAGTACATGAAGGGTATGAAGAATTCACAATGGTTGGGAGAAGAGCAACAGCTATTC
TCAGAAAGGCAACCAGGAGATTGATCCAATTGATAGTAAGCGGGAGAGACGAGCAGTCAATTGCTGAGGC
AATAATTGTGGCCATGGTATTCTCACAAGAGGATTGCATGATCAAGGCAGTTAGGGGCGACCTGAACTTT
GTCAATAGGGCAAACCAGCGACTGAACCCCATGCACCAACTCTTGAGGCATTTCCAAAAAGATGCAAAAG
TGCTTTTCCAGAACTGGGGAATTGAATCCATCGACAGTGTGATGGGAATGATCGGAATACTGCCCGACAT
GACCCCAAGCACGGAGATGTCGCTGAGAGGGATAAGAGTCAGCAAGATGGGGGTGGATGAATACTCCAGC
ACGGAGAGAGTGGTAGTGAGTATTGACCGATTTTTAAGGGTTAGAGATCAAAGAGGGAACGTACTATTGT
CTCCCGAAGAAGTCAGTGAAACGCAAGGAACTGAGAAGTTGACAATAACTTATTCGTCATCAATGATGTG
GGAGATCAATGGCCCTGAGTCAGTGCTAGTCAACACTTATCAATGGATAATTAGGAACTGGGAAATTGTG
AAAATTCAATGGTCACAAGATCCCACAATGTTATACAACAAAATGGAATTTGAACCATTTCAGTCTCTTG
TCCCTAAGGCAACCAGAAGCCGGTACAGTGGATTCGTAAGGACACTATTCCAGCAAATGCGGGATGTGCT
TGGGACATTTGACACTGTCCAAATAATAAAACTTCTCCCCTTTGCTGCTGCTCCACCAGAACAGAGTAGG
ATGCAATTTTCCTCATTGACTGTGAATGTGAGAGGATCAGGGTTGAGGATACTGGTAAGAGGCAATTCTC
CAGTATTCAATTACAACAAGGCAACCAAACGACTTACAGTTCTTGGAAAGGATGCAGGTGCATTGACTGA
AGATCCAGATGAAGGCACATCTGGGGTGGAGTCTGCTGTCCTGAGAGGATTTCTCATTTTAGGCAAAGAA
GACAAGAGATATGGCCCAGCATTAAGCATCAATGAACTGAGCAATCTTGCAAAAGGAGAGAAAGCTAATG
TGCTAATTGGGCAAGGGGACGTAGTGTTGGTAATGAAACGAAAACGGGACTCTAGCATACTTACTGACAG
CCAGACAGCGACCAAAAGAATTCGGATGGCCATCAATTAG
