# Supplementary material for: A computational method for predicting regulation of human microRNAs on the influenza virus genome
Source: BMC Syst Biol. 2013 Oct 14;7(Suppl 2):S3. doi: 10.1186/1752-0509-7-S2-S3 (PMC3851852; doi:10.1186/1752-0509-7-S2-S3)
Supplement: Additional File 12 — 58 miRNAs that were used as the test dataset, including positive samples and negative samples. [file 1752-0509-7-S2-S3-S12.PDF]

miRNA of Test Dataset

The sequences of miRNA which are used as test datasets are totally from miRBase, and this additional file gives 11 miRNA of Human used as positive samples in FASTA format, and 47 miRNA of Human, Mus, Drosophila used as negative samples in FASTA format

Positive samples:

>hsa-miR-1 MIMAT0000416 Homo sapiens miR-1
UGGAAUGUAAAGAAGUAUGUAU
>hsa-miR-124-5p MIMAT0004591 Homo sapiens miR-124-5p
CGUGUUCACAGCGGACCUUGAU
>hsa-miR-124-3p MIMAT0000422 Homo sapiens miR-124-3p
UAAGGCACGCGGUGAAUGCC
>hsa-miR-17-5p MIMAT0000070 Homo sapiens miR-17-5p
CAAAGUGCUUACAGUGCAGGUAG
>hsa-miR-17-3p MIMAT0000071 Homo sapiens miR-17-3p
ACUGCAGUGAAGGCACUUGUAG
>hsa-miR-206 MIMAT0000462 Homo sapiens miR-206
UGGAAUGUAAGGAAGUGUGUGG
>hsa-miR-208a MIMAT0000241 Hom0o sapiens miR-208a
AUAAGACGAGCAAAAAGCUUGU
>hsa-miR-21-5p MIMAT0000076 Homo sapiens miR-21-5p
UAGCUUAUCAGACUGAUGUUGA
>hsa-miR-21-3p MIMAT0004494 Homo sapiens miR-21-3p
CAACACCAGUCGAUGGGCUGU
>hsa-miR-140-5p MIMAT0000431 Homo sapiens miR-140-5p
CAGUGGUUUUACCCUAUGGUAG
>hsa-miR-140-3p MIMAT0004597 Homo sapiens miR-140-3p
UACCACAGGGUAGAACCACGG

Negative Sapmles:

>dme-miR-278-5p MIMAT0020805 Drosophila melanogaster
CCGGAUGAUGGUUCACAACGACC
>dme-miR-278-3p MIMAT0000339 Drosophila melanogaster
UCGGUGGGACUUUCGUCCGUUU
>dme-miR-317-5p MIMAT0020843 Drosophila melanogaster
UGGGAUACACCCUGUGCUCGCU
>dme-miR-317-3p MIMAT0000409 Drosophila melanogaster
UGAACACAGCUGGUGGUAUCCAGU
>dme-miR-318-5p MIMAT0020844 Drosophila melanogaster
GGAUACACACAGUUCAGUUUUG
>dme-miR-318-3p MIMAT0000410 Drosophila melanogaster
UCACUGGGCUUUGUUUAUCUCA

>hsa-let-7b-5p MIMAT0000063 Homo sapiens
UGAGGUAGUAGGUUGUGUGGUU
>hsa-let-7b-3p MIMAT0004482 Homo sapiens
CUAUACAACCUACUGCCUUCCC
>hsa-miR-1 MIMAT0000416 Homo sapiens
UGGAAUGUAAAGAAGUAUGUAU
>hsa-miR-124-5p MIMAT0004591 Homo sapiens
CGUGUUCACAGCGGACCUUGAU
>hsa-miR-124-3p MIMAT0000422 Homo sapiens
UAAGGCACGCGGUGAAUGCC
>hsa-miR-126-5p MIMAT0000444 Homo sapiens
CAUUAUUACUUUUGGUACGCG
>hsa-miR-126-3p MIMAT0000445 Homo sapiens
UCGUACCGUGAGUAAUAAUGCG
>hsa-miR-128 MIMAT0000424 Homo sapiens
UCACAGUGAACCGGUCUCUUU
>hsa-miR-138-5p MIMAT0000430 Homo sapiens
AGCUGGUGUUGUGAAUCAGGCCG
>hsa-miR-138-2-3p MIMAT0004596 Homo sapiens
GCUAUUUCACGACACCAGGGUU
>hsa-miR-141-5p MIMAT0004598 Homo sapiens
CAUCUUCCAGUACAGUGUUGGA
>hsa-miR-141-3p MIMAT0000432 Homo sapiens
UAACACUGUCUGGUAAAGAUGG
>hsa-miR-145-5p MIMAT0000437 Homo sapiens
GUCCAGUUUUCCCAGGAAUCCCU
>hsa-miR-145-3p MIMAT0004601 Homo sapiens
GGAUUCCUGGAAAUACUGUUCU
>hsa-miR-155-5p MIMAT0000646 Homo sapiens
UUAAUGCUAAUCGUGAUAGGGGU
>hsa-miR-155-3p MIMAT0004658 Homo sapiens
CUCCUACAUAUUAGCAUUAACA
>hsa-miR-15a-5p MIMAT0000068 Homo sapiens
UAGCAGCACAUAAUGGUUUGUG
>hsa-miR-15a-3p MIMAT0004488 Homo sapiens
CAGGCCAUAUUGUGCUGCCUCA
>hsa-miR-16-5p MIMAT0000069 Homo sapiens
UAGCAGCACGUAAAUAUUGGCG
>hsa-miR-16-1-3p MIMAT0004489 Homo sapiens
CCAGUAUUAACUGUGCUGCUGA
>hsa-miR-19a-5p MIMAT0004490 Homo sapiens
AGUUUUGCAUAGUUGCACUACA
>hsa-miR-19a-3p MIMAT0000073 Homo sapiens
UGUGCAAAUCUAUGCAAAACUGA

>hsa-miR-19b-1-5p MIMAT0004491 Homo sapiens
AGUUUUGCAGGUUUGCAUCCAGC
>hsa-miR-19b-3p MIMAT0000074 Homo sapiens
UGUGCAAAUCCAUGCAAAACUGA
>hsa-miR-19b-2-5p MIMAT0004492 Homo sapiens
AGUUUUGCAGGUUUGCAUUUCA
>hsa-miR-200a-5p MIMAT0001620 Homo sapiens
CAUCUUACCGGACAGUGCUGGA
>hsa-miR-200a-3p MIMAT0000682 Homo sapiens
UAACACUGUCUGGUAACGAUGU
>hsa-miR-29c-5p MIMAT0004673 Homo sapiens
UGACCGAUUUCUCCUGGUGUUC
>hsa-miR-29c-3p MIMAT0000681 Homo sapiens
UAGCACCAUUUGAAAUCGGUUA
>hsa-miR-302a-5p MIMAT0000683 Homo sapiens
ACUUAAACGUGGAUGUACUUGCU
>hsa-miR-302a-3p MIMAT0000684 Homo sapiens
UAAGUGCUUCCAUGUUUUGGUGA
>hsa-miR-375 MIMAT0000728 Homo sapiens
UUUGUUCGUUCGGCUCGCGUGA
>hsa-miR-429 MIMAT0001536 Homo sapiens
UAAUACUGUCUGGUAAAACCGU
>mmu-miR-141-5p MIMAT0004533 Mus musculus
CAUCUUCCAGUGCAGUGUUGGA
>mmu-miR-141-3p MIMAT0000153 Mus musculus
UAACACUGUCUGGUAAAGAUGG
>mmu-miR-200a-5p MIMAT0004619 Mus musculus
CAUCUUACCGGACAGUGCUGGA
>mmu-miR-200a-3p MIMAT0000519 Mus musculus
UAACACUGUCUGGUAACGAUGU
>mmu-miR-215-5p MIMAT0000904 Mus musculus
AUGACCUAUGAUUUGACAGAC
>mmu-miR-215-3p MIMAT0017169 Mus musculus
UCUGUCAUUCUGUAGGCCAAU
>mmu-miR-429-5p MIMAT0017178 Mus musculus
GUCUUACCAGACAUGGUUAGA
>mmu-miR-429-3p MIMAT0001537 Mus musculus
UAAUACUGUCUGGUAAUGCCGU
