# Supplementary material for: A computational method for predicting regulation of human microRNAs on the influenza virus genome
Source: BMC Syst Biol. 2013 Oct 14;7(Suppl 2):S3. doi: 10.1186/1752-0509-7-S2-S3 (PMC3851852; doi:10.1186/1752-0509-7-S2-S3)
Supplement: Additional File 13 — A list of test datasets including positive samples and negative samples. [file 1752-0509-7-S2-S3-S13.PDF]

List of Test Dataset

This additional file gives two tables, Table1 is the list of positive samples; and Table2 is the list of negative samples we used as test dataset.

Table1: The list of positive samples.

No.	miRNA	mRNA	No.	miRNA	mRNA	No.	miRNA	mRNA
1	hsa-miR-1	NM_020347	62	hsa-miR-124	NM_021814	123	hsa-miR-124	NM_032139
2	hsa-miR-1	NM_005715	63	hsa-miR-124	NM_001980	124	hsa-miR-124	NM_032236
3	hsa-miR-1	NM_145257	64	hsa-miR-124	NM_002293	125	hsa-miR-124	NM_145648
4	hsa-miR-1	NM_002822	65	hsa-miR-124	NM_002958	126	hsa-miR-124	NM_152237
5	hsa-miR-1	NM_004520	66	hsa-miR-124	NM_003909	127	hsa-miR-124	NM_172390
6	hsa-miR-1	NM_018362	67	hsa-miR-124	NM_003945	128	hsa-miR-124	NM_000104
7	hsa-miR-1	NM_016271	68	hsa-miR-124	NM_004339	129	hsa-miR-124	NM_000183
8	hsa-miR-1	NM_000362	69	hsa-miR-124	NM_004670	130	hsa-miR-124	NM_000611
9	hsa-miR-1	NM_001111	70	hsa-miR-124	NM_004817	131	hsa-miR-124	NM_001099678
10	hsa-miR-1	NM_014445	71	hsa-miR-124	NM_004945	132	hsa-miR-124	NM_001259
11	hsa-miR-1	NM_000891	72	hsa-miR-124	NM_005327	133	hsa-miR-124	NM_001921
12	hsa-miR-1	NM_017542	73	hsa-miR-124	NM_005498	134	hsa-miR-124	NM_003060
13	hsa-miR-1	NM_005324	74	hsa-miR-124	NM_005730	135	hsa-miR-124	NM_004239
14	hsa-miR-1	NM_015271	75	hsa-miR-124	NM_005754	136	hsa-miR-124	NM_004422
15	hsa-miR-1	NM_019004	76	hsa-miR-124	NM_014447	137	hsa-miR-124	NM_004815
16	hsa-miR-1	NM_019063	77	hsa-miR-124	NM_014481	138	hsa-miR-124	NM_005397
17	hsa-miR-1	NM_015455	78	hsa-miR-124	NM_015516	139	hsa-miR-124	NM_006410
18	hsa-miR-1	NM_014904	79	hsa-miR-124	NM_015627	140	hsa-miR-124	NM_014170
19	hsa-miR-1	NM_000161	80	hsa-miR-124	NM_018214	141	hsa-miR-124	NM_014300
20	hsa-miR-1	NM_003203	81	hsa-miR-124	NM_019054	142	hsa-miR-124	NM_014397
21	hsa-miR-1	NM_019027	82	hsa-miR-124	NM_020179	143	hsa-miR-124	NM_014452
22	hsa-miR-1	NM_152282	83	hsa-miR-124	NM_022075	144	hsa-miR-124	NM_018370
23	hsa-miR-1	NM_024738	84	hsa-miR-124	NM_024792	145	hsa-miR-124	NM_018719
24	hsa-miR-1	NM_145341	85	hsa-miR-124	NM_024944	146	hsa-miR-124	NM_018845
25	hsa-miR-1	NM_175866	86	hsa-miR-124	NM_031942	147	hsa-miR-124	NM_019027
26	hsa-miR-1	NM_000219	87	hsa-miR-124	NM_152261	148	hsa-miR-124	NM_019895
27	hsa-miR-1	NM_170735	88	hsa-miR-124	NM_153367	149	hsa-miR-124	NM_022152
28	hsa-miR-1	NM_014918	89	hsa-miR-124	NM_172037	150	hsa-miR-124	NM_032156
29	hsa-miR-1	NM_018448	90	hsa-miR-124	NM_172105	151	hsa-miR-124	NM_144578
30	hsa-miR-1	NM_004815	91	hsa-miR-124	NM_000966	152	hsa-miR-124	NM_152792
31	hsa-miR-1	NM_000274	92	hsa-miR-124	NM_001951	153	hsa-miR-124	NM_153186
32	hsa-miR-1	NM_014937	93	hsa-miR-124	NM_002211	154	hsa-miR-124	NM_173607
33	hsa-miR-1	NM_014408	94	hsa-miR-124	NM_002473	155	hsa-miR-124	NM_175866
34	hsa-miR-1	NM_013412	95	hsa-miR-124	NM_002508	156	hsa-miR-124	NM_206894
35	hsa-miR-1	NM_005034	96	hsa-miR-124	NM_002633	157	hsa-miR-124	NM_213636
36	hsa-miR-1	NM_002639	97	hsa-miR-124	NM_002742	158	hsa-miR-124	NM_000075
37	hsa-miR-1	NM_018319	98	hsa-miR-124	NM_002819	159	hsa-miR-124	NM_002632
38	hsa-miR-1	NM_000270	99	hsa-miR-124	NM_002835	160	hsa-miR-124	NM_004402
39	hsa-miR-1	NM_018413	100	hsa-miR-124	NM_003051	161	hsa-miR-124	NM_030935
40	hsa-miR-1	NM_006367	101	hsa-miR-124	NM_003068	162	hsa-miR-124	NM_178507
41	hsa-miR-1	NM_003564	102	hsa-miR-124	NM_003870	163	hsa-miR-124	NM_001084
42	hsa-miR-1	NM_022075	103	hsa-miR-124	NM_004099	164	hsa-miR-124	NM_004706
43	hsa-miR-1	NM_001829	104	hsa-miR-124	NM_004125	165	hsa-miR-124	NM_006289
44	hsa-miR-1	NM_001042351	105	hsa-miR-124	NM_004364	166	hsa-miR-124	NM_004710
45	hsa-miR-1	NM_024792	106	hsa-miR-124	NM_004685	167	hsa-miR-124	NM_145204
46	hsa-miR-1	NM_172020	107	hsa-miR-124	NM_004781	168	hsa-miR-124	NM_178439
47	hsa-miR-1	NM_152450	108	hsa-miR-124	NM_004862	169	hsa-miR-17-5p	NM_006534
48	hsa-miR-1	NM_173505	109	hsa-miR-124	NM_005720	170	hsa-miR-206	NM_007124
49	hsa-miR-1	NM_018290	110	hsa-miR-124	NM_005903	171	hsa-miR-206	NM_007085
50	hsa-miR-1	NM_005345	111	hsa-miR-124	NM_006016	172	hsa-miR-208a	NM_005121
51	hsa-miR-1	NM_021913	112	hsa-miR-124	NM_006111	173	hsa-miR-21	NM_000366
52	hsa-miR-1	NM_022071	113	hsa-miR-124	NM_006320	174	hsa-miR-140-3p	NM_006037
53	hsa-miR-1	NM_001194	114	hsa-miR-124	NM_006467	175	hsa-miR-1	NM_000165

54	hsa-miR-1	NM_005477	115	hsa-miR-124	NM_006496	176	hsa-miR-1	NM_003769
55	hsa-miR-1	NM_003271	116	hsa-miR-124	NM_014320	177	hsa-miR-1	NM_006148
56	hsa-miR-1	NM_018330	117	hsa-miR-124	NM_014445	178	hsa-miR-1	NM_003417
57	hsa-miR-1	NM_015318	118	hsa-miR-124	NM_014762	179	hsa-miR-1	NM_006454
58	hsa-miR-1	NM_002999	119	hsa-miR-124	NM_018226	180	hsa-miR-1	NM_007081
59	hsa-miR-1	NM_016291	120	hsa-miR-124	NM_020360	181	hsa-miR-1	NM_003290
60	hsa-miR-1	NM_016004	121	hsa-miR-124	NM_021961	182	hsa-miR-1	NM_019114
61	hsa-miR-124	NM_152237	122	hsa-miR-124	NM_022365	183	hsa-miR-124	NM_000696

Table2: The list of negative samples.

No.	miRNA	mRNA	No.	miRNA	mRNA
1	hsa-let-7b	NM_001614	15	hsa-miR-16	NM_001241
2	hsa-let-7b	NM_001237	16	hsa-miR-19a	NM_019102
3	hsa-miR-1	NM_021109	17	hsa-miR-19b	NM_138973
4	hsa-miR-1	NM_001111285	18	hsa-miR-200a	NM_030751
5	hsa-miR-124	NM_004781	19	hsa-miR-29c	NM_054016
6	hsa-miR-124	NM_001621	20	hsa-miR-302a	NM_003182
7	hsa-miR-124	NM_024551	21	hsa-miR-375	NM_130439
8	hsa-miR-126	NM_033102	22	hsa-miR-375	NM_0021961
9	hsa-miR-128	NM_003176	23	hsa-miR-429	NM_030751
10	hsa-miR-138	NM_033102	24	mmu-miR-141	NM_014795
11	hsa-miR-141	NM_001730	25	mmu-miR-200a	NM_030751
12	hsa-miR-145	NM_014547	26	mmu-miR-215	NM_014795
13	hsa-miR-155	NM_024900	27	mmu-miR-429	NM_030751
14	hsa-miR-15a	NM_138973			
