# Supplementary material for: A computational method for predicting regulation of human microRNAs on the influenza virus genome
Source: BMC Syst Biol. 2013 Oct 14;7(Suppl 2):S3. doi: 10.1186/1752-0509-7-S2-S3 (PMC3851852; doi:10.1186/1752-0509-7-S2-S3)
Supplement: Additional File 14 — The coding sequence of the gene fragments which were used as test datasets including positive samples and negative samples. [file 1752-0509-7-S2-S3-S14.PDF]

# mRNA of Test Dataset

This additional file gives 211 mRNA sequences which are used as test dataset in FASTA format, which are extracted from the University of California, Santra Cruz (UCSC) Genome bioinformatics site, and 183 of them are positive samples, and 28 of them are used as negative samples.

**Positive samples:**

>NM_020347 2

gttagcaaccagaggcttctaaacaacctaccctccttccccattttctg
tggtccaactaccctcggcgatcccaggcttggcggggcaccgcctggcc
tctcccgttcctttaggctgccgccgctgcctgccgccatggcagagttg
ggcctaaatgagcaccatcaaaatgaagttattaattatatgcgttttgc
tcgttcaaagagaggcttgagactcaaaactgtagattcctgcttccaag
acctcaaggagagcaggctggtggaggacaccttcaccatagatgaagtc
tctgaagtcctcaatggattacaagctgtggttcatagtgaggtggaatc
tgagctcatcaacactgcctataccaatgtgttacttctgcgacagctgt
ttgcacaagctgagaagtggtatcttaagctacagacagacatctctgaa
cttgaaaaccgagaattattagaacaagttgcagaatttgaaaaagcaga
gattacatcttcaaacaaaaagcccatcttagatgtcacaaagccaaaac
ttgctccacttaatgaaggtggaacagcagaactcctaaacaaggaaatt
ttaagacttcaagaagagaatgagaaattgaagtcaaggttgaagaccat
tgaaatacaggctacaaatgcactggatgaaaagtcaaaactagaaaaag
cactgcaagatttacagcttgatcaaggaaatcaaaaggattttataaag
gcccaagacttaagtaacttagaaaacactgtcgctgccttaaagagtga
gtttcagaagacacttaatgacaagacagaaaaccagaagtcactggagg
agaatctggcgacagccaagcacgatctactcagggttcaggagcagctg
cacatggctgaaaaggaattagaaaagaaatttcagcaaacagcagctta
tcgaaacatgaaagagattcttaccaagaagaatgaccaaatcaaagatc
tgaggaaaagactggcacaatatgaacctgaagattaaaactgaagattt
cctctggaagctaccacatgcaaacatacaagcagtctcacctcaggcat
gtattttgaaaagcattttgtcatatcccctctccttatttttctaatat
ttagactttgaatatttagaactagagttcctattttcagctagttgaaa
acagagaaaacctagtgattctgtctggtcatcccacacacctctctact
gagtgctccagagtcttagctcacatgcactgagaattctttacagcaaa
gaagagatttttagagtgggaagaagaggcttatccttctgcattagtat
aaagagtctgtatttttaataaatgttaaataagctaacaatgtttgtac
ttatgcattccccatgggtaaaaataaataatagcattgttaaagatagt
tattaccaaaaaaagagagttattacaaataaatatgtctctttattttt

aaaaatgaaatcttaattcatttactctatttgatgataaactataaatt
cattgaaaatgtgaattctattatgggtagcctttttaccaattataagg
aaaatttacagcagtgaacatgaacattcacttagcttcctcagtctctc
catcttaaagatcatttatcagaggaggttcagcatttttgcagcataa
cttttcatgagtctgtattactaatggataagtcaaatccatcctgcact
tctacagtttagaaagtatctggactcagaataaatgtaatatttatact
tgtttccagaatgttattttacattttatgttcaataagaacacttttta
aaagacgtatattcaacataaaatcagctatcagacttcagattagactt
tatttatgtgggtctataataattgtattttcaagaggttttcactatat
ttgtattggcctggttttctcagacgattttggacaaatcattagaaact
gggcatcatatccacagttatgtaaggcagtgatatactataaggataaa
caaagtcaagtccataaagcaataatccctcagaaggaaagtccttactt
ttcacatattaatatttagtaatttttcctgcttctaaaagtgagagtat
cacaccctaaatgaacactgtctactaagagacatcattccatttccaca
aatgaagattttattccaagaaacgagtttactgattggagcatagggct
tgttgttatttttattcaagcttttagtaatagccttgaatttattattt
ttcttataggctttttgttaaaatagtgaaggaacaaatgttaaagggta
agataatttccctgcaaaaggacacagaaggcagtcttaagaagatgaat
ggatgagagaagggagagaataaaatgcaataacgagccagcatttacta
tgtattttctcctcacctgtctctccatatttaggtcacttaccagtttc
tgtgcccttttggagcttttgttgagggcttcattctcaccctgtatttc
tttagccctaaattgacactctctccaaaaatccattccattgtctgtgg
accaagatgttctatgtaattcagaagcagaactcttggctaaagggcta
gtgtggccttcagaaaccattcaattattttctccctacacctttgtcag
tttgaaaccagtgaggaaaaaaggtatgttgataagaaacctatattgct
aggtagaatttgtacttgttttcttggtagcagttttgaaatattctgta
cagtacgttcctattgtttaataataaattcaaaaatatttctaaaacct
taaaaccaactatgccaagcattaagataaacaaatatgatgttctttga
cgtaaatcaacgtgatgattctttcacatgtaaacacattttagtgtttc
tggtttgtcatttttgttgttgttgttgttgttgttatttactctatacc
ctttagcaaaatacagttttaaatttttattgtttttagtagtttcccaa
ctttaagacttatctaatttaactgagaaagaaagcctttttcatatata
tatatattggatttctaaggatggtggtttgagccttgattagacttttg
atgtgctaagccagacaggcagtctgtacattgatggccatcacaatgca
gctttggtttaatttaattcaggcctgctgctgagttatgcacagacttt
ttgttgaccaaaataaaatataaagggttttcttctgtttgacatttgtg
ttcatttttctctttatgtattacattttaacctatattaaataaatgt
ttaaatgataatttgcttatgtcttataaaactcagcataagaaaaat

>NM_005715 2
ggcgcggcggggcgcggggcgtggggacgctagcgggcgccggacgggcg
cggcgccccgtcacgggcagcgccccgaaccggggccggacacctcggcc
gctcgggccgcggcggcggggaccatgccgaagaaagtctcctgagcccg
gcaacttcggcccctccccgcccccacccggctgccctccgcgcggccct
ccccatgtgcagccggccagccgggctctcctcctcgcggcggatgggtg
accttttcctggcacgggcaggctgtgggaggcagcggagcaggcgatga

agaagaagcagcagcatcccggcggcggcgcggatccctggccccatggg
gcccctatggggggcgcccctccgggcctgggcagctggaagcgtcgggt
gcccctgctgcctttcctgcgcttctccctccgggactacggcttctgca
tggccaccctgctggtcttctgcctgggctccctcctctatcagctcagc
gggggacccccctcgcttcctgctcgacctgcggcagtacttgggaaattc
cacttacttggatgaccatggaccacctcctagtaaggtactacctttcc
caagccaggtggtgtacaacagggtaggcaagtgtgggagccgtactgtg
gtcttgcttctgagaatcttgtcggagaagcacggatttaatttggtcac
atcagacattcacaacaaaaccaggcttactaaaaatgaacaaatggaac
tgattaaaaatataagtactgccgaacaaccctatttattcactcgacat
gttcatttcctcaacttctcaaggtttggaggagaccagcctgtctacat
caacatcattagagaccccgtcaaccggttcttatccaactatttttcc
gtcgctttggagactggagaggggaacaaaatcacatgatccgcaccccc
agcatgaggcaggaggagcgctacctggatatcaatgagtgtattcttga
aaactatcccgagtgctccaaccccaggttattttacatcattccgtact
tttgtggacagcatcccagatgcagggagcctggtgaatgggcccttgag
agagcaaagctgaacgtgaatgaaaacttcctgctcgtggggattcttga
agagttggaagatgtgctgctgttactggaaagatttttacctcattact
tcaagggcgtgctcagtatctacaaagacccagagcacaggaagcttgga
aacatgactgtgacggtgaagaagactgtcccctctcctgaggctgtgca
gatcctctaccagcggatgagatacgagtacgagttttaccactacgtca
aagagcagttccacctgctgaagcgcaagtttggacttaagtctcacgtc
agcaagcccccccctgaggccacacttctttatcccaactccactggaaac
cgaggagccaatcgacgatgaagaacaggatgatgaaaagtggctggaag
atatttataagaggtgatgtgactgtgttgcctctatggctttatctccc
ttttccagaaagttctttgtttggggaagtaaaatccttaagggactaaa
ttaatgcttgggtgcattaaaaagaacaaaacattcccacatgttggggt
cattgggagatgcccggttttgcgggttttatttgtttaattttattctg
tgttttctcttggctctttgggtctttcccgggtacactagatggctcca
tcccaaggcatcttgtcataaaacagctttcccccaccccatatcatggg
aaaagggggagaaatatagcccctagcctaataacttatcatttgtaaaa
tgacttataaaaatattacctcaatggtaggagacatccagacttgtata
tttcagtggaaatacaaaaccacttcagagaccagggtatctcctctgga
aggatctaagagaaggtaagacagattaggacatcgaaaaggaggatgga
gccaggtgccatggcttgagcctataatccgaggctgaggtgggaggatc
acttgagcccaggagtttgaggttgcagtgagctgtgatcacaccactgc
actccagcctgggtgacagagtgagactctgtctcaattaatttttttt
tttaaaggaggaggatctccatgggtaagtggtttctacccgcatgggta
gagttctgcctctggtccttctcagggggcactttcaccaagagcagtgt
aattatctctgaaagagcaagtcagcttgtgccgcatccccaaccaatcc
acagcctggagtacctttcaaggtcaaagtgcatggccagctccattgag
acattccatttcaaagcaccgtgctgacagatatcaaagtactctagcag
ggaaaataatttgtttgctgtgtaaggaagaatgtagacaagacagataa
atctgaaggtcatgtggcatcagggaaagggcatggctgtgtcttttgca
cccaatatgaaacatcttctcccaacactgctttaatggaagttctagga
accaatttagctcaggcatttgactcctacagcagaagttctgagcctga

ccacagatggtgtgtaatctatcaaacacacccctggccaagttgggtcc
tataggacctggtactatgtactattgtaacttctagttccctaagaggt
acctgttttcagtaaaaaggggtcctgagttctgtgcaggtggaagagct
acccgagaactacctgagttctgtgcaggtagagtcccatttcttatggg
acctgtgtgctcctgagaactcttacttgagacatcaaaaagaagcagca
agagcttctgggacagagactgcttggccagctttgtaagtaagtggctg
cctccaatgtgatgtgagtacatgttgggcagtctcactgtcctaaggta
tgtcttctttccacctcccactgcccctcccctgccacctatcaatgatg
ccttggttcagtcattagaaatctgttgctttgagttctgaaatattttc
accttaaaaaaaatgctgaaaatacacattctcctgggaagacgataaac
agctagctaagaagccgaggttcagtggtggcagcaggaaggacactgcc
acaaattttgtctatttcatatttgtcccctagagccagccctagcaaat
gtgtgagttgggagtagttaatagtaaataagactctgactttacacaag
ctacacattttatacttttcataaaccacaaagtctctctagaatttttt
ctgccttcactaaaattggactgtagccaagatataaagcaagtcatttg
gaacctgccgagtgagcactgaagctactttatcatgagatgtgtgttaa
gaaggctgcagcccacaggagtccagggaaggcggggaccacagaggcac
agagtccagcacttggccgctcatgggccttctttctgcctcagaggacg
ggggcagagaagtgatgaagggaaatgttcttagaggaggaaatatcctt
tgtcctgttcagagagaccagggccctaccattaggcatactttcagaag
caacctggagaacagctatcaatcatattcaaaaccagtacaagaactgc
tgcctggtaccctgtgagtcatttctatgaaattccatataaagaatgat
gataagtttacacactgtgcaatctcacaatctgaaaataaagttgagtt
ggctgtgttttctctgctcttgtcagaacattgggacaattggtcgttca
aaaacattcatcctcttactgcaagtttatctgggtacttttacctgtgt
gttcaaaggcatttcttttcagcagtgatcattataacttcacaaaaaaa
gatgctgacggatttacttacagggccttaatgttattttgtcccagcca
acaccctctaggtcctaaaagtcaaggtacttcagtttatttggcaaaca
tgacaacatttttttggccctgggcccaacagtttgtacttcatgaaac
atattgtacattttacatagtttaatttaaaaaataccttttaagctagt
tgatctttgactgtcttatttattataacctttcagcacattccaaggtt
ttagttactcaggaaggagttaattaaaatgattttattttggtctgatg
gatgtttttttaaaaggaaaattattattatgaaccttcagcctactttct
tgagtgccgtaaaagtgcttgtaaatcttttttttttttaagaagaaag
aaaaaaatggtgtttgacgttgatggaaattcaaaaatatatatggaact
gaaacattaacttagctaaaataaaagcaatctgtgtttgaaaaaa

>NM_145257 3
agaggatcggccgggcgcggcgggaaggaggctgcgggcagcgaggagcg
gctggcgctcgaggcggcggacggcaccatgtccccggggagcggggtga
agagcgagtacatgaagcgctaccaggagccgcgctgggaggagtacggg
ccgtgctaccgcgagctgctgcactaccgcctaggccgccggctgctgga
gcaggcgcacgcgccctggctctgggacgactggggcccggccggctcct
cggaggactcggcgtcgtcagagtcgtcgggcgccgggggccccgcaccc
cggtgcgccccgccctcgcccccgccgcccgtagagccggcgacccagga
ggaggcggaacggcgggcgcgcggggccccggaggagcaggacgcggagg

ccggggacgcggaggccgaggacgcggaggacgcggctctgccagcactg
ccagtgaaagatgtagaagataaacctgaacaacaaaccagaacaagaga
gactgacaaatcacccaccagtactgagcctcgacagcaaccaagtgcct
tatttgctagaggaaacaggaaagcggtcaaaagtccccaaagatcatcg
agtaaaataaaagaaaacaagcatccatttgctctttatggctggggaga
aaaacagaccgatacaggaagccagaagactcacaacgtctgtgcgtccg
ctcctgtgcacgagattcatgaatcagcattacgagccaagaacagaaga
caggtggaaaaaaggaaactggttgctcaaaggcagcgagctcactctgt
ggatgtggagaagaacagaaagatgaaggcttcctcctcagagaacccgt
ggatgacagaatacatgaggtgctattcggcaagagcttaaagaaacact
tgcgtggacagcctcttttaaaaagtgtaaatgactgaaaggaaaaacaa
aacaaaaaccatcaaaagaaacggacacaggtttaagaaaccaactgatt
atgcaaggtttttttagggaatttgtaaaagattgttttattttgatga
atattggtcacctacctcggcagtagggcagacagttgaagccatagaca
tttggttatttatgaagataattccctaaatctttgatattcttataagg
tttttgttttaaagcatcttaatcttttaagatactgacaccaaatgcct
ttaaatggcaacagatgcttacagttcagtattcttttcataagcttagg
tagagcctattatcatcttgttctaaataactttccagattccatagcta
taagatcattccatcctacagcataagactcgttttccttatatgccgtt
ttgtttgtgaaagaatatcaagtcaaaaatgagtgtcagcactactactg
attccatgtataatgaaagtagaactttgctagttcctgaaaatttttaa
cttatttgtatttcagttcagcagcatctttatgtagattgtgatattta
agaattatctgggctgggcgagggtctctttttctctttcagtgatcatc
taggcagttattatttaatagtttaatagctcaagtacattccaatagac
gaagtgcacacaacacaatatgtgtgtacagtagtagtaaagtttctttt
gagtagtcaaagactcactttttttattgcctttttttttttttttaaag
aatacatactgtggattcagtcccttgtcacacttgaccttttggcatac
acccactgtggacttttgcctcttctgtaatggctggcaatgacatttca
aacttacaatctggaattgcacttggtacattggcattgcttgttccact
gggatggggaccagtgtgaagatgcctgttagatagactgcccacccccta
ctttctctttttctttatagcacttaacaataacaaagtcttgatgatgt
acagtattcaaactttaggttgaaatacgttactctttgattcctagcca
gtagatcttatctacactttaatgggagagaatggtggtgtgtgggtagg
cacaaatttatgtaaatagtgctccttctctttagtatgtttgctttggg
ggtagaaaaatggttttaacaaacactggtttccatcaaatgaatgatgt
cttctccatcctgtggagacaagaatctgctagaaggatatgtgctaagt
tccttataagagataatggtgctctgcctatgccagcttggcacccgaag
atgtgtgagtggacgtgaggctgagtattaccttagtatttttctctggg
tctttggaaaaccatagtcaatttttagaacatattgctttcattcccca
taaactcttcacacatgataactgtttaagctttgaaaacacatactgaa
gtattgtgagcttaaaaaaactttttaaatatttgcatagttttgaggtg
aatttgtttccttacagatctctcctaatcattgagatgtatatttcaaa
agaggaaattttacatgttgtccaaaacagccttgctagtaactggtgaa
ttttggtattaactattattaaagtctttaaacgacacaggtacctaaag
atcaccttaatgtggcaatttgtgatggtgtagctagctgattgtgaaaa
ctgttcctttaaagtcgcttcttgcatgttcggtgttagtcatccagctc

aggcttgtgttgcagctgacaatctaggaaagacggccttagagagtggt
gcaggccccacactgacggactgccttagaaacccgacttcctctagact
ttgaaccgccagacttttctcttgtttagaaaacaaacttatatttaatg
tacttactacttaaaactccagacagagatataatgtagaaggcaaatat
tggccaattttttcctctttttaagtggaagacaaatgaacgggattttt
aaagtgcttttaaagtgcatgaatggttaataaatcagtatgaattgtaa
gccttcatcttacatccaagtccttagttggttagggtttcttttctttc
ttttttaaagagtgtcaattaccttttgaacctgtgaaaatttgatagtt
gttaacagtctgatggtcctaattctttcttttcattctagaaatgaatg
tggttgtaatcatgttcctaattcttgggacaacctgcaagacagtgaga
cagtttaaaaattacctttcatgttgaaaaagtctgaaacagagaaccca
atgatatttaaaataaatgctacataaaactctttttaaaattttgattt
taacttaattaaaacaatgtcataaatatgctttttgattttgttactgc
ttttaatattaaagtaatagaatattgaagcaatattgtctagcactctg
ctggacattaagtccgcgggaggagaagtgaacaggaatcgattctttgt
cttttaactgcccttagttaggagatgttaaaatacttggcacctctggt
tatatgtatgttatgtgtgttctccccccctaaaatttctaagcacattta
ttcacttttaaaatgaatctttaaaagattatagttagtagttatagtta
atattctatttacttggaaaaaatgtgaataaatggatcttcaaaagattc
attttaaaaatgaataaatgtataataggctataggtgatcttacttgcg
tattaggtaggaggcacatatttataccatttcatatgtaatatctttgt
cattgtgtttcatcgaagatcaattgctagcaacttgaagggtatttata
cttgggtcacttgaactcagctgactaaattgtaagaacgagagcaagca
agatggctgttattggaagccataacttccagaagataattctgcacaat
tcgtaagttaaaaaaaaatctgtagggtcttccactatcctttttcaggtt
gataatgctgttctgggcacacactttgtaaatggaatgttatggtacag
tcgcctctcagtatccatggggcattggttccaggcctcccttaggatgc
caaactccatggatactcaaaccccttctataaaatggtgtagtatttgc
atatatcctagacacatcctcctgtatgctttaaatcatctctagattac
ttaaaatacctaatacaatgtaaatgctctgtaaatagttgttatactat
attgtttagggaataatgacaaggaaaaaaaagcctgtacatgttcagca
caagtgaaaccatcctttttgcccccaaatattttcaatttgtatttggt
tgaatccatggatgcagaactcacggatacagagggccgactgtactttc
tttaaagtgttcaaaagtattactagcaaagaggaggaggagcaaagcat
atatcagaagtaaaacaatttttcttgttgactgctttggtaaaaaacag
tttgatggatagttttacatttcactggactagataaaaaatggtgctaa
tatttatgtagcttgatgctatagttgctttggtatcaaacttaatacct
aacccatataagatccttattatataattttgtgatcagtaaaatgatat
tttaaagagtgatcttaaaaatatgacctggtcattgcacaacgtttgca
tttgaaatgaatttgtgtactatagggtggatatggagttattcagtgca
agtgtgtgcttaatatcaaaccctatgcaaggagctatgtctagattttt
ggtccaaatttgcctccttcaagcctactagtgtgagatggaaaaaaatc
gattgctcttttaatattatttccattttgaaattctcgacacttgaatg
aaggcagtagaggcctctttttggatttctcttctaataacaaaacttta
tttagggaaggtttccctgtgctatcgtaagtttgttttgagcactgcat
tcactttaaaattctggaggaacaaaggctgggcacataatcacaaagcc

caggccacacaataattcggggttgtattttctaagaactatgtattgtt
tttagcttatagttaatattgtatttacttggaaaaatgtgaataaagtt
aattaacattacaagctgaga

>NM_002822 4
gggcggggcggccgcgggtcgcgggcggatgacgcgccggggccggcgga
ggagcagccacttcctggggccgccggccggggccgctggctgcactcag
cgccggagccgggagctagcggccgccgccatgtcccaccagaccggcat
ccaagcaagtgaagatgttaaagagatctttgccagagccagaaatggaa
agtacagacttctgaaaatatctattgaaaatgagcaacttgtgattgga
tcatatagtcagccttcagattcctgggataaggattatgattcctttgt
tttacccctgttggaggacaaacaaccatgctatatattattcaggttag
attctcagaatgcccagggatatgaatggatattcattgcatggtctcca
gatcattctcatgttcgtcaaaaaatgttgtatgcagcaacaagagcaac
tctgaagaaggaatttggaggtggccacattaaagatgaagtatttggaa
cagtaaaggaagatgtatcattacatggatataaaaaatacttgctgtca
caatcttcccctgccccactgactgcagctgaggaagaactacgacagat
taaaatcaatgaggtacagactgacgtgggtgtggacactaagcatcaaa
cactacaaggagtagcatttcccatttctcgagaagcctttcaggctttg
gaaaaattgaataatagacagctcaactatgtgcagttggaaatagatat
aaaaaatgaaattataattttggccaacacaacaaatacagaactgaaag
atttgccaaagaggattcccaaggattcagctcgttaccatttctttctg
tataaacattcccatgaaggagactatttagagtccatagtttttattta
ttcaatgcctggatacacatgcagtataagagagcggatgctgtattcta
gctgcaagagccgtctgctagaaattgtagaaagacaactacaaatggat
gtaattagaaagatcgagatagacaatggggatgagttgactgcagactt
cctttatgaagaagtacatcccaagcagcatgcacacaagcaaagttttg
caaaaccaaaaggtcctgcaggaaaaagaggaattcgaagactaattagg
ggcccagcggaaactgaagctactactgattaaagtcatcacattaaaca
ttgtaatactagtttttaaaagtccagcttttagtacaggagaactgaa
atcattccatgttgatataaagtagggaaaaaaattgtactttttggaaa
atagcacttttcacttctgtgtgtttttaaaattaatgttatagaagact
catgatttctatttttgagttaaagctagaaaagggttcaacataatgtt
taattttgtcacactgttttcatagcgttgattccacacttcaaatactt
cttaaaatttatacagttgggccagttctagaaagtctgatgtctcaaa
gggtaaacttactactttcttgtgggacagaaagaccttaaaatattcat
attacttaatgaatatgttaaggaccaggctagagtattttctaagctgg
aaacttagtgtgccttggaaaaggccgcaagttgcttactccgagtagct
gtgctagctctgtcagactgtaggatcatgtctgcaacttttagaaatag
tgctttatattgcagcagtcttttatatttgactttttttaatagcatt
aaaattgcagatcagctcactctgaaactttaagggtaccagatattttc
tatactgcaggatttctgatgacattgaaagactttaaacagccttagta
aattatctttctaatgctctgtgaggccaaacatttatgttcagattgaa
atttaaattaatatcattcaaaaggaaacaaaaaatgttgagttttaaaa
atcaggattgactttttctccaaaaccatacatttatgggcaaattgtg
ttctttatcacttccgagcaaatactcagatttaaaattactttaaagtc

ctggtacttaacaggctaacgtagataaacaccttaataatctcagttaa
tactgtatttcaaaacacatttaactgttttctaatgctttgcattatca
gttacaacctagagagattttgagcctcatatttctttgatacttgaaat
agagggagctagaacacttaatgtttaatctgttaaacctgctgcaagag
ccataactttgaggcattttctaaatgaactgtggggatccaggatttgt
aatttcttgatctaaactttatgctgcataaatcacttatcggaaatgca
catttcatagtgtgaagcactcatttctaaaccttattatctaaggtaat
atatgcacctttcagaaatttgtgttcgagtaagtaaagcatattagaat
aattgtgggttgacagatttttaaaatagaatttagagtatttggggttt
tgtttgtttacaaataatcagactataatatttaaacatgcaaaataact
gacaataatgttgcacttgtttactaaagatataagttgttccatgggtg
tacacgtagacagacacacatacacccaaattattgcattaagaatcctg
gagcagaccatagctgaagctgttattttcagtcaggaagactacctgtc
atgaaggtataaaataatttagaagtgaatgtttttctgtaccatctatg
tgcaattatactctaaattccactacactacattaaagtaaatggacatt
ccagaatatagatgtgattatagtcttaaactaattattattaaaccaat
gattgctgaaaatcagtgatgcatttgttatagagtataactcatcgttt
acagtatgttttagttggcagtatcatacctagatggtgaataacatatt
cccagtaaatttatatagcagtgaagaattacatgccttctggtggacat
tttataagtgcattttatatcacaataaaaatttttttctctttaaaaaaa
aaaaaaaaaaaa

>NM_004520 4
gcctccccagcgtcggctccccggcgggcggtgcgggccctcccactcta
ccccgcgccgtctcacggccccggccctagcttcaccccgactacccggc
gtgcgcgtcctcctgccggcctgcaggcccggggcctccgcctgcttccc
cacagctgctccttgcggccccgcttgcgttcacgctgtcgcccgggccg
gcgcggccgcgggcaaccgctccccctcccacacctaccccgccccctcc
ccgccttttccgccctccggtcccccctccctcggcccgctgctgctgctc
cagatgaggtgatggcaacggccaacttcggcaagatccagatcgggatt
tacgtggagatcaagcgcagcgatggccgaatacatcaagcaatggtaac
atctttaaatgaagataatgaaagtgtaactgttgaatggatagaaaatg
gagatacaaaaggcaaagagattgacctggagagcatcttttcacttaac
cctgaccttgttcctgatgaagaaattgaacccagtccagaaacacctcc
acctccagcatcctcagccaaagtaaacaaaattgtaaagaatcgacgga
ctgtagcttctattaagaatgaccctccttcaagagataatagagtggtt
ggttcagcacgtgcacggcccagtcaatttcctgaacagtcttcctctgc
acaacagaatggtagtgtttcagatatatctccagttcaagctgcaaaaa
aggaatttggaccccccttcacgtagaaaatctaattgtgtgaaagaagta
gaaaaactgcaagaaaaacgagagaaaaggagattgcaacagcaagaact
tagagaaaaaagagcccaggacgttgatgctacaaacccaaattatgaaa
ttatgtgtatgatcagagactttagaggaagtttggattatagaccatta
acaacagcagatcctattgatgaacataggatatgtgtgtgtgtaagaaa
acgaccactcaataaaaaagaaactcaaatgaaagatcttgatgtaatca
caattcctagtaaagatgttgtgatggtacatgaaccaaaacaaaaagta
gatttaacaaggtacctagaaaaccaaacatttcgttttgattatgcctt

tgatgactcagctcctaatgaaatggtttacaggtttactgctagaccac
tagtggaaactatatttgaaaggggaatggctacatgctttgcttatggg
cagactggaagtggaaaaactcatactatgggtggtgacttttcaggaaa
gaaccaagattgttctaaaggaatttatgcattagcagctcgagatgtct
ttttaatgctaaagaagccaaactataagaagctagaacttcaagtatat
gcaaccttctttgaaatttatagtggaaaggtgtttgacttgctaaacag
gaaaacaaaattaagagttctagaagatggaaaacagcaggttcaagtgg
tgggattacaggaacgggaggtcaaatgtgttgaagatgtactgaaactc
attgacataggcaacagttgcagaacatccggtcaaacatctgcaaatgc
acattcatctcggagccatgcagtgtttcagattattcttagaaggaaag
gaaaactacatggcaaattttctctcattgatttggctggaaatgaaaga
ggagctgatacttccagtgcggacaggcaaactaggcttgaaggtgctga
aattaataaaagccttttagcactcaaggagtgcatcagagccttaggta
gaaataaacctcatactcctttccgtgcaagtaaactcactcaggtgtta
agagattctttcataggtgaaaactctcgtacctgcatgattgccacaat
ctctccaggaatggcatcctgtgaaaatactcttaatacattaagatatg
caaatagggtcaaagaattgactgtagatccaactgctgctggtgatgtt
cgtccaataatgcaccatccaccaaaccagattgatgacttagagacaca
gtggggtgtggggagttcccctcagagagatgatctaaaacttctttgtg
aacaaaatgaagaagaagtctctccacagttgtttactttccacgaagct
gtttcacaaatggtagaaatggaagaacaagttgtagaagatcacagggc
agtgttccaggaatctattcggtggttagaagatgaaaaggccctcttag
agatgactgaagaagtagattatgatgtcgattcatatgctacacaactt
gaagctattcttgagcaaaaaatagacattttaactgaactgcgggataa
agtgaaatctttccgtgcagctctacaagaggaggaacaagccagcaagc
aaatcaacccgaagagaccccgtgccctttaaaccggcatttgctgctaa
aggatacccagaaccctcactactgtaacatacaacggttcagctgtaag
ggccatttgaaagtttggaattttaagtgtctgtggaaaatgttttgtcc
ttcacctgaattacatttcaattttgtgaaacactcttttgtctacaaaa
tgcttctagtccaggaggcacaaccaagaactgggattaatgaagcattt
tgtttcatttacacaaatagtgatttacttttggagatccttgtcagttt
tattttctatttgatgaagtaagactgtggactcaatccagagccagata
gtagggggaagccacagcatttccttttaactcagttcaatttttgtagt
gagactgagcagttttaaatcctttgcgtgcatgcatacctcatcagtga
ttgtacataccttgcccactcctagagacagctgtgctcaccttttcctg
ctttgtgccttgattaaggctactgaccctaaatttctgaagcacagcca
agaaaaattacattccttgtcattgtaaattacctttgtgtgtacatttt
tactgtatttgagacattttttgtgtgtgactagttaattttgcaggatg
tgccatatcattgaacggaactaaagtctgtgacagtggatatagctgct
ggaccattccatcttatatgtaaagaaatctggaattattattttaaaac
catataacatgtgattataatttttcttagcattttctttgtaaagaact
acaatataaactagttggtgtataataaaaagtaatgaaattctgagaag
agttttatcttaggaaaatacatatatatgcagtgtgtgtgccagtgtgg
tattaacaagactaatagtgcagtttgatccttaccaatatcattactta
atttgaagtgttcattagcaccccaaaatatacctttttctatgtactgtt
aaaagaaattggcttctgatgcatgaacatttacatgtacattgaaagta

gtccataatagaagttagtttaagccaagtgtagacagtacattactccc
ttgaaaaagaattaagttgaaagagttgactttgccttaaaaggcagatc
taacccaagctccatccagtaccaaatgtgaaacttcattgttgtttggt
gagaatcgccaaattctcactaatacattggtatggtgtagggcatgcta
ctttttaaacagcagcacttatttttacagattgctactccaaggaagaa
aactggccacttttcatgtaaatattttgttcaaagatttgtatatctct
ctaggagttttccctcagttcccaggatggggtccaggagtagattaaca
gctaaaaatctcccaaggatatcttgttttgatttatttactccagggaa
ctatcagctccttcacaggagccaaaggggagctatgaatagagggtcac
atgagccagattctttactgttcataaaacccagagagtagttgtgaaag
atcctaatacaattgtgaaaagctctgtaaacatgaaatctaaaacaaat
gtagattttcacaatatgcttattaataaatgaagtctatggaataaaaaaaaaaaaa

>NM_018362 3
ctgtgggtctgtaggttaagggagaagatggcggcgctaggggaacccgt
gcggctggagagagatatttgtagagcaattgaattattggaaaaactac
aaaggagtggagaagtaccaccacagaaacttcaggctttgcaaagagtc
cttcaaagtgaattctgcaatgctgtgagagaggtatatgaacatgtcta
tgagactgtggacatcagtagcagtcctgaagtgagagcgaacgctactg
caaaggctactgttgctgcatttgctgccagtgaaggacattctcatcct
cgagttgttgagctaccaaaaacagaagagggccttggattcaatattat
gggaggcaaagaacaaaactctccaatctatatatcccgaataattccag
gtggaattgctgatagacatgggggcctcaaacgtggagatcaactcctc
tctgttaatggagtgagtgttgaaggagaacatcatgaaaaagctgtaga
actgctgaaagccgcacaaggaaaggttaaattagtggtacgatacacac
ccaaagtcttagaagaaatggagtcgcgctttgaaaaaatgagatcagca
aaacgcaggcaacagacctaatacatttcaaaacttgatatttcattttg
cgttttagctagagaagttttccttgtgacttactaatggctgcaatgcc
aatgattgtaagaaaacaaacaaatttatcatgaaattctccttgtcatt
ttataaatgcctattttaacatcatttatggttccagagatgcatacact
tttttctgacaagaaaaagtaaaaggtgatgagggcaattctgtcctact
gtttttacaggccttttttcaaatgcagattttgtcataaagttgttatag
attttttaaaatgcttttttaatattaaaatgtacttttacattcttaat
cttttttttagaaaggaaaagttttcttcatttagctgctgatttaaaagt
aaagttctccaattcttttttttttgcttactctatttttttttaacctgtg
aaatttctttacagttttccaagaaattaagcataacagtctcatctacg
gcagttcattacactgtcaatgttaacatcattgctgcattttgtacttt
gaacacacacataatatatataaccagtggtaaatcatgactcagtacag
tgcaagtttttttcacttttacttaaacataatatataatggatattcatt
tatactgatttatgaaagtaagtttttaaacactgaaacaatcattgcta
aaatacatattcttaatatttgagcattgaggcaagaggatgtaattgat
agcattacttattacatccctatttatgttcaaccgtacatcaaattata
aatgcaaaacaggttcagatttcatcttttgtgatttcttttaaatacta
ttcatttttatttaaatgcacagtatttcccctatattttagtccttcca
ttcctagagacaaaccagttatttggtggtgggaagtagctgaagcaaag
aaggaaaagtaatacctttaacctcactagcttcaagagtagacattctt

actagctcaatttaaataattgattttaaataggaagaaaagaggatata
tttaagatacatagaaattatgatgtgaagtattcatgagaatctgtaga
ttccatcaaaataagtaggaactcatactaaaattgttggatttaaagag
gcaacttttgtttatgattcaaatatgggaatttgagaaatatttcattt
gtccactggatgtcactattttactaaaaggcagctattagtgtgggact
gtgactgaggtcttaaagactgaaagagttggggttcattttctgttaca
acttacaggaacatttgtactttaagaaaaatttaagtgacaaatgttag
aagatgatggattgaaaaatataagtaatttggcatattgaccctatata
aaaaagtgggcattttgtaacattccttcaggaagaatagaattgtatgc
ttttttctgcatgtttgtgatcactgtgagtctcagatgattattccagt
tttcaatgatttttcaaaataaaagtcaatcagcattgttatttatcatt
aaggtattgaacactggattgcatggttgaatgtgtctttaatccactac
aaaatgtgcttgttattgataggatcatgttgttaaattgtaaattttca
aaattaattgatgttttaaaaatgttgagaccaaagtagtatagaagtat
ggacttaatttaattgtaaaattattagaggtatttgttgtagagcttta
tatattaaagagaggtattggtgcatataaaaacagtaatattattgtta
tgcttgtattcttgcatttcagggtaagtaaaccctgaagaaatcttatt
ttagtatagctacagctgcagtagcttttaagtatgtatcaacatttcat
tataaattatagcttcctggttctgtgtctcagctgtttcagaactttca
gccaagttaaatctttttttggtgttgaaacttttagtaagttgagtaagc
tatttttttccctacggtaatccacaatctgttctttgtgtgggttgaca
cctatttataattcttaaaataagggtagctcaaaggagccaagtacagt
agtttagcttgctactgttgagcatgaatagcagtaggttgttttcatta
atgcagttttcataatgtatcccagaattttaaatcttgaccatagaaaa
atacagtcattcattggcaaagatgtacagtggtggtttagtgcagtgag
gaaatgatgggaatttttatcttagaaatggatgattcgtgaataaaact
aggtacatagtaagtgtatgatagatgtttgatttgtaaattacaaatat
aaattatcacccccatttccatttattttcttgatatatcaaaatgtgtt
gacttagtgattctcaatcttagctacatattagcctggcagagctttaa
aaaattgatacctgggtcctattccataacaactaaatcaaaatcaccct
tttttttttttttgtaaagctttccaggtggttctaaaggacacctaggg
tcaataacccattataaaatgatctgattacactggacactcttaggaag
tatcaaaatagaacagttcaatttgcagctacttaatgattaattacatt
tcttaggttttttttttgttttttttttggtttgtttgttttttgtttta
aaagagacaggatcttgctttgtctcccaggctggaacacagtggtgtga
tcatagctcactgcagcctgaaactccttggcttccacctcagcctccca
aagcattaggattataggcgtgagccactgcacctggccaagtaatttct
tttaggaataaaaggtacttggcattccttatttgggaagtaattacagt
cttatcttttttttttcttttttcttttttttaataaaatctaccagtaat
ttaaatgtgagctttttatgtctgtcatcattctgcccccctttgccttta
aaacttacaaaaatacgtgtttgttatttgagaaagcatgtttaaaaatt
gcagatgagaaatattgtcgttaataattatgtactgtcttaatgtgggc
acttcatttgttagtataatactagataatagcagcctagttctttagga
tttgacgattcaaattttttttttgttttttgtttttgttttttttttgag
acagagtcttgctctgttgcccaggctggcgtgcagtggtgtgatctcgg
ctcacttcagcctccgccacctcaaggcagttcaagcaattctcctgcct

cagcctcccgagtagctaggactacaggcgtgtgccacctctcccggcta
attttttgtatttttagtagagacggggtttcaccgtgttagccaggat
gatctcgatctcctgacctcgtgattcgcccgcctcggcctcccaaagtg
ctggaattactggcgtgagccaccatgcccagcctcaaatatgtttttaa
aaaatatcattgtcctcctcctcttaagattttttaagttattttgctc
aagtacttaagtagtctggctcaagtactttgtttacaattaaaatggat
attatagcatttaatagaagaaatggttatggcttatccaaaaagaatgt
cagcatgacctggtgtagacttaaaaaactacatgtttgtgaatatttta
taatgtggaatgatcattgaaatatcaaggattctagtaattgtatttcc
tgaataaatgtatgtttatgaattttctaacttacattttgttgtgtcct
ctgccaacaatctggttttataattgttgtctaaagtttgctagtagtgt
ttcttcaaaggttattccaggtatggttaagtagtgagaaaaatcactta
ttaattttcaagaactttgagactaatttgtattgactcggtttttaaa
ataactggcaaatgctatgcttctacttttaaaatttgtggtaataccca
gaatggatgtaaaataacatttttcaatagattacttcatttatcaattt
aattctgattttaaggcttatttaaaataaatttgtatgagttatgtcct
cgttctttggatttgaaaaaagttgatataactacacaatagataacgtt
tatgaaaggtatgtataaaaaaacataattcctggtggctgctgaaatga
aactttgaatttctatattttgctttgtctttcataaaatttaaagaaaa
caaaaaaaaaaaaaaa

>NM_016271 4
gtcacaacccgcggaaggcgccgtgcgccggttgctatacaggccgcact
tcacaccccgtgcctccccgcctctcctggctcctccgcccagatccccg
gcgctcccgcgttcggtgacggccgggtaggctgtaggcagcgcaatgcc
aagacagagctgctggcggcggcgggcgaatctccctgcaccatgagcct
cggctccggccccgttaggggccgataagcacagcgcacgccgccctcca
tttgccccggggcctcggctgcgaagatagcggcggccggacaggaagct
cgaggaaagcgctgggccgggtctctacgaacacgtgaaggaaaagcagc
tccgtccacaacgccgcttcggggctcctagggagtcgggccccgggccg
ccaccgtcacctcggccgctgccgctgtcgccatcgccttgtttccccat
cccccgccatggccgaggacctctctgcggccacgtcctacaccgaagat
gatttctactgccccgtctgtcaggaggtgctcaaaacgcccgtgcggac
cacggcctgtcagcacgttttctgtagaaaatgtttcctgactgcaatga
gggaaagcggagcacattgtcccctatgtcgtggaaatgtgactagaaga
gagagagcatgtcctgaacgggccttagaccttgaaaatataatgaggaa
gttttctggtagctgcagatgctgtgcaaaacagattaaattctatcgca
tgagacatcattacaaatcttgtaagaagtatcaggatgaatatggtgtt
tcttctatcattccaaactttcagatctctcaagattcagtagggaacag
caataggagtgaaacatccacatctgataacacagaaacttaccaagaga
atacaagttcttctggtcatcctacttttaagtgtcccctgtgtcaagaa
tcaaattttaccagacagcgtttactggatcactgtaacagtaatcacct
atttcagatagttcctgtgacatgtcctatttgtgtgtctcttccttggg
gagatcctagccagattaccagaaatttcgttagtcatctaaatcagaga
catcaatttgattatggagaatttgtgaatcttcagctagatgaagaaac
ccaataccaaactgctgttgaagaatcttttcaagtaaacatctgaaggc

tgtagacatctctgcatctttgtacctgcaagtgccatctttaaggggga
aactacatgaagtcaccgttacagtaacttgatgtgtatattaataaaag
taattcagtcattttagtttttgattgaaaataaaggtagggcttctaaa
aacttcatcatcttgataagttaaaaaatgaaagttatgacattagcttt
aaaggtgtaaaaaagatgtttcactaatgtaacggtgaaagagaatccct
gttgtactttatctttttgtaatattatttttgaatttttcattattatg
ttgcttttgaaatttgatgcattcctcccatttactttattattgtacac
atttaacacacagtagcaaattttgaacgatgtgattgatataacctaac
aaatctgagccagttattattagagttgcagaatagaaacttgaagtgct
aaatggaataatccaaaggaaattttttaaatgcaggttctagctgaaaa
attcaactataagaaaattgtatttatataacatttactatttttgaaga
ctagtgagatttctgtaataattttaattctttaaaaagtgaaagcttgt
tgtaaagatattttctttttgttattagaaggaaatacaaagagaaaaat
ttctttctttcatgggcatttgataatttcagtctttgactgatttgtaa
gcctagaatatactaagctgaataacagctctttggcctcagaattttca
gtagccagtatttctgattaactaagttgaaactcttattagaaactttc
agttggtgatattgtattctagaagatataaatgagaggtttggcttcat
ctcagtttagaaatttattcaaagctaaagatgtatatatacatatactt
ttgtgtgtatatatacacatatgtgtgtatgcagtttgtcaggttatata
tagaatttctattaaggatttttttaaatggacaagcaatagggggttgaa
gtgtttatctgatttgtttaaaattttgtatatcaccaaatttttaaaaa
gtgatagtcacagtgctaagttatctagttggctactattacaccttaaa
aattgagtttacacacacacaattacctgtttatatggtgctcatttgtt
attctcaaatataatgtgtgaccgtgatatagtgagaaagattctaccaa
ccactgtttcactactttttagttaaaattgggtatgttcttaatattca
ttagtgagaatcacaaagtattttgtagaaggcccaaatcacagaataaa
ggactaagagtggatttgctgacattccatactaatatacattgtttatg
ctttctttaaaataactagaagaacataaaagaaagagaatctcagaagt
agtttgctgctaatatatacatatattgtataaaaaggtatattttggtt
ttgttaaaacccttgttgacttttctacactgaacattttttttaacttg
atttaataaaaatgttaattttggaagtgcagttttgtaaaaaccttttt
cagtcaaacagtaaagactttatttatggattgtaataacaaccacaaga
aaagccatacatcttaatgaacatacgcctttgttctgtcattttaaaga
gtggtgtttactatgtgtgtgcttgagcattttctttcagttacatgggt
gtaatttaaaaaacagctcctttttaagacttttgaccatagtgttttcc
agtttaaagcaataactttcatagtttctttcaaagtttaatacatccaa
tatgtcaagttaaaccattctgggatttgcaaagtttaatacatccaata
tgtcaagttaaaccattctgggatttgcaaagtttaatacatccaatatg
tcaagttaaaccattctgggatttgggaacttctttaaaggaaaattgta
gtagaggtcatcttgatggaattgtgttttaaagttttgtgaatgagtta
aatatcattgatagtcttgtgtgtctcacatatcagctttttcataagga
aaatactttatttgcttttatctgagaatttctcagtatgaaaatgattg
tttaaaatttcccctctttttgtcagtgcattgggaatagggatagactt
tacagatttatggaaattaaatttatggggaaaagtttatttttaatatt
acttatagctgagtcagaattaaaggagggaattgtattgatactccaat
acctaaatttcacaataccacttaggaaagctatttcaacaaattacagt

tttattgttaaaacagagtcttatttgagatgtattgctttatttttcaa
ttaaaagtggttttctcctaaaaaaaaaaaaaaa

>NM_000362 4
ctgtggcttgccccagagctgatccttgtctttgtccacttctcagcgag
gatggcacttcagggagcccttcccttactatcgcagagagagcaggccc
tccccagtcatgtccaacccagaactctgttttgttttcttcatagccct
agcatcacagaaaatcaccctgtgcattcatggatgtccacgggggcaag
ggctttgtgttgcttaacccagcatcctgaaccgtgtttgttgaatgaat
acagaaccccgtttgctctgggagagcacagaaaacagtcttctatcata
tatcatagccagctgcaaacagcagatggcttcccatatcccagagagta
agaaccagagagagagagaaagagagagagtttgggtctttctcctctgt
gcctgctctctccagagaaactggaggggtagcagttagcattcccccgc
tggttccaccaagcacagtcaaggtctctaggacatggccacccctcacc
tgtggaagcggtcctgctggggtgggtgggtgttagttggttctggtttg
ggtcagagacacccagtggcccaggtgggcgtggggccagggcgcagacg
agaaggggcacgagggctccgctccgaggacccagcggcaagcaccggtc
ccgggcgcgccccagcccacccactcgcgtgcccacggcggcattattcc
ctataaggatctgaacgatccgggggcggccccgccccgttaccccttgc
ccccggccccgcccccttttggagggccgatgaggtaatgcggctctgc
cattggtctgagggggcgggccccaacagcccgaggcggggtccccgggg
gcccagcgctatatcactcggccgcccaggcagcggcgcagagcgggcag
caggcaggcggcgggcgctcagacggcttctcctcctcctcttgctcctc
cagctcctgctccttcgccgggaggccgcccgccgagtcctgcgccagcg
ccgaggcagcctcgctgcgccccatcccgtcccgccgggcactcggaggg
cagcgcgccggaggccaaggttgccccgcacggcccggcgggcgagcgag
ctcgggctgcagcagccccgccggcggcgcgcacggcaactttggagagg
cgagcagcagccccggcagcggcggcagcagcggcaatgaccccttggct
cgggctcatcgtgctcctgggcagctggagcctgggggactggggcgccg
aggcgtgcacatgctcgcccagccacccccaggacgccttctgcaactcc
gacatcgtgatccgggccaaggtggtggggaagaagctggtaaaggaggg
gcccttcggcacgctggtctacaccatcaagcagatgaagatgtaccgag
gcttcaccaagatgccccatgtgcagtacatccatacggaagcttccgag
agtctctgtggccttaagctggaggtcaacaagtaccagtacctgctgac
aggtcgcgtctatgatggcaagatgtacacggggctgtgcaacttcgtgg
agaggtgggaccagctcaccctctcccagcgcaaggggctgaactatcgg
tatcacctgggttgtaactgcaagatcaagtcctgctactacctgccttg
ctttgtgacttccaagaacgagtgtctctggaccgacatgctctccaatt
tcggttaccctggctaccagtccaaacactacgcctgcatccggcagaag
ggcggctactgcagctggtaccgaggatgggcccccccggataaaagcat
catcaatgccacagacccctgagcgccagaccctgccccacctcacttcc
ctcccttcccgctgagcttcccttggacactaactcttcccagatgatga
caatgaaattagtgcctgttttcttgcaaatttagcacttggaacattta
aagaaaggtctatgctgtcatatggggtttattgggaactatcctcctgg
ccccaccctgccccttctttttggttttgacatcattcatttccacctgg
gaatttctggtgccatgccagaaagaatgaggaacctgtattcctcttct

tcgtgataatataatctctatttttttaggaaaacaaaaatgaaaaacta
ctccatttgaggattgtaattcccacccctcttgcttcttccccacctca
ccatctcccagaccctcttccctttgcccttctcctccaatacataaagg
acacagacaaggaacttgctgaaaggccaaccatttcaggatcagtcaaa
ggcagcaagcagatagactcaaggtgtgtgaaagatgttatacaccagga
gctgccactgcatgtcccaaccagactgtgtctgtctgtgtctgcatgta
agagtgagggagggaaggaaggaactacaagagagtcggagatgatgcag
cacacacacaattccccagcccagtgatgcttgtgttgaccagatgttcc
tgagtctggagcaagcacccaggccagaataacagagctttcttagttgg
tgaagacttaaacatctgcctgaggtcaggaggcaatttgcctgccttgt
acaaaagctcaggtgaaagactgagatgaatgtctttcctctccctgcct
cccaccagacttcctcctggaaaacgctttggtagatttggccaggagct
ttcttttatgtaaattggataaatacacacaccatacactatccacagat
atagccaagtagatttgggtagaggatactatttccagaatagtgtttag
ctcacctagggggatatgtttgtatacacatttgcatatacccacatggg
gacataagctaatttttttacaggacacagaattctgttcaatgctgtta
aatatgccaatagtttaatctcttctattttgttgtcgttgcttgtttga
agaaaatcatgacattccaagttgacatttttttttcattttaattaaaa
tttgaaattctgaacaccgtcagcaccctctcttccctatcatgggtcat
ctgacccctgtccgtctccttgtccctgcttcatgtttgggggcctttct
ttaactgccttcctggcttagctcagatggcagatgagagtgtagtcaag
ggcctgggcacaggagggagagctgcagagtgtcctgcctgccttggctg
gagggacacctctcctgggtgtggagacagcttggttccctttccctagc
tccctggtgggtgaatgccacctcctgagatcctcacctcttggaattaa
aattgttggtcactggggaaagcctgagtttgcaaccagttgtagggttt
ctgttgtgtttttttttttttttgaaataaaactataatataaattct
cctattaaataaaattattttaagttttagtgtcaaaagtgagatgctga
gagtaggtgataatgtatattttacagagtgggggttggcaggatggtga
cattgaacatgattgctctctgtctcttttttcagcttatgggtatttat
cttctattagtatttgtatcttcagttcattccactttaggaaacagagc
tgccaattgaaacagaagaagaaaaaaaaaaaaagcagcagacaacacac
tgtagagtcttgcacacacacaagtgcccaggcaaggtgcttggcagaac
cgcagagtgggaagagagtaccggcatcgggtttccttgggatcaatttc
attaccgtgtacctttcccattgtggtcatgccatttggcagggggagaa
tgggaggcttggccttctttgtgaggcagtgtgagcagaagctgatgcca
gcatgtcactggttttgaagggatgagcccagacttgatgttttgggatt
gtccttattttaacctcaaggtctcgcatggtggggcccctgaccaacct
acacaagttccctcccacaagtggacatcagtgtcttctctgtgaggcat
ctggccattcgcactccctggtgtggtcagcctctctcacacaaggagga
acttgggtgaaggctgagtgtgaggcacctgaagtttccctgcggagtcg
ataaattagcagaaccacatccccatctgttaggccttggtgaggaggcc
ctgggcaaagaagggtctttcgcaaagcgatgtcagagggcggttttgag
ctttctataagctatagctttgtttatttcacccgttcacttactgtata
atttaaaatcatttatgtagctgagacacttctgtatttcaatcatatca
tgaacattttattttgctaaatcttgtgtcatgtgtaggctgtaatatgt
gtacattgtgtttaagagaaaaatgaaacccacatgccgccattttcctg

aatcaaattctgcagtggaatggagaggaaaatacttctaggcaagcagc
tagactggtgaattgggggaaatagaaggaactagtaactgagactcctc
cagcctcctccctattggaatcccaatggctcctggagtaggaaaaaagt
ttaaactacattcatgttcttgttctgtgtcactcggccctgggtagtct
accatttacttcaccccaagtcctgctgcccatccagttgggaagccatg
attttcctaagaatccagggccatgggagatacaattccaagttctcgct
tcctcctttgggcatctcttctgcctcccaatcaaggaagctccatgctc
aggctctcagctctcgggccagtgctctgctctgtccagggtaggtaata
ctgggagactcctgtcttttaccctcccctcgttccagacctgcctcatg
gtggcaacatggttcttgaacaattaaagaaacaaatgactttttggaat
agccctgtctagggcaaactgtggcccccaggagacactacccttccatg
ccccagacctctgtcttgcatgtgacaattgacaatctggactaccccaa
gatggcacccaagtgtttggcttctggctacctaaggttaacatgtcact
agagtatttttatgagagacaaacattataaaaatctgatggcaaaagca
aaacaaaatggaaagtaggggaggtggatgtgacaacaacttccaaattg
gctctttggaggcgagaggaaggggagaacttggagaatagtttttgctt
tgggggtagaggcttcttagattctcccagcatccgcctttccctttagc
cagtctgctgtcctgaaacccagaagtgatggagagaaaccaacaagaga
tctcgaaccctgtctagaaggaatgtatttgttgctaaatttcgtagcac
tgtttacagttttcctccatgttatttatgaattttatattccgtgaatg
tatattgtcttgtaatgttgcataatgttcactttttatagtgtgtcctt
tattctaaacagtaaagtggttttatttctatcacaaaaaaaaaaa

>NM_001111 4
gcccctcctcttggccaaactttccggaggggaaggctttccgaggaaac
gaaagcgaaattgaaccggagccatcttgggcccggcgcgcagacccgcg
gagtttcccgtgccgacgccccggggccacttccagtgcggagtagcgga
ggcgtgggggcctcgaggggctggcgcggcccagcggtcgggccagggtc
gtgccgccggcgggtcgggccgggcaatgcctcgcgggcgcaatgaatcc
gcggcaggggtattccctcagcggatactacacccatccatttcaaggct
atgagcacagacagctcaggtaccagcagcctgggccaggatcttccccc
agtagtttcctgcttaagcaaatagaatttctcaaggggcagctcccaga
agcaccggtgattggaaagcagacaccgtcactgccaccttccctcccag
gactccggccaaggtttccagtactacttgcctccagtaccagaggcagg
caagtggacatcaggggtgtccccaggggcgtgcatctcggaagtcaggg
gctccagagagggttccagcatccttcaccacgtggcaggagtctgccac
agagaggtgttgattgcctttcctcacatttccaggaactgagtatctac
caagatcaggaacaaaggatcttaaagttcctggaagagcttggggaagg
gaaggccaccacagcacatgatctgtctgggaaacttgggactccgaaga
aagaaatcaatcgagttttatactccctggcaaagaagggcaagctacag
aaagaggcaggaacacccccctttgtggaaaatcgcggtctccactcaggc
ttggaaccagcacagcggagtggtaagaccagacggtcatagccaaggag
ccccaaactcagacccgagtttggaaccggaagacagaaactccacatct
gtctcagaagatcttcttgagccttttattgcagtctcagctcaggcttg
gaaccagcacagcggagtggtaagaccagacagtcatagccaaggatccc
caaactcagacccaggtttggaacctgaagacagcaactccacatctgcc

ttggaagatcctcttgagtttttagacatggccgagatcaaggagaaaat
ctgcgactatctcttcaatgtgtctgactcctctgccctgaatttggcta
aaaatattggccttaccaaggcccgagatataaatgctgtgctaattgac
atggaaaggcagggggatgtctatagacaagggacaacccctcccatatg
gcatttgacagacaagaagcgagagaggatgcaaatcaagagaaatacga
acagtgttcctgaaaccgctccagctgcaatccctgagaccaaaagaaac
gcagagttcctcacctgtaatatacccacatcaaatgcctcaaataacat
ggtaaccacagaaaaagtggagaatgggcaggaacctgtcataaagttag
aaaacaggcaagaggccagaccagaaccagcaagactgaaaccacctgtt
cattacaatggcccctcaaaagcagggtatgttgactttgaaaatggcca
gtgggccacagatgacatcccagatgacttgaatagtatccgcgcagcac
caggtgagtttcgagccatcatggagatgccctccttctacagtcatggc
ttgccacggtgttcaccctacaagaaactgacagagtgccagctgaagaa
ccccatcagcgggctgttagaatatgcccagttcgctagtcaaacctgtg
agttcaacatgatagagcagagtggaccaccccatgaacctcgatttaaa
ttccaggttgtcatcaatggccgagagtttcccccagctgaagctggaag
caagaaagtggccaagcaggatgcagctatgaaagccatgacaattctgc
tagaggaagccaaagccaaggacagtggaaaatcagaagaatcatcccac
tattccacagagaaagaatcagagaagactgcagagtcccagaccccccac
cccttcagccacatccttcttttctgggaagagccccgtcaccacactgc
ttgagtgtatgcacaaattggggaactcctgcgaattccgtctcctgtcc
aaagaaggccctgcccatgaacccaagttccaatactgtgttgcagtggg
agcccaaactttccccagtgtgagtgctcccagcaagaaagtggcaaagc
agatggccgcagaggaagccatgaaggccctgcatggggaggcgaccaac
tccatggcttctgataaccagcctgaaggtatgatctcagagtcacttga
taacttggaatccatgatgcccaacaaggtcaggaagattggcgagctcg
tgagatacctgaacaccaaccctgtgggtggccttttggagtacgcccgc
tcccatggctttgctgctgaattcaagttggtcgaccagtccggacctcc
tcacgagcccaagttcgtttaccaagcaaaagttgggggtcgctggttcc
cagccgtctgcgcacacagcaagaagcaaggcaagcaggaagcagcagat
gcggctctccgtgtcttgattggggagaacgagaaggcagaacgcatggg
tttcacagaggtaaccccagtgacaggggccagtctcagaagaactatgc
tcctcctctcaaggtccccagaagcacagccaaagacactccctctcact
ggcagcaccttccatgaccagatagccatgctgagccaccggtgcttcaa
cactctgactaacagcttccagccctccttgctcggccgcaagattctgg
ccgccatcattatgaaaaaagactctgaggacatgggtgtcgtcgtcagc
ttgggaacagggaatcgctgtgtgaaaggagattctctcagcctaaaagg
agaaactgtcaatgactgccatgcagaaataatctcccggagaggcttca
tcaggtttctctacagtgagttaatgaaatacaactcccagactgcgaag
gatagtatatttgaacctgctaagggaggagaaaagctccaaataaaaaa
gactgtgtcattccatctgtatatcagcactgctccgtgtggagatggcg
ccctctttgacaagtcctgcagcgaccgtgctatggaaagcacagaatcc
cgccactaccctgtcttcgagaatcccaaacaaggaaagctccgcaccaa
ggtggagaacggagaaggcacaatccctgtggaatccagtgacattgtgc
ctacgtgggatggcattcggctcggggagagactccgtaccatgtcctgt
agtgacaaaatcctacgctggaacgtgctgggcctgcaaggggcactgtt

gacccacttcctgcagcccatttatctcaaatctgtcacattgggttacc
ttttcagccaagggcatctgacccgtgctatttgctgtcgtgtgacaaga
gatgggagtgcatttgaggatggactacgacatccctttattgtcaacca
ccccaaggttggcagagtcagcatatatgattccaaaaggcaatccggga
agactaaggagacaagcgtcaactggtgtctggctgatggctatgacctg
gagatcctggacggtaccagaggcactgtggatgggccacggaatgaatt
gtcccgggtctccaaaaagaacatttttcttctatttaagaagctctgct
ccttccgttaccgcagggatctactgagactctcctatggtgaggccaag
aaagctgcccgtgactacgagacggccaagaactacttcaaaaaaggcct
gaaggatatgggctatgggaactggattagcaaaccccaggaggaaaaga
acttttatctctgcccagtatagtatgctccagtgacagatggattaggg
tgtgtcatactagggtgtgagagaggtaggtcgtagcattcctcatcaca
tggtcaggggatttttttttctcctttttttttctttttaagccataatt
ggtgatactgaaaactttgggttcccatttatcctgctttctttgggatt
gctaggcaaggtctggccaggcccccctttttcccccaagtgaagaggc
agaaacctaagaagttatcttttctttctacccaaagcatacatagtcac
tgagcacctgcggtccatttcctcttaaaagttttgttttgatttgtttc
catttcctttccctttgtgtttgctacactgacctcttgcggtcttgatt
aggtttcagtcaactctggatcatgtcagggactgataatttcatttgtg
gattacgcagacccctctacttcccctctttcccttctgagattctttcc
ttgtgatctgaatgtctccttttccccctcagagggcaaagaggtgaaca
taaaggatttggtgaaacatttgtaagggtaggagttgaaaactgcagtt
cccagtgccacggaagtgtgattggagcctgcagataatgcccagccatc
ctcccatcctgcactttagccagctgcagggcgggcaaggcaaggaaagc
tgcttccctggaagtgtatcactttctccggcagctgggaagtctagaac
cagccagactgggttaagggagctgctcaagcaatagcagaggtttcacc
cggcaggatgacacagaccacttcccagggagcacgggcatgccttggaa
tattgccaagcttccagctgcctcttctcctaaagcattcctaggaatat
tttccccgccaatgctgggcgtacaccctagccaacgggacaaatcctag
agggtataaaatcatctctgctcagataatcatgacttagcaagaataag
ggcaaaaaatcctgttggcttaacgtcactgttccacccggtgtaatatc
tctcatgacagtgacaccaagggaagttgactaagtcacatgtaaattag
gagtgttttaaagaatgccatagatgttgattcttaactgctacagataa
cctgtaattgagcagatttaaaattcaggcatacttttccatttatccaa
gtgctttcatttttccagatggcttcagaagtaggctcgtgggcagggcg
cagacctgatctttatagggttgacatagaaagcagtagttgtgggtgaa
agggcaggttgtcttcaaactctgtgaggtagaatcctttgtctatacct
ccatgaacattgactcgtgtgttcagagcctttggcctctctgtggagtc
tggctctctggctcctgtgcattctttgaatagtcactcgtaaaaactgt
cagtgcttgaaactgtttcctttactcatgttgaagggactttgttggct
tttagagtgttggtcatgactccaagagcagagcagggaagagcccaagc
atagacttggtgccgtggtgatggctgcagtccagttttgtgatgctgct
tttacgtgtccctcgataacagtcagctagacacactcaggaggactact
gaggctctgcgaccttcaggagctgagcctgcctctctcctttagatgac
agaccttcatctgggaacgtgctgagccagcaccctcagatgatttccct
ccaaactgctgactaggtcatcctctgtctggtagagacattcacatctt

tgcttttattctatgctctctgtacttttgaccaaaaattgaccaaagta
agaaaatgcaagttctaaaaatagactaaggatgcctttgcagaacacca
aagcatcccaaggaactggtagggaagtggcgcctgtctcctggagtgga
agaggcctgctccctggctctgggtctgctgggggcacagtaaatcagtc
ttggcacccacatccagggcagagaggtctgtggttctcagcatcagaag
gcagcgcagcccctctcctcttcaggctacagggttgtcacctgctgagt
cctcaggttgtttggcctctctggtccatcttgggcattaggttctccag
cagagctctggccagctgcctcttctttaactgggaacacaggctctcac
aagatcagaacccccactcacccccaagatcttatctagcaagcctgtag
tattcagtttctgttgtaggaagagagcgaggcatccctgaattccacgc
atctgctggaaacgagccgtgtcagatcgcacatccctgcgcccccatgc
ccctctgagtcacacaggacagaggaggcagagcttctgcccactgttat
cttcactttctttgtccagtcttttgtttttaataagcagtgaccctccc
tactcttctttttaatgatttttgtagttgatttgtctgaactgtggcta
ctgtgcattccttgaataatcacttgtaaaaattgtcagtgcttgaagct
gtttcctttactcacattgaagggacttcgttggtttttggagtcttgg
ttgtgactccaagagcagagtgaggaagacccccaagcatagactcgggt
actgtgatgatggctgcagtccagttttatgattctgcttttatgtgtcc
cttgataacagtgacttaacaatatacattcctcataaataaaaaaaaaa
caagaatctgaattcttagaaaaaaaaaaaaaaaaaaaaaa

>NM_014445 3
aaacgcgcacgcgcaaatctagggcgacgcttgacagagcttgggagggt
gcgcctgctttcgccctccttctccagcgggaggggcgcgcacttccgcg
gggcggagtccgtctagtgctgacgttggcagccgaacccaaagtagatc
gaggcggcgggctgcacattcccgttgttgcgttgcgtttccttcctctt
tcactccgcgctcacggcggcggccaaagcggcggcgacggcggcgcgag
aacgacccggcggccagttctcttcctcctgcgcacctgccccgctcggt
cagtcagtcggcggccggcgcccggcttgtgctcagacctcgcgcttgcg
gcgcccaggcccagcggccgtagctagcgtctggcctgagaacctcggcg
ctccggcggcgcgggcaccacgagccgagcctcgcagcggctccagagga
ggcaggcgagtgagcgagtccgaggggtggccggggcaggtggtggcgcc
gcgaagatggtcgccaagcaaaggatccgtatggccaacgagaagcacag
caagaacatcacccagcgcggcaacgtcgccaagacctcgagaaatgccc
ccgaagagaaggcgtctgtaggaccctggttattggctctcttcattttt
gttgtctgtggttctgcaattttccagattattcaaagtatcaggatggg
catgtgaagtgactgaccttaagatgtttccattctcctgtgaattttaa
cttgaactcattcctgatgtttgataccctggttgaaaacaattcagtaa
agcatcctgcctcagaatgactttcctatcatgcttcatgtgtcattcca
aggtttcttcatgagtcattccaagttttctagtccataccacagtgcct
tgcaaaaaacaccacatgaataaagcaataaaatttgattgttaagatac
agtagtggaccctacttattcagtcaattaagagtaagtttttttatgtg
gttattaaaacagtatgaacaattagtctaactctgcatagacagggtct
agattttgttaacccaaatgtataactgcagttagcttaaattacaattt
gaagtcttgtggtttttatatagctaggcactttattactcttttgaact
gaaagcacactcccttataggttcatgtaactgtcctgtaataaggtgct

tataaatggaacaactacacagcctagttttgccacaacctttagcatct
aaaaagttttaaaagcttctaaatgtctaatataaagggagatgcttata
gccacaacatctattttaccaatattgtttccattacactaccttggatt
ttgcatgagtgagtatagtaacccaagatgccataaaaaaaaaacttgat
cgttttctgacttaatcagttactgtggtttcactaaaagctaccgtggt
ggagtgaagtcagtcagggaaggtttgtttatgttacatttatttcacca
gaactattttaatatatcaaaggggtttactatgccaaacaaaattctag
ggaaaaatactgctaaaaatggatgcctcatcagaacatgctgttgagtc
caatgtgccataagacattttagcatgttaaatagcacttttaatagcaa
aaaaaggcacatcaactgcgaagttatccttagtttgcaaatgctttttc
tagattaatgatttttcaatcattagggtactagacacatcagcctaaag
tggcatctggaattgaatggatttactgataatgatcagtctttagtctt
ccctttgttatatgactttataggttatgattgatcaaatttacgtttta
ctaatggtaagggtgagggtcatagggcaggttttgggttttctagtact
gttgaaaactgcaagtattggctatttgtatacttagccataacttggtg
aaaaaaaacctgagcagtgtctatgtattaatgcgttggaaagaaagctg
cttgtgtttgctttgttaattgcctcaggatatttcttttaaaataagct
gttttaagaggaacagaagggaaatctgctacctagtctatacacagcgt
gaacctcacaggggggcttctgataccctcaaacatggagaacagtaaggg
agcagagtggttaaggactttcaggaacttaactattctggaataaggaa
tgaatcaactgaccttgggccagcaggtttttaactaaattgttacttgc
ctttctcacccagttaatcagtctctgtacttgtttccctttttgaaaca
agtgtcttggttaactaattctgttttatggttgtgctaaattcatagca
ggtgccttattctttgcttttagtcaaaccattccatatcagaattttcc
ttggtttactatagatatttggctttaagttgttgtttgtgtttttttaat
gtacaatgttctgataaatttgactgttaaattgctatagctagcaatca
ttttacatatgtaaaattgcattccctttgtatttcatgtgtaattcacc
aattaagtgcagtttatattcaggttggattatgcatgtttaggtaaacg
aaagctgtgtcttacttgatttattctttaaaaataaagttccctgaata
tttgatgcttttcttctaaacggaaatgattttacagttatctgagtgta
ccttttatagttagtagaaaatgattttaaagaatgtttagtattgtact
taaatggtatgcagaggcacagatgtaaggtttataactggaaataggtg
gtaagaaaaatatatagaaagcacaatgatttgaatatttttccacttag
gatttcctaatctccttgtcatccaattcaagctcaagatgaagcacaat
tctttgatctccctttgccagttgaattttatagatcatctaatgttgag
cacagtatgagaataaatattggggttgtcaacattactcagttactctt
tgtggtttaactctaacatttcaacaagttgtcaattaattgtatctgtt
gggttgtatataatgttgctcaaaataattaagtggacttccaaaaataa
gatttccattgtaacaggatgcattgtgatgggctttgacttacattaaa
gaaatgtggatagtcaactgcaaaaaaaaaa

>NM_000891 2
gcgcactggagccctggccagcgcgcagccttcccggcgccggcgggctg
ggtcttgggaattctggtttgctttggctcactcgctttttacaaaccac
tggatcttacatgcctctgtaccccccacttccactccatgtccccatgc
tcctgcgccagcaacaggacatgttctctggatgtcagctgagtcattaa

agtaactctgcatgtcagtagacagaccttggtagaaccacaaggctccc
agagacacccatctctcctcatttttttggtgtgtgtgtcttcaccgaac
attcaaaactgtttctccaaagcgttttgcaaaaactcagactgttttcc
aaagcagaagcactggagtccccagcagaagcgatgggcagtgtgcgaac
caaccgctacagcatcgtctcttcagaagaagacggtatgaagttggcca
ccatggcagttgcaaatggctttgggaacgggaagagtaaagtccacacc
cgacaacagtgcaggagccgctttgtgaagaaagatggccactgtaatgt
tcagttcatcaatgtgggtgagaaggggcaacggtacctcgcagacatct
tcaccacgtgtgtggacattcgctggcggtggatgctggttatcttctgc
ctggctttcgtcctgtcatggctgtttttggctgtgtgttttggttgat
agctctgctccatggggacctggatgcatccaaagagggcaaagcttgtg
tgtccgaggtcaacagcttcacggctgccttcctcttctccattgagacc
cagacaaccataggctatggtttcagatgtgtcacggatgaatgcccaat
tgctgttttcatggtggtgttccagtcaatcgtgggctgcatcatcgatg
ctttcatcattggcgcagtcatggccaagatggcaaagccaaagaagaga
aacgagactcttgtcttcagtcacaatgccgtgattgccatgagagacgg
caagctgtgtttgatgtggcgagtgggcaatcttcggaaaagccacttgg
tggaagctcatgttcgagcacagctcctcaaatccagaattacttctgaa
ggggagtatatccctctggatcaaatagacatcaatgttgggtttgacag
tggaatcgatcgtatatttctggtgtccccaatcactatagtccatgaaa
tagatgaagacagtcctttatatgatttgagtaaacaggacattgacaac
gcagactttgaaatcgtggtcatactggaaggcatggtggaagccactgc
catgacgacacagtgccgtagctcttatctagcaaatgaaatcctgtggg
gccaccgctatgagcctgtgctctttgaagagaagcactactacaaagtg
gactattccaggttccacaaaacttacgaagtccccaacactcccctttg
tagtgccagagacttagcagaaaagaaatatatcctctcaaatgcaaatt
cattttgctatgaaaatgaagttgccctcacaagcaaagaggaagacgac
agtgaaaatggagttccagaaagcactagtacggacacgccccctgacat
agaccttcacaaccaggcaagtgtacctctagagcccaggcccttacggc
gagagtcggagatatgactgactgattccttctctggaatagttacttta
caacacggtctgttggtcagaggcccaaaacagttatacagatgacggta
ctggtcaagatgggtcaagcaagcggccacaagggactgaggcaagcaca
atggtttcaaagaaagactgtaagctccatgattagcataaagcactaac
catgtctccatgtgacccgatggcacatagatgttgtagaataagttatg
ggtttttatgttttgttttgtgtttttccaaaacttgaacttgcaggcaa
gccttggttgggtatttgatttatccagaatgcttctctttagggaacaa
ggatgtttttaatggcataacaaaggcaagactctgccttaatttttgaa
aagctgctaactacatgaacacaaatgtgtatttttgttgcagtgtagtt
ttccttttgtgtaattttaaagtcagtgttgaattttattgaaagctcat
gatgcgcttcaaagtggcaagtatttggctattaactgccaaaacaagag
cctgatttttttgaggccagtaattcgtttgctagaattgattttttttct
ctctctctttgttacataagggcattatgtaacactagccgaatggtagc
ctctgggttgttgtttttttcttttcctccatgatgttaatgggttatct
caaattttaagttaaactacctaaaataaataccaaagataatgcatatt
tttgcacagtggagcttacacttaaaagaaaacaaagccccatgggctgc
cttgaaatcaagagacaataactttgaacctcagcaagaccttgaaccgc

cggttcattttgcaccttattcagaaaatagagcatcatactcaccgagt
ctagtcagtgtagtgcttttaaaaattttgtcctttcatgtaactttttt
attttaagaggaagaagaagaaaggggcacacacacacaataccgacgtc
tatcctttcctgctaggcagtgctggccaggctcatgtgtagtgtgcgag
atggtgatgtactcttatatttttctgggcttttccttttgcacattcca
aaattcatttcataagacaagatcttcataggacctccttggcatcctgg
cattctcaaaactgagccatccagcatgaaagataaatgggtttaaaccc
ttgctgctgaatttattgcctggactgtcaggacatcaccagcccacctt
caccttagggaagatgccacacctggcctccacacttgctcttctgatca
gtctgtctggattgagtcctacagtgtcagatagggcggcaaatgccaaa
gcagggaaacagggaggtgtggacaagccagtttgatgcagcacttcaga
tcaagtgcttaggaaggagaggaaacttgcctttttatggcagaggata
gtaatgaaaatgtctcagtattttagggtcaatgagagccataaaaatat
aacataatcacaagtaaaggagataatggtctaaaacagctatttccctt
ttctgtgtgcatacttatgactgaatgtgagctaagcattttctcctgtg
gagccctagagcaggttactaaggaaggacacattgttttccagaagcct
cccctgcctggctgactgccttgctagaaacataattttttttttctcac
tgaagctcaataatggaactcttttttttttttttttaatttaaagttc
cctatttgtgaattctgggattactgacttttctttttaattggagtctc
aaaatcaactctcttatggtattatatctctgtatgccattaaaaaacag
cttgttctagaatcatgtattttgtaaactgatgtttgtgatggtctctg
gttcttgaacagccatatctgaatgccgtgcctgcaaaactatgacaatt
tttgctgttttcagccttcagatttgatggcttgggaaactgaggtgtta
ttttcaatgaaacaaagaaagagatgttaagcaagtggttgttttagatc
caaatgtaaaggcaggtttgggaaggtgtttaaagagttggaggaattgg
ggattgagttgtaaagaaaacttacagaagaggcaacaatttggttcttg
acagtgagaggatattgagggcttcagctgctgctattatgatgttttgc
aaaggaaaataatcaaaccaaagagtattcagtgatatgtaaattaaatg
aagatacagtggagaatgggggtgaccacaaaagaggctccccctaaaca
cacagtgctgccacttaaaaagacttgagaaatttgaaagggggtgggta
tggggggggcaagaaagagggagggaaatctttcaacttatttctgaaaa
agagaaaaaaatataaaatttctggtgcacaggtttgtttttttcaagaaa
attttgcagaagctatgtttttaaagtgtacattttataaagtttatcag
atattttcatatttaaagccaaatgtaaatagaggtctgtaaagaaaaat
aattgccatagaaagtataatttcagtgcagtaatttctgagagctagta
cctatatgctaccggttagcatggttttagcaaatatataccagccttat
aaggttcgtattgctatgttcttctgttatttatttcagcatggactgtt
catttgaaacctttttctagttattagcgttttaacagttacaagcttta
aatggcaattttttttttttttttttttttttttttttttttgtcaagag
ccaagacacaggtaatgcacgacattgattgctgcattttaccttcaaaa
tatttgtccttattgactgggtctccttaattaatgtacacatgtcatta
gaatgcagacggaggggactcaccatgaatatctggggttgattcccaga
tgtgtgttgcttctctattgcaagcagattccctgttggatttacttcgg
atttattcccttttaaagaatttttgcccatatctggaagggcactatat
ttttgggaggagccatagattcctggttatcctatttttaaacaaaatgt
agacaaagtgaactctattttgattattgagaaaggagtagttttctatc

cctctaagagtatacttgaatcagacattttaaggatgtcactatggcac
tgttgtcatttccaaattcctagaaaagtttgttttactttgtttttatt
ctgttaatgcattctttcttctctttacttcctttcttaccagtacactc
ctatctcaactctgtttatttgatgagttctgtcccgtaaatcatatttc
ccttacaattaataaatgtcacttcatattttataataaaccactcagta
aaagcaaaagcttgtcctgagaagtagagtgagttctttttcactctgtg
tctaataatgttaaggtgggaaaaaaaaaagtgtggcatagctacctgcc
catccccaaccctcagcaaagtagaatctcttttctggtaattttgggtt
tccgctctgggctctggcaagttgaacaatcctagccattgacaatcgtg
atagttattattttcccatttgctgtctttttgtatctaaagtcttccta
ttgtactgcacaaaccatggattgtacatatttttatatattatgtctta
ttttattatttctaaataaaaaaattaaaaattgaaaaaaaaaaaa

>NM_017542 3
cgctccctcccgaggggccgcgcgcacgggaggacggagaggcggaaagg
atggcgctgtgacagccgggccggagccctcgcgtccccaccccgcgccc
tggccgctgggcccggcgagatggagtccacagcctaccctctcaatttg
agcctgaaagaagaggaagaggaagaagagattcagagccgggaactaga
ggacggcccggcagacatgcagaaagtacgaatctgctctgagggcggat
gggtaccggccctatttgatgaggtggccatatatttttccgatgaggaa
tgggaagttttgacggagcaacaaaaggccctctaccgggaagtcatgag
gatgaattatgaaactgtcctgtccctggaattcccattccctaagccag
acatgatcacccgtttggaaggggaggaggagtctcagaattctgacgag
tggcagctccaaggaggcacctctgcagaaaatgaagaatctgacgtaaa
gcctccagactggccaaacccaatgaatgctacctcccagtttcctcagc
ctcagcactttgacagctttggcctccgtctgcctcgggatatcacagag
ctgcccgagtggagtgaggggtaccccttctacatggccatgggcttccc
agggtatgacctctcggctgatgacatagctgggaagtttcagttcagcc
ggggcatgcgccgcagttacgacgcagggttcaagctgatggtagtggaa
tatgctgagagtaccaacaactgccaggctgccaagcagtttggagtatt
ggaaaaaaacgttcgagactggcgcaaagtgaagccacagcttcaaaacg
cccacgccatgcggcgggcattccgaggccccaagaatgggaggtttgct
ctggtggaccagcgtgtggccgaatatgtcagatacatgcaggccaaagg
ggaccccatcacccgggaggcgatgcagctgaaagctctcgaaatcgccc
aggaaatgaacattccagagaaagggttcaaggcaagcttgggttggtgt
cgaagaatgatgagaaggtatgacctgtctctgaggcataaagtgcccgt
gccccagcacctgccggaagacctgactgagaaactcgtcacttaccagc
gcagtgtcctggctctgcgcagggcgcatgactatgaggtagctcagatg
gggaatgcagatgagacgcccatttgtttagaggtgccatcacgggtaac
tgttgataaccagggcgaaaagcctgtcttggtcaagacaccaggcaggg
aaaaactgaaaatcacagcaatgcttggtgtcttggctgatgggaggaag
ttaccaccgtacatcattttgaggggaacatatatcccccggggaagtt
tcccagtgggatggaaattcgctgccaccggtatgggtggatgactgaag
acttgatgcaggactggttggaagtggtgtggagacggaggacaggagca
gtgcccaagcagcgagggatgctgatcttgaatggcttccggggccatgc
cacagattccgtgaagaactccatggaaagcatgaacactgacatggtga

tcatcccagggggtctgacctcacagcttcaggtgctggatgtcgtggtc
tacaagccactgaatgacagtgtgcgggcccagtactccaactggcttct
ggctgggaacctggcgctgagcccaaccgggaatgctaagaagccacccc
tgggcctctttctggagtgggtcatggtcgcgtggaatagcatctcaagt
gagtccatcgtccaagggttcaagaagtgccatatctccagcaacttgga
ggaggaagacgatgtcctgtgggaaatcgagagtgagttgccaggaggag
gagaaccaccaaaagattgtgacaccgaaagcatggctgagagcaactga
agggaaagggaaagcaaatggaactctgatttaaacagctggggatgaaa
ttcctcaagatgattattcctgaaagtgtggatgcgctggatgcgcaggg
aacatcaggaaaaggccacggggctctgaacagccccggtccagacagca
gcctgtacatccatcccaggacacagcccagcccctccccacaccataca
aggtatcagaaaagtctaggacctatcatttcatcagagacatgatcaga
aaagaaactgcttctgccccatttcttgttttggagattactccatctgt
ccatcaaaagaaacctgtaaatatgaaagaacaaaggttatttcctggag
aaaagacaatttattcaacaccaacgagggactcatcatatgggcacaac
tctggtgtccttctatggagaaaacctcaagtaaagttttattctgcctt
tgaaaatgcttccaaaagtagaccctgtccccacacaggtcaagactaca
gagaaggctttgtagaaatgtgtcacctatgtacacctgctacttacaca
tttcctcttttggaaaaatgagatacttagaataacaagaaaattaagac
atactggcctggtgccagcagatggcttttctatagacaaactaggttag
tgtggaagatataggttaaaataaactatgctgttttatttatcttccca
acctgattggcagctagactttttttagggtctcatttaatggccctgttt
ttttcattattatatttaatgatagggcaggatttcgtatgcaagctctt
gtttctcaggctgcctgcagaagaagtcgctataaattatctgttgtcta
catggtacaaggcccattgactcatctgatgcttgttttgttaatttctt
taatatttttatcacggggcagtgggagggcttgggcttttagccacagc
tgttttaagacttctgatctcctgccctgcaggaataggtgggaagtcat
tgaatttttacactatagtaatttgcattcccacataagtttgagtgtta
cgaaaacattcctttaaagggatctgtgctacacaaaatatgccaggacc
tcacagacaaagccattgctagaaatgtcattccaatgatcagatctgga
aacaggctgccataaccacttttccttcttgtagactcagctcacctgta
tatttaaactgttcttggcatcttgaaacacctatttctactcaggtact
cattgtcctgttactgattcacctttctgatccttttcaaccagttttcc
cccaagggggggaaattttacttaacctctagtatttgaacaactcaatat
ttgaattgttgccccatttgcttttacctgtactgtattcttggtcatct
caaatggcgtctaaacccagctactttgcattccagaagtttccattccc
tccaattccacctaatttttcatctgtcctagttactggctctttcttca
tgtcttatttctcttgctttgggagcttaaaagattttacaagacctaat
tttgggttccttccttggagccatagttaccctgccaagaagagtagaaa
atgggttcaactcctgtttcgctccaccaacacctctgtgagtctcatca
tcagctgagcgatgatgccttacaggttgcatagcactggaactttccta
gagtaacggctctgctgccagggtttctctgggctcattcttccactgac
ttaattatgatctatgcctaacagagccccagtacaactattttgcagaa
tggctgttaccctagaattactatagcacatattgagatatagttgtact
ccctagtagataggaactgaccccaacaataaactttgataataaagaca
at

>NM_005324 3
gagcgcagagcggtttggtcgttcgttgggcggtgctggtttttcgctcg
tcgactgcggctcttcctcgggcagcggaagcggcgcggcggtcggagaa
gtggcctaaaacttcggcgttgggtgaaagaaaatggcccgaaccaagca
gactgctcgtaagtccaccggtgggaaagcccccgcaaacagctggcca
cgaaagccgccaggaaaagcgctccctctaccggcggggtgaagaagcct
catcgctacaggcccgggaccgtggcgcttcgagagattcgtcgttatca
gaagtcgaccgagctgctcatccggaagctgcccttccagaggttggtga
gggagatcgcgcaggatttcaaaaccgacctgaggtttcagagcgcagcc
atcggtgcgctgcaggaggctagcgaagcgtacctggtgggtctgttcga
agataccaacctgtgtgccatccacgctaagagagtcaccatcatgccca
aagacatccagttggctcgccggatacggggagagagagcttaagtgaag
gcagtttttatggcgttttgtagtaaattctgtaaaatactttggtttaa
tttgtgactttttttgtaagaaattgtttataatatgttgcatttgtact
taagtcattccatctttcactcaggatgaatgcgaaaagtgactgttcac
agacctcagtgatgtgagcactgttgctcaggagtgacaagttgctaata
tgcagaagggatgggtgatacttcttgcttctcatgatgcatgtttctgt
atgttaatgacttgttgggtagctattaaggtactagagttgataaatgt
gtacagggtccttttgcaataaaactggttatgacttgatccaagtgttt
aacaattggggctgttaagtctgaccatacatcactgtgatagaatgtgg
gctttttcaagggtgaagatacaagtcttaaccacagtgtaacttacagt
ttcctttaaaaaaaaaaaaagtaaacctggcagctatagaatacactatg
tgcatttataatagctattttatatattgtagtatcaacatttttaaatt
aaatgttttacattcacaagtggtggggagtcttgtcattaaggtgtgtg
taatttagagtccagttggttttcttctgactgcacttgttctcatagta
gtaaaatgctatgcgcatttataccttgcataagtcctcattctaccaca
tgttaaccctctagctgataatgcaaacactaactgggggattttattta
taagggctctagaaaaaacgagttattcacaccagcatcatcttaactaa
cattctgaactagttagtgcagcttttcattgtgttgtgtggttggtctc
ataactaggttgagtttttctcctctgctgaggaaacagtaccgaagttc
tttttcttgtggcatttgtattataaaaacttggtgtgggggaggagcac
aaaactccagcccactgaacctctgccaattaagatggtgttgggttagg
ttacatctggttactgtcctgggaaaatcatttttatagagatggccttc
caagtggttttaaaatttactgaagtttttaggtcaattatgtatgttga
ctaaatttacaaataaacttgtttatccaactaagtgtccaaaacctaaa
ttgaatgtactaagttttcacatgtcccattatctaggtccttgtatact
aatgttttgaacttagatcatttcaggtgttgtttggtggataaaggaac
cttttatttataaagatactgtagaaagcatgtgaacagctctctgcttg
attaagatgccataatagtgctgtatttgcagtgtgggctaagacaaagt
atattaataagcttttcagccccccccactcccgttccgtagtgtagaagc
ccacaggtgtagaactcagtcttaaacttcagtatgaaaccagtttcctt
gtgcgatgatggccactaaagcatagtacgtggatgtcagtgagacagca
tgagagccagcagtcatcaaagcgttccacgtttgaagttagcaactgct
taaagttatgccctattaaaattgctttctcaaaagtttgggttagtttc
aaatgtgatattttggagggaaggtaaagtaggtatctttcaggtcgtga

taatgagctcctatgaaaggatgcaatataatgacccgcttttctagaaa
gttcataatcagctctggaacaagcacacttgattcctcactgtgcttca
gaatgagattaagatcagatgttggaacgtgctatgctgtagcgtgtctg
gaaacaaagtacacaaacctggctacggtgatgagttagcttctgcttac
tacctgtgacaacccaagtgggtgacactagtgaaccttctccagtctgc
aggctggcatagaaggctcttagattatattgggcagcttgcaatctgcc
gaagcagtgacttgcatttccacacttggcttgagcactcaacccagaag
gcgaagatagcttttggttgtaggcggcttcctgtatgggatatccctcg
gtaagggtaaaggagcagaggcaaaggagaaaagcagaagttgcagctga
tgcaggtatcctatgcccttgatggatgagactaaaataaaatttttgaa
gttaaaaaaaaaaaaaaaaaaaaaaaaaaaaaaaaaaaaaaaaaaaaaaa
aaa

>NM_015271 3
agcttgatgactataatgggcccagttgtctgcgggctgcggggagctaa
gtccccagattggaggaggctggctctggtcttcgatgcacaggagtggc
cgttatggaacgcagcagcagcgtgcagggtcaaagacagccggcccccc
atgtcagtggtctaggatggccagtgaaggcaccaacatcccaagtcctg
tggtgcgccagattgacaagcagtttctgatttgcagtatatgcctggaa
cggtacaagaatcccaaggttctcccctgtctgcacactttctgcgagag
gtgcctgcagaactacattcctgcccacagtttaaccctctcctgcccag
tgtgccgccagacctccatcctgcccgagaaaggggtggccgcgctccag
aacaatttcttcatcacaaacctgatggacgtgctgcagcgaactccagg
cagcaacgctgaggagtcttccatcctggagacagtcactgctgtggctg
cgggaaagcctctctcttgcccaaaccacgatgggaatgtgatggaattt
tactgccagtcctgtgagactgccatgtgtcgggagtgcacggaggggga
gcacgcagagcaccccacagttccactcaaggatgtggtggaacagcaca
aggcctcgctccaggtccagctggatgctgtcaacaaaaggctcccagaa
atagattctgctcttcagttcatctctgaaatcattcatcagttaaccaa
ccaaaaggccagcatcgtggatgacattcattccacctttgatgagctcc
agaagactttaaatgtgcgcaagagtgtgctgcttatggaattggaggtc
aactatggcctcaaacacaaagtcctccagtcgcagctggatactctgct
ccaggggcaggagagcattaagagctgcagcaacttcacagcgcaggccc
tcaaccatggcacggagaccgaggtcctactggtgaagaagcagatgagc
gagaagctgaacgagctggccgaccaggacttccccttgcacccgcggga
gaacgaccagctggatttcatcgtggaaaccgaggggctgaagaagtcca
tccacaacctcgggacgatcttaaccaccaacgccgttgcctcagagaca
gtggccacgggcgaggggctgcggcagaccatcatcgggcagcccatgtc
cgtcaccatcaccaccaaggacaaagacggtgagctgtgcaaaaccggca
acgcctacctcaccgccgaactgagcacccccgacgggagcgtggcagac
ggggagatcctggacaacaagaacggcacctatgagtttttgtacactgt
ccagaaggaaggggactttaccctgtctctgagactctatgaccagcaca
tccgaggcagcccgtttaagctgaaagtgatccgatccgctgatgtgtct
cccaccacagaaggcgtgaagaggcgcgttaagtccccggggagcggcca
cgtcaagcagaaagctgtgaaaagacccgcaagcatgtacagcactggaa
aacgaaaagagaatcccatcgaagacgatttgatctttcgagtgggtacc

aaaggaagaaataaaggagagtttacaaatcttcagggggtagctgcatc
tacaaatggaaagatattaattgcagacagtaacaaccaatgtgtgcaga
tattttccaatgatggccagttcaaaagtcgttttggcatacggggacgc
tctccggggcagctgcagcggcccacaggagtggctgtacatcccagtgg
ggacataatcattgccgattatgataataaatgggtcagcattttctcct
ccgatgggaaatttaagacaaaaattggatcaggaaagctgatgggaccc
aaaggagtttctgtggaccgcaatgggcacattattgttgtggacaacaa
ggcgtgctgcgtgtttatcttccagccaaacgggaaaatagtcaccaggt
ttggtagccgaggaaatggggacaggcagtttgcaggtccccattttgca
gctgtaaatagcaataatgagattattattacagatttccataatcattc
tgtcaaggtgtttaatcaggaaggagaattcatgttgaagtttggctcaa
atggagaaggaaatgggcagtttaatgctccaacaggtgtagcagtggat
tcaaatggaaacatcattgtggccgactggggaaacagcaggatccaggt
ttttgatgggagtggatcatttttgtcctacattaacacatctgctgacc
cactctatggcccccaaggcctggccctaacttcagatggtcatgttgtg
gttgcagactctggaaatcactgtttcaaagtctatcgatacttacagta
atggtgggcaggtggatacccgcttccatggtcttgcactataaactgga
atggatttctcaatgcgggaccagattatgactagagtttttatgccaga
aggaatcattggtgaactttccaaggttatttctgaatgtaacaatttcc
ttaaaaatgacttatccaatttctgtatttcacctttagggttaaaaaaa
actcttctactgaatctataaaaactgcagttttacatctgtgaactatg
gcttaagggacaggatttatgtagctaaactaattttgcaaatcaaacag
acacttaaaaaactagcatatgtaaaggtattcgttaatcctgtgaatgg
tagcttttgcacagaacttccaaaagcaaaacaaaaacaaaatctattgt
agttatatacttcatttaacctaggtcacaagacccagggaatcttctaa
cctcacttttacagtaggtattactcttgtgacatttttttggttatcaa
caactaaatataaattactttggaaaaagtaaggctgtcttgcaaaatga
tcccagctctgattagcagccctctggagttcagaacttaagtatcagtg
caaatttctcaacctttctgggttagacaaagatcctttttgtgtgttc
ttttcaccacccctttggctcaccttgtatcagcaaacaaagtacttctt
cagggaaacctgaaatttctaatgccttgaaaagcatattacaaaagtaa
tgctaccttttgggaaacaaactgccccgttaactccagatcattgcact
ggaatgtaatcaagaaagttagtcatgttttatgtaccatgttttcacac
gtgtctcttctcttcgacttcctgaaagcgaaagctttacctcctgcaaa
tgtcagcacatgtagtaggacaccagtatcctaggacagagagccataag
tagccctttggaggactgatggtgtcaaccaaaggcatgtgattgattaa
tgattccccccttagaaagcaagtgttaccaaagttgtgttatcttgaaag
cattacaggtaagggcatgttatggttatttatcattgtttaatgaatag
tagaggtgtcaagggactatgtatacatgattagggtaagatagaatgta
ttatatatatatatatacacacacacatatatatagctgaatctttgg
tgtattgaaataggcagcactctgaaagacagaagcttcgtccagccact
cttcagcacattcctttactaagcagtttaaagccgtcctagtggagcaa
gccctaaagcagatttaatttttgccattttccaagaatgacggtggtgg
cttttagtcagaaaatggccttctgtgctttcaaaaaaaaaaaacaaaaaa
aaaaccacacacacacataaaaaacccaacaggtcaaaataaaagttgaa
cttgagttacatttaatttaaatataaatgcattttgagaaatgttaaga

acaatttagtcaatcgttcatctgtcattggtactgtaaaataagctgtg
gtctatttccactgtttaattttctactcagttctaccaaataggatgtc
atgtttgacatttttgatagtgactttggggtcttcttcactgaaagcac
cttagaactgtactataagaaaacatttcccctatgtataattatatgaa
tgtgatgtttattgcttattaatttataattcagtcattctctatatagg
acttcttaaaatttagaagggaaatctagctacttcaaattgtctgttaa
atttattatgcccaaatcaacctctgaaaaaaggtttttccaggaagatt
tacatttaggtttaatattttttagttaggtagagttttaaaaaatact
tgagcctgtccgtgataaagctataaaattcaataactttttagaatgtt
aaatgaagacactgtttcctaacatcagtgagatacatctttgaatttaa
acattcatatttactgagtacctactaggtaccaagtactcttttaggca
ctggaaatacagtgatggacaaaacaggtaaaaaatcgctgccccctcag
agctgacattctggggtgggaatttcattttgccacgtactaacgttctg
cacaaaagacaggctagactcttgtctagattgtttaaaagaaacttttc
aaattggttacattaattttagtttattttcacaagtaaaaatggctttt
tatttagattctttctgtcccaggctgttgatcttaaaactagttgattt
aaagagtttttttgcacaacatttcaattatatttgtgaacttagaaatt
aacttacaatctaaccagccatcatatcatatcctatcaggctagatatc
tcaatagtagactgaatacaaagctaattttttttacatgtcaatattgg
cacaaactggaatgaaagaatagtttgattcagacctgctccactatgtg
ttgctaaaacacatgctatgagcactccaggaaacactatattttttcca
aaaaatatgtgattatatatgttaaagtatagataacatttcacacttgg
atacatatgtgcatttactgtatttcttggtaagcatatttttgggggaa
agtgctgctgatatgatacaagtagacaaaatttaaatgaaattttgtca
cattctatggaaaatggtttctggtaaactgagaaggatattaaaataag
tggctttttttctgggctaccattattgtttgatttctctttgtcaagtgt
atagaacctgtcatacattcatgataagtagcactgaaaaattactcatt
caaatttcccctgggcacgtaaggcaaaatattgccggttgggatttcaa
ggtcagtgacgacgcatttcctcccagtacagacccccccagcccccccttg
ctggacatggggaggcagagagtcacttgaccatccagaaatacatgact
acaagtcctttatgactgtttgccatttttttttaatggtacttagtattt
tgatcaaactttagtctccagaactaaacaagtccctaagtttccttatt
ttaatttactgtgactagatttgaagcaaataaatactccagatccatgc
agctagaacacacttgcttccactactaaatatacagggtatgtcctaac
atggagttaactggaatagcagtacactagcaagtatctgtgaatcctta
gcactgacgggttaacagaaatgctttggtaatacctacttagttaattg
gaggaagtagtaaataaacattaggtaatctgcagattacttcaaatggg
aaaaatctttttgtagactctatagtaccctctctattcactagcttctg
aaaagggaggagtatttttagtttgacaatttaataatttaaaaacaaga
catctccaggtaggaaaaaatgaaagctatttcatgcaaacattatctaa
tttagcttaaaagtgaaagtggtaatactgttggtttctgtaaatgttgc
agggttttaaactttataattactttaatatttttgataactagaaatct
agtattgccataaaggaaactaagtgcccatcaaagatttgtttggtata
aataaagaattatttgttttgttttcaatgacagtaagctacaaatcatg
atgcttaaaaactttctaaagatgaattgtgtggcagtgattggtctgtt
tgtggagaatgtatgaaagctattaatattctagaatagattaataaatt

ggctatgttgttccaatgaatgtacagcacttccattaacttttgaaagc
aacacagccttaaactcaatgcttttgctttatgacatgggaatgttctg
tcatcaatggagtgtattcttgtaatagaattctttatatcgttctcaat
tctatagactttcaagcctatgtatgaatatgaaggggttttttttttt
gctttgttttctttttagattttgtacattccatctttataggtctgttt
catatgttttatgtatagaacactaagtcttgcactctctgacattgata
ctgatatattctcgtcatttgttcttttatgaatcaaaatgttgactgcc
tatttaaagaaaagaatgaacgctgtgcatcaaagtgtttgtatgttcgt
agctacatacgtaccacagtattttggatgctttagtctacaatgaaact
ttcaattaattctgtcttgaaacataggagaaacaggattcatgtgtatc
tctttaccatgcacaaaatctcaaatcattataataaagcttgttttctc
catttgc

>NM_019004 1
cgcagcggccggagagggatgggggcgcccacccagtctgagcctcgcc
gcgggcgccttcggctcacgcagcgcttcccgcgggccgccagtgcgcac
cctcggccacatcagcctccgcctggcgggtgcacccgggccggcgagga
aggggcaaccgtccgggtgggtggggcgggctgggtacctgaggtcacca
gctcggctgtagaggcaggggcggcggaggcggaactgcggagttgctgg
gtccaccgacccttaccctcagcgagagaagtaaccaatgtgaaagatgt
tgcagaagtgttccagaagtggctgaagatagaaggaaaaaagtgccact
gcctatcagaaaaaacaaaacaaaacatgggaaatacaaccaccaaattc
cgtaaagcactcatcaatggtgatgaaaacctggcctgccaaatatatga
aaacaatcctcagctaaaagaatctcttgatccaaatacatcttatgggg
agccctaccagcacaatactccattacattatgctgctagacatggaatg
aataaaatattagggacttttcttggtagagatggaaatccaaataaacg
gaatgtgcacaatgaaacatctatgcatttgttgtgtatgggacctcaaa
ttatgatatctgaaggagcccttcatcctcgcttggcacgccccacagaa
gatgatttcagaagagcagattgtctgcagatgatcttaaaatggaaagg
agcaaaacttgaccagggtgaatatgagagagcagctattgatgctgttg
ataacaaaaaaaacacacccttgcactatgctgctgcctcagggatgaaa
gcctgtgtagagcttttagtaaaacatggaggagacttgtttgctgagaa
tgaaaataaagatactccttgtgattgtgctgaaaagcaacaccacaaag
atttggccctcaatctggaatctcaaatggtattctcacgggatcccgag
gctgaagaaatagaagctgaatatgctgcattagacaaacgagagccata
tgaaggattaaggcctcaggatcttcgtagactaaaagatatgcttattg
tggaaactgcagacatgcttcaggctcctctctttactgctgaagcttta
cttcgagctcatgactgggacagggagaaattacttgaagcttggatgtc
caacccggagaactgctgccaacgatcaggtgttcaaatgccaactccac
caccaagtgggtataatgcctgggacacgctcccatctccaagaactcca
aggactacacgctcttctgtcacctccccagatgaaatcagcttatctcc
tggggatttagacaccagtttgtgtgacatttgtatgtgcagtatctctg
tatttgaagaccctgtggatatgccctgtggacatgacttttgtagagga
tgttgggagtcgtttttgaatctgaaaattcaagaaggtgaagctcacaa
catttttttgccctgcatatgattgcttccaacttgtacctgtggatatca
tagaaagtgtagtttcaaaggagatggacaaacgatacctacagtttgat

attaaggcctttgttgaaaataatcctgccattaaatggtgtcctactcc
aggctgtgacagagcagtaagactaacgaaacaagggtcaaatacatctg
gatctgatacactcagcttcccattgctgagagctcctgctgttgattgt
ggaaaaggacacctcttctgctgggagtgccttggtgaagcacatgagcc
ttgtgactgccaaacatggaagaattggctgcaaaaaataaccgaaatga
aaccagaagaacttgtgggagttagtgaagcctacgaggatgccgccaat
tgtctctggttattaactaactccaagccttgtgccaactgtaagtctcc
aatacagaagaatgaaggctgcaatcacatgcagtgtgctaagtgcaagt
atgacttttgctggatttgccttgaagagtggaaaaaacatagttcgtcc
actggaggttattacagatgtactcgctatgaagtcattcaacacgtgga
ggagcaatccaaggaaatgactgtggaggctgagaaaaaacacaaacgat
ttcaggaacttgacagatttatgcactattatacaagatttaaaaaccat
gagcatagttatcagctagaacaacgccttcttaaaacagccaaagaaaa
gatggagcaattgagcagagctctcaaagaaactgaaggaggctgtccag
ataccactttcattgaagatgcagttcatgtgctcttaaaaactcggcgc
attctcaagtgttcttatccatatggattttcttggaacctaaaagcac
aaagaaagaaatttttgaactaatgcaaacagacctagaaatggtcactg
aagaccttgcccagaaagtcaataggccttaccttcgcacaccccgccac
aagatcatcaaagcagcatgccttgtacagcagaagaggcaagaattcct
ggcatctgtggctcggggagtagctcctgcagactcaccagaagctccaa
ggcgcagctttgctggtggaacatgggattgggaatatttaggatttgca
tcaccagaggaatatgctgaatttcagtatcggaggaggcacagacaacg
tcgtcgaggagatgttcacagtctactcagtaatcctccagaccctgatg
agccaagtgaaagcactttagatattccagaaggcggcagcagcagccgc
aggcctggcacatccgtggtaagttctgcatctatgagtgtgctgcacag
ctcttccctgcgtgactacacccctgccagtcgctctgaaaaccaggact
ctcttcaggctctgagttccttggatgaagacgatcccaatatacttctt
gcaatacagttatcactgcaagagtctgggctggccctcgatgaagaaac
tagagacttcctcagtaatgaagcatccttaggtgcgataggcacttctt
taccttccaggctggactctgtccccagaaatacagatagccctcgggct
gcattgagcagctctgagcttttggaacttggtgacagcctcatgagact
aggagcagagaatgacccattttcaactgacaccctgagctcacaccctc
tcagtgaggcaagaagtgatttctgtccctcatctagtgatcctgactca
gctggccaggaccccaacatcaatgacaatcttctcggcaacatcatggc
ttggtttcatgacatgaaccctcagagtattgccctgattcctccagcaa
ctacagaaatcagtgcagattcccagctcccctgtatcaaagatgggtca
gaaggtgtgaaggatgtggaactggtgctgccagaagattcaatgtttga
agatgccagtgtcagtgaaggtagaggaacccagatagaagaaaatcctt
tggaagaaaatattctggcgggggaagcagcatctcaagctggtgacagt
ggtaacgaggcagccaacagaggagatggttcagatgtttcaagtcaaac
acctcaaacctcaagtgactggcttgaacaagtacatttagtgtgaactg
cacacatctgggctctaaatgaattacaggtacagatggtatgctaggtg
gagtatgcttgatagagactttgattcacttaattccaactcagtgataa
accactgacattagggttgaatacagagaagttcccttgaatggtagctt
catttttattttaaccttacagggaatttcctttgtacttaattgaata
gcttttcccctttttgctgacaaaaagaagagcaagagaaagagaaacaa

aaatgaaataaataagttgtattccacactctaagaaaatgcagtcctct
atttagcctaggcttgacaatacttaaattgaacatttaaactaaaggct
tactccctaatctttgggtggctttcctttaaaaaaaaaaaaaaagtttt
cttcattctagaaatttattttggataaatccgataacatatatgtcctc
aatctctttgtgctcttccataacttacttcctttttgtctgagcaatgt
gaattgaagtctctttagtaccacatctaccatagtgtaattagttttaa
ttttcacatgaatcaaaggtttcctttcatgtctatttacagtccaattg
tgccaaactcttacttgtgtgctgactaacaaggcatttaggtgtgcagc
atcctagagtgctccagggcagtgtcagcgttctcgggagtaaaaggtgc
cacttggtagcaatgatattccagaattaaatgggtttttgttgccatgg
agactgcatttatataaatgtagcctgtagcttaagttaactaaacctaa
tgctgctgttaaaaacagtttattttaatattaaaatacagttgattagc
aacagcggtgctgtattttaagagacactttattggaagtgcaatcatag
ttatttgttttcacaattttacagtgcattctaattactgatgggtgcaa
ttacttttaatcgtgttttataaaatagaaaaaaagtggagttttcatga
gttatagtaaatcccacgattattaagaaattcagtaaaacatcctgcgc
aacatgttaccgtgcctttgcctaacctaaatggatagttgccagttaaa
taagtgagtaattcaaatttcaatgtctcttctgaagtaactatgctatg
aattgcaaagacctccataaaaccacccatggccttgcttttacactaac
tataacaactaaatgttcaatcagtttgtttgcctaactagcaaatgctg
acatgtgtttgttctactgcgcaatactcatttgctgtgtgattactgtt
tagtgttgaaaaaaatcaacttcctagttatcagtgtcttactgtgaaga
aaatactggtcttagttgtaattaggatacaatggtacagtgtgtaatta
aaactagagtaaactgttggaatggctgttttacttaaatattatcaaaa
ctagcataacataagcaaaatagataagtacaacactccatttagtgttt
tgccagattgttaccagaagtctacagataccaaactttcagttctgagt
ttgtacaggcaagtcctgggctgggtaaaaagttatattaatattgttat
ccacaagagatgtgattatgggttttgattactttttttttttccaaacc
ctgcttttgaaatatccttgtacttaaaattcatattgctaagacactgt
attagaatatttaatattccccagatcctcttaggataaactgtgggaat
cctcctatgccatggatatcaaaggtccacattagtttttatttctccag
tgatcagaaacattgatatcaatccctattaaattagtggggggaatatt
aactttatctacagtgtattactgtatattaaactgaaatagtccattaa
aggatttttttataaatttattttggattaaaaatatcaacaccaataag
tttttagaccaagttgtaatttttccaatatagagtctttgcatcacact
gaggcatcttgcacagctgcagttaaggtgagaaagaatgctctgtgtga
agacagtgtacacaatgggttccggtttccttgcaccttgtgcagtatcc
tttatttctgtgctgttctctcctgagcatgaaaaatgatcattatccaa
tttgtatttccttggtacatattttaaaaacaacacagtcattgacttta
caattcagtaatgaagtttggcaaagcctattttgtaaacaagttaattt
tataatgtaaaaaaaaaaagttaatctaaccttgacttgttatttgcact
ttcatagtctatacttgatacattcccactttatatacagtaggattcta
caaacgtgtagatgtttggccaaatgaatgctgttaataatatgtaaaat
tctttgattaaacatttattacttaaactac

>NM_019063 3

ggcgcggcgctcgcggctgctgcctgggagggaggccgggcaggcggctg
agcggcgcggctctcaacgtgacggggaagtggttcgggcggccgcggct
tactaccccagggcgaacggacggacgacggaggcgggagccggtagccg
agccgggcgacctagagaacgagcgggtcaggctcagcgtcggccactct
gtcggtccgctgaatgaagtgcccgcccctctaagcccggagcccggcgc
tttccccgcaagatggacggtttcgccggcagtctcgatgatagtatttc
tgctgcaagtacttctgatgttcaagatcgcctgtcagctcttgagtcac
gagttcagcaacaagaagatgaaatcactgtgctaaaggcggctttggct
gatgttttgaggcgtcttgcaatctctgaagatcatgtggcctcagtgaa
aaaatcagtctcaagtaaaggccaaccaagccctcgagcagttattccca
tgtcctgtataaccaatggaagtggtgcaaacagaaaaccaagtcatacc
agtgctgtctcaattgcaggaaaagaaactctttcatctgctgctaaaag
tggtacagaaaaaaagaaagaaaaaccacaaggacagagagaaaaaaaag
aggaatctcattctaatgatcaaagtccacaaattcgagcatcaccttct
ccccagccctcttcacaacctctccaaatacacagacaaactccagaaag
caagaatgctactcccaccaaaagcataaaacgaccatcaccagctgaaa
agtcacataattcttgggaaaattcagatgatagccgtaataaattgtcg
aaaataccttcaacacccaaattaataccaaaagttaccaaaactgcaga
caagcataaagatgtcatcatcaaccaagaaggagaatatattaaaatgt
ttatgcgcggtcggccaattaccatgttcattccttccgatgttgacaac
tatgatgacatcagaacggaactgcctcctgagaagctcaaactggagtg
ggcatatggttatcgaggaaaggactgtagagctaatgtttaccttcttc
cgaccggggaaatagtttatttcattgcatcagtagtagtactatttaat
tatgaggagagaactcagcgacactacctgggccatacagactgtgtgaa
atgccttgctatacatcctgacaaaattaggattgcaactggacagatag
ctggcgtggataaagatggaaggcctctacaaccccacgtcagagtgtgg
gattctgttactctatccacactgcagattattggacttggcacttttga
gcgtggagtaggatgcctggatttttcaaaagcagattcaggtgttcatt
tatgtgttattgatgactccaatgagcatatgcttactgtatgggactgg
cagaagaaagcaaaaggagcagaaataaagacaacaaatgaagttgtttt
ggctgtggagtttcacccaacagatgcaaataccataattacatgcggta
aatctcatattttcttctggacctggagcggcaattcactaacaagaaaa
cagggaatttttgggaaatatgaaaagccaaaatttgtgcagtgtttagc
attcttggggaatggagatgttcttactggagactcaggtggagtcatgc
ttatatggagcaaaactactgtagagcccacacctgggaaaggacctaaa
ggtgtatatcaaatcagcaaacaaatcaaagctcatgatggcagtgtgtt
cacactttgtcagatgagaaatgggatgttattaactggaggagggaaag
acagaaaaataattctgtgggatcatgatctgaatcctgaaagagaaata
gaggttcctgatcagtatggcacaatcagagctgtagcagaaggaaaggc
agatcaatttttagtaggcacatcacgaaactttattttacgaggaacat
ttaatgatggcttccaaatagaagtacagggtcatacagatgagctttgg
ggtcttgccacacatcccttcaaagatttgctcttgacatgtgctcagga
caggcaggtgtgcctgtggaactcaatggaacacaggctggaatggacca
ggctggtagatgaaccaggacactgtgcagattttcatccaagtggcaca
gtggtggccataggaacgcactcaggcaggtggtttgttctggatgcaga
aaccagagatctagtttctatccacacagacgggaatgaacagctctctg

tgatgcgctactcaatagatggtaccttcctggctgtaggatctcatgac
aactttatttacctctatgtagtctctgaaaatggaagaaaatatagcag
atatggaaggtgcactggacattccagctacatcacacaccttgactggt
ccccagacaacaagtatataatgtctaactcgggagactatgaaatattg
tactgggacattccaaatggctgcaaactaatcaggaatcgatcggattg
taaggacattgattggacgacatatacctgtgtgctaggatttcaagtat
ttggtgtctggccagaaggatctgatgggacagatatcaatgcactggtg
cgatcccacaatagaaaggtgatagctgttgccgatgacttttgtaaagt
ccatctgtttcagtatccctgctccaaagcaaaggctcccagtcacaagt
acagtgcccacagcagccatgtcaccaatgtcagttttactcacaatgac
agtcacctgatatcaactggtggaaaagacatgagcatcattcagtggaa
acttgtggaaaagttatctttgcctcagaatgagactgtagcggatacta
ctctaaccaaagcccccgtctcttccactgaaagtgtcatccaatctaat
actcccacaccgcctccttctcagcccttaaatgagacagctgaagagga
aagtagaataagcagttctcccacacttctggagaacagcctggaacaaa
ctgtggagccaagtgaagaccacagcgaggaggagagtgaagagggcagc
ggagaccttggtgagcctctttatgaagagccatgcaacgagataagcaa
ggagcaggccaaagccacccttctggaggaccagcaagacccttcgccct
cgtcctaacaccctggcttcagtgcaactcttttccttcagctgcatgtg
attttgtgataaagttcaggtaacaggatgggcagtgatggagaatcact
gttgattgagattttggtttccatgtgatttgttttcttcaatagtctta
ttttcagtctctcaaatacagccaacttaaagttttagtttggtgtttat
tgaaaattaaccaaacttaatactaggagaagactgaatcattaatgatg
tctcacaaattactgtgtacctaagtggtgtgatgtaaatactggaaaca
aaaacagcagttgcattgattttgaaaacaaaccccccttgttatctgaac
atgttttcttcaggaacaaccagaggtatcacaaacactgttactcatct
actggctcagactgtactactttttttttttttttcctgaaaaagaaac
cagaaaaaaatgtactcttactgagataccctctcaccccaaatgtgtaa
tggaaaatttttaattaagaaaaacttcagttttgccaagtgcaatggtg
ttgccttctttaaaaaatgccgttttcttacactaccagtggatgtccag
acatgctcttagtctactagagaggtgctgcctttttctaagtcataatga
ggaacagtcccttaatttcttgtgtgcaactctgttttatcctagaacta
agagagcattggtttgttaaagagctttcaatgtatattaaaaccttcaa
tactcagaaatgatggattcctccaaggagtcctttactagcctaaacat
tctcaaatgtttgagattcaagtgaatggaaggaaaaccacatgcctttta
aaactaaactgtaataattacctggctaatttcagctaagccttcatcat
aatttgttccctcagtaataggagaaatataaatacagtaagtttagatt
attgaattggtgcttgaaatttattggttttgttgtaattttatacagat
tatatgagggataagatactcatcaaattgcaaattcttttttttacaga
agtgtgggtaacagtcacagcagtttttttttaccaacagcatacttaaca
gacttgctgtgtagcagtttttttctggtggagttgctgtaagtcttgta
agtctaatgtggctatcctactcttttgggcaatgcatgtattatgcatt
ggaaaggtattttttttaagttctgttggctagctatggttttcagtaca
tttcctactttaagagtaattactgacaaatatgtatttcctatatgttt
atactttgattataaaaaagtattttgttttgattttttaacttgctgca
ttgttttgatactttctattttttggtcaaatcatgtttagaaactttg

gatgagttaagaagtcttaagtatgcaggcgtttacgtgattgtgccatt
ccaaagtgcatcagaactgtcattcccttctaatatcttctcaggagtaa
tacaaatcaggtatttcatcatcatttggtaatatgaaaactccagtgaa
ctcccaaggacatttacaacatttatattcacacgctgtatggaagggtg
tgggtgtgtgtgaaggggcgagtggagacactgtgtgtatctctagataa
gaagatatgcaccacgttgaaaatactcagtgtagatctctatgtgtata
ggtatctgtatatctttccttttgtttacaactgttaaaaaacctcaaaa
tagttctcttcaaaagaagagagattccaagcaacccatctttcttcagt
atgtatgttctgtacatacttatcggagcgcgccagtaagtatcaggcat
atatatctgtctgttagcaatgattattacatcatcagatcagcatgtgc
tatactccctgcaagaaatatactgacatgaacaggcagttcttggagaa
gaaagagcatttctttaagtacctggggaatacagctctcagtgatcagc
agggagtttatttgaggacatcagtcacctttggggttgccatgtacaat
gagatttataatcatgatactcttcggtggtagtttcaaaagacactact
aatacgcaggaagcgttccagctatttaatgctggcaactactgtttaat
ggtcagttaaatctgtgataatggttggaagtgggtggggttatgaaatt
gtagatgtttttagaaaaacttgtgaatgaaaatgaatccaagtgtttca
tgtgaagatgttgagccattgctatcatgcattcctgtctcatggcagaa
aattttgaagattaaaaaataaaataatcaaaatgtttcctctttctaaa
aaaaaaaaaaaaaaa

>NM_015455 3
caatgaaaacgagggggcgcggaggaggaggcggcggcgtcggtggcgg
cggcgacggcggcgcggaggcgaaggcagcggcgggcgcagcgaggaggg
cgaggccgggggccgagagggcgggagggcgtagtggcggcccgtcgggg
cggctgaggcgggcagccgaagcagtggctctcggagggggaacaaagag
cagcgactaaggcggcagaggagcggcggcggtggcggcgctgcagcagc
gggcgggactggtatggtggttccacagggcagaccccgctgcactcaca
gggaggaggaggcggcagcggcggaggaaggcggcgcaccccgagaggca
tgcccaaagaaaaatacgagccccctgaccctcggaggatgtatacaatt
atgtcttctgaggaagcagcaaatggaaagaaatcccactgggcagagct
tgaaataagtggaaaagtaagaagcttaagcgcatctttgtggtcactaa
ctcacctgacagctttgcatttgagtgacaattccctgtcccgaattcct
tcagacattgccaagcttcacaatctggtgtatttggacctgtcatctaa
taaaattcgtagcttacccgcagaactcggaaacatggtatcactcaggg
agctccatttaaataacaacctgttacgagttctaccttttgagctggga
aaactgtttcagttgcagactttaggcctgaaaggaaatccccttaccca
ggatatattgaacctttatcaggaaccagatggaacaagacggctgctga
actatttgcttgataatttgtcaggtactgcaaaaagaattacaacagaa
caaccacctccaaggtcttggattatgttacaagaaccagataggacaag
gccaactgccttgttttctgtcatgtgctataatgttctttgtgataaat
atgcgacccggcagttatacggctactgtccatcatgggcgctaaactgg
gactacaggaaaaaggccattattcaagaaatcttgagctgcaatgctga
tatcgtaagtcttcaggaggttgaaacggaacagtattacagttttttc
tggtagagctgaaagaacgtggctataatggattcttcagtcctaagtct
agagctaggacaatgtcagaacaagaaaggaaacatgttgatggctgtgc

aatattcttcaagacagaaaaatttactttggttcagaaacacactgttg
aatttaatcagctagccatggcaaattctgaggggtctgaagctatgctg
aacagagtcatgacaaaagataacattggggttgcagtactgctagaact
tcggaaggaatcgattgaaatgccgtctggaaagccacatcttggaacag
aaaaacaacttattcttgtggctaacgcccacatgcattgggaccctgaa
tactctgatgtgaagttggtacaaactatgatgttcctctcagaagtgaa
gaacattattgataaagcctctcgcaacctcaaatccagtgttttgggag
aatttggaactattccacttgtgttatgtgcagatcttaattctttgcca
gactctggtgttgtagaatatttgagcacaggtggagtagaaacaaatca
caaagactttaaggagttgaggtataatgaaagtctcacaaacttcagct
gtcatgggaagaatggaaccaccaatggaaggatcactcatggtttcaag
ttacagagtgcctatgagagtggcctgatgccttacacgaattacacatt
tgatttcaagggtataatagactacattttctattctaaacctcagctga
acaccttaggcatcctgggccctctggaccaccactggctggttgagaat
aacatcagtggctgcccgcacccccctcatcccctctgaccacttctcact
ttttgcacaactggagctcttactgcctttcctgccccaagtcaacggca
tccaccttcctggcaggaggtagtcaagcaccttcagaggacagccttga
ttcacttgtaaacttgtgaaaatctgaacataggggagtgaggtatggcc
actgaggatttttgcttgcttaagaatgatttggactttcaatctgatta
tttgataaggatatagtatgaaagccaggtgctagcaacagacaaattct
gagcccaatatgctttatactgctagacagggattggtgtgtttgcacct
gtctttcatttgtcataagagattttcctatttcttctacccaatagaat
attttcatgcctggaaataggaaaatgtgtgaacagcgtattctcttcac
acagaaatctgtagcactgctttttttgaggccagtagcaacatccagaga
tcattcttccatactttactccctccttttttcagacttgtttgtaaagta
cagaatctgaatttagcctttatgattgtatatgatccacagaagacctg
atttatgaaattttgtactaaaatcatttggaaatgattgtattgtaaac
tgaggctaaattttttttttaaacctgtttcatgtgttataaaggccagct
tgtaaaagaagctgcaacagactttctctgctcatgatttgcactcttag
ggttttgttagcccttttgtactactttctttttaaattgagaacatggt
tctttacatataaatctgcttcaaccttaggatgttttcagaccagaggc
aacttattcatgaatttttatgaaaactatctactaggacagataagctg
aacagtgatgatctgtagacatttatggactgaatgtaatggttgatata
tgtacattctgatatttttaaatctttaactttttaaagttaaaaaccta
cagctgcttaggtccagcttcttaactctttttgagacacttcctgtcct
atctccactgtgcctgcctaaatttgttctcaccaagcactgcctgtgca
tgcagagaaaatctgtgcatcctcttttatatttttaaaatactgtttaa
catttgtgagaattttatgaaaatgcttttgtatgagctgtggctttttc
ccattgtgaagcattgaatatcacattttggaacatgttatagggtgagt
ccctggatctttgctcaccagatccaagcactgcttctcggtgtcattgc
agtgtgctgcttgtcacccagaatactactatcatgtgaattctttttgt
cgtcagtgtcttttccttagtctttttgttgttgttgttgttgttgtttt
aatcattcctttttttaagaagaagtaattttccatttatgaagcagtatg
aattagatgtattttcaaaacaggtccctaagacaattcttcagatcatt
tttaaaatgactaagtcattttagtatgtcaagcaagataaaaattacat
cataccgtctattttgcccatagtgccatttagagatgaaaaccagcttt

aactttgcaaagtgaacatgtacatggtctgctctcatttattcattctt
ctctcaaaagtcaaatgaatgcagagggagcttggtcaaactgcttttgt
ttcaactgcaggcaggggagcaaaaggacgccatgtgagcattaggaaaa
aaaatactcactcttactaacaatttttatacagaaaatgagtcattttg
gaaatgattcttatgtttttttttttctccttttagaaaattctccaaa
aggttttgatgttgaatcttggtcttggacctgtttttcctttgagggt
tttttgtttttgtttttctaggatttcattgtgatgttttggttttgt
tttttgctttttgtttaagttgtgctgacaccaaacacatccagtttata
atcagtacattggaaagctggtattgatgtagaaccagtgcataactttt
tatggggttttgttattggtttttttttgtaaagtgtgaataaaaggtat
gtttactcatttttcctgaacactgtgttggtaatgtgcatcatgacaat
ttccagtgaaggtgagctggagctggttggactaatgagactgaggaagc
agcttttcctacgatctgcattatgtaatcacaggtccagagagctttat
ggaagcgggagaggaggagcacttactcatgttgtatttgttaatggagg
atgtcatcttttcatagatgctggaactagagtgcacttgttagatgcta
aaggtttgagctttacacaaaatgtcttcatctgtatttgttattgtcta
caatatatttgaatttggggcagcatattaagatgtaatggcctgttatg
tcttgaaaatacttgttttgcctcttccaggcatactgcattctgtggat
cagtttgaacagcttctccaccttatttggacagtgataaattgaaccaa
gagtgtagatttacaagtgtaaccttcaaaagaggaagaactatttgggg
tctgtaggtaatgaacagtcacaccaaaatagactatgatgcttttgtta
agaaaggtttcatgttttagatattttccgtgtcctaaataattttcaat
aatctataatccctaaaatgcaataaaaactagtatgttttcacggtgta
tatttttttcaaaaattctgtaacaaatatatttaatgtgttgccataa
tgtcatttcagtatttttgctggataaatccattttagaaatgtcttaca
aaaagtttagggcatgttttctgttttaaagaccagaagtttgaaggcac
ccaaatccttgtcgccaatctcctgaagtacagctttaccatattagtgg
tcattatttatgctttgagagtttcaatattgttcgtcaagttaacttat
ggcataatttaagctaaagttgtagaaatgaattgttgagtgcaactttc
atagtcccaaagataggcagagaacatctccagaaatgtgtgtgtgtgtg
tgtgtgtgtgtgtgtgtgtttaatatgatttagtgacaaccaggaaaact
tacttgggattattagcactttcaaacttagggacattattaaattggat
ggaatgcacttctaaatgtttaaattaaaatttcaaagctctttcgagat
tcaggttctcaataataatattcaagttttagagtttcactttgtactat
ttaaatttatggaactagtttccatactgacttgtaaggttttggggtca
tatataaggactaaagccaagctaggcaaaacaatgacagcaccgttttt
tcagtgaagctctcagaatgtccagtacagatgttgcagataattccaag
aactccttcagcaggtgttcttcaccattcgtaaacagtattttaagatg
ttcactttgctcttctttttggttacatacattttaataactggtatgtt
gaagtagtgtctaaaagtatctagtctttattcacagtacattatattgt
gtgatgcactggactaactcttgtttaccattcatgtaacaaaacccagc
acattatctgcactataagctcaaagaatgtcacatccgcccagacagct
ctttgatgagggtgatgggaactgaataaaccatacggactggcagtaac
aagggcttttactgttctgttcagtggaaccttcttggtcaaattgtaat
tagctagcattatatttatatacagggtctttttctttaccgatgtatt
ttcctactcatagccaaccaataaattcagtatctgtttcaaatatttta

gaagtgtagtttgtatagctgtagtactgaggtttgaggaaaaataaatg
ttgatcagcagaccacgtatttaaaattctgaatcttctgggacagggtt
gtaactcagccttccaaagggaagagtgcaggggg acggggccatgatat
ggggaaatggtgtaaactaatgtatttctttattggctgttattctgtat
aacactcatatctttgccaaagttcaattttatatttaggcaactgatgg
tccttttgcatttaggattttcgttgttgttaccttatacctcatgatat
aaggaatgggctcatgtgtcttccgtcttttggaaggaggttgacatatt
ttaaataaatgcttttaaatacagtaaa

>NM_014904 2
gggcgggcgggaggcgcggaggcagcggaggctgctcggccgcaaggagg
caggcgggaggccaggggtgcagaattggggcgggggctcccttcacccc
cgcagccctccgcccgggtccgtgtggccaggcctcacgtgccccgcagg
cctggggaaggtggagacaggggcgggggtccgcgcagaggcctgcacgg
cgcggccaggtgatggtgggcctctgtgtttctccagacgaaaggggacc
cccggagcggccgtgcccctgggctgcccccgcgaggcgttttgacatca
gccatagggaagtcggggagccctgagcctgcctgtttccgttaagtgtc
cttaggatgcagtgattaaggaattaggctctccctccgggaggggcact
gcctgtgctagggaactcgggggcagagaaacaggacaggatgatgctgt
ccgagcaagcccaaaagtggtttccaacccacgtgcaggtcacagtgctc
caagccaaagatctgaagccaaaaggcaaaagtggtaccaatgacacata
cactataattcagctgggcaaggaaaagtactccacctctgtagctgaga
aaacccttgagccagtttggaaggaggaggcctctttcgagctacctgga
ttgctaattcagggaagtccagagaaatacattcttttccttatagttat
gcacaggtccctggtgggtctggataaatttttagggcaggtggcaatca
atctcaatgacatctttgaggacaaacaaagaaggaaaacagagtggttt
agattagaatccaaacaaggaaaacgaatcaaaaacaggggtgagataaa
ggtcaatattcagtttatgaggaacaatatgaccgcaagtatgtttgact
tatcaatgaaggacaaaaccagatctccttttgcaaagttaaaagataag
atgaagggtagaaaaaatgatggaacattttctgatacgtcttctgcaat
cattccaagtactcacatgcccgatgccaatagtgaattttcaagtggtg
aaatacagatgaaatccaaaccaaaaaagccttttctcttgggtcctcag
cgactctcgtcagcgcattcaatgtctgatttatctgggtcccatatgtc
ttctgagaaactgaaggctggcaccataggtcaaacacatcttctcggac
accagttagattcctttggaacagttccagaaagtggaagtctcaaatct
ccacacagaagaacattaagctttgatacttctaaaatgaaccaacctga
cagcattgtggatgaaggtgaattgtgtttcggaagacaaaatgacccat
ttacaaatgtgactgcttcattaccccaaaaatttgcaacactgccaagg
aagaaaaatccatttgaagaaagcagcgaaacatgggacagcagcatgaa
tttattttcaaaaccaattgaaataagaaaagaaaataaaagagagaaaa
gggagaaagttagcctgtttgaaagagtgactggaaaaaaagatagcaga
agatctgataaacttaacaatgggggatctgatagcccttgtgacttgaa
atcacctaatgcatttagtgaaaatcgccaggactattttgattatgagt
caaccaatccatttacagcaaaattcagggcttcaaatataatgccatct
tcaagttttcatatgagtccaacaagcaatgaagacctcaggaaaatccc
ggacagcaaccccctttgatgccactgcagggtatcgtagtctgacctatg

aagaggttctacaggagctggtgaaacacaaagaactccttaggaggaaa
gacacccacatccgggaactcgaggactacatcgacaacctccttgtaag
ggtaatggaagaaacgcccagtattctcagagtgccgtatgaaccatcca
ggaaagctggcaaattctctaacagttaataaagccaattgtattgtatt
gataattggacaaagagagagaaagaaagaaggaaggaaggaaaaacttg
ttactgaaagagactacactatcaggtttcattacatattctcctttatt
gtgaagagtagttttacctgtaaatattagcagggactatcaagagtgac
ctttgaagcaagctaaatcatttaaatgtttaaattgaaaatgggccaga
ttctcaatgaataagtggttccttaggactcactcaggtgctggtactga
cccactaatctgccataggcccagaaatatcttcagaagtacgtgagttg
ttcctcaggaatagatttttcataaagcagaattgatcctgtggctggtt
tctcagaaatcagtttgtagaaatatttagcttcaggaatactgctcgtt
ataaacactactaaaaaaacagtttatattttatatacacatatgtacat
gaatatatatatattcatgtatcttgtatataaaatataggcatatacct
catatatacatgtgtttttagaacatttagaaacaattgattgcctctaa
agttaggctaccaatttcttgatttgaaaatgtgtgtgctctattgatgc
aagacaagtcatccttcctactgtcatggtttgttatagagaagctgctt
caaatcactcttgactacatggaccttggtttcaaagtgagctgctatat
attattctagcatcaaagtttttctctttccccaaaagtgatgatctcaa
gtcctttttttaaagaatgacaaattttaggagacttcattaacttcataa
cagcatgaatttatttgaattaattaacatttgagtttgtttaaggtata
agcagaagataagctaatatagcttgttttgttgaaattctgcatgcctt
taaatcactagcaaagcaaatattataaagttatatctgaaaataattgt
gggaagtatgtgattgcctggtgtgtctgataacccaaagcagcgggcag
attccaggaatgaagaattaggcttggtttcaggttacataaataataga
gcagatggttgcctttgggtcttgaaccaaaataagacttactaaaggaa
gagtgagtttttagaagagaactttacgagcgtgacattatgcattactc
cacatccaccctttaagaattgatttattacaaaattatgttctatttta
aagtccactcccccaaaatactgatttcttccatggattatttttatatg
atttgtacaaatttttctgaaatttaaattgacaagcaatgttataaaa
atattgctaatcttctgcttatttttcagcctttaaagtctgaaatacta
taaaacactgagagtatctatgtctttctgaatttaaaatgaaaattcat
gtccatatggagacttacatattccccaatctttccatgaattgcatttt
tttctaatacttatctctgttaagactccattgaaagttaaacaaaaagc
cgatataatagtttgggttttttttttttgcttattttagtattttgaat
cagagaatacttggccaagctcgtatttgtaaactaaaaccccttgttat
ttgtatcttctgcatttcagttttactgtaagaacatacattttgaaatt
atgaacatttttttcataaaaattaaatgctttaagaaatggagaaggaa
aaatagcttttatcaattttttctcctcaagattctgtaataaacctca
aagtctggtgtagaatggtattttcccgtttgaggtcttggtagtttttt
tttttttttaaacatggtgccacttttaattaactaaatgttacaaaat
tctcagcatttgtttttgtctggtagtattattgagtcttttcatacact
tcttaaaatgtgaaaatctatgttttcagtattatgacagtatctgcaga
tactataataatatgctttggccaaatttgctgtttatagagtgaaccaa
gatgtcagttttgtattggggatatgttttacataaatagaactcattta
aaagtatggttcaagtactgctcttctttttatggggaaattgattttta

aggagcgattcttttcctatataaaagctattgaggcttagctttattca
tacatgagtgatttggttataactcaatcaaaatttttatctttaaatct
gagacaagtattatatttaaaactagatctattaatacattcatcactgc
ctgaaaatttgctgttctaatgtttgtgaattttcactcattacctagag
taaacttattgcatattttcataatcagtcaaaataataaaactaaaaac
aaaaagtaaaaattagttcccaagtaaatatatcagtgtttcctgtgttt
gggcagaagtgcaaagtagttttaatttctcacgttgtagaaaaagaggc
caaaaaggtcaccttagtttatttttccaaaacataactttaggtattct
aatgtattttgttagttgcttagaaatggaaataaaatatatgtgcttat
taaataatgcacagtacactagattatagagtttatttttgcttttatgc
tttaaagaatgcatgcccaaaacactactaccaacaagaggatttttttaa
aatcacagaattgatatttatctcaagactatcttaagctaatgtacttc
tgttacataattaaactatttctgctattatttttaaagcagtattattt
tataaccaattctaaaattcagctttaaaaatattgaacataccttccta
ccacttacgcttcaagtttcatttattgaataccagggtcatatcttaga
attgtatataaaacaacatgtaacccattcatgaaaaacgtttttaagta
ttgctttttgctgctatgttctgcattgagcctagttttgccattgtccc
ctgaatgtagtattatctatagattatacataactacatctattgctttt
ggtggtaaatcaagatttagttgtaattttccagctgtgaaaatgttgcc
ttgtatttaaaagggtttcatgaatggaaacctaagtaaaactaagctca
ttagtgacagacttgttttcttcttgttattcctccagcaactccctcac
caccacgcctccctgcctaccatccccggaagggtgcttattctttaaca
aagagaatctaaaaaaaaaaacaaaaaactgttgtctaatatatctacag
taaatgttacactagagcttttattttcctctgtagcttttcagaagaag
caatttaccttggatttgtaaatcaagctgaaactttttttgatgttcatg
tactcatatgtcttgtaggtggtaggtgtcacttgtgtgtatgtgtaagt
gaagtatgatgcatgctcattcacttggtccctttgaaataagggaaagg
atcacagtgctgctgggccatgatggtatgtagaagggtctgtcagcaca
catggaaagcccccatttgcacagatgtagaactttaataatccagatgc
aatcattggaagtatttaatatgcaattggtagcaccttagctaattaat
ctgcatatgaagcattttctgatatatttaataagaggtatagaagctct
tcaagcattaaaatagagttgatgtttcattccatttgttttatccatca
aaaaatgcactctcacagtggagttgaagtctaatacatgatacatgtgg
gcctgttagtcttcgaagcttccagatggtttgtgttttgaggtacaaaa
aaatgctgcagatctagaaatgcatatatttagcgggtatttgaaagata
gaagcattatttataactgaagagtataacacttatcttcttaagtaaat
gctctcataacttgtatacatttttgcaggtacaaattcaaaagtatgta
agcattgtataatgtgacaattgtataatttatgtaatctgatgcatgaa
tcagaatttttatataatatatgattattgacttatagttgattaaagtg
tttaaacttaaaaaaaaaaaaaaaaa

>NM_000161 2
cctgtggccgctcccggctcggagtgtgatctaagcaggttgcgtacctt
cctcaggtgactccggccacagcccattgtccgcggccaccggcggagtt
tagccgcagacctcgaagcgccccggggtccttcccgaacggcagcggct
gcggcgggtccatggagaagggccctgtgcgggcaccggcggagaagccg

cggggcgccaggtgcagcaatgggttccccgagcgggatccgccgcggcc
cgggcccagcaggccggcggagaagcccccgcggcccgaggccaagagcg
cgcagcccgcggacggctggaagggcgagcggccccgcagcgaggaggat
aacgagctgaacctccctaacctggcagccgcctactcgtccatcctgag
ctcgctgggcgagaacccccagcggcaagggctgctcaagacgccctgga
gggcggcctcggccatgcagttcttcaccaagggctaccaggagaccatc
tcagatgtcctaaacgatgctatatttgatgaagatcatgatgagatggt
gattgtgaaggacatagacatgttttccatgtgtgagcatcacttggttc
catttgttggaaaggtccatattggttatcttcctaacaagcaagtcctt
ggcctcagcaaacttgcgaggattgtagaaatctatagtagaagactaca
agttcaggagcgccttacaaaacaaattgctgtagcaatcacggaagcct
tgcggcctgctggagtcggggtagtggttgaagcaacacacatgtgtatg
gtaatgcgaggtgtacagaaaatgaacagcaaaactgtgaccagcacaat
gttgggtgtgttccgggaggatccaaagactcgggaagagttcctgactc
tcattaggagctgagcttcattcagtgtgtgtgcgttggttgccgatcgt
actgccagtagcattgtctgtctgtccggtcttgtttgtacattccattt
tcaattgttacagatgtgaactttattccttgtcactaattatatttaaa
attatttctaggaagtcaaataaatataataaagggttgagccctctact
ttcttcttgccaccttttttgtggcaatattaaagtgaactgctaatagtg
taagtacgtgcacaaaaccactgccagataaccagaggggcctgggaagg
gagaagaattagtgtattttttttcaaatagtacagtaatttgcctcataa
gcataggagcattgggaatgagagggaactgtgcccagtatactgttttt
tttcttcctccaataaaagtggtgtagtgccgaaagtgctaaaatattta
gtgcggtattgctctgtgaattcaagttcaacagacttcactttggtcat
gtttattaaaccaccagtgacatttaaaaatatatttttagcagtcgtaa
tgttagtcaccaagggaaggtggtggaatgtctatgtttttgattttact
gtgagttaaaaaggcacatttctaccttctattgtttttaaattcaagaa
tagggaattagttcctggtgttgtttacgagtgtattctcgtgtcaacat
acagggatttagacatttaactctctgtgccttgataagaatatcattta
gagtgtagatacttttgcctttttaaaaaagccattattttatgagactt
agtactcacactgcaaataactagtcagctcagttttaactttataggtt
tattgagtttcctttgtgtgatccatgtagatgcctcaaaatgtttcttc
ttcttctttttttaatcttataagatatttttctaagtatttccagaaa
catttgagagtgcccatcattttcaggtctgcagaaccatagcttccacg
cacctgaacgagcacagaatgaactgacggtggaagacattatgagctgt
gtccaacgttttaaccaaagcgtatcgtaccaacgatctgtgaaaatgca
ctggaagcttctggtcccggtttcctttgtggtctatgtgggtcttgtcc
tcattgtaactccgtatagatggtataggtattttaatcctggaagctgt
tgccttattaatgattatcttaaaatttcctccattggggcagcgtgggc
caaattaaaacaaacaaaaccgcaactcctccacagaaacacaaacacag
ttattccatgaagtttagtatttggttgacatagtgctcttcaaattcat
cccattaccctaaaagtaataactttgatgcttgctttaactttagtccc
atctctgccactttgatgctatttgggttatgatggggcaagatggcaga
ggtattgggtttttttgttttttccattcctctctacttctgtttccta
gctttttctttctggagtttaagtacagtgatggttggcttgagtacctt
tttaaatctagcccagtataaacattagcctgcttaatatttagacattt

ataggtagaattctgagcactcaactcatgtttggcattttaaagtaaaa
acaagtgtgacttcgaggaccaaagaaattgtcagctatacatttatctt
tatgaactcatttatattccttttaatgactcgttgttctaacatttcc
tagaagtgttcttataaaggtctaatgtatccacaggctgttgtcttatt
agtaaatgcaaagtaatgactttgtctgttttactctagtctttagtact
tcaaaattaccttttcatatccatgatcttgagtccatttgggggatttt
taagaatttgatgtatttcaatacactgttcaaaattaaattgtttaatt
ttatgtatgagtatgtatgttcctgaagttggtcctatttaaattattaa
actattgtaactttgttcttgtgaaaaaaaaaaaaaaaaaa

>NM_003203 4
agaaggcaggcgggacgggcgcgcgcactcggcggccaggcgcggcggga
gcgcgccctagggggcgggcactgaagctgcggcttgcggttcagcgggt
tctagggcgccgggcgctcgggcctcggccatggctcacaggccgaaaag
gacttttcggcagcgcgcggctgattccagcgacagcgatggcgccgagg
agtcgcctgctgagcctggggcgccgagggaacttccggtcccgggttct
gcggaggaagagccgccctctggaggaggccgcgcgcaggtggcgggact
gccccaccgggttcggggccctcgtggccggggccgggtctgggcgagct
cccggcgtgccaccaaagcggctccccgcgcggacgaaggctcagaatcc
agaacccttgatgtgtccacagatgaagaggataaaatacatcactcctc
agaaagtaaggatgatcagggtttgtcttctgacagttctagctctcttg
gagaaaaagaactttcatcaacagttaagatcccagatgcagcttttatt
caggcagcccgcagaaaacgtgaattggccagggcccaagatgactatat
ttctttggatgtacaacatacctcctccatctctggtatgaagagagaga
gcgaagatgaccctgagagtgagcctgatgaccatgaaaagagaatacca
tttactctaagacctcaaacacttagacaaaggatggctgaggaatcaat
aagcagaaatgaagaaacaagtgaagaaagtcaggaagatgaaaagcaag
atacttgggaacaacagcaaatgaggaaagcagttaaaatcatagaggaa
agagacatagatctttcctgtggcaatggatcttcaaaagtgaagaaatt
tgatacttccatttcatttccgccagtaaatttagaaattataaagaagc
aattaaatactagattaacattactacaggaaactcaccgctcacacctg
agggagtatgaaaaatacgtacaagatgtcaaaagctcaaagagtaccat
ccagaacctagagagttcatcaaatcaagctctaaattgtaaattctata
aaagcatgaaaatttatgtggaaaatttaattgactgccttaatgaaaag
attatcaacatccaagaaatagaatcatccatgcatgcactccttttaaa
acaagctatgacctttatgaaacgcaggcaagatgaattaaaacatgaat
caacgtatttacaacagttatcacgcaaagatgagacatccacaagtgga
aacttctcagtagatgaaaaaactcagtggattttagaagagattgaatc
tcgaaggacaaaaagaagacaagcaagggtgctttctgggaattgtaacc
atcaggaaggaacatctagtgatgatgaactgccttcagcagagatgatt
gacttccaaaaaagccaaggtgacattttacagaaacagaagaaagtttt
tgaagaagtgcaagatgatttttgtaacatccagaatattttgttgaaat
ttcagcaatggcgagagaaagtttcctgactcctattatgaagctttcatt
agtttatgcataccaaagcttttaaatcccctaatacgagttcagttgat
tgattggaatcctcttaagttggaatccacaggtttaaaagagatgccat
ggttcaaatctgtagaagaatttatggatagcagtgtggaagattcaaag

aaggaaagtagttcagataaaaaagtcttgtctgcaatcatcaacaaaac
aattattccccgacttacagactttgtagaattcctttgggatcctttgt
caacctcacagacaacaagtttaataacacattgcagagtgattcttgaa
gaacattccacttgtgaaaatgaagttagtaaaagcagacaggatttact
taaatccattgtttcaagaatgaaaaaggcagtagaagatgatgtttta
ttcctctgtatccaaagagtgctgtagaaaacaaaacatcacctcattca
aagttccaagaaagacagttctggtcaggcctaaagctcttccgcaatat
tcttctttggaatggactccttacagatgacaccttgcaagaactaggac
tagggaagctgctaaatcgttaccttattatagcacttctcaatgccaca
cctgggccagatgtggttaaaaagtgcaaccaggtagcagcatgtctacc
agaaaaatggtttgaaaattctgccatgaggacatctattccacagctag
aaaacttcattcagtttttattgcagtctgcacataaattatctagaagt
gaattcagggatgaagtcgaagaaataattcttattttggtgaaaataaa
agctttgaatcaagcagaatccttcataggagagcatcacctagaccatc
ttaaatcactaattaaagaagattgaataaactttattggaaaatgctaa
aattttaatatagttacactcagttcctttgtttgagaagaagctggtgc
ctctctcttctttattccctgtaatagaaggtaggatttgaaaaaaagca
ggactccacctctgtattcccccgtgctttaccttctggcatcatgaaaa
gctgccatgattctgtggtgttctaaggaattaaatgcactggagcttta
agagctcaacgtgtttccctttgattcttttggctggtctttcaattttc
ttaagaggggaactggagaaaccttgctaaaggttttagcttaagtgagc
atgctactaagtcttctgtcctgaaccacaataatatcttttagttctct
ttaactacctttatttaaaaataagtcaatatgaaaatctcagttggagt
gaattatttgctaagtattaaatgattaaggcatgtgccgattgtcaaat
tatccttcagaaatctgtaccaatttaatgtaccaacataagcataagtg
agtgccaatgaatccctaccagcagtagttattatccttttttaaaaatgc
attgaaaatttgaaaggtaaaaatcttattttaatagcgtattttgtgtt
ggctattagagagtaaacatattggttttggccatttgtaattatttctg
aaatatccatctttttcttatggactttaagacctttatatttttaaaga
tattgatttttttgcccatgcttgtggcagaagttttccttatttgccatt
tttcttttattttttttttaattgtattttttgagacagggtctggctct
gtcacccaggctggagagtagtggcacaatctcggctcactgcaacctct
gccttctgggctcaagcaatcctcccacttcaacctcccaagtagctggg
actataggcatgcgccaccatgcccagctgatttttgtaatttttttgtag
agatggggtttcgtgacattgcccaggctggtctcgaactcttgagctca
agcagtctacccaccttgacctcccaaagtgctgggattacaggcttgag
caaccatgtttggcctttcttttaaatttgttgatgttttttgaagcatt
aagtcattacttattagcaagttactattttgtttttttgtcattggtgg
taggactaagaaggaaacagatattttaaatactcacaacatgtgattca
ttttctgataaaatatttgtctaataaaacagaggcaagataatttaatc
aaaagaaattaagaaaattttccaaaaatgttagtttactagtaattttt
ctaagtctacttattaaataacttaagggaaactgtaaatatattttgca
aattgttgttcaaaagataattggaaaaaattgaatatattgtaggattt
gaatatacagcctacttttatgtcacctctaccttcaaaactacatataa
tttattttaggagttggatttgttatgtctgttgcagtgaggacttgtga
ttcactggtatgaagaatttttttgatgtttatattagtaacaaaattata

caaggatagaatctcatctagaaaatgcaaactattgttgaaaagcagtt
agcatttccacacaaccgctgtccccagagataactgctgttttcatttt
aatgttatccttttagaccttttctctatttacaaaaatgttacgtgttt
ctatgtgtacatagttttgggtttttttttttttttacgtaagtggcag
acactttattgcacatgaaatttgtaatttttttctatttaagtacacat
ttggaaattatcctctgacttttggatttttaactaaatatgttggacgt
gttcctttatatatttaagttcagcacagaaactttcctaccttttttgtg
gaagataaaatattaaaactgctttcaaaactacaaaaaaaaaaaaaaaa
aaaaa

>NM_019027 3
agtcagacccaggaggagtgaggtcaattgccctttagtcccaggactaa
ccggaagcttctgcaacaggaggacattgaaaataagatggaacccatcc
acataaggatttgcctcaaagggcactgcaaaaattgaacagaggaatcc
caaggaagctgcctgaatttgcctgtatactctcgttctgcgacttataa
aggaccagacaaatcaaattagtggttttggtttccgccagctgtggatg
cctttgacattatgaccgcagaggattccaccgcagccatgagcagtgac
tcggccgccgggtcctccgccaaggtgcccgagggcgtggcgggcgcgcc
caacgaggcagcactgctggcgctgatggagcgcacgggctacagcatgg
tgcaagagaacgggcagcgcaagtacggcggcccaccgcccggctgggag
ggcccgcacccgcagcgtggctgcgaggtcttcgtgggcaagatcccgcg
cgacgtgtacgaggacgagctggtgcccgtgttcgaggccgtgggccgca
tctacgagctgcgcctcatgatggactttgacggcaagaaccgcggctac
gccttcgtcatgtactgccacaagcacgaggccaagcgcgcagtgcgtga
gctcaacaactacgagatccgcccgggccgcctgctcggcgtgtgctgca
gcgtggacaactgccgcctcttcatcggcgggatccccaagatgaagaag
cgcgaggaaatcctggaggagattgccaaggtcaccgagggcgtgctgga
cgtgatcgtctacgccagcgcggccgacaagatgaagaaccgcggcttcg
ccttcgtggagtacgagagccaccgcgcggctgccatggctcgccgcaag
ctcatgcctggccgcatccagctgtggggccaccagatcgccgtggactg
ggccgaacctgagatcgacgtggacgaggacgtgatggagaccgtgaaga
tcctctacgtgcgcaacctcatgatcgagaccaccgaggacaccatcaag
aagagcttcggccagttcaaccccggctgcgtggagcgcgtcaagaagat
ccgcgactacgccttcgtgcacttcaccagccgcgaggatgccgtgcatg
ccatgaacaacctcaacggcactgagctggagggctcgtgcctggaggtc
acgctggccaagcccgtggacaaggagcagtactcgcgctaccagaaggc
agccaggggcggcggcgcggctgaggcagcgcagcagcccagctacgtgt
actcctgcgacccctacacactggcctactacggctacccctacaacgcg
ctcattgggcccaacagggactactttgtgaaagtagccatccctgccat
tggggctcagtattccatgtttccagcagctccagcccctaaaatgattg
aagatggcaaaatccacacagtggagcacatgatcagccccattgctgtg
cagccagacccagccagtgctgctgccgccgcagccgcggccgcagccgc
cgcagccgctgtcattcccactgtgtcgacgccaccacctttccagggcc
gcccaataactccagtatacacggtggctccaaacgttcagagaattcct
actgccgggatctacggggccagttacgtgccatttgctgctccagctac
agccacgatcgccacactacagaagaacgcggcagccgcggccgccatgt

atggaggatacgcaggctacatacctcaggccttccctgctgctgccatt
caggtccccatccccgacgtctaccagacatactgaggctggtgaccagc
acgaagacagaccacacaaacaccactgaaggaacgcttgactatttatg
aagaaggaacatgttggattcacacatgcaacctgaaagtgaagaatgtt
agcagatttatttctgaattattttatatacatgaagttttcactagttt
tttaagactattttcaacttagcatgcctacgttcatacatttccaaaag
acttgcaatggttcgtgccttcattccatcttttaaaaatttgtatgctg
tactacatttgtatagaggtttttgttgttgtttttttaaggatatattt
tcagtatgaaggttattttcttaacttctgcactccagagatttctattt
tgtagtaccttcaataatatatcaactatatattaaaaaagcacacttga
ggagctagggaactattttgaaaaatatatacaatatttaaagatacaaa
cagtagtgcttaaaaatactacataaagcattattttaaaggttatactg
gaaagtgcaattttaaaatgagtaaaacctctgtatttctgctggcatta
agggttgatggtgttaccatgtatcatcatggcggtactatttttttaaaa
gaaattaaacactggatctctccttaagccaacattgaaaagacttgccg
cacttctgagtccaaacactggaaagctctcctttgccaccgttagccgg
ggctcattctccatgtgccttagccttaaacatgcccccactcccacatc
tctcaccctgtcccctcctccccagattcccaatcccaccgcaatgtttg
gcaagcctaggactgataagtagctctgatagaggagctggtggctttta
tacttcttcctgggtttttgttggggtttgttgtttcgttgtttttttgtt
ttttttttgtttggttggggaagtattgtcttctacgtgtgctattttc
agtagcagagtaagcacaaggttttaatcgagttgcataagacacctttg
catagctatttaattgcccaatgtaaaactttaatgccatttctaatgct
tttattcatttttgaagtatgagtttgtagggacaaagaatgtatgttat
cgtagacaagacccccagagactcttttcagcagaaagttatgcttctag
ttgccttaccatgtttcttgcaaaactgtccatggtcctcaagggtgttg
gaaacattatgtttattaaatgggcctctcttcctttgctgtgcacttga
tgggtgaactggattggggtgtgcacatccaggaggaggaggagagacct
gtagaagtttaaagatagtttgtaaatatcttctaatgcttgtttttagt
ccttttatgttggagaagttcatggtatgtagtttaatgcaaaatgaaac
cattttatttcaatgttattaaaaaggtttgttttattaggaagttaatg
tattgttgcagtgttttgtgcctgtttaaaggcttttgtttagcagagtg
aatgtaaaatacagtaaaatgttaagattgtcatctactttttaaaaaaa
aatatcaacttggaattgtttttttaaaggctcaatcaaggaagtgaggtg
tgcaataaggtagcaagtaaaacgcagttgcgtttttatgtcatgttaga
gatccatacaattttccactcacgggatttttgttgatggctgaattctt
gtggattcataagaggatcatgcccttagcaagtacttttgttttgtttt
aaattaagagattcccaaatgccttttttccccctcatcttgaaatgagat
gagtttttatgtgtaagcaatatttatttaactattctataaaattattg
agtgcctactgaggcctttaagcaccgctaacattcctttccatcattct
tttaaatgacataaaataattgtgcaatgttcctgatgatgtaccccaca
agctgcattcaaactcaaatctgtgggaatgagtgactcgacaaaatgta
attcggatcagatcctcatcccctgactgtgtgaaaaaagtactctcctt
ctagtgaaggattgtcacagagtttcactggatgaaactatgacccagta
ttcttactgtattttacatatgcctgtaaattatttgcaaaaaagaagaa
gaagaggaagaaagaaaagaaagaaaagaaagaaagaaagaaagaaagaa

agaaagaaagaaagaaagaaagaaagaaagaaagaaaaagaaagaaagaa
taaaaaaaaatgataacatggcaaccaccaaactcccttaaaaacaaaca
ttggtgacatggccaattaagtatcagtgagcctcctatctgggacttat
ctgttcctattgtttcaaaaccatttcatgtacactactgaggtaagttt
ataacttgaaatgtgaacttttttttttttagtgttaagaacaaactat
ataaatgtaaaaaaaaagttttagagttctgttttcaaacattgctagtg
gtttagttaactttagctgctttatatttgaaaagcttttatttagatct
tgctccatttacagtacattcttaggatgtatgtagtaaataaagctttc
tttaaagcaatcgcaagaccttattttaaattgtgaaaaaaaaaaaaaaa
aaaa

>NM_152282 3
gggaggagctggcggcgagcgccgagccgggcgcgcagcgacggagctgg
ggccggcctgggaccatgggcgtgagtgcaatctacggatcagtctctga
tggtgggtcgttaacctcagtggggactccaagatttccatgaagaaaat
cagttgtcttcattcaagaattggggtctggctcagaattcctgcagctg
gtgaaaatctgttttctagaagaggtttaattaatgcctgcagtctgaca
tgttcccgatttgaggtgaaaccatgaagagaaaatagaatacttaataa
tgcttttccgcaaccgcttcttgctgctgctggccctggctgcgctgctg
gcctttgtgagcctcagcctgcagttcttccacctgatcccggtgtcgac
tcctaagaatggaatgagtagcaagagtcgaaagagaatcatgcccgacc
ctgtgacggagccccctgtgacagaccccgtttatgaagctcttttgtac
tgcaacatccccagcgtggccgagcgcagcatggaaggtcatgccccgca
tcattttaagctggtctcagtgcatgtgttcattcgccacggagacaggt
acccactgtatgtcattcccaaaacaaagcgaccagaaattgactgcact
ctggtggctaacaggaaaccgtatcacccaaaactggaagctttcattag
tcacatgtcaaaaggatccggagcctctttcgaaagccccttgaactcct
tgcctctttacccaaatcacccattgtgtgagatgggagagctcacacag
acaggagttgtgcagcatttgcagaacggtcagctgctgagggatatcta
tctaaagaaacacaaactcctgcccaatgattggtctgcagaccagctct
atttagagaccactgggaaaagccggaccctacaaagtgggctggccttg
ctttatggctttctcccagattttgactggaagaagatttatttcaggca
ccagccaagtgcgctgttctgctctggaagctgctattgcccggtaagaa
accagtatctggaaaaggagcagcgtcgtcagtacctcctacgtttgaaa
aacagccagctggagaagacctacggggagatggccaagatcgtggatgt
ccccaccaagcagcttagagctgccaaccccatagactccatgctctgcc
acttctgccacaatgtcagctttccctgtaccagaaatggctgtgttgac
atggagcacttcaaggtaattaagacccatcagatcgaggatgaaaggga
aagacgggagaagaaattgtacttcgggtattctctcctgggtgcccacc
ccatcctgaaccaaaccatcggccggatgcagcgtgccaccgagggcagg
aaagaagagctctttgccctctactctgctcatgatgtcactctgtcacc
agttctcagtgccttgggcctttcagaagccaggttcccaaggtttgcag
ccaggttgatctttgagctttggcaagacagagaaaagcccagtgaacat
tccgtccggattctttacaatggcgtcgatgtcacattccacacctcttt
ctgccaagaccaccacaagcgttctcccaagcccatgtgcccgcttgaaa
acttggtccgctttgtgaaaagggacatgtttgtagccctgggtggcagt

ggtacaaattattatgatgcatgtcacagggaaggattctaaaaggtatg
cagtacagcagtatagaatccatgccaatacagagcatagggaaaggtcc
acttctagttttgtctgttactaagggtagaagattattgctttttaaag
gctaaatattgtttgtgggaaccacagatggttggggttgaacagtaagc
acattgctgcaatgtggtacgtgaattgcttggtacaaaatggccagttc
acagaggaatagaaggtactttatcatagccagacttcgcttagaatgcc
agaataatatagttcaagacctgaagttgccaatccaagtttgcactctt
ctggcctgccccatgttactatgtgatggaaccagcacacctcaaccaaa
atttttttaatcttagacatttttaccttgtccttgttaagaatttcttg
aagtgatttatctaaaataaaggttggcaaactttttctgtaaagggcca
gattgtaaatatttcagactgtgtggaccaaaaggccacatacagtctct
gtcataactactcaactctgtttctgaagcaggaaagccaccacagacag
tacataaaggaatatgtgtagctgggttcccaggccggacaaaacagatg
gtgaccagatttggcccctgggctgtagtttgctgacccctcatctaaaa
aataggctatactacaattgcacttccagcactttgagaacgagttgaat
accaagaattattcaatggttcctccagtaacttctgctagaaacacaga
atttggtctgtatctgacactagaacaaaacttgagggtaaataaacatt
gaattagaatgaatcatagaaaactgattagaagaatacttgatgtttat
gatgattgtggtacaagatagttttaagtatgttctaaatatttgtctgc
tgtagtctatttgctgtatatgctgaaatttttgtatgccatttagtatt
tttatagtttaggaaaatattttctaagaccagttttagatgactcttat
tcctgtagtaatattcaatttgctgtacctgcttggtggttagaaggagg
ctagaagatgaattcaggcactttcttccaataaaactaattatggctca
ttccctttgacaagctgtagaactggattcatttttaaaccattttcatc
agtttcaaatggtaaattctgattgatttttaaatgcgtttttggaagaa
ctttgctattagatagtttacagatctttataaggtgttttatatattag
aagcaattataattacatctgtgatttctgaactaatggtgctaattcag
agaaatggaaagtgaaagtgagattctctgttgtcatcggcattccaact
ttttctctttgtttttgtccagtgttgcatttgaatatgtctgtttctat
aaataaatttttaagaatacctatactataaaaaaaaaaaaaaaaaa

>NM_024738 1
agcagactgggctgcgggcggcggggccggcgcctgcctggagggatggg
gctgccgggcgcgtaggggccatgccgcccgggacccgggcctgccgcgt
tccgcgccccggccgccgcgccccacgtccgcgccgggatggtgaacctg
gcggccatggtgtggcgccggcttctgcggaagaggtgggtgctcgccct
ggtcttcgggctgtcgctcgtctacttcctcagcagcaccttcaagcagg
aggagagggcagtgagagataggaatctcctccaggttcatgaccataat
cagcccatcccgtggaaagtgcagtttaacttgggcaatagcagtcgtcc
gagcaatcagtgccgcaactccattcaagggaagcacctcatcacggatg
aactcggctacgtttgcgagaggaaggatttgctggtaaatggctgctgt
aatgtcaacgtccctagcacgaagcagtactgctgtgatggctgctggcc
caacggctgctgcagcgcctatgagtactgtgtctcctgctgcctgcagc
ccaacaagcaacttctcctggagcgcttcctcaaccgggcagccgtggca
ttccagaacctcttcatggcagtcgaagatcactttgagttgtgcctggc
caaatgcaggacctcatctcagagcgtgcagcatgagaacacctaccggg

accccatagcaaagtattgctatggagaaagcccgcccgagctcttcccc
gcttgacgggtgcagcggacttgctccagcctgggtgaggaggccccgct
gaagaactcgcctcctgggacccagcttcagccatcgggccaggctgcag
gaagaagacaaaggcagcgtgaggaaaccttggctttgacccccttctcgt
gttgtcatctttggcttcgctcaccacccgggcttaccagatggaactct
tctgtaaagcagcttggcccctccagccagtcccattcgggaaagatgaa
accggaggccgggctcacggtggtggtggagttcttggatgactcagccc
tgggacctgcacagggacctgtgacttgtgttcatcgggggccggtgtca
cttccagttttgatccaggctctttcactgtaaaattatttattggattc
ctttggagtaatgggaacattctaatgttttatgtaggaaaatgccttgc
cattctagttgaatatgttcaaggaagttatttttgttgttgttctgtgt
tctcgagtttcaggagttaaatcattcttccacccagatacaacattttc
tcttttaggacgtgaatattctctctaggcagttatttttgtttgtattt
cgacagtatcaagcataggccctgaacgtgacctgttagccatatcctga
cgtgtaaaattatctaaaaactcagacactcttccattctaatctaactg
cacgatttctaacagtgggcaccatgtgcctgccctcaggttattttcca
gtggtagtcgaatgtgctcatataccctatggagagcactgttttagcag
aaatctaatttctcttcctggaggaatttgttctcatttcttttgccact
taaaattaactgtgggctactcagccagggtacagtgggagcctcaggaa
ggtcagaggcaacctcctcccctgttctatcaatagaaacccaacgttga
ggcaattcctaaacagacgcacctcgtagcttgctgtatgtgtttattct
ttattgctttcagctttggggctgtaacaggtacaaatatttggtttccc
tatgatttatagagaagaagaagaaacccagctttctatcagagcactgc
aagagaagagtcttacacctgccctcagtgggagatgagaatggtcatta
tgacttagagaatgctacacgtgtaggttgctggtgtgtcctgaatccac
aggcataaagcactccccatttttcctactgtaatgcagattctccggct
caaggtctagaatatttgatcctaagatcaagacatcatgcccttcgaat
agtactgctctttgttttcaggagtcacgtgaacacacaactctcctata
ttcctcacaaacctcaggattgagcaaggtctttgtaattttttttggttc
actttattgacctgggagcaaggtgctaattctgtggtcagtattcaatg
ttttttttcagtggagctttttctttgggccatatttgccttctaatacat
tcctgcaatatgtagtggtgatttcccttagcttcctcctactacctctt
atactcatctccccaaattatttgcctcccttaaataagttttcctagaa
ggtaagctggtcaggcaatttgaaaaatattagatcccaagaaatctatt
ccgtttgcattggacttctcggattccatgtgtttgcagcaggactacat
cgaactctgatgtgccggattgtggcatgtctgcatgtctcatccatcta
ttgtttttggtaactcagtttggaatttcagtgtctgtcttccctgggtt
gacattggaatcagcctctcctttgagcttattttaactcttgagcaaca
taacatagatttaatgtgaacagtttataccaaagggcagcctgtgcctg
tttatggatcctctctgcctttgtacttgaagagcgcattttacatttcc
agtcctttcacagacaggagctccaaccttacgatggagaattaaacttg
cttgtatttccactttgtggatgagaaaaaaaaaaaaaaaaaaaaaaa

>NM_145341 3
cttttcctcctcagctccggctccgccgccacgattggccagccgaccac
ccggcctcggccaataagcgccgccctctcgcccccgtgttactgggtag

aagaaaacaaaaacaaacagagcgagaagggccagagactctccgaggcg
gcggcagagacagaagagcggggtcggggccggctgaccaggaacctggg
cgagcagcggcgggggcccgagggattctgaaggaagatttccattaggt
aatttgtttaatcagtgcaagcgaaattaagggaaaatggatgtagaaaa
tgagcagatactgaatgtaaaccctgcagggtattttccctaattctcca
tggtgcttcaatagcatgttattatcataaaaatgaacagttttgtggaa
tagatgaccaaatatcctgataacttaagtgactctctcttttccggtga
tgaagaaaatgctgggactgaggaaataaagaatgaaataaatggaaatt
ggatttcagcatcctccattaacgaagctagaattaatgccaaggcaaaa
aggcgactaaggaaaaactcatcccgggactctggcagaggcgattcggt
cagcgacagtgggagtgacgcccttagaagtggattaactgtgccaacca
gtccaaagggaaggttgctggataggcgatccagatctgggaaaggaagg
ggactaccaaagaaaggtggtgcaggaggcaaaggtgtctggggtacacc
tggacaggtgtatgatgtggaggaggtggatgtgaaagatcctaactatg
atgatgaccaggagaactgtgtttatgaaactgtagttttgcctttggat
gaaagggcatttgagaagactttaacaccaatcatacaggaatattttga
gcatggagatactaatgaagttgcggaaatgttaagagatttaaatcttg
gtgaaatgaaaagtggagtaccagtgttggcagtatccttagcattggag
gggaaggctagtcatagagagatgacatctaagcttctttctgacctttg
tgggacagtaatgagcacaactgatgtggaaaaatcatttgataaattgt
tgaaagatctacctgaattagcactggatactcctagagcaccacagttg
gtgggccagtttattgctagagctgttggagatggaattttatgtaatac
ctatattgatagttacaaaggaactgtagattgtgtgcaggctagagctg
ctctggataaggctaccgtgcttctgagtatgtctaaaggtggaaagcgt
aaagatagtgtgtggggctctggaggtgggcagcaatctgtcaatcacct
tgttaaagagattgatatgctgctgaaagaatatttactctctggagaca
tatctgaagctgaacattgccttaaggaactggaagtacctcattttcac
catgagcttgtatatgaagctattataatggttttagagtcaactggaga
aagtacatttaagatgattttggatttattaaagtccctttggaagtctt
ctaccattactgtagaccaaatgaaaagaggttatgagagaatttacaat
gaaattccggacattaatctggatgtcccacattcatactctgtgctgga
gcggtttgtagaagaatgttttcaggctggaataatttccaaacaactca
gagatctttgtccttcaaggggcagaaagcgttttgtaagcgaaggagat
ggaggtcgtcttaaaccagagagctactgaatataagaactcttgcagtc
ttagatgttataaaaatatatatctgaattgtaagagttgttagcacaag
ttttttttttttttttttttaagcacttgttttgggtacaaggcatttct
gacattttataaacctacatttaaggggaatttttaaaggaaatgttttt
tcttttttttttgtttttcgagggggcaaggagggacagaaaagtaacct
cttcttaagtggaatattctaataagctacctttttgtaagtgccatgttt
attatctaatcattccaagttttgcattgatgtctgactgccactccttt
ctttcaaggacagtgtttttttgtagtaaaatcactggtttatacaaagct
ttatttagggggtaaagttaagctgctaaaaccccatgttggctgctgct
gttgagatactgtgctttgggagtaaaaaaagaaagttatttctttgtct
taaagaatttttaaaaaattagtcatgagacttattcatctttccaggga
acatactgattggtcttaaaagactagacagttaagtaaaaggtggctgg
aacatctatttttctacaaaactggaaaaatgaacctggttctagaagaa

tgtacaccaaaataaaacatgtgaagcagtattgattctttattgggagt
acatttttttaggtctcttaaactttaatttcacacagtaaattttgaat
ctcataaggaagcatatttgaacctagtcaatttaatcttagtgttccct
tgaaaactttttttccctacaaaattttaagtgaaaaatacaatagtaaa
ttaagattacactggggaaaaaaatgcaggtatcactttactccattgtt
atctgacctagagcttaattaagttttagaaatatgtaataccttccatc
attccatcatccttaaattctgttaccaaataatggctaatgttacaaaa
agttatactccagagacccaaagcttgacatttacctaatgtatgagaaa
atattaccaattaacaataaagaatgatcatatttttaacctcttttaca
tagcctaataactcagcaaggcctcaacgtctgtgctaatttaaactgcc
aaatattgactgcagcaaacaagaattatattcagaatttatgagggtac
tgttaggagtatactgcttacaggtttagatatagtctgttagaattaaa
accaagtttagtgttcatatttacctcatgggctttatcaagcccatatt
acctcagcttatatatagttaccatttttaggtttttaattgtttgacac
ttggatgataaatgcagtcattttattctcaagtgcttaaaattaatgta
attaaaagcttagctgactacagaataggtgagggtttcttaaaaatgag
atttaagggctgggcacggtggctcatgcctgtaatcccagcactttggg
aggccgaggtgggcggatcacttgaggttgggagttcatgaccagcttga
ccaacatgaagaaaccctgtctctattaaaaatacaaaagtagccaggca
tggtggcgcatgcgtgtaatcccagctacttgggaggctgaggcaggaga
attgcttgaacctgggaggcagaggttgcagtgagtcgagatggtgccat
tgctctcgtttgggcaacaagagtgaaactcttgtctcaaaaaaaaaaaa
aaatgaggtttaagacagttttgtcattactggtgggatctggtcacaca
agatagcattaaacgtgacatggcacataaaattggttaaaaaattttgt
tttttaattacgtaatgtaaaagcccaacaaacactttatgcaagattgg
aatgtatcttcaaattcagatttaataaacatgtaaagatcctctgtaaa
aaaaaaaaaaaaaaaaaaaaaaaaaa

>NM_175866 4
atttccggcttctgggactcgggtgcaccacggcttccggtgtcatggct
gcttgaagtcccgggagtcggtgaggcggctgcaggtccctccctgcgga
gccgctggtccggctggcggagatgtgaccgcgggcccggccggcctgcc
tcaggcgtcgcgtcagctcccgtgtccgtgcccttaacccacaccgatgg
cgggatccggctgcgcctggggcgcggagccgccgcgttttctggaggcc
ttcgggcggctgtggcaggtacagagccgtctgggtagcggctcctccgc
ctcggtgtatcgggttcgctgctgcggcaaccctggctcgcccccggcg
ccctcaagcagttcttgccgccaggaaccaccggggctgcggcctctgcc
gccgagtatggtttccgcaaagagagggcggcgctggaacagttgcaggg
tcacagaaacatcgtgactttgtatggagtgtttacaatccacttttctc
caaatgtgccatcacgctgtctgttgcttgaactcctggatgtcagtgtt
tcggaattgctcttatattccagtcaccagggttgttccatgtggatgat
acagcattgtgcccgagatgttttggaggcccttgcttttcttcatcatg
agggctatgtccatgcggacctcaaaccacgtaacatattgtggagtgca
gagaatgaatgttttaaactcattgactttggacttagcttcaaagaagg
caatcaggatgtaaagtatattcagacagacgggtatcggctccagaag
cagaattgcaaaattgcttggcccaggctggcctgcagagtgatacagaa

tgtacctcagctgttgatctgtggagcctaggaatcattttactggaaat
gttctcaggaatgaaactgaaacatacagtcagatctcaggaatggaagg
caaacagttctgctattattgatcacatatttgccagtaaagcagtggtg
aatgccgcaattccagcctatcacctaagagaccttatcaaaagcatgct
tcatgatgatccaagcagaagaattcctgctgaaatggcattgtgcagcc
cattctttagcattccttttgcccctcatattgaagatctggtcatgctt
cccactccagtgctaagactgctgaatgtgctggatgatgattatcttga
gaatgaagaggaatatgaagatgttgtagaagatgtaaaagaggagtgtc
aaaaatatggaccagtggtatctctacttgttccaaaggaaaatcctggc
agaggacaagtctttgttgagtatgcaaatgctggtgattccaaagctgc
gcagaaattactgactggaaggatgtttgatgggaagtttgttgtggcta
cattctacccgctgagtgcctacaagaggggatatctgtatcaaaccttg
ctttaatcagtaacctaaggactgtttccttttctcctcttccatttct
tgggttattccacatatgaatgcaggactaccccttaccattttaagaa
ggtactttatacatttatttaatcctactaatgtgcagccattgcccaag
cagtgactgcgttgcatacatttggcactgagtaggacaagacctctcag
ctatacattgaggggttttagagcatccatgtgggcaacccttttttgtg
cgggagagcaggtgttgctcttcagtatgtagcctaaaaaaatcttaatt
atttcatggatcatgaagcaaggatgaataatatcatgtcttggtaaata
ctaacaaatttgttaggtttggtgacatcatttacagattatttctttat
gttgtccagtggttcttccttattgttgatatccataagctggcactgga
tgctctcagtaatgttaagtaattgtcaagcagcagttacctactgtgtt
cttaacactgagttgtgaatttttttcttaaagcagtactgtagtactgaa
tattcctttaaaggaactgcagtgagcctatctaagtttttttaaattaa
ggcttttaaaatagaaagctgatgcttgatcttgcacaatttttatgtct
agtatgtatgcttgagtgaatgtgcgagtatgaatgattagagaaaattt
gagtcagtgtactttatagtgtgaatcctgtgagctaatacagtctatac
ttatttcttccctacctgtttcacatccgtaagatttaagatatacattt
tttgagaggtagtctgtctgatacaatgtaaatgacaaaacataattcct
gagaggcccagaacaaactggagtctagcctggagttaaattgagacttc
taaaatgattggaacaaagactaagttgtgccagatgtaaatcaacccct
cttttagtttactttagactttgtattagctcatctttttttgtagtaaat
ctatagttttaaggtttctcaagatgtggctctacctactatgatgaaaa
ttgaagtgggtcaaaagaattagatgtacagtgaagggaaaagaaaaaaa
atgggcgaagagagggtggaaaataaaaggattcttttttcttcctttct
gtttctcgtatccctgctccctttttcctccccttccctcattctttgcc
tctatccttagctgaagacaaactagaggagcagcatcccaggtagtttg
gcttttgactgcaaggtagttaagaaaggtgtagatataatgaggtagaa
gtagaaaggaagaaaaactcaaagaattcttaaaaggattcatagcaaca
taatgtgtccctgagtagaggatgctgctatgcgtgagttcatggacaca
agttgattacatggtttttagaattataattatggattcttcttatttca
tggtaggttgtctttaaagatataaaaattaggatgcctttatgaagcac
tgattataccaaaaaaaaaaagacaagttatatacaggttattaattttt
tatttatttattttttcctaaggaaaaagtcttctatctttccctgctgg
aagcctcctcatatttccttatgtttgccatgcaggttgctgagagtcca
gttagaatttgcattttacagaatgaaatactttaccccattcaaacaat

tattgtttgacattttagttatttataattgtcaaattcaggactctcct
ttaatgtttattatgaaaccaaatttggcataaggaggctgatttatgaa
ttaccaaagggtcttgtggcatgttccccaatacatgcccttagaaggaa
gaactattatttttattttggccctttcaggagttgattataaattggt
ttgttttcaagtcagaattcaagtgggcagaacccgtattgtgaagacct
aaactttcctaaatgttcatatgggtagcagattttgtggtgattagaaa
catcaggtccttaaatacgatgaacatgggatacaaaggaattctttata
agggcaagtatcctaagttagcacatttacttttctctcccctccgcccc
caaaagaaaaatccttacaaataaactgcaggtaggcttctaagcctagt
cctgcagtatgctgctaacatcttgatgccaatcttcacagcattctttg
attgtcatcttattgctgatacattcatacatatatttagtgcttgacac
tgtagaattttgttacagaagatggttactagattttaagggagctgagg
gaataattgatgagccttgaattaaccatgcatttaattagatttttttgt
tgttgttgttgttgttttgagatgatgtcttgctctgttgcccaggctgg
agtgcagtggctcaatctcggctcactgcaacctctgcctcgcaggtttg
agcgattctcctgtctcagcctccccagtagctgggtttacaggcgcttg
ccaccacacctggctagatgttttatattaaagccagagaattgaaaaag
aaatgcctcctgtgattgaaattattttatagctcttagcccgttctacc
aaagatcacattagcagattacccctccctcctgtgatattatctttctt
aaacctgaaccaaatatcaaaagatacaagtttcatgattaggatgaaat
atatacatagggattttggtttattgaagttattgaaagacctagaaatt
ttctttttgagagaaagggtaaagaggtgagggttcatgtcacttaaccc
ctacccttttgggcaaattttgtctcaggtagtgtttcattccctaaatt
aatacctgtgcttccttgcctctcaccttcccagttacttctttggggta
ttactgtggcacattgtacacccagaaaaggcacatacagtgattatttt
gtgggaaatacttgattcttttgaaatatatttggagggggtagctagct
agccactgtcaaaactaatgctgtatttacaggacaaaaacaagtagagt
tggacacctcagtaagagacagcattaactaaaagtactgactgcctttt
aaaggaatttttaaaactattctcttgcaataagagatccgcatttatta
ccactggatgtcttcctttttttgtggccaccaatatagcagctctcttta
gagggattccctttctgctatgccttgagccaaactggctgcttttagaa
caaaaaccataagtatggttttactcgaacttctctttgtttttattgag
agaaatattgccttctttttggtactccagctgctaaagagtgctgagat
atgttaaattatttttaaaagaaaatctccaaatacttatttaacttgt
cttctagattcatgttgcttatattttaactattgagcaagtttattata
aggaatgctgccatatagagaaagctgaagagaaaccatgggcagaaggc
tttttctgtcttattacactggaccttcactctggtaagggcatcagtt
acttttctggaataaaaggcggagttagagcacttatcagatgctgtttc
atggcttgttttagttaatttatcatgcagataactaacatttgggtatc
tttttgttttaggtcttattttctgactgttaggcctatcttacacataa
gttctactatcagtatacttggcagcatgacttttttttttttttacagt
taatttaatgaaaatcagcaccattgtaaatgatagtaattgtaacaaca
tagttactgggtgaaggagtaggggagacactcagtaaccatctatttac
ctagacacttatgcaggggtacatgctaggtggctttattcagattcggg
tacattggaatcacatcatgactttgagtctgatggctgtgtagataaag
atttaagcaagtgccttgtgtttgctggaaaatattaaaactcatttggg

tgaaagcttcccagatgatgataattaatagcacttctgcctttttcctg
gagtaacttttagattgagtcacatatgatagtataaaagtccaaaattt
tctgggtttacctttgttttatttttcatatatatattcactgtctgcct
ggaaatagccttggtttaccttattttttgtatatgtaaaactattcat
atttgattttattccaatattatatatgatggaaaagccagaattgtctg
gcagaatttaattcttccttggatctctagttctgtttttaatatcaggt
tccttcatcaaattttactactgtagctctgtgatataatttcatttctt
gtcaattatggtcacatatagacaagtacttggtcacaattagctcaaaa
gaatccccatttcttaaggtgctcagtcaatcacctttattttgagatgg
tagtttttcaagttagtattacttactagctttcttgatgaatataaaaa
tgttgtctccatcatacagagctgagcctcttgtggtatattaagataag
tcccattcttaatcacagaagaacatggatattttagtttttggagtacc
cagaattgtaaagattttgtttcattttgttttaggatagcttctctgcc
taatactataggatatgtcatgttcgttattgtagccacctccctcagat
gtttttcactatttttaaattatataatgaccagtagattcacagtagca
tgtacttgacaagtgccatggatatttaagaagaaggtaaataattttaa
tattttttgctgtaattttataggagcagtggactgtaagagaaatatct
ataaatcagagagtatttaggaaactttgtattttataggtggtcattaa
agtgatcttcaggatataagatagtttacacaaactttattctcctgata
ggaaatttttattcattttcctaccactcagcacttttgttctgccttgt
actgcttatgaggattaatgttttaaatttgctttatttgtgcttttcag
agagttgtcataattgtggcagttgtgtttgtggcactgtaaatgctgaa
attcatgggaaacttaccaaatgactgcttgagtgttatttcccattaaa
ttgggtagctgtcacaataaccagaagaaatcttgtggcccttttgaaga
aagccatgtatgttttgttacctaggttcatagtattttaatttaaaggc
aaagagaagcattctacttatttgtcttataaaccattaatcgtttttttg
tgcagaagagagcttattttgttatactgctttgtataatggaaataagt
cacattctgggggaaaaaaactggaattgtttgtattttgtttttttggg
gggggatctttatgtgaaaaatcagagctacttgttaccataagccctta
ctatcaacaagataattatttgtaatcactttttttatcccaggttggaat
tgctttccccttctaagttatcttcccttaataatatttatgataccagg
acagtgagggtataagagcaaatgtagtgaggtattcaaaaatcctgcat
atatggactcaaaagttctttagttatttgaattatatatagctatatta
ttttattagcttgggttgtcagaagattgccaattttaagagtaaagagg
agagagataagtaataaaaatagaggaggggaagaaaatggctcccttac
tggtcttagtaatcttctatatagttaatgagctaaaaaatgatacttaa
agttccaggtttggtaccgctagcatagaaacattgcctttcctacacat
tcctcatcattgtttcctcagaaaattattttaagagtttgtgaaaccag
attaaatatctatattcagtttggcctaatttttaaaaataaatatttat
actccagcttttgtgtatttggtgtacatcaccacttatgcaaatcaagg
atcagaaaactggaggttagccatctccattatttccttttgcacattgg
gtacagtgggtggcattagtatgcactagctgcaaagtcacagcacctta
tggaaataagtatgtttattataataaaaaaaagttaagctgcatctctg
tagattatttactttgcagactgtaaagctgccctatcttttccagcaga
atttactcttccattcttaattctttttttgaaatatcttaaataatttaa
cattcctttataacttcttaacagtgtcaaaactggggtagaagggattt

tattttttcccaaaagggttccatctttgctatctgttgatcagccttag
aaaatctaagtatgatcaataaattttaatggttgatggcatcctgtgtc
agctggagtagttggttgtgaatattaaaacctagtactattttccctca
gtaacatgtaattgctacatttttataagaaggtatggttagaaaaaaa
tgtgaaagatcacttaaaccaaagccagttacaaggagtaatctctcctg
ttggtttaccttcacctcagaactacaagaatattacaatacatagtgaa
tagttgtctgtaacatttctaccagttgtttcagtagcatattggtcttg
gcatttcttggcactgtggttctgctgtattatttgtgatgtcttattgt
ttgtgagcttttgtttttttttaaagaaaaaacaaaaactaagtgggac
tatgtatgtagatgtgtgtcagtaccaaaaagttattttctcaaatccct
aaatttctgatttccattcaagtcttttaaactctttcctgctgaatagc
aaagaacttttattttccactttcctatattcctaaaaagtgtgaacaat
gcgtttcagttgactgtattgcatactttttgctgaagactttttctgt
aaacacaattgccttgttcagttttgttgtaaactgacttaccataagat
gcactgttgataatgctttctgatgtgtgtttgataagagtgataaaata
aaagcttaaaaataaaggagttgctttttaatttc

>NM_000219 3
caaccaccaaacagacaagcccttcggcctgtcctggagggcgttgaatg
gcatggcctggagctcaaccaggagaaccgtgctcaggaggaagagacca
gaaggataactcaaaaagttctgagaagttcctaagaccacctgaagaga
aggagcctgctgccaatggtgtggacaccgcagtgtgcttgaggagactt
cagaaacgagaactggaaaaatccctctgctttctctggccagtttcaca
caatcatcaggtgagccgaggatccattggaggaaggcattatctgtatc
cagaggaaatagccaaggatattcagaggtgtgcctgggaagtttgagct
gcagcagtggaaccttaatgcccaggatgatcctgtctaacaccacagcg
gtgacgccctttctgaccaagctgtggcaggagacagttcagcagggtgg
caacatgtcgggcctggcccgcaggtccccccgcagcagtgacggcaagc
tggaggccctctacgtcctcatggtactgggattcttcggcttcttcacc
ctgggcatcatgctgagctacatccgctccaagaagctggagcactcgaa
cgacccattcaacgtctacatcgagtccgatgcctggcaagagaaggaca
aggcctatgtccaggcccgggtcctggagagctacaggtcgtgctatgtc
gttgaaaaccatctggccatagaacaacccaacacacaccttcctgagac
gaagccttccccatgaaccccaccactggctaaaactggacacatcctgc
ctggcaacctgattttctaatcacattcctctcatactctttattgtgat
ggataccactggatttctttttggctgttgtaaggggtgaggggtggatt
aatgacactgtttcactgtttctctaaaatcacgttcttttgtgatagac
tgtcagtggttcccccatatctgtccctgccttgctaaatttagcagaat
ccctgaggacatggcctctgagaatagcagctgcatttcccagactccct
tgcagctagcaaggttgtgtgactaagccctggccagtaggcatggaagt
gaagactgtaatgtccaagtaatccttggaaagaaaagaacgtgccctta
actaactttgtcctgcttcccagtggctggatgtggaggaggtggagagc
agttatgagactgggaaagaacggggcactcaaagagccacacacatctg
ggcctgggcgacgtggatcctccttaccacccaccaggccagatttacag
gagagagaaatccactccactcttccttaagccactgttattctgatctc
tgttaaggtcgcagaatcaatgcccttactgatacacctaccttatagga

ctgaacctaaaggcatgacatttccatacttgtcacaagcacacactgat
tctgcccttgtcacttctgtgctcactcttgtggctctatcctcctcctg
cccttccgccttccactcctcccttgcacccatcctgcacacatctccct
gaaaacacacaggcacatacactcatatacatagacacacatacacacct
caatctagaaagaacttgctttgtacagggctgagatggaggagaaaaaa
atgccccccttcagaatgcataccaaggggaaggtgctcggtcactgtggg
agcagggaaaggtgcccccactccccgagagccaggggaaggagtggctc
tgggcagagagggacacatagcactggggtggcaggtccttttgaggtga
tgggccggttttgtgagatgaattgtatcccccaaaaagacaggtacctt
caatgtgacctaattgggaaatagagtctttgcagatgatctagttgaga
tgaggtcattggggtgggccctcacccaatatgactggagtccttatcgg
aagagggaaattcagacacagatgcatagggaggacaccatgccgtgaca
gaggcagagggtgcagtgacacagccacaaaccaaggaaggccgaggatg
gatgcgcatccccatcccaagaagtcgggaagaagccaggaaggctcctc
tcccacaggtttcagaggaagcacagccctgcttgaattcaaacttttgg
cctccagaactgtgagtcagtacctgttgttgaagccacccagcttagga
tactctggcagcctactgccatacagtattgggatactatagtgagccca
tgcagcacctctcacccacccagagatggagctgctctgccttccagcgg
ggcacccggagggctgccccagcagatagagagggcctccgttctgccac
ctgccttgaaagggtctccagctgccatatgtagcattggagtcctctgc
aatgcgacatcctgaaagctcagctgcctgggcattcctgaagagtatgg
aatatttaaatgaaacatattttttaaaacctgcgcataagataaaagc
agcccgtgtgcatcttgggccatcctcaaatggacagacttggtcttgtg
aggttccagtccttgtttcacataataaacactggcatggctcagcccct
gagttaccacagtccttgagatgagtggttctttgggttacaaagtcctc
tgaaagtctagtgagagctgtgatctttgccccacccgaataatgcatat
ggacaccacaccttgcctgccgtgtccaggattcatgaccagtagcagcc
cagctatgcctgccacgtctcatggcccctgtgtaagccagacccttctt
aggcagttgcatattcccagactgaggcagggcaggtttgcagagagaga
cccagagtgcacgtgacccgcagtgtgatccctggcacgcactgactttg
atattccaggcacacggactggctatttatcaccacttctttttccccac
taagattcctgtgccttttaaggcagagggagatccctatggcgttagtc
ttcccaggccttaaagggcccttgtcttcactcacaaacctcttatctct
tcttctccttcctctacattttaaagggggagagggaaaagtaaccggga
gacaaattgagccacatattttcagacacttgttaccatattttaaaatc
tggcttcacatacacagagtctttgctatgcaccatgtactgttctaagc
ttcttaaaaatagaatctcaattattattttgcaggcaatactctatgca
ttcattagctaggacaacaatgcatttgcagtagtgagatttcgctaaaa
aattaaagccatttactatgaaaaaaaaaaaaaaaaaa

>NM_170735 5
cacacacacacacacacacagagagaacatctctagtaaaaagaaaagtt
gagctttcttagctagatgtgtgtattagccagaaaaagccaaggagtga
agggttttagagaactggaggagataaagtggagtctgcatatgggaggc
atttgaaatggacttaaatgtctttttaatgctgactttttcagttttct

ccttaccagacacattgttttcatgacattagccccaggcatagacacat
cattaaaatgaacatgtcaaaaaatgatttctgtttagaaataagcaaaa
catttt cagttgtgaccacccaggtgtagaataaagaacagtggaattgg
gagccctgagttctaacataaactttcttcatgacataaggcaagtcttc
tatggcctttggtttccttacctgtaaaacaggatggctcaatgaaatta
tctttcttctttgctataatagagtatctctgtgggaagaggaaaaaaaa
agtcaatttaaaggctccttatagttccccaactgctgttttattgtgct
attcatgcctagacatcacatagctagaaaggcccatcagacccctcagg
ccactgctgttcctgtcacacattcctgcaaaggaccatgttgctaactt
gaaaaaaattactattaattacacttgcagttgttgcttagtaacattta
tgattttgtgtttctcgtgacagcatgagcagagatcattaaaaattaaa
cttacaaagctgctaaagtgggaagaaggagaacttgaagccacaatttt
tgcacttgcttagaagccatctaatctcaggttatatgctagatcttggg
ggcaaacactgcatgtctctggtttatattaaaccacatacagcacacta
ctgacactgatttgtgtctggtgcagctggagtttatcaccaagacataa
aaaaaccttgaccctgcagaatggcctggaattacaatcagatgggccac
atggcatcccggtgaaagaaagccctaaccagttttctgtcttgtttctg
ctttctccctacagttccaccaggtgagaagagtgatgaccatccttttc
cttactatggttatttcatactttggttgcatgaaggctgcccccatgaa
agaagcaaacatccgaggacaaggtggcttggcctacccaggtgtgcgga
cccatgggactctggagagcgtgaatgggcccaaggcaggttcaagaggc
ttgacatcattggctgacactttcgaacacgtgatagaagagctgttgga
tgaggaccagaaagttcggcccaatgaagaaaacaataaggacgcagact
tgtacacgtccagggtgatgctcagtagtcaagtgcctttggagcctcct
cttctctttctgctggaggaatacaaaaattacctagatgctgcaaacat
gtccatgagggtccggcgccactctgaccctgcccgccgaggggagctga
gcgtgtgtgacagtattagtgagtgggtaacggcggcagacaaaaagact
gcagtggacatgtcgggcgggacggtcacagtccttgaaaaggtccctgt
atcaaaaggccaactgaagcaatacttctacgagaccaagtgcaatccca
tgggttacacaaaagaaggctgcaggggcatagacaaaaggcattggaac
tcccagtgccgaactacccagtcgtacgtgcgggcccttaccatggatag
caaaaagagaattggctggcgattcataaggatagacacttcttgtgtat
gtacattgaccattaaaaggggaagatagtggatttatgttgtatagatt
agattatattgagacaaaaattatctatttgtatatatacataacagggt
aaattattcagttaagaaaaaaataattttatgaactgcatgtataaatg
aagtttatacagtacagtggttctacaatctatttattggacatgtccat
gaccagaagggaaacagtcatttgcgcacaacttaaaaagtctgcattac
attccttgataatgttgtggtttgttgccgttgccaagaactgaaaacat
aaaaagttaaaaaaaataataaattgcatgctgctttaattgtgaattga
taataaactgtcctctttcagaaaacagaaaaaaacacacacacacacaa
caaaaatttgaaccaaaacattccgtttacattttagacagtaagtatct
tcgttcttgttagtactatatctgttttactgcttttaacttctgatagc
gttggaattaaaacaatgtcaaggtgctgttgtcattgctttactggctt
aggggatgggggatggggggtatatttttgtttgttttgtgttttttttt
cgtttgtttgttttgtttttttagttcccacagggagtagagatggggaaa
gaattcctacaatatatattctggctgataaaagatacatttgtatgttg

tgaagatgtttgcaatatcgatcagatgactagaaagtgaataaaaatta
aggcaactgaacaaaaaaatgctcacactccacatcccgtgatgcacctc
ccaggccccgctcattctttgggcgttggtcagagtaagctgcttttgac
ggaaggacctatgtttgctcagaacacattctttcccccctccccctct
ggtctcctctttgttttgttttaaggaagaaaaatcagttgcgcgttctg
aaatattttaccactgctgtgaacaagtgaacacattgtgtcacatcatg
acactcgtataagcatggagaacagtgattttttttagaacagaaaaca
acaaaaaataaccccaaaatgaagattatttttatgaggagtgaacatt
tgggtaaatcatggctaagcttaaaaaaaactcatggtgaggcttaacaa
tgtcttgtaagcaaaaggtagagccctgtatcaacccagaaacacctaga
tcagaacaggaatccacattgccagtgacatgagactgaacagccaaatg
gaggctatgtggagttggcattgcatttaccggcagtgcgggaggaattt
ctgagtggccatcccaaggtctaggtggaggtggggcatggtatttgaga
cattccaaaacgaaggcctctgaaggacccttcagaggtggctctggaat
gacatgtgtcaagctgcttggacctcgtgctttaagtgcctacattatct
aactgtgctcaagaggttctcgactggaggaccacactcaagccgactta
tgcccaccatcccacctctggataattttgcataaaattggattagcctg
gagcaggttgggagccaaatgtggcatttgtgatcatgagattgatgcaa
tgagatagaagatgtttgctacctgaacacttattgctttgaaactagac
ttgaggaaaccagggtttatcttttgagaacttttggtaagggaaaaggg
aacaggaaaagaaaccccaaactcaggccgaatgatcaaggggacccata
ggaaatcttgtccagagacaagacttcgggaaggtgtctggacattcaga
acaccaagacttgaaggtgccttgctcaatggaagaggccaggacagagc
tgacaaaattttgctccccagtgaaggccacagcaaccttctgcccatcc
tgtctgttcatggagagggtccctgcctcacctctgccattttgggttag
gagaagtcaagttgggagcctgaaatagtggttcttggaaaaatggatcc
ccagtgaaaactagagctctaagcccattcagcccatttcacacctgaaa
atgttagtgatcaccacttggaccagcatccttaagtatcagaaagcccc
aagcaattgctgcatcttagtagggtgagggataagcaaaagaggatgtt
caccataacccaggaatgaagataccatcagcaaagaatttcaatttgtt
cagtctttcatttagagctagtctttcacagtaccatctgaatacctctt
tgaaagaaggaagactttacgtagtgtagatttgttttgtgttgtttgaa
aatattatctttgtaattatttttaatatgtaaggaatgcttggaatatc
tgctatatgtcaactttatgcagcttccttttgagggacaaatttaaaac
aaacaaccccccatcacaaacttaaaggattgcaagggccagatctgtta
agtggtttcataggagacacatccagcaattgtgtggtcagtggctcttt
tacccaataagatacatcacagtcacatgcttgatggtttatgttgacct
aagatttattttgttaaaatctctctctgttgtgttcgttcttgttctgt
tttgttttgtttttaaagtcttgctgtggtctctttgtggcagaagtgt
ttcatgcatggcagcaggcctgttgctttttatggcgattcccattgaa
aatgtaagtaaatgtctgtggccttgttctctctatggtaaagatattat
tcaccatgtaaaacaaaaaacaatatttattgtattttagtatatttata
taattatgttattgaaaaaaattggcattaaaacttaaccgcatcagaac
ctattgtaaatacaagttctatttaagtgtactaattaacatataatata
tgttttaaatatagaatttttaatgtttttaaatatattttcaaagtaca
taaaa

>NM_014918 4
ggcgagctaagccggaggatgtgcagctgcggcggcggcgccggctacga
agaggacggggacaggcgccgtgcgaaccgagcccagccagccggaggac
gcgggcagggcgggacgggagcccggactcgtctgccgccgccgtcgtcg
ccgtcgtgccggccccgcgtccccgcgcgcgagcgggaggagccgccgcc
acctcgcgcccgagccgccgctagcgcgcgccgggcatggtcccctctta
aaggcgcaggccgcggcggcgggggcgggcgtgcggaacaaagcgccggc
gcggggcctgcgggcggctcgggggccgcgatgggcgcggcgggcccgcg
gcggcggcggcgctgcccgggccgggcctcgcggcgctagggcgggctgg
cctccgcgggcgggggcagcgggctgagggcgcgcggagcctgcggcggc
ggcccggcgggcggagcggcgcgggcatggccgcgcgcggccggcgcgcc
tggctcagcgtgctgctcgggctcgtcctgggcttcgtgctggcctcgcg
gctcgtcctgccccgggcttccgagctgaagcgagcgggcccacggcgcc
gcgccagccccgagggctgccggtccgggcaggcggcggcttcccaggcc
ggcggggcgcgcggcgatgcgcgcggggcgcagctctggccgcccggctc
ggacccagatggcggcccgcgcgacaggaactttctcttcgtgggagtca
tgaccgcccagaaatacctgcagactcgggccgtggccgcctacagaaca
tggtccaagacaattcctgggaaagttcagttcttctcaagtgagggttc
tgacacatctgtaccaattccagtagtgccactacggggtgtggacgact
cctacccgccccagaagaagtccttcatgatgctcaagtacatgcacgac
cactacttggacaagtatgaatggtttatgagagcagatgatgacgtgta
catcaaaggagaccgtctggagaacttcctgaggagtttgaacagcagcg
agcccctctttcttgggcagacaggcctgggcaccacggaagaaatggga
aaactggccctggagcctggtgagaacttctgcatgggggggcctggcgt
gatcatgagccgggaggtgcttcggagaatggtgccgcacattggcaagt
gtctccgggagatgtacaccacccatgaggacgtggaggtgggaaggtgt
gtccggaggtttgcaggggtgcagtgtgtctggtcttatgagatgcagca
gctttttatgagaattacgagcagaacaaaaaggggtacattagagatc
tccataacagtaaaattcaccaagctatcacattacaccccaacaaaaac
ccaccctaccagtacaggctccacagctacatgctgagccgcaagatatc
cgagctccgccatcgcacaatacagctgcaccgcgaaattgtcctgatga
gcaaatacagcaacacagaaattcataaagaggacctccagctgggaatc
cctccctccttcatgaggtttcagccccgccagcgagaggagattctgga
atgggagtttctgactggaaaatacttgtattcggcagttgacggccagc
cccctcgaagaggaatggactccgcccagagggaagccttggacgacatt
gtcatgcaggtcatggagatgatcaatgccaacgccaagaccagagggcg
catcattgacttcaaagagatccagtacggctaccgccgggtgaacccca
tgtatggggctgagtacatcctggacctgctgcttctgtacaaaaagcac
aaagggaagaaaatgacggtccctgtgaggaggcacgcgtatttacagca
gactttcagcaaaatccagtttgtggagcatgaggagctggatgcacaag
agttggccaagagaatcaatcaggaatctggatccttgtcctttctctca
aactccctgaagaagctcgtcccctttcagctccctgggtcgaagagtga
gcacaaagaacccaaagataaaaagataaacatactgattcctttgtctg
ggcgtttcgacatgtttgtgagatttatgggaaactttgagaagacgtgt
cttatccccaatcagaacgtcaagctcgtggttctgcttttcaattctga

ctccaaccctgacaaggccaaacaagttgaactgatgagagattaccgca
ttaagtaccctaaagccgacatgcagattttgcctgtgtctggagagttt
tcaagagccctggccctggaagtaggatcctcccagtttaacaatgaatc
tttgctcttcttctgcgacgtcgacctcgtgtttactacagaattccttc
agcgatgtcgagcaaatacagttctgggccaacaaatatattttccaatc
atcttcagccagtatgacccaaagattgtttatagtgggaaagttcccag
tgacaaccattttgcctttactcagaaaactggcttctggagaaactatg
ggtttggcatcacgtgtatttataagggagatcttgtccgagtgggtggc
tttgatgtttccatccaaggctgggggctggaggatgtggaccttttcaa
caaggttgtccaggcaggtttgaagacgtttaggagccaggaagtaggag
tagtccacgtccaccatcctgtcttttgtgatcccaatcttgaccccaaa
cagtacaaaatgtgcttggggtccaaagcatcgacctatgggtccaccca
gcagctggctgagatgtggctggaaaaaaatgatccaagttacagtaaaa
gcagcaataataatggctcagtgaggacagcctaatgtccagctttgctg
gaaaagacgtttttaattatctaatttatttttcaaaaattttttgtatg
atcagtttttgaagtccgtatacaaggatatattttacaagtggttttct
tacataggactcctttaagattgagctttctgaacaagaaggtgatcagt
gtttgcctttgaacacatcttcttgctgaacattatgtagcagacctgct
taactttgacttgaaatgtacctgatgaacaaaactttttttaaaaaaatg
ttttcttttgagaccctttgctccagtcctatggcagaaaacgtgaacat
tcctgcaaagtattattgtaacaaaacactgtaactctggtaaatgttct
gttgtgattgttaacattccacagattctaccttttgtgttttgtttttt
ttttttacaattgttttaaagccatttcatgttccagttgtaagataagg
aaatgtgataatagctgtttcatcattgtcttcaggagagctttccagag
ttgatcatttcctctcatggtactctgctcagcatggccacgtaggtttt
ttgtttgttttgttttgttcttttttgagacggagtctcactctgttac
ccaggctggaatgcagtggcgcaatcttggctcactttaacctccacttc
cctggttcaagcaattcccctgcctttgcctcccgagtagctgggattac
aggcacacaccaccacgcccagctagttttttttgtatttttagtagagac
ggggtttcaccatgcaagcccagctggccacgtaggttttaaagcaaggg
gcgtgaagaaggcacagtgaggtatgtggctgttctcgtggtagttcatt
cggcctaaatagacctggcattaaatttcaagaaggatttggcattttct
cttcttgacccttctctttaaagggtaaaatattaatgtttagaatgaca
aagatgaattattacaataaatctgatgtacacagactgaaacatacaca
catacaccctaatcaaaacgttggggaaaaatgtatttggttttgttcct
ttcatcctgtctgtgttatgtgggtggagatggttttcattctttcatta
ctgttttgttttatccttttgtatctgaaatacctttaatttatttaatat
ctgttgttcagagctctgccatttcttgagtacctgttagttagtattat
ttatgtgtatcgggagtgtgtttagtctgttttatttgcagtaaaccgat
ctccaaagatttccttttggaaacgctttttcccctccttaatttttata
ttccttactgttttactaaatattaagtgttctttgacaattttggtgct
catgtgttttggggacaaaagtgaaatgaatctgtcattataccagaaag
ttaaattctcagatcaaatgtgccttaataaatttgttttcatttagatt
tcaaacagtgatagacttgccattttaatacacgtcattggagggctgcg
tatttgtaaatagcctgatgctcatttggaaaaataaaccagtgaacaat
atttttctattgtacttttcgaaccattttgtctcattattcctgtttta

gctgaagaattgtattacatttggagagtaaaaaacttaaacacgattca
aaaaaaaaaaaaaaaa

>NM_018448 3
gtgaactctgaccttagctttccgtagcgcccgcgtctgccgcccccgcc
cccggagcgaaggaggcgggctttggccttttgccctagggagcgagtgc
ggagcgagtgggagcgagacggccctgagtggaagtgtctggctccccgt
agaggcccttctgtacgccccgccgcccatgagctcgttctcacgcgaac
agcgccgtcgttaggctggctctgtagcctcggcttaccccgggacaggc
ccacgcctcgccagggagggggcagcccgtcgaggcgcctccctagtcag
cgtcggcgtcgcgctgcgaccctggaagcgggagccgccgcgagcgagag
gaggagctccagtggcggcggcggcggcggcagcggcagcgggcagcagc
tccagcagcgccagcaggcgggatcgaggccgtcaacatggcgagcgcct
cgtaccacatttccaatttgctggaaaaaatgacatccagcgacaaggac
tttaggtttatggctacaaatgatttgatgacggaactgcagaaagattc
catcaagttggatgatgatagtgaaaggaaagtagtgaaaatgattttga
agttattggaagataaaaatggagaggtacagaatttagctgtcaaatgt
cttggtcctttagtgagtaaagtgaaagaataccaagtagagacaattgt
agataccctctgcactaacatgctttctgataaagaacaacttcgagaca
tttcaagtattggtcttaaaacagtaattggagaacttcctccagcttcc
agtggctctgcattagctgctaatgtatgtaaaaagattactggacgtct
tacaagtgcaatagcaaaacaggaagatgtctctgttcagctagaagcct
tggatattatggctgatatgttgagcaggcaaggaggacttcttgttaat
ttccatccttcaattctgacctgtctacttccccagttgaccagccctag
acttgcagtgaggaaaagaaccattatcgctcttggccatctggttatga
gctgtggaaatatagtttttgtagatcttattgaacatctgttgtcagag
ttgtccaaaaatgattctatgtcaacaacaagaacctacatacaatgtat
tgctgctattagtaggcaagctggtcatagaataggtgaataccttgaga
agataattcctttggtggtaaaattttgcaatgtagatgatgatgaatta
agagagtactgtattcaagcctttgaatcatttgtaagaagatgtcctaa
ggaagtatatcctcatgtttctaccattataaatatttgtcttaaatatc
ttacctatgatccaaattataattacgatgatgaagatgaagatgaaaat
gcaatggatgctgatggtggtgatgatgatgatcaagggagtgatgatga
atacagtgatgatgatgacatgagttggaaagtgagacgtgcagctgcga
agtgcttggatgctgtagttagcacaaggcatgaaatgcttccagaattc
tacaagaccgtctctcctgcactaatatccagatttaaagagcgtgaaga
gaatgtaaaggcagatgtttttcacgcatacctttctcttttgaagcaaa
ctcgtcctgtacaaagttggctatgtgaccctgatgcaatggagcaggga
gaaacacctttaacaatgcttcagagtcaggttcccaacattgttaaagc
tcttcacaaacagatgaaagaaaaaagtgtgaagacccgacagtgttgtt
ttaacatgttaactgagctggtaaatgtattacctggggccctaactcaa
cacattcctgtacttgtaccaggaatcattttctcactgaatgataaatc
aagctcatcgaatttgaagatcgatgctttgtcatgtctatacgtaatcc
tctgtaaccattctcctcaagtcttccatcctcacgttcaggctttggtt
cctccagtggtggcttgtgttggagacccattttacaaaattacatctga
agcacttcttgttactcaacagcttgtcaaagtaattcgtcctttagatc

agccttcctcgtttgatgcaactccttatatcaaagatctatttacctgt
accattaagagattaaaagcagctgacattgatcaggaagtcaaggaaag
ggctatttcctgtatgggacaaattatttgcaaccttggagacaatttgg
gttctgacttgcctaatacacttcagattttcttggagagactaaagaat
gaaattaccaggttaactacagtaaaggcattgacactgattgctgggtc
acctttgaagatagatttgaggcctgttctgggagaaggggttcctatcc
ttgcttcatttcttagaaaaaaccagagagctttgaaactgggtactctt
tctgcccttgatattctaataaaaaactatagtgacagcttgacagctgc
catgattgatgcagttctagatgagctcccacctcttatcagcgaaagtg
atatgcatgtttcacaaatggccatcagttttcttaccactttggcaaaa
gtatatccctcctccctttcaaagataagtggatccattctcaatgaact
tattggacttgtgagatcacccttattgcaggggggagctcttagtgcca
tgctagactttttccaagctctggttgtcactggaacaaataatttagga
tacatggatttgttgcgcatgctgactggtccagtttactctcagagcac
agctcttactcataagcagtcttattattccattgccaaatgtgtagctg
cccttactcgagcatgccctaaagagggaccagctgtagtaggtcagttt
attcaagatgtcaagaactcaaggtctacagattccattcgtctcttagc
tctactttctcttggagaagttgggcatcatattgacttaagtggacagt
tggaactaaaatctgtaatactagaagctttctcatctcctagtgaagaa
gtcaaatcagctgcatcctatgcattaggcagcattagtgtgggcaacct
tcctgaatatctgccgtttgtcctgcaagaaataactagtcaacccaaaa
ggcagtatcttttacttcattccttgaaggaaattattagctctgcatca
gtggtgggccttaaaccatatgttgaaaacatctgggccttattactaaa
gcactgtgagtgtgcagaggaaggaaccagaaatgttgttgctgaatgtc
taggaaaactcactctaattgatccagaaactctccttccacggcttaag
gggtacttgatatcaggctcatcatatgcccgaagctcagtggttacggc
tgtgaaatttacaatttctgaccatccacaacctattgatccactgttaa
agaactgcataggtgatttcctaaaaactttggaagacccagatttgaat
gtgagaagagtagccttggtcacatttaattcagcagcacataacaagcc
atcattaataagggatctattggatactgttcttccacatctttacaatg
aaacaaaagttagaaaggagcttataagagaggtagaaatgggtccattt
aaacatacggttgatgatggtctggatattagaaaggcagcatttgagtg
tatgtacacacttctagacagttgtcttgatagacttgatatctttgaat
ttctaaatcatgttgaagatggtttgaaggaccattatgatattaagatg
ctgacatttttaatgttggtgagactgtctaccctttgtccaagtgcagt
actgcagaggttggaccgacttgttgagccattacgtgcaacatgtacaa
ctaaggtaaaggcaaactcagtaaagcaggagtttgaaaaacaagatgaa
ttaaagcgatctgccatgagagcagtagcagcactgctaaccattccaga
agcagagaagagtccactgatgagtgaattccagtcacagatcagttcta
accctgagctggcggctatctttgaaagtatccagaaagattcatcatct
actaacttggaatcaatggacactagttagatgtttgttcaccatgggga
ccattacatatgaccatacaatgcactgaattgacaggttaatcataaga
catggaaagagaagtgtctaaaagcttcaaaatgttccactttttttcc
ttcatggagactgtttgtttggctttcttccattgttgtttttgtagcat
ttatttcagaaatgtgtatttccataatccagaggttgtaaaaccactag
tgttttagtggttacagcaacatttgaaatggaaactaaaagttaggatt

ttatggagtatggagatagggtccagtatctatttaccctgtaatgttta
ggattaaaatgttaaaattttgtgaccatgaatttctttcttttataaat
tttctcatttaaaaatcaaaaatcttgcaaaacaaaaaccatgtttcttt
ttcttgtataactttttgttttcagcaacataaattgatttttagctggc
agacaagaatatccatataagatttgttaaccatttcagagagtttggca
atttttaaaagataataaggtatcatttttaagtatgaaaattaacaata
tccctgttgcgcacactaattttgcatgagtaagtttacaaatatgtatc
gtctgtaaagcagcatgtgcagattattcataatatagaagttaaaataa
gtattagtgcaattttcagatatttatttttgcacagaaaacacattatc
tggagagaaagaaaggagaatttttgagacttgggttttcttaatgccag
tgtgaatttgcagatgttttcagaaaatcaagtcacagtaacaatttgcc
actttttctattataaatcttcttacttaaattttgaatatttagtttt
tctcagttacccatttgtgtgtgtgtgattccacttagaaattcttaaaa
ccagatttttctttcattccgtttggatgtctacattccttatcaaagga
tataaatactgtgtatgcttttgaattttatttttaggaaaattctgaag
ccagctatcacaggtttgttagctaataatagtattttcttttagttgag
ttaggtttttccccatctcctgtagagcgaatttacatattgtattgggt
aagtgttcactacttttcctgattaagggatctgtgctggggaacaaagc
ttttgcagtaccttatattgtagttaaaattttatttaacatatccttca
gtgagctcatttcacactgtagcctcttccttaaaatttgtggtgctcct
gtaacagtaagaactaattctgaaataaaagacatctcctaatgctgtgc
aaacatagtttacatgtattgaaggaggcagttgttaaattgagtgacca
atttaagcaatcagatatttgaaaactgcaccctttagttttgaaactgt
gaattagaaacacttttcctgctgtattactacctgctttaacatccaaa
tatacagtgattttaaatgataacatactgtggttattagattaacagct
tgattttgaatgttcagatgataatgcagaagacatcacttctagtaagg
attttgactagtgcattgatgttgaagttggtgccatttcaaaatgtggc
aggtgataatcttttaccataatttgcataaaactgtaatagaagtttat
tttgagatgttagtatattatgtactatgcatttctgtggtatagatgtt
gtggatatatttaagtatttggttacatggttttacaataaattacaata
ctgcaggcaaaaaaaaaaaaaaaaaaaaaaaaaaagaaaaaaaaaaaaaa
aaaaag

>NM_004815 3
gcgcaactgacggtcgcgttctgcgcgcgagctagttgcctcccgtacct
gccgcggtcgccggccccgcccccgggagcgcgggccaatgggctgggct
ccagggggcggggctggcgggcgggcggtgcggccgtggcggtagctgca
ggggcggtggcggctgcagtggtggtggtgcctgtggctgtggctgcggc
tgcggctgcggctgagatttggccgggcgtccgcaggccgtgggggatgg
gggcagcgagctccagccctcggcggtggcggcggccgtaggtgtggggc
gggcgtccgcgtccggcacgcgagatggagcgccgtggatttcagttttt
ctgactgttacatgaaaggatgattgctcacaaacagaaaaagacaaaga
aaaaacgtgcttgggcatcaggtcaactctctactgatattacaacttct
gaaatggggctcaagtccttaagttccaactctatttttgatccggatta
catcaaggagttggtgaatgatatcaggaagttctcccacatgttactat
atttgaaagaagccatattttcagactgttttaaagaagttattcatata

cgtctagaggaactgctccgtgttttaaagtctataatgaataaacatca
gaacctcaattctgttgatcttcaaaatgctgcagaaatgctcactgcaa
aagtgaaagctgtgaacttcacagaagttaatgaagaaaacaaaaacgat
ctcttccaggaagtgttttcttctattgaaactttggcatttacctttgg
aaatatccttacaaacttccttatgggagatgtaggcaatgattcattat
tgcgactgcctgtttctcgagaaactaagtcgtttgaaaatgtttctgtg
gaatcagtggactcatccagtgaaaaaggaaatttttcccctttagaact
agacaacgtgctgttaaagaacactgactctatcgagctggctttgtcat
atgctaaaacttggtcaaaatatactaagaacatagtttcatgggttgaa
aaaaagcttaacttggaattggagtccactagaaatatggtcaagttggc
agaggcaactagaactaacattggaattcaggagttcatgccactgcagt
ctctgtttactaatgctcttcttaatgatatagaaagcagtcacctttta
caacaaacaattgcagctctccaggctaacaaatttgtgcagcctctact
tggaaggaaaaatgaaatggaaaaacaaaggaaagaaataaaagagcttt
ggaaacaggagcaaaataaaatgcttgaagcagagaatgctctcaaaaag
gcaaaattattatgcatgcaacgtcaagatgaatatgagaaagcaaagtc
ttccatgtttcgtgcagaagaggagcatctgtcttcaagtggcggattag
caaaaaatctcaacaagcaactagaaaaaaagcgaaggttggaagaggag
gctctccaaaaagtagaagaagcaaatgaactttacaaagtttgtgtgac
aaatgttgaagaaagaagaaatgatctagaaaataccaaaagagaaattt
tagcacaactccggacacttgttttccagtgtgatcttacccttaaagct
gtaacagttaacctcttccacatgcagcatctgcaggctgcttcccttgc
agacagtttacagtctctctgtgatagtgccaaactctatgacccaggcc
aagagtacagtgaatttgtcaaggccacaaattcaactgaagaagaaaaa
gttgatggaaatgtaaataaacatttaaatagttcccaaccttcaggatt
tggacctgccaactctttagaggatgttgtacgccttcctgacagttcta
ataaaattgaagaggacagatgctctaacagtgcagatataacaggtcct
tcctttataagatcatggacatttgggatgtttagtgattctgagagcac
tggagggagcagcgaatctagatctctggattcagaatctataagtccag
gagactttcatcgaaaacttccacgaacaccatccagtggaactatgtcc
tctgcagatgatctagatgaaagagagccaccttccccttcagaaactgg
acccaattcccttggaacatttaagaaaacattgatgtcaaaggcagctc
tcacacacaagtttcgcaaattgagatcccccacgaaatgtagggattgt
gaaggcattgtagtgttccaaggtgttgaatgtgaagagtgtctccttgt
ttgtcatcgaaagtgtttggaaaatttagtcattatttgtggtcatcaga
aacttccaggaaaaatacacttatttggagcagaattcacacaagttgca
aaaaaggaaccagatggtatcccttttatactcaaaatatgtgcctcaga
gattgaaaatagagctttgtgtctacagggaatttatcgtgtgtgtggaa
acaaaataaaaactgaaaaattgtgtcaagctttggaaaatggaatgcac
ttggtagatatttcagaatttagttcacatgatatctgtgacgtcttgaa
attataccttcggcagctcccagaaccatttattttatttcgattgtaca
aggaatttatagaccttgcaaaagagatccaacatgtaaatgaagaacaa
gagacaaaaaagaatagtcttgaagacaaaaaatggccaaatatgtgtat
agaaataaaccgaattcttctaaaaagcaaagaccttctaagacaattgc
cagcatcaaattttaacagtcttcatttccttatagtacatctaaagcgg
gtagtagatcatgcagaagaaaacaagatgaactccaaaaacttggggt

gatatttggaccaagtctcattaggccaaggcccacaactgctcctatca
ccatctcctcccttgcagagtattcaaatcaagcacgcttggtagagttt
ctcattacttactcacagaagatcttcgatgggtccctacaaccacaaga
tgttatgtgtagcataggtgttgttgatcaaggctgttttccaaagcctc
tgttatcaccagaagaaagagacattgaacgttccatgaagtcactattt
ttttcttcaaaggaagatatccatacttcagagagtgaaagcaaaatttt
tgaacgagctacatcatttgaggaatcagaacgcaagcaaaatgcgttag
gaaaatgtgatgcatgtctcagtgacaaagcacagttgcttctagaccaa
gaggctgaatcagcatcccaaaagatagaagatggtaaaacccctaagcc
actttctctgaaatctgataggtcaacaaacaatgtggagaggcatactc
caaggaccaagattagacctgtaagtttgcctgtagatagactacttctt
gcaagtcctcctaatgagagaaatggcagaaatatgggaaatgtaaattt
agacaagttttgcaagaatcctgcctttgaaggagttaatagaaaagacg
ctgctactactgtttgttccaaatttaatggctttgaccagcaaactcta
cagaaaattcaggacaaacagtatgaacaaaacagcctaactgccaagac
tacaatgatcatgcccagtgcactccaggaaaaaggagtgacaacaagcc
tccagattagtggggaccattctatcaatgccactcaacccagtaagcca
tatgcagagccagtcaggtcagtgagagaggcatctgagagacggtcttc
agattcctaccctctcgctcctgtcagagcacccagaacactgcagcctc
aacattggacaacattttataaaccacatgctcccatcatcagtatcagg
gggaatgaggagaagccagcttcaccctcagcagcagtgcctcctggcac
agatcacgatccccacggtctcgtggtgaagtcaatgccagacccagaca
aagcatcagcttgtcctgggcaagcaactggtcaacctaaagaagactct
gaggagcttggcttgcctgatgtgaatccaatgtgtcagagaccaaggct
aaaacgaatgcaacagtttgaagacctcgaaggtgaaattccacaatttg
tgtagggatgtcaaatttcagggtttttttgttgttgttgtgttattttg
tggtattgtgcttgttttgtgaaagaatgttttgacagggcctcttttgt
ataggactgccaaatcatgggttttgccttttgttgttgtatttatcctc
tgttggtaatactgaatggtagaatgttttgatagggtcacatttgtgcc
tcactggaattatctttaaattctgtatttttaaagttgtgaataagata
ggtggattcgtatttttttaaagttcagttgactttccccaccaaatggtc
catttgaatgcatccctaatatatgatatagtctcaactaataggtgcaa
tttgggaaaatcaggtttattttttggagtggaactgttataagtgctta
tttataaaaggaatgtttctgaatgcaagtgcctaaaaagatctttgttg
gtatgcatatgttttgtcacacaattttatagtgcatctttcaccatttg
tgctttttttaagatacgtatgtaagctcttatttttcaattggcaattca
gttaatttttaaatgtttacataatggccagaaggcttgcaaatctgtat
ttaattgcattttaattaattgccagtttttacatgtgatagtcagttgt
acaaagaaaatgcacttaaacctgtttctaaattatatattcagttatat
tatatttggctttagatggttttaatacatttgatagtttttcacccctt
ggctttattttatataaacttttgtttttcagcagttctgaactttttag
tattttataaatggtccaaaaaatgcctgtttcagaagtttttgaattca
gtgcatttcctcttgatttgtctgggttaaaaccattccttttgtatgaa
atgttttgacttaggaatcattttatgtacttgttctacctggattgtca
acaactgaaagtacatatttcatccaaatcaagctaaaatttatttaagt
tgattctgagagtacaggtcagtaagcctcattatttggaatttgagaga

aggtataggtgatcggatctgtttcatttataaaaggtccagtttttagg
actagtacattcctgttattttctgggttttatcattttgcctaaaatag
gatataaaagggacaaaaaataagtagactgtttttatgtgtgaattata
tttctactaaatgtttttgtatgactgtgttatacttgataatatatata
tatatatatatcaacttgttaaattatttcatgttcccgtggcttctttt
cagttgttgcctattacagtatgagagtttaaggttattaaccattggct
tagaagtcaacacctaggatcatacatccgttgactacaatgtggaatga
attttatggaaacctgttttatagttctatgtgatgtaaaggtctaggga
gcaataaccagcccttttttttctgaaggcttgtttttagtcctcattgt
acaaaatataaaataatcatattgctatgaatacctaaaaagaaaattag
agcccatgtgtattgagccttctctgtgtgccaggaactttgccaaggtg
ctacatagattattttatttcaagttcacagcaacctataatgggtgggt
aagcatgttattatttctgtgttacagataaggaaactaaggcttagtta
aatgacttgcttcagtgttccacaggtgtaacaggtggagtcaaatctca
aacatagggctgtcagattttgatggttagtgctcttaatgactgtgatt
actaaataatatctggtagtttttaacaatagaaaatgcaactttaaaaa
atttttattgtggtaaaatacacataacatttaccatttttaagtatatt
gtttgaaccatttttaagcatatagttcagtggcattaagtatattcaca
ttgttttgcaaccatgagcacctattcatctccagaacattatcatcatc
ctatactaaaacttagaaaatgtacttttaaacttcttcagttctctttt
aatatatggagcccacccaaaggttatatcatgtaataaactcatttctc
tgttttccctcacagcttaaaaatgagaatttcacttttgggtcctttct
gcctttagggctccctggcactttgtccagcgtagagccttaagcctcta
gatgctttctagtttcctggggccctgaggcaagtatctcttgagaagag
gttgctatttctagaggatccacagtgtgcactgtgttgtgctatctaat
agtgtggtggaaatcatttatccagaagttgtttttttgcaacatggaaag
atacgtgaccaaaaggaaagggcaacagcaggagccctgttagtgctgcc
agaacaccaggaagccttgtgggaggcgtattgtccaagatgatgcgtat
tgtccaaacgactcagaagaagtcatttctgaagggttgatcataacttc
cctagccatgttttacctacagagaacttagttagaatttatgagtacag
tatgttaaattacttttagtgtaccttaggcagtgtatttgttttgatac
agagacaaagactatatgatccctgagacttgttgcctagtgaactcaca
agcagatagaattgtacctcagttatttgggtgatttaaagatggtacta
tggctacactattcccagctatcgttgtgttacagtatttatacaactgg
aaacatctctctaaatgacaaccattgtatattgttaaataacttatagt
cagtggaattctgtgggtttttctttcctacaggcaggaaattatggtga
tgtgttgatatgattatatatgtagactattcagttaacatctaggagtt
agcaaatttgctacattcacaatcagtatgtaagtcagttgtattcctag
atatcagcaactaacagaaatggaagtttttagaaagatctcttcaatat
cattaaaaaatacaaaatactctacctaagaataagtctaacaaaagatg
tacaagatcttgatggagaaaatgttagaccatgtcaggaagcatttaag
gagatcttaaataaatgtagtactccataatgtacacgaattagaagact
cagtcgtgtaaagatgttcatttacatttgtagattcaatgtaatcccaa
ccaaaatatttttttgcattattacctctatctactgtgtgtttccttaag
acaagggcattcctacgtaaccatagtacaaccatcaaacttaggagatt
gacattgacatcatgatatctaatccatagaccacattcaatctgctgat

tttgattgcctcagtaatgttcctgtgtttcagcatcccaatccaggacc
acatagtgcatttagttgccctatctcttgtttcattcagtccagaacat
cttttcagacttgtctttgaccttgacacttgaggaggattggccagttt
gtagaaagttcctcagttttggtttatcattagataagacccataaggtt
taggatagacatttttgtccaatactacagaaatgatgctctatactcaa
atgtgtcatgccaggatgcacacaatgtcaatttgtcttatacctgatag
tattaattttggtcacttggttcagatagtatataccaggtttctccatt
gtaaaattagaattaggaagtaatttgtgaggagattcttaaagattgta
aatgtattgtttctcatcgaattgtcacgggctagtctattgacaattct
tgactgaaccaattactattttggttgtcaaatggtgagtccgtggcttc
tgccttggttggcatcctactactgtaaggaagagctcttccttctctat
tccttcattcacttaaatcagtatagattcatgaatgcctgctttattca
gtgacagtgtattattgtcacttattttcatgctgaaattttccccgatt
tggtttttgggagtctcttcaagctggcttctgtgttccttagagatgcc
tcattgctttttcccctcttattttttttacgagctcatcttacactttt
tcctactccctaccccaagtctagaattagcccttttttccaaggagccc
tggtttctttaaatgggaaatggtatatggaaatgaagacctgagaaagg
ctcactgtcttattggtgaacagctcaaaggaaaacgtgtgtctacacac
attcaagaatagtcacctacttctatatctatttatatacatacgtattg
aaaactttgagttcacattgacaccttcaattcaatcctttgaggttaat
ctagtctccctttccatctttgtaacttccttctctcatagaaacctgac
tcccataatcctaaataatttgttcattcttcctccctccctcaacctct
tgacatgccagctgctgtctcatcacacccagtcccaaatatgccaaata
aatccgcagtcatagcagcgctggtcctgaccacaccaatccctaacatc
ctccccagcctggagcctggaccatggcagctgaatctttgaccccagtg
atgtcagtcctgagcacaccattggaatcctaggctcctgtcatctggct
ggaagagagacaaattcacaaccttttgataatgagcaattattgaatac
tcactgaatttatcagagaaagtttaaaagtgtacgattatacatattta
ccaggaatgtttaaacatagacatttatgaaatttatagaaaatattgaa
ttgtggttctcactttttggtcttggctgtaacaatttttaaatattaat
acttcctacaataactgtatcatgctatttttaagtgtcctcacactcaa
ccaatctactctgcatccataaaataagcatgcagttcagaaaatagatt
aaacgactaattggctaaaaa

>NM_000274 3
ttgggggcggggcagaatcagcctttaagttgcagtgactctccggcgtc
actgttgcgcttcatagacgccgcgtgtacccggttgtcctcaggcgctg
tcagatctgtggtttttctacttgaaggacacaatgttttccaaactagc
acatttgcagaggtttgctgtacttagtcgcggagttcattcttcagtgg
cttctgctacatctgttgcaactaaaaaaacagtccaaggccctccaacc
tctgatgacatttttgaaagggaatataagtatggtgcacacaactacca
tcctttacctgtagccctggagagaggaaaaggtatttacttatgggatg
tagaaggcagaaaatattttgacttcctgagttcttacagtgctgtcaac
caagggcattgtcaccccaagattgtgaatgctctgaagagtcaagtgga
caaattgaccttaacatctagagctttctataataacgtacttggtgaat

atgaggagtatattactaaacttttcaactaccacaaagttcttcctatg
aatacaggagtggaggctggagagactgcctgtaaactagctcgtaagtg
gggctataccgtgaagggcattcagaaatacaaagcaaagattgtttttg
cagctgggaacttctggggtaggacgttgtctgctatctccagttccaca
gacccaaccagttacgatggttttggaccatttatgccgggattcgacat
cattccctataatgatctgcccgcactggagcgtgctcttcaggatccaa
atgtggctgcgttcatggtagaaccaattcagggtgaagcaggcgttgtt
gttccggatccaggttacctaatgggagtgcgagagctctgcaccaggca
ccaggttctctttattgctgatgaaatacagacaggattggccagaactg
gtagatggctggctgttgattatgaaaatgtcagacctgatatagtcctc
cttggaaaggccctttctgggggcttataccctgtgtctgcagtgctgtg
tgatgatgacatcatgctgaccattaagccaggggagcatgggtccacat
acggtggcaatccactaggctgccgagtggccatcgcagcccttgaggtt
ttagaagaagaaaaccttgctgaaaatgcagacaaattgggcattatctt
gagaaatgaactcatgaagctaccttctgatgttgtaactgccgtaagag
gaaaaggattattaaacgctattgtcattaaagaaaccaaagattgggat
gcttggaaggtgtgtctacgacttcgagataatggacttctggccaagcc
aacccatggcgacattatcaggtttgcgcctccgctggtgatcaaggagg
atgagcttcgagagtccattgaaattattaacaagaccatcttgtctttc
tgagggtagccagctgttttcagtggtccctgggagccagctggagacag
gtggtcctgtaaaagctttattcctaatgtgggcacattccactcccatg
agtcttcaaaaactttttttttgaatatatttttttcagttgatacataa
tagaacaacgtttatgaacctgccgtttgctttgtaacgtaactaaataa
tgtaatggcatctatattcagttgaagtgttttgatgtgcatgtgtactt
cctaaggtgaaatgcatctatatacagacagcctctaaatcaagtccttc
agtataattgatatatgtttttataatttcctcactggtataagtgtttc
atatttgaaaaagttatctctgggtattgcataaaaggcttcatcttata
aagtgaaatcattgttattgaattttaggaaggattaatggttaagtgta
tataaaatactaatattaagtaaacttcatattggccaacaccagggttg
tattctatggatgtcattattttgaattaagaattagtgtttaacattcc
taaattgttttgagtgcttgattataatttgtaaaaaatgtttattttca
atacttctttaaatttaaaataaagcttatatttcaaatgtcaaaaaaaa
aa

>NM_014937 3
ggggagaggcctctacggccgccgctgccgccgccgctgccggggcgcgt
tctcctcctaccggtcgggtgccccggggcgttccctctgccgctgcttc
tcggcgcggttcctacccggccgctccccgaggcgcgggctctggcggcc
tcgaccgactaggacgccccgtgcgccgcccgcgggccgccgcctccctg
ggcgcgcggggccagcatggagctcttccaagccaaggaccactacatcc
tgcagcagggcgagcgcgcgctgtggtgcagccgccgcgacggcggcctc
cagctccgacccgctactgatctacttcttgcctggaatcccatttgttt
ggggttggtagaaggtgttattgggaaaattcaacttcattcagatcttc
catggtggcttattctaattcggcagaaagcattggtgggcaaactccca
ggagaccatgaggtctgtaaagttaccaaaattgctgtgctctcactttc

tgaaatggaacctcaggatcttgagctagagctctgtaagaagcatcatt
ttggtattaacaaaccagagaagatcataccatctcctgatgactcaaag
tttctactgaagacctttacgcatattaaatccaatgtgtctgctcctaa
taaaaagaaagttaaggaaagtaaagagaaggagaagttggagaggagat
tacttgaagagttgctgaagatgttcatggactcagaatccttttattat
agcttgacctatgacctgaccaattccgtgcagaggcagagcactgggga
gagggacggtcggcccctctggcagaaggttgatgaccgatttttttgga
ataaatacatgatacaagatcttactgagattggtactccagatgtggac
ttttggattatccccatgatccaaggttttgtgcagattgaagaacttgt
ggttaattataccgaatcatctgatgatgagaaaagcagcccagagaccc
cccctcaggagtccacctgtgtagatgatattcacccacgatttctagtg
gctctcatttcacgccgaagtaggcacagagcaggaatgcgctataaacg
aagaggagtggataaaaatggaaatgttgccaattatgtggagactgagc
agttgattcatgttcataatcataccctgtcatttgttcaaacacgaggc
tctgtgcctgtcttttggagccaggttgggtatcgatataacccaagacc
gcggctggacagaagtgaaaaggaaactgttgcctatttctgtgcccatt
tcgaagaacaactgaacatttacaaaaaacaggttattattaacttggta
gaccaggcaggaagagagaagattattggcgatgcttacctgaagcaagt
gttgcttttcaacaactcacacctcacttacgtttcgtttgacttccatg
agcactgccgaggaatgaagtttgagaatgttcagacactaacagatgcc
atttatgacattattcttgatatgaagtggtgttgggttgatgaagctgg
ggtaatatgtaagcaggaagggatttttcgtgttaattgtatggactgcc
tggatcgcaccaacgtggtccaagctgccatcgcgagagtggtcatggaa
cagcagctgaaaaaattaggtgtgatgcccccggaacagccattacctgt
gaaatgtaatcgcatctaccagataatgtgggccaataatggtgactcca
ttagcagacagtatgctgggacagctgctctgaagggtgactttacaagg
acaggagaaaggaagttagcaggagttatgaaagatggagtgaactcagc
aaacagatattacctcaaccgatttaaggatgcttataggcaagctgtta
tagatttgatgcaaggcattccagtgacagaagatctttattccatattt
accaaggagaaagaacatgaagctttgcataaggaaaatcagagaagcca
ccaggaactaattagccagctcttacaaagttacatgaagttactactgc
ctgatgatgagaagttccatgggggctgggccctcattgactgtgaccct
agcctcattgatgctactcacagagacgtggatgtgctgttactgctttc
taactctgcctactacgtggcctattatgatgatgaagttgataaagtaa
accagtatcaacgactaagtctagaaaacctggaaaaaattgaaataggc
cctgaacccactctttttggtaagccaaagttctcctgcatgcgactgca
ctacagatacaaagaagcgagtggctatttccacacattgcgagctgtaa
tgcgtaatcctgaagaggatggaaaagatacccttcagtgcattgcagag
atgctgcagatcaccaagcaagccatgggatcggatttacccataattga
gaagaaacttgagaggaagagcagtaaacctcacgaagacatcattggta
tcaggtctcaaaaccaaggttctttggcccagggaaagaatttttaatg
agcaaattttcatctctaaatcaaaaagtgaagcagaccaaatccaatgt
aaatattggcaacctccgaaagctaggaaactttaccaaacctgaaatga
aagttaactttctaaaaccaaacttaaaagtaaatctttggaaatcagat
agtagtcttgaaactatggaaaacacaggagtgatggataaggttcaggc
agagtctgatggggacatgtcttcagataatgactcataccactctgatg

aattccttacaaattctaagtctgatgaagacaggcagctagctaactca
ttagagagtgtagggccaatagattacgttcttcctagttgtggtattat
tgcctcagcgcctcgattgggcagtcggtcccagtctcttagcagcacag
atagtagcgttcatgctccttcagagattactgttgctcatgggagtggg
cttggaaaaggccaggagtctcctttgaagaaaagtccttctgctggcga
cgtacacatattgactggctttgccaagcctatggatatttactgccaca
gatttgtgcaagatgcacagaacaaagtgacccacctatcagagaccaga
tctgtgtctcagcaggctagtcaggaaagaaatcaaatgaccaatcaagt
ttcaaatgaaacccaatcagaatcaacagaacagacaccttctcggccat
cgcaattagatgtctctctttctgcaacaggcccacagtttttgtcagtt
gagccagcgcattcagttgcatctcaaaaaacccccacctccgcttccag
catgcttgaacttgagacagggcttcatgtaactccttctccttcagaga
gcagtagcagcagagcagtctctccctttgccaagattcgaagttccatg
gtccaggttgctagtattacccaagctggattaacccatgggataaactt
tgcagtgtcaaaagttcagaagagtcctccagaacctgaaatcattaatc
aagtccagcaaaatgaacttaaaaagatgtttatacaatgccagacacgg
ataattcagatttagcttttagccataagaatccttccatggcttttatt
taaaaatatgaaattttcacctcttggggtattttaattgtactgtctga
acccagggatcacaaattctgttcattggaaagggttttaaacggagtcg
gaacctgagtagatttccaaattttacagccaggactacagaagtgcatc
attctagaatgtgtagacctgagtagcttatacactacagagcactttgc
ttatttgaaagtaattcagcaacaggtcactttgggatataacctgaacc
tttttttggagtggggtgggtagactacagtagacacaagggctggacat
gcagatgcttaggggattagcgtttttcataatttgttctgtttgtcagt
tcattcctgtgtgttcttacctctacaaagtaaattacacatttagtttt
tagtgactttaacatgttactgaagcatttgaatataaagctattttagt
tttgatggcttaactgttccctgaggagttgagggttattgacactagag
aaatgaattctcatttgatcctaattttccccgtattctacttgaacaca
ttaaaaatactctgctgcctatacaatgtaaacctaggagcattaagact
tgtcacacagtaaacctgatacatcagaggtgaataccagcacctattag
gtttcattttgctgttttcaggaatgtaagaacacccatattggctactg
gaaattctagaagtcagtcaggttttaatttattccaggaggggcatcct
cgacatcttatgtagatgatccacaacttcaaaatttagtctgggcctag
tgcagtggctcacacctataatcccaacactttgggaggccaggagtttt
gagaccagcctgggaaacatctgtctctacaaaaaaatacaaaaattagc
tgggcatagtggtgcatgcctgtgtttctagctacgcaggaggattgctt
gagcccatgagattgaggctgcagtgagctgtgatcgtgccactgacctc
cagcctgggggacagagcaagaccgtgtctcaaaaacaatttagtctgaa
acacaattgtgctgaatctgtctgactataactctgaccacacagaacca
gggctgcccctgtaatccccacagtaagaaagttgtatggcatattccaa
caagtattggttcgtctggtgtctttagagctttactctgttgaagtgac
tgattctcaactgaacattatgtcgttactttgataagcattccactttt
gttatttattagtgctatcttttttttttacgtgttaaatcttgtgatta
ttaaaataaagtaccattgtaatttaaagtgacaaa

>NM_014408 3

gctgaggggcagcggcttaggctccggcgtctgcaggggtcgccgagcta
acccgtggctaggcgagtggggcggggcggccggcaccatgtcgaggcag
gcgaaccgtggcaccgagagcaagaaaatgagctctgagctcttcaccct
gacctatggtgccctggtcacccagctatgtaaggactatgaaaatgatg
aagatgtgaataaacagctggacaaaatgggctttaacattggagtccgg
ctgattgaagatttcttggctcggtcaaatgttgggaggtgccatgactt
tcgggaaactgcggatgtcattgccaaggtggcgttcaagatgtacttgg
gcatcactccaagcattactaattggagcccagctggtgatgaattctcc
ctcattttggaaaataaccccttggtggactttgtggaacttcctgataa
ccactcatcccttatttattccaatctcttgtgtggggtgttgcggggag
ctttggagatggtccagatggctgtggaggccaagtttgtccaggacacc
ctgaaaggagacggtgtgacagaaatccggatgagattcatcaggcggat
tgaggacaatcttccagctggagaggaataaccatccctacaactcgagg
atagccatcaggagcactgttggaatcagcaggcctctgtgctccctctg
ccctccagaactcagtgactcttgaacatggatgttatatattcttataa
cctgtttccattctccattcaaataaagagcagactgcgatatagtccat
ttaccccatgtgtgcacattcaggagcgacagtctctgcccccattccct
tgagaggggctggatgtaatcacctttggttggactagaaagagctcaaa
ccattttacattcctgtttgaatttttccaaagcaaaactcactttgacc
ccattaagaggcaagcctggcacatctatccctgggcctttagaaagcca
tttgcctcaaatggctatagggttgtggggtggagggaggaagggctggg
agggagtggggaggaattgctagctgtagtgtgacacattgtagtgtttg
ccaggaaaggagccagtcatgccggaaacactgacttctgggaagccacc
caggtctcattcctccctgctgttggaggcaacatctcctctttttacag
agggtacatcctttttttcttacaaattcttcaataaagacacattcttga
gtgaaatcccaaaaaaaaaaaaaaaaaa

>NM_013412 2
gcagctgcgaggacgcggggcgagccggaagtggagtgcgctgcggtgcg
agctgggccggcggggtggttcgagagcgcgcagagtccagactggcggc
agggcccgaggggccgacccgcagcgtccctggtctctccagccctcact
cggaaccgcactgacaataccctcccctcccttgggctggacccctctct
acagctaggagccaatggcagaagacaaaaccaaaccgagtgagttggac
caagggaagtatgatgctgatgacaacgtgaagatcatctgcctgggaga
cagcgcagtgggcaaatccaaactcatggagagatttctcatggatggct
ttcagccacagcagctgtccacgtacgccctgaccctgtacaagcacaca
gccacggtagatggcaagaccatccttgtggacttttgggacacggcagg
ccaggagcggttccagagcatgcatgcctcctactaccacaaggcccatg
cctgcatcatggtgtttgatatacagaggaaagtcacctataggaacctg
agcacctggtatacagagcttcgggagttcaggccagagatcccatgcat
cgtggtggccaataaaattgatgacataaacgtgacccaaaaaagcttca
attttgccaagaagttctccctgcccctgtatttcgtctcggctgctgat
ggtaccaatgttgtgaagctcttcaatgatgcaattcgattagctgtgtc
ttacaaacagaactcccaggacttcatggatgagatttttcaggagctcg
agaacttcagcttggagcaggaagaggaggacgtgccagaccaggaacag
agcagcagcatcgagaccccatcagaggaggtggcctctccccacagctg

aggggctggggctaggggtgggtggagcccttttaaataccсttccctt
caacaactctccagctctgaatggagaaactctctaggccatcccctctt
ctacctcctgcaacccacccatcctattagcctcccacattcaaggcccg
tgatacagggatgaggtcagcaccagcaaactctggactggtggaagaat
tccccaccagatctccttgaagcagaattagggatcagcatcattaacac
cttccccacccccctccccccaggcagacagtgaagagaatcagaaaacat
gattatgtgtcactttaatacaggaaatttaggtgtttttggtgttttt
gtttttgttttctttccaaagctcacctcggggacaattccttgggcttc
tcctgaggtaatgattacccccccacccacagctgagtctgtgaggcccc
atcctttccctacgttttctcccatctttttcctcttcaatctcccagt
catctggtttgtttgtttctttgttcgtcctgagacggagtctcgctctg
tcgccaggctggagtgcagtggcgcagtctcggctcgctgcaacctctga
ctccctggttcaaacgattctcctgcctcagcctcccgagtggctggcat
caccacgcccagctaatttttgtatttttagtagagacggggtttcacca
tgttgcccaggatggtctcgatctcctcacctcgtgatccgcccgcctcg
gcctcccaaagtgctgggattacaggcatgagccaccgcgcccggcccca
atcatctgtttttaaacaatcgtttttgagcagatagctattcattccag
atttccgtgtacccactctgtttcaggagctcttctaggtaaagctgaga
tcacaggaacagcaggtgacaggcctagctatagttaggaatacacaagc
ggtaaaatcgagtccttacagccataccacaaggtacgtccatttggact
acaagaagagcttcctttaaagttcctatttcagcataaagaggctgtcc
ttttttttaggaatagtttggaccttgtgcctcctgtgggaggctgagg
actgcaagaggagagctagcagatatgcctgttcacccctctctggtact
tgtggcttgctagtatgtttttatgataatctcgggcattgtttgcattg
tgtttattaatagggttttgtttttattgtttcctttttacagtaaagg
ctgaatgacataaacattgaaaaaaaaaaaaaaaaaa

>NM_005034 3
gcactgtgcgtcatgtaccagcgccggaagttggtctcgacacctggact
agccgggttgtatttggaaacgcggagtgagtttttccgtgctgtgtagg
ggctaacaatggacacccagaaggacgttcaacctccaaagcagcaacca
atgatatatatctgtggagagtgtcacacagaaaatgaaataaaatctag
ggatccaatcagatgcagagaatgtggatacagaataatgtacaagaaaa
ggactaaaagattggtcgtttttgatgctcgatgaatgctgggaattcag
aggaatgtcttcacttatacttggatttgctctcttcccatttctgattg
ttgtatagctttcgattttgcttacagtagttccccсttatcttcgggag
atacattccaaggcccccagtgaactcctgaaacctcaaacagtaccaaa
cctttatacactgtttttccatatatatatacctatgataaagtataat
gtataaattaagcatagcaagagattaataataatgtaatagaacaatga
taacatactataataaaagttatgtgaatgtggttggtctctcttgcttt
caaaatatcttcttgtacagtactcacctattttagaatgtggttgacta
caggtaaccaaaaccacagaaagggaaactttggatgagggggcactac
tgtacttaggaatacaactatatacatatgattttatttttaagaccata
ttatatttgggtatctactaatattttgtataaagcaatttttgttcca
ttacgtgactttttgttttattgtatatgtaatttaacacacaataaagg
gtaaagttgcttccccaaaccacacttttaatcaaaacctagaatcatct

gcagtccttgttaaaaatgcaggtttctagaaccctctgaagttctgatt
aaataaatttattgcaaacca

>NM_002639 4
agtgggcgtggcggtgctgcccaggtgagccaccgctgcttctgcccaga
cacggtcgcctccacatccaggtctttgtgctcctcgcttgcctgttcct
tttccacgcattttccaggataactgtgactccaggcccgcaatggatgc
cctgcaactagcaaattcggcttttgccgttgatctgttcaaacaactat
gtgaaaaggagccactgggcaatgtcctcttctctccaatctgtctctcc
acctctctgtcacttgctcaagtgggtgctaaaggtgacactgcaaatga
aattggacaggttcttcattttgaaaatgtcaaagatgtaccctttggat
ttcaaacagtaacatcggatgtaaacaaacttagttccttttactcactg
aaactaatcaagcggctctacgtagacaaatctctgaatctttctacaga
gttcatcagctctacgaagagaccgtatgcaaaggaattggaaactgttg
acttcaaagataaattggaagaaacgaaaggtcagatcaacaactcaatt
aaggatctcacagatggccactttgagaacattttagctgacaacagtgt
gaacgaccagaccaaaatccttgtggttaatgctgcctactttgttggca
agtggatgaagaaattttctgaatcagaaacaaaagaatgtcctttcaga
gtcaacaagacagacaccaaaccagtgcagatgatgaacatggaggccac
gttctgtatgggaaacattgacagtatcaattgtaagatcatagagcttc
cttttcaaaataagcatctcagcatgttcatcctactacccaaggatgtg
gaggatgagtccacaggcttggagaagattgaaaaacaactcaactcaga
gtcactgtcacagtggactaatcccagcaccatggccaatgccaaggtca
aactctccattccaaaatttaaggtggaaaagatgattgatcccaaggct
tgtctggaaaatctagggctgaaacatatcttcagtgaagacacatctga
tttctctggaatgtcagagaccaagggagtggccctatcaaatgttatcc
acaaagtgtgcttagaaataactgaagatggtggggattccatagaggtg
ccaggagcacggatcctgcagcacaaggatgaattgaatgctgaccatcc
ctttatttacatcatcaggcacaacaaaactcgaaacatcattttctttg
gcaaattctgttctccttaagtggcatagcccatgttaagtcctccctga
cttttctgtggatgccgatttctgtaaactctgcatccagagattcattt
tctagatacaataaattgctaatgttgctggatcaggaagccgccagtac
ttgtcatatgtagccttcacacagatagaccttttttttttttccaattc
tatcttttgtttccttttttcccataagacaatgacatacgcttttaatg
aaaaggaatcacgttagaggaaaaatatttattcattatttgtcaaattg
tccggggtagttggcagaaatacagtcttccacaaagaaaattcctataa
ggaagatttggaagctcttcttcccagcactatgctttccttctttggga
tagagaatgttccagacattctcgcttccctgaaagactgaagaaagtgt
agtgcatgggacccacgaaactgccctggctccagtgaaacttgggcaca
tgctcaggctactataggtccagaagtccttatgttaagccctggcaggc
aggtgtttattaaaattctgaattttggggattttcaaaagataatattt
tacatacactgtatgttatagaacttcatggatcagatctggggcagcac
cctataaatcaacaccttaatatgctgcaacaaaatgtagaatattcaga
caaaatggatacataaagactaagtagcccataaggggtcaaaatttgct
gccaaatgcgtatgccaccaacttacaaaaacacttcgttcgcagagctt
ttcagattgtggaatgttggataaggaattatagacctctagtagctgaa

atgcaagaccccaagaggaagttcagatcttaatataaattcactttcat
ttttgatagctgtcccatctggtcatttggttggcactagactggtggca
ggggcttctagctgacttgcacagggattctcacaatagccgatatcaga
atttgtgttgaaggaacttgtctcttcatctaatatgatagcgggaaaag
gagaggaaactactgcctttagaaaatataagtaaagtgattaaagtgct
cacgttaccttgacacatagtttttcagtctatgggtttagttactttag
atggcaagcatgtaacttatattaatagtaatttgtaaagttggttggat
aagctatccgtgttgcaggttcatggattacttctctataaaaaatatgt
atttaccaaaaattttgtgacattccttctcccatctcttccttgacctg
cattgtaaataggttcttcttgttctgagattcaatattgaatttttcct
atgctattgacaataaaatattattgaactaca

>NM_018319 3
actgcgcgcatgcgcggcggccgccgaaggggcgggatccgaggcaagcg
ttggttctgtgcgcctcagagttggagcacacagctgtattaaaaaggca
aatcgaaggccgggcgcggtgactcacgcctgtcatcctagcactttggg
aggccgaggcggctgaatcacttgaggttaggagtttgagatcagcccgg
gcaacatggtgaaaccccgtctctacaaaaatagaaaaattagccgagcg
tgatggtggatgcctgtaatcctagctcctcgggaggctaaggagtataa
tgtctcaggaaggcgattatgggaggtggaccatatctagtagtgatgaa
agtgaggaagaaaagccaaaaccagacaagccatctacctcttctcttct
ctgtgccaggcaaggagcagcaaatgagcccaggtacacctgttccgagg
cccagaaagctgcacacaagaggaaaatatcacctgtgaaattcagcaat
acagattcagttttacctcccaaaaggcagaaaagcggttcccaggagga
cctcggctggtgtctgtccagcagtgatgatgagctgcaaccagaaatgc
cgcagaagcaggctgagaaagtggtgatcaaaaaggagaaagacatctct
gctcccaatgacggcactgcccaaagaactgaaaatcatggcgctcccgc
ctgccacaggctcaaagaggaggaagacgagtatgagacatcaggggagg
gccaggacatttgggacatgctggataaagggaaccccttccagttttac
ctcactagagtctctggagttaagccaaagtataactctggagccctcca
catcaaggatattttatctcctttatttgggacgcttgtttcttcagctc
agtttaactactgctttgacgtggactggctcgtaaaacagtatccacca
gagttcaggaagaagccaatcctgcttgtgcatggtgataagcgagaggc
taaggctcacctccatgcccaggccaagccttacgagaacatctctctct
gccaggcaaagttggatattgcgtttggaacacaccacacgaaaatgatg
ctgctgctctatgaagaaggcctccgggttgtcatacacacctccaacct
catccatgctgactggcaccagaaaactcaaggaatatggttgagcccct
tatacccacgaattgctgatggaacccacaaatctggagagtcgccaaca
cattttaaagctgatctcatcagttacttgatggcttataatgccccttc
tctcaaggagtggatagatgtcattcacaagcacgatctctctgaaacaa
atgtttatcttattggttcaaccccaggacgctttcaaggaagtcaaaaa
gataattggggacattttagacttaagaagcttctgaaagaccatgcctc
atccatgcctaacgcagagtcctggcctgtcgtaggtcagttttcaagcg
ttggctccttgggagccgatgaatcaaagtggttatgttctgagtttaaa
gagagcatgctgacactggggaaggaaagcaagactccaggaaaaagctc
tgttcctctttacttgatctatccttctgtggaaaatgtgcggaccagtt

tagaaggatatcctgctgggggctctcttccctatagcatccagacagct
gaaaaacagaattggctgcattcctattttcacaaatggtcagctgagac
ttctggccgcagcaatgccatgccacatattaagacatatatgaggcctt
ctccagacttcagtaaaattgcttggttccttgtcacaagcgcaaatctg
tccaaggctgcctggggagcattggagaagaatggcacccagctgatgat
ccgctcctacgagctcggggtccttttcctcccttcagcatttggtctag
acagtttcaaagtgaaacagaagttcttcgctggcagccaggagccaatg
gccacctttcctgtgccatatgatttgcctccagaactgtatggaagtaa
agatcggccatggatatggaacattccttatgtcaaagcaccggatacgc
atgggaacatgtgggtgccctcctgagaatcttgaggcactgtgaaattt
aagtgtaagacattgagccacaaacatggaatctcttctttgtactggat
gtccacttcccttaaagtcttatttgcacccttacaaaatctttccaaag
gtcactcttatgaatggatgttggttatacttttaatggacattaacatt
cctaataaagtattagtttcttaattcacttttatatgttttggaaagaa
aattagtgaacttctctatgttaaaaatacgtactgcttgagtatcccct
gtctgaaatgcttgggaccagaagtgtttcagcttttggatttttttgaa
ttttggaatatttgcatagcataatgagatatcttgggaatgggacccaa
atctaaacacaaaattcatttatgtttcatatacaccttatatacaataa
cctaaaggtgattttatatgatattttgagtaattttatgcatgaaacaa
agttttgacaggcttttgaccgtgattcatcacatgagttcaggcatgga
aattttcatttggagcatcatgtcagcactcaaaaagttctggatcttgg
agcagttcagattttcagattagggatgctcaaatctatatagatataaa
attatcctcacagtaacatagaatctcttggtgctgtcagctgttgggaa
ttgaagattgactttgtgcttccaccctccatccagaaaggcacccttca
ttccaccagaactttacccaggaagaacacgatcatttcctttttcaccg
atgccctctctcagctttctgagtacgtctcttggggtcgctggaggtga
tcctaggatctgtctctgagaccaatgtgctgtttcagccccctgcagct
aagaattgtattgactgtcctcacagcggcttttcatagctttcagcttc
agctttacgaggcttctcctctctccctggcaccctgctggctgcctcac
tgcttacagacaggtcccaccaaacccaaacacctgcctagggtaaatgg
gtctctcttctatccccagaaactttcagaggaagcagctcatagaaaca
tacaaaagcacacaagtattttgggaaaaaatcctaaaaggtgacttaat
ttgatgccttaaattcacaagtgaggaagctaaggcctagaaggttaagg
atgtccccagggtcacacagtgagcggggctcagagcttgagtgtctttg
tgctttgtgtacattgtgttctccctagggtgctttagaccctgtttgtt
ttcttctgcatgaggctgatttccagtttgtcatcaacctctttatctta
taatttaggatagagttgaacgttagtcttgaaagattttctaaagtagt
ctttcaaactgttcctcagaggcctaggattttccaaaagtaccttagga
accttgtaggctgcagtgggggtgtggcgatagagcaggaggcagggaga
cagggctgcagggcctcccaccttccaacagacaggctctgctgtatctg
ttgtacatactgggattctgtaaaggacattatctggggtgttgtatgta
tttttgtgtgttctgctttttttaaataaacttgaaaagctactgaaaaa
aaaaaaaaaaaaa

>NM_000270 3
ataagccagagcctagaccagtgagccaactgtgcgaaccagacccggca

gccttgctcagttcagcatagcggagcggatccgatcggatcggagcgga
tcggagcacaccggagcaggctcatcgagaaggcgtctgcgagaccatgg
agaacggatacacctatgaagattataagaacactgcagaatggcttctg
tctcacactaagcaccgacctcaagttgcaataatctgtggttctggatt
aggaggtctgactgataaattaactcaggcccagatctttgactacggtg
aaatccccaactttccccgaagtacagtgccaggtcatgctggccgactg
gtgtttgggttcctgaatggcagggcctgtgtgatgatgcagggcaggtt
ccacatgtatgaagggtacccactctggaaggtgacattcccagtgaggg
ttttccaccttctgggtgtggacaccctggtagtcaccaatgcagcagga
gggctgaaccccaagtttgaggttggagatatcatgctgatccgtgacca
tatcaacctacctggtttcagtggtcagaaccctctcagagggcccaatg
atgaaaggtttggagatcgtttccctgccatgtctgatgcctacgaccgg
actatgaggcagagggctctcagtacctggaaacaaatgggggagcaacg
tgagctacaggaaggcacctatgtgatggtggcaggccccagctttgaga
ctgtggcagaatgtcgtgtgctgcagaagctgggagcagacgctgttggc
atgagtacagtaccagaagttatcgttgcacggcactgtggacttcgagt
ctttggcttctcactcatcactaacaaggtcatcatggattatgaaagcc
tggagaaggccaaccatgaagaagtcttagcagctggcaaacaagctgca
cagaaattggaacagtttgtctccattcttatggccagcattccactccc
tgacaaagccagttgacctgccttggagtcgtctggcatctcccacacaa
gacccaagtagctgctaccttctttggccccttgctggagtcatgtgcct
ctgtccttaggttgtagcagaaaggaaaagattcctgtccttcacctttc
ccactttcttctaccagacccttctggtgccagatcctcttctcaaagct
gggattacaggtgtgagcatagtgagaccttggcgctacaaaataaagct
gttctcattcctgttctttcttacacaagagctggagcccgtgccctacc
acacatctgtggagatgcccaggatttgactcgggccttagaactttgca
tagcagctgctactagctctttgagataatacattccgaggggctcagtt
ctgccttatctaaatcaccagagaccaaacaaggactaatccaatacctc
ttggattttatttaatgtcataatgttgtcagaataaagagaaagatgaa
ataatttcatttttttgtgtaacttggtatggggctggggcacagaccaa
gattgacatgaaaggatgtgagatcgcatgtcttgtgtgactatctgctt
ctcagacaagcagttaggaactgagatgagatagtatgtgagggcagcaa
aggatgaagaagggcaaaatgatgaaaggtgaggtggaaagaggttatga
gatggtaaagaaaagttaacttctggcacttgattgccacttctgtcagg
ctggtcctgcctctctcccttgccttctgattgtttcatttcctgtttat
ttgatcatatctgaattagttcactggttagcctcttccttagttcccac
ttccttaccaaagccctaattatatttcctcttgtttgccttttctctcc
tactcttctctaacatctgcagccacactctccattcactccatgctgac
aaggcagtggcaaacacttttctctgctgccagccactccactgttgact
ggattgctgccagccccaggcaaacctgtgaagttgtttcatactctgct
tctctttgagtgcctccttctctccttcctcttcctttctgggctccagt
ctttctcttcacttgtgcttgtcagaacctccctgtgatactgcctccag
gcatttcccccatgttggctcaccgcactattatctttgcttatcaactt
gcattcagctggctggcatgtttcaaaaccacactgccctcccaggcctg
tgtgccttttgagaaagaccagtgctggatgagcctctagtaatgacaac
attttagttgttagtggtataatacggaagagatattttgcacaggctgc

tttggagaactttcaaattatcctttgtttggtaactgacctacttaact
gcccaatacaaagaaaaagcaaaaaaaaaaaaaaaaa

>NM_018413 5
aggcgccctcccctcgccgggggccgcgagttgcatttggtaaaacccag
ccccggaatatatagatcgttggagcgcaatgaagtagcctttggagaga
agggagagggcccgtcggacagccacagcggccagcgcagcggcagcggc
ggcggcaccaccatcaccgctcgcaccccagccgcccggcccgcgaccag
gcagcggcggccgccggcgggatcggaggaggcggcggagcggcgaggag
gaggagcaggagcgcgcagccagcgggtccacgcatctcagcacttccag
accaactccggcaccttccacacccctgcccgggctgggggctccgagag
cggccgcgaagcgactccgatcctccctctgagccttgctcagctctgcc
ccgcgcctcccgggctccggtccgcgcggcggggtccctgctcctgcgcc
ccgggcgcgcttcccggacaccccggtccccgcagccaggacaaagccat
gaagccagcgctgctggaagtgatgaggatgaacagaatctgccggatgg
tgctggccacttgcttgggatcctttatcctggtcatcttctatttccaa
agtatgttgcacccagtcatgcggaggaatccctttggtgtggacatctg
ctgccggaaggggtcccgaagccccctgcaggaactctacaacccaatcc
agctggagctctcaaacactgctgtcctgcaccagatgcggcgggaccag
gtgacagacacgtgccgagccaacagcgccacaagccgtaagcggagggt
gctgacccccaacgacctgaagcacttggtggtggatgaggaccacgagc
tcatctactgctacgtgcccaaggtggcctgcaccaactggaagcggctc
atgatggtcctgaccgggcgggggaagtacagcgaccccatggagatccc
ggccaacgaggcacacgtctccgccaacctgaagaccctgaaccagtaca
gcatcccagaaatcaaccaccgcttgaaaagctacatgaagttcctgttt
gtccgggagcccttcgagaggctagtgtccgcctaccgcaacaagttcac
ccagaagtacaacatctccttccacaagcggtacggcaccaagatcatca
aacgccagcggaagaacgccacccaggaggccctgcgcaaaggggacgat
gtcaaattcgaggagtttgtggcctatctcatcgacccacacacccagcg
ggaggagcctttcaacgaacactggcaaaccgtctactcactctgccatc
cctgccacatccactatgacctcgtgggcaagtacgagacactggaagag
gattctaattacgtcctgcagctggcaggagtgggcagctacctgaagtt
ccccacctatgcaaagtctacgagaactactgatgaaatgaccacagaat
tcttccagaacatcagctcagagcaccaaacgcagctgtacgaagtctac
aaactcgatttttaatgttcaattactcagtgccaagctacctgaaatt
ggaataaagggggtggggagagggagagaatcatgctttttaatttaaga
tttttatttgtcaaaagaattatatggatattgggttattttgtaaatta
atatttctttggggatgatgctgcgagcagcatagtgagaattatttaaa
atccttcgtagggaaggacagctgtctttgcaggggaaataggatgggtc
gtccttgtctgtagaagtgaatactgcaacactgtctcaaaggtttcttg
tgttctggtgaattccatgaattgtgcattccataaattctaattaatat
tatttatagttatttaaacatggtctcattatttctttttgtggcagcaa
aattctaacatctttccagaagaaatctatgattcctgctttgcttgcta
cagctcatttgggggcatgaatgttctccttactaacctctcatggagtc
atgtgataacagccattgtcatgtgtatggatgccaccattcttgaattt
ctgctagaaataagtaggttgctattgcacttagagtcatctttgcaagg

gactcattccaaaccaattccaatcgacctgaaagtcccttgcactaaag
aaatgtgattttttttaacccaaggagaaagttggttgtaacaattttt
caccagaattagcaatctgatagaataggctttttaacaggaattttaa
actggcggtgccagttgcagtgaaggtgaagtggctattgctttatattc
gacaaatatttattgagcatctactatatactaggccctgaagatagagc
gttgaacaaacccaatagtcttggccctcaaaaagctttatgtgacatag
agcatccagctcgacaattagcaaacccctgatattattcatggacttga
catgaggtttggactattcatgagcaacagcagaaggtccatgacccta
caaatttacatttttctatggcccatatttgaaaacctcacattcggagc
aggccactttatattcgtgagtgatcccaagctcactgaccaacagaaaa
gatgctaaaaaaatgtgtcctatcatgtgcctgctccaactgatgggtaa
tgtctgcctttaagccccagaatggattctccaggcacagtgtggaagag
tgctgtggttgattagcaatgtctgccttgggtgaggaaaggggaacact
cctcaagtgcctcctactgtcatctccattcagcctagcaccacatcccc
atgtagaagtgttgtcttctactgcttgccacgtacaagtgtgaaatgcc
aaggtgagactcgtacacttggggatttgcaatggtttttacccctcatg
aaggtcagaggtggactatttgtacacatacacacatgcacaccctctca
cgtcctccagaggctgaaagactatcagcatcttggtaactgccatgcat
gagcagcaaggacagccttccataatccatgccccatttcctctggaagc
ctggaaacttttcatttcctgttccttgctaattaacatctaaacatgct
tctcatttccagccattgctgatgcttctcagttgaagtttgagccacat
ccctcttacagctagtgaatgagttggtagcagctactgtatataataat
aataatagttttaataataggggagggtgggatggggtgtgggtaagttt
tgccttttgttttgtttttgatgccgtatatagtgaaggggtgaggatat
tctaaacaaacaaaaattgaataatttattcacagaaactattaagattg
tattgtaaagctcacagcaagctcagtgggcagcagctgcaacatctcac
cggggaaattattttatttaacgtgagtgagatgtgggccggagaaggta
gctgaagctatttataaaacgttgtgtaccttcttccgagctctctcccc
tttgtgaagggcgcagcaactatacccttgatggatggagatttacgcaa
tgtgttttactgggtagagtgacagaccttggctgtccctggaattgaga
atctggaccttatttccaggcagagaacactgtcctcagaaatgggactt
ctgataatgaaacaggttgtccaacattttctaatacgcattctacagaa
cacaggttccaggaggcatcaggttggtgtgatgcagactaagggttttg
tggtcaaatatccttaagaaacaaagttaagtcagtttcctttctgcaag
gacttttcagccttcaatgtgtgctgtgcatggtgaatccccaaaaataa
tccttgagtctgtagcatttcctgaacttacatgaccaggatactctatt
ttgcagatcaccccacagagctagggttccaccgagtatactttgcttag
gttgacttagcatatctgaggtcttcaaggataaaaactgccaccccaac
acccttccattaaaaaaaaaatacaaaatagcaccacagtctccatctgg
tttatagcaacagaggtactttatttaatgaagcaatggttctaatcctg
gatactgccacggactacaattctatccctcccagagggagtggaggaag
tcttgggtggtgtggacagaaggaagagaggagagggtgagtggggtata
gggcccagggtggctccctactcctcaggctcaaaaggatgctcagtggg
aacagatgatctcttgatgagtgcttcttcagtttcatagtttggaatcg
ttcactgtgtgctttttgggggggttttcaatggaaattcacgttgctttg
catttctgtgtccgtctttggtcagttgtgcaagcctgctcactgtcatg

tgaagatggcctttcatctggcttctctctcttaagtgagaaagattgtc
cttcaggggacatgacatcaataggtttctggaatgagggactctttctc
cccgtgttttgctttgtgttcacattttcttttctaatggcattgaaact
ttaaaaaaaatggattcaactgtttttgcagaatgtagaaagtattctgt
gtccttggttaaagaaatccacttgtgaagtgtgcctggaaaatgaaagt
ttgtgtttttaaagaggaatatttgaaactgctttctatgcatgcttag
ctggagaaaagtacaggcaggcgtcccatctcccagccacttctcaaagg
tgctgctgtgttttaaagaccaggtacagccagggcagtatttgcaagga
cattcctgcttactttatccctttggttggaaagctctagatgattcccg
cagctcctccagaccccgcctccctgccctccccagctggtctgggaaga
ggtggtctgctgacctgtggtatctcagaggggacgttcctcctcctccc
tgtgcaccaggtgggctgcaccctcctgcctattcaggatgtggatgcca
caggagagcagcaggcagtggaaacttcagttgcactggttctcctggtg
gcaaaggcatgaagcacaggggtcgattaatccaggctactagaaagctc
cagagcaaagtgtgcgggtcccacaaatgcttggctggtggggtctggat
cagtgctgagatagagttggcagaagaagcagaggcactctgcttgcttt
cctagccagtcctcccctacacacacacacacacacacacacacacacac
acacaatctcagctgcgccattctgtgcaatcccagtgaccaaatccctt
ccttgcccacctctatgtcagcaggactgaccacatcactcccccgagtt
cccaccaccagcatttcctccaacctttttccatcacaaccagttagaac
cctacaggcaacaaggccttctagaatccgcttaacccttggctgataac
aggcaaatttcagtctgctacactttgttaggtccagaaggagctgccca
tactactttcttatgagcatgctcagtatggcatatggacatgtaatgtc
acatctttgtggagtgtgattttcttttttcacatatttgtatgcagtag
agagcctgttgtagaaaacgctccctgtatcttgctgtactgttaaagaa
agctgaattccacattgccaacaaaagcgtgaaaatgttcatgaaccttc
ctccaggaaaagccattcaagcctgattatttttctaagtaacttcaatt
aaattgaagaaaaaagaa

>NM_006367 3
tctccggggcgtggcgaagaggggcggagtcacgagcggggcggtgagac
ttcctgcccagtcgcgggccagcctagcgcttcagccggcggctcatttc
cggtgggggcgccgcgcccagtgagggcccggaagtgggtcgcgcggaga
ttgctgggcggttcttgccggaagcggagagcggctgatcgcagtccgga
ggtgaggcggaactctgaggtggtccattatggctgacatgcaaaatctg
gtagaaagattggagagggcagtgggccgcctggaggcagtatctcatac
ctctgacatgcaccgtgggtatgcagacagtccttcaaaagcaggagcag
ctccatatgtgcaggcatttgactcgctgcttgctggtcctgtggcagag
tacttgaagatcagtaaagagattgggggagacgtgcagaaacatgcgga
gatggtccacacaggtttgaagttggagcgagctctgttggttacagctt
ctcagtgtcaacagccagcagaaaataagctttccgatttgttggcaccc
atctcagagcagatcaaagaagtgataacctttcgggagaagaaccgagg
cagcaagttgtttaatcacctgtcagctgtcagcgaaagtatccaggccc
tgggctgggtggctatggctcccaagcctggcccttatgtgaaagaaatg
aatgatgccgccatgttttatacaaaccgagtcctcaaagagtacaaaga
tgtggataagaagcatgtagactgggtcaaagcttatttaagtatatgga

cagagctgcaggcttacattaaggagttccataccaccggactggcctgg
agcaaaacggggcctgtggcaaaagaactgagcggactgccatctggacc
ctctgccggatcaggtcctcctcccctccaccaggccccccctcctcccc
cagtctctaccagttcaggctcagatgagtctgcttcccgctcagcactg
ttcgcgcagattaatcagggggagagcattacacatgccctgaaacatgt
atctgatgacatgaagactcacaagaaccctgccctgaaggctcagagtg
gtccagtacgcagtggccccaaaccattctctgcacctaaaccccaaacc
agcccatcccccaaacgagccacaaagaaggagccagctgtacttgaact
ggagggcaagaagtggagagtggaaaatcaggaaaatgtttccaacctgg
tgattgaggacacagagctgaaacaggtggcttacatatacaagtgtgtc
aacacgacattgcaaatcaagggcaaaattaactccattacagtagataa
ctgtaagaaacttggcctggtattcgatgacgtggtgggcattgtggaga
taatcaacagtaaggatgtcaaagttcaggtaatgggtaaagtgccaacc
atatccatcaacaaaacagatggctgccatgcttacctgagcaagaattc
cctggattgtgaaatagtcagtgccaaatcttccgagatgaatgtcctca
ttcctacagaaggcggtgactttaatgaattcccagttcctgagcagttc
aagaccctatggaacgggcagaagttggtcaccacagtgacagaaattgc
tggataagcgaagtgccactgggttctttgccctcccttcacaccatggg
ataaatctgtatcaagacggttcttttctagatttcctctaccttttttgc
tcttaaaactgcttctctgctctgagaagcacagctacctgccttcactg
aaatatacctcaggctgaaatttggggtgggatagcaggtcagttgatct
tctgcaggaaggtgcagcttttccatatcagctcaaccacgccgccagtc
cattcttaaggaactgccgactaggactgatgatgcattttagctttgag
cttttggggggttattctaccaacaaacagtccattggaaagaaaacagtc
cctggaattaacagatcagaatgttcacactggttaatcttttttttaaca
atgagcatgaaggtagcagaagctggtgtgtttccagatggttcttctaa
ccaaactaatttttcactgttgacaagcgaggcaagggttgcactggacc
aaaggctgaggcttggccatctagcattccatacaaaattgtttcctata
agcattccttttattctctattctatcctgggtctgcctcaaccgtgaga
taggagagtctctggtactagctgctgtagcagtgcccttcatccagggc
agttaatggagtcttggaccctttctttctctgggatccctgcccagcac
cttcctatagagatgactttaaaaggaaaaaaaaaaaaaaaaaaaacccac
atgatttcaaggagtctggcattcctgaatccttcttccctgccaggtgc
ctgtcacctgtcttcactgcctccttttccctgtcatgctcatcagctta
tggcttctgtctaagcacctgaacagaggactgaaacctccactgcaggc
tggttttaggtcttgaattatgtaagaatcttgcacagcactgctaatgt
aaatttcagttgtttttccctctaggacaaacacttaccaaaatatgcaa
cttttttttggtgggaagagagattgtcctgtgatttctacccatttcct
gaggcctgtggaaataaacctttatgtacttaaagttatacagaaaatag
aataaagttaataccaaacttgcaaaaaaaaaaaaaaaaaaaaaaaaaa

>NM_003564
gcccttgccttgagtcagtgcgctgctctccagcccgcttgaacgctccccgcagccaccgccacccatt
ggaatggccaacaggggacctgcatatggcctgagccgggaggtgcagcagaagattgagaaacaat
atgatgcagatctggagcagatcctgatccagtggatcaccacccagtgccgaaaggatgtgggccgg

ccccagcctggacgcgagaacttccagaactggctcaaggatggcacggtgctatgtgagctcattaat
gcactgtaccccgaggggcaggccccagtaaagaagatccaggcctccaccatggccttcaagcagat
ggagcagatctctcagttcctgcaagcagctgagcgctatggcattaacaccactgacatcttccaaact
gtggacctctgggaaggaaagaacatggcctgtgtgcagcggacgctgatgaatctgggtgggctggc
agtagcccgagatgatgggctcttctctggggatcccaactggttccctaagaaatccaaggagaatcc
tcggaacttctcagataaccagctgcaagagggcaagaacgtgatcgggttacagatgggcaccaacc
gcggggcgtctcaggcaggcatgactggctacgggatgccacgccagatcctctgatcccaccccagg
ccttgcccctgccctcccacgaatggttaatatatatgtagatatatattttagcagtgacattcccagag
agccccagagctctcaagctcctttctgtcagggtgggggggttcagcctgtcctgtcacctctgaggtgc
ctgctggcatcctctcccccatgcttactaatacattcccttccccatagccatcaaaactggaccaactg
gcctcttcctttcccctgggaccaaaatttaggggcctcagtccctcaccgccatgccctggcctattctgt
ctctccttcttcccccctggcctgttctgtctctgagctctgtgtcctccgttcattccatggctgggagtcact
gatgctgcctctgccttctgatgctggactggccttgcttctacaagtatgcttctcccacagctgtggctg
caggaacttaatttatagggaggagcctgtggcagctgctgccccagccacagctgcactgactgtgct
caccacacatctggggcagccttccctggcaggggccctcgtggcttctcattttccattcccttcactgt
ggctaaggggtggggtgaggggatggagagggagggctgcctaccatggtctggggcttgaggaaga
tgagtttgttgatttaaataaagaatttgtcatttttg

>NM_006367 3
tctccggggcgtggcgaagaggggcggagtcacgagcggggcggtgagac
ttcctgcccagtcgcgggccagcctagcgcttcagccggcggctcatttc
cggtgggggcgccgcgcccagtgagggcccggaagtgggtcgcgcggaga
ttgctgggcggttcttgccggaagcggagagcggctgatcgcagtccgga
ggtgaggcggaactctgaggtggtccattatggctgacatgcaaaatctg
gtagaaagattggagagggcagtgggccgcctggaggcagtatctcatac
ctctgacatgcaccgtgggtatgcagacagtccttcaaaagcaggagcag
ctccatatgtgcaggcatttgactcgctgcttgctggtcctgtggcagag
tacttgaagatcagtaaagagattgggggagacgtgcagaaacatgcgga
gatggtccacacaggtttgaagttggagcgagctctgttggttacagctt
ctcagtgtcaacagccagcagaaaataagctttccgatttgttggcaccc
atctcagagcagatcaaagaagtgataacctttcgggagaagaaccgagg
cagcaagttgtttaatcacctgtcagctgtcagcgaaagtatccaggccc
tgggctgggtggctatggctcccaagcctggcccttatgtgaaagaaatg
aatgatgccgccatgttttatacaaaccgagtcctcaaagagtacaaaga
tgtggataagaagcatgtagactgggtcaaagcttatttaagtatatgga
cagagctgcaggcttacattaaggagttccataccaccggactggcctgg
agcaaaacggggcctgtggcaaaagaactgagcggactgccatctggacc
ctctgccggatcaggtcctcctccccctccaccaggcccccctcctcccc
cagtctctaccagttcaggctcagatgagtctgcttcccgctcagcactg
ttcgcgcagattaatcagggggagagcattacacatgccctgaaacatgt
atctgatgacatgaagactcacaagaaccctgccctgaaggctcagagtg
gtccagtacgcagtggccccaaaccattctctgcacctaaaccccaaacc
agcccatcccccaaacgagccacaaagaaggagccagctgtacttgaact
ggagggcaagaagtggagagtggaaaatcaggaaaatgtttccaacctgg
tgattgaggacacagagctgaaacaggtggcttacatatacaagtgtgtc
aacacgacattgcaaatcaagggcaaaattaactccattacagtagataa

ctgtaagaaacttggcctggtattcgatgacgtggtgggcattgtggaga
taatcaacagtaaggatgtcaaagttcaggtaatgggtaaagtgccaacc
atatccatcaacaaaacagatggctgccatgcttacctgagcaagaattc
cctggattgtgaaatagtcagtgccaaatcttccgagatgaatgtcctca
ttcctacagaaggcggtgactttaatgaattcccagttcctgagcagttc
aagaccctatggaacgggcagaagttggtcaccacagtgacagaaattgc
tggataagcgaagtgccactgggttctttgccctcccttcacaccatggg
ataaatctgtatcaagacggttcttttctagatttcctctaccttttgc
tcttaaaactgcttctctgctctgagaagcacagctacctgccttcactg
aaatatacctcaggctgaaatttggggtgggatagcaggtcagttgatct
tctgcaggaaggtgcagcttttccatatcagctcaaccacgccgccagtc
cattcttaaggaactgccgactaggactgatgatgcattttagctttgag
cttttggggggttattctaccaacaaacagtccattggaaagaaaacagtc
cctggaattaacagatcagaatgttcacactggttaatctttttttaaca
atgagcatgaaggtagcagaagctggtgtgtttccagatggttcttctaa
ccaaactaatttttcactgttgacaagcgaggcaagggttgcactggacc
aaaggctgaggcttggccatctagcattccatacaaaattgtttcctata
agcattccttttattctctattctatcctgggtctgcctcaaccgtgaga
taggagagtctctggtactagctgctgtagcagtgcccttcatccagggc
agttaatggagtcttggaccctttctttctctgggatccctgcccagcac
cttcctatagagatgactttaaaaggaaaaaaaaaaaaaaaaaaaacccac
atgatttcaaggagtctggcattcctgaatccttcttccctgccaggtgc
ctgtcacctgtcttcactgcctccttttccctgtcatgctcatcagctta
tggcttctgtctaagcacctgaacagaggactgaaacctccactgcaggc
tggttttaggtcttgaattatgtaagaatcttgcacagcactgctaatgt
aaatttcagttgtttttccctctaggacaaacacttaccaaaatatgcaa
cttttttttggtgggaagagagattgtcctgtgatttctacccatttcct
gaggcctgtggaaataaacctttatgtacttaaagttatacagaaaatag
aataaagttaataccaaacttgcaaaaaaaaaaaaaaaaaaaaaaaaa

>NM_022075 4
agcgcaaggtgacgggcgtggacgcggctgtggggccgggagcggagggt
tggggtctggcctcccgcgccggggcgaaggggcagccgcagcgcagagg
cccgccccgccctcccctcccgtcacgcccagcctcccggcccttgggct
gctcgcggcctttttttcccggctgggctcgggctcagctcgactgggct
cggcgggcggcggcggcggcgccggcggctggcggaggagggagggcgag
ggcgggcgcgggccggcgggcgggcggaagagggaggagaggcgcgggga
gccaggcctcggggcctcggagcaaccacccgagcagacggagtacacgg
agcagcggccccggccccgccaacgctgccgccgggatgctccagacctt
gtatgattacttctggtgggaacgtctgtggctgcctgtgaacttgacct
gggccgatctagaagaccgagatggacgtgtctacgccaaagcctcagat
ctctatatcacgctgcccctggccttgctcttcctcatcgttcgatactt
ctttgagctgtacgtggctacaccactggctgccctcttgaacataaagg
agaaaactcggctgcgggcacctcccaacgccaccttggaacatttctac
ctgaccagtggcaagcagcccaagcaggtggaagtagagcttttgtcccg
gcagagcgggctctctggccgccaggtagagcgttggttccgtcgccgcc

gcaaccaggaccggcccagtctcctcaagaagttccgagaagccagctgg
agattcacattttacctgattgccttcattgccggcatggccgtcattgt
ggataaaccctggttctatgacatgaagaaagtttgggagggatatccca
tacagagcactatcccttcccagtattggtactacatgattgaactttcc
ttctactggtccctgctcttcagcattgcctctgatgtcaagcgaaagga
tttcaaggaacagatcatccaccatgtggccaccatcattctcatcagct
tttcctggtttgccaattacatccgagctgggactctaatcatggctctg
catgactcttccgattacctgctggagtcagccaagatgtttaactacgc
gggatggaagaacacctgcaacaacatcttcatcgtcttcgccattgttt
ttatcatcacccgactggtcatcctgcccttctggatcctgcattgcacc
ctggtgtacccactggagctctatcctgccttctttggctattacttctt
caattccatgatgggagttctacagctgctgcatatcttctgggcctacc
tcattttgcgcatggcccacaagttcataactggaaagctggtagaagat
gaacgcagtgaccgggaagaaacagagagctcagagggggaggaggctgc
agctgggggaggagcaaagagccggcccctagccaatggccaccccatcc
tcaataacaaccatcgtaagaatgactgaaccattattccagctgcctcc
cagattaatgcataaagccaaggaactaccccgctccctgcgctataggg
tcactttaagctctggggaaaaaggagaaagtgagaggagagttctctgc
atcctccctccttgcttgtcacccagttgcctttaaaccaaattctaacc
agcctatccccaggtagggggacgttggttatattctgttagagggggac
ggtcgtattttcctccctacccgccaagtcatcctttctactgcttttga
ggccctccctcagctctctgtgggtaggggttacaattcacattccttat
tctgagaatttggccccagctgtttgcctttgactccctgacctccagag
ccagggttgtgccttattgtcccatctgtgggcctcattctgccaaagct
ggaccaaggctaacctttctaagctccctaacttgggccagaaaccaaag
ctgagcttttaactttctccctctatgacacaaatgaattgagggtagga
ggagggtgcacataacccttaccctacctctgccaaaaagtgggggctgt
actggggactgctcggatgatctttcttagtgctacttctttcagctgtc
cctgtagcgacaggtctaagatctgactgcctcctcctttctctggcctc
ttccccccttccctcttctcttcagctaggctagctggtttggagtagaat
ggcaactaattctaatttttatttattaaatatttggggttttggtttta
aagccagaattacggctagcacctagcatttcagcagagggaccatttta
gaccaaaatgtactgttaatgggtttttttaaaaattaaaagattaaat
aaaaaatattaaataaaacatggcaataagtgtcagactattaggaattg
agaaggggggatcaactaaataaacgaagagagtctttcttatgccttcct
tgca

>NM_001829 3
gtgacgtcacgcgtcgacgctggggcgtacctttcgggctcctgactcct
gccgcttctcttccccttccgtgggtcagggccggtccggtccggaacct
gcagcccctttcccagtgttctagttcgcccgtgacccggaataatgagc
aaggagggtgtggtgggttgaaagccatcctactttactcccgagttaga
gcatggattcagttttagtcttaagggggaagtgagattggagattttta
tttttaattttgggcagaagcaggttgactctagggatctccagagcgag
aggatttaacttcatgttgctcccgtgtttgaaggaggacaataaaagtc
ccaccgggcaaaattttcgtaacctctgcggtagaaaacgtcaggtatct

tttaaatcgcgatagttttcgctgtgtcaggctttcttcggtggagctcc
gagggtagctaggttctaggtttgaaacagatgcagaatccaaaggcagc
gcaaaaaacagccaccgattttgctatgtctctgagctgcgagataatca
gacagctaaatggagtctgagcagctgttccatagaggctactatagaaa
cagctacaacagtataacaagtgcaagtagtgatgaggaacttttagatg
gagcaggtgttattatggactttcaaacatctgaagatgacaatttatta
gatggtgacactgcagttggaactcattatacaatgacaaatggaggcag
cattaacagttctacacatttactggatcttttggatgaaccaattccag
gtgttggtacatatgatgatttccatactattgattgggtgcgagaaaaa
tgtaaagacagagaaaggcatagacggatcaacagcaaaaagaaagaatc
agcatgggaaatgacaaaaagtttgtatgatgcgtggtcaggatggctag
tagtaacactaacaggattggcatcaggggcactggccggattaatagac
attgctgccgattggatgactgacctaaaggagggcatttgccttagtgc
gttgtggtacaaccacgaacagtgctgttggggatctaatgaaacaacat
ttgaagagagggataaatgtccacagtggaaaacatgggcagaattaatc
ataggtcaagcagagggtcctggttcttatatcatgaactacataatgta
catcttctgggccttgagttttgcctttcttgcagtttccctggtaaagg
tatttgctccatatgcctgtggctctggaattccagagattaaaactatt
ttaagtggattcatcatcagaggttacttgggaaaatggactttaatgat
taaaaccatcacattagtcctggctgtggcatcaggtttgagtttaggaa
aagaaggtcccctggtacatgttgcctgttgctgcggaaatatcttttcc
tacctctttccaaagtatagcacaaacgaagctaaaaaaagggaggtgct
atcagctgcctcagctgcaggggtttctgtagcttttggtgcaccaattg
gaggagttctttttagcctggaagaggttagctattattttcctctcaaa
actttatggagatcatttttttgctgctttagtggctgcatttgttttgag
gtccatcaatccatttggtaacagccgtctggtcctttttttatgtggagt
atcatacaccatggtacctttttgaactgtttccttttattcttctaggg
gtatttggagggctttggggagcctttttcattagggcaaatattgcctg
gtgtcgtcgacgcaagtccacgaaatttggaaagtatcccgttctggaag
tcattattgttgcagccattactgctgtgatagccttccctaatccatac
actaggctaaacaccagtgaactgatcaaagagctttttacagactgtgg
tcccctggaatcctcttctctttgtgactacagaaatgacatgaatgcca
gtaaaattgtcgatgacattcctgatcgtccagcaggcattggagtatat
tcagctatatggcagttatgcctggcactcatatttaaaatcataatgac
agtattcacttttggcatcaaggttccatcaggcttgttcatccccagca
tggccattggagcgatcgcaggaaggattgtggggattgcggtggagcag
cttgcctactatcaccacgactggtttatctttaaggagtggtgtgaggt
cggggctgattgcattacacctggcctttatgccatggttggtgctgctg
catgcttaggtggtgtgacaagaatgactgtctccctggtggttattgtt
tttgagcttactggaggcttggaatatattgttccccttatggctgcagt
catgaccagtaaatgggttggagatgcctttggcagggaaggcatttatg
aagcacacatccgattaaatggataccctttcttggatgcaaaagaagaa
ttcactcataccaccctggctgctgacgttatgagacctcgaaggaatga
tcctcccttagctgtcctgacacaggacaatatgacagtggatgatatag
aaaacatgattaatgaaaccagctacaatggatttcctgtcataatgtca
aaagaatctcagagattagtgggatttgccctcagaagagacctgacaat

tgcaatagaaagtgccaggaaaaaacaagaaggtatcgttggcagttctc
gggtgtgttttgcacagcacaccccatctcttccagcagaaagtcctcgg
ccattgaagcttcgaagcattcttgacatgagcccttttacagtgacaga
ccacaccccaatggagatcgtggtggatattttccgaaagctgggactga
ggcagtgccttgtaactcacaatgggcgcctccttggcattataacaaaa
aaagatatcctccggcatatggcccagacggcaaaccaagaccccgcttc
aataatgttcaactgaatctcacagatgaggagagagaagaaacggaaga
ggaagtttatttgttgaatagcacaactctttaacctgagggagtcatct
actttttttcctcctttacaaaaaaagaaaggaaatataaaagccgggt
ttttgcaacatggtttgcaaataatgctggtggaatggaggagttgtttg
gggagggaaaggagagagaaggaaaggagtgaggtatttcccgtctaaca
gaaagcagcgtatcaactcctattgttctgcactggatgcattcagctga
ggatgtgcctgatagtgcaggcttgcgcctcaacagagatgacagcagag
tcctcgagcacctggcctgttgctccaacattgcaaagacacattatcag
tccctatttctagagggattactttgaattgagccatctataaaactgca
aggtcttgccctttttttaatcaaaactgttctgtttaattcatgaatt
gtatagttaagcattacctttctacattccagaagagcctttatttctct
ctctctctctctctctctctctctactgagctgtaacaaagcctct
ttaaatcggtgtatccttttgaagcagtcctttctcatattgagatgtac
tgtgattttactgaggtttcatcacaagaagggagtgtttcttgtgccat
taaccatgtagtttgtaccatcactaaatgcttggaacagtacacatgca
ccacaacaaaggctcatcaaacaggtaaagtctcgaaggaagcgagaacg
aaatctctcattgtgtgccgtgtggctcaaaaccgaaaacaatgaagctt
ggttttaaaggataaagttttctttttgttttcctctcagactttatgg
ataatgtgaccgggtcttatgcaaattttctatttctaaaactactacta
tgatatacaagtgctgttgagcataattaaataaaatgctgctgctttga
cagtaaagagaaggaagtattctgattagctgtatctggtattaattgca
tgttaaaacactggaatttttaaaattgaaattagatcagtcattctttt
cttttctcaagatatctcatggctgacactgaagaagaaatgtaattcat
aacttgcactaaatgtatatttttttcttaaaaatttaccattcttatt
tatatttttatggattaaaatttataaaatacagatcagttaatattgca
cttaagtaattttaccttttaatgtgatttttatagaataattcagact
tacaaatacagagatatgaacaaagtttacagtgggaacaaaggtttaaa
aaaaggttgtggttctctctctgtgatccagtgtgcacataaacctttct
ctgatctttcactgccatcctctggattatgtcttctgacctgtccattt
tgacccattaactggaaagttgaaaaactacattaactggaaagttgaaa
aactacattactttggagaataaaaccgaaagttcgtgtataccttctta
aaaaaaaaatcaaaccaaaaatgtgaaaacaatagaattgcaaagatagc
agttaaaattttaatctgaaaataacctttgaatctcgggctaggttacg
tccatatttgaagtggtcagtgatggtttgaacatttttttgcaggatgag
tgaaaatgcactggattatatttgggatttttgtttttggaattgtctgt
tttaatcacagccttaattcacaattggcaaaggcagtttactcaaagga
ctgggctaaatattctgtaattatgcatttttgataggaaaatgaaattt
ttgcaaacagacattttcttttttttggctggagtgcagtggggcatgg
tcttggctcactgcagcgttgaccacctgggctcaagtgatactcccgcc
tcagccacccaagtagctggcactacgggcacacgccaccatgcccagct

aattttttgtatttttagtagagatggggttttgccatgctgcccaggc
tggtctcaactcctcagctcaagcaatctgcctgcgtgagcctcccaaag
tggtggaattacaggcgtgggccactgcgcctggcccagacagacatttt
ctgaaacacaactggcaatgagctgtttttacattttgaaagtgattctt
cacttcctagttcttaattatagtatacctattaagatctgtaagatcct
gaagacataagatcatgaagccatataagaatgaggattgaaagttgagc
aaaattttcgggattttgggaaacattcttagctgtgctatctgcctaaa
attattccttattacttctctcctttgacagacttcaagttttcttcata
gcccttcaaagtttttgagccatccagagtaaaatcatttctaaatga
tagttctgtatatctccaactcgtcttaagtgtatttgcctgtgtgcaac
gtattgctagactatgaactcctcagcatggctgctggataacttaattg
tcctgagttaatagccttcaaaggacaaatcggtttctttgcagatagct
tcgtaaaacttcacatggagtttattttatcatatttcccttttttattt
ctgctcctcctttaattgcccatcttgcttcagagactgacatttcaggg
tggatattaattaaagcattaattttgttttttggtatatttctatccct
agtatttctatcttactgctaaaatacaggaaaagtgccgtatttttaat
gcatttagtggttttctttggtgttatctgttccatttttctttttcata
cattgaagtgtgtctccttttcaaccaaaataatgaaatagtggagacca
tgaaattgttgtgcctggctaattggcaaattaatttaccaatataataa
gtgtagcgcccttgtttgaataccctttttgagaaggtatgatgagaatgg
gcaagggtgtcagcatctcttcttcttaataattaattgttttcagtttt
ggttcacgaagaatgcttagttaatctgtaatgttgcctagagctgtatt
tatctgtttttatttatactagtgtagtaaagctgcatatcattacagta
aaaacgactactgtgatgagttaatcagaaaatctattaaaatctatatg
acaatgaaaaaaaaaaaaaaaaaa

>NM_001042351 1
cgagaactcgggaagccggcgagaagtgtgaggccgcggtagggccgcat
cccgctccggagagaagtctgagtccgccaggctctgcaggcccgcggaa
gctcgacagcgtcatggcagagcaggtggccctgagccggacccaggtgt
gcgggatcctgcgggaagagcttttccagggcgatgccttccatcagtcg
gatacacacatattcatcatcatgggtgcatcgggtgacctggccaagaa
gaagatctaccccaccatctggtggctgttccgggatggccttctgcccg
aaaacaccttcatcgtgggctatgcccgttcccgcctcacagtggctgac
atccgcaaacagagtgagcccttcttcaaggccaccccagaggagaagct
caagctggaggacttctttgcccgcaactcctatgtggctggccagtacg
atgatgcagcctcctaccagcgcctcaacagccacatgaatgccctccac
ctggggtcacaggccaaccgcctcttctacctggccttgcccccgaccgt
ctacgaggccgtcaccaagaacattcacgagtcctgcatgagccagatag
gctggaaccgcatcatcgtggagaagcccttcgggagggacctgcagagc
tctgaccggctgtccaaccacatctcctccctgttccgtgaggaccagat
ctaccgcatcgaccactacctgggcaaggagatggtgcagaacctcatgg
tgctgagatttgccaacaggatcttcggccccatctggaaccgggacaac
atcgcctgcgttatcctcaccttcaaggagccctttggcactgagggtcg
cgggggctatttcgatgaatttgggatcatccgggacgtgatgcagaacc

acctactgcagatgctgtgtctggtggccatggagaagcccgcctccacc
aactcagatgacgtccgtgatgagaaggtcaaggtgttgaaatgcatctc
agaggtgcaggccaacaatgtggtcctgggccagtacgtggggaaccccg
atggagagggcgaggccaccaaagggtacctggacgaccccacggtgccc
cgcgggtccaccaccgccacttttgcagccgtcgtcctctatgtggagaa
tgagaggtgggatggggtgcccttcatcctgcgctgcggcaaggccctga
acgagcgcaaggccgaggtgaggctgcagttccatgatgtggccggcgac
atcttccaccagcagtgcaagcgcaacgagctggtgatccgcgtgcagcc
caacgaggccgtgtacaccaagatgatgaccaagaagccgggcatgttct
tcaaccccgaggagtcggagctggacctgacctacggcaacagatacaag
aacgtgaagctccctgacgcctacgagcgcctcatcctggacgtcttctg
cgggagccagatgcacttcgtgcgcagcgacgagctccgtgaggcctggc
gtattttcaccccactgctgcaccagattgagctggagaagcccaagccc
atcccctatatttatggcagccgaggccccacggaggcagacgagctgat
gaagagagtgggtttccagtatgagggcacctacaagtgggtgaaccccc
acaagctctgagccctgggcacccacctccacccccgccacggccaccct
ccttcccgccgcccgaccccgagtcgggaggactccgggaccattgacct
cagctgcacattcctggccccgggctctggccaccctggcccgcccctcg
ctgctgctactacccgagcccagctacattcctcagctgccaagcactcg
agaccatcctggcccctccagaccctgcctgagcccaggagctgagtcac
ctcctccactcactccagcccaacagaaggaaggaggagggcgcccattc
gtctgtcccagagcttattggccactgggtctcactcctgagtggggcca
gggtgggagggagggacaagggggaggaaaggggcgagcacccacgtgag
agaatctgcctgtggccttgcccgccagcctcagtgccacttgacattcc
ttgtcaccagcaacatctcgagccccctggatgtcccctgtcccaccaac
tctgcactccatggccaccccgtgccacccgtaggcagcctctctgctat
aagaaaagcagacgcagcagctgggacccctcccaacctcaatgccctgc
cattaaatccgcaaacagcc

>NM_024792 1
agccacgcggcgccagcgaggcggccggacccgcagccccgatgctgctg
acgctggccgggggcgcgctcttcttcccggggctcttcgcgctctgcac
ctgggcgctgcgccgctcccagcccggatggagccgcaccgactgcgtga
tgatcagcaccaggctggtttcctcggtgcacgccgtgctggccaccggc
tcggggatcgtcatcattcgctcctgcgacgacgtgatcaccggcaggca
ctggcttgcccgggaatatgtgtggtttctgattccatacatgatctatg
actcgtacgccatgtacctctgtgaatggtgccgaaccagagaccagaac
cgtgcgccctccctcactcttcgaaacttcctaagtcgaaaccgcctcat
gatcacacatcatgcggtcattctctttgtccttgtgccagtcgcacaga
ggctccggggagaccttggggacttctttgtcggctgcatcttcacggca
gaactgagcactccgtttgtgtcgctgggcagggttctgattcagctaaa
gcagcagcacacccttctgtacaaggtgaatggaatcctcacgctggcca
ccttcctttcctgccggatccttctcttccccttcatgtactggtcctat
ggccgccagcagggactaagcctgctccaagtacccttcagcatcccatt
ctactgcaacgtggccaatgccttcctcgtagctcctcagatctactggt

tctgtctgctgtgcaggaaggcagtccggctctttgacactccccaagcc
aaaaaggatggctaaatgctcctgggagtcaggcgcagcctcacaccagc
tgcctcctccactcagcattccatggaccaaattgtgccctgggtagcct
cagactttgggtattgataagccgatggatttgagtttttctaaagaata
ttcatattacctccttcttctaacttgccctatttgcaaaagcacttttg
tagtaacaactattgggtcctgtcagacctccacggacagcaaagtggtt
ttaatgcaagcccaaggatccttcttaaggtcttatctcaagagctctgg
gaggtggaagcatggggtgggatcggtggaccagggtggtaagtgtctgc
acatctgcctgtccctgtatcagcggctacccaccttccaaaccactcag
gacagtacccgtggcactgggcccgcagaagcaagggatgacttggttct
tggaagtaatgtcgtcttgtgacattggcctgggacaatcattgtgggta
ggtagttattgatcgtttactagataacccattggttctttgcctcatcc
tctcatccatgggtcagagttgaattcttatgtctatagacttccaatca
gaagtctcactggtggggctgggggtgggggcaggcaggaggcatggatg
ggaacctgagtaggtagtgtggccaagagatcagcacaacctttgcaggc
tgacttgctaagtctgacagtgacaaacttgtgagcttactgcagtcagt
cacagaggctgttctttttcacacaccccttcatgcccggctttccccat
atccacatgcagagggcgagctcataaaactacagggaagcgtgaaatga
tggctttggtagctgtttactgggtaaccccactgtgacactgtcctttt
catgtgatgtggaaacctacttctgtcctccaaaccatgaaatgtgtcat
ctagactgcagagtacttgagtgctttgcctcccgatatgccagagcttg
tggtccaaagcccattcctgtgtgtccgtcctgccatttagccacagaag
gctgcggagtgaggcggcagctagcctggccagtggctgtcccgtggacc
gacacctgcgccccttctgcaagcaggattttctggtgccaacactcat
tcatcattcccgatcaactaggatgaatttaagactgtgctaccatgtgt
tctcaagtggtagtttaaaaagtggatttttaaagtgcctttcaattgtc
tgtgaacgtctaaaggactgatttgtctcaaaaaaaaaaaaaaaaa

>NM_172020 3
cttatgaggtggcaacatcttgtacttcggagatctgaagccgagcaact
tgcccaagtccttcttcttttcccattaacaagatatgatatcaccaagc
caaacgtcatcattaagttggagcagggagaggagctgtggataacggga
ggtgaatttccatgtcaacatagtccagggattgtgggactttaccaaat
cggtttgtaataacacctagaagacgctatccgatccatcaggcccagta
ttcctgtctgggggtacttcccaccgtgtgctggaatggttatcacaaga
aggctgtgctgtcccctcgcaactccaggatggtgtgtagcccagtgact
gtgaggatcgcccctcctgacagaagattttcgcgttctgcgataccaga
gcagataatcagctcaacactgtcctcaccatcaagtaacgccccagacc
catgtgcaaaggagacagtactgagtgccctcaaagagaaggagaagaaa
aggacagtggaggaagaagaccaaatattccttgatggccaggaaaataa
aagaaggcgccatgatagcagtggcagtggacattcagcatttgagcccc
tggtggccaatggagtccccgcttcttttgtgcctaagcctgggtctctg
aagagaggcctcaattctcagagctcagatgaccacttgaataagagatc
ccgaagctcttccatgagctccttgacaggcgcttacgcaagtggcatcc
ctagctccagccgcaatgccattaccagttcctacagctccactcgaggc
atctcacagctctggaagagaaatggccccagttcatcacccttctctag

cccagcctcctcccgctcccagacaccggagaggccagcaaagaaaataa
gagaagaggagctgtgtcatcattccagttcttcaactccattggcagca
gacagggagtcccagggagaaaaggctgcagatacaaccccaaggaagaa
acaaaactcgaattctcagtctacacctggcagctctgggcagcgtaagc
ggaaagttcagctgctgccttctcggcgaggggaacagctgaccttgcct
ccacctccccagcttggctattcgatcactgccgaggacctagacttaga
gaagaaggcttcattacagtggttcaaccaggccttggaggacaagagcg
atgctgcctcgaactctgtcactgagaccccacctatcactcagccttca
tttacctttaccctgcctgctgctgcacctgcctccccacccacctccct
cctggccccaagcaccaacccactgttagagagcttgaagaagatgcaga
ctcccccgagcctgccaccctgcccagaatctgctggagcagcaaccact
gaggccctctcacctccaaagacacccagcctcctacccccgctgggttt
atcacagtcagggccgccagggctgctccccagcccctcctttgactcca
aacccccgaccactttgctggggctgatccctgctccatccatggtacca
gccactgacaccaaggcacctccaacccttcaggcagagacggctaccaa
accccaagccacatctgccccgtcccccgcccccaagcaaagcttcctgt
ttggaacacagaacacctcaccttccagccctgccgcccctgctgcatct
tcagcacctcccatgttcaagcccattttcacggctccacccaagagtga
gaaggaaggccccacaccgcctggcccttcagtcacagccacagcgccct
ccagctcctccctccccacgaccaccagcaccacagccccgaccttccag
cctgtctttagcagcatggggccacctgcatctgtgcccttgcctgctcc
cttcttcaagcagacaactactcccgccactgctcccaccacaactgccc
cgctcttcactggcctggccagcgccacctctgctgtggctcccatcacc
tctgccagtccatccacagactctgcttcgaagcctgcgtttggctttgg
cataaacagtgtgagcagcagcagtgtgagtaccacgaccagcaccgcca
ctgccgcctcacagcctttcctcttcggggcgccccaggcctctgctgcc
agcttcaccccggccatgggctccatattccagtttggcaaacctcctgc
cttgcccacaaccaccacagtcaccaccttcagccagtccctgcacactg
ccgtgccaacggccaccagcagcagcgctgccgactttagtggttttggc
agcaccctcgccacctccgccccggccaccagcagccagcccactctgac
gttcagtaacacgagcacccccacgttcaacattccctttggctcaagcg
ccaagtccccgctcccatcatatccgggagccaacccccagcccgcattt
ggggccgctgaggggcagccaccgggggccgccaagccggcccttgcccc
cagctttggcagctctttcacttttggaaactctgcagccccggctgctg
cacccacacctgcacctccgtccatgatcaaggtcgtgcctgcgtacgtg
cctacgcccatccatcctatctttggcggtgccacgcactcggcgtttgg
gttgaaagccacggcttcggccttcggcgctcccgccagctcacagcccg
cctttggcggctccactgctgtcttcttcggtgcagccaccagctccggc
tttggagccaccacccagaccgccagcagcgggagcagcagctcggtgtt
tggcagcacaacaccatcacccttcacgtttgggggttcggcagcccccg
ctggcagtgggagctttgggatcaatgtggccaccccaggctccagcacc
accaccggagctttcagctttggagcaggacagagtgggagcacagccac
ctccacccccttcgcaggggggcttaggtcagaacgccctgggcaccaccg
gccagagcacaccgtttgccttcaacgtgagcagcacaactgagagcaaa
cctgtgtttggaggcaccgccacccccacctttggtctgaacacccctgc
gcctggagtgggcacatcaggcagcagcctctcctttggggcatcctcag

cacccgcccaaggctttgttggtgttgcacctttcggatcggcggccctt
tcattttccattggtgcgggatccaagaccccaggggctcgacagcgact
gcaggcccgaaggcagcacacccgcaaaaagtagcctttgtcccctgtcc
ctgttcccccaccccttccctaaatctggaccttggcacctgctaggaa
gagccttggacccttccagttgcgtaaagcaaacctaccccggatctctg
gcttcagccgccagggggcagtggcagccctggggccctttcccttctgg
aggaagcacaagcctcagggaaggggaagcaggatgcggagggccaaagc
ccgggacctctacttgaacagttctactggggaggctggagaactaagga
aacacctgtacatagtgtccgctgccctgactcccgcttagcacaccctt
aggcaggcgccccttccacctttccccgagaccgtcgtcgctggaggggg
cagggtccagcccgcctggatcggtggtgtgcacctgatgggatttggga
aatgggctatccgtaaagctttatcttgcttggcttagctgtgagaagtg
gttctcttcctctggtcccttctggggactctgtttccccatttcttgct
gctgtgtccctcaccagttccttgcaggattccttcgtttttaaatgccc
ttgaatctagctttgccttggagaccccagtgggtgctgctcctgccgtt
ttcttcctgccaagcctgaatcaatgtttcatctccaaccctctgccagt
ttggcccctcagagcttggtggctcaagactgttagcctggcagagccag
gggtgaagggagaagctcttggagcaggcaggatgcccaccgctgcttca
gctgcctcctcgcccagctaccctttggccccattgggccctcgtctgcc
tctccaggattgtatgtttcaagccttgtcctgtgttcctttgtctgacg
ctctgtgtattgctctttgaatcgagtttggaggaagagttgagttgtat
gagtggcggcatgttggtagtgccggacttcctgtttcaagttttctggg
gcctcgctaattgaatgtggaaagtagcaccacttgacggctacaagtgc
cgactcctgaattttcccatggtgttctgacttcaagggctggcagccag
ggagaatgggcccaggggaagcaaagacctcttccctctgccgtttctgt
cccacttaactgacctcactggaggctacatcacccaaagtagatgttag
aaaacctaaattaatgaaccatatttttaaaatcctatttttcccaaaca
gggccctctgcagcccatcctttccttccgtccttctgaaaccacatacc
ccaggcccaagcgccttgctgccacgcccaacctctttgggagaagtatg
aatgcgtgtgtctaaattaaaagaaaaaaatatttaaacgtttttttaaca
aaaatttatttttgtatttaagctaaattgccttttaaattccttcaagc
ttggttcattgaggtggttaagtataaatgctattaactaggaattagct
gtatagttaagttatgcctgtgcaaagaagaggctcaaatgctgtccccg
gcagctttcctgggggactagagctccttctggccatgttatatgaaatg
taattcttattttataaataatgtgatgtaaatgtaactggtgccccctc
cccgatgtgactgagggtgagtgagtggtggcggggctgctccttcccac
ccctcagaacagctctgatcctcgttaatacctggctgcgtgtgcagctg
aggagggagtgaacctcaagcctaaatacctgttaggattggagggtctg
ggtgggcctgggcctagcaatcaagcttctacctgtaccttatgtaaggt
agaccctcctagtgtcagtacctgagctagtttacctcagttccgcaggc
aggacagccggtccgggaaccctgagtgagaatgagtgtggatgtgtaca
gtacacgcactggacggcagcgggaggctgggactttccattacaaatag
agacttcattcctgttgagtctagttggaatttttagtatgaatgtgaga
tttttctcctgcttgtgacattaagaataaaaaactgtgatctatcgtag
agtaaaaaaaaaaaaaaaaaa

>NM_152450 2
gcggcgcgggcccgcagccgggcgccctgctcagccagcgccagcggccg
ccgcacccagccgcgccggcgaggacatgggcagccgcggcgcgcccacc
ccccgcgccgatgtgaattattaaaaagaaaatggcccaacggagcactg
tatttccttctcgtgtcaccaaggaaaggtataatatatggaaaatatgc
atctaaggcgagtgagaaccatgccccgacacagccagtccctgaccatg
gcaccatactcatctgtaagcctcgtggagcagctggaagacaggatcct
ctgccatgagaaaaccaccgccgccctcgtagagcacgcctttcggatta
aagatgacattgtcaacagtttgcagaaaatgcaaaacaaagggggaggt
gaccgcttggccaggcttttcttggaggagcatatcagaaacataactgc
catagtgaagcaacttaatcgggatatcgaggtactccaggagcagattc
gtgcccgggacaacattagctatggaactaattctgccttaaagaccctg
gagatgcgccagctctccggtttgggagatcttcgaggaagagtggcaag
atgtgatgccagcatagctagactttctgcagagcacaaaacgacctatg
aggggctccagcacttgaacaaagaacagcaggctgccaaacttatcttg
gaaacgaaaatcaaagatgcagagggacagatttctcagcttttgaacag
agtggacttgtcaatatcagagcagagcaccaaactgaagatgtctcaca
gagacagtaaccaccagcttcagcttttggacactaaatttaaaggtaca
gttgaggaactcagtaaccagatattatctgcacggagttggttgcaaca
ggaacaagaacggatagaaaaagagcttttacagaaaattgatcagcttt
ccttgattgttaaggaaaacagtggagccagtgaaagggatatggagaag
aagctcagccagatgtcagccaggcttgacaaaatagaagagggtcaaaa
gaagacttttgatggtcagagaacaaggcaagaagaggagaagatgcacg
ggcgaatcaccaagctggagttacagatgaaccagaacatcaaggaaatg
aaagcagaagttaatgctgggtttacagccgtctatgaaagcataggatc
cctcaggcaagttctcgaggccaagatgaagctggacagggaccagctac
agaagcaaatccagctgatgcagaagccagagacccccatgtgaagggag
ctgggacaaggtcctaaaagacagttttgccagtggggctaggagccgga
tacctctgtagccaggccatcgctgcattcaggattgttccatccatggc
gtgcatgtgccaagaaatgtgtttttatgggtctaaatgtttaccttgag
tcttgaaaatactcttttgttaaaagtatgaaatacagtttttaccagtt
tatttcacttctctaaattcaatggaaatcccccgccctggattttgaaa
ggcttttatcttcttcattttacgaatggaaagacgacaatttttcttca
atgcttgatgcactaatgaagactgtttactattttgaaaaatgtcatgg
ggattttttttaattaagaaactaatgaatcatcacaggaatgtgttgc
tcctcaccctaaattaagagaatgtcccagtagattagacttcaaccttt
gagtccaatttggattttattatcgttgtctatgcacttcttatattggt
tatcttcttgtaaatcttctgtcttttgtaaggggaaaggatttaacatt
tagaataaaccccaccatttatgtaatggaaatagtttaaaaattgctaa
ctgccatgtggattgcaaattaaatggaaacttatttagataacgtaagg
ctcaatatctgcgttgaccacctagatattacaggttttaatatttaaaa
ctatttttgaattatccacaacctgtatagtgatagccatatatttaata
atggaatggtggttaacagtctatttactgcacaattaattgttcactaa
tcaaatagaatgtggtaatttttcagactttatgatctgtttccaaaatt
ggcacaaagtgctagggtttatatacacttatcgtaactgtatttttgtg
ccttggttttatcatgtcaatgcactgtactctgtaaaagttttgcagac

aaaatagaaagtatgataatccgtcagaagtatgatgtaaaactggaatc
ctctgtattttttaaatgttctaaaaattttatcgctgttaaggtattaa
tcattcagtattactaatggaatagaaattcatacttttgtatggacaac
aattcaaattgatattgcatttatagcactgtaagaaactttcatcttga
gcaactttgtagatgatgggtgttttattttcaatcgccatatttgatca
gtcattgaaaattggccccagtgctgtttgttcatctctgtatgtaaaaa
ctgacagtgagacacaacgttctgaactgtgagggtgtcccaggaaaaag
aaaaacaggaatactttaacaattaaaaagaaaaaaatgtttttgtttg
ccaaggactcaggaaaataaaaagcattttctatttttaggacaaatcac
aaatgaagtgtctaactggctattactgtttacccatataaaatatgctg
ctaaagtacatattttgctgtcaatggcttgacaattttttttttcaaat
ttggacatgagaggttatatagggactatattatccaacacatattttct
tattttgccacaaatttccacttaacaaataaaaaaaggcgaatgctgtt
ttgcaatcagaaagtgaatttcttttgtggtagcgtacacgtggttcatg
tggttctccacgtttaagcacaaaccacagcacaggaagccacacccccct
ccagcatctctgtcttgtggggtctttgaggagaaggaatcagaacatcg
acacctgggaagagggaggcggggaacaccagctggactctggccaagga
gtgatgtaagcaggtgacacttggctgtcagctgagcttctcaggtgctt
ctgacattgccagccccttgagatgagccaccgcaggtgtgctcaccttg
gctacatgttgaaatcatccagaggagttgggaaatctactgatgccccg
ctgtgcctcccagaccgattaagtcagaacctctgggaggtgggtcgcag
gcatcagtgcgtgcccgaggcctccgcgtgtttgggtgtgcagccgaggt
ggtgagctgtcgagccacagtgcagttctcttctcaccaaagctcaaatg
agaatcagcttccatgttctttcctttaccagaaatttgcaataaaaaga
aaaataaaattttcacat

>NM_173505 2
gggtgcggcggctgctgccgggccgggcggcgggagcgcgcggcgtcgga
ggccgcccctctgcggaacgccgagagccccgggagagtgaagggccggg
gaggacaaggcgccccgggcccaaagcgcgccggctcccgctcgggcggc
cgcggacatgtgcaggatgtccttcaagaaggaaactccacttgccaatg
ctgcattctgggcagccaggagaggaaacctggcgctgctgaagctgctg
ttgaacagcggccgggtggacgtggactgcagagacagccatggcaccac
actcctgatggttgctgcctacgctggccacatagactgtgtgagggaac
tggttctgcaaggagcagacatcaatctccagagagagtcaggtacaact
gccctattctttgccgcccagcaaggccataatgatgtcgtgagatttct
ctttggatttggagcatccactgaatttaggaccaaagacgggggcaccg
ccctgttggctgccagtcagtacgggcacatgcaggtggtggagaccttg
ctgaagcacggagcaaacatccatgaccaactttatgatggagccactgc
cctcttcctagctgcccaaggtggttacttggatgttattcgattactgc
tggcttcaggagcaaaagtcaaccagccaaggcaggacgggacagcgccc
ctgtggatcgcgtcccagatgggccacagcgaggtggtgcgggtgatgct
gctgcgcggagccgaccgcgacgctgcgcggaacgatggcacaacagcat
tattgaaagcagccaacaaagggtataatgatgtcataaaagagttgctt
aaattctcacccactcttggtattttgaagaatgggacatcagcgctcca
tgcagcagtgctcagtggaaacattaaaacagttgcgctgctcctagaag

caggggcagacccatccctgagaaacaaggccaatgaacttccggcagaa
ctaaccaaaaatgaacgtatattgcgtctcctgagaagtaaagaaggtcc
cagaaagagctaacttagctccatatttgacagaaagatagaaagcttaa
ccacattgtccaaaaagaaattgcatttcaagcagtgttggaaattcttt
tatgaaaaaaaaagatgcccagaatgcccatcctgtgggtccctgacaaa
gaagagctacgctctgtgcacgaagtcaagaaccaaacagctcagggacc
ctcttgccccttcaccatggacttctcatggtgtcctgtaactcatctcc
cggggggcctggcatgttcacagattccacagaaactcattttcaacaat
gctaacttggacctgtcagttaaactctaaggtggacagggttctcagta
ctaagcaaggagacagaatgctttgttcctttaaaagactgaaaagctga
ccttcaatggattgaggcacttttgcttttgtgttaaatgtagatgtgct
aaaatatatagatctatcatattttacctacatatgtatgtcattccagt
ataaaacattctcctctacccaagaaccatagccatgattgttataaatc
aatgaagtgtaaacatacattattaaaaaaccacttctgacattccatta
tgtgctattcaaagatggactattgaactatagaaaagacagactgtgca
tttgttcgttgatcctcatcttattcctgacatgtaaaaatcaattttac
gtagagtcaacattgtaggtaggttaaaataccagtggcaaatttggaaa
ttcagaaacttataaaccacgagaaatatataggcttgtctctttggtct
tttattttggctctattgttgggaatctatttcctattctataagtaagt
atacctaacatgctgtggaatcttgagtttccaacaccgtgctgcttgat
agaatgactttgaggtccttggataaaatgtgatatatgcaagtacagta
tgttgctattactattgcaggaatataaataataaaagactgttattagc
acttagtaagtcttcatctatgcatgtttttgagttgactgattccaaga
atgaaatatgaggtttattgaattattcctttgaaagggatcaaaactta
tattcaatgcactttataattaatggtgtctaaatgcctcagtcagtgcc
taactgcacatacaaaaataaaaccttctttctgtaatctaccaaaataa
acgcaatggtatttctgctatttaaaaaaaaaaaaaaaaa

>NM_018290 3
cacgtcttggggccgggccggaaggcagatctcaccgcctgcttccctct
gcagcggtagcacaagctcagcgatggcggctccagaaggcagcggtcta
ggcgaggacgcccggctggaccaggagaccgcccagtggctgcgctggga
caagaattccttaactttggaggcagtgaaacgactaatagcagaaggta
ataaagaagaactacgaaaatgttttggggcccgaatggagtttgggaca
gctggcctccgagctgctatgggacctggaatttctcgtatgaatgactt
gaccatcatccagactacacagggattttgcagatacctggaaaaacaat
tcagtgacttaaagcagaaaggcatcgtgatcagttttgacgcccgagct
catccatccagtgggggtagcagcagaaggtttgcccgacttgctgcaac
cacatttatcagtcaggggattcctgtgtacctcttttctgatataacgc
caacccccttttgtgcccttcacagtatcacatttgaaactttgtgctgga
atcatgataactgcatctcacaatccaaagcaggataatggttataaggt
ctattgggataatggagctcagatcatttctcctcacgataaagggattt
ctcaagctattgaagaaaatctagaaccgtggcctcaagcttgggacgat
tctttaattgatagcagtccacttctccacaatccgagtgcttccatcaa
taatgactactttgaagaccttaaaaagtactgtttccacaggagcgtga
acagggagacaaaggtgaagtttgtgcacacctctgtccatggggtgggt

catagctttgtgcagtcagctttcaaggcttttgaccttgttcctcctga
ggctgttcctgaacagaaagatccggatcctgagtttccaacagtgaaat
acccgaatcccgaagaggggaaaggtgtcttgactttgtcttttgctttg
gctgacaaaaccaaggccagaattgttttagctaacgacccggatgctga
tagacttgctgtggcagaaaagcaagacagtggtgaatggagggtgtttt
caggcaatgagttgggggccctcctgggctggtggctttttacatcttgg
aaagagaagaaccaggatcgcagtgctctcaaagacacgtacatgttgtc
cagcaccgtctcctccaaaatcttgcgggccattgccttaaaggaaggtt
ttcattttgaggaaacattaactggctttaagtggatgggaaacagagcc
aaacagctaatagaccaggggaaaactgttttatttgcatttgaagaagc
tattggatacatgtgctgcccttttgttctggacaaagatggagtcagtg
ccgctgtcataagtgcagagttggctagcttcctagcaaccaagaatttg
tctttgtctcagcaactaaaggccatttatgtggagtatggctaccatat
tactaaagcttcctattttatctgccatgatcaagaaaccattaagaaat
tatttgaaaacctcagaaactacgatggaaaaaataattatccaaaagct
tgtggcaaatttgaaatttctgccattagggaccttacaactggctatga
tgatagccaacctgataaaaaagctgttcttcccactagtaaaagcagcc
aaatgatcaccttcacctttgctaatggaggcgtggccaccatgcgcacc
agtgggacagagcccaaaatcaagtactatgcagagctgtgtgccccacc
tgggaacagtgatcctgagcagctgaagaaggaactgaatgaactggtca
gtgctattgaagaacatttttttccagccacagaagtacaatctgcagcca
aaagcagactaaaatagtccagccttgggtatacttgcatttacctacaa
ttaagctgggtttaacttgttaagcaatatttttaagggccaaatgattc
aaaacatcacaggtatttatgtgttttacaaagacctacattcctcattg
tttcatgtttgacctttaaggtgaaaaaagaaaatggccaaacccaacaa
actaacattcctactaaaaagttgagcttggacatattttgaatttttgt
aagtgaagatttttaaactgactaacttaaaaaaatagattgtaattgat
gtgccttaatttgcataaatcataaatgtatgtcctctctgtaattgttt
taatgtgtgcttgaaatatccagaaaacctatggagttagtaaattctgg
gctgtcatatgtaggatagccacttttttaggtatatgtacatttatattt
ctatcaattccttagaaagtaaaataaatgaatagatcaaatgttgtgtt
catgtttggggaaaatataatttgcagaaacctatgaagtagagcaaaga
tgctttaaaaagataagttttttttgaactaaattttttttagttctaata
atgcacataggatattagtacatcgtacacgtgctaggaaaaaacagctt
cagtgtctttgtttaatgtgttgaaactcatctttttaaatcttgaaaaa
ccaattgtttacttgaaacttgaaagtagcatatttttctgtttttttggt
tgtttgttcatttgtattagcacaatttaatgtaattcctggtttggagg
cagcaagacctatgagcaagaactatttacttgaccctcgtttttttctc
ttgttcttgtgtggtctgaaatctaaaactagactttattatgatagatt
tcctataagccaatttctaataacaaatagatttattatttaatctgtac
cttctatcttctcataattcgtggtcttacagccttccaaaataactcca
gttgggcacccatgagctaggatcaaactttctttatatactttatatat
tttacattatttctgatttttaaagcaaatgattgccattatgattacac
tcaacctaaatagttatgaacagtttcagaacaatgaaaaattacaatac
tatgtgatagtattgtaactatttttctattttagtcatatgtcgcttat
atcctaccagaactcttaaatctataatattcgatatattctacaaactg

ctttattgtagaagccatatttatgtttattttataatgttttctagtgt
caaactgtactgtggagaaaagaaatgttagatctgtg

>NM_005345 5
ataaaagcccaggggcaagcggtccggataacggctagcctgaggagctg
ctgcgacagtccactacctttttcgagagtgactcccgttgtcccaaggc
ttcccagagcgaacctgtgcggctgcaggcaccggcgcgtcgagtttccg
gcgtccggaaggaccgagctcttctcgcggatccagtgttccgtttccag
cccccaatctcagagcggagccgacagagagcagggaaccggcatggcca
aagccgcggcgatcggcatcgacctgggcaccacctactcctgcgtgggg
gtgttccaacacggcaaggtggagatcatcgccaacgaccagggcaaccg
caccacccccagctacgtggccttcacggacaccgagcggctcatcgggg
atgcggccaagaaccaggtggcgctgaacccgcagaacaccgtgtttgac
gcgaagcggctgattggccgcaagttcggcgacccggtggtgcagtcgga
catgaagcactggcctttccaggtgatcaacgacggagacaagcccaagg
tgcaggtgagctacaaggggggagaccaaggcattctaccccgaggagatc
tcgtccatggtgctgaccaagatgaaggagatcgccgaggcgtacctggg
ctacccggtgaccaacgcggtgatcaccgtgccggcctacttcaacgact
cgcagcgccaggccaccaaggatgcgggtgtgatcgcggggctcaacgtg
ctgcggatcatcaacgagcccacggccgccgccatcgcctacggcctgga
cagaacgggcaaggggggagcgcaacgtgctcatctttgacctgggcgggg
gcaccttcgacgtgtccatcctgacgatcgacgacggcatcttcgaggtg
aaggccacggccggggacacccacctgggtggggaggactttgacaacag
gctggtgaaccacttcgtggaggagttcaagagaaaacacaagaaggaca
tcagccagaacaagcgagccgtgaggcggctgcgcaccgcctgcgagagg
gccaagaggaccctgtcgtccagcacccaggccagcctggagatcgactc
cctgtttgagggcatcgacttctacacgtccatcaccagggcgaggttcg
aggagctgtgctccgacctgttccgaagcaccctggagcccgtggagaag
gctctgcgcgacgccaagctggacaaggcccagattcacgacctggtcct
ggtcgggggctccacccgcatccccaaggtgcagaagctgctgcaggact
tcttcaacgggcgcgacctgaacaagagcatcaaccccgacgaggctgtg
gcctacggggcggcggtgcaggcggccatcctgatgggggacaagtccga
gaacgtgcaggacctgctgctgctggacgtggctcccctgtcgctggggc
tggagacggccggaggcgtgatgactgccctgatcaagcgcaactccacc
atccccaccaagcagacgcagatcttcaccacctactccgacaaccaacc
cggggtgctgatccaggtgtacgagggcgagagggccatgacgaaagaca
acaatctgttggggcgcttcgagctgagcggcatccctccggcccccagg
ggcgtgccccagatcgaggtgaccttcgacatcgatgccaacggcatcct
gaacgtcacggccacggacaagagcaccggcaaggccaacaagatcacca
tcaccaacgacaagggccgcctgagcaaggaggagatcgagcgcatggtg
caggaggcggagaagtacaaagcggaggacgaggtgcagcgcgagagggt
gtcagccaagaacgccctggagtcctacgccttcaacatgaagagcgccg
tggaggatgaggggctcaagggcaagatcagcgaggcggacaagaagaag
gtgctggacaagtgtcaagaggtcatctcgtggctggacgccaacacctt
ggccgagaaggacgagtttgagcacaagaggaaggagctggagcaggtgt
gtaaccccatcatcagcggactgtaccagggtgccggtggtcccgggcct

gggggcttcggggctcagggtcccaagggagggtctgggtcaggccccac
cattgaggaggtagattaggggcctttccaagattgctgtttttgttttg
gagcttcaagactttgcatttcctagtatttctgtttgtcagttctcaat
ttcctgtgtttgcaatgttgaaatttttttggtgaagtactgaacttgctt
tttttccggtttctacatgcagagatgaatttatactgccatcttacgac
tatttcttctttttaatacacttaactcaggccattttttaagttggtta
cttcaaagtaaataaactttaaaattcaaaaaaaaaaaaaaaaaa

>NM_021913 3
gggaaggaggcaggggtgctgagaaggcggctgctgggcagagccggtgg
caagggcctcccctgccgctgtgccaggcaggcagtgccaaatccgggga
gcctggagctgggggggagggccggggacagcccggccctgccccctcccc
cgctgggagcccaacaacttctgaggaaagtttggcacccatggcgtggc
ggtgccccaggatgggcagggtcccgctggcctggtgcttggcgctgtgc
ggctgggcgtgcatggcccccaggggcacgcaggctgaagaaagtccctt
cgtgggcaacccagggaatatcacaggtgcccggggactcacgggcaccc
ttcggtgtcagctccaggttcagggagagccccccgaggtacattggctt
cgggatggacagatcctggagctcgcggacagcacccagacccaggtgcc
cctgggtgaggatgaacaggatgactggatagtggtcagccagctcagaa
tcacctccctgcagctttccgacacgggacagtaccagtgtttggtgttt
ctgggacatcagaccttcgtgtcccagcctggctatgttgggctggaggg
cttgccttacttcctggaggagcccgaagacaggactgtggccgccaaca
ccccccttcaacctgagctgccaagctcagggaccccccagagcccgtggac
ctactctggctccaggatgctgtccccctggccacggctccaggtcacgg
cccccagcgcagcctgcatgttccagggctgaacaagacatcctctttct
cctgcgaagcccataacgccaagggggtcaccacatcccgcacagccacc
atcacagtgctcccccagcagccccgtaacctccacctggtctcccgcca
acccacggagctggaggtggcttggactccaggcctgagcggcatctacc
ccctgacccactgcaccctgcaggctgtgctgtcagacgatgggatgggc
atccaggcgggagaaccagacccccccagaggagcccctcacctcgcaagc
atccgtgccccccatcagcttcggctaggcagcctccatcctcacaccc
cttatcacatccgcgtggcatgcaccagcagccagggcccctcatcctgg
acccactggcttcctgtggagacgccggagggagtgcccctgggcccccc
tgagaacattagtgctacgcggaatgggagccaggccttcgtgcattggc
aagagccccgggcgcccctgcagggtaccctgttagggtaccggctggcg
tatcaaggccaggacaccccagaggtgctaatggacatagggctaaggca
agaggtgaccctggagctgcagggggacgggtctgtgtccaatctgacag
tgtgtgtggcagcctacactgctgctggggatggaccctggagcctccca
gtacccctggaggcctggcgcccagggcaagcacagccagtccaccagct
ggtgaaggaaccttcaactcctgccttctcgtggccctggtggtatgtac
tgctaggagcagtcgtggccgctgcctgtgtcctcatcttggctctcttc
cttgtccaccggcgaaagaaggagacccgttatggagaagtgtttgaacc
aacagtggaaagaggtgaactggtagtcaggtaccgcgtgcgcaagtcct
acagtcgtcggaccactgaagctaccttgaacagcctgggcatcagtgaa
gagctgaaggagaagctgcgggatgtgatggtggaccggcacaaggtggc
cctggggaagactctgggagagggagagtttggagctgtgatggaaggcc

agctcaaccaggacgactccatcctcaaggtggctgtgaagacgatgaag
attgccatctgcacgaggtcagagctggaggatttcctgagtgaagcggt
ctgcatgaaggaatttgaccatcccaacgtcatgaggctcatcggtgtct
gtttccagggttctgaacgagagagcttcccagcacctgtggtcatctta
cctttcatgaaacatggagacctacacagcttcctcctctattcccggct
cggggaccagccagtgtacctgcccactcagatgctagtgaagttcatgg
cagacatcgccagtggcatggagtatctgagtaccaagagattcatacac
cgggacctggcggccaggaactgcatgctgaatgagaacatgtccgtgtg
tgtggcggacttcgggctctccaagaagatctacaatggggactactacc
gccagggacgtatcgccaagatgccagtcaagtggattgccattgagagt
ctagctgaccgtgtctacaccagcaagagcgatgtgtggtccttcggggt
gacaatgtgggagattgccacaagaggccaaaccccatatccgggcgtgg
agaacagcgagatttatgactatctgcgccagggaaatcgcctgaagcag
cctgcggactgtctggatggactgtatgccttgatgtcgcggtgctggga
gctaaatccccaggaccggccaagttttacagagctgcgggaagatttgg
agaacacactgaaggccttgcctcctgcccaggagcctgacgaaatcctc
tatgtcaacatggatgagggtggaggttatcctgaacccccctggagctgc
aggaggagctgacccccccaacccagccagaccctaaggattcctgtagct
gcctcactgcggctgaggtccatcctgctggacgctatgtcctctgccct
tccacaacccctagccccgctcagcctgctgataggggctccccagcagc
cccagggcaggaggatggtgcctgagacaaccctccacctggtactccct
ctcaggatccaagctaagcactgccactggggaaaactccaccttcccac
tttcccaccccacgccttatccccacttgcagccctgtcttcctacctat
cccacctccatcccagacaggtccctccccttctctgtgcagtagcatca
ccttgaaagcagtagcatcaccatctgtaaaaggaaggggttggattgca
atatctgaagccctcccaggtgttaacattccaagactctagagtccaag
gtttaaagagtctagattcaaaggttctaggtttcaaagatgctgtgagt
ctttggttctaaggacctgaaattccaaagtctctaattctattaaagtg
ctaaggttctaaggcctactttttttttttttttttttttttttttttt
gcgatagagtctcactgtgtcacccaggctggagtgcagtggtgcaatct
cgcctcactgcaaccttcacctaccgagttcaagtgattttcctgccttg
gcctcccaagtagctgggattacaggtgtgtgccaccacacccggctaat
tttatatttttagtagagacagggtttcaccatgttggccaggctggtc
taaaactcctgacctcaagtgatctgcccacctcagcctcccaaagtgct
gagattacaggcatgagccactgcactcaaccttaagacctactgttcta
aagctctgacattatgtggttttagattttctggttctaacatttttgat
aaagcctcaaggttttaggttctaaagttctaagattctgattttaggag
ctaaggctctatgagtctagatgtttattcttctagagttcagagtcctt
aaaatgtaagattatagattctaaagattctatagttctagacatggagg
ttctaaggcctaggattctaaaatgtgatgttctaaggctctgagagtct
agattctctggctgtaaggctctagatcataaggcttcaaaatgttatct
tctcaagttctaagattctaatgatgatcaattatagtttctgaggcttt
atgataatagattctcttgtataagatcctagatcctaagggtcgaaagc
tctagaatctgcaattcaaaagttccaagagtctaaagatggagtttcta
aggtccggtgttctaagatgtgatattctaagacttactctaagatctta
gattctctgtgtctaagattctagatcagatgctccaagattctagatga

ttaaataagattctaacggtctgttctgtttcaaggcactctagattcca
ttggtccaagattccggatcctaagcatctaagttataagactctcacac
tcagttgtgactaactagacaccaaagttctaataatttctaatgttgga
cacctttaggttctttgctgcattctgcctctctaggaccatggttaaga
gtccaagaatccacatttctaaaatcttatagttctaggcactgtagttc
taagactcaaatgttctaagtttctaagattctaaaggtccacaggtcta
gactattaggtgcaatttcaaggttctaaccctatactgtagtattcttt
ggggtgcccctctccttcttagctatcattgcttcctcctccccaactgt
gggggtgtgccccccttcaagcctgtgcaatgcattagggatgcctccttt
cccgcaggggatggacgatctcccacctttcgggccatgttgcccccgtg
agccaatccctcaccttctgagtacagagtgtggactctggtgcctccag
aggggctcaggtcacataaaactttgtatatcaacgaaaaaaa

>NM_022071 3
ctggagagcctccaggcgcccgggtggggccgcgattgcgccccgccagt
caccccggcatgtgggcgcggcggggcggcgcgggcgggacggctacaag
agctgttttgcgtccgggccggagtatttgctcagcccgcctgcgccgct
tgggacgcctctgcctttccctccctcccttccccgacggcttctggcgg
ccaagtggatgtggcgggtgatcgagccaccctgcccaggggcgcccagc
actggttcagtgaacagcattttggacaggacatttggtgccaggtctga
gtagccagtttgctgaattattgtcccagtcagccaggattgtgagctgt
ttgggaagtttcgtggaaacgcccaagtgccagcacaggtggagggacac
ctggaggccagtttcaggaacttttgccacaagtataaaagacttcagaa
gtgcaaagatgctgaaacagatactgtcggagatgtacatagatcctgat
ctactggcagagctcagcgaagaacagaaacagatcctgttcttcaagat
gagagaggaacagatccgacgatggaaagaaagagaagcagctatggaaa
gaaaggagtccctgccagtgaaacccagaccaaagaaagagaatggcaaa
tcggttcattggaaacttggagctgataaggaagtctgggtatgggtgat
gggcgaacaccatctagataaaccctatgatgtgctctgtaatgaaatta
ttgctgagagggcccggctgaaagcagaacaggaggcagaagagcccaga
aaaactcactctgaagaattcaccaatagcttgaaaacaaaatcacagta
ccatgatctgcaggctccggataaccagcagactaaagacatctggaaga
aagtggcagaaaaggaggaactggagcaaggatcgaggccagcaccaacc
ctggaagaagagaaaatccgatcactctccagttcttcaagaaatattca
acaaatgttggcagattcaatcaatcgtatgaaggcatatgcatttcacc
agaagaaagaatctatgaagaaaaaacaagatgaagaaataaatcaaata
gaagaagagagaacgaagcagatttgtaagagctggaaagaagactcgga
atggcaggcatctctgcgaaaatccaaagcagctgatgagaagagacgct
ccttggctaaacaagcacgagaagactacaagaggttatccctcggggcc
cagaaaggaagaggcggtgagaggctgcaaagccccttgcgtgttccgca
gaaaccagaaagacctccccttccacccaagcctcagttcctaaactcag
gggcatatcctcaaaaacctcttagaaatcagggagtggtgaggacactg
tccagctctgcccaagaggacatcatccggtggtttaaagaggagcagct
accacttcgagcgggctaccagaaaacctcagacaccatagccccctggt
tccatggaattctcacactcaagaaagcaaatgaacttcttctgagcaca
ggcatgcccggcagttttctcatccgagtcagtgaaaggatcaaaggcta

tgccctgtcctatctgtcggaggacggctgtaaacatttcctcatcgatg
cctctgcagacgcctacagcttcctgggcgtggaccagctacagcatgcc
accttggcggatttggtggaatatcacaaggaggaacccatcacttccct
ggggaaggagctccttctctatccctgtggtcagcaggaccagctgcctg
actacctggagctgtttgagtgacagcctccatcagggtcatcctacagc
ctccaagcgggctttcccctggacaaatgccactgcaacatttatgtgtg
aagccaaaatcaccctgcagcagagccaatactgatcaactgaaagtaaa
gtatccatggagtcctcattgacacctcttttctgcacaaatactggaat
tcaatgtcaagagaaaatgacctctgctcaaaagggagaagagtctcaat
ttcagcaagtacctgtcatgaagggtatgaccttaatgatgtacataaaa
taaaacaaatgaagaaatggaaaacttttagaaattaaggtgtacttgaa
aacgaatatctatcatatgaccccctgcactccctctgtatcatctcagga
ggtttcaggggcctgttgacatgaagtttcgaagtttcatgttggctttg
gaatggtagcaaaagcctttcctggctgagatgatgcttaaaacacacct
cacttattgtacatgttggaaccaggacatgagagacatagaaaaacaga
agtcatgaatgtaaattgaatgagaggcttaacatgcatgaaaatacaga
tggacctgcaggaaagtgagcaaacatcgctgagtttgttttcttgttcg
ggagaatggggccggggctggcctggcctcccctggatatactctatagt
gcaccaaaaggataaagcatctgtacatgtattttttatttttttatcag
aagtgcttagacaagaacagaataagcaggctgtttggatgctacttgtg
gttgaattgtgttcccccaaaatatatggtgaagtcttaaccccccatccc
cgtgaatgggaccttgtttggaaatagggtctttgcagatatagtcaaga
tgaggtcacattggattagggtgggccccaaatccaatgactggcatcct
taggagaagagagagttttggtaatagacacaaatgcagtgggaagaaga
ccaggggacaagaggcaagttggagtgatgcagccggaagggaagggaca
ccaaggatctccggccaccagcagaagccagcagagaggcatgggacagg
ttccccacaagccttagaaggaagcatggccctgacttcagaattccaga
ctccagaactggaagaataaatgtctgttgttttaagctgcttagttcat
gctgagttcatgctgacttgttactatagccccagaaagctaatacagtc
gtttatgtaattacataacctgacacacaagatcgacccattcactgctg
cccagtccaccattttcataatgaagtagaaatgggaggtaagaaaaaca
ttccagccagttctgtttagccctggcacacatatttgtcccgtcaggaa
tcttatgccctcctggaacccccgcccacctcagtccagtcccagtcagg
cgaacggcctctggacagggactgaggtggctctgagccactggagatca
tttttcttggaggatggagattggctagtacctctggcctaactgtgtag
gtcaatactcttttacattgccttctaataaaagcagaatgatacagcaa
aaaaaaaaaaaaaaaa

>NM_001194 3
ggccggcggcggcggcggcggctccgctccgcactgcccggcgccgcctc
gccatggacgcgcgcgggggcggcgggcggcccggggagagcccgggcgc
gacccccgcgccggggccgccgccgccgccgccgcccgcgcccccccaac
agcagccgccgccgccgccgccgcccgcgcccccccccgggccccgggccc
gcgcccccccagcacccgccccgggccgaggcgttgcccccggaggcggc
ggatgagggcggcccgcggggccggctccgcagccgcgacagctcgtgcg
gccgccccggcaccccgggcgcggcgagcacggccaagggcagcccgaac

ggcgagtgcgggcgcggcgagccgcagtgcagccccgcggggcccgaggg
cccggcgcgggggcccaaggtgtcgttctcgtgccgcggggcggcctcgg
ggcccgcgccggggccggggccggcggaggaggcgggcagcgaggaggcg
ggcccggcgggggagccgcgcggcagccaggccagcttcatgcagcgcca
gttcggcgcgctcctgcagccgggcgtcaacaagttctcgctgcggatgt
tcggcagccagaaggccgtggagcgcgagcaggagcgcgtcaagtcggcg
ggggcctggatcatccacccgtacagcgacttcaggttctactgggactt
caccatgctgctgttcatggtgggaaacctcatcatcatcccagtgggca
tcaccttcttcaaggatgagaccactgccccgtggatcgtgttcaacgtg
gtctcggacaccttcttcctcatggacctggtgttgaacttccgcaccgg
cattgtgatcgaggacaacacggagatcatcctggaccccgagaagatca
agaagaagtatctgcgcacgtggttcgtggtggacttcgtgtcctccatc
cccgtggactacatcttccttatcgtggagaagggcattgactccgaggt
ctacaagacggcacgcgccctgcgcatcgtgcgcttcaccaagatcctca
gcctcctgcggctgctgcgcctctcacgcctgatccgctacatccatcag
tgggaggagatcttccacatgacctatgacctggccagcgcggtgatgag
gatctgcaatctcatcagcatgatgctgctgctctgccactgggacggct
gcctgcagttcctggtgcctatgctgcaggacttcccgcgcaactgctgg
gtgtccatcaatggcatggtgaaccactcgtggagtgaactgtactcctt
cgcactcttcaaggccatgagccacatgctgtgcatcgggtacggccggc
aggcgcccgagagcatgacggacatctggctgaccatgctcagcatgatt
gtgggtgccacctgctacgccatgttcatcggccacgccactgccctcat
ccagtcgctggactcctcgcggcgccagtaccaggagaagtacaagcagg
tggagcagtacatgtccttccacaagctgccagctgacttccgccagaag
atccacgactactatgagcaccgttaccagggcaagatgtttgacgagga
cagcatcctgggcgagctcaacgggcccctgcgggaggagatcgtcaact
tcaactgccggaagctggtggcctccatgccgctgttcgccaacgccgac
cccaacttcgtcacggccatgctgaccaagctcaagttcgaggtcttcca
gccgggtgactacatcatccgcgaaggcaccatcgggaagaagatgtact
tcatccagcacggcgtggtcagcgtgctcactaagggcaacaaggagatg
aagctgtccgatggctcctacttcggggagatctgcctgctcacccgggg
ccgccgcacggcgagcgtgcgggctgacacctactgccgcctctattcgc
tgagcgtggacaacttcaacgaggtgctggaggagtaccccatgatgcgg
cgcgccttcgagacggtggccatcgaccgcctggaccgcatcggcaagaa
gaattccatcctcctgcacaaggtgcagcatgacctcaactcgggcgtat
tcaacaaccaggagaacgccatcatccaggagatcgtcaagtacgaccgc
gagatggtgcagcaggccgagctgggtcagcgcgtgggcctcttcccgcc
gccgccgccgccgccgcaggtcacctcggccatcgccacgctgcagcagg
cggcggccatgagcttctgcccgcaggtggcgcggccgctcgtggggccg
ctggcgctcggctcgccgcgcctcgtgcgccgcccgcccccggggcccgc
acctgccgccgcctcacccgggcccccgcccccgccagcccccgggcg
cgcccgccagcccccgggcaccgcggacctcgccctacggcggcctgccc
gccgccccccttgctgggcccgccctgcccgcgcgccgcctgagccgcgc
gtcgcgcccactgtccgcctcgcagccctcgctgcctcacggcgcccccg
gccccgcggcctccacacgcccggccagcagctccacaccgcgcttgggg
cccacgcccgctgcccgggccgccgcgcccagcccggaccgcagggactc

ggcctcacccggcgccgccggcggcctggacccccaggactccgcgcgct
cgcgcctctcgtccaacttgtgaccctcgccgaccgccccgcgggcccag
gcgggccgggggcggggccgtcatccagaccaaagccatgccattgcgct
gccccggccgccagtccgcccagaagccatagacgagacgtaggtagccg
tagttggacggacgggcagggccggcggggcagccccctccgcgcccccg
gccgtccccccctcatcgccccgcgcccacccccatcgcccctgcccccgg
cggcggcctcgcgtgcgagggggctcccttcacctcggtgcctcagttcc
cccagctgtaagacagggacggggcggcccagtggctgagaggagccggc
tgtggagccccgcccgccccccaccctctaggtggcccccgtccgaggag
gatcgttttctaagtgcaatacttggcccgccggcttcccgctgcccccca
tcgcgctcacgcaataaccggcccggcccccgtccgcgcgcgtcccccgg
tgacctcggggagcagcaccccgcctccctccagcactggcaccgagagg
caggcctggctgcgcagggcgcgggggggaggctggggtcccgccgccgt
gatgaatgtactgacgagccgaggcagcagtgcccccaccgtggcccccc
acgccccattaacccccacacccccattccgcgcaataaacgacagcatt
ggcgccaaaaaaaaaaaaaaaaaaaaaaaaaaaaaaaaaaaaaaaaaaaa
aaaaaaaaa

>NM_005477 2
caaaaatgccagggaaaggcgagcccagagcttggtgatggagaaattgg
gaagccaccccccacccttcaatcttaggatggggaattcgcaactgaag
ccggagcttcagacttggggcgcactcccagcttagcccaggaaagagat
ttaagggcgcagcagtgtggatacctctcaccccggccccgaaggtctag
cgagggtctaacctgggccccttgccaggcccgccccccgcccctttcca
gcccccggcccgtgcgccgctgcccctttaagaagcccaggtaggcaggc
ccggctgctggagccgctcctatggcaacccgcgagctgcggcggcttca
tgaatattccggggcgcgggagcccgagcgctgccggagggcgcttcggg
ggaggcggccgctgatgtaagcccggcgggtcgctgggctccgctcggtt
gcggcgggagccccgggacgggccggacgggccggggcagaggaggcgag
gcgagctcgcgggtggccagccacaaagcccgggcggcgagacagacgga
cagccagccctcccgcgggacgcacgcccgggacccgcgcgggccgtgcg
ctctgcactccggagcggttccctgagcgccgcggccgcagagcctctcc
ggccggcgcccattgttccccgcgggggcggggcgcctggagccgggcgg
cgcgccgcgcccctgaacgccagagggagggagggaggcaagaagggagc
gcggggtccccgcgcccagccgggcccgggaggaggtgtagcgcggcgag
cccggggactcggagcgggactaggatcctccccgcggcgcgcagcctgc
ccaagcatgggcgcctgaggctgcccccacgccggcggcaaaggacgcgt
ccccacgggcggactgaccggcgggcggacctggagcccgtccgcggcgc
cgcgctcctgcccccggcccggtccgaccccggcccctggcgccatggac
aagctgccgccgtccatgcgcaagcggctctacagcctcccgcagcaggt
gggggccaaggcgtggatcatggacgaggaagaggacgccgaggaggagg
gggccgggggccgccaagaccccagccgcaggagcatccggctgcggcca
ctgccctcgccctccccctcggcggccgcgggtggcacggagtcccggag
ctcggccctcggggcagcggacagcgaagggccggcccgcggcgcgggca
agtccagcacgaacggcgactgcaggcgcttccgcgggagcctggcctcg
ctgggcagccggggcggcggcagcggcggcacggggagcggcagcagtca

cggacacctgcatgactccgcggaggagcggcggctcatcgccgagggcg
acgcgtcccccggcgaggacaggacgcccccaggcctggcggccgagccc
gagcgccccggcgcctcggcgcagcccgcagcctcgccgccgccgcccca
gcagccaccgcagccggcctccgcctcctgcgagcagccctcggtggaca
ccgctatcaaagtggagggaggcgcggctgccggcgaccagatcctcccg
gaggccgaggtgcgcctgggccaggccggcttcatgcagcgccagttcgg
ggccatgctccaacccggggtcaacaaattctccctaaggatgttcggca
gccagaaagccgtggagcgcgaacaggagagggtcaagtcggccggattt
tggattatccacccctacagtgacttcagattttactgggacctgaccat
gctgctgctgatggtgggaaacctgattatcattcctgtgggcatcacct
tcttcaaggatgagaacaccacaccctggattgtcttcaatgtggtgtca
gacacattcttcctcatcgacttggtcctcaacttccgcacagggatcgt
ggtggaggacaacacagagatcatcctggacccgcagcggattaaaatga
agtacctgaaaagctggttcatggtagatttcatttcctccatccccgtg
gactacatcttcctcattgtggagacacgcatcgactcggaggtctacaa
gactgcccgggccctgcgcattgtccgcttcacgaagatcctcagcctct
tacgcctgttacgcctctcccgcctcattcgatatattcaccagtgggaa
gagatcttccacatgacctacgacctggccagcgccgtggtgcgcatcgt
gaacctcatcggcatgatgctcctgctctgccactgggacggctgcctgc
agttcctggtacccatgctacaggacttccctgacgactgctgggtgtcc
atcaacaacatggtgaacaactcctgggggaagcagtactcctacgcgct
cttcaaggccatgagccacatgctgtgcatcggctacgggcggcaggcgc
ccgtgggcatgtccgacgtctggctcaccatgctcagcatgatcgtgggt
gccacctgctacgccatgttcattggccacgccactgccctcatccagtc
cctggactcctcccggcgccagtaccaggaaaagtacaagcaggtggagc
agtacatgtcctttcacaagctcccgcccgacacccggcagcgcatccac
gactactacgagcaccgctaccagggcaagatgttcgacgaggagagcat
cctgggcgagctaagcgagcccctgcgggaggagatcatcaactttaact
gtcggaagctggtggcctccatgccactgtttgccaatgcggaccccaac
ttcgtgacgtccatgctgaccaagctgcgtttcgaggtcttccagcctgg
ggactacatcatccgggaaggcaccattggcaagaagatgtacttcatcc
agcatggcgtggtcagcgtgctcaccaagggcaacaaggagaccaagctg
gccgacggctcctactttggagagatctgcctgctgacccggggccggcg
cacagccagcgtgagggccgacacctactgccgcctctactcgctgagcg
tggacaacttcaatgaggtgctggaggagtaccccatgatgcgaagggcc
ttcgagaccgtggcgctggaccgcctggaccgcattggcaagaagaactc
catcctcctccacaaagtccagcacgacctcaactccggcgtcttcaact
accaggagaatgagatcatccagcagattgtgcagcatgaccgggagatg
gcccactgcgcgcaccgcgtccaggctgctgcctctgccaccccaacccc
cacgcccgtcatctggaccccgctgatccaggcaccactgcaggctgccg
ctgccaccacttctgtggccatagccctcacccaccaccctcgcctgcct
gctgccatcttccgccctcccccaggatctgggctgggcaacctcggtgc
cgggcagacgccaaggcacctgaaacggctgcagtccctgatcccttctg
cgctgggctccgcctcgcccgccagcagcccgtcccaggtggacacaccg
tcttcatcctccttccacatccaacagctggctggattctctgcccccgc
tggactgagcccactcctgccctcatccagctcctccccacccccgggg

cctgtggctccccctcggctcccacaccatcagctggcgtagccgccacc
accatagccgggtttggccacttccacaaggcgctgggtggctccctgtc
ctcctccgactctcccctgctcaccccgctgcagccaggcgcccgctccc
cgcaggctgcccagccatctcccgcgccacccggggcccggggaggcctg
ggactcccggagcacttcctgccacccccaccctcatccagatccccgtc
atctagccccgggcagctgggccagcctcccggggagttgtccctaggtc
tggccactggcccactgagcacgccagagacacccccacggcagcctgag
ccgccgtcccttgtggcaggggcctctgggggggcttcccctgtaggctt
tactccccgaggaggtctcagccccccctggccacagcccaggcccccccaa
gaaccttcccgagtgccccgccccgggcctctggctcccacggatccttg
ctcctgccacctgcatccagccccccaccaccccaggtcccccagcgccg
gggcacaccccccgctcaccccccggccgcctcacccaggacctcaagctca
tctccgcgtctcagccagccctgcctcaggacggggcgcagactctccgc
agagcctccccgcactcctcaggggagtccatggctgccttcccgctctt
ccccagggctgggggtggcagcgggggcagtgggagcagcgggggcctcg
gtccccctgggaggccctatggtgccatccccggccagcacgtcactctg
cctcggaagacatcctcaggttctttgccaccccctctgtctttgtttgg
ggcaagagccacctcttctgggggggcccccctctgactgctggaccccaga
gggaacctggggccaggcctgagccagtgcgctccaaactgccatccaat
ctatgagctgggcccttccttccctcttctttcttcttttctctcccttc
cttcttccttcaggtttaactgtgattaggagatataccaataacagtaa
taattatttaaaaaaccacacacaccagaaaaacaaaagacagcagaaaa
taaccaggtattcttagagctatagatttttggtcacttgcttttataga
ctattttaatactcagcactagagggagggagggggagggaggagggagc
aggcaggtcccaaatgcaaaagccagagaaaggcagatggggtctccggg
gctgggcaggggtgggagtggccagtgttggcggttcttagagcagatgt
gtcattgtgttcatttagagaaacagctgccatcagcccgttagctgtaa
cttggagctccactctgcccccagaaaggggctgccctggggtgtgccct
ggggagcctcagaagcctgcgaccttgggagaaaagggccagggccctga
gggcctagcatttttttctactgtaaacgtagcaagatctgtatatgaata
tgtatatgtatatgtatgtaagatgtgtatatgtatagctatgtagcgct
ctgtagagccatgtagatagccactcacatgtgcgcacacgtgtgcggtc
tagtttaatcccatgttgacaggatgcccaggtcaccttacacccagcaa
cccgccttggcccacaggctgtgcactgcatggtctagggacgttctctc
tccagtcctcagggaagaggaccccaggacttcgcagcaggccccctctc
tccccatctctggtctcaaagccagtcccagcctgacctctcaccacacg
gaagtggaagactccccctttcctagggcctcaagcacacaccgccacctc
tggggccgtcagtttgcccatctgtacagtgggaggtgagcggaacttct
gtttattgagtctgctctgtgccaagcactggtttcgcactttacacaca
ttaactccttcagtttcacaaagaccatggggtgggtactttgattctcc
ccatttagcagaggaagaaacagttttgggtaatttttccagaatcatgt
aactaggagtggcagagtggggactgatttgaggttcgagtccacgcctc
cttgaggcccaagtctgtgttccttccatcagaaaactgtgttgaggggg
gctgaggtagatggtccccaagcatggtacagaaggaagacaccagattt
tggcagcagtcaggcctgggtttgaatcccagccctgccacttcttagct
gtatgatcttgggcaagttatctgacctttctgtcacctcatttgtaaaa

tgggaataattatggtactgcctcacaaggacctatgaggaccagatgag
aaaaatctatatgtgaaatgcccagcccagcgcctggcacataccatggt
aggtgctcaataaaaaatcacatttcttctgcccctcatatgcccagcct
attgctccagcaaactatgtgagagcccagggagctttggctgagggctc
caagacttaaaatctcaggactcaggaggtggctgggcctccctaagggc
ccaaggaaggtgtgtggccagaggtgggtgggagccaggccttgagaagt
gggaagacttcaacagggagagagggagggaatggtgggtgggatggagt
gtatggtggggagattcctgaggtggatgtggagtggtggatcagggctt
tgggaggggatccccaggctgaggggtcagagggacggccttgggtgata
gggtaagggattgtctgggcttagtcctggcaactaggagccataagcag
gttccagattgcgggaacgagaaagcagctcagatgcctttggaggcacc
atcctccctcctcccagatgggatcttgccagagccaaggtcaggggtct
gcccctgcctatagggccagagcaggtatggctgcaatccccaagtaatg
agaagggctggtcccacattatccatccagaaccttccatgctccaagcc
agaatgttggcaagatcgggttttgccttgagctatcctgggatgtgaga
caaaccgatttctccatagatgggctgcagggagtgggaggcagtactcc
aggagagaagtgggtgaaggttcctgggatcttaggtaaagactagacgc
cgcctagtactggtctctactgtgctggctcaggagttctgagaactgga
aggacttagcctcaacctgagttctgcacacaccccttccccttaaggaa
ggcagctctgagaggcagcaggacttgatccaaacccacagtcttgtcct
ggaggcagcaggggtgaaggtggagggtccagggccatgaggagcccccct
tgccatcagagcctggcctaaccaccctcttctctacttacacacacatg
cattttataatagctctgacccaacctggccactctgcagagactgggac
agacaggtgcaggcaatgggccctcccacacccagtcacctacaaggaat
tttcaaatccacttttaaaacagaaaccggtaaatgcgccgtattgtata
ttttatttaaataaaaaaaattccagcaaaaaaaaaaaaaaaaaa

>NM_022098 3
ggaggtgcggctggcccggtttcctggcgacgcggccctgcaggcggttg
cgttccccgtcgttaccctctttctcttcccgacgcgtgagttaggccgt
aatgccttggctgctctcagcccccaagctggttcccgctgtagcaaacg
tccgcggcctctcaggatgtatgttgtgttcacagcgaaggtactcccttt
cagcctgtcccagaaaggaggattccaaaccgatacttaggccagcccag
ccccttttacacacccacacctcctcagaccaggggaggtaactccaggac
tatctcaggtggaatatgcacttcgcagacacaaactaatgtctctgatc
cagaaggaagctcaagggcagagtgggacagaccagacagtggttgtgct
ctccaaccctacatactacatgagcaacgatattccctatactttccacc
aagacaacaatttcctgtacctatgtggattccaagagcctgatagcatt
cttgtccttcagagcctccctggcaaacaattaccatcacacaaagccat
actttttgtgcctcggcgagatcccagtcgagaactttgggatggtccgc
gatctggcactgatggagcaatagctctaactggagtagacgaagcctat
acgctagaagaatttcaacatcttctaccaaaaatgaaagctgagacgaa
catggtttggtatgactggatgaggccctcacatgcacagcttcactctg
actatatgcagcccctgactgaggccaaagccaagagcaagaacaaggtt
cggggtgttcagcagctgatacagcgcctccggctgatcaagtctcctgc
agaaattgaacgaatgcagattgctgggaagctgacatcacaggctttca

tagaaaccatgttcaccagtaaagcccctgtggaagaagcctttctttat
gctaagtttgaatttgaatgccgggctcgtggcgcagacattttagccta
tccacctgtggtggctggtggtaatcggtcaaacactttgcactatgtga
aaaataatcaactcatcaaggatggggaaatggtgcttctggatggaggt
tgtgagtcttcctgctatgtgagtgacatcacacgtacgtggccagtcaa
tggcaggttcaccgcacctcaggcagaactctatgaagccgttctagaga
tccaaagagattgtttggccctctgcttccctgggacaagcttggagaac
atctacagcatgatgctgaccctgataggacagaagcttaaagacttggg
gatcatgaagaacattaaggaaaataatgccttcaaggctgctcgaaaat
actgtcctcatcatgttggccactacctcgggatggatgtccatgacact
ccagacatgccccgttccctccctctgcagcctgggatggtaatcacaat
tgagcccggcatttatattccagaggatgacaaagatgccccagagaagt
ttcggggtcttggtgtacgaattgaggatgatgtagtggtgactcaggac
tcacctctcatcctttctgcagactgtcccaaagagatgaatgacattga
acagatatgcagccaggcttcttgaccttcactgcggcccacatgcacct
caggttcaaaatgggtgtcttctggcagccctgcacgtgtgctttctgag
tgtctctgtgtgtgcattaatatatgcattccatttgggagcatagcagc
tgtgtgaatgtatgtaattgtgtgtggggggtttttttgttttaagtagtt
agaagtctgggaaaatgaatttttgaatagtatgttactgcagctttggt
aacattaattctatagaattaatgatcagagcaagtttaatttttaaaca
taaaggtcttggttacacatgtccatgcattccagttaacacatttaaac
atatagaaaattcacttcttctttcaagtccttccctttctatacttttg
ctgagatcaacccaatatcataagaagtttgtgtgatgcctgtattttta
gctaattaccaaggtctctgcctatgcaaaaattcatctttcggtgattg
tgggcggccaatacataccttctgtaactcctccctacctattctagatc
ctgcatatctactggagccatatgagccctcaggggcactggtgtcctgc
agtcttactagtcagccacagtccaggttccagcaacactagctacctac
caaacaaaacacttctgagcttttcttgccatttggttgcctgggacctg
gaaagtgtttagattcttgataaagatggaaatggaagagaaagaaaatt
atatatgtatatactaaagatccagacatcagggctgggcacagcggttc
cgcctgtaatcccagcactttgggaggtccaggcaggtggatctcttgag
accaggaggtcaagaccagcctggccaacgtggtgaaacactgtctctac
taaaaatacaaaaatgagctgggtgtgatgatacatgcctgtaatcctgg
ctacttgggaggctgaggcatgagaattgcttgaacctgggaggtggagg
ttgcagtgagctgagctcatgcgactgcactccagcctgggtgactgagc
aagactctgcctcaaaaaaacaaataaataaataaaatcctgacatcctg
ccatcttttctcttcaatttaaagttgatttcttaatgatgacttcaaaa
attaataaaaagaaaagaaaaaaatgatttctggccgggcgtggtggctc
aagcctgtaatcccagcactttgggaggccgaggtgggcggatcacgagg
tcaggagattgagacctcctggctaacacggtgaaaccccgtctttacta
aaaatacaaaaaaaattagctgggcgtggtggcggatgcctgtagtccca
gctactcaggaggctgaggcaggagaatggcgtgaacccagaaggcagag
cttgcagtgagccaagatcgcgccactgcactccagcctgggtgacagag
cgagactccgtctcaaaaaaaaaaaaaaaaaaagaaaaaaaaaaagaaagat
ttctttcgcagatatccctgctccctctatagattcttaatgcactgacc
ctcacaggttaccttttagctgttgtgctgcgcaggagcagatcctcta

cttacgtaccctgtcagagtcacagtagtagcgcccagttttcagagaag
taccccctgttatcattctgtgatttcctctagtgctaatagaaacaaaat
atggaatctaccaagtacccgtaaatatcctatgattcttatctttcttt
gatactagcactgggagtgcagtggtaaagagtcatacttcttttttttt
ttttttttggagacagagtctcgctctgtcgcctgggctggatggagtg
cagtggcgtaatctcagctcactacaacctccaccacctgggttcaggca
attctcctgcctcagcctcccaagtaactgggactacaggtgcacgctgc
catgcccagctaatttttttgtattttagtagagacagggtttcaccctgt
tgcccaggccggtctcaaactcctgagctccggcaatccgcccacctcag
cctcccaaagtgctaggattacaggcatgagccactgcgcccggtgtaag
agtcatattttttttaaacacaaacattggatacaattatacagcaactca
gtttgcaagatgaacagggctagattaccacagatagcttatttaggagc
gtctgtgatagagggaagctttaagaaccagtgtgttcacctcctttaaa
cctggatatgaggtcaagatgtataaaacatgccaattgttagaacacaa
atttgagggtttcttaaaaaaacaatttgtattagggaagcacaagctag
gggattaacaatctctgtaatgctggaagtttgcctatcttatcagggaa
acccagggtacagagtgaaaagcagacaggcagtgacttcctcaagagac
agataatgtgaaaacaaaagtgatacaaatcatatcctgacagtgctagc
ctaggaatgcctggctttgcgcaccttatctaagcagctctcagaatgga
ggctgcacagctggagtctccatcccccatacccaccttcccacctccct
ccaatttctcaatccagagctttttaaaaaaaggggagggaaataactcc
acagctctggctgctcacttcccaggctctgtgcccccaacagccctttc
cttggattgtgtttcctttgttcatttgtaatccaggaataaggaatttc
cttttcttcttgtatcattttcttttcttttcttttttttttttttttg
agacggagtctcacactgtcaccggggctggtgtgcaatagcgagacctc
agctccctgcaacctccatctcccaggttcaagcgattctcctgcctcag
cctcccgaatagctaggaatacaggcgcctgccaccacacccggctattt
ttttgtattttaaatagagacgaggtttcactgtgttggccaggctggtc
tcgaactcctgacctcatgatctgcccgcctcggcctccctaagtgctgc
gattacagacgtgagccaccgcacctcctgtatcattttctatggtctaa
acttttctagctaaaagcctaggtagcttgtatgtatagcagcatacttg
gacatgaaacacacttctggttagattccagtcaagttctataggtgtta
ttggtaggcagaaaggctgttgtagaattcttctggtgattttcagtccc
tgacccaggtatattgtcctttgagtcccagattaactaactatagcagc
taagcatttgaatcagacttctcatagcaatgttatgggctgtctgatat
attcaggatttgttgagcagataagctgtgtgtgatcttactcattctca
gccatgccgcagacatacccatttccctttagtaatttttttaatacagag
aatgctattaactgttactggatatcaaataattttatttttctaatagt
attttccaaatatttcttaaaattcttaaaatttaggttaaagtttgctg
gtctcttacatttaataaagctgggacttgaagacttaccatagttttca
actgcctttgcaagttcataaacttctaagggtaaaaagtgaataagata
aattcagagttttaaggtaaaggctttatattagcttttttttttttaa
aggtttttttgtgggggtttttttgtttttttttttttgagatggagtctc
gctctgtcacccaggctggagtgcagtggcacgatcttggctcactgcaa
cctccatctcccgggttcaggcaattcttctgcctcagcctcctgagtag
ctgggactacaggcgcgtgccaccatgcccggctagtttattgtattttt

agtagagatggggttttactgtgttagccaggatggtctcaatctcctga
cctcataatctgcccacctcagcctcccaaagtgctgggattacaggcgt
gagccactgcacccagccttttttaaagtttttaattgacatataattgta
cgtatttacagggtaaataatgatgttttgatacatatataatgatcaga
tcagaacaattagcatatccatcacattgaacatttatcatttatttgtg
ttgagcctttagttcttttttttttttgagatagagtctcactctgtcac
ccaggctggagtacagtggtgtgatcttggctcactgcaacctccaccca
ccggcttcaaccaattctcctgcctcagcctccctagtaactgggattac
aggcacctgccaccacacccaatttttgtatttttagtagagacggggtt
tcaccatcttggccaggccaggctggtcttgatctcctgaccttgtgatc
cacccacctcagcttcccaaactgctgggattacaggcatgagccacggc
ccccagccagagacagtcttgctttgtcgccaggctggagtgtagtggcg
ccatcttggctcactgcaacctccaactccctggttcaagcagttctcct
gcctcagcctcccaagtagctgggattacaggcacgcaccaccatgccca
gctaatttttgtatttttagtagagacagggtttcaccatgttggccagg
atggtcttgatctcctgacctcgtgatctgcccactttggcctcccaaag
tgctgggattacaggtgtgagccaccgtgcccagccgagcctttagttct
aagtataataaataatatatataccaggcattcctattaaaaatcttaac
ttaaaaaaaattactcaaatgttactactggaatttttctccctgaattg
aagatacatggttagatcattaacaataccacctaaatgaaggaaacata
tctggatatccagtgtcacatacttttatgtatttatttcaggaattact
attttgtcaccataattaggaaacagactgtgttctttatttgaaacaca
acagtataaatactatagtaaagttgggtgcagtggatcacagctataat
cccagcactttgggagaccaaggagggatggtcacttgagcccaggagtt
tgagaccagcctaggcaacatagagagaccttgtctcaacaaaaaaattt
aaagttagccaggtgtggtggtttgcacctatagtcccagctactcagga
ggctgaggtgggaggatcgcttgagcttaggaggtcaaggctgcagtgag
ccatgattgtgccacttcaccctgggcaacagagaaagaccctgtctcaa
taaaaaagaagagaaaaactggagtgagaactctcctcctctcataatca
tagtatccacgttaggaaaatgcttctgagagtaacttaaagtactgtgt
gtgcatcttccttcaaaaccttgctttttctccgtttttgtaaggtgag
aaagggaaaaaccttcaccgtagaaatactgtcttgagattaagatacaa
ataaaacatgttaaacctccatctaaagacattgcaacttattaaaacag
aaaaatgtacctttgttatgtctttccaaatatccacacccacaccctgt
tcagctttgttaataatacagagatcagaaatgtgaagcacaaatgctgc
ttctccccctcctgttcccatgtcagagatggagaagaaagcacagtgag
ctgttcaccagtccagcggtgacctcggagtcctttgtaacagtgtttcc
aataacttctgccaataaaacagaatttgagttagaacctatctgctgtc
cctcaactagtgtctgatagctgagaatgtaattcgtttgtgtgtctatt
agaaaattaggcaggcaccacagacagtagcaacataaaggaacctgctt
agaagaggtttttatggtgtgattgtgttttttttttttaatttttgtt
tcctgaagtagaatccatagctcaaaacaagttttaagcctgagaaaata
taatttgaggtgtttttttatgctcctaatgaaaagatgaatgaatgcatc
attaaaccttaacagtagagcagaatttcactgttacaaaccacatgggt
tctcctggttgtgctgctgttctctacctgtgtgtcactgagtttgagca
tcattattgtttgaatcagaggacccactggaatcaaggcttcttggaat

tgcgcaagaatctgagaaaacttcaatctttaccagcatttcttaatgct
ttccatgacttttgtgcaattatataagttcagtttagacaaaatgctgt
tactttttaaggcattactaataactgaaaatagttacgtcattgcccat
caagggaaaagcacttgtaactgtcatttaaaaaaaataaaactataaaa
caatatagaaaaaaaaaaaaaaaaaa

>NM_003271 4
aaagctgcggcggcggttcgcgtttctcgtgtccgcttgactgacagctg
cgcggcgggagcgggcggcgcgagcgggaggcggcggcgcagagcttggg
gcttccttggtcgcacccaccacctgcctgcccactggtcagccttcagg
gaccctgagcaccgcctggtctctttcctgtggccagcccagaactgaag
cgctgcggcatggcgcgcgcctgcctccaggccgtcaagtacctcatgtt
cgccttcaacctgctcttctggctgggaggctgtggcgtgctgggtgtcg
gcatctggctggccgccacacaggggagcttcgccacgctgtcctcttcc
ttcccgtccctgtcggctgccaacttgctcatcatcaccggcgcctttgt
catggccatcggcttcgtgggctgcctgggtgccatcaaggagaacaagt
gcctcctgctcactttcttcctgctgctgctgctggtgttcctgctggag
gccaccatcgccatcctcttcttcgcctacacggacaagattgacaggta
tgcccagcaagacctgaagaaaggcttgcacctgtacggcacgcagggca
acgtgggcctcaccaacgcctggagcatcatccagaccgacttccgctgc
tgtggcgtctccaactacactgactggttcgaggtgtacaacgccacgcg
ggtacctgactcctgctgcttggagttcagtgagagctgtgggctgcacg
cccccggcacctggtggaaggcgccgtgctacgagacggtgaaggtgtgg
cttcaggagaacctgctggctgtgggcatctttgggctgtgcacggcgct
ggtgcagatcctgggcctgaccttcgccatgaccatgtactgccaagtgg
tcaaggcagacacctactgcgcgtaggccgcccaccgcccgcttctctgc
caaaaggacgcccacggggagatggccgcacccacagctgcctttcccac
caccagcctcggtgctctgccccatgctgggaggagggagggagggacag
gtgcctggagcccccggaaccctgtttctggaaggccctagctcaggtgg
cttcagggcctccggacccccctgggaggggtggccacgtgctggctgc
ggaacccagggcaggggtgggaggggcctccagcactttttatatttacg
tattctccaaagcagtgttcacacgggagccagcctgtggcccccagcct
cctggaaaacaggttggcgctggaggagccgggtcttggcatcctggagg
tggccccactggtcctggtgctccaggcggggccgtggacccctcaccta
cattccatagtgggcccgtggggctcctggtgcatcttaataaagtgtga
gcagcaaccttgaaaaaaaaaaaaaaaaa

>NM_018330 5
attgcgtcccgctctacctctgtggttctttgggagcgacccccgggaag
cgtccaaagtggagttcccacacacgctgcgaacccacagccggttttct
ctgaactcgcgtccctgagtccgggaggtggaggcggagaaaagggtgcg
gagcgaccccacgcagggccgccccccctcccaccagcgcgtcctgccgc
gccggcagccacaggctggcatagcggctgccgacccgccctcgttcctc
cacccccctgaacgggactgctgggcccgccccgccccgcctgcaggtgaa
gcggccgcagccgccgagtaggtgcgtggggatgatctcactcgcgcgct

ccgcgccaggaggaggaggagcgggagcggatccaacttccgggtagtgg
agccgcaagccaccggcatcttgctttttcttcccctcctcctgtgtgc
cccgcgccgctccctctttcccttttattcccggccccacccgccaaaat
gaacagctcggacgaagagaagcagctgcagctcattaccagtctgaagg
agcaagcaataggcgaatatgaagaccttagagcagagaaccagaaaaca
aaggagaagtgtgacaaaattaggcaagaacgagatgaagccgttaaaaa
actggaagaatttcagaaaatttctcacatggtcatagaggaagttaatt
tcatgcagaaccatcttgaaatagagaagacttgtcgagaaagtgctgaa
gctttggcaacaaagctaaataaagaaaataaaacgttgaaaagaatcag
catgttgtacatggccaagctgggaccagatgtaataactgaagagataa
acattgatgatgaagattcgactacagacacagacggtgccgccgagact
tgtgtctcagtacagtgtcagaagcaaattaaagaacttcgagatcaaat
tgtatctgttcaggaggaaaagaagattttagccattgagctggaaaatc
tcaagagcaaactcgtagaagtaattgaagaagtaaataaagttaaacaa
gaaaagactgttttaaattcagaagttcttgaacagagaaaagtcttaga
aaaatgcaatagagtgtccatgttagctgtagaagagtatgaggagatgc
aagtaaacctggagctggagaaggaccttcgaaagaaagcagagtcattt
gcacaagagatgttcattgagcaaaacaagctaaagagacaaagccacct
tctgctgcagagctccatccctgatcagcagcttttgaaagctttagacg
aaaatgcaaaactcacccagcaacttgaagaagagagaattcagcatcaa
caaaaggtcaaagaattagaagagcaactagaaaatgaaacactccacaa
agaaatacacaacctcaaacagcaactggagcttctagaggaagataaaa
aggaattggaattgaaatatcagaattctgaagagaaagccagaaattta
aagcactctgttgatgaactccagaaacgagtgaaccagtctgagaattc
agtacctccaccacctcctcctccaccaccacttcccctccacctccca
atcctatccgatccctcatgtccatgatccggaaacgatcccaccccagt
ggcagtggtgctaagaaagaaaaggcaactcaaccagaaacaactgaaga
agtcacagatctaaagaggcaagcagttgaagagatgatggatagaatta
aaaagggagttcatcttagacccgttaatcagacagccagaccgaagaca
aagccagaatcttcgaaaggctgcgaaagtgcagtggatgaactaaaagg
aatactggcctcccagtagcattggatgcaggaaaaaatacattgacggt
gaaaaacaagccgaaccagttgtagttttagatcctgtttctacacatga
accccaaaccaaagaccaggttgctgaaaaagatccaactcaacacaagg
aggatgaaggcgaaattcaaccagaaaacaaagaagacagcattgaaaac
gtgagagagacagacagctccaactgctgatccataaaccagaagcctga
catgtttggaagtccttttcaataagcacatgattagtgttgttatattg
gcaagggctgtagacattctgctctggtcactgtattcagaatacaggtt
cttttctggtgtcacttttgtaagtagcaactataaacataagtaagctg
tttagcaaaacacacattcctagtaggttttggtttttgatctttataa
agatgaggtttttttcctagttactgtattaagtatgacttcttttagaa
ggttacaaaaaaattcagatgttgataccttttaggaaatgtgcatacc
actcatcaaatggaatgctgaaagtttgaggtgcttgtatataatcggat
aaacaaaactgatcaacccaatgtgattttaaaagcccccaaagaagctt
ctgttttgggtctgatcctcttgatggagaaactgcagcagcatggaaat
tgttgggtactgtggcatacaagttattttctacagtagactgagataaa
ctgaaaactcaggagctggcatcaaactcgtagtcccatagtcagtgtta

attacacacattgttaactattggatgaaaaatacatgctattgattgtg
tccaaagcctcccgaggacctccgtggggatgctctggtagcctgaatac
agaactgaggtgaaagtccaaaccttgaattttacagtagtaagttggta
aaccatgtgctctgtgctatgagttaattatgttttcccaaatactaatg
tggcacaagtaccatattttatcagagttcttatgtacagtatggtgaag
ataagtgacaagcacacatttttcttgcttcactgctgttctatattaca
caggtttgttgttgtttttttaaaaaagaaattaagcagtagttagtct
ctaaaaatacaatgtttcaggctaccacagtgaataaatagaaatgtaat
cagggattaaaaaaaaaacttatgcagcttttcaaagttgattgtttcaa
aattggtgtttatttaaaataagtggtaatgtacttgaatgcacttttta
tgacaatgattcagtaatggtaattttactattaaagaaagtgaaaggtt
tagttttgttagcatggctcagcatgtagctgtcaggtgtttttcaccta
agggcaaaagaaaatgatagtaataattgcagtagttgtattgtattgta
tttttgcacgtgtggtaagcataggcttgaagaggtgggtaggcaggtac
atgtacttcctaaatttggagataattatctttctgtaagttcgttatgc
ttgactgtttccatgttctcccaataatgattttatagttacttatcact
ttactcatggagaattaaaacgtaatgtttttcaactgtatctttcttta
actggataatactgctatatgatatgcttactacagactgcattaattca
cgaaacgaattctgttatgctgtaatttgaactctcctcaccacaactta
ttaaaaaggcaccaatagtttcccattaaaaaaaaa

>NM_015318 3
ctcgcgcggcttcccgcttccggctcccagctgctagctactgtggatct
gggggggccggacggaggcatcggaggcggctgcgagagtggcagaggag
ctggcggagagcggcctgcgggcgatcgggccgagcctcgctcaaggagc
acccccggggcaccctcctgtccgatggcagcccggccctgtccaggaat
gtcggtatgacggtctctcagaaagggggtccccagccaacaccgagccc
ggctggccctgggacgcaactcggaccaatcacaggagagatggatgaag
ccgattctgcgtttttaaaatttaagcagacagctgatgactctctgtcc
cttacatctccaaacaccgagtccatttttgtagaagatccctacaccgc
ctcgctgaggagtgagattgagtcagacggccacgagtttgaagctgagt
cctggagcctcgccgtggatgcagcctacgccaagaagcaaaagagggag
gtggtgaaaagacaagatgtcctttatgagctgatgcagacagaggtgca
ccacgtgcggacgctcaagatcatgctgaaggtgtactccagggccctgc
aggaggagctgcagttcagcagcaaggccattggccgcctcttcccatgc
gctgacgacctgctggagacgcacagccacttcctcgctcggctcaagga
gcgccgccaggagtccctggaggagggcagtgaccggaattatgtcatcc
agaaaatcggcgacctcctggttcagcagttttcaggtgaaaatggggag
agaatgaaagaaaagtacggtgtgttttgtagtggccacaatgaagctgt
tagtcattacaagttgctgcttcagcaaaacaagaaatttcaaaacttga
tcaagaaaattggcaacttctccatcgtgcggcggcttggcgtgcaggag
tgcattctcctggttacacaacgcataaccaaatacccagtgctggtgga
gcgcatcatccagaacacggaagctggcactgaggactatgaagacctga
cccaggcccttgaacctcatcaaagatatcatctcacaagtggacgccaag
gtcagtgagtgtgagaagggccagcgcctcagggagatcgcagggaagat
ggacctgaagtcttccagcaaactcaagaacgggctcaccttccgcaagg

aagacatgcttcagcggcagctccacctggagggcatgctatgctggaag
accacatcagggcgcttgaaagatatcctggctatcctgctgaccgacgt
acttttgctgctacaagaaaaagatcagaaatacgtctttgcttctgtgg
actcaaagccacccgtcatctcgttacaaaagctcatcgtgagggaagtg
gccaacgaggagaaagcgatgtttctgatcagcgcctccttgcaagggcc
ggagatgtatgaaatctacacgagctccaaagaggacaggaacgcctgga
tggcccacatccaaagggctgtggagagctgccctgacgaggaggagggg
cccttcagcctgcccgaagaggaaaggaaggtggtcgaggcccgcgccac
gagactccgggactttcaagagcggttgagcatgaaagaccagctgatcg
cacagagcctcctagagaaacagcagatctacctggagatggccgagatg
ggcggcctcgaagacctgccccagccccgaggcctattccgtggaggga
cccatccgagaccctgcagggggagctaattctcaagtcggccatgagcg
agatcgagggcatccagagcctgatctgcaggcagctgggcagcgccaac
ggccaggcggaagacggaggcagctccacaggcccgcccaggagggctga
gaccttcgcgggctacgactgcacaaacagccccaccaagaatggcagtt
tcaagaagaaagtcagcagcactgaccccaggccccgagactggcgaggc
cccccaaacagcccggacttgaagctcagtgacagtgacattcctgggag
ctctgaggaatcgccgcaggtggtggaggcgccaggcacggaatccgatc
cccgtctgcccaccgtcctggagtcggagcttgtccagcggatccagaca
ctgtcccagctgctcctgaaccttcaggcggtaatcgcccaccaggacag
ctatgtggagacgcagcgggctgccatccaggagcgggagaagcagttcc
ggctgcagtcgacgcgtgggaacctgctgctggagcaggagcggcaacgc
aacttcgagaagcagcgggaggagcgcgcggccctggagaagctgcagag
ccagctgcggcacgagcagcagcgctgggagcgcgagcgccagtggcagc
accaggagctggagcgtgcgggcgcgcggctgcaggagcgcgagggcgag
gcgcggcagctacgcgagcggctggagcaggagcgggccgagctggagcg
ccagcgccaggcctaccagcacgacctggagcggctgcgcgaggcccagc
gtgccgtggagcgcgagcgggagcgcctggagctgctgcgccgcctcaag
aagcagaacaccgcgccaggcgcgctgccgcccgacacactggccgaggc
ccagcccccaagccaccctcccagcttcaacggggaagggctggagggcc
ctcgtgtgagcatgctgccatccggcgtggggccagagtacgcagagcgc
cccgaggtggctcgccgggacagcgcccccaccgagaaccggctggccaa
gagcgatgtgcccatccagctgctcagcgccaccaaccagttccagaggc
aggcggccgtgcagcagcagatccccaccaagctggcggcctccaccaag
ggtggcaaggacaagggcggcaagagcaggggctctcagcgctgggagag
ctcagcgtccttcgacctgaagcagcagctgctgctcaacaagctcatgg
ggaaagatgagagcacctcacggaaccgccgctcgctgagccctatcctg
cccggcagacacagtcctgcgcccccaccagaccctggcttccccgcccc
gagcccaccgccagctgacagcccctccgagggcttctctctcaaggccg
ggggcacagccctcctgcccgggcccccagctccctcgccactgccggcc
acaccactcagcgccaaggaggacgccagcaaagaagacgtcatcttctt
ctaaaagggccgtgactcaaggaaagtttttaatggaaagttgagccaga
actaaaccagggagctgtctgaaatcatagcaccccatccgggtggcggg
gagatcaactccgagctgtttttccgaggcagtgaggaacggtgccggct
ctgcacggagctgaggacaggacagaccttgctttgagaaggagctgccg
gccggggccacgctccacagccgccgcgcgacagtggagccaagggttag

ggcaccaggaggggccaggtggcgtcggcagcatctgtccccagaatcag
gcagaatccacttcccaaacagagccccacgcaggttcaccatgaacctc
agggtcagggaatgagccaggcacgggggcatgggcagagagggccacgg
ggcagggcccactgagggaacatcagtggccctccagtcaggttctgtgg
gtttggaagcccatcgtgaaaggggctgacctttgccccttttttacttgg
cattggttttgaaaccagctgtttcccaaactctgcttcccaagggcaac
cgttgctgttcacacgctcagcctgtctgggggagcgggcctctagcttc
agccagggcgggtacacaccctgggcacagggtcctcagcccccgggaaa
tgagctcccagggctggcgtcccaccttccaggtgggggctggcacatca
cagactgtcgagagcgccatgtcccagggcatgcagaggatgcacctaga
gacgttgcagcaagtggacaagtggccgctgtgcgggcccctcgcttgta
gtgagctgttgcagcttacggtccgttccctggaggggtggaggaaggag
gtgttgggcagcatcaaaggtgctgggacatcccagggtggtgagatcca
tccacgatccagctccggtggagaaagggcccatgtcaagccttgttctg
caccccaagcattggtggtaggactgggtcctggctgatcgtccttgttc
ccagtggggtacatgtgagcccctgccagggccaagtccttctcccgaac
ccagggtcctgggaactgcagatcccgggggggattcagcccttctcccac
tgtgctggcagaggcactcctgtgacgctgaatacagtgaacagggacat
tcccgccactcggggacagatgggcacaagggaggggaaactccatcagg
aagtgctcccctgggcagaggcgcccactgggtgctgtgggctcaggagg
gggcggggcaggagctggtgccaaccgggaaccagagccccacagccata
cagcccattggtgacaaggtcctgagaacacagtggccaggtgtccccag
gctcctggcccctccgacgacctcaactctgcccagcccggtccctggcc
atcagcgacgctgtccgcccccccgtcagatcccatgtgtgccatgtttat
catcagtgttttgtatttttgtactgagtatcggagcactttacagaagc
tgactgtacattcctgttctgttgtgaagagaacattcccagaccctggc
accctcctgagccggcgtgtgccggtccagccctccgagatgccacaatt
ccttggatgggggagaagttcaaggaatttctgctcggccacgcggtggg
aaccccgcgtccccgccatgtggcagaggggtctcagtcgtgctaggcat
cgggcggcagcgccgacagcccttccctcgccagtgcccctcggccactc
ctgggttggagcccgattttatttgtaaagttgacagtcgagcaaatgtt
cctattttcgtgggatctgcacacgtctttgtcagttgtggtcatgatct
tagtcacctgctaattatttttacaatgattacaacatttcctcactgcg
ggatatttctgacccgctttagaacttaagacctgattctagcaataaac
gtgtccgagatgagcggtgaaaaaaaaaaaaaaaaaaaaaaaaaaaaaaaa
aa

>NM_002999 3
actcgccgcagcctgcgcgccttctccagtccgcggtgccatggcccccg
cccgtctgttcgcgctgctgctgttcttcgtaggcggagtcgccgagtcg
atccgagagactgaggtcatcgacccccaggacctcctagaaggccgata
cttctccggagccctaccagacgatgaggatgtagtggggcccgggcagg
aatctgatgactttgagctgtctggctctggagatctggatgacttggaa
gactccatgatcggccctgaagttgtccatcccttggtgcctctagataa
ccatatccctgagagggcagggtctgggagccaagtccccaccgaaccca
agaaactagaggagaatgaggttatccccaagagaatctcacccgttgaa

gagagtgaggatgtgtccaacaaggtgtcaatgtccagcactgtgcaggg
cagcaacatctttgagagaacggaggtcctggcagctctgattgtgggtg
gcatcgtgggcatcctctttgccgtcttcctgatcctactgctcatgtac
cgtatgaagaagaaggatgaaggcagctatgacctgggcaagaaacccat
ctacaagaaagcccccaccaatgagttctacgcgtgaagcttgcttgtgg
gcactggcttggactttagcggggagggaagccaggggattttgaagggt
ggacattagggtagggtgaggtcaacctaatactgacttgtcagtatctc
cagctctgattacctttgaagtgttcagaagagacattgtcttctactgt
tctgccaggttcttcttgagctttgggcctcagttgccctggcagaaaaa
tggattcaacttggcctttctgaaggcaagactgggattggatcacttct
taaacttccagttaagaatctaggtccgccctcaagcccatactgaccat
gcctcatccagagctcctctgaagccagggggctaacggatgttgtgtgg
agtcctggctggaggtcctcccccagtggccttcctcccttcctttcaca
gccggtctctctgccaggaaatgggggaaggaactagaaccacctgcacc
ttgagatgtttctgtaaatgggtacttgtgatcacactacgggaatctct
gtggtatatacctggggccattctaggctctttcaagtgacttttggaaa
tcaaccttttttatttgggggggaggatggggaaaagagctgagagttta
tgctgaaatggatttatagaatatttgtaaatctatttttagtgtttgtt
cgtttttttaactgttcattcctttgtgcagagtgtatatctctgcctgg
gcaagagtgtggaggtgccgaggtgtcttcattctctcgcacatttccac
agcacctgctaagtttgtatttaatggtttttgtttttgtttttgtttgt
ttcttgaaaatgagagaagagccggagagatgatttttattaattttttt
ttttttttttttttttactatttatagctttagatagggcctcccttcc
cctcttctttctttgttctctttcattaaaccccttccccagtttttttt
ttatactttaaaccccgctcctcatggccttggccctttctgaagctgct
tcctcttataaaatagcttttgccgaaacatagtttttttttagcagatc
ccaaaatataatgaaggggatggtgggatatttgtgtctgtgttcttata
atatattattattcttccttggttctagaaaaatagataaatatattttt
ttcaggaaatagtgtggtgtttccagtttgatgttgctgggtggttgagt
gagtgaattttcatgtggctgggtgggtttttgcctttttctcttgccct
gttcctggtgccttctgatggggctggaatagttgaggtggatggttcta
ccctttctgccttctgtttgggacccagctggtgttctttggtttgcttt
cttcaggctctagggctgtgctatccaatacagtaaccacatgcggctgt
ttaaagttaagccaattaaaatcacataagattaaaaattccttcctcag
ttgcactaaccacgtttctagaggcgtcactgtatgtagttcatggctac
tgtactgacagcgagagcatgtccatctgttggacagcactattctagag
aactaaactggcttaacgagtcacagcctcagctgtgctgggacgaccct
tgtctccctgggtagggggggggggaatgggggagggctgatgaggcccca
gctggggcctgttgtctgggaccctccctctcctgagaggggaggcctgg
tggcttagcctgggcaggtcgtgtctcctcctgaccccagtggctgcggt
gaggggaaccaccctcccttgctgcaccagtggccattagctcccgtcac
cactgcaacccagggtcccagctggctgggtcctcttctgcccccagtgc
ccttccccttgggctgtgttggagtgagcacctcctctgtaggcacctct
cacactgttgtctgttactgatttttttgataaaaagataataaaacct
ggtactttctaaaaa

>NM_016291 3
gagccaatagtgaggggcaccgggagcgaggtgcagggggcggggctcgg
cctggccgcagccattttggtgcgagagaaacaataggacggaaacgccg
aggaacccggctgaggcggcagcagagcatcctggccagaacaagccaag
gagccaagacgagagggacacacggacaaacaacagacagaagacgtact
ggccgctggactccgctgcctcccccatctccccgccatctgcgcccgga
ggatgagcccagccttcagggccatggatgtggagccccgcgccaaaggc
gtccttctggagccctttgtccaccaggtcgggggcactcatgcgtgct
ccgcttcaatgagacaaccctgtgcaagcccctggtcccaagggaacatc
agttctacgagaccctccctgctgagatgcgcaaattcactccccagtac
aaaggtgtggtatctgtgcgctttgaagaagatgaagacaggaacttgtg
tctaatagcatatccattgaaaggggaccatggaattgtggacattgtag
ataattcagactgtgaaccaaaaagtaagctcctaaggtggacaacaaac
aaaaaacatcatgtcttagaaacagaaaagacccctaaggactgggtgcg
tcagcaccgtaaagaggagaaaatgaagagccataagttagaagaagaat
ttgagtggctaaagaaatctgaagtcttgtactacactgtagagaagaag
gggaatataagttcccagcttaaacactataacccttggagcatgaaatg
tcaccagcaacagttacagagaatgaaggagaatgcaaagcatcggaacc
agtacaaatttatcttactggaaaacctgacttcccgctacgaggtgcct
tgtgtccttgacctcaagatgggcacacgacaacatggtgatgatgcttc
agaggagaaggcagccaaccagatccgaaaatgtcagcagagcacatctg
cagtcattggtgtgcgtgtgtgtggcatgcaggtgtaccaagcaggcagt
gggcagctcatgttcatgaacaagtaccatggacggaagctatcggtgca
gggcttcaaggaggcacttttccagttcttccacaatgggcggtacctgc
gccgtgaactcctgggccctgtgctcaagaagctgactgagctcaaggca
gtgttggagcgacaggagtcctaccgcttctactcaagctccctgctggt
catttatgatggcaaggagcggcccgaagtggtcctggactcagatgctg
aggatttggaggacctgtcagaggaatcagctgatgagtctgctggtgcc
tatgcctacaaacccatcggcgccagctctgtagatgtgcgcatgatcga
ctttgcacacaccacctgcaggctgtatggcgaggacaccgtggtgcatg
agggccaggatgctggctatatcttcgggctccagagcctgatagacatt
gtcacagagataagtgaggagagtggggagtgagcttgctagctgctcca
gtacttgagagcgactctgtgtcccaggcacagctgtgctgcgtcaggga
ggaagccagtatggccaggtggtggctcctgcagcctggagctgatgtgc
agtggcctctgtgagccccagcctgagccagtcccagctgtgcttggagt
ctttatttattttaactatttcttcaacattccacatttgatgatgatac
ctctttcttccctgagtgtatatgttctaatacaaatctttttgtttatt
gtaaaaaaaaaaa

>NM_016004 2
gtcctgggccacgcctcccggcgcaccgacgcgcctctccggttactaag
cggccttggatacctggccgcgggatgctgggcggcgtcaggtgagcggt
ggtcgctgggcctcaggtaaccatggagaaagagctgcggagcaccattc
ttttcaatgcctacaaaaaggagatatttaccaccaacaatggctacaaa
tccatgcagaaaaaacttcggagtaattggaagattcagagcttaaaaga
tgaaatcacatctgagaagttaaatggagtaaaactgtggattacagctg

ggccaagggaaaaatttactgcagctgagtttgaaatcctgaagaaatat
cttgacactggtggagatgtctttgtgatgctaggagaaggtggagaatc
cagatttgacaccaatattaactttttactagaagaatatggaatcatgg
ttaataatgatgctgtggttagaaatgtatatcacaaatatttccatcct
aaagaagctctagtttccagtggagtcttgaacagggaaattagccgagc
tgcaggaaaggctgtgcctgggatcattgatgaggaaagcagtggaaaca
atgcccaggctctcacctttgtgtatccttttggtgccacattgagtgtc
atgaaaccagcagtggcggttctgtctacaggttctgtctgcttcccact
taacagacccattttggctttctatcactcaaagaaccaaggtgggaagc
tggcagtgcttggttcatgtcacatgttcagtgatcaatatttggacaaa
gaagaaaacagcaaaatcatggatgttgttttccagtggctcacgacagg
agacatccacctaaaccagattgatgctgaggacccagagatttctgact
acatgatgctgccctacacagccaccctatcaaagcggaatcgagagtgt
ctccaggagagtgatgagatcccaagggactttaccaccctcttcgacct
gtccatcttccagctggataccacctccttccacagcgtcatcgaggctc
acgagcagctaaatgtgaaacatgaaccactccagctcatccagcctcag
tttgagacgccgctgccaacccttcagcctgcggtttttcctcccagttt
ccgggagttaccacctcctcctctggagctatttgatttagatgaaacgt
tctcctctgagaaggcacggctggctcagattaccaataagtgtactgaa
gaagacctggaattttatgtcaggaagtgtggtgatattcttggagtaac
cagtaaactaccaaaggaccaacaggatgccaaacatatccttgagcacg
tcttcttccaagtggtggagttcaagaaattgaaccaggaacatgacatc
gatacaagtgaaacagcattccagaacaatttctgaagaccatgcctctt
gaagctttttctgcctcctgattctctctttgtaaactattttcaaattg
tttttcaactccttatcaaaattgtttatacactctttcctccatgagct
ctggaaggtatatgcatcttctgtaatactcagataggtataagattttt
cacaaaatccttatgtaagatacattccatttttaaaaattaaatgtatg
gttgcatctgtctttttataccccta

>NM_152237 1
agggaggcggggcggagacggcggcgaggaggaggccgcggcgcgggacg
catagagctgcggctcgggcggcgcctccctgcggcggcccggcccggct
ccggcccccgctggggcaatgctccccggggccgcgggatgagccagtga
ctcggccggtccgggcatggcagacccagtggcgggcatcgcgggctcgg
cggccaagagcgtgcggccatttcgctccagtgaggcctacgtggaggcc
atgaaggaggacctggccgagtggctcaatgccttgtacggcctgggtct
cccgggtggtggcgatggcttcctgacagggctggccacgggcacgaccc
tgtgccaacatgccaacgccgtgaccgaggctgcccgtgcattggcagcc
gcccgcccggcccgaggtgtggccttccaggcgcacagtgtagtgcctgg
ctccttcatggcgcgcgacaacgtggccaccttcatcggctggtgccgcg
tggagctgggtgtgccggaggtgctcatgtttgagactgaggacctggtg
ctgcgcaagaacgagaagagcgtggtgctgtgcctgctggaggtggcgcg
gcgtggggcacgcctgggcctgctggccccacgcctcgtgcagtttgagc
aggagattgagcgggagctgcgtgctgcacccccagcccccaacgcccct
gccgctggggaggacaccactgaaaccgcccccgcaccagggactcctgc
ccgcggcccccgcatgacacccagcgacctgcgcaacctcgacgagctgg

tgagggagattctgggccgctgcacctgccctgaccagtttcccatgatc
aaggtctcagaggggaagtaccgtgtgggggactcgagcctgctcatctt
tgtgcgggtgctgaggagccacgtgatggtgcgagtgggtggtggctggg
acacgctggagcattacctggacaagcacgacccgtgccgctgctcctcc
actgctcatcgcccaccccagccgagggtctgcaccttttctccacagag
ggtgtcgcccaccaccagtccccgccctgctagcccagtccctgggagtg
agcgccggggctcccggcctgagatgactcccgttagcttacgaagcaca
aaggaggggcccgagaccccacccaggtgagatgcaggaggacgaggagt
gaggggtccagagggtggggggcgtcagccctggcctgcatgatgggtt
gcctgtgcgccagagtgactcacaccctgggaaaagttcccgtgggtggg
ggcgggcctggcttcccatctccatggcaacccaaccaaaccaacaacac
agctggggcgggccctgtggctgggcggcagccagtccagctcttgcttc
ctctggcccgtcctgggaagggggtgtctagagcccaggaaacttcttc
ttccgtggcttttggggccctgggccgctggaggaagctgctcacttctc
cctggaagtccccaggacagaccgatgcccctgacagccccgtccaccag
ccacataccctgctgttcctctccctgcctgttctgcatgccagtccttc
cgtggtaccccatctgtctctattgtccccctgccccaggccccgggatc
agctgccccccatccccgctcccgccgctactccggggacagtgactcc
tcagcctcctccgcccagagcggccccttggtacccgcagtgatgacac
aggcactggcccccggagggagcgacccagccggcggctgaccacaggca
ccccggcctctccgagacggcctcctgccctgcgcagccagtcccgagac
cggctggatcgcggccggccccgggggccccaggaggcaggggagccca
gctgtcggtccccagccctgcccggcgggcccggagccagagccgcgagg
agcaggctgtgctgcttgtgcgcagggatcgagacgggcagcactcatgg
gtgccaaggggcaggggcagtgggggctcgggcaggagcaccccccagac
tccccgtgcccgcagccctgcagcaccccggctttcccgggtctccagcc
ccagtccagagttgggcaccacaccggccagcatcttccgcacaccccctg
cagctcgacccgcagcaggagcagcagctgttccggcgcctggaagagga
gttcctggccaatgcccgggcccttgaggctgttgctagcgtgaccccca
ctggaccagcccctgacccagctcgggcccccgaccctccagctcctgac
tctgcctattgttcctccagttcctcctcttcgtccctcagcgtcctggg
tggcaaatgtggccaacctggggactctggccggacggccaatgggctgc
ctgggccccgaagccaagccctttccagctcctccgatgaaggcagcccc
tgccctggcatgggggggccactagatgcacctgggagccccctggcttg
cactgaaccctcgaggacctgggcacggggtcggatggacacacagccag
accgtaaaccctcacgtatccccacgcctcggggcccccgccgcccctcc
ggacccgcagagctggggacatggcatgccctgcactcagtcaccccgag
ggctgagccagattcctggatgtgatggaccagctcagctgtccccagac
cccatcccttctccttttcctttgtggccttaacccttctgcatcaggga
gccccctctgcctcttgagtaccagacctcatgggaccagaccccttggg
accacatggcacaatgggacctctgttgtacattccggttgggggatgag
cgttgctatttaattactaatattattgaatgccttagaggaggccgggc
gagcccggtgttctgaagacctgtggcccagcagagcctctgacagtaaa
gttttgctccagc

>NM_021814 4

aaaaaaatttcccagagacacgtggaaccgaggggccaaccccggcctag
gctctccaccgcatcggattctggaatttacgatcacgaaagttctattg
tcccgcgattggctcccgggccgcatgacatcatagcgcttgattcatcc
ttcgggtcccgattggctggccgcgccattgtgacgtcacggtcagccca
cgttctgattgtagatagccggcgccttcctcttcccatcgcgcgggtcc
tagccaccggtgtctccttctacatccgcctctgcgccggctgccacccg
cgctccctccgccgccgccgccttgctgctgctcaaagctgctgccgccc
cttgggctaaaaggttttcaaatggaacattttgatgcatcacttagtac
ctatttcaaggcattgctaggccctcgagatactagagtaaaaggatggt
ttcttctggacaattatatacccacatttatctgctctgtcatatattta
ctaattgtatggctgggaccaaaatacatgaggaataaacagccattctc
ttgccgggggattttagtggtgtataaccttggactcacactgctgtctc
tgtatatgttctgtgagttagtaacaggagtatgggaaggcaaatacaac
ttcttctgtcagggcacacgcaccgcaggagaatcagatatgaagattat
ccgtgtcctctggtggtactacttctccaaactcatagaatttatggaca
ctttcttcttcatcctgcgcaagaacaaccaccagatcacggtcctgcac
gtctaccaccatgcctcgatgctgaacatctggtggtttgtgatgaactg
ggtcccctgcggccactcttattttggtgccacacttaatagcttcatcc
acgtcctcatgtactcttactatggtttgtcgtcagtcccttccatgcgt
ccatacctctggtggaagaagtacatcactcaggggcagctgcttcagtt
tgtgctgacaatcatccagaccagctgcggggtcatctggccgtgcacat
tccctcttggttggttgtatttccagattggatacatgatttccctgatt
gctctcttcacaaacttctacattcagacctacaacaagaaaggggcctc
ccgaaggaaagaccacctgaaggaccaccagaatgggtccatggctgctg
tgaatggacacaccaacagcttttcacccctggaaaacaatgtgaagcca
aggaagctgcggaaggattgaagtcaaagaattgaaaccctccaaaccac
gtcatctgattgtaagcacaatatgagttgtgccccaatgctcgttaaca
gctgctgtaactagtctggcctacaatagtgtgattcatgtaggacttct
ttcatcaattcaaaaccccctagaaaacgtatacagattatataagtaggg
ataagatttctaacatttctgggctctctgacccctgcgctagactgtgg
aaagggagtattattatagtatacaacactgctgttgccttattagttat
aacatgataggtgctgaattgtgattcacaatttaaaaacactgtaatcc
aaacttttttttttaactgtagatcatgcatgtgattgtaaatgtaaatt
tgtacaatgttgttatggtagagaaacacacatgccttaaaatttaaaaa
gcagggcccaaagcttattagtttaaattagggtatgtttcaagtttgta
ttaatttgtaatagctctgtttagaaaaaatcaaagaccatgatttatga
aactaatgtgacataatttccagtgacttgttgatgtgaaatcagacacg
gcaccttcagttttgtactattggctttgaatcaagcaggctcaaatcta
gtggaacagtcagtttaacttttaacagatcttattttttattttgag
tgccactattaatgtaaaaaggggggggctctacagcagtcgtgatgaaa
cttaaatatatattctttgtcctcgagattttaggaagggtgtagggtga
gtaggccatttttaatttctgaagtgctaagtgtttttatacagcaaaca
aaaagtcaattttgctttccaccagtgcgagagaggatgtatacttttca
agagagatgattgcctatttaccgtttgacagagtcccgtagatgagcaa
tggggaactggttgccagggtctaaatttggattgatttatgcactgtta
tctgttttgacacagatttccttgtaaaatgtgcctagtttaccaaaatt

aacaaagggggggaaaggaccttagaactttttaaggtaaaatcaaatat
agctacagcataagagaatcgagaaatttgatagaggtaacttgtttaat
gtaaatctaatagtacttgtaatttctttctgcttagaatctaaagatgt
gtttagaacctcttgtttaaaaataatagactgcttatcataaaatcaca
tctcacacatttgaggcagtggtcaaacaggtaaagcctatgatgtgtgt
cattttaaagtgtcggaatttagcctctgaataccttctccattgggggga
aagatattcttggaaccactcatgacatatcttagaaggtcattgacaat
gtataaactaattgttggtttgatatttatgtaaatatcagtttaccatg
ctttaattttgcacattcgtactatagggagcctattggttctctattag
tcttgtgggttttctgtttgaaaaggagtcatggcatctgtttacattta
ccttatcaaacctagaatgtgtatatttataaatgtatgtcttcattgct
aggtactaatttgcagatgtctttacatatttcaatacagaaactataac
attcaatagtgtgctgtcaaagtgtgcttagctcacctggatatacctac
attgttaaatgtctaaacagtaatcattaaaacatttttgattacctgtg
aaaaaaaaaaaaaaaaaaaaaaaaaaaaaaaaaaaa

>NM_001980 3
gggcatgcgcagagcgcgcggggcgcggttgccgtggcagcgccggctcc
agggagggctgctggcgccgggccgggaccgcggggcctgaggcggagac
cggagagcccgaggcccggccggaggcagctcgggacaggcttgagcggc
ggggcgcgctgcccggccggcggggatgcgggaccggctgccagacctga
cggcgtgtaggaagaatgatgatggagacacagttgttgtggttgagaaa
gatcatttcatggatgatttcttccatcaggtggaggagattagaaacag
tattgataaaataactcaatatgttgaagaagtaaagaaaaaccacagca
tcattctttctgcaccaaacccggaaggaaaaataaaagaagagcttgaa
gatctgaacaaagaaatcaagaaaactgcgaataaaattcgagccaagtt
aaaggctattgaacaaagttttgatcaggatgagagtgggaaccggactt
cagtggatcttcggatacgaagaacccagcattcggtgctgtctcggaag
tttgtggaagccatggcggagtacaatgaggcacagactctgtttcggga
gcggagcaaaggccgcatccagcgccagctggagataactgggagaacca
ccacagacgacgagctagaagagatgctggagagcgggaagccatccatc
ttcacttccgacattatatcagattcacaaattactagacaagctctcaa
tgaaatcgagtcacgtcacaaggacatcatgaagctggagaccagcatcc
gagagttgcatgagatgttcatggacatggctatgtttgtggagactcag
ggtgaaatgatcaacaacatagaaagaaatgttatgaatgccacagacta
tgtagaacacgctaaagaagaaacaaaaaaagctatcaaatatcagagca
aggcaagaaggaaattgatgttcattattatttgtgtaattgttttgctt
gtgatccttggaattatcctagcaacaacattgtcctagcaaccatatcc
caagagccatttatccttgagactcagaccacatctgcaaccaaatcagc
atcctgtcatttcgtgaatgaatctcagacgctgtaacccggcatcgagt
gctgaccttgtaatgtatgagttcacggaagaagcacaaccgaacgtctt
gttcagtgacagcgccgtaagcacagtgggtgtgatgcgtgaccctgaat
gacacactacagaatgtgagcagtgtgagacttctctcactggccttgaa
ataagaaaatattgaacattttaatattaaaaatatttcagtgttaacaa
atgtgcatgaagtttaattaggaaggaatgctttatgttatgaaggcttt
tttttttttttgagacggagtctcgctttgtcatccaggctggagtgc

agtggtgcgatctcagctcactgcaacctcggcctcccgggttcaagcaa
ttctcctgcctcagcctcccgagtagctgggattacaggcatgggccacc
acacccagctaatttttgtgttttctttttagtagagacggggttttac
catgctggccaggctggcctcaaactcctgaccttgtgatccacccacct
cagcctcccaaagtgctgggattacaggcgtgagtcaccgggcaggctgt
tctgaagcttttagatctcaggtctaaggcttcctttccctccctctccc
agctagtttgtgctaattaagagaccttttatactgttttattgcctgtt
tgaagaaataatttttatcacgtttttgtaagatatctataattttaaat
gtttataaattgtttaatttattagcatcttaatgtaccccatttttata
tactgaatgtggccttttgagtgaaataggaagcttcatggtgttggagc
cacctttgtacagttgtttaaagtttcccattgtcacggaaaacattggc
tgcaaagcccctcaaagccctcaagtgccttctgtgagtttaaatgtgct
ggtgccctccagaaaagcctcggcctcagctccgtttccgcctgttccct
cccccaggataatgaatggttactgcactgtaaagaccgtggtctctttt
cactaaataggagattcgagtttcccagtttacatgaatgaagtctgaat
ttaagacggtgatgaaactgaggttcagtactctcgggactcgaggaaat
tattcctgagacatggagtaattcttacaaatttaaactattgtacagat
ccacatacatgttgttaagtacctaatgttttgctgaacttttaaaagtt
aatttccaaaatgtatagggattcatgataattaaaccttttttattgctc
attttttagtagaagaatatcacttatttttagacttgtaaaatgtatg
aactggtgagcggacatctgttaagagagtcactagtcagaatgttaaag
gagtgcatgcaggatgccccaaatgtcgtgaactcttgttactcctgtat
gtagtagtgtaagcatgtgacttttaacaccatttggtttgaaactaatg
tagagatgcctgattccaaacaggtgtggagaatattgaatggctcagaa
gccgcgtcccttacttaacacaattccgaatctccctcatccatgatgcg
tccattggatcactcgctggtggtcactgtgtggcagttactaggggaat
tctgcctctgactgttctttttcttttggtctttaaacaccctgtcgtgg
gatgtgctcactgatttgtggctatgttgaaggtatcacttgtcttgagg
gttttcaatatttcaggatcatgctggtggcaaaaggactccacgcctct
gtggaatcatgtccacagggggacctgcctcccgtgatgtcccacctttc
cttcaaggtctgtcatatgagtcctccccttttacaacacttattatggt
atttttcaagttattcttcttagatttgcagtacctactgaaatttgtgt
ttttatagttgaagttaggaaaatgctatttgatttgtatttagatattt
aagtcacttgtccaatgatgtgtatgtctaagcctcatgtaccgatttga
agtcagacttaaaaatgtatttacagattcacttgagactttttaatcgg
ttcttcaaatatttcatgtttacattaaaaatttccagagaagcataaaa
gtattcactttcctgccttgtcatttctggaaagattttggggagatatt
ttattgcatattaattaataaattgttctactaggaaaaaaaaaaaaaa
a

>NM_002293 3
gtgcaggctgctcccggggtaggtgagggaagcgcggaggcggcgcgcgg
gggcagtggtcggcgagcagcgcggtcctcgctaggggcgcccacccgtc
agtctctccggcgcgagccgccgccaccgcccgcgccggagtcaggcccc
tgggcccccaggctcaagcagcgaagcggcctccgggggacgccgctagg
cgagaggaacgcgccggtgcccttgccttcgccgtgacccagcgtgcggg

cggcgggatgagagggagccatcgggccgcgccggccctgcggccccggg
ggcggctctggcccgtgctggccgtgctggcggcggccgccgcggcgggc
tgtgcccaggcagccatggacgagtgcacggacgagggcgggcggccgca
gcgctgcatgcccgagttcgtcaacgccgccttcaacgtgactgtggtgg
ccaccaacacgtgtgggactccgcccgaggaatactgtgtgcagaccggg
gtgaccggggtcaccaagtcctgtcacctgtgcgacgccgggcagcccca
cctgcagcacggggcagccttcctgaccgactacaacaaccaggccgaca
ccacctggtggcaaagccagaccatgctggccggggtgcagtaccccagc
tccatcaacctcacgctgcacctgggaaaagcttttgacatcacctatgt
gcgtctcaagttccacaccagccgcccggagagctttgccatttacaagc
gcacacgggaagacgggccctggattccttaccagtactacagtggttcc
tgtgagaacacctactccaaggcaaaccgcggcttcatcaggacaggagg
ggacgagcagcaggccttgtgtactgatgaattcagtgacatttctcccc
tcactgggggcaacgtggccttttctaccctggaaggaaggcccagcgcc
tataactttgacaatagccctgtgctgcaggaatgggtaactgccactga
catcagagtaactcttaatcgcctgaacacttttggagatgaagtgttta
acgatcccaaagttctcaagtcctattattatgccatctctgattttgct
gtaggtggcagatgtaaatgtaatggacacgcaagcgagtgtatgaagaa
cgaatttgataagctggtgtgtaattgcaaacataacacatatggagtag
actgtgaaaagtgtcttcctttcttcaatgaccggccgtggaggagggca
actgcggaaagtgccagtgaatgcctgccctgtgattgcaatggtcgatc
ccaggaatgctacttcgaccctgaactctatcgttccactggccatgggg
gccactgtaccaactgccaggataacacagatggcgcccactgtgagagg
tgccgagagaacttcttccgccttggcaacaatgaagcctgctcttcatg
ccactgtagtcctgtgggctctctaagcacacagtgtgatagttacggca
gatgcagctgtaagccaggagtgatgggggacaaatgtgaccgttgccag
cctggattccattctctcactgaagcaggatgcaggccatgctcttgtga
tccctctggcagcatagatgaatgtaatattgaaacaggaagatgtgttt
gcaaagacaatgtcgaaggcttcaattgtgaaagatgcaaacctggattt
tttaatctggaatcatctaatcctcggggttgcacaccctgcttctgctt
tgggcattcttctgtctgtacaaacgctgttggctacagtgtttattcta
tctcctctacctttcagattgatgaggatgggtggcgtgcggaacagaga
gatggctctgaagcatctctcgagtggtcctctgagaggcaagatatcgc
cgtgatctcagacagctactttcctcggtacttcattgctcctgcaaagt
tcttgggcaagcaggtgttgagttatggtcagaacctctccttctccttt
cgagtggacaggcgagatactcgcctctctgcagaagaccttgtgcttga
gggagctggcttaagagtatctgtacccttgatcgctcagggcaattcct
atccaagtgagaccactgtgaagtatgtcttcaggctccatgaagcaaca
gattacccttggaggcctgctcttaccccttttgaatttcagaagctcct
aaacaacttgacctctatcaagatacgtgggacatacagtgagagaagtg
ctggatatttggatgatgtcaccctggcaagtgctcgtcctgggcctgga
gtccctgcaacttgggtggagtcctgcacctgtcctgtgggatatggagg
gcagttttgtgagatgtgcctctcaggttacagaagagaaactcctaatc
ttggaccatacagtccatgtgtgctttgcgcctgcaatggacacagcgag
acctgtgatcctgagacaggtgtttgtaactgcagagacaatacggctgg
cccgcactgtgagaagtgcagtgatgggtactatggagattcaactgcag

gcacctcctccgattgccaaccctgtccgtgtcctggaggttcaagttgt
gctgttgttcccaagacaaaggaggtggtgtgcaccaactgtcctactgg
caccactggtaagagatgtgagctctgtgatgatggctactttggagacc
ccctgggtagaaacggccctgtgagactttgccgcctgtgccagtgcagt
gacaacatcgatcccaatgcagttggaaattgcaatcgcttgacgggaga
atgcctgaagtgcatctataacactgctggcttctattgtgaccggtgca
aagacggatttttggaaatcccctggctcccaatccagcagacaaatgc
aaagcctgcaattgcaatctgtatgggaccatgaagcagcagagcagctg
taaccccgtgacggggcagtgtgaatgtttgcctcacgtgactggccagg
actgtggtgcttgtgaccctggattctacaatctgcagagtgggcaaggc
tgtgagaggtgtgactgccatgccttgggctccaccaatgggcagtgtga
catccgcaccggccagtgtgagtgccagcccggcatcactggtcagcact
gtgagcgctgtgaggtcaaccactttgggtttggacctgaaggctgcaaa
ccctgtgactgtcatcctgagggatctctttcacttcagtgcaaagatga
tggtcgctgtgaatgcagagaaggctttgtgggaaatcgctgtgaccagt
gtgaagaaaactatttctacaatcggtcttggcctggctgccaggaatgt
ccagcttgttaccggctggtaaaggataaggttgctgatcatagagtgaa
gctccaggaattagagagtctcatagcaaaccttggaactggggatgaga
tggtgacagatcaagccttcgaggatagactaaaggaagcagagagggaa
gttatggacctccttcgtgaggcccaggatgtcaaagatgttgaccagaa
tttgatggatcgcctacagagagtgaataacactctgtccagccaaatta
gccgtttacagaatatccggaataccattgaagagactggaaacttggct
gaacaagcgcgtgcccatgtagagaacacagagcggttgattgaaatcgc
atccagagaacttgagaaagcaaaagtcgctgctgccaatgtgtcagtca
ctcagccagaatctacaggggacccaaacaacatgactcttttggcagaa
gaggctcgaaagcttgctgaacgtcataaacaggaagctgatgacattgt
tcgagtggcaaagacagccaatgatacgtcaactgaggcatacaacctgc
ttctgaggacactggcaggagaaaatcaaacagcatttgagattgaagag
cttaataggaagtatgaacaagcgaagaacatctcacaggatctggaaaa
acaagctgcccgagtacatgaggaggccaaaagggccggtgacaaagctg
tggagatctatgccagcgtggctcagctgagccctttggactctgagaca
ctggagaatgaagcaaataacataaagatggaagctgagaatctggaaca
actgattgaccagaaattaaaagattatgaggacctcagagaagatatga
gagggaaggaacttgaagtcaagaaccttctggagaaaggcaagactgaa
cagcagaccgcagaccaactcctagcccgagctgatgctgccaaggccct
cgctgaagaagctgcaaagaagggacgggataccttacaagaagctaatg
acattctcaacaacctgaaagattttgataggcgtgtgaacgataacaag
acggccgcagaggaggcactaaggaagattcctgccatcaaccagaccat
cactgaagccaatgaaaagaccagagaagcccagcaggccctgggcagtg
ctgcggcggatgccacagaggccaagaacaaggcccatgaggcggagagg
atcgcgagcgctgtccaaaagaatgccaccagcaccaaggcagaagctga
aagaacttttgcagaagttacagatctggataatgaggtgaacaatatgt
tgaagcaactgcaggaagcagaaaaagagctaaagagaaaacaagatgac
gctgaccaggacatgatgatggcagggatggcttcacaggctgctcaaga
agccgagatcaatgccagaaaagccaaaaactctgttactagcctcctca
gcattattaatgacctcttggagcagctggggcagctggatacagtggac

ctgaataagctaaacgagattgaaggcaccctaaacaaagccaaagatga
aatgaaggtcagcgatcttgataggaaagtgtctgacctggagaatgaag
ccaagaagcaggaggctgccatcatggactataaccgagatatcgaggag
atcatgaaggacattcgcaatctggaggacatcaggaagaccttaccatc
tggctgcttcaacaccccgtccattgaaaagccctagtgtctttagggct
ggaaggcagcatccctctgacagggggcagttgtgaggccacagagtgc
cttgacacaaagattacatttttcagaccccactcctctgctgctgtcc
atgactgtccttttgaaccaggaaaagtcacagagtttaaagagaagcaa
attaaacatcctgaatcgggaacaaagggttttatctaataaagtgtctc
ttccattcacgttgctaccttacccacactttcccttctgatttgcgtga
ggacgtggcatcctacgttactgtacagtggcataagcacatcgtgtgag
cccatgtatgctggggtagagcaagtagccctcccctgtctcatcgatac
cagcagaacctcctcagtctcagtactcttgtttctatgaaggaaaagtt
tggctactaacagtagcattgtgatggccagtatatccagtccatggata
aagaaaatgcatctgcatctcctacccctcttccttctaagcaaaaggaa
ataaacatcctgtgccaaaggtattggtcatttagaatgtcggtagccat
ccatcagtgcttttagttattatgagtgtaggacactgagccatccgtgg
gtcaggatgcaattatttataaaagtctccaggtgaacatggctgaagat
ttttctagtatattaataattgactaggaagatgaactttttttcagatc
tttgggcagctgataatttaaatctggatgggcagcttgcactcaccaat
agaccaaaagacatcttttgatattcttataaatggaacttacacagaag
aaatagggatatgataaccactaaaattttgttttcaaaatcaaactaat
tcttacagctttttttattagttagtcttggaactagtgttaagtatctgg
cagagaacagttaatccctaaggtcttgacaaaacagaagaaaaacaagc
ctcctcgtcctagtcttttctagcaaagggataaaacttagatggcagct
tgtactgtcagaatcccgtgtatccatttgttcttctgttggagagatga
gacatttgacccttagctccagttttcttctgatgtttccatcttccaga
atccctcaaaaaacattgtttgccaaatcctggtggcaaatacttgcact
cagtatttcacacagctgccaacgctatcgagttcctgcactttgtgatt
taaatccactctaaaccttccctctaagtgtagagggaagacccttacgt
ggagtttcctagtgggcttctcaacttttgatcctcagctctgtggtttt
aagaccacagtgtgacagttccctgccacacacccccttcctcctaccaa
cccacctttgagattcatatatagcctttaacactatgcaactttgtact
ttgcgtagcaggggcggggtggggggaaagaaactattatctgacacact
ggtgctattaattatttcaaatttatatttttgtgtgaatgttttgtgtt
ttgtttatcatgattatagaataaggaatttatgtaaatatacttagtcc
tatttctagaatgacactctgttcactttgctcaatttttcctcttcact
ggcacaatgtatctgaatacctccttccctcccttctagaattctttgga
ttgtactccaaagaattgtgccttgtgtttgcagcatctccattctctaa
aattaatataattgctttcctccacacccagccactgtaaagaggtaact
tgggtcctcttccattgcagtcctgatgatcctaacctgcagcacggtgg
ttttacaatgttccagagcaggaacgccaggttgacaagctatggtagga
ttaggaaagtttgctgaagaggatctttgacgccacagtgggactagcca
ggaatgagggagaaatgccctttctggcaattgttggagctggataggta
agttttataagggagtacattttgactgagcacttagggcatcaggaaca
gtgctacttactgatgggtagactgggagaggtggtgtaacttagttctt

gatgatcccacttcctgtttccatctgcttgggatataccagagtttacc
acaagtgttttgacgatatactcctgagctttcactctgctgcttctccc
aggcctcttctactatggcaggagatgtggcgtgctgttgcaaagttttc
acgtcattgtttcctggctagttcatttcattaagtggctacatcctaac
atatgcatttggtcaaggttgcagaagaggactgaagattgactgccaag
ctagtttgggtgaagttcactccagcaagtctcaggccacaatggggtgg
tttggtttggtttccttttaactttctttttgttatttgcttttctcctc
cacctgtgtggtatattttttaagcagaattttatttttttaaaataaaag
gttctttacaagatgataccttaattacactcccgcaacacagccattat
tttattgtctagctccagttatctgtattttatgtaatgtaattgacagg
atggctgctgcagaatgctggttgacacagggattattatactgctattt
ttccctgaattttttttcctttgaattccaactgtggaccttttatatgtg
ccttcactttagctgtttgccttaatctctacagccttgctctccggggt
ggttaataaaatgcaacacttggcatttttatgttttaagaaaaacagta
ttttatttataataaaatctgaatatttgtaacccttta

>NM_002958 3
cggctcggggctgtgagcggctcggggccgggggtgggcggcggtgcggc
gggcggccgacgctcctcttcggcggcggcggcggcggccatgcgtgggg
cggcgcggctggggcggccgggccggagttgcctcccgggggcccgcggc
ctgagggccccgccgccgccgccgctgctgcttctgcttgcgctgttgcc
gctgctgcccgcgcctggcgctgccgccgcccccgccccgcggcccccgg
agctgcagtcggcttccgcggggcccagcgtgagtctctacctgagcgag
gacgaggtgcgccggctgatcggtcttgatgcagaactttattatgtgag
aaatgaccttattagtcactacgctctatcctttagtctgttagtaccca
gtgagacaaatttcctgcacttcacctggcatgcgaagtccaaggttgaa
tataagctgggattccaagtggacaatgttttggcaatggatatgcccca
ggtcaacatttctgttcagggggaagttccacgcactttatcagtgtttc
gggtagagctttcctgtactggcaaagtagattctgaagttatgatacta
atgcagctcaacttgacagtaaattcttcaaaaaattttaccgtcttaaa
ttttaaacgaaggaaaatgtgctacaaaaaacttgaagaagtaaaaactt
cagccttggacaaaaacactagcagaactatttatgatcctgtacatgca
gctccaaccacttctacgcgtgtgttttatattagtgtaggggtttgttg
tgcagtaatatttctcgtagcaataatattagctgttttgcaccttcata
gtatgaaaaggattgaactggatgacagcattagtgccagcagtagttcc
caagggctgtctcagccatccacccagacgactcagtatctgagagcaga
cacgcccaacaatgcaactcctatcaccagttatcctaccttgcggatag
agaagaacgacttgagaagtgtcactcttttggaggccaaaggcaaggtg
aaggatatagcaatatccagagagaggataactctaaaagatgtactcca
agaaggtacttttgggcgtattttccatgggattttaatagatgaaaaag
atccaaataaagaaaaacaagcatttgtcaaaacagttaaagatcaagct
tctgaaattcaggtgacaatgatgctcactgaaagttgtaagctgcgagg
tcttcatcacagaaatcttcttcctattactcatgtgtgtatagaagaag
gagaaaagcccatggtgatattgccttacatgaattgggggaatcttaaa
ttgtttttacgacagtgcaagttagtagaggccaataatccacaggcaat
ttctcagcaagacctggtacacatggctattcagattgcctgtggaatga

gctacctggccagaagggaagtcatccacaaagacctggctgccaggaac
tgtgtcattgatgacacacttcaagttaagatcacagacaatgccctctc
cagagacttgttccccatggactatcactgtctgggggacaatgaaaaca
ggccagttcgttggatggctcttgaaagtctggttaataacgagttctct
agcgctagtgatgtgtgggcctttggagtgacgctgtgggaactcatgac
tctgggccagactccctacgtggacattgaccccttcgagatggccgcat
acctgaaagatggttaccgaatagcccagccaatcaactgtcctgatgaa
ttatttgctgtgatggcctgttgctgggccttagatccagaggagaggcc
caagtttcagcagctggtacagtgcctaacagagtttcatgcagccctgg
gggcctacgtctgactcctctccaatcccacaccatcaggaagaaggtgc
ctgtcggggctcacttgaagcctgtcagggatgctttgtatctaacacaa
cgccaacagaagcacatttgtcttccagaacaccgtgccttagaaatgct
ttagaatctgaactttttaagacagacttaataatgtggcatattttcta
gatatcacttttattaggttgaactgaaagggtttttgtaatttttttgg
ccaaaatttttaaaacatacttactttggactaggggtacattcttaca
aaataaataaacagtttttaaaattgtttagacacagatatttggaatta
gctatcttagtgccaactgctttttattttttttacttcatcaaggtgatg
taagtgactcacctttaaagttttttttagtgttatttttttatcactactc
tgggaaatggtttgtcttcaagatgcaatacttttcttagtaaaggaaaa
acagcataaaaagatacctggtctgccttgtacaagaaaaggcaatatta
gaggaagaaaatttaaagaaaagctagaggaaaaaaaaatttttttaaaa
atacttattagaagcaaactgcccttgcatggaaaactgtttattttttt
cagtgaaaaggaattctgctttcgtgtttttgggaaagcaggaactgagt
tcattacatctttaatttggcagaaattagcctttctgtgaaccagatgt
ggtttggggcagatctgtagtaaacaatggtgattttatttatttttact
ctctggaaaaggagataatacaattccagaaagtgaactcatatttctaa
ggttaagattcccttttattgcacctagaatagtgctatgcacagagcgg
gtgcttgagttgttgtcgtttttttgtttgtttttttaaatgtaaactggta
aattttgtgcttatcttcaaggctggcttaagtataaaattgttttttaa
acacttgaaaaattaaaggatttgttttatatt

>NM_003909 3
cttgctctgccagtccagccctaggttctcctgaatcctgcggtcccagc
gtcgctccggacgctgccaacctgttctccaccgtcgctcgacttccacc
tctaagactcccacgaaactcaggttgaataattcatcaaattacacaac
tgaactcaagacatggctgcccagtgtgtcacaaaggtggcgctgaatgt
ttcctgtgccaatcttttggataaagatatagggtcaaagtcagaccctt
tatgtgtgttgtttttgaatacaagtggtcaacagtggtatgaggttgag
cgcacagaaaggattaagaattgcttgaatccccaattttccaagacatt
tattattgattactactttgaagtggttcagaaattgaaatttggggttt
atgacatcgacaacaaaactattgagctgagtgatgatgacttcttaggg
gaatgtgaatgtacccttggacaaattgtttccagcaagaagctaactcg
accactggtgatgaaaactggcagacctgcaggaaaagggagcattacga
tttcagctgaagaaataaaagataatagagtggtcttgtttgaaatggaa
gccagaaaactggataataaggatctatttggaaagtcagacccatacct
ggaattccacaagcagacatctgatggaaactggctaatggttcatcgga

cagaggttgttaaaaacaacttgaatcctgtttggaggcctttcaagatc
tctcttaactcactgtgttacggagatatggacaaaaccattaaggtgga
gtgttatgattatgacaatgatgggtcacatgatctcattggaacatttc
agaccaccatgacaaaactgaaagaagcctccagaagctcacctgttgaa
tttgaatgcataaatgagaaaaaaaggcaaaagaaaaaaagctacaagaa
ttcaggtgttatcagtgtgaaacagtgtgagattacagtagaatgcacat
tccttgactatataatgggaggatgtcagctgaattttactgtgggagtg
gacttcactggctccaatggtgacccaaggtctccagactcccttcatta
catcagccccaatggcgttaatgagtatttgactgctctctggtctgtgg
gactggtcattcaagattatgatgctgataagatgtttccagcttttggt
tttggcgctcagatacctcctcagtggcaggtatcacatgaatttccaat
gaacttcaacccatccaatccctactgcaatggaatccaaggcattgtag
aggcgtatcggtcttgtcttcctcagataaaactctatggaccaactaat
ttttctccaatcataaatcacgtggccaggtttgctgctgcagccacgca
acagcagacagcttctcaatattttgtgcttttgattattactgatggtg
tgatcacagaccttgatgaaaccagacaagctatagttaatgcctccagg
ctgcctatgtccatcataattgttggagttggaggtgctgacttcagcgc
catggagtttctggatggtgatggtggaagtctccgctccccattgggcg
aagtggccatcagagatattgtccagtttgtgcctttcagacagttccag
aatgctccaaaagaagcacttgctcagtgtgtcttggcagagattcccca
gcaggtggtgggctacttcaatacatacaaactccttcctcccaagaacc
cagccacgaaacaacagaagcagtgaccacttcaacagaattcttttgtg
ttatgtggagcaatgccatctctcaccccaaatcgtgtatctgtcattct
acgtactttttacccccagcatttatgatgtaaatctctttctctatgga
ttatatctgtttaaagcattctttctaggttattttggggggacagtgcc
aagtccatctttgcccagtcaattcagtgattgatagcaatttacattaa
ttgcagtaaagctctttggattagaaattagtgtggggaaagcttattct
gttgttgtttttgtttactttcatatgatgaaaatgctgtgtttaagtgt
ttgtcaataggaagaatggaaaactgttgggatgatgtggtttgcaggtt
gctgtgcctgattcacagtgtatgttgtataagccaatgtccatacctga
ttatgagagcttcttaaattatatgatatcaaatttgttcctgtaactct
gtatacagtgcttttctgcaaggtaaaaataacctgtctatgcatctgat
ttttgctacagtttagacactgtggtttacaaaacagcatgcactcaact
tgggactttatgaaaagtactgaatgagcaggaaaaggcacatactcagt
tttttaaatgtacaatcaacaagtaaaaataacctcatgtaagtaagcca
tttttatttgcctttctagatattttattttattgtggaaaactgtaaac
atggtcagatttggcttttttttttcattaactgagcaagactttcaggat
attgtagatgcacagatggtaggttgtcctgaattctacattattagatt
actttaattgagatttgttaaaacggttaggactgttttgtccaggaaag
ataagaggaccaaacatataaggtgaaattcagaattccgtttccttcta
actaatgaaaaactgcttactaaaaaaaaattttatactttccttgctaa
ggtcccatatattgatttgtacagatccacttagtcattttctccttttt
ttaagaaccattttcatctgatttttaaactcacgataccagttatctgt
taatcaaaattgcattttacaatttaataatgtgatatttcctatgtcta
cagcataccttattaggtataaaacctactgcaacttagaaaaaggaaag
aaaaaagaaaacttttccaactgctgcattaagatagggtggattttatg

tgctttttttttttaagagttgaatttcttttcctgacttttacctttta
cagcgtattacttagtgaacattacattttcagaatagatcctaatattt
tattgagggcctatgtgctaaaaactatgcatatctatatattggccaat
tatctttaataatttaccttttgaaattgcatgtttatcatatatcctta
agtggacacatacagtgccatgttgatgtgcctctcagttttattgaaaa
gctgccccacagcccatgtctcttgttctctgcaatgcctcaagggagtg
agctctcaaccacagatagctgtggcttctcagaagcagctcattgccaa
ggccaggctgagaggggacctgcttgctgtggtggttgcctagcccagat
gagcatttacctaccaccttcccacttggctagctgtcctttggatatgt
gctgttaactggggaaggcatctaactagtagcctgctactccatagtat
ggctcaatagatgacacatcattttgacattatcaataggagaaaagaaa
actaacccttcttctgattgtttggagccatagttgtctcagatgttcta
attctctttgtatgcttggaaacagcatagatatgttgctgtggttttca
gaattttctcttttaatcacaagaagccttttaaaaaatgacttacacat
attctcaatgtacagtaaaacagacagaagtgagcttatctgtttgatgc
tgtggcagggtcccagtcactgggcatatcctccttctccttaaccagct
ccacagcagcccctgagtcacctgcacaaggtgcttgggaactgctggtt
atgagcattcctggttttcttcagccaaataacaggtaatcactgtcaat
tggatttggtcttcattattttatattctgattttatcagaattattcta
ttttaaaattgttttaaaatttaaaacatttaattcatgatcatgttcat
cagtagatgctattattcataagaactgtgattccagcaaactagggtaa
ttggtgcctttttacagttttgaataaaagcatttacaatttctaaatta
tcagttttcacagtttcagcactcaacctcatcatacgctgatttaatat
tgttttacattaaaatagtccttttccctgttgtgccaccattcatttaa
gtgctgtttgttcttaaaatgcatttaaagaaaaattacccatattgact
ttcacacctcatataatcagatctattacaaatatatatcggagtgacgg
tgcccaggatagatgtaatatttcttacagatgctggcacagaggaaata
atataccagctaatctagtcacctaaccttgtggttagaattgcaatttt
aagaccagaaaaatttgaagtctgatcagagatttacaactgttcattat
agtggtgccttaggcaatctttccaaagtaaattcagggccccattgcta
cttatgccatatttggacatacttttttttttcttcaattttgtaaacttc
ctggaaagctgtcttcactaagtatcccctagtctctatatatgtggtta
gtagtcatggaaatgacacataaagtacgccagaagtttgatggaacgtg
ttagaaactgttttgtgcttttatggatgtcatacttgacaatacatgtg
taagttactaatatatgaattgatgctaaatatatcttacatttgaattc
cttttggataaagttatttcttgatgtgacagagtagtgtgttttcattt
ttattctttacatgtgaccaaaacaatagaaaagttaaaaataaaatata
gtgttttaggtggcaaaaaaaaaaaaaaaaaaa

>NM_003945 3
aggcggggcttgcacacgctggtcacgcggtcagctattgacacttcctg
gtgggatccgagtgaggcgacggggtaggggttggcgctcaggcggcgac
catggcgtatcacggcctcactgtgcctctcattgtgatgagcgtgttct
ggggcttcgtcggcttcttggtgccttggttcatccctaagggtcctaac
cggggagttatcattaccatgttggtgacctgttcagtttgctgctatct
cttttggctgattgcaattctggcccaactcaaccctctctttggaccgc

aattgaaaaatgaaaccatctggtatctgaagtatcattggccttgagga
agaagacatgctctacagtgctcagtctttgaggtcacgagaagagaatg
ccttctagatgcaaaatcacctccaaaccagaccacttttcttgacttgc
ctgttttggccattagctgccttaaacgttaacagcacatttgaatgcct
tattctacaatgcagcgtgttttcctttgcctttttgcactttggtgaa
ttacgtgcctccataacctgaactgtgccgactccacaaaacgattatgt
actcttctgagatagaagatgctgttcttctgagagatacgttactctct
ccttggaatctgtggatttgaagatggctcctgccttctcacgtgggaat
cagtgaagtgtttagaaactgctgcaagacaaacaagactccagtggggt
ggtcagtaggagagcacgttcagagggaagagccatctcaacagaatcgc
accaaactatactttcaggatgaatttcttctttctgccatcttttggaa
taaatattttcctcctttctatggaaatctggaaaaaaaaaaaa

>NM_004339 3
atccgcgccgcgcaggcgcactgagcctcagccgctctcctcgctaccag
gctcctttcctctatcgcgcaggcgcactgaacctcagccgcgccgggag
gcgagcccgttggcgaggggcggggcagatggcgagggagcggggcgagc
tgctttttgaggctgcgcactcgcatggccccacctcgcaggtccacttc
cggcgccgcggctgttccgggcggagaccgcttgtgctggagtcggagtt
gtaacgctccactgactgatagagcgaccggccgaccatggcgcccggag
tggcccgcgggccgacgccgtactggaggttgcgcctcggtggcgccgcg
ctgctcctgctgctcatcccggtggccgccgcgcaggagcctcccggagc
tgcttgttctcagaacacaaacaaaacctgtgaagagtgcctgaagaacg
tctcctgtctttggtgcaacactaacaaggcttgtctggactacccagtt
acaagcgtcttgccaccggcttccctttgtaaattgagctctgcacgctg
gggagtttgttgggtgaactttgaggcgctgatcatcaccatgtcggtag
tcgggggaaccctcctcctgggcattgccatctgctgctgctgctgctgc
aggaggaagaggagccggaagccggacaggagtgaggagaaggccatgcg
tgagcgggaggagaggcggatacggcaggaggaacggagagcagagatga
agacaagacatgatgaaatcagaaaaaaatatggcctgtttaaagaagaa
aacccgtatgctagatttgaaaacaactaaagcgctccagcacatcagtc
ccgacgcttcctgtgaggtgcacgctccgcagcccagcccagccgggaga
ccacgtggccattgcggtctcctgaccttggccagtgaacctgccagcct
tccaggacaggcggccggagagctgcccctgaaggacagtcctctcgtct
tgcagactggtgaccttctattccctgttcatctctgtttctagatttag
tcacttgaaataagaaatctttgggggtttgggctttttatactcttctc
agtttgtgaaacgctaactgcacacgaagccgcctgacggcacccagcgc
tgtggctgtcattctcccagggcagaaccctgcgtttctctctgtccact
aacaagcttcacacgcaacacagggaagtcggtttgacttttgtcatgag
gagaactgaccagccctcatcattccccataaaaccacggacagcgtctg
tgtgcgcatcttgagtcttcacacctgttgactcacacggcttttgctga
tgacacggggctccagtacacagtctgataaggacttaacgtcctaacct
caattgtattaaatagcattggggaatagctaaaccttttttaaaaaaatt
tattggattttcctccctgcttaaaagatttcaccagaaaaccttcatat
aaaaattcaggccctttttggacaatttttaaaatttgtatctttactag
aacatgagaatctttttcccttggaagcttgaattataaatgtggtgttt

ggcctgcctcagcagcaccagttgactgctcgtgtgccagcggtgtgggg
aggacggggcaggacgctgcagctctctccagccctgttggcatcctcag
tgcctgcaggcctctcgctgcctgttgggctgtctggggggtggccattt
agggatcgtggggacggggtccaccccaagaagaaagaaaggcccgtcca
caggcccggctctgggccacgtgccccggaagcaggtgtgtccagagtca
gctgagggctctccccacaccacccagcaggcgctggtgctccttctgcc
tcatgggaccagtccagcttccagccgctctggctcgagggtggtctgac
cacttccttctgagtgggcttctctgggagctctccagtggcactgctgg
acctgcccacgtttctgtaaaatcaggatacgtggctttagtaagcagac
caagcgcttcgtggcagggaaagcagcgtgcggggaagtcactgaaaagt
gctgcctaaggaagtttggaaatagtccctgttccagattgccttgaatt
ttaaaacattttgctttgggaaagtaggtcagcagcacctaagatcaagg
atgcgttccattttcacacttcacagtcatgaaaactgagaagactgtct
tcagcgtgaactaaagttcacaggcagatcactgatccagaacacttcaa
gaactcgtcaaacagctcgataagcctttttgactgtgtacatctgtacc
gggaataacattcctaggctgaaatttccacaaagaatagaacctgtacc
cagttcttcaggctgatttccctgacctcttgggcatttgtatttgtagt
aaagtattgcagagattcctaagtattttatagcagccatcaaaattgga
ctttgtattgtttattcataaaagacacttggtaatagacttcagtgaac
tctgtatgaatgcagtagtgtgtgtgcaaaatccgcttcctgagcgtagg
gtgctgagctggcgctagggctcggttgtgaaatacagcgtagtcagccc
ttgcgctcagtgtagaaacccacgtctgtaaggtcggtcttcgtccatct
gctttttttctgaaatacactaagagcagccacaaaactgtaacctcaagg
aaaccataaagcttggagtgccttaatttttaaccagtttccaataaaac
ggtttactacctgcgtgtgtttagtaaaaaaaaaaaaaaaaaa

>NM_004670 3
ctaggcggcggcggccgggtccccaaggctgggcgctgcttgcggaaccg
acggggcggagaggagcgtggcgggaggaggagtaggagaagggggctgg
tcaagggaagtgcgacgtgtctgcggagcctttttatacctccttcccgg
gagtccggcagccgctgctgctgctgctgctgctgctgccgccgccgccg
ccgccgtccctgcgtccttcggtctctgctcccgggacccgggctccgcc
gcagccagccagcatgtcggggatcaagaagcaaaagacggagaaccagc
agaaatccaccaatgtagtctatcaggcccaccatgtgagcaggaataag
agagggcaagtggttggaacaaggggtgggttccgaggatgtaccgtgtg
gctaacaggtctctctggtgctggaaaaacaacgataagttttgccctgg
aggagtaccttgtctcccatgccatcccttgttactccctggatggggac
aatgtccgtcatggccttaacagaaatctcggattctctcctggggacag
agaggaaaatatccgccggattgctgaggtggctaagctgtttgctgatg
ctggtctggtctgcattaccagctttatttctccattcgcaaaggatcgt
gagaatgcccgcaaaatacatgaatcagcagggctgccattctttgaaat
atttgtagatgcacctctaaatatttgtgaaagcagagacgtaaaaggcc
tctataaaagggccagagctggggagattaaaggatttacaggtattgat
tctgattatgagaaacctgaaactcctgagcgtgtgcttaaaaccaattt
gtccacagtgagtgactgtgtccaccaggtagtggaacttctgcaagagc
agaacattgtaccctatactataatcaaagatatccacgaactctttgtg

ccggaaaacaaacttgaccacgtccgagctgaggctgaaactctcccttc
attatcaattactaagctggatctccagtgggtccaggttttgagcgaag
gctgggccactcccctcaaaggtttcatgcgggagaaggagtacttacag
gttatgcactttgacaccctgctagatgatggcgtgatcaacatgagcat
ccccattgtactgcccgtctctgcagaggataagacacggctggaagggt
gcagcaagtttgtcctggcacatggtggacggagggtagctatcttacga
gacgctgaattctatgaacacagaaaagaggaacgctgttcccgtgtttg
ggggacaacatgtacaaaacacccccatatcaaaatggtgatggaaagtg
gggactggctggttggtggagaccttcaggtgctggagaaaataagatgg
aatgatgggctggaccaataccgtctgacacctctggagctcaaacagaa
atgtaaagaaatgaatgctgatgcggtgtttgcattccagttgcgcaatc
ctgtccacaatggccatgccctgttgatgcaggacactcgccgcaggctc
ctagagaggggctacaagcacccggtcctcctactacaccctctgggcgg
ctggaccaaggatgacgatgtgcctctagactggcggatgaagcagcacg
cggctgtgctcgaggaaggggtcctggatcccaagtcaaccattgttgcc
atctttccgtctcccatgttatatgctggccccacagaggtccagtggca
ctgcaggtcccggatgattgcgggtgccaatttctacattgtggggaggg
accctgcaggaatgccccatcctgaaaccaagaaggatctgtatgaaccc
actcatgggggcaaggtcttgagcatggcccctggcctcacctctgtgga
aatcattccattccgagtggctgcctacaacaaagccaaaaaagccatgg
acttctatgatccagcaaggcacaatgagtttgacttcatctcaggaact
cgaatgaggaagctcgcccgggaaggagagaatcccccagatggcttcat
ggcccccaaagcatggaaggtcctgacagattattacaggtccctggaga
agaactaagcctttggctccagagtttctttctgaagtgctctttgatta
ccttttctatttttatgattagatgctttgtattaaattgcttctcaatg
atgcattttaatcttttataatgaagtaaaagttgtgtctataattaaaa
aaaaatatatatatacacacacacatatacatacaaagtcaaactgaa
gaccaaatcttagcaggtaaaagcaatattcttatacatttcataataaa
attagctctatgtattttctactgcacctgagcaggcaggtcccagattt
cttaaggctttgtttgaccatgtgtctagttacttgctgaaaagtgaata
tattttccagcatgtcttgacaacctgtactcttccaatgtcatttatca
gttgtaaaatatatcagattgtgtcctcttctgtacaattgacaaaaaaa
aaaatttttttttctcactctaaaagaggtgtggctcacatcaagattct
tcctgatattttacctcatgctgtacaaagccttaatgttgtaatcatat
cttacgtgttgaagacctgactggagaaacaaaatgtgcaataacgtgaa
ttttatcttagagatctgtgcagcctatttctgtcacaaaagttatattg
tctaataagagaagtcttaatggcctctgtgaataatgtaactccagtta
cacggtgacttttaatagcatacagtgatttgatgaaaggacgtcaaaca
atgtggcgatgtcgtggaaagttatctttcccgctctttgctgtggtcat
tgtgtcttgcagaaaggatggccctgatgcagcagcagcgccagctgtaa
taaaaaataattcacactatcagactagcaaggcactagaactggaaaag
accacagaaaacaaagaatccaaccctttcatcttacaggtgaacaaact
gtgatgatgcacatgtatgtgttttgtaagctgtgagcaccgtaacaaaa
tgtaaatttgccattattaggaagtgctggtggcagtgaagaagcaccca
ggccacttgactcccagtctggtgccctgtctacaccagacaacacagga
gctgggtcagattcccctcagctgcttaacaaagttcctcgaacagaaag

tgcttacaaagctgccttctcggatactgaaaggtcgagttttctgaact
gcactgattttattgcagttgaaaaaaaaaaaaagctattccaaagattt
caagctgttctgagacatcttctgatggctttacttcctgagaggcaatg
tttttactttatgcataattcattgttgccaaggaataaagtgaagaaac
agcaccttttaatatataggtctctctggaagagacctaaattagaaaga
gaaaactgtgacaattttcatattctcattcttaaaaaacactaatctta
actaacaaaagttcttttgagaataagttacacacaatggccacagcagt
ttgtctttaatagtatagtgcctatactcatgtaatcggttactcactac
tgcctttaaaaaaaaaaaccagcatatttattgaaaacatgagacaggat
tatagtgccttaaccgatatattttgtgacttaaaaaatacatttaaaac
tgctcttctgctctagtaccatgcttagtgcaaatgattatttctatgta
caactgatgcttgttcttattttaataaatttatcagagtgaaaaaaaaa
aaaaaaaaa

>NM_004817 3
gacgcggttcgccgcaggagcctcgaaggcgcggcgccggcgagcccttc
cccggcaggcgcgtgggtggtagcggccaatttgacagtttcccgggccg
ggcggccagcgcggaggcgccacgctcgggtcgggggcgggctgacgccg
ccgccgccgcgggaggagggacaaaggggtgggtccccgcgggtcggcac
cccggcggttgggctgcgggtcagagcactgtccggtggtgcccaggagg
agtaggagcaggagcagaagcagaagcggggtccggagctgcgcgcctac
gcgggacctgtgtccgaaatgccggtgcgaggagaccgcgggtttccacc
ccggcgggagctgtcaggttggctccgcgccccaggcatggaagagctga
tatgggaacagtacactgtgaccctacaaaaggattccaaaagaggattt
ggaattgcagtgtccggaggcagagacaacccccactttgaaaatggaga
aacgtcaattgtcatttctgatgtgctcccgggtgggcctgctgatgggc
tgctccaagaaaatgacagagtggtcatggtcaatggcacccccatggag
gatgtgcttcattcgtttgcagttcagcagctcagaaaaagtgggaaggt
cgctgctattgtggtcaagaggccccggaaggtccaggtggccgcacttc
aggccagccctcccctggatcaggatgaccgggcttttgaggtgatggac
gagtttgatggcagaagtttccggagtggctacagcgagaggagccggct
gaacagccatgggggggcgcagccgcagctgggaggacagcccggaaaggg
ggcgtccccatgagcgggcccggagccgggagcgggacctcagccgggac
cggagccgtggccggagcctggagcggggcctggaccaagaccatgcgcg
cacccgagaccgcagccgtggccggagcctggagcggggcctggaccacg
actttgggccatcccgggaccgggaccgtgaccgcagccgcggccggagc
attgaccaggactacgagcgagcctatcaccgggcctacgacccagacta
cgagcgggcctacagcccggagtacaggcgcggggcccgccacgatgccc
gctctcggggaccccgaagccgcagccgcgagcacccgcactcacggagc
cccagccccgagcctagggggcggccggggcccatcggggtcctcctgat
gaaaagcagagcgaacgaagagtatggtctccggcttgggagtcagatct
tcgtaaaggaaatgacccgaacgggtctggcaactaaagatggcaacctt
cacgaaggagacataattctcaagatcaatgggactgtaactgagaacat
gtctttaacggatgctcgaaaattgatagaaaagtcaagaggaaaactac
agctagtggtgttgagagacagccagcagaccctcatcaacatcccgtca
ttaaatgacagtgactcagaaatagaagatatttcagaaatagagtcaaa

ccgatcattttctccagaggagagacgtcatcagtattctgattatgatt
atcattcctcaagtgagaagctgaaggaaaggccaagttccagagaggac
acgccgagcagattgtccaggatgggtgcgacacccactccctttaagtc
cacaggggatattgcaggcacagttgtcccagagaccaacaaggaaccca
gataccaagaggacccccccagctcctcaaccaaaagcagccccgagaact
tttcttcgtcctagtcctgaagatgaagcaatatatggccctaataccaa
aatggtaaggttcaagaagggagacagcgtgggcctccggttggctggtg
gcaatgatgtcgggatatttgttgctggcattcaagaagggacctcggcg
gagcaggagggccttcaagaaggagaccagattctgaaggtgaacacaca
ggatttcagaggattagtgcgggaggatgccgttctctacctgttagaaa
tccctaaaggtgaaatggtgaccattttagctcagagccgagccgatgtg
tatagagacatcctggcttgtggcagaggggattcgtttttttataagaag
ccactttgaatgtgagaaggaaactccacagagcctggccttcaccagag
gggaggtcttccgagtggtagacacactgtatgacggcaagctgggcaac
tggctggctgtgaggattgggaacgagttggagaaaggcttaatccccaa
caagagcagagctgaacaaatggccagtgttcaaaatgcccagagagaca
acgctggggaccgggcagatttctggagaatgcgtggccagaggtctggg
gtgaagaagaacctgaggaaaagtcgggaagacctcacagctgttgtgtc
tgtcagcaccaagttcccagcttatgagagggttttgctgcgagaagctg
gtttcaagagacctgtggtcttattcggccccatagctgatatagcaatg
gaaaaattggctaatgagttacctgactggtttcaaactgctaaaacgga
accaaaagatgcaggatctgagaaatccactggagtggtccggttaaata
ccgtgaggcaaattattgaacaggataagcatgcactactggatgtgact
ccgaaagctgtggacctgttgaattacacccagtggttcccaattgtgat
ttttttcaacccagactccagacaaggtgtcaaaaccatgagacaaaggt
taaatccaacgtccaacaaaagttctcgaaagttatttgatcaagccaac
aagcttaaaaaaacgtgtgcacacctttttacagctacaatcaacctaaa
ttcagccaatgatagctggtttggcagcttaaaggacactattcagcatc
agcaaggagaagcggtttgggtctctgaaggaaagatggaagggatggat
gatgaccccgaagaccgcatgtcctacttaaccgccatgggcgcggacta
tctgagttgcgacagccgcctcatcagtgactttgaagacacggacggtg
aaggaggcgcctacactgacaatgagctggatgagccagccgaggagccg
ctggtgtcgtccatcacccgctcctcggagccggtgcagcacgaggagag
cataaggaaacccagcccagagccacgagctcagatgaggagggctgcta
gcagcgatcaacttagggacaatagcccgcccccagcattcaagccagag
ccgcccaaggccaaaacccagaacaaagaagaatcctatgacttctccaa
atcctatgaatataagtcaaacccctctgccgttgctggtaatgaaactc
ctggggcatctaccaaaggttatcctcctcctgttgcagcaaaacctacc
tttgggcggtctatactgaagccctccactcccatccctcctcaagaggg
tgaggaggtgggagagagcagtgaggagcaagataatgctcccaaatcag
tcctgggcaaagtcaaaatatttgagaagatggatcacaaggccaggtta
cagagaatgcaggagctccaggaagcacagaatgcaaggatcgaaattgc
ccagaagcatcctgatatctatgcagttccaatcaaaacgcacaagccag
accctggcacgccccagcacacgagttccagacccccctgagccacagaaa
gctccttccagaccttatcaggataccagaggaagttatggcagtgatgc
cgaggaggaggagtaccgccagcagctgtcagaacactccaagcgcggtt

actatggccagtctgcccgataccgggacacagaattatagatgtctgag
cacggactctcccaggcctgcctgcatggcatcagactagccactcctgc
caggccgccgggatggttcttctccagttagaatgcaccatggagacgtg
gtgggactccagctcgtgtgtcctcatggagaacccaggggacagctggt
gcaaattcagaactgagggctctgtttgtgggactgggttagaggagtct
gtggctttttgttcagaattaagcagaacactgcagtcagatcctgttac
ttgcttcagtggaccgaaatctgtattctgtttgcgtacttgtaatatgt
atattaagaagcaataactatttttcctcattaatagctgccttcaagga
ctgtttcagtgtgagtcagaatgtgaaaaaggaataaaaaatactgttgg
gctcaaactaaattcaaagaagtactttattgcaactcttttaagtgcct
tggatgagaagtgtcttaaattttcttcctttgaagctttaggcagagcc
ataatggactaaaacattttgactaagtttttataccagcttaatagctg
tagttttccctgcactgtgtcatcttttcaaggcatttgtctttgtaata
ttttccataaatttggactgtctatatcataactatacttgatagtttgg
ctataagtgctcaatagcttgaagcccaagaagttggtatcgaaatttgt
tgtttgtttaaacccaagtgctgcacaaaagcagatacttgaggaaaaca
ctatttccaaaagcacatgtattgacaacagttttataatttaataaaaa
ggaatacattgcaatccgtaatttt

>NM_004945 3
gaggtcgctcgggtcgggtgtcgcctgagaaccggatgaggcggcgaccg
tgaggccgagccgggagcgggcgtcttgccgaggcccgggcgggcgggga
gcaacggctacagacgccgcggggccaggtcgttgagggtcggcggcggg
cgaggagcgcagggcgctcgggccgggggccgccggcgccatgggcaacc
gcgggatggaagagctgatcccgctggtcaacaaactgcaggacgccttc
agctccatcggccagagctgccacctggacctgccgcagatcgctgtagt
gggcggccagagcgccggcaagagctcggtgctggagaacttcgtgggcc
gggacttccttccccgcggttcaggaatcgtcacccggcggcctctcatt
ctgcagctcatcttctcaaaaacagaacatgccgagtttttgcactgcaa
gtccaaaaagtttacagactttgatgaagtccggcaggagattgaagcag
agaccgacagggtcacggggaccaacaaaggcatctccccagtgcccatc
aaccttcgagtctactcgccacacgtgttgaacttgaccctcatcgacct
cccgggtatcaccaaggtgcctgtgggcgaccagcctccagacatcgagt
accagatcaaggacatgatcctgcagttcatcagccgggagagcagcctc
attctggctgtcacgcccgccaacatggacctggccaactccgacgccct
caagctggccaaggaagtcgatccccaaggcctacggaccatcggtgtca
tcaccaagcttgacctgatggacgagggcaccgacgccagggacgtcttg
gagaacaagttgctcccgttgagaagaggctacattggcgtggtgaaccg
cagccagaaggatattgagggcaagaaggacatccgtgcagcactggcag
ctgagaggaagttcttcctctcccacccggcctaccggcacatggccgac
cgcatgggcacgccacatctgcagaagacgctgaatcagcaactgaccaa
ccacatccgggagtcgctgccggccctacgtagcaaactacagagccagc
tgctgtccctggagaaggaggtggaggagtacaagaactttcggcccgac
gaccccacccgcaaaaccaaagccctgctgcagatggtccagcagtttgg
ggtggattttgagaagaggatcgagggctcaggagatcaggtggacactc
tggagctctccgggggcgcccgaatcaatcgcatcttccacgagcggttc

ccatttgagctggtgaagatggagtttgacgagaaggacttacgacggga
gatcagctatgccattaagaacatccatggagtcaggaccgggcttttca
ccccggacttggcattcgaggccattgtgaaaaagcaggtcgtcaagctg
aaagagccctgtctgaaatgtgtcgacctggttatccaggagctaatcaa
tacagttaggcagtgtaccagtaagctcagttcctacccccggttgcgag
aggagacagagcgaatcgtcaccacttacatccgggaacgggaggggaga
acgaaggaccagattcttctgctgatcgacattgagcagtcctacatcaa
cacgaaccatgaggacttcatcgggtttgccaatgcccagcagaggagca
cgcagctgaacaagaagagagccatccccaatcaggtgatccgcaggggc
tggctgaccatcaacaacatcagcctgatgaaaggcggctccaaggagta
ctggtttgtgctgactgccgagtcactgtcctggtacaaggatgaggagg
agaaagagaagaagtacatgctgcctctggacaacctcaagatccgtgat
gtggagaagggcttcatgtccaacaagcacgtcttcgccatcttcaacac
ggagcagagaaacgtctacaaggacctgcggcagatcgagctggcctgtg
actcccaggaagacgtggacagctggaaggcctcgttcctccgagctggc
gtctaccccgagaaggaccaggcagaaaacgaggatggggcccaggagaa
caccttctccatggacccccaactggagcggcaggtggagaccattcgca
acctggtggactcatacgtggccatcatcaacaagtccatccgcgacctc
atgccaaagaccatcatgcacctcatgatcaacaatacgaaggccttcat
ccaccacgagctgctggcctacctatactcctcggcagaccagagcagcc
tcatggaggagtcggctgaccaggcacagcggcgggacgacatgctgcgc
atgtaccatgccctcaaggaggcgctcaacatcatcggtgacatcagcac
cagcactgtgtccacgcctgtacccccgcctgtcgatgacacctggctcc
agagcgccagcagccacagccccactccacagcgccgaccggtgtccagc
atacacccccctggccggcccccagcagtgaggggccccactccagggcc
ccccctgattcctgttcccgtgggggcagcagcctccttctcggcgcccc
caatcccatcccggcctggaccccagagcgtgtttgccaacagtgacctc
ttcccagccccgcctcagatcccatctcggccagttcggatccccccagg
gattcccccaggagtgcccagcagaagacccccctgctgcgcccagccggc
ccaccattatccgcccagccgagccatccctgctcgactaggcctcgagg
ggggcgtgctctcgggggggcctcacgcacccgcggcgcaggagcttcag
tggtctggggccctccgccgcccctatgctgggaccaggctcccagtggg
cagccctggcctcttccttaacgctggccccggtccagggccggcccctg
tgcctggctggacaccgcactgcgcaaaggggccctggagctccaggcag
ggggcgctggggtgttgcactttgggggatggagtctcagggtggcagag
gggggaccagaacccttgacaccatcctgaatgaggggtccagcctgggg
gggactctaccaaggtcttcttgggctgggaaagcccatgtagggcaggc
cttctataagtgcgggcaccaagggcgcctacatccccaggccttgctgg
ggtgcaggggtatatcaacttcccattagcaggagctccccagcggcaag
cctggcccagtgggctcggtagtgcccagctggcaggcctgaggtgtaca
tagtccttcccggccatattaaccacacagcctgagcctggcccagcctc
ggctgccagaggtgcctttgctaggcccggagccgttggcccgggccggc
cttgccctattcctctcctcctcctcctcctgggtcccccagggtggctg
ggcttgggctatgtgggtggtggtggcgggggggtcttgggggcctctcag
ctcccgcccatgcctccctgatgggtgggcccagggcggcctctctctga
ggagacctcacccactcctcgctcagtttgaccactgtaagtgcctgcac

tctgtattctattaataaactaaaataaagggaagacgctgctggtggct
gctgaaaaaaaaaaaaaaaaaa

>NM_005327 4
cgtgtatacccgctcaacgctgggacgttacagccagggccaatgggcag
agcgggactcgaggccccgcccccgccttgtggcgtcacggggacgccgg
gggcgcgcgggctgcagggccgcgtaggtccccgcccccagagtctggct
ttccgcggctgcccgcctcgcgcgtcttccctgcccgggtctcctcgctg
tcgccgccgctgccacaccatggccttcgtcaccaggcagttcatgcgtt
ccgtgtcctcctcgtccaccgcctcggcctcggccaagaagataatcgtc
aagcacgtgacggtcatcggcggcgggctgatgggcgccggcattgccca
ggttgctgcagcaactggtcacacagtagtgttggtagaccagacagagg
acatcctggcaaaatccaaaaagggaattgaggaaagccttaggaaagtg
gcaaagaagaagtttgcagaaaaccctaaggccggcgatgaatttgtgga
gaagaccctgagcaccatagcgaccagcacggatgcagcctccgttgtcc
acagcacagacttggtggtggaagccatcgtggagaatctgaaggtgaaa
aacgagctcttcaaaaggctggacaagtttgctgctgaacatacaatctt
tgccagcaacacttcctccttgcagattacaagcatagctaatgccacca
ccagacaagaccgattcgctggcctccatttcttcaacccagtgcctgtc
atgaaacttgtggaggtcattaaaacaccaatgaccagccagaagacatt
tgaatctttggtagactttagcaaagccctaggaaagcatcctgtttctt
gcaaggacactcctgggtttattgtgaaccgcctcctggttccatacctc
atggaagcaatcaggctgtatgaacgaggtgacgcatccaaagaagacat
tgacactgctatgaaattaggagccggttaccccatgggcccatttgagc
ttctagattatgtcggactggatactacgaagttcatcgtggatgggtgg
catgaaatggatgcagagaacccattacatcagcccagcccatccttaaa
taagctggtagcagagaacaagttcggcaagaagactggagaaggatttt
acaaatacaagtgatgtgcagcttctccggctctgagaagaacacctgag
agcgctttccagccagtgccccgagtgcctgtgggaatgctctttggtca
gacattccctcacacagtacagtttaataaatgtgcattttgattgtaat
ctatcgaagtgattattacaccagttacagcagtaatagattctccatta
agaaataattcccttttttagtctgttcatttctgtgtattttctaaaca
gctttacaccccttggtgccttggagcaaacatgttttttgaaccttgtca
tttttgtgaagaattgcctagattccttctctcatcaacgggaaagtact
tcctctgagagtgcgagtgcaccatgctcactgttgctgcgtgggagagt
cacaagccactggcaagcaagtggtatagtctgtgaagcactgcagcgag
cagcacctggatcttgcctttataagaacatttactacctgcagctttg
agtcttgccctacattttgggcatgacataagatgtgtctttattcagct
cgtcgtgaagatgctgctgctgaatgggtcagcatatctctgtttgcatg
gtttgcaggaggtcggttttcatggtcattcagttccacagatctgaatg
attactgtctgtctgtgtcttttttccatgagaaatcactgttgcaaatt
gcctataaattgactctactaaaataacaatgtttcagtctgaaaatttg
aattgaaaaaaatgtataatataaaattgtaatacactcaaatgattata
aaagtaaaagttggtaatttaggcagaagctaaaaa

>NM_005498 4

ggcgcttccgcaggaagaaggaagcggcgccgccatcgcctcccggcgct
ccctccccgactcctaagtccttcggccgccaccatgtccgcctcggctg
tcttcattctggacgttaagggcaagccattgatcagccgcaactacaag
ggcgatgtggccatgagcaagattgagcacttcatgcctttgctggtaca
gcgggaggaggaaggcgccctggccccgctgctgagccacggccaggtcc
acttcctatggatcaaacacagcaacctctacttggtggccaccacatcg
aagaatgccaatgcctccctggtgtactccttcctgtataagacaataga
ggtattctgcgaatacttcaaggagctggaggaggagagcatccgggaca
actttgtcatcgtctacgagttgctggacgagctcatggactttggcttc
ccgcagaccaccgacagcaagatcctgcaggagtacatcactcagcagag
caacaagctggagacgggcaagtcacgggtgccacccactgtcaccaacg
ctgtgtcctggcgctccgagggtatcaagtataagaagaacgaggtcttc
attgatgtcatagagtctgtcaacctgctggtcaatgccaacggcagcgt
ccttctgagcgaaatcgtcggtaccatcaagctcaaggtgtttctgtcag
gaatgccagagctgcggctgggcctcaatgaccgcgtgctcttcgagctc
actggccgcagcaagaacaaatcagtagagctggaggatgtaaaattcca
ccagtgcgtgcggctctctcgctttgacaacgaccgcaccatctccttca
tcccgcctgatggtgactttgagctcatgtcataccgcctcagcacccag
gtcaagccactgatctggattgagtctgtcattgagaagttctcccacag
ccgcgtggagatcatggtcaaggccaaggggcagtttaagaaacagtcag
tggccaacggtgtggagatatctgtgcctgtacccagcgatgccgactcc
cccagattcaagaccagtgtgggcagcgccaagtatgtgccggagagaaa
cgtcgtgatttggagtattaagtctttcccgggggggcaaggagtacttga
tgcgagcccactttggcctccccagtgtggaaaaggaagaggtggagggc
cggccccccatcggggtcaagtttgagatcccctacttcaccgtctctgg
gatccaggtccgatacatgaagatcattgagaaaagtggttaccaggccc
tgccctgggttcgctacatcacccagagtggcgattaccaacttcgtacc
agctagaagggagaagagatgggggcttgaacacggggcttccttacagc
cccggatgcagattttagagggagggcaggtgcgggctgtgtgtgtctgt
gtgagggcaggtcctggacttggcagtttcttgctcccagcacccgcccc
ttcctcacctcttccttattccataggctgggagagaaactctctctgct
tccctcgcccttggagctttccccatccccctgattttatatgaagaaat
agaagaggggcttgaagtcctcctcgcgagtgccttcttgcaattacctg
ccttagcgggtgttgcgggtccctccttcacagccgctgagcccagaggt
cccgctggcccctcctctgaattttaggatgtcattaaaaagatgaatct
aaaaaa

>NM_005730 3
cgctttttccgtaacaaaatagcaaagctcccgactgtccgcagccccgg
ccgctcacagatggagggtcccagggcctaggacgcagcccccagcggga
agctccagctggccgtgaagaggccgagtcgagagccgggaggcgcgcgg
gggtgggcgctgcagccgcgggttgtttatctggagtacggaggggaggg
gggagcctggaaaccgccccgtgtcccatttcctcctcttgttttcgctc
cggattctccatgttggacccaaactgaggagcccggagctgccgctggg
ggatcggggccgggggcacccgggggagccgctgcccgggccgcccgccc
tttgtacaggccgcctcccttcccggtccggggaggaaacgagagggggg

atgtgaacagctgtggaagtcggagtctcgggagccggagcgggcccccg
cccaggcccccagcccagcccagcccgcgcgcccgcccgtcctcccgcc
cagccagcccgggcccgcgggattgttagatggaacacggctccatcatc
acccaggcgcggagggaagacgccctggtgctcaccaagcaaggcctggt
ctccaagtcctctcctaagaagcctcgtggacgtaacatcttcaaggccc
ttttctgctgttttcgcgcccagcatgttggccagtcaagttcctccact
gagctcgctgcgtataaggaggaagcaaacaccattgctaagtcggatct
gctccagtgtctccagtaccagttctaccagatcccagggacctgcctgc
tcccagaggtgacagaggaagatcaaggaaggatctgtgtggtcattgac
ctcgatgaaacccttgtgcatagctcctttaagccaatcaacaatgctga
cttcatagtgcctatagagattgaggggaccactcaccaggtgtatgtgc
tcaagaggccttatgtggatgagttcctgagacgcatgggggaactcttt
gaatgtgttctcttcactgccagcctggccaagtatgccgaccctgtgac
agacctgctggaccggtgtggggtgttccgggcccgcctattccgtgagt
cttgcgtgttccaccagggctgctacgtcaaggacctcagccgcctgggg
agggacctgagaaagaccctcatcctggacaactcgcctgcttcttacat
attccaccccgagaatgcagtgcctgtgcagtcctggtttgatgacatgg
cagacactgagttgctgaacctgatcccaatctttgaggagctgagcgga
gcagaggacgtctacaccagccttgggcagctgcgggccccttagcctgc
cctgcttccaagcgacggccatcccagtaggggactttcccacactgtgc
ctttacgatcagcgtgacagagtagaagctggagtgcctcaccacacggc
ccggaaacagcgggaagtaactggaaagagctttaggacagcttagatgc
cgagtgggcgaatgccagaccaatgatacccagagctacctgccgccaac
ttgttgagatgtgtgtttgactgtgagagagtgtgtgtttgtgtgtgtgt
tttgccatgaactgtggccccagtgtatagtgtttcagtgggggagaagc
tgaaagaccaagactcttcccaagttagcttgtctcctctcctgtcaccc
taagagccactgagttgtgtagggatgaagactattgaagactccattgc
caaaccatggcctttcctcagtgttgtaaggcctatgccaaggataaagg
aagggtatgcctttgggtactccaggcacacacctttctgaaatccttct
ccagccagctgctgcagacaaaagatcacatttctgggaagatgagaact
tgtttccagaccagcatccagtggccatcaggtcttgtggcccaaaggct
atgcttgcctccggctgagtgcctgggataggccttttctatgtctcccc
aaggctggggtgctgagcctgccttcctcaccacctagccatagtctcaa
acctgtggggaaggaggttttctccctgcccgggaagaggacagataact
gatttccgttcttttgactgtgttttaaaattctctttctaaacacagag
tgttgggcctggtttgtttctgacaaagttacagtcctgggcctgtaatg
aatgtcggcggcgctggggttgcagggaaaagacaaatcctcaaagcgtg
gacgtgtgtccccatggcttgtggatcagctaagctcgggatcatttcca
taagtctgcttttcagggattctctgctggtgctggtgcaaggacttctg
ttccaaaggctgggaaaaactaagctgtcccagcccctcccatttcttgg
gcagggctcttttcctgttgtgtcttcccccagggcctgtcctgtaccga
gctctgtctgttccagccttcatccttcctggctgttgcttttcctctta
agggcctcagaactcttgctcttcctgggctgagggggaatgagtgttct
tgccatttgccagcctaatgcgcatgctttctgcctctggtaacaggagt
gagtgagcccctcagacctgcactctgggtgtctcctgctttcaaaggtt
cttaatagtgaatgctttaaaattaaagtcatcacgaaatggaagttttc

ccagggtggaaaataagaggaagtgctgctgtaattgggagcacaagggg
cctcccaaaaaggagccccacctcagcatcactgccttaatcgtggcctc
cctggggtgggtggggttctctcctccctccctccctcctcctggggtgg
gagggcgctcctgttcccatctctgtgttccctggaggcaggtatcacaa
agcatttgtgaattgctttaggtgcagggacaccacccactcaggactct
tccccatcatcccttccattgccacaccctagatccagcctcaggaacta
acaagttttgagaaaagcaggtagtagagcagcagcttcgtgctctcagc
ggtggctggctggcatttttctctagcgttgtggtgccaccttcccttct
tgtcccaaggttataaggccttgtctttctctttggaatcataaagtgga
acagagtccccagaactcatgtggccatttccgccagcatcactccccgg
tgcctatggggtcccggtgtacctaaagggagaaggaccccatgtgctag
ccagaaatatactgtctcttgaaggaaagcaggagctcagactcttagag
ccagctgtggcttcggacccaaggcctgacctaggctgctatcctaatat
tgcaggaggggcctctcttccaagccccaccctaagggttagcccttggc
caaatcttttgccgtctaggcccagccaggcttttctgactaaataagca
ataagaggctctaagctgactgagttgcaaggaccctttccgccctccct
tggatctccatgtttttccagatggcggaagagcatgtgccacccccttt
cctaacagacttgtccaagtgcttggcgtgggacccatgaccaaagccca
ggatggcttggtgggagtgtccctgctgcatctgcatgaagcccctgctt
tttaggcctcactcccatcagaaccctgcctgcccacctgcaactccccc
ccaacaatgccattcccacttgccccagagaagctactcggccaaaccta
gccagggtctgttcttgtggaccagagccagcctagtcattatttgctgt
cgggtttccagtttcaccgtgtgttagggtgagggatgattgtaaaattt
gctcctcaaaggaatcaggccagactcaattttggagggcaagacaggga
ggaggccgcttcatcccagactctcttctagggcttcccaccatcagccc
ctcccacttgagactggtctttgggaggcaataggccaccatgcctggtc
agcaccaattcaagccatgccaggaatctgcctacctgccaggttcagtt
cttttaaggtgcctcttcagggacacagtgtgtctctctgattgggcttc
taaatcaaaagcctgatgttcgtgtccctctcataggggggagctttggac
acaggaccagtttggaaaagggtcaggtaagggtttccactctgcacatt
gtagagggaacactctgtaggcccatgggtcccttactagagaggttgag
tgaatttgccttcagttaacatgggaccttctgtttagcttcctcttgct
tcccaaagattttaagcattttgtaaatgtataaactcacctctggtaac
agtggcccagacgctgctttgtgctaaaagcatgggaaatgtaaaggcag
tctttctctgggaaatggatgctattctattctgctgcccctacctgttc
ctgaggcctcatttagaaagaaaatcccctcagaaggctgtctggcaccc
agtgtcctagccaggccaagtatatgagaaaggtaagtccattttcccct
tcaggtcctcagtggattacttaaccactgctgtccctcggtccctttt
cctaaacgggtttagttctgtctttttctcctttttctaaatgctggt
aaatatttacattcagccagggaagaggaggccagaggtcgggccagctg
ccccattcttttaacgttgtagggcctgcccatggagcggaccctcctct
ttgggcctcgtgagctttttgcttatcatgttccatttcgtgccgcttt
ccccccttcaagatgccatttggagggtaggggatctgcttcccactgtga
ctgggctatgggattctgactaccttgcttacagattcatggtttgataa
atttgttgtattcaaaaacttgaaatgcaggacgccattaagtgtctgtt
tatatttttggaatatttgtattacttacaattaattaataaaagtgggt

ttaaaaaacctttccaggaaaaaaaaaaaaaaaaaaa

>NM_005754 2
gagtggcagttatatagaccggcggcggagcacgcgtgtgtgcggacgca
gttgcgtgaggggtttgtactatcctcggtgctgtggtgcagagctagtt
cctctccagctcagccgcgtaggtttggacatatttgactcttttccccc
caggttgaattgaccaaagcaatggtgatggagaagcctagtcccctgct
ggtcgggcgggaatttgtgagacagtattacacactgctgaaccaggccc
cagacatgctgcatagattttatggaaagaactcttcttatgtccatggg
ggattggattcaaatggaaagccagcagatgcagtctacggacagaaaga
aatccacaggaaagtgatgtcacaaaacttcaccaactgccacaccaaga
ttcgccatgttgatgctcatgccacgctaaatgatggtgtggtagtccag
gtgatggggcttctctctaacaacaaccaggctttgaggagattcatgca
aacgtttgtccttgctcctgaggggtctgttgcaaataaattctatgttc
acaatgatatcttcagataccaagatgaggtctttggtgggtttgtcact
gagcctcaggaggagtctgaagaagaagtagaggaacctgaagaaagaca
gcaaacacctgaggtggtacctgatgattctggaactttctatgatcagg
cagttgtcagtaatgacatggaagaacatttagaggagcctgttgctgaa
ccagagcctgatcctgaaccagaaccagaacaagaacctgtatctgaaat
ccaagaggaaaagcctgagccagtattagaagaaactgcccctgaggatg
ctcagaagagttcttctccagcacctgcagacatagctcagacagtacag
gaagacttgaggacattttcttgggcatctgtgaccagtaagaatcttcc
acccagtggagctgttccagttactgggataccacctcatgttgttaaag
taccagcttcacagccccgtccagagtctaagcctgaatctcagattcca
ccacaaagacctcagcgggatcaaagagtgcgagaacaacgaataaatat
tcctccccaaaggggacccagaccaatccgtgaggctggtgagcaaggtg
acattgaaccccgaagaatggtgagacaccctgacagtcaccaactcttc
attggcaacctgcctcatgaagtggacaaatcagagcttaaagatttctt
tcaaagttatggaaacgtggtggagttgcgcattaacagtggtgggaaat
tacccaattttggttttgttgtgtttgatgattctgagcctgttcagaaa
gtccttagcaacaggcccatcatgttcagaggtgaggtccgtctgaatgt
cgaagagaagaagactcgagctgccagggaaggcgaccgacgagataatc
gccttcggggacctggaggccctcgaggtgggctgggtggtggaatgaga
ggccctccccgtggaggcatggtgcagaaaccaggatttggagtgggaag
gggcttgcgccacggcagtgaatcttcatggatcttcatgcagccatac
aaaccctggttccaacagaatggtgaattttcgacagcctttggtatctt
ggagtatgaccccagtctgttataaactgcttaagtttgtataattttac
tttttttgtgtgttaatggtgtgtgctccctctccctctcttccctttcc
tgacctttagtctttcacttccaattttgtggaatgatattttaggaata
acggacttttaaagaagcaaaaaaaaagactgaatttccttgcttacttt
gcatatacagactggatttttttttttttttacagccatttccccaaag
gaatgtcttgcatattactgacatttggtatgtttcattcattggaatat
ttcttattttctacgtgtttgaaaagcctgtaagaaatacaggatttgat
aatattttgaaggcaggaaaaacccaaattgtttcttctttgagagtcat
gactaccttctggtgtggagaaattgccattggaaaatttgacaattttg
attctcactggtatgtttaaaaactgaataaaaggaatagaatttttttt

tgataaaggatcacaaaacaattctaaaacctaactgtttttaccattga
aatttaaattgtgataataggttttaaatgtctagaatgcaactgatagg
cttttcttgaactgttagttttttgaagtagtttttcatgtttaattt
gtatttgtaaaaaaacaaaaagcaaaaaaattcccaaaacccagataaca
accagagcaaaactgttgtgccttctatttatctttgatttcagtcttgg
caattgtttaaaaaaaaaatctagatttgttttattaggttcagagtatg
tggggaattatagaatccctctttcatcactttgtgtatgtcttttgtta
acatatttgttatgccttattctaaaattgagtctcaaactggaatgcct
ttgaagacagatgcttctatagaggttctttgacctaaatagttcagcat
ttgtatttttattctggtatctaatcagattcctaatcatagcccgtaag
aaggaatgttactttaatattggactttgctcatgtgctcgtgtccgcat
tttttttttcttaaaatcatagccatatggtaaattttctattttgtta
tggttctcttttattgatgggcatgcagtgggtgttacttggaaatggcc
aatttttattaaaatatttctggaagaaaatttaaaaaaaaaaaaaaaa
aaaaa

>NM_014447 2
acttccggcttcgctgctcttggttctggttctggaggctgggttgagag
gtcgccggtccgactgtcctcggcggttggtcagtgtgaatttgtgacag
ctgcagttgctccccgcccccgagcagccgaggagtctaccatggctcaa
gaatctcccaaaaattcagcagcagaaattccagtgactagtaatggaga
agttgatgactctcgtgaacatagctttaatagggatttgaagcattcat
taccatctggacttggtctctcagaaacccaaattacatctcatggcttt
gacaataccaaagagggtgttattgaagcaggagcatttcaaggtggcca
gagaacacagacaaaaagtggaccagttattctagcagatgaaattaaaa
atcctgcaatggaaaagttagaacttgttagaaaatggagtctaaacacc
tataagtgtactcgacagattatctctgagaagctaggccgtggctcaag
aactgtggaccttgaacttgaagctcagattgatatattaagggataaca
agaaaaaatatgaaaatattttaaaactggctcaaacattgtcgacccag
cttttccagatggtacatacccaaaggcaacttggagatgcatttgctga
cctgagtttgaagtcactagaacttcatgaagaatttggctataatgccg
atacccagaaactgctggctaaaaatggagagactcttcttggggccatt
aattttttcattgctagtgtgaacactttggtgaataaaaccattgaaga
tacattaatgactgtgaaacagtatgaaagtgccaggattgaatatgatg
catatcgcactgatttggaagaactgaatcttggaccacgtgacgcaaac
actctgccaaagattgagcagtcacagcatctcttccaagcacataagga
aaaatatgataaaatgcgcaatgatgtttctgtcaaattgaaatttctag
aagaaaataaggttaaagtattgcacaatcagctggtccttttccacaat
gccattgccgcttactttgctgggaatcagaagcagcttgaacagacact
taaacagttccatatcaaattgaaaacccctggagtggatgccccatctt
ggcttgaagaacagtaaaatcacagcggaaaataaaaagaaagtcgcgtt
gttatatttctaaaccaacctaacaagaattaagcagagttgggggaagt
gggaggggtgacaagcattatagtgattcttgcacaaacagctttaattt
ttcccttttttcatactttaacaattgaactgttaagggtggttttaatgt
aaacatagtttctataatctgaacacaataattttcctttttgagaaatc
cttttgtattgtgagggttgatggctatttctgtagtttgaaaacatcta

atagagatttgcactgacaagataaaaattcaaggtttttggtcctggaa
atgagggccgattgtaacttttccaaagggaggtttacatgatttgtatc
aacatcagatattttatatgtgaatatattaaacatctttaagagattca
ttttcatgtattaaagaaaaaagaaactattttgcagagtaaagttatat
ggtgttcctgcagcgtccacacagaggcagcagctctaatgaatgattag
cttgtggagttgtggcaaataccttttttaaaaatgaatctgtaccactgt
aattttatttagactttttttttaaagacacagaaatcacactgtcatttc
tgtgaggcttttaagcttatgaatttgcttcacccgaagtcattggacag
ttacatttgcaatttcttttggttcataacaaaacctgcttaagagaatt
ttttttttgacaggtggacgtgggaatggaaattcttaatggtttctgg
agtagaaatctgtacttagaaaactagtttttgggtcagttccattttga
tagaagtgaatacatagacagcttttattgacttccatatgtaacaatac
cgtttaagccttaaatcacagatatgtgccttctcagatacagtaattct
tgttcaaccaatgtacaaaatccataaagaaacccctgttggataatgtt
tgatgtgtctattccttccatggaatggagcactatgtatgaatgttggg
tttctttgtacttgttaagccacatttgaggtttatggtaaaaatcatct
tttgagtttgctctttggtttttcttcattccttttgaggattgggaaaa
cagaaagattctttgatttgggtaatgaagaggtaatttgggacagtgtg
gtggtaccaggaagaaagaggattggaaaggccagtactgttttagttgc
tcggcactgttggttttgttttaatgtggttgccctgtccactacatggt
tctatcagtagtgtaatccattttcaatgtaaagctcttttagtttttgt
catagacataaattaatattttgagaggcatccctcacctgttcatttct
tctgtgttgaaatgaagtacttaaaattaccgttatacatgaactttgtg
gactgtaagatttgttatatatgttcaaatgccttttagctggcttttta
attaatatgcctgttttgagtgcttaatacaatgtaatgtgattgtaaat
catacctattttaaatcattccttcctgtatatttgtactcagagagcct
tattttattcttccagcagaattactacttgtgatagttcatttacattc
cccagaatgtgcctctagtgtgaattttgtattcttattaaacgtgtttg
ctgcagtaaa

>NM_014481 2
tggccaacttctgaacaggaagcagttcgctcgcgcctaggttggcgcgg
gctgggaggtgttccagccctttaagatgttgcgcgtggtgagctggaac
atcaatgggattcggagacccctgcaaggggtggcaaatcaggaacccag
caactgtgccgccgtggccgtggggcgcattttggacgagctggatgcgg
atatcgtctgtctccaggaaaccaaagtgaccagggatgcactgacagag
cccctggctatcgttgagggttataactcctatttcagcttcagccgcaa
ccgtagcggctattctggtgtagccaccttctgtaaggacaatgctaccc
cagtggctgctgaagaaggcctgagtggcctgtttgccacccagaatggg
gatgttggttgctatggaaacatggatgagtttacccaagaggaactccg
ggctctggatagtgagggcagggccctcctcacacagcataagatccgca
catgggaaggtaaggagaagaccttgaccctaatcaacgtgtactgcccc
catgcggaccctgggaggcctgagcggctagtctttaagatgcgcttcta
tcgtttgctgcaaatccgagcagaagccctcctggcggcaggcagccatg
tgatcattctgggtgacctgaatacagcccaccgccccattgaccactgg
gatgcagtcaacctggaatgctttgaagaggacccagggcgcaagtggat

ggacagcttgctcagtaacttggggtgccagtctgcctctcatgtagggc
ccttcatcgatagctaccgctgcttccaaccaaagcaggagggggccttc
acctgctggtcagcagtcactggcgcccgccatctcaactatggctcccg
gcttgactatgtgctgggggacaggaccctggtcatagacacctttcagg
cctctttcctgctgcctgaggtgatgggctctgaccactgccctgtgggt
gcagtcttgagtgtgtcctctgtgcctgcaaaacagtgcccacctctgtg
cacccgcttcctccctgagtttgcaggcacccagctcaagatccttcgct
tcctagttcctctcgaacaaagtcctgtgttggagcagtcgacgctgcag
cacaacaatcaaacccgggtacagacatgccaaaacaaagcccaagtgcg
ctcaaccaggcctcagcccagtcaggttggctctagcagaggccagaaaa
acctgaagagctactttcagccctcccctagctgtccccaagcctctcct
gacatagagctgcctagcctaccactgatgagcgccctcatgaccccgaa
gactccagaagagaaggcagtggccaaagtggtgaaggggcaggccaaga
cttcagaagccaaagatgagaaggagttacggacctcattctggaagtct
gtgctggcggggcccttgcgcacacccctctgtgggggccacagggagcc
atgtgtgatgcgtactgtgaagaagccaggacccaacttgggccgccgct
tctacatgtgtgccaggccccggggtcctcccactgacccctcctcccgg
tgcaacttcttcctctggagcaggcccagctgaaccaatggaggcctggg
gacatctggcatggtcacccctgcacatgatctgaggccagctccccttc
cctgagctgcctcctgcttctccctcaaagtctcctacccttctcttcct
cttttaagccctctcttcctcgctttccttcctacctagctccttgttgg
tgagcttcttgtgccttaatcctgtgacccagccccttacaccactttcc
accttcctgtccgaagtacacggacactagctgccccaggaagttgtgtg
attttaaatcacttctgtctttgctggaaagtgtatttgtgcataaataa
agtctgtgtatttgtttcagggttgcaaaaaaaaaaaaaa

>NM_015516 3
cgcggccggcccgagccgccacccggcgcccggcagggggggagcctggct
gcgggcggggaccgggggggcgggccgcgccgcgcggagaaaagcgctggc
cggagggcccgcggccgggccgccggggtgagcgtgccgaggcggctgtg
gcgcaggcttccagcccccaccatgccgtggcccctgctgctgctgctgg
ccgtgagtggggcccagacaacccggccatgcttccccgggtgccaatgc
gaggtggagaccttcggccttttcgacagcttcagcctgactcgggtgga
ttgtagcggcctgggcccccacatcatgccggtgcccatccctctggaca
cagcccacttggacctgtcctccaaccggctggagatggtgaatgagtcg
gtgttggcggggccgggctacacgacgttggctggcctggatctcagcca
caacctgctcaccagcatctcacccactgccttctcccgccttcgctacc
tggagtcgcttgacctcagccacaatggcctgacagccctgccagccgag
agcttcaccagctcacccctgagcgacgtgaaccttagccacaaccagct
ccgggaggtctcagtgtctgccttcacgacgcacagtcagggccgggcac
tacacgtggacctctcccacaacctcattcaccgcctcgtgccccacccc
acgagggccggcctgcctgcgcccaccattcagagcctgaacctggcctg
gaaccggctccatgccgtgcccaacctccgagacttgcccctgcgctacc
tgagcctggatgggaaccctctagctgtcattggtccgggtgccttcgcg
gggctgggaggccttacacacctgtctctggccagcctgcagaggctccc
tgagctggcgcccagtggcttccgtgagctaccgggcctgcaggtcctgg

acctgtcgggcaaccccaagcttaactgggcaggagctgaggtgttttca
ggcctgagctccctgcaggagctggacctttcgggcaccaacctggtgcc
cctgcctgaggcgctgctcctccacctcccggcactgcagagcgtcagcg
tgggccaggatgtgcggtgccggcgcctggtgcgggagggcacctacccc
cggaggcctggctccagccccaaggtggccctgcactgcgtagacacccg
ggattctgctgccaggggccccaccatcttgtgacaaatggtgtggccca
gggccacataacagactgctgtcctgggctgcctcaggtcccgagtaact
tatgttcaatgtgccaacaccagtggggagcccgcaggcctatgtggcag
cgtcaccacaggagttgtgggcctaggagaggctttggacctgggagcca
cacctaggagcaaagtctcacccctttgtctacgttgcttccccaaacca
tgagcagagggacttcgatgccaaaccagactcgggtcccctcctgcttc
ccttccccacttatcccccaagtgccttccctcatgcctgggccggcctg
acccgcaatgggcagagggtgggtgggacccccctgctgcagggcagagtt
caggtccactgggctgagtgtccccttgggcccatggcccagtcactcag
gggcgagtttcttttctaacatagccctttctttgccatgaggccatgag
gcccgcttcatcctttctatttccctagaaccttaatggtagaaggaat
tgcaaagaatcaagtccacccttctcatgtgacagatggggaaactgagg
ccttgagaaggaaaaaggctaatctaagttcctgcgggcagtggcatgac
tggagcacagcctcctgcctcccagcccggacccaatgcactttcttgtc
tcctctaataagccccaccctccccgcctgggctccccttgctgcccttg
cctgttccccattagcacaggagtagcagcagcaggacaggcaagagcct
cacaagtgggactctgggcctctgaccagctgtgcggcatgggctaagtc
actctgcccttcggagcctctggaagcttagggcacattggttccagcct
agccagtttctcaccctgggttggggtcccccagcatccagactggaaac
ctacccattttcccctgagcatcctctagatgctgccccaaggagttgct
gcagttctggagcctcatctggctgggatctccaaggggcctcctggatt
cagtccccactggccctgagcacgacagcccttcttaccctcccaggaat
gccgtgaaaggagacaaggtctgcccgacccatgtctatgctctaccccc
agggtagcatctcagcttccgaaccctgggctgtttccttagtcttcatt
ttataaaagttgttgcctttttaacggagtgtcactttcaaccggcctcc
cctacccctgctggccggggatggagacatgtcatttgtaaaagcagaaa
aaggttgcatttgttcacttttgtaatattgtcctgggcctgtgttgggg
tgttgggggaagctgggcatcagtggccacatgggcatcaggggctggcc
ccacagagaccccacagggcagtgagctctgtcttcccccacctgcctag
cccatcatctatctaaccggtccttgatttaataaacactataaaagtt
ctttgctttaaaaaaaaaaaaaaa

>NM_015627 2
ggagctggcgctgggaggggaggagcgcgcagcccgcgcgccgcagggcc
gggcggaaagtttttcctgacggagttttggctgcggcagcggcggcggc
ggccggagcgggccatggacgcgctcaagtcggcggggcgggcgctgatc
cggagccccagcttggccaagcagagctgggggggcggtggccggcaccg
caagctgcctgagaactggacagacacgcgggagacgctgctggaggggga
tgctgttcagcctcaagtacctgggcatgacgctagtggagcagcccaag
ggtgaggagctgtcggccgccgccatcaagaggatcgtggctacagctaa
ggccagtgggaagaagctgcagaaggtgactctgaaggtgtcgccacggg

gaattatcctgacagacaacctcaccaaccagctcattgagaacgtgtcc
atatacaggatctcctattgcacagcagacaagatgcacgacaaggtgtt
tgcatacatcgcccagagccagcacaaccagagcctcgagtgccacgcct
tcctctgcaccaagcggaagatggcacaggctgttaccctcaccgtagcc
caggccttcaaagtcgcctttgagttttggcaggtgtccaaggaagagaa
agagaagagggacaaagccagccaagagggaggggacgtcctgggggccc
gccaagactgcacccccctccttgaagagcttggtcgccactgggaacctg
ctggacttagaggagacagctaaggccccgctgtccacggtcagcgccaa
caccaccaacatggacgaggtgccgcggccacaagccttgagtggcagca
gtgttgtctgggagctggatgatggcctggatgaagcgttttcgaggctt
gcccagtctcggacaaaccctcaggtcctggacactggcctgacagccca
ggacatgcattacgcccagtgcctctcgcctgtcgactgggacaagcctg
acagcagcggcacagagcaggatgacctcttcagcttctgagggcccggg
gccagccggacacaagcggccctgacacgtgatggaccaaagccacctgc
tgcgggggagccagttctggggcccgcctgccacctctcccagccctcag
cattgtcagcctgaagatcagagctgcagccagtcaggcaggggagagat
ttttcttttaagccctgctctttctctgagaaccaaaagatgccttgaat
atttattcagtgacttctggcttatgctcagaagccagtctgcgtcaggc
acgtctcctgctgcgtgacatgtgcagtgctgtaatcggctcccgcttgc
tctcctggagcaagctctgccctggctgtgggtatcaggactgtgaccaa
agcatttctagtcccttctctctttctaaggacccaaatttccctggggg
catcctgcttcctgaaagctgttggatttcagtgatttttccccccaccc
cccagcacaggagagcacccacagccgcagaaggggaatgtgtcctcctg
ctctgcttcctcagggcccagcaggcgggggtttgagccctggaccccag
gctcttagagactaaggggcagctcctgaccaaagacgatacagcttggc
actttaaagcattaacagcaggtgtgaccctgagggctcctccatggtgc
tgcattgagtccagctttccttctgcccttcctccaggagaaggggccca
aggtccccgtggatggtctccacctgtgcttggaaccagtgtaactggct
gctccctgctcccagggactgacacggggatcatctctgtgaccgccctc
cgtcgggcccctgcctgccttctcccctccacgcaaggctgtgctcttcc
tctggtttctgtgtgtccgtttgagtgtctgcgccccgcctccccatact
tcctgggatgatgtgtgaaacctgacacctagatttatttggaaatattc
tatgaccactttacagatgaggaaacaggcctcaagcgtggaggggtaga
gtgaagagtagaacccaggtctgatgccaaagctgctttcttctctgcct
cctcctcacgcaactcacacctccttttcttctagctttgttgtcctccc
aggaaccaaaaaaccccagctattttctgaccaaaatgtgtttcataaca
aaccatctggtgcctttccacacagaactggcaggagcctcgtgtcctgc
tagctgtctctcttgttgatttccgtgaaaatgcaagtgtttgaagtctg
ctcattccgagggtgaaacaaaatccaaccctgtcagaatcatgctgttc
tctttgctgacactgtgaccctgggtcgggacagaccagcagcaatctgt
ctttagaatcgctttccttcctccccttttgcccccgtggggctcccggc
atcctgaaagccagcaaagcctccagcatcttttccatcctgaggtgcct
cccagtggcctggcttgtcggagcaagtttcatcagccctagggaaaaca
cggccctcctgggaacctccttacctggagtaaccggacaccttagacgg
aggtgcctgagggtggggtgggatttgcagggtcattatcagaacatgag
gataacttccttgcccctgctctgtagccacctccttggcaccggcctct

atttgtcataaggcggcgtgggcgaggcctgacacaggccagccttggca
cgagggggccaggggttctgagaagcgctgccctgtgagagccacgctg
gccttcgtctccatctctggttgacgggctgtccgtgtgcctcctgtgtg
tctgcagacaagtcttgctgtgctttatttgtgaaactttaatgaggaaa
aaacaaataataaatgttctcgttttgaaactcaa

>NM_018214 4
ggaagctccgcgcggcggcgggggcggcgacggcgactggcgggtgggag
tggaggcaccggctggcgggcgggggtacagggacggggcaggggctccc
gctccaggttccttgaagcacttccgaccgcgaagcccggcgcgagaagc
gagctaacccaagagccaacaacgagcgcggagagggcagcggactgagc
ggagccgccggccagagcgggctcggagcccgggtctccgccgctcggga
cccggctaggcggcggcgggggcggcgatgttccactgcatccccctgtg
gcggtgcaaccgtcatgtggagagcatcgacaagcgccactgctcgctgg
tctacgtccccgaggagatctaccgctatgcccggagcctggaggagctg
ctgctggacgccaaccagctccgcgagctgcccgagcaatttttccagct
agtcaaattacgaaagcttggacttagtgataatgaaattcagcggctcc
ctccagaaatagcaaacttcatgcagctggtggaactagatgtgtctcga
aatgagattcctgaaattccagaaagcatttcattctgtaaagcactgca
ggtagctgacttcagcggaaacccactgactaggttgccagaaagctttc
ctgaattacagaatttaacatgtctttctgtaaatgacatctcactacag
tctctacctgaaaatattggcaatctttataacctggcttcactggaact
gagagagaatcttcttacatatcttcctgactctcttacccagctgcgaa
gactagaagaacttgatttaggaaacaatgaaatatataatttgccagaa
tcaattggagccctcttacatctaaaagatctctggttggatggaaatca
actgtcagaattacctcaggaaataggaaatctgaagaacctgctgtgtt
tagatgtctctgaaaacaggttggaaagacttcctgaagaaatcagtggc
ctgacttcattaacggatttagtcatttcccagaacttattagaaacgat
tccggatggcattggaaaactaaagaaactgtcaatcttgaaggtggatc
agaatagactcacacagttgcctgaagcagttggggaatgtgaaagtctc
actgagttagttcttacagaaaatcagctcctgaccctgcctaaaagcat
tggaaaactaaagaagttgagcaacttgaatgcagacagaaataaattag
tgtccttaccaaaagagatcggcgggtgctgcagcctcactgtgttctgt
gtacgtgacaacagactaactcggatacctgcagaggtgtcacaggcaac
agaacttcatgtcctggatgtggcagggaacaggttgctgcatctacctt
tatccctgactgccttgaagttgaaggctctgtggctatctgacaaccag
tcccagcccctgcttacattccagacagacacagactacaccacaggaga
gaagattttaacctgtgtcttacttcctcagctgccttctgaacctactt
gtcaagagaatctgcctcgctgtggtgcactggagaacttggtaaatgat
gtctctgatgaagcctggaacgagcgtgctgtcaacagagtcagtgcgat
ccgatttgtggaggatgagaaagatgaagaagacaatgagacgagaacac
ttctaaggcgagccactccacacccaggggagttaaagcacatgaaaaag
acagtggagaatttacggaatgacatgaatgctgctaaaggactggactc
aaacaaaaacgaggtcaatcatgccattgaccgagtgaccacttctgtgt
agagtttcacctccaagttttacctcctgtgtcttcctctgctgtcgaga
cgttcctgtctgcttcccgggagcctcacgtgctccttgtcctaaccagc

ccccgcgcgccatcttcccgtggagtgtggggaagctgctgtctcccagg
aagtgccttactcatcccgcaaccagtcagcgcaccagtggtctcccggt
gtgattttttttttttttaatttcagttgtttgtaataagtagaatacac
tactgtaaacatacgacctttgtttttgtcttatgttggggtaaaggaaa
gcaggaaggggaatttttatcctcctcccttccgtaaagtgctgggatat
tttgaatcccccaagttcccttggacctactgatgagagatagttttatg
tatggggaaaaatggatactttttaaaccttttttggcagctcagatggt
gtaaattttaaaattttgtataggtatttcataacaaaaatatgtatttc
ttttttgttattttatcttgaaaacggtacatattttagtatttgtgcag
aaaaacaagtcctaaagtatttgtttttatttgtaccatccacttgtgcc
ttactgtatcctgtgtcatgtccaatcagttgtaaacaatggcatctttg
aacagtgtgatgagaataggaatgtggtgttttaaagcagtgttgcattt
taatcagtaatctacctggtggatttgtttttaaccaaaaagatgaatta
tcaatgatttgtaattatatcagttgattttttttgaaaagatgaaccaa
aggatttgactgctaatattttattccttacactttttttctgaataagt
ctctcataatgagtgcagtgtcagactgtgcctactctgatggtatgtgc
catttgtaaaataaaatagagcagaaaaacacaaaaagagaacactggtt
cagacattcagtgggcaagtaaattatggactgcaaaataatgattttta
ttcaagaaagctttaaaagttttatatccagatatacaaccacaataaag
caaaataacctactatcaaaatagaaatgttgctatctttataagtgcaa
tttaatttgtaaatagagtttgaatcaaagtatcacaaaatactgcttca
agatttaattttaaatctgctaatttaagggatattgggaaaagttttgg
tgtgtttctgttgatttcttttttgtatgctgtgataaaagagaaatgaa
aagtgccagtcactgtgtggtgtctaggaaaatcatatatttttttttct
ccaagaaataaattcatcctggacattggc

>NM_019054 2
acgggcggccggatttgcccggaggccgcacccgcctccggcggggctct
cagtgaaaaatgtactctgacctgaatcctgaattttccttgatggtcaa
aagattaagaaatcatgagtggaggatctcaagtccacattttttggggt
gctccaattgctccactgaaaatcacagtatcagaagacacagcttcttt
aatgtctgttgctgacccctggaaaaaaattcagcttttatacagtcaac
attctttatatctgaaggatgaaaaacagcacaaaaatcttgaaaactat
aaagtcccagaatctattggttctccagatcttagtggtcatttcttagc
aaactgtatgaatagacatgttcatgtgaaagatgactttgtacgttctg
tttctgaaacacagaatatagaatcccagaagattcactcctctagactg
agtgatataactagctctaatatgcaaatatgtggatttaaaagcacagt
tccgcatttcaccgaagaagaaaagtatcaaaagcttctcagtgaaaata
aaattagagatgaacagcctaaacatcagccagatatatgtggtaagaac
tttaacacaaatttgtttcagttgggccataaatgtgcagctgtgttgga
tttggtttgtagtactgaaaaaattaatatagggcctgaagtggtacaaa
gagagtgtgtgccaacagaatatcatgaaatacaaaaccagtgtttggga
ttattttcctcgaacgcagtagataagtcaaggtctgaagcagcagttag
gaaggtctcagaccttaaaatatcaactgatacagaatttctcagtataa
ttacctccagccaggttgcttttttagctcaaaagaaagataaaaggcgg
agtcctgtaaataaagggaatgtaaacatggagactgaaccaaaggcaag

ttacggggagataagaatacctgaagagaattcgattcagcttgatggtt
ttacagaagcatatgaaagtggacaaaaccaagcatattcccttgaactt
tttagtcctgtttgtcctaaaacagaaaatagccgcattcacataaactc
tgataaaggtcttgaagaacatacaggatctcaagaacttttcagttctg
aagatgaactgccaccaaatgagatacgtattgagttgtgtagctcagga
atactgtgttcccaactaaataccttccacaaaagtgctattaaaagaag
ctgtacctctgaagataaagtgggccagtctgaagctctatctagagtcc
ttcaagtagctaagaaaatgaagttgatttctaatggaggagattctgct
gtagaaatggatcggagaaatgtgtctgaatttaagagtattaaaaaaac
atcattaataaaaaactgtgattctaaaagccagaagtataattgtttag
tcatggtgctatctccatgccatgtgaaggaaataaacataaaattcgga
ccaaattctggctctaaagtgcctttagcaacagttacagtaattgatca
atcagaaactaagaagaaggtttttctgtggaggactgcagcattttggg
catttacagtgtttcttggagatataattttactcacagatgttgttatt
catgaggaccaatggattggcgagacagtactacaatcaacatttagcag
tcagttattaaatcttgggagttattcatctattcagcctgaagaatatt
ccagtgtagttagtgaagttgtacttcaagacttactggcatatgtgtcc
tcaaaacattcctacctcagagatcttcctccgaggcagcctcagagggt
gaacagtatagactttgtagaattggagcaccttcaacctgatgtattag
tccacgcagtactaagagttgttgatttcactatactgacagaggcagta
tacagttatagaggacagaagcagaaaaaagttatgttaacagtggaaca
ggcccaagatcaacattatgcgcttgtattatggggtcctggagcagcct
ggtaccctcaacttcaaaggaaaaaaggagtggttctgattaaagcccag
atttcagagctggcatttcctattacagcatctcagaagatagcgctaaa
tgctcacagttctctgaagagtattttttcttctcttcccaacatcgtat
atactggttgtgcaaaatgtggattggaactagaaacagatgagaacagg
atctacaaacaatgttttagctgcttgccatttactatgaagaaaatata
ttataggccagcgttaatgactgccattgatggaagacatgatgtttgta
tccgtgtagaatcaaagctgatagagaagattcttctcaacatttctgca
gactgcctcaacagagtgatagttccttcctcagagatcacctatgggat
ggtcgtggcagacctgttccactccttgttggcagtcagcgcagaacctt
gtgtattaaagattcagagcctttttgtgttagatgaaaacagctatcca
ttacaacaagatttctccctcctggatttttatcctgacattgtaaagca
tggagccaatgcccgtctctgaggccagaggaagaaattgcaggcatttc
aaggaagaagtactgaaatgatttgtcttttgaaataaatgaatgacagg
gcttttgctttggatttttttatgaaatatattttacaaagagaattgcac
tagatatataaattaaaacttttttctaagaaaatcctgtgaggtttaaa
aagattgtttttgtcttttggtttcttactttctcctggagaaatgatct
accagtcaaggcaatatgtagcagatccctgggaattaaaggtttgccca
tttgttcactgtatttagtccctgctacattccaggcattgtactaagta
tggggaaccacagagaagacattccctcagaaactgctgcagtgctttcg
cttatccctacctaaaaaaccgtcaatgtgaaatcatttccttgattata
actataatgataatggattagtttatataaacctatgtttagacaagttc
aagacaagcgtgtctttctataaaaagtattgaaaatgaaggaaatgaga
tcatgtttcaatttattaaagcagggaagagcgttctgtgcttagttcgt
gtctaggatttgagtgccttactgaatgcatttaccagcaaacatgtagc

aatctgttctctcattgtgattgtggaagaagctcatgtaaaatgatagt
cattaatgaggaagtatggcgtggtacttattctgtaagttcagagtatg
ctgagtgttaatagattatcatattgcctgaaaataaattcatgatgaca
tgaaaaaaaaaaaaaa

>NM_020179 2
cgcgggcgccgagaggagaggcgaggcggggagcgcaagcggcggcggcc
actgccacgtattcccggcagtggtggcggcggcggcggcggcgcccgcg
ggcaggaataactcaagtcacctgtactggaaatcagtttgctgaaatta
atcaacgattcttgaagttgaagaaaaggaggttccagccttggcaagag
gagtgtggcccttcctggaatccctctggacacaccctcctagcatcctc
taggaaagatgcggcagctcaaagggaagcccaagaaggagacctccaag
gacaagaaggagcggaagcaagccatgcaggaggcccggcagcagatcac
tacagtggtgctgcccacgctggccgtggtcgtgctcttgatcgtggtgt
ttgtgtacgtggccacgcgccccaccatcaccgagtgagccccgcagccg
gctgcggaccccatcggcagggagaggaggcgcgggagggggacgcaaac
aaaaaatggctttcatattcagagatgttcatgttgctgagctgtaagca
ggagcaccctgtcttctctggtctttgacttgattaaagtatctccgctt
tcttgggagggaataggggatgttttatcagtgaatgtgccatacacctt
atggtccacttcatgtgcctttcagacttcaaagcgcgcgcgcatgtgtg
tgtgtgtgtgtgtgtgtgtgtgtgtgtgtgtgtgcttctttttctctcct
aaaaatcgataagtagctccacctgaagagggatggaacctctgggtcag
gaaacagctggaatccacactcacctcattcccattgtttggatcatgct
tctttccaacacgtgttcacaatctccaaagggactgtatttcttctctg
tgcttaatgtgatttgaaatatgttgaatcaaagtgaaatatttattttt
tgaataaaggagataatagccttaaaaaa

>NM_022075 4
agcgcaaggtgacgggcgtggacgcggctgtggggccgggagcggagggt
tggggtctggcctcccgcgccggggcgaaggggcagccgcagcgcagagg
cccgccccgccctcccctcccgtcacgcccagcctcccggcccttgggct
gctcgcggcctttttttcccggctgggctcgggctcagctcgactgggct
cggcgggcggcggcggcggcgccggcggctggcggaggagggagggcgag
ggcgggcgcgggccggcgggcgggcggaagagggaggagaggcgcgggga
gccaggcctcggggcctcggagcaaccacccgagcagacggagtacacgg
agcagcggccccggccccgccaacgctgccgccgggatgctccagacctt
gtatgattacttctggtgggaacgtctgtggctgcctgtgaacttgacct
gggccgatctagaagaccgagatggacgtgtctacgccaaagcctcagat
ctctatatcacgctgcccctggccttgctcttcctcatcgttcgatactt
ctttgagctgtacgtggctacaccactggctgccctcttgaacataaagg
agaaaactcggctgcgggcacctcccaacgccaccttggaacatttctac
ctgaccagtggcaagcagcccaagcaggtggaagtagagcttttgtcccg
gcagagcgggctctctggccgccaggtagagcgttggttccgtcgccgcc
gcaaccaggaccggcccagtctcctcaagaagttccgagaagccagctgg
agattcacattttacctgattgccttcattgccggcatggccgtcattgt
ggataaaccctggttctatgacatgaagaaagtttgggagggatatccca

tacagagcactatcccttcccagtattggtactacatgattgaactttcc
ttctactggtccctgctcttcagcattgcctctgatgtcaagcgaaagga
tttcaaggaacagatcatccaccatgtggccaccatcattctcatcagct
tttcctggtttgccaattacatccgagctgggactctaatcatggctctg
catgactcttccgattacctgctggagtcagccaagatgtttaactacgc
gggatggaagaacacctgcaacaacatcttcatcgtcttcgccattgttt
ttatcatcacccgactggtcatcctgcccttctggatcctgcattgcacc
ctggtgtacccactggagctctatcctgccttctttggctattacttctt
caattccatgatgggagttctacagctgctgcatatcttctgggcctacc
tcattttgcgcatggcccacaagttcataactggaaagctggtagaagat
gaacgcagtgaccgggaagaaacagagagctcagagggggaggaggctgc
agctgggggaggagcaaagagccggcccctagccaatggccaccccatcc
tcaataacaaccatcgtaagaatgactgaaccattattccagctgcctcc
cagattaatgcataaagccaaggaactaccccgctccctgcgctataggg
tcactttaagctctggggaaaaaggagaaagtgagaggagagttctctgc
atcctccctccttgcttgtcacccagttgcctttaaaccaaattctaacc
agcctatccccaggtagggggacgttggttatattctgttagagggggac
ggtcgtattttcctccctacccgccaagtcatcctttctactgcttttga
ggccctccctcagctctctgtgggtaggggttacaattcacattccttat
tctgagaatttggccccagctgtttgcctttgactccctgacctccagag
ccagggttgtgccttattgtcccatctgtgggcctcattctgccaaagct
ggaccaaggctaacctttctaagctccctaacttgggccagaaaccaaag
ctgagcttttaactttctccctctatgacacaaatgaattgagggtagga
ggagggtgcacataacccttaccctacctctgccaaaaagtgggggctgt
actggggactgctcggatgatctttcttagtgctacttctttcagctgtc
cctgtagcgacaggtctaagatctgactgcctcctcctttctctggcctc
ttccccccttccctcttctcttcagctaggctagctggtttggagtagaat
ggcaactaattctaatttttatttattaaatatttggggttttggtttta
aagccagaattacggctagcacctagcatttcagcagagggaccatttta
gaccaaaatgtactgttaatgggttttttttaaaaattaaaagattaaat
aaaaaatattaaataaaacatggcaataagtgtcagactattaggaattg
agaagggggatcaactaaataaacgaagagagtctttcttatgccttcct
tgca

>NM_024792 1
agccacgcggcgccagcgaggcggccggacccgcagccccgatgctgctg
acgctggccgggggcgcgctcttcttcccggggctcttcgcgctctgcac
ctgggcgctgcgccgctcccagcccggatggagccgcaccgactgcgtga
tgatcagcaccaggctggtttcctcggtgcacgccgtgctggccaccggc
tcggggatcgtcatcattcgctcctgcgacgacgtgatcaccggcaggca
ctggcttgcccgggaatatgtgtggtttctgattccatacatgatctatg
actcgtacgccatgtacctctgtgaatggtgccgaaccagagaccagaac
cgtgcgccctccctcactcttcgaaacttcctaagtcgaaaccgcctcat
gatcacacatcatgcggtcattctctttgtccttgtgccagtcgcacaga
ggctccggggagaccttggggacttctttgtcggctgcatcttcacggca
gaactgagcactccgtttgtgtcgctgggcagggttctgattcagctaaa

gcagcagcacacccttctgtacaaggtgaatggaatcctcacgctggcca
ccttcctttcctgccggatccttctcttccccttcatgtactggtcctat
ggccgccagcagggactaagcctgctccaagtacccttcagcatcccatt
ctactgcaacgtggccaatgccttcctcgtagctcctcagatctactggt
tctgtctgctgtgcaggaaggcagtccggctctttgacactccccaagcc
aaaaaggatggctaaatgctcctgggagtcaggcgcagcctcacaccagc
tgcctcctccactcagcattccatggaccaaattgtgccctgggtagcct
cagactttgggtattgataagccgatggatttgagtttttctaaagaata
ttcatattacctccttcttctaacttgccctatttgcaaaagcacttttg
tagtaacaactattgggtcctgtcagacctccacggacagcaaagtggtt
ttaatgcaagcccaaggatccttcttaaggtcttatctcaagagctctgg
gaggtggaagcatggggtgggatcggtggaccagggtggtaagtgtctgc
acatctgcctgtccctgtatcagcggctacccaccttccaaaccactcag
gacagtacccgtggcactgggcccgcagaagcaagggatgacttggttct
tggaagtaatgtcgtcttgtgacattggcctgggacaatcattgtgggta
ggtagttattgatcgtttactagataacccattggttctttgcctcatcc
tctcatccatgggtcagagttgaattcttatgtctatagacttccaatca
gaagtctcactggtggggctgggggtgggggcaggcaggaggcatggatg
ggaacctgagtaggtagtgtggccaagagatcagcacaacctttgcaggc
tgacttgctaagtctgacagtgacaaacttgtgagcttactgcagtcagt
cacagaggctgttctttttcacacacccccttcatgcccggctttccccat
atccacatgcagagggcgagctcataaaactacagggaagcgtgaaatga
tggctttggtagctgtttactgggtaaccccactgtgacactgtcctttt
catgtgatgtggaaacctacttctgtcctccaaaccatgaaatgtgtcat
ctagactgcagagtacttgagtgctttgcctcccgatatgccagagcttg
tggtccaaagcccattcctgtgtgtccgtcctgccatttagccacagaag
gctgcggagtgaggcggcagctagcctggccagtggctgtcccgtggacc
gacacctgcgccccccttctgcaagcaggattttctggtgccaacactcat
tcatcattcccgatcaactaggatgaatttaagactgtgctaccatgtgt
tctcaagtggtagtttaaaaagtggatttttaaagtgcctttcaattgtc
tgtgaacgtctaaaggactgatttgtctcaaaaaaaaaaaaaaaaa

>NM_024944 2
gctgctgctgtgatccaggaccagggcgcaccggctcagcctctcacttg
tcagaggccggggaagagaagcaaagcgcaacggtgtggtccaagccggg
gcttctgcttcgcctctaggacatacacgggacccccctaacttcagtccc
ccaaacgcgcaccctcgaagtcttgaactccagccccgcacatccacgcg
cggcacaggcgcggcaggcggcaggtcccggccgaaggcgatgcgcgcag
ggggtcgggcagctgggctcgggcggcgggagtagggcccggcagggagg
cagggaggctgcagagtcagagtcgcgggctgcgccctgggcagaggccg
ccctcgctccacgcaacacctgctgctgccaccgcgccgcgatgagccgc
gtggtctcgctgctgctgggcgccgcgctgctctgcggccacggagcctt
ctgccgccgcgtggtcagcggccaaaaggtgtgttttgctgacttcaagc
atccctgctacaaaatggcctacttccatgaactgtccagccgagtgagc
tttcaggaggcacgcctggcttgtgagagtgagggaggagtcctcctcag
ccttgagaatgaagcagaacagaagttaatagagagcatgttgcaaaacc

tgacaaaacccgggacagggatttctgatggtgatttctggatagggctt
tggaggaatggagatgggcaaacatctggtgcctgcccagatctctacca
gtggtctgatggaagcaattcccagtaccgaaactggtacacagatgaac
cttcctgcggaagtgaaaagtgtgttgtgatgtatcaccaaccaactgcc
aatcctggccttgggggtccctacctttaccagtggaatgatgacaggtg
taacatgaagcacaattatatttgcaagtatgaaccagagattaatccaa
cagcccctgtagaaaagccttatcttacaaatcaaccaggagacacccat
cagaatgtggttgttactgaagcaggtataattcccaatctaatttatgt
tgttataccaacaatacccctgctcttactgatactggttgcttttggaa
cctgttgtttccagatgctgcataaaagtaaaggaagaacaaaaactagt
ccaaaccagtctacactgtggatttcaaagagtaccagaaaagaaagtgg
catggaagtataataactcattgacttggttccagaattttgtaattctg
gatctgtataaggaatggcatcagaacaatagcttggaatggcttgaaat
cacaaaggatctgcaagatgaactgtaagctccccttgaggcaaatatt
aaagtaatttttatatgtctattatttcatttaaagaatatgctgtgcta
ataatggagtgagacatgcttattttgctaaaggatgcacccaaacttca
aacttcaagcaaatgaaatggacaatgcagataaagttgttatcaacacg
tcgggagtatgtgtgttagaagcaattccttttatttctttcacctttca
taagttgttatctagtcaatgtaatgtatattgtattgaaatttacagtg
tgcaaaagtattttacctttgcataagtgtttgataaaaatgaactgttc
taatatttatttttatggcatctcatttttcaatacatgctcttttgatt
aaagaaacttattactgttgtcaactgaattcacacacacacaaatatag
taccatagaaaaagtttgttttctcgaaataattcatctttcagcttctc
tgcttttggtcaatgtctaggaaatctcttcagaaataagaagctatttc
attaagtgtgatataaacctcctcaaacattttacttagaggcaaggatt
gtctaatttcaattgtgcaagacatgtgccttataattatttttagctta
aaattaaacagattttgtaataatgtaactttgttaataggtgcataaac
actaatgcagtcaatttgaacaaaagaagtgacatacacaatataaatca
tatgtcttcacacgttgcctatataatgagaagcagctctctgagggttc
tgaaatcaatgtggtccctctcttgcccactaaacaaagatggttgttcg
gggtttgggattgacactggaggcagatagttgcaaagttagtctaaggt
ttccctagctgtatttagcctctgactatattagtatacaaagaggtcat
gtggttgagaccaggtgaatagtcactatcagtgtggagacaagcacagc
acacagacattttaggaaggaaaggaactacgaaatcgtgtgaaaatggg
ttggaacccatcagtgatcgcatattcattgatgagggtttgcttgagat
agaaaatggtggctcctttctgtcttatctcctagtttcttcaatgctta
cgccttgttcttctcaagagaaagttgtaactctctggtcttcatatgtc
cctgtgctccttttaaccaaataaagagttcttgtttctgaagaaaaaaa
aaaaaaaaaaaaaaaaaaaaaaaaaaaaaaaaaaaaaaaaaaaaaaaaaa
aaaaaaaaaaaaaaaaaaaaaaaa

>NM_031942 4
cctccgggcccgggtcggcgcgcccagcctgccagccgcgctgctgctgc
tcctcctgctgtgggaccgctgaccgcgcggctgctccgctctccccgct
ccaagcgccgatctgggcacccgccaccagcatggacgctcgccgcgtgc
cgcagaaagatctcagagtaaagaagaacttaaagaaattcagatatgtg

aagttgatttccatggaaacctcgtcatcctctgatgacagttgtgacag
ctttgcttctgataattttgcaaacacgaaacctaaattcaggtcagata
tcagtgaagaactggcaaatgtttttatgaggactctgataatgaatct
ttctgcggcttttcagaaagtgaggtgcaagatgtattagaccattgtgg
atttttacagaaaccaaggccagatgtcactaacgaactggccggtattt
ttcatgccgactctgacgatgaatcattttgcggtttctcagagagtgag
atacaagatggaatgaggctgcagtcagttcgggaaggctgtaggacccg
cagccagtgcaggcactctggacctctcagggtggcgatgaagtttccag
cgcggagtaccaggggagcaaccaacaaaaaagcagagtcccgccagccc
tcagagaattctgtgactgattccaactccgattcagaagatgaaagtgg
aatgaatttttggagaaaagggctttaaatataaagcaaaacaaagcaa
tgcttgcaaaactcatgtctgaattagaaagcttccctggctcgttccgt
ggaagacatcccctcccaggctccgactcacaatcaaggagaccgcgaag
gcgtacattcccgggtgttgcttccaggagaaaccctgaacggagagctc
gtcctcttaccaggtcaaggtcccggatcctcgggtcccttgacgctcta
cccatggaggaggaggaggaagaggataagtacatgttggtgagaaagag
gaagaccgtggatggctacatgaatgaagatgacctgcccagaagccgtc
gctccagatcatccgtgacccttccgcatataattcgcccagtggaagaa
attacagaggaggagttggagaacgtctgcagcaattctcgagagaagat
atataaccgttcactgggctctacttgtcatcaatgccgtcagaagacta
ttgataccaaaacaaactgcagaaacccagactgctggggcgttcgaggc
cagttctgtggcccctgccttcgaaaccgttatggtgaagaggtcaggga
tgctctgctggatccgaactggcattgcccgccttgtcgaggaatctgca
actgcagtttctgccggcagcgagatggacggtgtgcgactggggtcctt
gtgtatttagccaaatatcatggctttgggaatgtgcatgcctacttgaa
aagcctgaaacaggaatttgaaatgcaagcataatatctggaaaatttgc
tgcctgccttctacttctcaaatctttcttgtaaaagtttccaattttt
cactgaaacctgagttaaaaatcttgatgatcagcctgtttcataagaaa
ctccaatcaagttaatcttagcagacatgtgtttctggagcatcacagaa
ggtatattgctagttacactttgccctcctgcagtttcttctctgctccc
aacccccatctcacagcatccccctctatttccaatgctcctctccaacc
gcttagtttctgaatttcttttaaattacagttttatgaaagcatatttt
atttacttggtgttgaaatagccctcataaaacctaagcacttggaaaca
caataatagtattaactaactagatctattgaatttcagagaagagcctt
ctaacttgtttacacaaaaacgagtatgatttagcattcatactagttga
aatttttaatagaatcaaggcacaaaagtcttaaaaccatgtggaaaaat
taggtaattattgcagattgatgtctctcaatcccatgtattgcgcttat
gttacaagttgttgtcacagttgagacttaatttctcctaatttcttctg
cccgaagggtaagtggtgcgtccagcttacacaatcataattcaaaggtt
ggtgggcaatgtaatacttaattaaaataatgatggaagagctatctgga
gattatgagtaagctgatttgaattttcagtataaaactttagtataatt
gtagtttgcaaagtttatttcagttcacatgtaaggtattgcaaataaat
tcttggacaattttgtatggaaacttgatattaaaaactagtctgtggtt
ctttgcagtttcttgtaaatttataaaccaggcacaaggttcaagtttag
attttaagcacttttataacaatgataagtgcctttttggagatgtaact
tttagcagtttgttaacctgacatctctgccagtctagtttctgggcagg

tttcctgtgtcagtattccccctcctctttgcattaatcaaggtatttgg
tagaggtggaatctaagtgtttgtatgtccaatttacttgcatatgtaaa
ccattgctgtgccattcaatgtttgatgcataattggaccttgaatcgat
aagtgtaaatacagcttttgatctgtaatgcttttatacaaaagtttatt
ttaataataaaatgtttgttctaacttgtctgctttttaaaaataatct
tactgtacttaattctaatttttcctcatatttaaataaaaggccattt
ccaccttttcaaaaaaaaaaaaaaaaaaaaaaaaaaaaaa

>NM_152261 2
ggggcccgggctgcacgagcgcgcgtgcgcgtcggaactggagccgcggg
aggaggcgggagctgaggaggcggcggagggggcgggtcgcgtcgggcgc
cacgtccgcagccagaggcctccgcctccaccaccgccgccacagtcttc
cagctccacatcctgagaggacgcctctggagccgcgactgcccggggtt
gtgccggccgccgctgccgcccaggccgcctcagctctcctctgcgccgg
cccgctcactccgcccggccccagccctagcgctggccgcgaccccggcg
cctttgaaacttctgctggtgtgagtgccctcaggggttccccaggaata
tcgatacaacaccaacaggagatcatgaatcagacagataaaaatcaaca
agaaatcccatcataccttaatgatgaaccaccagaaggttcaatgaaag
atcacccacagcagcagccaggcatgttgtcccgtgtgactgggggtatc
ttcagtgttacaaagggagctgttggtgccaccattggtggtgtggcttg
gattggtggaaagagtctggaagtgaccaaaacagctgttacaactgtgc
cttccatgggaatagggctggtgaaaggggtgtctctgctgtggctgga
ggtgttacagctgttgggtctgctgttgtaaacaaagtgcccttaacagg
aaagaagaaagacaaatctgactgaaatatagagatacacttgcgctcca
cagcactgtaatgccagtggcattgaattgctaaattatggactacaacc
aagtcaactgttttggacgtttatcttctaaactgctgtgttgaaagtat
tgatgactggctttcatctaaaaagaagagaccaatacgagcacagtata
tgaaggtttctcatacttaagttccaggtttttatctggtaaaatgttac
acttactcggttgtaactgaagatatggtatgtttgaatatttactataa
gtctttcagtttgactaaaaatgtgaaagttgaatttagtagatgatctt
cacagttccatatgtataatgtgccaggtaactcacctgccccttaagaa
gggaaccttgaattacataagccgtacctttgatgtgcctaatagtttca
gatgtcttagtttttttataaccatagttgattaggccaagaggcattcat
ttcttatttaagctggcaaaattagcaggaattagagaagtttaaaaaga
taaatggttttatgataatgttaaccatcttttgttagtaaatatgcgtt
ctattattttaatcattgatgccttacaaaagaaaacatcttttctaata
ccctgaatatgtgctgttcttagaatcatctatggattcttttaaaggtt
gtttgtgaaattagttttcccttttttagaatctcaggagtagtggggtaa
agacatttcctgctgtcagtggataagacagagcttgttaactgttttgg
cagtagttaaaatcaaattgtacacttctcagcctgggttcatgcttcat
cattaatacacctcacagtgcctaaggaacatttacttactggtcagaag
gtattttggaagagtttcatattaaggaggaacaaataataattttaagt
tcttaaaaattacctaaaacaccccaaatataaaaagaagccttcacacc
tattctgtctttaggatgtcttaaattattagcagtactcctttttttaaa
aacactgtaaaagtaaccacaaatatgtgaggacttactattttaaatgg
aatggaatgagctccatagattagttttgaatataaagtatataaaagtg

catcagtggtttatataggctttaaaaacatgttatcttacagtccttta
aagcagccatagagtttgtatcatttttcaagccaatttcagtcagggat
ttgaattgtttgattatggatgataaatgtgtcatatcttattaatatgt
ctcatgtctcgttccttcttaatatgatttagctggaattcattttcttt
tcgtttcatgtttaatttcataaaacgtttaacaattggcatatatactt
ggcattcctgtccaccaaggattgtaatccaagcctgggaaaatcttaaa
tttctttttacttaaatctggaaatttgtctccattctgccaccttttttt
ttttttttttttatagtggaggggaggaaagggggggtgataccttgcag
taagtaagtcgaaatagcatgcctgaaaatttgaaacagaccattctaac
acccaaggcttgtttataaaatacttgagaattacattaatgtggaatca
acagatgcagaagaatataacataacttttaaaagctttcataaatacca
gcagcaattgtaagcaaatctacaaaggttcttgaacctttctattatat
acaaaactgaaaagtcattaaggagttcaactaatcaggaattaaatggt
catttatttcatgcagtatgatttaaggtatttcttgagattctggtcaa
atgtcataatcagcaaacgggattaaaaaaaaaactccaaaatcactaaa
taattatctaaataatggtattggagaacttgtttcctgctatttggaag
agattgttgcttcattgctagtttgtatttctaacttctacagttataga
ctccactgtgctttgtgtctgaatttctcagtatagacattttgtttact
gtatgcttgcatatttattttcaactttgtttgtctttaaaattgcttga
ggaaaaatggttgtaattaatttctgctacagaaaagccacctggtacgt
tttgtctcatcaggattgttttaaattctaaactataagtttgttcagag
gggcttttgcaatgatagcagaaaactgtacaaatgtacagttagttata
gaggttcttgttgaaatgaacttaccatctgatgatatgtatgtacagct
gtgtacttgagtctttttttagtttacttagaaagactagcagtttgacct
gttaaacaggactagttcaagtcaagaaactaaggttgttgtatacacct
ggaggcatctgttattcagcttatcctttgagtgggtatttggcacaatg
aggataaacttatgtgacccacttgaatggctgatctaataatgttgaca
ttatgcattctgtacttagtgaaatgtcagatgaaaataactgatgaata
atttttttgtattaaagggatgggaaaagaacacatgaatttgttaataa
agcactatgatctgcaaacgatggaatgtttcataaagatctaaagaaat
aaaggaaactttaaaacagaaaaaaaaaaaaaaaaa

>NM_153367 3
cgcagtcgggggggaggcggcggcagcggcagcggctgcgggcagcgcgcg
gggcgcagggcgcggggcgatcaccgtcggggctcgggaggcgggcagtg
ggcacaggctccccggtgcccgcccctccgctgcggagggggggccgcgag
cgggcggccggggaggggccggcaccgccgccacaaaatgagcctgctgt
cggccatcgacacgagcgccgcctcggtgtaccagcccgcccagctgctc
aactgggtctacctgtcgctgcaggacacgcaccaggctagcgccttcga
tgccttccggcccgagccgaccgccggcgccgcacccccggagctggcct
tcggcaagggccgccccgagcagctgggctcgcccctgcactccagctat
ctcaacagcttcttccagctgcagcgcggagaggcgctgagcaacagtgt
gtacaagggcgcctcaccctatggctccctcaacaacatcgccgatggcc
tcagctccctcaccgagcacttctcagacctgaccctcacctccgaggct
cgcaagcccagcaagcggcccccacccaactacctgtgccacctgtgctt

caacaaaggacactacatcaaggactgcccccaggcacgccccaaaggcg
agggcctgactccataccagggcaaaaagcgctgcttcggcgagtacaag
tgtcccaagtgcaagagaaaatggatgagcgggaactcctgggccaacat
ggggcaggagtgcatcaagtgccacatcaacgtgtatccacacaagcaga
gacccctggagaagcccgacggcctggacgtgtccgaccagagcaaggag
cacccgcagcacctctgcgagaagtgcaaggtcctgggctactactgccg
tcgcgtgcagtgacgggctgcccgcccgcacccagagccaccccccgcca
gcccgaggagacgctgcttccctgtgctactccgaggggctgcgtgtcgc
cctgtgcatggggtgccctcgcaggcctgcggggctgggccgggggctct
tagcgtccttgtcttgtgtgtgttgacaaacagtgttactgacattgctg
ccccccacaggccagggaaagcagggagtctggggctttttgcaggcggg
cctggggtctcagtggaggggacaaaggcaagcaaaagcccatgtccagg
agccctgggtgtccccacaggctcgcctctgagagcctctttggggtgag
cagccttgtattggccacaggtgcactaaattgactgtgaatcccaaacc
tccccagaccagccaggccgcctgcccccacccagaaccttccggtttgc
cctgtatggaaagccactctcagaaatccctctttcctgagtcagcaatc
gtggcaaggggacatgtgttccaacagcggctggggagtggacctctctg
tcccttgcccaccttaagccccaaatccagacccccctctgacatcactgg
cattgcacctgggtgtgcccccctccccacgctatggacccagataggag
gggttaggcatgggggaggcacagaatgctggagagatgcgtcctggtga
acgtggggcagcccctcccacgacccccaccagactgccttaggttttgt
cagccccactccctttccttcccctctgcctcccacccactctgggggtc
cacatcaagatagctgggcccagtgtggaagcccagcgtgtctgttccag
cagaaaggacacaagcctggtgttctggagacctcggctcaagtctttgc
cctgctgccgacgcattgtgtcaccttgggcaaggacttcgcatctccag
gcctcagtttccccattagttaaatgacagcataacactagagagcagag
ggcccatttcagctctgttgttctgggattcaggctggccggtgctgtgt
cctggagctttatttggggagtttcacccagaatggtgggagaaacctcc
caggtgccaggtaccccgcatcgtgacccttcacttggtgtcttaggaag
tcaagctgagggatgctgagtcctcccctgctggcccctgcagccccagc
cctgcttttcatcccccacccctgcaaacatggaggagcccccctccttct
cacctcggtctcctagcccctgacatggagaaccctgagacaagccacag
aacccctcttttctaaaatggagacaataatttcctacctcccaagggag
cagagaggcctcgtggcacgtccgtggccagggagcccactgtcctggct
ggcggcgggatcgtgcgctcctctgtctcccggatgagaagccccgtttc
catggtcttgacccttcctttctcccggctgtcagaactgggtctcttga
ttttgcccctacattatgcctctgtgggaaaaaaaaaaaaatcagaccaag
aaatgagcctgaaattcagtgtttaccatggctcaaggatgcccatctgg
tgtccagttgccttttgtattcaaatgaaaatgctttgtacaactgagga
gttacagtgaagtgttaaccaggggtccagggagcgagttgaaaagatgg
agtgagtgtatttgcagccagggagctgcagggtggatttgaggggccat
accctctgagcacttaaaaaaggtatttgctccaggccaggcagcaggct
gtggacacccttgccaccactggggactgccactgaggactccccgagca
cgttgttccccgtcttctccaaggtgttgaggtgagctggggttggcccc
ggcccaggcttctgtcccaaggagaagctgccactgacagtcatcctacc
gcactgctaaagagaatgttcgcagtggtgggcggcgtgcctgtgccaac

ccttccagggacccggccatgggggaccttggcccaaggatgcctggggc
ctgccagctgtgctgcaaaggtgggggcccacaccctaaaactaaccca
ggccccagaccactggaggccagggcttccctgcacgggctaaggggagt
tgggatatcaccccaaagtgaccttgccagtgagctgttcagcaggtagc
cactgccctgccatctgtgcagagccagccaccttgggggctggggttcc
cgctttgaggcccaccttccatactccccttgactcggctctggctgaac
tggggaactctcttgtggtcagcaaagcccctgccatgcaggccaggtgc
cattgagaattaagtgctcagagggccaggagcccaggggatgggaaagt
gtgtggttttagtacgttcaaaagggacaatcgcttgcagttggtagatc
tagcgatctagttgggagataatggtgtttaccccatatgaagtattcaa
tagttctacttgtgaatttgtatttattttgagttatacttgacacagaa
ttcctttttaaaaaaatatgtgtgtattttggaaaaaaaattcatagat
gttaaaatttctgcatggttaccagtttttctcacaacactgaatttggt
agcttttcccgaaaaaatcttcacagtaatttttgtctgtatatatttg
agggcctttttttaaaaaaaaaaaaagaaaagaaaaatataattgtttga
tttttgagattaaaacaaacaaaaagagaggcattttcaaaatttcagaa
ctttcaggagggcaagagaatatcaaacaaagatttctggaagtattttg
ccaaccttctggttgagctgcaagaaaatatttatggtgagaacttttct
gtttcccgttattgggtttttggttggtttttgtttgtttttttactatgc
tttggtctgtaaaaatatgcaactgaactacattcagaaggaaatattgt
ctacatagaatattatatgaagttggtacataattctgatgaggaaaaaa
aatctttgcaattctttaagccatattgttgtttttctgtgttgttttcc
ctggatgaaaatatcagtattaagtagacagcatattattcaagtgttta
gacttattaatatgttcttgtcctgtatttatacatatgtgtattttgga
aagtattgcctttttttaagggaagctataattcgatacatagtgaaaaag
ggaatggtgacccctttgtgcctcttccactgaggataacaaacagcatt
gtaatccattctcttgcaccttcttcttcttatcttgttattacggtttt
attaattttgtagagggacagggagtgggcaaggggaagaagcagcttat
ttgactaaccagcccctctgtggtccaccagcgtcttggcttggtgggag
ggctctcaatcagcagggccccaggagggaagaagaagtggggcaaagcc
tggcctcgccgctcgggagctttgccatctgagccacgcctcctccaggc
catgctccttgaacttggaaatgtcaaccggagcccttacaccagccctc
cagcatctaatagacttgaatctactctaaacgaatatttaatccaacct
cactacattgtagctcagtccaacgactaaccctgaaatgggggtgttcc
agccttcagcgagatggccaagcggtcccctgggggctgtggcagcgggc
ttatccttctctgttgccaaccttgccgtccgacctcctccgcccccatg
cggtgaccccgtccgtgtctgtgtctgtccatacgtgtgagtccagctaa
aaagacaaaacagaacccgtgggcccagctcggaaggtgcgtggagaagg
ctccgacgtctccgaagtgcagcccttgggatggcattccgttgtgtgcc
ttattcctggagaatctgtatacggctcgcctatagaaatatagcctctt
catgctgtattaaaaggacttttaaaagcaaaa

>NM_172037 4
agagccggcccggagcgctctgacttgcaagcgggctgcgctgcggagcc
cagtgcccgagtgacacccgcggagagtgcagggccggggaacgcgagcc
ctcgggggcagctgcaaggcgttgggcagcgcttgcctgcgccgagcgag

tctccccttcccggcgctccgccgccccgcaccccactctcccaccctct
cgcaacttgggtcgagttgacaactcccgcggcagcccgctggcccgtgc
cgcctccgctgcgcacccctcccccggggtgagagggagccggcgcgccg
gttccggggacgctcgggcggcagcagcttggccatgagggcagttcgag
tagtctaactcgcggctgtcaccgccactgcagcggagccggccggccgg
gcgctgcgggacgggcgggcggctgccggcaggaggcgccgagccgggtg
actgccgcggcgggcacagtccggggccacagcgccgagcccgggcggga
gtggccccgcgcaggcagggagcggcgccgcgcactccaacccggcgggc
acctcgggggcgggcgcggggcgcagccttctcgtcccggcctctgtgac
aagcgccccggagccgggagcccgattgccgggctcggggtgggcgcgga
cgcaggcactgggctcgtgcggggccccgggcgtcgcgatgaacatcgtg
gtggagttcttcgtggtcactttcaaagtgctctgggcgttcgtgctggc
cgcggcgcgctggctggtgcggcccaaggagaagagcgtggcgggccagg
tgtgcctcatcaccggcgccggcagcggcctgggccgcctcttcgcgctg
gagttcgcccggcgtcgggcgctgctggtgctgtgggacatcaacacgca
aagcaacgaggagacggctggcatggtgcgccacatctaccgcgacctgg
aggcggccgacgccgctgcgctgcaagctgggaatggtgaggaagaaatt
ctgccccactgtaacttgcaggtttttacctacacctgtgacgtggggaa
gagggagaacgtctacctgacggctgaaagagtccgcaaggaggttggcg
aagtctcagtcctggtcaataatgctggtgtggtctctgggcatcacctt
ctggaatgtcctgatgagctcattgagagaaccatgatggtcaattgcca
tgcacacttctggaccactaaggcttttcttcctacgatgctggagatta
atcatggtcatattgtgacagttgcaagttccttgggattgttcagtact
gccggagttgaggattactgtgccagtaaatttggagttgtgggttttca
tgaatccctgagccatgaactaaaggctgctgaaaaggatggaattaaaa
caaccttggtttgcccttatcttgtagacactggcatgttcagaggctgc
cgaatcaggaaagaaattgagccttttctgccacctctgaagcctgatta
ctgtgtgaagcaggccatgaaggccatcctcactgaccagcccatgatct
gcactccccgcctcatgtacatcgtgaccttcatgaagagcatcctacca
tttgaagcagttgtgtgcatgtatcggttcctaggagcggacaagtgtat
gtacccctttattgctcaaagaaagcaagccacaaacaataatgaagcaa
aaaatggaatctaagaatctttttgtatggaatattacttctatcagaag
atgatcaagatgtttcagtccagtgcacatcagcattgctgacattttat
ggattctaaacttgtgttgtttctttttaaatcaactttttaaaaaaat
aaagtgtaaattaaccgactagagtacttggaaaatgtgatcagtacaag
tgaacttaggttgttgccaacagggtccttttaggcagaacccagaaacc
agtcaaatctgtagagaagcagtgtgacatcttcaggttaccattatttt
ttaatgagcaggaagtctagaaatgataactagactgtatgtttcatgtg
tgtgatttttcagaattcccagagtttactcattcttgttattaaactct
agccagttgacatcttcgcaatttcaaggactgatagtgctgtattttct
cacgttttctaagtttccgttttgcaaggcctaggtgactttttcatggt
gtttgtatgtttagctcttttgaaaaggaattttgaaatctccatcaact
gaagtaaatgatgtctgagtgttacagtaaaggtgaccaagtctctttct
taaagtcacaatgactaaagtattagttgaattttttttttttttttttt
gatggagtctcgctctgtcaccaggctggagtgcagtagcacaatcacgg
ctcactgcaatctctgcctcccagtttcaagtgattctgctgtctcagcc

tcccaagtagctgggactacaggcatgcgccaccacgcccagctaatttt
tgtatttttagtagagacggggtttcaccatgttggtcaggatggtctcc
atctcttgacattgtgatccacctgcctcggcctcccaaagtgctgggat
tacaggcatgagccactgcacccagccttgaatttttaattttatctctg
atatacttcattaagtgtctggagacctaattatcctaaaagatcataca
ttttctacctatgaattttgctgcatacagaaagtgccctttcctcagga
agttgctgtgtttcatttctttggatggactcttatctagaatacatagc
agctctgcaaaggaacagtttttaaaaatgggaacttctacattgaaaag
tccccatttttgtgccaactatgattagtgagaggaagaaatcttattct
atggcatatgtatggaagggtgtaaagattcttttgaaaggtttattcac
attgtagaacagcaaatgacatttttacagtattttttgtaaagcaaac
tattttgtgccttgaatttggtatatgtgtattagtgaaacattgtaaag
gtgaacttctacctctgtatctaaatgtataccatccacttgtaaatgac
tataaactattatgtgattgcttttttttttagaatgtcttgtttaaata
gtggccaatgtttaaggctgttaaaataagccaacttttactaattgggg
agttttataaatgactgattaaatttaaagaattaacttacatgcaattg
tgtgattattagttatcagcagtgttgtaaggaaaattattgtgtttttt
tttatgatcattatcccactttaggtaaagaaaaatattggaatggaata
gtgttgggaaacagacattaacaacctagggtgcctgcactcaaatagcc
gatgttactgtccctagattagagacttgattaagggcttgtttgtacca
aaagtggggaaacaatgccatgacctgtgttttagtttggctgcaccaca
gatcaaatctgcactgtgtctacatataggaaaggtcctggtgtgtgcta
atgttcccaatgcaggacttgaggaagagctctgttatatgtttccattt
ctctttatcaaagataaccaaaccttatggcccttataacaatggaggca
ctggctgcctcttaattttcaatcatggacctaaagaagtactctgaagg
gtctcaacaatgccaggtggggacagatatactcagagattatccaggtc
tgcctcccagcgagcctggagtacaccagaccctcctagagaaatctgtt
ataatttaacaacccacttatccaccttaaaactgaggaaagtcgtcttt
acatctaattttattcttgtgtgttataacttaaacctatttctattttt
gtttgttattgcccttataagggtgtccatctccaagttcaataaactaa
ttcatttaactttaaaaaaaaaaaaaaaaaa

>NM_172105 3
gcaaaacttccatcctgtccacgtgaagttgtcgctgccttagagagggg
gaaagagctgcgggaaaagccggggagtgacgactgcggcggctgggcgc
gctctctcattttcttttcttctcctttccccccctgtcgcagtccggagt
tttggctcctctcctttcctcctccccctcggagccggcttctccctccg
ccccgcttctcccccgcttgtgtacgctatttgttgtggggtggccgaag
gggatgtcctgttttcaccagaggcacagcgcgaaggggaaacttcgaca
ctggaaggaacgagaataaatacttaattacggacgcactgaaccgcggc
tgggacagacacttcgggaacccgaggcggaccgggcgacgagatagtca
tttttacttgaaggaagctgcttctacttgggagtggcaggagaagtgag
aaaaccacatggaagactcccaggatttaaatgaacaatcagtaaagaaa
acgtgcacagaatcagatgtttcacaatctcagaattccaggtctatgga
aatgcaggacctagcaagtcctcatactcttgttggaggtggtgatactc
caggtagctccaaactggaaaaatctaatctcagcagcacatcagttact

acaaatgggacaggaggggaaaacatgactgttttaaacacagcagactg
gttgctgagttgcaacacccccctcttctgcaacaatgtctcttcttgcag
tcaaaacagagcccttgaacagcagtgaaaccacagccacgactggagat
ggagcgcttgacacttttactgggtcagtaattacaagtagtggctacag
ccccagatcagcacatcagtattccccacagctgtatccttccaagccct
atccacacattctttctacaccagcagctcaaacaatgtctgcctatgca
ggccagactcagtattcggggatgcagcagccagccgtctacacagccta
ctcacagacaggacagccctacagcttgcccacttacgatttgggtgtga
tgttgccagccatcaagacagagagtggactttcccaaactcagtcccca
ttacagagtggctgcctcagttacagcccagggttctctaccccacagcc
aggccagacaccttattcttaccaaatgccaggttctagttttgcaccat
catctactatttatgcaaataattcagtttccaattcaacgaatttcagt
ggttcacaacaggattatccatcctatacagcctttggccaaaaccagta
tgcacagtattattcagcatcaacgtatggagcgtatatgacatcgaata
acacagccgatggcacaccctcttcaacctctacttatcagttgcaggaa
tctctcccaggactgactaaccaaccaggagagttcgataccatgcagag
tccctccacacccatcaaagatcttgatgagagaacctgtaggagttctg
ggtcaaagtccagaggaagaggccggaaaaataatccctccccgcctcct
gatagtgacctggagcgtgtgtttgtctgggatttggatgaaaccatcat
tgtttttcactcactgctcaccgggtcttatgcacagaagtatggcaagg
atccccccatggctgtaacccttggactccgcatggaagaaatgattttt
aatcttgctgatactcatttgtttttttaatgatttagaggagtgtgatca
agttcatatagatgatgtttcctctgatgataatgggcaggacttaagta
cctacagttttgcaactgatggcttccatgcagctgcaagtagtgcaaac
ctttgtttgccaacaggtgtaagaggaggggttgactggatgaggaagtt
ggcttttcgttacagaagagtaaaagaattatataacacctacaagaaca
acgttggaggactccttggccctgccaagagggatgcctggctacagtta
agggcagagattgaaggtctgacagattcctggctaacaaatgcacttaa
gtctttatcaattattagcactaggagtaactgcataaatgtcttggtaa
cgacaactcaactgatcccagcacttgcgaaggttctactctatagttta
ggaggtgctttccccattgagaatatttacagtgcaactaaaataggcaa
ggaaagctgttttgagcgtatagtgtccagatttggcactaacataactt
atgttgtgattggagatggccgagatgaggagcatgccgctaaccagcac
aacatgcccttctggaggatatccagtcactcagacctcctggctctcca
ccaagcactggaattagagtatttgtaactgtgttctttagccggagatc
catttttatatttcaagtacactgaatttttatgtgtgattcaatgcct
ctggctctacacatataaattgtcttaatggatgaaatcatatttggaat
aaaaattccagaatgaagaattcagattgctgaatggagttaaactttag
tgctacagaaaagaaactctatggtcttatatttacaacactttaatggg
ttttttaaaaatctgtggaggttgctggtacacaccaaatgagtccaaac
tggaatgagcagctttagcaaagaactcttaccctggcaaagcagcaaca
cacatgctccgtctgacaaggtggtcaacaacattcctcaaaatgggaga
tcttctcagccctgaggtttgaatctgactttagcctacctaacccagaa
aatctgaattggaatgcactcagactgtataaggacagtcctatttagac
atgtaatttgtgtaaattattgatgaaaataatttactgtgactttatta
gcagctgactttcaaagtggatgcaatttttctttcttttgttggggagg

ggaatgggaggggaaatgggaatataatattgtctctttttttaagtttgg
caaacagaatgttcatactgatgtgttgtgccttaaagacaagacagcat
ttgtgtgttacaatgtaactttggttaaaatctctgtagataatgaaaaa
aaacaaaaaaaaaaaacctttgtgatgattcttaacatgaccaaatttaaa
agtcaagctctcagagcttaattaccgcatcagcaagaaactgagtattt
tttgcaataagaaaacaacaataataaaggaaagcttgtgtttcatttgg
gttcttaataattccaataattgtatgaggcaactatttgcgcatccaac
catgagtggaaggtttgggaaagactgtgggacctttacttagaaagtga
aatgtatgtagaagtctcaagtacccccttctacagttttactggagaaaa
ctaagagccatattcatgacaacttgcacagttttgaggttgagactttt
gatatgtgtaagttgcatagaggaggatattatcatgcaaatcatgagca
attatcacataaactttttttagaatgtgccatgaacatggcataaaattc
acattgagtgcacagggcttaaaataaagctaagtatgtttattcccaat
gccatggcaaaaatgataatatcatcagaaatggaaggcagttctcccag
atggtgtctaatgaaagcaatgagtctatgaaaattttacctagaatatc
atcataaattaaattagcaagtgcgctggatcttggcagcgctgctgaaa
tgacaacagtaaaataatacctggttctccatctgaatacatcaatgcag
gttctcctcgtaacagacttgcatatgtttgttagtttctgcctgtattg
tcactgcgcaacggatggcattcattacaagaagagcccatcatcgttgt
gtttgcatggtttttttccttgtgtgtagcccatgttgggaacacgatac
aggttctcctcttatttcctatgacacgatttcccttgtggaaatttaag
actttaagaactagagtatttttatggtgtctgcacctgcagttctgtgt
ttaaaatgtcataatgtggatcctggagtcaggctactagtcagtgcccc
tagccagaggctggttcatgagttcatcaactgaggcctctgtggcttca
ataaagtctacattttgctcacagatcacaacattcactgtggaaatatg
atttcatttctttaggctacaaacctgtatttctttactgaatgctaagg
ccatgtttatattgggtagaaagatattgagatcccaattttgtacaaga
ttgtgatttcattatctaaaccttaaacttaatcctttaaattttgtagc
ttttggctgcatctgccccaagtactattccaggcaaattaaagttggaa
tacctttaataatataaaaataatgatagtaaatcttatacttctgttgg
cccttagcttgaaaatagcagttaaaaaaatttaaatgttgccttgatta
tcagtacttaattatgttgtgcactaaaaccttaaatatttattactgtg
aataaaaacaaattatctttactgtatagctggtttctttaaatgttgat
agaattgtggcattacatctaaatttgtaagtcttttcatatcaaacaag
caaggctttttatgctgctaagtctgtgggtgcagaaagaaacacccctt
ggaagggcaaagagaagccggctggttgcatcaccccgtgcagtttctca
cacacatctctttttctgattctgtgttcagaagaggctgccggcataaa
acctaaatgcaaggttgacggagaacagcttgtctggcacaacaatggtg
caggcccacgagccagcatcacagcttggccatgggacgttgagtatgca
caaactagaactcttcccttcccaccttaggaatagaaaatcctcttcct
ttctaatctgaaaaacgaaaactgaacaaacacaaaaccaaccccttggc
agttcccacctcctattgacatatggaatattgtgccttattgtaaacca
gtttgaaaaatgttctgtaactaaagtgggttttcggctattatgtatac
agcttggtatattactacgaaagataaccaccttgtgttgcaccttaaaa
atatcaagaccatgtaatttccataacaaaataggtggcctggctatggt
attatagcatacaattaggacactatgcccctgcaaaattttgtaaatca

aattcagaggcaaaaacatattttagaatcatacagtttgcacacgacaa
gtaggtaataactgtctctaaaaatgttttctccttagtccgcaatgagc
taagattcagaatagtgtcaacagcatgctcctatatgaagttgtttttt
ttaaagcacatttgtttatgacaagcctacattctcagtgaatatggcat
ttagtatttcttttgaaaacaaacttaagcatcatgagccaaagttgcac
tttgactcccaactacggtagcattatggacatctcacaatgtcaagggt
ttctgttatgtattagtaaagtatagattattggggcctacataatttaa
aagaaatgtaaattaatattaaaagcttgtaaaaatatgtatatctttac
tatttctcaataaatacctttgcaattgttttcatttccttcaaaaa

>NM_000966 5
aggttgggggggggggtggggagggagaaaaagaaagaaaatattttccgt
gtccccgcctgcagagtcagtgtgcggtttgggagaaaatgtgtcggata
ttttggggcggtcacgtgggcgggcgggctccgagaggccccgggacagt
cccagcctagagccgtgcccccccaggagccccccagtacggcgagcccc
ggacattgcgacgctccatccaagagactgcccgacgccgggacctcggg
gctccgccgcctcccttccccctcccactccagcagctacggcccagttc
cctcaacctgacccagtatgtagaagccagtctctgcaggcggccagcgg
gacttttggaggcccagtgggcaggccaggcagggcgggtacggagcctc
ccaggctggggcagtgggcatgggcaggggctgtggctgaagacctcgcc
cgcccactgcagaccccaggggactctcacaccgcagctgccatggccac
caataaggagcgactctttgcggctggtgccctggggcctggatctggct
acccaggggcaggtttccccttcgccttcccaggggcactcagggggtct
ccgcctttcgagatgctgagccctagcttccggggcctgggccagcctga
cctccccaaggagatggcctctctgtcggtggagacacagagcaccagct
cagaggagatggtgcccagctcgccctcgccccctccgcctcctcgggtc
tacaagccatgcttcgtgtgcaatgacaagtcctctggctaccactatgg
ggtcagctcttgtgaaggctgcaagggcttctttcgccgaagcatccaga
agaacatggtgtacacgtgtcaccgcgacaaaaactgtatcatcaacaag
gtgaccaggaatcgctgccagtactgccggctacagaagtgcttcgaagt
gggcatgtccaaggaagctgtgcgaaatgaccggaacaagaagaagaaag
aggtgaaggaagaagggtcacctgacagctatgagctgagccctcagtta
gaagagctcatcaccaaggtcagcaaagcccatcaggagactttcccctc
gctctgccagctgggcaagtataccacgaactccagtgcagaccaccgcg
tgcagctggatctggggctgtgggacaagttcagtgagctggctaccaag
tgcatcatcaagatcgtggagtttgccaagcggttgcctggctttacagg
gctcagcattgctgaccagatcactctgctcaaagctgcctgcctagata
tcctgatgctgcgtatctgcacaaggtacaccccagagcaggacaccatg
accttctccgacgggctgaccctgaaccggacccagatgcacaatgccgg
cttcgggcccctcacagaccttgtctttgcctttgctgggcagctcctgc
ccctggagatggatgacaccgagacagggctgctcagcgccatctgcctc
atctgcggagaccgcatggacctggaggagcccgaaaaagtggacaagct
gcaggagccactgctggaagccctgaggctgtacgcccggcgccggcggc
ccagccagccctacatgttcccaaggatgctaatgaaaatcaccgacctc
cggggcatcagcactaagggagctgaaagggccattactctgaagatgga
gattccaggcccgatgcctcccttaatccgagagatgctggagaaccctg

aaatgtttgaggatgactcctcgcagcctggtccccaccccaatgcctct
agcgaggatgaggttcctgggggccagggcaaagggggcctgaagtcccc
agcctgaccagggcccctgacctccccgctgtgggggttggggcttcagg
cagcagactgaccatctcccagaccgccagtgactgggggaggacctgct
ctgccctctccccacccccttccaatgagctccttgtttttgccaaagttt
ctaggggtgcctctgtgttcatccccttcctgatctaaccggctccctcg
ccagtcccgggggcctgccctgctcccaccaggagagagggcaaagggat
gagcctgggtttggactctaaaatctcagcactgccccatgggtcctaga
cttcccagggcaagaggaagaccctgccattccacagccccttcctctgc
caggtgcttggctctctgagagcaaacaggaacactagagaccaaaaagg
ggacaaaggagaagggctgagcccaccttcttgctcctacccttggtgcc
taatgctgtgtgatgcacctgcagggtgtgtgctagcctctgtgccccgt
ccttgtgccaggtcaaggtgggggcaggctgggccctgcatttctggggc
aggaacagagggtgaaagggacagatagatgcaggtccattctgcacctc
ttggctcgggtgcagagttcaccctgtgccctccgttataagtccctccc
ccagccctgtcatgtgccttgggctcctcctgccctccatctcagccatt
ggggcagggaccctcctacactacagaggggccaggggatccctctctcc
ctagtgccttccacccttactccccagagcagcttggcccagggagggg
ggatgctgcttagctgatcccgccctgacccagaggaagcctctatttat
ttattagcttttgtttacaccgtggaattgaccccttcctccaggggtct
tgggtgggggagcccagggcccctgtgacccctcctttcttcctccaatc
cccagtttgtatttagctgccaaataagattcccattggctccctgtgtt
ctcttggggggtcagggtgctgtcccctcccctctgtttacatctcccct
ctaccccgctgtatcgcatattgctgagttttctatttttgcaaaataaa
gtgatggaaactcatgaaaaaaaaaaaaaaaaaaaaaaaaa

>NM_001951 3
gtgcgcgggggcccgaccaccgcggggccgggacgcgatggcggcggcag
agcccgcgagctcgggccagcaggcgccggcagggcaggggcagggccag
cggccgccgccgcagcctccgcaggcgcaagccccgcagccgcccccgcc
gccgcagctcgggggcgccgggggcggcagcagcaggcacgagaagagcc
tggggctgctcactaccaagttcgtgtcgctgctgcaggaggccaaggac
ggcgttctggatctcaaagcggctgctgatactttggctgtgaggcaaaa
aaggagaatttatgatatcaccaatgtcttagagggaattgacttgattg
aaaaaaagtcaaaaaacagtatccagtggaaaggtgtaggtgctggctgt
aatactaaagaagtcatagatagattaagatatcttaaagctgaaattga
agatctagaactgaaggaaagagaacttgatcagcagaagttgtggctac
agcaaagcatcaaaaatgtgatggacgattccattaataatagattttcc
tatgtaactcatgaagacatctgtaattgctttaatggtgatacactttt
ggccattcaggcaccttctggtacacaactggaggtacccattccagaaa
tgggtcagaatggacaaaagaaataccagatcaatctaaagagtcattca
ggacctatccatgtgctgcttataaataaagagtcgagttcatctaagcc
cgtggtttttcctgttcccccacctgatgacctcacacagccttcctccc
agtccttgactccagtgactccacagaaatccagcatggcaactcaaaat
ctgcctgagcaacatgtctctgaaagaagccaggctctgcagcagacatc
agctacagatatatcttcagcaggatctattagtggagatatcattgatg

agttaatgtcttctgacgtgtttcctctcttaaggctttctcctaccccg
gcagatgactacaactttaatttagatgataacgaaggagtttgtgatct
gtttgatgtccagatactaaattattagattccatggaaacttgggactg
ttatctacctctaactgtgtaacattttagacttcttaataacctaaata
tttaaaataatgaatgtaacacctttttttagttcactgattctgaagtgt
tcttccctaatactttctttacttcacaaaacttcaaccataaaaacaaa
gggctctgattgctttaggggataagtgatttaatatccacaaacgtccc
cactcccaaaagtaactatattctggatttcaacttttcttctaattgtg
aatccttctgtttttttcttcttaaggaggaaagttaaaggacactacagg
tcatcaaaaacaagttggccaaggactcattacttgtcttatatttttac
tgccactaaactgcctgtatttctgtatgtccttctatccaaacagacgt
tcactgccacttgtaaagtgaaggatgtaaacgaggatatataactgttt
cagtgaacagattttgtgaagtgccttctgttttagcactttaagtttat
cacattttgttgacttctgacattccactttcctaggttataggaaagat
ctgtttatgtagtttgtttttaaaatgtgccaatgcctgtacattaacaa
gatttttaaaaataaaattgtataaaacattcaaaaaaaaaaa

>NM_002211 3
atcagacgcgcagaggaggcggggccgcggctggtttcctgccgggggc
ggctctgggccgccgagtcccctcctcccgcccctgaggaggaggagccg
ccgccacccgccgcgcccgacacccgggaggccccgccagcccgcgggag
aggcccagcgggagtcgcggaacagcaggcccgagcccaccgcgccgggc
cccggacgccgcgcggaaaagatgaatttacaaccaattttctggattgg
actgatcagttcagtttgctgtgtgtttgctcaaacagatgaaaatagat
gtttaaaagcaaatgccaaatcatgtggagaatgtatacaagcagggcca
aattgtgggtggtgcacaaattcaacatttttacaggaaggaatgcctac
ttctgcacgatgtgatgatttagaagccttaaaaaagaagggttgccctc
cagatgacatagaaaatcccagaggctccaaagatataaagaaaaataaa
aatgtaaccaaccgtagcaaaggaacagcagagaagctcaagccagagga
tattactcagatccaaccacagcagttggttttgcgattaagatcagggg
agccacagacatttacattaaaattcaagagagctgaagactatcccatt
gacctctactaccttatggacctgtcttactcaatgaaagacgatttgga
gaatgtaaaaagtcttggaacagatctgatgaatgaaatgaggaggatta
cttcggacttcagaattggatttggctcatttgtggaaaagactgtgatg
ccttacattagcacaacaccagctaagctcaggaacccttgcacaagtga
acagaactgcaccagcccatttagctacaaaaatgtgctcagtcttacta
ataaaggagaagtatttaatgaacttgttggaaaacagcgcatatctgga
aatttggattctccagaaggtggtttcgatgccatcatgcaagttgcagt
ttgtggatcactgattggctggaggaatgttacacggctgctggtgtttt
ccacagatgccgggtttcactttgctggagatgggaaacttggtggcatt
gttttaccaaatgatggacaatgtcacctggaaaataatatgtacacaat
gagccattattatgattatccttctattgctcaccttgtccagaaactga
gtgaaaataatattcagacaatttttgcagttactgaagaatttcagcct
gtttacaaggagctgaaaaacttgatccctaagtcagcagtaggaacatt
atctgcaaattctagcaatgtaattcagttgatcattgatgcatacaatt
ccctttcctcagaagtcattttggaaaacggcaaattgtcagaaggcgta

acaataagttacaaatcttactgcaagaacggggtgaatggaacaggggaaaatggaagaaaatgttccaatatttccattggagatgaggttcaatttgaaattagcataacttcaaataagtgtccaaaaaaggattctgacagcttt
aaaattaggcctctgggctttacggaggaagtagaggttattcttcagta
catctgtgaatgtgaatgccaaagcgaaggcatccctgaaagtcccaagt
gtcatgaaggaaatgggacatttgagtgtggcgcgtgcaggtgcaatgaa
gggcgtgttggtagacattgtgaatgcagcacagatgaagttaacagtga
agacatggatgcttactgcaggaaagaaaacagttcagaaatctgcagta
acaatggagagtgcgtctgcggacagtgtgtttgtaggaagagggataat
acaaatgaaatttattctggcaaattctgcgagtgtgataatttcaactg
tgatagatccaatggcttaatttgtggaggaaatggtgtttgcaagtgtc
gtgtgtgtgagtgcaaccccaactacactggcagtgcatgtgactgttct
ttggatactagtacttgtgaagccagcaacggacagatctgcaatggccg
gggcatctgcgagtgtggtgtctgtaagtgtacagatccgaagtttcaag
ggcaaacgtgtgagatgtgtcagacctgccttggtgtctgtgctgagcat
aaagaatgtgttcagtgcagagccttcaataaaggagaaaagaaagacac
atgcacacaggaatgttcctattttaacattaccaaggtagaaagtcggg
acaaattaccccagccggtccaacctgatcctgtgtcccattgtaaggag
aaggatgttgacgactgttggttctattttacgtattcagtgaatgggaa
caacgaggtcatggttcatgttgtggagaatccagagtgtcccactggtc
cagacatcattccaattgtagctggtgtggttgctggaattgttcttatt
ggccttgcattactgctgatatggaagcttttaatgataattcatgacag
aagggagtttgctaaatttgaaaaggagaaaatgaatgccaaatgggaca
cgggtgaaaatcctatttataagagtgccgtaacaactgtggtcaatccg
aagtatgagggaaaatgagtactgcccgtgcaaatcccacaacactgaat
gcaaagtagcaatttccatagtcacagttaggtagctttagggcaatatt
gccatggttttactcatgtgcaggttttgaaaatgtacaatatgtataat
ttttaaaatgttttattattttgaaaataatgttgtaattcatgccaggg
actgacaaaagacttgagacaggatggttactcttgtcagctaaggtcac
attgtgcctttttgaccttttcttcctggactattgaaatcaagcttatt
ggattaagtgatatttctatagcgattgaaagggcaatagttaaagtaat
gagcatgatgagagtttctgttaatcatgtattaaaactgatttttagct
ttacaaatatgtcagtttgcagttatgcagaatccaaagtaaatgtcctg
ctagctagttaaggattgttttaaatctgttattttgctatttgcctgtt
agacatgactgatgacatatctgaaagacaagtatgttgagagttgctgg
tgtaaaatacgtttgaaatagttgatctacaaaggccatgggaaaaattc
agagagttaggaaggaaaaaccaatagctttaaaacctgtgtgccatttt
aagagttacttaatgtttggtaacttttatgccttcactttacaaattca
agccttagataaaagaaccgagcaattttctgctaaaaagtccttgattt
agcactatttacatacaggccatactttacaaagtatttgctgaatgggg
accttttgagttgaatttattttattatttttattttgtttaatgtctgg
tgctttctgtcacctcttctaatcttttaatgtatttgtttgcaattttg
gggtaagactttttttatgagtactttttctttgaagttttagcggtcaa
tttgcctttttaatgaacatgtgaagttatactgtggctatgcaacagct
ctcacctacgcgagtcttactttgagttagtgccataacagaccactgta
tgtttacttctcaccatttgagttgcccatcttgtttcacactagtcaca

ttcttgttttaagtgcctttagttttaacagttcactttttacagtgcta
tttactgaagttatttattaaatatgcctaaaatacttaaatcggatgtc
ttgactctgatgtattttatcaggttgtgtgcatgaaatttttatagatt
aaagaagttgaggaaaagcaaaaaaaaaa

>NM_002473 4
gagggcggggcgggaaggcggcgaggagccgagctgggtgcggtgaggcg
cgcagatcaccgcggttcctgggcagggcacggaaggctaagcaaggctg
acctgctgcagctcccgcctcgtgcgctcgccccacccggccgccgcccg
agcgctcgagaaagtcctctcgggagaagcagcgcctgttcccggggcag
atccaggttcaggtcctggctataagtcaccatggcacagcaagctgccg
ataagtatctctatgtggataaaaacttcatcaacaatccgctggcccag
gccgactgggctgccaagaagctggtatgggtgccttccgacaagagtgg
ctttgagccagccagcctcaaggaggaggtgggcgaagaggccatcgtgg
agctggtggagaatgggaagaaggtgaaggtgaacaaggatgacatccag
aagatgaacccgcccaagttctccaaggtggaggacatggcagagctcac
gtgcctcaacgaagcctcggtgctgcacaacctcaaggagcgttactact
cagggctcatctacacctattcaggcctgttctgtgtggtcatcaatcct
tacaagaacctgcccatctactctgaagagattgtggaaatgtacaaggg
caagaagaggcacgagatgccccctcacatctatgccatcacagacaccg
cctacaggagtatgatgcaagaccgagaagatcaatccatcttgtgcact
ggtgaatctggagctggcaagacggagaacaccaagaaggtcatccagta
tctggcgtacgtggcgtcctcgcacaagagcaagaaggaccagggcgagc
tggagcggcagctgctgcaggccaaccccatcctggaggccttcgggaac
gccaagaccgtgaagaatgacaactcctcccgcttcggcaaattcattcg
catcaactttgatgtcaatggctacattgttggagccaacattgagactt
atcttttggagaaatctcgtgctatccgccaagccaaggaagaacggacc
ttccacatcttctattatctcctgtctggggctggagagcacctgaagac
cgatctcctgttggagccgtacaacaaataccgcttcctgtccaatggac
acgtcaccatccccgggcagcaggacaaggacatgttccaggagaccatg
gaggccatgaggattatgggcatcccagaagaggagcaaatgggcctgct
gcgggtcatctcaggggttcttcagctcggcaacatcgtcttcaagaagg
agcggaacactgaccaggcgtccatgcccgacaacacagctgcccaaaag
gtgtcccatctcttgggtatcaatgtgaccgatttcaccagaggaatcct
caccccgcgcatcaaggtgggacgggattacgtccagaaggcgcagacta
aagagcaggctgactttgccatcgaggccttggccaaggcgacctatgag
cggatgttccgctggctggtgctgcgcatcaacaaggctctggacaagac
caagaggcagggcgcctccttcatcgggatcctggacattgccggcttcg
agatctttgatctgaactcgtttgagcagctgtgcatcaattacaccaat
gagaagctgcagcagctcttcaaccacaccatgttcatcctggagcagga
ggagtaccagcgcgagggcatcgagtggaacttcatcgactttggcctcg
acctgcagccctgcatcgacctcattgagaagccagcaggcccccccgggc
attctggccctgctggacgaggagtgctggttccccaaagccaccgacaa
gagcttcgtggagaaggtgatgcaggagcagggcacccaccccaagttcc
agaagcccaagcagctgaaggacaaagctgatttctgcattatccactat

gccggcaaggtggattacaaagctgacgagtggctgatgaagaacatgga
tcccctgaatgacaacatcgccacactgctccaccagtcctctgacaagt
ttgtctcggagctgtggaaggatgtggaccgcatcatcggcctggaccag
gtggccggcatgtcggagaccgcactgcccggggccttcaagacgcggaa
gggcatgttccgcactgtggggcagctttacaaggagcagctggccaagc
tgatggctacgctgaggaacacgaaccccaactttgtccgctgcatcatc
cccaaccacgagaagaaggccggcaagctggacccgcatctcgtgctgga
ccagctgcgctgcaacggtgttctcgagggcatccgtatctgccgccagg
gcttccccaacagggtggtcttccaggagtttcggcagagatatgagatc
ctgactccaaactccattcccaagggtttcatggacgggaagcaggcgtg
cgtgctcatgataaaagccctggagctcgacagcaatctgtaccgcattg
gccagagcaaagtcttcttccgtgccggtgtgctggcccacctggaggag
gagcgagacctgaagatcaccgacgtcatcatagggttccaggcctgctg
caggggctacctggccaggaaagcatttgccaagcggcagcagcagctta
ccgccatgaaggtcctccagcggaactgcgctgcctacctgaagctgcgg
aactggcagtggtggcggctcttcaccaaggtcaagccgctgctgcaggt
gagccggcaggaggaggagatgatggccaaggaggaggagctggtgaagg
tcagagagaagcagctggctgcggagaacaggctcacggagatggagacg
ctgcagtctcagctcatggcagagaaattgcagctgcaggagcagctcca
ggcagaaaccgagctgtgtgccgaggctgaggagctccgggcccgcctga
ccgccaagaagcaggaattagaagagatctgccatgacctagaggccagg
gtggaggaggaggaggagcgctgccagcacctgcaggcggagaagaagaa
gatgcagcagaacatccaggagcttgaggagcagctggaggaggaggaga
gcgcccggcagaagctgcagctggagaaggtgaccaccgaggcgaagctg
aaaaagctggaggaggagcagatcatcctggaggaccagaactgcaagct
ggccaaggaaaagaaactgctggaagacagaatagctgagttcaccacca
acctcacagaagaggaggagaaatctaagagcctcgccaagctcaagaac
aagcatgaggcaatgatcactgacttggaagagcgcctccgcagggagga
gaagcagcgacaggagctggagaagacccgccggaagctggagggagact
ccacagacctcagcgaccagatcgccgagctccaggcccagatcgcggag
ctcaagatgcagctggccaagaaagaggaggagctccaggccgccctggc
cagagtggaagaggaagctgcccagaagaacatggccctcaagaagatcc
gggagctggaatctcagatctctgaactccaggaagacctggagtctgag
cgtgcttccaggaataaagctgagaagcagaaacgggaccttggggaaga
gctagaggctctgaaaacagagttggaggacacgctggattccacagctg
cccagcaggagctcaggtcaaaacgtgagcaggaggtgaacatcctgaag
aagaccctggaggaggaggccaagacccacgaggcccagatccaggagat
gaggcagaagcactcacaggccgtggaggagctggcggagcagctggagc
agacgaagcgggtgaaagcaaacctcgagaaggcaaagcagactctggag
aacgagcgggggggagctggccaacgaggtgaaggtgctgctgcagggcaa
aggggactcggagcacaagcgcaagaaagtggaggcgcagctgcaggagc
tgcaggtcaagttcaacgagggagagcgcgtgcgcacagagctggccgac
aaggtcaccaagctgcaggtggagctggacaacgtgaccgggcttctcag
ccagtccgacagcaagtccagcaagctcaccaaggacttctccgcgctgg
agtcccagctgcaggacactcaggagctgctgcaggaggagaaccggcag
aagctgagcctgagcaccaagctcaagcaggtggaggacgagaagaattc

cttccgggagcagctggaggaggaggaggaggccaagcacaacctggaga
agcagatcgccaccctccatgcccaggtggccgacatgaaaaagaagatg
gaggacagtgtggggtgcctggaaactgctgaggaggtgaagaggaagct
ccagaaggacctggagggcctgagccagcggcacgaggagaaggtggccg
cctacgacaagctggagaagaccaagacgcggctgcagcaggagctggac
gacctgctggtggacctggaccaccagcgccagagcgcgtgcaacctgga
gaagaagcagaagaagtttgaccagctcctggcggaggagaagaccatct
ctgccaagtatgcagaggagcgcgaccgggctgaggcggaggcccgagag
aaggagaccaaggctctgtcgctggcccgggccctggaggaagccatgga
gcagaaggcggagctggagcggctcaacaagcagttccgcacggagatgg
aggaccttatgagctccaaggatgatgtgggcaagagtgtccacgagctg
gagaagtccaagcgggccctagagcagcaggtggaggagatgaagacgca
gctggaagagctggaggacgagctgcaggccaccgaagatgccaagctgc
ggttggaggtcaacctgcaggccatgaaggcccagttcgagcgggacctg
cagggccgggacgagcagagcgaggagaagaagaagcagctggtcagaca
ggtgcgggagatggaggcagagctggaggacgagaggaagcagcgctcga
tggcagtggccgcccggaagaagctggagatggacctgaaggacctggag
gcgcacatcgactcggccaacaagaaccgggacgaagccatcaaacagct
gcggaagctgcaggcccagatgaaggactgcatgcgcgagctggatgaca
cccgcgcctctcgtgaggagatcctggcccaggccaaagagaacgagaag
aagctgaagagcatggaggccgagatgatccagttgcaggaggaactggc
agccgcggagcgtgccaagcgccaggcccagcaggagcgggatgagctgg
ctgacgagatcgccaacagcagcggcaaaggagccctggcgttagaggag
aagcggcgtctggaggcccgcatcgcccagctggaggaggagctggagga
ggagcagggcaacacggagctgatcaacgaccggctgaagaaggccaacc
tgcagatcgaccagatcaacaccgacctgaacctggagcgcagccacgcc
cagaagaacgagaatgctcggcagcagctggaacgccagaacaaggagct
taaggtcaagctgcaggagatggagggcactgtcaagtccaagtacaagg
cctccatcaccgccctcgaggccaagattgcacagctggaggagcagctg
gacaacgagaccaaggagcgccaggcagcctgcaaacaggtgcgtcggac
cgagaagaagctgaaggatgtgctgctgcaggtggatgacgagcggagga
acgccgagcagtacaaggaccaggccgacaaggcatctacccgcctgaag
cagctcaagcggcagctggaggaggccgaagaggaggcccagcgggccaa
cgcctcccgccggaaactgcagcgcgagctggaggacgccactgagacgg
ccgatgccatgaaccgcgaagtcagctccctaaagaacaagctcaggcgc
ggggacctgccgtttgtcgtgccccgccgaatggcccggaaaggcgccgg
ggatggctccgacgaagaggtagatggcaaagcggatggggctgaggcca
aacctgccgaataagcctcttctcctgcagcctgagatggatggacagac
agacaccacagcctccccttcccagaccccgcagcacgcctctccccacc
ttcttgggactgctgtgaacatgcctcctcctgccctccgccccgtcccc
ccatcccgtttccctccaggtgttgttgagggcatttggcttcctctgct
gcatccccttccagctccctcccctgctcagaatctgataccaaagagac
agggcccgggcccaggcagagagcgaccagcaggctcctcagccctctct
tgccaaaaagcacaagatgttgaggcgagcagggcaggcccccggggagg
ggccagagttttctatgaatctatttttcttcagactgaggccttttggt
agtcggagcccccgcagtcgtcagcctccctgacgtctgccaccagcgcc

cccactcctcctcctttctttgctgtttgcaatcacacgtggtgacctca
cacacctctgccccttgggcctcccactcccatggctctgggcggtccag
aaggagcaggccctgggcctccacctctgtgcagggcacagaaggctggg
gtggggggaggagtggattcctccccaccctgtcccaggcagcgccactg
tccgctgtctccctcctgattctaaaatgtctcaagtgcaatgccccctc
ccctcctttaccgaggacagcctgcctctgccacagcaaggctgtcgggg
tcaagctggaaaggccagcagccttccagtggcttctcccaacactcttg
gggaccaaatatatttaatggttaagggacttgtcccaagtctgacagcc
agagcgttagaggggccagcggccctcccaggcgatcttgtgtctactct
aggactgggcccgagggtggtttacctgcaccgttgactcagtatagttt
aaaaatctgccacctgcacaggtatttttgaaagcaaaataaggttttct
tttttcccctttcttgtaataaatgataaaattccgagtctttctcactg
cctttgtttagaagagagtagctcgtcctcactggtctacactggttgcc
gaatttacttgtattcctaactgttttgtatatgctgcattgagacttac
ggcaagaaggcatttttttttttaaaggaaacaaactctcaaatcatga
agtgatataaaagctgcatatgcctacaaagctctgaattcaggtcccag
ttgctgtcacaaaggagtgagtgaaactcccaccctacccccttttttat
ataataaaagtgccttagcatgtgttgcagctgtcaccactacagtaagc
tggtttacagatgttttccactgagcatcacaataaagagaaccatgtgc
tacga

>NM_002508 2
gggaggcgggaggagagggggctgccaggggcgtccggttacatccccgc
cttcctctgtcctggccgcgggaccgggtttgcgggaccgcagttcggga
acatgttggcctcgagcagccggatccgggctgcgtggacgcgggcgctg
ctgctgccgctgctgctggcggggcctgtgggctgcctgagccgccagga
gctctttcccttcggccccggacagggggacctggagctggaggacgggg
atgacttcgtctctcctgccctggagctgagtggggcgctccgcttctac
gacagatccgacatcgacgcagtctacgtcaccacaaatggcatcattgc
tacgagtgaacccccggccaaagaatcccatcccgggctcttcccaccaa
cattcggtgcagtcgcccctttcctggcggacttggacacgaccgatggc
ctggggaaggtttattatcgagaagacttatccccctccatcactcagcg
agcagcagagtgtgtccacagagggttcccggagatctctttccagccta
gtagcgcggtggttgtcacttgggaatccgtggccccctaccaagggccc
agcagggacccagaccagaaaggcaagagaaacacgttccaggctgttct
agcctcctctgattccagctcctatgccattttcctttatcctgaggatg
gtctgcagttccatacgacattctcaaagaaggaaaacaaccaagttcct
gccgtggttgcattcagtcaaggttcagtgggattcttatggaagagcaa
cggagcttataacatatttgctaatgacagggaatcagttgaaaatttgg
ccaagagtagtaactctgggcagcagggtgtctgggtgtttgagattggg
agtccagccaccaccaatggcgtggtgcctgcagacgtgatcctcggaac
tgaagatggggcagagtatgatgatgaggatgaagattatgacctggcga
ccactcgtctgggcctggaggatgtgggcaccacgcccttctcctacaag
gctctgagaaggggaggtgctgacacatacagtgtgcccagcgtcctctc
cccgcgccgggcagctaccgaaaggccccttggacctcccacagagagaa
ccaggtctttccagttggcagtggagacttttcaccagcagcaccctcag

gtcatagatgtggatgaagttgaggaaacaggagttgttttcagctataa
cacggattcccgccagacgtgtgctaacaacagacaccagtgctcggtgc
acgcagagtgcagggactacgccacgggcttctgctgcagctgtgtcgct
ggctatacgggcaatggcaggcaatgtgttgcagaaggttccccccagcg
agtcaatggcaaggtgaaaggaaggatctttgtggggagcagccaggtcc
ccattgtctttgagaacactgacctccactcttacgtagtaatgaaccac
gggcgctcctacacagccatcagcaccattcccgagaccgttggatattc
tctgcttccactggccccagttggaggcatcattggatggatgtttgcag
tggagcaggacggattcaagaatgggttcagcatcaccgggggtgagttc
actcgccaggctgaggtgaccttcgtggggcacccgggcaatctggtcat
taagcagcggttcagcggcatcgatgagcatgggcacctgaccatcgaca
cggagctggagggccgcgtgccgcagattccgttcggctcctccgtgcac
attgagccctacacggagctgtaccactactccacctcagtgatcacttc
ctcctccacccgggagtacacggtgactgagcccgagcgagatggggcat
ctccttcacgcatctacacttaccagtggcgccagaccatcaccttccag
gaatgcgtccacgatgactcccggccagccctgcccagcacccagcagct
ctcggtggacagcgtgttcgtcctgtacaaccaggaggagaagatcttgc
gctatgctctcagcaactccattgggcctgtgagggaaggctcccctgat
gctcttcagaatccctgctacatcggcactcatgggtgtgacaccaacgc
ggcctgtcgccctggtcccaggacacagttcacctgcgagtgctccatcg
gcttccgaggagacgggcgaacctgctatgatattgatgaatgttcagaa
caaccctcagtgtgtgggagccacacaatctgcaataatcacccaggaac
cttccgctgcgagtgtgtggagggctaccagttttcagatgagggaacgt
gtgtggctgtcgtggaccagcgccccatcaactactgtgaaactggcctt
cataactgcgacataccccagcgggcccagtgtatctacacaggaggctc
ctcctacacctgttcctgcttgccaggcttttctggggatggccaagcct
gccaagatgtagatgaatgccagccaagccgatgtcaccctgacgccttc
tgctacaacactccaggctctttcacgtgccagtgcaaacctggttatca
gggagacggcttccgttgcgtgcccggagaggtggagaaaacccggtgcc
agcacgagcgagaacacattctcggggcagcgggggcgacagacccacag
cgacccattcctccggggctgttcgttcctgagtgcgatgcgcacgggca
ctacgcgcccacccagtgccacggcagcaccggctactgctggtgcgtgg
atcgcgacggccgcgaggtggagggcaccaggaccaggcccgggatgacg
cccccgtgtctgagtacagtggctcccccgattcaccaaggacctgcggt
gcctaccgccgtgatccccttgcctcctgggacccatttactctttgccc
agactgggaagattgagcgcctgcccctggagggaaataccatgaggaag
acagaagcaaaggcgttccttcatgtcccggctaaagtcatcattggact
ggcctttgactgcgtggacaagatggtttactggacggacatcactgagc
cttccattgggagagctagtctacatggtggagagccaaccaccatcatt
agacaagatcttggaagtccagaaggtatcgctgttgatcaccttggccg
caacatcttctggacagactctaacctggatcgaatagaagtggcgaagc
tggacggcacgcagcgccgggtgctctttgagactgacttggtgaatccc
agaggcattgtaacggattccgtgagagggaacctttactggacagactg
gaacagagataaccccaagattgaaacttcctacatggacggcacgaacc
ggaggatccttgtgcaggatgacctgggcttgcccaatggactgaccttc
gatgcgttctcatctcagctctgctgggtggatgcaggcaccaatcgggc

ggaatgcctgaaccccagtcagcccagcagacgcaaggctctcgaagggc
tccagtatccttttgctgtgacgagctacgggaagaatctgtatttcaca
gactggaagatgaattccgtggttgctctcgatcttgcaatttccaagga
gacggatgctttccaaccccacaagcagacccggctgtatggcatcacca
cggccctgtctcagtgtccgcaaggccataactactgctcagtgaacaat
ggcggctgcacccacctatgcttggccaccccagggagcaggacctgccg
ttgccctgacaacaccttgggagttgactgtatcgaacagaaatgaagac
aagagtgccttatttcctttccaagtatttcacagcaacactctacttga
agcaacttggtccagattgaaaagtgtcctctggctgagtggccactagg
cccagacccagcccagcctgagccccaacaacttttccctcactgttccc
caaaacatgcaccctggacttctctaatagaaaagtctccacccctacac
aaggacagaaccctccacccctacccccaaccctcagacagacttataca
cccctgagtgaggattacatgcccatcccagtgtcctaggaccttttccc
aatactagcccccagtggtgaacagaacctcccaaatttgagttgcacc
cttccctgtggccttatgagctcagcctcgctttgaggtacccaccgtcc
tgtcagctccttgacctatgagccggggcctgactaggaaaagttgggag
ttaaggaggaaattagcattccttaatgttttgttttggtgctctgaatt
tcttctttattatagtcctatagttttactcctcagttcctcaccatcat
catcttgtctaagacccccattataatattcatgcgctgctttttcatca
aaacctaccctgtcctagagatctatgggcatttggtggatgataatgag
cagcccctcccagatagaatgtcaatatttgagcagtaggatattggcat
ttgttagttaaaggcttaaatcaaaagaatgtccaatggtaggaatttca
aggtgtaggtcagatatttgagaataggggatttttttgatgtgccttaa
attataccaaagattactaattattcctctttgcccaaaatacttgcatc
caaggttctagtctctgttgctgtgctggtctttagccccactgcttgca
ctgatgtccctccttttcacggagacctatctgaggtacaggatggggct
ggcaccagatgatgtcccaccacagtccctcacctccggcctccacatga
cagaaccaatttacactcaaccatgacctcacccctccttggtttctccc
tcgatctgtggccctttttggatgtattcttatctaacaacacaatccgg
aaagactgaattgaatatttatactaatggttcatatcctttattgctca
atgatctaattaaagggatcattgccacatttcatgtttatatttctaca
atttgtttagaaaacatctcctgaccatatcagtagctcgtgttatcttt
ttatcaactgcttcccagagtcctaaaacaatagaaattttggattgaaa
agttcagcataaggagtttgagtcagtaaaggatgggataaaggagtcga
gatgattcaatgaaaagtatcacaaaaaagagattgatcaacaagagaaa
taaaaaagcccaagaggaagtggtaggggaaggaatttaagaacagcaat
aagtaaaactcttaagtaactccaaaaagaaaatggtacattttgccaaa
gaccacttatacttgagaacatggaagaatttgcctgatactctctttgg
ggaaaagagtctctcctcttttcctcaaaccccagtacactcagcctctc
tgccccaccttctcctgactttgtcctcacttgcttctgcagtacattgg
aacctgaattgaaagaaagtcttccttgaataattggagtttgtcttgag
aggcaaatatagccccaagaatcacaagattcgaggaccatgtaggtctt
ttacgtagcccaaatccataaattagtctcactttttgtatttatcgttt
catattaaaccctctatatcaaatgttcatcatgattttgtatgattttt
ataactattttattcattttattagatttattctaaaattttttaatggt
aaattcttaaactgtggaaaccactgaaggtgcttattaactgttctccc

agatttgtacaagtattggatgattccttgagtttacagctgtacaaata
gtgtggaaaataaacttttttaaaaaagaaaaaaaaaaaaaaaaaaaa
aaa

>NM_002633 2
cctttcccctcccgccggacctgccaggaggtgggctggcgcggagggag
ggccctgtcccctgtccctttaaggaggagggccaaacgccggcctagag
tgcggcgtagcccccacccgccgtgccctcaccccagagcagctgcagcc
tcagccggccgcccctccgccagccaagtccgccgctctgacccccggca
gcaagtcgccaccatggtgaagatcgtgacagttaagacccaggcgtacc
aggaccagaagccgggcacgagcgggctgcggaagcgggtgaaggtgttc
cagagcagcgccaactacgcggagaacttcatccagagtatcatctccac
cgtggagccggcgcagcggcaggaggccacgctggtggtgggcggggacg
gccggttctacatgaaggaggccatccagctcatcgctcgcatcgctgcc
gccaacgggatcggtcgcttggttatcggacagaatggaatcctctccac
ccctgctgtatcctgcatcattagaaaaatcaaagccattggtgggatca
ttctgacagccagtcacaacccaggggggccccaatggagattttggaatc
aaattcaatatttctaatggaggtcctgctccagaagcaataactgataa
aattttccaaatcagcaagacaattgaagaatatgcagtttgccctgacc
tgaaagtagaccttggtgttctgggaaagcagcagtttgacttggaaaat
aagttcaaacccttcacagtggaaattgtggattcggtagaagcttatgc
tacaatgctgagaagcatctttgatttcagtgcactgaaagaactacttt
ctgggccaaaccgactgaagatccgtattgatgctatgcatggagttgtg
ggaccgtatgtaaagaagatcctctgtgaagaactcggtgcccctgcgaa
ctcggcagttaactgcgttcctctggaggactttggaggccaccaccctg
accccaacctcacctatgcagctgacctggtggagaccatgaagtcagga
gagcatgattttggggctgcctttgatggagatggggatcgaaacatgat
tctgggcaagcatgggttctttgtgaacccttcagactctgtggctgtca
ttgctgccaacatcttcagcattccgtatttccagcagactggggtccgc
ggctttgcacggagcatgcccacgagtggtgctctggaccgggtggctag
tgctacaaagattgctttgtatgagaccccaactggctggaagtttttg
ggaatttgatggacgcgagcaaactgtccctttgtggggaggagagcttc
gggaccggttctgaccacatccgtgagaaagatggactgtgggctgtcct
tgcctggctctccatcctagccacccgcaagcagagtgtggaggacattc
tcaaagatcattggcaaaagtatggccggaatttcttcaccaggtatgat
tacgaggaggtggaagctgagggcgcaaacaaaatgatgaaggacttgga
ggccctgatgtttgatcgctcctttgtggggaagcagttctcagcaaatg
acaaagtttacactgtggagaaggccgataactttgaatacagcgaccca
gtggatggaagcatttcaagaaatcagggcttgcgcctcattttcacaga
tggttctcgaatcgtcttccgactgagcggcactgggagtgccggggcca
ccattcggctgtacatcgatagctatgagaaggacgttgccaagattaac
caggacccccaggtcatgttggcccccttatttccattgctctgaaagt
gtcccagctgcaggagaggacgggacgcactgcacccactgtcatcacct
aagaagacaggcctgatgtggtacgtccctccacccccggacccatccaa
gtcatctgattgaagagcatgacagaaacaaaatgtattcaccaagcatt
ttaggatttgactttttcactaaccagttgacgagcagtgcatttacaag

gcactgccaaacaagatgcccttgggagctgtgagggaaagaggacctgc
gggcttagatcaatctcaattccttttcatgccctcctgcattgctgctg
cgtgggtatttgtctccttagccatcaggtacagtttacactacaatgta
agctataggtggagcatcagcagtgagtgaggccattcttcatccttagg
atgtggcaatgaaatgatggtgcaagttcctttctcttttgtgaatcttt
ccccccatttcctgtttacatgtaacccaacaaaatgcaatttctagtgc
cttctgtccaatcagttctttcctctgagtgagacgtacttggctacaga
tttctgccttgttttgcgacattgtcccattcacacagatattttgggat
aataaaggaaaataagctacaaaaaaaaaaaaaaaa

>NM_002742 2
ccctcccctcccgatcctcatccccttgccctcccccagcccagggactt
ttccggaaagtttttattttccgtctgggctctcggagaaagaagctcct
ggctcagcggctgcaaaactttcctgctgccgcgccgccagcccccgccc
tccgctgcccggccctgcgccccgccgagcgatgagcgcccctccggtcc
tgcggccgcccagtccgctgctgcccgtggcggcggcagctgccgcagcg
gccgccgcactggtcccagggtccgggcccgggcccgcgccgttcttggc
tcctgtcgcggccccggtcgggggcatctcgttccatctgcagatcggcc
tgagccgtgagccggtgctgctgctgcaggactcgtccggggactacagc
ctggcgcacgtccgcgagatggcttgctccattgtcgaccagaagttccc
tgaatgtggtttctacggaatgtatgataagatcctgctttttcgccatg
accctacctctgaaaacatccttcagctggtgaaagcggccagtgatatc
caggaaggcgatcttattgaagtggtcttgtcagcttccgccacctttga
agactttcagattcgtccccacgctctctttgttcattcatacagagctc
cagctttctgtgatcactgtggagaaatgctgtgggggctggtacgtcaa
ggtcttaaatgtgaagggtgtggtctgaattaccataagagatgtgcatt
taaaatacccaacaattgcagcggtgtgaggcggagaaggctctcaaacg
tttccctcactggggtcagcaccatccgcacatcatctgctgaactctct
acaagtgcccctgatgagcccttctgcaaaaatcaccatcagagtcgtt
tattggtcgagagaagaggtcaaattctcaatcatacattggacgaccaa
ttcaccttgacaagattttgatgtctaaagttaaagtgccgcacacattt
gtcatccactcctacacccggcccacagtgtgccagtactgcaagaagct
tctgaaggggcttttcaggcagggcttgcagtgcaaagattgcagattca
actgccataaacgttgtgcaccgaaagtaccaaacaactgccttggcgaa
gtgaccattaatggagatttgcttagccctggggcagagtctgatgtggt
catggaagaagggagtgatgacaatgatagtgaaaggaacagtgggctca
tggatgatatggaagaagcaatggtccaagatgcagagatggcaatggca
gagtgccagaacgacagtggcgagatgcaagatccagacccagaccacga
ggacgccaacagaaccatcagtccatcaacaagcaacaatatcccactca
tgagggtagtgcagtctgtcaaacacacgaagaggaaaagcagcacagtc
atgaaagaaggatggatggtccactacaccagcaaggacacgctgcggaa
acggcactattggagattggatagcaaatgtattaccctctttcagaatg
acacaggaagcaggtactacaaggaaattcctttatctgaaattttgtct
ctggaaccagtaaaaacttcagctttaattcctaatggggccaatcctca
ttgtttcgaaatcactacggcaaatgtagtgtattatgtgggagaaaatg
tggtcaatccttccagcccatcaccaaataacagtgttctcaccagtggc

gttggtgcagatgtggccaggatgtgggagatagccatccagcatgccct
tatgcccgtcattcccaagggctcctccgtgggtacaggaaccaacttgc
acagagatatctctgtgagtatttcagtatcaaattgccagattcaagaa
aatgtggacatcagcacagtatatcagatttttcctgatgaagtactggg
ttctggacagtttggaattgtttatggaggaaaacatcgtaaaacaggaa
gagatgtagctattaaaatcattgacaaattacgatttccaacaaaacaa
gaaagccagcttcgtaatgaggttgcaattctacagaaccttcatcaccc
tggtgttgtaaatttggagtgtatgtttgagacgcctgaaagagtgtttg
ttgttatggaaaaactccatggagacatgctggaaatgatcttgtcaagt
gaaaagggcaggttgccagagcacataacgaagtttttaattactcagat
actcgtggctttgcggcaccttcattttaaaaatatcgttcactgtgacc
tcaaaccagaaaatgtgttgctagcctcagctgatccttttcctcaggtg
aaactttgtgattttggttttgcccggatcattggagagaagtctttccg
gaggtcagtggtgggtaccccgcttacctggctcctgaggtcctaagga
acaagggctacaatcgctctctagacatgtggtctgttggggtcatcatc
tatgtaagcctaagcggcacattcccatttaatgaagatgaagacataca
cgaccaaattcagaatgcagctttcatgtatccaccaaatccctggaagg
aaatatctcatgaagccattgatcttatcaacaatttgctgcaagtaaaa
atgagaaagcgctacagtgtggataagaccttgagccacccttggctaca
ggactatcagacctggttagatttgcgagagctggaatgcaaaatcgggg
agcgctacatcacccatgaaagtgatgacctgaggtgggagaagtatgca
ggcgagcaggggctgcagtaccccacacacctgatcaatccaagtgctag
ccacagtgacactcctgagactgaagaaacagaaatgaaagccctcggtg
agcgtgtcagcatcctctgagttccatctcctataatctgtcaaaacact
gtggaactaataaatacatacggtcaggtttaacatttgccttgcagaac
tgccattattttctgtcagatgagaacaaagctgttaaactgttagcact
gttgatgtatctgagttgccaagacaaatcaacagaagcatttgtatttt
gtgtgaccaactgtgttgtattaacaaaagttccctgaaacacgaaactt
gttattgtgaatgattcatgttatatttaatgcattaaacctgtctccac
tgtgcctttgcaaatcagtgtttttcttactggagcttcattttggtaag
agacagaatgtatctgtgaagtagttctgtttggtgtgtcccattggtgt
tgtcattgtaaacaaactcttgaagagtcgattatttccagtgttctatg
aacaactccaaaacccatgtgggaaaaaaatgaatgaggagggtagggaa
taaaatcctaagacacaaatgcatgaacaagttttaatgtatagttttga
atcctttgcctgcctggtgtgcctcagtatatttaaactcaagacaatgc
acctagctgtgcaagacctagtgctcttaagcctaaatgccttagaaatg
taaactgccatatataacagatacatttccctctttcttataatactctg
ttgtactatggaaaatcagctgctcagcaacctttcacctttgtgtattt
ttcaataataaaaaatattcttgtcaaaa

>NM_002819 4
tgcgggcgtctccgccattttgtgagtctataactcggagccgttgggtc
ggttcctgctattccggcgcctccactccgtcccccgcgggtctgctctg
tgtgccatggacggcattgtcccagatatagccgttggtacaaagcgggg
atctgacgagcttttctctacttgtgtcactaacggaccgtttatcatga
gcagcaactcggcttctgcagcaaacggaaatgacagcaagaagttcaaa

ggtgacagccgaagtgcaggcgtcccctctagagtgatccacatccggaa
gctccccatcgacgtcacggagggggaagtcatctccctggggctgccct
ttgggaaggtcaccaacctcctgatgctgaaggggaaaaaccaggccttc
atcgagatgaacacggaggaggctgccaacaccatggtgaactactacac
ctcggtgacccctgtgctgcgcggccagcccatctacatccagttctcca
accacaaggagctgaagaccgacagctctcccaaccaggcgcgggcccag
gcggccctgcaggcggtgaactcggtccagtcggggaacctggccttggc
tgcctcggcggcggccgtggacgcagggatggcgatggccgggcagagcc
ccgtgctcaggatcatcgtggagaacctcttctaccctgtgaccctggat
gtgctgcaccagattttctccaagttcggcacagtgttgaagatcatcac
cttcaccaagaacaaccagttccaggccctgctgcagtatgcggaccccg
tgagcgcccagcacgccaagctgtcgctggacgggcagaacatctacaac
gcctgctgcacgctgcgcatcgacttttccaagctcaccagcctcaacgt
caagtacaacaatgacaagagccgtgactacacacgcccagacctgcctt
ccggggacagccagccctcgctggaccagaccatggccgcggccttcggt
gcacctggtataatctcagcctctccgtatgcaggagctggtttccctcc
cacctttgccattcctcaagctgcaggcctttccgttccgaacgtccacg
gcgccctggccccctggccatcccctcggcggcggcggcagctgcggcg
gcaggtcggatcgccatcccgggcctggcgggggcaggaaattctgtatt
gctggtcagcaacctcaacccagagagagtcacaccccaaagcctcttta
ttcttttcggcgtctacggtgacgtgcagcgcgtgaagatcctgttcaat
aagaaggagaacgccctagtgcagatggcggacggcaaccaggcccagct
ggccatgagccacctgaacgggcacaagctgcacgggaagcccatccgca
tcacgctctcgaagcaccagaacgtgcagctgccccgcgagggccaggag
gaccagggcctgaccaaggactacggcaactcacccctgcaccgcttcaa
gaagccgggctccaagaacttccagaacatattcccgccctcggccacgc
tgcacctctccaacatcccgccctcagtctccgaggaggatctcaaggtc
ctgttttccagcaatgggggcgtcgtcaaaggattcaagttcttccagaa
ggaccgcaagatggcactgatccagatgggctccgtggaggaggcggtcc
aggccctcattgacctgcacaaccacgacctcggggagaaccaccacctg
cgggtctccttctccaagtccaccatctaggggcacaggcccccacggcc
gggccccctggcgacaacttccatcattccagagaaaagccactttaaaa
acagctgaagtgaccttagcagaccagagattttattttttaaagagaa
atcagtttacctgtttttaaaaaaattaaatctagttcaccttgctcacc
ctgcggtgacagggacagctcaggctcttggtgactgtggcagcgggagt
tcccggccctccacacccggggccagaccctcggggccatgccttggtgg
ggcctgtgtcgggcgtggggcctgcaggtgggcgccccgaccacgacttg
gcttccttgtgccttaaaaaacctgccttcctgcagccacacacccaccc
ggggtgtcctggggacccaaggggtgggggggtcacaccagagagaggca
gggggcctggccggctcctgcaggatcatgcagctggggcgcggcggccg
cggctgcgacaccccaaccccagccctctaatcaagtcacgtgattctcc
cttcaccccgcccccagggccttcccttctgcccccaggcgggctccccg
ctgctccagctgcggagctggtcgacataatctctgtattatatactttg
cagttgcagacgtctgtgcctagcaatatttccagttgaccaaatattct
aatctttttttcatttatatgcaaaagaaatagttttaagtaactttttat
agcaagatgatacaatggtatgagtgtaatctaaacttccttgtggtatt

accttgtatgctgttacttttattttattccttgtaattaagtcacaggc
aggacccagtttccagagagcaggcggggccgcccagtgggtcaggcaca
gggagccccggtcctatcttagagcccctgagcttcagggaaggggcggg
cgtgtcgccgcctctggcatcgcctccggttgccttacaccacgccttca
cctgcagtcgcctagaaaacttgctctcaaacttcagggtttttcttcc
ttcaaattttggaccaaagtctcatttctgtgttttgcctgcctctgatg
ctgggacccggaaggcgggcgctcctcctgtcttctctgtgctctttcta
ccgcccccgcgtcctgtcccgggggctctcctaggatcccctttccgtaa
aagcgtgtaacaagggtgtaaatatttataattttttatacctgttgtga
gacccgaggggcggcggcgcggtttttatggtgacacaaatgtatattt
tgctaacagcaattccaggctcagtattgtgaccgcggagccacaggga
ccccacgcacattccgttgccttacccgatggcttgtgacgcggagagaa
ccgattaaaaccgtttgagaaactcctcccttgtctagccctgtgttcgc
tgtggacgctgtagaggcaggttggccagtctgtacctggacttcgaata
aatcttctgtatcctcgctccgttccgccttaaaaaaaaaaaaaaaaaaa
aaaaaaaaaaaaaaaaaaaaaaaaaaaaaaaaaaaaaaa

>NM_002835 3
ggggaacgagctggggaagacggagcgggctctgtgccgggcgggcgggc
ggcgggggggccagcgaccgcagccgggggggacgcgggaggatggagcaa
gtggagatcctgaggaaattcatccagagggtccaggccatgaagagtcc
tgaccacaatggggaggacaacttcgcccgggacttcatgcggttaagaa
gattgtctaccaaatatagaacagaaaagatatatcccacagccactgga
gaaaaagaagaaaatgttaaaaagaacagatacaaggacatactgccatt
tgatcacagccgagttaaattgacattaaagactccttcacaagattcag
actatatcaatgcaaattttataaagggcgtctatgggccaaaagcatat
gtagcaactcaaggacctttagcaaatacagtaatagatttttggaggat
gatatgggagtataatgttgtgatcattgtaatggcctgccgagaatttg
agatgggaaggaaaaaatgtgagcgctattggcctttgtatggagaagac
cccataacgtttgcaccatttaaaatttcttgtgaggatgaacaagcaag
aacagactacttcatcaggacactcttacttgaatttcaaaatgaatctc
gtaggctgtatcagtttcattatgtgaactggccagaccatgatgttcct
tcatcatttgattctattctggacatgataagcttaatgaggaaatatca
agaacatgaagatgttcctatttgtattcattgcagtgcaggctgtggaa
gaacaggtgccatttgtgccatagattatacgtggaatttactaaaagct
gggaaaataccagaggaatttaatgtatttaatttaatacaagaaatgag
aacacaaaggcattctgcagtacaaacaaaggagcaatatgaacttgttc
atagagctattgcccaactgtttgaaaaacagctacaactatatgaaatt
catggagctcagaaaattgctgatggagtgaatgaaattaacactgaaaa
catggtcagctccatagagcctgaaaaacaagattctcctcctccaaaac
caccaaggacccgcagttgccttgttgaaggggatgctaaagaagaaata
ctgcagccaccggaacctcatccagtgccacccatcttgacaccttctcc
cccttcagcttttccaacagtcactactgtgtggcaggacaatgatagat
accatccaaagccagtgttgcatatggtttcatcagaacaacattcagca
gacctcaacagaaactatagtaaatcaacagaacttccagggaaaaatga
atcaacaattgaacagatagataaaaaattggaacgaaatttaagttttg

agattaagaaggtccctctccaagagggaccaaaaagttttgatgggaac
acacttttgaataggggacatgcaattaaaattaaatctgcttcaccttg
tatagctgataaaatctctaagccacaggaattaagttcagatctaaatg
tcggtgatacttcccagaattcttgtgtggactgcagtgtaacacaatca
aacaaagtttcagttactccaccagaagaatcccagaattcagacacacc
tccaaggccagaccgcttgcctcttgatgagaaaggacatgtaacgtggt
catttcatggacctgaaaatgccatacccatacctgatttatctgaaggc
aattcctcagatatcaactatcaaactaggaaaactgtgagtttaacacc
aagtcctacaacacaagttgaaacacctgatcttgtggatcatgataaca
cttcaccactcttcagaacacccctcagttttactaatccacttcactct
gatgactcagactcagatgaaagaaactctgatggtgctgtgacccagaa
taaaactaatatttcaacagcaagtgccacagtttctgctgccactagta
ctgaaagcatttctactaggaaagtattgccaatgtccattgctagacat
aatatagcaggaacaacacattcaggtgctgaaaaagatgttgatgttag
tgaagattcacctcctcccctacctgaaagaactcctgaatcgtttgtgt
tagcaagtgaacataatacacctgtaagatcggaatggagtgaacttcaa
agtcaggaacgatctgaacaaaaaaagtctgaaggcttgataacctctga
aaatgagaaatgtgatcatccagcgggaggtattcactatgaaatgtgca
tagaatgtccacctactttcagtgacaagagagaacaaatatcagaaaat
ccaacagaagccacagatattggttttggtaatcgatgtggaaaacccaa
aggaccaagagatccaccttcagaatggacatgattcagggagctagaag
acactttaagttatactggaaaattcaggtgccactgaaagccagattta
tagtattccatctttaatatgtgggactaacagcagtgtagattgttacc
ttaatattttttgctgggaccatctacctgccttatactacacttaggaa
aaagtattacatatggtttattttgaaacttcaagtattattgccttaat
gtctcttaaccctgttacacgctgcttgtagacatgttaatatagtaata
cctttatgatatattgagtttaaggactactctttttctgttttatcatg
tatgcattattttgtatatgtacagggcaagtaggtatataatttgataa
agttgcaattgaaatattattaacagaagatgtaagaaatttctgcatgg
tctaaatctttgtgtactttatttgtaaattatttgccctggagttttag
aaaatagtttctgaattttaaacttgctggattcatgcagccagctttgc
aggttatcagagatcaaagattgtaataataattttgtaaattgtaagca
aaaagttatttttatattatatacagtctaattgttcatcctaattgttc
ctgttttcatctagtcagagattcagtaagtgccttggaacaatattgaa
ttctcttagcttgtgtgtgtttctttaatatttgaactcaagtgggatta
gaagactatcaaaatacatgtatgtttcaggatatttgacctgtcattaa
aaaaaacaaacagttttacagtgccaaaaaaaaaaaaaaa

>NM_003051 3
agagggcgcgcgcggctgaaagcgtgtggaggcgcgggctgcagttcgga
tgtctgtgtggcggggagggggcggcggccgggagagacgactccgcccc
ctgcgcgcatgctccggccccggcgggttataaggcagcctcgctggccc
ggccagacaaagtggtgagctgcgacgtgactggctagctgcgtgggtac
tggaacaagcaaacgaggcagcgagcgaaggacgggagccggaccctggg
ccccgtggaactccagcctgcgccaccacgtcacgcacacgctcggcgct
gcgatccgcgcatataacgatatttggatttgacctgcattttggaattt

atctacacttaaaatgccaccagcagttggaggtccagttggatacaccc
ccccagatggaggctggggctgggcagtggtaattggagctttcatttcc
atcggcttctcttatgcatttcccaaatcaattactgtcttcttcaaaga
gattgaaggtatattccatgccaccaccagcgaagtgtcatggatatcct
ccataatgttggctgtcatgtatggtggaggtcctatcagcagtatcctg
gtgaataaatatggaagtcgtatagtcatgattgttggtggctgcttgtc
aggctgtggcttgattgcagcttctttctgtaacaccgtacagcaactat
acgtctgtattggagtcattggaggtcttgggcttgccttcaacttgaat
ccagctctgaccatgattggcaagtatttctacaagaggcgaccattggc
caacggactggccatggcaggcagccctgtgttcctctgtactctggccc
ccctcaatcaggttttcttcggtatctttggatggagaggaagctttcta
attcttgggggcttgctactaaactgctgtgttgctggagccctcatgcg
accaatcgggcccaagccaaccaaggcagggaaagataagtctaaagcat
cccttgagaaagctggaaaatctggtgtgaaaaaagatctgcatgatgca
aatacagatcttattggaagacaccctaaacaagagaaacgatcagtctt
ccaaacaattaatcagttcctggacttaaccctattcacccacagaggct
ttttgctatacctctctggaaatgtgatcatgtttttggactctttgca
cctttggtgtttcttagtagttatgggaagagtcagcattattctagtga
gaagtctgccttccttctttccattctggcttttgttgacatggtagccc
gaccatctatgggacttgtagccaacacaaagccaataagacctcgaatt
cagtatttctttgcggcttccgttgttgcaaatggagtgtgtcatatgct
agcacctttatccactacctatgttggattctgtgtctatgcgggattct
ttggatttgccttcgggtggctcagctccgtattgtttgaaacattgatg
gaccttgttggaccccagaggttctccagcgctgtgggattggtgaccat
tgtggaatgctgtcctgtcctcctggggccaccacttttaggtcggctca
atgacatgtatggagactacaaatacacatactgggcatgtggcgtcgtc
ctaattatttcaggtatctatctcttcattggcatgggcatcaattatcg
acttttggcaaaagaacagaaagcaaacgagcagaaaaaggaaagtaaag
aggaagagaccagtatagatgttgctgggaagccaaatgaagttaccaaa
gcagcagaatctccggaccagaaagacacagatggagggcccaaggagga
ggaaagtccagtctgaatccatggggctgaagggtaaattgagcagttca
tgacccaggatatctgaaaatattctactggcctgtaatctaccagtggt
gctcaatgcaaatagtagacatttgtgtggaaatcataccagttgttcat
tgatgggatttttgtttgactccttaccaatagcctgaatttgaggaggg
aatgattggtagcaaaggatgggggaaagaagtaggttctgttttgtttt
gttttaatcttagcttttaatagtgtcataaagattataatatgtgcctt
aagttttagtctttagaactctagagagccttaacttcttaaaccatttt
tgctgaattcatctatttcgagtgttgtgttaaaaggaaaaataacaact
aacttgtttgaggcaaatctaaaatttaaaattaatcttgcttcattgtt
acatgtaatatatttcagacattttcactggaagatttatgaacagaaat
attggttgaaagttagagattttacaaaatgctgacaaaaatattttcct
agcatcagtagatttctggcatatgtttctgctagctatatatttaggaa
attcaaagcataaaactttggcaacatcttggctgttctagacacagtgt
acttgtcaacccctctcaggtaccttttcttgggatgcttattagaagcc
aagtaaagtgcttaaggtttgttttcattaaattagctatttctgctccc
ctgttcaaagatgcattttgagtgtttatagatcactgcccttttgaaa

tcacctggtattatttttcttactggaaaagttagtattaaaatctacag
aactacatatttgtgcctccttggtaaatacaacacatctaattaaatgt
agacagatatttcaaacatcagctgaattcacttaagtttttccaaaacc
tcagttaaactgtgaagctattggaatttttttttcctggaatttttccc
ctttgattcacagtggtcccatttatatctgcttctagcttagtgctatg
tgtgagatatgtgtgtgtttggtgtttttgttttttgttttttttttt
taaggtttgcaaattaaaaagggccagaaaaatttggcaccaggcaaacg
aataaagataggattgggaaagaagttgctaagtgtgcttagttttaata
agtaattccttctctttttcagagaaggccttacagaaaattgttgtgc
ttagaattgctggatgcatttttaccctccacacaaacctaaaaattttg
tgacccctttcacttacctgaaaagtagagaaatggattcagtataagga
taaggagggaaggtggaccagaatgaaaactgtaaatattttttaacct
aatatcacttaaatcgaggcagaaagatacagacattcaatgaattatat
tcaatgcatttaaaataccactgtaattgacagagtaaaagtatagatac
aaaaccttgtgtaagaggctgacttttccaaataaacatttttaagaaa
acatttcttctcccaaatgtctattttcttgaggaaaatattgctgtgtc
ttcattttcattaccaggtttcattttgggccttgctaaattgattgaat
taaatcctccagcttttgaaccttgatatttgtgtatatgatttattttc
atttgaatttctcctttcctcttctttgctgtaaggcaaggaggaggga
attttaaaaccatcttatttgaactgagagcatccagagcagttaacctt
aaggaaacaatgaaaaactccctttgtatgcctgggcatcatggcagata
gaggaagagtgttagaggagaaaactgctgctgagagtattggcaggctt
ggcctcagtttggactctgtaattttctttggacccaagtctgtaacctc
tgcgtacttcttctctcttaccttctataaaaatgaggattactgttggt
gagggaataaggaatgtaagtaaaggaaatctgaaaaaataaaagtaaag
caagtataaaaaaaaaaaaaaaaaaa

>NM_003068 4
aaaacgggctcagttcgtaaaggagccgggtgacttcagaggcgccggcc
cgtccgtctgccgcacctgagcacggcccctgcccgagcctggcccgccg
cgatgctgtagggaccgccgtgtcctcccgccggaccgttatccgcgccg
ggcgcccgccagacccgctggcaagatgccgcgctccttcctggtcaaga
agcatttcaacgcctccaaaaagccaaactacagcgaactggacacacat
acagtgattatttccccgtatctctatgagagttactccatgcctgtcat
accacaaccagagatcctcagctcaggagcatacagccccatcactgtgt
ggactaccgctgctccattccacgcccagctacccaatggcctctctcct
ctttccggatactcctcatctttggggcgagtgagtccccctcctccatc
tgacacctcctccaaggaccacagtggctcagaaagccccattagtgatg
aagaggaaagactacagtccaagctttcagacccccatgccattgaagct
gaaaagtttcagtgcaatttatgcaataagacctattcaactttttctgg
gctggccaaacataagcagctgcactgcgatgcccagtctagaaaatctt
tcagctgtaaatactgtgacaaggaatatgtgagcctgggcgccctgaag
atgcatattcggacccacacattaccttgtgtttgcaagatctgcggcaa
ggcgttttccagaccctggttgcttcaaggacacattagaactcacacgg
gggagaagcctttttcttgccctcactgcaacagagcatttgcagacagg
tcaaatctgagggctcatctgcagacccattctgatgtaaagaaatacca

gtgcaaaaactgctccaaaaccttctccagaatgtctctcctgcacaaac
atgaggaatctggctgctgtgtagcacactgagtgacgcaatcaatgttt
actcgaacagaatgcatttcttcactccgaagccaaatgacaaataaagt
ccaaaggcattttctcctgtgctgaccaaccaaataatatgtatagacac
acacacatatgcacacacacacacacacacccacagagagagagctgcaa
gagcatggaattcatgtgtttaaagataatcctttccatgtgaagtttaa
aattactatatatttgctgatggctagattgagagaataaaagacagtaa
cctttctcttcaaagataaaatgaaaagcacattgcatcttttcttccta
aaaaaatgcaaagatttacattgctgccaaatcatttcaactgaaaagaa
cagtattgctttgtaatagagtctgtaataggatttcccataggaagaga
tctgccagacgcgaactcaggtgccttaaaaagtattccaagtttactcc
attacatgtcggttgtctggttgccattgttgaactaaagcctttttttg
attacctgtagtgctttaaagtatatttttaaaagggaggaaaaaaataa
caagaacaaaacacaggagaatgtattaaaagtatttttgttttgttttg
tttttgccaattaacagtatgtgccttgggggaggagggaaagattagct
ttgaacattcctggcgcatgctccattgtcttactattttaaaacatttt
aataatttttgaaaattaattaaagatgggaataagtgcaaaagaggatt
cttacaaattcattaatgtacttaaactatttcaaatgcataccacaaat
gcaataatacaataccccttccaagtgcctttttaaattgtatagttgat
gagtcaatgtaaatttgtgtttatttttatatgattgaatgagttctgta
tgaaactgagatgttgtctatagctatgtctataaacaacctgaagactt
gtgaaatcaatgtttctttttttaaaaaacaattttcaagtttttttttaca
ataaacagttttgatttaaaatctcgtttgtatactattttcagagactt
tacttgcttcatgattagtaccaaaccactgtacaaagaattgtttgtta
acaagaaaaaaa

>NM_003870 3
ggaccccggcaagcccgcgcacttggcaggagctgtagctaccgccgtcc
gcgcctccaaggtttcacggcttcctcagcagagactcgggctcgtccgc
catgtccgccgcagacgaggttgacgggctgggcgtggcccggccgcact
atggctctgtcctggataatgaaagacttactgcagaggagatggatgaa
aggagacgtcagaacgtggcttatgagtacctttgtcatttggaagaagc
gaagaggtggatggaagcatgcctaggggaagatctgcctcccaccacag
aactggaggaggggcttaggaatggggtctaccttgccaaactggggaac
ttcttctctcccaaagtagtgtccctgaaaaaaatctatgatcgagaaca
gaccagatacaaggcgactggcctccactttagacacactgataatgtga
ttcagtggttgaatgccatggatgagattggattgcctaagatttttttac
ccagaaactacagatatctatgatcgaaagaacatgccaagatgtatcta
ctgtatccatgcactcagtttgtacctgttcaagctaggcctggcccctc
agattcaagacctatatggaaaggttgacttcacagaagaagaaatcaac
aacatgaagactgagttggagaagtatggcatccagatgcctgcctttag
caagattgggggcatcttggctaatgaactgtcagtggatgaagccgcat
tacatgctgctgttattgctattaatgaagctattgaccgtagaattcca
gccgacacatttgcagctttgaaaaatccgaatgccatgcttgtaaatct
tgaagagcccttggcatccacttaccaggatatactttaccaggctaagc
aggacaaaatgacaaatgctaaaaacaggacagaaaactcagagagagaa

agagatgtttatgaggagctgctcacgcaagctgaaattcaaggcaatat
aaacaaagtcaatacattttctgcattagcaaatatcgacctggctttag
aacaaggagatgcactggccttgttcagggctctgcagtcaccagccctg
gggcttcgaggactgcagcaacagaatagcgactggtacttgaagcagct
cctgagtgataaacagcagaagagacagagtggtcagactgaccccctgc
agaaggaggagctgcagtctggagtggatgctgcaaacagtgctgcccag
caatatcagagaagattggcagcagtagcactgattaatgctgcaatcca
gaagggtgttgctgagaagactgttttggaactgatgaatcccgaagccc
agctgccccaggtgtatccatttgccgccgatctctatcagaaggagctg
gctaccctgcagcgacaaagtcctgaacataatctcacccacccagagct
ctctgtcgcagtggagatgttgtcatcggtggccctgatcaacagggcat
tggaatcaggagatgtgaatacagtgtggaagcaattgagcagttcagtt
actggtcttaccaatattgaggaagaaaactgtcagaggtatctcgatga
gttgatgaaactgaaggctcaggcacatgcagagaataatgaattcatta
catggaatgatatccaagcttgcgtggaccatgtgaacctggtggtgcaa
gaggaacatgagaggattttagccattggtttaattaatgaagccctgga
tgaaggtgatgcccaaaagactctgcaggccctacagattcctgcagcta
aacttgagggagtccttgcagaagtggcccagcattaccaagacacgctg
attagagcgaagagagagaaagcccaggaaatccaggatgagtcagctgt
gttatggttggatgaaattcaaggtggaatctggcagtccaacaaagaca
cccaagaagcacagaagtttgccttaggaatctttgccattaatgaggca
gtagaaagtggtgatgttggcaaaacactgagtgcccttcgctcccctga
tgttggcttgtatggagtcatccctgagtgtggtgaaacttaccacagtg
atcttgctgaagccaagaagaaaaaactggcagtaggagataataacagc
aagtgggtgaagcactgggtaaaaggtggatattattattaccacaatct
ggagacccaggaaggaggatgggatgaacctccaaattttgtgcaaaatt
ctatgcagctttctcgggaggagatccagagttctatctctggggtgact
gccgcatataaccgagaacagctgtggctggccaatgaaggcctgatcac
caggctgcaggctcgctgccgtggatacttagttcgacaggaattccgat
ccaggatgaatttcctgaagaaacaaatccctgccatcacctgcattcag
tcacagtggagaggatacaagcagaagaaggcatatcaagatcggttagc
ttacctgcgctcccacaaagatgaagttgtaaagattcagtccctggcaa
ggatgcaccaagctcgaaagcgctatcgagatcgcctgcagtacttccgg
gaccatataaatgacattatcaaaatccaggcttttattcgggcaaacaa
agctcgggatgactacaagactctcatcaatgctgaggatcctcctatgg
ttgtggtccgaaaatttgtccacctgctggaccaaagtgaccaggatttt
caggaggagcttgaccttatgaagatgcgggaagaggttatcaccctcat
tcgttctaaccagcagctggagaatgacctcaatctcatggatatcaaaa
ttggactgctagtgaaaaataagattacgttgcaggatgtggtttcccac
agtaaaaaacttaccaaaaaaaataaggaacagttgtctgatatgatgat
gataaataaacagaagggaggtctcaaggctttgagcaaggagaagagag
agaagttggaagcttaccagcacctgttttatttattgcaaaccaatccc
acctatctggccaagctcatttttcagatgccccagaacaagtccaccaa
gttcatggactctgtaatcttcacactctacaactacgcgtccaaccagc
gagaggagtacctgctcctgcggctctttaagacagcactccaagaggaa
atcaagtcgaaggtagatcagattcaagagattgtgacaggaaatcctac

ggttattaaaatggttgtaagtttcaaccgtggtgcccgtggccagaatg
ccctgagacagatcttggccccagtcgtgaaggaaattatggatgacaaa
tctctcaacatcaaaactgaccctgtggatatttacaaatcttgggttaa
tcagatggagtctcagacaggagaggcaagcaaactgccctatgatgtga
cccctgagcaggcgctagctcatgaagaagtgaagacacggctagacagc
tccatcaggaacatgcgggctgtgacagacaagtttctctcagccattgt
cagctctgtggacaaaatcccttatgggatgcgcttcattgccaaagtgc
tgaaggactcgttgcatgagaagttccctgatgctggtgaggatgagctg
ctgaagattattggtaacttgctttattatcgatacatgaatccagccat
tgttgctcctgatgcctttgacatcattgacctgtcagcaggaggccagc
ttaccacagaccaacgccgaaatctgggctccattgcaaaaatgcttcag
catgctgcttccaataagatgtttctgggagataatgcccacttaagcat
cattaatgaatatctttcccagtcctaccagaaattcagacggtttttcc
aaactgcttgtgatgtcccagagcttcaggataaatttaatgtggatgag
tactctgatttagtaaccctcaccaaaccagtaatctacatttccattgg
tgaaatcatcaacacccacactctcctgttggatcaccaggatgccattg
ctccggagcacaatgatccaatccacgaactgctggacgacctcggcgag
gtgcccaccatcgagtccctgataggggaaagctctggcaatttaaatga
cccaaataaggaggcactggctaagacggaagtgtctctcaccctgacca
acaagttcgacgtgcctggagatgagaatgcagaaatggatgctcgaacc
atcttactgaatacaaaacgtttaattgtggatgtcatccggttccagcc
aggagagaccttgactgaaatcctagaaacaccagccaccagtgaacagg
aagcagaacatcagagagccatgcagagacgtgctatccgtgatgccaaa
acacctgacaagatgaaaaagtcaaaatctgtaaaggaagacagcaacct
cactcttcaagagaagaaagagaagatccagacaggtttaaagaagctaa
cagagcttggaaccgtggacccaaagaacaaataccaggaactgatcaac
gacattgccagggatattcggaatcagcggaggtaccgacagaggagaaa
ggccgaactagtgaaactgcaacagacatacgctgctctgaactctaagg
ccaccttttatggggagcaggtggattactataaaagctatatcaaaacc
tgcttggataacttagccagcaagggcaaagtctccaaaaagcctaggga
aatgaaaggaaagaaaagcaaaaagatttctctgaaatatacagcagcaa
gactacatgaaaaaggagttcttctggaaattgaggacctgcaagtgaat
cagtttaaaaatgttatatttgaaatcagtccaacagaagaagttggaga
cttcgaagtgaaagccaaattcatgggagttcaaatggagacttttatgt
tacattatcaggacctgctgcagctacagtatgaaggagttgcagtcatg
aaattatttgatagagctaaagtaaatgtcaacctcctgatcttccttct
caacaaaaagttctacgggaagtaattgatcgtttgctgccagcccagaa
ggatgaaggaaagaagcacctcacagctcctttctaggtccttctttcct
cattggaagcaaagacctagccaacaacagcacctcaatctgatacactc
ccgatgccacatttttaactcctctcgctctgatgggacatttgttaccc
ttttttcatagtgaaattgtgtttcaggcttagtctgacctttctggttt
cttcattttcttccattacttaggaaagagtggaaactccactaaaattt
ctctgtgttgttacagtcttagaggttgcagtactatattgtaagctttg
gtgtttgtttaattagcaatagggatggtaggattcaaatgtgtgtcatt
tagaagtggaagctattagcaccaatgacataaatacatacaagacacac
aactaaaatgtcatgttattaacagttattaggttgtcatttaaaaataa

agttcctttatatttctgtcccatcaggaaaactgaaggatatggggaat
cattggttatcttccattgtgtttttctttatggacaggagctaatggaa
gtgacagtcatgttcaaaggaagcatttctagaaaaaaggagataatgtt
tttaaatttcattatcaaacttgggcaattctgtttgtgtaactccccga
ctagtggatgggagagtcccattgctaaaattcagctactcagataaatt
cagaatgggtcaaggcacctgcctgtttttgttggtgcacagagattgac
ttgattcagagagacaattcactccatccctatggcagaggaatgggtta
gccctaatgtagaatgtcattgtttttaaaactgttttatatcttaagag
tgccttattaaagtatagatgtatgtcttaaaatgtgggtgataggaatt
ttaaagatttatataatgcatcaaaagccttagaataagaaaagcttttt
ttaaattgctttatctgtatatctgaactcttgaaacttatagctaaaac
actaggatttatctgcagtgttcagggagataattctgcctttaattgtc
taaaacaaaaacaaaaccagccaacctatgttacacgtgagattaaaacc
aatttttccccatttttctcctttttctcttgctgcccacattgtgc
ctttattttatgagccccagttttctgggcttagtttaaaaaaaaaatca
agtctaaacattgcatttagaaagcttttgttcttggataaaaagtcata
cactttaaaaaaaaaaaaaactttttccaggaaaatatattgaaatcatg
ctgctgagcctctattttctttctttgatgttttgattcagtattctttt
atcataaatttttagcatttaaaaattcactgatgtacattaagccaata
aactgctttaatgaataacaaactatgtagtgtgtccctattataaatgc
attggagaagtatttttatgagactctttactcaggtgcatggttacagc
ccacagggaggcatggagtgccatggaaggattcgccactacccagacct
tgtttttgttgtattttggaagacaggtttttaaagaaacattttcct
cagattaaaagatgatgctattacaactagcattgcctcaaaaactggga
ccaaccaaagtgtgtcaaccctgtttccttaaaagaggctatgaatccca
aaggccacatccaagacaggcaataatgagcagagtttacagctccttta
ataaaatgtgtcagtaattttaaggtttatagttccctcaacacaattgc
taatgcagaatagtgtaaaatgcgcttcaagaatgttgatgatgatgata
tagaattgtggctttagtagcacagaggatgccccaacaaactcatggcg
ttgaaaccacacagttctcattactgttatttattagctgtagcattctc
tgtctcctctctctcctcctttgaccttctcctcgaccagccatcatgac
atttaccatgaatttacttcctcccaagagtttggactgcccgtcagatt
gttgctgcacatagttgcctttgtatctctgtatgaaataaaaggtcatt
tgttcatgttaaaaaaaaa

>NM_004099 4
gcctctggctcctcagggcattcccggcggctccgggtttggcaacgagg
acgggggagtgcgactgcgtctcgggcagcatggccgagaagcggcacac
acgggactccgaagcccagcggctccccgactccttcaaggacagcccca
gtaagggccttggaccttgcggatggattttggtggcgttctcattctta
ttcaccgttataactttcccaatctcaatatggatgtgcataaagattat
aaaagagtatgaaagagccatcatctttagattgggtcgcattttacaag
gaggagccaaaggacctggtttgtttttattctgccatgcactgacagc
ttcatcaaagtggacatgagaactatttcatttgatattcctcctcagga
gatcctcacaaaggattcagtgacaattagcgtggatggtgtggtctatt
accgcgttcagaatgcaaccctggctgtggcaaatatcaccaacgctgac

tcagcaacccgtcttttggcacaaactactctgaggaatgttctgggcac
caagaatctttctcagatcctctctgacagagaagaaattgcacacaaca
tgcagtctactctggatgatgccactgatgcctggggaataaaggtggag
cgtgtggaaattaaggatgtgaaactacctgtgcagctccagagagctat
ggctgcagaagcagaagcgtcccgcgaggcccgcgccaaggttattgcag
ccgaaggagaaatgaatgcatccagggctctgaaagaagcctccatggtc
atcactgaatctcctgcagcccttcagctccgatacctgcagacactgac
caccattgctgctgagaaaaactcaacaattgtcttccctctgcccatag
atatgctgcaaggaatcataggggcaaaacacagccatctaggctagtgt
agagatgagcgctagccttccaagcatgaagtcggggaccaaattagcct
ttaactcataaagagagggtagggcttttctttttccatatgtcaattgt
ggtgttcccagaatgtatagcagttataaaaataggtgaaagaattgtta
gcttgtaaatactgagagattggtgatttatataaggtaatctgttagtc
ttaaaatagttaaaagtttgtatttttagattattatgtagtaggttaga
tccctcttgttttgacttccactgactcattctgaacccccctaagcaccc
aggccagaggcaagaacctgggctgtaactgccacctgacaccgctgact
ggctaaatgctttgcagaaagtgatgaccttacaccacaaccagcttctc
caggtcatatgtgccttacctccagagagtcttttttttttttttctga
gatggagtttcactcttgttgcccaggctggagtgcaatagcatgatctc
ggctcactgcaacctccgcctcctgggttcaagagattctcctgcctcag
cctccccagtagctgggattacaggctcatgccaccatgcccagctaatt
tttgtattattattattgtttttagtagagacggggtttcaccatgttg
gccaggctagtcacgaactcctaacctcaggtgatccacccacctctgcc
tcccaaagtgctgggattacaggcatgagctaccacacctggtttggaga
gtcttaattaaggaaatttccctaatgttcatttattttctaaatccaga
ccgtgtttcagaataatccttacttgagagtagccattttcttgcctgta
cttgtcagaactagaggaaatagccaagactaatgaaaaagattactcta
acccttaaaagacttttaaattcactactagagtggtcattttaaaaata
catccatgttttaacttatttgagccttctttatgagtaaatgattcctc
cttgttctgtctttcaaaccagctaaatatttgtcacaaaagtgcttttt
tctcactgttgcctattttcatatatcaggttttaaatagttttaatttt
ttaataaaattttctctacgttctatatgcaattgttatatatctatttg
aatagctgaaggactaaaatactttttaagagataacttcaggaaacca
ttatattttactatctgcatgctgttaactgtggtacactgtgaaatatg
ttgattacaaacccattcattacatagtataaggaattcacagtatattg
actatatagtgtctaatgatcttgggcagatactgtcaaacttacaatat
ctatatagatgtaggtctttttaaatttacctagtcattcttctatcatg
tatattgatgctgaaagaggaactggtcagctcctctggacaacaaattc
ttagtctataatattaggagacatcttctgttttgcaaatgtctgtgaat
ctgagcaacctggcattctgcttactggccagaaagctggcgggtgacat
ttgtaacatttcctctttgagactctgagttcacctagagaagtctaagc
ataacagctttctttcccagcacgagcctttatagctctctttagctcaa
ccactctgtccatccagccaatggatgtcccttcccctgtaccccaattt
caagcttattttaggaagccttgaactaccatgtatcctggctcctagct
gagtttattagaggtatggagcagtgcaacttaaactcaagttgcactta
cattttgaattttaaaatgatggttttatctgttgtgtgaagtggttcac

ccttgaggaccaggagcctccatatcctgactgaaaccttttctgagac
ttagagtaacagtgcttttggttccttgagttctcctgtctccagatacc
aaatgaccttgacttttctgccttgtgaattcgtagtccaatcagctgaa
attaaatcacttgggagggacgcatagaaggagctctaggaacacagtgc
cagtgcagaagtttctccaggtggcctccctttccaacaatgtacataat
aaagtgtatgcactttcactaataaaaaaaaaaaaaaaaaaaaaaaaaaa
aaaaaaaa

>NM_004125 3
gcgcggctgagtgctgcagaatcgctggggtggcagagccgccagcgagg
ctggggatgggggcgccgctgctctctcccggctggggagccggggctgc
cggccggcgctggtggatgctgctggcgcccctgctgccggcgctgctgc
tggtgcggcccgcgggggccctggtggaggggctctactgcggcacgcgg
gactgctacgaggtgctgggcgtgagccgctcggcgggcaaggcggagat
cgcgcgggcctaccgccagctggcccggcgctaccaccctgaccgctacc
ggccccagcccggagacgagggccccgggcggacgccgcagagcgccgag
gaggctttcctgctggtggcaaccgcctacgagacactcaaggtctctca
ggcagctgcagagcttcaacagtactgtatgcagaatgcctgcaaggatg
ccctgctggtgggtgttccagctggaagtaaccccttccgggagcctaga
tcctgtgctttactctgaagactcgagagaagtttgctgaggaatgcctt
caagcacaaagtgatgaatgactgccttcaagtctcaagaaaacactttt
ccctaacttttagagatatttcagccctttcctgtggcctggtcctatag
ccaaaatcacagatattcatgagtttctacttgagtgagaaaactgggtg
aaggaatagaattttaaatagtaataactgcttgtttttttgtgcaagt
acttttatacataagataaacaaaaaccttaccaccaaacataccaaaat
gcacctctttcataagtgagttactaagatttctatacctggaatatcat
gtatgtttcatttactggatgtttacattttaggaaggaaaatagttttg
tttatttaaacaactgaatacttataaactgttgttcctggaagttattt
attccataaaaaatttgttcttttgtcatgaatttataattcctaaatga
agaccagaaagtacaaattgctgggaggaagaataggctttattaatcaa
ctgatgtcttgatttttctaaatgggaagattgctttatttttaacacta
attatgggagcagattcttagcaaacttctttggaaaagttaatgttatg
atgtgcattaggctgccccatcgtgtatataaatgaagcagatttgattt
ttgtattcttacgtttctctgctttgtagttgtggctgtacttaaagaaa
tacagaatttcatatatttaaaaatgtttaaaatgtgacccacagaacat
tgtaaatgattaaaaactaacatgaaaatattacaacctaaaagaattct
taacttcacaagtgttttacttcgacgatgtgcctttgatttaatttggg
acactttttagaaggatacattattcgtgtttgcaacggtctttgaaga
gcttggaaataaaatttctgcttaattaatcatttttctatgacagcaaa
aaaaaaaaaaaaaaa

>NM_004364 3
cggaggtgcgcgggcgcgggcgagcagggtctccgggtgggcggcggcga
cgccccgcgcaggctggaggccgccgaggctcgccatgccgggagaactc
taactcccccatggagtcggccgacttctacgaggcggagccgcggcccc
cgatgagcagccacctgcagagcccccgcacgcgcccagcagcgccgcc

ttcggctttccccggggcgcgggccccgcgcagcctcccgccccacctgc
cgccccggagccgctgggcggcatctgcgagcacgagacgtccatcgaca
tcagcgcctacatcgacccggccgccttcaacgacgagttcctggccgac
ctgttccagcacagccggcagcaggagaaggccaaggcggccgtgggccc
cacgggcggcggcggcggcggcgactttgactacccgggcgcgcccgcgg
gccccggcggcgccgtcatgcccgggggagcgcacgggcccccgcccggc
tacggctgcgcggccgccggctacctggacggcaggctggagcccctgta
cgagcgcgtcggggcgccggcgctgcggccgctggtgatcaagcaggagc
cccgcgaggaggatgaagccaagcagctggcgctggccggcctcttccct
taccagccgccgccgccgccgccgccctcgcacccgcacccgcacccgcc
gcccgcgcacctggccgccccgcacctgcagttccagatcgcgcactgcg
gccagaccaccatgcacctgcagcccggtcaccccacgccgccgcccacg
cccgtgcccagcccgcaccccgcgcccgcgctcggtgccgccggcctgcc
gggccctggcagcgcgctcaaggggctgggcgccgcgcaccccgacctcc
gcgcgagtggcggcagcggcgcgggcaaggccaagaagtcggtggacaag
aacagcaacgagtaccgggtgcggcgcgagcgcaacaacatcgcggtgcg
caagagccgcgacaaggccaagcagcgcaacgtggagacgcagcagaagg
tgctggagctgaccagtgacaatgaccgcctgcgcaagcgggtggaacag
ctgagccgcgaactggacacgctgcggggcatcttccgccagctgccaga
gagctccttggtcaaggccatgggcaactgcgcgtgaggcgcgcggctgt
gggaccgccctgggccagcctccggcggggacccagggagtggtttgggg
tcgccggatctcgaggcttgcccgagccgtgcgagccaggactaggagat
tccggtgcctcctgaaagcctggcctgctccgcgtgtcccctcccttcct
ctgcgccggacttggtgcgtctaagatgagggggccaggcggtggcttct
ccctgcgaggaggggagaattcttggggctgagctgggagcccggcaact
ctagtatttaggataaccttgtgccttggaaatgcaaactcaccgctcca
atgcctactgagtagggggagcaaatcgtgccttgtcattttatttggag
gtttcctgcctccttcccgaggctacagcagacccccatgagagaaggag
gggagcaggcccgtggcaggaggagggctcagggagctgagatcccgaca
agcccgccagccccagccgctcctccacgcctgtccttagaaaggggtgg
aaacatagggacttggggcttggaacctaaggttgttcccctagttctac
atgaaggtggagggtctctagttccacgcctctcccacctccctccgcac
acaccccaccccagcctgctataggctgggcttccccttggggcggaact
cactgcgatgggggtcaccaggtgaccagtgggagcccccaccccgagtc
acaccagaaagctaggtcgtgggtcagctctgaggatgtataccccctggt
gggagagggagacctagagatctggctgtggggcgggcatggggggtgaa
gggccactgggaccctcagccttgtttgtactgtatgccttcagcattgc
ctaggaacacgaagcacgatcagtccatcccagagggaccggagttatga
caagctttccaaatattttgctttatcagccgatatcaacacttgtatct
ggcctctgtgccccagcagtgccttgtgcaatgtgaatgtgcgcgtctct
gctaaaccaccattttatttggtttttgttttgttttggttttgctcgga
tacttgccaaaatgagactctccgtcggcagctgggggaagggtctgaga
ctccctttccttttggttttgggattacttttgatcctgggggaccaatg
aggtgaggggggttctcctttgccctcagctttccccagcccctccggcc
tgggctgcccacaaggcttgtcccccagaggccctggctcctggtcggga
agggaggtggcctcccgccaacgcatcactggggctgggagcagggaagg

acggcttggttctcttcttttggggagaacgtagagtctcactctagatg
ttttatgtattatatctataatataaacatatcaaagtcaa

>NM_004685 3
cagtaccggcctcctccagcagggggcgctgtgggatccgccgctgtccg
ggacgaaccagcaagccgctctcggtccacacacgctctgtctgcctgcc
ccgagtcccccgggaggccgcggggtttggggaagtgtttctaggagacg
gcgctcaccggctgcacctgcgccgttgacgccaccggggccggcagaca
gacccgcggcgctggctggtggagggagttcccgcttgctctctgtcgct
gtcaccgccctgtttctgtagccgtatggtacgcctgtgagaccggctgc
cggctgacgtctccttgcgatggagcatatccggacgaccaaggtcgaac
aagtaaaattacttgaccgattcagtaccagcaacaagtcattaacagga
acactgtatcttacggctacacatctattatttatcgactctcatcaaaa
agaaacctggatattacaccaccatattgcctcagtagagaaacttgctt
tgactacttctggatgccccttgtgatacagtgcaagaacttcagaact
gtgcatttcattgttcccagagaaagagattgccatgatatttacaactc
tttgctacaactgtcaaaacaagcaaaatatgaagatctctatgcatttt
cttataatcccaaacaaaatgattcagaacgactacaaggctggcagctc
attgatctcgctgaggaatataagaggatgggagtgccaaactcacactg
gcagttgtctgatgccaaccgggactacaagatttgtgaaacttacccca
gagaactttatgttccccggatagcaagcaaaccaataattgttggtagt
tccaagttccggagcaagggaagattcccagttctttcctactatcatca
agataaggaggctgccatttgtcgatgtagtcagccactctctggattca
gtgccaggtgcctggaggatgaacatttgcttcaagccattagtaaagcc
aatccagtcaatcgctatatgtacgtcatggataccaggccaaaactgaa
tgcaatggccaacagagcagctggaaaaggttatgaaaatgaagacaact
attccaatattagatttcagtttgttggaattgaaaatattcatgtcatg
aggtccagccttcagaaattattggaagtcaatggcactaaagggctttc
tgtcaatgatttctactccggtttggagagctcgggatggcttcgccata
tcaaagctgttatggatgctgcaatcttcttggccaaagcaataacagtt
gaaaatgcaagtgtgttggtgcattgttccgatggttgggataggacttc
ccaggtttgttccctgggttctcttttattggattcctactacaggacaa
tcaaaggattcatggttttaatagaaaaggattggatctcttttggacat
aaattttcagagaggtgtggccagttggatggtgacccaaaggaagtctc
accagtgtttactcagttcttggaatgtgtgtggcatttgaccgaacagt
ttccacaagcctttgaattcagtgaagcatttcttcttcagatccatgag
catattcattcatgccagtttggaaacttccttggaaattgtcagaagga
aagagaagagctcaagttgaaggagaagacttattccctgtggccatttc
ttttggaagaccaaaagaagtacttaaatcctctctacagttccgaatct
cacagatttacagttttggagccaaatacagtatctttcaattttaagtt
ttggaggaacatgtaccatcaatttgatcgaacactgcatcctaggcagt
ctgtatttaatataattatgaatatgaatgagcaaaataaacaattagag
aaagatattaaagacctagaatctaaaattaaacaacgcaaaaataagca
aacagatggcatcctcaccaaggaattgttacattcagttcatcctgaat
cacctaacctcaaaacttccctgtgttttaaagagcagactctgctaccc
gtaaatgatgctcttcgaactatagagggcagcagcccggcagataatcg

ttatagtgaatatgcagaagagttttctaaatcagaacctgctgtggtca
gcttagagtatggtgtggcaagaatgacttgttagactcatagagttttt
tctgcaatgattgcagtacaagaaaaggattattgtgaggatggtctgta
agcataaccaaaaggaatttgtctaataacaattttagggtttaacagta
ggctaatagttgaaggaaggataataactacccttgtgagagaaataagt
cattttaattgcatttccagcaaggaatgacattcagttctgtaagaaat
gagtggtatttgatgtatttactcaaaacacaatttgcactgtacactag
tgaattgacgtttatgatttatgttaattcagccaaacataaataacctt
ccttaagtacaatttaacttcaagaaaacaaaatttgacaacatagtttc
ttaataaatgatatggcatgtactttcaattatgtagctttgtaactatg
aatatttacatattttgccttttagtgatatttaatgttaaagtgccatg
aaaaatatttctaagaaagccttaaattcccagtggattctttaccctta
agttttacagcctacaacaagatttttttgttttgtttttcttctggtcag
ccttgttttgtttgtaaagaattgtgctctcattactgctgggggtgcat
gctacaatacttctatataaacacttgtagaagtacactgttcacgttta
gcctgccccacttttgtattcaaaattaatgaaactgaaggtttattctg
atcataatttgtttagtgctacatttgataatttattatttacagcttag
aatattgatttcttgaatacgtataagcacatttgactgtcttttatata
tggattactgcattccattgattcttattttgtggttggctttatttctt
tcacaactgtgtaagtttaaagagctaaagctctaaaactgttctgagaa
acaatgaatagtacatgtatatgtatatttttaaactgccttattgctca
atgagttgctgttcctggagtatctaactttcagtttactcaaaaacttt
tgggtaaataaaaaagggaagtacagatagtttaggttgtgcatggttgc
atgaattttgaagtcatttctatgtggagcattatttttcttcttgttaa
atatagaaaaaaaaagagattctagtacaaagttactgtttaacaaaagc
aacataaactctgggaaagatttcattttgccatgttatatttactgttt
attctgtgtactagtacatatctttaaattaccaaaaaacaagaaacaaa
acataaaaaccccaaaactatcacttggaattagcaatatcacccaactg
gctttaaaattgaaaatttaaataacatggtggcatgaagtacaattcga
gtattaggcaatttgcatagtgttcctcatgctactttctgttacacctc
tattatattagttttgaatataaacatctttttcagaccaaaaaaaactt
tattgtatgagagcttatcttatcctgtttatttttccaatgcttttctg
taatacattatgtaatttaaaaaatattcctttttaaacagcaacagaaa
tgcactataaaatatagtatgtgattaaccaatcctgcttccatatttaa
gcactgggaatggaaacttaatctctgtgactacaaagggaagtttttgt
gccttggtgttccagtcactgattgtggttttagaatcttctgtggctga
cttcttgtattcaactctggtttattatcttaatcttcaacttcagacat
atatttgtgtgtttatatgctcacaggtggactgagaaatcagttacatc
ttaagtgacctacagggtatatgttggcaaaagcagactgtgtatatgtc
ttataaagttgaatttatgttcagtgtgtttggaagtgtatagcatgtaa
attatttcatatatgatttaaaggtaattaaatgttcacattttactttg
aatgtttttcttctggataaaacaaatggaatcaagttgtagttgttttt
tttttcttctcacctaggcaagtagacttgcccattacattaacataaca
gcataaggtttgaggagtttattttccaaaagtttgttgataaataatac
ctgtttcatacaaaaataatttccagaatatttaaaataggtatagtggg
ctaaaaggcccatcagatgtaggtaataatgtcattaagttttcattttg

ttcctgtcacttgcttatattacaatcactattttttgtgcccttgcca
acttgatggaaggcagggtccattgagtacaaacattcgaggtgaaatgt
ctctccaaaggcagatctggatgtaaatatgaggaagaaaataactggct
gacaagaggataattttattgagggtgttttgaaaaggtcagctaggcta
gaagtagctcagggaatcttgagtaaagatgaagggaactcttttaaaac
aaattcaatttatttggtcgggtagcagagcagggaggtggtgtcagtca
agcctgttttgaaacatttttgcagaaggtcagctttaaaatattgaaa
aatttccagttgacatgctaagttggattaaatgctgatcatttataaaa
ctatatagttgctgcaaaagtaattggggcttttgccattgaaagtgatg
acaaaaaccgcaattacttttgcaccaacctaatactacagttgcagact
tttattagtgacgctgatcttcattagtcaagttttagttattttgttgg
cttttccatgtagaattccaagtgacattaacatactttgattaattaaa
gaaaatttggagactgtttaagatatatgtctagatatcaatgtaaacta
taagctttaggaacttacatctagaagaaagcctggaatctagaataatt
atctcaaaaaaaagtatatataccattgtacttgatataagaaaatgtaa
acatgaaaccttgacattttaataaacagtatttgtgctactctagttat
ttctaggaaagtaaaaaaaaaa

>NM_004781 3
agtgacgtctttgccccgcgccgcgccgtcccacccatctccctggcctc
cggtcccaacttcgcttctctgctgaccctctctcgtcgccgctgccgcc
gccgcagctgccaaaatgtctacaggtccaactgctgccactggcagtaa
tcgaagacttcagcagacacaaaatcaagtagatgaggtggtggacataa
tgcgagttaacgtggacaaggttctggaaagagaccagaagctctctgag
ttagacgaccgtgcagacgcactgcaggcaggcgcttctcaatttgaaac
gagcgcagccaagttgaagaggaaatattggtggaagaattgcaagatgt
gggcaatcgggattactgttctggttatcttcatcatcatcatcatcgtg
tgggttgtctcttcatgaagaaccagcggaactcaaaactgctgttcaag
aaacctcttcaagacttttgacttagaacctgctatattatcaagcttac
ctactgttatctctaaaattttttttgtgttaatgtaaagttgaatttct
aggaaacgtgcctttgtttttaatatgcactccaaattagaaggccggc
cccgtccacattttgcacagtgcctttacagatttacgtatgggctgatg
aagaggccttcttaagttccagagtgctataatctagatgtaatgttgtc
actaattaattgccattactcccagttagttacccttgtcatttggcatt
attttcagaaccacattttaaacctttgggtaatcagatttccaacttat
gccttccagaaaaaaacactactgcctaacacaaatctgtgataacaaca
ggctgtgccttattttgataattttctgattccctagaagagaaccctct
actttttgtaagcactactgactctcgctgtatttaagatgctggtgaag
agcttttgctcttgcattagatttgaagatgtttacattgttgttattgt
tatgtatcacttgctaaaaatattgttttaatcagagataacctctttaa
aaaaatttttaaagaactatggctatgaccaaagcttctattttgccaaa
aagttaaataccgataaaatggccttaagtgtattcctgacagttaaatt
cagaaacgtgccaaatggaactcaaggtgccccttcagaattaaaatcat
taccttgtgtgtgaaccttctacatcttcataggcctttcttccttttga
aaggctgtagacagtgtggctccccttctgattcagtattttgcatgggg
gttagagaaggtttgaggtagactctgaccgtctcataaaagagttctac

ccagcagttggcagattatcagctgtggactccagcatgtttctgataat
tatgcaagcaacaattctgtagcctcaagtaagaccacctgtgaacttga
tcattatctggcccaaatatgaagataaactataactttggagtttgttt
cctatttgtattcacattctgcttcctaaatcagttttctaaattatgcc
tgcaattaggcattggtcaggggtgaatggctcttttcacagagagtagc
caaccagagacctttgctttgatatcatcaactgcagagaatgctgttga
tgggaatgctggaagcagaaactttgtcatcggaaaaacttttcttgtat
gcatgagactcaacatcaggatccacagcttaaagatgggaattcaggta
tgaaagaaaacaggcaaggaggcactgagggagaaagacacagactttat
cgctctgtggctcattgttactggaatattctaaaactcttgttcacatg
ctattatgacttataaagcagcaacagctgaggcgcaccaggacacagct
tccatttctttaacgtctgttcccttaacatcgctgaaatgatttactgt
tgaagagatgccttgcggtgtggccagctgtgaggagaaagcagctggca
gtgttaggacattagtccaccttcagcgcagggtctctggccgggtctga
ctcagaaaccttggtactcgccccttggccacagtgcccagacccatgta
acccactggctcctgcattaacccagaaatacctcgcttctatctgtgca
cttagctgggaacttacccactgtaatcacctaaataaagtgtttataaa
catgaaaaaaaaaaaaaaaaaaaa

>NM_004862 3
gtttctctccctgcccccgcgacttcgcgcaagatccgggaaggacaccc
gaggcccctgggagaccctggggaggtgaaaatcagagagcgaagcgggc
cgtggcccctaggcctgacccctccccgcggggtaaggcgggcaccccgc
gagcgcaggggtcctcttactgctgatggcacccagctctgggcccagac
gccgctcaccgtccaccgccggtgctgggtaaaatgtcggttccaggacc
ttaccaggcggccactgggccttcctcagcaccatccgcacctccatcct
atgaagagacagtggctgttaacagttattaccccacacctccagctccc
atgcctgggccaactacggggcttgtgacggggcctgatgggaagggcat
gaatcctccttcgtattatacccagccagcgcccatccccaataacaatc
caattaccgtgcagacggtctacgtgcagcaccccatcacctttttggac
cgccctatccaaatgtgttgtccttcctgcaacaagatgatcgtgagtca
gctgtcctataacgccggtgctctgacctggctgtcctgcgggagcctgt
gcctgctggggtgcatagcgggctgctgcttcatcccccttctgcgtggat
gccctgcaggacgtggaccattactgtcccaactgcagagctctcctggg
cacctacaagcgtttgtaggactcagccagacgtggagggagccgggtgc
cgcaggaagtcctttccacctctcatccagcttcacgcctggtggaggtt
ctgccctggtggtctcacctctccagggggcccaccttcatgtcttcttt
tggggggaatacgtcgcaaaactaacaaatctccaaaccccagaaattgc
tgcttggagtcgtgcataggacttgcaaagacattccccttgagtgtcag
ttccacggtttcctgcctccctgagaccctgagtcctgccatctaactgt
gatcattgccctatccgaatatcttcctgtgatctgccatcagtggctct
tttttcctgcttccatgggcctttctggtggcagtctcaaactgagaagc
cacagttgccttatttttgaggctgttctgcccagagctcggctgaacca
gcctttagtgcctaccattatcttatccgtctcttcccgtccctgatgac
aaagatcttgccttacagactttacaggcttggctttgagattctgtaac
tgcagacttcattagcacacagattcactttaatttcttaattttttttt

taaatacaaggaggggggctattaacacccagtacagacatatccacaagg
tcgtaaatgcatgctagaaaaatagggctggatcttatcactgccctgtc
tccccttgtttctctgtgccagatcttcagtgccccttttccatacaggga
tttttttctcatagagtaattatatgaacagtttttatgacctccttttg
gtctgaaatacttttgaacagaatttcttttttttaaaaaaaaacagaga
tggggtcttactatgttgcccaggctggtgtcgaactcctgggctcaagc
gatccttctgccttggcctcccgaagtgctgggattgcaggcataagcta
ccatgctgggcctgaacataatttcaagaggaggatttataaaaccattt
tctgtaatcaaatgattggtgtcattttcccatttgccaatgtagtctca
cttaaaaaaaaaaaaaagaaaaagaaatggataatttcatctactgcctt
tacttggggttaatgtgattcttaaacaccttcatcatggaactctcaga
gtggggtccgttttggtttcctggtggtgggttttgaaagataagggaaa
gcacattttgagcatgtctgggtaccatggtgcggatgcttgggaaccag
aactgtttcagaggaatctaaagtctgattttagttttcagagacacagc
ttgttgtaaaacatgagaagacatgatttctaggactcaagcagcaagcc
aggattctaggttggctgctgtgtcatctttgaagtcaagacaaagctgg
gctcgaccttcaagggtcctcgttttgataatacttcagaatagggaact
catgtgaatactactatgtagaaataaaacctagaccttgagcgaacatc
tgtatattggttgaaaacgatagtggtaaccattgatccccccttcatttg
atgtttggaaaattccagtaattatcatttttgcaacgaatatggatacc
acatagtactttggtgttacctgcttttgaaaaataaagtctttggttca
cccggtgaactatttatgagttcttttggtgtgaagaaagggctcatgtt
gcatttccagccattgctacaaagaacctttatttgttcagtaacggtag
aaaatccttcccgattaaaaacttcagacttgctgaatatcctgcaatgt
caagatgaccgatgttgagttgggtggatttgctaacgagtcagatttga
acatgaggctattggaacccaataggcgtcattgatggcggcaagccata
gctttcaagttttaataaaatgcacaaaagagaaaaaaaaaa

>NM_005720 3
ggcaggccccgcccccgcccacgaggaagtggctgctgctccggcgcgg
agcccagagccggttcggcgcgtcgactgcccagagtccgcggccggggc
gcgggaggagccaagccgccatggcctaccacagcttcctggtggagccc
atcagctgccacgcctggaacaaggaccgcacccagattgccatctgccc
caacaaccatgaggtgcatatctatgaaaagagcggtgccaaatggacca
aggtgcacgagctcaaggagcacaacgggcaggtgacaggcatcgactgg
gccccgagagtaaccgtattgtgacctgcggcacagaccgcaacgccta
cgtgtggacgctgaagggccgcacatggaagcccacgctggtcatcctgc
ggatcaaccgggctgcccgctgcgtgcgctgggcccccaacgagaacaag
tttgctgtgggcagcggctctcgtgtgatctccatctgttatttcgagca
ggagaatgactggtgggtttgcaagcacatcaagaagcccatccgctcca
ccgtcctcagcctggactggcaccccaacaatgtgctgctggctgccggc
tcctgtgacttcaagtgtcggatcttttcagcctacatcaaggaggtgga
ggaacggccggcacccaccccgtggggctccaagatgccctttggggaac
tgatgttcgaatccagcagtagctgcggctgggtacatggcgtctgtttc
tcagccagcgggagccgcgtggcctgggtaagccacgacagcaccgtctg
cctggctgatgccgacaagaagatggccgtcgcgactctggcctctgaaa

cactaccactgctggcgctgaccttcatcacagacaacagcctggtggca
gcgggccacgactgcttcccggtgctgttcacctatgacgccgccgcggg
gatgctgagcttcggcgggcggctggacgttcctaagcagagctcgcagc
gtggcttgacggcccgcgagcgcttccagaacctggacaagaaggcgagc
tccgagggtggcacggctgcgggcgcgggcctagactcgctgcacaagaa
cagcgtcagccagatctcggtgctcagcggcggcaaggccaagtgctcgc
agttctgcaccactggcatggatggcggcatgagtatctgggatgtgaag
agcttggagtcagccttgaaggacctcaagatcaaatgacctgtgaggaa
tatgttgccttcatcctagctgctggggaagcggggagaggggtcaggga
ggctaatggttgctttgctgaatgtttctggggtaccaatacgagttccc
ataggggctgctccctcaaaaagggagggacagatggggagcttttctt
acctattcaaggaatacgtgcctttttcttaaatgctttcatttattgaa
aaaaaaaaaaaatgcccccaaagcactatgctggtcatgaactgcttcaa
aatgtggaggtaataaaatgcaactgtgtaaaaaaaaaaaaaaaaaaaa
a

>NM_005903 5
ccgggtcctgggcgagcgggcgccgtgcgcgtgtcccgcggccgagctgc
taataaagttgcagcgaggagaagcgcagcgacggcgtcgggagagcgcg
cctagccggctcgcgaaaaggaagctgttgaagttattgaagtacctgtt
gctatattctaagaaattaaaatgtccagaaatctgcctctgacttgacc
caatgaaagaagcatatggcacttgtgaagataaatgttactcctccctt
tttaattggaacttctgcttaggacctgtgtatgacgtttcacctgtgat
ctgttctttcggtagccactgactttgagttacaggaaggtctccgaaga
tttgtgtcaaatgacgtcaatggccagcttgttttcttttactagtccag
cagtaaagcgattgttgggctggaaacaaggtgatgaggaggagaaatgg
gcagaaaaggcagttgatgctttggtgaagaaactaaaaaagaaaaaggg
tgccatggaggaactggagaaagccttgagcagtccaggacagccgagta
aatgtgtcactattcccagatctttagatggacgcctgcaggtttctcac
agaaaaggcttaccccatgttatatattgtcgtgtttggcgctggccgga
tttgcagagtcatcatgagctaaagccgttggatatttgtgaatttcctt
ttggatctaagcaaaaagaagtttgtatcaacccataccactataagaga
gtggagagtccagtcttacctccagtattagtgcctcgtcataatgaatt
caatccacaacacagccttctggttcagtttaggaacctgagccacaatg
aaccacacatgccacaaaatgccacgtttccagattctttccaccagccc
aacaacactccttttcccttatctccaaacagcccttatccccccttctcc
tgctagcagcacatatcccaactccccagcaagttctggaccaggaagtc
catttcagctcccagctgatacgcctcctcctgcctatatgccacctgat
gatcagatgggtcaagataattcccagcctatggatacaagcaataatat
gattcctcagattatgcccagtatatccagcagggatgttcagcctgttg
cctatgaagagcctaaacattggtgttcaatagtctactatgaattaaac
aatcgtgttggagaagcttttcatgcatcttctactagtgtgttagtaga
tggattcacagatccttcaaataacaaaagtagattctgcttgggtttgt
tgtcaaatgttaatcgtaattcgacaattgaaaacactaggcgacatatt
ggaaaaggtgttcatctgtactatgttggtggagaggtgtatgcggaatg
cctcagtgacagcagcatatttgtacagagtaggaactgcaactttcatc

atggctttcatcccaccactgtctgtaagattcccagcagctgcagcctc
aaaatttttaacaatcaggagtttgctcagcttctggctcaatctgtcaa
ccatgggtttgaggcagtatatgagctcaccaaaatgtgtaccattcgga
tgagttttgtcaagggttggggagcagaatatcaccggcaggatgtaacc
agcaccccatgttggattgagattcatcttcatgggcctcttcagtggct
ggataaagtccttactcagatgggctcccctctgaaccccatatcttctg
tttcataatgcagaagtattcttttcaattatattgttagtggacttgtt
ttaattttagagaaactttgagtacagatactgtgagcttacattgaaaa
cagatattacagcttatttttttctacataattgtgaccaatacatttgt
attttgtgatgaatctacatttgtttgtattcatgttcatgtgattaact
cttagaagtgttgtaaaagatgcagagtaagtattatgccccagttcaga
aatttggcattgatcttaaactggaacatgcttttactttattgccctaa
caatttttattaaatttatttgaaaatgcatcacatgatgaaaaattat
agtagcttataagagggcatatacagtgaagagtaagttttccctcctac
tctcgatcttccagaagctgtacttttaccagtttctttgtcccaccaac
ttaaaaaaaaaaagtacaattcattgttttgcaaaagtgtatggtagggg
cttaaaagaaactataaagttttatttgaatgaacactatgcactgctgt
aactggtagtgttcagtaaaagcaaaatgatagttttctagatgacataa
aatttacatttaatacagataagtgttcttcagtgtaatgtgacttcatg
ctatatatcttttgtaagacatttcctttttaaaaaaatttttgcaaat
aactgatctcaagtatatgtcatttactcaaaatctgtcataagcattac
tttatagctagtgacagtgcatgcacagccttgttcaactatgtttgctg
cttttggacaatgttgcaagaactctatttttgacatgcattaatctttt
attttgcacttttatgggtgacagtttttagcataacctttgataaaata
cactcaagtgacttggacttagatgcttatccttacgtccttggtacctt
ttttgtattaacaaacactgcaatttatagattacatttgtaggaagtta
tgctttttctggtttttgttttactttcaacctaggttataagactgtt
attctatagctccaacttaaggtgcctttttaattccctacagttttatg
ggtgttatcagtgctggagaatcatgtagttaatcccattgctcttacaa
gtgtcagcttacttgtatcagcctccctacgcaaggacctatgcactgga
gccgtaggaggctcttcagttgggccccaaggataaggctactgatttga
tactaaatgaatcagcagtggatgtagggatagctgattttaaaacactc
ggctgggcacagtggctcacacctgtaatcccagcactttgggaggctga
ggcaggcagatcatgatgtcaggagtttgagaccagcctggccaatatgg
tgaaaccctgtctctacaaaaaatacaaaaattagctgggcatggtggtg
cgtgcctgaagtcccagctactcgggaagctgaggcagaagaatcacttg
aacctgggaggcggaggttgtggtgagccgagatcgcaccactgcactcc
agcctgggcgacagagcgagactctgcctcaaaaaacaaaacaaaacaaa
acactcacccatcaacgaatatagactcttctctcatttatcgatgatcc
tctttttccatttttttaagtacttatgtggaagctagtctcccaaaacac
aatctttagagagaaaagacatgaacgaactccaaaatatccatttaatc
aatcatgtttttggctttggataaagaactttgaaccagttttttttctca
ggagctgtcaaatggacacttaattatgacatgagaatgaagaaattatt
ttggaaaaaaaaaaatgacctaatttacctatcagtgaaagctttattttc
tggtgccttttgaaagtatatggagtcatatcattcttctgtttaaaatg
ttagtttggtttgactttccactttgtcctttctgctcttgtgaagaaaa

aaaaaagcattttcgaggaaagaattatgcaatttcttttgttttctgtg
tcattatttattgctttttcaatgtgcagccagtggatggttttagttct
ttcagatgaactgccatttgtgtttcagctcacagttctttgctgggtaa
aagaaatactttctgacagtcacctgagccttaaatgtaagtattacatg
acatgcattctgtttcttccagagttctgtctgccacacgaaagagaata
tttgcttacttgatagaactttggcattttcatcattcttttacttaacc
aggcttatggcatgatctctggaacaaatttgtaggaaaaaattactcca
attgaatgactgatgtatgtaatcaacttcattgggctgcagtaaactag
tggaaattagagagttgttttattggtgttttctactgtgagttaattaa
aaattgtttttatttggggtcattatgtcacagtcttgagttaacaagat
cttacgtgattggccttttctttgttttctcttaggagttgtgtctcatg
aatgacagtactaaagctattaacaactaagagtttgacagagaactata
agcctgttgtatctcctaaaagttgtcaactccccacccttggactttaa
atgaaaattttattcagtccagctattcttacagtccctaaggattttca
tatatctatgtataggagataaaatttgctagtaagatttttaaaaactg
gctagtgaaaggaaagtacctctgaaagaaaccattttagcaaattatgg
ttatatgttttaatttaatctacagaatgttttatagtaaaattctagca
ccactagaataatcacatagcatgtacaatatatttatgctggctgaaaa
gacagaatctgggaataataaaattgcaaccagtttggtaatgcaaacag
cagaatagaatgaaatctcagtaatgaattaaagcaacaaaaagatattg
attggcaaaaagcaagatataagagattcatttgcttaacatttctacat
aatatttatggtctggtcagtattggtctggtcagtattgcctggctgac
gtgaaatgtaaactagtaggcgtgttattgatctgctaaaactaaccctc
tttttaagaggagatttaaggaagacgtcaatcaaaatgtcaaatatgtg
tgtcagaatataaataatttttcacattgtattgttgctatataaaaaaa
ataatagaattggttgggtttctgaggtgaaatccagagtaagagtacta
gacagttcaacaagccacatctaatggcacagatagaggatgtagctatt
ttatacctttcataacatttgagagtaagatatccttcaggatgtgaagt
gattattaagtactcatacctgaaatctgttgtcaagattagaactgggg
ttcatgttaaaaaccttccatattacctgagggtacctgtggggaacagt
tccttcccctgtgtggtagtattttgttggaagagaatgtttatacaaaa
aatgaaattcttccaacagcagagaaactctaaaaagtttgatagtacct
atcaaagtgctgtacttctgtgatagagaacatctgatgtaccaatttag
atctatttctttatactttttctaatcaattgcttaatagtactttggat
gattatcacctttgccacttaaaatatataaatatcctttttacttcatg
aggaaggaagaatttttgataattactgagttcagccttttgtgatgac
ttatattttggacttacattttaactttaaagaatgtcagatcccttctt
tgtcttactagttaaatcctcacctaatctcttgggtatgaatataaatg
tgtgtcatcgttatattgttcagctagatgagcaagtatcttagggtagt
aggtagcctggtggttttagaagtgtttggtgatttttatggagagagtt
ttcctaagtggtggtttataggtggtatcagatattattagggcagcttt
ttggggagtaatctcaggtctcccagagcagcagcatttttctcattgat
ataagtaagattcttaggagcttttcttatcacacaagatgcctgaatcg
aatgtgagaattgaaggcatttcttctgcataaacaaagaattctacctg
ctggacagaaacctggaaagttctttggaattcgctgaattacagtttag
tatgtcctgattacagagtgacaatatttatcaagcctttgttatattgg

attatcttctctcttaaaatacaactgtattataattgaaatgacagccc
aaaattggatggtttaccaaaaccaatgaaagggatttcacacatcaatt
tttatttctgttttgaagagcacatgctatataataattgctagtagcaa
ctgcagtaaaacaggtgataagttattttctctgaaaagatccagtccta
gagcaggattcttcgatcattcatggcagagtgaaaaaggtttgtatggt
tcttgtccaaataactcagttcttaaaattcttaaaatgatcgtaaacca
ttatcctttaaaggtttatttgaagatgctgttaaagtacagaattttgt
gtacaggtagatttttccgtccctcattaatagtgccttcttaattaata
cagactggtgttagctataacaaaactccagtaaggccaaagaatcccaa
gttctttgtggaaaaaaaaaaaaaatcttttagggtcagattttcccttc
taatatcattgaagatgatgttgcattgatttattcataaagtattttaa
ctataggaactctagaagataatggttaggcaagtgattttttttttaaa
tatggttggcgtaagttgtattttgaaattcacttattttaaaatcgaag
aggattgtaatcatggaaatagaatgtttgtatctacctgcccacatttt
cttaaaaagatatttcatatacagataatgaagaccaagctagtggctgc
actgtaggtctgctgcttatttgtatttgttgtgcttctgtttatgttgt
agaagctgaaattctagcaacatgcttcaattctgttattttgatactta
tgaaaatgtattaggttttactatattgtgcttttgaaagccataactct
taagaactttgtttttgcatattgtttgctaattctttactttaataaac
ctcaaaacctgc

>NM_006016 4
atcgtccgagccccacctccctcctcttcgcccccgcacgccgaaaacag
gggcctctcacgtgacccctgcgcgctcccgcgggggagcgtagtctcgg
aggcggcgccgcaggggattgaggggttgactgagcgttgcgagccttag
ctttctcccgaacgccagcgctgaggacacgatgtcgcggctctcccgct
cactgctttgggccgccacctgcctgggcgtgctctgcgtgctgtccgcg
gacaagaacacgacccagcacccgaacgtgacgactttagcgcccatctc
caacgtaacctcggcgccggtgacgtccctcccgctggtcaccactccgg
caccagaaacctgtgaaggtcgaaacagctgcgtttcctgttttaatgtt
agcgttgttaatactacctgcttttggatagaatgtaaagatgagagcta
ttgttcacataactcaacagttagtgattgtcaagtggggaacacgacag
acttctgttccgtttccacggccactccagtgccaacagccaattctaca
gctaaacccacagttcagccctcccccttctacaacttccaagacagttac
tacatcaggtacaacaaataacactgtgactccaacctcacaacctgtgc
gaaagtctacctttgatgcagccagtttcattggaggaattgtcctggtc
ttgggtgtgcaggctgtaattttctttctttataaattctgcaaatctaa
agaacgaaattaccacactctgtaaacagacccattgaattaataaggac
tggtgattcatttgtgtaactcactgaagccaaaatactatcttttaaga
tgtcccacatggaagacgctattccaggatctttaaatttccatggatgc
atataggatgtttgggagcatcatccgtgaagaaaaaatcaattaaatca
ttgtgttcaacaggaatatttaaaatattctgcatgaatcctgtggctgt
cttattttaaatagctgctgctgtgggattatattttttttccttaacat
gccaaatataactttctgaaagtgatggaaaatgttgtcttgtgcagaca
acatcatggctcttggcagtttaaatttagtaattttaatttagtgaaca

gaattgagaagaacgtgccaaatgagaatcaattaggtggatttttggct
gtcatttcaaaagtggaataaatttattaatttagtagtactaaatggta
tccttagattaaaattttgtgcttgataacagctgtttttctacattag
aaataagatgccacacaaggaactacattccagatttaaagaaatgaaag
gataccattagtgtgtataacagattattgttcatacttgtaaagcatct
tatgtcattgagaatataaagaacagtgccttagaagacagtgaaaggta
agctctagcttaatgtctatgatttgttctttgacattaaggaaggtaag
gattggtcagaggatgtaacttgatgtgagcagtagtaaacctgttttag
atatcatactgttaatattttattgaaaatttatttcagagcggagaaac
ttaagctaaagtctgttatacagaattgaaagccttcgtatcttgaacct
cccaacatttttcttatggctgttgaaaagtatagagctaaattgattta
attacactttcctttgtactttaaaaaaaagtatgctagcactattgtac
cttgaaaggatttccaccagactgtcttgagtagtgacttctttggtgag
gcaagaaggatatacattattttagaatcatttactatttaaatgagaca
atcatattattttagaatcatttattttaaatgagacaatcattttaagt
tttaagataacagaagtgaccaatgtaatttcacaacacctaaggatttt
ttggttgatcaggttactgtagatttttactgattgtcctggatgaatag
actgtgctttttctttttctctcccttccttcttggtttcccatagtata
ataagcatgcatactttaacttctatagttttctcctttagagggtcgtc
ttcagttttagaggtttacttctcccttgcctttgactcattggactagt
gcagaggctttaagtagtttaaaatgggcttttgcttttctaggtcatta
acgtttttatttagtttctttagccaatagtggctgagtttcgcacttg
attttcaatattttatagtaagaaatgacaaactgctttgtttcatttca
taaacaaactctgcatttagataactattaaaggttgttaagatgaagat
ttactgtttctttgttactcgttggtacagctgtttgttttacttgcaca
tttgtacatatacttaatgttttcaagtgccttaattgtttaaaatctct
ggcttcaaagtttcttggggaaaggtcggtttacctcacatttttgttt
ccattagtaatattctaggtacctcacaaaatgtattatggtgccatggc
tgttagtttttagtgagtgctgtaggattaattcgaaaataggcagaatt
ccattcctcccaaggtggcaaaaattagctatactgatgtaattgtcatt
tacctgggtatgaattccctgacacacattcatgtcaacatatgtagcaa
attttgtgaaaacataacaatttgaagcttctgtaattttgagcactgct
ctaacaacaagcataatataaaattagttagattttgcaagtctacaaat
gagctcttgcaacagaactcacagcctttttactttttcccctaacttt
agcaatgtagtatcttgagccattaatttttgggtttttttaaaatccag
aaggtatatagaaaccttttcagatttttcatctgatttgttcttgcaga
tgttcttctatcaaataccttattttaccttacagatatttgttgcacag
gcagatactgctgtatttagacatttctatttcagttcattaaaaactgc
aaaaccaatctgtatcatgtaccaaactgacttaaaataaatctacatgt
ttattgaattaaaaaaaaaaaaaaaaa

>NM_006111 2
gtgtttagggtgttggcggagacaaaggggaagagtcatcgcctgtcggg
gctaggatatgatgggtgagaggtgtcaaaccaaattctctcggtttgga
aacggagaaaatctaaaaatgaggatgtgaggaaagagtccgctctcaag
gcgcgttgtggtctatccgagccccgtcccctgggctccctcgggctggg

gtgaggcgggcagcgctacgcgtggggtaggaccatcttacgctgggacc
ccgccaaggagccccaggaagtaggtgaaagggcaggggcgtggctctcg
gggcgccacccacgctcttgaaatctgggtgattgcgagcggccgctcag
cgtcccccacaccacagacccgcgccgccgacgacccagcagccgccatg
gctctgctccgaggtgtgtttgtagttgctgctaagcgaacgcccctttgg
agcttacggaggccttctgaaagacttcactgctactgacttgtctgaat
ttgctgccaaggctgcccttgtctgctggcaaagtctcacctgaaacagtt
gacagtgtgattatgggcaatgtcctgcagagttcttcagatgctatata
tttggcaaggcatgttggtttgcgtgtgggaatcccaaaggagaccccag
ctctcacgattaataggctctgtggttctggttttcagtccattgtgaat
ggatgtcaggaaatttgtgttaaagaagctgaagttgttttatgtggagg
aaccgaaagcatgagccaagctccctactgtgtcagaaatgtgcgttttg
gaaccaagcttggatcagatatcaagctggaagattctttatgggtatca
ttaacagatcagcatgtccagctccccatggcaatgactgcagagaatct
tgctgtaaaacacaaaataagcagagaagaatgtgacaaatatgccctgc
agtcacagcagagatggaaagctgctaatgatgctggctactttaatgat
gaaatggcaccaattgaagtgaagacaaagaaaggaaaacagacaatgca
ggtagacgagcatgctcggccccaaaccaccctggaacagttacagaaac
ttcctccagtattcaagaaagatggaactgttactgcagggaatgcatcg
ggtgtagctgatggtgctggagctgttatcatagctagtgaagatgctgt
taagaaacataacttcacaccactggcaagaattgtgggctactttgtat
ctggatgtgatccctctatcatgggtattggtcctgtccctgctatcagt
ggggcactgaagaaagcaggactgagtcttaaggacatggatttggtaga
ggtgaatgaagcttttgctccccagtacttggctgttgagaggagtttgg
atcttgacataagtaaaaccaatgtgaatggaggagccattgctttgggt
cacccactgggaggatctggatcaagaattactgcacacctggttcacga
attaaggcgtcgaggtggaaaatatgccgttggatcagcttgcattggag
gtggccaaggtattgctgtcatcattcagagcacagcctgaagagaccag
tgagctcactgtgacccatccttactctacttggccaggccacagtaaaa
caagtgaccttcagagcagctgccacaactggccatgccctgccattgaa
acagtgattaagtttgatcaagccatggtgacacaaaaatgcattgatca
tgaataggagcccatgctagaagtacattctctcagatttgaaccagtga
aatatgatgtatttctgagctaaaactcaactatagaagacattaaaaga
aatcgtattcttgccaagtaaccaccacttctgccttagataatatgatt
ataaggaaatcaaataaatgttgccttaacttcaaaaaaaaaaaaaaaaa
aa

>NM_006320 4
actttttcacactagaatttcaaggtgtgagaggctaaaagacgtccaag
tacatgaaacagaacctgcaaataagagtgaatgacgaatagaaagacca
cctcaacctgtccacttccctacggccgcagaagttatgattcaaggaat
aatgagactctaaatcctttaaaattctacaagagcagcctttgccaaac
ccagcggggagtggctgctcggtgccagcgcgaggagcacgtagactgcc
gatgccagagaagcccggtgcgggttccgtgctttctctccgttccagga
agaccccagctcgtggccaaatcttcttgccgcttcccggtctgcgcgcc

tacgctgccggtttcccgacaaaagcgacggcgccctggtgaaaagcaga
cgcccctcatcggtttgcgccttgcagctcaactatgccccagcctccca
caagccacctgcccgcgtcctccgccaggcgtccagcccggacctgcccc
cgcggccgccaaggcgcacgtgaagctcccgggcggggggagcgcaagga
ggcgggagtttgcggacgcgaacgcaggcctaggaagggaagcgattcca
gggcgcacgcgcagagcctggagactgggcggggcgtgcctcccgcaggg
gttacgaacgaaaggcgagcgcgtggcgctcaggagcccgcgcacgcgct
gtggtctcgccccgcccatgagcgtccgcgcggccgtcgtagcatctttt
cttcagacccgcccggctcgtctacttaacctagcggaggcttggagtga
gagcagggaagtgtggggcgaggtgggcggggcgttgcacgcgtgcgcgg
ccagcgaaagaagggagggggcgggagatccgagacaacgttaccccgtc
aacacccaatcaggagccggcctgaccccgccccctcttctcacgttatt
ggctgagaggcccccagatgggcggggccggccgtggggttggggagggc
agggggcggggaggaggaggaaggcgctggcgggcagtgatggcggctgg
tgatggggacgtgaagctaggcaccctggggagtggcagcgagagcagca
acgacggcggcagcgagagtccaggcgacgcgggagcggcagcggaaggg
ggaggctgggcggcggcggcgttggcgcttctgacggggggcggggaaat
gctgctgaacgtggcgctggtggctctggtgctgctgggggcctaccggc
tgtgggtgcgctgggggcggcggggtctgggggccggggccggggcgggc
gaggagagccccgccacctctctgcctcgcatgaagaagcgggacttcag
cttggagcagctgcgccagtacgacggctcccgcaacccgcgcatcctgc
tcgcggtcaatgggaaagtcttcgacgtgaccaaaggcagcaagttctac
ggcccggcgggtccatatggaatatttgctggtagggatgcctccagagg
actggccacattttgcctagataaagatgcacttagagatgaatatgatg
atctctcagatttgaatgcagtacaaatggagagtgttcgagaatgggaa
atgcagtttaaagaaaaatatgattatgtaggcagactcctaaaaccagg
agaagaaccatcagaatatacagatgaagaagataccaaggatcacaata
aacaggattgaactttgtaaacaaccaaagtcaggggccttcagaactgc
aattcttactccctttcacagactgtccggagtctttgggtttgattcac
ctgctgcgaaaaacattcaacaaattgtgtacaagataaattaatctcac
tatgaagatttgaataactagacattatttatgctgccaaactcatttgt
tgcagttgtttgtaatgtctagtggggcttcatcatcctgaaaagaagga
gacagggatttttttaaagagcaagaaagtcacaatattacttctttcct
tcctttttccttctttcctttcttctttctctttctttctttttaaaat
atattgaagacaaccagatatgtatttgctactcaagtgtacagatctcc
tcaagaaacatcaagggactcctgtgtcacatactgtgtttttattttaa
catgggtgagggaggcgacctgatcaggggaggtgggggtacacatcaat
ttgagttgttcaggctactgaaacattaaaatgtgaattcccaaactttt
ctttttggctttgtcagggaaaagaaaaatatctttataaagaaatcttt
ggaaattaggagaaggaatttcaggtgggtttaagtcagagctagttccc
caacagaaagatcatttgaaaccagtttttatcccttctctttccttccc
tttccctaaatcaaatcaatattaattgtgccttatttcacttaacatag
acttgaattatttttagggaaagcccctataatgaattcagaaatcacta
caagcagcattaagactgaagttggaatattctgttgaccataaaacctt
gatatcattctgtgtatatagaatgtaaaaggaatattacagtgttaact
gccatatatgtaatatacacaaactcaattagcattgtaatggccaaatg

cattcccccatgcttttctgttttcaaaaaaattgaaaaacaaatcaact
cttatccccaacagctgcctaattttaggagtctgaccctccacatctca
ctggtgtgggtgcatggggctgtggagtgggtgtcagtatggatgtgtct
gaatgtgtgaggccttggaagggactctttctgcagatactgtaaataca
agtaccattttaataaagcatgtacaataaaccaaaataagcttgagttg
gactttatatacagaactgtaagccagtgcattatgatacagttgtaaga
ttgtgcatttgattcaagataaggaaaaatcttggaaatgaaaagcaggc
actggttaaccaagttgtacacattgtaccacattcagcataactttagg
aagaaattccactttgtggaacattctccagaaatccaagattattcagg
taagaattggtatattaaatgtacatctttttactttctattttgatgcc
aactgattatactagacaattagcactccaggtggttattgaacacaaaa
cagtaaaagaatattgcactgatagatactaaattattattttattaggt
tgaaaaagcccttactaaaagcccctcatatatcaattactttatttcat
tatgactacttaggttccgggctggggacaagttcacttaaaaaggcaat
gttatttaacaggtcaccagttaagacttctgctttgtagatacatgcag
aagccatcaaacaagggggagcttttaactgcaacaataagctaaagtat
gtaaaatactacattctattcagtcttggagtgttttgtagaaagttatc
ttcagccaaatctttgctgaagactggttgtggagtgttggtaaatgctt
tgtgtttttatgtaaaatattttctaaacaaaaaaatgttaaaagtacatg
tcctctgtagtaaactgatatctatatatatgaatcattcaagcctaaag
tctagtaataaactgtacttgtgaatagagaaaccctaaatattcatgca
gaaaaaattatgcggtctgttaagaaaaaatgagtaatttgtgttttggac
ttgaaataaacagtgttctgtagataattcctcaacttca

>NM_006467 2
actccgcggtcctggtgccgcgtgcaggtcggtgcgcgcttctcccgagg
tggaacgggcggcagtcaagcgccggcgttctctgccgtcaccctttcct
tgccggccggcacttcggctgcagagttttgcccacgcttcgagacttag
ggagcagtgcctttcagaatttgcccactcatctggtataactggttctg
atggctgggaataaaggaagaggacgtgctgcttatacctttaatattga
ggctgttggatttagcaaaggtgaaaagttacctgatgtagtgttgaaac
cacccccactatttcctgatacagattataaaccagtgccactgaaaaca
ggagaaggtgaagaatatatgctggctttgaaacaggagttgagagaaac
aatgaaaagaatgccttattttattgaaacacctgaagaaagacaagata
ttgaaaggtatagtaaaagatacatgaaggtatacaaggaagaatggata
ccagattggagaagacttccaagagagatgatgccaagaaataaatgtaa
aaaagcaggcccaaaacccaaaaaggcaaaagacgcaggcaaaggcacac
cactcactaatactgaagatgtgttgaaaaaaatggaggaattggaaaaa
agaggtgatggtgaaaaatcagatgaggaaaatgaagagaaagaaggaag
caaagagaaaagtaaagaaggtgatgatgacgatgacgatgatgccgcag
aacaggaggaatatgatgaagaagagcaagaagaggaaaatgactacatt
aattcatactttgaagatggagatgattttggcgcagacagtgatgacaa
catggatgaggcaacctattaggcatgaaatttttcaaaaaatattttta
tgatgcagcttctgaacatttggacagacttgatttgtattttatttctg
ataaggaataagtacttgtttctgttgttttggacaaatagttgttacca
aaatattcaaaaccactttgagtttacatactagttaccttaaaaattat

tccctgacactctgacattccttctaaacacctctgccatctctcttatg
tactctcatgggtttttttgtatgatttgaatatgaatgtgcctaaagaa
tttttgctctcttaatctatgtatacatacttgaacaaatcattcttgct
taactgctgatcttttgtaaaactattgctagtcataatggatttctata
acctgaaagttgatataatacagcacgtggagcactttaaaaacaaagaa
acttgaaaataattttatttttttacctttacatggtgtgttctagactac
tgcattatgactacataaacctacctgagctcttacaagtacagaattgt
gagacagaggttttgtgatggaaaacatacacatagggaaaaataccaaa
ctgagcaatataaatttcacagctcacatcttagccagtacaaagaaact
ttcacatggggtgaatattataatacttgaatttgagacttaatactgta
tatgaattaatcatcccactgtaataactgacatcttcaaatagttgggc
ttatgaaaaatttgactaagacattttattttatactttatgtaaatgtg
aaataagttctggtcatataaatatgtagaatgcatgtgtgtgaactcta
tgaagtgaagtgtggtggccttttatgtttcagagtactcaagtgtacaa
ttgatttatataaaaatgcaaaatgaactcaggtcattttgaaattaata
ttaatatttttgttgtttaactttaaagctagctcccctaactttatata
ttttttgggaaaaaatacctaaaaacctcagcctacaaagactctcagaa
atgccaaagattttcaaggaaattcatattatatatttcaaaaatgattt
atcaatgttatctaccaaaagaaataattttatttttcccctttggggag
atattatcctctaatatgagaatcagatccctagattctatttctaccta
tactattaaactcaaccttgaacctgagcttggttgtttctgtgccttag
tttctccactcgtaaacaatttcctataaaaattgtttaaacaccaacca
aaatttaccatatgtattcattctcttgccttaaagatcacttcttaact
tacatttggctctcaccctctaaaaatttgtagtttggagagtaactgaa
agcacaattttaagaaacatgttttcctcacaaaatatattgtaatctga
ttttcctatcttgtattcatgcagaaaattggagaaaatgtgtattgtct
tgtgccattctgcaatatgaatttctcaggaaaaagatgtttcaatggca
atactttcttaaataattcaagatgggtgtgaggacctatcttattagtt
tagtgctgtgttaaccatttgtgaattacttcttgtttaaaggaagttgt
caaaaaattttaagattctaaatggaatacacgaataaagtaagccttag
aatagaaacctcacaaaaagttgtcatttgcggttgataattaacatagg
tgttttattctctcaactccttagtaccagagccaaatacaatttagttt
tcagaaatcttatcaatattgttctatttacctacttataaatctgcaga
taaccacttgaaccaggcaaatacactgaagataaaggttatttcttttt
ttagctttgaatttgtcatgaccattttagtcttgcagatagcagggcag
ccccttggggcaaggatttactctgggaggtaccgttaagagccttcttt
ccccctttgaaagatccttttacaatgttaaagtatactagttgcaagaa
caagcaggatttgcaggttgctttaccagcatgagtctcatttttctggc
ttaaaatctgggactgtgaaattattccataggaaagtgaatgttatttt
gcagaattagcctcttacataaaagtatttgttgaagtgtctttaaaatt
gctatcatgagcaaaactggttgctgtaatgcttgtttttctgtatttat
ttacacattaaattcttacaaaacaaaatgtgttcgtttgttttatagta
agatgttttattttgaacttatttaaatgtttatttgttagagaaaacta
atttgttaatatagatcttaactagtaactattaagcagggatgtttgtt
ccaaatattcttgaataaacaatcaccacaaacaa

>NM_006496 2
ggaagtgtcgtaaacgtcggatatccggttcttctgggcgctaagggagc
tgacggagagggccaccgcccagcaatagacggtgcctcagcctgccgag
ccgcagtttccgtggtgtgagtgagtccgggcccgtgtcccctctcccgc
cgccgccatgggctgcacgttgagcgccgaagacaaggcggcagtggagc
gaagcaagatgatcgaccgcaacttacgggaggacggggaaaaagcggcc
aaagaagtgaagctgctgctactcggtgctggagaatctggtaaaagcac
cattgtgaaacagatgaaaatcattcatgaggatggctattcagaggatg
aatgtaaacaatataaagtagttgtctacagcaatactatacagtccatc
attgcaatcataagagccatgggacggctaaagattgactttggggaagc
tgccagggcagatgatgcccggcaattatttgttttagctggcagtgctg
aagaaggagtcatgactccagaactagcaggagtgattaaacggttatgg
cgagatggtggggtacaagcttgcttcagcagatccagggaatatcagct
caatgattctgcttcatattatctaaatgatctggatagaatatcccagt
ctaactacattccaactcagcaagatgttcttcggacgagagtgaagacc
acaggcattgtagaaacacatttcaccttcaaagacctatacttcaagat
gtttgatgtaggtggccaaagatcagaacgaaaaaagtggattcactgtt
ttgagggagtgacagcaattatcttctgtgtggccctcagtgattatgac
cttgttctggctgaggacgaggagatgaaccgaatgcatgaaagcatgaa
actgtttgacagcatttgtaataacaaatggtttacagaaacttcaatca
ttctcttccttaacaagaaagacctttttgaggaaaaaataaagaggagt
ccgttaactatctgttatccagaatacacaggttccaatacatatgaaga
ggcagctgcctatattcaatgccagtttgaagatctgaacagaagaaaag
ataccaaggagatctatactcacttcacctgtgccacagacacgaagaat
gtgcagtttgtttttgatgctgttacagatgtcatcattaaaaacaactt
aaaggaatgtggactttattgagaagcatggatgttagtgaaagttacta
cagtgtggagtgttgagaccagacaccttttgctgtctcatggggcagct
acaagcatgaacgggaccagggaatggcagcagcatgcagaatcttagca
ctctttagcacaatattttgtattagggaacttttaattgacatgagatg
ctaaagtcagacattggaattggaagaactataaagtgtgattcgatcgt
caagacatcacttggatttcttaatcttaaatgcttatggaagatgtgaa
gttgaggtgctgcattctagaacttcaatatgtagcttactcttttttc
ccccccttcttaaaccaccagtggttcatttttaaggtttttcatcaaga
gaagaataactttactaaattttatttctttatttgcaaaagaatcttta
ttaaaacaaacaatcttaactatgcacatgatgtgaccagatcatcttga
aaatattcctctttagtaggaactctttgtttttaactcttggtatggtc
agaatataatacttccataattacttataattctttccggttactggggc
tataaatacaactttttaaatgaaatttatgttattatcctgctcaaagt
accattatggtttccatatggtaattacattggaaagttctgcaataaga
ttctagttctctcttttctttaagcttatggttgaaggttaaccttgttt
gtgcagattaccaaattactgctgatgtagtatgataaagtcagcttctt
ggatacagacacacccagggcagctgccaatcagaaatgcaaattgctaa
attccaaacagaaccagtgactttgctgctacatatttactaaattgacc
actttgattcttgcttgtttgtctcagttatagggagttttgtacatggt
gcctcttgttttccatcttcatggccttttctgttgagaaacatctaaag
tgtattaacagtgcatgcttttactttgtaaatgtggtgccaaatccctg

tttgccatcgtttttactgtaccgatgttaactgtagtaatccttagcca
gtatgttcttttgctgaaactgttgcattttgggactttttccactttg
tcatgtgtacatttttaatctgttatagttggcggcagtattaaaatggt
gagtaaagagcccaggtttgctcctgtttgtaactagtcttttctggaaa
tactgtcttctgtttccttttgcctttgcagaatgtgctttagcctttgt
ctgagaatgggtttaggtagaagggtttcctgaaagtctagtcttttggt
gtaatagtattgttgaaccttaggttttatgttatatctgcatatgagtg
atatgtgatcatgattcattttgcagatttacattttgcttgtgtggaaa
gttacgttcacttcaacctacagacccttttgtataatgtacagcaaatg
tcattaaatattgaatgctctattgggggaagacaaatgaagagaatgca
ttttgagcatttcaaatcagtagtttgggcagtgccttttggggtccaat
ctttttggattggattctatttcatcttttgatgtgacttttcactagtt
tacaaaaaaaaggttggtggtcaacaaaaaaaagcaagtcatagcagttca
gtgcctttgttggtagtttttaactttttttgtgattctttttggaaaat
catagcttacagatggtataactgtattatataatggaattttctttagg
tgtgggaaaacttgatctgaaagaaaattatcagcttcaattggcgattg
attcagtgcccacaatgtaaacagggttggtagttgttactcattttgaa
tataccttttccttattgtattctgtaatataggatcctggaaatgagac
ctggtggaatattattttcaactgtagtctttctatactgtggactaatg
aaatggtgttgttgataatctaaaataaccaataatccaaaaaagttcat
tttactttgaatttgtattttcatttgaatatctatctgatatgttaaat
ttttagcccttctgttaaagaaaaatagcaggtccccatttttctgcctc
tgtaccatttacatttgctctctgtttgttaacatgaagatcactcttat
ttcaccatcccaaataacttatctaaatgcacattccttgaggacacagg
gagacaatttaagtcagtagtttttaatatgacttaagtaaaaatcactt
ggacccatctaagggattgacttttggctggtgggctggcaatcgatttt
tttttttttttttttttttgagacggagtctcgctctgtcgcccaggct
ggagtgcagtggcgcgatctcggcttattgcaagctctgcctcccgggtt
catgctattctcctgctgggactaccaggttcctgccaccacgcccagct
aatttttttgtatgtcttagtagagacggggtttaaccgtgttagccagg
atggtcttgatcccctgacctcgtgatctgcctgcctcagcctcccaaag
tgctgggattacaggcgtgagccaccgcacctggctggcaatttataatt
tttaaaaggtgattctgattaccagccaggcttgcaaatcactggtttgt
attgaaataaaagatattttcttgtagcagggctaaaatgtgggatgaaa
cattaaatctactagcctgaaaaaaaaactttggcatttgtaatcaaagaa
gctgaaagttgccttgtgtgtttcctctgaagaatgctggagatttgtga
tggataatgtttcagaattcaggtgcctatgctttggaccatcaaagaag
ccattagtctctgtgtaaagaagaagtattcttaatggacagtcccccct
aaccttataaattcagctttgcaacagatttcacctcctagcatgaaaat
tccaaaaactttaacaattactcataagtggatcttgagatgacattttc
atcttggacagtttgaattctaaatctctagttcacagtgaatcgttttt
taaatttttactgatggatcgttatttttcacaaggtactacattcttac
aatgtaagactagatggaccaaaattttaaaaaaaacttcttgccttgtt
tttttaattttcatatatattctatttgttgtatatacacattagactat
tttaaaataacagctggtttttgtaattcattcattcaccctatattctt
atattctgctcattattctctcatttatagctgtgacattctttggacat

tacttgcctggcctgattagttacttaatataatttattcatttgcttag
catgtgtgacatagttctggaatttcctgttgcagaactccttgctgtat
tgcagtcatatataaggtaaagtcagtctcttgttcctattaaaaagcta
ccattgattattttccctttttacatgttc

>NM_014320 2
ggcgggccgaagcgaggtgcggctccctgtcgccgcggaggggcgggggc
aggacgcgcaactccggccggagctgtccggggtcgtgagccggccccgc
cttggtggcggcgccccctcgcggtccagaggcagacgcatcgggtgggc
tcgggtctccagcccggccgggaggagggaccgggtctgcggagcgggga
ctcggggcctcggcggggcgcgcacacgcaggcggggcggcccggggtgc
ggggcctctgcgcggctgaccaggctcccagagcgtcagcgccgcccatg
gccgagccgctccagccagaccccggggcggccgaggacgcggcggccca
agctgtggagacgccgggctggaaggccccggaggacgccggcccccagc
ccggaagttatgagatccgacactatggaccagccaagtgggtcagcacg
tccgtggagtctatggactgggattcagccatccagacgggctttacgaa
actgaacagctacattcaaggcaaaaacgagaaagagatgaaaataaaga
tgacagctccagtgacaagctacgtggagcctggttcaggtccttttagt
gagtctaccattaccatttccctgtatattccctctgaacagcaatttga
tccacccaggcctttagagtcagatgtcttcattgaagatagagccgaaa
tgactgtgtttgtacggtctttcgatggattttctagtgcccaaaagaat
caagaacaacttttgacattagcaagcattttaagggaagatggaaaagt
tttcgatgagaaggtttactacactgcaggctacaacagtcctgtcaaat
tgcttaatagaaataatgaagtgtggttgattcaaaaaaatgaacccacc
aaagaaaacgaatgagaaaaatgaaaggaagttctgctgtcagaggcaaa
acatctgtttatcatagacatcaacatgacctataagtaaagtgcgtgtc
tagtgtcttctattgagagtactactattaattaagcttatttccaatgt
gcctttttaatgcttgaagttttatctacatacacaggtaacagaggaca
gtagtctgtaaacatataaatcggtcataactatcgtggtctttatttct
gtgaggatctagggaaatttcatgtcacttccctccttcactgcatcaca
atcatattcccttttttttttcttggatttgtgtcagttggatgatatcc
cctccagatagtatcaataaaatgttaaaatt
>NM_014445 3
aaacgcgcacgcgcaaatctagggcgacgcttgacagagcttgggagggt
gcgcctgctttcgccctccttctccagcgggaggggcgcgcacttccgcg
gggcggagtccgtctagtgctgacgttggcagccgaacccaaagtagatc
gaggcggcgggctgcacattcccgttgttgcgttgcgtttccttcctctt
tcactccgcgctcacggcggcggccaaagcggcggcgacggcggcgcgag
aacgacccggcggccagttctcttcctcctgcgcacctgccccgctcggt
cagtcagtcggcggccggcgcccggcttgtgctcagacctcgcgcttgcg
gcgcccaggcccagcggccgtagctagcgtctggcctgagaacctcggcg
ctccggcggcgcgggcaccacgagccgagcctcgcagcggctccagagga
ggcaggcgagtgagcgagtccgaggggtggccggggcaggtggtggcgcc
gcgaagatggtcgccaagcaaaggatccgtatggccaacgagaagcacag
caagaacatcacccagcgcggcaacgtcgccaagacctcgagaaatgccc
ccgaagagaaggcgtctgtaggaccctggttattggctctcttcattttt

gttgtctgtggttctgcaattttccagattattcaaagtatcaggatggg
catgtgaagtgactgaccttaagatgtttccattctcctgtgaattttaa
cttgaactcattcctgatgtttgataccctggttgaaaacaattcagtaa
agcatcctgcctcagaatgactttcctatcatgcttcatgtgtcattcca
aggtttcttcatgagtcattccaagttttctagtccataccacagtgcct
tgcaaaaaacaccacatgaataaagcaataaaatttgattgttaagatac
agtagtggaccctacttattcagtcaattaagagtaagtttttttatgtg
gttattaaaacagtatgaacaattagtctaactctgcatagacagggtct
agattttgttaacccaaatgtataactgcagttagcttaaattacaattt
gaagtcttgtggtttttatatagctaggcactttattactcttttgaact
gaaagcacactcccttataggttcatgtaactgtcctgtaataaggtgct
tataaatggaacaactacacagcctagttttgccacaacctttagcatct
aaaaagttttaaaagcttctaaatgtctaatataaagggagatgcttata
gccacaacatctattttaccaatattgtttccattacactaccttggatt
ttgcatgagtgagtatagtaacccaagatgccataaaaaaaaaacttgat
cgttttctgacttaatcagttactgtggtttcactaaaagctaccgtggt
ggagtgaagtcagtcagggaaggtttgtttatgttacatttatttcacca
gaactattttaatatatcaaaggggtttactatgccaaacaaaattctag
ggaaaaatactgctaaaaatggatgcctcatcagaacatgctgttgagtc
caatgtgccataagacattttagcatgttaaatagcacttttaatagcaa
aaaaaggcacatcaactgcgaagttatccttagtttgcaaatgctttttc
tagattaatgatttttcaatcattagggtactagacacatcagcctaaag
tggcatctggaattgaatggatttactgataatgatcagtctttagtctt
ccctttgttatatgactttataggttatgattgatcaaatttacgtttta
ctaatggtaagggtgagggtcatagggcaggttttgggttttctagtact
gttgaaaactgcaagtattggctatttgtatacttagccataacttggtg
aaaaaaaacctgagcagtgtctatgtattaatgcgttggaaagaaagctg
cttgtgtttgctttgttaattgcctcaggatatttcttttaaaataagct
gttttaagaggaacagaagggaaatctgctacctagtctatacacagcgt
gaacctcacagggggcttctgataccctcaaacatggagaacagtaaggg
agcagagtggttaaggactttcaggaacttaactattctggaataaggaa
tgaatcaactgaccttgggccagcaggtttttaactaaattgttacttgc
ctttctcacccagttaatcagtctctgtacttgtttcccttttttgaaaca
agtgtcttggttaactaattctgttttatggttgtgctaaattcatagca
ggtgccttattctttgcttttagtcaaaccattccatatcagaattttcc
ttggtttactatagatatttggctttaagttgttgtttgtgtttttttaat
gtacaatgttctgataaatttgactgttaaattgctatagctagcaatca
ttttacatatgtaaaattgcattccctttgtatttcatgtgtaattcacc
aattaagtgcagtttatattcaggttggattatgcatgtttaggtaaacg
aaagctgtgtcttacttgatttattctttaaaaataaagttccctgaata
tttgatgcttttcttctaaacggaaatgattttacagttatctgagtgta
ccttttatagttagtagaaaatgattttaaagaatgtttagtattgtact
taaatggtatgcagaggcacagatgtaaggtttataactggaaataggtg
gtaagaaaaatatatagaaagcacaatgatttgaatatttttccacttag
gatttcctaatctccttgtcatccaattcaagctcaagatgaagcacaat
tctttgatctccctttgccagttgaattttatagatcatctaatgttgag

cacagtatgagaataaatattggggttgtcaacattactcagttactctt
tgtggtttaactctaacatttcaacaagttgtcaattaattgtatctgtt
gggttgtatataatgttgctcaaaataattaagtggacttccaaaaataa
gatttccattgtaacaggatgcattgtgatgggctttgacttacattaaa
gaaatgtggatagtcaactgcaaaaaaaaaa

>NM_014762 3
aatcgcgaggcggcgggcgatcccgggctccccgggctgtgggctacagg
cgcagagcgggccaggcgcggagctggcggcagtgacaggaggcgcgaac
ccgcagcgcttaccgcgcggcgccgcaccatggagcccgccgtgtcgctg
gccgtgtgcgcgctgctcttcctgctgtgggtgcgcctgaaggggctgga
gttcgtgctcatccaccagcgctgggtgttcgtgtgcctcttcctcctgc
cgctctcgcttatcttcgatatctactactacgtgcgcgcctgggtggtg
ttcaagctcagcagcgctccgcgcctgcacgagcagcgcgtgcgggacat
ccagaagcaggtgcgggaatggaaggagcagggtagcaagaccttcatgt
gcacggggcgccctggctggctcactgtctcactacgtgtcgggaagtac
aagaagacacacaaaaacatcatgatcaacctgatggacattctggaagt
ggacaccaagaaacagattgtccgtgtggagcccttggtgaccatgggcc
aggtgactgccctgctgacctccattggctggactctccccgtgttgcct
gagcttgatgacctcacagtgggggggcttgatcatgggcacaggcatcga
gtcatcatcccacaagtacggcctgttccaacacatctgcactgcttacg
agctggtcctggctgatggcagctttgtgcgatgcactccgtccgaaaac
tcagacctgttctatgccgtaccctggtcctgtgggacgctgggtttcct
ggtggccgctgagatccgcatcatccctgccaagaagtacgtcaagctgc
gtttcgagccagtgcggggcctggaggctatctgtgccaagttcacccac
gagtcccagcggcaggagaaccacttcgtggaagggctgctctactccct
ggatgaggctgtcattatgacaggggtcatgacagatgaggcagagccca
gcaagctgaatagcattggcaattactacaagccgtggttctttaagcat
gtggagaactatctgaagacaaaccgagagggcctggagtacattccctt
gagacactactaccaccgccacacgcgcagcatcttctgggagctccagg
acattatcccctttggcaacaaccccatcttccgctacctctttggctgg
atggtgcctcccaagatctccctcctgaagctgacccagggtgagaccct
gcgcaagctgtacgagcagcaccacgtggtgcaggacatgctggtgccca
tgaagtgcctgcagcaggccctgcacaccttccaaaacgacatccacgtc
taccccatctggctgtgtccgttcatcctgcccagccagccaggcctagt
gcaccccaaaggaaatgaggcagagctctacatcgacattggagcatatg
gggagccgcgtgtgaaacactttgaagccaggtcctgcatgaggcagctg
gagaagtttgtccgcagcgtgcatggcttccagatgctgtatgccgactg
ctacatgaaccgggaggagttctgggagatgtttgatggctccttgtacc
acaagctgcgagagaagctgggttgccaggacgccttccccgaggtgtac
gacaagatctgcaaggccgccaggcactgagctggagcccgcctggagag
acagacacgtgtgagtggtcaggcatcttcccttcactcaagcttggctg
ctttcctagatccacactttcaaagagaaacccctccagaactcccaccc
tgacagcccaacaccaccttcctcctggcttccagggggcagcccagtgg
aatggaaagaatgtgggatttggagtcagacaagcctgagtccagttccc
cgtttagaactcattagctgtgtgactctgggtgagtcccttaacccctc

tgagcccgggtctcttcattagttgaaagggatagtaatacctacttgca
ggttgttgtcatctgagttgagcactggtcacattgaaggtgctgggtaa
gtggtagctcttgttgcttcccgttcagcgtcacatctgcagtggagcct
gaaaaggctccacattaggtcacctgtgcacagccatggctggaatgatg
aaggggatacgctggagttgccctgccatcgcctccatcagccagacgag
gtcctcacaggagaaggacagctcttccccaccctgggatctcaggaggg
cagccacggagtggggaggccccagatgcgctgtgccaaagccaggtccg
aggccaaagttctccctgccatccttggtgccgtcctgccccttcctcct
tcatgcctgggcctgcaggcccaccccagccaccactgagtccactcgga
gtgccctgtgttcctggagaaggcattccagggttgaatcttgtcccagc
ctcagcctgggacacctaggtggagagagtggtctccgctctgaattgga
tccaggggacctgggctcattcttcttggctcaccaaccctgcaggcctc
atctttcccaaaacccactttgtcttggtgggagtgggtccgcgctgctc
tgcagcaggggctggggagtggacagcatcaggtgggaaagtggagtcca
ccctcatgtttctgtaggattctcaccgtggggctggaagaaaagagcat
cgacttgatttctccaaccactcatccctctttttctttcttccaccact
ccccaccccagctgtagttaatttcagtgccttacaaatcctaagctcag
agaaagttccatttccgttccagagggaagggaacctccctaggtccttc
cctggcttgttataacgcaaagcttggttgtttatgcaactctatcttaa
gaactgcccagcctcagctgaaaacccgaatctgagaaggaattgcgtca
tgtaagggaagctggaattaagggagctgagccagtcatggttgtggcgt
gtgagtcaggagacctaggtttcagcccctctctactgtcagcgagctgt
gcaacgtgggcaagtcattgtcctctgagctgcagtttcctcatctgtca
catcgctacagacaagacctccctggaacccttctgattgtcttagacac
tgtggttgcaaaacccacggaaagcctcatttgtgtggaaagtcagagga
aaaatgatccagtggacacttggggattatctgtcattcaagatccttcc
ttcaaccccaaggtcagctcccatctcatttccagaaaggctcatacctg
gcttgcagggaagcatctgtcttgtcattccaggtgccagaatcctctca
gagtcattgaagggtgttcacccatcccacccaaggcttggcacactgcc
agtgtcttagcagggtcttgtgagggctgggggcatccaggcactcagaa
ggcaaaggaaccaccctacccatttggcctctggagggggcagaagaaag
aaataaacctcatcctatattttacaaagcatgtgaattctggcattagc
tctcataggagacccatgtgcttccttgctcagtgcaaaactgatgattc
tacttgctgtagatgaatggttaacacgagctagttaaacagtgccattg
ttttgccagtgaagcctccaaccctaagccactgggacggtggccagaga
tgccagcagcctctgtcgcccttagtcatataaccaaaatccagacctta
tccacaacccggggcttggaaaggaaggtattttggaatcacaccctccg
gttatgttgctccagtaaaatcttgcctggaaagaggcagtcttcttagc
atggtgagctgagttcatggctttttttgtagccagtcctgtccctggc
catccatgtgatggttttggatggagttaaacttgatgccagtgggcagt
gcatgtggaaagtatcagagtaaggctctcccctccagagccctgagttt
cttggctgcatgaaggttttctttagaatcagaattgtagccagtttctt
tggccagaaggatgaatacttggatattactgaaagggaggggtggagat
gggtgtggcagtgtatggtgtgtgatttttattttcttctttggtcatgg
gggccaaggagaaaggcatgaatcttccctgtcaggctcttacagccaca
ggcactgtgtctactgtctggaagacatgtccccatggctgtggggccgc

tgcttctgtttaaataaaagtggcctggaagctggc

>NM_018226 4
atggccgcgcagtgctgctgccgccaggcgcccggcgccgaggccgcgcc
cgtccgcccgccgcccgagccgccgcccgccctggacgtggcctcggcct
ccagcgcgcagctcttccgcctccgccacctgcagctgggcctggagctg
cggcccgaggcgcgcgagttggccggctgcctggtgctcgagctgtgcgc
gctgcggcccgcgccccgcgcgctcgtgctcgacgcgcacccggctctgc
gcctgcactcagccgccttccgtcgcgcccccgccgccgccgccgagacg
ccctgcgccttcgccttctccgcccccgggccggggcccgcgccgccgcc
cccgctgcccgccttccccgaggcgcccggctccgagcccgcctgctgtc
cgctggccttcagggtggacccgttcaccgactacggctcctcgctcacc
gtcacgctgccgcccgagctgcaggcgcaccagcccttccaggtcatcct
gcggtacacctcgaccgacgcccccgccatctggtggctggacccagagc
tgacctatggctgcgccaagcccttcgtcttcacccagggccactccgtg
tgcaaccgctccttcttcccgtgcttcgacacacctgccgtgaagtgcac
ctactctgccgtcgtcaaggcgccatcgggggtgcaggtgctgatgagtg
ccacccggagtgcatacatggaggaagaaggcgtcttccacttccacatg
gagcaccccgtgcccgcctacctcgtggccctggtggccggagacctcaa
gccggcagacatcgggcccaggagccgcgtgtgggccgagccatgcctcc
tgcccacggccaccagcaagctgtcgggcgcagtggagcagtggctgagt
gcagctgagcggctgtatgggccctacatgtggggcaggtacgacattgt
cttcctgccaccctccttccccatcgtggccatggagaacccctgcctca
ccttcatcatctcctccatcctggagagcgatgagttcctggtcatcgat
gtcatccacgaggtggcccacagttggttcggcaacgctgtcaccaacgc
cacgtgggaagagatgtggctgagcgagggcctggccacctatgcccagc
gccgtatcaccaccgagacctacggtgctgccttcacctgcctggagact
gccttccgcctggacgccctgcaccggcagatgaagcttctgggagagga
cagcccggtcagcaaactgcaggtcaagctggagccaggagtgaatccca
gccacctgatgaacctgttcacctacgagaagggctactgcttcgtgtac
tacctgtcccagctctgcggagacccacagcgctttgatgactttctccg
agcctatgtggagaagtacaagttcaccagcgtggtggcccaggacctgc
tggactccttcctgagcttcttcccggagctgaaggagcagagcgtggac
tgccgggcagggctggaattcgagcgctggctcaatgccacaggcccgcc
gctggctgagccggacctgtctcagggatccagcctgacccggcccgtgg
aggcccttttccagctgtggaccgcagaacctctggaccaggcagctgcc
tcggccagcgccattgacatctccaagtggaggaccttccagacagcact
cttcctggaccggctcctggatgggtccccgctgccgcaggaggtggtga
tgagcctgtccaagtgctactcctccctgctggactcgatgaacgctgag
atccgcatccgctggctgcagattgtggtccgcaacgactactatcctga
cctccacagggtgcggcgcttcctggagagccagatgtcacgcatgtaca
ccatcccgctgtacgaggacctctgcaccggtgccctcaagtccttcgcg
ctggaggtcttctaccagacgcagggccggctgcaccccaacctgcgcag
agccatccagcagatcctgtcccagggcctgggctccagcacagagcccg
cctcagagcccagcacggagctgggcaaggctgaagcagacacagactcg
gacgcacaggccctgctgcttggggacgaggcccccagcagtgccatctc

tctcagggacgtcaatgtgtctgcctagccctgttggcgggctgaccctc
gacctcccagacaccacaattgtgccttctgtgggccaggcctgccatga
ctgcgtctcggctctggccatgagctctgcccaggcccacaagcccctcc
cctgggctctcccaggcagggagaatggggagagggacctccttgtgtct
ggcagagacctgtggacctggcctccccactcccagctctcttgcactgc
aggccctggggccagcccgcacacaccatgcctcctgtctcaacactgac
agctgtgcctagccccggatgccagcacctgccaggtgccgccccggggc
aagggccccagcagccctatggtgaccgccacactgtgccttaatgtctg
ccgggggcccaggctgtgctgtccctgcagcacgcctccttgcagggatc
tgagccaccctccccgcacagccctgcaccccgcccctggggttggcagc
ctcagttggcccctggcagaggaacaaggacacagacattccctcagtgt
ggggggcaggggacacagggagaggatggttgtccctggggagggccctc
tggccccaggcaaccttagcccctcagaacagggagtcccaggacccagg
gagagtgtggggacaggacagcctgtctcttgtagcttcctggggtggga
ggcacaggggcaaagcaataccccagggaaagtgggaggtggtgctggtg
ctctctccaggcccaccatgctgggagaggcggccagagcctggggcctc
cagcctgggactgctgtgatggggtatcacggtgatggtcccattaaact
tccactctgcaaacctgaaaaaaaaaaaaaaaaaaaaaaaaaaaaaaa

>NM_020360 3
gggccagcgcggcccgggccgccctcgcctcccacccagggcgctgagca
cgcacgccgccggcctggcggcgttcgcggggcccgggaccagccgccgc
cttctccaggcgctcctactctctgggcatctgcagtccgagcggaaggg
gcgggaacgtcggcgacgccgaggagcaaagcccacgatcggcttcccgg
ggcagcggacggctgccttcctcccggcgcaaacgcaccgcccaggaggg
ggcgggctcggcgggtcggggggcggggccggggtccgagctcgggcccg
cctccgcctccgccagctcctgtgagctgccgagtgctaggcacccgggc
tcttctgggggctccagaggcgccgcccaagagaccctgggccggcgccg
ggcgcagctgcctctccgtctttgtgtctgtctctgtgtctgtctggcta
tctccgagtttgcctccgcttccagaactaagccacccagacaccatcat
ctcgaaaaccccagcccttctcccatggcaggctacttgccccccaaagg
ctacgccccttcgcccccacctccctaccctgtcacccctgggtacccgg
agccggcgctacatcctgggcccgggcaggcgccagtgcccgcccaggta
cctgccccagctcccggcttcgccctcttcccctcgcctggccccgtggc
cttggggtctgctgccccccttcttgccactgccaggggtgccttctggcc
tcgaattcctggtgcagattgatcagattttgattcaccagaaggctgag
cgagtggaaacgttcctaggctgggagacctgtaatcggtatgaactgcg
ctctggggccgggcagcccctgggtcaggcggccgaggagagcaactgct
gcgcccgtctgtgctgtggcgcccgccggccgctgcgtgtccgcctggcc
gaccccggggaccgtgaggtgctgcgtttgctccgcccgctgcactgtgg
ctgcagctgctgcccctgtggcctccaggagatggaagtacaggctccac
caggcaccaccattggccacgtgctacagacctggcatcccttcctcccc
aagttctccatccaggatgccgatcgccagacagtcttgcgagtggtggg
gccctgctggacctgtggctgtggcacagacaccaactttgaggtgaaga
ctcgggatgaatcccgcagtgtgggccgcatcagcaagcagtgggggggc
ctggtccgagaagccctcacagatgcagatgactttggcctacagttccc

gctggacctggatgtgagggtgaaggctgtgctgctgggagccacattcc
tcattgactacatgttctttgagaagcgaggaggcgctgggccctctgcc
gtcaccagttagaggccaccatggtgtgaggagaccatcacctcgaccag
aactccagatggtcacctgccctggcccctcctctgggcagcccctttcc
tccatgtacactgcaggggacagaagggggccccatccctaccctactc
cctggccgcctgcccctgtggttcccaaggaggggtatgtatgagagccg
ctctcctgctacctcccaccactgtcccagcagtccctcggcacacaggc
atatcagctttcacactttccccatgcactctctcccacccccttccagg
gcctctgctccaaaggaggcctctggaacccaggactctggggttttaca
agagggctggggtgtggaagggcaagctgcaccaaagacggtggatatag
ccaccgcccccccgccgctgcctagcatctgcttggccaattagttcagc
ctcagaccatggcactttgagggggtctctacctccccatcaacagctgc
agggggaccccagtgccaacttcctctcccactagggccctgccttcagc
tggtgcttgctgcgattcctgtgccttatgtaactgcccttccttccctt
gccctaggaaaaaggctgcatctttatatgttacattcatataaactttg
taactttttggacattgaaaaaaaaaaaaaaaaaaaaaa

>NM_021961 5
ttccgaacattcttagcatcgctcgcgccgcgccgcgccgcctgagccga
gccgagcctctgctgccgccgccgcggccccgccgcccgccgcgggcgcc
caccaagcactttgcagactcgcttccaccctgcgggccattccgcgcgg
cggggcccgggcccggggcggccgcgtccaggcacaggccatgcagtgac
gcccccccacccctccacctttgcccggagcgcgggcagcagcccagcgc
gccagccggccccggggcaggagcggtgctaggcaggggtggggtggccg
ggcccagggaccgggagccggggagggagccgggcaccgagcagagggcg
ggggaagcggcgccgaagtttgcctcggactcgccgggcgctgcggtggc
tccctgggccgaggactgttgctgccgctgccgccgccgcttcattgcac
attcaagtggaaaattttcaggagtcagcagaaacattgtgtccaaaaaa
gactgagtcgcagttaccaccaaacccaggaggagactctccctggaaaa
cttcccttccctttcggtttattttcttgaaaaggctccaggcttcggct
tggaaaatcccaccgccaaaattgagcccagcagctggagcggcagtgag
agccctgccgaaaacatggaaaggatgagtgactctgcagataagccaat
tgacaatgatgcagaaggggtctggagccccgacatcgagcaaagctttc
aggaggccctggctatctatccaccatgtgggaggaggaaaatcatctta
tcagacgaaggcaaaatgtatggtaggaatgaattgatagccagatacat
caaactcaggacaggcaagacgaggaccagaaaacaggtgtctagtcaca
ttcaggttcttgccagaaggaaatctcgtgattttcattccaagctaaag
gatcagactgcaaaggataaggccctgcagcacatggcggccatgtcctc
agcccagatcgtctcggccactgccattcataacaagctggggctgcctg
ggattccacgcccgaccttcccaggggcgccggggttctggccgggaatg
attcaaacagggcagccaggatcctcacaagacgtcaagccttttgtgca
gcaggcctaccccatccagccagcggtcacagcccccattccagggtttg
agcctgcatcggccccagctccctcagtccctgcctggcaaggtcgctcc
attggcacaaccaagcttcgcctggtggaattttcagcttttctcgagca
gcagcgagacccagactcgtacaacaaacacctcttcgtgcacattgggc
atgccaaccattcttacagtgacccattgcttgaatcagtggacattcgt

cagatttatgacaaatttcctgaaaagaaaggtggcttaaaggaactgtt
tggaaagggccctcaaaatgccttcttcctcgtaaaattctgggctgatt
taaactgcaatattcaagatgatgctggggctttttatggtgtaaccagt
cagtacgagagttctgaaaatatgacagtcacctgttccaccaaagtttg
ctcctttgggaagcaagtagtagaaaaagtagagacggagtatgcaaggt
ttgagaatggccgatttgtataccgaataaaccgctccccaatgtgtgaa
tatatgatcaacttcatccacaagctcaaacacttaccagagaaatatat
gatgaacagtgttttggaaaacttcacaattttattggtggtaacaaaca
gggatacacaagaaactctactctgcatggcctgtgtgtttgaagtttca
aatagtgaacacggagcacaacatcatatttacaggcttgtaaaggactg
aacatggttatttatatatatagatatctgtatatacacacacacatatg
tgcacacacacactctctctccattatcgaacgactgactgtaaacctca
ccacacagggtggtgccctggccccgaggtcaccccgacttttctaaatc
ttgtttgagtgaagtcatttttttcatgtgttcatactatcattgtagctg
tgaagttctggtacagttgtaaaaagagaaattgagttgtttctctatgt
tcttcagatgtgcagcccacaattcctcgggaaaggtgaacctgaacaac
ccaagtctctctctgcagagccctgtttctaattgtggtagaaaatattg
agacagagcatttgccatgggacatttacagcctttatacaaatgtattt
agttctcttttttccaacataaaattcttgttttaagatacaagtaaaat
taatctttaaatataaatgtaaattagtacacaaaactaagaatctttag
acttatctttgtaactaattagggtggaagttatgaaagaatgtaattca
ctaaattatttttaaatgaaaccttttttttcttttttgaaaccaaatg
ttaaactatagccttaagaaatgcttggtagaagtgtcctaatgagacaa
atttgtacttttatcctcaaggttaacactaatctcctaatccattaaac
tcttgaacaggtattacaaaggaagaaaacttcaccccttatccttaaca
tatatagtatatttaaaaaatataaaattgtattgtactaatgtgatgat
ggattatttaatgaaaaagaaaaaatggctctttttgcaataagtagata
catactgaaaaaatctaaacttacaatgtttatagtcttgtgtgtgcagt
tatattttatatggacgaccaaattttttattaagatgagtaaatatttg
aaccactgaattttaataacaaaattttaaaattggcatgaatacggaat
actgcactgtgagatgcaaagtatacagaatctgtggctgggagaaaatt
tcatcaaatagacaagtaaaaggctcatcagttttagcatctctgctccc
cagaaaattgtaagcatcctcaccagcctgtggatacattctttatttct
agtgacccaatatgcatattaacctgctataactagggctatatgtgtag
gtatgtgtatacatatacacaaatgcacatatagagttaacacatttagt
gaacacttgtttagtgtcactcagtttgctaggtgctgatatgtacgtat
atctcaatgtgtctgtagacttagatacatcctcttgaagcacatccatt
tctttagcgtctctcagtaagttacagtacttgtttgacttaggtttaag
aggcccagctacctatctctgaccttttcaaataggctcatttgggagat
tcttttgccaggagagattcaactttccaatctaagtattccagagcatt
gcccaggcagagttggtttgatgtggccagatgttttgagttatttccct
taagtgtttcactggggagagaacagggagtgctcctccagcttcccaaa
gaaatatgtttttgtaagtggtaggaacatgtgcacacaatagaacatga
aataagttttttaacttgtaaaacatgtcaagatttttccaccaagctag
aaaataaaaaaacttagttctaccacatccaattaacttacacacccccctt
ccctgtctcaacacctgctttgaccctgcttttctattattacatcagtc

agcatcttgtggtccctaacatgaggatgtggctggctcgtgggaaacag
caaaacactaagcctgacctctcccaaattgggaagaccagaggagaaag
tgcaaaactgtccccatttggaatgcccattccttctagaaaccagttgg
acagtgctcctctgcccttcataaacagactactgttgggtccctgattc
caggctggcctgtgaaggattgccccaggtgtcccctttcacggttgtca
catttacagtgacttctgttgaacacccctcttagggatgtttcttttgc
tcttatttcctgcatctttccttaagggaagccccatcctctcccaggac
caggagtttatgaccaggcgagcacaaatggctaaaagccaagctgtcct
agaacttcagtgggagagctgtctggttcatattctacccaggaatggta
cttttcagtgcagccaggagggctcttgggatttcctttccaaagcacaa
aaatactgggacccaagaagaacagctagaggacaactctgttggcacag
agacggggacagcccagtctgctgacctcacagggtcagctgggcccccc
tggtgcttcaccacctgcatcctcttgctcagaatgcctttgcagttgag
ttttctgggtttctatgattgaccttgaggtttactccttgctcttacaa
catttctaaggatttttaaaagtttacttcttgtcttgttcttctaaagc
tttctccaggacagatattttccctgtcttaaccactggtccagtcatcc
cagtgggcttctctttgtctctcccagattagacctttgggtgagattgg
catcacaacatctaatctgagtctgtcttttgtccttcattctgtatggc
agtctccctttgttataaaagctttctaaagcatactaaagaagccttcc
cagagccccgtcttgcttctcttccaggtgctctatcccctcgagaccct
ctggtgccaggcttgcttcacggccatcttgtgttgtcactgcagagttt
ggaggccagttttccacagcctaaacagggaggagctgcagaatggggct
ctggtctctgggcattcatttccctcatagaggctgagaataaaacaagg
acttattcacacatgttctagaaccccagaatggcccaagttacctgaga
ccagggtttctcaaccttgacaccattgacattttggactgggtaattct
ttgttctgcagagctgtcctttgcactgtaggagatttactaatatccct
ggcctctacccagtagtaccactagcacctattccccacccagcgtgtct
ccagatattgtcaaatatcccatcgggtgcaaaatgatccctggtcaaga
tctgttgcccaagatgttacaggtcacaatgaccacatttgaaattgttt
tccctttcattttaccctgtgaaagcatctctcctagagccttgcaagag
gcaggtgacattgtgtccatatttcttcctgtttcagaacttctgtttca
caacaatttctctctcgctacaagtattctttcactcagcactggggaag
ttgggaacagctggtcaccatcatccctttaatcaactcacacctgttta
aagagtgtttctgatttgaccttcatcccttagtttactggcgttaaaaa
aagtctcagcaattttcattatttctcgtgggtctcattatcaaaccttt
acttatttcggcatatttcctctgggcttcttctagtttctgccttacaa
gcaatgctgttctgtaaatttattgaaacctctggaacatttcaccttta
gagatggaggatggaaggattggtaccagaagagggctaagatacgtttt
ctgtcttgagctgaaagcacagtctactctccttcgttttgtcgatgaga
aagttgaggccagaggggaggtgacatgtttagagtcacccagctggtta
gtgacagaaaaagcgtgagagttgtctaggattcctgccactttggtccc
tggcctctcctgggggaggctgctgttcttaggtgctctaagcttaatcc
ctcagaatgtgtggacaggtcagcttagaagagatggggagattcaggat
ccccctgtgccagagcacagcctcaccggatgctgcttcccacactgaag
tgtcctgtccgaccattgctatctgaggcatccacaagcaggtaggaaag
ctggcgagccattttacttcctgaggacaattccccagccacaggctctg

agtcaaatttctatttggtaagcatcctagcagcaaagtcctgcactcag
accagccaaaaaacagcccccattccaagtacttggtgtcaaaagtcccc
gaacgacttttaaacccaagtcttcttaaggtttcagtactgtggtggct
ttagcagttgtttttgtgcaactataaattatttaaatcatctgagatga
cagtcaattttacaaaccaggtacatattaatttgtataattttgtatat
gctctggtacactacctgaactaacgaagggtagaactaattctgtttgt
cagtgttcacacctgtaacattaggaggatatgtctgcattgcttatttc
tttatgttggtgtttctgtggcaaagccctgcacatggcatttctgaaaa
gccttaaatctttaagatgttgcatgtagggtatgcagtgcaaaaggctg
cctcagaactgtgagcccttttgtaagctggaagcatttctcttactact
gttacttttgtaggaagttttcaattcagagctgccaaagtgttcccgta
agcagtgccttagtaataccttagtcatgccgccagccttttcttacacc
aattcctaatgttcatttacgaattggcccaatattggaaacaaaacaag
caaaaattgtcttcatttttgttttgtaagcccatttttttctccagttct
ataggaaactgactgcttggtgtaaaatccgaaactggacacaagtcagt
tctttcaccacactcaaatgtatataccaaaacaaaaggttgcaacttca
tagtttactatgaaaagcaaattgtactttttaatgttgccttttaaatt
catgaccaaatacttagctatttgtgaatcttctgcactctagcatgaaa
gtgcctttggtttgagattccagcttagaaaagtgctgccataataacga
taatttgtagagagaccaaaaatattttgagatcaccgtaatgcctttgg
tttaccgggatgagtaaccaaccacaggcctctgttcacaagagcacgac
gtggtccccgcctgctgctagtctgtctgccactgggggcctcccaacat
ccatagcacacttcagcggaaggaccccagaaactgttgtgtttgtgtgt
gctgatgacctagtgtgtcatttcacctcgtcacccagccctgcgtccgg
atgaggggacttctgcacaaatgacagaatctcggctggtggacagatac
tacagctttctcctcctccttgtgttcgtgttcagtctctgtggagactt
tcttttccattcaaatgacagtgcgcacttatctggtttacacaatgata
ccattttgaaagttggaagcctcaaactgagacgacagtgcagaacaaaa
caaaagtgagttagggtcgttaaaattgaagtgttcttcttagggcaaac
atgttgactccgagtattgtgtatgaatgtgctacgagaaacttccaaag
agcaccattcacaatttggcattttcaaagaatgttccagccctcaaagg
ggcaactctttaaagtccttgttggcttttatccaaaccttgtagaaatt
gggaaagctgatagaggtaaggaagacgagtgaaaaggacaagaaggcca
aacaccagccaaaaagaaactaggaaaaaaagattttctttgctaatata
gatgtaaaaataacatcagacatctttgaaaattagcctctaaactctta
atacatacgttctgtgtgtctctacctggcgtctttaagaatatcctctc
tgggctctgaaattttaggagtgattcttatccactccaagttgtaagta
tttgtagaaatttgtgcaaacaaacaaaaactatcaaatgaaaagaaaat
gtactcaacctaacttatagttagcagctggaattctcaactcttccctg
ccagcactataccacagtgtggaagaaattagtcaaatgcttgttttcct
gcttctcttttcaactgttactgtgctttgtttgaaagtagttttctctc
tcaaagccgttgcttatatcgttaagaatgaaggtttgtgtttaaaattt
attgcattgcaaagggtagtttcactgaagtcatgcaccattaaataaga
tgaaatatttgtatttattgtcctacttcctaagccgtaacttcttttcc
tctgtgaatttgcattgagtcactcatgctacactacatcgctttagtat
ttgagatggcatttatgtttcctctcgtttatcatgaaatggggtcagat

tccatcagattccacctctgtcaggtggactcttgtctgccttccatgat
gagatttttttctccttcccctttctttaagagaggctgacagatctag
gtgtcaatcaattggaaaccagtctctgattttttttcattagttatttt
ctatcattagtttcactgtgtaaattagatatcaactgcacttctttaaa
aaaaaatacatctccctattacctccttgaaagatttacttctgtaggcc
tttttcaataggctcatgactgcagacaaggaaaaaaaaagtaaaaacaa
aaacagtatgtgcctgaaaatgacaaaaaaaaaatttgtaacatttaaaa
aagaaacctgaatagcctttaattctttaataatacacttaaattttatg
taaatcggttttcgccacgtgtgtttgttcacattctaaatgacttaatg
ggattctcacggtctgtgtctttgtgtcacgtgtataaaatgggcttgtg
atgtaagcgtttcatctggtcagtggttcctttgatattgtactgctgct
gggagtgggctgtggaacctgccttcgggtaactgggttcctcttgggta
gattggagagatgggggtgggcgtgggcaaattctcacacatgttttctt
aacctatttgcagaaactttcaaaaggcatttgattaaacctcttggcag
tacagtattcttgtatttgttaacgtctgtgtttaggtactggtaccttt
ttgttttaaaatgttctaagtgttggctttaaagtgaatttatctttagt
atgatagttatatgaaaattataggatttgtgtgcagagaatttttttat
aaagtgctttgtaaaaaaaaaaaaaatgtattctagcttttgcggtacata
tgtgtgataactttaatacccatgacagttaagtgcaattatttcatcac
tctaaaaatgctatttttgtgtcagttcctgcaggtgttttcatgtcttt
gcaaagtgacacattttgatgccttcttgataaagtggtagacattttgt
agctttctagaaactttgtattcatacggtatcaatgaaaaataaagaaa
atgaaagtgtgggtcaaaaaaaaaaaaaaaaaa

>NM_022365 3
cccaggcgccgggcgctgcctctacagctgtgtgtaggcctgggggcgag
ggtcttcggaacgtagcgctggctgcggccccgcccgcctacccacccgc
ccgtccggcagccggctcccgccgcctccgcgctctgtctggggccagcc
acctggcgggccgctccggtgcgcctgcccgcgcttttcactgacaggcg
ctgttccccacagccagcgccgcccgccacgtcccagctctcggccaacg
gagctgcgcggcgggtgacctttccgagcccagcgcgatgacggctcctt
gctcccagccggcgcagcttcctggacgccgccagctcgggctggtgccg
ttcccgccgccgccgccgcggacgccgctgctgtggctgctgctgctgct
gctggccgccgtggcgccggcgcgcggctgggagagcggagacctggagt
tgtttgacttagtggaggaggtgcagctcaacttctaccagttcctcggg
gtgcagcaggatgcatcatctgcagacatcagaaaagcatatcgtaagct
ttcactaactttacatccagacaagaataaagatgaaaatgcagaaactc
agtttagacaattggtggccatttatgaagttttaaaggatgatgaacga
aggcagaggtatgatgatattctgatcaatggacttccagattggcgaca
gcctgtattctactacaggcgggtgagaaaaatgagcaatgctgagctgg
cattactcttgttcattattctcacagtgggtcattatgctgtggtttgg
tcaatctacctggaaaaacaactggatgaactactaagtagaaaaaagag
agaaaagaaaaaaaagactggcagcaagagtgtggatgtatcaaaactcg
gtgcttcagaaaaaaatgaaagattgctgatgaaaccacagtggcatgat
ttgcttccatgcaaactggggatttggttttgccttacactaaaagcatt
acctcacctcatccaggatgctgggcagttttatgctaaatataaagaaa

caagattgaaggaaaaggaagatgcactgactagaactgaacttgaaaca
cttcaaaaacagaagaaagttaaaaaaccaaaacctgaatttcctgtata
cacacctttagaaactacatatattcagtcttatgatcatggaacttcca
tagaagaaattgaggaacaaatggatgattggttggaaaacaggaaccga
acacagaaaaaacaggcacctgaatggacagaagaggacctcagccaact
gacaagaagtatggttaagttcccaggagggactccaggtcgatgggaaa
agattgcccacgaattgggtcgatctgtgacagatgtgacaaccaaagcc
aagcaactgaaggattcagtgacctgctccccaggaatggttagactctc
cgaactcaaatcgacagttcagaattccaggcccatcaaaacggccacca
ccttgcccgatgacatgatcacccagcgagaggacgcagagggggtggca
gcggaggaggagcaggagggagactccggtgagcaggagaccggggccac
tgatgcccggcctcggaggcggaagccagccaggctgctggaggctacag
cgaagccggagccagaggagaagtccagagccaagcggcagaaggacttt
gacatagcagaacaaaacgagtccagcgacgaggagagcctgagaaaaga
gagagctcggtctgcagaggagccgtggactcaaaatcaacagaaacttc
tggaactggcgttgcagcagtacccaaggggatcctctgaccgctgggac
aaaatagccagatgtgtcccgtccaagagcaaggaagactgtatcgctag
gtacaagttgctggttgaactggtccaaaagaaaaaacaagctaaaagct
gaatattctgggagatgatgttcaccttcattttccaaaatgaatatctt
aaaaatcttatgcagaaatttgcattttgtacctcaatatttctacgtca
tgtgccttagtaaaaaaaaataataaataaataaaagatgaaaaaaaaaa
aaaaaaaaaaaaaaaaaaaaaaaaaaaaaaaaaaaaaaaaaaaaaaaaaa
aaaaaaaaaaaaaaaaaaaaaaaaaaaaaaaaaaaaaaaaaaaaaaaaaa
aaaaaaaaaaaaa

>NM_032139 2
gtaaggggtgggctatagggccagatcgccgcgcgagggtggtgggcatc
gaggtcccagcagcggacgagggaggtgccgccgtcgcccaggatgggct
gggaatgaagcgatgtagccttttaagagatttgctctgacccatctgaa
gtccatatggctctgtatgatgaagacctcctgaaaaatcctttctatct
ggctctgcaaaagtgccgccctgacttgtgcagcaaagtggcccaaatcc
atggcattgtcttagtaccctgcaaaggaagcctgtcgagcagcatccag
tctacttgtcagtttgagtcctacattttgatacctgtggaagagcattt
tcagaccttaaatggaaaggatgtctttattcaagggaacaggattaaat
taggagctggttttgcctgtcttctctcagtgcccattctctttgaagaa
actttctacaatgaaaaagaagagagtttcagcatcctgtgtatagccca
tcctttggaaaagagagagagttcagaagagcctttggcaccctcagatc
ccttttccctgaaaaccattgaagatgtgagagagttcttgggaagacac
tccgagcgatttgacaggaacatcgcctctttccatcgaacattccgaga
atgcgagagaaagagcctccgtcaccacatagactcagcgaatgctctct
acaccaaatgcctccagcagcttctgagggactctcacctgaaaatgctc
gccaagcaggaggcccagatgaacctgatgaagcaggcagtggagatata
cgtccatcatgaaatttacaacctgatctttaaatacgtggggaccatgg
aggcaagtgaggatgcggcctttaacaaaatcacaagaagccttcaagat
cttcagcagaaagatattggtgtgaaaccggagttcagctttaacatacc
tcgtgccaaaagagagctggctcagctgaacaaatgcacctccccacagc

agaagcttgtctgcttgcgaaaagtggtgcagctcattacacagtctcca
agccagagagtgaacctggagaccatgtgtgctgatgatctgctatcagt
cctgttatacttgcttgtgaaaacggagatccctaattggatggcaaatt
tgagttacatcaaaaacttcaggtttagcagcttggcaaaggatgaactg
ggatactgcctgacctcattcgaagctgccattgaatatattcggcaagg
aagcctctctgctaaaccccctgagtctgagggatttggagacaggctgt
tccttaagcagagaatgagcttactctctcagatgacttcgtctcccacc
gactgcctgtttaagcacattgcatcaggtaaccagaaagaagtggagag
acttctgagccaagaggaccatgataaagataccgtccaaaagatgtgtc
accctctctgcttctgcgatgactgtgagaaactcgtctctgggaggttg
aatgatccctcagttgtcactccattctccagagacgacagggggcacac
ccctctccatgtggctgctgtctgtgggcaggcatccctcatcgacctcc
tggtttccaagggcgccatggtaaatgccacagactaccatggagccact
ccgctccacctggcctgtcagaagggctaccagagcgtgacgctgctgct
gctgcactacaaggccagcgcggaagtgcaggacaacaatgggaatacgc
cactccacctggcctgcacctacggccacgaggactgtgtgaaggctctg
gtttactacgacgtggagtcgtgcagacttgacattggcaatgagaaagg
agacacccctctacacattgctgcccgctggggctaccaaggcgtcatag
agacattgctgcagaacggagcgtccaccgagatccagaacagactgaag
gagacgcccctcaagtgtgcattaaactcaaagattctgtctgtaatgga
agcctatcacctgtccttcgagaggaggcagaagtcgtccgaggcccctg
tgcagtccccgcagcgctccgtggactccatcagccaagagtcctccact
tccagcttctcctccatgtcagccagctcaaggcaggaggagaccaagaa
ggactacagagaggtagaaaaacttttgagagcagttgctgatggagatc
tagaaatggtgcgttacctgttggaatggacagaggaggacctggaggat
gcggaggacactgtcagtgcagcggaccccgaattctgtcacccgttgtg
ccagtgccccaagtgtgccccagctcagaagaggctggcgaaggttcctg
ccagtgggcttggtgtgaacgtgaccagccaggacggctcctccccgctg
catgtcgccgccctgcacggccgggcggacctcatccccctcctgctgaa
gcacggggccaacgcaggtgccaggaacgcagaccaagccgtcccgctcc
acctggcctgccagcagggccactttcaggtggtgaagtgtctgttagat
tcgaatgcaaaacccaataagaaggacctcagtggaaacacgcccctcat
ttacgcctgctccggtggccatcacgagcttgtggcactgctgctacagc
acggggcctccattaacgcttctaacaataagggcaacacagcgctgcac
gaggctgtgattgaaaagcacgtcttcgtggtagagctgcttctgctcca
cggagcgtcagttcaggtgctgaacaagcggcagcgcacggctgtagact
gtgctgaacagaattcaaaaataatggaattgcttcaggtggtaccaagc
tgtgttgcttcattagatgatgtggctgaaactgaccgcaaggagtatgt
cactgttaagatcaggaaaaaatggaactcaaaactgtatgatctaccag
atgagccttttacaagacagttttactttgtccactcagctggtcagttt
aagggaaagacttcaagggagattatggcaagagatagaagtgtccctaa
tttaaccgaaggttctttgcatgagccagggaggcaaagtgtcacactga
gacagaataacctgccagctcagagtggatctcatgctgctgagaaaggc
aacagcgactggccagagaggcctggactgacacagactggccctggaca
cagacggatgctgcggagacacacggtagaggatgcggtcgtgtcccagg
gcccggaggctgctggccccctctccactccccaagaggttagtgcttcc

cggtcctaacaggaatgaggagttgttgaacccactgctaggaagcaagg
atgcaacaagatgatgctgagcgtgaacacatctgagaactaaatgtgct
tccatgagactggcttgagaagtcttcagcaccaagttcctgaaagcttt
tctgtggcaggaaagaatgcaacaaaaaagttaaccaccaccatctctct
cctcttcaaagctaatgaatacaattgaaacagacaaaaattccagtagc
atccagatccttaagccagaggtgcatgcttctttttaagtatgagggtt
tgttggtcacagtgggagaggtttcaccaccgcattctgacctcctcctc
ccaaaaggtgctaaacctctctgacctgtgtacattcacaaaccacagct
agaattcctccacctaggattaagctggagagaagtaagtaatttaggtt
tcatggtactgtagaggccaggctgaaatgtcatatctgaaggaagaaag
cagcagctggacaatgtttctttgcaaagcaacactcgaaccaaaagatg
cctcaatcccattttgatattcattttagtgaaaggatgcatcagacctg
ttccacatcatgcacatgggaaagggtggttatcattttccttctaacaa
gtaggtacagatattcggttactacacgtgcacctgtagcagtatttcta
gaaacatccctttttgttgagaacctcccttgaatgtctgtcacactcac
acctgacgggatggttactggattagagagtagatttggcacatcttttc
ttagtcttttgattcaaattcaaaacttaacagcacaaaccaggtcagag
ttactttcggttagaatttattgccatttattccttttttataaatttcta
tagattatactgttatttttatgttattggcctagagctacacgtatatg
ggtttgtcctgagtccgttttcaaatgaccttgtgatagggaaatggttt
tgtccatgttcttggaaatacttgtgtatgtacagaaggaagggagggat
tatttttctacaaagtaatttatgatttctaattttctaatgtgccttgg
atatgtgccaaatgatggaaaagaaacagtaaactttatgattcttgact
gtgaaaaaaaa

>NM_032236 5
aacgtccgcgggcgcggggtgtgtcgggtgtcgacggcggcgctttgcgg
ccggtcgtgcgggtcgggcgcgggcgggcgcggcggcagtggcgcgcaca
ggtgattgactggccagctgcctgaaggagcgccaggtcctccttgctgg
caggtggcgaagcccattggggcggcggtgcagaccgcggcggcggctgc
ggcggtctggctcgggaggcgttcctggggccaaggccatggccccgcgg
ctgcagctggagaaggcggcctggcgctgggcggagacggtgcggcccga
ggaggtgtcgcaggagcacatcgagaccgcttaccgcatctggctggagc
cctgcattcgcggcgtgtgcagacgaaactgcaaaggaaatccgaattgc
ttggttggtattggtgagcatatttggttaggagaaatagatgaaaatag
ttttcataacatcgatgatcccaactgtgagaggagaaaaaagaactcat
ttgtgggcctgactaaccttggagccacttgttatgtcaacacatttctt
caagtgtggtttctcaacttggagcttcggcaggcactctacttatgtcc
aagcacttgtagtgactacatgctgggagacggcatccaagaagaaaaag
attatgagcctcaaacaatttgtgagcatctccagtacttgtttgccttg
ttgcaaaacagtaataggcgatacattgatccatcaggatttgttaaagc
cttgggcctggacactggacaacagcaggatgctcaagaattttcaaagc
tctttatgtctctattggaagatactttgtctaaacaaaagaatccagat
gtgcgcaatattgttcaacagcagttctgtggagaatatgcctatgtaac
tgtttgcaaccagtgtggcagagagtctaagcttttgtcaaaattttatg
agctggagttaaatatccaaggccacaaacagttaacagattgtatctcg

gaatttttgaaggaagaaaaattagaaggagacaatcgctatttttgcga
gaactgtcaaagcaaacagaatgcaacaagaaagattcgacttcttagcc
ttccttgcactctgaacttgcagctaatgcgttttgtctttgacaggcaa
actggacataagaaaaagctgaatacctacattggcttctcagaaatttt
ggatatggagccttatgtggaacataaaggtgggtcctacgtgtatgaac
tcagcgcagtcctcatacacagaggagtgagtgcttattctggccactac
atcgcccacgtgaaagatccacagtctggtgaatggtataagtttaatga
tgaagacatagaaaagatggaggggaagaaattacaactagggattgagg
aagatctagcagaaccttctaagtctcagacacgtaaacccaagtgtggc
aaaggaactcattgctctcgaaatgcatatatgttggtttatagactgca
aactcaagaaaagcccaacactactgttcaagttccagcctttcttcaag
agctggtagatcgggataattccaaatttgaggagtggtgtattgaaatg
gctgagatgcgtaagcaaagtgtggataaaggaaaagcaaaacacgaaga
ggttaaggagctgtaccaaaggttacctgctggagctgagccctatgagt
ttgtctctctggaatggctgcaaaagtggttggatgaatcaacacctacc
aaacctattgataatcacgcttgcctgtgttcccatgacaagcttcaccc
ggataaaatatcaattatgaagaggatatctgaatatgcagctgacattt
tctatagtagatatggaggaggtccaagactaactgtgaaagccctgtgt
aaggaatgtgtagtagaacgttgtcgcatattgcgtctgaagaaccaact
aaatgaagattataaaactgttaataatctgctgaaagcagcagtaaagg
gcagcgatggattttgggtggggaagtcctccttgcggagttggcgccag
ctagctcttgaacagctggatgagcaagatggtgatgcagaacaaagcaa
cggaaagatgaacggtagcaccttaaataaagatgaatcaaaggaagaaa
gaaaagaagaggaggaattaaattttaatgaagatattctgtgtccacat
ggtgagttatgcatatctgaaaatgaaagaaggcttgtttctaaagaggc
ttggagcaaactgcagcagtactttccaaaggctcctgagtttccaagtt
acaaagagtgctgttcacagtgcaagattttagaaagagaaggggaagaa
aatgaagccttacataagatgattgcaaacgagcaaaagacttctctccc
aaatttgttccaggataaaaacagaccgtgtctcagtaactggccagagg
atacggatgtcctctacatcgtgtctcagttctttgtagaagagtggcgg
aaatttgttagaaagcctacaagatgcagccctgtgtcatcagttgggaa
cagtgctcttttgtgtccccacgggggcctcatgtttacatttgcttcca
tgaccaaagaagattctaaacttatagctctcatatggcccagtgagtgg
caaatgatacaaaagctctttgttgtggatcatgtaattaaaatcacgag
aattgaagtgggagatgtaaacccttcagaaacacagtatatttctgagc
ccaaactctgtccagaatgcagagaaggcttattgtgtcagcagcagagg
gacctgcgtgaatacactcaagccaccatctatgtccataaagttgtgga
taataaaaaggtgatgaaggattcggctccggaactgaatgtgagtagtt
ctgaaacagaggaggacaaggaagaagctaaaccagatggagaaaaagat
ccagattttaatcaaagcaatggtggaacaaagcggcaaaagatatccca
tcaaaattatatagcctatcaaaagcaagttattcgccgaagtatgcgac
atagaaaagttcgtggtgagaaagcacttctcgtttctgctaatcagacg
ttaaaagaattgaaaattcagatcatgcatgcattttcagttgctccttt
tgaccagaatttgtcaattgatggaaagattttaagtgatgactgtgcca
ccctaggcacccttggcgtcattcctgaatctgtcattttattgaaggct
gatgaaccaattgcagattatgctgcaatggatgatgtcatgcaagtttg

tatgccagaagaagggtttaaaggtactggtcttcttggacattaatctt
tgaatacttgctgactgctaagaaatgaccagaggggaagaggagtttga
catgttagggcattaaagcaaaggtggatttaagaattaaaccattacat
gccccttccaaaaggcagaaatccattcaaacgtgactgtcccaaatgcc
ttatgtcaaataaagcagattgcactgatggacatcagacttgaaggaaa
tgtttccaattttatatttaaggggggtggtgggtgggagggggcaagta
aagacggaacaagtttagtagcagtaatagtaaatcatgtttacatatga
gatttatagtcgtgggaggggaataaagttctgttatatttccttgctcg
agtttcataccagatgcgttggtccataaaggattgtatcaagtagatgg
gacaacattctgctctgaacgaaaagtaattttagagacataacctgctt
accaatgcctgtctttgattcatattctactttcaataaagcatgaaagt
gaagaacttgtcctaagtgtggaaaagtgtcttcagatttagactcttct
ccatgtcagctgcagcgccacccgccttacacctgcccggccgtctgtct
cttggtattgggtaaaggagggggcacctgcatgtctcctgcaatgagca
aggaattatgtctcatgttttgacttcagaggctttttgctttggtgcat
ttcagaaaggatggagaacatttattatgtgtgaaagcatcctcttccgg
ttttgctgttattcaaaagtgggaaatgtacctggcacgtttgaaaataa
aaaatctgactacctatcagaagagtaaatcagactgaagtacatttgga
taacacaaggtttctataaaatttgttcttcctgtcctccatgtcactgt
ttcttggacctcagttctctttttgaaagcattattccaaaatgccctga
gagggtctcttagatcattgtttaaaaaaggaaaaaagtatatggatgtg
ctgtccatccaactcaggattatcattcttagcaacacgtaaccgaagca
atattcttaagaatattgaaggggttttttaattgaacttaagactgga
gtttttcctttgaaa

>NM_145648 3
tgggttctgggacaggtgacccggcggcggggcgaggcagctggcggcgt
cgcatggagggctctgggggcggtgcgggcgagcgggcgccgctgctggg
cgcgcggcgggcggcggcggccgcggcggcggctggggcgttcgcgggcc
ggcgcgcggcgtgcggggccgtgctgctgacggagctgctggagcgcgcc
gctttctacggcatcacgtccaacctggtgctattcctgaacggggcgcc
gttctgctgggagggcgcgcaggccagcgaggcgctgctgctcttcatgg
gcctcacctacctgggctcgccgttcggaggctggctggccgacgcgcgg
ctgggccgggcgcgcgccatcctgctgagcctggcgctctacctgctggg
catgctggccttcccgctgctggccgcgcccgccacgcgagccgcgctct
gcggttccgcgcgcctgctcaactgcacggcgcctggtcccgacgccgcc
gcccgctgctgctcaccggccaccttcgcggggctggtgctggtgggcct
gggcgtggccaccgtcaaggccaacatcacgcccttcggcgccgaccagg
ttaaagatcgaggtccggaagccactaggagatttttttaattggttttat
tggagcattaacctgggagcgatcctgtcgttaggtggcattgcctatat
tcagcagaacgtcagctttgtcactggttatgcgatccccactgtctgcg
tcggccttgcttttgtggtcttcctctgtggccagagcgttttcatcacc
aagcctcctgatggcagtgccttcaccgacatgttcaagatactgacgta
ttcctgctgttcccagaagcgaagtggagagcgccagagtaatggtgaag
gcattggagtctttcagcaatcttctaaacaaagtctgtttgattcatgt
aagatgtctcatggtgggccatttacagaagagaaagtggaagatgtgaa

agctctggtcaagattgtccctgttttcttggctttgataccttactgga
cagtgtatttccaaatgcagacaacatatgttttacagagtcttcatttg
aggattccagaaatttcaaatattacaaccactcctcacacgctccctgc
agcctggctgaccatgtttgatgctgtgctcatcctcctgctcatccctc
tgaaggacaaactggtcgatcccattttgagaagacatggcctgctccca
tcctccctgaagaggatcgccgtgggcatgttctttgtcatgtgctcggc
ctttgctgcaggaattttggagagtaaaaggctgaaccttgttaaagaga
aaaccattaatcagaccatcggcaacgtcgtctaccatgctgccgatctg
tcgctgtggtggcaggtgccgcagtacttgctgattgggatcagcgagat
ctttgcaagtatcgcaggcctggaatttgcatactcagctgcccccaagt
ccatgcagagtgccataatgggcttgttctttttcttctctggcgtcggg
tcgttcgtgggttctggactgctggcactggtgtctatcaaagccatcgg
atggatgagcagtcacacagactttggtaatattaacggctgctatttga
actattactttttcttctggctgctattcaaggagctaccctcctgctt
ttcctcattatttctgtgaaatatgaccatcatcgagaccatcagcgatc
aagagccaatggcgtgcccaccagcaggagggcctgaccttcctgaggcc
atgtgcggtttctgaggctgacatgtcagtaactgactggggtgcactga
gaacaggcaagactttaaattcccataaaatgtctgacttcactgaaact
tgcatgttgcctggattgatttcttctttccctctatccaaaggagcttg
gtaagtgccttactgcagcgtgtctcctggcacgctgggccctccgggag
gagagctgcagatttcgagtatgtcgcttgtcattcaaggtctctgtgaa
tcctctagctgggttccctttttttacagaaactcacaaatggagattgca
aagtcttggggaactccacgtgttagttggcatcccagtttcttaaacaa
atagtatcacctgcttcccatagccatatctcactgtaaaaaaaaaaatta
ataaactgttacttatatttaagaaagtgaggattttttttttttttaaag
ataaaagcatggtcagatgctgcaaggattttacataaatgccatattta
tggtttccttcctgagaacaatcttgctcttgccatgttctttgatttag
gctggtagtaaacacatttcatctgctgcttcaaaaagtacttacttttt
aaaccatcaacattacttttctttcttaaggcaaggcatgcataagagtc
atttgagaccatgtgtcccatctcaagccacagagcaactcacggggtac
ttcacaccttacctagtcagagtgcttatatatagctttattttggtacg
attgagactaaagactgatcatggttgtatgtaaggaaaacattcttttg
aacagaaatagtgtaattaaaaataattgaaagtgttaaatgtgaacttg
agctgtttgaccagtcacatttttgtattgttactgtacgtgtatctggg
gcttctccgtttgttaatactttttctgtatttgttgctgtatttttggc
ataactttattataaaaagcatctcaaatgcgaaatcca

>NM_152237 1
agggaggcggggcggagacggcggcgaggaggaggccgcggcgcgggacg
catagagctgcggctcgggcggcgcctccctgcggcggcccggcccggct
ccggcccccgctggggcaatgctccccggggccgcgggatgagccagtga
ctcggccggtccgggcatggcagacccagtggcgggcatcgcgggctcgg
cggccaagagcgtgcggccatttcgctccagtgaggcctacgtggaggcc
atgaaggaggacctggccgagtggctcaatgccttgtacggcctgggtct
cccgggtggtggcgatggcttcctgacagggctggccacgggcacgaccc
tgtgccaacatgccaacgccgtgaccgaggctgcccgtgcattggcagcc

gcccgcccggcccgaggtgtggccttccaggcgcacagtgtagtgcctgg
ctccttcatggcgcgcgacaacgtggccaccttcatcggctggtgccgcg
tggagctgggtgtgccggaggtgctcatgtttgagactgaggacctggtg
ctgcgcaagaacgagaagagcgtggtgctgtgcctgctggaggtggcgcg
gcgtggggcacgcctgggcctgctggccccacgcctcgtgcagtttgagc
aggagattgagcgggagctgcgtgctgcacccccagcccccaacgcccct
gccgctggggaggacaccactgaaaccgcccccgcaccagggactcctgc
ccgcggcccccgcatgacacccagcgacctgcgcaacctcgacgagctgg
tgagggagattctgggccgctgcacctgccctgaccagtttcccatgatc
aaggtctcagaggggaagtaccgtgtgggggactcgagcctgctcatctt
tgtgcgggtgctgaggagccacgtgatggtgcgagtgggtggtggctggg
acacgctggagcattacctggacaagcacgacccgtgccgctgctcctcc
actgctcatcgcccaccccagccgagggtctgcaccttttctccacagag
ggtgtcgcccaccaccagtccccgccctgctagcccagtccctgggagtg
agcgccggggctcccggcctgagatgactcccgttagcttacgaagcaca
aaggaggggcccgagaccccacccaggtgagatgcaggaggacgaggagt
gaggggtccagagggtgggggggcgtcagccctggcctgcatgatgggtt
gcctgtgcgccagagtgactcacaccctgggaaaagttcccgtgggtggg
ggcgggcctggcttcccatctccatggcaacccaaccaaaccaacaacac
agctggggcgggccctgtggctgggcggcagccagtccagctcttgcttc
ctctggcccgtcctgggaagggggtgtctagagcccaggaaacttcttc
ttccgtggcttttggggccctgggccgctggaggaagctgctcacttctc
cctggaagtccccaggacagaccgatgcccctgacagccccgtccaccag
ccacataccctgctgttcctctccctgcctgttctgcatgccagtccttc
cgtggtaccccatctgtctctattgtcccccctgccccaggccccgggatc
agctgcccccccatccccgctcccgccgctactccggggacagtgactcc
tcagcctcctccgcccagagcggccccccttggtacccgcagtgatgacac
aggcactggcccccggagggagcgacccagccggcggctgaccacaggca
ccccggcctctccgagacggcctcctgccctgcgcagccagtcccgagac
cggctggatcgcggccggccccggggggccccaggaggcaggggagccca
gctgtcggtccccagccctgcccggcgggcccggagccagagccgcgagg
agcaggctgtgctgcttgtgcgcagggatcgagacgggcagcactcatgg
gtgccaaggggcaggggcagtgggggctcgggcaggagcacccccccagac
tccccgtgcccgcagccctgcagcaccccggctttcccgggtctccagcc
ccagtccagagttgggcaccacaccggccagcatcttccgcacacccctg
cagctcgacccgcagcaggagcagcagctgttccggcgcctggaagagga
gttcctggccaatgcccgggcccttgaggctgttgctagcgtgacccccca
ctggaccagcccctgacccagctcgggcccccgaccctccagctcctgac
tctgcctattgttcctccagttcctcctcttcgtccctcagcgtcctggg
tggcaaatgtggccaacctggggactctggccggacggccaatgggctgc
ctgggccccgaagccaagccctttccagctcctccgatgaaggcagcccc
tgccctggcatggggggccactagatgcacctgggagccccctggcttg
cactgaaccctcgaggacctgggcacggggtcggatggacacacagccag
accgtaaaccctcacgtatccccacgcctcggggcccccgccgcccctcc
ggacccgcagagctggggacatggcatgccctgcactcagtcaccccgag
ggctgagccagattcctggatgtgatggaccagctcagctgtccccagac

cccatcccttctccttttcctttgtggccttaacccttctgcatcaggga
gccccctctgcctcttgagtaccagacctcatgggaccagacccccttggg
accacatggcacaatgggacctctgttgtacattccggttgggggatgag
cgttgctatttaattactaatattattgaatgccttagaggaggccgggc
gagcccggtgttctgaagacctgtggcccagcagagcctctgacagtaaa
gttttgctccagc

>NM_172390 1
ggcgggcgctcggcgactcgtccccggggccccgcgcgggcccgggcagc
aggggcgtgatgtcacggcagggagggggcgcgggagccgccgggccggc
ggggaggcgggggaggtgttttccagctttaaaaggcaggaggcagagc
gcggccctgcgtcagagcgagactcagaggctccgaactcgccggcggag
tcgccgcgccagatcccagcagcagggcgcgggcaccggggcgcgggcag
ggctcggagccaccgcgcaggtcctagggccgcggccgggccccgccacg
cgcgcacacgcccctcgatgactttcctccggggcgcgcggcgctgagcc
cggggcgagggctgtcttcccggagacccgaccccggcagcgcggggcgg
ccgcttctcctgtgcctccgcccgccgctccactccccgccgccgccgcg
cggatgccaagcaccagctttccagtcccttccaagtttccacttggccc
tgcggctgcggtcttcgggagaggagaaactttggggcccgcgccgcgcg
ccggcggcaccatgaagtcagcggaggaagaacactatggctatgcatcc
tccaacgtcagccccgccctgccgctccccacggcgcactccaccctgcc
ggccccgtgccacaaccttcagacctccacaccgggcatcatcccgccgg
cggatcacccctcggggtacggagcagctttggacggtgggcccgcgggc
tacttcctctcctccggccacaccaggcctgatggggcccctgccctgga
gagtcctcgcatcgagataacctcgtgcttgggcctgtaccacaacaata
accagtttttccacgatgtggaggtggaagacgtcctccctagctccaaa
cggtccccctccacggccacgctgagtctgcccagcctggaggcctacag
agacccctcgtgcctgagcccggccagcagcctgtcctcccggagctgca
actcagaggcctcctcctacgagtccaactactcgtacccgtacgcgtcc
ccccagacgtcgccatggcagtctccctgcgtgtctcccaagaccacgga
ccccgaggagggctttccccgcgggctgggggcctgcacactgctgggtt
ccccgcggcactccccctccacctcgccccgcgccagcgtcactgaggag
agctggctgggtgcccgctcctccagacccgcgtccccttgcaacaagag
gaagtacagcctcaacggccggcagccgccctactcaccccaccactcgc
ccacgccgtccccgcacggctccccgcgggtcagcgtgaccgacgactcg
tggttgggcaacaccacccagtacaccagctcggccatcgtggccgccat
caacgcgctgaccaccgacagcagcctggacctgggagatggcgtccctg
tcaagtcccgcaagaccaccctggagcagccgccctcagtggcgctcaag
gtggagcccgtcggggaggacctgggcagcccccgcccccggccgactt
cgcgcccgaagactactcctctttccagcacatcaggaagggcggcttct
gcgaccagtacctggcggtgccgcagcaccccctaccagtgggcgaagccc
aagcccctgtcccctacgtcctacatgagcccgaccctgcccgccctgga
ctggcagctgccgtcccactcaggcccgtatgagcttcggattgaggtgc
agcccaagtcccaccaccgagcccactacgagacggagggcagccggggg
gccgtgaaggcgtcggccggaggacaccccatcgtgcagctgcatggcta
cttggagaatgagccgctgatgctgcagcttttcattgggacggcggacg

accgcctgctgcgcccgcacgccttctaccaggtgcaccgcatcacaggg
aagaccgtgtccaccaccagccacgaggccatcctctccaacaccaaagt
cctggagatcccactcctgccggagaacagcatgcgagccgtcattgact
gtgccggaatcctgaaactcagaaactccgacattgaacttcggaaagga
gagacggacatcgggaggaagaacacacgggtacggctggtgttccgcgt
tcacgtcccgcaacccagcggccgcacgctgtccctgcaggtggcctcca
accccatcgaatgctcccagcgctcagctcaggagctgcctctggtggag
aagcagagcacggacagctatccggtcgtgggcgggaagaagatggtcct
gtctggccacaacttcctgcaggactccaaggtcattttcgtggagaaag
ccccagatggccaccatgtctgggagatggaagcgaaaactgaccgggac
ctgtgcaagccgaattctctggtggttgagatcccgccatttcggaatca
gaggataaccagccccgttcacgtcagtttctacgtctgcaacgggaaga
gaaagcgaagccagtaccagcgtttcacctaccttcccgccaacggtaac
gccatctttctaaccgtaagccgtgaacatgagcgcgtggggtgcttttt
ctaaagacgcagaaacgacgtcgccgtaaagcagcgtggcgtgttgcaca
tttaactgtgtgatgtcccgttagtgagaccgagccatcgatgccctgaa
aaggaaaggaaaagggaagcttcggatgcattttccttgatccctgttgg
gggtgggggggcgggggttgcatactcagatagtcacggttattttgcttc
ttgcgaatgtataacagccaaggggaaaacatggctcttctgctccaaaa
aactgagggggtcctggtgtgcatttgcaccctaaagctgcttacggtga
aaaggcaaataggtatagctattttgcaggcacctttaggaataaacttt
gcttttaagcctgtagtcctgatgtggtctttaaggatggtgaatgagct
ttgtgctgggcggacgtccccgagacacttctccaggggtaacttcatct
cctgggaccacgggcatccaggctggggcaccttctcctcaacctgcctg
cccctcacccgccgtgggaatggctccttttcactcatac

>NM_000104 3
aaaacccggaggagcgggatggcgcgctttgactctggagtgggagtggg
agcgagcgcttctgcgactccagttgtgagagccgcaagggcatgggaat
tgacgccactcaccgacccccagtctcaatctcaacgctgtgaggaaacc
tcgactttgccaggtccccaagggcagcggggctcggcgagcgaggcacc
cttctccgtccccatcccaatccaagcgctcctggcactgacgacgccaa
gagactcgagtgggagttaaagcttccagtgagggcagcaggtgtccagg
ccgggcctgcgggttcctgttgacgtcttgccctaggcaaaggtcccagt
tccttctcggagccggctgtcccgcgccactggaaaccgcacctccccgc
agcatgggcaccagcctcagcccgaacgacccttggccgctaaacccgct
gtccatccagcagaccacgctcctgctactcctgtcggtgctggccactg
tgcatgtgggccagcggctgctgaggcaacggaggcggcagctccggtcc
gcgcccccgggcccgtttgcgtggccactgatcggaaacgcggcggcggt
gggccaggcggctcacctctcgttcgctcgcctggcgcggcgctacggcg
acgttttccagatccgcctgggcagctgccccatagtggtgctgaatggc
gagcgcgccatccaccaggccctggtgcagcagggctcggccttcgccga
ccggccggccttcgcctccttccgtgtggtgtccggcggccgcagcatgg
ctttcggccactactcggagcactggaaggtgcagcggcgcgcagcccac
agcatgatgcgcaacttcttcacgcgccagccgcgcagccgccaagtcct
cgagggccacgtgctgagcgaggcgcgcgagctggtggcgctgctggtgc

gcggcagcgcggacggcgccttcctcgacccgaggccgctgaccgtcgtg
gccgtggccaacgtcatgagtgccgtgtgtttcggctgccgctacagcca
cgacgaccccgagttccgtgagctgctcagccacaacgaagagttcgggc
gcacggtgggcgcgggcagcctggtggacgtgatgccctggctgcagtac
ttccccaacccggtgcgcaccgttttccgcgaattcgagcagctcaaccg
caacttcagcaacttcatcctggacaagttcttgaggcactgcgaaagcc
ttcggcccggggccgcccccgcgacatgatggacgcctttatcctctct
gcggaaaagaaggcggccggggactcgcacggtggtggcgcgcggctgga
tttggagaacgtaccggccactatcactgacatcttcggcgccagccagg
acaccctgtccaccgcgctgcagtggctgctcctcctcttcaccaggtat
cctgatgtgcagactcgagtgcaggcagaattggatcaggtcgtggggag
ggaccgtctgccttgtatgggtgaccagcccaacctgccctatgtcctgg
ccttcctttatgaagccatgcgcttctccagctttgtgcctgtcactatt
cctcatgccaccactgccaacacctctgtcttgggctaccacattcccaa
ggacactgtggtttttgtcaaccagtggtctgtgaatcatgacccactga
agtggcctaacccggagaactttgatccagctcgattcttggacaaggat
ggcctcatcaacaaggacctgaccagcagagtgatgatttttcagtggg
caaaaggcggtgcattggcgaagaactttctaagatgcagctttttctct
tcatctccatcctggctcaccagtgcgatttcagggccaacccaaatgag
cctgcgaaaatgaatttcagttatggtctaaccattaaacccaagtcatt
taaagtcaatgtcactctcagagagtccatggagctccttgatagtgctg
tccaaaatttacaagccaaggaaacttgccaataagaagcaagaggcaag
ctgaaattttagaaatattcacatcttcggagatgaggagtaaaattcag
tttttttccagttcctcttttgtgctgcttctcaattagcgtttaaggtg
agcataaatcaactgtccatcaggtgaggtgtgctccatacccagcggtt
cttcatgagtagtgggctatgcaggagcttctgggagattttttttgagtc
aaagacttaaagggcccaatgaattattatatacatactgcatcttggtt
atttctgaaggtagcattctttggagttaaaatgcacatatagacacata
cacccaaacacttacaccaaactactgaatgaagcagtattttggtaacc
aggccatttttggtgggaatccaagattggtctcccatatgcagaaatag
acaaaaagtatattaaacaaagtttcagagtatattgttgaagagacaga
gacaagtaatttcagtgtaaagtgtgtgattgaaggtgataagggaaaag
ataaagaccagaaattccccttttcaccttttcaggaaaataacttagact
ctagtatttatgggtggatttatccttttgccttctggtatacttcctta
cttttaaggataaatcataaagtcagttgctcaaaaagaaatcaatagtt
gaattagtgagtatagtggggttccatgagttatcatgaattttaaagta
tgcattattaaattgtaaaactccaaggtgatgttgtacctcttttgctt
gccaaagtacagaatttgaattatcagcaaagaaaaaaaaaaaagccagc
caagctttaaattatgtgaccataatgtactgatttcagtaagtctcata
ggttaaaaaaaaaagtcaccaaatagtgtgaaatatattacttaactgtc
cgtaagcagtatattagtattatcttgttcaggaaaaggttgaataatat
atgccttgtataatattgaaaattgaaaagtacaactaacgcaaccaagt
gtgctaaaaatgagcttgattaaatcaaccacctatttttgacatggaaa
tgaagcagggtttcttttcttcactcaaattttggcgaatctcaaaatta
gatcctaagatgtgttcttatttttataacatctttattgaaattctatt
tataatacagaatcttgttttgaaaataacctaattaatatattaaaatt

ccaaattcatggcatgcttaaattttaactaaattttaaagccattctga
ttattgagttccagttgaagttagtggaaatctgaacattctcctgtgga
aggcagagaaatctaagctgtgtctgcccaatgaataatggaaaatgcca
tgaattacctggatgttctttttacgaggtgacaagagttggggacagaa
ctcccattacaactgaccaagtttctcttctagatgatttttttgaaagtt
aacattaatgcctgctttttggaaagtcagaatcagaagatagtcttgga
agctgtttggaaaagacagtggagatgaggtcagttgtgtttttttaagat
ggcaattactttggtagctgggaaagcataaagctcaaatgaaatgtatg
cattcacatttagaaaagtgaattgaagtttcaagttttaaagttcattg
caattaaacttccaaagaaagttctacagtgtcctaagtgctaagtgctt
attacattttattaagctttttggaatctttgtaccaaaattttaaaaaa
gggagtttttgatagttgtgtgtatgtgtgtgtggggtggggggatggta
agagaaaagagagaaacactgaaaagaaggaaagatggttaaacattttc
ccactcattctgaattaattaatttggagcacaaaattcaaagcatggac
atttagaagaaagatgtttggcgtagcagagttaaatctcaaataggcta
ttaaaaaagtctacaacatagcagatctgttttgtggtttggaatattaa
aaaacttcatgtaattttattttaaaatttcatagctgtacttcttgaat
ataaaaaatcatgccagtatttttaaaggcattagagtcaactacacaaa
gcaggcttgcccagtacatttaaattttttggcacttgccattccaaaat
attatgccccaccaaggctgagacagtgaatttgggctgctgtagcctat
ttttttagattgagaaatgtgtagctgcaaaaataatcatgaaccaatct
ggatgcctcattatgtcaaccaggtccagatgtgctataatctgttttta
cgtatgtaggcccagtcgtcatcagatgcttgcggcaaaaggaaagctgt
gtttatatggaagaaagtaaggtgcttggagtttacctggcttatttaat
atgcttataacctagttaaagaaaggaaaagaaaacaaaaaacgaatgaa
aataactgaatttggaggctggagtaatcagattactgctttaatcagaa
accctcattgtgtttctaccggagagagaatgtatttgctgacaaccatt
aaagtcagaagttttactccaggttattgcaataaagtataatgtttatt
aaatgcttcatttgtatgtcaaagctttgactctataagcaaattgcttt
tttccaaaacaaaaagatgtctcaggtttgttttgtgaattttctaaaag
ctttcatgtcccagaacttagcctttacctgtgaagtgttactacagcct
taatattttcctagtagatctatattagatcaaatagttgcatagcagta
tatgttaatttgtgtgtttttagctgtgacacaactgtgtgattaaaagg
tatactttagtagacatttataactcaaggataccttcttatttaatctt
ttcttatttttgtactttatcatgaatgcttttagtgtgtgcataatagc
tacagtgcatagttgtagacaaagtacattctggggaaacaacatttata
tgtagcctttactgtttgatataccaaattaaaaaaaaattgtatctcat
tacttatactgggacaccattaccaaaataataaaaatcactttcataat
cttgaaaaaa

>NM_000183 2
tcgctcctggctaacatggccgaaaggtcgtattctccgggggaggacgg
gggcccggagaggagggggtggagtgcctggttttccagtcaggcggccg
gagggcagccctcaagaacggccctgaccgcccgcggggtgaggggccct
ttctgggcaggacccgccccttggtcccgcagagccttggtacttggacc
tgaaccttgctccgagagggagtcctcgcggacgtcagccaagattccag

aatgactatcttgacttacccctttaaaaatcttcccactgcatcaaaat
gggccctcagattttccataagacctctgagctgttcctcccagctacga
gctgccccagctgtccagaccaaaacgaagaagacgttagccaaacccaa
tataaggaatgttgtggtggtggatggtgttcgcactccatttttgctgt
ctggcacttcatataaagacctgatgccacatgatttggctagagcagcg
cttacgggtttgttgcatcggaccagtgtccctaaggaagtagttgatta
tatcatctttggtacagttattcaggaagtgaaaacaagcaatgtggcta
gagaggctgcccttggagctggcttctctgacaagactcctgctcacact
gtcaccatggcttgtatctctgccaaccaagccatgaccacaggtgttgg
cttgattgcttctggccagtgtgatgtgatcgtggcaggtggtgttgagt
tgatgtccgatgtccctattcgtcactcaaggaaaatgagaaaactgatg
cttgatctcaataaggccaaatctatgggccagcgactgtctttaatctc
taaattccgatttaatttcctagcacctgagctccctgcggtttctgagt
tctccaccagtgagaccatgggccactctgcagaccgactggccgctgcc
tttgctgtttctcggctggaacaggatgaatatgcactgcgctctcacag
tctagccaagaaggcacaggatgaaggactcctttctgatgtggtaccct
tcaaagtaccaggaaaagatacagttaccaaagataatggcatccgtcct
tcctcactggagcagatggccaaactaaaacctgcattcatcaagcccta
cggcacagtgacagctgcaaattcttctttcttgactgatggtgcatctg
caatgttaatcatggcggaggaaaaggctctggccatgggttataagccg
aaggcatatttgagggattttatgtatgtgtctcaggatccaaaagatca
actattacttggaccaacatatgctactccaaaagttctagaaaaggcag
gattgaccatgaatgatattgatgcttttgaatttcatgaagctttctcg
ggtcagattttggcaaattttaaagccatggattctgattggtttgcaga
aaactacatgggtagaaaaaccaaggttggattgcctcctttggagaagt
ttaataactggggtggatctctgtccctgggacacccatttggagccact
ggctgcaggttggtcatggctgctgccaacagattacggaaagaaggagg
ccagtatggcttagtggctgcgtgtgcagctggagggcagggccatgcta
tgatagtggaagcttatccaaaataatagatccagaagaagtgacctgaa
gtttctgtgcaacactcacactaggcaatgccatttcaatgcattactaa
atgacatttgtagttcctagctcctcttaggaaaacagttcttgtggcct
tctattaaatagtttgcacttaagccttgccagtgttctgagcttttcaa
taatcagtttactgctctttcagggatttctaagccaccagaatctcaca
tgagatgtgtgggtggttgtttttggtctctgttgtcactaaagactaaa
tgagggtttgcagttgggaaagaggtcaactgagatttggaaatcatctt
tgtaatatttgcaaattatacttgttcttatctgtgtcctaaagatgtgt
tctctataaaatacaaaccaacgtgcctaattaattatggaaaaataatt
cagaatctaaacaccactgaaaacttataaaaaatgtttagatacataaa
tatggtggtcagcgttaataaagtggagaaatattggaaaaaaaaa

>NM_000611 5
ggggccggggggcggagccttgcgggctggagcgaaagaatgcgggggct
gagcgcagaagcggctcgaggctggaagaggatcttgggcgccgccaggt
tctgtggacaatcacaatgggaatccaaggagggtctgtcctgttcgggc
tgctgctcgtcctggctgtcttctgccattcaggtcatagcctgcagtgc
tacaactgtcctaacccaactgctgactgcaaaacagccgtcaattgttc

atctgattttgatgcgtgtctcattaccaaagctgggttacaagtgtata
acaagtgttggaagtttgagcattgcaatttcaacgacgtcacaacccgc
ttgagggaaaatgagctaacgtactactgctgcaagaaggacctgtgtaa
ctttaacgaacagcttgaaaatggtgggacatccttatcagagaaaacag
ttcttctgctggtgactccatttctggcagcagcctggagccttcatccc
taagtcaacaccaggagagcttctcccaaactccccgttcctgcgtagtc
cgctttctcttgctgccacattctaaaggcttgatattttccaaatggat
cctgttgggaaagaataaaattagcttgagcaacctggctaagatagagg
ggctctgggagactttgaagaccagtcctgtttgcagggaagccccactt
gaaggaagaagtctaagagtgaagtaggtgtgacttgaactagattgcat
gcttcctcctttgctcttgggaagaccagctttgcagtgacagcttgagt
gggttctctgcagccctcagattatttttcctctggctccttggatgtag
tcagttagcatcattagtacatctttggagggtggggcaggagtatatga
gcatcctctctcacatggaacgctttcataaacttcagggatcccgtgtt
gccatggaggcatgccaaatgttccatatgtgggtgtcagtcagggacaa
caagatccttaatgcagagctagaggacttctggcagggaagtggggaag
tgttccagatagcagggcatgaaaacttagagaggtacaagtggctgaaa
atcgagtttttcctctgtctttaaattttatatgggctttgttatcttcc
actggaaaagtgtaatagcatacatcaatggtgtgttaaagctatttcct
tgcctttttttattggaatggtaggatatcttggctttgccacacacag
ttacagagtgaacactctactacatgtgactggcagtattaagtgtgctt
attttaaatgttactggtagaaaggcagttcaggtatgtgtgtatatagt
atgaatgcagtggggacaccctttgtggttacagtttgagacttccaaag
gtcatccttaataacaacagatctgcaggggtatgttttaccatctgcat
ccagcctcctgctaactcctagctgactcagcatagattgtataaaatac
ctttgtaacggctcttagcacactcacagatgtttgaggctttcagaagc
tcttctaaaaaatgatacacacctttcacaagggcaaactttttcctttt
ccctgtgtattctagtgaatgaatctcaagattcagtagacctaatgaca
tttgtattttatgatcttggctgtatttaatggcataggctgacttttgc
agatggaggaatttcttgattaatgttgaaaaaaaacccttgattatact
ctgttggacaaaccgagtgcaatgaatgatgcttttctgaaaatgaaata
taacaagtgggtgaatgtggttatggccgaaaaggatatgcagtatgctt
aatggtagcaactgaaagaagacatcctgagcagtgccagctttcttctg
ttgatgccgttccctgaacataggaaaatagaaacttgcttatcaaaact
tagcattaccttggtgctctgtgttctctgttagctcagtgtctttcctt
acatcaataggttttttttttttttttggcctgaggaagtactgaccat
gcccacagccaccggctgagcaaagaagctcatttcatgtgagttctaag
gaatgagaaacaattttgatgaatttaagcagaaaatgaatttctgggaa
cttttttgggggcgggggggtggggaattcagccacactccagaaagcca
ggagtcgacagttttggaagcctctctcaggattgagattctaggatgag
attggcttactgctatcttgtgtcatgtacccactttttggccagactac
actgggaagaaggtagtcctctaaagcaaaatctgagtgccactaaatgg
ggagatggggctgttaagctgtccaaatcaacaagggtcatataaatggc
cttaaactttggggttgctttctgcaaaaagttgctgtgactcatgccat
agacaaggttgagtgcctggacccaaaggcaatactgtaatgtaaagaca
tttatagtactaggcaaacagcaccccaggtactccaggccctcctggct

ggagagggctgtggcaatagaaaattagtgccaactgcagtgagtcagcc
taggttaaatagagagtgtaagagtgctggacaggaacctccaccctcat
gtcacatttcttcaatgtgacccttctggcccctctcctcctgacagcgg
aacaatgactgccccgataggtgaggctggaggaagaatcagtcctgtcc
ttggcaagctcttcactatgacagtaaaggctctctgcctgctgccaagg
cctgtgactttctaacctggcctcacgctgggtaagcttaaggtagaggt
gcaggattagcaagcccacctggctaccaggccgacagctacatcctcca
actgaccctgatcaacgaagagggattcatgtgtctgtctcagttggttc
caaatgaaaccagggagcaggggagttaggaatcgaacaccagtcatgcc
tactggctctctgctcgagagccaataccctgtgccctccactcatctgg
atttacaggaactgtcatagtgttcagtattgggtggtgataagcccatt
ggattgtccccttgggggatgagctaggggtgcaaggaacacctgatga
gtagataagtggagctcatggtatttcctgaaagatgctaatctatttgc
caaacttggtcttgaatgtactgggggcttcaaggtatgggtatattttt
cttgtgtccttgcagttagcccccatgtcttatgtgtgtcctgaaaaaat
aagagcctgcccaagactttgggcctcttgacagaattaaccacttttat
acatctgagttctcttggtaagttctttagcagtgttcaaagtctactag
ctcgcattagtttctgttgctgccaacagatctgaactaatgctaacaga
tccccctgagggattcttgatgggctgagcagctggctggagctagtact
gactgacattcattgtgatgagggcagctttctggtacaggattctaagc
tctatgttttatatacattttcatctgtacttgcacctcactttacacaa
gaggaaactatgcaaagttagctggatcgctcaaggtcacttaggtaagt
tggcaagtccatgcttcccactcagctcctcaggtcagcaagtctacttc
tctgcctattttgtatactctctttaatatgtgcctagctttggaaagtc
tagaatgggtccctggtgcctttttactttgaagaaatcagtttctgcct
ctttttggaaaagaaaacaaagtgcaattgtttttttactggaaagttacc
caatagcatgaggtgaacaggacgtagttaggccttcctgtaaacagaaa
atcatatcaaaacactatcttcccatctgtttctcaatgcctgctacttc
ttgtagatatttcatttcaggagagcagcagttaaacccgtggattttgt
agttaggaacctgggttcaaaccctcttccactaattggctatgtctctg
gacaagtttttttttttttttttttttaaaccctttctgaactttcactt
tctatgtctacctcaaagaattgttgtgaggcttgagataatgcatttgt
aaagggtctgccagataggaagatgctagttatggatttacaaggttgtt
aaggctgtaagagtctaaaacctacagtgaatcacaatgcatttaccccc
actgacttggacataagtgaaaactagccagaagtctctttttcaaatta
cttacaggttattcaatataaaatttttgtaatggataatcttatttatc
taaactaaagcttcctgtttatacacactcctgttattctgggataagat
aaatgaccacagtaccttaatttctaggtgggtgcctgtgatggttcatt
gtaggtaaggacattttctctttttcagcagctgtgtaggtccagagcct
ctgggagaggagggggggtagcatgcacccagcaggggactgaactgggaa
actcaaggttctttttactgtggggtagtgagctgcctttctgtgatcgg
tttccctagggatgttgctgttcccctccttgctattcgcagctacatac
aacgtggccaaccccagtaggctgatcctatatatgatcagtgctggtgc
tgactctcaatagccccacccaagctggctataggtttacagatacatta
attaggcaacctaaaatattgatgctggtgttggtgtgacataatgctat
ggccagaactgaaacttagagttataattcatgtattagggttctccaga

gggacagaattagtaggatatatgtatatatgaaagggaggttattaggg
agaactggctcccacagttagaaggcgaagtcgcacaataggccgtctgc
aagctgggttagagagaagccagtagtggctcagcctgagttcaaaaacc
tcaaaactggggaagctgacagtgcagccagccttcagtctgtggccaaa
ggcccaagagcccctggcaaccaacccactggtgcaagtcctagattcca
aaggctgaagaacctggagtctgatgtccaagagcaggaagagtggaaga
aagccagaagactcagcaaacaaggtagacagtgtctaccaccatagtgg
ccataccaaagaggctaccgattccttcctgctacctggatccctgaagt
tgccctggtctctgcaccttctaaacctagttcttaagagctttccatta
catgagctgtctcaaagccctccaataaattctcagtgtaagcttctgtt
gcttgtggacagaaaattctgacagacctaccctataagtgttactgtca
ggataacatgagaacgcacaacagtaagtggtcactaagtgttagctacg
gttattttgcccaaggtagcatggctagttgatgccggttgatggggctt
aaacccagctccctcatcttccaggcctctgtactccctattccactaaa
ctacctctcaggtttattttttaaattcttactctgcaagtacatagga
ccacatttacctgggaaaacaagaataaaggctgctctgcatttttttaga
aactttttttgaaagggagatgggaatgcctgcacccccaagtccagacca
acacaatggttaattgagatgaataataaaggaaagactgttctgggctt
cccagaatagcttggtccttaaattgtggcacaaacaacctcctgtcaga
gccagcctcctgccaggaagaggggtaggagactagaggccgtgtgtgca
gccttgccctgaaggctagggtgacaatttggaggctgtccaaacaccct
ggcctctagagctggcctgtctatttgaaatgccggctctgatgctaatc
ggcgaccctcaggcaagttacttaaccttacatgcctcagttttctcatc
tggaaaatgagaaccctaggtttagggttgttagaaaagttaaatgagtt
aagacaagtgcctgggacacagtagcctcttgtgtgtgtttatcattatg
tcctcagcaggtcgtagaagcagcttctcaggtgtgaggctggcgcgatt
atctggagtgggttgggttttctaggatggaccccctgctgcattttcct
cattcatccaccagggcttaatggggaatcaaggaatccatgtgtaactg
tataataactgtagccacactccaatgaccacctactagttgtccctggc
actgcttatacatatgtccatcaaatcaatcctatgaagtagatactgtc
ttcattttatagatcagagacaattggggttcagagagctgatgtgattt
tcccagggtcacagagagtcccagattcaggcacaactcttgtattccaa
gacacaaccactacatgtccaaaggctgcccagagccaccgggcacggca
aattgtgacatatccctaaagaggctgagcacctggtcaggatctgatgg
ctgacagtgtgtccagatgcagagctggagtgggggaggggaagggggc
tccttgggacagagaaggctttctgtgctttctctgaagggagcagtctg
aggaccaagggaacccggcaaacagcacctcaggtactccaggccctcct
ggctggagagggctgtggcaatggaaaattagtgccaactgcaatgagtc
agcctcggttaaatagagagtgaagaatgctggacaggaacctccaccct
catgtcacatttcttcagtgtgacccttctggcccctctcctcctgacag
cggaacaatgactgccccgataggtgaggctggaggaagaatcagtcctg
tccttggcaagctcttcactatgacagtaaaggctctctgcctgctgcca
aggcctgtgactttctaacctggcctcacgctgggtaagcttaaggtaga
ggtgcaggattagcaagcccacctggctaccaggccgacagctacatctt
tcaactgaccctgatcaacgaagagggacttgtgtctctcagttggttcc
aaatgaaaccagggagcaggggcgttaggaagctccaacaggatggtact

taatggggcatttgagtggagaggtaggtgacatagtgctttggagccca
gggagggaaaggttctgctgaagttgaattcaagactgttctttcatcac
aaacttgagtttcctggacatttgtttgcagaaacaaccgtagggttttg
ccttaacctcgtgggtttattattacctcatagggactttgcctcctgac
agcagtttatgggtgttcattgtggcacttgagttttcttgcatacttgt
tagagaaaccaagtttgtcatcaacttcttatttaaccccctggctataa
cttcatggattatgttataattaagccatccagagtaaaatctgtttaga
ttatcttggagtaaggggggaaaaaatctgtaatttttttctcctcaactag
atatatacataaaaaatgattgtattgcttcatttaaaaaatataacgca
aaatctcttttccttctaaaaaaaaaaaaaaaaaa

>NM_001099678 1
ctcggttcaggttccggggcgccgcagagctcccggcctctggaccgcgc
gcggcgctctggggaatccggcgccacgcgccgtgcggtggccaggatgg
aggaggccggagcagcggtggtcacggccggggaggccgaactgaactgg
tcccgcctcagcgtgtccaccgagacgctggagtctgagctggaggcgcg
gggggaggagcggcgcggggcgcgggaggcgctgctgcggctgctgctgc
ctcacaaccgtctggtgtcgctgccacgggcgctgggcagcggcttcccg
cacctccagctgctggacgtgagcggcaacgcgttgaccgcgctcgggcc
ggagctgctcgctctgcgcggcctgcgcacgctgctggccaagaacaacc
ggctcggcgggcccagtgcgctgcccaagggcctggcccagtcgccgctc
tgccgcagcctccaggtgctcaacctcagcggcaactgtttccaggaggt
gcctgcctcgctcttagagctgcgcgcgctgcagaccctgagcctgggcg
gcaaccaactgcagagcatcccggctgagatcgagaacttgcagagttta
gagtgtttatatcttggaggaaatttcattaaagaaatcccaccagaatt
aggaaatctgccttctctgaattatttggtattatgtgacaacaaaatcc
aaagcatacctcctcaactttcacagttacattcacttcgttccctaagt
cttcacaataacttgctgacatatctgcctcgagagatcctcaaccttat
tcatttggaagagttgagtttacgaggaaatccattggttgttcgttttg
ttagagatttaacctatgatcctccaactctcctggaattagctgcacgg
accattaagattcgaaatatttcctacactccctatgatcttcctggaaa
tcttcttagatacttgggttcagccagcaattgcccaaacccaaagtgtg
gaggagtctactttgactgctgtgtcagacaaattaaatttgtggacttc
tgtgggaagtatcgcctcccactgatgcactacttgtgttcaccagaatg
ttcttccccttgcagttctgcctctcacagctccacttcccagagtgaat
ctgactcagaagatgaagctagtgttgctgcacgcagaatgcagaaagtt
cttcttggttgaacaggagtgcacagtgttgtgaaatacttaaaaaactg
agcaaaatggttagacaagaaaatagagcttgaagttcactagaaatctt
atcttcatcttaagtgttcatcaaagagtctgggatgatataaaacattt
tgtgtttcatctacccattcagcaagaatgagtccagtcccatgtttaga
ttattttaacataatttgtgaatgatagtttcacatttggctgatggttt
gcagttttcactcaagttttgcatgaatcacaaagcagaatggtaccttt
tgaagagcagttctgcaataagtcatgagtagtataacttttggacattg
agatggagaaagtgagaaattatcaaagctataattgccagagttgccct
tgggtttaggtagaacactgtataccatatatactatataccatatatac
tatatataccatatatattatatgccataaacaggattaaccctcccttt

tctaattgcctgtatgtctagattacagaactattcaaagaagctcacag
atggttacatgggacaaacctttctgacaaactaactggtaaagtaaggc
atataacatttacatgagatattaaaactttttcattattatcctttgtt
ctatatgattgctattaaccttatgtaatctagcaatgactgttatagtg
ccttaatccagcaaggatataaaaatgtattcaagaagtaatgccagagc
aacctataacctgacataccatgcagaacattgtaaaatattctttaaca
gaactaggtaaataactgtgtatctaatatccgatctcatggccctctta
acatttttttgtttaaatgtgcactcatcccttataagcacattactttt
acacttaatggtaggccacaatcttcatcaatctgttcatcataagacag
agtaagaatttttaacagaaaattctaaaagagatagaagctaggaaagc
ttctttatatcttgcattaaatgggtagggaaaaaatatgactctcaccc
atgtcatgtactggaccatctttctctactttttaaagaaaaactaatac
aactagaaaaacatcacaacaagccaaataaaaatggagaaaaggagtca
ttgtccttatacctaatggaacagtttccagaactactttaactatggaa
gcatttttgaaaataaagtattaataaaatagaaaaaaattattaaatat
attgtatccttaatgtatagcaggaaaatagtttcacaaattaattgcta
ttagaaattaccaaatttttattaagtactttaatatgcaatacaaattg
aataatagtacaactataagttgatataaagtaagtgcttactggtaatc
attaaataactaataacatgacttgttggttaatgttaaacctacagtaa
gaaatgttatacagatcagataggcattagaacatcaggtgtacctttttc
tgatttctggcattcatggcacaccatttcttaaaagcagattgttgtat
aataacatatacatgtattagggtttttttcatatatatttttttatttta
gaagaaactagcataattcagggtttatgtgtttatttgtatattaattc
atgaaacatgagagtttcatgaactgcagatgtgaaacgctgctatagaa
tgaatcccttcacctttaccttgttataatgtagtaagattttgttttcc
ttttgactcagaaattttctatgtctatagaggtaatctaatatgttcct
actgcctagaagagtaatacataggtgattatatcataaaatagaggtat
aaagtctttgcttacttctccatgtgggccacagcaagctgcagactgta
cagttatgaaaggtaaagatgacttaggctgtgcctaagtgacaaatgta
aaatgttttgtaaataagtgacttacctaagatacataagatattttcat
agaaaataatgtattcatagtaaaatactcaaattgataggaccaatttt
ttctggcctaaatgcttatgcctacttcctaatcaattaccagcttgact
taagtaactgcttgagtagtcttctaagagttagtcttcgtaaaataaat
ggataggtatgtaaatatataatcttacataccctttccagcttcctaga
aaggtctactgcttcatcttttacagatgtaaataggtccaaccagagac
agtttctggcctttgcatataagaatatagatgtataccacagtattatt
tcaacaaaatgttatggttctatgtatagttgaaaactctatgatcagca
tacaactgtcttttggtaatttttaatgaagtctacatttagccgtcggt
tttatctttcttaaatctttgacctaaacttttgtaagagcagctaataa
tttctaagattttccctgtttctttttcctacctttccttttctcataac
tgattggagaaattaatcagaagtcaaatacaagggacttcataatctag
aaaccagataatcagctcccaagtcatgtagctaactatgcatttaaaat
aagtctgtctctttagttcttaagtcatatatttgtttgttaattacagt
aaatgaactttaattggggtttttaaaagacagaattagcgaagtctaat
ttttataatgaaataagtttttgatattgctctacttggacgattttagt
gaccaaaactatggataaaactgcctaagcataacattaatatatttaga

atggcattcttcagtgctagtatttgaaattggaattagtacattgtgca
ttcttagtagtctttatccctagaatcaattctctcagcatcaccaaact
gaattggtgaaatagtgctaagattctgggcaataggaagattagtgaat
atgatacattgattccagggtgccagggattggctgacattaaggaccag
ctggctatggtcctcaggctacggtcctcagtattctagtatttgagtag
tggaggtaaatccaaaggcgactggttatattttacttctaaatgtgtgc
tgaagtgtctcttgttcaaaaattcttgatatttagagcttcaagttacc
acatttaaaagtaaagacttggcacggtggcacacacctataatcttagc
actttgggaggccgaagtggatggatcatatgcagtcaggaatttgagac
cagcctggccaacatggtgaaacctcatctttactaaaaatacaaaaatt
agccgggcgttgtgacgcacacccatagtcccagctaactccggaggcta
agacaagagaatcacttgaacttgggagacagaggttgcagtaatctgag
atcactccgctgcactccagcctgagtgagagtgagactgtgtctcaaac
aaacaaacaaaaaacaaacaaaaaaaatttgcattgtaaattgtacctcaa
atttgagcagtttttgtgtttgtgtaaattaaagtaaaaagcagtccaca
ataatgttttgtaatactgcacaccagttctaccagattttgtaactatc
agatttaaatgaaaaaagagaaggaagaggctgattgctggagtgttaaa
gaaatcttggccgggcgtggtggctcacgcttgtaatcccagcactttgg
gaggccgaggcgggtggatcacgaggtcaggagatcgagaccatcctggc
taacacggtgaaaccccgtctctactaaaaatacaaaaaaaaattagccg
ggcgtgatggcgggcgcctgtagtcccagctactcgggaggctgaggcaa
gagaatggcgtgaacccgggaggcggagcttgcagtgagccgagattgcg
ccactgcactcccgcctgggccacagagcgagactccgtctcaaaaaaaa
aaaaaaaaaaaaaagaaatctttagaagccacagggctagaagagaagtc
accaaacacctagggacagtcctaggcctagaaagccctttaaagtaata
gtgaattgttttaaaattaagttattgagtggcctactgactaccatcca
aaaacctttatcaccaaatttgttcagtgtttgctattgggagattgtcc
atatccctccgtaattaagggccagcaagtaatacgatcttcttacagcc
aaccttgctcttcccaaagggagaaagtaatacatcattaacctggaatt
tgtgatataacaggtagccttggttaatgtttaccttttcactatgtaaa
aaagaaaaaacacacagcaaatagtgcaaataaaatttggtcttttaaag
cattttagcagtagctatgtaaaaaaagttaatggttattattcctaaat
tttcataagacccttggtagacaaaactatcggttatattatgtatatat
gaggttagttaagctgcatttagttaggaacatttaatttttacctaaat
gatcctttttcatccagaggtagagattttattgtcattatgaatgtta
ctccccatttagtccaaaatagtgagcttttaagttgtgccaggtactta
acaatagtaaaagctattgtatagatttttattcaatgtgacctataata
catttctgattccttctgtattgaatgtcccagtttccatattaagtgct
atgccctgtataacattaatgaatttaatagtgtacaattttgggccagg
catggtggctcacgccagtaatcctggcactttgggaggcagaggcaggc
agatcacctgaggtcaggggttcgagatcagcctggccaacatggcaaaa
tcacatctctactaaaaatacaaaaattagccaggcgtggtggtgcatgc
ctgtagtcccagctactcaggaggctgaggcaggagaatcgcttgaaccc
aggaggcggaggttgcagtgagccgagatcatgccattgcactccagcct
gggagacagagcaagactctatctcaaaaataaaaataaaaaaaaaattg
cgtgcaattttgtattttcatagtcgtatctttttaaaggtatcatgatt

tcagttgtggtcaggaagtatgtgccttaaatcctctactctagacccaa
agtttggagagctatattatttaataagttgtttgtgacagccttgttac
ctttttcatttgatttgagggagaaagactgtgatcctgacagattcctt
ctcataaaatggcctaatgtgtatcagtctaggacttctggggagggaac
ctctaccatgcattctgtcccaggatgtcaaagtcataagaatcagggtc
ccctgaaataaaatcactgaaaagatatgttctgttatatattatttaaa
aaatttatctggtgccaccaaagaatgacagcagtttctaaccaacttca
tatttatagcatcttatgaagatattgtaaggcttagcatattttgccac
tggttttctttgtaatataggttgaaagtgagacatgtttgaatactttt
gtatgtaaatatctcccattctttttctatctcttcttggtctatattta
ctaagaattgatatttaaaaaacagttcactaatgaactctacatattat
tgaacactcacagggcaatattgatttgggtgctactagacttttaccta
acattagtctttctcaatagttgttgtaaaggatagtattcaatccagta
aatattaaagtgtattagtttaatgaaggttatttatatactgtcatacc
acaaacctatggtggaaagaacatctgcattcaccagaatgtacttgttc
ctttggctgtgaataaattggataagactttttattgtaagttccagct
gttggaagatacggggataagattgacattgctgttgcagtattgcaaaa
acatgactaaattggttaattatgtctaccgcttatgtttaagagaatcc
tttcactaacttaaattgttaacattgttgtgatattgagaaagaatatt
aacctaaacagtcactttacaacaatcatgtaaagacgtgtgcctgcagt
tgaggtttttttgcatttctgagcctgctttgtattcatgagaaacaaaaa
cataatgggagaaaagttttagataagcagcattgtaagtttttgtaaag
tttgggatgtcaaagtattaacgaagggtactgaaaacatacttttactt
gggtcaaattactttttatgatctgatttcttaattttctgtatttgaaa
tcttgcaaattaggaatatctacatctatagataaataagtaaaacttaa
tggtagaaataagtgtaattcagcaacatgattcaacaatttttatattt
aggataagttattgtttattatattaatatcaaatttatatattgccttg
taatgctaaatgctcttaaaagaatatatgggc

>NM_001259 6
aacctctccgcgcgaagacggcttcagccctgcagggaaagaaaagtgca
atgattctggactgagacgcgcttgggcagaggctatgtaatcgtgtctg
tgttgaggacttcgcttcgaggagggaagaggagggatcggctcgctcct
ccggcggcggcggcggcggcgactctgcaggcggagtttcgcggcggcgg
caccagggttacgccagccccgcggggaggtctctccatccagcttctgc
agcggcgaaagccccagcgcccgagcgcctgagccggcggggagcaagta
aagctagaccgatctccggggagccccggagtaggcgagcggcggccgcc
agctagttgagcgcacccccccgcccgccccagcggcgccgcggcgggcgg
cgtccaggcggcatggagaaggacggcctgtgccgcgctgaccagcagta
cgaatgcgtggcggagatcggggagggcgcctatgggaaggtgttcaagg
cccgcgacttgaagaacggaggccgtttcgtggcgttgaagcgcgtgcgg
gtgcagaccggcgaggagggcatgccgctctccaccatccgcgaggtggc
ggtgctgaggcacctggagaccttcgagcaccccaacgtggtcaggttgt
ttgatgtgtgcacagtgtcacgaacagacagagaaaccaaactaacttta
gtgtttgaacatgtcgatcaagacttgaccacttacttggataaagttcc

agagcctggagtgcccactgaaaccataaaggatatgatgtttcagcttc
tccgaggtctggactttcttcattcacaccgagtagtgcatcgcgatcta
aaaccacagaacattctggtgaccagcagcggacaaataaaactcgctga
cttcggccttgcccgcatctatagtttccagatggctctaacctcagtgg
tcgtcacgctgtggtacagagcacccgaagtcttgctccagtccagctac
gccacccccgtggatctctggagtgttggctgcatatttgcagaaatgtt
tcgtagaaagcctctttttcgtggaagttcagatgttgatcaactaggaa
aaatcttggacgtgattggactcccaggagaagaagactggcctagagat
gttgcccttcccaggcaggcttttcattcaaaatctgcccaaccaattga
gaagtttgtaacagatatcgatgaactaggcaaagacctacttctgaagt
gtttgacatttaacccagccaaaagaatatctgcctacagtgccctgtct
cacccatacttccaggacctggaaaggtgcaaagaaaacctggattccca
cctgccgcccagccagaacacctcggagctgaatacagcctgaggcctca
gcagccgccttaagctgatcctgcggagaacacccttggtggcttatggg
tccccctcagcaagccctacagagctgtggaggattgctatctggaggcc
ttccagctgctgtcttctggacaggctctgcttctccaaggaaaccgcct
agtttactgttttgaaatcaatgcaagagtgattgcagctttatgttcat
ttgtttgtttgtttgtctgtttgtttcaagaacctggaaaaattccagaa
gaagagaagctgctgaccaattgtgctgccatttgatttttctaaccttg
aatgctgccagtgtggagtgggtaatccaggcacagctgagttatgatgt
aatctctctgcagctgccgggcctgatttggtacttttgagtgtgtgtgt
gcatgtgtgtgtgtgtgtgtgtgtgtgtgtgtgtgtatgtgagagattct
gtgatcttttaaagtgttactttttgtaaacgacaagaataattcaattt
taaagactcaaggtggtcagtaaataacaggcatttgttcactgaaggtg
attcaccaaaatagtcttctcaaattagaaagttaaccccatgtcctcag
catttcttttctggccaaaagcagtaaatttgctagcagtaaaagatgaa
gttttatacacacagcaaaaaggagaaaaaattctagtatattttaagag
atgtgcatgcattctatttagtcttcagaatgctgaatttacttgttgta
agtctattttaaccttctgtatgacatcatgctttatcatttcttttgga
aaatagcctgtaagctttttattacttgctataggtttagggagtgtacc
tcagatagattttaaaaaaaagaatagaaagcctttatttcctggtttga
aattcctttcttccctttttttgttgttgttattgttgtttgttgttgtt
attttgtttttgtttttaggaatttgtcagaaactctttcctgttttggt
ttggagagtagttctctctaactagagacaggagtggccttgaaattttc
ctcatctattacactgtactttctgccacacactgccttgttggcaaagt
atccatcttgtctatctcccggcacttctgaaatatattgctaccattgt
ataactaataacagattgcttaagctgttcccatgcaccacctgtttgct
tgctttcaatgaacctttcataaattcgcagtctcagcttatggtttatg
gcctcgattctgcaaacctaacagggtcacatatgttctctaatgcagtc
cttctacctggtgtttacttttgttacctaaataatgagtaggatcttgt
tttgttttatcaccagcacacagattgctataaactgttactttgtgaat
tacatttttatagaagatattttcagtgtctttacctgagggtatgtctt
tagctatgttttagggccatacatttactctatcaaatgatcttttctcc
atcccccaggctgtgcttatttctagtgccttgtgctcactcctgctctc
tacagagccagcctggcctgggcattgtaaacagcttttcctttttctct
tactgttttctctacagtcctttatatttcataccatctctgccttataa

gtggtttagtgctcagttggctctagtaaccagaggacacagaaagtatc
ttttggaaagtttagccacctgtgctttctgactcagagtgcatgcaaca
gttagatcatgcaacagttagattatgtttagggttaggattttcaaaga
atggaggttgctgcactcagaaaataattcagatcatgtttatgcattat
taagttgtactgaattctttgcagcttaatgtgatatatgactatcttga
acaagagaaaaaactaggagatgtttctcctgaagagcttttggggttgg
gaactattcttttttaattgctgtactacttaacattgttctaattcagt
agcttgaggaacaggaacattgttttctagagcaagataataaaggagat
gggccatacaaatgttttctactttcgttgtgacaacattgattaggtgt
tgtcagtactataaatgcttgagatataatgaatccacagcattcaaggt
caggtctactcaaagtctcacatggaaaagtgagttctgcctttcctttg
atcgagggtcaaaatacaaagacatttttgctagggcctacaaattgaat
ttaaaaactcactgcactgattcatctgagctttttggttagtattcatg
gctagagtgaacatagctttagtttttgctgttgtaaaagtgttttcata
agttcactcaagaaaaatgcagctgttctgaactggaatttttcagcatt
ctttagaattttaaatgagtagagagctcaacttttattcctagcatctg
cttttgactcatttctaggcagtgcttatgaagaaaaattaaagcacaaa
cattctggcattcaatcgttggcagattatcttctgatgacacagaatga
aagggcatctcagcctctctgaactttgtaaaaatctgtccccagttctt
ccatcggtgtagttgttgcatttgagtgaatactctcttgatttatgtat
tttatgtccagattcgccatttctgaaatccagatccaacacaagcagtc
ttgccgttagggcattttgaagcagatagtagagtaagaacttagtgact
acagcttattcttctgtaacatatggtttcaaacatctttgccaaaagct
aagcagtggtgaactgaaaagggcatattgccccaaggttacactgaagc
agctcatagcaagttaaaatattgtgacagatttgaaatcatgtttgaat
ttcatagtaggaccagtacaagaatgtccctgctagtttctgtttgatgt
ttggttctggcggctcaggcattttgggaactgttgcacagggtggagtc
aaaacaacctacatataaaaagagaaaaagagaaacttgtccatttagct
ttcataagaaatcccatggcaaagggtaataaaaaggacctaatcttaaa
aatacaatttctaagcacttgtaagaacccagtgggttggagcctcccac
tttgtccctcctttgaagtggatgggaactcaaggtgcaaagaacctgtt
ttggaagaaagcttggggccatttcagccccctgtattctcatgattttc
tctcaggaagcacacactgtgaatggcagacttttcatttagccccaggt
gacttactaaaaatagttgaaaattattcacctaagaatagaatctcagc
attgtgttaaataaaaatgaaagctttagaaggcatgagatgttcctatc
ttaaataaagcatgtttcttttctatagagaaatgtatagtttgactctc
cagaatgtactatccatcttgatgagaaaactcttaaatagtaccaaaca
ttttgaactttaaattatgtatttaaagtgagtgtttaagaaactgtagc
tgcttcttttacaagtggtgcctattaaagtcagtaatggccattattgt
tccattgtggaaattaaattatgtaagcttcctaatatcataaacatatt
aaaattcttctaaaatattgcttttcttttaagtgacaatttgactattc
ttatgataagcacatgagagtgtcttacattttccaaaagcaggctttaa
ttgcatagttgagtctaggaaaaaataatgttaaaagtgaatatgccacc
ataattacttaattatgttagtatagaaactacagaatatttaccctgga
aagaaaatattggaatgttattataaactcttagatatttatataattca
aaagaatgcatgtttcacattgtgacagataaagatgtatgatttctaag

gctttaaaaattattcataaaacagtgggcaatagataaaggaaattctg
gagaaaatgaaggtatttaaagggtagtttcaaagctatatatattttga
aggatatattctttatgaacaaatatattgtaaaaatttatactaaggtc
atctggtaactgtgggattaatatggtcgaaaacaaatgttatggagaag
ctgtcccaagcaaactaaattacctgtactttttcccatttcaagggaa
gaggcaaccacatgaagcaatacttcttacacatgcctaagaacgttcat
tgaaaaaataaatttttaaaaggcatgtgtttcctatgccaccaatactt
ttgaaaaattgtgaaccttacccaaaaccatttatcatgtccattaagta
tatttgggtatataattaggaagatatttacatgttccatctccacagtg
gaaaaacttattgaggctaccaaagtgtgccaagaaatgtaagtccttag
agtaattagaaatgctgttttcctcaaaagcatgagaaactagcattttc
atttcttatttactccctttctatatcaatgcaattcacaacccaatttt
aatacatccctatatctcaagcatttctatcttgtactttttcagaaaat
aaaccaaaaataatcctttggtctctctatcttctgacctttgtaagcaa
cagaaatgtaaaaacagaaggggtccaatttttacacgtttttttctcaa
gtagcctttctggggatttttattttcttaatgaagtgccaatcagcttt
tcaaaatgttttctatttctcagcatttccaggaagtgataacgtttagc
taaatgagtagaagtggacttccttcaacatattgttaccttgtctagcc
ttaggaagaaaacaagagccacctgaaaataaatacaggctcttttcgag
catctgctgaaatactgttacagcaatttgaagttgatgtggtaggaaag
gaaggtgacttttcttgcaaaagtctttctaaacattcacactgtcctaa
gagatgagctttcttgttttattccggtatattccacaaggtggcacttt
tagagaaaaacaaatctgatgaagactaaagaggtacttctaaaagagat
ttcattctaactttatttttctgcgcatatttaactctttcctagcactt
gtttttgggatgattaatagtctctataatgttctgtaacttcaatatt
ttacttgttacctaggttctgaacaattgtctgcaaataaattgttctta
aggatggataatacacccattttgatcatttaagtaaagaaagcctagtc
attcattcagtcaagaaaaaatttttgaagtacccagttaccttactttt
ctagattaaaacaggcttagttactaaaaaggcagtcctcatctgtgaac
aggatagtttcgttagaagtataaaactcctttagtggccccagttaaaa
cacacataccctctctgctgctttcaaattccctagcatggtggcctttc
aacattgattaaattttaaaatcctaatttaaagatcaggtgagcaaaat
gagtagcacatcagtaattcagtagacaaaacttttgtctgaaaaattgc
tgtattgaaacagagccctaaaataccaaaagaccaggtaattttaacat
ttgtggaatcacaaatgtaaattcataagaagctctaattaaaaaaaaaa
agtctgaagtatatgagcataacaacttaggagtgtgtctacatacttaa
cttttgaagtttttggcaactttatatactttttttaaatttacaagtc
tacttaaagacttcttataccccaaatgattaagttaattttagaggtca
cctttctcacagcagtgtcacttgaaatttagtagggaaggatattgcag
tatttttcagtttccttagcacagcaccacagaaagcagcttattccttt
tgagtggcagacactcgacggtgcctgcccaactttcctcctgagtggca
agcagatgagtctcagtaattcatactgaaccaaaatgccacatacacta
ggggcagtcagaaactggctgagaaatcccccgcctcattcgcccctctg
ctcccaggaactagagtccagttaaagcccctatgcgaaaggccgaattc
caccccagggtttgttataacagtggccagtctgaaccccatttgctcgt
gctcaaaacttgattcccacttgaaagccttccgggcgcgctgcctcgtt

gccccgcccctttggcaggagagaggcagtgggcgaggccgggctggggc
cccgcctcccactcacctgccggtgcctgaaattatgtgcggccccgcgg
gctgctttccgaggtcagagtgccctgctgctgtctcagaggcatctgtt
ctgcaaatcttaggaagaaaaatgtccctagtagcaaacgggtgtcttct
gtgcataaataagtacaacacaattctccgaaagttcgggtaaaaagaga
tgcggtagcagctgccctgtgtgaagctgtctaccccgcatctctcaggc
gctaagctcagtttttgtttttgtttttgtttttttaaagaaaagatgta
taattgcaggaattttttttttattttttttattttccatcattctatatat
gtgatggtgaaagatatgcctggaaaagttttgttttgaaaagtttattt
tctgcttcgtcttcagttggcaaaagctctcaattctttagcttccagtt
tcttttctctctttttctttgttaggtaattaaaggtatgtaaacaaatt
atctcatgtagcaggggattttcatgttgagaggaatcttccgtgtgagt
tgtttggtcacacaaataaccctttctcaattttaggagtttggattgtc
aaatgtaggtttttctcaaagggggcatataactacatattgactgccaa
gaactatgactgtagcactaatcagcacacatagagccacacaattattt
aatttctaactctctgtggtccctagaaaaattccgttgatgtgcttagg
ttaaagttctgaagatacccgttgtacccttacttgaaagtttctaatct
taagttttatgaaatgcaataatatgtatcagctagcaatatttctgtga
tcaccaacaactctcagtttgatcttaaagtctgaataataaaacaaatc
ccagcagtaatacatttcttaaacctcacagtgcatgatatatcttttca
ttctgatcctgtgtttgcaaaaatatacacatgtatatcatagttcctca
ctttttattcatttgttttcctattacctgtagtaaatatattagttagt
acatggaatttatagcatcagctacccccaggaacagcacctgacaggcg
ggggattttttttcaagttgttctacatttgcataaattatttctattat
tattcatgtatgttatttatttctgaatcacactagtcctgtgaaagtac
aactgaaggcagaaagtgttaggattttgcatctaatgttcattatcatg
gtattgatggacctaagaaaataaaaattagactaagcccccaaataagc
tgcatgcatttgtaacatgattagtagatttgaatatatagatgtagtat
tttgggtatctaggtgttttatcattatgtaaaggaattaaagtaaagga
ctttgtagttgtttttattaaatatgcatatagtagagtgcaaaaatata
gcaaaaataaaaactaaaggtagaaaagcattttagatatgccttaattt
agaaactgtgccaggtggccctcggaatagatgccaggcagagaccagtg
cctgggtggtgcctcctcttgtctgccctcatgaagaagcttccctcacg
tgatgtagtgccctcgtaggtgtcatgtggagtagtgggaacaggcagta
ctgttgagaggagagcagtgtgagagtttttctgtagaagcagaactgtc
agcttgtgccttgaggcttccagaacgtgtcagatggagaagtccaagtt
tccatgcttcaggcaacttagctgtgtacagaagcaatccagtgtggtaa
taaaaagcaaggattgcctgtataatttattataaaataaaagggatttt
aacaaccaacaattcccaacacctcaaaagcttgttgcatttttttggtat
ttgaggtttttatctgaaggttaaagggcaagtgtttggtatagaagagc
agtatgtgttaagaaaagaaaaatattggttcacgtagagtgcaaattag
aactagaaagttttatacgattatcattttgagatgtgttaaagtaggtt
ttcactgtaaaatgtattagtgtttctgcattgccatagggcctggttaa
aactttctcttaggtttcaggaagactgtcacatacagtaagctttttttc
cttctgacttataatagaaaatgttttgaaagtaaaaaaaaaaaaatctaa
tttggaaatttgacttgttagtttctgtgtttgaaatcatggttctagaa

atgtagaaattgtgtatatcagatactcatctaggctgtgtgaaccagcc
caagatgaccaacatccccacacctctacatctctgtcccctgtatctct
tcctttctaccactaaagtgttccctgctaccatcctggcttgtccacat
ggtgctctccatcttcctccacatcatggaccacaggtgtgcctgtctag
gcctggccaccactcccaacttgacctagccacattcatctagagatggt
tcctgatgctgggcacagactgtgctcatggcacccattagaaatgcctc
tagcatctttgtatgcatcttgatttttaaaccaagtcattgtacagagc
attcagttttggctgtggtaccaagagaaaaactaatcaagaatataaac
cacattccaggctgctgttttctctccatctacaggccacacttttactg
tatttcttcatacttgaaattcattctgctattttcatatcagggtacag
acttataagggtgcatgttccttaaaggtgcataattattcttattccgt
ttgcttatattgctacagaatgctctgttttggtgctttgagttctgcag
acccaagaagcagtgtggaaattcactgcctgggacacagtcttataaga
atgttggcaggtgactttgtatcagatgttgcttctcttttctctgtaca
cagattgagagttaccacagtggcctgtcgggtccaccctgtgggtgcag
cacagctctctgaaagcaagaaccttcctacctattctaacgtttttgcc
ctctaagaaaaatggcctcaggtatggtatagacatagcaagaggggaag
ggctgtctcactctagcaaccatccctccattacacacagaaagccctct
tgaagcaaaagaagaagaaagaaagaaagcttatctctaaggctactgtc
ttcagaatgctctgagctgaatgctcttgctcctttcccaagaggcagat
gaaaatatagccagtttatctatacccttcctatctgaggaggagaatag
aaaagtagggtaaatatgtaacgtaaaatatgtcattcaaggaccaccaa
aactttaagtaccctatcattaaaaatctggttttaaaagtagctcaagt
aagggatgctttgtgacccagggtttctgaagtcagatagccattcttac
ctgccccttactctgacttattgggaaagggagaactgcagtggtgtttc
tgttgcagtggcaaaggtaacatgtcagaaaattcagagggttgcatacc
aataatcctttggaaactggatgtcttactgggtgctagaatgaaaatgt
aggtatttattgtcagatgatgaagttcattgtttttttcaaaattggtg
ttgaaatatcactgtccaatgtgttcacttatgtgaaagctaaattgaat
gaggcaaaaagagcaaatagtttgtatatttgtaataccttttgtatttc
ttacaataaaaatattggtagcaaataaaaataataaaaacaataacttt
aaactgctttctggagatgaattactctcctggctattttctttttact
ttaatgtaaaatgagtataactgtagtgagtaaaattcattaaattccaa
gttttagcagaaaaaaaaaaaaaaaaaa

>NM_001921 2
agggcttcgggcccccggggccagccgccgtcctgcgaacgcgcggcgcc
cgggccgctgccccttccggccccgcggcggccccgcctcctcccccga
cttccttccctgagcacggcggcggcggggacgagcaccggcctgcgcgc
ggagccggcaccggatgacccaacatgagtgaagtttcctgcaagaaacg
ggacgactatttggaatggccagagtattttatggctgtggccttcttat
cagcacagagaagcaaagatccaaattcccaggtcggcgcctgcatcgtg
aattcagaaaacaagattgtcgggattgggtacaatgggatgccaaatgg
gtgcagtgatgacgtgttgccttggagaaggacagcagagaataagctgg
acaccaaatacccgtacgtgtgccatgcggagctgaatgccatcatgaac
aaaaattcgaccgatgtgaaaggctgtagtatgtatgttgccttgttccc

ttgtaatgaatgcgctaagctcatcatccaggcaggtataaaagaagtga
ttttcatgtctgataaataccatgatagtgacgaggcaactgctgcgagg
ctcctgtttaatatggccggggtgacattccggaaattcataccgaagtg
cagcaagattgtcattgactttgattcaattaacagcagaccgagtcaaa
agcttcagtgagttacatctcattcaatctccagaagattgggattatcg
tcttctaagaggttgctaatgcctttcatcttgaagttacacataacttc
ttactagccagtatggcaaaagtaggcatctaaagaatataaagcctcaa
atcttccttactgtctctcttgtcacatggaatctacatgtgtttgaact
attgctttagggtttaaaatagggggagcctgtggtggcctggtgcacagg
gctagaacgagagtgcctccccttcttgtgtcctggctggctgggatgct
ggtggctcttcagaggagcatcagctgtctgtcatctgctgcgatccggc
agcctctcttcactgctacatgtgctggaaggacaaataaataattgtgg
ttgtgttcttaatggggacgagcagacacactgatctgaacatctggccc
aagtgaagcatggcatatagtgcccttggaagaaaattaggcctcaaatg
acagtagcattgaagtgtttgctgcagagttgagggaaacccccagccac
cctcccggaatccgagatagggtggcacatctgtcctgacagacgaggag
tgtaactgaaccaggaatatttcctccattcctgctctcccactgcacac
agggtggtggcacattatccctctggggggtggggacgcctgttgttttg
gctcaatttgggtttgttggtcacatggagctcttccatttcgtttagct
gaataatgagttgttcctagaggagacagcctgtctctccttgttgcccc
caaagcccatgccctgccgtggtggcagctggggctgtggatgggagggg
tccccaacatggatgtgttgcccctcctccgcatgccaacgcagttcatg
tacaaggcccctctgcaactggagagaaaattaattcctatcccgtgagt
ggattgtgagaaattccacccacgtggagacagcttactgcagcactgtt
ggtgttcggagctcttctgtgccctggctccatgctttcacctacacaag
catcaccttcctaatcaccgcggggcggggagcgtgtggctgtgcccctt
ctctttaatctcatttaatttttattaaacatgctcagtacctgtgttga
gaaaaggctttctttatcctaaagattattaccttttaaagtgctctta
tattttcatgagtttttattttgtctctgagattttgtattccacattct
agggtattctgtaatttggctccttaccaatattattaaaatcttattaa
aatctaccctaccaaagca

>NM_003060 3
cgccttcgccggcgccgctctgcctgccagcggggcgcgccttgcggccc
aggcccgcaaccttccctggtcgtgcgccctatgtaaggccagccgcggc
aggaccaaggcggcggtgtcagctcgcgagcctaccctccgcggacggtc
ttgggtcgcctgctgcctggcttgcctggtcggcggcgggtgccccgcgc
gcacgcgcaaagcccgccgcgttccccgaccccaggccgcgctctgtggg
cctctgagggcggcatgcgggactacgacgaggtgaccgccttcctgggc
gagtgggggcccttccagcgcctcatcttcttcctgctcagcgccagcat
catccccaatggcttcaccggcctgtcctccgtgttcctgatagcgaccc
cggagcaccgctgccgggtgccggacgccgcgaacctgagcagcgcctgg
cgcaaccacactgtcccactgcggctgcgggacggccgcgaggtgcccca
cagctgccgccgctaccggctcgccaccatcgccaacttctcggcgcttg
ggctggagccggggcgcgacgtggacctggggcagctggagcaggagagc
tgtctggatggctgggagttcagtcaggacgtctacctgtccaccattgt

gaccgagtggaacctggtgtgtgaggacgactggaaggccccactcacaa
tctccttgttcttcgtgggtgtgctgttgggctccttcatttcagggcag
ctgtcagacaggtttggccggaagaatgtgctgttcgtgaccatgggcat
gcagacaggcttcagcttcctgcagatcttctcgaagaattttgagatgt
ttgtcgtgctgtttgtccttgtaggcatgggccagatctccaactatgtg
gcagcatttgtcctggggacagaaattcttggcaagtcagttcgtataat
attctctacgttaggagtgtgcatattttatgcatttggctacatggtgc
tgccactgtttgcttacttcatccgagactggcggatgctgctggtggcg
ctgacgatgccgggggtgctatgcgtggcactctggtggttcatccctga
gtcccccgatggctcatctctcagggacgatttgaagaggcagaggtga
tcatccgcaaggctgccaaagccaatgggattgttgtgccttccactatc
tttgacccgagtgagttacaagacctaagttccaagaagcagcagtccca
caacattctggatctgcttcgaacctggaatatccggatggtcaccatca
tgtccataatgctgtggatgaccatatcagtgggctattttgggctttcg
cttgatactcctaacttgcatggggacatctttgtgaactgcttcctttc
agcgatggttgaagtcccagcatatgtgttggcctggctgctgctgcaat
atttgccccggcgctattccatggccactgccctcttcctgggtggcagt
gtccttctcttcatgcagctggtacccccagacttgtattatttggctac
agtcctggtgatggtgggcaagtttggagtcacggctgccttttccatgg
tctacgtgtacacagccgagctgtatcccacagtggtgagaaacatgggt
gtgggagtcagctccacagcatcccgcctgggcagcatcctgtctcccta
cttcgtttaccttggtgcctacgaccgcttcctgccctacattctcatgg
gaagtctgaccatcctgacagccatcctcaccttgtttctcccagagagc
ttcggtaccccactcccagacaccattgaccagatgctaagagtcaaagg
aatgaaacacagaaaaactccaagtcacacaaggatgttaaaagatggtc
aagaaaggcccacaatccttaaaagcacagccttctaacatcgcttccag
taagggagaaactgaagaggaaagactgtcttgccagaaatggccagctt
gtgcagactccgagtccttcagtgacaaaaggcctttgctgtttgtcctc
ttgacctgtgtctgacttgctcctggatgggcacccacactcagaggcta
catatggccctagagcaccaccttcctctagggacactggggctacctac
agacaacttcatctaagtcctaactattacaatgatggactcagcacctc
caaagcagttaatttttcactagaaccagtgagatctggaggaatgtgag
aagcatatgctaaatgtacattttaattttagactacttgaaaaggcccc
taataaggctagaggtctaagtcccccacccctttccccactcccctcta
gtggtgaactttagaggaaaaggaagtaattgcacaaggagtttgattct
taccttttctcagttacagaggacattaactggatcattgcttccccagg
gcaggagagcgcagagctagggaaagtgaaaggtaatgaagatggagcag
aatgagcagatgcagatcaccagcaaagtgcactgatgtgtgagctctta
agaccactcagcatgacgactgagtagacttgtttacatctgatcaaagc
actgggcttgtccaggctcataataaatgctccattgaatctactattct
tgttttccactgctgtggaaacctccttgctactatagcgtcttatgtat
ggtttaaaggaaatttatcaggtgagagagatgagcaacgttgtcttttc
tctcaaagctgtaatgtgggttttgttttattgtttatttgtttgttgtt
gtatccttttctccttgttatttgcccttcagaatgcacttgggaaaggc
tggttccttagcctcctggtttgtgtctttttttttttttttttaaaaca
gaatcactctggcaattgtctgcagctgccactggtgcaaggccttacca

gccctagcctctagcacttctctaagtgccaaaaacagtgtcattgtgtg
tgttcctttcttgatacttagtcatgggaggatattacaaaaaagaaatt
taaattgtgttcatagtctttcagagtagctcactttagtcctgtaactt
tattgggtgatattttgtgttcagtgtaattgtcttctctttgctgatta
tgttaccatggtactcctaaagcatatgcctcacctggttaaaaaagaac
aaacatgtttttgtgaaagctactgaagtgccttgggaaatgagaaagtt
ttaataagtaaaatgatttttaaataacaaaaaaaaaaaaaaaa

>NM_004239 3
gatccgctgccgctttccgagcgagtgtcatggcggccggcgtcgagttg
gcaggagtaacccacggaactgaggaaagtcattagagctgagaaagaag
tggcccaatctggacggtgggaattcgtgggaatgagcagaaggccctcc
gtaggtgactgtgtcactagaggcgggcccctggtaaaattccaggccag
gcctctgcgtttctaggcagaacctggagtcggccttgcctgagaaccca
gctttgtgttatcgtatcctgtctcgcgaaggcaggcgttcaaggatatt
tggtcggatcgcccggcggcgctaaacgttttctttttccgagcggacc
gggtcgttctctaaactcgccgcgatgtcgtcctggcttgggggcctcgg
ctccggattgggccagtctctgggtcaagtcgggggcagcctggcttccc
tcactggccagatatcaaactttacaaaggatatgctgatggagggcacg
gaggaagtggaagcagaattacctgattctaggacaaaggaaattgaagc
cattcatgcaatcttgagatcagagaatgaaaggcttaagaaactttgta
ctgatctagaagagaaacatgaagcatcagagattcaaataaagcagcaa
tctacaagttaccgaaatcaacttcaacaaaaagaggtagaaatcagcca
tcttaaagccagacagattgcactccaggatcagttgctgaaactgcagt
cagctgctcagtcagtaccttcaggagctggtgtaccagcaaccactgca
tcatcttcattcgcttatgggattagtcatcatccttcagctttccatga
cgatgacatggactttggtgatataatttcatcccaacaagaaataaacc
gactctcaaatgaagtttcaagacttgagtctgaagttggccattggagg
catattgctcagacttccaaagcacaaggaacagataactctgatcaaag
tgaaatatgtaaactacaaaatatcattaaggaactaaaacagaaccgaa
gtcaggaaattgatgaccatcaacatgaaatgtcagtactgcagaatgca
caccaacagaaattgacagaaataagtcgacgacatcgagaagaattaag
tgactatgaagaacgaattgaagaacttgaaaatctgttacaacaaggtg
gctctggagttatagaaactgatctctctaaaatctatgagatgcaaaaa
actattcaagttctacaaatagaaaaagtggagtctaccaaaaaaatgga
acaacttgaggataaaataaaagatataaataaaaaattatcttctgcag
aaaatgacagagatattttgaggagagaacaagaacagctaaatgtggaa
aagagacaaataatggaagaatgtgaaaacttgaaattggaatgtagtaa
attgcagccttctgctgtgaagcaaagtgatactatgacagaaaaggaaa
gaattcttgcccagagtgcatcagtggaagaagtgttcagactacaacaa
gcactgtctgatgccgaaaatgaaataatgagattgagtagtttaaacca
ggataacagtcttgctgaagacaatctgaaacttaaaatgcgtatcgaag
ttttagaaaaagagaagtcattactgagtcaagaaaaggaagaacttcag
atgtcacttttaaaattgaacaatgaatatgaagtaattaaaagtacagc
tacaagagacataagtttggattcagaattacatgacttaagacttaatt
tggaggcaaaggaacaagaactcaatcagagtattagtgaaaaggaaaca

ctgatagctgagatagaagaattggacagacagaatcaagaagctacaaa
gcacatgattttgataaaagatcagctatcaaaacaacaaaatgaaggag
atagcatcatcagtaaactgaaacaagatctaaatgatgaaaaaaagaga
gttcatcaacttgaagatgataaaatggacattactaaagagttagatgt
acagaaagaaaagctaattcaaagtgaagtggccctaaatgatttacatt
taaccaagcagaaacttgaggacaaagtagaaaatttagtagatcagcta
aataaatcacaagaaagtaatgtaagcatccagaaggagaatttagaact
taaggagcatattagacaaaatgaggaggagctttctagaataaggaatg
agttaatgcagtctctaaatcaagactctaatagtaattttaaggatacc
ttacttaaagaaagagaagctgaagttagaaacttaaagcaaaatctttc
agaattagaacagctcaatgaaaatttaaagaaagttgcttttgatgtca
aaatggaaaatgaaaagttagttttagcatgtgaagatgtgaggcatcag
ttagaagaatgtcttgctggtaacaatcagctttctctggaaaaaaacac
tattgtggagactctaaaaatggaaaaaggagagatagaggcagaattgt
gttgggctaaaaagaggctgttggaagaagcaaacaagtatgagaaaacc
attgaagaactgtcaaatgcacgtaatttgaatacctctgccttacagct
ggaacatgagcatttaattaaactcaatcaaaagaaagacatggaaatag
cagaactcaaaaagaatattgaacaaatggatactgaccataaagaaact
aaggacgttttgtcatctagtttagaagagcagaagcagttgacacaact
tataaacaagaaagaaatttttattgaaaagcttaaagaaagaagttcaa
agctgcaggaggaattggataaatattctcaggccttaagaaaaaatgaa
attttaagacagaccatagaggaaaaagaccgaagtcttggatccatgaa
agaggaaaataatcatctgcaagaagaattggaacgactcagggaagagc
agagtcgaaccgcacctgtggctgaccctaaacccttgatagtgttact
gaactagcatctgaggtatctcaactgaacacgatcaaggaacatcttga
agaggaaattaaacatcatcaaaagataattgaagatcaaaaccagagta
agatgcaactacttcagtctttacaagagcaaaagaaggaaatggatgag
tttagataccagcatgagcaaatgaacgccacacacacccagctcttttt
agagaaggatgaggaaattaagagtttgcaaaaaacaattgaacaaatca
aaacccagttgcatgaagaaagacaggacattcaaacagataactctgat
atttttcaagaaacaaaagttcagagccttaatatagaaaatggaagtga
aaagcatgatttatctaaagctgaaacggaaagattagtgaaaggaataa
aagagcgagaactggagattaaacttctaaatgaaaagaatatatcttta
actaaacagattgatcagttgtccaaagatgaagttggtaaactaactca
gattattcagcagaaagatttggagatacaagctcttcatgctagaattt
cttcaacttcccatactcaagatgttgtttaccttcaacagcaactgcag
gcttatgctatggaaagagaaaaggtatttgctgttttgaatgagaagac
tagggaaaatagccatctaaaaacagaatatcacaaaatgatggatattg
ttgctgccaaggaagcagctcttatcaaactgcaagatgaaaataaaaaa
ttgtccactagatttgaaagtagtggccaagatatgtttagagaaactat
tcagaatttatcacgtatcattcgagaaaaagacatcgaaatagatgcac
taagtcagaaatgtcagactttattggcagttttacaaacatccagcact
ggtaatgaggctggaggtgttaatagtaatcaatttgaggagcttctaca
ggaacgtgacaagttaaaacagcaagtaaagaaaatggaagagtggaagc
agcaggtgatgaccacagtacaaaatatgcaacacgagtcagcccagctt
caggaagagcttcaccaacttcaagcacaggttttggttgacagtgataa

taattctaaattacaagtggactatactggcctgatccaaagttatgagc
agaatgaaaccaaactcaaaaattttgggcaggaattagcacaagttcag
cacagcattgggcagctttgcaataccaaggatcttcttttaggaaaact
tgatattatttcaccccagctgtcttctgcatcattgcttactccccagt
ctgcagagtgtcttagagcaagtaagtctgaagtattgagtgaatcttct
gaattgcttcagcaagagttagaagagctaagaaaatcactacaggaaaa
agatgcaacaattagaactctccaggaaaataaccacagattgtctgatt
cgattgctgccacctcagagctagaaagaaaagaacacgaacaaaccgat
tcagaaatcaagcagctaaaggagaaacaagatgttttgcaaaagttact
taaggaaaaagacctcttaatcaaagccaaaagtgatcaactactttctt
ccaatgaaaatttcactaacaaagtaaatgaaaacgaacttttgaggcag
gcagtaacaaacctgaaggagagaatattaattctagagatggacattgg
caaactaaaaggagaaaatgaaaaaatagtggaaacatacaggggaaagg
aaacagaatatcaagcgttacaagagactaacatgaagttttctatgatg
ctgcgagaaaaagagtttgagtgccactcaatgaaggagaaggctcttgc
ttttgaacagctattgaaagagaaagaacagggcaagactggagagttaa
atcagcttttaaatgcagttaaatcaatgcaggagaagacagttgtgttt
caacaggagagagaccaagtcatgttggccctgaaacaaaaacaaatgga
aaatactgccctacagaatgaggttcaacgtttacgtgacaaagaatttc
gttcaaaccaagagctagagagattgcgtaatcatcttttagaatcagaa
gattcttatacccgtgaagctttggctgcagaagatagagaggctaaact
aagaaagaaagtcacagtattggaggaaaagctagtttcatcctctaatg
caatggaaaatgcaagccatcaagccagtgtgcaggtagagtcattgcaa
gaacagttgaatgtagtttccaagcaaagggatgaaactgcgctgcagct
ttctgtctctcaggaacaagtaaagcagtatgctctgtcactggccaacc
tgcagatggtactagagcatttccaacaagaggaaaaagctatgtattct
gctgaactcgaaaagcaaaaacagcttatagctgaatggaagaaaaacgc
agaaaatctggaaggaaaagtgatatcattacaggaatgtttggatgaag
caaatgctgcattggattcagcatcaagacttacagaacagttagatgta
aaagaagaacaaattgaagaacttaaaagacaaaatgagctccgacaaga
aatgctggatgatgtacaaaagaaattgatgagcttagcaaacagctcag
aaggaaaagtagacaaagtcctaatgagaaacctcttcattggtcatttc
cacacaccgaaaaatcagcgtcatgaagtgttacggttaatggggagcat
cctgggcgtcagaagggaggagatggagcagttgtttcatgacgatcagg
gcggtgttaccaggtggatgactgggtggcttggaggaggatcaaaaagt
gttcccaacacacctttgagaccaaatcagcaatctgtggttaatagttc
tttttcagaactttttgttaaatttctagaaacagaatctcatccatcca
ttccaccaccaaagctttctgttcatgatatgaaacctctggattcacca
ggaagaagaaaaagagatacaaatgcaccagaaagttttaaagatacagc
agaatccaggtctggtagaagaacagatgtaaatccgtttttggctcctc
gctcggcagctgtacctcttattaacccagctggacttggacctggtggg
cccgggcatcttcttctgaaacccatctcagatgttttgcccacatttac
acctttgccagcgttacctgacaacagtgctggggttgtgctgaaagacc
ttttaaagcaatagatgattctcaagccagagacaatctagcactttaaa
gaaaccatgaacactatatgtatgtactttatcacaaagtggcctttggg
gagaaagtcatgtatttgttcgcaattatgctttctctgaatttaataaa

aatattcctaatgcttttagaaactgcaggtgtcaggagcaagtgtttgc
tatataatctgctttattccttctgatttgacctaatttaagctaggaac
atttaatttatcttttaccttaatttacaaaaatacagacagacaaaaat
actagtagctatatggcagctttttatttccttttgtgatttccaagctc
atttaagctagagattgtggtttatcaagagagtgtttttttttttaaa
cgaagaataaaaaaatactagtagtggcttctctgcatagtgcagaaagt
tgatttgaagcatatattgatcatctttagaggactttgtggagacaggc
caacaagattgttgctggaaggtcataggttccctgaggtgaagtatgta
tttggtcctgtgcaatattatcatgtgtgtagattctttttttgtttgtt
ttaaatagagatggggtctcactatgttgccaggctggcctcaaactcct
gggctcaagtgatcctcccacctcagcctcccaaagtgctgggattaagg
catgagccactgcgcctggccctgtgtgtaaattcatgtgactaccacca
caggcaagacacggaagacttccattacaagcatccccctgtgttgccct
ctcataatcattgccacctctctccctggccctctccttaatccctgaca
accacttactgttctccatctcagtcattttgttattttgagaatgttat
ataaatggaatcaatatataccctcttgagactggcttttttcactcag
cataattcccttgagatccatccaaggctttggatgtatcactagtttgt
tcctttttattgttgagcatatagtgcagatatatcacttagttttaaca
gctaaggtatctatttaaagataccatcttaaaactaaatgttacacaaa
ttcatgcaactttcatataagcattaaaccatacagctgcatctacgtaa
gtataattagcaaaatcactgaacatgccacttcctgcctccgtctgcgg
tgttgacatatgaggtacagcaatccgagagtcaactgttatcacggtgg
ccactgagggcagtgaaatcactttgatagcaccagtacccagatgtttg
aaacttaagaaagtatttctttaatttttcataaacccatcataaaaaaa
aaatcccgacttgaaacttaactgtatctggatgttcatatttttattag
ttgttgaagttgtacatttaaaaagatatttcttgcaaattttgtaaatt
tctatatgcttttaaaatagaaacatttgtataacaaggaaggatgaaaa
taaaacaaattcaaatatgtgaatagtcctggctgaggccagaactaaat
ttggttatttgcaagttttactgtgattaactgtactttctaatgtattt
ggaagactggaagtcttaattccatgttgatgggtgaaacttgtttttct
gaaaaatgaagtaactgaaaatgctgcttcagaatgtcttgtatctcctt
agataactaatttatattgccattctttatttactatactttaaaggaag
ttgtcatgcaagttctatattagaactgtcaaactgccaaaaaaaaaaaa
aaaaaa

>NM_004422 2
agtcacgtgacatgaggagaggtgggcgggtacctggaggaagctcgcgg
cgtcggtggcggtggcgcgcggcggccgctgagaccggggctttgagtcg
caccccgcggcccgccccccgccgccaccctcgcagatccgtgctttttc
ccctttgcttctctcccgtactgggtcagtcctgtccgcgctcgcgcgtc
ggtttgcgggtgtgcgcaggcgcggcaggggccattagccctttgggtgg
gcggtggagcccgggagcgcgcgggcgagaccatggcgggtagcagcact
gggggcggtggggttggggagacgaaggtgatttaccacctggatgagga
agagactccctacctggtgaagatccctgtccccgccgagcgcatcaccc
tcggcgatttcaagagcgtcctgcagcggcccgcgggcgccaagtacttt
ttcaagtctatggatcaggatttcggggtggtgaaggaagaaatttcaga

tgacaacgcccgcctcccctgcttcaacggaagggtggtatcctggctgg
tgtcctcagataatccccaacccgagatggcccctccagtccatgagcct
cgggcagaactggcgcctccagccccacctttacctcctttgccacccga
gaggaccagcggcattggggactcaaggcctccatccttccaccctaatg
tgtccagcagccatgagaatctggagcctgagacagaaaccgagtcagta
gtgtcactgaggcgggagcggcctcgcaggagagacagcagtgagcatgg
cgctgggggccacaggactggtggcccctcaaggctggagcgccacctgg
ccggatacgagagctcctctaccctcatgaccagcgagctggagagtacc
agcctgggggactcggacgaggaggacaccatgagcaggttcagcagctc
cacggagcagagcagtgcctcccgcctccttaagcgccaccggcggcgaa
ggaagcagaggccaccccgcctggagaggacgtcatccttcagcagcgtc
acagattccacaatgtctctcaatatcatcacagtcacgctaaacatgga
gaagtacaacttcctgggtatctccattgttggccagagcaatgagcggg
gagacggaggcatctacattggctccatcatgaagggtggggctgtggcg
gccgacgggcgcattgagccaggggacatgcttttgcaggtgaatgacat
gaactttgagaacatgagcaacgatgacgctgtgcgggtgctgagggaca
ttgtgcacaagcctggccccattgtgctgactgtggccaagtgctgggat
ccctctcctcaggcctatttcactctcccccgaaatgagcccatccagcc
aattgaccctgctgcctgggtgtcccattccgcggctctgactggcacct
tcccagcctatccaggttcctcctccatgagcaccattacatctggatcg
tctttgcctgatggctgtgaaggccggggtctctccgtccatacggacat
ggcatcggtgaccaaggccatggcagctccagagtctggactggaagtcc
gggaccgcatgtggctcaagatcaccatccctaatgcctttctgggctcg
gatgtggttgactggctctaccatcacgtggagggctttcctgagcggcg
ggaggcccgcaagtatgccagcgggctgctcaaagcaggcctgatccgac
acaccgtcaacaagatcaccttctctgagcagtgctattacgtcttcgga
gacctcagtggtggctgtgagagctacctagtcaacctgtctctcaatga
caacgatggctccagtggggcttcagaccaggataccctggctcctctgc
ctggggccacccccctggcccctgctgcccactttctcctaccaataccct
gccccacacccctacagcccgcagcctccaccctaccatgagctttcatc
ttacacctatggtgggggcagtgccagcagccagcatagtgagggcagcc
ggagcagtgggtcgacacggagtgatgggggggcagggcgcacggggagg
cccgaggagcgggcccccgagtccaagtccggcagtggcagtgagtctga
gccctccagccgagggggcagccttcggcggggtggggaagcaagtggga
ctagcgatgggggccctcctccatccagaggctcaactgggggtgcccct
aatctccgagcccacccagggctccatccctatggaccgccccctggcat
ggccctcccctacaaccccatgatggtggtcatgatgcccccacctccac
ctccagtccctccagcagtgcagcctccgggggcccctccagtcagagac
ctgggctctgtgcccccagaactgacagccagccgccaaagcttccacat
ggccatgggcaatcccagcgagttctttgtggatgttatgtagcccactg
tggggccaggctgggccgggcgctcctggtgtgtgactgggtgtcctggc
cgtcatgtgcttgctcttacagtgcctgggctcagcctaccagctgctgc
catacaggagattgtggccactgtgactctcaccagcagtgcctggttcc
tccccccttccctcaggggtagacaagggacctttgattatttttagcttt
gtttttttataagcctttttgggggttaaaatagagtttcttacattttt
gggactttttttaataggcatttcctcttttatatgaagaattcccatcca

ttgggccccttctaaccccagaatgtgacctcctcctccagttacccaca
gccctgccctttgcagggttgggggtggtcagcggtaccccggggttagg
catcctagacagcagcctgaggaagctgggagatttgggccatgtagctg
cctttgttactctatttattttagtcacttgtataaaacaccaaataaag
caatagaggcaaactcaaaaaaaaaaaaaaaaaaaaaaaaaaaaa

>NM_004815 3
gcgcaactgacggtcgcgttctgcgcgcgagctagttgcctcccgtacct
gccgcggtcgccggccccgcccccgggagcgcgggccaatgggctgggct
ccagggggcggggctggcgggcgggcggtgcggccgtggcggtagctgca
ggggcggtggcggctgcagtggtggtggtgcctgtggctgtggctgcggc
tgcggctgcggctgagatttggccgggcgtccgcaggccgtgggggatgg
gggcagcgagctccagccctcggcggtggcggcggccgtaggtgtggggc
gggcgtccgcgtccggcacgcgagatggagcgccgtggatttcagttttt
ctgactgttacatgaaaggatgattgctcacaaacagaaaaagacaaaga
aaaaacgtgcttgggcatcaggtcaactctctactgatattacaacttct
gaaatggggctcaagtccttaagttccaactctatttttgatccggatta
catcaaggagttggtgaatgatatcaggaagttctcccacatgttactat
atttgaaagaagccatattttcagactgttttaaagaagttattcatata
cgtctagaggaactgctccgtgttttaaagtctataatgaataaacatca
gaacctcaattctgttgatcttcaaaatgctgcagaaatgctcactgcaa
aagtgaaagctgtgaacttcacagaagttaatgaagaaaacaaaaacgat
ctcttccaggaagtgttttcttctattgaaactttggcatttacctttgg
aaatatccttacaaacttccttatgggagatgtaggcaatgattcattat
tgcgactgcctgtttctcgagaaactaagtcgtttgaaaatgtttctgtg
gaatcagtggactcatccagtgaaaaaggaaatttttcccctttagaact
agacaacgtgctgttaaagaacactgactctatcgagctggctttgtcat
atgctaaaacttggtcaaaatatactaagaacatagtttcatgggttgaa
aaaaagcttaacttggaattggagtccactagaaatatggtcaagttggc
agaggcaactagaactaacattggaattcaggagttcatgccactgcagt
ctctgtttactaatgctcttcttaatgatatagaaagcagtcacctttta
caacaaacaattgcagctctccaggctaacaaatttgtgcagcctctact
tggaaggaaaaatgaaatggaaaaacaaaggaaagaaataaaagagcttt
ggaaacaggagcaaaataaaatgcttgaagcagagaatgctctcaaaaag
gcaaaattattatgcatgcaacgtcaagatgaatatgagaaagcaaagtc
ttccatgtttcgtgcagaagaggagcatctgtcttcaagtggcggattag
caaaaaatctcaacaagcaactagaaaaaaagcgaaggttggaagaggag
gctctccaaaaagtagaagaagcaaatgaactttacaaagtttgtgtgac
aaatgttgaagaaagaagaaatgatctagaaaataccaaaagagaaattt
tagcacaactccggacacttgttttccagtgtgatcttacccttaaagct
gtaacagttaacctcttccacatgcagcatctgcaggctgcttcccttgc
agacagtttacagtctctctgtgatagtgccaaactctatgacccaggcc
aagagtacagtgaatttgtcaaggccacaaattcaactgaagaagaaaaa
gttgatggaaatgtaaataaacatttaaatagttcccaaccttcaggatt
tggacctgccaactctttagaggatgttgtacgccttcctgacagttcta
ataaaattgaagaggacagatgctctaacagtgcagatataacaggtcct

tcctttataagatcatggacatttgggatgtttagtgattctgagagcac
tggagggagcagcgaatctagatctctggattcagaatctataagtccag
gagactttcatcgaaaacttccacgaacaccatccagtggaactatgtcc
tctgcagatgatctagatgaaagagagccaccttccccttcagaaactgg
acccaattcccttggaacatttaagaaaacattgatgtcaaaggcagctc
tcacacacaagtttcgcaaattgagatcccccacgaaatgtagggattgt
gaaggcattgtagtgttccaaggtgttgaatgtgaagagtgtctcccttgt
ttgtcatcgaaagtgtttggaaaatttagtcattatttgtggtcatcaga
aacttccaggaaaaatacacttatttggagcagaattcacacaagttgca
aaaaaggaaccagatggtatcccttttatactcaaaatatgtgcctcaga
gattgaaaatagagctttgtgtctacagggaatttatcgtgtgtgtggaa
acaaaataaaaactgaaaaattgtgtcaagctttggaaaatggaatgcac
ttggtagatatttcagaatttagttcacatgatatctgtgacgtcttgaa
attataccttcggcagctcccagaaccatttattttatttcgattgtaca
aggaatttatagaccttgcaaaagagatccaacatgtaaatgaagaacaa
gagacaaaaaagaatagtcttgaagacaaaaaatggccaaatatgtgtat
agaaataaaccgaattcttctaaaaagcaaagaccttctaagacaattgc
cagcatcaaattttaacagtcttcatttccttatagtacatctaaagcgg
gtagtagatcatgcagaagaaaacaagatgaactccaaaaacttgggggt
gatatttggaccaagtctcattaggccaaggcccacaactgctcctatca
ccatctcctcccttgcagagtattcaaatcaagcacgcttggtagagttt
ctcattacttactcacagaagatcttcgatgggtccctacaaccacaaga
tgttatgtgtagcataggtgttgttgatcaaggctgttttccaaagcctc
tgttatcaccagaagaaagagacattgaacgttccatgaagtcactattt
ttttcttcaaaggaagatatccatacttcagagagtgaaagcaaaatttt
tgaacgagctacatcatttgaggaatcagaacgcaagcaaaatgcgttag
gaaaatgtgatgcatgtctcagtgacaaagcacagttgcttctagaccaa
gaggctgaatcagcatcccaaaagatagaagatggtaaaaccccctaagcc
actttctctgaaatctgataggtcaacaaacaatgtggagaggcatactc
caaggaccaagattagacctgtaagtttgcctgtagatagactacttctt
gcaagtcctcctaatgagagaaatggcagaaatatgggaaatgtaaattt
agacaagttttgcaagaatcctgcctttgaaggagttaatagaaaagacg
ctgctactactgtttgttccaaatttaatggctttgaccagcaaactcta
cagaaaattcaggacaaacagtatgaacaaaacagcctaactgccaagac
tacaatgatcatgcccagtgcactccaggaaaaaggagtgacaacaagcc
tccagattagtggggaccattctatcaatgccactcaacccagtaagcca
tatgcagagccagtcaggtcagtgagagaggcatctgagagacggtcttc
agattcctaccctctcgctcctgtcagagcacccagaacactgcagcctc
aacattggacaacattttataaaccacatgctcccatcatcagtatcagg
gggaatgaggagaagccagcttcaccctcagcagcagtgcctcctggcac
agatcacgatccccacggtctcgtggtgaagtcaatgccagacccagaca
aagcatcagcttgtcctgggcaagcaactggtcaacctaaagaagactct
gaggagcttggcttgcctgatgtgaatccaatgtgtcagagaccaaggct
aaaacgaatgcaacagtttgaagacctcgaaggtgaaattccacaatttg
tgtagggatgtcaaatttcagggtttttttgttgttgttgtgttattttg
tggtattgtgcttgttttgtgaaagaatgttttgacagggcctcttttgt

ataggactgccaaatcatgggttttgccttttgttgttgtatttatcctc
tgttggtaatactgaatggtagaatgttttgatagggtcacatttgtgcc
tcactggaattatctttaaattctgtatttttaaagttgtgaataagata
ggtggattcgtatttttaaagttcagttgactttccccaccaaatggtc
catttgaatgcatccctaatatatgatatagtctcaactaataggtgcaa
tttgggaaaatcaggtttatttttggagtggaactgttataagtgctta
tttataaaaggaatgtttctgaatgcaagtgcctaaaaagatctttgttg
gtatgcatatgttttgtcacacaattttatagtgcatctttcaccatttg
tgctttttaagatacgtatgtaagctcttatttttcaattggcaattca
gttaatttttaaatgtttacataatggccagaaggcttgcaaatctgtat
ttaattgcattttaattaattgccagtttttacatgtgatagtcagttgt
acaaagaaaatgcacttaaacctgtttctaaattatatattcagttatat
tatatttggctttagatggttttaatacatttgatagtttttcacccctt
ggctttattttatataaactttttgtttttcagcagttctgaactttttag
tattttataaatggtccaaaaaaatgcctgtttcagaagtttttgaattca
gtgcatttcctcttgatttgtctgggttaaaaccattcctttgtatgaa
atgttttgacttaggaatcattttatgtacttgttctacctggattgtca
acaactgaaagtacatatttcatccaaatcaagctaaaatttatttaagt
tgattctgagagtacaggtcagtaagcctcattatttggaatttgagaga
aggtataggtgatcggatctgtttcatttataaaaggtccagtttttagg
actagtacattcctgttattttctgggttttatcattttgcctaaaatag
gatataaaagggacaaaaaataagtagactgtttttatgtgtgaattata
tttctactaaatgtttttgtatgactgtgttatacttgataatatatata
tatatatatatcaacttgttaaattatttcatgttcccgtggcttctttt
cagttgttgcctattacagtatgagagtttaaggttattaaccattggct
tagaagtcaacacctaggatcatacatccgttgactacaatgtggaatga
attttatggaaacctgttttatagttctatgtgatgtaaaggtctaggga
gcaataaccagcccttttttttctgaaggcttgtttttagtcctcattgt
acaaaatataaaataatcatattgctatgaatacctaaaaagaaaattag
agcccatgtgtattgagccttctctgtgtgccaggaactttgccaaggtg
ctacatagattattttatttcaagttcacagcaacctataatgggtgggt
aagcatgttattatttctgtgttacagataaggaaactaaggcttagtta
aatgacttgcttcagtgttccacaggtgtaacaggtggagtcaaatctca
aacatagggctgtcagattttgatggttagtgctcttaatgactgtgatt
actaaataatatctggtagtttttaacaatagaaaatgcaactttaaaaa
atttttattgtggtaaaatacacataacatttaccatttttaagtatatt
gtttgaaccatttttaagcatatagttcagtggcattaagtatattcaca
ttgttttgcaaccatgagcacctattcatctccagaacattatcatcatc
ctatactaaaacttagaaaatgtacttttaaacttcttcagttctctttt
aatatatggagcccacccaaaggttatatcatgtaataaactcatttctc
tgttttccctcacagcttaaaaatgagaatttcacttttgggtcctttct
gcctttagggctccctggcactttgtccagcgtagagccttaagcctcta
gatgctttctagtttcctggggccctgaggcaagtatctcttgagaagag
gttgctatttctagaggatccacagtgtgcactgtgttgtgctatctaat
agtgtggtggaaatcatttatccagaagttgtttttttgcaacatggaaag
atacgtgaccaaaaggaaagggcaacagcaggagccctgttagtgctgcc

agaacaccaggaagccttgtgggaggcgtattgtccaagatgatgcgtat
tgtccaaacgactcagaagaagtcatttctgaagggttgatcataacttc
cctagccatgttttacctacagagaacttagttagaatttatgagtacag
tatgttaaattacttttagtgtaccttaggcagtgtatttgttttgatac
agagacaaagactatatgatccctgagacttgttgcctagtgaactcaca
agcagatagaattgtacctcagttatttgggtgatttaaagatggtacta
tggctacactattcccagctatcgttgtgttacagtatttatacaactgg
aaacatctctctaaatgacaaccattgtatattgttaaataacttatagt
cagtggaattctgtgggtttttctttcctacaggcaggaaattatggtga
tgtgttgatatgattatatatgtagactattcagttaacatctaggagtt
agcaaatttgctacattcacaatcagtatgtaagtcagttgtattcctag
atatcagcaactaacagaaatggaagtttttagaaagatctcttcaatat
cattaaaaaatacaaaatactctacctaagaataagtctaacaaaagatg
tacaagatcttgatggagaaaatgttagaccatgtcaggaagcatttaag
gagatcttaaataaatgtagtactccataatgtacacgaattagaagact
cagtcgtgtaaagatgttcatttacatttgtagattcaatgtaatcccaa
ccaaaatatttttgcattattacctctatctactgtgtgtttccttaag
acaagggcattcctacgtaaccatagtacaaccatcaaacttaggagatt
gacattgacatcatgatatctaatccatagaccacattcaatctgctgat
tttgattgcctcagtaatgttcctgtgtttcagcatcccaatccaggacc
acatagtgcatttagttgccctatctcttgtttcattcagtccagaacat
cttttcagacttgtctttgaccttgacacttgaggaggattggccagttt
gtagaaagttcctcagttttggtttatcattagataagacccataaggtt
taggatagacatttttgtccaatactacagaaatgatgctctatactcaa
atgtgtcatgccaggatgcacacaatgtcaatttgtcttatacctgatag
tattaattttggtcacttggttcagatagtatataccaggtttctccatt
gtaaaattagaattaggaagtaatttgtgaggagattcttaaagattgta
aatgtattgtttctcatcgaattgtcacgggctagtctattgacaattct
tgactgaaccaattactattttggttgtcaaatggtgagtccgtggcttc
tgccttggttggcatcctactactgtaaggaagagctcttccttctctat
tccttcattcacttaaatcagtatagattcatgaatgcctgctttattca
gtgacagtgtattattgtcacttattttcatgctgaaattttccccgatt
tggtttttgggagtctcttcaagctggcttctgtgttccttagagatgcc
tcattgctttttcccctcttatttttttacgagctcatcttacactttt
tcctactccctaccccaagtctagaattagcccttttttccaaggagccc
tggtttctttaaatgggaaatggtatatggaaatgaagacctgagaaagg
ctcactgtcttattggtgaacagctcaaaggaaaacgtgtgtctacacac
attcaagaatagtcacctacttctatatctatttatatacatacgtattg
aaaactttgagttcacattgacaccttcaattcaatcctttgaggttaat
ctagtctccctttccatctttgtaacttccttctctcatagaaacctgac
tcccataatcctaaataatttgttcattcttcctccctccctcaacctct
tgacatgccagctgctgtctcatcacacccagtcccaaatatgccaaata
aatccgcagtcatagcagcgctggtcctgaccacaccaatccctaacatc
ctccccagcctggagcctggaccatggcagctgaatctttgaccccagtg
atgtcagtcctgagcacaccattggaatcctaggctcctgtcatctggct
ggaagagagacaaattcacaaccttttgataatgagcaattattgaatac

tcactgaatttatcagagaaagtttaaaagtgtacgattatacatattta
ccaggaatgtttaaacatagacatttatgaaatttatagaaaatattgaa
ttgtggttctcactttttggtcttggctgtaacaatttttaaatattaat
acttcctacaataactgtatcatgctatttttaagtgtcctcacactcaa
ccaatctactctgcatccataaaataagcatgcagttcagaaaatagatt
aaacgactaattggctaaaaa

>NM_005397 3
agccgcgcagacgccgcccaggacgcagccgccgccgccgctcctct
gccactggctctgcgccccagcccggctctgctgcagcggcagggaggaa
gagccgccgcagcgcgactcgggagccccgggccacagcctggcctccgg
agccacccacaggcctccccgggcggcgcccacgctcctaccgcccggac
gcgcggatcctccgccggcaccgcagccacctgctcccggcccagaggcg
acgacacgatgcgctgcgcgctggcgctctcggcgctgctgctactgttg
tcaacgccgccgctgctgccgtcgtcgccgtcgccgtcgccgtcgccctc
ccagaatgcaacccagactactacggactcatctaacaaaacagcaccga
ctccagcatccagtgtcaccatcatggctacagatacagcccagcagagc
acagtccccacttccaaggccaacgaaatcttggcctcggtcaaggcgac
caccccttggtgtatccagtgactcaccggggactacaaccctggctcagc
aagtctcaggcccagtcaacactaccgtggctagaggaggcggctcaggc
aaccctactaccaccatcgagagccccaagagcacaaaaagtgcagacac
cactacagttgcaacctccacagccacagctaaacctaacaccacaagca
gccagaatggagcagaagatacaacaaactctgggggaaaagcagccac
agtgtgaccacagacctcacatccactaaggcagaacatctgacgacccc
tcaccctacaagtccacttagcccccgacaacccacttcgacgcatcctg
tggccaccccaacaagctcgggacatgaccatcttatgaaaatttcaagc
agttcaagcactgtggctatccctggctacaccttcacaagcccggggat
gaccaccaccctaccgtcatcggttatctcgcaaagaactcaacagacct
ccagtcagatgccagccagctctacggccccttcctcccaggagacagtg
cagcccacgagcccggcaacggcattgagaacacctaccctgccagagac
catgagctccagccccacagcagcatcaactacccaccgataccccaaaa
caccttctcccactgtggctcatgagagtaactgggcaaagtgtgaggat
cttgagacacagacacagagtgagaagcagctcgtcctgaacctcacagg
aaacaccctctgtgcagggggcgcttcggatgagaaattgatctcactga
tatgccgagcagtcaaagccaccttcaacccggcccaagataagtgcggc
atacggctggcatctgttccaggaagtcagaccgtggtcgtcaaagaaat
cactattcacactaagctccctgccaaggatgtgtacgagcggctgaagg
acaaatgggatgaactaaaggaggcaggggtcagtgacatgaagctaggg
gaccaggggccaccggaggaggccgaggaccgcttcagcatgcccctcat
catcaccatcgtctgcatggcatcattcctgctcctcgtggcggccctct
atggctgctgccaccagcgcctctcccagaggaaggaccagcagcggcta
acagaggagctgcagacagtggagaatggttaccatgacaacccaacact
ggaagtgatggagacctcttctgagatgcaggagaagaaggtggtcagcc
tcaacggggagctgggggacagctggatcgtccctctggacaacctgacc
aaggacgacctggatgaggaggaagacacacacctctagtccggtctgcc
ggtggcctccagcagcaccacagagctccagaccaaccaccccaagtgcc

gtttggatggggaagggaaagactggggagggagagtgaactccgagggg
tgtcccctcccaatcccccagggccttaatttttccccttttcaacctga
acaaatcacattctgtccagattcctcttgtaaaataacccactagtgcc
tgagctcagtgctgctggatgatgagggagatcaagaaaaagccacgtaa
gggactttatagatgaactagtggaatcccttcattctgcagtgagattg
ccgagacctgaagagggtaagtgacttgcccaaggtcagagccacttggt
gacagagccaggatgagaacaaagattccatttgcaccatgccacactgc
tgtgttcacatgtgccttccgtccagagcagtcccgggcaggggtgaaac
tccagcaggtggctgggctggaaaggagggcagggctacatcctggctcg
gtgggatctgacgacctgaaagtccagctcccaagttttccttctcctac
cccagcctcgtgtacccatcttcccaccctctatgttcttacccctccct
acactcagtgtttgttcccacttactctgtcctggggcctctgggattag
cacaggttattcataaccttgaaccccttgttctggattcggattttctc
acatttgcttcgtgagatgggggcttaacccacacaggtctccgtgcgtg
aaccaggtctgcttaggggacctgcgtgcaggtgaggagagaaggggaca
ctcgagtccaggctggtatctcagggcagctgatgaggggtcagcaggaa
cactggcccattgcccctggcactccttgcagaggccacccacgatcttc
tttgggcttccatttccaccagggactaaaatctgctgtagctagtgaga
gcagcgtgttccttttgttgttcactgctcagctgatgggagtgattccc
tgagacccagtatgaaagagcagtggctgcaggagaggccttcccggggc
cccccatcagcgatgtgtcttcagagacaatccattaaagcagccaggaa
ggacaggctttcccctgtatatcataggaaactcagggacatttcaagtt
gctgagagttttgttatagttgttttctaacccagccctccactgccaaa
ggccaaaagctcagacagttggcagacgtccagttagctcatctcactca
ctctgattctcctgtgccacaggaaaagagggcctggaaagcgcagtgca
tgctgggtgcatgaagggcagcctgggggacagactgttgtgggaacgtc
ccactgtcctggcctggagctaggccttgctgttcctcttctctgtgagc
ctagtggggctgctgcggttctcttgcagtttctggtggcatctcagggg
aacacaaagctatgtctattccccaatataggacttttatgggctcggca
gttagctgccatgtagaaggctcctaagcagtgggcatggtgaggtttca
tctgattgagaagggggaatcctgtgtggaatgttgaactttcgccatgg
tctccatcgttctgggcgtaaattccctgggatcaagtaggaaaatgggc
agaactgcttaggggaatgaaattgccatttttcgggtgaaacgccacac
ctccagggtcttaagagtcaggctccggctgtagtagctctgatgaaata
ggctatccactcgggatggcttactttttaaaagggtagggggaggggct
ggggaagatctgtcctgcaccatctgcctaattccttcctcacagtctgt
agccatctgatatcctaggggaaaaggaaggccaggggttcacatagggc
cccagcgagtttcccaggagttagagggatgcgaggctaacaagttccaa
aaacatctgccccgatgctctagtgtttggaggtgggcaggatggagaac
agtgcctgtttgggggaaaacaggaaatcttgttaggcttgagtgaggtg
tttgcttccttcttgcccagcgctgggttctctccacccagtaggttttc
tgttgtggtcccgtgggagaggccagactggattattcctcctttgctga
tcctgggtcacacttcaccagccagggcttttgacggagacagcaaatag
gcctctgcaaatcaatcaaaggctgcaaccctatggcctcttggagacag
atgatgactggcaaggactagagagcaggagtgcctggccaggtcggtcc
tgactctcctgactctccatcgctctgtccaaggagaacccggagaggct

ctgggctgattcagaggttactgctttatattcgtccaaactgtgttagt
ctaggcttaggacagcttcagaatctgacaccttgccttgctcttgccac
caggacacctatgtcaacaggccaaacagccatgcatctataaaggtcat
catcttctgccacctttactgggttctaaatgctctctgataattcagag
agcattgggtctgggaagaggtaagaggaacactagaagctcagcatgac
ttaaacaggttgtagcaaagacagtttatcatcagctctttcagtggtaa
actgtggtttccccaagctgcacaggaggccagaaaccacaagtatgatg
actaggaagcctactgtcatgagagtggggagacaggcagcaaagcttat
gaaggaggtacagaatattctttgcgttgtaagacagaatacgggtttaa
tctagtctaggcaccagatttttttcccgcttgataaggaaagctagcag
aaagtttatttaaaccacttcttgagctttatctttttgacaatatact
ggagaaactttgaagaacaagttcaaactgatacatatacacatattttt
ttgataatgtaaatacagtgaccatgttaacctaccctgcactgctttaa
gtgaacatactttgaaaaagcattatgttagctgagtgatggccaagttt
tttctctggacaggaatgtaaatgtcttactggaaatgacaagtttttgc
ttgatttttttttttaaacaaaaaatgaaatataacaagacaaacttatg
ataaagtatttgtcttgtagatcaggtgttttgttttgtttttttaattt
taaaatgcaaccctgccccctccccagcaaagtcacagctccatttcagt
aaaggttggagtcaatatgctctggttggcaggcaaccctgtagtcatgg
agaaaggtatttcaagatctagtccaatctttttctagagaaaaagataa
tctgaagctcacaaagatgaagtgacttcctcaaaatcacatggttcagg
acagaaacaagattaaaacctggatccacagactgtgcgcctcagaagga
ataatcggtaaattaagaattgctactcgaaggtgccagaatgacacaaa
ggacagaattcctttcccagttgttaccctagcaaggctagggagggcat
gaacacaaacataagaactggtcttctacactttctctgaatcatttagg
tttaagatgtaagtgaacaattctttctttctgccaagaaacaaagtttt
ggatgagcttttatatatggaacttactccaacaggactgagggaccaag
gaaacatgatgggggaggcagagagggcaagagtaaaactgtagcatagc
ttttgtcacggtcactagctgatccctcaggtctgctgcaaacacagcat
ggaggacacagatgactctttggtgttggtctttttgtctgcagtgaatg
ttcaacagtttgcccaggaactgggggatcatatatgtcttagtggacag
gggtctgaagtacactggaatttactgagaaacttgtttgtaaaaactat
agttaataattattgcattttcttacaaaaatatattttggaaaattgta
tactgtcaattaaagtgtttttgtgtaaactggttcaaaaaaaaaaaaaa
aaaaaaaaaaa

>NM_006410 4
aggtcccgcccctgtgggcggggcgaggcagcgtcgccgcgaggccaccc
ggaagaccaagccggcatggccgaaacagaagccctgtcgaagcttcggg
aagacttcaggatgcagaataaatccgtctttattttgggcgccagcgga
gaaaccggcagagtgctcttaaaggaaatcctggagcagggcctgttttc
caaagtcacgctcattggccggaggaagctcaccttcgacgaggaagctt
ataaaaatgtgaatcaagaagtggtggactttgaaaagttggatgactac
gcctctgcctttcaaggtcatgatgttggattctgttgcctgggtaccac
cagagggaaagctggggcggagggatttgttcgtgttgaccgagattatg
tgctgaagtctgcagagctggcaaaagctggagggtgcaaacatttcaac

ttgctatcctctaaaggagctgataaatcaagcaatttttatatctaca
agttaagggagaagtagaagccaaggttgaagaattaaaatttgatcgtt
actctgtatttaggcctggagttctgttatgtgataggcaagaatctcgc
ccaggtgaatggctggttagaaagttctttggctccttaccagactcttg
ggccagtgggcattctgtgcctgtggtgaccgtggttagagcaatgctga
acaatgtggtgagaccaagagacaagcagatggaactgctggagaacaag
gccatccatgacctggggaaagcgcatggctctctcaagccatgaccaca
ttggagaaatggtttttattgtcaaccttaacacccatcaccaaatcggt
aatttcagggtctaaaaaaagtcagcatgttttaactttgttgttttact
atcctcaggcatccattccaatcaagaaatgatggtgctctgcatcagtg
gttcagagcctggttatacatatagatcactcagggagctttggaaaaat
aaagatttgtcagccctatctcaaacttgaatcaaaatttctggggtgtg
ggcacaataatctgtaattttctttgtttatacttcccctgatgccactg
gttccgatgccactggctgggggcctgctttgaaatgcttgtctgcaga
gtcacagcagccatgaaaaccttatgaccgtgcaaatgagctctgctcta
aaattgttgacattcatgtctctgagttacaaaagtgctaattcactaca
tgtaattgtgtaagtaaacattgtgcctttactacttctttatgtaatag
aagttatatacctaagcttatataatacatggggaggattaaataaagga
ataaagatgaatggacaactcctaaaaaaaaaaaaaaaaaaaaaaaaaa
aaaa

>NM_014170 2
caggccacaggagtaaaatctctctgcccgtcttctgggaagggagaatg
gcggcgcccgggctgcggctgggagcgggaagactctttgaaatgcctgc
ggtgctagagcgactgagccgctataatagcacgtcccaagcttttgctg
aggtgctgcggctgccgaagcagcagctgaggaagctgctgtacccgctg
caggaagtagagcggttcctcgcccccctacgggaggcaagaccttcacct
gcgtatctttgacccaagcccggaggacatagccagggcggacaacatct
tcacggccactgaacggaaccgcatcgactacgtcagctccgccgtccgt
atcgaccacgccccggaccttccgcggccagaggtgtgttttataggcag
aagcaatgttggaaaatcatctctaatcaaggctttattttcactggccc
ctgaggttgaagtcagagtctccaaaaaaccaggacacacaaagaaaatg
aattttttcaaagttggaaaacattttacagtggtggacatgccaggtta
tggctttagagcacctgaagattttgttgacatggtagagacctatctaa
aagaacgaaggaacttgaagagaacatttttattagtggatagcgttgtt
ggaattcaaaaaacagacaatattgccatagaaatgtgtgaagaatttgc
attaccttatgtgattgtattaacaaaaattgacaaatcttccaagggac
atcttttaaaacaagtgcttcagatccagaaatttgttaacatgaaaact
caaggatgttttcctcagttgtttcctgtaagtgctgtgaccttttctgg
aatccacctgttgagatgctttatagccagtgtaacaggaagtcttgact
aatggttcccggtttagctgaagattcaaaaaaaaaaaaaaaagctttat
gctaactggagttaaatacctagaagaatttcaacattgttttaaatgtt
gtgcatctgtaacttcaggaggatcacttgagctttaaaacctgtgcctt
ctcgaaacaagaatttgtgcctgaggtgaaaaaagtttgtaagttattga
attatggtgttcattagaacagctactagctgattcccctattttaacaa
actgacaagagcacatccataaaatgaaaacctgttacaactatgtacag

aagggtttgacgttttattgggcttttgtcttttaaagaatatgtctact
atgggtatttttttttaaatgttaaaatgggctaggtaaaagggggctg
cttttctgttaagcatcgataggtaagttgatggataaaagttactatgt
aagcctaaaaaaaa

>NM_014300 2
gaaggtggcagagcagcctccccaactaagcctgggtcgccgcctcggtg
aagggccgcggggccgagcgcgggaagtggcgtccagtcggtttttggca
ggcgagagccagagcgtgagaaccctcccacggcgaggccaagccctgag
actggactagcaaggtcgtgagtctctctgtcagctctccgcagcgaagg
ggcgcggggggcggggaccgtgcccgaccgtgccagctcccgctccggaa
gcggaagtgcaagcactgcggggtcctgtccagtgtgagcccgacccgag
ctccagtagttccgcccgctggtcatcgcgccctttcccctgccggtgtc
ctgctcgccgtccccgccatgctgtctctagactttttggacgatgtgcg
gcggatgaacaagcggcagctctattatcaagtcctaaattttggaatga
ttgtctcatcggcactaatgatctggaaggggttaatggtaataactgga
agtgaaagtccgattgtagtggtgctcagtggcagcatggaacctgcatt
tcatagaggagatcttctctttctaacaaatcgagttgaagatcccatac
gagtgggagaaattgttgtttttaggatagaaggaagagagattcctata
gttcaccgagtcttgaagattcatgaaaagcaaaatgggcatatcaagtt
tttgaccaaaggagataataatgcggttgatgaccgaggcctctataaac
aaggacaacattggctagagaaaaaagatgttgtggggagagccaggggga
tttgttccttatattggaattgtgacgatcctcatgaatgactatcctaa
atttaagtatgcagttctctttttgctgggtttattcgtgctggttcatc
gtgagtaagaagcctgccttgctgttcctgggaagatgccatagttttcg
ttactggatgtttggagtagatactggtctgtgattggtggaatggagaa
cacacgtgttggtgcttctgggtagcactggtttgcattagtttatgttt
ccatgccagagtttgtgtgggcgggcgcatgtgcaccacagagtgcactc
gaggggactttcagtcacaggatttcataattgtcattgtcacactttca
aatttttgtacatcagtgaatttttttatattaaaaggttgagccaaagc
ccccagtgtttgtattttgaagccaagcttcacttctaaagtgcctacag
agacttgtaaatgaaaatgcagctctgcacgagtttgaaaccgtcatacc
tccttctattaggaatggcatatactgaggtggtcgtaagtcttaacttc
taaaattttaaataaaagactttgcacattgaaaaaaaaaaaaaaaaaa

>NM_014397 5
gggcgggcgcgcgggcgcgcgggcccgcgcaggcggtggcggcggcggcg
gaaccgagctgacgggcgtgcggccgctgcgccgcaaactcgtgtgggac
gcaccgctccagccgcccgcgggccagcgcaccggtcccccagcggcagc
cgagcccgcccgcgcgccgttcgtgccctcgtgaggctggcatgcaggat
ggcaggacagcccggccacatgccccatggagggagttccaacaacctct
gccacaccctggggcctgtgcatcctcctgacccacagaggcatcccaac
acgctgtcttttcgctgctcgctggcggacttccagatcgaaaagaagat
aggccgaggacagttcagcgaggtgtacaaggccacctgcctgctggaca
ggaagacagtggctctgaagaaggtgcagatctttgagatgatggacgcc
aaggcgaggcaggactgtgtcaaggagatcggcctcttgaagcaactgaa

ccacccaaatatcatcaagtatttggactcgtttatcgaagacaacgagc
tgaacattgtgctggagttggctgacgcaggggacctctcgcagatgatc
aagtactttaagaagcagaagcggctcatcccggagaggacagtatggaa
gtactttgtgcagctgtgcagcgccgtggagcacatgcattcacgccggg
tgatgcaccgagacatcaagcctgccaacgtgttcatcacagccacgggc
gtcgtgaagctcggtgaccttggtctgggccgcttcttcagctctgagac
caccgcagcccactccctagtggggacgccctactacatgtcaccggaga
ggatccatgagaacggctacaacttcaagtccgacatctggtccctgggc
tgtctgctgtacgagatggcagccctccagagccccttctatggagataa
gatgaatctcttctccctgtgccagaagatcgagcagtgtgactaccccc
cactccccggggagcactactccgagaagttacgagaactggtcagcatg
tgcatctgccctgacccccaccagagacctgacatcggatacgtgcacca
ggtggccaagcagatgcacatctggatgtccagcacctgagcgtggatgc
accgtgccttatcaaagccagcaccactttgccttacttgagtcgtcttc
tcttcgagtggccacctggtagcctagaacagctaagaccacagggttca
gcaggttccccaaaaggctgcccagccttacagcagatgctgaaggcaga
gcagctgagggaggggcgctggccacatgtcactgatggtcagattccaa
agtcctttctttatactgttgtggacaatctcagctgggtcaataagggc
aggtggttcagcgagccacggcagccccctgtatctggattgtaatgtga
atctttagggtaattcctccagtgacctgtcaaggcttatgctaacagga
gacttgcaggagaccgtgtgatttgtgtagtgagcctttgaaaatggtta
gtaccgggttcagtttagttcttagtatcttttcaatcaagctgtgtgct
taatttactctgttgtaaagggataaagtggaaatcatttttttccgtgg
agtggtgattctgctaacatttttatctacgttttataacttggtgagtg
acgatgagagccctgcacctggccagagtgtcacaggcaaaaggcatcgg
gaagcaggagcatcttcttggcagccaggctgggccatcttctcctggac
acctgctgtgtaccaggaacttcgtcacctccttgaatgctggcggttca
tttcatgatcagtgttaagcattttcctccatgggaaggaagcatgggat
atagaaaagcgaagggctgtcctttacaaattctggttctgcaacttcct
agcgtgactttgggcttgggcaagtttcttagccgttctgagccttcatt
tcctcatctgtacaatgagattaatagtacctatcatctaccttcaggat
tgctgacagacagaatttgaaataaaatatgcaagttagctaatacaaaa
agtagatgatccaaaaatggtagccactcacccttcacaaactgaagtcc
atggaccacggaagtcgagaattaatgtacacctgtatcatgtgtaggaa
accagaaatgtgttccttatttcttgttcccaaacaggattaactgtgaa
gactaatttataaatgtgaacctaagaaaactccacctctgaaggaaatc
atttgaattttgtttttgtacgtaaagttaaccttccaattgtctgagct
gtcgtcactgacttcatgacagtctggccctccagacaagagcagcgctg
gcatcgggcaggtgattcctgacacctgctgcctgcaggcattcactgac
caggcctttcctggaggaaacacccagggccgggcggctgctgtttccac
acgtggactcggatctgctgtgacaccgtcagcccgacagtctctccata
tgcagcctttcctctgtacttttctccatggttgaaataaaacagggtga
ctgggagttacttagaattcatgaagattttaaaaaaaaaaaaaa

>NM_014452 3
gccaccacgtgtgtccctgcgcccggtggccaccgactcagtccctcgcc

gaccagtctgggcagcggaggagggtggttggcagtggctggaagcttcg
ctatgggaagttgttcctttgctctctcgcgcccagtcctcctccctggt
tctcctcagccgctgtcggaggagagcacccggagacgcgggctgcagtc
gcggcggcttctccccgcctgggcggccgcgccgctgggcaggtgctgag
cgcccctagagcctcccttgccgcctccctcctctgcccggccgcagcag
tgcacatggggtgttggaggtagatgggctcccggcccgggaggcggcgg
tggatgcggcgctgggcagaagcagccgccgattccagctgccccgcgcg
ccccgggcgcccctgcgagtccccggttcagccatggggacctctccgag
cagcagcaccgccctcgcctcctgcagccgcatcgcccgccgagccacag
ccacgatgatcgcgggctcccttctcctgcttggattccttagcaccacc
acagctcagccagaacagaaggcctcgaatctcattggcacataccgcca
tgttgaccgtgccaccggccaggtgctaacctgtgacaagtgtccagcag
gaacctatgtctctgagcattgtaccaacacaagcctgcgcgtctgcagc
agttgccctgtggggacctttaccaggcatgagaatggcatagagaaatg
ccatgactgtagtcagccatgcccatggccaatgattgagaaattacctt
gtgctgccttgactgaccgagaatgcacttgcccacctggcatgttccag
tctaacgctacctgtgccccccatacggtgtgtcctgtgggttggggtgt
gcggaagaaagggacagagactgaggatgtgcggtgtaagcagtgtgctc
ggggtaccttctcagatgtgccttctagtgtgatgaaatgcaaagcatac
acagactgtctgagtcagaacctggtggtgatcaagccggggaccaagga
gacagacaacgtctgtggcacactcccgtccttctccagctccacctcac
cttcccctggcacagccatctttccacgccctgagcacatggaaacccat
gaagtcccttcctccacttatgttcccaaaggcatgaactcaacagaatc
caactcttctgcctctgttagaccaaaggtactgagtagcatccaggaag
ggacagtccctgacaacacaagctcagcaagggggaaggaagacgtgaac
aagaccctcccaaaccttcaggtagtcaaccaccagcaaggcccccacca
cagacacatcctgaagctgctgccgtccatggaggccactgggggcgaga
agtccagcacgcccatcaagggccccaagaggggacatcctagacagaac
ctacacaagcattttgacatcaatgagcatttgccctggatgattgtgct
tttcctgctgctggtgcttgtggtgattgtggtgtgcagtatccggaaaa
gctcgaggactctgaaaaaggggccccggcaggatcccagtgccattgtg
gaaaaggcagggctgaagaaatccatgactccaacccagaaccgggagaa
atggatctactactgcaatggccatggtatcgatatcctgaagcttgtag
cagcccaagtgggaagccagtggaaagatatctatcagtttctttgcaat
gccagtgagagggaggttgctgctttctccaatgggtacacagccgacca
cgagcgggcctacgcagctctgcagcactggaccatccggggccccgagg
ccagcctcgcccagctaattagcgccctgcgccagcaccggagaaacgat
gttgtggagaagattcgtgggctgatggaagacaccacccagctggaaac
tgacaaactagctctcccgatgagccccagcccgcttagcccgagcccca
tccccagccccaacgcgaaacttgagaattccgctctcctgacggtggag
ccttccccacaggacaagaacaagggcttcttcgtggatgagtcggagcc
ccttctccgctgtgactctacatccagcggctcctccgcgctgagcagga
acggttcctttattaccaaagaaaagaaggacacagtgttgcggcaggta
cgcctggacccctgtgacttgcagcctatctttgatgacatgctccactt
tctaaatcctgaggagctgcgggtgattgaagagattccccaggctgagg
acaaactagaccggctattcgaaattattggagtcaagagccaggaagcc

agccagaccctcctggactctgtttatagccatcttcctgacctgctgta
gaacatagggatactgcattctggaaattactcaatttagtggcagggtg
gtttttaattttcttctgtttctgattttgttgtttggggtgtgtgtg
tgtgtttgtgtgtgtgtgtgtgtgtgtgtgtgtgtgtgtttaacagag
aatatggccagtgcttgagttctttctccttctctctctctcttttttt
ttaaataactcttctgggaagttggtttataagcctttgccaggtgtaac
tgttgtgaaatacccaccactaaagtttttaagttccatattttctcca
ttttgccttcttatgtattttcaagattattctgtgcactttaaatttac
ttaacttaccataaatgcagtgtgacttttcccacacactggattgtgag
gctcttaacttcttaaaagtataatggcatcttgtgaatcctataagcag
tctttatgtctcttaacattcacacctactttttaaaaacaaatattatt
actatttttattattgtttgtcctttataaattttcttaaagattaagaa
aatttaagaccccattgagttactgtaatgcaattcaactttgagttatc
ttttaaatatgtcttgtatagttcatattcatggctgaaacttgaccaca
ctattgctgattgtatggttttcacctggacaccgtgtagaatgcttgat
tacttgtactcttcttatgctaatatgctctgggctggagaaatgaaatc
ctcaagccatcaggatttgctatttaagtggcttgacaactgggccacca
aagaacttgaacttcaccttttaggatttgagctgttctggaacacattg
ctgcactttggaaagtcaaaatcaagtgccagtggcgccctttccataga
gaatttgcccagctttgctttaaaagatgtcttgtttttatatacacat
aatcaataggtccaatctgctctcaaggccttggtcctggtgggattcct
tcaccaattactttaattaaaaatggctgcaactgtaagaacccttgtct
gatatatttgcaactatgctcccatttacaaatgtaccttctaatgctca
gttgccaggttccaatgcaaaggtggcgtggactccctttgtgtgggtgg
ggtttgtgggtagtggtgaaggaccgatatcagaaaaatgccttcaagtg
tactaatttattaataaacattaggtgtttgttaaaaaaaaaaaaaaaaa
aaaaaaaaaaaa

>NM_018370 2
gagccagcgcggtagggccagagtgggaaggccagagcgggcgtccccgc
cagtgaccccacgccgcccgtccgcgcccaacccggcctccgccgagtgt
ccaaaccaaaagcgaaaggaacccgaccccgcgtcccctccggcggctcc
gtagtcgcgtccgcttggagctcgccgggcgcctccgaccctgccgggcc
gctttgtgacttcactcgtttcgcaacaagcccgggcagcccgcgcccca
cccactctggcccggcagcctcgccgcccgcagcctcgctccgctcctcg
cgcttcccctccctccggggctgggcctgccccggccgtcgcggagcctc
ccctcccaccgtccgtgagtgtacgcgcccggccgccgcctccaggcagc
ccggagcaacccggcgcccggccccgctgggcgcagcactccgtcggcgg
cggcggcggcgcgatgctgtgcttcctgaggggaatggctttcgtcccct
tcctcttggtgacctggtcgtcagccgccttcattatctcctacgtggtc
gccgtgctctccgggcacgtcaaccccttcctcccgtatatcagtgatac
gggaacaacacctccagagagtggtatttttggatttatgataaacttct
ctgcatttcttggtgcagccacgatgtatacaagatacaaaatagtacag
aagcaaaatcaaacctgctatttcagcactcctgtttttaacttggtgtc
tttagtgcttggattggtgggatgtttcggaatgggcattgtcgccaatt
ttcaggagttagctgtgccagtggttcatgacgggggcgctcttttggcc

tttgtctgtggtgtcgtgtacacgctcctacagtccatcatctcttacaa
atcatgtccccagtggaacagtctctcgacatgccacatacggatggtca
tctctgccgtttcttgcgcagctgtcatccccatgattgtctgtgcttca
ctaatttccataaccaagctggagtggaatccaagagaaaaggattatgt
atatcacgtagtgagtgcgatctgtgaatggacagtggcctttggtttta
ttttctacttcctaactttcatccaagatttccagagtgtcaccctaagg
atatccacagaaatcaatggtgatatttgaagaaagaagaattcagtctc
actcagtgaatgtcgcaggccatttctaaaagtgctacagaggacagaca
gggttttgaggccaccctgattattgggatgcatctgcagcacatccagg
acttgaatttcattacgagttcctaatagttgtatttctaaagatgtgtt
tcctagagaatgtacagccttatgacactgtagtgatgtttttataattt
tctaagtagatttttttatattaacaaattcatatacagaaaaaataagg
tgttacaaaaaatggagagctcttatttttgtacagattctgtcgttttt
gttttatttgtgtgagatttatggaaatacactaaatgagtaattcaggt
tcagtacatttattacaaagtgaaatcaggggatattcatttgtaaattt
tattcttagtgaatgaactgtataatttttttatcaggagagcacttat
aaaattcaatttataaagatcatatacccaaatcataaagatttagttga
tacattaacactaagatactctgatttttagccgaactaaacaaagtgct
tctactgagaggcctttataccaccatgtacagtaactctaagtgaatac
ggaagaccttggttttgaaattctgccaccttgtttctccctgctcatga
ggtcgcaccttttgctcttgctgctaattgcccattcgtagtgggtgtaa
tgccaggtggaatggtttcaacaagtcaggtgaaaaccatcctttattgt
tgctggcacaacttgatatatagtctgactcagaactgaagctcacatct
caaattcatttcatgccagtaaatgtggcaaagagaagaaaggcccaaga
gcgagacaagaagaatggagaagggggcagccaagaagaacttctgggtt
cagggtactgtttatttgctccttctcttcatgcctgtggctggatgtcc
cacaacactataagaaatataagtcaagccctttgtgttaagcaagaact
acagactccatcttttcacccaaatcatgaatgaccaataaaaagcaagt
tattccagaggaagaagcagcccttgaaatgttaaggcttaggcttgaaa
ggtgaagagcaggaattctctctttcaaatcctagagcataaacccatgt
gtggccaagtgagatcagccctcaagggcacatgccaagggcagagcagc
ccatgtagacagcttcggagggcatgggggtgtagggagttcggggtagc
tcctcattaactatttgttgggtgagtaaaggggtgaggctcagtggcag
gtacctctgcaatgacaagctgcctcccctctatgtgtttagcatatgtt
attagaacatgtccgacacccctaccgctgccatttgggccctttaataa
agccaagtagagaaatctggcaataaaaggcaaatgtaagcatgctttct
ttaagacgcatcataaatggttttctttaagtgaatggaagagtttgaca
gagatacacctttgtaagaaaacattaagaatgctggctggctgtggtgg
ctcacacctgtattcccagcactttgggaggcctaggcaggaggattgct
tgagcctgggacttcgagaccagactgggaaacatggcaaaatcccatct
ctacaacaaaaatacaaaaattagccaagtgcggtggtgtgcctgtagtc
ctagttacttgggaggctgaggtgggagaatcacctgagcccaggaggtg
gaggctgcagtgagccatgccaatgcactccagtctgggcaacagagtga
gaccctgtctcaaaaataaataaataaataaatgaataaagagaatgcta
atcatttctgggttcactgcgactcactgtagtgctggggatccccccttg
taacactggaactgaaagacagtgatgaaagctatgtcaagcattcatta

ttctgaagaggaggagaaatgccacatacctttcccatgggacctgtggt
ggaatgaatccatacttctgcctcacttcgagcagacttttgttctcggc
gctcctcacgatggagtttcatgcttcattttcacatctctctgcacaat
tagattgggagctccttgagggcagagtacgtgccttaatctttatcttt
gtaatgccacaatgaacagagtgcctcctggtacactgtaggagcttaag
aaatactcactgaatgcatgaatgaatgaatgaacaaatgaaggaatgac
taaggatgtttgtagtgctataatatagaatgggatttactctgctttac
cagttagtttcataataaacaaatagtctgtatcgcctggtaaaaaaaaa
aaa

>NM_018719 4
gtgctccgggcggagcgcgctagctgctcgggcgcggggcgttcttggtg
cgccgggccgtggtgagtccgggctcccgtggccgcgtgctgggaggaga
ctggagcccggttaggaagaatggagttggcgactcgctaccagatccct
aaagaagtggctgacatctttaacgcccccagtgatgatgaagagtttgt
tggcttccgagatgatgttcccatggaaaccctctcgtcagaggagagct
gcgatagttttgactcactagagtcagggaaacagcaggatgtgcgcttt
cattccaaatacttcacagaagagctaagaagaatttttatagaggacac
tgactcagagactgaggattttgcaggatttacgcagagtgatctgaatg
gaaagactaacccagaagtaatggtcgtggagtcagatttgagtgatgat
ggcaaagcatctttggtgagcgaggaagaggaagatgaagaagaagataa
ggctacccctagaagaagcaggtctagaagaagtagtattggtcttcgag
tagcctttcagttccccaccaagaagctggccaacaaaccagataaaaac
agttcttccgagcagttgttttctagcgcacgcttacagaatgagaaaaa
aacaattcttgaaagaaagaaagactgtagacaggtgatacaaagggaag
attctacctctgagtctgaggatgactctcgggatgagagccaggagagt
tcagatgctttgctgaaaaggaccatgaacatcaaggagaacaaagccat
gcttgcccagttattggcggaattgaactcgatgccagatttcttcccag
tacgaaccccaacctcagcttctaggaagaagacagtgaggcgggccttc
tcggagggacagatcacgcggcgtatgaacccaacccggagtgcgcggcc
tcctgagaagtttgctctagagaacttcactgtctcagccgctaaatttg
cggaagagttttacagcttccgaagaaggaagacaattgggggggaaatgc
cgggagtacagacgacgtcaccgtatatcttcttttcggccagtggagga
tatcaccgaagaggacttagaaaatgttgccataactgttcgagataaaa
tctatgataaagttctgggtaacacgtgccatcagtgtcgacaaaagacc
atcgacaccaagacagtgtgtcggaaccagggttgctgtggtgtgcgagg
acagttctgtggaccatgcctgcggaaccgctatggggaggatgtcagat
cggcattgctggacccggattgggtgtgtccccccctgtcgtgggatctgc
aattgcagctactgtcggaagcgtgacggccgctgtgccacaggaatcct
cattcatctggccaagttttatggttatgacaatgttaaggaatatctgg
agagcttacaaaaggagctggtagaagacaattaagaggaaaacaaacag
aaccagccacctcaccatagagtactccaacaagacatgcataccattgg
tgcctaagaaagatttttttacagttgtgtttttatacagaaattctttg
tagaaattactatttttgttaaagattgtttatatgcttacaaagattt
ctcaggaagacagcagagcagaggaatctatatagatgtatgcacagacc
tgtctgtatgctgaactttgttaaaaatatctgccagttattaaaaagca

cagtttaaatggggtggggttaaagttcaggtaagtaagttagagagaaa
acattgtatgatcagctcctgcacttgatctatctatctttggcttccca
aacagtaactcactccaggccaagtgtgccttagcacgagtgaccacagt
ttaatagaccacacacatcgtttaacctgctcttggtcattggaaattta
cactgaacaaagtgcaattaactgtagaacagttttatttttattaaaac
ttgactgaacaaaaggggaccatcaacattgtagacactggacactggag
gccttacagagtgctagcctcttccttcaggactcacctggggcctgctg
cttttatattttgaaagagtttaagggctaataatttaatttgttgtttg
ttaaaaattaaaatctccgttctttccggctgcattgcttttgcatgttc
acatatgatgtacttttatgatgtacttttttttttttgagatggagtct
tgttctgttgcccaggagggagtgcagtggtgcaacctcggctcactgca
acctttgcctcctaggttcaagtgattctcctacctcctgagtaactggg
attacagtcgtgtgccaccacgctcagccctgagttagtttctagtattt
ttttctgactttcactctaaggatagcgttgaaaaaagaatgcacgtgtg
agttactaaaaagttagaataatgtgcatgggaacagtgcagcaacatgc
tagaaaatcctcactgcactccagcagaggctagaggaatgccagtgtta
ccttacgcttctagaagcagagccactccagccagaacccattttgcagt
cttctcttcgctcttcagcctgaaggtccagatgtagctggggcctctca
gtttggttctatacacagggcactcgtaggtctgtttggtttcttgtctg
tccacgggggtggcttttgcaaagatgaccggcatagggcatgccagctc
cttgagacgggcttcaacaatggttcctgcttgggtgtcccagcgggcgc
ctatggcaaaaacacagcggttgcaattgtgtggcagcttgctactaatg
ataatgatcaagtaagttgtttgatgcaataaacattcaacattctggtt
ttgtcataaaaaaaaaaaaaaa

>NM_018845 3
cgcggggcggggctccagagccgcaggtctgggctgcagtaggtcccggc
aaccgcaggctcgcggcgggcgctgggcgcgggatccgactctagtcgta
atggaggcgggcggctttctggactcgctcatttacggagcatgcgtggt
cttcacccttggcatgttctccgccggcctctcggacctcaggcacatgc
gaatgacccggagtgtggacaacgtccagttcctgccctttctcaccacg
gaagtcaacaacctgggctggctgagttatggggctttgaagggagacgg
gatcctcatcgtcgtcaacacagtgggtgctgcgcttcagaccctgtata
tcttggcatatctgcattactgccctcggaagcgtgttgtgctcctacag
actgcaaccctgctaggggtccttctcctgggttatggctacttttggct
cctggtacccaaccctgaggcccggcttcagcagttgggcctcttctgca
gtgtcttcaccatcagcatgtacctctcaccactggctgacttggctaag
gtgattcaaactaaatcaacccaatgtctctcctacccactcaccattgc
tacccttctcacctctgcctcctggtgcctctatgggtttcgactcagag
atccctatatcatggtgtccaactttccaggaatcgtcaccagctttatc
cgcttctggcttttctggaagtacccccaggagcaagacaggaactactg
gctcctgcaaacctgaggctgctcatctgaccactgggcaccttagtgcc
aacctgaaccaaagagacctccttgtttcagctgggcctgctgtccagct
tcccaggtgcagtgggttgtgggaacaagagatgactttgaggataaaag
gaccaaagaaaaagctttacttagatgattgattggggcctaggagatga
aatcactttttatttttagagatttttttttttaattttggaggttggg

gtgcaatctttagaatatgccttaaaaggccgggcgcggtggctcacgcc
tgtaatcccagcactttgggaggccaaggtgggcggatcgcctgaggtca
ggagttcaagaccaacctgactaacatggtgaaaccccatctctactaaa
aatacaaaattagccaggcatgatggcacatgcctgtaatcccagatact
tgggaggctgaggcaggagaattgcttgaacccaggaggtggaggttgca
gtgagctgagatcgtgccattgtgatatgaatatgccttatatgctgata
tgaatatgccttaaaataaagtgttccccacccctgcccacgaaaaaaaa
aaaaaaaaaaaaa

>NM_019027 3
agtcagacccaggaggagtgaggtcaattgccctttagtcccaggactaa
ccggaagcttctgcaacaggaggacattgaaaataagatggaacccatcc
acataaggatttgcctcaaagggcactgcaaaaattgaacagaggaatcc
caaggaagctgcctgaatttgcctgtatactctcgttctgcgacttataa
aggaccagacaaatcaaattagtggttttggtttccgccagctgtggatg
cctttgacattatgaccgcagaggattccaccgcagccatgagcagtgac
tcggccgccgggtcctccgccaaggtgcccgagggcgtggcgggcgcgcc
caacgaggcagcactgctggcgctgatggagcgcacgggctacagcatgg
tgcaagagaacgggcagcgcaagtacggcggcccaccgcccggctgggag
ggcccgcacccgcagcgtggctgcgaggtcttcgtgggcaagatcccgcg
cgacgtgtacgaggacgagctggtgcccgtgttcgaggccgtgggccgca
tctacgagctgcgcctcatgatggactttgacggcaagaaccgcggctac
gccttcgtcatgtactgccacaagcacgaggccaagcgcgcagtgcgtga
gctcaacaactacgagatccgcccgggccgcctgctcggcgtgtgctgca
gcgtggacaactgccgcctcttcatcggcgggatccccaagatgaagaag
cgcgaggaaatcctggaggagattgccaaggtcaccgagggcgtgctgga
cgtgatcgtctacgccagcgcggccgacaagatgaagaaccgcggcttcg
ccttcgtggagtacgagagccaccgcgcggctgccatggctcgccgcaag
ctcatgcctggccgcatccagctgtggggccaccagatcgccgtggactg
ggccgaacctgagatcgacgtggacgaggacgtgatggagaccgtgaaga
tcctctacgtgcgcaacctcatgatcgagaccaccgaggacaccatcaag
aagagcttcggccagttcaaccccggctgcgtggagcgcgtcaagaagat
ccgcgactacgccttcgtgcacttcaccagccgcgaggatgccgtgcatg
ccatgaacaacctcaacggcactgagctggagggctcgtgcctggaggtc
acgctggccaagcccgtggacaaggagcagtactcgcgctaccagaaggc
agccaggggcggcggcgcggctgaggcagcgcagcagcccagctacgtgt
actcctgcgacccctacacactggcctactacggctaccccctacaacgcg
ctcattgggcccaacagggactactttgtgaaagtagccatccctgccat
tggggctcagtattccatgtttccagcagctccagcccctaaaatgattg
aagatggcaaaatccacacagtggagcacatgatcagccccattgctgtg
cagccagacccagccagtgctgctgccgccgcagccgcggccgcagccgc
cgcagccgctgtcattcccactgtgtcgacgccaccacctttccagggcc
gcccaataactccagtatacacggtggctccaaacgttcagagaattcct
actgccgggatctacggggccagttacgtgccatttgctgctccagctac
agccacgatcgccacactacagaagaacgcggcagccgcggccgccatgt
atggaggatacgcaggctacatacctcaggccttccctgctgctgccatt

caggtccccatccccgacgtctaccagacatactgaggctggtgaccagc
acgaagacagaccacacaaacaccactgaaggaacgcttgactatttatg
aagaaggaacatgttggattcacacatgcaacctgaaagtgaagaatgtt
agcagatttatttctgaattattttatatacatgaagttttcactagttt
tttaagactattttcaacttagcatgcctacgttcatacatttccaaaag
acttgcaatggttcgtgccttcattccatcttttaaaaatttgtatgctg
tactacatttgtatagaggtttttgttgttgttttttaaggatatattt
tcagtatgaaggttattttcttaacttctgcactccagagatttctattt
tgtagtaccttcaataatatatcaactatatattaaaaaagcacacttga
ggagctagggaactattttgaaaaatatatacaatatttaaagatacaaa
cagtagtgcttaaaaatactacataaagcattattttaaaggttatactg
gaaagtgcaattttaaaatgagtaaaacctctgtatttctgctggcatta
agggttgatggtgttaccatgtatcatcatggcggtactattttttaaaa
gaaattaaacactggatctctccttaagccaacattgaaaagacttgccg
cacttctgagtccaaacactggaaagctctcctttgccaccgttagccgg
ggctcattctccatgtgccttagccttaaacatgcccccactcccacatc
tctcaccctgtcccctcctccccagattcccaatcccaccgcaatgtttg
gcaagcctaggactgataagtagctctgatagaggagctggtggcttttta
tacttcttcctgggtttttgttggggtttgttgtttcgttgtttttttgtt
tttttttttgtttggttggggaagtattgtcttctacgtgtgctattttc
agtagcagagtaagcacaaggttttaatcgagttgcataagacacctttg
catagctatttaattgcccaatgtaaaactttaatgccatttctaatgct
tttattcatttttgaagtatgagtttgtagggacaaagaatgtatgttat
cgtagacaagacccccagagactcttttcagcagaaagttatgcttctag
ttgccttaccatgtttcttgcaaaactgtccatggtcctcaagggtgttg
gaaacattatgtttattaaatgggcctctcttcctttgctgtgcacttga
tgggtgaactggattggggtgtgcacatccaggaggaggaggagagacct
gtagaagtttaaagatagtttgtaaatatcttctaatgcttgtttttagt
ccttttatgttggagaagttcatggtatgtagtttaatgcaaaatgaaac
cattttatttcaatgttattaaaaaggtttgttttattaggaagttaatg
tattgttgcagtgttttgtgcctgtttaaaggcttttgtttagcagagtg
aatgtaaaatacagtaaaatgttaagattgtcatctactttttaaaaaaa
aatatcaacttggaattgtttttttaaaggctcaatcaaggaagtgaggtg
tgcaataaggtagcaagtaaaacgcagttgcgtttttatgtcatgttaga
gatccatacaattttccactcacgggatttttgttgatggctgaattctt
gtggattcataagaggatcatgcccttagcaagtacttttgttttgtttt
aaattaagagattcccaaatgcctttttcccccctcatcttgaaatgagat
gagtttttatgtgtaagcaatatttatttaactattctataaaattattg
agtgcctactgaggcctttaagcaccgctaacattcctttccatcattct
tttaaatgacataaaataattgtgcaatgttcctgatgatgtaccccaca
agctgcattcaaactcaaatctgtgggaatgagtgactcgacaaaatgta
attcggatcagatcctcatcccctgactgtgtgaaaaaagtactctcctt
ctagtgaaggattgtcacagagtttcactggatgaaactatgacccagta
ttcttactgtattttacatatgcctgtaaattatttgcaaaaaagaagaa
gaagaggaagaaagaaaagaaagaaaagaaagaaagaaagaaagaaagaa
agaaagaaagaaagaaagaaagaaagaaagaaagaaaaagaaagaaagaa

taaaaaaaaatgataacatggcaaccaccaaactcccttaaaaacaaaca
ttggtgacatggccaattaagtatcagtgagcctcctatctgggacttat
ctgttcctattgtttcaaaaccatttcatgtacactactgaggtaagttt
ataacttgaaatgtgaactttttttttttttagtgttaagaacaaactat
ataaatgtaaaaaaaaagttttagagttctgttttcaaacattgctagtg
gtttagttaactttagctgctttatatttgaaaagcttttatttagatct
tgctccatttacagtacattcttaggatgtatgtagtaaataaagctttc
tttaaagcaatcgcaagaccttattttaaattgtgaaaaaaaaaaaaaaa
aaaa

>NM_019895 2
tatcgccgcctcagacagctttcagtctgtccctcctacaactcccacaa
ggcccctcggccccgggccgcggcccggcccggagtgggggcgggcggag
gcgcgggagttatggagggggcgggctctgcagggaagtgcgtcagagga
ggcgcggggagagtagggtgctgtggtctgagctagagggtgaagctggc
ggagcaggaggatgggcgtatgcaggtgatagactagagaacaagacctc
tgtctccgtagcatcctggagcagtctgaatgccagaatggataaccgtt
ttgctacagcatttgtaattgcttgtgtgcttagcctcatttccaccatc
tacatggcagcctccattggcacagacttctggtatgaatatcgaagtcc
agttcaagaaaattccagtgatttgaataaaagcatctgggatgaattca
ttagtgatgaggcagatgaaaagacttataatgatgcactttttcgatac
aatggcacagtgggattgtggagacggtgtatcaccatacccaaaaacat
gcattggtatagcccaccagaaaggacagagtcatttgatgtggtcacaa
aatgtgtgagtttcacactaactgagcagttcatggagaaatttgttgat
cccggaaaccacaatagcgggattgatctccttaggacctatctttggcg
ttgccagttccttttaccttttgtgagtttaggtttgatgtgctttgggg
ctttgatcggactttgtgcttgcatttgccgaagcttatatcccaccatt
gccacgggcattctccatctccttgcaggtctgtgtacactgggctcagt
aagttgttatgttgctggaattgaactactccaccagaaactagagctcc
ctgacaatgtatccggtgaatttggatggtccttctgcctggcttgtgtc
tctgctcccttacagttcatggcttctgctctcttcatctgggctgctca
caccaaccggaaagagtacaccttaatgaaggcatatcgtgtggcatgag
caagaaactgcctgctttacaattgccattttttatttttttaaaataata
ctgatattttccccacctctcaattgtttttaatttttatttgtggatat
accattttattatgaaaatctattttatttatacacattcaccactaaat
acacacttaataccactaaaatttatgtggtttactttaagcgatgccat
ctttcaaataaactaatctaggtctagacagaaagaaatggatagagact
tgacacaaatttatgaaagaaaattgggagtaggaatgtgaccgaaaaca
agttgtgctaatgtctgttagacttttcagtaaaactaaagtaactgtat
ctgttcaactaaaaactctatattagtttctttgggaaacctctcatcgt
caaaactttatgttcactttgctgttgtagatagccagtcaaccagcagt
attagtgctgttttcaaagatttaagctctataaaattgggaaattatct
aagatcattttccctaagcattgacacatagcttcatctgaggtgagata
tggcagctgtttgtatctgcactgtgtctgtctacaaaaagtgaaaaata
cagtgtttacttgaaattttaactttgtaactgcaagaattccagttcag
ccgggcgaggattagtattatttttaactctccgtaagattttcagtacc

accaaattgttttggatttttttttctttcctcttcacataccagggttat
taaaagtgtgctttctttttacattatattacagttacaaggtaaaattc
ctcaactgctatttatttattccagcccagtactataaagaacgtttcac
cataatgaccctccagagctgggaaacctaccacaagatctaaagttctg
gctgtccattaacctccaactatggtctttatttcttgtggtaatatgat
gtgcctttccttgcctaaatcccttcctggtgtgtatcaacattatttaa
tgtcttctaattcagtcatttttttataagtatgtctataaacattgaac
tttaaaaaacttatttatttattccactactgtagcaattgacagattaa
aaaaatgtaacttcataatttcttaccataacctcaatgtctttttttaaa
aaataaaattaaaaatgaaaagagactcaattgtaaaaaaaaaa

>NM_022152 4
gggcgcggagccccagccgagcctagccctgcccggccccggaggacttg
caacactccgaggccaggaacgctccgtctggaacggcgcaggtcccagc
agctggggttcccctcagcccgtgagcagccatgtccaaccccagcgcc
ccaccaccatatgaagaccgcaacccctgtacccaggccctccgccccc
tgggggctatgggcagccatctgtcctgccaggagggtatcctgcctacc
ctggctacccgcagcctggctacggtcaccctgctggctacccacagccc
atgccccccacccacccgatgcccatgaactacggcccaggccatggcta
tgatggggaggagagagcggtgagtgatagcttcgggcctggagagtggg
atgaccggaaagtgcgacacacttttatccgaaaggtttactccatcatc
tccgtgcagctgctcatcactgtggccatcattgctatcttcacctttgt
ggaacctgtcagcgcctttgtgaggagaaatgtggctgtctactacgtgt
cctatgctgtcttcgttgtcacctacctgatccttgcctgctgccaggga
cccagacgccgtttcccatggaacatcattctgctgacccttttttacttt
tgccatgggcttcatgacgggcaccatttccagtatgtaccaaaccaaag
ccgtcatcattgcaatgatcatcactgcggtggtatccatttcagtcacc
atcttctgctttcagaccaaggtggacttcacctcgtgcacaggcctctt
ctgtgtcctgggaattgtgctcctggtgactgggattgtcactagcattg
tgctctacttccaatacgtttactggctccacatgctctatgctgctctg
ggggccatttgtttcaccctgttcctggcttacgacacacagctggtcct
ggggaaccggaagcacaccatcagccccgaggactacatcactggcgccc
tgcagatttacacagacatcatctacatcttcacctttgtgctgcagctg
atgggggatcgcaattaaggagcaagcccccattttcacccgatcctggg
ctctcccttccaagctagagggctgggccctatgactgtggtctgggctt
taggcccctttccttccccttgagtaacatgcccagtttcctttctgtcc
tggagacaggtggcctctctggctatggatgtgtgggtacttggtgggga
cggaggagctagggactaactgttgctcttggtgggcttggcagggacta
ggctgaagatgtgtcttctccccgccacctactgtatgacaccacattct
tcctaacagctggggttgtgaggaatatgaaaagagcctattcgatagct
agaagggaatatgaaaggtagaagtgacttcaaggtcacgaggttcccct
cccacctctgtcacaggcttcttgactacgtagttggagctatttcttcc
cccagcaaagccagagagctttgtccccggcctcctggacacataggcca
ttatcctgtattcctttggcttggcatcttttagctcaggaaggtagaag
agatctgtgcccatgggtctccttgcttcaatcccttcttgtttcagtga
catatgtattgtttatctgggttagggatgggggacagataatagaacga

gcaaagtaacctatacaggccagcatggaacagcatctcccctgggcttg
ctcctggcttgtgacgctataagacagagcaggccacatgtggccatctg
ctccccattcttgaaagctgctggggcctccttgcaggcttctggatctc
tggtcagagtgaactcttgcttcctgtattcaggcagctcagagcagaaa
gtaaggggcagagtcatacgtgtggccaggaagtagccagggtgaagaga
gactcggtgcgggcagggagaatgcctgggggtccctcacctggctaggg
agataccgaagcctactgtggtactgaagacttctgggttctttccttct
gctaacccagggagggtcctaagaggaaggtgacttctctctgtttgtct
taagttgcactggggatttctgacttgaggcccatctctccagccagcc
actgccttctttgtaatattaagtgccttgagctggaatggggaaggggg
acaagggtcagtctgtcgggtgggggcagaaatcaaatcagcccaaggat
atagttaggattaattacttaatagagaaatcctaactatatcacacaaa
gggatacaactataaatgtaataaagtttatgtctagaagttaaaaccca
aaaaaaaaaaaaaaaaaaaaaaaa

>NM_032156 3
gtcggtgcctgcagggctctccgtggccgctcgggagaggtgctcttcgc
tcaggctcccccggagacttgagggggtgcgcgctggcgtgcatggggcc
tgttctgtccttggtttggggatgctcctaagggggggagaaagggatacc
tcgggtgcttggaggtcgagagacggatacgtaagtgcgcaagggccact
gattagggcacgggaagggctcgctagaatgagctcttaaactggttggg
tgtaagcggttttgggctagtctaaaccaaacaactgagaataaacgcgg
ggttcctgcaggttgagagagtacctgtctgcaaatgtccagtagtgatg
gattgccttgtaggccccagagcaatcggggaagttagcattcttgttca
agaagctgctggttcacttagagctataaaagaatgttgtgagtttggat
tttcttccaaacgttcaggattgaggttagggctggtggtgagggatcat
gtaaagaggtttgagtggctcagccttaggtcccggtgtggaaagcttat
tcttttggtgtcatcacgtggccgtgggaattatttagaatgtgatctgc
cctcatgagaagaaaggagctctgaagccgttgcagttcttcctccctat
gtggaaaatcgaatttctggaaacatcctccgtattgggagattttgcct
atgtaaaggctatccttattggctagcggcactaattactttaaaagtag
gatggaagtacaagtatctcaagcatcattgggtttcgagctcacttctg
tggaaaagagtttaagggagtggtctagactttccagggaagttattgcc
tggctgtgtccctctagtcctaattttatacttaactttcccccacctcc
ttcagcctcatctgtttcaatggtgcaactcttttcatccccttttggtt
accagtcaccttcaggccattcagaggaggaaagagaggggaatatgaag
tcagccaagccccaagtgaaccacagtcagcatggggaaagccagcgggc
cttgagccccctgcagtctactctgagttctgctgcatctccttcccaag
cgtatgagacctatattgaaaatggactcatatgccttaaacacaaaatt
agaaacatcgagaaaaagaagctcaaactggaggattataaggatcgcct
gaaaagtggagagcatcttaatccagaccagttggaagctgtagagaaat
atgaagaagtgctacataatttggaatttgccaaggagcttcaaaaaacc
ttttctgggttgagcctagatctactaaaagcgcaaaagaaggcccagag
aagggagcacatgctaaaacttgaggctgagaagaaaaagcttcgaacta
tacttcaagttcagtatgtattgcagaacttgacacaggagcacgtacaa
aaagacttcaaaggggggtttgaatggtgcagtgtatttgccttcaaaaga

acttgactacctcattaagttttcaaaactgacctgccctgaaagaaatg
aaagtctgagtgttgaagaccagatggagcagtcatccttgtacttttgg
gaccttttggaaggtagtgagaaagcagtggtaggaacgacatacaaaca
cttgaaggatctactgtctaaattgctgaactcaggctattttgaaagta
tcccagttcccaaaaatgccaaggaaaaggaagtaccactggaggaagaa
atgctaatacaatcagagaaaaaaacacaattatcgaagactgaatctgt
caaagagtcagagtctctaatggaatttgcccagccagagatacaaccac
aagagtttcttaacagacgctatatgacagaagtagattattcaaacaaa
caaggcgaagagcaaccttgggaagcagattatgctagaaaaccaaatct
cccaaaacgttgggatatgcttactgaaccagatggtcaagagaagaaac
aggagtcctttaagtcctgggaggcttctggtaagcaccaggaggtatcc
aagcctgcagtttccttagaacagaggaaacaagacacctcaaaactcag
gtctactctgccggaagagcagaagaagcaggagatctccaaatccaagc
catctcctagccagtggaagcaagatacacctaaatccaaagcagggtat
gttcaagaggaacaaaagaaacaggagacaccaaagctgtggccagttca
gctgcagaaagaacaagatccaaagaagcaaactccaaagtcttggacac
cttccatgcagagcgaacagaacaccaccaagtcatggaccactcccatg
tgtgaagaacaggattcaaaacagccagagactccaaaatcctgggaaaa
caatgttgagagtcaaaaacactctttaacatcacagtcacagatttctc
caaagtcctggggagtagctacagcaagcctcataccaaatgaccagctg
ctgcccaggaagttgaacacagaacccaaagatgtgcctaagcctgtgca
tcagcctgtaggttcttcctctacccttccgaaggatccagtattgagga
aagaaaaactgcaggatctgatgactcagattcaaggaacttgtaacttt
atgcaagagtctgttcttgactttgacaaaccttcaagtgcaattccaac
gtcacaaccgccttcagctactccaggtagccccgtagcatctaaagaac
aaaatctgtccagtcaaagtgattttcttcaagagccgttacaggctact
tcttctccagttacttgtagctcaaatgcttgcttggttactaccgatca
ggcttcttctggatctgaaacagagtttatgacctcagagactcctgagg
cagcaattcccccaggcaagcaaccgtcttcactagcttctccaaatcct
cccatggcaaagggctctgaacagggcttccagtcacctccagcaagtag
tagttcagtaaccattaacacagcaccctttcaagccatgcagacagtat
ttaacgttaatgcacctctgcctccacgaaaagaacaagaaataaaagaa
tccccttattcacctggctacaatcaaagttttaccacagcaagtacaca
aacaccaccccagtgccaactgccatctatacatgtagaacaaactgtcc
attctcaagagactgcaaattatcatcctgatggaactattcaagtaagc
aatggtagccttgccttttacccagcacagacgaatgtgtttcccagacc
tactcagccatttgtcaatagccggggatctgttagaggatgtactcgtg
gtgggagattaataaccaattcctatcggtcccctggtggttataaaggt
tttgatacttatagaggactcccttcaatttccaatggaaattatagcca
gctgcagttccaagctagagagtattctggagcaccttattcccaaaggg
ataatttccagcagtgttataagcgaggagggacatctggtggtccacga
gcaaattcgagagctaactgcttcattatgagaaactcactgttgctaat
aaaacagcagggtggagtgattcttctcaggtgagcagcccagaaagaga
caacgaaacctttaacagtggtgactctggacaaggagactcccgtagca
tgacccctgtggatgtgccagtgacaaatccagcagccaccatactgcca
gtacacgtctaccctctgcctcagcagatgcgagttgccttctcagcagc

cagaacctctaatctggcccctggaactttagaccaacctattgtgtttg
atcttcttctgaacaacttaggagaaacttttgatcttcagcttggtaga
tttaattgcccagtgaatggcacttacgttttcatttttcacatgctaaa
gctggcagtgaatgtgccactgtatgtcaacctcatgaagaatgaagagg
tcttggtatcagcctatgccaatgatggtgctccagaccatgaaactgct
agcaatcatgcaattcttcagctcttccagggagaccagatatggttacg
tctgcacaggggagcaatttatggaagtagctggaaatattctacgtttt
caggctatcttctttatcaagattgaaagtcagtacagtattgacaataa
aaggatggtgttctaattagtgggattgaaggaaaagtagtctttgccct
catgactgattggtttaggaaaatgtttttgttcctagagggaggaggtc
cttactttttgttttccttcctgaggtgaaaaatcaagctgaatgacaa
ttagcactaatctggcactttataaattgtgatgtagcctcgctagtcaa
gctgtgaatgtatattgtttgcacttaatccttaactgtattaacgttca
gcttactaaactgactgcctcaagtccaggcaagttacaatgccttgttg
tgcctcaataaaaaagttacatgcacaaaaaaaaaaaaaaaaa

>NM_144578 3
ggcgcttcctgttccggcgccaggaggagccgcgcgctgctggtgctgtt
gccgccgctgctctagctgccgtcagtcaggctgcgcccgcgtcttcagg
gcccagtccctcggacccatcgccgcttctagaccctactgcggtctcgg
atattgccgggaaaatgtctgatgaattttcgttggcagatgcactacct
gaacactcccctgccaaaacctctgctgtgagcaatacaaaacctggcca
acctcctcaaggctggccaggctccaacccttggaataatccgagtgctc
catcttcagtgccatctggactcccaccaagtgcaacaccctccactgtg
ccttttggaccagcaccaacaggaatgtatccctccgtgcctcccaccgg
accacctccaggacccccagcaccctttcctccttccggaccatcatgtc
ccccacctggtggtccttatccagccccaactgtgccgggccctggcccc
acagggccatatcctacaccaaatatgccctttccagagctacccagacc
atatggtgcacccacagatccagctgcagctggtcctttaggtccatggg
gatccatgtcttctggaccttgggcgccaggaatgggagggcagtatcct
acccctaatatgccatatccatctccaggcccatatcccgctcctcctcc
tccccaagcccctggggcagcaccacctgttccatggggcaccgttccac
caggagcctggggaccaccagcaccatatcctgcccctacaggatcgtat
cccacaccaggactctatcctactcccagtaatcctttccaagtgccttc
aggaccttctggtgctccaccaatgcctggtggcccccattcttaccatt
aagttaacaatggacgaagagatgacgctttgctttttgaagtacatgta
tatgcacatgaatgcatatataaaaattgctggtttcactattagagggc
attcatgaaagaacaactcttgcacctctcagagaagataactgcctctt
gtacttggatgcgtagtacatcatatgtatacaatcagataaaagcatag
aagtaaatcattcggatgtgatttttatttggttttcatggaaagttaaa
gtgataaagtatattgaatagttctttgacagaatttgtttaaactatga
aactacacacttaaaaatctaagatgtggattattgttagaatctgcaac
ttcattggcaaattatttcaagtatttttctataatcactttccccttct
aaataaataaacttcgagaataacccatcataatccaaacaaatgatgcc
tcaacattttgagctgctctgtcggacaaataaacctggtcctcttgagg
ttatattttggatatacatttttaaactgtcagtaattattgtcagatgt

ggagttcaatagccagccagtgttcatttttatccttgagcttttagtaa
aaacttcctggttttatttttagtcattgggtcatacagcactaaagtct
gctatttatggaaactaactttttttgtttttaatccaggccaacatgtat
gtaaattaaatttttagataattgattatctctttgtactacttgagatt
tgattatgagatgtgcatattgctttgggaagagctcgaggaaggaaata
attctctcctttgttttgaacctcaaactagataaaccctaggaattgct
taactgcaacaagtaattttcattcccacaaaaacctgaggcagctcttt
tgcccagagcgttccctgtagccacccccaccccacttgcccttggttct
ttagaaggagcacacacatcccttgattcctccctgatgtggtaaactgg
cacactccaggggtctaaaacataaaacagttgtgtttagggaaccttaa
gtcatgcagacatgactgttctctttgtacaagtgtgaatcaaaatatgt
atctctttttcagagtctggttaagctatgtcattgtctactgcatagtt
tcctgagtctgtttgtaaagtgcttatggctaacagttcagttctgtatt
tgttgacaggtaaataagtggagttgagtgccatctttgaaaaaattacc
ctctagctctaacactgaaaataataataaattgtagatctctgcaacta
agtttaaagcagtgtgactgtgttgcttaaatatcaagtattgtttataa
ccaccaaaaaaaaaaagccctggtagtttttggcaccttatgtttaaat
cagattcttagatttggagtagacctgaccttgttatttattagataaca
ttttgaatgtatccattggatttctaaaatgtattgtgaatttctcagac
aaacaggatttatgctggagctctgttttgcttagaaataaaatatttag
tagtttatttctgctctaattaaaatgtcaagaatgccaaatgctgccag
ttttttggtttgatagctacctccttctaagaaagcaaaatggttacctt
tgagaggaacattcagtgtttaatcatcccttatgttaactagatgatag
attcaagcttttagaaatgagaaagtagaaactaatttgttaagatattt
tcagactgcggaatgttgttagctttttctttcacttctcttcaaggaca
ggtgttagctgtctacaatactgttgaactctgttgtcaaagtagccccc
ttagtctacaaggcaggtagccttggcttgaattatcaatatcaaaatgt
cagttaaccatggagggataaagtaatgtgaaaagtgagatggctgcaaa
gatagctctccttacagttattttggctgtcctacattgggataagctga
caaattagcagtatttagtttaacactggagcaaatataatttgagtagg
aagaagagatagcaggtttgggaatctataattatgaagtccattgattt
tgggagaaaatctgttgctaaaggatttgaagggccatgaacacaatttg
ggattattactccctataagtataataattttgctagtgacccatactgt
ccagtgtgccctaaatcatactgctattgtactccctttgttttcaagga
ctttgcaactggtatttgggggagattttttttttttttgagacggagt
ctcgctctgtcgcccatgctggagtgcagtggtgctatcttggttcactg
caagctccatctcccaggttcacaccattctcctgcctcagcctcccaag
cagctgggactacaggtgcccgccaccatgcccggctaatttttttttt
ttttttttttagtagagatggggtttcactgtgttagccaggatggtctc
gatctcctgacctcgtgatctgcccgccttggcttcccaaagtgctggga
ttacaggcgtgagccaccacgtccggccgatttttttttttttttttaat
gtaagaatggagataaaagggataatataatttgcttttatattgttatt
tttgtaaagcatcttttcttcaattcttgttggcattctgggccaaaata
tttcaggttggttcggtgtggagttaagaaaagcaggcgttttagtggag
aaatggggaacagcatcaagaaaggctttttttcctttttttcttttttttt
tggagacagagtcttgccctgtcacccaggctggagtgcaatggcgtgat

cttggctcgctgcaacctctgcctccaggttcaaacgattcttctgcctc
agcctcccaagtagctgggattacaggtgcccgccaccacacccattttt
gtatttttagtagagacggggtttcaccatgttggccagggtggtctga
aactcctgacctcgtgatccgcctgcctcagcctcccaaagtgctgagat
tacaggcgtgagccaccatgcgtgaccttttttctttttaaaagggaac
aatgttgctttcaaaacaagacatgctaggctgaaactgatttatggaaa
agactgcttgttagcaagtatatttggtcttgaggggatacagattata
gaatatgctgacatttgggcttcagaggaagaattttcaaatctaatgga
aatagttgaggtgttcaggaatgctgtttcttggagttggaagcttaggt
tttgaaatgttgaaaccaaaaagacaaaaattaaaacatagaccttaggt
cgtcattcacacccggttctcaagaatcaagtggagcacttcaaagacct
tggcttgtctgtcccatcctgccactttctcatcttttcatgcttttgaa
gacaccatttacagctctgactcagccctattttgtgtaaagtaatatat
tgattattcagaaatagacaatacatttttttaattacccaaggactgact
gttttgtgcattttactgttggttgtcttcagtagagaatagtaataggg
cagagaaaagtatatattttgcctcagtcagtcccaccaccacaatggac
tattgggatattttctaaaaaaccaatcaatttgcccatgattacctcac
aaataattagtgctacctggggtactctcaaatatacagcttttgaaact
gtagatgaaaaaagctctactcagagtttttgtcaagactgtgcctgggt
tgaatatcagtcaattgcctacacttctaaacaataagtgccaatgtctc
aattttctcaccctgaatgatagaagctagctttatcaaatgccaaggtt
agaaagcctggaaataaaacttaagcacagacattcaagtttttgaaaag
cataagcctaaattcagataaatcacactgatatattgtactatgcatag
aaagttgtaggtggcgttcagggaagactttgattttaataaagcaatat
ttagtattgaagacaaacactttttattttcagatttctgccaagtaaaa
cagaaattgccaataaaataatcagtattttgtaaatggcaggcaagctt
ctggctgtcgaaaacatctgagtcatttattcagtagacaatatgtcctt
gatccaggttctttgccagctataagggaatccctgtccttgagaggctc
atagtctataagtaacattacagaatttgttagcatacccattcattatt
agttttacctaaacgtgttaggatcactactggtggaaattgtaaccagc
ctttgggcatcttaaagggtgacatgtggcatgccttttttttttttaa
gaatttaatgtttttcaagattgtagtgttgatcagcgcaacaattcaag
tgtgcaaagtaacaggatagtttgcctcttcactttaccccctggataaag
gcactttcactgcctgtcactgatcagcagatactgacttgttgccatta
agtgaacttgacttcttatgtgtgctctatgagtttgttgtaattttctt
cttgaaattgtgatttttcactgacagtaatgacaaatttaatgtatgta
attgtctatgcattttaagttaaactgcctaaaatgtgatttgagacata
tacatatgtttgtattataaattgtaagcaatcagtttgagatactaggt
tttatcacctgctgctgtatttgtaaacaaagacaaatgttgctttaaga
agtaattataattaggaataggctatggatgtgatacttggtatttttta
agataaacttgtttgcttttgtgtattatacctggaaactttttttaaaa
aatgtattttcatggtttcacagatttttcatgttattttattctttagg
cccaattctgggcttctctgagcaagtccagagcctaattaactgtaaat
ttgttgtcaaaaaggaagaaaaaagggcctgagatacctctttgcatgtg
acctgcattcactaaggatatctggaaaccacccttcctccgcaaaccct
ctcagcaacatggtgtccattgtggtgatttctcttcttttaaggctag

gctactcttggtaaccagattatccgtatatatgataatatgaagtcagg
gaactttctctgtctgtccctactcccctcactcccccactttctgttat
gaaagatagttctacttttatcattaactgctacgcatttagtgagggtc
acattattaaacttggagtttaccattttcccacaggagatttcgctggc
attccttggaactcccaatttcagtagggcaatgaatgaatgaatacttt
gcagtgctacttttggaaggaatttctgctttttgccttatgattggaca
aaatgcagctgtaaaattttaaattgtttttgatatgttattcaatatcc
catgaaagtattcacctaaagtggagttatgaaatggatggtgaaataat
aagaccattctggagcagg

>NM_152792 2
gcagcagttgagtccatgtaagatgattgtgcacgtgtgtgtgtgtgtgt
gtgtgtgtgtgtgtgtcctcactcacttaaaaaagaccatttctgtgcat
tctttatgctggaaccctcctctgcctccctctccaggaaatcccatagg
atcccatacctgctttagaatgtgtgtgagacacttcccagtgcctggca
tgtggtaggagctcagtacatgtaaactgttttcctttgaccactcaaag
tagtcacttgttggggaggccaatggcggcttccccggtctctgagctga
tgggcgtctggtccctgcctccccttccttcactggctgatgacaaatac
ctgaatgtcatcacatcctccgtgtcctcatctgttattctggaacacag
gccagggagggtacagggtagtccccccttcaacagaggctggcctttctt
gggatgtgggctggctggtgttggtccaaggatggatgcagagggtgagg
cacccatcctgctagtccggccggatgctggcaggagggcggggtgagga
ggggcggagcttccagaacaaaggagaatggggagcccaggggccagcct
aggcatcaaaaaggctctgcagagtgaacaggccacagcactgcctgcct
ctgccccagcagtcagccagccgaccgcgcctgctccctcctgcttgccc
aaggccgggcaagtcatccccactctgcttcgagaggccccgttttccag
cgtgattgcgccgacactgctctgtgggtttctcttcttggcgtgggttg
ctgctgaggttccagaggagagcagcaggatggccgggagcggagccagg
agtgaggaaggccgccggcagcatgccttcgtcccggaaccttttgatgg
ggccaatgtcgtcccaaacctctggctgcacagctttgaagtcatcaatg
acctcaaccattgggaccatatcaccaagctaaggttcctgaaagagtcc
ctcagaggagaggccctgggtgtctacaataggctcagtccccaggacca
gggagactatgggactgtgaaagaggccctcctgaaggcctttggggtcc
ctggggctgcccccagccacctgcccaaagagatcgtctttgccaacagc
atgggtaagggctactatctcaaggggaagattggcaaagtgcccgtgag
gttcctggtggactctggggcccaggtctctgtggtccacccaaacttgt
gggaggaggtcactgatggcgatctggacaccctgcagccctttgagaat
gtggtaaaggtggccaatggtgctgaaatgaagatcctgggtgtctggga
tacagcggtgtccctaggcaagctgaagctgaaggcacagttcctagtgg
ccaatgcgagtgccgaggaagccatcattggcactgatgtgctccaggac
cacaatgctatcctggactttgagcaccgcacatgcaccctgaaagggaa
gaagtttcgccttctgcctgtgggagggtccctggaagatgagtttgacc
tggagctcatagaggaggacccctcctcagaagaagggcggcaggagcta
tcccactgagaagccaccttttctttaacctcctaaatattggtgggaag
acccaccgctgtggggggggttgcatatcctcatgggggtcactgggctt
ggccagtctgcttatcaactcttgctcttctctcccctttgcctccctct

gcaggggccttaatctgcccctggtaggggaggcttccactgaacaggca
caggtgagggagagcaggctggcttagagggacagggtccccatggtcat
caagctgctgttgatgacaaagactcaaaggctggaagagctcccaagga
agctagaaatgcttgtctttgaaagaactgtgggaccccttcagattccc
tgaggtatggcttggtcactctcaggtcctcaaagcctgtcttagttggg
ctgggtcctagctgcagggtctttgtgagggtcacagttgctctgggaca
cctccctgaagagcctttccacctgtacaatcgtattttctttctgtcat
ttgctttgaagcccattgtgccttatgccaataattcaattgctgcaaac
accaataaagattgattcatggaaaaaaaaaaaaaaaaaaaaaaaaaaaa
aaaaaaaaaaaaaaa

>NM_153186 4
tgattgtgcctctgcacctcctcaggtgaccgagggctgggcaggaggac
acagactctttcatgaagcagtgactagtataggaaaaacagagcctctc
tgtagagatgcttaaagtgctagctttatttaaaaatcagctggcaaagc
gggagtgaagaacacacattttcagaagataccggtttcctgttttcatt
aagaaaacagggaatgcggaacagctgagtgcggcgggggtggtgtcact
gcagccggagggagacctcgccggtgaaagctcagcctatggcatccgag
cgtggccggccaagtttacacgaatgttgaaacttctcctcacggggtga
ggatcgagggcgcccgaggccgggtccggctgagctggagcgagctgtgc
ggggcagcgcgggctggcggggagcgcggcgggcgccacgtggaaaagca
ggtgatattctcagtggagaccaggacaaggaacagaaagacccttactt
tgtggagacccccctatggttatcaactagacttagatttcctcaaatatg
tggatgacatacagaagggaaataccatcaaaagactgaacatccagaag
aggcggaagccgtccgtgccatgcccagaacccaggaccacatctggtca
gcaaggtatatggacttccactgaatccctctcatcctccaacagtgatg
acaacaagcagtgccccaacttcctcatagccagaagtcaagttacatca
actccaatctcaaagccacctcccctctggagacctcactccctttct
taccatcccagaaaatcgacagctgccacctccctcaccacaactcccaa
agcataaccttcatgtcaccaagacactgatggagacccggagaagactg
gaacaggagagagccaccatgcagatgacaccgggtgagttcagaaggcc
caggctggccagttttggaggcatgggcaccacaagctccctcccttctt
ttgtgggttctggaaaccacaatcctgccaagcaccagcttcagaatgga
taccaaggtaatggggattatggtagctatgccccagctgctcccaccac
ttcctccatggggagctccatccgccacagccccctgagctcagggatct
ccaccccagtgaccaacgtgagccccatgcacctgcagcacatccgcgag
cagatggccattgctctgaaacgcctgaaggagctggaggagcaggtgcg
aaccatccctgtgctccaggtaaagatctctgtcttgcaagaagagaaaa
ggcagttggtctcacagctgaaaaaccaaagggctgcatcccagatcaat
gtctgtggtgtgaggaagcggtcctatagtgcggggaacgcctcccagct
ggaacagctctcccgggcccgaagaagtggcggggaattatacattgact
atgaggaggaagaaatggagaccgtagaacagagcacgcagaggataaag
gagttccggcaacttacagcagacatgcaagccctggagcagaagatcca
ggacagcagctgtgaggcctcctcagagctcagggagaatggagagtgcc
ggtctgtggctgtgggtgccgaggagaacatgaacgacatcgtcgtgtac
cacagaggctccaggtcctgtaaggatgcagctgtagggacacttgttga

gatgagaaattgtggggtcagcgtgacagaggccatgcttggagtgatga
ctgaagctgacaaagaaattgagctgcaacagcagaccatagaatccttg
aaggaaaagatctatcgcctagaagtacagcttagagaaaccacccatga
ccgggagatgactaaactgaaacaagagctgcaggctgctggatcgagga
aaaaggttgacaaagccacgatggcccagccgcttgttttcagtaaggtg
gtggaggcagtggtgcagaccagagaccaaatggtcggcagtcacatgga
cctggtggacacgtgtgttgggacctccgtggaaacaaacagtgtaggca
tctcctgccagcctgaatgtaagaataaagtcgtagggcctgagctgcct
atgaattggtggattgttaaggagagggtggaaatgcatgaccgatgtgc
tgggaggtctgtggaaatgtgtgacaagagtgtgagtgtggaagtcagcg
tctgcgaaacaggcagcaacacagaggagtctgtgaacgacctcacactc
ctcaagacaaacttgaatctcaaagaagtgcggtctatcggttgtggaga
ttgttctgttgacgtgaccgtctgctctccaaaggagtgcgcctcccggg
gcgtgaacactgaggctgttagccaggtggaagctgccgtcatggcagtg
cctcgtactgcagaccaggacactagcacagatttggaacaggtgcacca
gttcaccaacaccgagacggccaccctcatagagtcctgcaccaacactt
gtctaagcactttggacaagcagaccagcacccagactgtggagacgcgg
acagtagctgtaggagaaggccgtgtcaaggacatcaactcctccaccaa
gacgcggtccattggtgttggaacgttgctttctggccattctgggtttg
acaggccatcagctgtgaagaccaaagagtcaggtgtggggcagataaat
attaacgacaactatctggttggtctcaaaatgaggactatagcttgtgg
gccaccacagttgactgtggggctgacagccagcagaaggagcgtggggg
ttggggatgaccctgtaggggaatctctggagaacccccagcctcaagct
ccacttggaatgatgactggcctggatcactacattgagcgtatccagaa
gctgctggcagaacagcagacactgctggctgagaactacagtgaactgg
cagaagctttcggggaacctcactcacagatgggctccctcaactctcag
ctcatcagcaccctgtcgtctatcaactctgtcatgaaatctgcaagcac
tgaagagctgaggaaccctgacttccagaaaaccagtctgggtaaaatca
caggcaattatttgggatatacctgtaagtgtgggggccttcagtcagga
agtcccttaagctcccagacatcccagcctgagcaagaagtggggacctc
agaaggaaagccaatcagcagcctggatgccttccccactcaggaaggta
cgctgtctccagtgaacctgacagacgaccagatcgccgctggcctctat
gcatgtacaaacaatgaaagtacactgaagtccatcatgaagaagaaaga
tggtaacaaagattcaaatggcgcaaaaaagaatcttcagtttgttggca
ttaatggagggtatgaaacaacttcaagtgatgattccagctcagatgaa
agctcttcttccgagtcagatgacgagtgtgatgtcattgagtatcctct
tgaagaagaggaggaggaggaggatgaagacactcggggaatggcagaag
ggcaccatgcagttaatattgaaggtttgaagtctgccagggtggaagat
gaaatgcaggttcaagaatgtgaacctgagaaggtggaaatcagagagag
gtatgaattaagtgaaaagatgttgtctgcatgcaacttactgaaaaata
ctataaatgaccccaaagctttgaccagcaaagatatgaggttctgtctg
aacaccctccagcacgagtggttccgcgtgtccagtcagaagtcagccat
tccagccatggtgggggactacatagctgcttttgaggccatttccccag
atgtcctccgctatgtcatcaacttggcagacggcaacggcaacacagcc
ctccattacagcgtgtcccactccaacttcgagattgtgaagctgctgtt
agatgccgatgtgtgtaatgtggatcaccagaacaaggcaggctacaccc

ccatcatgttggcggccctcgccgctgtggaagcagagaaggacatgcgg
attgtggaagaactcttcggctgtggggatgtgaatgccaaagctagtca
ggcgggacagacggccctcatgctggcggtcagtcacggacggatagaca
tggtgaagggccttctggcctgtggggctgatgtcaacatccaggatgac
gagggctccacggccctcatgtgtgccagcgagcacgggcacgtggagat
tgtcaagctgctgctggcccagcccggctgcaacggtcacctagaggaca
acgatggcagcactgcgctctcaatcgccctggaagcaggacacaaggac
atcgctgttcttctgtatgcccatgtcaactttgcaaaagcccagtctcc
gggcacccctaggcttggaaggaagacgtctcctggccccacccaccgag
gttcatttgattgattgtatgcaaatagccctttatttacatgccactat
taagctgctaattgttcctgttggggtgacagatactgaatgtatacgta
ttgtgcctgagctcaccagcaaacagaagcatcaagcccaggggtaaagg
ctgaagctttcacagtgcagagactgctagcctgggcacacacacctcct
ttctggccgtcttctgtgtagggcacactttaacccagtctctgttgctg
ttgagtctctgctccgttttgtacagtcacagggaattctgatctgaagg
ggcaccttctgttcactcccacaaagtggtgtctggttctcactgagacg
ttttaagatttttccacaaatatttatatgtactaaatgtggaaccatta
gaaagttcttccaaaatctcattccagcatagttttggatttttcttttg
tcttattttaaaataaggaagtcgagatgactttgatcattggtaacttg
ggcctgggccagacaaagtataaaacttacaaaagaatattctcatttgg
tcttaactaggtagatgtaatatatgactttttataaaaagggtatctat
atgaacttgacacagtattttcagcttttgtattccatactaaagccatg
aagaactacacgtaacatcatcatttgtattaattgcacaactccaatgc
taaaggttggattgtgttagaggaatcggctctgtatttgcctctagaga
aacacagtgttctctttgtatttatggattcctttttaccgtgtcacatt
tactttggtcctctatgtatttaaatgtttgaagtgccttagactcttgc
catattttcaaaataaaattccattaagctcttttaaaaaaaaaaaaaaa
aa
>NM_173607 3
cgggtgagtcccacttcccgacagcctggctcggccagcgactgggcggg
gagaccaaggatggaagtgggcttaccggccattaccctctttctcacca
gcgccagcagccctgtggtggcgacgacgatggaccaggagccagtgggc
ggtgtggaacgaggagaagccgtcgcagcctcgggagctgcggccgccgc
ggcattcggggaatctgcagggcagatgagtaacgaaagaggctttgaaa
atgtagaactgggagtcataggaaaaaagaagaaagtcccaaggagagtc
atccactttgttagtggtgaaacaatggaagaatatagcacagatgaaga
cgaagttgatggcctggagaagaaagatgttttgcctactgttgatccga
caaaacttacctggggtccctacttatggttttacatgcttcgggctgct
acatcaactctctcagtgtgtgacttccttggagagaagattgcatctgt
tttgggtatcagcaccccaaagtaccaatatgccattgatgaatattatc
ggatgaagaaggaggaagaagaagaagaagaagaaaacaggatgtctgaa
gaagcagaaaaacaatatcaacagaataaattgcagactgattccattgt
tcagacagatcaaccagagacagtgatatccagctcatttgtgaatgtca
attttgaaatggagggagacagtgaagtaattatggaaagcaagcaaaat
ccagtctctgtcccaccataaaatgaaatgactatcaagcttcaaactct
taagttttttttttttaatacaaaaactttcacattctttattcagtggg

acttaatacaattatttatattttaaattattaaagtatctggaaaggga
aaatgttttcttcatttttaggatctatctagcaaaagccagatctgaaa
ttcagatatttgtactgtttttactgtgtatagaaattagtgctttggtt
ttaaaatgatcttttaaaaaagttaaggacatcctagagccttaatagtt
aagaagagttaaattatcaagcctatttgtgcatttgcttttttgaaaa
aggtaagttgctgattaagtctaattggaattgataattccatagtctta
gattaaaatgaggatattttctcctagattttctcatgttatgccatgca
tttatatatctaaccattaatttcacactaaggatgcttcaccatataat
aaaaggagcaagatggaagcactttgaattttctttcattgagaataact
gttttatgtaagaatctgtatttataacaccagatattaagataggcttc
catttttaatgcaagccacttacttaatcttgtattctttttcaggact
caaataactagctttgaacataatattaaaacactacttatagaatagat
ttattaatgttaatacctagtgaatatccatgtggcatcctggttatgtt
atcggttcagcgttaatcctatagaaaagtggtttggaggggattggggg
atagtgggacaggtatagatttaatccatcaggagcaattagatattgta
taaggtgcaatgatagcctaatgaaattacccgtcattcatcatttagaa
gtagcaacagtgaagactggacagtttacttgaatctggttggccactcc
tctacctacttggttatttgtaaaccttacaaatgtatatattgtgaagc
taattttgaaaatattcctaaatatggccaggtacggtgctcacacctgt
aatcccagcactttgggaggctgaagtgggcagattacttgaggtcagga
gtttgagaccagcctggccaacgtagtgaaaccccgtctctacaaaaata
caaaaattagccaggcgtggtggtgcacgtctgtaatcccagctgctcgg
gaggctgaggcaggagaatcacttgaaccagggaggtggaggttgcagtg
agccaagattgcaccactgcactccagcctgggcgacagagtgagactcc
atcttgggggggaaaaaagtatatatatatacacacacacagacacacaca
cacacacatatatctctaaatgtgtgtatagaaccttttatcagtataac
attgatttataattaaatgtgggtgaggaagaatgtgtggagtgtttcag
aaattttgatcttaaaagccttttcagaaactcaaagctttcagaaatta
atagttatattaatagccttctaaacagcattaagttttcaattttaata
tatctatataaaatatagtgtcaaagaaagtaacatcaatatagtgggtc
tgtaatggaaatctgtcaatgaacatgaactgagttcttcaactacaggt
tgaaggataattaaacttttcattaggagtgttcagctacatggattcat
ggaatggtggttcatagtaagtgatggtaatctttttattcatgggtgta
aaccctttataacttagtgcttaaaatattaatcttttgattatataatg
tcccatgaattatagtttaccaatagttctaaaacatgctaacctgacct
tttccttctaatttgtttttaacttcattcgagatagtacctctcactca
cagatatttatttggttatcaagtgaagataggtgtgtctaaaagtgatc
ttctgaatcctgtctccctagaggtactagtatctagagtttacccagaa
aattttatgattgtaacaaaaggaagtagtgacttatgaaggttttgttt
cttgaattttacttttgctacttgtccaatagtggctagtttatgtttat
caatatagttattcactgtgccttaagtttatactttgtttatgcaaact
ataaaatttcccataaatgtattcaaaaaaaaaaaaaaaaaaaaaa

>NM_175866 4
atttccggcttctgggactcgggtgcaccacggcttccggtgtcatggct
gcttgaagtcccgggagtcggtgaggcggctgcaggtccctccctgcgga

gccgctggtccggctggcggagatgtgaccgcgggcccggccggcctgcc
tcaggcgtcgcgtcagctcccgtgtccgtgcccttaacccacaccgatgg
cgggatccggctgcgcctggggcgcggagccgccgcgttttctggaggcc
ttcgggcggctgtggcaggtacagagccgtctgggtagcggctcctccgc
ctcggtgtatcgggttcgctgctgcggcaaccctggctcgcccccggcg
ccctcaagcagttcttgccgccaggaaccaccggggctgcggcctctgcc
gccgagtatggtttccgcaaagagagggcggcgctggaacagttgcaggg
tcacagaaacatcgtgactttgtatggagtgtttacaatccacttttctc
caaatgtgccatcacgctgtctgttgcttgaactcctggatgtcagtgtt
tcggaattgctcttatattccagtcaccagggttgttccatgtggatgat
acagcattgtgcccgagatgttttggaggcccttgcttttcttcatcatg
agggctatgtccatgcggacctcaaaccacgtaacatattgtggagtgca
gagaatgaatgttttaaactcattgactttggacttagcttcaaagaagg
caatcaggatgtaaagtatattcagacagacgggtatcgggctccagaag
cagaattgcaaaattgcttggcccaggctggcctgcagagtgatacagaa
tgtacctcagctgttgatctgtggagcctaggaatcattttactggaaat
gttctcaggaatgaaactgaaacatacagtcagatctcaggaatggaagg
caaacagttctgctattattgatcacatatttgccagtaaagcagtggtg
aatgccgcaattccagcctatcacctaagagaccttatcaaaagcatgct
tcatgatgatccaagcagaagaattcctgctgaaatggcattgtgcagcc
cattctttagcattccttttgcccctcatattgaagatctggtcatgctt
cccactccagtgctaagactgctgaatgtgctggatgatgattatcttga
gaatgaagaggaatatgaagatgttgtagaagatgtaaaagaggagtgtc
aaaaatatggaccagtggtatctctacttgttccaaaggaaaatcctggc
agaggacaagtctttgttgagtatgcaaatgctggtgattccaaagctgc
gcagaaattactgactggaaggatgtttgatgggaagtttgttgtggcta
cattctacccgctgagtgcctacaagaggggatatctgtatcaaaccttg
ctttaatcagtaacctaaggactgtttcctttttctcctcttccatttct
tgggttattccacatatgaatgcaggactaccccccttaccattttaagaa
ggtactttatacatttatttaatcctactaatgtgcagccattgcccaag
cagtgactgcgttgcatacatttggcactgagtaggacaagacctctcag
ctatacattgaggggttttagagcatccatgtgggcaacccttttttgtg
cgggagagcaggtgttgctcttcagtatgtagcctaaaaaaatcttaatt
atttcatggatcatgaagcaaggatgaataatatcatgtcttggtaaata
ctaacaaatttgttaggtttggtgacatcatttacagattatttctttat
gttgtccagtggttcttccttattgttgatatccataagctggcactgga
tgctctcagtaatgttaagtaattgtcaagcagcagttacctactgtgtt
cttaacactgagttgtgaatttttttcttaaagcagtactgtagtactgaa
tattcctttaaaggaactgcagtgagcctatctaagtttttttaaattaa
ggcttttaaaatagaaagctgatgcttgatcttgcacaatttttatgtct
agtatgtatgcttgagtgaatgtgcgagtatgaatgattagagaaaattt
gagtcagtgtactttatagtgtgaatcctgtgagctaatacagtctatac
ttatttcttccctacctgtttcacatccgtaagatttaagatatacattt
tttgagaggtagtctgtctgatacaatgtaaatgacaaaacataattcct
gagaggcccagaacaaactggagtctagcctggagttaaattgagacttc
taaaatgattggaacaaagactaagttgtgccagatgtaaatcaacccct

cttttagtttactttagactttgtattagctcatctttttgtagtaaat
ctatagttttaaggtttctcaagatgtggctctacctactatgatgaaaa
ttgaagtgggtcaaaagaattagatgtacagtgaagggaaaagaaaaaaa
atgggcgaagagagggtggaaaataaaaggattctttttcttcctttct
gtttctcgtatccctgctccctttttcctccccttccctcattctttgcc
tctatccttagctgaagacaaactagaggagcagcatcccaggtagtttg
gcttttgactgcaaggtagttaagaaaggtgtagatataatgaggtagaa
gtagaaaggaagaaaaactcaaagaattcttaaaaggattcatagcaaca
taatgtgtccctgagtagaggatgctgctatgcgtgagttcatggacaca
agttgattacatggtttttagaattataattatggattcttcttatttca
tggtaggttgtctttaaagatataaaaattaggatgcctttatgaagcac
tgattataccaaaaaaaaaagacaagttatatacaggttattaatttttt
tatttatttatttttcctaaggaaaaagtcttctatctttccctgctgg
aagcctcctcatatttccttatgtttgccatgcaggttgctgagagtcca
gttagaatttgcattttacagaatgaaatactttaccccattcaaacaat
tattgtttgacattttagttatttataattgtcaaattcaggactctcct
ttaatgtttattatgaaaccaaatttggcataaggaggctgatttatgaa
ttaccaaagggtcttgtggcatgttccccaatacatgcccttagaaggaa
gaactattatttttattttggccctttcaggagttgattataaattggt
ttgttttcaagtcagaattcaagtgggcagaacccgtattgtgaagacct
aaactttcctaaatgttcatatgggtagcagattttgtggtgattagaaa
catcaggtccttaaatacgatgaacatgggatacaaaggaattctttata
agggcaagtatcctaagttagcacatttacttttctctcccctccgcccc
caaaagaaaaatccttacaaataaactgcaggtaggcttctaagcctagt
cctgcagtatgctgctaacatcttgatgccaatcttcacagcattctttg
attgtcatcttattgctgatacattcatacatatatttagtgcttgacac
tgtagaattttgttacagaagatggttactagattttaagggagctgagg
gaataattgatgagccttgaattaaccatgcatttaattagattttttgt
tgttgttgttgttgttttgagatgatgtcttgctctgttgcccaggctgg
agtgcagtggctcaatctcggctcactgcaacctctgcctcgcaggtttg
agcgattctcctgtctcagcctccccagtagctgggtttacaggcgcttg
ccaccacacctggctagatgttttatattaaagccagagaattgaaaaag
aaatgcctcctgtgattgaaattattttatagctcttagcccgttctacc
aaagatcacattagcagattacccctccctcctgtgatattatctttctt
aaacctgaaccaaatatcaaaagatacaagtttcatgattaggatgaaat
atatacatagggattttggtttattgaagttattgaaagacctagaaatt
ttctttttgagagaaagggtaaagaggtgagggttcatgtcacttaaccc
ctacccttttgggcaaattttgtctcaggtagtgtttcattccctaaatt
aatacctgtgcttccttgcctctcaccttcccagttacttctttggggta
ttactgtggcacattgtacacccagaaaaggcacatacagtgattatttt
gtgggaaatacttgattcttttgaaatatatttggagggggtagctagct
agccactgtcaaaactaatgctgtatttacaggacaaaaacaagtagagt
tggacacctcagtaagagacagcattaactaaaagtactgactgcctttt
aaaggaatttttaaaactattctcttgcaataagagatccgcatttatta
ccactggatgtcttcctttttgtggccaccaatatagcagctctcttta
gagggattccctttctgctatgccttgagccaaactggctgcttttagaa

caaaaaccataagtatggttttactcgaacttctctttgtttttattgag
agaaatattgccttctttttggtactccagctgctaaagagtgctgagat
atgttaaattatttttaaaagaaaatctccaaatacttatttaacttgt
cttctagattcatgttgcttatattttaactattgagcaagtttattata
aggaatgctgccatatagagaaagctgaagagaaaccatgggcagaaggc
tttttctgtcttattacactggaccttcactctggtaagggcatcagtt
acttttctggaataaaaggcggagttagagcacttatcagatgctgtttc
atggcttgttttagttaatttatcatgcagataactaacatttgggtatc
tttttgttttaggtcttattttctgactgttaggcctatcttacacataa
gttctactatcagtatacttggcagcatgacttttttttttttttacagt
taatttaatgaaaatcagcaccattgtaaatgatagtaattgtaacaaca
tagttactgggtgaaggagtaggggagacactcagtaaccatctatttac
ctagacacttatgcaggggtacatgctaggtggctttattcagattcggg
tacattggaatcacatcatgactttgagtctgatggctgtgtagataaag
atttaagcaagtgccttgtgtttgctggaaaatattaaaactcatttggg
tgaaagcttcccagatgatgataattaatagcacttctgcctttttcctg
gagtaacttttagattgagtcacatatgatagtataaaagtccaaaattt
tctgggtttacctttgttttatttttcatatatatattcactgtctgcct
ggaaatagccttggtttaccttattttttgtatatgtaaaactattcat
atttgattttattccaatattatatatgatggaaaagccagaattgtctg
gcagaatttaattcttccttggatctctagttctgtttttaatatcaggt
tccttcatcaaattttactactgtagctctgtgatataatttcatttctt
gtcaattatggtcacatatagacaagtacttggtcacaattagctcaaaa
gaatccccatttcttaaggtgctcagtcaatcacctttattttgagatgg
tagtttttcaagttagtattacttactagctttcttgatgaatataaaaa
tgttgtctccatcatacagagctgagcctcttgtggtatattaagataag
tcccattcttaatcacagaagaacatggatattttagtttttggagtacc
cagaattgtaaagattttgtttcattttgttttaggatagcttctctgcc
taatactataggatatgtcatgttcgttattgtagccacctccctcagat
gtttttcactatttttaaattatataatgaccagtagattcacagtagca
tgtacttgacaagtgccatggatatttaagaagaaggtaaataattttaa
tattttttgctgtaattttataggagcagtggactgtaagagaaatatct
ataaatcagagagtatttaggaaactttgtattttataggtggtcattaa
agtgatcttcaggatataagatagtttacacaaactttattctcctgata
ggaaatttttattcattttcctaccactcagcacttttgttctgccttgt
actgcttatgaggattaatgttttaaatttgctttatttgtgcttttcag
agagttgtcataattgtggcagttgtgtttgtggcactgtaaatgctgaa
attcatgggaaacttaccaaatgactgcttgagtgttatttcccattaaa
ttgggtagctgtcacaataaccagaagaaatcttgtggcccttttgaaga
aagccatgtatgttttgttacctaggttcatagtattttaatttaaaggc
aaagagaagcattctacttatttgtcttataaaccattaatcgtttttttg
tgcagaagagagcttattttgttatactgctttgtataatggaaataagt
cacattctgggggaaaaaaactggaattgtttgtattttgttttttttggg
gggggatctttatgtgaaaaatcagagctacttgttaccataagcccctta
ctatcaacaagataattatttgtaatcactttttttatcccaggttggaat
tgctttccccttctaagttatcttcccttaataatatttatgataccagg

acagtgagggtataagagcaaatgtagtgaggtattcaaaaatcctgcat
atatggactcaaaagttctttagttatttgaattatatatagctatatta
ttttattagcttgggttgtcagaagattgccaattttaagagtaaagagg
agagagataagtaataaaaatagaggaggggaagaaaatggctcccttac
tggtcttagtaatcttctatatagttaatgagctaaaaaatgatacttaa
agttccaggtttggtaccgctagcatagaaacattgcctttcctacacat
tcctcatcattgtttcctcagaaaattattttaagagtttgtgaaaccag
attaaatatctatattcagtttggcctaatttttaaaaataaatatttat
actccagcttttgtgtatttggtgtacatcaccacttatgcaaatcaagg
atcagaaaactggaggttagccatctccattatttccttttgcacattgg
gtacagtgggtggcattagtatgcactagctgcaaagtcacagcacctta
tggaaataagtatgtttattataataaaaaaaagttaagctgcatctctg
tagattatttactttgcagactgtaaagctgccctatcttttccagcaga
atttactcttccattcttaattctttttgaaatatcttaaataatttaa
cattcctttataacttcttaacagtgtcaaaactggggtagaagggattt
tattttttcccaaaagggttccatctttgctatctgttgatcagccttag
aaaatctaagtatgatcaataaattttaatggttgatggcatcctgtgtc
agctggagtagttggttgtgaatattaaaacctagtactattttccctca
gtaacatgtaattgctacatttttataagaaggtatggttagaaaaaaa
tgtgaaagatcacttaaaccaaagccagttacaaggagtaatctctcctg
ttggtttaccttcacctcagaactacaagaatattacaatacatagtgaa
tagttgtctgtaacatttctaccagttgtttcagtagcatattggtcttg
gcatttcttggcactgtggttctgctgtattatttgtgatgtcttattgt
ttgtgagcttttgttttttttttaaagaaaaaacaaaaactaagtgggac
tatgtatgtagatgtgtgtcagtaccaaaaagttattttctcaaatccct
aaatttctgatttccattcaagtcttttaaactctttcctgctgaatagc
aaagaacttttattttccactttcctatattcctaaaaagtgtgaacaat
gcgtttcagttgactgtattgcatactttttgctgaagactttttctgt
aaacacaattgccttgttcagttttgttgtaaactgacttaccataagat
gcactgttgataatgctttctgatgtgtgtttgataagagtgataaaata
aaagcttaaaaataaaggagttgctttttaatttc

>NM_206894 3
aatagaaagatcgcggaaccttccaccatcaagggaccgcagggccggct
ctgctttcccagcacgctgcctcaccaagaaagaatccagagaaggactg
gtggacttggaatgttaagtcatggcccatttgatgatgttcagggatgt
ggctgtagatttctctcaggaggagtgggagtgcctggacctggaacaga
gggatttatatagagatgtgatgttggagaactacagcaacatggtctca
ctgggtttttgcatttatcagccagaagcgttctctttattggagaaagg
gaaagagccctggaagattttgagggatgagacaagaggaccttgcccag
acatgcagtcgaggtgtcagaccaagaagttattaccaaaaaatggcatt
tttgagagagaaatagcccaattggaaataatgagaatttgtaaaaacca
cagccttgactgtttatgttttagaggtgactgggaaggcaacactcagt
ttcaaacacttcaagataatcaagaggaatgcttcaagcaggtgatacgc
acctgtgaaaaaaggcccacttttaaccagcatacagtgtttaatctaca
ccagagacttaatacaggagacaaactgaatgaatttaaagaactgggga

aagcctttatttctggttcagatcatactcaacatcagttaattcacaca
agtgagaaattctgtggagataaagaatgtgggaatacctttcttcctga
ttcagaagttattcaatatcagacagttcacactgttaagaaaacatatg
aatgtaaagaatgtgggaagtcttttagtttacgttcgagtcttactggt
cataagagaattcataccggtgagaaaccttttaaatgtaaggattgtgg
gaaagcctttagatttcattcacaacttagtgtccataagcgaattcata
ctggtgagaaatcttatgaatgtaaggaatgtgggaaggcctttagttgt
ggctcagatcttactcgacatcagagaattcatactggtgaaaaacccta
tgaatgtaatgaatgtagaaaggcctttagtcagcgatcacatcttatta
aacatcagagaattcacactggtgaaaaaccttatgaatgtaaggagtgt
gggaaagcttttactcgtggatcacacctaactcagcatcagagaattca
tactggtgagaaatctcatgagtgtaaggaatgtggaaaagcctttattc
gtggttcaaatcttgctcaacatcagaatgttcatgttggtaggaaacct
tataaatgtgagaaatgtgggaaagcctatatttggagctcacaccttgc
tcgacatcagcgaattcatactggcaggaaaccttatgaatgtaagcaat
gcgggaagacttttacttgggcttcgtatcttgctcaacatgagaaaatt
cacaatgagaggaaatcctatgaatgtaaggaatgtggaaagacctttct
tcatggctcagagtttaatcgacatcagaaaattcatactggtgagagaa
actatgaatgtaaggaatgtggaaagacctttttcgtggttcagaactt
aatcgacaccagaaaattcatactggaaagaggccatatgaatgtgaaga
atgtggaaaagcctttctctggggttcacaacttactcgacatcagagaa
tgcatactggtgaggaaccttacgtatgtaaagaatgtgggaaatctttt
atctggggttcacagcttacacgacataagaaaattcatactgatgcaga
accttatggatgcaagaaaagtagccacatctttagtcaccattcatatt
ttactgaacaaaaaattcataatagtgcaaatctctgtgaatggacagac
tatgggaacacctttagtcatgagtcaaactttgctcaacaccagaatat
ttacacttttgagaaatcctatgaatttaaagattttgagaaagcatttt
cttcaagctctcacttcatttcactcttgtgaaatattatataaaagtgt
aggaagggctttattcatgactcattaattgatgtcatttattttttct
cttgaggaacaccacaaatgtaagtaatattggaaggcatttt

>NM_213636 1
agaacactggcggccgatcccaacgaggctccctggagcccgacgcagag
cagcgccctggccgggccaagcaggagccggcatcatggattccttcaaa
gtagtgctggaggggccagcaccttggggcttccggctgcaagggggcaa
ggacttcaatgtgcccctctccatttcccggctcactcctgggggcaaag
cggcgcaggccggagtggccgtgggtgactgggtgctgagcatcgatggc
gagaatgcgggtagcctcacacacatcgaagctcagaacaagatccgggc
ctgcggggagcgcctcagcctgggcctcagcagggcccagccggttcaga
gcaaaccgcagaaggcctccgcccccgccgcggaccctccgcggtacacc
tttgcacccagcgtctccctcaacaagacggcccggccctttggggcgcc
cccgcccgctgacagcgccccgcagcagaatggacagccgctccgaccgc
tggtcccagatgccagcaagcagcggctgatggagaacacagaggactgg
cggccgcggccggggacaggccagtcgcgttccttccgcatccttgccca
cctcacaggcaccgagttcatgcaagacccggatgaggagcacctgaaga

aatcaagggaaaagtatgtcctggagctgcagagcccacgctacacccgc
ctccgggactggcaccaccagcgctctgcccacgtgctcaacgtgcagtc
gtagcccggccctctccagccggctgccctctctgcctccctctttctgt
tcctcctgcccagggcaccccttagtgcctccagcttctgcctacctca
ccccccctttcgtgcccctggcctgagcctcctgctggcctggccctggc
cgcccacctgggttcatctgacactgccttccctctttgccctgtggtac
tgctgtctgccaggtctgtgctgccttgggcatggaataaacattctcag
ccctg

>NM_000075 3
cacctcctgtccgcccctcagcgcatgggtggcggtcacgtgcccagaac
gtccggcgttcgccccgccctcccagtttccgcgcgcctctttggcagct
ggtcacatggtgagggtgggggtgagggggcctctctagcttgcggcctg
tgtctatggtcgggccctctgcgtccagctgctccggaccgagctcgggt
gtatggggccgtaggaaccggctccggggccccgataacgggccgccccc
acagcaccccgggctggcgtgagggtctcccttgatctgagaatggctac
ctctcgatatgagccagtggctgaaattggtgtcggtgcctatgggacag
tgtacaaggcccgtgatccccacagtggccactttgtggccctcaagagt
gtgagagtccccaatggaggaggaggtggaggaggccttcccatcagcac
agttcgtgaggtggctttactgaggcgactggaggcttttgagcatccca
atgttgtccggctgatggacgtctgtgccacatcccgaactgaccgggag
atcaaggtaaccctggtgtttgagcatgtagaccaggacctaaggacata
tctggacaaggcacccccaccaggcttgccagccgaaacgatcaaggatc
tgatgcgccagtttctaagaggcctagatttccttcatgccaattgcatc
gttcaccgagatctgaagccagagaacattctggtgacaagtggtggaac
agtcaagctggctgactttggcctggccagaatctacagctaccagatgg
cacttacacccgtggttgttacactctggtaccgagctcccgaagttctt
ctgcagtccacatatgcaacacctgtggacatgtggagtgttggctgtat
ctttgcagagatgtttcgtcgaaagcctctcttctgtggaaactctgaag
ccgaccagttgggcaaaatctttgacctgattgggctgcctccagaggat
gactggcctcgagatgtatccctgccccgtggagcctttcccccagagg
gccccgcccagtgcagtcggtggtacctgagatggaggagtcgggagcac
agctgctgctggaaatgctgacttttaacccacacaagcgaatctctgcc
tttcgagctctgcagcactcttatctacataaggatgaaggtaatccgga
gtgagcaatggagtggctgccatggaaggaagaaaagctgccatttccct
tctggacactgagagggcaatctttgcctttatctctgaggctatggagg
gtcctcctccatctttctacagagattactttgctgccttaatgacattc
ccctcccacctctcctttgaggcttctccttctccttcccatttctcta
cactaaggggtatgttccctcttgtccctttccctacctttatatttggg
gtcctttttatacaggaaaaacaaaacaaagaaataatggtcttttttt
tttttaatgtttcttcctctgtttggctttgccattgtgcgatttgga
aaaaccacttggaagaagggactttcctgcaaaaccttaaagactggtta
aattacagggcctaggaagtcagtggagccccttgactgacaaagcttag
aaaggaactgaaattgcttctttgaatatggattttaggcggggcgtggt
ggctcacgcctataatcccagcacgttgggaggccaacgcgggtggatca
cctgaggtcaggagttcgagaccagcctgactaacatggtgaaaccctgt

ctctactaaaaatacaaaattagtcaggcgtggtggtgcacacctgtaat
cccagctacttgggagactgaggcaggaggatcgcttgaacccgggaggc
agaggttgcggtgagccgagatcatgccattgcactccagcctgggcaac
agagcaagactctgtgtcaaaaaaaaaaaaagaatatagatttttaaatg
gcaaaaaaaaaaaaaaaaaa

>NM_002632 5
cctcgcacgcactgcgggctccggcgctgcgggctggccgggcgctgcgg
gctgaccgggcgctccgggaactcggctcgggaacctcgtctgcggtggg
cggggccggcccggagccccgccccggctcagtccctgaaacccaggcgc
ggaccggctgcagtctcagaagggagctgctgtctgcggaggaaactgca
tcgacggacggccgcccagctacgggaggacctggagtggcactgggcgc
ccgacggaccatccccgggacccgcctgcccctcggcgccccgccccgcc
gggccgctccccgtcgggttccccagccacagccttacctacgggctcct
gactccgcaaggcttccagaagatgctcgaaccaccggccggggcctcgg
ggcagcagtgagggaggcgtccagcccccactcagctcttctcctcctg
tgccaggggctccccgggggatgagcatggtggttttccctcggagcccc
ctggctcgggacgtctgagaagatgccggtcatgaggctgttcccttgct
tcctgcagctcctggccgggctggcgctgcctgctgtgcccccccagcag
tgggccttgtctgctgggaacggctcgtcagaggtggaagtggtaccctt
ccaggaagtgtggggccgcagctactgccgggcgctggagaggctggtgg
acgtcgtgtccgagtaccccagcgaggtggagcacatgttcagcccatcc
tgtgtctccctgctgcgctgcaccggctgctgcggcgatgagaatctgca
ctgtgtgccggtggagacggccaatgtcaccatgcagctcctaaagatcc
gttctggggaccggccctcctacgtggagctgacgttctctcagcacgtt
cgctgcgaatgccggcctctgcgggagaagatgaagccggaaaggaggag
acccaagggcagggggaagaggaggagagagaagcagagacccacagact
gccacctgtgcggcgatgctgttccccggaggtaacccaccccttggagg
agagagaccccgcacccggctcgtgtatttattaccgtcacactcttcag
tgactcctgctggtacctgccctctatttattagccaactgtttccctgc
tgaatgcctcgctcccttcaagacgaggggcagggaaggacaggaccctc
aggaattcagtgccttcaacaacgtgagagaaagagagaagccagccaca
gacccctgggagcttccgctttgaaagaagcaagacacgtggcctcgtga
ggggcaagctaggccccagaggccctggaggtctccaggggcctgcagaa
ggaaagaaggggccctgctacctgttcttgggcctcaggctctgcacag
acaagcagcccttgctttcggagctcctgtccaaagtagggatgcggatc
ctgctggggccgccacggcctggctggtgggaaggccggcagcgggcgga
ggggatccagccacttcccctcttcttctgaagatcagaacattcagct
ctggagaacagtggttgcctgggggcttttgccactccttgtcccccgtg
atctcccctcacactttgccatttgcttgtactgggacattgttctttcc
ggccaaggtgccaccaccctgcccccccctaagagacacatacagagtggg
ccccgggctggagaaagagctgcctggatgagaaacagctcagccagtgg
ggatgaggtcaccaggggaggagcctgtgcgtcccagctgaaggcagtgg
caggggagcaggttccccaagggccctggcacccccacaagctgtccctg
cagggccatctgactgccaagccagattctcttgaataaagtattctagt
gtggaaacgct

>NM_004402 2
ccgctactctggccctcagggttgtgtagtttccggggccacggcacctg
ggcacaccaggtggcgtcgcgcctttgctttcctgagccttctgagtaag
gtaatgtggtgtccgtggggcgacgcctgcgcacaagaccgcgtcgggcc
tcacttttcccaggccttctgggtaatgtagtttccgggatcggcacccg
gcctgtgccagcttgcagagctcaccaggtgcagacccctgcggccaggg
cgaggacggatctgagcagctgggcagcaggtgccaccgcctgtgggacc
cagagggcttgaggacatctgcaatgctccagaagcccaagagcgtgaag
ctgcgggccctgcgcagcccgaggaagttcggcgtggctggccggagctg
ccaggaggtgctgcgcaagggctgtctccgcttccagctccctgagcgcg
gttcccggctgtgcctgtacgaggatggcacggagctgacggaagattac
ttccccagtgttcccgacaacgccgagctggtgctgctcaccttgggcca
ggcctggcagggctatgtgagcgacatcaggcgcttcctcagtgcatttc
acgagccacaggtggggctcatccaggccgcccagcagctgctgtgtgat
gagcaggccccacagaggcagaggctgctggctgacctcctgcacaacgt
cagccagaacatcgcggccgagacccgggctgaggacccgccgtggtttg
aaggcttggagtcccgatttcagagcaagtctggctatctgagatacagc
tgtgagagccggatccggagttacctgagggaggtgagctcctacccctc
cacggtgggtgcggaggctcaggaggaattcctgcgggtcctcggctcca
tgtgccagaggctccggtccatgcagtacaatggcagctacttcgacaga
ggagccaagggcggcagccgcctctgcacaccggaaggctggttctcctg
ccagggtccctttgacatggacagctgcttatcaagacactccatcaacc
cctacagtaacagggagagcaggatcctcttcagcacctggaacctggat
cacataatagaaaagaaacgcaccatcattcctacactggtggaagcaat
taaggaacaagatggaagagaagtggactgggagtatttttatggcctgc
tttttacctcagagaacctaaaactagtgcacattgtctgccataagaaa
accacccacaagctcaactgtgacccaagcagaatctacaaaccccagac
aaggttgaagcggaagcagcctgtgcggaaacgccagtgacacgtacaca
ccacgtcctggtctttgtttgaggcctgacgtgggcatcattttaacagg
tgcctttttttgtttttttgtttttcgtttttttggtcactccagtagctc
ctggaaaaaaccttaaaaaatgtttcctccaaatctgatttcattacatt
tctgaattgttggggttttttttgttgttttgttttgttttgtagatgga
gtttcacttttgttgcccaggctggagtgtagtggcgcgatctcggctca
gcctcccgagtagctgggattacaggcatgtgccaccacgcccggctaat
gtttgtatttttagtagagacggggtttcaccatgttggtcaggctggtc
tcaaactcctgacctcaggtgatccgcccacctcagcctcccaaagtgct
gggatgacaggtgtgagccactgcgcccagcctgaatcatttcttatacc
ttctgacagcccaacttccagaggacagctctggggtactcgttggatgt
ctgtgagtacctggtcatacgggtcagtagggataagaattgtctctggg
ctgaggaattcttctgttctctggtttcaccagcgttgggtttgctcatg
taatgtggtcaccatactcaaatggtgtcatggctgaagttggccacctt
gcttgagggacaagttgtttatgtatcagctctctgctgggtctcccttt
ccatggcaaatgggcagctccatcctcttgactcttctaaatgcccaaaa
gaggtgtcatgctttgggggtacgatgtttatactccgtaaagaacatac
aaggacattcactgctgatttttttttttgtttgtttgagacagggtctc

actctgtcgctcaggctggagtgcagtgatgcaatcttggctcactgcaa
cctccgcctctcaggttcaagtggttctcctgcctcagcctcccaagtag
ctgggattacaggcacctaccaccagggccagctaatttttgtatgttta
gtagaaacggggtttcaccatgttggccaggctgttctcgaactcctgac
ctcaggtgatctgcccgcctcggtctcccaaagtgctgggattacaggca
tgagccactgcacctgacctgctgaattgtttataatggcaagaaatagg
aaacccccaatgtctgttgaacagctatcacgttgaaccacgtgaaact
gctgttttctaggccaaaaatggtgagcgatcatttatttcatgattcaa
cctgatacatttacatagtgcaaaactgtgtcacagtttcaggcttttat
gaggaaagcgtttctgtgtagaaactggaagctgttcagggcatcggcag
ctgaaccctgctccgttggtcagcgttactatcatctcggatcatatgga
gctcatgtcagccgtgtgggtggcgggtgcacagagacggtctggaagga
aacacgcggatctgaacagcagtaatcctgggggatacgggggttgggct
agattacagagggctcattttctacgtcatgtattttatgatacttgaat
tttttgaaatgggcatttattttataacatgttaaaatgtactttttaaa
ttaagtcattttgtaatatttgaatttttacatttgttgtacaatcagga
aaagcaataaagatttttcaaaaatagaaaaaaa

>NM_030935 3
ggaggaggaggaggaggagaggagcagtcagcaggcccgaggaggcagga
cttcctggtgtgggggttgtcaacagcccagaaagagaaagacggagagg
cagagacccaggggagccgaggagcggagacacggacccagcagggtcac
gcgggaggagactcgggacacccactcgctggctgccaccgggagcagag
gggggctcggccgcccctcccacatcagtctccagacccttggggcttc
agaggccgctcctaggctcctcggaggaggcgcgggttccttcttttccg
gagagcaaactttcctcggagatgcctggcaggatgagccgcggcccccg
gagaggacgggacagcctctgatcgccgcctgcgtgccccgccactgccc
atggccaggaggccccagggaacggacagcccgacctcttaggcgccggg
accctcaggctcagatcagtggccgaggccccagagtttgcaaactcagc
tctggagttcagcagcaacagcagcaggaaaaacctgcccctgctccccc
ctcccgccacctcccctctcctcttctcccctcacccagcaggcaccccc
ggttcccgccaggccctcctgccatgtcggacccagacgtccccaggggc
tcggatgtccccgccatgtggccccccttgttccaggggtgcctgagcccc
ttcaaggagccccaacccacccccaaccttggcccagccctgagccccag
ggaccatgagcgggggcaagaagaagagtagtttccaaatcaccagcgtc
accacggactatgagggccctgggagcccaggggcttcggatccccctac
cccacagcccccaaccgggcccccgccccgcctgcccaatggggagccca
gccccgatccgggggcaagggcacccccggaatggctccccaccacct
ggggccccttcctcccgtttccgggtggtgaagctgccccacggcctggg
agagccttatcgccgcggtcgctggacgtgtgtggatgtttatgagcgag
acctggagccccacagcttcggcggactcctggagggaattcgaggggcc
tcagggggcgccgggggcagatctttggattccaggttggagctggccag
cctcggcctgggcgcccccaccccaccgtcaggcctgtctcagggcccca
cctcctggctccgtccacccccacctctcctggacctcaggcccgctcc
ttcactgggggactgggccagctggtggtgcccagcaaagccaaggcaga
gaaacccccactgtcggcctcctcaccccagcagcgcccccagagcctg

agaccggtgagagtgcgggcacatcccgggctgccacgcccctgccctct
ctgagggtggaagcggaggctgggggctcaggggccaggacccctccact
gtcccggaggaaagctgtagacatgcggctgcggatggagttgggtgctc
cagaagagatggggcaggtgcccccacttgactctcgccccagctccccg
gccctctacttcacccacgatgccagcctggttcacaaatctccagaccc
cttcggagcagtagcagctcagaagttcagcctggcccactccatgttgg
ccatcagtggtcacctagacagcgacgatgatagtggctccggaagcctg
gttggcattgacaacaaaatcgagcaagccatggacttggtgaagtccca
cctcatgtttgcggtccgggaggaggtggaggtgctgaaggagcagatcc
gggaactggcggagcggaacgctgcgctggagcaggagaatgggctgctg
cgcgccctggccagcccggagcagctggctcagctgccctcctcgggggt
cccacggcttgggccccctgcgcccaatgggccctccgtctgagcctccc
ttcccttacaatgtgcctttggggctgcccggccttgcgtcagccgcctg
ccccctcttcctatgcagctttaatgtccccgtgtccccggggtgggagt
tcaaggctcagtaatggcctggtcccccggcccctgccccatctcctcat
catccccagccttgatggaggagggagggcttcaggacggggcgtcagag
ggagccccctctgggagggaaccaacccccaccctccctcctgggacccc
ccagcagtagacggcttgggggagtcggaggctccccggcagacacccca
cccccatcttgttcccttgaggtgcctcctctcctctgcccaggggaggg
agtgtggacagtatctggaagttctgggattcaggttgttattaaaataa
taataataattaaaaactctgaagaaacttgaaaaaaaaaaaaaaaaaa

>NM_178507 2
cccggggccgcggagccgggccggggcagcgccgtctccgcctcggggcc
gccgggggcgccctgctgagcgctacccacgtgcgtccgcgccacctcgc
gggcgaccccgcggccaaggcccccggcggagcggctcccgggcgccccg
aactagcccccaactttgggcgaagtttgcctgcgcctctccccgccccc
acgcggcgcgccggggccgcggacggcagcggcccccggggatgcgcctt
cccggggtaccccctggcgcgccctgcgctgctgctgctgctgccgctgct
cgcgccgctgctgggaacgggtgcgccggccgagctgcgggtccgcgtgc
ggctgccggacggccaggtgaccgaggagagcctgcaggcggacagcgac
gcggacagcatcagcctcgagctgcgcaagcccgacggcaccctcgtctc
cttcaccgccgacttcaagaaggatgtgaaggtcttccgggccctgatcc
tgggggagctggagaaggggcagagtcagttccaggccctctgctttgtc
acccagctgcagcacaatgagatcatccccagtgaggccatggccaagct
ccggcagaaaaatccccgggcagtgcggcaggcggaggaggttcggggtc
tggagcatctgcacatggatgtcgctgtcaacttcagccagggggccctg
ctgagcccccatctccacaacgtgtgtgccgaggccgtggatgccatcta
cacccgccaggaggatgtccggttctggctggagcaaggtgtggacagtt
ctgtgttcgaggctctgcccaaggcctcagagcaggcggagctgcctcgc
tgcaggcaggtgggggaccacgggaagccctgcgtctgccgctatggcct
gagcctggcctggtacccctgcatgctcaagtactgccacagccgcgacc
ggcccacgccctacaagtgtggcatccgcagctgccagaagagctacagc
ttcgacttctacgtgccccagaggcagctgtgtctctgggatgaggatcc
ctacccaggctagggtgggagcaacctggcgggtggctgctctgggccca
ctgctcttcaccagccactagagggggtggcaacccccacctgaggcctt

atttccctccctccccactcccctggccctagagcctgggcccctctggc
cccatctcacatgactgtgaagggggtgtggcatggcagggggtctcatg
aaggcacccccattcccaccctgtgccttccttgcgggcagagagggaga
gaagggctccccagatctacacccctccctcctgcatctcccctggagtg
ttcacttgcaagctgccaaaacatgatggcctctggttgttctgttgaac
tccttgaacgtttagaccctaaaaggagtctatacctggacacccacctc
cccagacacaactcccttccccatgcacacatctggaaggagctggcccc
tcagtcccttcctactccccaacaaggggctcactatccccaaagaagga
gctgttggggacccacgacgcagcccctgtactggattacagcatattct
catctctggccccgaggctgcctgtggggcgagtggagacctcccatcac
tgagacagatcacagaccacgagtgcctttcccggacctggacgttgcct
ccagagcaggcaccagctctttccctctctacacagaaatatttttgtaa
ggttctggggcagggagggagcatgaagtacgaggaaaacttgaattcca
gatttttagtgcaaagtatttatcatttctaccagaaataaacgttttaa
gtttttacttgaaaaaaaaaaaaaaaaaaaaaaaaaaaaaaaaaaaaaaa
aaaaaaaaaaaaaaaaaaaaaaaaaaaaaaaaaaaaaaaaaaaaaaaaaa
aaaaaaa

>NM_001084 4
taggcttcctccctccctgtcgccgccagcctctcgctctccccgtcttc
cgcccgcactctctgggcagggctgcggccaggacccgcccccgcgtccc
gcccgaccctccgccaggggtcacttcccctgtccaggtttcagcttcca
catgtgtcaagcggctggctcagcccagagtccctgtctcccgcccgccg
gcccgagccgccgcccctcccccgcctcccgtgcgcccgggacaatcctc
gccttgtctgtggcgccggcatctggagctttctgtagcctccggatacg
ccttttttttcagggcgtagccccagccaagctgctccccgcggcggccgc
acagcagcccgagcgcccccctttccagagctcccctccggagctgggatc
caggcgcgtagcggagatcccaggatcctgggtgctgtctgggcccgctc
cccaccatgacctcctcggggcctggaccccggttcctgctgctgctgcc
gctgctgctgccccctgcggcctcagcctccgaccggccccggggccgag
acccggtcaacccagagaagctgctggtgatcactgtggccacagctgaa
accgaggggtacctgcgtttcctgcgctctgcggagttcttcaactacac
tgtgcggaccctgggcctgggagaggagtggcgagggggtgatgtggctc
gaacagttggtggaggacagaaggtccggtggttaaagaaggaaatggag
aaatacgctgaccgggaggatatgatcatcatgtttgtggatagctacga
cgtgattctggccggcagccccacagagctgctgaagaagttcgtccaga
gtggcagccgcctgctcttctctgcagagagcttctgctggcccgagtgg
gggctggcggagcagtaccctgaggtgggcacggggaagcgcttcctcaa
ttctggtggattcatcggttttgccaccaccatccaccaaatcgtgcgcc
agtggaagtacaaggatgatgacgacgaccagctgttctacacacggctc
tacctggacccaggactgagggagaaactcagccttaatctggatcataa
gtctcggatctttcagaacctcaacggggctttagatgaagtggttttaa
agtttgatcggaaccgtgtgcgtatccggaacgtggcctacgacacgctc
cccattgtggtccatggaaacggtcccactaagctgcagctcaactacct
gggaaactacgtccccaatggctggactcctgagggaggctgtggcttct
gcaaccaggaccggaggacactcccgggggggcagcctccccccgggtg

tttctggccgtgtttgtggaacagcctactccgtttctgccccgcttcct
gcagcggctgctactcctggactatcccccgacagggtcaccctttttcc
tgcacaacaacgaggtcttccatgaaccccacatcgctgactcctggccg
cagctccaggaccacttctcagctgtgaagctcgtggggccggaggaggc
tctgagcccaggcgaggccagggacatggccatggacctgtgtcggcagg
accccgagtgtgagttctacttcagcctggacgccgacgctgtcctcacc
aacctgcagaccctgcgtatcctcattgaggagaacaggaaggtgatcgc
ccccatgctgtcccgccacggcaagctgtggtccaacttctggggcgccc
tgagccccgatgagtactacgcccgctccgaggactacgtggagctggtg
cagcggaagcgagtgggtgtgtggaatgtaccatacatctcccaggccta
tgtgatccggggtgataccctgcggatggagctgccccagagggatgtgt
tctcgggcagtgacacagacccggacatggccttctgtaagagctttcga
gacaagggcatcttcctccatctgagcaatcagcatgaatttggccggct
cctggccacttccagatacgacacggagcacctgcaccccgacctctggc
agatcttcgacaaccccgtcgactggaaggagcagtacatccacgagaac
tacagccgggccctggaaggggaaggaatcgtggagcagccatgcccgga
cgtgtactggttcccactgctgtcagaacaaatgtgtgatgagctggtgg
cagagatggagcactacggccagtggtcaggcggccggcatgaggattca
aggctggctggaggctacgagaatgtgcccaccgtggacatccacatgaa
gcaggtggggtacgaggaccagtggctgcagctgctgcggacgtatgtgg
gccccatgaccgagagcctgtttcccggttaccacaccaaggcgcgggcg
gtgatgaactttgtggttcgctaccggccagacgagcagccgtctctgcg
gccacaccacgactcatccaccttcaccctcaacgttgccctcaaccaca
agggcctggactatgagggaggtggctgccgcttcctgcgctacgactgt
gtgatctcctccccgaggaagggctgggcactcctgcaccccggccgcct
cacccactaccacgaggggctgccaacgacctggggcacacgctacatca
tggtgtcctttgtcgacccctgacactcaaccactctgccaaacctgccc
tgccattgtgcctttttagggggcctggcccccgtcctgggagttggggg
atgggtctctctgtctccccacttcctgagttcatgttccgcgtgcctga
actgaatatgtcaccttgctcccaagacacggccctctcaggaagctccc
ggagtccccgcctctctcctccgcccacaggggttcgtgggcacagggct
tctggggactccccgcgtgataaattattaatgttccgcagtctcactct
gaataaaggacagtttgtaagtcttgaaaaaaaaaaaaaaaaaaa

>NM_004706 3
agagccaggaagcgggagccgggacccagggcccgggatcgccgagcccg
acctcgggcgccccgccggtcacctccgcgcggacaccagccctgcagag
cccagggagatggaagacttcgcccgaggggcggcctccccaggcccctc
ccggcctggcctggttcccgtcagcatcatcggggctgaggatgaggatt
ttgagaacgagctggagacaaactcagaagagcaaaacagccagttccag
agcctggagcaggtgaagcggcgcccagcccacctcatggccctcctgca
gcacgtggccctgcagtttgagccaggaccccctgctttgctgtctgcatg
ccgacatgctgggctcactgggccccaaggaggccaagaaggccttcctg
gacttctaccacagcttcctggagaagacagcggttctccgggtgccggt
ccctcccaacgtcgcctttgaacttgaccgcactagggctgacctcatct
ccgaggatgtccagcggcggttcgtgcaggaggtggtgcaaagccagcag

gtagccgtgggccggcagctggaggacttccgttccaagcggctcatggg
catgacgccctgggagcaggagctggcccagctggaggcttgggttgggc
gggaccgagccagctacgaggcccgggagcggcacgtggcggagcggctg
ctcatgcacctggaggagatgcaacataccatctctaccgacgaagaaaa
gagtgctgccgtggtcaacgccattggcctgtacatgcgccaccttgggg
tgcggaccaagagtggagacaagaagtcggggaggaacttcttccggaaa
aaggtgatggggaaccggcggtcggacgagcctgccaagaccaagaaggg
gctgagcagcatcctggatgccgcccgctggaaccggggagagccccagg
ttccagattttcgacacctcaaagcagaggttgatgccgagaagccaggt
gctacagaccggaagggaggcgtggggatgccctctcgggaccggaatat
cggggctcctgggcaggacacccctggagtctctctgcaccctctgtccc
tggacagcccagaccgggaaccaggtgctgacgccccctggagctgggg
gactcatccccgcagggcccaatgagcctggagtccttggcgcccccaga
gagtaccgacgagggggccgaaaccgagagccccgagcctggagatgagg
gggagccggggcggtcgggactggagcttgaaccagaagagcctcccggc
tggcgggaactcgtcccccagacaccctgcacagcctgcccaagagcca
ggtgaagcggcaggaggtcatcagcgagctgctggtgacagaggcggccc
acgtgcgcatgctgcgggtgctgcacgacctcttcttccagcccatggca
gaatgcctgttcttccccttggaggagctgcagaacatcttccccagcct
ggacgagctcatcgaggtgcattccctgttcctcgatcgcctgatgaagc
ggaggcaggagagtggctacctcatcgaggagatcggagacgtgctgctg
gcccggtttgatggtgctgagggctcctggttccagaaaatctcctcccg
cttctgcagccgccagtcatttgccttagagcagctcaaagccaagcaac
gcaaggaccctcggttctgtgccttcgtgcaggaagctgagagccgcccg
cggtgccgccgcctgcagctgaaggacatgatccccacggagatgcagcg
gctgaccaagtacccccctgctcctgcagagcatcgggcagaacacagaag
agcccacagaacgggagaaagtggagctggcagccgagtgctgccgggaa
attctacaccacgtcaaccaagccgtgcgtgacatggaggacctgctgag
gctcaaggactatcagcggcgcctggacttgtcccaccttcggcagagca
gcgaccctatgctgagcgagttcaagaacctggacatcaccaagaagaaa
ttggtccacgagggcccactgacgtggcgggtgactaaggacaaggcagt
ggaggtgcatgtgctgctgctggacgacctgctgctgctgctccagcgcc
aggacgagcggctgctgctcaagtcccatagccggacactgacgcccacg
cccgatggcaagaccatgctgcggcccgtgctgcggctcacctccgccat
gacccgcgaggtggccaccgatcacaaagccttctacgtcctttttacct
gggaccaggaggcccagatatacgagctggtggcacagactgtgtcggag
cggaaaaactggtgtgctctcatcactgagactgccggatccctgaaagt
ccctgcccctgcctctcgccctaagccccggcccagcccgagcagcaccc
gagaacccctcctcagcagctctgagaacggcaatggtggccgagagacg
tctccagctgatgcccggaccgagagaatcctcagtgacctcctgccctt
ctgcagaccaggccccgagggccagctcgctgccacggcccttcggaaag
tgctgtccctgaagcagcttctgtttccggcggaggaagacaatggggcg
gggcctcctcgagatggggatggggtcccagggggcggcccccctgagccc
agcacggacccaggaaatccaggagaacctgctcagcttggaggagacca
tgaagcagctggaggagttggaggaggaattttgccgcctgagacccctc
ctgtctcagcttgggggggaactctgtcccccagcctggctgcacttgagg

ttcccgcccaggaaggccttttgcaagaaggagaggaatgggggagagga
cgtgagggaccacccccacccacacagctgccgcagcatctcacaccccg
agggcctgaggagagggagctgtgggccacgcctgggaggggcccagctg
gggttactggccccgcatgagcctcggccatctctccctcctgccctctg
cttgggggactcagggctccattctggagggcaccacggtgacccgggcc
atctcagtattgcctgtgggggccacccctccacccccacccccaagtgc
cttcgctctgtttttataccctgaattggaggtttattttttaatatata
ttatctaagaagaaaaaaaaaaaaaaaaaaaaaaa

>NM_006289 3
ggaagagttctagcctgaaagggaactcgggcgtcgtcctggcgtcctct
ccggattgcgccgcaccctcgcttcctgccagggaggggctgccgggctt
tggcggctcccgagcatcgagaacggggccagagcagcttcctgcctgcc
ccccgcgaccatagagcgcgggcccagggcgccgcccgcgggtgggggac
gttcccaggacggaagtggccgagagagtgtcgaagggagggcgaggccg
gagcccgagggcgacccgagaagcggcggggcggcgggccggcgggcggg
gcgcagagccaggcagcgcaggtatagccaggctggagaaaagaagctgc
caccatggttgcactttcactgaagatcagcattgggaatgtggtgaaga
cgatgcagtttgagccgtctaccatggtgtacgacgcctgccgcatcatt
cgtgagcggatcccagaggccccagctggtcctcccagcgactttgggct
ctttctgtcagatgatgaccccaaaaagggtatatggctggaggctggga
aagctttggactactacatgctccgaaatggggacactatggagtacagg
aagaaacagagacccctgaagatccgtatgctggatggaactgtgaagac
gatcatggtggatgactctaagactgtcactgacatgctcatgaccatct
gtgcccgcattggcatcaccaatcatgatgaatattcattggttcgagag
ctgatggaagagaaaaaggaggaaataacagggaccttaagaaaggacaa
gacattgctgcgagatgaaaagaagatggagaaactaaagcagaaattgc
acacagatgatgagttgaactggctggaccatggtcggacactgagggag
cagggtgtagaggagcacgagacgctgctgctgcggaggaagttctttta
ctcagaccagaatgtggattcccgggaccctgtacagctgaacctcctgt
atgtgcaggcacgagatgacatcctgaatggctcccaccctgtctccttt
gacaaggcctgtgagtttgctggcttccaatgccagatccagtttgggcc
ccacaatgagcagaagcacaaggctggcttccttgacctgaaggacttcc
tgcccaaggagtatgtgaagcagaagggagagcgtaagatcttccaggca
cacaagaattgtgggcagatgagtgagattgaggccaaggtccgctacgt
gaagctagcccgttctctcaagacttacggtgtctccttcttcctggtga
aggaaaaaatgaaagggaagaacaagctagtgcccaggcttctgggcatc
accaaggagtgtgtgatgcgagtggatgagaagaccaaggaagtgatcca
ggagtggaacctcaccaacatcaaacgctgggctgcgtctcccaaaagct
tcaccctggattttggagattaccaagatggctattactcagtacagaca
actgaaggggagcagattgcacagctcattgccggctacatcgatatcat
cctgaagaagaaaaaaagcaaggatcactttgggctggaaggagatgagg
agtctactatgctggaggactcagtgtcccccaaaaagtcaacagtcctg
cagcagcaatacaaccgggtggggaaagtggagcatggctctgtggccct
gcctgccatcatgcgctctggagcctctggtcctgagaatttccaggtgg
gcagcatgccccctgcccagcagcagattaccagcggccagatgcaccga

ggacacatgcctcctctgacttcagcccagcaggcactcactggaaccat
taactccagcatgcaggccgtgcaggctgcccaggccaccctggatgact
ttgacactctgccgcctcttggccaggatgctgcctctaaggcctggcgt
aaaaacaagatggatgaatcaaagcatgagatccactctcaggtagatgc
catcacagctggtactgcgtctgtggtgaacctgacagcaggggaccctg
ctgagacagactataccgcagtgggctgtgcagtcaccacaatctcctcc
aacctgacggagatgtcccgtggggtgaagctgctggctgccttgctgga
ggacgaaggcggcagtggtcggcccctgttgcaggcagcaaagggccttg
cgggagcagtgtcagaactgctgcgcagtgcccaaccagccagtgctgag
ccccgtcagaacctgctgcaagcagctgggaacgtgggccaggccagtgg
ggagctgttgcaacaaattggggaaagtgatactgacccccacttccagg
atgcgctaatgcagctcgccaaagctgtggcaagtgctgcagctgccctg
gtcctcaaggccaagagtgtggcccagcggacagaggactcgggacttca
gacccaagttattgctgcagcaacacagtgtgccctatccacttcccaac
tagtggcctgtactaaggtggtggcacctacaatcagctcacctgtctgc
caagagcaactggtggaggctggacgactggtagccaaagccgtggaggg
ctgtgtgtctgcctcccaggcagctacagaggatgggcaactgttgcgag
gggtaggagcagcagccacagctgtcacccaggccctaaatgagctgctg
cagcatgtgaaagcccatgccacaggggctgggcctgctggccgttatga
ccaggctactgacaccatcctaaccgtcactgagaacatctttagctcca
tgggtgatgctggggagatggtgcgacaggcccgcatcctggcccaagcc
acatctgacctggtcaatgccatcaaggctgatgctgagggggaaagtga
tctggagaactcccgcaagctcttaagtgctgccaagatcctagctgatg
ccacagccaagatggtagaggctgccaagggagcagctgcccaccctgac
agtgaggagcagcagcagcggctgcgggaggcagctgaggggctgcgcat
ggccaccaatgcagctgcgcagaatgccatcaagaaaaagctggtgcagc
gcctggagcatgcagccaagcaggctgcagcctcagccacacagaccatc
gctgcagctcagcacgcagcctctacccccaaggcctctgccggcccca
gcccctgctggtgcagagctgcaaggcagtggcagagcagattccactgc
tggtgcagggcgtccgaggaagccaagcccagcctgacagccccagcgct
cagcttgccctcattgctgccagccagagcttcctgcagccaggtgggaa
gatggtggcagctgcaaaggcctcagtgccaacgattcaggaccaggctt
cagccatgcagctgagtcagtgtgccaagaacctgggcaccgcgctggct
gaactccggacggctgcccagaaggctcaggaagcatgtggacctttgga
gatggattctgcactgagtgtggtacagaatctagagaaagatctacagg
aagtgaaggcagcagctcgagatggcaagcttaaacccttacctggggag
acaatggagaagtgtacccaggacctgggcaacagcaccaaagccgtgag
ctcagccatcgcccagctactgggagaggttgcccagggcaatgagaatt
atgcaggtattgcagctcgggatgtggcaggtgggctgcggtcactggcc
caggccgctaggggagtcgctgcactgacgtcagatcctgcagtgcaggc
cattgtacttgatacggccagtgatgtgctggacaaggccagcagcctca
ttgaggaggcgaaaaaggcagctggccatccaggggaccctgagagccag
cagcggcttgcccaggtggctaaagcagtgacccaggctctgaaccgctg
tgtcagctgcctacctggccagcgcgatgtggataatgccctgagggcag
ttggagatgccagcaagcgactcctgagtgactcgcttcctcctagcact
gggacatttcaagaagctcagagccggttgaatgaagctgctgctgggct

gaatcaggcagccacagaactggtgcaggcctctcggggaacccctcagg
acctggctcgagcctcaggccgatttggacaggacttcagcaccttcctg
gaagctggtgtggagatggcaggccaggctccgagccaggaggaccgagc
ccaagttgtgtccaacttgaagggcatctccatgtcttcaagcaaacttc
ttctggctgccaaggccctgtccacggaccctgctgcccctaacctcaag
agtcagctggctgcagctgccagggcagtaactgacagcatcaatcagct
catcactatgtgcacccagcaggcacccggccagaaggagtgtgataacg
ccctgcgggaattggagacggtccgggaactcctggagaacccagtccag
cccatcaatgacatgtcctactttggttgcctggacagtgtaatggagaa
ctcaaaggtgctgggcgaggccatgactggcatctcccaaaatgccaaga
acggaaacctgccagagtttggagatgccatttccacagcctcaaaggca
ctttgtggcttcaccgaggcagctgcacaggctgcatatctggttggtgt
ctctgaccccaatagccaagctggacagcaagggctagtggagcccacac
agtttgcccgtgcaaaccaggcaattcagatggcctgccagagtttggga
gagcctggctgtacccaggcccaggtgctctctgcagccaccattgtggc
taaacacacctctgcactgtgtaacagctgtcgcctggcttctgcccgta
ccaccaatcctactgccaagcgccagtttgtacagtcagccaaggaggtg
gccaacagcacagctaatcttgtcaagaccatcaaggcgctagatggggc
cttcacagaggagaaccgtgcccagtgccgagcagcaacagcccctctgc
tggaggctgtggacaatctgagtgcctttgcgtccaaccctgagttctcc
agcattcctgcccagatcagccctgagggtcgggctgccatggagcccat
tgtgatctctgccaagacaatgttagagagtgccgggggactcatccaga
cagcccgggccctcgcagtcaatccccgggacccccgagctggtcggtg
ctggccggccactcccgtactgtctcagactccatcaagaagctaattac
aagcatgagggacaaggctccagggcagctggagtgtgaaacggccattg
cagctctgaacagttgtctacgggacctagaccaggcttccctcgctgca
gtcagccagcagcttgctccccgtgagggaatctctcaagaggccttgca
cactcagatgctcactgcagtccaagagatctcccatctcattgagccgc
tggccaatgctgcccgggctgaagcctcccagctgggacacaaggtgtcc
cagatggcgcagtactttgagccgctcaccctggctgcagtgggtgctgc
ctccaagaccctgagccacccgcagcagatggcactcctggaccagacta
aaacattggcagagtctgccctgcagttgctatacactgccaaggaggct
ggtggtaacccaaagcaagcagctcacacccaggaagccctggaggaggc
tgtgcagatgatgaccgaggccgtagaggacctgacaacaaccctcaacg
aggcagccagtgctgctggggtcgtgggtggcatggtggactccatcacc
caggccatcaaccagctagatgaaggaccaatgggtgaaccagaaggttc
cttcgtggattaccaaacaactatggtgcggacagccaaggccattgcag
tgaccgttcaggagatggttaccaagtcaaacaccagcccagaggagctg
ggccctcttgctaaccagctgaccagtgactatggccgtctggcctcgga
ggccaagcctgcagcggtggctgctgaaaatgaagagataggttcccata
tcaaacaccgggtacaggagctgggccatggctgtgccgctctggtcacc
aaggcaggcgccctgcagtgcagccccagtgatgcctacaccaagaagga
gctcatagagtgtgcccggagagtctctgagaaggtctcccacgtcctgg
ctgcgctccaggctgggaatcgtggcacccaggcctgcatcacagcagcc
agcgctgtgtctggtatcattgctgacctcgacaccaccatcatgttcgc
cactgctggcacgctcaatcgtgagggtactgaaactttcgctgaccacc

gggagggcatcctgaagactgcgaaggtgctggtggaggacaccaaggtc
ctggtgcaaaacgcagctgggagccaggagaagttggcgcaggctgccca
gtcctccgtggcgaccatcacccgcctcgctgatgtggtcaagctgggtg
cagccagcctgggagctgaggaccctgagacccaggtggtactaatcaac
gcagtgaaagatgtagccaaagccctgggagacctcatcagtgcaacgaa
ggctgcagctggcaaagttggagatgaccctgctgtgtggcagctaaaga
actctgccaaggtgatggtgaccaatgtgacatcattgcttaagacagta
aaagccgtggaagatgaggccaccaaaggcactcgggccctggaggcaac
cacagaacacatacggcaggagctggcggttttctgttccccagagccac
ctgccaagacctctaccccagaagacttcatccgaatgaccaagggtatc
accatggcaaccgccaaggccgttgctgctggcaattcctgtcgccagga
agatgtcattgccacagccaatctgagccgccgtgctattgcagatatgc
ttcgggcttgcaaggaagcagcttaccacccagaagtggcccctgatgtg
cggcttcgagccctgcactatggccgggagtgtgccaatggctacctgga
actgctggaccatgtactgctgaccctgcagaagccaagcccagaactga
agcagcagttgacaggacattcaaagcgtgtggctggttccgtcactgag
ctcatccaggctgctgaagccatgaagggaacagaatgggtagacccaga
ggaccccacagtcattgctgagaatgagctcctgggagctgcagccgcca
ttgaggctgcagccaaaaagctagagcagctgaagccccgggccaaaccc
aaggaggcagatgagtccttgaactttgaggagcagatactagaagctgc
caagtccattgcagcagccaccagtgcactggtaaaggctgcgtcggctg
cccagagagaactagtggcccaagggaaggtgggtgccattccagccaat
gcactggacgatgggcagtggtcccagggcctcatttctgctgcccggat
ggtggctgcggccaccaacaatctgtgtgaggcagccaatgcagctgtac
aaggccatgccagccaggagaagctcatctcatcagccaagcaggtagct
gcctccacagcccagctccttgtggcctgcaaggtcaaggctgaccagga
ctcggaggcaatgaaacgacttcaggctgctggcaacgcagtgaagcgag
cctcagataatctggtgaaagcagcacagaaggctgcagcctttgaagag
caggagaatgagacagtggtggtgaaagagaagatggttggcggcattgc
ccagatcatcgcagcacaggaagaaatgcttcggaaggaacgagagctgg
aagaggcgcggaagaaactggcccagatccggcagcagcagtacaagttt
ctgccttcagagcttcgagatgagcactaaagaagcctcttctatttaat
gcagacccggcccagagactgtgcgtgccactaccaaagccttctgggct
gtcggggcccaacctgcccaaccccagcactccccaaagtgcctgccaaa
ccccagggcctggccccgcccagtcccgcagtacatcccctgtcccctcc
ccaaccccaagtgccttcatgccctagggcccccaagtgcctgcccctc
cccagagtattaacgctccaagagtattattaacgctgctgtacctcgat
ctgaatctgccggggccccagcccactccaccctgccagcagcttccggc
cagtccccacagcctcatcagctctcttcaccgtttttgatactatctt
cccccacccccagctacccataggggctgcagagttataagccccaaaca
ggtcatgctccaataaaaatgattctacctacaa

>NM_004710 3
ggcggcggcagcggcggcgacggcgacatggagagcggggcctacggcgc
ggccaaggcgggcggctccttcgacctgcggcgcttcctgacgcagccgc
aggtggtggcgcgcgccgtgtgcttggtcttcgccttgatcgtgttctcc

tgcatctatggtgagggctacagcaatgcccacgagtctaagcagatgta
ctgcgtgttcaaccgcaacgaggatgcctgccgctatggcagtgccatcg
gggtgctggccttcctggcctcggccttcttcttggtggtcgacgcgtat
ttcccccagatcagcaacgccactgaccgcaagtacctggtcattggtga
cctgctcttctcagctctctggaccttcctgtggtttgttggtttctgct
tcctcaccaaccagtgggcagtcaccaacccgaaggacgtgctggtgggg
gccgactctgtgagggcagccatcaccttcagcttcttttccatcttctc
ctggggtgtgctggcctccctggcctaccagcgctacaaggctggcgtgg
acgacttcatccagaattacgttgaccccactccggaccccaacactgcc
tacgcctcctacccaggtgcatctgtggacaactaccaacagccacccctt
cacccagaacgcggagaccaccgagggctaccagccgccccctgtgtact
gagcggcggttagcgtgggaagggggacagagagggccctcccctctgcc
ctggactttcccatgagcctcctggaactgccagcccctctctttcacct
gttccatcctgtgcagctgacacacagctaaggagcctcatagcctggcg
ggggctggcagagccacaccccaagtgcctgtgcccagagggcttcagtc
agccgctcactcctccagggcacttttaggaaagggtttttagctagtgt
ttttcctcgcttttaatgacctcagccccgcctgcagtggctagaagcca
gcaggtgcccatgtgctactgacaagtgcctcagcttccccccggcccgg
gtcaggccgtgggagccgctattatctgcgttctctgccaaagactcgtg
ggggccatcacacctgccctgtgcagcggagccggaccaggctcttgtgt
cctcactcaggtttgcttcccctgtgcccactgctgtatgatctgggggc
caccaccctgtgccggtggcctctgggctgcctcccgtggtgtgagggcg
gggctggtgctcatggcacttcctccttgctcccacccctggcagcaggg
aagggctttgcctgacaacacccagctttatgtaaatattctgcagttgt
tacttaggaagcctggggagggcaggggtgccccatggctcccagactct
gtctgtgccgagtgtattataaaatcgtgggggagatgcccggcctggga
tgctgtttggagacggaataaatgttttctcattcagtctccagtcattg
gttgagccacagcctaggggttggaggaagactccactctgggtacaccc
ttaggggctggctttatggaacttgtagtttgaacaaggcagtggcaatc
cgccccctccagcctgcctggctggccccttccctctgtctggggtcgc
attccgcacaagcctttcatcaacatcttaaaatagtaactgtg

>NM_145204 3
tttttcttcttcaccttacggcagagcagggaactcagctttagattct
ccctgtactggaaagggatgtcgcaaggttggggacgaaacctctgagtt
tgcgaccgtagctgtcgctttccggccaacacagaggtgcctgaaggctg
gttggggtggtgaggcccgaggcagctcttgttcagcttctggaatttct
gagcagccctcgtcagtacaagatggaccccgtagtcttgagttacatgg
acagtctactgcggcaatcagatgtctcactattggatccgccaagctgg
ctcaatgaccatattattgggtttgcgtttgagtactttgccaacagtca
gtttcatgactgctctgatcacgtcagtttcatcagccctgaagtcaccc
agttcatcaagtgcactagcaacccagcagagattgccatgttccttgaa
ccactggacctccccaacaagagagttgtatttttagccatcaatgataa
ctccaaccaggcagctggaggaacccactggagtttattggtctacctcc
aagataaaaaatagctttttttcattatgattcccatagcaggagcaactca
gttcacgcaaagcaggtagcagagaaactggaggctttcttaggcagaaa

aggagacaaactggcctttgtggaagagaaagcccctgcccaacaaaaca
gctatgactgtgggatgtacgtgatatgtaacactgaggccttgtgtcag
aacttctttaggcaacagacagaatcactgctgcagctactcacccctgc
atacatcacaaagaagaggggagaatggaaagatctcattaccacacttg
ctaaaaagtagctattgaagtatatttgcgacttttgaaggctcctcttt
ctgcccttccccatttgttggatggctgcaatctcagtgcctgagggaag
atgcctagtagaggaaagcttaatactctttttcctgaaagaatatcatc
ctctgcattatccccatggaacgtttcactttaaccctgactggggagca
atatgttctgtgaaaatatcttgaaattgtacaccaaaaccttacaacca
acttatttgaacatttattacacacagggtttacgtaagacttttcttat
tggtatataattaatttcctttggtctcccttatccacattggcttattc
tggaggaaaagcagtgatctgtaaaacaaatcaagaatatattaaatcta
gaggaatgcagagaagaaaactataaaacagaaccaaaaacttgttgcac
agcctacataattaagagatcaactggctggaagcagatcaaggcctaac
ttcattcaagacctaaatattatgagactcagttattcggttttatgtga
catctcttccattcaccatgcacaggcttttccagctatctatataatgt
ttgcaaatatttgataaagatgatgttaccctatcttcctccatctgatt
cctggaatgcttgaagaaaggggaaatcttgagtaacctcattaaaatta
atgtctggtggacctctca

>NM_178439 3
ggtgctgcggtgctagagcgcggcgcgaccggacgctgcgggcggggaag
aggatggagactgtggcgtccgctgcaacggttggggctgcgcgtgagaa
ggtggcggtgtaggcacctgcgctcggggaaggctggcggcggcggccga
gccatggcgggagaccccccttctctgggctccctgaagtctcggggagcc
gtgacccatgggatcgttgagcagccgggtgctgcgccagccaagaccag
cccttgcccagcaggcgcagggtgccagggcggggggctcggcccggagg
ccggacactggagacgatgcggcgggccacggattctgttactgtgcggg
cagccacaagcgcaagcggagcagcgggtccttctgctactgtcaccctg
actcggagacggacgaggatgaggaggagggggacgagcagcagcggctc
ctcaacacccctcgaaggaaaaaattaaagagtacatctaaatatattta
tcaaacattatttttgaatggtgaaaacagtgacattaagatttgtgctc
taggagaagaatggagcttacacaaaatatatttatgtcaatctggctac
ttttctagtatgttcagtggttcttggaaagaatccagcatgaatattat
tgaactggagattcctgaccagaacattgatgtagaagcactgcaggttg
catttggttcactgtatcgagatgatgtcttgataaagcccagtcgagtt
gttgccattttggcagcagcttgtttgctgcagttggacggtttaataca
gcagtgtggtgagacaatgaaggaaacagttaatgtgaaaactgtatgtg
gctattacacatcagcagggacctatggattagattctgtaaagaaaaag
tgccttgaatggcttctaaacaatttgatgactcaccagaatgttgaact
ttttaaagaactcagtataaatgtcatgaaacagctcattggttcatcta
acttatttgtgatgcaagtggagatggatatatacactgctctaaaaaag
tggatgttccttcaacttgtgccttcttggaatggatctttaaaacagct
tttgacagaaacagatgtctggttttctaaacagaggaaagattttgaag
gtatggcctttcttgaaactgaacaaggaaaaccatttgtgtcagtattc
agacatttaaggttacaatatattatcagtgatctggcttctgcaagaat

tattgaacaagatgctgtagtaccttcagaatggctctcttctgtgtata
aacagcagtggtttgctatgctgcgggcagaacaggacagtgaggtgggg
cctcaagaaatcaataaagaagaactagagggaaacagcatgaggtgtgg
tagaaagcttgccaaagatggtgaatactgctggcgttggacaggtttta
acttcggcttcgacctacttgtaacttacaccaatcgatacatcattttc
aaacgcaatacactgaatcagccatgtagcggatctgtcagtttacagcc
tcgaaggagcatagcatttagattacgtttggcttcttttgatagtagtg
gaaaactaatatgtagtagaacaactggctatcaaatacttacacttgaa
aaggatcaggaacaagtggtgatgaacttggacagcaggcttctgatctt
ccctttatatatctgctgtaacttcttgtatatatcaccagaaaaaaaga
attgaaaataatcgtcacccagaaaatccagaaaactgaagatttcatca
gttggaaacagtagcactttgaaaactttttaggccagctttaatttaat
ggccctactgatattcacatcgaaggtgactaacaatgacaaaggcctta
tgaactgtacagacaatacagaagattattcttatcctcattgcatttct
atgcatatgcgtaagaacattttaaagccaagaaaatatctgtcaaacca
tttctgttagaacgatgtcaattcatgcttttaatttagcatcaatagaa
aattgctgtaggtaaatctcacatttatctgcaacaaaatatagatttaa
tttttagcttaaactttgtttctaccttatgttagtggacctcagttatc
catctgtaaatttctttttatttggctaaaataatctaaaagaataattt
ggttggccaattagaaatgcctttttcagttggtgtattgaaagctttcc
tttaacattttcacctgctcattgtgattcctccttttagtctaatatct
ttccaggtcatacttgtttttaatcattaaatattttcttcctggtaaaa
aaaaaaaaaaaaaaaaaaaaaaaaaaaaaaaaaaaaaaaaaaaaaaaaaa
aa

>NM_006534 3
atatcccagtggcccccgtgcggcgactttagctgctgctgtctcagccg
ctccacagcgacggcggcggctgcggcttagtcggtggcggccggcggcg
gctgcgggctgagcggcgagtttccgatttaaagctgagctgcgaggaaa
atggcggcgggaggatcaaaatacttgctggatggtggactcagagacca
ataaaaataaactgcttgaacatcctttgactggttagccagttgctgat
gtatattcaagatgagtggattaggagaaaacttggatccactggccagt
gattcacgaaaacgcaaattgccatgtgatactccaggacaaggtcttac
ctgcagtggtgaaaaacggagacgggagcaggaaagtaaatatattgaag
aattggctgagctgatatctgccaatcttagtgatattgacaatttcaat
gtcaaaccagataaatgtgcgattttaaaggaaacagtaagacagatacg
tcaaataaaagagcaaggaaaaactatttccaatgatgatgatgttcaaa
aagccgatgtatcttctacagggcagggagttattgataaagactcctta
ggaccgcttttacttcaggcattggatggtttcctatttgtggtgaatcg
agacggaaacattgtatttgtatcagaaaatgtcacacaatacctgcaat
ataagcaagaggacctggttaacacaagtgtttacaatatcttacatgaa
gaagacagaaaggattttcttaagaatttaccaaaatctacagttaatgg
agtttcctggacaaatgagacccaaagacaaaaaagccatacatttaatt
gccgtatgttgatgaaaacaccacatgatattctggaagacataaacgcc
agtcctgaaatgcgccagagatatgaaacaatgcagtgctttgccctgtc

tcagccacgagctatgatggaggaaggggaagatttgcaatcttgtatga
tctgtgtggcacgccgcattactacaggagaaagaacatttccatcaaac
cctgagagctttattaccagacatgatctttcaggaaaggttgtcaatat
agatacaaattcactgagatcctccatgaggcctggctttgaagatataa
tccgaaggtgtattcagagattttttagtctaaatgatgggcagtcatgg
tcccagaaacgtcactatcaagaagcttatcttaatggccatgcagaaac
cccagtatatcgattctcgttggctgatggaactatagtgactgcacaga
caaaaagcaaactcttccgaaatcctgtaacaaatgatcgacatggcttt
gtctcaacccacttccttcagagagaacagaatggatatagaccaaaccc
aaatcctgttggacaagggattagaccacctatggctggatgcaacagtt
cggtaggcggcatgagtatgtcgccaaaccaaggcttacagatgccgagc
agcagggcctatggcttggcagaccctagcaccacagggcagatgagtgg
agctaggtatgggggttccagtaacatagcttcattgacccctgggccag
gcatgcaatcaccatcttcctaccagaacaacaactatgggctcaacatg
agtagcccccacatgggagtcctggtcttgccccaaaccagcagaatat
catgatttctcctcgtaatcgtgggagtccaaagatagcctcacatcagt
tttctcctgttgcaggtgtgcactctcccatggcatcttctggcaatact
gggaaccacagcttttccagcagctctctcagtgccctgcaagccatcag
tgaaggtgtggggacttcccttttatctactctgtcatcaccaggcccca
aattggataactctcccaatatgaatattacccaaccaagtaaagtaagc
aatcaggattccaagagtcctctgggcttttattgcgaccaaaatccagt
ggagagttcaatgtgtcagtcaaatagcagagatcacctcagtgacaaag
aaagtaaggagagcagtgttgagggggcagagaatcaaaggggtcctttg
gaaagcaaaggtcataaaaaattactgcagttacttacctgttcttctga
tgaccggggtcattcctccttgaccaactccccccctagattcaagttgta
aagaatcttctgttagtgtcaccagcccctctggagtctcctcctctaca
tctggaggagtatcctctacatccaatatgcatgggtcactgttacaaga
gaagcaccggattttgcacaagttgctgcagaatgggaattcaccagctg
aggtagccaagattactgcagaagccactgggaaagacaccagcagtata
acttcttgtggggacggaaatgttgtcaagcaggagcagctaagtcctaa
gaagaaggagaataatgcacttcttagatacctgctggacagggatgatc
ctagtgatgcactctctaaagaactacagccccaagtggaaggagtggat
aataaaatgagtcagtgcaccagctccaccattcctagctcaagtcaaga
gaaagaccctaaaattaagacagagacaagtgaagagggatctggagact
tggataatctagatgctattcttggtgatctgactagttctgacttttac
aataattccatatcctcaaatggtagtcatctggggactaagcaacaggt
gtttcaaggaactaattctctgggtttgaaaagttcacagtctgtgcagt
ctattcgtcctccatataaccgagcagtgtctctggatagccctgtttct
gttggctcaagtcctccagtaaaaaatatcagtgctttccccatgttacc
aaagcaacccatgttgggtgggaatccaagaatgatggatagtcaggaaa
attatggctcaagtatgggtgggccaaaccgaaatgtgactgtgactcag
actccttcctcaggagactggggcttaccaaactcaaaggccggcagaat
ggaacctatgaattcaaactccatgggaagaccaggaggagattataata
cttctttacccagacctgcactgggtggctctattcccacattgcctctt
cggtctaatagcataccaggtgcgagaccagtattgcaacagcagcagca
gatgcttcaaatgaggcctggtgaaatccccatgggaatgggggctaatc

cctatggccaagcagcagcatctaaccaactgggttcctggcccgatggc
atgttgtccatggaacaagtttctcatggcactcaaaataggcctcttct
taggaattccctggatgatcttgttgggccaccttccaacctggaaggcc
agagtgacgaaagagcattattggaccagctgcacactcttctcagcaac
acagatgccacaggcctggaagaaattgacagagctttgggcattcctga
acttgtcaatcagggacaggcattagagcccaaacaggatgctttccaag
gccaagaagcagcagtaatgatggatcagaaggcaggattatatggacag
acatacccagcacaggggcctccaatgcaaggaggctttcatcttcaggg
acaatcaccatcttttaactctatgatgaatcagatgaaccagcaaggca
attttcctctccaaggaatgcacccacgagccaacatcatgagaccccgg
acaaacacccccaagcaacttagaatgcagcttcagcagaggctgcaggg
ccagcagtttttgaatcagagccgacaggcacttgaattgaaaatggaaa
accctactgctggtggtgctgcggtgatgaggcctatgatgcagccccag
cagggttttcttaatgctcaaatggtcgcccaacgcagcagagagctgct
aagtcatcacttccgacaacagagggtggctatgatgatgcagcagcagc
agcagcagcaacagcagcagcagcagcagcagcagcagcaacagcaacag
caacagcaacagcagcaacagcagcaaacccaggccttcagcccacctcc
taatgtgactgcttcccccagcatggatgggcttttggcaggacccacaa
tgccacaagctcctccgcaacagtttccatatcaaccaaattatggaatg
ggacaacaaccagatccagcctttggtcgagtgtctagtcctcccaatgc
aatgatgtcgtcaagaatgggtccctcccagaatcccatgatgcaacacc
cgcaggctgcatccatctatcagtcctcagaaatgaagggctggccatca
ggaaatttggccaggaacagctccttttcccagcagcagtttgcccacca
ggggaatcctgcagtgtatagtatggtgcacatgaatggcagcagtggtc
acatgggacagatgaacatgaaccccatgcccatgtctggcatgcctatg
ggtcctgatcagaaatactgctgacatctctgcaccaggacctcttaagg
aaaccactgtacaaatgacactgcactaggattattgggaaggaatcatt
gttccaggcatccatcttggaagaaaggaccagctttgagctccatcaag
ggtattttaagtgatgtcatttgagcaggactggattttaagccgaaggg
caatatctacgtgtttttccccctccttctgctgtgtatcatggtgttc
aaaacagaaatgtttttttggcattccacctcctagggatataattctgga
gacatggagtgttactgatcataaaacttttgtgtcactttttttctgcct
tgctagccaaaatctcttaaatacacgtaggtgggccagagaacattgga
agaatcaagagagattagaatatctggtttctctagttgcagtattggac
aaagagcatagtcccagccttcaggtgtagtagttctgtgttgaccttt
gtccagtggaattggtgattctgaattgtcctttactaatggtgttgagt
tgctctgtccctattatttgccctaggctttctcctaatgaaggttttca
tttgccattcatgtcctgtaatacttcacctccaggaactgtcatggatg
tccaaatggctttgcagaaaggaaatgagatgacagtatttaatcgcagc
agtagcaaacttttcacatgctaatgtgcagctgagtgcactttatttaa
aaagaatggataaatgcaatattcttgaggtcttgagggaatagtgaaac
acattcctggtttttgcctacacttacgtgttagacaagaactatgattt
ttttttttaaagtactggtgtcaccctttgcctatatggtagagcaataa
tgctttttaaaaataaacttctgaaaacccaaggccaggtactgcattct
gaatcagaatctcgcagtgtttctgtgaatagattttttttgtaaatatga
cctttaagatattgtattatgtaaaatatgtatataccttttttttgtagg

tcacaacaactcatttttacagagtttgtgaagctaaatatttaacattg
ttgatttcagtaagctgtgtggtgaggctaccagtggaagagacatccct
tgacttttgtggcctgggggaggggtagtgctccacagcttttccttccc
caccccccagccttagatgcctcgctcttttcaatctcttaatctaaatg
ctttttaaagagattatttgtttagatgtaggcattttaatttttttaaaa
attcctctaccagaactaagcactttgttaatttggggggaaagaataga
tatggggaaataaacttaaaaaaaaaatcaggaatttaaaaaaacgagcaa
tttgaagagaatcttttggattttaagcagtccgaaataatagcaattca
tgggctgtgtgtgtgtgtgtatgtgtgtgtgtgtgtgtatgtttaatt
atgttaccttttcatcccctttaggagcgttttcagattttggttgctaa
gacctgaatcccatattgagatctcgagtagaatccttggtgtggtttct
ggtgtctgctcagctgtcccctcattctactaatgtgatgctttcattat
gtccctgtggattagaatagtgtcagttatttcttaagtaactcagtacc
cagaacagccagttttactgtgattcagagccacagtctaactgagcacc
ttttaaacccctccctcttctgccccctaccacttttctgctgttgcctc
tctttgacacctgttttagtcagttgggaggaagggaaaaatcaagttta
attccctttatctgggttaattcatttggttcaaatagttgacggaattg
ggtttctgaatgtctgtgaatttcagaggtctctgctagccttggtatca
ttttctagcaataactgagagccagttaattttaagaatttcacacattt
agccaatctttctagatgtctctgaaggtaagatcatttaatatctttga
tatgcttacgagtaagtgaatcctgattatttccagacccaccaccagag
tggatcttattttcaaagcagtatagacaattatgagtttgccctctttc
ccctaccaagttcaaaatatatctaagaaagattgtaaatccgaaaactt
ccattgtagtggcctgtgcttttcagatagtatactctcctgtttggaga
cagaggaagaaccaggtcagtctgtctctttttcagctcaattgtatctg
acccttctttaagttatgtgtgtggggagaaatagaatggtgctcttatc
tttcttgactttaaaaaaattattaaaaacaaaaaaaaaaataaattttttt
tgcaatcctttcctcagacctggctccaggctaactggaaggcagcactc
cctttttatatagtagaaaaatgaagtttattataagtttttatatttt
ctacttgttcatttggtgcaaactcaagatttcttttaataggtgcagtc
tttgagataatttgtttttacctgtattgccctttatctttttttaggtaa
ttctttgtactcctgctgtctacctctcctcacaccccagcaccccccat
tttttcaaaccttggtatctgttgggtgaacagtataatcttttcatctg
cttttagaatgtgggatatttccagtacctactttttttttttttttttg
ctgaatccaaagatatataaataaaatatatatattttataaagatcaga
atgatataaaggagatacatgtttcttcctttaaaaaataaacggaagtt
acattgttaatgttcatattatgatgccacttttctaaactgcatctgga
ttgaaaggtgtaaatatcaataacagtgctacttagttatcagtatttaa
tatctgaggtgagttgggggtatctatattaggggtagggtattacagaa
gataattggcttgatgtcctagaagttctttgatccagaggtgggtgcag
ctgaaagtaaacagaatggattgccagttacatgtatgcctgcccagttc
cctttttatttgcagaagctgtgagttttgttcacaattaggttcctagg
agcaaaacctcaaggattgatttattgttttcaactccaaggcacactgt
taataaacgagcagggtgttttctctcttcctttctaatatatggagttt
cgaagaataaaatatgagagcaatatttaaattctcaggaattgacttat
actcttgagaatgaattcagtttcaatcaagtttacattatgttgcttaa

aaaaatagaaattattctttatcttgcaaagaattgaaaccacatgaaat
gacttatgggggatggtgagctgtgactgctttgctgaccattttggatg
tcattgtaaataaaggtttctatttaaaattgga

>NM_007124 2
gtattgatgtcaagctgaaccatcgtaggaagttgaaagccttagaaaga
ggacttggtaaagtttttggattatcttgaaactctggcaagatggccaa
gtatggagaacatgaagccagtcctgacaatgggcagaacgaattcagtg
atatcattaagtccagatctgatgaacacaatgacgtacagaagaaaacc
tttaccaaatggataaatgctcgattttcaaagagtgggaaaccacccat
caatgatatgttcacagacctcaaagatggaaggaagctattggatcttc
tagaaggcctcacaggaacatcactgccaaaggaacgtggttccacaagg
gtacatgccttaaataacgtcaacagagtgctgcaggttttacatcagaa
caatgtggaattagtgaatatagggggaactgacattgtggatggaaatc
acaaactgactttggggttactttggagcatcattttgcactggcaggtg
aaagatgtcatgaaggatgtcatgtcggacctgcagcagacgaacagtga
gaagatcctgctcagctgggtgcgtcagaccaccaggccctacagccaag
tcaacgtcctcaacttcaccaccagctggacagatggactcgcctttaat
gctgtcctccaccgacataaacctgatctcttcagctgggataaagttgt
caaaatgtcaccaattgagagacttgaacatgccttcagcaaggctcaaa
cttatttgggaattgaaaagctgttagatcctgaagatgttgccgttcag
cttcctgacaagaaatccataattatgtatttaacatctttgtttgaggt
gctacctcagcaagtcaccatagacgccatccgtgaggtagagacactcc
caaggaaatataaaaaagaatgtgaagaagaggcaattaatatacagagt
acagcgcctgaggaggagcatgagagtccccgagctgaaactcccagcac
tgtcactgaggttgacatggatctggacagctatcagattgcgttggagg
aagtgctgacctggttgctttctgctgaggacactttccaggagcaggat
gatatttctgatgatgttgaagaagtcaaagaccagtttgcaacccatga
agcttttatgatggaactgactgcacaccagagcagtgtgggcagcgtcc
tgcaggcaggcaaccaactgataacacaaggaactctgtcagacgaagaa
gaatttgagattcaggaacagatgaccctgctgaatgctagatgggaggc
tcttagggtggagagtatggacagacagtcccggctgcacgatgtgctga
tggaactgcagaagaagcaactgcagcagctctccgcctggttaacactc
acagaggagcgcattcagaagatggaaacttgcccccctggatgatgatgt
aaaatctctacaaaagctgctagaagaacataaaagtttgcaaagtgatc
ttgaggctgaacaggtgaaagtaaattcactaactcacatggtggtcatt
gttgatgaaaacagtggtgagagtgctacagctatcctagaagaccagtt
acagaaacttggtgagcgctggacagcagtatgccgttggactgaagaac
gctggaataggttacaagaaatcaatatattgtggcaggaattattggaa
gaacagtgcttgttgaaagcttggttaaccgaaaaagaagaggctttaaa
taaagtccagacaagcaacttcaaagaccaaaaggaactaagtgtcagtg
ttcgacgtctggctattttgaaggaagacatggaaatgaagcgtcaaaca
ttggatcagctgagtgagattggccaggatgtgggacaattacttgataa
ttccaaggcatctaagaagatcaacagtgactcagaggaactgactcaaa
gatgggattctttggttcagagactagaagattcctccaaccaggtgact
caggctgtagcaaagctggggatgtctcagattcctcagaaggaccttt

ggagactgttcgtgtaagagaacaagcaattacaaaaaaatctaagcagg
aactgcctcctcctcctcccccaaagaagagacagatccatgtggatatt
gaagctaagaaaaagtttgatgctataagtgcagagctgttgaactggat
tttgaaatggaaaactgccattcagaccacagagataaaagagtatatga
agatgcaagacacttccgaaatgaaaaagaagttgaaggcattagaaaaa
gaacagagagaaagaatccccagagcagatgaattaaaccaaactggaca
aatccttgtggagcaaatgggaaaagaaggccttcctactgaagaaataa
aaaatgttctggagaaggtttcatcagaatggaagaatgtatctcaacat
ttggaagatctagaaagaaagattcagctacaggaagatataaatgctta
tttcaagcagcttgatgagcttgaaaaggtcatcaagacaaaggaggagt
gggtaaaacacacttccatttctgaatcttcccggcagtccttgccaagc
ttgaaggattcctgtcagcgggaattgacaaatcttcttggccttcaccc
caaaattgaaatggctcgtgcaagctgctcggccctgatgtctcagcctt
ctgccccagattttgtccagcggggcttcgatagctttctgggccgctac
caagctgtacaagaggctgtagaggatcgtcaacaacatctagagaatga
actgaagggccaacctggacatgcatatctggaaacattgaaaacactga
aagatgtgctaaatgattcagaaaataaggcccaggtgtctctgaatgtc
cttaatgatcttgccaaggtggagaaggccctgcaagaaaaaaagaccct
tgatgaaatccttgagaatcagaaacctgcattacataaacttgcagaag
aaacaaaggctctggagaaaaatgttcatcctgatgtagaaaaattatat
aagcaagaatttgatgatgtgcaaggaaagtggaacaagctaaaggtctt
ggtttccaaagatctacatttgcttgaggaaattgctctcacactcagag
cttttgaggccgattcaacagtcattgagaagtggatggatggcgtgaaa
gacttcttaatgaaacagcaggctgcccaaggagacgacgcaggtctaca
gaggcagttagaccagtgctctgcatttgttaatgaaatagaaacaattg
aatcatctctgaaaaacatgaaggaaatagagactaatcttcgaagtggt
ccagttgctggaataaaaacttgggtgcagacaagactaggtgactacca
aactcaactggagaaacttagcaaggagatcgctactcaaaaaagtaggt
tgtctgaaagtcaagaaaaagctgcgaacctgaagaaagacttggcagag
atgcaggaatggatgacccaggccgaggaagaatatttggagcgggattt
tgagtacaagtcaccagaagagcttgagagtgctgtggaagagatgaaga
gggcaaaagaggatgtgttgcagaaggaggtgagagtgaagattctcaag
gacaacatcaagttattagctgccaaggtgccctctggtggccaggagtt
gacgtctgagctgaatgttgtgctggagaattaccaacttctttgtaata
gaattcgaggaaagtgccacacgctagaggaggtctggtcttgttggatt
gaactgcttcactatttggatcttgaaactacctggttaaacactttgga
agagcggatgaagagcacagaggtcctgcctgagaagacggatgctgtca
acgaagccctggagtctctggaatctgttctgcgccacccggcagataat
cgcacccagattcgagagcttggccagactctgattgatgggggatcct
ggatgatataatcagtgagaaactggaggctttcaacagccgatatgaag
atctaagtcacctggcagagagcaagcagatttctttggaaaagcaactc
caggtgctgcgggaaactgaccagatgcttcaagtcttgcaagagagctt
gggggagctggacaaacagctcaccacatacctgactgacaggatagatg
ctttccaagttccacaggaagctcagaaaatccaagcagagatctcagcc
catgagctaaccctagaggagttgagaagaaatatgcgttctcagcccct
gacctccccagagagtaggactgccagaggaggaagtcagatggatgtgc

tacagaggaaactccgagaggtgtccacaaagttccagcttttccagaag
ccagctaacttcgagcagcgcatgctggactgcaagcgtgtgctggatgg
cgtgaaagcagaacttcacgttctggatgtgaaggacgtagaccctgacg
tcatacagacgcacctggacaagtgtatgaaactgtataaaactttgagt
gaagtcaaacttgaagtggaaactgtgattaaaacaggaagacatattgt
ccagaaacagcaaacggacaacccaaaagggatggatgagcagctgactt
ccctgaaggttctttacaatgacctgggcgcacaggtgacagaaggaaaa
caggatctggaaagagcatcacagttggcccggaaaatgaagaaagaggc
tgcttctctctctgaatggctttctgctactgaaactgaattggtacaga
agtccacttcagaaggtctgcttggtgacttggatacagaaatttcctgg
gctaaaaatgttctgaaggatctggaaaagagaaaagctgatttaaatac
catcacagagagtagtgctgccctgcaaaacttgattgagggcagtgagc
ctattttagaagagaggctctgcgtccttaacgctgggtggagccgagtt
cgtacctggactgaagattggtgcaataccttgatgaaccatcagaacca
gctagaaatatttgatgggaacgtggctcacataagtacctggctttatc
aagctgaagctctattggatgaaattgaaaagaaaccaacaagtaaacag
gaagaaattgtgaagcgtttagtatctgagctggatgatgccaacctcca
ggttgaaaatgtccgcgatcaagcccttattttgatgaatgcccgtggaa
gctcaagcagggagcttgtagaaccaaagttagctgagctgaataggaac
tttgaaaaggtgtctcaacatatcaaaagtgccaaattgctaattgctca
ggaaccattataccaatgtttggtcaccactgaaacatttgaaactggtg
tgcctttctctgacttggaaaaattagaaaatgacatagaaaatatgtta
aaatttgtggaaaaacacttggaatccagtgatgaagatgaaaagatgga
tgaggagagtgcccagattgaggaagttctacaaagaggagaagaaatgt
tacatcaacctatggaagataataaaaaagaaaagatccgtttgcaatta
ttacttttgcatactagatacaacaaaattaaggcaatccctattcaaca
gaggaaaatgggtcaacttgcttctggaattagatcatcacttcttccta
cagattatctggttgaaattaacaaaattttactttgcatggatgatgtt
gaattatcgcttaatgttccagagctcaacactgctatttacgaagactt
ctcttttcaggaagactctctgaagaatatcaaagaccaactggacaaac
ttggagagcagattgcagtcattcatgaaaaacagccagatgtcatcctt
gaagcctctggacctgaagccattcagatcagagatacacttactcagct
gaatgcaaaatgggacagaattaatagaatgtacagtgatcggaaaggtt
gttttgacagggcaatggaagaatggagacagttccattgtgaccttaat
gacctcacacagtggataacagaggctgaagaattactggttgatacctg
tgctccaggtggcagcctggacttagagaaagccaggatacatcagcagg
aacttgaggtgggcatcagcagccaccagcccagttttgcagcactaaac
cgaactggggatgggattgtgcagaaactctcccaggcagatggaagctt
cttgaaagaaaaactggcaggtttaaaccaacgctgggatgcaattgttg
cagaagtgaaggataggcagccaaggctaaaaggagaaagtaagcaggtg
atgaagtacaggcatcagctagatgagattatctgttggttaacaaaggc
tgagcatgctatgcaaaagagatcaaccaccgaattgggagaaaacctgc
aagaattaagagacttaactcaagaaatggaagtacatgctgaaaaactc
aaatggctgaatagaactgaattggagatgctttcagataaaagtctgag
tttacctgaaagggataaaatttcagaaagcttaaggactgtaaatatga
catggaataagatttgcagagaggtgcctaccaccctgaaggaatgcatc

caggagcccagttctgtttcacagacaaggattgctgctcatcctaatgt
ccaaaaggtggtgctagtatcatctgcgtcagatattcctgttcagtctc
atcgtacttcggaaatttcaattcctgctgatcttgataaaactataaca
gaactagccgactggctggtattaatcgaccagatgctgaagtccaacat
tgtcactgttggggatgtagaagagatcaataagaccgtttcccgaatga
aaattacaaaggctgacttagaacagcgccatcctcagctggattatgtt
tttacattggcacagaatttgaaaaataaagcttccagttcagatatgag
aacagcaattacagaaaaattggaaagggtcaagaaccagtgggatggca
cccagcatggcgttgagctaagacagcagcagcttgaggacatgattatt
gacagtcttcagtgggatgaccatagggaggagactgaagaactgatgag
aaaatatgaggctcgactctatattcttcagcaagcccgacgggatccac
tcaccaaacaaatttctgataaccaaatactgcttcaagaactgggtcct
ggagatggtatcgtcatggcgttcgataacgtcctgcagaaactcctgga
ggaatatgggagtgatgacacaaggaatgtgaaagaaaccacagagtact
taaaaacatcatggatcaatctcaaacaaagtattgctgacagacagaac
gccttggaggctgagtggaggacggtgcaggcctctcgcagagatctgga
aaacttcctgaagtggatccaagaagcagagaccacagtgaatgtgcttg
tggatgcctctcatcgggagaatgctcttcaggatagtatcttggccagg
gaactcaaacagcagatgcaggacatccaggcagaaattgatgcccacaa
tgacatatttaaaagcattgacggaaacaggcagaagatggtaaaagctt
tgggaaattctgaagaggctactatgcttcaacatcgactggatgatatg
aaccaaagatggaatgacttaaaagcaaaatctgctagcatcagggccca
tttggaggccagcgctgagaagtggaacaggttgctgatgtccttagaag
aactgatcaaatggctgaatatgaaagatgaagagcttaagaaacaaatg
cctattggaggagatgttccagccttacagctccagtatgaccattgtaa
ggccctgagacgggagttaaaggagaaagaatattctgtcctgaatgctg
tcgaccaggcccgagttttcttggctgatcagccaattgaggcccctgaa
gagccaagaagaaacctacaatcaaaaacagaattaactcctgaggagag
agcccaaaagattgccaaagccatgcgcaaacagtcttctgaagtcaaag
aaaaatgggaaagtctaaatgctgtaactagcaattggcaaaagcaagtg
gacaaggcattggagaaactcagagacctgcagggagctatggatgacct
ggacgctgacatgaaggaggcagagtccgtgcggaatggctggaagcccg
tgggagacttactcattgactcgctgcaggatcacattgaaaaaatcatg
gcatttagagaagaaattgcaccaatcaactttaaagttaaaacggtgaa
tgatttatccagtcagctgtctccacttgacctgcatccctctctaaaga
tgtctcgccagctagatgaccttaatatgcgatggaaacttttacaggtt
tctgtggatgatcgccttaaacagcttcaggaagcccacagagattttgg
accatcctctcagcattttctctctacgtcagtccagctgccgtggcaaa
gatccatttcacataataaagtgccctattacatcaaccatcaaacacag
accacctgttgggaccatcctaaaatgaccgaactctttcaatcccttgc
tgacctgaataatgtacgtttttctgcctaccgtacagcaatcaaaatcc
gaagactacaaaaagcactatgtttggatctcttagagttgagtacaaca
aatgaaattttcaaacagcacaagttgaaccaaaatgaccagctcctcag
tgttccagatgtcatcaactgtctgacaacaacttatgatggacttgagc
aaatgcataaggacctggtcaacgttccactctgtgttgatatgtgtctc
aattggttgctcaatgtctatgacacgggtcgaactggaaaaattagagt

gcagagtctgaagattggattaatgtctctctccaaaggtctcttggaag
aaaaatacagatatctctttaaggaagttgcagggccaacagaaatgtgt
gaccagaggcagctgggcctgttacttcatgatgccatccagatcccccg
gcagctaggtgaagtagcagcttttggaggcagtaatattgagcctagtg
ttcgcagctgcttccaacagaataacaataaaccagaaataagtgtgaaa
gagtttatagattggatgcatttggaaccacagtccatggtttggctccc
agttttacatcgagtggcagcagcggagactgcaaaacatcaggccaaat
gcaacatctgtaaagaatgtccaattgtcgggttcaggtatagaagcctt
aagcattttaactatgatgtctgccagagttgtttcttttcgggtcgaac
agcaaaaggtcacaaattacattacccaatggtggaatattgtatacct a
caacatctggggaagatgtacgagacttcacaaaggtacttaagaacaag
ttcaggtcgaagaagtactttgccaaacaccctcgacttggttacctgcc
tgtccagacagttcttgaaggtgacaacttagagactcctatcacactca
tcagtatgtggccagagcactatgacccctcacaatctcctcaactgttt
catgatgacacccattcaagaatagaacaatatgccacacgactggccca
gatggaaaggactaatgggtcttttctcactgatagcagctccaccacag
gaagtgtggaagacgagcacgccctcatccagcagtattgccaaacactc
ggaggagagtccccagtgagccagccgcagagcccagctcagatcctgaa
gtcagtagagagggaagaacgtggagaactggagaggatcattgctgacc
tggaggaagaacaaagaaatctacaggtggagtatgagcagctgaaggac
cagcacctccgaagggggctccctgtcggttcaccgccagagtcgattat
atctccccatcacacgtctgaggattcagaacttatagcagaagcaaaac
tcctcaggcagcacaaaggtcggctggaggctaggatgcagattttagaa
gatcacaataaacagctggagtctcagctccaccgcctccgacagctgct
ggagcagcctgaatctgattcccgaatcaatggtgtttccccatgggctt
ctcctcagcattctgcactgagctactcgcttgatccagatgcctccggc
ccacagttccaccaggcagcgggagaggacctgctggccccaccgcacga
caccagcacggatctcacggaggtcatggagcagattcacagcacgtttc
catcttgctgcccaaatgttcccagcaggccacaggcaatgtgaagtatt
catccggccaaccaatgtttcctgacgtacagtgttgcccttttcagcaa
atgccaattccaagttccattaaatcagaagctccatggctccttggccc
acgatgttgagtgctgactgtgtgttctactgaaagagtaaaacactgac
tatccaaagagaaatggatattttgtttttataataaccatatattattg
ttttcttcttccctttctatgcaagtgtaaattaatgaacagagaggtat
ttggaaatggtaatacatttgtcacggatttgtataatgtatacagcatt
gggaaagtgggtgggggctttctaatatgataccgtctttttaataacta
tgacaaagcttacataagaattagaagaccactttacatttttacattcc
ttctgctgttcatattaaccttgcacaattacttcatttttttctttgact
cttttaccacaatgttttggttatttataatttatcagccatatgtttat
cagccatataaccaactagatcccaaatagatccatgtatttgtttccgt
gatttggccacattaataaattcataaatttcaatcaaatatcttatata
tacacacatatggtttaagctacagccctgtgtatgccgtttaactttat
ttgacgttgcccacttacttctttgctgaccacttggataaccgtaataa
aaatcctataagcctaaatggcatttcttttgggatatttttcctgcatt
ttattccctttttatataagtaggaattaattatttattttatgtcttaa
tctatttgataaagaagactacattataataatctcaaagatcatattac

caaaggttgcccacttgagcatattttcattttgacacagaaacaaaatt
tagtacaacctttcctagttcccatgtcttgattttcatcattacatgca
cagcagacctttacctattgtgataccagaacacatcattgtctttggtt
cccttcaaagagaattttattgttgttttgtattttcaagtccttaatag
ttcttgaaactcctagttgttttcttgttgaaagcagacacacatttagt
gcacggcttattttacctttcgggtgaaagatcagatgtttttataccct
tcacttgatcaatatatttggaaagaatgtttatcaaaagtctatgtcac
tgcttctacagaagaatgaaattaatgcttaggtgatggtacctccacct
acatctttttgagtgcattcaattatgtattttggtttagcttctgattt
aacatttaattgattcagtttaaacatgttacttaattagcaaatgtaga
ggaaccaaaaaaaggtgaaaataatatgttttgattcaaacctaaagaca
taaaaacataaagacattttaactttgggttctctttagctgggatctgg
ccagaaggaggcttaaagttagaaattgctattattttagaataggttgg
gtgggttgggggcaagggtgtctatttgcagcagagatattttgaaaag
aagaaaattgttttatataaaaaggaaagccatgaccacctttctacctc
agatccatcttcatccattgcattggaaactgctttatgctgctgcagtc
tgcaaagtctagagcttttatcaggccatgtcatacccaagaaagcacct
atttaaagaaaaaacaattccctgagctctcaactccaagttgtagattt
ggtgtcttccttgttcttactttaaaaagtcatgtgttaattttttttct
gcctgtatttgtatgcaaaatgtcctctatctgctattaaagaaaagcta
cgtaaaacactacattgtaaccttctaagtaataataaataaaaagaaat
atattgcagtaacaatgggaagtaagtatgtagttcttttgaaatatgtg
gtaaagaactaatcacagactatcatctaatctggttacatattgtattt
ttcatcctgaataaaagtaattttaacacaaaaaaa

>NM_007085 4
aggagaatggggaggagctgggggggcaggcaggcggggaaggagaggtc
ttaaggggcggcgaggggaggtcgcatttcctccgaggctggcgatcggc
ggagctcccacctccgcttacagctcgctgccgccgtcctgccccgcgcc
cccaggagacctggaccagaccacgatgtggaaacgctggctcgcgctcg
cgctcgcgctggtggcggtcgcctgggtccgcgccgaggaagagctaagg
agcaaatccaagatctgtgccaatgtgttttgtggagccggccgggaatg
tgcagtcacagagaaaggggaacccacctgtctctgcattgagcaatgca
aacctcacaagaggcctgtgtgtggcagtaatggcaagacctacctcaac
cactgtgaactgcatcgagatgcctgcctcactggatccaaaatccaggt
tgattacgatggacactgcaaagagaagaaatccgtaagtccatctgcca
gcccagttgtttgctatcagtccaaccgtgatgagctccgacgtcgcatc
atccagtggctggaagctgagatcattccagatggctggttctctaaagg
cagcaactacagtgaaatcctagacaagtattttaagaactttgataatg
gtgattctcgcctggactccagtgaattcctgaagtttgtggaacagaat
gaaactgccatcaatattacaacgtatccagaccaggagaacaacaagtt
gcttaggggactctgtgttgatgctctcattgaactgtctgatgaaaatg
ctgattggaaactcagcttccaagagtttctcaagtgcctcaacccatct
ttcaaccctcctgagaagaagtgtgccctggaggatgaaacgtatgcaga
tggagctgagaccgaggtggactgtaaccgctgtgtctgtgcctgtggaa
attgggtctgtacagccatgacctgtgacggaaagaatcagaagggggcc

cagacccagacagaggaggagatgaccagatatgtccaggagctccaaaa
gcatcaggaaacagctgaaaagaccaagagagtgagcaccaaagagatct
aatgaggaggcacagaccagtgtctggatcccagcatcttctccacttca
gcgctgagttcagtatacacaagtgtctgctacagtcgccaaatcaccag
tatttgcttatatagcaatgagttttattttgtttatttgttttgcaata
aaggatatgaaggtggctggctaggaagggaagggccacagccttcattt
ctaggagtgctttaagagaaactgtaaatggtgctctggggctggaggct
agtaaggaaactgcatcacgattgaaagaggaacagacccaaatctgaac
ctcttttgagtttactgcatctgtcagcaggctgcagggagtgcacacga
tgccagagagaacttagcagggtgtccccggaggagaggtttgggaagct
ccacggagaggaacgctctctgcttccagcctctttccattgccgtcagc
atgacagacctccagcatccacgcatctcttggtcccaataactgcctct
agatacatagccatactgctagttaacccagtgtccctcagacttggatg
gagtttctgggagggtacacccaaatgatgcagatacttgtatactttga
gccccttagcgacctaaccaaattttaaaaatactttttaccaaaggtgc
tatttctctgtaaaacacttttttttggcaagttgactttattcttcaat
tattatcattatattattgtttttaatattttattttcttgactaggta
ttaagcttttgtaattatttttcagtagtcccaccacttcataggtggaa
ggagtttggggttcttcctggtgcaggggctgaaataacccagatgcccc
caccctgccacatactagatgcagcccatagttggccccccctagcttcca
gcagtccactatctgccagaggagcaagggtgccttagaccgaagccagg
ggaagaagcatcttcataaaaaaactttcaagatccaaacattaatttgtt
tttatttattctgagaagttgaggcaaatcagtattcccaaggatggcga
caagggcagccaagcagggcttaggatatcccagcctaccaatatgctca
ttcgactaactaggagggtgagttggccctgtctcttctttttttctggac
ctcagtttcctcagtgagctggtaagaatgcactaaccttttgatttgat
aagttataaattctgtggttctgatcattggtccagaggggagataggtt
cctgtgatttttccttcttctctatagaataaatgaaatcttgttactag
aacaagaaatgtcagatggccaaaaacaagatgaccagatttgatctcag
cctgatgaccctacaggtcgtgctatgatatggagtcctcatgggtaaag
caggaagagagtgggaaagagaaccaccccactctgtcttcatatttgca
tttcatgtttaacctccggctggaaatagaaagcattcccttagagatga
ggataaaagaaagtttcagattcaacagggggaagaaaatggagatttaa
tcctaaaactgtgacttggggaggtcagtcatttacagttagtcctgtgt
ctttcgacttctgtgattattaaccccactcactaccctgtttcagatgc
atttggaataccaaagattaaatccttgacataagatctcatttgcagaa
agcagattaaagaccatcagaaggaaattatttaggttgtaatgcacagg
caactgtgagaaactgttgtgccaaaaatagaattccttctagtttttct
tgttctcatttgaaaggagaaaattccactttgtttagcatttcaagctt
ttatgtatccatcccatctaaaaactcttcaaactccacttgttcagtct
gaaatgcagctccctgtccaagtgccttggagaactcacagcagcacgcc
ttaatcaaaggttttaccagcccttggacactatgggaggagggcaagag
tacaccaatttgttaaaagcaagaaaccacagtgtctcttcactagtcat
ttagaacatggttatcatccaagactactctaccctgcaacattgaactc
ccaagagcaaatccacattcctcttgagttctgcagcttctgtgtaaata
gggcagctgtcgtctatgccgtagaatcacatgatctgaggaccattcat

ggaagctgctaaatagcctagtctggggagtcttccataaagttttgcat
ggagcaaacaaacaggattaaactaggtttggttccttcagccctctaaa
agcatagggcttagcctgcaggcttccttgggctttctctgtgtgtgtag
ttttgtaaacactatagcatctgttaagatccagtgtccatggaaacatt
cccacatgccgtgactctggactatatcagtttttggaaagcagggttcc
tctgcctgctaacaagcccacgtggaccagtctgaatgtctttcctttac
acctatgtttttaagtagtcaaacttcaagaaacaatctaaacaagtttc
tgttgcatatgtgtttgtgaacttgtatttgtatttagtaggcttctata
ttgcatttaacttgtttttgtaactcctgattcttccttttcggatacta
ttgatgaataaagaaattaaagtgatggttttattggtttcctttccccc
aattaaggccaaataaagtcgtgagaacattacccattta

>NM_005121 2
gtttctctctctggtcggaggcggcggtaatggcggatggtgggttgtgg
cgccggcggcggctgctgtgagggacgatgagtgcctccttcgtgccgaa
cggggccagcctggaagattgtcactgtaacctcttctgcctggctgact
tgacaggaattaagtggaaaaaatatgtatggcaaggcccaacttctgcc
cctattctgtttcctgtgacagaagaagaccccattttgagcagttttag
tcgctgccttaaggcagatgtacttggtgtttggcggcgagatcaaagac
ctggaagaagagaattgtggatattttggtggggtgaagaccccagtttt
gctgaccttattcaccatgacttatcagaagaagaagatggagtgtggga
gaatggactttcctatgaatgccgtactctgcttttcaaagcagttcaca
atctattggaacggtgtttaatgaacaggaattttgtacgtattggcaag
tggtttgtaaagccttatgaaaaagatgaaaaacctataaataaaagtga
acacttgtcctgctccttcaccttttcttgcatggagacagcaatgttt
gtaccagtgtggaaattaaccaacatcaacctgtataccttctcagtgaa
gagcatatcacccttgctcaacagtctaatagcccatttcaagttatctt
atgcccatttggactaaatggcactctcacaggacaggcattcaagatgt
ctgattcagctacaaaaaaattaattggtgaatggaaacagttctatcct
atctcatgttgcttgaaggagatgtctgaagaaaaacaggaagatatgga
ttgggaagatgattctttagctgcagtagaagttcttgttgctggtgtcc
gaatgatctacccagcatgctttgttctagtccctcagtcagacattcct
actcctagccctgtgggatccactcactgttcatcttcttgcttgggtgt
ccaccaagtgcctgcttccacaagagatcctgctatgtcttcggttacgc
ttacaccacctacgtctcctgaggaagtccaaacagttgatcctcagtct
gtccagaagtgggtcaaattttcttcagtatctgatggcttcaactccga
tagtactagccaccatggtgggaaaatacccagaaaattagcaaatcatg
tggtggatagagtttggcaagaatgcaatatgaacagagcacagaacaag
aggaagtattctgcttcatcaggtggtctatgcgaagaagcgacagctgc
taaagtggcatcctgggattttgttgaagccacacaaagaacaaattgca
gttgtttgaggcacaaaaatctcaagtcaagaaatgctggacaacaagga
caggcaccatctttaggtcagcaacaacaaatacttcctaagcacaagac
caatgagaagcaagaaaagagtgaaaagccacagaaacgccccttgactc
cttttcaccatcgtgtgtctgttagtgatgatgttggcatggacgcagat
tcagccagccaaagacttgtgatctctgctccagacagtcaagtgagatt
ttcaaatatccgaactaatgatgtagcaaagactcctcagatgcatggca

ccgaaatggcaaattcacctcaaccacccccacttagtcctcacccttgt
gatgtggttgatgaaggagtgactaaaacaccttcaactcctcagagtca
acatttttatcaaatgccaacaccagatcccttggttccttctaaaccaa
tggaagataggatagacagtttgtcccagtctttcccacctcaatatcag
gaagctgtagaacctacagtatatgttggtacagcagtaaacttggaaga
agatgaagccaatatagcctggaagtattacaagttcccaaagaaaaaag
atgtagagtttttaccacctcaacttccaagtgataaattcaaggatgat
ccagttggaccttttggacaggaaagtgtaacatcagttacagagttaat
ggtgcaatgtaagaaacctttaaaagtttctgatgaattagtgcagcaat
atcaaattaaaaaccagtgtctttcagcaatagcatctgatgcagaacaa
gaacctaaaattgatccatatgcatttgttgaaggagatgaggaattcct
ttttcctgataaaaaagatagacaaaatagtgagagagaagctggaaaaa
aacacaaggtagaagatgggacatctagtgtaacagtgttatcacatgaa
gaagatgctatgtcattatttagtccctctatcaagcaagatgctccacg
ccctactagtcatgcccgtcctccatcaacaagtttgatttatgactcag
acctggctgtctcttatactgaccttgataatctcttcaattctgatgaa
gatgaactaacacctggatctaaaaaatcagcaaatggatcagatgataa
agccagctgcaaggaatcaaagacaggaaatctggacccgttatcttgca
taagcactgcagatcttcataaaatgtatcctacaccaccatcattggaa
caacatattatgggattttccccaatgaatatgaataataaagaatatgg
tagtatggatacaacacctggaggaactgttctagaaggaaatagttcta
gtataggagcgcagttcaaaattgaggttgatgagggattctgtagcccc
aaaccttctgaaattaaagatttttcttatgtctataagcctgaaaattg
tcaaattctagtgggatgttccatgtttgcacctctaaaaactctaccaa
gccaatatctgcccccctatcaaattgccagaagagtgtatttaccgtcag
agttggactgttggaaaattggaattgctttcttcagggccttcaatgcc
attcatcaaagagggtgatggaagtaatatggatcaagaatatggcactg
cttatacacctcaaactcatacttcttttgggatgcctcctagcagtgca
cctcctagtaacagcggagcaggaattcttccttctccatccacccctcg
gtttccaactccaaggactccaaggactcctcggactcctcgtggagctg
gtggacctgctagtgctcaaggttcagtcaaatatgaaaattcagacttg
tattcaccagcttctaccccatctacatgcagaccccttaattctgttga
acctgcaactgtcccttccatccctgaagcacacagtctttatgtaaacc
tcatcctttcagaatcagttatgaatttgtttaaagactgtaactttgat
agttgttgcatctgtgtttgcaacatgaacatcaagggtgccgatgttgg
agtttacattccagatccaacgcaggaagcacaatataggtgtacctgtg
gcttcagtgctgtcatgaacagaaaatttggaaacaattcaggattattt
cttgaagatgaactagatatcataggacgcaatacagactgtggcaaaga
agcagaaaaacgttttgaagctctcagggctacctctgctgaacatgtta
atggaggactaaaggaatctgaaaaattatctgatgatttgatattattg
ctacaagatcagtgcactaatttattttcaccctttggagcagcagacca
agatccttttcctaaaagtggtgtaattagcaattgggtacgtgttgaag
agcgtgactgttgcaatgactgctaccttgcattagaacatgggcgtcag
ttcatggataacatgtcaggaggaaaagttgatgaagcacttgtgaaaag
ttcatgcttacacccctggtccaaaagaaacgatgtgagtatgcagtgct
cacaggatatacttcgaatgctcctctctcttcagccagttcttcaggat

gccattcagaaaaaaagaacagtaagaccttggggtgttcagggtcctct
cacttggcaacaatttcataaaatggctggccgaggctcttatggaactg
atgaatccccagaaccactgccaatccccacatttttgttgggttatgat
tatgattatctggtgctttctccatttgctcttccttattgggagagact
tatgctggaaccctatggatctcaaagagatatagcctatgttgtactgt
gtccagaaaatgaagccttgttaaatggagcaaaaagctttttagagat
cttactgcaatatatgagtcctgtcgattaggtcaacatagacctgtttc
tcgactgttaacagatgggatcatgagagttggatctactgcatcaaaga
aactatcagaaaagttggtagcagaatggttttctcaggcagctgacggt
aacaatgaagcattttctaaactcaagctttatgcacaagtctgcagata
tgacctaggtccttatcttgcttccctgccattggacagctctctacttt
cccagccaaatttagttgcccctacaagtcagtctttgattactccacct
cagatgacaaatactggaaatgctaatactccatctgccaccttagcatc
tgcagcgagcagcactatgacagtgacttcaggtgttgccatatctactt
cagttgccacagctaattcaactttgaccacagcttcaacttcatcttca
tcatcctccaacttgaatagtggagtatcatcaaataaactaccttcgtt
tccaccctttggcagtatgaacagtaatgctgcaggatccatgtctacac
aagcaaatacagttcagagtggtcagctaggagggcaacagacatcagct
ctacagacagctgggatttctggagaatcatcttcacttcccactcagcc
gcatcctgatgtgtctgaaagcacgatggatcgggataaagtgggaatcc
ccacagatggtgattcacatgcagtcacgtatccacctgcaattgttgtt
tatataattgatccttttacatacgaaaatacagacgagagcactaactc
ttctagtgtgtggacattggggctacttcgatgctttctagaaatggtcc
agactcttcctcctcatatcaagagtactgtttctgtacagattattcct
tgtcagtacctgttgcaacctgtgaagcatgaagatagagaaatctatcc
ccagcatttaaaatccctggctttttcggcctttacccagtgtcggaggc
cacttccaacatcaaccaatgtgaaaacattgactggctttggtccaggt
ttagccatggaaactgcccttagaagtcctgatagaccagagtgtattcg
actttatgcacctccttttattctggctccagtgaaggacaaacagacag
agctaggagaaacatttggagaagctggacagaaatataatgttcttttt
gtgggatactgtttatcacatgatcaaaggtggattcttgcatcttgcac
agatctatatggagaacttttagaaacttgtatcattaacatcgatgttc
caaatagggctcgtcggaaaaaaagttctgctagaaaatttggtctacag
aaactttgggagtggtgcttaggacttgtacaaatgagttcattgccatg
gagagttgtaattggtcgtctaggaaggattggtcatggagaattgaaag
attggagctgtttgctgagtcgtcgaaacttgcagtctctaagtaaaagg
ctcaaagacatgtgtagaatgtgtggtatatctgctgcagactcccctag
cattctcagtgcttgcttggtggcaatggagccgcaaggctcttttgtta
ttatgccagattctgtgtcaactggttctgtatttggaagaagcacgact
ctaaatatgcagacatctcagctaaataccccacaggatacatcatgtac
tcatatacttgtgtttcctacttctgcttctgtgcaagtagcttcagcta
cttataccactgaaaatttggatttagctttcaatcccaacaatgatgga
gcagatggaatgggtatctttgatttgttagacacaggagatgatcttga
ccctgatatcattaatatccttcctgcttctccaactggttctcctgtac
attctccaggatctcattacccccatggaggtgatgcgggcaagggtcag
agtactgatcggctactatcaacagaacctcatgaggaagtacctaatat

tcttcagcaaccattggcccttggttactttgtatcaactgccaaagcag
gtccattacctgactggttctggtcagcatgtcctcaagcacaatatcag
tgtccccttttcttaaggcctctttgcacctccacgtgccttcagtgca
atctgacgagctgcttcacagtaaacactcccacccacttgactcaaatc
agacttcagatgtcctcaggtttgttttggaacagtacaatgcactctcc
tggctaacctgtgaccctgcaacccaggacagacgctcatgtctcccaat
tcattttgtggtgctgaatcagttatataactttattatgaatatgctgt
gatcttcatttgatggaactgtgcaagaaaagaacaaggaaaaatggatg
tttcgctgcaggattaagttacaattatcttctcagtgaaggtcatttgt
gatggggtctaattcttattacttcaacaaatattgttttgacttggggg
gaggggctataaccctgctatttttcattgactctattgaactctttagg
atgatgactgatcatacaaaacgtattataacattttcgtagcaaaatta
acctttttttttccagtcacagtatttgtgaaaagtaatgagccatagt
acccagtcatgttaaatgaatattaaaagcatggagaggaaacatgagga
acaatgaatttcaacatatggcttcagaacatgaagatgttcttgtatgg
attatagtatctagtattcaaaaatgcctgcatctcttctcttatttatt
gtaagtttttaaatgtataaattgtcttatatttcttaacctcttttata
aaaattttcctagaaggtttatactgccttcttgctttaaagcaattggt
ctaaaatatatgtaatcgtcttaattaaaaagttgcagtagggttgcttt
tagagtattattttttgtaagggggtgggtgggacagtaaatttgtatt
gtctcgatgtacagtttaacggggatagagggggaataatgtccatacca
ttgtgtgtggaggatttacagctaagctgtagttgcagagtacatgtaca
gtaatgaagttcactgtgtttataaattgaaaaggtaccaggtcttacag
cattttatatatcacatctttacagaataacatgatggcaatatacaagt
ggtattgttaggtggtttaacttagaataaaatgagaattcttcagttat
attttgtactatggtttagggctatgactaatatttcaggccatttccgg
tgaaagaaacttagttttacaagaaaaaccatttgctactgaatgcttaa
actaattttagtgtttaatgttacatgcttaaattttttcagttttaac
agtggcatatttaggcatggaaatattattatgaaatttattttcaggat
ctgctataaggttgaaatttagcccagctctaggcattttacaaattatt
tttcaagcagtcattcttgattgtttgacttttttttttaaattaaagat
tgggaatgtatgtgagagtatgcatatgtatgggtgtgtgtgtgtgcgcg
caatcaaactgtggtgtaaatagattctcagtgaattctggtattcagac
tctattccactagtgaaagaaccatttttttaaacttcccttgcctttttt
atttatttaattttcttggtttggagatgtcagtcccaaacaccagagtc
tgtacttttctataacacagctcagattaaggtagggcatatgccaagga
ggttctcacctccctaaagaagggacttgaattttagggactttaattca
cccctccttcaatacaactttccccccttcttgtttgcacatgccaagata
actgcttttatgcaggctgtaccccccttgaaaaatcctttctacagtgct
gctcacaaaagagcccaagttcgcctcctacctgcattgctgacttgaat
tcacagtcgccgagtctacctagctttcttggaagcagtctagcaaaatt
tctatttgtacgttcactaattatctacaaggacaaaatcagttgtattt
acaaaactctacttcagtgtttgttttagtttttttttttactgaaactt
gtttttgtgaatactctgtgcttagaattaaatatcactttcttatgaac
aacataacttcttcagattgtgtatatgaaaacattagcaagtcttgttt
tttctatgaagcaaacacaattggtgacaaaggttgtcaatcatttcttc

aaaattataatgcagttctaatggtcagcatattttgatattaaatttaa
agatcacctctctgcatttgtttttaaattatgctaatacaccacacatt
atgttggtatgttttgttctgtactttctttaaaaaaaaaaaaaaaaactt
gtctgagatttgaaggaaaatgtgcttatttggaatttccataaaaagag
tatcctttttatacacttaatagtgactttacaaaataaaagttatattc
tcagttgtttaaaatcactaacctatgataaccacacctcaatttgaaag
tagatttaaaattattccctgacaggttatttaatatggagccataagga
gggaacccagtacacaattatttttatttgggaatcagggaatagttcc
caaatatacaggatttattgataagatttttttcttcccttcatatatc
cattcaaactcaatggaaagttattaaataaccattagaaaagctcagta
gacttatttgagaaattaagccttgtgcaggatgatggatttgacttact
aatgtactgtcacagacaaatatgggtagttttgtttaaataggtaagca
aaatattatactttatagcagtggattaccaacaccttgacttctttgtt
acagtgctaacatcttttttttgtgcaggtatccatgattattaagcag
ggtggaagttcagtattttgtcatttaaaaagattagttatataatgtct
gcttccagccagtgagaaacatctagccatacctttcttatgcaagccat
tgagttatcaggactgtgaattaacactgtatgaataaatttctgtacac
cttattgtttggccagaaggccaccaagtgtacttatatgtaatccttaa
attttaaagtagctgtaatttttaaatatttctaaacttttcttaaacca
ctaaaattaagctcttactacttagtcaactatcctcagctgtattcgta
ctcaattgtcagtatggcacagattactgtattaaaatattctcctttcg
tcttcatatttaccttctgaggtaatttttaacttaatgtgttactaca
aagatttgcagatctttaatcaagcactatgttaatactgtaatatcaga
atactatgttgcattatttaaaatgttcaaattgaatagattaaaaagtt
tttaaatgctattgcatcatataatttgctattcatccactatggcattg
catatcaatcagttaatacacttaatgttgcatgagtgatattttggtct
gggtttcctcttaagattttagtttgtctgaattaaggaaaaatgttttt
aatatacattcttattttgtcccacccctccagaaataagctggaaatct
taactttttggggggtctttttttggtgttttaatgggcccagaactgtgg
tttaaatttttatgtatgtattttctttttttgtggagtataaatttaaaa
actggatttgggacctaaaatactcctcaggttgatgtattcatgaagtt
ttaaaacatctttagttttcaaagtaaactggatatgtggaccttaaagt
tattgagtttaagctacaaattgtaacgtcattactggacatgtcagcat
caaccctctcaaaatagcttggtcactttatgaaggggcgttttaaagtt
gttgtttagcagtgacatttaatatggtccaattgcttttctttttaacg
tgacaaaaagagaataaggaacaaacactattgctgccgaatgccataac
actgagttgtacaaattgtgattgaggaaatgaaaaggtttatacttttt
aaaaaaaaaaaaaacaaaaacaaaaaacaaaacttcaaatggaataaatta
ttcatgaagccttcaaaaaaaaaa

>NM_000366 5
ggaaggggggcaggagaaaaaagcttttccaaaaaagtattggctgtctt
gaggaatgcggtcgccccccttgggaaagtacatatctgggagaagcaggc
ggctccgcgctcgcactcccgctcctccgcccgaccgcgcgctcgccccg
ccgctcctgctgcagccccagggcccctcgccgccgccaccatggacgcc
atcaagaagaagatgcagatgctgaagctcgacaaggagaacgccttgga

tcgagctgagcaggcggaggccgacaagaaggcggcggaagacaggagca
agcagctggaagatgagctggtgtcactgcaaaagaaactcaagggcacc
gaagatgaactggacaaatactctgaggctctcaaagatgcccaggagaa
gctggagctggcagagaaaaaggccaccgatgctgaagccgacgtagctt
ctctgaacagacgcatccagctggttgaggaagagttggatcgtgcccag
gagcgtctggcaacagctttgcagaagctggaggaagctgagaaggcagc
agatgagagtgagagaggcatgaaagtcattgagagtcgagcccaaaaag
atgaagaaaaaatggaaattcaggagatccaactgaaagaggccaagcac
attgctgaagatgccgaccgcaaatatgaagaggtggcccgtaagctggt
catcattgagagcgacctggaacgtgcagaggagcgggctgagctctcag
aaggccaagtccgacagctggaagaacaattaagaataatggatcagacc
ttgaaagcattaatggctgcagaggataagtactcgcagaaggaagacag
atatgaggaagagatcaaggtcctttccgacaagctgaaggaggctgaga
ctcgggctgagtttgcggagaggtcagtaactaaattggagaaaagcatt
gatgacttagaagacgagctgtacgctcagaaactgaagtacaaagccat
cagcgaggagctggaccacgctctcaacgatatgacttccatgtaaacgt
tcatccactctgcctgcttacaccctgccctcatgctaatataagtttct
ttgcttcacttctcccaagactccctcgtcgagctggatgtcccacctct
ctgagctctgcatttgtctattctccagctgaccctggttctctctctta
gcatcctgccttagagccaggcacacactgtgctttctattgtacagaag
ctcttcgtttcagtgtcaaataaacactgtgtaagctaaaaaaa

>NM_006037 3
ggaggttgtggggccgccgccgcggagcaccgtccccgccgccgcccgag
cccgagcccgagcccgcgcacccgcccgcgccgccgccgccgccgcccga
acagcctcccagcctgggcccccggcggcgccgtggccgcgtcccggctg
tcgccgcccgagcccgagcccgcgcgccggcgggtggcggcgcaggctga
ggagatgcggcgcggagcgccggagcagggctagagccggccgccgccgc
ccgccgcggtaagcgcagccccggcccggcgcccgcgggccattgtccgc
cgcccgccccgcgccccgcgcagcctgcaggccttggagcccgcggcagg
tggacgccgccggtccacacccgccccgcgcgcggccgtgggaggcgggg
gccagcgctggccgcgcgccgtgggacccgccggtccccagggccgcccg
gccccttctggacctttccacccgcgccgcgaggcggcttcgcccgccgg
ggcgggggcgcgggggtgggcacggcaggcagcggcgccgtctcccggtg
cggggcccgcgcccccgagcaggttcatctgcagaagccagcggacgcc
tctgttcaacttgtgggttacctggctcatgagaccttgccggcgaggct
cggcgcttgaacgtctgtgacccagccctcaccgtcccggtacttgtatg
tgttggtgggagtttggagctcgttggagctatcgtttccgtggaaattt
tgagccatttcgaatcacttaaaggagtggacattgctagcaatgagctc
ccaaagccatccagatggactttctggccgagaccagccagtggagctgc
tgaatcctgcccgcgtgaaccacatgcccagcacggtggatgtggccacg
gcgctgcctctgcaagtggcccccctcggcagtgcccatggacctgcgcct
ggaccaccagttctcactgcctgtggcagagccggccctgcgggagcagc
agctgcagcaggagctcctggcgctcaagcagaagcagcagatccagagg
cagatcctcatcgctgagttccagaggcagcacgagcagctctcccggca
gcacgaggcgcagctccacgagcacatcaagcaacaacaggagatgctgg

ccatgaagcaccagcaggagctgctggaacaccagcggaagctggagagg
caccgccaggagcaggagctggagaagcagcaccgggagcagaagctgca
gcagctcaagaacaaggagaagggcaaagagagtgccgtggccagcacag
aagtgaagatgaagttacaagaatttgtcctcaataaaaagaaggcgctg
gcccaccggaatctgaaccactgcatttccagcgaccctcgctactggta
cgggaaaacgcagcacagttcccttgaccagagttctccaccccagagcg
gagtgtcgacctcctataaccacccggtcctgggaatgtacgacgccaaa
gatgacttccctcttaggaaaacagcttctgaaccgaatctgaaattacg
gtccaggctaaagcagaaagtggccgaaagacggagcagccccctgttac
gcaggaaagacgggccagtggtcactgctctaaaaaagcgtccgttggat
gtcacagactccgcgtgcagcagcgccccaggctccggacccagctcacc
caacaacagctccgggagcgtcagcgcggagaacggtatcgcgcccgccg
tccccagcatcccggcggagacgagtttggcgcacagacttgtggcacga
gaaggctcggccgctccacttcccctctacacatcgccatccttgcccaa
catcacgctgggcctgcctgccaccggcccctctgcgggcacggcgggcc
agcaggacgccgagagactcacccttcccgccctccagcagaggctctcc
cttttccccggcacccacctcactccctacctgagcacctcgcccttgga
gcgggacggagggggcagcgcacagccctcttctgcagcacatggtcttac
tggagcagccgccggcacaagcacccctcgtcacaggcctgggagcactg
cccctccacgcacagtccttggttggtgcagaccgggtgtccccctccat
ccacaagctgcggcagcaccgcccactggggcggacccagtcggccccgc
tgccccagaacgcccaggctctgcagcacctggtcatccagcagcagcat
cagcagtttctggagaaacacaagcagcagttccagcagcagcaactgca
gatgaacaagatcatccccaagccaagcgagccagcccggcagccggaga
gccacccggaggagacggaggaggagctccgtgagcaccaggctctgctg
gacgagccctacctggaccggctgccggggcagaaggaggcgcacgcaca
ggccggcgtgcaggtgaagcaggagcccattgagagcgatgaggaagagg
cagagcccccacgggaggtggagccgggccagcgccagcccagtgagcag
gagctgctcttcagacagcaagccctcctgctggagcagcagcggatcca
ccagctgaggaactaccaggcgtccatggaggccgccggcatccccgtgt
ccttcggcggccacaggcctctgtcccgggcgcagtcctcacccgcgtct
gccaccttccccgtgtctgtgcaggagcccccccaccaagccgaggttcac
gacaggcctcgtgtatgacacgctgatgctgaagcaccagtgcacctgcg
ggagtagcagcagccaccccgagcacgccgggaggatccagagcatctgg
tcccgcctgcaggagacgggcctccggggcaaatgcgagtgcatccgcgg
acgcaaggccaccctggaggagctacagacggtgcactcggaagcccaca
ccctcctgtatggcacgaacccccctcaaccggcagaaactggacagtaag
aaacttctaggctcgctcgcctccgtgttcgtccggctcccttgcggtgg
tgttggggtggacagtgacaccatatggaacgaggtgcactcggcggggg
cagcccgcctggctgtgggctgcgtggtagagctggtcttcaaggtggcc
acaggggagctgaagaatggctttgctgtggtccgcccccctggacacca
tgcggaggagagcacgcccatgggcttttgctacttcaactccgtggccg
tggcagccaagcttctgcagcagaggttgagcgtgagcaagatcctcatc
gtggactgggacgtgcaccatggaaacgggacccagcaggctttctacag
cgaccccagcgtcctgtacatgtccctccaccgctacgacgatgggaact
tcttcccaggcagcggggctcctgatgaggtgggcacagggcccggcgtg

ggtttcaacgtcaacatggctttcaccggcggcctggaccccccatggg
agacgctgagtacttggcggccttcagaacggtggtcatgccgatcgcca
gcgagtttgccccggatgtggtgctggtgtcatcaggcttcgatgccgtg
gagggccaccccacccctcttgggggctacaacctctccgccagatgctt
cgggtacctgacgaagcagctgatgggcctggctggcggccggattgtcc
tggccctcgagggaggccacgacctgaccgccatttgcgacgcctcggaa
gcatgtgtttctgccttgctgggaaacgagcttgatcctctcccagaaaa
ggttttacagcaaagacccaatgcaaacgctgtccgttccatggagaaag
tcatggagatccacagcaagtactggcgctgcctgcagcgcacaacctcc
acagcggggcgttctctgatcgaggctcagacttgcgagaacgaagaagc
cgagacggtcaccgccatggcctcgctgtccgtgggcgtgaagcccgccg
aaaagagaccagatgaggagcccatggaagaggagccgcccctgtagcac
tccctcgaagctgctgttctcttgtctgtctgtctctgtcttgaagctca
gccaagaaactttcccgtgtcacgcctgcgtcccaccgtggggctctctt
ggagcacccagggacacccagcgtgcaacagccacgggaagcctttctgc
cgcccaggcccacaggtctcgagacgcacatgcacgcctgggcgtggcag
cctcacagggaacacgggacagacgccggcgacgcgcagacacacggaca
cgcggaagccaagcacactctggcgggtcccgcaagggacgccgtggaag
aaaggagcctgtggcaacaggcggccgagctgccgaattcagttgacacg
aggcacagaaaacaaatatcaaagatctaataatacaaaacaaacttgat
taaaactggtgcttaaagtttattacccacaactccacagtctctgtgta
aaccactcgactcatcttgtagcttatttttttttaaagaggacgttttc
tacggctgtggcccgcctctgtgaaccatagcggtgtgcggcggggggtc
tgcacccgggtgggggacagagggacctttaaagaaaacaaaactggaca
gaaacaggaatgtgagctgggggagctggcttgagtttctcaaaagccat
cggaagatgcgagtttgtgcctttttttttattgctctggtggatttttg
tggctgggttttctgaagtctgaggaacaatgccttaagaaaaaacaaac
agcaggaatcggtgggacagtttcctgtggccagccgagcctggcagtgc
tggcaccgcgagctggcctgacgcctcaagcacgggcaccagccgtcatc
tccggggccaggggctgcagcccggcggtccctgttttgctttattgctg
tttaagaaaaatggaggtagttccaaaaaagtggcaaatcccgttggagg
ttttgaagtccaacaaattttaaacgaatccaaagtgttctcacacgtca
catacgattgagcatctccatctggtcgtgaagcatgtggtaggcacact
tgcagtgttacgatcggaatgctttttattaaaagcaagtagcatgaagt
attgcttaaattttaggtataaataaatatatatatgtataatatatatt
ccaatgtattccaagctaagaaacttacttgattcttatgaaatcttgat
aaaatatttataatgcatttatagaaaaagtatatatatatatataaaat
gaatgcagattgcgaaggtccctgcaaatggatggcttgtgaatttgctc
tcaaggtgcttatggaaagggatcctgattgattgaaattcatgttttct
caagctccagattggctagatttcagatcgccaacacattcgccactggg
caactaccctacaagtttgtactttcattttaattattttctaacagaac
cgctcccgtctccaagccttcatgcacatatgtacctaatgagtttttat
agcaaagaatataaatttgctgttgatttttgtatgaatttttttcacaaa
aagatcctgaataagcattgttttatgaattttacatttttcctcaccat
ttagcaattttctgaatggtaataatgtctaaatctttttcctttctgaa
ttcttgcttgtacatttttttttacctttcaaaggtttttaattattttt

gtttttatttttgtacgatgagttttctgcagcgtacagaattgttgctg
tcagattctattttcagaaagtgagaggagggaccgtaggtcttttcgga
gtgacaccaacgattgtgtctttcctggtctgtcctaggagctgtataaa
gaagcccaggggctctttttaactttcaacactagtagtattacgagggg
tggtgtgtttttcccctccgtggcaagggcagggagggttgcttaggatg
cccggccaccctgggaggcttgccagatgccgggggcagtcagcattaat
gaaactcatgtttaaacttctctgaccacatcgtcaggatagaattctaa
cttgagttttccaaagaccttttgagcatgtcagcaatgcatggggcaca
cgtggggctctttacccacttgggtttttccactgcagccacgtggccag
ccctggattttggagcctgtggctgcaaggaacccagggacccttgttgc
ctggtgaacctgcagggagggtatgattgcctgaccaggacagccagtct
ttactctttttctcttcaacagtaactgacagtcacgttttactggtaac
ttattttccagcacatgaagccaccagtttcattccaaagtgtatattgg
gttcagacttgggggcagaagttcagacacaccgtgctcaggagggaccc
agagccgagtttcggagtttggtaaagtttacagggtagcttctgaaatt
aactcaaacttttgaccaaatgagtgcagattcttggattcacttggtca
ctgggctgctgatggtcagctctgagacagtggtttgagagcaggcagaa
cggtcttgggacttgtttgactttcccctccctggtggccactctttgct
ctgaagcccagattggcaagaggagctggtccattccccattcatggcac
agagcagtggcagggcccagctagcaggctcttctggcctccttggcctc
attctctgcatagccctctggggatcctgccacctgccctcttaccccgc
cgtggcttatggggaggaatgcatcatctcacttttttttttttaagcaga
tgatgggataacatggactgctcagtggccaggttatcagtgggggggact
taattctaatctcattcaaatggagacgccctctgcaaaggcctggcagg
gggaggcacgtttcatctgtcagctcactccagcttcacaaatgtgctga
gagcattactgtgtagccttttctttgaagacacactcggctcttctcca
cagcaagcgtccagggcagatggcagaggatctgcctcggcgtctgcagg
cgggaccacgtcagggagggttccttcatgtgttctccctgtgggtcctt
ggacctttagcctttttcttcctttgcaaaggccttgggggcactggctg
ggagtcagcaagcgagcactttatatccctttgagggaaaccctgatgac
gccactgggcctcttggcgtctgccctgccctcgcggcttcccgccgtgc
cgcagcgtgcccacgtgcccacgccccaccagcaggcggctgtcccggag
gccgtggcccgctgggactggccgcccctccccagcgtcccagggctctg
gttctggagggccactttgtcaaggtgtttcagtttttctttacttcttt
tgaaaatctgtttgcaaggggaaggaccatttcgtaatggtctgacacaa
aagcaagtttgatttttgcagcactagcaatggactttgttgtttttctt
tttgatcagaacattccttctttactggtcacagccacgtgctcattcca
ttcttctttttgtagactttgggcccacgtgttttatgggcattgataca
tatataaatatatagatataaatatatatgaatatatttttttaagtttc
ctacacctggaggttgcatggactgtacgaccggcatgactttatattgt
atacagattttgcacgccaaactcggcagctttggggaagaagaaaaatg
cctttctgttcccctctcatgacatttgcagatacaaaagatggaaattt
ttctgtaaaacaaaaccttgaaggagaggagggcggggaagtttgcgtct
tattgaacttattcttaagaaattgtactttttattgtaagaaaaataaa
aaggactacttaaacatttgtcatattaagaaaaaaagtttatctagcac
ttgtgacataccaataatagagtttattgtatttatgtggaaacagtgtt

ttagggaaactactcagaattcacagtgaactgcctgtctctctcgagtt
gatttggaggaattttgttttgttttgttttgtttgtttccttttatctc
cttccacgggccaggcgagcgccgcccgccctcactggccttgtgacggt
ttattctgattgagaactgggcggactcgaaagagtccccttttccgcac
agctgtgttgactttttaattacttttaggtgatgtatggctaagatttc
actttaagcagtcgtgaactgtgcgagcactgtggtttacaattatactt
tgcatcgaaaggaaaccatttcttcattgtaacgaagctgagcgtgttct
tagctcggcctcactttgtctctggcattgattaaaagtctgctattgaa
agaaaaagaaagcgaacagtttttgtttgtttttttgccgtgtgtgctc
catagtggaggcgctattttccaattgatgagaatgacaaacatatataa
tctatctatctatctatctatctatctatctatacagggggcttgaacct
tactcacccaagagcttcttacggaatgtggtagaaaaccaagttgtaac
gacactgtaacctacctgatgcctgttcgcgcccgccgtgagctgcgcac
tggccgtggccaccattcacctctgtaatttaatccgtttctcttggatt
gtctggacgtgcccgatggttctttcttttgctcagtggagttggaggtt
ttgtgtttggttttctcattcttgtcttgttttgtgtgggtggattttca
ccgaccaatgatatcctcttctgacggtcaccttctttccacttcactgg
agtccagtattctgtaccacatcacgcaacgtgttatcttgtggtgtaaa
taaagactgcgttacttgccctcccaaaaa

>NM_000165 3
gagtcagtggcttgaaacttttaaaagctctgtgctccaagttacaaaaa
agcttttacgaggtatcagcacttttctttcattagggggaaggcgtgag
gaaagtaccaaacagcagcggagttttaaactttaaatagacaggtctga
gtgcctgaacttgccttttcattttacttcatcctccaaggagttcaatc
acttggcgtgacttcactacttttaagcaaaagagtggtgcccaggcaac
atgggtgactggagcgccttaggcaaactccttgacaaggttcaagccta
ctcaactgctggagggaaggtgtggctgtcagtacttttcattttccgaa
tcctgctgctggggacagcggttgagtcagcctggggagatgagcagtct
gcctttcgttgtaacactcagcaacctggttgtgaaaatgtctgctatga
caagtctttcccaatctctcatgtgcgcttctgggtcctgcagatcatat
ttgtgtctgtacccacactcttgtacctggctcatgtgttctatgtgatg
cgaaaggaagagaaactgaacaagaaagaggaagaactcaaggttgccca
aactgatggtgtcaatgtggacatgcacttgaagcagattgagataaaga
agttcaagtacggtattgaagagcatggtaaggtgaaaatgcgaggggggg
ttgctgcgaacctacatcatcagtatcctcttcaagtctatctttgaggt
ggccttcttgctgatccagtggtacatctatggattcagcttgagtgctg
tttacacttgcaaaagagatccctgcccacatcaggtggactgtttcctc
tctcgccccacggagaaaaccatcttcatcatcttcatgctggtggtgtc
cttggtgtccctggccttgaatatcattgaactcttctatgttttcttca
agggcgttaaggatcgggttaagggaaagagcgacccttaccatgcgacc
agtggtgcgctgagccctgccaaagactgtgggtctcaaaaatatgctta
tttcaatggctgctcctcaccaaccgctcccctctcgcctatgtctcctc
ctgggtacaagctggttactggcgacagaaacaattcttcttgccgcaat
tacaacaagcaagcaagtgagcaaaactgggctaattacagtgcagaaca
aaatcgaatggggcaggcgggaagcaccatctctaactcccatgcacagc

cttttgatttccccgatgataaccagaattctaaaaaactagctgctgga
catgaattacagccactagccattgtggaccagcgaccttcaagcagagc
cagcagtcgtgccagcagcagacctcggcctgatgacctggagatctaga
tacaggcttgaaagcatcaagattccactcaattgtggagaagaaaaaag
gtgctgtagaaagtgcaccaggtgttaattttgatccggtggaggtggta
ctcaacagccttattcatgaggcttagaaaacacaaagacattagaatac
ctaggttcactgggggtgtatggggtagatgggtggagagggaggggata
agagaggtgcatgttggtatttaaagtagtggattcaaagaacttagatt
ataaataagagttccattaggtgatacatagataagggctttttctcccc
gcaaacacccctaagaatggttctgtgtatgtgaatgagcgggtggtaat
tgtggctaaatatttttgttttaccaagaaactgaaataattctggccag
gaataaatacttcctgaacatcttaggtcttttcaacaagaaaaagacag
aggattgtccttaagtccctgctaaaacattccattgttaaaatttgcac
tttgaaggtaagctttctaggcctgaccctccaggtgtcaatggacttgt
gctactatatttttttattcttggtatcagtttaaaattcagacaaggcc
cacagaataagattttccatgcatttgcaaatacgtatattctttttcca
tccacttgcacaatatcattaccatcactttttcatcattcctcagctac
tactcacattcatttaatggtttctgtaaacatttttaagacagttggga
tgtcacttaacattttttttttgagctaaagtcagggaatcaagccatgc
ttaatatttaacaatcacttatatgtgtgtcgaagagtttgttttgtttg
tcatgtattggtacaagcagatacagtataaactcacaaacacagatttg
aaaataatgcacatatggtgttcaaatttgaacctttctcatggattttt
gtggtgtgggccaatatggtgtttacattatataattcctgctgtggcaa
gtaaagcacactttttttttctcctaaaatgtttttccctgtgtatccta
ttatggatactggttttgttaattatgattctttattttctctccttttt
ttaggatatagcagtaatgctattactgaaatgaatttcctttttctgaa
atgtaatcattgatgcttgaatgatagaattttagtactgtaaacaggct
ttagtcattaatgtgagagacttagaaaaaatgcttagagtggactatta
aatgtgcctaaatgaattttgcagtaactggtattcttgggttttcctac
ttaatacacagtaattcagaacttgtattctattatgagtttagcagtct
tttggagtgaccagcaactttgatgtttgcactaagattttatttggaat
gcaagagaggttgaaagaggattcagtagtacacatacaactaatttatt
tgaactatatgttgaagacatctaccagtttctccaaatgcctttttaa
aactcatcacagaagattggtgaaaatgctgagtatgacacttttcttct
tgcatgcatgtcagctacataaacagttttgtacaatgaaaattactaat
ttgtttgacattccatgttaaactacggtcatgttcagcttcattgcatg
taatgtagacctagtccatcagatcatgtgttctggagagtgttctttat
tcaataaagttttaatttagtataaacata

>NM_003769 2
cgccgacccccttcgcttccgctccgcgttcccacaatgcagtgcggctga
gcgcctcggagcccgcggggacgctgcgggggacccgtgctgaggcggc
ggcggcgacgtgggctgcggcgggcccgcggcgtcgggcggtgcggatgt
cgggctgggcggacgagcgcggcggcgagggcgacgggcgcatctacgtg
gggaaccttccgaccgacgtgcgcgagaaggacttggaggacctgttcta
caagtacggccgcatccgcgagatcgagctcaagaaccggcacggcctcg

tgcccttcgccttcgtgcgcttcgaggacccccgagatgcagaggatgct
atttatggaagaaatggttatgattatggccagtgtcggcttcgtgtgga
gttccccaggacttatggaggtcggggtgggtggccccgtggtgggagga
atgggcctcctacaagaagatctgatttccgagttcttgtttcaggactt
cctccgtcaggcagctggcaggacctgaaggatcacatgcgagaagctgg
ggatgtctgttatgctgatgtgcagaaggatggagtggggatggtcgagt
atctcagaaaagaagacatggaatatgccctgcgtaaactggatgacacc
aaattccgctctcatgagggtgaaacttcctacatccgagtttatcctga
gagaagcaccagctatggctactcacggtctcggtctgggtcaaggggcc
gtgactctccataccaaagcaggggttccccacactacttctctcctttc
aggccctactgagacaggtgatgggaatttttctttatttttttaggtta
actgagctgctttgtgctcagaatctacattccagattgaggatttagtg
tcttaggaaatttttttaattttttttttttaaagaagaaaaaaaactac
ataatttctaccagggccatattagcagtgaaacattttaaactgcagaa
attgtggttttggttcagaaacaagttgtatatttttcacccctgattat
gggaaaaaaatcagttctgtctttgtgggttgctctactatggagatcaa
cagttactgtgactgagtcggcccattctgtttagaaatatattttaaat
gtttagtaattgaaaaaaaaaaaaaaaaaaaaaaaaaaaaaaaaaaaaaa
aaaa

>NM_006148 2
gcagtcagcctgagagcgctgaggcgccagcaccgcgggccccgccgtcc
ccccaggaacccgcccccgcgcagccccacgtcgcccggagagcgcgcct
cgctgcgcgtgcccttgcccgcgcccgaggcgcgtgcccgccccagtgca
gcccctcctccccgctgtgtttattaggggaaggagggcggaggcggagg
ccagttccccagctccagccgccgtcgctgctgcctgtgtagttgcagcc
gcggccgcctcccgccagctcgcctcggggaacaggacgcgcgtgagctc
aggcgtccccgccccagcttttctcggaaccatgaaccccaactgcgccc
ggtgcggcaagatcgtgtatcccacggagaaggtgaactgtctggataag
ttctggcataaagcatgcttccattgcgagacctgcaagatgacactgaa
catgaagaactacaagggctacgagaagaagccctactgcaacgcacact
accccaagcagtccttcaccatggtggcggacaccccggaaaaccttcgc
ctcaagcaacagagtgagctccagagtcaggtgcgctacaaggaggagtt
tgagaagaacaagggcaaaggtttcagcgtagtggcagacacgcccgagc
tccagagaatcaagaagacccaggaccagatcagtaacataaaataccat
gaggagtttgagaagagccgcatgggccctagcgggggcgagggcatgga
gccagagcgtcgggattcacaggacggcagcagctaccggcggcccctgg
agcagcagcagcctcaccacatcccgaccagtgccccggtttaccagcag
ccccagcagcagccggtggcccagtcctatggtggctacaaggagcctgc
agccccagtctccatacagcgcagcgccccaggtggtggcgggaagcggt
accgcgcggtgtatgactacagcgccgccgacgaggacgaggtctccttc
caggacggggacaccatcgtcaacgtgcagcagatcgacgacggctggat
gtacgggacggtggagcgcaccggcgacacggggatgctgccggccaact
acgtggaggccatctgaacccgcagcgcccccatctgtcttcagcacatt
ccacggcatcgcatccgtcctgggcgtgacccgtccattcttcagtgtct
ctgtttttaaaacctgcgacagcttgtgattcctacccctcttccagct

tcttttgccaactgaagccttcttctgccacttctgcgggctccctcctc
tggcaggcttccccccttgatcgacttcttggttttctctctggatggaac
gggcatgggcctctctgggggaggcagggctggaatgggagacctgttgg
cctgtgggcctcacctgcccctctgttctctcccctcacatcctcctgcc
cagctcctcacatacccacacattccagggctggggtgagcctgactgcc
aggaccccaggtcaggggctccctacattccccagagtgggatccacttc
ttggttcctgggatggcgatggggactctgccgctgtgtagggaccagtg
ggatgggctctacctctctttctcaaagagggggctctgcccacctgggg
tctctctccctacctccctcctcaggggcaacaacaggagaatggggttc
ctgctgtggggcgaattcatcccctccccgcgcgttccttcgcacactgt
gattttgccctcctgcccacgcagacctgcagcgggcaaagagctcccga
ggaagcacagcttgggtcaggttcttgcctttcttaattttagggacagc
taccggaaggaggggaacaaggagttctcttccgcagcccctttccccac
gcccacccccagtctccagggacccttgcctgcctcctaggctggaagcc
atggtcccgaagtgtagggcaagggtgcctcaggaccttttggtcttcag
cctccctcagcccccaggatctgggttaggtggccgctcctccctgctcc
tcatgggaagatgtctcagagccttccatgacctcccctccccagcccaa
tgccaagtggacttggagctgcacaaagtcagcagggaccactaaatctc
caagacctggtgtgcggaggcaggagcatgtatgtctgcaggtgtctgac
acgcaagtgtgtgagtgtgagtgtgagagatggggcgggggtgtgtctgt
aggtgtctctgggcctgtgtgtgggtggggttatgtgagggtatgaagag
ctgtcttcccctgagagtttcctcagaacccacagtgagaggggagggct
cctggggcagagaagttccttaggttttctttggaatgaaattcctcctt
ccccccatctctgagtggaggaagcccaccaatctgccctttgcagtgtg
cagggtggaaggtaagaggttggtgtggagttggggctgccatagggtct
gcagcctgctggggctaagcggtggaggaaggctctgtcactccaggcat
atgtttccccatctctgtctggggctacagaatagggtggcagaagtgtc
accctgtgggtgtctccctcgggggctcttcccctagacctccccctcac
ttacataaagctcccttgaagcaagaaagagggtcccagggctgcaaaac
tggaagcacagcctcggggatggggagggaaagacggtgctatatccagt
tcctgctctctgctcatgggtggctgtgacaaccctggcctcacttgatt
catctctggttttcttgccaccctctgggagtccccatcccattttcatc
ctgagcccaaccaggccctgccattggcctcttgtcccttggcacacttg
tacccacaggtgaggggcaggacctgaaggtattggcctgttcaacaatc
agtcatcatgggtgtttttgtcaactgcttgttaattgatttggggatgt
ttgccccgaatgagaggttgaggaaaagactgtgggtggggaggccctgc
ctgacccatcccttttcctttctggccccagcctaggtggaggcaagtgg
aatatcttatattgggcgatttgggggctcggggaggcagagaatctctt
gggagtcttgggtggcgctggtgcattctgtttcctcttgatctcaaagc
acaatgtggatttggggaccaaaggtcagggacacatccccttagaggac
ctgagtttgggagagtggtgagtggaagggaggagcagcaagaagcagcc
tgttttcactcagcttaattctccttcccagataaggcaagccagtcatg
gaatcttgctgcaggccctccctctactcttcctgtcctaaaaataggggg
ccgttttcttacacacccccagagagaggagggactgtcacactggtgct
gagtgaccgggggctgctgggcgtctgttctttaccaaaaccatccatcc
ctagaagagcacagagccctgaggggctgggctgggctgggctgagcccc

tggtcttctctacagttcacagaggtctttcagctcatttaatcccagga
aagaggcatcaaagctagaatgtgaatataacttttgtggaccaatacta
agaataacaagaagcccagtggtgaggaaagtgcgttctcccagcactgc
ctcctgttttctccctctcatgtccctccagggaaaatgactttattgct
taatttctgcctttccccctcacacatgcacttttgggccttttttttat
agctggaaaaaacaaaataccaccctacaaacctgtatttaaaaagaaac
agaaatgaccacgtgaaatttgcctctgtccaaacatttcatccgtgtgt
atgtgtatgtgtgtgagtgtgtgaagccgccagttcatctttttatatgg
ggttgttgtctcattttggtctgttttggtcccctccctcgtgggcttgt
gctcgggatcaaacctttctggcctgttatgattctgaacatttgacttg
aaccacaagtgaatctttctcctggtgactcaaataaaagtataattttt
acctgcgga

>NM_003417 4
attttcttctgcacttctgtctggagaggtctgtagccactgagggcccc
ggtcggggccgctttgcaggtccctagtcaggaccgagcaggggagtagg
ataggaatccccgccgcacctttgtacgagcctgaccccttccgtgggtt
tgttcctgggtcgccgtcaagctgcggtctctcctcccccgcccttcagc
cccgcggtctccaggggcggcgccctgggtctggaacgcggttgccaccg
aggaggcggcggccctgcgtctggaacgccgttgccaccgaggaggcggc
ggccccgagcgcgcctggaagccccgggcaaccggccagggtcgggcaca
ggtggggtccgtcaggccgcccggggctcctctgtcccagctctgcggcc
cagggggtgacgtgatggcggcagcggtgctgacggaccgggcccaggtg
tctgtgacctttgatgatgtggctgtgactttcaccaaggaggagtgggg
gcagctggacctagctcagcggaccctgtaccaggaggtgatgctggaaa
actgtgggctcctggtgtctctggggtgtcctgttcccaaagctgagctg
atctgccacctagagcatgggcaggagccatggaccaggaaggaagacct
ctcccaagacacctgtccaggcgacaaaggaaaacctaagaccacagaac
ctaccacttgtgagccagccttgtcagagggaatctcacttcagggacaa
gtgacacaaggaaactcagtggactcacagttggggcaagccgaggatca
ggatgggctatcagaaatgcaggaaggacacttcagaccaggaatagatc
cccaggagaagtctcctgggaagatgagccctgaatgtgatggtttaggg
acagctgatggtgtgtgttcaaggattggacaggagcaagtctctccagg
agatagagtccgtagccataactcatgtgagtcaggtaaagatcccatga
ttcaggaagaggaaaataactttaaatgcagtgaatgtggaaaagtattt
aacaagaaacacctccttgctggacatgagaaaattcactctggagttaa
gccctatgaatgcacagaatgtgggaaaacctttattaagagcacacatc
tcctgcaacatcacatgatccacactggggagaggccctatgagtgcatg
gagtgtggaaaggccttcaaccgcaagtcataccttacccagcaccagcg
gattcacagtggagagaagccttacaagtgcaatgaatgcggaaaggcct
tcacccaccgctccaattttgtcttgcataacaggagacacactggagaa
aaatcctttgtgtgcacagaatgtggccaagtctttcgacataggccagg
ctttctccggcactatgttgtccacagtggtgagaatccctatgagtgct
tggagtgtggcaaggtcttcaaacacaggtcatatctcatgtggcaccag
cagactcataccggggagaagccctatgagtgcagtgaatgtgggaaggt
cttcttggagagtgcagccctgattcaccactatgtcatccacactggag

agaagccctttgagtgcctcgagtgtgggaaggctttcaaccaccgatcc
tacctcaagaggcaccagcggattcacactggggagaagcccttcgtgtg
cagtgaatgtggaaaggccttcacccactgctctacttttatcttgcata
aaagggcccacactggagaaaagcctttcgagtgcaaagagtgtgggaaa
gcctttagcaatcggaaggacctcattcgccacttcagcatccacactgg
agagaagccctatgagtgcgtggagtgtggaaaggccttcacccgcatgt
cgggcctcacgaggcacaagcggattcatagtggagagaagccctatgaa
tgtgttgagtgtgggaaatcgttttgctggagcacaaacctcattcgaca
tgccattatccacactggagagaagccctataaatgtagtgaatgtggaa
aggccttcagtcgcagctcgtccctcactcagcatcaaaggatgcatact
gggaaaaatcccatcagtgtaacagatgtgggaagaccttttacaagtgg
acaaacctcagttacccttcgagaacttcttttagggaaggactttttga
atgtaaccactgaggcaaatattttgccagaggaaacatcttcctctgca
tctgatcaaccataccaaagagaaaccccacaagtgtcttcactgtgaga
aaaccttctgttgctgaatattacttgtcatctgaagagtcatattagaa
attcgttcagtctagagccttattctccatctgataatttatcctggaga
gagacccagtggttattgtgcacataggagaaccttcagctgcatctttc
tccttagtttacagtgcaattttatctcaggaattatttttaaaaggagg
aggggacatagaaaaaatgaaatgcaagcacacatcttttcaggcttctc
tgccaagcctatggcgctttgtcatggatttcttagtgtatttgggggaa
gggaaatgtttcaaggtaaagaaccttgaccctttatgtgcttgtatgta
catttattgctatccagtgtcagaacagttagtttaggaaaagtatgcaa
actttaatcgcacatcttctgtattccacaatagtacttactcttgagaa
gcataactttatgacaacttagggggcttgagccatgaaatactcacgtt
taagtcagtaaggatacacatgttaacattcaggcttttgtcttgatgcc
cgtcttttggtttacgtcatcattgtagccatatggtaaatttttatttg
ttaaatttttaaaaattacttagctcaaaatgtcagtggtaaatttttaa
attgagtaaagtgattcttctttgctcttatttaaaatcgacagcatttc
tagttcctttgacattccatataatttttaggattagtttgtacttattt
acaaaacagtcttgttcagatttttacagaaattgagttaagtctgtgga
ttaatctgttaagaattggtatctttactatgttgggtcttgtagttcat
gagtgcaggagtaggcctcttaatttataatagttaagcattccttaaag
tatttcattaatgttaaaaattttcagcgtatagatccctatgcattttt
gttagaatttgcatcctagggtgctttttttgtttgtgtgtgtgtgtaatt
tgagtgattgtaaatggcattgatgtttgaactttgaattgcacacaatt
tttggtagtatacagaaatataatatttttggacattgttttgctaccct
gcaaccttattgagttcacttattatttttagctatttctttcgacagaa
tccttggaattttctatatagaaatgtcatctgcaaatacagaccatttt
ttatctttcatcttacctgtaatatacctttttatttcctttttgtttgtc
ttattgcattgcacaagctgggacttccattatagcgttgaataggaata
gtgagaaaggccatccttacctggttcctgatagtagggggggacaatgtt
cagttttttgtttttttgtttttttgtttgagacagggtctcgctctgtca
cccaggctggagtgcagtggcacgatcttgtctcaccatagccttaagtt
cctgggctcaagtgatcccccccacctcagccctcaccaccccccagtagc
tggtactacaagcatgcaccaccagtcccagctaatttttgtatttttttg
gtagagacagggtttcaccatggtgcccaggctggtcttgaactcctggg

ctcaagcaacctgcttgcctgggtctcccaaagtgctgggattacaggcg
tgagccacagtgcacagcatgttcagtttttcatcatgaagtataatgta
agttttataaaacatatgtcctctataagggtaaaaaatttcctctgttc
ccagttttctgagactctgtgttgctatgaataagtgtggaattttgttc
agtactttttctccaccagttgatgtcattatgctattttccttctacat
cctattgatggaatgtattgctttgatcaagttttgaatattgagccagc
cttgcatccttagaataaaccatacttgtttgtagagtataattgttttt
atatatcatttgacatcattccctttgtttgtattttgttgaggaaatat
tcctctaatttattggctaataatgtgtagttctttttttcttttgtttct
tatgttctaaatgtcttatgtgtggttttgatgtctaggtgatcttgacc
tttcaacatgaattggaaggagttctcttctaatttttggaagactgtgt
agaattggtgttatttgttcttgtcatgtttgatgacctctagtcaacca
ttgggccagagatttttcttctggaggcttttaaattacatatttatttt
attggataggtagaactattcgaagtatctatttcttgtgtaataaattt
tgatagtctgtcctgttgaaggaatatttcatctgtatttgtagcactcc
cttattcttttgatagcaggatctgcacttctttttatttttaatattta
aaatgtgcatactcattttatgattgagtcttcttagacatttatcagtt
ttattgcgcatcatgctgaaagatgtaatttttgcttcatctctcacttc
agagaactagcactttatattaatgatttgtctctattcttgatttcttt
gatttctgctctgatcggtattatattccacttgctttggttgtatttgg
ctctttctaacttttcaaggtggggttttagattattgatttgagatttt
tttgcttttttaatgtaagcattacattgtataaatttacctcatatcac
tattggtttgatcccacaagttttaatgttttcattacattttaattcta
atagtttttatttttttgagacattctctcttagccatgaattatttaata
gtatgcttaattttctggtgtttggcatatttgttactgatttctaattt
gttccatgtttgatctgactgtttaaatgtgttaacattttttttatgacc
caggatagggtctatcttgtttcgtatagctgcttgaaaagaatgggtgt
tctgctgttactgggtggagtttctataaatgtctctatgatcctgctca
ttgttattcaagtcagctatgtccttgctgattttctgtccagcatttct
gtcagttgctgagaggggtgttgaagtcctgaacataggccgggtgcagt
ggctcacgcctgtaatcctagcactttgagaccactaaggcaggtggatc
acctgaggccaggagttcgagaccagcctggccaacatggcaaaaccctg
tctactaaaaatacaaaaattagccaggcatggtggcgtgtgcctgtaat
cccagctgctgggggggctgaggcaggaggatcagttgaacgtgggaggca
gaggttgcagtgagctgagatctcaccactgcactccagcctgggcaaca
gagcgagactctgtctcaacaacaacaacaaaaagtcctgaacatgattg
tggaagtgtgttgctctttcaagttctatcactttttgtttgcaaagttc
aaagctgtattgtttggtacatatacatgtaggtttgccaagtctttgtg
gtgaattgactcttctgtcattatgtgatgtcattttttttgccttttaat
agtcttgtcaatactttacctgatgttctcatagtgactcctgcatattt
tgattaatgtttgcatggttaatatttcttcattttattttaaagcttac
ctgtatcattacttatgaagtcagtttctttgaacagcatatactcaggc
catgctttttttattcattctgcatatgtctctcttaattggtatgttga
aatgatttacattaaaataattattgatattttagggcttaagtgtgccc
ttaaatgatttttgtgttctttttttattgttcctctgttatttggggttg
tttactagtcttcctataggttacttcacctttttttttttttaataattt

gatttgatacatatgtagtgtttttttagtatagatcctccttgacttatg
atggggttatgtcacaataaacccattgcaagttgaaaatactatgtcaa
atatgcatttaatacacctaccctgctgaacatcatagccgatcttgcct
tcagaatgctcagaaaatttacattagcctgcagttgggcaaaatcatct
aacacaaggcctactttataataaagttactgcaaagaattttaaataaa
aattcaagtgtggtttctactgaatgcatgtcgcattcgcaccattgtaa
agtcaagtagtaagtcgaaccatcctaagtcagggactgtctgtatatat
ctgcatttttagtgattgttctactattacagtgtaaatatataactta
tgacagtttaataagttatcagcaatttagcactttacttccattaggtt
cctttagcttacctactaatgtaactatcttaagtaatagtaattcctct
tcaaaaattgagcgctatgttgcaagatgtaatttttcttttcctttttt
ttgagaccaagtctcactctgtcacccacgctggagtgctgtgatgcgat
ctcggctctctgcaacctctgcctcccgggttcaagcaattaactgcctc
agcttccctagtaatggattacaggcgcccgccaccacgcctggctaatt
tttgtatttttagtagagacggggtttcaccatcttggccaggttggtct
tgaactcttgacctcatgatccacccgcctcggcccccccaaagtgctggg
gttacaggtgtgagccactgcacccggccacaagatgtaatttttacttt
atctctcacacgtattttacaaaacgtcatgagaaacattgtctcttgac
ctttttttttttttttttttttaaagagacagagtctcactctgtcacc
caggctggagtgcagtggcacgatcttggctcactgcaacctccgcctcc
tgggttcaagcgattctcctgcctcagcctcctgagtagctgggattaca
ggtgtgcgccgccacacccagctaattttgtatttttagtagagacgggg
tttcaccatgttgctcaggctggtctcaaactcctgaccttgtgatccgc
caaccttggcctgtcgacctctttacccattccattgttcattcttcttt
cttgaaatcccaaaccttctattaacatttcttttcagttatacactgaa
ctttactgtagcctttcttctagagtaacaaatgttctttgttttccttc
ctctaaagatgtctttatgtttccttcattcccaaagaatatttttgtgg
aatataagattcagagttggcagttgttttcttttagacttcagagatgt
atctctctgttctgtacattattgtttaatataagaaacctactagcatt
caaataatcattccctattttacaatgcatcagttctgtcaagcttcatt
caaagtgtttttcattgtctctagtgttcagaagtttggctgtgatgtgc
gtggcatggaagtttttgggtgtattctatttggcgctccctggtgcttg
cccagctttttgatctgtaggattatgccttttgcaaaatttggggaact
ttcaactattatttcttcaaatattttttcacccccccagtcttgtctttt
ttagggacttcaataacatgagtggcagatcttgttttacactcccatgg
gtccttcaggctctcatctttttttcttttttccagtctattttctgtcttg
ttaatattgattaatttttattgaccttccatggtcctcactgattgttt
tctttgtcatacctaatctgttgagtttgtgcagtgagttttcattttgg
ttttgtattttccagttgtttaatttccattgggtgggttcttttgtaca
ccttctgtttctttgcttatttttttaacgccaaagaaagactctcagaga
atagacaactatattccaaagtcatggttctctggtggtttgtcttgaca
tttgaatagaaatgttaaactatctgggggaatagaaagcccacagtctt
ctgagttgtgctacaccaatatttctatgaacagatcttacaactgagag
tgatctgcagatttttcagagtcatgttctccatggaatgtttgtaaaat
tccctagctctctgcactgagctgagatcgtgccactgcactccagcctg
ggcaacagagcgagactccatctcaaaaaaaaaaaaaaattctctagctct

ctatgccttgttgtatcctcaggaaggagtcacctctcttccaggatcac
tcttgccttttgttattggacacattttcctacctcctgcccagtctttt
ttaggctctctgtcatacttcctcgcctagtctttaacttgtctctgata
accagatttttccccataatctgtatctgtattcaagtctctatcctca
tccccaaatggatttcaccccgcttgtccatcagtctctgcccatttctt
ctgtctcccacagacatggtctgtgtagttgttctgcacactacccgtcc
tcgccccttctcatttccctcactccacggacagctacaggagggcttcc
atcatttgtcctcttacttccttgttagaactttgtgctgctgaccacca
ctctgaaccaaaacttctttcttggttcttgaaagttgctgtctttctcc
tccagagggtctgtgtcttccttccaactcttaaacattttgtttccca
gaatccatcattggccattgctttttcacttgccatgagctctgtgaatg
agatggttcctgctgaagacatttactttcaatgatctacagaatttccc
aaatctatgtctccagactggatgtttctcacgtctgtggcccactgggc
atcttctgcactttctctgcatttgtaaacactgagcctgtatgcgtgtc
tatgtggccatggccctggccgtggggatgcacccataattataatgaga
gcaggtgaatgtcatgtggctatgtatgagacattgttgtgaatgtttta
catgcatcagctctttcaacatttacagcatctgtgtgtggtaggtaatg
ttgtttccttcattttgcagagcagaatatagataggctcaggttaagaa
tttgcccaaggttacacagttatgaagtttggatttaggaatgggaggcc
acgtagactggcttcagaattcatgtctttaaccactttattgcttctca
tgttgggggggtttgttttctggtcactatttttactaaagttgattcaac
aatgctataatctgttactctcattcgacaatacatcttgactgtttctt
caacttgaggaaggtaaaccaaagccactctttaatgggtgcaaatatct
gttgtaactatgtagaagatacagtgtctgggtttgaggacaggggagtg
ggaaaactgagtagggaagcggtaagacatgcatctggctttaggagtgt
ttgaagtgcctaaagatcagggtgaatttcccaaatttgaatcttgttgc
ccaagccacattgcaaccacctctttttctcttacctcgtccccacatcc
aggcactgccccattctgttcattccaccttttttttttagtagaccctcc
atatatgtgtgtgttttggtgaatggccacacacagcaaagttattttta
ttgcaattttgttgttgttgtttgagacacagagtctcatactctgccac
ccagactgaagtgcagtggtgcgatctcagttcactgcaacctccatccc
cccatgttcaagcaattctcctgcctcagcctcccaagtagctgggatta
caggcatgggccaccatgcccggctaatttttgtatttttttgtagacacg
gggtttcaccatgttggccaggctggtctcacactcctgacctcaggtga
tccacccgcctcggcctcccgaagtgctggcattacaggcatgagccacc
acacccagcctttattgcaatttttggttccctcacagaatgtttaccat
gaagctgtgcgtataggtggtaccatgtatgacataccttgacctcaaag
ataatctacctgggcttttttttgtttgtttgttttgagatggagtctcac
tctgtcacccatgctggtgtgcagtggcgtgatcttggctcacagcagcc
tctgcctcccgggttcaagcgattctcctgcctcagcctcctgagtagct
gggactacaggtgcctgcgaccacacccagctaatttttgtatttttagt
agtagtgggatttcaccatgttggccaggatggcctccacctcctgactt
catgatccacccgcctcggcctcccaaagtgctgggattacaggcgtgag
ccaccgcacctggcctacctgggctttttttggacagatgtacatgatttt
agaaattgttatgaatgactgtcttgggatcctcaagaacacccccaggt
ttgacgatttgctgagaagactcaggtctcagcattctgtcatactcaac

agctatgatttactgcagcaaaaaaatacaaagcaaatagtaaagggaaa
aagccaaaattcagagaaaaccaggcacaatcttctgagtcttgtttcag
tgaagtcacaggatgtgccttatttaagttccatctggtacttatgacaa
cacatgctatatgtccactgggaagcttattagagactcagtgccagggt
ttttattgggcatgttttctagcacatcccacaattctggacttccagaa
ggttagcaggggttcagcttttcagctgaaaacgttatccccctttcagc
tggggggaaaaaaaggcaccgaaaaacagggcagtgagctactcctgttt
tgggtgggtggagcagtagtaagcctcccaaaatctaaggtcccacactc
cagcaagggtgaaccttgcaaactcaaaaggatagcagtctccaacctgc
tgttttatacagtgacttaagtggtactaaaatgctatataagtcaaatt
tacgtaacattaagcaaataatttttttcagcaaaaaaataatggtcat
gactttgtggtgaaggcttatgtaatgattgtaataaattcaggtggctc
acgcctgtgataccagcacttttgggaggctgaggcgggtggatcacttg
aggtcaggagtttgagaccagcctagccaacatggtgaaatcccatctct
acaaaaatgaaaaattagccgggcgtagtggtacatgcatttagtcctag
ctacttgggaggctgaggcaggaaaatcgcttgaacctgggaggcagagg
ttgcagtgagccgagatcatgccactgcactccagcctgggtgacagagt
gagactgtgtctcaaaaaaaaaaaaaaaaaaaaaaatgcctcagttttgtt
aagaggaaaaagcaaatcctaagttacagtggaacttatgaacaccctag
cctagagatttaatttagtgatgaaaaggtacagagagagtctttcaaga
aaggacctagtctgctcgcgttctctctctctctctctatatatatat
atgtatatgtgtgtgtacatatggaccacttcaagctacacacacacaca
cacatatacacgtgtatgaatatatatatatggctataagtggtgcgact
tgcaggtactccctttgttcacctttgtaaagatgttattgccagttcag
tgtgtatttctatagtatatatgtaaacaatttgtcatccttttgttgct
gcttttgaaactagttcatggcttcagtgggggagagatttaaataatat
gggtttataatttctgcattattaaatgcagataacttgtgattaaagag
tatgttattggaaaaaaaaaaa

>NM_006454 2
ccgtcccggggcggacgggcgcgggcgggaggatggagctgaactccctg
ctgatcctgctggaggcggccgagtacctggagcgcagggatcgagaggc
cgagcacggctacgcctcggtgctgcccttcgacggcgacttcgccaggg
agaaaacaaaggcggccggcctggtgcgcaaggccccgaacaacaggtct
tcacacaacgagctagaaaagcacagacgagccaaactcaggctgtacct
tgagcagctcaagcaactggtgcccctgggccccgacagcacccgccaca
ccacgctgagcctcctgaagcgggccaaggtgcacatcaagaaactggag
gagcaggaccgccgggcactgagcatcaaggagcagctgcagcaggagca
tcgtttcctgaagcggcgcctggagcagctgtcggtgcagagcgtggagc
gcgtgcgcacagatagcacgggctctgctgtctccacggacgactcagag
caagaagtggacatagagggcatggagtttggccctggtgagctggacag
tgttggcagcagcagtgacgcggacgaccactacagcctgcagagtggca
ccggcggcgacagtggcttcgggccccactgccggcggctgggccgcccc
gccctctcgtaggcccgtgccctctgctccttggcctgcctgcccgccag
ccacgcgtgtcagccctccagttctccttcagttgacgccagcctctcca
caggcccactgctgtgccattctggaagctccagctgctgctgggctgcc

tggcactgcccgcttgccggtcagggcctgccgagctgcctgccccttcc
agctgggcagagtcccctgcaaggaggcagggcccagcttccacatccgg
agccctggtcagcatagccgcccacggtctgttctcagattcctaatcat
tccagaagtattaaacgtcattgctgcaaacctcggcaggtgccgtgtga
ggggcttaatgaccaccacagggagctcagaccccaaccctggatcccag
gagaaaggagtggaccgaggaaggaaggaaggcaaggctgtctgtccatc
cgtccgtctgtccacctacctgtcagtccacataggctcctggcgtggac
aaggggtctgtgaagggcgggaactgggtgagcacctggggcaggtgggt
ggttaaggtccttcccacttcgcaggtgtcagaacctagggctgggctct
cggggccaggcaggccagcccagcccacgccgagctgggcagcgtctgct
ctggtaggactgtcagacgcacacgcgcacgcacctagacacacccactc
atgtacatgctcacacatgcagacacacctgggcgtcccgaggtcacatg
ttctggggatgatggccttcaggggtcatctggcaaacagcccctgggct
gtgcctggatccccctctagctcctgctcacccacgcccacccagtagtc
ctgcctgtctgcacaggagaggggcttctcttcctggctggggctggggt
gaactggaggctggttaagttgcagccgctgggtcctcgggggcttactc
atctcccttttttaaacaaaaagcaaaaaagtaaaatgctgcactgccca
gcagcccggttagggctcctggagccaccttaggaaagggcttctcatga
gctctgctgcggcagcttcagctggcagagaggctttcccagaaaaaaaa
aaaaaaccatttttaaaaagaagaaagcctaaagactctcggcctaggga
cgtccgtgtgtgccgcctctgtttcctgtaccagatttttgtattctatt
ttcctagctgttgttgcgtccttgtttgctgaggggtgggagccacccag
cgtctcagggacctgtccctccgtcacgtcgtcaaagtgtgccttgtgtc
ttgtgtcaggccttgcccttcccaccagcatgtccctcgtggctcagggt
gccccaggcctgcccagctagtgctgtcctcccatctcctgtgggcagcc
cctcccggcagccagggcttcctggaggcgatgcagccaggcccctgtgg
gtggcacggaggggctgtgacctggtccccagtgttgccctccccagtgg
ctggcaggggcctgcttgctcactagagagatggattctcacctgtcacc
tgactcgagccccctgcttcctggcctaggcgagggttccaggtttcaga
cactggcagccaatgaagactgtgctcgctgggtggtgcaggcctggcac
caggaggcttgcacccgcctttccttcctgacgtcctctgtccttggggc
tggcccatagcagtgcctgccgtgcctctggtacatctgtagccaattcc
catatcatggggaaaattcgtgtctattttcagtcgtacgcatagacgcc
ccaggatgggggcccactgtggcggaagggggtccctggaaacaactct
ggcacagaacctgccctgcaggctgtagggggcatggtgcctggagctga
ggggcatccgaacgcgttgcgggtggttgtgaggaggcctgtctgcatct
ccttccggccccactggggtccaggggtgcccagaaaggagcttcccctg
cctgcctgagtctgtccccccaggcttcatttcaaacaccgtggcacctc
cgagcaaggcgggccgtgtgtaaaagcttgcttccccagccagcactgca
gggccctgaggtggtcgtgtccctgccctcaattcttgaagcaccagctc
cctgccccaccctccagtgcctgaggcagctaggggcttctgctctcatc
tctgaccagcagaatccacccggtgaccagtggtggcccctcagcccacc
ctcccggcagctcagcctgtggctcttgaggccgtggttcccacgtggac
tgggaggcagtctcagccacccggggtgctgttcagctgcccctccctgc
catcagcaggtgggtgaggggttgcccactgggtgggggcccgtgctagg
agtcaccacatgctccaacctcccactgctccctgtcaggggcccaggct

gccatcactggaggctgcagggaccaagaggccatcaccgtgtctataga
gagcagacagaagcagaacagagcccggggctcctgagcctctgcgtgtg
ccctcccagcccacaccagtgctctcggccactgagcacccagactcagg
cttgggttccccagccttattggaaggcagctcccgcataccaggataac
ccccgcaaaccacatagcagacccccgccatcctcgcagagtgggagagg
ctgcagcaaggctttgcctctgcagaccccatcttagtggcacggtgctt
gggcctgtgtccccgggtggtggaaccctgtaccggtctgtggccctagg
gtccctgctctgtctgccccggcccgtgctgtccgctgggtgaggcaggc
tcccccgtgccctgcctccctctgtcagggaacctgggacccccctcccca
ctgcctgcacagaggaccctgaccctcggccagcagggtggccccaggtc
catgttgggcactagggcaggttccgtgccagagtcgggggccacacgag
ggcctggtgccggtgagggggggcgtgcgctagaggggggaaaggggccccc
ggccacctgtccaccgtgtgggccgtgctgtgtccttatgtcattgtaat
ataaatacagatttttatatctc

>NM_007081 2
gctgcgaggacgcgggccgagccggaagtggagtgcgctgcggcgcgagc
tgggccggcgggcgtggttcgagagcgcgcagagtccagactggcggcag
ggcccgaggggccgacccgcagcgtccctggtctctccagccctcactcg
gaaccgcactgacaataccctcccctcccttgggctggacccctctctac
agctaggagccaatggcagaagacaaaaccaaaccgagtgagttggacca
agggaagtatgatgctgatgacaacgtgaagatcatctgcctgggagaca
gcgcagtgggcaaatccaaactcatggagagatttctcatggatggcttt
cagccacagcagctgtccacgtacgccctgaccctgtacaagcacacagc
cacggtagatggaaggaccatccttgtggacttttgggacacggcaggcc
aggagcggttccagagcatgcatgcctcctactaccacaaggcccacgcc
tgcatcatggtgtttgatgtacagaggaaagtcacctataggaacctgag
cacctggtatacagagcttcgggagttcaggccagagatcccatgcatcg
tggtggccaataaaattgatgacataaacgtgacccaaaaaagcttcaat
tttgccaagaagttctccctgcccctgtatttcgtctcggctgctgatgg
taccaatgttgtgaagctcttcaatgatgcaattcgattagctgtgtctt
acaaacagaactcccaggacttcatggatgagatttttcaggagctcgag
aacttcagcttggagcaggaagaggaggacgtgccagaccaggaacagag
cagcagcatcgagaccccatcagaggaggcggcctctccccacagctgag
gggctggggctaggggtgggtggagcccttttaaaatacccttcccttca
acaactctccagctctgaatggagaaactctctaggccatcccctcttct
acctcctgcaacccacccatcctattagcctcccacattctaggcccgtg
atacagggatgaggtcagcaccagcaaactctggactggtggaagaattc
cccaccagatctccttgaagcagaagtagggatcagcatcattaacacct
tccccacccccctccccgcaggcagacagtgaagagaatcagaaaacatga
ttatgtgtcactttaatacaggaaatttaggtgtttttggtgtttttgt
ttttgtttttgttttctttccaaagctcacctcggggacaattccttggg
cttctcctgaggtaatgatttaccccccacccacagctgagtctgtgag
gccccatcctttccctacgttttctcccatctttttcctcttcagtctc
ccagtcatctggtttgtttgtttctttgttcgttctgagacggagtctcg
ctctgtcgccaggctggagtgcagtggcgcagtctcggctcgctgcaacc

tctgactccctggttcaaacgattctcctgcctcagcctcccgagtggct
ggcatcaccacgcccagctaatttttgtatttttagtagagacggggttt
caccatgttgcccaggatggtctcgatctcctcacctcgtgatccgcccg
cctcggcctcccaaagtgctgggattacaggcatgagccaccgcgcccgg
ccccaatcatctgtttttaaacaatcgtttttgagcagatagctattcat
tccagattttcgtgtacccattctgtttcaggagctcttctaggtaaagc
tgagatcacaggaacagcaggtgacaggcctagctatagttaggaataca
caagccgtaaaatcgagtccttacagccataccacaaggtacgtccattt
ggactacaagaagagcttcctttaaagttcctatttcagcataaagaggc
tgtcctttttttttgaggaatagtttggaccttgtgcctcctgtgggaggc
tgaggactgcaagaggagagctagcagatatgcctgttcacccctctctg
gtacttgtggcttgctagtatgtttttatgataatctcgggcattgtttg
cattgtgtttattaatagggttttgtttttgttgtttcctttttttacagt
aaaggctgaattacataaacattgagtaaatgt

>NM_003290 2
tttccagcagctgtggccagcggtgccgacgtcaggccctcccccagcgg
tgctgacgtcggcggtccggccgggtgacctcatcgccccgacggcagcc
ggcccggggggcggggagaggcgggggcggcccccgcgcaggcaaaggct
tggggggccggggcgcggctgtgcagctctcgccggagccgagcccagcc
gagcgtccgccgctgcccgtgcgcctctgcgcctccgcgccatggccggc
ctcaactccctggaggcggtgaaacgcaagatccaggccctgcagcagca
ggcggacgaggcggaagaccgcgcgcagggcctgcagcgggagctggacg
gcgagcgcgagcggcgcgagaaagctgaaggtgatgtggccgccctcaac
cgacgcatccagctcgttgaggaggagttggacagggctcaggaacgact
ggccacggccctgcagaagctggaggaggcagaaaaagctgcagatgaga
gtgagagaggaatgaaggtgatagaaaaccgggccatgaaggatgaggag
aagatggagattcaggagatgcagctcaaagaggccaagcacattgcgga
agaggctgaccgcaaatacgaggaggtagctcgtaagctggtcatcctgg
agggtgagctggagagggcagaggagcgtgcggaggtgtctgaactaaaa
tgtggtgacctggaagaagaactcaagaatgttactaacaatctgaaatc
tctggaggctgcatctgaaaagtattctgaaaaggaggacaaatatgaag
aagaaattaaacttctgtctgacaaactgaaagaggctgagacccgtgct
gaatttgcagagagaacggttgcaaaactggaaaagacaattgatgacct
ggaagagaaacttgcccaggccaaagaagagaacgtgggcttacatcaga
cactggatcagacactaaacgaacttaactgtatataagcaaaacagaag
agtcttgttccaacagaaactctggagctccgtgggtctttctcttctct
tgtaagaagttccttttgttattgccatcttcgctttgctggaaatgtca
agcaaattatgaatacatgaccaaatattttgtatcggagaagctttgag
caccagttaaatctcattccttccctttttttttcaaatggcaccagctt
tttcagctctcttattttttccttaagtagcatttattcctaaggtaggc
agggtatttcctagtaagcatactttcttaagacggaggccatttggttc
ctgggagaataggcagccccacactttgaagaatacagaccccagtatct
agtcgtggatataattaaaacgctgaagaccataaccttttgggtcaact
gttggtcaaactataggagagaccagggaccatcacatgggtagggattt
tccatccagagccaataaaaggactggtgggggccgggggtggctattgt

gggaagtcataacccacagatagatcaacctaagaatcctggcccttctc
cactctccaccatgcaggacaaacatcttctcaagcagtcaacgtagaat
gcttgggaaatagtcataattacccacatatagtaattaatagatggtaa
ttaattgatccttgatgtgatgttcttttgcatatttccttcattctaaa
gttgttccctggccgggagcgttggctttcgcctgtaatcccaacacttt
gggaggccaggacagatcacttgaggtcaggagttcgagaccagcccagc
caacatggcgaaaccatgtctctactaaaaatacaaaaattatggtgacg
cctgcctgtagtcccagctactcgggaggctgaggcaggaggatcgcttg
aacccaggaagtggagactgcagtgagccgatatcgcaccacagcgctcc
agcctggtcgacagagtgagactccatctcaagaaaaaataaaaataaag
ttgttctctgaagagcaaatgtctcattccagtaatgacccactcagcag
gaatatggtggagttcagtccaattcaggtcagccatatccaaaagacca
caagtcattactaagttgagcaaaagagtttttatctattagcagaaagg
gcctctctggcagcagagattaaaaactggcccaacttcatttccatact
tcagggaacagcaaattgaggatttacttatctaggacttgaattccttc
tttgggaccaagttaataaaagaccaagaaactcctgattaaactggata
atgaaggattctgtagacagggctgcacgtatcggctttgtttgacttct
cttttctcagttaacatctcagagctagaacattccacattccccagcag
cgtgtgggggctgactaaagtttacaattccaactaaaaatcaccctgct
tctggcttatctgaatcccttacccaccccaccccaccaccctactccta
tttattcagcaccacactacccaggaaatacactagcaaattgtgcaatg
gaataaaatccacactttagattcttgcaactgtatcatatgtaatagta
tcactttttctacattttggtcaaataaatttttacataaactac

>NM_019114 3
caggccgccggcccgggagagcgcgcggcggcggcggcggcgggacggcc
ccgagcgcgccctcccgcctgcggccactcgcagccggcgctggccgggc
cggcgcgccccgcaggcggctagagcgcggcctcggcctcggcgcgggct
tgccccgggccgtagcccgcgagaggcggcgcgggcggccgagggcactg
acggcgtcgcgggacgctcccgggcggcggcgcagcggcagcggcagcgg
cagcggcagcgcagggggcggaggcagggggcgcccccagccaggatgct
gcggttcctgcgccggacctttggccgccgctccatgcagcgctacgcgc
ggggcgcggcggggcgcggggccgccgggctgggggacgagcgcgatggg
gggccacgggggggcccggccgccgccgcctcctcctcggcgctgcccgc
cgcgcccgggggcagcgtgttcccggcgggcggcgggcccctgctcaccg
gcggcgcggccgtgcacatctccgccgccggcgccgccaaggccaccctc
tactgccgcgtcttcctgctcgacgggaccgaagtgagcgtggacctgcc
gaaacatgccaaaggccaggatttgtttgatcagattgtgtaccacttgg
accttgtggaaacagattactttggcctccagttcctcgactctgcccag
gttgcgcactggctggatcatgccaaacccataaaaaagcagatgaaaat
tggacctgcttatgctttacactttcgagttaaatactattcttcagaac
caaacaaccttcgtgaggagtttacaaggtacctgtttgttttacaactc
aggcatgacattctttctggaaaattgaaatgcccttatgaaacagctgt
ggaattagctgctctctgtctacaagcggagcttggggagtgcgagcttc
cagaacacacaccagagcttgtgtctgagtttcggttcattccaaatcag
acagaagcaatggaatttgatatcttccagagatggaaagagtgcagggg

aaagagccctgcccaggcggaactctcctatctgaataaagcgaagtggc
tggaaatgtatggggtagacatgcacgttgtcaggggaagagatggctgt
gaatattctcttggactgaccccgacaggcatattaatctttgaaggagc
taacaaaataggcttattcttttggcctaaaattaccaaaatggatttta
aaaagagcaaattgacactcgtggtggtcgaggatgatgatcagggacgt
gagcaagagcacacgtttgtgttccggttagacagtgccaggacctgcaa
acacctttggaagtgtgcagttgagcaccacgcattcttccgactgcgga
cgccaggaaacagcaaatccaatagatccgactttatcaggctgggctct
cgcttcagattcagtgggcggacagaatatcaagctacacatggctccag
gttacgaagaaccagcacctttgagaggaagcctagtaaacgttatccat
cccggagacattcaacgttcaaagcaagcaacccagtgatagcagcccag
ctctgctctaaaacaaatccagaagtccataattaccagcctcaatatca
tcctaatatccatcccagccagccccggtggcatcctcactctccaaatg
tcagctacccgctcccttccccagtgcttagcagctcggaccggttgcct
tttggcattgaggagaatgggggcacaccgttcctcaccgcagcttcagg
aaggcatcaccaccagcaccagcatcagcatcagcaccagcaccactcaa
actacagcctctcactgaccctggagaacaaagaggggcctctgaggtcc
ccaaactccagcagcaagtcccttacaaaactgagtccaggaacacctgc
cttgttcagtgaagccgctgcccatctaaagaagctggaactggaaactg
tgaaggctgctggaccctggcctcctctgcacatcaacataaacaaggct
gaagaaaagaaagtctcggagaaaactcttcagactccacttttgccttc
ccctgttgcggatcatgtgaagtgtaacattctgaaagcccagttggaaa
atgcttcccgagtgaacatccagggtggaaaggaggaatcaccgtttgta
aatatcaataagaaatccagtcttcaggacgctagtgtaagaagtcctat
tcctattcgtgtggaaactgcccagccagctgtggaaaagccggaaatca
agcctccccgagtgaggaagttaacaagacagtatagttttgatgaagac
gacctccctccagacctggccgaggcagtgggagtgaccacatctacaac
cacaaacaccacaacggccgccacacaagtctccgtgccgctgccgtccc
ccaaggtccagaatgtcagctcgcctcacaagtcagaaggcaaaggcctg
ctgtcccctggggccaagagcccctctgaccgaggaggtgcctttaccct
ggagccgggtgatcttctgatggatttcacagaagccactcctctggcag
agcccgccagcaacccccactgtgcccactctcgctgttctcctccactc
tctctccccatgaaggaagagaccactggagtttgcatgtaccctccaat
caaaacgaggctgataaaaacattcccggttgatacaatgaacccgtttc
ctgatactttcaccacagggccacagtttactgcagacttcagagacagt
aaattacagtgctgtcctggcccgacttccccgctgatcccagcagcgac
cctgaggcctttgacagagaccgtctccacagtgcagaccatttacacca
cccggaaacctgtttctctggcagccagtgcagagacactccggcaggaa
ctggagagagagaagatgatgaaaagactgttgatgaccgaactgtgaaa
ttctccccttgtcacctggaagatggcatggtgccttctgtccgtcttct
ttcttcgggctttgtgtgctcactctagcacagcatacaagtgtgtgctc
tgttcgcccaggtctccatggttagttgaagccaatttctggcttgactt
ttatgggaaaagttattttatgtctcctaagcattagagtttttctatta
ctctatgtagttgagacaggatttgataagtctaggaaaagaaagatggg
aaaacgggattccttttcagaagtacctgtgtgtatctgttaataaccac
aggggttaatatgatgtaggatcttttactatcaatttcaaccatttgat

tttgtatgattgaaacttgcaccgagctttgactgtttgttaaagagtca
tttttaatgaaagaataattctttattgctggtttttcatttacactgat
aaatacacagatcttataaagtctttaacattcatttgtattcagatgtg
agtagaagaactaaaaaaagaaagttacatatcactatgactgaaggtac
ttcagcttaatctgaaatataatttaacttgtgaactccttggatatgat
attatttggaataaacagaatttatcattgaacccaaagtaggaaatgat
agcttacattgtctaaaaatccttacaaggttaagatgattcaatatcaa
gaagattcagaaaattatttctaaagttgatcgattcatgtcgtattgat
agaatcttgaccagaagaaattttgctctttttatatagtttcaagaaat
gtgtttttaaatttttattaatgcacttgaacaactttgcaggaataaag
caaccccctaaccacaaaatatccctctaaattagttccctagctttctc
aatgaatacacacatatttttacatagctatgatcgttgtgtacattctc
ctttgttttacttctcggcctaacacttgtctcctcttgtcaacacagat
tctactctcaccaatttaaatgtctttatatccatgtaacatgggtaacc
tcacttcaccccattattagatatttgagttatatctaatttttcactct
tataaatagtgctgctatgaatgtctgtaaaaaaaaaaaaactgctccttc
ttttggattattcccttaggaatatctccaaagagggattacaaggtcaa
agagcatgaagtattttatagctcttgttttatattgccagattgctttc
tagaaagatccaatctttgggttggaaggaccttaaaggtcatctagttt
agcctccccaccccctctgaatgcttgaatcccctcgacaatttatgatg
ccaccagcaatgtataagcatttctgtttaccaatagctctgccagtatt
gggttttgccatttttatttatttttgctagtttaataggtatgtatagt
tgttcttgaagagttgttttatttcattaattgctagcaaggctgagcac
ttttccatgtgatgatttactagttgtatttccttgtgtgtaaaatgttc
attcatttcttatgaccacttgttaagaggaactgatctcatatatttgt
atcagaactgtatttttatgttatattgtatagtttgctctcctgcccct
ctccttaaaactgaatggtgccaataatttgatactaatgactacaaaaa
aaggtaatgcctcatttactagtattgttgtaaaatgaggaatgtatgtg
aatattcagataaccgaggattaaccctttaagtgctgaatctttaaaat
tttaatatatttttttttgagggaaatctttctaaaatgtattacgcact
tccctgccttagtaaacagagtatactggagagtatttaaccttttcttg
atgagtcatggtcatgattataaacatcagccccttttataccttggtac
ggtgcagtgatatcattaagagctatcaatatgtgtagggcttggcttgg
ccttttataggatattatgctgttctcactgatggttttttactgctctc
tgctctgtcagtggagctatccggggcaattgtagcgtttgggtcctttt
acccctatgtcccccggctatacttttaaaacagctttagctgttcttta
tcttgtgcacatgatacaaaatatgttcccgtacaatatggggctgtcac
ttcttgccaacccagcaccctcttcctcttctaacctgctttctgaggct
tctgctcttcacctcctgctcgctgatggaaacctccagggcaaagctga
aggtttcttggggaagccaggaaagccagtatttcctatgtgtcagatct
gcttggcttccaagaagggatgcatgggctttttggccagtgtttccagg
aggctctgggcttcctgcttcttccccgcttcccccagagttcacagatg
ttgaagtttctgaaggttgacgtcactggaagtctgaccacaaacaagtt
ggctgttactgtatttgaaacccagtacctttggcagctcacctctaacc
agtaaaataagaggattccatggtttca

>NM_000696 3
ctgcccctaaaatctcctagaaccgatcccgcggccccgcccctcccgcg
gccccgcccctcccgcggcccgtcagcctctgccgcggagctgcgtccgc
cactcatgtttctccgagcaggcctggccgcgctctccccgcttcttcgc
agtcttcggccctctcctgtcgccgccatgagcactggcaccttcgtcgt
gtcgcagccgctcaattaccgcggcggggcccgcgtggagccggcggacg
cctccggtaccgagaaagctttcgagccagcaaccggccgagtgatagct
actttcacatgttcaggagaaaaggaagtaaatttggctgttcaaaatgc
aaaggctgcttttaaaatatggagtcaaaaatctggcatggagcgttgcc
gaatccttttggaggctgccaggataataagggaacgggaggatgaaatt
gctactatggagtgcatcaacaatggcaagtccatctttgaggcccgctt
ggacattgacatttcctggcagtgcctggagtattatgcgggcttggctg
catccatggctggtgaacacatccagctcccaggtggatcgtttggttat
accagaagagaaccacttggggtatgtgtgggaataggagcatggaacta
ccccttttcagattgcctcttggaagtcggctccagcattagcctgtggta
atgccatggtctttaaaccttctccctttacacctgtttctgcattgcta
ctggctgaaatctacagtgaggctggtgtacctcctgggctcttcaatgt
ggtgcagggaggggctgccacaggccagtttctgtgtcagcatcccgatg
tggccaaagtctccttcactggaagtgtgcccactggcatgaagatcatg
gagatgtcagctaaaggaatcaaacctgttaccttggaacttggaggcaa
atctccactcatcatcttctcagactgtgatatgaacaatgctgtaaagg
gggcgctgatggccaacttcctcacacaaggccaggtttgctgtaatggc
acaagagtatttgtgcagaaagaaattcttgataaatttacagaggaagt
ggtgaaacagacccaaaggattaaaattggagatccccttctggaagata
caaggatgggtccactcatcaaccgaccacacctggagcgagtccttggg
tttgtcaaagtggcaaaggagcagggtgctaaagtgttatgtggtggaga
tatatatgtacctgaagatcccaaattaaaggatggatattacatgagac
cttgtgtattaactaattgcagagacgacatgacctgtgtgaaggaagag
atctttgggcctgttatgtccattttatcatttgacactgaagctgaggt
tctagaaagagccaatgataccacttttggactagcagctggcgtcttta
ccagggacatccaacgggctcatagagtggtagctgagcttcaggctggg
acgtgcttcattaacaactataacgtcagcccagtggagttgccctttgg
tggatataagaagtcaggatttggcagagagaacggccgtgtgacaatcg
aatattattcacagctgaagactgtgtgtgtggagatgggtgatgtggaa
tctgctttttgaaaacctgcagtgaaacctattgacatggccacgctgtg
gaatgatgtgaattggccctgtttacagaggcagtacaactgaatgttat
tttacatccagaattttggcgttcagtataagagaatggttcatgttact
ctttctctctccatcagcttcctcactgaaaatgtgcattaagtgccttg
tagatactaatcaagaaagctgtgattctcctcaaagcgtatttttgtga
aatcttttaagagccagtaacatacttctagagaacaggaaaagagacta
ggataatacatcttccacacatttggcccactgataatgttaattctctg
gcgtatttcaaagaacttgttcctggctgatccaagtgcagtggtattta
caactaattgatcacaaccagtttgtagatttctttgttccttctccatt
cccactgcttcacttgcctagtcttgaagaaaaaaaacaaaaaacaaaaa
aaaaccttgttcctttataggttcctggtagaatcagtagagatgatttc
agctcattgacattttttaagctatatccccttgtcattccattgagaa

agctgacaactgggatagggaggggattagataatagatggggtcaaatt
ctgtgtgaatgtgaacttgcctagtaagcactttgtctctgttcactact
gcgatagaggaaatctatctccctatcttgggtccttgaactacagcctg
ctgtcttacaccagtggagctaccctttaaatgtacaaattatttgtatg
ctaatgtaatatggtgaaattaaaataaatcacactgttaattgttttcc

**Negative samles:**

>NM_001237 3

ccatttcaatagtcgcgggatacttgaactgcaagaacagccgccgctcc
ggcgggctgctcgctgcatctctgggcgtctttggctcgccacgctgggc
agtgcctgcctgcgcctttcgcaacctcctcggccctgcgtggtctcgag
ctgggtgagcgagcgggcgggctggtaggctggcctgggctgcgaccggc
ggctacgactattctttggccgggtcggtgcgagtggtcggctgggcaga
gtgcacgctgcttggcgccgcaggctgatcccgccgtccactcccgggag
cagtgatgttgggcaactctgcgccggggcctgcgacccgcgaggcgggc
tcggcgctgctagcattgcagcagacggcgctccaagaggaccaggagaa
tatcaacccggaaaaggcagcgcccgtccaacaaccgcggacccgggccg
cgctggcggtactgaagtccgggaacccgcggggtctagcgcagcagcag
aggccgaagacgagacgggttgcacccccttaaggatcttcctgtaaatga
tgagcatgtcaccgttcctccttggaaagcaaacagtaaacagcctgcgt
tcaccattcatgtggatgaagcagaaaaagaagctcagaagaagccagct
gaatctcaaaaaatagagcgtgaagatgccctggcttttaattcagccat
tagtttacctggacccagaaaaccattggtccctcttgattatccaatgg
atggtagttttgagtcaccacatactatggacatgtcaattgtattagaa
gatgaaaagccagtgagtgttaatgaagtaccagactaccatgaggatat
tcacacataccttagggaaatggaggttaaatgtaaacctaaagtgggtt
acatgaagaaacagccagacatcactaacagtatgagagctatcctcgtg
gactggttagttgaagtaggagaagaatataaactacagaatgagaccct
gcatttggctgtgaactacattgataggttcctgtcttccatgtcagtgc
tgagaggaaaacttcagcttgtgggcactgctgctatgctgttagcctca
aagtttgaagaaatatacccccagaagtagcagagtttgtgtacattac
agatgatacctacaccaagaaacaagttctgagaatggagcatctagttt
tgaaagtccttacttttgacttagctgctccaacagtaaatcagtttctt
acccaatactttctgcatcagcagcctgcaaactgcaaagttgaaagttt
agcaatgtttttgggagaattaagtttgatagatgctgacccatacctca
agtatttgccatcagttattgctggagctgcctttcatttagcactctac
acagtcacgggacaaagctggcctgaatcattaatacgaaagactggata
taccctggaaagtcttaagccttgtctcatggaccttcaccagacctacc
tcaaagcaccacagcatgcacaacagtcaataagagaaaagtacaaaaat
tcaaagtatcatggtgtttctctcctcaacccaccagagacactaaatct
gtaacaatgaaagactgcctttgttttctaagatgtaaatcactcaaagt
atatggtgtacagtttttaacttaggttttaattttacaatcatttctga
atacagaagttgtggccaagtacaaattatggtatctattactttttaaa
tggttttaatttgtatatcttttgtatatgtatctgtcttagatatttgg
ctaattttaagtggttttgttaaagtattaatgatgccagctgtcaggat
aataaattgatttggaaaactttgcaagtcaaatttaacttcttcaggat
tttgcttagtaaagaagtttacttggtttactatataatgggaagtgaaa
agccttcctctaaaattaaagtaggtttaggaaaacagaccctcaaattc
tgacattcattttcctaagcaactggatcaatttgctgacttgggcataa
tctaatctaagcatatctgaatacagtattcagagatagatacagtagag
attccccagactttttcgctctttgtaaaacctgtttgtttaggttttgc
gaggtaaactcaacagaggttgggagtggaagagggtgggaagcttatat

gcaaattaacagacgagaaatgctccagaaggtttattattttaaagcac
attaaaaacaaaaaactatttttaaaatcctgctagattttataatggat
ttgtgaataaaaaatacccagggttctcagaatggaataaatatcccttt
taatagttatatatacagatatacaactgttagctttaattggcagctct
cttcttttttcttcttttcactggctttttacttggtgctttttcttgtt
ttgcactggtggtctgtgttctgtgaataaagcaaagtaagaatttacta
agagtatgttaagttttggattattgaaataagaggcatttcttagtttt
ccagtaggatctaaaatgtgtcagctatgagtaagactggcatccaagaa
gtttatattatagatttaggtcctaatttttataaatcacaaggtaaaaa
aatcacagaacagatggatctctaatgaaaaagggatgtctttttgttta
tagtcatgtggcaagatgagagtaaaaccagagagcaaacctctataagt
gttgagtatatgtatacatttgaaataaaccagaaatttgttaccttaaa
aaaaaaaaaaa

>NM_021109 3
gacaactcggtggtggccactgcgcagaccagacttcgctcgtactcgtg
cgcctcgcttcgcttttcctccgcaaccatgtctgacaaacccgatatgg
ctgagatcgagaaattcgataagtcgaaactgaagaagacagagacgcaa
gagaaaaatccactgccttccaaagaaacgattgaacaggagaagcaagc
aggcgaatcgtaatgaggcgtgcgccgccaatatgcactgtacattccac
aagcattgccttcttattttacttcttttagctgtttaactttgtaagat
gcaaagaggttggatcaagtttaaatgactgtgctgcccctttcacatca
aagaactactgacaacgaaggccgcgcctgcctttcccatctgtctatct
atctggctggcagggaaggaaagaacttgcatgttggtgaaggaagaagt
ggggtggaagaagtggggtgggacgacagtgaaatctagagtaaaaccaa
gctggcccaaggtgtcctgcaggctgtaatgcagtttaatcagagtgcca
ttttttttttgttcaaatgattttaattattggaatgcacaattttttt
aatatgcaaataaaaagtttaaaaacttaaaaaaaaaaaaaaaaaaaaa
aaaaaaa

>NM_001111285 1
ttttgtagataaatgtgaggattttctctaaatccctcttctgtttgcta
aatctcactgtcactgctaaattcagagcagatagagcctgcgcaatgga
ataaagtcctcaaaattgaaatgtgacattgctctcaacatctcccatct
ctctggatttctttttgcttcattattcctgctaaccaattcattttcag
actttgtacttcagaagcaatgggaaaaatcagcagtcttccaacccaat
tatttaagtgctgcttttgtgatttcttgaaggtgaagatgcacaccatg
tcctcctcgcatctcttctacctggcgctgtgcctgctcaccttcaccag
ctctgccacggctggaccggagacgctctgcggggctgagctggtggatg
ctcttcagttcgtgtgtggagacaggggcttttatttcaacaagcccaca
gggtatggctccagcagtcggagggcgcctcagacaggcatcgtggatga
gtgctgcttccggagctgtgatctaaggaggctggagatgtattgcgcac
ccctcaagcctgccaagtcagctcgctctgtccgtgcccagcgccacacc
gacatgcccaagacccagaagtatcagcccccatctaccaacaagaacac
gaagtctcagagaaggaaaggttggccaaagacacatccaggaggggaac
agaaggaggggacagaagcaagtctgcagatcagaggaaagaagaaagag

cagaggagggagattggaagtagaaatgctgaatgcagaggcaaaaaagg
aaaatgaaggacaggaggattaaacagacagaggcaaggatgatgagaga
ggagcagacagcaagaatgaaaagcagaaaatacaatagaggaaatgaag
aaaagtaggcctgctggagctagatgatgatgtgatggaaatagaagta

>NM_004781 3
agtgacgtctttgccccgcgccgcgccgtcccacccatctccctggcctc
cggtcccaacttcgcttctctgctgaccctctctcgtcgccgctgccgcc
gccgcagctgccaaaatgtctacaggtccaactgctgccactggcagtaa
tcgaagacttcagcagacacaaaatcaagtagatgaggtggtggacataa
tgcgagttaacgtggacaaggttctggaaagagaccagaagctctctgag
ttagacgaccgtgcagacgcactgcaggcaggcgcttctcaatttgaaac
gagcgcagccaagttgaagaggaaatattggtggaagaattgcaagatgt
gggcaatcgggattactgttctggttatcttcatcatcatcatcatcgtg
tgggttgtctcttcatgaagaaccagcggaactcaaaactgctgttcaag
aaacctcttcaagacttttgacttagaacctgctatattatcaagcttac
ctactgttatctctaaaattttttttgtgttaatgtaaagttgaatttct
aggaaacgtgcctttgtttttaatatgcactccaaattagaaggccggc
cccgtccacattttgcacagtgcctttacagatttacgtatgggctgatg
aagaggccttcttaagttccagagtgctataatctagatgtaatgttgtc
actaattaattgccattactcccagttagttacccttgtcatttggcatt
attttcagaaccacattttaaacctttgggtaatcagatttccaacttat
gccttccagaaaaaaacactactgcctaacacaaatctgtgataacaaca
ggctgtgccttattttgataattttctgattccctagaagagaaccctct
actttttgtaagcactactgactctcgctgtatttaagatgctggtgaag
agcttttgctcttgcattagatttgaagatgtttacattgttgttattgt
tatgtatcacttgctaaaaatattgttttaatcagagataacctctttaa
aaaaatttttaaagaactatggctatgaccaaagcttctattttgccaaa
aagttaaataccgataaaatggccttaagtgtattcctgacagttaaatt
cagaaacgtgccaaatggaactcaaggtgccccttcagaattaaaatcat
taccttgtgtgtgaaccttctacatcttcataggcctttcttccttttga
aaggctgtagacagtgtggctccccttctgattcagtattttgcatgggg
gttagagaaggtttgaggtagactctgaccgtctcataaaagagttctac
ccagcagttggcagattatcagctgtggactccagcatgtttctgataat
tatgcaagcaacaattctgtagcctcaagtaagaccacctgtgaacttga
tcattatctggcccaaatatgaagataaactataactttggagtttgttt
cctatttgtattcacattctgcttcctaaatcagttttctaaattatgcc
tgcaattaggcattggtcaggggtgaatggctcttttcacagagagtagc
caaccagagacctttgctttgatatcatcaactgcagagaatgctgttga
tgggaatgctggaagcagaaactttgtcatcggaaaaacttttcttgtat
gcatgagactcaacatcaggatccacagcttaaagatgggaattcaggta
tgaaagaaaacaggcaaggaggcactgagggagaaagacacagactttat
cgctctgtggctcattgttactggaatattctaaaactcttgttcacatg
ctattatgacttataaagcagcaacagctgaggcgcaccaggacacagct
tccatttctttaacgtctgttcccttaacatcgctgaaatgatttactgt
tgaagagatgccttgcggtgtggccagctgtgaggagaaagcagctggca

gtgttaggacattagtccaccttcagcgcagggtctctggccgggtctga
ctcagaaaccttggtactcgccccttggccacagtgcccagacccatgta
acccactggctcctgcattaacccagaaatacctcgcttctatctgtgca
cttagctgggaacttacccactgtaatcacctaaataaagtgtttataaa
catgaaaaaaaaaaaaaaaaaaaa

>NM_001621 4
agtggctggggagtcccgtcgacgctctgttccgagagcgtgccccggac
cgccagctcagaacaggggcagccgtgtagccgaacggaagctgggagca
gccgggactggtggcccgcgcccgagctccgcaggcgggaagcaccctgg
atttaggaagtcccgggagcagcgcggcggcacctccctcacccaagggg
ccgcggcgacggtcacggggcgcggcgccaccgtgagcgacccaggccag
gattctaaatagacggcccaggctcctcctccgcccgggccgcctcacct
gcgggcattgccgcgccgcctccgccggtgtagacggcacctgcgccgcc
ttgctcgcgggtctccgcccctcgcccaccctcactgcgccaggcccagg
cagctcacctgtactggcgcgggctgcggaagcctgcgtgagccgaggcg
ttgaggcgcggcgcccacgccactgtcccgagaggacgcaggtggagcgg
gcgcggcttcgcggaacccggcgccggccgccgcagtggtcccagcctac
accgggttccggggacccggccgccagtgcccggggagtagccgccgccg
tcggctgggcaccatgaacagcagcagcgccaacatcacctacgccagtc
gcaagcggcggaagccggtgcagaaaacagtaaagccaatcccagctgaa
ggaatcaagtcaaatccttccaagcggcatagagaccgacttaatacaga
gttggaccgtttggctagcctgctgcctttcccacaagatgttattaata
agttggacaaactttcagttcttaggctcagcgtcagttacctgagagcc
aagagcttctttgatgttgcattaaaatcctcccctactgaaagaaacgg
aggccaggataactgtagagcagcaaatttcagagaaggcctgaacttac
aagaaggagaattcttattacaggctctgaatggctttgtattagttgtc
actacagatgctttggtcttttatgcttcttctactatacaagattatct
agggtttcagcagtctgatgtcatacatcagagtgtatatgaacttatcc
ataccgaagaccgagctgaatttcagcgtcagctacactgggcattaaat
ccttctcagtgtacagagtctggacaaggaattgaagaagccactggtct
cccccagacagtagtctgttataacccagaccagattcctccagaaaact
ctcctttaatggagaggtgcttcatatgtcgtctaaggtgtctgctggat
aattcatctggttttctggcaatgaatttccaagggaagttaaagtatct
tcatggacagaaaaagaaagggaaagatggatcaatacttccacctcagt
tggctttgtttgcgatagctactccacttcagccaccatccatacttgaa
atccggaccaaaaattttatctttagaaccaaacacaaactagacttcac
acctattggttgtgatgccaaaggaagaattgttttaggatatactgaag
cagagctgtgcacgagaggctcaggttatcagtttattcatgcagctgat
atgctttattgtgccgagtcccatatccgaatgattaagactggagaaag
tggcatgatagttttccggcttcttacaaaaaacaaccgatggacttggg
tccagtctaatgcacgcctgctttataaaaatggaagaccagattatatc
attgtaactcagagaccactaacagatgaggaaggaacagagcatttacg
aaaacgaaatacgaagttgccttttatgtttaccactggagaagctgtgt
tgtatgaggcaaccaacccttttcctgccataatggatcccttaccacta
aggactaaaaatggcactagtggaaaagactctgctaccacatccactct

aagcaaggactctctcaatcctagttccctcctggctgccatgatgcaac
aagatgagtctatttatctctatcctgcttcaagtacttcaagtactgca
ccttttgaaaacaactttttcaacgaatctatgaatgaatgcagaaattg
gcaagataatactgcaccgatgggaaatgatactatcctgaaacatgagc
aaattgaccagcctcaggatgtgaactcatttgctggaggtcacccaggg
ctctttcaagatagtaaaaacagtgacttgtacagcataatgaaaaacct
aggcattgattttgaagacatcagacacatgcagaatgaaaaatttttca
gaaatgatttttctggtgaggttgacttcagagacattgacttaacggat
gaaatcctgacgtatgtccaagattctttaagtaagtctcccttcatacc
ttcagattatcaacagcaacagtccttggctctgaactcaagctgtatgg
tacaggaacacctacatctagaacagcaacagcaacatcaccaaaagcaa
gtagtagtggagccacagcaacagctgtgtcagaagatgaagcacatgca
agttaatggcatgtttgaaaattggaactctaaccaattcgtgcctttca
attgtccacagcaagacccacaacaatataatgtctttacagacttacat
gggatcagtcaagagttcccctacaaatctgaaatggattctatgcctta
tacacagaactttatttcctgtaatcagcctgtattaccacaacattcca
aatgtacagagctggactaccctatggggagttttgaaccatccccatac
cccactacttctagtttagaagattttgtcacttgtttacaacttcctga
aaaccaaaagcatggattaaatccacagtcagccataataactcctcaga
catgttatgctggggccgtgtcgatgtatcagtgccagccagaacctcag
cacacccacgtgggtcagatgcagtacaatccagtactgccaggccaaca
ggcatttttaaacaagtttcagaatggagttttaaatgaaacatatccag
ctgaattaaataacataaataacactcagactaccacacatcttcagcca
cttcatcatccgtcagaagccagaccttttcctgatttgacatccagtgg
attcctgtaattccaagcccaattttgaccctggtttttggattaaatta
gtttgtgaaggattatggaaaaataaaactgtcactgttggacgtcagca
agttcacatggaggcattgatgcatgctattcacaattattccaaaccaa
attttaatttttgcttttagaaaagggagtttaaaaatggtatcaaaatt
acatatactacagtcaagatagaaagggtgctgccacggagtggtgaggt
accgtctacatttcacattattctgggcaccacaaaatatacaaaacttt
atcagggaaactaagattcttttaaattagaaaatattctctatttgaat
tatttctgtcacagtaaaaataaaatactttgagttttgagctactggat
tcttattagttccccaaatacaaagttagagaactaaactagtttttcct
atcatgttaacctctgcttttatctcagatgttaaaataaatggtttggt
gctttttataaaaagataatctcagtgctttcctccttcactgtttcatc
taagtgcctcacatttttttctacctataacactctaggatgtatatttt
atataaagtattctttttctttttaaattaatatctttctgcacacaaa
tattatttgtgtttcctaaatccaaccattttcattaattcaggcatatt
ttaactccactgcttacctactttcttcaggtaaagggcaaataatgatc
gaaaaaataattatttattacataatttagttgtttctagactataaatg
ttgctatgtgccttatgttgaaaaaatttaaaagtaaaatgtctttccaa
attatttcttaattattataaaaatattaagacaatagcacttaaattcc
tcaacagtgttttcagaagaaataaatataccactctttacctttattga
tatctccatgatgatagttgaatgttgcaatgtgaaaaatctgctgttaa
ctgcaaccttgtttattaaattgcaagaagctttatttctagctttttaa
ttaagcaaagcacccatttcaatgtgtataaattgtctttaaaaactgtt

ttagacctataatccttgataatatattgtgttgactttataaatttcgc
ttcttagaacagtggaaactatgtgtttttctcatatttgaggagtgtta
agattgcagatagcaaggtttggtgcaaagtattgtaatgagtgaattga
atggtgcattgtatagatataatgaacaaaattatttgtaagatatttgc
agtttttcattttaaaaagtccataccttatatatgcacttaatttgttg
gggctttacatactttatcaatgtgtctttctaagaaatcaagtaatgaa
tccaactgcttaaagttggtattaataaaaagacaaccacatagttcgtt
taccttcaaactttaggttttttaatgatatactgatcttcattaccaa
taggcaaattaatcaccctaccaactttactgtcctaacatggtttaaaa
gaaaaaatgacaccatcttttattctttttttttttttttttgagagag
agtcttactctgccgcccaaactggagtgcagtggcacaatcttggctca
ctgcaacctctacctcctgggttcaagtgattctcttgcctcagcctccc
gagttgctgggattacaggcatgtgccaccatgcccagctaatttttgta
tttttagtagaaacgggtttcaccatgttggccagactggtctcaaactc
ctgacctcaggtgagcctcccaccttggcctcccaaagtgctgggattac
aggcgtgagccactgcattcagctcttcttttctttagatatgagagctg
aagagcttagacacattttgcatgtattatttgaaaatctgatggaatcc
caaactgagatgtattaaaatacaatttttggccgggtgcagtggctcac
gcctgtaatcccagcacttggggagggcgaggagggtggatcacgaggtc
aagagatggagaccatcctgaccaacatggtgaaaccctgtctctactaa
aaatacagaaattagctgggcatggtggcgtgagcctgtagtcctagcta
ctcaggaggctgaggcaggagaatagcctgaacctgggaatcggaggttg
cagagccaagatcgccccactgcactccagcctggcaatagaccgagact
ccgtctccaaaaaaaaaaaaaaatacaatttttatttcttttactttttt
agtaagttaatgtatataaaaatggcttcggacaaaatatctctgagttc
tgtgtattttcagtcaaaactttaaacctgtagaatcaatttaagtgttg
gaaaaaatttgtctgaaacatttcataatttgtttccagcatgaggtatc
taaggatttagaccagaggtctagattaatactctatttttacatttaaa
ccttttattataagtcttacataaaccatttttgttactctcttccacat
gttactggataaattgtttagtggaaaataggctttttaatcatgaatat
gatgacaatcagttatacagttataaaattaaaagtttgaaaagcaatat
tgtatatttttatctatataaaataactaaaatgtatctaagaataataa
aatcacgttaaaccaaatacacgtttgtctgtattgttaagtgccaaaca
aaggatacttagtgcactgctacattgtgggatttatttctagatgatgt
gcacatctaaggatatggatgtgtctaatttagtcttttcctgtaccagg
tttttcttacaatacctgaagacttaccagtattctagtgtattatgaag
ctttcaacattactatgcacaaactagtgtttttcgatgttactaaattt
taggtaaatgctttcatggctttttttcttcaaaatgttactgcttacata
tatcatgcatagatttttgcttaaagtatgatttataatatcctcattat
caaagttgtatacaataatatataataaaataacaaatatgaataat

>NM_001730 3
tagtcgcggggcaggtacgtgcgctcgcggttctctcgcggaggtcggcg
gtggcgggagcgggctccggagagcctgagagcacggtggggcggggcgg
gagaaagtggccgcccggaggacgttggcgtttacgtgtggaagagcgga
agagttttgcttttcgtgcgcgccttcgaaaactgcctgccgctgtctga

ggagtccacccgaaacctcccctcctccgccggcagccccgcgctgagct
cgccgacccaagccagcgtgggcgaggtgggaagtgcgcccgacccgcgc
ctggagctgcgcccccgagtgcccatggctacaagggtgctgagcatgag
cgcccgcctgggacccgtgccccagccgccggcgccgcaggacgagccgg
tgttcgcgcagctcaagccggtgctgggcgccgcgaatccggcccgcgac
gcggcgctcttccccggcgaggagctgaagcacgcgcaccaccgcccgca
ggcgcagcccgcgcccgcgcaggccccgcagccggcccagccgcccgcca
ccggcccgcggctgcctccagaggacctggtccagacaagatgtgaaatg
gagaagtatctgacacctcagcttcctccagttcctataattccagagca
taaaaagtatagacgagacagtgcctcagtcgtagaccagttcttcactg
acactgaagggttaccttacagtatcaacatgaacgtcttcctccctgac
atcactcacctgagaactggcctctacaaatcccagagaccgtgcgtaac
acacatcaagacagaacctgttgccattttcagccaccagagtgaaacga
ctgcccctcctccggccccgacccaggccctccctgagttcaccagtata
ttcagctcacaccagaccgcagctccagaggtgaacaatattttcatcaa
acaagaacttcctacaccagatcttcatctttctgtccctacccagcagg
gccacctgtaccagctactgaatacaccggatctagatatgcccagttct
acaaatcagacagcagcaatggacactcttaatgtttctatgtcagctgc
catggcaggccttaacacacacacctctgctgttccgcagactgcagtga
aacaattccagggcatgccccccttgcacatacacaatgccaagtcagttt
cttccacaacaggccacttactttcccccgtcaccaccaagctcagagcc
tggaagtccagatagacaagcagagatgctccagaatttaaccccacctc
catcctatgctgctacaattgcttctaaactggcaattcacaatccaaat
ttacccaccaccctgccagttaactcacaaaacatccaacctgtcagata
caatagaaggagtaaccccgatttggagaaacgacgcatccactactgcg
attaccctggttgcacaaaagtttataccaagtcttctcatttaaaagct
cacctgaggactcacactggtgaaaagccatacaagtgtacctgggaagg
ctgcgactggaggttcgcgcgatcggatgagctgacccgccactaccgga
agcacacaggcgccaagcccttccagtgcggggtgtgcaaccgcagcttc
tcgcgctctgaccacctggccctgcatatgaagaggcaccagaactgagc
actgcccgtgtgacccgttccaggtcccctgggctccctcaaatgacaga
cctaactattcctgtgtaaaaacaacaaaaacaaacaaaagcaagaaaac
cacaactaaaactggaaatgtatattttgtatatttgagaaaacagggaa
tacattgtattaataccaaagtgtttggtcattttaagaatctggaatgc
ttgctgtaatgtatatggctttactcaagcagatctcatctcatgacagg
cagccacgtctcaacatgggtaaggggtgggggtggaggggagtgtgtgc
agcgtttttacctaggcaccatcatttaatgtgacagtgttcagtaaaca
aatcagttggcaggcaccagaagaagaatggattgtatgtcaagatttta
cttggcattgagtagttttttttcaatagtaggtaattccttagagataca
gtatacctggcaattcacaaatagccattgaacaaatgtgtgggtttta
aaaattatatacatatatgagttgcctatatttgctattcaaaattttgt
aaatatgcaaatcagctttataggtttattacaagtttttttaggattctt
ttggggaagagtcataattcttttgaaaataaccatgaatacacttacag
ttaggatttgtggtaaggtacctctcaacattaccaaaatcatttcttta
gagggaaggaataatcattcaaatgaactttaaaaaagcaaatttcatgc
actgattaaaataggattattttaaatacaaaaggcattttatatgaatt

ataaactgaagagcttaaagatagttacaaaatacaaaagttcaacctct
tacaataagctaaacgcaatgtcatttttaaaaagaaggacttagggtgt
cgttttcacatatgacaatgttgcatttatgatgcagtttcaagtaccaa
aacgttgaattgatgatgcagttttcatatatcgagatgttcgctcgtgc
agtactgttggttaaatgacaatttatgtggattttgcatgtaatacaca
gtgagacacagtaattttatctaaattacagtgcagtttagttaatctat
taatactgactcagtgtctgcctttaaatataaatgatatgttgaaaact
taaggaagcaaatgctacatatatgcaatataaaatagtaatgtgatgct
gatgctgttaaccaaagggcagaataaataagcaaaatgccaaaaggggt
cttaattgaaatgaaaatttaattttgtttttaaaatattgtttatcttt
atttattttgtggtaatatagtaagtttttttagaagacaattttcataa
cttgataaattatagttttgtttgttagaaaagttgctcttaaaagatgt
aaatagatgacaaacgatgtaaataatttgtaagaggcttcaaaatgtt
tatacgtggaaacacacctacatgaaaagcagaaatcggttgctgttttg
cttctttttccctcttatttttgtattgtggtcatttcctatgcaaataa
tggagcaaacagctgtatagttgtagaatttttttgagagaatgagatgtt
tatatattaacgacaatttttttttggaaaataaaaagtgcctaaaaga

>NM_001241 3
gtgaatgaaggagcgggcggaggaggaagtgtcatggcgtcgggccgtgg
agcttcttctcgctggttctttactcgggaacagctggagaacacgccga
gccgccgctgcggagtggaggcggataaagagctctcgtgccgccagcag
gcggccaacctcatccaggagatgggacagcgtctcaatgtctctcagct
tacaataaacactgcgattgtttatatgcacaggttttatatgcaccatt
ctttcaccaaattcaacaaaaatataatatcgtctactgcattatttttg
gctgcaaaagtggaagaacaggctcgaaaacttgaacatgttatcaaagt
agcacatgcttgtcttcatcctctagagccactgctggatactaaatgtg
atgcttaccttcaacagactcaagaactggttatacttgaaaccataatg
ctacaaactctaggttttgagatcaccattgaacacccacacacagatgt
ggtgaaatgtacccagttagtaagagcaagcaaggatttggcacagacat
cctatttcatggctaccaacagtctgcatcttacaaccttctgtcttcag
tacaaaccaacagtgatagcatgtgtatgcattcatttggcttgcaaatg
gtccaattgggagatccctgtatcaactgatggaaagcattggtgggaat
atgtggatcctacagttactctagaattattagatgagctaacacatgag
tttctacaaatattggagaaaacgcctaataggttgaagaagattcgaaa
ctggagggctaatcaggcagctaggaaaccaaaagtagatggacaggtat
cagagacaccacttcttggttcatctttggtccagaattccattttagta
gatagtgtcactggtgtgcctacaaacccaagttttcagaaaccatctac
atcagcattccctgcgccagtacctctaaattcaggaaatatttctgttc
aagacagccatacatctgataatttgtcaatgctagcaacaggaatgcca
agtacttcatacggtttatcatcacaccaggaatggcctcaacatcaaga
ctcagcaaggacagaacagctatattcacagaaacaggagacatctttgt
ctggtagccagtacaacatcaacttccagcagggaccttctatatcactg
cattcaggattacatcacagacctgacaaaatttcagatcattcttctgt
taagcaagaatatactcataaagcagggagcagtaaacaccatgggccaa
tttccactactccaggaataattcctcagaaaatgtctttagataaatat

agagaaaagcgtaaactagaaactcttgatctcgatgtaagggatcatta
tatagctgcccaggtagaacagcagcacaaacaagggcagtcacaggcag
ccagcagcagttctgttacttctcccattaaaatgaaaatacctatcgca
aatactgaaaaatacatggcagacaaaaaggaaaagagtgggtcactgaa
attacggattccaataccacccactgataaaagcgccagtaaagaagaac
tgaaaatgaaaataaaagtttcttcttcagaaagacacagctcttctgat
gaaggcagtgggaagagcaaacattcaagcccacatattagcagagacca
taaggagaagcacaaggagcatccttcaagccgccaccacaccagcagcc
acaagcattcccactcgcatagtggcagcagcagcggtggcagtaaacac
agtgccgacggaataccacccactgttctgaggagtcctgttggcctgag
cagtgatggcatttcctctagctccagctcttcaaggaagaggctgcatg
tcaatgatgcatctcacaaccaccactccaaaatgagcaaaagttccaaa
agttcaggtgggctacggacatctcagcaccctcgtgaaactggacaaga
agccagtggagaccaacggtcctgatgccaatcacgagtacagtacaagc
agccagcatatggactacaaagacacattcgacatgctggactcactgtt
aagtgcccaaggaatgaacatgtaataatttgtttaggtcaatttttcct
ttactttttaatttaaaaattgttagaatggaaaaattccttctgatct
agcagtggtaacccctgctgttgctgccactgcttcaatatttgtaagtg
ctgctttattcttcattctgaaaagaagagattatagtaaacaagtcttt
atctccacatatgatagtgttataaatactgtaaaagcatggaaggtgca
aaactcagtatttctacaattgcagctaagaacattaggatgaatggctg
gctgcttctaggaatataagatgcctcaagcattcattatttatgatttg
aatactgtagctatttttttgttgcttggcttttgaatgagtgtaaattgt
tttcttttgtgtatttatacttgtatgtatgatttgcatgtttcaatgat
aaagggataaaacagtatactgacaactgtttacaagaaagtggagaaaa
atgtacatacatttttgtatgtttagatattaccgtaaatactcaggatt
ggagctgcttgtaagtataacaatatacagaataactttattttatcttg
tcagagtccatcactaatctaaaacaaaggtggcactttttttatgttaac
cttaaactctaggccttactggaagccactgataggggacacttcactac
cagatgtgtgcagtgcaacagatggtcatatacactgtgaggcactgaaa
ttttgccttcagaggttctgaccagattggctgctgaaatagcccctaac
tttctgaaggcttgaagaggaaaaaataaagtttacatactcttgatgtg
aagtgcatttaaatgtttgttggcttgttgcagttctatgaaacagagct
gttaataatggttatgtggattactgtgatttgaaaactaaattcacaat
aacttacctagtagagatttagtgagttgtttcctttaaagaattttaca
ctacatattttaatagtaaacagggatcacttttcctttagcattcagaa
tgacaccatattcttaaatatactccttccctgaagcgtgtttgtgtgtg
atgccatatttctttttcaggtaaatgtagtcttccttataaaaatgaaa
ttaaacctatgctctcaattcttttatattctaacaataaataaaaaaga
aaagattactgactgtgcattgtacctgtatttatagtttatggttatca
ggaagctctgtaagaaagaaaaggtcagcctcccaggcaaaccagtagtg
gaggttttacatttgtttgcacatctcagtatatttctgttgaggtaaag
tttgcacagtcatctgacttctgatcaagcattagattttaacttgttta
gattttgtcttaaacaccagtaatatggctcttgtttatcagctaatctt
gaatttattctgtggtaaatcttttgagttgttgagtatatttgagattg
attggattcgacctcttgttgaactgaaaacttaatttttctctgtatt

tttgttacaaagccactgatacgtgcacaattgtaattaagtatgttgca
gttgtaaatattagagtttaatctcatgctctacctttatttagcaatta
cctaatttgccagtagctttataatttttaaagataattgttcattattt
tgtcaatgttatttgaacttggggtacttaggagcctctttgtagggact
gtgcctaggtagcatgtcctaacatttgttctggtcttgcataacttcag
taatctttgtcattatatgtaactttgttgctctgtatggcataatattg
tatccataaacatggtaattttgatacagttatacttttacagtggtaca
taatccaaggactagtatagaattaagctgagtgcaagatgagggaggga
agggctttcttggtaatttagatgtgaaacctctacagagctatcatgta
aaaactacataaggtggttgtgctactgtataattgggggtgataatacc
aggaattttaataagattttgtaaagaatatccagaaaagtagtgaactt
attttcagtagacatagaaaacaatgtgaatatttaaggtctgtgactat
agttaaacttcactaagaatttgcagaattgttttgagatgtgtgaataa
aggtaattttattgaatcttcattggtgctaatgatggacagttaaaaag
atagctagtgtatattgttatgggtcagtacttattagtacttccaaaat
tgaatttgaaatgctatgtattcacttttcactctgtaaatgtaattctt
tacaatgactttatttattaaagggcagccagttgtcatttttacaggat
tgtgtgagctattcaaactcttcaacccctgaacagggtattaagcttcc
aaaataatgatggggataaatatggaaatcctttttaagttgtatttcca
ttaaacaaaaacccttataattcatactatcatgaatttgctttatccat
ctcatttgcataacagttcatctgtctggtcccattaggctctaccaaag
aaagactctgatgagtggacattattactgtgactcttgtaagtagccat
aaataaaccaaaatagtatcaaatttaggtatgaaattccacatgtgcaa
agtactgttaaggtgataacatttttgatgagatttaaaaaaatatttga
aatattaaaaagcattatctttaaacatcctataaaagagtgagtcattt
ttaagttgtgttttccctagatcactgatttgttacctttacacagaagc
acattgcaaagaaaggctttatttctacctcttccattcagttttccaat
attgagctgtgtccctctgaatatttgccttagctatttcaaaataggta
ttttcagaaccaaagcaaagggtggtttgttcactgaaacattcttagag
gcctacttaacgggcggcagtatccggtagtggtaaaacctcacagtttc
ctaattgcaggtatgtaaataccaaaaaagtcctgttgtcagccctaggt
ttatctttgaaagcagttaaatgtgcaccttggtcttcctccgtgtgttc
agattccttctggttaaagttcttattcagagtcatctaaaagaaatttc
taacatgatgtatggtttcttgaatgtcatgagtggctgcgtatattgct
ttgtagcaacagattgtattaataattaatagaatacctaattttttgg
cctatcctctttgaactagaaatgactacttgagggctatttcagagaac
caatatgaaatgtttttgttagtacaaaataccattgccttaaaaggttt
caagcacattttcttttctatttgagaacttaaattgctgacttgtattc
gttaagcaaccttagaaataaaccactgcttatatctgcatgaatattat
gcatttcatgagtacagttagttgattagatatttcagggttttgcattt
tctatagacatggggactttttaacatggtaacgtcttaggaaaaccat
tctctcaaggtattacaccatttccaattttatccaaatgaaaagcatat
ctgcttaaatttaggatgtctttgctgagaatatttggaggctcttgtta
ggaagaatcatggattcagtatttctaacctgattactgcctcagaggct
gtcttgaagggagacagtaggattcactgactacctttacccagatgagg
gatcaacaaatctcacacttaaactaaatgagttgacaggacaataggtg

ctaatctcatgtgccctaagatggacttatttagatagggatttttgcat
tggaaaagcagcacattctctgaccttgggtaccaagctatgatactaat
ttgagaatcacattctgatacgttaatatcagaaatcagtgagcaaccta
attgctgtaagctgacatacatggaagccgaaatcaaaaggttgcttttg
ttagtttccctggagcatctatttttgtaatacattttgccagtgggact
gaaaagctcaaaatgttttagacctaatatcatacattaattttctgctc
tgcagtgtcaatttgggcacagccagtttttacctctgtggcaccttctt
tataatttactgttacctattctgcctgacagattgattaagtaggttga
taagatccatcgaagattgaatggctaggagtaattctgttctcgtatag
gcaacttaacttcactgtggaatccatttatctcaaagtttgagggctgt
ctactttattttcaagtttggggcggaaactcaaaatcccgagcattgcc
actttagtagtttgtttgatttgcctattgtttaataatgagtacattct
tattaagtgatgagtacattcttaattttaaggtatgtttttttcaagca
gcttcattttattaggaaaaatacatactcatttgttgtaaacagtgatt
gtggggtttcattttctgaatgtactcaaaactcccaacaacaattgaac
aggatgtttgctaatagaaaatctaaaacaggaatatgtgtatatggcat
gaattagtcattgactttaagcttcttatccatacaaggaactaataaaa
tgtttattgacaacataaatcc

>NM_003182 2
cacgcaagcgaaaggagaggaggcggctaattaaatattgagcagaaagt
cgcgtggggagaatgtcacgtgggtctggaggctcaaggaggctgggata
aataccgcaaggcactgagcaggcgaaagagcgcgctcggacctccttcc
cggcggcagctaccgagagtgcggagcgaccagcgtgcgctcggaggaac
cagagaaactcagcaccccgcgggactgtccgtcgcaaaatccaacatga
aaatcctcgtggccttggcagtctttttcttgtctccactcagctgttt
gcagaagaaataggagccaatgatgatctgaattactggtccgactggta
cgacagcgaccagatcaaggaggaactgccggagccctttgagcatcttc
tgcagagaatcgcccggagacccaagcctcagcagttctttggattaatg
ggcaaacgggatgctgattcctcaattgaaaaacaagtggccctgttaaa
ggctctttatggacatggccagatctctcacaaaagacataaaacagatt
cctttgttggactaatgggcaaaagagctttaaattctgtggcttatgaa
aggagtgcaatgcagaattatgaaagaagacgttaataaactacctaaca
ttatttattcagcttcatttgtgtcaatgggcaatgacaggtaaattaag
acatgcactatgaggaataattatttatttaataacaattgtttggggtt
gaaaattcaaaaagtgtttatttttcatattgtgccaatatgtattgtaa
acatgtgttttaattccaatatgatgactcccttaaaatagaaataagtg
gttatttctcaacaaagcacagtgttaaatgaaattgtaaaacctgtcaa
tgatacagtccctaaagaaaaaaaatcattgctttgaagcagttgtgtca
gctactgcggaaaaggaaggaaactcctgacagtcttgtgcttttcctat
ttgttttcatggtgaaaatgtactgagattttggtattacactgtatttg
tatctctgaagcatgtttcatgttttgtgactatatagagatgtttttaa
aagtttcaatgtgattctaatgtcttcatttcattgtatgatgtgttgtg
atagctaacattttaaataaaagaaaaaatatcttgaa

>NM_024551 2

cgcggcggcggcagcggcggcggctacaccgggcttggccccctccctcc
tccgttccccccctcctccccccctcccctcagcggtggctcccaagaagtc
cgagacacgcggatcaactcactatcctgaaggtccattctcccaagaag
aggggacagaaagacagatctatttgtaagaaaggcttgggtatcccatg
aacgagccaacagaaaaccgattggggtgcagcaggactccagagccaga
tataaggctcagaaaagggcaccaactggatggtacacgaagaggtgata
atgacagccaccaaggagatttggagcccattttagaggcatctgttcta
tcttcccatcataaaaaaagctctgaggaacatgaatacagtgatgaagc
tcctcaggaagatgagggctttatgggcatgtcccctctcttacaagccc
atcatgctatggaaaaaatggaagaatttgtttgtaaggtatgggaaggt
cggtggcgagtgatccctcatgatgtactaccagactggctcaaggataa
tgacttcctcttgcatggacaccggcctcctatgccttctttccgggcct
gttttaagagcattttcagaatacacacagaaacaggcaacatttggaca
catctcttaggttgtgtattcttcctgtgcctggggatcttttatatgtt
tcgcccaaatatctcctttgtggcccctctgcaagagaaggtggtctttg
gattatttttcttaggagccattctctgcctttctttttcatggctcttc
cacacagtctactgccactcagagggggtctctcggctcttctctaaact
ggattactctggtattgctcttctgattatgggaagttttgttccttggc
tttattattctttctactgtaatccacaaccttgcttcatctacttgatt
gtcatctgtgtgctgggcattgcagccattatagtctcccagtgggacat
gtttgccacccctcagtatcggggagtaagagcaggagtgtttttgggcc
taggcctgagtggaatcattcctaccttgcactatgtcatctcggagggg
ttccttaaggccgccaccatagggcagataggctggttgatgctgatggc
cagcctctacatcacaggagctgccctgtatgctgcccggatccccgaac
gctttttccctggcaaatgtgacatctggtttcactctcatcagctgttt
catatctttgtggttgctggagcttttgttcacttccatggtgtctcaaa
cctccaggagtttcgtttcatgatcggcgggggctgcagtgaagaggatg
cactgtgatacctaccagtctccagggactatgaccctaaaccagggcct
gcggcacttgcgggcctccctgctggctactgatgccagtaccagaggag
ccccaaaactttgacagcctcgtgggctttgtgacggcccaggggctctg
cgtggtacatgactgagaagagaaaaacaaaaataaatcatacctcaaag
gatggagtgcatcaattgggagaaaaggagacatagcccaaaccctggct
tattcttgggatctactgattgcgggctctgcaagacccttggcaaactg
gcttctgatccatatcatatttatttgtagaagatggcgaaacagtttag
ctggtggttctttcttctccctttctctctctctatgacaataatacaaa
ccaatttaagtgaacatttatatccgataaggggtgggagtgtgatttta
aatgctcttttgggagaacaaagaaattaatgtaaataagatttctaact
gtttaaataagactttatataaatgtttaaaacataggggtaagggaggg
agggagaatttttgtatagaatgaaacatgcaagtaccacacactgtttg
aattttgcacaaaaagtgactgtaggatcaggtgatagccccggaatgta
cagtgtcttggtgcaccaagatgccttctaaaggctgacataccttggac
cctaatggggcagagagtatagccctagcccagtggtgacatgaccactc
cctttgggaggcctgaggtagaggggagtggtatgtgttttctcagtgga
agcagcacatgagtgggtgacaggatgttagataaaggctctagttaggg
tgtcattgtcatttgagagactgacacactcctagcagctggtaaagggg
tgctggaggccatggaggagctctagaaacattagcatgggctgatctga

ttacttcctggcatcccgctcacttttatgggaagtcttattagagggat
gggacagttttccatatccttgctgtggagctctggaacactctctaaat
ttccctctattaaaaatcactgccctaactacacttcctccttgagggaa
tagaaatggacctttctctgacatagttcttggcatgggagccagccaca
aatgagattctgacgtgtccaggtttctcctgagctcatctacatagatt
ggtagacccttcctttggattaggaaagatgagttttacctctggtacac
tgtcttggtaagcctggatgtgacagacacctcggctctccttgaataag
aaagccagcagaactcttaaagccagttgtagtacggagttgtcagcact
cactgaacctcactttacagggataagagtggtgtggcattttaaataca
atggtatgttattgccagggagtgaggtacaagacgatggctcatgtcac
aggcctacctgatacggtgtcagagaaagtggtggggaaaggatctggtt
catggaattctgatcttggcccataggtgaaccaccaaaatagtgctcga
gtcttaggttactgtcatcaaagacttgggatgactccattatatcctgg
ggttgtgggtattagaactaaatatggaggtcctgagcatggggactggc
gtcctcagtaggtgtttgggaatatgggaagggtctcctatttattcaat
agagttttctcagttattttcctcccttgcccttgcaatctccagcaaaa
ggtgggatctaggaagaaagaatccagtgtagaagttgagaagaacttga
acgttttggttctggataaggtcactgtcctaggtgctaggtggaccgag
caaaagactcagtggatgaactggtgcagtgcctgacagaataaagaaca
gtattaatccctttgagaaagcatagtccagcaggacagtggccatttgg
acagaagcccacttagtttcttgggagcaacagcacgtatcagaagccag
acttgctcttcggtcatgcactttgggatacagcgtataggtgcagccct
gtcacaacaccaacagaagtagcagcctctgggtgcagtcacccacaccc
caaagctggaaggatctggttcaacatagcacaaacccttaggaaaaatg
aaattaacatcactgatgtgtaatccagtaaaatctcccttttttcgggtg
tgtatgtgggcatgtgcccatttctatgtgtgtgtctacgtgcagctcac
taccaacagcctcatgtgcacttgacctgacagtgctcgctgagaactct
caccaggttggcgcctgaatgccttactctcagcagtcagaggcttgctt
gctctgtgcagatttttaattttctttttggccctaggctggttgggac
ctctacagcttcattctttcaccattaaatagtggccttttttcagtattt
tccctcttcccctttataaattatgctaaagccacaaagcacatttttgg
ggatcatagaaggttggggttccagaaaggcatctgtgtgatggttccat
tgatgtgggatttccctacttgctgtattctcagtttctaataaaaagaa
ccaaatgaaatatgaaaaaaaaaaaaaaaaaa

>NM_001614 3
gcgcgccgccgggccgcgcgggcgcgccgcttccgcttaaataacggcgg
gggaggccgcggtcggtctcagtcgccgctgccagctctcgcactctgtt
cttccgccgctccgccgtcgcgtttctctgccggtcgcaatggaagaaga
gatcgccgcgctggtcattgacaatggctccggcatgtgcaaagctggtt
ttgctggggacgacgctccccgagccgtgtttccttccatcgtcgggcgc
cccagacaccagggcgtcatggtgggcatgggccagaaggactcctacgt
gggcgacgaggcccagagcaagcgtggcatcctgaccctgaagtacccca
ttgagcatggcatcgtcaccaactgggacgacatggagaagatctggcac
cacaccttctacaacgagctgcgcgtggccccggaggagcacccagtgct
gctgaccgaggccccccctgaaccccaaggccaacagagagaagatgactc

agattatgtttgagaccttcaacaccccggccatgtacgtggccatccag
gccgtgctgtccctctacgcctctgggcgcaccactggcattgtcatgga
ctctggagacggggtcacccacacggtgcccatctacgagggctacgccc
tcccccacgccatcctgcgtctggacctggctggccgggacctgaccgac
tacctcatgaagatcctcactgagcgaggctacagcttcaccaccacggc
cgagcgggaaatcgtgcgcgacatcaaggagaagctgtgctacgtcgccc
tggacttcgagcaggagatggccaccgccgcatcctcctcttctctggag
aagagctacgagctgcccgatggccaggtcatcaccattggcaatgagcg
gttccggtgtccggaggcgctgttccagccttccttcctgggtatggaat
cttgcggcatccacgagaccaccttcaactccatcatgaagtgtgacgtg
gacatccgcaaagacctgtacgccaacacggtgctgtcgggcggcaccac
catgtacccgggcattgccgacaggatgcagaaggagatcaccgccctgg
cgcccagcaccatgaagatcaagatcatcgcacccccagagcgcaagtac
tcggtgtggatcggtggctccatcctggcctcactgtccaccttccagca
gatgtggattagcaagcaggagtacgacgagtcgggcccctccatcgtcc
accgcaaatgcttctaaacggactcagcagatgcgtagcatttgctgcat
gggttaattgagaatagaaatttgcccctggcaaatgcacacacctcatg
ctagcctcacgaaactggaataagccttcgaaaagaaattgtccttgaag
cttgtatctgatatcagcactggattgtagaacttgttgctgattttgac
cttgtattgaagttaactgttccccttggtatttgtttaataccctgtac
atatctttgagttcaacctttagtacgtgtggcttggtcacttcgtggct
aaggtaagaacgtgcttgtggaagacaagtctgtggcttggtgagtctgt
gtggccagcagcctctgatctgtgcagggtattaacgtgtcagggctgag
tgttctgggatttctctagaggctggcaagaaccagttgttttgtcttgc
gggtctgtcagggttggaaagtccaagccgtaggacccagtttcctttct
tagctgatgtctttggccagaacaccgtgggctgttacttgctttgagtt
ggaagcggtttgcatttacgcctgtaaatgtattcattcttaatttatgt
aaggtttttttgtacgcaattctcgattctttgaagagatgacaacaaa
ttttggttttctactgttatgtgagaacattaggccccagcaacacgtca
ttgtgtaaggaaaaataaaagtgctgccgtaaccaaaaaaaaaaaaaaaa
aaaa

>NM_033102 2
aacctggagatttaaaagccgccggctggcgcgcgtgggggggcaaggaag
ggggggcggaaccagcctgcacgcgctggctccgggtgacagccgcgcgc
ctcggccaggatctgagtgatgagacgtgtccccactgaggtgccccaca
gcagcaggtgttgagcatgggctgagaagctggaccggcaccaaagggct
ggcagaaatgggcgcctggctgattcctaggcagttggcggcagcaagga
ggagaggccgcagcttctggagcagagccgagacgaagcagttctggagt
gcctgaacggcccccctgagccctacccgcctggcccactatggtccagag
gctgtgggtgagccgcctgctgcggcaccggaaagcccagctcttgctgg
tcaacctgctaacctttggcctggaggtgtgtttggccgcaggcatcacc
tatgtgccgcctctgctgctggaagtgggggtagaggagaagttcatgac
catggtgctgggcattggtccagtgctgggcctggtctgtgtcccgctcc
taggctcagccagtgaccactggcgtggacgctatggccgccgccggccc
ttcatctgggcactgtccttgggcatcctgctgagcctctttctcatccc

aagggccggctggctagcagggctgctgtgcccggatcccaggcccctgg
agctggcactgctcatcctgggcgtggggctgctggacttctgtggccag
gtgtgcttcactccactggaggccctgctctctgacctcttccgggaccc
ggaccactgtcgccaggcctactctgtctatgccttcatgatcagtcttg
ggggctgcctgggctacctcctgcctgccattgactgggacaccagtgcc
ctggccccctacctgggcacccaggaggagtgcctctttggcctgctcac
cctcatcttcctcacctgcgtagcagccacactgctggtggctgaggagg
cagcgctgggccccaccgagccagcagaagggctgtcggccccctccttg
tcgccccactgctgtccatgccgggcccgcttggctttccggaacctggg
cgccctgcttccccggctgcaccagctgtgctgccgcatgccccgcaccc
tgcgccggctcttcgtggctgagctgtgcagctggatggcactcatgacc
ttcacgctgttttacacggatttcgtgggcgaggggctgtaccagggcgt
gcccagagctgagccgggcaccgaggcccggagacactatgatgaaggcg
ttcggatgggcagcctggggctgttcctgcagtgcgccatctccctggtc
ttctctctggtcatggaccggctggtgcagcgattcggcactcgagcagt
ctatttggccagtgtggcagctttccctgtggctgccggtgccacatgcc
tgtcccacagtgtggccgtggtgacagcttcagccgccctcaccgggttc
accttctcagccctgcagatcctgccctacacactggcctccctctacca
ccgggagaagcaggtgttcctgcccaaataccgaggggacactggaggtg
ctagcagtgaggacagcctgatgaccagcttcctgccaggccctaagcct
ggagctcccttccctaatggacacgtgggtgctggaggcagtggcctgct
cccacctccacccgcgctctgcggggcctctgcctgtgatgtctccgtac
gtgtggtggtgggtgagcccaccgaggccagggtggttccgggccggggc
atctgcctggacctcgccatcctggatagtgccttcctgctgtcccaggt
ggccccatccctgtttatgggctccattgtccagctcagccagtctgtca
ctgcctatatggtgtctgccgcaggcctgggtctggtcgccatttacttt
gctacacaggtagtatttgacaagagcgacttggccaaatactcagcgta
gaaaacttccagcacattggggtggagggcctgcctcactgggtcccagc
tccccgctcctgttagccccatggggctgccgggctggccgccagtttct
gttgctgccaaagtaatgtggctctctgctgccaccctgtgctgctgagg
tgcgtagctgcacagctgggggctggggcgtccctctcctctctccccag
tctctagggctgcctgactggaggccttccaaggggggtttcagtctggac
ttatacagggaggccagaagggctccatgcactggaatgcggggactctg
caggtggattacccaggctcagggttaacagctagcctcctagttgagac
acacctagagaagggtttttgggagctgaataaactcagtcacctggttt
cccatctctaagccccttaacctgcagcttcgtttaatgtagctcttgca
tgggagtttctaggatgaaacactcctccatgggatttgaacatatgaaa
gttatttgtaggggaagagtcctgaggggcaacacacaagaaccaggtcc
cctcagcccacagcactgtctttttgctgatccaccccccctcttacctttt
tatcaggatgtggcctgttggtccttctgttgccatcacagagacacagg
catttaaatatttaacttatttatttaacaaagtagaagggaatccattg
ctagcttttctgtgttggtgtctaatatttgggtagggtgggggatcccc
aacaatcaggtcccctgagatagctggtcattgggctgatcattgccaga
atcttcttctcctggggtctggccccccaaaatgcctaacccaggacctt
ggaaattctactcatcccaaatgataattccaaatgctgttacccaaggt
tagggtgttgaaggaaggtagagggtggggcttcaggtctcaacggcttc

cctaaccacccctcttctcttggcccagcctggttcccccacttccact
cccctctactctctctaggactgggctgatgaaggcactgcccaaaattt
cccctacccccaactttcccctacccccaactttccccaccagctccaca
accctgtttggagctactgcaggaccagaagcacaaagtgcggtttccca
agcctttgtccatctcagcccccagagtatatctgtgcttggggaatctc
acacagaaactcaggagcacccccctgcctgagctaagggaggtcttatct
ctcaggggggggtttaagtgccgtttgcaataatgtcgtcttatttattta
gcggggtgaatattttatactgtaagtgagcaatcagagtataatgttta
tggtgacaaaattaaaggctttcttatatgttaaaaaaaaaaaaaaaa

>NM_014547 4
gggagtgcgcacgcgcgtcggaggcgaaggaggaacccaccgcacctaca
gggcggtcgagtgagggagctgctggcgggtgggtctggcaactctttgg
gaggccgacgcgggcggaccggcgggtgctgggaaccgagcctcggcttg
cggccggcagtttccgtgggtctgtgaagaggtcggcggcccctgcgggc
gccagtcagctttaagaaaaagtagagatcacttctgactgtactgaaca
gcaaaaattaagtgacttgctgccctgcacatcatggcactgccattccg
taaggacttagaaaagtacaaagaccttgatgaagatgagctccttggga
atctgtcagaaacagaactgaaacaactggaaactgttttggatgatctt
gaccccgagaatgcccttctgcctgcagggttccggcagaagaaccagac
atcaaagtccaccacagggccatttgatagagagcatctcctttcatatc
tggagaaagaagcattggagcataaagacagggaagactatgtgccctac
actggagaaaaaaaagggaaaatatttatccccaaacagaaacctgtaca
gacttttacagaagaaaaagtgtctcttgatccagaattagaagaagctt
tgacaagtgcttctgatacagaattgtgtgacctcgcagcaattcttggg
atgcacaatttgataacgaatacaaagttctgtaatataatgggaagtag
taatggtgttgaccaagaacatttttcaaatgtggtcaaaggtgaaaaga
ttcttccggtatttgatgagccaccaaatccaaccaatgtagaagagagt
ttgaagagaactaaagaaaacgatgctcatcttgttgaagttaatttgaa
taatataaagaatatcccaattccaaccctaaaagattttgcaaaggctt
tggaaaccaacacacacatgtgaaatgtttcagtcttgcagccacccggagc
aatgaccctgttgctactgcttttgcagaaatgctgaaagtgaacaaaac
tttgaagagcttaaatgtggagtccaactttatcacgggagttgggattc
tggcactgattgatgcgttaagagataatgaaaccctggcagagctcaag
attgacaatcagaggcagcagttggggacagctgtagaattggaaatggc
caagatgcttgaggaaaatacaaatatccttaaatttggatatcagttta
cacagcagggaccacgaaccagagcagctaatgctataacaaaaaacaat
gacttagtgcgtaagagacgagttgaaggagatcaccagtaagtctgcaa
aggtgtaatctttggaagacttcagaagatcaccaagggctcatgttggt
gacatcatgtaaaattttcctgggtagaagggaaaagactggaaaaattt
ttttagtgacatgcattttttttttagttgttatcaaattgtaaaatcag
taatgtgatattttatattctgaaacatttctactttctgctaaaatcaa
ttttaatttagtttaattgaatgatttatgatgaatcttgggcaaaaaaa
tacaactgtaaaaaatttcacaggtcatttgtgtagaataatttgaacat
tgtgaggaccaatctttttttaaatcaaaagggatgttgctggtatcagaa
ttgttattgcttcatttagacataaaacacttaagtgttttcttcactcc

gtgacctggagagttttccattttttaaaatacatgaacttggcaagggt
gtgatttttctttatcaagagaacaaaatgccagaatgttaaaacagtta
actcaaaatctagtcatctgtgtaggagctaaagcaggtctccagggaga
gagggtggtcgtctgtgcattccaggttactgcacttgtctgatgtgacg
tagcaacacccaggtcttcttaaaacaaagtcatcagctttgccaggtta
catactttatttaaaagtatagtgaggtcgaagttcattccagtgtttca
ttttaataatgtgggcattattaaatttttgtggaagctaatagtaaaaa
ataaaagctgttttcaccacctccctccccaccccccaaaaaaggcgtac
agccaactcttagttatttagggtaagagaaaaaagaaaactgttaaaca
atgaaatcaaagataataaatatgatagatttccagtatgaggcacttgc
aaaacagtcatttaaactgatggttactgtaagtcagttggaagctggca
tgtatgtaaattacttggtgttacaccatatccgaagaagttcctatgtg
cagtgcagaattgcataaacagtatttaatcactgctacaaatcttccaa
ccaagaaggaaaactgctattcttcataaatctaatttttagtatatgct
tcctttcatcttatacttttatcaatatttataaaagtcatttctataat
aaaagcagtctttcttgctcaaagtattaaggtgaacaattgaatagagt
actgtggtcgggagactgatttgagactgcagagctgatgctgggtagag
ggtctggacttgtattcatgttctgtctcagggcagcccctggagcagga
gatggcagaggcatttacagctgcagaaaacagggaggaatggaatctga
ggtagccctggcctcaaaattcaggcctggctgtatcatttacagagatt
tttctggagggaaaagtctcatttctgaggaaggcaaggtggctaatcat
tattaatttttttaaactttttgggccgggcgcagtggctcacgcctgt
aatcccagcacttttgggaagccaaagtgggtggatcacttaaggtcagg
agttcaagaccagcctggccaacatggtgaaaccctgtctctactaaaaa
tcaaaaaagtagccgggtgtggtggtgcacacctgtaatcccagctactc
aggaggctgaggcaggagaattgcttgaacctgggagatggagattgcag
tgagctgagatcgtaccactgcactgcagtctgggtgacaaagcaagact
ctgtctcaaaaaacaaacaaacaaacaaactttttgaaattgaattgcaa
tatgtctgctactttggcttgggctggacagtttatctaataagcaaggc
agcttcttgtgtgctatggtaataagccttcttaacaggctaagcttccc
tcataagaactgtggtgtttctcttttttttttttgttttggtttgttt
tggtttttggttttgttttgttttgtttttaaatcagtatccaatgtt
ttatagggggccaaaggttaactttgcactattccctgtcagttaaaggcc
actgatatttttctaagttagcaaggctcacttgcttgctttcttgcctc
ccttgcctccatccctctcccttcctaccttctctatttacctatttctc
tccctccctctcttctcctctcttttttttttccccgcatatgcagctttt
tgattgtacttgattttatagagactgcacagttccagcaagattgggag
tcaggcatggagcaggcatctcaggctaccagaaagaattggtcacctag
actttcagtcaggcatcctcgtttgcattgtcctgtaagtcaattagttg
ataaatagttccccccttcatcccttaagttttgtttttgtttttgttttt
aatataggtaagtgggactctacctaaaattttgcatcatacttatgggt
aatatctttttcatatattatttatcaaagtatgaagttgagtattttgc
ttgtaccatttcaattctgcattatagtagttcattgtataactgaaaga
aatgatttcttcataagtgacattaaatatgaacattcatccaattgaat
ttacaaaatctttccaaaattataagggagaaaaatcgggtttacttcaa
tctttaaaaatctggcacctcttagtaactttcagtatttctaaattgct

ctaaaattttataataaatagattagggttttgcaatagtcttaattttt
aagccaaaggttttctgagaccttaggttttgtagtctaaccagcttatg
tggttatttaaaagaatattctttgtgtttttaaattgctatttttaaaa
aagctttttattacccattttataaaatgttatactcatatttgtctgaa
tttttccagtattcctacatgaaattgtatgtattaaaaactcaatatta
gtggcaaatattaatactattaaaatggattttggtgctatattcttgta
gctaaagcctagtagaattctcaagaataggtgaaatacactttcaaagt
tgcctgtcatttaaaagaccaaataagcattttgtattaaataaattagc
agatggttagagacttcaaggagagttagcttggtcatttaattgcgact
tcatgttatttgattgaaataacccacatccttgctttgtataagatatt
cgctctggagaagttacatgtaaatagaattctataaattgttttccagt
tgaccgagtatctgttgtgtttttgtttaaaaagaggattccatgacata
ataaaaattattttaaaaaaaaaaaaaaaaa

>NM_024900 3
gttttggcaagggattaaagtgctccccctgtggcagcagtgacccaga
aatgagtttgattcacatagtccttcctccataacaagccaaacgccaga
ccgagagtgcctccgtgcgcgagtgcccggtgtgtgcgcgccggcgagag
caggggcccgcccggctccccgcccgccgcggcccgaactcatgcagctc
cgagcgagcgagcggcgcccagcccagcgcctcggccgaacccctccgca
gcaggctgcctgctgtttcccggggagatcatgaaacgaggtcgccttcc
cagcagcagtgaggattctgacgacaatggcagcctgtcaactacttggt
cccagaattcccgatcccagcataggagaagctcctgctccagacatgaa
gatcgaaagccttcagaggtgtttaggacagacctgatcactgccatgaa
gttgcatgactcctaccagctgaatccggatgagtactatgtgttggcag
atccctggagacaggaatgggagaaaggggtccaggtgcctgtgagcccg
gggaccatccctcagcctgtggccagggttgtgtctgaagagaaatccct
catgttcatcaggcccaagaagtacatcgtgtcatcaggctctgagcctc
ccgagttgggctatgtggacatccggacgctggctgacagcgtgtgtcgc
tatgacctcaatgacatggatgctgcatggctggaactgaccaatgaaga
atttaaggagatgggaatgcctgaactagatgaatacaccatggagaggg
tcctagaggaatttgagcagcgatgctacgacaatatgaatcatgccata
gagactgaggaaggcctggggatcgaatatgatgaagatgttgtctgtga
tgtctgccagtctcctgatggtgaggacggcaatgagatggtgttctgtg
acaaatgcaacatctgtgtgcaccaggcctgttatggaatcctcaaggta
ccagagggcagctggctgtgccggacatgtgccctgggggttcagccaaa
atgtctgctgtgtccgaagaagggtggagctatgaagcccacccgtagcg
gaaccaagtgggtccacgttagctgtgctctgtggatccctgaggtgagc
attggcagcccagagaagatggagcccatcaccaaggtgtcacacattcc
cagcagccggtgggcgctagtgtgcagcctctgcaatgagaagtttgggg
cctctatacagtgctctgtgaagaactgccgcacagccttccatgtgacc
tgtgcttttgaccggggcctggagatgaagaccatcttagcagagaatga
tgaagtcaagttcaagtcctattgcccaaagcacagctcacataggaaac
ccgaggagagtcttggcaagggggctgcacaggagaatggggcccctgag
tgttccccccggaatccgctggagccctttgccagccttgagcagaaccg
ggaggaggcccaccgggtgagtgtccgtaagcagaagctgcagcagttgg

aggatgagttctacaccttcgtcaacctgctggatgttgccagggctctg
cggctgcctgaggaagtagtggatttcctgtaccagtactggaagttgaa
gaggaaggtcaacttcaacaagcccctgatcaccccaaagaaagatgaag
aggacaatctagccaagcgggagcaggatgtcttatttaggaggctgcag
ctgttcacgcacctgcggcaggacctggagagggtaatgattgacactga
caccttatagtgacttagagaagaagatgcaaagaggcgaacgctcgccc
agagcaagaaatgatagccagtcatatactctcagacccttgtacacacc
acagcatgaggttgtgttggttaaaaatatttatgggctggtaaactcat
tgtacatatgtgcaaaactgctactgagtggggagcttctttgtggtttt
ttttttttaaaaacactttcccattaatctttactgttttgtaagatca
aatggggtgtgtcttccccacccccattccttcattctagagctagagtg
aatgagccccaagaaaatgacccaaggagttgactcaggatggtttacag
actgatttagaaaaccagaacggatttcatttctaatggagggggccaga
gatgggaaaatttcttgttcagtccggggaaacacacctaggtgctggtg
atgggcttatgaaggaagctaagcacggctgctcactggcccccactttg
tttcttgggtaattcacaggggaattcccagtactgtcatggagcagagc
aggcagtgggtgctgatgtgtgtgcatgagctgtatgtacacatgcatat
atctgttacagaagatactcctggcagtgaggtgctaagtcatcactgag
gctgtgtgtgtgtgtgtgtgtgcgcgtgcccgtgtccatccatgtctctg
ttgtgtgtctgtgtgcgggtatgggtgggattcctggtggacaggggtgt
cagatctgtctgaggagccccagtcatgctcagcacgctacagatgtgtt
gtttgtcacactgagattgctgaatgtcgtggctgttggctgccgagcct
cagctgctggcatttccttctgctgtttgctgcttttgtgcctcccccac
tttccatcacctctggagtcccgtctggacgtcccttcctgctacaggaa
taatgaggcgtgggctgcctcccgctagggcctcctgctccctgtaggta
gtttctggctgaggcttgctaattggggatgcttcttagagcatcttcca
catcaactcccctggctgctggctaccgattaaattcattagtgtgaaag
aggtgggagtgaggttttctggcctgaagcagtctgcactgaaaggtacc
caagtggcctgaaacagtgtagggaaagacctgggaaacactggaccaaa
aaagcctgatctcatggagacctgcatggccctgttagagatggcgtaga
agtgaaagtcttaaagggagcattagagatccttttaatacacgactgag
tgccagcttatttgtgatgccccttcccagaccaggttaggattcctggg
aaggccgcggattccggccctggaagaggcaggatcctggagcagttttg
tgaggcttttgtgctcccatacgccccctggtggtgagtgtaaagaagac
tttgcctctcacaactacatgtatgtgtggcatttttgttagagatgaga
aaaggattgagaaggataaactggaatcctggtaagaagcctttatgcca
gcccgacacctgctgtaattggggtgcatgagctatggagtcagatagtt
gttgggagggggaggacaagaagtctattgtttggactgtgtttgtctca
caatcaccacaaaataaaagtgtagaaaatgcttgtggtgtactaactct
tttctgtacttaagttactcagattaacatgcacttctaattctaaacat
tttttttggagtcatcactgtcgctgtttaagtagaacaatgccaacgat
gtatgagaaacagggaataaatcaggatttgtgtgtgtatgtgtgtggca
ttacttacatttactttattaaaaagcctaaggaactttaaagaatttgt
attcagtttatttttattttttcaagacagagtctcactttgttgcccag
gctggagtgcagtggcgtgatctcggctcactgcagcctccgcctcccgg
gttcaagtgattctcctgcctcggtctcatgagtagctgggattacaggc

gtgcgccaccatgcctggctaatttttgtatttttagtagagacggtttc
accatgttggtcaggttggtcttgaactcctgacctcaagtgatctacct
gtcttggcctcccagagtgttgggattacgccactgtacccagccttaga
ctcagtttaaaatgggcttattgtaatgaaaaaaaatacgctacccaaaa
tttgccatgatgcctgtgatggtgaaggcattaaacaattgaaatctttg
ccttgcccagagagttgcagttatttgtaattataaacagtaagtgtatc
agggcaggggtgacaataaaaaaccaatcattactaccaa

>NM_138973 3
acaagtctttccgcctccccagcccgcccgggagctgcgagccgcgagct
ggattatggtggcctgagcagccaacgcagccgcaggagcccggagccct
tgcccctgcccgcgccgccgcccgccgggggggaccagggaagccgccacc
ggcccgccatgcccgcccctcccagccccgccgggagcccgcgcccgctg
cccaggctggccgccgccgtgccgatgtagcgggctccggatcccagcct
ctcccctgctcccgtgctctgcggatctcccctgaccgctctccacagcc
cggacccgggggctggcccagggccctgcaggccctggcgtcctgatgcc
cccaagctccctctcctgagaagccaccagcaccacccagacttgggggc
aggcgccagggacggacgtgggccagtgcgagcccagagggcccgaaggc
cggggcccaccatggcccaagccctgccctggctcctgctgtggatgggc
gcgggagtgctgcctgcccacggcacccagcacggcatccggctgcccct
gcgcagcggcctgggggggcgcccccctggggctgcggctgccccgggaga
ccgacgaagagcccgaggagcccggccggagggggcagctttgtggagatg
gtggacaacctgagggggcaagtcggggcagggctactacgtggagatgac
cgtgggcagccccccgcagacgctcaacatcctggtggatacaggcagca
gtaactttgcagtgggtgctgccccccaccccttcctgcatcgctactac
cagaggcagctgtccagcacataccgggacctccggaagggtgtgtatgt
gccctacacccagggcaagtgggaaggggagctgggcaccgacctgcttt
gtggtgctggcttcccccctcaaccagtctgaagtgctggcctctgtcgga
gggagcatgatcattggaggtatcgaccactcgctgtacacaggcagtct
ctggtatacacccatccggcgggagtggtattatgaggtgatcattgtgc
gggtggagatcaatggacaggatctgaaaatggactgcaaggagtacaac
tatgacaagagcattgtggacagtggcaccaccaaccttcgtttgcccaa
gaaagtgtttgaagctgcagtcaaatccatcaaggcagcctcctccacgg
agaagttccctgatggtttctggctaggagagcagctggtgtgctggcaa
gcaggcaccaccccttggaacattttcccagtcatctcactctacctaat
gggtgaggttaccaaccagtccttccgcatcaccatccttccgcagcaat
acctgcggccagtggaagatgtggccacgtcccaagacgactgttacaag
tttgccatctcacagtcatccacgggcactgttatgggagctgttatcat
ggagggcttctacgttgtctttgatcgggcccgaaaacgaattggctttg
ctgtcagcgcttgccatgtgcacgatgagttcaggacggcagcggtggaa
ggcccttttgtcaccttggacatggaagactgtggctacaacattccaca
gacagatgagtcaaccctcatgaccatagcctatgtcatggctgccatct
gcgccctcttcatgctgccactctgcctcatggtgtgtcagtggcgctgc
ctccgctgcctgcgccagcagcatgatgactttgctgatgacatctccct
gctgaagtgaggaggcccatgggcagaagatagagattcccctggaccac
acctccgtggttcactttggtcacaagtaggagacacagatggcacctgt

ggccagagcacctcaggaccctccccacccaccaaatgcctctgccttga
tggagaaggaaaaggctggcaaggtgggttccagggactgtacctgtagg
aaacagaaaagagaagaaagaagcactctgctggcgggaatactcttggt
cacctcaaatttaagtcgggaaattctgctgcttgaaacttcagccctga
acctttgtccaccattcctttaaattctccaacccaaagtattcttcttt
tcttagtttcagaagtactggcatcacacgcaggttaccttggcgtgtgt
ccctgtggtaccctggcagagaagagaccaagcttgtttccctgctggcc
aaagtcagtaggagaggatgcacagtttgctatttgctttagagacaggg
actgtataaacaagcctaacattggtgcaaagattgcctcttgaattaaa
aaaaaaaactagattgactatttatacaaatgggggcggctggaaagagg
agaaggagagggagtacaaagacagggaatagtgggatcaaagctaggaa
aggcagaaacacaaccactcaccagtcctagttttagacctcatctccaa
gatagcatcccatctcagaagatgggtgttgttttcaatgttttcttttc
tgtggttgcagcctgaccaaaagtgagatgggaagggcttatctagccaa
agagctctttttagctctcttaaatgaagtgcccactaagaagttccac
ttaacacatgaatttctgccatattaatttcattgtctctatctgaacca
ccctttattctacatatgataggcagcactgaaatatcctaaccccctaa
gctccaggtgccctgtgggagagcaactggactatagcagggctgggctc
tgtcttcctggtcataggctcactctttcccccaaatcttcctctggagc
tttgcagccaaggtgctaaaaggaataggtaggagacctcttctatctaa
tccttaaaagcataatgttgaacattcattcaacagctgatgccctataa
cccctgcctggatttcttcctattaggctataagaagtagcaagatcttt
acataattcagagtggtttcattgccttcctaccctctctaatggcccct
ccatttatttgactaaagcatcacacagtggcactagcattataccaaga
gtatgagaaatacagtgctttatggctctaacattactgccttcagtatc
aaggctgcctggagaaaggatggcagcctcagggcttccttatgtcctcc
accacaagagctccttgatgaaggtcatctttttcccctatcctgttctt
cccctccccgctcctaatggtacgtgggtacccaggctggttcttgggct
aggtagtggggaccaagttcattacctccctatcagttctagcatagtaa
actacggtaccagtgttagtgggaagagctgggttttcctagtataccca
ctgcatcctactcctacctggtcaacccgctgcttccaggtatgggacct
gctaagtgtggaattacctgataagggagagggaaatacaaggagggcct
ctggtgttcctggcctcagccagctgcccacaagccataaaccaataaaa
caagaatactgagtcagtttttttatctgggttctcttcattcccactgca
cttggtgctgctttggctgactgggaacaccccataactacagagtctga
caggaagactggagactgtccacttctagctcggaacttactgtgtaaat
aaactttcagaactgctaccatgaagtgaaaatgccacattttgctttat
aatttctacccatgttgggaaaaactggctttttcccagccctttccagg
gcataaaactcaacccccttcgatagcaagtcccatcagcctattattttt
ttaaagaaaacttgcacttgtttttctttttacagttacttccttcctgc
cccaaaattataaactctaagtgtaaaaaaaaagtcttaacaacagcttct
tgcttgtaaaaatatgtattatacatctgtatttttaaattctgctcctg
aaaaatgactgtcccattctccactcactgcatttggggcctttcccatt
ggtctgcatgtcttttatcattgcaggccagtggacagagggagaaggga
gaacaggggtcgccaacacttgtgttgctttctgactgatcctgaacaag
aaagagtaacactgaggcgctcgctcccatgcacaactctccaaaacact

tatcctcctgcaagagtgggctttccagggtctttactgggaagcagtta
agccccctcctcacccccttccttttttctttctttactcctttggcttca
aaggattttggaaaagaaacaatatgctttacactcattttcaatttcta
aatttgcaggggatactgaaaaatacggcaggtggcctaaggctgctgta
aagttgaggggagaggaaatcttaagattacaagataaaaaacgaatccc
ctaaacaaaaagaacaatagaactggtcttccattttgccacctttcctg
ttcatgacagctactaacctggagacagtaacatttcattaaccaaagaa
agtgggtcacctgacctctgaagagctgagtactcaggccactccaatca
ccctacaagatgccaaggaggtcccaggaagtccagctccttaaactgac
gctagtcaataaacctgggcaagtgaggcaagagaaatgaggaagaatcc
atctgtgaggtgacaggcaaggatgaaagacaaagaaggaaaagagtatc
aaaggcagaaaggagatcatttagttgggtctgaaaggaaaagtctttgc
tatccgacatgtactgctagtacctgtaagcattttaggtcccagaatgg
aaaaaaaaatcagctattggtaatataataatgtcctttccctggagtca
gtttttttaaaaagttaactcttagtttttacttgtttaattctaaaaga
gaagggagctgaggccattccctgtaggagtaaagataaaaggataggaa
aagattcaaagctctaatagagtcacagctttcccaggtataaaacctaa
aattaagaagtacaataagcagaggtggaaaatgatctagttcctgatag
ctacccacagagcaagtgatttataaatttgaaatccaaactactttctt
aatatcactttggtctccatttttcccaggacaggaaatatgtcccccc
taactttcttgcttcaaaaattaaaatccagcatcccaagatcattctac
aagtaattttgcacagacatctcctcaccccagtgcctgtctggagctca
cccaaggtcaccaaacaacttggttgtgaaccaactgccttaaccttctg
ggggagggggattagctagactaggagaccagaagtgaatgggaaagggt
gaggacttcacaatgttggcctgtcagagcttgattagaagccaagacag
tggcagcaaaggaagacttggcccaggaaaaacctgtgggttgtgctaat
ttctgtccagaaaatagggtggacagaagcttgtggggtacatggaggaa
ttgggacctggttatgttgttattctcggactgtgaattttggtgatgta
aaacagaatattctgtaaacctaatgtctgtataaataatgagcgttaac
acagtaaaatattcaataagaagtcaaactactagggttaaaaaaaaaaa
aaaaaaa

>NM_019102 3
gggtgctatagacgcacaaacgaccgcgagccacaaatcaagcacacata
tcaaaaaacaaatgagctcttattttgtaaactcattttgcggtcgctat
ccaaatggcccggactaccagttgcataattatggagatcatagttccgt
gagcgagcaattcagggactcggcgagcatgcactccggcaggtacggct
acggctacaatggcatggatctcagcgtcggccgctcgggctccggccac
tttggctccggagagcgcgcccgcagctacgctgccagcgccagcgcggc
gcccgccgagcccaggtacagccagccggccacgtccacgcactctcctc
agcccgatccgctgccctgctccgccgtggccccctcgcccggcagcgac
agccaccacggcgggaaaaactccctaagcaactccagcggcgcctcggc
cgacgccggcagcacccacatcagcagcagagagggggttggcacggcgt
ccggagccgaggaggacgcccctgccagcagcgagcaggcgagtgcgcag
agcgagccgagcccggcgccgcccgcccaaccccagatctacccctggat
gcgcaagctgcacataagtcatgacaacataggcggcccggaaggcaaaa

gggcccggacggcctacacgcgctaccagaccctggagctggagaaggag
ttccacttcaaccgttacctgacccgcagaaggaggattgaaatagcaca
tgctctttgcctctccgagagacaaattaaaatctggttccaaaaccgga
gaatgaagtggaaaaaagataataagctgaaaagcatgagcatggccgcg
gcaggaggggccttccgtccctgagtatctgagcgtttaaagtactgagc
agtattagcggatcccgcgtagtgtcagtactaaggtgactttctgaaac
tcccttgtgttccttctgtgaagaagccctgttctcgttgccctaattca
tcttttaatcatgagcctgtttattgccattatagcgcctgtataagtag
atctgctttctgttcatctctttgtcctgaatggctttgtcttgaaaaaa
aatagatgttttaacttatttatatgaagcaagctgtgttacttgaagta
actataacaaaaaaagaaaagagaaaaaaaaacacacaaaaagtcccccct
tcaatctcgtttagtgccaatgttgtgtgttgcactcaagttgtttaact
gtgcatgtgcgtggaagtgttcctgtctcaatagctccaagctgttaaag
atatttttattcaaactacctatattccttgtgtaattaatgctgttgta
gaggtgacttgatgagacacaacttgttcgacgtgtagtgactagtgact
ctgtgatgaaaactgtgactccaagcggtgtgtccctgcgtgcctttata
ggaccctttgcacgaactctggaagtggctcttataagcgcagcttcagt
gatgtatgtttttgtgaacaaagttacaaatattgtccaagtctggctgt
tttaagcaaactgtgatcagcttttttttttttttttttttttttgtatt
tgtttttaaggaaaaaatactgactggaacaaaaaataaactttctattg
taagttc

>NM_138973 3
acaagtctttccgcctccccagcccgcccgggagctgcgagccgcgagct
ggattatggtggcctgagcagccaacgcagccgcaggagcccggagccct
tgcccctgcccgcgccgccgcccgccgggggggaccagggaagccgccacc
ggcccgccatgcccgcccctcccagccccgccgggagcccgcgcccgctg
cccaggctggccgccgccgtgccgatgtagcgggctccggatcccagcct
ctcccctgctcccgtgctctgcggatctcccctgaccgctctccacagcc
cggacccgggggctggcccagggccctgcaggccctggcgtcctgatgcc
cccaagctccctctcctgagaagccaccagcaccacccagacttgggggc
aggcgccagggacggacgtgggccagtgcgagcccagagggcccgaaggc
cggggcccaccatggcccaagccctgccctggctcctgctgtggatgggc
gcgggagtgctgcctgcccacggcacccagcacggcatccggctgcccct
gcgcagcggcctgggggcgccccccctggggctgcggctgccccgggaga
ccgacgaagagcccgaggagcccggccggaggggcagctttgtggagatg
gtggacaacctgaggggcaagtcggggcagggctactacgtggagatgac
cgtgggcagccccccgcagacgctcaacatcctggtggatacaggcagca
gtaactttgcagtgggtgctgccccccaccccttcctgcatcgctactac
cagaggcagctgtccagcacataccgggacctccggaagggtgtgtatgt
gccctacacccagggcaagtgggaaggggagctgggcaccgacctgcttt
gtggtgctggcttcccccctcaaccagtctgaagtgctggcctctgtcgga
gggagcatgatcattggaggtatcgaccactcgctgtacacaggcagtct
ctggtatacacccatccggcgggagtggtattatgaggtgatcattgtgc
gggtggagatcaatggacaggatctgaaaatggactgcaaggagtacaac
tatgacaagagcattgtggacagtggcaccaccaaccttcgtttgcccaa

gaaagtgtttgaagctgcagtcaaatccatcaaggcagcctcctccacgg
agaagttccctgatggtttctggctaggagagcagctggtgtgctggcaa
gcaggcaccaccccttggaacattttcccagtcatctcactctacctaat
gggtgaggttaccaaccagtccttccgcatcaccatccttccgcagcaat
acctgcggccagtggaagatgtggccacgtcccaagacgactgttacaag
tttgccatctcacagtcatccacgggcactgttatgggagctgttatcat
ggagggcttctacgttgtctttgatcgggcccgaaaacgaattggctttg
ctgtcagcgcttgccatgtgcacgatgagttcaggacggcagcggtggaa
ggcccttttgtcaccttggacatggaagactgtggctacaacattccaca
gacagatgagtcaaccctcatgaccatagcctatgtcatggctgccatct
gcgccctcttcatgctgccactctgcctcatggtgtgtcagtggcgctgc
ctccgctgcctgcgccagcagcatgatgactttgctgatgacatctccct
gctgaagtgaggaggcccatgggcagaagatagagattcccctggaccac
acctccgtggttcactttggtcacaagtaggagacacagatggcacctgt
ggccagagcacctcaggaccctccccacccaccaaatgcctctgccttga
tggagaaggaaaaggctggcaaggtgggttccagggactgtacctgtagg
aaacagaaaagagaagaaagaagcactctgctggcgggaatactcttggt
cacctcaaatttaagtcgggaaattctgctgcttgaaacttcagccctga
acctttgtccaccattcctttaaattctccaacccaaagtattcttcttt
tcttagtttcagaagtactggcatcacacgcaggttaccttggcgtgtgt
ccctgtggtaccctggcagagaagagaccaagcttgtttccctgctggcc
aaagtcagtaggagaggatgcacagtttgctatttgctttagagacaggg
actgtataaacaagcctaacattggtgcaaagattgcctcttgaattaaa
aaaaaaaactagattgactatttatacaaatgggggcggctggaaagagg
agaaggagagggagtacaaagacagggaatagtgggatcaaagctaggaa
aggcagaaacacaaccactcaccagtcctagttttagacctcatctccaa
gatagcatcccatctcagaagatgggtgttgttttcaatgttttcttttc
tgtggttgcagcctgaccaaaagtgagatgggaagggcttatctagccaa
agagctctttttagctctcttaaatgaagtgcccactaagaagttccac
ttaacacatgaatttctgccatattaatttcattgtctctatctgaacca
ccctttattctacatatgataggcagcactgaaatatcctaaccccctaa
gctccaggtgccctgtgggagagcaactggactatagcagggctgggctc
tgtcttcctggtcataggctcactctttcccccaaatcttcctctggagc
tttgcagccaaggtgctaaaaggaataggtaggagacctcttctatctaa
tccttaaaagcataatgttgaacattcattcaacagctgatgccctataa
cccctgcctggatttcttcctattaggctataagaagtagcaagatcttt
acataattcagagtggtttcattgccttcctaccctctctaatggcccct
ccatttatttgactaaagcatcacacagtggcactagcattataccaaga
gtatgagaaatacagtgctttatggctctaacattactgccttcagtatc
aaggctgcctggagaaaggatggcagcctcagggcttccttatgtcctcc
accacaagagctccttgatgaaggtcatctttttcccctatcctgttctt
cccctccccgctcctaatggtacgtgggtacccaggctggttcttgggct
aggtagtggggaccaagttcattacctccctatcagttctagcatagtaa
actacggtaccagtgttagtgggaagagctgggttttcctagtatacccа
ctgcatcctactcctacctggtcaacccgctgcttccaggtatgggacct
gctaagtgtggaattacctgataagggagagggaaatacaaggagggcct

ctggtgttcctggcctcagccagctgcccacaagccataaaccaataaaa
caagaatactgagtcagttttttatctgggttctcttcattcccactgca
cttggtgctgctttggctgactgggaacaccccataactacagagtctga
caggaagactggagactgtccacttctagctcggaacttactgtgtaaat
aaactttcagaactgctaccatgaagtgaaaatgccacattttgctttat
aatttctacccatgttgggaaaaactggctttttcccagccctttccagg
gcataaaactcaacccccttcgatagcaagtcccatcagcctattattttt
ttaaagaaaacttgcacttgtttttctttttacagttacttccttcctgc
cccaaaattataaactctaagtgtaaaaaaaagtcttaacaacagcttct
tgcttgtaaaaatatgtattatacatctgtatttttaaattctgctcctg
aaaaatgactgtcccattctccactcactgcatttggggcctttcccatt
ggtctgcatgtcttttatcattgcaggccagtggacagagggagaaggga
gaacaggggtcgccaacacttgtgttgctttctgactgatcctgaacaag
aaagagtaacactgaggcgctcgctcccatgcacaactctccaaaacact
tatcctcctgcaagagtgggctttccagggtctttactgggaagcagtta
agccccctcctcacccccttcctttttctttctttactcctttggcttca
aaggattttggaaaagaaacaatatgctttacactcattttcaatttcta
aatttgcaggggatactgaaaaatacggcaggtggcctaaggctgctgta
aagttgaggggagaggaaatcttaagattacaagataaaaaacgaatccc
ctaaacaaaaagaacaatagaactggtcttccattttgccacctttcctg
ttcatgacagctactaacctggagacagtaacatttcattaaccaaagaa
agtgggtcacctgacctctgaagagctgagtactcaggccactccaatca
ccctacaagatgccaaggaggtcccaggaagtccagctccttaaactgac
gctagtcaataaacctgggcaagtgaggcaagagaaatgaggaagaatcc
atctgtgaggtgacaggcaaggatgaaagacaaagaaggaaaagagtatc
aaaggcagaaaggagatcatttagttgggtctgaaaggaaaagtctttgc
tatccgacatgtactgctagtacctgtaagcattttaggtcccagaatgg
aaaaaaaaatcagctattggtaatataataatgtcctttccctggagtca
gtttttttaaaaagttaactcttagtttttacttgtttaattctaaaaga
gaagggagctgaggccattccctgtaggagtaaagataaaaggataggaa
aagattcaaagctctaatagagtcacagctttcccaggtataaaacctaa
aattaagaagtacaataagcagaggtggaaaatgatctagttcctgatag
ctacccacagagcaagtgatttataaatttgaaatccaaactactttctt
aatatcactttggtctccatttttcccaggacaggaaatatgtccccccc
taactttcttgcttcaaaaattaaaatccagcatcccaagatcattctac
aagtaattttgcacagacatctcctcaccccagtgcctgtctggagctca
cccaaggtcaccaaacaacttggttgtgaaccaactgccttaaccttctg
ggggagggggattagctagactaggagaccagaagtgaatgggaaagggt
gaggacttcacaatgttggcctgtcagagcttgattagaagccaagacag
tggcagcaaaggaagacttggcccaggaaaaacctgtgggttgtgctaat
ttctgtccagaaaatagggtggacagaagcttgtggggtacatggaggaa
ttgggacctggttatgttgttattctcggactgtgaattttggtgatgta
aaacagaatattctgtaaacctaatgtctgtataaataatgagcgttaac
acagtaaaatattcaataagaagtcaaactactagggttaaaaaaaaaaa
aaaaaaa

>NM_030751 5
gggggggaagggggagggagggggaggaggtgactcgagcatttagacac
aagcgagaggatcatggcggatggccccaggtgtaagcgcagaaagcagg
cgaacccgcggcgcaataacgttacaaattataatactgtggtagaaaca
aattcagattcagatgatgaagacaaactgcatattgtggaagaagaaag
tgttacagatgcagctgactgtgaaggtgtaccagaggatgacctgccaa
cagaccagacagtgttaccagggaggagcagtgaaagagaagggaatgct
aagaactgctgggaggatgacagaaaggaagggcaagaaatcctggggcc
tgaagctcaggcagatgaagcaggatgtacagtaaaagatgatgaatgcg
agtcagatgcagaaaatgagcaaaaccatgatcctaatgttgaagagttt
ctacaacaacaagacactgctgtcatttttcctgaggcacctgaagagga
ccagaggcagggcacaccagaagccagtggtcatgatgaaaatggaacac
cagatgcattttcacaattactcacctgtccatattgtgatagaggctat
aaacgctttacctctctgaaagaacacattaaatatcgtcatgaaaagaa
tgaagataactttagttgctccctgtgcagttacacctttgcatacagaa
cccaacttgaacgtcacatgacatcacataaatcaggaagagatcaaaga
catgtgacgcagtctgggtgtaatcgtaaattcaaatgcactgagtgtgg
aaaagctttcaaatacaaacatcacctaaaagagcacttaagaattcaca
gtggagagaagccatatgaatgcccaaactgcaagaaacgcttttcccat
tctggctcctatagctcacacataagcagtaagaaatgtatcagcttgat
acctgtgaatgggcgaccaagaacaggactcaagacatctcagtgttctt
caccgtctctttcagcatcaccaggcagtcccacacgaccacagatacgg
caaaagatagagaataaaccccttcaagaacaactttctgttaaccaaat
taaaactgaacctgtggattatgaattcaaacccatagtggttgcttcag
gaatcaactgttcaacccctttacaaaatggggttttcactggtggtggc
ccattacaggcaaccagttctcctcagggcatggtgcaagctgttgttct
gccaacagttggtttggtgtctcccataagtatcaatttaagtgatattc
agaatgtacttaaagtggcggtagatggtaatgtaataaggcaagtgttg
gagaataatcaagccaatcttgcatccaaagaacaagaaacaatcaatgc
ttcacccatacaacaaggtggccattctgttatttcagccatcagtcttc
ctttggttgatcaagatggaacaaccaaaattatcatcaactacagtctt
gagcagcctagccaacttcaagttgttcctcaaaatttaaaaaaagaaaa
tccagtcgctacaaacagttgtaaaagtgaaaagttaccagaagatctta
ctgttaagtctgagaaggacaaaagctttgaaggggggggtgaatgatagc
acttgtcttctgtgtgatgattgtccaggagatattaatgcacttccaga
attaaagcactatgacctaaagcagcctactcagcctcctccactccctg
cagcagaagctgagaagcctgagtcctctgtttcatcagctactggagat
ggcaatttgtctcctagtcagccacctttaaagaacctcttgtctctcct
aaaagcatattatgctttgaatgcacaaccaagtgcagaagagctctcaa
aaattgctgattcagtaaacctaccactggatgtagtaaaaaagtggttt
gaaaagatgcaagctggacagatttcagtgcagtcttctgaaccatcttc
tcctgaaccaggcaaagtaaatatccctgccaagaacaatgatcagcctc
aatctgcaaatgcaaatgaaccccaggacagcacagtaaatctacaaagt
cctttgaagatgactaactccccagttttaccagtgggatcaaccaccaa
tggttccagaagtagtacaccatccccatcacctctaaacctttcctcat
ccagaaatacacagggttacttgtacacagctgagggtgcacaagaagag

ccacaagtagaacctcttgatctttcactaccaaagcaacagggagaatt
attagaaaggtcaactatcactagtgtttaccagaacagtgtttattctg
tccaggaagaacccttgaacttgtcttgcgcaaaaaaggagccacaaaag
gacagttgtgttacagactcagaaccagttgtaaatgtaatcccaccaag
tgccaaccccataaatatcgctatacctacagtcactgcccagttaccca
caatcgtggccattgctgaccagaacagtgttccatgcttaagagcgcta
gctgccaataagcaaacgattctgattccccaggtggcatacacctactc
aactacggtcagccctgcagtccaagaaccacccttgaaagtgatccagc
caaatggaaatcaggatgaaagacaagatactagctcagaaggagtatca
aatgtagaggatcagaatgactctgattctacaccgcccaaaaagaaaat
gcggaagacagaaaatggaatgtatgcttgtgatttgtgtgacaagatat
tccaaaagagtagttcattattgagacataaatatgaacacacaggtaaa
agacctcatgagtgtggaatctgtaaaaaggcatttaaacacaaacatca
tttgattgaacacatgcgattacattctggagaaaagccctatcaatgtg
acaaatgtggaaagcgcttctcacactctgggtcttattctcaacacatg
aatcatcgctactcctactgtaagagagaagcggaagaacgtgacagcac
agagcaggaagaggcagggcctgaaatcctctcgaatgagcacgtgggtg
ccagggcgtctccctcacagggcgactcggacgagagagagagtttgaca
agggaagaggatgaagacagtgaaaaagaggaagaggaggaggataaaga
gatggaagaattgcaggaagaaaaagaatgtgaaaaaccacaaggggatg
aggaagaggaggaggaggaggaagaagtggaagaagaagaggtagaagag
gcagagaatgagggagaagaagcaaaaactgaaggtctgatgaaggatga
cagggctgaaagtcaagcaagcagcttaggacaaaaagtaggcgagagta
gtgagcaagtgtctgaagaaaagacaaatgaagcctaatcgtttttctag
aaggaaaataaattctaattgataatgaatttcgttcaatattatccttg
cttttcatggaaacacagtaacctgtatgctgtgattcctgttcactact
gtgtaaagtaaaaactaaaaaaatacaaaatacaaaacacacacacacac
acacacacacacacacacacacacaaaataaatccgggtgtgcctgaa
cctcagacctagtaatttttcatgcagttttcaaagttaggaacaagttt
gtaacatgcagcagattagaaaaccttaatgactcagagagcaacaatac
aagaggttaaaggaagctgattaattagatatgcatctggcattgtttta
tcttatcagtattatcactcttatgttggtttattcttaagctgtacaat
tgggagaaattttataatttttattggtaaacatatgctaaatccgctt
cagtattttattatgtttttaaaatgtgagaacttctgcactacaaaat
tcccttcacagagaagtataatgtagttccaacccgtgctaactaccttt
tataaattcagtctagaaggtagtaatttctaatatttagatgtcttagt
agagcgtattatcatttaaagtgtattgttagccttaagaaagcagctga
tagaagaactgaagtttcttactcacgtggtttaaaatggagttcaaaag
attgccattgagttctgattgcagggactaacaatgttaatctgataagg
acagcaaaatcatcagaatcagtgtttgtgattgtgtttgaatatgtggt
aacatatgaaggatatgacatgaagctttgtatctcctttggccttaagc
aagacctgtgtgctgtaagtgccatttctcagtattttcaaggctctaac
ccgccttcatccaatgtgtggcctacaataactagcatttgttgatttgt
ctcttgtatcaaaattcccaaataaaacttaaaaccactgactctgtcag
agaaactgaaacactgggacatttcatccttcaattcctcggtattgatt
ttatgttgattgattttcagaatttctctacagaaacgaaagggaaattt

tctaatctgctttatccatgtacttgcatttcagacatggacatgctatt
gttatttggctcataactgtttccaaatgttagttattatggacccaatt
tattaacaacattagctgattttacctatcagtattattttatttcttt
tagtttatagatctgtgcaacatttttgtactgtatgtcttcaaacctgg
cagtattaatacccttcttactgacatatgtactttagttttagaaaac
ttttatatttatgtgtcttatttttatatttctttatttattacacagtg
tagtgtataatactgtagtttgtattaatacaataatatattttagtatg
aaaatttggaaagttgataagatttaaagtagagatgcaattggttctcc
tgcattgagatttgatttaacagtgttatgttaacatttatacttgcctt
ggactgtagaacagaacttaaatgggaatgtattagttttacaactacaa
tcaagtcattttacctttacccagtttttaatataaaacttaaattttga
aattcactgtgtgactaatagcatgatgctctgcagttttattaagaaat
cagcctaaccatacaactctcatttccttagtaagccaaattaggattaa
cttctataaacagtgttgggaacaatgtttaacattttgtgccaatttgt
tcctgtattcatgtatgtaagttacagatctgactcttcatttttaagtt
ccttgttacatcatggtcattttctagtttttttaccagactcccatctca
caataaaatgcatcaacaagcctgaactgctgtcattcttttcatcatta
tcagtattttctttggaaaactgtgaaatggggtacattgtcatcctgca
tttgattcatcttgagctgaatttgggtaacactaaatgttttagacatt
ctccactaaattatggattttcttgtggctaaatgtttctggagaggtca
gagttgacaaaacctcttcacaggttgctccttcttcctgaaatccttaa
tcctccgcatttcatgcttcaggtcatttcagggaagcctgggtttagat
gcctttctgactctcagctcctgcacttctgtcatcatacctctgatact
attatttatattccttccccactaggaacaggaaccacatttgtcatagt
cactctcacattcctcactgcctaacagggtgcctggcataagttgggac
aacagatatttgttgaataaaaatataatttgcatgtttatggagctcag
ctatgttctcactttttttgcttctaattccagaatatatgttaaatgat
ctaataatttgattattttcttataagtcttattaaacactagtcataat
agacacaataaattatgccttctttttctattgccttaaaaaaaaaaa

>NM_054016 2
ggacgcctggtgttctcgcgggaaagaaccgttgcgcccgactttgcgcg
gaggtaggagggggaagtggaggcgggagtgaagtctcgcgagaagagtc
ggttgccgtagcagagccctctagctgtgtgtgtctgaggctcggccgcc
tgagccgcggacggtttgctgagcccgttagtgcgcccggccgagacacg
ccgccgccatgtcccgctacctgcgtccccccaacacgtctctgttcgtc
aggaacgtggccgacgacaccaggtctgaagacttgcggcgtgaatttgg
tcgttatggtcctatagttgatgtgtatgttccacttgatttctacactc
gccgtccaagaggatttgcttatgttcaatttgaggatgttcgtgatgct
gaagacgctttacataatttggacagaaagtggatttgtggacggcagat
tgaaatacagtttgcccaggggggatcgaaagacaccaaatcagatgaaag
ccaaggaagggaggaatgtgtacagttcttcacgctatgatgattatgac
agatacagacgttctagaagccgaagttatgaaaggaggagatcaagaag
tcggtcttttgattacaactatagaagatcgtatagtcctagaaacagta
gaccgactggaagaccacggcgtagcagaagccattccgacaatgataga
ttcaaacaccgaaatcgatctttttcaagatctaaatccaattcaagatc

acggtccaagtcccagcccaagaaagaaatgaaggctaaatcacgttcta
ggtctgcatctcacaccaaaactagaggcacctctaaaacagattccaaa
acacattataagtctggctcaagatatgaaaaggaatcaaggaaaaaaga
accacctagatccaaatctcagtcaagatcacagtctaggtctaggtcaa
aatctagatcaaggtcttggactagtcctaagtccagtggccactgatag
tataaaccatggtcatttttaggcatgtatcattcatttactcatagttt
ggtttacttaaattatcaggaatacaatgttgcaatgatgcttaaaaaac
acttgttagttttccctgtaccaggcaatggttataattaaaatgatatg
ctgttgagaagccactcttaagagtccagtttgtttaatgttatgggcag
ctaccaatttgtggtgtctctgtatatttttgtaaagattctcatttttt
atgcttgaagtatttggtgaaaagatgttggttgaccataatttgcaaca
ttgtctcattaaaaataaactttcatattcatatttggtagaactgttaa
cctagaaatgtagcttgctaataagatagaatgatacaaaagtgaagtag
tagccacagtacaacactgactgctcagacacatttaggttcagggtgga
cctttatgtcttgtcaagatgtctaggcccggctgggcgtggtggctcac
acctgtaatcccagcactttgggaggccgaggcgggcggatcacgaggtc
aggagttcgagaccagcctgaccaacacggtgaaaccccgtctctactaa
aaatacaaaaattatccgggcatggtggcacatgcctgtaatctcagcta
ctcaggaggctgaggcaagagaatcgcttgaacctgggaggtagaagttg
cagtgagccaaaatcacgccactgcactccagcctgggcaacagagtgag
actccgtctcaaaaaaaaaaaaaaaaccggatgtctaggccaatgataatt
atttttgatgcagtgtggattagttcttttgttaaccccactgtcttggg
gaatgatgccagctgggaaattgagtttttgactgaaacatggagccttc
actgctttttttctggttcctatgaagatttggaacatagaaaacacaaa
aactcaccttaaaatttgagcaggtcgttgatggcaaaaataattttaag
gaaaaaggaatattcttatgtagttattctaaagtttaaggagcgttgtt
gaccataatattgcttagttttcttactgctgttaagtaagtaaattgtt
tcaaagtaggttttgtgtgtgtgtgcctagtgtaaaagaactgaaatttt
gatgcttacagcacttggctcgtgcatttgtatcaaaatttgcctgcctc
tttatgagggaggcctgcttttcacacctcagtttatttaatacgaggca
agttgtaagacaacactcattctaggtgattctgtggtgccatgaaattt
aaggtaatttggggaaaaggattagtcagttttaagcaagagtcacatct
tttgagctttcgattatcagtgtagtacctgactaaaaatgaagtaatac
ccttaaaccatttataatttctagtatttctctgaaagatcgttttgggg
acaaaagtgacttgacatgtccaatttcatttcagaataaaaagctagca
tctttaaaaatctcagattgcttgcttacagatacaagtacgaattatgg
acaaacgattccttttagaggattactttttcaatttcggttttagtaa
tctaggctttgcctgtaaagaatacaacgatggattttaaatactgtttg
tggaatgtgtttaaaggattgattctagaacctttgtatatttgatagta
tttctaactttcatttctttactgtttgcagttaatgttcatgttctgct
atgcaatcgtttatatgcacgtttctttaattttttagattttcctgga
tgtatagtttaaacaacaaaaagtctatttaaaactgtagcagtagttta
cagttctagcaaagaggaaagttgtggggttaaactttgtattttctttc
ttatagaggcttctaaaaaggtatttttatatgttctttttaacaaatat
tgtgtacaacctttaaaacatcaatgtttggatcaaaacaagacccagct
tattttctgcttgctgtaaattaagcaaacatgctataataaaaacaaaa

tgaagg

>NM_130439 3
atacacacagactcacagcgagaccgacacacactcccatacactcacac
acacaactgcaggcagcgaggctcgggaagtcaggccggcttttcgcccc
ggcgccttctctgctccagccggccgggtctccctgggggcccggagctc
ggccgggccgcgcagccccgttagaggacgagctcggcggacccccgctc
ctccatgggcaaacgcgggcggccgcgcaaggaggcgcgctgcgagggcg
cggggctggccccgccgcgcccccggctgtgccccccgccgtggccgcg
ccccagcccccggccctgcccgaggaccccgctggggccaagcccaggtg
ccccttctcagacattttcaacaccagcgagaactcgatggagaagcaca
tcaacacttttctgcagaacgtgcagattctgctcgaggccgccagctac
ctggagcagatcgagaaagaaaacaaaaagtgtgaacatggctacgcctc
ttcattcccgtccatgccgagcccccgactgcagcattcaaagcccccac
ggaggttgagccgggcacagaaacacagcagcgggagcagcaacaccagc
actgccaacagatctacacacaatgagctggaaaagaatcgacgagctca
tctgcgcctttgtttagaacgcttaaaagttctgattccactaggaccag
actgcacccggcacacaacacttggtttgctcaacaaagccaaagcacac
atcaagaaacttgaagaagctgaaagaaaaagccagcaccagctcgagaa
tttggaacgagaacagagatttttaaagtggcgactggaacagctgcagg
gtcctcaggagatggaacgaatacgaatggacagcattggatcaactatt
tcttcagatcgttctgattcagagcgagaggagattgaagtggatgttga
aagcacagagttctcccatggagaagtggacaatataagtaccaccagca
tcagtgacattgatgaccacagcagcctgccgagtattgggagtgacgag
ggttactccagtgccagtgtcaaactttcattcacttcatagaacccagc
atgacataacagtgcagggcaaaatattcactgggccaattcaatacaaa
caatctcttaaattgggttcatgatgcagtctcctctttaaaacaaaaca
aaacaaaacaaaactatacttgaacaaaagggtcagaggacctgtattta
agcaaatacttagcaaaaagtggggcagagcctcccaaggagaacaaata
ttcagaatattcatattggaaaaatcacaatttttaatggcagcagaaaa
cttgtgtgaaattttcttgatttgagttgattgagaagaggacattggag
atgccatcctctttctcttttctagtttgctcatactacattgagtagac
acatttaaggatggggttatgaacccttcctgagctttatggtcctaaaa
gcaaaataaaaactattcgaatgaaaagacaagaaaatcaggtattaatc
ttggatagctaataatgagctattaaaactcagcctgggacagtttatca
tgaagcctgtggatgatcaatcctttattattatttttttttttgaaaa
aagctcatttcatgctctgcaaaaggagagactcccatgaagccttttga
aagggatcatcatgcagctcaactttctgttggattccatgctaagcaag
ctaaccttatcctgcattgttagcactaggcacccagctgccacctctcc
atcctgctgcccttaggccacatgggagcagtccatgcatgacagcctct
atcctacaaggcctatgagtatggattgggggggccaaaaggaaaaagct
ccatgtgcctctttgtctgcgtgggtcagaagagttgtgcacgcagatta
gcaggccaaggtctgagccacagcagcatttttatttcagattttgataa
ctgtttatatgtgttgaaaaccaaaatgacatctttttaaagcttatcca
taaaaaaaaatagatgtcttttatagtggaaaaacacatggggaaaaaaa
tcatctattttgatgcagcatttgataatgataaaacacctcacacctca

ctctttatagtgcacaaaatgaatgaggtctgggctaggtagaaaaaggg
tcaatgctatttttgtttttagaatcattaccttttaccagcttttaacc
atctgatatctatagtagacacactatcatagttaacatagtaagttcag
cacttgtctcattttaatgtaaagatttgcttccattttcctacaggcag
tctctctcttcctcacagtcccactgtgcaggtgctattgttactcttac
gaatattttcagtaatgttattttcttctaagtgaaatttctagcctgca
ctttgatgtcatgtgttccctttgtctttcaaactccaaggttcccttgt
ggccctctcccttaccctgggaaggcctcttggagaccttaccccctggct
gtttggactttgtatactttaaataatttaactacccttaattacttaaa
aaaaaaaaaaagctttatgattttcataacttattgctgattttaatgga
ttgttaatttcagtcctgtagttttattttatgtttagatagggctgggc
aaggaaaaagaaaataaagacaaccatatttagcagtgcagttgagttgt
gtgttaatgttagactatccctttgtgagtgacactttaacagcattcac
tgcttctatatatagtgtaccatcttggtcatacattacgcctcaacata
tacttgtgctcttcctttgcctccagaagaagtttttccttgattgtgct
atgtttcagtggaagaaattctttgaagtagatgtgagtgaaaaactgca
tgcctttagaagcccagtatcagaacttgctacgtttcaggtgctaggga
cttaatgaaaaacaggacaaaacaattcctttttgtggcccaggtaaatt
atttctggtttcacttataattactaatggctgagtcaagatgttgtctc
tgtgtttgcttactcttgatcaagtgtgagacagtttgaagactgtgcta
ccatacaaagtgaatgaagccagtgactaagcttctgtttgttttgttat
tctcatggccttcgcttgcattatttgggccttcattcagatgaacttga
ggtgccattttgttgcatatgtacaggattatgggctggaaagcatttgt
tataaacctatagtgcacattttaactgccccctaaattacccttccctg
ggtttgttttccttggggtggtgtagattgtatgagtaagaagtattaat
tttttaaaagacaaatcaactttgaagacacaaaagttaattggaagaaa
taaaaactgtgaacgaagaa

>NM_014795 3
atttcatttcttccactaaagcgtttgcggagacttcaaggtataatcta
tcccagatcctttcccagagagaaacttggcgatcacgttttcacatgat
gctcacgctcagggcgcttcaattatccctccccacaaagataggtggcg
cgtgtttcagggtctctcgtctctctcctacagaaaagaaaaagaaaaaa
atgtcattagaagaggcgtaacacgtcagtccgtccccaggtttgtgttt
cctggagtggccgaaagagatcagttctaacctgctctgcaggaataacg
gtcctgcctcccgacactcttggcgaggtttttgtacagtttgctccggg
agctgtttcttcgcttccacctttttctcccccacacttcgcggcttctt
catgctttttcttctcaccatttctggccaaaactacaaacaagacttcg
cagatcgagcctgcgtgctgccgaagcagggcgccgagtccatgcgaact
gccatctgatccgctcttatcaatgaagcagccgatcatggcggatggcc
cccggtgcaagaggcgcaaacaagccaatcccaggaggaaaaacgtggtg
aactatgacaatgtagtggacacaggttctgaaacagatgaggaagacaa
gcttcatattgctgaggatgacggtattgccaaccctctggaccaggaga
cgagtccagctagtgtgcccaaccatgagtcctccccacacgtgagccaa
gctctgttgccaagagaggaagaggaagatgaaataagggagggtggagt
ggaacacccctggcacaacaacgagattctacaagcctctgtagatggtc

cagaagaaatgaaggaagactatgacactatggggccagaagccacgatc
cagaccgcaattaacaatggtacagtgaagaatgcaaattgcacatcaga
ttttgaggaatactttgccaaaagaaaactggaggaacgcgatggtcatg
cagtcagcatcgaggagtaccttcagcgcagtgacacagccattatttac
ccagaagcccctgaggagctgtctcgccttggcacgccagaggccaatgg
gcaagaagaaaatgacctgccacctggaactccagatgcttttgcccaac
tgctgacctgcccctactgcgaccggggctacaagcgcttgacatcactg
aaggagcacatcaagtaccgccacgagaagaatgaagagaacttttcctg
ccctctctgtagctacacgtttgcctaccgcacccagctcgagcggcata
tggtgacacacaagccagggacagatcagcaccaaatgctaacccaagga
gcaggtaatcgcaagttcaaatgcacagagtgtggcaaggccttcaaata
taaacaccatctgaaagaacacctgcgaattcacagtggtgaaaaacctt
acgagtgcccaaactgcaagaaacgtttctcccattctggttcctacagt
tcgcacatcagcagcaagaaatgtattggtttaatctctgtaaatggccg
aatgagaaacaatatcaagacgggttcttcccctaattctgtttcttctt
ctcctactaattcagccattacccagttaagaaacaagttggagaatgga
aaaccacttagtatgtctgaacagacaggcttacttaaaattaaaacaga
accactagacttcaatgactataaagttcttatggctacacacgggttta
gtggcactagtccctttatgaatggtgggcttggagccaccagcccttta
ggagttcatccatctgctcagagtccaatgcagcacttaggtgtagggat
ggaagcccctttacttgggtttcccaccatgaatagtaatttaagtgagg
tacaaaaggttctacagattgtggacaatactgtttccaggcaaaaaatg
gactgcaaggctgaagaaatttcaaagttgaaaggttatcacatgaagga
tccatgctctcaacctgaggaacaaggagttacttctcctaatattccgc
ctgtcggtcttccggtagtgagtcataatggtgccactaaaagtattatt
gactatacgttggaaaaagtcaatgaagccaaagcttgcctccagagctt
gactactgactcaaggagacagatcagtaatataaagaaagagaagctac
gtactttaatagatttggtcactgatgacaaaatgattgagaaccacaac
atatccactccattttcatgccagttctgtaaagaaagttttcctggccc
catccctttgcatcagcatgaacgttacctttgtaagatgaatgaagaga
tcaaggcggtcctgcagcctcatgaaaacatagtccccaacaaagccgga
gtttttgttgataataaagccctcctcttgtcatctgtactttctgagaa
aggaatgacaagccccatcaacccatacaaggaccacatgtctgtactca
aagcatactatgctatgaacatggagcccaactccgatgaactgctgaaa
atttccattgctgtgggccttcctcaggaatttgtgaaggaatggtttga
acaacgaaaagtctaccagtactcaaattccaggtccccatccctggaaa
gaagctccaagccgttagctcccaacagtaaccctcccacaaaagactct
ttattacccaggtctcctgtaaaacctatggactccataacatcaccatc
tatagcagaactccacaacagtgttacgaattgtgatcctcctctcaggc
taacaaaaccttcccattttaccaatattaaaccagttgaaaaattggac
cactccaggagtaatactccttctcccttaaatctttcctccacatcttc
taaaaactcccacagtagttcatacactccaaacagcttctcttctgagg
agctccaggctgagcctttagacttgtcattaccaaaacaaatgaaagaa
cccaaaagtattatagccacaaagaacaaaacaaaagctagtagcatcag
tttagatcataacagtgtttcttcctcatctgaaaactcagatgagcctc
tgaacttgacttttatcaagaaggaattttcaaattcaaataatctggac

aacaaaagcactaacccagtgttcagcatgaacccatttagtgccaaacc
tttatacacagctcttccacctcaaagcgcatttccccctgctactttca
tgccaccagtccagaccagtattcctgggctacgaccatacccaggactg
gatcagatgagcttcctaccacatatggcctacacctacccaactggagc
agctacttttgctgatatgcagcaaaggagaaagtaccagcggaaacaag
gatttcagggagaattgcttgatggagcacaagactacatgtcaggccta
gatgatatgacagactccgactcctgtctgtctcgcaaaaagatcaagaa
gacagagagtggcatgtatgcatgtgacttatgtgacaagacattccaga
aaagcagttcccttctgcgacataaatacgaacacacaggaaaaagacca
catcagtgtcagatttgtaagaaagcgtttaaacacaagcaccaccttat
cgagcactcaaggcttcactcgggcgagaagccctatcagtgtgataaat
gtggcaagcgcttctcacactcgggctcgtactcgcagcacatgaatcac
aggtattcctactgcaagcgggaggcggaggagcgggaagcggcggagcg
cgaggcgcgcgagaaagggcacttggaacccaccgagctgctgatgaacc
gggcttacttgcagagcattacccctcaggggtactctgactcggaggag
agggagagtatgccgagggatggcgagagcgagaaggagcacgagaaaga
aggcgaggatggctacgggaagctgggcagacaggatggcgacgaggagt
tcgaggaggaagaggaagaaagtgaaaataaaagtatggatacggatccc
gaaacgatacgagatgaagaagagactggagatcactccatggacgatag
ttcggaggatgggaaaatggaaaccaaatcagaccacgaggaagacaata
tggaagatggcatgtaataaactactgcattttaagcttcctattttttt
ttccagtagtattgttacctgcttgaaaacactgctgtgttaagctgttc
atgcacgtgcctgacgcttccaggaagctgtagagagggacagaaggggc
ggttcagccaagacagatgtagacggagttggagctgggtattgttaaaa
actgcattatgcaaaaattttgtacagtgttaaggcctaaaaactgtgtg
gttcagagactaattcctgtgtttaatagcatttatactttaagcacaac
tagaaaattgtaagaattgcactctacttatgtatcactacaaactttaa
aaaactatgtctaatttatattaatacattttaaaaaggtgcccgcacta
ccatacatcagtatttttattattattattgttattccttttttaatttaa
tgtgctcgcactacaatgcatcagtattatgattcctctgtactttcctt
tcgctattcatcaatttcccattttttttttcagcttaagtaaccacaca
attttaggcctcaatttttttttttttctgtgaaggaacttgaagtgatg
catgtgtgaatttaagataccgaagtcttaaagtgacctggacgtgaagg
aaaaagtaagatgagaaataaagaaagcctttgtaaggtggttttaaaag
ccttatatgcaaaccttttaatctgtgtttctgcaagtgccatccttgta
cagtgttaagagggtaacatgggttacctttgcaccagcttcagtgttaa
gctcaccctgttctttgaagcacccatgtcagtattagaagaataggcag
cagttccttagtttacatatgtttgtgcaattattttctgtactttttttg
ttcattaattttgtcagtattacaccaaactgtttttgcaacaaaaaaat
ttttttgcattcatttaattttaggtcaaataacattttatttatgtgg
ctcattttatatttcctaattttatttatttcatactgtagtgtacagta
ttatagttcttcaatatatagatatattttagtaaaaaaggaacatgacg
ttgatcatttgggcaaattttacgtaaagagaagagcatttattgtgttt
tggaacattaattgtgagatgggatttttcaattttattattttattttt
gtttttttccaattactggaaattccaaatttgggaacttttgatacgat
cttgtgaaaacactgtattttcgactgaaaattccactttcttcatcttg

ttttttagctaaaaagagggactgttaaatacaatgtatgataccatgac
aaaaatctttcctgaattgtctttgtaaaagtattattgaattttcaatt
tgtaatttcttttgaaaatgaccatgctcgaataaaaatgtagccaaact
aagaatgtagttaatgagttctgtacttttagagagttttccttcaatga
ccattaacatgtaacatgctttatgcttataataatgctaattatgtttt
tttcatataattttagtttagcaataattttgactggtaccaataactgt
tttttaaaattccatacctatgtacagcaattttacagcttttctcaact
gatcctgattccagattgtgtatttttatgtgaggttatattattcaaat
ttagtctatttactttacagacatttctacttttgcattacgagtattta
gagattatgtgttaaaaattcacttctctgtccaaggggtctttgtgatt
tattcaaaaaaaaagtctaatttcaaaaagacagctattattcagtgttat
ttataatatgtaaccttttttaaaggattgggatagtttatctcactttt
tgaaatgcagacagtagtttaccgtttatctgaaactagaaggcgtgggt
gggagaggaaaagctaaaagcaaatgctaacaaaaataaccgtgattttc
taagacagtttttcagtttttacaagatgaccctaatattcagaatatga
atgtattcgtaggttttacataatgacttttatcaagaaactagattctg
cttcttaaatctaattgccaagtgaagaataacagaaaaaacagattacc
ttatcaaatttacagctcttgaatatacagaactataatatagtagctgt
ccatgtattttttctactttagaatcaaagaagaaaagcatcattttgct
attaaatttgctaaaattttgagtatgatatttccagttggcaagaacaa
catatttatatttattccttagccataataccactttcctaaatttcaca
aaagtcattctttgcaacttgaaactcaatagaaagtgtgtatgtgtgtg
tgtgtatatatatatatatacacacacacacatacacagaaaggatgt
aatgaagatacagtaatagttgagcagaccttttttagaaaaacatgtttt
tagctctatcttcaaactttctggcagagggggtgggggggggcaggggga
ggagtggcatcaaaatgctatgcctcctgttatccacagcctagagtttt
tatatttggaaagtttagaaaattctatcctcgtttctccttctttgaat
ggcacaaataaatacactacataaatttttctggtttgaaaggctctagg
cgataactttattaattcaacctgaaaatatcaagccattaaattttgtc
cgggtagaataaatccctgtggcctcttttaaagcaatgtaggtctctgt
tgcccatggggcatatctgtgtcccaatccacaagagataggaccaacaa
acaatgaatgtgcaacctaactctttctccttggaaagaagaaagtgtgc
acgaagtagaggagggtgggcagaccctgccttgcccctcctgttacccc
cttctctgtcatttgttcctaactccatttcataggcaggctcagaatac
ctgagtctgaaaatatcaggataacacttgtgaattgtgacaatcactac
aatgtcccatatctgaggagttttttttaatgctatttatccgctggaca
cgattgcacattagggctgcataatcctctaactctagggaaaaataaaa
acttttgatttgtcttaagattcttctccaaggtcgcaaacaagaaattc
ccctccacaaccaagagatgtgcattttagtaacatcagatgtgttcttc
tgttttatcaactacttactcttcccacacgcttagttctaaatctaacc
tttccccccctcgaatagggggcaggggaggatgaggaaacactggaacaa
ctgaacacccctgcccattttctccaagagccttttgtattctagcatat
ctgtgcaatcttttctttttcttcacatgacactgtaagcttaggcctg
aaataactgggaagagagatgcgtatcagaatttctccgcaagagctaaa
caaaacatacatcttccttagcatgaattggactgggggcggagtgggag
ggcttggaggaaaggggaaagaagggactatatttgaataaatatgaata

aatgtattagatacttttcacaatcagataacttttaaaaaggtcatttt
ttatctttctaataatgtaagccttaataaaagcaaatcttagtcacaaa
tttgaggagactgcccaataataagtttacatgtatttgaactgaaaaat
tgttaaccatgcttttgctccaagatgtgtgaggccattcaggggctgta
gggccctggatatacacacaaacaagtgtgtgtatatctggagccccaca
cattgtaataaacacagctgcatttatttgactatgtgatcccatgtaca
tgtaaaaacattcaaacaaacacactcagcggatttatttattgtgcaat
ggggcaattattcaaataaacatgctcaatgcaattatttgaatctcaca
ttgcatgttcatcaatcatagcactaaaaaaagagggggaaaaaacacca
aagaattcacatggggaaaaaatatatatatgaaaaccaccttattatag
attttatagggcagctgaggttatggctcccttcttaactgtaactcaac
tattctgtattcaatgacatttgtttctaatgattaattggttcactcac
ttgatcatataatagcaaactttataaacctgtattgtgtagagatgtga
aatctctatatttcaagagcagaagagttctttctagacaccttacatca
agggacactggtccaattattatcgcttatataagcactcctataaattc
tgaaaaattttatacatgcaacaaaacattcctacatttgaagacattaa
gaaaaatcacaggtgactcatctgatcattctatatattaataaatatta
tgacatatatgtgaacacatcacaaatcatattggtgtaccaagaggcaa
tttatgcctctcttaagtatgtactgacataacctaatatactaaaatgg
gaaggggcttttagtcactgaaatatgcatcgtgtaacaaagatgaagaa
aatacatggcttgtgcccatcataaaaaaagattcagactgaaggcttag
ctttggtttttcaattaaattgttaaactgtgcacagtgattttttttt
agaacttgagacatttgtgatgttggctgtttaaatctttgttaccttcg
ctgtgaattgaaattgtacatatttagtaaatcatgcagacaaaacaaac
tttttagacaatatttttattggagagttttcttttcctgtatccatgtt
aaaaaaaaaaaagacctcctttcccaaaataaaaatgtcaatactaaatt
taaagaagtataaaggaatgattgcttcctttagagcaaaatatttaaat
aaacatggagataattggcaacatgttctttttgggctagtaggctgtgt
ccaatttttgggtctgatgtttcagagggcctctgtttcagggttgaag
atgatatattaatctcggaattaaacaaatgctattaaataac

>NM_030751 5
gggggggaagggggagggagggggaggaggtgactcgagcatttagacac
aagcgagaggatcatggcggatggccccaggtgtaagcgcagaaagcagg
cgaacccgcggcgcaataacgttacaaattataatactgtggtagaaaca
aattcagattcagatgatgaagacaaactgcatattgtggaagaagaaag
tgttacagatgcagctgactgtgaaggtgtaccagaggatgacctgccaa
cagaccagacagtgttaccagggaggagcagtgaaagagaagggaatgct
aagaactgctgggaggatgacagaaaggaagggcaagaaatcctggggcc
tgaagctcaggcagatgaagcaggatgtacagtaaaagatgatgaatgcg
agtcagatgcagaaaatgagcaaaaccatgatcctaatgttgaagagttt
ctacaacaacaagacactgctgtcatttttcctgaggcacctgaagagga
ccagaggcagggcacaccagaagccagtggtcatgatgaaaatggaacac
cagatgcattttcacaattactcacctgtccatattgtgatagaggctat
aaacgctttacctctctgaaagaacacattaaatatcgtcatgaaaagaa
tgaagataactttagttgctccctgtgcagttacacctttgcatacagaa

cccaacttgaacgtcacatgacatcacataaatcaggaagagatcaaaga
catgtgacgcagtctgggtgtaatcgtaaattcaaatgcactgagtgtgg
aaaagctttcaaatacaaacatcacctaaaagagcacttaagaattcaca
gtggagagaagccatatgaatgcccaaactgcaagaaacgcttttcccat
tctggctcctatagctcacacataagcagtaagaaatgtatcagcttgat
acctgtgaatgggcgaccaagaacaggactcaagacatctcagtgttctt
caccgtctctttcagcatcaccaggcagtcccacacgaccacagatacgg
caaaagatagagaataaaccccttcaagaacaactttctgttaaccaaat
taaaactgaacctgtggattatgaattcaaacccatagtggttgcttcag
gaatcaactgttcaacccctttacaaaatggggttttcactggtggtggc
ccattacaggcaaccagttctcctcagggcatggtgcaagctgttgttct
gccaacagttggtttggtgtctcccataagtatcaatttaagtgatattc
agaatgtacttaaagtggcggtagatggtaatgtaataaggcaagtgttg
gagaataatcaagccaatcttgcatccaaagaacaagaaacaatcaatgc
ttcacccatacaacaaggtggccattctgttatttcagccatcagtcttc
ctttggttgatcaagatggaacaaccaaaattatcatcaactacagtctt
gagcagcctagccaacttcaagttgttcctcaaaatttaaaaaaagaaaa
tccagtcgctacaaacagttgtaaaagtgaaaagttaccagaagatctta
ctgttaagtctgagaaggacaaaagctttgaagggggggtgaatgatagc
acttgtcttctgtgtgatgattgtccaggagatattaatgcacttccaga
attaaagcactatgacctaaagcagcctactcagcctcctccactccctg
cagcagaagctgagaagcctgagtcctctgtttcatcagctactggagat
ggcaatttgtctcctagtcagccacctttaaagaacctcttgtctctcct
aaaagcatattatgctttgaatgcacaaccaagtgcagaagagctctcaa
aaattgctgattcagtaaacctaccactggatgtagtaaaaaagtggttt
gaaaagatgcaagctggacagatttcagtgcagtcttctgaaccatcttc
tcctgaaccaggcaaagtaaatatccctgccaagaacaatgatcagcctc
aatctgcaaatgcaaatgaaccccaggacagcacagtaaatctacaaagt
cctttgaagatgactaactccccagttttaccagtgggatcaaccaccaa
tggttccagaagtagtacaccatccccatcacctctaaacctttcctcat
ccagaaatacacagggttacttgtacacagctgagggtgcacaagaagag
ccacaagtagaacctcttgatctttcactaccaaagcaacagggagaatt
attagaaaggtcaactatcactagtgtttaccagaacagtgtttattctg
tccaggaagaacccttgaacttgtcttgcgcaaaaaaggagccacaaaag
gacagttgtgttacagactcagaaccagttgtaaatgtaatcccaccaag
tgccaaccccataaatatcgctatacctacagtcactgcccagttaccca
caatcgtggccattgctgaccagaacagtgttccatgcttaagagcgcta
gctgccaataagcaaacgattctgattccccaggtggcatacacctactc
aactacggtcagccctgcagtccaagaaccacccttgaaagtgatccagc
caaatggaaatcaggatgaaagacaagatactagctcagaaggagtatca
aatgtagaggatcagaatgactctgattctacaccgcccaaaaagaaaat
gcggaagacagaaaatggaatgtatgcttgtgatttgtgtgacaagatat
tccaaaagagtagttcattattgagacataaatatgaacacacaggtaaa
agacctcatgagtgtggaatctgtaaaaaggcatttaaacacaaacatca
tttgattgaacacatgcgattacattctggagaaaagccctatcaatgtg
acaaatgtggaaagcgcttctcacactctgggtcttattctcaacacatg

aatcatcgctactcctactgtaagagagaagcggaagaacgtgacagcac
agagcaggaagaggcagggcctgaaatcctctcgaatgagcacgtgggtg
ccagggcgtctccctcacagggcgactcggacgagagagagagtttgaca
agggaagaggatgaagacagtgaaaaagaggaagaggaggaggataaaga
gatggaagaattgcaggaagaaaaagaatgtgaaaaaccacaaggggatg
aggaagaggaggaggaggaggaagaagtggaagaagaagaggtagaagag
gcagagaatgagggagaagaagcaaaaactgaaggtctgatgaaggatga
cagggctgaaagtcaagcaagcagcttaggacaaaaagtaggcgagagta
gtgagcaagtgtctgaagaaaagacaaatgaagcctaatcgtttttctag
aaggaaaataaattctaattgataatgaatttcgttcaatattatccttg
cttttcatggaaacacagtaacctgtatgctgtgattcctgttcactact
gtgtaaagtaaaaactaaaaaaatacaaaatacaaaacacacacacacac
acacacacacacacacacacacacaaaataaatccgggtgtgcctgaa
cctcagacctagtaatttttcatgcagttttcaaagttaggaacaagttt
gtaacatgcagcagattagaaaaccttaatgactcagagagcaacaatac
aagaggttaaaggaagctgattaattagatatgcatctggcattgtttta
tcttatcagtattatcactcttatgttggtttattcttaagctgtacaat
tgggagaaattttataatttttattggtaaacatatgctaaatccgctt
cagtattttattatgttttttaaaatgtgagaacttctgcactacaaaat
tcccttcacagagaagtataatgtagttccaacccgtgctaactaccttt
tataaattcagtctagaaggtagtaatttctaatatttagatgtcttagt
agagcgtattatcatttaaagtgtattgttagccttaagaaagcagctga
tagaagaactgaagtttcttactcacgtggtttaaaatggagttcaaaag
attgccattgagttctgattgcagggactaacaatgttaatctgataagg
acagcaaaatcatcagaatcagtgtttgtgattgtgtttgaatatgtggt
aacatatgaaggatatgacatgaagctttgtatctcctttggccttaagc
aagacctgtgtgctgtaagtgccatttctcagtattttcaaggctctaac
ccgccttcatccaatgtgtggcctacaataactagcatttgttgatttgt
ctcttgtatcaaaattcccaaataaaacttaaaaccactgactctgtcag
agaaactgaaacactgggacatttcatccttcaattcctcggtattgatt
ttatgttgattgattttcagaatttctctacagaaacgaaagggaaattt
tctaatctgctttatccatgtacttgcatttcagacatggacatgctatt
gttatttggctcataactgtttccaaatgttagttattatggacccaatt
tattaacaacattagctgatttttacctatcagtattattttatttcttt
tagtttatagatctgtgcaacatttttgtactgtatgtcttcaaacctgg
cagtattaatacccttcttactgacatatgtacttttagttttagaaaac
ttttatatttatgtgtcttatttttatatttctttatttattacacagtg
tagtgtataatactgtagtttgtattaatacaataatatattttagtatg
aaaatttggaaagttgataagatttaaagtagagatgcaattggttctcc
tgcattgagatttgatttaacagtgttatgttaacatttatacttgcctt
ggactgtagaacagaacttaaatgggaatgtattagttttacaactacaa
tcaagtcattttacctttacccagtttttaatataaaacttaaattttga
aattcactgtgtgactaatagcatgatgctctgcagttttattaagaaat
cagcctaaccatacaactctcatttccttagtaagccaaattaggattaa
cttctataaacagtgttgggaacaatgtttaacattttgtgccaatttgt
tcctgtattcatgtatgtaagttacagatctgactcttcatttttaagtt

ccttgttacatcatggtcattttctagtttttttaccagactcccatctca
caataaaatgcatcaacaagcctgaactgctgtcattcttttcatcatta
tcagtattttctttggaaaactgtgaaatggggtacattgtcatcctgca
tttgattcatcttgagctgaatttgggtaacactaaatgttttagacatt
ctccactaaattatggattttcttgtggctaaatgtttctggagaggtca
gagttgacaaaacctcttcacaggttgctccttcttcctgaaatccttaa
tcctccgcatttcatgcttcaggtcatttcagggaagcctgggtttagat
gcctttctgactctcagctcctgcacttctgtcatcatacctctgatact
attatttatattccttccccactaggaacaggaaccacatttgtcatagt
cactctcacattcctcactgcctaacagggtgcctggcataagttgggac
aacagatatttgttgaataaaaatataatttgcatgtttatggagctcag
ctatgttctcactttttttgcttctaattccagaatatatgttaaatgat
ctaataatttgattattttcttataagtcttattaaacactagtcataat
agacacaataaattatgccttctttttctattgccttaaaaaaaaaaa
