# Supplementary material for: A computational method for predicting regulation of human microRNAs on the influenza virus genome
Source: BMC Syst Biol. 2013 Oct 14;7(Suppl 2):S3. doi: 10.1186/1752-0509-7-S2-S3 (PMC3851852; doi:10.1186/1752-0509-7-S2-S3)
Supplement: Additional File 15 — 204 miRNAs that were used as the training datasets including positive samples and negative samples. [file 1752-0509-7-S2-S3-S15.PDF]

miRNA of Training Dataset

The sequences of miRNA which are used as trainging dataset are totally from miRBase, and this additional file gives 137 miRNA of Human used as positive samples in FASTA format, and 67 miRNA of Human, Mus, Rat used as negative samples in FASTA format

Positive samples:

>hsa-let-7a-5p MIMAT0000062 Homo sapiens
UGAGGUAGUAGGUUGUAUAGUU
>hsa-let-7a-3p MIMAT0004481 Homo sapiens
CUAUACAAUCUACUGUCUUUC
>hsa-let-7a-2-3p MIMAT0010195 Homo sapiens
CUGUACAGCCUCCUAGCUUUCC
>hsa-let-7b-5p MIMAT0000063 Homo sapiens
UGAGGUAGUAGGUUGUGUGGUU
>hsa-let-7b-3p MIMAT0004482 Homo sapiens
CUAUACAACCUACUGCCUUCCC
>hsa-let-7c MIMAT0000064 Homo sapiens
UGAGGUAGUAGGUUGUAUGGUU
>hsa-let-7e-5p MIMAT0000066 Homo sapiens
UGAGGUAGGAGGUUGUAUAGUU
>hsa-let-7e-3p MIMAT0004485 Homo sapiens
CUAUACGGCCUCCUAGCUUUCC
>hsa-let-7g-5p MIMAT0000414 Homo sapiens
UGAGGUAGUAGUUUGUACAGUU
>hsa-let-7g-3p MIMAT0004584 Homo sapiens
CUGUACAGGCCACUGCCUUGC
>hsa-miR-101-5p MIMAT0004513 Homo sapiens
CAGUUAUCACAGUGCUGAUGCU
>hsa-miR-101-3p MIMAT0000099 Homo sapiens
UACAGUACUGUGAUAACUGAA
>hsa-miR-103a-2-5p MIMAT0009196 Homo sapiens
AGCUUCUUUACAGUGCUGCCUUG
>hsa-miR-103a-3p MIMAT0000101 Homo sapiens
AGCAGCAUUGUACAGGGCUAUGA
>hsa-miR-103b MIMAT0007402 Homo sapiens
UCAUAGCCCUGUACAAUGCUGCU
>hsa-miR-106a-5p MIMAT0000103 Homo sapiens
AAAAGUGCUUACAGUGCAGGUAG
>hsa-miR-106a-3p MIMAT0004517 Homo sapiens
CUGCAAUGUAAGCACUUCUUAC
>hsa-miR-107 MIMAT0000104 Homo sapiens
AGCAGCAUUGUACAGGGCUAUCA

>hsa-miR-10a-5p MIMAT0000253 Homo sapiens
UACCCUGUAGAUCCGAAUUUGUG
>hsa-miR-10a-3p MIMAT0004555 Homo sapiens
CAAAUUCGUAUCUAGGGGAAUA
>hsa-miR-10b-5p MIMAT0000254 Homo sapiens
UACCCUGUAGAACCGAAUUUGUG
>hsa-miR-10b-3p MIMAT0004556 Homo sapiens
ACAGAUUCGAUUCUAGGGGAAU
>hsa-miR-122-5p MIMAT0000421 Homo sapiens
UGGAGUGUGACAAUGGUGUUUG
>hsa-miR-122-3p MIMAT0004590 Homo sapiens
AACGCCAUUAUCACACUAAAUA
>hsa-miR-125a-5p MIMAT0000443 Homo sapiens
UCCCUGAGACCCUUUAACCUGUGA
>hsa-miR-125b-5p MIMAT0000423 Homo sapiens
UCCCUGAGACCCUAACUUGUGA
>hsa-miR-125b-1-3p MIMAT0004592 Homo sapiens
ACGGGUUAGGCUCUUGGGAGCU
>hsa-miR-125b-2-3p MIMAT0004603 Homo sapiens
UCACAAGUCAGGCUCUUGGGAC
>hsa-miR-129-5p MIMAT0000242 Homo sapiens
CUUUUUGCGGUCUGGGCUUGC
>hsa-miR-130a-5p MIMAT0004593 Homo sapiens
UUCACAUUGUGCUACUGUCUGC
>hsa-miR-130a-3p MIMAT0000425 Homo sapiens
CAGUGCAAUGUUAAAAGGGCAU
>hsa-miR-133a MIMAT0000427 Homo sapiens
UUUGGUCCCCUUCAACCAGCUG
>mmu-miR-133b-5p MIMAT0017083 Mus musculus
GCUGGUCAAACGGAACCAAGUC
>mmu-miR-133b-3p MIMAT0000769 Mus musculus
UUUGGUCCCCUUCAACCAGCUA
>hsa-miR-133b MIMAT0000770 Homo sapiens
UUUGGUCCCCUUCAACCAGCUA
>hsa-miR-137 MIMAT0000429 Homo sapiens
UUAUUGCUUAAGAAUACGCGUAG
>hsa-miR-140-5p MIMAT0000431 Homo sapiens
CAGUGGUUUUACCCUAUGGUAG
>hsa-miR-141-5p MIMAT0004598 Homo sapiens
CAUCUUCCAGUACAGUGUUGGA
>hsa-miR-141-3p MIMAT0000432 Homo sapiens
UAACACUGUCUGGUAAAGAUGG
>hsa-miR-145-5p MIMAT0000437 Homo sapiens
GUCCAGUUUUCCCAGGAAUCCCU

>hsa-miR-145-3p MIMAT0004601 Homo sapiens
GGAUUCCUGGAAAUACUGUUCU
>hsa-miR-147a MIMAT0000251 Homo sapiens
GUGUGUGGAAAUGCUUCUGC
>hsa-miR-147b MIMAT0004928 Homo sapiens
GUGUGCGGAAAUGCUUCUGCUA
>hsa-miR-148a-5p MIMAT0004549 Homo sapiens
AAAGUUCUGAGACACUCCGACU
>hsa-miR-148a-3p MIMAT0000243 Homo sapiens
UCAGUGCACUACAGAACUUUGU
>hsa-miR-148b-5p MIMAT0004699 Homo sapiens
AAGUUCUGUUAUACACUCAGGC
>hsa-miR-148b-3p MIMAT0000759 Homo sapiens
UCAGUGCAUCACAGAACUUUGU
>hsa-miR-155-5p MIMAT0000646 Homo sapiens
UUAAUGCUAAUCGUGAUAGGGGU
>hsa-miR-155-3p MIMAT0004658 Homo sapiens
CUCCUACAUAUUAGCAUUAACA
>hsa-miR-15a-5p MIMAT0000068 Homo sapiens
UAGCAGCACAUAAUGGUUUGUG
>hsa-miR-15a-3p MIMAT0004488 Homo sapiens
CAGGCCAUAUUGUGCUGCCUCA
>hsa-miR-15b-5p MIMAT0000417 Homo sapiens
UAGCAGCACAUCAUGGUUUACA
>hsa-miR-15b-3p MIMAT0004586 Homo sapiens
CGAAUCAUUAUUUGCUGCUCUA
>hsa-miR-16-5p MIMAT0000069 Homo sapiens
UAGCAGCACGUAAAUAUUGGCG
>hsa-miR-16-1-3p MIMAT0004489 Homo sapiens
CCAGUAUUAACUGUGCUGCUGA
>hsa-miR-16-2-3p MIMAT0004518 Homo sapiens
CCAAUAUUACUGUGCUGCUUUA
>hsa-miR-17-5p MIMAT0000070 Homo sapiens
CAAAGUGCUUACAGUGCAGGUAG
>hsa-miR-17-3p MIMAT0000071 Homo sapiens
ACUGCAGUGAAGGCACUUGUAG
>hsa-miR-181a-5p MIMAT0000256 Homo sapiens
AACAUUCAACGCUGUCGGUGAGU
>hsa-miR-181a-3p MIMAT0000270 Homo sapiens
ACCAUCGACCGUUGAUUGUACC
>hsa-miR-181a-2-3p MIMAT0004558 Homo sapiens
ACCACUGACCGUUGACUGUACC
>hsa-miR-181b-5p MIMAT0000257 Homo sapiens
AACAUUCAUUGCUGUCGGUGGGU

>hsa-miR-181b-3p MIMAT0022692 Homo sapiens
CUCACUGAACAAUGAAUGCAA
>hsa-miR-182-5p MIMAT0000259 Homo sapiens
UUUGGCAAUGGUAGAACUCACACU
>hsa-miR-182-3p MIMAT0000260 Homo sapiens
UGGUUCUAGACUUGCCAACUA
>hsa-miR-193a-3p MIMAT0000459 Homo sapiens
AACUGGCCUACAAAGUCCCAGU
>hsa-miR-196a-5p MIMAT0000226 Homo sapiens
UAGGUAGUUUCAUGUUGUUGGG
>hsa-miR-196a-3p MIMAT0004562 Homo sapiens
CGGCAACAAGAAACUGCCUGAG
>hsa-miR-199b-5p MIMAT0000263 Homo sapiens
CCCAGUGUUUAGACUAUCUGUUC
>hsa-miR-19a-5p MIMAT0004490 Homo sapiens
AGUUUUGCAUAGUUGCACUACA
>hsa-miR-19a-3p MIMAT0000073 Homo sapiens
UGUGCAAAUCUAUGCAAAACUGA
>hsa-miR-200a-5p MIMAT0001620 Homo sapiens
CAUCUUACCGGACAGUGCUGGA
>hsa-miR-200a-3p MIMAT0000682 Homo sapiens
UAACACUGUCUGGUAACGAUGU
>hsa-miR-200b-5p MIMAT0004571 Homo sapiens
CAUCUUACUGGGCAGCAUUGGA
>hsa-miR-200b-3p MIMAT0000318 Homo sapiens
UAAUACUGCCUGGUAAUGAUGA
>hsa-miR-200c-5p MIMAT0004657 Homo sapiens
CGUCUUACCCAGCAGUGUUUGG
>hsa-miR-200c-3p MIMAT0000617 Homo sapiens
UAAUACUGCCGGGUAAUGAUGGA
>hsa-miR-205-5p MIMAT0000266 Homo sapiens
UCCUUCAUUCCACCGGAGUCUG
>hsa-miR-205-3p MIMAT0009197 Homo sapiens
GAUUUCAGUGGAGUGAAGUUC
>hsa-miR-206 MIMAT0000462 Homo sapiens
UGGAAUGUAAGGAAGUGUGUGG
>hsa-miR-20a-5p MIMAT0000075 Homo sapiens
UAAAGUGCUUAUAGUGCAGGUAG
>hsa-miR-20a-3p MIMAT0004493 Homo sapiens
ACUGCAUUAUGAGCACUUAAAG
>hsa-miR-21-5p MIMAT0000076 Homo sapiens
UAGCUUAUCAGACUGAUGUUGA
>hsa-miR-21-3p MIMAT0004494 Homo sapiens
CAACACCAGUCGAUGGGCUGU

>hsa-miR-210 MIMAT0000267 Homo sapiens
CUGUGCGUGUGACAGCGGCUGA
>hsa-miR-212-5p MIMAT0022695 Homo sapiens
ACCUUGGCUCUAGACUGCUUACU
>hsa-miR-212-3p MIMAT0000269 Homo sapiens
UAACAGUCUCCAGUCACGGCC
>hsa-miR-214-5p MIMAT0004564 Homo sapiens
UGCCUGUCUACACUUGCUGUGC
>hsa-miR-214-3p MIMAT0000271 Homo sapiens
ACAGCAGGCACAGACAGGCAGU
>hsa-miR-218-5p MIMAT0000275 Homo sapiens
UUGUGCUUGAUCUAACCAUGU
>hsa-miR-218-1-3p MIMAT0004565 Homo sapiens
AUGGUUCCGUCAAGCACCAUGG
>hsa-miR-218-2-3p MIMAT0004566 Homo sapiens
CAUGGUUCUGUCAAGCACCGCG
>hsa-miR-22-5p MIMAT0004495 Homo sapiens
AGUUCUUCAGUGGCAAGCUUUA
>hsa-miR-22-3p MIMAT0000077 Homo sapiens
AAGCUGCCAGUUGAAGAACUGU
>hsa-miR-221-5p MIMAT0004568 Homo sapiens
ACCUGGCAUACAAUGUAGAUUU
>hsa-miR-221-3p MIMAT0000278 Homo sapiens
AGCUACAUUGUCUGCUGGGUUUC
>hsa-miR-222-5p MIMAT0004569 Homo sapiens
CUCAGUAGCCAGUGUAGAUCCU
>hsa-miR-222-3p MIMAT0000279 Homo sapiens
AGCUACAUCUGGCUACUGGGU
>hsa-miR-223-5p MIMAT0004570 Homo sapiens
CGUGUAUUUGACAAGCUGAGUU
>hsa-miR-223-3p MIMAT0000280 Homo sapiens
UGUCAGUUUGUCAAAUACCCCA
>hsa-miR-23a-5p MIMAT0004496 Homo sapiens
GGGGUUCCUGGGGAUGGGAUUU
>hsa-miR-23a-3p MIMAT0000078 Homo sapiens
AUCACAUUGCCAGGGAUUUCC
>hsa-miR-24-1-5p MIMAT0000079 Homo sapiens
UGCCUACUGAGCUGAUAUCAGU
>hsa-miR-24-3p MIMAT0000080 Homo sapiens
UGGCUCAGUUCAGCAGGAACAG
>hsa-miR-24-2-5p MIMAT0004497 Homo sapiens
UGCCUACUGAGCUGAAACACAG
>hsa-miR-26a-5p MIMAT0000082 Homo sapiens
UUCAAGUAAUCCAGGAUAGGCU

>hsa-miR-26a-1-3p MIMAT0004499 Homo sapiens
CCUAUUCUUGGUUACUUGCACG
>hsa-miR-27b-5p MIMAT0004588 Homo sapiens
AGAGCUUAGCUGAUUGGUGAAC
>hsa-miR-27b-3p MIMAT0000419 Homo sapiens
UUCACAGUGGCUAAGUUCUGC
>hsa-miR-29a-5p MIMAT0004503 Homo sapiens
ACUGAUUUCUUUUGGUGUUCAG
>hsa-miR-29a-3p MIMAT0000086 Homo sapiens
UAGCACCAUCUGAAAUCGGUUA
>hsa-miR-29b-1-5p MIMAT0004514 Homo sapiens
GCUGGUUUCAUAUGGUGGUUUAGA
>hsa-miR-29b-3p MIMAT0000100 Homo sapiens
UAGCACCAUUUGAAAUCAGUGUU
>hsa-miR-29b-2-5p MIMAT0004515 Homo sapiens
CUGGUUUCACAUGGUGGCUUAG
>hsa-miR-29c-5p MIMAT0004673 Homo sapiens
UGACCGAUUUCUCCUGGUGUUC
>hsa-miR-29c-3p MIMAT0000681 Homo sapiens
UAGCACCAUUUGAAAUCGGUUA
>hsa-miR-34a-5p MIMAT0000255 Homo sapiens
UGGCAGUGUCUUAGCUGGUUGU
>hsa-miR-34a-3p MIMAT0004557 Homo sapiens
CAAUCAGCAAGUAUACUGCCCU
>hsa-miR-370 MIMAT0000722 Homo sapiens
GCCUGCUGGGGUGGAACCUGGU
>hsa-miR-372 MIMAT0000724 Homo sapiens
AAAGUGCUGCGACAUUUGAGCGU
>hsa-miR-373-5p MIMAT0000725 Homo sapiens
ACUCAAAAUGGGGGCGCUUUCC
>hsa-miR-373-3p MIMAT0000726 Homo sapiens
GAAGUGCUUCGAUUUUGGGGUGU
>hsa-miR-424-5p MIMAT0001341 Homo sapiens
CAGCAGCAAUUCAUGUUUUGAA
>hsa-miR-424-3p MIMAT0004749 Homo sapiens
CAAAACGUGAGGCGCUGCUAU
>hsa-miR-433 MIMAT0001627 Homo sapiens
AUCAUGAUGGGCUCCUCGGUGU
>hsa-miR-504 MIMAT0002875 Homo sapiens
AGACCCUGGUCUGCACUCUAUC
>hsa-miR-520g MIMAT0002858 Homo sapiens
ACAAAGUGCUUCCCUUUAGAGUGU
>hsa-miR-520h MIMAT0002867 Homo sapiens
ACAAAGUGCUUCCCUUUAGAGU

>hsa-miR-7-5p MIMAT0000252 Homo sapiens
UGGAAGACUAGUGAUUUUGUUGU
>hsa-miR-7-1-3p MIMAT0004553 Homo sapiens
CAACAAAUCACAGUCUGCCAUA
>hsa-miR-7-2-3p MIMAT0004554 Homo sapiens
CAACAAAUCCCAGUCUACCUAA
>hsa-miR-9-5p MIMAT0000441 Homo sapiens
UCUUUGGUUAUCUAGCUGUAUGA
>hsa-miR-9-3p MIMAT0000442 Homo sapiens
AUAAAGCUAGAUAACCGAAAGU
>hsa-miR-96-5p MIMAT0000095 Homo sapiens
UUUGGCACUAGCACAUUUUUGCU
>hsa-miR-96-3p MIMAT0004510 Homo sapiens
AAUCAUGUGCAGUGCCAAUAUG
>hsa-miR-98 MIMAT0000096 Homo sapiens
UGAGGUAGUAAGUUGUAUUGUU

Negative Sapmles:
>hsa-let-7g-5p MIMAT0000414 Homo sapiens
UGAGGUAGUAGUUUGUACAGUU
>hsa-let-7g-3p MIMAT0004584 Homo sapiens
CUGUACAGGCCACUGCCUUGC
>hsa-let-7i-5p MIMAT0000415 Homo sapiens
UGAGGUAGUAGUUUGUGCUGUU
>hsa-let-7i-3p MIMAT0004585 Homo sapiens
CUGCGCAAGCUACUGCCUUGCU
>hsa-miR-1 MIMAT0000416 Homo sapiens
UGGAAUGUAAAGAAGUAUGUAU
>hsa-miR-103b MIMAT0007402 Homo sapiens
UCAUAGCCCUGUACAAUGCUGCU
>hsa-miR-1245b-5p MIMAT0019950 Homo sapiens
UAGGCCUUUAGAUCACUUAAA
>hsa-miR-1245b-3p MIMAT0019951 Homo sapiens
UCAGAUGAUCUAAAGGCCUAUA
>hsa-miR-132-5p MIMAT0004594 Homo sapiens
ACCGUGGCUUUCGAUUGUUACU
>hsa-miR-132-3p MIMAT0000426 Homo sapiens
UAACAGUCUACAGCCAUGGUCG
>hsa-miR-137 MIMAT0000429 Homo sapiens
UUAUUGCUUAAGAAUACGCGUAG
>hsa-miR-138-5p MIMAT0000430 Homo sapiens
AGCUGGUGUUGUGAAUCAGGCCG
>hsa-miR-138-2-3p MIMAT0004596 Homo sapiens
GCUAUUUCACGACACCAGGGUU

>hsa-miR-138-5p MIMAT0000430 Homo sapiens
AGCUGGUGUUGUGAAUCAGGCCG
>hsa-miR-138-2-3p MIMAT0004596 Homo sapiens
GCUAUUUCACGACACCAGGGUU
>hsa-miR-143-5p MIMAT0004599 Homo sapiens
GGUGCAGUGCUGCAUCUCUGGU
>hsa-miR-143-3p MIMAT0000435 Homo sapiens
UGAGAUGAAGCACUGUAGCUC
>hsa-miR-145-5p MIMAT0000437 Homo sapiens
GUCCAGUUUUCCCAGGAAUCCCU
>hsa-miR-145-3p MIMAT0004601 Homo sapiens
GGAUUCCUGGAAAUACUGUUCU
>hsa-miR-153 MIMAT0000439 Homo sapiens
UUGCAUAGUCACAAAAGUGAUC
>hsa-miR-155-5p MIMAT0000646 Homo sapiens
UUAAUGCUAAUCGUGAUAGGGGU
>hsa-miR-155-3p MIMAT0004658 Homo sapiens
CUCCUACAUAUUAGCAUUAACA
>hsa-miR-15a-5p MIMAT0000068 Homo sapiens
UAGCAGCACAUAAUGGUUUGUG
>hsa-miR-15a-3p MIMAT0004488 Homo sapiens
CAGGCCAUAUUGUGCUGCCUCA
>hsa-miR-190a MIMAT0000458 Homo sapiens
UGAUAUGUUUGAUAUAUUAGGU
>hsa-miR-190b MIMAT0004929 Homo sapiens
UGAUAUGUUUGAUAUUGGGUU
>hsa-miR-191-5p MIMAT0000440 Homo sapiens
CAACGGAAUCCCAAAAGCAGCUG
>hsa-miR-191-3p MIMAT0001618 Homo sapiens
GCUGCGCUUGGAUUUCGUCCCC
>hsa-miR-196b-5p MIMAT0001080 Homo sapiens
UAGGUAGUUUCCUGUUGUUGGG
>hsa-miR-196b-3p MIMAT0009201 Homo sapiens
UCGACAGCACGACACUGCCUUC
>mmu-miR-196b-5p MIMAT0001081 Mus musculus
UAGGUAGUUUCCUGUUGUUGGG
>mmu-miR-196b-3p MIMAT0017170 Mus musculus
UCGACAGCACGACACUGCCUUC
>rno-miR-196b MIMAT0001082 Rattus norvegicus
UAGGUAGUUUCCUGUUGUUGGG
>rno-miR-196b* MIMAT0017171 Rattus norvegicus
UCGACAGCACGACACUGCCUUCA
>hsa-miR-200a-5p MIMAT0001620 Homo sapiens
CAUCUUACCGGACAGUGCUGGA

>hsa-miR-200a-3p MIMAT0000682 Homo sapiens
UAACACUGUCUGGUAACGAUGU
>hsa-miR-203 MIMAT0000264 Homo sapiens
GUGAAAUGUUUAGGACCACUAG
>hsa-miR-205-5p MIMAT0000266 Homo sapiens
UCCUUCAUUCCACCGGAGUCUG
>hsa-miR-205-3p MIMAT0009197 Homo sapiens
GAUUUCAGUGGAGUGAAGUUC
>hsa-miR-22-5p MIMAT0004495 Homo sapiens
AGUUCUUCAGUGGCAAGCUUUA
>hsa-miR-22-3p MIMAT0000077 Homo sapiens
AAGCUGCCAGUUGAAGAACUGU
>hsa-miR-221-5p MIMAT0004568 Homo sapiens
ACCUGGCAUACAAUGUAGAUUU
>hsa-miR-221-3p MIMAT0000278 Homo sapiens
AGCUACAUUGUCUGCUGGGUUUC
>hsa-miR-222-5p MIMAT0004569 Homo sapiens
CUCAGUAGCCAGUGUAGAUCCU
>hsa-miR-222-3p MIMAT0000279 Homo sapiens
AGCUACAUCUGGCUACUGGGU
>hsa-miR-23a-5p MIMAT0004496 Homo sapiens
GGGGUUCCUGGGGAUGGGAUUU
>hsa-miR-23a-3p MIMAT0000078 Homo sapiens
AUCACAUUGCCAGGGAUUUCC
>hsa-miR-23b-5p MIMAT0004587 Homo sapiens
UGGGUUCCUGGCAUGCUGAUUU
>hsa-miR-23b-3p MIMAT0000418 Homo sapiens
AUCACAUUGCCAGGGAUUACC
hsa-miR-24-1-5p MIMAT0000079 Homo sapiens
UGCCUACUGAGCUGAUAUCAGU
>hsa-miR-24-3p MIMAT0000080 Homo sapiens
UGGCUCAGUUCAGCAGGAACAG
>hsa-miR-24-2-5p MIMAT0004497 Homo sapiens
UGCCUACUGAGCUGAAACACAG
>hsa-miR-26a-5p MIMAT0000082 Homo sapiens
UUCAAGUAAUCCAGGAUAGGCU
>hsa-miR-26a-1-3p MIMAT0004499 Homo sapiens
CCUAUUCUUGGUUACUUGCACG
>hsa-miR-26b-5p MIMAT0000083 Homo sapiens
UUCAAGUAAUUCAGGAUAGGU
>hsa-miR-26b-3p MIMAT0004500 Homo sapiens
CCUGUUCUCCAUUACUUGGCUC
>hsa-miR-27a-5p MIMAT0004501 Homo sapiens
AGGGCUUAGCUGCUUGUGAGCA

>hsa-miR-27a-3p MIMAT0000084 Homo sapiens
UUCACAGUGGCUAAGUUCCGC
>hsa-miR-29a-5p MIMAT0004503 Homo sapiens
ACUGAUUUCUUUUGGUGUUCAG
>hsa-miR-29a-3p MIMAT0000086 Homo sapiens
UAGCACCAUCUGAAAUCGGUUA
>hsa-miR-29b-1-5p MIMAT0004514 Homo sapiens
GCUGGUUUCAUAUGGUGGUUUAGA
>hsa-miR-29b-3p MIMAT0000100 Homo sapiens
UAGCACCAUUUGAAAUCAGUGUU
>hsa-miR-29b-2-5p MIMAT0004515 Homo sapiens
CUGGUUUCACAUGGUGGCUUAG
>hsa-miR-30c-5p MIMAT0000244 Homo sapiens
UGUAAACAUCCUACACUCUCAGC
>hsa-miR-30c-2-3p MIMAT0004550 Homo sapiens
CUGGGAGAAGGCUGUUUACUCU
>hsa-miR-30d-5p MIMAT0000245 Homo sapiens
UGUAAACAUCCCCGACUGGAAG
>hsa-miR-345-5p MIMAT0000772 Homo sapiens
GCUGACUCCUAGUCCAGGGCUC
>hsa-miR-345-3p MIMAT0022698 Homo sapiens
GCCCUGAACGAGGGGUCUGGAG
