# Supplementary material for: A computational method for predicting regulation of human microRNAs on the influenza virus genome
Source: BMC Syst Biol. 2013 Oct 14;7(Suppl 2):S3. doi: 10.1186/1752-0509-7-S2-S3 (PMC3851852; doi:10.1186/1752-0509-7-S2-S3)
Supplement: Additional File 16 — The list of training datasets including positive samples and negative samples. [file 1752-0509-7-S2-S3-S16.PDF]

List of Training Dataset

This additional file gives two tables, one Table1 is the list of positive samples; and Table2 is the list of negative samples we used as test dataset.

Table1: The list of positive samples.

No.	miRNA	Refseq Id	No.	miRNA	Refseq Id	No.	miRNA	Refseq Id
1	hsa-let-7a	NM_001039111	93	hsa-miR-15a	NM_018307	185	hsa-miR-221	NM_004064
2	hsa-let-7a	NM_181833	94	hsa-miR-15a	NM_032124	186	hsa-miR-222	NM_000222
3	hsa-let-7a	NM_004985	95	hsa-miR-15a	NM_002228	187	hsa-miR-222	NM_004064
4	hsa-let-7a	NM_024674	96	hsa-miR-15a	NM_003930	188	hsa-miR-223	NM_005595
5	hsa-let-7a	NM_003483	97	hsa-miR-15a	NM_003376	189	hsa-miR-23a	NM_024909
6	hsa-let-7a	NM_002524	98	hsa-miR-15a	NM_015948	190	hsa-miR-23a	NM_004575
7	hsa-let-7b	NM_024674	99	hsa-miR-15a	NM_002687	191	hsa-miR-23a	NM_005524
8	hsa-let-7b	NM_001259	100	hsa-miR-15a	NM_003359	192	hsa-miR-24	NM_000791
9	hsa-let-7b	NM_003483	101	hsa-miR-15a	NM_145243	193	hsa-miR-24	NM_004302
10	hsa-let-7b	NM_201567	102	hsa-miR-15a	NM_018263	194	hsa-miR-26a	NM_005900
11	hsa-let-7c	NM_001039111	103	hsa-miR-15a	NM_145341	195	hsa-miR-26a	NM_002655
12	hsa-let-7c	NM_003483	104	hsa-miR-15a	NM_173473	196	hsa-miR-26a	NM_003242
13	hsa-let-7c	NM_002467	105	hsa-miR-15a	NM_182763	197	hsa-miR-27b	NM_000104
14	hsa-let-7e	NM_006306	106	hsa-miR-15a	NM_014999	198	hsa-miR-29a	NM_175848
15	hsa-let-7e	NM_003483	107	hsa-miR-15a	NM_000657	199	hsa-miR-29a	NM_022552
16	hsa-let-7g	NM_003483	108	hsa-miR-15b	NM_000633	200	hsa-miR-29a	NM_003118
17	hsa-let-7g	NM_006546	109	hsa-miR-16	NM_003376	201	hsa-miR-29b	NM_175848
18	hsa-miR-101	NM_005378	110	hsa-miR-16	NM_016140	202	hsa-miR-29b	NM_022552
19	hsa-miR-101	NM_152998	111	hsa-miR-16	NM_000633	203	hsa-miR-29b	NM_021960
20	hsa-miR-103	NM_015640	112	hsa-miR-16	NM_003387	204	hsa-miR-29b	NM_021966
21	hsa-miR-106a	NM_000321	113	hsa-miR-16	NM_014333	205	hsa-miR-29c	NM_175848
22	hsa-miR-107	NM_012104	114	hsa-miR-16	NM_005736	206	hsa-miR-29c	NM_022552
23	hsa-miR-107	NM_015640	115	hsa-miR-16	NM_005324	207	hsa-miR-29c	NM_003211
24	hsa-miR-10a	NM_153620	116	hsa-miR-16	NM_032303	208	hsa-miR-29c	NM_001855
25	hsa-miR-10b	NM_002148	117	hsa-miR-16	NM_021925	209	hsa-miR-29c	NM_000090
26	hsa-miR-122	NM_003045	118	hsa-miR-16	NM_001006605	210	hsa-miR-29c	NM_002293
27	hsa-miR-125a-5p	NM_024674	119	hsa-miR-16	NM_000365	211	hsa-miR-29c	NM_001845
28	hsa-miR-125a-5p	NM_001982	120	hsa-miR-16	NM_152729	212	hsa-miR-29c	NM_000089
29	hsa-miR-125a-5p	NM_004448	121	hsa-miR-16	NM_005316	213	hsa-miR-29c	NM_000138
30	hsa-miR-125b	NM_024674	122	hsa-miR-16	NM_019083	214	hsa-miR-29c	NM_001846
31	hsa-miR-125b	NM_003901	123	hsa-miR-16	NM_024759	215	hsa-miR-29c	NM_000088
32	hsa-miR-125b	NM_001806	124	hsa-miR-16	NM_022173	216	hsa-miR-34a	NM_001949
33	hsa-miR-125b	NM_175709	125	hsa-miR-16	NM_018263	217	hsa-miR-34a	NM_000633

34	hsa-miR-125b	NM_005324	126	hsa-miR-16	NM_002228	218	hsa-miR-34a	NM_003376
35	hsa-miR-125b	NM_173473	127	hsa-miR-16	NM_003930	219	hsa-miR-34a	NM_005618
36	hsa-miR-125b	NM_203339	128	hsa-miR-16	NM_015948	220	hsa-miR-34a	NM_017617
37	hsa-miR-125b	NM_001982	129	hsa-miR-16	NM_032124	221	hsa-miR-34a	NM_005378
38	hsa-miR-125b	NM_002644	130	hsa-miR-16	NM_018307	222	hsa-miR-370	NM_005204
39	hsa-miR-125b	NM_182511	131	hsa-miR-16	NM_002687	223	hsa-miR-372	NM_014572
40	hsa-miR-125b	NM_199332	132	hsa-miR-16	NM_022353	224	hsa-miR-372	NM_003376
41	hsa-miR-125b	NM_173843	133	hsa-miR-16	NM_145243	225	hsa-miR-373	NM_015455
42	hsa-miR-125b	NM_005105	134	hsa-miR-16	NM_003359	226	hsa-miR-373	NM_152835
43	hsa-miR-125b	NM_022121	135	hsa-miR-16	NM_145341	227	hsa-miR-373	NM_015076
44	hsa-miR-125b	NM_001188	136	hsa-miR-16	NM_182763	228	hsa-miR-373	NM_016603
45	hsa-miR-125b	NM_002167	137	hsa-miR-16	NM_173473	229	hsa-miR-373	NM_021914
46	hsa-miR-125b	NM_000499	138	hsa-miR-16	NM_000657	230	hsa-miR-373	NM_004896
47	hsa-miR-125b	NM_003538	139	hsa-miR-16	NM_014999	231	hsa-miR-373	NM_153042
48	hsa-miR-125b	NM_001007156	140	hsa-miR-17	NM_001754	232	hsa-miR-373	NM_152261
49	hsa-miR-125b	NM_014373	141	hsa-miR-17	NM_181659	233	hsa-miR-373	NM_016343
50	hsa-miR-125b	NM_004973	142	hsa-miR-17	NM_003376	234	hsa-miR-373	NM_006282
51	hsa-miR-125b	NM_001013398	143	hsa-miR-181a	NM_005523	235	hsa-miR-373	NM_004427
52	hsa-miR-125b	NM_213609	144	hsa-miR-181b	NM_003385	236	hsa-miR-373	NM_152345
53	hsa-miR-125b	NM_181353	145	hsa-miR-181b	NM_021966	237	hsa-miR-373	NM_153369
54	hsa-miR-125b	NM_004448	146	hsa-miR-182	NM_015270	238	hsa-miR-373	NM_013446
55	hsa-miR-129-5p	NM_177422	147	hsa-miR-182	NM_198159	239	hsa-miR-373	NM_178818
56	hsa-miR-129-5p	NM_015215	148	hsa-miR-193a-3p	NM_198256	240	hsa-miR-373	NM_016019
57	hsa-miR-130a	NM_019102	149	hsa-miR-193a-3p	NM_182763	241	hsa-miR-373	NM_004233
58	hsa-miR-130a	NM_005461	150	hsa-miR-193a-3p	NM_005607	242	hsa-miR-373	NM_003764
59	hsa-miR-130a	NM_005924	151	hsa-miR-196a	NM_019558	243	hsa-miR-373	NM_003981
60	hsa-miR-130a	NM_000757	152	hsa-miR-196a	NM_022658	244	hsa-miR-373	NM_004404
61	hsa-miR-133a	NM_172057	153	hsa-miR-199b-5p	NM_005562	245	hsa-miR-373	NM_003223
62	hsa-miR-133a	NM_000218	154	hsa-miR-19a	NM_000314	246	hsa-miR-373	NM_014051
63	hsa-miR-133b	NM_005029	155	hsa-miR-200a	NM_014795	247	hsa-miR-373	NM_144596
64	hsa-miR-137	NM_001259	156	hsa-miR-200b	NM_014795	248	hsa-miR-373	NM_019555
65	hsa-miR-137	NM_198256	157	hsa-miR-200b	NM_030751	249	hsa-miR-373	NM_145048
66	hsa-miR-137	NM_006540	158	hsa-miR-200c	NM_030751	250	hsa-miR-373	NM_144563
67	hsa-miR-140-5p	NM_003376	159	hsa-miR-203	NM_003722	251	hsa-miR-373	NM_013448
68	hsa-miR-141	NM_004898	160	hsa-miR-203	NM_003955	252	hsa-miR-373	NM_016133
69	hsa-miR-145	NM_024615	161	hsa-miR-205	NM_003376	253	hsa-miR-373	NM_016433
70	hsa-miR-145	NM_005544	162	hsa-miR-205	NM_014795	254	hsa-miR-373	NM_003816
71	hsa-miR-147	NM_003376	163	hsa-miR-206	NM_003182	255	hsa-miR-373	NM_005573
72	hsa-miR-148a	NM_175850	164	hsa-miR-206	NM_000165	256	hsa-miR-373	NM_015853

73	hsa-miR-148a	NM_175849	165	hsa-miR-20a	NM_005225	257	hsa-miR-373	NM_018421
74	hsa-miR-148a	NM_033013	166	hsa-miR-21	NM_014456	258	hsa-miR-373	NM_173647
75	hsa-miR-148b	NM_175850	167	hsa-miR-21	NM_001018005	259	hsa-miR-373	NM_025205
76	hsa-miR-155	NM_032049	168	hsa-miR-21	NM_144949	260	hsa-miR-373	NM_021633
77	hsa-miR-155	NM_206866	169	hsa-miR-21	NM_005596	261	hsa-miR-373	NM_018492
78	hsa-miR-15a	NM_000633	170	hsa-miR-21	NM_181869	262	hsa-miR-373	NM_014572
79	hsa-miR-15a	NM_014333	171	hsa-miR-21	NM_181359	263	hsa-miR-373	NM_0010013 92
80	hsa-miR-15a	NM_003387	172	hsa-miR-21	NM_170709	264	hsa-miR-424	NM_005595
81	hsa-miR-15a	NM_005736	173	hsa-miR-21	NM_018593	265	hsa-miR-433	NM_019851
82	hsa-miR-15a	NM_005324	174	hsa-miR-21	NM_001259	266	hsa-miR-504	NM_003376
83	hsa-miR-15a	NM_021925	175	hsa-miR-21	NM_001204	267	hsa-miR-520g	NM_003376
84	hsa-miR-15a	NM_001006605	176	hsa-miR-21	NM_002639	268	hsa-miR-520h	NM_004827
85	hsa-miR-15a	NM_022173	177	hsa-miR-21	NM_005734	269	hsa-miR-520h	NM_003376
86	hsa-miR-15a	NM_152729	178	hsa-miR-21	NM_014454	270	hsa-miR-7	NM_003749
87	hsa-miR-15a	NM_032303	179	hsa-miR-210	NM_004952	271	hsa-miR-7	NM_005228
88	hsa-miR-15a	NM_005316	180	hsa-miR-212	NM_175610	272	hsa-miR-7	NM_005544
89	hsa-miR-15a	NM_024759	181	hsa-miR-214	NM_000314	273	hsa-miR-9	NM_0010071 56
90	hsa-miR-15a	NM_019083	182	hsa-miR-218	NM_001017402	274	hsa-miR-96	NM_015270
91	hsa-miR-15a	NM_000365	183	hsa-miR-22	NM_000125	275	hsa-miR-96	NM_198159
92	hsa-miR-15a	NM_022353	184	hsa-miR-221	NM_000222	276	hsa-miR-98	NM_003483

Table2: The list of negative samples.

No.	miRNA	Refseq ID	No.	miRNA	Refseq ID	No.	miRNA	Refseq ID
1	hsa-let-7g	NM_005546	72	hsa-miR-155	NM_152869	143	hsa-miR-23b	NM_000191
2	hsa-let-7g	NM_014716	73	hsa-miR-155	NM_004683	144	hsa-miR-23b	NM_002488
3	hsa-let-7i	NM_001152	74	hsa-miR-15a	NM_001003941	145	hsa-miR-23b	NM_000435
4	hsa-let-7i	NM_014716	75	hsa-miR-15a	NM_002541	146	hsa-miR-23b	NM_001163
5	hsa-let-7i	NM_004456	76	hsa-miR-15a	NM_006254	147	hsa-miR-23b	NM_017533
6	hsa-let-7i	NM_152998	77	hsa-miR-15a	NM_212539	148	hsa-miR-23b	NM_005001
7	hsa-miR-1	NM_002812	78	hsa-miR-15a	NM_000803	149	hsa-miR-24	NM_004750
8	hsa-miR-1	NM_002078	79	hsa-miR-15a	NM_000552	150	hsa-miR-24	NM_002019
9	hsa-miR-1	NM_000461	80	hsa-miR-15a	NM_006412	151	hsa-miR-24	NM_017459
10	hsa-miR-1	NM_000481	81	hsa-miR-15a	NM_000286	152	hsa-miR-24	NM_002403
11	hsa-miR-1	NM_003613	82	hsa-miR-15a	NM_001024382	153	hsa-miR-24	NM_002084
12	hsa-miR-103	NM_003165	83	hsa-miR-15a	NM_000190	154	hsa-miR-24	NM_001124
13	hsa-miR-103	NM_016453	84	hsa-miR-15a	NM_001040031	155	hsa-miR-24	NM_004995
14	hsa-miR-103	NM_184231	85	hsa-miR-15a	NM_001002027	156	hsa-miR-24	NM_002964
15	hsa-miR-103	NM_000067	86	hsa-miR-15a	NM_005175	157	hsa-miR-24	NM_004429
16	hsa-miR-103	NM_014575	87	hsa-miR-15a	NM_002134	158	hsa-miR-26a	NM_000719
17	hsa-miR-103	NM_001094	88	hsa-miR-15a	NM_005675	159	hsa-miR-26a	NM_005257

18	hsa-miR-132	NM_016310	89	hsa-miR-15a	NM_002250	160	hsa-miR-26a	NM_018222
19	hsa-miR-132	NM_000320	90	hsa-miR-15a	NM_018696	161	hsa-miR-26a	NM_004684
20	hsa-miR-132	NM_001819	91	hsa-miR-15a	NM_005529	162	hsa-miR-26b	NM_004684
21	hsa-miR-132	NM_004505	92	hsa-miR-190	NM_013445	163	hsa-miR-26b	NM_017426
22	hsa-miR-132	NM_182470	93	hsa-miR-190	NM_003350	164	hsa-miR-26b	NM_018222
23	hsa-miR-137	NM_005574	94	hsa-miR-190	NM_003430	165	hsa-miR-26b	NM_000210
24	hsa-miR-137	NM_005539	95	hsa-miR-190	NM_006409	166	hsa-miR-27a	NM_005545
25	hsa-miR-137	NM_002436	96	hsa-miR-191	NM_003846	167	hsa-miR-27a	NM_201526
26	hsa-miR-137	NM_003763	97	hsa-miR-191	NM_001014444	168	hsa-miR-27a	NM_00100139<br>2
27	hsa-miR-137	NM_001001433	98	hsa-miR-191	NM_001888	169	hsa-miR-27a	NM_00100139<br>0
28	hsa-miR-137	NM_024663	99	hsa-miR-191	NM_002126	170	hsa-miR-27a	NM_00100139<br>1
29	hsa-miR-138	NM_014110	100	hsa-miR-191	NM_002109	171	hsa-miR-27a	NM_00100138<br>9
30	hsa-miR-138	NM_003592	101	hsa-miR-191	NM_022003	172	hsa-miR-27a	NM_000610
31	hsa-miR-138	NM_003325	102	hsa-miR-191	NM_004420	173	hsa-miR-27a	NM_198177
32	hsa-miR-138	NM_002109	103	hsa-miR-196b	NM_014164	174	hsa-miR-27a	NM_198159
33	hsa-miR-138	NM_002738	104	hsa-miR-196b	NM_144779	175	hsa-miR-27a	NM_198158
34	hsa-miR-138	NM_002695	105	hsa-miR-196b	NM_001870	176	hsa-miR-27a	NM_000248
35	hsa-miR-138	NM_004564	106	hsa-miR-196b	NM_003120	177	hsa-miR-27a	NM_198178
36	hsa-miR-143	NM_001185	107	hsa-miR-200a	NM_004988	178	hsa-miR-27a	NM_198256
37	hsa-miR-143	NM_004126	108	hsa-miR-200a	NM_002674	179	hsa-miR-29a	NM_001849
38	hsa-miR-143	NM_021910	109	hsa-miR-200a	NM_021804	180	hsa-miR-29a	NM_005157
39	hsa-miR-143	NM_000867	110	hsa-miR-200a	NM_030765	181	hsa-miR-29a	NM_001856
40	hsa-miR-145	NM_005978	111	hsa-miR-200a	NM_002020	182	hsa-miR-29a	NM_002403
41	hsa-miR-145	NM_015392	112	hsa-miR-200a	NM_182925	183	hsa-miR-29a	NM_017459
42	hsa-miR-145	NM_000299	113	hsa-miR-200a	NM_014384	184	hsa-miR-29a	NM_021738
43	hsa-miR-145	NM_003008	114	hsa-miR-200a	NM_004170	185	hsa-miR-29a	NM_003174
44	hsa-miR-145	NM_005795	115	hsa-miR-205	NM_005978	186	hsa-miR-29a	NM_014296
45	hsa-miR-145	NM_004755	116	hsa-miR-205	NM_003890	187	hsa-miR-29a	NM_014211
46	hsa-miR-145	NM_004672	117	hsa-miR-205	NM_001886	188	hsa-miR-29a	NM_016831
47	hsa-miR-145	NM_000350	118	hsa-miR-205	NM_030960	189	hsa-miR-29b	NM_201539
48	hsa-miR-145	NM_004532	119	hsa-miR-205	NM_004103	190	hsa-miR-29b	NM_201540
49	hsa-miR-145	NM_004785	120	hsa-miR-205	NM_173174	191	hsa-miR-29b	NM_201535
50	hsa-miR-145	NM_021910	121	hsa-miR-205	NM_173176	192	hsa-miR-29b	NM_201537
51	hsa-miR-145	NM_001039550	122	hsa-miR-205	NM_030770	193	hsa-miR-29b	NM_182764
52	hsa-miR-153	NM_005165	123	hsa-miR-205	NM_030775	194	hsa-miR-29b	NM_002811
53	hsa-miR-153	NM_001092	124	hsa-miR-22	NM_005855	195	hsa-miR-29b	NM_000067
54	hsa-miR-153	NM_015185	125	hsa-miR-22	NM_022449	196	hsa-miR-29b	NM_005175
55	hsa-miR-153	NM_004644	126	hsa-miR-22	NM_001013436	197	hsa-miR-29b	NM_00100202<br>7
56	hsa-miR-153	NM_005648	127	hsa-miR-22	NM_004306	198	hsa-miR-29b	NM_002079

57	hsa-miR-153	NM_006158	128	hsa-miR-22	NM_005700	199	hsa-miR-30c	NM_003891
58	hsa-miR-153	NM_001201	129	hsa-miR-22	NM_005029	200	hsa-miR-30c	NM_002084
59	hsa-miR-153	NM_000320	130	hsa-miR-22	NM_000017	201	hsa-miR-30c	NM_000124
60	hsa-miR-153	NM_005271	131	hsa-miR-22	NM_001144	202	hsa-miR-30c	NM_003569
61	hsa-miR-153	NM_005010	132	hsa-miR-22	NM_005258	203	hsa-miR-30c	NM_005956
62	hsa-miR-153	NM_001037132	133	hsa-miR-221	NM_024663	204	hsa-miR-30c	NM_000192
63	hsa-miR-153	NM_176795	134	hsa-miR-221	NM_014303	205	hsa-miR-30c	NM_181486
64	hsa-miR-153	NM_005343	135	hsa-miR-221	NM_000251	206	hsa-miR-30c	NM_005550
65	hsa-miR-153	NM_006176	136	hsa-miR-222	NM_000251	207	hsa-miR-30c	NM_002633
66	hsa-miR-153	NM_016237	137	hsa-miR-222	NM_004541	208	hsa-miR-30c	NM_000727
67	hsa-miR-153	NM_003367	138	hsa-miR-222	NM_014820	209	hsa-miR-30c	NM_000696
68	hsa-miR-153	NM_207291	139	hsa-miR-23a	NM_003890	210	hsa-miR-30c	NM_002631
69	hsa-miR-153	NM_004128	140	hsa-miR-23a	NM_005720	211	hsa-miR-30c	NM_002560
70	hsa-miR-155	NM_000850	141	hsa-miR-23a	NM_003255	212	hsa-miR-345	NM_018962
71	hsa-miR-155	NM_001122	142	hsa-miR-23a	NM_018961			
