# Supplementary material for: A computational method for predicting regulation of human microRNAs on the influenza virus genome
Source: BMC Syst Biol. 2013 Oct 14;7(Suppl 2):S3. doi: 10.1186/1752-0509-7-S2-S3 (PMC3851852; doi:10.1186/1752-0509-7-S2-S3)
Supplement: Additional File 17 — The coding sequence of the gene fragments which were used as training datasets including positive samples and negative samples. [file 1752-0509-7-S2-S3-S17.PDF]

mRNA of Training Dataset

This additional file gives 488 mRNA sequences which are used as test dataset in FASTA format, which are extracted from the University of California, Santra Cruz (UCSC) Genome bioinformatics site, and 276 of them are positive samples, and 212 of them are used as negative samples.

Positive samples:

>NM_001039111 1
gctctctcctcctcctcctcctcttcctctctggtctcctccctcctccg
ggctgggttgcaaatggcttcgttccccgagaccgatttccagatctgct
tgctgtgcaaggagatgtgcggctcgccggcgccgctctcctccaactcg
tccgcgtcgtcgtcctcctcgcagacgtccacgtcgtcgggggggcggcgg
cgggggccctggggcggcggcgcgccgcctacacgtcctgccctgcctgc
acgccttctgccgcccctgcctcgaggcgcaccggctgccggcggcgggc
ggcggcgcggcgggagagccgctcaagctgcgctgccccgtgtgcgacca
gaaagtagtgctagccgaggcggcgggtatggacgcgctgccttcgtccg
ccttcctgcttagcaacctgctcgacgcggtggtggccactgccgacgag
ccgccgcccaagaacgggcgcgccggcgctccggcgggagcgggcggcca
cagcaaccaccggcaccacgctcaccacgcgcacccgcgcgcgtccgcct
ccgcgccgccactcccgcaggcgccgcagccgcccgcgccttcccgctcg
gcacccggcggccctgccgcttccccgtcggcgctgctgctccgccgtcc
tcacggctgcagctcgtgcgatgagggcaacgcagcttcttcgcgctgcc
tcgactgccaggagcacctgtgcgacaactgcgtccgagcgcaccagcgc
gtgcgcctcaccaaggaccactacatcgagcgcggcccgccgggtcccgg
tgccgcagcagcggcgcagcagctcgggctcgggccgccctttcccggcc
cgcccttctccatcctctcagtgtttcccgagcgcctcggcttctgccag
caccacgacgacgaggtgctgcacctgtactgtgacacttgctctgtacc
catctgtcgtgagtgcacaatgggccggcatgggggccacagcttcatct
acctccaggaggcactgcaggactcacgggcactcaccatccagctgctg
gcagatgcccagcagggacgacaggcaatccagctgagcatcgagcaggc
ccagacggtggcggaacaggtggagatgaaggcgaaggttgtgcagtcgg
aggtcaaagccgtgacggcgaggcataagaaagccctggaggaacgcgag
tgtgagctgctgtggaaggtagaaaagatccgccaggtgaaagccaagtc
tctgtacctgcaggtggagaagctgcggcaaaacctcaacaagcttgaga
gcaccatcagtgccgtgcagcaggtcctggaggagggtagagcgctagac
atcctactggcccgagaccggatgctggcccaggtgcaggagctgaagac
cgtgcggagcctcctgcagccccaggaagacgaccgagtcatgttcacac
cccccgatcaggcactgtaccttgccatcaagtcttttggctttgttagc
agcggggcctttgccccactcaccaaggccacaggcgatggcctcaagcg
tgccctccagggtaaggtggcctccttcacagtcattggttatgaccacg
atggtgagccccgcctctcaggaggcgacctgatgtcggctgtggtcctg

ggccctgatggcaacctgtttggtgcagaggtgagtgatcagcagaatgg
gacatacgtggtgagttaccgaccccagctggagggtgagcacctggtat
ctgtgacactgtgcaaccagcacattgagaacagccctttcaaggtggtg
gtcaagtcaggccgcagctacgtgggcattgggctcccgggcctgagctt
cggcagtgagggtgacagcgatggcaagctctgccgcccttggggtgtga
gtgtagacaaggagggctacatcattgtcgccgaccgcagcaacaaccgc
atccaggtgttcaagccctgcggcgccttccaccacaaattcggcaccct
gggctcccggcctgggcagttcgaccgaccagccggcgtggcctgtgacg
cctcacgcaggatcgtggtggctgacaaggacaatcatcgcatccagatc
ttcacgttcgagggccagttcctcctcaagtttggtgagaaaggaaccaa
gaatgggcagttcaactacccttgggatgtggcggtgaattctgagggca
agatcctggtctcagacacgaggaaccaccggatccagctgtttgggcct
gatggtgtcttcctaaacaagtatggcttcgaggggggctctctggaagca
ctttgactccccacggggtgtggccttcaaccatgagggccacttggtgg
tcactgacttcaacaaccaccggctcctggttattcaccccgactgccag
tcggcacgctttctgggctcggagggcacaggcaatgggcagttcctgcg
cccacaaggggtagctgtggaccaggaagggcgcatcattgtggcggatt
ccaggaaccatcgggtacagatgtttgaatccaacggcagcttcctgtgc
aagtttggtgctcaaggcagcggctttgggcagatggaccgcccttccgg
catcgccatcacccccgacggaatgatcgttgtggtggactttggcaaca
atcgaatcctcgtcttctaattgcatttcctaggtttctgtgtttggggt
gtgtgtgcgtgtctctctctctctctctctctttctctttctctctcttt
ttgaatttcaaagaagaaacagtctcagggaaatttctttttctttttt
ttttttaaagagaacaagaaaagtacaacattgcttaagtcctacctcat
ctttattttttacagatgaatgtacttatcttttctgcagggattgagc
ctgtgaagtgataatttctatctacctcataaatctttacatttccttct
gcaacaggccctcttcccctcctcagtggagtttgcatttccctcttccc
ctgcgtggggcatgatatgcacaagcctggcatctgtatggctgggaggg
cactggatgtgtgtggtggggtgtattctgtagattgagccaaggaaaca
caaaaaaaaactactaagtaaaaaaacaaaaaactata
>NM_181833 2
tgcgcttcccgcgggcgcgcggagtgaggacggtgacagccacgcgcgcg
cgtacgcgcccgatgcagcgcggccccgtgaccctagtcggccgctgaga
ggcgcgcggagtctgggccgctgccgtctaggggtcccgtcccgaggcgt
ccccggcatctccggcccgaatcccggagtgccgggtcgcgcctgcaccg
aaggtcccggctcctgtgccctccctgcagccgtcagggcccgtcccca
actccccttccgctcaggcagggtcctcgcggcccatgctggccgctgg
ggacccgcgcagcccagaccgttcccgggccgggcagccggccaccatgg
tggccctgaggcctgtgcagcaactccaggggggctaaagggctcagagt
gcaggccgtggggcgcgagggtcccgggcctgagccccgcgccatggccg
gggccatcgcttcccgcatgagcttcagctctctcaagaggaagcaaccc
aagacgttcaccgtgaggatcgtcaccatggacgccgagatggagttcaa
ttgcgagatgaagtggaaagggaaggacctctttgatttggtgtgccgga
ctctggggctccgagaaacctggttctttggactgcagtacacaatcaag
gacacagtggcctggctcaaaatggacaagaaggtactggatcatgatgt
ttcaaaggaagaaccagtcacctttcacttcttggccaaattttatcctg
agaatgctgaagaggagctggttcaggagatcacacaacatttattcttc
ttacaggtaaagaagcagattttagatgaaaagatctactgccctcctga
ggcttctgtgctcctggcttcttacgccgtccaggccaagctcaccttgc

agagcgccaagtcccgagtggccttctttgaagagctctagcaggtgacc
cagccaccccaggacctgccacttctcctgctaccgggaccgcgggatgg
accagatatcaagagagccatccatagggagctggctgggggtttccgtg
ggagctccagaactttccccagctgagtgaagagcccagcccctcttatg
tgcaattgccttgaactacgaccctgtagagatttctctcatggcgttct
agttctctgacctgagtctttgttttaagaagtatttgtcttcctttgtc
taatgtgggattcctgactcccttcgtccaaggcaccggtgtgtgtgtgt
cttgcactccagagctgacctccaccgcccagcctgggaagtcattgtag
ggagtgagacactgaagccctgagaagccagtgccatcatccccaccccg
cccagggttccggaacattcattcccccaccggtgaggacctggcatgca
gcgaagcagcccagcccggcggatcccaggccagcacgcctgccggcttc
tcatcgtcagggagcccgcccagagctcgtgacgagcaagtgctgggtcc
ccgccaggcaccccgaggcggcgctctggctggcagctggtggggaatag
gcagggcagctgtggctggggagagactttaggcagaagctgtgatgcag
gctgactgccagccgaggggctgggtagtgccgtgcgggagctgatggta
cagggcactcgctgtccccctccggccaccctagaccagggtccgagagg
caggcaggagccactcatgtcttccccattgcccgacgcccatagacgct
ccttcctgtgtggggctggggtactccctggtcgtactgcagtcagcacc
cgtaacccggctatgaccagggatctgtaagccctgtggctccacaggtg
ctgcttctcactggcccagactctgagctccaccggcccagtctgcacgg
cccatctgcttcaccttccctcccagccacgtgccagtggccacagccca
cttcccaacccactgttgtacccaggcctcactttgctgttgcccttgtc
cctcttcgggccctgaattttctgttccctgggggccagccagggcccctt
tgtgcccctcccagcacaggcctgatgcaggtgtccactcacaggtggcg
ctcacctaggctgtcacaggacccacctccatgccaggcaacagagggcc
acagaaccacccccacggctcactccttggtctggggccaccttcttgcc
ctttctttttttttttttttttttttccgagatggagtctctctctgt
cacccaggtgggagtgtggtggcacaatctcggctcactgcaacctccac
ctcctgagttcaagcaattctcctgcctcagcctcccaagtagctgggac
tacaggcatgcaccaccacacttggccaatgttctgtatttttaataggg
acggggttttgccatgttagctaggctggtctcaaactcacacctgggat
tacaggcatgagccactgcacccagccccttcttgccctttcttttctcc
atggctgatgctgctgtggccagccagggcccttgagatccttccagttt
ggctgttatgcaaagcaggtgatttgtcttaatcagataaaagatagagg
ctatgggggcctcaagatttttggagagcagaggtggtctctggcaattc
catctggttttgagaaacttagcagctcacagagcacagagatcctgcct
tcttcctactatcaggctgacctaatggggttgggctgctcggcaactgc
ttgggtcaccttgccccaaggaaaccagccctgggtgccacccagccact
tagggtctacagggtgggactccagacctagagcgtaagtatggatgttg
tggccctgtgtcttcctagtgtgacccagccaggagcggaagcttcaggc
gtttgtaaagtgaggtctggctctgcctcctccgttttttttttttttct
gtttctgtttctgtttttttttttgagatggagtctcgctctgtcgcccag
gctggagtgcagtgtcacgatctcagctcactgcaacctccgcctcccag
gtacaagagattctcctgcctcagcctcccgagtagctgggactacaggc
gtgtgccaccatgcctggctaatttttgtatttttagtagagatggggtt
tcgccatgttagccagactggtctcgaactcctgacctcaggtgatcctc
ccaccccggcttcccaaagttctgggattataggcgtgagccaccatgcc
cggtctcttctcagtcttgaagcccatccctggatttccaccaggagtta
ctttcctcctgacctgtaaatttgttctttaacaatggctgcaggtggga

gcatatggtggtttataaaaacgctgtcgggctttgtttccttctttagc
tgccgtgtctacttctgaagtctgggaagtgccaagccacgcggcctcaa
gggagctggctggtgtttgagctgtggcagaagcacctggggctccaggg
agcaggctgggaactgcaggaccttgctcagccaggagcacttccccctc
cttgaggcaggaatactgaggtgcctccccacagatggagaaggtggaga
ggaggatgggcctcaggagcatctcaagccccagtagcaggagaaagaaa
gaaagagatgcctggttttcacagactggttcctgtggctgggatgactg
catccttttttttttttttttgagacggagtttttgcctttgtcgcccag
gctggagtgcaatggcgtgatctcggctcaccgcaacctccgcctcccgg
attcaagcaattctcctgcctcagcctcccgagtagctgggattacaggc
acgcacctccacgtccggctaattttgtatttttagtggagacggggttt
ctccatgtcggtcaggctggtctcgaactcccgacctcaggtgatctgcc
cacctcggcctcccaaagtgctgggattacaggcatgagccaccgcgctt
ggccagactgcgtcctttttaagcgaacattttagggcctgggagtttgt
caagtaaggaagtctcaagcccaaagagcagcgtcctgaccatggtggtt
tcattacgagcccttctgctggctctcaggcagaagccccacagcaccgg
gaccattcatgaggtcactgcccagctcatgatgtccgtgaggctgtcct
tttggccagtagccgtgtgcagctgtgtggcacagatggcttcgttcatc
ctgatcaaggccccacctcagccacagcagtcccccccaacctgtgttgtc
caccctattattcatgtacctgccaggccctgctagatagcaccccgtgg
cattacataacacttcatgagtggctgtgtcttgtaattttggggacagg
tttctctctttccctctctttttttttgtcaaaagcccagagactgacaac
cagctgcagtgtctaagtgttcctcactgacagggtggggcctcaccacc
cctggagggagcagcgttggcagggagacagcctggcccagtgaccctgg
gcccaagccagcccctccagggctttcagggaagcgccatccattttcaa
agatgtcaaacgtcacttcttcctgtagggcccgagtcctgcctcctatc
agggccagatcatagaaggctattttctattctggggaacgattataact
taaatgattgttttaataaaaattctaagctggaaaataaaaaaaaaaaa
aaaaaa
>NM_004985 3
ggccgcggcggcggaggcagcagcggcggcggcagtggcggcggcgaagg
tggcggcggctcggccagtactcccggcccccgccatttcggactgggag
cgagcgcggcgcaggcactgaaggcggcggcggggccagaggctcagcgg
ctcccaggtgcgggagagaggcctgctgaaaatgactgaatataaacttg
tggtagttggagctggtggcgtaggcaagagtgccttgacgatacagcta
attcagaatcattttgtggacgaatatgatccaacaatagaggattccta
caggaagcaagtagtaattgatggagaaacctgtctcttggatattctcg
acacagcaggtcaagaggagtacagtgcaatgagggaccagtacatgagg
actggggagggctttctttgtgtatttgccataaataatactaaatcatt
tgaagatattcaccattatagagaacaaattaaaagagttaaggactctg
aagatgtacctatggtcctagtaggaaataaatgtgatttgccttctaga
acagtagacacaaaacaggctcaggacttagcaagaagttatggaattcc
ttttattgaaacatcagcaaagacaagacagggtgttgatgatgccttct
atacattagttcgagaaattcgaaaacataaagaaaagatgagcaaagat
ggtaaaaagaagaaaaagaagtcaaagacaaagtgtgtaattatgtaaat
acaatttgtactttttttcttaaggcatactagtacaagtggtaatttttg
tacattacactaaattattagcatttgttttagcattacctaattttttt
cctgctccatgcagactgttagcttttaccttaaatgcttattttaaaat
gacagtggaagttttttttttcctctaagtgccagtattcccagagttttg

gtttttgaactagcaatgcctgtgaaaaagaaactgaatacctaagattt
ctgtcttggggtttttggtgcatgcagttgattacttcttatttttctta
ccaattgtgaatgttggtgtgaaacaaattaatgaagcttttgaatcatc
cctattctgtgttttatctagtcacataaatggattaattactaatttca
gttgagaccttctaattggtttttactgaaacattgagggaacacaaatt
tatgggcttcctgatgatgattcttctaggcatcatgtcctatagtttgt
catccctgatgaatgtaaagttacactgttcacaaaggttttgtctcctt
tccactgctattagtcatggtcactctccccaaaatattatattttttct
ataaaaagaaaaaaatggaaaaaaattacaaggcaatggaaactattata
aggccatttccttttcacattagataaattactataaagactcctaatag
cttttcctgttaaggcagacccagtatgaaatggggattattatagcaac
cattttggggctatatttacatgctactaaatttttataataattgaaaa
gattttaacaagtataaaaaattctcataggaattaaatgtagtctccct
gtgtcagactgctctttcatagtataactttaaatcttttcttcaacttg
agtctttgaagatagttttaattctgcttgtgacattaaaagattatttg
ggccagttatagcttattaggtgttgaagagaccaaggttgcaaggccag
gccctgtgtgaacctttgagctttcatagagagtttcacagcatggactg
tgtccccacggtcatccagtgttgtcatgcattggttagtcaaaatgggg
agggactagggcagtttggatagctcaacaagatacaatctcactctgtg
gtggtcctgctgacaaatcaagagcattgcttttgtttcttaagaaaaca
aactctttttttaaaaattacttttaaatattaactcaaaagttgagattt
tggggtggtggtgtgccaagacattaattttttttttaaacaatgaagtg
aaaaagttttacaatctctaggtttggctagttctcttaacactggttaa
attaacattgcataaacacttttcaagtctgatccatatttaataatgct
ttaaaataaaaataaaaacaatcctttttgataaatttaaaatgttactta
ttttaaaataaatgaagtgagatggcatggtgaggtgaaagtatcactgg
actaggaagaaggtgacttaggttctagataggtgtcttttaggactctg
attttgaggacatcacttactatccatttcttcatgttaaaagaagtcat
ctcaaactcttagttttttttttttacaactatgtaatttatattccatt
tacataaggatacacttatttgtcaagctcagcacaatctgtaaattttt
aacctatgttacaccatcttcagtgccagtcttgggcaaaattgtgcaag
aggtgaagtttatatttgaatatccattctcgttttaggactcttcttcc
atattagtgtcatcttgcctccctaccttccacatgccccatgacttgat
gcagttttaatacttgtaattcccctaaccataagatttactgctgctgt
ggatatctccatgaagttttcccactgagtcacatcagaaatgccctaca
tcttatttcctcagggctcaagagaatctgacagataccataaagggatt
tgacctaatcactaattttcaggtggtggctgatgctttgaacatctctt
tgctgcccaatccattagcgacagtaggatttttcaaacctggtatgaat
agacagaaccctatccagtggaaggagaatttaataaagatagtgctgaa
agaattccttaggtaatctataactaggactactcctggtaacagtaata
cattccattgttttagtaaccagaaatcttcatgcaatgaaaaatacttt
aattcatgaagcttacttttttttttggtgtcagagtctcgctcttgtc
acccaggctggaatgcagtggcgccatctcagctcactgcaacctccatc
tcccaggttcaagcgattctcgtgcctcggcctcctgagtagctgggatt
acaggcgtgtgccactacactcaactaatttttgtatttttaggagagac
ggggtttcaccctgttggccaggctggtctcgaactcctgacctcaagtg
attcacccaccttggcctcataaacctgttttgcagaactcatttattca
gcaaatatttattgagtgcctaccagatgccagtcaccgcacaaggcact
gggtatatggtatccccaaacaagagacataatcccggtccttaggtagt

gctagtgtggtctgtaatatcttactaaggcctttggtatacgacccaga
gataacacgatgcgtattttagttttgcaaagaaggggtttggtctctgt
gccagctctataattgttttgctacgattccactgaaactcttcgatcaa
gctactttatgtaaatcacttcattgttttaaaggaataaacttgattat
attgtttttttatttggcataactgtgattcttttaggacaattactgta
cacattaaggtgtatgtcagatattcatattgacccaaatgtgtaatatt
ccagttttctctgcataagtaattaaaatatacttaaaaattaatagttt
tatctgggtacaaataaacaggtgcctgaactagttcacagacaaggaaa
cttctatgtaaaaatcactatgatttctgaattgctatgtgaaactacag
atctttggaacactgtttaggtagggtgttaagacttacacagtacctcg
tttctacacagagaaagaaatggccatacttcaggaactgcagtgcttat
gaggggatatttaggcctcttgaatttttgatgtagatgggcattttttt
aaggtagtggttaattacctttatgtgaactttgaatggtttaacaaaag
atttgtttttgtagagattttaaagggggagaattctagaaataaatgtt
acctaattattacagccttaaagacaaaaatccttgttgaagttttttta
aaaaaagctaaattacatagacttaggcattaacatgtttgtggaagaat
atagcagacgtatattgtatcatttgagtgaatgttcccaagtaggcatt
ctaggctctatttaactgagtcacactgcataggaatttagaacctaact
tttataggttatcaaaactgttgtcaccattgcacaattttgtcctaata
tatacatagaaactttgtggggcatgttaagttacagtttgcacaagttc
atctcatttgtattccattgattttttttttcttctaaacattttttctt
caaacagtatataactttttttaggggattttttttttagacagcaaaaac
tatctgaagatttccatttgtcaaaaagtaatgatttcttgataattgtg
tagtaatgtttttttagaacccagcagttaccttaaagctgaatttatatt
tagtaacttctgtgttaatactggatagcatgaattctgcattgagaaac
tgaatagctgtcataaaatgaaactttctttctaaagaaagatactcaca
tgagttcttgaagaatagtcataactagattaagatctgtgttttagttt
aatagtttgaagtgcctgtttgggataatgataggtaatttagatgaatt
taggggaaaaaaaaagttatctgcagatatgttgagggcccatctctcccc
ccacacccccacagagctaactgggttacagtgttttatccgaaagtttc
caattccactgtcttgtgttttcatgttgaaaatacttttgcatttttcc
tttgagtgccaatttcttactagtactatttcttaatgtaacatgtttac
ctggaatgtattttaactatttttgtatagtgtaaactgaaacatgcaca
ttttgtacattgtgctttcttttgtgggacatatgcagtgtgatccagtt
gttttccatcatttggttgcgctgacctaggaatgttggtcatatcaaac
attaaaaatgaccactcttttaattgaaattaacttttaaatgtttatag
gagtatgtgctgtgaagtgatctaaaatttgtaatatttttgtcatgaac
tgtactactcctaattattgtaatgtaataaaaatagttacagtgacaaa
aaaaaaaaaaaa
>NM_024674 4
gtgcgggggaagatgtagcagcttcttctccgaaccaaccctttgccttc
ggacttctccggggccagcagccgcccgaccaggggcccggggccacggg
ctcagccgacgaccatgggctccgtgtccaaccagcagtttgcaggtggc
tgcgccaaggcggcagaagaggcgcccgaggaggcgccggaggacgcggc
ccgggcggcggacgagcctcagctgctgcacggtgcgggcatctgtaagt
ggttcaacgtgcgcatggggttcggcttcctgtccatgaccgcccgcgcc
ggggtcgcgctcgaccccccagtggatgtctttgtgcaccagagtaagct
gcacatggaagggttccggagcttgaaggagggtgaggcagtggagttca
cctttaagaagtcagccaagggtctggaatccatccgtgtcaccggacct

ggtggagtattctgtattgggagtgagaggcggccaaaaggaaagagcat
gcagaagcgcagatcaaaaggagacaggtgctacaactgtggaggtctag
atcatcatgccaaggaatgcaagctgccaccccagcccaagaagtgccac
ttctgccagagcatcagccatatggtagcctcatgtccgctgaaggccca
gcagggccctagtgcacagggaaagccaacctactttcgagaggaagaag
aagaaatccacagccctaccctgctcccggaggcacagaattgagccaca
atgggtgggggctattcttttgctatcaggaagttttgaggagcaggcag
agtggagaaagtgggaatagggtgcattggggctagttggcactgccatg
tatctcaggcttgggttcacaccatcaccctttcttccctctaggtgggg
ggaaagggtgagtcaaaggaactccaaccatgctctgtccaaatgcaagt
gagggttctgggggcaaccaggagggggggaatcaccctacaacctgcata
ctttgagtctccatccccagaatttccagcttttgaaagtggcctggata
gggaagttgttttcctttaaagaaggatatataataattcccatgccag
agtgaaatgattaagtataagaccagattcatggagccaagccactacat
tctgtggaaggagatctctcaggagtaagcattgttttttttcacatct
tgtatcctcatacccacttttgggatagggtgctggcagctgtcccaagc
aatgggtaatgatgatggcaaaaagggtgtttgggggaacagctgcagac
ctgctgctctatgctcacccccgccccattctgggccaatgtgattttat
ttatttgctcccttggatactgcaccttgggtcccactttctccaggatg
ccaactgcactagctgtgtgcgaatgacgtatcttgtgcattttaacttt
ttttccttaatataaatattctggttttgtatttttgtatattttaatct
aaggccctcatttcctgcactgtgttctcaggtacatgagcaatctcagg
gatagccagcagcagctccaggtctgcgcagcaggaattactttttgttg
tttttgccaccgtggagagcaactatttggagtgcacagcctattgaact
acctcatttttgccaataagagctggcttttctgccatagtgtcctcttg
aaaccccctctgccttgaaaatgttttatgggagactaggttttaactgg
gtggccccatgacttgattgccttctactggaagattgggaattagtcta
aacaggaaatggtggtacacagaggctaggagaggctgggcccggtgaaa
aggccagagagcaagccaagattaggtgagggttgtctaatcctatggca
caggacgtgctttacatctccagatctgttcttcaccagattaggttagg
cctaccatgtgccacagggtgtgtgtgtgtttgtaaaactagagttgcta
aggataagtttaaagaccaataccccctgtacttaatcctgtgctgtcgag
ggatggatatatgaagtaaggtgagatccttaacctttcaaaattttcgg
gttccagggagacacacaagcgagggttttgtggtgcctggagcctgtgt
cctgccctgctacagtagtgattaatagtgtcatggtagctaaaggagaa
aaagggggtttcgtttacacgctgtgagatcaccgcaaacctaccttact
gtgttgaaacgggacaaatgcaatagaacgcattgggtggtgtgtgtctg
atcctgggttcttgtctcccctaaatgctgccccccaagttactgtattt
gtctgggctttgtaggacttcactacgttgattgctaggtggcctagttt
gtgtaaatataatgtattggtctttctccgtgttctttgggggttttgtt
tacaaacttctttttgtattgagagaaaaatagccaaagcatctttgaca
gaaggttctgcaccaggcaaaaagatctgaaacattagtttggggggccc
tcttcttaaagtggggatcttgaaccatcctttcttttgtattccccttc
ccctattacctattagaccagatcttctgtcctaaaaacttgtcttctac
cctgccctcttttctgttcacccccaaaagaaaacttacacacccacaca
catacacatttcatgcttggagtgtctccacaactcttaaatgatgtatg
caaaaatactgaagctaggaaaaccctccatcccttgttcccaacctcct
aagtcaagaccattaccatttctttctttcttttttttttttttttaaaa
tggagtctcactgtgtcacccaggctggagtgcagtggcatgatcggctc

actgcagcctctgcctcttgggttcaagtgattctcctgcctcagcctcc
tgagtagctgggatttcaggcacccgccacactcagctaatttttgtatt
tttagtagagacggggtttcaccatgttgtccaggctggtctggaactcc
tgacctcaggtgatctgcccaccttggcttcccaaagtgctgggattaca
ggcatgagccaccatgctgggccaaccatttcttggtgtattcatgccaa
acacttaagacactgctgtagcccaggcgcggtggctcacacctgtaatc
ccagcactttggaaggctgaggcgggcggatcacaaggtcacgagttcaa
aactatcctggccaacacagtgaaaccccgtctctactaaaatacaaaaa
aattagccgggtgtggtggtgcatgcctttagtcctagctattcaggagg
ctgaggcaggggaatcgcttgaacccgagaggcagaggttgcagtgagct
gagatcgcaccactgcactccagcctggttacagagcaagactctgtctc
aaacaaaacaaaacaaaacaaaaacacactactgtattttggatggatca
aacctccttaattttaatttctaatcctaaagtaaagagatgcaattggg
ggccttccatgtagaaagtggggtcaggaggccaagaaagggaatatgaa
tgtatatccaagtcactcaggaacttttatgcaggtgctagaaactttat
gtcaaagtggccacaagattgtttaataggagacgaacgaatgtaactcc
atgtttactgctaaaaaccaaagctttgtgtaaaatcttgaatttatggg
gcgggagggtaggaaagcctgtacctgtctgtttttttcctgatcctttt
ccctcattcctgaactgcaggagactgagcccctttgggctttggtgacc
ccatcactggggtgtgtttatttgatggttgattttgctgtactgggtac
ttcctttcccattttctaatcatttttttaacacaagctgactcttccctt
cccttctcctttccctgggaaaatacaatgaataaataaagacttattgg
tacgcaaactgtca
>NM_003483 4
cttgaatcttggggcaggaactcagaaaacttccagcccgggcagcgcgc
gcttggtgcaagactcaggagctagcagcccgtcccccctccgactctccg
gtgccgccgctgcctgctcccgccaccctaggaggcgcggtgccacccac
tactctgtcctctgcctgtgctccgtgcccgaccctatcccggcggagtc
tccccatcctcctttgctttccgactgcccaaggcactttcaatctcaat
ctcttctctctctctctctctctctctctctctctctctctctctctctc
tctctctctcgcagggtgggggggaagaggaggaggaattctttccccgcc
taacatttcaagggacacaattcactccaagtctcttccctttccaagcc
gcttccgaagtgctcccggtgcccgcaactcctgatcccaacccgcgaga
ggagcctctgcgacctcaaagcctctcttccttctccctcgcttccctcc
tcctcttgctacctccacctccaccgccacctccacctccggcacccacc
caccgccgccgccgccaccggcagcgcctcctcctctcctcctcctcctc
ccctcttctctttttggcagccgctggacgtccggtgttgatggtggcag
cggcggcagcctaagcaacagcagccctcgcagcccgccagctcgcgctc
gccccgccggcgtccccagccctatcacctcatctcccgaaaggtgctgg
gcagctccggggcggtcgaggcgaagcggctgcagcggcggtagcggcgg
cgggaggcaggatgagcgcacgcggtgagggcgcggggcagccgtccact
tcagcccagggacaacctgccgccccagcgcctcagaagagaggacgcgg
ccgccccaggaagcagcagcaagaaccaaccggtgagccctctcctaaga
gacccaggggaagacccaaaggcagcaaaaacaagagtccctctaaagca
gctcaaaagaaagcagaagccactggagaaaaacggccaagaggcagacc
taggaaatggccacaacaagttgttcagaagaagcctgctcaggaggaaa
ctgaagagacatcctcacaagagtctgccgaagaggactagggggcgcca
acgttcgatttctacctcagcagcagttggatcttttgaagggagaagac
actgcagtgaccacttattctgtattgccatggtctttccactttcatct

ggggtggggtggggtggggtgggggaggggggggtggggtggggagaaat
cacataaccttaaaaaggactatattaatcaccttctttgtaatcccttc
acagtcccaggtttagtgaaaaactgctgtaaacacaggggacacagctt
aacaatgcaacttttaattactgttttctttttcttaacctactaatag
tttgttgatctgataagcaagagtgggcgggtgagaaaaaccgaattggg
tttagtcaatcactgcactgcatgcaaacaagaaacgtgtcacacttgtg
acgtcgggcattcatataggaagaacgcggtgtgtaacactgtgtacacc
tcaaataccaccccaacccactccctgtagtgaatcctctgtttagaaca
ccaaagataaggactagatactactttctctttttcgtataatcttgtag
acacttacttgatgatttttaactttttatttctaaatgagacgaaatgc
tgatgtatcctttcattcagctaacaaactagaaaaggttatgttcattt
ttcaaaaagggaagtaagcaaacaaatattgccaactcttctatttatgg
atatcacacatatcagcaggagtaataaatttactcacagcacttgtttt
caggacaacacttcattttcaggaaatctacttcctacagagccaaaatg
ccatttagcaataaataacacttgtcagcctcagagcatttaaggaaact
agacaagtaaaattatcctctttgtaatttaatgaaaaggtacaacagaa
taatgcatgatgaactcacctaattatgaggtgggaggagcgaaatctaa
atttcttttgctatagttatacatcaatttaaaaagcaaaaaaaaaaaag
gggggggcaatctctctctgtgtctttctctctctctcttcctctccctc
tctcttttcattgtgtatcagtttccatgaaagacctgaataccacttac
ctcaaattaagcatatgtgttacttcaagtaatacgttttgacataagat
ggttgaccaaggtgcttttcttcggcttgagttcaccatctcttcattca
aactgcacttttagccagagatgcaatatatccccactactcaatactac
ctctgaatgttacaacgaatttacagtctagtacttattacatgctgcta
tacacaagcaatgcaagaaaaaaacttactgggtaggtgattctaatcat
ctgcagttctttttgtacacttaattacagttaaagaagcaatctcctta
ctgtgtttcagcatgactatgtatttttctatgttttttaattaaaaat
ttttaaaatacttgtttcagcttctctgctagatttctacattaacttga
aaattttttaaccaagtcgctcctaggttcttaaggataattttcctcaa
tcacactacacatcacacaagatttgactgtaatatttaaatattaccct
ccaagtctgtacctcaaatgaattctttaaggagatggactaattgactt
gcaaagacctacctccagacttcaaaaggaatgaacttgttacttgcagc
attcatttgtttttcaatgtttgaaatagttcaaactgcagctaaccct
agtcaaaactatttttgtaaaagacatttgatagaaaggaacacgttttt
acatacttttgcaaaataagtaaataataaataaaataaaagccaacctt
caaagaaacttgaagctttgtaggtgagatgcaacaagccctgcttttgc
ataatgcaatcaaaaatatgtgtttttaagattagttgaatataagaaaa
tgcttgacaaatattttcatgtattttacacaaatgtgatttttgtaata
tgtctcaaccagatttattttaaacgcttcttatgtagagtttttatgcc
tttctctcctagtgagtgtgctgactttttaacatggtattatcaactgg
gccaggaggtagtttctcatgacggcttttgtcagtatggcttttagtac
tgaagccaaatgaaactcaaaaccatctctcttccagctgcttcagggag
gtagtttcaaaggccacatacctctctgagactggcagatcgctcactgt
tgtgaatcaccaaaggagctatggagagaattaaaactcaacattactgt
taactgtgcgttaaataagcaaataaacagtggctcataaaaataaaagt
cgcattccatatctttggatgggccttttagaaacctcattggccagctc
ataaaatggaagcaattgctcatgttggccaaacatggtgcaccgagtga
tttccatctctggtaaagttacacttttatttcctgtatgttgtacaatc
aaaacacactactacctcttaagtcccagtatacctcatttttcatactg

aaaaaaaaagcttgtggccaatggaacagtaagaacatcataaaattttt
atatatatagtttatttttgtgggagataaattttataggactgttcttt
gctgttgttggtcgcagctacataagactggacatttaacttttctacca
tttctgcaagttaggtatgtttgcaggagaaaagtatcaagacgtttaac
tgcagttgactttctccctgttcctttgagtgtcttctaactttattctt
tgttctttatgtagaattgctgtctatgattgtactttgaatcgcttgct
tgttgaaaatatttctctagtgtattatcactgtctgttctgcacaataa
acataacagcctctgtgatccccatgtgttttgattcctgctctttgtta
cagttccattaaatgagtaataaagtttggtcaaaacagaaaaaaaaaaa
>NM_002524 4
gaaacgtcccgtgtgggagggggcgggtctgggtgcggcctgccgcatgac
tcgtggttcggaggcccacgtggccggggcggggactcaggcgcctgggg
cgccgactgattacgtagcgggcggggccggaagtgccgctccttggtgg
gggctgttcatggcggttccggggtctccaacatttttcccggctgtggt
cctaaatctgtccaaagcagaggcagtggagcttgaggttcttgctggtg
tgaaatgactgagtacaaactggtggtggttggagcaggtggtgttggga
aaagcgcactgacaatccagctaatccagaaccactttgtagatgaatat
gatcccaccatagaggattcttacagaaaacaagtggttatagatggtga
aacctgtttgttggacatactggatacagctggacaagaagagtacagtg
ccatgagagaccaatacatgaggacaggcgaaggcttcctctgtgtattt
gccatcaataatagcaagtcatttgcggatattaacctctacagggagca
gattaagcgagtaaaagactcggatgatgtacctatggtgctagtgggaa
acaagtgtgatttgccaacaaggacagttgatacaaaacaagcccacgaa
ctggccaagagttacgggattccattcattgaaacctcagccaagaccag
acagggtgttgaagatgcttttacacactggtaagagaaatacgccagt
accgaatgaaaaactcaacagcagtgatgatgggactcagggttgtatg
ggattgccatgtgtggtgatgtaacaagatacttttaaagttttgtcaga
aaagagccactttcaagctgcactgacaccctggtcctgacttccctgga
ggagaagtattcctgttgctgtcttcagtctcacagagaagctcctgcta
cttccccagctctcagtagtttagtacaataatctctatttgagaagttc
tcagaataactacctcctcacttggctgtctgaccagagaatgcacctct
tgttactccctgttatttttctgccctgggttcttccacagcacaaacac
acctctgccaccccaggtttttcatctgaaaagcagttcatgtctgaaac
agagaaccaaaccgcaaacgtgaaattctattgaaaacagtgtcttgagc
tctaaagtagcaactgctggtgatttttttttctttttactgttgaact
tagaactatgctaatttttggagaaatgtcataaattactgttttgccaa
gaatatagttattattgctgtttggtttgtttataatgttatcggctcta
ttctctaaactggcatctgctctagattcataaatacaaaaatgaatact
gaattttgagtctatcctagtcttcacaactttgacgtaattaaatccaa
ctttcacagtgaagtgcctttttcctagaagtggtttgtagacttccttt
ataatatttcagtggaatagatgtctcaaaaatccttatgcatgaaatga
atgtctgagatacgtctgtgacttatctaccattgaaggaaagctatatc
tatttgagagcagatgccattttgtacatgtatgaaattggttttccaga
ggcctgttttggggctttcccaggagaaagatgaaactgaaagcacatga
ataatttcacttaataatttttacctaatctccactttttcataggtta
ctacctatacaatgtatgtaatttgtttcccctagcttactgataaacct
aatattcaatgaacttccatttgtattcaaatttgtgtcataccagaaag
ctctacatttgcagatgttcaaatattgtaaaactttggtgcattgttat
ttaatagctgtgatcagtgattttcaaacctcaaatatagtatattaaca

aattacattttcactgtatatcatggtatcttaatgatgtatataattgc
cttcaatccccttctcaccccaccctctacagcttcccccacagcaatag
gggcttgattatttcagttgagtaaagcatggtgctaatggaccagggtc
acagtttcaaaacttgaacaatccagttagcatcacagagaaagaaattc
ttctgcatttgctcattgcaccagtaactccagctagtaattttgctagg
tagctgcagttagccctgcaaggaaagaagaggtcagttagcacaaaccc
tttaccatgactggaaaactcagtatcacgtatttaaacatttttttttc
ttttagccatgtagaaactctaaattaagccaatattctcatttgagaat
gaggatgtctcagctgagaaacgttttaaattctctttattcataatgtt
ctttgaagggtttaaaacaagatgttgataaatctaagctgatgagtttg
ctcaaaacaggaagttgaaattgttgagacaggaatggaaaatataatta
attgatacctatgaggatttggaggcttggcattttaatttgcagataat
accctggtaattctcatgaaaaatagacttggataacttttgataaaaga
ctaattccaaaatggccactttgttcctgtctttaatatctaaatactta
ctgaggtcctccatcttctatattatgaattttcatttattaagcaaatg
tcatattaccttgaaattcagaagagaagaaacatatactgtgtccagag
tataatgaacctgcagagttgtgcttcttactgctaattctgggagcttt
cacagtactgtcatcatttgtaaatggaaattctgcttttctgtttctgc
tccttctggagcagtgctactctgtaattttcctgaggcttatcacctca
gtcatttctttttttaaatgtctgtgactggcagtgattctttttcttaaa
aatctattaaatttgatgtcaaattagggagaaagatagttactcatctt
gggctcttgtgccaatagcccttgtatgtatgtacttagagttttccaag
tatgttctaagcacagaagtttctaaatggggccaaaattcagacttgag
tatgttctttgaataccttaagaagttacaattagccgggcatggtggcc
cgtgcctgtagtcccagctacttgagaggctgaggcaggagaatcacttc
aacccaggaggtggaggttacagtgagcagagatcgtgccactgcactcc
agcctgggtgacaagagagacttgtctccaaaaaaaaaagttacacctagg
tgtgaattttggcacaaaggagtgacaaacttatagttaaaagctgaata
acttcagtgtggtataaaacgtggtttttaggctatgtttgtgattgctg
aaaagaattctagtttacctcaaaatccttctctttccccaaattaagtg
cctggccagctgtcataaattacatattccttttggttttttttaaaggtt
acatgttcaagagtgaaaataagatgttctgtctgaaggctaccatgccg
gatctgtaaatgaacctgttaaatgctgtatttgctccaacggcttacta
tagaatgttacttaatacaatatcatacttattacaatttttactatagg
agtgtaataggtaaaattaatctctattttagtgggcccatgtttagtct
ttcaccatcctttaaactgctgtgaattttttttgtcatgacttgaaagca
aggatagagaaacactttagagatatgtggggtttttttaccattccaga
gcttgtgagcataatcatatttgctttatatttatagtcatgaactccta
agttggcagctacaaccaagaaccaaaaaatggtgcgttctgcttcttgt
aattcatctctgctaataaattataagaagcaaggaaaattagggaaaat
attttatttggatggtttctataaacaagggactataattcttgtacatt
atttttcatctttgctgtttctttgagcagtctaatgtgccacacaatta
tctaaggtatttgttttctataagaattgttttaaaagtattcttgttac
cagagtagttgtattatatttcaaaacgtaagatgatttttaaaagcctg
agtactgacctaagatggaattgtatgaactctgctctggagggaggga
ggatgtccgtggaagttgtaagacttttatttttttgtgccatcaaatat
aggtaaaaataattgtgcaattctgctgtttaaacaggaactattggcct
ccttggccctaaatggaagggccgatattttaagttgattattttattgt
aaattaatccaacctagttctttttaatttggttgaatgttttttcttgt

taaatgatgtttaaaaaataaaaactggaagttcttggcttagtcataat
tctt
>NM_001259 6
aacctctccgcgcgaagacggcttcagccctgcagggaaagaaaagtgca
atgattctggactgagacgcgcttgggcagaggctatgtaatcgtgtctg
tgttgaggacttcgcttcgaggagggaagaggagggatcggctcgctcct
ccggcggcggcggcggcggcgactctgcaggcggagtttcgcggcggcgg
caccagggttacgccagccccgcggggaggtctctccatccagcttctgc
agcggcgaaagccccagcgcccgagcgcctgagccggcggggagcaagta
aagctagaccgatctccggggagccccggagtaggcgagcggcggccgcc
agctagttgagcgcacccccgcccgccccagcggcgccgcggcgggcgg
cgtccaggcggcatggagaaggacggcctgtgccgcgctgaccagcagta
cgaatgcgtggcggagatcggggagggcgcctatgggaaggtgttcaagg
cccgcgacttgaagaacggaggccgtttcgtggcgttgaagcgcgtgcgg
gtgcagaccggcgaggagggcatgccgctctccaccatccgcgaggtggc
ggtgctgaggcacctggagaccttcgagcaccccaacgtggtcaggttgt
ttgatgtgtgcacagtgtcacgaacagacagagaaaccaaactaacttta
gtgtttgaacatgtcgatcaagacttgaccacttacttggataaagttcc
agagcctggagtgcccactgaaaccataaaggatatgatgtttcagcttc
tccgaggtctggactttcttcattcacaccgagtagtgcatcgcgatcta
aaaccacagaacattctggtgaccagcagcggacaaataaaactcgctga
cttcggccttgcccgcatctatagtttccagatggctctaacctcagtgg
tcgtcacgctgtggtacagagcacccgaagtcttgctccagtccagctac
gccacccccgtggatctctggagtgttggctgcatatttgcagaaatgtt
tcgtagaaagcctctttttcgtggaagttcagatgttgatcaactaggaa
aaatcttggacgtgattggactcccaggagaagaagactggcctagagat
gttgcccttcccaggcaggcttttcattcaaaatctgcccaaccaattga
gaagtttgtaacagatatcgatgaactaggcaaagacctacttctgaagt
gtttgacatttaacccagccaaaagaatatctgcctacagtgccctgtct
cacccatacttccaggacctggaaaggtgcaaagaaaacctggattccca
cctgccgcccagccagaacacctcggagctgaatacagcctgaggcctca
gcagccgccttaagctgatcctgcggagaacacccttggtggcttatggg
tccccctcagcaagccctacagagctgtggaggattgctatctggaggcc
ttccagctgctgtcttctggacaggctctgcttctccaaggaaaccgcct
agtttactgttttgaaatcaatgcaagagtgattgcagctttatgttcat
ttgtttgtttgtttgtctgtttgtttcaagaacctggaaaaattccagaa
gaagagaagctgctgaccaattgtgctgccatttgatttttctaaccttg
aatgctgccagtgtggagtgggtaatccaggcacagctgagttatgatgt
aatctctctgcagctgccgggcctgatttggtacttttgagtgtgtgtgt
gcatgtgtgtgtgtgtgtgtgtgtgtgtgtgtgtgtatgtgagagattct
gtgatcttttaaagtgttactttttgtaaacgacaagaataattcaattt
taaagactcaaggtggtcagtaaataacaggcatttgttcactgaaggtg
attcaccaaaatagtcttctcaaattagaaagttaaccccatgtcctcag
catttcttttctggccaaaagcagtaaatttgctagcagtaaaagatgaa
gttttatacacacagcaaaaaggagaaaaaattctagtatattttaagag
atgtgcatgcattctatttagtcttcagaatgctgaatttacttgttgta
agtctattttaaccttctgtatgacatcatgctttatcatttcttttgga
aaatagcctgtaagctttttattacttgctataggtttagggagtgtacc
tcagatagatttaaaaaaaagaatagaaagcctttatttcctggtttga

aattcctttcttcccttttttttgttgttgttattgttgtttgttgttgtt
attttgtttttgtttttaggaatttgtcagaaactctttcctgttttggt
ttggagagtagttctctctaactagagacaggagtggccttgaaattttc
ctcatctattacactgtactttctgccacacactgccttgttggcaaagt
atccatcttgtctatctcccggcacttctgaaatatattgctaccattgt
ataactaataacagattgcttaagctgttcccatgcaccacctgtttgct
tgctttcaatgaacctttcataaattcgcagtctcagcttatggtttatg
gcctcgattctgcaaacctaacagggtcacatatgttctctaatgcagtc
cttctacctggtgtttacttttgttacctaaataatgagtaggatcttgt
tttgttttatcaccagcacacagattgctataaactgttactttgtgaat
tacatttttatagaagatattttcagtgtctttacctgagggtatgtctt
tagctatgttttagggccatacatttactctatcaaatgatcttttctcc
atcccccaggctgtgcttatttctagtgccttgtgctcactcctgctctc
tacagagccagcctggcctgggcattgtaaacagcttttcctttttctct
tactgttttctctacagtcctttatatttcataccatctctgccttataa
gtggtttagtgctcagttggctctagtaaccagaggacacagaaagtatc
ttttggaaagtttagccacctgtgctttctgactcagagtgcatgcaaca
gttagatcatgcaacagttagattatgtttagggttaggattttcaaaga
atggaggttgctgcactcagaaaataattcagatcatgtttatgcattat
taagttgtactgaattctttgcagcttaatgtgatatatgactatcttga
acaagagaaaaaactaggagatgtttctcctgaagagcttttggggttgg
gaactattctttttaattgctgtactacttaacattgttctaattcagt
agcttgaggaacaggaacattgttttctagagcaagataataaaggagat
gggccatacaaatgttttctactttcgttgtgacaacattgattaggtgt
tgtcagtactataaatgcttgagatataatgaatccacagcattcaaggt
caggtctactcaaagtctcacatggaaaagtgagttctgcctttcctttg
atcgagggtcaaaatacaaagacatttttgctagggcctacaaattgaat
ttaaaaactcactgcactgattcatctgagctttttggttagtattcatg
gctagagtgaacatagctttagtttttgctgttgtaaaagtgttttcata
agttcactcaagaaaaatgcagctgttctgaactggaatttttcagcatt
ctttagaattttaaatgagtagagagctcaacttttattcctagcatctg
cttttgactcatttctaggcagtgcttatgaagaaaaattaaagcacaaa
cattctggcattcaatcgttggcagattatcttctgatgacacagaatga
aagggcatctcagcctctctgaactttgtaaaaatctgtccccagttctt
ccatcggtgtagttgttgcatttgagtgaatactctcttgatttatgtat
tttatgtccagattcgccatttctgaaatccagatccaacacaagcagtc
ttgccgttagggcattttgaagcagatagtagagtaagaacttagtgact
acagcttattcttctgtaacatatggtttcaaacatctttgccaaaagct
aagcagtggtgaactgaaaagggcatattgccccaaggttacactgaagc
agctcatagcaagttaaaatattgtgacagatttgaaatcatgtttgaat
ttcatagtaggaccagtacaagaatgtccctgctagtttctgtttgatgt
ttggttctggcggctcaggcattttgggaactgttgcacagggtggagtc
aaaacaacctacatataaaaagagaaaaagagaaacttgtccatttagct
ttcataagaaatcccatggcaaagggtaataaaaaggacctaatcttaaa
aatacaatttctaagcacttgtaagaacccagtgggttggagcctcccac
tttgtccctcctttgaagtggatgggaactcaaggtgcaaagaacctgtt
ttggaagaaagcttggggccatttcagccccctgtattctcatgattttc
tctcaggaagcacacactgtgaatggcagacttttcatttagccccaggt
gacttactaaaaatagttgaaaattattcacctaagaatagaatctcagc

attgtgttaaataaaaatgaaagctttagaaggcatgagatgttcctatc
ttaaataaagcatgtttcttttctatagagaaatgtatagtttgactctc
cagaatgtactatccatcttgatgagaaaactcttaaatagtaccaaaca
ttttgaactttaaattatgtatttaaagtgagtgtttaagaaactgtagc
tgcttcttttacaagtggtgcctattaaagtcagtaatggccattattgt
tccattgtggaaattaaattatgtaagcttcctaatatcataaacatatt
aaaattcttctaaaatattgcttttcttttaagtgacaatttgactattc
ttatgataagcacatgagagtgtcttacattttccaaaagcaggctttaa
ttgcatagttgagtctaggaaaaaataatgttaaaagtgaatatgccacc
ataattacttaattatgttagtatagaaactacagaatatttaccctgga
aagaaaatattggaatgttattataaactcttagatatttatataattca
aaagaatgcatgtttcacattgtgacagataaagatgtatgatttctaag
gctttaaaaattattcataaaacagtgggcaatagataaaggaaattctg
gagaaaatgaaggtatttaaagggtagtttcaaagctatatatattttga
aggatatattctttatgaacaaatatattgtaaaaatttatactaaggtc
atctggtaactgtgggattaatatggtcgaaaacaaatgttatggagaag
ctgtcccaagcaaactaaattacctgtactttttcccatttcaagggaa
gaggcaaccacatgaagcaatacttcttacacatgcctaagaacgttcat
tgaaaaaataaatttttaaaaggcatgtgtttcctatgccaccaatactt
ttgaaaaattgtgaaccttacccaaaaccatttatcatgtccattaagta
tatttgggtatataattaggaagatatttacatgttccatctccacagtg
gaaaaacttattgaggctaccaaagtgtgccaagaaatgtaagtccttag
agtaattagaaatgctgttttcctcaaaagcatgagaaactagcattttc
atttcttatttactccctttctatatcaatgcaattcacaacccaatttt
aatacatccctatatctcaagcatttctatcttgtactttttcagaaaat
aaaccaaaaataatcctttggtctctctatcttctgacctttgtaagcaa
cagaaatgtaaaaacagaaggggtccaatttttacacgtttttttctcaa
gtagcctttctggggatttttattttcttaatgaagtgccaatcagcttt
tcaaaatgttttctatttctcagcatttccaggaagtgataacgtttagc
taaatgagtagaagtggacttccttcaacatattgttaccttgtctagcc
ttaggaagaaaacaagagccacctgaaaataaatacaggctcttttcgag
catctgctgaaatactgttacagcaatttgaagttgatgtggtaggaaag
gaaggtgacttttcttgcaaaagtctttctaaacattcacactgtcctaa
gagatgagctttcttgttttattccggtatattccacaaggtggcacttt
tagagaaaaacaaatctgatgaagactaaagaggtacttctaaaagagat
ttcattctaactttatttttctgcgcatatttaactctttcctagcactt
gtttttgggatgattaatagtctctataatgttctgtaacttcaatatt
ttacttgttacctaggttctgaacaattgtctgcaaataaattgttctta
aggatggataatacacccattttgatcatttaagtaaagaaagcctagtc
attcattcagtcaagaaaaaatttttgaagtacccagttaccttactttt
ctagattaaaacaggcttagttactaaaaaggcagtcctcatctgtgaac
aggatagtttcgttagaagtataaaactcctttagtggccccagttaaaa
cacacataccctctctgctgctttcaaattccctagcatggtggcctttc
aacattgattaaattttaaaatcctaatttaaagatcaggtgagcaaaat
gagtagcacatcagtaattcagtagacaaaacttttgtctgaaaaattgc
tgtattgaaacagagccctaaaataccaaaagaccaggtaattttaacat
ttgtggaatcacaaatgtaaattcataagaagctctaattaaaaaaaaaa
agtctgaagtatatgagcataacaacttaggagtgtgtctacatacttaa
cttttgaagtttttggcaactttatatactttttttaaatttacaagtc

tacttaaagacttcttataccccaaatgattaagttaattttagaggtca
cctttctcacagcagtgtcacttgaaatttagtagggaaggatattgcag
tatttttcagtttccttagcacagcaccacagaaagcagcttattccttt
tgagtggcagacactcgacggtgcctgcccaactttcctcctgagtggca
agcagatgagtctcagtaattcatactgaaccaaaatgccacatacacta
ggggcagtcagaaactggctgagaaatcccccgcctcattcgcccctctg
ctcccaggaactagagtccagttaaagcccctatgcgaaaggccgaattc
caccccagggtttgttataacagtggccagtctgaaccccatttgctcgt
gctcaaaacttgattcccacttgaaagccttccgggcgcgctgcctcgtt
gccccgcccctttggcaggagagaggcagtgggcgaggccgggctggggc
cccgcctcccactcacctgccggtgcctgaaattatgtgcggccccgcgg
gctgctttccgaggtcagagtgccctgctgctgtctcagaggcatctgtt
ctgcaaatcttaggaagaaaaatgtccctagtagcaaacgggtgtcttct
gtgcataaataagtacaacacaattctccgaaagttcgggtaaaaagaga
tgcggtagcagctgccctgtgtgaagctgtctaccccgcatctctcaggc
gctaagctcagtttttgtttttgtttttgtttttttaaagaaaagatgta
taattgcaggaattttttttattttttttattttccatcattctatatat
gtgatggtgaaagatatgcctggaaaagttttgttttgaaaagtttattt
tctgcttcgtcttcagttggcaaaagctctcaattctttagcttccagtt
tcttttctctctttttctttgttaggtaattaaaggtatgtaaacaaatt
atctcatgtagcaggggattttcatgttgagaggaatcttccgtgtgagt
tgtttggtcacacaaataaccctttctcaattttaggagtttggattgtc
aaatgtaggtttttctcaaagggggcatataactacatattgactgccaa
gaactatgactgtagcactaatcagcacacatagagccacacaattattt
aatttctaactctctgtggtccctagaaaaattccgttgatgtgcttagg
ttaaagttctgaagatacccgttgtacccttacttgaaagtttctaatct
taagttttatgaaatgcaataatatgtatcagctagcaatatttctgtga
tcaccaacaactctcagtttgatcttaaagtctgaataataaaacaaatc
ccagcagtaatacatttcttaaacctcacagtgcatgatatatcttttca
ttctgatcctgtgtttgcaaaaatatacacatgtatatcatagttcctca
ctttttattcatttgttttcctattacctgtagtaaatatattagttagt
acatggaatttatagcatcagctacccccaggaacagcacctgacaggcg
ggggattttttttcaagttgttctacatttgcataaattatttctattat
tattcatgtatgttatttatttctgaatcacactagtcctgtgaaagtac
aactgaaggcagaaagtgttaggattttgcatctaatgttcattatcatg
gtattgatggacctaagaaaataaaaattagactaagcccccaaataagc
tgcatgcatttgtaacatgattagtagatttgaatatatagatgtagtat
tttgggtatctaggtgttttatcattatgtaaaggaattaaagtaaagga
ctttgtagttgtttttattaaatatgcatatagtagagtgcaaaaatata
gcaaaaataaaaactaaaggtagaaaagcattttagatatgccttaattt
agaaactgtgccaggtggccctcggaatagatgccaggcagagaccagtg
cctgggtggtgcctcctcttgtctgccctcatgaagaagcttccctcacg
tgatgtagtgccctcgtaggtgtcatgtggagtagtgggaacaggcagta
ctgttgagaggagagcagtgtgagagtttttctgtagaagcagaactgtc
agcttgtgccttgaggcttccagaacgtgtcagatggagaagtccaagtt
tccatgcttcaggcaacttagctgtgtacagaagcaatccagtgtggtaa
taaaaagcaaggattgcctgtataatttattataaaataaaagggatttt
aacaaccaacaattcccaacacctcaaaagcttgttgcatttttggtat
ttgaggtttttatctgaaggttaaagggcaagtgtttggtatagaagagc

agtatgtgttaagaaaagaaaaatattggttcacgtagagtgcaaattag
aactagaaagttttatacgattatcattttgagatgtgttaaagtaggtt
ttcactgtaaaatgtattagtgtttctgcattgccatagggcctggttaa
aactttctcttaggtttcaggaagactgtcacatacagtaagctttttc
cttctgacttataatagaaaatgttttgaaagtaaaaaaaaaaaatctaa
tttggaaatttgacttgttagtttctgtgtttgaaatcatggttctagaa
atgtagaaattgtgtatatcagatactcatctaggctgtgtgaaccagcc
caagatgaccaacatccccacacctctacatctctgtcccctgtatctct
tcctttctaccactaaagtgttccctgctaccatcctggcttgtccacat
ggtgctctccatcttcctccacatcatggaccacaggtgtgcctgtctag
gcctggccaccactcccaacttgacctagccacattcatctagagatggt
tcctgatgctgggcacagactgtgctcatggcacccattagaaatgcctc
tagcatctttgtatgcatcttgatttttaaaccaagtcattgtacagagc
attcagttttggctgtggtaccaagagaaaaactaatcaagaatataaac
cacattccaggctgctgttttctctccatctacaggccacacttttactg
tatttcttcatacttgaaattcattctgctattttcatatcagggtacag
acttataagggtgcatgttccttaaaggtgcataattattcttattccgt
ttgcttatattgctacagaatgctctgttttggtgctttgagttctgcag
acccaagaagcagtgtggaaattcactgcctgggacacagtcttataaga
atgttggcaggtgactttgtatcagatgttgcttctcttttctctgtaca
cagattgagagttaccacagtggcctgtcgggtccaccctgtgggtgcag
cacagctctctgaaagcaagaaccttcctacctattctaacgtttttgcc
ctctaagaaaaatggcctcaggtatggtatagacatagcaagaggggaag
ggctgtctcactctagcaaccatccctccattacacacagaaagccctct
tgaagcaaaagaagaagaaagaaagaaagcttatctctaaggctactgtc
ttcagaatgctctgagctgaatgctcttgctcctttcccaagaggcagat
gaaaatatagccagtttatctataccccttcctatctgaggaggagaatag
aaaagtagggtaaatatgtaacgtaaaatatgtcattcaaggaccaccaa
aactttaagtaccctatcattaaaaatctggttttaaaagtagctcaagt
aagggatgctttgtgacccagggtttctgaagtcagatagccattcttac
ctgccccttactctgacttattgggaaagggagaactgcagtggtgtttc
tgttgcagtggcaaaggtaacatgtcagaaaattcagagggttgcatacc
aataatcctttggaaactggatgtcttactgggtgctagaatgaaaatgt
aggtatttattgtcagatgatgaagttcattgtttttttcaaaattggtg
ttgaaatatcactgtccaatgtgttcacttatgtgaaagctaaattgaat
gaggcaaaaagagcaaatagtttgtatatttgtaataccttttgtatttc
ttacaataaaaatattggtagcaaataaaaataataaaaacaataacttt
aaactgctttctggagatgaattactctcctggctattttcttttttact
ttaatgtaaaatgagtataactgtagtgagtaaaattcattaaattccaa
gttttagcagaaaaaaaaaaaaaaaaaaa
>NM_201567 1
gaacagcgaagacagcgtgagcctgggccgttgcctcgaggctctcgccc
ggcttctcttgccgacccgccacgtttgtttggatttaatcttcaggttg
ccggcgcccgcccgcccgctggcctcgcggtgtgagagggaagcacccgt
gcctgtggctggtggctggcgcctggagggtccgcacacccgcccggccg
cgccgcttgcccgcggcagccgcgtccctgaaccgcggagtcgtgtttgt
gtttgacccgcgggcgccggtggcgcgcggccgaggccggtgtcggcggg
gcggggcggtcgcggcggaggcagaggaagagggagcgggagctctgcga
ggccgggcgccgccatggaactgggcccggagcccccgcaccgccgccgc

ctgctcttcgcctgcagccccccctcccgcgtcgcagcccgtcgtgaaggc
gctatttggcgcttcagccgccgggggactgtcgcctgtcaccaacctga
ccgtcactatggaccagctgcagggtctgggcagtgattatgagcaacca
ctggaggtgaagaacaacagtaatctgcagagaatgggctcctccgagtc
aacagattcaggtttctgtctagattctcctgggccattggacagtaaag
aaaaccttgaaaatcctatgagaagaatacattccctacctcagaagctg
ttgggatgtagtccagctctgaagaggagccattctgattctcttgacca
tgacatctttcagctcatcgacccagatgagaacaaggaaaatctttcct
caaatgaaagagatagcagtgaaccagggaatttcattcctctttttaca
ccccagtcacctgtgacagccactttgtctgatgaggatgatggcttcgt
ggaccttctcgatggagagaatctgaagaatgaggaggagacccccctcgt
gcatggcaagcctctggacagctcctctcgtcatgagaactacaaacctt
gacaaccgatgcaagctgtttgactccccttccctgtgtagctccagcac
tcggtcagtgttgaagagaccagaacgatctcaagaggagtctccacctg
gaagtacaaagaggaggaagagcatgtctggggccagccccaaagagtca
actaatccagagaaggcccatgagactcttcatcagtctttatccctggc
atcttcccccaaaggaaccattgagaacattttggacaatgacccaaggg
accttataggagacttctccaagggttatctctttcatacagttgctggg
aaacatcaggatttaaaatacatctctccagaaattatggcatctgtttt
gaatggcaagtttgccaacctcattaaagagtttgttatcatcgactgtc
gatacccatatgaatacgagggaggccacatcaagggtgcagtgaacttg
cacatggaagaagaggttgaagacttcttattgaagaagcccattgtacc
tactgatggcaagcgtgtcattgttgtgtttcactgcgagttttcttctg
agagaggtccccgcatgtgccggtatgtgagagagagagatcgcctgggt
aatgaataccccaaactccactaccctgagctgtatgtcctgaaggggggg
atacaaggagttctttatgaaatgccagtcttactgtgagccccctagct
accggcccatgcaccacgaggactttaaagaagacctgaagaagttccgc
accaagagccggacctgggcaggggagaagagcaagagggagatgtacag
tcgtctgaagaagctctgagggcggcaggaccagccagcagcagcccaag
cttccctccatccccctttaccctctttgctgcagagaaacttaagcaaa
ggggacagctgtgtgacatttggagagggggcctgggacttccatgcctt
aaacctacctcccacactcccaaggttggagcccagggcatcttgctggc
tacgcctcttctgtccctgttagacgtcctccgtccatatcagaactgtg
ccacaatgcagttctgagcaccgtgtcaagctgctctgagccacagtggg
atgaaccagccggggccttatcgggctccagccatctcatgaggggagag
gagacggaggggagtagagaagttacacagaaatgctgctggccaaatag
caaagacaacctgggaaggaaaggtctttgtgggataatccatatgttta
atttattcaacttcatcaatcactttattttattttttttctaactcct
ggagacttattttactgcttcattaggttgaaatactgccattctaggta
gggttttattatcccagggactacctcggcttttaatttaaaaaaaaaaa
agaagtgggtaagaaaatgcaaacctgttataagttatcggacagaaagc
taggtgctctgtcacccccaggaggcgctgtggtactggggctgctgcta
tttaagccaagaactgaggtcctggtgagagcgttggacccaggcttggc
tgcctgacataagctaaatctcccagacccaccactggctaccgatatct
atttggtgggaggtgtggccctgttcttcctcaccccagttccatgacat
tggctggtataggagccacagtcaggaaagcacttgaggcagcatctgtt
gggccacccccggctcagtgctggaatgttgcagtgtaggtttcccaggg
aagggggggtgggggtaggtgggctccacaggatgggggaggagcatgtcc
actgagtatcttccttatgttgctgtgatattgatagcttttattttcta

atttttaaaaaatggtcatattatgagtcaaagagtatcaaatcagtgtt
ggatggaccacccaagggtgaggagaggggctggaagccctgggcattag
gagaagggagtgggtgctggcatggacatgactggatagaattttctcag
gagggagcttggtggattttgaaggtaaaactttctgggtttatcatgtt
ttaattttagagacagggagtgatgaatcatcaccggttgtccccttatc
taactccataaaagtgggaatttcaaaagaacacctcatccaaggagctg
gggcagacttcattgattctagagagacctgtttcagtgcctactcatcc
ctgccctctggtgccagcctccttaccatcacggcttcactgaggtgtag
gtgggtttttcttaaacaggagacagtctctcccctcttacctcaacttc
ttggggtgggaatcagtgatactggagatggctagttgctgtgttacggg
tttgagttacatttggctataaaacaatcttgttgggaaaaatgtggggg
agaggacttcttcctacacgcgcattgagacagattccaactggttaatg
atattgtttgtaagaaagagattctgttggttgactgcctaaagagaaag
gtgggatggccttcagattataccagcttagctagcattactaaccaact
gttggaagctctgaaaataaaagatcttgaacccataaaaaaaaaaa
>NM_006306 2
cgcgcgggctacctcagttctcgggcgtacggcgcggcctgtcctactgc
cgccggcgccgcggccgtcatggggttcctgaaactgattgagattgaga
actttaagtcgtacaagggtcgacagattatcggaccatttcagaggttc
accgccatcattggacccaatggctctggtaagtcaaatctcatggatgc
catcagctttgtgctaggtgaaaaaaccagcaacctgcgggtaaagaccc
tgcgggacctgatccatggagctcctgtgggcaagccagctgccaaccgg
gcctttgtcagcatggtctactctgaggagggtgctgaggaccgtacctt
tgcccgtgtcattgtaggaggttcttctgagtacaagatcaacaacaaag
tggtccaactacatgagtacagtgaggaattagagaagttgggcattctc
atcaaagctcgtaacttcctcgttttccagggtgctgtggaatctattgc
catgaagaaccccaaagagaggacagctctatttgaagagattagtcgtt
ctggggagctggcgcaggagtatgacaagcgaaagaaggaaatggtgaag
gctgaagaggacacacagtttaattaccatcgcaagaaaaatattgcggc
tgaacgcaaggaagcaaagcaggagaaagaagaggctgaccggtaccagc
gcctgaaggatgaggtagtacgggctcaggtacagctgcagctctttaag
ctttaccataatgaagtggaaattgagaagctcaacaaggaactggcctc
aaagaacaaggagatcgagaaggacaagaagcgtatggacaaggtggagg
atgaactgaaggagaagaagaaggagctgggcaaaatgatgcgggagcag
cagcagattgagaaggagatcaaggagaaggactcagaattgaaccagaa
gcggcctcagtacatcaaagccaaggagaacacctcccacaaaatcaaga
agctggaagcagccaagaagtctctgcagaatgctcagaagcactacaag
aagcgtaaaggtgacatggatgagctggagaaggagatgctgtcagtgga
gaaggctcggcaggagtttgaagaacggatggaagaagagagtcagagtc
agggcagagatttgacgttggaggagaatcaggtgaagaaataccaccgg
ttgaaagaagaagccagcaagagagcagctaccctggcccaggagctgga
gaaattcaatcgagaccagaaagctgaccaggaccgtctggatctggaag
aacggaagaaagtagagacagaggccaagatcaagcaaaagctgcgggaa
attgaagagaatcagaagcggattgagaaactggaggaatacatcaccac
tagcaagcagtccctagaagagcagaagaagctagagggggagctgacag
aggaggtggagatggccaagcggcgtattgatgaaatcaataaggagctg
aaccaggtgatggagcagctaggggatgcccgcatcgaccgccaggagag
cagccgccagcagcgaaaggcagagataatggaaagcatcaagcgccttt
accctggctctgtgtacggccgcctcattgacctatgccagcccacacaa

aagaagtatcagattgctgtaaccaaggttttgggcaagaacatggatgc
cattattgtggactcggagaagacaggccgggactgtattcagtatatca
aggagcagcgtggggagcctgagaccttcttgcctcttgactacctggag
gtgaagcctacagatgagaaactccgggagctgaaggggccaagctagt
gattgatgtgattcgctatgagccacctcatatcaaaaaggccctgcagt
atgcttgtggcaatgcccttgtctgtgacaacgtggaagatgcccgccgc
attgcctttggaggccaccagcgccacaagacagtggcactggatggaac
cctattccagaagtcaggagtgatctctggtggggccagtgacctgaagg
ccaaggcacggcgctgggatgagaaagcagtagacaagttgaaagagaag
aaggagcgcttgacagaggagctgaaagagcagatgaaggcaaaacggaa
agaggcagagctgcgtcaggtgcagtctcaggcccatggactgcagatgc
ggctcaagtactcccagagtgacctagaacagaccaagacacgacatcta
gccctgaatctgcaggaaaaatccaagctggagagtgagctagccaactt
tgggcctcgcattaatgatatcaagaggatcattcagagccgagagaggg
aaatgaaagacttgaaggagaagatgaaccaggtagaggatgaggtgttt
gaagagttttgtcgggagattggtgtgcgcaacatccgggagtttgagga
agaaaaggtgaaacggcagaatgaaatcgccaagaagcgtttggagtttg
agaatcagaagactcgcttgggcattcagttggattttgaaaagaaccaa
ctgaaggaggaccaagataaagtacacatgtgggagcagacagtgaaaaa
agatgaaaatgagatagaaaagctcaaaaaggaggaacaaagacacatga
agatcatagatgagaccatggctcagctacaagacctgaagaatcagcat
ctggccaagaagtcggaagtgaatgacaagaatcatgagatggaggagat
tcgtaagaaactcgggggcgccaacaaggaaatgacccatttacagaagg
aggtgacagccattgagaccaagcttgaacagaagcgcagtgaccgtcac
aacttgctacaggcctgtaagatgcaggacattaagttgccactgtcaaa
aggcaccatggatgatattagtcaggaagagggtagctcccagggggagg
actcagtgagtggttcacagagaatttccagtatctatgcacgagaggcc
ctcattgagattgactacggtgatctgtgtgaggatctgaaggatgccca
ggctgaggaagagatcaagcaagagatgaacacactgcagcagaagctga
atgagcagcagagtgtgcttcagcgtattgccgcccccaacatgaaggcc
atggaaaagctggaaagtgtccgagacaagttccaggagacctcagatga
gtttgaagcagcccgaaagcgagcaaagaaggccaagcaggcattcgaac
agatcaagaaggagcgctttgaccgcttcaatgcttgttttgaatctgtg
gctaccaacattgatgagatctataaggccctgtcccgcaatagcagtgc
ccaggcattcctgggccctgagaaccctgaagagccctacttggatggca
tcaactacaactgtgtggctcctgggaaacgcttccggcctatggacaac
ttgtcaggcggggagaagacagtggcagctctggccctgctctttgccat
ccacagctacaagccagccccttcttcgtcctggatgagattgatgctg
ccttggataacaccaacattggcaaggtggcaaattacatcaaggagcag
tcgacttgcaacttccaggccatcgtcatctctctcaaggaggagttcta
caccaaggccgagagcctcattggagtctatcctgagcaaggggactgtg
tgatcagcaaagtcctgaccttcgacctcaccaagtacccagatgccaac
cccaaccccaatgagcagtagcagtatttttgccctcccgccctgtctgg
atccctaagctgtccctctcccaatctctggatatttgactcccaacctt
ccccctacctcctggccctttttggtgtagtcatgggatttaggcactgc
taatcaagcatgaagaggaacagaggtgatgttaggtctggagcaaaaat
tcctgaacgacagggagtattctggcctctgaaaggaggtgctgagctga
acagggccatctgttcatcacacacaccccccttcctccccctcatcaccc
ataatcgtgggccccttgggcctcttgcccactgtgtgtgtgggtatgta

tgtgtgtatgtatgtatccgcatgtgtgcatgtgagtatgtttgcaaaat
aataaaggatattggagacctgttttagaaggagcctaggctgaatttga
ttccaagagagcttaggatgacagcacccctgagctgggcaaaggtactc
aggacctcataggagtcttaggcagttacctgaaactgccttcattcact
catttgtgtattcattcatttatgtattcatcagacacataccgaacacc
ctctatttgtcaggctctgtgcttggaatacagagttgaatcagacatga
tctctaccctcctagtaaggagatacagtgggttcatgaatgactatagt
tagctgaatgtcatatgtactttgaatttgagaagtgggtgatcccctct
aggcttcctggaggtcacatttaagctagaccttgacaaattggtaggat
ttggtcaggcactaggagtggagcatgagctctggggacagacagttatg
ggttctggtcccactttttatcacttactagttgtttgaccttgggcaag
tcatttgaccttctgtgcctcagtttcctcatctgtaaaatggggctaac
aatattacctacctcataggatttaatgatgtcaagctcctcactggagg
ccttatcccttcgtggagcccactaggtgccgacccctcagaatataacc
ctcatgcctggacccctgagagcttctgatcccagctattagggacagaa
gaagcctccaaatctggaaggtgctgaatgccctgctgactgggaaagtt
tcagggcactgatggggtctacctggtaagcggagggcctgaggaaacct
gtagcttcaatcatgtctggtaaccgggtgcctgagccccaatctgggtt
gtgaggaaataggggagaggtatcctgggccacatcccagcctaacacct
gtgaggttcattttaggaactaacctcattagctataaggatcatgcaga
ggcagcaaagccgggtgcgatgagctcagcctttactcattcacatacac
catcacactttaattccaatctgtatattgctttttaaaagttaagtcca
ttctaattacccaaatatgcatgaattcattctccttttgagaagttaga
ttgttaaagatagtctcattcagctaccaaccactccttgatccttccct
tcttagtggctgttgtttgttgtacttccgtttagactttgttttaatgc
ttgtacgtacatatgtgaactcattggaaatattgtgtgtttaatgcaaa
tgatatattgaattgtttagcaatttgttttctttgcttaacgatgtttt
tgagatctgtgcatgttacttaatgtagctcaatccatcttctgtaattg
ctgtatagattgtcatcatatgattaccacattttacttacgcatttctt
ttgtgatggacattaagactgtttttaggttttgctattacaaaatacta
cacaggagcatcactatgcctgtgtgaaagtatatgtatgaaagtttacc
tagggttgattcctagaagtggaattgcaaagtcataggatatttatata
ttggtttttaataatacttccaaattgccctcctgtactatttactcagt
atttttcttgaggttgatctgaggtctaacattgttatcctatatcattt
tcatcccaagtagtgatatctgtgaaatcacaggtttgatgtgtgctaat
tatgtattcttctaatacatattaaaagacataactatcaaaacaaaata
aatttgtctgttttcaaccaaagaagtcacgtaccactggtggtactgtg
tgccataatttggcaaatgctggcctttatggacgagcacaattcggggg
tcagacctggttcaaattctagctgtagaaacttgtgcaagttacttcac
ctctgagcctaagtttccacatctgtaaaaggagataataaacacctacc
ttgcagtagtgaagcaaagagaaaattaaatatatatgaagcaatttggc
tggcatctagatcattcacagccctttaaaggtcacctttgctgttctcc
ccactttacagataaggaaactgaggcccaaaaaggtttgaacccaggtc
ttccaagtcattcaagtgctttctccactgtacaggtggttatcaacctt
ggctgcgcatcagaatcgtttgtaaagctttttctttttcctttttaaaa
agtaaagcaatatatacacaggtaaaaaaataaaatagtacagaagggct
tataatgagaagcagcagttccctgcttgcacccccacatccaaaggatg
tggagctctttaaaaataaattgctctggtcccacctctggaaatctgat
tcagccagcatggataataacccagataactaacccctacctcacaggat

aaaaaggattacatgagatgccttaggctaaggccctggcacacaggaac
acatgtgctacaaaggagctttggggacttaagtcctgaggatccaggag
gtgaggtgacttgtccaagattccactggtttagtggcagagcctagact
tccactcggatctatttagtgcttgccccctgctctctcctgtcgtgccc
caccacctcctggcatcacagggcaaccgttgtcaaggctatgctcacgg
gaggctgggcaccacagtgtttccaagagcaagctggatccgagtagatt
ccctagggcttgttggaggaactagtttgactcccttatactgtggacgc
agtagccttgctgtagggagttgaagagtactccacaacagtatcttaag
tttaactgggcacttccctctggaaatcacagtgttgtgcaccaggaaca
caaagatgagtcaaatctttatcctgcctttgaggagctcactgtttagt
tggggaaaccatttgtaaaacagccattaaccatacagtgtgatcaacac
tgacaggagcacaggaaaaacatctagcttatgtgaagattcagagaagg
catcctgtagtctaggtggtgatacctgaactgagtcttgagggacgggt
aggaattagccagttgaggaagtagaaggaatttccagatattggaaaca
gtatgcatgaagacatgaaggcaagaaacagcaaaacaaatactgaagca
tgaagattcctggggtggggggaaagcagcaagaaaaggtagagaggaac
cagattggaagagggtcgtaaatgcatggctacagaattcagatttgttt
tgtaggacagtgtggttcccaaactggctgtataccacaaacaggtacgg
cattctgggccccggccccctaaaacattcattaagtctggggtgaagatt
tggaatcttgaatgcttataaaggttaccacatgactagggtacagccag
atttggaaaccatagcttgaaggcagtgagggagccatgaaatggttttt
aatagggggactccagatcagatgtgaacttaacctgtttctggctggct
agccaaccagcatggaaaacagattaggttagatgttcatgctgtatgtg
cccgtgcctgtagcttccctgttaatcagcttcttacactactatatttg
cttattttgtctctgaataagctttaggcaccacaagggtgggcctgggg
atattttgcttaccagtatagcccctgcaaaaaagcacagtgcctgacac
aaaacaggcacccagtaaagtttttgaatgaatgaatgcatgagtgaatc
catttgtgagagagcgaatggagatgacaagattagctaggagactggaa
aaagaccaggaggcctgcactagggcaaaggccagtaggaatagattgga
ggtgttaaggtgtgaactgttaaggtaagatgataacttaatgactgatt
attggatgtggagggtgactgagaggatagaatgagtacccatgaatagc
catgattcctaccctgtcccagtcatctctttccttatccatctctgaaa
caatctgcttacatcctcctcagcaactggaattcctcaagttagttaga
cattctgtgtgctgtgtggtctctcactgcccccccactccccacccctc
cacaagccattgattcattcatccagttcaataaatcttggctaagcacc
tccagtgtgcagtaaggctcttccaagccaggactctgactccctctttc
ctacctcaagagatgtttttgagggctttcccaggtaagagtcacatctc
ttatacaataacttatagtgagatacccagaatgtcagacttgtaaggga
agactgcccaaacccccttctgaggtcctcagaggggaattaacttcctaa
ggtccgactgctaggaagtgttggagccagaaatggaacctaggtttcct
ttctatgtcatctctggagtcttgatcttgatctatcccattgtagatca
ggacaggcagaggtggtcagggagaaggtgggacttaggttgaaccttga
aggtcaatgtattggacaggtcaaacaagatggttgccaattacactgcc
cccttctggaaacccttagcaaacctgccatgcttgcagtcccttctaag
gggtttccttagcataagttgccatgctctgtaccatgtgacctcacaat
cctggccacagatagctagatgtggatagtgtctggttcaagggcaacca
atctctaggctggccagtggcctgttagctggactggcataaggacttca
ccttacaggggtggcatgtatcaaatggcaaatgtatgaaacaaccagat
ctttcagggaggcagaatgtgagctattcagaagaagtgaacgttaatta

gaatttaatgaggcattagtggtggtggatgaggggtggccagaaactaa
acagcaaaagcaaagagaaagctgcagaaaccataagtaagcagaggtca
tgagacatttgtataatgagatcacggagccacagggtggcagaagccat
gaagcagcaaggcaacaatgggctagaagccatgaagcaataggagccac
gaggaacagaaaccgtgagacaaaactgactatgagatccacaaagcagc
agaaggcttgaatagataagatcatgagacagtagaagcgatgagactgc
aagaaccacaaggtagccagaaccatgtggcaacatggcaacaggaatgg
aagaggcagcaggagctacaatgcagaaaagccatggattaataggaact
gaagcgccgggagccatgaagctgcaggacccatgaggcagaaaaagcca
tgggctagcatcgagggggcagaaagaagttagtcagtagcagtaggag
gagtataaatacagccagaaaggagttgagtcaccaatttgggaagcact
agagaagggagcaacagatgcctgcagctgaggggtgacaagataagcc
aggctctagagctgctttggatcatgaaccattttcaagtttctgttctt
ccatgaggctgcctgtgtagctgttcttgtcttccttatttccctgtgaa
tgctttaataaatccccatcactaa
>NM_006546 3
atttagaggcggcgccagggcggccgcggagaaacgtgacacaccagccc
tctcggaggggtttcggaccgaagggaagaagctgcgccgtgtcgtccgt
ctccctgcgcgccgcgggcacttctcctgggctctccccgaactctcccg
cgacctctgcgcgccctcaggccgccttccccgccctgggctcgggacaa
cttctggggtggggtgcaaagaaagtttgcggctcctgccgccggcctct
ccgcctcttggcctaggaggctcgccgcccgcgcccgctcgttcggcctt
gcccgggaccgcgtcctgccccgagaccgccaccatgaacaagctttaca
tcggcaacctcaacgagagcgtgacccccgcggacttggagaaagtgttt
gcggagcacaagatctcctacagcggccagttcttggtcaaatccggcta
cgccttcgtggactgcccggacgagcactgggcgatgaaggccatcgaaa
ctttctccgggaaagtagaattacaaggaaaacgcttagagattgaacat
tcggtgcccaaaaaacaaaggagccggaaaattcaaatccgaaatattcc
accccagctccgatgggaagtactggacagcctgctggctcagtatggta
cagtagagaactgtgagcaagtgaacaccgagagtgagacggcagtggtg
aatgtcacctattccaaccgggagcagaccaggcaagccatcatgaagct
gaatggccaccagttggagaaccatgccctgaaggtctcctacatccccg
atgagcagatagcacagggacctgagaatgggcgccgagggggctttggc
tctcggggtcagccccgccagggctcacctgtggcagcgggggccccagc
caagcagcagcaagtggacatccccccttcggctcctggtgcccacccagt
atgtgggtgccattattggcaaggagggggccaccatccgcaacatcaca
aaacagacccagtccaagatagacgtgcataggaaggagaacgcaggtgc
agctgaaaaagccatcagtgtgcactccacccctgagggctgctcctccg
cttgtaagatgatcttggagattatgcataaagaggctaaggacaccaaa
acggctgacgaggttcccctgaagatcctggcccataataactttgtagg
gcgtctcattggcaaggaaggacggaacctgaagaaggtagagcaagata
ccgagacaaaaatcaccatctcctcgttgcaagaccttaccctttacaac
cctgagaggaccatcactgtgaagggggccatcgagaattgttgcagggc
cgagcaggaaataatgaagaaagttcgggaggcctatgagaatgatgtgg
ctgccatgagcctgcagtctcacctgatccctggcctgaacctggctgct
gtaggtcttttcccagcttcatccagcgcagtcccgccgcctcccagcag
cgttactggggctgctccctatagctcctttatgcaggctcccgagcagg
agatggtgcaggtgtttatccccgcccaggcagtgggcgccatcatcggc
aagaaggggcagcacatcaaacagctctcccggtttgccagcgcctccat

caagattgcaccacccgaaacacctgactccaaagttcgtatggttatca
tcactggaccgccagaggcccaattcaaggctcagggaagaatctatggc
aaactcaaggaggagaacttctttggtcccaaggaggaagtgaagctgga
gacccacatacgtgtgccagcatcagcagctggccgggtcattggcaaag
gtggaaaaacggtgaacgagttgcagaatttgacggcagctgaggtggta
gtaccaagagaccagacccctgatgagaacgaccaggtcatcgtgaaaat
catcggacatttctatgccagtcagatggctcaacggaagatccgagaca
tcctggcccaggttaagcagcagcatcagaagggacagagtaaccaggcc
caggcacggaggaagtgaccagcccctccctgtcccttcgagtccaggac
aacaacgggcagaaatcgagagtgtgctctccccggcaggcctgagaatg
agtgggaatccgggacacctgggccgggctgtagatcaggtttgcccact
tgattgagaaagatgttccagtgaggaaccctgatctctcagccccaaac
acccacccaattggcccaacactgtctgcccctcggggtgtcagaaattc
tagcgcaaggcacttttaaacgtggattgtttaaagaagctctccaggcc
ccaccaagagggtggatcacacctcagtgggaagaaaaataaaatttcct
tcaggttttaaaaacatgcagagaggtgttttaatcagccttaaaggatg
gttcatttcttgaccttaatgtttttccaatcttcttcccccctacttggg
taattgattaaaatacctccatttacggcctctttctatatttacactaa
ttttttatctttattgctaccagaaaaaaaatgcgaacgaatgcattgct
ttgcttacagtattgactcaagggaaaagaactgtcagtatctgtagatt
aattccaatcactccctaaccaataggtacaatacggaatgaagaagagg
ggaaaatggggagaaagatggttaaaatacataataatccacgtttaaaa
ggagcgcacttgtggctgatctatgccagatcaccatcttcaaattggca
caactgaaatttccccactctgttggggcttccccaccacattcatgtcc
ctctcccgtgtaggtttcacattatgtccaggtgcacataggtggtattg
aatgctcagcagggtaggggctgaccactgtccctgattcccatcgttct
caggcggattttatattttttaaagtctattttaatgattggatatgag
cactgggaaggggacgctaactccccttgataaagtctcggttccatgga
ggacttgagtggccccaaaggctgccacggtgccctcaccccagcccatg
tgctcccataagggctggttcctagaggcaggggttgtggggcactccca
gccacggcactgttaccttggtggtgggacttggaacccaaccctgagct
cccgataaagctaaagtccatcatctggcaaattcagtaaattggagagt
acttgcttctgtttgtatctgagaggaatttttaactgacggcttctgtc
tccatgaatcattatcagcatgatgaaaggtgtgtctaaaaaacaattca
gaataccagcagcattgtacagcaaggggtaaataagcttaatttattaa
tttaccaggcttaattaagatcccatggagtgtttagcccttgtgggaga
cagaagccatcagttaaatgaggttaggcctctcctcctaatatactgat
tgacaatgcatattagccaggtaatgcactttagctaccctggacaatgc
tatcaagtgtgctgggaagggaggaaggcctctctacatatggaaaagcc
catgcgtggagttcccctcctttcaacattgcaacaacagtaacaacaag
acaaccgcaacatgtgggcgtagtcaggcaatgctgtgtgcgaagtaaac
tacctcaaggtatgaagttacctcagcaattattttcctttttgttcccc
ccaaccccattaaaaaaattttttttgatttttgtttttttgcagcttg
ctgatattttatataaaaaagaaaagcaaagcaaaagagaagctgatagt
cttgaatattttattttttaatgaaaagaaaaaacaagaaagttatgtt
tcataatttcttacaacatgagccagtaaccctttaggaactctctatgg
agaacaggcctggtgggaaaggctttgggggctgccccccttaggaggagg
ctagtgctaagagggaaggcccaggtttgagagagcccagaggggcagag
cccagagccttgtttggccctgatctctgacttctagagccccagctgct

ggcggctgctggaatatcctacctgataggattaaaaggcctagtggagc
tgggggctctcagtggttaaacaatgcccaacaaccaaccagctggccct
tggtctcctctctttcctcctttggttaaagagcatctcagccagctttt
cccaccagtggtgctgttgagatattttaaaatattgcctccgttttatc
gaggagagaaataataactaaaaaatataccctttaaaaaaacctatatt
tctctgtctaaaaatatgggagctgagattccgttcgtggaaaaaagaca
aggccaccctctcgccctcagagaggtccacctggtttgtcattgcaatg
cttttcattttttttttttgttattgtttcatttcagttccgtcttgcta
ttcttcctaatctatatccatagatctaaggggcaaacagatactagtta
actgcccccacctctgtctccctgtcttctttagatcggtctgattgatt
ttaaaagtggacccaaacttagggaattcttgatttagggtggctggtgg
caaggaggggcaggggatatggggacgtgactgggacaggttcctgcctt
atcattttctccctaggacattcccttgtagcccccagaattgtctggcc
caaattgaatagaagcagaaaaacatttagggataacatcaggccagtag
aattaagcctctccacctgtcccaaccataaaaagggtctcccagctttc
catctctggctctatatgctttatcccaaaacaaagcagataacgttcag
acgtcggccatttagtaatttaaagcgaatttccagcagcaagcatgctt
tgatatctggttcagactatcatcaggaagaaaaaaaaatcccacagtac
ctgaaatgtgattgttgcagtgttcagtttccttgggggcctgctccctt
cacaccttgagcccaagtccttttccgttggctgattcagctcccagaag
agacgaggaagtgtgtggcaagggactggaaaacttcacttgcttggatt
aggcaaggctccactcattgttgatatttgcccagcaggaaaatcatgta
agttataccaccagaaagcaaaaggagcatggtttggtggttaaggttta
gtgggatgaaggacctgtcttggtgggccgggccctcttgtgccccgtag
gctaggtcttagggcaactccttgccctcctgctcagcacctccatttcc
ccatccttggtgagataacaagctatcgcgaaaagcacttgggagatttg
gatgatttgagaagagtgacttaaaaaaaatgcttctgtgctctaagata
tatatgtgtgtgtgtgtgctacatatatatttttaagaaaggaccatctc
tttaggatatatttttaaattctttgaaacacataaccaaaatggtttga
ttcactgactgactttgaagctgcatctgccagttacaccccaaatggct
ttaatcccctctcgggtctggttgccttttgcagtttgggttgtggactc
agctcctgtgaggggtctggttaggagagagccatttttaaggacaggga
gttttatagcccttttctactttcctcccctcctcccagtccttatcaat
cttttttcctttttcctgacccccctccttctggaggcagttgggagctat
ccttgtttatgcctcactattggcagaaaagaccccatttaaaacccaga
gaacactggagggggatgctctagttggttctgtgtccattttcctctgt
gccaaagacagacagacagaggctgagagaggctgttcctgaatcaaagc
aatagccagctttcgacacatacctggctgtctgaggaggaaggcctcct
ggaaactgggagctaagggcgaggcccttcccttcagaggctcctggggg
attagggtgtggtgtttgccaagccaaggggtagggagccgagaaattgg
tctgtcggctcctggttgcactttggggaaggagaggaagtttggggctc
caggtagctccctgttgtgggactgctctgtcccctgcccctactgcaga
gatagcactgccgagttcccttcaggcctggcagacgggcagtgaggagg
ggcctcagttagctctcaagggtgccttcccctcctcccaacccagacat
accctctgccaaactgggaaccagcagtgctagtaactacctcacagagc
cccagagggcctgcttgagccttcttgctccacaggagaagctggtgcct
ctaggcaacccccttcctcccacctctcatcaggggtgggggttctccttt
ctttcccctgaagtgtttatggggagatcctagtggctttgccattcaaa
ccactcgactgtttgcctgtttcttgaaaaccagtagaagggaaacagca

cagcctgtcacagtaattgcaggaagattgaagaaaaatcctcatcaatg
ccaggggacataaaagccatttcccttccaaatactcgacaatttagatg
cagaacatttctctgtattcagacttagagtaacaccagctgaaaactgc
agtttctttcctttggatacataaggcttctctatcggggtacgggacag
ggaggaggcctcatgtctgaagggggatttaggggcgagagccccagccc
tgaccctcggtcctgtgcaccgctttggggcacagtctgatggcgcccttt
gctggcgccttagtatggttgactccggatggacaaaagaaaaaaaattt
tttttcttgaatgaaatagcaggaagctcctcgggagcatgtgttttgat
taaccgcaggtgatggatgctacgagtataaatggattaactacctcaat
ccttacagtaagattggaactaagggcagggactcatgcataagggtatg
aatcccagccaggacaagtgagttgaggcttgtgccacaaaaggtttgtc
cttggggaacaggcaggcctgccaggatccccccccatatcgattgggctg
ggagggctggccatgaggtccccactttctgctttccttgcccatgtgtc
accccctttggcctccagcttgtccctctctcactttctatagctttgttg
gaccagatggtgaggaaaggaatggcctcttcccttctagagggggctgg
ctggagtgagacctggggcttggcctggaacccaccacacagccccaaag
tcaggaagcctggggaaaccagagctgagacctcttcaacagggtttctt
tgagatcctacacctccattgggcccttttttcagtcttcaatgggggccc
agttggctctagaaggagaagaggtgaagcaggatcctttgccctggggg
agtctgagggcgcggtccttggactcattcaggccgtctttgtagttggg
ggagttccactgggcgatcccagcccctccccacccaccctctaatggac
ctcctcatagaagccccatttcacttttgttttatctacctcttagcaaa
acaatagataaattaggtagtggcagctccacttgcttaggttagggggg
gaaaaagatttctttttccaaaggaaaaaaatattaccttgagaatactt
tccaaaaaataaaattaaaaaaaaaaaaaaccaaaaaaaaaaaatttttttt
taaaagggagacattttccagtgaccactggattgttttaatttcccaag
ctttttttttcccccataaataagtttcactctttggcgattttcttcact
tgtttaagataacgtgctagctattccaacaggtaacagctttcacagtc
tgcccctggcctgtctcaccccatcccccaccctattcctgccagtgagt
ccttcctgtgcttctctcccttctcccctcccagccagctgacttcagtc
acccctgtcccccctcccctgccaataagctcccccaggaataaaggctt
tgttttggggatgcttaaatcttgactggcacttcccggctgtgggggct
ggggagccacttgtaacatttctgtgcagattttatgttagccactgcta
tgtaaaagcacgttcaaaatgaatttcagcagattatgtgttaccataat
gaataaacgtcctctatcaccatttggagtctcccttttctccaggatct
tgatcctggtccccaaaaccagagtgaatcaaaagagcttcctcccctga
ggcaaagtggatttgtaagcagttctgaaacatcacttactcagaagagg
gaacgatgtattttgatgagtgcaaattgggaagagctggaggcctactg
cttgggacagttttttttttttttttttttttaaatatgagtgctagctt
attctgtaattgcggcaactttgaaaattgtattttactggaaatctgcc
agccatcaccacccgattttgattgtatccttcctcccatcctttaatct
gttcattgctttgggggaggtggggcagctggctcacacgttggagtttg
ttctttgatggatgaacgaacactccagttttctttcccgtgaaggttgt
ttcagccacaaaccacttcattttgctgtttcaatttcaaaataaaagga
aacttatattgaaagacaa
>NM_005378 4
gtcatctgtctggacgcgctgggtggatgcggggggctcctgggaactgt
gttggagccgagcaagcgctagccaggcgcaagcgcgcacagactgtagc
catccgaggacacccccgcccccccggcccacccggagacacccgcgcag

aatcgcctccggatcccctgcagtcggcgggagtgttggaggtcggcgcc
ggcccccgccttccgcgccccccacgggaaggaagcacccccggtattaa
aacgaacggggcggaaagaagccctcagtcgccggccgggaggcgagccg
atgccgagctgctccacgtccaccatgccgggcatgatctgcaagaaccc
agacctcgagtttgactcgctacagccctgcttctacccggacgaagatg
acttctacttcggcggccccgactcgacccccccgggggaggacatctgg
aagaagtttgagctgctgcccacgcccccgctgtcgcccagccgtggctt
cgcggagcacagctccgagcccccgagctgggtcacggagatgctgcttg
agaacgagctgtggggcagcccggccgaggaggacgcgttcggcctgggg
ggactgggtggcctcacccccaacccggtcatcctccaggactgcatgtg
gagcggcttctccgcccgcgagaagctggagcgcgccgtgagcgagaagc
tgcagcacggccgcgggccgccaaccgccggttccaccgcccagtccccg
ggagccggcgccgccagccctgcgggtcgcgggcacggcggggctgcggg
agccggccgcgccggggccgccctgcccgccgagctcgcccacccggccg
ccgagtgcgtggatcccgccgtggtcttcccctttcccgtgaacaagcgc
gagccagcgcccgtgcccgcagccccggccagtgccccggcggcgggccc
tgcggtcgcctcgggggcgggtattgccgccccagccggggccccggggg
tcgcccctccgcgcccaggcggccgccagaccagcggcggcgaccacaag
gccctcagtacctccggagaggacaccctgagcgattcagatgatgaaga
tgatgaagaggaagatgaagaggaagaaatcgacgtggtcactgtggaga
agcggcgttcctcctccaacaccaaggctgtcaccacattcaccatcact
gtgcgtcccaagaacgcagccctgggtcccgggagggctcagtccagcga
gctgatcctcaaacgatgccttcccatccaccagcagcacaactatgccg
cccccctctccctacgtggagagtgaggatgcacccccacagaagaagata
aagagcgaggcgtccccacgtccgctcaagagtgtcatccccccaaaggc
taagagcttgagcccccgaaactctgactcggaggacagtgagcgtcgca
gaaaccacaacatcctggagcgccagcgccgcaacgaccttcggtccagc
tttctcacgctcagggaccacgtgccggagttggtaaagaatgagaaggc
cgccaaggtggtcattttgaaaaaggccactgagtatgtccactccctcc
aggccgaggagcaccagcttttgctggaaaaggaaaaattgcaggcaaga
cagcagcagttgctaaagaaaattgaacacgctcggacttgctagacgct
tctcaaaactggacagtcactgccactttgcacattttgattttttttt
aaacaaacattgtgttgacattaagaatgttggtttactttcaaatcggt
cccctgtcgagttcggctctgggtgggcagtaggaccaccagtgtggggt
tctgctgggaccttggagagcctgcatcccaggatgctgggtggccctgc
agcctcctccacctcacctccatgacagcgctaaacgttggtgacggttg
ggagcctctggggctgttgaagtcaccttgtgtgttccaagtttccaaac
aacagaaagtcattccttctttttaaaatggtgcttaagttccagcagat
gccacataaggggtttgccatttgataccccctggggaacatttctgtaaa
taccattgacacatccgccttttgtatacatcctgggtaatgagaggtgg
cttttgcggccagtattagactggaagttcatacctaagtactgtaataa
tacctcaatgtttgaggagcatgttttgtatacaaatatattgttaatct
ctgttatgtactgtactaattcttacactgcctgtatactttagtatgac
gctgatacataactaaatttgatacttatattttcgtatgaaaatgagtt
gtgaaagttttgagtagatattactttatcactttttgaactaagaaact
tttgtaaagaaatttactatatatatatgcctttttcctagcctgtttct
tcctgttaatgtatttgttcatgtttggtgcatagaactgggtaaatgca
aagttctgtgtttaatttcttcaaaatgtatatatttagtgctgcatctt
atagcactttgaaatacctcatgtttatgaaaataaatagcttaaaatta

aatgaaaaaaaaa
>NM_152998 2
ggcggcgcttgattgggctggggggggccaaataaaagcgatggcgattgg
gctgccgcgtttggcgctcggtccggtcgcgtccgacacccggtgggact
cagaaggcagtggagccccggcggcggcggcggcggcgcgcgggggcgac
gcgcgggaacaacgcgagtcggcgcgcgggacgaagaataatcatgggcc
agactgggaagaaatctgagaagggaccagtttgttggcggaagcgtgta
aaatcagagtacatgcgactgagacagctcaagaggttcagacgagctga
tgaagtaaagagtatgtttagttccaatcgtcagaaaattttggaaagaa
cggaaatcttaaaccaagaatggaaacagcgaaggatacagcctgtgcac
atcctgacttctgtgagctcattgcgcgggactagggaggtggaagatga
aactgttttacataacattccttatatgggagatgaagttttagatcagg
atggtactttcattgaagaactaataaaaaattatgatgggaaagtacac
ggggatagagaatgtgggtttataaatgatgaaatttttgtggagttggt
gaatgcccttggtcaatataatgatgatgacgatgatgatgatggagacg
atcctgaagaaagagaagaaaagcagaaagatctggaggatcaccgagat
gataaagaaagccgcccacctcggaaatttccttctgataaaatttttga
agccatttcctcaatgtttccagataagggcacagcagaagaactaaagg
aaaaatataaagaactcaccgaacagcagctcccaggcgcacttcctcct
gaatgtacccccaacatagatggaccaaatgctaaatctgttcagagaga
gcaaagcttacactcctttcatacgcttttctgtaggcgatgttttaaat
atgactgcttcctacatccttttcatgcaacacccaacacttataagcgg
aagaacacagaaacagctctagacaacaaaccttgtggaccacagtgtta
ccagcatttggagggagcaaaggagtttgctgctgctctcaccgctgagc
ggataaagaccccaccaaaacgtccaggaggccgcagaagaggacggctt
cccaataacagtagcaggcccagcacccccaccattaatgtgctggaatc
aaaggatacagacagtgatagggaagcagggactgaaacggggggagaga
acaatgataaagaagaagaagagaagaaagatgaaacttcgagctcctct
gaagcaaattctcggtgtcaaacaccaataaagatgaagccaaatattga
acctcctgagaatgtggagtggagtggtgctgaagcctcaatgtttagag
tcctcattggcacttactatgacaatttctgtgccattgctaggttaatt
gggaccaaaacatgtagacaggtgtatgagtttagagtcaaagaatctag
catcatagctccagctcccgctgaggatgtggatactcctccaaggaaaa
agaagaggaaacaccggttgtgggctgcacactgcagaaagatacagctg
aaaaaggacggctcctctaaccatgtttacaactatcaaccctgtgatca
tccacggcagccttgtgacagttcgtgcccttgtgtgatagcacaaaatt
tttgtgaaaagttttgtcaatgtagttcagagtgtcaaaaccgctttccg
ggatgccgctgcaaagcacagtgcaacaccaagcagtgcccgtgctacct
ggctgtccgagagtgtgaccctgacctctgtcttacttgtggagccgctg
accattgggacagtaaaaatgtgtcctgcaagaactgcagtattcagcgg
ggctccaaaaagcatctattgctggcaccatctgacgtggcaggctgggg
gatttttatcaaagatcctgtgcagaaaaatgaattcatctcagaatact
gtggagagattatttctcaagatgaagctgacagaagagggaaagtgtat
gataaatacatgtgcagctttctgttcaacttgaacaatgattttgtggt
ggatgcaacccgcaagggtaacaaaattcgttttgcaaatcattcggtaa
atccaaactgctatgcaaaagttatgatggttaacggtgatcacaggata
ggtatttttgccaagagagccatccagactggcgaagagctgttttttga
ttacagatacagccaggctgatgccctgaagtatgtcggcatcgaaagag
aaatggaaatcccttgacatctgctacctcctcccccctcctctgaaaca

gctgccttagcttcaggaacctcgagtactgtgggcaatttagaaaaaga
acatgcagtttgaaattctgaatttgcaaagtactgtaagaataatttat
agtaatgagtttaaaaatcaactttttattgccttctcaccagctgcaaa
gtgttttgtaccagtgaatttttgcaataatgcagtatggtacatttttc
aactttgaataaagaatacttgaacttgtccttgttgaatc
>NM_015640 3
cggatgtgtgcctggcgccggaagagaagacggcccccctctctcggccc
ggccatcttgtgggaagagctgaagcaggcgctcttggctcggcgcggcc
cgctgcaatccgtggaggaacgcgccgccgagccaccatcatgcctgggc
acttacaggaaggcttcggctgcgtggtcaccaaccgattcgaccagtta
tttgacgacgaatcggaccccttcgaggtgctgaaggcagcagagaacaa
gaaaaaagaagccggcgggggcggcgttgggggccctggggccaagagcg
cagctcaggccgcggcccagaccaactccaacgcggcaggcaaacagctg
cgcaaggagtcccagaaagaccgcaagaacccgctgcccccccagcgttgg
cgtggttgacaagaaagaggagacgcagccgcccgtggcgcttaagaaag
aaggaataagacgagttggaagaagacctgatcaacaacttcagggtgaa
gggaaaataattgatagaagaccagaaaggcgaccacctcgtgaacgaag
attcgaaaagccacttgaagaaaagggtgaaggaggcgaattttcagttg
atagaccgattattgaccgacctattcgaggtcgtggtggtcttggaaga
ggtcgagggggccgtggacgtggaatgggccgaggagatggatttgattc
tcgtggcaaacgtgaatttgataggcatagtggaagtgatagatctggcc
tgaagcacgaggacaaacgtggaggtagcggatctcacaactggggaact
gtcaaagacgaattaactgacttggatcaatcaaatgtgactgaggaaac
acctgaaggtgaagaacatcatccagtggcagacactgaaaataaggaga
atgaagttgaagaggtaaaagaggagggtccaaaagagatgactttggat
gagtggaaggctattcaaaataaggaccgggcaaaagtagaatttaatat
ccgaaaaccaaatgaaggtgctgatgggcagtggaagaagggatttgttc
ttcataaatcaaagagtgaagaggctcatgctgaagattcggttatggac
catcatttccggaagccagcaaatgatataacgtctcagctggagatcaa
ttttggagaccttggccgcccaggacgtggcggcaggggaggacgaggtg
gacgtgggcgtggtgggcgcccaaaccgtggcagcaggaccgacaagtca
agtgcttctgctcctgatgtggatgacccagaggcattcccagctctggc
ttaactggatgccataagacaaccctggttcctttgtgaacccttctgtt
caaagcttttgcatgcttaaggattccaaacgactaagaaattaaaaaaa
aaaagactgtcattcataccattcacacctaaagactgaattttatctgt
tttaaaaatgaacttctcccgctacacagaagtaacaaatatggtagtca
gttttgtatttagaaatgtattggtagcagggatgttttcataattttca
gagattatgcattcttcatgaatacttttgtattgctgcttgcaaatatg
catttccaaacttgaaatataggtgtgaacagtgtgtaccagtttaaagc
tttcacttcatttgtgtttttttaattaaggatttagaagttcccccaatt
acaaactggttttaaatattggacatactggttttaatacctgctttgca
tattcacacatggtcaactgggacatgttaaactttgatttgtcaaattt
tatgctgtgtggaatactaactatatgtattttaacttagttttaatatt
ttcatttttggggaaaaatctttttcacttctcatgatagctgttatat
atatatgctaaatctttatatacagaaatatcagtacttgaacaaattca
aagcacatttggtttattaacccttgctccttgcatggctcattaggttc
aaattataactgatttacattttcagctatatttactttttaaatgcttg
agtttcccattttaaaatctaaactagacatcttaattggtgaaagttgt
ttaaactacttattgttggtaggcacatcgtgtcaagtgaagtagtttta

taggtatgggtttttctccccttcaccagggtgggtggaataagttga
tttggccaatgtgtaatatttaaactgttctgtaaaataagtgtctggcc
atttggtatgatttctgtgtgtgaaaggtcccaaaatcaaaatggtacat
ccataatcagccaccatttaacccttccttgttctaaaacaaaaaccaaa
gggcgctggttggtagggtgaggtgggggagtattttaatttttggaatt
tgggaagcagacagctttactttgtaaggttggaacagcagcactataca
tgaaatataaaccaaaaacctttactgtttctaaatttcctagattgcta
ttatttggttgtaagttgagtattccacagaaagtggtaattatctcttc
tctcttcctccattagaaaattaggtaaataatggattcctataatggga
gcatcaccacttattaaaacacacatagaatgatgaattaaaaaagtttt
ctaggattgtcttttattctgccacatttattgataaacagtgaaggaat
ttttaaaaaatttttaagaattgtttgtcacgtcatttttagaaatgttc
tacctgtatatggtaatgtccagttttaaaaatattggacatcttcaatc
ttaaacatttctatttagctgattggttctcacatatacttctaaaagaa
acttttatgttataagagttactttttggataagatttattaatctcagt
tacctactattctgacattttaggaaggaggtaattgtttttaatgatgg
ataaacttgtgctggtgttttggatcttatgatgctgagcatgttctgca
ctggtgctaatgtctaatataattttatatttacacacatacgtgctacc
cagagattaatttagtccatatgaactattgacccattgttcattgagac
agcaacatacgcactcctaaatcagtgtgtttagacttttcaagtatcta
actcatttccaaacatgtaccatgttttataaacctcttgatttccagca
acatactatagaaaacacctgctactcaaaacacaacttctcagtgtcat
ccattgctgtcgtgagagacaacatagcaatatctggtatgttgcaagct
ttcaagatagcctgaacttaaaaagttggtgcattagttgtatctgatgg
atataaatttgcctcctagttcactttgtgtcaagagctaaaactgtgaa
cctaactttctcttattggtgggtaataactgaaaataaagatttatttt
catgctcacttcttaaaagtcataaaaacaatcaaataggatcatgttta
ttgtcatgtgtttcctggtttctgacctgtgtgcacacccctgtgtgttt
ataatttttaaattgaattttatatggggtttttatttgctaaaaaccag
gctgttgaatcacatttgggaagggtacttatcttaatgactaatgactt
aattgggaaagttgaattcttgtaaaatacaaaatccaaggacttcttgg
atttaatctgattgtcacttcttagcagatcactttttgataatgaaag
ttaagcatactgaatgctacttttgattgacaaactggctataatagtct
aggggaaaaatccctaaacagataaagattcctaaagtaatggtggcagc
tgatgtttcagtgaacttttatcttgatgcgtttaaatggaagtaatgcc
agacctgagatttttaaggcatttttacagcttgtattgaaatgattgga
gacatggtttctttattagctattttgagacctgtggagttaagcaagac
ttttaaaaattggcaccatatacatctagttagttcctttactcttattt
ttttaaataaaagtagtacacatcatttccagggttgtaaatatatttgg
ggcttgtttttggtatggatttaaaaggaggatattaagtattcattcta
attttgttatttttctagttgccagagatggttgcactgaaatagaacag
ggagttgcatacaaagcctaaatgtgtattggatttcgaaaatactaggt
tggtgcaattggttttgtaccaacctaacatgtctttaggaaagtaccat
catgtggaaggaaacaacaggtgttaaaaggttcaaaggaatgagaaata
ggaagttactagaacctaactgatgttgaccttagaggtaagattattca
ggtatattagtggacctccagtccaatggtatagcaaattccagggatct
caggtgcatgcaattttactttctaaagtaaacacttagaaaatagatta
taacccagacgttttggattatactgagacaaatatgtaaataagtttta
gcaagtctgaacatgtaccagcgagatcttcaggttaactaagaaaagcc

cagaaacttcattatttactgtgctttgtatggcataactggtaacaagg
cagtaaaatgatacatatttgaactggaccatagtaattaaatgatttat
caatatcatttgcaagataattgtcaggttgagttaatagtaagtggcag
cttcccagaaatttgggttatttggcctaagctgtgccctgggattacct
cttcatcttccttgacttttaagttcaaatttggaggttatgtgaagtga
ttgaaataaatctttcaggctgaggaagtcggtaatttcaagaatatagt
gaaaacaaggttgtaatctaaacatgagaagcttaagtttaggaaatggt
tagaatataaattgctaaagccatcatgattttggccacagatgaaaata
tgaacactggaaatgagcgccatttaaatgagatgctgtatgtaagccag
gggtcagcaaagttcagcctgtgacctggtgtgtgtgggggtgtgtgtgt
gtgtgtgtgtgtgtgtgtgtgtgtccgtgtccctgagttaagaatggttt
ttaggcttgtataggattaaaaaaaaaaaaaacccacagaaaaatgtagg
cagcccagcataagataccgcctttcacagaaatgtttgctgaccccgga
actgtcactctcgggttacaagagtttgtttctttgaaacagtctggctc
tgtcacccaggctggagtgcagtggcatgatctcaggtcactgcagcctc
cacctcccgggttcaagcaattctgcctcagcctcctgagtagctgggat
tacaggtgtgcaccaccacagacagctaattttacaggtgtacaccacca
cacccaagaatcacttgaacccgggaggtgggggttgcagtgagccaaga
tcatagcactgtactccagtctgggtgacacactgtctgaaaaataaaag
tttagttctaggtacaaattaagtggtctacccaacaggaagctgtgaaa
ttagaagtaattttaaagcatactttacacctctatataatcaaagtaag
ggcaagataaaacttgttagtaaaacatgttttattatttttagatagg
tagcctgcaacataaaatatatttgaaagctgttaaggggtgtataatc
tgttaaaaaataccatgattagtatttcacatttagtgactcagatccct
ttgtgtaagtcctttgcgttgtgcaaagaggtttttctatgagatgagaa
ttatgaacacttcatataggtaaattccataggttgagctggggcagaaa
ttaatggctcctggagattgcatcttttttttttttttttgagcttaga
attcccatttggacagaatttagcttttgtgctgttgattacttctgtac
taaactggtagccaattgttcacttctaaatggaagggaaaaagttaccc
acacaagttagaaaatgatttaccgaattattttttgagttagtatgta
tatactgttttaaatcagtaatgtttgttccttttgctgcccatttggga
gtatgtggcaattcctagtgctcttgtatgacattactcttcttgagact
tgtatatgcaagaaagtatatataaaagatgcctggtggtaactttgtat
ctgagttctgattcattatggacttttagaaatctatttagataattggt
tcatactaggtgctcaattactctgaccttgtgtggcttaatccttaaat
agttgctttaggtgttactgggtggaagcatggaaagtgaagggctaata
ttaaggatttttatgtattactgagtagggaaagtgggaagtagcttatc
tttggacagttgtattactttgcactaatcaccttgtttgcagttgtagc
tgattcagttctagattttaggaccaaaaagatctctaatttaaagaaac
agtttttatatacctagaatgaagattctgtttaaagcggttttggttaa
cttggttcttgacatttgcctaaaattttgttggattgggactaaaatgt
tctattagccaatgaataacgtccaagtaaatttttgttttattttggga
a
>NM_000321 2
gctcagttgccgggcgggggagggcgcgtccggtttttctcaggggacgt
tgaaattatttttgtaacgggagtcgggagaggacggggcgtgccccgac
gtgcgcgcgcgtcgtcctccccggcgctcctccacagctcgctggctccc
gccgcggaaaggcgtcatgccgcccaaaaccccccgaaaaacggccgcca
ccgccgccgctgccgccgcggaaccccggcaccgccgccgccgcccccct

cctgaggaggacccagagcaggacagcggcccggaggacctgcctctcgt
caggcttgagtttgaagaaacagaagaacctgattttactgcattatgtc
agaaattaaagataccagatcatgtcagagagagagcttggttaacttgg
gagaaagtttcatctgtggatggagtattgggaggttatattcaaaagaa
aaaggaactgtggggaatctgtatctttattgcagcagttgacctagatg
agatgtcgttcacttttactgagctacagaaaaacatagaaatcagtgtc
cataaattctttaacttactaaaagaaattgataccagtaccaaagttga
taatgctatgtcaagactgttgaagaagtatgatgtattgtttgcactct
tcagcaaattggaaaggacatgtgaacttatatatttgacacaacccagc
agttcgatatctactgaaataaattctgcattggtgctaaaagtttcttg
gatcacatttttattagctaaaggggaagtattacaaatggaagatgatc
tggtgatttcatttcagttaatgctatgtgtccttgactattttattaaa
ctctcacctcccatgttgctcaaagaaccatataaaacagctgttatacc
cattaatggttcacctcgaacacccaggcgaggtcagaacaggagtgcac
ggatagcaaaacaactagaaaatgatacaagaattattgaagttctctgt
aaagaacatgaatgtaatatagatgaggtgaaaaatgtttatttcaaaaa
ttttataccttttatgaattctcttggacttgtaacatctaatggacttc
cagaggttgaaaatctttctaaacgatacgaagaaatttatcttaaaaat
aaagatctagatgcaagattatttttggatcatgataaaactcttcagac
tgattctatagacagttttgaaacacagagaacaccacgaaaaagtaacc
ttgatgaagaggtgaatgtaattcctccacacactccagttaggactgtt
atgaacactatccaacaattaatgatgattttaaattcagcaagtgatca
accttcagaaaatctgatttcctattttaacaactgcacagtgaatccaa
aagaaagtatactgaaaagagtgaaggatataggatacatctttaaagag
aaatttgctaaagctgtgggacagggttgtgtcgaaattggatcacagcg
atacaaacttggagttcgcttgtattaccgagtaatggaatccatgctta
aatcagaagaagaacgattatccattcaaaattttagcaaacttctgaat
gacaacatttttcatatgtctttattggcgtgcgctcttgaggttgtaat
ggccacatatagcagaagtacatctcagaatcttgattctggaacagatt
tgtctttcccatggattctgaatgtgcttaatttaaaagcctttgatttt
tacaaagtgatcgaaagttttatcaaagcagaaggcaacttgacaagaga
aatgataaaacatttagaacgatgtgaacatcgaatcatggaatcccttg
catggctctcagattcacctttatttgatcttattaaacaatcaaaggac
cgagaaggaccaactgatcaccttgaatctgcttgtcctcttaatcttcc
tctccagaataatcacactgcagcagatatgtatctttctcctgtaagat
ctccaaagaaaaaaggttcaactacgcgtgtaaattctactgcaaatgca
gagacacaagcaacctcagccttccagacccagaagccattgaaatctac
ctctctttcactgttttataaaaaagtgtatcggctagcctatctccggc
taaatacactttgtgaacgccttctgtctgagcacccagaattagaacat
atcatctggacccttttccagcacaccctgcagaatgagtatgaactcat
gagagacaggcatttggaccaaattatgatgtgttccatgtatggcatat
gcaaagtgaagaatatagaccttaaattcaaaatcattgtaacagcatac
aaggatcttcctcatgctgttcaggagacattcaaacgtgttttgatcaa
agaagaggagtatgattctattatagtattctataactcggtcttcatgc
agagactgaaaacaaatattttgcagtatgcttccaccaggccccctacc
ttgtcaccaatacctcacattcctcgaagcccttacaagtttcctagttc
acccttacggattcctggagggaacatctatatttcacccctgaagagtc
catataaaatttcagaaggtctgccaacaccaacaaaaatgactccaaga
tcaagaatcttagtatcaattggtgaatcattcgggacttctgagaagtt

ccagaaaataaatcagatggtatgtaacagcgaccgtgtgctcaaaagaa
gtgctgaaggaagcaaccctcctaaaccactgaaaaaactacgctttgat
attgaaggatcagatgaagcagatggaagtaaacatctcccaggagagtc
caaatttcagcagaaactggcagaaatgacttctactcgaacacgaatgc
aaaagcagaaaatgaatgatagcatggatacctcaaacaaggaagagaaa
tgaggatctcaggaccttggtggacactgtgtacacctctggattcattg
tctctcacagatgtgactgtataactttcccaggttctgtttatggccac
atttaatatcttcagctctttttgtggatataaaatgtgcagatgcaatt
gtttgggtgattcctaagccacttgaaatgttagtcattgttatttatac
aagattgaaaatcttgtgtaaatcctgccatttaaaaagttgtagcagat
tgtttcctcttccaaagtaaaattgctgtgctttatggatagtaagaatg
gccctagagtgggagtcctgataacccaggcctgtctgactactttgcct
tcttttgtagcatataggtgatgtttgctcttgtttttattaatttatat
gtatattttttaatttaacatgaacacccttagaaaatgtgtcctatct
atcttccaaatgcaatttgattgactgcccattcaccaaaattatcctga
actcttctgcaaaaatggatattattagaaattagaaaaaaattactaat
tttacacattagattttattttactattggaatctgatatactgtgtgct
tgttttataaaattttgcttttaattaaataaaagctggaagcaaagtat
aaccatatgatactatcatactactgaaacagatttcatacctcagaatg
taaaagaacttactgattattttcttcatccaacttatgtttttaaatga
ggattattgatagtactcttggtttttataccattcagatcactgaattt
ataaagtacccatctagtacttgaaaaagtaaagtgttctgccagatctt
aggtatagaggaccctaacacagtatatcccaagtgcactttctaatgtt
tctgggtcctgaagaattaagatacaaattaattttactccataaacaga
ctgttaattataggagccttaattttttttcatagagatttgtctaatt
gcatctcaaaattattctgccctccttaatttgggaaggtttgtgttttc
tctggaatggtacatgtcttccatgtatcttttgaactggcaattgtcta
tttatcttttattttttaagtcagtatggtctaacactggcatgttcaa
agccacattatttctagtccaaaattacaagtaatcaagggtcattatgg
gttaggcattaatgtttctatctgattttgtgcaaaagcttcaaattaaa
acagctgcattagaaaaagaggcgcttctcccctcccctacacctaaagg
tgtatttaaactatcttgtgtgattaacttatttagagatgctgtaactt
aaaataggggatatttaaggtagcttcagctagcttttaggaaaatcact
ttgtctaactcagaattatttttaaaaagaaatctggtcttgttagaaaa
caaaattttattttgtgctcatttaagtttcaaacttactattttgacag
ttattttgataacaatgacactagaaaacttgactccatttcatcattgt
ttctgcatgaatatcatacaaatcagttagtttttaggtcaagggcttac
tatttctgggtcttttgctactaagttcacattagaattagtgccagaat
tttaggaacttcagagatcgtgtattgagatttcttaaataatgcttcag
atattattgctttattgctttttgtattggttaaaactgtacatttaaa
attgctatgttactattttctacaattaatagtttgtctattttaaaata
aattagttgttaagagtcttaa
>NM_012104 4
acaagtctttccgcctccccagcccgcccgggagctgcgagccgcgagct
ggattatggtggcctgagcagccaacgcagccgcaggagcccggagccct
tgcccctgcccgcgccgccgcccgccgggggggaccagggaagccgccacc
ggcccgccatgcccgcccctcccagccccgccgggagcccgcgcccgctg
cccaggctggccgccgccgtgccgatgtagcgggctccggatcccagcct
ctcccctgctcccgtgctctgcggatctcccctgaccgctctccacagcc

cggacccgggggctggcccagggccctgcaggccctggcgtcctgatgcc
cccaagctccctctcctgagaagccaccagcaccacccagacttgggggc
aggcgccagggacggacgtgggccagtgcgagcccagagggcccgaaggc
cggggcccaccatggcccaagccctgccctggctcctgctgtggatgggc
gcgggagtgctgcctgcccacggcacccagcacggcatccggctgcccct
gcgcagcggcctgggggcgccccccctggggctgcggctgccccgggaga
ccgacgaagagcccgaggagcccggccggaggggcagctttgtggagatg
gtggacaacctgaggggcaagtcggggcagggctactacgtggagatgac
cgtgggcagcccccgcagacgctcaacatcctggtggatacaggcagca
gtaactttgcagtgggtgctgccccccaccccttcctgcatcgctactac
cagaggcagctgtccagcacataccgggacctccggaagggtgtgtatgt
gccctacacccagggcaagtgggaaggggagctgggcaccgacctggtaa
gcatcccccatggccccaacgtcactgtgcgtgccaacattgctgccatc
actgaatcagacaagttcttcatcaacggctccaactgggaaggcatcct
ggggctggcctatgctgagattgccaggcctgacgactccctggagcctt
tctttgactctctggtaaagcagacccacgttcccaacctcttctccctg
cagctttgtggtgctggcttcccctcaaccagtctgaagtgctggcctc
tgtcggagggagcatgatcattggaggtatcgaccactcgctgtacacag
gcagtctctggtatacacccatccggcgggagtggtattatgaggtgatc
attgtgcgggtggagatcaatggacaggatctgaaaatggactgcaagga
gtacaactatgacaagagcattgtggacagtggcaccaccaaccttcgtt
tgcccaagaaagtgtttgaagctgcagtcaaatccatcaaggcagcctcc
tccacggagaagttccctgatggtttctggctaggagagcagctggtgtg
ctggcaagcaggcaccaccccttggaacattttcccagtcatctcactct
acctaatgggtgaggttaccaaccagtccttccgcatcaccatccttccg
cagcaatacctgcggccagtggaagatgtggccacgtcccaagacgactg
ttacaagtttgccatctcacagtcatccacgggcactgttatgggagctg
ttatcatggagggcttctacgttgtctttgatcgggcccgaaaacgaatt
ggctttgctgtcagcgcttgccatgtgcacgatgagttcaggacggcagc
ggtggaaggcccttttgtcaccttggacatggaagactgtggctacaaca
ttccacagacagatgagtcaaccctcatgaccatagcctatgtcatggct
gccatctgcgccctcttcatgctgccactctgcctcatggtgtgtcagtg
gcgctgcctccgctgcctgcgccagcagcatgatgactttgctgatgaca
tctccctgctgaagtgaggaggcccatgggcagaagatagagattcccct
ggaccacacctccgtggttcactttggtcacaagtaggagacacagatgg
cacctgtggccagagcacctcaggaccctccccacccaccaaatgcctct
gccttgatggagaaggaaaaggctggcaaggtgggttccagggactgtac
ctgtaggaaacagaaaagagaagaaagaagcactctgctggcgggaatac
tcttggtcacctcaaatttaagtcgggaaattctgctgcttgaaacttca
gccctgaacctttgtccaccattcctttaaattctccaacccaaagtatt
cttcttttcttagtttcagaagtactggcatcacacgcaggttaccttgg
cgtgtgtccctgtggtaccctggcagagaagagaccaagcttgtttccct
gctggccaaagtcagtaggagaggatgcacagtttgctatttgctttaga
gacagggactgtataaacaagcctaacattggtgcaaagattgcctcttg
aattaaaaaaaaaaactagattgactatttatacaaatgggggcggctgg
aaagaggagaaggagagggagtacaaagacagggaatagtgggatcaaag
ctaggaaaggcagaaacacaaccactcaccagtcctagttttagacctca
tctccaagatagcatcccatctcagaagatgggtgttgttttcaatgttt
tcttttctgtggttgcagcctgaccaaaagtgagatgggaagggcttatc

tagccaaagagctctttttagctctcttaaatgaagtgcccactaagaa
gttccacttaacacatgaatttctgccatattaatttcattgtctctatc
tgaaccaccctttattctacatatgataggcagcactgaaatatcctaac
cccctaagctccaggtgccctgtgggagagcaactggactatagcagggc
tgggctctgtcttcctggtcataggctcactctttcccccaaatcttcct
ctggagctttgcagccaaggtgctaaaaggaataggtaggagacctcttc
tatctaatccttaaaagcataatgttgaacattcattcaacagctgatgc
cctataacccctgcctggatttcttcctattaggctataagaagtagcaa
gatctttacataattcagagtggtttcattgccttcctaccctctctaat
ggcccctccatttatttgactaaagcatcacacagtggcactagcattat
accaagagtatgagaaatacagtgctttatggctctaacattactgcctt
cagtatcaaggctgcctggagaaaggatggcagcctcagggcttccttat
gtcctccaccacaagagctccttgatgaaggtcatctttttcccctatcc
tgttcttcccctccccgctcctaatggtacgtgggtacccaggctggttc
ttgggctaggtagtggggaccaagttcattacctccctatcagttctagc
atagtaaactacggtaccagtgttagtgggaagagctgggttttcctagt
atacccactgcatcctactcctacctggtcaacccgctgcttccaggtat
gggacctgctaagtgtggaattacctgataagggagagggaaatacaagg
agggcctctggtgttcctggcctcagccagctgcccacaagccataaacc
aataaaacaagaatactgagtcagtttttttatctgggttctcttcattcc
cactgcacttggtgctgctttggctgactgggaacaccccataactacag
agtctgacaggaagactggagactgtccacttctagctcggaacttactg
tgtaaataaactttcagaactgctaccatgaagtgaaaatgccacatttt
gctttataatttctacccatgttgggaaaaactggctttttcccagccct
ttccagggcataaaactcaacccccttcgatagcaagtcccatcagcctat
tattttttaaagaaaacttgcacttgtttttctttttacagttacttcc
ttcctgccccaaaattataaactctaagtgtaaaaaaaagtcttaacaac
agcttcttgcttgtaaaaatatgtattatacatctgtatttttaaattct
gctcctgaaaaatgactgtcccattctccactcactgcatttggggcctt
tcccattggtctgcatgtcttttatcattgcaggccagtggacagaggga
gaagggagaacaggggtcgccaacacttgtgttgctttctgactgatcct
gaacaagaaagagtaacactgaggcgctcgctcccatgcacaactctcca
aaacacttatcctcctgcaagagtgggctttccagggtctttactgggaa
gcagttaagccccctcctcacccccttccttttttctttctttactccttt
ggcttcaaaggattttggaaaagaaacaatatgctttacactcattttca
atttctaaatttgcaggggatactgaaaaatacggcaggtggcctaaggc
tgctgtaaagttgaggggagaggaaatcttaagattacaagataaaaaac
gaatcccctaaacaaaaagaacaatagaactggtcttccattttgccacc
tttcctgttcatgacagctactaacctggagacagtaacatttcattaac
caaagaaagtgggtcacctgacctctgaagagctgagtactcaggccact
ccaatcaccctacaagatgccaaggaggtcccaggaagtccagctcctta
aactgacgctagtcaataaacctgggcaagtgaggcaagagaaatgagga
agaatccatctgtgaggtgacaggcaaggatgaaagacaaagaaggaaaa
gagtatcaaaggcagaaaggagatcatttagttgggtctgaaaggaaaag
tctttgctatccgacatgtactgctagtacctgtaagcattttaggtccc
agaatggaaaaaaaaatcagctattggtaatataataatgtcctttccct
ggagtcagttttttttaaaaagttaactcttagtttttacttgtttaattc
taaaagagaagggagctgaggccattccctgtaggagtaaagataaaagg
ataggaaaagattcaaagctctaatagagtcacagctttcccaggtataa

aacctaaaattaagaagtacaataagcagaggtggaaaatgatctagttc
ctgatagctacccacagagcaagtgatttataaatttgaaatccaaacta
ctttcttaatatcactttggtctccatttttcccaggacaggaaatatgt
cccccctaactttcttgcttcaaaaattaaaatccagcatcccaagatc
attctacaagtaattttgcacagacatctcctcaccccagtgcctgtctg
gagctcacccaaggtcaccaaacaacttggttgtgaaccaactgccttaa
ccttctgggggagggggattagctagactaggagaccagaagtgaatggg
aaagggtgaggacttcacaatgttggcctgtcagagcttgattagaagcc
aagacagtggcagcaaaggaagacttggcccaggaaaaacctgtgggttg
tgctaatttctgtccagaaaatagggtggacagaagcttgtggggtacat
ggaggaattgggacctggttatgttgttattctcggactgtgaattttgg
tgatgtaaaacagaatattctgtaaacctaatgtctgtataaataatgag
cgttaacacagtaaaatattcaataagaagtcaaactactagggttaaaa
aaaaaaaaaaaaaa
>NM_153620 2
attcatatcatttttcttctccggccccatggaggaagtgagaaagttgg
cacagtcacgccgggcttcgcaggaccaggtcactcagtgacagatggac
aatgcaagaatgaactccttcctggaataccccatacttagcagtggcga
ctcggggacctgctcagcccgagcctacccctcggaccataggattacaa
ctttccagtcgtgcgcggtcagcgccaacagttgcggcggcgacgaccgc
ttcctagtgggcagggggggtgcagatcggttcgccccaccaccaccacca
ccaccaccatcaccacccccagccggctacctaccagacttccgggaacc
tgggggtgtcctactcccactcaagttgtggtccaagctatggctcacag
aacttcagtgcgccttacagcccctacgcgttaaatcaggaagcagaccc
accaagaagcctgtcgctcccccgcatcggagacatcttctccagcgcag
acttttgactggatgaaagtcaaaagaaaccctcccaaaacagggaaagt
tggagagtacggctacctgggtcaacccaacgcggtgcgcaccaacttca
ctaccaagcagctcacggaactggagaaggagttccacttcaacaagtac
ctgacgcgcgcccgcagggtggagatcgctgcatccctgcagctcaacga
gacccaagtgaagatctggttccagaaccgccgaatgaagcaaaagaaac
gtgagaaggagggtctcttgcccatctctccggccaccccgccaggaaac
gacgagaaggccgaggaatcctcagagaagtccagctcttcgccctgcgt
tccttccccggggtcttctacctcagacactctgactacctcccactgag
gcggctccagccccagacaacagcccaggcatctccttgggctgggactt
cttacccaaagcacatgcttagcttatctttctttccatttacagtctct
ttcttcctttctaatcctatctggggagctcctggccaggataatatatt
tgcagataattctggaccagagacttggtgcggggttaacaccttcatcc
agattgggtgccagcatacattttctggtgggccttaacatccctcctgc
ttttaggagaattcacagaacctactgttcctttcagatgaccttttgga
aaatagttccctttgccaacagaaacatgccagaaggaatcttctcatct
tttatctaactatatgtacagctctcccctcccttgtccttgaaagtagg
atatagcgaaaggcgagtccaggagctcaggaagaagagatgcactatat
gtttacacaattaattcatcccttaatttaagtcattttcatgtgtgtga
gtttgctggttgtaaatactttgtcctaagagatttatctttatacagat
tttctagaaatgtttaggttactaaaacagggtgggcaaactctctaaac
tggtacaattttataggtgaaagaaaaaaattccctcatttaaacccaatc
agatgcctcagagggtagccttgatttgttcttacagttaagaagccctg
cagagcacaaacttcagaaacccggcttcctgtgctaagtctttcccaat
ctctacccctttcttctcgggccaccctctgtttaaaatttgtgctgggt

tattcagaacctaaaagtattattcaaaccaatttcttccttccacagtt
atcttagctggatataatgtattttcagctcaattgttaatgtgatggat
ggcacaatgaatgtatattttgtgttattcgtgaatagtcttttgcatgt
cgcacaatgtttgatgtccccaaagtaccacactgagttctatcagttat
cctttgtgagcctatgatattccccatttcctgtacaatcatgaacagct
ctgagatcctggagtgatatgatccagagcagagtttacgggtcttagga
tgtctgtaataaataaatatactcaagtttcaggtatgcttaagcatccg
tgtatttggctgggctacaatttgttaattcctatgaagttggcacattt
catgaggggaaagggagaagggtggtaaatattttcaaagagatgggcct
tttcttgaataaaagttaataacagctcctttattataatcaaagctcat
aatggaaaaaaagactgatgaagaaatttatgaagcagatttatttttga
aacaaacatggatacttcctgggtcaagtgctaaccttttcacctccaac
tggatgttgacgtatatataaacagaactcccttcaaaagccaaaaaaaa
aaaaaaaa
>NM_002148 3
cggggaatgttttcctagagatgtcagcctacaaaggacacaatctctct
tcttcaaattcttccccaaaatgtcctttcccaacagctctcctgctgct
aatactttttttagtagattccttgatcagtgcctgcaggagtgacagttt
ttattccagcagcgccagcatgtacatgccaccacctagcgcagacatgg
ggacctatggaatgcaaacctgtggactgctcccgtctctggccaaaaga
gaagtgaaccaccaaaatatgggtatgaatgtgcatccttatatacctca
agtagacagttggacagatccgaacagatcttgtcgaatagagcaacctg
ttacacagcaagtccccacttgctccttcaccaccaacattaaggaagaa
tccaattgctgcatgtattctgataagcgcaacaaactcatttcggccga
ggtcccttcgtaccagaggctggtccctgagtcttgtcccgttgagaacc
ctgaggttcccgtccctggatattttagactgagtcagacctacgccacc
gggaaaacccaagagtacaataatagccccgaaggcagctccactgtcat
gctccagctcaaccctcgtggcgcggccaagccgcagctctccgctgccc
agctgcagatggaaaagaagatgaacgagcccgtgagcggccaggagccc
accaaagtctcccaggtggagagccccgaggccaaaggcggccttcccga
agagaggagctgcctggctgaggtctccgtgtccagtcccgaagtgcagg
agaaggaaagcaaagaggaaatcaagtctgatacaccaaccagcaattgg
ctcactgcaaagagtggcagaaagaagaggtgcccttacactaagcacca
aacgctggaattagaaaaagagttcttgttcaatatgtacctcacccgcg
agcgccgcctagagatcagtaagagcgttaacctcaccgacaggcaggtc
aagatttggtttcaaaaccgccgaatgaaactcaagaagatgagccgaga
gaaccggatccgagaactgaccgccaacctcacgttttcttaggtctgag
gccggtctgaggccggtcagaggccaggattggagagggggcaccgcgtt
ccagggcccagtgctggaggactgggaaagcggaaacaaaaccttcaccg
ctctttgtttgttgttttgttgtattttgttttcctgctagaatgtgact
ttggggtcattatgttcgtgctgcaagtgatctgtaatccctatgagtat
atatatatatatatatatatatatataaaaacttagcacgtgtaatttat
tattttttcatcgtaatgcagggtaactattattgcgcattttcatttgg
gtcttaacttattggaactgtagagcatccatccatccatccatccagca
atgtgactttttcatgtctttcctaacacaaaaggtctatgtgtgtggtt
agtccatgaactcatggcattttgaatacatccagtactttaaaaatgac
atatatatttaaaaaaaaaaagattaagaaaacccacaagttggagggagg
gggacttaaaaagcacattacaatgtatcttttcacaaatgaatttagca
gttgtccttggtgagatgggatattggcgatttatgccttgtagcctttc

ccttgtggtgcatctgtggtttggtagaagtacaacagcaacctgtcctt
tctgtgcatgttctggtcgcatgtataatgcaataaactctggaaatgag
ttcaaaaaaaaaaa
>NM_003045 4
gcactgctgatgaaacctggcgccggaacccgccagccctcggcgcccat
tcagtccgcgcaggcaggtgtgagcagcgggtcaactacctggcaggcgc
gcacgcggccgcgggctcccgctaaccgcagcctccactcctctccccgc
gcgccgcgcccccgccccgccccgccccgcccggtctcgccggccgagcg
tccgttggtccttgagcgcgtccgacagtctgtctgttcgcgatcctgcc
ggagccccgccgccgccggcttggattctgaaaccttccttgtatccctc
ctgagacatctttgctgcaagatcgaggctgtcctctggtgagaaggtgg
tgaggcttcccgtcatattccagctctgaacagcaacatggggtgcaaag
tcctgctcaacattgggcagcagatgctgcggcggaaggtggtggactgt
agccgggaggagacgcggctgtctcgctgcctgaacacttttgatctggt
ggccctcggggtgggcagcacactgggtgctggtgtctacgtcctggctg
gagctgtggcccgtgagaatgcaggccctgccattgtcatctccttcctg
atcgctgcgctggcctcagtgctggctggcctgtgctatggcgagtttgg
tgctcgggtccccaagacgggctcagcttacctctacagctatgtcaccg
ttggagagctctgggccttcatcaccggctggaacttaatcctctcctac
atcatcggtacttcaagcgtagcgagggcctggagcgccaccttcgacga
gctgataggcagacccatcggggagttctcacggacacacatgactctga
acgcccccggcgtgctggctgaaaaccccgacatattcgcagtgatcata
attctcatcttgacaggacttttaactcttggtgtgaaagagtcggccat
ggtcaacaaaatattcacttgtattaacgtcctggtcctgggcttcataa
tggtgtcaggatttgtgaaaggatcggttaaaaactggcagctcacggag
gaggattttgggaacacatcaggccgtctctgtttgaacaatgacacaaa
agaagggaagcccggtgttggtggattcatgcccttcgggttctctggtg
tcctgtcgggggcagcgacttgcttctatgccttcgtgggctttgactgc
atcgccaccacaggtgaagaggtgaagaacccacagaaggccatccccgt
ggggatcgtggcgtccctcttgatctgcttcatcgcctactttggggtgt
cggctgccctcacgctcatgatgccctacttctgcctggacaataacagc
cccctgcccgacgcctttaagcacgtgggctgggaaggtgccaagtacgc
agtggccgtgggctccctctgcgctctttccgccagtcttctaggttcca
tgtttcccatgcctcgggttatctatgccatggctgaggatggactgcta
tttaaattcttagccaacgtcaatgataggaccaaaacaccaataatcgc
cacattagcctcgggtgccgttgctgctgtgatggccttcctctttgacc
tgaaggacttggtggacctcatgtccattggcactctcctggcttactcg
ttggtggctgcctgtgtgttggtcttacggtaccagccagagcagcctaa
cctggtataccagatggccagtacttccgacgagttagatccagcagacc
aaaatgaattggcaagcaccaatgattcccagctggggtttttaccagag
gcagagatgttctctttgaaaaccatactctcacccaaaaacatggagcc
ttccaaaatctctgggctaattgtgaacatttcaaccagccttatagctg
ttctcatcatcaccttctgcattgtgaccgtgcttggaagggaggctctc
accaaaggggcgctgtgggcagtctttctgctcgcagggtctgccctcct
ctgtgccgtggtcacgggcgtcatctggaggcagcccgagagcaagacca
agctctcatttaaggttcccttcctgccagtgctccccatcctgagcatc
ttcgtgaacgtctatctcatgatgcagctggaccagggcacctgggtccg
gtttgctgtgtggatgctgataggcttcatcatctactttggctatggcc
tgtggcacagcgaggaggcgtccctggatgccgaccaagcaaggactcct

gacggcaacttggaccagtgcaagtgacgcacagccccgccccccggagg
tggcagcagccccgagggacgcccccagaggaccgggaggcaccccaccc
tccccaccagtgcaacagaaaccacctgcgtccacaccctcactgcagcc
aaaggtgcaattacttgacctgcagccccagcccaccctcggctctgcag
ccggttctccgggccctggtcacctccagacagctgcctggccggggcca
ctaggctgcggctggccactgtgtctcctcacttctctgaacaaagcagt
tcctcccctaccagctcagccccgagctgccgcagcctcaggcagaacgg
aggtcaccttctctccttatcttgggaaccaggccttcctcccggggact
gttctgggattgaaattgtgcatactccaaactttcgcagccatcttccc
gctcagccccagacacccagcaatcaagccagatgagtaccacaaaacag
tgtgtccccagcagctccccaccccagagccaaatgacagtagtgcactt
aaaaaggaaaatcaggcctgttgtccttctccggttgcattcagatgggt
cattagggccggaccctgcctgccccttggcttctcagggctttgctctg
acaccatgacagctgcccggggctgagggcagctggctccactcaaatga
ggaagaagggatcactcccattagggcctgctttgcttatgcatgtgtgt
gcacatgcatgtaaaccagggaccttcagctcacggcctccaggcctggg
ccagttcttgctgctcctgccgtctcccccgactggctgtgtcctgagta
actggaacatgagacagtatctgcaggactggccccatggtggccgagtc
agaagtctgtttcctgtgagtcgccaccgttcactcagtcttgccctccc
atgctttggagccagtctggtggctcctgtaaggttctcaaggctggtgg
cagctcagtctggggtcaggacatgtcggggtcatgcgtttctggccctg
acataagctgtctggcctctctgtgacatgatgaaattgaaatcaatcca
cagtccatgaaattgtgacactccaccagattaagttagggcataacatt
aacttggaaatggccatgtcatcacccctgcggctgtcctatagctgaga
tgcgtgggtcgcaggggaggtgatttctaggcatattgctgtccctttttg
tgtatctgtcatccggatgcttcggaccccacgcctctgcaagtgggaga
gacccgagcatcctccccacccccatagctccagtgcacgccacccccgt
cttgcctgggtcggggcctgcggccagcaccatttcacacacactccttg
tagatgggagccagaggaaacctgaacgtgggtggagcgttccactgagt
ctacttcaggagacagaaggcccatgctgatgggggaggaggagggatgt
gggcattttggacaccaggggaaatggaaatgctgctttcaaaacttagt
ttcctttccatttcttcctagtctggcctttgacacaaatctggtagaaa
gaagcctgataaattgagggcacttgtaccctccctgtgcccccagaagg
ttcttggagagaagtgcaagaatttgtgaacacggcggtggagggcgggt
ggatggccatgggctgagcctccgtatcaggcctgctcaccttgctggga
gctttattctgatctcattttgaatgttccagagggagcatcataagagc
ccagagctccgatttccaaagagtgatattgacatttatggagattggtg
ttgtaacatattttgataaatactaacttattttgttggggttttggttg
tctcttgtcttaggacctggtagttatttgcttgattttttttccgtta
ttttctacataggcaaagagaattcgagggatagacagtctccaagaaaa
gtgaagtggtgggagagaattgctttttcttttttttcttttctctagt
ttttctttctggctgagatttccgtgcaagacagcacccaatagactatt
tagagttgacatttgacattttaatgggcgccatggctcattttgtagat
tgagaaggtgcgtctcccctgctccaagtctcatcatgacagcgtgctga
cagctgggagtctgtggccttcctcacgcagaggccttaaagctggacac
agaagcacgcctaggctgggcagggatgggacccatgccccctccttaga
ggacgggcttcctggttaggaaaggacacgtgggggtgccttgcataata
gttcactggtcaccgtgcttttatgagtagtgtttttgtgcacttgccag
gggttttctctctgtgtgagaggggagtgatttaagcaatggtgtctgga

gtaagccttacaattttaatagactttttcttatcatatccctcatttct
ttccctgaaataaaaatacacacaagcaaaaaaaaaatgatagtttcaca
tctcttagttcccttgcccaaacaagaatattcttagttccactggccag
gattttcctacatagtcagaacttacacattactagaggcacacccacca
aggagtattgtgtctacttttatctgtgcaccagccacaaatacccacat
tggaaagacccatttgtgatgggtaaacatcccttcctgtctcccacaac
ccctgtgactgccctgcatgtgttcatgacctccgaaggcccaaattcat
gaagcagcaaacccagcagatctccaccccctgcctcaggacctctgct
gaagaggggatgaagtgggtctccagggaggcagtgggggccttgttgg
cagctggctcgggagccggcttacaggagggcagctctgcagttgggagg
ggcaccgtccggaggagaccaggcctctacacacccccactctacttat
catccctgctcacacacccttgtccaaggctttatgcatcggatttattt
ttccaaatcaagaggacagtgatagatgcattttccccaggctgtctcag
aaaggtcgctaaatgtatactgttgtcagaattgctgagatctcccccca
cttttggtttttgcagcagtaaaaactctttccactgtgacttattttct
ctctcaggcagccagccacctggtcccttgtgctgactctagcacagtgg
ccaggatccaatacgagtccaggggtgaccgcaggatggtgggggcagcg
ggcttctccacctaccccagccaccaaggccctgacgcactgcctcctgc
accttcagcacatccctgtgcacagctggaagggtgcatggcccgctcac
ctttgttcagatgggtggaaacgctgatgataccagctcctccctgccgt
gcccctgccacggagcaggcattgtgaactggctggtgtttgcagtccca
cgtggcatggcctccagcccaacccacagtggagactggagacagggcaa
tgagtctggtggggggcacgtggacatgccccatagggggccccacccaga
cttaacaggcaaggtcctgggcattgcgcgacgcaggactcaatgctaaa
gcaagcctgcctggctctgtgccagggcccctcttctgattcacacatcc
catttttacacagacccttccttcttaataaaggctgacagttctgttgg
cagccaagaacccacaccatgaagacagggagtgaggggcctttgtgccc
aactccagcacagctgcgttctggggtgtgtgagaggcatgttcgtgtct
gtgcgctggtggtctcgtgagacagttccgaggacggggaaattgcaggg
tggtgggggcgtgaggcttatatgtggaactgatgcagagttcgcctgca
gacggatctggatatacactatgtataattgttacgtgtaatttaaaata
tatctgtttgccatcgtcatgagaagattatatgtaaggctctgaaggga
gagggagatgtacattctgccaggctcctggggaccttatccgagtcatg
aaattgattactgttgatccagtggtgcaagaagctacactccatgtgtc
atcacgcttatgactcctaatgtatttttaaggcaaaaaatgtcagccga
ctccatcttcacccctcgattcctcgagtccagcctttctgtgccagtgc
ttcactgagccacaacgctctcgccatcgggacccggctgggcctggagt
ctcggggcacagttgccatggagccctcctgggtcattctacaaatgtgc
tgagtgccagctgaaaaccccacaggagatggagtaccttggccaagctt
aaagagaagattttctcagggtatttattagtgtgtccagcagggtcagg
aagcaggatggaaagatgcactcagactgttaatttattaacaaggcaaa
tgattttgtgtttcttgatgacagactattaagtttgggacttattttcc
catttgagaagttataatatatatttaagatgataagtttcctgcttaag
ttgtgcctttcagcttcaatgagtttaaggagcactaagggtaatgatac
caatgagggttggtttattatcaaacctgaatagctgtggtttctccagt
aaatattttcttctactgaacatggagccattattaagagttgtgtgttt
tttattatgtacatttgtatattttttttgcttgtttgatgttctattttt
ctaatagttttcttttagtttcttaaagttgtgatactagatttagattc
tgatgctaactgcaaatcaggttggtctctgctgggtctctcctgctttt

attttactttaaggacaagtgtagttgtcgtccaccacctttcaaaaaat
gtgaaactgccctgcctcccctttttgctgacaacactgtgtacattgac
cacttcctaccatactttatgttgtaaaatcaaactcttttgtggtacat
tatctcatgcttctgcaaattcgaataaattctatggcttccaaaaaaaa
aaaaaaa
>NM_001982 3
actccagcctcgcgcgggagggggcgcggccgtgactcaccccccttccct
ctgcgttcctccctccctctctctctctctcacacacacacaccccctc
ccctgccatccctccccggactccggctccggctccgattgcaatttgca
acctccgctgccgtcgccgcagcagccaccaattcgccagcggttcaggt
ggctcttgcctcgatgtcctagcctaggggcccccgggccggacttggct
gggctcccttcaccctctgcggagtcatgagggcgaacgacgctctgcag
gtgctgggcttgcttttcagcctggcccggggctccgaggtgggcaactc
tcaggcagtgtgtcctgggactctgaatggcctgagtgtgaccggcgatg
ctgagaaccaataccagacactgtacaagctctacgagaggtgtgaggtg
gtgatggggaaccttgagattgtgctcacgggacacaatgccgacctctc
cttcctgcagtggattcgagaagtgacaggctatgtcctcgtggccatga
atgaattctctactctaccattgcccaacctccgcgtggtgcgagggacc
caggtctacgatgggaagtttgccatcttcgtcatgttgaactataacac
caactccagccacgctctgcgccagctccgcttgactcagctcaccgaga
ttctgtcagggggtgtttatattgagaagaacgataagctttgtcacatg
gacacaattgactggagggacatcgtgagggaccgagatgctgagatagt
ggtgaaggacaatggcagaagctgtccccccctgtcatgaggtttgcaagg
ggcgatgctggggtcctggatcagaagactgccagacattgaccaagacc
atctgtgctcctcagtgtaatggtcactgctttgggcccaaccccaacca
gtgctgccatgatgagtgtgccgggggctgctcaggccctcaggacacag
actgctttgcctgccggcacttcaatgacagtggagcctgtgtacctcgc
tgtccacagcctcttgtctacaacaagctaactttccagctggaacccaa
tccccacaccaagtatcagtatggaggagtttgtgtagccagctgtcccc
ataactttgtggtggatcaaacatcctgtgtcagggcctgtcctcctgac
aagatggaagtagataaaaatgggctcaagatgtgtgagccttgtggggg
actatgtcccaaagcctgtgagggaacaggctctgggagccgcttccaga
ctgtggactcgagcaacattgatggatttgtgaactgcaccaagatcctg
ggcaacctggactttctgatcaccggcctcaatggagacccctggcacaa
gatccctgccctggacccagagaagctcaatgtcttccggacagtacggg
agatcacaggttacctgaacatccagtcctggccgccccacatgcacaac
ttcagtgtttttttccaatttgacaaccattggaggcagaagcctctacaa
ccggggcttctcattgttgatcatgaagaacttgaatgtcacatctctgg
gcttccgatccctgaaggaaattagtgctgggcgtatctatataagtgcc
aataggcagctctgctaccaccactctttgaactggaccaaggtgcttcg
ggggcctacggaagagcgactagacatcaagcataatcggccgcgcagag
actgcgtggcagagggcaaagtgtgtgacccactgtgctcctctgggga
tgctggggcccaggccctggtcagtgcttgtcctgtcgaaattatagccg
aggaggtgtctgtgtgacccactgcaactttctgaatggggagcctcgag
aatttgcccatgaggccgaatgcttctcctgccacccggaatgccaaccc
atggagggcactgccacatgcaatggctcgggctctgatacttgtgctca
atgtgcccattttcgagatgggccccactgtgtgagcagctgcccccatg
gagtcctaggtgccaagggcccaatctacaagtacccagatgttcagaat
gaatgtcggccctgccatgagaactgcacccaggggtgtaaaggaccaga

gcttcaagactgtttaggacaaacactggtgctgatcggcaaaacccatc
tgacaatggctttgacagtgatagcaggattggtagtgattttcatgatg
ctgggcggcacttttctctactggcgtgggcgccggattcagaataaaag
ggctatgaggcgatacttggaacggggtgagagcatagagcctctggacc
ccagtgagaaggctaacaaagtcttggccagaatcttcaaagagacagag
ctaaggaagcttaaagtgcttggctcgggtgtctttggaactgtgcacaa
aggagtgtggatccctgagggtgaatcaatcaagattccagtctgcatta
aagtcattgaggacaagagtggacggcagagttttcaagctgtgacagat
catatgctggccattggcagcctggaccatgcccacattgtaaggctgct
gggactatgcccagggtcatctctgcagcttgtcactcaatatttgcctc
tgggttctctgctggatcatgtgagacaacaccgggggcactggggcca
cagctgctgctcaactggggagtacaaattgccaagggaatgtactacct
tgaggaacatggtatggtgcatagaaacctggctgcccgaaacgtgctac
tcaagtcacccagtcaggttcaggtggcagattttggtgtggctgacctg
ctgcctcctgatgataagcagctgctatacagtgaggccaagactccaat
taagtggatggcccttgagagtatccactttgggaaatacacacaccaga
gtgatgtctggagctatggtgtgacagtttgggagttgatgaccttcggg
gcagagccctatgcagggctacgattggctgaagtaccagacctgctaga
gaagggggagcggttggcacagccccagatctgcacaattgatgtctaca
tggtgatggtcaagtgttggatgattgatgagaacattcgcccaaccttt
aaagaactagccaatgagttcaccaggatggcccgagacccaccacggta
tctggtcataaagagagagagtgggcctggaatagcccctgggccagagc
cccatggtctgacaaacaagaagctagaggaagtagagctggagccagaa
ctagacctagacctagacttggaagcagaggaggacaacctggcaaccac
cacactgggctccgccctcagcctaccagttggaacacttaatcggccac
gtgggagccagagccttttaagtccatcatctggatacatgcccatgaac
cagggtaatcttggggagtcttgccaggagtctgcagtttctgggagcag
tgaacggtgcccccgtccagtctctctacacccaatgccacggggatgcc
tggcatcagagtcatcagaggggcatgtaacaggctctgaggctgagctc
caggagaaagtgtcaatgtgtaggagccggagcaggagccggagcccacg
gccacgcggagatagcgcctaccattcccagcgccacagtctgctgactc
ctgttaccccactctccccacccgggttagaggaagaggatgtcaacggt
tatgtcatgccagatacacacctcaaaggtactccctcctcccgggaagg
caccctttcttcagtgggtctcagttctgtcctgggtactgaagaagaag
atgaagatgaggagtatgaatacatgaaccggaggagaaggcacagtcca
cctcatccccctaggccaagttcccttgaggagctgggttatgagtacat
ggatgtggggtcagacctcagtgcctctctgggcagcacacagagttgcc
cactccaccctgtacccatcatgcccactgcaggcacaactccagatgaa
gactatgaatatatgaatcggcaacgagatggaggtggtcctgggggtga
ttatgcagccatgggggcctgcccagcatctgagcaagggtatgaagaga
tgagagcttttcaggggcctggacatcaggccccccatgtccattatgcc
cgcctaaaaactctacgtagcttagaggctacagactctgcctttgataa
ccctgattactggcatagcaggcttttccccaaggctaatgcccagagaa
cgtaactcctgctccctgtggcactcagggagcatttaatggcagctagt
gcctttagagggtaccgtcttctccctattccctctctctcccaggtccc
agccccttttccccagtcccagacaattccattcaatctttggaggcttt
taaacattttgacacaaaattcttatggtatgtagccagctgtgcacttt
cttctctttcccaaccccaggaaaggttttccttattttgtgtgctttcc
cagtcccattcctcagcttcttcacaggcactcctggagatatgaaggat

tactctccatatcccttcctctcaggctcttgactacttggaactaggct
cttatgtgtgcctttgtttcccatcagactgtcaagaagaggaaagggag
gaaacctagcagaggaaagtgtaattttggtttatgactcttaacccccct
agaaagacagaagcttaaaatctgtgaagaaagaggttaggagtagatat
tgattactatcataattcagcacttaactatgagccaggcatcatactaa
acttcacctacattatctcacttagtcctttatcatccttaaaacaattc
tgtgacatacatattatctcattttacacaaagggaagtcgggcatggtg
gctcatgcctgtaatctcagcactttgggaggctgaggcagaaggattac
ctgaggcaaggagtttgagaccagcttagccaacatagtaagaccccccat
ctctttaaaaaaaaaaaaaaaaaaaaaaaaaaaactttagaactgggtgc
agtggctcatgcctgtaatcccagccagcactttgggaggctgagatggg
aagatcacttgagcccagaattagagataagcctatggaaacatagcaag
acactgtctctacaggggaaaaaaaaaaaagaaactgagccttaaagaga
tgaaataaattaagcagtagatccaggatgcaaaatcctcccaattcctg
tgcatgtgctcttattgtaaggtgccaagaaaaactgatttaagttacag
cccttgtttaaggggcactgtttcttgtttttgcactgaatcaagtctaa
ccccaacagccacatcctcctatacctagacatctcatctcaggaagtgg
tggtgggggtagtcagaaggaaaaataactggacatctttgtgtaaacca
taatccacatgtgccgtaaatgatcttcactccttatccgagggcaaatt
cacaaggatccccaagatccacttttagaagccattctcatccagcagtg
agaagcttccaggtaggacagaaaaaagatccagcttcagctgcacacct
ctgtccccttggatggggaactaagggaaaacgtctgttgtatcactgaa
gttttttgttttgtttttatacgtgtctgaataaaaatgccaaagttttt
tttcagcaaaaaaaa
>NM_004448 2
ggaggaggtggaggaggagggctgcttgaggaagtataagaatgaagttg
tgaagctgagattcccctccattgggaccggagaaaccaggggagccccc
cgggcagccgcgcgccccttcccacggggccctttactgcgccgcgcgcc
cggcccccacccctcgcagcaccccgcgccccgcgccctcccagccgggt
ccagccggagccatggggccggagccgcagtgagcaccatggagctggcg
gccttgtgccgctgggggctcctcctcgccctcttgccccccggagccgc
gagcacccaagtgtgcaccggcacagacatgaagctgcggctccctgcca
gtcccgagacccacctggacatgctccgccacctctaccagggctgccag
gtggtgcagggaaacctggaactcacctacctgcccaccaatgccagcct
gtccttcctgcaggatatccaggaggtgcagggctacgtgctcatcgctc
acaaccaagtgaggcaggtcccactgcagaggctgcggattgtgcgaggc
acccagctctttgaggacaactatgccctggccgtgctagacaatggaga
cccgctgaacaataccacccctgtcacaggggcctccccaggaggcctgc
gggagctgcagcttcgaagcctcacagagatcttgaaaggaggggtcttg
atccagcggaacccccagctctgctaccaggacacgattttgtggaagga
catcttccacaagaacaaccagctggctctcacactgatagacaccaacc
gctctcgggcctgccacccctgttctccgatgtgtaagggctcccgctgc
tggggagagagttctgaggattgtcagagcctgacgcgcactgtctgtgc
cggtggctgtgcccgctgcaaggggccactgcccactgactgctgccatg
agcagtgtgctgccggctgcacgggccccaagcactctgactgcctggcc
tgcctccacttcaaccacagtggcatctgtgagctgcactgcccagccct
ggtcacctacaacacagacacgtttgagtccatgcccaatcccgagggcc
ggtatacattcggcgccagctgtgtgactgcctgtccctacaactacctt
tctacggacgtgggatcctgcaccctcgtctgccccctgcacaaccaaga

ggtgacagcagaggatggaacacagcggtgtgagaagtgcagcaagccct
gtgcccgagtgtgctatggtctgggcatggagcacttgcgagaggtgagg
gcagttaccagtgccaatatccaggagtttgctggctgcaagaagatctt
tgggagcctggcatttctgccggagagctttgatggggacccagcctcca
acactgccccgctccagccagagcagctccaagtgtttgagactctggaa
gagatcacaggttacctatacatctcagcatggccggacagcctgcctga
cctcagcgtcttccagaacctgcaagtaatccggggacgaattctgcaca
atggcgcctactcgctgaccctgcaagggctgggcatcagctggctgggg
ctgcgctcactgagggaactgggcagtggactggccctcatccaccataa
cacccacctctgcttcgtgcacacggtgccctgggaccagctctttcgga
acccgcaccaagctctgctccacactgccaaccggccagaggacgagtgt
gtgggcgagggcctggcctgccaccagctgtgcgcccgagggcactgctg
gggtccagggcccacccagtgtgtcaactgcagccagttccttcggggcc
aggagtgcgtggaggaatgccgagtactgcaggggctccccagggagtat
gtgaatgccaggcactgtttgccgtgccaccctgagtgtcagccccagaa
tggctcagtgacctgttttggaccggaggctgaccagtgtgtggcctgtg
cccactataaggaccctcccttctgcgtggcccgctgccccagcggtgtg
aaacctgacctctcctacatgcccatctggaagtttccagatgaggaggg
cgcatgccagccttgccccatcaactgcacccactcctgtgtggacctgg
atgacaagggctgccccgccgagcagagagccagccctctgacgtccatc
atctctgcggtggttggcattctgctggtcgtggtcttgggggtggtctt
tgggatcctcatcaagcgacggcagcagaagatccggaagtacacgatgc
ggagactgctgcaggaaacggagctggtggagccgctgacacctagcgga
gcgatgcccaaccaggcgcagatgcggatcctgaaagagacggagctgag
gaaggtgaaggtgcttggatctggcgcttttggcacagtctacaagggca
tctggatccctgatggggagaatgtgaaaattccagtggccatcaaagtg
ttgagggaaaacacatcccccaaagccaacaaagaaatcttagacgaagc
atacgtgatggctggtgtgggctccccatatgtctcccgccttctgggca
tctgcctgacatccacggtgcagctggtgacacagcttatgccctatggc
tgcctcttagaccatgtccgggaaaaccgcggacgcctgggctcccagga
cctgctgaactggtgtatgcagattgccaaggggatgagctacctggagg
atgtgcggctcgtacacagggacttggccgctcggaacgtgctggtcaag
agtcccaaccatgtcaaaattacagacttcgggctggctcggctgctgga
cattgacgagacagagtaccatgcagatgggggcaaggtgcccatcaagt
ggatggcgctggagtccattctccgccggcggttcacccaccagagtgat
gtgtggagttatggtgtgactgtgtgggagctgatgacttttggggccaa
accttacgatgggatcccagcccgggagatccctgacctgctggaaaagg
gggagcggctgccccagcccccatctgcaccattgatgtctacatgatc
atggtcaaatgttggatgattgactctgaatgtcggccaagattccggga
gttggtgtctgaattctcccgcatggccagggacccccagcgctttgtgg
tcatccagaatgaggacttgggcccagccagtcccttggacagcaccttc
taccgctcactgctggaggacgatgacatgggggacctggtggatgctga
ggagtatctggtaccccagcagggcttcttctgtccagaccctgccccgg
gcgctgggggcatggtccaccacaggcaccgcagctcatctaccaggagt
ggcggtggggacctgacactagggctggagccctctgaagaggaggcccc
caggtctccactggcaccctccgaaggggctggctccgatgtatttgatg
gtgacctgggaatgggggcagccaaggggctgcaaagcctccccacacat
gaccccagccctctacagcggtacagtgaggaccccacagtacccctgcc
ctctgagactgatggctacgttgcccccctgacctgcagcccccagcctg

aatatgtgaaccagccagatgttcggccccagccccccttcgccccgagag
ggccctctgcctgctgcccgacctgctggtgccactctggaaaggcccaa
gactctctccccagggaagaatggggtcgtcaaagacgtttttgcctttg
ggggtgccgtggagaaccccgagtacttgacaccccagggaggagctgcc
cctcagccccaccctcctcctgccttcagcccagccttcgacaacctcta
ttactgggaccaggacccaccagagcggggggctccacccagcaccttca
aagggacacctacggcagagaacccagagtacctgggtctggacgtgcca
gtgtgaaccagaaggccaagtccgcagaagccctgatgtgtcctcaggga
gcagggaaggcctgacttctgctggcatcaagaggtgggagggccctccg
accacttccaggggaacctgccatgccaggaacctgtcctaaggaacctt
ccttcctgcttgagttcccagatggctggaaggggtccagcctcgttgga
agaggaacagcactggggagtctttgtggattctgaggccctgcccaatg
agactctagggtccagtggatgccacagcccagcttggccctttccttcc
agatcctgggtactgaaagccttagggaagctggcctgagaggggaagcg
gccctaagggagtgtctaagaacaaaagcgacccattcagagactgtccc
tgaaacctagtactgccccccatgaggaaggaacagcaatggtgtcagta
tccaggctttgtacagagtgcttttctgtttagtttttactttttttgtt
ttgtttttttaaagatgaaataaagacccagggggagaatgggtgttgta
tggggaggcaagtgtgggggggtccttctccacacccactttgtccatttg
caaatatattttggaaaacagcta
>NM_003901 3
atttccgggaggggcgaggccggcggctgccgggcctccaatctcggcgg
cggcggcggcaacaggggagcctgggtctcgcggcctgcgagtccgtcgc
gtgctgagggagacgcaggaggtggagccggccgggtgctcgagggaagg
agactggaagctggttccggcgtgaggagagtctgaaaaaggggagcgcg
gagaggaggctggaagaggaagatgcctagcacagaccttctgatgttga
aggcctttgagccctacttagagattttggaagtatactccacaaaagcc
aagaattatgtaaatggacattgcaccaagtatgagccctggcagctaat
tgcatggagtgtcgtgtggaccctgctgatagtctggggatatgagtttg
tcttccagccagagagtttatggtcaaggtttaaaaagaaatgttttaag
ctcaccaggaagatgcccattattggtcgtaagattcaagacaagttgaa
caagaccaaggatgatattagcaagaacatgtcattcctgaaagtggaca
aagagtatgtgaaagctttaccctcccagggtctgagctcatctgctgtt
ttggagaaacttaaggagtacagctctatggacgccttctggcaagaggg
gagagcctctggaacagtgtacagtggggaggagaagctcactgagctcc
ttgtgaaggcttatggagattttgcatggagtaacccccctgcatccagat
atcttcccaggactacgcaagatagaggcagaaatcgtgaggatagcttg
ttccctgttcaatgggggaccagattcgtgtggatgtgtgacttctgggg
gaacagaaagcatactgatggcctgcaaagcatatcgggatctggcctttt
gagaaggggatcaaaactccagaaattgtggctccccaaagtgcccatgc
tgcatttaacaaagcagccagttactttgggatgaagattgtgcgggtcc
cattgacgaagatgatggaggtggatgtgcgggcaatgagaagagctatc
tccaggaacactgccatgctcgtctgttctaccccacagtttcctcatgg
tgtaatagatcctgtccctgaagtggccaagctggctgtcaaatacaaaa
taccccttcatgtcgacgcttgtctgggaggcttcctcatcgtctttatg
gagaaagcaggatacccactggagcacccatttgatttccgggtgaaagg
tgtaaccagcatttcagctgacacccataagtatggctatgccccaaaag
gctcatcattggtgttgtatagtgacaagaagtacaggaactatcagttc
ttcgtcgatacagattggcagggtggcatctatgcttccccaaccatcgc

aggctcacggcctggtggcattagcgcagcctgttgggctgccttgatgc
acttcggtgagaacggctatgttgaagctaccaaacagatcatcaaaact
gctcgcttcctcaagtcagaactggaaaatatcaaaggcatctttgtttt
tgggaatccccaattgtcagtcattgctctgggatcccgtgattttgaca
tctaccgactatcaaacctgatgactgctaaggggtggaacttgaaccag
ttgcagttcccacccagtattcatttctgcatcacattactacacgcccg
gaaacgagtagctatacaattcctaaaggacattcgagaatctgtcactc
aaatcatgaagaatcctaaagcgaagaccacaggaatgggtgccatctat
ggcatggcccagacaactgttgacaggaatatggttgcagaattgtcctc
agtcttcttggacagcttgtacagcaccgacactgtcacccagggcagcc
agatgaatggttctccaaaaccccactgaacttggaccctttctagtctc
aaggggattccagccttcagaaggttcttgggatatggaacaggccgtgc
acaactttgacatctggtcttgctccatagagcacaactcaagatagacc
atgagacagcttgagcctcaggattcttgttcttcctcttatcttccttt
tgtggtttttaatttgaagaccccagagaattccattacataatgatttt
gcccttgttataaatgttaccctaggaattgttttaaccatttccttttc
taaactctctagctttcaactttacttaaacattgtgtggtagctctgac
ctgtcctgattctttagagaagctggggtacagtttatgagatagctaga
gcttctttgttatctcaggcaggaggcgtttacataacagatgtttcctc
agctgggtgtgaggtatactctaagcaggaggctttttcagccttctctc
tctttttttttttttttttttttttgagatggaattttgctcttttgcc
cagtctggagtgcagtggcatgatctcagctcactgcaacctccacccac
tgggttcaagcgattcttctgcctcagcctcccgagtagctgggattacc
ggcacccaccaccacgcctggctaatttttcaattttctttttcagtaga
gacgggttcaccgtgttggccaggctggtcttgaactcctgacctcaggt
gatacccgcccccccgcctcagcctcccaaagtgctgggattacaggcgt
gagccaccgtgcctggccctgtctctcttaagagtaggttcattgtctgt
cttagagtcacttctattgcaactcattttctttttccagggcacagatc
gaccaagctgccgttccctattctgcaggacaggactattctagcatacc
tgcttcgtccacccaggcagggtttggggtggtctcttctgtgcctgcag
tccccatttgacacttggttgccaccatctttggagattattgtttggaa
tgatgcttccattggctttttcttgttaccatggactaggaagaaaacat
ggtttccaaataatctgggagcttttggccatggtgccgccttcctgaat
tggcagtggtcagagcacacctgaaccctatcctgggctggtgatgagca
gaaatcagacctttttctatgctttttgaatatcagagtaggatgaaca
cccagattcaaatatgtcaccaaagttggtggtggtccttccctgcaccc
ttgcgttaagccattatgtaatgaaaatgtgtttgcttgaaggaacagct
caaagcaccttcacaagttgccttgacttaccctaggtgggtgtgaaaga
gcacccgtagcaaggaaaattttctctattagtgtgttcttctgcctctt
ccccccttgattcagctttcagaggtactatggcagttttgcctcaggtgc
tgaacatttctcagccctggctaaaagggagcagcacagggagagaaaca
ggataggaaagcagaatggcgagcagcctatggcccagggcctgtaatcc
cttcccaagactagctgctcagggtggtgcagggacaggaccagaccctg
cgcctatttcctgccttctttcccctatagggaactctgtaggctgagcc
actgtcctgctcttatgacattatatcttgtgcctttctcctcagcagtg
agcagtgagctactcctggcccaggccctaggggaaatggatcagtcttt
gaggtttctatttggggaggggagtacttaagatgagtcaaaagacactt
tcctctgttccattccccatctcagggactcctgaatattcagcctctcc
aggctggtgtcttctagtttcccccactgggaatgctggctgggagagcc

atgactaccagacttttcctcaggctccttggcatgttagtctgaattgt
tcttgagcactgtactactgacccaacaactgtgactagctggccacgcc
attcagggctggtgtggcatttatgtgtgtgtgtgtgtgtgtgtgtgttt
ttcctgtttgcccagcagtgcattgtgggttccaagagtgggtagtgtgt
gtatgtgtgtgtgtcagagggagacctggcaggcacctctttgagagtag
ctgtggtcagagctgtttggtcagtgcattatgttgaatgaggtccagga
acccagagccacccagcagacaccactgtggcttgccagctgccaagatg
gagaagcatgtgcccctgtagagcgtctccccagaaccagaccccgagcc
actcgcttcctctgtgctgtgacaacattggtgccaggggagatggtgtt
tttcaaagggacctactgtagccactttaatttacaattaagagccttag
tttgacttaacacttttgtaggcttttcattgtgtatttttgtgtatgtg
tgcatatagcagctactctgtagcagaggtgggtagagacacttaatagt
atcatgtcgcatgcagatgtcacatcggcctctgcaaaaactgtactgtc
ttgtttctgcattagacttaagtagtcatgtgaatatactgctatgtcac
ttttaatattacgagttttatacttggaaaatggtacttgcttcttttaa
atctctgtcttctctaacctccccttcccatttcaatgctcccttccta
atttcagcaataatctcaaaaagcaattaaatagttaaatgaccctaatt
gtaattactgtggatggttgcattcatttgattacttgggcacacacgag
atgacaaatggggcagtggccatgcttgaatgggctcctggtgagagatt
gccccctggtggtgaaacaatcgtgtgtgcccactgataccaagaccaat
gaaagagacacagttaagcagcaatccatctcatttccaggcacttcaat
aggtcgctgattggtccttgcaccagcagtggtagtcgtacctatttcag
agaggtctgaaattcaggttcttagtttgccagggacaggccctatctta
tattttttccatcttcatcatccacttctgcttacagtttgctgcttac
aataacttaatgatggattgagttatctgggtggtctctagccatctggg
cagtgtggttctgtctaaccaaagggcattggcctcaaaccctgcatttg
gtttaggggctaacagagctcctcagataatcttcacacacatgtaactg
ctggagatcttattctattatgaataagaaacgagaagtttttccaaagt
gttagtcaggatctgaaggctgtcattcagataacccagcttttcctttt
ggcttttagcccattcagactttgccagagtcaagccaaggattgctttt
ttgctacagttttctgccaaatggcctagttcctgagtacctggaaacca
gagagaaagaggatccaggatgtacttggatgaggaggcctggcttatct
aggaagtcgtgtctggggtgcttattgctgctccatacagctgtacgtca
gccccttggccttctctgtaggttcttggcagcaatgagcagctttcact
cagtgacacaagtaattactgagtcctaatttgatagccaccaactgtac
ctgggtaggcaaagtcagatttttgagaaccttttcctgatttgaagtt
ttaattaccttattttcttttatgctttcctctgtcttgtaatcttttct
cttcttaatatccttccctataatttcaattatttggattaattttagaa
taaacctatttatttctaaaaaaaaaaaagaaaa
>NM_001806 3
gcaggccggcgctcccattggccgggatggcggcggcggcgcgcgcggcc
ccggcgagcaggggaagccggtggccgcggctgcggaacgggcggaggct
gccggtttcgtaaccgtcgctcctcctcgctgactcgcgggctgtgaggc
ctgggtcggctcgggccgcaccgcgcggggccgctcggagtggaggccgc
ctgggggcaggcgggctagaggagcaggtacatgtgaagatttttggca
gcttagcgtggaaaccattgatcaccctgctctcatttctacctgttctg
tgttggcaagggagagtgcccaaatgagcaagatatcgcagcaaaacagc
actccaggggtgaacggaattagtgttatccatacccaggcacatgccag
cggcttacagcaggttcctcagctggtgcctgctggccctgggggaggag

gcaaagctgtggctcccagcaagcagagcaaaaagagttcgcccatggat
cgaaacagtgacgagtatcggcaacgccgagagaggaacaacatggctgt
gaaaaagagccggttgaaaagcaagcagaaagcacaagacacactgcaga
gagtcaatcagctcaaagaagagaatgaacggttggaagcaaaaatcaaa
ttgctgaccaaggaattaagtgtactcaaagatttgtttcttgagcatgc
acacaaccttgcagacaacgtacagtccattagcactgaaaatacgacag
cagatggcgacaatgcaggacagtagacctcaccctttccagactttaga
gcttgtggcttgaatgttaaaggtgtgaccaccgacaccactcatgtcaa
tggctgaaagttgtccatttccatgactcaaagacccattggaggctatt
ttctgggatcagcactgaagagttgattagctaaaaatgttagccttgta
attcgaatatctggttttaaatgatagaggtttttgtgggaatcaaaatc
ccccaaatgttaaggtatatggtaaaaaaagaaatatctgggatcccgat
gttcttaataaatcctgacttcccaagaaatgcttctttttttaagttgac
aaaaggaatggggaactggcaggccgcgcagaaggttcttggttttaatg
gataggctgaattggattaagaaaagttgaatgccacctatggtaatcta
tttgtgattttcttctaaattatgtattataaattcgtagagctatagaa
agcaatgagtgtgtaatttggagtgattttatatatggcataaactttgt
tttaacataattagtactgttttcccccaaaagtacaagtttttgagtag
caatgtcaggttaagtaaagaaacttcatcacatcttataggtagtgtgt
ggccaattgacttaaaaaatacaaataacatttaggaagcaaatagatta
aacacaaaaataaaactaaagcataggaattatgtttttgagataccttt
gggcttagattggcattgttttattctaaaaacccaactcagtggtgtag
agaaacttgtgtaccaaaattttagtttctgcagatgctagtgttttttt
ggatacaattttgacaaccaagttagtaaacaaaatatcttaacagtttg
atgacacaagctactgatgagggtttggaatattaattcagaaggtagtt
tctcttgtgttcaaaatagctgccatggggctgttacttttaaagtcaaa
attttcttctgaaggctcattttggtatttgatcttaaccaagtgattat
tagagaaatgtatcaactccatgccatctcccaaaataattgtctaagaa
aacttgaaagtgtaaggttttaacctttaatttatttctcttaaatacat
cttttgatattgttgttgtgacatttctttttctggttagtgggctttcc
agactttgtaccactgcttctgtttattcatttatatgcttttgtgtccc
ataaattatttcagaaaatgctgataaaactcaggatattgacatttttg
ttgagactaaaaaatggcagtcgctaaagtagggactctagagtctggct
tacgtcagtgttggtagtttagattgtctttgtcaacgtttttttcttctc
tcttttgctttcttttctttctctttttttcttagcacagttctagctca
aatttgtgtattttttgtgtgcctgggctggagatgagagactgagtcat
aactgatttaaaagtttgtgttatcaggtatcttatttgaacatggtcat
ttttggccacattgctgtttcatactaggacttgggatgatgtagccaga
ataaaactcaagttgcaccctccggttggtggaagattgctgaccgtgcc
gtttctgggcaggagaagacatcatggtgtccagcaactcagcaaagcca
ttcttaagagtcgtgaggtccttctgaatgtaaaactggagcccaggaga
agctgtcccaggagggctgttaactccctatagagccaggagacaggata
ggggtttctagggtccaacaccagcttaccttggagtatgaatctaccca
tgaaggatgagagatgttttgaaaaactagccaggacacacccacaggat
cctactggctccttagcagctgattggtgttacataattaacttaattgg
agatgcattaggtcacttgaatgtataagcaagcacctatggtaggcgct
acagacatttaaatctcttgggaattcgatgctcccatggaatttatacc
agttatatgaattgacttaagtatcttgaaaaagaaactttagagaaagc
atcaggggtgtgtactcagtatttcaaatcagaacacaagattggaactt

ttggaaaaatgggttcaagctttcctattagccatggaaatgcaaagttt
agcagaagcaagcaattaggcagagaacaaaaatgttaagcatggtgttg
tctatcttattgaagtggttggaaatgaaagcttttaatttgatagattt
atcagtataaaattagggaaaccacgtgtggggaatgaatcaatttagag
cttcgggaattgtgaggtgacttttgtaacttttgttctgtgtgtgacct
gtgaaccactaggatgtgatctgcccttgtgggcaggtccagcatagtta
ggagttaggctttagcataaatttctagctgcatctgagtctcctgggat
gggtgctctttggctggttttggctgcggatggtgagatcagagcagctc
ttcctgctgctggcccctgcaatcagttgttgggatgccagtgcagatca
ctaagtagtaagattttaatcaaacacgaccaggtccgaaatgcaggtca
tgagtgtgaaattctcaaatttacataaaaagtagaagtatagacagttt
aacatttggtattaaaggagaggaaattgtagcagcttttcacgtttccc
agtccccattagagggcttgagaccttgtacctgaacaacccattttgca
ctcagtgctttctgatgccttaggagaattgttttgtttcacaaaagctg
ggaaggaagaagtccattctgcagctgttagatctgcctctcaggaaaaa
gtactaacttgttctttttgttcctggctttcatcagtttgtgagatttc
tctattttttttaaatataattttatttctttcaacaaatataaaataaa
aaacaactttggaacaatgaaaaaaaaaaaaaaa
>NM_175709 3
ccagccccagcatcgcgcgccgcagccgcggccccgcagctccgcccccg
gcccggcccggccccgggcccgctcgcccgccgccccgcatggagctgtc
agccatcggcgagcaggtgttcgccgtggagagcatccggaagaagcgcg
tgcggaagggtaaagtcgagtatctggtgaagtggaaaggatggcccccca
aagtacagcacgtgggagccagaagagcacatcttggacccccgcctcgt
catggcctacgaggagaaggaggagagagaccgagcatcggggtatagga
agagaggtccgaaacccaagcggcttctgctgcagcggctgtacagcatg
gacctgcggagctcccacaaggccaagggcaaggagaagctctgcttctc
cctgacgtgcccactcggcagcgggagccctgagggggtggtcaaggcgg
gggcacctgagctggtggacaagggccccttggtgcccaccctgcccttc
ccgctccgcaagccccgaaaggcccacaagtacctgcggctctcgcgcaa
gaagttcccgccccgcgggcccaacctggagagccacagccatcgacggg
agctcttcctgcaggagccaccggccccagacgtcctgcaggcggctggc
gagtgggagcctgctgcgcagccccctgaagaggaggcagatgccgacct
ggccgaggggcccccctccctggacacctgcgctccccctcaagtgaggtga
ccgtgaccgacatcaccgccaactccatcaccgtcaccttccgcgaggcc
caggcagctgagggcttcttccgagaccgcagtgggaagttctgaatcac
cgtttttactcttcttaaactgttttcttttgggcttggggtgggacttc
cagagatagggatgggttgggggcggggtaattattttatttaaaaaaat
accgagcagcaaaaggggagaagatcccactactctcccaccacctgccc
tttctctgagggacgtttaccacgaggcctcaggctggggatggagagag
ttgctctgggagttggggtaccacccccagggcaggatggggacaggatc
acctgcccgggacaccaccattatcattctcctctagtgacgcagcagct
ggttctgggagttaaaggagcattggaaggcccaaaccctctcccttgag
tggccaccccagcctggttggctggttttccccttttctcttgtttcaat
tgggtctttaccttgaactctcctctctggctttgcggtgggctgtggag
gctggttttgaccaaaagtgagtggggcgggaggaaggggcaggaggaag
ggttgaggttacttggggcgagtcccttccccttcagagaggcttctatc
cttcccagggaggaggcgccgctgagacccttctgctgagagctctgccc
tcccctcatcacctggcctgtgcagaaacgctcatgcacacctggctgca

caggtgtgcacgcattacccttcgcgtgtacgttcccatgtgccccgtga
aagcatgtgtggctgcagacgtgtccacatgggccttgcgaacctgggtt
agaaaccctggccaggcgaacgtggggtgattcacagcacaaaagacctc
accaccacacctgcactcaccccaccttgcatgcaccttgctacctgctt
gcggctttcagtggagggcaggggtctggcacaggtgcgatggcacccca
tgctccaggcatacagatgtggtttctcggctgcaccgggccaggctgcg
ggtgtgcaggcgtctgctaagttgtgtgatgtatcagcacaggctttgag
acgtctggaccctgtccttcctcccgtgaggggttcttgttctttctgac
tcaggtgacttttcagcccttccaattcccctctttttctgccctcccct
ccaactcagccaacccaggtgtgggcagtcagggagggagggagtgtccc
accacgttctcagggcagcccttgactcctaagccccttcctccttccat
tctgcatcccctccccatccaacctaaatgccacagctggggctgagctg
tattcctgtggagggacctctgccgtgcctctctgaggtcaggctgtgct
gtgtgatgggcaggctttgccccagcccacccctggcaaggtgcacttgt
tttctggtttgtacaaggtgtcctgggggcccgtggcttccctgccagtg
aggagtgacttctccctctcttccagtcctgtaggggagacaaaaccaga
ttgggggcccaaggggagcatggaaaaggccggctcccctgtctttcct
tggctgtcagagtcagggtaacacacaccaagagtggagtgcggccagca
agtttgagacctgcccgccctcctcgcagctctgctctgtgtcctcagga
agtcacagagtctactgaggcaaggagagggtgattctttccccaaatcc
cttcttccctggttcccaaaccaaagacagcctgcagccctttctgcatg
gggtgctctgttgacaggcttcccagatccctgagtctctctttccttcc
tcctcgatctttagttgtccacggtcaattcagtgcttccattgggggac
agtcccctccgggatgacctgattcacctccagcccagggaatggaatct
agaggaatacgtggggtgggtctggacaaggagcggcaggaatcaccacc
catctccagctgtggagccctgtggaggggaaggggaagcttggggttca
gaggggactcttccaggagaggggtgcccagcggaggtaaagatgataga
gggttgtggggggtctctagttgaatgttttggcccatgactttggaaca
tggctggcagcttccagcagaagtcacgctccccatcccccaggggacat
aggacctttttcctgcttcctggtcactttcaaagaactatttgcgcaat
ctgtgggtctgtggattcacggggctttctgtgtgggtgctgcagttgct
tttgtctgcagcagcaggacacatctttcctcttactcagccctttatgg
cccatggggaactccgtggctcagggagagctgaactccaggggtgtgac
ctgggacgggtgggcctgaggtgcccagctcagggcagccaggtggctca
tgggctgtagtgagccagctccctgggggaaaaggctgtgggccgttagg
accatcctccaggacaggtgacctctatgaggtcacctacggctgtggcc
gtgcaggcctccttccagcccagagtggcccagtagagcaaggcagacag
tgacctccacccccgcagccctcttaaaaggccagtactcttgggggtgg
ggggagggtttagaaagcatttgcccatctgcctttctttcccccagccc
ccacccgctttgaatgtagagacccgtgggcacttttccttttgtggtgg
ggggtgcggaggaggtacccccacccctggcacagccgcctggaatgcag
gactgtcactgctgttcgggtgatgacctcgttgccaagctcctcctgtc
cccttgttctgggggcaggcgctgtgcttctgtgaggtggtttagctttt
gctttcgaagtggccagctgcggccaccaggtctcagcacaagagcgctt
cctttgcacagaatgagcttcgagctttgttcagactaaatgaatgtatc
tgggaggggtcgggggcacgagttgattccaagcacatgcctttgctgag
tgtgtgtgtgctgggagagtcagagtggatgtagagcgcggttttatttt
tgtactgacattggtaagagactgtatagcatctatttatttagatgatt
tatctggtaaatgaggcaaaaaaattattaaaaatacattaaagatgatt

taaaaaaaagaaaa
>NM_005324 3
gagcgcagagcggtttggtcgttcgttgggcggtgctggtttttcgctcg
tcgactgcggctcttcctcgggcagcggaagcggcgcggcggtcggagaa
gtggcctaaaacttcggcgttgggtgaaagaaaatggcccgaaccaagca
gactgctcgtaagtccaccggtgggaaagccccccgcaaacagctggcca
cgaaagccgccaggaaaagcgctccctctaccggcggggtgaagaagcct
catcgctacaggcccgggaccgtggcgcttcgagagattcgtcgttatca
gaagtcgaccgagctgctcatccggaagctgcccttccagaggttggtga
gggagatcgcgcaggatttcaaaaccgacctgaggtttcagagcgcagcc
atcggtgcgctgcaggaggctagcgaagcgtacctggtgggtctgttcga
agataccaacctgtgtgccatccacgctaagagagtcaccatcatgccca
aagacatccagttggctcgccggatacggggagagagagcttaagtgaag
gcagtttttatggcgttttgtagtaaattctgtaaaatactttggtttaa
tttgtgactttttttgtaagaaattgtttataatatgttgcatttgtact
taagtcattccatctttcactcaggatgaatgcgaaaagtgactgttcac
agacctcagtgatgtgagcactgttgctcaggagtgacaagttgctaata
tgcagaagggatgggtgatacttcttgcttctcatgatgcatgtttctgt
atgttaatgacttgttgggtagctattaaggtactagagttgataaatgt
gtacagggtccttttgcaataaaactggttatgacttgatccaagtgttt
aacaattggggctgttaagtctgaccatacatcactgtgatagaatgtgg
gctttttcaagggtgaagatacaagtcttaaccacagtgtaacttacagt
ttcctttaaaaaaaaaaaaagtaaacctggcagctatagaatacactatg
tgcatttataatagctattttatatattgtagtatcaacatttttaaatt
aaatgttttacattcacaagtggtggggagtcttgtcattaaggtgtgtg
taatttagagtccagttggttttcttctgactgcacttgttctcatagta
gtaaaatgctatgcgcatttataccttgcataagtcctcattctaccaca
tgttaaccctctagctgataatgcaaacactaactggggggattttattta
taagggctctagaaaaaacgagttattcacaccagcatcatcttaactaa
cattctgaactagttagtgcagcttttcattgtgttgtgtggttggtctc
ataactaggttgagtttttctcctctgctgaggaaacagtaccgaagttc
tttttcttgtggcatttgtattataaaaacttggtgtgggggaggagcac
aaaactccagcccactgaacctctgccaattaagatggtgttgggttagg
ttacatctggttactgtcctgggaaaatcatttttatagagatggccttc
caagtggttttaaaatttactgaagtttttaggtcaattatgtatgttga
ctaaatttacaaataaacttgtttatccaactaagtgtccaaaacctaaa
ttgaatgtactaagttttcacatgtcccattatctaggtccttgtatact
aatgttttgaacttagatcatttcaggtgttgtttggtggataaaggaac
cttttatttataaagatactgtagaaagcatgtgaacagctctctgcttg
attaagatgccataatagtgctgtatttgcagtgtgggctaagacaaagt
atattaataagcttttcagcccccccactcccgttccgtagtgtagaagc
ccacaggtgtagaactcagtcttaaacttcagtatgaaaccagtttcctt
gtgcgatgatggccactaaagcatagtacgtggatgtcagtgagacagca
tgagagccagcagtcatcaaagcgttccacgtttgaagttagcaactgct
taaagttatgccctattaaaattgctttctcaaaagtttgggttagtttc
aaatgtgatattttggagggaaggtaaagtaggtatctttcaggtcgtga
taatgagctcctatgaaaggatgcaatataatgacccgcttttctagaaa
gttcataatcagctctggaacaagcacacttgattcctcactgtgcttca
gaatgagattaagatcagatgttggaacgtgctatgctgtagcgtgtctg

gaaacaaagtacacaaacctggctacggtgatgagttagcttctgcttac
tacctgtgacaacccaagtgggtgacactagtgaaccttctccagtctgc
aggctggcatagaaggctcttagattatattgggcagcttgcaatctgcc
gaagcagtgacttgcatttccacacttggcttgagcactcaacccagaag
gcgaagatagcttttggttgtaggcggcttcctgtatgggatatccctcg
gtaagggtaaaggagcagaggcaaaggagaaaagcagaagttgcagctga
tgcaggtatcctatgcccttgatggatgagactaaaataaaatttttgaa
gttaaaaaaaaaaaaaaaaaaaaaaaaaaaaaaaaaaaaaaaaaaaaaaa
aaa
>NM_173473 3
acgggcgcgcgcacgccgccttcgccgctggctccgtctgttggggggcg
aacacgccgcggtcctcgtcgtggtgagcgcagccactcaggctggtcct
gggggtggggctgtaggggaaagtgctaaagccgctgagtgaagtaagaa
ctctgctagagaggaaatggctgcttcatcatcatcctcctcagctggtg
gggtcagtggaagttctgtcactggatctggtttcagtgtctcagacctt
gccccaccacggaaagcccttttcacctaccccaaaggagctggagagat
gttagaagatggctctgagagattcctctgcgaatctgtttttagctatc
aagtggcatccacgcttaaacaggtgaaacatgatcagcaagttgctcgg
atggaaaaactagctggtttggtagaagagctggaggctgacgagtggcg
gtttaagcccatcgagcagctgctgggattcacccccctcttcaggttgat
actgcctggatggtcacctctggtgcgcagcaagtgcaaagccagtgggg
gactttctcacagcttacatagccatccagagatccacagctacgtcact
gaattgttaatgcacatttgtacttggtttctctgtatctattcacaggc
aacaaatacttatatgtgtgatctttcagggaatgttttgtttatttgtt
tttaaaagtattgggaatcagattaagacaatcagtttcagagaaccagg
aggtttggggttaagagatactcaaaaattttcacaagccaagtagggca
tatatcagatttggccaactgaatggcgtctgtcctgtcatccatatggt
gcctggaaatatttaccagtcaaggtcaaggtcagcatctgtggttaaaa
atatagcattctgacctaaaaaagttattttgcagatgaatgtgttttca
actcaggacctatccaaatgaggaatttttaaatattcttttttttttcc
tatttttagacatcaattctatagattctgactttttctaacctcttata
gacatgccaaatgctggcaaaaagaagtgctttttggatatggcagcact
tgtaaaaataaagcagtaagcaaaatccttttaaacacagaaatcctgag
ttcttctcattggtggactcaagcaattctgtagcaaataaatcctttga
aagagctccaaattggtggcattatcctttcaaaatctcagggatttggg
atgaagtgaagaggtcaaattactttttagaagaagggaatctaaaaacc
atctctcctaaacaaatggtagactggctttacttaagggatatttgtct
ttataggagtacataaatttatcttaatgatatttaagtagttttttttt
ttttttgagacgaagttttgctcttgttgcccaggctgtatcgcaatggc
gcgatctctgctcactgcaacatccacctcccaggttcaagcaattctcc
tgcctcagcctcccaagtagctgggattacaggcacccgccaccacaccc
agctaatttttttgtatatttagtagagacggggtttcaccatgttgacca
ggctagtctcgaacttctgacctcaggtgatccacctgcctcagcctccc
aaattgctgggattacaggcgtgagccaccttgcccagccaattttttttt
taaagagacatggtcttgctatgttgcccaggttggtcttgaactcctgg
cctcaagagatccccccaaccttggcctccaaagtgctgggattacaggc
atgagccaccgcgcctggcctcaagtaagtttttataccatgatagggga
aaacctggaaggctaagagatgtgctgttttccttcatgggaccccactt
tccacagtaaacaactggtataatcatttattacgttgtatgcaaatttt

gccttagacaacatttgtctggttccttattaataacttgcatctgatct
gcttagagttgcttaattataagtaaatataatcccagtagcatttagcc
ttaatctcaagagttcaaaatgtttaacacattctacctgacgggaggtg
gtgacctcattcagtcggttaagagcctgagccctgtgaagttaaacacc
tgctcatggtcacatagcccaggcagaagaaaatctcggacttggaatag
atcccagttgcctgataattcatctcattattgcctctaaagcaaattgg
agactgggcacagtggctcccacctttaatcccagcacctggggaggctg
aggtgagtggatcactagaggtcaggagttcaagaccagtctggccgaca
tggtgaaactcagtctctattgaaaatacaaaaattagctcagtgtggtg
gcacacacttgtaatcccagctactcaggaggctgaggcatgagaatcgc
ttaaacccaggaggcggaggttgcagtgagctgagatcacaccattgcac
tctagtctgggtgacagagcgagactctgtctcaaaaaaaaaaaaataaa
gccgggcgtggtggctcacgcctgtaatcccggcactttgggaggctgag
gcaggcggattgcctgagatcgggagttcgagaccagcctggccaacatg
gtgaaaccttgtctctactaaaactacaaaaaaaattagccaggcgtggt
ggcgggcgcctgtaatcccagctactcaggaggctgaggcagcagaatcg
tttgaacccgggaggcggaggttgcagtgagtggagatcgtgcctgtgtg
ctccagcctgagcaacaagagcgaaactgtctcaaaaaaaagaagaaaat
aaataaataaaaatcagagcacaagaagtaccagtccaccaactcacatt
ttctatattaataattcttaactaaaagaagttccatattcgacagcatt
ttagttttttaatagtacttattagcatcaaccatcttgataatcaccaa
gggaaaaattaaaaatacctagactgcaataaacctttaaattggatttt
cagtgagaatttagaagcaatcactacctttctcgtttcatttgtgtaac
catgcagcataggcactgtgttacatcatctttacgctttggtatttgaa
cttattacagaattttttctctaaattggatgtttctttatatgaccttg
gaaactgaagtgatcttagcattaaacaaaaatgctttttgaaaaaaaaa
aa
>NM_002644 3
gagcagagtttcagttttggcagcagcgtccagtgccctgccagtagctc
ctagagaggcaggggttaccaactggccagcaggctgtgtccctgaagtc
agatcaacgggagagaaggaagtggctaaaacattgcacaggagaagtcg
gcctgagtggtgcggcgctcgggacccaccagcaatgctgctcttcgtgc
tcacctgcctgctggcggtcttcccagccatctccacgaagagtcccata
tttggtcccgaggaggtgaatagtgtggaaggtaactcagtgtccatcac
gtgctactacccacccacctctgtcaaccggcacacccggaagtactggt
gccggcagggagctagaggtggctgcataaccctcatctcctcggagggc
tacgtctccagcaaatatgcaggcagggctaacctcaccaacttcccgga
gaacggcacatttgtggtgaacattgcccagctgagccaggatgactccg
ggcgctacaagtgtggcctgggcatcaatagccgaggcctgtcctttgat
gtcagcctggaggtcagccagggtcctgggctcctaaatgacactaaagt
ctacacagtggacctgggcagaacggtgaccatcaactgccctttcaaga
ctgagaatgctcaaaagaggaagtccttgtacaagcagataggcctgtac
cctgtgctggtcatcgactccagtggttatgtaaatcccaactatacagg
aagaatacgccttgatattcagggtactggccagttactgttcagcgttg
tcatcaaccaactcaggctcagcgatgctgggcagtatctctgccaggct
ggggatgattccaatagtaataagaagaatgctgacctccaagtgctaaa
gcccgagcccgagctggtttatgaagacctgaggggctcagtgaccttcc
actgtgccctgggccctgaggtggcaaacgtggccaaatttctgtgccga
cagagcagtggggaaaactgtgacgtggtcgtcaacaccctggggaagag

ggccccagcctttgagggcaggatcctgctcaaccccccaggacaaggatg
gctcattcagtgtggtgatcacaggcctgaggaaggaggatgcagggcgc
tacctgtgtggagcccattcggatggtcagctgcaggaaggctcgcctat
ccaggcctggcaactcttcgtcaatgaggagtccacgattccccgcagcc
ccactgtggtgaagggggtggcaggaggctctgtggccgtgctctgcccc
tacaaccgtaaggaaagcaaaagcatcaagtactggtgtctctgggaagg
ggcccagaatggccgctgcccccctgctggtggacagcgaggggtgggtta
aggcccagtacgagggccgcctctccctgctggaggagccaggcaacggc
accttcactgtcatcctcaaccagctcaccagccgggacgccggcttcta
ctggtgtctgaccaacggcgatactctctggaggaccaccgtggagatca
agattatcgaaggagaaccaaacctcaaggtaccagggaatgtcacggct
gtgctgggagagactctcaaggtcccctgtcactttccatgcaaattctc
ctcgtacgagaaatactggtgcaagtggaataacacgggctgccaggccc
tgcccagccaagacgaaggccccagcaaggccttcgtgaactgtgacgag
aacagccggcttgtctccctgaccctgaacctggtgaccagggctgatga
gggctggtactggtgtggagtgaagcagggccacttctatggagagactg
cagccgtctatgtggcagttgaagagaggaaggcagcggggtcccgcgat
gtcagcctagcgaaggcagacgctgctcctgatgagaaggtgctagactc
tggttttcgggagattgagaacaaagccattcaggatcccaggctttttg
cagaggaaaaggcggtggcagatacaagagatcaagccgatgggagcaga
gcatctgtggattccggcagctctgaggaacaaggtggaagctccagagc
gctggtctccaccctggtgcccctgggcctggtgctggcagtgggagccg
tggctgtgggggtggccagagcccggcacaggaagaacgtcgaccgagtt
tcaatcagaagctacaggacagacattagcatgtcagacttcgagaactc
cagggaatttggagccaatgacaacatgggagcctcttcgatcactcagg
agacatccctcggaggaaaagaagagtttgttgccaccactgagagcacc
acagagaccaaagaacccaagaaggcaaaaaggtcatccaaggaggaagc
cgagatggcctacaaagacttcctgctccagtccagcaccgtggccgccg
aggcccaggacggcccccaggaagcctagacggtgtcgccgcctgctccc
tgcacccatgacaatcaccttcagaatcatgtcgatcctggggccctcag
ctcctggggaccccactccctgctctaacacctgcctaggtttttcctac
tgtcctcagaggcgtgctggtcccctcctcagtgacatcaaagcctggcc
taattgttcctattggggatgagggtggcatgaggaggtcccacttgcaa
cttctttctgttgagagaacctcaggtacggagaagaatagaggtcctca
tgggtcccttgaaggaagagggaccagggtgggagagctgattgcagaaa
ggagagacgtgcagcgcccctctgcacccttatcatgggatgtcaacaga
atttttccctccactccatccctccctcccgtccttcccctcttcttctt
tccttccatcaaaagatgtatttgaattcatactagaattcaggtgcttt
gctagatgctgtgacaggtatgccaccaacactgctcacagcctttctga
ggacaccagtgaaagaagccacagctcttcttggcgtatttatactcact
gagtcttaacttttcaccaggggtgctcacctctgcccctattgggagag
gtcataaaatgtctcgagtcctaaggccttaggggtcatgtatgatgagc
atacacacaggtaattataaacccacattcttaccatttcacacataaga
aaattgaggtttggaagagtgaagcgtttttctttttctttttttttttt
gagacggagtctctcactgtcgcccaggctggagtgcagtggcgcaatct
cggctcactgcaacctccgcctcccaggttgacaccattctcctgcctca
ccctcccaagtagctgggactacaggcgcctgccagcacgcctggctaat
tttttgtatttttagtagagacagggtttcaccgtgttagccaggatggt
ctcgatctcctgacctcgtgatccgcctgcctctgcctcccaaagtgctg

ggattacaggcgtgagccaccgcgtccggcctctttttttcttttctttt
ttttgagacaaagtctcactgtgtcacccagactggaatgcagtgacaca
atctcggctcactgaaacctctgccttccaggttcaagctattctcatgc
ctcagcctctcaagtagctgggactacagatgtgggccaccatgtctggc
taattttttttttttttttttttttttgtagagacagggtttcgccatgt
tgacgagactggtctcgaactcctggcctcaagtgatctgccgcctcagc
ttctcaaagtactgggattatataggcatgagccactgagcctggccctg
aagcgtttttctcaaaggccctcagtgagataaattagatttggcatctc
ctgtcctgggccagggatctctctacaagagcccctgcccctctgttgga
ggcacagttttagaataaggaggaggagggagaagagaaaatgtaaagga
gggagatctttcccaggccgcaccatttctgtcactcacatggacccaag
ataaaagaatggccaaaccctcacaacccctgatgtttgaagagttccaa
gttgaagggaaacaaagaagtgtttgatggtgccagagaggggctgctct
ccagaaagctaaaatttaatttctttttcctctgagttctgtacttcaa
ccagcctacaagctggcacttgctaacaaatcagaaatatgacaattaat
gattaaagactgtgattgccaccaaaaaaaaaaaaaaaaaaaaa
>NM_182511 3
acccgcaggtggcgccccacgtccgtgtttgcgccctcagccgagctcca
ggcgcagctgggaaacccagcgcgtctccccgggtcctttgagcgagcgc
ggacaccgcaatttaataaataagcgcaagcgccagccgctccaccagtc
cgaggtccagaggagcagacacacagacccgggccggaggcccctcttct
agccctgcgggaaccggacagttccccaactgggactctggaaccacagc
tcctaaatcatcaaattctcaagcttttttttccctctcttcgtcccag
ccatcccagtcttcttcttctttttttttttttaacttattgttttttt
cgctcctgtcattatgaaagtggtcacgccattcaatattaagacttgga
gggaattggggaaagaaaagaaagaatctaaaagaagagaagcgaccggt
gcttttaagggtgtctaattttcaaaagagacgtctgggagtattttgct
ctgggcgtttggagcaacttcgcggacagcggagctcgcccagcatggat
gttccaggttcacaggcgcctttcttctgagaacgaccctggccttgaac
gtcagagccggggacgaaggcccccggaggctgctgcgagctccgcgcgt
tccttcgcgcccttccgcgccgctcgcgccggcgccggcctccacccccg
cgcgccgcctcccaccagtcccgatgcaggcgcccggccgggggccactc
gggctgcggctgatgatgcccgggcgccggggggcgctgcgcgagccggg
cggctgcggatcctgcctgggggtggcgctggccctgctgttgctgctac
tgcccgcctgctgccccgtgcgggcgcagaacgacacggagcccatcgtg
ctggagggcaagtgcctggtggtgtgcgactccagcccgtcggcggacgg
cgccgtcacctcctccctaggcatctccgtgcgctccggcagcgccaagg
tggccttctccgccacgcggagcaccaaccacgagccgtccgagatgagc
aaccgcaccatgaccatctatttcgaccaggtattagtaaatattggcaa
ccactttgatcttgcttccagtatatttgtagcaccgagaaaaggattt
atagcttcagcttccacgtggtcaaagtgtataacagacaaaccatccag
gtcagtttaatgcagaatggctacccagtgatctcggcctttgcaggaga
ccaggatgtcaccagagaagctgctagcaatggcgtgctgctgctcatgg
aaagggaagacaaagtgcatctcaaacttgagagaggcaacctcatgggg
ggctggaaatactccacattctcgggcttcttggtgtttcctctataaac
acagagccccctagatggtgggggaatggcaaactggacccaggactccg
ccctttaaaacaccctgaacttactggaattggacaccttgtttccaacc
tccgtcagactgttgcagtagaagaatgatttcctttgaaacctccagta
cttttgtttttgtttttggaatactgacaattcctcgggaacctggcct

ctaattagttttagatgacaaggtcttaaggagaaatgaaattatcgatt
tgagcaatttgtacctgtgattgtaaagtcaatatcggattttattgttg
ggaccatggacctcttttgtttgtatgttgtattgtcgtcccaacggaag
gagagctcctgactccaggatgggctgcaggttgcagtcagggcttgaag
taggagcccagcaaagaaccacctgctggacagtccttgacatgtgttct
gtgtgtgtctgtatagccttaagaaaaagaatggcttcactttcattctg
tattcttcccccaccatgtggctgggaggacttgggagggggatgggga
cattgggaacctgtcaagaagtgctttatccagagaagcaaattttgcac
gattggactgcaatttttgttttgtattgtttgtgttttttcttgaaaag
ctttacttttctttccacactcagctctccctcctcaaccccacttttat
ttttcttgctggggttgaggagagaaaatatagaattcctggataagacc
aaacaaaacaaaacattaaaatacctgtatgttttgttttagacgagacc
aaactaaacaaaaagtatctgtttatcaaagtaaaagtaacacaatggac
aattctgcttattctctcaaagagattctaagatgcacctttagaactat
taatagcaacctgcatttttttttaatttatacttcagaatcctttaaga
acctggtgttcctgagtggtcctgaatcatataagttggtaatggaagct
gtaatgaccaagtcccctaaacatactatgtctttgccacgtgtgctgtg
acttctctgtgggtgatttaatttatttggatccacctctgagtgagcgc
acagtgatcaggtgcttcaaagccaacagaccagctcctcttcctccgga
tcctcttttgatctgcccaggaaagggatgcattgacactctcctgcatg
cacctggcgagaagccacctgaaagtcactgtggttaaagatattggtgg
aggtaccccaggagcactgttacaaatccttcttgttttggcatctcgta
caacattattaagacacagctgagagttgatgggtgtgtaatgcatatgc
caaggaaatgtcactaatcccaaagcaatcaaaaaggagacctcaaacca
gatgttaatttgttctttgtgtaacaatgtaaccaaaatattgatgataa
aagtcataatttaagattcagaataaatgggtttgatgtctggcaaaaaa
aaaaaaaaaa
>NM_173843 2
gggcagctccaccctgggagggactgtggcccaggtactgcccgggtgct
actttatgggcagcagctcagttgagttagagtctggaagacctcagaag
acctcctgtcctatgaggccctccccatggctttagggggattataaaac
taatcatcaaagccaagaaggcaagagcaagcatgtaccgctgaaaacac
aagataactgcataagtaatgactttcagtgcagattcatagctaaccca
taaactgctggggcaaaaatcatcttggaaggctctgaacctcagaaagg
attcacaagacgatctgccgaccctctgggagaaaatccagcaagatgca
agccttcagaatctgggatgttaaccagaagaccttctatctgaggaaca
accaactagttgctggatacttgcaaggaccaaatgtcaatttagaagaa
aagatagatgtggtacccattgagcctcatgctctgttcttgggaatcca
tggagggaagatgtgcctgtcctgtgtcaagtctggtgatgagaccagac
tccagctggaggcagttaacatcactgacctgagcgagaacagaaagcag
gacaagcgcttcgccttcatccgctcagacagtggccccaccaccagttt
tgagtctgccgcctgccccggttggttcctctgcacagcgatggaagctg
accagcccgtcagcctcaccaatatgcctgacgaaggcgtcatggtcacc
aaattctacttccaggaggacgagtagtactgcccaggcctgcctgttcc
cattcttgcatggcaaggactgcagggactgccagtccccctgccccagg
gctcccggctatgggggcactgaggaccagccattgaggggtggaccctc
agaaggcgtcacaacaacctggtcacaggactctgcctcctcttcaactg
accagcctccatgctgcctccagaatggtctttctaatgtgtgaatcaga
gcacagcagcccctgcacaaagcccttccatgtcgcctctgcattcagga

tcaaaccccgaccacctgcccaacctgctctcctcttgccactgcctctt
cctccctcattccaccttcccatgccctggatccatcaggccacttgatg
acccccaaccaagtggctcccacaccctgttttacaaaaaagaaaagacc
agtccatgagggaggtttttaagggtttgtggaaaatgaaaattaggatt
tcatgatttttttttttcagtccccgtgaaggagagcccttcatttggag
attatgttctttcggggagaggctgaggacttaaaatattcctgcatttg
tgaaatgatggtgaaagtaagtggtagcttttcccttctttttcttcttt
ttttgtgatgtcccaacttgtaaaaattaaaagttatggtactatgttag
ccccataatttttttttcctttttaaaacacttccataatctggactcct
ctgtccaggcactgctgcccagcctccaagctccatctccactccagatt
ttttacagctgcctgcagtactttacctcctatcagaagtttctcagctc
ccaaggctctgagcaaatgtggctcctggggggttctttcttcctctgctg
aaggaataaattgctccttgacattgtagagcttctggcacttggagact
tgtatgaaagatggctgtgcctctgcctgtctcccccaccgggctgggag
ctctgcagagcaggaaacatgactcgtatatgtctcaggtccctgcaggg
ccaagcacctagcctcgctcttggcaggtactcagcgaatgaatgctgta
tatgttgggtgcaaagttccctacttcctgtgacttcagctctgttttac
aataaaatcttgaaaatgcctaaaaaaaaaaaaaaaaaaaaaaaaaaaaa
aaaaaaaaaaaaaaaaaaaaaa
>NM_005105 3
agagttagcctttgattggtcagcttgactggcgacctttcccctctgcg
acagtttcccgaggtacctagtgtctgagcggcacagacgagatctcgat
cgaaggcgagatggcggacgtgctagatcttcacgaggctgggggcgaag
atttcgccatggatgaggatggggacgagagcattcacaaactgaaagaa
aaagcgaagaaacggaagggtcgcggctttggctccgaagaggggtcccg
agcgcggatgcgtgaggattatgacagcgtggagcaggatggcgatgaac
ccggaccacaacgctctgttgaaggctggattctctttgtaactggagtc
catgaggaagccaccgaagaagacatacacgacaaattcgcagaatatgg
ggaaattaaaaacattcatctcaacctcgacaggcgaacaggatatctga
aggggtatactctagttgaatatgaaacatacaaggaagcccaggctgct
atggagggactcaatggccaggatttgatgggacagcccatcagcgttga
ctggtgttttgttcggggtccaccaaaaggcaagaggagaggtggccgaa
gacgcagcagaagtccagaccggagacgtcgctgacaggtcctctgttgt
ccaggtgttctcttcaagattccatttgaccatgcagccttggacaaata
ggactggggtggaacttgctgtgtttatatttaatctcttaccgtatatg
cgtagtatttgagttgcgaataaatgttccatttttgttttctacattta
atgttactttcctgtcctaaaattgaaagttctaaagcatagcaaggctg
tatggatcattgtgaagatacttctagggactgaactctatgtatttctt
ttttttctttttttttgagatagagtcttgctgtgttacccagggtggatt
gcagctgatcatagctcactgcagcttcaaactcttgggctcaagccatc
cttctgcctcactgtccctagtagttgggattacaggcacatgccaccat
gcccagctaaatttttaatatttttgtagagatggggtcttgctgtgtta
cctgggctagttatgtgagtttctatattagacatagtctcaagtttcag
gtagggtttaaagtagagacactggtcagtatttcttttttgggggggaac
taggagagcaggagtagaagtgagatgttaagatcttatggcactaaaga
cttactattctgttcctacatactctgttaggatcagatagatgttatag
aaatgcctttttgtttctcctgcccttcttgatgtcacagtttttttgtact
tccagctgtctaaaggcatgaatctcctcttggagcattctcccagaccc
ttctttagaagagtctatactaagttcttggtgccctcttcggcagccaa

gggtgaaggccccatagaggagaggatccaaaggagcattgaggaggccc
aagaggaaaaggatgtggctcaggctgggagggacttcagttagcatggt
gggggagaaccagtaccacatacccagtaggtaataaggtgtccagcaga
ggatgaaggtcagcaagataagcagggccagtctcagggcccggagacga
acacggggacaattgtcaaaggagcgggggagggcaaattcaccagcagg
ggctaggaatttagaaaatatactgtaattcagacactcagcttctgatc
tgagtatagggtgaattgatggaggggcatagctagtgagacagagctca
cctcctacaaggaggagaatgttgcaaaccgttttccccttcccaacctg
ggactatatgatttcttaccccccagggattatgatagaaatatgaagcca
ccaagtctagacttgatggtgttcaagaataaataatactgattgcctcc
ctagtccttgtccagctaactcagctgtttataattgaagggattcaaca
aaattatctctagcatcaggtgctagacatggttagaatctcaccatggt
ttagtgactggtagatagctattaggtaggtagataaataaatgatgcta
gaggcaacaggtctagggttaaggattaaggcctgggaattggagtctca
ccatggctccccttccttgtctggggcctggacacactgaggacaatgcg
gctatagcagatggccatggcagtcagtggcagcagaaagaggcagcaga
aggtgaagaggttataggtggtctcttgccattgagccttgaagctgcct
ttggtgacacactgagtgaaagggactgggccagctcagtggaccgtgtg
gaacaggaacagctggagtggagttgaggactattagaactggttcccct
caccacccaacctacccacctatgtcatactgtctcctcccaattcatcc
ttaattccaagtgaagcagcacagtgctgagaaacagttcatccatggtg
ccatgttaaagaagttggaaatatatcttgaaaatcctatcttcctttta
ggcttgaatatgatgctgaacagtaagtttgttaaatcttggaacttaaa
acaatcctgctttctcaagtactattctaacattgcgctttataagggat
gatatttctaccacctcactcatatttttagctgaaatgattttcctggt
atgtctgttattttgtggaaaaagaaatattgtgtaaaatgggtgctgcc
aaaattccaggccattttgcagggactctgaagtgacctttagtagtaat
agtcttatgtgcagtaactataatggtaaagaatgttaaataataaaatt
taacattttccaaatgctattgggctgcccctcccccttttttgttaaatt
gctgggttttccaactgaatcagtaaaaactatttctgtttagagctaca
aggttaaagtgcctgctttccagtaatggagattgagtcactattaattt
gataaaaggtaagctcagtaggcatcagattcctagatacaaggcatttg
ggaaagtgattttagcagacatgagggacatttaggaaagatgaatagtt
tcagcctaagagaattttgtgaactgtttggagttacgatcaggctactc
tgagctagttgggaaatggtctttcctcttcccatctcttgcattcatat
atttctaagttttttttttttttttgtttttgtgctctgcctaagaagtg
cttgagaattgtgaggagtataaaaatagtcaaagctggctgggcgcggt
ggctcacgcctgtaatcccagcactttgggaggctgaggcgggcggatca
cgaggttaggagatggagaccatcctggctaacacagtgaaaccctgtct
gtactaaagatacaaaaaactagccgggcgtggtggtaggtgcctgtagt
cccagctacttgggaactcgggaggctgaggcaggagaatgacctgaacc
caggaggcggagcttgcagtgagcagagattgcgccattgcactccagcc
tgggcgacagagtaagactgtctccaaaaaaaaaaaaaaataataatcaaa
gctcttggatttatagtttggtccacagccttgttttgatctttccttta
tcctgttttattgccatttaccacgtactgtagaaacatccctttcaact
gctgataacttggaaacaagcctacaaaaataagtaatttctaactactc
ctaatactacctataactaccccctaagcccttaccactctaacgtgacat
tattaaatttttttattttattaacactaatattttaactacaattacagc
atatgggcaatacagaatttacctaaaaggatactaatttggaacaaaaa

aaatcacctttcgcacatgtatcatgtcacaaccagtttgccattgaaac
aaatagaggttgcaaatattgtcagattgtcaggctgtaagaaaggatga
aattcatttcccattgcatcatcttgtggcccatggatttcaagtgcctt
agccaaaatcatatagctagttagcagtagagccgagactcagaaaaaaa
caaagtaaaacaggcagactgaaacaaaaagtcttctaattcccagtcca
catgtaaaatttgcttcatataaacaaacctaattgtaaatggcactgta
gcaacaggcttctttttaacacttggattggtaaaggtcttgtttgcaac
atattagaagtattatttttctctttccccccaccccacccccaacaga
gtctggctctgccgcccacgccggagtgcagtggtgcagtcttggctcac
tgcagcctccacttcccaggttcaagcaattctcgtgcctcagcctcctg
agtagttgggattacaggtgctcaccactatacccggctaatttttgtat
ttttagtagagatggggtttcgtcatgttggccaggttggtcttaaactc
ctgacctcaagtgatccacccaccttggccttccaaaatgctgggattac
aggcttgagccaccaggcctttctttgttcttaggagtatagtcagacta
acttctagtagttatatttctaataattgaggatgtaagtaaggatcaaa
tcttaaatcagtataatgcattgtcattccagagataaatcctagaccct
tcttggcctccttctgacataattctaatcctacagtctcagagatgctg
ttgtatcctgccccccaaccccatgatagtgatagtggtttttgccttga
aggaattgctttgtatttagcttttccccctctagatttctagttccttt
tcagtattggattggatttgagatttgattaacctagtactcaggttcag
atgctcgcctctttgcaattttaacactcattcgacaataaagtcagtaa
aaaacacaaaaaaaaaaaaaaaa
>NM_022121 4
ctctgagtcaccggaatctaggtggggccgcccggagcggcgtcctcggg
agccgcctccccgcggcctcttcgcttttgtggcggcgcccgcgctcgca
ggccactctctgctgtcgcccgtcccgcgcgctcctccgacccgctccgc
tccgctccgctcggccccgcgccgcccgtcaacatgatccgctgcggcct
ggcctgcgagcgctgccgctggatcctgcccctgctcctactcagcgcca
tcgccttcgacatcatcgcgctggccggccgcggctggttgcagtctagc
gaccacggccagacgtcctcgctgtggtggaaatgctcccaagagggcgg
cggcagcgggtcctacgaggagggctgtcagagcctcatggagtacgcgt
ggggtagagcagcggctgccatgctcttctgtggcttcatcatcctggtg
atctgtttcatcctctccttcttcgccctctgtggaccccagatgcttgt
cttcctgagagtgattggaggtctccttgccttggctgctgtgttccaga
tcatctccctggtaatttaccccgtgaagtacacccagaccttcaccctt
catgccaaccctgctgtcacttacatctataactgggcctacggctttgg
gtgggcagccacgattatcctgattggctgtgccttcttcttctgctgcc
tccccaactacgaagatgaccttctgggcaatgccaagcccaggtacttc
tacacatctgcctaacttgggaatgaatgtgggagaaaatcgctgctgct
gagatggactccagaagaagaaactgtttctccaggcgactttgaaccca
ttttttggcagtgttcatattattaaactagtcaaaaatgctaaaataat
ttgggagaaaatatttttaagtagtgttatagtttcatgtttatctttt
attatgttttgtgaagttgtgtcttttcactaattacctatactatgcca
atatttccttatatctatccataacatttatactacatttgtaagagaat
atgcacgtgaaacttaacactttataaggtaaaaatgaggtttccaagat
ttaataatctgatcaagttcttgttatttccaaatagaatggactcggtc
tgttaagggctaaggagaagaggaagataaggttaaaagttgttaatgac
caaacattctaaaagaaatgcaaaaaaaaagtttattttcaagccttcga
actatttaaggaaagcaaaatcatttcctaaatgcatatcatttgtgaga

atttctcattaatatcctgaatcattcattttagctaaggcttcatgttg
actcgatatgtcatctaggaaagtactatttcatggtccaaacctgttgc
catagttggtaaggctttcctttaagtgtgaaatatttagatgaaatttt
ctcttttaaagttctttatagggttagggtgtgggaaaatgctatattaa
taaatctgtagtgttttgtgtttatatgttcagaaccagagtagactgga
ttgaaagatggactgggtctaatttatcatgactgatagatctggttaag
ttgtgtagtaaagcattaggagggtcattcttgtcacaaaagtgccacta
aaacagcctcaggagaataaatgacttgcttttctaaatctcaggtttat
ctgggctctatcatatagacaggcttctgatagtttgcaactgtaagcag
aaacctacatatagttaaaatcctggtctttcttggtaaacagattttaa
atgtctgatataaaacatgccacaggagaattcggggatttgagtttctc
tgaatagcatatatatgatgcatcggataggtcattatgatttttttacca
tttcgacttacataatgaaaaccaattcattttaaatatcagattattat
tttgtaagttgtggaaaaagctaattgtagttttcattatgaagttttcc
caataaaccaggtattctaaacttgtttccagtttgtagtttttccattt
ttcaaatctggggaaaggaattaaaaaaaaaatgggtaataagaacatgg
gatataatgaaaagtggtttttgtttgtttttttgtttgaagttttaagg
gccttgctcattttaggtgtccaaaaccaatttttgagtggagattaatg
aattctaatagtctattccctgaacttttcctcaatgaacaataccctag
acacacattaaacaatttctctgcagtgctatcaaccagaggaaaatgga
ctaagagatttctggcaggttcagacacccgggggacatgtgtgcagtgt
agctgaagcctcctccttgtgctggggtccccttccattcaggtggtggg
gtagcagtctctctattttccccttgccctccttcccattttatcatttg
ttattttttttcccaccataagtcatatgttacttccactatggtgtatg
tcattgtgaggatgggtgcagagaggctgggtgggagaacggaaatatat
ctccctagggctactgttggccagctagtccttggcagtgaatttttcta
tgcttttcaaaatgcgaggtgaatgtttctcatagagaaatgtaatctgg
gtgattataccaaaattgaaaagaaaaacccacacaactatgccgtggct
ggtggagaatttgaagtggtcattaaaaatgttaaaaatcccatctttta
aagtgataccacagctcattcaagaagatactggatatctagagattaag
aaacgtggtctcctgttaaacatgaaaatgactccgtttataagcttctc
taccacatgcacttgtctttgcatgatttcccatccagccttcttcccct
cctcaatcacacaataccttaacggcgcacatttaggaaaaatgcaacct
cctgggaccaacgagcctgatataatagaaccatgtcaacctaaagtatt
tatgacaaagataaactcttattttgcagaaatggtctgcttccttcagc
cttgttctagtatagagatctgccattccttgttgatccagattcaccaa
gacagataccttttatgtcataacagaagggaagttccagaggattctgga
gagtaatgaagaattgggctgagaaaccacctgaaggctaacagtgcatt
gcatgagatttcccacagtaaagctgaggtgctttttggttcagtaatta
aatattgagttcccaccctttaaataagcagttctaggttcctaagcaat
tatttcactctgtaagtagccagacatgctaagtggcacttactgctgat
tgtaacaaagaagtaatatatcaaggtctttccatgttcacacaaggtag
cttgtgtgtaataacttagcttcaaaaccatagactgcagaactcacaag
ttcaacagcctttcctttttaaggaaatgaaaacaatggaaaatatagt
catcataacttaattcggtttattttttttttctgtaaactccccctgaa
agacattcctattaatacagtaaatgtgaacactgacttgtttttataag
cacatctgaaagggcatatttgagtctcatcccaactttggtccttgcta
tctgtgcaggcttgggcaggtcatctccctgctggtctcaatatcctcac
ctgtaaaatgattgtaaatgatccccctaccttcaagattctctgattga

tagaattttttctttaattaaaaaattttaaatattccttgagttggaag
cactgatcaataagtggattgcttagggaggttggaacgaatagattcag
tcccaacttcctcttttaaattccctcttcctcactcttcctgcaacact
tatttttacagttgagttttaaaaataagtaatatataaaataatttctg
tagtgtggtttcagatttaaaaattcctgcagacaggctgggcttgcaac
cccatcagtcgatggtcagagccctttgctttttgagaccatttttaggt
gagcttggcttgcctggatacagtgtgcagtgcattcttcctgaattttg
caattctggtatctgggtgtattttctaggtgtgtcagggtgagtgtaat
ccacctagggtgtggaaaaagccaagaaagggaaattaaaagaggttcct
atccagtcatgttaatgatcttccacttgtactatcctgtgcttcgttgt
taacctcgaaaacatactttgttggctgcaaaaataaacaaagggaaact
caaaaaaaaaaaaaaaaa
>NM_001188 3
gtctgcatccggtggccacagagcaacttcctctagagggagctgattgg
agccgggtgccgctggcacctctatgatcactggagtctcgcgggtccct
cgggctgcacagggacaagtaaaggctacatccagatgccgggaatgcac
tgacgcccattcctggaaactgggctcccactcagcccctgggagcagca
gccgccagcccctcgggacctccatctccaccctgctgagccacccgggt
tgggccaggatcccggcaggctgatcccgtcctccactgagacctgaaaa
atggcttcggggcaaggcccaggtcctcccaggcaggagtgcggagagcc
tgccctgccctctgcttctgaggagcaggtagcccaggacacagaggagg
ttttccgcagctacgtttttaccgccatcagcaggaacaggaggctgaa
ggggtggctgcccctgccgacccagagatggtcaccttacctctgcaacc
tagcagcaccatggggcaggtgggacggcagctcgccatcatcggggacg
acatcaaccgacgctatgactcagagttccagaccatgttgcagcacctg
cagcccacggcagagaatgcctatgagtacttcaccaagattgccaccag
cctgtttgagagtggcatcaattggggccgtgtggtggctcttctgggct
tcggctaccgtctggccctacacgtctaccagcatggcctgactggcttc
ctaggccaggtgacccgcttcgtggtcgacttcatgctgcatcactgcat
tgcccggtggattgcacagaggggtggctgggtggcagccctgaacttgg
gcaatggtcccatcctgaacgtgctggtggttctgggtgtggttctgttg
ggccagtttgtggtacgaagattcttcaaatcatgactcccaagggtgcc
ctttggggtcccggttcagacccctgcctggacttaagcgaagtctttgc
cttctctgttcccttgcaggggtccccccctcaagagtacagaagctttag
caagtgtgcactccagcttcggagggcccctgcgtgggggccagtcaggc
tgcagaggcacctcaacattgcatggtgctagtgggccctctctctgggc
ccaggggctgtggccgtctcctccctcagctctctgggacctccttagcc
ctgtctgctaggcgctggggagactgataacttggggaggcaagagactg
ggagccacttctccccagaaagtgtttaacggttttagctttttataata
cccttgtgagagcccattcccaccattctacctgaggccaggacgtctgg
ggtgtggggattggtgggtctatgttccccaggattcagctattctggaa
gatcagcaccctaagagatgggactaggacctgagcctggtcctggccgt
ccctaagcatgtgtcccaggagcaggacctactaggagagggggggccaag
gtcctgctcaactctacccctgctcccattcctccctccggccatactgc
ctttgcagttggactctcagggattctgggcttggggtgtggggtggggt
ggagtcgcagaccagagctgtctgaactcacgtgtcagaagcctccaagc
ctgcctcccaaggtcctctcagttctctcccttcctctctccttatagac
acttgctcccaacccattcactacaggtgaaggctctcacccccatccct
gggggccttgggtgagtggcctgctaaggctcctccttgcccagactaca

gggcttaggacttggtttgttatatcagggaaaaggagtagggagttcat
ctggagggttctaagtgggagaaggactatcaacaccactaggaatccca
gaggtgggatcctccctcatggctctggcacagtgtaatccaggggtgta
gatgggggaactgtgaatacttgaactctgttcccccaccctccatgctc
ctcacctgtctaggtctcctcagggtggggggtgacagtgccttctctat
tgggcacagcctagggtcttgggggtcagggggggagaagttcttgattca
gccaaatgcagggagggggaggcagatggagcccataggccaccccctatc
ctctgagtgtttggaaataaactgtgcaatcccctcaccctgaaaaaaaa
aaa
>NM_002167 4
gatctggggtgctgccaggaaaaagcaaattctggaagttaatggttttg
agtgatttttaaatccttgctggcggagaggcccgcctctccccggtatc
agcgcttcctcattctttgaatccgcggctccgcggtcttcggcgtcaga
ccagccggaggaagcctgtttgcaatttaagcgggctgtgaacgcccagg
gccggcgggggcagggccgaggcgggccattttgaataaagaggcgtgcc
ttccaggcaggctctataagtgaccgccgcggcgagcgtgcgcgcgttgc
aggtcactgtagcgggacttcttttggttttctttctctttggggcacct
ctggactcactccccagcatgaaggcgctgagcccggtgcgcggctgcta
cgaggcggtgtgctgcctgtcggaacgcagtctggccatcgcccggggcc
gagggaagggcccggcagctgaggagccgctgagcttgctggacgacatg
aaccactgctactcccgcctgcgggaactggtacccggagtcccgagagg
cactcagcttagccaggtggaaatcctacagcgcgtcatcgactacattc
tcgacctgcaggtagtcctggccgagccagcccctggacccccctgatggc
ccccaccttcccatccagacagccgagctcactccggaacttgtcatctc
caacgacaaaaggagcttttgccactgactcggccgtgtcctgacacctc
cagaacgcaggtgctggcgcccgttctgcctgggaccccgggaacctctc
ctgccggaagccggacggcagggatgggccccaacttcgccctgcccact
tgacttcaccaaatcccttcctggagactaaacctggtgctcaggagcga
aggactgtgaacttgtggcctgaagagccagagctagctctggccaccag
ctgggcgacgtcaccctgctcccaccccacccccaagttctaaggtctct
tcagagcgtggaggtgtggaaggagtggctgctctccaaactatgccaag
gcggcggcagagctggtcttctggtctccttggagaaaggttctgttgcc
ctgatttatgaactctataatagagtatataggttttgtacctttttac
aggaaggtgactttctgtaacaatgcgatgtatattaaactttttataaa
agttaacattttgcataataaacgatttttaaacacttgaaaaaaaaaaa
aa
>NM_000499 3
ctcaccctgaaggtgacagttccttggaaccttccctgatccttgtgatc
ccaggctccaagagtccacccttcccagctcagctcagtacctcagccac
ctccaagatccctacactgatcatgcttttcccaatctccatgtcggcca
cggagtttcttctggcctctgtcatcttctgtctggtattctgggtaatc
agggcctcaagacctcaggtccccaaaggcctgaagaatccaccagggcc
atggggctggcctctgattgggcacatgctgaccctgggaaagaacccgc
acctggcactgtcaaggatgagccagcagtatggggacgtgctgcagatc
cgaattggctccacacccgtggtggtgctgagcggcctggacaccatccg
gcaggccctggtgcggcagggcgatgatttcaagggccggcccgacctct
acaccttcaccctcatcagtaatggtcagagcatgtccttcagcccagac
tctggaccagtgtgggctgcccgccggcgcctggcccagaatggcctgaa
aagtttctccattgcctctgacccagcctcctcaacctcctgctacctgg

aagagcatgtgagcaaggaggctgaggtcctgataagcacgttgcaggag
ctgatggcagggcctgggcactttaacccctacaggtatgtggtggtatc
agtgaccaatgtcatctgtgccatttgctttggccggcgctatgaccaca
accaccaagaactgcttagcctagtcaacctgaataataatttcggggag
gtggttggctctggaaacccagctgacttcatccctattcttcgctacct
acccaacccttccctgaatgccttcaaggacctgaatgagaagttctaca
gcttcatgcagaagatggtcaaggagcactacaaaacctttgagaagggc
cacatccgggacatcacagacagcctgattgagcactgtcaggagaagca
gctggatgagaacgccaatgtccagctgtcagatgagaagatcattaaca
tcgtcttggacctctttggagctgggtttgacacagtcacaactgctatc
tcctggagcctcatgtatttggtgatgaaccccagggtacagagaaagat
ccaagaggagctagacacagtgattggcaggtcacggcggccccggctct
ctgacagatcccatctgccctatatggaggccttcatcctggagaccttc
cgacactcttccttcgtccccttcaccatcccccacagcacaacaagaga
cacaagtttgaaaggcttttacatccccaaggggcgttgtgtctttgtaa
accagtggcagatcaaccatgaccagaagctatgggtcaacccatctgag
ttcctacctgaacggtttctcacccctgatggtgctatcgacaaggtgtt
aagtgagaaggtgattatctttggcatgggcaagcggaagtgtatcggtg
agaccattgcccgctgggaggtctttctcttcctggctatcctgctgcaa
cgggtggaattcagcgtgccactgggcgtgaaggtggacatgacccccat
ctatgggctaaccatgaagcatgcctgctgtgagcacttccaaatgcagc
tgcgctcttaggtgcttgagagccctgaggcctagactctgtctacctgg
tctggttgggcagccagaccagcaggctggcctatgtggtctaaggttca
gcctgaaactcatagacactgatctggctgcagttttgctatctgggctg
tgggcaagcctaagggatcctgcctgccccctaccctggacttgcctctgc
acaccctccagagacaacaggtaaaacagggccacatagatgctgatgga
gccttcccaagttgtgcttgagccaggaggcctgctagggttaggaggtc
cttaggcctctgagaagctctgaagaactctctggaagcccctgggccca
gtacctagctggctctgtgagggtgctgactggcttcagcaagttagaac
tagccaaaccaggaccctgtccaatctttgacaattgggagctgccaaga
gtgaagggaagagacagcccaggatactggcacagaggtagtctcactgc
ttgaactaggctgagcaatctgaccctatgggtctaggacacagttcctg
ggaacatcacattcctctgcccttcctgcaggcaggaacaaacagggctg
ccttctggccttgtaagacccttattgctgtcctggaggggctggggact
tgtgtctgcggggatcagagcgcacagggagtgcacatatccaggcacca
ggactagggctggagtgagggggggggtatttcaattaccttctattggtc
tcccttctctacactcttgtaataaaatgtctatttttaatgtttgtaca
caacaatccttctattctagcctgcattgagcttgcatgcttgcataaga
gcttaagaaccattgatttaatgtaatagggaaaattctaacccaggtat
ccaaaaatgtgtaagaacaactacctgagctaaataaagatattgttcag
aaatccta
>NM_003538 3
atgtctggacgtggtaagggcgggaagggtttgggtaagggggggtgccaa
gcgccaccgcaaggtgttgcgtgacaacatccagggcatcaccaagccgg
ccatccggcgtctggcccggcgtggcggtgtgaagcggatctctggtctg
atctacgaggagactcgcggggtgctcaaggtgtttttggagaacgtgat
ccgtgacgctgtcacctatacggagcacgccaagcgcaagacagtcactg
ccatggacgtggtctacgcgcttaagcgccagggacgcaccctttatggc
tttggcggttaaggttgctgatttctccacagcttgcatttctgaaccaa

aggccccttttcagggccgccca
>NM_001007156 2
acatttctgcagccgcgcggcgagccattcgcggcggctgctgcagctcc
tactgcatcttccttctcttcctttcctcgggctccggtctcggagtcgg
agagcgcgcctcgcttccagagcccccggacccggcgagtcagcgatcgc
cgagccggccaccatgcccggcagaccgcgccactaggcgctcctcgcgg
ctcccacccggcggcggcggcggcggcggcggcgtccgcgatggtttcag
acgctgaaggattttgcatctgatcgctcggcgtttcaaagaagcagcga
tcggagatggatgtctctctttgcccagccaagtgtagtttctggcggat
tttcttgctgggaagcgtctggctggactatgtgggctccgtgctggctt
gccctgcaaattgtgtctgcagcaagactgagatcaattgccggcggccg
gacgatgggaacctcttccccctcctggaagggcaggattcagggaacag
caatgggaacgccagtatcaacatcacggacatctcaaggaatatcactt
ccatacacatagagaactggcgcagtcttcacacgctcaacgccgtggac
atggagctctacaccggacttcaaaagctgaccatcaagaactcaggact
tcggagcattcagcccagagcctttgccaagaacccccatttgcgttata
taaacctgtcaagtaaccggctcaccacactctcgtggcagctcttccag
acgctgagtcttcgggaattgcagttggagcagaactttttcaactgcag
ctgtgacatccgctggatgcagctctggcaggagcagggggaggccaagc
tcaacagccagaacctctactgcatcaacgctgatggctcccagcttcct
ctcttccgcatgaacatcagtcagtgtgaccttcctgagatcagcgtgag
ccacgtcaacctgaccgtacgagagggtgacaatgctgttatcacttgca
atggctctggatcaccccttcctgatgtggactggatagtcactgggctg
cagtccatcaacactcaccagaccaatctgaactggaccaatgttcatgc
catcaacttgacgctggtgaatgtgacgagtgaggacaatggcttcaccc
tgacgtgcattgcagagaacgtggtgggcatgagcaatgccagtgttgcc
ctcactgtctactatcccccacgtgtggtgagcctggaggagcctgagct
gcgcctggagcactgcatcgagtttgtggtgcgtggcaaccccccaccaa
cgctgcactggctgcacaatgggcagcctctgcgggagtccaagatcatc
catgtggaatactaccaagagggagagatttccgagggctgcctgctctt
caacaagcccacccactacaacaatggcaactataccctcattgccaaaa
acccactgggcacagccaaccagaccatcaatggccacttcctcaaggag
ccctttccagagagcacggataactttatcttgtttgacgaagtgagtcc
cacacctcctatcactgtgacccacaaaccagaagaagacacttttgggg
tatccatagcagttggacttgctgcttttgcctgtgtcctgttggtggtt
ctcttcgtcatgatcaacaaatatggtcgacggtccaaatttggaatgaa
gggtcccgtggctgtcatcagtggtgaggaggactcagccagcccactgc
accacatcaaccacggcatcaccacgccctcgtcactggatgccgggccc
gacactgtggtcattggcatgactcgcatccctgtcattgagaaccccca
gtacttccgtcagggacacaactgccacaagccggacacgtgggtctttt
caaacatagacaatcatgggatattaaacttgaaggacaatagagatcat
ctagtcccatcaactcactatatatatgaggaacctgaggtccagagtgg
ggaagtgtcttacccaaggtcacatggtttcagagaaattatgttgaatc
caataagccttcccggacattccaagcctcttaaccatggcatctatgtt
gaggatgtcaatgtttatttcagcaaaggacgtcatggcttttaaaaact
ccttttaagcctccttgttttgatgtcaccttggtaggctgggccctctg
agaggttggaagctctaggcattgttctctttggatccagggatgctaag
tagaaactgcatgagccaccagtgccccggcaccctttaacaccaccaga
tgggtgttttcccccatccaccactggcagggttgccccttccctccaat

catcactgtgctccttttttcccggcctacgaggcagctcctgccactat
ctttagagccaataaagagaattaaaaacctgtgcaccaggagcatcttt
taaatacactagccattctcttgctttacaaaaacaacctaaccatcaca
agaaagcctgatgaagtccagccgtgctccagcctcactttccctgcttg
gaagcgtggggtctccctggctctcccaggataccatgctgtcctcttag
tgacctcgtcgccctgcaacctccagtggggaagagtcacagagagcacc
taagcagaggtggagacggcgcggtaagaggagggggagccaggctcaag
tattggcaccaagttaggtctcagaggaaagaatggaaaccaatcacttt
acatttttatttttattttcggtggaaaaatcatcctttttttgggacata
cttgccccctacttcctcttctctctggaacggctcacaatgagtgtgac
attagaaaactccttgcagaggagagtttctccaggctcttcctgggccc
ttagatctgcagttccgacaagctttggctgcaggaggttttacccatga
actggccatcctactaggaccacaagggaccaagggaatcagggacaaag
gcccttcctgccagcccatgatcccgggattggctctcttcccctacttc
cacttattcttgactctgagaacttttggaacccaatggaatcagcattt
caaggtcaagatgaactgaaggggaagagaagtaaaacttggcctcctcc
agcccctctcatggcaccaatggaagtgtcctcctgttctctggtcaata
tgtgtgtttactttgcttgctttgactcatgccttactccatggccaccc
tctcccaaagagggggctcgcttcccccattttcaacttgatccactgag
gagagggaagggggtgactttcccttcttcagtaggaaaggcacatttgt
agggcctgaaactctcccgtatttgctgactcattggtggagtagacttc
tggctcccagctccactggcccatggggcctccattgtatgaagtcagca
taggctgcccacctaatggtggagagcatgaaactgggagcatcctgtgg
ggggcttgtgggggaaaaaggtggttgttttaacccaccgttgttttggg
gtggtgttgcacactagtagagaatagagtctatgcctttggcaaattta
actgggagtttggattcccacttaagggttttacttcttgggtcctgtgg
atggtggttcttcgtgtcaggatcccagcccgattctgcaaatgcctcca
tggggtttaaaaacatgaggctttccaagttcttgcccagtatctggggc
agcctccagagtatcacctgggagttcaggttctctccagggctccaggt
gtgtgtttatctcgccccctccagctctcctcatcctgctccccattgct
ccatgtcaggctgttccccattgtgccctgctgatgctttgggtccaggg
cctcctcccaagtgtggctttaaggagtaagcttgaggatgatgtttttt
aattattgtaaatcattacctcatttccagcctcccaggctccatccatc
ccagcatcttttattctgccattttcctcaccttgtgctatgacaatggg
gcgttgtgtttccacagagacttataggagtgttcagtgtatagtttctt
aataaacactttattttctaatgaaaaaaaaaaaaaaaaaa
>NM_014373 2
ggcccggacgggacgtgcgcgctcaaaggttgcccgtctctgacgcccgc
atttcctggtctggagccggctgagccacagcagggtcgccgcggggtcc
cggggccgtgctcccctgcccctcccgggagcgcgcggggcggggcgggg
cggggcgggaccaggcgggcgagctgggccctcgcccctccctcgggcgg
tcacctgggcacgggcgctgcaggtgtcggggcctcaaccttgcggagcc
gacagccatcgatcctcgggtggcctcgaggtggtggcagggccgccccc
tgcagtccggagacgaacgcacggaccgggcctccggaggcaggttcggc
tggaaggaaccgctctcgcttcgtcctacacttgcgcaaatgtctccgag
cttactcacatagcatattggtatatcaaaatgaaatgcaaggaaccaaa
aataacataattgaaggcagtaaaagtgaaattaaataggaagatcatca
gtcaaggaagacccactggagaggacagaaaatgaagcagtgttttatca
tgtgtatttcagcaggtcttcttgaaatttaactaaaaatatgactgctc

tctcttcagagaactgctcttttcagtaccagttacgtcaaacaaaccag
cccctagatgttaactatctgctattcttgatcatacttgggaaaatatt
attaaatatccttacactaggaatgagaagaaaaaacacctgtcaaaatt
ttatggaatatttttgcatttcactagcattcgttgatcttttacttttg
gtaaacatttccattatattgtatttcagggattttgtacttttaagcat
taggttcactaaataccacatctgcctatttactcaaattatttccttta
cttatggctttttgcattatccagttttcctgacagcttgtatagattat
tgcctgaatttctctaaaacaaccaagctttcatttaagtgtcaaaaatt
attttatttctttacagtaattttaatttggatttcagtccttgcttatg
ttttgggagacccagccatctaccaaagcctgaaggcacagaatgcttat
tctcgtcactgtcctttctatgtcagcattcagagttactggctgtcatt
tttcatggtgatgattttatttgtagctttcataacctgttgggaagaag
ttactactttggtacaggctatcaggataacttcctatatgaatgaaact
atcttatattttcctttttcatcccactccagttatactgtgagatctaa
aaaaatattcttatccaagctcattgtctgttttctcagtacctggttac
catttgtactacttcaggtaatcattgttttacttaaagttcagattcca
gcatatattgagatgaatattccctggttatactttgtcaatagttttct
cattgctacagtgtattggtttaattgtcacaagcttaatttaaaagaca
ttggattacctttggatccatttgtcaactggaagtgctgcttcattcca
cttacaattcctaatcttgagcaaattgaaaagcctatatcaataatgat
ttgttaatattattaattaaaagttacagctgtcataagatcataatttt
atgaacagaaagaactcaggacatattaaaaaataaactgaactaaaaca
acttttgccccctgactgatagcatttcagaatgtgtcttttgaagggct
atgataccagttattaaatagtgttttattttaaaaacaaaataattcca
agaagtttttatagttattcagggacactatattacaaatattactttgt
tattaacacaaaaagtgataagagttaacatttggctatactgatgtttg
tgttactcaaaaaaactactggatgcaaactgttatgtaaatctgagatt
tcactgacaactttaagatatcaacctaaacatttttattaaatgttcaa
atgaaagcaagaaaaaaaaaa
>NM_004973 2
gttttactaaagtgaatttttttttgtttgcttcgttcgtctttggctct
ttttttttccttcccaatttcggatttatttcaaggcgaatctggctttg
ggggaagaggaagaaaagtcggattacaagatcaaccaccaccaacaaca
ataaaaaccaccaggatattttttgcaaatttctgacggctttaaattc
atgaagcaattgtccccttttgcaatcagcatttggatctcagaatgagc
aaggaaagacccaagaggaatatcattcagaagaaatacgatgacagtga
tgggattccgtggtcagaagaacgggtggtacgtaaagtcctttatttgt
ctctgaaggagttcaagaattcccagaagaggcagcatgcggaaggcatt
gctgggagcctgaaaactgtgaatgggctccttggtaatgaccagtctaa
gggattaggaccagcatcagaacagtcagagaatgaaaaggacgatgcat
cccaagtgtcctccactagcaacgatgttagttcttcagattttgaagaa
gggccgtcgaggaaaaggcccaggctgcaagcacaaaggaagtttgctca
gtctcagccgaatagtcccagcacaactccagtaaagatagtggagccat
tgctacccccctccagctactcagatatcagacctctctaaaaggaagcct
aagacagaagattttcttacctttctctgccttcgaggttctcctgcgct
gcccaacagcatggtgtattttggaagctctcaggatgaggaggaagtcg
aggaggaagatgatgagacagaagacgtcaaaacagccaccaacaatgct
tcatcttcatgccagtcgacccccaggaaaggaaaaacccacaaacatgt
tcacaacgggcatgttttcaatggttccagcaggtcaacacgggagaagg

aacctgttcaaaaacacaaaagcaaagaggccactcccgcaaaggagaag
cacagcgatcaccgggctgacagccgccgggagcaggcttcagctaacca
ccccgcagcggcccccctccacgggttcctcggccaaggggcttgctgcca
cccatcaccacccccctctgcatcggtcggctcaggacttacggaaacag
gtttctaaggtaaacggagtcactcgaatgtcatctctgggtgcaggtgt
aaccagtgccaaaaagatgcgcgaggtcagaccttcaccatccaaaactg
tgaagtacactgccacggtgacgaagggggctgtcacatacaccaaagcc
aagagagaactggtcaaggacaccaaacccaatcaccacaagcccagttc
cgctgtcaaccacacaatctcagggaaaactgaaagtagcaatgcaaaaa
cccgcaaacaggtgctatccctcggggggcgtccaagtccactgggccc
gccgtcaatggcctcaaggtcagtggcaggttgaacccaaagtcatgcac
taaggaggtgggggggcggcagctgcgggagggcctgcagctgcgggagg
ggctgcggaactccaagaggagactggaagaggcacaccaggcggagaag
ccgcagtcgcccccccaagaagatgaaaggggcggctggccccgccgaagg
ccctggcaagaaggccccggccgagagaggtctgctgaacggacacgtga
agaaggaagtgccggagcgcagtctggagaggaatcggccgaagcgggcc
acggccgggaagagcacgccaggcagacaagcacatggcaaggcggacag
cgcctcctgtgaaaatcgttctacctcgcaaccggagtccgtgcacaagc
cgcaggactcgggcaaggccgagaagggcggcggcaaggccgggtgggcg
gccatggacgagatccccgtcctcaggccctccgccaaggagttccacga
tccgctcatctacatcgagtcggtccgcgctcaggtggagaagttcggga
tgtgcagggtgatcccccctccggactggcggcccgagtgcaagctcaac
gatgagatgcggtttgtcacgcagattcagcacatccacaagctgggccg
gcgctggggccccaacgtgcagcggctggcctgcatcaagaagcacctca
aatctcagggcatcaccatggacgagctcccgctcataggggggctgtgag
ctcgacctggcctgctttttccggctgattaatgagatgggcggcatgca
gcaagtgactgacctcaaaaaatggaacaaactagcagacatgctgcgca
tccccagaactgcccaggaccggctggccaagctgcaggaggcctactgc
cagtacctactctcctacgactccctgtccccagaggagcaccggcggct
ggagaaggaggtgctgatggagaaggagatcctggagaagcgcaaggggc
cgctggaaggccacacagagaacgaccaccacaagttccaccctctgccc
cgcttcgagcccaagaatgggctcatccacggcgtggcccccaggaacgg
cttccgcagcaagctcaaggaggtgggccaggcccagttgaagactggcc
ggcggcgactcttcgctcaggaaaaagaagtggtcaaggaagaggaggag
gacaaaggcgtcctcaatgacttccacaagtgcatctataagggaaggtc
tgtttctctaacaactttttatcgaacagcgaggaatatcatgagcatgt
gtttcagcaaggagcctgccccagccgaaatcgagcaagagtactggagg
ctagtggaagagaaggactgccacgtggcagtgcactgcggcaaggtgga
caccaacactcacggcagtggattcccagtaggaaaatcagaacccttttt
cgaggcatggatggaacctcaccgtcctccccaataacacagggtccatc
ctgcgtcacctcggtgctgtgcctggagtgactattccctggctaaatat
tggcatggtcttttctacctcatgctggtctcgagaccaaaatcaccttc
catacattgactacttacacactggtgctgactgcatttggtattgcatt
cctgctgaggaggagaacaagctggaagatgtggtccacaccctgctgca
agccaatggcaccccagggctgcagatgctggaaagcaacgtcatgatct
ccccggaggtgctgtgcaaagaggggatcaaggtgcacaggaccgtgcag
cagagtggccagtttgtcgtctgcttcccgggatcctttgtgtccaaagt
gtgctgtgggtacagcgtgtctgaaaccgtgcactttgctaccacccagt
ggacaagtatgggctttgagaccgccaaggaaatgaagcgtcgccatata

gctaagccattctccatggagaagttactctaccagattgcacaagcaga
agcaaaaaaagaaaacggtcccactctcagtaccatctcagccctcctgg
atgagctcagggatacagagctgcggcagcgcaggcagctgttcgaggct
ggcctccactcctccgcacgctatggcagccacgatggcagcagcacggt
ggcggacgggaagaaaaagcctcgaaagtggctgcagttggagacgtcag
agaggaggtgtcagatctgccagcacctgtgctacctgtccatggtggta
caagagaacgaaaacgtcgtgttctgtctggagtgtgctctgcgccacgt
ggagaaacagaagtcctgccgagggctgaagttgatgtaccgctacgatg
aggaacagattatcagtctggtcaatcagatctgcggcaaagtgtctggt
aaaaacggcagcattgagaactgtctcagtaaacccacaccaaaaagagg
tccccgcaagagagcgacagtggacgtgccccctcccgtctgtcagcct
ccagttcatccaaaagtgcttcgagctcatcatgaagatgccaacgcccg
tggtcgatttatatatattttttgtaattattatattctagtttggagt
acttgctgtaggattcaagctgtctttgcactagctctaaagaagatttt
cttctggttttagagaactaattttgttttagcattaaactgttgaactt
ttttttgtacttagaaaacctagatactgcagtcagattttggaaactgc
cgtatagtcactgttttaaaaaccccggaggggctgtattaatttgtatt
gccccatggctgacaaaagccttttttttggttttgattttttttttt
tgtaactgttggggggaaaaaggctttttaacccatttttgaagagggtg
aagtttggagaacaaatttaaaaaccatcagtcatgtgagcagatttttt
agaagggataggagacacacgcgcacacacacacacacacgaaacttgaa
atggctttgctttggctgtcgtcttctgccgtgtgccagatgagcttgtg
atctgggaagccggggcacccccgttttgtttctctgggcggttgtggca
gctgaaggcggacgttgtttcctaaccataggtggaacgaggagacggga
gcgagtgggctctccaccagcacatcactatgcatctgttccaggaaaga
agaaaagcgagcgaggaagacggaaaagactgcctgccttggaggggtca
catgagggagacctgtgcctgatttcattaggaaatccattctgttattt
tttggtgctgttggctactttatcaaaaaacccttcaatagcatccttaa
gatttaaaaaaaaaaaaaaaaaaaaaggaaaaaaaagtgatggaagccgta
agtgcttctttgtcatcgacgtgcaatctttctaacattccatctccatc
tcaccgcttcttgtttgacaccttcacaagtcagcattaatctttctttt
aaaacttgtttcatttatgatcatgtagagagccactaggaggcctgcag
ttatttttgaatgtgaaaatgcatttgcgttcatcttgtctattttttct
cttcatgttgtaacaaaaaggaaaaaagaaaaaaaaatcccatccctttt
gtacatatgcctgtaaattgttttaaatacttgagcctttttctcggtgg
ggggtggggaggggggtgagaagacaagatgaagaaaagccttacatttc
agtttcttcatcggttggattggatgcttacagggtttttcttgtaacat
ttataagtgctgcttacatcactgaacaacaacaaaaaaataataatgga
gtagctgttgcccttctccggttgtgtgtacagtatgtgtggaataaaaa
agggaaactgttttcacaagctgttctttgtttcataattggattcatca
atcccgtagctacccatattgcactgagcttgccagtggtgactgccagg
aacgtcctatgatccactttgttggttgttgttgcagaagactgaactgt
tttggaatatttaacaattacagaaacagtcaagtgttttccaatgtggt
tgtccggtttctatggccttgctgtgtactttccctctttttgacagtaa
acttctgcctatggcttacagtttgacatttaatttattagcgctgctct
gcacccctcccttgggagggagacttcatgtggtttattgcgagtttttt
gtttacttttcaggtttgtactacaaggtttaataataaaaacaaagttt
tttgga
>NM_001013398 1

agatgcgagcactgcggctgggcgctgaggatcagccgcttcctgcctgg
attccacagcttcgcgccgtgtactgtcgccccatccctgcgcgcccagc
ctgccaagcagcgtgccccggttgcaggcgtcatgcagcgggcgcgaccc
acgctctgggccgctgcgctgactctgctggtgctgctccgcgggccgcc
ggtggcgcgggctggcgcgagctcggcgggcttgggtcccgtggtgcgct
gcgagccgtgcgacgcgcgtgcactggcccagtgcgcgcctccgcccgcc
gtgtgcgcggagctggtgcgcgagccgggctgcggctgctgcctgacgtg
cgcactgagcgagggccagccgtgcggcatctacaccgagcgctgtggct
ccggccttcgctgccagccgtcgcccgacgaggcgcgaccgctgcaggcg
ctgctggacggccgcgggctctgcgtcaacgctagtgccgtcagccgcct
gcgcgcctacctgctgccagcgccgccagctccaggtgagccgcccgcgc
caggaaatgctagtgagtcggaggaagaccgcagcgccggcagtgtggag
agcccgtccgtctccagcacgcaccgggtgtctgatcccaagttccaccc
cctccattcaaagataatcatcatcaagaaagggcatgctaaagacagcc
agcgctacaaagttgactacgagtctcagagcacagatacccagaacttc
tcctccgagtccaagcgggagacagaatatggtccctgccgtagagaaat
ggaagacacactgaatcacctgaagttcctcaatgtgctgagtcccaggg
gtgtacacattcccaactgtgacaagaagggattttataagaaaaagcag
tgtcgcccttccaaaggcaggaagcggggcttctgctggtgtgtggataa
gtatgggcagcctctcccaggctacaccaccaaggggaaggaggacgtgc
actgctacagcatgcagagcaagtagacgcctgccgcaaggttaatgtgg
agctcaaatatgccttattttgcacaaaagactgccaaggacatgaccag
cagctggctacagcctcgatttatatttctgtttgtggtgaactgatttt
ttttaaaccaaagtttagaaagaggtttttgaaatgcctatggtttcttt
gaatggtaaacttgagcatcttttcactttccagtagtcagcaaagagca
gtttgaattttcttgtcgcttcctatcaaaatattcagagactcgagcac
agcacccagacttcatgcgcccgtggaatgctcaccacatgttggtcgaa
gcggccgaccactgactttgtgacttaggcggctgtgttgcctatgtaga
gaacacgcttcacccccactccccgtacagtgcgcacaggctttatcgag
aataggaaaacctttaaaccccggtcatccggacatcccaacgcatgctc
ctggagctcacagccttctgtggtgtcatttctgaaacaagggcgtggat
ccctcaaccaagaagaatgtttatgtcttcaagtgacctgtactgcttgg
ggactattggagaaaataaggtggagtcctacttgtttaaaaaatatgta
tctaagaatgttctagggcactctgggaacctataaaggcaggtatttcg
ggccctcctcttcaggaatcttcctgaagacatggcccagtcgaaggccc
aggatggcttttgctgcggccccgtggggtaggagggacagagagacagg
gagagtcagcctccacattcagaggcatcacaagtaatggcacaattctt
cggatgactgcagaaaatagtgttttgtagttcaacaactcaagacgaag
cttatttctgaggataagctctttaaaggcaaagctttattttcatctct
catcttttgtcctccttagcacaatgtaaaaaagaatagtaatatcagaa
caggaaggaggaatggcttgctggggagcccatccaggacactgggagca
catagagattcacccatgtttgttgaacttagagtcattctcatgctttt
ctttataattcacacatatatgcagagaagatatgttcttgttaacattg
tatacaacatagccccaaatatagtaagatctatactagataatcctaga
tgaaatgttagagatgctatatgatacaactgtggccatgactgaggaaa
ggagctcacgcccagagactgggctgctctcccggaggccaaacccaaga
aggtctggcaaagtcaggctcagggagactctgccctgctgcagacctcg
gtgtggacacacgctgcatagagctctccttgaaaacagaggggtctcaa
gacattctgcctacctattagcttttctttattttttaactttttgggg

ggaaaagtatttttgagaagtttgtcttgcaatgtatttataaatagtaa
ataaagtttttaccattaaaaaaatatctttccctttgttattgaccatc
tctgggctttgtatcactaattattttattttattatataataattattt
tattataataaaatcctgaaaggggaaaataaaaaaaa
>NM_213609 3
gatactttagagcggagggatgtattgaaacagactactgctacttacag
caccgtatagcagccctgctcctacattttgctgccttactctgccccga
atgcactggagtggggatggtccatcggcaactataaactgattctcatc
aggaaactgcacattatctccccatcacttcaaaggtctcgtcaggcaga
ggtgacgccaggagatgatttaaaggtgaaaatgacaaggtttccacccc
tcaaaccttggctccttttctgacaatacagtctgaatgaacccgatgtc
tttttttttactgtggaaataggatcggaagagagtaacattttttttt
tttaatcctgataaagaagattgttgggaagctctttgaaaaaaaatttt
aaattgtggcacagatggattttaaaaagtgttagatctttccaatgaac
actaatagagtactctgctcttggctggatttttcagagaatggcaatgg
tctctgcgatgtcctgggtcctgtatttgtggataagtgcttgtgcaatg
ctactctgccatggatcccttcagcacactttccagcagcatcacctgca
cagaccagaaggagggacgtgtgaagtgatagcagcacaccgatgttgta
acaagaatcgcattgaggagcggtcacaaacagtaaagtgttcctgtcta
cctggaaaagtggctggaacaacaagaaaccggccttcttgcgtcgatgc
ctccatagtgattgggaaatggtggtgtgagatggagccttgcctagaag
gagaagaatgtaagacactccctgacaattctggatggatgtgcgcaaca
ggcaacaaaattaagaccacgagaattcacccaagaacctaacagaagca
tttgtggtagtaaaggaaaaccaaccctctggaaaatacattttgagaat
ctcaaacatctcacatatatacaagccaaatggatttcttacttgcactt
tgactggctaccagataatcacagtgcgtttactgtgtgtaacgaaatat
cctacagtgagaagacacagcgttttggcaacaccatggaaagtgggctt
aaaaaagggttttctcagtgaaatttttgggcatcatgaagaacgatcaa
ctatcttctaatttgaatctatagttactttgtaccatttgaaatatatg
tatatatatatatataatattttgaaatattatctattctcttcaagaaa
tgaacagtaccacagtttgagacggctggtgtaccccttttgagttttgga
tgttttgtctgttttgctttgttttgttagtcatttctttttctaacggc
aaggaagatatgtgcccttttgagaattcaagatggcactgacacgggaa
ggccagctacaggtggactcctggaatttgaggcatcataatgatactga
atcaagaacttccttctgcttctaccagatggcccaaggaagcacatcgt
cctgttttattgctttctaccctgtgcaatattagcatgcaagcttggct
tacatagtcatactttatattcaattgatatataataaccgttctaacct
cttccaggaaaatatttttagaactactagcttttccacttagaagaaaa
tgaggattcttaagggagccactccaccatgctattaagactctggcaga
gttatgggtaggatatggatccctacatgaataagtcctgtaaatacaat
gtcttaaggctttgtatagctgtcctagactgcagaaatgtcctctgatt
aaatccaaagtctggcatcgttaactacatagtgctgtagcaacaagtct
tatcatggcatctctttctatgtttggtttgctttttccaagagtattca
ggtctcctcttgtgagataggaaggccatgaaaacaattagatttcaaga
tgatctatgtgaccaaatgttggacagccctattaaagtggtaaacaact
tctttctaaaaaaaaaaaaaaaaaaaaaaaaa
>NM_181353 2
actctcattccacgttcttaactgttccattttccgtatctgcttcgggc
ttccacctcattttttcgctttgcccattctgtttcagccagtcgccaa

gaatcatgaaagtcgccagtggcagcaccgccaccgccgccgcgggcccc
agctgcgcgctgaaggccggcaagacagcgagcggtgcgggcgaggtggt
gcgctgtctgtctgagcagagcgtggccatctcgcgctgcgccgggggcg
ccggggcgcgcctgcctgccctgctggacgagcagcaggtaaacgtgctg
ctctacgacatgaacggctgttactcacgcctcaaggagctggtgcccac
cctgccccagaaccgcaaggtgagcaaggtggagattctccagcacgtca
tcgactacatcagggaccttcagttggagctgaactcggaatccgaagtt
ggaacccccgggggccgagggctgccggtccgggctccgctcagcaccct
caacggcgagatcagcgccctgacggccgaggtgagatccagatccgacc
actagatcatccttataccgacggggaaacggaggccagagagggcgtgg
gcgcttgcaccacttccgtcccatccttgcgggtacctggctatgcgggg
gtgcctaaggagcctggaaaaagcgctcccccgtcgtgcttcctggggaa
gggggcgttcgctgcgctcggagcggcgtcccttccaacccgccggtctc
atttcttctcgttttcacaggcggcatgcgttcctgcggacgatcgcatc
ttgtgtcgctgaagcgcctcccccagggaccggcggaccccagccatcca
gggggcaagaggaattacgtgctctgtgggtctcccccaacgcgcctcgc
cggatctgagggagaacaagaccgatcggcggccactgcgcccttaactg
catccagcctggggctgaggctgaggcactggcgaggagagggcgctcct
ctctgcacacctactagtcaccagagactttagggggtgggattccactc
gtgtgtttctattttttgaaaagcagacattttaaaaaatggtcacgttt
ggtgcttctcagatttctgaggaaattgctttgtattgtatattacaatg
atcaccgactgaaaatattgttttacaatagttctgtggggctgtttttt
tgttattaaacaaataatttagatggtggtaaaaaaaaa
>NM_177422 2
gagcttccggggcggccccgggcaggtcggcggcggcggcccgcagtcgt
ggaggagcggtgggagcgtcggcggccgcgggcgatgcaacttccggacg
ggactcccctctgtccgcgcctcacatctccccttcctctcgcctagtcc
tgtgccgttttccgtccgcgactcttccggcccagagctttcggagtgcg
gttgctcaggggaagccgtcgccgcccccgcctcggggccgagtgagagt
gcccgtcgcgtcgcgccgcgtcgcccccgggccgcctccttgccgccag
tggcgggctccgttctccctcgaagcactccccccagctccatgaatgga
aatcggctccgcaggacccgctggggcccagcccctactcatggtgccca
gaagacctggctatggcaccatgggcaaacccattaaactgctggctaac
tgttttcaagttgaaatcccaaagattgatgtctacctctatgaggtaga
tattaaaccagacaagtgtcctaggagagtgaacaggtagatttagacgt
tactttacctggggaaggtggaaaagatcgacctttcaaggtgtcaatca
aatttgtctctcgggtgagttggcacctactgcatgaagtactgacagga
cggaccttgcctgagccactggaattagacaagccaatcagcactaaccc
tgtccatgccgttgatgtggtgctacgacatctgccctccatgaatttct
gccactgccttctacaaagcacaacctgtaattcagttcatgtgtgaagt
tcttgatattcataatattgatgagcaaccaagacctctgactgattctc
atcgggtaaaattcaccaaagagataaaaggtttgaaggttgaagtgact
cattgtggaacaatgagacggaaataccgtgtttgtaatgtaacaaggag
gcctgccagtcatcaaacctttcctttacagttagaaaacggccaaactg
tggagagaacagtagcgcagtatttcagagaaaagtatactcttcagctg
aagtacccgcaccttccctgtctgcaagtcgggcaggaacagaaacacac
ctacctgccactagaagtctgtaatattgtggcagggcaacgatgtatca
agaagctaacagacaatcagacttccactatgatcaaggcaacagcaaga
tctgcaccagatagacaagaggaaattagcagattggtaagaagtgcaaa

ttatgaaacagatccatttgttcaggagtttcaatttaaagttcgggatg
aaatggctcatgtaactggacgcgtacttccagcacctatgctccagtat
ggaggacggaatcggacagtagcaacaccgagccatggagtatgggacat
gcgagggaaacaattccacacaggagttgaaatcaaaatgtgggctatcg
cttgttttgccacacagaggcagtgcagagaagaaatattgaagggtttc
acagaccagctgcgtaagatttctaaggatgcagggatgcccatccaggg
ccagccatgcttctgcaaatatgcacagggggcagacagcgtagagccca
tgttccggcatctcaagaacacatattctggcctacagcttattatcgtc
atcctgccggggaagacaccagtgtatgcggaagtgaaacgtgtaggaga
cacacttttgggtatggctacacaatgtgttcaagtcaagaatgtaataa
aaacatctcctcaaactctgtcaaacttgtgcctaaagataaatgttaaa
ctcggagggatcaataatattcttgtacctcatcaaagaccttctgtgtt
ccagcaaccagtgatctttttgggagccgatgtcactcatccacctgctg
gtgatggaaagaagccttctattgctgctgttgtaggtagtatggatgca
cacccaagcagatactgtgccacagtaagagttcagagaccccgacagga
gatcatccaggacttggcctccatggtccgggaacttcttattcaatttt
ataagtcaactcggttcaagcctactcgtatcatcttttatcgggatggt
gtttcagaggggcagtttaggcaggtattatattatgaactactagcaat
tcgagaagcctgcatcagtttggagaaagactatcaacctggaataacct
acattgtagttcagaagagacatcacactcgattattttgtgctgatagg
acagaaagggttggaagaagtggcaatatcccagctggaacaacagttga
tacagacattacacacccatatgagttcgatttttacctctgtagccatg
ctggaatacagggtaccagtcgtccttcacactatcatgttttatgggat
gataactgctttactgcagatgaacttcagctgctaacttaccagctctg
ccacacttacgtacgctgtacacgatctgtttctatacctgcaccagcgt
attatgctcacctggtagcatttagagccagatatcatcttgtggacaaa
gaacatgacagtgctgaaggaagtcacgtttcaggacaaagcaatgggcg
agatccacaagctcttgccaaggctgtacagattcaccaagataccttac
gcacaatgtacttcgcttaaatagtccaagtatattctctgagaggaagt
actgaaagatgaattgacatacaacgtatgtttccagtgaagtcaattga
gtaaggacacctccagccatacagaaaccaacactgtgtgggggccaagg
tctgatccttatgttaatacaaggaagattgtttacttcatcaaggaaca
cagcatcattatgcaatatgaaaccagccaactgctttttgtgcggtctc
ctataggaagtatcgcaattgttttgttttcatttcttgtagtctaaccc
ttttaatgcctttacctcaagttgcttggcagcacaactatctttgcaaa
aaaaagtaaagaaaaagtaaatgatggtttaaaaaatacacaccttcatg
aataatcaaagtgatttttcagaattatgtgtgcaaaaaattaatgtgca
ttcatatattcttgtaaaaggtgtctgtgtatttttaaaatatatacatc
catacttcatatgcatatatatctagatctggattgataatagatatata
tgtgtctgttatatattttagagttcattccattggggaattttctttcc
cttttattctaccccactaccgcctttatttctctatttcccttgcctt
catcacctacatttttttcccagtcctaccagtgacattcaaatgttgat
gtatctggttcgtttgaatataaaatatggcaaactaaaaaaaaaaaaaa
aaaaaaaaa
>NM_015215 2
gacgctcctcccggagagtagtgagacccctggtgcggggcgattggcgg
cgggagcgatgagtggcagccgcacggcccaacgggagctgtgcgtgggc
cgcggggcggggccagggcgggtgcgcggcggcggcggggtggctgggcc
ggcggcggcggcggtacgaggcgcgcgctcggggtcccggtcgcgaggag

gaggaggatgtggcgcgcggaggggaaatggctgccgaaaacaagccgga
agagcgtttcccaaagtgtattctgcggaactagcacctactgtgttctc
aacaccgtgccacctatagaagatgatcatgggaacagcaatagtagtca
tgtaaaaatctttttaccgaaaaagctgcttgaatgtctgccgaaatgtt
caagtttaccaaaagagaggcaccgctggaacactaatgaggaaattgca
gcttatttaataacatttgagaaacacgaagaatggctaaccacctcccc
taagacaagaccacagaatggctcaatgatactctacaacaggaagaaag
tgaaatacaggaaagatgggtattgctggaaaaagaggaaagatgggaaa
acgaccagagaggaccacatgaaactcaaggtccagggagtggagtgctt
gtacggctgctatgtccattcctccatcatccccaccttccaccggaggt
gctactggctccttcagaaccccgacatcgtcctggtgcactacctgaac
gtgccggccatcgaggactgcggcaagccttgcggccccatcctctgctc
catcaacaccgacaagaaggagtgggcgaaatggacgaaagaagagctca
tcgggcagctgaaacccatgttccatggcatcaagtggacctgcagcaat
gggaacagcagctcaggcttctcggtggaacagctggtgcagcagatcct
cgacagccaccagaccaagccccagccgcggacccacaactgcctctgca
ccggcagcctgggagctggcggcagcgtgcatcacaagtgtaacagcgcc
aaacaccgcatcatctcgcccaaggtggagccacggacagggggtacgg
gagccactcggaggtgcagcacaatgacgtgtcggagggcaagcacgagc
acagccacagcaagggctccagccgtgagaagaggaacggcaaggtggcc
aagcccgtgctcctgcaccagagcagcaccgaggtctcctccaccaacca
ggtggaagtccccgacaccacccagagctcccctgtgtccatcagcagcg
ggctcaacagcgacccggacatggtggacagcccggtggtcacaggtgtg
tccggtatggcggtggcctctgtgatggggagcttgtcccagagcgccac
ggtgttcatgtcagaggtcaccaatgaggccgtgtacaccatgtcccca
ccgctggccccaaccaccacctcctctcacctgacgcctctcagggcctc
gtcctggccgtgagctctgatggccacaagttcgcctttcccaccacggg
cagctcggagagcctgtccatgctgcccaccaacgtgtccgaagagctgg
tcctctccaccaccctcgacggtggccggaagattccagaaaccaccatg
aactttgaccccgactgtttccttaataacccaaagcagggccagacgta
cgggggtggaggcctgaaagccgagatggtcagctccaacatccggcact
cgccacccggggagcggagcttcagctttaccaccgtcctcaccaaggag
atcaagaccgaggacacctccttcgagcagcagatggccaaagaagcgta
ctcctcctccgcggcggctgtggcagccagctccctcaccctgaccgccg
gctccagcctcctgccgtcgggcggcggcctgagtcccagcaccaccctg
gagcagatggacttcagcgccatcgactccaacaaggactacacgtccag
cttcagccagacgggccacagcccccacatccaccagacccccctccccga
gcttcttcctgcaggacgccagcaaacccctccccgtcgagcagaacacc
cacagcagcctgagtgactctgggggcaccttcgtgatgcccacggtgaa
aacggaggcctcgtcccaaaccagctcctgcagcggtcacgtggagacgc
ggatcgagtccacttcctccctccacctcatgcagttccaggccaacttc
caggccatgacggcagaaggggaggtcaccatggagacctcgcaggcggc
ggaagggagcgaggtcctgctcaagtctggggagctgcaggcttgcagct
ctgagcactacctgcagccggagaccaacggggtaatccgaagcgccggc
ggcgtccccatcctcccgggcaacgtggtgcagggactctaccccgtggc
ccagcccagcctcggcaacgcctccaacatggagctcagcctggaccact
ttgacatctccttcagcaaccagttctccgacctgatcaacgacttcatc
tccgtggagggggcagcagcaccatctatgggcaccagctggtgtcggg
ggacagcacggcgctctcacagtcagaggacggggcgcgggccccccttca

cccaggcagagatgtgcctcccctgctgtagcccccagcagggtagcctg
cagctgagcagctcggagggcggggccagcaccatggcctacatgcacgt
cgccgaggtggtctcggccgcctcggcccagggcaccctaggcatgctgc
agcagagcggacgggtgttcatggtgaccgactactccccagagtggtct
tacccagagggaggagtgaaggtcctcatcacaggcccgtggcaagaagc
cagcaataactacagctgcctgtttgaccagatctcagtgcctgcatccc
tgattcagcctggggtgctgcgctgctactgcccagcccatgacactggt
cttgtgaccctacaagttgccttcaacaaccagatcatctccaactcggt
ggtgtttgagtacaaagcccgggctctgcccacgctcccttcctcccagc
acgactggctgtcgttggacgataaccagttcaggatgtccatcctggaa
cgactggagcagatggagaggaggatggccgagatgacggggtcccagca
gcacaaacaggcgagcggaggcggcagcagtggaggcggcagcgggagcg
ggaatggagggagccaggcacagtgtgcttctgggactggggccttgggg
agctgctttgagagccgtgtggtcgtggtatgcgagaagatgatgagccg
agcctgctgggcgaagtccaagcacttgatccactcaaagactttccgcg
gaatgaccctactccacctggccgctgcccagggctatgccaccctaatc
cagaccctcatcaaatggcgtacaaagcacgcggatagcattgacctgga
actggaagttgaccccttgaatgtggaccacttctcctgtactcctctga
tgtgggcgtgtgccctagggcacttggaagctgccgtcgtgctgtacaag
tgggaccgtcgggccatctcgattcccgactctctaggaaggctgccttt
gggaattgccaggtcacggggtcatgtgaaattagcagagtgtctggagc
acctgcagagagatgagcaggctcagctgggacagaaccccagaatccac
tgtcctgcaagcgaagagcccagcacagagagctggatggcccagtggca
cagcgaagccatcagctctccagaaatacccaagggagtcactgttattg
caagcaccaacccagagctgagaagacctcgttctgaaccctctaattac
tacagcagtgagagccacaaagattatccggctcccaaaaagcataaatt
gaaccctgagtacttccagacaaggcaggagaagctgcttcccactgcac
tgagtctggaagagccaaatatcaggaagcaaagccctagttctaagcag
tctgtccccgagacactcagccccagtgaaggagtgagggacttcagccg
ggaactctcccctcccactccagagactgcagcatttcaagcctctggat
ctcagcctgtaggaaagtggaattccaaagatctttacattggtgtgtct
acagtacaggtgactggaaatccgaaggggaccagtgtaggaaaggaggc
agcaccttcacaggtgcgtccacgggaaccaatgagtgtcctgatgatgg
ctaacagagaggtggtgaatacagagctggggtcctaccgtgatagtgca
gaaaatgaagaatgcggccagcccatggatgacatacaggtgaacatgat
gaccttggcagaacacattattgaagccacacctgaccgaatcaagcagg
agaattttgtgcccatggagtcctcaggattggaaagaacagaccctgcc
accattagcagtacaatgagctggctggccagttatctagcggatgctga
ctgccttcccagtgctgcccagatccgaagtgcatataacgagcctctaa
ccccttcttctaataccagcttgagccctgttggctctcccgtcagtgaa
atcgctttcgagaaacctaaccttccctccgccgcggattggtcagaatt
cctgagtgcatctaccagtgagaaggtagagaatgagtttgctcagctca
ctctgtctgatcatgaacagagagaactctatgaggctgccaggcttgtc
cagacagctttccggaaatacaagggccgacccttgcgggaacagcaaga
agtagctgctgctgttattcagcgttgttacagaaaatataaacagtacg
cactttataaaaagatgacacaggctgccatccttatccagagcaaattc
cgaagttactatgaacaaaaaaaattccagcagagccgacgggctgctgt
gctcatccaaaagtactaccgaagttataagaaatgtggcaaaagacggc
aggctcgccggacggctgtgattgtacaacagaaactcaggagcagtttg

ctaaccaaaaagcaggatcaagctgctcgaaaaataatgaggtttcttcg
ccgctgtcgccacagccccctggtggaccataggctgtacaaaaggagtg
aaagaattgaaaaaggccaaggaacttgaagacatacagcagcatccctt
agcaatgtgacattgcttttcagactgttttcatttctgtttttagcaga
gacatgcaacaacaacacacacgcacacacgcacacacacacacgtacac
acacatacaaaatccctctgcagttttggggagatcagctgcaggatttt
aacaggaatgttttggtcattgcatttgcactttcatggacaacttttaa
tttgatcagcaagacatcttggaactcaatcttctgttggatcacgggaa
atcaagacacccaggaggaattgaaagaggcttcctcttctcaggaagaa
gccatttccttctcatatagggctgtattcaaacatcgtgtggaactgta
caaatatttataccaaaaatatagataagaaaaggtggggctatactagc
aacaaaaaaagaatgctgttcctgcacctgccggttatttccaagaagct
gaatctttgggactgattctcagtggagggcttagatcatacaaaaatct
ttattgggtccgtgtgttctcatttccttcactgtttatttttgtttgtt
tgtttgtttgttttaatctctacagcacatttaatgcaacttttgaaatc
tgcaggtttttaatgtcttgtggaaatttgcagaggggcaggtgtgtggt
aaacgggtaatgcatgggaaataatgagaagcagctcacagagtttaaac
tattttcttgtccccaccaccttccaagaacctgcgagggtagtaatcat
cttgtccccttttttcatgttcagcactttaattttttgccttactttca
tgtgcaatgagaattacttaagaattggtaacgcatgtagcctttttag
taaccttggaagctgtagtaattctaaggaatcatgaaccttgcctggac
atttgccacctaaacgatcagtgtggtgctgcgttctggccagtaaattc
catgtttttggctatatctcatccaaactgagcagtttctgtgtatatat
agaaggtagaaatgaaaagtgagaaaatatttgaaagggattatattaat
tgctaaatattttattcacaaaggtcaataacatggcaagataaaattat
ttgtatagttttgtctgaatgagcgagaaaaatgtggatgtactgtttgt
atatattgtatatattaaaacagagatatgtgcatgaaatcaagaaaaaa
gaaatgaacaaaagcaaagcattagtggctatggtctgtaaaatgaaaca
aaaaaactttatttcactataagagtactttattttaaatgttctttagg
agaacattttgctaaagcatgactaaactgcaaaaaaaaaaaaaagagcta
ctgtatttagacttaggaaaaaaggcagagtaacattacttaaaaaaaaa
aggatatgtttacatttaattttggctaccaggagtttagtttattttat
ttaaaattttttgccaatggtgccaagtaatgtgaatgctaatactgct
taagaaaattaagttacttttgcaaaacagataatcataagatgaatcag
tatgtagcttaacaccatccactcactccaacaaagaacacttagaatga
taaaaaaaaaaaaaaaaaaacctgacaaaagaaatagtatgaaaagtagaa
aaatgtcacgtttccatatctcctgctggaaatcagaaaatataataaaa
ttgcacaaaaaaaaaaaatgaaaaagatgcagactggtcttttagagacgg
catggtatattactatttccacataatgaggagccaaagaaatctgatgt
ttttaacaattaaactgctaatgttaaattgagagaataaagttcgtatt
tgctgatgccagtttaaaattcccaggttacgtctgaggatcagttggtg
taaagctgagatgttttttcttggttctggctgccaactgtgagttaaaa
ctcaaggcttgttgtgaagcctaaaaatattcacaaataagcttttaaac
tggtgtctttggaaggaaggtagatacaaaaagattgtggtaaaaactgg
ggtcagtgctcttggtgccttttctataattgtacttgtttttttaattac
ttcctttcactgccaacctcgaattactgtacagtatatgtctttctgct
tgtgatcagctttgacaacagtgacagccccacaactagtagccacctgt
acatttgtaaactgacctgactccattttgtttttaaatgtgtgggttat
gttgcagctgttgcagtcccccagatacctattattttacacaatttgac

ctataggaggacactgagtaatttacaaacacaactgcattcataaatgg
gaatagaacgtgaaagccagctctttcagaatatcctctattaatactga
atttagatatctttattccatttattatggtacaaataactgatgtttta
accagagtaatgacctcagtggatttgctttaaccctcacatttttttt
taatgtttcacatgttacattattagctgaatacgttagaaaatgacaga
tggtagagacttccatagaattaagagggttctcatggagggataggaa
gtaggtttaaagcctaccagtgtaacctaccagtacaactgtgaatccta
ggcaaggcaaaaatgcacttccactgaaacgaagcatttctgaccgcttt
ttcttggttatgaatcttaatttcgaatataagatgataggtaagcgcag
ctttcttggataatctgaattcccaagtgccaggagtaggatttcattat
aaaattaatagctaatcttattctatctcctgaagatttaattgctattg
ttacccattcgaaatcagctgtactgtgtgaacgaaataaagacaataat
acgaacccctctctggctgcacggtcgcttatggcagttccacacagtag
ttggcgcccaatggggggtccctgagacttgcatgtaaaactagtctaga
tgtcttcttttttgtaagttttatttttgtaattgtgcatgtaaagcat
cattgaatcaatggatctgttgaagaactcagctgctggaaaccatgcaa
aatgttttgaattgcccttaaaatatggaaaatgttttctgaatgcttta
tattctttctgctgtaaattaaaaatgaagaaaatttccccata
>NM_019102 3
gggtgctatagacgcacaaacgaccgcgagccacaaatcaagcacacata
tcaaaaaacaaatgagctcttattttgtaaactcattttgcggtcgctat
ccaaatggcccggactaccagttgcataattatggagatcatagttccgt
gagcgagcaattcagggactcggcgagcatgcactccggcaggtacggct
acggctacaatggcatggatctcagcgtcggccgctcgggctccggccac
tttggctccggagagcgcgcccgcagctacgctgccagcgccagcgcggc
gcccgccgagcccaggtacagccagccggccacgtccacgcactctcctc
agcccgatccgctgccctgctccgccgtggccccctcgcccggcagcgac
agccaccacggcgggaaaaactccctaagcaactccagcggcgcctcggc
cgacgccggcagcacccacatcagcagcagagagggggttggcacggcgt
ccggagccgaggaggacgcccctgccagcagcgagcaggcgagtgcgcag
agcgagccgagcccggcgccgcccgcccaaccccagatctaccccctggat
gcgcaagctgcacataagtcatgacaacataggcggcccggaaggcaaaa
gggcccggacggcctacacgcgctaccagaccctggagctggagaaggag
ttccacttcaaccgttacctgacccgcagaaggaggattgaaatagcaca
tgctctttgcctctccgagagacaaattaaaatctggttccaaaaccgga
gaatgaagtggaaaaaagataataagctgaaaagcatgagcatggccgcg
gcaggaggggccttccgtccctgagtatctgagcgtttaaagtactgagc
agtattagcggatcccgcgtagtgtcagtactaaggtgactttctgaaac
tcccttgtgttccttctgtgaagaagccctgttctcgttgccctaattca
tcttttaatcatgagcctgtttattgccattatagcgcctgtataagtag
atctgctttctgttcatctctttgtcctgaatggctttgtcttgaaaaaa
aatagatgttttaacttatttatatgaagcaagctgtgttacttgaagta
actataacaaaaaaagaaaagagaaaaaaaaacacacaaaaagtccccct
tcaatctcgtttagtgccaatgttgtgtgttgcactcaagttgtttaact
gtgcatgtgcgtggaagtgttcctgtctcaatagctccaagctgttaaag
atatttttattcaaactacctatattccttgtgtaattaatgctgttgta
gaggtgacttgatgagacacaacttgttcgacgtgtagtgactagtgact
ctgtgatgaaaactgtgactccaagcggtgtgtccctgcgtgcctttata
ggaccctttgcacgaactctggaagtggctcttataagcgcagcttcagt

gatgtatgtttttgtgaacaaagttacaaatattgtccaagtctggctgt
tttaagcaaactgtgatcagcttttttttttttttttttttttttgtatt
tgtttttaaggaaaaaatactgactggaacaaaaaataaactttctattg
taagttc
>NM_005461 3
acagctgcaccgccgagctgcgagcggctgcgagcgagagagcgtaagag
caagagagctagagagcgagcaacgggcactcgccccacgcctcccctca
gccccaccgcgcgctccgcttgcctctccaccccgcccgactctacccgg
cccggtccctgcgcgggcacagcccagagctctggggcggtgcaggcagc
ctcgggactctccggcgcgccgccgcgtccccagacaaaggcttggccgg
cggccccggcccgctgcgccctcgctccccgcctccccagctcttctccg
ctcttcccccccgcgcttggctcggcgcgctccggccggccgcaaagttt
cccgggcggcagcggcggctgcgcctcgcttcagcgatggccgcggagct
gagcatggggccagagctgcccaccagcccgctggccatggagtatgtca
acgacttcgacctgctcaagttcgacgtgaagaaggagccactggggcgc
gcggagcgtccgggcaggccctgcacacgcctgcagccagccggctcggt
gtcctccacaccgctcagcactccgtgtagctccgtgccctcgtcgccca
gcttcagcccgaccgaacagaagacacacctcgaggatctgtactggatg
gcgagcaactaccagcagatgaaccccgaggcgctcaacctgacgcccga
ggacgcggtggaagcgctcatcggctcgcacccagtgccacagccgctgc
aaagcttcgacagctttcgcggcgctcaccaccaccaccatcaccaccac
cctcacccgcaccacgcgtacccgggcgccggcgtggcccacgacgagct
gggcccgcacgctcacccgcaccatcaccatcatcaccaagcgtcgccgc
cgccgtccagcgccgctagcccggcgcaacagctgcccactagccacccc
gggcccgggccgcacgcgacggcctcggcgacggcggcgggcggcaacgg
cagcgtggaggaccgcttctccgacgaccagctcgtgtccatgtccgtgc
gcgagctgaaccgccacctgcggggcttcaccaaggacgaggtgatccgc
ctgaagcagaagcggcggaccctgaagaaccggggctacgcccagtcttg
caggtataaacgcgtccagcagaagcaccacctggagaatgagaagacgc
agctcattcagcaggtggagcagcttaagcaggaggtgtcccggctggcc
cgcgagagagacgcctacaaggtcaagtgcgagaaactcgccaactccgg
cttcagggaggcgggctccaccagcgacagcccctcctctcccgagttct
ttctgtgagtcgtggccggtcctggccccgcccttgccccggcccggac
tccctgtcccacgtccctagtcccagactaccccggaccctgtccctgcc
gcggccccagccttgacctgtttgacttgagcgagagggaggaagggcgc
gcgggccgcgggcgacgggcgggtgcgcgggcgggcaggggaccttggct
aaggcgagagtagcgcacgccagcgccgcctcctagactcgagcagagcc
ggagagagagacgagagggtgggaggtcccggagtaacttctctccaggc
tgaagggcggcgaggcatagtcccgagaagtcaccaaggccatctggaga
ctcctggctttctgaactttgcgcgttaagccgggacagctgctttgctg
cccggagagtagtccgcgccaggaagagagcaacgaggaaaggagaggga
ctctggcgtcccggcaggcgagaggcgaggctgagcgaaagaaggaagga
cagacggacctgtctgtcagagttcggagaacactggctctcagccctga
gacacaggcctcagttaggacgctcggcgcccaaatctcatcagttttat
tgcctgctcgattatatagaaaaatacaaaaaatctgcattaaaaatatt
aatcctgcatgctggacatgtatggtaataatttctattttgtaccattt
tcttgtttaactttagcatgttgttgatcatggatcatactccccttgtt
tctttgggtgagaagggatcgcagtttggaaactccggcggctgcgtgcg
gggtttcagtcccagctgtaggcttgtaaatacccgccccgccaaaccgc

atagagaacgtggcagcaagctgagggtctttgtttgggtttattattac
ggtatttttgtttgtaagttaaaaagaaaaaaaaaaaagaaaaagttccgg
gcattttgcatcagaaaacaactttgtcttggggcacacttggaagttgc
atgttttctttccttcccttatccccattcggtcctctttttcctctctc
gctttagttttcaaccttgttggtgctgagagagagaaccgagaggtccc
agtacaagggcagggcagggcagggaagctgccaagctccgcaccccaga
ggagtgttctggactacagccttgtcttatggtcaaattgatacccttaa
taagaaaggaaaggaaaggaaaacagatcctcccctctgctttttattgt
aaccagaatcaccctgaggtcccttctgaaccctctgggcctgcgctaat
tgtaggagccacagcgctcctagggtgagaggcttagccatccctgaccc
tggcagtgcactggtaagcagacactgcactgaaccaactgctatgctca
gaatgtaccagaaacccaaacattggcaagtaattttgcaactttcaagt
gcgttctttagaccaatgcattgcgtttctttccctgcttttgagatagt
aggaagagttcttggtggtgtcccccccttcaattcttcagttgtatag
tagttatagggaagatatgggtgtttttctttattattactttttttttt
ctgcaggtcagtaaaaggatttaagttgcactgacaaaaataccaaaata
aaagtgtatttttaagttcccatttgaaattgctggcgctgctggccgga
tgcatttttgagtttgtattagttgataaattaacagtaataacaagatt
gtatgaaccgcatggtgcttgcagttttaaatattgtggatatttgtcct
gcatcagaaacgagctttggtttttacagattcaactgtgttgaaatcaa
acctgccgcaacagaaattgtttttatttcatgtaaaataagggatcaat
ttcaaaccctgcttatgatatgaaaatattaaaacctagtctattgtagt
tttattcagactggtttctgtttttttggttattaaaatggtttcctattt
tgcttattaaaaaaaaaaaaaaaaaaaa
>NM_005924 4
gaaagcagttctctgggaccaccttcttttggcttcaacctctcccactc
ttgacatctgagtagctcagggaagctcttccaggtccgactgttcatat
gtaaaggagactggccgctggggctcaggaccgggattatccgagctctg
cagaagtgcaccgctattgctttgggaggttaaaaaaaaaaatcacacggt
ttccagtgaaaaagtgacagagggtggtggcctttggaaccgccgtgaag
tcttctgcctggaacccgaaacttgcatgctatggaacacccgctctttg
gctgcctgcgcagccctcacgccacggcgcaaggcttgcacccgttctcc
caatcctctctcgccctccatggaagatctgaccatatgtcttaccccga
gctctctacttcttcctcatcttgcataatcgcgggataccccaacgaag
agggcatgtttgccagccagcatcacagggggcaccaccaccaccaccac
caccaccaccatcaccaccatcagcagcagcagcaccaggctctgcaaac
caactggcacctcccgcagatgtcttccccaccgagtgcggctcggcaca
gcctctgcctccagcccgactctggagggcccccagagttggggagcagc
ccgcccgtcctgtgctccaactcttccagcttgggctccagcaccccgac
tggggccgcgtgcgcgccgggggactacggccgccaggcactgtcacctg
cggaggcggagaagcgaagcggcggcaagaggaaaagcgacagctcagac
tcccaggaaggaaattacaagtcagaagtcaacagcaaacccaggaaaga
aaggacagcatttaccaaagagcaaatcagagaacttgaagcagaatttg
cccatcataattatctcaccagactgaggcgatacgagatagcagtgaat
ctggatctcactgaaagacaggtgaaagtctggttccaaaacaggcggat
gaagtggaagagggtaaagggtggacagcaaggagctgcggctcgggaaa
aggaactggtgaatgtgaaaaagggaacacttctcccatcagagctgtcg
ggaattggtgcagccaccctccagcaaacaggggactctatagcaaatga
agacagtcacgacagtgaccacagctcagagcatgcgcacttatgatata

aacagaggaccagctccattctcaggaaagaaatgttgtgatggcaagcc
ttacccaaatatcgtttacacagagagatgactatggcagtgatgtttaa
tattattaaatccaggcatttcgaatctgtttttcatgatttatagaggg
tttacacaaagtgccacttattaaagagcttccacagtgaagatggagaa
ggtgaacttgctttgaatattccagatgtgtttggtcgtgcgtatggcag
tgagcaggtatgtgtttgcttttgcttgcactgaaaattaaattgctatc
aagagcaaactatgaacggtttttattcaagatgtctccagagtgaaga
tgccgaggatgaacttgcattgaacattccagatgtgtgagatcatgtgt
attacagtgggcaggtatttgcttttgcttgcactgaaaattaaattgct
atcaagaataaaccatgaaacattttatcctgaacagccacagtgcctga
attcactcaagtggataaaaagtgtattttaactctgtatatattaccct
taagtcattttcctgtcttcactaatttagcaatgcattcatattagctg
atgaaaataggcactcacaatgacaaccagagccagtttcttgtcttttt
tatacattttgtcatcccagagacaatcagtatgtgcttacctgtgttca
agtagagaaaaatacagtagagtctgataggacatattcttgtaccacag
acaaaacaaatcttatgttgcatttactatcaactgctgctaatacgtta
ttataaaacttacctagctcctgaattcttcctatcttatagcttaaaac
aattaggatcataggcaaatcagttaccttgcagaaagagctttgtatga
cagacattgtcttattttatttctgtaaaatattagctgtatgaatatga
tttaattaacaagaaaacatttcttcctgattgacaacagtgttagacaa
ggtgcaaagcgaaactggttgctcaagttgatagaaaacaaaattctgaa
tatcttcaaattaaattcggtaaaaacacattatttttcatatgtgatg
tattcatgcagaacaactatctttgtattttgtttttaaaatgtgtttaa
taaatgatcctttgtaaataaaaaaaaaaaaaa
>NM_000757 5
agtgcagcgcagaagacagagggtgactaggaagacgcgcgagcggggct
ggccggccggcgggtgggggaggggaggcgggggaaggcggctgagtggg
cctctggagtgtgtgtgtctgtgtcagtgtgtgtgtgtgtgtgtatgt
gtgtgtctggcgcctggccagggtgatttcccataaaccacatgcccccc
agtcctctcttaaaaggctgtgccgagggctggccagtgaggctcggccc
ggggaaagtgaaagtttgcctgggtcctctcggcgccagagccgctctcc
gcatcccaggacagcggtgcggccctcggccggggcgcccactccgcagc
agccagcgagcgagcgagcgagcgagggcggccgacgcgcccggccggga
cccagctgcccgtatgaccgcgccgggcgccgccgggcgctgccctccca
cgacatggctgggctccctgctgttgttggtctgtctcctggcgagcagg
agtatcaccgaggaggtgtcggagtactgtagccacatgattgggagtgg
acacctgcagtctctgcagcggctgattgacagtcagatggagacctcgt
gccaaattacatttgagtttgtagaccaggaacagttgaaagatccagtg
tgctaccttaagaaggcatttctcctggtacaagacataatggaggacac
catgcgcttcagagataacacccccaatgccatcgccattgtgcagctgc
aggaactctctttgaggctgaagagctgcttcaccaaggattatgaagag
catgacaaggcctgcgtccgaactttctatgagacacctctccagttgct
ggagaaggtcaagaatgtctttaatgaaacaaagaatctccttgacaagg
actggaatattttcagcaagaactgcaacaacagctttgctgaatgctcc
agccaagatgtggtgaccaagcctgattgcaactgcctgtaccccaaagc
catccctagcagtgacccggcctctgtctcccctcatcagcccctcgccc
cctccatggcccctgtggctggcttgacctgggaggactctgagggaact
gagggcagctccctcttgcctggtgagcagcccctgcacacagtggatcc
aggcagtgccaagcagcggccacccaggagcacctgccagagctttgagc

cgccagagaccccagttgtcaaggacagcaccatcggtggctcaccacag
cctcgcccctctgtcggggccttcaaccccgggatggaggatattcttga
ctctgcaatgggcactaattgggtcccagaagaagcctctggagaggcca
gtgagattcccgtaccccaagggacagagctttccccctccaggccagga
gggggcagcatgcagacagagcccgccagacccagcaacttcctctcagc
atcttctccactccctgcatcagcaaagggccaacagccggcagatgtaa
ctggtaccgccttgcccagggtgggccccgtgaggcccactggccaggac
tggaatcacacccccccagaagacagaccatccatctgccctgctcagaga
ccccccggagccaggctctcccaggatctcatcactgcgcccccagggcc
tcagcaacccctccaccctctctgctcagccacagctttccagaagccac
tcctcgggcagcgtgctgccccttggggagctggagggcaggaggagcac
cagggatcggaggagccccgcagagccagaaggaggaccagcaagtgaag
gggcagccaggcccctgccccgttttaactccgttcctttgactgacaca
ggccatgagaggcagtccgagggatcctccagcccgcagctccaggagtc
tgtcttccacctgctggtgcccagtgtcatcctggtcttgctggccgtcg
gaggcctcttgttctacaggtggaggcggcggagccatcaagagcctcag
agagcggattctcccttggagcaaccagagggcagccccctgactcagga
tgacagacaggtggaactgccagtgtagagggaattctaagctggacgca
cagaacagtctctccgtgggaggagacattatggggcgtccaccaccacc
cctccctggccatcctcctggaatgtggtctgccctccaccagagctcct
gcctgccaggactggaccagagcagccaggctggggcccctctgtctcaa
cccgcagacccttgactgaatgagagaggccagaggatgctccccatgct
gccactatttattgtgagccctggaggctcccatgtgcttgaggaaggct
ggtgagcccggctcaggaccctcttccctcaggggctgcaccctcctctc
actcccttccatgccggaacccaggccagggacccaccggcctgtggttt
gtgggaaagcagggtggacgctgaggagtgaaagaaccctgcacccagag
ggcctgcctggtgccaaggtatcccagcctggacaggcatggacctgtct
ccagagagaggagcctgaagttcgtggggcgggacagcgtcggcctgatt
tcccgtaaaggtgtgcagcctgagagacgggaagaggaggcctctggacc
tgctggtctgcactgacagcctgaagggtctacaccctcggctcacctaa
gtgccctgtgctggttgccaggcgcagaggggaggccagccctgccctca
ggacctgcctgacctgccagtgatgccaagagggggatcaagcactggcc
tctgcccctcctccttccagcacctgccagagcttctccaggaggccaag
cagaggctcccctcatgaaggaagccattgcactgtgaacactgtacctg
cctgctgaacagcctgcccccgtccatccatgagccagcatccgtccgtc
ctccactctccagcctctccccagcctcctgcactgagctggcctcacca
gtcgactgagggagcccctcagccctgaccttctcctgacctggcctttg
actccccggagtggagtggggtgggagaacctcctgggccgccagccaga
gccggtctttaggctgtgttgttcgcccaggtttctgcatcttgcacttt
gacattcccaagagggaagggactagtgggagagagcaagggagggggagg
gcacagacagagaggctacagggcgagctctgactgaagatgggcctttg
aaatataggtatgcacctgaggttgggggagggtctgcactcccaaaccc
cagcgcagtgtcctttccctgctgccgacaggaacctggggctgaacagg
ttatccctgtcaggagccctggactgggctgcatctcagccccacctgca
tggtatccagctcccatccacttctcacccttctttcctcctgaccttgg
tcagcagtgatgacctccaactctcacccacccccctctaccatcacctct
aaccaggcaagccagggtgggagagcaatcaggagagccaggcctcagct
tccaatgcctggagggcctccactttgtggccagcctgtggtggtggctc
tgaggcctaggcaacgagcgacagggctgccagttgcccctgggttcctt

tgtgctgctgtgtgcctcctctcctgccgccctttgtcctccgctaagag
accctgccctacctggccgctgggccccgtgactttcccttcctgcccag
gaaagtgagggtcggctggccccaccttccctgtcctgatgccgacagct
tagggaagggcagtgaacttgcatatggggcttagccttctagtcacagc
ctctatatttgatgctagaaaacacatatttttaaatggaagaaaaataa
aaaggcattccccttcatccccctaccttaaacatataatattttaaag
gtcaaaaaagcaatccaacccactgcagaagctctttttgagcacttggt
ggcatcagagcaggaggagccccagagccacctctggtgtccccccaggc
tacctgctcaggaacccccttctgttctctgagaagtcaagagaggacatt
ggctcacgcactgtgagattttgtttttatacttggaagtggtgaattat
tttatataaagtcatttaaatatctatttaaaagataggaagctgcttat
atatttaataataaaagaagtgcacaagctgccaaaaaaaaaaaaaaa
>NM_172057 2
ggccggctggaggagctgaggttccgagtgcggccgctgctgggctggcg
ggcgggcagagcacggcaccctggcagcagggcccacgccacggggccat
gggcagctcgagccaggcaggctgctgcccacgcttactgccagggtgac
cccagccctggggcccagccacaaccaccctggcttcatgccaggggctg
ctctggttgccagtcggccagcctcgggggtgcagcctgggctgggactg
ctgctggggtgcaggtgaggcagtggccgggccctcaggccccagggcag
gcaggctgcagggagccaagtcctccatggcggccccagccgggaaggcg
agcaggacaggggctctgcggcccagggcccagaaaggccgggtgaggcg
ggccgtgcgcatctccagcctcgtggcccaggaggtcctgtccctgggcg
ccgacgtgctgcctgagtacaagctgcaggcaccgcgcatccaccgctgg
accatcctgcattacagccccttcaaggccgtgtgggactggctcatcct
gctgctggtcatctacacggctgtcttcacaccctactcggctgccttcc
tgctgaaggagacggaagaaggcccgcctgctaccgagtgtggctacgcc
tgccagccgctggctgtggtggacctcatcgtggacatcatgttcattgt
ggacatcctcatcaacttccgcaccacctacgtcaatgccaacgaggagg
tggtcagccaccccggccgcatcgccgtccactacttcaagggctggttc
ctcatcgacatggtggccgccatcccttcgacctgctcatcttcggctc
tggctctgaggagctgatcgggctgctgaagactgcgcggctgctgcggc
tggtgcgcgtggcgcggaagctggatcgctactcagagtacggcgcggcc
gtgctgttcttgctcatgtgcacctttgcgctcatcgcgcactggctagc
ctgcatctggtacgccatcggcaacatggagcagccacacatggactcac
gcatcggctggctgcacaacctgggcgaccagataggcaaaccctacaac
agcagcggcctgggcggcccctccatcaaggacaagtatgtgacggcgct
ctacttcaccttcagcagcctcaccagtgtgggcttcggcaacgtctctc
ccaacaccaactcagagaagatcttctccatctgcgtcatgctcattggc
tccctcatgtatgctagcatcttcggcaacgtgtcggccatcatccagcg
gctgtactcgggcacagcccgctaccacacacagatgctgcgggtgcggg
agttcatccgcttccaccagatccccaatcccctgcgccagcgcctcgag
gagtacttccagcacgcctggtcctacaccaacggcatcgacatgaacgc
ggtgctgaagggcttccctgagtgcctgcaggctgacatctgcctgcacc
tgaaccgctcactgctgcagcactgcaaacccttccgaggggccaccaag
ggctgccttcgggccctggccatgaagttcaagaccacacatgcaccgcc
aggggacacactggtgcatgctggggacctgctcaccgccctgtacttca
tctcccggggctccatcgagatcctgcggggcgacgtcgtcgtggccatc
ctggggaagaatgacatctttggggagcctctgaacctgtatgcaaggcc
tggcaagtcgaacggggatgtgcgggccctcacctactgtgacctacaca

agatccatcgggacgacctgctggaggtgctggacatgtaccctgagttc
tccgaccacttctggtccagcctggagatcaccttcaacctgcgagatac
caacatgatcccgggctcccccggcagtacggagttagagggtggcttca
gtcggcaacgcaagcgcaagttgtccttccgcaggcgcacggacaaggac
acggagcagccaggggaggtgtcggccttggggccgggccgggcgggggc
agggccgagtagccggggccggccgggggggccgtgggggagagcccgt
ccagtggcccctccagccctgagagcagtgaggatgagggcccaggccgc
agctccagcccctccgcctggtgcccttctccagccccaggcccccgg
agagccgccgggtggggagcccctgatggaggactgcgagaagagcagcg
acacttgcaacccccctgtcaggcgccttctcaggagtgtccaacattttc
agcttctggggggacagtcggggccgccagtaccaggagctccctcgatg
ccccgcccccacccccagcctcctcaacatcccctctccagcccgggtc
ggcggccccggggcgacgtggagagcaggctggatgccctccagcgccag
ctcaacaggctggagacccggctgagtgcagacatggccactgtcctgca
gctgctacagaggcagatgacgctggtcccgcccgcctacagtgctgtga
ccaccccggggcctggccccacttccacatccccgctgttgcccgtcagc
cccctccccaccctcaccttggactcgctttctcaggtttcccagttcat
ggcgtgtgaggagctgcccccgggggccccagagcttccccaagaaggcc
ccacacgacgcctctccctaccgggccagctgggggccctcacctcccag
cccctgcacagacacggctcggacccgggcagttagtggggctgcccagt
gtggacacgtggctcacccagggatcaaggcgctgctgggccgctcccct
tggaggccctgctcaggaggccctgaccgtggaaggggagaggaactcga
aagcacagctcctcccccagcccttgggaccatcttctcctgcagtcccc
tgggccccagtgagaggggcaggggcagggccggcagtaggtggggcctg
tggtccccccactgccctgagggcattagctggtctaactgcccggaggc
acccggccctgggccttaggcacctcaaggacttttctgctatttactgc
tcttattgttaaggataataattaaggatcatatgaataattaatgaaga
tgctgatgactatgaataataaataattatcctgaggagactccaaaaaa
aaaaaaaaaaaa
>NM_000218 2
gcggcggggctggcagcagtggctgcccgcactgcgcccgggcgctcgcc
ttcgctgcagctcccggtgccgccgctcgggccggccccccggcaggccc
tcctcgttatggccgcggcctcctccccgcccagggccgagaggaagcgc
tggggttggggccgcctgccaggcgcccggcggggcagcgcgggcctggc
caagaagtgccccttctcgctggagctggcggagggcggcccggcgggcg
gcgcgctctacgcgcccatcgcgcccggcgccccaggtcccgcgcccccct
gcgtccccggccgcgcccgccgcgcccccagttgcctccgaccttggccc
gcggccgccggtgagcctagacccgcgcgtctccatctacagcacgcgcc
gcccggtgttggcgcgcacccacgtccagggccgcgtctacaacttcctc
gagcgtcccaccggctggaaatgcttcgtttaccacttcgccgtcttcct
catcgtcctggtctgcctcatcttcagcgtgctgtccaccatcgagcagt
atgccgccctggccacggggactctcttctggatggagatcgtgctggtg
gtgttcttcgggacggagtacgtggtccgcctctggtccgccggctgccg
cagcaagtacgtgggcctctgggggcggctgcgctttgcccggaagccca
tttccatcatcgacctcatcgtggtcgtggcctccatggtggtcctctgc
gtgggctccaaggggcaggtgtttgccacgtcggccatcaggggcatccg
cttcctgcagatcctgaggatgctacacgtcgaccgccagggaggcacct
ggaggctcctgggctccgtggtcttcatccaccgccaggagctgataacc
accctgtacatcggcttcctgggcctcatcttctcctcgtactttgtgta

cctggctgagaaggacgcggtgaacgagtcaggccgcgtggagttcggca
gctacgcagatgcgctgtggtgggggtggtcacagtcaccaccatcggc
tatggggacaaggtgccccagacgtgggtcgggaagaccatcgcctcctg
cttctctgtctttgccatctccttctttgcgctcccagcggggattcttg
gctcggggtttgccctgaaggtgcagcagaagcagaggcagaagcacttc
aaccggcagatcccggcggcagcctcactcattcagaccgcatggaggtg
ctatgctgccgagaaccccgactcctccacctggaagatctacatccgga
aggcccccggagccacactctgctgtcacccagccccaaacccaagaag
tctgtggtggtaaagaaaaaaaagttcaagctggacaaagacaatggggt
gactcctggagagaagatgctcacagtcccccatatcacgtgcgaccccc
cagaagagcggcggctggaccacttctctgtcgacggctatgacagttct
gtaaggaagagcccaacactgctggaagtgagcatgccccatttcatgag
aaccaacagcttcgccgaggacctggacctggaaggggagactctgctga
cacccatcacccacatctcacagctgcgggaacaccatcgggccaccatt
aaggtcattcgacgcatgcagtactttgtggccaagaagaaattccagca
agcgcggaagccttacgatgtgcgggacgtcattgagcagtactcgcagg
gccacctcaacctcatggtgcgcatcaaggagctgcagaggaggctggac
cagtccattgggaagccctcactgttcatctccgtctcagaaaagagcaa
ggatcgcggcagcaacacgatcggcgcccgcctgaaccgagtagaagaca
aggtgacgcagctggaccagaggctggcactcatcaccgacatgcttcac
cagctgctctccttgcacggtggcagcacccccggcagcggcggcccccc
cagagagggcggggcccacatcacccagccctgcggcagtggcggctccg
tcgaccctgagctcttcctgcccagcaacaccctgcccacctacgagcag
ctgaccgtgcccaggaggggccccgatgaggggtcctgaggaggggatgg
ggctggggggatgggcctgagtgagaggggaggccaagagtggccccacct
ggccctctctgaaggaggccacctcctaaaaggcccagagagaagagccc
cactctcagaggccccaataccccatggaccatgctgtctggcacagcct
gcacttgggggctcagcaaggccacctcttcctggccggtgtgggggccc
cgtctcaggtctgagttgttaccccaagcgccctggcccccacatggtga
tgttgacatcactggcatggtggttgggacccagtggcagggcacagggc
ctggcccatgtatggccaggaagtagcacaggctgagtgcaggcccaccc
tgcttggcccagggggcttcctgaggggagacagagcaacccctggaccc
cagcctcaaatccaggaccctgccaggcacaggcagggcaggaccagccc
acgctgactacagggccgccggcaataaaagcccaggagcccatttggag
ggcctgggcctggctccctcactctcaggaaatgctgacccatgggcagg
agactgtggagactgctcctgagcccccagcttccagcaggagggacagt
ctcaccatttccccagggcacgtggttgagtgggggggaacgcccacttcc
ctgggttagactgccagctcttcctagctggagaggagccctgcctctcc
gcccctgagcccactgtgcgtggggctcccgcctccaacccctcgcccag
tcccagcagccagccaaacacacagaaggggactgccacctccccttgcc
agctgctgagccgcagagaagtgacggttcctacacaggacaggggttcc
ttctgggcattacatcgcatagaaatcaataatttgtggtgatttggatc
tgtgttttaatgagtttcacagtgtgattttgattattaattgtgcaagc
ttttcctaataaacgtggagaatcacaggctgggctgggcactgctctca
ccttggttcctggggcatccatggggtctctcacagacaggaccccctgca
gttcccctggaagcagtgcccaggtggctgtggaataggaacgctaaaaa
aaaaaaaaaaaa
>NM_005029 3
ggagcgcccgagcggagaggcggcccgggagcagggggcggcccccact

ccggccgggtgcccggcccctggcccctgcctgccctctagatcgccgcc
gcagccgccgctactgggagtctgcctgttgcaggacgcactagccctcc
ctccatggagttcggcctgctcagcgaggcagaggcccggagccctgccc
tgtcgctgtcagacgctggcactccgcacccccagctcccagagcacggc
tgcaagggccaggagcacagcgactcagaaaaggcctcggcttcgctgcc
cggcggctccccagaggacggttcgctgaaaaagaagcagcggcggcagc
gcacgcacttcaccagccagcagctacaggagctagaggcgaccttccag
aggaaccgctaccccgacatgagcacgcgcgaggagatcgccgtgtggac
caacctcaccgaggcccgcgtgcgggtgtggttcaagaaccggcgcgcca
aatggcggaagcgcgagcgcagccagcaggccgagctatgcaaaggcagc
ttcgcggcgccgctcgggggggctggtgccgccctacgaggaggtgtaccc
cggctactcgtacggcaactggccgcccaaggctcttgccccgccgctcg
ccgccaagacctttccattcgccttcaactcggtcaacgtggggcctctg
gcttcgcagcccgtcttctcgccacccagctccatcgccgcctccatggt
gccctccgccgcggctgccccgggcaccgtgccagggcctggggccctgc
agggcctgggcgggggcccccccgggctggctccggccgccgtgtcctcc
ggggccgtgtcctgcccttatgcctcggccgccgccgccgccgcggctgc
cgcctcttcccccctacgtctatcgggacccgtgtaactcgagcctggcca
gcctgcggctcaaagccaaacagcacgcctccttcagctaccccgctgtg
cacgggccgcccccggcagccaaccttagtccgtgccagtacgccgtgga
aaggcccgtatgagcggccccgcccgtagatcatccccgagggcgggggc
aacgattcacagcctccgcggactggggtcattttgactggcttgctccc
gccccagggtctgaaaggggtgtttgggcagctgggggggcaccggctcag
gagagggccttcccctcccagccctgaggggtggactaggccctacacac
agaccgcgcccctgggactaaagccaggaacagggaccagctccccgggg
gccaactcacccttggcccatcccgccttctccaggcttcccctccctcg
ttttcaaagataaatgaaataaacgtgcgcggactgtcaaaaaaaaaaaa
aaaaaaa
>NM_198256 2
agagggggcggtgtactgcgcatgcgggaagatggcgggccgggcgact
tgagatccgcgggtctccctcctctttttccgtctgcgtcgggagctccc
gggcacgtgaggccgtgccgcgtttactggcgggcgggacggcctagccg
ggcggcgcctcggaggaagccgcggaccccttaggtgctgggcccttgga
aatcggcgcgtgggggggcggtgctcgagctgagcgcgagagggcgggaga
gctcgtggggtgcgaggggagcaggacgcccggccgggcagcatgagtca
gcagcggccggcgaggaagttacccagtctcctcctggacccgacggagg
agacggttcgccgtcggtgccgagaccccatcaacgtggagggcctgctg
ccatcaaaaataaggattaatttagaagataatgtacaatatgtgtccat
gagaaaagctctaaaagtgaagagacctcgttttgatgtatcgctggttt
atttaactcgaaaatttatggatcttgtcagatctgctcccgggggtatt
cttgacttaaacaaggttgcaacgaaactgggagtccgaaagcggagagt
gtatgacatcaccaatgtcttagatggaatcgacctcgttgaaaagaaat
ccaagaaccatattagatggataggatctgatcttagcaattttggagca
gttccccaacaaaagaagctacaggaggaactttctgacttatcagcaat
ggaagatgctttggatgagttaattaaggattgtgctcagcagctgtttg
agttaacagatgacaaagaaaatgaaagactagcatatgtgacctatcaa
gacattcatagcattcaggccttccatgaacagatcgtcattgcagttaa
agctccagcagaaaccagattggatgttccagctcccagagaagactcta
tcacagtgcacataaggagcaccaacggacctatcgatgtctatttgtgt

gaagtggagcagggtcagaccagtaacaaaaggtctgaaggtgtcgggac
ctcttcatctgagagcactcatccagaaggccctgaggaagaagaaaatc
ctcagcaaagtgaagaattgcttgaagtaagcaactgatggcatttgaga
atttatgtatcactgagtttttgggaatatcttcgtggagaattacgca
tcaaatttgattctcagagcaataaattatccatgaagtgctctcgttct
cagtagcggcatcatggccagtagtgtctttgaggagttcaccacttaga
ttactgagtaattgtggtttccacatttgaaaacaactccttttataatt
attcactgctttttgtcagtgaaatagacatcttgcctcctgaagtagct
tcatcacagagtgtcatgaagacagacagtcaggctgaaatggacagttc
tttgtggactctacccttcccttcaaggagtatgtcatatatcacaaaag
aaattgccttacactggttcatgtttgcagttactgttgtacattgcata
gatgtacacacgaatttaaatgtgatgtctttgtatatatctgtataatg
ttgagattacttacgaaatatgtctgagtgacacttttcacccttgtaca
gccaaaataatgtatatatggaaagtgacagacaaattctctaatctctt
tggtacctataacttattagaatcctctggatgagggttagaagagactt
tttccaaacttctacatgtagaagtatcataaatgtgctacacatttatg
tttgtggatttaattaaagtattttaatatggttttcagtgctaaaattg
gagtcagatacttcttggttttaagctgtctacctaattgctgtctccca
gcagactggtggcatgcccagtggctttgggggcaaggatagaaatgcca
tcaggaaatagctgaattcattgtgaaacatgaattcagtcatggtgata
attggaaactcctttcaggtttttgcaagtagattttgtaatgtttgtgt
atgcagccttgctgttgagtcagtccaaggggttttacttaggacaagtt
gtaccttgccctctctccagctctgctcccacattttcacatacctagct
gtttctacctcattgggtaagtcatttaccactctgtgcctcagtttact
ctgtagtttaccattagactgtgagctccttgagggactttgtcataatc
actgttacatcccagtgcctcacaccatgcctggcccttaagaagtgctc
aataaatgtctgaacaaataagtgagtggagtgagtgaattgtaagatca
gaataataatatttggtttgtctatcgtacaagattcctgtatcgtttga
atattgcttttaaagaaatatttgaagcgacttcaaattcagactgtgtt
taaaaagatttaaaatcggaatccaaaatcaaagtattaaaaaataatgc
tgagtgtttaggattcacatacctctgagctctggtggcttttgccagtc
ctgtccctctgctgagcaccagcccttcctgcagcagtcctgccggtgtg
tttctgtgcacctggggatgaagtgtgctcctcccccagcggttagcctc
aatgcttttcctccctctgttttattatttgaaattagccaaagaaaagc
tgcagggggcactgctgatcatcactggactggtgggtcgtactgtttga
cagaatagggaatacaggaggaggagcaagcctagggtaaaacttaggtt
tgggacatgcttgatgaaagcaagagttgagcaggttgttgtgtgtatgt
aggtcaggtgctcagcggaaaagtctgagctgtgtagtacgtcaggcaga
tgtcagtttagatgcgatagtttaagccacaagagtaggagaggtcatct
aaagagtacagcaggcctaagggcatctatatttaaggagaaatacagaa
gaagtctcctgagaaggaataatggggcgggtggggagggagaaggcagt
gtcccaaaaaccaacaaaggttttaccaaaggtaataccatcatttctga
gcagcagttccttaacttgtgaatgtggcaaatgttctcctttacaaatt
gttctgaaggtaaaatgagatcatgtaaacgaaaatcctttgtaaattac
agatgtgtgttataaatgaagtatctctcgagtcactcca
>NM_006540 2
ggcggccgcagcctcggctacagcttcggcggcgaaggtcagcgccgacg
gcagccggcacctgacggcgtgaccgacccgagccgatttctcttggatt
tggctacacacttatagatcttctgcactgtttacaggcacagttgctga

tatgtgttcaagatgagtgggatgggagaaaatacctctgacccctccag
ggcagagacaagaaagcgcaaggaatgtcctgaccaacttggacccagcc
ccaaaaggaacactgaaaaacgtaatcgtgaacaggaaaataaatatata
gaagaacttgcagagttgatttttgcaaattttaatgatatagacaactt
taacttcaaacctgacaaatgtgcaatcttaaaagaaactgtgaagcaaa
ttcgtcagatcaaagaacaagagaaagcagcagctgccaacatagatgaa
gtgcagaagtcagatgtatcctctacagggcagggtgtcatcgacaagga
tgcgctggggcctatgatgcttgaggcccttgatgggttcttctttgtag
tgaacctggaaggcaacgttgtgtttgtgtcagagaatgtgacacagtat
ctaaggtataaccaagaagagctgatgaacaaaagtgtatatagcatctt
gcatgttggggaccacacggaatttgtcaaaaacctgctgccaaagtcta
tagtaaatgggggatcttggtctggcgaacctccgaggcggaacagccat
accttcaattgtcggatgctggtaaaacctttacctgattcagaagagga
gggtcatgataaccaggaagctcatcagaaatatgaaactatgcagtgct
tcgctgtctctcaaccaaagtccatcaaagaagaaggagaagatttgcag
tcctgcttgatttgcgtggcaagaagagttcccatgaaggaaagaccagt
tcttccctcatcagaaagttttactactcgccaggatctccaaggcaaga
tcacgtctctggataccagcaccatgagagcagccatgaaaccaggctgg
gaggacctggtaagaaggtgtattcagaagttccatgcgcagcatgaagg
agaatctgtgtcctatgctaagaggcatcatcatgaagtactgagacaag
gattggcattcagtcaaatctatcgtttttccttgtctgatggcactctt
gttgctgcacaaacgaagagcaaactcatccgttctcagactactaatga
acctcaacttgtaatatctttacatatgcttcacagagagcagaatgtgt
gtgtgatgaatccggatctgactggacaaacgatggggaagccactgaat
ccaattagctctaacagccctgcccatcaggccctgtgcagtgggaaccc
aggtcaggacatgaccctcagtagcaatataaattttcccataaatggcc
caaaggaacaaatgggcatgcccatgggcaggtttggtggttctgggga
atgaaccatgtgtcaggcatgcaagcaaccactcctcagggtagtaacta
tgcactcaaaatgaacagcccctcacaaagcagccctggcatgaatccag
gacagcccacctccatgctttcaccaaggcatcgcatgagccctggagtg
gctggcagccctcgaatcccacccagtcagttttcccctgcaggaagctt
gcattcccctgtgggagtttgcagcagcacaggaaatagccatagttata
ccaacagctccctcaatgcacttcaggccctcagcgaggggcacggggtc
tcattagggtcatcgttggcttcaccagacctaaaaatgggcaatttgca
aaactccccagttaatatgaatcctcccccactcagcaagatgggaagct
tggactcaaaagactgttttggactatatggggagccctctgaaggtaca
actggacaagcagagagcagctgccatcctggagagcaaaaggaaacaaa
tgaccccaacctgcccccggccgtgagcagtgagagagctgacgggcaga
gcagactgcatgacagcaaagggcagaccaaactcctgcagctgctgacc
accaaatctgatcagatggagccctcgcccttagccagctctttgtcgga
tacaaacaaagactccacaggtagcttgcctggttctgggtctacacatg
gaacctcgctcaaggagaagcataaaattttgcacagactcttgcaggac
agcagttcccctgtggacttggccaagttaacagcagaagccacaggcaa
agacctgagccaggagtccagcagcacagctcctggatcagaagtgacta
ttaaacaagagccggtgagccccaagaagaaagagaatgcactacttcgc
tatttgctagataaagatgatactaaagatattggtttaccagaaataac
ccccaaacttgagagactggacagtaagacagatcctgccagtaacacaa
aattaatagcaatgaaaactgagaaggaggagatgagctttgagcctggt
gaccagcctggcagtgagctggacaacttggaggagattttggatgattt

gcagaatagtcaattaccacagcttttcccagacacgaggccaggcgccc
ctgctggatcagttgacaagcaagccatcatcaatgacctcatgcaactc
acagctgaaaacagccctgtcacacctgttggagcccagaaaacagcact
gcgaatttcacagagcacttttaataacccacgaccagggcaactgggca
ggttattgccaaaccagaatttaccacttgacatcacattgcaaagccca
actggtgctggacctttcccaccaatcagaaacagtagtccctactcagt
gatacctcagccaggaatgatgggtaatcaagggatgataggaaaccaag
gaaatttagggaacagtagcacaggaatgattggtaacagtgcttctcgg
cctactatgccatctggagaatgggcaccgcagagttcggctgtgagagt
cacctgtgctgctaccaccagtgccatgaaccggccagtccaaggaggta
tgattcggaacccagcagccagcatccccatgaggcccagcagccagcct
ggccaaagacagacgcttcagtctcaggtcatgaatatagggccatctga
attagagatgaacatggggggacctcagtatagccaacaacaagctcctc
caaatcagactgccccatggcctgaaagcatcctgcctatagaccaggcg
tcttttgccagccaaaacaggcagccatttggcagttctccagatgactt
gctatgtccacatcctgcagctgagtctccgagtgatgagggagctctcc
tggaccagctgtatctggccttgcggaattttgatggcctggaggagatt
gatagagccttaggaatacccgaactggtcagccagagccaagcagtaga
tccagaacagttctcaagtcaggattccaacatcatgctggagcagaagg
cgcccgttttcccacagcagtatgcatctcaggcacaaatggcccagggt
agctattctcccatgcaagatccaaactttcacaccatgggacagcggcc
tagttatgccacactccgtatgcagcccagaccgggcctcaggcccacgg
gcctagtgcagaaccagccaaatcaactaagacttcaacttcagcatcgc
ctccaagcacagcagaatcgccagccacttatgaatcaaatcagcaatgt
ttccaatgtgaacttgactctgaggcctggagtaccaacacaggcaccta
ttaatgcacagatgctggcccagagacagagggaaatcctgaaccagcat
cttcgacagagacaaatgcatcagcaacagcaagttcagcaacgaacttt
gatgatgagaggacaagggttgaatatgacaccaagcatggtggctccta
gtggtatgccagcaactatgagcaaccctcggattccccaggcaaatgca
cagcagtttccatttcctccaaactacggaataagtcagcaacctgatcc
aggctttactggggctacgactccccagagcccacttatgtcaccccgaa
tggcacatacacagagtcccatgatgcaacagtctcaggccaacccagcc
tatcaggccccctccgacataaatggatgggcgcaggggaacatgggcgg
aaacagcatgttttcccagcagtccccaccacactttgggcagcaagcaa
acaccagcatgtacagtaacaacatgaacatcaatgtgtccatggcgacc
aacacaggtggcatgagcagcatgaaccagatgacaggacagatcagcat
gacctcagtgacctccgtgcctacgtcagggctgtcctccatgggtcccg
agcaggttaatgatcctgctctgaggggaggcaacctgttcccaaaccag
ctgcctggaatggatatgattaagcaggagggagacacaacacggaaata
ttgctgacactgctgaagccagttgcttcttcagctgaccgggctcactt
gctcaaaacacttccagtctggagagctgtgtctatttgtttcaacccaa
ctgacctgccagccggttctgctagagcagacaggcctggccctggttcc
cagggtggcgtccactcggctgtggcaggaggagctgcctcttctcttga
cagtctgaagctcgcatccagacagtcgctcagtctgttcactgcattca
ccttagtgcaacttagatctctcctgcaaaagtaaatgttgacaggcaaa
tttcatacccatgtcagattgaatgtatttaaatgtatgtatttaaggag
aaccatgctcttgttctgttcctgttcggttccagacactggtttcttgc
tttgttttccctggctaacagtctagtgcaaaagattaagattttatctg
ggggaaagaaaagaatttttaaaaaattaaactaaagatgttttaagct

aaagcctgaatttgggatggaagcaggacagacaccgtggacagcgctgt
atttacagacacacccagtgcgtgaagaccaacaaagtcacagtcgtatc
tctagaaagctctaaagaccatgttggaaagagtctccagttactgaaca
gatgaaaaggagcctgtgagagggctgttaacattagcaaatattttttc
cttgtttttctttgttaaaaccaaactggttcacctgaatcatgaattg
agaagaaataattttcatttctaaattaagtccctttagtttgatcaga
cagcttgaatcagcatctcttcttccctgtcagcctgactcttcccttcc
cctctctcattccccatactccctattttcattcctttttaaaaaataa
tataagctacagaaaccaggtaagccctttatttccttaaatgttttgcc
agccacttaccaattgctaagtattgaatttcagaaaaaaaaaaatgcatt
tactggcaaggagaagagcaaagttaaggcttgataccaatcgagctaag
gatacctgctttggaagcatgtttattctgttccccagcaactctggcct
ccaaaatgggagaaaacgccagtgtgtttaaattgatagcagatatcacg
acagatttaacctctgccatgtgtttttattttgtttttagcagtgct
gactaagccgaagttttgtaaggtacataaaatccaatttatatgtaaac
aagcaataatttaagttgagaacttatgtgttttaattgtataatttttg
tgaggtatacatattgtggaattgactcaaaaatgaggtacttcagtatt
aaattagatatcttcatagcaatgtctcctaaaggtgttttgtaaaggat
atcaatgccttgattagacctaatttgtagacttaagactttttattttc
taaaccttgtgattctgcttataagtcatttatctaatctatatgatatg
cagccgctgtaggaaccaattcttgatttttatatgtttatattctttct
taatgaaccttagaaagactacatgttactaagcaggccacttttatggt
tgttttt
>NM_004898 2
gagcgagagcgcgaaggaaatctggccgccgccgccgcgagcgctcccga
atttttacttgttcctgcaaagctgctggagctcagaagctgattctatc
acattgtaagatgcctttggataattctacagtcctcttaaatgaatctt
tagaacttggcaagtctcactagataccttcaatcatcattttgagctca
aagaattctgagacttatggttggtcatatagaagagtaccttgaaccta
tagtttcctgaagaatcagtttaaaagatccaaggagtacaaaaggagaa
gtacaaatgtctactacaagacgaaaacgtagtatgttatgttgtttacc
gtaagctgtagtaaaatgagctcgattgttgacagagatgacagtagtat
ttttgatgggttggtggaagaagatgacaaggacaaagcgaaaagagtat
ctagaaacaaatctgaaaagaaacgtagagatcaatttaatgttctcatt
aaagaactgggatccatgcttcctggtaatgctagaaagatggacaaatc
tactgttctgcagaaaagcattgatttttacgaaaacataaagaaatca
ctgcacagtcagatgctagtgaaattcgacaggactggaaacctacattc
cttagtaatgaagagtttacacaattaatgttagaggctcttgatggttt
tttttagcaatcatgacagatggaagcataatatatgtgtctgagagtg
taacttcattacttgaacatttaccatctgatcttgtggatcaaagtata
tttaattttatcccagaaggggaacattcagaggtttataaaatactctc
tactcatctgctggaaagtgattcattaaccccagaatatttaaaatcaa
aaaatcagttagaattctgttgtcacatgctgcgaggaacaatagaccca
aaggagccatctacctatgaatatgtaaaatttataggaaatttcaaatc
tttaaacagtgtatcctcttcagcacacaatggttttgaaggaactatac
aacgcacacataggccatcttatgaagatagagtttgttttgtagctact
gtcaggttagctacacctcagttcatcaaggaaatgtgcactgttgaaga
acccaatgaagagtttacatctagacatagtttagaatggaagtttctgt
ttctagatcacagggcaccacccataatagggtatttgccatttgaagtt

ctgggaacatcaggctatgattactatcatgtggatgacctagaaaattt
ggcaaaatgtcatgagcacttaatgcaatatgggaaaggcaaatcatgtt
attataggttcctgactaaggggcaacagtggatttggcttcagactcat
tattatatcacttaccatcagtggaattcaaggccagagtttattgtttg
tactcacactgtagtaagttatgcagaagttagggctgaaagacgacgag
aacttggcattgaagagtctcttcctgagacagctgctgacaaaagccaa
gattctgggtcagataatcgtataaacacagtcagtctcaaggaagcatt
ggaaaggtttgatcacagcccaaccccttctgcctcttctcggagttcaa
gaaaatcatctcacacggccgtctcagacccttcctcaacaccaaccaag
atcccgacggatacgagcactccacccaggcagcatttaccagctcatga
gaagatggtgcaaagaaggtcatcatttagtagtcagtccataaattccc
agtctgttggttcatcattaacacagccagtgatgtctcaagctacaaat
ttaccaattccacaaggcatgtcccagtttcagttttcagctcaattagg
agccatgcaacatctgaaagaccaattggaacaacggacacgcatgatag
aagcaaatattcatcggcaacaagaagaactaagaaaaattcaagaacaa
cttcagatggtccatggtcaggggctgcagatgtttttgcaacaatcaaa
tcctgggttgaattttggttccgttcaactttcttctggaaattcatcta
acatccagcaacttgcacctataaatatgcaaggccaagttgttcctact
aaccagattcaaagtggaatgaatactggacacattggcacaactcagca
catgatacaacaacagactttacagagtacatcaactcagagtcaacaaa
atgtactgagtgggcacagtcagcaaacatctctacccagtcagacacag
agcactcttacagccccactgtataacactatggtgatttctcagcctgc
agccggaagcatggtccagattccatctagtatgccacaaaacagcaccc
agagtgctgcagtaactacattcactcaggacaggcagataagattttct
caaggtcaacaacttgtgaccaaattagtgactgctcctgtagcttgtgg
ggcagtcatggtacctagtactatgcttatgggccaggtggtgactgcat
atcctacttttgctacacaacagcaacagtcacagacattgtcagtaacg
cagcagcagcagcagcagagctcccaggagcagcagctcacttcagttca
gcaaccatctcaggctcagctgacccagccaccgcaacaatttttacaga
cttctaggttgctccatgggaatccctcaactcaactcattctctctgct
gcatttcctctacaacagagcaccttccctcagtcacatcaccagcaaca
tcagtctcagcaacagcagcaactcagccggcacaggactgacagcttgc
ccgacccttccaaggttcaaccacagtagcacacgtgcttcctctcttga
catcaagggaggaaggggatggcccattaagagttactcagatgacctga
ggaaaggagggaaagttccagcagtttcatgagatgcagtattgagtgtt
ctagttcctggaattagttggcagagaaaatgctgcctagtgctacagat
gtacattaaataccagccagcaggaggtgatcataggggcatagccagtt
ctgacagtgttttaggtgcctggatatttttgatggaaaaagaatatat
tgccaaatattaagaagctcagctatgaaatgacctccagggaatcagaa
aggcactaatgatgttagtaacttttagtggttctgtgcctcttatcaag
tgttacagaggacataccactgccatgtcaggggtttgcttacagtgatg
ccatgaagacagtccagtagacttggtagcgacccccctcccccaacccct
ctccctttcagataatgatggaacagtaattactttcagaatgttgtgt
gggttcaaattctctatgtacagatgatgtaaaaatatgtatatgtctag
ataaaaggagagaaagcaaaacattttgtatgctgcatgaaagcgttatc
tcttccttacaggtgtgagcacctttcctgaaattctgacaccatgtgca
aactgatccatcctgtttttccttttgtttacaacacagtagtgttctgt
tcacttttccggggcacaagttttttgttcatactttggctgtgatgtc
acagtttgttcagtgaggtatgatgtgctgctgggaatggattttttttt

tcaggttaaattattgatacaacaggattttcaagttattcagaaatatc
cctcatttcattatttttcaattatgtttgaaaataggatttgcactgct
ttattttaggtggctgggagttttgattgcatatttgttatagttcata
gttggaaatatttgcgtaaatggttttcaacaagcctgaaagtaatttca
agaatgtttcagttatagaggtaaaatttgcacacaaaacatcttaggca
ctttttaacattctcaatcatgggaattttaacttttgggatttgttgaa
atctttttattatccttcacaatttcaatgcttcttttagtcagaaatg
attcagggttatttgaggggaaaaaaccccatagtgccttgattttaatt
caggtgataactcaccatcttgaagtcattgtccggtttccgtagcagtt
ttgaaaccttagtaccttttttaacagcatgtgggtgtcagtgtcattatt
agtctcctaataagttcctctgaagactgctatcagtctcttggactgga
ggtacaaataatttagaaataaaagatgataacctaacactatcatagtt
attaatgtgatcctaaaattgtttcctaaatcagcatttttctttagtca
tttaagaatttaccagaaatatttgctcaatatgatcttgatattcctac
aaagaaaaaagaaggggtagggatttggctatgccttcactacaacatta
gaatattgtaactcacatgccttctaaacgtgaactaagatttcctttgg
caatatcatattctaaaagtaataaattccaatacaagttacatacattt
aaaaaacattttacagattttatggtactaatgaaatttacagtgataga
acaaaagaggattagtagaaaatacattattagaatataaaaaatgttat
tactgaggaaagggaggagaggacaagtgtaataaatcaaaattgacctc
aaaagaaaatgtgtaacagagttgaggttgttaaaacagaaaaggttctg
aataatgaagattaacctaatgcagaattgctaggtaaagaggtcagggg
aatgctaagccagttcttaagacttctctgtcctctgctttgctgttatc
cttaaggcatatactttgtctttctgcagaaaattctacctggctacaat
tactttgaacattaatgttgaaaaagaaaacaaccaaagaaaattggtac
ttacccttctacaaaagaagtgtgactagatatcaatcagtaattaacat
atcaaggagctcttctagctaaatgaccatccagtagagatttcccacat
tcccatgaatatcaagaatagttgtcagaatatgtatgtacctgagcata
tgtacacagacaaggggggatgttgtggaatatggcaatagcattgttctt
ctccccttcaaattgcctttcttgaccttatgccattccatatatatct
gagttgtgcctcatttatttattggcaatacctagtgatacggatttagc
taacaaaagatatgaagaactattatattgaggcctgtcctctacatacc
acacttaaaagatggtgaactgtgagtactacttaggttgacagcaacaa
agcataagacaagccccaggtaaacgtctaaactgtttactcacattgtc
ctactccagccccttcaattatttcccatctccacaaatagtcgggggaa
aaaattaaaattttcctttatgattcttactgttcttcgcagctcatctt
ttcctgcttagaattaaccattgctaatttaaaggagcagctagctgctt
ttctgtcagtctgaagcgtagtagtggaagaggtagtaagcaccagctgc
ctctttgctgctttgttttcctcctgattctcttaaatttgggttgcaaa
gctatcccgccccccaccctgccccatgaaacttgagcattcaaatgaag
attcagcagtgtctgttcttcatttctatagccaaagctgttagttaaaa
tcccaaatctatagcatttaaagataccaaatagaaacaccttccagctt
t
>NM_024615 3
ttctctcacccaggatcacttccgaaaccacttcgccttcagcccctgcc
tcggccagaggtttcatttttaactgaatatttacgaaagctggaagcgt
gcgagggggggtggggtggggtggaaatagcggctgcttcttttccaggga
tttatttaatggggatgtgttcaaggcaagagcgaattcagaaggatatc
gacgtcgtgatccagaagtccagagctgagaaggactgcctgtttgcaga

tttcagatactctgactccacctttacttttacctacgttggcggcccca
gaagtgtatcctactcagtacatgtatctgaagattacccagataataca
tatgtgtcaagttcagagaatgatgaagatgtgctagttactacagagcc
aataccagtaatttttcatagaatagcaacagaattaagaaaaacaaatg
acattaactgttgcttatccataaaatccaaattacaaaaggaaaatggg
gaggaatcaagacagaatagtacagtggaggaagattctgaaggtgacaa
tgattccgaagaattttattacggagggcaggtgaactatgatggggaac
tgcacaagcacccacaactggaagctgatttgtcagcagttagagagata
tatgggccacatgcagtttctctcagggaatatggagccattgatgatgt
agatattgatctgcatatcgatgttagctttcttgatgaggagattgctg
tggcttgggaagtaattcgaacagaacctataattgttcgactacactgt
tcacttacacagtatttaaatggcccagtgcccactgttgatgtctttca
gatttccacaaaagagcgatttggattgggacatcagctgaaaaaaatca
tgcagacatttgttacacagcagtggaaacagagcaaagaaaaatccaat
tgcctgcacaataaaaagttgtcagagaagaaagtgaagtctcccctgca
tttattttctactttgcgcaggtcgccaagttatcctccccctggttgtg
gcaaaagcaaatccaaactgaaatctgagcaggacggaatctccaaaacg
cataagctgctgcggaggacttgttccagcacagtcaagactgatgatgt
gtgtgtcacaaagtcacacaggacctttggccgctccttgtccagcgatc
ccagggcggagcaggctatgacagcaattaaatcgcacaaacttttgaac
cgtccttgccctgcagctgttaagtcagaggaatgcctaactctaaagtc
gcatagactattgactcgatcttgttctggagatccacgatgtgagcaca
acacaaacttgaagccccataaactgttaagcaggtcttactctagtaat
ctcagaatggaagaattatatggactgaaaaatcacaaattgctcagcaa
gtcctactccagtgcccccaagtcatccaaaactgagcttttcaaggaac
ctaacgcagagggcaggaggctctctcttacctcagggcttattggtatc
ctaacaccatcttcatcttcatcttctcagcttgctccaaatggtgcaaa
atgcattccagtacgagaccgtggcttcctggtgcagacaattgagtttg
ctgaacagcggatccctgtattaaatgaatattgtgtggtttgtgatgag
ccacatgtgtttcaaaatggccctatgcttaggcctaccgtatgtgaacg
ggagctgtgtgtgtttgcttttcaaaccctgggagtaatgaatgaagctg
ctgatgaaatagcaactggagctcaggtggtagatctactagtatccatg
tgtaggtctgcgttggaatctcctagaaaagttgtgattttcgagccata
tccttctgtggtagatcctaatgatcctcagatgttggccttcaacccca
ggaaaaagaactatgatcgagtaatgaaagcactggatagcataacttct
atcagagaaatgacacaagcaccatatctggaaatcaagaagcaaatgga
taaacaggaccccccttgctcatcccttactgcaatgggttatatcaagta
atagatcacatattgtgaaactgccagttaacaggcaattgaagtttatg
catactccacatcagttccttcttctcagcagtccaccagccaaagaatc
caattttagagctgctaaaaaactctttggaagcacctttgcatttcatg
gctcacacattgaaaactggcactccatcctgaggaatggtctggttgtt
gcttctaatacacgattgcagctccatggtgcaatgtatggaagtggaat
ctatcttagtccaatgtcaagcatatcatttggttactcagggatgaaca
agaaacagaaggtgtcagccaaggacgagccagcttcaagcagtaaaagc
agcaatacatcacagtcacagaaaaaaggacagcaatcccaattcctgca
aagccgtaacttaaaatgcatagccttatgtgaagtgatcacctcatctg
acctgcacaaacatggagagatatgggttgtccccaatactgaccatgtc
tgcacacgattctttttcgtctatgaagacggccaagtgggagatgcaaa
tattaatacacaagaaggaggcattcacaaagagatcctccgagtaattg

gtaatcaaactgctactggttaaaggaccaccatttaattaacatgattc
gaaagccttcctcgggttcaaagctggattttgaactgaagaagattata
aaattatttattgttattataaacaaaattaaccctttgaatactgattt
tttttcttagtatttctaagtatctcattaaatacctaaaatggtataag
atttatcaattgtagggttatggaatctagtaataaaatttcaacagcac
ttaaactgaagtttgggttgctcatacaataaacagattgaaaaaactgt
tttgtgctgatatttttatactaaactctttaactggatatttggtatta
tataccgacattttaggttaccaaaatagaattgagaacattttataatg
gcatctaaatctatcagatactttgcaataagtatactgtttgcacattc
tgatgtgctatatcattagtagtatgggcattctaatgttttggaagggg
tgttccctattctatttttcttcataaaaaaagaagtaggtcttagaagc
agtgctttgccaaaaagtatgtaatatacacatctatacattcatgcatc
aaaatgtgtggttgtgaatatatttgtgtatattcacacgtatgttttga
caagaaagatggcttagcttcacaattctatcctcgtttaatggaaagaa
gataatatttagtaagcaataaatttggacataaatcaagatatgagcca
gatttaccaccattcattcactgaaaatatttgcagacaatttttctgtt
tgtctgaagaaattcagcccttttaaaatgttatctaaaagtcataattt
ggggaacatacatggcctggtagaccgaatctatattcaaatgattatgt
atttaaaagaaagacctcagaatttaaagaaaccatatatattttttatt
tactacaaaaaaattatacaattttatttatgccacaccagcatttttat
atttgttcatctaatatctctgcttattttttcatattgagaaagaaca
caaacttgatgcttcttttatattaaaagaccacaaataagtttatagag
aacagtaacaccagctctcagcatttcctttcaagagtcataatttcat
caaaatcatttcttattctttgagtcctagagttcttattcatcaagttt
taattgacagtgtttattttcccacatgttgtctttggaataaggccatt
catgaaagcagtttaattagtgagtctgccactctattcaaaccctgaat
caaggctctatctgattttaatttatgtaaaaaaaaataaaaatgtatag
tcatttgcaagtataatgtgataactgctgtgtttatttcctttgtctgg
cataaaaggtgagaaaaaggtcatacatgttgttaagggtagcagtttag
gaagtgttttctaataatttgttttgttatattgcaaataattgatatgc
cacttatttctgcaagaaaaataggcttgaagtggttgggatattaatca
ttttgatagtatctacttaaagcattgatctgttttgacttgtggctctg
aaccctgggctaggctgagggataatagtagtggagtagggcaaagatat
aatatcctatccagtgttttgtgaccacttatttgaagattagtgtggga
catgaggaccttgctactttttgccaagatgctgcaagtctcccaaatc
acttcaactgtgtttttaaaacatgtttaagcaacagaattatttttttg
aaattagtctatacgttagacagtctatacatgacaaacatgaagatcta
atatcaagatttgggagacgggatcttggaactccacctgtttgaccact
aggaccactgaggcacctccaataagctctgtagagtgcctccagcataa
ttcaaaatccactgacctaatccattatggctttacttaacattggagtg
atccgtaaagaatatagtattggagtctgaactggaacggtaactcgtaa
gactcaagaaagaccgtttaaggaaaattggcataatacaaactgttcct
gttgacttaagaataacagtaggttcacctatctgaggaaattaagtagt
tgatagtttaatgctaggcatcttttatgataataaagtaagatgtcttc
ttatgacagttaattgagttgtatcatgatacagaatgacagattttgtt
tcaaacaattgcgtcagaacctttgtaagcaaatattctaaggagaatca
atcttattttttaattgaactttttgaaattagggatactataataactg
ataaagatgaatttctgtgtttctaaagaatctaatttcaattactacca
ttaacatataaatgtgcttccaaaatccaagcaggtccatacaatttaga

gatttgaacactgttatcatgctttaattcagtcattcattaaaatatta
tctattcacttatttgatgattccaaacatttgatattgtattgtgtttt
tataattatttgaaatctgttcaccaatgctttgtaatggttttattaat
tgtgtagccattgctacttagatagtaacccactgagcaaaggcagtcat
ttattttcatccttgaagtaggggaagaaaattattaatttattagttct
actttttcctgcattgcttattttgtaaacattttactatgaataatctg
ttctctttcactgtattaaaaggagaagaaaatgaacatttgagaatgga
aaaaagtaataatatacaaataatctagtgctataaaatgcattatagta
agacacttttggtgtcttcgagtgcactatgttggaataaattttatact
tgttgcttagtaaatctcagtcatacgtttggtttgaagctgtttttttt
tttaatttgaagtttaattttaaaagtgccacattgtaaaagttaccgtt
aaatactatgtagtataaaaaaccaacccaagctattgttaatgctctga
atgtttttctttgaaaatttaagtttagagaagaaattccttcatttttc
tccccttttgctaaaagaaagaaaatctataatatgtaagtctatgaatct
ttaaataactgaaggaattttagcagtactttgtataataaaaatatttt
ttaaactataacttgccttagagcatggggtttcagaaagttttgacgaa
aaactcatcttcattaaagagtctggataacttcatcagatggtaattta
atatgtcatgaaggggacactcaacattgggtcatctaccgtctaaataa
atacggatttcatggtggcatttgaagcacattttgcaaacattatgatg
ttctattgtcttgctgaattctttattataacactacctcaagacaaatg
atagactgttggtaaggtaaattgggttttcaatgtcaggaacaaataaa
attttgcaaatagaagtcatcaaataattcaacaggaagaccatactgga
cacagatgttgtcagttacttctggacaatgtggtaacatggatatttca
gtcactggtaacaatgtcagggttaacacccaatgaattatttgaaacaa
tttgggttgcaaaatttgaaattaattagtctattttgagattgattttc
tacgtgggccaattttatgtccattacctaaagccacaatctcccgctct
aaggagttaaaggatgaaaacacccaactgctacactggaatggcagact
ggatattcaagcattttggcatttaatgcctgctaaatttttgtccgatt
atatatagatttgatgtatattattacccagtaaattcttcttgggaatt
ttgtatacattatggaatattaggataagtttttgtaggtcagaagaatt
tcaagtagtttttagacaaaacatatccatgagtgtgaaaaaagctgtgt
tgacgaatagattacatttcacatttaccagcaagtcagtaaaaaatagt
gcttatttacatagtcaatataatttaatgttctaaaaataatatcttcg
atctgcccaatatttaatgtatcatttgagatttttaaaaatgcatccgc
tccattatgtaaacattaagatatgcctatgtttctttaactatacagcc
tctttacaataaatttcttgatttttgtgcacaggatagtattgcaacct
gctatttagcctttggtgccttagaattattataaatatttaacaatatg
tacataatgtaaatactgccaagagatcagtaaggccaaatattttctct
attcactttttattgctcttgctttctattgctactaaagcctcttttat
ccagctttgtaatagttccaacattgtagcgaatgtaaatgtttactttc
aataaatctgaacttgttcaacaagta
>NM_005544 2
ggttgtttttcggagcctccctctgctcagcgttggtggtggcggtggca
gcatggcgagccctccggagagcgatggcttctcggacgtgcgcaaggtg
ggctacctgcgcaaacccaagagcatgcacaaacgcttcttcgtactgcg
cgcggccagcgaggctgggggcccggcgcgcctcgagtactacgagaacg
agaagaagtggcggcacaagtcgagcgcccccaaacgctcgatccccctt
gagagctgcttcaacatcaacaagcgggctgactccaagaacaagcacct
ggtggctctctacacccgggacgagcactttgccatcgcggcggacagcg

aggccgagcaagacagctggtaccaggctctcctacagctgcacaaccgt
gctaagggccaccacgacggagctgcggccctcggggcgggaggtggtgg
gggcagctgcagcggcagctccggccttggtgaggctggggaggacttga
gctacggtgacgtgcccccaggacccgcattcaaagaggtctggcaagtg
atcctgaagcccaagggcctgggtcagacaaagaacctgattggtatcta
ccgcctttgcctgaccagcaagaccatcagcttcgtgaagctgaactcgg
aggcagcggccgtggtgctgcagctgatgaacatcaggcgctgtggccac
tcggaaaacttcttcttcatcgaggtgggccgttctgccgtgacggggcc
cggggagttctggatgcaggtggatgactctgtggtggcccagaacatgc
acgagaccatcctggaggccatgcgggccatgagtgatgagttccgccct
cgcagcaagagccagtcctcgtccaactgctctaaccccatcagcgtccc
cctgcgccggcaccatctcaacaatcccccgcccagccaggtggggctga
cccgccgatcacgcactgagagcatcaccgccacctccccggccagcatg
gtgggcgggaagccaggctccttccgtgtccgcgcctccagtgacggcga
aggcaccatgtcccgcccagcctcggtggacggcagccctgtgagtccca
gcaccaacagaacccacgcccaccggcatcggggcagcgcccggctgcac
cccccgctcaaccacagccgctccatccccatgccggcttcccgctgctc
gccttcggccaccagcccggtcagtctgtcgtccagtagcaccagtggcc
atggctccacctcggattgtctcttcccacggcgatctagtgcttcggtg
tctggttcccccagcgatggcggtttcatctcctcggatgagtatggctc
cagtccctgcgatttccggagttccttccgcagtgtcactccggattccc
tgggccacaccccaccagcccgcggtgaggaggagctaagcaactatatc
tgcatgggtggcaaggggccctccaccctgaccgcccccaacggtcacta
cattttgtctcggggtggcaatggccaccgctgcaccccaggaacaggct
tgggcacgagtccagccttggctggggatgaagcagccagtgctgcagat
ctggataatcggttccgaaagagaactcactcggcaggcacatcccctac
cattacccaccagaagaccccgtcccagtcctcagtggcttccattgagg
agtacacagagatgatgcctgcctacccaccaggaggtggcagtggaggc
cgactgccgggacacaggcactccgccttcgtgcccacccgctcctaccc
agaggagggtctggaaatgcaccccttggagcgtcgggggggcaccacc
gcccagacagctccaccctccacacggatgatggctacatgcccatgtcc
ccaggggtggccccagtgcccagtggccgaaagggcagtggagactatat
gcccatgagccccaagagcgtatctgccccacagcagatcatcaatccca
tcagacgccatccccagagagtggaccccaatggctacatgatgatgtcc
cccagcggtggctgctctcctgacattggaggtggccccagcagcagcag
cagcagcagcaacgccgtcccttccgggaccagctatggaaagctgtgga
caaacggggtagggggccaccactctcatgtcttgcctcaccccaaaccc
ccagtggagagcagcggtggtaagctcttaccttgcacaggtgactacat
gaacatgtcaccagtgggggactccaacaccagcagcccctccgactgct
actacggccctgaggacccccagcacaagccagtcctctcctactactca
ttgccaagatcctttaagcacacccagcgccccggggagccggaggaggg
tgcccggcatcagcacctccgcctttccactagctctggtcgccttctct
atgctgcaacagcagatgattcttcctcttccaccagcagcgacagcctg
ggtgggggatactgcggggctaggctggagcccagccttccacatcccca
ccatcaggttctgcagccccatctgcctcgaaaggtggacacagctgctc
agaccaatagccgcctggcccggcccacgaggctgtccctgggggatccc
aaggccagcaccttacctcgggcccgagagcagcagcagcagcagcagcc
cttgctgcaccctccagagcccaagagcccgggggaatatgtcaatattg
aatttgggagtgatcagtctggctacttgtctggcccggtggctttccac

agctcaccttctgtcaggtgtccatcccagctccagccagctcccagaga
ggaagagactggcactgaggagtacatgaagatggacctggggccgggcc
ggagggcagcctggcaggagagcactggggtcgagatgggcagactgggc
cctgcacctcccggggctgctagcatttgcaggcctacccgggcagtgcc
cagcagccggggtgactacatgaccatgcagatgagttgtccccgtcaga
gctacgtggacacctcgccagctgcccctgtaagctatgctgacatgcga
acaggcattgctgcagaggaggtgagcctgcccagggccaccatggctgc
tgcctcctcatcctcagcagcctctgcttccccgactgggcctcaagggg
cagcagagctggctgcccactcgtccctgctggggggcccacaaggacct
gggggcatgagcgccttcacccgggtgaacctcagtcctaaccgcaacca
gagtgccaaagtgatccgtgcagacccacaagggtgccggcggaggcata
gctccgagactttctcctcaacacccagtgccacccgggtgggcaacaca
gtgcccttggagcgggggcagcagtagggggcggtggcggtagcagcag
cagcagcgaggatgtgaaacgccacagctctgcttcctttgagaatgtgt
ggctgaggcctggggagcttgggggagcccccaaggagccagccaaactg
tgtggggctgctgggggtttggagaatggtcttaactacatagacctgga
tttggtcaaggacttcaaacagtgccctcaggagtgcacccctgaaccgc
agcctcccccacccccacccccctcatcaacccctgggcagcggtgagagc
agctccacccgccgctcaagtgaggatttaagcgcctatgccagcatcag
tttccagaagcagccagaggaccgtcagtagctcaactggacatcacagc
agaatgaagacctaaatgacctcagcaaatcctcttctaactcatgggta
cccagactctaaatatttcatgattcacaactaggacctcatatcttcct
catcagtagatggtacgatgcatccatttcagtttgtttactttatccaa
tcctcaggatttcattgactgaactgcacgttctatattgtgccaagcga
aaaaaaaaaatgcactgtgacaccagaataatgagtctgcataaacttca
tcttcaaccttaaggacttagctggccacagtgagctgatgtgcccacca
ccgtgtcatgagagaatgggtttactctcaatgcattttcaagatacatt
tcatctgctgctgaaactgtgtacgacaaagcatcattgtaaattatttc
atacaaaactgttcacgttgggtggagagagtattaaatatttaacatag
gttttgatttatatgtgtaattttttaaatgaaaatgtaacttttcttac
agcacatcttttttttggatgtgggatggaggtatacaatgttctgttgt
aaagagtggagcaaatgcttaaaacaaggcttaaaagagtagaatagggt
atgatccttgttttaagattgtaattcagaaaacataatataagaatcat
agtgccatagatggttctcaattgtatagttatatttgctgatactatct
cttgtcatataaacctgatgttgagctgagttccttataagaattaatct
taattttgtattttttcctgtaagacaataggccatgttaattaaactga
agaaggatatatttggctgggtgttttcaaatgtcagcttaaaattggta
attgaatggaagcaaaattataagaagaggaaattaaagtcttccattgc
atgtattgtaaacagaaggagatgggtgattccttcaattcaaaagctct
ctttggaatgaacaatgtgggcgtttgtaaattctggaaatgtctttcta
ttcataataaactagatactgttgatcttttcttctgtcccctccccccca
ccacttctgtaagtttcctgctctattcccaccattttttttctgtgcaca
cattatgatatatttcatttcctgcattgtcttgagaaagatggtaaggc
aagtgagctgttgctaaccagaaattaaaattccagtaagtgtttttcat
tatgaccagggctatgtgtcaccttccctaagactcttaccttatctcat
attttttgagaacttccagtgttacattatttaactgaatgtaattggcc
catttgccttggtgggtgctggcctattagtgattagttaacaaaacaca
gcgtacagagagcacagaaaagcttaatgacctgctactgaaacacctag
ccagcagtgaaaatgttaattcttttcttgtttggaaagtatacacgtct

tggaattttttccacgtgaaaaacaaatggcaatgaatgcatttaaagat
attgccgacagatttttaaatctttttaccaggaaacttcctaaaggtta
aatgaattaatgcaaatatcaggctccctctgagtctgtgggagcctcta
tctctctatcaggaattcgcatccctactattgggaggagcaacatttta
tttctctgaacgcctaagctccctgggtgggagtggggactacaaggtag
gggccagggttggagggcattgtagtggctgctgcctcctgatgaactgt
ttggggaccccagctctactcaaaagggagcggagataaatggaacccct
cacactgctgaggcccgtgttactgttcattcagccaggtggcatgtacc
tcacagactgttgtgcagtgtccgttattgcagattttaatcatttgcat
gctcatcaatttctaagataaatagggtctagagtcataagaatccattg
ttttcaaggaacttgcagaattacatcatttcctattagtagagagcact
acccattttgaaaatctgatatgaaagttgtttttttactcttgtaaaaaa
agactttcttagtcaaactaacttttcatattttcaagcattctgattca
tactcttgctagtggaagaagagagcaagctgccctgctcttttcctttg
aggactgaaatagttaaagagaaatcaatgaacaaagtcactcccaacca
ttttcctgtaaagctggttattatttctcaaggaacctacactttgaata
tgtgttaccgagatacctctacatgtggaattatcaacatgttttgaatg
agagcagaaatgaacagactggaaaaatctatctcttggtttctatttct
ctgactttttgagtcgaaaagcataaaggtagaaattcttatttaagctg
cttctagtggtgcctgagctggttttgatggtggcatcaaactaccgatt
taaaactggaagttgctggtactcaaaccaaaagttcatactctggcgac
acgaagggtttcctttgagcaacgtcagctgctgagtccttgtgttcagt
tcccattgaggagagttggcttttatccatttcaaagcatttgtaggcca
gccaagggctttcattattgaggcttctagtggcctctggttaacctaga
agttagtgggtttttcttgatgacaccaacctctcacagcgtttttcctt
agagacttaagcagagttttaaaatcctcttttgcgaaaagaacaaatat
gttttatgactttgatgatatcttcattctgggcaaaagaatggccctag
aaccagctagaagtgaagagaatccattaatgatcaaccacctgagtcaa
taatgagaaatcagtactgatgttacatttgtggctatttcttgctgact
ttcaaaggtcaaggactcttgactaatccagtgactgcaaaaatggatct
actaaaagtcatctagccagaagtagagatttttaacctttctttccctg
gcttttgtcttctagctatcatttaaatttgagacatttgaagtattaag
aaacaatttttctgtatggtaagaaacagtattttacaatactgaagccc
tgttttattcaatcttgcatcttgaatacaatataccacaaagtcggaaa
ctttatatttatttactgcaggtggttaaaaaaagggggggaaagggtttc
accatccactgacaacgagagccatgacaaatagtatccatgtgcagtct
tccaactgctggtgacaatgaccccatatttggtctcatgctgcttttgc
agagcactctgtaggttagtccatcacacaaggaggccctgaatccagac
actgtgaattaagaccttggcggggagagatgtgacccttttggtaggaa
tgggaagaagaatgggtggaagccaatatgaaatttcttctttgcaatga
cttgacaggggagttaatgttcctaggatgccatgaatgatgaatgttag
ttggaggtaatgctgtatatgtgtgtgtgtgtgtgtgtgtgtgtttgtgt
gtgtgtatatatatgtgtgtgtgtatatatatgtgacatgtgtgtgtctt
tgtgtgtgtgtatatatatgtgacgtgtgtgtgggatgtgtgtgtgtata
tatagatgaatatatacaaatatatagatatatacacacatatatagata
tatatacacatatatagatatatatacacacatatatagatacacatatg
tgtgtgtgtgtatatatatatatatatatatatagatgtataggcttgtg
agaaacttgagaggaagaagcatgctcttctaggaatgtgaggaaatatg
accttgccaagactaaaagacctctagactgtgagctcagttatggagaa

caaaaacagcttcatagtgagtagaacaccgaggataaacactggggcca
tgggtcctttctgaggcagcgccacagaagatctttgtggtccttccgta
gttctgtaagtctgtctcctaagtatgggtagagaatatgtagcctgttg
tgtgtctcccactacttgtaaacagagcatcacattaggggcagggagga
ggtggaatgatattggaggtgcttaaccctactcgaggaattaattatga
ataaagagcttataattagctaacatgactagaaaacacatgacttaggt
ggagagttagctttcttttctagtttgtgtatgacttgccatttgtgacg
tatacaccaaaagatctggtgttttagacttctgccattcacttggcatt
taaatctctctttgcttatgctgttaacgagtatgccataggataggaca
aattcagtaaacaggaaaacttgtccatatttgcatagacatttgtaggg
tttttttttctttttctttttagaacttcaccattggcttaagaatgta
gttcccaaaacaatttttcttgcaaagtactttccttacacctcttggc
tacagggtgggccaaattaaacatatatgtattttcatttaatgtatgtg
cagtttggtttatcatcttaagatggtggtgctgccccggtgctacttca
tctgtgtacacaaagaccaatgcatggtctgtattgctaccaaaacattt
actgtatatatgtttataacatgtattatgtatatatgtaatgggtgcca
ggccaggtatatattttttatttagaagtgtttcacttttccaagttttc
tttatagtgttatgcttattttcaatttttttttttcctgattctgtctgg
tacttagaattgtagtgtcttcatcatcaattaaagaaaactgtctaaat
gaattcatggatgtaaatattagtggtccttaatgtctttgattgctgga
catgaaacaaactgccaattaaattttgcggagacaaaaaaaa
>NM_175850 2
gaagggagagagcaaacaaaggggtcaggagggaaaataatgcactggct
tcctgagcccctgcagaggctgagcagggagagggggccagggccagagg
ggacagaggctggcggcagacgggccgggacaggcaggtcctaaatggca
ttgtttgaaggggccggctaattgcacagagcagtctgagcctgagaccc
cagccctggcctccccactctgtcctgggtgctggcgtctgagccttcgg
gacagcctgtccacatggaaccaagtcctgagcctccaagcttggaaagc
atgaagggagacaccaggcatctcaatggagaggaggacgccggcgggag
ggaagactcgatcctcgtcaacggggcctgcagcgaccagtcctccgact
cgcccccaatcctggaggctatccgcaccccggagatcagaggccgaaga
tcaagctcgcgactctccaagagggaggtgtccagtctgctaagctacac
acaggacttgacaggcgatggcgacggggaagatggggatggctctgaca
ccccagtcatgccaaagctcttccgggaaaccaggactcgttcagaaagc
ccagctgtccgaactcgaaataacaacagtgtctccagccgggagaggca
caggccttccccacgttccacccgaggccggcagggccgcaaccatgtgg
acgagtcccccgtggagttcccggctaccaggtccctgagacggcgggca
acagcatcggcaggaacgccatggccgtcccctcccagctcttaccttac
catcgacctcacagacgacacagaggacacacatgggacgccccagagca
gcagtacccccctacgcccgcctagcccaggacagccagcagggggcatg
gagtccccgcaggtggaggcagacagtggagatggagacagttcagagta
tcaggatgggaaggagtttggaataggggacctcgtgtggggaaagatca
agggcttctcctggtggcccgccatggtggtgtcttggaaggccacctcc
aagcgacaggctatgtctggcatgcggtgggtccagtggtttggcgatgg
caagttctccgaggtctctgcagacaaactggtggcactggggctgttca
gccagcactttaatttggccaccttcaataagctcgtctcctatcgaaaa
gccatgtaccatgctctggagaaagctagggtgcgagctggcaagacctt
ccccagcagccctggagactcattggaggaccagctgaagcccatgttgg
agtgggcccacgggggcttcaagcccactgggatcgagggcctcaaaccc

aacaacacgcaaccagagaacaagactcgaagacgcacagctgacgactc
agccacctctgactactgccccgcacccaagcgcctcaagacaaattgct
ataacaacggcaaagaccgaggggatgaagatcagagccgagaacaaatg
gcttcagatgttgccaacaacaagagcagcctggaagatggctgtttgtc
ttgtggcaggaaaaaccccgtgtccttccaccctctctttgaggggggc
tctgtcagacatgccgggatcgcttccttgagctgttttacatgtatgat
gacgatggctatcagtcttactgcactgtgtgctgcgagggccgagagct
gctgctttgcagcaacacgagctgctgccggtgtttctgtgtggagtgcc
tggaggtgctggtgggcacaggcacagcggccgaggccaagcttcaggag
ccctggagctgttacatgtgtctcccgcagcgctgtcatggcgtcctgcg
gcgccggaaggactggaacgtgcgcctgcaggccttcttcaccagtgaca
cggggcttgaatatgaagcccccaagctgtaccctgccattcccgcagcc
cgaaggcggcccattcgagtcctgtcattgtttgatggcatcgcgacagg
ctacctagtcctcaaagagttgggcataaaggtaggaaagtacgtcgctt
ctgaagtgtgtgaggagtccattgctgttggaaccgtgaagcacgagggg
aatatcaaatacgtgaacgacgtgaggaacatcacaaagaaaaatattga
agaatggggcccatttgacttggtgattggcggaagcccatgcaacgatc
tctcaaatgtgaatccagccaggaaaggcctgtatgagggtacaggccgg
ctcttcttcgaattttaccacctgctgaattactcacgccccaaggaggg
tgatgaccggccgttcttctggatgtttgagaatgttgtagccatgaagg
ttggcgacaagagggacatctcacggttcctggagtgtaatccagtgatg
attgatgccatcaaagtttctgctgctcacagggcccgatacttctgggg
caacctacccgggatgaacaggcccgtgatagcatcaaagaatgataaac
tcgagctgcaggactgcttggaatacaataggatagccaagttaaagaaa
gtacagacaataaccaccaagtcgaactcgatcaaacaggggaaaaacca
acttttccctgttgtcatgaatggcaaagaagatgttttgtggtgcactg
agctcgaaaggatctttggctttcctgtgcactacacagacgtgtccaac
atgggccgtggtgcccgccagaagctgctgggaaggtcctggagcgtgcc
tgtcatccgacacctcttcgcccctctgaaggactactttgcatgtgaat
agttccagccaggccccaagcccactggggtgtgtggcagagccaggacc
caggaggtgtgattcctgaaggcatccccaggccctgctcttcctcagct
gtgtgggtcataccgtgtacctcagttccctcttgctcagtgggggcaga
gccacctgactcttgcaggggtagcctgaggtgccgcctccttgtgcaca
aatcagacctggctgcttggagcagcctaacacggtgctcattttttctt
ctcctaaaactttaaaacttgaagtaggtagcaacgtggctttttttttt
tcccttcctgggtctaccactcagagaaacaatggctaagataccaaaac
cacagtgccgacagctctccaatactcaggttaatgctgaaaaatcatcc
aagacagttattgcaagagtttaatttttgaaaactggctactgctctgt
gtttacagacgtgtgcagttgtaggcatgtagctacaggacatttttaag
ggcccaggatcgtttttttcccagggcaagcagaagagaaaatgttgtata
tgtcttttacccggcacattccccttgcctaaatacaagggctggagtct
gcacgggacctattagagtattttccacaatgatgatgatttcagcaggg
atgacgtcatcatcacattcagggctatttttttcccccacaaacccaagg
gcaggggccactcttagctaaatccctccccgtgactgcaatagaaccct
ctggggagctcaggaaggggtgtgctgagttctataatataagctgccat
atattttgtagacaagtatggctcctccatatctccctcttccctaggag
aggagtgtgaagcaaggagcttagataagacaccccctcaaacccattcc
ctctccaggagacctaccctccacaggcacaggtccccagatgagaagtc
tgctaccctcatttctcatctttttactaaactcagaggcagtgacagca

gtcagggacagacatacatttctcataccttccccacatctgagagatga
cagggaaaactgcaaagctcggtgctccctttggagattttttaatcctt
ttttattccataagaagtcgtttttagggagaacgggaattcagacaagc
tgcatttcagaaatgctgtcataatggtttttaacaccttttactcttct
tactggtgctattttgtagaataaggaacaacgttgacaagttttgtggg
gctttttatacactttttaaaatctcaaacttctatttttatgtttaacg
ttttcattaaaattttttttgtaactggagccacgacgtaacaaatatgg
ggaaaaaactgtgccttgtttcaacagtttttgctaatttttaggctgaa
agatgacggatgcctagagtttaccttatgtttaattaaaatcagtattt
gtctaaaaaaaaaaaaaaaaaa
>NM_175849 1
acccactcccgctgccccgtccggcccgcgccgcttcctcgcagcagctg
ctcccggctccgcggccgcagcccgcgtggacgctccgagcgccccccga
cggacgggaccggctccctggcggtcgggcgagcgggcggcaacgctgcc
cggccggcagcgctggggttaagtggcccaagtaaacctagctcggcgat
cggcgccggagattcgcgagcccagcgccctgcacggccgccagccggcc
tcccgccagccagccccgacccgcggctccgccgcccagccgcgccccag
ccagccctgcggcaggaaagcatgaagggagacaccaggcatctcaatgg
agaggaggacgccggcgggagggaagactcgatcctcgtcaacggggcct
gcagcgaccagtcctccgactcgcccccaatcctggaggctatccgcacc
ccggagatcagaggccgaagatcaagctcgcgactctccaagagggaggt
gtccagtctgctaagctacacacaggacttgacaggcgatggcgacgggg
aagatggggatggctctgacaccccagtcatgccaaagctcttccgggaa
accaggactcgttcagaaagcccagctgtccgaactcgaaataacaacag
tgtctccagccgggagaggcacaggccttccccacgttccacccgaggcc
ggcagggccgcaaccatgtggacgagtcccccgtggagttcccggctacc
aggtccctgagacggcgggcaacagcatcggcaggaacgccatggccgtc
ccctcccagctcttaccttaccatcgacctcacagacgacacagaggaca
cacatgggacgccccagagcagcagtacccccctacgcccgcctagcccag
gacagccagcagggggggcatggagtccccgcaggtggaggcagacagtgg
agatggagacagttcagagtatcaggatgggaaggagtttggaataggggg
acctcgtgtggggaaagatcaagggcttctcctggtggcccgccatggtg
gtgtcttggaaggccacctccaagcgacaggctatgtctggcatgcggtg
ggtccagtggtttggcgatggcaagttctccgaggtctctgcagacaaac
tggtggcactggggctgttcagccagcactttaatttggccaccttcaat
aagctcgtctcctatcgaaaagccatgtaccatgctctggagaaagctag
ggtgcgagctggcaagaccttccccagcagccctggagactcattggagg
accagctgaagcccatgttggagtgggcccacgggggcttcaagcccact
gggatcgagggcctcaaacccaacaacacgcaaccagagaacaagactcg
aagacgcacagctgacgactcagccacctctgactactgccccgcaccca
agcgcctcaagacaaattgctataacaacggcaaagaccgaggggatgaa
gatcagagccgagaacaaatggcttcagatgttgccaacaacaagagcag
cctggaagatggctgtttgtcttgtggcaggaaaaaccccgtgtccttcc
accctctctttgaggggggggctctgtcagacatgccgggatcgcttcctt
gagctgttttacatgtatgatgacgatggctatcagtcttactgcactgt
gtgctgcgagggccgagagctgctgctttgcagcaacacgagctgctgcc
ggtgtttctgtgtggagtgcctggaggtgctggtgggcacaggcacagcg
gccgaggccaagcttcaggagccctggagctgttacatgtgtctcccgca
gcgctgtcatggcgtcctgcggcgccggaaggactggaacgtgcgcctgc

aggccttcttcaccagtgacacggggcttgaatatgaagcccccaagctg
taccctgccattcccgcagcccgaaggcggcccattcgagtcctgtcatt
gtttgatggcatcgcgacaggctacctagtcctcaaagagttgggcataa
aggtaggaaagtacgtcgcttctgaagtgtgtgaggagtccattgctgtt
ggaaccgtgaagcacgaggggaatatcaaatacgtgaacgacgtgaggaa
catcacaaagaaaaatattgaagaatggggcccatttgacttggtgattg
gcggaagcccatgcaacgatctctcaaatgtgaatccagccaggaaaggc
ctgtatgagggtacaggccggctcttcttcgaattttaccacctgctgaa
ttactcacgccccaaggagggtgatgaccggccgttcttctggatgtttg
agaatgttgtagccatgaaggttggcgacaagagggacatctcacggttc
ctggagtgtaatccagtgatgattgatgccatcaaagtttctgctgctca
cagggcccgatacttctggggcaacctacccgggatgaacaggatctttg
gctttcctgtgcactacacagacgtgtccaacatgggccgtggtgcccgc
cagaagctgctgggaaggtcctggagcgtgcctgtcatccgacacctctt
cgcccctctgaaggactactttgcatgtgaatagttccagccaggcccca
agcccactggggtgtgtggcagagccaggacccaggaggtgtgattcctg
aaggcatccccaggccctgctcttcctcagctgtgtgggtcataccgtgt
acctcagttccctcttgctcagtgggggcagagccacctgactcttgcag
gggtagcctgaggtgccgcctccttgtgcacaaatcagacctggctgctt
ggagcagcctaacacggtgctcatttttttcttctcctaaaactttaaaac
ttgaagtaggtagcaacgtggcttttttttttttcccttcctgggtctacc
actcagagaaacaatggctaagataccaaaaccacagtgccgacagctct
ccaatactcaggttaatgctgaaaaatcatccaagacagttattgcaaga
gtttaatttttgaaaactggctactgctctgtgtttacagacgtgtgcag
ttgtaggcatgtagctacaggacatttttaagggcccaggatcgtttttt
cccagggcaagcagaagagaaaatgttgtatatgtcttttacccggcaca
ttccccttgcctaaatacaagggctggagtctgcacgggacctattagag
tattttccacaatgatgatgatttcagcagggatgacgtcatcatcacat
tcagggctattttttcccccacaaacccaagggcaggggccactcttagc
taaatccctccccgtgactgcaatagaaccctctggggagctcaggaagg
ggtgtgctgagttctataatataagctgccatatattttgtagacaagta
tggctcctccatatctccctcttccctaggagaggagtgtgaagcaagga
gcttagataagacaccccctcaaacccattccctctccaggagacctacc
ctccacaggcacaggtccccagatgagaagtctgctaccctcatttctca
tctttttactaaactcagaggcagtgacagcagtcagggacagacataca
tttctcataccttccccacatctgagagatgacagggaaaactgcaaagc
tcggtgctccctttggagatttttaatcctttttttattccataagaagt
cgtttttagggagaacgggaattcagacaagctgcatttcagaaatgctg
tcataatggtttttaacaccttttactcttcttactggtgctattttgta
gaataaggaacaacgttgacaagttttgtggggctttttatacacttttt
aaaatctcaaacttctatttttatgtttaacgttttcattaaaatttttt
ttgtaactggagccacgacgtaacaaatatggggaaaaaactgtgccttg
tttcaacagtttttgctaatttttaggctgaaagatgacggatgcctaga
gtttaccttatgtttaattaaaatcagtatttgtctaaaaaaaaaaaaaa
aaaa
>NM_033013 2
ttcttaacccttccagctttcccaccctctttggctttagccatggcct
tctgatctgtgtttctcaggggacctgcaggccccagatatagccccatg
ctgtcctcctaccccagagcacactgttcaggctacttccactggtactg

aaatccagtatttcacttactctttttctttccaatatcctcatgacatt
caatatttcacttactctaggtcctccctgcctaaggcccaagtcaactt
tctgtccagtgggatttgtaatccaatacctcctagccctagcagaatcc
catgtggataatcagaaatgtgactggaaaaaggacagagctctatggct
gtgggtcccagtccccactgctggcagtaagtccccagcagtgagctgtg
taagcaccttacattctgcgcttggttgaaaacagcaaggcaagcatcca
cttgagaaatgtcaacccctaggaaatcccagcctcaagtctttctcatc
ccttgggaagtgcaaattggatagagaagaaaccaattaaaaacaaaaca
aacaaatcatacttagatattctggcttttctcaccagggctggattaaa
gcatgtacttcaaaataataacaacttaagtcaataaataaatgtaagga
agtccaaatgttcacctgaagacaactgtggtcatttttggcaatccca
ggttctcttttctacctgtttgctcaatcgtggtctccctctccctctct
tgttggggcccatgcccctgctttactgttgccagaggcttgtacttgtt
tgccttttaggtaggagcagttacttccactcccctcacctgccataaag
catctttataaacaaagcaagtagaagaaacacatcctggtatccaccac
attcggcttttgttgattctgttcacttgggagcacctgctgctagggaa
taagaaggttgaggctgaagagtgaggactcttcagctcccctctggcag
gacccgggagaggaaagagccctcagctggtccatcctccccactcctgg
tcagccttctgttctgagatcaaagtggtggggtcacattctcgagaact
gtgctcagccccctcatctcacaccctttccctctccctgtgtgcctgcc
cccctcttacataaccatgctggtgattggcaccgtcataaatcaatact
ttgctcactttcacatcaagtaacactatccagggaggtggtttcaacaa
aggaggaagtataaggagatctaggttcaaattaatgttgcccctagtgg
taaaggacagagaccctcagactgatgaaatgcactcagaattacttaga
caaagcggatatttgccactctcttcccctttcctgtgtttttgtagtg
aagagacctgaaagaaaaaagtagggagaacataatgagaacaaatacgg
taatctcttcatttgctagttcaagtgctggacttgggacttaggagggg
caatggagccgcttagtgcctacatctgacttggactgaaatataggtga
gagacaagattgtctcatatccggggaaatcataacctatgactaggacg
ggaagaggaagcactgcctttacttcagtgggaatctcggcctcagcctg
caagccaagtgttcacagtgagaaaagcaagagaataagctaatactcct
gtcctgaacaaggcagcggctccttggtaaagctactccttgatcgatcc
tttgcaccggattgttcaaagtggaccccaggggagaagtcggagcaaag
aacttaccaccaagcagtccaagaggcccagaagcaaacctggaggtgag
acccaaagaaagctggaaccatgctgactttgtacactgtgaggacacag
agtctgttcctggaaagcccagtgtcaacgcagatgaggaagtcggaggt
ccccaaatctgccgtgtatgtggggacaaggccactggctatcacttcaa
tgtcatgacatgtgaaggatgcaagggctttttcaggagggccatgaaac
gcaacgcccggctgaggtgccccttccggaagggcgcctgcgagatcacc
cggaagacccggcgacagtgccaggcctgccgcctgcgcaagtgcctgga
gagcggcatgaagaaggagatgatcatgtccgacgaggccgtggaggaga
ggcgggccttgatcaagcggaagaaaagtgaacggacagggactcagcca
ctgggagtgcaggggctgacagaggagcagcggatgatgatcagggagct
gatggacgctcagatgaaaacctttgacactaccttctcccatttcaaga
atttccgggtctctctgcagctgcggggggaggatggcagtgtctggaac
tacaaacccccagccgacagtggcgggaaagagatcttctccctgctgcc
ccacatggctgacatgtcaacctacatgttcaaaggcatcatcagctttg
ccaaagtcatctcctacttcagggacttgcccatcgaggaccagatctcc
ctgctgaagggggccgctttcgagctgtgtcaactgagattcaacacagt

gttcaacgcggagactggaacctgggagtgtggccggctgtcctactgct
tggaagacactgcaggtggcttccagcaacttctactggagcccatgctg
aaattccactacatgctgaagaagctgcagctgcatgaggaggagtatgt
gctgatgcaggccatctccctcttctccccagaccgcccaggtgtgctgc
agcaccgcgtggtggaccagctgcaggagcaattcgccattactctgaag
tcctacattgaatgcaatcggccccagcctgctcataggttcttgttcct
gaagatcatggctatgctcaccgagctccgcagcatcaatgctcagcaca
cccagcggctgctgcgcatccaggacatacacccctttgctacgcccctc
atgcaggagttgttcggcatcacaggtagctgagcggctgcccttgggtg
acacctccgagaggcagccagacccagagccctctgagccgccactcccg
ggccaagacagatggacactgccaagagccgacaatgccctgctggcctg
tctccctagggaattcctgctatgacagctggctagcattcctcaggaag
gacatgggtgcccccacccccagttcagtctgtagggagtgaagccaca
gactcttacgtggagagtgcactgacctgtaggtcaggaccatcagagag
gcaaggttgccctttccttttaaaaggccctgtggtctggggagaaatcc
ctcagatcccactaaagtgtcaaggtgtggaagggaccaagcgaccaagg
atgggccatctggggtctatgcccacatacccacgtttgttcgcttcctg
agtcttttcattgctacctctaatagtcctgtctcccacttcccactcgt
tcccctcctcttccgagctgctttgtgggctccaggcctgtactcatcgg
caggcgcatgagtatctgtgggagtcctctagagagatgagaagccagga
ggcctgcaccaaatgtcagaagcttggcatgacctcattccggccacatc
attctgtgtctctgcatccatttgaacacattattaagcaccgataatag
gtagcctgctgtggggtatacagcattgactcagatatagatcctgagct
cacagagtttatagttaaaaaaacaaacagaaacacaaacaatttggatc
aaaaggagaaatgataagtgacaaaagcagcacaaggaatttccctgtgt
ggatgctgagctgtgatggcgggcactgggtacccaagtgaaggttcccg
aggacatgagtctgtaggagcaagggcacaaactgcagctgtgagtgcgt
gtgtgtgatttggtgtaggtaggtctgtttgccacttgatggggcctggg
tttgttcctggggctggaatgctgggtatgctctgtgacaaggctacgct
gacaatcagttaaacacaccggagaagaaccatttacatgcaccttatat
ttctgtgtacacatctattctcaaagctaaagggtatgaaagtgcctgcc
ttgtttatagccacttgtgagtaaaaatttttttgcattttcacaaatta
tactttatataaggcattccacacctaagaactagttttgggaaatgtag
ccctgggtttaatgtcaaatcaaggcaaaaggaattaaataatgtacttt
tggctaaaaaaaaaaaaaaaaaaaaaaaaaaaaa
>NM_032049 2
atgaagaaaatgaatcacaagtcaactgacagtccaaaggctccacagct
cagaggagggcatgccattgcaagagaatgctcagccatgttcatctggg
gacctgctcctggtagagcaataggatctgtgtgcccagcaccactgccc
acctagctgtcctggcctgtgcccaatgctgaccttcacctcagagtgtg
gctgtaccacattcggtgtatttgatatagtgtttgcaacaaattcgacc
caggtgatcaaaatgattctcaactcttctactgaagatggtattaaaag
aatccaagatgattgtcccaaagctggaaggcataattacatatttgtca
tgattcctactttatacagtatcatctttgtggtgggaatatttggaaac
agcttggtggtgatagtcatttacttttatatgaagctgaagactgtggc
cagtgtttttcttttgaatttagcactggctgacttatgctttttactga
ctttgccactatgggctgtctacacagctatggaataccgctggcccttt
ggcaattacctatgtaagattgcttcagccagcgtcagtttcaacctgta
cgctagtgtgtttctactcacgtgtctcagcattgatcgatacctggcta

ttgttcacccaatgaagtcccgccttcgacgcacaatgcttgtagccaaa
gtcacctgcatcatcatttggctgctggcaggcttggccagtttgccagc
tataatccatcgaaatgtatttttcattgagaacaccaatattacagttt
gtgctttccattatgagtcccaaaattcaaccctcccgatagggctgggc
ctgaccaaaaatatactgggtttcctgtttccttttctgatcattcttac
aagttatactcttatttggaaggccctaaagaaggcttatgaaattcaga
agaacaaaccaagaaatgatgatatttttaagataattatggcaattgtg
cttttctttttcttttcctggattccccaccaaatattcacttttctgga
tgtattgattcaactaggcatcatacgtgactgtagaattgcagatattg
tggacacggccatgcctatcaccatttgtatagcttattttaacaattgc
ctgaatcctctttttatggctttctggggaaaaaatttaaaagatattt
tctccagcttctaaaatatattcccccaaaagccaaatcccactcaaacc
tttcaacaaaaatgagcacgctttcctaccgcccctcagataatgtaagc
tcatccaccaagaagcctgcaccatgttttgaggttgagtgacatgttcg
aaacctgtccataaagtaattttgtgaaagaaggagcaagagaacattcc
tctgcagcacttcactaccaaatgagcattagctacttttcagaattgaa
ggagaaaatgcattatgtggactgaaccgacttttctaaagctctgaaca
aaagcttttctttccttttgcaacaagacaaagcaaagccacattttgca
ttagacagatgacggctgctcgaagaacaatgtcagaaactcgatgaatg
tgttgatttgagaaattttactgacagaaatgcaatctccctagcctgct
tttgtcctgttatttttatttccacataaaggtatttagaatatattaa
atcgttagaggagcaacaggagatgagagttccagattgttctgtccagt
ttccaaagggcagtaaagttttcgtgccggttttcagctattagcaactg
tgctacacttgcacctggtactgcacattttgtacaaagatatgctaagc
agtagtcgtcaagttgcagatctttttgtgaaattcaacctgtgtcttat
aggtttacactgccaaaacaatgcccgtaagatggcttatttgtataatg
gtgttactaaagtcacatataaaagttaaactacttgtaaaggtgctgca
ctggtcccaagtagtagtgtcttcctagtatattagtttgatttaatatc
tgagaagtgtatatagtttgtggtaaaaagattatatatcataaagtatg
ccttcctgtttaaaaaaagtatatattctacacatatatgtatatgtata
tctatatctctaaactgctgttaattgattaaaatctggcaaagttatat
ttactttaaaataaaataattttattgcaaaaaaaa
>NM_206866 1
gtctactcagcccggtggctgtcgcgcgtggaatcgcgtaagaaaagccg
agtttgtggctggggagagaaggccaccgtgctgagctggatttagcgaa
gactggttttggggaccggagagcccaggactccctttgttggagttttg
cccacgcgttgtaattaagcctcgcacaatatggttgatgataattagaa
gcatgctttccactgaacttcccgacaacatttgttatgcagaatgtctc
tgagtgagaactcggtttttgcctatgaatcttctgtgcatagcaccaat
gttttactcagccttaatgaccagcggaagaaagatgtgctgtgcgatgt
caccatctttgtggagggacagcggttccgcgctcaccggtccgtgctgg
cggcatgcagcagttacttccactcaagaatcgtaggccaggctgatgga
gagctgaacattactcttccagaagaggtgacagttaaaggatttgaacc
tttaattcagtttgcctacactgctaaactgattttaagtaaagagaatg
tggatgaagtgtgcaaatgtgtggagtttttaagtgtacataatattgag
gaatcctgctttcagtttctgaaatttaagtttttggactccactgcaga
ccagcaagaatgcccaagaaaaaaatgcttttcatcacactgtcagaaaa
cagaccttaaactttcacttttggaccagagggatctagaaactgatgaa
gtggaggaatttctggaaaataaaaatgttcagactcctcagtgtaaact

ccgcaggtatcaaggaaatgcaaaagcctcacctcctctacaagacagtg
ccagtcagacatatgagtccatgtgcttagagaaggatgctgctctggcc
ttgccttctttatgccccaaatacagaaaattccaaaaagcatttggaac
tgacagagtccgtactggggaatctagtgtcaaagacattcatgcttctg
ttcagccaaatgaaaggtctgaaaatgaatgcctgggaggagtcccggag
tgtagagatttgcaggtgatgttaaaatgtgacgaaagtaaattagcaat
ggaacctgaagaaacgaagaaagatcctgcttctcagtgcccaactgaaa
aatcagaagtgactcctttcccccacaattcttccatagaccctcatgga
ctttattctttgtctcttttacacacatatgaccaatatggtgacttgaa
ttttgctggtatgcaaaacacaacagtgttaacagaaaagcctttgtcag
gtacagacgtccaagaaaaaacatttggtgaaagtcaggatttacctttg
aaatccgacttgggcaccagggaagatagtagtgttgcatctagtgatag
gagtagtgtggagcgagaagtggcagaacacctagcaaaaggcttctgga
gtgacatttgcagcacggacactccttgccaaatgcagttatcacctgct
gtggccaaagatggctcagaacagatctcacagaaacggtctgagtgtcc
gtggttaggtatcaggattagtgagagcccagaaccaggtcaaaggactt
tcacaacattaagttctgtcaactgcccttttataagtactctgagtact
gaaggctgttcaagcaatttggaaattggaaacgatgattatgtttcaga
accccagcaagaaccttgcccatatgcttgtgtcattagcttgggagacg
actctgagacggacaccgaaggagacagtgaatcctgttcagccagagaa
caagaatgtgaggtaaaactgccattcaatgcacaacggataatttcact
gtctcgaaatgattttcagtccttgttgaaaatgcacaagcttactccag
aacagctggattgtatccatgatattcgaagaagaagtaaaaacagaatt
gctgcacagcgctgtcgcaagagaaaacttgactgtatacagaatcttga
atcagaaattgagaagctgcaaagtgaaaaggagagcttgttgaaggaaa
gagatcacattttgtcaactctgggtgagacaaagcagaacctaactgga
ctttgccagaaagtttgtaaagaagcagctctgagtcaagaacaaataca
gatactcgccaagtactcagctgcagattgcccactttcatttttaattt
ctgaaaaagataaaagtactcctgatggtgaactggcgttaccatcaatt
ttcagtttatctgaccggcctccagcagtgctgcctccctgtgccagagg
aaacagtgagcctggctacgcgcgagggcaggagtcccagcagatgtcca
cagccacctctgagcaagctgggcctgcggaacagtgtcgtcagagtggt
gggatctcagatttctgtcagcagatgactgataaatgtactactgatga
gtaaacttgcattcacttccttcaaaccatctaattttctcctgaagttt
tggcagcgtcttgaaagcctaatatgaccatctgttgctcaacaatactg
tttttttcctttagtagtttaccataagggaatttcctttaagtcaacca
tgatttctccttgatttctacaagagacaaagaaatgattttgcctcctg
gatatcagaaaaatccatgtgaaaatgtagtaaacctttaaaactcatgt
tttaaagaataataactctagtaataactcttcctgctattcagaataag
taggagaatgaaaactgcagcatatcagacagcaatttaacagcttgaaa
catctacagatagttcctactaaaagaagtggcctgcagaagtttaataa
tttgactttttttctaatattttagtttgaaagaaaatttcttcccaagca
atgctaatagagttctattcttagaagcagggtgtcagctactgggaata
tttttgtagagctgcattgtgaaaaaaagatggtcttacctgaatcttag
ggctttgttcttcggctcctaaaatcaggctttaagctacattgggaaga
tttagtaaataggcaagtggttggcctaagacgggggctgcttctcctct
tcagtatggactctagaaagtctggctacatgaatagatttaagtgtcac
tttccctccctgccccccgcttcagtctctaccatatctggtcccatcat
ggacttcctatttcctggcatttttgtccctttggaagaagaaataggac

tcagaatacagtggcatgagtgattacactggcagcattatctcaggctc
cctagaatctggagagcttaccaacatgtaaagctgttcatttttccacc
gtgggtcaccaatgccagaaaaccagacatcacggggaaagaatgttgct
tactttttaccaggagtgcagttcatttttttcaccctgtttttgaagtc
gtattattcacttgtaaaaatgattgtaacagataaaaaatgtatctgca
gcaactctgcaggtttgtgaaataggatgaaactcaatctttttctattg
tgggtttgcatttgaaaagcaggttgaatccttgctctcttctccaaatt
tggtgtggtataaagacacacaaatcattttaacttggacatttaaagat
cagtcttagtgtttgttcagtcctgttacaaaatagataactgagcacct
atcgcataacattttgcggtggcttttagccatgctggggttagatgtgt
ttgagagtcaaatgaaagctatggatcttctcagcaattaaaaaaaatgc
atatattcacattcacagaaacattggcagaacccagttttaatggtaca
gaggagtagtttatagtgttgatttcaccaaaatcagagggctgaaagag
acacttctatagactgcatcctgagcctagtgcagggcttgtctagctaa
tgtgggcagccaccacccactgtgtatgaacaagtctgaagcaagttggc
cttgcccttgagagtatatggggaccagtcttcatgtcttggagtaattt
gtcaaatgttacccttttgatcagggtgtagggggaggatattgctagt
atattttcagtggtttgtatgttctctctgtcactgacttatttgtaaga
gaaaattagttggacttgtttattttctagtagcttttataagtacactc
aagaatttgtcagggagaataattctgatagtgcatcccatactgcaaaa
gaatttgtgtgtgtgtgtgtgtgtgtgtgtgtgtgtgtgtatgtgtatgt
atacatatatatctctccatataggtatttctttgatacttgtaattta
aatttcagcttcacgatataaaataatataagaacttctggtttacaaaa
tgtaaaatcttaagccaatggaacccttgatttcctacctcagtgtacac
ccaactattggttgtatcagtttgtgtatgtgcaaatgtcaaataatctt
ttgctttaattgctactgtacttgctttgaaagattacctactattttat
gataaaatgtagttgtctccagagcttaaatataatttgtaaagcacttg
gtttaaatttctctctacctataaacagtttagcattaagggtttctatt
aatgacacagaattattggccaagtgtaatttcttaaaatttagcattac
tttaaatagccagcatgtaatacaagtaactacactacctcatatctaca
tgattttcaagttgtaatgcagatggacagataaaaaagattttacgttt
gtcttttggccataagtgggaaagttttctgtatattgcatagcattaca
catttatgcctattttaacattaacttctaaagaagtttttctaagaaa
atgtttcaaggcaatatttttttgaggctgccgaagacaaatgacagga
ttatgagtatacagtgtatgccttttccttcatgcagaattttgaaatgt
tttcagtttgtatattgcatattcacatgatcattgttcactattttatg
aactggccttctcaatgtttgatgatttttaaaagctgttatgttgaat
tcagtaaaataacattaccttattttttttcttattcaaattctggaact
atagcaaataattcgttaaattgtcatattcaaaacaaatgtggatacag
tcttggttctccatctgtaatttttttaacagtttgctatagcttactg
cttaactaattttaaataaggaaataagtatgttagatgcagtagacgat
acaggttgcatgtggacactcagtcacattaacaacttgggaaaaaaatg
gcaatgttacggtgaattctcaggtgaactttttcagttataaaacatc
tattttgaatctgtaaatattttaaatgttttattaaggcatgtaataaa
ctattctttgaaacttgttgggtagaatgaaaattaaagccataatggta
gaagatggcatactgattataaaagaagcagaaaaacattgattttttta
tatctttcataatataattttctaacaatgcaataaaaccactaaacttt
tgtgtccatattttactgagaccatgtttcattaaaagcatagtttcata
gtatttaatttacatttctccctaatgttcttacccaaatgtacctgaac

taaaaaatgttagatgttggtgataagttgacagttaaataaaattctct
aaaattgcttctaattgaaa
>NM_000633 2
tttctgtgaagcagaagtctgggaatcgatctggaaatcctcctaatttt
tactccctctccccgcgactcctgattcattgggaagtttcaaatcagct
ataactggagagtgctgaagattgatgggatcgttgccttatgcatttgt
tttggttttacaaaaaggaaacttgacagaggatcatgctgtacttaaaa
aatacaacatcacagaggaagtagactgatattaacaatacttactaata
ataacgtgcctcatgaaataaagatccgaaaggaattggaataaaaattt
cctgcatctcatgccaagggggaaacaccagaatcaagtgttccgcgtga
ttgaagacaccccctcgtccaagaatgcaaagcacatccaataaaatagc
tggattataactcctcttctttctctgggggccgtggggtgggagctggg
gcgagaggtgccgttggcccccgttgcttttcctctgggaaggatggcgc
acgctgggagaacagggtacgataaccgggagatagtgatgaagtacatc
cattataagctgtcgcagaggggctacgagtgggatgcgggagatgtggg
cgccgcgcccccgggggccgcccccgcaccgggcatcttctcctcccagc
ccgggcacacgccccatccagccgcatcccgggacccggtcgccaggacc
tcgccgctgcagaccccggctgcccccggcgccgccgcggggcctgcgct
cagcccggtgccacctgtggtccacctgaccctccgccaggccggcgacg
acttctcccgccgctaccgccgcgacttcgccgagatgtccagccagctg
cacctgacgcccttcaccgcgcggggacgctttgccacggtggtggagga
gctcttcagggacggggtgaactgggggaggattgtggccttctttgagt
tcggtggggtcatgtgtgtggagagcgtcaaccgggagatgtcgcccctg
gtggacaacatcgccctgtggatgactgagtacctgaaccggcacctgca
cacctggatccaggataacggaggctgggatgcctttgtggaactgtacg
gccccagcatgcggcctctgtttgatttctcctggctgtctctgaagact
ctgctcagtttggccctggtgggagcttgcatcaccctgggtgcctatct
gggccacaagtgaagtcaacatgcctgccccaaacaaatatgcaaaaggt
tcactaaagcagtagaaataatatgcattgtcagtgatgtaccatgaaac
aaagctgcaggctgtttaagaaaaaataacacacatataaacatcacaca
cacagacagacacacacacacacaacaattaacagtcttcaggcaaaacg
tcgaatcagctatttactgccaaagggaaatatcatttattttttacatt
attaagaaaaaaagatttatttatttaagacagtcccatcaaaactcctg
tctttggaaatccgaccactaattgccaagcaccgcttcgtgtggctcca
cctggatgttctgtgcctgtaaacatagattcgctttccatgttgttggc
cggatcaccatctgaagagcagacggatggaaaaaggacctgatcattgg
ggaagctggctttctggctgctggaggctggggagaaggtgttcattcac
ttgcatttctttgccctgggggctgtgatattaacagagggagggttcct
gtggggggaagtccatgcctccctggcctgaagaagagactctttgcata
tgactcacatgatgcatacctggtgggaggaaaagagttgggaacttcag
atggacctagtacccactgagatttccacgccgaaggacagcgatgggaa
aaatgcccttaaatcataggaaagtattttttaagctaccaattgtgcc
gagaaaagcattttagcaatttatacaatatcatccagtaccttaagccc
tgattgtgtatattcatatattttggatacgcacccccccaactcccaata
ctggctctgtctgagtaagaaacagaatcctctggaacttgaggaagtga
acatttcggtgacttccgcatcaggaaggctagagttacccagagcatca
ggccgccacaagtgcctgcttttaggagaccgaagtccgcagaacctgcc
tgtgtcccagcttggaggcctggtcctggaactgagccggggccctcact
ggcctcctccagggatgatcaacagggcagtgtggtctccgaatgtctgg

aagctgatggagctcagaattccactgtcaagaaagagcagtagaggggt
gtggctgggcctgtcaccctggggccctccaggtaggcccgttttcacgt
ggagcatgggagccacgacccttcttaagacatgtatcactgtagaggga
aggaacagaggccctgggcccttcctatcagaaggacatggtgaaggctg
ggaacgtgaggagaggcaatggccacggcccattttggctgtagcacatg
gcacgttggctgtgtggccttggcccacctgtgagtttaaagcaaggctt
taaatgactttggagagggtcacaaatcctaaaagaagcattgaagtgag
gtgtcatggattaattgacccctgtctatggaattacatgtaaaacatta
tcttgtcactgtagtttggttttatttgaaaacctgacaaaaaaaaagtt
ccaggtgtggaatatggggggttatctgtacatcctggggcattaaaaaaa
aaatcaatggtggggaactataaagaagtaacaaaagaagtgacatcttc
agcaaataaactaggaaatttttttttcttccagtttagaatcagccttg
aaacattgatggaataactctgtggcattattgcattatataccatttat
ctgtattaactttggaatgtactctgttcaatgtttaatgctgtggttga
tatttcgaaagctgctttaaaaaaatacatgcatctcagcgttttttgt
ttttaattgtatttagttatggcctatacactatttgtgagcaaaggtga
tcgttttctgtttgagatttttatctcttgattcttcaaaagcattctga
gaaggtgagataagccctgagtctcagctacctaagaaaaacctggatgt
cactggccactgaggagctttgtttcaaccaagtcatgtgcatttccacg
tcaacagaattgtttattgtgacagttatatctgttgtccctttgacctt
gtttcttgaaggtttcctcgtccctgggcaattccgcatttaattcatgg
tattcaggattacatgcatgtttggttaaacccatgagattcattcagtt
aaaaatccagatggcaaatgaccagcagattcaaatctatggtggtttga
cctttagagagttgctttacgtggcctgtttcaacacagacccacccaga
gccctcctgccctccttccgcgggggctttctcatggctgtccttcaggg
tcttcctgaaatgcagtggtgcttacgctccaccaagaaagcaggaaacc
tgtggtatgaagccagacctccccggcgggcctcagggaacagaatgatc
agacctttgaatgattctaatttttaagcaaaatattattttatgaaagg
tttacattgtcaaagtgatgaatatggaatatccaatcctgtgctgctat
cctgccaaaatcattttaatggagtcagtttgcagtatgctccacgtggt
aagatcctccaagctgctttagaagtaacaatgaagaacgtggacgtttt
taatataaagcctgttttgtcttttgttgttgttcaaacgggattcacag
agtatttgaaaaatgtatatatattaagaggtcacgggggctaattgctg
gctggctgccttttgctgtggggttttgttacctggttttaataacagta
aatgtgcccagcctcttggccccagaactgtacagtattgtggctgcact
tgctctaagagtagttgatgttgcattttccttattgttaaaaacatgtt
agaagcaatgaatgtatataaaagcctcaactagtcattttttctcctc
ttcttttttttcattatatctaattattttgcagttgggcaacagagaac
catccctattttgtattgaagagggattcacatctgcatcttaactgctc
tttatgaatgaaaaaacagtcctctgtatgtactcctctttacactggcc
agggtcagagttaaatagagtatatgcactttccaaattggggacaaggg
ctctaaaaaaagccccaaaaggagaagaacatctgagaacctcctcggcc
ctcccagtccctcgctgcacaaatactccgcaagagaggccagaatgaca
gctgacagggtctatggccatcgggtcgtctccgaagatttggcaggggc
agaaaactctggcaggcttaagatttggaataaagtcacagaattaagga
agcacctcaatttagttcaaacaagacgccaacattctctccacagctca
cttacctctctgtgttcagatgtggccttccatttatatgtgatctttgt
tttattagtaaatgcttatcatctaaagatgtagctctggcccagtggga
aaaattaggaagtgattataaatcgagaggagttataataatcaagatta

aatgtaaataatcagggcaatcccaacacatgtctagctttcacctccag
gatctattgagtgaacagaattgcaaatagtctctatttgtaattgaact
tatcctaaaacaaatagtttataaatgtgaacttaaactctaattaattc
caactgtacttttaaggcagtggctgtttttagactttcttatcacttat
agttagtaatgtacacctactctatcagagaaaaacaggaaaggctcgaa
atacaagccattctaaggaaattagggagtcagttgaaattctattctga
tcttattctgtggtgtcttttgcagcccagacaaatgtggttacacactt
tttaagaaatacaattctacattgtcaagcttatgaaggttccaatcaga
tctttattgttattcaatttggatctttcagggatttttttttaaatta
ttatgggacaaaggacatttgttggaggggtgggagggaggaagaatttt
taaatgtaaaacattcccaagtttggatcagggagttggaagttttcaga
ataaccagaactaagggtatgaaggacctgtattggggtcgatgtgatgc
ctctgcgaagaaccttgtgtgacaaatgagaaacattttgaagtttgtgg
tacgacctttagattccagagacatcagcatggctcaaagtgcagctccg
tttggcagtgcaatggtataaatttcaagctggatatgtctaatgggtat
ttaaacaataaatgtgcagttttaactaacaggatatttaatgacaacct
tctggttggtagggacatctgtttctaaatgtttattatgtacaatacag
aaaaaaattttataaaattaagcaatgtgaaactgaattggagagtgata
atacaagtcctttagtcttacccagtgaatcattctgttccatgtctttg
gacaaccatgaccttggacaatcatgaaatatgcatctcactggatgcaa
agaaaatcagatggagcatgaatggtactgtaccggttcatctggactgc
cccagaaaaataacttcaagcaaacatcctatcaacaacaaggttgttct
gcataccaagctgagcacagaagatgggaacactggtggaggatggaaag
gctcgctcaatcaagaaaattctgagactattaataaataagactgtagt
gtagatactgagtaaatccatgcacctaaaccttttggaaaatctgccgt
gggccctccagatagctcatttcattaagtttttccctccaaggtagaat
ttgcaagagtgacagtggattgcatttcttttggggaagctttcttttgg
tggttttgtttattataccttcttaagttttcaaccaaggtttgcttttg
ttttgagttactggggttatttttgttttaaataaaaataagtgtacaat
aagtgtttttgtattgaaagcttttgttatcaagattttcatacttttac
cttccatggctctttttaagattgatacttttaagaggtggctgatattc
tgcaacactgtacacataaaaaaatacggtaaggatactttacatggttaa
ggtaaagtaagtctccagttggccaccattagctataatggcactttgtt
tgtgttgttggaaaaagtcacattgccattaaactttccttgtctgtcta
gttaatattgtgaagaaaaataaagtacagtgtgagatactg
>NM_014333 3
ggttgggctcgcggcgctgtgattggtctgcccggactccgcctccagcg
catgtcattagcatctcattagctgtccgctcgggctccggaggcagcca
acgccgccagtctgaggcaggtgcccgacatggcgagtgtagtgctgccg
agcggatcccagtgtgcggcggcagcggcggcggcggcgcctcccgggct
ccggctccggcttctgctgttgctcttctccgccgcggcactgatcccca
caggtgatgggcagaatctgtttacgaaagacgtgacagtgatcgaggga
gaggttgcgaccatcagttgccaagtcaataagagtgacgactctgtgat
tcagctactgaatcccaacaggcagaccatttatttcagggacttcaggc
ctttgaaggacagcaggtttcagttgctgaatttttctagcagtgaactc
aaagtatcattgacaaacgtctcaatttctgatgaaggaagatacttttg
ccagctctataccgatcccccacaggaaagttacaccaccatcacagtcc
tggtcccaccacgtaatctgatgatcgatatccagaaagacactgcggtg
gaaggtgaggagattgaagtcaactgcactgctatggccagcaagccagc

cacgactatcaggtggttcaaagggaacacagagctaaaaggcaaatcgg
aggtggaagagtggtcagacatgtacactgtgaccagtcagctgatgctg
aaggtgcacaaggaggacgatggggtcccagtgatctgccaggtggagca
ccctgcggtcactggaaacctgcagacccagcggtatctagaagtacagt
ataagcctcaagtgcacattcagatgacttatcctctacaaggcttaacc
cgggaaggggacgcgcttgagttaacatgtgaagccatcgggaagcccca
gcctgtgatggtaacttgggtgagagtcgatgatgaaatgcctcaacacg
ccgtactgtctgggcccaacctgttcatcaataacctaaacaaaacagat
aatggtacataccgctgtgaagcttcaaacatagtggggaaagctcactc
ggattatatgctgtatgtatacgatccccccacaactatccctcctccca
caacaaccaccaccaccaccaccaccaccaccaccaccatccttaccatc
atcacagattcccgagcaggtgaagaaggctcgatcagggcagtggatca
tgccgtgatcggtggcgtcgtggcggtggtggtgttcgccatgctgtgct
tgctcatcattctggggcgctattttgccagacataaaggtacatacttc
actcatgaagccaaaggagccgatgacgcagcagacgcagacacagctat
aatcaatgcagaaggaggacagaacaactccgaagaaaagaaagagtact
tcatctagatcagcctttttgtttcaatgaggtgtccaactggccctatt
tagatgataaagagacagtgatattggaacttgcgagaaattcgtgtgtt
tttttatgaatgggtggaaaggtgtgagactgggaaggcttgggatttgc
tgtgtaaaaaaaaaaaaaatgttctttggaaagtacactctgctgtttga
cacctctttttcgtttgtttgtttgtttaatttttatttcttcctacca
agtcaaacttggatacttggatttagtttcagtagattgcagaaaattct
gtgccttgtttttgtttgtttgttgcgttcctttcttttccccctttgt
gcacatttatttcctccctctaccccaatttcggatttttccaaaatct
cccattttggaatttgcctgctgggattccttagactcttttccttccct
tttctgttctagtttttactttgtttatttttatggtaactgctttct
gttccaaattcagtttcataaaaggagaaccagcacagcttagatttcat
agttcagaatttagtgtatccataatgcattcttctctgttgtcgtaaag
atttgggtgaacaaacaatgaaaactctttgctgctgcccatgtttcaaa
tacttagagcagtgaagactagaaaattagactgtgattcagaaaatgtt
ctgtttgctgtggaactacattactgtacagggttatctgcaagtgaggt
gtgtcacaatgagattgaatttcactgtctttaattctgtatctgtagac
ggctcagtatagataccctacgctgtccagaaaggtttggggcagaaagg
actcctcctttttccatgccctaaacagacctgacaggtgaggtctgttc
cttttatataagtggacaaattttgagttgccacaggaggggaagtaggg
agggggaaatacagttctgctctggttgtttctgttccaaatgattcca
tccacctttcccaatcggccttacttctcactaatttgtaggaaaaagca
agttcgtctgttgtgcgaatgactgaatgggacagagttgattttttttt
ttttttcctttgtgcttagttaggaaggcagtaggatgtggcctgcatgt
actgtatattacagatatttgtcatgctgggatttccaactcgaatctgt
gtgaaactttcattccttcagatttggcttgacaaaggcaggaggtacaa
aagaagggctggtattgttctcacactggtctgctgtcgctctcagttct
cgataggtcagagcagaggtggaaaaacagcatgtacggattttcagtta
cttaatcaaaactcaaatgtgagtgtttttatctttttacctttcataca
ctagccttggcctctttcctcagccttaagaaccatctgccaaaaattac
tgatcctcgcatgatggcagccatagtgcatagctactaaaatcagtgac
cttgaacatatcttagatggggagcctcgggaaaaggtagaggagtcacg
ttaccatttacatgttttaaagaaagaagtgtggggattttcactgaaac
gtctaggaaatctagaagtagtcctgaaggacagaaactaaactcttacc

atatgtttggtaagactccagactccagctaacagtccctatggaaagat
ggcatcaaaaaagatagatctatatatatatataaatatatattctatta
cattttcagtgagtaattttggattttgcaaggtgcatttttactattgt
tacattatgtggaaaacttatgctgatttatttaagggggaaaaagtgtc
aactctttgttatttgaaaacatgtttatttttcttgtctttattttaac
ctttgatagaaccattgcaatatgggggcctttgggaacggactggtat
gtaaaagaaaatccattatcgagcagcattttatttacccctcccctatc
cctaggcacttaaccaagacaaaaagccacaatgaacatcccttttttcaa
tgaattttataatctgcagctctattccgagcccttagcacccattccga
ccatagtataatcatatcaaagggtgagaatcatttagcatgttgttgaa
aggtttttttcagttgttctttttagaaaaaaagaaaaacaaaaacaaa
aacaaaaaaaaaaaatcacaccattgctcacagaattggcatctcatttt
tgggacctcccatctttctgttttgaaaagtgtacagtagtgcagtgttc
ctgatgtaactttatggcttacaatgttgacatgtctcaggttcatgtgt
tgcgattggtgttttccgtctcaggtagattgcaaagtgtaggccccaca
cattggaaaaaataataataaaacaaagcaaaaacaggaaattatggatt
ttagttgtatattggtttatgtattttttcttaagtatacagtgcactgt
ttgaaatgtattgttgagtattactttgtacaggttgatcactttttta
gagtgaagaaagaacaaacttgtttttttgtgtttttttaaaggaatataaa
ataatgaaggatgtataattgatgccaaataagcttgttctttagtcaca
ccgacgtcttatttttccctttaggccagttctgtttttaaggtgtacat
ggacaatgttacagtgtaagaaactccatatccatatgttcccattcgca
ttttgtattggttcatgtataccatttttacaaaaaaaaaaaagaaaaaaa
agaagtactataaaatatctgtcttcttaataaaaaaaaattaatgttac
aaagtgaaaaaaaaaaaaaaaaaa
>NM_003387 4
ggctagaagacagcaggggaactcgagaagttggttgttttcagcagatt
aaaacaatacagatttatcagcaagactgttgaacgcataactgcccaag
atgcctgtccctcccccctccagcacccccgccgcccccgacgtttgcact
ggccaatacagagaagcctaccttgaataagacagagcaggctgggagaa
atgctctcctttctgatatcagcaaagggaagaaactaaagaagacggtc
accaatgacagaagtgcaccaatactggacaaacctaaaggagctggtgc
tggaggcggtggtggtggctttggtggaggcggcggatttggcggaggag
gtggtggcggaggcggtggaagttttggaggggggcggacctccaggtctg
ggaggattgttccaggctggaatgccgaagctgagatccacggccaacag
ggataatgattctggaggaagccgaccaccattgttgccaccgggaggaa
gatccacatctgcgaaacccttttcacccccaagtggcccagggaggttt
cctgtgccttctccaggccacagaagtggtcccccagagcctcagaggaa
ccgaatgccgcccccaaggcccgacgtgggctcaaagcctgatagcattc
ctcctccagtacctagtactccaagacccattcaatcaagtccgcacaac
cgggggtccccaccagtgcccggaggccccaggcagcccagccccgggcc
cactcctcccccttttccctggaaaccgcggcactgctttgggaggaggct
caatacgtcagtccccccttgagctcctcctcgcccttctccaaccggcct
cccctgccgcctacccccagcagggccttggatgacaaacccccctccacc
acctcctccagtgggcaacaggccctccatccacagggaagcggttcccc
ctcctcctcctcagaacaacaagcctccagtgccttccactccgcggcct
tcggcctcctcacaggccccacctccgccgccacctcccagcaggcccgg
gccgcctcctctgcctccaagttccagcggcaatgacgaaaccccaagac
tcccacagcggaatctgtccctcagttcgtccacgcccccgttaccttcg

ccaggacgttcaggtcctcttcctcccccgcccagtgagagacccccacc
tccagtgagggacccgccaggccgatcaggccccctcccaccacctcctc
cagtaagcagaaacggcagcacatctcgggccctgcctgctacccctcag
ttgccatccaggagtggagtagacagtcccaggagtggacccaggcctcc
ccttcctcctgataggcccagtgctggggcacctcccccacctccaccat
caacatctattagaaatggcttccaagactctccatgtgaagatgagtgg
gaaagcagattctacttccatccgatttccgatttgccacctccagagcc
atatgtacaaacgaccaaaagttatcccagcaaactggcaagaaacgaaa
gccggagtggatccaaccgaagagaaaggggtgctccaccactccctccc
atcccgaggtgatctttgcctgctcttctctacccaagctcaagagctgc
ttctgttgctatctaagaactgcataccctcctccctgcttcttcccttg
tgcctcatgtatgggcaggaggaaaggtgggagggggagtgggaatatgc
gtgtgtgggtgggaatcggtaagaaatgcacctagcttttcatattgtgt
ttattctccaggctattgcttgcttcagctgcagcctgcctgtgctggct
gctggggtcgataggcttttgtcgtaataggcagagatgacttgcatccc
agctttccaccaaccaaattcaaacattcactgcttatttgttacagact
gtaattattaaagtccctgagagctgttttctcccgttcctttttcgcat
gcttggcctcctctctgtttctatgaaccacagaccacctaagcaagctg
ctgagtaagggctcactggaaacttgcagtcacaggatgtccaatctttg
gcagtccgagcttggctctaggacagagctgtccaatagaaatataatgt
gagccccatatacaatttttacatttctaatatattttaaacaagtgaag
ttaatatgcatccaaaatatttcaacctgtaatcaacataaaattttaat
gagatattttatattattttttggtactgaatcttcaaaatccagagtgt
attttacacttaccgcacatctccattcagactagtcacatttttaagtg
ctcagtagccacatgtggctggtggctactggattagacagcacgagtct
ggaagatggaagctagtgcagaaacctcttgtttaaaaaacaaaaaaggc
aagatgggcttgagcgattcaagaggcaactaaaaataaaattaggaccc
agcaccttgtttgacacacagtttgaccttcgattttcctcccttaactt
ccctcttcccttaatatctgtatacaagtgttgcttcaaagtaccaaggt
cagaaattgattcagtacggtttactaaagtcatgtggaataaagccatt
ggaaacaaatggaaagcctgtcgggacttctgggctcagaaccagctggc
tcacgcactccacttgtcagctggacttctgccttgtgaaatggaagcag
cctttgttcctttctggctgagcaagctcctgaggctgggagagactagg
aaggcttggtaggaggggaaaaaagtcaggaaaagatatcaaatcagaaa
catggaagaagaagggaaccgatttgagttggtgggcaaaactctaaaaa
tctaaatctgatgcttatgtaagggttgagcgaattagggagattgctag
tggaaattggagggaatttgttttgcatcatttgtctaggatctatgcaa
atatagctccactaaaggaccatagggaagagccagccttgccttttctt
atatgattttgtttacaaaattttactgggacttttaaatctagctatag
agttgggaaaaaatatttccacttagatattttacatggttttgtttaaa
attaccattacttgtttttttaaaaacacatgaccacatatgtatatgtat
atctacctaaacattgtatcatggtttcagtatgttattcatgtattact
gggagatgctaccaagaaaccaacccaaagaaaattctgaaaaatacatt
tctatttatagaataaatgtttcatttatataaaagcaaaagaacttaga
gttctaataaatgggatgtctaataaattatgaagttactgatttgaata
tattatatttttataacttccttgccaaagtcctgatttagtacattaga
gaacctgtgtttcctctctcctctaccattcatctctcttccatacagtc
atttgggctttttactcaaagagaatcaagaaataataaggtataacaag
cttggcaaagtgttggctttttaaaaaaaaatttttttaatctctagcag

tttggtaatttagcagcatcatttatttgggattcttttatctgatttca
acagtgaaaaacatccctatgataaagcctaatgacccatttcacaaaag
atggaatttgcccttcctagaaaatatgacggagaaaagtctgactcaga
gaaagtgagtctgaattttataaggggtagtaagaattggacaattcctt
tgcatatctgaacttggcaggtaccgttctaaatctgaaacagggtgata
gctcaaagttgccattcatccagaatagattgttttagaatgtagtgttt
aagtgactgtttcattaatacacctacaccctttctttgaaagtttgcaa
cctaattgcatctaaaactatgaataagttctgtggtaaaatcttaaact
atggaaaattacaaaaatgaatttttcttccctgaaatcagagcttacat
gtgtgtttttttataacattttcagataaatgtattcaacatgtaataca
gtattttaacattcacctcttattttatattgaaatgtattacagtatta
aaactcagtgttcagtatttatttcactatgcattttatttagtaaaagc
caggagaaatgtttaatccaatggtgccttactttgtgatttaaaagaaa
tcaactttttttatgtctaagtagtagattatttgcatatttgtaaaaa
ctgttaggtctttatattttaaagtgtaataccagttttgttattttagt
agcagaaatgggatgattgttaaagttccccaaaaatgttggcatgaaat
taatttttccctccttatagtcaaggaccgtagaggaagaaaaactttttt
tttcataccatgcactatgtaaacagacacattttgctatctgtgtcatc
aggatagtgtaagtggtagggtagagactaccctagacatctgcatcttt
gtaagttagccagacaataaagaaaagcagaatgaaaaaaaaaaaaaaa
>NM_005736 3
gctccctcgccgccctgaaccggcggctagactgcgcatgcgtgtcagtg
gcgctagcggcggacccggctgggcagttccttccccagaaggagagatt
cctctgccatggagtcctacgatgtgatcgccaaccagcctgtcgtgatc
gacaacggatccggtgtgattaaagctggttttgctggtgatcagatccc
caaatactgctttccaaactatgtgggccgacccaagcacgttcgtgtca
tggcaggagcccttgaaggcgacatcttcattggccccaaagctgaggag
caccgagggctgctttcaatccgctatcccatggagcatggcatcgtcaa
ggattggaacgacatggaacgcatttggcaatatgtctattctaaggacc
agctgcagactttctcagaggagcatcctgtgctcctgactgaggcgcct
ttaaacccacgaaaaaaccgggaacgagctgccgaagttttcttcgagac
cttcaatgtgcccgctcttttcatctccatgcaagctgtactcagccttt
acgctacaggcaggaccacaggggtggtgctggattctggggatggagtc
acccatgctgtgcccatctatgagggctttgccatgccccactccatcat
gcgcatcgacatcgcgggccgggacgtctctcgcttcctgcgcctctacc
tgcgtaaggagggctacgacttccactcatcctctgagtttgagattgtc
aaggccataaaagaaagagcctgttacctatccataaaccccccaaaagga
tgagacgctagagacagagaaagctcagtactacctgcctgatggcagca
ccattgagattggtccttcccgattccgggcccctgagttgctcttcagg
ccagatttgattggagaggagagtgaaggcatccacgaggtcctggtgtt
cgccattcagaagtcagacatggacctgcggcgcacgcttttctctaaca
ttgtcctctcaggaggctctaccctgttcaaaggttttggtgacaggctc
ctgagtgaagtgaagaaactagctccaaaagatgtgaagatcaggatatc
tgcacctcaggagagactgtattccacgtggattgggggctccatccttg
cctccctggacacctttaagaagatgtgggtctccaaaaaggaatatgag
gaagacggtgcccgatccatccacagaaaaaccttctaatgtcgggacat
catcttcacctctctctgaagttaactccactttaaaactcgctttcttg
agtcggagtgtttgcgaggaactgcctgtgtgtgagtgcgtgtgtggata
tgagtgtgtgtgcacatgcgagtgccgtgtggccctgggaccctgggccc

agaaaggacgatgaactacctgcagtggtgatggcctgaggcctggggtt
gaccactaactggctcctgacagggaagagcgctggcagaggctgtgctc
cctcctcaggtggcctctggctggctgtgggggactccgtttactaccac
agggagacagagggaggtaagccatcccccgggagaccttgctgctgacc
atcctaggctgggctggccccaccctcacccccacccccagggtgccctg
aggccccaggcagctgctgcctccactatcgatgcctcctgactgcacac
tgaggactgggactggggttgagttctgtctggttttgttgccattttgg
tttgggaggctggaaaagcaccccaagagctattacagagactggagtca
ggagagagcaggaggccctcatgttcaccagggaacaggaccacaccggc
cactggaggagggcaggagcagtcctcactctgaatggctgcagagttaa
tgttcccagcccagtcccctttcgggggccttgggagagtttaaggcacc
tgctggttccaggacctcgctttccatctgttcttgttgcaatgccatct
tcaaaccgttttatttattgaagtgtttgttcagttaggggctggagaga
gggagcttgctgcctcctgccttgctacactaatgtttacagcacctaag
cttagcctccagggccccacctctcccagctgatggtgagctgacagtgt
ccacaggttccaggaccatttgagattggaagctacactcaaagacactc
ccaccaggctctttctcccttttcctcttgctcactgccctggaatcaac
aggctggttgctggttagattttctgaaacaggaggtaaaatttttcttt
ggcagaggcccctaagcaagggaggggtgttggagagccagtgcccttaa
gactggagaaagctgcaatttaccaagttgccttttgccactgtagctga
ccaggggactaggttgtagaggtgggaaggccccctctgggctgatcttg
tgccattcttgaccttggacctgcttggttaaggagggagtgggccagac
cagagtgccaggagctaatggagccaggcctgactcctaggagtggtcca
aaggccttcagcctagatggtgcaaagctggggccagcctgtcttcaccg
gcaccctcacctgtgacaccaagacccaccccaatcccagacttcacaca
gtattctcccccacgccgtcctatgaccaaaggcccctgccaggtgtggg
ccacagcagcaggtatgtgtgaaagcaacgtagcgccccgcggactgcag
tgcgcttaaccaactcacctcccttctcttagcccaagcctgtccctcgc
acagcctcgcacaaaccacattgcctggtggggcccagtgtactgaaata
aagtcgttccgatagacacgtcaaaaaaaaaaaaaaaaaa
>NM_021925 2
agggagagcgggcagccttccaggtggacagaaatggactcagaactcaa
ggaagaaattcctgtgcatgaggaattcattttgtgtggtggagccgaaa
cccaggttctaaaatgtgggccctggacagacctctttcatgatcaaagt
gtcaaaaggcctaagctgcttattttcattattcctggtaacccaggttt
ttctgccttttatgtgccatttgcaaaggctttatactctttgacaaaca
gacgctttccagtttggactatcagtcatgctgggcatgcgttggctccc
aaagacaagaagattcttacaacatcagaggattcaaacgctcaagaaat
taaggacatttatggactaaatggacaaatagagcacaaactagctttcc
tgagaactcatgtgccaaaggacatgaaacttgtgctcattggccattca
ataggcagctatttcacacttcagatgctgaagcgagtccctgagctccc
ggtaattcgtgcctttctgctctttccaacaattgaacgaatgtctgagt
cacccaatggcagaattgccactccacttttgtgctggtttcgatatgtt
ctctatgttactggctacttattattgaaaccgtgtcctgagacaatcaa
gtccttgctaatcagaaggggccttcaagtaatgaacctagagaatgaat
tttcaccattgaatatattagaaccattctgccttgctaatgctgcctac
cttgggggccaagaaatgatggaggtggtgaagagagatgacgaaaccat
aaaggagcatttatgtaagcttacattttattatggtactatagatcctt
ggtgtccaaaagagtactatgaagacattaagaaggattttccagaagga

gacattcgactctgtgagaaaaacatacctcatgctttcatcacccattt
taaccaggaaatggcagacatgattgctgactccctaaaggatgacttgt
ccaaaatgtaaattggcctgaggaacgagcccccactgccagtacatgga
ggcagtcagtgtactagacttagtaggtaaatgtttaattttgaagactg
atattagaaatgaagaaagtgagaacctttgtcttacaaaccaactctcc
gctcgccatgttataggctgaagtaaacacagttgatgaatcattccata
ggtttaaccatacattttccaagactcagggaacacagtgatctacacag
agtcttgtgtttgcacaagatgcccagtggcaccatatggtttattttgg
taggcaggatctttgcagatgaaaaaaaaatctacatgtacttgatttta
attgagttacattgtagaataggctcctctggaggaaattatgaaatacc
tactagaaaatgtaaaataaatcagtgaatgttaagagtatagttagata
tgtgaagtgtatgagattatgacaaggatacactcatgttccaggagcag
gaagtgaacctgggtctcctgtaagacagaagatgaagatgagcccaggc
taacttagcacagatcttggctgagatcatcaatgtgacgtctaatgtac
ctgcactagacagagaataaagttcaccagacattactctggtcagctaa
ccagataaagaattgttgaaggaccccaactgtgcctcctgccacaggac
aaccagcaagttctatgctgagccttagcctcccaggttataagctccct
gcaggctcctcctctccagagccaggatggagaggcactgggctgtccca
aagcaggcttggatgtgccaacgtacagttgctccttctgtaattcttgc
actaaaactccattaaagaccatcaatgagccaaatagtgtgctagccat
tgaggatggagttcttagctacttaatgattcttttcttctggaaatctt
ctagtgaataatttttaaaagcttcttttaaactgctcagtaaaacattg
ccatacattaactctccatttctgcattaacttcatttgctggaaagaaa
ttattttgttaatgaaccaaacccagccattaagttatgtgaattcacct
tctatagacacattgaggtatggcagagaagacacatattccctggccta
gagtgggctgacaatattgctgcctcccctaaattgactcaatcttgaga
accacatactgcagtgacagttgatcatggatcagaaatattcctttttc
tcctagaggaaatccctcatggcattatcattccctaaggacaatgagat
gtccctccgctcaaatcctaaagccccctgatgaaagatttggctctgat
ttgctgccaacttattggtaagtgctgaatgagttactgtgctttggcat
agcttctacatcacagcaagtgacttctcagaggggaaaagccaagctcc
agacaaggagtgctgttagcccaaggggaagagctaatagatacataggc
cgtaagcacagggctgtacacagatcctttattggcatctgcatgagagg
cctagaaatcggatgttagaagctaggaatcatggctctgagcgccttgc
atacaggagatactcactgatactaaatcaaggcatgtaaacaatactgg
gtaaatcatacctaccagaataaagagtaacattcagagcttttctttta
tctgttattttaaatgttactccatatttcaaaagtctgcacgtatgaaa
ttgtgtcttatttctggtttttagagcctaagaaagatactcattcttaa
ttcctatttatcatctgaaaaccaaatgaacttttgaaataaatgtttta
ccagcaaaaaaaaaaaaaaaaaaaaa
>NM_001006605 4
agggggcggggaggcggggggaggcggggagcccggccgccagcgctcgg
gtccgcctctgactgcagcgcggcggggcgatgtgtgattaccatggcga
ggagtctctgtccgggggcctggctaaggaaaccctattacctccaggct
cgcttctcatatgtgcggatgaaatatcttttcttttcctggttagtggt
ttttgttggaagctggattatatatgtgcagtattctacctatacagaat
tatgcagaggaaaggactgtaagaaaataatatgtgacaagtacaagact
ggagttattgatgggcctgcatgtaacagcctttgtgttacagaaactct
ttactttggaaaatgtttatccaccaagcccaacaatcagatgtatttag

ggatttgggataatctaccaggtgttgtgaaatgtcaaatggaacaagcg
cttcatcttgattttggaactgaattggaaccaagaaaagaaatagtgct
atttgataagccaactagaggaactactgtacaaaaatttaaagaaatgg
tctatagtctctttaaggcaaaattgggtgaccaaggaaacctctctgaa
ctggttaatctcatcttgacggtggctgatggagacaaagatggccaggt
ttccttgggagaagcaaagtcggcatgggcacttcttcaactgaatgaat
ttcttctcatggtgatacttcaagataaagaacatacccccaaattaatg
ggattctgtggtgacctctatgtgatggaaagtgttgaatatacctctct
ttatggaataagccttccttgggtcattgaacttttttattccatctgggt
tcagaagaagcatggatcagctgttcacaccatcatggccaagaaaggcc
aaaatagccataggacttctagaatttgtggaagatgttttccatggccc
ctacggaaatttcctcatgtgcgatactagtgccaaaaacctaggatata
atgataagtatgatttgaaaatggtggatatgagaaaaattgtgccagag
acaaacctgaaagaacttattaaggatcgtcactgtgagtctgatttgga
ctgtgtctatggcacagattgtagaactagctgtgatcagagtacaatga
agtgtacttcagaagtgatacaaccaaacttggcaaaagcttgtcagtta
ctcaaagactacctactgcgtggtgctccaagtgaaattcgtgaagaatt
agaaaagcagctttattcttgtattgctctcaaagtcacagcaaatcaaa
tggaaatggaacattctttgatactaaataacctaaaaacattattgtgg
aagaaaatttcctacactaatgactcttagttcatttggacataattacc
attttaagaaacctgccacttttaaagaacaattttgagcattaaaaaaa
aatggcttcaaattccggccagttacacaaaactccttccccccaggcct
gagaagccatcagtatgtgatcactgaagtaatggcaggtgtaggatcaa
caggtccccaagatgtcattcctgcccttttagaagccctgttacatctc
cgaagtacattcattgtgtaactattttgactgactttaaaaaccaatgc
tgtgaaaagcttcattccataaacatcaacagtgagtgatttgtagattt
accttagccaaaataccaatgctggaagcattgtgtttgcattgaagctg
ctgttcaacaagaaaatttataaatttactaatgtcttagcatggtaaag
tttgcacattaacagaaattaagactgcaaagcaggttaaacttgcttct
ttataaaacagatgttgggttaatagcatggtttactgtattaaagactt
atacacccatttttaacctcattcagacatcaagttatgtgtagcttcac
aatggttcaagtggcttacttcaagaaatcttatacttgacagtacacca
attttattgactaaaaatggatgaactttcctaaagattcaaagggccca
tcttagtatcacgcagctgactgagcccttcaaaactgacatcttaaggc
ccaatcaagatccacatatcctgattttgaactatgtgaaagtgggactg
taagtgcaagactaaaataaattatagcagactttttagtaataactttc
cattttcaaacagtatatcctgtgggccaaagggctatttcttaaagagg
catgtaaatgtatttatttatctaatgtttttttccccatgtaaacttga
tatacaaggtttagtatttgctcctctttcatattattttcacacgtata
ctcagatttggcatgtacctttcaacatctccataaaattaaacaccttt
tggagaaaagaaccactattttctgctcaaaggtttcgcctacctaaagt
ggaacatgttaaaaatctatgtgaccatcactggacagctttctctcaaa
actttccttcaacgccatggattagcaccagttttgtttactttaaggta
cttttcccattcatcatctggttataataaatggatggaagaaatatttc
cca
>NM_022173 2
gctcctaggctcccggctcgccgccatcttgtattggggtttcattgttc
ccgctgggccgggcggtttagtgtaattgccgccggaggaggaggcggag
taacctctggtcagccgagaaaccccactatcctgtagccataaccgctt

aaacgatttgggaggtagtgaagggcagggagctggacctggaggcgccg
ccgcgacagcagcagccatggaggacgagatgcccaagactctatacgtc
ggtaacctttccagagatgtgacagaagctctaattctgcaactctttag
ccagattggaccttgtaaaaactgcaaaatgattatggatacagctggaa
atgatccctattgttttgtggagtttcatgagcatcgtcatgcagctgca
gcattagctgctatgaatggacggaagataatgggtaaggaagtcaaagt
gaattgggcaacaacccctagcagtcaaaagaaagatacaagcagtagta
ccgttgtcagcacacagcgttcacaagatcatttccatgtctttgttggt
gatctcagcccagaaattacaactgaagatataaaagctgcttttgcacc
atttggaagaatatcagatgcccgagtggtaaaagacatggcaacaggaa
agtctaagggatatggctttgtctcctttttcaacaaatgggatgctgaa
aacgccattcaacagatgggtggccagtggcttggtggaagacaaatcag
aactaactgggcaacccgaaagcctcccgctccaaagagtacatatgagt
caaataccaaacagctatcatatgatgaggttgtaaatcagtctagtcca
agcaactgtactgtatactgtggaggtgttacttctgggctaacagaaca
actaatgcgtcagactttttcaccatttggacaaataatggaaattcgag
tctttccagataaaggatattcatttgttcggttcaattcccatgaaagt
gcagcacatgcaattgtttctgttaatggtactaccattgaaggtcatgt
tgtgaaatgctattggggcaaagaaactcttgatatgataaatcccgtgc
aacagcagaatcaaattggatatccccaaccttatggccagtggggccag
tggtatggaaatgcacaacaaattggccagtatatgcctaatggttggca
agttcctgcatatggaatgtatggccaggcatggaaccagcaaggattta
atcagacacagtcttctgcaccatggatgggaccaaattatggagtgcaa
ccgcctcaagggcaaaatggcagcatgttgcccaatcagccttctgggta
tcgagtggcagggtatgaaacccagtgaataaggactccagaatctaaag
ccagtggcttgaggctacagggagtgtagtaaagccgttgtttacttaaa
gatttatcaaatcagtcagtgcaaatgtcagatacaatgtatttatttaa
aagattcatttttaatcatgaaattacttatcatccacattgttttaaaa
agaaacaagatgctggatgtctgccaatttttgccttcattaccttttttt
gataaagtttctcagatccttgtttcaaacacaaatgcagggattgctgc
cactttttaactattaagaggcagaaaattgcacaatattgaactttttt
ccactgaagtagtgtgcagttctagtttgcattcctgatatgatttaaaa
catgtaatataaagatgttaaaaaaaaaaaccaaaactgtgcagagtcta
gaagttgtttgtcatcttcagcttgtgcacaattctgttttaggttaaaa
aaaggcattgtttgagctgtcccatctccactgttatccctttggggttt
tttaatataaattattagtttacatcatttttgtatctacatctttttc
acaaatttgtcttgccttattaaagttctgtaaaatatacttaaatggaa
aaaatgatgttcatttagattgaaaacttttctcagatggattgataatt
gcattcatcttgtgttttatatgagaaggtgcctcaagaatttcctgttg
gatttgtttaaaaggatttttatctttcgtgataaactttgctgtgtacc
aggaactataaaaacaaaaacttgttactaaagaaaatatctgaaatgtg
ataagttcttatgccatgttaatttcatgtgtcaacttcaacatttacat
gtattatttcattatgtaaaatgttttagcaatttaatattttgcacagt
tagcaaactttgtatgtcatttccttcaaggcatcatgcagagttgacat
gagatttataaggttttaagttgtttgcatgtgaaaatcaaatacatact
ttggtagtctttgaatacaaagtcatctgctcttgtttttcaagaatttt
gagacacaaagttgtatgtaaaggaatatattaatttgccgttttctagg
tagatttgctcaaaaagagtgaatcaacttaatatgtacaaatgatagct
gtgaaactgtagaatatctttgtgtcaggcttggagttcattgtgacctc

caaattttgcctgaaggaccagctgggcaaagcatcttttaaatgttcag
aggccaaaagataaacaaaaaaaaaaccttaaaatcctacctctttaaac
agccttcagataagagaatcctcagtgcaatcattattttgattcgtttg
gtacctgttttcctggagttcccgattttattattttggggtggctccaa
gcattaagaggtttaatctttgatggcattgttctagttttgaaatttct
agtatatttcagagtctcttagaagacttgtgtgggaagtttcactttgt
tttcagtgaagatcacaaacctccttcttcctttactcaagaggaaaggt
cccagtatacatatttgaatggttgatggttttcaagaccttcagggagc
tccctgcattttacctagaaacagaaaaggcccgcaaaatcttaagtttc
ctggcctgcatttcccgggtaggggcaaatgactccaagctggtctctaa
gccaatacccttataaaccagagcccaggaaagacagctcgagtgtataa
ttctctggagctcaattctatgcagttgtgctgatatttcattaagtcac
tgtgtatttttaagtgttgatacattaaaagtcgctttatggaagatgag
taaatttttaaatacttggaaattttatttccttgttaacttctacaga
tcagggcatgcaaccaaaagcagcttaaatgaaatattttaaaataaaat
atcaggaagctatttttagatttcttctggcttatgtttctactttagga
ccctcattgttctcttattaaaaaaaattatttcctgtgcatctcatgga
ctgcagggtaaattatttgggcataaataatttaaatagttttctttcat
tttgactatctccagtaataacagtttttattatccagcatattggctta
ttgcacaaatcttaaaatgtacattgactactttctgagaagaaagtggt
atcagtactcatgatgaaaaggttactactgaacaaattcacatttcagg
aacacctctatctttggtttaaatcttactcttagtttttccgtctaaaa
atcatactggtattagtatcaggtaaggaaattaaagtttttaaaatggt
ttcattctctgcaatatgcaaaatttagattttactttctggtactgtaa
agaacctgaagtgatttacacttaatgggtgattaatccagtattcttta
ccctgaatgtttggatattaaagttcctttatgttttctataacctgtgg
gatcttcttgcagtgattattgtgtgtgagattttttttctttttggtct
atccatattgttatattcactcaggtatttttttttaatcttattccag
aatcagtggtttatattgggttactgtttaacaccaaatggaattggcat
tctgcagatttaattaattatgaaaccagggtctcattttccttgctgat
acttgttgaaaatgagattcacattctagtctttattttcctcctgtttt
gtccctgtgcttgtacatcttccttttatttgtgtgttatagttctattc
catttgagaaggcagttggtaagaactagattgcatgtacaaagacaggt
ttactaagtgctgtacagtggtcctgaggttacagttgaattagaaaaac
gaaatgtacttacaggaaataagaaagcaaacctttcaaatgagagtgat
gatttctttaaaaaaaatcagtttttttctctcaaataatgttctttatt
tcacgaaatcgtcaatcttaagcatgagcagggataaacaactcctagaa
ggaactcaattcattcttcctggattttctctgttgttaaatcacaaaaa
tgatagtccccaatcgtttctttataggaggttattacatttcattacag
tcactgcattttgactgttgtgtttagaatttgaatgtacatccaaaatg
atgagtttcaatttaagagccttaataaaatgtgtgagtgtgtctcaatt
gaa
>NM_152729 2
gccgcgtccggcccggtcctgtcccgcagcgtcccgccagccagctcctt
gcacccttcgcggccgaggcgctccctggtgctccccgcgcagccatggc
tcagcacttctccctggccgcctgcgacgtggtcggattcgacctggacc
acactctgtgtcgctacaacctgcccgagagcgccccgctcatttataat
agctttgcccagttcctagttaaggagaaagggtacgataaggaattgct
caatgtgaccccagaggattgggatttctgttgcaaaggtttggcattgg

atctagaagatgggaacttccttaaacttgcaaataatggcactgttctc
agggcaagccatggcaccaagatgatgactccagaggtgctggcagaggc
atatggcaagaaagagtggaagcacttcttgtcggacactggaatggctt
gccgctcaggaaagtattacttttacgacaactactttgacctgccagga
gctcttctgtgtgccagggtggtggactatttaacaaaactgaacaatgg
tcaaaaaacatttgatttttggaaggatatagttgctgctatacaacaca
attataaaatgtcagcttttaaggaaaactgtggaatatattttccagaa
ataaaaagagatccaggcagatatttacatagttgtcctgaatctgtgaa
aaaatggcttcgacagctaaagaatgctgggaaaattcttctgttaatta
ccagttctcacagtgattactgtagacttctctgcgaatatattcttggg
aatgattttacagacctttttgacattgtgattacaaatgcattgaagcc
tggtttcttctcccacttaccaagtcagagacctttccggacactcgaga
atgatgaggagcaggaggcactgccatctctggataaacctggctggtac
tcccaagggaacgctgtccacctctatgaacttctgaagaaaatgactgg
caaacctgaacccaaggttgtttattttggtgacagcatgcattcagata
ttttcccagctcgtcactatagtaattgggagacagtcctcatcctggaa
gaactcagaggggatgaaggcacgaggagtcagaggcctgaggagtcaga
gcctctagagaagaaaggaaaatatgagggaccaaaagcaaaacctttaa
atacttcatctaaaaaatggggctctttttttattgattcagttttggga
ctggaaaatacagaagactccttggtttatacatggtcttgtaagagaat
cagtacttacagcactattgcaattccaagtattgaagcaatcgcagaat
tacctctggactacaaatttacaagattctcttcaagcaattcaaaaaca
gctggctactatccaaatcctccactggtcttatcaagtgatgagacact
gatatccaaataagttgtctttactgaaaaatgaagtgaagacccatata
tgcagttaaaaaaaagttaattttcaaaaaatactgtaaaagactttaag
gaacaagttttattgaccaataagttgatatttgtccataggtctccttt
ctataaatcatcttgatgtttaacaactcttattatattaaaatctcagt
atcctaaaacttagaaccttattgatattttctatacagtagttttgtga
ttagaattcacctggggacacacactcacacgcacagtcactcttacaca
tatgcctagtccagtggttctcaaagtgtgatccatagactaatagcata
gcattacctgagaacttgataatgcatatttgcaggccccacgcaaaacc
tgctgaatcagaagctctgagagtaaggcctaccaatctggtttaacaag
cccgtcaggtgattctgatttacagtacagtttgagacccactggctaga
acatatcctccagagacttggcttaattggtctgggtaaatatggctgag
cgttgttatcttttgttatcttttatatgccctaggaaagtacaaccaga
attgaaaaccaactgatctataccagttctttcaattaatagtcaagaac
attgagtccagtgagattaagtgatttttgcaggctatgaggttttatgc
tattcctgtgttctccaaaacaagaaaataaaaagacatgacttattctc
tgttcatacagcaagtatggaaaaaagatgatctagaagtcaactatctc
aacctccattgttacccactaggtttttttctaataatgttaaaggctat
ttaatttatttaatcaactttaagcattacattctcaattggggacaaaa
attggttcttggggtgaaaaaatattagatatggcttgtggacttccaat
atcatcctatccaacaaaatcttattccttagtattaatttatctcattg
ggaagaaatttgtagttaaaactatttaatgtaatttgaatttaatttct
gtaattgggaaggagggtaataatgaacaaaaagtttgagaaaaaaatgc
tttaatgggttttacaggcatgctacaatccaggactgtggtgttctatg
tgccgtgtatggtcatattagtagctatttgttgcaagatacttgaagcc
aaatgtttgcagtataggttcctggctactgtttatgctgtgatttcact
ctgtctatcatgggcagtattagaaaacacactggttttcactatctggt

ttacaaacactatcattattttacctggattgaagtgcagaatttggcat
atcgatgcatgcaatggaaagaaaaaaaatttgttaaaatttgtaaatgt
tcagctaaatctcagatatactgtgggatgagtatctcagggtaagtaac
aaaaaaacaaacaaaaaaaatcactgaaatttttccctcacagtcagtag
ctttgtttgatatttaacaataagtatatggtgatatacttgaaatttat
gtccagaatgtcactggaatattacaatgagcataaataatcaagcttag
gactaaatgaaagatactatctcatgtatgctttaaatagagaatttatt
tcttttaaaaataaagctatttttccttaaaaaaaaaaaaaaaaaaaaaa
aaaaaaaaaaaaaaaaaaaaaaaaaaaaaaaaaaaaaaaaaaaaaa
>NM_032303 4
aaagcgcctgcgcggagagcctgcaatcagggaccaggcttgggaggcga
cgaagaggaacgctgggcgcaggaggcccactgcatacgcaggcgcactc
ggcgatcccggcttttccgcgcgcagttcccgcacaggcatcgggggggg
caggggcggggcaggggcggggataaatgcggagggacggtccagcttta
gctctctgctcgccgccgccgctgtcgccgccacctcctctgatctacga
aagtcatgttacccaacaccgggaggctggcaggatgtacagtttttatc
acaggtgcaagccgtggcattggcaaagctattgcattgaaagcagcaaa
ggatggagcaaatattgttattgctgcaaagaccgcccagccacatccaa
aacttctaggcacaatctatactgctgctgaagaaattgaagcagttgga
ggaaaggccttgccatgtattgttgatgtgagagatgaacagcagatcag
tgctgcagtggagaaagccatcaagaaatttggaggaattgatattctgg
taaataatgccagtgccattagtttgaccaatacattggacacacctacc
aagagattggatctgatgatgaacgtgaacaccagaggcacctaccttgc
atctaaagcatgtattccttatttgaaaaagagcaaagttgctcatatcc
tcaatatcagtccaccactgaacctaaatccagtttggttcaaacagcac
tgtgcttataccattgctaagtatggtatgtctatgtatgtgcttggaat
ggcagaagaatttaaaggtgaaattgcagtcaatgcattatggcctaaaa
cagccatacacactgctgctatggatatgctgggaggacctggtatcgaa
agccagtgtagaaaagttgatatcattgcagatgcagcatattccatttt
ccaaaagccaaaaagttttactggcaactttgtcattgatgaaaatatct
taaaagaagaaggaatagaaaattttgacgtttatgcaattaaaccaggt
catcctttgcaaccagatttcttcttagatgaatacccagaagcagttag
caagaaagtggaatcaactggtgctgttccagaattcaaagaagagaaac
tgcagctgcaaccaaaaccacgttctggagctgtggaagaaacatttaga
attgttaaggactctctcagtgatgatgttgttaaagccactcaagcaat
ctatctgtttgaactctccggtgaagatggtggcacgtggtttcttgatc
tgaaaagcaagggtgggaatgtcggatatggagagccttctgatcaggca
gatgtggtgatgagtatgactactgatgactttgtaaaaatgttttcagg
gaaactaaaaccaacaatggcattcatgtcagggaaattgaagattaaag
gtaacatggccctagcaatcaaattggagaagctaatgaatcagatgaat
gccagactgtgaaggaaaatataaaaaaaaagtcgactgctatgctcaaa
aagtaaaaaaagctcaacagttaaaatctaatgtttgttttctttcctgt
tatattataaggatatgcacgtttgttctggaaaagatagaatttgtctc
taaaagacttgaaattgtaattaaaatggcaagctaatcaaacataagct
tcattaagtgggattctaagacagtctgtgtttttatatttcaagggttt
aaccctttgagccttacatctcattcactgtctttctccaagaaaagtat
tttgggcggacagtcagatcaagcagtaaaattagctctttcaaatcttc
ttgtcatgtaaaatgaagctagtctgttttaaaatttttagttttggatt
gtatactaatgaaaatcttaatgatgtttttgattttatatacttattt

taaagaaaatcttatatagtacattttacaaaaattataaaaaatgaatt
agtactggcgaggactaaatgaaacaataatttttcattttgataactag
ctttccaggtggacttagccataggaaaatattactaatgtaatttaaca
aattgctgcatgtattccatttaaaaatatgtttaaattgtcctaaaaca
aaataattttctccctaggagtatgcatttggctacagtgttttgaaaca
gaaaccttagaataggtcattggtatgggctgaactgtgtatccccaat
tcatttgttgaggtcctaactcccatttcttttgaatgtgactgttcgga
gatgaggcctttaaagaggtgacttaagttcaaaggaggctgttagtcta
atccaacatggtgtcctttggacataagagataccagcaatgtgtgcaca
gaacaaagaccaggagaggacacagtgagaaggcagttatctgcaagcaa
agagagaggcttcagaagaaacaaaatcaccagcaccttgatctttgact
tctaatctccagaatagtgagaaataaatttctgttgttaagccgtccac
tgtgggaggccgacgcaggaggattgcttgaggccaggagttcaaggcca
gcctggacaacatagtaagaccctatctctaccccccctaataaattaatt
taaaaagcccccaatctgtggtattttattatggcagccctagcaagct
aatacagtggtttgagaggctgggagggttgaggggaagataaactttta
aaaagctcttatctttcatttcaatcagttaaaaatacttgctcagtgta
acaattttgcttctcagcttccactctaatattgttgtgccattaagcaa
tttagctaatcctgacatttcttagattcataatgttaggagcatttaat
ctgtattttacaagttaggaagcagaggatcagagatgggaaaggactag
cccaaggccaacattaacaagccctctaacaaaaactttacaatacattt
atgttgaatggaactccaagatctcacctctccatccaggaatggagtcc
atgtaatcaaagtgaacttaaaaataggacagtttcaacaagtcaggaga
ttcacagcaactgatcaaagggagtccagtcaacgtgagcaagcgtgatt
atgatgaggaagccccctctgctttaatccacacaaggaacgtaacctga
agtaacctgatgttaaccaatctgctgtgtctactatgctgtttccttgt
tcctgctagtgctgctttacaaatgcagaccattctatcatacctggcgg
ggcttctgttttattttgtaggctggatgctacccagttcatgaatcgct
aataaaagccaattagatctttaaaaaaaaaaaaaaaaaaaaa
>NM_005316 3
ctccttccgcgtggtccctccccctcaggccgcggtcgcgattacgctct
ctacggcctgcgaccgcagggccgttgcgggctggagacacggcgccgac
tggaaccggaggagctctaggccaaatggttgggccagccaggatcccag
gacccttcgccgctcgagaccggagagaggaaacgaaacaggcgggaacc
cgtgggggagggagggaactagcggaaggtgtcatggcggccgcgctctt
gagtcacgtgcccagggcccgccttgctacttccggtcacgtgccctcag
actcctcgcagccagcgatggaggcgagaccccctagtaacagaggcggt
ggctactgctgcggccactgggtttcggcctcttcccagcagcggctcta
agaagcgcagcggaactcgaccggatccaacccagttagttacttcctgt
ctagagttgtagcttccacctgcaccttctagccaccatggcaacctcat
ctgaagaagttttgctgattgtaaagaaagtgcgtcaaaagaagcaggat
ggagctctgtacctcatggcagaaagaattgcttgggcacctgaaggcaa
agatagatttacaatcagccatatgtatgcagatattaaatgccagaaaa
ttagtccagaaggaaaagctaaaattcagcttcagctggtcctacatgca
ggggacacaactaacttccatttttccaatgaaagcacagcagtgaaaga
gcgagatgcagtaaaagaccttcttcagcagctgctgcccaaattcaaga
ggaaagcaaataaagaactggaagagaagaacagaatgctgcaagaagat
cctgttttgtttcagctttataaagaccttgttgtgagtcaagtgatcag
tgctgaggaattctgggccaatcgtttaaatgtgaatgcaacagatagtt

cttccacatccaatcataagcaggatgttggcatttctgctgcatttctg
gctgatgtccggccccaaactgatggctgtaacggtctaagatataattt
aacttctgatatcattgagtccatatttaggacctatccagcagtaaaaa
tgaaatatgcagaaaatgttccccacaacatgacagagaaggaattctgg
acacgtttttccagtcccattattttcacagggatcggctgaatacagg
gtcaaaggatctctttgcagaatgtgccaaaatagatgaaaaaggcctaa
aaacaatggtttcattaggagtgaaaaacccactactagatttaacagct
ttggaagataaaccattagatgagggctatggcatttcctctgtgccatc
tgcttccaattctaaatccataaaagagaatagtaatgctgccatcatca
agagatttaaccatcacagtgccatggtcctggcagctggactcagaaaa
caagaagcacaaaatgaacaaactagtgagcccagcaacatggatggaaa
ttccggagatgcagactgctttcagccagcagtcaaaagggcgaaattac
aagagtccattgaatatgaagacttggggaaaaataattctgtaaaaacg
attgcactaaacctcaagaagtcagataggtattatcatggtccaactcc
aatccagtcactacagtatgcaacaagtcaggacattattaattcttttc
aaagtattagacaagaaatggaagcttatacacccaagttaactcaggtt
ctctcaagtagtgctgccagtagtaccatcacagcactgtcacctggagg
ggcacttatgcagggaggaacacagcaagccataaaccagatggtgccaa
atgatattcaatctgaattgaaacacttatatgtagctgttggagaactt
ctacgacatttctggtcctgctttcctgttaatacgccattcctagaaga
aaaggtagtgaaaatgaaaagtaatttggaacgattccaagttacgaagc
tctgtccattccaagaaaagattcggagacagtatttaagcacaaatttg
gtaagtcacatagaagagatgctccagacagcctacaacaagctccacac
atggcagtcacggcgtctgatgaagaaaacgtgaggtggccatgatgctt
acaggttttgtgagattgagagaactatgacctgcagcaactctggaaac
ctggcctgacagacaagcagatgacctcacaggagtgataagaaacatct
gctccacgccaactcccagagctgatgctattgtacttgcacattggaga
ctgaaaggaaagaagggactaaatgctggggaggtaaattaagacagaac
caaatgagctaagttgcaaatatatatatatacacacacacacatatatg
tacatgtgtatgtacatatatattttaaaagactgtttactgcagttgct
caggaactgcttttgattcacattaagctgctttcagaaattaaaaaaac
actttttaaagggtgcattgataaaatctgaggtttttggttgtcgttt
ttttctgtgtacatttttttcctaagtttatggcacagggtagaccttaa
gtattcctcctccatccttcattcttcaccctccattggatcctcaagtt
ttaatgaattccaattataccttacatcagcaagttaaaaaaagtacttt
aaaataaagcaaagggagactgttgctcaaccatcaggaaacagttgtca
gaagacatcattggttctgtgtttcctacggaaataagaaacgataaata
ttgcactgaatgtttgtggtttggagtccctgaataataaagagggaata
tatttgcagaaagtcgcatagggtttttaatgcagaattttgtcagaag
acaatggcgctgcatgtttttctttgagtgcaaatgtacattgctaagat
ttttttaagatggcatgtgctttgaaaagaagatattgcatttttaagag
tttaaaaatcttatgagtgagaaatattaaaaaaatcttattttcacctc
tttagaagaaataaaagatgtttctcctatctccttttctctagtatttg
actgttactgtccttggcgaatcgataatcattgcatagtgactgaaaag
cctaagtgcaaaaaaaaaaaaaaaagatgttcttgtttctgaacttcgtg
ccatattttgttcctgatgggatcaacttaatgtttaagactttagatgt
cttgtattaaaaattacacaaaaaaagtaaaactttttatacttacccttt
ttaactct
>NM_024759 1

agctggacggccccgggaggccgcagaccgccgggctccccgaggacacc
tcgcaccggaggaggagaggaggcagcgcccggccaggctgggagcacct
acggccgcgcgggggcgggagccaggtggcctcggcgcgcccgcctcgcc
cgggcaccgagcaggaagtggctgcggcgcggcctcctcgcggtgcaaca
gggcggggaggcggccgcagcccgagccggagcccgagcgccgggagcga
gacctcatggcagcggtggcgcccgcgggccccggggactccgcctcggc
cgccctggacgagctgtcactgaatttcacgtacggggcaccaggcgccg
gcaacggctccctctcgggcgactggtaccgcaggaaccagattcacctt
tttggagttttgctggctattttaggaaacttggtgatcagtatttctct
aaatattcagaaatattctcaccttcagctggcacaacaagagcacccaa
ggccatacttcaagagtgtgctgtggtggggtggtgtcctgctgatggcc
gtgggagagacggggaactttgcagcctatggatttgctcccattactct
gatcgctccgttaggctgtgtgtctgttacaggtagtgccattatttctg
ttacatttctgaaagacaatttgagagcctcagacttactcggtacgaca
ctggcatttgcaggaacatatttactggtgaactttgctccaaatataac
tcaggcaatctcagcaagaacagtacagtattaccttgtcggatggcagt
tcctgatctatgtgattttagaaatattaattttctgcattctcctgtat
ttctataaaagaaaaggaatgaagcatatggtgattctgctaaccctggt
ggcaattctagcctcattgactgttatttcagtaaaggccgtctcaggca
tgatcactttttctgtgatggataaaatgcaactaacttaccccattttc
tatatcatgtttatcatcatgatagcatcttgtgttttccaagtcaagtt
cctgaatcaagccacgaaactctacaatacgacaacagtggtgccagtta
atcatattttctttacaatcagtgccatcattgcaggtatcatattttat
caggaattccttggtgctccttttctcactgtatttatatatctttttgg
gtgttttctgtcattccttggtgtatttttggtcacaagaaatcgagaaa
aggaacatctgcaacagtcttatattgattttggaaatattcctgacaca
accccagagagaaaggcttggagggaaacaaatgttggacaaaatacaac
cagattcacatagcttatcctatggaactttgcctgatggaagtgactca
acaaagagccaaagtggagagaagaaagaggtctaaatgctgagaaggat
ggctgttggcctgttattcgataccacctttttaaaaaattgcacatgtt
caatttgtgtcagcagctcttttataagctaacatgtgctagcattcatt
tcagtcctttcccccacctcagcgtctatggacaatcggggacttcctaa
gctctgacagtctaatatttccatggaatgtgatttggagtgttccccac
accctggacctctccctagtgattatctagccagctacaccttactcaga
gcccggctgctctagcaggaatgttgcacagaaacatacatttgcggttt
ggccacaggtcgcataggtggcttctcccacgggtagtgtcagttgcttc
actttaaaaagtcacttcagccccacaacctcactcccaaaagaaaggtt
tgagagcgctcattttttttctaagacaccttttataagcctccctataa
aaatctcacctttcaagtactaagttagaattgcaagtcatcttttctga
aaactaatgctatcaaagtcctcctttgaaaattaagggtctctttaaaa
ttagaatttataaatggcattcagtgatagtgtacaccaaaattaaaaga
ccttgactggtgggctttttacgtgtaaggaagaggaagtttaagaagta
ggactctgcttgtttcagtagcccatgtactaaaataggaacaatgcaga
gattagcacagcccctgcacaaggctgacacataaattcatgaagcgtta
gagacaaaaagtaggactaatgtttgtttattttttaataaaaatctaga
atggt
>NM_019083 2
ggaagcgagccctagaattatggcgacctccgcgacgtcgccgcacgcgc
ctggttttccagctgagggtagatgcggttactatgtggaaaagaagaaa

cggttctgcaggatggtggtggccgcagggaaaagattttgtggtgaaca
cgctggagccgcggaggaagaagatgctcggaaaagaatcctgtgtcctt
tagatccaaaacacacagtatatgaagatcaactagcaaagcatttgaaa
aaatgtaactcaagagagaaaccaaaacctgatttctatattcaagatat
taatgcaggcttaagagatgaaacagaaatacctgaacaattagttccaa
tttcttctctatctgaagagcagttggaaaagttaattaagaaattgaga
aaagcaagtgaaggcttgaattctacacttaaagatcatattatgtccca
tccagcattacacgatgcacttaatgaccctaaaaatggcgattctgcaa
ccaagcacctgaaacagcaggcttctattttaggtaacattgaaaattta
aagttacttggtccaagaagatgctttgttgagtttggagcgggaaaggg
aaaattatctcattgggttgatattgccttaaaagatgctgaaaaagttc
acttcatcctagtggaaaaggtgaccacaagattcaaggtggatggaaaa
cacagaaagaaaaattcagtgtttgaaagacttcaaattgatattcaaca
cttgtgtttgaacaagattcctgtgctaagagaagaaaaactacctgtgg
taggaattggaaagcatctgtgtggtatggcaacagatcttgcattacga
tgtttggttgaaacctatgctgccagttttgaggaaaggaatgaagaacc
tttagccaaacgcataaagaatgataaaacagaaaaagaaatttacactt
tggccaaggaaggaaatgaaaaaaatgtcccagagaagtggaaccctgtg
gctggcattgttattgcactctgttgtcaccacaggtgtgattggagaca
ttatgtgggcaaagaatatttcagggctctaggccttggagcagtggaat
tccattatttccagcgaatgagtagttgggcaacttgtgggatgcggaaa
acatctttggaaacctcaaatagtaccacaaagaggcaagataatcagaa
tgatgatagtgaagagcatgatgatggaggatacagaatcacagatgatg
gcgctgattgtttgcctgggcttcttagtgttgaagaaaagaagaaaata
gggcatctttgtaaattgctgattgaccaaggtcgaatccagtatttgca
gcagaagggattcagtcctgctttgcagtactatacagaccctctggtgt
ctttggaaaatgttttgttaactgctttaccaaatcattcttcatcacca
gaaacaactgcttaatggaaaagaaatttgagcatcatctgtcttccacc
aaaaaaaatttttttaattatatttttatatcaaaaaaaatatatactttaa
atagcaaataatatgaactttaaaaaatgctgtggcctcattaaactttg
gtaatagcttttctttttacttcagaaatccaaacattagagaattcacc
aaagtaatcctctttagaagggcatcttgaattatattatccatccgtat
ttatggagattggtaaagtagttgaaccgttgacttggtgatctgaaaca
tacataacatgtcaacacataattagcgtatttctgtttgtattaatttt
ggaaatttatcttcctttgattttatttaatattttttatttcttaagtt
caagaggtagtgattgattgtaacaagggccaatctgagaatttgactta
tatgtggacatttttccctacagatctggaaagcacaattgtgattttct
caaatgcagacaattcagagatattcacaattaataaacacaattaatta
atgaagtcaccttcaaatttccagagccatacatgtatatattgtcagaa
tctgtccatgacaaacacaaaactgaagagctgttttcaaagaaaaaaga
ttatttcacttaattattttgttggataattgtctagttagaacttccaa
aaagatacttacaaacataattcacaaatttgaaataatttctgaagttg
attagctatctcatatcttttatcaggtcatttttttatatatgtaggcac
aaacaataagtatgttctcttctgtttgggaaaataatttggaataaaat
agaaattagataatgaaaccaaaaaccttctcaaatttaggccttattta
actcatgactggtttctatgcacaagaaaatactaaaccaaatatatggt
aggactgttattctcttctgcatcttatccctattttgtttctgccttta
tttgtaaaaattgtcttaattgatggtacatttgccaagacaaaggttca
gaattactaattttagatattatgatattctgaaataactatttttatcc

tgtagttctatgattatatgatttgtaaataagaagcctaaccaatttaa
aattcctgactttagtctcatatatcttgtcagactgtctcggttcaaat
tccaaacctaccatcttcagttgtgcgaccttgggcatgctacctaatct
ctttgtgcctcaatttcctccttttaaaatggggatgatgatgataacaa
taatacctacctcacaaggttgttgtgagcatcaaatgagataacacaag
taaaatgcatagaacagttccaagcacagagtaattcaataaatattaac
tagtaatagtagtggtagtaactcgtgaatctttttaataacataatagg
ctttgattttattatctctttaagttgttaactttttttcccttgttataa
gttttatgtcaagtaaggtagtttgtttaagttagttacccatgtcccca
atcaagggaacctaaatgaagatatatcattaaataattaacctttttttt
attgtgtcaagcaatactaatttgtgtcataaaatttgttttgcatcatt
caacaatttatgcccagaaataaacaatattctacaacaggcaaaaaaaa
aaaaaaa
>NM_000365 5
gcgcagacactgaccttcagcgcctcggctccagcgccatggcgccctcc
aggaagttcttcgttgggggaaactggaagatgaacgggcggaagcagag
tctgggggagctcatcggcactctgaacgcggccaaggtgccggccgaca
ccgaggtggtttgtgctcccccctactgcctatatcgacttcgcccggcag
aagctagatcccaagattgctgtggctgcgcagaactgctacaaagtgac
taatggggcttttactggggagatcagccctggcatgatcaaagactgcg
gagccacgtgggtggtcctggggcactcagagagaaggcatgtctttggg
gagtcagatgagctgattgggcagaaagtggcccatgctctggcagaggg
actcggagtaatcgcctgcattggggagaagctagatgaaagggaagctg
gcatcactgagaaggttgttttcgagcagacaaaggtcatcgcagataac
gtgaaggactggagcaaggtcgtcctggcctatgagcctgtgtgggccat
tggtactggcaagactgcaacaccccaacaggcccaggaagtacacgaga
agctccgaggatggctgaagtccaacgtctctgatgcggtggctcagagc
acccgtatcatttatggaggctctgtgactggggcaacctgcaaggagct
ggccagccagcctgatgtggatggcttccttgtgggtggtgcttccctca
agcccgaattcgtggacatcatcaatgccaaacaatgagccccatccatc
ttccctacccttcctgccaagccagggactaagcagcccagaagcccagt
aactgccctttccctgcatatgcttctgatggtgtcatctgctccttcct
gtggcctcatccaaactgtatcttcctttactgtttatatcttcaccctg
taatggttgggaccaggccaatcccttctccacttactataatggttgga
actaaacgtcaccaaggtggcttctccttggctgagagatggaaggcgtg
gtgggatttgctcctgggttccctaggccctagtgagggcagaagagaaa
ccatcctctcccttcttacaccgtgaggccaagatcccctcagaaggcag
gagtgctgccctctcccatggtgcccgtgcctctgtgctgtgtatgtgaa
ccacccatgtgagggaataaacctggcactaggtcttgtggtttgtctgc
cttcactggacttgcccagataatcttcctttttgaggcagctatataaa
tgatcatttgtgcaagaaaaaaaaaaaaacaagaacaggtttctataaca
aaaaaaaaaaaaaaaa
>NM_022353 2
agattccatcctatttctccgatgaaagtatcaggtacctcacccctaag
tttagatttgatgatagttcccttaaaaatgaatgacgaataatctaccc
catccttttctcacagtagcaaccaaattccagccaagggcaaaaaaaaa
atttctttttaatgtagcttagtgtttggaacttgatgttgtgtagtcag
acaaacctgtattcggcctccgctggatcagtcactagctgtgtaatttt
acagaactctcatcacctgcaaatctgggggaaatgcaggtgcgcacaga

gagcgttttgggcaaagacggcctttaagcttttcttcactaagcatgcc
gctcgctagcggaacagcagccagctctggacgggaccttgcacagttcc
gatgacatcacttccgggcgccaggttcgggctttctcctgcagcgataa
gggcagtcgacagtctttagtagggaaaggagacaagtgctagctactgc
cgcccaagtggaaggaattatctatagagtaagtatgctaatcttgacta
agactgcaggagtttttttaaaccatcaaaaaggaaagtttatgaattt
ttaagaagttttaattttcatcctggaacactatttcttcataaaatagt
attgggaattgaaactagttgtgatgatacagcagctgctgtggtggatg
aaactggaaatgtgttgggagaagcaatacattcccaaactgaagttcat
ttaaaaacaggtgggattgttcctccagcagctcaacagcttcacagaga
aaatattcaacgaatagtacaagaagctctttctgccagtggagtctctc
caagtgacctctcagcaattgcaactaccataaaaccaggacttgcttta
agcctgggagtgggcttatcatttagcttacagctggtaggacagttaaa
aaagccattcattcccattcatcatatggaggctcatgcacttactatta
ggttgaccaataaagtagaatttcctttttagttcttttgatttctgga
ggtcactgtctgttggcattagttcaaggagtttcagattttctgcttct
tggaaagtctttggacatagcaccaggtgacatgcttgacaaggtggcaa
gaagactttctttaataaaacatccagagtgctccaccatgagtggtggg
aaagccatagaacatttggccaaacaaggaaatagatttcattttgacat
caaacctcccttgcatcatgctaaaaattgtgatttttcttttactggac
ttcaacacgttactgataaaataataatgaaaaaggaaaaagaggaaggt
attgagaaggggcaaatcctgtcttcagcagcagacattgctgccacagt
acagcacacaatggcatgtcatcttgtgaaaagaacacatcgggctattc
tgttttgtaagcagagagacttgttacctcaaaataatgcagtactggtt
gcatctggtggtgtcgcaagtaacttctatatccgcagagctctggaaat
tttaacaaatgcaacacagtgcactttgttgtgtcctcctcccagactat
gcactgataatggcattatgattgcatggaatggtattgaaagactacgt
gctggcttgggcattttacatgacatagaaggcatccgctatgaaccaaa
atgtcctcttggagtagacatatcaaaagaagttggagaagcttccataa
aagtaccacaattaaaaatggagatatgatttctgctgttcaaaaaagtc
cctaaagagacaggctcttgccatgttgcccgggctggtcctgaactgct
gaattcaagtgatcctcccaccttggcctccagaagtgctgggattatgg
gtgtgagccaccatgcctagccaaaatgtttcttaaggtatacattttgg
gtcttagaagacttatacatttgtaatatttattactaaatatctcaaag
tattacaataaatgttaccatgtgagctactttgaatcaggcttcttgca
caccaatttaaaaatgttaactcttgatatatacactagttataccactc
atgtcagtcaataaattttaaggtttaagtgcaggcctttgtttacagaa
atcctaatttttgaaaccataactctgacctgacactaaattcctgtag
acatgctaaggaaaatctgcttagtatcgagatcaagaactttcattcaa
aaagattattcagttatgttatttgcatattaccattattaaaaataaaa
aaatttttaaaagatgaaaaaaaaaaaaaaaaaaaaaaa
>NM_018307 3
gccgccgccgccgccgccgccgccgccgccgccgccgccgccgccgccac
agcccgctgggccggaggaggcggagctggcgctgtcccggctctcttgc
ggggaagcaactgagggggcggcgcggcgggccccggcggccgaagaggc
tggcaggtggcgccgtggggtgggtgctcctggtgagaggagtccactcc
gtgcgtgcgggcggaggccggcccccgagagccgccgacatgaagaaaga
cgtgcggatcctgctggtgggagaacctagagttgggaagacatcactga
ttatgtctctggtcagtgaagaatttccagaagaggttcctccccgggca

gaagaaatcaccattccagctgatgtcaccccagagagagttccaacaca
cattgtagattactcagaagcagaacagagtgatgaacaacttcatcaag
aaatatctcaggctaatgtcatctgtatagtgtatgccgttaacaacaag
cattctattgataaggtaacaagtcgatggattcctctcataaatgaaag
aacagacaaagacagcaggctgcctttaatattggttgggaacaaatctg
atctggtggaatatagtagtatggagaccatccttcctattatgaaccag
tatacagaaatagaaacctgtgtggagtgttcagcgaaaaacctgaagaa
catatcagagctcttttattacgcacagaaagctgttcttcatcctacag
ggcccctgtactgcccagaggagaaggagatgaaaccagcttgtataaaa
gcccttactcgtatatttaaaatatctgatcaagataatgatggtactct
caatgatgctgaactcaacttctttcagaggatttgtttcaacactccat
tagctcctcaagctctggaggatgtcaagaatgtagtcagaaaacatata
agtgatggtgtggctgacagtgggttgaccctgaaaggttttctcttttt
acacacactttttatccagagagggagacacgaaactacttggactgtgc
ttcgacgatttggttatgatgatgacctggatttgacacctgaatatttg
ttccccctgctgaaaatacctcctgattgcactactgaattaaatcatca
tgcatatttatttctccaaagcacctttgacaagcatgatttggatagag
actgtgctttgtcacctgatgagcttaaagatttatttaaagttttccct
tacataccttgggggccagatgtgaataacacagtttgtaccaatgaaag
aggctggataacctaccagggattcctttcccagtggacgctcacgactt
atttagatgtacagcggtgcctggaatatttgggctatctaggctattca
atattgactgagcaagagtctcaagcttcagctgttacagtgacaagaga
taaaaagatagacctgcagaaaaaacaaactcaaagaaatgtgttcagat
gtaatgtaattggagtgaaaaactgtgggaaaagtggagttcttcaggct
cttcttggaagaaacttaatgaggcagaagaaaattcgtgaagatcataa
atcctactatgcgattaacactgtttatgtatatggacaagagaaatact
tgttgttgcatgatatctcagaatcggaatttctaactgaagctgaaatc
atttgtgatgttgtatgcctggtatatgatgtcagcaatcccaaatcctt
tgaatactgtgccaggatttttaagcaacactttatggacagcagaatac
cttgcttaatcgtagctgcaaagtcagacctgcatgaagttaaacaagaa
tacagtatttcacctactgatttctgcaggaaacacaaaatgcctccacc
acaagccttcacttgcaatactgctgatgcccccagtaaggatatctttg
ttaaattgacaacaatggccatgtatccgcacgtgacacaagctgacctc
aagagctccacgttttggcttcgagcaagttttggtgctactgtttttgc
agttttgggctttgctatgtacaaagcattattgaaacagcgatgatata
aaaagaaatactgtccctaccaaaaacaaatacttttatgtacattctga
atgctttaagttctgctagaattattgagatatttatacatgcagagtta
ctttattaatatttgtaattcatgcataagagtattttaatgatagttat
aactgcagtattggctagcatatggaaagaaaacagctaacagccaaact
aaaatggctaaattccagaggccaaaagggaatattttgtaaatatatgt
acatattcaggcaagatatggtctcccaagctgagttctagaaatgatgt
ttctagacatttctaagtggtattgttagtgctcacttggctcactcttc
taggtttaagttagcccagagattgtatttactcatggatcactttattt
atttcacatttactcagaatgatcctttgggttctataaggacataaggt
acaatttgccattgtctctccatttttaaaaacatacaagtcagtgtcag
cttaccaacatgacattttttcagtcagttgtggtaggccagccttgaag
ccatcgcacagtctagaaacttgtgtagctgagtgtgcagctcaccttta
agggtgaagttaggtaaaagcaattagcagaggcgttatctatgtgatta
tgttgcttccttgtcagtatgttgaattttatagccctttcaatgaaata

aaaaaaaaatttgtatattaccaatgtttttagtttaaataaagagtcac
ccttactactgttgaatttcatcccaagtgtaaatcattctataatggct
gtgtctgttatagtatattacagtaactgcatgtgtcaccaagtgttcta
tatcaggctaggataacctagaggcagtaatttttaaatgataaaataa
atctaatgaatataaactctcatgataaacctatttttccatcatcagc
cttttcaagtatttaaataaataactgctgtgtactgtg
>NM_032124 4
aacttccggcgtccgtcgggcagcagcggggctgtctatcccggctgagg
acccgcggccagtgcgggtggctggctttgccattagcgggggcctttcc
tgaggacggcgtacggagtgtggggaatgaaggatggcagcatgccgtgc
attaaaagctgttttggtagatctcagtggcacacttcacattgaagatg
cagctgtgccaggcgcacaggaagctcttaaaaggttacgtggtgcttct
gtaatcattaggtttgtgaccaatacaaccaaagagagcaagcaagacct
gttagaaaggttgagaaaattggaatttgatatctctgaagatgaaatat
tcacatctctgactgcagccagaagtttactagagcggaaacaagtcaga
cccatgctgctagttgatgatcgggcactacctgatttcaaaggaataca
aacaagtgatcctaatgctgtggtcatgggattggcaccagaacattttc
attatcaaattctgaatcaagcattccggttactcctggatggagcacct
ctgatagcaatccacaaagccaggtattacaagaggaaagatggcttagc
cctggggcctggaccatttgtgactgctttagagtatgccacagatacca
aagccacagtcgtggggaaaccagagaagacgttctttttggaagcattg
cggggcactggctgtgaacctgaggaggctgtcatgataggagatgattg
cagggatgatgttggtggggctcaagatgtcggcatgctgggcatcttag
taaagactgggaaatatcgagcatcagatgaagaaaaaattaatccacct
ccttacttaacttgtgagagtttccctcatgctgtggaccacattctgca
gcacctattgtgaagcaatgtgtgcatctgaagcaacttgaaatgcagct
tcttattgtctggaatgaatcccttaccaactcagtgccagcatcggtag
acaccagtcagtgctgatcgctttttaaccctcttttgttgtgcattaat
tagaaagaaaggtattgaattgcggctagccagtaagccttgctaatctc
ttttattttgtaactgaagatgagacccaaagaaagggaaagctgagatt
ttgtgccattccttttaaaatattcatcaggttaggtggggctgtggggg
aaaagctactacagggaagagtgttctctgctgtctcttcactggaaaac
agggagggggggatttcagactgtgaagaaagttgaatggtggtttttaaa
ttataaagtaatgtattaaaaggtgcattaggctgtagttctaatattga
gttcaactgtgaaatccatcagatgtgccaaatggagaagacagaaagca
acaaagtgaattgttctttagcccaagtggtacagtgaatttgctttaac
agatgttgaaaactaaattttctactgtattcccagcacgggtgacttct
ttttctcttcattagccagagatgactaatttaaatttagaaccagattt
taatttaaattaatatttccattaataacctattcattgcagatacctat
tatactgtgtaacagttgttttggaaattttatgtaaaattaaaactatc
agtattttacagatgttttaattagacattgttattaacaggaacagtgc
agaaactagaatcaagccttataatatcttatagaccatgcatttttgaa
gttagtgtccactagggtcctattaactgtacatttgcaagatttcatta
tttttgcctctgacactatgggaaaaatttttttagaagctattgggacag
attcaagcttttatgcacttggttactacagctgtaaaatgaaatctcgt
cttgtagcatggattattcttctcatgttaaacccaccaaaataaagggg
actaaataggtaatgattttcctagtgcatttgcatactgtgataatcct
gggccttgcaatagttctacagggctcttgggcattgaattattaggatg
taattgtacatcattgtagtgttcaccttattgaagctcactctgatgtt

aatgagcttcgggttttgatgcttgtttagagatcagcagtcttggatgg
gagggaacaaagctaaataaatgttagtttggtgaaaaaaaaaaaaaaaa
aaaaaaaaaaaaaaaaaaaaaaaaaaaaaaaaaaaaaaaaaaa
>NM_002228 3
gacatcatgggctatttttaggggttgactggtagcagataagtgttgag
ctcgggctggataagggctcagagttgcactgagtgtggctgaagcagcg
aggcgggagtggaggtgcgcggagtcaggcagacagacagacacagccag
ccagccaggtcggcagtatagtccgaactgcaaatcttattttcttttca
ccttctctctaactgcccagagctagcgcctgtggctcccgggctggtgt
ttcgggagtgtccagagagcctggtctccagccgcccccgggaggagagc
cctgctgcccaggcgctgttgacagcggcggaaagcagcggtacccacgc
gcccgccgggggaagtcggcgagcggctgcagcagcaaagaactttcccg
gctgggaggaccggagacaagtggcagagtcccggagcgaacttttgcaa
gcctttcctgcgtcttaggcttctccacggcggtaaagaccagaaggcgg
cggagagccacgcaagagaagaaggacgtgcgctcagcttcgctcgcacc
ggttgttgaacttgggcgagcgcgagccgcggctgccgggcgccccctcc
ccctagcagcggaggaggggacaagtcgtcggagtccgggcggccaagac
ccgccgccggccggccactgcagggtccgcactgatccgctccgcgggga
gagccgctgctctgggaagtgagttcgcctgcggactccgaggaaccgct
gcgcccgaagagcgctcagtgagtgaccgcgacttttcaaagccgggtag
cgcgcgcgagtcgacaagtaagagtgcgggaggcatcttaattaaccctg
cgctccctggagcgagctggtgaggagggcgcagcggggacgacagccag
cgggtgcgtgcgctcttagagaaactttccctgtcaaaggctccgggggg
cgcgggtgtcccccgcttgccagagccctgttgcggccccgaaacttgtg
cgcgcagcccaaactaacctcacgtgaagtgacggactgttctatgactg
caaagatggaaacgaccttctatgacgatgccctcaacgcctcgttcctc
ccgtccgagagcggaccttatggctacagtaaccccaagatcctgaaaca
gagcatgaccctgaacctggccgacccagtggggagcctgaagccgcacc
tccgcgccaagaactcggacctcctcacctcgcccgacgtggggctgctc
aagctggcgtcgcccgagctggagcgcctgataatccagtccagcaacgg
gcacatcaccaccacgccgacccccacccagttcctgtgccccaagaacg
tgacagatgagcaggagggcttcgccgagggcttcgtgcgcgccctggcc
gaactgcacagccagaacacgctgcccagcgtcacgtcggcggcgcagcc
ggtcaacggggcaggcatggtggctcccgcggtagcctcggtggcagggg
gcagcggcagcggcggcttcagcgccagcctgcacagcgagccgccggtc
tacgcaaacctcagcaacttcaacccaggcgcgctgagcagcggcggcgg
ggcgccctcctacggcgcggccggcctggcctttcccgcgcaaccccagc
agcagcagcagccgccgcaccacctgccccagcagatgcccgtgcagcac
ccgcggctgcaggccctgaaggaggagcctcagacagtgcccgagatgcc
cggcgagacaccgcccctgtcccccatcgacatggagtcccaggagcgga
tcaaggcggagaggaagcgcatgaggaaccgcatcgctgcctccaagtgc
cgaaaaaggaagctggagagaatcgcccggctggaggaaaaagtgaaaac
cttgaaagctcagaactcggagctggcgtccacggccaacatgctcaggg
aacaggtggcacagcttaaacagaaagtcatgaaccacgttaacagtggg
tgccaactcatgctaacgcagcagttgcaaacattttgaagagagaccgt
cgggggctgaggggcaacgaagaaaaaaaataacacagagagacagactt
gagaacttgacaagttgcgacggagagaaaaaagaagtgtccgagaacta
aagccaagggtatccaagttggactgggttgcgtcctgacggcgcccccca
gtgtgcacgagtgggaaggacttggcgcgccctcccttggcgtggagcca

gggagcggccgcctgcgggctgccccgctttgcggacgggctgtccccgc
gcgaacggaacgttggacttttcgttaacattgaccaagaactgcatgga
cctaacattcgatctcattcagtattaaagggggggagggggagggggtta
caaactgcaatagagactgtagattgcttctgtagtactccttaagaaca
caaagcggggggagggttggggaggggcggcaggagggaggtttgtgaga
gcgaggctgagcctacagatgaactctttctggcctgccttcgttaactg
tgtatgtacatatatatatttttaatttgatgaaagctgattactgtca
ataaacagcttcatgcctttgtaagttatttcttgtttgtttgtttgggt
atcctgcccagtgttgtttgtaaataagagatttggagcactctgagttt
accatttgtaataaagtatataattttttatgttttgtttctgaaaatt
ccagaaaggatatttaagaaaatacaataaactattggaaagtactcccc
taacctcttttctgcatcatctgtagatactagctatctaggtggagttg
aaagagttaagaatgtcgattaaaatcactctcagtgcttcttactatta
agcagtaaaaactgttctctattagactttagaaataaatgtacctgatg
tacctgatgctatggtcaggttatactcctcctccccagctatctatat
ggaattgcttaccaaaggatagtgcgatgtttcaggaggctggaggaagg
ggggttgcagtggagagggacagcccactgagaagtcaaacatttcaaag
tttggattgtatcaagtggcatgtgctgtgaccatttataatgttagtag
aaattttacaataggtgcttattctcaaagcaggaattggtggcagattt
tacaaaagatgtatccttccaatttggaatcttctctttgacaattccta
gataaaaagatggcctttgcttatgaatatttataacagcattcttgtca
caataaatgtattcaaataccaaaaaaaaaaaaaaaaa
>NM_003930 3
ggaaaaccgaggaatacacatgcgcagttggacccctcaggcccttcgtg
tcccttcccacccatctccccgccccggccctctgggcggggctgggccg
acagtccagctgcagctcgctggagattcagtgacttccttgttgtgagc
cccggcccggcagtgtcccgactcgtagccccgctgttcttaatccgggc
cgctagcctgagtctaggtcgcagccgcagccccaccccgtcggtcacct
tttcagcgcaggtcctttccccgcacgccctgcgctccctaacatgccca
accccagcagcacctcctctccctacccctccctgaggaaattaggaac
ctgttggcagatgttgaaacatttgtagcagatatactgaaaggagaaaa
tttatccaagaaagcaaaggaaaagagagaatcccttattaagaagataa
aagatgtaaagtctatctatcttcaggaatttcaagacaaaggtgatgca
gaagatggggaagaatatgatgacccttttgctgggcctccagacactat
ttcattagcctcagaacgatatgataaagacgatgaagccccctctgatg
gagcccagtttcctccaattgcagcacaagaccttccttttgttctaaag
gctggctaccttgaaaaacgcagaaaagatcacagctttctgggatttga
atggcagaaacggtggtgtgctctcagtaaaacggtattctattattatg
gaagtgataaagacaaacaacagaaaggtgaatttgcaatagatggctac
agtgtcagaatgaataacactctaagaaaggatggaaagaaagattgctg
ttttgaaatctctgctcctgataaacgtatatatcagtttacagcagctt
ctcccaaagatgctgaagaatgggtacagcagctgaaatttgtattgcaa
gatatggaatctgatattattcctgaggattatgatgagagaggagaatt
atatgatgatgttgatcatcctctaccaataagcaatccactaacaagca
gtcaaccaatagatgatgaaatttatgaagaacttccagaagaagaagag
gacagtgctccagtgaaagtggaagaacaaaggaagatgagtcaggatag
tgtccatcacacctcaggggataagagcactgattatgctaatttttacc
agggattgtgggattgtactggagctttttctgatgagttgtcatttaag
cgtggtgatgtgatttacattcttagcaaggaatacaatagatatggctg

gtgggtaggagaaatgaagggagccattggcttggtgcctaaagcctaca
taatggagatgtatgatatttgagagtcctggaaaaggaaaattcttctg
cttgtctgcaaatgctttggatttagaagcgtcatgaaagcacgagtgac
agctcctaacctctccttgttttattaaacattacttatctttgactgtt
attttatgcagtcgctcattaaaatattcctctgatgtgaaattaaatga
aggatattaatgtaaattagatgcaaccagttaagttatacctgttgcta
ttttgcaaagaaataattatagtttttatttacccatttgatttgtgtga
agaattcatcactattttatacgtaacatatagtctactatagcatagta
tgctactattgctacttctggtgtgatttgtaatgtttcttaatcattgg
acatcaattatttttagagagtaatgtataatttcatagcattttaaatt
tagtgtatcatgcgagttttttttggtagatgctgaagaatgtggttgct
aaacaaagaatgctaaagaatgttcaaactttatagataactttattgtt
attatttattttcacacatttaattcctattaagtacagccgcaagaaag
aaaaaatgatgaagttgcaaatggcagtgtgctgtcacctgcaacagaag
tgcgtaccagaagtatgactcatgcaaagcattttaccgtacaaatatcc
tggtgcgatgatgggcctggcaacattttctactgtacttttgtttaaat
ttaatgaaacaaaaaattcctaagaaataccaccctaccactaacaaaat
ggtataaagaatctcccagccaggccaacatggtgaaaccctgtctctac
taaaaattagccaagtgtggtaatgttcacctgcagtcccagctacttgg
gaggccaaggcacgagaattgcttgaacaaggtaggcagaggttgcagtg
agccaagatcgcgccactgcactccagcctgggcgacagagtgagactcc
atctcagaaaaaaaaaaaaaaaaaaacagaatctcagtgtattctcaaag
taaaaaggcataaccaagcactctcttatcttgccttattgctatactat
ttacatcccactgcagaacagcagatttgaggcttttctatatacttctc
agagcactgaaaagaaaggaagggtttgcagaggagagtgtagaaatccc
agtggtagcatgtaccaacaggtgagtagaaaggtagtgttagcctgaca
tttgagttatactctgtgctgcctgagcaagatttgtagaatcatataat
tacctttttcatgtatatttgaaatcagaggtgtttaaaatacctatgaga
taccaatgtagccttaacatatgtcaaaatgcattgctggttagataatt
atttggactacacataaactcctaatattgaatcattacctatcaggtat
tatctttatggaacttttcaatatctttgctttataaagattctaaacat
gtatctgagctggtaatattttaaaatctccattatttgtgtaaaactgt
ttataagcagtgtttgagagggtctgctttaccattacccccctcaatatc
atgatcatccaatctcaaatgtgaaaaaaaaaaagaaatttgattttagg
tattgtgagtaaacaagtttatatagagagacattgtgaagttaaagttt
tcagaagttacatttgtgcagttcttacctttcctcatatagtgccatt
gaaatagactgaaattatcttggcaaaagttagacaaccaaagacgactt
tagtggactggttttcaaaacttgagcagctgaaaagcaaaagccgttgt
ttcccatgacaatgtagcctttgtggatttgggtttgtgctttgggttga
aaagaagtttttagtcctaggccagtagatggcagcagcttttcattgca
gacaaaacctcttgaaacccttcccccatggcacaaactcgcccatgatg
gaaagcatctagatttctgcctccttttacagttaatccaggagagggag
tcctttgccaactgatgaccaacagttccaagccagatagtctcgtgaac
agtgacaatacagaaataaggtgttatttctgttcagatctccaccggcc
tttgttcttttaaaacttgaatataggtgggagacataagaaaggaaaga
aaagacttaaaactggagtgacaggacaaataatcattactttcaattca
tgactgctttatattcatttgatgaaatcatttgtatacaaaccagggag
agttttctttacacccttgacaatatatcacatactcttcaagatcataa
taatatcattaatataaatttaaacaacatggcttgttagaaaatatgct

aattgctatggtctcattatgtttgcttagcttttatttgtttttctgtg
aacagttagagagctaattttttttcaaaggtgattgtaagtcatatttta
tatagcattttgcttgattatttgctctgtactgaatttgtactctattg
ccattagatcttacaataatgttccactctgcaaatttttaaggttcaaa
taaagtttaattgtttgcaaaaaaaaaaaaaaa
>NM_015948 3
gcacgcgcgcagggacacataggcggaagtggcctgcgggcgccggagtt
gagagtgctttccggggaggcgaccgccgcggtggaaagtgcaggagtgg
gcgaggaggaggaggaagaggaggtgatggcgacggaccaggggaggggg
agatgctccgtctagctcagacgccacgcaggctgccgtcacccttccgg
gtgacataccggccttggggtacgcgtgccttcggggcctttcctgtgtt
tgggacccagatctcctctcctccagaaaccttgacactgcccatcttc
ctcatccagaaggagcgttcgaggaatggatgaggtcgggatggaagaaa
cgggtgattccctttttgaaagagcggaaaccggccgggcggtttccag
ggaggagcatctgttcctcctcgcccgtttttcgccctcgggctggcgag
aactccctcaggtcccaatccgtggactcgcaggggccatgattgattcc
ggagcgcagttaatcaaggcataatggcatggacttgacacagcaagcaa
aagacatacagaacataacagtccaggaaaccaacaaaaataactctgaa
agcattgaatgcagcaaaataacaatggatctcaagttcaacaattccag
gaaatatatttctatcactgtgccatccaaaacccaaacaatgtcaccac
acatcaagtcagttgacgacgttgtggtacttggcatgaatctcagcaag
tttaacaaacttactcagtttttcatatgtgttgctggagtttttgtatt
ttacctaatttatgggtatttacaggaattaatattttcagtggagggtt
ttaagtcctgtggctggtaccttaccttagtgcagtttgccttttactcc
atatttggcctaatagaacttcagcttattcaggacaaaaggaggagaat
accaggaaaaacctacatgataatagcttttctaactgtgggtactatgg
ggttatcaaacacttccttgggctacctgaattaccctacccaagtcatc
ttcaagtgctgcaaattgattcctgttatgctaggaggagtttttattca
aggaaagcgttataatgttgcagatgtgtctgctgccatatgtatgagcc
ttggcctgatatggtttaccctcgctgacagcacaactgcaccaaatttc
aacctgacgggtgtggtgcttatttccctggcactatgtgcagatgccgt
cattggaaatgttcaagagaaagctatgaaacttcataatgcttctaatt
ctgaaatggtattgtattcgtattcaattggttttgtatacattttactg
ggattgacatgcactagtggattaggccctgcagtaacattttgtgcaaa
gaatccagttcggacctatggttatgcgttcctttttccctcactggat
attttggaatctcctttgttctggctttgattaaaatttttggtgcactt
attgctgtaacagtgacaacaggaagaaaagcaatgaccattgtactttc
gtttatattctttgctaaaccattcacgtttcagtatgtatggtctggtt
tgttagttgtccttggtatatttcttaatgtttacagcaaaaaatatggat
aaaataagactaccatcactgtatgatttgataaacaaatcagtggaagc
aagaaagtcaaggacgctggcacagactgtatagacagtgattgtcctat
ttaaaatagaattttaagggaacaatcatcaattaattaactttccaaag
ggactgataaaaaccaaaggatctggaggcattgctatcccatttgtgga
cagatttcatatgaagttgttttgcggtgtcagccttttcttcagagcat
ttgtttgactgacttccaaagcaatcaagagagccacgtctagcagactt
tacaataaaatgtcaatatgaaggactgtaattcctagcagtttattgag
aatttcactggaaatggaccatgtgttgcaagactaattggctataatta
tatcctatcaaagaaatcgatacgtaatagcagattgttttatattcatt
ccattttgatggtgttatttaaattgattctctgttataagagtaaactg

atgagttgaagtctggagagaataacattcattataaataaaattattct
gtgatctttttttcaaaaaaaaaaaaaaaaaaaaaaaa
>NM_002687 3
attggctgagcccggctgtcagtcctttcgcgcctcggcggcgcggcata
gcccggctcggcctgtaaagcagtctcaagcctgccgcagggagaagatg
gcggtcgccgtgagaactttgcaggaacagctggaaaaggccaaagagag
tcttaagaacgtggatgagaacattcgcaagctcaccgggcgggatccga
atgacgtgaggcccatccaagccagattgctggccctttctggtcctggt
ggaggtagaggacgtggtagtttattactgaggcgtggattctcagatag
tggaggaggacccccagccaaacagagagaccttgaaggggcagtcagta
ggctgggcggggagcgtcggaccagaagagaatcacgccaggaaagcgac
ccggaggatgatgatgttaaaaagccagcattgcagtcttcagttgtagc
tacctccaaagagcgcacacgtagagaccttatccaggatcaaaatatgg
atgaaaagggaaagcaaaggaaccggcgaatatttggcttgttgatgggt
acccttcaaaaatttaaacaagaatccactgttgctactgaaaggcaaaa
gcggcgccaggaaattgaacaaaaacttgaagttcaggcagaagaagaga
gaaagcaggttgaaaatgaaaggagagaactgtttgaagagaggcgtgct
aaacagacagaactgcggcttttggaacagaaagttgagcttgcgcagct
gcaagaagaatggaatgaacataatgccaaaataattaaatatataagaa
ctaagacaaagccccatttgttttatattcctggaagaatgtgtccagct
acccaaaaactaatagaagagtcacagagaaaaatgaacgctttatttga
aggtagacgcatcgaatttgcagaacaaataaataaaatggaggctaggc
ctagaagacaatcaatgaaggaaaaagagcatcaggtggtgcgtaatgaa
gaacagaaggcggaacaagaagagggtaaggtggctcagcgagaggaaga
gttggaggagacaggtaatcagcacaatgatgtagaaatagaggaagcag
gagaggaagaggaaaaggaaatagcgattgttcatagtgatgcagagaaa
gaacaggaggaggaagaacaaaaacaggaaatggaggttaagatggagga
ggaaactgaggtaagggaaagtgagaagcagcaggatagtcagcctgaag
aagttatggatgtgctagagatggttgagaatgtcaaacatgtaattgct
gaccaggaggtaatggaaactaatcgagttgaaagtgtagaaccttcaga
aaatgaagctagcaaagaattggaaccagaaatggaatttgaaattgagc
cagataaagaatgtaaatccctttctcctgggaaagagaatgtcagtgct
ttagacatggaaaaggagtctgaggaaaaagaagaaaaagaatctgagcc
ccaacctgagcctgtggctcaacctcagcctcagtctcagccccagcttc
agcttcaatcccagtcccaaccagtactccagtcccagcctccctctcag
cctgaggatttgtcattagctgttttacagccaacaccccaagttactca
ggagcaagggcatttactacctgagaggaaggattttcctgtagagtctg
taaaactcactgaggtaccagtagagccagtcttgacagtacatccagag
agcaagagcaaaaccaaaactaggagcagaagtagaggtcgagctagaaa
taaaacaagcaagagtagaagtcgaagcagtagcagtagcagttctagta
gcagttcaaccagtagcagcagtggaagtagttccagcagtggaagtagt
agcagtcgcagtagttccagtagcagctccagtacaagtggcagcagcag
cagagatagtagcagtagcactagtagtagtagtgagagtagaagtcgga
gtaggggccggggacataatagagatagaaagcacagaaggagcgtggat
cggaagagaagggatacttcaggactagaaagaagtcacaaatcttcaaa
aggtggtagtagtagagatacaaaaggatcaaaggataagaattcccggt
ccgacagaaagaggtctatatcagagagtagtcgatcaggcaaaagatct
tcaagaagtgaaagagaccgaaaatcagacaggaaagacaaaaggcgtta
atggaagaagccaggctttcttagccattctttgcagcagaagatttctt

gataaaaaaggattacctttccttgtaaagaggatgctgccttaagaatt
gcatgttgtaaaaaatctttttggaaaatacagactgtttgtttaccaga
cattcttgtactttttgcataattttgtaagagttatttatcaaaattat
gtgaggttccaaaatatgtaaaaatgataataataaaaaaagattaacat
cccttgtcatctttttaaatatcctatactcttcagtaagaatctgtat
attttaataggcaaatctttaagtctgttcccttctaattctgtatcata
cattgcttttgtagaaataaatgtgtgtttatttcattatttttgggatg
tcctcgttgacacttgtataataaatatcctctttatcattttcagcttt
taacactagatactgcacgtgattagaatgtttttgaaggtttcctcgtt
tttatttgccttggacagtttttagttgtcagagtcagagctttgcagct
ttgagggggaacagttctctttaaaatcatttgctattttctattctccc
ttgttattttaatctaagcattttcccccgtttctcatattttaaccata
tgttcggtagataactaaacagtatgatctggttggcattttcttcctga
tgatgggagcgtcattcttttgtcttcatggttacttgtgtgatataaca
tacatctgttaaagaaaatcacttctttctaggggagggaggtagaaaag
tatctttcaaacttggtttttgagtttgtgtcttgtcttaactttgtgtt
ggctctaacttaaacatgctgatatgtgttttcaagaattttgtttaagg
aagtattgtatggaagtccacaaaatgaaggaagttcatctaggttttaa
tatgtaagcaagataacacacaagtgtaccaagtgattattaactttgtt
gttttacaaatttgtatgaacttggagtatctgttggccattactataca
tgtgcaaataaatgtggcttagacttgtgtgactgcttaagactagtact
tgtattaactttctgatgctttatacaagagagcacttaaattgcatcct
tctttgagtttaacactgtagctttagccttgactttgaatattcatttt
gccttcccttgacaagtaaatggttacagtgaaaatgtgaagaagtattt
ccagttagtttttgtgtgtatataactcacttgtgtaagtggatgtgtta
aaagatctctttttaaaaatccttttattggagtaattattaaaacagta
aatgca
>NM_003359 3
gtgaaggaaatagggacctggccctgggccttgtgtagcgggaggggggag
ctaggaagcagctgagggcagaatccaggagggcctggctgcgggggaat
gaagcctccgccttcgcaggcaaaagcctttaaatacgggctcaggcccg
ggactcagagtgtaacgcgtggcagcctgagggaggggcgtgcgccgaga
gggagctcagatcgagcggggcgcgggtggagaagctgcggcggcgcggc
ccgtaggaaggtgctgtccgaacgatcgggataggagcggtccctgcgct
tgctgctgggaagtggtacaatcatgtttgaaattaagaagatctgttgc
atcggtgcaggctatgttggaggacccacatgtagtgtcattgctcatat
gtgtcctgaaatcagggtaacggttgttgatgtcaatgaatcaagaatca
atgcgtggaattctcctacacttcctatttatgagccaggactaaaagaa
gtggtagaatcctgtcgaggaaaaaatctttttttttctaccaatattga
tgatgccatcaaagaagctgatcttgtatttatttctgtgaatactccaa
caaaaacctatggaatggggaaaggccgggcagcagatctgaagtatatt
gaagcttgtgctagacgcattgtgcaaaactcaaatgggtacaaaattgt
gactgagaaaagcacagttccagtgcgggcagcagaaagtatccgtcgca
tatttgatgcaaacacaaaacccaacttgaatttacaggtgctgtccaac
cctgagtttctggcagagggaacagccatcaaggacctaaagaacccaga
cagagtactgattggaggggatgaaactccagagggccagagagctgtgc
aggccctgtgtgctgtatatgagcactgggttcccagagaaaagatcctc
accactaatacttggtcttcagagctttccaaactggcagcaaatgcttt
tcttgcccagagaataagcagcattaactccataagtgctctgtgtgaag

caacaggagctgatgtagaagaggtagcaacagcgattggaatggaccag
agaattggaaacaagtttctaaaagccagtgttgggtttggtgggagctg
tttccaaaaggatgttctgaatttggtttatctctgtgaggctctgaatt
tgccagaagtagctcgttattggcagcaggtcatagacatgaatgactac
cagaggaggaggtttgcttcccggatcatagatagtctgtttaatacagt
aactgataagaagatagctattttgggatttgcattcaaaaaggacactg
gtgatacaagagaatcttctagtatatatattagcaaatatttgatggat
gaaggtgcacatctacatatatatgatccaaaagtacctagggaacaaat
agttgtggatctttctcatccaggtgtttcagaggatgaccaagtgtccc
ggctcgtgaccatttccaaggatccatatgaagcatgtgatggtgcccat
gctgttgttatttgcactgagtgggacatgtttaaggaattggattatga
acgcattcataaaaaaatgctaaagccagcctttatcttcgatggacggc
gtgtcctggatgggctccacaatgaactacaaaccattggcttccagatt
gaaacaattggcaaaaaggtgtcttcaaagagaattccatatgctccttc
tggtgaaattccgaagtttagtcttcaagatccacctaacaagaaaccta
aagtgtagagattgccatttttatttgtgattttttttttttttttttgg
tacttcaggatagcaaatatctatctgctattaaatggtaaatgaaccaa
gtgtttttttttgtttttttttgagacagagtctcactgttgcccaggc
tggagtgcagtggtgcaatctcggctcactgcaagctctgcttcccaggt
tcacgccattctcctggctcagcctcccaagtagctgggactacaggcac
ccgccacagtgcctggctaatttttgtatttttagtagagacagggttt
caccatgtgagccaggatggtctcaatctcctgaccttgtgaaccacccg
tctcggcctcccaaagtgctgggattacaggtgtgagccaccacgcctgg
cccatgaaccaagtgtttttaaggaaacaaaactattttttaatcatca
gatttatactagctatatggatattagcatatctggtaattatgaatcta
gaatttttttacatattttataatactgttagctcagttattggatgag
tgaaagataatcatgttggttttaatagtgtcaatttttgtaaaataaaa
attaaacttcaaactctttactttataaattgtccataggccacacttta
atatcacattataaagggaaggacagtcttcattcctcctggttattggt
ttgtttgtcattaaagatatattttgaatccatgaaattgctatgctaaa
cagcctttacatgtatggtctggttaaagttcctttgttccttttgtttt
aataaaatgtgtcactgatttttagctcaaaatcatcactgttaatttc
cagtcaccccaaatatggttaaaagattttttttaatcatgaagagaa
aattagtagcatttctttctctccccattatttattggttttcctcacta
atctttttttttttagtccaaaagccaaaaatatttatcttggttttaca
ttttaatttccattcttaattgtaatttttttctttaaataaggaaacca
atataatctcatgtataaaaacttaaatattttacaagttacatatagca
tcattctaaaataagaatttttttgttttctgtctgctttttcttatg
tctcttgttgagttttatattttcagtggttatttttgcttgtgttagat
cattattaaaatatatccaatgtccctttgatacttgtgctctgctgaga
atgtacagtttgcattaaacatcccaggtctcatccttcaggaattttgc
agttcaatgagaagagggagacaaatataaagatgaggacagaagcatct
ctacagatgaaaattacataaataaaacattctccatcaacaact
>NM_145243 3
cgggcaggaagtgcgggtgcgcgcctgcgcataggtcgggtctgcggtgt
caccgctttcgcttctgcttgagtaatcaagtgaaaaaatgagcttcatc
tgtggattgcagtctgctgctagaaaccatgttttcttccgatttaattc
actgtctaactggagaaaatgtaacacattagcatccacctcacggggct
gtcatcaagtacaagttaaccatatagtaaataagtatcagggactggga

gtaaatcagtgtgacaggtggagttttctgcctggaaactttcattttta
tagtacttttaacaacaaaagaacaggaggcctctcaagtaccaaaagta
aggaaatttggaggattaccagcaaatgtactgtatggaatgatgctttt
tcaagacagctgctaataaaagaagttacagcagtccctagtctgtcagt
attgcatcctctaagccctgcttccataagagctattaggaatttccata
cttctccacggtttcaagctgctccggttcctctcttgttgatgattctt
aaaccagtacagaagttatttgcaatcattgtaggcaggggcataaggaa
atggtggcaggcacttcctcctaacaagaaggaagtagttaaagaaaata
taaggaagaataaatggaagctattccttggtttgagtagttttggattg
ctctttgtggtgttttattttactcacctggaagtaagtccaatcacagg
aaggagcaagctactattattggggaaagagcagttcagacttttatcgg
aactggaatatgaagcatggatggaagaatttaaaaatgatatgctaact
gagaaagatgcccgatacctggctgttaaagaagtgctttgtcatctaat
tgaatgcaataaagatgttccagggatctctcagatcaattgggttattc
atgtggttgattccccaattattaatgccttcgtgcttccaaatggacaa
atgtttgttttcactggatttttaaatagtgtaaccgatattcatcaact
ttctttccttctgggccatgaaatagcacatgcagtacttgggcatgctg
cagaaaaggctggcatggttcatttgttggatttcctaggtatgattttc
ctcacaatgatttgggccatttgtcctcgagatagcttggcacttttgtg
ccagtggatacagtctaaattgcaggagtatatgtttaatagaccataca
gcagaaaattggaggccgaagctgacaaaattggactactgcttgctgca
aaggcttgtgcagacataagagccagttcagtgttttggcagcaaatgga
gttcgttgatagcctgcatggccaacccaagatgccagaatggttatcta
cacacccttctcatggcaatcgagttgagtacttggatagacttatacct
caggctctcaaaattagagagatgtgtaattgtccaccactgtctaatcc
agaccctcgattactattcaaactcagcacgaagcattttcttgaagaat
cagagaaagaagacctaaatatcacgaagaaacagaaaatggatactctt
cctattcaaaaacaggagcaaataccattaacatacatagttgagaaaag
aacgggcagttgaattaaaatttatgagacacaagatatatgaagaatgt
tgcagtccttatcattttatgttactttttaaaaaatgatgtttgaagtg
aaaaaaaaaaggatattcagggtcaaatcatgtacattacagatattatc
taaattcttctagaatttatttttcatgaaatattgatgtattttaatct
atgttaaaatatcttcaatgaggaaaatgtcacagaataaatttatatta
cacattttaaaaaaaaaaaaaaaaaaaa
>NM_018263 4
agcggcggtagcagtggcaacggcagcaagaggaccggccgccccataga
ccccccgcgcaccacccccgccgcttcctctgcttgggtgcccccccgg
tctgacttccctttctcccacctttcccggcaccgcgggaaaagcagcg
cagggcagagcaggcagggagggcggccggagcccggacacgggagcctc
ccagtcagttcaagacccgacatgagggaaaagggacgtaggaagaaggg
caggacctgggcggaggccgccaagacggtcttagaaaaataccccaata
cacccatgagtcataaagaaattcttcaagttatccagagagaaggacta
aaagaaatcagaagtgggacttctcctcttgcatgcctgaatgcaatgct
tcacacaaactccagaggtgaagagggcatcttctataaggttccaggta
gaatgggagtatatactttgaagaaagatgtgccggatggggtgaaagag
ctgtcagaaggttcagaagaaagcagtgatggtcagtcagattcccagag
ttctgagaacagcagcagcagcagtgatggtggcagcaacaaggagggaa
aaaagagcaggtggaaaaggaaagtatcgtcgtcctccccgcagtcaggc
tgcccatcacccaccattccagcaggtaaagtcatttctccatcacagaa

gcacagcaagaaggcactaaagcaggcgctaaagcagcaacagcagaaga
agcagcagcagcaatgcaggccaagcatatccatctcctccaaccagcat
ctctcactaaagactgtcaaagcagccagtgactctgtacctgccaaacc
tgcaacatgggaaggaaagcaatctgatggacagacaggcagccctcaaa
actcaaactccagcttttcttcctcagttaaagtggaaaatactttacta
ggcttggggaagaagtcattccagagatctgagagactccataccagaca
aatgaaaagaactaaatgtgctgacattgacgttgagacaccggactcca
ttctggttaatacaaatctgcgagcactgatcaacaagcacacattttca
gtccttcctggagattgccagcaacgactgcttttactactcccagaggt
agatcgacaggttggtccagatggtttaatgaagttaaatggctcagccc
ttaacaatgaattcttcacttcagcagcccaaggctggaaggaaagactc
tcagaaggtgagtttacacctgagatgcaggtgagaattcgacaagagat
tgagaaggagaaaaaagtggagccatggaaagaacaattctttgaaagct
actatgggcagagttctggcctgagccttgaagattctaagaaattgaca
gcttctcccagtgatcccaaagtaaagaaaaccccagctgaacaaccaaa
atccatgcctgtgtcagaggcctctcttatcagaatagttccagtagtct
cccagtcagagtgtaaagaagaagcattgcaaatgtcatcaccaggcaga
aaagaagagtgtgaaagccaaggtgaagtgcagccgaacttctccacatc
ttcagagcccctgctttcctcagctctcaatacacatgagcttagcagca
ttcttcccatcaagtgcccaaaggatgaggatctcttggagcagaagcca
gtcacctctgctgaacaggaatctgagaagaaccatctcaccacagcttc
taattataacaaaagtgaaagccaagaatctttagttacatcgccaagca
aacccaagagtcctggggttgaaaaaccaatagtgaagcccacagcagga
gcgggtccacaggagactaatatgaaagaacctctagcaactcttgttga
tcagagcccagaaagcctcaagaggaagtcttccctcacccaagaagagg
cccctgtgagctgggagaagaggccacgtgtcactgagaatcgccagcac
cagcagccatttcaggtctcaccacagccctttctcaatagaggggacag
aatccaggtgcgaaaagtaccacctctcaagatcccggtctccagaatct
cccccatgccgtttcatccatcgcaggtctctcccagggctcgttttcca
gtctccatcactagtcctaacagaacaggagccagaactcttgcagacat
caaagcaaaagcccaactggtcaaagcacagagggcagcagctgccgctg
ccgccgcagctgctgcagccgcctcagttggagggaccattccaggacct
ggcccagggggtggacaaggtccaggagagggtggtgaagggcagactgc
tagaggaggcagtccaggctcagacagagtcagtgaaactggaaagggcc
ccacactggaactggcaggaactggaagcaggggaggtacgagagagctt
ttaccctgtggtccagagactcagccccagtctgagaccaagaccacccc
aagccaggcacagcctcatagtgtctctggagcacaactacagcaaaccc
ccccagtgcctccaacacctgccgtcagtggagcatgcacaagtgtccca
tcaccagcccacatagagaaattggataatgaaaaactgaaccccaccag
agcaacagccacagtggcctctgtcagccatccacaagggcccagtagtt
gcagacaggagaaagcaccttctccaacaggtcctgctctaatctcaggt
gcctcacctgttcattgtgcagctgatggcacagttgagctcaaagcagg
tcctagtaagaatatacctaacccttcagcctcatcaaagacagatgcta
gtgtgccagtggctgtaactccctccccttttaacatctttattgaccaca
gccactttagaaaagcttcctgtaccccaggtcagtgcaactacagcacc
tgctggatcagctccaccctcgagcactttgccagcagcttctagcctta
aaaccccaggaacttctttaaacatgaatggacccactttaagaccaacc
tctagtatccctgctaataatcctttagtgactcagctgcttcaaggcaa
agatgttcccatggagcaaattctgcctaaacctctcaccaaagttgaaa

tgaaaacggttccactgactgcaaaagaggaaagggggatgggagcgctc
atagctaccaacacaacagaaaatagcaccagagaggaagttaatgagag
acagtcccatccagctacgcagcagcagctgggcaaaaccttgcaaagta
agcagctcccccaggttccaaggccccttcagctcttttcagctaaggag
ctgagggactccagcattgacacacaccaataccacgaaggactaagtaa
agcaacccaagatcagatccttcagactctcattcagagggttcggaggc
agaatcttctctcagttgtgccgccctcacagttcaacttcgctcactca
ggtttccagctggaagacatctccacaagccagaggttcatgctgggttt
tgctggcagaaggacatccaaacctgcaatggcagggcactacttactga
atatttctacctacggccggggctcagagagctttaggaggacccattct
gtaaaccctgaagatcgtttttgtctaagcagccccactgaagccttgaa
aatgggatatacagactgtaaaaatgcaacaggagagagtagcagcagca
aagaagatgacactgatgaggaaagtactggtgatgagcaggaatctgtc
acagtgaaagaggagccccaggtttcccagagtgctggcaagggtgacac
aagttcaggacctcacagcagggaaactctatctaccagtgattgcttag
ctagcaagaatgtgaaggctgagataccattgaatgagcaaaccacttta
agtaaggagaattacctgttcactagaggccaaacatttgatgaaaagac
cctagccagagatttaattcaggcagcacagaagcagatggctcatgcag
tgagaggtaaggcaatccgtagcagccccgagcttttcagttctactgtt
cttcctctgcctgcagacagccccacccaccagcccctactccttccacc
cctgcaaaccccgaagttgtatggaagccccacccagatagggccaagct
atagaggcatgatcaatgtctccacctcatctgacatggaccataactct
gctgtaccaggtagccaggtatctagcaatgtaggtgatgtcatgtcatt
ttcagtgactgtcactaccatccctgctagccaagctatgaatcccagca
gccatggccagaccattcctgttcaggcgttctccgaagagaacagcata
gagggcacgccttcgaaatgttactgccgcttgaaagccatgatcatgtg
caaaggctgtggcgctttctgccatgatgattgcatcggcccctccaaac
tgtgcgtctcctgccttgtcgttcggtaatgagactagaaagagatacac
tgtaaaggagggggaagggaagggttgaccagttgggtttttgcgtcct
tttggaatcacagaaataaacaagtaggtttcagttgtcttcagagacaa
atgttacttaatcaggaatacaaagtacagatttttacattttagagga
agagcaccttgtatagtaagtctaagtcaagcaagactttgtgaatttgt
gacaagtttgcacttacaaaatcatgtaaatatgtcacattttgtttaac
attatttttccaagtgtttaggtaataatgtattactttgaattcagatt
aatgttctactacttgtgactgagaaaagtttaatgccggcatggtaaaa
ggaagctgtctcccttctgacatttcctaggaagctaggaaaaggaaagt
gaaggaatggtctaaagaaatgaccattcacactgattttgtctggacag
tctggccgcaggttattcatagattattcagcctttgcaggacttggatt
cagggttttactcagtccctttaccttaggtggaatcttcttaaattgaa
atttttggtaaggaattcatttcacaggtagtgtttcagactctgaaagc
cctgacttggttcttggcttctactgtactagttactagttactggtact
gttgccaagcaatctgcttggaatttgtggatccctgctgttccctaatc
ccaccccctgccctgagacagtgaatgtagtccgtgaagggagtgcctct
ctgggacccccctgtgttgttacaggctgtgcagtgcaacaattccagcaa
aaataccctatccccgcacttagtcattcgtggtaactaacaattttgaa
atactcatataaaatgaacaggaaagtgttagtgattgggtgatttgagc
tacatcttgttgagcaaggggagtgaaaccacagaaactgttaaattgaa
cgtaaataactttggaaaacagttttgacactagaacaaggtcgcttctc
ttttcctcttctctcccaacctgttaacctagtgctagatagtcagctgc

tgttgcaggtagaaaagctcaccaatgccacatttgttcattctgttcaa
cctgttttgacctcccaggcatctgaacaggatgacatcatcttattata
tcttactttacacatacgtgtatgtacacacacacacacaatacaagaca
tttttcctgttttagaaaatatagcccttgtttggattactgctgtctga
gcactagaaatttctaatggaaagaggcctctgaatggctaagggaacat
ctggaaaggaaggaaaatgagccctaaaggttttttggttgcttgttttt
gtttctttttctttcttgtttcctttttgtttttaaatccttctctagt
gactgaagaaattgtgagtacataatatttaggtcatggttaaggaacat
taaaataaacctgcagtttaacagaattttgtattaaatgggggagtgtc
aatttgcatcatttgcatattgatttcaagtctcctaatttggaatgggt
aaagtacaaatgttaatgcaacatagtacagttgtatttgtccttcatat
tcaggatgtgatatttacaaaatgcagaacagtttaaacttgttaagtca
cattccagattaggagaaaatgggcaataatcatctctgttttgcccca
ttctactacccaccactgcccaccccacccccacatcacacactcttcga
tgctttcccacagcccctgctccgctcctctccgtaagaaactcacttca
ctgtttttcacttttcacttctggctttttgtggcccaaggccaatata
ggattaatatctgtgttccagcttcagccagaacgatatgtctgtccatc
tttctcctttcttccagcacacacagctaagagataagaaggctgcaagg
attggggataaggagtatattttcaagccaagaaccagtagaaaacagaa
ataatggtgctcccaaatattcagtaccttgggcaatccaggaagggttt
aaagagacctgttgctgcatagaactctccctgccctaccccttaactcc
accttagtgccagggccgagggctggtctgcatgctggaagaggttgaga
gttttggaattgattccatgctaaatgtcagttaccagtttggattgatg
aaagtgatttttctgctttacctttttcctgtcagaaaggttttcctctg
tgctacccttaacttagggatgaacagactataactaggaaaggaggaaa
ggtatcagggatttttaagtccgtttagagagcaaaccatattgtgcctc
ttcatcttgacccaggctcttatagataggtcctaaacattgctgttcac
tagattatcttaacaaataatactcagctaaatatttgcatttaggaata
ctcctaaaacagatattgccaactagatacactgcctcaatcttgaccag
acaagagaacagttaagttctctcagttgggtagtaacttgaataccagt
ctatttatcagttggtttcaacacagaggtttcaagtggaaagccattca
tcttaaaagctgaacagaaaaatt
>NM_145341 3
cttttcctcctcagctccggctccgccgccacgattggccagccgaccac
ccggcctcggccaataagcgccgccctctcgcccccgtgttactgggtag
aagaaaacaaaaacaaacagagcgagaagggccagagactctccgaggcg
gcggcagagacagaagagcggggtcggggccggctgaccaggaacctggg
cgagcagcggcgggggcccgagggattctgaaggaagatttccattaggt
aatttgtttaatcagtgcaagcgaaattaagggaaaatggatgtagaaaa
tgagcagatactgaatgtaaaccctgcagggtattttccctaattctcca
tggtgcttcaatagcatgttattatcataaaaatgaacagttttgtggaa
tagatgaccaaatatcctgataacttaagtgactctctcttttccggtga
tgaagaaaatgctgggactgaggaaataaagaatgaaataaatggaaatt
ggatttcagcatcctccattaacgaagctagaattaatgccaaggcaaaa
aggcgactaaggaaaaactcatcccgggactctggcagaggcgattcggt
cagcgacagtgggagtgacgcccttagaagtggattaactgtgccaacca
gtccaaagggaaggttgctggataggcgatccagatctgggaaaggaagg
ggactaccaaagaaaggtggtgcaggaggcaaaggtgtctggggtacacc
tggacaggtgtatgatgtggaggaggtggatgtgaaagatcctaactatg

atgatgaccaggagaactgtgtttatgaaactgtagttttgcctttggat
gaaagggcatttgagaagactttaacaccaatcatacaggaatattttga
gcatggagatactaatgaagttgcggaaatgttaagagatttaaatcttg
gtgaaatgaaaagtggagtaccagtgttggcagtatccttagcattggag
gggaaggctagtcatagagagatgacatctaagcttctttctgacctttg
tgggacagtaatgagcacaactgatgtggaaaaatcatttgataaattgt
tgaaagatctacctgaattagcactggatactcctagagcaccacagttg
gtgggccagtttattgctagagctgttggagatggaattttatgtaatac
ctatattgatagttacaaaggaactgtagattgtgtgcaggctagagctg
ctctggataaggctaccgtgcttctgagtatgtctaaaggtggaaagcgt
aaagatagtgtgtggggctctggaggtgggcagcaatctgtcaatcacct
tgttaaagagattgatatgctgctgaaagaatatttactctctggagaca
tatctgaagctgaacattgccttaaggaactggaagtacctcattttcac
catgagcttgtatatgaagctattataatggttttagagtcaactggaga
aagtacatttaagatgattttggatttattaaagtccctttggaagtctt
ctaccattactgtagaccaaatgaaaagaggttatgagagaatttacaat
gaaattccggacattaatctggatgtcccacattcatactctgtgctgga
gcggtttgtagaagaatgttttcaggctggaataatttccaaacaactca
gagatctttgtccttcaaggggcagaaagcgttttgtaagcgaaggagat
ggaggtcgtcttaaaccagagagctactgaatataagaactcttgcagtc
ttagatgttataaaaatatatatctgaattgtaagagttgttagcacaag
ttttttttttttttttttttaagcacttgttttgggtacaaggcatttct
gacattttataaacctacatttaaggggaatttttaaaggaaatgttttt
tcttttttttttgtttttcgagggggcaaggagggacagaaaagtaacct
cttcttaagtggaatattctaataagctaccttttgtaagtgccatgttt
attatctaatcattccaagttttgcattgatgtctgactgccactccttt
ctttcaaggacagtgtttttttgtagtaaaatcactggtttatacaaagct
ttatttagggggtaaagttaagctgctaaaaccccatgttggctgctgct
gttgagatactgtgctttgggagtaaaaaaagaaagttatttctttgtct
taaagaatttttaaaaaattagtcatgagacttattcatctttccaggga
acatactgattggtcttaaaagactagacagttaagtaaaaggtggctgg
aacatctatttttctacaaaactggaaaaatgaacctggttctagaagaa
tgtacaccaaaataaaacatgtgaagcagtattgattctttattgggagt
acattttttaggtctcttaaactttaatttcacacagtaaattttgaat
ctcataaggaagcatatttgaacctagtcaatttaatcttagtgttccct
tgaaaacttttttccctacaaaattttaagtgaaaaatacaatagtaaa
ttaagattacactggggaaaaaaatgcaggtatcactttactccattgtt
atctgacctagagcttaattaagttttagaaatatgtaataccttccatc
attccatcatccttaaattctgttaccaaataatggctaatgttacaaaa
agttatactccagagacccaaagcttgacatttacctaatgtatgagaaa
atattaccaattaacaataaagaatgatcatatttttaacctcttttaca
tagcctaataactcagcaaggcctcaacgtctgtgctaatttaaactgcc
aaatattgactgcagcaaacaagaattatattcagaatttatgagggtac
tgttaggagtatactgcttacaggtttagatatagtctgttagaattaaa
accaagtttagtgttcatatttacctcatgggctttatcaagcccatatt
acctcagcttatatatagttaccatttttaggtttttaattgtttgacac
ttggatgataaatgcagtcattttattctcaagtgcttaaaattaatgta
attaaaagcttagctgactacagaataggtgagggtttcttaaaaatgag
atttaagggctgggcacggtggctcatgcctgtaatcccagcactttggg

aggccgaggtgggcggatcacttgaggttgggagttcatgaccagcttga
ccaacatgaagaaaccctgtctctattaaaaatacaaaagtagccaggca
tggtggcgcatgcgtgtaatcccagctacttgggaggctgaggcaggaga
attgcttgaacctgggaggcagaggttgcagtgagtcgagatggtgccat
tgctctcgtttgggcaacaagagtgaaactcttgtctcaaaaaaaaaaaa
aaatgaggtttaagacagttttgtcattactggtgggatctggtcacaca
agatagcattaaacgtgacatggcacataaaattggttaaaaaattttgt
tttttaattacgtaatgtaaaagcccaacaaacactttatgcaagattgg
aatgtatcttcaaattcagatttaataaacatgtaaagatcctctgtaaa
aaaaaaaaaaaaaaaaaaaaaaaaaaaa
>NM_182763 2
gcgcaaccctccggaagctgccgcccctttccccttttatgggaatactt
tttttaaaaaaaaagagttcgctggcgccaccccgtaggactggccgccc
taaaaccgtgataaaggagctgctcgccacttctcacttccgcttccttc
cagtaaggagtcggggtcttccccagttttctcagccaggcggcggcggc
gactggcaatgtttggcctcaaaagaaacgcggtaatcggactcaacctc
tactgtgggggggccggcttgggggccggcagcggcggcgccacccgccc
gggagggcgacttttggctacggagaaggaggcctcggcccggcgagaga
tagggggaggggaggccggcgcggtgattggcggaagcgccggcgcaagc
cccccgtccaccctcacgccagactcccggagggtcgcgcggccgccgcc
cattggcgccgaggtccccgacgtcaccgcgacccccgcgaggctgcttt
tcttcgcgcccacccgccgcgcggcgccgcttgaggagatggaagccccg
gccgctgacgccatcatgtcgcccgaagaggagctggacgggtacgagcc
ggagcctctcgggaagcggccggctgtcctgccgctgctggagttggtcg
gggaatctggtaataacaccagtacggacgggtcactaccctcgacgccg
ccgccagcagaggaggaggaggacgagttgtaccggcagtcgctggagat
tatctctcggtaccttcgggagcaggccaccggcgccaaggacacaaagc
caatgggcaggtctggggccaccagcaggaaggcgctggagaccttacga
cgggttggggatggcgtgcagcgcaaccacgagacggccttccaaggatg
ggtttgtggagttcttccatgtagaggacctagaaggtggcatcaggaat
gtgctgctggcttttgcaggtgttgctggagtaggagctggtttggcata
tctaataagatagccttactgtaagtgcaatagttgacttttaaccaacc
accaccaccaccaaaaccagtttatgcagttggactccaagctgtaactt
cctagagttgcaccctagcaacctagccagaaaagcaagtggcaagagga
ttatggctaacaagaataaatacatgggaagagtgctccccattgattga
agagtcactgtctgaaagaagcaaagttcagtttcagcaacaaacaaact
ttgtttgggaagctatggaggaggacttttagatttagtgaagatggtag
ggtggaaagacttaatttccttgttgagaacaggaaagtggccagtagcc
aggcaagtcatagaattgattacccgccgaattcattaatttactgtagt
gttaagagaagcactaagaatgccagtgacctgtgtaaaagttacaagta
atagaactatgactgtaagcctcagtactgtacaagggaagcttttcctc
tctctaattagctttcccagtatacttcttagaaagtccaagtgttcagg
acttttatacctgttatactttggcttggtttccatgattcttactttat
tagcctagtttatcaccaataatacttgacggaaggctcagtaattagtt
atgaatatggatatcctcaattcttaagacagcttgtaaatgtatttgta
aaaattgtatatatttttacagaaagtctatttctttgaaacgaaggaag
tatcgaatttacattagttttttcatacccttttgaactttgcaacttc
cgtaattaggaacctgtttcttacagcttttctatgctaaactttgttct
gttcagttctagagtgtatacagaacgaattgatgtgtaactgtatgcag

actggttgtagtggaacaaatctgataactatgcaggtttaaattttctt
atctgattttggtaagtattccttagataggtttttctttgaaaacctgg
gattgagaggttgatgaatggaaattctttcacttcattatatgcaagtt
ttcaataattaggtctaagtggagttttaaggttactgatgacttacaaa
taatgggctctgattgggcaatactcatttgagttccttccatttgacct
aatttaactggtgaaatttaaagtgaattcatgggctcatctttaaagct
tttactaaaagattttcagctgaatggaactcattagctgtgtgcatata
aaaagatcacatcaggtggatggagagacatttgatcccttgtttgctta
ataaattataaaatgatggcttggaaaagcaggctagtctaaccatggtg
ctattattaggcttgcttgttacacacacaggtctaagcctagtatgtca
ataaagcaaatacttactgttttgtttctattaatgattcccaaaccttg
ttgcaagtttttgcattggcatctttggatttcagtcttgatgtttgttc
tatcagacttaaccttttatttcctgtccttccttgaaattgctgattgt
tctgctccctctacagatatttatatcaattcctacagctttcccctgcc
atccctgaactctttctagcccttttagattttggcactgtgaaacccct
gctggaaacctgagtgaccctccctccccaccaagagtccacagaccttt
catctttcacgaacttgatcctgttagcaggtggtaataccatgggtgct
gtgacactaacagtcattgagaggtgggaggaagtcccttttccttggac
tggtatcttttcaactattgttttatcctgtctttgggggcaatgtgtca
aaagtcccctcaggaattttcagaggaaagaacattttatgaggctttct
ctaaagtttcctttgtataggagtatgctcacttaaatttacagaaagag
gtgagctgtgttaaacctcagagtttaaaagctactgataaactgaagaa
agtgtctatattggaactagggtcatttgaaagcttcagtctcggaacat
gacctttagtctgtggactccatttaaaaataggtatgaataagatgact
aagaatgtaatggggaagaactgccctgcctgcccatctcagagccataa
ggtcatctttgctagagctatttttacctatgtatttatcgttcttgatc
ataagccgcttatttatatcatgtatctctaaggacctaaaagcacttta
tgtagtttttaattaatcttaagatctggttacggtaactaaaaaagcct
gtctgccaaatccagtggaaacaagtgcatagatgtgaattggtttttag
gggccccacttcccaattcattaggtatgactgtggaaatacagacaagg
atcttagttgatattttgggcttggggcagtgagggcttaggacacccca
agtggtttgggaaaggaggaggggagtggtgggtttatagggggaggagg
aggcaggtggtctaagtgctgactggctacgtagttcgggcaaatcctcc
aaaagggaaagggaggatttgcttagaaggatggcgctcccagtgactac
tttttgacttctgtttgtcttacgcttctctcagggaaaaacatgcagtc
ctctagtgtttcatgtacattctgtggggggtgaacaccttggttctggt
taaacagctgtacttttgatagctgtgccaggaagggttaggaccaacta
caaattaatgttggttgtcaaatgtagtgtgtttccctaactttctgttt
ttcctgagaaaaaaaaataaatcttttattcaaatacagggaaaaaaaaa
aaaaaaaaa
>NM_014999 2
cgccattagagggaggcagagagggatcgttcttcgcttttcctccggtg
cctgacgtggtgggctggggcccttcattctcggactttccctcagccct
tccaggcctcgcccgaggagggcgtggggacagcgcccgggtcggcgggg
ccggggcggtgggggctgagccggccgtggctgtgaaggcgctgccgcgg
ctgtcgggagggcggcgcgacactcgggctcgggcggccgggaagcgacg
ggatggctgcggccggcggcggcggcggcggggcggcggcggcgggccga
gcctactcgttcaaggtggtgctgctgggggaaggctgcgtggggaagac
gtcgctggtgctgcgctactgcgagaacaagtttaacgacaagcacatca

ccactctgcaggcatcattcttaacaaagaagttaaatattggtgggaaa
agagtaaaccttgccatatgggatacggcaggtcaagagagattccatgc
attgggtccaatttactacagagattcaaatggagcgattttagtttatg
acataacagatgaagattcttttcagaaggtaaaaaactgggtcaaagaa
ttacggaaaatgttgggaaatgaaatctgtttatgtatagttggtaataa
aatagacttggaaaaggagagacatgtttccattcaagaagcagagtcgt
atgcagaatctgtgggagcaaaacattatcatacttcagccaaacagaac
aaaggaattgaggaactctttcttgacctttgtaaaaggatgatagaaac
agcacaagtggatgagagagcaaaaggcaatggctctagtcagccgggaa
ctgcaaggcgaggtgtacagattattgatgatgaacctcaagcccagacc
agtggtggagggtgctgttcttctggataactgttcacgcctaagaaatt
aaaagacagaacaaaactgtggatcattgccctcaacatgaagactgcca
tattccaagtcacattattttaccaatggaattatagaattaacagtatt
ttaaattacgtttataacactgcagagaccttaagtgctaaacttagtgg
agtttgtgaccagagaattggcattttctacaaatgtttttttttgtttt
ttttttgtttttttttgttttttttgagacggagtctcgctctgtcacc
caggctggagtgcactggcttgatctcggctcactgcaagctccacctcc
caggttcacgccattctcctgcctcagcctcccgagtagctgggactata
ggcgcccaccacgacgcccggctaatttttgtatttttagtagagacgg
ggtttcaccaggttagccaggatggtctcgatctcctgaccttgtgatcc
gcccgcctcggcctcccaaagtgctgggattacaggcgtgagccaccgtg
cccggcctacaaatgttaacaaagcaattaccaatggccttttttacatat
tttttctttaatgaggaataatatgcatgtagaaaagacctacttaaagt
cttcatttatattctttcaaatcaaatctttatttaataacttatatatg
ttgttggaatggttacatttttgcagccctttgtattttgtggtagttga
aattgtatcacttctaaacagcaaactgttttgtttttaattagcagat
atctgaagtactatttttgcagggtttgcacaggcccgtaactgtctact
actttgatataatctgtatacatcctgtatgctgagctggtaaaatacat
tgtaaattacatattaaatattttatctgcttttacaagcgcaaggtgca
aaaatatatacaatagtctcattgatgactgtaaagtgaattaacatttg
gtgattatgcctagctttttttgactaatataaagatcatagctccccttc
acttctgtcttaacttgaacatggcgtgtttaaattttcatacatacttt
acttgaattattgctgttgtcacatatttttgcctctgtgagttcatctg
atgattgagcagtagcatttgccttttgggtttttttgtttgttattatag
aagagatgacttctgctgattttgctttagaatggttaccttagaagaat
ttgggtggctcatgttgaatttcacttctgcaatagctttcattttctca
taggctttataagagatgggttcagtggtatgagcagaggaagagatccc
agatagtagccagttaaccaagactcattcatatagcacgtagtttatgt
tcctgaggcagcacttttagatcctttgtgagcaagttctatttgttcat
tgcttgccagagatgaacacagaatgttctgtttcattttacaagaacta
tcctgagtttctgtggatggaaacattacatgtaatgcagatatagtgaa
cactggaaagatttattaaagaattatatttgtgtatactttataaatta
gtccctcattagatttttttttcttaagcataagactgaacttaaatgtg
ttaattttagtagaatcaggcactgctcgcagaaggaacacagattgtag
agattaacataaattgttcttgttcta
>NM_000657 2
tttctgtgaagcagaagtctgggaatcgatctggaaatcctcctaatttt
tactccctctccccgcgactcctgattcattgggaagtttcaaatcagct
ataactggagagtgctgaagattgatgggatcgttgccttatgcatttgt

tttggttttacaaaaaggaaacttgacagaggatcatgctgtacttaaaa
aatacaacatcacagaggaagtagactgatattaacaatacttactaata
ataacgtgcctcatgaaataaagatccgaaaggaattggaataaaaattt
cctgcatctcatgccaaggggaaacaccagaatcaagtgttccgcgtga
ttgaagacaccccctcgtccaagaatgcaaagcacatccaataaaatagc
tggattataactcctcttctttctctgggggccgtggggtgggagctggg
gcgagaggtgccgttggcccccgttgcttttcctctgggaaggatggcgc
acgctgggagaacagggtacgataaccgggagatagtgatgaagtacatc
cattataagctgtcgcagaggggctacgagtgggatgcgggagatgtggg
cgccgcgcccccgggggccgcccccgcaccgggcatcttctcctcccagc
ccgggcacacgccccatccagccgcatcccgggacccggtcgccaggacc
tcgccgctgcagaccccggctgcccccggcgccgccgcggggcctgcgct
cagcccggtgccacctgtggtccacctgaccctccgccaggccggcgacg
acttctcccgccgctaccgccgcgacttcgccgagatgtccagccagctg
cacctgacgcccttcaccgcgcggggacgctttgccacggtggtggagga
gctcttcagggacggggtgaactgggggaggattgtggccttctttgagt
tcggtggggtcatgtgtgtggagagcgtcaaccgggagatgtcgcccctg
gtggacaacatcgccctgtggatgactgagtacctgaaccggcacctgca
cacctggatccaggataacggaggctgggtaggtgcacttggtgatgtga
gtctgggctgaggccacaggtccgagatgcgggggttggagtgcgggtgg
gctcctggggcaatgggaggctgtggagccggcgaaataaaatcagagtt
gttgcta
>NM_016140 2
ggcggagccgcctgggctgcagtcccacccgggagccggcagggagcgga
gctgcggagccgcctggtctcccgcgtccatcggtccattcctgcgtcgt
tctgtccttccgaacgcacacttcaggagcagccgcgaggagtgagcact
gcacgcaggatccgcccagtgcttgggccaagcaccctgtggcatccaag
ctcccctggacgttacccttggtgaaccaaggtggcatggcagcgagcac
agacatggctgggctggaggagagcttccgcaagtttgccatccatggtg
accccaaggccagtgggcaagagatgaatggcaagaactgggccaagctg
tgcaaggactgcaaggtggctgacggaaagtccgtgacagggaccgatgt
ggacatcgtcttctccaaagtcaaggggaagtctgctcgggtcatcaact
atgaggagttcaagaaggccctggaagagctggcgaccaagagattcaag
gggaagagcaaggaggaggccttcgatgccatctgccagctggtggcagg
caaagagccagccaatgtgggcgtcactaaagcaaaaacagggggtgctg
tagaccggctgacggacaccagcagatacacgggctcccacaaggagcgc
ttcgatgagagcggcaagggcaagggcattgcgggacggcaggacatcct
ggacgacagtggctacgtgagcgcctacaagaatgcaggcacctacgatg
ccaaggtgaagaagtgaggcttgggaagaccgccctgccaagtgcggctg
cccctgccagaggctcaggcctgggtctaaggggcacgtggagcaagaga
tcctggtcccctccctgctggacctgccacccagagcttcctgcctagtc
ccactgggctggcccaccaggcctctgacccaggctgctctgcggcccct
tcctcctcctcttcctgctccaacttctgtccacctggggacagtctgtg
cctgtagcctcatgaccccaacccagccccaggcatggctaacccctgac
tgcttgcctcatatttaagctgctgctctggccaagtgcctaattttaac
ccagacctcaataaagacaccttttgtaccaaaaaaaaaaaaaaaa
>NM_001754 4
ctttgggcctcataaacaaccacagaaccacaagttgggtagcctggcag
tgtcagaagtctgaacccagcatagtggtcagcaggcaggacgaatcaca

ctgaatgcaaaccacagggtttcgcagcgtggtaaaagaaatcattgagt
cccccgccttcagaagagggtgcattttcaggaggaagcgatggcttcag
acagcatatttgagtcatttccttcgtacccacagtgcttcatgagagaa
tgcatacttggaatgaatccttctagagacgtccacgatgccagcacgag
ccgccgcttcacgccgccttccaccgcgctgagcccaggcaagatgagcg
aggcgttgccgctgggcgccccggacgccggcgctgccctggccggcaag
ctgaggagcggcgaccgcagcatggtggaggtgctggccgaccacccggg
cgagctggtgcgcaccgacagccccaacttcctctgctccgtgctgccta
cgcactggcgctgcaacaagaccctgcccatcgctttcaaggtggtggcc
ctaggggatgttccagatggcactctggtcactgtgatggctggcaatga
tgaaaactactcggctgagctgagaaatgctaccgcagccatgaagaacc
aggttgcaagatttaatgacctcaggtttgtcggtcgaagtggaagaggg
aaaagcttcactctgaccatcactgtcttcacaaacccaccgcaagtcgc
cacctaccacagagccatcaaaatcacagtggatgggccccgagaacctc
gaagacatcggcagaaactagatgatcagaccaagcccgggagcttgtcc
ttttccgagcggctcagtgaactggagcagctgcggcgcacagccatgag
ggtcagcccacaccacccagcccccacgcccaaccctcgtgcctccctga
accactccactgcctttaaccctcagcctcagagtcagatgcaggataca
aggcagatccaaccatccccaccgtggtcctacgatcagtcctaccaata
cctgggatccattgcctctccttctgtgcacccagcaacgcccatttcac
ctggacgtgccagcggcatgacaaccctctctgcagaactttccagtcga
ctctcaacggcacccgacctgacagcgttcagcgacccgcgccagttccc
cgcgctgccctccatctccgacccccgcatgcactatccaggcgccttca
cctactccccgacgccggtcacctcgggcatcggcatcggcatgtcggcc
atgggctcggccacgcgctaccacacctacctgccgccgccctaccccgg
ctcgtcgcaagcgcagggaggcccgttccaagccagctcgccctcctacc
acctgtactacggcgcctcggccggctcctaccagttctccatggtgggc
ggcgagcgctcgccgccgcgcatcctgccgccctgcaccaacgcctccac
cggctccgcgctgctcaaccccagcctcccgaaccagagcgacgtggtgg
aggccgagggcagccacagcaactcccccaccaacatggcgccctccgcg
cgcctggaggaggccgtgtggaggccctactgaggcgccaggcctggccc
ggctgggccccgcgggccgccgccttcgcctccgggcgcgcgggcctcct
gttcgcgacaagcccgccgggatcccgggccctgggcccggccaccgtcc
tggggccgagggcgcccgacggccaggatctcgctgtaggtcaggcccgc
gcagcctcctgcgcccagaagcccacgccgccgccgtctgctggcgcccc
ggccctcgcggaggtgtccgaggcgacgcacctcgagggtgtccgccggc
cccagcacccaggggacgcgctggaaagcaaacaggaagattcccggagg
gaaactgtgaatgcttctgatttagcaatgctgtgaataaaaagaaagat
tttatacccttgacttaactttttaaccaagttgtttattccaaagagtg
tggaattttggttggggtgggggggagaggagggatgcaactcgccctgtt
tggcatctaattcttatttttaatttttccgcaccttatcaattgcaaaa
tgcgtatttgcatttgggtggtttttatttttatatacgtttatataaat
atatataaattgagcttgcttctttcttgctttgaccatggaaagaaata
tgattccctttttctttaagttttatttaacttttcttttggacttttggg
tagttgtttttttttgttttgttttgtttttttgagaaacagctacagct
ttgggtcatttttaactactgtattcccacaaggaatccccagatattta
tgtatcttgatgttcagacatttatgtgttgataatttttttaattattta
aatgtacttatattaagaaaaatatcaagtactacattttcttttgttct
tgatagtagccaaagttaaatgtatcacattgaagaaggctagaaaaaaa

gaatgagtaatgtgatcgcttggttatccagaagtattgtttacattaaa
ctccctttcatgttaatcaaacaagtgagtagctcacgcagcaacgtttt
taataggatttttagacactgagggtcactccaaggatcagaagtatgga
attttctgccaggctcaacaagggtctcatatctaacttcctccttaaaa
cagagaaggtcaatctagttccagagggttgaggcaggtgccaataatta
catctttggagaggatttgatttctgcccagggatttgctcaccccaagg
tcatctgataatttcacagatgctgtgtaacagaacacagccaaagtaaa
ctgtgtaggggagccacatttacataggaaccaaatcaatgaatttaggg
gttacgattatagcaatttaagggcccaccagaagcaggcctcgaggagt
caatttgcctctgtgtgcctcagtggagacaagtgggaaaacatggtccc
acctgtgcgagacccccctgtcctgtgctgctcactcaacaacatctttgt
gttgctttcaccaggctgagaccctaccctatggggtatatgggcttttta
cctgtgcaccagtgtgacaggaaagattcatgtcactactgtccgtggct
acaattcaaaggtatccaatgtcgctgtaaattttatggcactattttta
ttggaggatttggtcagaatgcagttgttgtacaactcataaatactaac
tgctgattttgacacatgtgtgctccaaatgatctggtggttatttaacg
tacctcttaaaattcgttgaaacgatttcaggtcaactctgaagagtatt
tgaaagcaggacttcagaacagtgtttgatttttattttataaatttaag
cattcaaattaggcaaatctttggctgcaggcagcaaaaacagctggact
tatttaaaacaacttgtttttgagttttcttatatatatattgattattt
gttttacacacatgcagtagcactttggtaagagttaaagagtaaagcag
cttatgttgtcaggtcgttcttatctagagaagagctatagcagatctcg
gacaaactcagaatatattcactttcatttttgacaggattccctccaca
actcagtttcatatattattccgtattacatttttgcagctaaattacca
taaaatgtcagcaaatgtaaaaatttaatttctgaaaagcaccattagcc
catttcccccaaattaaacgtaaatgtttttttttcagcacatgttaccat
gtctgacctgcaaaaatgctggagaaaaatgaaggaaaaaattatgtttt
tcagtttaattctgttaactgaagatattccaactcaaaaccagcctcat
gctctgattagataatcttttacattgaacctttactctcaaagccatgt
gtggagggggcttgtcactattgtaggctcactggattggtcatttagag
tttcacagactcttaccagcatatatagtatttaattgtttcaaaaaaaa
tcaaactgtagttgttttggcgataggtctcacgcaacacatttttgtat
gtgtgtgtgtgcgtgtgtgtgtgtgtgtgaaaaattgcattcattg
acttcaggtagattaaggtatctttttattcattgccctcaggaaagtta
aggtatcaatgagacccttaagccaatcatgtaataactgcatgtgtctg
gtccaggagaagtattgaataagccatttctactgcttactcatgtccct
atttatgatttcaacatggatacatatttcagttctttctttttctcact
atctgaaaatacatttccctccctctcttccccccaatatctccctttttt
ttctctcttcctctatcttccaaaccccactttctccctcctccttttcc
tgtgttctcttaagcagatagcacatacccccacccagtaccaaatttca
gaacacaagaaggtccagttcttccccccttcacataaaggaacatggttt
gtcagcctttctcctgtttatgggtttcttccagcagaacagagacattg
ccaaccatattggatctgcttgctgtccaaaccagcaaactttcctgggc
aaatcacaatcagtgagtaaatagacagcctttctgctgccttgggtttc
tgtgcagataaacagaaatgctctgattagaaaggaaatgaatggttcca
ctcaaatgtcctgcaatttaggattgcagatttctgccttgaaatacctg
tttctttgggacattccgtcctgatgattttatttttgttggtttttat
ttttggggggaatgacatgtttgggtcttttatacatgaaaatttgtttg
acaataatctcacaaaacatatttacatctgaacaaaatgcctttttgt

ttaccgtagcgtatacatttgttttgggatttttgtgtgtttgttgggaa
ttttgtttttagccaggtcagtattgatgaggctgatcatttggctcttt
ttttccttccagaagagttgcatcaacaaagttaattgtatttatgtatg
taaatagattttaagcttcattataaaatattgttaatgcctataacttt
ttttcaattttttgtgtgtgtttctaaggactttttcttaggtttgcta
aatactgtagggaaaaaaatgcttctttctactttgtttattttagactt
taaaatgagctacttcttattcacttttgtaaacagctaatagcatggtt
ccaattttttttaagttcacttttttgttctaggggaaatgaatgtgca
aaaaaagaaaaagaactgttggttatttgtgttattctggatgtataaaa
atcaatggaaaaaaataaactttcaaattgaaatgacggtataacacatc
tactgaaaaagcaacgggaaatgtggtcctatttaagccagcccccacct
agggtctatttgtgtggcagttattgggtttggtcacaaaacatcctgaa
aattcgtgcgtgggcttctttctccctggtacaaacgtatggaatgcttc
ttaaaggggaactgtcaagctggtgtcttcagccagatgacatgagagaa
tatcccagaaccctctctccaaggtgtttctagatagcacaggagagcag
gcactgcactgtccacagtccacggtacacagtcgggtgggccgcctccc
ctctcctgggagcattcgtcgtgcccagcctgagcagggcagctggactg
ctgctgttcaggagccaccagagccttcctctctttgtaccacagtttct
tctgtaaatccagtgttacaatcagtgtgaatggcaaataaacagtttga
caagtacatacaccata
>NM_181659 2
atatcccagtggcccccgtgcggcgactttagctgctgctgtctcagccg
ctccacagcgacggcggcggctgcggcttagtcggtggcggccggcggcg
gctgcgggctgagcggcgagtttccgatttaaagctgagctgcgaggaaa
atggcggcgggaggatcaaaatacttgctggatggtggactcagagacca
ataaaaataaactgcttgaacatcctttgactggttagccagttgctgat
gtatattcaagatgagtggattaggagaaaacttggatccactggccagt
gattcacgaaaacgcaaattgccatgtgatactccaggacaaggtcttac
ctgcagtggtgaaaaacggagacgggagcaggaaagtaaatatattgaag
aattggctgagctgatatctgccaatcttagtgatattgacaatttcaat
gtcaaaccagataaatgtgcgattttaaaggaaacagtaagacagatacg
tcaaataaaagagcaaggaaaaactatttccaatgatgatgatgttcaaa
aagccgatgtatcttctacagggcagggagttattgataaagactcctta
ggaccgcttttacttcaggcattggatggtttcctatttgtggtgaatcg
agacggaaacattgtatttgtatcagaaaatgtcacacaatacctgcaat
ataagcaagaggacctggttaacacaagtgtttacaatatcttacatgaa
gaagacagaaaggattttcttaagaatttaccaaaatctacagttaatgg
agtttcctggacaaatgagacccaaagacaaaaaagccatacatttaatt
gccgtatgttgatgaaaacaccacatgatattctggaagacataaacgcc
agtcctgaaatgcgccagagatatgaaacaatgcagtgctttgccctgtc
tcagccacgagctatgatggaggaagggggaagatttgcaatcttgtatga
tctgtgtggcacgccgcattactacaggagaaagaacatttccatcaaac
cctgagagctttattaccagacatgatctttcaggaaaggttgtcaatat
agatacaaattcactgagatcctccatgaggcctggctttgaagatataa
tccgaaggtgtattcagagattttttagtctaaatgatgggcagtcatgg
tcccagaaacgtcactatcaagaagcttatcttaatggccatgcagaaac
cccagtatatcgattctcgttggctgatggaactatagtgactgcacaga
caaaaagcaaactcttccgaaatcctgtaacaaatgatcgacatggcttt
gtctcaacccacttccttcagagagaacagaatggatatagaccaaaccc

aaatcctgttggacaagggattagaccacctatggctggatgcaacagtt
cggtaggcggcatgagtatgtcgccaaaccaaggcttacagatgccgagc
agcagggcctatggcttggcagaccctagcaccacagggcagatgagtgg
agctaggtatgggggttccagtaacatagcttcattgacccctgggccag
gcatgcaatcaccatcttcctaccagaacaacaactatgggctcaacatg
agtagccccccacatgggagtcctggtcttgccccaaaccagcagaatat
catgatttctcctcgtaatcgtgggagtccaaagatagcctcacatcagt
tttctcctgttgcaggtgtgcactctcccatggcatcttctggcaatact
gggaaccacagcttttccagcagctctctcagtgccctgcaagccatcag
tgaaggtgtggggacttccctttatctactctgtcatcaccaggcccca
aattggataactctcccaatatgaatattacccaaccaagtaaagtaagc
aatcaggattccaagagtcctctgggcttttattgcgaccaaaatccagt
ggagagttcaatgtgtcagtcaaatagcagagatcacctcagtgacaaag
aaagtaaggagagcagtgttgagggggcagagaatcaaaggggtcctttg
gaaagcaaaggtcataaaaaattactgcagttacttacctgttcttctga
tgaccggggtcattcctccttgaccaactccccctagattcaagttgta
aagaatcttctgttagtgtcaccagcccctctggagtctcctcctctaca
tctggaggagtatcctctacatccaatatgcatgggtcactgttacaaga
gaagcaccggattttgcacaagttgctgcagaatgggaattcaccagctg
aggtagccaagattactgcagaagccactgggaaagacaccagcagtata
acttcttgtggggacggaaatgttgtcaagcaggagcagctaagtcctaa
gaagaaggagaataatgcacttcttagatacctgctggacagggatgatc
ctagtgatgcactctctaaagaactacagccccaagtggaaggagtggat
aataaaatgagtcagtgcaccagctccaccattcctagctcaagtcaaga
gaaagaccctaaaattaagacagagacaagtgaagagggatctggagact
tggataatctagatgctattcttggtgatctgactagttctgacttttac
aataattccatatcctcaaatggtagtcatctggggactaagcaacaggt
gtttcaaggaactaattctctgggtttgaaaagttcacagtctgtgcagt
ctattcgtcctccatataaccgagcagtgtctctggatagccctgtttct
gttggctcaagtcctccagtaaaaaatatcagtgctttccccatgttacc
aaagcaacccatgttgggtgggaatccaagaatgatggatagtcaggaaa
attatggctcaagtatgggtgggccaaaccgaaatgtgactgtgactcag
actccttcctcaggagactggggcttaccaaactcaaaggccggcagaat
ggaacctatgaattcaaactccatgggaagaccaggaggagattataata
cttctttacccagacctgcactgggtggctctattcccacattgcctctt
cggtctaatagcataccaggtgcgagaccagtattgcaacagcagcagca
gatgcttcaaatgaggcctggtgaaatccccatgggaatgggggctaatc
cctatggccaagcagcagcatctaaccaactgggttcctggcccgatggc
atgttgtccatggaacaagtttctcatggcactcaaaataggcctcttct
taggaattccctggatgatcttgttgggccaccttccaacctggaaggcc
agagtgacgaaagagcattattggaccagctgcacactcttctcagcaac
acagatgccacaggcctggaagaaattgacagagctttgggcattcctga
acttgtcaatcagggacaggcattagagcccaaacaggatgctttccaag
gccaagaagcagcagtaatgatggatcagaaggcaggattatatggacag
acatacccagcacaggggcctccaatgcaaggaggctttcatcttcaggg
acaatcaccatcttttaactctatgatgaatcagatgaaccagcaaggca
attttcctctccaaggaatgcacccacgagccaacatcatgagaccccgg
acaaacacccccaagcaacttagaatgcagcttcagcagaggctgcaggg
ccagcagtttttgaatcagagccgacaggcacttgaattgaaaatggaaa

accctactgctggtggtgctgcggtgatgaggcctatgatgcagccccag
gtgagctcccagcagggttttcttaatgctcaaatggtcgcccaacgcag
cagagagctgctaagtcatcacttccgacaacagagggtggctatgatga
tgcagcagcagcagcagcagcaacagcagcagcagcagcagcagcagcag
caacagcaacagcaacagcaacagcagcaacagcagcaaacccaggcctt
cagcccacctcctaatgtgactgcttcccccagcatggatgggcttttgg
caggacccacaatgccacaagctcctccgcaacagtttccatatcaacca
aattatggaatgggacaacaaccagatccagcctttggtcgagtgtctag
tcctcccaatgcaatgatgtcgtcaagaatgggtccctcccagaatccca
tgatgcaacacccgcaggctgcatccatctatcagtcctcagaaatgaag
ggctggccatcaggaaatttggccaggaacagctccttttcccagcagca
gtttgcccaccaggggaatcctgcagtgtatagtatggtgcacatgaatg
gcagcagtggtcacatgggacagatgaacatgaaccccatgcccatgtct
ggcatgcctatgggtcctgatcagaaatactgctgacatctctgcaccag
gacctcttaaggaaaccactgtacaaatgacactgcactaggattattgg
gaaggaatcattgttccaggcatccatcttggaagaaaggaccagctttg
agctccatcaagggtattttaagtgatgtcatttgagcaggactggattt
taagccgaagggcaatatctacgtgtttttccccctccttctgctgtgt
atcatggtgttcaaaacagaaatgtttttggcattccacctcctaggga
tataattctggagacatggagtgttactgatcataaaacttttgtgtcac
ttttttctgccttgctagccaaaatctcttaaatacacgtaggtgggcca
gagaacattggaagaatcaagagagattagaatatctggtttctctagtt
gcagtattggacaaagagcatagtcccagccttcaggtgtagtagttctg
tgttgaccctttgtccagtggaattggtgattctgaattgtcctttacta
atggtgttgagttgctctgtccctattatttgccctaggctttctcctaa
tgaaggttttcatttgccattcatgtcctgtaatacttcacctccaggaa
ctgtcatggatgtccaaatggctttgcagaaaggaaatgagatgacagta
tttaatcgcagcagtagcaaacttttcacatgctaatgtgcagctgagtg
cactttatttaaaaagaatggataaatgcaatattcttgaggtcttgagg
gaatagtgaaacacattcctggtttttgcctacacttacgtgttagacaa
gaactatgatttttttttttaaagtactggtgtcaccctttgcctatatg
gtagagcaataatgctttttaaaaataaacttctgaaaacccaaggccag
gtactgcattctgaatcagaatctcgcagtgtttctgtgaatagattttt
ttgtaaatatgacctttaagatattgtattatgtaaaatatgtatatacc
tttttttgtaggtcacaacaactcatttttacagagtttgtgaagctaaa
tatttaacattgttgatttcagtaagctgtgtggtgaggctaccagtgga
agagacatcccttgacttttgtggcctgggggagggggtagtgctccacag
cttttccttccccacccccccagccttagatgcctcgctcttttcaatctc
ttaatctaaatgctttttaaagagattatttgtttagatgtaggcatttt
aatttttttaaaaattcctctaccagaactaagcactttgttaatttgggg
ggaaagaatagatatggggaaataaacttaaaaaaaaatcaggaatttaa
aaaaacgagcaatttgaagagaatcttttggattttaagcagtccgaaat
aatagcaattcatgggctgtgtgtgtgtgtgtatgtgtgtgtgtgtgt
gtatgtttaattatgttaccttttcatcccctttaggagcgttttcagat
tttggttgctaagacctgaatcccatattgagatctcgagtagaatcctt
ggtgtggtttctggtgtctgctcagctgtcccctcattctactaatgtga
tgctttcattatgtccctgtggattagaatagtgtcagttatttcttaag
taactcagtacccagaacagccagttttactgtgattcagagccacagtc
taactgagcacctttttaaacccctccctcttctgccccctaccacttttc

tgctgttgcctctctttgacacctgttttagtcagttgggaggaagggaa
aaatcaagtttaattccctttatctgggttaattcatttggttcaaatag
ttgacggaattgggtttctgaatgtctgtgaatttcagaggtctctgcta
gccttggtatcattttctagcaataactgagagccagttaattttaagaa
tttcacacatttagccaatctttctagatgtctctgaaggtaagatcatt
taatatctttgatatgcttacgagtaagtgaatcctgattatttccagac
ccaccaccagagtggatcttattttcaaagcagtatagacaattatgagt
ttgccctctttcccctaccaagttcaaaatatatctaagaaagattgtaa
atccgaaaacttccattgtagtggcctgtgcttttcagatagtatactct
cctgtttggagacagaggaagaaccaggtcagtctgtctctttttcagct
caattgtatctgacccttctttaagttatgtgtgtggggagaaatagaat
ggtgctcttatctttcttgactttaaaaaaattattaaaaacaaaaaaaa
aataaattttttgcaatcctttcctcagacctggctccaggctaactgg
aaggcagcactcccttttttatatagtagaaaaatgaagtttattataag
tttttatattttctacttgttcatttggtgcaaactcaagatttctttta
ataggtgcagtctttgagataatttgtttttacctgtattgccctttatc
ttttttaggtaattctttgtactcctgctgtctacctctcctcacacccc
agcacccccccatttttcaaaccttggtatctgttgggtgaacagtataa
tcttttcatctgcttttagaatgtgggatatttccagtacctactttttt
ttttttttttgctgaatccaaagatatataaataaaatatatatatttt
ataaagatcagaatgatataaaggagatacatgtttcttcctttaaaaaa
taaacggaagttacattgttaatgttcatattatgatgccacttttctaa
actgcatctggattgaaaggtgtaaatatcaataacagtgctacttagtt
atcagtatttaatatctgaggtgagttgggggtatctatattaggggtag
ggtattacagaagataattggcttgatgtcctagaagttctttgatccag
aggtgggtgcagctgaaagtaaacagaatggattgccagttacatgtatg
cctgcccagttcccttttttatttgcagaagctgtgagttttgttcacaat
taggttcctaggagcaaaacctcaaggattgatttattgttttcaactcc
aaggcacactgttaataaacgagcagggtgttttctctcttcctttctaa
tatatggagtttcgaagaataaaatatgagagcaatatttaaattctcag
gaattgacttatactcttgagaatgaattcagtttcaatcaagtttacat
tatgttgcttaaaaaaatagaaattattctttatcttgcaaagaattgaa
accacatgaaatgacttatgggggatggtgagctgtgactgctttgctga
ccattttggatgtcattgtaaataaaggtttctatttaaaattgga
>NM_005523 5
cttcaaagaggcagctgcagtggagaatcatgttaagctcggctactgcg
gagagcccaaggtagcccaataatggattttgatgagcgtggtccctgct
cctctaacatgtatttgccaagttgtacttactacgtctcgggtccagat
ttctccagcctcccttcttttctgccccagaccccgtcttcgcgcccaat
gacatactcctactcctccaacctgccccaggtccaacccgtgcgcgaag
tgaccttcagagagtacgccattgagcccgccactaaatggcacccccgc
ggcaatctggcccactgctactccgcggaggagctcgtgcacagagactg
cctgcaggcgcccagcgcggccggcgtgcctggcgacgtgctggccaaga
gctcggccaacgtctaccaccaccccacccccgcagtctcgtccaatttc
tatagcaccgtgggcaggaacggcgtcctgccacaggctttcgaccagtt
tttcgagacagcctacggcaccccggaaaacctcgcctcctccgactacc
ccggggacaagagcgccgagaagggcccccggcggccacggcgacctcc
gcggcggcggcggcggctgcaacgggcgcgccggcaacttcaagttcgga
cagcggcggcggcggcggctgccgggagacggcggcggcagcagaggaga

aagagcggcggcggcgccccgagagcagcagcagccccgagtcgtcttcc
ggccacactgaggacaaggccggcggctccagtggccaacgcacccgcaa
aaagcgctgcccctataccaagtaccagatccgagagctggaacgggagt
tcttcttcagcgtctacattaacaaagagaagcgcctgcaactgtcccgc
atgctcaacctcactgatcgtcaagtcaaaatctggtttcagaacaggag
aatgaaggaaaaaaaaattaacagagaccgtttacagtactactcagcaa
atccactcctctaagactccagcggctggaattgggtgggggcttcata
cacatgagataatatgcagattttgcccttgacaaagtcaagccacatgg
tgacttttgaaaagaggtgtgcaagagagggatgcatggagatagcccca
caggaggtggtctgggactctcttgattaagatctcagtggttaagattc
ctaataatcattggattctgagagctgtgcatcagctagaatgacaggtt
tgggacccctggtggttcactcttggagcctgcagagctgcgggctgggt
gtggtctccactggggattgggcccctgccagacccccctggagactaacc
ccaccacaccctccctctactgggagcctacccacccccaggacccctga
gtaaaaaagctgtgtgctctccaagcccagttcagcttggggacaggggc
aggaggaaggggtaggattactaggtgcccagaatgaggctgctttccaa
agccaatgtgaacagcggctggacttggaggtagctttgaggtggaagag
ggctgcaaatccttgtgggaaaagaaatctatgattccaggtggcatcag
tgtctttccactcctcctagccacccaccacactgatccagccctgagtt
cctagccaccgcctcctacagcccacctggcttttctttctaccaaatga
gggtcttggttccagcctgccactcaggcccaaagcctcgacacagagtg
gactgttccctgaggtgggagatgtggaaaagccaagaggctgcagccag
gccactggcccctgagatctctgcaggaaatggctgtggagtgtggcagt
ttggcaaactctccaccacacgtaatgaaacttggatttgctcagtgtct
ggctgcagagcagtgggcctggccagcaggtccccagctttggctatgag
ggccttgagtcccccaaaacaccgggttccagcaccacactcagccctca
ttggctcttgaactgagcttggaagcttctggtgaccttccaagagcctg
agagtgaggtggaattattttaaaagataaatattatattatatatatat
atatttccctgaaggaaccaaagcgaattttaaaagatgcaatgtagagg
ggaaaagagatgatgaaaatatttaaaggccctatctgtttacagtgttc
cgtggttaaactcgctcactgctaagaatatttgaatgtatgcttcatac
agggatggtgttcaaaaaacttgtaaataaaggaaccataatcaattttc
ttttctttctttctctttttctttttcttttgccattagttgatttcct
ttagggtgttggagggggtggaaaaggtattgagaatggtctttttaatc
tcttgcaacatttggaaagagttagggaaatgctcagaggcagtcggcct
ggccggcctggggatctcatctgggaaagccaggcaccctcccattgaat
ctcctttgcctccctgtgttaagaaatgtctgttggctccatttgtactg
ggagtgttggcctgtcctcaattctggttcttacccaccgtgtgtgttgc
agcacttatacaggcaactgggcacaaggaaaataaagacggtggaaatt
tga
>NM_003385 4
aggcggcttttggtcacaggctcccgagttctcctagctggggctgcgga
gctgggggagggaagagaggaaaggggagggggtgcctggagaggcgga
ggctcgcgcgcctgcgcatccagctccagggaccctaggttttctatggg
attcccaatctgcagcagagatttacccgagcgtgttgcggcagcggctg
ggcttgcaaggcgcgatccaagagggatttaagcagcccagagctccaga
gaaaaagagagcgagagagaaccacacacagagacggcttaagcgtttac
ccgaattaaatatatatttttaaaaagaactgttgagttttatcattttc
gttaagtgaccgtgcgcagcgctgtaactgcaggatggggaagcagaata

gcaaactggcccctgaagtgatggaggacctggtgaagagcacagagttt
aatgagcatgaactcaagcagtggtacaaaggatttctcaaggactgtcc
aagtgggaggctaaatctcgaggaatttcagcagctctatgtgaagttct
ttccttatggagacgcctccaagtttgcccagcatgccttccgaaccttc
gacaagaatggggacggcaccattgacttccgagagttcatctgcgctct
gtccatcacctccaggggcagctttgagcagaagctgaactgggccttca
atatgtatgacctggatggtgatggcaagatcacccgagtggagatgctg
gagatcatcgaggctatctacaaaatggtaggcactgtgatcatgatgaa
aatgaatgaggatggcctgacgcctgagcagcgagtagacaagattttca
gcaagatggataagaacaaagatgaccagattacactggatgaattcaaa
gaagctgcaaagagcgacccttccattgtattacttctgcagtgcgacat
ccagaaatgagctgatgtcaatgctatggactgcacaaaagtctcaatgt
tccattcagtctgcagctattcacacacacacacacacacacacacacac
acacacacacacacacaaatattgcttggactacctataaatggacttgc
ttcttgtgtttgaaacactcgtgtgcatgagaatgtcatttgctaatgaa
ttttaaaagcatatataaaacaaaacaaacaacctgccacaatgtgatat
gtgtaatatcatttcataaaaatccctcttcctccaaagcctgggcagaa
atgtgctgcaaagagttatatgacttcttgttcatgttttgctaatgctc
gtatctccttgattacataatgttagtagcactgagacccccatggtaat
gtaacttaattataagctatgtcactaccctcctgtaaaatactattgga
cagacacagagggacccttggctcctgtgtctggtccacacaccacagaa
gcttgtattatcagtgaatataaatgtactacatttgcatgccttttggg
tttgccttaattcttacctcatttgcatcctatcgatctggaaagagctg
ttttggatgaatgcagtataaaatgtaaaaaccctgctaaatgacttatt
gattaagtatatctatctatatatacatatacacaaagatattatttatc
gaaagtaaaaaagatggaagtgtattggtttctgtttgaattttcaaagg
cttccaatgtggtggcaataaatgtcccaaataaatttataacaattgat
tttccccctaattcttattttataattttaaaattgcagcagttgctagc
aacaacttactaaatctactcttaaatatacaactttggaatttgaagaa
ttaatgacaacaaaagggaaaaaagcaactttccaacttttcatccaggc
tcccaaaagagggacaacgaacatggcatgtgaaaagtaaaacagatttg
ttcattccgaaaaaaaaaatgttcattctatgacaataaattttatctcag
tgtgaaaaaaaaaa
>NM_021966 2
gtcctcccgccccgccgcttggtggcggccgcatgctgcccggatataaa
gggtcggccccacatcccagggaccagcgagcggccttgagaggctctgg
ctcttgcttcttaggcggcccgaggacgccatggccgagtgcccgacact
cggggaggcagtcaccgaccacccggaccgcctgtgggcctgggagaagt
tcgtgtatttggacgagaagcagcacgcctggctgcccttaaccatcgag
ataaaggataggttacagttacgggtgctcttgcgtcgggaagacgtcgt
cctggggaggcctatgacccccacccagataggcccaagcctgctgccta
tcatgtggcagctctaccctgatggacgataccgatcctcagactccagt
ttctggcgcttagtgtaccacatcaagattgacggcgtggaggacatgct
tctcgagctgctgccagatgactgatgtatggtcttggcagcacctgtct
cctttcaccccagggcctgagcctggccagcctacaatggggatgttgtg
tttctgttcaccttcgtttactatgcctgtgtcttctccaccacgctggg
gtctgggaggaatggacagacagaggatgagctctacccaggcctgcag
gacctgcctgtagcccactctgctcgccttagcactaccactcctgccaa
ggaggattccatttggcagagcttcttccaggtgcccagctatacctgtg

cctcggcttttctcagctggatgatggtcttcagcctctttctgtccctt
ctgtccctcacagcactagtatttcatgttgcacacccactcagctccgt
gaacttgtgagaacacagccgattcacctgagcaggacctctgaaaccct
ggaccagtggtctcacatggtgctacgcctgcatgtaaacacgcctgcaa
acgctgcctgccggtaaacacgcctgcaaacgctgcctgcccgtaaacac
gcctgcaaacgctgcctgcccacacaggttcacgtgcagctcaaggaaag
gcctgaaaggagcccttatctgtgctcaggactcagaagcctctgggtca
gtggtccacatcccgggacgcagcaggaggccaggccggcgagccctgtg
gatgagccctcagaacccttggcttgcccacgtggaaaagggatagaggt
tgggtttcccccctttttatagatggtcacgcacctgggtgttacaaagtt
gtatgtggcatgaatactttttgtaatgattgattaaatgcaagatagtt
tatctaacttcgtgcggaatcagcttctatccttgacttagattctggtg
gagagaagtgagaataggcagcccccaaataaaaaatattcatggaaaaa
aaaaaaaaaa
>NM_198159 2
gtaaactccccgcgctggggcgggcggccgcgagccggcgagcgggcaga
gctcggcactgcgccggggcgcacggctcgggggacccaggcccagctac
cttccctccgcccccgggctctgttctcactttccagcagtggaaggacg
ggaagcgggagccatgcagtccgaatcggggatcgtgccggatttcgaag
tcggggaggagtttcatgaagagcccaaaacctattacgaactcaaaagt
caaccgctgaagagcagcagttccgccgagcatcctggggcctccaagcc
tccgataagctcctccagtatgacatcacgcatcttgctacgccagcaac
tcatgcgtgagcagatgcaggagcaggagcgcagggagcagcagcagaag
ctgcaggcggcccagttcatgcaacagagagtgcccgtgagtcagacacc
agccataaacgtcagtgtgcccaccacccttccctctgccacgcaggtgc
cgatggaagtccttaaggtgcagacccacctcgaaaaccccaccaagtac
cacatacagcaagcccaacggcagcaggtaaagcagtacctttctaccac
tttagcaaataaacatgccaaccaagtcctgagcttgccatgtccaaacc
agcctggcgatcatgtcatgccaccggtgccggggagcagcgcacccaac
agccccatggctatgcttacgcttaactccaactgtgaaaaagagggatt
ttataagtttgaagagcaaaacagggcagagagcgagtgcccaggcatga
acacacattcacgagcgtcctgtatgcagatggatgatgtaatcgatgac
atcattagcctagaatcaagttataatgaggaaatcttgggcttgatgga
tcctgctttgcaaatggcaaatacgttgcctgtctcgggaaacttgattg
atctttatggaaaccaaggtctgcccccaccaggcctcaccatcagcaac
tcctgtccagccaaccttcccaacataaaaagggagctcacagagtctga
agcaagagcactggccaaagagaggcagaaaaaggacaatcacaacctga
ttgaacgaagaagaagatttaacataaatgaccgcattaaagaactaggt
actttgattcccaagtcaaatgatccagacatgcgctggaacaagggaac
catcttaaaagcatccgtggactatatccgaaagttgcaacgagaacagc
aacgcgcaaaagaacttgaaaaccgacagaagaaactggagcacgccaac
cggcatttgttgctcagaatacaggaacttgaaatgcaggctcgagctca
tggactttcccttattccatccacgggtctctgctctccagatttggtga
atcggatcatcaagcaagaacccgttcttgagaactgcagccaagacctc
cttcagcatcatgcagacctaacctgtacaacaactctcgatctcacgga
tggcaccatcaccttcaacaacaacctcggaactgggactgaggccaacc
aagcctatagtgtccccacaaaaatgggatccaaactggaagacatcctg
atggacgacaccctttctcccgtcggtgtcactgatccactcctttcctc
agtgtcccccggagcttccaaaacaagcagccggaggagcagtatgagca

tggaagagacggagcacacttgttagcgaatcctccctgcactgcattcg
cacaaactgcttcctttcttgattcgtagatttaataacttacctgaagg
ggttttcttgataattttcctttaatatgaaattttttttcatgctttat
caatagcccaggatatattttatttttagaattttgtgaaacagacttgt
atattctattttacaactacaaatgcctccaaagtattgtacaaataagt
gtgcagtatctgtgaactgaattcaccacagactttagctttctgagcaa
gaggattttgcgtcagagaaatgtctgtccatttttattcaggggaaact
tgatttgagatttttatgcctgtgacttccttggaaatcaaatgtaaagt
ttaattgaaagaatgtaaagcaaccaaaaagaaaaaaaaaaagaaagaaa
gaggaaaagaaatccatactaacccttttccattttataaatgtattgat
tcattggtactgccttaaagatacagtacccctctagctttgtttagtct
ttatactgcaaactatttaaagaaatatgtattctgtaaaagaaaaaaaa
aatgcggccttttcatgaggatcgtctggttagaaaacataactgatacc
aaccgaaactgaagggagttagaccaaggctctgaaatataaagtctaat
cttgctctcttttattctgtgctgttacagttttcttcatcaatgagtgt
gatccagtttttcataagatattttattttgaaatggaaattaatgtcct
ctcaaagtaaaatattgaggagcactgaaagtatgttttactttttttt
attttatttttgcttttgataagaaaaccgaactgggcatatttctaatt
ggctttactatttttatttttaaattatgttttactgttcatttgatttg
tacagattctttattatcattgttcttttcaatatatttgtattaatttg
taagaatatgcatcttaaaatggcaagttttccatatttttacaactcac
tggtggttttccgcattctttgtacacccatgaaagaaaacttttatgca
aggtcttgcatttaaaagacagctttgcgaatattttgtaaattacagtc
tcactcagaactgtttttggacacatttaaggtgtagtattaataggtta
aaaccaggctttctagaaagaataaacttacatatttatttttaggacat
gaaaatagcaatattcttggagattgataaccatagcattaatacgccca
ttatggtcatttaaattggggtttatttcagcaaacttgttgaatttatt
tttaagaaagaaatactgtattgggaagttactgttacttgataacaatg
ttttaacaagaagcaatgttataaagttagtttcagtgcattatctactt
gtgtagtcctatgcaataacagtagtgttacatgtatcaagcctagatgt
tttatacagatgccatatagtgttatgagccaggctgttgaatggaattt
ctcagtagcagcctacaactgaatagcaagtggcataaagcatatccatt
cagaatgaagtgccttaaatatagcagtagtctttttttggactagcactg
actgaactgtaatgtaggggaaagtttcatgatggtatctatagtcaaga
cgaacatgtagcatggtgcctatgtagacaatataagagcttccaatttt
ccttcagatatttttaatattaaatatattttagtgacagagtgccaact
tctttcatcaggaaaccttattcaggagggtttttaaaaagtgtttaaat
gtcaaatgtgaattggtgatgggtgatggagggttcagagaggagtgatc
gtcagatgtgtgaatggacggtttaggtgaaaataatcaactgcatagtt
cccatgcacgctgggcaatgagaatccttggaaacattggtgatgctatc
agttttatagctttatttcttaagggggtagggaaaattagttcccattc
tttcaaccccccttaactgtatagctcttttcctagaatagtgacgcaaat
ctgcatgaacagctaattgtaccatagtgttcattgatacaatcatagca
ttgtctatttttctcttcatatttatatgggggggagggcgctggatgca
aaagttgaagatcgtgatgctatgatgttagttttccttagctgattttg
agggtttttaaaaataaagcaaggttgactaacctacggccacgggaaca
ggaccatggttaagcaaccatatagaaagctttgttgaaagaaagtatgg
catcttgtaccactgccctgactgtcacaactcctaaccttgccattgcc
tgcctccccctccccttctccttaagagacaatttctgcaggtggcaggt

gagcaagcccaggagaatgctgcaatcttgggggtggttttatttatttc
ttttttgccaaatagagtgtggattcatttcaggggctagctaagccaag
aggcagtggtttgggcttgttgtttgtaacaagaaaatgatccacaccac
tcccccgattcccgggtgcagaattgtaactcggggttgggcctctatat
ggagtgaccaaaatgccaaaattgtccatctgcctctgagtagggcaatg
gaaataccaaaccttctgactttgccaaaaagcatacaagcaacctggtc
atacataggatgacaaaattctttctggttgtttttaaacaataaagcaa
taagaacaaatacaatacataggaagttaaaagcacaaaggaatgaactt
attaatatttttgaaaaatgcactgggaaaaagttgatgtcaataacagt
ataaaacagccctatttcttgataaaaaatgacaaatgactgtctcttgc
ggatgcttggtactgtaatgttaataatagtcacctgctgttggatgcag
caataatttctgtatggtccatagcactgtatattatggatcgatattaa
tgtatccaatgaaataatcgacttgttcttgatagcctcattaaagcatt
tggtttttcacatag
>NM_005607 4
gcgcacgcgcgcgggcccgcgccgacgcagcacggcctcgagggcgcgag
cccgcgccgccgccgccgccgccggtcccggaccactgtgagcccgcggc
gtgaggcgtgggaggaagcgcggctgctgtcgcccagcgccgccccgtcg
tcgtctgccttcgcttcacggcgccgagccgcggtccgaagtcttgctgt
gtcacccaggctgccaggctggagtggagtggcatgatctcggctgactg
caacctctgcctcccagaatatgacagatacctagcatctagcaaaataa
tggcagctgcttaccttgaccccaacttgaatcacacaccaaattcgagt
actaagactcacctgggtactggtatggaacgttctcctggtgcaatgga
gcgagtattaaaggtctttcattattttgaaagcaatagtgagccaacca
cctgggccagtattatcaggcatggagatgctactgatgtcaggggcatc
attcagaagatagtggacagtcacaaagtaaagcatgtggcctgctatgg
attccgcctcagtcacctgcggtcagaggaggttcactggcttcacgtgg
atatgggcgtctccagtgtgagggagaagtatgagcttgctcacccacca
gaggagtggaaatatgaattgagaattcgttatttgccaaaaggatttct
aaaccagtttactgaagataagccaactttgaatttcttctatcaacagg
tgaagagcgattatatgttagagatagctgatcaagtggaccaggaaatt
gctttgaagttgggttgtctagaaatacggcgatcatactgggagatgcg
gggcaatgcactagaaaagaagtctaactatgaagtattagaaaaagatg
ttggtttaaagcgatttttcctaagagtttactggattctgtcaaggcc
aaaacactaagaaaactgatccaacaaacatttagacaatttgccaacct
taatagagaagaaagtattctgaaattctttgagatcctgtctccagtct
acagatttgataaggaatgcttcaagtgtgctcttggttcaagctggatt
atttcagtggaactggcaatcggcccagaagaaggaatcagttacctaac
ggacaagggctgcaatcccacacatcttgctgacttcactcaagtgcaaa
ccattcagtattcaaacagtgaagacaaggacagaaaaggaatgctacaa
ctaaaaatagcaggtgcacccgagcctctgacagtgacggcaccatccct
aaccattgcggagaatatggctgacctaatagatgggtactgccggctgg
tgaatggaacctcgcagtcatttatcatcagacctcagaaagaaggtgaa
cgggctttgccatcaataccaaagttggccaacagcgaaaagcaaggcat
gcggacacacgccgtctctgtgtcagaaacagatgattatgctgagatta
tagatgaagaagatacttacaccatgccctcaaccagggattatgagatt
caaagagaaagaatagaacttggacgatgtattggagaaggccaatttgg
agatgtacatcaaggcatttatatgagtccagagaatccagctttggcgg
ttgcaattaaaacatgtaaaaactgtacttcggacagcgtgagagagaaa

tttcttcaagaagccttaacaatgcgtcagtttgaccatcctcatattgt
gaagctgattggagtcatcacagagaatcctgtctggataatcatggagc
tgtgcacacttggagagctgaggtcatttttgcaagtaaggaaatacagt
ttggatctagcatctttgatcctgtatgcctatcagcttagtacagctct
tgcatatctagagagcaaaagatttgtacacagggacattgctgctcgga
atgttctggtgtcctcaaatgattgtgtaaaattaggagactttggatta
tcccgatatatggaagatagtacttactacaaagcttccaaaggaaaatt
gcctattaaatggatggctccagagtcaatcaattttcgacgttttacct
cagctagtgacgtatggatgtttggtgtgtgtatgtgggagatactgatg
catggtgtgaagccttttcaaggagtgaagaacaatgatgtaatcggtcg
aattgaaaatggggaaagattaccaatgcctccaaattgtcctcctaccc
tctacagccttatgacgaaatgctgggcctatgaccccagcaggcggccc
aggtttactgaacttaaagctcagctcagcacaatcctggaggaagagaa
ggctcagcaagaagagcgcatgaggatggagtccagaagacaggccacag
tgtcctgggactccggagggtctgatgaagcaccgcccaagcccagcaga
ccgggttatcccagtccgaggtccagcgaaggattttatcccagcccaca
gcacatggtacaaaccaatcattaccaggtttctggctaccctggttcac
atggaatcacagccatggctggcagcatctatccaggtcaggcatctctt
ttggaccaaacagattcatggaatcatagacctcaggagatagcaatgtg
gcagcccaatgtggaggactctacagtattggacctgcgagggattgggc
aagtgttgccaacccatctgatggaagagcgtctaatccgacagcaacag
gaaatggaagaagatcagcgctggctggaaaaagaggaaagatttctgaa
acctgatgtgagactctctcgaggcagtattgacagggaggatggaagtc
ttcagggtccgattggaaaccaacatatatatcagcctgtgggtaaacca
gatcctgcagctccaccaaagaaaccgcctcgccctggagctcccggtca
tctgggaagccttgccagcctcagcagccctgctgacagctacaacgagg
gtgtcaagcttcagccccaggaaatcagccccccctcctactgccaacctg
gaccggtcgaatgataaggtgtacgagaatgtgacgggcctggtgaaagc
tgtcatcgagatgtccagtaaaatccagccagccccaccagaggagtatg
tccctatggtgaaggaagtcggcttggccctgaggacattattggccact
gtggatgagaccattcccctcctaccagccagcacccaccgagagattga
gatggcacagaagctattgaactctgacctgggtgagctcatcaacaaga
tgaaactggcccagcagtatgtcatgaccagcctccagcaagagtacaaa
aagcaaatgctgactgctgctcacgccctggctgtggatgccaaaaactt
actcgatgtcattgaccaagcaagactgaaaatgcttgggcagacgagac
cacactgagcctcccctaggagcacgtcttgctaccctcttttgaagatg
ttctctagccttccaccagcagcgaggaattaaccctgtgtcctcagtcg
ccagcacttacagctccaactttttgaatgaccatctggttgaaaaatc
tttctcatataagtttaaccacactttgatttgggttcatttttttgtttt
gtttttttcaatcatgatattcagaaaaatccaggatccaaaatgtggcg
tttttctaagaatgaaaattatatgtaagcttttaagcatcatgaagaac
aatttatgttcacattaagatacgttctaaagggggatggccaaggggtg
acatcttaattcctaaactaccttagctgcatagtggaagaggagagcat
gaagcaaagaattccaggaaacccaagaggctgagaattcttttgtctac
catagaattattatccagactggaatttttgtttgttagaacacccttca
gttgcaatatgctaatcccactttacaaagaatataaaagctatattttg
aagacttgagttatttcagaaaaaactacagcccttttttgtcttacctgc
cttttactttcgtgtggatatgtgaagcattgggtcgggaactagctgta
gaacacaactaaaaactcatgtctttttcacagaataatgtgccagttt

tttgtagcaatgttatttctcttggaagcagaaatgctttgtaccagagc
acctccaaactgcattgaggagaagttccagaaccatcccctttttccat
ttttatataatttataaagaaagattaaagccatgttgactattttacag
ccactggagttaactaacccttccttgtatctgtcttcccaggagagaat
gaagcaaaacaggaatttggttttcttttgatgtccagttacaccatcca
ttctgttaattttgaaaaaatataccctccctttagtttgttgggggata
taaattattctcaggaagaatataatgaactgtacagttactttgaccta
ttaaaaaggtgttaccagtaaagttcttgttgtaatatccttaaaaaaaa
>NM_019558 3
tccgtgcagtgctgtggtgcgaaaatgcctcgccggtgcgcaccgggtcg
gcagcctcggcggcgggggcgagattggcgggagggggcgcggggggggg
cgcggtaagaggtggcggcgggcagagggtgttttttttcttttccctcc
agagccggggtttgtaaaccgaggccagagtgtccccgtgggccgagcgc
actttttttcttgtccgggtgcgctcagtcactggtgcctgagaggaaaca
gtggaggcagcggggcaggtcgcctggggcgtcggcgattatattgcggc
cgagccggggcgcgccgggaaaggccgggagggcggcggcgcgcgggggc
tgggcgaggccccgcgacccgcgagggaggcggcgcgaagccgaggcggc
gggcgcaagagccgggcatgagcgcccagtagctgagcgcccgcggctgc
ctggcctcagaagcgacgcgcgagcgcgggcgggcggcagcagcgacgta
gcccggcggtcccggcggcgagagcagccgccccacaggcccccgcggca
gtgcggccgagtcgaggctcgctctctggctgcttagcgccgcccgcccg
cccggggccgccgccgctgacgccccaatgagttcgtacttcgtgaaccc
gctgtactccaagtacaaggcggcggctgcggcggcggcggcggcgggcg
aggccatcaatcccacttactacgactgtcacttcgcgcccgaggtcggc
ggccgtcacgccgccgccgcagcagccctgcagctctatggcaacagcgc
cgccggcttcccgcacgcgcccccgcaggcgcacgcgcacccgcacccgt
ccccgccgccctccgggactgggtgcggcggtagggaaggccggggccag
gagtacttccaccccggcgggggcagcccggccgctgcctaccaggccgc
ccccccctcctcctccgcatcctccgcctccgccgccacctccccccctgcg
gcgggattgcctgtcacggggagcccgcgaagttttacggatacgataac
ttacagagacagccgatttttacgacccagcaagaggccgagctggtaca
atatcctgactgtaaatcgtccagtggtaatattggcgaggacccagacc
acttaaatcagagctcgtctccttctcaaatgtttccgtggatgagacca
caagcagctcctggtagacgaagaggaagacaaacctacagtcgcttcca
aactctagagttggaaaaggaatttctttttaacccctatctgaccagga
aaagaagaatcgaggtttcccacgccctagccctcaccgagagacaggta
aaaatctggttccagaacaggagaatgaaatggaaaaaggaaaacaacaa
ggacaaatttcccgtttcccggcaggaggtgaaggacggggaaacgaaaa
aggaagcccaagagctggaggaagacagagccgaaggcctgacaaattaa
cttctacctttaaaatttaccacagactattaaaactaataatcaccata
tgctgtggacaccacctattttctttgttggaaaggaccttacctgtgtt
tcaagctaccttcatgtcactgctcttgaggttttctgtgctttgagagg
gatttgggtgtttaaaaaagtttctagtatcacatagaagctgtccttga
gctgtcctatggaagggtaatttgatactgaccttgtagctatattttta
taatggtttttaatgtctgagctagtgatttgcctcaacaacgtaaactt
cctaatgattagcacttaataattgcatataaaatgctttattaattaaa
caagtgcacttgaacattttaatatttgtggtgagtaaattaaaaggagt
ttattaattaaaaaaaaattatgtctgcagaatactttatattatttgatt
acaatgtattatttatggatttttttattctttcctttataatgaatagtt

cgggtgcgttttgtttactcctaaaaggtttctttgcgtattttctaaat
gtaatatctcggggaaaatattagaaaagcacgtattagctgaagaatgt
aacttgtagtccagctctgcagcttccttaaacttaagaaaaagattggg
ccagtgacaagaatttaaagacaatgtccaagttgacaattatttttcta
tagtccatacaaattaaataatctggcaactctggcaaatcgccttgtaa
aatgcgtctcatttttaacttgctttcgttttgaaccgcccttgtaatc
gcctgaaatcgctagttctttatgcggtggctgccgctgtgttccgttat
tttcagtaggtgtcatatttatttgtattgcctttgttctgttcgccgct
ggttttaaaccagcttgctgtgtgcatctcagacgtcggttggtacgtcc
tccgctgttcttcaggaaagcgatagcctcacctatttgaaacaagccct
gagaggaaacgcagaaaaacctgagtgtaaacaactccggaatgtcgcta
gctccttagtaaataaatgaatctctttctggaaaaaaaaaaaaaaaa
>NM_022658 3
gccgagctcagcaccgaggcgcccccaacctgcccagcccccagcccac
cagcccagcccagtcccggggagccagctggcctggggttcggtcccggg
gggaggggagtttcgggggtactgggcggggtactcgtgagccagagggg
agggggccgcgggttttcatgtacccagcatgagctcctacttcgtcaac
cccctgttctccaaatacaaagccggcgagtccctggaaccggcctatta
cgactgccggttccctcagagcgtgggcaggagccatgcgctggtgtacg
ggcccggcggctcggcgcccggcttccagcacgcttcgcaccacgttcaa
gacttcttccaccacggcacctccggcatctccaactcaggctaccagca
gaacccgtgctcgcttagctgccacggagacgcctccaaattctatggct
acgaggcgctccccagacagtccctttatggggctcagcaagaggcgagc
gtggtgcaatatcccgactgtaaatcctccgccaacactaacagtagcga
aggacaaggccacttaaatcaaaactcgtctcccagcctcatgtttccat
ggatgagaccccacgctccggggaggcgcagtggacggcaaacttacagc
cggtatcagaccttggaactagaaaaggagtttctctttaatccttattt
gacacgaaaacgtcggattgaagtctctcatgccctgggactgaccgaga
gacaagtgaagatctggttccagaaccgaaggatgaagtggaaaaaggag
aacaacaaggataaactgccgggagcccgagatgaggagaaggtggagga
agaaggaaatgaggaagaggagaaagaagaggaggaaaaggaagaaaaca
aggactaagcaaaaaagaaagacccccccccccttagcaactcccttgaa
gtttcgttttatggtagcagataaattgagaagtttacgactgtcatttg
cttttatagagaatagaatgacactcacaactctaactacctgtcagata
cttgcagctctggttttattacctttggacttcccccactctttatttgt
ttgggggctggagggggggagacggagaaacagtgaaaagttcggactctc
tgtctcactccttgccccacacacacttgtccctgcccccacccttctga
gtccttcctggattttaaggtctgagacctggcctccgtgctcctcggtc
tgtctctcaccacactcccacctccctgcttctctggtatttattttaga
ggggagccccctcgaaatgcagaaaaggacttgtggttttgtttttatgc
taaggctagtgtactagatgtactttttctaaaaaggaaaggacagaaaa
aaatgaagaaaggaaaggaagacaaatgtaaagaaataaggaaaaaagca
aggaatatcccctccccctccccctccccctccccagggctccctgctta
gaaaaacccccttgactttctctaggaacctgatggaaacctgaaggaga
tgtgggtctttcccctcccccatttccagaagggtagataggagcctgc
agtcgcctctaaaatcctacctaaccatcccatggtcactcgggcccatg
ccttcctctccttcgctgtttgatttctattctgttgggcccgccttcct
ctgagctgcattagtgttagtgctcagaaatcaccataatcacgaaaata
ataataataaatctttaacatactacctaaagggaacctgcaataatctt

gaaaaagaaaaagagaaaaattttaaaatcctgctataggagaaaaaaag
agaaaaaaataaaaatcaaaaaaaaaaaaaagaaagaaagaaacctccag
cgtattttatcactacctatagaaagaaatcctgctttgagagtatttgt
aatgcggttttgttgtcgtttgttgctgcttatttcactaagaaaaccca
acaactgagactgcctagcccgccggtcctgtgcgcttttattgtgcttc
taaccccagtagagtagaactaaattgcactgaatgtatagttaactctg
tcttgaattctctgtttatgcaatgtgctcgaaagaaaaaaatgttaaaa
atatatctataataataattttttgtcatttgtctttatgtccagctatg
aatgtagattttgtgtcccgacagccctgttcctggtccaagtactttgt
attgtatacgtgagtcataataataaaaagaagaagaaaa
>NM_005562 2
cttggcccgggccaggtgtgcgccctcctcgctgcgagggggagcgggcg
gctgcggggagcgattttccagcccggtttgtgctctgtgtgtttgtctg
cctctggagggctgggtcctccttattcacaggtgagtcacaccctgaaa
cacaggctctcttcctgtcaggactgagtcaggtagaagagtcgataaaa
ccacctgatcaaggaaaaggaaggcacagcggagcgcagagtgagaacca
ccaaccgaggcgccgggcagcgacccctgcagcggagacagagactgagc
ggcccggccccgccatgcctgcgctctggctgggctgctgcctctgcttc
tcgctcctcctgcccgcagcccgggccacctccaggagggaagtctgtga
ttgcaatgggaagtccaggcagtgtatctttgatcgggaacttcacagac
aaactggtaatggattccgctgcctcaactgcaatgacaacactgatggc
attcactgcgagaagtgcaagaatggcttttaccggcacagagaaaggga
ccgctgtttgccctgcaattgtaactccaaaggttctcttagtgctcgat
gtgacaactccggacggtgcagctgtaaaccaggtgtgacaggagccaga
tgcgaccgatgtctgccaggcttccacatgctcacggatgcggggtgcac
ccaagaccagagactgctagactccaagtgtgactgtgacccagctggca
tcgcagggccctgtgacgcgggccgctgtgtctgcaagccagctgtcact
ggagaacgctgtgataggtgtcgatcaggttactataatctggatggggg
gaaccctgagggctgtacccagtgtttctgctatgggcattcagccagct
gccgcagctctgcagaatacagtgtccataagatcacctctacctttcat
caagatgttgatggctggaaggctgtccaacgaaatgggtctcctgcaaa
gctccaatggtcacagcgccatcaagatgtgtttagctcagcccaacgac
tagaccctgtctattttgtggctcctgccaaatttcttgggaatcaacag
gtgagctatggtcaaagcctgtcctttgactaccgtgtggacagaggagg
cagacacccatctgcccatgatgtgattctggaaggtgctggtctacgga
tcacagctcccttgatgccacttggcaagacactgccttgtgggctcacc
aagacttacacattcaggttaaatgagcatccaagcaataattggagccc
ccagctgagttactttgagtatcgaaggttactgcggaatctcacagccc
tccgcatccgagctacatatggagaatacagtactgggtacattgacaat
gtgaccctgatttcagcccgccctgtctctggagccccagcaccctgggt
tgaacagtgtatatgtcctgttgggtacaaggggcaattctgccaggatt
gtgcttctggctacaagagagattcagcgagactggggccttttggcacc
tgtattccttgtaactgtcaagggggaggggcctgtgatccagacacagg
agattgttattcaggggatgagaatcctgacattgagtgtgctgactgcc
caattggtttctacaacgatccgcacgacccccgcagctgcaagccatgt
ccctgtcataacgggttcagctgctcagtgatgccggagacggaggaggt
ggtgtgcaataactgccctcccggggtcaccggtgcccgctgtgagctct
gtgctgatggctactttggggacccctttggtgaacatggcccagtgagg
ccttgtcagccctgtcaatgcaacaacaatgtggaccccagtgcctctgg

gaattgtgaccggctgacaggcaggtgtttgaagtgtatccacaacacag
ccggcatctactgcgaccagtgcaaagcaggctacttcggggacccattg
gctcccaacccagcagacaagtgtcgagcttgcaactgtaaccccatggg
ctcagagcctgtaggatgtcgaagtgatggcacctgtgtttgcaagccag
gatttggtggccccaactgtgagcatggagcattcagctgtccagcttgc
tataatcaagtgaagattcagatggatcagtttatgcagcagcttcagag
aatggaggccctgatttcaaaggctcagggtggtgatggagtagtacctg
atacagagctggaaggcaggatgcagcaggctgagcaggcccttcaggac
attctgagagatgcccagatttcagaaggtgctagcagatcccttggtct
ccagttggccaaggtgaggagccaagagaacagctaccagagccgcctgg
atgacctcaagatgactgtggaaagagttcgggctctgggaagtcagtac
cagaaccgagttcgggatactcacaggctcatcactcagatgcagctgag
cctggcagaaagtgaagcttccttgggaaacactaacattcctgcctcag
accactacgtggggccaaatggctttaaaagtctggctcaggaggccaca
agattagcagaaagccacgttgagtcagccagtaacatggagcaactgac
aagggaaactgaggactattccaaacaagccctctcactggtgcgcaagg
ccctgcatgaaggagtcggaagcggaagcggtagcccggacggtgctgtg
gtgcaagggcttgtggaaaaattggagaaaaccaagtccctggcccagca
gttgacaagggaggccactcaagcggaaattgaagcagataggtcttatc
agcacagtctccgcctcctggattcagtgtctcggcttcagggagtcagt
gatcagtcctttcaggtggaagaagcaaagaggatcaaacaaaaagcgga
ttcactctcaagcctggtaaccaggcatatggatgagttcaagcgtacac
agaagaatctgggaaactggaaagaagaagcacagcagctcttacagaat
ggaaaaagtgggagagagaaatcagatcagctgctttcccgtgccaatct
tgctaaaagcagagcacaagaagcactgagtatgggcaatgccactttt
atgaagttgagagcatccttaaaaacctcagagagtttgacctgcaggtg
gacaacagaaaagcagaagctgaagaagccatgaagagactctcctacat
cagccagaaggtttcagatgccagtgacaagacccagcaagcagaaagag
ccctggggagcgctgctgctgatgcacagagggcaaagaatggggccggg
gaggccctggaaatctccagtgagattgaacaggagattgggagtctgaa
cttggaagccaatgtgacagcagatggagccttggccatggaaaagggac
tggcctctctgaagagtgagatgagggaagtggaaggagagctggaaagg
aaggagctggagtttgacacgaatatggatgcagtacagatggtgattac
agaagcccagaaggttgataccagagccaagaacgctggggttacaatcc
aagacacactcaacacattagacggcctcctgcatctgatggaccagcct
ctcagtgtagatgaagaggggctggtcttactggagcagaagctttcccg
agccaagacccagatcaacagccaactgcggcccatgatgtcagagctgg
aagagagggcacgtcagcagaggggccacctccatttgctggagacaagc
atagatgggattctggctgatgtgaagaacttggagaacattagggacaa
cctgcccccaggctgctacaatacccaggctcttgagcaacagtgaagct
gccataaatatttctcaactgaggttcttgggatacagatctcagggctc
gggagccatgtcatgtgagtgggtgggatggggacatttgaacatgttta
atgggtatgctcaggtcaactgacctgaccccattcctgatcccatggcc
aggtggttgtcttattgcaccatactccttgcttcctgatgctgggcaat
gaggcagatagcactgggtgtgagaatgatcaaggatctggaccccaaag
aatagactggatggaaagacaaactgcacaggcagatgtttgcctcataa
tagtcgtaagtggagtcctggaatttggacaagtgctgttgggatatagt
caacttattctttgagtaatgtgactaaaggaaaaaactttgactttgcc
caggcatgaaattcttcctaatgtcagaacagagtgcaacccagtcacac

tgtggccagtaaaatactattgcctcatattgtcctctgcaagcttcttg
ctgatcagagttcctcctacttacaacccagggtgtgaacatgttctcca
ttttcaagctggaagaagtgagcagtgttggagtgaggacctgtaaggca
ggcccattcagagctatggtgcttgctggtgcctgccaccttcaagttct
ggacctgggcatgacatcctttcttttaatgatgccatggcaacttagag
attgcatttttattaaagcatttcctaccagcaaagcaaatgttgggaaa
gtatttactttttcggtttcaaagtgatagaaaagtgtggcttgggcatt
gaaagaggtaaaattctctagatttattagtcctaattcaatcctacttt
tcgaacaccaaaaatgatgcgcatcaatgtattttatcttattttctcaa
tctcctctctctttcctccacccataataagagaatgttcctactcacac
ttcagctgggtcacatccatccctccattcatccttccatccatctttcc
atccattacctccatccatccttccaacatatatttattgagtacctact
gtgtgccaggggctggtgggacagtggtgacatagtctctgccctcatag
agttgattgtctagtgaggaagacaagcatttttaaaaaataaatttaaa
cttacaaactttgtttgtcacaagtggtgtttattgcaataaccgcttgg
tttgcaacctctttgctcaacagaacatatgttgcaagaccctcccatgg
gggcacttgagttttggcaaggctgacagagctctgggttgtgcacattt
ctttgcattccagctgtcactctgtgcctttctacaactgattgcaacag
actgttgagttatgataacaccagtgggaattgctggaggaaccagaggc
acttccaccttggctgggaagactatggtgctgccttgcttctgtatttc
cttggattttcctgaaagtgtttttaaataaagaacaattgttagatgcc
tggggtcagtttctgatttactaaactctaagcctatgatggtaccaaag
taatggaaatgaaaatgccacagaaatggcaataggtcctagagttagca
tcttcagttcccacttctgagcatcttacttcgcccattccaaccataca
ggattgatgatgctgtcaatgagatttttatgaggtttaactgatgctgt
cttctaagcataaagctttattt
>NM_000314 4
cctcccctcgcccggcgcggtcccgtccgcctctcgctcgcctcccgcct
cccctcggtcttccgaggcgcccgggctcccggcgcggcggcggaggggg
cgggcaggccggcgggcggtgatgtggcgggactctttatgcgctgcggc
aggatacgcgctcggcgctgggacgcgactgcgctcagttctctcctctc
ggaagctgcagccatgatggaagtttgagagttgagccgctgtgaggcga
ggccgggctcaggcgagggagatgagagacggcggcggccgcggcccgga
gcccctctcagcgcctgtgagcagccgcgggggcagcgccctcggggagc
cggccggcctgcggcggcggcagcggcggcgtttctcgcctcctcttcgt
cttttctaaccgtgcagcctcttcctcggcttctcctgaaagggaaggtg
gaagccgtgggctcgggcgggagccggctgaggcgcggcggcggcggcgg
cacctcccgctcctggagcggggggggagaagcggcggcggcggcggccgc
ggcggctgcagctccagggagggggtctgagtcgcctgtcaccatttcca
gggctgggaacgccggagagttggtctctccccttctactgcctccaaca
cggcggcggcggcggcggcacatccagggacccgggccggttttaaacct
cccgtccgccgccgccgcaccccccgtggcccgggctccggaggccgccg
gcggaggcagccgttcggaggattattcgtcttctccccattccgctgcc
gccgctgccaggcctctggctgctgaggagaagcaggcccagtcgctgca
accatccagcagccgccgcagcagccattacccggctgcggtccagagcc
aagcggcggcagagcgaggggcatcagctaccgccaagtccagagccatt
tccatcctgcagaagaagccccgccaccagcagcttctgccatctctctc
ctcctttttcttcagccacaggctcccagacatgacagccatcatcaaag
agatcgttagcagaaacaaaaggagatatcaagaggatggattcgactta

gacttgacctatatttatccaaacattattgctatgggatttcctgcaga
aagacttgaaggcgtatacaggaacaatattgatgatgtagtaaggtttt
tggattcaaagcataaaaaccattacaagatatacaatctttgtgctgaa
agacattatgacaccgccaaatttaattgcagagttgcacaatatccttt
tgaagaccataacccaccacagctagaacttatcaaacccttttgtgaag
atcttgaccaatggctaagtgaagatgacaatcatgttgcagcaattcac
tgtaaagctggaaagggacgaactggtgtaatgatatgtgcatatttatt
acatcggggcaaatttttaaaggcacaagaggccctagatttctatgggg
aagtaaggaccagagacaaaaagggagtaactattcccagtcagaggcgc
tatgtgtattattatagctacctgttaaagaatcatctggattatagacc
agtggcactgttgtttcacaagatgatgtttgaaactattccaatgttca
gtggcggaacttgcaatcctcagtttgtggtctgccagctaaaggtgaag
atatattcctccaattcaggacccacacgacgggaagacaagttcatgta
ctttgagttccctcagccgttacctgtgtgtggtgatatcaaagtagagt
tcttccacaaacagaacaagatgctaaaaaaggacaaaatgtttcacttt
tgggtaaatacattcttcataccaggaccagaggaaacctcagaaaaagt
agaaaatggaagtctatgtgatcaagaaatcgatagcatttgcagtatag
agcgtgcagataatgacaaggaatatctagtacttactttaacaaaaaat
gatcttgacaaagcaaataaagacaaagccaaccgatacttttctccaaa
ttttaaggtgaagctgtacttcacaaaaacagtagaggagccgtcaaatc
cagaggctagcagttcaacttctgtaacaccagatgttagtgacaatgaa
cctgatcattatagatattctgacaccactgactctgatccagagaatga
accttttgatgaagatcagcatacacaaattacaaaagtctgaattttt
tttatcaagagggataaaacaccatgaaaataaacttgaataaactgaaa
atggacctttttttttttaatggcaataggacattgtgtcagattaccag
ttataggaacaattctcttttcctgaccaatcttgttttaccctatacat
ccacagggttttgacacttgttgtccagttgaaaaaaggttgtgtagctg
tgtcatgtatatacctttttgtgtcaaaaggacatttaaaattcaattag
gattaataaagatggcactttcccgttttattccagttttataaaaaagtg
gagacagactgatgtgtatacgtaggaatttttttccttttgtgttctgtc
accaactgaagtggctaaagagctttgtgatatactggttcacatcctac
ccctttgcacttgtggcaacagataagtttgcagttggctaagagaggtt
tccgaagggttttgctacattctaatgcatgtattcgggttaggggaatg
gagggaatgctcagaaaggaaataattttatgctggactctggaccatat
accatctccagctatttacacacacctttctttagcatgctacagttatt
aatctggacattcgaggaattggccgctgtcactgcttgttgtttgcgca
ttttttttaaagcatattggtgctagaaaaggcagctaaaggaagtgaa
tctgtattggggtacaggaatgaaccttctgcaacatcttaagatccaca
aatgaaggatataaaaataatgtcataggtaagaaacacagcaacaatg
acttaaccatataaatgtggaggctatcaacaaagaatgggcttgaaaca
ttataaaaattgacaatgatttattaaatatgttttctcaattgtaacga
cttctccatctcctgtgtaatcaaggccagtgctaaaattcagatgctgt
tagtacctacatcagtcaacaacttacacttattttactagttttcaatc
ataatacctgctgtggatgcttcatgtgctgcctgcaagcttcttttttc
tcattaaatataaaatattttgtaatgctgcacagaaattttcaatttga
gattctacagtaagcgtttttttttctttgaagatttatgatgcacttatt
caatagctgtcagccgttccaccccttttgaccttacacattctattacaa
tgaattttgcagttttgcacatttttttaaatgtcattaactgttagggaa
ttttacttgaatactgaatacatataatgtttatattaaaaaggacattt

gtgttaaaaaggaaattagagttgcagtaaactttcaatgctgcacacaa
aaaaaaagacatttgatttttcagtagaaattgtcctacatgtgctttatt
gatttgctattgaaagaatagggtttttttttttttttttttttttt
ttaaatgtgcagtgttgaatcatttcttcatagtgctcccccgagttggg
actagggcttcaatttcacttcttaaaaaaaatcatcatatatttgatat
gcccagactgcatacgattttaagcggagtacaactactattgtaaagct
aatgtgaagatattattaaaaaggtttttttttccagaaatttggtgtct
tcaaattataccttcaccttgacatttgaatatccagccattttgtttct
taatggtataaaattccattttcaataacttattggtgctgaaattgttc
actagctgtggtctgacctagttaatttacaaatacagattgaataggac
ctactagagcagcatttatagagtttgatggcaaatagattaggcagaac
ttcatctaaaatattcttagtaaataatgttgacacgttttccatacctt
gtcagtttcattcaacaatttttaaatttttaacaaagctcttaggattt
acacatttatatttaaacattgatatatagagtattgattgattgctcat
aagttaaattggtaaagttagagacaactattctaacacctcaccattga
aatttatatgccaccttgtctttcataaaagctgaaaattgttacctaaa
atgaaaatcaacttcatgttttgaagatagttataaatattgttctttgt
tacaatttcgggcaccgcatattaaaacgtaactttattgttccaatatg
taacatggagggccaggtcataaataatgacattataatgggcttttgca
ctgttattatttttcctttggaatgtgaaggtctgaatgagggttttgat
tttgaatgtttcaatgtttttgagaagccttgcttacattttatggtgta
gtcattggaaatggaaaaatggcattatatatattatatatataaatata
tattatacatactctccttactttatttcagttaccatccccatagaatt
tgacaagaattgctatgactgaaaggttttcgagtcctaattaaaacttt
atttatggcagtattcataattagcctgaaatgcattctgtaggtaatct
ctgagtttctggaatattttcttagactttttggatgtgcagcagcttac
atgtctgaagttacttgaaggcatcacttttaagaaagcttacagttggg
ccctgtaccatcccaagtcctttgtagctcctcttgaacatgtttgccat
acttttaaaagggtagttgaataaatagcatcaccattctttgctgtggc
acaggttataaacttaagtggagtttaccggcagcatcaaatgtttcagc
tttaaaaaataaaagtagggtacaagtttaatgtttagttctagaaattt
tgtgcaatatgttcataacgatggctgtggttgccacaaagtgcctcgtt
tacctttaaatactgttaatgtgtcatgcatgcagatggaaggggtggaa
ctgtgcactaaagtgggggctttaactgtagtatttggcagagttgcctt
ctacctgccagttcaaaagttcaacctgttttcatatagaatatatatac
taaaaaatttcagtctgttaaacagccttactctgattcagcctcttcag
atactcttgtgctgtgcagcagtggctctgtgtgtaaatgctatgcactg
aggatacacaaaaataccaatatgatgtgtacaggataatgcctcatccc
aatcagatgtccatttgttattgtgtttgttaacaaccctttatctctta
gtgttataaactccacttaaaactgattaaagtctcattcttgtcaaaaa
aaaaaaaaaaaaaaaaaaaaa
>NM_014795 3
atttcatttcttccactaaagcgtttgcggagacttcaaggtataatcta
tcccagatcctttcccagagagaaacttggcgatcacgttttcacatgat
gctcacgctcagggcgcttcaattatccctccccacaaagataggtggcg
cgtgtttcagggtctctcgtctctctcctacagaaaagaaaaagaaaaaa
atgtcattagaagaggcgtaacacgtcagtccgtccccaggtttgtgttt
cctggagtggccgaaagagatcagttctaacctgctctgcaggaataacg
gtcctgcctcccgacactcttggcgaggtttttgtacagtttgctccggg

agctgtttcttcgcttccaccttttctcccccacacttcgcggcttctt
catgctttttcttctcaccatttctggccaaaactacaaacaagacttcg
cagatcgagcctgcgtgctgccgaagcagggcgccgagtccatgcgaact
gccatctgatccgctcttatcaatgaagcagccgatcatggcggatggcc
cccggtgcaagaggcgcaaacaagccaatcccaggaggaaaaacgtggtg
aactatgacaatgtagtggacacaggttctgaaacagatgaggaagacaa
gcttcatattgctgaggatgacggtattgccaaccctctggaccaggaga
cgagtccagctagtgtgcccaaccatgagtcctccccacacgtgagccaa
gctctgttgccaagagaggaagaggaagatgaaataagggagggtggagt
ggaacaccccctggcacaacaacgagattctacaagcctctgtagatggtc
cagaagaaatgaaggaagactatgacactatggggccagaagccacgatc
cagaccgcaattaacaatggtacagtgaagaatgcaaattgcacatcaga
ttttgaggaatactttgccaaaagaaaactggaggaacgcgatggtcatg
cagtcagcatcgaggagtaccttcagcgcagtgacacagccattatttac
ccagaagcccctgaggagctgtctcgccttggcacgccagaggccaatgg
gcaagaagaaaatgacctgccacctggaactccagatgcttttgcccaac
tgctgacctgcccctactgcgaccggggctacaagcgcttgacatcactg
aaggagcacatcaagtaccgccacgagaagaatgaagagaacttttcctg
ccctctctgtagctacacgtttgcctaccgcacccagctcgagcggcata
tggtgacacacaagccagggacagatcagcaccaaatgctaacccaagga
gcaggtaatcgcaagttcaaatgcacagagtgtggcaaggccttcaaata
taaacaccatctgaaagaacacctgcgaattcacagtggtgaaaaacctt
acgagtgcccaaactgcaagaaacgtttctcccattctggttcctacagt
tcgcacatcagcagcaagaaatgtattggtttaatctctgtaaatggccg
aatgagaaacaatatcaagacgggttcttcccctaattctgtttcttctt
ctcctactaattcagccattacccagttaagaaacaagttggagaatgga
aaaccacttagtatgtctgaacagacaggcttacttaaaattaaaacaga
accactagacttcaatgactataaagttcttatggctacacacgggttta
gtggcactagtccctttatgaatggtgggcttggagccaccagcccttta
ggagttcatccatctgctcagagtccaatgcagcacttaggtgtagggat
ggaagcccctttacttgggtttcccaccatgaatagtaatttaagtgagg
tacaaaaggttctacagattgtggacaatactgtttccaggcaaaaaatg
gactgcaaggctgaagaaatttcaaagttgaaaggttatcacatgaagga
tccatgctctcaacctgaggaacaaggagttacttctcctaatattccgc
ctgtcggtcttccggtagtgagtcataatggtgccactaaaagtattatt
gactatacgttggaaaaagtcaatgaagccaaagcttgcctccagagctt
gactactgactcaaggagacagatcagtaatataaagaaagagaagctac
gtactttaatagatttggtcactgatgacaaaatgattgagaaccacaac
atatccactccattttcatgccagttctgtaaagaaagttttcctggccc
catccctttgcatcagcatgaacgttacctttgtaagatgaatgaagaga
tcaaggcggtcctgcagcctcatgaaaacatagtccccaacaaagccgga
gtttttgttgataataaagccctcctcttgtcatctgtactttctgagaa
aggaatgacaagccccatcaacccatacaaggaccacatgtctgtactca
aagcatactatgctatgaacatggagcccaactccgatgaactgctgaaa
atttccattgctgtgggccttcctcaggaatttgtgaaggaatggtttga
acaacgaaaagtctaccagtactcaaattccaggtccccatccctggaaa
gaagctccaagccgttagctcccaacagtaaccctcccacaaaagactct
ttattacccaggtctcctgtaaaacctatggactccataacatcaccatc
tatagcagaactccacaacagtgttacgaattgtgatcctcctctcaggc

taacaaaaccttcccattttaccaatattaaaccagttgaaaaattggac
cactccaggagtaatactccttctcccttaaatctttcctccacatcttc
taaaaactcccacagtagttcatacactccaaacagcttctcttctgagg
agctccaggctgagcctttagacttgtcattaccaaaacaaatgaaagaa
cccaaaagtattatagccacaaagaacaaaacaaaagctagtagcatcag
tttagatcataacagtgtttcttcctcatctgaaaactcagatgagcctc
tgaacttgacttttatcaagaaggaattttcaaattcaaataatctggac
aacaaaagcactaacccagtgttcagcatgaacccatttagtgccaaacc
tttatacacagctcttccacctcaaagcgcatttccccctgctactttca
tgccaccagtccagaccagtattcctgggctacgaccatacccaggactg
gatcagatgagcttcctaccacatatggcctacacctacccaactggagc
agctacttttgctgatatgcagcaaaggagaaagtaccagcggaaacaag
gatttcagggagaattgcttgatggagcacaagactacatgtcaggccta
gatgatatgacagactccgactcctgtctgtctcgcaaaaagatcaagaa
gacagagagtggcatgtatgcatgtgacttatgtgacaagacattccaga
aaagcagttcccttctgcgacataaatacgaacacacaggaaaaagacca
catcagtgtcagatttgtaagaaagcgtttaaacacaagcaccaccttat
cgagcactcaaggcttcactcgggcgagaagccctatcagtgtgataaat
gtggcaagcgcttctcacactcgggctcgtactcgcagcacatgaatcac
aggtattcctactgcaagcgggaggcggaggagcgggaagcggcggagcg
cgaggcgcgcgagaaagggcacttggaacccaccgagctgctgatgaacc
gggcttacttgcagagcattacccctcaggggtactctgactcggaggag
agggagagtatgccgagggatggcgagagcgagaaggagcacgagaaaga
aggcgaggatggctacgggaagctgggcagacaggatggcgacgaggagt
tcgaggaggaagaggaagaaagtgaaaataaaagtatggatacggatccc
gaaacgatacgagatgaagaagagactggagatcactccatggacgatag
ttcggaggatgggaaaatggaaaccaaatcagaccacgaggaagacaata
tggaagatggcatgtaataaactactgcattttaagcttcctattttttt
ttccagtagtattgttacctgcttgaaaacactgctgtgttaagctgttc
atgcacgtgcctgacgcttccaggaagctgtagagagggacagaaggggc
ggttcagccaagacagatgtagacggagttggagctgggtattgttaaaa
actgcattatgcaaaaattttgtacagtgttaaggcctaaaaactgtgtg
gttcagagactaattcctgtgtttaatagcatttatactttaagcacaac
tagaaaattgtaagaattgcactctacttatgtatcactacaaactttaa
aaaactatgtctaatttatattaatacattttaaaaaggtgcccgcacta
ccatacatcagtatttttattattattattgttattcctttttaatttaa
tgtgctcgcactacaatgcatcagtattatgattcctctgtactttcctt
tcgctattcatcaatttcccattttttttttcagcttaagtaaccacaca
attttaggcctcaattttttttttttttctgtgaaggaacttgaagtgatg
catgtgtgaatttaagataccgaagtcttaaagtgacctggacgtgaagg
aaaaagtaagatgagaaataaagaaagcctttgtaaggtggttttaaaag
ccttatatgcaaaccttttaatctgtgtttctgcaagtgccatccttgta
cagtgttaagagggtaacatgggttacctttgcaccagcttcagtgttaa
gctcaccctgttctttgaagcacccatgtcagtattagaagaataggcag
cagttccttagtttacatatgtttgtgcaattattttctgtactttttttg
ttcattaattttgtcagtattacaccaaactgtttttgcaacaaaaaaat
ttttttgcattcatttaattttaggtcaaataacattttatttatgtgg
ctcattttatatttcctaattttatttatttcatactgtagtgtacagta
ttatagttcttcaatatatagatatattttagtaaaaaaggaacatgacg

ttgatcatttgggcaaattttacgtaaagagaagagcatttattgtgttt
tggaacattaattgtgagatgggatttttcaattttattattttattttt
gtttttttccaattactggaaattccaaatttgggaacttttgatacgat
cttgtgaaaacactgtattttcgactgaaaattccactttcttcatcttg
ttttttagctaaaaagagggactgttaaatacaatgtatgataccatgac
aaaaatctttcctgaattgtctttgtaaaagtattattgaattttcaatt
tgtaatttcttttgaaaatgaccatgctcgaataaaaatgtagccaaact
aagaatgtagttaatgagttctgtacttttagagagttttccttcaatga
ccattaacatgtaacatgctttatgcttataataatgctaattatgtttt
tttcatataattttagtttagcaataattttgactggtaccaataactgt
tttttaaaattccatacctatgtacagcaattttacagcttttctcaact
gatcctgattccagattgtgtatttttatgtgaggttatattattcaaat
ttagtctatttactttacagacatttctacttttgcattacgagtattta
gagattatgtgttaaaaattcacttctctgtccaaggggtctttgtgatt
tattcaaaaaaaaagtctaatttcaaaaagacagctattattcagtgttat
ttataatatgtaaccttttttaaaggattgggatagtttatctcactttt
tgaaatgcagacagtagtttaccgtttatctgaaactagaaggcgtgggt
gggagaggaaaagctaaaagcaaatgctaacaaaaataaccgtgattttc
taagacagtttttcagtttttacaagatgaccctaatattcagaatatga
atgtattcgtaggttttacataatgacttttatcaagaaactagattctg
cttcttaaatctaattgccaagtgaagaataacagaaaaaacagattacc
ttatcaaatttacagctcttgaatatacagaactataatatagtagctgt
ccatgtatttttctactttagaatcaaagaagaaaagcatcattttgct
attaaatttgctaaaattttgagtatgatatttccagttggcaagaacaa
catatttatatttattccttagccataataccactttcctaaatttcaca
aaagtcattctttgcaacttgaaactcaatagaaagtgtgtatgtgtgtg
tgtgtatatatatatatatacacacacacacatacacagaaaggatgt
aatgaagatacagtaatagttgagcagacctttttagaaaaacatgtttt
tagctctatcttcaaactttctggcagagggggtgggggggcaggggga
ggagtggcatcaaaatgctatgcctcctgttatccacagcctagagtttt
tatatttggaaagtttagaaaattctatcctcgtttctccttctttgaat
ggcacaaataaatacactacataaatttttctggtttgaaaggctctagg
cgataactttattaattcaacctgaaaatatcaagccattaaattttgtc
cgggtagaataaatccctgtggcctcttttaaagcaatgtaggtctctgt
tgcccatggggcatatctgtgtcccaatccacaagagataggaccaacaa
acaatgaatgtgcaacctaactctttctccttggaaagaagaaagtgtgc
acgaagtagaggagggtgggcagaccctgccttgcccctcctgttacccc
cttctctgtcatttgttcctaactccatttcataggcaggctcagaatac
ctgagtctgaaaatatcaggataacacttgtgaattgtgacaatcactac
aatgtcccatatctgaggagtttttttaatgctatttatccgctggaca
cgattgcacattagggctgcataatcctctaactctagggaaaaataaaa
acttttgatttgtcttaagattcttctccaaggtcgcaaacaagaaattc
ccctccacaaccaagagatgtgcattttagtaacatcagatgtgttcttc
tgttttatcaactacttactcttcccacacgcttagttctaaatctaacc
tttccccccctcgaatagggggcaggggaggatgaggaaacactggaacaa
ctgaacacccctgcccattttctccaagagccttttgtattctagcatat
ctgtgcaatcttttctttttcttcacatgacactgtaagcttaggcctg
aaataactgggaagagagatgcgtatcagaatttctccgcaagagctaaa
caaaacatacatcttccttagcatgaattggactgggggcggagtgggag

ggcttggaggaaaggggaaagaagggactatatttgaataaatatgaata
aatgtattagatacttttcacaatcagataacttttaaaaaggtcatttt
ttatctttctaataatgtaagccttaataaaagcaaatcttagtcacaaa
tttgaggagactgcccaataataagtttacatgtatttgaactgaaaaat
tgttaaccatgcttttgctccaagatgtgtgaggccattcaggggctgta
gggccctggatatacacacaaacaagtgtgtgtatatctggagccccaca
cattgtaataaacacagctgcatttatttgactatgtgatcccatgtaca
tgtaaaaacattcaaacaaacacactcagcggatttatttattgtgcaat
ggggcaattattcaaataaacatgctcaatgcaattatttgaatctcaca
ttgcatgttcatcaatcatagcactaaaaaaagaggggaaaaaacacca
aagaattcacatggggaaaaaatatatatatgaaaaccaccttattatag
attttatagggcagctgaggttatggctcccttcttaactgtaactcaac
tattctgtattcaatgacatttgtttctaatgattaattggttcactcac
ttgatcatataatagcaaactttataaacctgtattgtgtagagatgtga
aatctctatatttcaagagcagaagagttctttctagacaccttacatca
agggacactggtccaattattatcgcttatataagcactcctataaattc
tgaaaaattttatacatgcaacaaaacattcctacatttgaagacattaa
gaaaaatcacaggtgactcatctgatcattctatatattaataaatatta
tgacatatatgtgaacacatcacaaatcatattggtgtaccaagaggcaa
tttatgcctctcttaagtatgtactgacataacctaatatactaaaatgg
gaaggggcttttagtcactgaaatatgcatcgtgtaacaaagatgaagaa
aatacatggcttgtgcccatcataaaaaaagattcagactgaaggcttag
ctttggtttttcaattaaattgttaaactgtgcacagtgattttttttt
agaacttgagacatttgtgatgttggctgtttaaatctttgttaccttcg
ctgtgaattgaaattgtacatatttagtaaatcatgcagacaaaacaaac
tttttagacaatatttttattggagagttttcttttcctgtatccatgtt
aaaaaaaaaaaaagacctcctttcccaaaataaaaatgtcaatactaaatt
taaagaagtataaaggaatgattgcttcctttagagcaaaatatttaaat
aaacatggagataattggcaacatgttctttttgggctagtaggctgtgt
ccaatttttgggtctgatgtttcagagggcctctgtttcagggttgaag
atgatatattaatctcggaattaaacaaatgctattaaataac
>NM_030751 5
gggggggaagggggagggagggggaggaggtgactcgagcatttagacac
aagcgagaggatcatggcggatggccccaggtgtaagcgcagaaagcagg
cgaacccgcggcgcaataacgttacaaattataatactgtggtagaaaca
aattcagattcagatgatgaagacaaactgcatattgtggaagaagaaag
tgttacagatgcagctgactgtgaaggtgtaccagaggatgacctgccaa
cagaccagacagtgttaccagggaggagcagtgaaagagaagggaatgct
aagaactgctgggaggatgacagaaaggaagggcaagaaatcctggggcc
tgaagctcaggcagatgaagcaggatgtacagtaaaagatgatgaatgcg
agtcagatgcagaaaatgagcaaaaccatgatcctaatgttgaagagttt
ctacaacaacaagacactgctgtcatttttcctgaggcacctgaagagga
ccagaggcagggcacaccagaagccagtggtcatgatgaaaatggaacac
cagatgcattttcacaattactcacctgtccatattgtgatagaggctat
aaacgctttacctctctgaaagaacacattaaatatcgtcatgaaaagaa
tgaagataactttagttgctccctgtgcagttacacctttgcatacagaa
cccaacttgaacgtcacatgacatcacataaatcaggaagagatcaaaga
catgtgacgcagtctgggtgtaatcgtaaattcaaatgcactgagtgtgg
aaaagctttcaaatacaaacatcacctaaaagagcacttaagaattcaca

gtggagagaagccatatgaatgcccaaactgcaagaaacgcttttcccat
tctggctcctatagctcacacataagcagtaagaaatgtatcagcttgat
acctgtgaatgggcgaccaagaacaggactcaagacatctcagtgttctt
caccgtctctttcagcatcaccaggcagtcccacacgaccacagatacgg
caaaagatagagaataaacccccttcaagaacaactttctgttaaccaaat
taaaactgaacctgtggattatgaattcaaacccatagtggttgcttcag
gaatcaactgttcaacccctttacaaaatggggttttcactggtggtggc
ccattacaggcaaccagttctcctcagggcatggtgcaagctgttgttct
gccaacagttggtttggtgtctcccataagtatcaatttaagtgatattc
agaatgtacttaaagtggcggtagatggtaatgtaataaggcaagtgttg
gagaataatcaagccaatcttgcatccaaagaacaagaaacaatcaatgc
ttcacccatacaacaaggtggccattctgttatttcagccatcagtcttc
ctttggttgatcaagatggaacaaccaaaattatcatcaactacagtctt
gagcagcctagccaacttcaagttgttcctcaaaatttaaaaaaagaaaa
tccagtcgctacaaacagttgtaaaagtgaaaagttaccagaagatctta
ctgttaagtctgagaaggacaaaagctttgaaggggggggtgaatgatagc
acttgtcttctgtgtgatgattgtccaggagatattaatgcacttccaga
attaaagcactatgacctaaagcagcctactcagcctcctccactccctg
cagcagaagctgagaagcctgagtcctctgtttcatcagctactggagat
ggcaatttgtctcctagtcagccacctttaaagaacctcttgtctctcct
aaaagcatattatgctttgaatgcacaaccaagtgcagaagagctctcaa
aaattgctgattcagtaaacctaccactggatgtagtaaaaaagtggttt
gaaaagatgcaagctggacagatttcagtgcagtcttctgaaccatcttc
tcctgaaccaggcaaagtaaatatccctgccaagaacaatgatcagcctc
aatctgcaaatgcaaatgaaccccaggacagcacagtaaatctacaaagt
cctttgaagatgactaactccccagttttaccagtgggatcaaccaccaa
tggttccagaagtagtacaccatccccatcacctctaaacctttcctcat
ccagaaatacacagggttacttgtacacagctgagggtgcacaagaagag
ccacaagtagaacctcttgatctttcactaccaaagcaacagggagaatt
attagaaaggtcaactatcactagtgtttaccagaacagtgtttattctg
tccaggaagaacccttgaacttgtcttgcgcaaaaaaggagccacaaaag
gacagttgtgttacagactcagaaccagttgtaaatgtaatcccaccaag
tgccaaccccataaatatcgctatacctacagtcactgcccagttaccca
caatcgtggccattgctgaccagaacagtgttccatgcttaagagcgcta
gctgccaataagcaaacgattctgattccccaggtggcatacacctactc
aactacggtcagccctgcagtccaagaaccacccttgaaagtgatccagc
caaatggaaatcaggatgaaagacaagatactagctcagaaggagtatca
aatgtagaggatcagaatgactctgattctacaccgcccaaaaagaaaat
gcggaagacagaaaatggaatgtatgcttgtgatttgtgtgacaagatat
tccaaaagagtagttcattattgagacataaatatgaacacacaggtaaa
agacctcatgagtgtggaatctgtaaaaaggcatttaaacacaaacatca
tttgattgaacacatgcgattacattctggagaaaagccctatcaatgtg
acaaatgtggaaagcgcttctcacactctgggtcttattctcaacacatg
aatcatcgctactcctactgtaagagagaagcggaagaacgtgacagcac
agagcaggaagaggcagggcctgaaatcctctcgaatgagcacgtgggtg
ccagggcgtctccctcacagggcgactcggacgagagagagagtttgaca
agggaagaggatgaagacagtgaaaaagaggaagaggaggaggataaaga
gatggaagaattgcaggaagaaaaagaatgtgaaaaaccacaaggggatg
aggaagaggaggaggaggaggaagaagtggaagaagaagaggtagaagag

gcagagaatgagggagaagaagcaaaaactgaaggtctgatgaaggatga
cagggctgaaagtcaagcaagcagcttaggacaaaaagtaggcgagagta
gtgagcaagtgtctgaagaaaagacaaatgaagcctaatcgtttttctag
aaggaaaataaattctaattgataatgaatttcgttcaatattatccttg
cttttcatggaaacacagtaacctgtatgctgtgattcctgttcactact
gtgtaaagtaaaaactaaaaaaatacaaaatacaaaacacacacacacac
acacacacacacacacacacacacacaaaataaatccgggtgtgcctgaa
cctcagacctagtaatttttcatgcagttttcaaagttaggaacaagttt
gtaacatgcagcagattagaaaaccttaatgactcagagagcaacaatac
aagaggttaaaggaagctgattaattagatatgcatctggcattgtttta
tcttatcagtattatcactcttatgttggtttattcttaagctgtacaat
tgggagaaattttataattttttattggtaaacatatgctaaatccgctt
cagtattttattatgttttttaaaatgtgagaacttctgcactacaaaat
tcccttcacagagaagtataatgtagttccaacccgtgctaactaccttt
tataaattcagtctagaaggtagtaatttctaatatttagatgtcttagt
agagcgtattatcatttaaagtgtattgttagccttaagaaagcagctga
tagaagaactgaagtttcttactcacgtggtttaaaatggagttcaaaag
attgccattgagttctgattgcagggactaacaatgttaatctgataagg
acagcaaaatcatcagaatcagtgtttgtgattgtgtttgaatatgtggt
aacatatgaaggatatgacatgaagctttgtatctcctttggccttaagc
aagacctgtgtgctgtaagtgccatttctcagtattttcaaggctctaac
ccgccttcatccaatgtgtggcctacaataactagcatttgttgatttgt
ctcttgtatcaaaattcccaaataaaacttaaaaccactgactctgtcag
agaaactgaaacactgggacatttcatccttcaattcctcggtattgatt
ttatgttgattgattttcagaatttctctacagaaacgaaagggaaattt
tctaatctgctttatccatgtacttgcatttcagacatggacatgctatt
gttatttggctcataactgtttccaaatgttagttattatggacccaatt
tattaacaacattagctgatttttacctatcagtattattttatttcttt
tagtttatagatctgtgcaacatttttgtactgtatgtcttcaaacctgg
cagtattaatacccttcttactgacatatgtacttttagttttagaaaac
ttttatatttatgtgtcttatttttatatttctttatttattacacagtg
tagtgtataatactgtagtttgtattaatacaataatatattttagtatg
aaaatttggaaagttgataagatttaaagtagagatgcaattggttctcc
tgcattgagatttgatttaacagtgttatgttaacatttatacttgcctt
ggactgtagaacagaacttaaatgggaatgtattagttttacaactacaa
tcaagtcattttacctttacccagtttttaatataaaacttaaattttga
aattcactgtgtgactaatagcatgatgctctgcagttttattaagaaat
cagcctaaccatacaactctcatttccttagtaagccaaattaggattaa
cttctataaacagtgttgggaacaatgtttaacattttgtgccaatttgt
tcctgtattcatgtatgtaagttacagatctgactcttcatttttaagtt
ccttgttacatcatggtcattttctagttttttaccagactcccatctca
caataaaatgcatcaacaagcctgaactgctgtcattcttttcatcatta
tcagtattttctttggaaaactgtgaaatggggtacattgtcatcctgca
tttgattcatcttgagctgaatttgggtaacactaaatgttttagacatt
ctccactaaattatggattttcttgtggctaaatgtttctggagaggtca
gagttgacaaaacctcttcacaggttgctccttcttcctgaaatccttaa
tcctccgcatttcatgcttcaggtcatttcagggaagcctgggtttagat
gcctttctgactctcagctcctgcacttctgtcatcatacctctgatact
attatttatattccttccccactaggaacaggaaccacatttgtcatagt

cactctcacattcctcactgcctaacagggtgcctggcataagttgggac
aacagatatttgttgaataaaaatataatttgcatgtttatggagctcag
ctatgttctcactttttttgcttctaattccagaatatatgttaaatgat
ctaataatttgattattttcttataagtcttattaaacactagtcataat
agacacaataaattatgccttctttttctattgccttaaaaaaaaaaa
>NM_003722 4
cccggctttatatctatatatacacaggtatatgtgtatattttatataa
ttgttctccgttcgttgatatcaaagacagttgaaggaaatgaattttga
aacttcacggtgtgccaccctacagtactgccctgacccttacatccagc
gtttcgtagaaaccccagctcatttctcttggaaagaaagttattaccga
tccaccatgtcccagagcacacagacaaatgaattcctcagtccagaggt
tttccagcatatctgggattttctggaacagcctatatgttcagttcagc
ccattgacttgaactttgtggatgaaccatcagaagatggtgcgacaaac
aagattgagattagcatggactgtatccgcatgcaggactcggacctgag
tgaccccatgtggccacagtacacgaacctggggctcctgaacagcatgg
accagcagattcagaacggctcctcgtccaccagtccctataacacagac
cacgcgcagaacagcgtcacggcgccctcgccctacgcacagcccagctc
caccttcgatgctctctctccatcacccgccatcccctccaacaccgact
acccaggcccgcacagtttcgacgtgtccttccagcagtcgagcaccgcc
aagtcggccacctggacgtattccactgaactgaagaaactctactgcca
aattgcaaagacatgccccatccagatcaaggtgatgaccccacctcctc
agggagctgttatccgcgccatgcctgtctacaaaaaagctgagcacgtc
acggaggtggtgaagcggtgccccaaccatgagctgagccgtgaattcaa
cgagggacagattgcccctcctagtcatttgattcgagtagaggggaaca
gccatgcccagtatgtagaagatcccatcacaggaagacagagtgtgctg
gtaccttatgagccaccccaggttggcactgaattcacgacagtcttgta
caatttcatgtgtaacagcagttgtgttggagggatgaaccgccgtccaa
ttttaatcattgttactctggaaaccagagatgggcaagtcctgggccga
cgctgctttgaggcccggatctgtgcttgcccaggaagagacaggaaggc
ggatgaagatagcatcagaaagcagcaagtttcggacagtacaaagaacg
gtgatggtacgaagcgcccgtttcgtcagaacacacatggtatccagatg
acatccatcaagaaacgaagatccccagatgatgaactgttatacttacc
agtgaggggccgtgagacttatgaaatgctgttgaagatcaaagagtccc
tggaactcatgcagtaccttcctcagcacacaattgaaacgtacaggcaa
cagcaacagcagcagcaccagcacttacttcagaaacagacctcaataca
gtctccatcttcatatggtaacagctccccacctctgaacaaaatgaaca
gcatgaacaagctgccttctgtgagccagcttatcaaccctcagcagcgc
aacgccctcactcctacaaccattcctgatggcatgggagccaacattcc
catgatgggcacccacatgccaatggctggagacatgaatggactcagcc
ccacccaggcactccctcccccactctccatgccatccacctcccactgc
acacccccacctccgtatcccacagattgcagcattgtcagtttcttagc
gaggttgggctgttcatcatgtctggactatttcacgacccaggggctga
ccaccatctatcagattgagcattactccatggatgatctggcaagtctg
aaaatccctgagcaatttcgacatgcgatctggaagggcatcctggacca
ccggcagctccacgaattctcctccccttctcatctcctgcggaccccaa
gcagtgcctctacagtcagtgtgggctccagtgagacccggggtgagcgt
gttattgatgctgtgcgattcaccctccgccagaccatctctttcccacc
ccgagatgagtggaatgacttcaactttgacatggatgctcgccgcaata
agcaacagcgcatcaaagaggagggggagtgagcctcaccatgtgagctc

ttcctatccctctcctaactgccagcccccctaaaagcactcctgcttaat
cttcaaagccttctccctagctcctccccttcctcttgtctgatttctta
ggggaaggagaagtaagaggctacctcttacctaacatctgacctggcat
ctaattctgattctggctttaagccttcaaaactatagcttgcagaactg
tagctgccatggctaggtagaagtgagcaaaaaagagttgggtgtctcct
taagctgcagagatttctcattgacttttataaagcatgttcacccttat
agtctaagactatatatataaatgtataaatatacagtatagatttttgg
gtggggggcattgagtattgtttaaaatgtaatttaaatgaaagaaaatt
gagttgcacttattgaccatttttaatttacttgttttggatggcttgt
ctatactccttcccttaaggggtatcatgtatggtgataggtatctagag
cttaatgctacatgtgagtgacgatgatgtacagattctttcagttcttt
ggattctaaatacatgccacatcaaacctttgagtagatccatttccatt
gcttattatgtaggtaagactgtagatatgtattcttttctcagtgttgg
tatattttatattactgacatttcttctagtgatgatggttcacgttggg
gtgatttaatccagttataagaagaagttcatgtccaaacgtcctcttta
gtttttggttgggaatgaggaaaattcttaaaaggcccatagcagccagt
tcaaaaacacccgacgtcatgtatttgagcatatcagtaacccccttaaa
tttaataccagataccttatcttacaatattgattgggaaaacatttgct
gccattacagaggtattaaaactaaatttcactactagattgactaactc
aaatacacatttgctactgttgtaagaattctgattgatttgattgggat
gaatgccatctatctagttctaacagtgaagttttactgtctattaatat
tcagggtaaataggaatcattcagaaatgttgagtctgtactaaacagta
agatatctcaatgaaccataaattcaactttgtaaaaatcttttgaagca
tagataatattgtttggtaaatgtttcttttgtttggtaaatgtttcttt
taaagaccctcctattctataaaactctgcatgtagaggcttgtttacct
ttctctctctaaggtttacaataggagtggtgatttgaaaaatataaaat
tatgagattggttttcctgtggcataaattgcatcactgtatcattttct
tttttaaccggtaagagtttcagtttgttggaaagtaactgtgagaaccc
agtttcccgtccatctcccttagggactacccatagacatgaaaggtccc
cacagagcaagagataagtctttcatggctgctgttgcttaaaccactta
aacgaagagttcccttgaaactttgggaaaacatgttaatgacaatattc
cagatctttcagaaatataacacattttttgcatgcatgcaaatgagct
ctgaaatcttcccatgcattctggtcaagggctgtcattgcacataagct
tccattttaattttaaagtgcaaaagggccagcgtggctctaaaaggtaa
tgtgtggattgcctctgaaaagtgtgtatatattttgtgtgaaattgcat
actttgtattttgattattttttttttcttcttgggatagtgggatttcc
agaaccacacttgaaacctttttttatcgtttttgtattttcatgaaaat
accatttagtaagaataccacatcaaataagaaataatgctacaatttta
agaggggagggaagggaaagtttttttttattattttttttaaaattttgt
atgttaaagagaatgagtccttgatttcaaagttttgttgtacttaaatg
gtaataagcactgtaaacttctgcaacaagcatgcagctttgcaaaccca
ttaaggggaagaatgaaagctgttccttggtcctagtaagaagacaaact
gcttcccttactttgctgagggtttgaataaacctaggacttccgagcta
tgtcagtactattcaggtaacactagggccttggaaattcctgtactgtg
tctcatggatttggcactagccaaagcgaggcacccttactggcttacct
cctcatggcagcctactctccttgagtgtatgagtagccagggtaagggg
taaaaggatagtaagcatagaaaccactagaaagtgggcttaatggagtt
cttgtggcctcagctcaatgcagttagctgaagaattgaaaagtttttgt
ttggagacgtttataaacagaaatggaaagcagagttttcattaaatcct

tttaccttttttttttcttggtaatcccctaaaataacagtatgtgggat
attgaatgttaaagggatattttttctattatttttataattgtacaaa
attaagcaaatgttaaaagttttatatgctttattaatgttttcaaaagg
tattatacatgtgatacatttttaagcttcagttgcttgtcttctggta
ctttctgttatgggcttttggggagccagaagccaatctacaatctcttt
ttgtttgccaggacatgcaataaaatttaaaaaataaataaaaactaatt
aagaaattgaaaaaaaaaaaaaaaaaa
>NM_003955 3
ggctccgacttggactccctgctccgctgctgccgcttcggccccgcacg
cagccagccgccagccgcccgcccggcccagctcccgccgcggcccttg
ccgcggtccctctcctggtcccctcccggttggtccgggggtgcgcaggg
ggcagggcgggcgcccaggggaagctcgagggacgcgcgcgcgaaggctc
ctttgtggacttcacggccgccaacatctgggcgcagcgcgggccaccgc
tggccgtctcgccgccgcgtcgccttggggacccgaggggggctcagcccc
aaggacggagacttcgattcgggaccagcccccgggatgcggtagcggc
cgctgtgcggaggccgcgaagcagctgcagccgccgccgcgcagatccac
gctggctccgtgcgccatggtcacccacagcaagtttcccgccgccggga
tgagccgccccctggacaccagcctgcgcctcaagaccttcagctccaag
agcgagtaccagctggtggtgaacgcagtgcgcaagctgcaggagagcgg
cttctactggagcgcagtgaccggcggcgaggcgaacctgctgctcagtg
ccgagcccgccggcacctttctgatccgcgacagctcggaccagcgccac
ttcttcacgctcagcgtcaagacccagtctgggaccaagaacctgcgcat
ccagtgtgagggggggcagcttctctctgcagagcgatccccggagcacgc
agcccgtgccccgcttcgactgcgtgctcaagctggtgcaccactacatg
ccgccccctggagcccccctccttcccctcgccacctactgaaccctcctc
cgaggtgcccgagcagccgtctgcccagccactccctgggagtcccccca
gaagagcctattacatctactccgggggcgagaagatccccctggtgttg
agccggcccctctcctccaacgtggccactcttcagcatctctgtcggaa
gaccgtcaacggccacctggactcctatgagaaagtcacccagctgccgg
ggcccattcgggagttcctggaccagtacgatgccccgctttaaggggta
aagggcgcaaagggcatgggtcgggagaggggacgcaggcccctctcctc
cgtggcacatggcacaagcacaagaagccaaccaggagagagtcctgtag
ctctgggggaaagagggcggacaggcccctccctctgccctctccctgc
agaatgtggcaggcggacctggaatgtgttggagggaagggggagtacca
cctgagtctccagcttctccggaggagccagctgtcctggtgggacgata
gcaaccacaagtggattctccttcaattcctcagcttcccctctgcctcc
aaacaggggacacttcgggaatgctgaactaatgagaactgccagggaat
cttcaaactttccaacggaacttgtttgctctttgatttggtttaaacct
gagctggttgtggagcctgggaaaggtggaagagagagaggtcctgaggg
ccccagggctgcgggctggcgaaggaaatggtcacaccccccgcccaccc
caggcgaggatcctggtgacatgctcctctccctggctccggggagaagg
gcttggggtgacctgaagggaaccatcctggtaccccacatcctctcctc
cgggacagtcaccgaaaacacaggttccaaagtctacctggtgcctgaga
gcccagggcccttcctccgttttaagggggaagcaacatttggaggggat
ggatgggctggtcagctggtctccttttcctactcatactataccttcct
gtacctgggtggatggagcgggaggatggaggagacgggacatctttcac
ctcaggctcctggtagagaagacaggggattctactctgtgcctcctgac
tatgtctggctaagagattcgccttaaatgctccctgtcccatggagagg
gacccagcataggaaagccacatactcagcctggatgggtggagaggctg

agggactcactggagggcaccaagccagcccacagccagggaagtgggga
gggggggcggaaacccatgcctcccagctgagcactgggaatgtcagccc
agtaagtattggccagtcaggcgcctcgtggtcagagcagagccaccagg
tcccactgccccgagccctgcacagccctccctcctgcctgggtgggggа
ggctggaggtcattggagaggctggactgctgccaccccgggtgctcccg
ctctgccatagcactgatcagtgacaatttacaggaatgtagcagcgatg
gaattacctggaacagtttttgtttttgtttttgtttttgtttttgtgg
gggggggcaactaaacaaacacaaagtattctgtgtcaggtattgggctg
gacagggcagttgtgtgttggggtggtttttttctctatttttttgtttg
tttcttgtttttaataatgtttacaatctgcctcaatcactctgtcttt
tataaagattccacctccagtcctctctcctccccсctactcaggccctt
gaggctattaggagatgcttgaagaactcaacaaaatcccaatccaagtc
aaactttgcacatatttatatttatattcagaaaagaaacatttcagtaa
tttataataaagagcactatttttaatgaaaaaaaaaaaaaaaaa
>NM_003182 2
cacgcaagcgaaaggagaggaggcggctaattaaatattgagcagaaagt
cgcgtggggagaatgtcacgtgggtctggaggctcaaggaggctgggata
aataccgcaaggcactgagcaggcgaaagagcgcgctcggacctccttcc
cggcggcagctaccgagagtgcggagcgaccagcgtgcgctcggaggaac
cagagaaactcagcaccccgcgggactgtccgtcgcaaaatccaacatga
aaatcctcgtggccttggcagtctttttcttgtctccactcagctgttt
gcagaagaaataggagccaatgatgatctgaattactggtccgactggta
cgacagcgaccagatcaaggaggaactgccggagccctttgagcatcttc
tgcagagaatcgcccggagacccaagcctcagcagttctttggattaatg
ggcaaacgggatgctgattcctcaattgaaaaacaagtggccctgttaaa
ggctctttatggacatggccagatctctcacaaaagacataaaacagatt
cctttgttggactaatgggcaaaagagctttaaattctgtggcttatgaa
aggagtgcaatgcagaattatgaaagaagacgttaataaactacctaaca
ttatttattcagcttcatttgtgtcaatgggcaatgacaggtaaattaag
acatgcactatgaggaataattatttatttaataacaattgtttggggtt
gaaaattcaaaaagtgtttatttttcatattgtgccaatatgtattgtaa
acatgtgttttaattccaatatgatgactcccttaaaatagaaataagtg
gttatttctcaacaaagcacagtgttaaatgaaattgtaaaacctgtcaa
tgatacagtccctaaagaaaaaaaatcattgctttgaagcagttgtgtca
gctactgcggaaaaggaaggaaactcctgacagtcttgtgcttttcctat
ttgttttcatggtgaaaatgtactgagattttggtattacactgtatttg
tatctctgaagcatgtttcatgttttgtgactatatagagatgtttttaa
aagtttcaatgtgattctaatgtcttcatttcattgtatgatgtgttgtg
atagctaacattttaaataaaagaaaaaatatcttgaa
>NM_000165 3
gagtcagtggcttgaaacttttaaaagctctgtgctccaagttacaaaaa
agcttttacgaggtatcagcacttttctttcattaggggggaaggcgtgag
gaaagtaccaaacagcagcggagttttaaactttaaatagacaggtctga
gtgcctgaacttgccttttcattttacttcatcctccaaggagttcaatc
acttggcgtgacttcactacttttaagcaaaagagtggtgcccaggcaac
atgggtgactggagcgccttaggcaaactccttgacaaggttcaagccta
ctcaactgctggagggaaggtgtggctgtcagtacttttcattttccgaa
tcctgctgctggggacagcggttgagtcagcctggggagatgagcagtct
gcctttcgttgtaacactcagcaacctggttgtgaaaatgtctgctatga

caagtctttcccaatctctcatgtgcgcttctgggtcctgcagatcatat
ttgtgtctgtacccacactcttgtacctggctcatgtgttctatgtgatg
cgaaaggaagagaaactgaacaagaaagaggaagaactcaaggttgccca
aactgatggtgtcaatgtggacatgcacttgaagcagattgagataaaga
agttcaagtacggtattgaagagcatggtaaggtgaaaatgcgaggggggg
ttgctgcgaacctacatcatcagtatcctcttcaagtctatctttgaggt
ggccttcttgctgatccagtggtacatctatggattcagcttgagtgctg
tttacacttgcaaaagagatccctgcccacatcaggtggactgtttcctc
tctcgccccacggagaaaaccatcttcatcatcttcatgctggtggtgtc
cttggtgtccctggccttgaatatcattgaactcttctatgttttcttca
agggcgttaaggatcgggttaagggaaagagcgacccttaccatgcgacc
agtggtgcgctgagccctgccaaagactgtgggtctcaaaaatatgctta
tttcaatggctgctcctcaccaaccgctcccctctcgcctatgtctcctc
ctgggtacaagctggttactggcgacagaaacaattcttcttgccgcaat
tacaacaagcaagcaagtgagcaaaactgggctaattacagtgcagaaca
aaatcgaatggggcaggcgggaagcaccatctctaactcccatgcacagc
cttttgatttccccgatgataaccagaattctaaaaaactagctgctgga
catgaattacagccactagccattgtggaccagcgaccttcaagcagagc
cagcagtcgtgccagcagcagacctcggcctgatgacctggagatctaga
tacaggcttgaaagcatcaagattccactcaattgtggagaagaaaaaag
gtgctgtagaaagtgcaccaggtgttaattttgatccggtggaggtggta
ctcaacagccttattcatgaggcttagaaaacacaaagacattagaatac
ctaggttcactgggggtgtatggggtagatgggtggagagggaggggata
agagaggtgcatgttggtatttaaagtagtggattcaaagaacttagatt
ataaataagagttccattaggtgatacatagataagggctttttctcccc
gcaaacaccccctaagaatggttctgtgtatgtgaatgagcgggtggtaat
tgtggctaaatatttttgttttaccaagaaactgaaataattctggccag
gaataaatacttcctgaacatcttaggtcttttcaacaagaaaaagacag
aggattgtccttaagtccctgctaaaacattccattgttaaaatttgcac
tttgaaggtaagctttctaggcctgaccctccaggtgtcaatggacttgt
gctactatatttttttattcttggtatcagtttaaaattcagacaaggcc
cacagaataagattttccatgcatttgcaaatacgtatattctttttcca
tccacttgcacaatatcattaccatcactttttcatcattcctcagctac
tactcacattcatttaatggtttctgtaaacatttttaagacagttggga
tgtcacttaacattttttttttgagctaaagtcagggaatcaagccatgc
ttaatatttaacaatcacttatatgtgtgtcgaagagtttgttttgtttg
tcatgtattggtacaagcagatacagtataaactcacaaacacagatttg
aaaataatgcacatatggtgttcaaatttgaacctttctcatggattttt
gtggtgtgggccaatatggtgtttacattatataattcctgctgtggcaa
gtaaagcacactttttttttctcctaaaatgtttttccctgtgtatccta
ttatggatactggttttgttaattatgattctttattttctctccttttt
ttaggatatagcagtaatgctattactgaaatgaatttcctttttctgaa
atgtaatcattgatgcttgaatgatagaattttagtactgtaaacaggct
ttagtcattaatgtgagagacttagaaaaaatgcttagagtggactatta
aatgtgcctaaatgaattttgcagtaactggtattcttgggttttcctac
ttaatacacagtaattcagaacttgtattctattatgagtttagcagtct
tttggagtgaccagcaactttgatgtttgcactaagattttatttggaat
gcaagagaggttgaaagaggattcagtagtacacatacaactaatttatt
tgaactatatgttgaagacatctaccagtttctccaaatgcctttttaa

aactcatcacagaagattggtgaaaatgctgagtatgacacttttcttct
tgcatgcatgtcagctacataaacagttttgtacaatgaaaattactaat
ttgtttgacattccatgttaaactacggtcatgttcagcttcattgcatg
taatgtagacctagtccatcagatcatgtgttctggagagtgttctttat
tcaataaagttttaatttagtataaacata
>NM_005225 2
ttggcgcgtaaaagtggccgggactttgcaggcagcggcggccgggggcg
gagcgggatcgagccctcgccgaggcctgccgccatgggcccgcgccgcc
gccgccgcctgtcacccgggccgcgcgggccgtgagcgtcatggccttgg
ccggggcccctgcgggcggcccatgcgcgccggcgctggaggccctgctc
ggggccggcgcgctgcggctgctcgactcctcgcagatcgtcatcatctc
cgccgcgcaggacgccagcgccccgccggctcccaccggccccgcggcgc
ccgccgccggcccctgcgaccctgacctgctgctcttcgccacaccgcag
gcgccccggcccacacccagtgcgccgcggcccgcgctcggccgcccgcc
ggtgaagcggaggctggacctggaaactgaccatcagtacctggccgaga
gcagtgggccagctcggggcagaggccgccatccaggaaaaggtgtgaaa
tccccgggggagaagtcacgctatgagacctcactgaatctgaccaccaa
gcgcttcctggagctgctgagccactcggctgacggtgtcgtcgacctga
actgggctgccgaggtgctgaaggtgcagaagcggcgcatctatgacatc
accaacgtccttgagggcatccagctcattgccaagaagtccaagaacca
catccagtggctgggcagccacaccacagtgggcgtcggcggacggcttg
aggggttgacccaggacctccgacagctgcaggagagcgagcagcagctg
gaccacctgatgaatatctgtactacgcagctgcgcctgctctccgagga
cactgacagccagcgcctggcctacgtgacgtgtcaggaccttcgtagca
ttgcagaccctgcagagcagatggttatggtgatcaaagcccctcctgag
acccagctccaagccgtggactcttcggagaactttcagatctcccttaa
gagcaaacaaggcccgatcgatgttttcctgtgccctgaggagaccgtag
gtgggatcagccctgggaagaccccatcccaggaggtcacttctgaggag
gagaacagggccactgactctgccaccatagtgtcaccaccaccatcatc
tccccccctcatccctcaccacagatcccagccagtctctactcagcctgg
agcaagaaccgctgttgtcccggatgggcagcctgcgggctcccgtggac
gaggaccgcctgtccccgctggtggcggccgactcgctcctggagcatgt
gcgggaggacttctccggcctcctccctgaggagttcatcagcctttccc
caccccacgaggccctcgactaccacttcggcctcgaggagggcgagggc
atcagagacctcttcgactgtgactttggggacctcacccccctggattt
ctgacagggcttggagggaccagggtttccagagatgctcaccttgtctc
tgcagccctggagccccctgtccctggccgtcctcccagcctgtttggaa
acatttaatttatacccctctcctctgtctccagaagcttctagctctgg
ggtctggctaccgctaggaggctgagcaagccaggaagggaaggagtctg
tgtggtgtgtatgtgcatgcagcctacacccacacgtgtgtaccgggggt
gaatgtgtgtgagcatgtgtgtgtgcatgtaccggggaatgaaggtgaac
atacacctctgtgtgtgcactgcagacacgccccagtgtgtccacatgtg
tgtgcatgagtccatgtgtgcgcgtgggggggctctaactgcactttcgg
cccttttgctctgggggtcccacaaggcccagggcagtgcctgctcccag
aatctggtgctctgaccaggccaggtggggaggctttggctggctgggcg
tgtaggacggtgagagcacttctgtcttaaaggtttttctgattgaagc
tttaatggagcgttatttatttatcgaggcctctttggtgagcctgggga
atcagcaaaggggaggagggggtgtggggttgataccccaactccctctac
ccttgagcaagggcaggggtccctgagctgttcttctgccccatactgaa

ggaactgaggcctgggtgatttatttattgggaaagtgagggagggagac
agactgactgacagccatgggtggtcagatggtggggtgggccctctcca
gggggccagttcagggccccagctgccccccaggatggatatgagatggg
agaggtgagtgggggaccttcactgatgtgggcaggaggggtggtgaagg
cctcccccagcccagaccctgtggtccctcctgcagtgtctgaagcgcct
gcctccccactgctctgccccaccctccaatctgcactttgatttgcttc
ctaacagctctgttccctcctgctttggttttaataaatattttgatgac
gtttgggccgggttttgggactctgttgggaacatttcggggcgggagag
gccaaggttgctggggaaatgcccattctccacttcccttctccctgtcc
gtgcccgatttgatttgagcctcataactcgaagaaaggtcagcttcctc
gctgttttggtcctaactcaaaagcagatccagtaaaggtttttgttgta
aaaaaaaaaaaaaaaaaaaaa
>NM_014456 4
cttttcctcctcagctccggctccgccgccacgattggccagccgaccac
ccggcctcggccaataagcgccgccctctcgcccccgtgttactgggtag
aagaaaacaaaaacaaacagagcgagaagggccagagactctccgaggcg
gcggcagagacagaagagcggggtcggggccggctgaccaggaacctggg
cgagcagcggcgggggcccgagggattctgaaggaagatttccattaggt
aatttgtttaatcagtgcaagcgaaattaagggaaaatggatgtagaaaa
tgagcagatactgaatgtaaaccctgcagatcctgataacttaagtgact
ctctcttttccggtgatgaagaaaatgctgggactgaggaaataaagaat
gaaataaatggaaattggatttcagcatcctccattaacgaagctagaat
taatgccaaggcaaaaaggcgactaaggaaaaactcatcccgggactctg
gcagaggcgattcggtcagcgacagtgggagtgacgcccttagaagtgga
ttaactgtgccaaccagtccaaagggaaggttgctggataggcgatccag
atctgggaaaggaaggggactaccaaagaaaggtggtgcaggaggcaaag
gtgtctggggtacacctggacaggtgtatgatgtggaggaggtggatgtg
aaagatcctaactatgatgatgaccaggagaactgtgtttatgaaactgt
agttttgcctttggatgaaagggcatttgagaagactttaacaccaatca
tacaggaatattttgagcatggagatactaatgaagttgcggaaatgtta
agagatttaaatcttggtgaaatgaaaagtggagtaccagtgttggcagt
atccttagcattggaggggaaggctagtcatagagagatgacatctaagc
ttctttctgacctttgtgggacagtaatgagcacaactgatgtggaaaaa
tcatttgataaattgttgaaagatctacctgaattagcactggatactcc
tagagcaccacagttggtgggccagtttattgctagagctgttggagatg
gaattttatgtaatacctatattgatagttacaaaggaactgtagattgt
gtgcaggctagagctgctctggataaggctaccgtgcttctgagtatgtc
taaaggtggaaagcgtaaagatagtgtgtggggctctggaggtgggcagc
aatctgtcaatcaccttgttaaagagattgatatgctgctgaaagaatat
ttactctctggagacatatctgaagctgaacattgccttaaggaactgga
agtacctcattttcaccatgagcttgtatatgaagctattataatggttt
tagagtcaactggagaaagtacatttaagatgattttggatttattaaag
tccctttggaagtcttctaccattactgtagaccaaatgaaaagaggtta
tgagagaatttacaatgaaattccggacattaatctggatgtcccacatt
catactctgtgctggagcggtttgtagaagaatgttttcaggctggaata
atttccaaacaactcagagatctttgtccttcaaggggcagaaagcgttt
tgtaagcgaaggagatggaggtcgtcttaaaccagagagctactgaatat
aagaactcttgcagtcttagatgttataaaaatatatatctgaattgtaa
gagttgttagcacaagtttttttttttttttttttaagcacttgttttg

ggtacaaggcatttctgacattttataaacctacatttaaggggaatttt
taaaggaaatgtttttcttttttttttgtttttcgagggggcaaggagg
gacagaaaagtaacctcttcttaagtggaatattctaataagctaccttt
tgtaagtgccatgtttattatctaatcattccaagttttgcattgatgtc
tgactgccactcctttctttcaaggacagtgtttttgtagtaaaatcac
tggtttatacaaagctttatttagggggtaaagttaagctgctaaaaccc
catgttggctgctgctgttgagatactgtgctttgggagtaaaaaaagaa
agttatttctttgtcttaaagaatttttaaaaaattagtcatgagactta
ttcatctttccagggaacatactgattggtcttaaaagactagacagtta
agtaaaaggtggctggaacatctatttttctacaaaactggaaaaatgaa
cctggttctagaagaatgtacaccaaaataaaacatgtgaagcagtattg
attctttattgggagtacattttttaggtctcttaaactttaatttcac
acagtaaattttgaatctcataaggaagcatatttgaacctagtcaattt
aatcttagtgttcccttgaaaactttttttccctacaaaattttaagtga
aaaatacaatagtaaattaagattacactggggaaaaaaatgcaggtatc
actttactccattgttatctgacctagagcttaattaagttttagaaata
tgtaataccttccatcattccatcatccttaaattctgttaccaaataat
ggctaatgttacaaaaagttatactccagagacccaaagcttgacattta
cctaatgtatgagaaaatattaccaattaacaataaagaatgatcatatt
tttaacctcttttacatagcctaataactcagcaaggcctcaacgtctgt
gctaatttaaactgccaaatattgactgcagcaaacaagaattatattca
gaatttatgagggtactgttaggagtatactgcttacaggtttagatata
gtctgttagaattaaaaccaagtttagtgttcatatttacctcatgggct
ttatcaagcccatattacctcagcttatatatagttaccatttttaggtt
tttaattgtttgacacttggatgataaatgcagtcattttattctcaagt
gcttaaaattaatgtaattaaaagcttagctgactacagaataggtgagg
gtttcttaaaaatgagatttaagggctgggcacggtggctcatgcctgta
atcccagcactttgggaggccgaggtgggcggatcacttgaggttgggag
ttcatgaccagcttgaccaacatgaagaaaccctgtctctattaaaaata
caaaagtagccaggcatggtggcgcatgcgtgtaatcccagctacttggg
aggctgaggcaggagaattgcttgaacctgggaggcagaggttgcagtga
gtcgagatggtgccattgctctcgtttgggcaacaagagtgaaactcttg
tctcaaaaaaaaaaaaaaaatgaggtttaagacagttttgtcattactggt
gggatctggtcacacaagatagcattaaacgtgacatggcacataaaatt
ggttaaaaaattttgtttttttaattacgtaatgtaaaagcccaacaaaca
ctttatgcaagattggaatgtatcttcaaattcagatttaataaacatgt
aaagatcctctgtaaaaaaaaaaaaaaaaaaaaaaaaaaaaaaaa
>NM_001018005 1
ggaagggggggcaggagaaaaaagcttttccaaaaaagtattggctgtctt
gaggaatgcggtcgccccccttgggaaagtacatatctgggagaagcaggc
ggctccgcgctcgcactcccgctcctccgcccgaccgcgcgctcgccccg
ccgctcctgctgcagccccagggcccctcgccgccgccaccatggacgcc
atcaagaagaagatgcagatgctgaagctcgacaaggagaacgccttgga
tcgagctgagcaggcggaggccgacaagaaggcggcggaagacaggagca
agcagctggaagatgagctggtgtcactgcaaaagaaactcaagggcacc
gaagatgaactggacaaatactctgaggctctcaaagatgcccaggagaa
gctggagctggcagagaaaaaggccaccgatgctgaagccgacgtagctt
ctctgaacagacgcatccagctggttgaggaagagttggatcgtgcccag
gagcgtctggcaacagctttgcagaagctggaggaagctgagaaggcagc

agatgagagtgagagaggcatgaaagtcattgagagtcgagcccaaaaag
atgaagaaaaaatggaaattcaggagatccaactgaaagaggccaagcac
attgctgaagatgccgaccgcaaatatgaagaggtggcccgtaagctggt
catcattgagagcgacctggaacgtgcagaggagcgggctgagctctcag
aaggcaaatgtgccgagcttgaagaagaattgaaaactgtgacgaacaac
ttgaagtcactggaggctcaggctgagaagtactcgcagaaggaagacag
atatgaggaagagatcaaggtcctttccgacaagctgaaggaggctgaga
ctcgggctgagtttgcggagaggtcagtaactaaattggagaaaagcatt
gatgacttagaagacgagctgtacgctcagaaactgaagtacaaagccat
cagcgaggagctggaccacgctctcaacgatatgacttccatataagttt
ctttgcttcacttctcccaagactccctcgtcgagctggatgtcccacct
ctctgagctctgcatttgtctattctccagctgaccctggttctctctct
tagcatcctgccttagagccaggcacacactgtgctttctattgtacaga
agctcttcgtttcagtgtcaaataaacactgtgtaagctaaaaaaa
>NM_144949 2
ggaaacgggtgaagaaggggaggcggcagggaagggggtgggggcctggc
gggggcatccggcggagctggggtccccgggctccgtccggaggaagcga
cgctgcgctcgctgggcagtcggaggggacgggacgcaccggagggcagg
cggactcgccctgtcggtgactgcgccgtccgggcccgtcctgcctggcc
gcaggtgccctggatgaggccgccccgcgcgccccaaacgattttataat
caatggataaagtgggaaaaatgtggaataacttcaaatacaggtgtcag
aatctcttcggtcatgagggaggaagccgtagtgaaaatgtggacatgaa
ctccaacagatgtttgtctgtcaaagagaaaaacatcagcataggagact
caactcctcagcaacaaagcagtcccttaagagaaaatattgccttacaa
ctgggattaagcccttcgaagaattcttcaaggagaaatcaaaattgtgc
cacagaaatccctcaaattgttgaaataagcatcgaaaaggataatgatt
cttgtgttaccccaggaacaagacttgcacgaagagattcctactctcga
catgctccatggggtgggaagaaaaaacattcctgttctacaaagaccca
gagttcattggatgctgataaaaagtttggtagaactcgaagtggacttc
aaaggagagagaggcgctacggcgtaagttctgtacacgacatggacagt
gtttccagcagaactgtaggaagtcgctctctaagacagaggttgcagga
tactgtgggcttgtgttttcccatgagaacttacagcaagcagtcaaagc
ctctcttttccaataaaagaaaaatccatctctctgaattaatgcttgag
aaatgcccttttcctgctggctcagatttagcccaaaaatggcatttgat
taaacagcatacagctcctgtgagcccacattcaacatttttgatacat
ttgatccatctttggtttctacagaagatgaagaagataggcttagagag
agaaggcggcttagtattgaagaaggggttgatccccctcccaatgcaca
aatacatacatttgaagctactgcacaggttaatccattatataaactgg
gaccaaaattagctcctggaatgactgaaataagtggggacagttctgca
attccacaagctaattgtgactcggaagaggatacaaccaccctgtgttt
gcagtcacggaggcagaagcagcgtcagatatctggagacagccataccc
atgttagcagacagggagcttggaaagtccacacacagattgattacata
cactgcctcgtgcctgatttgcttcaaattacagggaatccctgttactg
gggagtgatggaccgttatgaagcagaagcccttctcgaagggaaacctg
aaggcacgtttttgctcagggactctgcgcaagaggactacctcttctct
gtgagcttccgccgctacaacagatccctgcatgcccgaattgagcagtg
gaatcacaactttagtttcgacgcccatgacccgtgtgtatttcactcct
ccactgtaacgggacttttagaacattataaagatcccagttcgtgcatg
ttttttgaaccattgcttactatatcactaaataggactttccctttttag

cctgcagtatatctgtcgcgcggtaatctgcaggtgcactacgtatgatg
gaattgatgggctccctctaccctcaatgttacaggatttttaaaagag
tatcattataaacaaaaagttagagttcgctggttggaacgagaaccagt
caaggcaaagtaaactctccggtccccaaaggttgttaactaggtccgct
ttcatgtgcatcagacagtacacctatagcaagcacacgtagcagtgtta
ggctttttcatacagtatgtaagcttagtgttagtatctgtcagatgcta
cctgctgttacttattcagataaacatggtgcctattggaacaatagcgg
atagagctacaggtgttcagtaagactacaaaaacattttgcctatttcg
ctaacagtttggtttttaatggctgtggtatttgagtgaggcaactctgg
ggcatttgttatgaagaattctatttcttactgaagaacaaattattaat
attggatgagtatttcaacagtgtgactaatgtttgaaattattttttct
aagagtttttctataaccttccaaaagtcgtgatgtttgtagttactata
aatcaagctttggaagtccaaaaagaataaaagactgccttccttttaga
aaaaaatgcaattttctggccacaagggcatagtgcagttcacttacgtg
ttgatgtagtttataatcagacgccttttctcttctgcaaaaggtactgt
taagtaaaccagattttctaaataggcattcttaaaatttcagacttaca
aagctagtagtagaattttattgaaaggcctaggtattaatttttttaaat
gagtgctttaacttaaaacaggcgtttggaatagctgctgcaatgtagtc
ttgtgtgtgattttttttttaagttgatgtgcagtctaattgttgtttcat
aaaagttggatctgttcctatgcccaggatgattttgtgaaccgtgaagt
acgtgagactagaagacgcccaaacaagtcagataatagtaactacaatg
gttgctgatgttgagattattgttgaactataattaataatttggatggc
agaatttatctctttttttgtaaactctcataactgaattgcttaagtata
atttatagaatttcagtgcagttcattcttaatggaaaatctgaaaccta
aattgcagatttaaaaggtactgtacaaccattatatctgtaaataactt
agcaccttttttgtcacttagaataatatgtactactacttgagtgagcgc
ttttggaagttatatcaagttctagtgtttgcttcttagtaactgaactg
aatttacagttctgtcctagacattttgcactaaagtagccgaatccact
ctcatgtcttttcgttaatgtgctctgtaccactggtgagtgctccatag
tttccttacctgctgctacagaatgttattttacatccctatggctattg
ccaaggctacaaaaaaaggaaagctatatttgtatgcaacactaacctttt
gactgctaatgtatgtttctgcttgctgtgccttgttatggctgcttttt
ttgtgctaataaagtatgtttggtgttctccttgtatatctgctgtttta
tacatttgcaacaatttctcttgtaaatggaatggtttggggtttttaaa
taagcattaactaacaacctttctatagttaatgcagagttaatgaacag
tctaatattgacttatcagaataagctaactctaaatttaatgctctaca
tcttatcagtcataattatatatactgtggaacagtatctgtagttactg
caaattactgtacagtttaggttataacagaaaactgacagagaagtaat
aaacctattgatttctctgcttataaatgaaagattgaaactatccaatg
acatattatagtaaatgagtatctgtaacctcccactgcatcagaagcag
gttaaatgaagtcttgtgaatttgtaatagatcagtaccatttattggtt
tggggaccatcttaattaaaaataaatgcccaaaatgtagaactttaacc
aaagacttgtcccttttaaagcaaaatggggattgaagggacttataatt
tctgttgtttctaattaaagtccctgaagatcatataccaaagtgtttga
gaacttcatccaaacctactttaaagcattatgtgcaattaagttgttat
gacataattatattgcctaattgttgggtctttttttcttgagcttataat
gtacctggaaaataaacctcttgagaaaaagaaaagttcatactgattat
tggaaaaggactatatatgtgagcaagattgtgttttagagaggaaactt
gaaactccaagaaagcacttgatgtttttatatgcttgtagcaaattgat

gttctaactgtagttttatagaaagtattaatgcttttatgtatttcaaa
actttcatatgttaaatggaaattgttttaaatgtgtttgagtttatgta
agcatgtatacactgtgctaaaagtcacatgtttcagtttgtgtataata
ttaatatgcaatttttggtttaaatttttgtcttaaaatattagtggctt
acattttaaaaaagaaaaatcaccagcatgaacttgcacctaagtctata
ttcactgtgtccttttctgaatcccattgtagcctgtcaactaaatttga
gtgttaacggtctttttaaagtgcatttaaatacaaaccaggaatttctt
tagaagttga
>NM_005596 3
gggctgtaaccttgaactttcccagcgcggtgacacattctccccgctct
ccctcccgcccgcccgctcgccctcctgcgccctcccgcgcccccctccc
cgcctttttttgaaaaagcattttaccaccaaccaccaccccaatccaacc
cacaccgaaccttcgcgcacccccctacaccccaacaacaacaacaactgc
aaaatagaaaacaaatccccaaacccaggcgaaaagcagccaacaccggc
ggcggcggcggcctcggcaagcacggccagcgcgctcggactgcaagagg
gttaaaagtgtagattggatttcacccctggaaatctagcacgccgagtg
aacttgaatctttggctatttaaggaggactgggtttgttgtgaagttgc
ggtgatccagcgcagagccccgtcctgattgatcgcatcgcggggctcag
atgactgtaaaatgaatagatgaaattcttgcttctcgaagattttcttg
ggcatctcccggaaagtgcgttttaaggcgaagtcatgatgtattctccc
atctgtctcactcaggatgaatttcacccattcatcgaggcacttcttcc
acatgtccgtgcaattgcctatacttggttcaacctgcaggctcgaaaac
gcaagtactttaaaaagcatgagaagcgaatgtcaaaggatgaagaaaga
gcagtcaaagatgagcttctcagtgaaaagcctgaaatcaaacagaagtg
ggcatccaggctccttgccaaactgcgcaaagatattcgccaggagtatc
gagaggactttgtgctcaccgtgactggcaagaagcacccgtgctgtgtc
ttatccaatcccgaccagaagggtaagattaggagaatcgactgcctgcg
acaggcagacaaagtctggcgtctggatctagtcatggtgatcctgttca
aaggcatccccttggaaagtaccgatggagagcggctcatgaaatcccca
cattgcacaaacccagcactttgtgtccagccacatcatatcacagtatc
agttaaggagcttgatttgtttttggcatactacgtgcaggagcaagatt
ctggacaatcaggaagtccaagccacaatgatcctgccaagaatcctcca
ggttaccttgaggatagttttgtaaaatctggagtcttcaatgtatcaga
acttgtaagagtatccagaacgcccataacccagggaactggagtcaact
tcccaattggagaaatcccaagccaaccatactatcatgacatgaactcg
ggggtcaatcttcagaggtctctgtcttctccaccaagcagcaaaagacc
caaaactatatccatagatgaaaatatggaaccaagtcctacaggagact
tttacccctctccaagttcaccagctgctggaagtcgaacatggcacgaa
agagatcaagatatgtcttctccgactactatgaagaagcctgaaaagcc
attgttcagctctgcatctccacaggattcttccccaagactgagcactt
tcccccagcaccaccatcccggaatacctggagttgcacacagtgtcatc
tcaactcgaactccacctccaccttcaccgttgccatttccaacacaagc
tatccttcctccagccccatcgagctacttttctcatccaacaatcagat
atcctccccacctgaatcctcaggatactctgaagaactatgtaccttct
tatgacccatccagtccacaaaccagccagtcctggtacctgggctagct
tggttcctttccaagtgtcaaataggacacccatcttaccggccaatgtc
caaaattacggtttgaacataattggagaacctttccttcaagcagaaac
aagcaactgagggaaaaagaaacacaacaatagtttaagaaatttttttt
ttaaataaaaaaaaaggaaaagaggaagactggacaaaacaacacaaagg

cagaaaggaaagaaactgaagaaagaagataatagaccagcaattgcagc
acttacaatcactaattcccttaaggttgaaactgtaatgacataaaaag
ggtcgatgatatttcactgatggtagatcgcagcccctgcaacgtagcct
ttgttacatgaagtccgctgggaaatagatgttctgtctctatgacaata
tattttaactgactttctagatgccttaatatttgcatgataagctagtt
ttattggtttagtattcttgttgtttacgcatggaatcactattcctggt
tatctcaccaacgaaggctaggaggcggcgtcagaggtgctgggtgacag
agccatgagccagccattttataagcactctgatttctaaaagttaaaaa
aaatatatgaaatctctgtagcctttagttatcagtacagatttattaaa
tttcggcccttaacccagccttttccagtgtgtaacccagtttgaaatct
taaaaaaagaaaaaatgaaaaaaaaaggaaaaaaagaaaaaaggaaaaaa
acagtttgaacacaaaggctctatggaagaaatgcctctatgtaggtgaa
gtgttctctctgcatgcaacagtaaaaattaatataatattttccccaca
aaagaaacacttaacagaggcaagtgcaatttataaatttatatctaaag
gggaatcatgattataagtccttcagcccttggactctaaattgaggga
ttaaaaagaatttaaaataattttgaacgaatttattttcccctcagttt
ttgagggcattaaaaaggcattaaatcaagacaaatcatgtgcttgagaa
aaataaaattaatgaaaacacagcacttatgttggtttagctgcagcctc
cttggaggtagaatttatttatttaaaattactggttgcatcaagaaccc
atagggtgtacaaaaggttctataaaatctgcattatagagacaaagagg
caggcaaatccatgtcacaagggtaaagcttacagtttacaaactgggaa
cgccagggtgtaggatataaaaacgcactcttgagaaaacaaatgtaatc
agggtgctgaaaacttgcatggtgctttcagacattagccttgttcaaca
aatttcttgtattgacagatccatagtgtgcatgggcagacacattttgc
ctctatgtctcttaaaattttaattaaaaatactctttccagtaatccta
atttgcacgaagatataatgtccacattacgtgccttgccttgaaatcta
aaaaacaaaaaacaaaaaaaaaaaaaacaaaaaaatacaacaaagtgacat
cactacacttgttttgctgcatttattatcattttaaatctttaccattt
ttatgacaaaatattttgtactccagacgaagaaaaatgtgtgacatcat
ggatttttagacagttatacctttatctcacatttataaagcatatcat
ggctgtgtatagttgccgcttaaaaattgtaatcgaccagcaatattttc
agtattttggtgtttttttctattaacctttcatgtttttcatcttccaa
ttaatatttgggggggaggggtttcaaatttatacgaattatgcaatacc
aagttttgcctatgtaggtagtgcttttagctgtattggttattataggt
aagtacacagatttaaaaaaaaaataatgtatgctttttgtttgtttgt
ttgttttaattgaccaaagtgggtactgctatttttgcagtgtgatgagg
tccttttgtgtactgagagatggacaggggattttttttaatatacatat
atatatattctggggtgggtgggaggatttttaacactttgcagtgtagc
tgtgaagcagtgcaccctgagatgggcctgggctgcaaagcgactgttct
gcctactgtgacaaacttcaacttacacaggttcccctctctaacttccc
acctgggttgcaagctgaactcattactggttttcataacaacacaatag
taagaacaagcaaacacaacaaattctcctggaggcagacttggcttaaa
aaggcagacttggcttggtgatagtttttcttgaaagttccagatccaca
gtggagagtgagcctgtctcatatttggcaaaaatatttgttgaaatgtc
cacataggggatgttggatgtttaacacttttgagagtttaacacatgaa
tattctttctcctagaaaacacattagacctgttggagggagtctcccgt
attccttttctgccacttttcgtccccatttcatttcattaatgatagga
tatgatttacctgtgacttactacttcaaatggatggcagtgcacttgga
ttttttttaatatccagaagattgaacagagggttgctattgttgaatg

tatttggactgatagattaaaatcaaagttcaatttttaaggaacaaaaa
agtaaatcctgttttcattttatctccccttttaaaactgagaaccagag
cagaagggaaatatagaattttaagcaattaatcttcctgtggatgaatt
aaacccattagatgctgatgggatttttttaaggaatggtaccttaacta
tatatttgatttcgtttcccctgagggctagaggctgaatggaggctggt
tttattttgcctttccctcaccgcccagtcccattgagtgtattcattac
tagaaggaaaatctttcagaattggtgacacatggtaggctgtcttaagg
agtcccctggccccccttcccctaggccatggcctaataaaataaactgtc
aattgttctcacagcatatcatttaataatgaatactttagaacaatgct
tatgggctggagaattgtatttgattagcccattcagtttgatagcccaa
atgctgaacagcacagcgggatcctagcagtgcaagttcaaaagtaagtc
caatcatttctgtgatactcgccctggtagcaaacagatcatctcagcca
agctcttcatgtatctttgacctattaggtgaacaaatgaacctcacagg
acacacagtattttttaaaggcagactcgctctctttttttgccagtgagc
agttctagctaaccaagttacacactgtgggtattcctgcctgcctcttg
aatacaaaggcctagttcaagtgttgcttttttttatttcaaatcaatttt
ttcttctttcctttttgagataaaactattaaaagtactactatatatat
aaaatctcaaatcaacttttcggcctcctcctcgtgtaccaggaagtata
ttctgacgaagggccccacttttgcaggtcttgcacgcccctcccttacc
cagaactgcagagcttcaggatggcgaaggtcacccaagggcatgagtag
ggagtggtgtctccaaccatcagttccgtggcactgttcagcctttgtgt
gctgccctgccacccaccactcacagtgcctctgaagcgtgttacccctg
gagtgacgtgagcatttgaggcttgtctaaggaaaaaaataaaaggcagt
gaaggagactgtacataaagacatggcaaaaatcttaattatagcaatat
agttatcgggtaatgttcgggtgggcagctccattaaaaaatatgtgaat
gaatctgtgaagctgcaagtagcgagaagagcgaaaggtcttcttaatga
accgcctaccttgtagacagtaatttgtacactgtatagttttgttaaga
atttttttaaattaaaattcccatgtttgtaaagctaactttttaacaa
ttataatggaactatatgttgtttccatttttaaagtaaacaagaatatt
ccttgtttagagactggacttgagttaaaactctccagtctcttaagtta
tgtattaaaaagaaaatctgtccatgttaggagttatttcacagattcct
gtgcttgaaaagcataggatactaatcctttaaaaaagtgtaaatggaga
aaagttatattttatgaaggttattttgttgtatttagtattggaaaagt
tggtttccagagcatttcagaatgtcgaagcaccactgtctttttattag
tatatacggcctttagcaaaagtttttgtgattgttacgtgatggtattt
aaggttaagtttcacagagcattcaggataggcagaaaactaaaacagtg
ctatgtctcacataacgtgtcctcagggagcagaatcttggatttgtgac
ttgtagcttcataaggactcaacgaaagagattgcacagggacatcttca
gcggtgtgacagcaggacatgttctttacctagattcaaattctatgtac
tgtgtgaaatgatgaaggctgcagaaagttatcccatattcagtgtacag
tattcatttttaatgaaacaactctacaatattgctggcagataggcccc
aagcatgacattcaatatagtttacatgttcctgtcaaggtcttttgtta
acattaaccagctgcatgctttctggactttaagaaattgggtttctata
gaaaactttttttttttttttttttaatgtgcaggctattcaagttca
atagtaaaagctcaaaaatgaatgttctactccatgctgaaggagctgaa
agctgccttcttcatattttgcactttctggtagttcccctgttttttct
aattccctaaaattgtgtgggtggagtggagccctgcagttggggggtaa
catggaccactgattttgccctttgaccctgcacaatgacctttgcatca
gccaaactcattgccatgacaactctttgtactgtgtccgtgccacagat

ctgttggtcacattgttaatagtaaaggggacaagttggagacggtcaat
ttttacatttttgttgcaatttttcttcaatggttgtaagtagttttt
ttttttttttaataataaaagggttcactagttaatactctagaaatatc
tgtgtgttgcaattcaaatgtatgttgagattgtgaaaagcgcttcagtg
ccactagcttaccggtacactagactaagcccttgatgacttattgcatg
atacagtaccaggaacaacaggtggcctaaatacatgaaaagcagtgtaa
gctagtgacactaaagccagtcttgtattactgtatttttgacagaatgg
ttttgaaaactgtgctacagggactgatgtggcaaatatatctctttatg
cagaaggaagtctttttttttcttttttttttttttaagaagtatggctt
tttatgcatccttcatcgagggcattgaagttgcatggactgataaaagt
tgatgcaaaacaagaaagaaacaaacaaaaaaaaaaaaaccagcaaaatgt
ttaccaaaaaactcaaacaaatgagcagtgcctgttcaatttcacagtct
ctgttgagttcagttgtaaatatgtttcaaatgacattttcttgggaaaa
aaaatctctacaacattgtagaatgtgaggggtaactacatcccaggcat
aggtttctcaaagctgcagtagattatgtcttcatcaagctgttaatttg
tgcttatatcatatagaacttttagcatcctgggaagagctgcccccacc
tcaatgatatttctctgagaacaacttttgtaggactgtgtgtttcttta
gatacatttagtacaactgtaggtgacgagtagtcagttattgcttgcta
gctacacaccagggttgatccattttaaaacttttggcattttgtcctca
tgggccataaatacagaaccttgtattttaattaaatttttttacaaaag
gaggcacatgcacaatctccatgtaacaaacctttagcagtaggatgtat
tatacgacagttacttaatttctagagttcaggcctctgggatcaacccc
agactgggccagaatgttagtgaaggttttattgtgcccggttggaggat
aacgttctttgggtactttttgtgggttgcaaatgaactcaattgccaca
agttttaaactggtgtaaatcaagcttgacttaatgtgattgttactgtt
atatccagcctatactgctagcagctgctcatactgcagtcaattactgg
aagcggatatatttcctatgcaaaaactgtttaaacaataaaatgagcta
tgctacagactctgaaaaaaaaaaaaaaaaaaaaa
>NM_181869 1
aagaagaggtagcgagtggacgtgactgctctatcccgggcaaaagggat
agaaccagaggtggggagtctgggcagtcggcgacccgcgaagacttgag
gtgccgcagcggcatccggagtagcgccgggctccctccggggtgcagcc
gccgtcgggggaagggcgccacaggccgggaagacctcctccctttgtgt
ccagtagtggggtccaccggagggcggcccgtgggccgggcctcaccgcg
gcgctccgggactgtggggtcaggctgcgttgggtggacgcccacctcgc
caaccttcggaggtccctgggggtcttcgtgcgccccggggctgcagaga
tccaggggaggcgcctgtgaggcccggacctgccccggggcgaagggtat
gtggcgagacagagccctgcacccctaattcccggtggaaaactcctgtt
gccgtttccctccaccggcctggagtctcccagtcttgtcccggcagtgc
cgccctccccactaagacctaggcgcaaaggcttggctcatggttgacag
ctcagagagagaaagatctgagggaagatggatgcaaaagctcgaaattg
tttgcttcaacatagagaagctctggaaaaggacatcaagacatcctaca
tcatggatcacatgattagtgatggatttttaacaatatcagaagaggaa
aaagtaagaaatgagcccactcaacagcaaagagcagctatgctgattaa
aatgatacttaaaaaagataatgattcctacgtatcattctacaatgctc
tactacatgaaggatataaagatcttgctgcccttctccatgatggcatt
cctgttgtctcttcttccagtggtaaagattcagttagtggaataacttc
gtatgtaaggacagtcctgtgtgaaggtggagtaccacagaggccagttg
tttttgtcacaaggaagaagctggtgaatgcaattcagcagaagctctcc

aaattgaaaggtgaaccaggatgggtcaccatacatggaatggcaggctg
tgggaagtctgtattagctgcagaagctgttagagatcattccctttag
aaggttgtttcccagggggagtgcattgggtttcagttgggaaacaagac
aaatctgggcttctgatgaaactgcagaatctttgcacacggttggatca
ggatgagagtttttcccagaggcttccacttaatattgaagaggctaaag
accgtctccgcattctgatgcttcgcaaacacccaaggtctctcttgatc
ttggatgatgtttgggactcttgggtgttgaaagcttttgacagtcagtg
tcagattcttcttacaaccagagacaagagtgttacagattcagtaatgg
gtcctaaatatgtagtccctgtggagagttccttaggaaaggaaaaagga
cttgaaattttatcccttttgttaatatgaagaaggcagatttgccaga
acaagctcatagtattataaaagaatgtaaagtggtggaacgttgtcact
ggggaatcctcacagaccttctacacaaatggaaccaatcttaagaaaat
acacgtgtcccctgacttcaaaacatatgtgactgtggataatcttggta
ttttatatattttacagactttagaataaaatagttaagcattaatgtag
ttgaactttttaaatttttgaattggaaaaaaattctaatgaaaccctga
tatcaactttttataaagctcttaattgttgtgcagtattgcattcatta
caaaagtgtttgtggttggatgaataatattaatgtagctttttcccaaa
tgaacatacctttaatcttgtttttcatgatcatcattaacagtttgtcc
ttaggatgcaaatgaaaatgtgaatacataccttgttgtactgttggtaa
aattctgtcttgatgcattcaaaatggttgacataattaatgagaagaat
ttggaagaaattggtattttaatactgtctgtatttattactgttatgca
ggctgtgcctcagggtagcagtggcctgctttttgaaccacacttacccc
aagggggttttgttctcctaaatacaatcttagaggttttttgcactctt
taaatttgctttaaaaatattgtgtctgtgtgcatagtctgcagcatttc
ctttaattgactcaataagtgagtcttggatttagcaggcccccccacct
ttttttttgtttttggagacagagtcttgctttgttgccaggctggagt
gcagtggcgcgatctcggctcaccacaatcgctgcctcctgggttcaagc
aattctcctgcctcagcctcccgagtagctgggactacaggtgtgcgcac
atgccaggctaatttttgtatttttagtagagacggggtttcaccatgtt
ggccgggatggtctcgatctcttgacctcatgatctacccgccttggcct
cccaaagtgctgagattacaggcgtgagccaccgtgcctggccaggcccc
ttctcttttaatggagacagggtcttgcactatcacccaggctggagtgc
agtggcataatcatacctcattgcagcctcagactcctgggttcaagcaa
tcctcctgcctcagcctcccaagtagctgagactacaggcacgagccacc
acacccagctaatttttaagttttcttgtagagacagggtctcactatgt
tgtctaggctggtcttgaactcttggcctcaagtaatcctcctgcctcag
cctcccaaagtgttgggattgcagatatgagccactggcctggccttcag
cagttctttttgtgaagtaaaacttgtatgttggaaagagtagattttat
tggtctacccttttctcactgtagctgctggcagccctgtgccatatctg
gactctagttgtcagtatctgagttggacactattcctgctccctcttgt
ttcttacatatcagacttcttacttgaatgaaacctgatctttcctaatc
ctcactttttttctttttaaaaagcagtttctccactgctaaatgttagt
cattgaggtggggccaattttaatcataagccttaataagatttttctaa
gaaatgtgaaatagaacaattttcatctaattccatttacttttagatga
atggcattgtgaatgccattcttttaatgaatttcaagagaattctctgg
ttttctgtgtaattccagatgagtcactgtaactctagaagattaacctt
ccagccaacctattttcctttcccttgtctctctcatcctcttttccttc
cttctttcctttctcttcttttatctccaaggttaatcaggaaaaatagc
ttttgacaggggaaaaaactcaataactagctatttttgacctcctgatc

aggaactttagttgaagcgtaaatctaaagaaacattttctctgaaatat
attattaagggcaatggagataaattaatagtagatgtggttcccagaaa
atataatcaaaattcaaagatttttttgtttctgtaactggaactaaat
caaatgattactagtgttaatagtagataacttgtttttattgttggtgc
atattagtataactgtggggtaggtcggggagagggtaagggaatagatc
actcagatgtattttagataagctatttagcctttgatggaatcataaat
acagtgaatacaatcctttgcattgttaaggaggtttttgtttttaaat
ggtgggtcaaggagctagtttacaggcttactgtgatttaagcaaatgtg
aaaagtgaaaccttaattttatcaaaagaaatttctgtaaatggtatgtc
tccttagaatacccaaatcataattttatttgtacacactgttaggggct
catctcatgtaggcagagtataaagtattaccttttggaattaaaagcca
ctgactgttataaagtataacaacacacatcaggttttaaaaagccttga
atggcccttgtcttaaaaagaaattaggagccaggtgcggtggcacgtgc
ctgtaatcccagctccttgggaggctaagacaggaggattccttgagccc
tggagtttgagtccagcctgggtgacatagcaagaccctgtcttaaaaga
aaaatgggaagaaagacaaggtaacatgaagaaagaagagatacctagta
tgatggagctgcaaatttcatggcagttcatgcagtcggtcaagaggagg
attttgttttgtagtttgcagatgagcatttctaaagcattttcccttgc
tgtattttttgtattataaattacattggacttcatatatataattttt
ttttacattatatgtctcttgtatgttttgaaactcttgtatttatgata
tagcttatatgatttttttgccttggtatacattttaaaatatgaattta
aaaaatttttgtaaaaataaaattcacaaaattgttttgaaaaacaaaaa
aaaaaaaaa
>NM_181359 2
ggcggtcccctgttctccccgctcaggtgcggcgctgtggcaggaagcca
ccccctcggtcggccggtgcgcggggctgttgcgccatccgctccggctt
tcgtaaccgcaccctgggacggcccagagacgctccagcgcgagttcctc
aaatgttttcctgcgttgccaggaccgtccgccgctctgagtcatgtgcg
agtgggaagtcgcactgacactgagccgggccagagggagaggagccgag
cgcggcgcggggccgagggactcgcagtgtgtgtagagagccgggctcct
gcggatgggggctgcccccggggcctgagcccgcctgcccgcccaccgcc
ccgccccgcccctgccacccctgccgcccggttcccattagcctgtccgc
ctctgcgggaccatggagtggtagccgaggaggaagcatgctggccgtcg
gctgcgcgctgctggctgccctgctggccgcgccgggagcggcgctggcc
ccaaggcgctgccctgcgcaggaggtggcgagaggcgtgctgaccagtct
gccaggagacagcgtgactctgacctgcccgggggtagagccggaagaca
atgccactgttcactgggtgctcaggaagccggctgcaggctcccacccc
agcagatgggctggcatgggaaggaggctgctgctgaggtcggtgcagct
ccacgactctggaaactattcatgctaccgggccggccgcccagctggga
ctgtgcacttgctggtggatgttcccccgaggagccccagctctcctgc
ttccggaagagcccctcagcaatgttgtttgtgagtggggtcctcggag
caccccatccctgacgacaaaggctgtgctcttggtgaggaagtttcaga
acagtccggccgaagacttccaggagccgtgccagtattcccaggagtcc
cagaagttctcctgccagttagcagtcccggagggagacagctctttcta
catagtgtccatgtgcgtcgccagtagtgtcgggagcaagttcagcaaaa
ctcaaacctttcagggttgtggaatcttgcagcctgatccgcctgccaac
atcacagtcactgccgtggccagaaacccccgctggctcagtgtcacctg
gcaagacccccactcctggaactcatctttctacagactacggtttgagc
tcagatatcgggctgaacggtcaaagacattcacaacatggatggtcaag

gacctccagcatcactgtgtcatccacgacgcctggagcggcctgaggca
cgtggtgcagcttcgtgcccaggaggagttcgggcaaggcgagtggagcg
agtggagcccggaggccatgggcacgccttggacagaatccaggagtcct
ccagctgagaacgaggtgtccacccccatgcaggcacttactactaataa
agacgatgataatattctcttcagagattctgcaaatgcgacaagcctcc
caggttcaagaagacgtggaagctgcgggctctgaaggaaggcaagacaa
gcatgcatccgccgtactctttggggcagctggtcccggagaggcctcga
cccaccccagtgcttgttcctctcatctccccaccggtgtcccccagcag
cctggggtctgacaatacctcgagccacaaccgaccagatgccagggacc
cacggagcccttatgacatcagcaatacagactacttcttccccagatag
ctggctgggtggcaccagcagcctggaccctgtggatgataaaacacaaa
cgggctcagcaaaagatgcttctcactgccatgccagcttatctcagggg
tgtgcggcctttggcttcacggaagagccttgcggaaggttctacgccag
gggaaaatcagcctgctccagctgttcagctggttgaggtttcaaacctc
cctttccaaatgcccagcttaaaggggctagagtgaacttgggccactgt
gaagagaaccatatcaagactctttggacactcacacggacactcaaaag
ctgggcaggttggtgggggcctcggtgtggagaagcggctggcagcccac
ccctcaacacctctgcacaagctgcaccctcaggcaggtgggatggattt
ccagccaaagcctcctccagccgccatgctcctggcccactgcatcgttt
catcttccaactcaaactcttaaaacccaagtgccttagcaaattctgtt
tttctaggcctggggacggcttttacttaaaccgccaaggctgggggaag
aagctctctcctccctttcttccctacagttgaaaaacagctgagggtga
gtgggtgaataatacagtatctcagggcctggtcgttttcaacagaatta
taattagttcctcattagcattttgctaaatgtgaatgatgatcctaggc
atttgctgaatacagaggcaactgcattggctttgggttgcaggacctca
ggtgagaagcagaggaaggagaggagaggggcacagggtctctaccatcc
cctgtagagtgggagctgagtgggggatcacagcctctgaaaaccaatgt
tctctcttctccacctcccacaaaggagagctagcagcagggagggcttc
tgccatttctgagatcaaaacggttttactgcagctttgtttgttgtcag
ctgaacctgggtaactagggaagataatattaaggaagacaatgtgaaaa
gaaaaatgagcctggcaagaatgtgtttaaacttggtttttaaaaaactg
ctgactgttttctcttgagagggtggaatatccaatattcgctgtgtcag
catagaagtaacttacttaggtgtgggggaagcaccataactttgtttag
cccaaaaccaagtcaagtgaaaaaggaggaagagaaaaaatattttcctg
ccaggcatggtggcccacgcacttcgggaggtcgaggcaggaggatcact
tgagtccagaagtttgagatcagcctgggcaatgtgataaaaccccatct
ctacaaaaagcataaaaattagccaagtgtggtagagtgtgcctgaagtc
ccagatacttggggggctgaggtgggaggatctcttgagcctgggaggtc
aaggctgcagtgagccgagattgcaccactgcactccagcctgggtgaca
gagcaagtgagaccctgtctcaaaaaaagaaaaagaaaaagaaaaaatat
tttccctattagagaagagattgtggtttcattctgtattttgtttttgt
cttaaaaagtggaaaaatagcctgcctcttctctactctagggaaaaacc
agcgtgtgactactcccccaggtggttatggagagggtgtccggtccctg
tcccagtgccgagaaggaagcctcccacgactgcccggcagggtcctaga
aattccccaccctgaaagccctgagctttctgctatcaaagaggttttaa
aaaaatcccatttaaaaaaaatcccttacctcggtgccttcctcttttta
tttagttccttgagttgattcagctctgcaagaattgaagcaggactaaa
tgtctagttgtaacaccatgattaaccacttcagctgacttttctgtccg
agctttgaaaattcagtggtgttagtggttacccagttagctctcaagtt

atcagggtattccagagtggggatatgatttaaatcagccgtgtaaccat
ggacccaatatttaccagaccacaaaacttttctaatactctaccctctt
agaaaaaccaccaccatcaccagacaggtgcgaaaggatgaaagtgacca
tgttttgtttacggttttccaggtttaagctgttactgtcttcagtaagc
cgtgattttcattgctgggcttgtctgtagattttagaccctattgctgc
ttgaggcaactcatcttaggttggcaaaaaggcaggatggccgggcgcgg
tggctcacgcctgtaatcctagcactttgggaggccaaggtgggaggatt
gcttgagctcaggagtttgagaccaacctgggtaacatagtgagacacca
tctctattatgaacaataacagttaagaaaaaaaaaggcaggcaggcggt
tatggtggttccctcccatcccaccacataaagtttctgagacttgagaa
cagcaaaatgctgttaaagggaaatattaagaatgagaatctgcagtaag
ggtgattctgtgcccacagttcttcaattctttataccgttttacccaca
tgtggtgttaccaaagccgggcagaaccatgctagcggaagatgtgaaat
ccagatagctcattattgccaagagctaggcagctttgatctccaaattg
ttattgctttcatttttattgtaatggaattgctttgttttgttttttttg
tttttgtattgaagagggttgttttccctttatttttcataagctaatgt
aaatgaagaaaaaaatgtcttctctgggctgtaggcctggctcagcgtaca
caggtatacatcctaagctctctatgttctctaatctgtggtgactgaac
atgtgtctcaatgcacggggcatttctacctgtgtttctgcagcaccccc
actgccttgagtccccagcagtgctgttatttgcctaacacctgtagcca
tctgccacgcagccagacgtgaaacgctgagacagagaccatttaggtta
aatacgacagcttatcctgctgggtggggaaagtaaaaaatatgctggtt
caaggcctaaagtaaaatgatcaataatgtttgtagcattaatgaaatat
tttcaagaaatgtgtccaggggtagcactggctatgttgacgaggcctttt
ggtaactcagagagctcttggccctgatggggacttgcccttacgctttc
tttatcaggctctgagttcacacggagcctctggcacttccctgctgtct
tgggagaaaggaaactggttgccgcggcaggttgtggaatctgttgctgg
aaccaggctggaagcccacctggtagtgaacagggcccagtggggcaggc
tgggcatgttgtggtctatgggtttgtttcctggagaatgttcaggaatg
tcttcccagctgctttggtgctgagctctattatctcacagcacgtccag
aaggctaacccaggtggggaggatgctgacaccagctccaggtggagttg
gtggtcttaatttggagatgcaggggcaacctgtgaccctttgaggcaag
agccctgcacccagctgtcccgtgcagccgtgggcaggggctgcacacgg
aggggcaggcgggccagttcagggtccgtgccaggccctcctcagtgccc
tgtgaaggcctcctgtcctccgtgcggctgggcaccagcaccagggagtt
tctatggcaaccttagtgattattaaggaacactgtcagttttatgaaca
tatgctcaaatgaaattctactttaggaggaaaggattggaacagcatgt
cacaaggctgttaattaacagagagaccttattggatggagatcacatct
gttaaatagaatacctcaactctacgttgttttcttggagataaataata
gtttcaagtttttgtttgtttgttttacctaattacctgaaagcaaatac
caaaggctgatgtctgtatatggggcaaagggtcagtatatttttcagtg
tttttttttctaccagctattttgcatttaaagtgaacattgtgtttgga
ataaatactcttaaaaaataaaaaaaaaaaaaaa
>NM_170709 2
ggggcctgcaggtttggcccccgcagggagcgcagctggcgccgctggga
gctggtggcgcggcgcaggtcccggccgagtgtggcgcagcagtggcggc
gcttcccattcgccatgcgccgggggtgggtgcccgaaggttgcatgatg
gaatttgaacattacttcaagaggttttgtattttggattagttaattgg
gtttgtcctctgctgactgtttcttcggatgcatttttggtgtgctctt

gagggattaaatgcaaagagatcacaccatggactacaaggaaagctgcc
caagtgtaagcattcccagctccgatgaacacagagagaaaaagaagagg
tttactgtttataaagttctggtttcagtgggaagaagtgaatggtttgt
cttcaggagatatgcagagtttgataaactttataacactttaaaaaaac
agtttcctgctatggccctgaagattcctgccaagagaatatttggtgat
aattttgatccagattttattaaacaaagacgagcaggactaaacgaatt
cattcagaacctagttaggtatccagaactttataaccatccagatgtca
gagcattccttcaaatggacagtccaaaacaccagtcagatccatctgaa
gatgaggatgaaagaagttctcagaagctacactctacctcacagaacat
caacctgggaccgtctggaaatcctcatgccaaaccaactgactttgatt
tcttaaaagttattggaaaaggcagctttggcaaggttcttcttgcaaaa
cggaaactggatggaaaattttatgctgtcaaagtgttacagaaaaaaat
agttctcaacagaaaagagcaaaaacatattatggctgaacgtaatgtgc
tcttgaaaaatgtgaaacatccgtttttggttggattgcattattccttc
caaacaactgaaaagctttattttgttctggattttgttaatggaggga
gctttttttccacttacaaagagaacggtcctttcctgagcacagagcta
ggttttacgctgctgaaattgctagtgcattgggttacttacattccatc
aaaatagtatacagagacttgaaaccagaaaatattcttttggattcagt
aggacatgttgtcttaacagattttgggctttgtaaagaaggaattgcta
tttctgacaccactaccacattttgtgggacaccagagcctccttttat
tgccgagatgttgctgaaatgtatgacaatatccttcacaaacccctaag
tttgaggccaggagtgagtcttacagcctggtccattctggaagaactcc
tagaaaaagacaggcaaaatcgacttggtgccaaggaagactttcttgaa
attcagaatcatcctttttttgaatcactcagctgggctgaccttgtaca
aaagaagattccaccaccatttaatcctaatgtggctggaccagatgata
tcagaaactttgacacagcatttacagaagaaacagttccatattctgtg
tgtgtatcttctgactattctatagtgaatgccagtgtattggaggcaga
tgatgcattcgttggtttctcttatgcacctccttcagaagacttatttt
tgtgagcagtttgccattcagaaaccattgagcaaaataagtctatagat
gggactgaaacttctatttgtgtgaatatattcaaatatgtataactagt
gcctcatttttatatgtaatgatgaaaactatgaaaaaatgtattttctt
ctatgtgcaagaaaaatagggcatttcaaagagctgttttgattaaaatt
tatattcttgtttaataagcttatttttaaacaatttaaaagctattatt
cttagcattaacctatttttaaagaaaccttttttgctattgactgtttt
ttccctctaagtttacactaacatctacccaagatagactgtttttaac
agtcaatttcagttcagctaacatatattaataccttttgtaactctttgc
tatggcttttgttatcacaccaaaactatgcaattggtacatggttgttt
aagaagaaaccgtatttttccatgataaatcactgtttgaaatatttggt
tcatggtatgatcgaaatgtaaaagcataattaacacattggctgctagt
taacaattggaataactttattctgcagatcatttaagaagtaacaggcc
gggcgcggtggctcacgcctgtaatcccagcactttgggaggctgaggcg
ggcagatcacctgaggtcaggagttggagaccagcctgaccaacatggac
aaaccccgtctctactaaaaatacaaaattggcagggtgtggtggcacat
gcctataatcccagctacttgggaggctaaggcaggagaatcgcttgaac
ccgggaggcggaggttgcagtgagccgagatcgcaccattgcactcctgc
ctgggcaacaagagtgaaactccatctccaaaaaaaaaaaagaaaaagtaa
caaaaggaaattatttgtttttgaaataccagttcaactttgtggattat
ttttcctctgaaggaaaagaaaaggcttaatggttaggatttttaagtat
tcccaaagatctgaagggtaataaaatgtactggatttttttaaggtggta

ccaaaaatgaatgtctgtcatatatttatattacaaatacattatattta
tgttctattcatcttttgaatgtttagtatgctattaagtcattctgaat
ctttgtatttgcttttgcaaataggtatttcaaagctcttttcctaactg
gttaagtaaaataaaaaattgagctttctagaatatttgcctaattggga
attaaaaagtaaaataataggccaggcatggtggctcatgcctataagca
ccctgggaggccgaggcaggcagattatttgagctcaggagtttgagacc
atcctgggcaacatggcgaaaccctatctctacaaaaaatacaaaaatta
gccagacatggtggcacatgcctttagtcccagctactctggaggctgaa
gttggaggatggcttgagcccacgagatggaagttgcagtgaactgaaat
tgtgccactgcacttttcagcctgggtgccacagcgagaccctagttaag
agaaaaaaaaagtaaaaacaaattgtgggtcaaagtaaatgtatacagtt
ttattacaatgtaacaaaagttgaaaatcaggcagatgtgtattcagtat
ccaattcaatatatcttagaaaaagcacaggaaacagaccttaaaattgt
aacctaccaactaacttacatgcttataaaagtaaaggagaataactggc
cgggcacggtggctcatgcctataaaattccagcactttgggaggccaag
gcaggaggatcacttgagcctatgagttcaagaccagcctaggcaatgta
gtgacacctcatttctatttattttaaaaaaaagagagagtaactacaga
agaactttaaaaaataaaaaataagcttaccttggattcttggcttagag
tagaggttttttttaagttatggaggaaacatttttgtaaaagtttaatg
acccactttagatgctccaagaacaagcatcccttccatgtatgtcttga
gaaagaaatcacagaagcatttctcaccaatactctttggcttaaaatgt
tcagcagaattgggcagtgggggtgacttttcttatattaataatattta
catccaatacactgaatcttcctttagaggtaagactttaatatctatac
tgtaaatatttggtttatttggcactactgtaagttttgtttttcacaaa
gctcttattatgaagcaaaataaaaattctagtttcttgtatgatttttt
gtactcattcattcctgttaagctgccaaaaattaaagtgcaatattgta
tatttttaagaacaaatttaaaatagaattttgatgtttctcagatcaca
agaaatacaaatctatatagtataataaaatcagcaaaaagatcaaaaaa
aaaaaaaaaa
>NM_018593 4
gcggccgcctgcgcgctggccgcctgcgcgctgccagcccgcccgcccgc
caggggctccgccgccctcgcctcggcctcgttagcccgccaggagcccc
gcagctcctccgggagcccgctggtaactcgcgtccctcgcgcttctccg
gcgcctgaggggcccgcctcgggccatggtgctctcccaggaggagccgg
actccgcgcggggcacgagcgaggcgcagccgctcggccccgcgcccacg
ggggccgctccgccgcccggcccgggaccctcggacagccccgaggcggc
tgtcgagaaggtggaggtggagctggcggggccggcgaccgcggagcccc
atgagcccccgaacccccgagggcggctggggctggctggtgatgctg
gcggccatgtggtgcaacgggtcggtgttcggcatccagaacgcttgcgg
ggtgctcttcgtgtccatgctggaaaccttcggctccaaagacgatgaca
agatggtctttaagacagcatgggtaggttctctctccatggggatgatt
ttcttttgctgcccaatagtcagcgtcttcacagacctatttggttgtcg
gaaaacagctgtcgtgggtgctgctgttggatttgttgggctcatgtcca
gttcttttgtaagttccatcgagcctctgtaccttacctatggaatcata
tttgcctgcggctgctcctttgcataccagccttcattggtcattttggg
acactatttcaagaagcgccttggactggtgaatggcattgtcactgctg
gcagcagtgtcttcacaatcctgctgcctttgctcttaagggttctgatt
gacagcgtgggcctcttttacacattgagggtgctctgcatcttcatgtt
tgttctctttctggctggctttacttaccgacctcttgctaccagtacca

aagataaagagagtggaggtagcggatcctccctcttttccaggaaaaag
ttcagtcctccaaaaaaaattttcaattttgccatcttcaaggtgacagc
ttatgcagtgtgggcagttggaataccacttgcactttttggatactttg
tgccttatgttcacttgatgaaacatgtaaatgaaagatttcaagatgaa
aaaaataaagaggttgttctcatgtgcattggcgtcacttcaggagttgg
acgactgctctttggccggattgcagattatgtgcctggtgtgaagaagg
tttatctacaggtactctcctttttcttcattggtctgatgtccatgatg
attcctctgtgtagcatctttggggccctcattgctgtgtgcctcatcat
gggtctcttcgatggatgcttcatttccattatggctcccatagcctttg
agttagttggtgcccaggatgtctcccaagcaattggatttctgctcgga
ttcatgtctatacccatgactgttggcccacccattgcagggttacttcg
tgacaaactgggctcctatgatgtggcattctacctcgctggagtccctc
cccttattggaggtgctgtgctttgttttatcccgtggatccatagtaag
aagcaaagagagatcagtaaaaccactggaaaagaaaagatggagaaaat
gttggaaaaccagaactctctgctgtcaagttcatctggaatgttcaaga
aagaatctgactctattatttaatatcttacatacctccaccagactgga
cttgctttttgaattttaagcaagtttcctttccttttatacaaattgca
aatttcatattttttaatcacatcctaggaatagcacaataattgggaa
atagaacccttatcactagaagaaccattttctgccactaaatatctctg
atgtttccatgagtctgagggcagagactctggtatatgaaaacgtctga
aagtcacatattgtgaaaatttgaagctatctcagtaaaaagcagctttg
gaaactgtgaatgatctttagcttgtacaaatgtttaaaaatacctcagg
ctatactgaaagggttgcagtttggttaggagtggaaatattttgtttgt
taatgatgtcttcagttctggtacctctgttttactttcttatgctcttt
ggaaactttttgcaaaatttaagcctgggttctagataataccagatcta
cctaaacctcaagtctatgttaaagttgctttcctgctgttaaataagct
atgatattaagatattctgacttgctccagtgtcaagggaccttctggga
gcaggtgctaacatagtgttcagaatcaatatgtgagatgaaaaggatcc
cctccaggaggatcctgagctgttcagaaatcatttaagtttacagcgtt
gttccctttgcgtttgcagtgcgttttactcaagtagccagaaacacccc
acgtttctgaatttgtttaaactgtaacaataaagtaaaatagaatgcat
gaaagatattctggcgattgtaacttagaatttttctgacttctggattt
gttggcactagaacctgatatttaaagtcttactgagcagctatcaagtg
gcagttacaggcacaaattggtggaggctggaggatggggaggggagcaa
aaccctttatatttgtgaagaaaatatctgtagctgatagaaataattgc
ttaaattggtttatgaaattaatgagtctgaaaaggttaaaagcacttat
aaaaagaaccaagtcctacatttccagaactttctggcaaaaatttgcac
tcatattatttatcctatgaacattcccattgtttttttttgctatttat
atacagattatcataagaaagctctcagtttgaggacccaaaaaaaaaaa
aaaaaaaaaaaaaaaaaa
>NM_002639 4
agtgggcgtggcggtgctgcccaggtgagccaccgctgcttctgcccaga
cacggtcgcctccacatccaggtctttgtgctcctcgcttgcctgttcct
tttccacgcattttccaggataactgtgactccaggcccgcaatggatgc
cctgcaactagcaaattcggcttttgccgttgatctgttcaaacaactat
gtgaaaaggagccactgggcaatgtcctcttctctccaatctgtctctcc
acctctctgtcacttgctcaagtgggtgctaaaggtgacactgcaaatga
aattggacaggttcttcatttttgaaaatgtcaaagatgtaccctttggat
ttcaaacagtaacatcggatgtaaacaaacttagttccttttactcactg

aaactaatcaagcggctctacgtagacaaatctctgaatctttctacaga
gttcatcagctctacgaagagaccgtatgcaaaggaattggaaactgttg
acttcaaagataaattggaagaaacgaaaggtcagatcaacaactcaatt
aaggatctcacagatggccactttgagaacattttagctgacaacagtgt
gaacgaccagaccaaaatccttgtggttaatgctgcctactttgttggca
agtggatgaagaaattttctgaatcagaaacaaaagaatgtcctttcaga
gtcaacaagacagacaccaaaccagtgcagatgatgaacatggaggccac
gttctgtatgggaaacattgacagtatcaattgtaagatcatagagcttc
cttttcaaaataagcatctcagcatgttcatcctactacccaaggatgtg
gaggatgagtccacaggcttggagaagattgaaaaacaactcaactcaga
gtcactgtcacagtggactaatcccagcaccatggccaatgccaaggtca
aactctccattccaaaatttaaggtggaaaagatgattgatcccaaggct
tgtctggaaaatctagggctgaaacatatcttcagtgaagacacatctga
tttctctggaatgtcagagaccaagggagtggccctatcaaatgttatcc
acaaagtgtgcttagaaataactgaagatggtggggattccatagaggtg
ccaggagcacggatcctgcagcacaaggatgaattgaatgctgaccatcc
ctttatttacatcatcaggcacaacaaaactcgaaacatcattttctttg
gcaaattctgttctccttaagtggcatagcccatgttaagtcctccctga
cttttctgtggatgccgatttctgtaaactctgcatccagagattcattt
tctagatacaataaattgctaatgttgctggatcaggaagccgccagtac
ttgtcatatgtagccttcacacagatagaccttttttttttttccaattc
tatcttttgtttcctttttcccataagacaatgacatacgcttttaatg
aaaaggaatcacgttagaggaaaaatatttattcattatttgtcaaattg
tccggggtagttggcagaaatacagtcttccacaaagaaaattcctataa
ggaagatttggaagctcttcttcccagcactatgctttccttctttggga
tagagaatgttccagacattctcgcttccctgaaagactgaagaaagtgt
agtgcatgggacccacgaaactgccctggctccagtgaaacttgggcaca
tgctcaggctactataggtccagaagtccttatgttaagccctggcaggc
aggtgtttattaaaattctgaattttggggattttcaaaagataatattt
tacatacactgtatgttatagaacttcatggatcagatctggggcagcac
cctataaatcaacaccttaatatgctgcaacaaaatgtagaatattcaga
caaaatggatacataaagactaagtagcccataaggggtcaaaatttgct
gccaaatgcgtatgccaccaacttacaaaaacacttcgttcgcagagctt
ttcagattgtggaatgttggataaggaattatagacctctagtagctgaa
atgcaagaccccaagaggaagttcagatcttaatataaattcactttcat
ttttgatagctgtcccatctggtcatttggttggcactagactggtggca
ggggcttctagctgacttgcacagggattctcacaatagccgatatcaga
atttgtgttgaaggaacttgtctcttcatctaatatgatagcgggaaaag
gagaggaaactactgcctttagaaaatataagtaaagtgattaaagtgct
cacgttaccttgacacatagtttttcagtctatgggtttagttactttag
atggcaagcatgtaacttatattaatagtaatttgtaaagttggttggat
aagctatccgtgttgcaggttcatggattacttctctataaaaaatatgt
atttaccaaaaattttgtgacattccttctcccatctcttccttgacctg
cattgtaaataggttcttcttgttctgagattcaatattgaatttttcct
atgctattgacaataaaatattattgaactaca
>NM_005734 3
ccgggaaggaagatgagggagacgggcccggcgcttagcagccagagcag
cagcagcagcagcagcggtcgggggagggtgtttcgccgtttcctctcag
ccgccaggacaagatggcagcggccgcggagaggggctgagcccgggctg

ggtggtgccgcctgctgaagcgcctggctcccggtccccggcacggccct
gcgccccaccccggacatgctcagggctgcggccgcccgaagaggagaga
gcgcgggcctctaggaaggtatggcctcacaagtcttggtctacccacca
tatgtttatcaaactcagtcaagtgccttttgtagtgtgaagaaactcaa
agtagagccaagcagttgtgtattccaggaaagaaactatccacggacct
atgtgaatggtagaaactttggaaattctcatcctcccactaagggtagt
gcttttcagacaaagataccatttaatagacctcgaggacacaacttttc
attgcagacaagtgctgttgttttgaaaaacactgcaggtgctacaaagg
tcatagcagctcaggcacagcaagctcacgtgcaggcacctcagattggg
gcgtggcgaaacagattgcatttcctagaaggcccccagcgatgtggatt
gaagcgcaagagtgaggagttggataatcatagcagcgcaatgcagattg
tcgatgaattgtccatacttcctgcaatgttgcaaaccaacatgggaaat
ccagtgacagttgtgacagctaccacaggatcaaaacagaattgtaccac
tggagaaggtgactatcagttagtacagcatgaagtcttatgctccatga
aaaatacttacgaagtccttgattttcttggtcgaggcacgtttggccag
gtagttaaatgctggaaaagagggacaaatgaaattgtagcaatcaaaat
tttgaagaatcatccttcttatgcccgtcaaggtcaaatagaagtgagca
tattagcaaggctcagtactgaaaatgctgatgaatataactttgtacga
gcttatgaatgctttcagcaccgtaaccatacttgtttagtctttgagat
gctggaacaaaacttgtatgactttctgaaacaaaataaatttagtcccc
tgccactaaaagtgattcggcccattcttcaacaagtggccactgcactg
aaaaaattgaaaagtcttggtttaattcatgctgatctcaagccagagaa
tattatgttggtggatcctgttcggcagccttacagggttaaagtaatag
actttgggtcggccagtcatgtatcaaagactgtttgttcaacatatcta
caatctcggtactacagagctccagagattatattggggttgccattttg
tgaagccatagacatgtggtcattgggatgtgtgattgcagaattatttc
ttggatggccgctctacccaggagccttggagtatgatcagattcgatac
atttctcagactcaaggtttgccaggagaacagttgttaaatgtgggtac
taaatccacaagatttttttgcaaagaaacagatatgtctcattctggtt
ggagattaaagacattggaagagcatgaggcagagacaggaatgaagtct
aaagaagccagaaaatacattttcaacagtctggatgatgtagcgcatgt
gaacacagtgatggatttggaaggaagtgatcttttggctgagaaagctg
atagaagagaatttgttagtctgttgaagaaaatgttgctgattgatgca
gatttaagaattactccagctgagaccctgaaccatccttttgttaatat
gaaacatcttctagatttccctcatagcaaccatgtaaagtcctgttttc
atattatggatatttgtaagtcccacctaaattcatgtgacacaaataat
cacaacaaaacttcacttttaagaccagttgcttcaagcagtactgctac
actgactgcaaattttactaaaatcggaacattaagaagtcaggcattga
ccacatctgctcattcagttgtgcaccatggaatacctctgcaggcagga
actgctcagtttggttgtggtgatgcttttcagcagacattgattatctg
tcccccagctattcaaggtattcctgcaacacatggtaaacccaccagtt
attcaataagggtagataatacagttccacttgtaactcaggccccagct
gtgcagccactacagatccgaccaggagttctttctcagacgtggtctgg
tagaacacagcagatgctggtgcctgcctggcaacaggtgacacccctgg
ctcctgctactactacactaacttctgagagtgtggctggttcacacagg
cttggagactgggggaagatgatttcatgcagcaatcattataactcagt
gatgccgcagcctcttctgaccaatcagataactttatctgcccctcagc
cagttagtgtggggattgcacatgttgtctggcctcagcctgccactacc
aagaaaaataaacagtgccagaacagaggtattttggtaaaactaatgga

atgggagccaggaagagaggaaataaatgctttcagttggagtaattcat
tacagaataccaatatcccacattcagcatttatttctccaaagataatt
aatgggaaagatgtcgaggaagtaagttgtatagaaacacaggacaatca
gaactcagaaggagaggcaagaaattgctgtgaaacatctatcagacagg
actctgattcatcagtttcagacaaacagcggcaaaccatcattattgcc
gactccccgagtcctgcagtgagtgtcatcactatcagcagtgacactga
tgaggaagagacttcccagagacattcactcagagaatgtaaaggtagtc
tagattgtgaagcttgccagagcactttgaatattgatcggatgtgttca
ttaagtagtcctgatagtactctgagtaccagctcctcagggcagtccag
cccatccccctgcaagagaccgaatagtatgtcagatgaagagcaagaaa
gtagttgtgatacggtggatggctctccgacatctgactcttccgggcat
gacagtccatttgcagagagcacttttgtggaggacactcatgaaaacac
agaattggtatcctctgctgacacagaaaccaagccagctgtctgttctg
ttgtggtgccaccagtggaactagaaaatggcttaaatgccgatgagcat
atggcaaacacagattctatatgccagccattaataaaaggacgatctgc
ccctggaagattaaaccagccttctgcagtgggtactcgtcagcaaaaat
tgacatcagcattccagcagcagcatttgaacttcagtcaggttcagcac
tttggatctgggcatcaagagtggaatggaaactttgggcacagaagaca
gcaagcttatattcctactagtgttaccagtaatccattcactctttctc
atggaagtcccaatcacacagcagtgcatgcccacctggctggaaataca
cacctcggaggacagcctactctacttccatacccatcatcagccaccct
cagtagtgctgcaccagtggcccacctgttagcctctccgtgtacctcaa
gacctatgttacagcatccaacttataatatctcccatcccagtggcata
gttcaccaagtcccagtgggcttaaatccccgtctgttaccatccccaac
cattcatcagactcagtacaaaccaatcttcccaccacattcttacattg
cagcatcacctgcatatactggatttccactgagtccaacaaaactcagc
cagtatccatatatgtgaaaaacagtatattggggaagctcaatgataca
aacatttgattaaaaataaaaacatggtatttaatattagccatggcaca
agaaaattatttttgaatcatgtagacttgggtgcaatttaaacaacttt
gagctttaaaaactcacttttgatgtgttttgcacatttggtataacttg
tctttggtcatgttatcttcttatgtagtaactctagacaggtgacttat
gggagcagaagtccagttttgctcctgctatttttttataaattgccttct
aactagtgcaagacacgtctacatttgggaagccattctgtgtacagact
tagagcaacagatgcacatatgtcagaattacagcatacaagtgaattgt
attatccgtgtcttagtgtataaatgttgggtcacttacctaagaaattg
agctattgttctttacatttgcatgtgtcttttgcatgggcaaaatgttg
cctagactttgctcttaaatgttgttctaataatctcagctgcattgtaa
accgttcctacacatagtgccttaaatatttgaggttgttaatgttatta
cctatatataaatgttgaggactgcagcacttaaaattcagacctactat
ttagtttccttttgatagcgtaatgttcatttttgtttttgtgtggtatg
atttcaggtagtagctgtttttttccttattaagagggcagcatgtttgc
tatagctgaattctgctgtctgatttttcagaatgatctagcttcaagaa
aagcaagcagttagtagtgcttaagaaaaattgattcagtatc
>NM_014454 2
gaacccggtggctgcacagacaaaaaagccccgaatggctggagggcgtt
cagctgttaacagccttttggggcagagcacggatttgacagctccacaa
cgtgaggatatccactgaccccgcgagacggaggagaacgcttccccgaa
attctctgcccaccaaagccagcgctgcaaggttgcaactttcaaacttt
gtttttccagaaagaagactgccctttcgtgtacaaggagagggtgagag

ggtgacctagcttgtagatcggctgaaggcaccagtggttccaaatgtca
cccagatgtgtgttttcatgacgatttgatttctctgattttatttttac
atttttcattttaaaaatacaaagcaattttttttggggcatgctgaaagg
taactgaagaccgcaaaggaaaaactattgtcatggctgaaggagagaat
gaagtgagatgggatggactctgcagcagagattcaactactagggagac
agcattggaaaacattaggcaaaccattttgaggaaaaccgagtatcttc
gttcggtgaaagaaacacctcatcgtccatcagacgggctttcaaatacc
gagtcttcggatgggttgaataagctacttgctcatctgcttatgctttc
taagaggtgtcccttcaaagatgtgagagagaaaagtgagtttattctga
agagcatccaggaacttggcattagaattcctcgaccactaggacaggga
ccaagcagattcatcccagaaaaggagatcctccaagtggggagtgaaga
cgcacagatgcatgctttatttgcagattcttttgctgctttgggccgtt
tggataacattacgttagtgatggttttccacccacaatatttagaaagt
ttcttaaaaactcagcactatctactgcaaatggatgggccgttacccct
acattatcgtcactacattggaataatggctgcggcaagacatcagtgct
cctacttagtgaacctgcatgtaaatgatttccttcatgttggtggggac
cccaagtggctcaatggtttagagaatgctcctcaaaaactacagaattt
aggagaacttaacaaagtgttagcccatagaccttggcttattaccaaag
aacacattgagggacttttaaaagctgaagagcacagctggtcccttgcg
gaattggtacatgcagtagttttactcacacactatcattctcttgcctc
attcacattcggctgtggaatcagtccagaaattcattgtgatggtggcc
acacattcagacctccttctgttagcaactactgcatctgtgacattaca
aatggcaatcacagtgtggatgagatgccggtcaactcagcagaaaatgt
ttctgtaagtgattctttctttgaggttgaagccctcatggaaaagatga
ggcagttacaggaatgtcgagatgaagaagaggcaagtcaggaagagatg
gcttcacgttttgaaatagaaaaaagagagagtatgtttgtcttctcttc
agatgatgaagaagttacaccagcaagagctgtatctcgtcattttgagg
atactagttatggctataaagatttctctagacatgggatgcatgttcca
acatttcgtgtccaggactattgctgggaagatcatggttattctttggt
aaatcgcctttatccagatgtgggacagttgattgatgaaaaatttcaca
ttgcttacaatcttacttataatacaatggcaatgcacaaagatgttgat
acctcaatgcttagacgggcaatttggaactatattcactgcatgtttgg
aataagatatgatgattatgactatggtgaaattaaccagctattggatc
gtagctttaaagtttatatcaaaactgttgtttgcactcctgaaaaggtt
accaaaagaatgtatgatagcttctggaggcagttcaagcactctgagaa
ggttcatgttaatctgcttcttatagaagctaggatgcaagcagaactcc
tttatgctctgagagccattacccgctatatgacctgatgcctttccttc
attaaagatgattctggaatgatcagcagatatagtctacaagggggaag
gtactaagccccaggaccaatggtagacaaaataattcagaaatccattg
tgccatgattcctttagtttctgctatttttctgtggaaaaccactgctg
gcacaagcagtgactgtttggcagcttcaagtttagagctgtgaagacag
gctgccattcacagtattttgctttttgacagtacaagatgctgtgtaac
tgttttaatacagcaaatagtaactctccaaatcctgttgcttttatgtt
aaataagataacaagaattggagcatgcaaagaatgggacttggataatg
acttaagctttatatgtaaagaattttagaagatcttggtgctgctattc
ctgctggaggaatgaatagatggctgtttcagttaagctattagtaataa
aagtgaacattgctactatctgagcctacatacataacttgtgtgatttc
aaattaaacttgcattatgtgttaattttcttgcatctaaaaaagcatag
aattcctactcacacagctcagcaacaaccattttgatggtaacagttaa

tttctttcattagttttttaaattcagggttctggatattaaattaaaat
ggcattcttaaagattttcttcaaaaagcaatcctaaatgaaagtgtgta
aattataagaagctggcgatcttttgatatgctgtttcacaggatcctga
cactggagggcagctgtcttgtgcattacttgtgtttccagcaccaaagt
tgtgggacatgttgctgtagactgctgcgcagtcctgggtgcattcagtc
tctctgcctctgcctgcctcctggtccccactttaaaggctgtgcagctc
cttaaataataaagctggaaaatatttttagtcgggttatcaaatttgat
ttacaaaaacgctaactttgtttgaaatgcaaacaggtttgaaaatatgt
attaagtactttgtattctggaagcgtgaattgcttttgaagtctgtcag
tattactggtatttttaaataaagaagaatttttctccaattttaaaaaa
aaaaaaaaaaaa
>NM_004952 4
ggagctgggaagcggagaagccgggagcgcggggctcagtcgggggcgg
cggcggcggcggctccggggatggcggcggctccgctgctgctgctgctg
ctgctcgtgcccgtgccgctgctgccgctgctggcccaagggcccggagg
ggcgctgggaaaccggcatgcggtgtactggaacagctccaaccagcacc
tgcggcgagagggctacaccgtgcaggtgaacgtgaacgactatctggat
atttactgcccgcactacaacagctcgggggtgggccccggggcgggacc
ggggcccggaggcggggcagagcagtacgtgctgtacatggtgagccgca
acggctaccgcacctgcaacgccagccagggcttcaagcgctgggagtgc
aaccggccgcacgccccgcacagccccatcaagttctcggagaagttcca
gcgctacagcgccttctctctgggctacgagttccacgccggccacgagt
actactacatctccacgcccactcacaacctgcactggaagtgtctgagg
atgaaggtgttcgtctgctgcgcctccacatcgcactccggggagaagcc
ggtccccactctcccccagttcaccatgggccccaatgtgaagatcaacg
tgctggaagactttgagggagagaaccctcaggtgcccaagcttgagaag
agcatcagcgggaccagccccaaacgggaacacctgcccctggccgtggg
catcgccttcttcctcatgacgttcttggcctcctagctctgccccctcc
cctgggggggggagagatggggcggggcttggaaggagcagggagcctttg
gcctctccaagggaagcctagtgggcctagacccctcctcccatggctag
aagtggggcctgcaccatacatctgtgtccgccccctctaccccttcccc
ccacgtagggcactgtagtggaccaagcacggggacagccatgggtcccg
ggcggccttgtggctctggtaatgtttggtaccaaacttgggggccaaaa
agggcagtgctcaggactccctggcccctggtacctttccctgactcctg
gtgccctctccctttgtccccccagagagacatatgcccccagagagagc
aaatcgaagcgtgggaggcacccccattgctctcctccaggggcagaaca
tggggaggggactagatgggcaaggggcagcactgcctgctgcttccttc
ccctgtttacagcaataagcacgtcctcctcccccactcccacttccagg
attgtggtttggattgaaaccaagtttacaagtagacaccccctgggggg
cgggcagtggacaaggatggcaaggggtgggcattggggtgccaggcagg
catgtacagactctatatctctatatataatgtacagacagacagagtcc
cttccctctttaacccccctgacctttcttgacttccccttcagcttcaga
ccccttccccaccaggctaggcccccacacctgggggacccccctggccc
ctcttttgtcttctgtgaagacaggacctatgcaacgcacagacactttt
ggagaccgtaaaacaacaacgcccctcccttccagccctgagccgggaa
ccatctcccaggaccttgccctgctcaccctatgtggtcccacctatcct
cctgggcctttttcaagtgctttggctgtgactttcatactctgctctta
gtctaaaaaaaataaactggagataaaaataa
>NM_175610 2

cgggcatgctcagtgggccgggccggcaggtttgcgtggccgctgagttg
ccggcgccggctgagccagcggacgccgcgttccttggcggccgccggtt
cccgggaagttacgtggcgaagccggcttccgaggagacgccgggaggcc
acgggtgctgctgacgggcgggcgaccgggcgaggccgacgtggccgggc
tgcgaaagctgcgggaggccgagtgggtggccgcgctcggagggaggtgc
cggtcgggcgcgccccgtggagaagacccgggcggggcgggcgcttcccg
gacttttgtccgagttgaattccctcccctgggccgggcccttccggcc
gcccccgcccgtgccccgctcgctctcgggagatgtttatttgggctgtg
gcgtgaggagcgggcgggccagcgccgcggagtttcgggtccgaggagcc
tcgcgcggcgctggagagagacaagatgtccgccagagctgcggccgcca
agagcacagcaatggaggaaacagctatatgggaacaacatacagtgacg
cttcacagggctcctggatttggatttggaattgcaatatctggtggacg
agataatcctcattttcagagtggggaaacgtcaatagtgatttcagatg
tgctgaaaggaggaccagctgaaggacagctacaggaaaatgaccgagtt
gcaatggttaacggagtttcaatggataatgttgaacatgcttttgctgt
tcagcaactaaggaaaagtgggaaaaatgcaaaaattacaattagaagga
agaagaaagttcaaataccagtaagtcgtcctgatcctgaaccagtatct
gataatgaagaagatagttatgatgaggaaatacatgatccaagaagtgg
ccggagtggtgtggttaacagaaggagtgagaagatttggccgagggata
gaagtgcaagtagagagaggagcttgtccccgcggtcagacaggcggtca
gtggcttccagccagcctgctaaacctactaaagtcacactggtgaaatc
ccggaaaaatgaagaatatggtcttcgattggcaagccatatatttgtta
aggaaatttcacaagatagtttggcagcaagagatggcaatattcaagaa
ggtgatgttgtattgaagataaatggtactgtgacagaaaatatgtcatt
gacagatgcaaagacattgatagaaaggtctaaaggcaaattaaaaatgg
tagttcaaagagatgaacgggctacgctattgaatgtccctgatctttct
gacagcatccactctgctaatgcctctgagagagacgacatttcagaaat
tcagtcactggcatcagatcattctggtcgatcacacgataggcctcccc
gccgcagccggtcacgatctcctgaccagcggtcagagccttctgatcat
tccaggcactcgccgcagcagccaagcaatggcagtctccggagtagaga
tgaagagagaatttctaaacctggggctgtctcaactcctgtaaagcatg
ctgatgatcacacacctaaaacagtggaagaagttacagttgaaagaaat
gagaaacaaacaccttctcttccagaaccaaagcctgtgtatgcccaagt
tgggcaaccagatgtggatttacctgtcagtccatctgatggtgtcctac
ctaattcaactcatgaagatgggattcttcggcccagcatgaaattggta
aaattcagaaaaggagatagtgtgggtttgcggctggctggtggaaatga
tgttggaatatttgtagctggcgttctagaagatagccctgcagccaagg
aaggcttagaggaaggtgatcaaattctcagggtaaacaacgtagatttt
acaaatatcataagagaagaagccgtccttttcctgcttgacctccctaa
aggagaagaagtgaccatattggctcagaagaagaaggatgtttatcgtc
gcattgtagaatcagatgtaggagattctttctatattagaacccatttt
gaatatgaaaaggaatctccctatggacttagttttaacaaaggagaggt
gttccgtgttgtggataccttgtacaatggaaaactgggctcttggcttg
ctattcgaattggtaaaaatcataaggaggtagaacgaggcatcatccct
aataagaacagagctgagcagctagccagtgtacagtatacacttccaaa
aacagcaggcggagaccgtgctgacttctggagattcagaggtcttcgca
gctccaagagaaatcttcgaaaaagcagagaggatttgtccgctcagcct
gttcaaacaaagtttccagcttatgaaagagtggttcttcgagaagctgg
atttctgaggcctgtaaccatttttggaccaatagctgatgttgccagag

aaaagctggcaagagaagaaccagatatttatcaaattgcaaagagtgaa
ccacgagacgctggaactgaccaacgtagctctggcattattcgcctgca
tacaataaagcaaatcatagatcaagacaaacatgctttattagatgtaa
caccaaatgcagttgatcgtcttaactatgcccagtggtatccaattgtt
gtatttcttaaccctgattctaagcaaggagtaaaaacaatgagaatgag
gttatgtccagaatctcggaaaagtgccaggaagttatacgagcgatctc
ataaacttcgtaaaaataatcaccatctttttacaactacaattaactta
aattcaatgaatgatggttggtatggtgcgctgaaagaagcaattcaaca
acagcaaaaccagctggtatgggtttccgagggaaaggcggatggtgcta
caagtgatgaccttgatttgcatgatgatcgtctgtcctacctgtcagct
ccaggtagtgaatactcaatgtatagcacggacagtagacacacttctga
ctatgaagacacagacacagaaggcggggcctacactgatcaagaactag
atgaaactcttaatgatgaggttgggactccaccggagtctgccattaca
cggtcctctgagcctgtaagagaggactcctctggaatgcatcatgaaaa
ccaaacatatcctccttactcaccacaagcgcagccacaaccaattcata
gaatagactcccctggatttaagccagcctctcaacaggtgtatagaaag
gatccatatcccgaggaaatgatgaggcagaaccatgttttgaaacagcc
agccgttagtcacccagggcacaggccagacaaagagcctaatctgacct
atgaaccccaactcccatacgtagagaaacaagccagcagagacctcgag
cagcccacatacagatacgagtcctcaagctatacggaccagttttctcg
aaactatgaacatcgtctgcgatacgaagatcgcgtccccatgtatgaag
aacagtggtcatattatgatgacaaacagccctacccatctcggccacct
tttgataatcagcactctcaagaccttgactccagacagcatcccgaaga
gtcctcagaacgagggtactttccacgttttgaagagccagcccctctgt
cttacgacagcagaccacgttacgaacaggcacctagagcatccgccctg
cggcacgaagagcagccagctcctgggtatgacacacatggtagactcag
accggaagcccagccccacccttcagcagggcccaagcctgcagagtcca
agcagtattttgagcaatattcacgcagttacgagcaagtaccaccccaa
ggatttacctctagagcaggtcattttgagcctctccatggtgctgcagc
tgtccctccgctgataccttcatctcagcataagccagaagctctgcctt
caaacaccaaaccactgcctccacccccaactcaaaccgaagaagaggaa
gatccagcaatgaagccacagtctgtactcaccagagttaagatgtttga
aaacaaaagatctgcatccttagagaccaagaaggatgtaaatgacactg
gcagttttaagcctccagaagtagcatctaaaccttcaggtgctcccatc
attggtcccaaacccacttctcagaatcaattcagtgaacatgacaaaac
tctgtacaggatcccagaacctcaaaaacctcaactgaagccacctgaag
atattgttcggtccaatcattatgaccctgaagaagatgaagaatattat
cgaaaacagctgtcatactttgaccgaagaagttttgagaataagcctcc
tgcacacattgccgccagccatctctccgagcctgcaaagccagcgcatt
ctcagaatcaatcaaatttttctagttattcttcaaagggaaagcctcct
gaagctgatggtgtggatagatcatttggcgagaaacgctatgaacccat
ccaggccactcccccctcctcctccattgccctcgcagtatgcccagccat
ctcagcctgtcaccagcgcgtctctccacatacattctaagggagcacat
ggtgaaggtaattcagtgtcattggattttcagaattccttagtgtccaa
accagacccacctccatctcagaataagccagcaactttcagaccaccaa
accgagaagatactgctcaggcagctttctatccccagaaaagttttcca
gataaagccccagttaatggaactgaacagactcagaaaacagtcactcc
agcatacaatcgattcacaccaaaaccatatacaagttctgcccgaccat
ttgaacgcaagtttgaaagtcctaaattcaatcacaatcttctgccaagt

gaaactgcacataaacctgacttgtcttcaaaaactcccacttctccaaa
aactcttgtgaaatcgcacagtttggcacagcctcctgagtttgacagtg
gagttgaaactttctctatccatgcagagaagcctaaatatcaaataaat
aatatcagcacagtgcctaaagctattcctgtgagtccttcagctgtgga
agaggatgaagatgaagatggtcatactgtggtggccacagcccgaggca
tatttaacagcaatgggggcgtgctgagttccatagaaactggtgttagt
ataattatccctcaaggagccattcccgaaggagttgagcaggaaatcta
tttcaaggtctgccgggacaacagcatccttccacctttagataaagaga
aaggtgaaacactgctgagtcctttggtgatgtgtggtccccatggcctc
aagttcctgaagcctgtggagctgcgcttaccacactgtgatcctaaaac
ctggcaaaacaagtgtcttcccggagatccaaattatctcgttggagcaa
actgtgtttctgtccttattgaccacttttaactcttgaaatataggaac
ttaaataatgtgaaactggattaaacttaatctaaatggaaccactctat
caagtattatacctttttagagttgatactacagtttgttagtatgagg
catttgtttgaactgataaagatgagtgagcatgcccctgaaccatggtc
ggaaaacatgctacacactgcatgtttgtgattgacgggactgttggtat
tggctagaggttcaaagatattttgctttgtgatttttgtaattttttta
tcgtcactgcttaacttcacatattgatttccgttaaaataccagccagt
aaatgggggtgcatttgaggtctgttctttccaaagtacactgtttcaaa
ctttactatggccctggcctagcatacgtacacattttattttattatgc
atgaagtaatatgcacacatttttaaatgcacctggaatatataaccag
tgttgtggatttaacagaaatgtacagcaaggagatttacaactggggga
gggtgaagtgaagacaatgacttactgtacatgaaaacacatttttctta
gggaaggatacaaaagcatgtgagactggttccatggcctcttcagatct
ctaacttcaccatattaccacagacatactaaccagcagaaatgccttac
cctcatgttcttaattcttagctcattctccttgtgttactaagtttta
tggcttttgtgcattatctagatactgtatcatgacaaagactgagtacg
ttgtgcatttggtggtttcagaaatgtgttatcacccagaagaaaatagt
ggtgtgatttggggatattttttcttttcttttcttttcttttttttt
tttttgacaaggggcagtggtggttttctgttctttctggctatgcattt
gaaaattttgatgttttaaggatgcttgtacataatgcgtgcataccact
tttgttcttggtttgtaaattaacttttataaactttacctttttatac
ataaacaagaccacgtttctaaaggctacctttgtattctctcctgtacc
tcttgagccttgaactttgacctctgcagcaataaagcagcgtttctatg
acacatgcaaggtcattttttttaagaaaaaggatgcacagagttgttac
atttttaagtgctgcatttaaaagatacagttactcagaattctctagtt
tgattaaattcttgcaaagtatccctactgtaatttgtgatacaatgctg
tgccctaaagtgtattttttttactaatagacaatttattatggcacatca
gcacgatttctgtttagataatacaccactacattctgttaatcattagg
tgtgactgaatttcttttgccgttattaaaaatctcaaatttctaaatct
ccaaaataaaactttttaaaataaa
>NM_001017402 1
cttttctcctgcaaagggtccagccttttcctgctccccacgttgtcctt
acggctgtgtggggtagggcagggtccacactccttcccatccattttag
aggaggaagctggagtctgggaagggatgggattttcccagggcaccctg
tgagtcacatgccacttgagacaagggtctagagctccagcattttccaa
gctacaaatgtatctgctgctccaagtgtcctagatcaggaccctaggta
aagacttctgaaaaatacttgagtctagaaacagaaaagaaaaagggcta
gtgtgctctaggggtttgcactgttgtacccagtttgctttgctgtgtcc

taggaaaggtcctttctggggatcaccccattggctgaagatgagaccat
tcttcctcttgtgttttgccctgcctggcctcctgcatgcccaacaagcc
tgctcccgtggggcctgctatccacctgttggggacctgcttgttgggag
gacccggtttctccgagcttcatctacctgtggactgaccaagcctgaga
cctactgcacccagtatggcgagtggcagatgaaatgctgcaagtgtgac
tccaggcagcctcacaactactacagtcaccgagtagagaatgtggcttc
atcctccggccccatgcgctggtggcagtcacagaatgatgtgaaccctg
tctctctgcagctggacctggacaggagattccagcttcaagaagtcatg
atggagttccaggggcccatgcccgccggcatgctgattgagcgctcctc
agacttcggtaagacctggcgagtgtaccagtacctggctgccgactgca
cctccaccttccctcgggtccgccagggtcggcctcagagctggcaggat
gttcggtgccagtccctgcctcagaggcctaatgcacgcctaaatggggg
gaaggtccaacttaaccttatggatttagtgtctgggattccagcaactc
aaagtcaaaaaattcaagaggtgggggagatcacaaacttgagagtcaat
ttcaccaggctggcccctgtgccccaaaggggctaccaccctcccagcgc
ctactatgctgtgtcccagctccgtctgcaggggagctgcttctgtcacg
gccatgctgatcgctgcgcacccaagcctggggcctctgcaggcccctcc
accgctgtgcaggtccacgatgtctgtgtctgccagcacaacactgccgg
cccaaattgtgagcgctgtgcacccttctacaacaaccggccctggagac
cggcggagggccaggacgcccatgaatgccaaaggtgcgactgcaatggg
cactcagagacatgtcactttgaccccgctgtgtttgccgccagccaggg
ggcatatggaggtgtgtgtgacaattgccgggaccacaccgaaggcaaga
actgtgagcggtgtcagctgcactatttccggaaccggcgcccgggagct
tccattcaggagacctgcatctcctgcgagtgtgatccggatggggcagt
gccaggggctccctgtgacccagtgaccgggcagtgtgtgtgcaaggagc
atgtgcagggagagcgctgtgacctatgcaagccgggcttcactggactc
acctacgccaacccgcagggctgccaccgctgtgactgcaacatcctggg
gtcccggagggacatgccgtgtgacgaggagagtgggcgctgcctttgtc
tgcccaacgtggtgggtcccaaatgtgaccagtgtgctccctaccactgg
aagctggccagtggccagggctgtgaaccgtgtgcctgcgacccgcacaa
ctccctcagcccacagtgcaaccagttcacagggcagtgcccctgtcggg
aaggctttggtggcctgatgtgcagcgctgcagccatccgccagtgtcca
gaccggacctatggagacgtggccacaggatgccgagcctgtgactgtga
tttccggggaacagagggcccgggctgcgacaaggcatcaggccgctgcc
tctgccgccctggcttgaccgggccccgctgtgaccagtgccagcgaggc
tactgtaatcgctacccggtgtgcgtggcctgccacccttgcttccagac
ctatgatgcggacctccgggagcaggccctgcgctttggtagactccgca
atgccaccgccagcctgtggtcagggcctgggctggaggaccgtggcctg
gcctcccggatcctagatgcaaagagtaagattgagcagatccgagcagt
tctcagcagccccgcagtcacagagcaggaggtggctcaggtggccagtg
ccatcctctccctcaggcgaactctccagggcctgcagctggatctgccc
ctggaggaggagacgttgtcccttccgagagacctggagagtcttgacag
aagcttcaatggtctccttactatgtatcagaggaagagggagcagtttg
aaaaaataagcagtgctgatccttcaggagccttccggatgctgagcaca
gcctacgagcagtcagcccaggctgctcagcaggtctccgacagctcgcg
ccttttggaccagctcagggacagccggagagaggcagagaggctggtgc
ggcaggcgggaggaggaggaggcaccggcagccccaagcttgtggccctg
aggctggagatgtcttcgttgcctgacctgacacccaccttcaacaagct
ctgtggcaactccaggcagatggcttgcaccccaatatcatgccctggtg

agctatgtccccaagacaatggcacagcctgtggctcccgctgcaggggt
gtccttcccagggccggtggggccttcttgatggcggggcaggtggctga
gcagctgcggggcttcaatgcccagctccagcggaccaggcagatgatta
gggcagccgaggaatctgcctcacagattcaatccagtgcccagcgcttg
gagacccaggtgagcgccagccgctcccagatggaggaagatgtcagacg
cacacggctcctaatccagcaggtccgggacttcctaacagaccccgaca
ctgatgcagccactatccaggaggtcagcgaggccgtgctggccctgtgg
ctgcccacagactcagctactgttctgcagaagatgaatgagatccaggc
cattgcagccaggctccccaacgtggacttggtgctgtcccagaccaagc
aggacattgcgcgtgcccgccggttgcaggctgaggctgaggaagccagg
agccgagcccatgcagtggagggccaggtggaagatgtggttgggaacct
gcggcaggggacagtggcactgcaggaagctcaggacaccatgcaaggca
ccagccgctcccttcggcttatccaggacagggttgctgaggttcagcag
gtactgcggccagcagaaaagctggtgacaagcatgaccaagcagctggg
tgacttctggacacggatggaggagctccgccaccaagcccggcagcagg
gggcagaggcagtccaggcccagcagcttgcggaaggtgccagcgagcag
gcattgagtgcccaagagggatttgagagaataaaacaaaagtatgctga
gttgaaggaccggttgggtcagagttccatgctgggtgagcagggtgccc
ggatccagagtgtgaagacagaggcagaggagctgtttggggagaccatg
gagatgatggacaggatgaaagacatggagttggagctgctgcggggcag
ccaggccatcatgctgcgctcagcggacctgacaggactggagaagcgtg
tggagcagatccgtgaccacatcaatgggcgcgtgctctactatgccacc
tgcaagtgatgctacagcttccagcccgttgccccactcatctgccgcct
ttgcttttggttgggggcagattgggttggaatgctttccatctccagga
gactttcatgcagcctaaagtacagcctggaccaccccctggtgtgtagct
agtaagattaccctgagctgcagctgagcctgagccaatgggacagttac
acttgacagacaaagatggtggagattggcatgccattgaaactaagagc
tctcaagtcaaggaagctgggctgggcagtatcccccgcctttagttctc
cactggggaggaatcctggaccaagcacaaaaacttaacaaaagtgatgt
aaaaatgaaaagccaaataaaaatctttggaaaagagcctggaggttcaa
cgaggaaaaaaaaaaaaaaaaaaaaaaaaaaaaaaaa
>NM_000125 3
aggagctggcggagggcgttcgtcctgggactgcacttgctcccgtcggg
tcgcccggcttcaccggacccgcaggctcccggggcagggccggggccag
agctcgcgtgtcggcgggacatgcgctgcgtcgcctctaacctcgggctg
tgctctttttccaggtggcccgccggtttctgagccttctgccctgcggg
gacacggtctgcaccctgcccgcggccacggaccatgaccatgaccctcc
acaccaaagcatctgggatggccctactgcatcagatccaagggaacgag
ctggagcccctgaaccgtccgcagctcaagatccccctggagcggcccct
gggcgaggtgtacctggacagcagcaagcccgccgtgtacaactaccccg
agggcgccgcctacgagttcaacgccgcggccgccgccaacgcgcaggtc
tacggtcagaccggcctcccctacggccccgggtctgaggctgcggcgtt
cggctccaacggcctgggggggtttccccccactcaacagcgtgtctccga
gcccgctgatgctactgcacccgccgccgcagctgtcgcctttcctgcag
ccccacggccagcaggtgccctactacctggagaacgagcccagcggcta
cacggtgcgcgaggccggcccgccggcattctacaggccaaattcagata
atcgacgccagggtggcagagaaagattggccagtaccaatgacaagga
agtatggctatggaatctgccaaggagactcgctactgtgcagtgtgcaa
tgactatgcttcaggctaccattatggagtctggtcctgtgagggctgca

aggccttcttcaagagaagtattcaaggacataacgactatatgtgtcca
gccaccaaccagtgcaccattgataaaaacaggaggaagagctgccaggc
ctgccggctccgcaaatgctacgaagtgggaatgatgaaaggtgggatac
gaaaagaccgaagaggagggagaatgttgaaacacaagcgccagagagat
gatggggagggcaggggtgaagtggggtctgctggagacatgagagctgc
caacctttggccaagcccgctcatgatcaaacgctctaagaagaacagcc
tggccttgtccctgacggccgaccagatggtcagtgccttgttggatgct
gagcccccatactctattccgagtatgatcctaccagacccttcagtga
agcttcgatgatgggcttactgaccaacctggcagacagggagctggttc
acatgatcaactgggcgaagagggtgccaggctttgtggatttgaccctc
catgatcaggtccaccttctagaatgtgcctggctagagatcctgatgat
tggtctcgtctggcgctccatggagcacccagggaagctactgtttgctc
ctaacttgctcttggacaggaaccagggaaaatgtgtagagggcatggtg
gagatcttcgacatgctgctggctacatcatctcggttccgcatgatgaa
tctgcagggagaggagtttgtgtgcctcaaatctattattttgcttaatt
ctggagtgtacacatttctgtccagcaccctgaagtctctggaagagaag
gaccatatccaccgagtcctggacaagatcacagacactttgatccacct
gatggccaaggcaggcctgaccctgcagcagcagcaccagcggctggccc
agctcctcctcatcctctcccacatcaggcacatgagtaacaaaggcatg
gagcatctgtacagcatgaagtgcaagaacgtggtgcccctctatgacct
gctgctggagatgctggacgcccaccgcctacatgcgcccactagccgtg
gaggggcatccgtggaggagacggaccaaagccacttggccactgcgggc
tctacttcatcgcattccttgcaaaagtattacatcacgggggaggcaga
gggtttccctgccacggtctgagagctccctggctcccacacggttcaga
taatccctgctgcattttaccctcatcatgcaccactttagccaaattct
gtctcctgcatacactccggcatgcatccaacaccaatggctttctagat
gagtggccattcatttgcttgctcagttcttagtggcacatcttctgtct
tctgttgggaacagccaaagggattccaaggctaaatctttgtaacagct
ctctttccccttgctatgttactaagcgtgaggattcccgtagctcttc
acagctgaactcagtctatgggttggggctcagataactctgtgcattta
agctacttgtagagacccaggcctggagagtagacattttgcctctgata
agcactttttaaatggctctaagaataagccacagcaaagaatttaaagt
ggctcctttaattggtgacttggagaaagctaggtcaagggtttattata
gcaccctcttgtattcctatggcaatgcatccttttatgaaagtggtaca
ccttaaagcttttatatgactgtagcagagtatctggtgattgtcaattc
attcccccctataggaatacaaggggcacacagggaaggcagatcccctag
ttggcaagactattttaacttgatacactgcagattcagatgtgctgaaa
gctctgcctctggctttccggtcatgggttccagttaattcatgcctccc
atggacctatggagagcagcaagttgatcttagttaagtctccctatatg
agggataagttcctgatttttgtttttatttttgtgttacaaaagaaagc
cctccctccctgaacttgcagtaaggtcagcttcaggacctgttccagtg
ggcactgtacttggatcttcccggcgtgtgtgtgccttacacaggggtga
actgttcactgtggtgatgcatgatgagggtaaatggtagttgaaaggag
caggggccctggtgttgcatttagccctggggcatggagctgaacagtac
ttgtgcaggattgttgtggctactagagaacaagagggaaagtagggcag
aaactggatacagttctgaggcacagccagacttgctcagggtggccctg
ccacaggctgcagctacctaggaacattccttgcagaccccgcattgccc
tttgggggtgccctgggatccctggggtagtccagctcttcttcatttcc
cagcgtggccctggttggaagaagcagctgtcacagctgctgtagacagc

tgtgttcctacaattggcccagcaccctggggcacgggagaagggtgggg
accgttgctgtcactactcaggctgactggggcctggtcagattacgtat
gcccttggtggtttagagataatccaaaatcagggtttggtttggggaag
aaaatcctccccttcctcccccgccccgttccctaccgcctccactcct
gccagctcatttccttcaatttcctttgacctataggctaaaaaagaaag
gctcattccagccacagggcagccttccctgggcctttgcttctctagca
caattatgggttacttccttttcttaacaaaaaagaatgtttgatttcc
tctgggtgaccttattgtctgtaattgaaaccctattgagaggtgatgtc
tgtgttagccaatgacccaggtgagctgctcgggcttctcttggtatgtc
ttgtttggaaaagtggatttcattcatttctgattgtccagttaagtgat
caccaaaggactgagaatctgggagggcaaaaaaaaaaaaaaagtttta
tgtgcacttaaatttggggacaattttatgtatctgtgttaaggatatgt
ttaagaacataattcttttgttgctgtttgtttaagaagcaccttagttt
gtttaagaagcaccttatatagtataatatatatttttttgaaattacat
tgcttgtttatcagacaattgaatgtagtaattctgttctggatttaatt
tgactgggttaacatgcaaaaaccaaggaaaaatatttagtttttttttt
tttttttgtatacttttcaagctaccttgtcatgtatacagtcatttatg
cctaaagcctggtgattattcatttaaatgaagatcacatttcatatcaa
cttttgtatccacagtagacaaaatagcactaatccagatgcctattgtt
ggatactgaatgacagacaatcttatgtagcaaagattatgcctgaaaag
gaaaattattcagggcagctaattttgcttttaccaaaatatcagtagta
atatttttggacagtagctaatgggtcagtgggttctttttaatgtttat
acttagattttcttttaaaaaaattaaaataaaacaaaaaaaaaatttcta
ggactagacgatgtaataccagctaaagccaaacaattatacagtggaag
gttttacattattcatccaatgtgtttctattcatgttaagatactacta
catttgaagtgggcagagaacatcagatgattgaaatgttcgcccagggg
tctccagcaactttggaaatctctttgtatttttacttgaagtgccacta
atggacagcagatattttctggctgatgttggtattgggtgtaggaacat
gatttaaaaaaaaactcttgcctctgctttcccccactctgaggcaagtt
aaaatgtaaaagatgtgatttatctggggggctcaggtatggtggggaag
tggattcaggaatctggggaatggcaaatatattaagaagagtattgaaa
gtatttggaggaaaatggttaattctgggtgtgcaccagggttcagtaga
gtccacttctgccctggagaccacaaatcaactagctccatttacagcca
tttctaaaatggcagcttcagttctagagaagaaagaacaacatcagcag
taaagtccatggaatagctagtggtctgtgtttcttttcgccattgccta
gcttgccgtaatgattctataatgccatcatgcagcaattatgagaggct
aggtcatccaaagagaagaccctatcaatgtaggttgcaaaatctaaccc
ctaaggaagtgcagtctttgatttgatttccctagtaaccttgcagatat
gtttaaccaagccatagcccatgccttttgagggctgaacaaataaggga
cttactgataatttacttttgatcacattaaggtgttctcaccttgaaat
cttatacactgaaatggccattgatttaggccactggcttagagtactcc
ttcccctgcatgacactgattacaaatactttcctattcatactttccaa
ttatgagatggactgtgggtactgggagtgatcactaacaccatagtaat
gtctaatattcacaggcagatctgcttggggaagctagttatgtgaaagg
caaatagagtcatacagtagctcaaaaggcaaccataattctctttggtg
caggtcttgggagcgtgatctagattacactgcaccattcccaagttaat
cccctgaaaacttactctcaactggagcaaatgaactttggtcccaaata
tccatcttttcagtagcgttaattatgctctgtttccaactgcatttcct
ttccaattgaattaaagtgtggcctcgtttttagtcatttaaaattgttt

tctaagtaattgctgcctctattatggcacttcaattttgcactgtcttt
tgagattcaagaaaaatttctattcttttttttgcatccaattgtgcctg
aacttttaaaatatgtaaatgctgccatgttccaaacccatcgtcagtgt
gtgtgtttagagctgtgcaccctagaaacaacatattgtcccatgagcag
gtgcctgagacacagacccctttgcattcacagagaggtcattggttata
gagacttgaattaataagtgacattatgccagtttctgttctctcacagg
tgataaacaatgctttttgtgcactacatactcttcagtgtagagctctt
gttttatgggaaaaggctcaaatgccaaattgtgtttgatggattaatat
gcccttttgccgatgcatactattactgatgtgactcggttttgtcgcag
ctttgctttgtttaatgaaacacacttgtaaacctcttttgcactttgaa
aaagaatccagcgggatgctcgagcacctgtaaacaattttctcaaccta
tttgatgttcaaataaagaattaaactaaa
>NM_000222 2
tctgggggctcggctttgccgcgctcgctgcacttgggcgagagctggaa
cgtggaccagagctcggatcccatcgcagctaccgcgatgagaggcgctc
gcggcgcctgggattttctctgcgttctgctcctactgcttcgcgtccag
acaggctcttctcaaccatctgtgagtccaggggaaccgtctccaccatc
catccatccaggaaaatcagacttaatagtccgcgtgggcgacgagatta
ggctgttatgcactgatccgggctttgtcaaatggacttttgagatcctg
gatgaaacgaatgagaataagcagaatgaatggatcacggaaaaggcaga
agccaccaacaccggcaaatacacgtgcaccaacaaacacggcttaagca
attccatttatgtgtttgttagagatcctgccaagcttttccttgttgac
cgctccttgtatgggaaagaagacaacgacacgctggtccgctgtcctct
cacagacccagaagtgaccaattattccctcaaggggtgccaggggaagc
ctcttcccaaggacttgaggtttattcctgaccccaaggcgggcatcatg
atcaaaagtgtgaaacgcgcctaccatcggctctgtctgcattgttctgt
ggaccaggagggcaagtcagtgctgtcggaaaaattcatcctgaaagtga
ggccagccttcaaagctgtgcctgttgtgtctgtgtccaaagcaagctat
cttcttagggaaggggaagaattcacagtgacgtgcacaataaaagatgt
gtctagttctgtgtactcaacgtggaaaagagaaaacagtcagactaaac
tacaggagaaatataatagctggcatcacggtgacttcaattatgaacgt
caggcaacgttgactatcagttcagcgagagttaatgattctggagtgtt
catgtgttatgccaataatacttttggatcagcaaatgtcacaacaacct
tggaagtagtagataaaggattcattaatatcttccccatgataaacact
acagtatttgtaaacgatggagaaaatgtagatttgattgttgaatatga
agcattccccaaacctgaacaccagcagtggatctatatgaacagaacct
tcactgataaatgggaagattatcccaagtctgagaatgaaagtaatatc
agatacgtaagtgaacttcatctaacgagattaaaaggcaccgaaggagg
cacttacacattcctagtgtccaattctgacgtcaatgctgccatagcat
ttaatgtttatgtgaatacaaaaccagaaatcctgacttacgacaggctc
gtgaatggcatgctccaatgtgtggcagcaggattcccagagcccacaat
agattggtatttttgtccaggaactgagcagagatgctctgcttctgtac
tgccagtggatgtgcagacactaaactcatctgggccaccgtttggaaag
ctagtggttcagagttctatagattctagtgcattcaagcacaatggcac
ggttgaatgtaaggcttacaacgatgtgggcaagacttctgcctatttta
actttgcatttaaaggtaacaacaaagagcaaatccatccccacaccctg
ttcactcctttgctgattggtttcgtaatcgtagctggcatgatgtgcat
tattgtgatgattctgacctacaaatatttacagaaacccatgtatgaag
tacagtggaaggttgttgaggagataaatggaaacaattatgtttacata

gacccaacacaacttccttatgatcacaaatgggagtttcccagaaacag
gctgagttttgggaaaaccctgggtgctggagctttcgggaaggttgttg
aggcaactgcttatggcttaattaagtcagatgcggccatgactgtcgct
gtaaagatgctcaagccgagtgcccatttgacagaacgggaagccctcat
gtctgaactcaaagtcctgagttaccttggtaatcacatgaatattgtga
atctacttggagcctgcaccattggagggcccaccctggtcattacagaa
tattgttgctatggtgatcttttgaattttttgagaagaaaacgtgattc
atttatttgttcaaagcaggaagatcatgcagaagctgcactttataaga
atcttctgcattcaaaggagtcttcctgcagcgatagtactaatgagtac
atggacatgaaacctggagtttcttatgttgtcccaaccaaggccgacaa
aaggagatctgtgagaataggctcatacatagaaagagatgtgactcccg
ccatcatggaggatgacgagttggccctagacttagaagacttgctgagc
ttttcttaccaggtggcaaagggcatggctttcctcgcctccaagaattg
tattcacagagacttggcagccagaaatatcctccttactcatggtcgga
tcacaaagatttgtgattttggtctagccagagacatcaagaatgattct
aattatgtggttaaaggaaacgctcgactacctgtgaagtggatggcacc
tgaaagcattttcaactgtgtatacacgtttgaaagtgacgtctggtcct
atgggatttttctttgggagctgttctctttaggaagcagcccctatcct
ggaatgccggtcgattctaagttctacaagatgatcaaggaaggcttccg
gatgctcagccctgaacacgcacctgctgaaatgtatgacataatgaaga
cttgctgggatgcagatcccctaaaaagaccaacattcaagcaaattgtt
cagctaattgagaagcagatttcagagagcaccaatcatatttactccaa
cttagcaaactgcagccccaaccgacagaagcccgtggtagaccattctg
tgcggatcaattctgtcggcagcaccgcttcctcctcccagcctctgctt
gtgcacgacgatgtctgagcagaatcagtgtttgggtcacccctccagga
atgatctcttcttttggcttccatgatggttattttcttttctttcaact
tgcatccaactccaggatagtgggcaccccactgcaatcctgtctttctg
agcacactttagtggccgatgatttttgtcatcagccaccatcctattgc
aaaggttccaactgtatatattcccaatagcaacgtagcttctaccatga
acagaaaacattctgatttggaaaaagagagggaggtatggactgggggc
cagagtcctttccaaggcttctccaattctgcccaaaaatatggttgata
gtttacctgaataaatggtagtaatcacagttggccttcagaaccatcca
tagtagtatgatgatacaagattagaagctgaaaacctaagtcctttatg
tggaaaacagaacatcattagaacaaaggacagagtatgaacacctgggc
ttaagaaatctagtatttcatgctgggaatgagacataggccatgaaaaa
aatgatccccaagtgtgaacaaaagatgctcttctgtggaccactgcatg
agcttttatactaccgacctggtttttaaatagagtttgctattagagca
ttgaattggagagaaggcctccctagccagcacttgtatatacgcatcta
taaattgtccgtgttcatacatttgaggggaaaacaccataaggtttcgt
ttctgtatacaaccctggcattatgtccactgtgtatagaagtagattaa
gagccatataagtttgaaggaaacagttaataccatttttttaaggaaaca
atataaccacaaagcacagtttgaacaaaatctcctcttttagctgatga
acttattctgtagattctgtggaacaagcctatcagcttcagaatggcat
tgtactcaatggatttgatgctgtttgacaaagttactgattcactgcat
ggctcccacaggagtgggaaaacactgccatcttagtttggattcttatg
tagcaggaaataaagtataggtttagcctccttcgcaggcatgtcctgga
caccgggccagtatctatatatgtgtatgtacgtttgtatgtgtgtagac
aaatatttggaggggtatttttgccctgagtccaagagggtcctttagta
cctgaaaagtaacttggctttcattattagtactgctcttgtttcttttc

acatagctgtctagagtagcttaccagaagcttccatagtggtgcagagg
aagtggaaggcatcagtccctatgtatttgcagttcacctgcacttaagg
cactctgttatttagactcatcttactgtacctgttccttagaccttcca
taatgctactgtctcactgaaacatttaaattttaccctttagactgtag
cctggatattattcttgtagtttacctctttaaaaacaaaacaaaacaaa
acaaaaaactccccttcctcactgcccaatataaaaggcaaatgtgtaca
tggcagagtttgtgtgttgtcttgaaagattcaggtatgttgcctttatg
gtttccccccttctacatttcttagactacatttagagaactgtggccgtt
atctggaagtaaccatttgcactggagttctatgctctcgcacctttcca
aagttaacagattttggggttgtgttgtcacccaagagattgttgtttgc
catactttgtctgaaaaattcctttgtgtttctattgacttcaatgatag
taagaaaagtggttgttagttatagatgtctaggtacttcaggggcactt
cattgagagttttgtcttggatattcttgaaagtttatatttttataatt
ttttcttacatcagatgtttctttgcagtggcttaatgtttgaaattatt
ttgtggcttttttgtaaatattgaaatgtagcaataatgtcttttgaat
attcccaagcccatgagtccttgaaaatatttttatatatacagtaact
ttatgtgtaaatacataagcggcgtaagtttaaaggatgttggtgttcca
cgtgttttattcctgtatgttgtccaattgttgacagttctgaagaattc
taataaaatgtacatatataaatcaaaaaaaaaaaaaaaa
>NM_004064 3
cttcttcgtcagcctcccttccaccgccatattgggccactaaaaaaagg
gggctcgtcttttcggggtgtttttctccccctcccctgtccccgcttgc
tcacggctctgcgactccgacgccggcaaggtttggagagcggctgggtt
cgcgggacccgcgggcttgcacccgcccagactcggacgggctttgccac
cctctccgcttgcctggtcccctctcctctccgccctcccgctcgccagt
ccatttgatcagcggagactcggcggccgggccggggcttccccgcagcc
cctgcgcgctcctagagctcgggccgtggctcgtcggggtctgtgtcttt
tggctccgagggcagtcgctgggcttccgagaggggttcgggctgcgtag
gggcgctttgttttgttcggttttgtttttttgagagtgcgagagaggcg
gtcgtgcagacccgggagaaagatgtcaaacgtgcgagtgtctaacggga
gccctagcctggagcggatggacgccaggcaggcggagcaccccaagccc
tcggcctgcaggaacctcttcggcccggtggaccacgaagagttaacccg
ggacttggagaagcactgcagagacatggaagaggcgagccagcgcaagt
ggaatttcgattttcagaatcacaaacccctagagggcaagtacgagtgg
caagaggtggagaagggcagcttgcccgagttctactacagacccccgcg
gccccccaaaggtgcctgcaaggtgccggcgcaggagagccaggatgtca
gcgggagccgcccggcggcgcctttaattggggctccggctaactctgag
gacacgcatttggtggacccaaagactgatccgtcggacagccagacggg
gttagcggagcaatgcgcaggaataaggaagcgacctgcaaccgacgatt
cttctactcaaaacaaaagagccaacagaacagaagaaaatgtttcagac
ggttccccaaatgccggttctgtggagcagacgcccaagaagcctggcct
cagaagacgtcaaacgtaaacagctcgaattaagaatatgtttccttgtt
tatcagatacatcactgcttgatgaagcaaggaagatatacatgaaaatt
ttaaaaatacatatcgctgacttcatggaatggacatcctgtataagcac
tgaaaaacaacaacacaataacactaaaattttaggcactcttaaatgat
ctgcctctaaaagcgttggatgtagcattatgcaattaggtttttcctta
tttgcttcattgtactacctgtgtatatagtttttaccttttatgtagca
cataaactttggggaagggagggcagggtggggctgaggaactgacgtgg
agcggggtatgaagagcttgctttgatttacagcaagtagataaatattt

gacttgcatgaagagaagcaattttggggaagggtttgaattgttttctt
taaagatgtaatgtccctttcagagacagctgatacttcatttaaaaaaa
tcacaaaaatttgaacactggctaaagataattgctatttatttttacaa
gaagtttattctcatttgggagatctggtgatctcccaagctatctaaag
tttgttagatagctgcatgtggctttttaaaaaagcaacagaaacctat
cctcactgccctccccagtctctcttaaagttggaatttaccagttaatt
actcagcagaatggtgatcactccaggtagtttggggcaaaaatccgagg
tgcttgggagttttgaatgttaagaattgaccatctgcttttattaaatt
tgttgacaaaattttctcattttcttttcacttcgggctgtgtaaacaca
gtcaaaataattctaaatccctcgatatttttaaagatctgtaagtaact
tcacattaaaaaatgaaatatttttaatttaaagcttactctgtccatt
tatccacaggaaagtgttatttttcaaggaaggttcatgtagagaaaagc
acacttgtaggataagtgaaatggatactacatctttaaacagtatttca
ttgcctgtgtatggaaaaaccatttgaagtgtacctgtgtacataactct
gtaaaaacactgaaaaattatactaacttatttatgttaaaagatttttt
ttaatctagacaatatacaagccaaagtggcatgttttgtgcatttgtaa
atgctgtgttgggtagaataggttttcccctcttttgttaaataatatgg
ctatgcttaaaaggttgcatactgagccaagtataatttttttgtaatgtg
tgaaaaagatgccaattattgttacacattaagtaatcaataaagaaaac
ttccatagctatt
>NM_024909 2
ggggcggaagtgacgtcgtgtggggcgggtccgaccgcgcacaatgggcc
atggagttcccgttcgatgtggacgcgctgttcccggagcggatcacggt
gctggaccagcacctgaggcccccagcccgccgacccggaaccacaacgc
cggcccgtgttgatctacagcagcaaattatgaccattatagatgaactg
ggcaaggcttctgccaaggcccagaatctttccgctcctatcactagtgc
atcaaggatgcagagtaaccgccatgttgtttatattctcaaagacagtt
cagcccgaccggctggaaaaggagccattattggtttcatcaaagttgga
tacaagaagctctttgtactggatgatcgtgaggctcataatgaggtaga
accactttgcatcctggacttttacatccatgagtctgtgcaacgccatg
gccatgggcgagaactcttccagtatatgttgcagaaggagcgagtggaa
ccgcaccaactggcaattgaccgaccctcacagaagctgctgaaattcct
gaataagcactacaatctggagaccacagtcccacaggtgaacaactttg
tgatctttgaaggcttctttgcccatcaacatcggccccctgctccctct
ctgagggcaactcgacactctcgtgctgctgcagtcgatcccacgcccgc
tgctccagcaaggaagctgccacccaagagagcagagggagacatcaagc
catactcctctagtgaccgagaatttctgaaggtagctgtggagcctcct
tggcccctaaacagggcccctcgccgcgccacacctccagcccacccacc
cccccgctccagcagcctgggaaactcaccagaacgaggtcccctccgcc
cctttgtgccagagcaggagctgctgcgttccttgcgcctctgcccccca
caccctaccgcccgccttctgttggctgctgaccctgggggcagcccagc
tcaacgtcgtcgcaccagctcccttccccgctctgaggagagtcgatact
aacagctaccctctccctgccctgggagacctggggtgggcagggaaccc
ctccctgagaacctcagacccactcttccattgcatcctgtaggacccag
tggaacctgacagagcccataggattccctcttctactttcttagacagc
agggatgtcagggtctcaaactgcctaacactttgtagcttttcttaaca
caaaagcaccccttctctcctaacttgggctctgaatactttcccaacag
gaagtctgatctgttgccagacttcttggttagatggctcatacatttat
ctagagaagcacactcttgcttgctgtcaaactttagaccaccatggaag

gtctaagggcatcctgtgccagggaaactttttaaggaattttatctatg
ggataaaccccatattccctctagtgtctactggtggctctaatactgct
ttgtgctgcctgccacacttgccctttgagcctgcgaatggccgctagtg
agcaagctctgcttcagagcagtctagttaggtagaacagggacttacca
gcttcccaaagggatctactcaccattgccaaactcttcatttccacatt
ttgtgtaggtgtcagggaaccccaaactggtgttgctttggggtctctaa
aggagattggctgacaccaccatttcccccagatccagattctctgaggg
aggttgtttcttgagagtagatccagagtgtcaaggatctgttagatcct
ggaatcccttcttgcatccatccctccctggtagctaggtcccgatatac
tcctgtcttgtgagattgtcgagatgagatgggggaccactcttcctctg
tccttcctctctcctttcctccatagcaaggacgaccttccctgctccat
gcccagagtatagctagatcccttcccctccctaccctctgaatgtgtgc
tagatcaggtgccccactgtgtttcctgaaatccttgggagccggatctc
cccatctcccctactcactcttcccttttcttctctcagtgttgtctgaa
taaagtgtgaaatcttttgtgttttctaaattgacattttcaatgaaaaa
aagaatcacaaaaaaaaaaaaaaaaaaaaaaaaaaaaaaaaaaaaa
>NM_004575 2
agtttcgggtgccgaggtctgcagctagcggcaagcggagtcaggcatcc
gttcagactgacagcagaggcggcgaaggagcgcgtagccgagatcaggc
gtacagagtccggaggcggcggcgggtgagctcaacttcgcacagccctt
cccagctccagccccggctggcccggcacttctcggagggtcccggcagc
cgggaccagtgagtgcctctacggaccagcgccccggcgggcgggaagat
gatgatgatgtccctgaacagcaagcaggcgtttagcatgccgcacggcg
gcagcctgcacgtggagcccaagtactcggcactgcacagcacctcgccg
ggctcctcggctcccatcgcgccctcggccagctcccccagcagctcgag
caacgctggtggtggcggcggcggcggcggcggcggcggcggcggcggag
gccgaagcagcagctccagcagcagtggcagcagcggcggcgggggctcg
gaggctatgcggagagcctgtcttccaaccccaccgagcaatatattcgg
cgggctggatgagagtctgctggcccgcgccgaggctctggcagccgtgg
acatcgtctcccagagcaagagccaccaccaccatccaccccaccacagc
cccttcaaaccggacgccacctaccacactatgaataccatcccgtgcac
gtcggccgcctcttcttcatcggtgcccatctcgcacccttccgcgttgg
cgggcacgcaccaccaccaccaccatcaccaccaccaccaccaccaaccg
caccaggcgctggagggcgagctgctggagcacctgagtcccgggctggc
cctgggcgctatggcgggccccgacggcgctgtggtgtccacgccggctc
acgcgccgcacatggccaccatgaaccccatgcaccaagcagcgctcagc
atggcccacgcgcacgggctgccgtcgcacatgggctgcatgagcgacgt
ggacgccgacccgcgggacctggaggcattcgccgagcgcttcaagcagc
gacgcatcaagctgggggtgacccaggcagatgtgggctccgcgctggcc
aacctcaagatccccggcgtgggctcgcttagccagagcaccatctgcag
gttcgagtccctcacactgtcccacaataatatgatcgcgctcaaaccca
tcctgcaggcatggctcgaggaggccgagaagtcccaccgcgagaagctc
accaagcctgaactcttcaatggcgcggagaagaagcgcaagcgcacgtc
catcgctgcgccagagaagcgctcgctcgaagcctactttgccattcagc
ctcggccctcctctgaaaagatcgccgccatcgcggagaagctggacctg
aagaaaaacgtggtgcgcgtctggttctgcaaccagaggcagaaacagaa
aagaatgaaatattccgccggcatttagaagactcttggcctctccagag
acgcccctttcctcgtccgctcttttctctcctctcttctgcctcttttc
acttttggcgactagaaacaattccagtaaatgtgaatctcgacaaatcg

aggactgaagagggagcgaacgagcgaacaactgagcccaagccggtgag
aatgtgaaacagtttctcaaaggaaagaataacaaaagatggtatttgtc
tgttgtagcaaagttgtccctttgaaccccacctcggcttcttcagagga
agtgtggagatggctgtttgcaggaaggcagacgagacagtgtttaaaaa
gtccacaagaatgatcaagtaagatttgtttttattcttacagacatcac
ccgtgttcaagtttaaaagtacactttgcaactatttttcagaaatagaa
attgattcaggactaaaactttaaactagagttgatgcttaatgtgatag
agacatctctaaagtattttgaattttaaaaaaagatggcagattttctg
catttacactgtatattatatatatatttttattgtggttcttaccccct
tttccttctctgaagtgttaatgcttaagaaaagagttgcgcctgctgtg
ttcactgatcttgaaagctattattagattattgcagaacaaccctctgt
aaattattaatttatctctctagcaacttaattttgtgcacattctaatt
aattaaacttcttccgtctaaaaaaagtgggggaaatgtatagctagtaa
cgttcaaaaaattttgtttgatgagtttaccgaatttttacagctttcct
cctatactgtgttccttttgacccatttgtatattctcacttgaatgaag
attgtttttttctttgtttttactggtagtgttctgatttgtgagtcgac
actcagtaatggatgtcttaatcgtgtagacctgattcactgtctgaagt
attgtttacttcgttacatatttaatggggattcccacattgtccccatg
acacatgagcgctctcacttacccttacacacacacacacacacacacac
acctctaacagaagggaagaagcagttggaagcatgaccgatgcaccatt
ttctagttttaggtgcatttgccacttggtgtttgcccttcagattttag
atttcaccaaggtatttcagtcttccagttttcaattgctttgttggcta
catgttaatatttataggaatacttcagtttttccttttggaggtttgtt
tgtagaaaaactaatttgaactataagaaagacagtgcactgcttgtaaa
ttcacattgtttggaaaaattcttttggaacaaaaaaattaggtacatgat
aactggtaccttatctactgtaaatatttcattaaaaatgatgcacacat
agatatattcttacaaattttgctgtattgctgttctctttgaggctctc
caaagtcttgagttctgtatatggcctggtttcttgtttttattaataga
tggtttatttactatggtaatgtattaatttatttttggtgttgttcgat
tgtctttcattgaagagataattttaatgttttattggcaacgtatgctg
ctttttcattaaaatatgctattaaaattaaatggctttta
>NM_005524 3
gggatcacacaggatccggagctggtgctgataacagcggaatcccccgt
ctacctctctccttggtcctggaacagcgctactgatcaccaagtagcca
caaaatataataaaccctcagcacttgctcagtagttttgtgaaagtctc
aagtaaaagagacacaaacaaaaaattctttttcgtgaagaactccaaaa
ataaaattctctagagataaaaaaaaaaaaaaaaggaaaatgccagctga
tataatggagaaaaattcctcgtccccggtggctgctaccccagccagtg
tcaacacgacaccggataaaccaaagacagcatctgagcacagaaagtca
tcaaagcctattatggagaaaagacgaagagcaagaataaatgaaagtct
gagccagctgaaaacactgattttggatgctctgaagaaagatagctcgc
ggcattccaagctggagaaggcggacattctggaaatgacagtgaagcac
ctccggaacctgcagcgggcgcagatgacggctgcgctgagcacagaccc
aagtgtgctggggaagtaccgagccggcttcagcgagtgcatgaacgagg
tgacccgcttcctgtccacgtgcgagggcgttaataccgaggtgcgcact
cggctgctcggccacctggccaactgcatgacccagatcaatgccatgac
ctaccccgggcagccgcaccccgccttgcaggcgccgccaccgcccccac
cgggacccggcggcccccagcacgcgccgttcgcgccgccgccgccactc
gtgcccatccccgggggcgcggcgccccctcccggcggcgccccctgcaa

gctgggcagccaggctggagaggcggctaaggtgtttggaggcttccagg
tggtaccggctcccgatggccagtttgctttcctcattcccaacggggcc
ttcgcgcacagcggccctgtcatccccgtctacaccagcaacagcggcac
ctccgtgggccccaacgcagtgtcaccttccagcggcccctcgcttacgg
cggactccatgtggaggccgtggcggaactgagggggctcaggccacccc
tcctcctaaactccccaacccacctctcttccctccggactctaaacagg
aacttgaatactgggagagaagaggactttttgattaagtggttacttt
gtgtttttttaatttctaagaagttactttttgtagagagagctgtatta
agtgactgaccatgcactatatttgtatatattttatatgttcatattgg
attgcgcctttgtattataaaagctcagatgacatttcgtttttttacacg
agatttctttttatgtgatgccaaagatgtttgaaaatgctcttaaaat
atcttcctttggggaagtttatttgagaaaatataataaaagaaaaaagt
aaaggcttttaaaaaaaaaaaaaaa
>NM_000791 3
tcccagacagaacctactatgtgcggcggcagctggggcgggaaggcggg
agctgggggcgctgggggcgctgcggccgctgcggccgctgcagccgctg
cagcgccagggtccacctggtcggctgcacctgtggaggaggaggtggat
ttcaggcttcccgtagactggaagaatcggctcaaaaccgcttgcctcgc
aggggctgagctggaggcagcgaggccgcccgacgcaggcttccggcgag
acatggcagggcaaggatggcagcccggcggcagggcctggcgaggagcg
cgagcccgcggccgcagttcccaggcgtctgcgggcgcgagcacgccgcg
accctgcgtgcgccggggcgggggggcggggcctcgcctgcacaaatggg
gacgagggggggcggggcggccacaatttcgcgccaaacttgaccgcgcgt
tctgctgtaacgagcgggctcggaggtcctcccgctgctgtcatggttgg
ttcgctaaactgcatcgtcgctgtgtcccagaacatgggcatcggcaaga
acggggacctgccctggccaccgctcaggaatgaattcagatatttccag
agaatgaccacaacctcttcagtagaaggtaaacagaatctggtgattat
gggtaagaagacctggttctccattcctgagaagaatcgacctttaaagg
gtagaattaatttagttctcagcagagaactcaaggaacctccacaagga
gctcattttctttccagaagtctagatgatgccttaaaacttactgaaca
accagaattagcaaataaagtagacatggtctggatagttggtggcagtt
ctgtttataaggaagccatgaatcacccaggccatcttaaactatttgtg
acaaggatcatgcaagactttgaaagtgacacgtttttttccagaaattga
tttggagaaatataaacttctgccagaatacccaggtgttctctctgatg
tccaggaggagaaaggcattaagtacaaatttgaagtatatgagaagaat
gattaatatgaaggtgttttctagtttaagttgttcccccctccctctgaa
aaaagtatgtatttttacattagaaaaggtttttttgttgactttagatct
ataattatttctaagcaactagtttttattccccactactcttgtctcta
tcagataccatttatgagacattcttgctataactaagtgcttctccaag
accccaactgagtccccagcacctgctacagtgagctgccattccacacc
catcacatgtggcactcttgccagtccttgacattgtcgggcttttcaca
tgttggtaatatttattaaagatgaagatccacatacccttcaactgagc
agtttcactagtggaaataccaaaagcttcctacgtgtatatccagaggt
ttgtagataaatgttgccaccttgtttgtaacagtgaaaaattgaaaaca
acctggaagtccagtgatgggaaaatgagtatgtttctgtcttagattgg
ggaacccaaagcagattgcaagactgaaatttcagtgaaagcagtgtatt
tgctaggtcataccagaaatcatcaattgaggtacggagaaactgaactg
agaaggtaagaaaagcaatttaaagtcagcgagcaggttctcattgataa
caagctccatactgctgagatacagggaaatggagggggaaagctggag

tattgatcccgccccccctccttggttgtcagctccctgtcctgtgtgtgg
gcggaacatagtccagctgctctatagcaagtctcaggtgtttgcagtaa
gaagctgctggcatgcacgggaacagtgaatgccaaacacttaaagcaat
tcgatgtttaagtatgtaagttcttttttttttagacagcgtttcgctct
tgttgcccaggctagcatgcaatggtgtgacctcggcttactgcaacctc
cgccttcccagattcaagcgattctcctgcctcaggctcccaagtagcta
ggaccaggtgcgcgccaccacgcccggctaatttttgtattttgtatttt
tagtagagatggggtttcaccatgttggtcaggctagtctcgaactcgtg
accgcaagcgattcacccacctcagcctcccaaagtgctgggattaccgg
cttgagccaccacacccggcacatcttcattctttttatgtagtaaaaag
tataaggccacacatggtttatttgaagtattttataatttaaaaaaata
cagaagcaggaaaaccaattataagttcaagtgagggatgatggttgctt
gaaccaaagggttgcatgtagtaagaaattgtgatttaagatatatttta
aagttataagtagcaggatattctgatggagtttgactttggttttgggc
ccagggagtttcagatgcctttgagaaatgaatgaagtagagagaaaata
aaagaaaaaccagccaggcacagtggctcacacctgtaatcccagcgctt
tgggaggctaaggcaggcagatcacttgagaccagcttgggcaacatggc
aaagccccatctctacaaaaaacacaaaaattagctgggcattgtggcgc
acacctgtattcccatctagtcaggaagctgagatggaagaattaattga
gcccacgagttcaaggctgcagtgagtcgtgattgtgccactgcactcca
gccggggtgacagaagagaccttgtctcgaaaaggaatctgaaaacaatg
gaaccatgccttcataattctagaaagttattttcaactgataaatctat
attcacccaaataatcaagggtgaaggtaaaataatacatttttagacaa
gcaaagactcaggggttacctccatgtgccctttttagggaagctgttgg
agaaaatactccagcaaaatgaaggagtacacaaaccagagaatgacatg
aatccagcaaataggatccaacacaggcaatattccagctatggagctag
ctttaaaaaggaacagtaaaaatattaatcggttagctgggtggaatggc
ccatgcctgtagtcccagctactcaggaggctcagcagcaggacgacttg
agcccaagagttccagaccagcctggccaccttagtgagatcccttctct
taaaaataataacttattgccagatttggggcatttggaaagaagttcat
tgaagataaagcaaaagtaaaaaaaaaaaaaaaaaaaacaaggggaaagg
gttggttaggcaatcattctagggcagaaagaagtacaggataggaagag
cataatacactgtttttctcaacaaggagcagtatgtacacagtcataat
gatgtgactgcttagcccctaaatatggtaactactctgggacaatatgg
gaggaaaagtgaagattgtgatggtgtaagagctaaatcctcatctgtca
tatccagaaatcactatataatatataataatgaaatgactaagttatgt
gaggaaaaaaacagaagacattgctaaaagagttaaaagtcattgctctg
gagaattaggagggatggggcaggggactgttaggatgcattataaactg
aaaagcctttttaaaattttatgtattaatatatgcattcacttgaaaaa
ctaaaaaaaaacaataatttggaaaaacccatgaaggtaactaacggaag
gaaaaactaagagaatgaaaagtatttgcctctggaaagaacaactggca
ggactgttgttttcattgtaagacttttggagccatttaattgtacttaa
ccattttcatctatttctttaataagaacaattccatcttaataaagagt
tacacttgttaataagtaaaaaaaaaaaaaaa
>NM_004302 4
ggggaggcgccgggggcgcgcgcgcgcgctgggcgctgctgggctgcg
gcggcggcggcggcggtggttactatggcggagtcggccggagcctc
ctccttcttccccccttgttgtcctcctgctcgccggcagcggcgggtccg
ggccccgggggtccaggctctgctgtgtgcgtgcaccagctgcctccag

gccaactacacgtgtgagacagatggggcctgcatggtttccattttcaa
tctggatgggatggagcaccatgtgcgcacctgcatccccaaagtggagc
tggtccctgccgggaagcccttctactgcctgagctcggaggacctgcgc
aacacccactgctgctacactgactactgcaacaggatcgacttgagggt
gcccagtggtcacctcaaggagcctgagcacccgtccatgtggggcccgg
tggagctggtaggcatcatcgccggcccggtgttcctcctgttcctcatc
atcatcattgttttccttgtcattaactatcatcagcgtgtctatcacaa
ccgccagagactggacatggaagatccctcatgtgagatgtgtctctcca
aagacaagacgctccaggatcttgtctacgatctctccacctcagggtct
ggctcagggttacccctctttgtccagcgcacagtggcccgaaccatcgt
tttacaagagattattggcaagggtcggtttggggaagtatggcggggcc
gctggaggggtggtgatgtggctgtgaaaatattctcttctcgtgaagaa
cggtcttggttcagggaagcagagatataccagacggtcatgctgcgcca
tgaaaacatccttggatttattgctgctgacaataaagataatggcacct
ggacacagctgtggcttgtttctgactatcatgagcacgggtccctgttt
gattatctgaaccggtacacagtgacaattgaggggatgattaagctggc
cttgtctgctgctagtgggctggcacacctgcacatggagatcgtgggca
cccaagggaagcctggaattgctcatcgagacttaaagtcaaagaacatt
ctggtgaagaaaaatggcatgtgtgccatagcagacctgggcctggctgt
ccgtcatgatgcagtcactgacaccattgacattgccccgaatcagaggg
tggggaccaaacgatacatggcccctgaagtacttgatgaaaccattaat
atgaaacactttgactcctttaaatgtgctgatatttatgccctcgggct
tgtatattgggagattgctcgaagatgcaattctggaggagtccatgaag
aatatcagctgccatattacgacttagtgccctctgacccttccattgag
gaaatgcgaaaggttgtatgtgatcagaagctgcgtcccaacatccccaa
ctggtggcagagttatgaggcactgcgggtgatggggaagatgatgcgag
agtgttggtatgccaacggcgcagcccgcctgacggccctgcgcatcaag
aagaccctctcccagctcagcgtgcaggaagacgtgaagatctaactgct
ccctctctccacacggagctcctggcagcgagaactacgcacagctgccg
cgttgagcgtacgatggaggcctacctctcgtttctgcccagccctctgt
ggccaggagccctggcccgcaagagggacagagcccgggagagactcgct
cactcccatgttgggtttgagacagacaccttttctatttacctcctaat
ggcatggagactctgagagcgaattgtgtggagaactcagtgccacacct
cgaactggttgtagtgggaagtcccgcgaaacccggtgcatctggcacgt
ggccaggagccatgacaggggcgcttgggaggggccggaggaaccgaggt
gttgccagtgctaagctgccctgagggtttccttcggggaccagcccaca
gcacaccaaggtggcccggaagaaccagaagtgcagcccctctcacaggc
agctctgagccgcgctttcccctcctccctgggatggacgctgccgggag
actgccagtggagacggaatctgccgctttgtctgtccagccgtgtgtgc
atgtgccgaggtgcgtcccccgttgtgcctggttcgtgccatgcccttac
acgtgcgtgtgagtgtgtgtgtgtgtctgtaggtgcgcacttacctgctt
gagctttctgtgcatgtgcaggtcgggggtgtggtcgtcatgctgtccgt
gcttgctggtgcctcttttcagtagtgagcagcatctagtttccctggtg
cccttccctggaggtctctccctcccccagagcccctcatgccacagtgg
tactctgtgtctggcaggctactctgcccaccccagcatcagcacagctc
tcctcctccatctcagactgtggaaccaaagctggcccagttgtccatga
caaaagaggcttttgggccaaaatgtgagggtggtgggtgggatggcag
ggaaggaatcctggtggaagtcttgggtgttagtgtcagccatgggaaat
gagccagcccaagggcatcatcctcagcagcatcgaggaagggccgagga

atgtgaagccagatctcgggactcagattggaatgttacatctgtctttc
atctcccagatcctggaaacagcagtgtatatttttggtggtggtgggtt
tggggtggggaagggaagggcgggcaaggagtggggagggagtctggggt
gggagggaggcatctgcatgggtcttcttttactggactgtctgatcagg
gtggagggaaggtgagaggtttgcatccacttcaggagccctactgaagg
gaacagcctgagccgaacatgttatttaacctgagtatagtatttaacga
agcctagaagcacggctgtgggtggtgatttggtcagcatatcttaggta
tataataactttgaagccataacttttaactggagtggtttgatttcttt
ttttaattttattgggagggtttggattttaacttttttttaatgttgtta
aatattaagtttttgtaaaaggaaaaccatctctgtgattacctctcaat
ctatttgtttttaaagaaatccctaaaaaaaaaaaattatccaattgaacg
cacatagctcaatcacactggaaatgtttgtccttgcacctgagcctgtt
cccactcagcagtgagagttcctctttgccctgaggctcagtctctctcg
tattttgtccccacccccaattccttgagtggtttttgctctagggccct
ttcttgcactgtccagctggttgtaccctctccaggcatttattcaacaa
atgtgggtgaagtgcctgctgggtgccaggtgctgggaatacatctgtgg
acaagacatgcttgggtcctactcctggagcactgtaaaaagagctgatt
caagtaagtagatgcctgttttgagaccagaaggtttcataattggttct
acgacccttttgagcctagaattattgttcttatataagatcactgaaga
aagaggaacccccacaaccccctccacaaagagaccaggggcgggtgatg
agacctggggtttagaaccccaggtgagacctcaaatcactgcattcatt
ctgagccccccttcctgtccccaggggaggtgtattgtgtatgtagcctta
gagcatctctgcctccaacccagcagttctctgccaaagcttgtggagga
gggagagccctgtccctgccctcaggctccccagtgctcctggcccttct
atttatttgactgattattgcttctttccttgcattaaaggagatcttcc
cctaacctttgggccaatttactggccactaatttcgtttaaataccatt
gtgtcattggggggaccgtctttacccctgctgacctcccacctatccgc
cctgcagcagaaccttggcggtttataggtaatgatggaacttagactcc
tcttcccagagtcacaagtagcctctgggatctgccaacacacgtccact
cccaagccactagcccactccccagttggcccttctgcccttaccccaca
cacagtccaactcttccacctctggggaagatggagcaggtctttgggaa
gctcccacacccacctctgccactcttaacactaagtgagagttggggag
aaactgaagccgtgtttttggcccccgaggctaaccctgatccatagtg
ctacctgcacctctggattctggattcacagaccaagtccaagcccgttc
ttacgtcgccataaaggcccccgaacggcattctcggtacttctgtttgt
ttttgtacattttattagaaaggactgtaaaatagccacttagacacttt
acctcttcagtatgcaaatgtaaataaattgtaatataggaaatcttttg
ttttaatataagaatgagcctgtccaatttctgctgtacattattaaaag
ttttattcacagag
>NM_005900 2
ggcagctgaggagtggaggctgggcagctccgactccctgacgccagcgc
gaccagatcaatccaggctccaggagaaagcaggcgggcgggcggagaaa
ggagaggccgagcggctcaacccgggccgaggctcggggagcggagagtg
gcgcagcgcccggccgtccggacccgggccgcgagaccccgctcgcccgg
ccactcgtgctcccacacggacgggcgcgccgccaacccggtgctgactg
ggttactttttaaacactaggaatggtaatttctactcttctggacttc
aaactaagaagttaaagagacttctctgtaaataaacaaatctcttctgc
tgtccttttgcatttggagacagctttatttcaccatatccaaggagtat
aactagtgctgtcattatgaatgtgacaagtttattttcctttacaagtc

cagctgtgaagagacttcttgggtggaaacagggcgatgaagaagaaaaa
tgggcagagaaagctgttgatgctttggtgaaaaaactgaagaaaaagaa
aggtgccatggaggaactggaaaaggccttgagctgcccagggcaaccga
gtaactgtgtcaccattccccgctctctggatggcaggctgcaagtctcc
caccggaagggactgcctcatgtcatttactgccgtgtgtggcgctggcc
cgatcttcagagccaccatgaactaaaaccactggaatgctgtgagtttc
cttttggttccaagcagaaggaggtctgcatcaatccctaccactataag
agagtagaaagccctgtacttcctcctgtgctggttccaagacacagcga
atataatcctcagcacagcctcttagctcagttccgtaacttaggacaaa
atgagcctcacatgccactcaacgccacttttccagattctttccagcaa
cccaacagccacccgtttcctcactctcccaatagcagttacccaaactc
tcctgggagcagcagcagcacctaccctcactctcccaccagctcagacc
caggaagccctttccagatgccagctgatacgcccccacctgcttacctg
cctcctgaagaccccatgacccaggatggctctcagccgatggacacaaa
catgatggcgcctccccctgccctcagaaatcaacagaggagatgttcagg
cggttgcttatgaggaaccaaaacactggtgctctattgtctactatgag
ctcaacaatcgtgtgggtgaagcgttccatgcctcctccacaagtgtgtt
ggtggatggtttcactgatccttccaacaataagaaccgtttctgccttg
ggctgctctccaatgttaaccggaattccactattgaaaacaccaggcgg
catattggaaaaggagttcatctttattatgttggaggggaggtgtatgc
cgaatgccttagtgacagtagcatctttgtgcaaagtcggaactgcaact
accatcatggatttcatcctactactgtttgcaagatccctagtgggtgt
agtctgaaaatttttaacaaccaagaatttgctcagttattggcacagtc
tgtgaaccatggatttgagacagtctatgagcttacaaaaatgtgtacta
tacgtatgagctttgtgaagggctggggagcagaataccaccgccaggat
gttactagcacccccctgctggattgagatacatctgcacggcccccctcca
gtggctggataaagttcttactcaaatgggttcacctcataatcctattt
catctgtatcttaaatggccccaggcatctgcctctggaaaactattgag
ccttgcatgtacttgaaggatggatgagtcagacacgattgagaactgac
aaaggagccttgataatacttgacctctgtgaccaactgttggattcaga
aatttaaacaaaaaaaaaaaaaaacacacacaccttggtaacatactgtt
gatatcaagaacctgtttagtttacattgtaacattctattgtaaaatca
actaaaattcagacttttagcaggactttgtgtacagttaaaggagagat
ggccaagccagggacaaattgtctattagaaaacggtcctaagagattct
ttggtgtttggcactttaaggtcatcgttgggcagaagtttagcattaat
agttgttctgaaacgtgttttatcaggtttagagcccatgttgagtcttc
ttttcatgggttttcataatattttaaaactatttgtttagcgatggttt
tgttcgtttaagtaaaggttaatcttgatgatatacataataatctttct
aaaattgtatgctgaccatacttgctgtcagaataatgctaggcatatgc
tttttgctaaatatgtatgtacagagtatttggaagttaagaattgatta
gactagtgaatttaggagtatttgaggtgggtgggggaagagggaaatg
acaactgcaaatgtagactatactgtaaaaattcagtttgttgctttaaa
gaaacaaactgatacctgaattttgctgtgtttccatttttttagagattt
ttatcattttttctctctcggcattctttttctcatactcttcaaaaa
gcagttctgcagctggttaattcatgtaactgtgagagcaaatgaataat
tcctgctattctgaaattgcctacatgtttcaataccagttatatggagt
gcttgaatttaataagcagtttttacggagtttacagtacagaaataggc
tttaattttcaagtgaattttttgccaaacttagtaactctgttaaatat
ttggaggatttaaagaacatcccagtttgaattcatttcaaactttttaa

attttttgtactatgtttggttttattttccttctgttaatcttttgta
ttcacttatgctctcgtacattgagtacttttattccaaaactagtgggt
tttctctactggaaattttcaataaacctgtcattattgcttactttgat
taaaaa
>NM_002655 2
agctgcaagttgggctgcaggggcagcgcatacactacaatggctgctgg
aaagaggcgtaaggaaacaatttccaggcccgccgcgtccagcccgaaat
atgagaaaaaaattattagaaattccgcgggcggtgtagaggcggcggac
gggccggagggaggatgttaaagccccgcggttgcctcttggtgctgcct
tggccgtatttggcacccagaatgcttcattctgtgacggtctattaata
aggttgccttgctagagtttggagcagggcctcagattggccaaaatggg
aaggattggattccactctcttccacgaagagtcaatgggactggctaag
atcaaagtctgaggctttttccatcagtaatcagtcccttttttgctttct
tttacgaccacatgaaacttgagaagccacctaaagctatatcatttagt
ggagttgggcagttcccaagtgtccaacaagaaggcctggtttaggctgc
gatggccactgtcattcctggtgatttgtcagaagtaagagatacccaga
aagtcccttcagggaaacgtaagcgtggtgaaaccaaaccaagaaaaaac
tttccttgccaactgtgtgacaaggcctttaacagtgttgagaaattaaa
ggttcactcctactctcacacaggagagaggccctacaagtgcatacaac
aagactgcaccaaggcctttgtttctaagtacaaattacaaaggcacatg
gctactcattctcctgagaaaacccacaagtgtaattattgtgagaaaat
gtttcaccggaaagatcatctgaagaatcacctccatacacacgaccta
acaaagagacgtttaagtgcgaagaatgtggcaagaactacaataccaag
cttggatttaaacgtcacttggccttgcatgccgcaacaagtggtgacct
cacctgtaaggtatgtttgcaaacttttgaaagcacgggagtgcttctgg
agcaccttaaatctcatgcaggcaagtcgtctggtggggttaaagaaaaa
aagcaccagtgcgaacattgtgatcgccggttctacacccgaaaggatgt
ccggagacacatggtggtgcacactggaagaaaggacttcctctgtcagt
attgtgcacagagatttgggcgaaaggatcacctgactcgacatatgaag
aagagtcacaatcaagagcttctgaaggtcaaaacagaaccagtggattt
ccttgacccatttacctgcaatgtgtctgtgcctataaaagacgagctcc
ttccggtgatgtccttaccttccagtgaactgttatcaaagccattcaca
aacactttgcagttaaacctctacaacactccatttcagtccatgcagag
ctcgggatctgcccaccaaatgatcacaactttacctttgggaatgacat
gcccaatagatatggacactgttcatccctctcaccacctttctttcaaa
tatccgttcagttctacctcatatgcaatttctattcctgaaaaagaaca
gccattaaaggggggaaattgagagttacctgatggagttacaaggtggcg
tgccctcttcatcccaagattctcaagcatcgtcatcatctaagctaggg
ttggatcctcagattgggtccctagatgatggtgcaggagacctctccct
atccaaaagctctatctccatcagtgacccccctaaacacaccagcattgg
atttttctcagttgtttaatttcatacctttaaatggtcctccctataat
cctctatcagtggggagccttggaatgagctattcccaggaagaagcaca
ttcttctgtttcccagctccccccacaaacacaggatcttcaggatcctg
caaacactatagggcttgggtctctgcactcactgtcagcagctttcacc
agcagtttaagcacaagtaccaccctcccacgtttccatcaagcttttca
gtaggattctgggacatggattcattacagaaatgtatgtgtagctgtgc
cctagatgaccatttttattttagtgcctactttaaaacagtataaaaat
ttctgcttttgtataatacaaattttcattaagccagtataaaatagaaa
ctagcttttaaactgagctttggaaccatttgtgttcagttaagtttacc

tgggtattttgtcctgattcactgccaattgtcacattttaagacttttt
tttttccatataggaaagccattattagtagtaaacttttacaaatccca
ttttcaaattacttttagatcttaaaattttcatttttgtctaataacag
tggctctaccttttgacatctggctcattaaaaaatttagcaatagaatg
taaattgtataaaaagtttgtgaataactcaagggtttaaattttcttac
tagcttctaaatggattaataatcaagtgcttcaaatgaattaagagtcc
agtttcggaagataataaatgtttgttagatacaccataatttcagatca
gtatattctgaagactccctgttgtctggctaaaatatttgccatcttta
ttatgagcctttaaggaaaacaaaccctaaacacaaagcatcagtattta
tagcaaaaagagactctgttaggtgacatggcatttcgtgtcacttaata
gttggccctaaattagtacacaggatattttgtcgtgtttcatccttctt
aacatgctatcttttcatttaataatagtaatagtgtatggcattggggt
cttcagagtcgatatataggtagatctctttagtcttttccacctttcac
atccaaggggtgggtcaagtgcagccagcaatttattttcattgttggcc
cacggttagtccataatctagagccattgtggaactgcagccatgaggtg
tgtttatcccacagtggattgactcagcctctgtgggtgacagacttcta
agcaggaagatagacgtgaagcacatggttacatttgggaacttgtgtag
ggatcatggcccctgtagccagggttaaaaactggacttttagaagtaa
agtaaaagcatatcgcttatatcatttcttgctgaatttgatatgttttt
ctttcccttaagaatcaaaagcagaaaacaaaaacaacagtcctactccg
atgttatctttctgattcaatgtgaatccatctttccttgcaatattttg
gatggagaatttgaagttaaatgcattagaaaactacctgatgaactacc
acaaagttttaagtgactagaaatatatacagtaaaatcccactttcatg
catctctgggaaatgataggagtattgcaaataagttgagtttgtagagg
gtaacaaagtaaagtaaaacaaacctatcttggttaacatgaaaataaca
attgagaatatattatattcactgaataattataggcttttcctcacatt
agacaaccaacataatcttcttaaaggtctaattaatatatttttctaag
ggtcagttgggacattaacctaagaaacatatctattaagcacttgttaa
caccttattttaggaccctttccgttggggatgggggcaagggtgggagg
tttttagaagagtatatatctctttaaaaaaaaacagaaagaaaaatatt
tctgagcactcattagccctatatggaaacttctttcctttttgtagggc
cagttatcactgcagattgcaatgtttaccaagaatttctaaaaatgagt
gcagattactgaatataatacattatttaaaatatttgggagtagtataa
tttgtggagaaatgtaaattgtaataatgtaaatgggggcttcaatatat
atatataatacacacacacacacacatgcacacataccgcacttcataga
atcaaagttgctctctgaaggagctttggctcctgatattttatcatgct
cctatattttttaatccttggagcagtagtttttatacttatgtattta
aattttattatgaaaaattacatttattaaaaaagtgtgttccaaaggca
ttaaaattatatatgttaataaggaagtacatttttaaatttttcaaact
gctcctagcttttgattaggagaatattttttctgaaagtaggcttttcg
ctctgcttcattactgcttcctttagtttctatgaaacagattgcttacc
taaatctttagttgaatgattagtgttcaatattgctttaatcaccatat
aaaaggaaaaaaattggtgacagagcacaaatagaaaacctatttttaaa
tagaaatcacaaatagcaagtgtggaagcactactttattctgtttaaaa
tgtacttaagaagtcatcaaattagtgaactgagacattggccttagtag
gctgtattcactgctaatttaaaaaagggagtaccaggatttattaagta
aagcattttggaaatggggaatagcgccatatatgtatgtatgtgtatgt
gtgtgtgtgtgtgtgtatatatacacacacacacacatacttaaatcttg
ccctgcatgaaattcaaatacatggaggcacatcttcagggcaccagtgt

taaaattttggagtcttaattttcatgtgtacacctctttgcctgttccc
acccccagacttgaaataacacttcagagtaagagggaattcagctaatt
tgtttttaaaattgactgtagtggtcactaaacccttttttgagagaattt
ctattaaagatgaggcagactcgcttatttgaattgcacaatgttctaac
aaggatgtaacacagaattggcttttttttccctagaaaaagattgtttg
tttctatgtcaactagatatgattaaaaataagtattgccaatgctgttt
tcattctctagtggccagaatcattatccttgaaatttctggtagtgcct
tagcttggttaaaaaaaaaaaaaaaaaaaaaaaaaagggattaacattaa
ataaaagtagtttagaatttgggcctcagacaagatattgaacctcattc
agtttcacttccacatgtatgtacaagttaggtcaccaaacacggaagtg
tgagtgtggaaggatcttggcactgtaagcaatgctatccattgatgtat
acaagtacctttatagttatcgatcactgttaaaactttcattttaaaat
cctattaccaagttcagtttttttaaaacttcaattgtcctggctgattat
gcatcactctgtgtgcaactttttttatttcatttagtgtttctttcaagc
tgtgtatttttgcctatttgttgcttgtgctttatttttcttagtcattt
gtggaatatagtgatatattgtgttaatttggacagtagcggtttttaaa
aaccatatactgactgaaacatgagccagagccgattgctttattaagct
aataatgaatgttaaagagtacatattttcaggatcgttcatctagtgag
caatacacatattataggccaatatttttttaaaaaatagagcttggtca
acctctatactacacatattacaagatatagcactttcaaaatgaatcta
aacctttacagaaactttcttataggttatgccttttattttaagactta
ttataattcaagtgccattagatgatatatatgtaggcctttgatatata
atgctttgtgtacaaaaatggtagatggtattttaaacaggtacattttt
acagtgttttcttatcaatttgctatattgcacagaatcagtgtgtgtct
tttcataaggttttacaatggtttattttttttacaaggtttacgtgtctc
aaagcacactgtcttcccagtacgtaagttaaaaaataccagttcaccca
agttgcttctagcctactgagatccatgtgacattggaggagatctttta
aatgtttagtattcgtcattagcaatggctggctgttagttctggtaaat
gtgtgcctaagttgaatttgtcttgtttttctcacactgtgtcagcagcc
atgtctacaacacagataagtctgttgtgatcacatagatctacataagt
tgtgcagttttgtgctaaaaacccatagggagctcctttgggatcataga
aaagaagatcatgcaaccagcattggtgaaggcacactcagattgcactt
agggcctttctatgatgttgtcaaccctctgaggatggaaggcagtgtct
tttgatgttatctagcctagaaatgacacagaactattgctaatgtataa
aacacttcattatataagcttcagtggtacagatgaaccagaatgaatgt
ttatcttctcagaaacactccttcaatattatattggatcatgctgctaa
tgtaacttgggctacaactcttcatggtgctacaaacttctctgtctcat
tcagtcgtattttttttatccatagaaaaaggactacattaggtgtaaaag
tgtacaatatatttttatactgtgacttaatttgtcattaacaaactttt
acaccaccacaatgtattcatgtgcacttgcaaaaggagatctcggacat
gcaaatgttaccagaacaaacccagcttttgtccacaaggtgactgtaac
tcagaatggaaagtgggctttataatagggtgtggagtgaagaacatgct
gtatgttactaacagccctttgaatttaacaaaaactgggaatccattag
gaaacggattgcatcatacctgaacataagctggactgctgaaattgtat
ttttagctaatgaaaaagtgtttggactagtactctaaaaatgttctaat
gataaagttttgagtcaaaatagaaaagaaaaaaatctgcattccaggcc
gaattttgtatatttttattgcatttaaaattgctattctgtaatattgg
gaaatcaagtggcttatcatgtatatcgtgtacttaaaatgtattcacaa
actactgttgtatttgtataaaatatagacaaagatcatattttttgtgt

gtgtataagctctgtaaaatagcaatcacattatgaagctgcagtgatac
tacattttaaacattcacatccaaagaagcagactatttattgtccatat
accagatttaaaatattaatttgctgctaattaaataatagtactgcagc
ttcttgtggcctacagtgttatgtttgctgtaagaataagatatgtgaat
tccacaaaatatatgaataaaattatagaatggcttta
>NM_003242 5
ggagagggagaaggctctcgggcggagagaggtcctgcccagctgttggc
gaggagtttcctgtttcccccgcagcgctgagttgaagttgagtgagtca
ctcgcgcgcacggagcgacgacacccccgcgcgtgcacccgctcgggaca
ggagccggactcctgtgcagcttccctcggccgccgggggcctccccgcg
cctcgccggcctccaggccccctcctggctggcgagcgggcgccacatct
ggcccgcacatctgcgctgccggcccggcgcggggtccggagagggcgcg
gcgcggaggcgcagccaggggtccgggaaggcgccgtccgctgcgctggg
ggctcggtctatgacgagcagcggggtctgccatgggtcgggggctgctc
aggggcctgtggccgctgcacatcgtcctgtggacgcgtatcgccagcac
gatcccaccgcacgttcagaagtcggttaataacgacatgatagtcactg
acaacaacggtgcagtcaagtttccacaactgtgtaaattttgtgatgtg
agattttccacctgtgacaaccagaaatcctgcatgagcaactgcagcat
cacctccatctgtgagaagccacaggaagtctgtgtggctgtatggagaa
agaatgacgagaacataacactagagacagtttgccatgaccccaagctc
ccctaccatgactttattctggaagatgctgcttctccaaagtgcattat
gaaggaaaaaaaaaagcctggtgagactttcttcatgtgttcctgtagct
ctgatgagtgcaatgacaacatcatcttctcagaagaatataacaccagc
aatcctgacttgttgctagtcatatttcaagtgacaggcatcagcctcct
gccaccactgggagttgccatatctgtcatcatcatcttctactgctacc
gcgttaaccggcagcagaagctgagttcaacctgggaaaccggcaagacg
cggaagctcatggagttcagcgagcactgtgccatcatcctggaagatga
ccgctctgacatcagctccacgtgtgccaacaacatcaaccacaacacag
agctgctgcccattgagctggacaccctggtggggaaaggtcgctttgct
gaggtctataaggccaagctgaagcagaacacttcagagcagtttgagac
agtggcagtcaagatctttccctatgaggagtatgcctcttggaagacag
agaaggacatcttctcagacatcaatctgaagcatgagaacatactccag
ttcctgacggctgaggagcggaagacggagttggggaaacaatactggct
gatcaccgccttccacgccaagggcaacctacaggagtacctgacgcggc
atgtcatcagctgggaggacctgcgcaagctgggcagctccctcgcccgg
gggattgctcacctccacagtgatcacactccatgtgggaggcccaagat
gcccatcgtgcacagggacctcaagagctccaatatcctcgtgaagaacg
acctaacctgctgcctgtgtgactttgggctttccctgcgtctggaccct
actctgtctgtggatgacctggctaacagtgggcaggtgggaactgcaag
atacatggctccagaagtcctagaatccaggatgaatttggagaatgttg
agtccttcaagcagaccgatgtctactccatggctctggtgctctgggaa
atgacatctcgctgtaatgcagtgggagaagtaaaagattatgagcctcc
atttggttccaaggtgcgggagcacccctgtgtcgaaagcatgaaggaca
acgtgttgagagatcgagggcgaccagaaattcccagcttctggctcaac
caccagggcatccagatggtgtgtgagacgttgactgagtgctgggacca
cgacccagaggcccgtctcacagcccagtgtgtggcagaacgcttcagtg
agctggagcatctggacaggctctcggggaggagctgctcggaggagaag
attcctgaagacggctccctaaacactaccaaatagctcttctggggcag
gctgggccatgtccaaagaggctgcccctctcaccaaagaacagaggcag

caggaagctgcccctgaactgatgcttcctggaaaaccaagggggtcact
cccctccctgtaagctgtggggataagcagaaacaacagcagcagggagt
gggtgacatagagcattctatgcctttgacattgtcataggataagctgt
gttagcacttcctcaggaaatgagattgatttttacaatagccaataaca
tttgcactttattaatgcctgtatataaatatgaatagctatgttttata
tatatatatatatatctatatatgtctatagctctatatatatagccata
ccttgaaaagagacaaggaaaaacatcaaatattcccaggaaattggttt
tattggagaactccagaaccaagcagagaaggaagggacccatgacagca
ttagcatttgacaatcacacatgcagtggttctctgactgtaaaacagtg
aactttgcatgaggaaagaggctccatgtctcacagccagctatgaccac
attgcacttgcttttgcaaaataatcattccctgcctagcacttctcttc
tggccatggaactaagtacagtggcactgtttgaggaccagtgttcccgg
ggttcctgtgtgcccttatttctcctggacttttcatttaagctccaagc
cccaaatctggggggctagtttagaaactctccctcaacctagtttagaa
actctaccccatctttaataccttgaatgttttgaaccccactttttacc
ttcatgggttgcagaaaaatcagaacagatgtccccatccatgcgattgc
cccaccatctactaatgaaaaattgttcttttttcatctttcccctgca
cttatgttactattctctgctcccagccttcatccttttctaaaaaggag
caaattctcactctaggctttatcgtgtttactttttcattacacttgac
ttgattttctagttttctatacaaacaccaatgggttccatctttctggg
ctcctgattgctcaagcacagtttggcctgatgaagaggatttcaactac
acaatactatcattgtcaggactatgacctcaggcactctaaacatatgt
tttgtttggtcagcacagcgtttcaaaaagtgaagccactttataaatat
ttggagattttgcaggaaaatctggatccccaggtaaggatagcagatgg
ttttcagttatctccagtccacgttcacaaaatgtgaaggtgtggagaca
cttacaaagctgcctcacttctcactgtaaacattagctctttccactgc
ctacctggaccccagtctaggaattaaatctgcacctaaccaaggtccct
tgtaagaaatgtccattcaagcagtcattctctgggtatataatatgatt
ttgactaccttatctggtgttaagatttgaagttggccttttattggact
aaaggggaactcctttaagggtctcagttagcccaagtttcttttgctta
tatgttaatagttttaccctctgcattggagagaggagtgctttactcca
agaagctttcctcatggttaccgttctctccatcatgccagccttctcaa
cctttgcagaaattactagagaggatttgaatgtgggacacaaaggtccc
atttgcagttagaaaatttgtgtccacaaggacaagaacaaagtatgagc
tttaaaactccataggaaacttgttaatcaacaaagaagtgttaatgctg
caagtaatctctttttttaaaactttttgaagctacttattttcagccaaa
taggaatattagagagggactggtagtgagaatatcagctctgtttggat
ggtggaaggtctcattttattgagatttttaagatacatgcaaaggtttg
gaaatagaacctctaggcaccctcctcagtgtgggtgggctgagagttaa
agacagtgtggctgcagtagcatagaggcgcctagaaattccacttgcac
cgtagggcatgctgataccatcccaatagctgttgcccattgacctctag
tggtgagtttctagaatactggtccattcatgagatattcaagattcaag
agtattctcacttctgggttatcagcataaactggaatgtagtgtcagag
gatactgtggcttgttttgtttatgtttttttttcttattcaagaaaaaa
gaccaaggaataacattctgtagttcctaaaaatactgactttttcact
actatacataaagggaaagttttattcttttatggaacacttcagctgta
ctcatgtattaaaataggaatgtgaatgctatatactctttttatatcaa
aagtctcaagcacttatttttattctatgcattgtttgtcttttacataa
ataaaatgtttattagattgaataaagcaaaatactcaggtgagcatcct

gcctcctgttcccattcctagtagctaaa
>NM_000104 3
aaaacccggaggagcgggatggcgcgctttgactctggagtgggagtggg
agcgagcgcttctgcgactccagttgtgagagccgcaagggcatgggaat
tgacgccactcaccgacccccagtctcaatctcaacgctgtgaggaaacc
tcgactttgccaggtccccaagggcagcggggctcggcgagcgaggcacc
cttctccgtccccatcccaatccaagcgctcctggcactgacgacgccaa
gagactcgagtgggagttaaagcttccagtgagggcagcaggtgtccagg
ccgggcctgcgggttcctgttgacgtcttgccctaggcaaaggtcccagt
tccttctcggagccggctgtcccgcgccactggaaaccgcacctccccgc
agcatgggcaccagcctcagcccgaacgacccttggccgctaaacccgct
gtccatccagcagaccacgctcctgctactcctgtcggtgctggccactg
tgcatgtgggccagcggctgctgaggcaacggaggcggcagctccggtcc
gcgcccccgggcccgtttgcgtggccactgatcggaaacgcggcggcggt
gggccaggcggctcacctctcgttcgctcgcctggcgcggcgctacggcg
acgttttccagatccgcctgggcagctgccccatagtggtgctgaatggc
gagcgcgccatccaccaggccctggtgcagcagggctcggccttcgccga
ccggccggccttcgcctccttccgtgtggtgtccggcggccgcagcatgg
ctttcggccactactcggagcactggaaggtgcagcggcgcgcagcccac
agcatgatgcgcaacttcttcacgcgccagccgcgcagccgccaagtcct
cgagggccacgtgctgagcgaggcgcgcgagctggtggcgctgctggtgc
gcggcagcgcggacggcgccttcctcgacccgaggccgctgaccgtcgtg
gccgtggccaacgtcatgagtgccgtgtgtttcggctgccgctacagcca
cgacgaccccgagttccgtgagctgctcagccacaacgaagagttcgggc
gcacggtgggcgcgggcagcctggtggacgtgatgccctggctgcagtac
ttccccaacccggtgcgcaccgttttccgcgaattcgagcagctcaaccg
caacttcagcaacttcatcctggacaagttcttgaggcactgcgaaagcc
ttcggcccggggccgcccccgcgacatgatggacgcctttatcctctct
gcggaaaagaaggcggccggggactcgcacggtggtggcgcgcggctgga
tttggagaacgtaccggccactatcactgacatcttcggcgccagccagg
acaccctgtccaccgcgctgcagtggctgctcctcctcttcaccaggtat
cctgatgtgcagactcgagtgcaggcagaattggatcaggtcgtggggag
ggaccgtctgccttgtatgggtgaccagcccaacctgccctatgtcctgg
ccttcctttatgaagccatgcgcttctccagctttgtgcctgtcactatt
cctcatgccaccactgccaacacctctgtcttgggctaccacattcccaa
ggacactgtggtttttgtcaaccagtggtctgtgaatcatgacccactga
agtggcctaacccggagaactttgatccagctcgattcttggacaaggat
ggcctcatcaacaaggacctgaccagcagagtgatgatttttcagtggg
caaaaggcggtgcattggcgaagaactttctaagatgcagctttttctct
tcatctccatcctggctcaccagtgcgatttcagggccaacccaaatgag
cctgcgaaaatgaatttcagttatggtctaaccattaaacccaagtcatt
taaagtcaatgtcactctcagagagtccatggagctccttgatagtgctg
tccaaaatttacaagccaaggaaacttgccaataagaagcaagaggcaag
ctgaaattttagaaatattcacatcttcggagatgaggagtaaaattcag
tttttttccagttcctcttttgtgctgcttctcaattagcgtttaaggtg
agcataaatcaactgtccatcaggtgaggtgtgctccatacccagcggtt
cttcatgagtagtgggctatgcaggagcttctgggagatttttttgagtc
aaagacttaaagggcccaatgaattattatatacatactgcatcttggtt
atttctgaaggtagcattctttggagttaaaatgcacatatagacacata

cacccaaacacttacaccaaactactgaatgaagcagtattttggtaacc
aggccatttttggtgggaatccaagattggtctcccatatgcagaaatag
acaaaaagtatattaaacaaagtttcagagtatattgttgaagagacaga
gacaagtaatttcagtgtaaagtgtgtgattgaaggtgataagggaaaag
ataaagaccagaaattcccttttcaccttttcaggaaaataacttagact
ctagtatttatgggtggatttatccttttgccttctggtatacttcctta
cttttaaggataaatcataaagtcagttgctcaaaaagaaatcaatagtt
gaattagtgagtatagtggggttccatgagttatcatgaattttaaagta
tgcattattaaattgtaaaactccaaggtgatgttgtacctcttttgctt
gccaaagtacagaatttgaattatcagcaaagaaaaaaaaaaaagccagc
caagctttaaattatgtgaccataatgtactgatttcagtaagtctcata
ggttaaaaaaaaaagtcaccaaatagtgtgaaatatattacttaactgtc
cgtaagcagtatattagtattatcttgttcaggaaaaggttgaataatat
atgccttgtataatattgaaaattgaaaagtacaactaacgcaaccaagt
gtgctaaaaatgagcttgattaaatcaaccacctatttttgacatggaaa
tgaagcagggtttcttttcttcactcaaattttggcgaatctcaaaatta
gatcctaagatgtgttcttatttttataacatctttattgaaattctatt
tataatacagaatcttgttttgaaaataacctaattaatatattaaaatt
ccaaattcatggcatgcttaaattttaactaaattttaaagccattctga
ttattgagttccagttgaagttagtggaaatctgaacattctcctgtgga
aggcagagaaatctaagctgtgtctgcccaatgaataatggaaaatgcca
tgaattacctggatgttctttttacgaggtgacaagagttggggacagaa
ctcccattacaactgaccaagtttctcttctagatgattttttgaaagtt
aacattaatgcctgctttttggaaagtcagaatcagaagatagtcttgga
agctgtttggaaaagacagtggagatgaggtcagttgtgttttttaagat
ggcaattactttggtagctgggaaagcataaagctcaaatgaaatgtatg
cattcacatttagaaaagtgaattgaagtttcaagttttaaagttcattg
caattaaacttccaaagaaagttctacagtgtcctaagtgctaagtgctt
attacattttattaagctttttggaatctttgtaccaaaattttaaaaaa
gggagtttttgatagttgtgtgtatgtgtgtgtggggtggggggatggta
agagaaaagagagaaacactgaaaagaaggaaagatggttaaacattttc
ccactcattctgaattaattaatttggagcacaaaattcaaagcatggac
atttagaagaaagatgtttggcgtagcagagttaaatctcaaataggcta
ttaaaaaagtctacaacatagcagatctgttttgtggtttggaatattaa
aaaacttcatgtaattttattttaaaatttcatagctgtacttcttgaat
ataaaaaatcatgccagtatttttaaaggcattagagtcaactacacaaa
gcaggcttgcccagtacatttaaattttttggcacttgccattccaaaat
attatgccccaccaaggctgagacagtgaatttgggctgctgtagcctat
ttttttagattgagaaatgtgtagctgcaaaaataatcatgaaccaatct
ggatgcctcattatgtcaaccaggtccagatgtgctataatctgtttttta
cgtatgtaggcccagtcgtcatcagatgcttgcggcaaaaggaaagctgt
gtttatatggaagaaagtaaggtgcttggagtttacctggcttatttaat
atgcttataacctagttaaagaaaggaaaagaaaacaaaaaacgaatgaa
aataactgaatttggaggctggagtaatcagattactgctttaatcagaa
accctcattgtgtttctaccggagagagaatgtatttgctgacaaccatt
aaagtcagaagttttactccaggttattgcaataaagtataatgtttatt
aaatgcttcatttgtatgtcaaagctttgactctataagcaaattgcttt
tttccaaaacaaaaagatgtctcaggtttgttttgtgaattttctaaaag
ctttcatgtcccagaacttagcctttacctgtgaagtgttactacagcct

taatattttcctagtagatctatattagatcaaatagttgcatagcagta
tatgttaatttgtgtgtttttagctgtgacacaactgtgtgattaaaagg
tatactttagtagacatttataactcaaggataccttcttatttaatctt
ttcttatttttgtactttatcatgaatgcttttagtgtgtgcataatagc
tacagtgcatagttgtagacaaagtacattctggggaaacaacatttata
tgtagcctttactgtttgatataccaaattaaaaaaaaattgtatctcat
tacttatactgggacaccattaccaaaataataaaaatcactttcataat
cttgaaaaaa
>NM_175848 1
acccactcccgctgccccgtccggcccgcgccgcttcctcgcagcagctg
ctcccggctccgcggccgcagcccgcgtggacgctccgagcgccccccga
cggacgggaccggctccctggcggtcgggcgagcgggcggcaacgctgcc
cggccggcagcgctggggttaagtggcccaagtaaacctagctcggcgat
cggcgccggagattcgcgagcccagcgccctgcacggccgccagccggcc
tcccgccagccagccccgacccgcggctccgccgcccagccgcgccccag
ccagccctgcggcaggaaagcatgaagggagacaccaggcatctcaatgg
agaggaggacgccggcgggagggaagactcgatcctcgtcaacggggcct
gcagcgaccagtcctccgactcgcccccaatcctggaggctatccgcacc
ccggagatcagaggccgaagatcaagctcgcgactctccaagagggaggt
gtccagtctgctaagctacacacaggacttgacaggcgatggcgacgggg
aagatggggatggctctgacaccccagtcatgccaaagctcttccgggaa
accaggactcgttcagaaagcccagctgtccgaactcgaaataacaacag
tgtctccagccgggagaggcacaggccttccccacgttccacccgaggcc
ggcagggccgcaaccatgtggacgagtcccccgtggagttcccggctacc
aggtccctgagacggcgggcaacagcatcggcaggaacgccatggccgtc
ccctcccagctcttaccttaccatcgacctcacagacgacacagaggaca
cacatgggacgccccagagcagcagtacccccctacgcccgcctagcccag
gacagccagcaggggggcatggagtccccgcaggtggaggcagacagtgg
agatggagacagttcagagtatcaggatgggaaggagtttggaatagggg
acctcgtgtggggaaagatcaagggcttctcctggtggcccgccatggtg
gtgtcttggaaggccacctccaagcgacaggctatgtctggcatgcggtg
ggtccagtggtttggcgatggcaagttctccgaggtctctgcagacaaac
tggtggcactggggctgttcagccagcactttaatttggccaccttcaat
aagctcgtctcctatcgaaaagccatgtaccatgctctggagaaagctag
ggtgcgagctggcaagaccttccccagcagccctggagactcattggagg
accagctgaagcccatgttggagtgggcccacgggggcttcaagcccact
gggatcgagggcctcaaacccaacaacacgcaaccagagaacaagactcg
aagacgcacagctgacgactcagccacctctgactactgccccgcaccca
agcgcctcaagacaaattgctataacaacggcaaagaccgaggggatgaa
gatcagagccgagaacaaatggcttcagatgttgccaacaacaagagcag
cctggaagatggctgtttgtcttgtggcaggaaaaaccccgtgtccttcc
accctctctttgaggggggggctctgtcagacatgccgggatcgcttcctt
gagctgttttacatgtatgatgacgatggctatcagtcttactgcactgt
gtgctgcgagggccgagagctgctgctttgcagcaacacgagctgctgcc
ggtgtttctgtgtggagtgcctggaggtgctggtgggcacaggcacagcg
gccgaggccaagcttcaggagccctggagctgttacatgtgtctcccgca
gcgctgtcatggcgtcctgcggcgccggaaggactggaacgtgcgcctgc
aggccttcttcaccagtgacacggggcttgaatatgaagcccccaagctg
taccctgccattcccgcagcccgaaggcggcccattcgagtcctgtcatt

gtttgatggcatcgcgacaggctacctagtcctcaaagagttgggcataa
aggtaggaaagtacgtcgcttctgaagtgtgtgaggagtccattgctgtt
ggaaccgtgaagcacgaggggaatatcaaatacgtgaacgacgtgaggaa
catcacaaagaaaaatattgaagaatggggcccatttgacttggtgattg
gcggaagcccatgcaacgatctctcaaatgtgaatccagccaggaaaggc
ctgtatgagggtacaggccggctcttcttcgaattttaccacctgctgaa
ttactcacgccccaaggagggtgatgaccggccgttcttctggatgtttg
agaatgttgtagccatgaaggttggcgacaagagggacatctcacggttc
ctggagtgtaatccagtgatgattgatgccatcaaagtttctgctgctca
cagggcccgatacttctggggcaacctacccgggatgaacaggcccgtga
tagcatcaaagaatgataaactcgagctgcaggactgcttggaatacaat
aggatagccaagttaaagaaagtacagacaataaccaccaagtcgaactc
gatcaaacaggggaaaaaccaacttttccctgttgtcatgaatggcaaag
aagatgttttgtggtgcactgagctcgaaaggatctttggctttcctgtg
cactacacagacgtgtccaacatgggccgtggtgcccgccagaagctgct
gggaaggtcctggagcgtgcctgtcatccgacacctcttcgcccctctga
aggactactttgcatgtgaatagttccagccaggccccaagcccactggg
gtgtgtggcagagccaggacccaggaggtgtgattcctgaaggcatcccc
aggccctgctcttcctcagctgtgtgggtcataccgtgtacctcagttcc
ctcttgctcagtgggggcagagccacctgactcttgcaggggtagcctga
ggtgccgcctccttgtgcacaaatcagacctggctgcttggagcagccta
acacggtgctcatttttcttctcctaaaactttaaaacttgaagtaggt
agcaacgtggcttttttttttcccttcctgggtctaccactcagagaaa
caatggctaagataccaaaaccacagtgccgacagctctccaatactcag
gttaatgctgaaaaatcatccaagacagttattgcaagagtttaattttt
gaaaactggctactgctctgtgtttacagacgtgtgcagttgtaggcatg
tagctacaggacatttttaagggcccaggatcgtttttcccagggcaag
cagaagagaaaatgttgtatatgtcttttacccggcacattccccttgcc
taaatacaagggctggagtctgcacgggacctattagagtattttccaca
atgatgatgatttcagcagggatgacgtcatcatcacattcagggctatt
ttttcccccacaaacccaagggcaggggccactcttagctaaatccctcc
ccgtgactgcaatagaaccctctggggagctcaggaaggggtgtgctgag
ttctataatataagctgccatatattttgtagacaagtatggctcctcca
tatctccctcttccctaggagaggagtgtgaagcaaggagcttagataag
acaccccctcaaacccattccctctccaggagacctaccctccacaggca
caggtccccagatgagaagtctgctaccctcatttctcatctttttacta
aactcagaggcagtgacagcagtcagggacagacatacatttctcatacc
ttccccacatctgagagatgacagggaaaactgcaaagctcggtgctccc
tttggagatttttaatcctttttattccataagaagtcgtttttaggg
agaacgggaattcagacaagctgcatttcagaaatgctgtcataatggtt
tttaacaccttttactcttcttactggtgctattttgtagaataaggaac
aacgttgacaagttttgtggggctttttatacactttttaaaatctcaaa
cttctatttttatgtttaacgttttcattaaaattttttttgtaactgga
gccacgacgtaacaaatatggggaaaaaactgtgccttgtttcaacagtt
tttgctaatttttaggctgaaagatgacggatgcctagagtttaccttat
gtttaattaaaatcagtatttgtctaaaaaaaaaaaaaaaaaa
>NM_022552 4
cggcggcggcgagagcagaggacgagccgggacgcggcgccgcggcacca
gggcgcgcagccgggccggcccgaccccaccggccatacggtggagccat

cgaagcccccacccacaggctgacagaggcaccgttcaccagagggctca
acaccgggatctatgtttaagttttaactctcgcctccaaagaccacgat
aattccttccccaaagcccagcagcccccagccccgcgcagccccagcc
tgcctcccggcgcccagatgcccgccatgccctccagcggccccggggac
accagcagctctgctgcggagcgggaggaggaccgaaaggacggagagga
gcaggaggagccgcgtggcaaggaggagcgccaagagcccagcaccacgg
cacggaaggtggggcggcctgggaggaagcgcaagcacccccggtggaa
agcggtgacacgccaaaggaccctgcggtgatctccaagtccccatccat
ggcccaggactcaggcgcctcagagctattacccaatggggacttggaga
agcggagtgagccccagccagaggaggggagccctgctgggggcagaag
ggcggggccccagcagagggagagggtgcagctgagaccctgcctgaagc
ctcaagagcagtggaaaatggctgctgcacccccaaggagggccgaggag
cccctgcagaagcgggcaaagaacagaaggagaccaacatcgaatccatg
aaaatggagggctcccggggccggctgcggggtggcttgggctgggagtc
cagcctccgtcagcggcccatgccgaggctcaccttccaggcgggggacc
cctactacatcagcaagcgcaagcgggacgagtggctggcacgctggaaa
agggaggctgagaagaaagccaaggtcattgcaggaatgaatgctgtgga
agaaaaccaggggcccggggagtctcagaaggtggaggaggccagccctc
ctgctgtgcagcagcccactgaccccgcatcccccactgtggctaccacg
cctgagcccgtggggtccgatgctggggacaagaatgccaccaaagcagg
cgatgacgagccagagtacgaggacggccggggctttggcattggggagc
tggtgtgggggaaactgcggggcttctcctggtggccaggccgcattgtg
tcttggtggatgacgggccggagccgagcagctgaaggcacccgctgggt
catgtggttcggagacggcaaattctcagtggtgtgtgttgagaagctga
tgccgctgagctcgttttgcagtgcgttccaccaggccacgtacaacaag
cagcccatgtaccgcaaagccatctacgaggtcctgcaggtggccagcag
ccgcgcggggaagctgttcccggtgtgccacgacagcgatgagagtgaca
ctgccaaggccgtggaggtgcagaacaagcccatgattgaatgggccctg
gggggcttccagccttctggccctaagggcctggagccaccagaagaaga
gaagaatccctacaaagaagtgtacacggacatgtgggtggaacctgagg
cagctgcctacgcaccacctccaccagccaaaaagccccggaagagcaca
gcggagaagcccaaggtcaaggagattattgatgagcgcacaagagagcg
gctggtgtacgaggtgcggcagaagtgccggaacattgaggacatctgca
tctcctgtgggagcctcaatgttaccctggaacacccccctcttcgttgga
ggaatgtgccaaaactgcaagaactgctttctggagtgtgcgtaccagta
cgacgacgacggctaccagtcctactgcaccatctgctgtgggggccgtg
aggtgctcatgtgcggaaacaacaactgctgcaggtgcttttgcgtggag
tgtgtggacctcttggtggggccgggggctgcccaggcagccattaagga
agacccctggaactgctacatgtgcgggcacaagggtacctacgggctgc
tgcggcggcgagaggactggccctcccggctccagatgttcttcgctaat
aaccacgaccaggaatttgaccctccaaaggtttacccacctgtcccagc
tgagaagaggaagcccatccgggtgctgtctctctttgatggaatcgcta
cagggctcctggtgctgaaggacttgggcattcaggtggaccgctacatt
gcctcggaggtgtgtgaggactccatcacggtgggcatggtgcggcacca
ggggaagatcatgtacgtcggggacgtccgcagcgtcacacagaagcata
tccaggagtggggcccattcgatctggtgattgggggcagtccctgcaat
gacctctccatcgtcaaccctgctcgcaagggcctctacgagggcactgg
ccggctcttctttgagttctaccgcctcctgcatgatgcgcggcccaagg
agggagatgatcgccccttcttctggctctttgagaatgtggtggccatg

ggcgttagtgacaagagggacatctcgcgatttctcgagtccaaccctgt
gatgattgatgccaaagaagtgtcagctgcacacagggcccgctacttct
ggggtaaccttcccggtatgaacaggccgttggcatccactgtgaatgat
aagctggagctgcaggagtgtctggagcatggcaggatagccaagttcag
caaagtgaggaccattactacgaggtcaaactccataaagcagggcaaag
accagcattttcctgtcttcatgaatgagaaagaggacatcttatggtgc
actgaaatggaaagggtatttggtttcccagtccactatactgacgtctc
caacatgagccgcttggcgaggcagagactgctgggccggtcatggagcg
tgccagtcatccgccacctcttcgctccgctgaaggagtattttgcgtgt
gtgtaagggacatgggggcaaactgaggtagcgacacaaagttaaacaaa
caaacaaaaaacacaaaacataataaaacaccaagaacatgaggatggag
agaagtatcagcacccagaagagaaaaaggaatttaaaacaaaaaccaca
gaggcggaaataccggagggctttgccttgcgaaaagggttggacatcat
ctcctgatttttcaatgttattcttcagtcctatttaaaaacaaaaccaa
gctcccttcccttcctccccttccctttttttcggtcagaccttttat
tttctactcttttcagaggggttttctgtttgtttgggttttgtttcttg
ctgtgactgaaacaagaaggttattgcagcaaaaatcagtaacaaaaaat
agtaacaataccttgcagaggaaaggtgggagagaggaaaaaaggaaatt
ctatagaaatctatatattgggttgtttttttttttgtttttttgtttttt
ttttttgggtttttttttttactatatatcttttttttgttgtctctagc
ctgatcagataggagcacaagcaggggacggaaagagagagacactcagg
cggcagcattccctcccagccactgagctgtcgtgccagcaccattcctg
gtcacgcaaaacagaacccagttagcagcagggagacgagaacaccacac
aagacatttttctacagtatttcaggtgcctaccacacaggaaaccttga
agaaaatcagtttctagaagccgctgttacctcttgtttacagtttatat
atatatgatagatatgagatatatatatataaaaggtactgttaactactgt
acaacccgacttcataatggtgctttcaaacagcgagatgagtaaaaaca
tcagcttccacgttgccttctgcgcaaagggtttcaccaaggatggagaa
agggagacagcttgcagatggcgcgttctcacggtgggctcttccccttg
gtttgtaacgaagtgaaggaggagaacttgggagccaggttctccctgcc
aaaaaggggggctagatgaggtggtcgggcccgtggacagctgagagtggg
attcatccagactcatgcaataaccctttgattgttttctaaaaggagac
tccctcggcaagatggcagagggtacggagtcttcaggcccagtttctca
ctttagccaattcgagggctccttgtggtgggatcagaactaatccagag
tgtgggaaagtgacagtcaaaaccccacctggagcaaataaaaaaacata
caaaacgtactggtgctttcctgt
>NM_003118 3
gggagaaggaggaggccgggggaaggaggagacaggaggaggagggacca
cggggtggaggggagatagacccagcccagagctctgagtggtttcctgt
tgcctgtctctaaacccctccacattcccgcggtccttcagactgcccgg
agagcgcgctctgcctgccgcctgcctgcctgccactgagggttcccagc
accatgagggcctggatcttctttctcctttgcctggccgggagggcctt
ggcagcccctcagcaagaagccctgcctgatgagacagaggtggtggaag
aaactgtggcagaggtgactgaggtatctgtgggagctaatcctgtccag
gtggaagtaggagaatttgatgatggtgcagaggaaaccgaagaggaggt
ggtggcggaaaatccctgccagaaccaccactgcaaacacggcaaggtgt
gcgagctggatgagaacaacacccccatgtgcgtgtgccaggaccccacc
agctgcccagcccccattggcgagtttgagaaggtgtgcagcaatgacaa
caagaccttcgactcttcctgccacttctttgccacaaagtgcaccctgg

agggcaccaagaagggccacaagctccacctggactacatcgggccttgc
aaatacatcccccccttgcctggactctgagctgaccgaattccccctgcg
catgcgggactggctcaagaacgtcctggtcaccctgtatgagagggatg
aggacaacaaccttctgactgagaagcagaagctgcgggtgaagaagatc
catgagaatgagaagcgcctggaggcaggagaccaccccgtggagctgct
ggcccgggacttcgagaagaactataacatgtacatcttccctgtacact
ggcagttcggccagctggaccagcaccccattgacgggtacctctcccac
accgagctggctccactgcgtgctcccctcatccccatggagcattgcac
cacccgctttttcgagacctgtgacctggacaatgacaagtacatcgccc
tggatgagtgggccggctgcttcggcatcaagcagaaggatatcgacaag
gatcttgtgatctaaatccactccttccacagtaccggattctctcttta
accctccccttcgtgtttcccccaatgtttaaaatgtttggatggtttgt
tgttctgcctggagacaaggtgctaacatagatttaagtgaatacattaa
cggtgctaaaaatgaaaattctaacccaagacatgacattcttagctgta
acttaactattaaggccttttccacacgcattaatagtcccatttttctc
ttgccatttgtagctttgcccattgtcttattggcacatgggtggacacg
gatctgctgggctctgccttaaacacacattgcagcttcaacttttctct
ttagtgttctgtttgaaactaatacttaccgagtcagactttgtgttcat
ttcatttcagggtcttggctgcctgtgggcttccccaggtggcctggagg
tgggcaaagggaagtaacagacacacgatgttgtcaaggatggttttggg
actagaggctcagtggtgggagagatccctgcagaacccaccaaccagaa
cgtggtttgcctgaggctgtaactgagagaaagattctggggctgtgtta
tgaaaatatagacattctcacataagcccagttcatcaccatttcctcct
ttacctttcagtgcagtttcttttcacattaggctgttggttcaaacttt
tgggagcacggactgtcagttctctgggaagtggtcagcgcatcctgcag
ggcttctcctcctctgtcttttggagaaccagggctcttctcaggggctc
tagggactgccaggctgtttcagccaggaaggccaaaatcaagagtgaga
tgtagaaagttgtaaaatagaaaaagtggagttggtgaatcggttgttct
ttcctcacatttggatgattgtcataaggtttttagcatgttcctccttt
tcttcaccctcccctttttcttctattaatcaagagaaacttcaaagtt
aatgggatggtcggatctcacaggctgagaactcgttcacctccaagcat
ttcatgaaaaagctgcttcttattaatcatacaaactctcaccatgatgt
gaagagtttcacaaatccttcaaaataaaaagtaatgacttagaaactgc
cttcctgggtgatttgcatgtgtcttagtcttagtcaccttattatcctg
acacaaaaacacatgagcatacatgtctacacatgactacacaaatgcaa
acctttgcaaacacattatgcttttgcacacacacacctgtacacacaca
ccggcatgtttatacacagggagtgtatggttcctgtaagcactaagtta
gctgttttcatttaatgacctgtggtttaacccttttgatcactaccacc
attatcagcaccagactgagcagctatatccttttattaatcatggtcat
tcattcattcattcattcacaaaatatttatgatgtatttactctgcacc
aggtcccatgccaagcactggggacacagttatggcaaagtagacaaagc
atttgttcatttggagcttagagtccaggaggaatacattagataatgac
acaatcaaatataaattgcaagatgtcacaggtgtgatgaagggagagta
ggagagaccatgagtatgtgtaacaggaggacacagcattattctagtgc
tgtactgttccgtacggcagccactacccacatgtaactttttaagattt
aaatttaaattagttaacattcaaaacgcagctccccaatcacactagca
acatttcaagtgcttgagagccatgcatgattagtggttaccctattgaa
taggtcagaagtagaatcttttcatcatcacagaaagttctattggacag
tgctcttctagatcatcataagactacagagcacttttcaaagctcatgc

atgttcatcatgttagtgtcgtattttgagctggggttttgagactcccc
ttagagatagagaaacagacccaagaaatgtgctcaattgcaatgggcca
catacctagatctccagatgtcatttcccctctcttattttaagttatgt
taagattactaaaacaataaaagctcctaaaaaatcaaactgtattctgg
tgttctcttctacacagtgggagggcgagcagtaggagagattggcccat
ttggtgctggccatttgaggaatgcaagcccagcactagtctcataatct
ctaggaatctgtagagagaggaattgaagtaaatttcagcattggctcat
tcagtcattcggcgacattcatcaggtacctgcaatgtgttaggggatct
tatgagtaggcagcgtgcgtgatccttgctcccctggagctttctaacat
tctagcaggcagaccacacataaatttgcaatactgtttctgataaaaac
gtgctgtaaaggaaataaagcagagaactatcatggaaaaaaaaaaaaaa
aaaa
>NM_021960 4
gcgcaaccctccggaagctgccgcccctttccccttttatgggaatactt
tttttaaaaaaaaagagttcgctggcgccaccccgtaggactggccgccc
taaaaccgtgataaaggagctgctcgccacttctcacttccgcttccttc
cagtaaggagtcggggtcttccccagttttctcagccaggcggcggcggc
gactggcaatgtttggcctcaaaagaaacgcggtaatcggactcaacctc
tactgtggggggggccggcttgggggccggcagcggcggcgccacccgccc
gggagggcgacttttggctacggagaaggaggcctcggcccggcgagaga
taggggggaggggaggccggcgcggtgattggcggaagcgccggcgcaagc
cccccgtccaccctcacgccagactcccggagggtcgcgcggccgccgcc
cattggcgccgaggtccccgacgtcaccgcgacccccgcgaggctgcttt
tcttcgcgcccacccgccgcgcggcgccgcttgaggagatggaagccccg
gccgctgacgccatcatgtcgcccgaagaggagctggacgggtacgagcc
ggagcctctcgggaagcggccggctgtcctgccgctgctggagttggtcg
gggaatctggtaataacaccagtacggacgggtcactaccctcgacgccg
ccgccagcagaggaggaggaggacgagttgtaccggcagtcgctggagat
tatctctcggtaccttcgggagcaggccaccggcgccaaggacacaaagc
caatgggcaggtctggggccaccagcaggaaggcgctggagaccttacga
cgggttggggatggcgtgcagcgcaaccacgagacggccttccaaggcat
gcttcggaaactggacatcaaaaacgaagacgatgtgaaatcgttgtctc
gagtgatgatccatgttttcagcgacggcgtaacaaactggggcaggatt
gtgactctcatttcttttggtgcctttgtggctaaacacttgaagaccat
aaaccaagaaagctgcatcgaaccattagcagaaagtatcacagacgttc
tcgtaaggacaaaacgggactggctagttaaacaaagaggctgggatggg
tttgtggagttcttccatgtagaggacctagaaggtggcatcaggaatgt
gctgctggcttttgcaggtgttgctggagtaggagctggtttggcatatc
taataagatagccttactgtaagtgcaatagttgacttttaaccaaccac
caccaccaccaaaaccagtttatgcagttggactccaagctgtaacttcc
tagagttgcaccctagcaacctagccagaaaagcaagtggcaagaggatt
atggctaacaagaataaatacatgggaagagtgctccccattgattgaag
agtcactgtctgaaagaagcaaagttcagtttcagcaacaaacaaacttt
gtttgggaagctatggaggaggacttttagatttagtgaagatggtaggg
tggaaagacttaatttccttgttgagaacaggaaagtggccagtagccag
gcaagtcatagaattgattacccgccgaattcattaatttactgtagtgt
taagagaagcactaagaatgccagtgacctgtgtaaaagttacaagtaat
agaactatgactgtaagcctcagtactgtacaagggaagcttttcctctc
tctaattagctttcccagtatacttcttagaaagtccaagtgttcaggac

ttttatacctgttatactttggcttggtttccatgattcttactttatta
gcctagtttatcaccaataatacttgacggaaggctcagtaattagttat
gaatatggatatcctcaattcttaagacagcttgtaaatgtatttgtaaa
aattgtatatatttttacagaaagtctatttctttgaaacgaaggaagta
tcgaatttacattagttttttcatacccttttgaactttgcaacttccg
taattaggaacctgtttcttacagcttttctatgctaaactttgttctgt
tcagttctagagtgtatacagaacgaattgatgtgtaactgtatgcagac
tggttgtagtggaacaaatctgataactatgcaggtttaaattttcttat
ctgattttggtaagtattccttagataggtttttctttgaaaacctggga
ttgagaggttgatgaatggaaattctttcacttcattatatgcaagtttt
caataattaggtctaagtggagttttaaggttactgatgacttacaaata
atgggctctgattgggcaatactcatttgagttccttccatttgacctaa
tttaactggtgaaatttaaagtgaattcatgggctcatctttaaagcttt
tactaaaagattttcagctgaatggaactcattagctgtgtgcatataaa
aagatcacatcaggtggatggagagacatttgatcccttgtttgcttaat
aaattataaaatgatggcttggaaaagcaggctagtctaaccatggtgct
attattaggcttgcttgttacacacacaggtctaagcctagtatgtcaat
aaagcaaatacttactgttttgtttctattaatgattcccaaaccttgtt
gcaagtttttgcattggcatctttggatttcagtcttgatgtttgttcta
tcagacttaaccttttatttcctgtccttccttgaaattgctgattgttc
tgctccctctacagatatttatatcaattcctacagctttcccctgccat
ccctgaactctttctagcccttttagattttggcactgtgaaacccctgc
tggaaacctgagtgaccctccctccccaccaagagtccacagacctttca
tctttcacgaacttgatcctgttagcaggtggtaataccatgggtgctgt
gacactaacagtcattgagaggtgggaggaagtcccttttccttggactg
gtatcttttcaactattgttttatcctgtctttgggggcaatgtgtcaaa
agtcccctcaggaattttcagaggaaagaacattttatgaggctttctct
aaagtttcctttgtataggagtatgctcacttaaatttacagaaagaggt
gagctgtgttaaacctcagagtttaaaagctactgataaactgaagaaag
tgtctatattggaactagggtcatttgaaagcttcagtctcggaacatga
cctttagtctgtggactccatttaaaaataggtatgaataagatgactaa
gaatgtaatggggaagaactgccctgcctgcccatctcagagccataagg
tcatctttgctagagctatttttacctatgtatttatcgttcttgatcat
aagccgcttatttatatcatgtatctctaaggacctaaaagcactttatg
tagtttttaattaatcttaagatctggttacggtaactaaaaaagcctgt
ctgccaaatccagtggaaacaagtgcatagatgtgaattggtttttaggg
gccccacttcccaattcattaggtatgactgtggaaatacagacaaggat
cttagttgatattttgggcttggggcagtgagggcttaggacaccccaag
tggtttgggaaaggaggaggggagtggtgggtttatagggggaggaggag
gcaggtggtctaagtgctgactggctacgtagttcgggcaaatcctccaa
aagggaaagggaggatttgcttagaaggatggcgctcccagtgactactt
tttgacttctgtttgtcttacgcttctctcagggaaaaacatgcagtcct
ctagtgtttcatgtacattctgtggggggtgaacaccttggttctggtta
aacagctgtacttttgatagctgtgccaggaagggttaggaccaactaca
aattaatgttggttgtcaaatgtagtgtgtttccctaactttctgttttt
cctgagaaaaaaaaataaatcttttattcaaatacagggaaaaaaaaaaa
aaaaaaa
>NM_003211 4
gatttggctccgaggaggcggaagtgcagcacagaaagggggtccgtggg

ggacggtagaagcctggaggaggagcttgagtccagccactgtctgggta
ctgccagccatcgggcccaggtctctggggttgtcttaccgcagtgagta
ccacgcggtactacagagaccggctgcccgtgtgcccggcaggtggagcc
gcccgcatcagcggcctcggggaatggaagcggagaacgcgggcagctat
tcccttcagcaagctcaagctttttatacgtttccatttcaacaactgat
ggctgaagctcctaatatggcagttgtgaatgaacagcaaatgccagaag
aagttccagccccagctcctgctcaggaaccagtgcaagaggctccaaaa
ggaagaaaaagaaaacccagaacaacagaaccaaaacaaccagtggaacc
caaaaaacctgttgagtcaaaaaaatctggcaagtctgcaaaatcaaaag
aaaaacaagaaaaaattacagacacatttaaagtaaaaagaaaagtagac
cgttttaatggtgtttcagaagctgaacttctgaccaagactctccccga
tattttgaccttcaatctggacattgtcattattggcataaacccgggac
taatggctgcttacaaagggcatcattaccctggacctggaaaccatttt
tggaagtgtttgtttatgtcagggctcagtgaggtccagctgaaccatat
ggatgatcacactctaccagggaagtatggtattggatttaccaacatgg
tggaaaggaccacgcccggcagcaaagatctctccagtaaagaatttcgt
gaaggaggacgtattctagtacagaaattacagaaatatcagccacgaat
agcagtgtttaatggaaaatgtatttatgaaatttttagtaaagaagttt
ttggagtaaaggttaagaacttggaatttgggcttcagccccataagatt
ccagacacagaaactctctgctatgttatgccatcatccagtgcaagatg
tgctcagtttcctcgagcccaagacaaagttcattactacataaaactga
aggacttaagagatcagttgaaaggcattgaacgaaatatggacgttcaa
gaggtgcaatatacatttgacctacagcttgcccaagaggatgcaaagaa
gatggctgttaaggaagaaaaatatgatccaggttatgaggcagcatatg
gtggtgcttacggagaaaatccatgcagcagtgaaccttgtggcttctct
tcaaatgggctaattgagagcgtggagttaagaggagaatcagctttcag
tggcattcctaatgggcagtggatgacccagtcatttacagaccaaattc
cttcctttagtaatcactgtggaacacaagaacaggaagaagaaagccat
gcttaagaatggtgcttctcagctctgcttaaatgctgcagttttaatgc
agttgtcaacaagtagaacctcagtttgctaactgaagtgttttattagt
attttactctagtggtgtaattgtaatgtagaacagttgtgtggtagtgt
gaaccgtatgaacctaagtagtttggaagaaaaagtagggtttttgtata
ctagcttttgtatttgaattaattatcattccagctttttatatactata
tttcatttatgaagaaattgattttcttttgggagtcacttttaatctgt
aattttaaaatacaagtctgaatatttatagttgattcttaactgcataa
acctagatataccattatcccttttatacctaagaagggcatgctaataa
ttaccactgtcaaagaggcaaaggtgttgatttttgtatatgaagttaag
cctcagtggagtctcatttgttagtttttagtggtaactaagggtaaact
cagggttccctgagctatatgcacactcagacctctttgctttaccagtg
gtgtttgtgagttgctcagtagtaaaaactggcccttacctgacagagcc
ctggctttgacctgctcagccctgtgtgttaatcctctagtagccaatta
actactctggggtggcaggttccagagaatgcagtagaccttttgccact
catctgtgttttacttgagacatgtaaatatgatagggaaggaactgaat
ttctccattcatatttataaccattctagttttatcttccttggctttaa
gagtgtgccatggaaagtgataagaaatgaacttctaggctaagcaaaaa
gatgctggagatatttgatactctcatttaaactggtgctttatgtacat
gagatgtactaaaataagtaatatagaatttttcttgctaggtaaatcca
gtaagccaataattttaaagattctttatctgcatcattgctgtttgtta
ctataaattaaatgaacctcatggaaaggttgaggtgtatacctttgtga

ttttctaatgagttttccatggtgctacaaataatccagactaccaggtc
tggtagatattaaagctgggtactaagaaatgttatttgcatcctctcag
ttactcctgaatattctgatttcatacgtacccagggagcatgctgtttt
gtcaatcaatataaaatatttatgaggtctcccccacccccaggaggtta
tatgattgctcttctctttataataagagaaacaaattcttattgtgaat
cttaacatgctttttagctgtggctatgatggattttatttttttcctagg
tcaagctgtgtaaaagtcatttatgttatttaaatgatgtactgtactgc
tgtttacatggacgttttgtgcgggtgctttgaagtgccttgcatcaggg
attaggagcaattaaattatttttttcacgggactgtgtaaagcatgtaac
taggtattgctttggtatataactattgtagctttacaagagattgtttt
atttgaatggggaaaatacccttaaattatgacggacatccactagaga
tgggtttgaggattttccaagcgtgtaataatgatgtttttcctaacatg
acagatgagtagtaaatgttgatatatcctatacatgacagtgtgagact
ttttcattaaataatattgaaagattttaaaattcatttgaaagtctgat
ggcttttacaataaaagatattaagaattgttatccttaacttaaaaaaa
a
>NM_001855 3
ggagtcgggtttcagagcgcgggtgactcggggcgcgggccgggagccgg
gattctgcccgccgccgccgctgccgagcgccgcctttgttccctgcagg
aagggcgagcgcggcggccagcgctcagcgacccttcgtcctccgctaag
ctccaacgctctgctcgactagccgcgcgccttccggggctccgcagacc
cgcgagatggcaccaaggaggaacaacgggcagtgctggtgtctgctgat
gctgctctcggtctccacgcccctccctgctgtcacccagacccgcggtg
cgacagagactgcttcccagggtcacctggacctcacgcagctcatcggt
gtcccgctgccctcgtccgtatcctttgtcacaggctatggtggcttccc
ggcctacagtttcgggcctggtgccaatgttggccgcccagccaggactc
tcatcccatccaccttcttcagggacttcgccatcagcgtcgtggtgaag
cccagcagcacccgtggtggcgtgctcttcgccatcactgacgccttcca
gaaggtcatctacctgggcctgcggctctcaggtgtggaggacggccacc
agcggatcatcctctactacacggagccaggctcccatgtgtcccaagag
gctgctgccttctcggtgcctgtgatgacccacaggtggaaccgcttcgc
catgattgtccagggtgaggaagtgaccctcctcgtgaactgtgaggagc
acagccgcatccccttccagcggtcctcccaggctttggcttttgagtcc
agcgctggaatcttcatgggcaatgcaggagctacagggctcgagagatt
cactggctccctccagcagctcaccgtgcaccccgaccccaggactcccg
aggagctgtgtgaccctgaagagtcctcggcatctggagagaccagtggg
ctgcaggaggcagacggagtagctgagatcttagaagccgtcacctacac
tcaagcctcgcccaaagaagcaaaagttgaacccataaacacacctccaa
ctccatcctccccctttgaagacatggaactttctggtgaacctgtaccc
gaggggaccctggaaaccaccaacatgagcatcatccagcacagcagccc
caaacaagggtctggtgagatcctgaatgacacactggaggggggttcatt
ctgtggatggtgaccccattactgacagcggctcaggggctggggccttc
cttgacattgctgaagaaaagaatttagcagcaacagcagcggggctggc
cgaggtgcccatcagcactgctggagaagcagaggccagcagtgtgccca
ccggggggaccaaccctctctatgtccacggagaacccagaggaaggggtc
actccaggtccagataatgaagagcgtttagcagcaacagcagcaggggga
ggccgaggcactcgccagcatgcctggggaagtggaggccagtggtgtgg
cccccggggagctggacctctccatgtccgcccagagcctcggggaagag
gccactgtgggtccaagcagtgaagacagtttaacaacagctgcagctgc

aaccgaagtgtccctcagtacttttgaggatgaggaagccagtggggtcc
ccacagatggcctggctcccctcacagccaccatggcccctgagcgggca
gtcacttctggtcctggtgatgaagaagacttggcagcagccacaacaga
ggagcccctcatcacagctgggggtgaagagtccggcagccctccccctg
atgggccaccgctgcccctgcccacagtggctcctgaaagatggatcact
ccagctcaaagagaacatgtgggaatgaaaggacaggctgggcccaaagg
agaaaagggtgatgctggggaggagcttcctggccctcctgaaccttctg
ggcctgttggacccacggcaggagcagaagcagagggctctggcctaggc
tggggctcggacgtcggctctggctctggtgacctggtgggcagtgagca
gctgctgagaggtcctccaggacccccagggccacctggcttacctggga
ttccaggaaaaccaggaactgatgttttcatgggaccccctggatctcct
ggagaggatggacctgctggtgaacctgggcccccgggccctgagggaca
gcctggagttgatggagccaccggccttcccgggatgaaaggggagaagg
gagcaagagggcctaatggctcagttggtgaaaagggtgaccctggcaac
agaggcttacctggaccccggggaaaaagggacaagctggccctcctgg
ggtcatgggacccccagggcctcctggacccccctgggcccccaggccctg
gatgcacaatgggacttggattcgaggataccgaaggctctggaagcacc
cagctattgaatgaacccaaactctccagaccaacggctgcaattggtct
caaaggagagaaaggagaccggggacccaagggagaaagggggatggatg
gagccagtattgtgggaccccctgggccgagagggccacctgggcacatc
aaggtcttgtctaattccttgatcaatatcacccatggattcatgaattt
ctcggacattcctgagctggtggggcctccggggccggacgggttgcctg
ggctgccaggatttccaggtcctagaggaccaaaaggtgacactggttta
cctggctttccaggactaaaaggagaacagggcgagaagggagagccggg
tgccatcctgacagaggacattcctctggaaaggctgatggggaaaaagg
gtgaacctggaatgcatggagccccaggaccaatggggcccaaaggacca
ccaggacataaaggagaatttggccttcccgggcgacctggtcgcccagg
actgaatggcctcaagggtaccaaaggagatccaggggtcattatgcagg
gcccacctggcttacctggccctccaggcccccctgggccacctggagct
gtgattaacatcaaaggagccattttcccaatacccgtccgaccacactg
caaaatgccagttgatactgctcatcctgggagtccagagctcatcactt
ttcacggtgttaaaggagagaaaggatcctggggtcttcctggctcaaag
ggagaaaaaggcgaccagggagcccagggaccaccaggtcctccacttga
tctagcttacctgagacactttctgaacaacttgaagggggagaatggag
acaaggggttcaaaggtgaaaaaggagaaaaaggagacattaatggcagc
ttccttatgtctgggcctccaggcctgcccggaaatccaggcccggctgg
ccaaaaaggggagacagtcgttgggccccaaggacccccaggtgctcctg
gtctgcctgggccacctggctttggaagacctggtgatcctgggccaccg
gggcccccggggccaccaggacctccagctatcctgggagcagctgtggc
ccttccaggtcccccctggccctccaggacagccagggcttcccggatcca
gaaacctggtcacagcattcagcaacatggatgacatgctgcagaaagcg
catttggttatagaaggaacattcatctacctgagggacagcactgagtt
tttcattcgtgttagagatggctggaaaaaattacagctgggagaactga
tccccattcctgccgacagccctccaccccctgcgctttccagcaaccca
catcagcttctgcctccaccaaaccctatttcaagtgccaattatgagaa
gcctgctctgcatttggctgctctgaacatgccattttctggggacattc
gagctgattttcagtgcttcaagcaggccagagctgcaggactgttgtcc
acctaccgagcattcttatcttcccatttgcaagatctgtccaccattgt
gaggaaagcagagagatacagccttcccatagtgaacctcaagggccaag

tactttttaataattgggactcaatttttctggccacggaggtcagttc
aatatgcatattccaatatactcctttgatggtcgagacataatgacaga
tccttcttggccccagaaagtcatttggcatggctccagcccccatggcg
tccgccttgtggataactactgtgaagcatggcgaaccgcggacacagcg
gtcacgggacttgcctccccgctgagcacggggaagattctggaccagaa
agcatacagctgtgctaatcggctaattgtcctatgtatcgaaaacagtt
tcatgacagacgctaggaagtaatggccttctgatgattcttaaagagtt
ttcaatttttcttatgtgaagagttgacactgaaatctaaaatgtttaa
ttgttgtaaatattacagtttttttttttactacatattctttacaaca
gcaaccaaagaaaacatacctcaatacactcaaaactgaagacatagagg
actcagatcaaagacaaaatctgatccatatattggtgctagattctgca
ggaaaccccagcagtgtgaacgcatcccaacataggttaagagcaagttg
aaaacaaaggccatggcattctgccactgcatccttcagacagttatatc
ctccttttaaaccattgttgttgagtgtaagatgtccttcatgttttctt
ataaagtcagtgtttagaaatgttaccctttctaagttatatacagatca
aatgctttttctttcacgtacatccatcatttgcaactgctgttcgtac
acagaaacaggactgctcaaatgatcctatttgtattttctgatgctatc
agactctaatgtttttttccctaaaatattattgccatcatgctttagga
attttatatttttacacaatcatattttagtatggtgtctgtttatgtaa
ctctgacttgctggaaaagttgaaactccaaataatctgaaactagaaaa
gaaatagcacataattactaccttccccttggcggctctcctccccaacc
cccaccccacaattttatgacttccatttggcaattgttgaattataact
gcgactgaaacaaacaggttcatagagatgaattttctgagaaacatata
tctacatgttgtataattggatttttttccatgtaagtgaacataaaaa
catcttttccgggtgctttcttc
>NM_000090 3
ggctgagttttatgacgggcccggtgctgaagggcagggaacaacttgat
ggtgctactttgaactgcttttcttttctcctttttgcacaaagagtctc
atgtctgatatttagacatgatgagctttgtgcaaaaggggagctggcta
cttctcgctctgcttcatcccactattattttggcacaacaggaagctgt
tgaaggaggatgttcccatcttggtcagtcctatgcggatagagatgtct
ggaagccagaaccatgccaaatatgtgtctgtgactcaggatccgttctc
tgcgatgacataatatgtgacgatcaagaattagactgccccaacccaga
aattccatttggagaatgttgtgcagtttgcccacagcctccaactgctc
ctactcgccctcctaatggtcaaggacctcaaggccccaagggagatcca
ggccctcctggtattcctgggagaaatggtgaccctggtattccaggaca
accagggtcccctggttctcctggccccctggaatctgtgaatcatgcc
ctactggtcctcagaactattctccccagtatgattcatatgatgtcaag
tctggagtagcagtaggaggactcgcaggctatcctggaccagctggccc
cccaggccctcccggtcccctggtacatctggtcatcctggttcccctg
gatctccaggataccaaggacccctggtgaacctgggcaagctggtcct
tcaggccctccaggacctcctggtgctataggtccatctggtcctgctgg
aaaagatggagaatcaggtagacccggacgacctggagagcgaggattgc
ctggacctccaggtatcaaaggtccagctgggatacctggattccctggt
atgaaaggacacagaggcttcgatggacgaaatggagaaaagggtgaaac
aggtgctcctggattaaagggtgaaaatggtcttccaggcgaaaatggag
ctcctggacccatgggtccaagaggggctcctggtgagcgaggacggcca
ggacttcctggggctgcaggtgctcggggtaatgacggtgctcgaggcag
tgatggtcaaccaggccctcctggtcctcctggaactgccggattccctg

gatcccctggtgctaagggtgaagttggacctgcagggtctcctggttca
aatggtgcccctggacaaagaggagaacctggacctcagggacacgctgg
tgctcaaggtcctcctggccctcctgggattaatggtagtcctggtggta
aaggcgaaatgggtcccgctggcattcctggagctcctggactgatggga
gcccggggtcctccaggaccagccggtgctaatggtgctcctggactgcg
aggtggtgcaggtgagcctggtaagaatggtgccaaaggagagcccggac
cacgtggtgaacgcggtgaggctggtattccaggtgttccaggagctaaa
ggcgaagatggcaaggatggatcacctggagaacctggtgcaaatgggct
tccaggagctgcaggagaaaggggtgcccctgggttccgaggacctgctg
gaccaaatggcatcccaggagaaaagggtcctgctggagagcgtggtgct
ccaggccctgcagggcccagaggagctgctggagaacctggcagagatgg
cgtccctggaggtccaggaatgaggggcatgcccggaagtccaggaggac
caggaagtgatgggaaaccagggcctcccggaagtcaaggagaaagtggt
cgaccaggtcctcctgggccatctggtccccgaggtcagcctggtgtcat
gggcttccccggtcctaaaggaaatgatggtgctcctggtaagaatggag
aacgaggtggccctggaggacctggccctcagggtcctcctggaaagaat
ggtgaaactggacctcagggacccccagggcctactgggcctggtggtga
caaaggagacacaggacccccctggtccacaaggattacaaggcttgcctg
gtacaggtggtcctccaggagaaaatggaaaacctggggaaccaggtcca
aagggtgatgccggtgcacctggagctccaggaggcaagggtgatgctgg
tgcccctggtgaacgtggacctcctggattggcaggggccccaggactta
gaggtggagctggtccccctggtcccgaaggaggaaagggtgctgctggt
cctcctgggccacctggtgctgctggtactcctggtctgcaaggaatgcc
tggagaaagaggaggtcttggaagtcctggtccaaagggtgacaagggtg
aaccaggcggtccaggtgctgatggtgtcccagggaaagatggcccaagg
ggtcctactggtcctattggtcctcctggcccagctggccagcctggaga
taagggtgaaggtggtgcccccggacttccaggtatagctggacctcgtg
gtagccctggtgagagaggtgaaactggccctccaggacctgctggtttc
cctggtgctcctggacagaatggtgaacctggtggtaaaggagaaagagg
ggctccgggtgagaaaggtgaaggaggccctcctggagttgcaggacccc
ctggaggttctggacctgctggtcctcctggtccccaaggtgtcaaaggt
gaacgtggcagtcctggtggacctggtgctgctggcttccctggtgctcg
tggtcttcctggtcctcctggtagtaatggtaacccaggacccccaggtc
ccagcggttctccaggcaaggatgggcccccaggtcctgcgggtaacact
ggtgctcctggcagccctggagtgtctggaccaaaaggtgatgctggcca
accaggagagaagggatcgcctggtgcccagggcccaccaggagctccag
gcccacttgggattgctgggatcactggagcacggggtcttgcaggacca
ccaggcatgccaggtcctaggggaagccctggccctcagggtgtcaaggg
tgaaagtgggaaaccaggagctaacggtctcagtggagaacgtggtcccc
ctggaccccagggtcttcctggtctggctggtacagctggtgaacctgga
agagatggaaaccctggatcagatggtcttccaggccgagatggatctcc
tggtggcaagggtgatcgtggtgaaaatggctctcctggtgcccctggcg
ctcctggtcatccaggcccacctggtcctgtcggtccagctggaaagagt
ggtgacagaggagaaagtggccctgctggccctgctggtgctcccggtcc
tgctggttcccgaggtgctcctggtcctcaaggcccacgtggtgacaaag
gtgaaacaggtgaacgtggagctgctggcatcaaaggacatcgaggattc
cctggtaatccaggtgccccaggttctccaggccctgctggtcagcaggg
tgcaatcggcagtccaggacctgcaggccccagaggacctgttggaccca
gtggacctcctggcaaagatggaaccagtggacatccaggtcccattgga

ccaccagggcctcgaggtaacagaggtgaaagaggatctgagggctcccc
aggccacccagggcaaccaggccctcctggacctcctggtgcccctggtc
cttgctgtggtggtgttggagccgctgccattgctgggattggaggtgaa
aaagctggcggttttgccccgtattatggagatgaaccaatggatttcaa
aatcaacaccgatgagattatgacttcactcaagtctgttaatggacaaa
tagaaagcctcattagtcctgatggttctcgtaaaaaccccgctagaaac
tgcagagacctgaaattctgccatcctgaactcaagagtggagaatactg
ggttgaccctaaccaaggatgcaaattggatgctatcaaggtattctgta
atatggaaactggggaaacatgcataagtgccaatcctttgaatgttcca
cggaaacactggtggacagattctagtgctgagaagaaacacgtttggtt
tggagagtccatggatggtggttttcagtttagctacggcaatcctgaac
ttcctgaagatgtccttgatgtgcagctggcattccttcgacttctctcc
agccgagcttcccagaacatcacatatcactgcaaaaatagcattgcata
catggatcaggccagtggaaatgtaaagaaggccctgaagctgatggggt
caaatgaaggtgaattcaaggctgaaggaaatagcaaattcacctacaca
gttctggaggatggttgcacgaaacacactggggaatggagcaaaacagt
ctttgaatatcgaacacgcaaggctgtgagactacctattgtagatattg
caccctatgacattggtggtcctgatcaagaatttggtgtggacgttggc
cctgtttgctttttataaaccaaactctatctgaaatcccaacaaaaaaa
atttaactccatatgtgttcctcttgttctaatcttgtcaaccagtgcaa
gtgaccgacaaaattccagttatttatttccaaaatgtttggaaacagta
taatttgacaaagaaaaatgatacttctcttttttgctgttccaccaaa
tacaattcaaatgctttttgttttattttttaccaattccaatttcaaa
atgtctcaatggtgctataataaataaacttcaacactctttatgataac
aacactgtgttatattctttgaatcctagcccatctgcagagcaatgact
gtgctcaccagtaaaagataacctttctttctgaaatagtcaaatacgaa
attagaaaagccctccctattttaactacctcaactggtcagaaacacag
attgtattctatgagtcccagaagatgaaaaaaattttatacgttgataa
aacttataaatttcattgattaatctcctggaagattggtttaaaaagaa
aagtgtaatgcaagaatttaaagaaatatttttaaagccacaattatttt
aatattggatatcaactgcttgtaaaggtgctcctctttttcttgtcat
tgctggtcaagattactaatatttgggaaggctttaaagacgcatgttat
ggtgctaatgtactttcacttttaaactctagatcagaattgttgacttg
cattcagaacataaatgcacaaaatctgtacatgtctcccatcagaaaga
ttcattggcatgccacaggggattctcctccttcatcctgtaaaggtcaa
caataaaaaccaaattatggggctgcttttgtcacactagcatagagaat
gtgttgaaatttaactttgtaagcttgtatgtggttgttgatcttttttt
tccttacagacacccataataaaatatcatattaaaattc
>NM_002293 3
gtgcaggctgctcccggggtaggtgagggaagcgcggaggcggcgcgcgg
gggcagtggtcggcgagcagcgcggtcctcgctaggggcgcccacccgtc
agtctctccggcgcgagccgccgccaccgcccgcgccggagtcaggcccc
tgggcccccaggctcaagcagcgaagcggcctccgggggacgccgctagg
cgagaggaacgcgccggtgcccttgccttcgccgtgacccagcgtgcggg
cggcgggatgagagggagccatcgggccgcgccggccctgcggccccggg
ggcggctctggcccgtgctggccgtgctggcggcggccgccgcggcgggc
tgtgcccaggcagccatggacgagtgcacggacgagggcgggcggccgca
gcgctgcatgcccgagttcgtcaacgccgccttcaacgtgactgtggtgg
ccaccaacacgtgtgggactccgcccgaggaatactgtgtgcagaccggg

gtgaccggggtcaccaagtcctgtcacctgtgcgacgccgggcagcccca
cctgcagcacggggcagccttcctgaccgactacaacaaccaggccgaca
ccacctggtggcaaagccagaccatgctggccggggtgcagtaccccagc
tccatcaacctcacgctgcacctgggaaaagcttttgacatcacctatgt
gcgtctcaagttccacaccagccgcccggagagctttgccatttacaagc
gcacacgggaagacgggccctggattccttaccagtactacagtggttcc
tgtgagaacacctactccaaggcaaaccgcggcttcatcaggacaggagg
ggacgagcagcaggccttgtgtactgatgaattcagtgacatttctcccc
tcactgggggcaacgtggcctttctaccctggaaggaaggcccagcgcc
tataactttgacaatagccctgtgctgcaggaatgggtaactgccactga
catcagagtaactcttaatcgcctgaacacttttggagatgaagtgttta
acgatcccaaagttctcaagtcctattattatgccatctctgattttgct
gtaggtggcagatgtaaatgtaatggacacgcaagcgagtgtatgaagaa
cgaatttgataagctggtgtgtaattgcaaacataacacatatggagtag
actgtgaaaagtgtcttcctttcttcaatgaccggccgtggaggagggca
actgcggaaagtgccagtgaatgcctgccctgtgattgcaatggtcgatc
ccaggaatgctacttcgaccctgaactctatcgttccactggccatgggg
gccactgtaccaactgccaggataacacagatggcgcccactgtgagagg
tgccgagagaacttcttccgccttggcaacaatgaagcctgctcttcatg
ccactgtagtcctgtgggctctctaagcacacagtgtgatagttacggca
gatgcagctgtaagccaggagtgatgggggacaaatgtgaccgttgccag
cctggattccattctctcactgaagcaggatgcaggccatgctcttgtga
tccctctggcagcatagatgaatgtaatattgaaacaggaagatgtgttt
gcaaagacaatgtcgaaggcttcaattgtgaaagatgcaaacctggattt
tttaatctggaatcatctaatcctcggggttgcacaccctgcttctgctt
tgggcattcttctgtctgtacaaacgctgttggctacagtgtttattcta
tctcctctacctttcagattgatgaggatgggtggcgtgcggaacagaga
gatggctctgaagcatctctcgagtggtcctctgagaggcaagatatcgc
cgtgatctcagacagctactttcctcggtacttcattgctcctgcaaagt
tcttgggcaagcaggtgttgagttatggtcagaacctctccttctccttt
cgagtggacaggcgagatactcgcctctctgcagaagaccttgtgcttga
gggagctggcttaagagtatctgtacccttgatcgctcagggcaattcct
atccaagtgagaccactgtgaagtatgtcttcaggctccatgaagcaaca
gattacccttggaggcctgctcttaccccttttgaatttcagaagctcct
aaacaacttgacctctatcaagatacgtgggacatacagtgagagaagtg
ctggatatttggatgatgtcaccctggcaagtgctcgtcctgggcctgga
gtccctgcaacttgggtggagtcctgcacctgtcctgtgggatatggagg
gcagttttgtgagatgtgcctctcaggttacagaagagaaactcctaatc
ttggaccatacagtccatgtgtgctttgcgcctgcaatggacacagcgag
acctgtgatcctgagacaggtgtttgtaactgcagagacaatacggctgg
cccgcactgtgagaagtgcagtgatgggtactatggagattcaactgcag
gcacctcctccgattgccaaccctgtccgtgtcctggaggttcaagttgt
gctgttgttcccaagacaaaggaggtggtgtgcaccaactgtcctactgg
caccactggtaagagatgtgagctctgtgatgatggctactttggagacc
ccctgggtagaaacggccctgtgagactttgccgcctgtgccagtgcagt
gacaacatcgatcccaatgcagttggaaattgcaatcgcttgacgggaga
atgcctgaagtgcatctataacactgctggcttctattgtgaccggtgca
aagacggatttttggaaatcccctggctcccaatccagcagacaaatgc
aaagcctgcaattgcaatctgtatgggaccatgaagcagcagagcagctg

taaccccgtgacggggcagtgtgaatgtttgcctcacgtgactggccagg
actgtggtgcttgtgaccctggattctacaatctgcagagtgggcaaggc
tgtgagaggtgtgactgccatgccttgggctccaccaatgggcagtgtga
catccgcaccggccagtgtgagtgccagcccggcatcactggtcagcact
gtgagcgctgtgaggtcaaccactttgggtttggacctgaaggctgcaaa
ccctgtgactgtcatcctgagggatctctttcacttcagtgcaaagatga
tggtcgctgtgaatgcagagaaggctttgtgggaaatcgctgtgaccagt
gtgaagaaaactatttctacaatcggtcttggcctggctgccaggaatgt
ccagcttgttaccggctggtaaaggataaggttgctgatcatagagtgaa
gctccaggaattagagagtctcatagcaaaccttggaactggggatgaga
tggtgacagatcaagccttcgaggatagactaaaggaagcagagagggaa
gttatggacctccttcgtgaggcccaggatgtcaaagatgttgaccagaa
tttgatggatcgcctacagagagtgaataacactctgtccagccaaatta
gccgtttacagaatatccggaataccattgaagagactggaaacttggct
gaacaagcgcgtgcccatgtagagaacacagagcggttgattgaaatcgc
atccagagaacttgagaaagcaaaagtcgctgctgccaatgtgtcagtca
ctcagccagaatctacaggggacccaaacaacatgactcttttggcagaa
gaggctcgaaagcttgctgaacgtcataaacaggaagctgatgacattgt
tcgagtggcaaagacagccaatgatacgtcaactgaggcatacaacctgc
ttctgaggacactggcaggagaaaatcaaacagcatttgagattgaagag
cttaataggaagtatgaacaagcgaagaacatctcacaggatctggaaaa
acaagctgcccgagtacatgaggaggccaaaagggccggtgacaaagctg
tggagatctatgccagcgtggctcagctgagccctttggactctgagaca
ctggagaatgaagcaaataacataaagatggaagctgagaatctggaaca
actgattgaccagaaattaaaagattatgaggacctcagagaagatatga
gagggaaggaacttgaagtcaagaaccttctggagaaaggcaagactgaa
cagcagaccgcagaccaactcctagcccgagctgatgctgccaaggccct
cgctgaagaagctgcaaagaagggacgggataccttacaagaagctaatg
acattctcaacaacctgaaagattttgataggcgtgtgaacgataacaag
acggccgcagaggaggcactaaggaagattcctgccatcaaccagaccat
cactgaagccaatgaaaagaccagagaagcccagcaggccctgggcagtg
ctgcggcggatgccacagaggccaagaacaaggcccatgaggcggagagg
atcgcgagcgctgtccaaaagaatgccaccagcaccaaggcagaagctga
aagaacttttgcagaagttacagatctggataatgaggtgaacaatatgt
tgaagcaactgcaggaagcagaaaaagagctaaagagaaaacaagatgac
gctgaccaggacatgatgatggcagggatggcttcacaggctgctcaaga
agccgagatcaatgccagaaaagccaaaaactctgttactagcctcctca
gcattattaatgacctcttggagcagctggggcagctggatacagtggac
ctgaataagctaaacgagattgaaggcaccctaaacaaagccaaagatga
aatgaaggtcagcgatcttgataggaaagtgtctgacctggagaatgaag
ccaagaagcaggaggctgccatcatggactataaccgagatatcgaggag
atcatgaaggacattcgcaatctggaggacatcaggaagaccttaccatc
tggctgcttcaacaccccgtccattgaaaagccctagtgtctttagggct
ggaaggcagcatccctctgacaggggggcagttgtgaggccacagagtgc
cttgacacaaagattacatttttcagacccccactcctctgctgctgtcc
atgactgtccttttgaaccaggaaaagtcacagagtttaaagagaagcaa
attaaacatcctgaatcgggaacaaagggttttatctaataaagtgtctc
ttccattcacgttgctaccttacccacactttcccttctgatttgcgtga
ggacgtggcatcctacgttactgtacagtggcataagcacatcgtgtgag

cccatgtatgctggggtagagcaagtagccctcccctgtctcatcgatac
cagcagaacctcctcagtctcagtactcttgtttctatgaaggaaaagtt
tggctactaacagtagcattgtgatggccagtatatccagtccatggata
aagaaaatgcatctgcatctcctacccctcttccttctaagcaaaaggaa
ataaacatcctgtgccaaaggtattggtcatttagaatgtcggtagccat
ccatcagtgcttttagttattatgagtgtaggacactgagccatccgtgg
gtcaggatgcaattatttataaaagtctccaggtgaacatggctgaagat
ttttctagtatattaataattgactaggaagatgaactttttttcagatc
tttgggcagctgataatttaaatctggatgggcagcttgcactcaccaat
agaccaaaagacatcttttgatattcttataaatggaacttacacagaag
aaatagggatatgataaccactaaaattttgttttcaaaatcaaactaat
tcttacagctttttattagttagtcttggaactagtgttaagtatctgg
cagagaacagttaatccctaaggtcttgacaaaacagaagaaaaacaagc
ctcctcgtcctagtcttttctagcaaagggataaaacttagatggcagct
tgtactgtcagaatcccgtgtatccatttgttcttctgttggagagatga
gacatttgacccttagctccagttttcttctgatgtttccatcttccaga
atccctcaaaaaacattgtttgccaaatcctggtggcaaatacttgcact
cagtatttcacacagctgccaacgctatcgagttcctgcactttgtgatt
taaatccactctaaaccttccctctaagtgtagagggaagacccttacgt
ggagtttcctagtgggcttctcaacttttgatcctcagctctgtggtttt
aagaccacagtgtgacagttccctgccacacacccccttcctcctaccaa
cccacctttgagattcatatatagcctttaacactatgcaactttgtact
ttgcgtagcaggggcggggtgggggaaagaaactattatctgacacact
ggtgctattaattatttcaaatttatatttttgtgtgaatgttttgtgtt
ttgtttatcatgattatagaataaggaatttatgtaaatatacttagtcc
tatttctagaatgacactctgttcactttgctcaatttttcctcttcact
ggcacaatgtatctgaatacctccttccctcccttctagaattctttgga
ttgtactccaaagaattgtgccttgtgtttgcagcatctccattctctaa
aattaatataattgctttcctccacacccagccactgtaaagaggtaact
tgggtcctcttccattgcagtcctgatgatcctaacctgcagcacggtgg
ttttacaatgttccagagcaggaacgccaggttgacaagctatggtagga
ttaggaaagtttgctgaagaggatctttgacgccacagtgggactagcca
ggaatgagggagaaatgccctttctggcaattgttggagctggataggta
agttttataagggagtacattttgactgagcacttagggcatcaggaaca
gtgctacttactgatgggtagactgggagaggtggtgtaacttagttctt
gatgatcccacttcctgtttccatctgcttgggatataccagagtttacc
acaagtgttttgacgatatactcctgagctttcactctgctgcttctccc
aggcctcttctactatggcaggagatgtggcgtgctgttgcaaagttttc
acgtcattgtttcctggctagttcatttcattaagtggctacatcctaac
atatgcatttggtcaaggttgcagaagaggactgaagattgactgccaag
ctagtttgggtgaagttcactccagcaagtctcaggccacaatggggtgg
tttggtttggtttccttttaactttctttttgttatttgcttttctcctc
cacctgtgtggtatattttttaagcagaattttattttttaaaataaaag
gttctttacaagatgataccttaattacactcccgcaacacagccattat
tttattgtctagctccagttatctgtattttatgtaatgtaattgacagg
atggctgctgcagaatgctggttgacacagggattattatactgctattt
ttccctgaattttttttcctttgaattccaactgtggaccttttatatgtg
ccttcactttagctgtttgccttaatctctacagccttgctctccggggt
ggttaataaaatgcaacacttggcatttttatgttttaagaaaaacagta

ttttatttataataaaatctgaatatttgtaacccttta
>NM_001845 4
gcttggagccgccgcacccgggacggtgcgtagcgctggaagtccggcct
tccgagagctagctgtccgccgcggcccccgcacgccgggcagccgtccc
tcgccgcctcgggcgcgccaccatggggccccggctcagcgtctggctgc
tgctgctgcccgccgcccttctgctccacgaggagcacagccgggccgct
gcgaagggtggctgtgctggctctggctgtggcaaatgtgactgccatgg
agtgaagggacaaaagggtgaaagaggcctcccggggttacaaggtgtca
ttgggtttcctggaatgcaaggacctgaggggccacagggaccaccagga
caaaagggtgatactggagaaccaggactacctggaacaaaagggacaag
aggacctccgggagcatctggctaccctggaaacccaggacttcccggaa
ttcctggccaagacggcccgccaggcccccaggtattccaggatgcaat
ggcacaaagggggagagagggccgctcgggcctcctggcttgcctggttt
cgctggaaatcccggaccaccaggcttaccagggatgaagggtgatccag
gtgagatacttggccatgtgcccgggatgctgttgaaaggtgaaagagga
tttcccggaatcccagggactccaggcccaccaggactgccagggcttca
aggtcctgttgggcctccaggatttaccggaccaccaggtcccccaggcc
ctcccggccctccaggtgaaaagggacaaatgggcttaagttttcaagga
ccaaaaggtgacaagggtgaccaaggggtcagtgggcctccaggagtacc
aggacaagctcaagttcaagaaaaaggagacttcgccaccaagggagaaa
agggccaaaaaggtgaacctggatttcaggggatgccaggggtcggagag
aaaggtgaacccggaaaaccaggacccagaggcaaacccggaaaagatgg
tgacaaaggggaaaaagggagtcccggttttcctggtgaacccgggtacc
caggactcataggccgccagggcccgcagggagaaaagggtgaagcaggt
cctcctggcccacctggaattgttataggcacaggacctttgggagaaaa
aggagagaggggctaccctggaactccggggccaagaggagagccaggcc
caaaaggtttcccaggactaccaggccaacccggacctccaggcctccct
gtacctgggcaggctggtgcccctggcttccctggtgaaagaggagaaaa
aggtgaccgaggatttcctggtacatctctgccaggaccaagtggaagag
atgggctcccgggtcctcctggttcccctgggcccccctgggcagcctggc
tacacaaatggaattgtggaatgtcagcccggacctccaggtgaccaggg
tcctcctggaattccagggcagccaggatttataggcgaaattggagaga
aaggtcaaaaaggagagagttgcctcatctgtgatatagacggatatcgg
gggcctcccgggccacagggacccccgggagaaataggtttcccagggca
gccaggggccaagggcgacagaggtttgcctggcagagatggtgttgcag
gagtgccaggccctcaaggtacaccagggctgataggccagccaggagcc
aagggggagcctggtgagttttatttcgacttgcggctcaaaggtgacaa
aggagacccaggctttccaggacagcccggcatgacagggagagcgggtt
ctcctggaagagatggccatccgggtcttcctggccccaagggctcgccg
ggttctgtaggattgaaaggagagcgtggcccccctggaggagttggatt
cccaggcagtcgtggtgacaccggcccccctgggcctccaggatatggtc
ctgctggtcccattggtgacaaaggacaagcaggctttcctggaggccct
ggatccccaggcctgccaggtccaaagggtgaaccaggaaaaattgttcc
tttaccaggcccccctggagcagaaggactgccggggtccccaggcttcc
caggtccccaaggagaccgaggctttcccggaaccccaggaaggccaggc
ctgccaggagagaagggcgctgtgggccagccaggcattggatttccagg
gccccccggccccaaaggtgttgacggcttacctggagacatggggccac
cggggactccaggtcgcccgggatttaatggcttacctgggaacccaggt
gtgcagggccagaagggagagcctggagttggtctaccgggactcaaagg

tttgccaggtcttcccggcattcctggcacacccggggagaaggggagca
ttggggtaccaggcgttcctggagaacatggagcgatcggaccccctggg
cttcaggggatcagaggtgaaccgggacctcctggattgccaggctccgt
ggggtctccaggagttccaggaataggccccccctggagctaggggtcccc
ctggaggacagggaccaccggggttgtcaggccctcctggaataaaagga
gagaagggtttccccggattccctggactggacatgccgggccctaaagg
agataaaggggctcaaggactccctggcataacgggacagtcggggctcc
ctggccttcctggacagcagggggctcctgggattcctgggtttccaggt
tccaagggagaaatgggcgtcatggggacccccgggcagccgggctcacc
aggaccagtgggtgctcctggattaccgggtgaaaaaggggaccatggct
ttccgggctcctcaggacccaggggagaccctggcttgaaaggtgataag
ggggatgtcggtctccctggcaagcctggctccatggataaggtggacat
gggcagcatgaagggccagaaaggagaccaaggagagaaaggacaaattg
gaccaattggtgagaagggatcccgaggagaccctgggaccccaggagtg
cctggaaaggacgggcaggcaggacagcctgggcagccaggacctaaagg
tgatccaggtataagtggaaccccaggtgctccaggacttccgggaccaa
aaggatctgttggtggaatgggcttgccaggaacacctggagagaaaggt
gtgcctggcatccctggcccacaaggttcacctggcttacctggagacaa
aggtgcaaaaggagagaaagggcaggcaggcccacctggcataggcatcc
cagggctgcgaggtgaaaagggagatcaagggatagcgggtttcccagga
agccctggagagaagggagaaaaaggaagcattgggatcccaggaatgcc
agggtccccaggccttaaagggtctcccgggagtgttggctatccaggaa
gtcctgggctacctggagaaaaaggtgacaaaggcctcccaggattggat
ggcatccctggtgtcaaaggagaagcaggtcttcctgggactcctggccc
cacaggcccagctggccagaaaggggagccaggcagtgatggaatcccgg
ggtcagcaggagagaagggtgaaccaggtctaccaggaagaggattccca
gggtttccaggggccaaaggagacaaaggttcaaagggtgaggtgggttt
cccaggattagccgggagcccaggaattcctggatccaaaggagagcaag
gattcatgggtcctccggggccccagggacagccggggttaccgggatcc
ccaggccatgccacggaggggcccaaaggagaccgcggacctcagggcca
gcctggcctgccaggacttccgggacccatggggcctccagggcttcctg
ggattgatggagttaaaggtgacaaaggaaatccaggctggccaggagca
cccggtgtcccagggcccaagggagaccctggattccagggcatgcctgg
tattggtggctctccaggaatcacaggctctaagggtgatatggggcctc
caggagttccaggatttcaaggtccaaaaggtcttcctggcctccaggga
attaaaggtgatcaaggcgatcaaggcgtcccgggagctaaaggtctccc
gggtcctcctggccccccaggtccttacgacatcatcaaaggggagcccg
ggctccctggtcctgagggcccccagggctgaaagggcttcagggactg
ccaggcccgaaaggccagcaaggtgttacaggattggtgggtatacctgg
acctccaggtattcctgggtttgacggtgcccctggccagaaaggagaga
tgggacctgccgggcctactggtccaagaggatttccaggtccaccaggc
cccgatgggttgccaggatccatggggcccccaggcaccccatctgttga
tcacggcttccttgtgaccaggcatagtcaaacaatagatgacccacagt
gtccttctgggaccaaaattctttaccacgggtactctttgctctacgtg
caaggcaatgaacgggcccatggccaggacttgggcacggccggcagctg
cctgcgcaagttcagcacaatgcccttcctgttctgcaatattaacaacg
tgtgcaactttgcatcacgaaatgactactcgtactggctgtccacccct
gagcccatgcccatgtcaatggcacccatcacggggaaaacataagacc
atttattagtaggtgtgctgtgtgaggcgcctgccatggtgatggccg

tgcacagccagaccattcagatcccaccgtgccccagcgggtggtcctcg
ctgtggatcggctactcttttgtgatgcacaccagcgctggtgcagaagg
ctctggccaagccctggcgtcccccggctcctgcctggaggagtttagaa
gtgcgccattcatcgagtgtcacggccgtgggacctgcaattactacgca
aacgcttacagcttttggctcgccaccatagagaggagcgagatgttcaa
gaagcctacgccgtccaccttgaaggcaggggagctgcgcacgcacgtca
gccgctgccaagtctgtatgagaagaacataatgaagcctgactcagcta
atgtcacaacatggtgctacttcttcttcttttgttaacagcaacgaac
cctagaaatatatcctgtgtacctcactgtccaatatgaaaaccgtaaag
tgccttataggaatttgcgtaactaacacaccctgcttcattgacctcta
cttgctgaaggagaaaaagacagcgataagctttcaatagtggcatacca
aatggcacttttgatgaaataaaatatcaatattttctgcaatccaatgc
actgatgtgtgaagtgagaactccatcagaaaaccaaagggtgctaggag
gtgtgggtgccttccatactgtttgcccattttcattcttgtattataat
taattttctacccccagagataaatgtttgtttatatcactgtctagctg
tttcaaaatttaggtcccttggtctgtacaaataatagcaatgtaaaaat
ggtttttgaacctccaaatggaattacagactcagtagccatatcttcc
aaccccccagtataaatttctgtctttctgctatgtgtggtactttgcag
ctgcttttgcagaaatcacaattttcctgtggaataaagatggtccaaaa
atagtcaaaaattaaatatatatatatattagtaatttatatagatgtca
gcaattaggcagatcaaggtttagtttaacttccactgttaaaataaagc
ttacatagttttcttcctttgaaagactgtgctgtcctttaacataggtt
tttaaagactaggatattgaatgtgaaacatccgttttcattgttcactt
ctaaaccaaaaattatgtgttgccaaaaccaaacccaggttcatgaatat
ggtgtctattatagtgaaacatgtactttgagcttattgtttttattctg
tattaaatattttcagggttttaaacactaatcacaaactgaatgacttg
acttcaaaagcaacaaccttaaaggccgtcatttcattagtattcctcat
tctgcatcctggcttgaaaaacagctctgttgaatcacagtatcagtatt
ttcacacgtaagcacattcgggccatttccgtggtttctcatgagctgtg
ttcacagacctcagcagggcatcgcatggaccgcaggagggcagattcgg
accactaggcctgaaatgacatttcactaaaagtctccaaaacatttcta
agactactaaggccttttatgtaatttctttaaatgtgtatttcttaaga
attcaaatttgtaataaaactatttgtataaaaattaagcttttattaat
ttgttgctagtattgccacagacgcattaaaagaaacttactgcacaagc
tgctaataaatttgtaagctttgcataccttaaaaaaaaaaaaaaaaaa
>NM_000089 3
gtgtcccatagtgtttccaaacttggaaagggcgggggagggcgggagga
tgcggagggcggaggtatgcagacaacgagtcagagtttccccttgaaag
cctcaaaagtgtccacgtcctcaaaaagaatggaaccaatttaagaagcc
agccccgtggccacgtcccttcccccattcgctccctcctctgcgccccc
gcaggctcctcccagctgtggctgcccgggcccccagccccagccctccc
attggtggaggcccttttggaggcaccctagggccagggaaacttttgcc
gtataaatagggcagatccgggctttattattttagcaccacggcagcag
gaggtttcggctaagttggaggtactggccacgactgcatgcccgcgccc
gccaggtgatacctccgccggtgacccaggggctctgcgacacaaggagt
ctgcatgtctaagtgctagacatgctcagctttgtggatacgcggacttt
gttgctgcttgcagtaaccttatgcctagcaacatgccaatctttacaag
aggaaactgtaagaaagggcccagccggagatagaggaccacgtggagaa
aggggtccaccaggcccccccaggcagagatggtgaagatggtcccacagg

ccctcctggtccacctggtcctcctggccccctggtctcggtgggaact
ttgctgctcagtatgatggaaaaggagttggacttggccctggaccaatg
ggcttaatgggacctagaggcccacctggtgcagctggagccccaggccc
tcaaggtttccaaggacctgctggtgagcctggtgaacctggtcaaactg
gtcctgcaggtgctcgtggtccagctggccctcctggcaaggctggtgaa
gatggtcaccctggaaaacccggacgacctggtgagagaggagttgttgg
accacagggtgctcgtggtttccctggaactcctggacttcctggcttca
aaggcattaggggacacaatggtctggatggattgaagggacagcccggt
gctcctggtgtgaagggtgaacctggtgcccctggtgaaaatggaactcc
aggtcaaacaggagcccgtgggcttcctggtgagagaggacgtgttggtg
cccctggcccagctggtgcccgtggcagtgatggaagtgtgggtcccgtg
ggtcctgctggtcccattgggtctgctggccctccaggcttcccaggtgc
ccctggccccaagggtgaaattggagctgttggtaacgctggtcctgctg
gtcccgccggtccccgtggtgaagtgggtcttccaggcctctccggcccc
gttggacctcctggtaatcctggagcaaacggccttactggtgccaaggg
tgctgctggccttcccggcgttgctggggctcccggcctccctggacccc
gcggtattcctggccctgttggtgctgccggtgctactggtgccagagga
cttgttggtgagcctggtccagctggctccaaaggagagagcggtaacaa
gggtgagcccggctctgctgggccccaaggtcctcctggtcccagtggtg
aagaaggaaagagaggccctaatggggaagctggatctgccggccctcca
ggacctcctgggctgagaggtagtcctggttctcgtggtcttcctggagc
tgatggcagagctggcgtcatgggccctcctggtagtcgtggtgcaagtg
gccctgctggagtccgaggacctaatggagatgctggtcgccctggggag
cctggtctcatgggacccagaggtcttcctggttcccctggaaatatcgg
ccccgctggaaaagaaggtcctgtcggcctccctggcatcgacggcaggc
ctggcccaattggcccagctggagcaagaggagagcctggcaacattgga
ttccctggacccaaaggccccactggtgatcctggcaaaaacggtgataa
aggtcatgctggtcttgctggtgctcggggtgctccaggtcctgatggaa
acaatggtgctcagggacctcctggaccacagggtgttcaaggtggaaaa
ggtgaacagggtccccctggtcctccaggcttccagggtctgcctggccc
ctcaggtcccgctggtgaagttggcaaaccaggagaaaggggtctccatg
gtgagtttggtctccctggtcctgctggtccaagaggggaacgcggtccc
ccaggtgagagtggtgctgccggtcctactggtcctattggaagccgagg
tccttctggacccccagggcctgatggaaacaagggtgaacctggtgtgg
ttggtgctgtgggcactgctggtccatctggtcctagtggactcccagga
gagaggggtgctgctggcatacctggaggcaagggagaaaagggtgaacc
tggtctcagaggtgaaattggtaaccctggcagagatggtgctcgtggtg
ctcctggtgctgtaggtgcccctggtcctgctggagccacaggtgaccgg
ggcgaagctggggctgctggtcctgctggtcctgctggtcctcggggaag
ccctggtgaacgtggtgaggtcggtcctgctggccccaatggatttgctg
gtcctgctggtgctgctggtcaacctggtgctaaaggagaaagaggagcc
aaagggcctaagggtgaaaacggtgttgttggtcccacaggccccgttgg
agctgctggcccagctggtccaaatggtcccccggtcctgctggaagtc
gtggtgatggaggccccccctggtatgactggtttccctggtgctgctgga
cggactggtcccccaggaccctctggtatttctggccctcctggtccccc
tggtcctgctgggaaagaagggcttcgtggtcctcgtggtgaccaaggtc
cagttggccgaactggagaagtaggtgcagttggtccccctggcttcgct
ggtgagaagggtccctctggagaggctggtactgctggacctcctggcac
tccaggtcctcagggtcttcttggtgctcctggtattctgggtctccctg

gctcgagaggtgaacgtggtctaccaggtgttgctggtgctgtgggtgaa
cctggtcctcttggcattgccggccctcctggggcccgtggtcctcctgg
tgctgtgggtagtcctggagtcaacggtgctcctggtgaagctggtcgtg
atggcaaccctgggaacgatggtcccccaggtcgcgatggtcaacccgga
cacaagggagagcgcggttaccctggcaatattggtcccgttggtgctgc
aggtgcacctggtcctcatggccccgtgggtcctgctggcaaacatggaa
accgtggtgaaactggtccttctggtcctgttggtcctgctggtgctgtt
ggcccaagaggtcctagtggcccacaaggcattcgtggcgataagggaga
gcccggtgaaaaggggcccagaggtcttcctggcttaaagggacacaatg
gattgcaaggtctgcctggtatcgctggtcaccatggtgatcaaggtgct
cctggctccgtgggtcctgctggtcctaggggccctgctggtccttctgg
ccctgctggaaaagatggtcgcactggacatcctggtacagttggacctg
ctggcattcgaggccctcagggtcaccaaggccctgctggcccccctggt
ccccctggccctcctggacctccaggtgtaagcggtggtggttatgactt
tggttacgatggagacttctacagggctgaccagcctcgctcagcacctt
ctctcagacccaaggactatgaagttgatgctactctgaagtctctcaac
aaccagattgagacccttcttactcctgaaggctctagaaagaacccagc
tcgcacatgccgtgacttgagactcagccacccagagtggagcagtggtt
actactggattgaccctaaccaaggatgcactatggatgctatcaaagta
tactgtgatttctctactggcgaaacctgtatccgggcccaacctgaaaa
catcccagccaagaactggtataggagctccaaggacaagaaacacgtct
ggctaggagaaactatcaatgctggcagccagtttgaatataatgtagaa
ggagtgacttccaaggaaatggctacccaacttgccttcatgcgcctgct
ggccaactatgcctctcagaacatcacctaccactgcaagaacagcattg
catacatggatgaggagactggcaacctgaaaaaggctgtcattctacag
ggctctaatgatgttgaacttgttgctgagggcaacagcaggttcactta
cactgttcttgtagatggctgctctaaaaagacaaatgaatggggaaaga
caatcattgaatacaaaacaaataagccatcacgcctgcccttccttgat
attgcacctttggacatcggtggtgctgaccaggaattctttgtggacat
tggcccagtctgtttcaaataaatgaactcaatctaaattaaaaaagaaa
gaaatttgaaaaaactttctctttgccatttcttcttcttctttttaac
tgaaagctgaatccttccatttcttctgcacatctacttgcttaaattgt
gggcaaaagagaaaaagaaggattgatcagagcattgtgcaatacagttt
cattaactccttcccccgctcccccaaaaatttgaattttttttcaaca
ctcttacacctgttatggaaaatgtcaacctttgtaagaaaaccaaaata
aaaattgaaaaataaaaaccataaacatttgcaccacttgtggcttttga
atatcttccacagagggaagtttaaaacccaaacttccaaaggtttaaac
tacctcaaaacactttcccatgagtgtgatccacattgttaggtgctgac
ctagacagagatgaactgaggtccttgttttgttttgttcataatacaaa
ggtgctaattaatagtatttcagatacttgaagaatgttgatggtgctag
aagaatttgagaagaaatactcctgtattgagttgtatcgtgtggtgtat
tttttaaaaaatttgatttagcattcatattttccatcttattcccaatt
aaaagtatgcagattatttgcccaaatcttcttcagattcagcatttgtt
ctttgccagtctcattttcatcttcttccatggttccacagaagctttgt
ttcttgggcaagcagaaaaattaaattgtacctattttgtatatgtgaga
tgtttaaataaattgtgaaaaaaatgaaataaagcatgtttggttttcca
aaagaacatat
>NM_000138 4
agtatttctctcgcgagaaaccgctgcgcggacgatacttgaagaggtgg

ggaaaggagggggctgcgggagccgcggcagagactgtgggtgccacaag
cggacaggagccacagctgggacagctgcgagcggagccgagcagtggct
gtagcggccacgactgggagcagccgccgccgcctcctcgggagtcggag
ccgccgcttctccactggcaggggccgcctgaagtgggagcagcgcctgg
agaaggcgggaggagcccggcccgggggacgggcggcgggatagcgggac
cccggcggcgcggtgcgcttcagggcgcagcggcggccgcagaccgagcc
ccgggcgcggcaagaggcggcgggagccggtggcggctcggcatcatgcg
tcgagggcgtctgctggagatcgccctgggatttaccgtgcttttagcgt
cctacacgagccatggggcggacgccaatttggaggctgggaacgtgaag
gaaaccagagccagtcgggccaagagaagaggcggtggaggacacgacgc
gcttaaaggacccaatgtctgtggatcacgttataatgcttactgttgcc
ctggatggaaaaccttacctggcggaaatcagtgtattgtccccatttgc
cggcattcctgtggggatggattttgttcgaggccaaatatgtgcacttg
cccatctggtcagatagctccttcctgtggctccagatccatacaacact
gcaatattcgctgtatgaatggaggtagctgcagtgacgatcactgtcta
tgccagaaaggatacatagggactcactgtggacaacctgtttgtgaaag
tggctgtctcaatggaggaaggtgtgtggccccaaatcgatgtgcatgca
cttacggatttactggaccccagtgtgaaagagattacaggacaggccca
tgttttactgtgatcagcaaccagatgtgccagggacaactcagcgggat
tgtctgcacaaaaacgctctgctgtgccacagtcggccgagcctggggcc
acccctgtgagatgtgtcctgcccagcctcacccctgccgccgtggcttc
attccaaatatccgcacgggagcttgtcaagatgtggatgaatgccaggc
catccccgggctctgtcagggaggaaattgcattaatactgttgggtctt
ttgagtgcaaatgccctgctggacacaaacttaatgaagtgtcacaaaaa
tgtgaagatattgatgaatgcagcaccattcctggaatctgtgaaggggg
tgaatgtacaaacacagtcagcagttacttttgcaaatgtccccctggtt
tttacacctctccagatggtaccagatgcatagatgttcgcccaggatac
tgttacacagctctgacaaacgggcgctgctctaaccagctgccacagtc
cataaccaaaatgcagtgctgctgtgatgccggccgatgctggtctccag
gggtcactgtcgcccctgagatgtgtcccatcagagcaaccgaggatttc
aacaagctgtgctctgttcctatggtaattcctgggagaccagaatatcc
tcccccaccccttggccccattcctccagttctccctgttcctcctggct
ttcctcctggacctcaaattccggtccctcgaccaccagtggaatatctg
tatccatctcgggagccaccaagggtgctgccagtaaacgttactgatta
ctgccagttggtccgctatctctgtcaaaatggacgctgcattccaactc
ctgggagttaccggtgtgagtgcaacaaagggttccagctggacctccgt
ggggagtgtattgatgttgatgaatgtgagaaaaaccccctgtgctggtgg
tgagtgtattaacaaccagggttcgtacacctgtcagtgccgagctggat
atcagagcacactcacgcggacagaatgccgagacattgatgagtgttta
cagaatggccggatctgcaataatggacgctgcatcaacacagatggcag
ttttcattgcgtgtgtaatgcgggctttcatgttacacgagatgggaaga
actgtgaagatatggatgaatgcagcataaggaacatgtgccttaatgga
atgtgtatcaatgaagatggcagttttaaatgtatttgcaaacctggatt
ccagctggcatcagatggacgttattgcaaagacattaacgagtgtgaaa
cccctgggatctgcatgaatgggcgttgcgtcaacactgatggctcctac
agatgtgaatgcttccctggactggctgtgggtctggatggccgtgtgtg
tgttgacacacacatgcggagcacatgctatggtggatacaagagaggcc
agtgtatcaaacctttgtttggtgctgtcactaaatctgaatgctgttgc
gccagcactgagtatgcatttggggaaccttgccagccgtgtcctgcaca

gaattcagcggaatatcaggcactctgcagcagtgggccaggaatgacgt
cagcaggcagtgatataaatgaatgtgcactagatcctgatatttgccca
aatggaatctgtgaaaaccttcgtgggacctataaatgtatatgcaattc
aggatatgaagtggattcaactgggaaaaactgcgttgatattaatgaat
gtgtactgaacagtctcctttgtgacaatggacaatgtagaaatactcct
ggaagttttgtctgtacctgccccaagggatttatctacaaacctgatct
aaaaacatgtgaagacattgatgaatgcgaatcaagtccttgcattaatg
gagtctgcaagaacagcccaggctcttttatttgtgaatgttcttctgaa
agtactttggatccaacaaaaaccatctgcatagaaaccatcaagggcac
ttgctggcagactgtcattgatgggcgatgtgagatcaacatcaatggag
ccaccttaaagtcccagtgctgctcctccctcggtgctgcgtggggaagc
ccgtgcaccctatgccaagttgatcccatatgtggtaaagggtactcaag
aattaaaggaacacaatgtgaagatatagatgaatgtgaagtgttcccag
gagtgtgtaaaaatggcctgtgtgttaacactagggggtcattcaagtgt
cagtgtcccagtggaatgactttggatgccacaggaaggatctgtcttga
tatccgcctggaaacctgcttcctgaggtacgaggacgaggagtgcaccc
tgcctattgctggccgccaccgcatggacgcctgctgctgctccgtcggg
gcagcctggggtactgaggaatgcgaggagtgtcccatgagaaatactcc
tgagtacgaggagctgtgtccgagaggacccggatttgccacaaaagaaa
ttacaaatggaaagcctttcttcaaagatatcaatgagtgcaagatgata
cccagcctctgcacccacggcaagtgcagaaacaccattggcagctttaa
gtgcaggtgtgacagcggctttgctcttgattctgaagaaaggaactgca
cagacattgacgaatgccgcatatctcctgacctctgtggcagaggccag
tgtgtgaacacccctggggactttgaatgcaagtgtgacgaaggctatga
aagtggattcatgatgatgaagaactgcatggatattgatgagtgtcaga
gagatcctctcctatgccgaggtggtgtttgccataacacagagggaagt
taccgctgtgaatgcccgcctggccatcagctgtcccccaacatctccgc
gtgtatcgacatcaatgaatgtgagctgagtgcacacctgtgccccaatg
gccgttgcgtgaacctcatagggaagtatcagtgtgcctgcaaccctggc
taccattcaactcccgataggctattttgtgttgacattgatgaatgcag
cataatgaatggtggttgtgaaaccttctgcacaaactctgaaggcagct
atgaatgtagctgtcagccgggatttgcactaatgcctgaccagagatca
tgcaccgacatcgatgagtgtgaagataatcccaatatctgtgatggtgg
tcagtgcacaaatatccctggagagtacaggtgcttgtgttatgatggat
tcatggcatctgaagacatgaagacttgtgtagatgtcaatgagtgtgac
ctgaatccaaatatctgcctaagtgggacctgtgaaaacacgaaaggctc
atttatctgccactgtgatatgggctactccggcaaaaaaggaaaaactg
gctgtacagacatcaatgaatgtgaaattggagcacacaactgtggcaaa
catgctgtatgtaccaatacagcaggaagcttcaaatgtagctgcagtcc
cgggtggattggagatggcattaagtgcactgatctggacgaatgttcca
atggaacccatatgtgcagccagcatgcagactgcaagaataccatggga
tcttaccgctgtctgtgcaaggaaggatacacaggtgatggcttcacttg
tacagaccttgatgagtgctctgagaacctgaatctctgtggcaatggcc
agtgcctcaatgcaccaggaggataccgctgtgaatgcgacatgggcttc
gtgcccagtgctgacgggaaagcctgtgaagatattgatgagtgctccct
tccgaacatctgtgtctttggaacttgccacaacctccctggcctgttcc
gctgtgagtgtgagataggctacgaactggacagaagcggcgggaactgc
acagatgtgaatgaatgcctggatccaaccacgtgcatcagtgggaactg
tgtcaacactccaggcagctatatctgtgactgcccacctgattttgaac

tgaacccaactcgagttggctgtgttgatacccgctctggaaattgctat
ttggatattcgacctcgaggagacaatggagatacagcctgcagcaatga
aattggagttggtgtttccaaagcttcctgctgctgttctctgggtaaag
cctggggtactccttgtgagatgtgtcctgctgtgaacacatccgagtac
aaaattctttgtcctggaggggaaggtttccgaccaaatcctatcaccgt
tatattggaagatattgatgagtgccaggagctaccagggctgtgccaag
gaggaaaatgtatcaacacctttgggagtttccagtgccgctgtccaacc
ggctactacctgaatgaagatacacgagtgtgtgatgatgtgaatgaatg
tgagactcctggaatctgtggtccagggacatgttacaacaccgttggca
actacacctgtatctgtcctccagactacatgcaagtgaatgggggaaat
aattgcatggatatgagaagaagtttgtgctacagaaactactatgctga
caaccagacctgtgatggagaattgttattcaacatgaccaagaagatgt
gctgctgttcctacaacattggccgggcgtggaacaagccctgtgaacag
tgtcccatcccaagtacagatgagtttgctacactctgtggaagtcaaag
gccaggctttgtcatcgacatttataccggtttacccgttgatattgatg
agtgccgggagatcccaggggtctgtgaaaatggagtgtgtatcaacatg
gttggcagcttccgatgtgaatgtccagtgggattcttctataatgacaa
gttgttggtttgtgaagatattgacgagtgtcagaacggcccagtgtgcc
agcgcaacgccgaatgcatcaacactgcaggcagctaccgctgtgactgt
aagcccggctaccgcttcacctccacaggacagtgcaatgatcgtaatga
atgtcaagaaatccccaatatatgcagtcatgggcagtgcattgacacag
ttggaagcttttattgcctttgccacactggttttaaaacaaatgatgac
caaaccatgtgcttggacataaatgaatgtgaaagagatgcctgtgggaa
tggaacttgccggaacacaattggttccttcaactgccgctgcaatcatg
gtttcatcctttctcacaacaatgactgtatagatgttgatgaatgtgca
agtggaaatgggaatctttgcagaaatggccaatgcattaatacagtggg
gtctttccagtgccagtgcaatgaaggctatgaggtggctccagatggga
ggacctgtgtggatatcaatgaatgtcttctagaacccagaaaatgtgca
ccaggtacctgtcaaaacttggatgggtcctacagatgcatttgcccacc
tggatacagtcttcaaaatgagaagtgtgaagatattgatgagtgtgtcg
aagagccagaaatttgtgccctgggcacatgcagtaacactgaaggcagc
ttcaaatgtctgtgtccagaagggttttccttgtcctccagtggaagaag
gtgccaagatttgcgaatgagctactgttatgcgaagtttgaaggaggaa
agtgttcatcacccaaatccagaaatcactccaagcaggaatgctgctgt
gccttgaagggagaaggctggggagacccctgcgagctctgccccacgga
acctgatgaggccttccgccagatatgtccttatggaagtgggatcatcg
tgggacctgatgattcagcagttgatatggacgaatgcaaagaacccgat
gtctgtaaacatggacagtgcatcaatacagatggttcctatcgctgcga
gtgtccctttggttatattctagcagggaatgaatgtgtagatactgatg
aatgttctgttggcaatccttgtggaaatggaacctgcaagaatgtgatt
ggaggttttgaatgcacctgcgaggagggatttgagcccggtccaatgat
gacatgtgaagatataaatgaatgtgcccagaatcctctgctctgtgcct
tccgatgtgtgaacacttatgggtcatatgaatgcaaatgtcccgtggga
tatgtgctcagagaagaccgtaggatgtgcaaagatgaggatgagtgtga
agagggaaaacatgactgtactgaaaaacaaatggaatgcaagaacctca
ttggcacatatatgtgcatctgtggacccgggtatcagcggagacctgat
ggagaaggctgtgtagatgagaatgaatgtcagacgaagccagggatctg
tgagaatgggcgctgcctcaacacccgtgggagctacacctgtgagtgta
atgatgggtttaccgccagccccaaccaggacgagtgccttgacaatcgg

gaagggtactgcttcacagaggtgctacaaaacatgtgtcagatcggctc
cagcaacaggaaccccgtcaccaaatcggaatgctgctgtgacggaggga
gaggctggggtccccactgtgagatctgccctttccaggggactgtggct
ttcaagaaactctgtccccatggccgaggattcatgaccaatggagcaga
tatcgatgaatgcaaggttattcacgatgtttgccgaaatggggaatgtg
tcaatgacagaggatcatatcattgcatttgtaaaactgggtacactcca
gatataactgggacttcctgtgtagatctgaacgagtgcaaccaggctcc
caaaccctgcaattttatctgcaaaaacacagaagggagttaccagtgtt
catgcccgaaaggctacattctgcaagaggatggaaggagctgcaaagat
cttgatgagtgtgcaaccaagcaacacaactgccagttcctatgtgttaa
caccattggcggcttcacatgcaaatgtcctcccggatttacccaacacc
atacgtcctgcattgataacaatgaatgcacctctgacatcaatctgtgc
gggtctaagggcatttgccagaacactcctggaagcttcacctgtgaatg
ccagcggggattctcacttgatcagaccggctccagctgtgaagacgtgg
acgagtgtgagggtaaccaccgctgccagcatggctgccagaacatcatt
gggggctacaggtgcagctgcccccagggctacctccagcactaccagtg
gaaccagtgtgttgatgaaaacgaatgcctcagcgctcacatctgcggag
gagcctcctgtcacaacaccctggggagctacaagtgcatgtgtcccgcc
ggcttccagtatgaacagttcagtggaggatgccaagacatcaatgaatg
tggctctgcgcaggccccctgcagctatggctgttccaataccgagggcg
gttacctgtgtggctgtccacctggttacttccgcataggccaagggcac
tgtgtttctggaatgggcatgggccgaggaaacccagagccacctgtcag
tggtgaaatggatgacaattcactctccccagaggcttgttacgagtgta
agatcaatggctaccccaaacggggcaggaaacggagaagcacaaacgaa
actgatgcctccaatatcgaggatcagtctgagacagaagccaatgtgag
tcttgcaagttgggatgttgagaagacagccatctttgctttcaatattt
cccacgtcagtaacaaggttcgaatcctagaactccttccagctcttaca
actctgacgaatcacaacagatacttgatcgaatctggaaatgaagatgg
cttctttaaaatcaaccaaaaggaagggatcagctacctccacttcacaa
agaagaagccagtggctggaacctattcattacaaatcagtagtactcca
ctttataaaaagaaagaacttaaccaactagaagacaaatatgacaaaga
ctacctcagtggtgaactgggtgataatctgaagatgaaaatccaggttt
tgcttcattaattcaccatccagagaccaaataattaaaagaaaaacaaa
tatagataggtagaactatattttcccccaatcagaatcatcatatcata
ggtacaatctttcaccaagtaaatttgtataaataagcactattctttgt
attaccaaagcaaggtacaggtgactaccctagttcaaaacaaccacttt
ctcaggcttctcatgtgtgtagctaagctaccttgtcatatgtgttgatt
cttgaaaactgggacgtgtatttccattgggggttggccatttatgctga
catgccatccttccagcaaacgtacgggaatgtgctttcaattgatggac
tactctattttttgcaaatttgtaaactttgcttctccaaatacaagtac
taggttgtccatttatggtacctatttggtgctagtaaattttcaaacta
gatttataaatgcactgtaatatgtacacaacttagaaaccaaattacaa
gtattcagttccaatacttcattaatttcaatcaaccaaagttagttcag
tagcttatctcagttatgagtataatacattacatgtaaattaagtgtgt
gtatactgtaatcgtgctattttttatcattgaaacatttataaactaga
ataataatgcccttaatgtgagggtttgtaatggtgcttattaagaccaa
agacttgttaaatgtatacaccaagtggtaatgaaatttcggtgactggc
ccacacgtgcatagaggtctgggaggaccaggaaacagcctcagtggcca
gaggatcaccagtgcatccttcatcacagcatgtgcaatatgccaagatt

accctcggtcattcctgtcaacaaggggtcaatgtcataaatgtcacaat
aaaacaatctcttcttttttttagtttaccccttggctttgtgttcttgc
atggatttggggttggaggggccattccggaggctaaataaagtctcctg
gatttaaattatcctgggtctcttacttatggcttatgaaagtaccaaat
gtataaccactagaagaaaatttaacatatgagtcgatcccttgttttat
ccattgaaagtagcagagtctggtgtcattaacctgacttgcttgtgaga
aatttagattgtagagtcatttctgaaacatgacctaattcatcttgtga
cttttaaatagtcttaaataccaagttcagtcattgtcttagagcacatg
aatttcattataatagatttatcatgccccccctctcaaatatacacagtt
ttggcaagccttaggtgttctgttccattttttttttcccctaaacatctt
tcgttagtcaatgctcatctaattacaaagggataatcccagactgtatc
caattgctgtaacttttggtttcttaatgtcataatttttaaagtctgtt
ttattttaagtgcaatattgagtatttagctgttaggctcaatccgtcga
tatgaaataatttttaaatccctaagggcaggaaagcatttcgtggtag
tgaaaataagaggaaataagatggcatgaaggtggtgggcggagaaacta
ggtaggacacaggaaagtgctctcaaaaatctttgaagagctcagctgaa
aaaaatggagtagatttggctcatactattccggaaggcaaaaccagggt
cagctgatgtcagccccagtttaatacacacggtcccaattatagagcta
ctcactgaaagaatgggtttccttgcattgtggtgagctccctgtcacaa
gatagaagagtttcagtctaggcttaatggcaaccattggacaaagatgc
tttcttccacctaacaggccattaacatcttaaaggtatttttgtatctc
taattttgtttataataggtgctcaacagaatgagctgaatggctgttac
aaagggggtttgtaccttgggtaagagattaaaatataactcaaaatttc
cttctaacgctgcacctatggaaccatgtgatagaggtgtattaaaattg
ttatcgaagaatatatagcatatggtaaacaacagtttgcatatggaaaa
tgtctttgataatttaaccagaactgcattatattcaataacggattttc
tttataacaaacaacaggggaaaatggagttggcacacagtggatcactt
tgatatttttaatagtccaagtctggattttatttattcctgagccaaca
attttgaacagcatattttccatgtttctgactgtaacaaaacattttcc
tcattgttccattgtaaatattcctcttgttggaactctttttaatcctg
agatttaaacctgtacctttcaattgtctgtgacctttcaatttcacttt
caatagttgaagaacttggctttgtaaatctctcagaagcttgaaaatat
cttgtctctacccccctcagcccatttcatttgccaataattattttgtaa
gtagggttgaaatgaactcagctggccttgtgaaatgtttaaacttgcac
aaacaactacatttttgttcaacaaatagcagtttactcagccaaaatca
ctttggatattgccattacaaatactgttaaacttcagaaatcatgtctg
taaattagatgagccaaaataaaggacaattgggttgatgctgca
>NM_000088 3
tcgtcggagcagacgggagtttctcctcggggtcggagcaggaggcacgc
ggagtgtgaggccacgcatgagcggacgctaacccccctccccagccacaa
agagtctacatgtctagggtctagacatgttcagctttgtggacctccgg
ctcctgctcctcttagcggccaccgccctcctgacgcacggccaagagga
aggccaagtcgagggccaagacgaagacatcccaccaatcacctgcgtac
agaacggcctcaggtaccatgaccgagacgtgtggaaacccgagccctgc
cggatctgcgtctgcgacaacggcaaggtgttgtgcgatgacgtgatctg
tgacgagaccaagaactgccccggcgccgaagtccccgagggcgagtgct
gtcccgtctgccccgacggctcagagtcacccaccgaccaagaaaccacc
ggcgtcgagggacccaagggagacactggcccccgaggcccaaggggacc
cgcaggcccccctggccgagatggcatccctggacagcctggacttcccg

gacccccggacccccggacctcccggacccctggcctcggaggaaac
tttgctccccagctgtcttatggctatgatgagaaatcaaccggaggaat
ttccgtgcctggccccatgggtccctctggtcctcgtggtctccctggcc
cccctggtgcacctggtccccaaggcttccaaggtccccctggtgagcct
ggcgagcctggagcttcaggtcccatgggtccccgaggtcccccaggtcc
ccctggaaagaatggagatgatggggaagctggaaaacctggtcgtcctg
gtgagcgtgggcctcctgggcctcagggtgctcgaggattgcccggaaca
gctggcctccctggaatgaagggacacagaggtttcagtggtttggatgg
tgccaagggagatgctggtcctgctggtcctaagggtgagcctggcagcc
ctggtgaaaatggagctcctggtcagatgggcccccgtggcctgcctggt
gagagaggtcgccctggagcccctggccctgctggtgctcgtggaaatga
tggtgctactggtgctgccgggccccctggtcccaccggccccgctggtc
ctcctggcttccctggtgctgttggtgctaagggtgaagctggtccccaa
gggccccgaggctctgaaggtccccagggtgtgcgtggtgagcctggccc
ccctggccctgctggtgctgctggccctgctggaaaccctggtgctgatg
gacagcctggtgctaaaggtgccaatggtgctcctggtattgctggtgct
cctggcttccctggtgcccgaggcccctctggaccccagggccccggcgg
ccctcctggtcccaagggtaacagcggtgaacctggtgctcctggcagca
aaggagacactggtgctaagggagagcctggccctgttggtgttcaagga
ccccctggccctgctggagaggaaggaaagcgaggagctcgaggtgaacc
cggacccactggcctgcccggacccccctggcgagcgtggtggacctggta
gccgtggtttccctggcgcagatggtgttgctggtcccaagggtcccgct
ggtgaacgtggttctcctggccctgctggccccaaaggatctcctggtga
agctggtcgtcccggtgaagctggtctgcctggtgccaagggtctgactg
gaagccctggcagccctggtcctgatggcaaaactggccccccctggtccc
gccggtcaagatggtcgccccggacccccaggcccacctggtgcccgtgg
tcaggctggtgtgatgggattccctggacctaaaggtgctgctggagagc
ccggcaaggctggagagcgaggtgttcccggacccccctggcgctgtcggt
cctgctggcaaagatggagaggctggagctcagggacccccctggccctgc
tggtcccgctggcgagagaggtgaacaaggccctgctggctcccccggat
tccagggtctccctggtcctgctggtcctccaggtgaagcaggcaaacct
ggtgaacagggtgttcctggagaccttggcgcccctggcccctctggagc
aagaggcgagagaggtttccctggcgagcgtggtgtgcaaggtccccctg
gtcctgctggtccccgaggggccaacggtgctcccggcaacgatggtgct
aagggtgatgctggtgcccctggagctcccggtagccagggcgcccctgg
ccttcagggaatgcctggtgaacgtggtgcagctggtcttccagggccta
agggtgacagaggtgatgctggtcccaaaggtgctgatggctctcctggc
aaagatggcgtccgtggtctgactggccccattggtcctcctggccctgc
tggtgcccctggtgacaagggtgaaagtggtcccagcggccctgctggtc
ccactggagctcgtggtgcccccggagaccgtggtgagcctggtcccccc
ggccctgctggctttgctggcccccctggtgctgacggccaacctggtgc
taaaggcgaacctggtgatgctggtgctaaaggcgatgctggtccccctg
gccctgccggacccgctggacccccctggccccattggtaatgttggtgct
cctggagccaaaggtgctcgcggcagcgctggtccccctggtgctactgg
tttccctggtgctgctggccgagtcggtcctcctggcccctctggaaatg
ctggacccccctggccctcctggtcctgctggcaaagaaggcggcaaaggt
ccccgtggtgagactggccctgctggacgtcctggtgaagttggtccccc
tggtccccctggccctgctggcgagaaaggatcccctggtgctgatggtc
ctgctggtgctcctggtactcccgggcctcaaggtattgctggacagcgt

ggtgtggtcggcctgcctggtcagagaggagagagaggcttccctggtct
tcctggcccctctggtgaacctggcaaacaaggtccctctggagcaagtg
gtgaacgtggtccccctggtcccatgggccccccctggattggctggaccc
cctggtgaatctggacgtgaggggggctcctggtgccgaaggttcccctgg
acgagacggttctcctggcgccaagggtgaccgtggtgagaccggccccg
ctggaccccctggtgctcctggtgctcctggtgcccctggccccgttggc
cctgctggcaagagtggtgatcgtggtgagactggtcctgctggtcccgc
cggtcctgtcggccctgttggcgcccgtggccccgccggaccccaaggcc
cccgtggtgacaagggtgagacaggcgaacagggcgacagaggcataaag
ggtcaccgtggcttctctggcctccagggtccccctggccctcctggctc
tcctggtgaacaaggtccctctggagcctctggtcctgctggtccccgag
gtccccctggctctgctggtgctcctggcaaagatggactcaacggtctc
cctggccccattgggccccctggtcctcgcggtcgcactggtgatgctgg
tcctgttggtcccccccggccctcctggacctcctggtccccctggtcctc
ccagcgctggtttcgacttcagcttcctgccccagccacctcaagagaag
gctcacgatggtggccgctactaccgggctgatgatgccaatgtggttcg
tgaccgtgacctcgaggtggacaccaccctcaagagcctgagccagcaga
tcgagaacatccggagcccagagggcagccgcaagaaccccgcccgcacc
tgccgtgacctcaagatgtgccactctgactggaagagtggagagtactg
gattgaccccaaccaaggctgcaacctggatgccatcaaagtcttctgca
acatggagactggtgagacctgcgtgtaccccactcagcccagtgtggcc
cagaagaactggtacatcagcaagaaccccaaggacaagaggcatgtctg
gttcggcgagagcatgaccgatggattccagttcgagtatggcggccagg
gctccgaccctgccgatgtggccatccagctgaccttcctgcgcctgatg
tccaccgaggcctcccagaacatcacctaccactgcaagaacagcgtggc
ctacatggaccagcagactggcaacctcaagaaggccctgctcctccagg
gctccaacgagatcgagatccgcgccgagggcaacagccgcttcacctac
agcgtcactgtcgatggctgcacgagtcacaccggagcctggggcaagac
agtgattgaatacaaaaccaccaagacctcccgcctgcccatcatcgatg
tggccccccttggacgttggtgccccagaccaggaattcggcttcgacgtt
ggccctgtctgcttcctgtaaactccctccatcccaacctggctccctcc
cacccaaccaactttccccccaacccggaaacagacaagcaacccaaact
gaaccccctcaaaagccaaaaaatgggagacaatttcacatggactttgg
aaaatattttttcctttgcattcatctctcaaacttagtttttatcttt
gaccaaccgaacatgaccaaaaaccaaaagtgcattcaaccttaccaaaa
aaaaaaaaaaaaaaaagaataaataaataactttttaaaaaaggaagcttg
gtccacttgcttgaagacccatgcgggggtaagtccctttctgcccgttg
ggcttatgaaaccccaatgctgccctttctgctcctttctccacaccccc
cttggggcctcccctccactccttcccaaatctgtctccccagaagacac
aggaaacaatgtattgtctgcccagcaatcaaaggcaatgctcaaacacc
caagtggcccccaccctcagcccgctcctgcccgcccagcacccccaggc
cctgggggacctggggttctcagactgccaaagaagccttgccatctggc
gctcccatggctcttgcaacatctccccttcgtttttgagggggtcatgc
cgggggagccaccagcccctcactgggttcggaggagagtcaggaagggc
cacgacaaagcagaaacatcggatttggggaacgcgtgtcaatcccttgt
gccgcagggctgggcgggagagactgttctgttccttgtgtaactgtgtt
gctgaaagactacctcgttcttgtcttgatgtgtcaccggggcaactgcc
tgggggcggggatgggggcagggtggaagcggctccccattttataccaa
aggtgctacatctatgtgatgggtggggtggggagggaatcactggtgct

atagaaattgagatgccccccaggccagcaaatgttccttttgttcaa
agtctatttttattccttgatatttttcttttttttttttttttttgtg
gatggggacttgtgaatttttctaaaggtgctatttaacatgggaggaga
gcgtgtgcggctccagcccagcccgctgctcactttccaccctctctcca
cctgcctctggcttctcaggcctctgctctccgacctctctcctctgaaa
ccctcctccacagctgcagcccatcctcccggctccctcctagtctgtcc
tgcgtcctctgtccccgggtttcagagacaacttcccaaagcacaaagca
gtttttccccctaggggtgggaggaagcaaaagactctgtacctattttg
tatgtgtataataatttgagatgtttttaattattttgattgctggaata
aagcatgtggaaatgacccaaacataa
>NM_001949 4
gccatttttcagctgccggccgcagcacccgggctgccgccgccgcctcg
caatccgttgcatcggccgcccccgacgcctccatccccgcttggggccc
gatatccgtgcggccgggaccctcctctctccagagccccgattattttt
ggcccccggggcctgtgcggtgcggaaaaataaaaagaaaagagagagag
ggggctcggaagcgccgggcggggaggagagaaggaggagagacttggaa
actccgactgcaaataataaagaaattgaaaacaatacattaatatacca
taacactaaaaagagcaggagcgagagatgagaaagggaatccagcccgc
tctggagcagtacctggtgaccgccgggggtggggagggggcggctgtcg
tcgccgccgccgctgcagcctccatggacaaaagggcactgctagccagc
cccggcttcgccgccgccgccgccgctgccgccgccccgggcgcgtacat
ccagatcctcaccacgaacacttccaccacctcctgttcctcctccctcc
aaagcggcgccgtagccgccggcccccctcctccccagtgcccccggcgcg
gagcagaccgccggcagcctcctctacaccacgccgcacggaccctccag
cagagccgggctgctgcagcagccaccagcgctgggacgcggcggcagcg
gcggcggcggcggccctccggcaaagcgaaggctggagctaggagaaagc
ggtcatcagtacctctcagatggtttaaaaacccccaagggcaaaggaag
agctgcactacgaagtccagatagtccaaaaactccaaaatctccctcag
aaaaaacgcggtatgatacgtctcttggtctgctcaccaagaagttcatt
cagctcctgagccagtcacccgatggggtattggatttgaacaaggcagc
agaagtgctaaaagtgcaaaagagaaggatttatgatatcaccaacgttc
tggaaggcatccacctcattaagaagaagtctaaaaacaacgtccaatgg
atgggctgcagtctgtctgaggatgggggcatgctggcccagtgtcaagg
cctgtcaaaagaagtgaccgagctcagtcaggaagagaagaaattagatg
aactgatccaaagctgcaccctggacctcaaactgttaaccgaggattca
gagaatcaaaggttagcttatgttacatatcaagatattcgaaaaattag
tggccttaaagaccaaactgttatagttgtgaaagcccctccagaaacaa
gacttgaagtgcctgactcaatagagagcctacaaatacatttggcaagt
acccaagggcccattgaggtttacttatgtccagaagagactgaaacaca
cagtccaatgaaaacaaacaaccaagaccacaatgggaatatccctaaac
ccgcttccaaagacttggcttcaaccaactcaggacatagcgattgctca
gtttctatgggaaacctttctcctctggcctccccagccaacctcttaca
gcagactgaggaccaaattccttccaacctagaaggaccgtttgtgaact
tactgcctcccctgctgcaagaggactatctcctgagcctcggggaggag
gaaggcatcagcgatctcttcgatgcttacgatttggaaaagctcccact
ggtggaagacttcatgtgtagttgattatgcttcgtgtgaactctcctta
aaaaccgatattttttatcatggaaccagaacatctgtcatgcagtgtt
gtcccttcctaccttcttcctccaagagagtatcatgaagtaaactacaa
acttcagaagaaagctgacattttaatgaatttttaaaaaattaataaa

caaattgtctaaacgcacagttgcaggctcccttgggaaagccctgcttt
gctccaggctccaagatctcctggctaagtcagcaagtgagaaaatgtgc
aatcaggtgtctctcaccccgaattgtccttcctccttcctccccggatt
ggcttgctgtgcctgacggatgggctgtagaatggggtctggccacctgg
cctgctgggaaacagcaatcttccttaatagcatttcaagccgtgccttc
tccgcagaatgcatgtctttgaggtctgctaatatggaatggaactgcag
caaatgcaaacttgaagtcatgcaaaagtatgaaatggatttcttcagct
cttcttaggaatatttaaattactgtcataattcagtttaagctatgaac
tgtgtgtcccagtaggaggtcaagaaaacctccacagccttctggatgaa
gaacctgttttcaaatatacttgttgcagatacagaagactagtagagtt
ctgccactctaagctgttgtggattttcctgtctccatgaaccactccca
ttcccccgtccccaatgtgtttgtgagtttccagttgatttgtagcaaat
gcctacttagttctttgtggattgttctagacttttaattttttttagctg
ccatttaagcattcctgtggcacccatcaccatttcaatttaattgttta
ctttgaagcggtttttgcaaattcatattacttaagcagagggagagaac
ctctactgatcagagcatctaaacctgtgtgatctaaggtttatcagcct
ctgcaaggagctttgtcccatcgtgcttccattcccaggagggggagctt
ggagcgagtcagtcctggggcttgctgacatgggtggcccattggaaagg
agaaccaggtcagatgatgtaacagccccaaggagcagcaggcatgggtc
cctccatccttgggcttcccgggcccctgtgacaggggaaagggctctct
tacaccgcactcagggagaccacttctcaggatggggtcagatggagaga
cctctagggagaaagacatccccattgtgtgagtggcatttccttaagct
ggcaggaacagggagcagccctgtgtcgggggctggaatagttctggcca
gaccccgttccccttcctctatgaaggaataagttggaccaagggaagtc
ggggacgtaaaaaatgaagcaaaacaatgccagggtgtctcccgctttac
tcttcaggaatggtgtcccaagttggaggctttgtgtcagctgcaaatcc
taccagttatgtccaagaatggctttccctcgggcaggtggcagcggcca
tctcccactgggaatatggcgtagtatctccggtccattccttggatgct
aaggactgcgggaatgagggagtcagataaagaacaaacctcgaaacgaa
cagttaaattgaaatgctatgtgcctgacccaatggtaggcacatagtag
gcactcaactcatatgtttaattgaattgaaaatatcccttaggaaaaaa
aaaaaacacacaaaaaaccacaagagccccagccagtttactccaggtag
atttccacaatatgcaaagtggtggtggggtcaagacagatgacaccagc
actttaaactctttgtgtgggtatgcgtgggtgtatgtttgggaagaaaa
acaaaggtgcagactatcttcctttttttcttcttcagcctccatccctg
gcctcctcccctcacacacactggacttggtacaaaatgtcggtgtggtc
ctagatgaagcattggggtgggggagggagagggagctttgtgttaagtg
cctactggaaatgcactgtggggttttttcctgtatgggaaaccatttat
gccaagcttttccccatttcccatatttatctcatctggttagctgcctc
tgcttccagctttgtgtaattctctttgccagctgcacaaagctgatttt
ttccaaagtctaaagactgagctcacctggctagattgttgtgtgttttg
ttgaatttttttcataatgtaatgccgtatttattgtttttaaaatgaaag
gaatactaataagtcttaaaagttccttcatgcataagattttttttccag
ttactgggtttaactggtgtacattaattagatgtccatactgtattttg
tttgcattaagtaattttcttttttgacttagtatccggcacacaaagtgg
gttagtactacagtatttgcgttactttaagtactaagtatgcaggtttc
ctggtaccattgagttgctgctattaaagctcacacacgaaatggctaaa
agttacaagtgtgcaaattatgactgcgtgagccttagaaaataaaatgt
ataaagggcaacacatgagctgtcaaacagtgttaggagtgtgtttatat

gtacagagttgtgcatagcaatcgttttatttaagttgatatgtagtcta
ctcacattttcattatttagcaattttgtacaaaaatagcaattaatttg
taaacactgccagaatactttctagctgctttgtaatttttaagagtgt
tattttgtttttgtttttctgttctttgttgtggctcttgttttcatttt
tgttgtacgtgtagatctgtaaataaaattgcagtatttaaagcttaagc
tttcaggaaaaagaaaataagaattcagtgtgtgcatgacaactcgtgtg
tatgagaaggagggatatgaaggaagatggcttgcagagtaagtcgggtg
gcaattgtcagggtgtgggaatttcttttcctacggggtacgtgattttg
taaaaaggaagtatttctcccaaaattgggagtaggcaaactactaatca
gtttagctttgtgttgtatgctagtttaaaaaagaaaatatgtaatataa
tgtaaaaaaaaacaaaaaaaagcttttatgatggattttgtaaatagatt
tgttacagggtgacctgttctctagctgtgatcttaccacttcaaatggg
tgtaatttgaataaattttgtatggtaaaggatcaataaaatgatttttt
ttaagagttaaaaaaaaaaaaaaaaa
>NM_005618 3
cgtgggatttccagaccgcggctttctaatcggctcgggaggaagctctg
cagctctcttgggaattaagctcaatctctggactctctctctttctctt
tctccccctccctctcctgcgaagaagctcaagacaaaaccaggaagccg
gcgaccctcacctcctcgggggctgggaggaaggaggaaaacgaaagtcg
ccgccgccgcgctgtcccccgagagctgcctttcctcgggcatccctggg
gctgccgcgggacctcgcagggcggatataaagaaccgcggccttgggaa
gaggcggagaccggcttttaaagaaagaagtcctgggtcctgcggtctgg
ggcgaggcaagggcgcttttctgcccacgctccccgtggcccatcgatcc
cccgcgcgtccgccgctgttctaaggagagaagtgggggccccccaggct
cgcgcgtggagcgaagcagcatgggcagtcggtgcgcgctggccctggcg
gtgctctcggccttgctgtgtcaggtctggagctctggggtgttcgaact
gaagctgcaggagttcgtcaacaagaaggggctgctggggaaccgcaact
gctgccgcgggggcgcggggccaccgccgtgcgcctgccggaccttcttc
cgcgtgtgcctcaagcactaccaggccagcgtgtcccccgagccgccctg
cacctacggcagcgccgtcacccccgtgctgggcgtcgactccttcagtc
tgcccgacggcgggggcgccgactccgcgttcagcaaccccatccgcttc
cccttcggcttcacctggccgggcaccttctctctgattattgaagctct
ccacacagattctcctgatgacctcgcaacagaaaacccagaaagactca
tcagccgcctggccacccagaggcacctgacggtgggcgaggagtggtcc
caggacctgcacagcagcggccgcacggacctcaagtactcctaccgctt
cgtgtgtgacgaacactactacggagagggctgctccgttttctgccgtc
cccgggacgatgccttcggccacttcacctgtggggagcgtggggagaaa
gtgtgcaaccctggctggaaagggccctactgcacagagccgatctgcct
gcctggatgtgatgagcagcatggattttgtgacaaaccaggggaatgca
agtgcagagtgggctggcagggccggtactgtgacgagtgtatccgctat
ccaggctgtctccatggcacctgccagcagccctggcagtgcaactgcca
ggaaggctgggggggccttttctgcaaccaggacctgaactactgcacac
accataagccctgcaagaatggagccacctgcaccaacacgggccagggg
agctacacttgctcttgccggcctgggtacacaggtgccacctgcgagct
ggggattgacgagtgtgaccccagcccttgtaagaacggagggagctgca
cggatctcgagaacagctactcctgtacctgcccacccggcttctacggc
aaaatctgtgaattgagtgccatgacctgtgcggacggcccttgctttaa
cgggggtcggtgctcagacagccccgatggagggtacagctgccgctgcc
ccgtgggctactccggcttcaactgtgagaagaaaattgactactgcagc

tcttcaccctgttctaatggtgccaagtgtgtggacctcggtgatgccta
cctgtgccgctgccaggccggcttctcggggaggcactgtgacgacaacg
tggacgactgcgcctcctccccgtgcgccaacgggggcacctgccgggat
ggcgtgaacgacttctcctgcacctgcccgcctggctacacgggcaggaa
ctgcagtgcccccgtcagcaggtgcgagcacgcaccctgccacaatgggg
ccacctgccacgagaggggccaccgctatgtgtgcgagtgtgcccgaggc
tacgggggtcccaactgccagttcctgctccccgagctgcccccgggccc
agcggtggtggacctcactgagaagctagagggccagggcgggccattcc
cctgggtggccgtgtgcgccggggtcatccttgtcctcatgctgctgctg
ggctgtgccgctgtggtggtctgcgtccggctgaggctgcagaagcaccg
gcccccagccgacccctgccgggggggagacggagaccatgaacaacctgg
ccaactgccagcgtgagaaggacatctcagtcagcatcatcggggccacg
cagatcaagaacaccaacaagaaggcggacttccacggggaccacagcgc
cgacaagaatggcttcaaggcccgctacccagcggtggactataacctcg
tgcaggacctcaagggtgacgacaccgccgtcagggacgcgcacagcaag
cgtgacaccaagtgccagccccagggctcctcaggggaggagaaggggac
cccgaccacactcaggggtggagaagcatctgaaagaaaaaggccggact
cgggctgttcaacttcaaaagacaccaagtaccagtcggtgtacgtcata
tccgaggagaaggatgagtgcgtcatagcaactgaggtgtaaaatggaag
tgagatggcaagactcccgtttctcttaaaataagtaaaattccaaggat
atatgccccaacgaatgctgctgaagaggagggaggcctcgtggactgct
gctgagaaaccgagttcagaccgagcaggttctcctcctgaggtcctcga
cgcctgccgacagcctgtcgcggcccggccgcctgcggcactgccttccg
tgacgtcgccgttgcactatggacagttgctcttaagagaatatatattt
aaatgggtgaactgaattacgcataagaagcatgcactgcctgagtgtat
attttggattcttatgagccagtcttttcttgaattagaaacacaaacac
tgcctttattgtcctttttgatacgaagatgtgctttttctagatggaaa
agatgtgtgttatttttggatttgtaaaaatatttttcatgatatctgt
aaagcttgagtattttgtgatgttcgtttttataatttaaattttggta
aatatgtacaaaggcacttcgggtctatgtgactatatttttttgtatat
aaatgtatttatggaatattgtgcaaatgttatttgagtttttttactgtt
ttgttaatgaagaaattccttttaaaatatttttccaaaataaatttta
tgaatgacaaaaaaaaaaaaaaaaaaaaaaaaaaaaaaaaaaaaaaaaa
aaaaaaaaaaaaaaa
>NM_017617 3
atgccgccgctcctggcgcccctgctctgcctggcgctgctgcccgcgct
cgccgcacgaggcccgcgatgctcccagcccggtgagacctgcctgaatg
gcgggaagtgtgaagcggccaatggcacggaggcctgcgtctgtggcggg
gccttcgtgggcccgcgatgccaggaccccaacccgtgcctcagcacccc
ctgcaagaacgccgggacatgccacgtggtggaccgcagaggcgtggcag
actatgcctgcagctgtgccctgggcttctctgggcccctctgcctgaca
cccctggacaatgcctgcctcaccaacccctgccgcaacgggggcacctg
cgacctgctcacgctgacggagtacaagtgccgctgcccgcccggctggt
cagggaaatcgtgccagcaggctgacccgtgcgcctccaacccctgcgcc
aacggtggccagtgcctgcccttcgaggcctcctacatctgccactgccc
acccagcttccatggccccacctgccggcaggatgtcaacgagtgtggcc
agaagcccgggctttgccgccacggaggcacctgccacaacgaggtcggc
tcctaccgctgcgtctgccgcgccacccacactggccccaactgcgagcg
gccctacgtgccctgcagcccctcgccctgccagaacgggggcacctgcc

gccccacgggcgacgtcacccacgagtgtgcctgcctgccaggcttcacc
ggccagaactgtgaggaaaatatcgacgattgtccaggaaacaactgcaa
gaacgggggtgcctgtgtggacggcgtgaacacctacaactgccgctgcc
cgccagagtggacaggtcagtactgtaccgaggatgtggacgagtgccag
ctgatgccaaatgcctgccagaacggcgggacctgccacaacacccacgg
tggctacaactgcgtgtgtgtcaacggctggactggtgaggactgcagcg
agaacattgatgactgtgccagcgccgcctgcttccacggcgccacctgc
catgaccgtgtggcctccttctactgcgagtgtccccatggccgcacagg
tctgctgtgccacctcaacgacgcatgcatcagcaacccctgtaacgagg
gctccaactgcgacaccaaccctgtcaatggcaaggccatctgcacctgc
ccctcggggtacacgggcccggcctgcagccaggacgtggatgagtgctc
gctgggtgccaacccctgcgagcatgcgggcaagtgcatcaacacgctgg
gctccttcgagtgccagtgtctgcagggctacacgggcccccgatgcgag
atcgacgtcaacgagtgcgtctcgaacccgtgccagaacgacgccacctg
cctggaccagattggggagttccagtgcatctgcatgcccggctacgagg
gtgtgcactgcgaggtcaacacagacgagtgtgccagcagcccctgcctg
cacaatggccgctgcctggacaagatcaatgagttccagtgcgagtgccc
cacgggcttcactgggcatctgtgccagtacgatgtggacgagtgtgcca
gcacccccctgcaagaatggtgccaagtgcctggacggacccaacacttac
acctgtgtgtgcacggaagggtacacggggacgcactgcgaggtggacat
cgatgagtgcgaccccgacccctgccactacggctcctgcaaggacggcg
tcgccaccttcacctgcctctgccgcccaggctacacgggccaccactgc
gagaccaacatcaacgagtgctccagccagccctgccgccacgggggcac
ctgccaggaccgcgacaacgcctacctctgcttctgcctgaaggggacca
caggacccaactgcgagatcaacctggatgactgtgccagcagcccctgc
gactcgggcacctgtctggacaagatcgatggctacgagtgtgcctgtga
gccgggctacacagggagcatgtgtaacatcaacatcgatgagtgtgcgg
gcaacccctgccacaacgggggcacctgcgaggacggcatcaatggcttc
acctgccgctgccccgagggctaccacgaccccacctgcctgtctgaggt
caatgagtgcaacagcaacccctgcgtccacggggcctgccgggacagcc
tcaacgggtacaagtgcgactgtgaccctgggtggagtgggaccaactgt
gacatcaacaacaatgagtgtgaatccaacccttgtgtcaacggcggcac
ctgcaaagacatgaccagtggctacgtgtgcacctgccgggagggcttca
gcggtcccaactgccagaccaacatcaacgagtgtgcgtccaacccatgt
ctgaaccagggcacgtgtattgacgacgttgccgggtacaagtgcaactg
cctgctgccctacacaggtgccacgtgtgaggtggtgctggccccgtgtg
cccccagcccctgcagaaacggcggggagtgcaggcaatccgaggactat
gagagcttctcctgtgtctgccccacgggctggcaagggcagacctgtga
ggtcgacatcaacgagtgcgttctgagcccgtgccggcacggcgcatcct
gccagaacacccacggcggctaccgctgccactgccaggccggctacagt
gggcgcaactgcgagaccgacatcgacgactgccggcccaacccgtgtca
caacgggggctcctgcacagacggcatcaacacggccttctgcgactgcc
tgcccggcttccggggcactttctgtgaggaggacatcaacgagtgtgcc
agtgacccctgccgcaacggggccaactgcacggactgcgtggacagcta
cacgtgcacctgccccgcaggcttcagcgggatccactgtgagaacaaca
cgcctgactgcacagagagctcctgcttcaacggtggcacctgcgtggac
ggcatcaactcgttcacctgcctgtgtccacccggcttcacgggcagcta
ctgccagcacgatgtcaatgagtgcgactcacagccctgcctgcatggcg
gcacctgtcaggacggctgcggctcctacaggtgcacctgcccccagggc

tacactggccccaactgccagaaccttgtgcactggtgtgactcctcgcc
ctgcaagaacggcggcaaatgctggcagacccacacccagtaccgctgcg
agtgccccagcggctggaccggcctttactgcgacgtgcccagcgtgtcc
tgtgaggtggctgcgcagcgacaaggtgttgacgttgcccgcctgtgcca
gcatggagggctctgtgtggacgcgggcaacacgcaccactgccgctgcc
aggcgggctacacaggcagctactgtgaggacctggtggacgagtgctca
cccagcccctgccagaacggggccacctgcacggactacctgggcggcta
ctcctgcaagtgcgtggccggctaccacggggtgaactgctctgaggaga
tcgacgagtgcctctcccacccctgccagaacgggggcacctgcctcgac
ctccccaacacctacaagtgctcctgcccacggggcactcagggtgtgca
ctgtgagatcaacgtggacgactgcaatcccccgttgaccccgtgtccc
ggagccccaagtgctttaacaacggcacctgcgtggaccaggtgggcggc
tacagctgcacctgcccgccgggcttcgtgggtgagcgctgtgaggggga
tgtcaacgagtgcctgtccaatccctgcgacgcccgtggcacccagaact
gcgtgcagcgcgtcaatgacttccactgcgagtgccgtgctggtcacacc
gggcgccgctgcgagtccgtcatcaatggctgcaaaggcaagccctgcaa
gaatgggggcacctgcgccgtggcctccaacaccgcccgcgggttcatct
gcaagtgccctgcgggcttcgagggcgccacgtgtgagaatgacgctcgt
acctgcggcagcctgcgctgcctcaacggcggcacatgcatctccggccc
gcgcagccccacctgcctgtgcctgggccccttcacgggccccgaatgcc
agttcccggccagcagcccctgcctgggcggcaacccctgctacaaccag
gggacctgtgagcccacatccgagagccccttctaccgttgcctgtgccc
cgccaaattcaacgggctcttgtgccacatcctggactacagcttcgggg
gtggggccgggcgcgacatcccccgccgctgatcgaggaggcgtgcgag
ctgcccgagtgccaggaggacgcgggcaacaaggtctgcagcctgcagtg
caacaaccacgcgtgcggctgggacggcggtgactgctccctcaacttca
atgacccctggaagaactgcacgcagtctctgcagtgctggaagtacttc
agtgacggccactgtgacagccagtgcaactcagccggctgcctcttcga
cggctttgactgccagcgtgcggaaggccagtgcaacccctgtacgacc
agtactgcaaggaccacttcagcgacgggcactgcgaccagggctgcaac
agcgcggagtgcgagtgggacgggctggactgtgcggagcatgtacccga
gaggctggcggccggcacgctggtggtggtggtgctgatgccgccggagc
agctgcgcaacagctccttccacttcctgcgggagctcagccgcgtgctg
cacaccaacgtggtcttcaagcgtgacgcacacggccagcagatgatctt
cccctactacggccgcgaggaggagctgcgcaagcaccccatcaagcgtg
ccgccgagggctgggccgcacctgacgccctgctgggccaggtgaaggcc
tcgctgctccctggtggcagcgagggtgggcggcggcggagggagctgga
ccccatggacgtccgcggctccatcgtctacctggagattgacaaccggc
agtgtgtgcaggcctcctcgcagtgcttccagagtgccaccgacgtggcc
gcattcctgggagcgctcgcctcgctgggcagcctcaacatcccctacaa
gatcgaggccgtgcagagtgagaccgtggagccgcccccgccggcgcagc
tgcacttcatgtacgtggcggcggccgcctttgtgcttctgttcttcgtg
ggctgcggggtgctgctgtcccgcaagcgccggcggcagcatggccagct
ctggttccctgagggcttcaaagtgtctgaggccagcaagaagaagcggc
gggagcccctcggcgaggactccgtgggcctcaagcccctgaagaacgct
tcagacggtgccctcatggacgacaaccagaatgagtggggggacgagga
cctggagaccaagaagttccggttcgaggagcccgtggttctgcctgacc
tggacgaccagacagaccaccggcagtggactcagcagcacctggatgcc
gctgacctgcgcatgtctgccatggcccccacaccgccccagggtgaggt

tgacgccgactgcatggacgtcaatgtccgcgggcctgatggcttcaccc
cgctcatgatcgcctcctgcagcgggggcggcctggagacgggcaacagc
gaggaagaggaggacgcgccggccgtcatctccgacttcatctaccaggg
cgccagcctgcacaaccagacagaccgcacgggcgagaccgccttgcacc
tggccgcccgctactcacgctctgatgccgccaagcgcctgctggaggcc
agcgcagatgccaacatccaggacaacatgggccgcaccccgctgcatgc
ggctgtgtctgccgacgcacaaggtgtcttccagatcctgatccggaacc
gagccacagacctggatgcccgcatgcatgatggcacgacgccactgatc
ctggctgcccgcctggccgtggagggcatgctggaggacctcatcaactc
acacgccgacgtcaacgccgtagatgacctgggcaagtccgccctgcact
gggccgccgccgtgaacaatgtggatgccgcagttgtgctcctgaagaac
ggggctaacaaagatatgcagaacaacagggaggagacacccctgtttct
ggccgcccgggagggcagctacgagaccgccaaggtgctgctggaccact
ttgccaaccgggacatcacggatcatatggaccgcctgccgcgcgacatc
gcacaggagcgcatgcatcacgacatcgtgaggctgctggacgagtacaa
cctggtgcgcagcccgcagctgcacggagccccgctgggggggcacgccca
ccctgtcgcccccgctctgctcgcccaacggctacctgggcagcctcaag
cccggcgtgcagggcaagaaggtccgcaagcccagcagcaaaggcctggc
ctgtggaagcaaggaggccaaggacctcaaggcacggaggaagaagtccc
aggacggcaagggctgcctgctggacagctccggcatgctctcgcccgtg
gactccctggagtcaccccatggctacctgtcagacgtggcctcgccgcc
actgctgccctccccgttccagcagtctccgtccgtgcccctcaaccacc
tgcctgggatgcccgacacccacctgggcatcgggcacctgaacgtggcg
gccaagcccgagatggcggcgctgggtgggggcggccggctggcctttga
gactggcccacctcgtctctcccacctgcctgtggcctctggcaccagca
ccgtcctgggctccagcagcggaggggccctgaatttcactgtgggcggg
tccaccagtttgaatggtcaatgcgagtggctgtcccggctgcagagcgg
catggtgccgaaccaatacaaccctctgcgggggagtgtggcaccaggcc
ccctgagcacacaggccccctccctgcagcatggcatggtaggcccgctg
cacagtagccttgctgccagcgccctgtcccagatgatgagctaccaggg
cctgcccagcacccggctggccacccagcctcacctggtgcagacccagc
aggtgcagccacaaaacttacagatgcagcagcagaacctgcagccagca
aacatccagcagcagcaaagcctgcagccgccaccaccaccaccacagcc
gcaccttggcgtgagctcagcagccagcggccacctgggccggagcttcc
tgagtggagagccgagccaggcagacgtgcagccactgggccccagcagc
ctggcggtgcacactattctgccccaggagagccccgccctgcccacgtc
gctgccatcctcgctggtcccacccgtgaccgcagcccagttcctgacgc
ccccctcgcagcacagctactcctcgcctgtggacaacacccccagccac
cagctacaggtgcctgagcacccccttcctcaccccgtcccctgagtcccc
tgaccagtggtccagctcgtccccgcattccaacgtctccgactggtccg
agggcgtctccagccctcccaccagcatgcagtcccagatcgcccgcatt
ccggaggccttcaagtaaacggcgcgccccacgagaccccggcttccttt
cccaagccttcgggcgtctgtgtgcgctctgtggatgccagggccgacca
gaggagcctttttaaaacacatgtttttatacaaaataagaacgaggatt
ttaattttttttagtatttatttatgtacttttattttacacagaaacac
tgcctttttatttatatgtactgttttatctggccccaggtagaaacttt
tatctattctgagaaaacaagcaagttctgagagccagggttttcctacg
taggatgaaaagattcttctgtgtttataaaatataaacaaagattcatg
atttataaatgccatttatttattgattcctttttcaaaatccaaaaag

aaatgatgttggagaagggaagttgaacgagcatagtccaaaaagctcct
ggggcgtccaggccgcgccctttccccgacgcccacccaaccccaagcca
gcccggccgctccaccagcatcacctgcctgttaggagaagctgcatcca
gaggcaaacggaggcaaagctggctcaccttccgcacgcggattaatttg
catctgaaataggaaacaagtgaaagcatatgggttagatgttgccatgt
gttttagatggtttcttgcaagcatgcttgtgaaaatgtgttctcggagt
gtgtatgccaagagtgcacccatggtaccaatcatgaatctttgtttcag
gttcagtattatgtagttgttcgttggttatacaagttcttggtccctcc
agaaccaccccggcccccctgcccgttcttgaaatgtaggcatcatgcatg
tcaaacatgagatgtgtggactgtggcacttgcctgggtcacacacggag
gcatcctaccctttttctggggaaagacactgcctgggctgaccccggtgg
cggccccagcacctcagcctgcacagtgtcccccaggttccgaagaagat
gctccagcaacacagcctgggccccagctcgcgggacccgaccccccgtg
ggctcccgtgttttgtaggagacttgccagagccgggcacattgagctgt
gcaacgccgtgggctgcgtcctttggtcctgtccccgcagccctggcagg
gggcatgcggtcgggcaggggctggagggaggcgggggctgcccttgggc
cacccctcctagtttgggaggagcagattttgcaataccaagtatagcc
tatggcagaaaaaatgtctgtaaatatgtttttaaaggtggattttgttt
aaaaaatcttaatgaatgagtctgttgtgtgtcatgccagtgagggacgt
cagacttggctcagctcggggagccttagccgcccatgcactggggacgc
tccgctgccgtgccgcctgcactcctcagggcagcctcccccggctctac
gggggccgcgtggtgccatccccagggggcatgaccagatgcgtcccaag
atgttgatttttactgtgttttataaaatagagtgtagtttacagaaaaa
gactttaaaagtgatctacatgaggaactgtagatgatgtattttttttca
tcttttttgttaactgatttgcaataaaaatgatactgatggtgaaaaaa
aaaaaaaaa
>NM_005204 3
accctgcaaccgggcagtctctttctgtttacggagagaaaggggaaatg
gaaaagtcggggaggcggtggctggcgtccgctgcgccgcccctgggcag
gctcagacgccgtgagtcaggggcagagcagggcggtctgagcgtgcggg
cgacgcgggtctcactcgtccgctccgctctggactgcgcgccacgctct
ggggtccggcgccctggttcctgcttctgccgctgccgccgccggatccc
agtggcccggcgtgctcggctcccacaggcctgcagccagcatcgcaccg
aaccttcgggggggccgcggctggagcgctcggccggcgtgggagcgccaa
ggccgcagatgcaatcttcttaccgcgaagaagccaggggaataggtagc
cacatcttgtttgcagataagaaaggaagctaacgcagtatctgcaaagc
caggagtctgactcagtacttttctcactcatgcatacaaagcagctaaa
aatgacacagcttatttaccatgcccctgacactgcactgagcactttat
gagcttgaactctgttaatcctcacgaccacctcatgagactctccagaa
agagcaacagtaatggagtacatgagcactggaagtgacaataaagaaga
gattgatttattaattaaacatttaaatgtgtctgatgtaatagacatta
tggaaaatctttatgcaagtgaagagccagcagtttatgaacccagtcta
atgaccatgtgtcaagacagtaatcaaaacgatgagcgttctaagtctct
gctgcttagtggccaagaggtaccatggttgtcatcagtcagatatggaa
ctgtggaggatttgcttgcttttgcaaaccatatatccaacactgcaaag
catttttatggacaacgaccacaggaatctggaattttattaaacatggt
catcactccccaaaatggacgttaccaaatagattccgatgttctcctga
tcccctggaagctgacttacaggaatattggttctgattttattcctcgg
ggcgcctttggaaaggtatacttggcacaagatataaagacgaagaaaag

aatggcgtgtaaactgatcccagtagatcaatttaagccatctgatgtgg
aaatccaggcttgcttccggcacgagaacatcgcagagctgtatggcgca
gtcctgtggggtgaaactgtccatctctttatggaagcaggcgagggagg
gtctgttctggagaaactggagagctgtggaccaatgagagaatttgaaa
ttatttgggtgacaaagcatgttctcaagggacttgattttctacactca
aagaaagtgatccatcatgatattaaacctagcaacattgttttcatgtc
cacaaaagctgttttggtggattttggcctaagtgttcaaatgaccgaag
atgtctattttcctaaggacctccgaggaacagagatttacatgagccca
gaggtcatcctgtgcaggggccattcaaccaaagcagacatctacagcct
gggggccacgctcatccacatgcagacgggcaccccaccctgggtgaagc
gctaccctcgctcagcctatccctcctacctgtacataatccacaagcaa
gcacctccactggaagacattgcagatgactgcagtccagggatgagaga
gctgatagaagcttccctggagagaaaccccaatcaccgcccaagagccg
cagacctactaaaacatgaggccctgaacccgcccagagaggatcagcca
cgctgtcagagtctggactctgccctcttggagcgcaagaggctgctgag
taggaaggagctggaacttcctgagaacattgctgattcttcgtgcacag
gaagcaccgaggaatctgagatgctcaagaggcaacgctctctctacatc
gacctcggcgctctggctggctacttcaatcttgttcggggaccaccaac
gcttgaatatggctgaaggatgccatgtttgctctaaattaagacagcat
tgatctcctggaggctggttctgctgcctctacacaggggccctgtacag
tgaatggtgccattttcgaaggagcagtgtgacctcctgtgacccatgaa
tgtgcctccaagcggccctgtgtgtttgacatgtgaagctatttgatatg
caccaggtctcaaggttctcatttctcaggtgacgtgattctaaggcagg
aatttgagagttcacagaaggatcgtgtctgctgactgtttcattcactg
tgcactttgctcaaaattttaaaaataccaatcacaaggataatagagta
gcctaaaattactattcttggttcttatttaagtatggaatattcatttt
actcagaatagctgttttgtgtatattggtgtatattatataactctttg
agcctttattggtaaattctggtatacattgaattcattataatttgggt
gactagaacaacttgaagattgtagcaataagctggactagtgtcctaaa
aatggctaactgatgaattagaagccatctgacagcaggccactagtgac
agtttcttttgtgttcctatggaaacattttatactgtacatgctatgct
gaagacattcaaaacgtgatgttttgaatgtggataaaactgtgtaaacc
acataatttttgtacatcccaaaggatgagaatgtgacctttaagaaaaa
tgaaaacttttgtaaattattgatgattttgtaattcttatgactaaatt
ttcttttaagcatttgtatattaaaatagcatactgtgtatgttttatat
caaatgccttcatgaatctttcatacatatatatatttgtaacattgtaa
agtatgtgagtagtcttatgtaaagtatgtttttacattatgcaaataaa
acccaatacttttgtccaatgtggttggtcaaatcaactgaataaattca
gtattttgcctta
>NM_014572 2
gcccgtggaatgccaacaatgtagcgaatgtcccacttgggtctgcgctt
tggaaccgcggcgtgagcgccccgggaagatggagcagtcgccgtccacg
ccaccgccgccgcccggggctcccccgtccctgcggggccagcagcagct
ccagccaccagtgcccggtctcccggcgcgagaggcccgggagccgccgg
ccaggacgcccccgagggtgtagaccgcgcccctggagagagtgataatc
ttcaaaatgaagactttggaaaattttaggttctctataggaactacaaa
aatggaaggaaagaacattttcaaaaggaaattattttgaaagtatgttt
acaacaaactgatactattgacagttttttttttaaataataaaacact
ttaagaagattgtatttatggtaaaaggaaactggactaacaatgaggcc

aaagacttttcctgccacgacttattctggaaatagccggcagcgactgc
aagagattcgtgaggggttaaaacagccatccaagtcttcggttcagggg
ctacccgcaggaccaaacagtgacacttccctggatgccaaagtcctggg
gagcaaagatgccaccaggcagcagcagcagatgagagccaccccaaagt
tcggaccttatcagaaagccttgagggaaatcagatattccttgttgcct
tttgctaatgaatcgggcacctctgcagctgcagaagtgaaccggcaaat
gctgcaggaactggtgaacgcaggatgcgaccaggagatggctggccgag
ctctcaagcagactggcagcaggagcatcgaggccgccctggagtacatc
agcaagatgggctacctggacccgaggaatgagcagattgtgcgggtcat
taagcagacctccccaggaaaggggctcatgccaaccccagtgacgcgga
ggcccagcttcgaaggaaccggcgattcgtttgcgtcctaccaccagctg
agcggtacccсctacgagggcccaagcttcggcgctgacggccccacggc
gctggaggagatgccgcggccgtacgtggactaccttttccccggagtcg
gcccccacgggcccggccaccagcaccagcacccacccaagggctacggt
gccagcgtagaggcagcaggggcacacttcccgctgcagggcgcgcacta
cgggcggccgcacctgctggtgcctggggaacccctgggctacggagtgc
agcgcagcccctccttccagagcaagacgccgccggagaccgggggttac
gccagcctgcccacgaagggccagggaggaccgccaggcgccggcctcgc
tttcccacccсctgccgccgggctctacgtgccgcacccacaccacaagc
aggccggtcccgcggcccaccagctgcatgtgctgggctcccgcagccag
gtgttcgccagcgacagcccccсgcagagcctgctcactccctcgcggaa
cagcctcaacgtggacctgtatgaattgggcagcacctccgtccagcagt
ggccggctgccaccctggcccgccgggactccctgcagaagccgggcctg
gaggcgccgccgcgcgcgcacgtggccttccggcctgactgcccagtgcc
cagcaggaccaactccttcaacagccaccagccgcggcccggtccgcctg
gcaaggccgagccctccctgcccgcccсcaacaccgtgacggctgtcacg
gccgcgcacatcttgcacccggtgaagagcgtgcgtgtgctgaggccgga
gccgcagacggctgtggggccctcgcaccccgcctgggtgcccgcgcctg
ccccggccccсgcccccgcccccgccccggctgcggagggcttggacgcc
aaggaggagcatgccctggcgctgggcggcgcaggcgccttcccgctgga
cgtggagtacggaggcccagaccggaggtgcccgcctccgccctacccga
agcacctgctgctgcgcagcaagtcggagcagtacgacctggacagcctg
tgcgcaggcatggagcagagcctccgtgcgggccccaacgagcccgaggg
cggcgacaagagccgcaaaagcgccaagggggacaaaggcggaaaggata
aaaagcagattcagacctctcccgttcccgtccgcaaaaacagcagagac
gaagagaagagagagtcacgcatcaagagctactcgccatacgcctttaa
gttcttcatggagcagcacgtggagaatgtcatcaaaacctaccagcaga
aggttaaccggaggctgcagctggagcaagaaatggccaaagctggactc
tgtgaagctgagcaggagcagatgcggaagatcctctaccagaaagagtc
taattacaacaggttaaagagggccaagatggacaagtctatgtttgtca
agatcaaaaccctggggatcggtgcctttggagaagtgtgccttgcttgt
aaggtggacactcacgccctgtacgccatgaagaccctaaggaaaaagga
tgtcctgaaccggaatcaggtggcccacgtcaaggccgagagggacatcc
tggccgaggcagacaatgagtgggtggtcaaactctactactccttccaa
gacaaagacagcctgtactttgtgatggactacatccctggtggggacat
gatgagcctgctgatccggatggaggtcttccctgagcacctggcccggt
tctacatcgcagagctgactttggccattgagagtgtccacaagatgggc
ttcatccaccgagacatcaagcctgataacattttgatagatctggatgg
tcacattaaactcacagatttcggcctctgcactgggttcaggtggactc

acaattccaaatattaccagaaagggagccatgtcagacaggacagcatg
gagcccagcgacctctgggatgatgtgtctaactgtcggtgtggggacag
gctgaagaccctagagcagagggcgcggaagcagcaccagaggtgcctgg
cacattcactggtggggactccaaactacatcgcacccgaggtgctcctc
cgcaaagggtacactcaactctgtgactggtggagtgttggagtgattct
cttcgagatgctggtggggcagccgccctttttggcacctactcccacag
aaacccagctgaaggtgatcaactgggagaacacgctccacattccagcc
caggtgaagctgagccctgaggccagggacctcatcaccaagctgtgctg
ctccgcagaccaccgcctggggcggaatggggccgatgacctgaaggccc
acccccttcttcagcgccattgacttctccagtgacatccggaagcagcca
gccccctacgttcccaccatcagccaccccatggacacctcgaatttcga
ccccgtagatgaagaaagcccttggaacgatgccagcgaaggtagcacca
aggcctgggacacactcacctcgcccaataacaagcatcctgagcacgca
ttttacgaattcaccttccgaaggttctttgatgacaatggctacccctt
tcgatgcccaaagccttcaggagcagaagcttcacaggctgagagctcag
atttagaaagctctgatctggtggatcagactgaaggctgccagcctgtg
tacgtgtagatgggggccaggcacccccaccactcgctgcctcccaggtc
agggtcccggagccggtgccctcacaggccaatagggaagccgagggctg
ttttgttttaaattagtccgtcgattacttcacttgaaattctgctcttc
accaagaaaacccaaacaggacacttttgaaaacaggactcagcatcgct
ttcaataggcttttcaggaccttcactgcattaaaacaatatttttgaaa
atttagtacagtttagaaagagcacttattttgtttatatccattttttc
ttactaaattatagggattaactttgacaaatcatgctgctgttattttc
tacatttgtattttatccatagcacttattcacatttaggaaaagacata
aaaactgaagaacattgatgagaaatctctgtgcaataatgtaaaaaaaa
aaaaagataacactctgctcaatgtcacggagaccattttatccacacaa
tggtttttgtttttttatttttcccatgtttcaaaattgtgatataatga
tataatgttaaaagctgcttttttggctttttgcatatctagtataata
ggaagtgtgagcaaggtgatgatgtggctgtgatttccgacgtctggtgt
gtggagagtactgcatgagcagagttcttctattataaaattaccatatc
ttgccattcacagcaggtcctgtgaatacgtttttactgagtgtctttaa
atgaggtgttctagacagtgtgctgataatgtattgtgcgggtgacctct
tcgctatgattgtatctcttactgttttgttaaagaaatgcagatgtgta
actgagaagtgatttgtgtgtgtgtcttggttgtgattggattctttggg
gggggggaactgaaacatttgtcatatactgaacttatatacatcaaaag
ggattaatacagcgatgccaaaaagtttaatcacggacacatgtccgttt
ctgtagtccgtatgctctttcattcttggtagagctggtatgtggaatgc
catacctctgaccctactacttacctttttactgacagactgcccacact
gaaagcttcagtgaatgttcttagtcctgttttcttctgttactgtcagg
aaactgagtgatctaatggttctctcactttttttttgttcttttagtgt
actttgaagtatcaaatcttaacttggtttaaacaatacatattcctaac
ctttgtaaaaaagcaaagattcttcaaaatgacattgaaataaaaagtaa
gccatacgtattttcttagaagtatagatgtatgtgcgtgtatacacaca
cacacacacacacagagataaacacaatattccttatttcaaattagtat
gattcctatttaaagtgatttatatttgagtaaaaagttcaattcttttt
tgctttttaaaaaaatctgatgcttcataattttcattatattattccaca
tatttttccttgaagttcttagcataatgtatccattacttagtatatat
ctaggcaacaacacttagaagtttatcagtgtttaaactaaaaaaataaa
gattcctgtgtactggtttacatttgtgtgagtggcatactcaagtctgc

tgtgcctgtcgtcgtgactgtcagtattctcgctattttatagtcgtgcc
atgttgttactcacagcgctctgacatactttcatgtggtaggttctttc
tcaggaactcagtttaactattatttattgatatatcattacctttgaaa
agcttctactggcacaatttattattaaaattttgaatccaaaaaaaaaa
aaaaaaaa
>NM_015455 3
caatgaaaacgagggggcgcggaggaggaggcggcggcgtcggtggcgg
cggcgacggcggcgcggaggcgaaggcagcggcgggcgcagcgaggaggg
cgaggccgggggccgagagggcgggagggcgtagtggcggcccgtcgggg
cggctgaggcgggcagccgaagcagtggctctcggagggggaacaaagag
cagcgactaaggcggcagaggagcggcggcggtggcggcgctgcagcagc
gggcgggactggtatggtggttccacagggcagaccccgctgcactcaca
gggaggaggaggcggcagcggcggaggaaggcggcgcaccccgagaggca
tgcccaaagaaaaatacgagccccctgaccctcggaggatgtatacaatt
atgtcttctgaggaagcagcaaatggaaagaaatcccactgggcagagct
tgaaataagtggaaaagtaagaagcttaagcgcatctttgtggtcactaa
ctcacctgacagctttgcatttgagtgacaattccctgtcccgaattcct
tcagacattgccaagcttcacaatctggtgtatttggacctgtcatctaa
taaaattcgtagcttacccgcagaactcggaaacatggtatcactcaggg
agctccatttaaataacaacctgttacgagttctaccttttgagctggga
aaactgtttcagttgcagactttaggcctgaaaggaaatccccttaccca
ggatatattgaacctttatcaggaaccagatggaacaagacggctgctga
actatttgcttgataatttgtcaggtactgcaaaaagaattacaacagaa
caaccacctccaaggtcttggattatgttacaagaaccagataggacaag
gccaactgccttgttttctgtcatgtgctataatgttctttgtgataaat
atgcgacccggcagttatacggctactgtccatcatgggcgctaaactgg
gactacaggaaaaaggccattattcaagaaatcttgagctgcaatgctga
tatcgtaagtcttcaggaggttgaaacggaacagtattacagtttttttc
tggtagagctgaaagaacgtggctataatggattcttcagtcctaagtct
agagctaggacaatgtcagaacaagaaaggaaacatgttgatggctgtgc
aatattcttcaagacagaaaaatttactttggttcagaaacacactgttg
aatttaatcagctagccatggcaaattctgaggggtctgaagctatgctg
aacagagtcatgacaaaagataacattggggttgcagtactgctagaact
tcggaaggaatcgattgaaatgccgtctggaaagccacatcttggaacag
aaaaacaacttattcttgtggctaacgcccacatgcattgggaccctgaa
tactctgatgtgaagttggtacaaactatgatgttcctctcagaagtgaa
gaacattattgataaagcctctcgcaacctcaaatccagtgttttgggag
aatttggaactattccacttgtgttatgtgcagatcttaattctttgcca
gactctggtgttgtagaatatttgagcacaggtggagtagaaacaaatca
caaagactttaaggagttgaggtataatgaaagtctcacaaacttcagct
gtcatgggaagaatggaaccaccaatggaaggatcactcatggtttcaag
ttacagagtgcctatgagagtggcctgatgccttacacgaattacacatt
tgatttcaagggtataatagactacattttctattctaaacctcagctga
acaccttaggcatcctgggccctctggaccaccactggctggttgagaat
aacatcagtggctgcccgcacccccctcatcccctctgaccacttctcact
ttttgcacaactggagctcttactgcctttcctgccccaagtcaacggca
tccaccttcctggcaggaggtagtcaagcaccttcagaggacagccttga
ttcacttgtaaacttgtgaaaatctgaacataggggagtgaggtatggcc
actgaggatttttgcttgcttaagaatgatttggactttcaatctgatta

tttgataaggatatagtatgaaagccaggtgctagcaacagacaaattct
gagcccaatatgctttatactgctagacagggattggtgtgtttgcacct
gtctttcatttgtcataagagattttcctatttcttctacccaatagaat
attttcatgcctggaaataggaaaatgtgtgaacagcgtattctcttcac
acagaaatctgtagcactgctttttttgaggccagtagcaacatccagaga
tcattcttccatactttactccctcctttttcagacttgtttgtaaagta
cagaatctgaatttagcctttatgattgtatatgatccacagaagacctg
atttatgaaattttgtactaaaatcatttggaaatgattgtattgtaaac
tgaggctaaatttttttttaaacctgtttcatgtgttataaaggccagct
tgtaaaagaagctgcaacagactttctctgctcatgatttgcactcttag
ggttttgttagcccttttgtactactttctttttaaattgagaacatggt
tctttacatataaatctgcttcaaccttaggatgttttcagaccagaggc
aacttattcatgaatttttatgaaaactatctactaggacagataagctg
aacagtgatgatctgtagacatttatggactgaatgtaatggttgatata
tgtacattctgatatttttaaatctttaactttttaaagttaaaaaccta
cagctgcttaggtccagcttcttaactctttttgagacacttcctgtcct
atctccactgtgcctgcctaaatttgttctcaccaagcactgcctgtgca
tgcagagaaaatctgtgcatcctcttttatatttttaaaatactgtttaa
catttgtgagaattttatgaaaatgcttttgtatgagctgtggctttttc
ccattgtgaagcattgaatatcacattttggaacatgttatagggtgagt
ccctggatctttgctcaccagatccaagcactgcttctcggtgtcattgc
agtgtgctgcttgtcacccagaatactactatcatgtgaattctttttgt
cgtcagtgtcttttccttagtctttttgttgttgttgttgttgttgtttt
aatcattcctttttttaagaagaagtaattttccatttatgaagcagtatg
aattagatgtattttcaaaacaggtccctaagacaattcttcagatcatt
tttaaaatgactaagtcattttagtatgtcaagcaagataaaaattacat
cataccgtctattttgcccatagtgccatttagagatgaaaaccagcttt
aactttgcaaagtgaacatgtacatggtctgctctcatttattcattctt
ctctcaaaagtcaaatgaatgcagagggagcttggtcaaactgcttttgt
ttcaactgcaggcaggggagcaaaaggacgccatgtgagcattaggaaaa
aaaatactcactcttactaacaatttttatacagaaaatgagtcattttg
gaaatgattcttatgtttttttttttctccttttagaaaattctccaaa
aggttttgatgttgaatcttggtcttggacctgtttttcctttgagggt
tttttgttttttgttttttctaggatttcattgtgatgttttggttttgt
tttttgctttttgtttaagttgtgctgacaccaaacacatccagtttata
atcagtacattggaaagctggtattgatgtagaaccagtgcataactttt
tatggggttttgttattggttttttttttgtaaagtgtgaataaaaggtat
gtttactcatttttcctgaacactgtgttggtaatgtgcatcatgacaat
ttccagtgaaggtgagctggagctggttggactaatgagactgaggaagc
agcttttcctacgatctgcattatgtaatcacaggtccagagagctttat
ggaagcgggagaggaggagcacttactcatgttgtatttgttaatggagg
atgtcatcttttcatagatgctggaactagagtgcacttgttagatgcta
aaggtttgagctttacacaaaatgtcttcatctgtatttgttattgtcta
caatatatttgaatttggggcagcatattaagatgtaatggcctgttatg
tcttgaaaatacttgttttgcctcttccaggcatactgcattctgtggat
cagtttgaacagcttctccaccttatttggacagtgataaattgaaccaa
gagtgtagatttacaagtgtaaccttcaaaagaggaagaactatttgggg
tctgtaggtaatgaacagtcacaccaaaatagactatgatgcttttgtta
agaaaggtttcatgttttagatattttccgtgtcctaaataattttcaat

aatctataatccctaaaatgcaataaaaactagtatgttttcacggtgta
tattttttttcaaaaattctgtaacaaatatatttaatgtgttgccataa
tgtcatttcagtatttttgctggataaatccattttagaaatgtcttaca
aaaagtttagggcatgttttctgttttaaagaccagaagtttgaaggcac
ccaaatccttgtcgccaatctcctgaagtacagctttaccatattagtgg
tcattatttatgctttgagagtttcaatattgttcgtcaagttaacttat
ggcataatttaagctaaagttgtagaaatgaattgttgagtgcaactttc
atagtcccaaagataggcagagaacatctccagaaatgtgtgtgtgtgtg
tgtgtgtgtgtgtgtgtgtttaatatgatttagtgacaaccaggaaaact
tacttgggattattagcactttcaaacttagggacattattaaattggat
ggaatgcacttctaaatgtttaaattaaaatttcaaagctctttcgagat
tcaggttctcaataataatattcaagttttagagtttcactttgtactat
ttaaatttatggaactagtttccatactgacttgtaaggttttggggtca
tatataaggactaaagccaagctaggcaaaacaatgacagcaccgttttt
tcagtgaagctctcagaatgtccagtacagatgttgcagataattccaag
aactccttcagcaggtgttcttcaccattcgtaaacagtattttaagatg
ttcactttgctcttctttttggttacatacattttaataactggtatgtt
gaagtagtgtctaaaagtatctagtctttattcacagtacattatattgt
gtgatgcactggactaactcttgtttaccattcatgtaacaaaacccagc
acattatctgcactataagctcaaagaatgtcacatccgcccagacagct
ctttgatgagggtgatgggaactgaataaaccatacggactggcagtaac
aagggcttttactgttctgttcagtggaaccttcttggtcaaattgtaat
tagctagcattatatttatatacagggtctttttctttaccgatgtatt
ttcctactcatagccaaccaataaattcagtatctgtttcaaatatttta
gaagtgtagtttgtatagctgtagtactgaggtttgaggaaaaataaatg
ttgatcagcagaccacgtatttaaaattctgaatcttctgggacagggtt
gtaactcagccttccaaagggaagagtgcagggggacggggccatgatat
ggggaaatggtgtaaactaatgtatttctttattggctgttattctgtat
aacactcatatctttgccaaagttcaattttatatttaggcaactgatgg
tccttttgcatttaggattttcgttgttgttaccttatacctcatgatat
aaggaatgggctcatgtgtcttccgtcttttggaaggaggttgacatatt
ttaaataaatgcttttaaatacagtaaa
>NM_152835 4
gtaagggggtgtgtccgcgcgcaccacgggggcgcgcgccggctgctgac
tggaggcggcggcagcggaggcgcgagctgcccgataatggcggcctgca
gagcccatgagagggagaagcggcagcgtctaccctgagaaacctcgacc
ttgaagatggtgagtagccagccaaagtacgatctaatacgggaggtagg
ccgaggtagttacggtgttgtgtatgaagcagtcatcagaaagacctctg
cacgggtggcagtgaagaaaattcgatgtcacgcacctgaaaatgttgaa
ctagcccttcgtgagttctgggcactaagcagtatcaagagccaacatcc
aaatgtgattcacttggaggaatgcatcctacaaaaggatgggatggtgc
aaaagatgtcccacggctctaattcttccctttatttacagcttgtagaa
acttcattaaaaggagaaattgcctttgatcccagaagcgcctattattt
gtggtttgtgatggatttttgtgacggaggagatatgaatgagtatctgt
tgtccaggaaacccaatcgtaaaactaacaccagcttcatgcttcagctg
agcagtgccctggctttcttgcataaaaaccagatcatccaccgagatct
taagcctgataacatcctgatttctcaaaccaggttggataccagtgact
tggaacctaccctcaaagtggctgattttggtctaagtaaagtttgttca
gcctctgggcagaacccagaagaacctgtcagtgtaaacaagtgtttcct

ttccacagcatgtggaacagatttttacatggctcctgaagtttgggaag
gacattacacagcaaaagctgacatctttgctctggggattatcatctgg
gcaatgctggaaaggatcacattcatagacacagagacaaagaaggaact
cttggggagttatgtaaaacaaggaactgagattgtgcctgttggggagg
cacttctggaaaatcccaaaatggaacttctcattcctgtgaagaaaaaa
tctatgaatgggcgaatgaaacaactgattaaggaaatgctggctgcaaa
ccctcaggatcgtccagatgcttttgaactagaactcagattagtacaaa
ttgcatttaaagatagcagctgggaaacgtgacacatattatttgcaaat
accatggatgatatgctgcttctgtttaacagtgatgcaacattatgtgg
ctgaaaaagaatataaaaagctagactctaccctctaagggtttagattt
tttgtgggattttttttttcctcatttttcttaaatccaagttggccgtt
ttattagtatgtttcaaatgtgtattaccaatgtgggtgtaaatttttaa
aaaatgattattgatagaagtttggcaggaaaattctttaagagctaaca
agagaagagagtccagttttctggaaatatgtctttaagtattttagaca
ttcctcgtcagtattaggaatttccatgggaaaagaggtttgcatgctgg
taatgcaacctttgaaactttgtaaaggaaacatatatgtatatatttat
gtatatgtaagtatgtgaatgtgcgcattttgcattccatatgaaaaaaa
tgccacgtctgtttaaattatttgatgtaggtttgggtttttgagatttg
ctggtgaagtcagtgacgaaaaataaaccttcccttatcttcctactctg
cccctccccctaatgaaatcatattaagttgtttttttttttttttgtaa
tatacagcttttttttttaaggcatcattttcgagggtctaaaattatctg
gtaaaacaaatgaaattaagtgatccaaagctgctgaagtatgtttgaac
tctccagtgccctatagctgcaagagttgaattagtcatgcagtcatatg
gcagcaggttggtgattcagtccgaccttctttaggtaaccgagcattta
tttaaccaactataccactgcattgggacgtctgcacttgagcttctaca
atctgatataaatctgaatacaggattattctgtttgaaaactttgctat
gtgtgaactgtaatacttgatacatttttggtttgaagactttgtctaag
cctcattatactgaagttgaagaaaccaaatgagctccaccctcactttg
cctgccctgatatgatcacttgttgagtcactggagttcctttcattttg
gcactgatgctgttttctcttgtctgatttgaaacagtactggtaataat
atggttgttgaaaatgtgatcacctttatctaaagtgggagaagatacat
acacagtatggcaaatggataaaatattgtggcatcaactacttttaaag
tagaaaagctatccattttatacaatcactgatacagtctatgtcaaaaa
accaacagaccattattcctcttgaagttagtttgattttatctttgtat
ggttgtctacaatttgttttcctgtaaatgcagatagttaaaatctaaag
gatagaatgctttcctaaaacagaacattagcctttaaggttaaatagac
tttacattgtatctaagttaatctttataaagcactagaatttttggtca
aaagtcaaaatagtattagaatattgaaattattgatagctttcataatc
tcagctttttgaacttccacaatttttggaatatgtcataatttttttc
cctgtttcaaatgtgttcagaggaaatagaatgtaaaacatcacaaaaaa
aattgcagtacataaaatttctggactatgttagttacttgatctggaag
tttaaaatttggactaaaaggttttattgaaataaagcctcttaaaatga
aaaatcatatacttttaacatcacttttaagctaataaatataagaattc
ttttggaaattagaggtggaaacacactggaacctcatgataattgaccc
tctggatcaccagattagtttaatttaacagatatgtccaaggaagttga
gctatagggagtaagaaaaagcatcatgataacgtctagtcaataacatg
gcacttcagcagagattcatagatgatggtatgcccatgcatcatttgct
cttcaaatatctatgtgcttggagccacagtttcctaagcgcatagtttt
tggtcctgcctcatgtaaaatagttaatgattatcatttacttggaaaga

ttttacctgtgaagtgaaattctttcattgggggtaagggtatgaggtga
tcaggaagtttcttctggaattttatcaagtccttgtattctaaaatagc
caagcaaaatgatagtaaattctttgtctttgttctttcagtcagaggta
tttattaagtacctactgtgtgctcagcactaaaggtttgttgataaaat
gacaattaaaaaatggcatttagccaaaggccttttcagccaggagtaag
aaaagatggttagtgatatttggaaatacctggatatttgctagaggaaa
aatgaaagcaaaacttcgtacacagtgccttgcccataataggtactcaa
taagcaattgttcctcatagagtcttttgccatgatttatggtgtttttt
gaaatttgtcttaattgaaaaaagaccaaatatttcaaatgagcttatag
gcagctgtggccagtgaaaatatggggttgtacagttagttcctagaatg
ttattttgcactagttgtcacttagaaagatggggttcctgtagattttt
tggtgctaagggatactttgtcattatgatgaagtaagtgttaagtgtca
gataaatagcacacagaatagttctttctgctggtctgttttttctcttc
gttgttgttgtttttaattgtaaattgttatcaatcaaactattgtagca
gctacaaagtaattcaccagcatgacaaaatggtcaaaaaataagtcatc
agcacttaagaaatgaaagcaacttttgaaattttctgttaaaattgtca
aatatattttatgaagaatttataaaaggggaaaatgattgtaatttatt
gttcatgactttttttttaaataaagtgagtttcaacaaaaaaaaaact
gaa
>NM_015076 3
cgcgggggaggaggaggagggactgagcggcggcggcccccgcgtcccg
tgcctctatgggggaagcagacaatggattatgatttcaaggcgaagctg
gcggcggagcgggagcgggtggaggatttgtttgagtacgaagggtgcaa
agtgggacgcggcacctacggtcacgtctacaaggcgaggcggaaagatg
gaaaagatgaaaaggaatatgcattgaagcaaattgaaggcacaggaata
tccatgtcggcttgtagagagattgcacttttgcgagaattgaagcaccc
taatgtgattgcattgcagaaggtgttcctttctcacagtgacaggaagg
tatggctgctgtttgattatgcagagcatgacttgtggcatattattaag
tttcaccgtgcatcaaaagcaaataaaaagcccatgcagttgccaagatc
tatggttaaatccttactttaccagattcttgatggtatccattacctcc
atgcaaattgggtgcttcacagagacttgaaaccagcaaatatcctagta
atgggagaaggtcctgagaggggggagagtcaaaatagctgacatgggttt
tgccagattattcaattctcctctaaagccactagcagatttggatccag
tagttgtgacattttggtatcgggctccagaacttttgcttggtgcaagg
cattatacaaaggccattgatatatgggcaataggttgtatatttgctga
attgttgacttcggaacctatttttcactgtcgtcaggaagatataaaaa
caagcaatccctttcatcatgatcaactggatcggatatttagtgtcatg
gggtttcctgcagataaagactgggaagatattagaaagatgccagaata
tcccacacttcaaaaagactttagaagaacaacgtatgccaacagtagcc
tcataaagtacatggagaaacacaaggtcaagcctgacagcaaagtgttc
ctcttgcttcagaaactcctgaccatggatccaaccaagagaattacctc
ggagcaagctctgcaggatccctattttcaggaggaccctttgccaacat
tagatgtatttgccggctgccagattccataccccaaacgagaattcctt
aatgaagatgatcctgaagaaaaaggtgacaagaatcagcaacagcagca
gaaccagcatcagcagcccacagcccctccacagcaggcagcagcccctc
cacaggcgcccccaccacagcagaacagcacccagaccaacgggaccgca
ggtggggctggggccggggtcgggggcaccggagcagggttgcagcacag
ccaggactccagcctgaaccaggtgcctccaaacaagaagccacggctag
ggccttcaggcgcaaactcaggtggacctgtgatgccctcggattatcag

cactccagttctcgcctgaattaccaaagcagcgttcagggatcctctca
gtcccagagcacacttggctactcttcctcgtctcagcagagctcacagt
accacccatctcaccaggcccaccggtactgaccagctcccgttgggcca
ggccagcccagcccagagcacaggctccagcaatatgtctgcattgaaaa
gaaccaaaaaaatgcaaactatgatgccatttaaaactcatacacatggg
aggaaaaccttatatactgagcattgtgcaggactgatagctcttcttta
ttgacttaaagaagattcttgtgaagtttccccagcaccccttccctgca
tgtgttccattgtgacttctctgataaagcgtctgatctaatcccagcac
ttctgtaaccttcagcatttctttgaaggatttcctggtgcacctttctc
atgctgtagcaatcactatggtttatcttttcaaagctcttttaatagga
ttttaatgttttagaaacaggattccagtggtgtatagttttatacttca
tgaactgatttagcaacacaggtaaaaatgcaccttttaaagcactacgt
tttcacagacaataactgttctgctcatggaagtcttaaacagaaactgt
tactgtcccaaagtactttactattacgttcgtatttatctagtttcagg
gaaggtctaataaaaagacaagcggtgggacagagggaacctacaaccaa
aaactgcctagatctttgcagttatgtgctttatgccacgaagaactgaa
gtatgtggtaatttttatagaatcattcatatggaactgagttcccagca
tcatcttattctgaatagcattcagtaattaagaattacaattttaacct
tcatgtagctaagtctaccttaaaaagggtttcaagagctttgtacagtc
tcgatggcccacaccaaaacgctgaagagagtaacaactgcactaggatt
tctgtaaggagtaattttgatcaaaagacgtgttacttccctttgaagga
aaagtttttagtgtgtattgtacataaagtcggcttctctaaagaaccat
tggtttcttcacatctgggtctgcgtgagtaactttcttgcataatcaag
gttactcaagtagaagcctgaaaattaatctgcttttaaaataaagagca
gtgttctccattcgtatttgtattagatatagagtgactatttttaaagc
atgttaaaaatttaggttttattcatgtttaaagtatgtattatgtatgc
ataattttgctgttgttactgaaacttaattctatcaagaatctttttca
ttgcactgaatgatttcttttgcccctaggagaaaacttaataattgtgc
ctaaaaactatgggcggatagtataagactatactagacaaagtgaatat
ttgcatttccattatctatgaattagtggctgagttctttcttagctgct
ttaaggagcccctcactccccagagtcaaaaggaaatgtaaaaacttaga
gctcccattgtaatgtaaggggcaagaaatttgtgttcttctgaatgcta
ctagcagcaccagccttgttttaaatgttttcttgagctagaagaaatag
ctgattattgtatatgcaaattacatgcatttttaaaaactattctttct
gaacttatctacctggttatgatactgtgggtccatacacaagtaaaata
agattagacagaagccagtatacattttgcactattgatgtgatactgta
gccagccaggaccttactgatctcagcataataatgctcactaataatga
agtctgcatagtgacactcatcaagactgaagatgaagcaggttacgtgc
tccattggaaggagtttctgatagtctcctgctgttttaccccttccatt
ttttaaaataagaaattagcagccctctgcataatgtagctgcctatatg
cagttttatcctgtgccctaaagcctcactgtccagagctgttggtcatc
agatgcttattgcaccctcaccatgtgcctggtgccctgctgggtagaga
acacagaggacagggcatacttcttgtccttaaggagcttgtgatctgtg
acagtaagccctcctgggatgtctgtgccatgtgattgacttacaagtga
aactgtcttataatatgaaggtctttttgtttacttctaaacccacttgg
gtagttactatccccaaatctgttctgtaaataatattatggaagggttt
ctatgtcagtctaccttagagaaagccagtgattcaatatcacaaaaggc
attgacgtatctttgaaatgttcacagcagccttttaacaacaactgggt
ggtccttgtaggcagaacatactctcctaagtggttgtaggaaattgcaa

ggaaaatagaaggtctgttcttgctctcaaggaggttacctttaataaaa
gaagacaaacccagatagatatgtaaaccaaaatactatgccccttaata
ctttataagcagcattgttaaatagttcttacgcttatacattcacagaa
ctaccctgttttccttgtatataatgacttttgctggcagaactgaaata
taaactgtaaggggatttcgtcagttgctcccagtatacaatatcctcca
ggacatagccagaaatctccattccacacatgactgagttcctatccctg
cactggtactggctcttttctcctctttccttgcctcagggttcgtgcta
cccactgattccctttacccttagtaataattttggatcattttctttcc
tttaaaggggaacaaagcctttttttttttgagacggagtgttgctctg
tcacccaagctggagtgcagtggcacgatcttggctcactccaacctcca
ccttccaggttcaagtgattctcctgcctcagcctcccgagtagctggga
ctacgggcacgcaccaccacgtctggctaatttttgtatttttagtagag
atggggtttcaccctattggtcaggctggtcttgaattcctcacctcagg
tcatccgcctgtctcggcctcccgaagtgctgggattataggtgtgagcc
accgcacccagttgggaacaaagcctttttaacacacgtaagggccctca
aaccgtgggacctctaaggagacctttgaagctttttgagggcaaacttt
acctttgtggtccccaaatgatggcatttctctttgaaatttattagata
ctgttatgtcccccaagggtacaggaggggcatccctcagcctatgggaa
cacccaaactaggaggggttattgacaggaaggaatgaatccaagtgaag
gctttctgctcttcgtgttacaaaccagtttcagagttagctttctgggg
aggtgtgtgtttgtgaaaggaattcaagtgttgcaggacagatgagctca
aggtaaggtagctttggcagcagggctgatactatgaggctgaaacaatc
cttgtgatgaagtagatcatgcagtgacatacaaagaccaaggattatgt
atatttttatatctctgtggttttgaaactttagtacttagaattttggc
cttctgcactactcttttgctcttacgaacataatggactcttaagaatg
gaaagggatgacatttacctatgtgtgctgcctcattcctggtgaagcaa
ctgctacttgttctctatgcctctaaaatgatgctgttttctctgctaaa
ggtaaaagaaaagaaaaaaatagttggaaaataagacatgcaacttgatg
tgcttttgagtaaatttatgcagcagaaactatacaatgaaggaagaatt
ctatggaaattacaaatccaaaactctatgatgatgtcttcctagggagt
agagaaaggcagtgaaatggcagttagaccaacagaggcttgaaggattc
aagtacaagtaatattttgtataaaacatagcagtttaggtccccataat
cctcaaaaatagtcacaaatataacaaagttcattgttttagggttttta
aaaaacgtgttgtacctaaggccatacttactcttctatgctatcactgc
aaaggggtgatatgtatgtattatataaaaaaaaaaaacccttaatgcact
gttatctcctaaatatttagtaaattaatactatttaattttttaaaga
tttgtctgtgtagacactaaaagtattacacaaaatctggactgaaggtg
tcctttttaacaacaatttaaagtactttttatatatgttatgtagtata
tcctttctaaactgcctagtttgtatattcctataattcctatttgtgaa
gtgtacctgttcttgtctctttttttcagtcattttctgcacgcatccccc
tttatatggttatagagatgactgtagcttttcgtgctccactgcgaggt
ttgtgctcagagccgctgcaccccagcgaggcctgctccatggagtgcag
gacgagctactgctttggagcgagggtttcctgcttttgagttgacctga
cttccttcttgaaatgactgttaaaactaaaataaattacattgcattta
ttttatattcttggttgaaataaaatttaattgactttg
>NM_016603 2
acgcacgtcacgtccggcccggcctccgcccgccagccgctctcctcccg
ctcgccgttagggaggctctgcacctcagccgccgcctctgtcgccactc
tctccccctgttccgcgtctgcgtcgccaccaccgccgggggtatccggg

gggtcgcttaccgggctggccccgcgccacagccgtagctttaacctccc
gtcagcggccgccccaggaagaggcttctcggccgcggagccacttgggg
cctcagttctcgccctcttgccgtcgccgcgggtcctggagaagcggccg
cggccagggaacggggcatcgcggttccaacgattaactgctgaagtact
gatcgagttctgcatttcttcaatgaggaactacaggctgatcttctgcc
ataatctcaaacagccataaatgacaaaagaatgcttgctcagtgagggt
agctggtgcagaagccatttttaaacttaggtatttaagtactgaaaga
aaagacagctttgatttctggctgcaaaaaagatatgaggaagagctcct
ccccttccttgagtaactgcaactccgttcttgctaacaaaatatttgga
attccacttgatgagctgcagcagggaggacatccagacaatgaggttcc
attcatagtccgccacgttgtggactatattgaggaacatggaggtctgg
agcaacaaggactttttcaagtcaatggaaatgctgagacagtggagtgg
cttcggcagagatacgacagcggagaagaggtggatttggttaaggaagc
agatgttccctcagctattagccttcttagatttttttcttcaagaacttc
ctgaacctgttatccctggcagtttacatattcacttgatgcagctttct
caagattataataatgaagatgaatttggaagaaagttgaggttcctctt
gcaacagcttccacctgttaattatagtttgttaaagtttctgtgtagat
ttttagccaatgtagcatcacatcatgaagaaatttggtccgcaaattct
ttggctgctgtctttggtccagatgtcttccacatttacacagatgtgga
agacatgaaagagcaagaaatagtgagcaggataatggctggacttctgg
aaaactactatgagtttttgagaatgaagaggaagatttttcatctaat
gatttgagttcaattactgaacaggttaatgaactttctgaggaagaaga
ggaagatgaaaagctggaacatattgaagaacttccagaagagggtgcag
aaaaatcaaatgacatgccagaggtggtacaattaaggatgactgaaaac
atcctggaatcaaatagtgttacggcaacaagcacccatatatctcccat
cagcatcctaccagcctctacagatattttagaaagaacaattagagcag
ctgtggaacagcacctttttgatcttcagagcagcatagatcatgatctt
aagaatttacaacagcaaagtgtggtgtgtaataatgaagcagaaagtat
tcattgtgatggggaaggatctaataaccagattgatattgctgatgata
ttattaatgccagtgaaagtaacagagactgttcaaaacctgtggctagc
actaatttagacaatgaagctatgcagcaagattgtgtatttgagaatga
agaaaatacccagtctgtaggtatattgttagagccatgcagtgaccgtg
gtgatagtgaagatggctgtcttgagagggaagaatatttgttatttgac
agtgataaattgtcacacttgattctggattctagtagcaagatatgtga
tttgaatgccaacactgaatcagaagtaccaggaggtcagagtgttggtg
ttcaaggggaagcagcgtgtgtcagtattccacatttagatctgaagaat
gtttctgatggtgataaatgggaagagccatttcctgcttttaagtcttg
gcaggaggactctgagtctggagaagctcagctgtctccacaagctggaa
gaatgaatcatcaccccttggaagaggactgtcctccagtattatcacac
cgcagtttagattttggtcaaagccagcgtttcctacatgatccagaaaa
gttggattcctcatctaaagcactgtcttttactagaattcgaagatcat
cctttagttcaaaagatgaaaagagagaggacagaacaccttatcagctg
gtcaagaaacttcagaagaaaatcagacaatttgaggaacagtttgaaag
ggaaagaaatagcaagccctcctacagtgatattgctgccaatccaaagg
tattaaaatggatgacagagcttacaaaactgcggaagcaaattaaagat
gcaaaacacaaaaattctgatggagaatttgtacctcagacacgtccacg
tagtaacacacttccaaaaagctttggctcttctctagaccatgaagatg
aagagaatgaagatgaacccaaggtcattcagaaggagaaaaaaccatct
aaagaagcaacccttgaacttattcttaaaagactgaaagaaaaacgtat

tgagaggtgtcttccagaagatatcaagaaaatgaccaaagatcatttgg
tagaagagaaagcttctcttcagaaaagtcttctttactatgaaagtcaa
catggaaggccggtgaccaaggaagaaaggcacattgttaaacctctcta
tgatagatacaggcttgtaaaacaaatgctgacaagagctagcatcactc
ctgtccttggatctccatccaccaagcgaaggggtcagatgttacagcca
atcatagaaggagaaactgcacatttttttgaagaaatcaaggaggaaga
agaagatggtgttaatctgtcctctgagttaggtgatatgttgaaaactg
cagtacaggtacagtcttcattagaaaactctgaatctgatgttgaagaa
aatcaagaaaaactggctctggatctccgattgtcaagttctcgagcagc
ttctatgcctgaattattggaacaactttggaaagccagagctgaaaaaa
agaaactacgcaaaacgttgcgggaatttgaagaagcattttatcaacaa
aatggaaggaatgcccagaaagaggatcgtgttccagtgcttgaggagta
cagagagtacaagaaaattaaagccaagcttaggcttcttgaagttctta
taagcaaacaagattcttcaaaatccatataagatacgttccttgaaaat
tcatatcataaagtcagttgtattccttgaagctatcatcgtgtgtactt
ggctggtacttaaattcgttttgtcaggcactcagagaatctcattttgt
acttggttgtggtgccactaaacaaaacaaatgatgggagagtaagtagg
ccctctcaagggagcgtaagtgatgttccatattaaccacagactgtcct
ctccacctttagcaaacatatggtttacttcattgttttgtactacagat
acatccttttagcaaaagactggaattttatcctttcaacttcaagaact
ttggaaatgcaagctttttgttattaccaactttttgtttctcattactg
aagaaaattggaggaaaatcttcatactgaattcttctgttgcctttttc
ttacagaatgactgaaaatttgaaagaaaagcctataaaagtgcacatac
gcaactgtgttctctgttttcctagaaaaccaaaatcaaactgagatttc
aagtcgtactatggaacacctttctgtttaattcccaaagtgacatcttc
tcacaggtagtatgttagtaaaattttgcaattcgctggtcacacagctc
ccggatgaagataacagttggtagaccaaaaggtgacctcgcttagtata
ttgaaaacacacaccacacatcacacacagaactgaatcataccacatgc
tgcctatgggacttcagttactgtttcctgagttactgtttatccgctgg
caatggtgtgatttgtctggatcagatccgcgctattccccaacccacct
ccagataatgtgagaaaatagccatgtcagtatgtaggaactctgatggt
gctcagatttgtgtgttcaatcaatgggcttaaatcagcaaaatattatg
cctaaagtaaatctgttcttaacaagggctctaccactgcattttcacat
gtaccttcaggggttatctttttttttttcccctcctattttaatcagc
aaaatccataccaaaacaacgacaacaacgccctcttaagggaccaccct
ttggtcaatcataacatgctgtttaaagcagctgtttacaggatgtgtag
tggtatgccccttgtcatatactcttagcatatcttttttcctttggct
ttgcatggcttttcttcaggtactgtctcggtatcattctgctaatcatt
gttacagaatggtgacttcatttgtgctaacagtacaacagcagatttgg
gtcaggcttaatctaagtgttaactttttttctggtgctttttttggatt
gatgactgtctcactttgactatacccatgttttgcatgcaatgactcat
gcatggttttcttaactagctaatattaacaatttattccatataaaat
ggaattttgcaacatcctttaataaggtgagggaagcatgaacctcagac
ttctggcactattacatagtaagcacatgaagtagtttgataataaatag
cagttctagtacttcacatttcacccgtgtgtgcaatgccttttctggg
gggtgggggtgagggaaaacctggtagtgaatgtgtagttggggaataa
agaaaagcactaaatcctgcccttttgtgtggtttccttttgatacaac
taggttattcataatgtatacctagaaaagtgaaattgaaaataccaaaa
gatgtatcattttatttgaatccatcatgcagtgtacatttcagataat

ttccttcagtctccagataggagtgtatccaaacatctaattttatgtgc
actgtgtatcttatatgaatgttttattttatataccacatgcaaaaatg
tccatatgcactatttaaatgttttaaataatatattccttctttataat
gctaaatctatatgagtaccatatttttataagtcagtggtctgactggt
ttcattttagaattaacagctgcttcaatatgttattcaatgttaatgtt
tggctgtgagtagaatatgtaaaagtggcatggcagcacttatgctctgt
gacagtattgtgtgtcatagttgagcagtagctggtagaattaggcagtt
ggtgatagttttactttggtacaaataaaaactgtatatctatatacaaa
taatatatagatatatatgtccaccagtataatggcattgctgtgtctgg
cacttcattgtacagacttttataataaaagaacttgaaagttctaagtc
attgaaaa
>NM_021914 7
ggatgggacaacttgtatttgtccctttcgcttccacgtccaaaccccctt
taagaaggatgaatgggcaggatgagttagactccttcgctgtatcgtct
actgattcttaaaatgtgacaaatctgattggacgacttacatggcttct
ggagttacagtgaatgatgaagtcatcaaagtttttaatgatatgaaagt
aaggaaatcttctacacaagaggagatcaaaaagagaaagaaagcagttc
tcttctgtttaagcgatgacaaaagacaaataattgtagaggaagcaaag
cagatcttggtgggtgacattggtgatactgtagaggacccctacacatc
ttttgtgaagttgctacctctgaatgattgccgatatgctttgtacgatg
ccacatacgaaacaaaagagtctaagaaagaagacctagtatttatattc
tgggctcctgaaagtgcacctttaaaaagcaagatgatttatgctagctc
taaagatgccattaaaaagaaatttacaggtattaaacatgagtggcaag
taaatggcttggatgatattaaggaccgttcgacacttggagagaaattg
ggaggcaatgtagtagtttcacttgaaggaaaaccattataaaatgacag
tcaagtgccatctggatcttaaggagcttccatttctccagctcagtcca
ttggaatagtattaggttttggtttttgttgtatttccccctttccact
gggcccttccaacacaatgaatgaaggaaatatcatttatttaagcagcc
tatcagtgattgccattagactgttgaatactgttacttttatatagaac
ccaaggaatgccttcctgtcatattttagccaaaacaactggttatatgc
ctcccttgcagcaagcactacaatgtatgtgatcgtcaatgtgaatagct
tagaatactgcaaaggataagctaattgaatgccttgaaagtattatcca
ctggtcagatggtcaactttttttcagtattatttatagttggcacttgat
tgcagttctgtgaggcttgagcattcatacacctcacctgccttggcaag
cctattttagtgatatggcagcacggatataacactatgcattaaaagca
ctttttgtaataagtttaatatcctaaaaggaatgccaattaagttttgt
taactgtgtcatcaacttatcctagtacctcagtgttcattcctgttacc
tgcatatcttcttaaaagaaatagctgttattaatgccttttttgttttcc
attgagtgtacactactgaataagtgtaggagttttatgtttaccatgtg
agtcctgcaacactaaagatattttgaatatcagtcatgatggcaatttc
tgtataaaagagccttaaatggaacattgttttgagatcaaactccccac
cctcacaaaaatggccacgttgcaataaaaaattgtggcatattacagaac
gttgccttgttttccttggaaattttgcaaaatgttatgtgaaacaactt
ctagggtaaaaacagctattactaatctctgcactggtcatttgagaatt
ttttttgtacagcattcatgtgtgatattttccagatttgttggatctat
ttggtttaaaaagtattctatcttaaggccaactaatataaaataccatt
gttaaagaatggtacttttataaacattagtgtatttatttcctatgtgt
taatatgaagatcagaaattattttttgcactttggcataaatacttttc
aatatctgatttgttctctggataaattagcatagttattttttttattca

catttacatttctaagtagttgtatagtagaagcaggaagctcttattgc
ttatttggtcgtaatgaaaataatttgtaaaatgtcctttaaaagtttaa
tgatacttctgatgtttcggaacagtcatttcacctactatttctgaata
tattttgcaaattgaattggaataggaattgataatagcagtcttaaaca
ttagtagtgggatttggctatggtccagactgtgctccttatagagaatt
tgatctgctcagtgtgagcggtttgctgttagccagggctatttatggca
aacacatgcttttgtatcttgtcatagttatccacaaatggcaaaactgg
acttgattctactggtatgcaaaacaggcatgctagtaagcagtcagtcg
tggctcagaacttaaccccatagctcagaggaatgcttttagcagaaaac
aggaaagaaaatatcccttaaaaatttttttgaatgtgtggaagtaatt
ttagtataattagatttttccatatttttgaaagatttttcagatgtga
acattaaaaatagggattaaatgtctaggcttccatttaaaattatatga
atggtttgggatctttttgcactgagcaattttatttcaggcttccagct
gtccctgtgagttatcctggacatttcgatggtttttggtaaggccaaac
tctgataagcaaaacagagaatactgacgtatacttaaccatatgtgtaa
ctgatacttggcaccatggaatttttcattgagttatttcctcattcttt
taaaaaataagggactataaatcagttatgtagtatcttttgtttttgta
gctgattccttaactttcttgtatgcctctagtaatttcagagattaaat
attgctttaaactgtgatactttgatttgctagattgacaaaactgatac
taatataattaagttcatctttgaaatacatctttgtgcgtagagccaaa
aaaagagataaaattaataatagttcacttgttatttgagattaatttgg
catttgaaatgatcattttattttacaatcatttataatgaatcaatgtt
ccagttagctttaaaaggtatacggtgctaattagtaaaatattgaaggc
aatattttactgctagcttgcaaagttatgagagtttaaaaaataaaata
tatgaaaatatgtaaagctgttgagatgtgtttacttatacttcagaaca
ttaaaagtttaaaaactggtatttc
>NM_004896 3
gagcggtcacgtgtgaggggcggcccgaggtcacgtgacggagcgccgga
gcggagggagccggggctgggagttctcctgagggaagaggagtggagta
ggggggacgcggcggcggcgttgacaatgagttttcttggaggctttttt
ggtccaatttgtgagatcgatattgttcttaatgatggggaaaccaggaa
aatggcagaaatgaaaactgaagatggcaaagtagaaaaacactatctct
tctatgacggagaatccgtttcaggaaaggtaaacctagcctttaagcaa
cctggaaagaggctagaacaccaaggaattagaattgaatttgtaggtca
aattgaacttttcaatgacaagagtaatactcatgaatttgtaaacctag
tgaaagaactagccttacctggagaactgactcagagcagaagttatgat
tttgaatttatgcaagttgaaaagccatatgaatcttacatcggtgccaa
tgtccgcttgaggtattttcttaaagtgacaatagtgagaagactgacag
atttggtaaaagagtatgatcttattgttcaccagcttgccacctatcct
gatgttaacaactctattaagatggaagtgggcattgaagattgtctaca
tatagaatttgaatataataaatcaaagtatcatttaaaggatgtgattg
ttggaaaaatttacttcttattagtaagaataaaaatacaacatatggag
ttacagctgatcaaaaaagagatcacaggaattggacccagtaccacaac
agaaacagaaacaatcgccaaatatgaaataatggatggtgcaccagtaa
aaggtgaatcaattccaataaggctatttttagcaggatatgacccaact
ccaacaatgagagatgtgaacaaaaaattttcagtaaggtactttttgaa
tttagtgcttgttgatgaggaagaccggaggtacttcaaacagcaggaga
taattttatggagaaaagctcctgaaaaactgaggaaacagagaacaaac
tttcaccagcgatttgaatctccagaatcacaggcatctgccgaacagcc

tgaaatgtgaactgaacaggagaaaaaaagaaaagcaaaaaactcctgta
acccttgagattaagttcagcaggttaaagatggttgcagctggaggggg
cggaaaaaggccaaaactccatatatgttagtcttcctttatcttacagc
gcagcatttattttatgatataatgaaatgttcgttcatgtatatacatt
tttaaaagtgctttctttgaaacactggaactttgttaagctgccttttt
ttttttaacttcctactttgatgataagcactcagatatatatcagcgta
aacatgaaaaattttcatgtgagtaggctggtatttgtaattttgctttc
tttctgcataatgttgattataaatcctctcttttcaggctaatgattac
ctcttattctctacatgcaaaaaattaaatattttgtgttcaaataaaat
tagaaaacctgagtggcctcttgtgtcctgcagagatttaaaacatggca
tctcaatatttttgagaactacatttgtttttaacatatgtgtttgagaa
aagcatatggagtgtttcaccgcaggcacttctgagtaccattccatggc
ttccagaattttatcctctttgaggtcttctgtgctatgaatattagatt
tctttccccaagggattatgtggcaggtcattatggccttcttttttttg
gccatattaagtaacagttttgctatattccagtagtaccgttgtgtgtt
ttctgcaatgtggagttgacttagcttggcattttagatttgttaaaact
attttttccataaatactttgaaacatatttatatatttcaatttaagga
atctttttgccatgtgtatgcaaatattattttcttcatacattcatttt
cttttcaggggaaaattttgggatgggggactcaggaggacctgtgaagc
atgtagttatctagatctgggtaatttcatgtttattaaactcgaacttt
ggctagttaaactcatattgaaacttcatctagtctcttaatttttttaac
actaaattcaagtcatttgttttaagtctctaaaaaagaagattgcagtc
atccattcatatgcatggggtctgatcgcaaatacactaaatgtggagtg
taggaaccaaaatgaaacctgctgtatggaaactactttcacttatggtt
cattggtttttgtaccaatatttttttatgcacttcagtgcaagtcttgtc
agttaaccttactttatgagtaagctaaataacccaaattacatttcttt
aaacctgttttactactatggcactttgataaaatggtcaggaaccaact
ttactggcaaaagggtccatgtaccaccatgtgctggagcatctgttcta
catgtggatatctatgaatggtaatgttttccttcatgtaagtgcctatt
cagagtttcagaattttaaaatgccaaatattttcatggtcatttgcatg
tagtaagccagaaaatattcaaagagattttgaaaaccaattgtatttaa
ccagcctcaaattgtgcaaccatgatgtataataaagaatttgaaacaga
aaaaaaa
>NM_153042 3
agtcctgtcggctcctcccaccccgggtcacgccgtgacaggggcggaag
cggcggcggcggcggcggccgagaagaggctggggctcgcggcgcggctg
cagccgtcctgtgcgcgcggcgcgcggctccggagaggcgcccgcagtcc
agggcggcgcgcaccgcctcgctggcgctcagagcggtgccttttccccg
agactcccggcacctcttcagcgcaaagattatttaatgtaatggcaact
ccacgggggaggacaaagaaaaaagcatcttttgatcattctccggatag
ccttcctttgaggagctccggtaggcaggcgaagaagaaagcaacagaga
caacagatgaggatgaagatggtggctcagagaagaagtacaggaaatgt
gaaaaggcaggctgtacggcaacatgtcctgtgtgctttgcaagtgcttc
tgaaagatgtgccaaaaatggctacacctcccgatggtatcatctctcct
gtggggaacatttctgtaatgaatgctttgaccattactacagaagccat
aaggatggatatgacaaatatactacatggaaaaaaatatggactagcaa
tggcaaaaccgaacctagtcccaaagctttcatggcagaccagcaactcc
cctactgggttcagtgtacaaaacctgagtgtagaaaatggaggcagctt
accaaggaaatccagcttactccacagatagccaagacttatcgatgcgg

tatgaaaccaaatactgctattaagcctgagacctcagatcattgttccc
tcccagaggatctagaagctcttactcctcagaaatgtattcctcacatc
atcgtccggggtctcgtgcgtattcgatgcgttcaggaagtggagagaat
actgtattttatgaccagaaaaggtctcatcaacactggagttctcagcg
tgggagccgaccagtatcttctccctaaggactaccacaataaatcagtc
atcattatcggggctggtccagcaggattagcagctgctaggcaactgca
taactttggaattaaggtgactgtcctggaagccaaagacagaattggag
gccgagtctgggatgataaatcttttaaaggcgtcacagtgggaagagga
gctcagattgtcaatgggtgtattaacaacccagtagcattaatgtgtga
acaagtatctgctcgctcgtgggaccacaatgaattctttgcccagtttg
ctggtgaccacactctgctaactcccgggtactcggtgataattgaaaaa
ctggcagaagggcttgacattcaactcaaatctccagtgcagtgtattga
ttattctggagatgaagtgcaggttaccactacagatggcacagggtatt
ctgcacaaaaggtattagtcactgtaccactggctttactacagaaaggt
gccattcagtttaatccaccgttgtcagagaagaagatgaaggctatcaa
cagcttaggcgcaggcatcattgaaaagattgccttgcaatttccgtata
gattttgggacagtaaagtacaaggggctgactttttggtcacgttcct
cccagtgccagcaagcgagggctttttgccgtgttctatgacatggatcc
ccagaagaagcacagcgtgctgatgtctgtgattgccggggaggctgtcg
catccgtgaggaccctggatgacaaacaggtgctgcagcagtgcatggcc
acgctccgggagctgttcaaggagcaggaggtcccagatcccacaaagta
ttttgtcactcggtggagcacagacccatggatccagatggcatacagtt
ttgtgaagacaggtggaagtggggaggcctacgatatcattgctgaagac
attcaaggaaccgtctttttcgctggtgaggcaacaaacaggcatttccc
acaaactgttacaggggcatatttgagtggcgttcgagaagcaagcaaga
ttgcagcattttaagaattcggtggacccagctttcttctgtaccccaga
tggggaaatttgaatcacatgttaaacctcagttttataagagggggaaa
aaaccgtctctacatagtaaaactgaaatgtttctaaggcgatatgataa
tgcaaacctatttcatcactctaaaagcactgacctcaaaaaaccttata
agcacttagatttaattgcattttccataggttcaactactgctgaaagt
ctggatttcagaataaagcagaatgtaagtttcagttgaggccatggatt
tgattgttccatggctggaagttccctttagatttcacattttatatggc
tgatcaattttcatacattgagaaaccaagtcaatcaagcaggaatcatt
taaaaaccagataaagccatgtttttcttctgtgacaatttatcagtatc
tttaccaatgagccttaatttttatataggtccaatattgagcttttact
taaaatttagatagaacttttttttggatacagcacaaactccagttgac
agtaaaatgaagcttctaggtattttgtattgtacatatttcctcctact
gggtgttcaaaagaaatttaaattcaagtaccttttgtgataaaatgttt
tagatttgtgcacccattggcaaaacaggaaagtttccagataggtattg
tatcattgagaatgcagcacagatagtgtgggcttcacactatagacaca
gaatatagctttttcttaaagccaaatttgggtgataggacactttaaat
atccttaattttggcaaccactagcaaaaaaacttgtcagaataatttaa
ccaagcccctctccacttcttttatttaaaagcactgattcaattgctag
gaatatttttgcagatttttctttacagtattccataggcaggtccactg
gaaaactgcagaaaaatgtgagctctcctggtaaatagtatacattttat
aagctatattttaaaggcctaagaacatggcaagtatttacttttatctt
ttttttaaaaacactcatgacagaaaacagtttaataatatctcattcta
aaataaaacactggttgcagggtcttcaggatgcctattttgccaagaaa
cttcagtatacaggttagaaatatgcttttgtttttgaacaataatatac

tggtttgctttaaagaagggactaaatatgactttaaagagacttcaaaa
tattgagtattttaaaaatttaaaagtaggtcagtttataacgagtaaat
acctaacacaccaagaatgtgcagtgaacctcaggcatttaagacacctc
ccccaccgcccgccccccgccccccccaatcaaagtgtggtcccaaaaca
agccaacagctgtatatctcaaaagttaacccaagacaactctgatattt
aggttatttgttgagactcattggtactgactggcaagtattctgcttta
aagtatcatgtattaaaatgtttagacagcatgtgttttaaagtgataaa
tgcaaaatgttaagtttgaaatggttaacagtaaattattatgttagttt
ccaggcacttgaactgtgctacaagtaggggaaaacctactttaaagtat
ggtaaatgtgtgttttaaacttcctatcaagtgacatacttcatttgatt
ttttgtttaagaagccatggtactttttcttgagttactttggatatgt
tttttcaatgccatctgaagattttgtaattgagtagcagtaaatataca
gatttacaatgttttaactacagttcatgaatagctggttgtgtaaaact
aataaaaaactagactttcacatgt
>NM_152261 2
ggggcccgggctgcacgagcgcgcgtgcgcgtcggaactggagccgcggg
aggaggcgggagctgaggaggcggcggagggggcgggtcgcgtcgggcgc
cacgtccgcagccagaggcctccgcctccaccaccgccgccacagtcttc
cagctccacatcctgagaggacgcctctggagccgcgactgcccggggtt
gtgccggccgccgctgccgcccaggccgcctcagctctcctctgcgccgg
cccgctcactccgcccggccccagccctagcgctggccgcgaccccggcg
cctttgaaacttctgctggtgtgagtgccctcaggggttccccaggaata
tcgatacaacaccaacaggagatcatgaatcagacagataaaaatcaaca
agaaatcccatcataccttaatgatgaaccaccagaaggttcaatgaaag
atcacccacagcagcagccaggcatgttgtcccgtgtgactgggggtatc
ttcagtgttacaaagggagctgttggtgccaccattggtggtgtggcttg
gattggtggaaagagtctggaagtgaccaaaacagctgttacaactgtgc
cttccatgggaatagggctggtgaaagggggtgtctctgctgtggctgga
ggtgttacagctgttgggtctgctgttgtaaacaaagtgcccttaacagg
aaagaagaaagacaaatctgactgaaatatagagatacacttgcgctcca
cagcactgtaatgccagtggcattgaattgctaaattatggactacaacc
aagtcaactgttttggacgtttatcttctaaactgctgtgttgaaagtat
tgatgactggctttcatctaaaaagaagagaccaatacgagcacagtata
tgaaggtttctcatacttaagttccaggtttttatctggtaaaatgttac
acttactcggttgtaactgaagatatggtatgtttgaatatttactataa
gtctttcagtttgactaaaaatgtgaaagttgaatttagtagatgatctt
cacagttccatatgtataatgtgccaggtaactcacctgccccttaagaa
gggaaccttgaattacataagccgtacctttgatgtgcctaatagtttca
gatgtcttagttttttataaccatagttgattaggccaagaggcattcat
ttcttatttaagctggcaaaattagcaggaattagagaagtttaaaaaga
taaatggttttatgataatgttaaccatcttttgttagtaaatatgcgtt
ctattattttaatcattgatgccttacaaaagaaaacatcttttctaata
ccctgaatatgtgctgttcttagaatcatctatggattcttttaaaggtt
gtttgtgaaattagttttccctttttagaatctcaggagtagtggggtaa
agacatttcctgctgtcagtggataagacagagcttgttaactgttttgg
cagtagttaaaatcaaattgtacacttctcagcctgggttcatgcttcat
cattaatacacctcacagtgcctaaggaacatttacttactggtcagaag
gtattttggaagagtttcatattaaggaggaacaaataataattttaagt
tcttaaaaattacctaaaacaccccaaatataaaaagaagccttcacacc

tattctgtctttaggatgtcttaaattattagcagtactcctttttaaa
aacactgtaaaagtaaccacaaatatgtgaggacttactattttaaatgg
aatggaatgagctccatagattagttttgaatataaagtatataaaagtg
catcagtggtttatataggctttaaaaacatgttatcttacagtccttta
aagcagccatagagtttgtatcatttttcaagccaatttcagtcagggat
ttgaattgtttgattatggatgataaatgtgtcatatcttattaatatgt
ctcatgtctcgttccttcttaatatgatttagctggaattcattttcttt
tcgtttcatgtttaatttcataaaacgtttaacaattggcatatatactt
ggcattcctgtccaccaaggattgtaatccaagcctgggaaaatcttaaa
tttctttttacttaaatctggaaatttgtctccattctgccaccttttttt
tttttttttttatagtggaggggaggaaaggggggtgataccttgcag
taagtaagtcgaaatagcatgcctgaaaatttgaaacagaccattctaac
acccaaggcttgtttataaaatacttgagaattacattaatgtggaatca
acagatgcagaagaatataacataacttttaaaagctttcataaatacca
gcagcaattgtaagcaaatctacaaaggttcttgaacctttctattatat
acaaaactgaaaagtcattaaggagttcaactaatcaggaattaaatggt
catttatttcatgcagtatgatttaaggtatttcttgagattctggtcaa
atgtcataatcagcaaacgggattaaaaaaaaaactccaaaatcactaaa
taattatctaaataatggtattggagaacttgtttcctgctatttggaag
agattgttgcttcattgctagtttgtatttctaacttctacagttataga
ctccactgtgctttgtgtctgaatttctcagtatagacattttgtttact
gtatgcttgcatatttattttcaactttgtttgtctttaaaattgcttga
ggaaaaatggttgtaattaatttctgctacagaaaagccacctggtacgt
tttgtctcatcaggattgttttaaattctaaactataagtttgttcagag
gggcttttgcaatgatagcagaaaactgtacaaatgtacagttagttata
gaggttcttgttgaaatgaacttaccatctgatgatatgtatgtacagct
gtgtacttgagtctttttttagtttacttagaaagactagcagtttgacct
gttaaacaggactagttcaagtcaagaaactaaggttgttgtatacacct
ggaggcatctgttattcagcttatcctttgagtgggtatttggcacaatg
aggataaacttatgtgacccacttgaatggctgatctaataatgttgaca
ttatgcattctgtacttagtgaaatgtcagatgaaaataactgatgaata
atttttttgtattaaagggatgggaaaagaacacatgaatttgttaataa
agcactatgatctgcaaacgatggaatgtttcataaagatctaaagaaat
aaaggaaactttaaaacagaaaaaaaaaaaaaaaaa
>NM_016343 3
gagaccagaagcgggcgaattgggcaccggtggcggctgcgggcagtttg
aattagactctgggctccagcccgccgaagccgcgccagaactgtactct
ccgagaggtcgttttcccgtccccgagagcaagtttatttacaaatgttg
gagtaataaagaaggcagaacaaaatgagctgggctttggaagaatggaa
agaagggctgcctacaagagctcttcagaaaattcaagagcttgaaggac
agcttgacaaactgaagaaggaaaagcagcaaaggcagtttcagcttgac
agtctcgaggctgcgctgcagaagcaaaaacagaaggttgaaaatgaaaa
aaccgagggtacaaacctgaaaagggagaatcaaagattgatggaaatat
gtgaaagtctggagaaaactaagcagaagatttctcatgaacttcaagtc
aaggagtcacaagtgaatttccaggaaggacaactgaattcaggcaaaaa
acaaatagaaaaactggaacaggaacttaaaaggtgtaaatctgagcttg
aaagaagccaacaagctgcgcagtctgcagatgtctctctgaatccatgc
aatacaccacaaaaaatttttacaactccactaacaccaagtcaatatta
tagtggttccaagtatgaagatctaaaagaaaaatataataaagaggttg

aagaacgaaaaagattagaggcagaggttaaagccttgcaggctaaaaaa
gcaagccagactcttccacaagccaccatgaatcaccgcgacattgcccg
gcatcaggcttcatcatctgtgttctcatggcagcaagagaagaccccaa
gtcatctttcatctaattctcaaagaactccaattaggagagatttctct
gcatcttacttttctggggaacaagaggtgactccaagtcgatcaacttt
gcaaatagggaaaagagatgctaatagcagtttctttgacaattctagca
gtcctcatcttttggatcaattaaaagcgcagaatcaagagctaagaaac
aagattaatgagttggaactacgcctgcaaggacatgaaaaagaaatgaa
aggccaagtgaataagtttcaagaactccaactccaactggagaaagcaa
aagtggaattaattgaaaaagagaaagttttgaacaaatgtagggatgaa
ctagtgagaacaacagcacaatacgaccaggcgtcaaccaagtatactgc
attggaacaaaaactgaaaaaattgacggaagatttgagttgtcagcgac
aaaatgcagaaagtgccagatgttctctggaacagaaaattaaggaaaaa
gaaaaggagtttcaagaggagctctcccgtcaacagcgttctttccaaac
actggaccaggagtgcatccagatgaaggccagactcacccaggagttac
agcaagccaagaatatgcacaacgtcctgcaggctgaactggataaactc
acatcagtaaagcaacagctagaaaacaatttggaagagtttaagcaaaa
gttgtgcagagctgaacaggcgttccaggcgagtcagatcaaggagaatg
agctgaggagaagcatggaggaaatgaagaaggaaaacaacctccttaag
agtcactctgagcaaaaggccagagaagtctgccacctggaggcagaact
caagaacatcaaacagtgtttaaatcagagccagaattttgcagaagaaa
tgaaagcgaagaatacctctcaggaaaccatgttaagagatcttcaagaa
aaaataaatcagcaagaaaactccttgactttagaaaaactgaagcttgc
tgtggctgatctggaaaagcagcgagattgttctcaagaccttttgaaga
aaagagaacatcacattgaacaacttaatgataagttaagcaagacagag
aaagagtccaaagccttgctgagtgctttagagttaaaaaagaaagaata
tgaagaattgaaagaagagaaaactctgttttcttgttggaaaagtgaaa
acgaaaaacttttaactcagatggaatcagaaaaggaaaacttgcagagt
aaaattaatcacttggaaacttgtctgaagacacagcaaataaaaagtca
tgaatacaacgagagagtaagaacgctggagatggacagagaaaacctaa
gtgtcgagatcagaaaccttcacaacgtgttagacagtaagtcagtggag
gtagagacccagaaactagcttatatggagctacagcagaaagctgagtt
ctcagatcagaaacatcagaaggaaatagaaaatatgtgtttgaagactt
ctcagcttactgggcaagttgaagatctagaacacaagcttcagttactg
tcaaatgaaataatggacaaagaccggtgttaccaagacttgcatgccga
atatgagagcctcagggatctgctaaaatccaaagatgcttctctggtga
caaatgaagatcatcagagaagtcttttggcttttgatcagcagcctgcc
atgcatcattcctttgcaaatataattggagaacaaggaagcatgccttc
agagaggagtgaatgtcgtttagaagcagaccaaagtccgaaaaattctg
ccatcctacaaaatagagttgattcacttgaattttcattagagtctcaa
aaacagatgaactcagacctgcaaaagcagtgtgaagagttggtgcaaat
caaaggagaaatagaagaaaatctcatgaaagcagaacagatgcatcaaa
gttttgtggctgaaacaagtcagcgcattagtaagttacaggaagacact
tctgctcaccagaatgttgttgctgaaaccttaagtgcccttgagaacaa
ggaaaaagagctgcaacttttaaatgataaggtagaaactgagcaggcag
agattcaagaattaaaaaagagcaaccatctacttgaagactctctaaag
gagctacaacttttatccgaaaccctaagcttggagaagaaagaaatgag
ttccatcatttctctaaataaaagggaaattgaagagctgacccaagaga
atgggactcttaaggaaattaatgcatccttaaatcaagagaagatgaac

ttaatccagaaaagtgagagttttgcaaactatatagatgaaagggagaa
aagcatttcagagttatctgatcagtacaagcaagaaaaacttattttac
tacaaagatgtgaagaaaccggaaatgcatatgaggatcttagtcaaaaa
tacaaagcagcacaggaaaagaattctaaattagaatgcttgctaaatga
atgcactagtctttgtgaaaataggaaaaatgagttggaacagctaaagg
aagcatttgcaaaggaacaccaagaattcttaacaaaattagcatttgct
gaagaaagaaatcagaatctgatgctagagttggagacagtgcagcaagc
tctgagatctgagatgacagataaccaaaacaattctaagagcgaggctg
gtggtttaaagcaagaaatcatgactttaaaggaagaacaaaacaaaatg
caaaaggaagttaatgacttattacaagagaatgaacagctgatgaaggt
aatgaagactaaacatgaatgtcaaaatctagaatcagaaccaattagga
actctgtgaaagaaagagagagtgagagaaatcaatgtaattttaaacct
cagatggatcttgaagttaaagaaatttctctagatagttataatgcgca
gttggtgcaattagaagctatgctaagaaataaggaattaaaacttcagg
aaagtgagaaggagaaggagtgcctgcagcatgaattacagacaattaga
ggagatcttgaaaccagcaatttgcaagacatgcagtcacaagaaattag
tggccttaaagactgtgaaatagatgcggaagaaaagtatatttcagggc
ctcatgagttgtcaacaagtcaaaacgacaatgcacaccttcagtgctct
ctgcaaacaacaatgaacaagctgaatgagctagagaaaatatgtgaaat
actgcaggctgaaaagtatgaactcgtaactgagctgaatgattcaaggt
cagaatgtatcacagcaactaggaaaatggcagaagaggtagggaaacta
ctaaatgaagttaaaatattaaatgatgacagtggtcttctccatggtga
gttagtggaagacataccaggaggtgaatttggtgaacaaccaaatgaac
agcaccctgtgtctttggctccattggacgagagtaattcctacgagcac
ttgacattgtcagacaaagaagttcaaatgcactttgccgaattgcaaga
gaaattcttatctttacaaagtgaacacaaaattttacatgatcagcact
gtcagatgagctctaaaatgtcagagctgcagacctatgttgactcatta
aaggccgaaaatttggtcttgtcaacgaatctgagaaactttcaaggtga
cttggtgaaggagatgcagctgggcttggaggaggggctcgttccatccc
tgtcatcctcttgtgtgcctgacagctctagtcttagcagtttgggagac
tcctccttttacagagctcttttagaacagacaggagatatgtctctttt
gagtaatttagaaggggctgtttcagcaaaccagtgcagtgtagatgaag
tattttgcagcagtctgcaggaggagaatctgaccaggaaagaaacccct
tcggccccagcgaagggtgttgaagagcttgagtccctctgtgaggtgta
ccggcagtccctcgagaagctagaagagaaaatggaaagtcaagggatta
tgaaaaataaggaaattcaagagctcgagcagttattaagttctgaaagg
caagagcttgactgccttaggaagcagtatttgtcagaaaatgaacagtg
gcaacagaagctgacaagcgtgactctggagatggagtccaagttggcgg
cagaaaagaaacagacggaacaactgtcacttgagctggaagtagcacga
ctccagctacaaggtctggacttaagttctcggtctttgcttggcatcga
cacagaagatgctattcaaggccgaaatgagagctgtgacatatcaaaag
aacatacttcagaaactacagaaagaacaccaaagcatgatgttcatcag
atttgtgataaagatgctcagcaggacctcaatctagacattgagaaaat
aactgagactggtgcagtgaaacccacaggagagtgctctggggaacagt
ccccagataccaattatgagcctccaggggaagataaaacccaggctct
tcagaatgcatttctgaattgtcattttctggtcctaatgctttggtacc
tatggatttcctggggaatcaggaagatatccataatcttcaactgcggg
taaaagagacatcaaatgagaatttgagattacttcatgtgatagaggac
cgtgacagaaaagttgaaagtttgctaaatgaaatgaaagaattagactc

aaaactccatttacaggaggtacaactaatgaccaaaattgaagcatgca
tagaattggaaaaaatagttggggaacttaagaaagaaaactcagattta
agtgaaaaattggaatatttttcttgtgatcaccaggagttactccagag
agtagaaacttctgaaggcctcaattctgatttagaaatgcatgcagata
aatcatcacgtgaagatattggagataatgtggccaaggtgaatgacagc
tggaaggagagatttcttgatgtggaaaatgagctgagtaggatcagatc
ggagaaagctagcattgagcatgaagccctctacctggaggctgacttag
aggtagttcaaacagagaagctatgtttagaaaaagacaatgaaaataag
cagaaggttattgtctgccttgaagaagaactctcagtggtcacaagtga
gagaaaccagcttcgtggagaattagatactatgtcaaaaaaaaaccacgg
cactggatcagttgtctgaaaaaatgaaggagaaaacacaagagcttgag
tctcatcaaagtgagtgtctccattgcattcaggtggcagaggcagaggt
gaaggaaaagacggaactccttcagactttgtcctctgatgtgagtgagc
tgttaaaagacaaaactcatctccaggaaaagctgcagagtttggaaaag
gactcacaggcactgtctttgacaaaatgtgagctggaaaaccaaattgc
acaactgaataaagagaaagaattgcttgtcaaggaatctgaaagcctgc
aggccagactgagtgaatcagattatgaaaagctgaatgtctccaaggcc
ttggaggccgcactggtggagaaaggtgagttcgcattgaggctgagctc
aacacaggaggaagtgcatcagctgagaagaggcatcgagaaactgagag
ttcgcattgaggccgatgaaaagaagcagctgcacatcgcagagaaactg
aaagaacgcgagcgggagaatgattcacttaaggataaagttgagaacct
tgaaagggaattgcagatgtcagaagaaaaccaggagctagtgattcttg
atgccgagaattccaaagcagaagtagagactctaaaaacacaaatagaa
gagatggccagaagcctgaaagtttttgaattagaccttgtcacgttaag
gtctgaaaaagaaaatctgacaaaacaaatacaagaaaaacaaggtcagt
tgtcagaactagacaagttactctcttcatttaaaagtctgttagaagaa
aaggagcaagcagagatacagatcaaagaagaatctaaaactgcagtgga
gatgcttcagaatcagttaaaggagctaaatgaggcagtagcagccttgt
gtggtgaccaagaaattatgaaggccacagaacagagtctagacccacca
atagaggaagagcatcagctgagaaatagcattgaaaagctgagagcccg
cctagaagctgatgaaaagaagcagctctgtgtcttacaacaactgaagg
aaagtgagcatcatgcagatttacttaagggtagagtggagaaccttgaa
agagagctagagatagccaggacaaaccaagagcatgcagctcttgaggc
agagaattccaaaggagaggtagagaccctaaaagcaaaaatagaaggga
tgacccaaagtctgagaggtctggaattagatgttgttactataaggtca
gaaaaagaaaatctgacaaatgaattacaaaaagagcaagagcgaatatc
tgaattagaaataataaattcatcatttgaaaatattttgcaagaaaaag
agcaagagaaagtacagatgaaagaaaaatcaagcactgccatggagatg
cttcaaacacaattaaaagagctcaatgagagagtggcagccctgcataa
tgaccaagaagcctgtaaggccaaagagcagaatcttagtagtcaagtag
agtgtcttgaacttgagaaggctcagttgctacaaggccttgatgaggcc
aaaaataattatattgttttgcaatcttcagtgaatggcctcattcaaga
agtagaagatggcaagcagaaactggagaagaaggatgaagaaatcagta
gactgaaaaatcaaattcaagaccaagagcagcttgtctctaaactgtcc
caggtggaaggagagcaccaactttggaaggagcaaaacttagaactgag
aaatctgacagtggaattggagcagaagatccaagtgctacaatccaaaa
atgcctctttgcaggacacattagaagtgctgcagagttcttacaagaat
ctagagaatgagcttgaattgacaaaaatggacaaaatgtcctttgttga
aaaagtaaacaaaatgactgcaaaggaaactgagctgcagagggaaatgc

atgagatggcacagaaaacagcagagctgcaagaagaactcagtggagag
aaaaataggctagctggagagttgcagttactgttggaagaaataaagag
cagcaaagatcaattgaaggagctcacactagaaaatagtgaattgaaga
agagcctagattgcatgcacaaagaccaggtggaaaaggaagggaaagtg
agagaggaaatagctgaatatcagctacggcttcatgaagctgaaaagaa
acaccaggctttgcttttggacacaaacaaacagtatgaagtagaaatcc
agacataccgagagaaattgacttctaaagaagaatgtctcagttcacag
aagctggagatagaccttttaaagtctagtaaagaagagctcaataattc
attgaaagctactactcagattttggaagaattgaagaaaaccaagatgg
acaatctaaaatatgtaaatcagttgaagaaggaaaatgaacgtgcccag
gggaaaatgaagttgttgatcaaatcctgtaaacagctggaagaggaaaa
ggagatactgcagaaagaactctctcaacttcaagctgcacaggagaagc
agaaaacaggtactgttatggataccaaggtcgatgaattaacaactgag
atcaaagaactgaaagaaactcttgaagaaaaaaccaaggaggcagatga
atacttggataagtactgttccttgcttataagccatgaaaagttagaga
aagctaaagagatgttagagacacaagtggcccatctgtgttcacagcaa
tctaaacaagattcccgagggtctcctttgctaggtccagttgttccagg
accatctccaatcccttctgttactgaaaagaggttatcatctggccaaa
ataaagcttcaggcaagaggcaaagatccagtggaatatgggagaatggt
agaggaccaacacctgctaccccagagagcttttctaaaaaaagcaagaa
agcagtcatgagtggtattcaccctgcagaagacacggaaggtactgagt
ttgagccagagggacttccagaagttgtaaagaaagggtttgctgacatc
ccgacaggaaagactagcccatatatcctgcgaagaacaaccatggcaac
tcggaccagcccccgcctggctgcacagaagttagcgctatccccactga
gtctcggcaaagaaaatcttgcagagtcctccaaaccaacagctggtggc
agcagatcacaaaaggtcaaagttgctcagcggagcccagtagattcagg
caccatcctccgagaacccaccacgaaatccgtcccagtcaataatcttc
ctgagagaagtccgactgacagccccagagagggcctgagggtcaagcga
ggccgacttgtccccagccccaaagctggactggagtccaacggcagtga
gaactgtaaggtccagtgaaggcactttgtgtgtcagtacccctgggagg
tgccagtcattgaatagataaggctgtgcctacaggacttctctttagtc
agggcatgctttattagtgaggagaaaacaattccttagaagtcttaaat
atattgtactctttagatctcccatgtgtaggtattgaaaaagtttggaa
gcactgatcacctgttagcattgccattcctctactgcaatgtaaatagt
ataaagctatgtatataaagctttttggtaatatgttacaattaaaatga
caagcactatatcacaatctctgtttgtatgtgggttttacactaaaaaa
atgcaaaacacattttattcttctaattaacagctcctaggaaaatgtag
acttttgctttatgatattctatctgtagtatgaggcatggaatagtttt
gtatcgggaatttctcagagctgagtaaaatgaaggaaaagcatgttatg
tgtttttaaggaaaatgtgcacacatatacatgtaggagtgtttatcttt
ctcttacaatctgttttagacatctttgcttatgaaacctgtacatatgt
gtgtgtgggtatgtgtttatttccagtgagggctgcaggcttcctagagg
tgtgctataccatgcgtctgtcgttgtgctttttttctgtttttagaccaa
ttttttacagttctttggtaagcattgtcgtatctggtgatggattaaca
tatagcctttgttttctaataaaatagtcgccttcgttttctgtaaaaaa
aaaaaaaaaaaaaaaa
>NM_006282 2
gcggaagtgtgggagggtctgcggggcgggctcaggaggtccgcgggagg
atggagcagtgagcgggtctgggcggctgctggcagcgccatggagacgg

tacagctgaggaacccgccgcgccggcagctgaaaaagttggatgaagat
agtttaaccaaacaaccagaagaagtatttgatgtcttagagaaacttgg
agaagggtcctatggcagcgtatacaaagctattcataaagagaccggcc
agattgttgctattaagcaagttcctgtggaatcagacctccaggagata
atcaaagaaatctctataatgcagcaatgtgacagccctcatgtagtcaa
atattatggcagttattttaagaacacagacttatggatcgttatggagt
actgtggggctggttctgtatctgatatcattcgattacgaaataaaacg
ttaacagaagatgaaatagctacaatattacaatcaactcttaagggact
tgaataccttcattttatgagaaaaatacaccgagatatcaaggcaggaa
atattttgctaaatacagaaggacatgcaaaacttgcagattttggggta
gcaggtcaacttacagataccatggccaagcggaatacagtgataggaac
accattttggatggctccagaagtgattcaggaaattggatacaactgtg
tagcagacatctggtccctgggaataactgccatagaaatggctgaagga
aagccccccttatgctgatatccatccaatgagggcaatcttcatgattcc
tacaaatcctcctcccacattccgaaaaccagagctatggtcagataact
ttacagattttgtgaaacagtgtcttgtaaagagccctgagcagagggcc
acagccactcagctcctgcagcacccatttgtcaggagtgccaaaggagt
gtcaatactgcgagacttaattaatgaagccatggatgtgaaactgaaac
gccaggaatcccagcagcgggaagtggaccaggacgatgaagaaaactca
gaagaggatgaaatggattctggcacgatggttcgagcagtgggtgatga
gatgggcactgtccgagtagccagcaccatgactgatggagccaatacta
tgattgagcacgatgacacgttgccatcacaactgggcaccatggtgatc
aatgcagaggatgaggaagaggaaggaactatgaaaagaagggatgagac
catgcagcctgcgaaaccatcctttcttgaatattttgaacaaaaagaaa
aggaaaaccagatcaacagctttggcaagagtgtacctggtccactgaaa
aattcttcagattggaaaataccacaggatggagactacgagtttcttaa
gagttggacagtggaggaccttcagaagaggctcttggccctggacccca
tgatggagcaggagattgaagagatccggcagaagtaccagtccaagcgg
cagcccatcctggatgccatagaggctaagaagagacggcaacaaaactt
ctgagcaaggccaggctgtgagggccccagctccacccaggctttgggtg
aattctggatggcttgcctcatgtttgttagccagcacttctgctctgtc
gtctctccacagcacctttgtgaactcaggaatgtgcgccagtgggaagg
gctctcttgacagtcagcgtgccatcttgatgtgtgtatgtacattggtc
aggtatattatctcaaaggatttatattggcgcttttaactcagagtttt
aaaccccaggaacagagactcctagttgagtgatagctgggaaagtttta
cattgtctgtttttcttctcccaatagctttcaattgttctttctggaag
acttttaaaaaaatataaatatgcatatatatatataaattataaataga
ttccccacgcagtgtggtggcatctctgtacaggtacagttttaaacggt
ttgcctcttttctgtaagattatggtactgtggaacatgagggcagagga
caccgggaggctgttagggggtcactgaatcccaggagccaacctccccc
tttgcagggctgcatttaaaaattaggtttgggacagttcttgtaccgtg
gtttcagccttgtgtggtcatcactggcttctggagctattggtgatgtc
caagggaaagctttgagagtttatgtttactctttgagtcccaggagaag
cctggcaccctctttgcaaattggcctttgctctttcaatgcctttcatc
catctccactctctcaactgcctaaagtcacagcacagatactgcccagt
gccttaagaggagacatgatctctaccagggactctcagcaaacacggga
ctgtgttcagtccacaaaggaaaagcgtttttgaagctctcattgttcat
gtaaaaatcatacacgtggcatgttgctccacattccttacacacagggg
tagaggggattgcttttgtgacccacgttcaaatatgtgactgttttctt

ttctcttttactgctaagcagcctggaaaggataaatgaatattagacta
agatttgttttccaggaggctcaatctgaacacacagaatgtcagagctg
gaagggactatagagatcatctgatctgatcctcttgtacggatgatcgc
aaaactgaggtgtagagaggggaatggccaaaatcacaaagcaagttagc
gttaagagctgagactagaattcagggtcctcactcccaggccaccgaac
catgcagccccttctttggggggaagagacctgtgtcagtcttggttaatt
gttccagggaaccttgctaacagaaacttgctcttgccttggctcttcag
tagatgacctggctgtaaagagattccctggacgagccagatcattcagt
ttcagcgagtccttgagctccacaacatctaccagatatagcagacaagc
acccatggaggcaggtttcgggcctgaagcagatcagagggctttgcaaa
agacagcatagagccatcttcctgcaactttacctctttccctcagatgg
ggagccatgactgggttgcacctcaggatactgtaatttgactccataat
tgcttttgctcctgaaacctgggaatcaatggaaaggcagggaatgtgcc
tcttctgtggccagattctgttatttgcaattaaagcaagtttttaaaaa
atgcaagaggcagttgttagtcttcagggcttggcaactgaaatagctat
gtggcggatacggaaaacagaggacaatttgaggatcttgctggaataat
aaatgacagctaccatttgttgagcacctattatatatcaggcactgagc
tgggtaggctctaaacttcacaataaccctgtgacttaactactttatct
ccattttgtagttgaagaaataagttcagagagaaagattccttcccaag
gtcatgcagctagtaaatgatagaatcaggattcatagcatcactatagg
gggtcaatatttacacaaaaaaaggaaagtcacaagcctgtttaaaatgaa
gtgaccaccttttcttgcatagactaaataactcgaactggcatttttag
gttggaaagacagctgaattagtagttaagtctgatagccaagtaagttt
taaaaaccaaagcatccaggatgcacacccctgcaccatttgctgtgcga
attaatagttctgtctctctctctctttcttttttcttttattctttga
gatggattttcgctcttgtcgcccaggctggagtacaatggcacgatctt
ggctcactgcaacctccgcctcccgggttcaagcgattcttctgctggga
ttacagcatatgccaccatgcccagattattttttttgtatttgtagtaga
gacggggtttcaccatgtcagtcaggctggtcttgaactcctgacctcag
gtgatccacccgcctcagcctcccacactgctgggattacaggcatgagc
caccgctcctggcctctctttctttttttaaacaaagaactttgcacttgg
ccagagaggaggagaaagcccattttctcccttcctaagctagatccaaa
taaaagaaagttcagttttcccccataactattcttgggtcatgaacttt
gatctggagtttgttttgtttcaggaatgtgtgcacccagcttgctgatc
caacaaagtctattgcttaccagtctagcttgatgaagccttttggccag
aagtcaatttgttttggatcagagaaatttcctgacaaggtatatttgtt
ttctagtgacagaaaggcaaaggaacaagtcctagttgttgttgttgttg
ttgaatactaaatttaagatatgtcagcttgctttcaatgagccttgggc
ttctgttattgcttgagcatttggaactcgagcttccagagaaatttgag
gtcctcgcttgttctctgccttcaagaaacaatgacctgattctgtcttt
aaaaaaaaaaatctcagaattctttttttgtttgtgtttttttttttttt
tgagacagagtctcactctgttgcccaggctggagtgcagtggcgccatc
tcggctcactgcaacctccgcctcccaggttcaagcaattctcctgcctc
agcctcccaggtagctgccactacaggtgctgcaccaccacgcccggcta
atttttgtatttttagtagagacagggtttcaccatattagccaggtggg
tcttgaactcctgaccttgtgatccacccgcctcggcctcccaaagtgct
gggattacaggcgtgagccaccttgcctggccaaaaatctcagaattctt
taagactgttttaattgctccatcagtaattttgaagcactttccttttt
tttttttttccccttttttgtccctttccccaagccaccaattggatgga

tgaatgtttgacggggaagaggaagggtaggaggatgcatggatgagtgg
atgagtggatcgatggatgtattgataaatagatagaaccagtcatctga
agcaacttaagaattgtagccttgactccttgagactgtagatttcgatc
caggaaacatttatttagcacctgccagatgccagaaatttataccattt
aaaactcagtaagtcttttaaatatcaggaaggagagaagcgacatcatg
atacatcctatgggtattaaaaagccaatagaatattatgaataattta
tgctaataaatttaacaacttcaacatcataaacaaattccttgaaaaat
aaaaagtaccaaaattcattcaagaagaaatagataccagcctgagcaac
atggcaaaatcccatctctacaaaacatcaaaaaaaaaaaaaaattagtcg
ggcatggtggtgcacacctgtaatcccagcttgtcaggaggctgaagtgg
gaggatcacctgagcccagggaggtcaaggatgcagtgagccatggtctc
accactgcactctagcctgggtgacagaatgagaccccgtctcaaaaaaa
aagaagaagtagataatctgaatagccctatatctatagaaacttaatag
tgctgggagatataggtattattatcctcattttacagatgtgaaaattg
aggctcagagaagtaaagtctattgctcaaggtcatgtggctagaatatg
gcagagccatgattcagatccaggtcttctgattcttattccagtgtcct
ttctagcataccatgttgcctctaaagattgcagctccttatttactaga
aaattgttcctgcccaatctacatctccacctcaccccatcttttcttaa
gcactatgtttgtgtttttatcagtattatattcattgtctttggaatac
atgttcttgtttgtgtttggaaaaaaaatctcttttaccagcttgcactc
ggaccaacttggaaaaaaaaaaagcttaaatgtttttgctatgtacagttt
aaaaatgtgaagtttgtagctttaactttttgtaagaaaatctaataaca
ctggcttaagtgctgacttgaaatgctattttgtaaggtttggatgtaag
taatcaattgaggtcagcagtttgtatgagacatagcttcctccattgcc
cccactcctttttctttttaagtttgagatgcttcctgtgtttttatg
ttagaattgttgttctccttcttttcttcttcctatacctcatcacgttt
gttttaaataaactgtcctttggaccacaaaaaaaaaaaaaaaa
>NM_004427 3
cattgtctccgcggcggctgcagccctcgagcgcccgccgcgcgcgcg
cgcaaccccggccgccgcccgcgctcccgccccggcctcgcgcccccgtc
ccggcctcgcgccccggccgccctttgttgacgccggccaggccggtgcg
gtcggatgcgccgcggcagccccgggccccggctcggaggctcccggcgg
agaggaggcggcccgcccgggcccgggaccccgcgcgagtcggcgcccgg
ccgaggggctgcgtaggccccgcccggccaggcccagccgggccctggac
agagacagggcagggcattgttcatgcactgaccgacctcagcagccccg
gcatgacctcagggaacggaaactctgcctccagcatcgccggcactgcc
ccccagaatggtgagaataaaccaccacaggccattgtgaaaccccaaat
cctgacgcatgttatcgaagggtttgtgatccaggagggggcggagcctt
tcccggtgggacgctcgtccctgctggtggggaatctcaagaagaagtat
gcacaggggttcctgcctgagaaacttccacagcaggatcacaccaccac
cactgactcggagatggaggagccctatctgcaagaatccaaagaggagg
gtgctcccctcaaactcaagtgtgagctctgtggccgggtggactttgcc
tataagttcaagcgttccaagcgcttctgttccatggcttgtgcaaagag
gtacaacgtgggatgcaccaaacgggtgggacttttccactcagaccgga
gcaagctgcagaaggcaggagctgcgacccacaaccgccgtcgggccagc
aaagccagtctgccaccacttaccaaggataccaagaagcagccaacagg
cactgtgccccttttcggttactgctgctttgcagctaacacacagccagg
aagactccagccgttgctcagataactcaagctatgaggaacccttgtca
cccatctcagccagctcatctacttcccgccggcgacaaggccagcggga

cctggagctccccgacatgcatatgcgggacctggtgggcatgggacacc
acttcctgccaagtgagcccaccaagtggaatgtagaagacgtctacgaa
ttcatccgctctctgccaggctgccaggagatagcagaggaattccgtgc
ccaggaaatcgacgggcaagccctgctgctgctcaaggaggaccacctga
tgagcgccatgaacatcaagctggggcccgccctgaagatctacgcccgc
atcagcatgctcaaggactcctagggctggtggcagccaggattctggcc
cagggcgcctcctcccgactgagcagagccagacagacattcctgagggg
cccagaaatggggccggttggagggcaggggctctccctaggggcatagc
tggtgaggaggtctgggcacctcctccatggctctcaggggcctttcatt
tctgtgggaggggcagagaggtaggtggcacagaagatggggctttatgc
ttgtaaatattgatagcactggcttcctccaaagtcccaatactctagcc
ccgctctcttcccctctttctgtcccccattttccagggggtatatggtc
agggctccccaacctgagttgggttacttcaagggcagccagcaggcctg
gatggaggcctagaaagcccttgccttccttcctcccacttctttctcca
ggcctggttaactcttccgttgtcagcttctccccttcagcctgtttct
gcagcagccagggttctccccctacaccctctgcaggtggagagagaga
agctgggcccagccgggccgtgcctgctggcacagacgccttaacgctgt
gtgtatgactgtgtgactgtgtgggagcctggactgacagataggccaag
ggctactctctggcatctccaggtgttttgtagcaaacagccacttagtg
ctttgtcctggactccactcagcctcaggatggggaatagccaagaatgg
cagcctcagcgcagaggcaaggtcagaaagagacggcgcttcagagtttc
ctttccagacacccctccccgcactgtgaagttcccctgaccgccctcct
ggttcacaaagagcattaagaaagctgcggtggtctgagcaacatagccc
aaagggctgagcctcctggcctgcctgcccgcccaccctgggagtcccag
tggtgaggctcagagaactgctaaggggaaagaacagctggagtttctgt
tgatgtgaagaaggcagctcttggcctcccactcccacacttctttgcct
ataaatcttcctagcagcaatttgagctacctgaggaggaggcagggcag
aaagggcgagggcctgcctctgacctgccgtgtcctttgcaggaaggagg
taggcacctttctgagcttattctattccccacccacacccccaggcagg
gttggaaatgaaggactttttaacctttgttttgtttttaaaaataaa
tctgtaaaatctgtct
>NM_152345 4
gcagccccgcgagcaggcagcgccggcccccgccccgcggccccgggcc
ccggctccggcgccgtccctcctccccggccgggcgccgcggccccggca
tgaggagcgggcgatgatccccgccaacgcctccgccaggaaggggcccg
agggcaagtatccgctgcactacctcgtgtggcacaaccgccaccgcgag
ctggagaaggaggtccgcgcgggccaggtggacatcgagcagctggatcc
ccgcggccggactcccctgcacctggccaccacgctggggcaccttgagt
gtgcccgtgtgctcctggcgcacggcgcagacgtgggcagggagaatcgc
agcggctggacagtgctccaggaggctgtgagtacccgggacctggagct
ggtgcagctggtgcttcggtaccgggactaccagcgggtggtgaagcggc
tggcgggcatccccgtgctcctggagaagctgcgcaaggcccaggacttc
tacgtggagatgaaatgggagttcactagctgggtgcccctggtgtccaa
gatctgccctagtgacacctacaaagtgtggaagagcggccagaacctga
gggtagacaccacactcctgggctttgaccacatgacctggcagcgaggg
aaccgcagctttgtcttcaggggccaagacacaagcgccgtggtcatgga
gattgaccacgaccgccgggtggtgtacacagagactctggcactggctg
ggcaggaccgggagctgctgctggctgctgctcagcccactgaggaacag
gtgctgagccggcttaccgcgcccgtcgtcaccactcagcttgacaccaa

gaatatctcctttgagaggaacaagactggcatcctgggctggcgcagtg
aaaagacggagatggtgaatgggtatgaagctaaggtgtatggggcatct
aacgtggagctcatcacccgcacacggacagaacatctttcagaacagca
caagggcaaggtcaaaggctgtaagacacctttgcagtccttcctgggaa
tcgctgagcagcacgggggcccccaaaatgggaccctgatcactcagact
ctgagccaagccaaccccactgccatcactgcagaagaatacttcaaccc
caactttgagctgggcaaccgtgatatgggccgccccatggaactgacca
ccaagacacagaagttcaaggccaagctgtggctgtgtgaggagcatccc
ctgtccctgtgtgagcaggtggcccccatcattgacctcatggccgtcag
caatgcgctttttgccaagctccgggacttcatcaccctgcgtctgcctc
ctggcttcccagttaagattgaaatcccgatcttccacatcctcaacgcc
cgcatcaccttcgggaacctcaacggctgcgacgaaccggtgccatcggt
gcgaggcagccccagcagcgagacgccttccccaggcagcgactcctcca
gcgtcagcagctccagctccacgacctcctgccgcggctgcgagatctcc
ccagcgttgttcgaggccccgcgcggctacagcatgatgggcggccagcg
ggaggcggcgacccgggacgacgacgacgacctgctgcaattcgccatcc
agcagagcctgcttgaggcgggcagtgagtatgaccaggtcaccatctgg
gaggcgctaaccaacagcaagccaggcacccaccccatgtcctacgaggg
tcgccgacaggacaggagcgccccgcccacgccgcagcgccagcctgcgc
ccccggcgtcagtgcccagccctcggcccagctcagggccaggttccggc
ggccacgtgttccggagctacgacgagcagctgcggctggcgatggaact
gtcggcgcaggagcaggaggagaggcggcggcgcgcgcgccaggaggagg
aggagctggagcgcatcctgaggctctcactgaccgagcagtagcgcccc
ctgccgggaccctcgccagcgccacgcgcgccacgcccagggccaggagc
cagacaaaccccggcctgcgcgcctgcagagcggcggctggagactggag
ccaccgcctcgcgggtgcagcagcacagcaggcacggttcggggagggat
tcggcatggccgcggggtaccttcccaggccagggccctggaggcaactg
gcacggcctggtccccctgctttgctgtattctgattccccaacccgctc
ccctgggctcagatctgtcctgtcctagggcggagccaggcggtcctgag
ggggagatgaatccttagaggagcgctgtccctatcccttgctccttctg
gccggtccgcatttctgttcactaaccccactccaggacactgcccctga
agcctttgcatctctgctcttcactcctgggggcagctgagctccccgc
gtgctgtgtcactgctttgttccagacacaaaccagcacgtctagggccc
agcccctcccccaccccggcatttcagcgtcaagtgcacttagcggggta
cccggctcccccagcccccacacccgtcctgggtctcagggtggttccag
cttctccttgggcagccagaagttggagtcccatcccccaaggcacattt
tttttttttttttttgtgatcagggagtgtgtccaacgtagccccctgg
cctgtgcaagccctgactccctcatggtgcctcggagagtggggagcata
ttgggctggggtaagcactagacccaagtagactggacacaaagggctcg
cccagggccctggcgccacccccaccccttcccaccagctgctgctagcc
tctgtggttgtacatcccacttgcccccacacggagactgactctaaaac
ccttcatccaatggtgctaacccccggctctcccctgccccacctcaccc
acccagagaagcacagaccccgccaggggcaggggcccaccgcacaccct
tgtcccgggcctgtctgggactggccttcccggctcagccagtgaggctc
agaagggacacaaagagggatggaagaaaagaacaaagagaaactgttcc
tcccaccccccttccctgatgccaggggcaccagactgattctgaggcaca
aataaaagaggcttcataccggaaaaaaaaaaaaaaaaaaaaaaaaaaaa
aaa
>NM_153369 2

gatagcggttggcgagaggccacatttccgccacgtgacccgcgcatgcg
cctgcttgctggagagcgagcgtcttttgccacgaacacctgcggcgtgc
cgaagttcctcttctctgccccggttccgggcggtccgctgggaggtggc
gcccggagggacacccgtgcctggggtcggggccagtgctcttctcggag
ctccagaagttctgctgagaagcgcggcggcagcaagacgacttctccgg
agccgccgagctggagttagaggtggagctccgtggggccgggcccccgg
ctgcggggcagcggctcctgcaggcggaggccccggcggagaatgagccg
gagccggaggtggtggtggtctcctggcagagcggtgggaccgggagcaa
gctgcggtggttcaccaccttgatgctgtgtgcctccttcctggggctgg
gattgagtgttgctatagtgggacccacgtttcaagatttggcaacaaac
gtgaaccgaaatatcagtagtctgtctttcatttttgtgggtcgtgcctt
gggatatttgagtggctctgtgattggtggatttcttgtcgatgtcatga
attattttttacttttgggaatctcaatgtcggctaccaccgttggtctt
tatcttgttcctttttgcaagacagcaatattactcactgtcatgatgtc
tatcttcggtgtttcaattggcattctggatacaggtggtaacgtcctta
tcttggctatttggggggacaaaggagccccacatatgcaggccttacac
ttctcttttgccttgggtgcctttttggctccactgctagctaaactggc
tttgggtccgacagcgtctgctgaaaaccacacagagtctgacttccatc
ctgcactcaaccaatcatctgatgctgactcagaagctctgtttggagta
cctaatgataagaatttactgtgggcttatgctgttatcggtacttacat
gttcttagtttctgtcattttttttgtctgtttttaaagaatagctcaa
agcaagaaaaagcaagagcatctgctgagacatttcgaagagcaaaatat
cacaacgcccttctttgtctcctttttctgttcttcttttttatgttgg
agctgaggtaacatatggctcttatgttttctcatttgcaaccacccatg
ctggcatgaaagaaagtgaagctgctgggttgaactccatcttctggggg
acatttgcagcctgcaggggcctggcaatctttttgctacctgtttaca
gcctggaaccatgattgtgttgagcaacattggcagcctgacttcatctt
tatttctggtgctttttgacaagaacccaatttgtctctggatagcaact
tcagtgtatggggcttcaatggcaaccacatttccgagtggtgtttcttg
gattgagcagtacacgaccatccatgggaaatctgcagcatttttttgtaa
ttggtgcttccctgggagaaatggctattcctgcagtcattggaattctt
caaggaaaataccctgatttgcctgtagttctgtatacctctttgggagc
atcaatagctactggtattttatttcctgtgctatataaattagccactt
cacctcttgatcgccagcgaaaagaagacagaaagagtgaggaccagaaa
gctctgctctctagctccgggctaaatgaatatgaggaagagaatgaaga
ggaggatgcagaaaaatggaatgaaatggattttgaaatgattgaaacga
atgatacaatgaggcattctataatagagacatctagaagtagtctgacg
gagcccacagctgaagtctataatcaatacccatcaaatgcactggtgtt
tgagtcttctccttttaatactggcagtgcccatgtgaagcacttgccag
aaaccaggacaaaagggactaacgtttagagaagatggattactcactga
catctttgaataactgccacttctaaggacgccattcagagcagagcatt
agcagaatttcaccatgctttggggattaaaatttttaggtccatattat
agcaaatgctaaaaatttttgaaatgttctgtaattcccagagtcttcca
taaataaccaaatggtctcatacacgtacaataggatcttgttataacgc
tatcatttgacatgtgactgattaggaaatattttatggtctccacccct
cagagcacccaaagaaggcattgtttttgagaagggggggtattctcagaa
tgagaccatgtaggagttgaattgattggttgaacaaattatcaggtatc
aatatttttccaggaaagttgaggagaatagtttcataaaagagaaatgg
gccgggtgcagtggctaaagcctgtaatcccagcactttgggaggccgag

acgggcagatcacgaggtcaggagatcgagaccatcctggctaacacagt
gaaaccctgtctctactaaaaatacaaaaaattagccaggcgtggtggca
ggcacctgtagtcccagctacttgggaggctgaggcaggagaatggcgtg
aacccgggaggtggagcttgcagtgagccgagatcatgccacaccgtact
ccagcctgggcgacatctagactccgtctcaaaaaagaaatggagaggta
tttttattaattgctttaccttatctgtagggttggatcaaagaatgtaa
tgactcatttgatttctaatttcaatgaagagaacacagaacagggccca
aggggtcgggcttcaagccttgacttcttctagccctgcactctgtcact
taacgtaggccttgtttatctcgcctcctgatctgtgattctagaaatga
aaatgagatgatttccaaattgggaatctgtaagttttaaagtttgtgca
aaatgtctgctagaaattagtacaagtgacagcatgttcttcatttcaaa
tgagaagtgaaaccaaaatgataaatagctttaagaatgtgctaatgata
aatgattacatgtcaatttaatgtacttaatgtttaataccttatttgaa
taattacctgaagaatatatttttagtactgcatttcattgattctaag
ttgcactttttacccccatactgttaacatatctgaaatcagaatgtgtc
ttacaatcagtgatcgtttaacattgtgacaaagtttaatggacagtttt
ttcccatatgtatatataaaataatgtgttttacaatcagtggcttagat
tcagtgaaatacagtaattcattcaattatgatagtatctttacagacat
tttaaaaataagttatttttatatgctaatattctatgttcaagtggaat
ttggagacactatgttctaattttaagaactgtttcagtgttattagatt
agtagtagggctgtagatggggtctttcgaggtcctttgcctacctcagt
aacagaggctgggtctgagccttgctcgagattagctcttcaccaggtct
gtgtggtaatcatatcgtagttaccgtaggtcacgctgcaagtgtaattt
acagtgaggcatagtcttttccagtgattggaatacttgtgtcaccgatc
cagggtttctctttcctgagtctgaaatacctgggaaaccagttagaagg
tgactgggtagcctaggagtcaaggatcagaaagggtacttcatatatat
gtggcagaattggtgcaggtgatcctttgaaaaatgacaagaattgatga
aattttgtttacttcctccaatttttttgctgaataacatctcctttctac
ctgattggagtaggcttgctttctcttacttaatttctataaatttgcca
ttgttttgtctcaagtaaaaaaaaaaaaaa
>NM_013446 3
accaatgagcggcgccgtgggcgggccgcgggcgcgcatgctctgggccc
gcagttgaagtggcgagagcgctcagatacgcgacgcgtagcaggcgggg
accgaacgggtgcctcagtgtccttcccctcccctcgcctggcctcgccg
tcctctccccgcagccggaccggaactatgtgatcccggaagttccgggg
cctttgctgtgtgggataaacagtaatggcggaggctgcaactcccggaa
caacagccacaacatcaggagcaggagcggcagcggcgacggcggcagca
gcctcccccaccccgatccccacagtcaccgccccgtccctgggggcggg
cggagggggcggcggcagcgacggcagcggcggcggctggactaaacagg
tcacctgcaggtattttatgcatggggtttgtaaggaaggagacaactgt
cgctactcgcatgacctctctgacagtccgtatagtgtagtgtgcaagta
ttttcagcgagggtactgtatttatggagaccgctgcagatatgaacata
gcaaaccattgaaacaggaagaagcaactgctacagagctaactacaaag
tcatcccttgctgcttcctcaagtctctcatcgatagttggaccacttgt
tgaaatgaatacaggcgaagctgagtcaagaaattcaaactttgcaactg
taggagcaggttcagaggactgggtgaatgctattgagtttgttcctggg
caaccctactgtggccgtactgcgccttcctgcactgaagcacccctgca
gggctcagtgaccaaggaagaatcagagaaagagcaaaccgccgtggaga
caaagaagcagctgtgcccctatgctgcagtgggagagtgccgatacggg

gagaactgtgtgtatctccacggagattcttgtgacatgtgtgggctgca
ggtcctgcatccaatggatgctgcccagagatcgcagcatatcaaatcgt
gcattgaggcccatgagaaggacatggagctctcatttgccgtgcagcgc
agcaaggacatggtgtgtgggatctgcatggaggtggtctatgagaaagc
caaccccagtgagcgccgcttcgggatcctctccaactgcaaccacacct
actgtctcaagtgcattcgcaagtggaggagtgctaagcaatttgagagc
aagatcataaagtcctgcccagaatgccggatcacatctaactttgtcat
tccaagtgagtactgggtggaggagaaagaagagaagcagaaactcattc
tgaaatacaaggaggcaatgagcaacaaggcgtgcaggtattttgatgaa
ggacgtgggagctgcccatttggagggaactgtttttacaagcatgcgta
ccctgatggccgtagagaggagccacagagacagaaagtgggaacatcaa
gcagataccgggcccaacgaaggaaccacttctgggaactcattgaggaa
agagagaacagcaacccctttgacaacgatgaagaagaggttgtcacctt
tgagctgggcgagatgttgcttatgcttttggctgcaggtggggacgacg
aactaacagactctgaagatgagtgggacttgtttcatgatgagctggaa
gatttttatgacttggatctatagcaaccttgcgtggcgtgtgaactggt
ctgctgacctcagacagcagctgtcccctgtggtggtgtggcagtgcctg
tgttctctcctaggcaggcctctcaactccaggtgctgtcctaagaattt
ttacccagggcctgtcttctcaacccctcacctttccctgaggagtgtgt
tgttttccctgttgaaaaaagttacaaaaataaatcttaaagttagtttt
ttgtaacacgaatttaactgtcagacagttagtgtaggtgtgttgcgtca
tctgttttcaaccagattgcatttatggacttttcacacactcattttga
ggaccccaggttcaaaagtaaaagcagtggccctgctttggggtccaaga
ataggagtgatgggtgaagggacctaagctggccaatagccctctgcccc
agacatgggatgtggatccttgaggtttctggtgaaatctgcacatctgt
gtttttatatctgttccctaccctgtaatccctaccacgtgcacttgttc
tgtggttttggtctcttgtttaattgcacacaagtaatactactgggtaa
ccagaatcaggtgtgaatgtgttgagatttttttactgttttgcatgatag
gaaaattgagaaagaatacgtataaaagatagagaggcataacatcaatg
cagagttggaagttggctcccaagggctgacatggtgtgagtgtgtgggt
gtgtgataagcttctcatccctgcatagatgcagtattcttagccttagt
agaaaaacctggtttagtggtttaagccttgtgtggcagatagatcttaa
agggcaaagcagtatattggtagttgtcaatatagcagtgctagctctgt
ctatataaatagagaaatggggttagccatagaggttaaaactacctggt
tatcccatataataacacaaactgggtcttggatacacagttgtatttaa
tgttttacgatctagcctttccagtacaggcactttctgagaaacctttg
tcctcacttgaggcattttgttgtcgggtttttgtgtttgtttttgtggg
tatttgcctcattccacccctgagctttcaggtagacagacgtgattcaa
aactctgttctaaggtgtttattgtagtggagtaatgggtttgcagtgat
aagtcatacttttccaccgaaagggagggcttgggaatccctgagattag
ctaaagttaagttgttggaagaattccttgattggaaattgtacctttgt
gttttgttgctctgtttcctgaaaataactcggggatgctcctggtttgt
ccatctactgctttgattccttggatcccacccattctttcactttaaga
aaaaacaaataattgttgcagaggtctctgtattttgcagctgcccttttt
gtaagaagcacttttcccaaataaaacaattaaaaaaaaagttggctgtt
caaaaaaa
>NM_178818 2
agcggaccgagccgcagccgcagccgggcggcgggcgagaggcggcggcg
gcgggcgggccgcgggcagtcagtcgggcggcggcggcggcggcggc

ggcgatgcggcggccccgctgagtccgcccgctcctggcgccgggagcca
gccgcgcgaggcggcccgggccgggcggcagcatgcggagcggcgaggag
ctggacggcttcgagggcgaggcctcgagcacctccatgatctcgggcgc
cagcagcccgtaccagcccaccaccgagccggtgagccagcgccgcgggc
tggccggcctgcgctgcgaccccgactacctgcgcggcgcgctcggccgc
ctcaaggtcgcccaagtgatcttggccctgattgcattcatctgcataga
gaccatcatggcatgctccccgtgtgaaggcctctactttttgagtttg
tgagctgcagtgcgtttgtggtgactggcgtcttgctgattatgttcagt
ctcaacctgcacatgaggatcccccagatcaactggaatctgacagattt
ggtcaacactggactcagcgctttccttttctttattgcttcaatcgtac
tggctgctttaaaccatagagccggagcagaaattgctgccgtgatattt
ggcttcttggcgactgcggcatatgcagtgaacacattcctggcagtgca
gaaatggagagtcagcgtccgccagcagagcaccaatgactacatccgag
cccgcacggagtccagggatgtggacagtcgccctgagatccagcgcctg
gacactttttcctactccacaaacgtaacagtaaggaaaaaatcacccac
aaacctgctgagtttgaatcactggcaacttgcctagaaaagttcagatg
atgggtccttgaactgtgtatctatttcatagggtaagtttgttgtctat
gtttgtttttttatgctgaggctttaattcaggtgacttgctatctagac
acctggggtggcgtgttttctttgggatgcataatacacaaaggccaggg
agcctttggcttggacccccagggagctattgaaacttttaaagtttgag
tttaggagtaagaacatatctcagaccaccttagtcagctttgctgatgg
gatttagggccaggtcagagtttcttttcataactgcctgaaaagagaga
tggtgtgaaacaagattctgataatggtttgtgtgagatttgatcatagt
ctaaaactatcacgtctgagttgccttaggatgacagtgctgacacccag
taggaagtatcccatttttatcaggaaagtcagtcacgcgtagggatggt
gaggagacgcgtagggatggtgaggaggggagaggagggagacctgctgg
tgcccttgcaccagggtgaggcctgactcacgctgcttccccccacaggc
cctgctttgcttgcctgctttttccagaatcgattttgcaagcttcaaga
ttctgttcccctcttcgcagaagtgaggaaggcaaatactcagggtttga
agggagacctggccggcctgagggctggcagatgtgagggcaggacacct
gggatggactcgtaggctgacccaggcccaaagggggctgcctgttccca
actctttcactctgtaacccattttaaaatgagtttttgaatcttgcctc
aaattgacctacttggataaaatcagtgcttttcctaacttgattttgtt
tgacgtggttccctctaagagaatggtaggaattgaaactatttgtatat
gttgaaatttgtaggggttcaggaacccatggcagaaacactaaactatt
tatttacaagtatgactatttttttttcaaagtaggcaattctttgtata
ttttaaggcaaataatcacttcacctctggtgccttccatgcgtcgcata
agcactcttgtcaatcatggaattggaaaaaaagatgtacatttagttaa
acaagataacactatttttgtagcatgattttagattgtgtccttttaat
attgttttcaacaatggtagttttgaggttttggccattttctctttaca
cttcgtatgtattttaaagaagaaaaaaaacaaaaaaaattccctggcta
ttcctctttcctctctgctgctgcttctcctgcatcttgttcctgcttat
ttaaatagtctgagagtaagagtatatttctgtgaaataattataagtca
gtataattgcatttggcatttttttaaccacttgctcagtttggagccact
gctcacctcaaacagtacaaagaaagtggttccatattttacaaacagaa
gagtagcatcagagccctgacctcctgtctttgggtgtaaaatggtgaga
ggccggtgtccggctccttcccctgtggtttgggctggtgaactgagccc
aagtgtattctctggcctgggccccttccagaatcagtgcgagcgtcctg
cagaggcatggccttcctgccactgcagagtgggaagcttcctgggggac

ctcagccagctccggggctctcatcttccactcggatctcagattggttc
gttgtggatgctctggctaggctcggcggccttttccaggcttcaggcct
gaacaaaacaggctgtttgctgtgaggagctagatgacaccaagacacag
acacgtactcttcaccggctccttttgcttctccttcctgcaggacgtga
gttatgtctggtcccaaaacccttgagtaaactagctgagccattgcaaa
tgaaggtacagatggattgaaacataaaattgtagcttttggtgtccttc
tgaagggtcagaatatgtaattatttgataaccaaggaagtcaagtcaga
tggctaagtagtaactttccaatatgatttctccgtggtacttcacctgg
tggcacttttggggggcaatgttaacaactatcacattttactcatcaag
tctctttcatttcggcagtttaaagacacaactgtgggctttctgaggcg
atgcccgttgggcaaacttaggtttatgtgtttggtttttcagctgacgt
ggaagccctgggtgggcgtcccctggctggcctggcttttgggttcaagt
ctgtatgtatggagtgggttggggccccaaagctttgctgctattgattt
gtccccgacagcaggcaaagcctcacattgccctcaattttccccctttc
atttgaaggaccagtttttcatggtacttgtcttgatctttttcatagtg
ttcaaatctgtatttttgtatgtagagggaaataatttctcaacactaa
tcgttaataactattgtattaaattgtcatgaaggaacttgtttaataaa
tggacgtgtaaggaaaaaaaaaaaaaaaaa
>NM_016019 3
gcgggaaacgactgagcgcgaggagcgtgcgcgctcccgtcgtggcgacg
gtggcggcgagcggcgtcagagcttgaggggggggttgacggcttctggcg
ggtggcggtgttgaaggcgagagcttgcttggcccgtgtcgcttctgtcc
caagaaccggacggagagtgagggcacgagggtcgctgtcgggggctgtc
gtcttccacgtacacgtcgtcgtgaggagcgcagtccggactcttcccgc
aacccctccggctccctttccgcacgcctcgaggcggcggcggccaccga
gacagcagcgcaccttccccccatcccttccccttatcccccagcccaaaa
gggcccggtctgcgccccacccccgcccgtccgcccgctacgccgccgcc
atgtcggcgcaggcccagatgcgcgcgatgctggaccagttgatgggcac
ctcccgggacggagatacaactcgtcaacgaatcaaattcagtgatgaca
gagtatgcaagagtcaccttctcaactgttgtcctcatgatgtcctttct
ggaactagaatggatcttggagaatgtctgaaagtccatgacctggcttt
aagagcggattatgaaattgcatccaaagaacaagatttttttctttgaac
ttgatgccatggatcatctgcagtcattcattgcagattgtgatcgtaga
acagaagtggccaagaaaagattagcagaaactcaagaagagattagtgc
tgaagtagcagcaaaggcagaacgtgttcatgagttaaatgaagaaattg
gtaaattgttagccaaggtggaacaactaggagctgaagggaatgtggag
gaatcccagaaagtaatggatgaagtagagaaagcacgggcaaagaaaag
agaagcagaggaagtttatcggaattctatgccagcttccagttttcagc
agcagaaacttcgagtctgtgaagtctgctctgcctatttaggacttcat
gataatgacagacgactggctgatcattttggggggtaaactgcacctggg
atttattgaaataagagagaagcttgaagaattaaagagagtcgtagctg
agaagcaggagaaaagaaaccaggaacggctgaaacgaagagaagagaga
gagagagaagaaagggagaagctgaggaggtcccgatcacacagcaagaa
tccaaaaaagatccaggtccagagagcatcgcagacatcgatctcgctcca
tgtcacgtgaacgcaagaggagaactcgatccaaatctcgggagaaacgc
catcgccacaggtcccgctccagcagccgtagccgcagccgtagccacca
gagaagtcggcacagttctagagataggagcagagaacgatccaagagga
gatcctcaaaagaaagattcagagaccaagacttagcatcatgtgacaga
gacaggagttcaagagacagatcacctcgtgacagagatcggaaagataa

gaagcggtcctatgagagtgctaatggcagatcagaagacaggaggagct
ctgaagagcgcgaagcaggggagatctaactagctgtgtacatttcttca
gtccttaagcttcctacggagttacgtactattgtttagttcacagctgt
tcagggtgacagtgagcagatccagacaccagatccagctaggctagatg
tacagtatctaacttgatctgaactgaacctgttttccttgatgatgcct
aaaactacatccatagtttctggtgaacctgtaatacagttctgaaagta
cagttttatataataagatgctgatctctttattctttcaagtaagagtg
ctagtgaacaaattgtgttacttgccttgggatttttgaacgtttgtaa
aatgctgtcttcctagtccaaacagctgcagctttgggcatttttctttt
taattattcttcctctgactttgtatcccttaatacctacactctccaat
tgtaagagaaagggggcagggaagcaatatagcttccattctaaggctgt
attcccgttatgaattactagctgattacagttcagagcattgatcctgg
aatgtgtgctggagaaatttaaaatactggggtttttttgtttaatggtgc
ctatttagagttggaagttgaacagctgttgcattacatacttttgcttt
tttattgaaattttgaaatcaaacgtcttgatttttctgttctgttgaat
tgctatgttcaggatgttctagggggtgggggcagggactcttttcgtaa
taagcacttgttttattttgtgtgtgtggagtataaaggctacacccctta
ttgtaaaaaaataataataataaaatgaaagaaacaatcaccaccaccat
tattaaactgtaacttgtctagtaaattgtcactagaactatttgctgtg
ggcatttcctctattctgttacgtcattaacagtgccttactgtgcaagg
caccaaaggacataaatacgtacttgcaaagattccagatgagctgttca
tgtgggccccttgctgactatattccagccacttcagggttgtttgtaat
gaccgttatagagaagggctcgacctgcagaagaaactggggtgttttta
ttctcattcaacaaacccaaataaaaacagtatatgtaaccaacat
>NM_004233 3
gacgggggcgccccggcctaagcgggactaggagggcgcgccacccgctt
ccgctgcccgccggggaatcccccgggctggcgcgcagggaagttcccga
acgcgcgggcataaaagggcagccggcgcccgcgcgccacagctctgcag
ctcgtggcagcggcgcagcgctccagccatgtcgcgcggcctccagcttc
tgctcctgagctgcgcctacagcctggctcccgcgacgccggaggtgaag
gtggcttgctccgaagatgtggacttgccctgcaccgccccctgggatcc
gcaggttccctacacggtctcctgggtcaagttattggagggtggtgaag
agaggatggagacaccccaggaagaccacctcaggggacagcactatcat
cagaaggggcaaaatggttctttcgacgcccccaatgaaaggccctattc
cctgaagatccgaaacactaccagctgcaactcggggacatacaggtgca
ctctgcaggacccggatgggcagagaaacctaagtggcaaggtgatcttg
agagtgacaggatgccctgcacagcgtaaagaagagacttttaagaaata
cagagcggagattgtcctgctgctggctctggttattttctacttaacac
tcatcattttcacttgtaagtttgcacggctacagagtatcttcccagat
ttttctaaagctggcatggaacgagcttttctcccagttacctccccaaa
taagcatttagggctagtgactcctcacaagacagaactggtatgagcag
gatttctgcaggttcttcttcctgaagctgaggctcaggggtgtgcctgt
ctgttacactggaggagagaagaatgagcctacgctgaagatggcatcct
gtgaagtccttcacctcactgaaaacatctggaaggggatcccaccccat
tttctgtgggcaggcctcgaaaaccatcacatgaccacatagcatgaggc
cactgctgcttctccatggccaccttttcagcgatgtatgcagctatctg
gtcaacctcctggacatttttttcagtcatataaaagctatggtgagatgc
agctggaaaagggtcttgggaaatatgaatgcccccagctggcccgtgac
agactcctgaggacagctgtcctcttctgcatcttggggacatctctttg

aattttctgtgttttgctgtaccagcccagatgttttacgtctgggagaa
attgacagatcaagctgtgagacagtgggaaatatttagcaaataatttc
ctggtgtgaaggtcctgctattactaaggagtaatctgtgtacaaagaaa
taacaagtcgatgaactattccccagcagggtcttttcatctgggaaaga
catccataaagaagcaataaagaagagtgccacatttatttttatatcta
tatgtacttgtcaaagaaggtttgtgtttttctgcttttgaaatctgtat
ctgtagtgagatagcattgtgaactgacaggcagcctggacatagagagg
gagaagaagtcagagagggtgacaagatagagagctatttaatggccggc
tggaaatgctgggctgacggtgcagtctgggtgctcgcccacttgtccca
ctatctgggtgcatgatcttgagcaagttccttctggtgtctgctttctc
cattgtaaaccacaaggctgttgcatgggctaatgaagatcatatacgtg
aaaattatttgaaaacatataaagcactatacagattcgaaactccattg
agtcattatccttgctatgatgatggtgttttggggatgagagggtgcta
tccatttctcatgttttccattgtttgaaacaaagaaggttaccaagaag
cctttcctgtagccttctgtaggaattcttttggggaagtgaggaagcca
ggtccacggtctgttcttgaagcagtagcctaacacactccaagatatgg
acacacgggagccgctggcagaagggacttcacgaagtgttgcatggatg
ttttagccattgttggctttcccttatcaaacttgggcccttcccttctt
ggtttccaaaggcattttattgcttgagttatatgttcactgtcccccta
atattagggagtaaaacggataccaagttgatttagtgtttttacctctg
tcttggctttcatgttattaaacgtatgcatgtgaagaaagggtgttttt
ctgttttatattcaactcataagactttgggataggaaaaatgagtaatg
gttactaggcttaatacctgggtgattacataatctgtacaatgaacccc
catgatgtaagtttacctatgtaacaaacctgcacttatacccatgaact
taaaatgaaagttaaaaataaaaaacatatacaaataaaaaaatcccgac
tttgggatgagtgctaggatgttgtaaa
>NM_003764 3
agatgcggccgcggcggcgcggagctcgggcggccgtggaggaactcagc
ctcggccgcaggaggcgccgggagcggagccgccgggagtcgcgcaacag
gtttccttctccatcgctgcgcccacaggggacgcgcgccctgccgggag
aggggcttctcggttcgcactctcgctcccagtccaggcaaaatgaaaga
ccggctagcagaacttctggacttgtccaagcaatatgaccagcagttcc
cagacggggacgatgagtttgactcgccccacgaggacatcgtgttcgag
acggaccacatcctggagtccctgtaccgagacatccgggacattcagga
tgaaaaccagctgctggtggccgacgtgaagcggctgggaaagcagaacg
cccgcttcctcacgtccatgcggcgcctcagcagcatcaagcgcgacacc
aactccatcgccaaggccatcaaggcccggggcgaggtcatccactgcaa
gctgcgcgccatgaaggagctgagcgaggcggctgaggcccagcacggcc
cgcactcggcagtggcgcgcatttcgcgggcgcagtacaacgcgctcacc
ctcaccttccagcgcgccatgcacgactacaaccaggccgagatgaagca
gcgcgacaactgcaagatccgcatccagcgccagctggagatcatgggca
aggaagtctcgggcgaccagatcgaggacatgttcgagcagggtaagtgg
gacgtgttttccgagaacttgctggccgacgtgaagggcgcgcgggccgc
cctcaacgagatcgagagccgccaccgcgaactgctgcgcctggagagcc
gcatccgcgacgtacacgagctcttcttgcagatggcggtgctggtggag
aagcaggccgacaccctgaacgtcatcgagctcaacgtacaaaagacggt
cgactacaccggccaggccaaggcgcaggtgcggaaggccgtgcagtacg
aggagaagaaccccctgccggaccctctgctgcttctgctgtccctgcctc
aagtagcaggccggcccgggccgccaccgcccatcccagaccatggagcg

cgctgggaaggacgcaccaaagccgggagctctgccctgcagggagttgc
cccaacccttccggaactcagtctttagaaaagaaacgccaggttcaag
aattgcaaaccagcctgtgcttggaaagatggttagttgataccgtccga
tgattcttcagtaaagatagattcccacaaagttgtgcaatgtcattata
tgacaccttgcactcttaccgtcttgacagaagccaagtaaggaactgaa
gttgtatctgactgtagggtgaatgtctgaggcctgcctcctaataaaga
ctcaaggaggaagtcaattgggcatctgctaatagaatgaactcatgatg
gaaacttcagttcatttactttgtcctgaaaattccctggttctgttcca
ttttgagcgaaattggccttgggaaaaaccacgttcttcctttccgattc
ttcatccggtctacgctatgcaattcctccccaaatatagatcttatttc
tgctcatttcccctacttattaaaatcacaccaaacacttactattttct
tatctctttcactttttaaatatctttcaccaggttatattttggtatta
tttttccaaacatttttaagcactgaatatcgaacaagcactcaaattga
agtatcagtcatgttttgtgtatttttcgctgataaaaattatttaacat
ttatatttttacttgattacatatgcacatgtatgtaaatgtaaaatact
aatattcactaatatatgtacataatgatcaattggtttaacttctttta
tgtaagtatggtatataaatttcaagacgaacacttttctggctcttggt
attggtttgcttgttttgagtttgtttcactccagtttgccccttcctag
tccagtttgggtcaaacttcatgttaaacaactctgcattggttatggcg
gtagacatatggcggtagaaaatgtatacggagctagagacaactaacat
tcttggaaatactgcttttgttttactgtggaccattccttccatgcatt
gaaatggagaaattcaaagtaaaagaattctgtttttcaagcaagcttaa
taaacattacattatacacatatttttatacatttctggcttgaccattt
agtttactttctcaattattgttaaaatttttctttttcctttttttttt
tttttttttgagatggagtctcactgtgttgcccaggctggagtgcagtg
gcaggatcttggctcattgcaacctctgtctcccaggttcgagcgattct
cctgactcagcctcctgagaagttgggactctgggcgcgtgccacaatgt
ctggctaattttttatgtttttagtaaagacggtgtttcaccgtgttagc
caggatggtcttgatctcctgacctcgtgatccgcccgcctcagcctccc
atagtgctgggattacaggtgtgagccaccatgcctggcctttttttcccc
ccttttgagacagggtctccctttgtcacccaggctgaagtgcagtggca
taatcatggcttactgcagccttaaactcccaggctcaagtgatcctccc
acctcagcctacaaatagctgagactacagatatgtgccaccatgcccgg
ctaatttttgtattttctgtagagacagggtttgccatgtggcccaggct
ggtctcaaacttgtgagcttgagcaatccgcccaccttggcctcccaaag
tgctgagattacaggcctgagccactgaccctggccaaattttttttcta
ctagctactgaggctgccacatctggatggaactgagtggagggggaaaa
gaatgaaaaactcaaaagaattcccatgagggtgtcttgctttctctcct
gagttacaatactttagcaaaatcatgaggctttagagatatggtgtagt
ctgcaaacttcttaatgcccttacccacatttaccatgtttcctggcctt
cctctgtgtcaactcttagctcttcctaatcattatttaatacatgagtg
agtttagtagtgatcatatttctcaggtcctttagaagctggaattttaa
aagaattagaaggaggagtatgtgaattctttggagctcactgcctgact
tgcttatgaccaggaaaatctatcccctgtatctaattttaatttcatgg
ttaaatttgagaattgtggaaaccaagttccacaaggctattctcatatt
tctcccaatttctttttcagccaactccaaggatatgtatcacctttgac
ttaatttgctttctctaagggaaaggggaaaaaaatgttcacatagctcca
ctgcaatgtttttttataatagaggagagatattgtaaatagagactgcca
gccagtttccacaaaaaaacgaagagttcataaatttgacatgtttgaac

ccataaagcattttctttgcttggaaccattataaaagtaagtgagtttt
caggctctatatacattttaattcctcacgttttatattggagagttcgg
tacagactgtccattactgcaccaaaagaatgagtgaactgttacctata
gggaaagaacacttcttcttcctgctgtttgggaaccatctcagtgtggc
gtaatggttaggagtacagattccagatcctgtttcttagatttaaatct
tgactctgccacatactagctgtctgactgaaccttggtttttctgtgct
tcagtttcctcatctgtaaaacggagataacagtacttacctcatagagc
tgttgtgaaaagtgatgactgaatatgtaaaagcacctagaacagtgcct
ggcacatgctaagtgctttgttcattattgttgttattatgtaattttct
ctcagactgagagcactgttagtgacccaagtaaatttatagtttttaag
tacagaggaaaaataaagcctatttttttgttaacagtcttaataaataat
aaaatggaataaagaaaccaagaccccatcttctgtgaatattagggctt
tttttttttgacagtcataaagatgttttcactatggcatttctatccc
tgtgtatatccaaacatgtcctgaagaagaaatgagatgttccaccaaaa
acacgtaagcaggaagcagctgttctgctcagcttggcaggtgttctttc
ctaattcttcccaagctgtgagtcagaaagtcctggaaggagttgtagga
agttgtagaggctgggtcactgacctaagagaaggcatcatttggcccac
tgcacgtcctggcctattcaccaaagcccttcctggctctgactgccaca
ccaggcagtgggtgaaatgctggctttttccttaagaaattgtgttctag
tgccaccaagagatgctgtagagctggctttaccaatctcatgatgcttg
cttggcaactctgaaaggtgactttggccaagaagaccttgtggcaattc
tgcaaattttatacactcatatcttttagggtacaaaatgaaagaacaaa
tcacaaagaacaatagatccttcaggagctgaaggtaagaatcttttata
gctattttaacatatacagtgactactttctactagccaaatatcaaatt
ttacaactaccaccaagccacagattataggtggtaacaactccagaaat
gtcctaactaggaaaggtgctcatctagtatgcatcggtatccaggataa
tatgagttagaattttaaaaatgtcagtcattcaaaaatatttgaactgt
gacatcacagaagtaattttatggccttttaaggtaacaacttaaaaaga
gaacagtactctttttatatcaatgcctttacatttatttaaaaacagtc
ctaatgctttatagttaaatgtcatatgcagatatgttcaggctctaaca
tataaagttcctaacttgacaggaaactactgaagattgtgtacagctta
aaaaaaaaaatagggtaactatagtcttgatttttatgtataaattctat
cattctatattttaccatcagacatatttctactcctttctttgaagtat
gcgaagtatctccaactgcagcatgcaactcattcatttgtaatcaagac
gatagtttgaaacacccaattgtaatcagagcaacagttgacttcctttt
gatagcggagttgaaaatcattgcaattaataaaatggggctattagaaa
tggaaaacgaataggatctagaatgtaacttcatcatataaatgatgagt
gtctttgttatcaacacgttattaagaatgggcaagatgtccttatatac
tagaagcttttgtaaagtcatgtgtctattgataataaagattttcggaa
ctgaaaaaa
>NM_003981 2
gcttcgccccgtggcgcggtttgaaattttgcggggctcaacggctcgcg
gagcggctacgcggagtgacatcgccggtgtttgcgggtggttgttgctc
tcggggccgtgtggagtaggtctggacctggactcacggctgcttggagc
gtccgccatgaggagaagtgaggtgctggcggaggagtccatagtatgtc
tgcagaaagccctaaatcaccttcgggaaatatgggagctaattgggatt
ccagaggaccagcggttacaaagaactgaggtggtaaagaagcatatcaa
ggaactcctggatatgatgattgctgaagaggaaagcctgaaggaaagac
tcatcaaaagcatatccgtctgtcagaaagagctgaacactctgtgcagc

gagttacatgttgagccatttcaggaagaaggagagacgaccatcttgca
actagaaaaagatttgcgcacccaagtggaattgatgcgaaaacagaaaa
aggagagaaaacaggaactgaagctacttcaagagcaagatcaagaactg
tgcgaaattctttgtatgccccactatgatattgacagtgcctcagtgcc
cagcttagaagagctgaaccagttcaggcaacatgtgacaactttgaggg
aaacaaaggcttctaggcgtgaggagtttgtcagtataaagagacagatc
atactgtgtatggaagaattagaccacaccccagacacaagctttgaaag
agatgtggtgtgtgaagacgaagatgccttttgtttgtctttggagaata
ttgcaacactacaaaagttgctacggcagctggaaatgcagaaatcacaa
aatgaagcagtgtgtgaggggctgcgtactcaaatccgagagctctggga
caggttgcaaatacctgaagaagaaagagaagctgtggccaccattatgt
ctgggtcaaaggccaaggtccggaaagcgctgcaattagaagtggatcgg
ttggaagaactgaaaatgcaaaacatgaagaaagtgattgaggcaattcg
agtggagctggttcagtactgggaccagtgcttttatagccaggagcaga
gacaagcttttgcccctttctgtgctgaggactacacagaaagtctgctc
cagctccacgatgctgagattgtgcggttaaaaaactactatgaagttca
caaggaactctttgaaggtgtccagaagtgggaagaaacctggaggcttt
tcttagagtttgagagaaaagcttcagatccaaatcgatttacaaaccga
ggaggaaatcttctaaaagaagaaaaacaacgagccaagctccagaaaat
gctgcccaagctggaagaagagttgaaggcacgaattgaattgtgggaac
aggaacattcaaaggcatttatggtgaatgggcagaaattcatggagtat
gtggcagaacaatgggagatgcatcgattggagaaagagagagccaagca
ggaaagacaactgaagaacaaaaaacagacagagacagagatgctgtatg
gcagcgctcctcgaacacctagcaagcggcgaggactggctcccaataca
ccgggcaaagcacgtaagctgaacactaccaccatgtccaatgctacggc
caatagtagcattcggcctatctttggagggacagtctaccactcccccg
tgtctcgacttcctccttctggcagcaagccagtcgctgcttccacctgt
tcagggaagaaaacaccccgtactggcaggcatggagccaacaaggagaa
cctggagctcaacggcagcatcctgagtggtgggtaccctggctcggccc
ccctccagcgcaacttcagcattaattctgttgccagcacctattctgag
tttgcgaaggatccgtccctctctgacagttccactgttgggcttcagcg
agaactttcaaaggcttccaaatctgatgctacttctggaatcctcaatt
caaccaacatccagtcctgagaagccctgatcagtcaaccagctgtggct
tcctgtgcctagactggacctaattatatgggggtgactttagtttttct
tcagcttaggcgtgcttgaaaccttggccaggttccatgaccatgggcct
aacttaaagatgtgaatgagtgttacagttgaaagcccatcataggttta
gtggtcctaggagacttggttttgacttatatacatgaaaagtttatggc
aagaagtgcaaattttagcatatggggcctgacttctctaccacataatt
ctacttgctgaagcatgatcaaagcttgttttatttcaccactgtaggaa
aatgattgactatgcccatccctgggggtaattttggcatgtatacctgt
aactagtaattaacatctttttttgtttaggcatgttcaattaatgctgta
gctatcatagctttgctcttacctgaagccttgtccccaccacacaggac
agccttcctcctgaagagaatgtctttgtgtgtccgaagttgagatggcc
tgccctactgccaaagaggtgacaggaaggctgggagcagctttgttaaa
ttgtgttcagttctgttacacagtgcattgccctttgttgggggtatgca
tgtatgaacacacatgcttgtcggaacgctttctcggcgtttgtcccttg
gctctcatctcccccattcctgtgcctactttgcctgagttcttctaccc
ccgcagttgccagccacattgggagtctgtttgttccaatgggttgagct
gtctttgtcgtggagatctggaactttgcacatgtcactactggggaggt

gttcctgctctagcttccacgatgaggcgccctctttacctatcctctca
atcactactcttcttgaagcactattatttattcttccgctgtctgcctg
cagcagtactactgtcaacatagtgtaaatggttctcaaaagcttaccag
tgtggacttggtgttagccacgctgtttactcatacagtacgtgtcctgt
ttttaaaatatacaattattcttaaaaataaattaaaatctgtatactta
catttcaaaaagaaaaaaaaaaaaaaaa
>NM_004404 3
aatccgcctgcgcgctgggcggggcggggcgggctggggcgggctgtgag
cggaccgcgagcgctgggcgggtccgcggcgcggtcggtcggcgcctgtt
ctcgggctgtttggcggacgaagcttcacaaaagatgtctaagcaacagc
caactcagtttataaatccagaaacacctggctatgttggatttgcaaac
ctccccaatcaagttcaccgaaaatcagtgaaaaaaggttttgagttcac
actgatggtggtcggtgaatcaggtctaggaaaatcgactctcataaaca
gcctattcctaactgatctgtacccagaaagagtcatacctggagcagca
gaaaaaattgaaagaactgtccagattgaggcttcaactgttgaaattga
agagcgaggggtcaagctacgcctgacagtggtagataccccctggctatg
gtgacgctatcaactgcagagattgttttaagacaattatctcctatatt
gatgagcaatttgagaggtacctgcatgacgagagcggcttgaacaggcg
gcacatcattgataatagggtgcattgttgcttttactttatttcacctt
ttggacatggacttaagcccttagatgtggcgtttatgaaggcaatacac
aacaaggtgaatattgtgcctgtcattgcaaaagctgacactctcaccct
gaaggaacgggagcggctgaagaaaaggattctggatgaaattgaagaac
ataacatcaaaatctatcacttacctgatgcagaatcagatgaagatgaa
gattttaaagagcagactagacttctcaaggctagcatcccattctctgt
ggttggatccaatcagttgattgaagccaaaggaaagaaggtcagaggcc
gcctctaccccctggggtgttgtggaagtggagaacccagagcacaatgac
tttctgaagctgagaaccatgctcatcacccacatgcaggatctccagga
ggtgacccaggaccttcattatgaaaacttccgttctgagagactcaaga
gaggcggcaggaaagtggagaatgaggacatgaataaagaccagatcttg
ctggaaaaagaagctgagctccgccgcatgcaagagatgattgcaaggat
gcaggcgcagatgcagatgcagatgcagggcggggatggcgatggcgggg
ctctcgggcaccacgtgtaaggtgatgtgcacatatcaagaagtcagaga
aaacactttcctggataaaaaagaaaacattccagatgcatgatccagct
gtgtgttttcaatccttgggagggtgccatccacattttaacagtacctg
tgcctgagaatttaatttttaaaagactttgatgtgtttttgtatgaagt
acttttaacgtatgtatttcattgctgtgtcacactctgtgttttgtgag
gtgaatgtcttccttttctttctccctaaccactaatgttagaattgatt
tccaagaatcggcatgtatacttaatactgaatttctttgatttaactga
cttaacaactgactaaccattgatgagcactcctgatttttatctagaac
attcagatttaccataatgttccttagtggtagaggtgtgtgcctagtga
tgtagaaagatacactgacttggtgcaaggccatctgcttaccacatcac
accacttggagatctttgcttccttgcttttatgtttgtacacaacacct
aaaaccagttttgctgctataattctatactgttgattcgtctgcgattt
tatctgttaaccaaataaaacataatagaatttcctaatgagatatatct
ttatacttaaacagctttttttagaggtgagttttaaagaagtctcttaat
tctgatgctaggttgtttttaaaaccactatgcaaagaactcaccacaag
ccaccttttgtagtgttctccactaatactggttatcctgtgctacagag
aaaatcaaagcagtcataagctccagttttcgtattgcaaataagactct
tacctacaaaatgagattcagtgaactaatttggtttttactcaaccaaa

ttaaaaattttttaaggaaaattagcagttggtctattcagaatcaaac
ctttttatattttatactgcactttagtgtattttctgtcactgtaggta
tagaagatctgcctcccctgtggaaattggggtctgttggtgggcgtgcc
cctgaagcctggcttgggttgaaaagtgttcccgccctaaggccttggtg
ccctgaacctctgatgcctaccgggttctcctgatttgagtttcctttaa
atactcccttttttgagtaattttctgatgggaggaaagtagcagtcatca
tctttttgtgtgcaggctgtctcatttatttttagccattgtcgtttcat
tcattttgtgtaatataaaccgtgtgtcatgtcaaagtgaaagacatttc
aaatctgtagcataggctagtgggcaggtccgcacagtcgaagccacacc
tggtctgttttctgtgcactgtagccttagtgtcacctttcttcttgtgt
ctccttatggtacactccagcggttgcctttttttatcatttctactgaag
ttgggaaattcaaccccagaaattgacagatgaaaggagacaatggttgt
gtagggagatggagaaaatgcttaatctgaggatgagacagggtttttttc
atttttgtgggggctagaaaaaacataaaatgaggcagttaaataataat
agttaatgaaggtgtgctacagaaaataatctggtgttcttgctaacttt
gcccttcactgttgcttaattgtgaacagccaaaagctatatgttatggc
ttattgtgtgaaggtaactaagaagtggtgttccatgacttcagagtaca
tccatgcggagtccattatttgagtttgacatttaataactttgctggaa
aatctgtaaaaaagaaaaacaagtttgctagtgactaagccccgcatatg
tgagtgaaagtacttcaggcacgctgcctcctggtaacagctatgcaggg
agggaggacccacactgctacacttctgatcccctttggttttactaccc
aaatctaaatagatacttttgataatagataactgctcttttactaagac
atagtctctacctatagaaatgtattttgaaaacacttattttacacagc
aattttgtatccatttaaactaaccttttatcaataaagcactattgttt
agatattaaaa
>NM_003223 2
cacacacacacacacacacacacacacacacacacacacacacacataca
cacgcccggggaggcaggccggagagacctccctcccgcccctcccgccc
gcctccctcccctcgccgccgccgccgccgccagcatctgggaccggccg
attctgcacctccgtccggcgctgccctttgattcggatttccatcttgc
attctccggctgatcgcgggacctggctcgtgcagaggaggggggccgat
cgctatggagtatttcatggtgcccactcagaaggtgccctctttgcaac
atttcaggaaaacagagaaagaagtgataggagggctctgtagccttgcc
aacattccactaacccccgagactcagcgggaccaggagcggcggattcg
gcgggagatcgccaacagcaacgagcggagacgcatgcagagcatcaacg
cgggattccagtccctcaagaccctcatcccccacacagacggagagaag
ctcagcaaggcagccattctccagcagacagccgagtacatcttctccct
ggagcaggagaagaccaggctcttgcagcagaacacacagctcaagcgct
tcatccaggagctgagcggctcgtcccccaagcgacggcgggcagaggac
aaggacgaaggcataggctccccggacatctgggaggacgagaaggcgga
ggacctgcggcgggagatgattgagctgcggcagcagctggacaaggagc
gctcggtgcgcatgatgctggaggagcaggtgcgctcgctggaggcccac
atgtacccggaaaagctcaaggtgattgcgcagcaggtgcagctgcagca
gcagcaggaacaggtgaggctgctgcaccaggagaagctggagcgggaac
agcagcagctgcggacccagcttctgccccctccggcccccacccaccac
cccacggtgatcgtgccagcaccgcctcctcctccctcccaccacatcaa
tgtcgtcaccatgggcccctcctcggtcatcaactctgtttccacatccc
ggcaaaatctggacaccatcgtgcaggcaatccagcacatcgagggcacc
caggaaaagcaggagctggaggaggagcagcggcgagctgtcatcgtgaa

gcctgtccgcagctgcccggaggcccccacctctgacaccgcctccgact
ccgaggcctcagacagtgacgccatggaccagagccgggaggagccgtcg
ggggacggggagcttccctgactacccccccagccctcctctcccttctg
ggggctggagggagccggggcagccacagggagagacatgggcgaatgag
tgagaaatttttacaaaattacgatgtcatttgggtctcttttatgacct
ctttttcaatactgtaaatcgacctttgaacgaagccactcaacccgagg
tcccggggctggggtgtcgcagagcgtgtgggagcatcggcaccccaggg
cggggcctcggcccggggggctggaggaagctgacacggagatgcctggc
ctctctctgccaaaaagcatttttttcctttaaatatgttttttaagaaca
gggaaaattaaacaaaaccccaggttatttcttccctgcccagagccagc
ctgggattgtcagccttcaatccccttttccttcctctttttgggttttct
tctttctcctttaagcacttacatggttgggggtaagactaggctggggc
attctgggggcccggaggtctccgttgcttcttggttggggtttgctgct
gctgtgcccccctccccttccccatctcggcactagaattcgccactct
cccaccccccagcccccacctctgcctccaggtctcatcttccaccccaa
aaatgtctgtctctctctttttgttttgtttgttgttggttttttatttc
tttttggtttgctttctgtttttgttttgtttttcttttttttcttctt
tttttttttttttacaattttgaggtcttcgtgttcaaggagaagctat
tatattttgttaagaaagtggggagaaaaaaaccaagaggccaccgtgcc
tttgtaaagaaacaaaataaagtttgtactttgttttttaaaaaaaa
>NM_014051 3
gtttccaggagggagcggcctttgctcagcgcgagacggctgggcgccga
gtgggacagcgctggtgcggagactgcttccggactccaggtaccgcgct
tggcggcagctggccccagacttctgtcttttcagctgcagtgaaggctc
ggggctgcagaattgcaaccttgccaatggacctgatcggttttggttat
gcagccctcgtgacatttggaagcatttttggatataagcggagaggtgg
tgttccgtctttgattgctggtctttttgttggatgtttggccggctatg
gagcttaccgtgtctccaatgacaaacgagatgtaaaagtgtcactgttt
acagctttcttcctggctaccataatgggtgtgagatttaagaggtccaa
gaaaataatgcctgctggtttggttgcaggtttaagcctcatgatgatcc
tgagacttgtcttgttgctgctctgagcatctggaggaacagaaaactaa
gttcatgtcatcctgctgtaatgggcagagcatattttttttgtatttaa
aagataaacttcaatatggaatgctagaaacacaaatagcactgtcacct
ctaatatgaacattagtttgaggtagtttttttctaaagcaaaaattta
actgttttctaattgtcaagcactattttcattaaaagtgtctaatgaat
catgatatactcttccatttgttgtgtctattttttatatatttggtatt
ttttgaaaattccaaatactcatgtctcaagtaagcttaaactacaactt
gtcacataaaggaagtcttaagtggagttcacagaatgataatgtatcta
tttgtcatttgtgttatatttgaaattattagaaattatgctttttccat
tttaattgtattgctgccagtgctattttttttctttaaaaaattttattc
ttagcacactgttatgtcctaactgaatgtattcagtattcaaataaaag
acattttggttcaaa
>NM_144596 2
gacgccgccagctcttcactccacgcccacctctctcctggagcgctggg
ccttcgctggccgcaccggcagccatgagctcggagatggagccgctgct
cctggcctggagctattttaggcgcaggaagttccagctctgcgccgatc
tatgcacgcagatgctggagaagtccccttatgaccaggaaccagatcct
gaattgccagtgcatcaggcagcttggatcttaaaagcaagagcgctaac
agaaatggtatacatagatgaaattgatgtagatcaggaaggaattgcag

aaatgatgctggatgaaaatgctatagctcaagttccacgccctggaacg
tctttgaaactccctggaactaatcagacaggagggcctagccaggccgt
taggccaatcacacaagctggaagacccattacaggtttcctcaggccca
gcacgcagagtggaaggccaggcactatggaacaggctatcagaacaccc
agaaccgcctacacagcccgccctatcaccagctcctccggaagatttgt
caggctgggaacggcttccatgcttacaagtcctgatggaccatttataa
atttatctaggctgaatttaacaaagtattcccagaaacctaagttggca
aaggctttgtttgagtatatctttcatcatgaaaatgatgttaagactgc
tttggatctggctgccctctccacagaacattctcagtacaaggactggt
ggtggaaagtacagattggaaaatgttactacaggttgggaatgtatcgt
gaagcagaaaaacagtttaaatcagccctgaagcagcaggaaatggtaga
tacatttctgtacttggcaaaagtttatgtctcattggatcaacctgtga
ctgctttaaatcttttcaaacaaggcttagataagtttccaggagaagta
accctgctctgtggaattgcaagaatctatgaggaaatgaacaatatgtc
atcagcagcagaatattacaaagaagttttgaaacaagacaatactcatg
tggaagccatcgcatgcattggaagcaaccacttctattctgatcagcca
gaaatagctctccggttttacaggcggctgctgcagatgggcatttataa
cggccagctttttaacaatctggggctgtgttgcttctatgcccagcagt
atgatatgactctgacctcatttgaacgtgccctttctttggctgaaaat
gaagaagaggcagctgatgtctggtacaacttgggacatgtagctgtggg
aataggagatacaaatttggcccatcagtgcttcaggctggctctggtca
acaacaacaaccacgccgaggcctacaacaacctggctgtgctggagatg
cggaagggccacgttgaacaggcaagggcactattacaaactgcatcatc
attagcaccccatatgtatgaaccgcattttaattttgcaacaatctctg
ataagattggagatctgcagagaagctatgttgctgcgcagaagtctgaa
gcagcatttccagaccatgtggacacacaacatttaattaaacaattaag
gcagcattttgctatgctctgattgttccttagaccacatatgttcttat
gaagcagcattatgcaaggggaaaaaagcactatgtctgtgtatgtatgt
atatagtgtaatacgtatattttaacaaacctgtccttgatattagttaa
ggtgacacataagggtgacacagaatgtgtaatgcaaatttcatagtaat
agtaactttataaaataatattataaaatacaggatttaaacctttctaa
atagatcctgaaactgtctctcacattatatagtagatgtttgtttataa
tgtttacaaaacattttggtgaatttcctcaatgttttataaatgtacat
tttttaagtccttaagctgactcttagccatcatgtagcttaaggagtct
gaaatctgccattaaaactgcacctttaagccaggtgtggtagcatgtgc
ctatagtcccagctacttgggaggtggaggtgggaggattataaatagag
actttccttaagactttaaaaatgtatttaaaactatttttttattaaata
ctttgtgatttcctattaagctttaaaataaatcattgtgtaaaacacca
tcaaaaaaaaaaaaaaaaaaaaaaaaaaaaaaaaaaaaaaaaaaaaaaa
>NM_145048 3
attgcgcggcgcgaccaggttcaggggcgggccgcgcggagcctcatttc
cccaaacgcaggcgctcggtggcggtagccgcggttgttggccgaccgag
tgccggtcataagcccccccggtggggggcagctggtgtgcggatcgcg
gcgggagagaggcgcggtaggaacgggtccccggagccgtgaaccgcggg
tacaggtgtcctgtctgcgctctctgccaagccggcttgcttcctgatct
gttgctagggccgctggaccccgttgctaaggacccttgagatcgtgagc
gcttggagtgtacccctcctttcctggggtagagggtcagtcgagagtag
ccttcgttaaccttaattgaaactggagtgaaagggaagcaatgcagaaa
tcagagggctctggaggtacacagttgaaaaacagagcaacaggtaacta

tgatcaaaggacatcatcaagcacacagttaaaacacaggaatgcagttc
agggaagcaaatcctcattgtcaaccagttctccagagtctgcaagaaaa
cttcatcctagaccaagtgataaactgaaccctaaaacaattaatccgtt
tggtgaacagtcacgagtgccttctgcatttgcagctatttactctaaag
gaggtattccttgcagattggtacatggttcagtaaaacacagattacag
tgggaatgtcctcctgaaagtctttcatttgatccacttcttattacttt
agctgagggtctgagagagactaagcatccatacacttttgtgtcaaagg
agggttttagagaattacttttggtcaaaggtgctcctgaaaaagctatt
cctttgctacctagactgattcctgtgctaaaggcagctctggtccattc
ggatgatgaagtgtttgaaagaggattgaatgctctagttcagctaagtg
tcgttgttggtccttctctaaacgaccatctgaagcatctgcttacaagc
gggagccttagcatcatcaaatctaaaattccaacatactgctccatatg
ctgttgaagaagggagccaacaaaaattgtttttctacctgttgacgtg
tcaagcactaactgtgggtactcatttattttattcttttgtaaatcaca
gccaccattcattatttactaggttaagatgaatagacactgaatcaaag
ttattcatcaacaaaaaagaacagttactaatggaaagttattaaaataa
gttctataagagtacaattttgaggtttatttttgtatttttattattt
gtgcgaagaaccattattgagtttgcaagataagatgtatttgtattttc
tagtgtctttttatttataacaacactttttggcaatcagtttgtagct
gtgcccttatttttatagatagtttgccatttcttgggtttaatgtttaa
caaggctatagaagtttaaaatagtggacaaatgtttgtgaatacacttt
taaaaaattgttttaaaagagaaaagcggtcttgctttgttgcccagact
ggagtgcagtggcataatcatagctcaccgcaagcctcaaatttttgtgc
tcagatgatccttttcatctcagcctcctgattagctgggactataggcg
tgtgccaccatgcccagctaattttgaaatttgcttgtagagatgggatc
tcactctgttgtccaggctggtctcagaattctggtgagtgatcctccca
cagtggacttccaaagtgctgggattacaggtgtgagccaccatgcctgg
ccccatgaatacactactaatattatttgtaaatatgtattttataatgt
acaatgcttggaaaattttcagagtattctagtttaaaaaattttttcta
attctgtaaattctattacaagtatgttgtttattacaactcattacaac
tacctaaattttaggtgttaactgtatttaagtaaatgtgttatttttgc
atttcatagccagaaaatactgatgttcacttaaagatattcagcaatta
aaatgttaaaacttgtacatgcaaaaaaaaaaaaaaaaaa
>NM_144563 2
cgggggcgggacttcagcggaggccggagcgaggcgtcgggatgcagcgc
cccgggcccttcagcaccctctacgggcgggtcttggccccgctgcccgg
gagggccgggggcgcggcctccggcggaggagggaacagctgggacctcc
cgggttcccacgtgcggctgccggggcgtgcacagtctgggacccgtggc
ggtgctggcaacacaagcaccagctgcggggactccaacagcatctgccc
ggccccctccacgatgtccaaggccgaggaggccaagaagctggcgggcc
gcgcggctgtggagaaccacgtgaggaataaccaagtgctgggaattgga
agtggttctacaattgtccatgctgtgcagcgaatagctgaaagggtgaa
gcaagagaatctgaacctcgtctgtattcccacttccttccaggcccgcc
agctcatcctgcagtatggcttgaccctcagtgatctggatcgacaccca
gagatcgaccttgccatcgatggtgctgatgaagtagatgctgatctcaa
tctcatcaagggtggcggaggctgcctgacccaggagaagattgtggctg
gctatgctagtcgcttcatcgtgatcgctgatttcaggaaagattcgaag
aatctcggggatcagtggcacaagggaatccccatcgaggtcatcccaat
ggcctatgtcccagtgagccgagctgtgagccagaagtttgggggcgtgg

ttgaacttcgaatggctgtcaacaaggctggtcctgtggtgacagataat
gggaattttatcttggactggaagtttgaccgggtacacaaatggagtga
agtgaatacagctatcaaaatgatcccaggtgtggtggacacaggcctat
tcatcaacatggctgagagagtctactttgggatgcaggatggctcagtg
aacatgagggagaagcctttctgttgaccctgcaaggagcagagtgtgtt
caccttgagtctccagcccacagccaaggtggacgtacctctccaggagc
ctttgccttaatgtatctctgcctggacaacttgtggtggggggtggggg
gaagagtgggagggggagttaaatccagtcttatgaagtattgttattaa
atgtctttttaaaaagagaaatataaacatatatttttactattaaaata
ttcagtttttttaaatgaagtagaacttgagttcatgttttatatgaaata
tttaccaaaaaaaaaaaaatgaggtaaactgtatttaaaacctttgacttg
agtctgctggtaaagcttctgaatattgagtttgctgagaaataaaaatc
aaaacttctttaagctggtaaagtgaggggcccaccagcagtgatctcct
gatgccttactggaaactttgtttacttgtctgctaccctctgatttgtt
tttagttagtttttattgtgagcacacatagtacctagttacatcttaag
atcaggtttataaaactgtggagtggagcggtatggtatggaatgacttg
gaatgtaagctgtcagggagaaaatgttgttacacttttgctaagatctg
ggggtttcttcatattcctgctgttggaagcagttgaccagaaatgcttg
ccagtactgccaaagcactgctgtgaaatgtgaagtactttgttttttta
tttttaatgattttctttttgttattaatatttttctctgttcctttgtt
attacttgcatggtttggcgtcagaagtccttacctctttatattgtttg
caggtttaaataaaacagtgtggtgccattttga
>NM_013448 2
cttttcccatcgtgtagtcaagagtctgtgccagacttgaaggctttact
ttgttagccatgtgtttatgaacccccagcgctttccctagatcttttgg
ctgataatctcaaacatggaggatgcttctgaatcttcacgaggggttgc
tccattaattaataatgtagttctcccaggctctccgctgtctcttcctg
tatcagtgacaggctgtaaaagtcatcgagtagccaataaaaaggtagaa
gcgaggagtgaaaagctcctcccaacagctcttcctccttcagagccgaa
agtagatcagaaacttcccaggagctccgagaggcggggaagtggcggtg
ggacgcaattccccgcgcggagtcgggcagtggcagcgggagaagcggca
gccaggggcgcggcggggccggagagaggcggtcccctgggaggacgggg
tctcccctcgttgcctttgtagtggagaaggtggacaagtggcagtcggc
gtgatcgcagggaagcggggccggcgcgggcggccgagggtccaggcgag
cccgcgggcggacgggagatgccgctgctacaccgaaagccgtttgtgag
acagaagccgcccgcggacctgcggcccgacgaggaagttttctactgta
aagtcaccaacgagatcttccgccactacgatgactttttgaacgaacc
attctgtgcaacagccttgtgtggagttgtgctgtgacgggtagacctgg
actgacgtatcaggaagcacttgagtcagaaaaaaaagcaagacagaatc
ttcagagttttccagaaccactaattattccagttttatacttgaccagc
cttacccatcgttcgcgcttacatgaaatttgtgatgatatctttgcata
tgtcaaggatcgatattttgtcgaagaaactgtggaagtcattaggaaca
atggtgcaaggttgcagtgtaggattttggaagtcctccctccatcacat
caaaatggttttgctaatggacatgttaacagtgtggatggagaaactat
tatcatcagtgatagtgatgattcagaaacacaaagctgttcttttcaaa
atgggaagaaaaaagatgcaattgatcccttactattcaagtataaagtg
caacccactaaaaaagaattacatgagtctgctattgttaaagcaacaca
aatcagccggagaaaacacctattttctcgtgataaactaaagctttttc
tgaagcaacactgtgaaccacaagatggagtcattaaaataaaggcatca

tctctttcaacgtataaaatagcagaacaagatttttcttatttcttccc
tgatgatccacccacatttatcttcagtcctgctaacagacgaagaggga
gacctcccaaacgaatacatattagtcaagaggacaatgttgctaataaa
cagactcttgcaagttataggagcaaagctactaaagaaagagataaact
tttgaaacaagaagaaatgaagtcactggcttttgaaaaggctaaattaa
aaagagaaaaagcagatgccctagaagcgaagaaaaaagaaaaagaagat
aaagagaaaaagagggaagaattgaaaaaaattgttgaagaagagagact
aaagaaaaaagaagaaaaagagaggcttaaagtagaaagagaaaaggaaa
gagagaagttacgtgaagaaaagcgaaagtatgtggaatacttaaaacag
tggagtaaacctagagaagatatggaatgtgatgaccttaaggaacttcc
agaaccaacaccagtgaaaactagactacctcctgaaatctttggtgatg
ctctgatggttttggagttccttaatgcatttggggaactttttgatctt
caagatgagtttcctgatggagtaaccctagaagtattagaggaagctct
tgtaggaaatgacagtgaaggcccactgtgtgaattgctttttttcttcc
tgactgcaatcttccaggcaatagctgaagaagaagaggaagtagccaaa
gagcaactaactgatgctgacaccaaagatttaacagaggctttggatga
agatgcagaccccacaaaatctgcactgtctgcagttgcatctttggcag
ctgcatggccacagttacaccagggctgcagtttgaaaagtttggatctt
gatagctgcactctttcagaaatcctcagactgcacatcttagcttcagg
tgctgatgtaacatcagcaaatgcaaagtatagatatcaaaaacgaggag
gatttgatgctacagatgatgcttgtatggagcttcgtttgagcaatccc
agtctagtgaagaaactgtcaagcacctcagtgtatgatttgacaccagg
agaaaaaatgaagatactccatgctctctgtggaaagctactgaccctag
tttcaactagggattttattgaagattatgttgatatattacgacaggca
aagcaggagttccgggaattaaaagcagaacaacatcgaaaagagaggga
agaagcagctgccagaattcgtaaaaggaaggaagaaaaacttaaggagc
aagaacaaaaaatgaaagagaaacaagaaaaactgaaagaagatgagcaa
agaaattcaacggcagatatatctattggggaggaagaaagggaagattt
tgatactagcattgagagcaaagacacagagcaaaaggaattagatcaag
atatggtcactgaagatgaagatgacccaggatcacataaaagaggcaga
agggggaaaagaggacaaaatggatttaaagaatttacaaggcaagaaca
gatcaactgtgtaacaagagagcctcttactgctgatgaggaagaagcat
taaaacaggaacaccaacgaaaagagaaagagctcttagaaaaaatccaa
agtgccatagcctgtaccaatatctttcccttgggtcgcgaccgcatgta
tagacgatactggattttcccttctattcctggactctttattgaagagg
attattctggtcttactgaagacatgctgttgcctagaccttcatcattt
cagaataatgtacagtctcaagatcctcaggtatccactaaaactggaga
gcctttgatgtctgaatctacctccaacattgaccaaggtccacgtgacc
attctgtgcagctgccaaaaccagtgcataagccaaatcggtggtgcttt
tacagttcttgtgaacagctagaccagcttattgaagctcttaattctag
aggacatagagaaagtgccttaaaagaaactttgttacaagagaaaagca
gaatatgtgcacagctagcccgtttttctgaagagaaatttcatttttca
gacaaacctcagcctgatagcaaaccaacatatagtcggggaagatcttc
caatgcatatgatccatctcagatgtgtgcagaaaagcaacttgaactaa
ggctgagagattttcttttagatattgaagatagaatctaccaaggaaca
ttaggagccatcaaggttacagatcgacatatctggagatcagcattaga
aagtggacggtatgagctgttaagtgaggaaaacaaggaaaatgggataa
ttaaaactgtgaatgaagacgtagaagagatggaaattgatgaacaaaca
aaggtcatagtaaaagacagacttttggggataaaaacagaaactccaag

tactgtatcaacaaatgcaagtacaccacaatcagtgagcagtgtggttc
attatctggcaatggcactctttcaaatagagcagggcattgagcggcgt
tttctgaaagctccacttgatgccagtgacagtgggcgttcttataaaac
agttctggaccgttggagagagtctctcctttcttctgctagtctatccc
aagtttttcttcacctatccaccttggatcgtagcgtgatatggtctaaa
tctatactgaatgcgcgttgcaagatatgtcgaaagaaaggcgatgctga
aaacatggttctttgtgatggctgtgataggggtcatcatacctactgtg
ttcgaccaaagctcaagactgtgcctgaaggagactggttttgtccagaa
tgtcgaccaaagcaacgttctagaagactctcctctagacagagaccatc
cttggaaagtgatgaagatgtggaagacagtatgggaggtgaggatgatg
aagttgatggcgatgaagaagaaggtcaaagtgaggaggaagagtatgag
gtagaacaagatgaagatgactctcaagaagaggaagaagtcagcctacc
caaacgaggaagaccacaagttagattgccagttaaaacaagagggaaac
ttagctcttctttctcaagtcgtggccaacaacaagaacctggaagatac
ccttcaaggagtcagcagagcacacccaaaacaactgtttcttctaaaac
tggtagaagcctaagaaagataaactctgctcctcctacagaaacaaaat
ctttaagaattgccagtcgttctactcgccacagtcatggcccactgcaa
gcagatgtatttgtggaattgcttagtcctcgtagaaaacgcagaggcag
gaaaagtgctaataatacaccagaaaatagtcccaacttccctaacttca
gagtcattgccacaaagtcaagtgaacagtcaagatctgtaaatattgct
tcaaaactttctctccaagagagtgaatccaaaagaagatgcagaaaaag
acaatctccagagccatcgcctgtgacactgggtcgaaggagttctggcc
gacagggaggagttcatgaattgtctgcttttgaacaacttgttgtagaa
ttggtacgacatgatgacagctggcctttttgaaacttgtttctaaaat
ccaggtcccagactactatgacatcatcaaaaagcccattgccttaaata
taattcgtgaaaaagtgaataagtgtgaatataaattagcatctgagttt
attgatgacattgagttaatgttttcgaactgctttgaatacaaccctcg
taacacaagtgaagcaaaagctggaactaggcttcaagcatttttcata
ttcaggctcaaaagcttggactccacgtcacacccagtaatgtggaccaa
gttagcacaccaccggctgcgaaaaagtcacgaatctgactttgtccttc
taaaggatatatttgaagaaaaacaaattgttcatgaaaatggaacatta
aatcatgctgtataaagcaataacaattgattgaccacatgaaagtgtgg
cctgcactatattctcaattttaatattaagcactcaggagaatgtagga
aagatatcctttgctacagttttgttcagtatctaataagtttgatagat
gtattggatacagtactggtttacagaggtttttgtacatttttgagatc
attcatgtgtccagagatcttggaaaatattttttcacccacgatttatt
ttgttattgatgatttttttttaaagtggtggtattaagggagagttatc
tacatggatgagtcttccgctatagcacagtttagaaaaggtgtttatgt
cttaattaattgtttgagtacattctttcaacactacacatgaatgaatc
caatcttataaccttgaagtgctgtaccagtgctggctgcaggtattaag
tccaagtttattaactagatatttatttagtattgagagtaatttgtgaa
tttgttttgtatttataaaatttatacctgaaaaatgttccttaatgttt
taaaccttttactgtgtttttattcctctaacttccttaatgatcaatca
aaaaaagtaacaccctccctttttcctgacagttctttcagctttacaga
actgtattataagtttctatgtataactttttaactgtacaaataaaata
acattttttcaaataaaaaaaaaaaaaaaaa
>NM_016133 2
gtgggagtggaggaggaagaggcggtagggggtacgggggctggtcccag
aagatggcggaggcgggggatttctggtaggtcctactttaggacaagat

gtggtaccgttgaagcgtcagtctttgattcacagacagttgagcttttc
agctgggaagcctttccattttttttttttttaacggctttctgaacctat
gaaaccatggcagaaggagagacagagtcacctgggcccaaaaagtgtgg
cccatatatttcatctgtcactagccagagtgtgaacttgatgattcgag
gagtagtgctattttttattggagtatttcttgcattagtgttaaattta
cttcagattcagagaaatgtgacgctctttccacctgatgtgattgcaag
catcttttcttctgcatggtgggtacccccatgctgtggcacggcttcag
ctgtgattgggttattatacccctgcattgacagacatctaggagaacca
cataaatttaaaagagagtggtccagtgtaatgcggtgtgtagcagtctt
tgttggtataaatcatgccagtgctaaagtggatttcgataacaacatac
agttgtctctcacactggctgcactatccattggactgtggtggactttt
gatagatctagaagtggttttggccttggagtaggaattgccttcttggc
aactgtggtcactcaactgctagtatataatggtgtttaccaatatacat
ctccagatttcctctatgttcgttcttggttaccatgtatattttttgct
ggaggcataacaatgggaaacattggtcgacaactggcaatgtacgaatg
taaagttatcgcagaaaaatctcatcaggaatgaagaaggcaaaaaatat
cttttgtacagaaaagcaagatgaaaaggatgtgaaatggtagatatacc
aacaaaacttcagactgtaaaattgccaggatgcagttttccccttgatt
ggcgtgtgtgtatatatggataaatatatatatacacacacacatattac
tgcaatctgtgattgcttcatctgtaaatcagttgtaaacctttacatat
ttgacttaaataactgtaagatatatatgtactacattaaaaagtgttga
ttaatagatgaaatttttaaattaatttttttaaaacatgccatacattgt
atcacaatgttaatgtgccaagatattgttcctgtcatgcagagtataag
aatgctttgaacaatttgtagacttagtgaaataaaataagaggaaagcc
aaaaacaaacaaacaaaaagcatatggggagctggtattttctctttagc
ttactgttgtgccttttttatttttctaatcacagcagtatgagttatgag
tgccctaatttgtggttagtttctaatttaatgttgtttcatagagtttg
gagtgttttgatacagggtgaaaatgaacttctggtttcaaacctgcgtt
actggagacagcccaaagagtaattttctgttttgacaggttttactgga
agtatatgtgatgagcagaagaggttatcagcattaaattgttttggttc
taaatttggaacagtatatataattaaaagtaaggaacattagaggattt
aattagaataaatacatgttttggaaatacagtgacctcttgcagtgtca
caaaagtgcaaagtgatattagctgtcatctgcaatacagaatctcattg
cttttgcacatggagcatataggaaactccaaacagatcacaatgaggtt
tctaaatctgttgggttctgtcttctattgggttctgtgaagcaaaccac
tgtagctttagctgggttcagtcatatgactcgttggtggaatgcctagg
tttttcatcttacatgcagtcttgggggtggatgaatacataatttctta
tgtattcgtgtatccattagtgaatagttcaagtctgtttaagagtgtat
tgagatggcattctctgcatgttaaagatcttaatggcaaccagcacctc
ttaagtatggtttaaacatattcttagctaatttttttccattagtttttg
aaattggtggcagttgtctgatccacaagggcaagatcttctgagtactc
tggggtgtgagtatgtgtgcacacgtgtgtgttggagtgagtgagagaat
gtgtctgtgcatgtggccatgctttcctagaatgtcaagtagatattttt
acactttgagttttaaagcaattactatcagactgagatcttgtatgcca
aactttaatctgcttttatgttttcaggctgaaggtgtgaaaatcctaag
aggatttcatattgaatatgtgtacacaatcttaactatcgtggtggaaa
acatactactataatttattattatatcttccagataatgttattcattt
agaacaaataaggtatatttttttagaatcaactttgtaagcactataaaa
tctttaataagttataaggtctatgatgtgtttactttaaaaattgctgt

taaaagcaacacgtattaaatatgtaattatcaaaaaaaaaa
>NM_016433 3
ccggagcctgcggcgggcggctgtgacggccccggcgctgacggcggcgg
ccggggggggcggtgtcgacgcccgcggcgccggctggggcatcaccgcgg
gcctcgaccccgaaatggcgctgctggccgaacacttgctgaagccgctg
cccgcggacaagcagatcgagaccgggcccttcctcgaggcggtgtccca
cctgccgcccttcttcgattgccttgggtccccagtgtttactcccatca
aggcagacataagcggcaacatcacgaaaatcaaagctgtgtacgacacc
aacccagccaagttccggaccctgcagaacatcctggaggtggagaaaga
aatgtatggagcagagtggcccaaagtaggggccacactggcgctgatgt
ggctgaaaagaggcctccgcttcatccaggtcttcctccagagcatctgc
gacggggagcgggacgagaaccaccccaacctcatccgtgtcaacgccac
caaggcctacgagatggccctcaagaagtaccatggctggatcgtgcaga
agatcttccaggcagcactgtacgcagcaccctataagtctgacttcctg
aaagcgctctccaaggggcagaatgttacggaggaggagtgcctggagaa
gatccgcctcttcctagtcaactacacggcgaccatcgatgtcatctacg
agatgtacacccagatgaacgctgagcttaactacaaggtgtaggcatgc
ccactgctggacacgtccccgactcgtggccacatggagaaacaggcaaa
ccagaatcactgtgaatcaagtcggtgaactgcccctggcctgtgtcccc
cagagcagcccagggtcctgccagagtctgcaggggacagccctcgtttt
ttaagtctttttttttttttttttggtctattctaaaggaccagtaaat
aatgatcttacttccaaatctccttggaatttcacgacagcacagactga
ctttataccttcatttcagcgtggtaaaaaccgattaacacttctaatga
gtcaagtcctagggttttttggttttgttttgttgccaacgaggaacaca
gctctgggggaatggtgtcatccacctcgctttaaaaataagcacatgat
ggctgggcaccgtggctcacgcctgtaatcccagcactttgggaggctga
ggcgggtggatcacctgaggtcgggagtttgagaccagcctggccaacat
ggtgaaaccccatcgctactaaaaatataaaaaattagctgggcatggtg
gcgcacgcctgtagttccagctactcaggaggctgaggcaggagaatcgc
ttgaacccgggaggtggaggttgcagtgagctgagatcgcaccattgcac
tccagcctgggcaacaagagcgaaactctgtctcaaaaaaaaaaaaaaga
ggtgggtggattacttgaggtcagggtttgagatcagcctgaccaacatg
gtgaaaccctatctctactaaaaatatagaattagccaggcatggtagcg
cacgcctgtaatcccatcttcttgggaggctgaggcaggagaatcgctag
aaactgggaggtggaggttacagtagccgagatcgcgccactgcattcca
gcctgggcaacaaaagcgaaactctgtctcaaaaaagaaaaaaaagaaaa
aaaagcacgtgaccttatgaggctctcgctgtattgttattttaaggact
ataaagagtttgatttaaaattatgcagggcccctatgtgggatttttta
aaaagcaaactggtgtgtattctcatgtggtttgcacagcccagcctcac
agcactattgtaaaccctgctctttctgtctcgctagacagattttttg
tttgttttctttttctggttgtttttgttgttgttgttgttgttttac
agctgaaaccaaccagcaagcccttgatgaccaagaggcgtttctttcaa
agctatagggcacaaacaattgaccatagatgactccgtttgcattcttc
tgcagaattatttccttcagggacagattttccaacctaagaaactacct
accgtgtgtattctcttgacggggagagatgaacccttcagctgctaaga
tccaagaaaacgcctcactgccttaaccttaactgttcttcctggcgcta
aaaagagctgtatttttaaagtgctggggcaaacaaagcaaccccaaaa
gagttgatgtgtgttttaaaagaaaaaacccaatgaggaacaattggaga
tttttatgcagaaactaaataatccttaataaataaatctctattttgga

atcacaaaaaaaaaaaaaaa
>NM_003816 2
cggcagggttggaaaatgatggaagaggcggaggtggaggcgaccgagtg
ctgagaggaacctgcggaatcggccgagatggggtctggcgcgcgctttc
cctcggggacccttcgtgtccggtggttgctgttgcttggcctggtgggc
ccagtcctcggtgcggcgcggccaggctttcaacagacctcacatctttc
ttcttatgaaattataactccttggagattaactagagaaagaagagaag
cccctaggccctattcaaaacaagtatcttatgttattcaggctgaagga
aaagagcatattattcacttggaaaggaacaaagaccttttgcctgaaga
ttttgtggtttatacttacaacaaggaagggactttaatcactgaccatc
ccaatatacagaatcattgtcattatcggggctatgtggagggagttcat
aattcatccattgctcttagcgactgttttggactcagaggattgctgca
tttagagaatgcgagttatgggattgaacccctgcagaacagctctcatt
ttgagcacatcatttatcgaatggatgatgtctacaaagagcctctgaaa
tgtggagtttccaacaaggatatagagaaagaaactgcaaaggatgaaga
ggaagagcctcccagcatgactcagctacttcgaagaagaagagctgtct
tgccacagacccggtatgtggagctgttcattgtcgtagacaaggaaagg
tatgacatgatgggaagaaatcagactgctgtgagagaagagatgattct
cctggcaaactacttggatagtatgtatattatgttaaatattcgaattg
tgctagttggactggagatttggaccaatggaaacctgatcaacatagtt
gggggtgctggtgatgtgctggggaacttcgtgcagtggcgggaaaagtt
tcttatcacacgtcggagacatgacagtgcacagctagttctaaagaaag
gttttggtggaactgcaggaatggcatttgtgggaacagtgtgttcaagg
agccacgcaggcgggattaatgtgtttggacaaatcactgtggagacatt
tgcttccattgttgctcatgaattgggtcataatcttggaatgaatcacg
atgatgggagagattgttcctgtggagcaaagagctgcatcatgaattca
ggagcatcgggttccagaaactttagcagttgcagtgcagaggactttga
gaagttaactttaaataaaggaggaaactgccttcttaatattccaaagc
ctgatgaagcctatagtgctccctcctgtggtaataagttggtggacgct
ggggaagagtgtgactgtggtactccaaaggaatgtgaattggacccttg
ctgcgaaggaagtacctgtaagcttaaatcatttgctgagtgtgcatatg
gtgactgttgtaaagactgtcggttccttccaggaggtactttatgccga
ggaaaaaccagtgagtgtgatgttccagagtactgcaatggttcttctca
gttctgtcagccagatgtttttattcagaatggatatccttgccagaata
acaaagcctattgctacaacggcatgtgccagtattatgatgctcaatgt
caagtcatctttggctcaaaagccaaggctgcccccaaagattgtttcat
tgaagtgaattctaaaggtgacagatttggcaattgtggtttctctggca
atgaatacaagaagtgtgccactgggaatgctttgtgtggaaagcttcag
tgtgagaatgtacaagagatacctgtatttggaattgtgcctgctattat
tcaaacgcctagtcgaggcaccaaatgttggggtgtggatttccagctag
gatcagatgttccagatcctgggatggttaacgaaggcacaaaatgtggt
gctggaaagatctgtagaaacttccagtgtgtagatgcttctgttctgaa
ttatgactgtgatgttcagaaaaagtgtcatggacatggggtatgtaata
gcaataagaattgtcactgtgaaaatggctgggctcccccaaattgtgag
actaaaggatacggaggaagtgtggacagtggacctacatacaatgaaat
gaatactgcattgagggacggacttctggtcttcttcttcctaattgttc
cccttattgtctgtgctatttttatcttcatcaagagggatcaactgtgg
agaagctacttcagaaagaagagatcacaaacatatgagtcagatggcaa
aaatcaagcaaacccttctagacagccggggagtgttcctcgacatgttt

ctccagtgacacctcccagagaagttcctatatatgcaaacagatttgca
gtaccaacctatgcagccaagcaacctcagcagttcccatcaaggccacc
tccaccacaaccgaaagtatcatctcagggaaacttaattcctgcccgtc
ctgctcctgcacctcctttatatagttccctcacttgattttttaacct
tctttttgcaaatgtcttcagggaactgagctaatacttttttttttct
tgatgttttcttgaaaagcctttctgttgcaactatgaatgaaaacaaaa
caccacaaaacagacttcactaacacagaaaaacagaaactgagtgtgag
agttgtgaaatacaaggaaatgcagtaaagccagggaatttacaataaca
tttccgtttccatcattgaataagtcttattcagtcatcggtgaggttaa
tgcactaatcatggatttttgaacatgttattgcagtgattctcaaatt
aactgtattggtgtaagatttttgtcattaagtgtttaagtgttattctg
aattttctaccttagttatcattaatgtagttcctcattgaacatgtgat
aatctaatacctgtgaaaactgactaatcagctgccaataatatctaata
tttttcatcatgcacgaattaataatcatcatactctagaatcttgtctg
tcactcactacatgaataagcaaatattgtcttcaaaagaatgcacaaga
accacaattaagatgtcatattattttgaaagtacaaaatatactaaaag
agtgtgtgtgtattcacgcagttactcgcttccatttttatgacctttca
actataggtaataactcttagagaaattaatttaatattagaatttctat
tatgaatcatgtgaaagcatgacattcgttcacaatagcactattttaaa
taaattataagctttaaggtacgaagtatttaatagatctaatcaaatat
gttgattcatggctataataaagcaggagcaattataaaatcttcaatca
attgaacttttacaaaaccacttgagaatttcatgagcactttaaaatct
gaactttcaaagcttgctattaaatcatttagaatgtttacatttactaa
ggtgtgctgggtcatgtaaaatattagacactaatattttcatagaaatt
aggctggagaaagaaggaagaaatggttttcttaaatacctacaaaaaag
ttactgtggtatctatgagttatcatcttagctgtgttaaaaatgaattt
ttactatggcagatatggtatggatcgtaaaattttaagcactaaaaatt
ttttcataacctttcataataaagtttaataataggtttattaactgaat
ttcattagttttttaaaagtgtttttggtttgtgtatatatacatataca
aatacaacatttacaataaataaaatacttgaaattctcttttgtgtctc
ctagtagcttcctactcaactatttataatctcattaattaaaaagttat
aattttagataaaaattctagtcaaatttttacagatattatctcactaa
ttttcagacttttgccaaagtgtgcacaatggctttttgttaataaagaa
cagattagttttgaagaaggcaaaaatttcagttttctgaagacagcatg
ttattttaacaatcaagtatacatattaaaaattgtgagcaatctcaaaa
aaaaaaaaaaa
>NM_005573 3
ggagcgggggataaggttaccacgcccgcggtggccggggacactctgag
tttcgcgtgtggcttttagggacgtttatatttgaatttccctgaaccgc
cgagtgtgggcggtggcgcagatccgtcccggaaacctccgggctccttc
ccgcctttctcaggcccggcccctccaaggggtccccgcggggcggcggg
agggccctgggcccagagccgcgcgggtgggcagtcccaggcgtccttcc
ttacagccctgagcctggtccgggaaccgcccagccgggagggccgagct
gacggttgcccaagggccagattttaaatttacaggcccggcccccgaac
cgccgaagcgcgctgcctgctccccattggcccatggtagtcacgtggag
gcgccggggcgtgccggccatgttggggagtgcggcgccgcggcccgcgc
cacctccgccccccgcggcttgcctccagcccgcccctcccggccctcct
ccccccgcccgccgctccgtgcagcctgagaggaaacaaagtgctgcgag
caggagacggcggcggcgcgaaccctgctgggcctccagtcaccctcgtc

ttgcattttcccgcgtgcgtgtgtgagtgggtgtgtgtgttttcttacaa
agggtatttcgcgatcgatcgattgattcgtagttcccccccgcgcgcct
ttgccctttgtgctgtaatcgagctcccgccatcccaggtgcttctccgt
tcctctaaacgccagcgtctggacgtgagcgcaggtcgccggtttgtgcc
ttcggtccccgcttcgccccctgccgtcccctccttatcacggtcccgct
cgcggcctcgccgccccgctgtctccgccgcccgccatggcgactgcgac
ccccgtgccgccgcggatgggcagccgcgctggcggccccaccacgccgc
tgagccccacgcgcctgtcgcggctccaggagaaggaggagctgcgcgag
ctcaatgaccggctggcggtgtacatcgacaaggtgcgcagcctggagac
ggagaacagcgcgctgcagctgcaggtgacggagcgcgaggaggtgcgcg
gccgtgagctcaccggcctcaaggcgctctacgagaccgagctggccgac
gcgcgacgcgcgctcgacgacacggcccgcgagcgcgccaagctgcagat
cgagctgggcaagtgcaaggcggaacacgaccagctgctcctcaactatg
ctaagaaggaatctgatcttaatggcgcccagatcaagcttcgagaatat
gaagcagcactgaattcgaaagatgcagctcttgctactgcacttggtga
caaaaaaagtttagagggagatttggaggatctgaaggatcagattgccc
agttggaagcctccttagctgcagccaaaaaacagttagcagatgaaact
ttacttaaagtagatttggagaatcgttgtcagagccttactgaggactt
ggagtttcgcaaaagcatgtatgaagaggagattaacgagaccagaagga
agcatgaaacgcgcttggtagaggtggattctgggcgtcaaattgagtat
gagtacaagctggcgcaagcccttcatgagatgagagagcaacatgatgc
ccaagtgaggctgtataaggaggagctggagcagacttaccatgccaaac
ttgagaatgccagactgtcatcagagatgaatacttctactgtcaacagt
gccagggaagaactgatggaaagccgcatgagaattgagagcctttcatc
ccagctttctaatctacagaaagagtctagagcatgtttggaaaggattc
aagaattagaggacttgcttgctaaagaaaaagacaactctcgtcgcatg
ctgacagacaaagagagagagatggcggaaataagggatcaaatgcagca
acagctgaatgactatgaacagcttcttgatgtaaagttagccctggaca
tggaaatcagtgcttacaggaaactcttagaaggcgaagaagagaggttg
aagctgtctccaagcccttcttcccgtgtgacagtatcccgagcatcctc
aagtcgtagtgtacgtacaactagaggaaagcggaagagggttgatgtgg
aagaatcagaggcgagtagtagtgttagcatctctcattccgcctcagcc
actggaaatgtttgcatcgaagaaattgatgttgatgggaaatttatccg
cttgaagaacacttctgaacaggatcaaccaatgggaggctgggagatga
tcagaaaaattggagacacatcagtcagttataaatatacctcaagatat
gtgctgaaggcaggccagactgttacaatttgggctgcaaacgctggtgt
cacagccagccccccaactgacctcatctggaagaaccagaactcgtggg
gcactggcgaagatgtgaaggttatattgaaaaattctcagggagaggag
gttgctcaaagaagtacagtctttaaaacaaccatacctgaagaagagga
ggaggaggaagaagcagctggagtggttgttgaggaagaacttttccacc
agcagggaaccccaagagcatccaatagaagctgtgcaattatgtaaaat
tttcaactgtcttcctcaaaataaagaagtatggtaatctttacctgtat
acagtgcagagccttctcagaagcacagaatatttttatatttcctttat
gtgaatttttaagctgcaaatctgatggccttaatttccttttttgacact
gaaagttttgtaaaagaaatcatgtccatacactttgttgcaagatgtga
attattgacactgaacttaataactgtgtactgttcggaaggggttcctc
aaattttttgactttttttgtatgtgtgttttttcttttttttttaagttc
ttatgaggaggggagggtaaataaaccactgtgcgtcttggtgtaatttg
aagattgccccatctagactagcaatctcttcattattctctgctatata

taaaacggtgctgtgagggaggggaaaagcatttttcaatatattgaact
tttgtactgaatttttttgtaataagcaatcaaggttataattttttta
aaatagaaattttgtaagaaggcaatattaacctaatcaccatgtaagca
ctctggatgatggattccacaaaacttggttttatggttacttcttctct
tagattcttaattcatgaggagggtgggggagggaggtggagggagggaa
gggtttctctattaaaatgcattcgttgtgtttttttaagatagtgtaact
tgcttaaatttcttatgtgacattaacaaataaaaaagctcttttaatat
tgaaaaaaaaaaaaaaaaaa
>NM_015853 3
ggctgctatagagccgggtgagagagcgagcgcccgtcggcgggtgtcga
gggcgggttgcctcgcgctgacccttcccgccctccttctcgtcacacac
caggtccccgcggaagccgcggtgtcggcgccatggcggagctgacggct
cttgagagtctcatcgagatgggcttccccaggggacgcgcggagaaggc
tctggccctcacagggaaccagggcatcgaggctgcgatggactggctga
tggagcacgaagacgaccccgatgtggacgagcctttagagactcccctt
ggacatatcctgggacgggagcccacttcctcagagcaaggcggccttga
aggatctggttctgctgccggagaaggcaaacccgctttgagtgaagagg
aaagacaggaacaaactaagaggatgttggagctggtggcccagaagcag
cgggagcgtgaagaaagagaggaacgggaggcattggaacggaacggca
gcgcaggagacaagggcaagagttgtcagcagcacgacagcggctacagg
aagatgagatgcgccgggctgctgaggagaggcggagggaaaaggccgag
gagttagcagccagacaaagagttagagaaaagatcgagagggacaaagc
agagagagccaagaagtatggtggcagtgtgggctctcagccacccccag
tggcaccagagccaggtcctgttccctcttctcccagccaggagcctccc
accaagcgggagtatgaccagtgtcgcatacaggtcaggctgccagatgg
gacctcactgacccagacgttccgggcccgggaacagctggcagctgtga
ggctctatgtggagctccaccgtggggaggaactaggtgggggccaggac
cctgtgcaattgctcagtggcttccccagacgggccttctcagaagctga
catggagcggcctctgcaggagctgggtatggctgcaagactagaaacca
ggactagaaactgggggagtagggaggcatgcctaggaaaaggagggatg
caaagagaaggggctttgtgaacatggtgcaaggccaggaattttgggag
caaaaaccaagtatccttgtggttcaagcctgttttcttccatcttcagg
actcgtgccttctgctgttctcattgtggccaagaaatgtcccagctgag
ggcctttgtcccattgtccctctgtgacccccttcatctttgataaagcac
tgacatctccttcctaataaatagaccctgagttctgtaaaaaaaaaaaa
aaaaaaa
>NM_018421 3
atctttgaggcgcgcgcaccgcacccggtcccgactctgtggcttcctgg
gggcgggttcgccgtcggccccgcccccgcccaggtgtctccctttggga
agctgcccgccgagtctccgagatttgtccctggtggtcccgcggacccc
tcgtccctccgcagtctccggctggcagcgatggagggcgctggggagaa
cgccccggagtccagctcctctgcccctgggtccgaagagtctgccaggg
atccacaggtgccgcctccggaggaagaatcgggggactgcgcccggtcc
ctggaggcggtccccaagaaactctgtgggtatttaagtaagttcggcgg
caaagggcccatccggggctggaaatcccgctggttcttctacgacgaaa
ggaaatgtcagctgtattactcgcggaccgctcaggatgccaatcccttg
gacagcatcgacctctccagtgcagtgtttgactgtaaggcggacgctga
ggaggggatcttcgaaatcaagactcccagccgggttattaccctgaagg
ccgccaccaagcaagcgatgctgtactggctgcagcagctgcagatgaag

cgctgggaattccacaacagcccgccggcacctcctgccacccctgatgc
cgccctggctgggaatgggcccgtcctgcacctcgagctagggcaagaag
aggcagagctggaggagttcctgtgccctgtgaaaacacccccctgggcta
gtgggcgtggcagctgccttgcagcccttccctgcccttcagaatatttc
cctcaagcacctggggactgaaatacagaacacaatgcacaacatccgtg
gcaacaagcaggcccagggaacaggccatgaacctccaggggaagattct
ccacagagtggggagcctcagagggaggagcagcccttggcctctgacgc
cagcaccccagggagagagccagaggattctccaaagcctgcacccaagc
cttctctgaccatcagtttcgctcagaaagccaagcgccagaacaacacc
ttcccattcttttctgaaggaatcacacggaaccgaactgcccaggagaa
agtggcagccttggagcaacaggttctgatgctcaccaaggagttaaagt
ctcagaaggagctagtgaagatcctgcacaaggcactggaggccgcccag
caggagaagcgggcgtccagcgcatacctggcggcggctgaggacaagga
ccggctggagctggtgcggcacaaagtgcggcagatcgcggagctgggcc
ggcgggtggaggccctggagcaggagcgggagagcctggcgcacacagcg
agcctgcgggagcagcaggtgcaggagctacagcagcacgtgcagctgct
tatggacaagaaccacgccaagcagcaggtcatctgcaagctctctgaga
aggtcacccaggacttcacgcacccccctgaccagtctcctttgcgcccc
gacgctgccaacagggacttcctgagccagcaggggaagatagagcacct
gaaggatgacatggaagcttaccggacccagaactgcttcctcaactccg
agatccaccaggtcacaaagatctggagaaaggtggctgagaaggagaag
gcccttctgacgaagtgcgcctacctccaagccagaaactgccaggtgga
aagcaagtacctggccggtctgagaaggctgcaggaggccctgggggacg
aagccagcgagtgctcagagctgctgaggcagcttgtccaggaggcactg
cagtgggaagctggggaggcctcatctgacagcatcgagctgagccccat
cagtaagtatgatgagtacggcttcctgacggtgcccgactatgaggtgg
aagacctgaagctgctggccaagatccaggcattggagtcacgatcccac
cacctgctgggcctcgaggctgtggatcggccgctgagggagcgctgggc
tgccctgggcgatcttgtgccctcagccgagctcaagcagctactgcggg
caggagtaccccgtgaacaccggcctcgtgtctggaggtggctggtccac
ctccgtgtccagcacctgcacactccaggctgctaccaggaactgctgag
ccggggccaggcccgcgagcaccctgctgcccgccagattgagctggacc
tgaaccggaccttccccaacaacaaacacttcacctgccccacctccagc
ttccccgacaagctccgccgggtgctgctggccttctcctggcagaaccc
caccatcggctactgccagggcctgaacaggctggcggccattgccctgc
tggtcctagaggaggaggagagcgccttctggtgcctggtggccattgtg
gagaccatcatgcccgctgattactactgcaacacgctgacggcatccca
ggtggaccagcgggtgctccaggacctgctctcggagaagctgcccaggc
tgatggcccatctggggcagcaccacgtggatctctccctcgtcaccttc
aactggttcctcgtggtctttgcggacagtctcattagcaacatcctcct
tcgggtctgggatgccttcctgtacgaggggacgaagtacaacgagaagg
agatcttgaggctacagaatggcctggaaatctaccagtacctgcgcttc
ttcaccaagaccatctccaacagccggaagctgatgaacatcgccttcaa
tgacatgaacccc ttccgcatgaaacagctgcggcagctgcgcatggtcc
accgggagcggctggaggctgagctgcgggagctggagcagcttaaggca
gagtacctggagaggcgggcatcccggcgcagagctgtgtccgagggctg
tgccagcgaggacgaggtggagggggaagcctgacttggccacctcccct
ccccacagccttcctcacccttggctggcagacccactggaggtcaggca
cggaccagtggcccagccctgggtgtcccatcaccatgtgaccttggaca

tgtcccttcccctctctggccctcagtttccccactgggacattgtgtgc
tgcaaagccattggttgggctacttcttcataggcacttacttacccagg
gatgccacccttcgtcacctcttccacagagcactttggcatgtaaaca
agcaagagcactgcctctatagggtaacctggaacattctctaggttata
tcaatataaaacaatgtaaatggtggaaatcattcataagctttggaact
taaacagttctcagttaaaaaaaaaaaaaa
>NM_173647 3
gtgcgcagcggccccgaccccgcctcagtcttccagggcggcggtgggtg
tccgcttctctctgctcttcgactgcaccgcactcgcgcgtgaccctgac
tccccctagtcagctcagcggtgctgccatggcgtggcggcggcgcgaag
ccagcgtcggggctcgcggcgtgttggctctggcgttgctcgccctggcc
ctgtgcgtgcccggggcccggggccgggctctcgagtggttctcggccgt
ggtaaacatcgagtacgtggacccgcagaccaacctgacggtgtggagcg
tctcggagagtggccgcttcggcgacagctcgcccaaggagggcgcgcat
ggcctggtgggcgtcccgtgggcgcccggcggagacctcgagggctgcgc
gcccgacacgcgcttcttcgtgcccgagcccggcggccgaggggccgcgc
cctgggtcgccctggtggctcgtgggggctgcaccttcaaggacaaggtg
ctggtggcggcgcggaggaacgcctcggccgtcgtcctctacaatgagga
gcgctacgggaacatcaccttgcccatgtctcacgcgggaacaggaaata
tagtggtcattatgattagctatccaaaaggaagagaaattttggagctg
gtgcaaaaaggaattccagtaacgatgaccatagggggttggcacccggca
tgtacaggagttcatcagcggtcagtctgtggtgtttgtggccattgcct
tcatcaccatgatgattatctcgttagcctggctaatattttactatata
cagcgtttcctatatactggctctcagattggaagtcagagccatagaaa
agaaactaagaaagttattggccagcttctacttcatactgtaaagcatg
gagaaaagggaattgatgttgatgctgaaaattgtgcagtgtgtattgaa
aatttcaaagtaaaggatattattagaattctgccatgcaagcatatttt
tcatagaatatgcattgacccatggcttttggatcaccgaacatgtccaa
tgtgtaaacttgatgtcatcaaagccctaggatattggggagagcctggg
gatgtacaggagatgcctgctccagaatctcctcctggaagggatccagc
tgcaaatttgagtctagctttaccagatgatgacggaagtgatgacagca
gtccaccatcagcctcccctgctgaatctgagccacagtgtgatcccagc
tttaaaggagatgcaggagaaaatacggcattgctagaagccggcaggag
tgactctcggcatggaggacccatctcctagcacacgtgcccactgaagt
ggcaccaacagaagtttggcttgaactaaaggacattttattttttttac
tttagcacataatttgtatatttgaaaataatgtatattattttacctat
tagattctgatttgatatacaaaggactaagatattttcttcttgaagag
acttttcgattagtcctcatatatttatctactaaaatagagtgtttacc
atgaacagtgtgttgcttcagactattacaaagacaactggggcaggtac
tctaatataaaggacaggtggtgtttctaaataattggctgctatggttc
tgtaaaaaccagttaattctatttttcaaggtttttggcaaagcacatca
atgttagactagttgaagtggaattgtataattcaattcgataattgatc
tcatgggctttccctggaggaaaggttttttttgttgtttttttttaag
aacttgaaacttgtaaactgagatgtctgtagcttttttgcccatctgta
gtgtatgtgaagatttcaaaacctgagagcactttttctttgtttagaat
tatgagaaaggcactagatgactttaggatttgcatttttccctttattg
cctcatttcttgtgacgccttgttggggagggaaatctgtttattttttc
ctacaaataaaaagctaagattctatatcgcacatgagcattaagttctt
cattgccttgttaaggaaaatgagtaggcagactcagaatctgttatatt

gatttcagttacaatttaatctttacaattaaaagggcgaaagatgtaga
attttagtttttgttgttgactcgaaataaccagttttcttgattagagt
ttaagcagatttaataccatgaccttgcttaaccgtttcttctttttttac
ttgcttgctgttcttttgggtcaaaggagcaggctaatgcaaagcttttg
gagactgctaagtgttaaaaagtgattaaacacacactctgctatttttt
cacttcttggaggtagaagtcgagtatgaggcagttatttttttagagtgt
ggaattatagtctttccttgctcctagttattctgtatatctttactttg
taggtaaaaataaatgtttatttaaaacaatttttaaaattataaattta
tttttatagccatatgtaggatataaagatttatatagattattttctca
agctacttaatgctttaattctagctactcatcatgaaatagtaaacagt
tttactgaaataaactctacagacagatgcagtatgaggagctattgaag
tagaaaatgtatttcgctttgcacactgagttggttttagcagacaacct
caagtaggtttccataacagccattacctttgaaatctaccttctgtagt
ttattataaataggaaaatacatcctgattctgtagcaagtagattgttt
gctctttgtctttaagaaatactaggagggccgggcgcggtggctcacgc
ctgtaatcccagcactttgggaggccgaggcgggcggatcacgaggtcag
gagatcgagaccatcccggctaaaacggtgaaaccccgtctctactaaaa
atacaaaaaattagccgg
>NM_025205 3
ggcacagcaaacatggcggctccactagggggtatgttttctgggcagcc
acccggtccccctcaggccccgccgggccttccgggccaagcttcgcttc
ttcaggcagctccaggcgctcctagaccttccagcagtactttggtggac
gagttggagtcatctttcgaggcttgctttgcatctctggtgagtcagga
ctatgtcaatggcaccgatcaggaagaaattcgaaccggtgttgatcagt
gtatccagaagtttctggatattgcaagacagacagaatgttttttctta
caaaaaagattgcagttatctgtccagaaaccagagcaagttatcaaaga
ggatgtgtcagaactaaggaatgaattacagcggaaagatgcactagtcc
agaagcacttgacaaagctgaggcattggcagcaggtgctggaggacatc
aacgtgcagcacaaaaagcccgccgacatccctcagggctccttggccta
cctggagcaggcatctgccaacatccctgcacctctgaagccaacgtgag
caaagggcagaggcagttggcctatgagtgggctgatgcgtgaggttggc
cacacattccttcctgtggacttgacattttggaagaactctttgccaga
taatgagttcattttagttttatgctcccattgaaaaattttccactatt
tttataagctgttaatttcttgagtactttataacatgtctgtagcttgg
ataaaccaagtaagtattttttttttgtctttagcaaagtttagactgtg
aatatgatgacacagattctttttatggtggctttgcttgttttaaatt
tttgcatgacttttcatctttttatgtgtgtttcctgtagtttgatccga
aggaaaagagtatagtagcctgagaatcaggagatgggagttttagtcgt
aggccttatgataattaccccgcggtggtgtgtagaaaagtatgtaaatt
tgctctgttttaagactttgaactacctcaagaagaggaatctaatacaa
tatttgtaatgtttccagagctctcagaatgaggattttttttgtaaatag
gtcagaagacgatggaactgtcctgggttagtatagtaatcttacagtag
gatccttaggttgatgctgacttctgtttggggtatgtttatattttatg
tggtgtttactttttttttttgacataaaaggatatagtgggagcagtga
tacgctaacattcattacattctgcagtaatgaatctgaaaaaaaaaaaa
aaaaaaaaaaaaaaaaaaaaaaaaaaaaaaaaaaaaaaaaaaaaaaaaaa
aaaaaaaaaaaaa
>NM_021633 2
gcaggggccagacccggacggctccagagcctccagagcctccgggtctg

ggcggcgcttcggctcctcccgagccgcctgctagccccgcgccgcactc
catccccacaggctggggacgggcccggtgcggctgtgtgggttcgggag
cggagttgcagaatccaaggacccattttgttctttctccgcactgcttt
atgggaggcattatggcccccaaagacataatgacaaatactcatgctaa
atccatcctcaattcaatgaactccctcaggaagagcaataccctctgtg
atgtgacattgagagtagagcagaaagacttccctgcccatcggattgtg
ctggctgcctgtagtgattacttctgtgccatgttcactagtgagctctc
agagaaggggaaaccttatgttgacatccaaggtttgactgcctctacca
tggaaattttattggactttgtgtacacagaaacggtacatgtgacagtg
gagaatgtacaagaactgcttcctgcagcctgtctgcttcagttgaaagg
tgtgaaacaagcctgctgtgagttcttagaaagtcagttggacccttcta
attgcctgggtattagggattttgctgaaacccacaattgtgttgacctg
atgcaagcagctgaggtttttagccagaagcattttcctgaagtggtaca
gcatgaagagttcattcttctgagtcaaggagaggtggaaaagctaatca
agtgcgacgaaattcaggtggattctgaagagccagtctttgaggctgtc
atcaactgggtgaagcatgccaagaaagagcgggaagaatccttgcctaa
cctgctacagtatgtgcggatgcccctactaacccccaggtatatcacag
atgtaatagatgctgagcctttcatccgctgtagtttacaatgcagggat
ctggttgatgaagcaaagaagtttcatctgaggcctgaacttcggagtca
gatgcagggacccaggacaagggctcgcctaggagccaatgaagtgcttt
tggtggttgggggctttggaagccagcagtctcccattgatgtggtagag
aaatatgaccccaagactcaggagtggagctttttgccaagcatcactcg
taagagacgttatgtggcctcagtgtcccttcatgaccggatctacgtca
ttggtggctatgatggccgttcccgccttagttcagtggaatgtctagac
tacacagcagatgaggatggggtctggtattctgtggcccctatgaatgt
ccgacgaggtcttgctggagccaccaccctgggagatatgatctatgtct
ctggaggctttgatggaagcaggcgtcacaccagtatggagcgctatgat
ccaaacattgaccagtggagcatgctgggggatatgcagacagcccggga
aggtgccggactcgtagtggccagtggagtgatctactgtctaggaggat
atgacggcttgaatatcttaaattcagttgagaaatacgaccctcataca
ggacattggactaatgttacaccaatggccaccaagcgttctggtgcagg
agtagccctgctgaatgaccatatttatgtggtgggggggatttgatggta
cagcccacctttcttccgttgaagcatacaacattcgcactgattcctgg
acaactgtcaccagtatgaccactccacgatgctatgtaggggccacagt
gcttcgggggagactctatgcaattgcaggatatgatggtaattccctgc
taagtagcattgaatgttatgaccctatcatcgacagctgggaagtcgtg
acatccatgggaacccagcgctgtgatgctggtgtttgtgttctccgcga
gaagtgaccattgttggagcaccatccagagctagtgaccagtccagtgg
acagttagtgggagtatcaaaaatcctttccagaatgtctgtttctcact
atgtgcaccgggtgattacaggcaccagtgcagtgatgattgtacttatt
tgacacatactccccgtcgtcctggttcttgttcctgagaagggtgggta
acagatattccaggaaaaagaatgcacattgaatggatgtgagagaccac
attgcctctcccactgctttggggagcactttcctgtcatttctaactta
ccacatgcttggtgtactatatgtacgttgtgcctcatatgttgcaaaga
actaaggtgagtatagcctactagatatgggcaatatccagcctagatga
ttggaaagataccagtttaagtaaacttggtaaaatccaagtcttttttt
ttttttccaggaacaactacattttctcatatacaggtagctaggggcaa
cacagttccattctagagggaaacaaaagggagagccccacaaaactttg
gggacaagggagagagagactcatctgacacttcttttggaggtcaggat

ttgtatatcagaattgaagttagaattaagtgaattaaactgaatttgat
tgtgagtgaacctagaacagcactgaagtattacataacctggaagactg
agaagggtatattatttgaaggatctttttatttccccgaggtctttcgc
actggagacagcataaaagagtgaacaaatgttgggatgagagaagatga
catcaatgtgggagttcagtataactggggataaactagaagaacctgtg
attttacagtcatcttattacctgccagggctcatctagccatggcaatg
tttgccttgaatggggggtgaaagcctttctttgttggatcaaatactact
acactattacacttccacactatttatttggggatgggctgggagtgaca
gtagcctagtagttcagctacctgattactgccccattcttttagaagca
catgtctgccaaggagtggtttgtactgctgtgtttggtacatctagtct
tttttctgctataagttttccttacctgtcctttagtgtagattttattc
atcacaggacagaataatcaaggacaaccaaaatccttttgttagtttca
gtacctcagctatcaacatttctgagctaccattcaatgttcctctgtgt
catggagtgaaattcttgttttgtgggtattaggagtgtgggaatgtgat
aacctaaacaacctttgctctgaaattccatttttccctctttccctgag
ttgtattgacctacagagttaatttcctttgtattttttaagaaaatat
taaaaatcaacggtctcaaatgccgagagtttgtggct
>NM_018492 2
agcgcgcgactttttgaaagccaggagggttcgaattgcaacggcagctg
ccgggcgtatgtgttggtgctagaggcagctgcagggtctcgctgggggc
cgctcgggaccaattttgaagaggtacttggccacgacttattttcacct
ccgacctttccttccaggcggtgagactctggactgagagtggctttcac
aatggaagggatcagtaatttcaagacaccaagcaaattatcagaaaaaa
agaaatctgtattatgttcaactccaactataaatatcccggcctctccg
tttatgcagaagcttggctttggtactggggtaaatgtgtacctaatgaa
aagatctccaagaggtttgtctcattctccttgggctgtaaaaaagatta
atcctatatgtaatgatcattatcgaagtgtgtatcaaaagagactaatg
gatgaagctaagattttgaaaagccttcatcatccaaacattgttggtta
tcgtgcttttactgaagccaatgatggcagtctgtgtcttgctatggaat
atggaggtgaaaagtctctaaatgacttaatagaagaacgatataaagcc
agccaagatccttttccagcagccataattttaaaagttgctttgaatat
ggcaagagggttaaagtatctgcaccaagaaaagaaactgcttcatggag
acataaagtcttcaaatgttgtaattaaaggcgattttgaaacaattaaa
atctgtgatgtaggagtctctctaccactggatgaaaatatgactgtgac
tgaccctgaggcttgttacattggcacagagccatggaaacccaaagaag
ctgtggaggagaatggtgttattactgacaaggcagacatatttgccttt
ggccttactttgtgggaaatgatgactttatcgattccacacattaatct
ttcaaatgatgatgatgatgaagataaaacttttgatgaaagtgattttg
atgatgaagcatactatgcagcgttgggaactaggccacctattaatatg
gaagaactggatgaatcataccagaaagtaattgaactcttctctgtatg
cactaatgaagaccctaaagatcgtccttctgctgcacacattgttgaag
ctctggaaacagatgtctagtgatcatctcagctgaagtgtggcttgcgt
aaataactgtttattccaaaatatttacatagttactatcagtagttatt
agactctaaaattggcatatttgaggaccatagtttcttgttaacatatg
gataactatttctaatatgaaatatgcttatattggctataagcacttgg
aattgtactgggttttctgtaaagttttagaaactagctacataagtact
ttgatactgctcatgctgacttaaaacactagcagtaaaacgctgtaaac
tgtaacattaaattgaatgaccattacttttattaatgatctttcttaaa
tattctatattttaatggatctactgacattagcactttgtacagtacaa

aataaagtctacatttgtttaaaacactgaaccttttgctgatgtgttta
tcaaatgataactggaagctgaggagaatatgcctcaaaaagagtagctc
cttggatacttcagactctggttacagattgtcttgatctcttggatctc
ctcagatctttggtttttgctttaatttattaaatgtattttccatactg
agtttaaaatttattaatttgtaccttaagcatttcccagctgtgtaaaa
acaataaaactcaaataggatgataaagaataaaggacactttgggtacc
agaaaaaaaaaaaaaaaaaaaaaaaaaaaaaaaaaaaaaaaaaaaaaaaa
>NM_001001392 1
gagaagaaagccagtgcgtctctgggcgcaggggccagtggggctcggag
gcacaggcaccccgcgacactccaggttccccgacccacgtccctggcag
ccccgattatttacagcctcagcagagcacggggcgggggcagaggggcc
cgcccgggagggctgctacttcttaaaacctctgcgggctgcttagtcac
agccccccttgcttgggtgtgtccttcgctcgctccctccctccgtctta
ggtcactgttttcaacctcgaataaaaactgcagccaacttccgaggcag
cctcattgcccagcggaccccagcctctgccaggttcggtccgccatcct
cgtcccgtcctccgccggcccctgccccgcgcccagggatcctccagctc
ctttcgcccgcgccctccgttcgctccggacaccatggacaagttttggt
ggcacgcagcctggggactctgcctcgtgccgctgagcctggcgcagatc
gatttgaatataacctgccgctttgcaggtgtattccacgtggagaaaaa
tggtcgctacagcatctctcggacggaggccgctgacctctgcaaggctt
tcaatagcaccttgcccacaatggcccagatggagaaagctctgagcatc
ggatttgagacctgcagtttgcattgcagtcaacagtcgaagaaggtgtg
ggcagaagaaaaagctagtgatcaacagtggcaatggagctgtggaggac
agaaagccaagtggactcaacggagaggccagcaagtctcaggaaatggt
gcatttggtgaacaaggagtcgtcagaaactccagaccagtttatgacag
ctgatgagacaaggaacctgcagaatgtggacatgaagattggggtgtaa
cacctacaccattatcttggaaagaaacaaccgttggaaacataaccatt
acagggagctgggacacttaacagatgcaatgtgctactgattgtttcat
tgcgaatctttttttagcataaaattttctactctttttgttttttgtgtt
ttgttctttaaagtcaggtccaatttgtaaaaacagcattgctttctgaa
attagggcccaattaataatcagcaagaatttgatcgttccagttcccac
ttggaggcctttcatccctcgggtgtgctatggatggcttctaacaaaaa
ctacacatatgtattcctgatcgccaacctttcccccaccagctaaggac
atttcccagggttaatagggcctggtccctgggaggaaatttgaatgggt
ccattttgcccttccatagcctaatccctgggcattgctttccactgagg
ttgggggttggggtgtactagttacacatcttcaacagaccccctctaga
aatttttcagatgcttctgggagacacccaaagggtgaagctatttatct
gtagtaaactatttatctgtgtttttgaaatattaaaccctggatcagtc
ctttgatcagtataattttttaaagttactttgtcagaggcacaaaaggg
tttaaactgattcataataaatatctgtacttcttcgatcttcaccttttt
gtgctgtgattcttcagtttctaaaccagcactgtctgggtccctacaat
gtatcaggaagagctgagaatggtaaggagactcttctaagtcttcatct
cagagaccctgagttcccactcagacccactcagccaaatctcatggaag
accaaggagggcagcactgtttttgtttttgtttttgtttttttttt
tgacactgtccaaaggttttccatcctgtcctggaatcagagttggaagc
tgaggagcttcagcctcttttatggtttaatggccacctgttctctcctg
tgaaaggctttgcaaagtcacattaagtttgcatgacctgttatccctgg
ggccctatttcatagaggctggccctattagtgatttccaaaaacaatat
ggaagtgccttttgatgtcttacaataagagaagaagccaatggaaatga

aagagattggcaaaggggaaggatgatgccatgtagatcctgtttgacat
ttttatggctgtatttgtaaacttaaacacaccagtgtctgttcttgatg
cagttgctatttaggatgagttaagtgcctggggagtccctcaaaaggtt
aaagggattcccatcattggaatcttatcaccagataggcaagtttatga
ccaaacaagagagtactggctttatcctctaacctcatattttctcccac
ttggcaagtcctttgtggcatttattcatcagtcagggtgtccgattggt
cctagaacttccaaaggctgcttgtcatagaagccattgcatctataaag
caacggctcctgttaaatggtatctcctttctgaggctcctactaaaagt
catttgttacctaaacttatgtgcttaacaggcaatgcttctcagaccac
aaagcagaaagaagaagaaaagctcctgactaaatcagggctgggcttag
acagagttgatctgtagaatatctttaaaggagagatgtcaactttctgc
actattcccagcctctgctcctccctgtctaccctctcccctccctctct
ccctccacttcaccccacaatcttgaaaaacttcctttctcttctgtgaa
catcattggccagatccattttcagtggtctggatttctttttattttct
tttcaacttgaaagaaactggacattaggccactatgtgttgttactgcc
actagtgttcaagtgcctcttgttttcccagagatttcctgggtctgcca
gaggcccagacaggctcactcaagctctttaactgaaaagcaacaagcca
ctccaggacaaggttcaaaatggttacaacagcctctacctgtcgcccca
gggagaaaggggtagtgatacaagtctcatagccagagatggttttccac
tccttctagatattcccaaaaagaggctgagacaggaggttattttcaat
tttattttggaattaaatactttttccctttattactgttgtagtccct
cacttggatatacctctgttttcacgatagaaataagggaggtctagagc
ttctattccttggccattgtcaacggagagctggccaagtcttcacaaac
ccttgcaacattgcctgaagtttatggaataagatgtattctcactccct
tgatctcaagggcgtaactctggaagcacagcttgactacacgtcatttt
taccaatgattttcaggtgacctgggctaagtcatttaaactgggtcttt
ataaaagtaaaaggccaacatttaattattttgcaaagcaacctaagagc
taaagatgtaatttttcttgcaattgtaaatcttttgtgtctcctgaaga
cttcccttaaaattagctctgagtgaaaaatcaaaagagacaaaagacat
cttcgaatccatatttcaagcctggtagaattggcttttctagcagaacc
tttccaaaagttttatattgagattcataacaacaccaagaattgatttt
gtagccaacattcattcaatactgttatatcagaggagtaggagagagga
aacatttgacttatctggaaaagcaaaatgtacttaagaataagaataac
atggtccattcacctttatgttatagatatgtctttgtgtaaatcatttg
ttttgagttttcaaagaatagcccattgttcattcttgtgctgtacaatg
accactgttattgttactttgacttttcagagcacacccttcctctggtt
tttgtatatttattgatggatcaataataatgaggaaagcatgatatgta
tattgctgagttgaaagcacttattggaaaatattaaaaggctaacatta
aaagactaaaggaaacagaaaaaaaaaaaaaaaaa
>NM_005595 4
ggccgcggaggctcgggacccggctggccgcgcggcgccgcagccgcccc
ctcccccacacccccctccccccgcggcggcggcgcgagcgggcggcggc
tgtgcggtgcggtgcagagcggaggcggaggcgggcgcgcgggcagctcg
cgggcacccggccgggccggcgcgggagcgggaaagggtgcgctatgcct
ttaacacccgcgtacagtaggcatgtatagtggagtgtagggaaactcta
ggcggggttaaagttcagctcatggagcggcaatagcgctggctggctgg
ctgcagttgagccgacttggaaatgtgaacgcaagaagcaggcttgattt
ttttttctccccccttctctctctctctctctctctcttcctctctcc
ctctttctcctctctcacccacactcacgcacacctccaaaccgcacacc

cagacgcacacgcataccccagcgcccggcagttatgtattctccgctct
gtctcacccaggatgaatttcatcctttcatcgaagcacttctgccccac
gtccgagcctttgcctacacatggttcaacctgcaggcccgaaaacgaaa
atacttcaaaaaacatgaaaagcgtatgtcaaaagaagaagagagagccg
tgaaggatgaattgctaagtgaaaaaccagaggtcaagcagaagtgggca
tctcgacttctggcaaagttgcggaaagatatccgacccgaatatcgaga
ggattttgttcttacagttacagggaaaaaacctccatgttgtgttcttt
ccaacccagaccagaaaggcaagatgcgaagaattgactgcctccgccag
gcagataaagtctggaggttggaccttgttatggtgattttgtttaaagg
tattccgctggaaagtactgatggcgagcgccttgtaaagtccccacaat
gctctaatccagggctctgtgtccaaccccatcacataggggtttctgtt
aaggaactcgatttatatttggcatactttgtgcatgcagcagattcaag
tcaatctgaaagtcccagccagccaagtgacgctgacattaaggaccagc
cagaaaatggacatttgggcttccaggacagttttgtcacatcaggtgtt
tttagtgtcactgagctagtaagagtgtcacagacaccaatagctgcagg
aactggcccaaatttttctctctcagatttggaaagttcttcatactaca
gcatgagtccaggagcaatgaggaggtctttacccagcacatcctctacg
agctccacaaagcgcctcaagtctgtggaggatgaaatggacagtcctgg
tgaggagccattttatacaggccaagggcgctccccaggaagtggcagtc
agtcaagtggatggcatgaagtggagccaggaatgccatctccaaccaca
ctgaagaagtcggagaagtctggtttcagcagcccctccccttcacagac
ctcctccctgggaacggcgttcacacagcatcaccgacctgtcattacag
gacccagagcaagtccgcatgcaacaccatcgactcttcatttcccgaca
tcacccattatccagcagcctgggccttacttctcacacccagccatccg
ctatcaccctcaggagacgctgaaagaatttgtccaacttgtctgccctg
atgctggtcagcaggctggacaggtggggttcctcaatcccaatgggagc
agccaaggcaaggtgcacaacccattccttcccaccccaatgttgccacc
gccaccgccaccaccgatggccaggcctgtgcctctgccggtgccagaca
caaagcctccaaccacgtcaacagaaggaggtgcagcctcccccacgtca
ccaatcctggtacctgggataaaagttgcagcgtcccaccatccaccaga
cagaccacctgaccccttctcaactctgtaacatggacgcaacctcaacc
cagcgcagttacaacttcactatcagcggaaggggagaaaaaccgattca
aatcaacttgtacatggaaacagcaagcattatggtcaaacagcaaaggc
cataacctttgggatttttttttttttaaaatactttagggactgttgt
aatttctcatatggtgctggaaatggttgggctttgtaacatttgaagtg
tttccatggtagcgtgagcattaggtgacgtggctagcggaggactaccc
ttgctcactgacttcctgttgtaacacactttccttacggagcctggctg
tttcacagtatttcatgaatttacccacacaggtgtgatcctccttgagc
attgaggaggcacatggagaactaaatcttttgtagtagctgagatctgc
aatatataacgggacagtcaaagggcaatgtttttctgtaacatattgga
aaaagaaaatgcagttatattcctttttatttgttcctttagtttgttt
tggttcagcagtcagcagttaagtatataacatggcccgcaaggacaatg
aatccactcacattgcagaacaattccgaaaatggcaaactactactact
actgttcagttttttaaaagttttgaaatgctgcacttacatttaaaaaa
acaacaacaacatttttttcaacaatttcaacaatgacacaaaaattcaca
tggaaatggggaagatggtctgttttgacagaaactgacaggaatcaatc
aaaacaatcgaattttgaattgagtaaagtgcaatttcattggatagcta
aatatctttgtaagatagagattgttgaaaattctatttttgtttttcta
gtcctttcaccccaggactctaaattattggggtaaaaaacagccttgca

agaaaaaggggagctatttttgctttttatgtttttattgttaaacttg
tatccctttaaaaactgaaggaaattaaaaaaaaaaaacaaaaaaacaaa
tctaatggtgcttttaccacaatatgttaactacattaaatgctaattaa
ttattttctgttatcaaagcacatgactaaaatgaaatcatggtatctgt
taattttataagctagaagtcactataatggattacgccaattctaaaaa
attttacacctatctggcatcataggatttatcagttatcagacacctca
ttgtaccagagattgtccagaagttttaaagacctttgcatccctgaact
gggctatgggaaataataatagtaataataataataataataatgatgaa
accaatactgacacaaatgctggtgcccattcagatcaagggtacttgtt
agggaaaaaaaaaaaaagtttgcacccccaaacgtcctgtatcttatgaaa
aaaaaaacaaaaaacaaaaacaaaaaaaaaacacaaaaaaccacagaaac
aaaaacaaaaaaaagtgcaagtgattttctaccagacagcgaagcaccc
ctttgcttcccatgcgacttcaagaaggtttcctatactatacatatata
tacgttctggttggcaagccctgctgatcagagaaagtctctgcatgttc
tagtgttagtaactaattttatatagttaatgtaggataaagtagagtg
cattaagacacaatattgtaatccctactctaggcacttgcctttaaact
atgtttttcagcccttcagaagggttctactactgtcctatacaatcaag
taactgaaattcttgggaagacactttgctcctcatctttctccccgaaa
caatgttgttttgttttgtttttttttccttaatttgcacgaaaacaaaaa
ttccatatcaatgtgccttgccctggatagcgattatttgtggaattgtt
gcacatgctcctctattgaaaggggtttttccctagtcaagcatttggag
acactttttgtaaatgtgacttttatgtcagccatcgtcagtttcaacat
ctagaactaaatagaaagctagttgttccgcagataggagtagtctttat
tgtcctgtacggtcggtggcagtgctattctgagatctgtagatgcttag
aatatcagtattttggatgttgctgcattttacaatttatttggagtctt
cctttattttcccccagatatatgaaaatatgcaatacctgcttatatca
tgtagaaaagcttagcaattattaatttttcttttattttttttatttg
accaaagtcggtgctgcacttgacgcagtgtgttttaggtgtttgtcttt
gtactttttgtgatttttgaatgcacgtgcgcaggaagggctcctctta
gagaagcagtcaaactgtgaagcactaagctgaccctgcttcaagcaatt
ttgtttttacaactgttcctttcacaagcaagccttaaaaaaaaaaaaga
caacttcctttttcttcagctcccacaccccatttttcttagcagactgc
agtcaatccacattcaataaaaagtatataatgcccatttttatatgcac
gtttttaaacttccaagttctgaaaattgtttactggttatctctattta
aggaaaaaaaaataaaataaaacattttggattttcatatgtgtctgata
agtggttgaatagtcgtttggcgctgttgtatggtgtgattgtcagtgta
tggtgtcacttcctatagccagccagcatactttgccttcccctatagca
cttagctgggcattactttattatgacatatgtgcactaaaaaatgaaaa
aaaggaaaaaaagaaaaaaaaaaagaaaaaatagcagctttcagtgcttc
acagtgaagggaaaaaagcctagacaaacattttgtcagaaccttgcaat
aagccaaggtattaccagtaaattggttgtatatacaataaaattgcacc
cttttttaaacaaaacaaactaagcaatagtttgggcagttttagttgtt
tttagtgagcatgttgtagtcatgactgcaaagagagagaataaactgcc
cgctcagaagatatgtaatttgtattgttgtatagttttattgattacac
tgatttattctaccctattttataatgcaggacttttgtaatgttgttta
aatgaggaaaaatttctgtcaaattagcctagtaaaatttctgatcgttc
attataaaggcagcgttcatagaattgcttttctttctttttacccccc
tttgggaactggatttaagtttaaaactttcctgtttcctttttttttt
tttttgtaagtatttaaatacaattattttttctctcaatggtatagc

atattcctatgcttgagaagtataggtctactgaaaaaccattgtaaatg
gacgttacaggtatgctgtatttttgaaggtattttgttgtattaagttt
gatgaagctaaaattagggaactctgaacagatttgcaggaaaaaatgtt
ttaaaggctttaaaacattagggaggcagtctagggtgataacgaacagg
ggttaagtattaaatacacgaagttacatttttgttcatgtttcattgtc
cagaaagcagcaggaaactattcagttgtgatcaagcaggaaaaaagaaa
caccaacagttgccagtgtttttgctttttagcttaaaagcatagtgaag
atgcttgaggaagactttgctacctggggtgtgtagacagacagactgag
agctatcagcatttgaaggcccagcccttgactctgagacacatttgaat
tttttctttcccatcaaatggcattaacaagattgggcaaagatgagtcc
ctcaaatttctgtgttttttgtttgtttgtttgtttgtttttttctttggg
aactgaagtcagaggcacgaacactaactcttagcatttttctgtagact
ttttcttctggcccttgtccctgccagcaaaacgccccttttctgatcat
tcgtgcgcagagggcctcccagtaatgccacgctctccatgctagagagc
cttctctttcctctgaggtttgaactgatgttctgtgtcttcacaccctg
gcatgacagttacgtgtggtcagcccgctccccaggcccgtccctgccgc
cgccaggtgtgggctctaggcaggccgacaaggttacacctcccagagct
tgtgatcttcattttctgacagtcaaagtgtgaaggaacccagacttccc
cgagccacggtgttcagtcagcccacaggaatatgcaagacccatctcca
aaagtttgtctttgattttttccaagcccttagccccataagctttgaat
cctgtagttacagtggcataaaggactgacaaaacctggataaggaaaaa
cctttttttttctatgaattttttttgtttttttaggggaaagggattctaa
gaatgtcatttaatgtactttgcatcatgtctctagaaatatctttgtcc
atagtggtggtggagtctctctctctctctctctttttttgtttgcttctgt
tttctttcttgtcttcattctttcttttcttttttatttctggtagcagg
cctccatagaacaaatctaaaacacaaccaccatagtaatgtaaggagag
cttcagtggcacctcaaaacccacccttcgagatctgtccaaagacagtc
tcagaaagctgcactgcccaccggctcagctttcattcaaaaaggcttcc
aaggccaattctgtcttgaagtcaatgcatgtatttactgtttgacagta
aacccgctctgccttctccacgtccaaggctgtgcattcgtctaattagc
gtcgtgtatgttttccttttatttttccaataaaaaagcagtgggatga
aaattgctttgatatatagcaggtaacattgaagctattccatagcactt
aactgtagtgaatactgtgtcaccaattttgaaatcaatttaatgtttaa
tgcaaatccattacatggtgctattataggctgacaaaatgatttacaca
aatgtgacaacttgggctcaattcactctgctttccaacagtgtaaatgc
atagcagtgtttatctgcatgagaactatgcactaatctatctgaagaaa
aaaactatatcaactttggtatctactttccgtttacttcaatccttgcc
tttttggtcattgttataatgccagctttaggacagaaagaattataaga
aaaccagcataatacctgatatattaaaatgtagtgcctgtgaaatctgt
attatattgctcttctgaagtaagatttttctacaccggtagccttcgct
gtctgtcagtcaggaccttctggtataggtgatgtaaaataaccgtacaa
tattaatgcatgcgattccataatgcttagtgaactgtatgaatattact
caaagttatgttagtctttttttccgacttggttcttgtcagctaggttt
aaaggtatttcactgagaacgcaaattctgtcttttcttgatttcggctg
ttttcagtattttggaggtatacatttacttaaattcagtattactcgtg
ttttgtttttgtttttgttttttgttttctttttcctaggggacaagcat
gggtgtttgatttcagaaatcagtacctggcgagatttttgtctcaaaac
gactatttgaatttcaagaactgtgctgcgaagacactctgagaacattt
gcaagtcaggggcattttccttgacccttgactgatgctatgcggagact

gatacattttcttaatggacaatgttcaagccaggtacccatgcttgatc
tgtcttcacaccagacctcctcatattaaaaggaaaaataagaaaaaaaa
tgtaagaaatcacatggctatttagtttcatgcacagttgcaatattttc
ttcaaaaataaaactctgtacaaactttgggcccgattcataagaaaaag
aagtttgctattaacacgggatttttttaatatactttttttggtctaaa
tttgaaattacttgcttcccaaattaaataaatttcatctcatttttttc
cctaaaccagcacccatctgccttttattccccaaagagttacctttccc
agattagggggatggtatgtggggagcagatagcggaaatgcttagaaag
ataagggggaccacccacagctggtcgtgagaacagggagacagtgtgtg
ggggtgggacctcatctgtgtgcctggtatcctgagttttacatgtagat
gcattcgcctatttgattcagaaaaataaactttcccaaaatgtgtctga
accacaagagcatacagtggaagtgctacctctaatctaaccagagcacc
ttcatggtggaagacacccaccaggtcatacaatgtgaacttttgtatct
ctgcagtggtttcaaggacaaatagtgtccaatgtattgggccatttttc
ctgctgtttttatactcaacttctcaaaatgaaaaaagcttttatttttc
ctttgacttatttgtgttgttcttattttttaaatttttattttttgata
atagtctgtaagttagcctttttgggttttttttttttttttttggcttt
ttttttgtttgtttttttttcttttgacattgcaaccgaaggtcataag
gccgctagctccgctgggacagaggcttgagagaactaacggctcggtgc
cttctccctggtctcagaccatcgtctctgcactgcgaaggcatttggta
gcctcgccactgagatactaactagacctagactaggagctttatcaggt
tctaggaggtcctttaggaagactctcaaaggcaaatccctgatcccccg
ccccacccttagccctgccctctcaccagagcaaaattcactggggactt
ttcccaccacacatggaaatctgtccactcggaatacctctgttttccat
ttcaaattgtagggggaggggatggaacacttccagtgatggtaagagat
ctgttatgaaacgaaacaccccccgtgttaataacttggtctgaaatctg
tttttatgagccgggccccctgtgcctctagtatacttgtattgactctc
atagttacccttttagttttactgtgttctgtgaaaatttgtaattggtt
gagaatcactgtgggcgtccattcttattcaactaaatctccacaggttt
tttgagctggtgtggattagtttaactcttgtattcaaccattagtgcta
ccaccttctcacattacaatacaattactggaagcaagtactgcatttcc
tatgcaacaaaaaaggaaaaataaaaaattgctaatgctaaaaaaaaaaa
aaa
>NM_019851 2
agcgacctcagaggagtaaccgggccttaactttttgcgctcgttttgct
ataatttttctctatccacctccatcccacccccacaacactctttactg
ggggggtcttttgtgttccggatctcccctccatggctcccttagccga
agtcgggggctttctgggcggcctggagggcttgggccagcaggtgggtt
cgcatttcctgttgcctcctgccggggagcggccgccgctgctgggcgag
cgcaggagcgcggcggagcggagcgcgcgcggcgggccgggggctgcgca
gctggcgcacctgcacggcatcctgcgccgccggcagctctattgccgca
ccggcttccacctgcagatcctgcccgacggcagcgtgcagggcacccgg
caggaccacagcctcttcggtatcttggaattcatcagtgtggcagtggg
actggtcagtattagaggtgtggacagtggtctctatcttggaatgaatg
acaaaggagaactctatggatcagagaaacttacttccgaatgcatcttt
agggagcagtttgaagagaactggtataacacctattcatctaacatata
taaacatggagacactggccgcaggtattttgtggcacttaacaaagacg
gaactccaagagatggcgccaggtccaagaggcatcagaaatttacacat
ttcttacctagaccagtggatccagaaagagttccagaattgtacaagga

cctactgatgtacacttgaagtgcgatagtgacattatggaagagtcaaa
ccacaaccattctttcttgtcatagttcccatcataaaataatgacccaa
ggagacgttcaaaatattaaagtctattttctactgagagactggatttg
gaaagaatattgagaaaaaaaaccaaaaaaaattttgactagaaatagat
catgatcactctttatatgtggattaagttcccttagatacattggatta
gtccttaccagtagac
>NM_004827 2
gtcagcgctgcctgagctcgtcccctggatgtccgggtctccccaggcgg
ccacccgccggctcccatcgtgacctccagccgcagcgcctcccacgccg
gccgccgcgcgaggggagcgctcgggcgcgccgggtgtggttgggggaag
gggttgtgccgcgcgcgggctgcgtgctgtgcccactcaaaaggttccgg
gcgcgcaggagggaagaggcagtgcccgccactcccactgagattgagag
acgcggcaaggaggcagcctgtggaggaactgggtaggatttaggaacgc
accgtgcacatgcttggtggtcttgttaagtggaaactgctgctttagag
tttgtttggaaggtccgggtgactcatcccaacatttacatccttaattg
ttaaagcgctgcctccgagcgcacgcatcctgagatcctgagcctttggt
taagaccgagctctattaagctgaaaagataaaaactctccagatgtctt
ccagtaatgtcgaagtttttatcccagtgtcacaaggaaacaccaatggc
ttccccgcgacagcttccaatgacctgaaggcatttactgaaggagctgt
gttaagttttcataacatctgctatcgagtaaaactgaagagtggctttc
taccttgtcgaaaaccagttgagaaagaaatattatcgaatatcaatggg
atcatgaaacctggtctcaacgccatcctgggacccacaggtggaggcaa
atcttcgttattagatgtcttagctgcaaggaaagatccaagtggattat
ctggagatgttctgataaatggagcaccgcgacctgccaatttcaaatgt
aattcaggttacgtggtacaagatgatgttgtgatgggcactctgacggt
gagagaaaacttacagttctcagcagctcttcggcttgcaacaactatga
cgaatcatgaaaaaaacgaacggattaacagggtcattcaagagttaggt
ctggataaagtggcagactccaaggttggaactcagtttatccgtggtgt
gtctggaggagaaagaaaaaggactagtataggaatggagcttatcactg
atccttccatcttgttcttggatgagcctacaactggcttagactcaagc
acagcaaatgctgtccttttgctcctgaaaaggatgtctaagcagggacg
aacaatcatcttctccattcatcagcctcgatattccatcttcaagttgt
ttgatagcctcaccttattggcctcaggaagacttatgttccacgggcct
gctcaggaggccttgggatactttgaatcagctggttatcactgtgaggc
ctataataaccctgcagacttcttcttggacatcattaatggagattcca
ctgctgtggcattaaacagagaagaagactttaaagccacagagatcata
gagccttccaagcaggataagccactcatagaaaaattagcggagattta
tgtcaactcctccttctacaaagagacaaaagctgaattacatcaacttt
ccgggggtgagaagaagaagaagatcacagtcttcaaggagatcagctac
accacctccttctgtcatcaactcagatgggtttccaagcgttcattcaa
aaacttgctgggtaatccccaggcctctatagctcagatcattgtcacag
tcgtactgggactggttataggtgccatttactttgggctaaaaaatgat
tctactggaatccagaacagagctggggttctcttcttcctgacgaccaa
ccagtgtttcagcagtgtttcagccgtggaactctttgtggtagagaaga
agctcttcatacatgaatacatcagcggatactacagagtgtcatcttat
ttccttggaaaactgttatctgatttattacccatgaggatgttaccaag
tattatatttacctgtatagtgtacttcatgttaggattgaagccaaagg
cagatgccttcttcgttatgatgtttacccttatgatggtggcttattca
gccagttccatggcactggccatagcagcaggtcagagtgtggtttctgt

agcaacacttctcatgaccatctgttttgtgtttatgatgattttttcag
gtctgttggtcaatctcacaaccattgcatcttggctgtcatggcttcag
tacttcagcattccacgatatggatttacggctttgcagcataatgaatt
tttgggacaaaacttctgcccaggactcaatgcaacaggaaacaatcctt
gtaactatgcaacatgtactggcgaagaatatttggtaaagcagggcatc
gatctctcaccctggggcttgtggaagaatcacgtggccttggcttgtat
gattgttattttcctcacaattgcctacctgaaattgttatttcttaaaa
aatattcttaaatttccccttaattcagtatgatttatcctcacataaaa
aagaagcactttgattgaagtattcaatcaagttttttgttgttttctg
ttcccttgccatcacactgttgcacagcagcaattgttttaaagagatac
atttttagaaatcacaacaaactgaattaaacatgaaagaacccaagaca
tcatgtatcgcatattagttaatctcctcagacagtaaccatggggaaga
aatctggtctaatttattaatctaaaaaaggagaattgaattctggaaac
tcctgacaagttattactgtctctggcatttgtttcctcatctttaaaat
gaataggtaggttagtagcccttcagtcttaatactttatgatgctatgg
tttgccattatttaataaatgacaaatgtattaatgctatactggaaatg
taaaattgaaaatatgttggaaaaaagattctgtcttatagggtaaaaaa
agccaccgtgatagaaaaaaaatctttttgataagcacattaaagttaat
agaacttactgatattcctgtctagtggtataatatctcaggaatcttgg
ctgagggtttggaactgtgggtagagtagagggccaggagtccagtaata
gaattcttgcaccatttctggaacattctagctctgggaggtcacgtaac
cttcttggggtagttcagtggtttagtggtttataatccaggtgtgcgtc
agaatcatctgaggaactttgctaaaatacaaaaatctggcctaagtagc
tccagatctaccttcataaaggaatctgaccactcctggatttggtaatt
tccaagttctgaaaattttacttaggatttaataactattaacatctgtc
cctacataggttttctttcctacttatataccttatgttctcttcattct
aaccttcatcagtaatagggaaatgttttaattttattttttagttgaa
gggtaatgtaccaaaaaatatagttcagtgaattaaaatgaacacacatg
tgcaaccatcaattcaggtcaagaaatagaagattgtagcacacaaaagc
ctactcagccattctcccagtcactacttccttccttacccctgggttat
ttttgaaatgacacttgatgtatttccctctgttgctgttatgagaacat
tgctacagccaagtgttgtgtttctgtgtgcataggttgatacttaatta
tctccccactttttaataaacttttaatttggaaataattttagattgac
agaaaagttgcaaagatagtgaggaaagttcctgtctactctttgctcag
cttcccttaatgttaacattttatatagcaagatgcatttgtcaaagcta
acaagttaacattggtacaatcactgttaattaaactgcacacaatattc
agatttcaccacttttccactaatattctttcattgttctaggattcaat
tcaggagaccacatttcatctagccctctttttaaaagtaaatactttt
cagcacttacaggagttaactgagctggggcatcatggtgtatagacgcc
ctgacactggtcatcttggaattcatttagtttgtcagtgggtgccctga
cattctgtcacaacatcaatttgggaacatggcattatatttttatcttt
gaactttttttctttttggatgacatttgattaatgcgtcatcttggaaca
cattatctttttttcttggttatgtgatcaggaagattaatcagtttttcc
tgttcttggtataattcctgcttttcacatacctgtcccttacagttctc
tatatataccccttcccttattacacagagagaaatatctatctatacttt
ttacacaaaatatacttcaaaagaaacaaaacagccacaattattaactt
tttaaataaatgagaatttaattatatcctaaaaaaaaaaaaaaaa
>NM_003749 2
cggggaccgcgacgagcccgggtcgccgttggcagcagcagcagcaacac

cagcagcagcagcagccccggcggcggcgcggaccccgagcgcccgggcg
caccccggcttcccggagcgcgacgcggcggcagcagccccggtgcggcc
gcgcgcgccttaggctcggccccgcggctcggggaccccgactcccggcc
cagcgagcgcgtcccccggcgccgcccgagagcccgaggaggcagcggcc
gcaggcagccggggagggggcggccaccgcccgcgccgggcatcctcag
gagccccagagcgcggagggcgcggcgccgccgagcggtgctggcccccg
cgggcctccccggaccttccccaccgcctgggcccgagggacgcgtgatc
gggcgggcggccgggcgcaagggtgggagggagccgcccccgcccgcgcc
ccctccgcccctcgccccaacccctgggcgccgggcccgggccgcgcggc
ctgaagcgcccgcgatggcgagcccgccgcggcacgggccgcccgggccg
gcgagcggagacggccccaacctcaacaacaacaacaacaacaacaacca
cagcgtgcgcaagtgcggctacctgcgcaagcagaagcatggccacaagc
gcttcttcgtgctgcgcggacccggcgcgggcggcgacgaggcgacggcg
ggcggggggtcggcgccgcaaccgccgcggctcgagtactacgagagcga
gaaaaagtggcggagcaaggcaggcgcgccgaaacgggtgatcgctctcg
actgctgcctgaacatcaacaagcgcgccgacgccaagcacaagtacctg
atcgccctctacaccaaggacgagtacttcgccgtggccgccgagaacga
gcaggagcaggagggctggtaccgcgcgctcaccgacctggtcagcgagg
gccgcgcggccgccggagacgcgccccccgccgccgcgcccgccgcgtcc
tgcagcgcctccctgcccggcgccctgggcggctctgccggcgccgccgg
ggccgaggacagctacgggctggtggctcccgccacggccgcctaccgtg
aggtgtggcaggtgaacctgaagcccaagggtctgggccagagcaagaac
ctgacgggggtgtaccgtctgtgcctgtctgcgcgcaccatcggcttcgt
gaagctcaactgcgagcagccgtcggtgacgctgcagctcatgaacatcc
gccgctgcggccactcggacagcttcttcttcatcgaggtgggccgctcg
gccgtcacaggccccggcgagctgtggatgcaggcggacgactcggtggt
ggcgcagaacatccacgagaccatcctggaggccatgaaggcgctcaagg
agctcttcgagttccggccgcgcagtaagagccaatcgtcggggtcgtcg
gccacgcaccccatcagcgtccccggcgcgcgccgccaccaccacctggt
caacctgcccccagccagacgggcctggtgcgccgctcgcgcaccgaca
gcctggccgccaccccgccggcggccaagtgcagctcgtgccgggtgcgc
accgccagcgagggcgacggcggcgcggcggcgggagcggcggccgcggg
cgccaggccggtgtcggtggctgggagccccctgagccccgggccggtgc
gcgcgcccctgagccgctcgcacaccctgagcggcggctgcggcggccgc
gggagcaaggtggcgctgctgccggcagggggcgcgctgcaacacagccg
ctccatgtccatgcccgtggcgcactcgccgcccgccgccaccagccccg
gctccctgtcgtccagcagcggccacggctcgggctcctacccgccgccg
cccggcccgcacccgcctctgccgcatccgctgcaccacggccccggcca
gcggccctccagcggcagcgcctccgcctcgggctcccccagcgaccccg
gcttcatgtccctggacgagtacggctccagcccaggcgacctgcgcgcc
ttctgcagccaccgaagcaacacgcccgagtccatcgcggagacgccccc
ggcccgagacggcggcggcggcggtgagttctacgggtacatgaccatgg
acaggcccctgagccactgtggccgctcctaccgccgggtctcgggggac
gcggcccaggacctggaccgagggctgcgcaagaggacctactccctgac
cacgccagcccggcagcggccggtgccccagccctcctctgcctcgctgg
atgaatacaccctgatgcgggccaccttctcgggcagcgcgggccgcctc
tgcccgtcctgccccgcgtcctctcccaaggtggcctaccacccctaccc
agaggactacggagacatcgagatcggctcccacaggagctccagcagca
acctgggggcagacgacggctacatgcccatgacgcccggcgcggccctc

gcgggcagtgggagcggcagctgcaggagcgacgactacatgcccatgag
ccccgccagcgtgtccgcccccaagcagatcttgcagcccagggccgccg
ccgccgccgccgccgccgtgccttctgcggggcctgcggggccagcaccc
acctctgcggcgggcaggacattcccggcgagcgggggcggctacaaggc
cagctcgcccgccgagagctcccccgaggacagtgggtacatgcgcatgt
ggtgcggttccaagctgtccatggagcatgcagatggcaagctgctgccc
aacggggactacctcaacgtgtcccccagcgacgcggtcaccacgggcac
cccgcccgacttcttctccgcagccctgcaccccggcggggagccgctca
ggggcgttcccggctgctgctacagctccttgccccgctcctacaaggcc
ccctacacctgtggcggggacagcgaccagtacgtgctcatgagctcccc
cgtggggcgcatcctggaggaggagcgtctggagcctcaggccacgccag
ggcccagccaggcggccagcgccttcggggccggccccacgcagcccccct
caccctgtagtgccttcgcccgtgcggcctagcggcggccgcccggaggg
cttcttgggccagcgcggccgggcggtgaggcccacgcgcctgtccctgg
aggggctgcccagcctgcccagcatgcacgagtacccactgccaccggag
cccaagagccccggcgagtacatcaacatcgactttggcgagcccggggc
ccgcctgtcgccgcccgcgcctcccctgctggcgtcggcggcctcgtcct
cctcgctcttgtccgccagcagcccggcctcgtcgctgggctcaggcacc
ccgggcaccagcagcgacagccggcagcggtctccgctctccgactacat
gaacctcgacttcagctcccccaagtctcctaagccgggcgccccgagcg
gccaccccgtgggctccttggacggcctcctgtcccccgaggcctcctcc
ccgtatccgccgttgcccccgcgtccgtccgcgtccccgtcgtcgtctct
gcagccgccgccaccgccgccggccccgggggagctgtaccgcctgcccc
ccgcctcggccgttgccaccgcccagggcccgggcgccgcctcatcgttg
tcctcggacaccggggacaatggtgactacaccgagatggcttttggtgt
ggccgccaccccgccgcaacctatcgcggcccccccgaagccagaagctg
cccgcgtggccagcccgacgtcgggcgtgaagaggctgagcctcatggag
caggtgtcgggagtcgaggccttcctgcaggccagccagcccccggaccc
ccaccgcggcgccaaggtcatccgcgcagacccgcagggggggccgccgcc
gccacagttccgagaccttctcctccaccacgacggtcacccccgtgtcc
ccgtccttcgcccacaaccccaagcgccacaactcggcctccgtggaaaa
tgtctctctcaggaaaagcagcgagggcggcgtgggtgtcggccctggag
ggggcgacgagccgcccacctccccacgacagttgcagccggcgcccccct
ttggcaccgcagggccggccgtggaccccgggtcagcccgggggcttggt
cggttgtcctgggagcggtggatcgcccatgcgcagagagacctctgccg
gcttccagaatggtctcaactacatcgccatcgacgtgagggaggagccc
gggctgccaccccagccgcagccgccgccgccgccgcttcctcagccggg
agacaagagctcctggggccggacccgaagcctcgggggtctcatcagcg
ctgtgggcgtcggcagcaccggcggcgggtgcggggggccgggtcccggt
gccctgccccctgccaacacctacgccagcattgacttcttgtcccacca
cttgaaggaggccaccatcgtgaaagagtgaagatctgtctggctttatc
accaggatgtcacatgtcagagagtatcattaaaagaagacgctcagcac
tgtttcagcccgaagctgcttgcagttttcttttggatctgagcaatgac
tgtgtttggaaacatctgtggactctgttagatgaggcaccaacaaggca
aggtcacctgcctctttcccttgttcccggatggggcattcatcattgtg
ctgtttgcgttttgttttgttttgttttaacaaaattagctgaagaagtt
attctcaagaaaattggatgttttcattggccttcttaaattgtggccag
tgtcttttaatttcttcttttccttttggcaaagcagatataaccct
cagcatgctaggagagtgcacccgtacctatggaagtggtaaaatctggt

atttactggcttacactcaaaacgaccacagtcctacctcagttcaaggt
aaagccggatttccgtggcgggggtcccacaggacctcctgtagtagccc
ctgcgctgtgtgtctggagcgcggtcctcggccttattgaaatggtccaa
gtagacagctgcttgttggattccagtgcaggtacctgcgatgtttacgt
ccacaccgagcccagtgtgggactgacatttctcaatggaagtgaaattt
gggattggactttgaagacggattactaaataataattattatatgtaac
tgaagcaacctacttttgaaaatcaactgtattgggtagtgggaggtggg
agggaagggctttgggaaggggatgaatatctctttttacctttaacaga
cttgtttaatcttctcgatgtagatgtttatgtaggtacttcacattgca
aacgccttttattctatttacaagctcagatgtctctgctctcctgaatc
ttgggcatgcctttctgtaaccaaaaatccctgtaggcgtgctagcaatt
ccagggtggtccgggtttggcagatttgatttttaaaaaacgtattatct
ttaataaaatgttattatgtcaaccagtgaggctgccctgaacaaaaaaa
acaaaaagaaaaaaaaaaaaggaaagaaagaaactgataaaaagaggcat
tccagcccctatgttattgatggaaaaagaaaaagaagaaaagcaatctc
gcagtacatgttacttgtcgaaaaaattccggacaagactacccttgttt
tatgttttcagtattctgaaaataccagtgtgtggcagttctcgcagatg
ttacctaaaactgctgaacttgaccggcagaatgttctgccgttttctgc
tccctcgacacttgattggagggctgtcgacctctcctcccgtgggggct
tccccagtgcctatcttctctgatagtcatggagaggttacactaattca
ttggagatgtaagttgttggttttgttttgttttgtttttagaaaaatat
atataaatatataatagatatctatcgctatagaataatgcattaataaa
atgaggctttttttagaggaagaccaaaaaattcaatgtcttaaaaatata
tttaatggcaatgcaaaagtcttcctgcttccgtgctgaactttagaaca
gaggattgtattgcaagacaaagttgaatgtaaagtgatctccctgaaca
tttttaaggttttacttttctgaaattatacatcacagcagtgcataggc
catataatgttagctggaaggtcaatttcagtgtatgatatactttatta
agatgtataaaaatcctgaagtttttatttagttttgggaataggcatca
atgggtggtatttgctttgtaactccccccaggtacgatagggactgaat
atggaccctgctgaaagcagtgtattgacgcatatttaactcgccctcta
tccgtagagtagtcatgacactatacagatggttcgtgttcatactgcag
cttaaaacaagcaaaatacacagatgataatatgctaaattttcctctat
cctgtacatttcacaaaaaggcatatgcaatatttacatttttaatttag
tttacagaatggaaccaaaatgtataaatgttatgtttgctaaaacttca
caatgtatattgggtctttgtacattttgcctgacttaccttaaatttaa
aatattttttgctatataaactttaacagttattaaacagtgttttcttt
ttgggtacgtattgtttctggatatcaagatgttaaatatatttcttgct
attgtgatatgacaagagacttaacttatcttgctctgtcttccactgta
cacgctgtatataggggtcaatgtgatgctgctggagacgagaataaact
ggactagaatagtgcattgtatttagtctgtattgatcatggatgccctc
cttaatagccatatgcaataaaataaagtacattatttatgaaatgaaaa
aaaaaaaaaaaaaa
>NM_005228 3
ccccggcgcagcgcggccgcagcagcctccgcccccgcacggtgtgagc
gcccgacgcggccgaggcggccggagtcccgagctagccccggcggccgc
cgccgcccagaccggacgacaggccacctcgtcggcgtccgcccgagtcc
ccgcctcgccgccaacgccacaaccaccgcgcacggccccctgactccgt
ccagtattgatcgggagagccggagcgagctcttcggggagcagcgatgc
gaccctccgggacggccggggcagcgctcctggcgctgctggctgcgctc

tgcccggcgagtcgggctctggaggaaaagaaagtttgccaaggcacgag
taacaagctcacgcagttgggcacttttgaagatcattttctcagcctcc
agaggatgttcaataactgtgaggtggtccttgggaatttggaaattacc
tatgtgcagaggaattatgatctttccttcttaaagaccatccaggaggt
ggctggttatgtcctcattgccctcaacacagtggagcgaattcctttgg
aaaacctgcagatcatcagaggaaatatgtactacgaaaattcctatgcc
ttagcagtcttatctaactatgatgcaaataaaaccggactgaaggagct
gcccatgagaaatttacaggaaatcctgcatggcgccgtgcggttcagca
acaaccctgccctgtgcaacgtggagagcatccagtggcgggacatagtc
agcagtgactttctcagcaacatgtcgatggacttccagaaccacctggg
cagctgccaaaagtgtgatccaagctgtcccaatgggagctgctggggtg
caggagaggagaactgccagaaactgaccaaaatcatctgtgcccagcag
tgctccgggcgctgccgtggcaagtcccccagtgactgctgccacaacca
gtgtgctgcaggctgcacaggcccccgggagagcgactgcctggtctgcc
gcaaattccgagacgaagccacgtgcaaggacacctgcccccccactcatg
ctctacaaccccaccacgtaccagatggatgtgaaccccgagggcaaata
cagctttggtgccacctgcgtgaagaagtgtccccgtaattatgtggtga
cagatcacggctcgtgcgtccgagcctgtggggccgacagctatgagatg
gaggaagacggcgtccgcaagtgtaagaagtgcgaagggccttgccgcaa
agtgtgtaacggaataggtattggtgaatttaaagactcactctccataa
atgctacgaatattaaacacttcaaaaactgcacctccatcagtggcgat
ctccacatcctgccggtggcatttaggggtgactccttcacacatactcc
tcctctggatccacaggaactggatattctgaaaaccgtaaaggaaatca
cagggtttttgctgattcaggcttggcctgaaaacaggacggacctccat
gcctttgagaacctagaaatcatacgcggcaggaccaagcaacatggtca
gttttctcttgcagtcgtcagcctgaacataacatccttgggattacgct
ccctcaaggagataagtgatggagatgtgataatttcaggaaacaaaaat
ttgtgctatgcaaatacaataaactggaaaaaactgtttgggacctccgg
tcagaaaaccaaaattataagcaacagaggtgaaaacagctgcaaggcca
caggccaggtctgccatgccttgtgctcccccgagggctgctggggcccg
gagcccagggactgcgtctcttgccggaatgtcagccgaggcagggaatg
cgtggacaagtgcaaccttctggagggtgagccaagggagtttgtggaga
actctgagtgcatacagtgccacccagagtgcctgcctcaggccatgaac
atcacctgcacaggacggggaccagacaactgtatccagtgtgcccacta
cattgacggcccccactgcgtcaagacctgcccggcaggagtcatgggag
aaaacaacaccctggtctggaagtacgcagacgccggccatgtgtgccac
ctgtgccatccaaactgcacctacggatgcactgggccaggtcttgaagg
ctgtccaacgaatgggcctaagatcccgtccatcgccactgggatggtgg
gggccctcctcttgctgctggtggtggccctggggatcggcctcttcatg
cgaaggcgccacatcgttcggaagcgcacgctgcggaggctgctgcagga
gagggagcttgtggagcctcttacacccagtggagaagctcccaaccaag
ctctcttgaggatcttgaaggaaactgaattcaaaaagatcaaagtgctg
ggctccggtgcgttcggcacggtgtataagggactctggatcccagaagg
tgagaaagttaaaattcccgtcgctatcaaggaattaagagaagcaacat
ctccgaaagccaacaaggaaatcctcgatgaagcctacgtgatggccagc
gtggacaacccccacgtgtgccgcctgctgggcatctgcctcacctccac
cgtgcagctcatcacgcagctcatgcccttcggctgcctcctggactatg
tccgggaacacaaagacaatattggctcccagtacctgctcaactggtgt
gtgcagatcgcaaagggcatgaactacttggaggaccgtcgcttggtgca

ccgcgacctggcagccaggaacgtactggtgaaaacaccgcagcatgtca
agatcacagattttgggctggccaaactgctgggtgcggaagagaaagaa
taccatgcagaaggaggcaaagtgcctatcaagtggatggcattggaatc
aattttacacagaatctatacccaccagagtgatgtctggagctacgggg
tgaccgtttgggagttgatgacctttggatccaagccatatgacggaatc
cctgccagcgagatctcctccatcctggagaaaggagaacgcctccctca
gccacccatatgtaccatcgatgtctacatgatcatggtcaagtgctgga
tgatagacgcagatagtcgcccaaagttccgtgagttgatcatcgaattc
tccaaaatggcccgagacccccagcgctaccttgtcattcagggggatga
aagaatgcatttgccaagtcctacagactccaacttctaccgtgccctga
tggatgaagaagacatggacgacgtggtggatgccgacgagtacctcatc
ccacagcagggcttcttcagcagcccctccacgtcacggactcccctcct
gagctctctgagtgcaaccagcaacaattccaccgtggcttgcattgata
gaaatgggctgcaaagctgtcccatcaaggaagacagcttcttgcagcga
tacagctcagaccccacaggcgccttgactgaggacagcatagacgacac
cttcctcccagtgcctgaatacataaaccagtccgttcccaaaaggcccg
ctggctctgtgcagaatcctgtctatcacaatcagcctctgaaccccgcg
cccagcagagacccacactaccaggacccccacagcactgcagtgggcaa
ccccgagtatctcaacactgtccagcccacctgtgtcaacagcacattcg
acagccctgcccactgggcccagaaaggcagccaccaaattagcctggac
aaccctgactaccagcaggacttctttcccaaggaagccaagccaaatgg
catctttaagggctccacagctgaaaatgcagaatacctaagggtcgcgc
cacaaagcagtgaatttattggagcatgaccacggaggatagtatgagcc
ctaaaaatccagactctttcgatacccaggaccaagccacagcaggtcct
ccatcccaacagccatgcccgcattagctcttagacccacagactggttt
tgcaacgtttacaccgactagccaggaagtacttccacctcgggcacatt
ttgggaagttgcattcctttgtcttcaaactgtgaagcatttacagaaac
gcatccagcaagaatattgtccctttgagcagaaatttatctttcaaaga
ggtatatttgaaaaaaaaaaaaaagtatatgtgaggatttttattgattgg
ggatcttggagtttttcattgtcgctattgatttttacttcaatgggctc
ttccaacaaggaagaagcttgctggtagcacttgctaccctgagttcatc
caggcccaactgtgagcaaggagcacaagccacaagtcttccagaggatg
cttgattccagtggttctgcttcaaggcttccactgcaaaacactaaaga
tccaagaaggccttcatggccccagcaggccggatcggtactgtatcaag
tcatggcaggtacagtaggataagccactctgtcccttcctgggcaaaga
agaaacggaggggatggaattcttccttagacttacttttgtaaaaatgt
ccccacggtacttactccccactgatggaccagtggtttccagtcatgag
cgttagactgacttgtttgtcttccattccattgttttgaaactcagtat
gctgcccctgtcttgctgtcatgaaatcagcaagagaggatgacacatca
aataataactcggattccagcccacattggattcatcagcatttggacca
atagcccacagctgagaatgtggaatacctaaggatagcaccgcttttgt
tctcgcaaaaacgtatctcctaatttgaggctcagatgaaatgcatcagg
tcctttggggcatagatcagaagactacaaaaatgaagctgctctgaaat
ctcctttagccatcaccccaaccccccaaaattagtttgtgttacttatg
gaagatagttttctccttttacttcacttcaaaagctttttactcaaaga
gtatatgttccctccaggtcagctgcccccaaacccccctccttacgcttt
gtcacacaaaaagtgtctctgccttgagtcatctattcaagcacttacag
ctctggccacaacagggcattttacaggtgcgaatgacagtagcattatg
agtagtgtggaattcaggtagtaaatatgaaactagggtttgaaattgat

aatgctttcacaacatttgcagatgttttagaaggaaaaaagttccttcc
taaaataatttctctacaattggaagattggaagattcagctagttagga
gcccacctttttcctaatctgtgtgtgccctgtaacctgactggttaac
agcagtcctttgtaaacagtgttttaaactctcctagtcaatatccaccc
catccaatttatcaaggaagaaatggttcagaaaatattttcagcctaca
gttatgttcagtcacacacacatacaaaatgttccttttgcttttaaagt
aatttttgactcccagatcagtcagagcccctacagcattgttaagaaag
tatttgatttttgtctcaatgaaaataaaactatattcatttccactcta
aaaaaaaaaaaaaaaa
>NM_015270 3
ttttttttttttgagacggagtctcactctgtcacccaggctggagtgc
aatggcgcgatctttgctcactgcaacctctgccacccgggttcaagcaa
ttctcctgcctcagctcctgagtagctgggattacaggcgcccgccacca
cgcccggctaatttttgtatttttagtagagacgaggtttcaccatcttg
gccaggctggtcttgaactcctgaccttgtgatctacctgccagggcctt
ccaaagtgctgggattacggacgtgagccaccacgcctggcccaggctaa
ttttttgaaattttaatagaggcagggtcttgctgtattgcccaggttgc
tctcacattcctagccttaagccatcctcctatctcggcctcacaaagtg
ctgggattacaggtgtaagccactgtgcctggttttgggcaagtttcttg
tcctgactgtgtcttgggttctcacctgttgattggggataccccttgttg
cctacctccaagtaattttatgaggatgaaactcatagcttgtcagtggg
aagtgcttaggtaatgagctactagtggtggcagtggctgtgatggtggt
attaataccactcctactggatggtctctaatactatctccttccttccc
taccagcaacatgtcatggtttagtggcctcctggtccctaaagtggatg
aacggaaaacagcctggggtgaacgcaatgggcagaagcgttcgcggcgc
cgtggcactcgggcaggtggcttctgcacgccccgctatatgagctgcct
ccgggatgcagagccacccagccccacccctgcgggccccccctcggtgcc
cctggcaggatgacgccttcatccggagggggcggcccaggcaagggcaag
gagctggggctgcgggcagtggccctgggcttcgaggataccgaggtgac
aacgacagcgggcgggacggctgaggtggcgcccgacgcggtgcccagga
gtgggcgatcctgctggcgccgtctggtgcaggtgttccagtcgaagcag
ttccgttcggccaagctggagcgcctgtaccagcggtacttcttccagat
gaaccagagcagcctgacgctgctgatggcggtgctggtgctgctcacag
cggtgctgctggctttccacgccgcacccgcccgccctcagcctgcctat
gtggcactgttggcctgtgccgccgccctgttcgtggggctcatggtggt
gtgtaaccggcatagcttccgccaggactccatgtgggtggtgagctacg
tggtgctgggcatcctggcggcagtgcaggtcgggggcgctctcgcagca
gacccgcgcagcccctctgcgggcctctggtgccctgtgttctttgtcta
catcgcctacacgctcctccccatccgcatgcgggctgccgtcctcagcg
gcctgggcctctccaccttgcatttgatcttggcctggcaacttaaccgt
ggtgatgccttcctctggaagcagctcggtgccaatgtgctgctgttcct
ctgcaccaacgtcattggcatctgcacacactatccagcagaggtgtctc
agcgccaggcctttcaggagacccgcggttacatccaggcccggctccac
ctgcagcatgagaatcggcagcaggagcggctgctgctgtcggtattgcc
ccagcacgttgccatggagatgaaagaagacatcaacacaaaaaaagaag
acatgatgttccacaagatctacatacagaagcatgacaatgtcagcatc
ctgtttgcagacattgagggcttcaccagcctggcatcccagtgcactgc
gcaggagctggtcatgaccctgaatgagctctttgcccggtttgacaagc
tggctgcggagaatcactgcctgaggatcaagatcttgggggactgttac

tactgtgtgtcagggctgccggaggcccgggccgaccatgcccactgctg
tgtggagatgggggtagacatgattgaggccatctcgctggtacgtgagg
tgacaggtgtgaatgtgaacatgcgcgtgggcatccacagcgggcgcgtg
cactgcggcgtccttggcttgcggaaatggcagttcgatgtgtggtccaa
tgatgtgaccctggccaaccacatggaggcaggaggccgggctggccgca
tccacatcactcgggcaacactgcagtacctgaacggggactacgaggtg
gagccaggccgtggtggcgagcgcaacgcgtacctcaaggagcagcacat
tgagactttcctcatcctgggcgccagccagaaacggaaagaggagaagg
ccatgctggccaagctgcagcggactcgggccaactccatggaagggctg
atgccgcgctgggttcctgatcgtgccttctcccggaccaaggactccaa
ggccttccgccagatgggcattgatgattccagcaaagacaaccggggca
cccaagatgccctgaaccctgaggatgaggtggatgagttcctgagccgt
gccatcgatgcccgcagcattgatcagctgcggaaggaccatgtgcgccg
gtttctgctcaccttccagagagaggatcttgagaagaagtactcccgga
aggtggatccccgcttcggagcctacgttgcctgtgccctgttggtcttc
tgcttcatctgcttcatccagcttctcatcttcccacactccaccctgat
gcttgggatctatgccagcatcttcctgctgctgctaatcaccgtgctga
tctgtgctgtgtactcctgtggttctctgttccctaaggccctgcaacgt
ctgtcccgcagcattgtccgctcacgggcacatagcaccgcagttggcat
cttttccgtcctgcttgtgtttacttctgccattgccaacatgttcacct
gtaaccacacccccatacggagctgtgcagcccggatgctgaatttaaca
cctgctgacatcactgcctgccacctgcagcagctcaattactctctggg
cctggatgctcccctgtgtgagggcaccatgcccacctgcagctttcctg
agtacttcatcgggaacatgctgctgagtctcttggccagctctgtcttc
ctgcacatcagcagcatcgggaagttggccatgatctttgtcttggggct
catctatttggtgctgcttctgctgggtcccccagccaccatctttgaca
actatgacctactgcttggcgtccatggcttggcttcttccaatgagacc
tttgatgggctggactgtccagctgcagggagggtggccctcaaatatat
gacccctgtgattctgctggtgtttgcgctggcgctgtatctgcatgctc
agcaggtggagtcgactgcccgcctagacttcctctggaaactacaggca
acaggggagaaggaggagatggaggagctacaggcatacaaccggaggct
gctgcataacattctgcccaaggacgtggcggcccacttcctggcccggg
agcgccgcaatgatgaactctactatcagtcgtgtgagtgtgtggctgtt
atgtttgcctccattgccaacttctctgagttctatgtggagctggaggc
aaacaatgagggtgtcgagtgcctgcggctgctcaacgagatcatcgctg
actttgatgagattatcagcgaggagcggttccggcagctggaaaagatc
aagacgattggtagcacctacatggctgcctcagggctgaacgccagcac
ctacgatcaggtgggccgctcccacatcactgccctggctgactacgcca
tgcggctcatggagcagatgaagcacatcaatgagcactccttcaacaat
ttccagatgaagattgggctgaacatgggcccagtcgtggcaggtgtcat
cggggctcggaagccacagtatgacatctgggggaacacagtgaatgtct
ctagtcgtatggacagcacgggggtccccgaccgaatccaggtgaccacg
gacctgtaccaggttctagctgccaagggctaccagctggagtgtcgagg
ggtggtcaaggtgaagggcaagggggagatgaccacctacttcctcaatg
ggggccccagcagttaacagggcccagccacaaattcagctgaagggacc
aaggtgggcattgagtggactctgtgctcactgggtggagctgtggcagg
gggcactgagcctccagaccctgctaaccacaaaagggaacatcccagca
ggctgtgcttggatcatgctcgtctgccctcaagctggaaaacaaggggc
tacctaccgagaggattatgcaagtgactttctttcttacttggggtagg

gctgttccctctccaatcttccagcctttgggagcaggggaggggtcagt
agcagaagcagagggaggcctcttgcctgagggattaaaatggcagcttg
ccatgcctacccttccctgtctgtctgggcagcaggttcagggctgagcc
cttcttttccctctttttcctgggaatattttgtacaatattttgtaca
aagacaggcatgaggagtgcctattccatgcttgcctttgcaatacctgc
atccccagcactggtcctgggcacttccccaccccagccaggtgtccctc
ctatgcacagagcagaggagggagaagctctggggagccagctttggcca
tatttcaggagaatgtttccatgtgccaaatcttagtcccatgatctgtc
cccaaaggggaacaaagggacctctgacagcttagatttagccccagttc
ctgcacgctccagggaacggggtgtctggcctcactggtactgtgaaaaa
tgctcagagagcaagcctgtgtgtggggatgtcaggtcaggagctggaag
ttcacctgcaggtgccaaagagcaggccggccagggctggggcagtgcca
gactctgatctgaggaccccgtcggggtccagatcaggtcactctgcccc
agtgctctcttgctgtctgctgacaagggggcatggagcatctcttcctc
ttctgttgccaaatagaaaagggtcagggcatggagaaaggtgaccctga
tcccaaacctgccctcccaagtctctggtgttggggagggcccgtgtgtt
tgtgtaactgtgtgtgcatgttggtctttgtgtgcatatctgttttccag
gtctatgtgagtccttgtgctcctgctcctcagctctccaccccaggttg
cctctctcctgtgggcctctgtcttctgggaataaagcagggtttcctac
ttcaggggatgtagagagatgcccaggttgcacaggagtgggatggggtg
tggtagcaaaaggagggagaggagtccttttgtgccaaatccctaagtg
ccgttcgggggccatgtgtgcagcatgactctccctgtctgtggcaggga
cccaagcgcttgcttaagccccagtgctccatgccagcacttgaactgtc
tggggtttgatggacagaggctgaggaatttcctggcttccccagatagt
gtcctgggacatggtatgctttggggctggggtagcatggaatccctctg
aggacctggatactggtactacggggtggggaagaggaaccttaaacttg
gctttccccagccttcagcctgagtctagcatgtttctagctccccagtc
ccttgtgaagccttgagggctggcagaagggttaggaggttgaactctag
attcccttcctgtctttgccttccttttacccctttccctgcaacctcct
tgactctggcctgaatttgttggtgcctcagtttctctgtctgtacctat
ttaagccaaaggcactagcctgaattttgcttgaagatcactttgtcttg
gaaatgactagagaggcagaggagaagggtttccagagttgctaggtttg
ggagtggaaggggcaggcagtgcacttgcccctcctcatgccccttctga
caccagctccctgtggaggcctggtttctgggtaatgcctcccttgggca
tcttcatgcatcaaccaaatgggccatcaggtacttcattagtcatggca
gaaggaggggaaaagacttgttttccagacagaaaatctactcccctgtc
cccagccatatccctggataggaagggataggaagagactacttggtgcc
atggggtaggggtgagggtataagtagatcagagtgggaagacctcagcc
ttgggtggcttgtctctgcttcttgccaggtgggagggcctgtccacacc
ctggatccccgtaccacagtgccagccatgcccttcccctgggctaccat
tgtccctttcctcacccagttggtagaggagtcaggaggtgggaggccgt
gggctttggttttataatgtaaccactgtgggggtgggggaggatggtga
accatgtatttcagtgaaatatttaatatatttaaatatcaataaaatca
aactctttgtaaaattaaaaaaaaaaaaaaaaa

Negative samples:

>NM_005546 3
tgcattctttgccccaaaactctttcctttggttgtgctaagaggtgatg
cccaaggtgcaccacctttcaagaactggatcatgaacaactttatcctc
ctggaagaacagctcatcaagaaatcccaacaaaagagaagaacttctcc
ctcgaactttaaagtccgcttctttgtgttaaccaaagccagcctggcat
actttgaagatcgtcatgggaagaagcgcacgctgaaggggtccattgag
ctctcccgaatcaaatgtgttgagattgtgaaaagtgacatcagcatccc
atgccactataaatacccgtttcaggtggtgcatgacaactacctcctat
atgtgtttgctccagatcgtgagagccggcagcgctgggtgctggccctt
aaagaagaaacgaggaataataacagtttggtgcctaaatatcatcctaa
tttctggatggatgggaagtggaggtgctgttctcagctggagaagcttg
caacaggctgtgcccaatatgatccaaccaagaatgcttcaaagaagcct
cttcctcctactcctgaagacaacaggcgaccactttgggaacctgaaga
aactgtggtcattgccttatatgactaccaaaccaatgatcctcaggaac
tcgcactgcggcgcaacgaagagtactgcctgctggacagttctgagatt
cactggtggagagtccaggacaggaatgggcatgaaggatatgtaccaag
cagttatctggtggaaaaatctccaaataatctggaaacctatgagtggt
acaataagagtatcagccgagacaaagctgaaaaacttcttttggacaca
ggcaaagaaggagccttcatggtaagggattccaggactgcaggaacata
caccgtgtctgttttcaccaaggctgttgtaagtgagaacaatccctgta
taaagcattatcacatcaaggaaacaaatgacaatcctaagcgatactat
gtggctgaaaagtatgtgttcgattccatccctcttctcatcaactatca
ccaacataatggaggaggcctggtgactcgactccggtatccagtttgtt
ttgggaggcagaaagccccagttacagcagggctgagatacgggaaatgg
gtgatcgacccctcagagctcacttttgtgcaagagattggcagtgggca
atttgggttggtgcatctgggctactggctcaacaaggacaaggtggcta
tcaaaaccattcgggaaggggctatgtcagaagaggacttcatagaggag
gctgaagtaatgatgaaactctctcatcccaaactggtgcagctgtatgg
ggtgtgcctggagcaggcccccatctgcctggtgtttgagttcatggagc
acggctgcctgtcagattatctacgcacccagcggggactttttgctgca
gagaccctgctgggcatgtgtctggatgtgtgtgagggcatggcctacct
ggaagaggcatgtgtcatccacagagacttggctgccagaaattgtttgg
tgggagaaaaccaagtcatcaaggtgtctgactttgggatgacaaggttc
gttctggatgatcagtacaccagttccacaggcaccaaattcccggtgaa
gtgggcatccccagaggttttctctttcagtcgctatagcagcaagtccg
atgtgtggtcatttggtgtgctgatgtgggaagttttcagtgaaggcaaa
atcccgtatgaaaaccgaagcaactcagaggtggtggaagacatcagtac
cggatttcggttgtacaagccccggctggcctccacacacgtctaccaga
ttatgaatcactgctggaaagagagaccagaagatcggccagccttctcc
agactgctgcgtcaactggctgaaattgcagaatcaggactttagtagag
actgagtaccaggccacgggctgcagatcctgaatggaggaaggatatgt
cctcattccatagagcattagaagctgccaccagcccaggaccctccaga
ggcagcctggcctgtggcatcagtccctgagtcaccatggaagcagcatc
ctgaccacagctggcagtcaagccacagctggagggtcagccaccaagct
gggagctgagccagaacaggagtgatgtctctgcccttcctctagcctct
tgtcacatgtggtgcacaaacctcaacctgacagctttcagacagcattc
ttgcacttcttagcaacagagagagacatgagtaagacccagattgctat
ttttattgttatttttaacatgaatctaaagtttatggttccagggactt

tttatttgacccaacaacacagtatcccaggatatggaggcaaggggaac
aaagagcatgagtctttttccaagaaaactggtgagttaagtaagattag
agtgagtgtgctctgttgctgtgatgctgtcagccacagcttcctgccgt
agagaatgatagagcagctgctcacacaggaggccggatattctgagaag
cagctttatgaggttttacagagtatgctgctacctctctccttgaaggg
agcatggcgagacccattggatggattggggtgaacagttcaggtcccat
gcttggagcattgggtatctgatgtctgcaccagaacaagagaacctctg
acggtggagaaccatgtggtgcaagaagagatcttaggtctcttctttta
taccaagctcatcttttataccaagctgtgcaggtgactatgcctcctct
tctgcacagaatgcttccaccagcatcctgagaagaaatgattacttctg
aaaaacatcctttttccagcctctgggaatcagcccccctctctgcac
tatccgatcctcatcaacagagggcagcattgtgttggtcaatgttccct
tggcgagcaattgaaacttgtttaggccctagggttgagcaattttaagg
ttgagactccaagtctcctaaaattctaggagagaaataaagagtctgtt
tttgctcaaaccatcaggatggaaacagtcaggcactgactggggtgctt
ccaagaggcatgagagtgcctactctggcttgagcacttctatatgcaag
gtgaatatgtactgagctaggagacttccctgcaaaatctctgttcaccc
tgggttcacatccccatgaggtaatattattattcccattttacaaataa
tgtaactgaggctttaaaaagccaagacatctgcccaaagtgatggaact
agaaagtctagagctggtattctagcccaaatctgtctgaccgcaataca
cagattctttattcctattcgacactggcttctactgaaaatgaaacgga
ttgcagagggaataaatacaaagatggaaagccagtaaagaagtcagtat
agaaccactagcgaatagtgttgctctggcacagaccactgtggttgatg
gcatggccctccaacttggaataggattttccttttcctattctgtatcc
ttaccttggtcatgttaatgactttggagttattcagttaatgacccttt
aattctcacaaccaaccagtcatgttgcttgaagccatttatagacgagc
ttcaaagcaactttaaaagattcttctgtagaagtatgagttcttccttt
aattatcattccaactttcagctgtagtcttcttgaacacttcatgagga
gggacattccctgatataagagaggatggtgttgcaattggctctttcta
aatcatgtgacgttttgactggcttgagattcagatgcataatttttaat
tataattattgtgaagtggagagcctcaagataaaactctgtcattcaga
agatgattttactcagcttatccaaaattatctctgtttactttttagaa
ttttgtacattatcttttgggatccttaattagagatgatttctggaaca
ttcagtctagaaagaaaacattggaattgactgatctctgtggtttggtt
tagaaaattcccctgtgcatggtattacctttttcaagctcagattcatc
taatcctcaactgtacatgtgtacattcttcacctcctggtgccctatcc
cgcaaaatgggcttcctgcctggtttttctcttctcacatttttttaaatg
gtcccctgtgtttgtagagaactcccttatacagagttttggttctagtt
ttatttcgtagattttgcattttgtaccttttgagactatgtatttatat
ttggatcagatgcatatttattaatgtacagtcactgctagtgttcaaaa
taaaaatgttacaaat
>NM_014716 3
agcactgcctggaagtgtggggtgagagctcctcctaggacacccctttc
cccttggggaaagaattgtgcccccaggcccttccccgcggaggtccctc
tcctccttccccctcatctccccttcctgggacagaaagtgcctccacct
gcatccccaggggcccggcctccagggcccgctggccccacagcaggcaa
gctgagatgacggtcaagctggatttcgaggagtgtctcaaggactcacc
ccgtttccgagcctctattgagctggtggaagccgaagtgtcagaattgg
agacccgtctggaaaagctcctgaaactgggcactggtctcctggaaagt

gggcgccattaccttgctgccagccgcgccttcgttgtcggcatttgtga
cctggcccgcctgggtccaccagagcccatgatggcggagtgtctggaaa
aattcaccgtgagcctgaaccacaagctggacagccatgcggagcttcta
gatgccacccaacacacactgcagcagcagatccagaccctggtcaagga
aggtctgcggggtttccgagaggctcgccgggatttctggcggggggctg
agagcctggaggctgccctgacccacaacgcagaggttcccaggcgccgg
gcccaggaggcagaagaggcaggagctgctttgaggacggctcgagctgg
gtaccggggacgggcactggattatgccctgcagatcaacgtgattgagg
acaagaggaagtttgacatcatggagtttgtgctgcgtttggtggaggcc
caggctacccatttccagcagggccatgaggagctgagccggctgtccca
gtatcgaaaggagctgggcgcccagttgcaccagctggtcttgaattcag
cacgagagaagagggacatggagcagagacacgtgctgctgaaacagaag
gagctgggtggggaggagccagaaccaagcttaagagaggggcctggtgg
cctggtgatggaaggacatctcttcaaacgggccagcaacgcatttaaga
cctggagcagacgctggttcaccattcagagcaaccaactggtttaccag
aagaagtacaaggaccctgtgactgtggtggtggatgaccttcgtctctg
cacagtgaaactctgccctgactcagaaaggcggttctgctttgaggtgg
tgtccaccagcaagtcctgcctcctccaggctgactcagagcgcctcctg
cagctgtgggtcagtgctgtgcagagcagcattgcttctgccttcagtca
ggctcgccttgatgacagcccccggggtccaggccagggctcaggacacc
tggccataggctctgctgccaccctgggctctggtggaatggccagggga
agggagcctgggggagtcgggcacgtggtggcccaggtccagagtgtgga
tggcaatgcccagtgctgcgactgccgggagccagccccggagtgggcca
gcatcaaccttggtgtcaccctctgcattcagtgttccggcatccacagg
agccttggtgttcacttctccaaagtccggtctctgacccttgactcatg
ggagccagaactagtgaagctcatgtgtgagctgggaaatgtcatcatca
accagatctatgaggcccgcgtggaggccatggcagtgaagaaaccaggg
cccagctgctcccggcaggagaaggaggcctggattcacgctaaatacgt
ggagaagaagttcctgaccaagctgcctgagattcgagggcgaagaggtg
gccgggggcgcccaagggggcagcctcctgtgcccccaaagccttccatc
aggccccggccagggagcttgagatccaagccagagccccccctctgagga
cctgggaagcctgcaccctggggccctactgtttcgagcgtctgggcatc
ctccatctcttcccaccatggctgatgcccttgcccatggagctgatgtc
aactgggtcaatgggggccaagataatgccacaccgctgatccaggccac
agctgctaattctcttctggcctgtgagtttctcctccagaacggggcga
acgtgaaccaagcggacagtgcgggccggggcccgctgcaccacgcaacc
attcttggccacacggggctcgcctgcctgttcctgaaacggggagctga
tctgggggctcgagactctgaaggcagggaccctctgaccatcgccatgg
aaacagccaacgctgacatcgtcaccctgctacgactggcaaagatgagg
gaggctgaagcggcccaggggcaggcaggagatgagacgtatcttgacat
cttccgcgacttctccctcatggcgtcagacgacccggagaagctgagcc
gtcgcagtcatgacctccacacgctgtgacccgaggcccacggggcccgc
gcctgcctcccttccccgccaccgggccctctgccattaaagcctccgtg
cttcgctcaaaaaaaaaaaaaaa
>NM_001152 4
agctccggctcccccctatataaatcggccatttgcttcgctccgccccgc
agcgccggagtcaaagccggttcccggcccagtcccgtcctgcagcagtc
tgcctcctctttcaacatgacagatgccgctgtgtccttcgccaaggact
tcctggcaggtggagtggccgcagccatctccaagacggcggtagcgccc

atcgagcgggtcaagctgctgctgcaggtgcagcatgccagcaagcagat
cactgcagataagcaatacaaaggcattatagactgcgtggtccgtattc
ccaaggagcagggagttctgtccttctggcgcggtaacctggccaatgtc
atcagatacttccccacccaggctcttaacttcgccttcaaagataaata
caagcagatcttcctgggtggtgtggacaagagaacccagttttggctct
actttgcagggaatctggcatcgggtggtgccgcaggggccacatccctg
tgttttgtgtaccctcttgattttgcccgtacccgtctagcagctgatgt
gggtaaagctggagctgaaagggaattccgaggcctcggtgactgcctgg
ttaagatctacaaatctgatgggattaagggcctgtaccaaggctttaac
gtgtctgtgcagggtattatcatctaccgagccgcctacttcggtatcta
tgacactgcaaagggaatgcttccggatcccaagaacactcacatcgtca
tcagctggatgatcgcacagactgtcactgctgttgccgggttgacttcc
tatccatttgacactgttcgccgccgcatgatgatgcagtcagggcgcaa
aggaactgacatcatgtacacaggcacgcttgactgctggcggaagattg
ctcgtgatgaaggaggcaaagctttttttcaagggtgcatggtccaatgtt
ctcagaggcatgggtggtgcttttgtgcttgtcttgtatgatgaaatcaa
gaagtacacataagttatttcctaggatttttccccctgtgaacaggcat
gttgtattatataacatatcttgagcattcttgacagactcctggctgtc
agtttctcagtggcaactatttactggttgaaaatgggaagcaataatat
tcatctgaccagttttctcttaaagccatttccatgatgatgatgatggg
actcaattgtatttttttatttcagtcactcctgataaataacaaatttgg
agaaataaaaatatctaaaataaattttgtctgcagtatattttcatata
aaaatgcatatttgagtgctacattcgaataaatactaccttttagtga
a
>NM_014716 3
agcactgcctggaagtgtggggtgagagctcctcctaggacaccccttc
cccttggggaaagaattgtgcccccaggcccttccccgcggaggtccctc
tcctccttccccctcatctccccttcctgggacagaaagtgcctccacct
gcatccccaggggcccggcctccagggcccgctggccccacagcaggcaa
gctgagatgacggtcaagctggatttcgaggagtgtctcaaggactcacc
ccgtttccgagcctctattgagctggtggaagccgaagtgtcagaattgg
agacccgtctggaaaagctcctgaaactgggcactggtctcctggaaagt
gggcgccattaccttgctgccagccgcgccttcgttgtcggcatttgtga
cctggcccgcctgggtccaccagagcccatgatggcggagtgtctggaaa
aattcaccgtgagcctgaaccacaagctggacagccatgcggagcttcta
gatgccacccaacacacactgcagcagcagatccagaccctggtcaagga
aggtctgcggggtttccgagaggctcgccgggatttctggcggggggctg
agagcctggaggctgccctgacccacaacgcagaggttcccaggcgccgg
gcccaggaggcagaagaggcaggagctgctttgaggacggctcgagctgg
gtaccggggacgggcactggattatgccctgcagatcaacgtgattgagg
acaagaggaagtttgacatcatggagtttgtgctgcgtttggtggaggcc
caggctacccatttccagcagggccatgaggagctgagccggctgtccca
gtatcgaaaggagctgggcgcccagttgcaccagctggtcttgaattcag
cacgagagaagagggacatggagcagagacacgtgctgctgaaacagaag
gagctgggtggggaggagccagaaccaagcttaagagaggggcctggtgg
cctggtgatggaaggacatctcttcaaacgggccagcaacgcatttaaga
cctggagcagacgctggttcaccattcagagcaaccaactggtttaccag
aagaagtacaaggaccctgtgactgtggtggtggatgaccttcgtctctg
cacagtgaaactctgccctgactcagaaaggcggttctgctttgaggtgg

tgtccaccagcaagtcctgcctcctccaggctgactcagagcgcctcctg
cagctgtgggtcagtgctgtgcagagcagcattgcttctgccttcagtca
ggctcgccttgatgacagcccccggggtccaggccagggctcaggacacc
tggccataggctctgctgccaccctgggctctggtggaatggccaggga
agggagcctgggggagtcgggcacgtggtggcccaggtccagagtgtgga
tggcaatgcccagtgctgcgactgccgggagccagccccggagtgggcca
gcatcaaccttggtgtcaccctctgcattcagtgttccggcatccacagg
agccttggtgttcacttctccaaagtccggtctctgacccttgactcatg
ggagccagaactagtgaagctcatgtgtgagctgggaaatgtcatcatca
accagatctatgaggcccgcgtggaggccatggcagtgaagaaaccaggg
cccagctgctcccggcaggagaaggaggcctggattcacgctaaatacgt
ggagaagaagttcctgaccaagctgcctgagattcgagggcgaagaggtg
gccgggggcgcccaagggggcagcctcctgtgcccccaaagccttccatc
aggccccggccagggagcttgagatccaagccagagcccccctctgagga
cctgggaagcctgcaccctggggccctactgtttcgagcgtctgggcatc
ctccatctcttcccaccatggctgatgcccttgcccatggagctgatgtc
aactgggtcaatgggggccaagataatgccacaccgctgatccaggccac
agctgctaattctcttctggcctgtgagtttctcctccagaacggggcga
acgtgaaccaagcggacagtgcgggccggggcccgctgcaccacgcaacc
attcttggccacacggggctcgcctgcctgttcctgaaacggggagctga
tctgggggctcgagactctgaaggcagggaccctctgaccatcgccatgg
aaacagccaacgctgacatcgtcaccctgctacgactggcaaagatgagg
gaggctgaagcggcccaggggcaggcaggagatgagacgtatcttgacat
cttccgcgacttctccctcatggcgtcagacgacccggagaagctgagcc
gtcgcagtcatgacctccacacgctgtgacccgaggcccacggggcccgc
gcctgcctcccttccccgccaccgggccctctgccattaaagcctccgtg
cttcgctcaaaaaaaaaaaaaaa
>NM_004456 4
ggcggcgcttgattgggctgggggggccaaataaaagcgatggcgattgg
gctgccgcgtttggcgctcggtccggtcgcgtccgacacccggtgggact
cagaaggcagtggagccccggcggcggcggcggcggcgcgcgggggcgac
gcgcgggaacaacgcgagtcggcgcgcgggacgaagaataatcatgggcc
agactgggaagaaatctgagaagggaccagtttgttggcggaagcgtgta
aaatcagagtacatgcgactgagacagctcaagaggttcagacgagctga
tgaagtaaagagtatgtttagttccaatcgtcagaaaattttggaaagaa
cggaaatcttaaaccaagaatggaaacagcgaaggatacagcctgtgcac
atcctgacttctgtgagctcattgcgcgggactagggagtgttcggtgac
cagtgacttggattttccaacacaagtcatcccattaaagactctgaatg
cagttgcttcagtacccataatgtattcttggtctcccctacagcagaat
tttatggtggaagatgaaactgttttacataacattccttatatgggaga
tgaagttttagatcaggatggtactttcattgaagaactaataaaaaatt
atgatgggaaagtacacggggatagagaatgtgggtttataaatgatgaa
atttttgtggagttggtgaatgcccttggtcaatataatgatgatgacga
tgatgatgatggagacgatcctgaagaaagagaagaaaagcagaaagatc
tggaggatcaccgagatgataaagaaagccgcccacctcggaaatttcct
tctgataaaatttttgaagccatttcctcaatgtttccagataagggcac
agcagaagaactaaaggaaaaatataaagaactcaccgaacagcagctcc
caggcgcacttcctcctgaatgtacccccaacatagatggaccaaatgct
aaatctgttcagagagagcaaagcttacactcctttcatacgcttttctg

taggcgatgttttaaatatgactgcttcctacatcgtaagtgcaattatt
cttttcatgcaacacccaacacttataagcggaagaacacagaaacagct
ctagacaacaaaccttgtggaccacagtgttaccagcatttggagggagc
aaaggagtttgctgctgctctcaccgctgagcggataaagaccccaccaa
aacgtccaggaggccgcagaagaggacggcttcccaataacagtagcagg
cccagcacccccaccattaatgtgctggaatcaaaggatacagacagtga
tagggaagcagggactgaaacgggggagagaacaatgataaagaagaag
aagagaagaaagatgaaacttcgagctcctctgaagcaaattctcggtgt
caaacaccaataaagatgaagccaaatattgaacctcctgagaatgtgga
gtggagtggtgctgaagcctcaatgtttagagtcctcattggcacttact
atgacaatttctgtgccattgctaggttaattgggaccaaaacatgtaga
caggtgtatgagtttagagtcaaagaatctagcatcatagctccagctcc
cgctgaggatgtggatactcctccaaggaaaaagaagaggaaacaccggt
tgtgggctgcacactgcagaaagatacagctgaaaaaggacggctcctct
aaccatgtttacaactatcaaccctgtgatcatccacggcagccttgtga
cagttcgtgcccttgtgtgatagcacaaaatttttgtgaaaagttttgtc
aatgtagttcagagtgtcaaaaccgctttccgggatgccgctgcaaagca
cagtgcaacaccaagcagtgcccgtgctacctggctgtccgagagtgtga
ccctgacctctgtcttacttgtggagccgctgaccattgggacagtaaaa
atgtgtcctgcaagaactgcagtattcagcggggctccaaaaagcatcta
ttgctggcaccatctgacgtggcaggctgggggatttttatcaaagatcc
tgtgcagaaaaatgaattcatctcagaatactgtggagagattatttctc
aagatgaagctgacagaagagggaaagtgtatgataaatacatgtgcagc
tttctgttcaacttgaacaatgattttgtggtggatgcaacccgcaaggg
taacaaaattcgttttgcaaatcattcggtaaatccaaactgctatgcaa
aagttatgatggttaacggtgatcacaggataggtatttttgccaagaga
gccatccagactggcgaagagctgtttttgattacagatacagccaggc
tgatgccctgaagtatgtcggcatcgaaagagaaatggaaatcccttgac
atctgctacctcctcccccctcctctgaaacagctgccttagcttcagga
acctcgagtactgtgggcaatttagaaaaagaacatgcagtttgaaattc
tgaatttgcaaagtactgtaagaataatttatagtaatgagtttaaaaat
caactttttattgccttctcaccagctgcaaagtgttttgtaccagtgaa
tttttgcaataatgcagtatggtacatttttcaactttgaataaagaata
cttgaacttgtccttgttgaatc
>NM_152998 2
ggcggcgcttgattgggctgggggggccaaataaaagcgatggcgattgg
gctgccgcgtttggcgctcggtccggtcgcgtccgacacccggtgggact
cagaaggcagtggagccccggcggcggcggcggcggcgcgcgggggcgac
gcgcgggaacaacgcgagtcggcgcgcgggacgaagaataatcatgggcc
agactgggaagaaatctgagaagggaccagtttgttggcggaagcgtgta
aaatcagagtacatgcgactgagacagctcaagaggttcagacgagctga
tgaagtaaagagtatgtttagttccaatcgtcagaaaattttggaaagaa
cggaaatcttaaaccaagaatggaaacagcgaaggatacagcctgtgcac
atcctgacttctgtgagctcattgcgcgggactagggaggtggaagatga
aactgttttacataacattccttatatgggagatgaagttttagatcagg
atggtactttcattgaagaactaataaaaaattatgatgggaaagtacac
ggggatagagaatgtgggtttataaatgatgaaatttttgtggagttggt
gaatgcccttggtcaatataatgatgatgacgatgatgatgatggagacg
atcctgaagaaagagaagaaaagcagaaagatctggaggatcaccgagat

gataaagaaagccgcccacctcggaaatttccttctgataaaatttttga
agccatttcctcaatgtttccagataagggcacagcagaagaactaaagg
aaaaatataaagaactcaccgaacagcagctcccaggcgcacttcctcct
gaatgtacccccaacatagatggaccaaatgctaaatctgttcagagaga
gcaaagcttacactcctttcatacgcttttctgtaggcgatgttttaaat
atgactgcttcctacatccttttcatgcaacacccaacacttataagcgg
aagaacacagaaacagctctagacaacaaaccttgtggaccacagtgtta
ccagcatttggagggagcaaaggagtttgctgctgctctcaccgctgagc
ggataaagaccccaccaaaacgtccaggaggccgcagaagaggacggctt
cccaataacagtagcaggcccagcacccccaccattaatgtgctggaatc
aaaggatacagacagtgatagggaagcagggactgaaacgggggagaga
acaatgataaagaagaagaagagaagaaagatgaaacttcgagctcctct
gaagcaaattctcggtgtcaaacaccaataaagatgaagccaaatattga
acctcctgagaatgtggagtggagtggtgctgaagcctcaatgtttagag
tcctcattggcacttactatgacaatttctgtgccattgctaggttaatt
gggaccaaaacatgtagacaggtgtatgagtttagagtcaaagaatctag
catcatagctccagctcccgctgaggatgtggatactcctccaaggaaaa
agaagaggaaacaccggttgtgggctgcacactgcagaaagatacagctg
aaaaaggacggctcctctaaccatgtttacaactatcaaccctgtgatca
tccacggcagccttgtgacagttcgtgcccttgtgtgatagcacaaaatt
tttgtgaaaagttttgtcaatgtagttcagagtgtcaaaaccgctttccg
ggatgccgctgcaaagcacagtgcaacaccaagcagtgcccgtgctacct
ggctgtccgagagtgtgaccctgacctctgtcttacttgtggagccgctg
accattgggacagtaaaaatgtgtcctgcaagaactgcagtattcagcgg
ggctccaaaaagcatctattgctggcaccatctgacgtggcaggctgggg
gatttttatcaaagatcctgtgcagaaaaatgaattcatctcagaatact
gtggagagattatttctcaagatgaagctgacagaagagggaaagtgtat
gataaatacatgtgcagctttctgttcaacttgaacaatgattttgtggt
ggatgcaacccgcaagggtaacaaaattcgttttgcaaatcattcggtaa
atccaaactgctatgcaaaagttatgatggttaacggtgatcacaggata
ggtatttttgccaagagagccatccagactggcgaagagctgtttttga
ttacagatacagccaggctgatgccctgaagtatgtcggcatcgaaagag
aaatggaaatcccttgacatctgctacctcctccccccctcctctgaaaca
gctgccttagcttcaggaacctcgagtactgtgggcaatttagaaaaaga
acatgcagtttgaaattctgaatttgcaaagtactgtaagaataatttat
agtaatgagtttaaaaatcaactttttattgccttctcaccagctgcaaa
gtgttttgtaccagtgaatttttgcaataatgcagtatggtacatttttc
aactttgaataaagaatacttgaacttgtccttgttgaatc
>NM_002812 4
ccaacttccggtcaccatcttgagtgacgacagaggcggagctccaactg
acatgttcattaagggcagggctccgagggcgccacctcgagagcgacgg
cgggctacccggggcgggctgaggcaggttgtagccccgccccgggcctt
gggctccacctctcggccccacttccgccgggcaagcgtttgtaggcggc
gctgccgtaaatcaggcggtctgcttgccgcatcacgcaagatggcggcc
gcggcggtgaacggggcggcaggcttctcgagctccgggcccgcggcaac
ctcgggcgctgttctgcaggccgcgaccggcatgtacgagcaactcaagg
gcgagtggaaccgtaaaagccccaatcttagcaagtgcggggaagagctg
ggtcgactcaagctagttcttctggagctcaacttcttgccaaccacagg
gaccaagctgaccaaacagcagctaattctggcccgtgacatactggaga

tcggggcccaatggagcatcctacgcaaggacatcccctccttcgagcgc
tacatggcccagctcaaatgctactactttgattacaaggagcagctccc
cgagtcagcctatatgcaccagctcttgggcctcaacctcctcttcctgc
tgtcccagaaccgggtggctgagttccacacggagttggagcggctgcct
gccaaggacatacagaccaatgtctacatcaagcacccagtgtccctgga
gcaatacctgatggagggcagctacaacaaagtgttcctggccaagggta
acatccccgccgagagctacaccttcttcattgacatcctgctcgacact
atcagggatgagatcgctgggtgcatcgagaaggcctacgagaaaatcct
tttcactgaggccacccggatcctcttcttcaacacacccaaaaagatga
cagactacgccaagaagcgagggtgggtcctgggccccaacaactactac
agttttgccagccagcagcagaagccggaagacaccaccattccctccac
agaactggccaaacaggtcatcgagtatgcccggcagctggagatgatcg
tctgagcccccgggcactgggtggggcagggcacgagttatttaaaaca
gttacactgcagggtttcgcccaataaaggtggactgacattccctcttc
caggcccttgtctccccagttgggacggcagagagacaagttcttatatc
tgaagaacttggaggttttggggcattcaggagttggagatagcctccaa
ctgggtcagcctctgtctggtgggcattgctcagggtctcaaacatggac
gcccactgtggggccccagcagcactgtggcctgcaggagggcatggccc
caggtagggggactgttctagccagctgtggacacataggaatgctggac
cagggtaccagatttttttcaacaaagggggtgaagtgtcctactaaaaag
aataaatgttggcagtgaattaaacaatttttcaaatgaaaaaaaaaaaa
aaaaaa
>NM_002078 4
acattgtgcttgtcccatccggtgctccgtagttagcctctgtgagctcc
acgtaggccgcgcaagcgcccttggcgcacagttcacctgctgccgttgt
cgtcgccgccgcggctcccggggctggatggggggccgaggccagccagt
ggcacccggaagaaagagacgcggcggcggcgacgccgacaccctcagga
cgagtgtccggacttgcccacagcctcaaggaggagacggcgaggcccgg
cccccgctgtccctggtgtaaagaagtcgccgtagccgtcgcggccggga
ctccccgggctctcgcccttcaggtttcgttgacactcaggaccgtacgt
acgctgcgccatgttcaagaaactgaagcaaaagatcagcgaggagcagc
agcagctccagcaggcgctggctcctgctcaggcgtcctccaattcttca
acaccaacaagaatgaggagcaggacatcttcatttacagagcaacttga
tgaaggtacacccaatagagagtcaggtgacacacagtcttttgcacaga
agctccagctccgggtgccctccgtggagtctttgtttcgaagtccgata
aaggaatctctattccggtcttcttctaaagagtctttggtacgaacatc
ttccagagaatccctgaatcgacttgacctggacagttctactgccagtt
ttgatccaccctctgatatggatagcgaggctgaagacttggtagggaat
tcagacagtctcaacaaagaacagttgattcagcggttgcgaagaatgga
acgaagcttaagtagctacaggggaaaatattctgagcttgttacagctt
atcagatgcttcagagagagaagaaaaagctacaaggtatattaagtcag
agtcaggataaatcacttcggagaatagcagaattaagagaggagctcca
aatggaccagcaggcaaagaaacatctgcaagaggagtttgatgcatctt
tagaggagaaagatcagtatatcagtgttctccaaactcaggtttctcta
ctgaaacaacgattacgaaatggcccgatgaatgttgatgtactgaaacc
acttcctcagctggaaccacaggctgaagtcttcactaaagaagagaatc
cagaaagtgatggagagccagtagtggaagatggaacttctgtaaaaaca
ctggaaacactccagcaaagagtgaagcgtcaagagaacctacttaagcg
ttgtaaggaaacaattcagtcacataaggaacaatgtacactattaacta

gtgaaaaagaagctctgcaagaacaactggatgaaagacttcaagaacta
gaaaagataaaggaccttcatatggccgagaagactaaacttatcactca
gttgcgtgatgcaaagaacttaattgaacagcttgaacaagataagggaa
tggtaatcgcagagacaaaacgtcagatgcatgaaaccctggaaatgaaa
gaagaagaaattgctcaactccgtagtcgcatcaaacagatgactaccca
gggagaggaattacgggaacagaaagaaaagtccgaaagagctgcttttg
aggaacttgaaaaagctttgagtacagcccaaaaaacagaggaagcacgg
agaaaactgaaggcagaaatggatgaacaaataaaaactatcgaaaaaac
aagtgaggaggaacgcatcagtcttcaacaggaattaagtcgggtgaaac
aggaggttgttgatgtaatgaaaaaatcctcagaagaacaaattgctaag
ctacagaagcttcatgaaaaggagctggccagaaaagagcaggaactgac
caagaagcttcagacccgagaaagggaatttcaggaacaaatgaaagtag
ctcttgaaaagagtcaatcagaatatttgaagatcagccaagaaaaagaa
cagcaagaatctttggccctagaagagttagagttgcagaaaaaagcaat
cctcacagaaagtgaaaataaacttcgggaccttcagcaagaagcagaga
cttacagaactagaattcttgaattggaaagttctttggaaaaaagctta
caagaaaacaaaaatcagtcaaaagatttggctgttcatctggaagctga
aaaaaataagcacaataaggagattacagtcatggttgaaaaacacaaga
cagaattggaaagccttaagcatcagcaggatgccctttggactgaaaaa
ctccaagtcttaaagcaacaatatcagactgaaatggaaaaacttaggga
aaagtgtgaacaagaaaaagaaacattgttgaaagacaaagagattatct
tccaggcccacatagaagaaatgaatgaaaagactttagaaaagcttgat
gtgaagcaaacagaactagaatcattatcttctgaactgtcagaagtatt
aaaagcccgtcacaaactagaagaggaactttctgttctgaaagatcaaa
cagataaaatgaagcaggaattagaggccaagatggatgaacagaaaaat
catcaccagcagcaagttgacagtatcattaaagaacacgaggtatctat
ccagaggactgagaaggcattaaaagatcaaattaatcaacttgagcttc
tcttgaaggaaagggacaagcatttgaaagagcatcaggctcatgtagaa
aatttagaggcagatattaaaaggtctgaaggggaactccagcaggcatc
tgctaagctggacgtttttcagtcttaccagagtgccacacatgagcaga
caaaagcatatgaggaacagttggcccaattgcagcagaagttgttggat
ttggaaacagaaagaattcttcttaccaaacaggttgctgaagttgaagc
acaaaagaaagatgtttgtactgagttagatgctcacaaaatccaggtgc
aggacttaatgcagcaacttgaaaaacaaaatagtgaaatggagcaaaaa
gtaaaatctttaacccaagtctatgagtccaaacttgaagatggtaacaa
agaacaggaacagacaaagcaaatcttggtggaaaaggaaaatatgattt
tacaaatgagagaaggacagaagaaagaaattgagatactcacacagaaa
ttgtcagccaaggaggacagtattcatattttgaatgaggaatatgaaac
caaatttaaaaaccaagaaaaaaagatggaaaaagttaagcagaaagcaa
aggagatgcaagaaacgttaaagaaaaaattactggatcaggaagccaaa
cttaagaaagagcttgaaaatactgctctagagcttagtcagaaagaaaa
acagtttaatgccaaaatgctggaaatggcacaggctaactcagctggaa
tcagtgatgcagtgtcaagactggaaacaaaccaaaaagaacaaatagaa
agtcttactgaggttcatcgacgagaactcaatgatgtcatatcaatctg
ggaaaagaaacttaatcagcaagctgaagaacttcaggaaatacatgaaa
tccaattacaggaaaaagaacaagaggtagcagaactgaaacaaaagatc
ctcctatttgggtgtgaaaaagaagagatgaacaaggaaataacatggct
gaaggaagaaggtgttaagcaggatacaacattaaatgaattacaggaac
agttaaagcagaagtctgcccatgtgaattctcttgcacaagatgaaact

aaactgaaagctcatcttgaaaagctagaggttgacttgaataagtctct
gaaggaaaatacttttcttcaagagcagctagttgaactgaagatgctgg
cagaagaagataagcggaaggtttctgagttgactagcaagttgaaaacc
acagatgaagaattccagagtttgaaatcttcacatgaaaaaagtaacaa
aagcctagaggacaagagcttggaatttaaaaaactgtctgaggaactag
cgattcagctagatatttgctgtaagaaaaccgaagccttattagaagct
aaaacaaatgagctaatcaacattagtagtagtaaaactaatgccattct
ttctaggatttctcattgtcagcaccgtacaactaaagttaaggaggcac
tgttaattaaaacttgcacagtttctgaattagaagcacaacttagacag
ttgacagaggagcaaaatacactaaatatttcttttcaacaggctactca
tcagttagaagaaaaagaaaatcaaattaagagcatgaaggctgatattg
aaagtcttgtaacagaaaaagaagccttacagaaggaaggaggcaatcag
caacaggctgcttctgaaaaggagtcttgtataacacagttgaagaaaga
gttatctgaaaacatcaatgctgtcacattgatgaaagaagagcttaaag
aaaaaaaagttgagattagcagtcttagtaaacaactaactgatttgaat
gttcagcttcaaaatagcatcagcctatccgaaaaagaagcagccatttc
atcactaagaaagcagtatgatgaagaaaaatgtgaattgctggatcagg
tgcaagatttatcttttaaagttgacactctgagtaaagagaaaatttct
gctcttgagcaggtagatgactggtccaataaattctcagaatggaagaa
gaaagcacagtcaagatttacacagcatcaaaacactgttaaagaattgc
agatccagcttgagttaaaatcaaaggaagcttatgaaaaggatgagcag
ataaatttattgaaggaagagcttgatcagcaaaataaaagatttgattg
tttaaagggtgaaatggaagacgacaagagcaagatggagaaaaaggagt
ctaatttagaaacagagttaaagtctcaaacagcaagaattatggaatta
gaggaccatattacccagaaaactattgaaatagagtccttaaatgaagt
tcttaaaaattacaatcaacaaaaggatattgaacacaaagaattggttc
agaaacttcaacattttcaagagttaggagaagaaaaggacaacagggtt
aaagaagctgaagaaaaaatcttaacacttgaaaaccaagtttattccat
gaaagctgaacttgaaactaagaagaaagaattagaacatgtgaatttaa
gtgtgaaaagcaaagaggaggagttaaaggcattggaagataggcttgag
tcagaaagtgctgcaaaattagcagagttgaagagaaaagctgaacaaaa
aattgctgccattaagaagcagttgttatctcaaatggaagagaaagaag
aacagtataaaaaaggtacagaaagccatttgagtgagctaaatacaaaa
ttgcaggaaagagaaagggaagttcacatcttggaagaaaaacttaagtc
agtggaaagttcacagtcagaaacattaattgtacccagatcagcaaaaa
atgtggcagcatatactgaacaagaagaagcagattcccaaggctgtgtg
cagaagacatatgaagaaaaaatcagtgttttacaaagaaacttaactga
aaaagaaaagctattgcagagggtagggcaggaaaaagaagagacagttt
cttctcattttgaaatgcgatgccaataccaggagcgcttaataaagcta
gaacatgctgaggcaaagcaacatgaagatcaaagtatgataggtcatct
tcaagaggagcttgaagaaaaaaacaagaaatattccttgatagtagccc
agcatgtggaaaaagaaggaggtaaaaataacatacaggcaaagcaaaac
ttggaaaatgtgtttgacgacgtccagaaaaccctccaggagaaggaact
aacctgtcagattttggagcaaaagataaaagagctggattcctgcttag
taagacagaaagaagtacatagagttgaaatggaagagttgacctcaaaa
tatgaaaaattacaggctttacaacagatggatggaagaaataaacccac
agaacttttggaagaaaacactgaagaaaagtccaaatcacatttggtcc
aacccaaattgcttagtaacatggaagcccagcacaatgatctggagttt
aaattagccggggcagaacgggagaaacagaaactgggcaaggagattgt

tagattgcagaaagaccttcgaatgttgagaaaggagcatcagcaagaat
tggaaatactaaagaaagaatatgatcaagaaagggaagagaaaatcaaa
caggagcaggaagatcttgaactgaagcacaattccacattaaaacagct
gatgagggagtttaatacacagctggcacaaaaggaacaagagctggaaa
tgaccataaaagaaactatcaataaggcccaggaggtggaggctgaactt
ttagaaagccatcaagaagagacaaatcagttacttaaaaaaattgctga
gaaagatgatgatctaaaacgaacagccaaaagatatgaagaaatccttg
atgctcgtgaagaagaaatgactgcaaaagtaagggacctgcagactcaa
cttgaggagctgcagaagaaataccagcaaaagctagagcaggaggagaa
ccctggcaatgataatgtaacaattatggagctacagacacagctagcac
agaagacgactttaatcagtgattcgaaattgaaagagcaagagttcaga
gaacagattcacaatttagaagaccgtttgaagaaatatgaaaagaatgt
atatgcaacaactgtggggacaccttacaaaggtggcaatttgtaccata
cggatgtctcactctttggagaacctaccgaatttgagtatttgcgaaaa
gtgctttttgagtatatgatgggtcgtgagactaagaccatggcaaaagt
tataaccaccgtactgaagttccctgatgatcagactcagaaaattttgg
aaagagaagatgctcggctgatgtttacttcacctcgcagtggtatcttc
tgagtaaaccatcagtctgtgcttagttaacatgtgtcatggctccgatc
ttcatcttgaagaagagtgacattgggtgactgctgcttggaaaactgtc
cacacttgctactctttgagaatgaagttgtcattcagggcccctcatgt
agccaaaagaccaagaaaaatctggcccacagataagttgcagactgcct
ttaaaatagattttatcagtggagaaatggtgatagtttttttcttcagtt
ttctcttgggaagagttttatgttgtttaaaagatattttgataacttaa
cctgctttatgggcttacataatattcctttcatccattctttttaaaga
acggcttacctttcctatttatttttagggtgatttttttaaaaagacttg
tgcaatacattttgaggtgaaacttagtggatttttttctgataaattaga
gcatttaattgactattttattcaggttgatctgttgaatatttgctaaa
gaccagttctttaagctaagacatgtaaaaaaatcccaaatggcagtacct
cattgtttacttagcttttgtacttatatttttcagaggaaaaaacacta
ctgtaaattgtgaatagccaatacataactgtattgtatgcaaatctgtg
attgttggcagtgtcatctctgagaaacagataaataaagtttatttact
atataacatttgtttattgtacttgttcctcagttgaaatctattttaaa
atgtttaagaatgcatattctatttggatgaatttagtgaggaattattt
gtcaatataactaaatgctgagcatttgaaaagaat
>NM_000461 4
cggcggggattaactttgcatgaataatgtgagtgcgcttggaaaagaga
cctcctgctccgcgggctcggggcaagagcccgcaggctaccttccccgg
gcaggggcgctcaacccaaccggctccagggcactggtaatttggctaga
ggaccgcgcggaggcagcgggatctgcgatttccttctggttggctgtcc
tgcgtgggtgccaagttccacacatgatttaatgaataagaaggagatgt
cagtgaaaaaaggatccagaatgattactaacctatgactcccaacagt
atgacagaaaatggccttacagcctgggacaaaccgaagcactgtccaga
ccgagaacacgactggaagctagtaggaatgtctgaagcctgcctacata
ggaagagccattcagagaggcgcagcacgttgaaaaatgaacagtcgtcg
ccacatctcatccagaccacttggactagctcaatattccatctggacca
tgatgatgtgaacgaccagagtgtctcaagtgcccagaccttccaaacgg
aggagaagaaatgtaaagggtacatccccagttacttagacaaggacgag
ctctgtgtagtgtgtggtgacaaagccaccgggtatcactaccgctgtat
cacgtgtgaaggctgcaagggtttctttagaagaaccattcagaaaaatc

tccatccatcctattcctgtaaatatgaaggaaaatgtgtcatagacaaa
gtcacgcgaaatcagtgccaggaatgtcgctttaagaaatgcatctatgt
tggcatggcaacagatttggtgctggatgacagcaagaggctggccaaga
ggaagctgatagaggagaaccgggagaaaagacggcgggaagagctgcag
aagtccatcgggcacaagccagagcccacagacgaggaatgggagctcat
caaaactgtcaccgaagcccatgtggcgaccaacgcccaaggcagccact
ggaagcaaaaacggaaattcctgccagaagacattggacaagcaccaata
gtcaatgccccagaaggtggaaaggttgacttggaagccttcagccattt
tacaaaaatcatcacaccagcaattaccagagtggtggattttgccaaaa
agttgcctatgttttgtgagctgccatgtgaagaccagatcatcctcctc
aaaggctgctgcatggagatcatgtcccttcgcgctgctgtgcgctatga
cccagaaagtgagactttaaccttgaatggggaaatggcagtgacacggg
gccagctgaaaaatgggggtcttggggtggtgtcagacgccatctttgac
ctgggcatgtctctgtcttctttcaacctggatgacactgaagtagccct
ccttcaggccgtcctgctgatgtcttcagatcgcccggggcttgcctgtg
ttgagagaatagaaaagtaccaagatagtttcctgctggcctttgaacac
tatatcaattaccgaaaacaccacgtgacacacttttggccaaaactcct
gatgaaggtgacagatctgcggatgataggagcctgccatgccagccgct
tcctgcacatgaaggtggaatgccccacagaactcttccccccctttgttc
ttggaagtgttcgaggattagactgactggattcattctcataattccta
cagcactactgggtgtcatttcattccattgcctagctctttttttgtttg
tttctttgtgttgggagggattatttgggagggaaaagggaagtagtcct
tggcatagacatggatgaaattgccccttgaatgcgggtacttgaaacta
ttgcatttcgttctccggtcctgtgatgtgaatgctctgaaggttttatg
gttgtggaggtggggtgggggacaatcattaactcaccagcaccaagcat
caccagctcccacccgtccctggtccaagacttgagtcagcaaaatggcg
ccacaggacactaaagaagccttaaaaccaagataatacgaccacctcca
cccaatcctgatgttcgcagggctgaagttaacagagcacagaccacctt
tagttagatgtggctttcagccttttagggaaagactcgaacaaattttc
atctattcaagagcatgctgtttgcagcactctttatggtctcctaggag
cataacttggaatgtacaggaatatttgtgtcctaaaaaaatcagctgcc
cttccaatatcatttttataacgttatggtttaattcaagttatacaaag
gtgtgactcaaggatgtgcaggtattgactgatggaggccaacaaggaaa
atatttttttcagtgagcccagagaaattagaaatcttcaccaatgtca
gtgactgcatggtttccaaaaaattcacatagtattacctcacagtaggaa
ggcttctttcccatttaggctcagagaacacttttacagaaatcaaacct
atttacagggtggcaccccttttctgtggcatctgatctgtcatctccca
agctcctgaagttctttatccaggttgtatctttgctgagctgacaacag
atttctctatggatcaaatattctaatcaaggaaagatacggtgaaactt
cagtcttcttctggagaaatgctgaggcgagattagtgcagctacattct
gagttgaaggaaatgcagtgttctgagggcccctttgtgtcctgatggct
ggaggacgcttcctcctggttagatcctcaagctccttgcctcattgaca
aatgtctcctagtccccttttttccctctgactctgaggaactaaatagct
tgactatctctaaacagagactggccctgccactgaccctcctgtccctc
tcatagcataccctgacagatactcaatagaaaacacctgcagaccatgt
gcacacttaactttcctgcccaacctgcctccccaacggcccatgctaga
atagcttgtgcccagaaaacaaacagtgacacttcatctcttcagcgtca
gaaaatgggcatagaatgtacccatgcaattgaatgtatctcattctcca
ctggcatagtagatataaactattcctttgcctaccttttactgcctta

cctggaaatattggggaaaataagccatatcagtgagatttacctaaaaa
caaaggaaggaaagtcactatggaaaccaagtagatgctttttgtaagtt
tggtggagcataactggcccagaagtctgggtgcctgagttctagttcgt
gctctgtcaactggtagatttgtaattctaggtcttggttttcttacctg
gtgaataagtcatgatctctaaaacccttcctgcccttaatattgtctga
atttcaaggggaaaaaaaaaaatctggttcctttaattattttttttaac
acttaaattcctaactttattagacaagaagtatggcccacttaacagtt
aaggaacgtaaatttgttattggctcgaaataataatttgacacaatact
agtcaaccatttagaggccttggaaaaagaagaggaacagcactgaaaat
tccaagtatgattggaaattatctgggagcggggaattaaatattcaatg
catgaaagcaattgtactggcttctgggaaaaaggaaacaagaacattgc
tatggaacttgttacccagagaaaggaagtgaaaagatttacaattataa
gcacgaaattcattggcagattacctgctttatctacagacagattgttt
tggggtttggttttgtctaggcagcctctggccaggctccttaattgctt
gctctgctaatatttcagcttcctctcttgactccttgaggaaccaaccg
ctctttggaagacaaacacttagccacctctgtgctccagggtaaccctg
agaagctctgctgtctggggcaagctttctttccaaagggaatgagagga
aggcaagcaggcagggtcctgcagcgtcaggccttctggggaaagtttcc
tcattttgtttgacagggtcctcagtacatcagacacccagatttgaata
taagttcagagcctgatctcaagctaaatcagaatgctttggggactaac
catgatcgagaattggccaaatagtatggcatataaacagccatccagcc
agccgcaagtaaaggaacgtttgatccttggcaccttaaatgattgattg
ccttcaataggcaaactgctggtctggaaatctcgttgaaggctgaatac
ttcatgcggagtaggacaagccttagcccaccttcaggttgaaaggaaag
gccataggactcaaaagataatgactctgaaactaaactccttccttctg
aagctgacagaggtacttagtgcatctcaagactccatctcataaaactc
atttgtgagggatttgctctagatttttctaaacatttttttcatctattg
tccagacttgcaaatccagtgtggacctgatggagatacccttcacacct
gcgctcccaccccctcggcagggagacgtctcagtagaggagaacacacc
cattatgctgggatttgtatttataaatcacttcccttttagggatgagc
ggtgtggggcgtgtgtgcgtgtgagtgtatgcgcgtgcgtgtgtgaccct
caagtgtactttgctcaatttagacgtgcagtatttccagaaacaatgaa
aaaagtaagatgttttcttctttatgattctgctcaaaacaccacgtgcc
gcctcagagctttttctagagcctcggtcctcaatgacgcgcttgacctg
ttgatgcctcagcccagcgaagcccctcacctgtgccccgggcgacagct
cttcagtgtgcagtgcaggcctggggtggactgccaggccttcgctctgt
gccagcaacgccgttcttgttgctcctccatgaacaggggtacaagccct
ggcccctcctcgacactaagccccccacagactcagcctccaaggaaccg
ctgagcacccttgaagcatgtcattgtcagtgataccttttttattctatg
gaactctaacctattcgtgtcatattgaccttttgctgcatgagtcataa
attatgaaatcagtcttacagtttttgaaatgtagccagcatttgtaagg
ctaaacctttttcatgaactgaatttaagtgaataaccaagccacagttc
ctcctcaaatggagagtgatgatcgacatttgaatctctttgccctttcc
aacggctatggcatcaggttctaaaataagctcgtaatttttcctgttat
tttaataatatggaaatattagcatagtgtttcttttgatagtgatagac
tataatccatatttaaattttatagagaagaaattttattgtactgtgat
gtagatatttattatccaggtaaggatttgcccggtgtgtatttttttaca
attgagacattttactttaatctttaaaaaaaatgcattaaaaacacact
caaaaaaaaaaaaaaaaaaaaaaaaaaaaaccagactggggaaaaaag

gtttggccttatcacagaatttttactgtgctgtataaatgtttgcaaat
gcgaaaagtgtgcatttatagtgtgtttccacattaattaatgccgcctt
caagagcaatattgtgcaggtaaaacgctgtttgtggctttctgttgcac
agttaaagctgacccaccctatgcacaagacaatcaaatataaatctgtc
ctcctcaggatgcagataatactcatgtaaattatggagcacaatgtaag
acaaaaggagacactttacatatgtattgttgatacagaaattatcaggg
catgaatgagctgatgtgacattatgccacttctttcttttggttctccc
ttattaacttggcatttttaatttgaccatagagccaaattagttctcca
tgctttcttcgtagaaaatcctttttttcccataatgttttcatgccct
gagaatcagaaaaatactacagaatagaaaaagatttacagtcattggaa
gatttgtgttttgcaaacagtgttaagtgatattgctagatggtgatatt
tttttcagcctaaaactctgttgaaatctgtgatcaaaatgttttaaact
atccaaaaaaaatttgtatatttgggaaaaatggatttttacatagcttt
tgtatgcagatataaaactgtaattatgaatatagtgggcgtagataaac
tcataagcttaaattctaaaaaagaaaaagcttataacagatatattttc
ctgtctctttctttaaatattattattttctacataggcaaagagttgcc
tgctcttctcacttcagctgctgccaaaagacggaaccacggatgtaagg
acatttatttcataaataaacatctactagaataagcaaatttacttgag
aagaaactatggagggagagaagaaactaagtaaaatgctgatggggggt
tacctctttggaaggggccacaaagatgaagggaggagagaggtgaagtt
ttgcagctcaaggacaacattcatcttctcatctcaaattattcaggaat
attactaatggaattatgttaacatcagctgccaacatatatctcaatct
ctgttgccctgaacacatgttaattgagattctttctatgacaagtaaca
aaaagctaactcaaactggcttaagcagaaagctaattattgactttaat
atctgggatctagggtattgcataccaaagcttcaggcatggctggatct
gggtgcacttactcagtattctcaggaagctcctttttttgtcattcatg
tagacttaacattcacatggcctttcaccttacggtggcctccaatagct
ccaggatgagctttacagctccaaatatagtgaaaggagaatttcttttc
tccaacaattctaatgaaaattctggcctaattctcactggcccaagttc
agtcatgtgcccattcctgagccaatcactgtggtgaggaaaacagatgc
ataaccaaccttatcctcaggaatcctggaactgggtgaggggaaagagg
gacagtcagcttcatctaaactatggactgagagttgaggcaagatgatt
cctgaaagaaaattctaagtcttgctgctataagtggaagagaatggtgg
gcaagcaaaagcaaccagtgcccacaccaaaggtaaatctaaaacaacat
cccatttcagtacagcaagcggttctccttcacacagaggaagctcatct
ccaagtttttatttggccaggacatgaaccaaataaacaccttccaaaga
aaaaaaaaaaaaaaaa
>NM_000481 3
gagctgccctgaagagtcagccgaaagagctggaggcttcttccctgtgg
ttgcgggttcctgcattctctgctctaaatcccagcctgcccttggggct
gcccacgccccсttcagatcctttgctccggagagagacctgtccgagca
gaggcctggactacatctcccggcgtgcctggcagtgtggtggcctctgt
gcgccgtctgcactcgttgcaggcgacgatgcagagggctgtaagtgtgg
tggcccgtctgggctttcgcctgcaggcattcccccggccttgtgtcgt
ccacttagttgcgcacaggaggtgctccgcaggacaccgctctatgactt
ccacctggcccacggcgggaaaatggtggcgtttgcgggttggagtctgc
cagtgcagtaccgggacagtcacactgactcgcacctgcacacacgccag
cactgctcgctctttgacgtgtctcatatgctgcagaccaagatacttgg
tagtgaccgggtgaagctgatggagagtctagtggttggagacattgcag

agctaagaccaaaccaggggacactgtcgctgtttaccaacgaggctgga
ggcatcttagatgacttgattgtaaccaatacttctgagggccacctgta
tgtggtgtccaacgctggctgctgggagaaagatttggccctcatgcagg
acaaggtcagggagcttcagaaccagggcagagatgtgggcctggaggtg
ttggataatgccctgctagctctgcaaggccccactgcagcccaggtact
acaggccggcgtggcagatgacctgaggaaactgcccttcatgaccagtg
ctgtgatggaggtgtttggcgtgtctggctgccgcgtgacccgctgtggc
tacacaggagaggatggtgtggagatctcggtgccggtagcgggggcagt
tcacctggcaacagctattctgaaaaacccagaggtgaagctggcagggc
tggcagccagggacagcctgcgcctggaggcaggcctctgcctgtatggg
aatgacattgatgaacacactacacctgtggagggcagcctcagttggac
actggggaagcgccgccgagctgctatggacttccctggagccaaggtca
ttgttccccagctgaagggcagggtgcagcggaggcgtgtggggttgatg
tgtgagggggcccccatgcgggcacacagtcccatcctgaacatggaggg
taccaagattggtactgtgactagtggctgcccctcccccctctctgaaga
agaatgtggcgatgggttatgtgccctgcgagtacagtcgtccagggaca
atgctgctggtagaggtgcggcggaagcagcagatggctgtagtcagcaa
gatgccctttgtgcccacaaactactataccctcaagtgaagctggctca
gggtggggctgtcccttccaggagttttgcccctacaaggggttagtcaa
gaagctgaggcagaactcactgggggtgggcagttaaggtggaggctgat
tctaattgtctggttgaggggccacaccacctattccccccacctaactc
atgccattccagcttccttcaggaccctgcttctgagtgacggaccagct
cacacaatgtcttgtttcagtccatgatcccactgacctactcttgcctg
ctggagggtaatgagaagctttggttctgccatctctcccactctgccag
gtgctggctgtggagcaaaggctcacctttgtggagaggataaaacctgc
ccaacctacctcaccatggtttttcacattgcaaagggtaataacatggg
cagtgcggacttaggctacccccctccagtttgctttccgtaaatgcaaat
tgtccttactgcaagtcaggaatgattgctgactcacagtagggctgcta
tgcctgtgtgtaaacttggggatggctgagggaacatagactcactcttc
cacattcccaagttggtctagtgtgctgcccagtagcaaaccatggcaga
ctcaccacctattctgagttccagggctgctgtagggcagggtgggcttc
ctcccagacttgccttaccctgggctgatctttgcccctggtatgcatta
atggactccactgaatcctgaaaaaaaaattaaacttccttcttacttgc
caaaaaaaaaaaaaa
>NM_003613 3
tatataaggccggcactggactgctggagaggcgaaggcttggaccccca
gctgaggagtcctgctcaagacacggtcactggatctgagaaacttccca
ggggaccgcattccagagtcagtgactctgtgaagcacccacatctacct
cttgccacgttcccacgggcttgggggaaagatggtggggaccaaggcct
gggtgttctccttcctggtcctggaagtcacatctgtgttggggagacag
acgatgctcacccagtcagtaagaagagtccagcctgggaagaagaaccc
cagcatctttgccaagcctgccgacaccctggagagccctggtgagtgga
caacatggttcaacatcgactacccaggcgggaagggcgactatgagcgg
ctggacgccattcgcttctactatggggaccgtgtatgtgcccgtcccct
gcggctagaggctcggaccactgactggacacctgcgggcagcactggcc
aggtggtccatggtagtccccgtgagggtttctggtgcctcaacagggag
cagcggcctggccagaactgctctaattacaccgtacgcttcctctgccc
accaggatccctgcgccgagacacagagcgcatctggagcccatggtctc
cctggagcaagtgctcagctgcctgtggtcagactggggtccagactcgc

acacgcatttgcttggcagagatggtgtcgctgtgcagtgaggccagcga
agagggtcagcactgcatgggccaggactgtacagcctgtgacctgacct
gcccaatgggccaggtgaatgctgactgtgatgcctgcatgtgccaggac
ttcatgcttcatggggctgtctcccttcccggaggtgccccagcctcagg
ggctgctatctacctcctgaccaagacgccgaagctgctgacccagacag
acagtgatgggagattccgaatccctggcttgtgccctgatggcaaaagc
atcctgaagatcacaaaggtcaagtttgcccccattgtactcacaatgcc
caagactagcctgaaggcagccaccatcaaggcagagtttgtgagggcag
agactccatacatggtgatgaaccctgagacaaaagcacggagagctggg
cagagcgtgtctctgtgctgtaaggccacagggaagcccaggccagacaa
gtatttttggtatcataatgacacattgctggatccttccctctacaagc
atgagagcaagctggtgctgaggaaactgcagcagcaccaggctggggag
tacttttgcaaggcccagagtgatgctggggctgtgaagtccaaggttgc
ccagctgattgtcatagcatctgatgagactccttgcaacccagttcctg
agagctatcttatccggctgccccatgattgctttcagaatgccaccaac
tccttctactatgacgtgggacgctgccctgttaagacttgtgcagggca
gcaggataatgggatcaggtgccgtgatgctgtgcagaactgctgtggca
tctccaagacagaggaaagggagatccagtgcagtggctacacgctaccc
accaaggtggccaaggagtgcagctgccagcggtgtacggaaactcggag
catcgtgcggggccgtgtcagtgctgctgacaatggggagcccatgcgct
ttggccatgtgtacatggggaacagccgtgtaagcatgactggctacaag
ggcactttcaccctccatgtcccccaggacactgagaggctggtgctcac
atttgtggacaggctgcagaagtttgtcaacaccaccaaagtgctacctt
tcaacaagaaggggagtgccgtgttccatgaaatcaagatgcttcgtcgg
aaagagcccatcactttggaagccatggagaccaacatcatccccctggg
ggaagtggttggtgaagaccccatggctgaactggagattccatccagga
gtttctacaggcagaatggggagccctacataggaaaagtgaaggccagt
gtgaccttcctggatccccggaatatttccacagccacagctgcccagac
tgacctgaacttcatcaatgacgaaggagacactttccccttcggacgt
atggcatgttctctgtggacttcagagatgaggtcacctcagagccactt
aatgctggcaaagtgaaggtccaccttgactcgacccaggtcaagatgcc
agagcacatatccacagtgaaactctggtcactcaatccagacacagggc
tgtgggaggaggaaggtgatttcaaatttgaaaatcaaaggaggaacaaa
agagaagacagaaccttcctggtgggcaacctggagattcgtgagaggag
gctctttaacctggatgttcctgaaagcaggcggtgctttgttaaggtga
gggcctaccggagtgagaggttcttgcctagtgagcagatccaggggggtt
gtgatctccgtgattaacctggagcctagaactggcttcttgtccaaccc
tagggcctggggccgctttgacagtgtcatcacaggccccaacggggcct
gtgtgcctgccttctgtgatgaccagtcccctgatgcctactctgcctat
gtcttggcaagcctggctggggaggaactgcaagcagtggagtcttctcc
taaattcaacccaaatgcaattggcgtccctcagccctatctcaacaagc
tcaactaccgtcggacggaccatgaggatccacgggttaaaaagacagct
ttccagattagcatggccaagccaaggcccaactcagctgaggagagcaa
tgggcccatctatgcctttgagaacctccgggcatgtgaagaggcaccac
ccagtgcagcccacttccggttctaccagattgagggggatcgatatgac
tacaacacagtccccttcaacgaagatgaccctatgagctggactgaaga
ctatctggcatggtggccaaagccgatggaattcagggcctgctatatca
aggtgaagattgtggggccactggaagtgaatgtgcgatcccgcaacatg
gggggcactcatcggcagacagtggggaagctgtatggaatccgagatgt

gaggagcactcgggacagggaccagcccaatgtctcagctgcctgtctgg
agttcaagtgcagtgggatgctctatgatcaggaccgtgtggaccgcacc
ctggtgaaggtcatcccccagggcagctgccgtcgagccagtgtgaaccc
catgctgcatgagtacctggtcaaccacttgccacttgcagtcaacaacg
acaccagtgagtacaccatgctggcacccttggacccactgggccacaac
tatggcatctacactgtcactgaccaggaccctcgcacggccaaggagat
cgcgctcggccggtgctttgatggcacatccgatggctcctccagaatca
tgaagagcaatgtgggagtagccctcaccttcaactgtgtagagaggcaa
gtaggccgccagagtgccttccagtacctccaaagcaccccagcccagtc
ccctgctgcaggcactgtccaaggaagagtgccctcgaggaggcagcagc
gagcgagcaggggtggccagcgccagggtggagtggtggcctctctgaga
tttcctagagttgctcaacagcccctgatcaactaagttttgtggtactt
caccctcttctgccctcatttcatgtgacagccattgtgagactgatgca
caaactgtcacttggttaatttaagcacttctgttttcgtgaatttgctt
gtttgtttcttcatgcctttacttactttgtcccatgctactgattggca
cgtggcccccacaatggcacaataaagccccttttgtgaaactgttcttta
aatgaaacacaagaaattggccactggtaaaactctgcagcttcaactgt
acttcatttaatgccattaatgcaaatatacttcctcttctttttgcatg
gttttgcccacctctgcaatagtgataatctgatgctgaagatcaaataa
ccaatataaagcatatttcttggccttgctccacaggacataggcaagcc
ttgatcatagttcatacatataaatggtggtgaaataaagaaataaaaca
caatacttttacttgaaatgtaaataacttatttatttctttgctaaatt
tggaattctagtgcacattcaaagttaagctattaaatatagggtgatca
tagttcctctaccaagtctggaaagaacatctcctggtatccacaattac
accaggttgctaactgtatttgtacatttccctttgcattcgcttctgtt
cttgctagaaacccagtgtagcccagggcagatgtcaataaatgcatact
ctgtatttcgattctgagaaaaaaaaaaaaaaaa
>NM_003165 3
cccgcgcgcgccggcggcggggcagcctcgctctggctcgcgccgcgccc
ccgcgcccagtccgcgcgtcagtcggtccctagcgcggctgcggggcgga
gagctgcggctggcccagcgcgcccacctgaggaggcggcggggtccgca
ggcgtcgcgggacgaggagatcggagccgggagactcgcgcagcgccatg
gcccccattggcctcaaagctgttgtcggagagaagattatgcatgatgt
gataaagaaggtcaagaagaagggggaatggaaggtgctggtggtggatc
agttaagcatgaggatgctgtcctcctgctgcaagatgacagacatcatg
accgagggcataacgattgtggaagatatcaataagcgcagagagccgct
ccccagcctggaggctgtgtatctcatcactccatccgagaagtccgtcc
actctctcatcagtgactttaaggacccgccgactgctaaataccgggct
gcacacgtcttcttcactgactcttgtccagatgccctgtttaatgaact
ggtaaaatcccgagcagccaaagtcatcaaaactctgacggaaatcaata
ttgcatttctcccgtatgaatcccaggtctattccttggactctgctgac
tctttccaaagcttctacagtccccacaaggctcagatgaagaatcctat
actggagcgcctggcagagcagatcgcgaccctttgtgccaccctgaagg
agtacccggctgtgcggtatcgggggaatacaaggacaatgccctgctg
gctcagctaatccaggacaagctcgatgcctataaagctgatgatccaac
aatgggggagggcccagacaaggcacgctcccagctcctgatcctggatc
gaggctttgaccccagctcccctgtgctccatgaattgacttttcaggct
atgagttatgatctgctgcctatcgaaaatgatgtatacaagtatgagac
cagcggcatcggggaggcacgggtgaaggaggtgctcctggacgaggacg

acgacctgtggatagcactgcgccacaagcacatcgcagaggtgtcccag
gaagtcacccggtctctgaaagatttttcttctagcaagagaatgaatac
tggagagaagaccaccatgcgggacctgtcccagatgctgaagaagatgc
ctcagtaccagaaagagctcagcaagtactccacccacctgcaccttgct
gaggactgtatgaagcattaccaaggcaccgtagacaaactctgccgagt
ggagcaggacctggccatgggcacagatgctgagggagagaagatcaagg
accctatgcgagccatcgtccccattctgctggatgccaatgtcagcact
tatgacaaaatccgcatcatccttctctacatctttttgaagaatggcat
cacggaggaaaacctgaacaaactgatccagcacgcccagataccccccgg
aggatagtgagatcatcaccaacatggctcacctcggcgtgcccatcgtc
accgattccacgctgcgtcgccggagcaagccggagcggaaggaacgcat
cagcgagcagacctaccagctctcacggtggactccgattatcaaggaca
tcatggaggacactattgaggacaaacttgacaccaaacactacccttat
atctctacccgttcctctgcctccttcagcaccaccgccgtcagcgcccg
ctatgggcactggcataagaacaaggccccaggcgagtaccgcagtggcc
cccgcctcatcattttcatccttgggggtgtgagcctgaatgagatgcgc
tgcgcctacgaggtgacccaggccaacggaaagtgggaggtgctgatagg
ttctactcacattcttactcccaccaaatttctcatggacctgagacacc
ccgacttcagggagtcctctagggtatcttttgaggatcaggctccaaca
atggagtgagagccaaagaaacaaagatccacacacatcctcaccccaca
gaaactgctggacacactgaagaaactgaataaaacagatgaagaaataa
gcagttaaaaaaataagtcgcccctccaaaacacgcccccatcccacagc
gctccgcagcttcccaccaccgcccgcctcagttcctttgcgtctgttgc
ctccccagccctgcacgccctggctggcactgttgccgctgcattctcgt
gttcagtgatgccctcttcttgtttgaaacaaaagaaaataatgcattgt
gtttttaaaaagagtatcttatacatgtatcctaaaaagagaagctcat
gtgcaattggtgcacagcaggagaaatttctggactgttaggatgaatgg
acgccttctccccgttatttaagatttgtgaccttgtacataaccctggg
tgacgtgcacattgcttgggtatggaacggtagaaatttgggtgtttta
aaaccttgtttggggttgttcctgtccttgttgagaatcatagagatgtc
tgtgttcttggagtatttcacactgaggactaatctgctatcttcattcc
agtccctacccctcagtgcctgctctcatccaaataacctgggaggtgac
aatcaggatatctcaggaggtccaaggtggaacagacctctttgcctttc
ccagcgtctcataccccggtagtgcagctgtgggtggaggctggggtgt
ctgcacgaagtcaggccagcgtcctcctccacagcctgtcactgcccct
ccccagcctgtgtccacagtgctgtgatcccgagggaagtcctccagtct
aagtcacagtgccctgacaggtgagaagcaaactcccgctggaagcctcc
atctctttggaaaaacagttagtctggagcctgtggcccaggcccttctg
tccccaggcatcatcccaacagctcattttccctagtccgccttcgttca
agggtcaggaatggaccagaacagatgggttctggaggcccctgaacaga
gggctatggctgtggagaaggttcttggcccgttggactcacacagaccc
tgtaccctctcggcaagcatcttcagtcagattatcctcagtttcagata
cttcataataccttgtgttgtgtggggtcatacatcatcgtgtttgtaag
agaagatggtcattttattctctgtataaaacttagctctaaagcagaaa
ctaaagcagcaaatgcaggaaggctgtctcgccatcctcaagactcagca
gctctcattctccagtggtgagcacaccatttgtgctgctgctgttgtcg
tgaaatataataacagtggaagtcacaaaaatgtcccctgcccagccccc
tcgccgcccttgacctcctgcaggccatgtgtgtattacttgtctagtga
tgtcctctcaaagtgctgtacgcgagctcggcgccacctccgcctccctt

tcagagcctgctccccgccctctctgctcgctgcattgtggtgttctctt
ctcaaggctttgaaatctccccttgcactgagattagtcgtcagatctct
ccccgtctccctcccaacttatacgacctgatttccttaggacggaaccg
caggcacctgcgccgggcgtcttactcccgctgcttgttctgtcccctcc
ctcggaccaaacagtgctcatgcttcaggaccttgtttgtcgaagatgtt
ggtttccctttctctgttatttatataaaaataatttatcaaaaggatat
tttaaaaaagctagtctgtcttgaaacttgtttaccttaaaattatcaga
atctcagtgtttgaaagtactgaagcacaaacatatatcatctctgtacc
attctgtactaaagcacttgagtctaataaataaagaaatcagcacccct
tcccggtgtccaggggggaaaaaaaaa
>NM_016453 2
ggcgggacaaccgcggcgcggctcggcgctcccggcctggcgttgtggcg
ccgcagcccttgccaccttccctgcacctgccctgcccggcctcatgtac
cgcgcgctgtacgcgttccgctcggcggagcccaacgcgctggcgttcgc
cgcgggcgagaccttcctggtgctagagcgaagcagcgcgcactggtggc
tggccgcgcgggcgcgcagtggtgagacgggctacgtgccgccagcctac
ctgcgccgcctgcagggcctggagcaggatgtcctccaggccattgaccg
ggccatcgaggctgtacacaacacagccatgcgggatggtggcaagtaca
gcctggaacagcgtggagtcctccagaagctgatccaccaccggaaagag
accctgtcacgcagaggcccttcagcctccagtgttgcagttatgacctc
atcaaccagtgaccaccacttggatgctgctgcagccaggcagcccaatg
gggtgtgtcgagctgggttcgagcggcagcacagcctacccagttctgag
catcttggggcagatggaggcctctaccagatcccacttccatcttccca
gatcccaccacagcctcgccgagcagcacccaccacaccgcccccaccag
tgaagcgccgagaccgcgaggccctgatggcctctgggagtggtggccac
aacaccatgccctccgggggtaactctgtgtccagcggctcctcagtcag
cagcacctccctggacacgctctataccagctccagcccatctgaaccag
gctccagctgctcacccacacccccacctgtgccccgccgaggcacccac
accaccgtgtcccaagtccagccccctccctccaaggcatcagcacctga
accccctgcagaagaagaagtggcaactggtacaacctcagcctctgatg
acctggaagccctgggtacactgagcctggggaccacagaggagaaggca
gcagctgaggcggctgtgcccaggaccattggggccgagctgatggagct
ggtgcggagaaacactggcctgagccacgaattatgccgggtggccatcg
gcatcatagtgggtcacatccaggcctcggtgccggccagctcaccagtc
atggagcaggtcctcctctcactcgtagagggcaaggacctcagcatggc
cctgccctcagggcaggtctgccacgaccagcagaggctggaggtgatct
ttgcagacctggctcgccggaaggacgacgcccagcagcgcagttgggca
ctatatgaggatgagggtgtcatccgctgctacctagaggagctgctgca
tattctgactgatgcagaccctgaagtttgcaagaaaatgtgcaagagaa
acgagttcgagtctgtcctggccttggtggcctattaccaaatggaacac
cgagcatcactgcggctgctgctcctcaagtgctttggcgccatgtgcag
cctggatgcagccatcatctccacgcttgtgtcatccgtgctgcctgtag
agctggcgagggacatgcagacagacacgcaggaccaccagaaactctgt
tactctgccctcatcctggccatggtcttctccatgggagaggcagtgcc
ctatgcacactatgagcacctgggcacgcctttcgcccagttcctactga
acatcgtcgaggatgggctgcccttggacaccacagagcagctgccggac
ctctgcgtgaacctgcttctggctctcaacctgcacctgccagctgctga
ccagaatgtcatcatggctgccctgagcaaacacgccaatgtcaagatct
tctccgagaagctgttgttgctcctgaacagaggggatgaccctgtgcgc

atcttcaaacatgagccacagccaccacactctgtcctcaagttcctgca
ggacgtgtttggcagcccggccacagctgccatcttctaccacacagaca
tgatggctctcattgacatcactgtgcggcacatcgcagacctgtcacca
ggagacaagctgcgcatggagtacctctccctgatgcatgctatagtccg
caccacaccctacctgcagcaccgccaccggctacccgacctgcaggcca
tactgcgacgcatcctgaatgaggaggagacctcaccccagtgccagatg
gaccgcatgattgtccgagagatgtgcaaggaattcctggtgctgggga
ggctcccagctagcaccttgctgtcctcccttccctgcagctccagtcag
tgtgcaggggactcaggggcttggccctaagaatgttttgcactgacagt
gggaggggataaggtggcagaaggaacctgagcaggacccccacctgaag
tagaacttaagagaggggccaagtgtaggattggggaccatggtctggga
gctgttctggggcagggggaatatgcaagctaaagcccctctataccctg
gtaggcttcctcttcagcatgcccccctcttcaccccacacccatgtgta
cactcagagtcctgctgccgctccaggctggaccagacccccatccccac
ccctcctggtctgtatcctgggcctcagcaagccatgcccctggggtgag
gggaggcatctcccaccagctctattctttgccttagctttgcagtgagt
gctgctcatgtcctcccctccacccgcacatggggggtcgctgcccttcac
tcctacccccagaggccttggccaagctgctgctttgaaagcgggatctt
ggagacaggccatccatcctggagcctcatggaacaggatgatggcactg
agaaagccaatgaccgaatctcttttctctgtaaaaatgtagactgaaaa
gccatgtgtattttcctatgtgctgtagctctcctttggaaataaatcac
aggcatctggaaaaaaaaaaaaaaaaaaaaaaaaaaaaaaaaaaaaaaaa
aaaaaaaaaaaaaaaaaaaaaa
>NM_184231 1
ggcgggacaaccgcggcgcggctcggcgctcccggcctggcgttgtggcg
ccgcagcccttgccaccttccctgcacctgccctgcccggcctcatgtac
cgcgcgctgtacgcgttccgctcggcggagcccaacgcgctggcgttcgc
cgcgggcgagaccttcctggtgctagagcgaagcagcgcgcactggtggc
tggccgcgcgggcgcgcagtggtgagacgggctacgtgccgccagcctac
ctgcgccgcctgcagggcctggagcaggatgtcctccaggccattgaccg
ggccatcgaggctgtacacaacacagccatgcgggatggtggcaagtaca
gcctggaacagcgtggagtcctccagaagctgatccaccaccggaaagag
accctgtcacgcagaggcccttcagcctccagtgttgcagttatgacctc
atcaaccagtgaccaccacttggatgctgctgcagccaggcagcccaatg
gggtgtgtcgagctgggttcgagcggcagcacagcctacccagttctgag
catcttggggcagatggaggcctctaccagatcccaccacagcctcgccg
agcagcacccaccacaccgcccccaccagtgaagcgccgagaccgcgagg
ccctgatggcctctgggagtggtggccacaacaccatgccctccgggggt
aactctgtgtccagcggctcctcagtcagcagcacctccctggacacgct
ctataccagctccagcccatctgaaccaggctccagctgctcacccacac
ccccacctgtgccccgccgaggcacccacaccaccgtgtcccaagtccag
ccccctccctccaaggcatcagcacctgaaccccctgcagaagaagaagt
ggcaactggtacaacctcagcctctgatgacctggaagccctgggtacac
tgagcctggggaccacagaggagaaggcagcagctgaggcggctgtgccc
aggaccattggggccgagctgatggagctggtgcggagaaacactggcct
gagccacgaattatgccgggtggccatcggcatcatagtgggtcacatcc
aggcctcggtgccggccagctcaccagtcatggagcaggtcctcctctca
ctcgtagagggcaaggacctcagcatggccctgccctcagggcaggtctg
ccacgaccagcagaggctggaggtgatctttgcagacctggctcgccgga

aggacgacgcccagcagcgcagttgggcactatatgaggatgagggtgtc
atccgctgctacctagaggagctgctgcatattctgactgatgcagaccc
tgaagtttgcaagaaaatgtgcaagagaaacgagttcgagtctgtcctgg
ccttggtggcctattaccaaatggaacaccgagcatcactgcggctgctg
ctcctcaagtgctttggcgccatgtgcagcctggatgcagccatcatctc
cacgcttgtgtcatccgtgctgcctgtagagctggcgagggacatgcaga
cagacacgcaggaccaccagaaactctgttactctgccctcatcctggcc
atggtcttctccatgggagaggcagtgccctatgcacactatgagcacct
gggcacgcctttcgcccagttcctactgaacatcgtcgaggatgggctgc
ccttggacaccacagagcagctgccggacctctgcgtgaacctgcttctg
gctctcaacctgcacctgccagctgctgaccagaatgtcatcatggctgc
cctgagcaaacacgccaatgtcaagatcttctccgagaagctgttgttgc
tcctgaacagaggggatgaccctgtgcgcatcttcaaacatgagccacag
ccaccacactctgtcctcaagttcctgcaggacgtgtttggcagcccggc
cacagctgccatcttctaccacacagacatgatggctctcattgacatca
ctgtgcggcacatcgcagacctgtcaccaggagacaagctgcgcatggag
tacctctccctgatgcatgctatagtccgcaccacaccctacctgcagca
ccgccaccggctacccgacctgcaggccatactgcgacgcatcctgaatg
aggaggagacctcaccccagtgccagatggaccgcatgattgtccgagag
atgtgcaaggaattcctggtgctgggggaggctcccagctagcaccttgc
tgtcctcccttccctgcagctccagtcagtgtgcaggggactcaggggct
tggccctaagaatgttttgcactgacagtgggaggggataaggtggcaga
aggaacctgagcaggacccccacctgaagtagaacttaagagaggggcca
agtgtaggattggggaccatggtctgggagctgttctggggcagggggaa
tatgcaagctaaagcccctctataccctggtaggcttcctcttcagcatg
ccccccctcttcaccccacacccatgtgtacactcagagtcctgctgccgc
tccaggctggaccagacccccatccccacccctcctggtctgtatcctgg
gcctcagcaagccatgcccctggggtgaggggaggcatctcccaccagct
ctattctttgccttagctttgcagtgagtgctgctcatgtcctcccctcc
acccgcacatgggggtcgctgcccttcactcctacccccagaggccttgg
ccaagctgctgctttgaaagcgggatcttggagacaggccatccatcctg
gagcctcatggaacaggatgatggcactgagaaagccaatgaccgaatct
cttttctctgtaaaaatgtagactgaaaagccatgtgtattttcctatgt
gctgtagctctcctttggaaataaatcacaggcatctggaaaaaaaaaaa
aaaaaaaaaaaaaaaaaa
>NM_000067 2
cctccccttgtcgcctaggtccacccgagcccccctcccccgggccgcccc
cgagcacgaagttggcgggagcctataaaagctggtgccggcgcgacccg
cggacacacagtgcaggcgcccaagccgccgccgccagatcggtgccgat
tcctgccctgccccgaccgccagcgcgaccatgtcccatcactgggggta
cggcaaacacaacggacctgagcactggcataaggacttccccattgcca
agggagagcgccagtcccctgttgacatcgacactcatacagccaagtat
gacccttccctgaagcccctgtctgtttcctatgatcaagcaacttccct
gaggatcctcaacaatggtcatgctttcaacgtggagtttgatgactctc
aggacaaagcagtgctcaagggaggacccctggatggcacttacagattg
attcagtttcactttcactggggttcacttgatggacaaggttcagagca
tactgtggataaaaagaaatatgctgcagaacttcacttggttcactgga
acaccaaatatggggattttgggaaagctgtgcagcaacctgatggactg
gccgttctaggtatttttttgaaggttggcagcgctaaaccgggccttca

gaaagttgttgatgtgctggattccattaaaacaaagggcaagagtgctg
acttcactaacttcgatcctcgtggcctccttcctgaatccttggattac
tggacctacccaggctcactgaccacccctcctcttctggaatgtgtgac
ctggattgtgctcaaggaacccatcagcgtcagcagcgagcaggtgttga
aattccgtaaacttaacttcaatggggagggtgaacccgaagaactgatg
gtggacaactggcgcccagctcagccactgaagaacaggcaaatcaaagc
ttccttcaaataagatggtcccatagtctgtatccaaataatgaatcttc
gggtgtttccctttagctaagcacagatctaccttggtgatttggaccct
ggttgctttgtgtctagttttctagacccttcatctcttacttgatagac
ttactaataaaatgtgaagactagaccaattgtcatgcttgacacaactg
ctgtggctggttggtgctttgtttatggtagtagtttttctgtaacacag
aatataggataagaaataagaataaagtaccttgactttgttcacagcat
gtagggtgatgagcactcacaattgttgactaaaatgctgcttttaaaac
ataggaaagtagaatggttgagtgcaaatccatagcacaagataaattga
gctagttaaggcaaatcaggtaaaatagtcatgattctatgtaatgtaaa
ccagaaaaaataaatgttcatgatttcaagatgttatattaaagaaaaac
tttaaaaattattatatatttatagcaaagttatcttaaatatgaattct
gttgtaatttaatgacttttgaattacagagatataaatgaagtattatc
tgtaaaaattgttataattagagttgtgatacagagtatatttccattca
gacaatatatcataacttaataaatattgtattttagatatattctctaa
taaaattcagaattct
>NM_014575 3
aagagataatctgtgaaagccacagagcctgatttctgctgtgataatct
tcagcagaccgaggctggggcgagaatgaggcagacctgtttacatggtg
tgtgcagtgccttgtggaattcttaactctattttattgtattattttta
atcgaaaagtagatcagatgatagcatgaagtgaatcggctgctttggac
taatgggatcttcttttctgcattaagatcaagaacttttctggaatcct
ctgttctccgagtagccttgtcttctctgtgtgttttatcaatttgaaga
acactggcaaaaatcagcaaacttcatctcccaaaaagctctcgctcttt
tttgccaatacttgaagatttctgctccaaaatctactttgagctgcttg
agcatggtggttgataaccgaagggcaaaagcatcacttatttagtgtgt
gttatagaaatttttagacaaccttactagctaacccaggtgaccctttt
acggatttctttcaaagccaatttgaataaacaactcttccctctcaaat
gggcaagagtaactcttcagcaccatggagaggtccgggcagcgtgttac
aacgtgggactgtgaccagggcaagcactctgacagtgattaccgagagg
atgggatggatctaggcagtgacgccggcagcagcagcagcagcagccgc
gccagttcacagtccaactccaccaaagtgaccccttgctccgagtgcaa
atcttcatcgtcgccggggggcagcctggacttggtgtctgccctggagg
actatgaggagcccttcccggtctaccagaagaaggtgattgatgagtgg
gcgccggaggaggacggggaggaggaggaagaggaggacgagcgcgacca
gcgagggtaccgggatgaccgctctccggcccgggaaccgggggacgtaa
gcgccaggacccgcagcggcggcggcgggggcaggagcgccaccaccgcc
atgccgcccccggtgcccaacggcaacctccaccagcacgacccccagga
cctcaggcacaatggcaacgtggtggtggctggccggccgagctgttccc
ggggcccccgccgggcgatccaaaagccccagccggctgggggccggcgc
agtggccgcggcccggcggctggggggctctgccttcagcccccagacgg
cgggacgtgcgtccccgaagagcccccggtgccacctatggattgggagg
cgctggagaagcatctggccgggctgcagttccgggagcaggaggtacgg
aaccagggccaggcgaggaccaactccacctccgcacagaaaaatgagag

agagtctatcagacagaagttggcacttggaagcttctttgatgatggcc
caggaatttataccagctgtagcaaaagtgggaagccaagcctttcctcc
cgactgcagagtgggatgaacttgcagatatgctttgtcaacgacagtgg
cagtgataaggacagtgatgctgatgacagtaagactgaaaccagcttgg
acacccccttgtctcccatgagcaaacagagttcttcctattctgataga
gacactactgaagaggagtctgaatccttggatgacatggacttccttac
aaggcaaaagaaattgcaagctgaagccaaaatggcccttgccatggcca
aaccaatggccaaaatgcaagtagaagtggagaaacagaacaggaaaaag
tctcccgtcgctgatcttctgccacacatgcctcatataagtgaatgctt
gatgaaaagaagtttaaaacccaccgacctgagagacatgactattgggc
agctacaagtgatagtcaatgatctccattcccagatagaaagcttgaat
gaagagttggtccagctgcttctcatccgagatgagctgcacacagagca
ggatgccatgctggtggacattgaagacttgaccagacatgctgaaagtc
agcagaagcacatggcagagaaaatgcctgcaaagtgaaaagaagccatt
caaccagagaacaagctagaatttattttgcttctgtggttgtaaaaatg
ctgttgctaaaggtggcgcagaaacaaatatcagtgttagtcattgataa
tgtctgaagcttaatgtccagtgattggcctttgcttcttaatttatttt
aattttttacttgtgccacttaatatcaggcattttaataaaatattgtt
acaaaaaatgtacagtactgacaccaccacaaatcatggttaataaaaga
gagtagttttaactttatttttatttgtttagagattttaagttggaaca
gtattttcccattgactacttttcattcttcactgtagttttaaagaaga
actgtaaatgacggtgctatacaagtcaaaaaatacatgcctgcctcgta
gtgaagttgtagctctccgtaatatgtatattttactcagttttcaacat
tttgtgaatgttgactacctgaagttcctttttagatgtgctattaacat
tctgttggattcagagggttccttgaaagttttatgtataaatatgtaaa
ataaaaattaaaactttgtttcatatcataaaaaaaaaaaaaaaaaa
>NM_001094 4
ctcttccacatcaccttggtgtctccctaaataaaaccagccctccttat
cgcctggaaaaaatcaagagctagagtttgaatgggttttatataaacac
tcacccctgtcagcgtgcggctgggctctcaggataaactcacagcatct
ggcgcgatgcttgccttgcgttctctcccctgaacgtcaaggtttaagca
gagcccgaggactgggagctcttctctgaaattcgatcaacctgaagcca
gttgcggaactgcacggggtcccgatggacctcaaggaaagccccagtga
gggcagcctgcaaccttctagcatccagatctttgccaacacctccaccc
tccatggcatccgccacatcttcgtgtatgggccgctgaccatccggcgt
gtgctgtgggcagtggccttcgtgggctctctgggcctgctgctggtgga
gagctctgagagggtgtcctactacttctcctaccagcatgtcactaagg
tggacgaagtggtggctcaaagcctggtcttcccagctgtgaccctctgt
aacctgaatggcttccggttctccaggctcaccaccaatgacctgtacca
tgctggggagctgctggccctgctggatgtcaacctgcagatcccggacc
cccatctggctgacccctccgtgctggaggccctgcggcagaaggccaac
ttcaagcactacaaacccaagcagttcagcatgctggagttcctgcaccg
tgtgggccatgacctgaaggatatgatgctctactgcaagttcaaagggc
aggagtgcggccaccaagacttcaccacagtgtttacaaaatatgggaag
tgttacatgtttaactcaggcgaggatggcaaacctctgctcaccacggt
caagggggggacaggcaacgggctggagatcatgctggacattcagcagg
atgagtacctgcccatctggggagagacagaggaaacgacatttgaagca
ggagtgaaagttcagatccacagtcagtctgagccacctttcatccaaga
gctgggctttggggtggctccagggttccagacctttgtggccacacagg

agcagaggctcacatacctgcccccaccgtggggtgagtgccgatcctca
gagatgggcctcgactttttcctgtttacagcatcaccgcctgtaggat
tgactgtgagacccgctacattgtggaaaactgcaactgccgcatggttc
acatgccaggggatgcccctttttgtaccccctgagcagcacaaggagtgt
gcagagcctgccctaggtctgttggcggaaaaggacagcaattactgtct
ctgcaggacaccctgcaacctaacccgctacaacaaagagctctccatgg
tgaagatccccagcaagacatcagccaagtaccttgagaagaaatttaac
aaatcagaaaaatatatctcagagaacatccttgttctggatatattttt
tgaagctctcaattatgagacaattgaacagaagaaggcgtatgaagttg
ctgccttacttggtgatattggtggtcagatgggattgttcattggtgct
agtatccttacaatactagagctctttgattatatttatgagctgatcaa
agagaagctattagacctgcttggcaaagaggaggacgaagggagccacg
atgagaatgtgagtacttgtgacacaatgccaaaccactctgaaaccatc
agtcacactgtgaacgtgcccctgcagacgaccctggggaccttggagga
gattgcctgctgacacccctcgagtcacccagcactccctccaaacagac
cttgaggcccaagacccaggacaaggaacagcaagctcaggtgggatggc
cccagtgctggaaagaagcaagagcccccctatgcacacattgcagactag
ctgcctagacctcgctccggccacgtccaacacgacgcatccttgggccc
cgccgtgcgtccctcttaggagagatgagtcacactctggaactgtccaa
gaacgaacctgccatcacatctcactgccagatgtataaagcacctgcat
gctcagacttcttgtggcgccacctccacgtctgtcttgtacatgacact
cctccacgcggtttccagtgtccacactgctgcccgtgcagtgggaccag
attccaggtccaaagtcaccatgaggccaccctggaatcagaactgcaca
atcaagagggaacccatgggactctctgctacattcagttcttgtgtcgt
ttgtgaaagttcttaacctgcccaaaaaccccccttttccccaagctgccc
atggggcttcggcgccaaaggtgacccgcgccaacctccctcccccccag
tgcctatgacggcggcacagcagccagcgggtgggggacgcctgtgttca
cccatggtgcccatgtcgttcttctctccctgtgacacagcttgtacagt
ctgattctttttatctggggtagggggggcttttatgtttgtccgatggag
atttgttttgttttgcttcattttatgctttttttattttagttttgatgt
tctgaggtttgctttggtttttccattttctttggcatttatttattcgt
gcttcaaatcacagtcatattaaaagctggtcttgtggaaaaaaaaaaaa
aaaaaaaaaaaaaaaaaaaaaaaaaaaaaaaaaaaaaaaaaaaaaaaaa
>NM_019555 2
cactcggctccggagcccagtcgagtcctgcccggctagaagccggctgt
cggtctccgtgtcgccgccgccgcccggcatcgtggagctggggccccct
tttgcctgggagttttgtagtcgcctagggtcagcggtgacatcccaaag
ggcaggcccggcagccgccatggtggccaaggattaccccttctacctca
cggtcaagagagcgaactgcagcctggagctacccccggccagcggtccg
gccaaggacgctgaggagcctagtaataaacgggtcaaacccctttcccg
agtcacgtcgctagcaaacctcatcccgcccgtgaaggccacgccattaa
agcgcttcagtcaaaccctgcagcgctccattagcttccgcagtgagagc
cgccctgacatcctcgccccccgaccctggtccagaaatgccgccccctc
gagcacgaaacggagagatagcaagctgtggagtgagaccttcgatgtgt
gcgtcaatcagatgcttacatccaaggaaatcaaacgtcaggaggcgatc
tttgagctttcccaaggagaagaagacttgatagaagacttgaaattagc
aaaaaaggcctatcatgaccccatgctgaaactctccataatgacagaac
aagagttgaatcaaatttttggaacactggactctctaattcctctacat
gaagagctccttagtcagcttcgagatgttaggaagcctgatggctcgac

tgaacatgttggtcccatcctcgtgggctggctcccttgcctcagctcct
atgatagctactgcagcaatcaagtagccgccaaagctctgctggaccac
aaaaagcaagatcaccgagtccaggatttcctacagcgatgtttagaatc
cccctttagccgcaaactagatctctggaatttcctcgatattccaagaa
gccgcctggtaaaataccctctgcttctccgagaaatcttgaggcacaca
ccaaatgataatccagatcagcagcacttggaagaagctataaatatcat
tcagggaattgtggcagaaatcaacaccaagactggtgaatctgaatgcc
gctattataaagagcggcttctttacttggaagaaggccagaaagactcc
ctgatcgacagctctcgagtcttgtgttgtcatggtgaactgaagaacaa
tcggggcgtgaaactgcatgttttcctgttccaagaagtgcttgtgatca
ctcgagccgtcacccacaatgagcagctttgctaccagctgtaccgtcag
ccaatccccgtgaaagacctcctgctggaagacctccaggatggagaagt
gaggctgggtggctccctgcgaggggcattcagcaacaatgagagaatta
aaaacttcttcagagtcagtttcaaaaatggatcccaaagtcagacccac
tcgctacaagccaatgacactttcaacaaacagcagtggcttaactgtat
tcgtcaagccaaagaaacagttttgtgtgctgccgggcaagctggggtgc
ttgactccgagggatcgttcctaaatcccaccaccgggagcagagagcta
cagggagaaacaaaacttgagcagatggaccaatcggacagtgagtcaga
ctgtagtatggacacgagtgaggtcagcctcgactgtgagcgcatggaac
agacagactcttcctgtggaaacagcaggcacggtgaaagtaacgtctga
cagaagcatgtgcacttcgggaagcaggcctgcatcttacctgtacagta
tttgcattccacagatggaacggtttggagaagcactttttcatactttt
gtgaaagtatacatgttggcccagtctctcgtatctgtacctttgtccct
agtactgtaactgccaatctgtctgtgtaagctggaatctgtggcaacta
ttaccctgtgttgtatttcccaagtgtctggatggatggagaggtactca
aacaagttactttcagttgtcctgctggattttaaaaaaatagaaaaaga
atctcaaaactactgttttacatagattgtttgaagagtccttcctcttg
tgcttctgtaccactttcccagctcttagatgtggtagctaaaggcacgg
aatttagacggccttgtaaatagggcatgaggaactcatctgtgtattgg
gatggtattagagagagaatcaggaaagaccaactcatgaagtgaacttg
gtttgatcttactcaactagaaagcttgaaaacatccctggggattctga
aggcttaattttgcaaaggaggatgcattgtctgaactttgcaacttcat
ccagtgcaagtttgatgcaagaatgtattaggacataaaatagaggctga
ccttaaaagggccaggacagaagcggctgccagctctgaatctttaactg
aaatgcacatggcaccaggaggtgtctctcatagttggttgctagcctaa
aacatcagaatagaacccaaagggcttaggaaggcctgccaggataacaa
gaaggccctgtattcattgtgtttcatctgcctaggcctactcattattt
tagagaatgaatgaagcaacaaggaagagagaccatgactctatcgatga
cactgtttatagaaacacaggagaggaagaatttggaatgaaaagcactt
cgtcagaaccttctgtgggagccattgagagaaaagcatggtccagtgcc
ttctgagaaaggccagagctttgggctttcctgctctgcttttgggtcgt
caatttgccatctctggttctgtgctataatcagaattgtaattatgttc
tccagaggccaatttcattaactctgattaattagaatcagctagccaga
ttagtaacctctttgtccagccttgatttacagtgcagggtaaagtgcag
accttaaaaacagctaagtacctagaagagctccctgcaagtgtaaatat
taaggatgacctgtgcaaaattatacccacaccagcactagtggtaatta
ttctaaattattgccaaaaagtttttttaatctgtctttcaagtttaca
gaaaagaaagcagtaaatgcattgatgtcattttattatgtacatatatc
atgtgcattcaagctgtgtgacaagatatatcaatataaaaacaaggtat

atactttattatttttgaaaacaaggatattgtgatcaattttaccctg
taaaacatatttctgtatttataggtcttaaacatgatgaatttttcta
ttacaagtttatttaaaactgctttctcaagtcgttattgatacagcaag
tgaacctgctgcagacagaagcagaggaaagccaagaacagcctttattg
gtgaagaaaagaatgaatgattctttgtaggcgccatcagccacttttag
aagccatcagccagtgtgttgggaaaagaggtttgtcaagtgttggccta
tgggaaggtggtcaatgaatgttttgatgaaatgaatgtttttgtataat
ggccttaaacttttctggaagtatttcaaataaattacattattaagtca
tcaaaaaaaaaa
>NM_016310 3
cggcagtgggagctgccgggagttggagcctgcggagttcgagaccatgc
tgctgttctgccccggctgcgggaacgggctgatcgtggaggagggacaa
cgctgccaccgcttctcctgcaacacgtgcccctacgtgcacaacatcac
ccgcaaggtaacaaatcggaagtacccaaaactgaaagaagtggatgatg
tgcttggtggagcagctgcctgggagaatgttgactctactgcagagtcg
tgtcccaaatgcgaacatcctcgtgcttacttcatgcagcttcagacccg
ctctgcagatgagccgatgaccaccttctacaagtgctgcaatgctcagt
gtggacaccgctggagggattagggccaggatggcccagctgccctagtg
tgtgcttgccttgtccctcggggtagatgcttagctggcagtatgagttg
tgtgtcctgagggtctttgctagtgtggtggaaagataaaccttttgagg
tgaagagccagggggtcaggaaatatggcctatctgccaggcagggtgga
tgaagtcatgaatgtctgggagtttttctgtgtggggaggagacagagac
ccataactaaatatgctctgtgtaaagtcctattctttcatcttccactt
tattggcagttgacattcccttactcccaatcaacactcttaaatatttg
tactgtttgtaaaacttagtacatgtccctaaatatttaactgttacttg
taaacttgtgtaatttattatttattttaatcaaaattctgaatatttca
tttaaatgaaagttggaatattgccctttctttccttctctccttcttt
atttgaggggggaagagtgctgtacaaagtcaaacaaacaagtttacagt
tgtaagtgcaatgacccgagtcctccacatgaccttgtgaattgtgtgct
gtccttctgtgctctgtgagaactcgtggtacttcagtgtccctccccct
gtattgtgacaaggtaattctgtggtatcagaataaaaggacttgatata
aacaacctaaaaaa
>NM_000320 2
acagtccctccgggtggcggggcgccccgagcgtggcagcgcgctaggcg
gcagcagcgggcgcggagcggggcgcggccgccgcgcgttccctcttggc
ggggttggccggccggggcggggcgcggcgctccggctcgaggcattcgg
agctgcgggagccgggctggcaggagcaggatggcggcggcggcggctgc
aggcgaggcgcgccgggtgctggtgtacggcggcaggggcgctctgggtt
ctcgatgcgtgcaggcttttcgggcccgcaactggtgggttgccagcgtt
gatgtggtggagaatgaagaggccagcgctagcatcattgttaaaatgac
agactcgttcactgagcaggctgaccaggtgactgctgaggttggaaagc
tcttgggtgaagagaaggtggatgcaattctttgcgttgctggaggatgg
gccgggggcaatgccaaatccaagtctctctttaagaactgtgacctgat
gtggaagcagagcatatggacatcgaccatctccagccatctggctacca
agcatctcaaggaaggaggcctcctgaccttggctggcgcaaaggctgcc
ctggatgggactcctggtatgatcgggtacggcatggccaagggtgctgt
tcaccagctctgccagagcctggctgggaagaacagcggcatgccgcccg
gggcagccgccatcgctgtgctcccggttaccctggataccccgatgaac
aggaaatcaatgcctgaggctgacttcagctcctggacacccttagaatt

cctagttgaaactttccatgactggatcacagggaaaaaccgaccgagct
caggaagcctaatccaggtggtaaccacagaaggaaggacggaactcacc
ccagcatatttttaggcctcatctcagtgcctatgaggggcctgccagaa
aagtcactaacctgtctcagtgtggccttgtccagccttgtgttttctgt
aacccctgtttgtggtacgagataatgagtcctatttttctctcacataa
tatgcatttgctctcctaggacagtgtaatacatttatgtgaagtaaaga
catgcgagactggtggcctgcaaatagcatccgttgatctgtgttaactg
catagggagggctctgcatagcacctgctatagcggtgtcatgttggatc
gcttttgtgactgttcatctgtccttgacagtggctgtcatcttgactac
tttgttgatttgttggtattggggacattttaaaggctgagttatttttg
aatgtcatgtttatgtcatagacgtagttttcgcatccttgaattaaact
gccttaactccttttgtggtataagcaaaactacatggactctgtcctgg
tatccttttcctgtgtggttgccccgtgtcctctggcctagggttaagtg
tgcaagataactactcgtgagtattcagaatgttgttcctaataaatgca
cttgttgtctgtcttctttaatcaaatcacatcttatatacagcagtcag
agatgagtatactagaatcatggattgctggaggtcttttaatctgatgt
tctcagaagggggtggatttaaatcctgaaataaatatttcaacacaaga
acaaaaaaaa
>NM_001819 2
agaccctgcacaccaggcgactggactggcgtaaatttccgcccatttgc
gtcgccgcctgggggtgctgcttcgtgacgtcagggctgagctagggggc
gcccgctcctggctgcccgctcctcccggccctgcggcccccgcctgccc
tttaaaagagcggggcctgcgccggccgcgccacaccgcggggaccagga
ggcacgctggttttccggggccgctccatcgcgccttcctcctgcgcctc
gcttctccggtccagccgccatcttcctttccgcacaggggccgccgagc
ggggccatgcagccaacgctgcttctcagcctcctgggagccgtggggct
ggcggctgtcaattccatgccagtggataacaggaaccacaatgaaggaa
tggtgactcgctgcatcattgaggtcctctcaaatgccttgtcgaagtcc
agcgctccacccatcaccccctgagtgccgccaagtcctgaagacgagtag
aaaagacgtcaaagacaaagagacaactgaaaatgaaaacacaaagtttg
aagtaagattgttaagagacccagctgatgcctcggaagcccacgagtcc
tccagcaggggagaggcaggagccccaggggaggaggacatccaaggccc
aacaaaggcagacacagagaaatgggcagagggaggcgggcacagccgag
agcgagcggatgagccccagtggagcctctatccctccgacagccaagtc
tctgaagaagtgaagacacgccattctgagaagagccagagagaggatga
ggaggaggaggagggagagaactatcaaaaaggggagcgaggggaagata
gcagtgaagagaaacaccttgaagagccaggagagacacaaaacgctttt
ctcaatgaaagaaagcaggcttcagctataaaaaaagaggagttagtggc
cagatcggaaacacatgctgccgggcattctcaggagaagacacatagcc
gagagaagagtagccaggagagtggagaggagacagggagccaggagaat
cacccccaggagtctaaaggccaaccccgaagccaggaagaatctgagga
aggtgaggaagatgccacctctgaggtggacaaacgacgcacgaggccca
gacaccaccacgggaggagcaggcccgacaggtcctctcaaggagggagt
cttccctctgaggaaaagggacacccccaggaggaatctgaggagtcaaa
cgtcagcatggccagtttaggggaaaagagggaccaccattcaacccact
acagggcttcagaggaagaacctgaatatggagaagaaataaagggttat
ccaggcgtccaggcccctgaggacctggagtgggagcgctataggggcag
aggaagtgaagaatacagggctccaagacctcagagtgaggagagttggg
atgaggaggacaagagaaactaccccagcttagagcttgataagatggca

catggatatggtgaagaaagtgaggaagagaggggccttgagccgggaaa
gggacgccatcacagaggcaggggaggggagccacgtgcctatttcatgt
ctgacaccagagaagagaaaaggttcttgggtgaaggacaccaccgtgtc
caagaaaaccagatggacaaggcaaggaggcatccacaaggtgcgtggaa
agagctggacagaaattatctcaactacggtgaggaaggagccccaggga
agtggcagcagcagggagacctgcaggacactaaagaaaacagggaggaa
gctaggtttcaagataaacaatatagctcccatcacacagctgaaaagag
gaagagattaggggaactgttcaacccatactacgaccctctccagtgga
agagcagccattttgaaagaagagacaacatgaatgacaattttctcgag
ggtgaggaggaaaatgagctgaccttgaacgagaagaatttcttcccaga
atacaactatgactggtgggagaaaaagcccttctctgaggatgtgaact
gggggtatgagaagagaaacctcgccagggtccccaagctggacctgaaa
aggcaatatgacagggtggcccaactggaccagctccttcactacaggaa
gaagtcagctgagtttccagacttctatgattctgaggagccggtgagca
cccaccaggaggcagaaaatgaaaaggacagggctgaccagacagtcctg
acagaggacgagaaaaaagaactcgaaaacttggctgcaatggatttgga
actacagaagatagctgagaaattcagccaaaggggctgactgtcattgg
agcggtgggcactgttaagaagcagccatcacatgatctgtttttcacca
cttcactgaaagacaccatttatctacccaagggcagaaagtagaactta
ctattcattaaatgtttgacacaattggaattgtctttaatttctgtcag
aatgctattgaaaatgtgaattgcatgacttgtagcatattcttttctgc
aaaatagacatattaacatgcttatgacaatgactgtgctactgtctttg
gaaaaatgtttgtctcagttggaaataataaaagattcacctgagaccaa
aaaaaaaaaaaaaaaa
>NM_004505 2
ctaaaaataccattaagtaatagtattagcttttgtattctgagattcaa
cagcagcagtcacttccctccactcctatgtgtatcccaggaccaccctg
ggcggggagggctgaggtcagggaggtctgaagctggtcctgggctccgg
gggtgacagtgatgaggaactgggtgcacacatgagtggggcagccgggc
ctggccagagaagcaacacacacgtgcacagacatgtttatccacataca
catgtgcacgcatgtgcacaaacacattgcaggcaggcatgttgacgcct
caggcagcggaggaccctgactctgggccctgctgacccaggcaaggccc
cattgtgatgcgtgccatgacctcagaatgtcactggtgcttagcaccta
tccgctctccagactgcgtctgtgttctacggcagttacacacacgcagt
ggtattcacaagcggttttgtggactcaaaggttttctccctgagaggca
taacccaggccagctgattcatcagaatcaggtgagtgtgacctgctctc
ttccctccaggctgacttggggacagtggctatggtatgggcggtgttgg
cctctgggcagctacagaggagggtcatccctgagcactcaccgggcgcc
cgttctacactgcccatgtagacgattttctctttcgtcttcatggtggc
ttcgtagagtgggtgctgttcccaaatgtacccattcgacaggtgagccg
tctggggtcagagaggcagtaactggcctgggaatccagacaagaccctg
ggttttgctctcagccctgctgtgtgccatgctagacttcaggcctcaac
cctgagacctccctgctctagatcccaaatctgcccagatttccgatccc
aatggggcagagcctggccctggcagagacactgggatggatccactgtg
ggtggggaggagggaagggtcctcagaacacacctggggcctaagctggg
tcttgatggtcactgtgggacccactggacacacacagtcccttgtctgg
gagtggcatggggagccttctgcccttgggcagttgtggaaagtgaagga
gccctggagagctggctgaggggagactatcttcccttgtgttcaaaggg
gtccaggcactggggctctccccaagtatttcttattctgtctggcctcg

ctttccttttgccctgagtattctcaggagggacggtccatctagatgtc
ctccaggagcaaggacccactgttcttcatcagtgacccaggaaaatgaa
gccccctcctgtggggacagctcagaatggtggagtccacagtccctccc
tgagagacatggtttccatgagcacagtggctgctttggagacagtaatc
attttcatccccaaaaccaaacacactcctgctcaaatggtgttattgct
aaagcagcttcactggttagactgaagggccatggtagcccaagtgatga
gcggggtagaatggagcagtcaggagagatcttgttccccgtaggaaact
gggcatctctgtggccctgaacatcccaggaggccgatcgtacagagacc
tctggtgcctgaccgcagttcacatccacatccctggaatagaccatcac
aggctcttcacccttggcaggtggacaccattcaacctgccggggcagga
tggacatggtagagaatgcagatagtttgcaggcacaggagcggaaggac
atacttatgaagtatgacaagggacaccgagctgggctgccagaggacaa
ggggcctgagcccgttggaatcaacagcagcattgatcgttttggcattt
tgcatgagacggagctgcctcctgtgactgcacgggaggcgaagaaaatt
cggcgggagatgacacgaacgagcaagtggatggaaatgctgggagaatg
ggagacatataagcacagtagcaaactcatagatcgagtgtacaagggaa
ttcccatgaacatccggggcccggtgtggtcagtcctcctgaacattcag
gaaatcaagttgaaaaaccccggaagataccagatcatgaaggagagggg
caagaggtcatctgaacacatccaccacatcgacctggacgtgaggacga
ctctccggaaccatgtcttctttagggatcgatatggagccaagcagagg
gaactattctacatcctcctggcctattcggagtataacccggaggtggg
ctactgcagggacctgagccacatcaccgccttgttcctcctttatctgc
ctgaggaggacgcattctgggcactggtgcagctgctggccagtgagagg
cactccctgccaggattccacagcccaaatggtgggacagtccaggggct
ccaagaccaacaggagcatgtggtacccaagtcacaacccaagaccatgt
ggcatcaggacaaggaaggtctatgcgggcagtgtgcctcgttaggctgc
cttctccggaacctgattgacgggatctctctcgggctcaccctgcgcct
gtgggacgtgtatttggtggaaggagaacaggtgttgatgccaataacca
gcattgctcttaaggttcagcagaagcgcctcatgaagacatccaggtgt
ggcctgtgggcacgtctgcggaaccaattcttcgatacctgggccatgaa
cgatgacaccgtgctcaagcatcttagggcctctacgaagaaactaacaa
ggaagcaaggggacctgccacccccagccaaacgcgagcaagggtccttg
gcacccaggcctgtgccggcttcacgtggtgggaagaccctctgcaaggg
gtataggcaggcccctccaggcccaccagcccagttccagcggcccattt
gctcagcttccccgccatgggcatctcgtttttccacgccctgtcctggt
ggggctgtccgggaagacacgtaccctgtgggcactcagggtgtgcccag
cctggccctggctcagggaggacctcagggttcctggagattcctggagt
ggaagtcaatgccccggctcccaacggacctggatatagggggcccttgg
ttcccccattatgattttgaatggagctgctgggtccgtgccatatccca
ggaggaccagctggccacctgctggcaggctgaacactgcggagaggttc
acaacaaagatatgagttggcctgaggagatgtcttttacagcaaatagt
agtaaaatagatagacaaaaggttcccacagaaaagggagccacaggtct
aagcaacctgggaaacacatgcttcatgaactcaagcatccagtgcgtta
gtaacacacagccactgacacagtattttatctcagggagacatctttat
gaactcaacaggacaaatcccattggtatgaaggggcatatggctaaatg
ctatggtgatttagtgcaggaactctggagtggaactcagaagagtgttg
ccccattaaagcttcggcggaccatagcaaaatatgctcccaagtttgat
gggtttcagcaacaagactcccaagaacttctggcttttctcttggatgg
tcttcatgaagatctcaaccgagtccatgaaaagccatatgtggaactga

aggacagtgatggccgaccagactgggaagtagctgcagaggcctgggac
aaccatctaagaagaaatagatcaattattgtggatttgttccatgggca
gctaagatctcaagtcaaatgcaagacatgtgggcatataagtgtccgat
ttgaccctttcaatttttttgtctttgccactaccaatggacagttacatg
gacttagaaataacagtgattaagttagatggtactacccctgtacggta
tggactaagactgaatatggatgaaaagtacacaggtttaaaaaaacagc
tgagggatctctgtggacttaattcagaacaaatcctactagcagaagta
catgattccaacataaagaactttcctcaggataaccaaaaagtacaact
ctcagtgagcggatttttgtgtgcatttgaaattcctgtcccttcatctc
caatttcagcttctagtccaacacaaatagatttctcctcttcaccatct
acaaatggaatgttcaccctaactaccaatggggacctacccaaaccaat
attcatccccaatggaatgccaaacactgttgtgccatgtggaactgaga
agaacttcacaaatggaatggttaatggtcacatgccatctcttcctgac
agcccctttacaggttacatcattgcagtccaccgaaaaatgatgaggac
agaactgtatttcctgtcacctcaggagaatcgccccagcctctttggaa
tgccattgattgttccatgcactgtgcatacccggaagaaagacctatat
gatgcggtttggattcaagtatcctggttagcaagaccactcccacctca
ggaagctagtattcatgcccaggatcgtgataactgtatgggctatcaat
atccattcactctacgagttgtgcagaaagatgggaactcctgtgcttgg
tgcccacagtatagattttgcagaggctgtaaaattgattgtggggaaga
cagagctttcattggaaatgcctatattgctgtggattggcaccccacag
cccttcaccttcgctatcaaacatcccaggaaagggttgtagataagcat
gagagtgtggagcagagtcggcgagcgcaagccgagcccatcaacctgga
cagctgtctccgtgctttcaccagtgaggaagagctaggggaaagtgaga
tgtactactgttccaagtgtaagacccactgcttagcaacaaagaagctg
gatctctggaggcttccacccttcctgattattcaccttaagcgatttca
atttgtaaatgatcagtggataaaatcacagaaaattgtcagatttcttc
gggaaagttttgatccgagtgctttttttggtaccacgagacccggccctc
tgccagcataaaccactcacaccccaggggatgagctctccaagcccag
gattctggcaagagaggtgaagaaagtggatgcgcagagttcggctggaa
aagaggacatgctcctaagcaaaagcccatcctcactcagcgctaacatc
agcagcagcccaaaaggttctccttcttcatcaagaaaaagtggaaccag
ctgtccctccagcaaaaacagcagccctaatagcagcccacggactttgg
ggaggagcaaagggaggctccggctgccccagattggcagcaaaaataag
ccgtcaagtagtaagaagaacttggatgccagcaaagagaatggggctgg
gcagatctgtgagctggctgacgccttgagccgagggcatatgcgggggg
gcagccaaccagagctggtcactcctcaggaccatgaggtagctttggcc
aatggattcctttatgagcatgaagcatgtggcaatggctgtggcgatgg
ctacagcaatggtcagcttggaaaccacagtgaagaagacagcactgatg
accaaagagaagacactcatattaagcctatttataatctatatgcaatt
tcatgccattcaggaattctgagtgggggccattacatcacttatgccaa
aaacccaaactgcaagtggtactgttataatgacagcagctgtgaggaac
ttcaccctgatgaaattgacaccgactctgcctacattcttttctatgag
cagcaggggatagactacgcacaatttctgccaaagattgatggcaaaaa
gatggcagacacaagcagtacggatgaagactctgagtctgattacgaaa
agtactctatgttacagtaaagctaccactctggctgctagacagcttgg
tggcgagggagatgactccttgtagctgatacttggcaaaagtgtcactg
aaagacaagctaaatgtagttattttatcctgttagaacaaaaattctaa
ttaaaatagttaacttgaagagtagaaacaattgtattttgaagtctcat

acaagctgtctgatagagaactttcaggcagatcccaccattagcctgta
aacaaaaggtgtggcaccagccacctgggaccaaataagaattgaattgt
gcttgtccagatatgaacaaatatgtagtgagtatagagtttaccaataa
tcataacaaatattaaagatttccttggagtcagaggaaaaaacaaacaa
ttataatgttgtctagggacgacatgatacgctacctccttttcctgaa
gttttattccattatattgacaagatggagaaagcaagatcatgaaggtg
tgcaaatgattcttacggcatggacaaggatttttcaatttattttttaa
actgtttccatacccttttcttttttcttgctttttgtttttgccattgtgt
ttacgtttgagacacaaccagtcattggtggcaggggcatagagtggtca
gtctgaaagggaggctctcttaagagctatgtgccttccaaccagaggga
gacccagtagaaagaaaaacatcctgggaaatccagctaccagggccctc
ccagtggaggcatcttacatttaggctacttcaagtatcctcagaaatgt
attctgcaccccccggccccgcccatgctgagggaaggggagcagttgcca
atatttgcaccatcttcacatgcacatgttgcaacaagagcttctgggaa
ggtaagcggcatcggagctagatcacgtttcacaattagtggttattctt
ttctgtgtttgttttgcactttaaaaaagagagaacacatgcaaatgaac
ttgcttgtgtgtatttgatggctctaagggctataaattacaaacaaaac
acatcccagacattaggagttcataagtatatttaatgaaattggtggtt
ttaggaagtcaactttagttttgctttgtttgcatgtccactggtttttt
tattttgatatttgtcttttttttaaattttacagtagtcattgaaagtta
tgtttctttgcttacttcattttttccctctaattatttaagattggaac
aaaagtataaatattatttatttgaggtagaattttttttcatgtagtttc
ttaatatatacttgaaggaaatgtttcaccttatttttggtctttgttta
ttcatttagaccctgcaagttgattctcattgccagattccattaccctt
tcttcctcataggtagtaattaccaatgtaactaagcatttgtgttctga
tatctgaggccagtaactattaatatctagttctcagagcatttggaaag
gttatcttaaatggctacctaaattgaaatccttttcagaaaaaaatataa
ttgcaagtaggtaggagtggcctaaattgtctaatgtaataaagtcagac
aaaatgcacactttatagtttcaagattttcagtaaataaaatctgtcca
ttcctacctggacatgtcccattaaaaagtggaagattttaaataatttc
tttacagatgttttatttaaacaggtagcacaatctactaatgttgtgtg
atttgtgttatactggttgtaattaattttttttaattcatgaactagcgg
aaaatttattaaattaactattaactacattcaccttgtaaattactgta
taaaacttgttgacaatgcactgactttagaaagatgttaatgtacataa
atagagtgtaaataaaatagtgttgatgtactgaaatatgaactgtatca
aaagtattggtaattgtatatggggtgtacctgtttatctgttaactatt
atccaaacaaattaaatactgtggttgcctctatgtgctgtttttcctca
tacaagtaaacacagaaagtc
>NM_182470 2
agtcccaccgaaagggcaacctgcccgcgcgttccgccaccgccgccgcg
cttcctcctgaaggtgactgcgcccgcggggacgcagggggcggggcccg
ggtcgcccggagccgggattgggcagagggcggggcggcggagggattgc
ggcggcccgcagcgggataaccttgaggctgaggcagtggctccttgcac
agcagctgcacgcgccgtggctccggatctcttcgtctttgcagcgtagc
ccgagtcggtcagcgccggaggtgagcggtgcaggaggctacgccatcag
tccccaccaagggccagtcgcccggctagtgcggaatcccggcgcgccgg
ccggccccgggcacgcaggcagggcggcgcaggatccagggcgtctggga
tgcagtggagctcagagagaggagaacggctcctcacgcctggggcctgc
tcttcagaagtccccagcgccgttccttccagatcaggacctcagcagcc

atgtcgaagccccatagtgaagccgggactgccttcattcagacccagca
gctgcacgcagccatggctgacacattcctggagcacatgtgccgcctgg
acattgattcaccacccatcacagcccggaacactggcatcatctgtacc
attggcccagcttcccgatcagtggagacgttgaaggagatgattaagtc
tggaatgaatgtggctcgtctgaacttctctcatggaactcatgagtacc
atgcggagaccatcaagaatgtgcgcacagccacggaaagctttgcttct
gaccccatcctctaccggcccgttgctgtggctctagacactaaaggacc
tgagatccgaactgggctcatcaagggcagcggcactgcagaggtggagc
tgaagaagggagccactctcaaaatcacgctggataacgcctacatggaa
aagtgtgacgagaacatcctgtggctggactacaagaacatctgcaaggt
ggtggaagtgggcagcaagatctacgtggatgatgggcttatttctctcc
aggtgaagcagaaaggtgccgacttcctggtgacggaggtggaaaatggt
ggctccttgggcagcaagaagggtgtgaaccttcctggggctgctgtgga
cttgcctgctgtgtcggagaaggacatccaggatctgaagtttggggtcg
agcaggatgttgatatggtgtttgcgtcattcatccgcaaggcatctgat
gtccatgaagttaggaaggtcctgggagagaagggaaagaacatcaagat
tatcagcaaaatcgagaatcatgagggggttcggaggtttgatgaaatcc
tggaggccagtgatgggatcatggtggctcgtggtgatctaggcattgag
attcctgcagagaaggtcttccttgctcagaagatgatgattggacggtg
caaccgagctgggaagcctgtcatctgtgctactcagatgctggagagca
tgatcaagaagccccgccccactcgggctgaaggcagtgatgtggccaat
gcagtcctggatggagccgactgcatcatgctgtctggagaaacagccaa
aggggactatcctctggaggctgtgcgcatgcagcacctgatagctcgtg
aggctgaggcagccatgttccaccgcaagctgtttgaagaacttgtgcga
gcctcaagtcactccacagacctcatggaagccatggccatgggcagcgt
ggaggcttcttataagtgtttagcagcagctttgatagttctgacggagt
ctggcaggtctgctcaccaggtggccagataccgcccacgtgcccccatc
attgctgtgacccggaatccccagacagctcgtcaggcccacctgtaccg
tggcatcttccctgtgctgtgcaaggacccagtccaggaggcctgggctg
aggacgtggacctccgggtgaactttgccatgaatgttggcaaggcccga
ggcttcttcaagaagggagatgtggtcattgtgctgaccggatggcgccc
tggctccggcttcaccaacaccatgcgtgttgttcctgtgccgtgatgga
ccccagagcccctcctccagcccctgtcccaccccttcccccagcccat
ccattaggccagcaacgcttgtagaactcactctgggctgtaacgtggca
ctggtaggttgggacaccagggaagaagatcaacgcctcactgaaacatg
gctgtgtttgcagcctgctctagtgggacagcccagagcctggctgccca
tcatgtggccccacccaatcaagggaagaaggaggaatgctggactggag
gcccctggagccagatggcaagagggtgacagcttcctttcctgtgtgta
ctctgtccagttcctttagaaaaaatggatgcccagaggactcccaaccc
tggcttggggtcaagaaacagccagcaagagttaggggccttagggcact
gggctgttgttccattgaagccgactctggccctggcccttacttgcttc
tctagctctctaggcctctccagtttgcacctgtccccaccctccactca
gctgtcctgcagcaaacactccaccctccaccttccattttcccccacta
ctgcagcacctccaggcctgttgctatagagcctacctgtatgtcaataa
acaacagctgaagcaccaaaaaaaaaaaaaaa
>NM_005574 3
gaattcgtccaaactgaggatcacaagtctccacattctgagtaggagga
tgagggtctgagttaggatttgggtcctgcagggcttgctaaggaatccc
ctgatggcctaggattccacgcagagcacatctggtgtgagagagctcgc

tgcaagggtgaaggctccgccctatcagatagacaaccaggccaccaaga
ggcccagccctccaaaccctggatttgcaacatcctcaaagaacagcaac
gggccttgagcagaattgagaaggaaatacccccacctgccctcagccgt
taagtgggctttgctattcacaagggcctctgggtgtcctggcagagagg
ggagatggcacaggcaccaggtgctagggtgccagggcctcccgagaagg
aacaggtgcaaagcaggcaattagcccagaaggtatccgtggggcaggca
gcctagatctgatgggggaagccaccaggattacatcatctgctgtaaca
actgctctgaaaagaagatatttttcaacctgaacttgcagtagctagtg
gagaggcaggaaaaaggaaatgaaaccagagacagagggaagctgagcga
aaatagaccttcccgagagaggaggaagcccggagagagacgcacggtcc
cctccccgccccctaggccgccgccccctctctgccctcggcggcgagcag
cgcgccgcgacccgggccgaaggtgcgaggggctccgggcggccgggcgg
gcgcacaccatccccgcgggcggcgcggagccggcgacagcgcgcgagag
ggaccgggcggtggcggcggcgggaccgggatggaagggagcgcggtgac
tgtccttgagcgcggaggggcgagctcgccggcggagcgccggagcaagc
ggaggcgcaggagcggcggcgacggcggcggcggcggcggcgcccgagca
cccgagggggtccgagccccggcagccggccagccccgcgccacaaaggg
agcgcccccgccgcccggcaccccgcctccctccccaatgtcctcggcca
tcgaaaggaagagcctggacccttcagaggaaccagtggatgaggtgctg
cagatccccccatccctgctgacatgcggcggctgccagcagaacattgg
ggaccgctacttcctgaaggccatcgaccagtactggcacgaggactgcc
tgagctgcgacctctgtggctgccggctgggtgaggtggggcggcgcctc
tactacaaactgggccggaagctctgccggagagactatctcaggctttt
tgggcaagacggtctctgcgcatcctgtgacaagcggattcgtgcctatg
agatgacaatgcgggtgaaagacaaagtgtatcacctggaatgtttcaaa
tgcgccgcctgtcagaagcatttctgtgtaggtgacagatacctcctcat
caactctgacatagtgtgcgaacaggacatctacgagtggactaagatca
atgggatgatataggcccgagtccccgggcatctttggggaggtgttcac
tgaagacgccgtctccatggcatcttcgtcttcactcttaggcactttgg
gggtttgagggtggggtaagggatttcttaggggatggtagacctttatt
gggtatcaagacatagcatccaagtggcataattcaggggctgacacttc
aaggtgacagaaggaccagcccttgagggagaacttatggccacagccca
tccatagtaactgacatgattagcagaagaaaggaacatttaggggcaag
caggcgctgtgctatcatgatggaatttcatatctacagatagagagttg
ttgtgtacagacttgttgtgactttgacgcttgcgaactagagatgtgca
attgatttcttttcttcctggctttttaactcccctgtttcaatcactgt
cctccacacaagggaaggacagaaaggagagtggccattcttttttctt
ggccccttcccaaggccttaagctttggacccaaggaaaactgcatgga
gacgcatttcggttgagaatggaaaccacaacttttaaccaaacaattat
ttaaagcaatgctgatgaatcactgtttttagacaccttcattttgaggg
gaggagttccacagattgtttctatacaaatataaatcttaaaaagttgt
tcaactattttattatcctagattatatcaaagtatttgtcgtgtgtaga
aaaaaaaacagctctgcaggcttaataaaaatgacagactgaaaaaaaaa
aaa
>NM_005539 3
gcgcgggccgctgtgaggcgcggcggcgagcgacgggcgcggggccgcgg
agcagcgagcgagcgagcgagcgcgaggccggagccccggccaggcccgg
ccgacccgccgagcccgcgatgcgccccggggccgcccccggcgcagct
gacgccccgcggccccgcgaagaccccggccggccggtcccggaggaagc

ggccgccgccgccgcccagcccagcgcccgcgccgcccgggcaccat
ggcggggaaggcggccgccccgggcaccgcggtgctgctggtcacggcca
acgtgggctcgctcttcgacgacccagaaaacctgcagaagaactggctt
cgggaattttaccaggtcgtgcacacacacaagccgcacttcatggcctt
gcactgtcaggagtttggagggaagaactacgaggcctccatgtcccacg
tggacaagttcgtcaaagaactattgtcgagtgatgcgatgaaagaatat
aacagggctcgagtctacctggatgaaaactacaaatcccaggagcactt
cacggcactaggaagcttttattttcttcatgagtccttaaaaaacatct
accagtttgactttaaagctaagaagtatagaaaggtcgctggcaaagag
atctactcggataccttagagagcacgcccatgctggagaaggagaagtt
tccgcaggactacttccccgagtgcaaatggtcaagaaaaggcttcatcc
ggacgaggtggtgcattgcagactgtgcctttgacttggtgaatatccat
cttttccatgatgcttccaatctggtcgcctgggaaacaagcccttccgt
gtactcgggaatccggcacaaggcactgggctacgtgctggacagaatca
ttgatcagcgattcgagaaggtttcctactttgtatttggtgatttcaac
ttccggctggattccaagtccgtcgtggagacgctctgcacaaaagccac
catgcagacggtccgggccgccgacaccaatgaagtggtgaagctcatat
ttcgtgagtcggacaacgaccggaaggttatgctccagttagaaaagaaa
ctcttcgactacttcaaccaggaggttttccgagacaacaacggcaccgc
gctcttggagtttgacaaggagttgtctgtctttaaggacagactgtatg
aactggacatctcgttccctcccagctacccgtacagtgaggacgcccgc
cagggtgagcagtacatgaacacccggtgcccagcctggtgtgaccgcat
cctcatgtccccgtctgccaaggagctggtgctgcggtcggagagcgagg
agaaggttgtcacctatgaccacattgggcccaacgtctgcatgggagac
cacaagcccgtgttcctggccttccgaatcatgcccggggcaggtaaacc
tcatgcccatgtgcacaagtgttgtgtcgtgcagtgacgtggtgggaaga
gatgccagcgccacgagaggacacttcgtgagcctccctgtagccgtgga
ccgaatacgcactcttgaaagctgcatcgagaacccgcccaagcgccacc
tgctagacggccagccccacacttcgcttcagcctccggaccattccgga
gcagcctcacatacctcactgtctcgtctgtctatgtgacattaagtaga
aatattggttttttttttttttttttaaataagtcacagtcctgttgtca
aaactctaatagacagcaaagagggtctgtaccgtagacttcacagtttt
cagtttttaatgattgccagtggaggggcttcttcagcacagagaccccc
cactgtgtccagggacccccctctgccaggtggaggtgtgtccaggggctg
gggaagccgagacgggcactccctctgccggccggcagcgtggccctgag
catggcaagggggtctgtctctgccgatgctccttccgcggcactgactc
tgcgccgtgtcacatggtttttgaatcacactgcagctgctttccatttt
tatatatatataaatatatatataaatatatactttttaaaaataatttata
aatcttaccaaaacttatgctaaatatactttccagtatgaacgcacagg
agagtcccatcagcaggcggcattggagtctaggagctcagctgtgtgtc
catcaacacacaaattcgtaaaaaacacacatggcctcgccatcgtgggt
aaaatcggccccacagcacgtctgcaccagcgggccgttactcccatgcc
gttcttctgtgtaatattaagaactgaatgtgaagtttatagctagcctg
ggtgtaccttttaagaattttgtaaaccgtttgtctgtcttttgttactg
ttttatggtgccaagtatcctacgttacaacaataatatcatgggagaaa
tagaaatagcctagtttgcttccaatagaaactgcttttaacatgggctg
tatataaaaatattaaagagaaacaaaactgtacatttcctcattgctcc
gctacagacaacccatgtcataaccttgttgcaaatatttttctcctata
gcagtaagtacagcattagaaggtgattagagagtctgttgatgaaacac

aaatgtatgttttattgattttactttagaacactacagagttcctggac
cgggtgaaggcattagctgggtgtttgtgtgggataaatactaccactgc
aagtgactgctgtccgctgcggaatctgttcttggtggaagcacaggtcc
gtgtcgctgctgtggttgccgctgtccgcggttcaacacggagtccgccc
cgcgggtttcagctgttggtcgttctgaggggcctttggaagtgaccggt
ctggttcctaagcaataaaattgaccgtggtgaaaata
>NM_002436 3
agctccgtccgcgccctcccggcgcaccgcctgcggggcggtgactggcc
cagccgcaccgcgtctcccgccttctccgcagccccgcaggccccgggcc
ctgtcattcccagcgctgccctgtcttgcgttccagtgttccagcttctg
cgagatgaccctcaaggcgagcgagggcgagagtgggggcagcatgcaca
cggcgctctccgacctctacctggagcatttgctgcagaagcgtagtcgg
ccagaggctgtatcgcatccattgaatactgtgaccgaggacatgtacac
caacgggtctcctgccccaggtagccctgcccaggtcaagggacaggagg
tgcggaaagtgcgactcatacagtttgagaaggtcacagaagagcccatg
ggaatcacgctgaagctgaatgaaaaacagtcctgtacggtggccagaat
tcttcatggtggcatgatccatagacaaggctcccttcacgtgggggatg
agatcctagaaatcaatggcacaaatgtgacaaatcattcagtggatcag
ctgcagaaggcgatgaaagaaaccaaaggaatgatctcattaaaagtaat
tcccaaccagcaaagccgtcttcctgcactacagatgttcatgagagcgc
agtttgactatgatcccaaaaaggacaatctgatcccttgcaaggaggcg
ggactgaagtttgctactggggacattatccagattatcaacaaggatga
cagcaattggtggcagggacgggtggaaggctcctccaaggagtcagcag
gattgatcccttcccctgagctgcaggaatggcgagtggcaagtatggct
cagtcagctcctagcgaagccccgagctgcagtccctttgggaagaagaa
gaagtacaaagacaaatatctggccaagcacagctcgatttttgatcagt
tggatgttgtttcctacgaggaagtcgttcggctccctgcattcaagagg
aagaccctggtgctgatcggagccagtggggtgggtcgcagccacattaa
gaatgccctgctcagccagaatccggagaagtttgtgtaccctgtcccat
atacaacacggccgccaaggaagagtgaggaagatgggaaggagtaccac
tttatctcaacggaggagatgacgaggaacatctctgccaatgagttctt
ggagtttggcagctaccaaggcaacatgtttggcaccaaatttgaaacag
tgcaccagatccataagcagaacaagattgccatccttgacattgagccc
cagaccctgaaaattgttcggacagcagaactttcgcctttcattgtgtt
cattgcacctactgaccagggcactcagacagaagccctgcagcagctgc
agaaggactctgaggccatccgcagccagtacgctcactactttgacctc
tcactggtcaataatggtgttgatgaaacccttaagaaattacaagaagc
cttcgaccaagcgtgcagttctccacagtgggtgcctgtctcctgggttt
actaagcttgtagaatgggggaacccactgtatgcccctctccagcattt
ggaattccacccgccttgctttaagacaaacagggctgctccaactagtt
ttgtgtcagcttccagctctctgcagctatcctaattcagccagtaaggt
tcagtcttcttgctcaggctcctgaagggttgattctcctgatagatggg
gccccactgatctggatttgaaaaggatttctagaaattgggggtaagaa
gtactaccaaaatgtaactgctaatcaagggtgatgcacagcaaaagcaa
tggaccccatccctctaaagcctgccctcctttgccttcaactgtatatg
ctgggtatttcatttgtctttttattttggagaaagcgtttttaactgca
actttctataatgccaaaatgacacatctgtgcaatagaatgatgtctgc
tctagggaaaccttcaaaagcaataaaaatgctgtgttgaaatgccaaaa
aaaaaaaaaaaaaaaa

>NM_003763 5
acggaaacggtccggggctgagctgcagtcagatccggggatggagggag
ccggagaagaggagtgagaaggccggggaggcaggccctgctgtcttcct
gcaggccggcagcctgctggcagtggaggaccaggccgtccaggaaagga
aggggtcaggaggtcgggcagggcctcagcaggttcagacaagtccccag
aagggagctgcaggcgccctcgagtccaggccccaagcctggctccggtt
gtccaaagagggcctaggccaggcctctggtgcggaaactgagtcacagc
cagacctcagggcacggcctagacgcttacgagccaaagttaggggtaga
gagcagagacctccggggggtctcagggagtagggggcttcagggcctcc
cagtctagagccggattggcgagttagacgcttgtagatccaaggttgga
ttggggtggggtctctgggacgttggatcgctacgcaaggattgggggga
ttcaagtgcttagagatcgaagtctgccctgggtagggggagtcagacaa
ttggaaagcctaggtagttatttggggaggggtctctacgccttggcgaa
gcgcacagctgcatctttttggcttgaggcctgaggccttgtcgagaagc
ttccgtgaaagggtgggccagccgggccacgagaaagaaagtgaataaat
caggaatataagtgggcggggggcccctgagaggggggtcgcaaagggtg
agacatggccaccaggcgtttaaccgacgctttcttgttgttgcggaata
attccatccaaaaccggcagctgttagccgagcaacttgctgatgaccgt
atggcactggtgtcaggcatcagcttagatccagaagcagcgattggtgt
gacaaaacggccacctcctaagtgggtggatggagtggatgaaattcagt
atgatgttggccggattaagcagaagatgaaagaattggccagccttcat
gacaagcatttaaacagacccaccctggatgacagcagcgaagaggaaca
tgccattgagataactacccaagagatcactcagctcttccacaggtgcc
agcgtgccgtgcaggccctgccgagccgggcccgggcctgctccgagcag
gaggggcggctgcttgggaacgtggtggcctcgctggcgcaggccctgca
ggaactctccaccagcttccggcacgcacagtcaggctacctcaaacgca
tgaagaatcgagaggaaagatcccagcatttttcgacacatcagtacca
ctaatggatgatggagacgataacactctttaccatcggggttttacaga
ggaccagttagttctggtggagcagaacacactgatggtggaagagcggg
aacgagagattcgccagattgtacagtccatttctgacctgaatgaaata
ttcagggacttaggggcgatgattgtagaacagggtacagtccttgacag
aattgactataacgttgaacagtcctgtatcaaaactgaagatggtttga
aacagcttcacaaggcagaacagtatcaaaagaagaatcggaagatgctt
gtgattttaatattatttgtcatcatcattgtgctcattgttgtcctcgt
tggcgtgaagtctcgataagtggcattgggttttcgtgtgtgccgcgcgt
gtggatctcccgggtgtgaggggcttggcctgcgccccgccagctgcccg
cagaggtgcagcctcgaggaatctgagggcgtcggggcagcgaacctttg
catccacggactcctccttccctagtcctgctcaagcggtccggggaatg
ggtttttgtttttccttcattgttgagaatttaaggacctttgatactgc
tgcacaatagagattgagttctgggatcagttataatatatatttttttt
agaaagagaaaatgagctgagagtttacaggcactgcagaagctgttcag
tattatatgtgtatatgctattttttttagcagccatgtctgagcagaatg
ttaacataatttttggaaaaaattttctttaaaccatctggtttttaagaa
atttggtaccaaaaattgatgagtagaggcaaaaatgccccttcatcttt
ctctatatattttccagttgaaaacaaactaagaagtcctttaagaattt
ataatccgttccccattaacttaatttagcttttccatcactgttaatga
tcagagtaacaaagtgtgaaaaataagaaaccatcattgatgttaattaa
cacatttagatgggccagttaaaacagcttcataagttttaaattaattg
taaatggaatattcctcatttaaaatttttgcactgcattttctactgta

aaccaaaagaggttactaagcaaaaccacctaaatttaagttggtgattt
aaatgaatctcacattaaaagaaagcttgacagtgttatgaaagccacca
gactcagccagtgtgtccccatgggtatccccagccatccttgctcaatc
cattacttatatactaactacataatgacctgttcaaaccagactctatt
taatgaactgtgaatttacacagaggccattttaaatgggtcaccccatt
taggattagtggatctcaaattattaaccaaacatcactccatttcaaag
taaaatattccaccagcgatttgatttattggctcttccattgccactga
gcaatgcccaggaagcaggcacattgccaaggactgggggcatcttgact
aggaagctcctcgtttgtgtgagcgtgggtcagaccgccaaagtaggact
tcatcgtttacctacctattatcaatatggtgcttgaatatattcctata
actgtagaacagtgtggaaagtgatggtaactttgaagttgttcatgttt
tattggctttgttttaattgacactagtagagttttaatggtctttgtgt
aaattttaatggcttttccattgtttttgcttctcttaaaaagtttaaga
agaatatgacctcattaaatgtgctgttttatttggaccagtcacacaaa
atgtctctctagagttgactttaaagttgtttacagaaatttaaactcaa
ttccagagattgaagttgtccaaacagctcatgggcttagtgtccaaacc
cctgcccagccttccctttccaagttggtgccacctccaggtagccattg
gtggttttcctattactgatgtggctgtggaatgataaggtcctagaggg
gccctggcatttcaggaagcggacctggtattccctgacaaacattgctt
aggaaagagggccggagaataagaagccagcacctcatcagtcagccctg
caatgtgagacttccgtagctgttgagagtcactgcaggtgttctttcat
cccatctgattttaacccatggttgagacgttccaagttagatctgctgc
tcttaggtgtggggctgtccacattagggaacttgcctcaaaaacagtcc
acagtggcaataccggacttctgttcaagctttttaaagtgctgagcctt
acaaacccctgaaggaaggtgaccctttaagagaggatcatcttttttta
tatttattttcaccaaatagggtgatgagggttggtttttgttccctcaa
cccatttgggtaaaataacggatctgattagaaactgttttaaggagggg
ctgggctcggtggctcacgcctgtaatcccagactttgagaggccaaggc
ggtcacgaggtcaggagttcgagaccagcctagccaacatggtgaaaccc
cgtctctacaaaaaatacaaaaattagccgggtgggacacgcctgtaatc
ccagctactcaagaggctgaggcaggagaatagcttgaacccgggaggca
gaggttgcagtgagccgagatcacaccactgcactccagcctgggtgaca
gagcgaggctccgtctcaaaaaaaaaaaaaaaaaaattgttttagcgctgg
gtttcccaaggtgggagagacagacccagcctggagctggcccctggcct
gtgtgctgacttcttggggtcctcaaaccactgtatttttctgttgagcc
tgtacttggggagagatcagtagcatttgaggaagtaagagaaaagaatc
atggtacctcagggtttctttccctttactcgctggcagccattgtctgt
gggcacctcatgtttttccacactctactgggccgtggaggtaacgatca
cccaggccagtctcctctgcctgggatgcgccctctgagaggaggcctag
cagggcaggctccctctgggcatccctggatgcagcctctggacacatgc
ctcctttaaagtgtccgggtgcagctcaggttgagtggaggtagaaggag
aaacagacatgtttaccacgcgttttccaaagctcctgatctttcccaag
attgtaactgaaaactgctgtctcttgttttgttcgttttgggggtggtg
gtgctggctgggccatgcttgtgaagtgatgtgtgtctctgatttaacgg
attcactgttttctctgctaattgagagagcgttatttacattatttatt
tgttttgacacaagtgctttcagtgttttatcctagctaatggcttctta
aaggtaataaaacccttccaacgtaattggtcagataaaacttttttttct
tgtatgcttaaataaagcaattagtgaagcacttctatccaaaatgactt
ttttgtcctttttaaaaccaatttactgttactggaaactttttgtaca

ataaagcaatcacgcagattaaagaacatcctgcaatcctagtattaatt
ttaaaaaaaaaaaaa
>NM_001001433 2
acggaaacggtccggggctgagctgcagtcagatccggggatggagggag
ccggagaagaggagtgagaaggccggggaggcaggccctgctgtcttcct
gcaggccggcagcctgctggcagtggaggaccaggccgtccaggaaagga
aggggtcaggaggtcgggcagggcctcagcaggttcagacaagtccccag
aagggagctgcaggcgccctcgagtccaggccccaagcctggctccggtt
gtccaaagagggcctaggccaggcctctggtgcggaaactgagtcacagc
cagacctcagggcacggcctagacgcttacgagccaaagttaggggtaga
gagcagagacctccggggggtctcagggagtaggggggcttcagggcctcc
cagtctagagccggattggcgagttagacgcttgtagatccaaggttgga
ttggggtggggtctctgggacgttggatcgctacgcaaggattgggggga
ttcaagtgcttagagatcgaagtctgccctgggtagggggagtcagacaa
ttggaaagcctaggtagttatttggggaggggtctctacgccttggcgaa
gcgcacagctgcatctttttggcttgaggcctgaggccttgtcgagaagc
ttccgtgaaagggtgggccagccgggccacgagaaagaaagtgaataaat
caggaatataagtgggcggggggcccctgagagggggggtcgcaaagggtg
agacatggccaccaggcgtttaaccgacgctttcttgttgttgcggaata
attccatccaaaaccggcagctgttagccgagcaagtgagtagtcacatc
acctccagccctctgcattcacgtagcattgctgcggagctggacgagct
tgctgatgaccgtatggcactggtgtcaggcatcagcttagatccagaag
cagcgattggtgtgacaaaacggccacctcctaagtgggtggatggagtg
gatgaaattcagtatgatgttggccggattaagcagaagatgaaagaatt
ggccagccttcatgacaagcatttaaacagacccaccctggatgacagca
gcgaagaggaacatgccattgagataactacccaagagatcactcagctc
ttccacaggtgccagcgtgccgtgcaggccctgccgagccgggcccgggc
ctgctccgagcaggaggggcggctgcttgggaacgtggtggcctcgctgg
cgcaggccctgcaggaactctccaccagcttccggcacgcacagtcaggc
tacctcaaacgcatgaagaatcgagaggaaagatcccagcattttttcga
cacatcagtaccactaatggatgatggagacgataacactctttaccatc
ggggttttacagaggaccagttagttctggtggagcagaacacactgatg
gtggaagagcgggaacgagagattcgccagattgtacagtccatttctga
cctgaatgaaatattcagggacttaggggcgatgattgtagaacagggta
cagtccttgacagaattgactataacgttgaacagtcctgtatcaaaact
gaagatggtttgaaacagcttcacaaggcagaacagtatcaaaagaagaa
tcggaagatgcttgtgattttaatattatttgtcatcatcattgtgctca
ttgttgtcctcgttggcgtgaagtctcgataagtggcattgggttttcgt
gtgtgccgcgcgtgtggatctcccgggtgtgaggggcttggcctgcgccc
cgccagctgcccgcagaggtgcagcctcgaggaatctgagggcgtcgggg
cagcgaacctttgcatccacggactcctccttccctagtcctgctcaagc
ggtccggggaatgggtttttgtttttccttcattgttgagaatttaagga
cctttgatactgctgcacaatagagattgagttctgggatcagttataat
atatattttttttagaaagagaaaatgagctgagagtttacaggcactgc
agaagctgttcagtattatatgtgtatatgctatttttttagcagccatg
tctgagcagaatgttaacataatttttggaaaaaattttctttaaaccatc
tggtttttaagaaatttggtaccaaaaattgatgagtagaggcaaaaatg
ccccttcatctttctctatatattttccagttgaaaacaaactaagaagt
cctttaagaatttataatccgttccccattaacttaatttagcttttcca

tcactgttaatgatcagagtaacaaagtgtgaaaaataagaaaccatcat
tgatgttaattaacacatttagatgggccagttaaaacagcttcataagt
tttaaattaattgtaaatggaatattcctcatttaaaatttttgcactgc
attttctactgtaaaccaaaagaggttactaagcaaaaccacctaaattt
aagttggtgatttaaatgaatctcacattaaaagaaagcttgacagtgtt
atgaaagccaccagactcagccagtgtgtccccatgggtatccccagcca
tccttgctcaatccattacttatatactaactacataatgacctgttcaa
accagactctatttaatgaactgtgaatttacacagaggccattttaaat
gggtcaccccatttaggattagtggatctcaaattattaaccaaacatca
ctccatttcaaagtaaaatattccaccagcgatttgatttattggctctt
ccattgccactgagcaatgcccaggaagcaggcacattgccaaggactgg
gggcatcttgactaggaagctcctcgtttgtgtgagcgtgggtcagaccg
ccaaagtaggacttcatcgtttacctacctattatcaatatggtgcttga
atatattcctataactgtagaacagtgtggaaagtgatggtaactttgaa
gttgttcatgttttattggctttgttttaattgacactagtagagtttta
atggtctttgtgtaaattttaatggcttttccattgtttttgcttctctt
aaaaagtttaagaagaatatgacctcattaaatgtgctgttttatttgga
ccagtcacacaaaatgtctctctagagttgactttaaagttgtttacaga
aatttaaactcaattccagagattgaagttgtccaaacagctcatgggct
tagtgtccaaacccctgcccagccttccctttccaagttggtgccacctc
caggtagccattggtggttttcctattactgatgtggctgtggaatgata
aggtcctagaggggccctggcatttcaggaagcggacctggtattccctg
acaaacattgcttaggaaagagggccggagaataagaagccagcacctca
tcagtcagccctgcaatgtgagacttccgtagctgttgagagtcactgca
ggtgttctttcatcccatctgattttaacccatggttgagacgttccaag
ttagatctgctgctcttaggtgtggggctgtccacattagggaacttgcc
tcaaaaacagtccacagtggcaataccggacttctgttcaagctttttaa
agtgctgagccttacaaacccctgaaggaaggtgaccctttaagagagga
tcatctttttttatatttattttcaccaaatagggtgatgagggttggtt
tttgttccctcaacccatttgggtaaaataacggatctgattagaaactg
ttttaaggaggggctgggctcggtggctcacgcctgtaatcccagacttt
gagaggccaaggcggtcacgaggtcaggagttcgagaccagcctagccaa
catggtgaaaccccgtctctacaaaaaatacaaaaattagccgggtggga
cacgcctgtaatcccagctactcaagaggctgaggcaggagaatagcttg
aacccgggaggcagaggttgcagtgagccgagatcacaccactgcactcc
agcctgggtgacagagcgaggctccgtctcaaaaaaaaaaaaaaaaaatt
gttttagcgctgggtttcccaaggtgggagagacagacccagcctggagc
tggcccctggcctgtgtgctgacttcttggggtcctcaaaccactgtatt
tttctgttgagcctgtacttggggagagatcagtagcatttgaggaagta
agagaaaagaatcatggtacctcagggtttctttccctttactcgctggc
agccattgtctgtgggcacctcatgtttttccacactctactgggccgtg
gaggtaacgatcacccaggccagtctcctctgcctgggatgcgccctctg
agaggaggcctagcagggcaggctccctctgggcatccctggatgcagcc
tctggacacatgcctcctttaaagtgtccgggtgcagctcaggttgagtg
gaggtagaaggagaaacagacatgtttaccacgcgttttccaaagctcct
gatctttcccaagattgtaactgaaaactgctgtctcttgttttgttcgt
tttgggggtggtggtgctggctgggccatgcttgtgaagtgatgtgtgtc
tctgatttaacggattcactgttttctctgctaattgagagagcgttatt
tacattatttatttgttttgacacaagtgctttcagtgttttatcctagc

taatggcttcttaaaggtaataaaacccttccaacgtaattggtcagata
aaactttttttcttgtatgcttaaataaagcaattagtgaagcacttcta
tccaaaatgactttttgtcctttttaaaaccaatttactgttactgga
aactttttgtacaataaagcaatcacgcagattaaagaacatcctgcaat
cctagtattaattttaaaaaaaaaaaa
>NM_024663 3
ggggcggtgccgaggccgggccggagcggggcgaagggggccgagcggcg
ggccgggccgggccgggcagggccggggcgtgggccggcaggaagatggc
gaacgtggggctgcagttccaggcgagcgcgggggactcggacccacaga
gccggcccctgctgctgctcgggcagctgcaccacctgcaccgcgtgccc
tggagccacgtccgcgggaagctgcagccccgggtcaccgaggagctctg
gcaggctgccctgagcacgctcaaccccaaccccacggacagctgtcccc
tctacctgaactacgccaccgtggctgccctgccctgcagggtgagccgg
cacaacagcccctcggccgcccacttcatcacgcggctggtgcggacctg
cctgccgcccggagcgcatcgctgcattgtgatggtctgcgagcagccgg
aggtctttgcttccgcctgtgccctggcccgggccttcccgctgttcacc
caccgctcaggtgcctctcggcgcttggagaagaagacggtcaccgtgga
gtttttcctggtgggacaagacaacgggccggtggaggtgtccacattgc
agtgcttagcgaatgccacagacggcgtgcggctagcagcccgcatcgtg
gacacaccctgcaatgagatgaacaccgacaccttcctcgaggagattaa
caaagttggaaaggagctggggatcatcccaaccatcatccgggatgagg
aactgaagacgagaggatttggaggaatctatggggttggcaaagccgcc
ctgcatcccccagccctggccgtcctcagccacaccccagatggagccac
gcagaccatcgcctgggtgggcaaaggcatcgtctatgacactggaggcc
tcagcatcaaagggaagactaccatgccggggatgaagcgagactgcggg
ggtgctgcggccgtcctgggggccttcagagccgcaatcaagcagggttt
caaagacaacctccacgctgtgttctgcttggctgagaactcggtggggc
ccaatgcgacaaggccagatgacatccacctgctgtactcagggaagacg
gtggaaatcaacaacacggatgccgagggcaggctggtgctggcagatgg
cgtgtcctatgcttgcaaggacctgggggccgacatcatcctggacatgg
ccaccctgaccggggctcagggcattgccacagggaagtaccacgccgcg
gtgctcaccaacagcgctgagtgggaggccgcctgtgtgaaggcgggcag
gaagtgtggggacctggtgcacccgctggtctactgccccgagctgcact
tcagcgagttcacctcagctgtggcggacatgaagaactcagtggcggac
cgagacaacagccccagctcctgtgctggcctcttcatcgcctcacacat
cggcttcgactggcccggagtctgggtccacctggacattgctgcaccgg
tgcatgctggtgagcgagccacaggcttcggtgtggccctcctgctggcg
ctcttcggccgtgcctctgaggaccctctgctgaacctggtgtccccact
gggctgtgaggtggatgtcgaggaggggggacctggggagggactccaaga
gacgcaggcttgtgtgagcctcctgcctcggccctgacaaacggggatct
tttacctcactttgcactgattaattttaagcaattgaaagattgccctt
catatgggttttggtttgtctttctggtcgtcagcgtggtggtggaaaca
gctgaagttttaggagacagcttagggtttggtgcgggccacggggaggg
gaccgggaagcgctggggcttgtttctgtttgttacttacaggactgaga
catcttctgtaaactgctaccccctggggccttctgcaccccggggtgagg
cctcctgcctgcctggtgccctgtcccagccccaggtcctgtgcagggca
cctgcgtggctgacagccaggctcttactccagccggggctgccagcgca
tccagccagcccagccctgtgaaagatggagctgacttgctgcaggggac
ctgatttatagggcaagagaagtcacactccggcctctcagaattcactt

gaggttcaattaaatacagtcacaccgccccctcaaaaaaaaaaaaaaa
aaaaaaaaaaaaaaaaa
>NM_014110 4
gtacgcacaggctgctgggatgccgcttttcccttctcggtcttccagtt
tcccggcgtgcttagggcgcgccaaatgggagggggagacgcaagatggc
ggcagccgcgaactccggctctagcctcccgctgttcgactgcccaacct
gggcaggtaagccccctcccggtttacatctggatgtagtcaaaggagac
aaactaattgagaaactgattattgatgagaagaagtattacttatttgg
gagaaaccctgatttgtgtgactttaccattgaccaccagtcttgctctc
gggtccatgctgcacttgtctaccacaagcatctgaagagagttttcctg
atagatctcaacagtacacacggcactttcttgggtcacattcggttgga
acctcacaagcctcagcaaattcccatcgattccacggtctcatttggcg
catccacaagggcatacactctgcgcgagaagcctcagacattgccatcg
gctgtgaaaggagatgagaagatgggtggagaggatgatgaactcaaggg
cttactggggcttccagaggaggaaactgagcttgataacctgacagagt
tcaacactgcccacaacaagcggatttctacccttaccattgaggaggga
aatctggacattcaaagaccaaagaggaagaggaagaactcacgggtgac
attcagtgaggatgatgagatcatcaacccagaggatgtggatccctcag
ttggtcgattcaggaacatggtgcaaactgcagtggtcccagtcaagaag
aagcgtgtggagggccctggctccctgggcctggaggaatcagggagcag
gcgcatgcagaactttgccttcagcggaggactctacgggggcctgcccc
ccacacacagtgaagcaggctcccagccacatggcatccatgggacagca
ctcatcggtggcttgcccatgccatacccaaaccttgcccctgatgtgga
cttgactcctgttgtgccgtcagcagtgaacatgaaccctgcaccaaacc
ctgcagtctataaccctgaagctgtaaatgaacccaagaagaagaaatat
gcaaaagaggcttggccaggcaagaagcccacaccttccttgctgatttg
atatttttggtcatggagaagggtgggattgggtgggaatggggtggaag
ggtgatggggagctaatgaactagggagaaaaactttccatgtgtgcggt
atcgtctttcagaatgtctcctggcatcctaaccatgtaatatgacaatt
gggggtggggttgaaatagcccataaagacctgtcttcacaacacttgca
ttgtagagaaaggcttcttatatccttttcaatagactgccctggctctt
tcctaggccttccactacctcctttctttctcccactttctaggatcatt
tttatgtaaagtcacatatcccaggccctcaggttgaatccagagctgta
gaggttacagtagcatcaccagccttgggggtccagagcctaatttatat
tcactatccttccaagtcccgggtagcagaagggttgccatagatctcag
tttgatcaaaaagaaggcttagaattctgcagttaagctgaggtttaaac
taaaaaatgtttccttgggtcagtggttttgaggtccagtagctaggctt
ttctcttttgtccttcctgttggaatgaaaacatttcgattttccttcat
ctgtgactggtgccatagacacaggtttatagttttaacttacagtattg
tttgaaatttacctgtttttcttgtcaaacctgagcactcctcctgctga
agtttcttatttaattccagagtactgtcctctactctaaggcattactt
ttaagtgtattatgaaggcagttttcaaaggatatgaccagttggggtaa
ttcaaattaaaaaggaaaagatttgtttggaagtaactggtgtctctaag
aggaatttttagatgtcagtttggaggctctttccccccctcaattgagag
ctcttgttattcagagctccaagactagacctggctaacaaacataggag
acaaagttaggaaacattgatacaagctttgtacagagatttgtacattt
gtgtaataggccttttcatgctttatgtgtagctttttacctgtaacctt
tattacattgtaaattaaacgtaacttttgtcatttgggtgcaggctgtg
aatttgtctctcagtcactgattgccactgccatctggaaatgtttgcta

aaggcacagtcactgggcttgggaggcaatgctccatccccattatatta
caaataaagatgccctaaatgagtgtg
>NM_003592 2
aggaggagggccgggcgggcaggggaggaggaggaggcgggcgccgtgtc
gcacgcagctccaggcggggcagccccggtagctgagggacgcagctaga
ccttggcgggacggggctttcgccggggcccaggcccagggaccaggcgg
aggcgtcgcgggagcctttggggcaccacagagatgcgggtttgcctgca
atgagatttcattctctacatttaaaggacatcctttctgagctgctgtg
aataaatttggaatggtactgtatattttcatctaatggagaactagctg
tactttgaataaggattgctgcactggacgactttagaacatccctcaca
atgtcgtcaacccggagccagaacccccacggcctgaagcagattggcct
ggaccagatctgggacgacctcagagccggcatccagcaggtgtacacac
ggcagagcatggccaagtccagatatatggagctctacactcatgtttat
aactactgtactagtgttcaccagtcaaaccaagcacgaggagctggagt
tcctccttctaagtcgaaaaaggggcagacacctggaggagctcagtttg
ttggcctggaattatataaacgacttaaggaatttttgaagaattacttg
acaaatcttcttaaggatggagaagatttgatggatgagagtgtactgaa
attctacactcaacaatgggaagattatcgattttcaagcaaagtgctga
atggaatttgtgcctacctcaatagacattgggttcgccgtgaatgtgac
gaaggacgaaaaggaatatatgaaatctattcgcttgcattggtgacttg
gagagactgtctgttcaggccactgaataaacaggtaacaaatgctgttt
taaagctgattgaaaaggaaaggaatggtgaaaccatcaatacaagattg
attagtggagttgtacagtcttacgtggaattggggctgaatgaagatga
tgcatttgcaaagggccctacgttaacagtgtataaagaatcctttgaat
ctcaatttttggctgacacagagagattttataccagagagagtactgaa
ttcttgcagcagaacccagttactgaatatatgaaaaaggcagaggctcg
tctgcttgaggaacaacgaagagttcaggtttaccttcatgaaagcacac
aagatgaattagcaaggaaatgtgaacaagtcctcattgaaaaacacttg
gaaattttccacacagaatttcagaatttattggatgctgacaaaaatga
agatttgggacgcatgtataatcttgtatctagaatccaggatggcctag
gagaattgaaaaaactgttggagacacacattcataatcagggtcttgca
gccattgaaaagtgtggagaagctgctttaaatgaccccaaaatgtatgt
acagacagtgcttgatgttcataaaaaatacaatgccctggtaatgtctg
cattcaacaatgacgctggctttgtggctgctcttgataaggcttgtggt
cgcttcataaacaacaacgcggttaccaagatggcccaatcatccagtaa
atcccctgagttgctggctcgatactgtgactccttgttgaagaaaagtt
ccaagaacccagaggaggcagaactagaagacacactcaatcaagtgatg
gttgtcttcaagtacatagaagacaaagacgtatttcagaagttctatgc
gaagatgctcgccaagaggctcgtccaccagaacagtgcaagtgacgatg
ccgaagccagcatgatctccaagttaaagcaagcttgcgggttcgagtac
acctctaaacttcagcgcatgtttcaagacattggcgtgagcaaagatct
gaacgagcaattcaaaaagcacttgacaaactcagaacccctagacttgg
atttcagcattcaagtgctgagctccgggtcctggcccttccagcagtct
tgtacatttgccttgccgtcagagttggaacgtagttatcagcgattcac
agctttctacgccagccgccacagtggccgaaaattgacgtggttatatc
agttgtctaaaggagaattggtaactaactgcttcaaaaacagatatact
ttgcaggcgtcgacattccagatggctatcctgcttcagtacaacacgga
agatgcctacactgtgcagcagctgaccgacagcactcaaattaaaatgg
acattttggcgcaagttttacagattttattaaagtcgaagctattggtc

ttggaagatgaaaatgcaaatgttgatgaggtggaattgaagccagatac
cttaataaaattatatcttggttataaaaataagaaattaagggttaaca
tcaatgtgccaatgaaaaccgaacagaagcaggaacaagaaaccacacac
aaaaacatcgaggaagaccgcaaactactgattcaggcggccatcgtgag
aatcatgaagatgaggaaggttctgaaacaccagcagttacttggcgagg
tcctcactcagctgtcctccaggttcaaacctcgagtccctgtgatcaag
aaatgcattgacattctaattgagaaagaatatttggagcgagtggatgg
tgaaaaggacacctacagttacttggcttaacccttctggaagggtctga
ctgtgtgacccgcagcaaatagttcatgttggaaagaatgaaaacaactc
aagttcatagcagccagcctgccgccattggacctcccttttaaaaactg
agaccaagactcccatcagctggtctcggatttacatcggaactgctcag
gattgatacatttcaagtctgtaaatacggacaccaacgccatttaccct
aatttaagaacagcggggactgaccctccgtgccgagggctgcatgctac
cgcactaagtcaatacatgggctccccgattcgcagctgtcgtcttggca
gcacttgtcacgttggcagcactttgagagcaagtctgagtggacccaca
tgtaacctgctatgaaaaccatttgtatagtgtgtttcattttttaatgt
gtgaaaataaagaaaattaaaggatttctgtacaagtcgcattgggtttt
gttttaagttttactaatttctatatgtaaataaaagatataatgattgt
gcaaatttaaaaaaaaaaaaaaaaaa
>NM_003325 3
gatgcggctgtggtggcggcggcggcggccgagcgcgggtggcggctgtg
gcggcggaggggggcgcgggccggcgatggcgcggcggccctgagggcgc
ggggcgggcggcggccggagggcgggtggcgcgggaggaagcggcggcgg
tggctccatggcccgggcgcgctgagggacccggcgctcgcctcagcccg
gcggcggcggcggccgaacaatgaagctcctgaagccgacctgggtcaac
cacaatggcaagccgatttttcagttgatattcaccctgacgggaccaa
gttcgcaactggaggacaagggcaggattctgggaaggttgtgatctgga
atatgtctccagtcctccaggaggatgacgagaaggatgaaaatattccc
aagatgctttgccagatggacaatcacttagcatgtgtgaactgtgtgcg
gtggtcaaacagtgggatgtatttagcttctgggggagatgacaaactga
ttatggtgtggaagcgggctacgtacatcggccccagcaccgtgttcggc
tccagtggtaagcttgccaatgtggagcagtggcggtgtgtctctatcct
ccggaatcattcaggcgatgtgatggatgtagcatggtctccccacgatg
cctggctagcctcatgcagcgtggataacactgtcgtcatctggaatgct
gtaaagttcccagaaattctagctactctgagaggtcattctggcttggt
caaagggttgacatgggaccctgttggtaaatacatagcttctcaagctg
atgaccgcagcctaaaggtgtggaggacgctggactggcagttggagacc
agcatcaccaagccttttgatgagtgtggaggaacgacccatgtgttgcg
gctcagctggtcacctgatgggcattacctggtgtctgcccatgccatga
acaactcaggccccactgcccagatcatcgaacgggagggatggaagacc
aacatggactttgttgggcaccggaaagctgtgactgtcgtgaaattcaa
cccaaaaatcttcaaaaagaagcagaagaatgggagttctgcgaagccta
gctgcccgtactgctgctgtgctgttggcagcaaggaccgctcgctttct
gtctggctcacatgtctgaaacggccgctggtggtcatccatgaactgtt
tgacaaatccatcatggatatttcctggactctgaatgggctgggcatct
tggtatgctctatggacggctctgtggcattcctcgacttctcccaggat
gagcttggcgatcccctgagcgaggaggagaagagccgcattcaccagtc
cacctatggcaagagcctagccatcatgaccgaggcccagctctccacag
ccgtcattgagaaccctgagatgctcaagtaccagcgaaggcagcagcag

cagcagctggaccagaagagtgctgcgaccagggagatgggctcagccac
ctcagtcgcaggcgttgtcaacggggagagtcttgaagatatcaggaaga
atcttttgaagaaacaagttgagactcggacagcagatggccggagaaga
atcacgcctctctgcatagcacagctggacactggggacttctccacggc
attctttaacagcatccccctctcgggctccctggcgggcaccatgctct
cttctcatagcagtccacagctactgccactggactccagtacccctaac
tccttcggcgcctcgaagccttgcacagagcctgtggtggctgccagtgc
cagacctgcaggcgattctgtcaataaagacagtatgaatgctacctcta
ctcctgctgcattgtcaccttctgtgttaacgaccccgtccaagatcgaa
cccatgaaagcgtttgactcccggttcacagagcggtccaaagccacacc
aggtgctcctgccctgaccagcatgactccgacagctgtggaaaggttaa
aagagcagaaccttgtgaaagagctgaggccccgagacctcctggagagc
agcagtgacagcgatgagaaagtccctttggctaaggcttcctcactgtc
caagcgaaaacttgagcttgaggtagagacagtagagaagaagaagaaag
ggcggcctcggaaggactctcgtctcatgcctgtgtctctgtctgtccag
tctccagctgccctaaccgcagagaaggaggccatgtgtctgtctgcacc
agcacttgcactgaagctgccaattccaagcccccagagagcattcaccc
tccaggtcagctccgatccttccatgtacattgaggtggagaatgaagtg
acagtggtggggggcgtgaagctgagccgcctgaagtgcaaccgggaagg
gaaggagtgggagacggtactcaccagccggatcctcactgctgcgggca
gctgtgacgtggtgtgtgtcgcctgtgaaaaaaggatgctgtcagtgttc
tccacctgtggtcgccgtctcctctctcccatcctcctgccatccccgat
ctctactttgcattgcacaggctcctacgtcatggcgctcaccgctgcag
ccacactctctgtctgggatgttcacagacaggtggttgtggtgaaagaa
gagtctctacactccatcctggcaggaagtgatatgacggtatcacagat
cttgctgacgcagcatggaatcccagtaatgaacctgtccgatgggaagg
cgtactgctttaatccgtcactttccacatggaacctggtttctgacaag
caggactcactggctcagtgtgcagactttaggagcagcctgccatccca
ggacgccatgctgtgctcaggaccgttagccataatccagggccgcacct
ccaactcgggaaggcaggctgcccggctcttctccgtgcctcatgtggtg
cagcaagagaccaccctggcctacctagagaaccaggtggcagcagcact
caccctgcagtccagccacgagtaccgccattggctcctcgtctacgcac
ggtacctcgtaaacgaagggtttgaataccgacttcgagaaatatgcaag
gacttactgggtccggttcactactccactggaagccagtgggagtcaac
agtagtgggtctgcggaagagggagctgctgaaggagctgctaccagtca
tcgggcagaacctccgattccagcgcctcttcaccgagtgtcaggaacag
ctcgacatcctgagggacaagtagcctgccccagcctgccctggctgcag
caagggcagggccacactctcgccgctgatgacatgcaggaccgcctctc
acctgaccaggctgtagggggaggagacactggcaggagatgtgctgtcc
tgcaccagcgccagcccagctccctgggcagatgtgccctgtgtcctggg
tctgacattgcctcaggagggggaagctcatccctccctccaagcccccg
atgcggagctagggctggagctctgcccaggtgccctggggccagcaagg
cagtcccaggcctgccgtctccagcacggccccaaggtggacactagccc
ctgctgctgcaggcaccatgctgcttcagcagtgacgattgagccatttg
tgagagagaatccggaagcgttaggtattacagcagactgacctaagacc
tatgtaaaaggaaagctgttccaacacacggactatttttgtactagaat
ttgctaacttgtaatattgaatttcctttggccgataatcaggatttccc
tataagtcacttggacattggtcacttgtaggaaatttaaactctaatta
tgacagctacactgaaaaataattgtactgaaattaacttgtctatcttc

atttggttttaatttttaaatgtttgtaaaaagagactgttttgggggaa
tggggcaaaggggtgggcgatttcttttgtaagtgtaaaataaatgaaca
cgcattgaatacc
>NM_002109 3
tcgatagccggaagtcatccttgctgaggctggggcaaccaccgcaggtc
gagacagcaggcggctcaagtggacagccgggatggcagagcgtgcggcg
ctggaggagctggtgaaacttcagggagagcgcgtgcgaggcctcaagca
gcagaaggccagcgccgagctgatcgaggaggaggtggcgaaactcctga
aactgaaggcacagctgggtcctgatgaaagcaaacagaaatttgtgctc
aaaacccccaagggcacaagagactatagtccccggcagatggcagttcg
cgagaaggtgtttgacgtaatcatccgttgcttcaagcgccacggtgcag
aagtcattgatacacctgtatttgaactaaaggaaacactgatgggaaag
tatggggaagactccaagcttatctatgacctgaaggaccagggcgggga
gctcctgtcccttcgctatgacctcactgttcctttttgctcggtatttgg
caatgaataaactgaccaacattaaacgctaccacatagcaaaggtatat
cggcgggataacccagccatgacccgtggccgataccgggaattctacca
gtgtgattttgacattgctgggaactttgatcccatgatccctgatgcag
agtgcctgaagatcatgtgcgagatcctgagttcacttcagataggcgac
ttcctggtcaaggtaaacgatcgacgcattctagatgggatgtttgctat
ctgtggtgtttctgacagcaagttccgtaccatctgctcctcagtagaca
agctggacaaggtgtcctgggaagaggtgaagaatgagatggtgggagag
aagggccttgcacctgaggtggctgaccgcattggggactatgtccagca
acatggtggggtatccctggtggaacagctgctccaggatcctaaactat
cccaaaacaagcaggccttggagggcctgggagacctgaagttgctcttt
gagtacctgaccctatttggcattgatgacaaaatctcctttgacctgag
ccttgctcgagggctggattactacactggggtgatctatgaggcagtgc
tgctacagaccccagcccaggcaggggaagagcccctgggtgtgggcagt
gtggctgctggaggacgctatgatgggctagtgggcatgttcgaccccaa
agggcgcaaggtgccatgtgtggggctcagcattggggtggagcggattt
tctccatcgtggaacagagactagaggctttggaggagaagatacggacc
acggagacacaggtgcttgtggcatctgcacagaagaagctgctagagga
aagactaaagcttgtctcagaactgtgggatgctgggatcaaggctgagc
tgctgtacaagaagaacccaaagctactgaaccagttacagtactgtgag
gaggcaggcatcccactggtggctatcatcggcgagcaggaactcaagga
tggggtcatcaagctccgttcagtgacgagcagggaagaggtggatgtcc
gaagagaagaccttgtggaggaaatcaaaaggagaacaggccagcccctc
tgcatctgctgaactgaacaaactatcagaggaaaggaagtgggactggc
actatttgaggttaagacaaactgcatatgtacttcaattgctttgcact
tttccgtttcagcggaagacctgaagagtggtcagaacagagcctttgat
ttttattatggttattttattgattattactggcaaaaacggccaggtac
aacaccttttttcatacaaggcccaggaggcttagtccagtctgtgctcct
gggctacaaggacccagcctgagatggtcccatctgcagggccccgcacc
agttggagcagatgcctccccaccaccaattgccaaaggtccaataaaat
gcctcaaccacggaaaaaaaaaaaaaaaaaa
>NM_002738 6
agctggacgagcggcagcagctgggcgagtgacagccccggctccgcgcg
ccgcggccgccagagccggcgcaggggaagcgcccgcggccccgggtgca
gcagcggccgccgcctcccgcgcctccccggcccgcagcccgcggtcccg
cggccccggggccggcacctctcgggctccggctccccgcgcgcaagatg

gctgacccggctgcggggccgccgccgagcgagggcgaggagagcaccgt
gcgcttcgcccgcaaaggcgccctccggcagaagaacgtgcatgaggtca
agaaccacaaattcaccgcccgcttcttcaagcagcccaccttctgcagc
cactgcaccgacttcatctggggcttcgggaagcagggattccagtgcca
agtttgctgctttgtggtgcacaagcggtgccatgaatttgtcacattct
cctgccctggcgctgacaagggtccagcctccgatgacccccgcagcaaa
cacaagtttaagatccacacgtactccagccccacgttttgtgaccactg
tgggtcactgctgtatggactcatccaccaggggatgaaatgtgacacct
gcatgatgaatgtgcacaagcgctgcgtgatgaatgttcccagcctgtgt
ggcacggaccacacggagcgccgcggccgcatctacatccaggcccacat
cgacagggacgtcctcattgtcctcgtaagagatgctaaaaaccttgtac
ctatggaccccaatggcctgtcagatccctacgtaaaactgaaactgatt
cccgatcccaaaagtgagagcaaacagaagaccaaaaccatcaaatgctc
cctcaaccctgagtggaatgagacatttagatttcagctgaaagaatcgg
acaaagacagaagactgtcagtagagatttgggattgggatttgaccagc
aggaatgacttcatgggatctttgtcctttgggatttctgaacttcagaa
agccagtgttgatggctggtttaagttactgagccaggaggaaggcgagt
acttcaatgtgcctgtgccaccagaaggaagtgaggccaatgaagaactg
cggcagaaatttgagagggccaagatcagtcagggaaccaaggtcccgga
agaaaagacgaccaacactgtctccaaatttgacaacaatggcaacagag
accggatgaaactgaccgattttaacttcctaatggtgctggggaaaggc
agctttggcaaggtcatgctttcagaacgaaaaggcacagatgagctcta
tgctgtgaagatcctgaagaaggacgttgtgatccaagatgatgacgtgg
agtgcactatggtggagaagcgggtgttggccctgcctgggaagccgccc
ttcctgacccagctccactcctgcttccagaccatggaccgcctgtactt
tgtgatggagtacgtgaatgggggcgacctcatgtatcacatccagcaag
tcggccggttcaaggagccccatgctgtattttacgctgcagaaattgcc
atcggtctgttcttcttacagagtaagggcatcatttaccgtgacctaaa
acttgacaacgtgatgctcgattctgagggacacatcaagattgccgatt
ttggcatgtgtaaggaaaacatctgggatggggtgacaaccaagacattc
tgtggcactccagactacatcgcccccgagataattgcttatcagcccta
tgggaagtccgtggattggtgggcatttggagtcctgctgtatgaaatgt
tggctgggcaggcaccctttgaaggggaggatgaagatgaactcttccaa
tccatcatggaacacaacgtagcctatcccaagtctatgtccaaggaagc
tgtggccatctgcaaagggctgatgaccaaacacccaggcaaacgtctgg
gttgtggacctgaaggcgaacgtgatatcaaagagcatgcatttttccgg
tatattgattgggagaaacttgaacgcaaagagatccagccccccttataa
gccaaaagcttgtgggcgaaatgctgaaaacttcgaccgatttttcaccc
gccatccaccagtcctaacacctcccgaccaggaagtcatcaggaatatt
gaccaatcagaattcgaaggattttcctttgttaactctgaatttttaaa
acccgaagtcaagagctaagtagatgtgtagatctccgtccttcatttct
gtcattcaagctcaacggctattgtggtgacatttttatgtttttcattg
ccaagttgcatccatgtttgattttctgatgagactagagtgacagtgtt
tcagaacccaaatgtcctcaggtagtttggagcatctctatgagatggga
ttatgcagatggcctatggaaaatgcagctgcataattaacacattatca
aagtcctcttacaatttattttccgcagcatgtcagctaagtagacccaa
tggggagagaaaatgcctgctttctttccctctttttctgcactgccata
ttcacccccaaccatccaatctgtggataattggatgttagcggtactct
tccacttccgggcctggagcttggcttgtatccaagtgtatggttgcttt

gcctaagaggaatccctctatttcacctgttctggaggcaccagaccttg
aaaagaacatgctcaaaataaaatgttatctgttatttttgtaaactcaa
agttaagatgatcaaagttctaaaattccaagaatgtgcttttagacggt
ctcaatctaaaagcacttcaaggggtcaaagggcaaccagcttgggtgct
acctcagtgttgtagtttctgatactttatgtctttgctcaccctcatcc
ccaaactacttgaaaagggcatttggcaccactctctgaaacaacacagt
cactctagcaaggcccccaaagggccctggttttacattacatttcaaac
tttatttgctttggggttttgtttctgttgttgttcaaatgcaaaaaaaa
gaaaaaaaaagaaaaaaaaaggtgactcacattgttacacatgctttaaa
atatgtattcaaatgttattaaccacaatgacgacctgctttgatttaac
caagaagacggctgcggagcctagcagactcaggcctgtgggaatgggat
ttgttacaaatctaggtttgttactggcttcagaaagctaattaagtgct
ctgaaaaagacaccgtttcttgaaacaaagatggttgtattcctcacttt
gatgttgttttgcaagatgtttgtggaaatgttcatttgtatctggatct
ctgttatgtgccatttttcttctagcatcgagatacaataaaaaaaaaaa
aaaagaaaagaagaagaaatactatttcaaggaaaactgctctttttgag
aaacgtggacctaaactacaaagtgggaactgaggagggaactcaggaga
aaggaactaactgcggagctttaatcttggccccagtgttcagccactcg
gaggggcgggggctgtggcccattcaggggctgctggtgggctgtagtgg
ggtgggatgacctggccagagccaacgaggatactggagcccaaagtcaa
gtttagagaccagctgggaacgtgaatggggctcttgattttcttatcaa
aatcaccactcctcccagcttggactaaatattctttctagcaagcagct
ttgtgagctccctgaagcccaaggaaacccttcggtgggagaaatttcat
ttctgtctgagaggattaaggcagcaggtgactccccctcctcgcctgcc
gtgtcctgctattctcaggcagctctaaggagaattcttatcacagttca
agtgatttccagaagttccagggcttctgagagaccatcaagggaacttt
aacaacttgacaaatgtccttgaagtaagatgcctcatctttagggaaaa
atggggtttggatttctgcttaggcaaagtctcctgcagttcatccttct
ctgtcctcttcttgcttcaggcttggggaccgtccctgctgtccccactg
tggtggcaatcaggacctaaggtgaagcaaacttgaagttctatctgaca
agtttaggcagtaagagaaggagggaaatcggagcaaagctccctcactt
tattgttgagaaactggcatctggaaagaggaaggaatttgcccaaagtc
agtcagctgggataaaaacctgggtgtcctgtccagaaagtgcagggtgc
tttctgctctgtagcaaggcagcagacatctctgagccaggcccaccaac
aggcccttatctggtggttggatcatgatcccattttgcttggacatgct
ctcaggaagataaaaaccatggagaaacactaggccattgacaaatgatc
tgagacaactttagaaaacaatgtaggatgaatggaaagagaaagaaagg
aaagaaagaagaaaaagaaagaaggaaagaaagaaagagaaaggaaggaa
ggaaagaaggaaggaaaagaaggaaggaaggaaggaatatagtgttataa
atactgcactcaacattttccaaattcttgccattatttttcaaaagttt
aatagtttgcagaaatagatactcaagccaaagtctgttttagagaaact
ttccatggaaagtcagaatttctaccacttcctttttctatccacatttcc
agtgcagaagaaactgagaaacagagctttttgaagagaggacagggcca
tagcaacaaggaccttcttgggggattaatgggaggtcagtagaattaat
aaccctccttggatgagtgctactgttttcacatggcttcagatgctatc
aacctcaaagaaatgatctcaacagagaagcttattctctcccaacttct
acggtaaaatccaggagtattttctctggggatctgcccacaggacaaag
tccataaaagcaagtcctgtctggaccatgtggttatctgaagcattagc
catcaccagcacaacaaacggggcagggctttccaaggtggggctggtca

gaagggaatctttgataagaggcccacaggcagggaaagcgaaatagggt
tgatgagaccaggggagacctaaaaaaaaggcagctttgtgtcttctagc
tccaaatatacctgccttttagctcacacactgtcctggagttctcagac
ctttaggggccctaacacagttcagttcatacaggggttcaaaagggaca
gtggcccatttgggagacctttaggatcaatgggaatcaattccattgtt
ttgcctcagagtaaagtttctggctcggggacaattataagttgcaaaaa
ggatagaggcatatcccaagtcttccttcattccacaaataattacaaac
aacctactgtgtgccaggcactattcttagcactggaaatacactagtga
agaagcagatgaggaccctgtttattgtttctctccaagaaattctccaa
gaatattgtttcttggagagaaataataaataaacaagacaatttctgaa
agcaataagtgcaatcaagataattaaaggatgctaaagtgtgacttgtg
gggattgggagagagatgcacagacaatattaaagaggaggcattcgagc
tttgttgtgaacaccggaagtaacatgccgagcgcctgggggatggaaac
tcctatagcaccccacaggctaacagcaagcaggacaagacaaaaagggc
aggtgggacatggtagagatggaccctacccaggaaacagctccatcagc
atcttagcctgccccactctagccacacatacccacgtgtgctcctgagt
tcagtgtgcccacctcactcccacaccctcacatagacttggcaagagta
aggagggaactccatagagacattttacctatctcaggggagcagccaca
aagaagcaagtcttgtaaaaggtcttttgcaaaggagagtgaacccagca
atgagagatccttaacagctagtgcccattagggggctaaacctaaagcc
tgggtggtgatggctcaaacgctaatgagtcagtgaatccttaccgaccc
cctggcctttataatctgaggcaactttggctgcagcccgggaatgtgca
gggcactagggaatacaaggccttcttccctggttgtcttgtaataaaac
agccatggggttgtccctccagtccgagagactgtgatgaggcctacata
gcagcgatgtggtcaggtaaaaatcaggaacccactgaaatcttgggcaa
gccaccctgcctgcttgtgcctcggttctctcatatgtcatatataggag
gtgaggactccagctccacctgccccaggtgggtgtggtgatgatgagga
aagacaagaggcttgcaaggaccctgaagaggtcggagcatcatacagat
tcctttattagcccacattctgatgttccctggtgagacttgccccaagc
aattgctagtaaatgggggttaatttcttctccacctccctactgaacaa
aaaaagaaatgccagacttactaggagaatcgagttgctttgagtttctt
ttgttttgttttgttttgttttgttttaaggctccccttacacaccctcc
tttaagctttgggttttctctcttatagtttgttgacacatgctaaaaat
gtctttggagagaacttctgcctgataaacacccaattctagactgtggg
tggattttcgagctgacggtggtcaattcctttcattaagcagtgatctg
atttctccacatggccattctgccttcttgggggcagagtagatgggcag
cagttcaccttttcagagaaagaggtcttctagccacctgggctgctact
gaatggttttctccaggacgctctacctaatgattatttctataacatta
agcatggtaataagtagcttccaattcaattcatcctaaagccaaagaaa
atacagcaacacacacacacacacacacacacacacacacacacacacac
acacaccactttatggcaattcttaactgacattcaatgacttacttctt
ttcttagaaaatttccaccacatttctatccccaagccaacatacaatgt
gaaatgaaagccagtgcgtggagtgcagctgctaaaaattttcagcacag
ggctctttctgactctgctcatgagatggtatcagccacccaatgactgg
cgtatcttggtcctgtgtctttcttcttacgctgtgttaatgtgtttact
ttccatttggcagagagacaagagagacacctccaacttcgacaaagagt
tcaccagacagcctgtggaactgacccccactgataaactcttcatcatg
aacttggaccaaaatgaatttgctggcttctcttatactaacccagagtt
tgtcattaatgtgtaggtgaatgcaaactccatcgttgagcctggggtgt

aagacttcaagccaagcgtatgtatcaattctagtcttccaggattcacg
gtgcacatgctggcattcaacatgtggaaagcttgtcttagagggctttt
ctttgtatgtgtagcttgctagtttgttttctacatttgaaaatgtttag
tttagaataagcgcattatccaattatagaggtacaattttccaaacttc
cagaaactcatcaaatgaacagacaatgtcaaaactactgtgtctgatac
caaaatgcttcagtatttgtaatttttcaagtcagaagctgatgttcctg
gtaaaagtttttacagttattctataatatcttctttgaatgctaagcat
gagcgatatttttaaaaattgtgagtaagctttgcagttactgtgaacta
ttgtctcttggaggaagttttttgtttaagaattgatatgattaaactga
attaatatatgcaa
>NM_002695 3
ggattcggcgcaggcgcagtccgcgcgggttctcgcgcggagaaggggtg
cgagcggcggcggcggcggaggctgccatggacgacgaggaggagacgta
ccggctctggaaaatccgcaagaccatcatgcagctgtgccacgaccgtg
gctatctggtgacccaggacgagcttgaccagaccctggaggagttcaaa
gcccaatttggggacaagccgagtgaggggcggccgcggcgcacggacct
caccgtgctggtggcccacaacgatgaccccaccgaccagatgtttgtgt
tctttccagaggagcccaaggtgggcatcaagaccatcaaggtgtactgc
cagcgcatgcaggaggagaacatcacacgggctctcatcgtggtgcagca
gggcatgacaccctccgccaagcagtccctggtcgacatggcccccaagt
acatcctggagcagtttctgcagcaggagctgctcatcaacatcacggag
cacgagctagtccctgagcacgtcgtcatgaccaaggaggaggtgacaga
gctgctggcccgatataagctccgagagaaccagctgcccaggatccagg
cgggggaccctgtggcgcgctactttgggataaagcgtgggcaggtggtg
aagatcatccggcccagtgagacggctggcaggtacatcacctaccggct
ggtgcagtagctaccgcctgacagcccctagaggcggacacacagcgacc
cccatccctgcaggacaaacgcccctgccctgccagaatccggcccccac
agctctcacggctgctgctcctctggactccccaaggcaggtggcctcca
cccacgttctcccgtcctggggtgaggcttcctgtggcccagcccgcccc
attcacctgtggatttgtgcgagatgcagcctcagaaggaacaaggcccc
cagagggaggtcacctgggggcagctggtgccgggtcttcacccagacca
cgctgggtcccctctgttgggggtttggggtccgggtctcccaccagcca
ctgcttcctcctgggccctcggccttccacccctcgtcttccctccctcg
ggggccctgatgcgtggcggcccccgcccggcctcggctctttactccat
tcacagccatgcacgcgctcaagccaccagggtgcgagatgccagctctg
gagttctcggttgttgtaggaggttgggtgttttcaaatggtaaagatgt
tttgagcaaataaatttgcttgatacagaaaccaggccgactaacaaggt
ttccagcaggtggccttgcctcaccccaagcacacgaggtcagcctggca
ggtgtgccctcctcgcgctggcttcaggggtcagtgtctccccaggccct
cgccctgccctcccacttcgtgctcctgggtaaggcgtgagcgtgtctgt
ctcttcccttcccgtgagggtgtggagttgttctctggcttgtgcagctg
tggtttgcggccagttcctcttacagacgtttgggatctggctctacttc
cccgtttgcttactgtctgatttcagaaggcctcgcctgcccaagtttaa
cctagccacgtggttctctcacagtagctttattaagatgtaattcaccc
tcgggcggtcggtggtctgtaacgcgcccactgttctgtgcagcagggtc
cccagatccccaggccgcggactgtgtgtgtgatggggacagtgcttccc
cttctctccaaatgtactttttttcaaaattgtcttggccaaagcaagcca
gacccaaagggacaaacactgatgtcactcctaggaggtcactagagtcg
tcagagactggaagtaggatgggggttcccgggactggggagttgggtgg

ggatgggggttcccagggctggggaggagggaggggacagagtgtgtcat
gtgcacagttttagtttgggaagatgagaaaattctggaggtggccgggc
gcggtggctcacgcctgtaatcccagcactttgggaggccgaggtgggcg
gatcacctgaggtcgggagttcgagaccagcctgaccaacatggcaaaat
cccatcactgcaaaaaatacaaaaattacctgggcgtggtggtgcatgcc
tgtaatcccagctacttgggagctaaggcaggagaatcacttgaacccag
gaagtggaggatcgcttgagctcaggagttaaaagaccagcccgggcaac
acagagaaacccccacctctacaaaaaactttaagattacctgggcaca
gtggctcacacctgtaatcccaacactttgggaggctgaggcgggaggat
catttgagcccaggaggccgaggctgcagtgagctacgattgtgctcctg
tactccagcctgggccacagggtcacaccctgtctcaaaaaaaaaaaaaa
atgcagttccacaggcgcatgggccacgtttccagtgcttggtggccatg
tggggctgacgtgcgaaaccttagagccgggggatacgtccgtcatcgag
aacgttttcctggactgccctgggctgcaggtggacgtgacgaggtcatt
cgatgcctgaccgtctgcacagcctgtggtgaagaccacccgggctccca
cctcgcggggccccgtggcggggggttcccttcctcacacatccctcccc
tagggggaggcccagggccacgccacggggctctctctgtcccctccctg
gccccttcttctaagcttgcagctgccacagaaaatcttctaagcttgca
gctgccacagaaaacacccgattaaaaactttttatttcagcaaaataaa
cgtgcctgtgaaagaa
>NM_004564 2
atagtgaggctgagtcacctgaccaagaccctggagttacaatggcggcg
cccatgctgcgctggggctgccgtggaagacgttgggctttcgcccgggt
tgacggtggttcttgccaccgaagaggggctccgactgggtccacatcca
accagattaggggagagagctcagtggctcagcagcccctccacacggcc
cagaagacgaggaaaggtgaacacaaatgggctgctgtggtaggtttgga
aattcatgcccagatttcctccaactctaaactcttctctggatctcaag
ttcgcttttcagcacctccaaattctttggtttctttttttgatgcatct
ctacctggaactttgccggttctcaacaggaggtgtgtagaagcggcggt
gatgacaggcctggctctgaactgccacataaacaagaagtccttgtttg
acaggaagcactacttctatgcagacctccctgcaggctaccaaattacc
cagcagaggctcccaattgctgtgaatgggagcttgatatatggcgtctg
tgcagggaagaagcagagtcaggtgatccccaagacggtgaggatcaagc
agatccagttggagcaagacagtggcaaaagcctccacgacaacctgagg
tctcagacgctcattgatttgaacagggcaggagtgggccttctggaggt
ggtcctggagcccgacatgtcctgtggagaagaggcggcaacagctgtca
gggagctgcagctgatccttcaagccctggggaccagccaggcgaacatg
gcagagggccagttgagagtggatgccaatatatccgtgcatcaccctgg
ggagcctttgggcgttcgaacggaagtgaagaatctcaacagcatcaggt
tcctggccaaagccatagactatgaaattcagaggcaaatcaatgaactt
gagaatggaggtgaaattctgaacgaaacacgctcatttcatcacaagct
ggggtgcaccatgtcaatgagagacaaagaaggaaaacaggactacaggt
tcatgccagaacccaacctgcctcccctggtgctctacgacgccacatct
ctgcccgcaggtgcagacccacagcaagtgatcaatattgaccagattcg
ggagacactcccggagctccccagtgtgacccgagagaagcttgtccaac
agtatgggatgctgctggaacacagcttcactttgctgaacgaagtcggc
ctactggagttcttccaaaatgtgataaaagaaactagggcagagccaaa
aaaggtgactagttgggtcctcaacacttttctgggctatttaaagcaac
agaacctcgctgtcagtgagagtcctgtcacaccctctgcactcgctgag

cttcttgacctgctggacagcagaacaatttcttcatcagcagctaaaca
ggtgtttgaggaactgtggaagagggaaggcaagactccagggcagattg
tttcagaaaagcagcttgaactgatgcaggaccagggggcactggagcag
ctctgccactctgtgatggaggcccatcctcaagtggtaatggatgtgaa
gaacagaaaccccagagctataaataaactgattgggttggtccggaaag
cgactcaaagccgagcagatccagtcatgataaaggagatcctggagaag
aagctgtcattgtgagatgtttgggatccccttgcccaagggacaacaac
aaacagtgcagcctgactgggaacaggatcctgtgaaagctgatgcccat
gtgccctgagagctgcctctcaatccctgtcccaagccacagctatggca
ttaatgtcaccagtgttctcaccctctaggccctgtgcctggaggtgcct
ccacagccgaccagcagccaccccgcctgcttcatccacatcaggagggt
ccggtgaggctgcagcagtggttaaggagtaacaccttcttgtattaagg
aattttaaactaaataaaatgtatgttggagatactgttacccattctaa
gaaaacttttcattctgcagttccttcaacaaatatttttgagcaactcc
taagggccagccattgtaggtgatgggcattagcagtggtcaagagagac
tgtaatttggatgtattgttttcagacctaaacacaggttaacaaagatg
aaaatacaaccaaataaactggcacagtataaa
>NM_001185 3
ccattggcctgtagattcacctcccctgggcagggccccaggacccagga
taatatctgtgcctcctgcccagaaccctccaagcagacacaatggtaag
aatggtgcctgtcctgctgtctctgctgctgcttctgggtcctgctgtcc
cccaggagaaccaagatggtcgttactctctgacctatatctacactggg
ctgtccaagcatgttgaagacgtccccgcgtttcaggcccttggctcact
caatgacctccagttctttagatacaacagtaaagacaggaagtctcagc
ccatgggactctggagacaggtggaaggaatggaggattggaagcaggac
agccaacttcagaaggccagggaggacatctttatggagaccctgaaaga
catcgtggagtattacaacgacagtaacgggtctcacgtattgcagggaa
ggtttggttgtgagatcgagaataacagaagcagcggagcattctggaaa
tattactatgatggaaaggactacattgaattcaacaaagaaatcccagc
ctgggtccccttcgacccagcagcccagataaccaagcagaagtgggagg
cagaaccagtctacgtgcagcgggccaaggcttacctggaggaggagtgc
cctgcgactctgcggaaatacctgaaatacagcaaaaatatcctggaccg
gcaagatcctccctctgtggtggtcaccagccaccaggccccaggagaaa
agaagaaactgaagtgcctggcctacgacttctacccagggaaaattgat
gtgcactggactcgggccggcgaggtgcaggagcctgagttacggggaga
tgttcttcacaatggaaatggcacttaccagtcctgggtggtggtggcag
tgcccccgcaggacacagcccccctactcctgccacgtgcagcacagcagc
ctggcccagcccctcgtggtgccctgggaggccagctaggaagcaagggt
tggaggcaatgtgggatctcagacccagtagctgcccttcctgcctgatg
tgggagctgaaccacagaaatcacagtcaatggatccacaaggcctgagg
agcagtgtgggggacagacaggaggtggatttggagaccgaagactggg
atgcctgtcttgagtagacttggacccaaaaaatcatctcaccttgagcc
cacccccaccccattgtctaatctgtagaagctaataaataatcatccct
ccttgcctagcataaaaaaaaaaaaaaaa
>NM_004126 3
gagaacacaaagtaaaacttctgctgttcccagtcctgcagccccagccc
agccgtcccccgcgggcgtggcaggtcctgagtcagggcctgtccgtggc
cagcatccctgctgggcgattgagcagcgggaagctgcttggacccagtc
tcaaacttaaccctcatctagcacccgggcaggcctcctgggttgcaggg

acttgagaaaaggcagagttctcaggtcctaggaagctggggcacgctgg
cgtgacaagcgtcccggagaaagccaagccctcggggagctggggaccgc
agcagggctgcagtcacatcctgcgcgggtgggcggcgggccaggccttc
agttgtttcgggacgcgccgagcttcgccgctcttccagcggctccgctg
ccagagctagcccgagcccggttctggggcgaaaatgcctgcccttcaca
tcgaagatttgccagagaaggaaaaactgaaaatggaagttgagcagctt
cgcaaagaagtgaagttgcagagacaacaagtgtctaaatgttctgaaga
aataaagaactatattgaagaacgttctggagaggatcctctagtaaagg
gaattccagaagacaagaacccctttaaagaaaaaggcagctgtgttatt
tcataaataacttgggagaaactgcatcctaagtggaagaactagtttgt
tttagttttcccagataaaaccaacatgctttttaaggaaggaagaatga
aattaaaaggagactttcttaagcaccatatagatagggttatgtataaa
agcatatgtgctactcatctttgctcactatgcagtctttttttaagagag
cagagagtatcagatgtacaattatggaaataagaacattacttgagcat
gacacttctttcagtatattgcttgatgcttcaaataaagttttgtcttg
ggaaaaaaaaaaaa
>NM_021910 2
cgctagcatcttctgctgatcctgaaattgtaccagcggcaagatgtggc
ctggaaggggactttaagttctccacaactgccagcaatccttccaccag
gcaaaacacatcatctaaggaaaagaagtgaggtttgcttagggcgtggc
agcttcggataaacgcaggactccgcctggcagcccgatttctcccggaa
cctctgctcagcctggtgaaccacacaggcccgagtttcacccagtcccc
actccacggtgcagctgcggcttatctctcagcccagcgagatgccagcc
ttcctgtcccgggccagcgctctgacatgcagaaggtgaccctgggcctg
cttgtgttcctggcaggctttcctgtcctggacgccaatgacctagaaga
taaaaacagtcctttctactatgactggcacagcctccaggttggcgggc
tcatctgcgctggggttctgtgcgccatgggcatcatcatcgtcatgagt
gagtggaggagctcgggggagcaggcgggccggggctggggctcccctcc
cctgaccactcagctctccccaacaggtgcaaaatgcaaatgcaagtttg
gccagaagtccggtcaccatccaggggagactccacctctcatcacccca
ggctcagcccaaagctgatgaggacagaccagctgaaattgggtggagga
ccgttctctgtccccaggtcctgtctctgcacagaaacttgaactccagg
atggaattcttcctcctctgctgggactcctttgcatggcagggcctcat
ctcacctctcgcaagagggtctctttgttcaatttttttaatctaaaat
gattgtgcctctgcccaagcagcctggagacttcctatgtgtgcattggg
gtggggcttggggcaccatgagaaggttggcgtgccctggaggctgacac
agaggctggcactgagcctgcttgttgggaaaagcccacaggcctgttcc
cttgtggcttgggacatggcacaggcccgccctctgcctcctcagccatg
ggaacctcatatgcaatttgggatttactagtagccaaaaggaatgaaag
agagctctaaccagatggaacactggaacattccagtggaccctggacca
ttccaggaaaactgggacataggatcgtcccgctatgatggaagtgttca
gacagtttataatagtaagcccctgtgaccctctcacttaccccgagacc
tcactttattacaagatctttccaaatacccaaatgtccctgcaagcccg
ttaaataattccctatgctacccttaataacatacaatgaccacatagtg
tgagaacttccaacaagcctcaaagtcccttgagactccccaatacctaa
taaggcatgcgaaatgttctcatgaactaccccacaacacgcctaaaact
caaaacacccaaaaatatctcctccaatgtcctgaaacatgaacccaaaa
agagacccacaataaactcgtgacttgtcccctcaaaaaaaaaa
>NM_000867 4

gtatttgtttcactgctttcaaccgcctgtgctggaggctcagaataagt
caatgggaggaggatttcagtcacagcagcaagcaagtctagtgaacaga
taagatgacatgctcagcaaaataacaacgaaaccagagggggaactctc
tggcatgcaagttcaaacacgactctacaactacggcagaaaaagagaga
gagagaaactaaaaatatatatatatcctattttttcacagctatcagt
ttctttcactgagctttcctaaatttaagcctctagaaaataataaatac
ttggatatcttacctacaaacatggacagatgtgtgtatgcgctcatttt
agagaacttgaattttttttttaaaggaaggtgtcaactttggcttttg
agtgtttggcatggttacaatgccttaaaaaaacagatgagcagcttagc
tactaaccatgctgaccactgttcggaacgggattgaatcacagaaaaac
agcaaatggctctctcttacagagtgtctgaacttcaaagcacaattcct
gagcacattttgcagagcacctttgttcacgttatctcttctaactggtc
tggattacagacagaatcaataccagaggaaatgaaacagattgttgagg
aacagggaaataaactgcactgggcagctcttctgatactcatggtgata
atacccacaattggtggaaatacccttgttattctggctgtttcactgga
gaagaagctgcagtatgctactaattactttctaatgtccttggcggtgg
ctgatttgctggttggattgtttgtgatgccaattgccctcttgacaata
atgtttgaggctatgtggcccctcccacttgttctatgtcctgcctggtt
atttcttgacgttctcttttcaaccgcatccatcatgcatctctgtgcca
tttcagtggatcgttacatagccatcaaaaagccaatccaggccaatcaa
tataactcacgggctacagcattcatcaagattacagtggtgtggttaat
ttcaataggcattgccattccagtccctattaaagggatagagactgatg
tggacaacccaaacaatatcacttgtgtgctgacaaaggaacgttttggc
gatttcatgctctttggctcactggctgccttcttcacacctcttgcaat
tatgattgtcacctactttctcactatccatgctttacagaagaaggctt
acttagtcaaaaacaagccacctcaacgcctaacatggttgactgtgtct
acagttttccaaagggatgaaacaccttgctcgtcaccggaaaaggtggc
aatgctggatggttctcgaaaggacaaggctctgcccaactcaggtgatg
aaacacttatgcgaagaacatccacaattgggaaaaagtcagtgcagacc
atttccaacgaacagagagcctcaaaggtcctagggattgtgtttttcct
ctttttgcttatgtggtgtcccttctttattacaaatataactttagttt
tatgtgattcctgtaaccaaactactctccaaatgctcctggagatattt
gtgtggataggctatgtttcctcaggagtgaatcctttggtctacaccct
cttcaataagacatttcgggatgcatttggccgatatatcacctgcaatt
accgggccacaaagtcagtaaaaactctcagaaaacgctccagtaagatc
tacttccggaatccaatggcagagaactctaagtttttcaagaaacatgg
aattcgaaatgggattaaccctgccatgtaccagagtccaatgaggctcc
gaagttcaaccattcagtcttcatcaatcattctactagatacgcttctc
ctcactgaaaatgaaggtgacaaaactgaagagcaagttagttatgtata
gcagaactggcagttgtcatcaaacataatgatgagtaagatgatgaatg
agatgtaaatgtgccaagaatatattatataaagaattttatgtcatata
tcaaatcatctctttaacctaagatgtaagtattaagaatatctaatttt
cctaatttggacaagattattccatgaggaaaataattttatatagctac
aaatgaaaacaatccagcactctggttaaattttaaggtattcgaatgaa
ataaagtcaaatcaataaatttcaggctttaaaaaaaaaa
>NM_005978 3
ctcccctcaccccggtccaggatgcccagtccccacgacacctcccactt
cccactgtggcctgggtgggctcaggggctgcccttgacctggcctagag
ccctcccccagctggtggtggagctggcactctctgggagggagggggct

gggagggaatgagtgggaatggcaagaggccagggtttggtgggatcagg
ttgaggcaggtttggtttccttaaaatgccaagttgggggccagtggggc
ccacatataaatcctcaccctgggagcctggctgccttgctctccttcct
gggtctgtctctgccacctggtctgccacagatccatgatgtgcagttct
ctggagcaggcgctggctgtgctggtcactaccttccacaagtactcctg
ccaagagggcgacaagttcaagctgagtaagggggaaatgaaggaacttc
tgcacaaggagctgcccagctttgtgggggagaaagtggatgaggagggg
ctgaagaagctgatgggcagcctggatgagaacagtgaccagcaggtgga
cttccaggagtatgctgttttcctggcactcatcactgtcatgtgcaatg
acttcttccagggctgcccagaccgaccctgaagcagaactcttgacttc
ctgccatggatctcttgggcccaggactgttgatgcctttgagttttgta
ttcaataaactttttttgtctgttgataatattttaattgctcagtgatg
ttccataacccggctggctcagctggagtgctgggagatgagggcctcct
ggatcctgctcccttctgggctctgactctcctggaaatctctccaaggc
cagagctatgctttaggtctcaattttggaatttcaaacaccagcaaaaa
attggaaatcgagataggttgctgacttttattttgtcaaataaagatat
taaaaaaggcaaaaaaaaaa
>NM_015392 3
gcgcaggcgcagtctccgcgtgcgcgcctcgccggcgcctccatcccgga
tccttgctgcagcgtcagcgccgccgcccgtgcctttcctcttcctcctc
ctcctccttggcatccgcctcttcttcctcctgcgtcctcccccgctgcc
tccgctgctcccgacgcggagcccggagcccgcgccgagcccctggcctc
gcggtgccatgctgccccggcggcggcgctgaaggatggcgacgccgctg
cctccgccctccccgcggcacctgcggctgctgcggctgctgctctccgg
cctcgtcctcggcgccgccctgcgtggagccgccgccggccacccggatg
tagccgcctgtcccgggagcctggactgtgccctgaagaggcgggcaagg
tgtcctcctggtgcacatgcctgtgggccctgccttcagcccttccagga
ggaccagcaagggctctgtgtgcccaggatgcgccggcctccaggcgggg
gccggccccagcccagactggaagatgagattgacttcctggcccaggag
cttgcccggaaggagtctggacactcaactccgcccctacccaaggaccg
acagcggctcccggagcctgccaccctgggcttctcggcacgggggcagg
ggctggagctgggcctcccctccactccaggaacccccacgcccacgccc
cacacctccctgggctcccctgtgtcatccgacccggtgcacatgtcgcc
cctggagccccggggagggcaaggcgacggcctcgcccttgtgctgatcc
tggcgttctgtgtggccggtgcagccgccctctccgtagcctccctctgc
tggtgcaggctgcagcgtgagatccgcctgactcagaaggccgactacgc
cactgcgaaggcccctggctcacctgcagctccccggatctcgcctgggg
accagcggctggcacagagcgcggagatgtaccactaccagcaccaacgg
caacagatgctgtgcctggagcggcataaagagccacccaaggagctgga
cacggcctcctcggatgaggagaatgaggacggagacttcacggtgtacg
agtgcccgggcctggccccgaccggggaaatggaggtgcgcaaccctctg
ttcgaccacgccgcactgtccgcgcccctgccggcccccagctcaccgcc
tgcactgccatgacctggaggcagacagacgcccacctgctccccgacct
cgaggcccccggggaggggcagggcctggagcttcccactaaaaacatgt
tttgatgctgtgtgcttttggctgggcctcgggctccaggccctgggacc
ccttgccagggagacccccgaacctttgtgccaggacacctcctggtccc
ctgcacctctcctgttcggtttagacccccaaactggagggggcatggag
aaccgtagagcgcaggaacgggtgggtaattctagagacaaaagccaatt
aaagtccatttcagacctgcggcttctgaaaaaaaaaaaaaaaaaaa

>NM_000299 3
gggtggtgcagggcaggggtggtatatcctgtctgacggagggcgggcct
cgccagtgccagagagggacgaaccagggtggaagcgccaggagcagctg
cagggagccctcacgcggaccacgcactctatggccgtagggagccgctg
agagcgagaagagcacgctcctgcccgcccgctgcaccgcacctcgcctc
gcctctctgctctcctaggccccggccgcgcgccacccgcctcccgccac
catgaaccactcgccgctcaagaccgccttggcgtacgaatgcttccagg
accaggacaactccacgttggctttgccgtcggaccaaaagatgaaaaca
ggcacgtctggcaggcagcgcgtgcaggagcaggtgatgatgaccgtcaa
gcggcagaagtccaagtcttcccagtcgtccaccctgagccactccaatc
gaggttccatgtatgatggcttggctgacaattacaactatgggaccacc
agcaggagcagctactactccaagttccaggcagggaatggctcatgggg
atatccgatctacaatggaaccctcaagcgggagcctgacaacaggcgct
tcagctcctacagccagatggagaactggagccggcactacccccggggc
agctgtaacaccaccggcgcaggcagcgacatctgcttcatgcagaaat
caaggcgagccgcagtgagcccgacctctactgtgacccacggggcaccc
tgcgcaagggcacgctgggcagcaagggccagaagaccacccagaaccgc
tacagcttttacagcacctgcagtggtcagaaggccataaagaagtgccc
tgtgcgcccgccctcttgtgcctccaagcaggaccctgtgtatatcccgc
ccatctcctgcaacaaggacctgtcctttggccactctagggccagctcc
aagatctgcagtgaggacatcgagtgcagtgggctgaccatccccaaggc
tgtgcagtacctgagctcccaggatgagaagtaccaggccattggggcct
attacatccagcatacctgcttccaggatgaatctgccaagcaacaggtc
tatcagctgggaggcatctgcaagctggtggacctcctccgcagccccaa
ccagaacgtccagcaggccgcggcaggggccctgcgcaacctggtgttca
ggagcaccaccaacaagctggagacccggaggcagaatgggatccgcgag
gcagtcagcctcctgaggagaaccgggaacgccgagatccagaagcagct
gactgggctgctctggaacctgtcttccactgacgagctgaaggaggaac
tcattgccgacgccctgcctgttctggccgaccgcgtcatcattcccttc
tctggctggtgcgatggcaatagcaacatgtcccgggaagtggtggaccc
tgaggtcttcttcaatgccacaggctgcttgagaaagagactgggcatgc
gggagcttctggctcttgttccgcaaagggccactagtagcagggtgaac
ctgagctcggccgatgcaggccgccagaccatgcgtaactactcagggct
cattgattccctcatggcctatgtccagaactgtgtagcggccagccgct
gtgacgacaagtctgtggaaaactgcatgtgtgttctgcacaacctctcc
taccgcctggacgccgaggtgcccacccgctaccgccagctggagtataa
cgcccgcaacgcctacaccgagaagtcctccactggctgcttcagcaaca
agagcgacaagatgatgaacaacaactatgactgccccctgcctgaggaa
gagaccaaccccaagggcagcggctggttgtaccattcagatgccatccg
cacctacctgaacctcatgggcaagagcaagaaagatgctaccctggagg
cctgtgctggtgccctgcagaacctgacagccagcaaggggctgatgtcc
agtggcatgagccagttgattgggctgaaggaaaagggcctgccacaaat
tgcccgcctcctgcaatctggcaactctgatgtggtgcggtccggagcct
ccctcctgagcaacatgtcccgccaccctctgctgcacagagtgatgggg
aaccaggtgttcccggaggtgaccaggctcctcaccagccacactggcaa
taccagcaactccgaagacatcttgtcctcggcctgctacactgtgagga
acctgatggcctcgcagccacaactggccaagcagtacttctccagcagc
atgctcaacaacatcatcaacctgtgccgaagcagtgcctcacccaaggc
cgcagaagctgcccggcttctcctgtctgacatgtggtccagcaaggaac

tgcagggtgtcctcagacagcaaggtttcgataggaacatgctgggaacc
ttagctggggccaacagcctcaggaacttcacctcccgattctaagaaga
gactgtccaagcaagttaggcttgcaggaagatatgacccagctgagaag
ccctcaggcctcgctggatggggttttctgtccatcctgtgcagtatttg
ggaaagttcacaagaaactgagaagaaacctaaaaactgtggatagtgga
aagatttttagattttttttttccttggggaaactggcaggcaatggggg
ttagggaggttggggcgggggggcctttcttgagttaaaggggcttatat
gtgatgtcaatatttcttcctctgagaaatggtatatatatgtgtataat
gtaagtgtgtgcatgcatgtgcgcgtgcatgtgtgtgtgtgagtgtct
taaagcataaccacaaactgcaaaaagctaggtaagctattttgttgcag
ctcataaggtggtgaaaaggactctcctgtgtttcttactcataggcaag
gacaacatgtgctttttggtgagctgctcataattcctgaaatgtgtggt
gccagggcaagggggccatcactgcagtcaggccctcagaggagtcctgc
aggcttcctaccagtggtctccaggggtgcaggagtaactggggctgggc
cagcctccccacttacaaggctgctttccaggaagggaggtctggtgtat
ctcatgggagaatctggggtgtctgtaatgtcacccctccagcagcgcca
caaggactgaggttgggtaggtgtggggttccagaggacagcaggacact
ctcgcatactttgccaaatgaggcctgctcagaggagtaggagctgaaag
atggtgccttccaccctcttgggctgtgtgcccatcagagcaggctcagc
ctgcaaaggccctgcattcagaggtcttgtaatctacttgttgcaggaga
aagaaggtaaaaaatgatttttttaagaaaagctattttattgcagctct
ttcccaagagctgttctgggaatggctggtcttcatattcccagtggaga
ggggaacaagtggggctgggcatatacctattccggcttctagtgggatg
gagttggggtatagaaattaaccaggaagatgtttccaccaagcctgctg
tgagtcaattgagggagtgtttggggtcccaggagacttggacgggggga
gtttgggtagactaggaaaggaaagtgccatatcagggtaccggtaccgg
caagctcacatctcagccaggggccatgccccacttcccctgaccccagc
tgtcttgtctccactctgtgaaacccacaggggatgtgataaacagggct
attaggggtatcagccacgtcgagcccccagactctgtgcacttcagacc
agcagcagcaggagggctcccgagggccttatgagaaaacctgtgtggac
atcccttggtgtacactaagacagagcagagcccagcgctcccaagcctt
cctccttccagcttctacctccatgctagcattgctggtgttagagagga
attaacttcctggtctgtgcccttctctagaagaatataagatgctcctc
ctcctcaccccttctcagcctcctcccaagtcttcctcttctgcaccacc
cccgagtccaaacccacctcttgccccagcattcaggctggaaaacactg
atgtggactcagtatgataactgagatgggggacgccagacatgtgagga
cgctgtcctccgagaggtgtccccggctgttagccagctgtgctgtggtg
ctgtgggtctgtcataccctcccttgcttctgttcacactgggaggccca
ctcctggctcacctctccctctcagggacccacgtgggagcctggatccc
tggactgtcctgggcataggtttcaggggcctcctttgttgtcatcagaa
cccagaggaattcttctcctaaaaaatacgtatggcataccaatctgtgc
ggggcagtgtcctaagcacttagactacatcagggaagaacacagaccac
atccctgtcctcatgcggcttatgttttctggaggaaagtggagacacaa
gtccttggctttagggctcccccggctgggggctgtgcagtccggtcagg
gcgggaggggaaatgcaccgctgcatgtgaaccttaccagcccaggcgga
tgccccttccccttagcactaccctggcctcctgcatcccctcgcctcat
gttcctcccaccttcaaagaatgaagagccccatgggcccagcccctgcc
ctgggaaccaggcagccttccagacctcaggggctgaggcagactattag
ggcagggctgactttggtgacactgcccattccctctcaggccagctcag

gtcacccgggcctctgacccaggcctgtcactttgagaggggcaaaactg
agaggggcttttcctagagaaagagaacaaggagcttgccaggcttcatg
tagccgacacacgtctcaggattttaagtccacattggcctcacactacc
agggccaatgcccaaaataaggagttccaatttggggccaaatgaggaag
gacacagactctgccctgggatctcctgtgctagcggccaatgacaaatc
cagtcattggccaccagccacctctgcagtggggaccacactagcagccc
tgactccacactcctcctggggacccaagaggcagtgttgctgtctgcat
gtccaccttggaatctggctgaactggctggcaggaccaagactgcggct
ggggtgggcagggaagggaagccgggggctgctgtgagggatcttggagc
ttccctgtagcccaccttccccttgcttcatgtttgtagaggaaccttgt
gccggccaggcccagtttccttgtgtgatacactaatgtatttgcttttt
ttggaaatagagaaaatcaataaattgctagtgtttctttgaacttt
>NM_003008 2
agacaagatttttcaagcaagatgaagtccatcatcctctttgtcctttc
cctgctccttatcttggagaagcaagcagctgtgatgggacaaaaaggtg
gatcaaaaggccaattgccaagcggatcttcccaatttccacatggacaa
aagggccagcactattttggacaaaaagaccaacaacatactaaatccaa
aggcagtttttctattcaacacacatatcatgtagacatcaatgatcatg
actggacccgaaaaagtcagcaatatgatttgaatgccctacataaggcg
acaaaatcaaaacaacacctaggtggaagtcaacaactgctcaattataa
acaagaaggcagagaccatgataaatcaaaaggtcattttcacatgatag
ttatacatcataaaggaggccaagctcatcatgggacacaaaatccttct
caagatcaggggaatagcccatctggaaagggattatccagtcaatgttc
aaacacagaaaaaaggctatgggttcatggactaagtaaagaacaagctt
cagcctctggtgcacaaaaaggtagaacacaaggtggatcccaaagcagt
tatgttctccaaactgaagaactagtagttaacaaacaacaacgtgagac
taaaaattctcatcaaaataaagggcattaccaaaatgtggttgacgtga
gagaggaacattcaagtaaactacaaacttcactccatcctgcacatcaa
gacagactccaacatggacccaaagacatttttactacccaagatgagct
cctagtatataacaagaatcaacaccagacaaaaaatctcagtcaagatc
aagagcatggccggaaggcacataaaatatcatacccgtcttcacgtaca
gaagaaagacaacttcaccatggagaaaagagtgtacagaaagatgtatc
caaaggcagcatttctatccaaactgaagagaaaatacatggcaagtctc
aaaaccaggtaacaattcatagtcaagatcaagagcatggccataaggaa
aataaaatatcataccaatcttcaagtacagaagaaagacatctcaactg
tggagaaaagggcatccagaaaggtgtatccaaaggcagtatttcgatcc
aaactgaagagcaaatacatggcaagtctcaaaaccaggtaagaattcct
agtcaagctcaagagtatggccataaggaaaataaaatatcataccaatc
ttcgagtacagaagaaagacgtctcaacagtggagaaaaggatgtacaga
aaggtgtatccaaaggcagtatttctatccaaactgaagagaaaatacat
ggcaagtctcaaaaccaggtaacaattcctagtcaagatcaagagcatgg
ccataaggaaaataaaatgtcataccaatcttcaagtacagaagaaagac
gactcaactatggaggaaagagcacgcagaaagatgtatcccaaagcagt
atttctttccaaattgaaaagctagtagaaggcaagtctcaaatccagac
accaaatcctaatcaagatcaatggtctggccaaaatgcaaaaggaaagt
ctggtcaatctgcagatagcaaacaagacctactcagtcatgaacaaaaa
ggcagatacaaacaggaatccagtgagtcacataatattgtaattactga
gcatgaggttgcccaagatgatcatttgacacaacaatataatgaagaca
gaaatccaatatctacatagccctgttgcttagcaaccacttgaaaagct

ggaccaatagcaaggtgtcacccgacctcagtgaagtctttgatgtttct
gagaggcagactcccatgtggtcccagatccttggtccatggatgacacc
accttcccatgcttccttgcattaggctttctaaacccggagccccttca
aacttccaataaagggatcattttctgcttta
>NM_005795 4
gctgctgatcacttacaatctgacaacacttacaatctactcagaacaac
ctctctctctccagcagagagtgtcacctcctgctttaggaccatcaagc
tctgctaactgaatctcatcctaattgcaggatcacattgcaaagctttc
actctttcccaccttgcttgtgggtaaatctcttctgcggaatctcagaa
agtaaagttccatcctgagaatatttcacaaagaatttccttaagagctg
gactgggtcttgaccccctgaatttaagaaattcttaaagacaatgtcaaa
tatgatccaagagaaaatgtgatttgagactggagacaattgtgcatatc
gtctaataataaaaacccatactagcctatagaaaacaatatttgaaaga
ttgctaccactaaaaagaaaactactacaacttgacaagactgctgcaaa
cttcaatttgtcaaccacaacttgacaaggttgctataaaacaagattgc
tacaacttctagtttatgttatacagcatatttcattttggcttaatgat
ggagaaaaagtgtaccctgtattttctggttctcttgccttttttttatga
ttcttgttacagcagaattagaagagagtcctgaggactcaattcagttg
ggagttactagaaataaaatcatgacagctcaatatgaatgttaccaaaa
gattatgcaagaccccattcaacaagcagaaggcgtttactgcaacagaa
cctgggatggatggctctgctggaacgatgttgcagcaggaactgaatca
atgcagctctgccctgattactttcaggactttgatccatcagaaaaagt
tacaaagatctgtgaccaagatggaaactggtttagacatccagcaagca
acagaacatggacaaattatacccagtgtaatgttaacacccacgagaaa
gtgaagactgcactaaatttgttttacctgaccataattggacacggatt
gtctattgcatcactgcttatctcgcttggcatattcttttatttcaaga
gcctaagttgccaaaggattaccttacacaaaaatctgttcttctcattt
gtttgtaactctgttgtaacaatcattcacctcactgcagtggccaacaa
ccaggccttagtagccacaaatcctgttagttgcaaagtgtcccagttca
ttcatctttacctgatgggctgtaattacttttggatgctctgtgaaggc
atttacctacacacactcattgtggtggccgtgtttgcagagaagcaaca
tttaatgtggtattattttcttggctggggatttccactgattcctgctt
gtatacatgccattgctagaagcttatattacaatgacaattgctggatc
agttctgatacccatctcctctacattatccatggcccaatttgtgctgc
tttactggtgaatcttttttttcttgttaaatattgtacgcgttctcatca
ccaagttaaaagttacacaccaagcggaatccaatctgtacatgaaagct
gtgagagctactcttatcttggtgccattgcttggcattgaatttgtgct
gattccatggcgacctgaaggaaagattgcagaggaggtatatgactaca
tcatgcacatccttatgcacttccagggtcttttggtctctaccattttc
tgcttctttaatggagaggttcaagcaattctgagaagaaactggaatca
atacaaaatccaatttggaaacagcttttccaactcagaagctcttcgta
gtgcgtcttacacagtgtcaacaatcagtgatggtccaggttatagtcat
gactgtcctagtgaacacttaaatggaaaaagcatccatgatattgaaaa
tgttctcttaaaaccagaaaatttatataattgaaaatagaaggatggtt
gtctcactgttttgtgcttctcctaactcaaggacttggacccatgactc
tgtagccagaagacttcaatattaaatgactttttgaatgtcataaagaa
gagccttcacatgaaattagtagtgtgttgataagagtgtaacatccagc
tctatgtgggaaaaaagaaatcctggtttgtaatgtttgtcagtaaatac
tcccactatgcctgatgtgacgctactaacctgacatcaccaagtgtgga

attggagaaaagcacaatcaacttttctgagctggtgtaagccagttcca
gcacaccattgcatgaattcacaaacaaatggctgtaaaactaaacatac
atgttgggcatgattctacccttattgccccaagagacctagctaaggtc
tataaacatgaagggaaaattagcttttagttttaaaactctttatccca
tcttgattggggcagttgactttttttttgcccagagtgccgtagtcctt
tttgtaactaccctctcaaatggacaataccagaagtgaattatccctgc
tggctttcttttctctatgaaaagcaactgagtacaattgttatgatcta
ctcatttgctgacacatcagttatatcttgtggcatatccattgtggaaa
ctggatgaacaggatgtataatatgcaatcctacttctatatcattagga
aaacatcttagttgatgctacaaaacaccttgtcaacctcttcctgtctt
accaaacagtgggagggaattcctagctgtaaatataaattttgtccctt
ccatttctactgtataaacaaattagcaatcattttatataaagaaaatc
aatgaaggatttcttattttcttggaattttgtaaaaagaaattgtgaaa
aatgagcttgtaaatactccattattttattttatagtctcaaatcaaat
acatacaacctatgtaatttttaaagcaaatatataatgcaacaatgtgt
gtatgttaatatctgatactgtatctgggctgatttttttaaataaaatag
agtctggaatgctatatttggtaaatattttaaagacaaccagatgccag
catcagaagtctgtttgagaactaagagaacagaaacatctatcataaga
tatatttattttaaaaacacaaggtcactattttattgaatatatttgtt
ttgataactcataccttaataataggtgtgtttgacatatttcttttttc
attttgacaatgaactcacattctaatccagaaattttaaacaactactg
tgataaataccaatctgctacttttatagattttaccccattaaaatatt
actttactgacttttactatgtgaagatatatagctttggaaatgtccca
ggctattcaagaaatataaaaaactagaaggatactatatataccatata
caatgctttaatattttaatagagctactgtatataatacaaattaggga
aatacttgaatatatcattgagaaaaaattattgtcagatcttactgaat
tattgtcagactttattaaataaagatagaagaaaaccttgctaatgaat
taaagtgaaatttgcatgggattcagtttctctaatgttattttccgctg
aaatctctaaagaacaagaatgacttcaattagtaaaagtcaattttggg
aaaagtcatgggtatctgtttttttaagtgtgtcaatctgattaaaatgga
tgaaacaaattactcatcataagttgtttcttaagctgtcaatatgtcaa
tagatggtgagttcagaacttatttcaaattgctaagacaaattatctaa
attcgtaagaattaacatatagaatggtctggtcagtacatttataattt
atctatgcatgaaaaagtattgttttgtttgaaacatgaatttcatagca
agctgccatagaaaggaacgcaggctgttctagaccttcaactgcctaaa
ttatacaaaaattcattttaataaactcaattattagctatttattattc
aaagacccatatttaaatcctttgctgaccatgttgacatatatcagcct
tcttctagacaaactgtcaactctcaaccatcttgacagtagaagtgaca
gtaaaaaaatgttgaatgatcagagattatattaaaataaacatgtaattt
tcaagtatttttgttgtgcttttataatattaattctagatcagatttat
tttatagccagggtttgtctgttgtagagtcttgaggcgtagcagtcatt
catgattaatcactgttagttttgtacccatatatttttagaatagtttt
aaatgttagatttctcaaaagctaaatgctacttaatatctttgtatcat
actcataaagcaaagtaaatctgacactttttttaaagcaaacttctttg
ctgtcaaaaaaataaatttggggaaatttctagcttttaaaatgtagatc
tgcattttactgtgattacttgtgaaagtcatattttaattttctaaatt
ctaatttgtcattttatttcctaaagttaatttccaatgcatttattcat
aaaatattcattctggaatgcagtgtttgtttaaatgtaatccaatgtat
atagaattagtggtggctgtagtgctgtatttattgcttataattttttt

taaatgtgaacttacttttaattttctcttggttttaatctgctagtaga
aaccactagttatctgtaaaaatatattcaagatattctgatcaattata
acaatttatgttatgcctagagtatatctctatttttgattgtatgaaa
atattaaagttatgagttaaagtttattttcactgatatttactacagtg
ccaaataatctaatttataaacataattcttacagtaatcaatgggatac
ttctcaaaattaacaaatctcttaacaaaatatatcttttgccctcttta
aagtcttcagtaaaccagtaaatgaattcaataaaccaattaagaaaaaa
aaaaaa
>NM_004755 2
ttggttgcgcggtactagcggtgcccgccgaagggggaggaggcgaggag
cgagccgtgcggccagagcgggaaagagactcgtctttgcgtccgagttc
tggagccgccgcaccccgactcctggggccgcggcagcggctgcgagggg
acgggcgtccgctgtctcctgggttcccctcgtagcgacccgcgggatcg
gaaaaaaaggagaagatggaggaggagggtggcagcagcggcggcgccgc
ggggaccagcgcggacggcggcgacggaggagagcagctcctcactgtca
agcacgagctgcggactgctaatttgacaggacatgctgagaaggtggga
atagaaaattttgagctcctgaaggtcctaggaactggagcttatggaaa
agtatttctagttcgtaaaataagtggccatgatactggaaagctgtatg
ccatgaaagttttgaaaaaggcaacaatcgttcaaaaggccaaaaccaca
gagcatacaaggacagaacgacaagtcctggaacacattaggcagtcgcc
atttttggtaacattacattatgctttccagacagaaaccaaacttcatc
tcattttagattatataaatggtggtgaactttttactcatctttctcaa
agagagcgtttcacagagcatgaggtgcagatttatgttggagagattgt
gcttgccctcgaacatctccacaagttggggattatatatcgtgatatta
agcttgagaatattctacttgattctaatggccatgtggtgctgacagat
tttggtctgagtaaggagtttgtggctgatgaaactgaaagagcatattc
cttttgtggaactattgaatacatggcaccagatattgtcagagggggag
attcaggacatgacaaggcagttgactggtggagtttgggtgttctaatg
tatgaattactaactggagcatctcctttcactgttgatggagaaaaaaa
ttcccaagctgagatatctaggagaatattaaaaagtgagcctccatatc
cccaagaaatgagtgctttagcgaaagacctaattcagcgtcttttgatg
aaagatcccaagaagagattgggatgtggtccacgtgatgcagatgaaat
caaagaacatctcttctttcagaaaataaattgggatgatttagccgcca
aaaaagtgcctgcaccatttaagccagtcattcgagatgaattagatgtg
agtaactttgcagaagagttcacagaaatggatcccacttattctcccgc
agccctgccccagagttctgagaagctgtttcagggctattcctttgttg
ctccttccatcctattcaagcgtaatgcagctgtcatagaccctcttcag
tttcacatgggagttgaacgtcctggagtgacaaatgttgccaggagtgc
aatgatgaaggactctccattctatcaacactatgacctagatttgaagg
acaaacccctgggagaaggtagtttttcaatttgtcgaaagtgtgtgcat
aaaaaaagtaaccaagcttttgcagtcaaaataatcagcaaaaggatgga
agccaatactcaaaaggaaataacagctctgaaactctgtgaaggacacc
ccaatattgtgaagttgcatgaagtttttcatgatcagcttcacacgttt
ctagtgatggaacttctgaatggaggagaactgtttgagcgcattaagaa
aaagaagcacttcagtgagacggaagccagctacatcatgaggaagcttg
tttcagctgtaagccacatgcatgatgttggagtggtgcacagggatctg
aaacctgagaatttattgttcaccgatgaaaatgacaatttggaaattaa
aataattgattttggatttgcacggctaaagccaccggataatcagcccc
tgaagactccatgcttcacccttcattatgccgccccagagctcttgaat

cagaacggctacgatgagtcctgtgacctgtggagcttgggcgtcatttt
gtacacaatgttgtcaggacaggttcccttccaatctcatgaccgaagtt
tgacgtgtaccagcgcggtggaaatcatgaagaaaattaaaaagggagat
ttctcctttgaaggagaagcctggaagaatgtatcccaagaggctaaaga
tttgatccaaggacttctcacagtagatccaaacaaaaggcttaaaatgt
ctggcttgaggtacaatgaatggctacaagatggaagtcagctgtcctcc
aatcctctgatgactccggatattctaggatcttccggagctgccgtgca
tacctgtgtgaaagcaaccttccacgcctttaacaaatacaagagagagg
ggttttgccttcagaatgttgataaggcccctttggctaagagaagaaaa
atgaaaaagactagcaccagtaccgagacgcgcagcagttccagtgagag
ttcccattcttcttcctctcattctcacggtaaaactacacccaccaaga
cactgcagcccagcaatcctgccgacagcaataacccggagaccctcttc
cagttctcggactcagtagcttaggcatggtaggagtgtatcagtgatcc
attgcacctttattccctcagcatatgcctgaggcgatcttttatgcttt
taaaaatgtttcccgttggtctcattggaatctgcctcctaatgattttt
ttcaggaaaacctgtttggttatcctcattcaaaagcactggacagagaa
tgttactgtgaatagagcacatattactctttttagcaacctagcatgat
gccaacaagactattcttgaaagagcaaaggttcctgtaaatttaattag
ggctagatttgagctgcttgtaagtcacaggttttccagatgtctgccaa
caagaaatgactcatactgtgatgatacctttttgctttgccttgtggaca
atgtgggtttttgaaatttgcacccttcaaacaatgatttatcagagaaa
ggggtctgttttcaaaaaagattctgtaatgaattttatgtgtggcatat
acttatttcttgagagaagattttaacttattgtttttattttatggtta
catatgatgataacctgctattattaaactttttctaaaaagtgaaaaaa
aaaaaaaaagatataagaactcaaggtcccatactctgtattcgggatcc
atctgagatgcatgctaagctatgtgtatgtttttaattttgcactgctc
tttcctggcaatttgttttaatggttattgcagaatattaaggtacatgt
ctctctgttttaagtaatattgcactttataaaaaagtatgaataaagca
aactattttataaagtgcactgtttaaagcatttgcactgtatttttgcc
atttattttcattttctactttaaatttgtcctcacattcctcttctact
ttgtatgcaacaagtagaatggggcatgttgtgtcatgtagtcagccact
tatgcaccaatgtgaggaaaacctaaagggaaattaaactaaacactgtg
cttcatatttgtacactgtgttgtactacagtgaggaatttcctcctgta
gtcatatattatgtacataatattttagaatcatacctatgacttgtttg
gaaatttttctgttaaattttaaatccagaaagcatattttataaactta
tgcagagcacttttattgctcaaaagttctgaattcatacagaaaacaag
tactatgtgatgaaaacatttcattaaaagattgctgcatttaaaaatac
aattaattcgttccctatgcaaaaaaaaaaaaa
>NM_004672 3
gccccagattcctgggacccaatcccggatttcagaacctcaggaccttg
caagtccgagctaggatttctccctggactttagattcctggaacgggac
gtcaggccgtattccggatccctagatcccgtgggagattctccgattcc
gaacggattccagatcccaagattcctgatctgccggcccaggctcctgc
cccgcccccggttcccagtgcgcggcgccccgcgcctgagcgcccccgca
tggcggggccgtgtccccggtccggggcggagcgcgccggcagctgctgg
caggacccgctggccgtggcgctgagccggggccggcagctcgcggcgcc
cccgggccggggctgcgcgcggagccggccgctcagcgtggtctacgtgc
tgacccgggagccgcagcccgggctcgagcctcgggagggaaccgaggcg
gagccgctgcccctgcgctgcctgcgcgaggcttgcgcgcaggtcccccg

gccgcggccgcccccgcagctgcgcagcctgcccttcgggacgctggagc
taggcgacaccgcggctctggatgccttctacaacgcggatgtggtggtg
ctggaggtgagcagctcgctggtacagccctccctgttctaccaccttgg
tgtgcgtgagagcttcagcatgaccaacaatgtgctcctctgctcccagg
ccgacctccctgacctgcaggccctgcgggaggatgttttccagaagaac
tcggattgcgttggcagctacacactgatcccctatgtggtgacggccac
tggtcgggtgctgtgtggtgatgcaggccttctgcggggcctggctgatg
ggctggtacaggctggagtggggaccgaggccctgctcactcccctggtg
ggccggcttgcccgcctgctggaggccacacccacagactcttgtggcta
tttccgggagaccattcggcgggacatccggcaggcgcgggagcggttca
gtgggccacagctgcggcaggagctggctcgcctgcagcggagactggac
agcgtggagctgctgagccccgacatcatcatgaacttgctgctctccta
ccgcgatgtgcaggactactcggccatcattgagctggtggagacgctgc
aggccttgcccacctgtgatgtggccgagcagcataatgtctgcttccac
tacacttttgccctcaaccggaggaacaggcctggggaccgggcgaaggc
cctgtctgtgctgctgccgctggtacagcttgagggctctgtggcgcccg
atctgtactgcatgtgtggccgtatctacaaggacatgttcttcagctcg
ggtttccaggatgctgggcaccgggagcaggcctatcactggtatcgcaa
ggcttttgacgtagagcccagccttcactcaggcatcaatgcagctgtgc
tcctcattgctgccgggcagcactttgaggattccaaagagctccggcta
ataggcatgaagctgggctgcctgctggcccgcaaaggctgcgtggagaa
gatgcagtattactgggatgtgggtttctacctgggagcccagatcctcg
ccaatgaccccacccaggtggtgctggctgcagagcagctgtataagctc
aatgcccccatatggtacctggtgtccgtgatggagaccttcctgctcta
ccagcacttcaggcccacgccagagccccctggagggccaccacgccgtg
cccacttctggctccacttcttgctacagtcctgccaaccattcaagaca
gcctgtgcccagggcgaccagtgcttggtgctggtcctggagatgaacaa
ggtgctgctgcctgcaaagctcgaggttcggggtactgacccagtaagca
cagtgaccctgagcctgctggagcctgagacccaggacattccctccagc
tggaccttcccagtcgcctccatatgcggagtcagcgcctcaaagcgcga
cgagcgctgctgcttcctctatgcactcccccggctcaggacgtccagc
tgtgcttccccagcgtagggcactgccagtggttctgcggcctgatccag
gcctgggtgacgaacccggattccacggcgcccgcggaggaggcggaggg
cgcgggggagatgttggagtttgattatgagtacacggagacgggcgagc
ggctggtgctgggcaagggcacgtatggggtggtgtacgcgggccgcgat
cgccacacgagggtgcgcatcgccatcaaggagatcccggagcgggacag
caggttctctcagcccctgcatgaagagatcgctcttcacagacgcctgc
gccacaagaacatagtgcgctatctgggctcagctagccagggcggctac
cttaagatcttcatggaggaagtgcctggaggcagcctgtcctccttgct
gcggtcggtgtggggacccctgaaggacaacgagagcaccatcagtttct
acacccgccagatcctgcagggacttggctacttgcacgacaaccacatc
gtgcacagggacataaaaggggacaatgtgctgatcaacaccttcagtgg
gctgctcaagatttctgacttcggcacctccaagcggctggcaggcatca
caccttgcactgagaccttcacaggaactctgcagtatatggccccagaa
atcattgaccagggcccacgcgggtatgggaaagcagctgacatctggtc
actgggctgcactgtcattgagatggccacaggtcgcccccccttccacg
agctcgggagcccacaggctgccatgtttcaggtgggtatgtacaaggtc
catccgccaatgcccagctctctgtcggccgaggcccaagcctttctcct
ccgaacttttgagccagacccccgcctccgagccagcgcccagacactgc

tggggggaccccttcctgcagcctgggaaaaggagccgcagccccagctcc
ccacgacatgctccacggccctcagatgccccttctgccagtcccactcc
ttcagccaactcaaccacccagtctcagacattcccgtgccctcaggcac
cctctcagcacccacccagccccccgaagcgctgcctcagttatgggggc
accagccagctccgggtgcccgaggagcctgcggccgaggagcctgcgtc
tccggaggagagttcggggctgagcctgctgcaccaggagagcaagcgtc
gggccatgctggccgcagtattggagcaggagctgccagcgctggcggag
aatctgcaccaggagcagaagcaagagcagggggcccgtctgggcagaaa
ccatgtggaagagctgctgcgctgcctcggggcacacatccacactccca
accgccggcagctcgcccaggagctgcgggcgctgcaaggacggctgagg
gcccagggccttgggcctgcgcttctgcacagaccgctgtttgccttccc
ggatgcggtgaagcagatcctccgcaagcgccagatccgtccacactgga
tgttcgttctggactcactgctcagccgtgctgtgcgggcagccctgggt
gtgctaggaccggaggtggagaaggaggcggtctcaccgaggtcagagga
gctgagtaatgaaggggactcccagcagagcccaggccagcagagcccgc
ttccggtggagcccgagcagggccccgctcctctgatggtgcagctgagc
ctcttgagggcagagactgatcggctgcgcgaaatcctggcggggaagga
acgggagtaccaggccctggtgcagcgggctctacagcggctgaatgagg
aagcccggacctatgtcctggccccagagcctccaactgctctttcaacg
gaccagggcctggtgcagtggctacaggaactgaatgtggattcaggcac
catccaaatgctgttgaaccatagcttcaccctccacactctgctcacct
atgccactcgagatgacctcatctacacccgcatcaggggagggatggta
tgccgcatctggagggccatcttggcacagcgagcaggatccacaccagt
cacctctggaccctgagagctgaatgagggcatcataggccagacaggcc
caaggatggatgaatggagaggacaaaggcagcttctgacacaccagccc
caggacctggggcgactggaggaagccaggcgagtggggcccaggactgg
ttccagtgagagaaaccaaccacaggcacccaagcactaccagacaaagc
gtattaaacagaacacttttgaacctttaaaaa
>NM_000350 2
aggacacagcgtccggagccagaggcgctcttaacggcgtttatgtcctt
tgctgtctgaggggcctcagctctgaccaatctggtcttcgtgtggtcat
tagcatgggcttcgtgagacagatacagcttttgctctggaagaactgga
ccctgcggaaaaggcaaaagattcgctttgtggtggaactcgtgtggcct
ttatctttatttctggtcttgatctggttaaggaatgccaacccgctcta
cagccatcatgaatgccatttccccaacaaggcgatgccctcagcaggaa
tgctgccgtggctccaggggatcttctgcaatgtgaacaatccctgtttt
caaagccccaccccaggagaatctcctggaattgtgtcaaactataacaa
ctccatcttggcaagggtatatcgagattttcaagaactcctcatgaatg
caccagagagccagcaccttggccgtatttggacagagctacacatcttg
tcccaattcatggacaccctccggactcacccggagagaattgcaggaag
aggaatacgaataagggatatcttgaaagatgaagaaacactgacactat
ttctcattaaaaacatcggcctgtctgactcagtggtctaccttctgatc
aactctcaagtccgtccagagcagttcgctcatggagtcccggacctggc
gctgaaggacatcgcctgcagcgaggccctcctggagcgcttcatcatct
tcagccagagacgcggggcaaagacggtgcgctatgccctgtgctccctc
tcccagggcaccctacagtggatagaagacactctgtatgccaacgtgga
cttcttcaagctcttccgtgtgcttcccacactcctagacagccgttctc
aaggtatcaatctgagatcttggggaggaatattatctgatatgtcacca
agaattcaagagtttatccatcggccgagtatgcaggacttgctgtgggt

gaccaggcccctcatgcagaatggtggtccagagacctttacaaagctga
tgggcatcctgtctgacctcctgtgtggctaccccgagggaggtggctct
cgggtgctctccttcaactggtatgaagacaataactataaggcctttct
ggggattgactccacaaggaaggatcctatctattcttatgacagaagaa
caacatccttttgtaatgcattgatccagagcctggagtcaaatccttta
accaaaatcgcttggagggcggcaaagcctttgctgatgggaaaaatcct
gtacactcctgattcacctgcagcacgaaggatactgaagaatgccaact
caacttttgaagaactggaacacgttaggaagttggtcaaagcctgggaa
gaagtagggccccagatctggtacttctttgacaacagcacacagatgaa
catgatcagagataccctggggaacccaacagtaaaagactttttgaata
ggcagcttggtgaagaaggtattactgctgaagccatcctaaacttcctc
tacaagggccctcgggaaagccaggctgacgacatggccaacttcgactg
gagggacatatttaacatcactgatcgcaccctccgcctggtcaatcaat
acctggagtgcttggtcctggataagtttgaaagctacaatgatgaaact
cagctcacccaacgtgccctctctctactggaggaaaacatgttctgggc
cggagtggtattccctgacatgtatccctggaccagctctctaccacccc
acgtgaagtataagatccgaatggacatagacgtggtggagaaaaccaat
aagattaaagacaggtattgggattctggtcccagagctgatcccgtgga
agatttccggtacatctggggcgggtttgcctatctgcaggacatggttg
aacaggggatcacaaggagccaggtgcaggcggaggctccagttggaatc
tacctccagcagatgccctacccctgcttcgtggacgattctttcatgat
catcctgaaccgctgtttccctatcttcatggtgctggcatggatctact
ctgtctccatgactgtgaagagcatcgtcttggagaaggagttgcgactg
aaggagaccttgaaaaatcagggtgtctccaatgcagtgatttggtgtac
ctggttcctggacagcttctccatcatgtcgatgagcatcttcctcctga
cgatattcatcatgcatggaagaatcctacattacagcgacccattcatc
ctcttcctgttcttgttggctttctccactgccaccatcatgctgtgctt
tctgctcagcaccttcttctccaaggccagtctggcagcagcctgtagtg
gtgtcatctatttcaccctctacctgccacacatcctgtgcttcgcctgg
caggaccgcatgaccgctgagctgaagaaggctgtgagcttactgtctcc
ggtggcatttggatttggcactgagtacctggttcgctttgaagagcaag
gcctggggctgcagtggagcaacatcgggaacagtcccacggaaggggac
gaattcagcttcctgctgtccatgcagatgatgctccttgatgctgctgt
ctatggcttactcgcttggtaccttgatcaggtgtttccaggagactatg
gaaccccacttccttggtactttcttctacaagagtcgtattggcttggc
ggtgaagggtgttcaaccagagaagaaagagccctggaaaagaccgagcc
cctaacagaggaaacggaggatccagagcacccagaaggaatacacgact
ccttctttgaacgtgagcatccagggtgggttcctggggtatgcgtgaag
aatctggtaaagatttttgagccctgtggccggccagctgtggaccgtct
gaacatcaccttctacgagaaccagatcaccgcattcctgggccacaatg
gagctgggaaaaccaccaccttgtccatcctgacgggtctgttgccacca
acctctgggactgtgctcgttgggggaagggacattgaaaccagcctgga
tgcagtccggcagagccttggcatgtgtccacagcacaacatcctgttcc
accacctcacggtggctgagcacatgctgttctatgcccagctgaaagga
aagtcccaggaggaggcccagctggagatggaagccatgttggaggacac
aggcctccaccacaagcggaatgaagaggctcaggacctatcaggtggca
tgcagagaaagctgtcggttgccattgcctttgtgggagatgccaaggtg
gtgattctggacgaacccacctctggggtggacccttactcgagacgctc
aatctgggatctgctcctgaagtatcgctcaggcagaaccatcatcatgt

ccactcaccacatggacgaggccgacctccttggggaccgcattgccatc
attgcccagggaaggctctactgctcaggcaccccactcttcctgaagaa
ctgctttggcacaggcttgtacttaaccttggtgcgcaagatgaaaaaca
tccagagccaaaggaaaggcagtgaggggacctgcagctgctcgtctaag
ggtttctccaccacgtgtccagcccacgtcgatgacctaactccagaaca
agtcctggatggggatgtaaatgagctgatggatgtagttctccaccatg
ttccagaggcaaagctggtggagtgcattggtcaagaacttatcttcctt
cttccaaataagaacttcaagcacagagcatatgccagccttttcagaga
gctggaggagacgctggctgaccttggtctcagcagttttggaatttctg
acactcccctggaagagatttttctgaaggtcacggaggattctgattca
ggacctctgtttgcgggtggcgctcagcagaaaagagaaaacgtcaaccc
ccgacacccctgcttgggtcccagagagaaggctggacagacaccccagg
actccaatgtctgctccccaggggcgccggctgctcacccagagggccag
cctcccccagagccagagtgcccaggcccgcagctcaacacggggacaca
gctggtcctccagcatgtgcaggcgctgctggtcaagagattccaacaca
ccatccgcagccacaaggacttcctggcgcagatcgtgctcccggctacc
tttgtgtttttggctctgatgctttctattgttatccctccttttggcga
ataccccgctttgacccttcacccctggatatatgggcagcagtacacct
tcttcagcatggatgaaccaggcagtgagcagttcacggtacttgcagac
gtcctcctgaataagccaggctttggcaaccgctgcctgaaggaagggtg
gcttccggagtacccctgtggcaactcaacaccctggaagactccttctg
tgtccccaaacatcacccagctgttccagaagcagaaatggacacaggtc
aacccttcaccatcctgcaggtgcagcaccagggagaagctcaccatgct
gccagagtgccccgagggtgccgggggcctcccgcccccccagagaacac
agcgcagcacggaaattctacaagacctgacggacaggaacatctccgac
ttcttggtaaaaacgtatcctgctcttataagaagcagcttaaagagcaa
attctgggtcaatgaacagaggtatggaggaatttccattggaggaaagc
tcccagtcgtccccatcacgggggaagcacttgttgggtttttaagcgac
cttggccggatcatgaatgtgagcgggggccctatcactagagaggcctc
taaagaaatacctgatttccttaaacatctagaaactgaagacaacatta
aggtgtggtttaataacaaaggctggcatgccctggtcagctttctcaat
gtggcccacaacgccatcttacgggccagcctgcctaaggacaggagccc
cgaggagtatggaatcaccgtcattagccaacccctgaacctgaccaagg
agcagctctcagagattacagtgctgaccacttcagtggatgctgtggtt
gccatctgcgtgattttctccatgtccttcgtcccagccagctttgtcct
ttatttgatccaggagcgggtgaacaaatccaagcacctccagtttatca
gtggagtgagccccaccacctactgggtgaccaacttcctctgggacatc
atgaattattccgtgagtgctgggctggtggtgggcatcttcatcgggtt
tcagaagaaagcctacacttctccagaaaaccttcctgcccttgtggcac
tgctcctgctgtatggatgggcggtcattcccatgatgtacccagcatcc
ttcctgtttgatgtccccagcacagcctatgtggctttatcttgtgctaa
tctgttcatcggcatcaacagcagtgctattaccttcatcttggaattat
ttgagaataaccggacgctgctcaggttcaacgccgtgctgaggaagctg
ctcattgtcttcccccacttctgcctgggccggggcctcattgaccttgc
actgagccaggctgtgacagatgtctatgcccggtttggtgaggagcact
ctgcaaatccgttccactgggacctgattgggaagaacctgtttgccatg
gtggtggaaggggtggtgtacttcctcctgaccctgctggtccagcgcca
cttcttcctctcccaatggattgccgagcccactaaggagcccattgttg
atgaagatgatgatgtggctgaagaaagacaaagaattattactggtgga

aataaaactgacatcttaaggctacatgaactaaccaagatttatccagg
cacctccagcccagcagtggacaggctgtgtgtcggagttcgccctggag
agtgctttggcctcctgggagtgaatggtgccggcaaaacaaccacattc
aagatgctcactggggacaccacagtgacctcaggggatgccaccgtagc
aggcaagagtattttaaccaatatttctgaagtccatcaaaatatgggct
actgtcctcagtttgatgcaattgatgagctgctcacaggacgagaacat
ctttacctttatgcccggcttcgaggtgtaccagcagaagaaatcgaaaa
ggttgcaaactggagtattaagagcctgggcctgactgtctacgccgact
gcctggctggcacgtacagtgggggcaacaagcggaaactctccacagcc
atcgcactcattggctgcccaccgctggtgctgctggatgagcccaccac
agggatggacccccaggcacgccgcatgctgtggaacgtcatcgtgagca
tcatcagagaagggagggctgtggtcctcacatcccacagcatggaagaa
tgtgaggcactgtgtacccggctggccatcatggtaaagggcgcctttcg
atgtatgggcaccattcagcatctcaagtccaaatttggagatggctata
tcgtcacaatgaagatcaaatccccgaaggacgacctgcttcctgacctg
aaccctgtggagcagttcttccaggggaacttcccaggcagtgtgcagag
ggagaggcactacaacatgctccagttccaggtctcctcctcctccctgg
cgaggatcttccagctcctcctctcccacaaggacagcctgctcatcgag
gagtactcagtcacacagaccacactggaccaggtgtttgtaaattttgc
taaacagcagactgaaagtcatgacctccctctgcaccctcgagctgctg
gagccagtcgacaagcccaggactgatctttcacaccgctcgttcctgca
gccagaaaggaactctgggcagctggaggcgcaggagcctgtgcccatat
ggtcatccaaatggactggccagcgtaaatgaccccactgcagcagaaaa
caaacacacgaggagcatgcagcgaattcagaaagaggtctttcagaagg
aaaccgaaactgacttgctcacctggaacacctgatggtgaaaccaaaca
aatacaaaatccttctccagaccccagaactagaaaccccgggccatccc
actagcagctttggcctccatattgctctcatttcaagcagatctgcttt
tctgcatgtttgtctgtgtgtctgcgttgtgtgtgattttcatggaaaaa
taaaatgcaaatgcactcatcacaaa
>NM_004532 5
ctcttttgtcctcttcccaggttccctggccccttcggagaaacgcactt
ggttcgggccagccgcctgaggggacgggctcacgtctgctcctcacact
gcagctgctgggccgtggagcttccccagggagccagggggacttttgcc
gcagccatgaagggggcacgctggaggagggtcccctgggtgtccctgag
ctgcctgtgtctctgcctccttccgcatgtggtcccaggaatgacaacac
cgtcactgaagacagacggtgggagacgcacagccacatcaccaccccc
acaacctcccagaccatcatttccaccattcccagcactgccatgcacac
ccgctccacagctgcccccatccccatcctgcctgagagaggagtttccc
tcttcccctatggggcaggcgccggggacctggagttcgtcaggaggacc
gtggacttcacctccccactcttcaagccggcgactggcttcccccttgg
ctcctctctccgtgattccctctacttcacagacaatggccagatcatct
tcccagagtcagactaccagattttctcctaccccaacccactcccaaca
ggcttcacaggccgggaccctgtggccctggtggctccgttctgggacga
tgctgacttctccactggtcgggggaccacattttatcaggaatacgaga
cgttctatggtgaacacagcctgctagtccagcaggccgagtcttggatt
agaaagatgacaaacaacgggggctacaaggccaggtgggccctaaaggt
cacgtgggtcaatgcccacgcctatcctgcccagtggaccctcgggagca
acacctaccaagccatcctctccacggacgggagcaggtcctatgccctg
tttctctaccagagcggtgggatgcagtgggacgtggcccagcgctcagg

caacccggtgctcatgggcttctctagtggagatggctatttcgaaaaca
gcccactgatgtcccagccagtgtgggagaggtatcgccctgatagattc
ctgaattccaactcaggcctccaagggctgcagttctacaggctacaccg
ggaagaaaggcccaactaccgtctcgagtgcctgcagtggctgaagagcc
agcctcggtggcccagctggggctggaaccaggtctcctgcccttgttcc
tggcagcagggacgacgggacttacgattccaacccgtcagcataggtcg
ctggggcctcggcagtaggcagctgtgcagcttcacctcttggcgaggag
gcgtgtgctgcagctacgggccctggggagagtttcgtgaaggctggcac
gtgcagcgtccttggcagttggcccaggaactggagccacagagctggtg
ctgccgctggaatgacaagccctacctctgtgccctgtaccagcagaggc
ggccccacgtgggctgtgctacatacaggcccccacagcccgcctggatg
ttcggggacccccacatcaccaccttggatggtgtcagttacaccttcaa
tgggctgggggacttcctgctggtcggggcccaagacgggaactcctcct
tcctgcttcagggccgcaccgcccagactggctcagcccaggccaccaac
ttcatcgcctttgcggctcagtaccgctccagcagcctgggccccgtcac
ggtccaatggctccttgagcctcacgacgcaatccgtgtcctgctggata
accagactgtgacatttcagcctgaccatgaagacggcggaggccaggag
acgttcaacgccaccggagtcctcctgagccgcaacggctctgaggtctc
ggccagcttcgacggctgggccaccgtctcggtgatcgcgctctccaaca
tcctccacgcctccgccagcctcccgcccgagtaccagaaccgcacggag
gggctcctgggggtctggaataacaatccagaggacgacttcaggatgcc
caatggctccaccattcccccagggagccctgaggagatgcttttccact
ttggaatgacctggcagatcaacgggacaggcctccttggcaagaggaat
gaccagctgccttccaacttcacccctgttttctactcacaactgcaaaa
aaacagctcctgggctgaacatttgatctccaactgtgacggagatagct
catgcatctatgacaccctggccctgcgcaacgcaagcatcggacttcac
acgagggaagtcagtaaaaactacgagcaggcgaacgccaccctcaatca
gtacccgccctccatcaatggtggtcgtgtgattgaagcctacaaggggc
agaccacgctgattcagtacaccagcaatgctgaggatgccaacttcacg
ctcagagacagctgcaccgacttggagctctttgagaatgggacgttgct
gtggacacccaagtcgctggagccattcactctggagattctagcaagaa
gtgccaagattggcttggcatctgcactccagcccaggactgtggtctgc
cattgcaatgcagagagccagtgtttgtacaatcagaccagcagggtggg
caactcctccctggaggtggctggctgcaagtgtgacgggggcaccttcg
gccgctactgcgagggctccgaggatgcctgtgaggagccgtgcttcccg
agtgtccactgcgttcctgggaagggctgcgaggcctgccctccaaacct
gactggggatgggcggcactgtgcggctctggggagctctttcctgtgtc
agaaccagtcctgccctgtgaattactgctacaatcaaggccactgctac
atctcccagactctgggctgtcagcccatgtgcacctgcccccccagcctt
cactgacagccgctgcttcctggctgggaacaacttcagtccaactgtca
acctagaacttcccttaagagtcatccagctcttgctcagtgaagaggaa
aatgcctccatggcagaagtcaacgcctcggtggcatacagactggggac
cctggacatgcgggcctttctccgcaacagccaagtggaacgaatcgatt
ctgcagcaccggcctcgggaagccccatccaacactggatggtcatctcg
gagttccagtaccgccctcggggcccggtcattgacttcctgaacaacca
gctgctggccgcggtggtggaggcgttcttataccacgttccacggagga
gtgaggagcccaggaacgacgtggtcttccagcccatctccggggaagac
gtgcgcgatgtgacagccctgaacgtgagcacgctgaaggcttacttcag
atgcgatggctacaagggctacgacctggtctacagcccccagagcggct

tcacctgcgtgtccccgtgcagtaggggctactgtgaccatggaggccag
tgccagcacctgcccagtgggccccgctgcagctgtgtgtccttctccat
ctacacggcctggggcgagcactgtgagcacctgagcatgaaactcgacg
cgttcttcggcatcttctttggggccctgggcggcctcttgctgctgggg
gtcgggacgttcgtggtcctgcgcttctggggttgctccggggccaggtt
ctcctatttcctgaactcagctgaggccttgccttgaaggggcagctgtg
gcctaggctacctcaagactcacctcatccttaccgcacatttaaggcgc
cattgcttttgggagactggaaaagggaaggtgactgaaggctgtcagga
ttcttcaaggagaatgaatactgggaatcaagacaagactataccttatc
cataggcgcaggtgcacaggggggaggccataaagatcaaacatgcatgga
tgggtcctcacgcagacacacccacagaaggacactagcctgtgcacgcg
cgcgtgcacacacacacacacacacacgagttcataatgtggtgatggcc
ctaagttaagcaaaatgcttctgcacacaaaactctctggtttacttcaa
attaactctatttaaataaagtctctctgactttttgtgtctccaaaaaa
aaaaaaaaaaa
>NM_004785 5
ggagcccgagccggatcccgaggcgacgggagccgaacaggagccgccgc
tgaagccaccgccgggtgcccagcgccgccgccgcccccgagctcccccg
cgcccctgcccgcgggcggccggtgggcagcgggcgccatggccgcgccg
gagccgctgcggccgcgcctgtgccgcttggtgcgcggagagcagggcta
cggcttccacctgcacggcgagaagggccgccgcgggcagttcatccggc
gcgtggaacccggttcccccgccgaggccgccgcgctgcgcgctggggac
cgcctggtcgaggtcaacggcgtcaacgtggagggcgagacgcaccacca
ggtggtgcaaaggatcaaggctgtggaggggcagactcggctgctggtgg
tggaccaggagacagatgaggagctccgccggcggcagctgacctgtacc
gaggagatggcccagcgagggctcccacccgcccacgacccctgggagcc
gaagccagactgggcacacaccggcagccacagctccgaagctggcaaga
aggatgtcagtgggcccctgagggagctgcgccctcggctctgccacctg
cgaaagggacctcagggctatgggttcaacctgcatagtgacaagtcccg
gcccggccagtacatccgctctgtggacccgggctcacctgccgcccgct
ctggcctccgcgcccaggaccggctcattgaggtgaacgggcagaatgtg
gagggactgcgccatgctgaggtggtggccagcatcaaggcacgggagga
cgaggcccggctgctggtcgtggaccccgagacagatgaacacttcaagc
ggcttcgggtcacacccaccgaggagcacgtggaaggtcctctgccgtca
cccgtcaccaatggaaccagccctgcccagctcaatggtggctctgcgtg
ctcgtcccgaagtgacctgcctggttccgacaaggacactgaggagagcg
gcctccacctgagccccacggcggccgaggccaaggagaaggctcgagcc
atgcgagtcaacaagcgcgcgccacagatggactggaacaggaagcgtga
aatcttcagcaacttctgagccccttcctgcctgtctcgggaccctggga
cccctcccgcacggaccttgggcctcagcctgccccgagctcccccagcc
tcagtggactggagggtggtcctgccattgcccagaaatcagccccagcc
ccggtgagccccccatcctgcccctgcccaccaggtactgggggcctgtgg
cagcaagataggggggagagagacccagagatgtgagagagagtcagagac
agagacagagagagagagagagagagacacagagagagacagagagagagcg
agcgagcgcgcggcagccgcggggcgagggcctttgctgctctgccgggg
cctgctgactgaaaggaatttgtgtttttgcttttttttccaaaaagatct
ccagctccacacatgtttccacttaataccagagacccccccccttcccc
tccccccttcccctccccccttgggacgcgctctaaataattgcaataaaac
aaacctttctctgcaaaccatttcctccccgccccctcccctcagcagcg

gccgtcctgagtgggagtccctgggacttcccagtggccaagttggggcg
cccagcctcttcgtggggaccttgggtaaggccagggaggcctgatgtgg
ccgtaggagctgcccctgcccacctgccctggtgtgggggtccctaggcc
acaccctgctccccacccagctaccctgtgcgcctgtgccctgctggggg
cctgggctctccgaggggcctgaggatggaggccccacgtccccgaggag
ggcggcctctggacaggcccctcattccgcgcggcagctcccaggcctgg
ggaacgtaggtgtgtgagagcggcacccgggaaggacgcctggcctctgg
ctcagccctgcttggcgggctcccccgtggacaccctgttgactttgcac
ttccctcccgggccccgcacccccgaaccgaccaccgatcgaccggcacc
gctgttgcctcgtaagccatagcgcatgcgcgctctcaggataaacaggc
cctgcctggga
>NM_021910 2
cgctagcatcttctgctgatcctgaaattgtaccagcggcaagatgtggc
ctggaaggggactttaagttctccacaactgccagcaatccttccaccag
gcaaaacacatcatctaaggaaaagaagtgaggtttgcttagggcgtggc
agcttcggataaacgcaggactccgcctggcagcccgatttctcccggaa
cctctgctcagcctggtgaaccacacaggcccgagtttcacccagtcccc
actccacggtgcagctgcggcttatctctcagcccagcgagatgccagcc
ttcctgtcccgggccagcgctctgacatgcagaaggtgaccctgggcctg
cttgtgttcctggcaggctttcctgtcctggacgccaatgacctagaaga
taaaaacagtcctttctactatgactggcacagcctccaggttggcgggc
tcatctgcgctggggttctgtgcgccatgggcatcatcatcgtcatgagt
gagtggaggagctcgggggagcaggcgggccggggctggggctcccctcc
cctgaccactcagctctccccaacaggtgcaaaatgcaaatgcaagtttg
gccagaagtccggtcaccatccaggggagactccacctctcatcacccca
ggctcagcccaaagctgatgaggacagaccagctgaaattgggtggagga
ccgttctctgtccccaggtcctgtctctgcacagaaacttgaactccagg
atggaattcttcctcctctgctgggactcctttgcatggcagggcctcat
ctcacctctcgcaagagggtctctttgttcaatttttttaatctaaaat
gattgtgcctctgcccaagcagcctggagacttcctatgtgtgcattggg
gtggggcttggggcaccatgagaaggttggcgtgccctggaggctgacac
agaggctggcactgagcctgcttgttgggaaaagcccacaggcctgttcc
cttgtggcttgggacatggcacaggcccgccctctgcctcctcagccatg
ggaacctcatatgcaatttgggatttactagtagccaaaaggaatgaaag
agagctctaaccagatggaacactggaacattccagtggaccctggacca
ttccaggaaaactgggacataggatcgtcccgctatgatggaagtgttca
gacagtttataatagtaagcccctgtgaccctctcacttaccccgagacc
tcactttattacaagatctttccaaatacccaaatgtccctgcaagcccg
ttaaataattccctatgctacccttaataacatacaatgaccacatagtg
tgagaacttccaacaagcctcaaagtcccttgagactccccaatacctaa
taaggcatgcgaaatgttctcatgaactaccccacaacacgcctaaaact
caaaacacccaaaaatatctcctccaatgtcctgaaacatgaacccaaaa
agagacccacaataaactcgtgacttgtcccctcaaaaaaaaaa
>NM_001039550 1
cccccacccccccaggggacaggcggagccggccttctccccgtctctag
tagtttccgcaccgctgacgcatgcctgcgcgcacagctgggccgggcgc
gtcctacgcagcagccgcgagccgggctgcgggtgccagacggttcccgg
cgggggggcaggggcggggcgccgcaggaggccgggactcctggcggagga
gccccaaggaggcccgcctgacgactgaccagttgccatggcatcctact

acgagatcctagacgtgccgcgaagtgcgtccgctgatgacatcaagaag
gcgtatcggcgcaaggctctccagtggcacccagacaaaaacccagataa
taaagagtttgctgagaagaaatttaaggaggtggccgaggcatatgaag
tgctgtctgacaagcacaagcgggagatttacgaccgctatggccgggaa
gggctgacagggacaggaactggcccatctcgggcagaagctggcagtgg
tgggcctggcttcaccttcaccttccgcagccccgaggaggtcttccggg
aattctttgggagtggagacccttttgcagagctctttgatgacctgggc
cccttctcagagcttcagaaccggggttcccgacactcaggccccttctt
taccttctcttcctccttccctgggcactccgatttctcctcctcatctt
tctccttcagtcctggggctggtgcttttcgctctgtttctacatctacc
acctttgtccaaggacgccgcatcaccacacgcagaatcatggagaacgg
gcaggagcgggtggaagtggaggaggatgggcagctgaagtcagtcacaa
tcaatggtgtcccagatgacctggcactgggcttggagctgagccgtcgc
gagcagcagccgtcagtcacttccaggtctgggggcactcaggtccagca
gacccctgcctcatgccccttggacagcgacctctctgaggatgaggacc
tgcagctggccatggcctacagcctgtcagagatggaggcagctgggaag
aaacccgcagatgtgttctgagctggatgccgggttccagaatcgctgca
cagttccaacaggacagcgccttccccccatgcgctgggaggggaccctcc
atttctccccctcacccatgctgagtgtagagccggggcctgggtggcgg
gtgggggccgggtgggaggtggcagtagtcttagcctgtgcactctcttc
cttgggtgtttggtgctggctcctggggactacaaatcccagagtgcggt
gtgcccggcctcatttctgatagatcccgcttgggggaggtggtgtatgg
ttacggagctgtgcatcttgggacatgtagtagcccaggtcggcttgtca
ctcgctgtgagatggggagattttgtcttttgatttatccctgtagggct
ggcagggttgtagatgaagggggaatgatctgagccttggttcccctgac
acgtcttgctagccccagggttagagtgggcagggcagagccgcgcagca
cctgggagcggtacctttcccttgggcagcctggggtcccaggaacaagc
cagggcgagtggcatgtctgcctgagcagggtgtggccccagaaagctga
ggagtgtgggctggcagagagcttcgagggcaaggccacccgcgggggcg
tgtgtgtggtggggcttggcatgtgatggcagctccaggcatgccgctgc
ttgtatggctttctttggcctctgaccctgctgcccattctttccaacat
cacagatgaactgcctctcctcctccctgcctggggagcccagtggccag
ggaggggagtggtggagccagtcgctgtaacactgagcctcagagacgaa
ccaaaaccagctgggctgagctcagatccagggggaaatgctggaagtca
ataaaactgagttttgagagcttgaaaa
>NM_005165 2
ccggaggagtcacgtagctctgcgacatccgcagcctcatttaccagagg
gagccagggctgcagcctcatctgtttgcggatcagaacccgagctgtgc
ttgtggctgcggctgctaactggctgcgcacagggagctgtcaccatgcc
tcactcgtacccagccctttctgctgagcagaagaaggagttgtctgaca
ttgccctgcggattgtagccccgggcaaaggcattctggctgcggatgag
tctgtaggcagcatggccaagcggctgagccaaattggggtggaaaacac
agaggagaaccgccggctgtaccgccaggtcctgttcagtgctgatgacc
gtgtgaaaaagtgcattggaggcgtcattttcttccatgagaccctctac
cagaaagatgataatggtgttcccttcgtccgaaccatccaggataaggg
catcgtcgtgggcatcaaggttgacaagggtgtggtgcctctagctggga
ctgatggagaaaccaccactcaagggctggatgggctctcagaacgctgt
gcccaatacaagaaggatggtgctgactttgccaagtggcgctgtgtgct
gaaaatcagtgagcgtacaccctctgcacttgccattctggagaacgcca

acgtgctggcccgttatgccagtatctgccagcagaatggcattgtgcct
attgtggaacctgaaatattgcctgatggagaccacgacctcaaacgttg
tcagtatgttacagagaaggtcttggctgctgtgtacaaggccctgagtg
accatcatgtatacctggaggggaccctgctcaagcccaacatggtgacc
ccgggccatgcctgtcccatcaagtataccccagaggagattgccatggc
aactgtcactgccctgcgtcgcactgtgcccccagctgtcccaggagtga
ccttcctgtctgggggtcagagcgaagaagaggcatcattcaacctcaat
gccatcaaccgctgcccccttccccgaccctgggcgcttaccttctccta
tgggcgtgccctgcaagcctctgcactcaatgcctggcgagggcaacggg
acaatgctggggctgccactgaggagttcatcaagcgggctgaggtgaat
gggcttgcagcccagggcaagtatgaaggcagtggagaagatggtggagc
agcagcacagtcactctacattgccaaccatgcctactgagtatccactc
cataccacagcccttggcccagccatctgcacccacttttgcttgtagtc
atggccagggccaaatagctatgcagagcagagatgccttcacctggcac
caacttgtcttcctttctctcttcccttcccctctctcattgctgcacct
gggaccataggatgggaggatagggagcccctcatgactgagggcagaag
aaattgctagaagtcagaacaggatggctgggtctccccctacctcttcc
agctcccacaattttcccatgatgaggtagcttctccctgggctctcctt
cttgcctgccctgtctcctgggatcagagggtagtacagaagccctgact
catgccttgagtacataccatacagcaaataaatggtagcaaaacattct
aaaaaaaaaaaaaaa
>NM_001092 3
gcgggctccggctgccatggaggaggaagaggaggcgataggcttgctgg
acaaggttctggaggacgaagatgtgtttctcctggaggagtgcgagctg
ggaaccccgaccagccccggctcagggtccccccttcctggtggctgtgaa
ggtggaagcagggaaaggcctggagatgaggaagctggttctctcggggt
tcttggccagcgaagagatctacattaaccagctggaagccctgttgctg
cccatgaaacccctgaaggccaccgccaccacctcccagcccgtgctcac
catccagcagatcgagaccatcttctacaagatccaggacatctatgaga
tccacaaggagttctatgacaacctgtgccccaaggtgcaacagtgggac
agccaggtcaccatgggccacctcttccagaagctggccagccagctcgg
tgtgtacaaagcgtttgtcgataactataaagtcgctctggagacagctg
agaagtgcagccagtccaacaaccagttccagaagatctcagaggaactc
aaagtgaaaggtcccaaggactccaaggacagccacacgtctgtcaccat
ggaagctctgctctacaagcccattgaccgggtcactcggagcaccctag
tcctacacgacctgctgaagcacacacctgtggaccaccccgactacccg
ctgctgcaggatgccctccgcatctcccagaacttcctgtccagcatcaa
cgaggacatcgacccccgccggactgcagtgacaacgcccaagggggaga
cgcgacagctggtgaaggacggcttcctggtggaagtgtcagagagctcc
cggaagctgcggcacgtcttcctctttacagatgtcctactgtgtgccaa
gctgaagaagacctctgcagggaagcaccagcagtatgactgtaagtggt
acatccccctggccgacctggtgtttccatcccccgaggagtctgaggcc
agcccccaggtgcacccccttcccagaccatgagctggaggacatgaagat
gaagatctctgccctcaagagtgaaatccagaaggagaaagccaacaaag
gccagagccgggccatcgagcgcctgaagaagaagatgtttgagaatgag
ttcctgctgctgctcaactcccccacaatcccgttcaggatccacaatcg
gaatggaaagagttacctgttcctactgtcctcggactacgagaggtcag
agtggagagaagcaattcagaaactacagaagaaggatctccaggccttt
gtcctgagctcagtggagctccaggtgctcacaggatcctgtttcaagct

taggactgtacacaacattcctgtcaccagcaataaagacgacgatgagt
ctccaggactctatggcttccttcatgtcatcgtccactctgccaaggga
tttaagcaatcagccaacctgtactgtaccctggaggtggattccttcgg
ctattttgtcagcaaagccaaaaccagggtgttccgggacacagcggagc
ccaagtgggatgaggagtttgagatcgagctggagggctcccagtccctg
aggatcctgtgctatgagaagtgctatgacaagaccaaggtcaacaagga
caacaatgagatcgtggacaagatcatgggcaaaggacagatccagctgg
acccacaaaccgtggagaccaagaactggcacacggacgtgattgagatg
aacgggatcaaagtggaattttccatgaaattcaccagccgagatatgag
cctgaagaggaccccgtccaaaaagcagaccggcgtcttcggtgtgaaga
tcagcgtggtgacgaagcgggagcgctccaaggtgccctacatcgtccgg
cagtgtgtggaggaggtggagaagaggggtatcgaggaggttggcatcta
caggatatcgggcgtggccacggacatccaggcgctcaaggccgtcttcg
atgccaataacaaggacatcctgctgatgctgagtgacatggacatcaac
gccatcgccgggacgctcaagctgtacttccgggaactgcccgagccgct
cctcacggaccgactctacccagccttcatggagggcatcgccctgtcag
accctgctgccaaggaaaactgcatgatgcacctgctccgctccctgccc
gaccccaacctcatcaccttcctcttcctgctggaacacttgaaaagggt
tgccgagaaggagcccatcaacaaaatgtcacttcacaacctggctaccg
tgtttggacccacgttactgagaccctcagaagtggagagcaaagcacac
ctcacctcggctgcggacatctggtcccatgacgtcatggcgcaggtcca
ggtcctcctctactacctgcagcacccccccatttccttcgcagaactca
agcggaacacactgtacttctccaccgacgtgtagcccgaggcagggtgg
ctgcgggcgggtggtggaaccagcccctccagcctggggtccaactcaga
cttgaaagactgcaatagaaaactcccaaacccagcactccagactcgag
ggaagccagcttccaagaactggaatgcgtacgtcttttgtgccaccttg
tacaaagccggctgcccagccccagcctcaccaccgcatcccacctcctg
ccctccatacctctagttgtgtctgatgctccgtgctgttcgggaattgt
tttatgtacacttgtcaggcagaaaaggtagtgaccggcccggcgtgggc
acacagacagcccgctttgttctttcatttcctccagcactttctttccg
cctgagtccagcccaaggccttttattttgcgctgtgtaactgctgccag
cttctctcttggccctgctcccagatggcggtctcctggcagcctcccct
cagtcttcctccacccgctcttccttcccagcctgcctgcatgcatgtgc
acccttggtcttcgctccatcgccttgaaagctctgaagaggccctgggt
tgccgcggcagcagtggtctgtttgatgctgccgtttgccgctgccggcc
cctcctcagactccgcctttgggagcacacctgctttgccttgctgcctg
tgcaaatgttggacaagcagacacactcacactcgtccccagcttagcac
agagctggagcgcccatttctggaattttccgtttgggaatctccacttc
tggggtttacctgttcggcctcctgtctatcagtgaggcatctctgactg
tttcttctactgcttttcagttcccttccctgctgttctatttcctttga
gtgtaaagactcacaggtgacctgctatcgagatagccagagggtcagga
gagaatgggggaggaggcggtcaggctgctgaggaaacaccacaggctga
acgggggaggaatgcacatgccacgctgggtgtcccgggtcgcggggagg
cagctcagctcttaggagcaagttgtgggggcttttcaagaggggccagg
cttcctggagggtgactgatgtggccgaagcaggtgtccaggcaggtagg
ctgcagccaggagctccctggcaccgcaggacctcgtggtactcttgcct
tagattttacacacactccacagccaagcactgccacggtcctccaggac
ctgggaagcaaaggcacaggcccacggtggccagccattgtggtgccgcc
ccagcttctggatacagccttttgggtaaacactgggaactccagaagtt

gtggggagagtggggaatcagacagccgcctctaggggctgggttctgct
ggggcctccttgttggtgctgtaggcacccgccagggagcagggacccga
cttgcagacgcattgcccggtactaggaaggagtgaggtgtgttcccacc
gtacacttcccacacgagctgcggctgccagcctcgggccatcagcctag
gagagcagatgcagctccaggggctcgacttatagccagttacagctccc
cggctcttctgtgtggcagagcgtcgtttccgggccctcagggctgggga
gctcagttcccattgcttgtgctcagggctgagtcttaaagaagggtttg
ccggccctaacgctgcagcgcgtgcgcggtgagaggccctttttgagcct
gtttactcctgtggccttgggcagaacagtaaatactctgtgcacggagg
aaagacatgcccaagaggaaggaagtactgaccatcggctgcctgtgagc
agcttagcaaggagcccttgctccctgggaaaggcggtgaacttgagtct
aaagatgcagtgcctggcccttcctaaggtccctgcctggcatccgagtg
tcggtgtgtggcacagaaggctcctgcttgcttccaaagtgatggacagg
aaggggcagagtgagtcacggcccagactgggcaccttcgcgtctcagcc
tcagggagccccacagccccaagctcgctgaggcaacgtgagaacaggct
atgggaaggctgcaaaggctgagaaatgcaaaggctcatatttataaatc
ccacccccagagtggggagggtcaggtgccagacctggactaaactgcac
caaggaaacacccagcagggtctcctgtgagccggggaccatgcagcccg
aaacctccagtcactgcgcccggcaggagtcaggagccagggactgtgca
gcctggaacctccagtcactgtgcccagcagggtgggctgtgcccagcag
gagtcaggctaagaaacgccaggtctgcctgttcttgctgggcaatggct
gatggctgccagtttctgctgatacacaggtaggatgggacccttcatga
atatctgactttaataagttggtaaggatatatttttttgtctatgttct
gtttcaacttatgtagattattataaattgatgtaaaccacgtgagagga
aaatgttaataaaaaatgcaaagccccatcatttgcacaaaactcaaaaa
aaaaaaaaaaaaaaaaaaaaaaaaaaaaaaaaaaaaaaaaaaaaaaaaaa
>NM_015185 2
aaaaaggcagcctatgggctgagcgggggatggggagagaagagggatag
ggccagcaaggcagggatcgaacgagtgtctggcagccgggagcccagcg
aagagagcgagcaagcttaggaaaacgagcgaagtaaagggagtagggga
gactgagactgaccggtagccaggcaggcggacggacgcacgcccggaca
gactgagcaggcgccggagaaccactcacaggttcccccgcctttccct
ttgaaagctaggattttgcctttcccgtggcgcccgagagagaatgctgg
actctgccgacttcagcgcaagctaagatttctcagctagggacaaacga
tcagcccaatcctgagaagggggaaccaagcaccccgtccccatccccc
tcccctcccccgactaaactcgggcgccaaacccagcccttctctaacca
ccctacttcctcctctcctttctagcatggtggctgtatggacagtctga
cagaacagagactgacatctcccaatctgccggccccccacctggaacac
tacagtgttctgcattgcaccatgaccctggatgtgcaaactgtagtcgt
ttttgccgtgattgtagtcctcctgcttgtcaatgtcatactcatgtttt
tcctgggaacgcgctgaatggagtccagccacctgagctgtcgcgaactc
tcgctttgatttcatcccgagagccaccgagaaaaaaaaaaaaatcacaga
cagagacagggaaagagagagaaagaacaagctttcttactcaggggga
aaacgttttgagcttcaacatggcctcgctgtgatatgtatgacgttgct
gatcactggagattccatcgttagtgctgaggcagtatgggatcacgtca
ccatggccaaccgggagttggcatttaaagctggcgacgtcatcaaagtc
ttggatgcttccaacaaggattggtggtggggccagatcgacgatgagga
gggatggtttcctgccagctttgtgaggctctgggtgaaccaggaggatg
aggtggaggaggggcccagcgatgtgcagaacggacacctggaccccaat

tcagactgcctctgtctggggcggccactacagaaccgggaccagatgcg
ggccaatgtcatcaatgagataatgagcactgagcgtcactacatcaagc
acctcaaggatatttgtgagggctatctgaagcagtgccggaagagaagg
gacatgttcagtgacgagcaactgaaggtaatctttgggaacattgaaga
tatctacagatttcagatgggctttgtgagagacctggagaaacagtata
acaatgatgacccccacctcagcgagataggaccctgcttcctagagcac
caagatggattctggatatactctgagtattgtaacaaccacctggatgc
ttgcatggagctctccaaactgatgaaggacagccgctaccagcacttct
ttgaggcctgtcgcctcttgcagcagatgattgacattgctatcgatggt
ttccttttgactccagtgcagaagatctgcaagtatcccttacagttggc
tgagctcctaaagtatactgcccaagaccacagtgactacaggtatgtgg
cagctgctttggctgtcatgagaaatgtgactcagcagatcaacgaacgc
aagcgacgtttagagaatattgacaagattgctcagtggcaggcttctgt
cctagactgggagggcgaggacatcctagacaggagctcggagctgatct
acactggggagatggcctggatctaccagccctacggccgcaaccagcag
cgggtcttcttcctgtttgaccaccagatggtcctctgcaagaaggacct
aatccggagagacatcctgtactacaaaggccgcattgacatggataaat
atgaggtagttgacattgaggatggcagagatgatgacttcaatgtcagc
atgaagaatgcctttaagcttcacaacaaggagactgaggagatacatct
gttctttgccaagaagctggaggaaaaaatacgctggctcagggctttca
gagaagagaggaaaatggtacaggaagatgaaaaaattggctttgaaatt
tctgaaaaccagaagaggcaggctgcaatgactgtgagaaaagtccctaa
gcaaaaaggtgtcaactctgcccgctcagttcctccttcctacccaccac
cgcaggacccgttaaaccacggccagtacctggtccccgacggcatcgct
cagtcgcaggtctttgagttcaccgaacccaagcgcagccagtcaccatt
ctggcaaaacttcagcaggttaaccccttcaaaaaatgatacctacagg
gaggcagataattttaaaataaagtaaataaaattatatttatagatgga
cctttttcggagaagcactgttgaaatttatacacacacacacacacag
agacccttgagtacacatacacacacacacacacagacacacacacacac
acacacacacacacacacagagagataaggaacaaaagtgttttctgt
tgttttggggaagtgaaatatgtggttggtaggaagaggtaccaatgact
tccaaacatgtgattccgtcttaaaagttttccatttttaccctgtcccc
cttccctttgctttcagaagttgacatttctattcattgcttttcttgtt
aagataatctctttactcccctgtgagtgattcactgccttgtcattatt
acgatagatgtgtttgtattgttttttttctgatgatactgatgttgatg
aatttttaattttatttgatgtggtagagttgggaggtttcagggttttt
tcccctcttttactttccattgaggaagggaatgagctcctttctcctct
ccttcagccaatcattatcaaatgttccttcagccctgcagttgccccaa
ataaccttttttcagcatcctctgtcctcagtcatgccagtctggacatg
ctctgttgtgccctgtgacaaaactgctcagtattcctattgcttttact
gtgttttaggtactgtgaagggatcaaaaaaccaaacagaagcaagggag
tatcagactatgatgatgctggagtggacttctgttcagggaacattttg
cattcaggctgtttcttctatcactggggtttcccatgttgcagcacttc
tgggtcgttgcaattttgcatctaggagttagtttgatcgagttattctc
ttttttcaagtcactttttgttataggtctccccctaggcctgtctctccc
ttagcccaaaagatctgaactggaagcagaggttgagattctgcctccca
ggagagggatttacctgccccctagtaccagataggtttagggcagtgat
ctctacagcaatcagttcagtgtcctggttgtccctgctcccatttacag
atgtttgggcagcattgatagaagtatggaggggttcaagacagagccca

cctgatcaagatcatcagctaccttcaaattattgacctggacagggtcc
aagtctgatagtaaccttttacaagaaagaacagggatgggaatggaaag
agatagccttgatccacagtattgtacctgcattttctaccaccctaaaa
ttgtgtgagacttctcccattgttaacagattgcatggacaatcttccct
ggcttctttctttccctctctctttcttctttctcctgccatcctagcac
aggaggatttttggtattgatatagttaaagctgttctggcactcaaaga
aggccgtgtttccaacatcctctcatcccaggacatttggggcaagtgag
ttaggggcccaggggcaattttccctctgaataacgtgtctgaggcaggg
atgctaccctcaggctcgcttttggccagctttttgcttgggaaaatcta
acttctttcacaaggaggcaggcttcctatggatgttggagtacctgttt
ttcctccacacatagcccttttcatggatagaccttgaacaacaaaaagg
gtataagggaataaggatgaactctgctgtgaagagcaagccactgtagt
gaggaatgtggagactgggagtctgtcctaaaccccatgggagaagactt
catcatgacaggacttcagcttaccaagcagcagccatagctgtgtggag
gcttcagcatagctagcatgtttactgctctatgcctcctgatccagacc
aggcattgcccagcctgggaatcttttctttgtgggaatcaaattacaag
ctatttaagtttatattccatcacaaccaagtcagacttgtattataagt
caaggatgagcctgatctggggagagggccggggctcgggactggccacc
actgttcagcacatgacctaactacgtaagcctctttggcaagggtcctg
gtgcccagcacccaggctaaaatatcctgtctggcagagtgttttggtag
ctatgcaggcctcccttcagtgtacctctttttccaacttctcactcctc
cttactaggcttggccttgacatgcttcttcgagggttggcagcacaccg
ggaggggatgcttggacaagtttctgggcctacatttcttgactaggccc
tctcatttcctccctccttggggcttctgcccagggctccaggatcaggg
atattacttctcaacccgcacttctcctctactgaacccactggcatcac
ctgatgccactaatttgtgaacaacaagaaatcatttccccattggttgg
agtattccctcagcctatagcatcaaagcagaccagtggccaacagcccc
aaggggagcccaattaaatacctgggttcagtatcctaacctgttatgtc
ctgacagcaatggtaaccccagtaattctgtaatgttgtaatttccgcat
ggccctgagctcccttttcctcaactcagtgaggccaggatttgctctcc
aaaaggctttgctagtgtgttcaatgggacctgctgtggggagtcctaag
acagacatctaattattctctctttttccccccctctctatgtgtatatt
tctaatggatctataagaacagcaacaagagagttctaacaattctagtg
tgaagccaaatagtgatcttttagtgctttggggatggggtgggctgggg
tggatggatgggcaacagtgactttgattacccttgctgctctgcatttg
ccagtttattcttttgtttcttttatctgactgactctgtcaaacaagtg
tcaaagttgtgtgttaaaaaatgtttaacaaaaaaaatgttgtaatgaca
caaagccttatgaaaatatttatggagttcaataaaagaagtaaaaagac
>NM_004644 3
ccctgattgagaaatccgcgcgcagcctccgccgctgcacactgcagcct
ctgctgaacccctgccgcgccgccgccttcgccagcccggaccggaccgg
cccgccttcccttcctccgctccagccggcctccggccccgcaacctcct
cctcggcgaagtctccctggccgccccatgtcggccgcccccgcctacag
cgaagacaagggcggctccgctggccccggggagcccgagtacggccacg
accccgcgagcggcggcatcttctcctccgactacaagcggcatgatgac
ctgaaggagatgctggacaccaacaaggattctctcaagctggaggccat
gaagaggattgtggcgatgattgcccgaggaaagaatgcttcagacctgt
ttcccgcggtggtgaagaacgtggcctgtaagaacatagaggtgaagaag
cttgtctatgtgtacctggtacgctacgctgaggagcagcaagacctggc

cctgctgtccatctccaccttccaacgtggcctaaaggatcccaaccagc
tgattcgtgccagtgccctccgtgtcctctctagcatccgtgtgcccatc
atagtgcccatcatgatgctagctatcaaggaagccgcctcggacatgtc
accctatgtgcggaaaacagctgcccacgccatccctaaactctacagtt
tggactctgaccagaaggatcagctgatagaagtcattgagaagcttctg
gctgacaagaccacgctggtggcgggcagtgtggtgatggcctttgagga
ggtctgcccggagcgcatcgacctgattcacaaaaactaccggaaactct
gtaacctgctgatcgacgtggaggagtggggccaggtggtcatcatcagc
atgctcacccgctacgcccgcacgcagttcctgagccccacccagaacga
atccctactagaggagaacgcggaaaaagccttctacggctcagaggagg
acgaggccaagggcgcggggtctgaggagacggccgccgcggccgccccc
tcccgaaagccctatgtcatggaccccgaccaccggctgctgctgcgcaa
caccaaacccctgctgcagagccgcagcgccgcggtggtgatggcggtgg
cgcagctctacttccacctggcgcccaaggcggaagtgggcgtcatcgcc
aaggcgctggtgcgcctgctgcgcagccacagtgaggtgcagtacgttgt
gctccagaacgtggccaccatgtccatcaagcgccggggtatgtttgagc
cctacctgaagagcttctacatcaggtccaccgaccccacccagattaag
atcctgaagctggaagtgctgaccaacctggccaatgagaccaacattcc
tactgtcctacgggaattccagacctatattcgcagcatggacaaggact
ttgtggcagccacaatccaggccattggacgctgtgcaactaacatcggc
cgagtccgtgacacctgcctcaatggcctggtgcagctgctgtccaaccg
tgatgagcttgtggttgcagagtcagtggtcgtcattaagaaattgctac
agatgcagccagcacaacatggagagatcatcaaacacttggcaaagctt
acagacaacatccaggtgcccatggcccgagccagcatcctgtggctcat
cggagagtactgtgagcatgtccccaggattgcacctgatgtcttaagaa
aaatggccaagtcattcacagcagaggaggatattgtcaagctgcaggtc
atcaacctggcagccaagctctacctgaccaactctaaacagaccaagct
gctgacccagtatgtgctgagtctggccaaatatgaccagaactatgata
ttcgcgaccgggcgcgcttcacccggcagctcatcgtcccttccgagcag
ggtggggccctcagccgccatgccaagaagctcttcctggcacccaaacc
agctccagtcttggagtcatccttcaaagaccgggaccacttccagctgg
gctcactgtcccacctgcttaatgccaaggccacaggctaccaggagctc
ccagactggccggaggaagccccagacccatctgtgcgcaacgtggaggt
acctgaatggaccaagtgctcaaatcgggagaagagaaaggagaaggaaa
aacccttctactcggactctgagggggagtcaggccccacggagtccgca
gacagtgaccctgagtctgagagtgaatcggacagtaagagcagcagtga
gagcggctctggggagtccagcagtgagtccgacaatgaagaccaggatg
aggatgaggagaaagggagaggcagtgagagtgaacagagtgaggaggat
ggtaagaggaagacaaagaagaaggtgccagagagaaaaggagaagcgtc
atcctctgatgagggcagcgattccagcagtagctcatcagagtccgaga
tgacatcggagtccgaggaggagcagttagaacctgcctcctggagcagg
aaaacacctcccagcagcaaaagtgctcctgcaaccaaggagatctccct
gcttgatctagaggatttcacccctcccagtgtccagcctgtgtctcccc
cagcaattgtgtctaccagtctggctgctgacctggagggcctgacactc
acagactccaccctggtaccgtcgcttctgagtccagtatcgggtgttgg
gcggcaggagctgctgcaccgggtagctggcgaggggctggctgtggact
acaccttcagccgccaacctttctccggggatccccacatggtgtccgtg
cacatccacttctccaacagctctgataccccatcaagggcctgcatgt
gggcactcccaaactgcctgctggcatcagcatccaagaatttcccgaaa

ttgagtccctggcacctggagaatctgccactgctgtaatgggcattaat
ttctgtgactcaacccaggcagccaacttccagctgtgcacccagacccg
acagttctacgtctccattcagccacctgttggggagctgatggcccctg
tgttcatgagtgaaaatgagtttaagaaggaacagggaaagctgatgggc
atgaatgagatcacagagaaactcatgctgccagacacctgtcggagtga
ccacattgtggtgcagaaagtgactgccactgccaacctgggtcgtgttc
cttgtgggacatctgatgagtacaggtttgcagggaggacactgactggt
ggaagcctcgttctgctgaccctggatgcccggccagctggagctgccca
gctgactgtcaacagcgagaaaatggtgattggcaccatgctggtaaagg
atgtgatacaggctctgacccagtgacttccaaatgctgtgacctgtttg
gctcccatctatacctccccatgacacctaggctgtcagtctctctcatc
tttctctctctctcatcatcctcctcatgccagatagcattcagggtg
tcctctctccctctggaggaccaagccctcccctaatgccctccccatgg
attccttagtgatctgtgcagagagagatggcagccactcccttcagtcc
tcccagatttctgtagctatttatgtagcaggctcaataaaatgtcttct
ctctaaaaaaaaaaaaaaaaaaaaaaaaaaaaaaaaaaaaaaaaaaa
>NM_005648 3
cagacggaagtgagcgacacactctgcgtcctcgcctcaccaggtacgga
tctcccgcgcgtcgccgaaagcggctctgacccagcggaagtaattcttt
cgactgccccggaacccaccggagcaggcagctgggggtgggggggcggc
cctgggataggggctgtggcagtacgcggggacccggctgcggtggctgc
gggactgacgagaaactactaaagttcctggggaagcaaagtagaatttc
ataagaacaaaatggatggagaggagaaaacctatggtggctgtgaagga
cctgatgccatgtatgtcaaattgatatcatctgatggccatgaatttat
tgtaaaaagagaacatgcattaacatcaggcacgataaaagccatgttga
gtggcccaggtcagtttgctgagaacgaaaccaatgaggtcaattttaga
gagataccttcacatgtgctatcgaaagtatgcatgtattttacgtacaa
ggttcgctacactaacagctccaccgagattcctgaattcccaattgcac
ctgaaattgcactggaactgctgatggctgcgaacttcttagattgttaa
ataaaataaattataataaactgttaactcttttcagtatttaatacctg
tagttcagttagtaactttttcatatatagcatgttgcctgtatgcagtt
gaactatataaagttcattgcaaagcagattatcttgtttttttgcatag
caatcaaagttgaaatttgtttgctacatcaacaaattaaggacattttc
acaaactgagaaataaacaaatatgccaattcgtaggtggttttgcctta
tcctttgaatgtgacttaaaatcagcaatgatgatatagtaaatactgaa
atttaggtgtaaatcaatacgttctacagggaaataatgaggctaagtat
ttttatgtttttagtggtttttttagaaacctaatcttatagccgccatta
gcattactagagttatgcaaataattgcattataaacatgtttataactt
agccaaaacattgatttttataactctccaagtatgagttgaaatttctt
atgtcttttgataaactgcagtattctttaagtgtactttgtccttttgt
ttattgctacaatttaagaactttatttaaaaacaattttggaaggttac
taggtacagcttttgcagaagaaactgaactgtagccagatggtcagcaa
gtaaactacatgacttagagttcttgtgcattatacattttgggtaattc
aaagtactgtatttgcattgactataaagcgtcgtttgcaatatttattc
ttccttcatagtcccttgttgtgattatacttccagttgtttaaatgtat
tttaaaagactcactattaactttattcagatcttaaaagctacaccttt
ttgaactattccagtctttctccatccaattagaatgttatggaataaca
tgtgaagagatacagtgaaaatcatattataatgcattgaagatatccta
catgaattatcttctattaaaatatgtcccaataaatatacccataatca

gtgggtcttctactgaaatcaagctcattcagcttttctattaagcagcc
aaaaaatttttgaaaaaatatgacagcatcattccttatgcatggggaa
agaagcactggagatgggtttagtattgtctttatctcagaggtggaaca
aagttgtcattatagggtctataaacttaagtctccagcttatttgagac
tggcttaatatggctgagcccggtggctcatgcctgtaatcccagcactt
tcagaggacgaggcgggtagatcacttgaggccaggagtttgagaccagc
ctggccaccatggcaaaaccccatctctaccaaaaatgcaaaaattagct
aggccatggtggcacacgcctgtaatcccagctacttgtgaggctgaggc
atgagaatcgcttgaacctgggaggtggaggtggcagtgagccgagatcg
caccactgcactccagcctgggtgacagagcgagactgtctc
>NM_006158 3
gagccgcacacagccatccatcctccccttccctctctcccctgtcctc
tctctccgggctcccaccgccgccgcgggccggggagccaccggccgcca
ccatgagttccttcagctacgagccgtactactcgacctcctacaagcgg
cgctacgtggagacgccccgggtgcacatctccagcgtgcgcagcggcta
cagcaccgcacgctcagcttactccagctactcggcgccggtgtcttcct
cgctgtccgtgcgccgcagctactcctccagctctggatcgttgatgccc
agtctggagaacctcgacctgagccaggtagccgccatcagcaacgacct
caagtccatccgcacgcaggagaaggcgcagctccaggacctcaatgacc
gcttcgccagcttcatcgagcgcgtgcacgagctggagcagcagaacaag
gtcctggaagccgagctgctggtgctgcgccagaagcactccgagccatc
ccgcttccgggcgctgtacgagcaggagatccgcgacctgcgcctggcgg
cggaagatgccaccaacgagaagcaggcgctccagggcgagcgcgaaggg
ctggaggagaccctgcgcaacctgcaggcgcgctatgaagaggaggtgct
gagccgcgaggacgccgagggccggctgatggaagcgcgcaaaggcgccg
acgaggcggcgctcgctcgcgccgagctcgagaagcgcatcgacagcttg
atggacgaaatctcttttctgaagaaagtgcacgaagaggagatcgccga
actgcaggcgcagatccagtacgcgcagatctccgtggagatggacgtga
ccaagcccgacctttccgccgcgctcaaggacatccgcgcgcagtacgag
aagctggccgccaagaacatgcagaacgctgaggaatggttcaagagccg
cttcaccgtgctgaccgagagcgccgccaagaacaccgacgccgtgcgcg
ccgccaaggacgaggtgtccgagagccgtcgtctgctcaaggccaagacc
ctggaaatcgaagcatgccggggcatgaatgaagcgctggagaagcagct
gcaggagctggaggacaagcagaacgccgacatcagcgctatgcaggaca
cgatcaacaaattagaaaatgaattgaggaccacaaagagtgaaatggca
cgatacctaaaagaataccaagacctcctcaacgtgaagatggctttgga
tattgagattgcagcttacaggaaactcttggaaggcgaggagacccgac
tcagtttcaccagcgtgggaagcataaccagtggctactcccagagctcc
caggtctttggccgatctgcctacggcggtttacagaccagctcctatct
gatgtccacccgctccttcccgtcctactacaccagccatgtccaagagg
agcagatcgaagtggaggaaaccattgaggctgccaaggctgaggaagcc
aaggatgagccccctctgaaggagaagccgaggaggaggagaaggacaa
ggaagaggccgaggaagaggaggcagctgaagaggaagaagctgccaagg
aagagtctgaagaagcaaaagaagaagaagaaggaggtgaaggtgaagaa
ggagaggaaaccaaagaagctgaagaggaggagaagaaagttgaaggtgc
tggggaggaacaagcagctaagaagaaagattgaaccccccatttccttaa
ttatttcaggaataattctcccgaaatcaggtcaaccccatcaccaacca
accaaccagttgagttccagattctatgtgaattaaaaagtcaatatatg
tataattctgagatgacttaggttggacattcaatgttgtgctatgaatt

tcctctttatgcagagtatctgtttgcttgcagagtggctttctggcttg
ctgccagcctgtgcatggaccacgcttatgagttcaggatctacggcaat
gtgaatcattcagatgtttacaataaaaaacaccacatgagtaaatgaat
tcactaatgttaatgttaaacttcatggaaaaatagtcctttgaaccttc
ggtggttagcaattaaagaccctgagttatgtgcaataaatagtaaataa
agttataccgaatgatgtattttttgctgtggttgttacttaattaaaat
accttaaagatggcaccaatataaagtatataccagtggactattgacct
ccaatttttaaaaagttgaaattttaacaattaccaatacttttttttc
ttccttcaattggaaattctgagggatacagttatgtcatgattacttgt
gaaatctctccccacaaaaattttactgataataaacatgagtaaatgag
aaagtctataaataacaatagactttgcctcataacacgtgttgtgttag
gttttgggatggctttctagggagcaattctgaaattacttgacagatac
actctttatggtaaacaggtcatttcagagtctagagtgctaaccattac
actatggaaccatgataaataggtcatttcaaactgatatgtcacaatgc
aatatggatagaatatgaatcccatacattcatccctataactgcatttt
aaaatatagtctaaatatcatgggaggaggttaaaacctgagaacatggt
acaataagaaggaataatttttatctatatataatttcaggagaaggga
ttaaaactaccaaaggttatttgccactccactgcaaacatgttgcctgc
aatcctgagctcatgatttagtgtgttgttaagcgtaattctgttgcctc
aagtaaataccacttaatgctacacatttttaaacctttaaaagctacag
acacaagtaatgaaattatgagtcacttaagggggggaggtaatatgcttg
tatgttacattgacattgctctgcatgtctctactgggagatatttggat
ttcaatgatagcttgactggttggacattgcatcaaatacacgccccatg
tatcctttctgtcgtaaagttaagaacgctcttgcatttgatgtggaata
aatatagatgatattactatctgctattctgcctttgttctgaaacaatt
gctttgtcagctaatttcattcaatatttgtttttagacgatctctata
agttattaatttaacctgaaaccaaagtggtctccatttaaagttactta
atccctttgtaccacctatttctagttaaatatatgttgctatgcaaata
ggtaaagtgcttccttgccatgatggtaatggattggaactatgaaggct
ctcagtgtattggcttctgtaaagatgaggcgtctcctcagaaacaaaac
ttttcacatttctgcttactagacctgggttgatgtacatggtaagtctc
aaacagatgcaagctatgtgcaaaaagtaactttagccaaatggaaatag
ctggatgctttgagaattacttggttgaagtaagaaaactgtaccatcct
ctatcctgtgtgccataataaaatagtaaaaaacctaaaaaaaaaaaaaa
aa
>NM_001201 2
agatcttgaaaacacccgggccacacacgccgcgacctacagctctttct
cagcgttggagtggagacggcgcccgcagcgccctgcgcgggtgaggtcc
gcgcagctgctggggaagagcccacctgtcaggctgcgctgggtcagcgc
agcaagtggggctggccgctatctcgctgcacccggccgcgtcccgggct
ccgtgcgccctcgccccagctggtttggagttcaaccctcggctccgccg
ccggctccttgcgccttcggagtgtcccgcagcgacgccgggagccgacg
cgccgcgcgggtacctagccatggctggggcgagcaggctgctctttctg
tggctgggctgcttctgcgtgagcctggcgcagggagagagaccgaagcc
acctttcccggagctccgcaaagctgtgccaggtgaccgcacggcaggtg
gtggcccggactccgagctgcagccgcaagacaaggtctctgaacacatg
ctgcggctctatgacaggtacagcacggtccaggcggcccggacaccggg
ctccctggagggaggctcgcagccctggcgccctcggctcctgcgcgaag
gcaacacggttcgcagctttcgggcggcagcagcagaaactcttgaaaga

aaaggactgtatatcttcaatctgacatcgctaaccaagtctgaaaacat
tttgtctgccacactgtatttctgtattggagagctaggaaacatcagcc
tgagttgtccagtgtctggaggatgctcccatcatgctcagaggaaacac
attcagattgatctttctgcatggaccctcaaattcagcagaaaccaaag
tcaactccttggccatctgtcagtggatatggccaaatctcatcgagata
ttatgtcctggctgtctaaagatatcactcaactcttgaggaaggccaaa
gaaaatgaagagttcctcataggatttaacattacgtccaagggacgcca
gctgccaaagaggaggttaccttttccagagccttatatcttggtatatg
ccaatgatgccgccatttctgagccagaaagtgtggtatcaagcttacag
ggacaccggaattttcccactggaactgttcccaaatgggatagccacat
cagagctgccctttccattgagcggaggaagaagcgctctactggggtct
tgctgcctctgcagaacaacgagcttcctggggcagaataccagtataaa
aaggatgaggtgtgggaggagagaaagccttacaagacccttcaggctca
ggcccctgaaaagagtaagaataaaaagaaacagagaaaggggcctcatc
ggaagagccagacgctccaatttgatgagcagaccctgaaaaaggcaagg
agaaagcagtggattgaacctcggaattgcgccaggagatacctcaaggt
agactttgcagatattggctggagtgaatggattatctcccccaagtcct
ttgatgcctattattgctctggagcatgccagttccccatgccaaagtct
ttgaagccatcaaatcatgctaccatccagagtatagtgagagctgtggg
ggtcgttcctgggattcctgagccttgctgtgtaccagaaaagatgtcct
cactcagtattttattctttgatgaaaataagaatgtagtgcttaaagta
taccctaacatgacagtagagtcttgcgcttgcagataacctggcaaaga
actcatttgaatgcttaattcaatcattagtttatttttatggacttctt
cctgttttttttttttttttttttgcactgccaatgcattttgtttcaaa
agattatttctatagtcagaggggaatgagcaaatagactgaagattgcc
accaaggaaaagaactgtatttgtttctgaatgtaacttaaagcaagatt
tttagtaaatatggacatctatttctctttttgtaatcaaacacaacaac
ttatcaaactgtttttagaactgttagagaacacactggtttatttttgt
aatgttctttgaaaacagaatggagaagcagcaatagcttgtcatttatc
tcatttaatgactaatgggaaatagagaacaatttcgcgttttgaattag
gcttattgccttagaatcctgagaaagtgctaaataatcaactctgatgt
ttttcttaagttcttgagactcttgtttatccttgtttttcctccacaag
tcattgtctaagtgtaatggaaagtttatgctgagcgttagtgtgtatgt
atgtgcgtacatgcgccaggtgcctgtgccctctgtaggatggtttgctt
aatatggttttataattcagtttacacaggattctttattttttttaatt
ttgtattttggcaaacaccattcagttataagaactttgccaaatatgat
agaataattcaagagcatatacagagagttaccacttgacccagctattt
aattgcaaatacagttgttttcatttcatttcctaccagaaaaaggaatc
agaaacctagtttttgaaaacacaagtgtaattcctcttttgtacttctt
tttcacaaatgcttttatttattctaaattgaatttaaaaatccttccta
aagccattaactctttaattctcctgatatgcctttacttcctatgaagt
tattggtagatgttgaggcccaaaaactggtagaatattgaagatcttct
taaatgaccaatttaaccataaccaaatattgaatatcattcttcagtca
catctaagtcaggcactttttcacatagatcagggcttttggctcagtca
cgaaatctacaagttagcaaagcttacaaaacattattcgtcaggtatgg
gaatcaaatatagacacttgtttgtctttgctttccatttctatgtgtca
catacatatatgtgtcctcttataactttagtcttcaaaattatttcaat
atccttcttctcactatatttatttgtgtgatggaaatgctttcaggccg
tagatcattgttggtgttaatctgtggttaatcctcattttagttccgtc

ttatctgatacttagaaatatctcagccattttggaggctgtgcagtatc
agaagacgtggagtttgttctgtctctgcctgtagctaattatggtggtt
cagtcatttaataaatatgttttgagcatctattttgtgaaaggcactgt
gttacctgtgtgtctttagtgtcctcactggtaaaatgaagaggctggcc
atgagctggaagggttaagtttataattccagctatttcacacccgtctt
ccttgaaggaatgatagtgatagatataaaaacactgtaagtccctcttt
taataaactaaatgaaagaacatcctatacttcgctgtttgtaaattagt
atggcattcgctttggtttaagtggtattttattgcaaacccattaaaag
aataactcatgaaaagaagctctttgacaccttggggtacacaaatgttg
gtgtgggtgtgtgttaattctgtgagtgagacacaccagttctaaaaaaa
atgagtgaagttctggtgcctgagttaccatgctttcttctagttcttac
agtagcataaaattaaagattcaaagtgagatggaggataaaattacttt
ttaatacatgttctcaaacatttgaaaataaaagtatatgatagaagggg
gccagagtgtggccaccatcctgatcgtactgtttttcaataaagaaaac
tttttcattggtagatttggtgaaattctaaatttaggttttttttctaga
gctgtatcaaccaaaacttctggcaattcccagtatcacttcttagcctt
cttatatccaaatgcctgtttattacctttcttaatttgaatcaatgcct
agttattacagattgcaccccacaatggccaaaaacccactacataataa
aatttacaggtactaactagttaagattatattttaagtagcaattgata
taaaattacaacacaatgaaagaacttgggtaatctcttagcaatggaaa
taggttttaaccagcagtttttctgggtgctttgtaactatcattttact
aatgaattgaggatgtattatggtttaaattggaagagttttattcccaa
agaataaagcaagattatctttcagtagtagagattgaagtaaatgtatt
aatattttaattaatacagatttactaagagtagttagaaaatttagtaa
gtgcctgttttacaaattgttaggtactagtttctgtataattcctacac
agaagctttagaaatctcctgatattaaattattaaattggcattcatga
aaagagaagctacaattataaactccatttgctaaatcatgcataatact
ctctctctctcttcccccacaagtaatctctctaccccatgcagtgtgca
cacacacacacacacagtcagttactgaaaaaaataattctttttctttt
ttttttaaatggagtttcactcttgtcgcccaggctggagtgcagtggc
gtgatctcggcccactgcaacctccacctcccaggttcaagcagttctcc
tgcctcagcctcctgagtagctgggattacaggtgtccgccaccatgcct
ggctaatttttttatttgataaaaagaattctttttctcaataactgttc
tcttgaattcaaattaagggactgccaaagtcaattagaatattttaaaa
atactttgttgtaacctgtgtaaataatatacaatttacaggatttggga
ttgtagaacttaaactggaagactggattcctcagatctcaggactataa
cattccagataaatttttacattccctttgctgtatattaactgatgatc
atttatatgttaagatttttttaccttaatatttctgaataaaactcttat
tgcccatttaatattttcataggcaatcaaatgtgagtaatactgctaag
agtctgatttattaaaaatatttgtataattcattcagtttagtttttca
gtttagtctttctgctttcacttttctctgtgctaacaagtaactaatgt
ctgggcattgacttcttattgaatcaaagttgggttaggcatagctatgc
acacctgatgtgtaagattaaagaagagattaaataagaaatcttgggta
agttggacttttctgtatagctcttttttcctctgagttgtattttaatg
tagtttataagtgataaaatgatccttgttttctaaaagccagtccttcc
cttcagctttccacagtttctgtaaatgtttaatacttgtacagtcaatg
gcaattttaaatatatatatatatataatatatgtatatggaaaaggttc
aaagatgcttttaatttatttaatgactattgccttcctataataataat
tttcatccttaattatgataatacttttagcaagaaaaattcctttttac

tacagtttttagatgcaaaatgcagtttggttctttagtcaaatccactt
agagggtatattgcagtgaaactgtgaaggatacttcactaccaatgtat
aagctttgttgaatttgtatcattttctttcagtaatgaaaagctattca
ttatacagtatggaaataaaaattgcttcattga
>NM_000320 2
acagtccctccgggtggcggggcgccccgagcgtggcagcgcgctaggcg
gcagcagcgggcgcggagcggggcgcggccgccgcgcgttccctcttggc
ggggttggccggccggggcggggcgcggcgctccggctcgaggcattcgg
agctgcgggagccgggctggcaggagcaggatggcggcggcggcggctgc
aggcgaggcgcgccgggtgctggtgtacggcggcaggggcgctctgggtt
ctcgatgcgtgcaggcttttcgggcccgcaactggtgggttgccagcgtt
gatgtggtggagaatgaagaggccagcgctagcatcattgttaaaatgac
agactcgttcactgagcaggctgaccaggtgactgctgaggttggaaagc
tcttgggtgaagagaaggtggatgcaattctttgcgttgctggaggatgg
gccgggggcaatgccaaatccaagtctctctttaagaactgtgacctgat
gtggaagcagagcatatggacatcgaccatctccagccatctggctacca
agcatctcaaggaaggaggcctcctgaccttggctggcgcaaaggctgcc
ctggatgggactcctggtatgatcgggtacggcatggccaagggtgctgt
tcaccagctctgccagagcctggctgggaagaacagcggcatgccgcccg
gggcagccgccatcgctgtgctcccggttaccctggataccccgatgaac
aggaaatcaatgcctgaggctgacttcagctcctggacacccttagaatt
cctagttgaaactttccatgactggatcacagggaaaaaccgaccgagct
caggaagcctaatccaggtggtaaccacagaaggaaggacggaactcacc
ccagcatatttttaggcctcatctcagtgcctatgaggggcctgccagaa
aagtcactaacctgtctcagtgtggccttgtccagccttgtgttttctgt
aacccctgtttgtggtacgagataatgagtcctatttttctctcacataa
tatgcatttgctctcctaggacagtgtaatacatttatgtgaagtaaaga
catgcgagactggtggcctgcaaatagcatccgttgatctgtgttaactg
catagggagggctctgcatagcacctgctatagcggtgtcatgttggatc
gcttttgtgactgttcatctgtccttgacagtggctgtcatcttgactac
tttgttgatttgttggtattggggacattttaaaggctgagttatttttg
aatgtcatgtttatgtcatagacgtagttttcgcatccttgaattaaact
gccttaactccttttgtggtataagcaaaactacatggactctgtcctgg
tatccttttcctgtgtggttgccccgtgtcctctggcctagggttaagtg
tgcaagataactactcgtgagtattcagaatgttgttcctaataaatgca
cttgttgtctgtcttctttaatcaaatcacatcttatatacagcagtcag
agatgagtatactagaatcatggattgctggaggtcttttaatctgatgt
tctcagaagggggtggatttaaatcctgaaataaatatttcaacacaaga
acaaaaaaaa
>NM_005271 3
gcgctgccgccagcgaggcccggggaggccgcggcggaggcggaggcccg
gcgccctgggcggcgccctgtccccgaagtccgtcctccccgttaggtgg
cgagcgcccgaggggaggggacagccgggcaagcaggaagctgcggctta
aaagggcaacccgcgcgggacccttcctccctagtcgcggggagtctgag
aaagcgcgcctgtttcgcgaccatcacgcacctcccctccgcttgtggcc
atgtaccgctacctgggcgaagcgctgttgctgtcccgggccgggcccgc
tgccctgggctcggcgtccgccgactcggccgcgttgctgggctgggccc
ggggacagcccgccgccgccccgcagccggggctggcattggccgcccgg
cgccactacagcgaggcggtggccgaccgcgaggacgaccccaacttctt

caagatggtggagggcttcttcgatcgcggcgccagcatcgtggaggaca
agctggtggaggacctgaggacccgggagagcgaggagcagaagcggaac
cgggtgcgcggcatcctgcggatcatcaagccctgcaaccatgtgctgag
tctctccttccccatccggcgcgacgacggctcctgggaggtcatcgaag
gctaccgggcccagcacagccagcaccgcacgccctgcaagggaggtatc
cgttacagcactgatgtgagtgtagatgaagtaaaagctttggcttctct
gatgacatacaagtgtgcagtggttgatgtgccgtttgggggtgctaaag
ctggtgttaagatcaatcccaagaactatactgataatgaattggaaaag
atcacaaggaggttcaccatggagctagcaaaaaagggctttattggtcc
tggcattgatgtgcctgctccagacatgagcacaggtgagcgggagatgt
cctggatcgctgatacctatgccagcaccatagggcactatgatattaat
gcacacgcctgtgttactggtaaacccatcagccaaggggggaatccatgg
acgcatctctgctactggccgtggtgtcttccatgggattgaaaatttca
tcaatgaagcttcttacatgagcattttaggaatgacaccagggtttgga
gataaaacatttgttgttcagggatttggtaatgtgggcctacactctat
gagatatttacatcgttttggtgctaaatgtattgctgttggtgagtctg
atgggagtatatggaatccagatggtattgacccaaaggaactggaagac
ttcaaattgcaacatgggtccattctgggcttccccaaggcaaagcccta
tgaaggaagcatcttggaggccgactgtgacatactgatcccagctgcca
gtgagaagcagttgaccaaatccaacgcacccagagtcaaagccaagatc
attgctgaaggtgccaatgggccaacaactccagaagctgacaagatctt
cctggagagaaacattatggttattccagatctctacttgaatgctggag
gagtgacagtatcttactttgagtggctgaagaatctaaatcatgtcagc
tatggccgtttgaccttcaaatatgaaagggattctaactaccacttgct
catgtctgttcaagagagtttagaaagaaaatttggaaagcatggtggaa
ctattcccattgtacccacggcagagttccaagacaggatatcgggtgca
tctgagaaagacatcgtgcactctggcttggcatacacaatggagcgttc
tgccaggcaaattatgcgcacagccatgaagtataacctgggattggacc
tgagaacagctgcctatgttaatgccattgagaaagtcttcaaagtgtac
aatgaagctggtgtgaccttcacatagatggatcatggctgacttcctca
ctatcctcttcacatgtaacttctgcagacctatcacaagtttacatgta
accacagaaatccctttctctcctgactcattaataatggataccattct
caacaagtcaatccaagtcagcccgttaaggagaaagaaattaaggttag
cggatcatgtacaagctgagtgtgaaagtagaaatcacctacaccagaga
gccattttggtattttgcctttaaataaaaagcctcctttatctggctgt
gcagccttgctctgtggcttttcccaacacaatcagtgctagtgctgggg
aggaacagtcaagagcagtcagttgcttgcttatttttctggatgagtc
tgggacacactgtaactttaacacatttaagaagtaggtgtgtggccttt
tcagaaggtggcatggtcctcaagtgagttcttagtattttatatcagca
aaataattcaattttgcaggttgcaaacaaatataaaacctgtttctgtt
tatgaatattattcttttagaatagaataagtacatgctgctgtaataaa
attgcctttaatcacttaacaagcctaaccttgactcaaacagtgaatgc
ctatagaaataataaatgaaaaaaactagtatttttatatcataaaacaa
tgtcatttatagcttatcattcatgtattgtccagcagacattaaaagcc
ctgtggataattaagttatcttcatacctgcaaaatggtggaggctattt
tcattaaaactgtcagaatttgcttactataattatgatacagtccaaag
aatgcagtcactttttatcatgttaactaattgttctcttttgaagatct
atggttgactaattaaacaataattcaagtagagtgtcccagaaaaaaac
cacttgggctccctgtttggagtctggctggctctgagcattgccaatgg

cccctactcacctgactttgtatcctctccttttagaggctttgcattct
gcacccagcttcactaacagtgggctgaaaacatccttgggttgagtgtt
tcatttgggagttatttggccagggcctttgaacagtagtgtccccatg
aagtgctagataatatatgtgtaagagtcagcttttttttttttttaac
tctaacacccttcagaaatttctaactactttgtaactgcatggcttaac
ctggtgataaaagcagttattaaaagtctacgttttccaaaacttacgtt
tcttttctgtgtttttacatgtggtagtttctcttttcataagttataat
actgcaattggatttctgaaatgtttatagcgaccacctgtataacattt
ccttccactttattgtgagctgcccagattttattcttgaattgtttttt
ttttttttgttcggtgctttacacgttcagagaaacttcccgagtaacga
actatagaaatgatccctgaaagcatagtctttattctcgaattattttg
tattttattaaataatatgaacagctaaaaaaaaaa
>NM_005010 4
agtccgagcctcagacgcgcgggcgcgggggacggcgcaggggccgcctg
gctggagcagccagaggggatagcttcgtcgagaacggaggacaccggcg
gtccagggtcctgggcagtcgcgccagagctgagcggagggcgcggcgcg
agaacgaatctttgtgacattctctctcagcattctttatcccctgtttg
ctgaagacttcgacaaaagctggtcttagctgttggcattctcctgagaa
aaggatagcttcagaaatcagaaaaacatttgggaggtgtctagcccagt
ggaccttctgaagagcaatgctaagaagacgtttggtttaaagaattaaa
aggaagaacaacttaagagcttcttcaaagtttcccgcatgaaaattact
taaacgttgcacacaacgtttcacaaaatcttttgtgaaagaagaaaagg
aaattcagtgtgtgagtctcagcaggagttaagctaatgcagcttaaaat
aatgccgaaaaagaagcgcttatctgcgggcagagtgcccctgattctct
tcctgtgccagatgattagtgcactggaagtacctcttgatctggtacag
cctccaaccatcacccaacagtctccaaaagattacattattgaccctcg
ggagaatattgtaatccagtgtgaagccaaagggaaaccgcccccaagct
tttcctggacccgtaatgggactcattttgacatcgataaagaccctctg
gtcaccatgaagcctggcacaggaacgctcataattaacatcatgagcga
agggaaagctgagacctatgaaggagtctatcagtgtacagcaaggaacg
aacgcggagctgcagtttctaataacattgttgtccgcccatccagatca
ccattgtggaccaaagaaaaacttgaaccaatcacacttcaaagtggtca
gtctttagtacttccctgcagacccccaattggattaccaccacctataa
tattttggatggataattcctttcaaagacttccacaaagtgagagagtt
tctcaaggtttgaatggggacctttatttttccaatgtcctcccagagga
cacccgcgaagactatatctgttatgctagatttaatcatactcaaacca
tacagcagaagcaacctatttctgtgaaggtgatttcagtggatgaattg
aatgacactatagctgctaatttgagtgacactgagttttatggtgctaa
atcaagtagagagaggccaccaacatttttaactccagaaggcaatgcaa
gtaacaaagaggaattaagaggaaatgtgctttcactggagtgcattgca
gaaggactgcctaccccaattatttactgggcaaaggaagatggaatgct
acccaaaaacaggacagtttataagaactttgagaaaaccttgcagatca
ttcatgtttcagaagcagactctggaaattaccaatgtatagcaaaaaac
gcattaggagccatccaccataccatttctgttagagttaaagcggctcc
atactggatcacagcccctcaaaatcttgtgctgtccccaggagaggatg
ggaccttgatctgcagagctaatggcaaccccaaacccagaattagctgg
ttaacaaatggagtcccaatagaaattgcccctgatgaccccagcagaaa
aatagatggcgataccattatttttcaaatgttcaagaaagatcaagtg
cagtctatcagtgcaatgcctctaatgaatatggatatttactggcaaac

gcatttgtaaatgtgctggctgagccaccacgaatcctcacacctgcaaa
cacactctaccaggtcattgcaaacaggcctgctttactagactgtgcct
tctttgggtctcctctcccaaccatcgagtggtttaaaggagctaaagga
agtgctcttcatgaagatatttatgttttacatgaaaatggaactttgga
aattcctgtggcccaaaaggacagtacaggaacttatacgtgtgttgcaa
ggaataaattagggatggcgaagaatgaagttcacttagaaatcaaagat
cctacatggatcgttaaacagcccgaatatgcagttgtgcaaagagggag
catggtgtcctttgaatgcaaagtgaaacatgatcacaccttatccctca
ctgtcctgtggctgaaggacaacagggaactgcccagtgatgaaaggttc
actgttgacaaggatcatctagtggtagctgatgtcagtgacgatgacag
cgggacctacacgtgtgtggccaacaccactctggacagcgtctccgcca
gcgctgtgcttagcgttgttgatgtcccaaatcctccctttgacttagaa
ctgacagatcaacttgacaaaagtgttcagctgtcatggaccccaggcga
tgacaacaatagccccattacaaaattcatcatcgaatatgaagatgcaa
tgcacaagccagggctgtggcaccaccaaactgaagtttctggaacacag
accacagcccagctgaagctgtctccttacgtgaactactccttccgcgt
gatggcagtgaacagcattgggaagagcttgcccagcgaggcgtctgagc
agtatttgacgaaagcctcagaaccagataaaaaccccacagctgtggaa
ggactgggatcagagcctgataatttggtgattacgtggaagcccttgaa
tggtttcgaatctaatgggccaggccttcagtacaaagttagctggcgcc
agaaagatggtgatgatgaatggacatctgtggttgtggcaaatgtatcc
aaatatattgtctcaggcacgccaacctttgttccatacctgatcaaagt
tcaggccctgaatgacatggggtttgcccccgagccagctgtagtcatgg
gacattctggagaagacctcccaatggtggctcctgggaacgtgcgtgtg
aatgtggtgaacagtaccttagccgaggtgcactgggacccagtacctct
gaaaagcatccgaggacacctacaaggctatcggatttactattggaaga
cccagagttcatctaaaagaaacagacgtcacattgagaaaaagatcctc
accttccaaggcagcaagactcatggcatgttgccggggctagagccctt
tagccactacacactgaatgtccgagtggtcaatgggaaaggggagggcc
cagccagccctgacagagtctttaatactccagaaggagtccccagtgct
ccctcgtctttgaagattgtgaatccaacactggactctctcactttgga
atgggatccaccgagccacccgaatggcattttgacagagtacaccttaa
agtatcagccaattaacagcacacatgaattaggccctctggtagatttg
aaaattcctgccaacaagacacggtggactttaaaaaatttaaatttcag
cactcgatataagttttatttctatgcacaaacatcagcaggatcaggaa
gtcaaattacagaggaagcagtaacaactgtggatgaagcgatggcaagc
cggcaggtggatattgcaactcagggctggttcattggtctgatgtgtgc
tgttgctctcccttatcttaattttgctgattgtttgcttcatcagaagaa
acaagggtggtaaatatccagttaaagaaaaggaagatgcccatgctgac
cctgaaatccagcctatgaaggaagatgatgggacatttggagaatacag
tgatgcagaagaccacaagcctttgaaaaaaggaagtcgaactccttcag
acaggactgtgaaaaaagaagatagtgacgacagcctagttgactatgga
gaaggggttaatggccagttcaatgaggatggctcctttattggacaata
cagtggtaagaaagagaaagagccggctgaaggaaacgaaagctcagagg
caccttctcctgtcaacgccatgaattcctttgtttaatttttaagctct
ttgccaatattccatttctctagaatgtttatcctaagcacttgtttgtc
agccctctcatactatgaacatatgggtagagagtatattttctgctgta
tgttagtattatgagaatagttacagcaaaaacataactcagtcaaatga
tatgttaatatgaactggaatgcaaagtgcatactttttcattcaaaatg

ggtattcttgatttcctcagaactgataaaaaataatgcaacatcaccaa
cagatcctgttatttcctctgcaggatacagttcaatatgatgcatgaaa
aatgctccacatttaaaggacatacccgtgtatgttatgaaaacatggtt
tgatactttgtttatactaccctcagctgaacccctatatatgaattccg
ttttcattgtcaagaatgttactgtagtattctctagaacttcaatgtct
ttgtggacattgttgtgaaattggtgactatgtatagctgtcgttagtct
ttttgggagactgttaggaacagtttgtacagtatatacttgctaaatga
gttcattatgacagtcacattgctgatgcttactgagaactattacctac
tcttggctcctgttactccgtaggcttcttaatcttccaggcattacagc
agcacagtgttctactttttacatcatttctatgttcggttgtttttagg
cataaacaatgtgtattgcagtgcatttcggcatttgtgccatactgaaa
gaatcaaaaacaaatcatccaaattaaatttcaaacattatttcagagaa
cacagggcaagacacatacagtgccttcagatattaagcattccacaaca
tcgtgcattctgtatcagctggtccagtccattctgggtcctagattact
gtcattgtctaaaagtaacttttaaaaagcagagttcatgaaaactgcaa
tgctgggaaaagaaggaaacatgaaaataaaaataagacagtttattaga
aatagcatttcctcataagcataaaaagaaaatctttgttgccaactgaa
gcacatgatgattttgtggtcctttatggtttctattacattcagtaaga
aagatgtcaacatgctagaaaattaattttaaaactaagttattccaaca
ctaaaagcatacaacagcatgccaacagtaatatattattctccaagact
ttacctatgtaagtgttcaaaactctgcagcattaaacaacgtgtatgca
aattgttatggatacatttcagaatctaagaaatcaggcaagtgcttaaa
aggccaacggtccaagggattacatctgcagtttaaaaagtaaatatata
ttctatcgtattcataaacaatatctatcaaatgggttacctccaaatat
gaaaatctataacaacctatggttgaaggaatgctcagtttcatttgcca
ataaattggtttctcataacttgcatcaagtttaattttaagtaaagctt
tttatatgtagatattttgttgaatttgtaaatacacttaaaatgtagat
gctatatgcttataggtgttacatacaaataaacatgcaatgtttatgtt
gtactgtataagaggtaagctaattaatgcagtgaatgggattggaaagc
atctacttaaatatctattgggttccccctcccccaccttttttgctg
tgaaactgaaatagtgaacttttctacgtattgacagcagatttttcgat
gaaatcttcagagctttgcctatggggcacagtaggcctagtaacctggc
atgtttgatatatgtaggtaaagcataatttaaagtaatcccaggtaaag
atggccctaaatactttcatgtctctatattcatttttcacagatccacc
tgtctcttgaaaatataaaaagacaaaacaggtttgccttggcatcagag
agcacaaagattaaaagttactttaaatttgccaatattttgggagaaca
ataaaactacatttttcctcttccatactggtagatgcgaaatttatct
gtgcatgaaagggtcacttctgtaatagtgcaacagatttggtattaaaa
attaaatgtggttttaaaagttcctctctcttttgtaatttatgttccca
attgagtgtgaatgtccaagtaatggtgtatgtaatggtacaggcaaatg
tgactggatttccctcaaaaaagtaactattaaacagtcttgatctcttt
gtgacttttaactttttcaaac
>NM_001037132 2
tttcccgcatgaaaattacttaaacgttgcacacaacgtttcacaaaatc
ttttgtgaaagaagaaaaggaaattcagtgtgtgagtctcagcaggagtt
aagctaatgcagcttaaaataatgccgaaaaagaagcgcttatctgcggg
cagagtgcccctgattctcttcctgtgccagatgattagtgcactggaag
tacctcttgatccaaaacttcttgaagacttggtacagcctccaaccatc
acccaacagtctccaaaagattacattattgaccctcgggagaatattgt

aatccagtgtgaagccaaagggaaaccgcccccaagcttttcctggaccc
gtaatgggactcattttgacatcgataaagaccctctggtcaccatgaag
cctggcacaggaacgctcataattaacatcatgagcgaagggaaagctga
gacctatgaaggagtctatcagtgtacagcaaggaacgaacgcggagctg
cagtttctaataacattgttgtccgcccatccagatcaccattgtggacc
aaagaaaaacttgaaccaatcacacttcaaagtggtcagtctttagtact
tccctgcagacccccaattggattaccaccacctataatattttggatgg
ataattcctttcaaagacttccacaaagtgagagagtttctcaaggtttg
aatggggacctttatttttccaatgtcctcccagaggacacccgcgaaga
ctatatctgttatgctagatttaatcatactcaaaccatacagcagaagc
aacctatttctgtgaaggtgatttcagtggatgaattgaatgacactata
gctgctaatttgagtgacactgagttttatggtgctaaatcaagtagaga
gaggccaccaacatttttaactccagaaggcaatgcaagtaacaaagagg
aattaagaggaaatgtgctttcactggagtgcattgcagaaggactgcct
accccaattatttactgggcaaaggaagatggaatgctacccaaaaacag
gacagtttataagaactttgagaaaaccttgcagatcattcatgtttcag
aagcagactctggaaattaccaatgtatagcaaaaaacgcattaggagcc
atccaccataccatttctgttagagttaaagcggctccatactggatcac
agcccctcaaaatcttgtgctgtccccaggagaggatgggaccttgatct
gcagagctaatggcaaccccaaacccagaattagctggttaacaaatgga
gtcccaatagaaattgcccctgatgaccccagcagaaaaatagatggcga
taccattatttttcaaatgttcaagaaagatcaagtgcagtctatcagt
gcaatgcctctaatgaatatggatatttactggcaaacgcatttgtaaat
gtgctggctgagccaccacgaatcctcacacctgcaaacacactctacca
ggtcattgcaaacaggcctgctttactagactgtgccttctttgggtctc
ctctcccaaccatcgagtggtttaaaggagctaaaggaagtgctcttcat
gaagatatttatgttttacatgaaaatggaactttggaaattcctgtggc
ccaaaaggacagtacaggaacttatacgtgtgttgcaaggaataaattag
ggatggcgaagaatgaagttcacttagaaatcaaagatcctacatggatc
gttaaacagcccgaatatgcagttgtgcaaagagggagcatggtgtcctt
tgaatgcaaagtgaaacatgatcacaccttatccctcactgtcctgtggc
tgaaggacaacagggaactgcccagtgatgaaaggttcactgttgacaag
gatcatctagtggtagctgatgtcagtgacgatgacagcgggacctacac
gtgtgtggccaacaccactctggacagcgtctccgccagcgctgtgctta
gcgttgttgctcctactccaactccagctcccgtttacgatgtcccaaat
cctccctttgacttagaactgacagatcaacttgacaaaagtgttcagct
gtcatggaccccaggcgatgacaacaatagccccattacaaaattcatca
tcgaatatgaagatgcaatgcacaagccagggctgtggcaccaccaaact
gaagtttctggaacacagaccacagcccagctgaagctgtctccttacgt
gaactactccttccgcgtgatggcagtgaacagcattgggaagagcttgc
ccagcgaggcgtctgagcagtatttgacgaaagcctcagaaccagataaa
aaccccacagctgtggaaggactgggatcagagcctgataatttggtgat
tacgtggaagcccttgaatggtttcgaatctaatgggccaggccttcagt
acaaagttagctggcgccagaaagatggtgatgatgaatggacatctgtg
gttgtggcaaatgtatccaaatatattgtctcaggcacgccaacctttgt
tccatacctgatcaaagttcaggccctgaatgacatggggtttgcccccg
agccagctgtagtcatgggacattctggagaagacctcccaatggtggct
cctgggaacgtgcgtgtgaatgtggtgaacagtaccttagccgaggtgca
ctgggacccagtacctctgaaaagcatccgaggacacctacaaggctatc

ggatttactattggaagacccagagttcatctaaaagaaacagacgtcac
attgagaaaaagatcctcaccttccaaggcagcaagactcatggcatgtt
gccggggctagagccctttagccactacacactgaatgtccgagtggtca
atgggaaaggggagggcccagccagccctgacagagtctttaatactcca
gaaggagtccccagtgctccctcgtctttgaagattgtgaatccaacact
ggactctctcactttggaatgggatccaccgagccacccgaatggcattt
tgacagagtacaccttaaagtatcagccaattaacagcacacatgaatta
ggccctctggtagatttgaaaattcctgccaacaagacacggtggacttt
aaaaaatttaaatttcagcactcgatataagttttatttctatgcacaaa
catcagcaggatcaggaagtcaaattacagaggaagcagtaacaactgtg
gatgaagctggtattcttccacctgatgtaggtgcaggcaaagttcaagc
agtaaatcccaggatcagcaatcttactgctgcagctgctgagacctatg
ccaatatcagttgggaatatgagggaccagagcatgtgaacttttatgtt
gaatatggtgtagcaggcagcaaagaagaatggagaaaagaaattgtaaa
tggttctcggagcttctttgggttaaagggtctaatgccaggaacagcat
acaaagttcgagttggtgctgtgggggactctggttttgtgagttcagag
gatgtgtttgagacaggcccagcgatggcaagccggcaggtggatattgc
aactcagggctggttcattggtctgatgtgtgctgttgctctccttatct
taattttgctgattgtttgcttcatcagaagaaacaagggtggtaaatat
ccagttaaagaaaaggaagatgcccatgctgaccctgaaatccagcctat
gaaggaagatgatgggacatttggagaatacagtgatgcagaagaccaca
agcctttgaaaaaaggaagtcgaactccttcagacaggactgtgaaaaaa
gaagatagtgacgacagcctagttgactatggagaaggggttaatggcca
gttcaatgaggatggctcctttattggacaatacagtggtaagaaagaga
aagagccggctgaaggaaacgaaagctcagaggcaccttctcctgtcaac
gccatgaattcctttgtttaatttttaagctctttgccaatattccattt
ctctagaatgtttatcctaagcacttgtttgtcagccctctcatactatg
aacatatgggtagagagtatattttctgctgtatgttagtattatgagaa
tagttacagcaaaaacataactcagtcaaatgatatgttaatatgaactg
gaatgcaaagtgcatactttttcattcaaaatgggtattcttgatttcct
cagaactgataaaaaataatgcaacatcaccaacagatcctgttatttcc
tctgcaggatacagttcaatatgatgcatgaaaaatgctccacatttaaa
ggacatacccgtgtatgttatgaaaacatggtttgatactttgtttatac
taccctcagctgaacccctatatatgaattccgttttcattgtcaagaat
gttactgtagtattctctagaacttcaatgtctttgtggacattgttgtg
aaattggtgactatgtatagctgtcgttagtctttttgggagactgttag
gaacagtttgtacagtatatacttgctaaatgagttcattatgacagtca
cattgctgatgcttactgagaactattacctactcttggctcctgttact
ccgtaggcttcttaatcttccaggcattacagcagcacagtgttctactt
tttacatcatttctatgttcggttgtttttaggcataaacaatgtgtatt
gcagtgcatttcggcatttgtgccatactgaaagaatcaaaaacaaatca
tccaaattaaatttcaaacattatttcagagaacacagggcaagacacat
acagtgccttcagatattaagcattccacaacatcgtgcattctgtatca
gctggtccagtccattctgggtcctagattactgtcattgtctaaaagta
acttttaaaaagcagagttcatgaaaactgcaatgctgggaaaagaagga
aacatgaaaataaaaataagacagtttattagaaatagcatttcctcata
agcataaaaagaaaatctttgttgccaactgaagcacatgatgattttgt
ggtcctttatggtttctattacattcagtaagaaagatgtcaacatgcta
gaaaattaattttaaaactaagttattccaacactaaaagcatacaacag

catgccaacagtaatatattattctccaagactttacctatgtaagtgtt
caaaactctgcagcattaaacaacgtgtatgcaaattgttatggatacat
ttcagaatctaagaaatcaggcaagtgcttaaaaggccaacggtccaagg
gattacatctgcagtttaaaaagtaaatatatattctatcgtattcataa
acaatatctatcaaatgggttacctccaaatatgaaaatctataacaacc
tatggttgaaggaatgctcagtttcatttgccaataaattggtttctcat
aacttgcatcaagtttaattttaagtaaagctttttatatgtagatattt
tgttgaatttgtaaatacacttaaaatgtagatgctatatgcttataggt
gttacatacaaataaacatgcaatgtttatgttgtactgtataagaggta
agctaattaatgcagtgaatgggattggaaagcatctacttaaatatcta
ttgggttcccccctccccccacctttttgctgtgaaactgaaatagtga
acttttctacgtattgacagcagatttttcgatgaaatcttcagagcttt
gcctatggggcacagtaggcctagtaacctggcatgtttgatatatgtag
gtaaagcataatttaaagtaatcccaggtaaagatggccctaaatacttt
catgtctctatattcatttttcacagatccacctgtctcttgaaaatata
aaaagacaaaacaggtttgccttggcatcagagagcacaaagattaaaag
ttactttaaatttgccaatattttgggagaacaataaaactacatttttt
cctcttccatactggtagatgcgaaatttatctgtgcatgaaagggtcac
ttctgtaatagtgcaacagatttggtattaaaaattaaatgtggttttaa
aagttcctctctcttttgtaatttatgttcccaattgagtgtgaatgtcc
aagtaatggtgtatgtaatggtacaggcaaatgtgactggatttccctca
aaaaagtaactattaaacagtcttgatctctttgtgacttttaacttttt
caaac
>NM_176795 3
tgccctgcgcccgcaacccgagccgcacccgccgcggacggagcccatgc
gcggggcgaaccgcgcgcccccgcccccgccccgccccggcctcggcccc
ggccctggccccgggggcagtcgcgcctgtgaacggtggggcaggagacc
ctgtaggaggaccccgggccgcaggcccctgaggagcgatgacggaatat
aagctggtggtggtgggcgccggcggtgtgggcaagagtgcgctgaccat
ccagctgatccagaaccattttgtggacgaatacgaccccactatagagg
attcctaccggaagcaggtggtcattgatggggagacgtgcctgttggac
atcctggataccgccggccaggaggagtacagcgccatgcgggaccagta
catgcgcaccggggagggcttcctgtgtgtgtttgccatcaacaacacca
agtcttttgaggacatccaccagtacagggagcagatcaaacgggtgaag
gactcggatgacgtgcccatggtgctggtggggaacaagtgtgacctggc
tgcacgcactgtggaatctcggcaggctcaggacctcgcccgaagctacg
gcatcccctacatcgagacctcggccaagacccggcagggcagccgctct
ggctctagctccagctccgggaccctctgggacccccgggacccatgtg
acccagcggcccctcgcgctggagtggaggatgccttctacacgttggtg
cgtgagatccggcagcacaagctgcggaagctgaaccctcctgatgagag
tggccccggctgcatgagctgcaagtgtgtgctctcctgacgcaggtgag
ggggactcccagggcggccgccacgcccaccggatgaccccggctccccg
cccctgccggtctcctggcctgcggtcagcagcctcccttgtgccccgcc
cagcacaagctcaggacatggaggtgccggatgcaggaaggaggtgcaga
cggaaggaggaggaaggaaggacggaagcaaggaaggaaggaagggctgc
tggagcccagtcaccccgggaccgtgggccgaggtgactgcagaccctcc
cagggaggctgtgcacagactgtcttgaacatcccaaatgccaccggaac
cccagcccttagctcccctcccaggcctctgtgggcccttgtcgggcaca
gatgggatcacagtaaattattggatggtcttgaaaaaaaaaaaaaaaa

a
>NM_005343 2
tgccctgcgcccgcaacccgagccgcacccgccgcggacggagcccatgc
gcggggcgaaccgcgcgcccccgcccccgccccgccccggcctcggcccc
ggccctggccccgggggcagtcgcgcctgtgaacggtggggcaggagacc
ctgtaggaggaccccgggccgcaggcccctgaggagcgatgacggaatat
aagctggtggtggtgggcgccggcggtgtgggcaagagtgcgctgaccat
ccagctgatccagaaccattttgtggacgaatacgaccccactatagagg
attcctaccggaagcaggtggtcattgatggggagacgtgcctgttggac
atcctggataccgccggccaggaggagtacagcgccatgcgggaccagta
catgcgcaccggggagggcttcctgtgtgtgtttgccatcaacaacacca
agtcttttgaggacatccaccagtacagggagcagatcaaacgggtgaag
gactcggatgacgtgcccatggtgctggtggggaacaagtgtgacctggc
tgcacgcactgtggaatctcggcaggctcaggacctcgcccgaagctacg
gcatcccctacatcgagacctcggccaagacccggcagggagtggaggat
gccttctacacgttggtgcgtgagatccggcagcacaagctgcggaagct
gaaccctcctgatgagagtggccccggctgcatgagctgcaagtgtgtgc
tctcctgacgcagcacaagctcaggacatggaggtgccggatgcaggaag
gaggtgcagacggaaggaggaggaaggaaggacggaagcaaggaaggaag
gaagggctgctggagcccagtcaccccgggaccgtgggccgaggtgactg
cagaccctcccagggaggctgtgcacagactgtcttgaacatcccaaatg
ccaccggaaccccagcccttagctcccctcccaggcctctgtgggccctt
gtcgggcacagatgggatcacagtaaattattggatggtcttgaaaaaaa
aaaaaaaaaaa
>NM_006176 2
gagcgcgggagaccggacccgagagcagagctgctgtttcggcgcgggtc
ggctggcggccgactgccccagagcccccacccggcaccacacagacccc
acccccgccctgcgccagccttcgtccccgcagaggacccccgacacca
gcatggactgctgcaccgagaacgcctgctccaagccggacgacgacatt
ctagacatcccgctggacgatcccggcgccaacgcggccgccgccaaaat
ccaggcgagttttcggggccacatggcgcggaagaagataaagagcggag
agcgcggccggaagggcccgggccctggggggcctggcggagctggggtg
gcccggggaggcgcgggcggcggccccagcggagactaggccagaactga
gcattttcaaagttcccgaggagagatggatgccgcgtccccttcgcagc
gacgagacttccctgccgtgtttgtgacccccctcctgcccagcaacctgc
cagctacaggagccccctgcgtcccagagactccctcacccaggcaggct
ccgtcgcggagtcgctgagtccgtgcccttttagttagttctgcagtcta
gtatggtccccatttgcccttccactccaccccaccctaaaccatgcgct
cccaatcttccttcttttgcttctcgcccacctcttcccgcacccagcat
gcagctctgcctccgcagcctcagtgcgctttcctgcgcgcactgcggag
ggcgccctaagcgtcacccaagcacactcacttaaagaaaaaacgagttc
tttcgttctgtgcgcagctaaaaggggcgccctacatctccgtgccactc
ccgccccagcctagccccaagactttggatccggggcgagatgaagggaa
gagggttgttttggtttcggacgacccttgctctgaccggaagagaagtc
cctatcccacacctgcctgtcacgttccctcccctttccccagcgcactg
ttgagggcagcctctccagctctcttgtttatgcaaacgccgagcgcctg
ggaggctcggtaggaggagtcttccacggccccgccccgcccctgtcggt
cccgccctcccccccgccgggctcctggggctgtggccgaaaggtttctg
atctccgtgtgtgcatgtgactgtgctgggttggaatgtgaacaataaag

aggaatgtccaagtgaaaaaaaaaaaaaaaaaaaa
>NM_016237 4
gcggatgtctgtttcctgggatattgtgagggcgggcggagatgtagacc
cggtagtgttgtgccttgtggtgacaactggcggcagcgcgccgcgggcc
cgagacttagtctcgggccgccatggccagcgtccacgagagcctctact
tcaatcccatgatgaccaatggggttgtgcacgccaatgtgttcggcatc
aaggactgggtgacgccgtacaagatcgcggtgctggtgctgctgaacga
gatgagccgcacaggcgagggcgccgtcagcctcatggagcggcggaggc
tcaaccagctgctcctgcccctgctgcagggcccagatattacactgtca
aaactttacaagttaattgaagagtcttgtccacagctggcaaattcagt
gcagatcagaatcaaactgatggctgaaggcgagttgaaggatatggaac
agtttttgatgacctttcagattctttctctggaactgaaccagaggtt
cacaaaacaagtgtagtaggtttgtttctgcgtcacatgatcttggccta
cagtaagctttctttcagccaagtgtttaaactgtacactgcccttcagc
agtacttccagaatggtgagaaaaagacagtggaggatgctgatatggaa
ctgaccagtagagatgagggtgaaagaaaaatggaaaaagaagaacttga
tgtatctgtaagagaagaggaggtatcttgcagtgggcctctgtcccaaa
aacaagcagaatttttctttctcaacaggcttctttgctaaagaatgat
gagactaaggccctcactccagcttccttgcagaaggaattaaacaattt
gttgaaatttaatcctgattttgctgaagcgcattatctcagctacttaa
acaacctccgtgtccaagatgttttcagttcaacacacagtctcctccat
tattttgatcgtctgattcttaccggagccgaaagcaaaagtaatgggga
agagggctatggccggagcttgagatacgccgctctgaatcttgccgccc
tgcactgccgcttcggtcactatcaacaggcagagctcgccctgcaggag
gcaattaggattgcccaggagtccaacgatcacgtgtgtctccagcactg
tttgagctggctttatgtgctggggcagaagagatccgatagctatgttc
tgctggagcattctgtgaagaaggcagtacattttgggttaccgtacctc
gcctccctgggaatacagtcccttgttcaacagagagcttttgctgggaa
gacggcaaacaagctgatggatgccctaaaggactccgacctcctgcact
ggaaacacagcctgtcagagctcatcgatatcagcatcgcacagaaaacg
gccatctggaggctgtatggccgcagcaccatggcactgcaacaggccca
gatgttgctgagcatgaacagcctggaggcggtgaatgcgggcgtgcagc
agaacaacacagagtcctttgctgtcgcactctgccacctcgcagagcta
cacgcggagcagggctgttttgctgcagcttctgaagtgttaaagcactt
gaaggaacgatttccgcctaatagtcagcacgcccagttatggatgctat
gtgatcaaaaaatacagtttgacagagcaatgaatgatggcaaatatcat
ttggctgattcacttgttacaggaatcacagctctcaatagcatagaggg
tgtttataggaaagcggttgtattacaagctcagaaccaaatgtcagagg
cacataagcttttacaaaaattgttggttcattgtcagaaactgaagaac
acagaaatggtgatcagtgtcctactgtccgtggcagagctgtactggcg
atcttcctcccctaccatcgcgctgcccatgctcctgcaggctctggccc
tctccaaggagtaccggttacagtacttggcctctgaaacagtgctgaac
ttggcttttgcgcagctcattcttggaatcccagaacaggccttaagtct
tctccacatggccatcgagcccatcttggctgacggggctatcctggaca
aaggtcgtgccatgttcttagtggccaagtgccaggtggcttcagcagct
tcctacgatcagccgaagaaagcagaagctctggaggctgccatcgagaa
cctcaatgaagccaagaactattttgcaaaggttgactgcaaagagcgca
tcagggacgtcgtttacttccaggccagactctaccatacccctggggaag
acccaggagaggaaccggtgtgcgatgctcttccggcagctgcatcagga

gctgccctctcatggggtacccttgataaaccatctctagagaggacatc
cctgctgggctgctgtgcagagtataagattttggacttgttcatgtccc
ctctctccctataaatgatgtatttgtgacaccctatcttgtcaataaac
agcattctgattagtttgtcttattttgttgctagtaactacgtatttgt
tttattccccttttcttcccttttggtagcaaaggacaccaactttttct
taataaatgggtattttgctaacaa
>NM_003367 2
gtgagcgccgggctcggggcccccccggccgcccgccccctcccctccct
ccctcccctcccctcccctccccccgggccccgcgcccccccgccccc
gccccccccatggacatgctggacccgggtctggatcccgctgcctcggc
caccgctgctgccgccgccagccacgacaagggacccgaggcggaggagg
gcgtcgagctgcaggaaggcggggacggcccaggagcggaggagcagaca
gcggtggccatcaccagcgtccagcaggcggcgttcggcgaccacaacat
ccagtaccagttccgcacagagacaaatggaggacaggtgacataccgcg
tagtccaggtgactgatggtcagctggacggccagggcgacacagctggc
gccgtcagcgtcgtgtccaccgctgccttcgcggggggggcagcaggctgt
gacccaggtgggtgtggacggggcagcccagcgcccgggccccgccgctg
cctctgtgcccccaggtcctgcagcgcccttcccgctggctgtgatccaa
aatcccttcagcaatggtggcagtccggcggccgaggctgtcagcgggga
ggcacgatttgcctatttcccagcgtccagtgtgggagatactacggctg
tgtccgtacagaccacagaccagagcttgcaggctggaggccagttctac
gtcatgatgacgccccaggatgtgcttcagacaggaacacagaggacgat
cgccccccggacacacccttactctccaaaaattgatggaaccagaacac
cccgagatgagaggagaagagcccagcacaacgaagtggagcggaggcgg
agggacaagatcaacaactggatcgtccagctttcgaaaatcattccaga
ctgtaacgcagacaacagcaagacgggagcgagtaaaggagggatcctgt
ccaaggcctgcgattacatccgggagttgcgccagaccaaccagcgcatg
caggagaccttcaaagaggccgagcggctgcagatggacaacgagctcct
gaggcagcagatcgaggagctgaagaatgagaacgccctgcttcgagccc
agctgcagcagcacaacctggagatggtgggcgagggcacccggcagtga
cgcccgccaccaccacgcagccgccgccgcccacgccggcctctgctgcc
cccttccccagcccttagcacagagagggacacatgcccctcccccagct
gcgtttttttatagtagatttttaacaaaaaacggggagaaataatgcat
ttctgtggatacagtgcccaccgccctcctccacttggaaacggtatcct
ccctgcccatccgtctgtctgtcgcccttctcccggccctcactaagccc
cggcacttctagtggtctcacctggaggcaagagggaggggacagaggcc
ctgccacgtcccgctgcctcctgctctctggaggtactgagacagggtgc
tgatgggaaggaggggagcctttggggggccacccggggcctggacctat
gcagggaggccacgtcccaccccacctcttgtttctgggtccctgctccc
ctttgggggtgtgtgtgtgtgttttaattttctttatggaaaaattgaca
aaaaaaaaatagagagagaggtatttaactgcaataaactggccccatgt
ggcccccgcaaaaaaaaaaaaaaaaaaaaaa
>NM_207291 1
gtgagcgccgggctcggggcccccccggccgcccgccccctcccctccct
ccctcccctcccctcccctccccccgggccccgcgcccccccgccccc
gccccccccatggacatgctggacccgggtctggatcccgctgcctcggc
caccgctgctgccgccgccagccacgacaagggacccgaggcggaggagg
gcgtcgagctgcaggaaggcggggacggcccaggagcggaggagcagaca
gcggtggccatcaccagcgtccagcaggcggcgttcggcgaccacaacat

ccagtaccagttccgcacagagacaaatggaggacaggctgtgatccaaa
atcccttcagcaatggtggcagtccggcggccgaggctgtcagcggggag
gcacgatttgcctatttcccagcgtccagtgtgggagatactacggctgt
gtccgtacagaccacagaccagagcttgcaggctggaggccagttctacg
tcatgatgacgccccaggatgtgcttcagacaggaacacagaggacgatc
gccccccggacacacccttactctccaaaaattgatggaaccagaacacc
ccgagatgagaggagaagagcccagcacaacgaagtggagcggaggcgga
gggacaagatcaacaactggatcgtccagctttcgaaaatcattccagac
tgtaacgcagacaacagcaagacgggagcgagtaaaggagggatcctgtc
caaggcctgcgattacatccgggagttgcgccagaccaaccagcgcatgc
aggagaccttcaaagaggccgagcggctgcagatggacaacgagctcctg
aggcagcagatcgaggagctgaagaatgagaacgccctgcttcgagccca
gctgcagcagcacaacctggagatggtgggcgagggcacccggcagtgac
gcccgccaccaccacgcagccgccgccgcccacgccggcctctgctgccc
ccttccccagcccttagcacagagagggacacatgcccctcccccagctg
cgttttttatagtagatttttaacaaaaaacggggagaaataatgcatt
tctgtggatacagtgcccaccgccctcctccacttggaaacggtatcctc
cctgcccatccgtctgtctgtcgcccttctcccggccctcactaagcccc
ggcacttctagtggtctcacctggaggcaagagggaggggacagaggccc
tgccacgtcccgctgcctcctgctctctggaggtactgagacagggtgct
gatgggaaggaggggagcctttgggggccacccggggcctggacctatg
cagggaggccacgtcccaccccacctcttgtttctgggtccctgctcccc
tttggggggtgtgtgtgtgttttaattttctttatggaaaaattgacaa
aaaaaaaatagagagagaggtatttaactgcaataaactggccccatgtg
gccccgcaaaaaaaaaaaaaaaaaaaaaaa
>NM_004128 2
gtgttcctcttttcctcggttcccagtgttctggcaggtaaggaacgccg
gctcttcgcctctcagcgcggcttgtcctttgttccggacgcccgctcct
cagccctgcggctcctggggtcgctgctgcatcccgcacgcctccaccgg
ctgcagacccatggccgagcgcggggaactcgacttgaccggcgccaaac
agaacacaggagtgtggctagtcaaggttcctaaatatttgtcacagcaa
tgggctaaagcctctggaagaggtgaagttgggaaactgcggattgccaa
gactcaaggaaggactgaggtgtcatttactttgaatgaggatcttgcaa
atattcatgatattggtggaaaaccagcttcagtcagtgctcctagagaa
catccatttgtcttgcaaagtgttggaggacagacattaacagtatttac
tgagagctcatcagataagctgtcattggaaggaatagtggtacaaagag
ctgaatgccgaccagctgccagtgaaaactacatgcgattaaaaagattg
caaatagaagagtcttccaaaccagtgaggctatcacaacagctggacaa
agttgtaacaaccaattacaaacctgttgctaatcatcaatacaatatcg
aatatgaaaggaaaaagaaagaagacggaaagcgagctcgagctgataaa
caacatgttttagacatgctattttcagcctttgagaaacatcaatacta
taatcttaaggacttggtggacatcacaaagcaacctgtggtgtacctga
aggaaatcttaaaagaaattggtgttcagaatgtaaaagggatccacaaa
aacacatgggagctgaagccagagtacagacactatcaaggagaagaaaa
gagtgactaagaagactcctagccagcatgctagtgaaacgactagcagc
gatgctatgcaaaaggcgctgatactggaaggcttgaacaccgtatgtta
atagggggttaagtgacagtactttgatttctctcggtaaattttttaaac
ctgtaattcttgtaaagtttcttaactgttttttgaggagaaagaacag
atttatttatagacttaacttgtattaaaccagattattcacagtaggaa

taggggttggaggattaagagggtttttcttaaatattagcttttaatgt
gtacaattaggaaattttttttaagtgaggactctctacccttgccgtatc
taaggagctgaggtaatacagatccaaggagaattgtatagcaagaaaaa
aacagtcaactacagaaactcttaaaaggaataaaaacccaaagttcctt
attttgaaattgtcaaagaacaaagcggtgtttttcttttaaacaggtga
tcacgtttcgtgttcatactcaacgttaataaaaggagagagtttgtagt
gaaaaaaaaaaaaaaaaaa
>NM_000850 4
aagctggcgaggccgagcccctcctagtgcttccggaccttgctccctga
acactcggaggtggcggtggatcttactccttccagccagtgaggatcca
gcaacctgctccgtgcctcccgcgcctgttggttggaagtgacgaccttg
aagatcggccggttggaagtgacgaccttgaagatcggcgggcgcagcgg
ggccgagggggcgggtctggcgctaggtccagcccctgcgtgccgggaac
cccagaggaggtcgcagttcagcccagctgaggcctgtctgcagaatcga
caccaaccagcatcatgtccatgacactggggtactgggacatccgcggg
ctggcccacgccatccgcctgctcctggaatacacagactcaagctacga
ggaaaagaagtatacgatgggggacgctcctgactatgacagaagccagt
ggctgaatgaaaaattcaagctgggcctggactttcccaatctgccctac
ttgattgatggggctcacaagatcacccagagcaacgccatcctgtgcta
cattgcccgcaagcacaacctgtgtggggagacagaagaggagaagattc
gtgtggacattttggagaaccaggctatggacgtctccaatcagctggcc
agagtctgctacagccctgactttgagaaactgaagccagaatacttgga
ggaacttcctacaatgatgcagcacttctcacagttcctggggaagaggc
catggtttgttggagacaagatcacctttgtagatttcctcgcctatgat
gtccttgacctccaccgtatatttgagcccaactgcttggacgcctttcc
aaatctgaaggacttcatctcccgctttgagggcttggagaagatctctg
cctacatgaagtccagccgcttcctcccaaaacctctgtacacaagggtg
gctgtctggggcaacaagtaatgccttgaaggccaggaggtgggagtgag
gagcccatactcagcctgctgcccaggctgtgcagcgcagctggactctg
catcccagcacctgcctcctcgttcctttctcctgtttattcccatcttt
acccccaagactttattgggcctcttcacttcccctaaacccctgtccca
tgcaggccctttgaagcctcagctacccactttccttcatgaacatcccc
ctcccaacactacccttccctgcactaaagccagcctgaccttccttcct
gttagtggttgtatctgctttgaagggcctacctggcccctcgcctgtgg
agctcagccctgagctgtccccgtgttgcatgacagcattgactggttta
caggccctgctcctgcagcatggcccctgccttaggcctacctgatcaaa
ataaagcctcagccacatttgctataaaaaaaaaaaaaaaa
>NM_001122 3
ccgagggtgacactcgggcttgggacagggcgtgctgccgcgggtcacgt
gctgcggaggcttggggagggggcggcgaggcggggtttatagcccgggcg
cccgcgggccccacgctttgaccgggtcgtggcagccggagtcgtcttcg
ggacgcgcctgctcttcgcctttcgctgcagtccgtcgatttctttctcc
aggaagaaaaatggcatccgttgcagttgatccacaaccgagtgtggtga
ctcgggtggtcaacctgcccttggtgagctccacgtatgacctcatgtcc
tcagcctatctcagtacaaaggaccagtatccctacctgaagtctgtgtg
tgagatggcagagaacggtgtgaagaccatcacctccgtggccatgacca
gtgctctgcccatcatccagaagctagagccgcaaattgcagttgccaat
acctatgcctgtaaggggctagacaggattgaggagagactgcctattct
gaatcagccatcaactcagattgttgccaatgccaaaggcgctgtgactg

gggcaaaagatgctgtgacgactactgtgactggggccaaggattctgtg
gccagcacgatcacaggggtgatggacaagaccaaaggggcagtgactgg
cagtgtggagaagaccaagtctgtggtcagtggcagcattaacacagtct
tggggagtcggatgatgcagctcgtgagcagtggcgtagaaaatgcactc
accaaatcagagctgttggtagaacagtacctccctctcactgaggaaga
actagaaaaagaagcaaaaaaagttgaaggatttgatctggttcagaagc
caagttattatgttagactgggatccctgtctaccaagcttcactcccgt
gcctaccagcaggctctcagcagggttaaagaagctaagcaaaaaagcca
acagaccatttctcagctccattctactgttcacctgattgaatttgcca
ggaagaatgtgtatagtgccaatcagaaaattcaggatgctcaggataag
ctctacctctcatgggtagagtggaaaaggagcattggatatgatgatac
tgatgagtcccactgtgctgagcacattgagtcacgtactcttgcaattg
cccgcaacctgactcagcagctccagaccacgtgccacaccctcctgtcc
aacatccaaggtgtaccacagaacatccaagatcaagccaagcacatggg
ggtgatggcaggcgacatctactcagtgttccgcaatgctgcctccttta
aagaagtgtctgacagcctcctcacttctagcaaggggcagctgcagaaa
atgaaggaatctttagatgacgtgatggattatcttgttaacaacacgcc
cctcaactggctggtaggtcccttttatcctcagctgactgagtctcaga
atgctcaggaccaaggtgcagagatggacaagagcagccaggagacccag
cgatctgagcataaaactcattaaacctgcccctatcactagtgcatgct
gtggccagacagatgacaccttttgttatgttgaaattaacttgctaggc
aaccctaaattgggaagcaagtagctagtataaaggccctcaattgtagt
tgtttccagctgaattaagagctttaaagtttctggcattagcagatgat
ttctgttcacctggtaagaaaagaatgataggcttgtcagagcctatagc
cagaactcagaaaaaattcaaatgcacttatgttctcattctatggccat
tgtgttgcctctgttactgtttgtattgaataaaaacatcttcatgtggg
ctggggtagaaactggtgtctgctctggtgtgatctgaaaaggcgtcttc
actgctttatctcatgatgcttgcttgtaaaacttgattttagtttttca
tttctcaaataggaatactacctttgaattcaataaaattcactgcagga
tagaccagttaaaaaaaaaaaaaaaaaaa
>NM_152869 2
gtgcccgagccaggccggcctccccgccccctccctggaaaggaaaggcc
ccggcgacaacagagccagacccgctcatcccgatctcccagaaggcgac
tgacagctgactgccagaaggagatcgcgccaggagactgactgctctgt
gcccacccggggacccgggcccgttcagccgggctggctggtgcgccctc
tgcaaagcctgcgccagggaggaggcaggctcaaccttcagattcccagg
gcctctctgtcgctgtcgccgtcgccgtcgcccgaggtcccagcggctct
accagattgttgtggaggcctctcacccgcacagataatttttctctgtc
ttctcccagttcgctggtcatggtctctggcctaaagtcccagcatagac
tgttcctctcctgtggagtctaacactctttgccaaactggcacggtgtc
ttcctgggaagcaagaaatcactgagggcctgtcagttcctgccagggtg
gttcgatgctgtccttttctccactccttccatggtctgcgttggaaaat
gtgtgttagttcgaattcagcgagttctcattggggtgaacatagtccta
aaaatgcagttcataaaaatcaaccacacagagaagaaaaataaataaat
cagtaaagcagctcagaaaaatgcaggtgtggcgggccctggcctctgca
ccctcatagaggggctcaacagcatcaacagaaggtgggggagcagaagg
tgccctgcagacacccagaggggccaccaatgagcaaactgccagctccc
tgacccgccctggggcccagggtattgacataaaggacggccttgcacta
tttcaacagccagttctctgagggtcacaaacaccaaggagtggaggtca

gagtgtcactttttttgttttctttttgaaagatcattcgagaaacacgtc
actgatctcccctgcgaccatgtcttccattaagattgagtgtgttttgc
cagagaactgccggtgtggtgagtctccagtatgggaggaagtgtccaac
tctctgctctttgtagacattcctgcaaaaaaggtttgccggtgggattc
attcaccaagcaagtacagcgagtgaccatggatgccccagtcagctccg
tggctcttcgccagtcgggaggctatgttgccaccattggaacaaagttc
tgtgctttgaactggaaagaacaatcagcagttgtcttggccacggtgga
taacgacaagaaaaacaatcgcttcaatgatgggaaggtggatcccgccg
ggaggtactttgctggcaccatggctgaggaaacagctccagcagttctt
gagcggcaccagggggccctgtactccctctttcctgatcaccacgtgaa
aaagtactttgaccaggtggacatttccaatggtttggattggtcgctag
accacaaaatcttctattacattgacagcctgtcctactccgtggatgcc
tttgactatgacctgcagacaggacagatctccaaccgcagaagtgttta
caagctagaaaaggaagaacaaatcccagatggaatgtgtattgatgctg
aggggaagctctgggtggcctgttacaatggaggaagagtgattcgttta
gatcctgtgacagggaaaagacttcaaactgtgaagttgcctgttgataa
aacaacttcatgctgctttggagggaagaattactctgaaatgtatgtga
cctgcgcccgggatgggatggaccccgagggtcttttgaggcaacctgaa
gctggtggaattttcaagataactggtctgggggtcaaaggaattgctcc
ctactcctatgcgggatgaggacaggtcttctttcctgccagagggagct
ctgaagacaactagagaattctgggcctgaaatttcaatctagttagaaa
gaaaaatgaggcaatgattttattaacagcgttaagttttaatttacaac
ttttaaaaggcagagcatttttaacaaggggtgacaggtggttttgataa
cacacttataaggctttctgtaaaaggtactatagaagggcgaagaatcg
ttcaactgtcaatcagcctcttgattctttgtaaattgccagggtgggtg
ggtacatatctcttcttgattctgcatttcatacttaactatattaaagc
ttcaaggaacaataaatagtaacctggtaatgaccaaaaaaaaaaaaaaa
aaaaa
>NM_004683 4
gtgcccgagccaggccggcctccccgccccctccctggaaaggaaaggcc
ccggcgacaacagagccagacccgctcatcccgatctcccagaaggcgac
tgacagctgactgccagaaggagatcgcgccaggagactgactgctctgt
gcccacccggggacccgggcccgttcagccgggctggctggtgcgccctc
tgcaaagcctgcgccagggaggaggcaggctcaaccttcagattcccagg
gcctctctgtcgctgtcgccgtcgccgtcgcccgaggtcccagcggctct
accagattgttgtggaggcctctcacccgcacagatctcccctgcgacca
tgtcttccattaagattgagtgtgttttgccagagaactgccggtgtggt
gagtctccagtatgggaggaagtgtccaactctctgctctttgtagacat
tcctgcaaaaaaggtttgccggtgggattcattcaccaagcaagtacagc
gagtgaccatggatgccccagtcagctccgtggctcttcgccagtcggga
ggctatgttgccaccattggaacaaagttctgtgctttgaactggaaaga
acaatcagcagttgtcttggccacggtggataacgacaagaaaaacaatc
gcttcaatgatgggaaggtggatcccgccgggaggtactttgctggcacc
atggctgaggaaacagctccagcagttcttgagcggcaccagggggccct
gtactccctctttcctgatcaccacgtgaaaaagtactttgaccaggtgg
acatttccaatggtttggattggtcgctagaccacaaaatcttctattac
attgacagcctgtcctactccgtggatgcctttgactatgacctgcagac
aggacagatctccaaccgcagaagtgtttacaagctagaaaaggaagaac
aaatcccagatggaatgtgtattgatgctgaggggaagctctgggtggcc

tgttacaatggaggaagagtgattcgtttagatcctgtgacagggaaaag
acttcaaactgtgaagttgcctgttgataaaacaacttcatgctgctttg
gagggaagaattactctgaaatgtatgtgacctgcgcccgggatgggatg
gaccccgagggtcttttgaggcaacctgaagctggtggaattttcaagat
aactggtctgggggtcaaaggaattgctccctactcctatgcgggatgag
gacaggtcttctttcctgccagagggagctctgaagacaactagagaatt
ctgggcctgaaatttcaatctagttagaaagaaaaatgaggcaatgattt
tattaacagcgttaagttttaatttacaacttttaaaaggcagagcattt
ttaacaaggggtgacaggtggttttgataacacacttataaggctttctg
taaaaggtactatagaagggcgaagaatcgttcaactgtcaatcagcctc
ttgattctttgtaaattgccagggtgggtgggtacatatctcttcttgat
tctgcatttcatacttaactatattaaagcttcaaggaacaataaatagt
aacctggtaatgaccaaaaaaaaaaaaaaaaaaaa
>NM_001003941 2
tgtaagaggggagggcattcttctccaggggagggctacgtgttgacgcc
atacgccggggcggggccgagagtttggagccccggagtggggtgtcggc
gcctcattcgggtggagctgagccggagacaggcagttgtgaaaaacttc
aggacaaaaatgtttcatttaaggacttgtgctgctaagttgaggccatt
gacggcttcccagactgttaagacattttcacaaaacagaccagcagcag
ctaggacatttcaacagattcggtgctattctgcacctgttgctgctgag
ccctttctcagtgggactagttcgaactatgtggaggagatgtactgtgc
ttggctggaaaaccccaaaagtgtacataagtcatgggacattttttttc
gcaacacgaatgccggagccccaccgggcactgcctaccagagtcccctt
cccctgagccgaggctccctggctgctgtggcccatgcacagtccctggt
agaagcacagcccaacgtggacaagctcgtggaggaccacctggcagtgc
agtcgctcatcagggcatatcagatacgagggcaccatgtagcacagctg
gaccccctggggattttggatgctgatctggactcctccgtgcccgctga
cattatctcatccacagacaaacttgggttctatggcctggatgagtctg
acctcgacaaggtcttccacttgcccaccaccactttcatcgggggacag
gaatcagcacttcctctgcgggagatcatccgtcggctggagatggccta
ctgccagcatattggggtggagttcatgttcatcaatgacctggagcagt
gccagtggatccggcagaagtttgagacccctgggatcatgcagttcaca
aatgaggagaaacggaccctgctggccaggcttgtgcggtccaccaggtt
tgaggagttcctacagcggaagtggtcctctgagaagcgctttggtctag
aaggctgcgaggtactgatccctgccctcaagaccatcattgacaagtct
agtgagaatggcgtggactacgtgatcatgggcatgccacacagagggcg
gctgaacgtgcttgcaaatgtcatcaggaaggagctggaacagatcttct
gtcaattcgattcaaagctggaggcagctgatgagggctccggagatgtg
aagtaccacctgggcatgtatcaccgcaggatcaatcgtgtcaccgacag
gaacattaccttgtccttggtggccaacccttcccaccttgaggccgctg
accccgtggtgatgggcaagaccaaagccgaacagttttactgtggcgac
actgaagggaaaaaggtaaggcccagagagaggcgtgcaaggcagatcgt
caaggccccatgttccagcatggagttccgctcaccaacataacccagag
ccctgggtgcatctagactttaaaaaaatatttaaagtcggccgggcgca
gtgtctcacgcctgtaatcccagcactttgggaggccgaggtgggcagat
cacctgagttcgggagttggagaccagcctgaccaacatggagaaactcc
atctctactaaaaatacaaaattagctgggcgtggtggcgcgcgcctgta
atcccagctactcaggaggctgaggcaggagaatcgcttgaacccgggag
gtggaggttgcagtgagccgagattacgccattgcactccagcctgggcc

aacaagagcgaaactctgtctcaaagaaaaaaataaataaataaaaaata
tatgtatatgtaaaaaaaaaaaaaaaaaa
>NM_002541 3
tgtaagaggggagggcattcttctccaggggagggctacgtgttgacgcc
atacgccggggcggggccgagagtttggagccccggagtggggtgtcggc
gcctcattcgggtggagctgagccggagacaggcagttgtgaaaaacttc
aggacaaaaatgtttcatttaaggacttgtgctgctaagttgaggccatt
gacggcttcccagactgttaagacattttcacaaaacagaccagcagcag
ctaggacatttcaacagattcggtgctattctgcacctgttgctgctgag
ccctttctcagtgggactagttcgaactatgtggaggagatgtactgtgc
ttggctggaaaaccccaaaagtgtacataagtcatgggacatttttttc
gcaacacgaatgccggagccccaccgggcactgcctaccagagtcccctt
cccctgagccgaggctccctggctgctgtggcccatgcacagtccctggt
agaagcacagcccaacgtggacaagctcgtggaggaccacctggcagtgc
agtcgctcatcagggcatatcagatacgagggcaccatgtagcacagctg
gaccccctggggattttggatgctgatctggactcctccgtgcccgctga
cattatctcatccacagacaaacttgggttctatggcctggatgagtctg
acctcgacaaggtcttccacttgcccaccaccactttcatcgggggacag
gaatcagcacttcctctgcgggagatcatccgtcggctggagatggccta
ctgccagcatattggggtggagttcatgttcatcaatgacctggagcagt
gccagtggatccggcagaagtttgagacccctgggatcatgcagttcaca
aatgaggagaaacggaccctgctggccaggcttgtgcggtccaccaggtt
tgaggagttcctacagcggaagtggtcctctgagaagcgctttggtctag
aaggctgcgaggtactgatccctgccctcaagaccatcattgacaagtct
agtgagaatggcgtggactacgtgatcatgggcatgccacacagagggcg
gctgaacgtgcttgcaaatgtcatcaggaaggagctggaacagatcttct
gtcaattcgattcaaagctggaggcagctgatgagggctccggagatgtg
aagtaccacctgggcatgtatcaccgcaggatcaatcgtgtcaccgacag
gaacattaccttgtccttggtggccaacccttcccaccttgaggccgctg
accccgtggtgatgggcaagaccaaagccgaacagttttactgtggcgac
actgaagggaaaaaggtcatgtccatcctgttgcatggggatgctgcatt
tgctggccagggcattgtgtacgagaccttccacctcagcgacctgccat
cctacacaactcatggcaccgtgcacgtggtcgtcaacaaccagatcggc
ttcaccaccgaccctcggatggcccgctcctcccccctaccccactgacgt
ggcccgagtggtgaatgcccccattttccacgtgaactcagatgaccccg
aggctgtcatgtacgtgtgcaaagtggcggccgagtggaggagcaccttc
cacaaggacgtggttgtcgatttggtgtgttaccggcgcaacggccacaa
cgagatggatgagcccatgttcacgcagccgctcatgtacaagcagatcc
gcaagcagaagcctgtgttacagaagtacgctgagctgctggtgtcgcag
ggtgtggtcaaccagcctgagtatgaggaggaaatttccaagtatgataa
gatctgtgaggaagcttttgccagatctaaagatgagaagatcttgcaca
ttaagcactggctggactctccctggcctggcttcttcaccctggacggg
cagcccaggagcatgtcctgcccctccacgggtctgacggaggatattct
gacacacatcgggaatgtggctagttctgtgcctgtggaaaactttacta
ttcatggagggctgagccggatcttgaagactcgtggggaaatggtgaag
aaccggactgtggactgggctctagcggagtacatggcgtttggctcgct
cctgaaggagggcatccacattcggctgagcggccaggacgtggagcggg
gcacattcagccaccgccaccatgtgctccatgaccagaatgtggacaag
agaacctgcatccccatgaaccatctctggcccaatcaggccccctatac

tgtgtgcaacagctcactgtctgagtacggcgtgctgggctttgagctgg
gcttcgccatggccagtcctaatgccctggtcctctgggaagcccaattt
ggtgacttccacaacacggcccagtgtatcatcgaccagttcatctgccc
gggacaagccaagtgggtgcggcagaatggcatcgtgttgctgctgcccc
atggcatggagggcatgggtccagaacattcctccgcccgcccagagcgg
ttcttgcagatgtgcaacgatgacccagatgtcctgccagaccttaaaga
agccaacttcgacatcaatcagctatatgactgcaattgggttgttgtca
actgctccactcctggcaacttcttccacgtgctacgacgccagatcctg
ctgccattccggaagccgttaattatcttcacccccaaatccctgttgcg
ccaccccgaggccagatccagctttgatgagatgcttccaggaacccact
tccagcgggtgatcccagaagatggccctgcagctcagaacccagaaaat
gtcaaaaggcttctcttctgcaccggcaaagtgtattatgacctcacccg
ggagcgcaaagcacgcgacatggtggggcaggtggccatcacaaggattg
agcagctgtcgccattcccctttgacctcctgctgaaggaggtgcagaag
taccccaatgctgagctggcctggtgccaggaggagcacaagaaccaagg
ctactatgactacgtgaagccaagacttcggaccaccatcagccgcgcca
agcccgtctggtatgccggccgggacccagcggctgctccagccaccggc
aacaagaagacccacctgacggagctgcagcgcctcctggacacggcctt
cgacctggacgtcttcaagaacttctcgtagatgctgcctagggttgctt
gggccactgccctctccacacccatgactgccccttgcttctcaactaaa
gaatagtgcctcagcgctgcccacaccaccgccctcctcgctgtgccacc
acccctccctctgctctcataggagttaggctgtcgtccccctccagtgc
ttggctgccccacaggccacacgctgcccaggctctgctgacttctgagc
agttttccaggaggccgggggagcaggaggaggaaaggtagcccccgag
ggatgtccttggggaggggtcagctctggccacaatcctccccaccagtc
tcacccactaggataggaactgggccttgtgtgctggcttccgctgtcac
ccagcaaggcacaggctcctgtatttgagactaggatagcttcatcttga
gcctgagccttagaatctgtagaggagcctggagtcggatctagccatgg
ctggcagaggtttctagggtgggccccagccgtggcgtgaactgaggatg
acccggggcagctggcaggagagagccttggcctgacctggcacagaaag
ggcagcttcagtctctgcagtgtccattatctgctgttccttcgagggtt
ccaggctgtgtgtggggcccaagcatgccccacccacccctcctgggccc
aggcagcacctggagcccacagagtctgtgtgtagccaggaagccccgct
caggtagccaccaccggggcactggctgctctgtcttggtcctgttaacc
ctccacctcctctcttggactccctccccaccccaaccactctttctttc
tcctttaacccaatggagactttctgatgcatcgttttctttgctgtgcc
aaagcaggtcagaagagggagaggaggggctgggggtgaggggccaggcc
atggccaaggggccagctgcccctcatttatcactctgaccttcacaggg
acagatctgatttatttattttggttaaaaaaaaaaaaaaaggaacagaaa
caactttgcattgcattggcttgacccataaactaagttatatccgtggg
caaaaaaaaaaaaaaaaa
>NM_006254 3
tcgcacttccgtgtgccgcggcgccggagcccgaggcggctgtagcccac
atctcccgagcgacccccggcgcccgcccgccgcgcggaggcccgggcca
cacctcactggccgcttggcccatcccagtcagcgccgcgccgaaccccg
tccgcgcgcgccggggagcggcgcccccgccgctgccgccgcgacccttg
gcgcctgcccctgcaacgggaggtctgcagggaactggccaggcaagggg
gcaggcccgtttctcctggtggttggtgcgttgtagcagcagcgggagcc
aggactaaggacaagcaggagctgggagccccagccccactgcaggcccc

accatggcgccgttcctgcgcatcgccttcaactcctatgagctgggctc
cctgcaggccgaggacgaggcgaaccagcccttctgtgccgtgaagatga
aggaggcgctcagcacagagcgtgggaaaacactggtgcagaagaagccg
accatgtatcctgagtggaagtcgacgttcgatgcccacatctatgaggg
gcgcgtcatccagattgtgctaatgcgggcagcagaggagccagtgtctg
aggtgaccgtgggtgtgtcggtgctggccgagcgctgcaagaagaacaat
ggcaaggctgagttctggctggacctgcagcctcaggccaaggtgttgat
gtctgttcagtatttcctggaggacgtggattgcaaacagtctatgcgca
gtgaggacgaggccaagttcccaacgatgaaccgccgcggagccatcaaa
caggccaaaatccactacatcaagaaccatgagtttatcgccaccttctt
tgggcaacccaccttctgttctgtgtgcaaagactttgtctggggcctca
acaagcaaggctacaaatgcaggcaatgtaacgctgccatccacaagaaa
tgcatcgacaagatcatcggcagatgcactggcaccgcggccaacagccg
ggacactatattccagaaagaacgcttcaacatcgacatgccgcaccgct
tcaaggttcacaactacatgagccccaccttctgtgaccactgcggcagc
ctgctctggggactggtgaagcagggattaaagtgtgaagactgcggcat
gaatgtgcaccataaatgccgggagaaggtggccaacctctgcggcatca
accagaagcttttggctgaggccttgaaccaagtcacccagagagcctcc
cggagatcagactcagcctcctcagagcctgttgggatatatcagggttt
cgagaagaagaccggagttgctggggaggacatgcaagacaacagtggga
cctacggcaagatctgggagggcagcagcaagtgcaacatcaacaacttc
atcttccacaaggtcctgggcaaaggcagcttcgggaaggtgctgcttgg
agagctgaagggcagaggagagtactttgccatcaaggccctcaagaagg
atgtggtcctgatcgacgacgacgtggagtgcaccatggttgagaagcgg
gtgctgacacttgccgcagagaatccctttctcacccacctcatctgcac
cttccagaccaaggaccacctgttctttgtgatggagttcctcaacgggg
gggacctgatgtaccacatccaggacaaaggccgctttgaactctaccgt
gccacgttttatgccgctgagataatgtgtggactgcagtttctacacag
caagggcatcatttacagggacctcaaactggacaatgtgctgctggacc
gggatggccacatcaagattgccgactttgggatgtgcaaagagaacata
ttcggggagagccgggccagcaccttctgcggcacccctgactatatcgc
ccctgagatcctacagggcctgaagtacacattctctgtggactggtggt
ctttcggggtccttctgtacgagatgctcattggccagtccccttccat
ggtgatgatgaggatgaactcttcgagtccatccgtgtggacacgccaca
ttatccccgctggatcaccaaggagtccaaggacatcctggagaagctct
ttgaaagggaaccaaccaagaggctgggagtgaccggaaacatcaaaatc
cacccccttcttcaagaccataaactggactctgctggaaaagcggaggtt
ggagccacctttcaggcccaaagtgaagtcacccagagactacagtaact
ttgaccaggagttcctgaacgagaaggcgcgcctctcctacagcgacaag
aacctcatcgactccatggaccagtctgcattcgctggcttctcctttgt
gaaccccaaattcgagcacctcctggaagattgaggttcctggacagatc
aggctagccctgccctccacccacacctgcccgctccccacgataagcac
cagtgggactgtggtgacttctgctgctggccccgcccctgcccccagag
cgtccttggctgccgtctggccgggctctcatggtacttcctctgtgaac
tgtgtgtgaatctgcttttcctctgccttcggagggaaattgtaaatcct
gtgtttcattacttgaatgtagttatctattgaaaatatatattatatac
atagacatatatatatatataataggctgtatatattgctcagtagagaa
aaaccatgggggactggtgatatgttgatctttttcaaaaaaatatatat
atgacaaaaaaaaaaaaaaaggagcacaagctgtttgaaccaccaggttt

atttgtgtgtctaaataaacaccaaatagtaccaaaaaaaaaaaaaaaa
>NM_212539 1
tcgcacttccgtgtgccgcggcgccggagcccgaggcggctgtagcccac
atctcccgagcgacccccggcgcccgcccgccgcgcggaggcccgggcca
cacctcactggccgcttggcccatcccagtcagcgccgcgccgaaccccg
tccgcgcgcgccggggagcggcgcccccgccgctgccgccgcgacccttg
gcgcctgcccctgcaacgggagccccactgcaggccccaccatggcgccg
ttcctgcgcatcgccttcaactcctatgagctgggctccctgcaggccga
ggacgaggcgaaccagcccttctgtgccgtgaagatgaaggaggcgctca
gcacagagcgtgggaaaacactggtgcagaagaagccgaccatgtatcct
gagtggaagtcgacgttcgatgcccacatctatgaggggcgcgtcatcca
gattgtgctaatgcgggcagcagaggagccagtgtctgaggtgaccgtgg
gtgtgtcggtgctggccgagcgctgcaagaagaacaatggcaaggctgag
ttctggctggacctgcagcctcaggccaaggtgttgatgtctgttcagta
tttcctggaggacgtggattgcaaacagtctatgcgcagtgaggacgagg
ccaagttcccaacgatgaaccgccgcggagccatcaaacaggccaaaatc
cactacatcaagaaccatgagtttatcgccaccttctttgggcaacccac
cttctgttctgtgtgcaaagactttgtctggggcctcaacaagcaaggct
acaaatgcaggcaatgtaacgctgccatccacaagaaatgcatcgacaag
atcatcggcagatgcactggcaccgcggccaacagccgggacactatatt
ccagaaagaacgcttcaacatcgacatgccgcaccgcttcaaggttcaca
actacatgagccccaccttctgtgaccactgcggcagcctgctctgggga
ctggtgaagcagggattaaagtgtgaagactgcggcatgaatgtgcacca
taaatgccgggagaaggtggccaacctctgcggcatcaaccagaagcttt
tggctgaggccttgaaccaagtcacccagagagcctcccggagatcagac
tcagcctcctcagagcctgttgggatatatcagggtttcgagaagaagac
cggagttgctggggaggacatgcaagacaacagtgggacctacggcaaga
tctgggagggcagcagcaagtgcaacatcaacaacttcatcttccacaag
gtcctgggcaaaggcagcttcgggaaggtgctgcttggagagctgaaggg
cagaggagagtactttgccatcaaggccctcaagaaggatgtggtcctga
tcgacgacgacgtggagtgcaccatggttgagaagcgggtgctgacactt
gccgcagagaatccctttctcacccacctcatctgcaccttccagaccaa
ggaccacctgttctttgtgatggagttcctcaacgggggggacctgatgt
accacatccaggacaaaggccgctttgaactctaccgtgccacgttttat
gccgctgagataatgtgtggactgcagtttctacacagcaagggcatcat
ttacagggacctcaaactggacaatgtgctgctggaccgggatggccaca
tcaagattgccgactttgggatgtgcaaagagaacatattcggggagagc
cgggccagcaccttctgcggcaccccctgactatatcgcccctgagatcct
acagggcctgaagtacacattctctgtggactggtggtctttcggggtcc
ttctgtacgagatgctcattggccagtccccttccatggtgatgatgag
gatgaactcttcgagtccatccgtgtggacacgccacattatccccgctg
gatcaccaaggagtccaaggacatcctggagaagctctttgaaagggaac
caaccaagaggctgggagtgaccggaaacatcaaaatccacccctcttc
aagaccataaactggactctgctggaaaagcggaggttggagccaccttt
caggcccaaagtgaagtcacccagagactacagtaactttgaccaggagt
tcctgaacgagaaggcgcgcctctcctacagcgacaagaacctcatcgac
tccatggaccagtctgcattcgctggcttctcctttgtgaaccccaaatt
cgagcacctcctggaagattgaggttcctggacagatcaggctagccctg
ccctccacccacacctgcccgctccccacgataagcaccagtgggactgt

ggtgacttctgctgctggccccgcccctgcccccagagcgtccttggctg
ccgtctggccgggctctcatggtacttcctctgtgaactgtgtgtgaatc
tgcttttcctctgccttcggagggaaattgtaaatcctgtgtttcattac
ttgaatgtagttatctattgaaaatatatattatatacatagacatatat
atatatataataggctgtatatattgctcagtagagaaaaaccatggggg
actggtgatatgttgatctttttcaaaaaaatatatatatgacaaaaaaa
aaaaaaaaggagcacaagctgtttgaaccaccaggtttatttgtgtgtct
aaataaacaccaaatagtaccaaaaaaaaaaaaaaaaa
>NM_000803 4
atttcactcagtgcttaccagagcgcgttgtctaccctgtaccgaagaca
gaggctgtggggacagcctaggggcctggatctattgcctacttagagag
aggccaactcagacacagccgtgtatgctcccagcagcaacggaggttca
gctccgcctgcagggacagaaagacatggtctggaaatggatgccacttc
tgctgcttctggtctgtgtagccaccatgtgcagtgcccaggacaggact
gatctcctcaatgtctgtatggatgccaagcaccacaagacaaagccagg
tcctgaggacaagctgcatgaccaatgcagtccctggaagaagaatgcct
gctgcacagccagcaccagccaggagctgcacaaggacacctcccgcctg
tacaactttaactgggaccactgcggcaagatggagcccgcctgcaagcg
ccacttcatccaggacacctgtctctatgagtgctcacccaacctggggc
cctggatccagcaggtgaatcagagctggcgcaaagaacgcttcctggat
gtgcccttatgcaaagaggactgtcagcgctggtgggaggattgtcacac
ctcccacacgtgcaagagcaactggcacagaggatgggactggacctcag
gagttaacaagtgcccagctggggctctctgccgcacctttgagtcctac
ttccccactccagctgccctttgtgaaggcctctggagtcactcatacaa
ggtcagcaactacagccgagggagcggccgctgcatccagatgtggtttg
attcagcccagggcaaccccaacgaggaagtggcgaggttctatgctgca
gccatgcatgtgaatgctggtgagatgcttcatgggactgggggtctcct
gctcagtctggccctgatgctgcaactctggctccttggctgagttcagt
cctcccagactacctgccctcagcttggataaccaggctgggctcagctc
agctcccacaaatgacagccccttaagcatgcttctattagtcacctaac
cctctgtcacccagtctgttgctgctccatggtggggccaagagtcactt
ctaataaacagactgttttctaataattccaaaaaaaaaaaaaaa
>NM_000552 3
agctcacagctattgtggtgggaaagggagggtggttggtggatgtcaca
gcttgggctttatctcccccagcagtggggactccacagcccctgggcta
cataacagcaagacagtccggagctgtagcagacctgattgagcctttgc
agcagctgagagcatggcctagggtgggcggcaccattgtccagcagctg
agtttcccagggaccttggagatagccgcagccctcatttgcaggggaag
atgattcctgccagatttgccggggtgctgcttgctctggccctcatttt
gccagggaccctttgtgcagaaggaactcgcggcaggtcatccacggccc
gatgcagccttttcggaagtgacttcgtcaacacctttgatgggagcatg
tacagctttgcgggatactgcagttacctcctggcagggggctgccagaa
acgctccttctcgattattggggacttccagaatggcaagagagtgagcc
tctccgtgtatcttggggaatttttgacatccatttgtttgtcaatggt
accgtgacacagggggaccaaagagtctccatgccctatgcctccaaagg
gctgtatctagaaactgaggctgggtactacaagctgtccggtgaggcct
atggctttgtggccaggatcgatggcagcggcaactttcaagtcctgctg
tcagacagatacttcaacaagacctgcgggctgtgtggcaactttaacat
ctttgctgaagatgactttatgacccaagaagggaccttgacctcggacc

cttatgactttgccaactcatgggctctgagcagtggagaacagtggtgt
gaacgggcatctcctcccagcagctcatgcaacatctcctctggggaaat
gcagaagggcctgtgggagcagtgccagcttctgaagagcacctcggtgt
ttgcccgctgccaccctctggtggaccccgagccttttgtggccctgtgt
gagaagactttgtgtgagtgtgctgggggggctggagtgcgcctgccctgc
cctcctggagtacgcccggacctgtgcccaggagggaatggtgctgtacg
gctggaccgaccacagcgcgtgcagcccagtgtgccctgctggtatggag
tataggcagtgtgtgtccccttgcgccaggacctgccagagcctgcacat
caatgaaatgtgtcaggagcgatgcgtggatggctgcagctgccctgagg
gacagctcctggatgaaggcctctgcgtggagagcaccgagtgtccctgc
gtgcattccggaaagcgctaccctcccggcacctccctctctcgagactg
caacacctgcatttgccgaaacagccagtggatctgcagcaatgaagaat
gtccaggggagtgccttgtcacaggtcaatcacacttcaagagctttgac
aacagatacttcaccttcagtgggatctgccagtacctgctggcccggga
ttgccaggaccactccttctccattgtcattgagactgtccagtgtgctg
atgaccgcgacgctgtgtgcacccgctccgtcaccgtccggctgcctggc
ctgcacaacagccttgtgaaactgaagcatggggcaggagttgccatgga
tggccaggacgtccagctcccccctcctgaaaggtgacctccgcatccagc
atacagtgacggcctccgtgcgcctcagctacggggaggacctgcagatg
gactgggatggccgcgggaggctgctggtgaagctgtcccccgtctatgc
cgggaagacctgcggcctgtgtgggaattacaatggcaaccagggcgacg
acttccttacccccctctgggctggcggagccccgggtggaggacttcggg
aacgcctggaagctgcacggggactgccaggacctgcagaagcagcacag
cgatccctgcgccctcaacccgcgcatgaccaggttctccgaggaggcgt
gcgcggtcctgacgtcccccacattcgaggcctgccatcgtgccgtcagc
ccgctgccctacctgcggaactgccgctacgacgtgtgctcctgctcgga
cggccgcgagtgcctgtgcggcgccctggccagctatgccgcggcctgcg
cggggagaggcgtgcgcgtcgcgtggcgcgagccaggccgctgtgagctg
aactgcccgaaaggccaggtgtacctgcagtgcgggacccccctgcaacct
gacctgccgctctctctcttacccggatgaggaatgcaatgaggcctgcc
tggagggctgcttctgccccccagggctctacatggatgagaggggggac
tgcgtgcccaaggcccagtgcccctgttactatgacggtgagatcttcca
gccagaagacatcttctcagaccatcacaccatgtgctactgtgaggatg
gcttcatgcactgtaccatgagtggagtccccggaagcttgctgcctgac
gctgtcctcagcagtcccctgtctcatcgcagcaaaaggagcctatcctg
tcggcccccccatggtcaagctggtgtgtcccgctgacaacctgcgggctg
aagggctcgagtgtaccaaaacgtgccagaactatgacctggagtgcatg
agcatgggctgtgtctctggctgcctctgcccccccgggcatggtccggca
tgagaacagatgtgtggccctggaaaggtgtccctgcttccatcagggca
aggagtatgcccctggagaaacagtgaagattggctgcaacacttgtgtc
tgtcgggaccggaagtggaactgcacagaccatgtgtgtgatgccacgtg
ctccacgatcggcatggcccactacctcaccttcgacgggctcaaatacc
tgttccccggggagtgccagtacgttctggtgcaggattactgcggcagt
aaccctgggacctttcggatcctagtggggaataagggatgcagccaccc
ctcagtgaaatgcaagaaacgggtcaccatcctggtggagggaggagaga
ttgagctgtttgacggggaggtgaatgtgaagaggcccatgaaggatgag
actcactttgaggtggtggagtctggccggtacatcattctgctgctggg
caaagccctctccgtggtctgggaccgccacctgagcatctccgtggtcc
tgaagcagacataccaggagaaagtgtgtggcctgtgtgggaattttgat

ggcatccagaacaatgacctcaccagcagcaacctccaagtggaggaaga
ccctgtggactttgggaactcctggaaagtgagctcgcagtgtgctgaca
ccagaaaagtgcctctggactcatcccctgccacctgccataacaacatc
atgaagcagacgatggtggattcctcctgtagaatccttaccagtgacgt
cttccaggactgcaacaagctggtggaccccgagccatatctggatgtct
gcatttacgacacctgctcctgtgagtccattggggactgcgcctgcttc
tgcgacaccattgctgcctatgcccacgtgtgtgcccagcatggcaaggt
ggtgacctggaggacggccacattgtgcccccagagctgcgaggagagga
atctccgggagaacgggtatgagtgtgagtggcgctataacagctgtgca
cctgcctgtcaagtcacgtgtcagcaccctgagccactggcctgccctgt
gcagtgtgtggagggctgccatgcccactgccctccagggaaaatcctgg
atgagcttttgcagacctgcgttgaccctgaagactgtccagtgtgtgag
gtggctggccggcgttttgcctcaggaaagaaagtcaccttgaatcccag
tgaccctgagcactgccagatttgccactgtgatgttgtcaacctcacct
gtgaagcctgccaggagccgggaggcctggtggtgcctcccacagatgcc
ccggtgagccccaccactctgtatgtggaggacatctcggaaccgccgtt
gcacgatttctactgcagcaggctactggacctggtcttcctgctggatg
gctcctccaggctgtccgaggctgagtttgaagtgctgaaggcctttgtg
gtggacatgatggagcggctgcgcatctcccagaagtgggtccgcgtggc
cgtggtggagtaccacgacggctcccacgcctacatcgggctcaaggacc
ggaagcgaccgtcagagctgcggcgcattgccagccaggtgaagtatgcg
ggcagccaggtggcctccaccagcgaggtcttgaaatacacactgttcca
aatcttcagcaagatcgaccgccctgaagcctcccgcatcaccctgctcc
tgatggccagccaggagccccaacggatgtcccggaactttgtccgctac
gtccagggcctgaagaagaagaaggtcattgtgatcccggtgggcattgg
gccccatgccaacctcaagcagatccgcctcatcgagaagcaggcccctg
agaacaaggccttcgtgctgagcagtgtggatgagctggagcagcaaagg
gacgagatcgttagctacctctgtgaccttgcccctgaagcccctcctcc
tactctgccccccgacatggcacaagtcactgtgggcccggggctcttgg
gggtttcgaccctggggcccaagaggaactccatggttctggatgtggcg
ttcgtcctggaaggatcggacaaaattggtgaagccgacttcaacaggag
caaggagttcatggaggaggtgattcagcggatggatgtgggccaggaca
gcatccacgtcacggtgctgcagtactcctacatggtgactgtggagtac
cccttcagcgaggcacagtccaaaggggacatcctgcagcgggtgcgaga
gatccgctaccagggcggcaacaggaccaacactgggctggccctgcggt
acctctctgaccacagcttcttggtcagccagggtgaccgggagcaggcg
cccaacctggtctacatggtcaccggaaatcctgcctctgatgagatcaa
gaggctgcctggagacatccaggtggtgcccattggagtgggccctaatg
ccaacgtgcaggagctggagaggattggctggcccaatgcccctatcctc
atccaggactttgagacgctcccccgagaggctcctgacctggtgctgca
gaggtgctgctccggagaggggctgcagatccccaccctctcccctgcac
ctgactgcagccagcccctggacgtgatccttctcctggatggctcctcc
agtttcccagcttcttattttgatgaaatgaagagtttcgccaaggcttt
catttcaaaagccaatatagggcctcgtctcactcaggtgtcagtgctgc
agtatggaagcatcaccaccattgacgtgccatggaacgtggtcccggag
aaagcccatttgctgagccttgtggacgtcatgcagcgggagggaggccc
cagccaaatcggggatgccttgggctttgctgtgcgatacttgacttcag
aaatgcatggtgccaggccgggagcctcaaaggcggtggtcatcctggtc
acggacgtctctgtggattcagtggatgcagcagctgatgccgccaggtc

caacagagtgacagtgttccctattggaattggagatcgctacgatgcag
cccagctacggatcttggcaggcccagcaggcgactccaacgtggtgaag
ctccagcgaatcgaagacctccctaccatggtcaccttgggcaattcctt
cctccacaaactgtgctctggatttgttaggatttgcatggatgaggatg
ggaatgagaagaggcccggggacgtctggaccttgccagaccagtgccac
accgtgacttgccagccagatggccagaccttgctgaagagtcatcgggt
caactgtgaccgggggctgaggccttcgtgccctaacagccagtcccctg
ttaaagtggaagagacctgtggctgccgctggacctgcccctgcgtgtgc
acaggcagctccactcggcacatcgtgacctttgatgggcagaatttcaa
gctgactggcagctgttcttatgtcctatttcaaaacaaggagcaggacc
tggaggtgattctccataatggtgcctgcagccctggagcaaggcagggc
tgcatgaaatccatcgaggtgaagcacagtgccctctccgtcgagctgca
cagtgacatggaggtgacggtgaatgggagactggtctctgttccttacg
tgggtgggaacatggaagtcaacgtttatggtgccatcatgcatgaggtc
agattcaatcaccttggtcacatcttcacattcactccacaaaacaatga
gttccaactgcagctcagccccaagacttttgcttcaaagacgtatggtc
tgtgtgggatctgtgatgagaacggagccaatgacttcatgctgagggat
ggcacagtcaccacagactggaaaacacttgttcaggaatggactgtgca
gcggccagggcagacgtgccagcccatcctggaggagcagtgtcttgtcc
ccgacagctcccactgccaggtcctcctcttaccactgtttgctgaatgc
cacaaggtcctggctccagccacattctatgccatctgccagcaggacag
ttgccaccaggagcaagtgtgtgaggtgatcgcctcttatgcccacctct
gtcggaccaacggggtctgcgttgactggaggacacctgatttctgtgct
atgtcatgcccaccatctctggtctacaaccactgtgagcatggctgtcc
ccggcactgtgatggcaacgtgagctcctgtggggaccatccctccgaag
gctgtttctgccctccagataaagtcatgttggaaggcagctgtgtccct
gaagaggcctgcactcagtgcattggtgaggatggagtccagcaccagtt
cctggaagcctgggtcccggaccaccagccctgtcagatctgcacatgcc
tcagcgggcggaaggtcaactgcacaacgcagccctgccccacggccaaa
gctcccacgtgtggcctgtgtgaagtagcccgcctccgccagaatgcaga
ccagtgctgccccgagtatgagtgtgtgtgtgacccagtgagctgtgacc
tgcccccagtgcctcactgtgaacgtggcctccagcccacactgaccaac
cctggcgagtgcagacccaacttcacctgcgcctgcaggaaggaggagtg
caaaagagtgtccccaccctcctgcccccgcaccgtttgcccacccttc
ggaagacccagtgctgtgatgagtatgagtgtgcctgcaactgtgtcaac
tccacagtgagctgtccccttgggtacttggcctcaactgccaccaatga
ctgtggctgtaccacaaccacctgccttcccgacaaggtgtgtgtccacc
gaagcaccatctaccctgtgggccagttctgggaggagggctgcgatgtg
tgcacctgcaccgacatggaggatgccgtgatgggcctccgcgtggccca
gtgctcccagaagccctgtgaggacagctgtcggtcgggcttcacttacg
ttctgcatgaaggcgagtgctgtggaaggtgcctgccatctgcctgtgag
gtggtgactggctcaccgcgggggggactcccagtcttcctggaagagtgt
cggctcccagtgggcctccccggagaacccctgcctcatcaatgagtgtg
tccgagtgaaggaggaggtctttatacaacaaaggaacgtctcctgcccc
cagctggaggtccctgtctgcccctcgggctttcagctgagctgtaagac
ctcagcgtgctgcccaagctgtcgctgtgagcgcatggaggcctgcatgc
tcaatggcactgtcattgggcccgggaagactgtgatgatcgatgtgtgc
acgacctgccgctgcatggtgcaggtgggggtcatctctggattcaagct
ggagtgcaggaagaccacctgcaacccctgcccccctgggttacaaggaag

aaaataacacaggtgaatgttgtgggagatgtttgcctacggcttgcacc
attcagctaagaggaggacagatcatgacactgaagcgtgatgagacgct
ccaggatggctgtgatactcacttctgcaaggtcaatgagagaggagagt
acttctgggagaagagggtcacaggctgcccaccctttgatgaacacaag
tgtctggctgagggaggtaaaattatgaaaattccaggcacctgctgtga
cacatgtgaggagcctgagtgcaacgacatcactgccaggctgcagtatg
tcaaggtgggaagctgtaagtctgaagtagaggtggatatccactactgc
cagggcaaatgtgccagcaaagccatgtactccattgacatcaacgatgt
gcaggaccagtgctcctgctgctctccgacacggacggagcccatgcagg
tggccctgcactgcaccaatggctctgttgtgtaccatgaggttctcaat
gccatggagtgcaaatgctcccccaggaagtgcagcaagtgaggctgctg
cagctgcatgggtgcctgctgctgcctgccttggcctgatggccaggcca
gagtgctgccagtcctctgcatgttctgctcttgtgcccttctgagccca
caataaaggctgagctcttatcttgcaaaaggc
>NM_006412 3
ccagccccgccgccctcgcaataaggggcctgagcgcgcgggggagaagc
gggagcgggagcgggagcgagctggcggcgccgtcgggcgccgggccggg
ccatggagctgtggccgtgtctggccgcggcgctgctgttgctgctgctg
ctggtgcagctgagccgcgcggccgagttctacgccaaggtcgccctgta
ctgcgcgctgtgcttcacggtgtccgccgtggcctcgctcgtctgcctgc
tgcgccacggcggccggacggtggagaacatgagcatcatcggctggttc
gtgcgaagcttcaagtacttttacgggctccgcttcgaggtgcgggaccc
gcgcaggctgcaggaggcccgtccctgtgtcatcgtctccaaccaccaga
gcatcctggacatgatgggcctcatggaggtccttccggagcgctgcgtg
cagatcgccaagcgggagctgctcttcctggggcccgtgggcctcatcat
gtacctcgggggcgtcttcttcatcaaccggcagcgctctagcactgcca
tgacagtgatggccgacctgggcgagcgcatggtcagggagaacctcaaa
gtgtggatctatcccgagggtactcgcaacgacaatggggacctgctgcc
ttttaagaagggcgccttctacctggcagtccaggcacaggtgcccatcg
tccccgtggtgtactcttccttctcctccttctacaacaccaagaagaag
ttcttcacttcaggaacagtcacagtgcaggtgctggaagccatccccac
cagcggcctcactgcggcggacgtccctgcgctcgtggacacctgccacc
gggccatgaggaccaccttcctccacatctccaagacccccccaggagaac
ggggccactgcggggtctggcgtgcagccggcccagtagcccagaccacg
gcagggcatgacctggggagggcaggtggaagccgatggctggaggatgg
gcagaggggactcctcccggcttccaaataccactctgtccggctccccc
agctctcactcagcccgggaagcaggaagccccttctgtcactggcctca
gacacaggcccctggtgtcccctgcaggggctcagctggaccctccccg
ggctcgagggcagggactcgcgcccacggcacctctgggagctgggatga
taaagatgaggcttgcggctgtggcccgctggtgggctgagccacaaggc
ccccgatggcccaggagcagatgggaggaccccgaggccagacgcacact
gtccgagccctctgctcagccgcctgggacccaccagggtgcagctgggc
tccagggtccagcccacaagctgcatcaggctctctgggagaggaggggc
ctggagggccaggagtcccagactcacgcaccctgggccacagggagccg
ggaatcggggcctgctgctcctgctggcctggaagactctgtggggtcag
cactgtactccgttgctgtttttttataaacacactcttggaagtggcaa
aaaaaaaaaaaaaaaaaaaaaaaaa
>NM_000286 2
tagagcgcctctcttccgccaggcatcccagaggtcctggtggtttcatt

tccgggtgcggcttctgtcataaagcggagacctcccttcaaacgtggcg
tcgtgggttgtttgcgcctcgcctggggtcagcgagcaaggacgggcgcg
ggcggggatactcaaagccaacagctggagtcagcccttgtgtcccgggc
tcacagtggcacgactgaatcctcagagtcggctggcttttgagctctca
cgattggggaggagggggcgtttctggttcgcagctccagaggattgcgt
tccttcccccatacctgtcccccacagtcacgctctgccctgacgtgcag
catttgacaagttacccccctcgccacatactacttccacccacgtccgag
ttaactttgttcttaaccttcttgagactaccctcggcctccaggtcttt
ttttcccagttcatttttgcccataagattgagtttcgagtttcagatat
catgcagaaagtttacctttaagactgagcacccatctgatactcttcct
cccgaaaaagttcatgctcacgagagagtttgtgggaaaagtgaaagcca
gtacacgcaggaaactatggctgagcacggggctcacttcacagctgctt
ctgtggccgatgaccagccatccatctttgaggtggtagcacaggacagt
ttaatgacagcagtgagacccgctcttcagcatgtggtcaaggttcttgc
agaatcaaatcccacccactatggcttcttgtggaggtggtttgatgaaa
tctttactctgctagatcttctgctccagcaacattatctgtctagaacc
agtgcctcattttctgaaaacttttacggcttaaagagaattgtaatggg
ggacactcacaagtctcagagattggctagtgctggtctcccaaagcagc
agctttggaaatctattatgttcctggttcttcttccctatctgaaagtg
aagctggagaagctggtttctagcctgagagaagaggatgaatattctat
tcatccccccttcttcccgctggaaacgattttacagagctttcctggcag
cctacccatttgtgaacatggcctgggaaggatggtttcttgtacaacaa
cttcgatacatcctaggaaaagctcagcatcactcaccactgctgaggct
ggctggagttcagctaggtcgactgacagttcaggatatacaagctctgg
agcacaaaccagctaaggccagcatgatgcagcaaccagccaggagtgtt
agtgagaagataaactcagctctgaagaaagctgtgggggtgttgcctt
atccctgtctactggcctttctgtgggtgtattcttcttgcagttccttg
actggtggtactcatctgaaaatcaagaaaccatcaagtcattgactgcc
ctgcctactccaccaccacctgtacacctagactataactctgattctcc
cctcttacccaaaatgaagactgtgtgcccactgtgtcgtaaaacccggg
tgaatgatactgttcttgccacctctggctatgtgttttgttaccgctgt
gtgtttcattatgtgaggagtcaccaagcttgtcccatcacaggttatcc
aacagaagtacaacatctgattaaactctactcccctgagaactgaaagg
gaatcatgtcttatcctcacaacaaaagtattgcgctgtaatgtcacaga
gctggtttcagcatggtattgagttgacacttctcagcctcaattcataa
ctatatgaactttaaatgattatatggatttctttaaaaggatatgccca
tttgatcctaaccttttagcctgctctctagaaccctacttacagttgac
caagctgcttaaagtagaaggatcaggactggcaggggatatgagggcca
gtgtagagattaaacagaataatgagtacacaagtttttgcctatccctt
tccttaaaatataagaaataaaggagctcttacgaaaaacccaggttaga
ccgcataaaaaataaagtgaacagtgaggtggtagcaagacttcttttta
gaaaagaaagcatttacctgcctgtctgtaaggtggaaatttcatcagtt
tgcaaacgataagaaatgcagacttgctcttgatagaaatgcttagaaac
actctgggggggaaaaaagctccttccatatactgtgagacatttgttaa
gtgacatctattgtttatcagcttttaaggataaaaaaggtattttaaaa
gttggatatttaggatatttgaggatattcctttatgagctctccatatc
cttcttgagaaactggttaaaaaaggaataggggttgagtgttacagaga
gtagtctgaagattcctgtgtaaaagcaaagctaacaagcaatgaagaca
tgaagcaaaatactaatctaattgtgtaaagaaaggatattttaataagt

tctttctgcttgctgctaagagtttgctaaagtgtcatgaattattctgg
ttattactaaagtttctatgaaacacttaagtagattttaagaataaatg
tttctggaaaagaactatgttatgattttgtagaaatgtaaagattactt
gaggtgtttaaaataaatttttcattcagactaaaaaaaaaa
>NM_001024382 1
agtgtcctggttactgcagcggcagcaacagcaggtcctactatcgcctc
cctctagtctctgcttctctggatccctgaggagggcagaaggaagaaaa
cagcccaaagatgagagtgattcgcgtgggtacccgcaagagccagcttg
ctcgcatacagacggacagtgtggtggcaacattgaaagcctcgtaccct
ggcctgcagtttgaaatcattgctatgtccaccacaggggacaagattct
tgatactgcactctctaagattggagagaaaagcctgtttaccaaggagc
ttgaacatgccctggagaagaatgaagtggacctggttgttcactccttg
aaggacctgcccactgtgcttcctcctggcttcaccatcggagccatctg
caagcgggaaaaccctcatgatgctgttgtctttcacccaaaatttgttg
ggaagaccctagaaaccctgccagagaagagtgtggtgggaaccagctcc
ctgcgaagagcagcccagctgcagagaaagttcccgcatctggagttcag
gagtattcggggaaacctcaacacccggcttcggaagctggacgagcagc
aggagttcagtgccatcatcctggcaacagctggcctgcagcgcatgggc
tggcacaaccgggtggggcagatcctgcaccctgaggaatgcatgtatgc
tgtgggccaggggggccttgggcgtggaagtgcgagccaaggaccaggaca
tcttggatctggtgggtgtgctgcacgatcccgagactctgcttcgctgc
atcgctgaaagggccttcctgaggcacctggaaggaggctgcagtgtgcc
agtagccgtgcatacagctatgaaggatgggcaactgtacctgactggag
gagtctggagtctagacggctcagatagcatacaagagaccatgcaggct
accatccatgtccctgcccagcatgaagatggccctgaggatgacccaca
gttggtaggcatcactgctcgtaacattccacgagggccccagttggctg
cccagaacttgggcatcagcctggccaacttgttgctgagcaaaggagcc
aaaaacatcctggatgttgcacggcagcttaacgatgcccattaactggt
ttgtggggcacagatgcctgggttgctgctgtccagtgcctacatcccgg
gcctcagtgccccattctcactgctatctggggagtgattaccccgggag
actgaactgcagggttcaagccttccagggatttgcctcaccttggggcc
ttgatgactgccttgcctcctcagtatgtgggggcttcatctctttagag
aagtccaagcaacagcctttgaatgtaaccaatcctactaataaaccagt
tctgaaggtgtaaaaaaaaaaaaaaaaa
>NM_000190 3
ccggaagtgacgcgaggctctgcggagaccaggagtcagactgtaggacg
acctcgggtcccacgtgtccccggtactcgccggccggagcccccggctt
cccggggccgggggaccttagcggcacccacacacagcctactttccaag
cggagccatgtctggtaacggcaatgcggctgcaacggcggaagaaaaca
gcccaaagatgagagtgattcgcgtgggtacccgcaagagccagcttgct
cgcatacagacggacagtgtggtggcaacattgaaagcctcgtaccctgg
cctgcagtttgaaatcattgctatgtccaccacaggggacaagattcttg
atactgcactctctaagattggagagaaaagcctgtttaccaaggagctt
gaacatgccctggagaagaatgaagtggacctggttgttcactccttgaa
ggacctgcccactgtgcttcctcctggcttcaccatcggagccatctgca
agcgggaaaaccctcatgatgctgttgtctttcacccaaaatttgttggg
aagaccctagaaaccctgccagagaagagtgtggtgggaaccagctccct
gcgaagagcagcccagctgcagagaaagttcccgcatctggagttcagga
gtattcggggaaacctcaacacccggcttcggaagctggacgagcagcag

gagttcagtgccatcatcctggcaacagctggcctgcagcgcatgggctg
gcacaaccgggtggggcagatcctgcaccctgaggaatgcatgtatgctg
tgggccagggggccttgggcgtggaagtgcgagccaaggaccaggacatc
ttggatctggtgggtgtgctgcacgatcccgagactctgcttcgctgcat
cgctgaaagggccttcctgaggcacctggaaggaggctgcagtgtgccag
tagccgtgcatacagctatgaaggatgggcaactgtacctgactggagga
gtctggagtctagacggctcagatagcatacaagagaccatgcaggctac
catccatgtccctgcccagcatgaagatggccctgaggatgacccacagt
tggtaggcatcactgctcgtaacattccacgagggccccagttggctgcc
cagaacttgggcatcagcctggccaacttgttgctgagcaaaggagccaa
aaacatcctggatgttgcacggcagcttaacgatgcccattaactggttt
gtggggcacagatgcctgggttgctgctgtccagtgcctacatcccgggc
ctcagtgccccattctcactgctatctggggagtgattaccccgggagac
tgaactgcagggttcaagccttccagggatttgcctcaccttggggcctt
gatgactgccttgcctcctcagtatgtgggggcttcatctctttagagaa
gtccaagcaacagcctttgaatgtaaccaatcctactaataaaccagttc
tgaaggtgtaaaaaaaaaaaaaaaaa
>NM_001040031 1
ttcctttctctctcagctctccgtctctctttctctctcagcctctttct
ttctccctgtctcccccactgtcagcacctcttctgtgtggtgagtggac
cgcttaccccactaggtgaagatgtcagcccaggagagctgcctcagcct
catcaagtcctcggcagcctgatcttctgcttcggcatctggatcctcat
tgacaagaccagcttcgtgtcctttgtgggcttggccttcgtgcctctgc
agatctggtccaaagtcctggccatctcaggaatcttcaccatgggcatc
gccctcctgggttgtgtgggggccctcaaggagctccgctgcctcctggg
cctgtattttgggatgctgctgctcctgtttgccacacagatcaccctgg
gaatcctcatctccactcagcgggcccagctggagcgaagcttgcgggac
gtcgtagagaaaaccatccaaaagtacggcaccaaccccgaggagaccgc
ggccgaggagagctgggactatgtgcagttccagctgcgctgctgcggct
ggcactacccgcaggactggttccaagtcctcatcctgagaggtaacggg
tcggaggcgcaccgcgtgccctgctcctgctacaacttgtcggcgaccaa
cgactccacaatcctagataaggtgatcttgccccagctcagcaggcttg
gacacctggcgcggtccagacacagtgcagacatctgcgctgtccctgca
gagagccacatctaccgcgagggctgcgcgcagggcctccagaagtggct
gcacaacaaccttatttccatagtgggcatttgcctgggcgtcggcctac
tcgagctcgggttcatgacgctctcgatattcctgtgcagaaacctggac
cacgtctacaaccggctcgctcgataccgttaggccccgccctccccaaa
gtcccgccccgcccccgtcacgtgcgctgggcacttccctgctgcctgta
aatatttgtttaatccccagttcgcctggagccctccgccttcacattcc
cctggggacccacgtggctgcgtgcccctgctgctgtcacctctcccacg
ggacctggggctttcgtccacagcttcctgtccccatctgtcggcctacc
accacccacaagattatttttcacccaaacctcaaataaatcccctgcgt
ttttggtaaaaaaaaaaaaaaaaaaaaaa
>NM_001002027 1
caggacacgtgggtgggggaagctgagcgctgagaccaagggctaaagct
gggagactgaaaaaatgcagaccgccggggcattattcatttctccagct
ctgatccgctgttgtaccaggggtctaatcaggcctgtgtctgcctcctt
cttgaatagcccagtgaattcatctaaacagccttcctacagcaacttcc
cactccaggtggccagacgggagttccagaccagtgttgtctcccgggac

attgacacagcagccaagtttattggtgctggggcagccacagttggtgt
ggctggttcaggggctggcattggaaccgtgtttggcagcttgatcattg
gctatgccaggaacccgtctctcaagcagcagctcttctcctatgccatt
cttggctttgccctgtctgaggccatggggcttttctgtttgatggtcgc
cttcctcatcctcttcgccatgtgaggctccatggggggtcaccggcctg
ttgctactgcaactccacaccattcttggtgctggggtgtgttaagcttt
accattaaacacaacgtttctctaaaaaaaaaaaaaaaaaaaa
>NM_005175 2
caggacacgtgggtgggggaagctgagcgctgagaccaagggctaaagct
gggaggtgagtctgtcaccttgagccgggcgagcgctgtgggccaagcag
gggttgcagggtagtaggagtgcagactgaaaaaatgcagaccgccgggg
cattattcatttctccagctctgatccgctgttgtaccaggggtctaatc
aggcctgtgtctgcctccttcttgaatagcccagtgaattcatctaaaca
gccttcctacagcaacttcccactccaggtggccagacgggagttccaga
ccagtgttgtctcccgggacattgacacagcagccaagtttattggtgct
ggggcagccacagttggtgtggctggttcaggggctggcattggaaccgt
gtttggcagcttgatcattggctatgccaggaacccgtctctcaagcagc
agctcttctcctatgccattcttggctttgccctgtctgaggccatgggg
cttttctgtttgatggtcgccttcctcatcctcttcgccatgtgaggctc
catggggggtcaccggcctgttgctactgcaactccacaccattcttggt
gctggggtgtgttaagctttaccattaaacacaacgtttctctaaaaaaa
aaaaaaaaaaaaa
>NM_002134 3
catctctaggccccgccccgcgctgcgtgcccacgttgcgccggcctcgc
gccagtccgctgggctgcagggactgcggcgcctgagggagtcgctgacg
ggcacgctgactggaggctggcggacaggcgacagcgacctgcggcagga
ccagaggagcgagagcagcaagaaccacacccagcagcaatgtcagcgga
agtggaaacctcagagggggtagacgagtcagaaaaaaagaactctgggg
ccctagaaaaggagaaccaaatgagaatggctgacctctcggagctcctg
aaggaagggaccaaggaagcacacgaccgggcagaaaacacccagtttgt
caaggacttcttgaaaggcaacattaagaaggagctgtttaagctggcca
ccacggcactttacttcacatactcagccctcgaggaggaaatggagcgc
aacaaggaccatccagcctttgcccctttgtacttccccatggagctgca
ccggaaggaggcgctgaccaaggacatggagtatttctttggtgaaaact
gggaggagcaggtgcagtgccccaaggctgcccagaagtacgtggagcgg
atccactacatagggcagaacgagccggagctactggtggcccatgcata
cacccgctacatggggggatctctcggggggccaggtgctgaagaaggtgg
cccagcgagcactgaaactccccagcacaggggaagggacccagttctac
ctgtttgagaatgtggacaatgcccagcagttcaagcagctctaccgggc
caggatgaacgccctggacctgaacatgaagaccaaagagaggatcgtgg
aggaggccaacaaggcttttgagtataacatgcagatattcaatgaactg
gaccaggccggctccacactggccagagagaccttggaggatgggttccc
tgtacacgatgggaaaggagacatgcgtaaatgccctttctacgctgctg
aacaagacaaaggtgccctggagggcagcagctgtcccttccgaacagct
atggctgtgctgaggaagcccagcctccagttcatcctggccgctggtgt
ggccctagctgctggactcttggcctggtactacatgtgaagcacccatc
atgccacaccggtaccctcctcccgactgaccactggcctacccctttct
ccagccctgactaaactaccacctcaggtgactttttaaaaaatgctggg
tttaagaaaggcaaccaataaaagccagatgctagagcctctgcctgaca

gcatcctctctatgggccatattccgcactgggcacaggccgtcaccctg
ggagcagtcggcacagtgcagcaagcctggcccccgacccagctctactc
caggcttccacacttctgggccctaggctgcttccggtagtccctgtttt
tgcagtacatgggtgactatctcccctgttggaggtgagtggcctgtaag
tccaagctgtgcgaggggggccttgctggatgctgctgtacaacttctggg
cctctcttggaccctgggagtgagggtgggtgtgggtggaagcctcagag
gccttgggagctcatccctctcacccagaatccctctaacccccttgggtg
cggtttgctcagccccagcttatctcctcctccgcgctgtgtaaatgctc
cagcactcaataaagtgggctttgcaagctaaaaaaaaaaaaaaaaaaa
aaaa
>NM_005675 4
cgcgaggcgcgccgcgatcggggactgtcctaagacgggcggggcgcgct
gcgctagggactgtcataaaaggggcgggacgcgccgcggtcgggatgac
gtgagctgggggcgctcgtcgctgcagccggcggctagcgggcgtccgcg
ccatggagcgctacgcgggcgccttggaggaggtggcggacggtgcccgg
cagcaggagcgacactaccagctgctgtcggcgttacagagcctggtgaa
ggagttgcccagctcattccagcagcgcttgtcctacaccacgctgagcg
acctggccctggcgcttctcgacggcaccgtgttcgaaatcgtgcagggg
ctactggagatccagcacctcaccgaaaagagcctgtacaaccagcgcct
gcgcctacagaacgagcatcgagtgctcaggcaggcgctgcggcagaagc
accaggaagcccagcaggcctgccggccccataacctgcctgtgcttcag
gcggctcagcagcgagaactagaggcggtggagcaccggatccgtgagga
gcagcgggcgatggaccagaagatcgtcctggagctggaccggaaggtgg
ctgaccagcagagcacactggagaaggcgggggtggctggcttctacgtg
accaccaacccacaggagctgatgctgcagatgaacctgctggaactcat
ccggaagctgcagcagaggggctgctgggcagggaaggcagccctggggc
taggaggtccctggcagttgcctgctgcccagtgtgaccagaaaggcagc
cctgtcccaccatagccacaggcagcagaagtctgggcagagttcatctt
cttgacctttggccactgccttcccagctgcccgcaggggggttccccctg
ctgaggagagaccaggtggaccccagctgcctgtcacccttcatctggga
cttgctgtcaaaccctaggatagtctcataaaggggaggctgggccagcc
tgctgctgtctgcttcagggccaggcagagagtgaggctgggggttctca
caccttactccaccgggcacatcccaacctgcactggggcccactcgagc
gcttgttctggtctcagccgctcccttggcagctgcagcccccatgcaga
agaggctcccaggcccaagctctgtgtgacccagagaaataaagatgcct
cagtgtggcccgcaaaaaaaaaaaaaaaaaaaaaaaaaaaaaaa
>NM_002250 2
gtccttcggtgtctgggtgtggtgagtagaggtgtgtgtcacaaagtaca
gaccattgtgtgtgacaaagcccatcgtgtgtctgtgtgtgtctttatcc
acgtggatggacgtctctttcttgctctgccccaagacacaccctagccc
ctccttattctcaaaagggggagctggggagcctccccctaccctggggc
ctcccctgcccctccccgccctgcctggccgtcaccactccccagagggc
acagggctctgctgtgcctcagagcaaaagtcccagagccagcagagcag
gctgacgacctgcaagccacagtggctgccctgtgcgtgctgcgaggtgg
gggaccctgggcaggaagctggctgagccccaagaccccgggggccatgg
gcggggatctggtgcttggcctgggggccttgagacgccgaaagcgcttg
ctggagcaggagaagtctctggccggctgggcactggtgctggcaggaac
tggcattggactcatggtgctgcatgcagagatgctgtggttcgggggggt
gctcgtgggcgctctacctgttcctggttaaatgcacgatcagcatttcc

accttcttactcctctgcctcatcgtggcctttcatgccaaagaggtcca
gctgttcatgaccgacaacgggctgcgggactggcgcgtggcgctgaccg
ggcggcaggcggcgcagatcgtgctggagctggtggtgtgtggctgcac
ccggcgcccgtgcggggcccgccgtgcgtgcaggatttaggggcgccgct
gacctccccgcagccctggccgggattcctgggccaaggggaagcgctgc
tgtccctggccatgctgctgcgtctctacctggtgccccgcgccgtgctc
ctgcgcagcggcgtcctgctcaacgcttcctaccgcagcatcggcgctct
caatcaagtccgcttccgccactggttcgtggccaagctttacatgaaca
cgcaccctggccgcctgctgctcggcctcacgcttggcctctggctgacc
accgcctgggtgctgtccgtggccgagaggcaggctgttaatgccactgg
gcacctttcagacacactttggctgatccccatcacattcctgaccatcg
gctatggtgacgtggtgccgggcaccatgtggggcaagatcgtctgcctg
tgcactggagtcatgggtgtctgctgcacagccctgctggtggccgtggt
ggcccggaagctggagtttaacaaggcagagaagcacgtgcacaacttca
tgatggatatccagtataccaaagagatgaaggagtccgctgcccgagtg
ctacaagaagcctggatgttctacaaacatactcgcaggaaggagtctca
tgctgcccgcaggcatcagcgcaagctgctggccgccatcaacgcgttcc
gccaggtgcggctgaaacaccggaagctccgggaacaagtgaactccatg
gtggacatctccaagatgcacatgatcctgtatgacctgcagcagaatct
gagcagctcacaccgggccctggagaaacagattgacacgctggcgggga
agctggatgccctgactgagctgcttagcactgccctggggccgaggcag
cttccagaacccagccagcagtccaagtagctggacccacgaggaggaac
caggctactttccccagtactgaggtggtggacatcgtctctgccactcc
tgacccagccctgaacaaagcacctcaagtgcaaggaccaaagggggccc
tggcttggagtgggttggcttgctgatggctgctggaggggacgctggct
aaagtgggtaggccttggcccacctgaggccccaggtgggaacatggtca
cccccactctgcataccctcatcaaaaacactctcactatgctgctatgg
acgacctccagctctcagttacaagtgcaggcgactggaggcaggactcc
tgggtccctgggaaagagggtactaggggcccggatccaggattctggga
ggcttcagttaccgctggccgagctgaagaactgggtatgaggctggggc
ggggctggaggtggcgccccctggtgggacaacaaagaggacaccatttt
tccagagctgcagagagcacctggtggggaggaagaagtgtaactcacca
gcctctgctcttatctttgtaataaatgttaaagccagaa
>NM_018696 2
gcgcacttccgcccgggcgcattgtagccccgcggacagctgggccaggg
tgcgggcctgcgcctccctcggctcctggcgcgggcctcggggagagggg
tggaagatgtctatggatgtgacattcctggggacgggtgcagcataccc
atctccaacccggggtgcctctgctgtggtccttcggtgtgaaggcgagt
gctggctctttgactgtggggagggaacacagacacagcttatgaaaagc
caacttaaagcagggagaattaccaagatcttcatcacacaccttcatgg
agaccatttctttggccttcctgggctcctctgcacaatcagcctgcaga
gtggctccatggtgtccaaacagcctattgaaatctatggccctgtaggg
cttcgggactttatctggcgaaccatggaactctctcacacggagctggt
cttccattatgtggttcatgaactggttcctacagcagatcaatgtcctg
cagaagaactaaaagaatttgcgcatgtgaatagagcagacagtcctccc
aaagaggaacaaggaagaactatcctgttagactcagaagaaaactcata
ccttctgtttgatgatgaacaatttgttgtaaaagcatttcgcctctttc
acagaattccctcatttgggttttcagtcgtggaaaagaaacgcccaggt
aaactcaatgcacagaaacttaaagaccttggtgttccaccaggtcctgc

ctatgggaagctgaaaaatggaatttctgttgttctggaaaatggggtta
caatttctccccaagatgtcttaaaaaagcctattgttggaagaaaaatc
tgcatattgggtgactgctctggggttgtgggtgatggaggagtaaaact
gtgctttgaagcagacctgttgatccacgaagcaaccctggatgatgccc
agatggacaaagcaaaggagcatggccacagcacaccacagatggcagca
acatttgcaaagttgtgccgtgcaaagaggctggttctgactcacttcag
tcagaggtacaaaccagttgccttggccagagaaggagaaacagatggca
ttgcagaactaaaaaagcaagctgaatcagtgttagatctccaagaagtg
actctagcagaagattttatggtgataagcattccaatcaagaaatgaaa
ccagtgttcctgagtgcacactgacatgtctgtgaatatgttactgaacc
tatagtccagtttttttatttcttgttttagtctgaaattatttgggccc
taataatcctaaaaagaatggagctgcattgatgaattggctcagtattt
aaagggagcaaactttttgataataaatctttttaagagaaaaaaaaccc
agcatcctttttgaagtccagatttgtcaaaatgatagactattcagtta
tacatcttattttgtgctactaccacagatagccaatattccatgcagtc
ctgggcttagcttctgcccagctttattgctgctattggcaaagagcaca
ggactcagccctcgtggctaaaaatggtattttggcagtttgtattgaat
ctgtttgtgttattaacagaagagggagaaatgtcatgagacgttggaca
ggcaggattgatgatagcatgaccatagctttgctggaatactgaatgca
gggtttggctaggtgtttattttaacattttattaaactttctatttggg
tcttaacccatggttctcagctggggtgacactgctcctctagaagcagg
tttgaaattatgggtatgttttcttgtcacagtgactgagtacggtggtg
gtagtggtggtactactactactggtatttagtcagcagggataagggat
gttagctgtcctgcaaagttttggtcagtcatatacaataaagaatcatc
tcaccccgaaatgcaaattgcaggcccattgagatacacagcaagttagt
ttatactcttgatgtgaatatagtttaccaaaattagtgacataatgagt
ggaattactaggtaagtcaaatcctaaagagtgaatgtgttagaattgca
gtgtcataccaaataacattcaaaagaaatgagccattaagtgaagctta
tgaacctggaggtaaaaaatgcttatctgttaaactgaggtttcattgac
gtgtccctattaataaacgttatttgccccaca
>NM_005529 5
gcgcggagcgagcgagcgagagagcggcgcgggccgggccatggggtggc
gggcggcgggcgcgctgctgctggcgctgctgctgcacgggcggctgctg
gcggtgacccatgggctgagggcatacgatggcttgtctctgcctgagga
catagagaccgtcacagcaagccaaatgcgctggacacattcgtaccttt
ctgatgatgaggacatgctggctgacagcatctcaggagacgacctgggc
agtggggacctgggcagcggggacttccagatggtttatttccgagccct
ggtgaatttcactcgctccatcgagtacagccctcagctggaggatgcag
gctccagagagttccgagaggtgtccgaggctgtggtagacacgctggag
tcggagtacttgaaaattcccggagaccaggttgtcagtgtggtgttcat
caaggagctggatggctgggtttttgtggagctggatgtgggctcggaag
ggaatgcggatggggctcagattcaggagatgctgctcagggtcatctcc
agcggctctgtggcctcctacgtcacctctccccagggattccagttccg
acgcctgggcacagtgccccagttcccaagagcctgcacggaggccgagt
ttgcctgccacagctacaatgagtgtgtggccctggagtatcgctgtgac
cggcggcccgactgcagggacatgtctgatgagctcaattgtgaggagcc
agtcctgggtatcagccccacattctctctccttgtggagacgacatctt
taccgccccggccagagacaaccatcatgcgacagccaccagtcacccac
gctcctcagcccctgcttcccggttccgtcaggcccctgccctgtgggcc

ccaggaggccgcatgccgcaatgggcactgcatccccagagactacctct
gcgacggacaggaggactgcgaggacggcagcgatgagctagactgtggc
cccccgccaccctgtgagcccaacgagttcccctgcgggaatggacattg
tgccctcaagctgtggcgctgcgatggtgactttgactgtgaggaccgaa
ctgatgaagccaactgccccaccaagcgtcctgaggaagtgtgcgggccc
acacagttccgatgcgtctctaccaacatgtgcatcccagccagcttcca
ctgtgacgaggagagcgactgtcctgaccggagcgacgagtttggctgca
tgccccccaggtggtgacacctccccgggagtccatccaggcttcccgg
ggccagacagtgaccttcacctgcgtggccattggcgtccccacccccat
catcaattggaggctcaactggggccacatcccctctcatcccagggtga
cagtgaccagcgagggtggccgtggcacactgatcatccgtgatgtgaag
gagtcagaccagggtgcctacacctgtgaggccatgaacgcccggggcat
ggtgtttggcattcctgacggtgtccttgagctcgtcccacaacgaggcc
cctgccctgacggccacttctacctggagcacagcgccgcctgcctgccc
tgcttctgctttggcatcaccagcgtgtgccagagcacccgccgcttccg
ggaccagatcaggctgcgctttgaccaacccgatgacttcaagggtgtga
atgtgacaatgcctgcgcagcccggcacgccacccctctcctccacgcag
ctgcagatcgacccatccctgcacgagttccagctagtcgacctgtcccg
ccgcttcctcgtccacgactccttctgggctctgcctgaacagttcctgg
gcaacaaggtggactcctatggcggctccctgcgttacaacgtgcgctac
gagttggcccgtggcatgctggagccagtgcagcggccggacgtggtcct
catgggtgccgggtaccgcctcctctcccgaggccacacacccacccaac
ctggtgctctgaaccagcgccaggtccagttctctgaggagcactgggtc
catgagtctggccggccggtgcagcgcgcggagctgctgcaggtgctgca
gagcctggaggccgtgctcatccagaccgtgtacaacaccaagatggcca
gcgtgggacttagcgacatcgccatggataccaccgtcacccatgccacc
agccatggccgtgcccacagtgtggaggagtgcagatgccccattggcta
ttctggcttgtcctgcgagagctgtgatgcccacttcactcgggtgcctg
gtgggccctacctgggcacctgctctggttgcaattgcaatggccatgcc
agctcctgtgaccctgtgtatggccactgcctgaattgccagcacaacac
ggaggggccacagtgcaacaagtgcaaggctggcttctttggggacgcca
tgaaggccacggccacttcctgccggccctgcccttgcccatacatcgat
gcctcccgcagattctcagacacttgcttcctggacacggatggccaagc
cacatgtgacgcctgtgccccaggctacactggccgccgctgtgagagct
gtgcccccggatacgagggcaaccccatccagcccggcgggaagtgcagg
cccgtcaaccaggagattgtgcgctgtgacgagcgtggcagcatggggac
ctccggggaggcctgccgctgtaagaacaatgtggtggggcgcttgtgca
atgaatgtgctgacggctctttccacctgagtacccgaaaccccgatggc
tgcctcaagtgcttctgcatgggtgtcagtcgccactgcaccagctcttc
atggagccgtgcccagttgcatggggcctctgaggagcctggtcacttca
gcctgaccaacgccgcaagcacccacaccaccaacgagggcatcttctcc
cccacgcccggggaactgggattctcctccttccacagactcttatctgg
accctacttctggagcctcccttcacgcttcctgggggacaaggtgacct
cctatggaggagagctgcgcttcacagtgacccagaggtcccagccgggc
tccacacccctgcacgggcagccgttggtggtgctgcaaggtaacaacat
catcctagagcaccatgtggcccaggagcccagccccggccagcccagca
ccttcattgtgcctttccgggagcaagcatggcagcggcccgatgggcag
ccagccacacgggagcacctgctgatggcactggcaggcatcgacaccct
cctgatccgagcatcctacgcccagcagcccgctgagagcagggtctctg

gcatcagcatggacgtggctgtgcccgaggaaaccggccaggaccccgcg
ctggaagtggaacagtgctcctgcccacccgggtaccgtgggccgtcctg
ccaggactgtgacacaggctacacacgcacgcccagtggcctctacctgg
gtacctgtgaacgctgcagctgccatggccactcagaggcctgcgagcca
gaaacaggtgcctgccagggctgccagcatcacacggagggccctcggtg
tgagcagtgccagccaggatactacggggacgcccagcgggggacaccac
aggactgccagctgtgcccctgctacggagaccctgctgccggccaggct
gcccacacttgttttctggacacagacggccaccccacctgtgatgcgtg
ctccccaggccacagtgggcgtcactgtgagaggtgcgcccctggctact
atggcaaccccagccagggccagccatgccagagagacagccaggtgcca
gggcccataggctgcaactgtgacccccaaggcagcgtcagcagccagtg
tgatgctgctggtcagtgccagtgcaaggcccaggtggaaggcctcactt
gcagccactgccggccccaccacttccacctgagtgccagcaacccagac
ggctgcctgccctgcttctgtatgggcatcacccagcagtgcgccagctc
tgcctacacacgccacctgatctccacccactttgcccctggggacttcc
aaggctttgccctggtgaacccacagcgaaacagccgcctgacaggagaa
ttcactgtggaacccgtgcccgagggtgcccagctctcttttggcaactt
tgcccaactcggccatgagtccttctactggcagctgccggagacatacc
agggagacaaggtggcggcctacggtgggaagttgcgatacaccctctcc
tacacagcaggcccacagggcagcccactctctgaccccgatgtgcagat
cacgggcaacaacatcatgctagtggcctcccagccagcgctgcagggcc
ctgagaggaggagctacgagatcatgttccgagaggaattctggcgccgg
cccgatgggcagccggccacacgcgagcacctcctgatggcactggccga
cctggatgagctcctgatccgggccacgttctcctccgtgccgctggcgg
ccagcatcagcgcagtcagcctggaggtcgcccagccggggccctcaaac
agaccccgcgccctcgaggtggaggagtgccgctgcccgccaggctacat
cggtctgtcctgccaggactgtgcccccggctacacgcgcaccgggagtg
ggctctacctcggccactgcgagctatgtgaatgcaatggccactcagac
ctgtgccacccagagactggggcctgctcgcaatgccagcacaacgccgc
aggggagttctgcgagctttgtgcccctggctactacggagatgccacag
ccgggacgcctgaggactgccagccctgtgcctgcccactgaccaaccca
gagaacatgttttcccgcacctgtgagagcctgggagccggcgggtaccg
ctgcacggcctgcgaacccggctacactggccagtactgtgagcagtgtg
gcccaggttacgtgggtaaccccagtgtgcaagggggccagtgcctgcca
gagacaaaccaagccccactggtggtcgaggtccatcctgctcgaagcat
agtgccccaaggtggctcccactccctgcggtgtcaggtcagtgggagcc
caccccactacttctattggtcccgtgaggatgggcggcctgtgcccagc
ggcacccagcagcgacatcaaggctccgagctccacttccccagcgtcca
gccctcggatgctggggtctacatttgcacctgccgtaatctccaccaat
ccaataccagccgggcagagctgctggtcactgaggctccaagcaagccc
atcacagtgactgtggaggagcagcggagccagagcgtgcgccccggagc
tgacgtcaccttcatctgcacagccaaaagcaagtccccagcctataccc
tggtgtggacccgcctgcacaacgggaaactgcccacccgagccatggat
ttcaatggcatcctgaccattcgcaacgtccagctgagtgatgcaggcac
ctacgtgtgcaccggctccaacatgtttgccatggaccagggcacagcca
ctctacatgtgcaggcctcgggcaccttgtccgcccccgtggtctccatc
catccgccacagctcacagtgcagcccgggcaactggcggagttccgctg
cagcgccacagggagccccacgcccaccctcgagtggacagggggccccg
gcggccagctccctgcgaaggcacaaatccacggcggcatcctgcgcctg

ccagctgtcgagcccacggatcaggcccagtacttgtgccgagcccacag
cagcgctgggcagcaggtggccagggctgtgctccacgtgcatgggggcg
gtgggcccagagtccaagtgagcccagagaggacccaggtccacgcaggc
cgcaccgtcaggctgtactgcagggctgcaggcgtgcctagcgccaccat
cacctggaggaaggaagggggcagcctcccaccacaggcccggtcagagc
gcacagacatcgcgacactgctcatcccagccatcacgactgctgacgcc
ggcttctacctctgcgtggccaccagccctgcaggcactgcccaggcccg
gatccaagtggttgtcctttcagcctcagatgccagcccaccgccggtca
agattgagtcctcatcgccttctgtgacagaagggcaaacactcgacctc
aactgtgtggtggcagggtcagcccatgcccaggtcacctggtacaggcg
agggggtagcctgcctccccacacccaggtgcacggctcccgtctgcggc
tcccccaggtctcaccagctgattctggagaatatgtgtgccgtgtggag
aatggatcgggccccaaggaggcctccattactgtgtctgtgctccacgg
cacccattctggccccagctacaccccagtgcccggcagcacccggccca
tccgcatcgagccctcctcctcacacgtggcggaagggcagaccctggat
ctgaactgcgtggtgcccgggcaggcccacgcccaggtcacgtggcacaa
gcgtgggggcagcctccctgcccggcaccagacccacggctcgctgctgc
ggctgcaccaggtgaccccggccgactcaggcgagtatgtgtgccatgtg
gtgggcacctccggcccccctagaggcctcagtcctggtcaccatcgaagc
ctctgtcatccctggacccatcccacctgtcaggatcgagtcttcatcct
ccacagtggccgagggccagaccctggatctgagctgcgtggtggcaggg
caggcccacgcccaggtcacatggtacaagcgtgggggcagcctccctgc
ccggcaccaggttcgtggctcccgcctgtacatcttccaggcctcacctg
ccgatgcgggacagtacgtctgccgggccagcaacggcatggaggcctcc
atcacggtcacagtaactgggacccagggggccaacttagcctaccctgc
cggcagcacccagcccatccgcatcgagccctcctcctcgcaagtggcgg
aagggcagaccctggatctgaactgcgtggtgcccgggcagtcccatgcc
caggtcacgtggcacaagcgtgggggcagcctccctgtccggcaccagac
ccacggctccctgctgagactctaccaagcgtcccccgccgactcgggcg
agtacgtgtgccgagtgttgggcagctccgtgcctctagaggcctctgtc
ctggtcaccattgagcctgcgggctcagtgcctgcacttggggtcacccc
cacggtccggatcgagtcatcgtcttcgcaagtggccgaggggcagaccc
tggacctgaactgcctcgttgctggtcaggcccatgcccaggtcacgtgg
cacaagcgcgggggcagcctcccggcccggcaccaggtgcatggctcgag
gctacgcctgctccaggtgaccccagctgattcaggggagtacgtgtgcc
gtgtggtcggcagctcaggtacccaggaagcctcagtccttgtcaccatc
cagcagcgccttagtggctcccactcccagggtgtggcgtaccccgtccg
catcgagtcctcctcagcctccctggccaatggacacaccctggacctca
actgcctggttgccagccaggctccccacaccatcacctggtataagcgt
ggaggcagcttacccagccggcaccagatcgtgggctcccggctgcggat
ccctcaggtgactccggcagactcgggcgagtacgtgtgtcacgtcagta
acggtgcaggctcccgggagacctcgctcatcgtcaccatccagggcagc
ggttcctcccacgtgcccagcgtctccccaccgatcaggatcgagtcgtc
ttcccccacggtggtggaagggcagaccttggatctgaactgcgtggtcg
ccaggcagccccaggctatcatcacatggtacaagcgtgggggcagcctt
ccctcccgacaccagacccatggctcccacctgcggttgcaccaaatgtc
tgtggctgactcgggcgagtatgtgtgccgggccaacaacaacatcgatg
ccctggaggcctccatcgtcatctccgtctcccctagcgccggcagcccc
tccgcccctggcagctccatgcccatcagaattgagtcatcctcctcaca

cgtggccgaaggggagaccctggatctgaactgcgtggtccccgggcagg
cccatgcccaggtcacttggcacaagcgtgggggcagcctccccagtcac
catcagacccgcggctcacggctgcggctgcaccatgtgtccccggccga
ctcgggtgaatacgtgtgccgggtgatgggcagctctggcccccctggagg
cctcagtcctggtcaccatcgaagcctctggctcaagtgctgtccacgtc
cccgccccaggtggagccccacccatccgcatcgagccctcctcctcccg
agtggcagaagggcagaccctggatctgaagtgcgtggtgcccgggcagg
cccacgcccaggtcacgtggcacaagcgtggaggaaacctccctgcccgg
caccaggtccacggcccactgctgaggctgaaccaggtgtccccggctga
ctctggcgagtactcgtgccaagtgaccggaagctcaggcaccctggagg
catctgtcctggtcacaattgagccctccagcccaggacccattcctgct
ccaggactggcccagcccatctacatcgaggcctcctcttcacacgtgac
tgaagggcagactctggatctgaactgtgtggtgcccgggcaggcccatg
cccaggtcacgtggtacaagcgcgggggcagcctccccgcccggcaccag
acccatggctcccagctgcggctccacctcgtctcccctgccgactcagg
cgagtatgtgtgtcgtgcagccagcggcccaggccctgagcaagaagcct
ccttcacagtcaccgtcccgcccagtgaggggtcttcctaccgccttagg
agcccggtcatctccatcgacccgcccagcagcaccgtgcagcagggcca
ggatgccagcttcaagtgcctcatccatgacggggcagcccccatcagcc
tcgagtggaagacccggaaccaggagctggaggacaacgtccacatcagt
cccaatggctccatcatcaccatcgtgggcacccggcccagcaaccacgg
tacctaccgctgcgtggcctccaatgcctacggtgtggcccagagtgtgg
tgaacctcagtgtgcacgggccccctacagtgtccgtgctccccgagggc
cccgtgtgggtgaaagtgggaaaggctgtcaccctggagtgtgtcagtgc
cggggagccccgctcctctgctcgttggacccggatcagcagcacccctg
ccaagttggagcagcggacatatgggctcatggacagccacgcggtgctg
cagatttcatcagctaaaccatcagatgcgggcacttatgtgtgccttgc
tcagaatgcactaggcacagcacagaagcaggtggaggtgatcgtggaca
cgggcgccatggccccaggggcccctcaggtccaagctgaagaagctgag
ctgactgtggaggctggacacacggccaccttgcgctgctcagccacagg
cagccccgcgcccaccatccactggtccaagctgcgttccccactgccct
ggcagcaccggctggaaggtgacacactcatcataccccgggtagcccag
caggactcgggccagtacatctgcaatgccactagccctgctgggcacgc
tgaggccaccatcatcctgcacgtggagagcccaccatatgccaccacgg
tcccagagcacgcttcggtgcaggcaggggagacggtgcagctccagtgc
ctggctcacgggacacccccactcaccttccagtggagccgcgtgggcag
cagccttcctgggagggcgaccgccaggaacgagctgctgcactttgagc
gtgcagcccctgaggactcaggccgctaccgctgccgggtcaccaacaag
gtgggctcagccgaggcctttgcccagctgctcgtccaaggccctcccgg
ctctctccctgccacctccatcccagcagggtccacgcccaccgtgcagg
tcacgcctcagctagagaccaagagcattggggccagcgttgagttccac
tgtgctgtgcccagcgaccggggtacccagctccgttggttcaaggaagg
gggtcagctgcctccgggtcacagcgtgcaggatggggtgctccgaatcc
agaacttggaccagagctgccaagggacgtatatatgccaggcccatgga
ccttgggggaaggcccaggccagtgcccagctggttatccaagccctgcc
ctcggtgctcatcaacatccggacctctgtgcagaccgtggtggttggcc
acgccgtggagttcgaatgcctggcactgggtgaccccaagcctcaggtg
acatggagcaaagttggagggcacctgcggccaggcattgtgcagagcgg
aggtgtcgtcaggatcgcccacgtagagctggctgatgcgggacagtatc

gctgcactgccaccaacgcagctggcaccacacaatcccacgtcctgctg
cttgtgcaagccttgccccagatctcaatgccccaagaagtccgtgtgcc
tgctggttctgcagctgtcttcccctgcatagcctcaggctaccccactc
ctgacatcagctggagcaagctggatggcagcctgccacctgacagccgc
ctggagaacaacatgctgatgctgccctcagtccgaccccaggacgcagg
tacctacgtctgcaccgccactaaccgccagggcaaggtcaaagcctttg
cccacctgcaggtgccagagcgggtggtgccctacttcacgcagacccc
tactccttcctaccgctgcccaccatcaaggatgcctacaggaagttcga
gatcaagatcaccttccggcccgactcagccgatgggatgctgctgtaca
atgggcagaagcgagtcccagggagccccaccaacctggccaaccggcag
cccgacttcatctccttcggcctcgtgggggggaaggcccgagttccggtt
cgatgcaggctcaggcatggccaccatccgccatcccacaccactggccc
tgggccatttccacaccgtgaccctgctgcgcagcctcacccagggctcc
ctgattgtgggtgacctggccccggtcaatgggacctcccagggcaagtt
ccagggcctggatctgaacgaggaactctacctgggtggctatcctgact
atggtgccatccccaaggcggggctgagcagcggcttcataggctgtgtc
cgggagctgcgcatccagggcgaggagatcgtcttccatgacctcaacct
cacggcgcacggcatctcccactgccccacctgtcgggaccggccctgcc
agaatggcggtcagtgccatgactctgagagcagcagctacgtgtgcgtc
tgcccagctggcttcaccgggagccgctgtgagcactcgcaggccctgca
ctgccatccagaggcctgtgggcccgacgccacctgtgtgaaccggcctg
acggtcgaggctacacctgccgctgccacctgggccgctcggggttgcgg
tgtgaggaaggtgtgacagtgaccaccccctcgctgtcgggtgctggctc
ctacctggcactgcccgccctcaccaacacacaccacgagctacgcctgg
acgtggagttcaagccactcgcccctgacggggtcctgctgttcagcggg
gggaagagcgggcctgtggaggacttcgtgtccctggcgatggtgggcgg
ccacctggagttccgctatgagttggggtcagggctggccgttctgcgga
gcgccgagccgctggccctgggccgctggcaccgtgtgtctgcagagcgt
ctcaacaaggacggcagcctgcgggtgaatggtggacgccctgtgctgcg
ctcctcgcccggcaagagccagggcctcaacctgcacaccctgctctacc
tggggggtgtggagccttccgtgccactgtccccggccaccaacatgagc
gctcacttccgcggctgtgtgggcgaggtgtcagtgaatggcaaacggct
ggacctcacctacagtttcctaggcagccagggcatcgggcaatgctatg
atagctccccatgtgagcgccagccttgccaacatggtgccacgtgcatg
cccgctggcgagtatgagttccagtgcctgtgtcgagatggattcaaagg
agacctgtgtgagcacgaggagaacccctgccagctccgtgaaccctgtc
tgcatgggggcacctgccagggcacccgctgcctctgcctccctggcttc
tctggcccacgctgccaacaaggctctggacatggcatagcagagtccga
ctggcatcttgaaggcagcgggggcaatgatgcccctgggcagtacggag
cctatttccacgatgatggcttcctcgccttccctggccatgtcttctcc
aggagcctgcccgaggtgcccgagaccatcgagctggaggttcggaccag
cacagccagtggcctcctgctctggcagggtgtggaggtgggagaggccg
gccaaggcaaggacttcatcagcctcgggcttcaagacgggcaccttgtc
ttcaggtaccagctgggtagtggggaggcccgcctggtctctgaggaccc
catcaatgacggcgagtggcaccgggtgacagcactgcgggagggccgca
gaggttccatccaagtcgacggtgaggagctggtcagcggccggtcccca
ggtcccaacgtggcagtcaacgccaagggcagcgtctacatcggcggagc
ccctgacgtggccacgctgaccgggggcagattctcctcaggcatcacag
gctgtgtcaagaacctggtgctgcactcggcccgacccggcgccccgccc

ccacagcccctggacctgcagcaccgcgcccaggccggggccaacacacg
cccctgcccctcgtaggcacctgcctgccccacacggactcccgggccac
gccccagcccgacaatgtcgagtatattattattaatattattatgaatt
tttgtaagaaaccgaggcgatgccacgctttgctgctaccgccctgggct
ggactggaggtgggcatgccaccctcacacacacagctgggcaaagccac
aaggctggccagcaaggcaggttggatgggagtgggcacctcagaaagtc
accaggacttggggtcaggaacagtggctgggtgggcccagaactgcccc
cactgtcccсctacccaccgatggagcccccagatagagctgggtggcct
gtttctgcagcccttgggcagttctcactcctaggagagccaacctcggc
ttgtgggctggtgccccacagctacctgagacgggcatcgcaggagtctc
tgccacccactcaggattgggaattgtctttagtgccggctgtggagcaa
aaggcagctcaccсctgggcaggcggtccccatccccaccagctcgtttt
tcagcacccccacccacctccacccagcccctggcacctcctctggcaga
ctcccсctcctaccacgtcctcctggcctgcattcccacccccctcctgcc
agcacacagcctggggtccctccctcaggggctgtaagggaaggcccacc
ccaactcttaccaggagctgctacaggcagagcccagcactgatagggcc
ccgcccaccgggccccgcccaccccaggccacatccccacccatctggaa
gtgaaggcccagggactcctccaacagacaacggacggacggatgccgct
ggtgctcaggaagagctagtgccttaggtgggggaaggcaggactcacga
ctgagagagagaggagggggatatgaccaccctgccccatctgcaggagc
ctgaagatccagctcaagtgccatcctgccagtggcccccagactgtggg
gttgggacgcctggcctctgtgtcctagaagggaccctcctgtggtcttt
gtcttgatttttcttaataaacggtgctatccccgccaaaaaaa
>NM_001243526 1
gtgatccctctctcgctaaccgtaggcgcttttcgtgaaggcccgggttt
ttacagcacttcgcttttctaaccacgaacagtgctcgttcgttcgcagg
gccagcaaggagagccccgcccccgcccgccgcccgccgcccgccgcccg
ccgcctttggatcccgcggactccgcccggcccggcctccccaggcatgg
cgccgctgcgcttctccgccaatctgtcctggctattccccgagctctcc
ggcctccccgcgcgggtgcgggccgcgggcagctcgggcttcgaggccgt
cgaggtggcctggccgtacgcggagacgcctgaggcgctggcgcgcgccg
cgcgagaagcggggctgcggcttgtactgatcaacacgcccccgggagac
caagagaagggggaaatggggctgggggccgtccccgggagacaggcggc
cttccgagagggactggagcaggccgtgcggagtgggcattgcttgatgg
gcaggaagttgagtgttccttgcaagggtgctgtggcaagaggaggcctg
gtgtatttggcagcgttcctgaggctggacatgatccacctgatggctgg
ccgagtaccccagggagctgatcgaatagcagtcaaggctgagatggagg
ccgtttttctggagaacctgaggcatgcagctggggttttggctcaggag
gacctcgtgggactgctggagcccatcaacacccgcatcactgacсccca
gtacttcctggacacgccccagcaggcggcagccatcttacagaaggtag
gaagacccaacctccaattacaaatggacatattccactggcagatcatg
gatgggaacctgacaggaaacatccgggagttcctgcccattgttgggca
tgtgcaggtggcacaggtcccaggccgaggggagcccagcagccccggag
agctgaatttcсccctatctgtttcaactgctggaagatgaaggctacaaa
ggcttcgtgggctgagacacagtagagggcttgagttggctacgttcata
ctgggataggcggggccacccagaggctggccagtgagggcccgcacacc
acccacgtgcctccagacagcgagtgacatcccatctcctcctctgcatt
aaagatgacctgctgaacattgtcatatgtctctgtaatggggtggtggg
acacatagtgtctaacacttcagtttctctgctgcttccctcccattgag

aagccagtgccagggttgctgtgaagatgggagagcttctgaacccacct
cattaaaggatgagaaacccagaaaa
>NM_013445 3
gcaaaaccgtgagctggatttataatcgccctataaagctccagaggcgg
tcaggcacctgcagaggagccccgccgctccgccgactagctgcccccgc
gagcaacggcctcgtgatttccccgccgatccggtccccgcctccccact
ctgcccccgcctaccccggagccgtgcagccgcctctccgaatctctctc
ttctcctggcgctcgcgtgcgagagggaactagcgagaacgaggaagcag
ctggaggtgacgccgggcagattacgcctgtcagggccgagccgagcgga
tcgctgggcgctgtgcagaggaaaggcgggagtgcccggctcgctgtcgc
agagccgaggtggcctgtttctgcgccggaccagtcgaggactctggaca
gtagaggccccgggacgaccgagctgatggcgtcttcgaccccatcttcg
tccgcaacctcctcgaacgcgggagcggaccccaataccactaacctgcg
ccccacaacgtacgatacctggtgcggcgtggcccatggatgcaccagaa
aactggggctcaagatctgcggcttcttgcaaaggaccaacagcctggaa
gagaagagtcgccttgtgagtgccttcaaggagaggcaatcctccaagaa
cctgctttcctgtgaaaacagcgaccgggatgcccgcttccggcgcacag
agactgacttctctaatctgtttgctagagatctgcttccggctaagaac
ggtgaggagcaaaccgtgcaattcctcctggaagtggtggacatactcct
caactatgtccgcaagacatttgatcgctccaccaaggtgctggactttc
atcacccacaccagttgctggaaggcatggagggcttcaacttggagctc
tctgaccaccccgagtccctggagcagatcctggttgactgcagagacac
cttgaagtatggggttcgcacaggtcatcctcgatttttcaaccagctct
ccactggattggatattattggcctagctggagaatggctgacatcaacg
gccaataccaacatgccatcagacatgagggagtgttggttgctacggtg
atggggctcagagcagaaccaaagcatgattgtgacctccagaggtgatg
gtaactgcacacatggtttccaagggtcttcctcctaaatttccaggggc
ctcccaaggaaaatggacatattctttttggaaataaaatacttctacca
acataaaaaaaaaaaaaaaaaa
>NM_003350 2
cgcgtcgggctgcaggagaagatggcggtctccacaggagttaaagttcc
tcgtaattttcgcttgttggaagaacttgaagaaggacaaaaaggagtag
gcgacggtacagttagctggggccttgaagatgatgaagatatgacactt
acaaggtggacaggcatgattattgggccaccaaggacaaattatgaaaa
cagaatatatagcctgaaagtagaatgtggacctaaatacccagaagctc
ctccgtcagttagatttgtaacaaaaattaatatgaacggaataaataat
tccagtgggatggtggatgcccggagcataccagtgttagcaaaatggca
aaattcatatagcattaaagttgtacttcaagagctaagacgtctaatga
tgtccaaagaaaatatgaagcttccacagccaccagaaggacaaacatac
aacaattaattttagtggatctcaaacttgtcttaaatcaacaaccttct
actcatgttaatgtcttgattaaatatcacaatgcaaaatacacattaag
taaaagaattccagctggtaaacatgacctggacatttgtaagaatatat
ttaatatatgtacacccattatgttttcaggtaacaggaggaaaaatgca
gcacaatttttttctcttgaaaggcactgtcatttaaacataaacctgg
agtactcgaaatagaattcaggtttacaagatgaaagcgtgtggagaagt
gtcagatggcagtggaagcatgtgtgtttctaaaaagtaaaaatctcaag
aaaacagaaatggcatgctttacccatcttacttagtgaaagagagctgc
agttgaaattgtttaaaaagtagcaggtacaatgaatattgtcacagatg
tgttaatttttgaagcaatgtgggtgctgactactagtagtatcaaaaat

atgttcaggattgttttgatacctgtatttataataaaaaatgttggggg
gagttgatgaattcctgttaaaagctgttcttgtgtgttacatgtaacag
acatggtaaatatttgtttacagtctttgtttaacaaaccatgcatttaa
gtttaagtgaagtcaacaaaaaggaaataggtgtatggatatgtgatttt
gagattaaagttagtcttaaaatgtaaataaaatgtgaaacgtgtcctca
gagactgtgccatttctattatgttgatgtatatgtacagtaccttgcca
gggaagcaaaaattggaattattgtagcttttcatgtatacacactttta
tttaccctattttgtgtacttcttgtgaattataatttgcagactatttc
agaaaagaaattatctagtttaatttcttctttggacaaggagtcctagg
tattatattttgagtttgatttcaccagaaataataatattaaaaagatc
tttgcattctggcagttcttttaggattataggttgcaaattatccaaat
atatatcccattttttaaagcataaaaaaaaaaaa
>NM_003430 2
ctcgctgccgccggagtttccaggtctcgacttcactgctctgtgtcctc
tgctccaggaggcccagcctgtgtggccctgtgacctgcaggtattggag
agccacagctaagatgccaggaacccctggaagcctagaaatgggactgt
tgacatttagggatgtggccatagaattctctccggaggagtggcaatgt
ctggacactgcacagcagaatttatataggaatgtgatgttagagaacta
cagaaacctggccttcctgggtattgctctctctaagccagacctgatta
cttatctggagcaaggaaaagagccctggaatatgaagcaacatgagatg
gtggatgaacccacaggtatatgtcctcattttcctcaagacttttggcc
agagcagagcatggaagattcttttcaaaaagtattactgagaaaatatg
aaaaatgtggacatgagaatttacagttaagaaaaggttgtaaaagtgtg
gatgagtgtaaggtgcacaaagaaggttataataaacttaaccagtgtct
cacaactgcccagagcaaagtatttcaatgtgggaaatatttgaaagtct
tctataaatttttaaattcaaacagacatacgataagacatactggaaag
aaatgcttcaaatgtaaaaaatgtgtcaagtcattttgcatccgtttaca
caaaacccaacataaatgcgtttatattacagagaagtcctgtaaatgta
aagaatgtgaaaaaaccttcattggtcctcaacccttactaatcataag
gaaattcatactgaagataaaccctacaaatgtgaagaatgtggcaaagc
ttttaagcagctctcaacccttactacacataaaataatctgtgctaaag
agaaaatctacaagtgtgaagaatgtggcaaagcatttctatggtcctca
accctaactagacataagaggatacacactggagagaaaccctacaaatg
tgaagaatgtggcaaagcttttagccattcttcaacccttgctaaacata
agagaattcatactggagagaaaccctacaaatgtgaagaatgtggcaaa
gcttttagccgttcttcaacccttgctaaacataagagaattcatactgg
agagaaaccctacaaatgtaaagaatgtggcaaagcttttagcaattcct
caacccttgctaatcataagataactcatactgaagagaaaccctacaaa
tgtaaagaatgtgacaaagcttttaagcgactctcaacccttactaaaca
taaaataatacatgctggagagaaactctacaaatgtgaagaatgtggca
aagcttttaatcgatcttcaaatcttactatacataagtttattcatact
ggagagaaaccttacaagtgtgaagaatgtggcaaagcatttaactggtc
ctcaagccttactaaacataaaagatttcatactagagagaaacccttca
aatgtaaagaatgtggcaaagcatttatatggtcttcaaccctaactaga
cataagaggatacacactggagagaagccctacaaatgtgaagaatgtgg
caaagcttttaggcaatcctcaacccttactaaacataagataattcata
ctggagagaaaccctacaaatttgaagaatgtggcaaagcttttagacaa
tccttaacccttaataaacataagataattcatagtagagagaaacccta
caaatgtaaagaatgtggcaaagcttttaagcaattctcaacccttacta

cacataaaataattcatgctggaaagaaactctacaaatgtgaagaatgt
ggcaaagcttttaatcattcctcaagtctttctacacataagataattca
tactggagagaagtcttacaagtgtgaagaatgtggcaaagcatttctat
ggtcctcaaccctaagaagacataagaggatacacactggagagaaaccc
tacaaatgtgaagaatgtggcaaagcttttagccattcttcagcccttgc
taaacataagagaattcatactggagagaaaccctacaaatgtaaagaat
gtggcaaagcttttagcaattcctcaacccttgctaatcataagataact
catactgaagagaaaccctacaaatgtaaagaatgtgacaaaacttttaa
gcgactctcaacccttactaaacataaaataatacatgctggagagaaac
tctacaaatgtgaagaatgtggcaaagcttttaatcgatcttcaaatctt
actatacataagtttattcatactggagagaaaccttacaagtgtgaaga
atgtggcaaagcatttaactggtcctcaagccttactaaacataaaagaa
ttcatactagagagaaacccttcaaatgtaaagaatgtggcaaagcattt
atatggtcttcaaccctaactagacataagaggatacacactggagagaa
gccctacaaatgtgaagaatgtggcaaagcttttagccgttcctcaaccc
ttactaagcataagacaattcatactggagagaaaccctacaaatgtaaa
gaatgtggcaaagcttttaagcactcctcagcccttgctaaacataaaat
aatacatgctggagagaaactctacaaatgtgaggaatgtggcaaagctt
ttaatcaatcttcaaatcttacgacacataagataattcatactaaagag
aaaccttccaagagtgaagaatgtgacaaagcatttatctggtcctcaac
ccttactgaacataagagaattcataccagagagaaaacctacaaatgtg
aagaatgtggcaaagcatttagccagccttcacaccttactacacataag
aggatgcacactggagagaaaccctacaaatgtgaagaatgtggcaaagc
ttttagccaatcctcaacccttactacacataagataattcatactggag
agaaaccctacaaatgtgaagaatgtggcaaagcttttaggaaatcttca
actcttactgaacataagataattcatactggagagaaaccctacaaatg
tgaagaatgtggcaaagcatttagccaatcctcaaccctaactagacata
cgaggatgcacactggagagaaaccatacaaatgtgaagaatgtggcaaa
gcttttaatcgatcctcaaagcttactacacataagataattcatactgg
agagaaaccttacaagtgtgaagaatgtggcaaagcatttatatcatcct
caaccctaaatggacataagagaattcatactagagagaaaccctacaaa
tgtgaagaatgtggcaaagcatttagccaatcttcaaccctaactagaca
taagaggttgcacaccggagagaaaccctacaaatgtggagaatgtggca
aagcctttaaagagtcctcagctcttactaaacataagataattcacact
ggagagaaaccctacaaatgtgaaaaatgtggcaaagcctttaaccagtc
ttcaatccttactaaccataagaaaattcatactatcacacctgtaatcc
cactactttgggaggccgaggcgggcggatcacgaggtcaggagatggag
accatcctggctaacacagtgaaacccccttctctactaaaaaatacaaaa
aattagccgggcgtggtggcgggcgcctgtagtcccagctatgcgagagg
ctgaggcaggagaatggcgtgaacccgggaggcggagcttgcagtgagcc
gagatcacgccactgcactcaagcctgggcgacagagcgagactccgtct
caaaaaaaaaaaaaagaaaattcatactagagagaaaccctacaaatata
aagaatgtggcaaatcttttaaccggtcttcaacctttactaaacataag
gtaattcatactggagtaaaactctacaaatgtgaagaatgtggcaaatc
ctttttctggtcctcagccctaaccagacataagaaaattcatactggac
agcaaccctacaaacaggaaaaatttggcaaagcctttaaccagttttca
caccttactacagataagataactcatattggagagaaatcttacaagtg
tgaataatgtggtaaagtctataaaaagtcctgaattcttaacagacata
atattattcatactggagagaaactctacaaacctgaaagatgtgccaac

gcttttgacaagtcaaacttttctaaacatcaagaaaatcatgctgctga
gaaatcctagaaatgtgaagaatgtgacaaagcctctaaatgattgtcac
acttgatcgtaggtaagataattcatactggagaaaactaccagtgtgaa
caatgtggccaagctttgaactaatgctcacaccttaatgcataggaaag
catttatgcttgagaaaaagtgaacaaatatagacaaaaagccattaata
cctgctcacatcgtactcaaattcagagcgttcatactaaataagaagtg
taattactgtcaaacgactgatcagaaaatatgtctttaaagtacagaag
agtattttgaaaaagcattacaaatatgaagaggcattatttatgacctt
ttctatggaaaggtaaggacattaaaaatgtaagatgcatgatgaaaatt
taagtagagaggatctttatggttaacttataatatcgagtgatgcatga
ggtagatgttcagattaatattctgcattattatgaaagaaaaacattct
taattttagttaaagtttagtatgatttattgtatttttatgaaatatat
gtagcatatttttaaattataaattgcatgtgaacttttcaaccaaacag
ttttaacatgttaaatactatcatgaattcaatgaagtgttattatgcca
gtaactttaacctattccaccttactcaagggtgtaggtaaaagatggta
aaaatacactatttggtaagataatggactgacatctctagtgattttt
ttgccagtgagttttaattgccattaagttaaagaatattgttcccatag
gctaaatttttatttttattttctcatttaaatgtatttgacttaatttt
tgtggatacctaatatgtgtacatattatgccatatgtggcatattttga
tacggacacacaatatataacaatcgcattagactaaatgcggtctccat
tacctctagcatttattctttgtattacaaataattcaattctacagttt
tagttatttttaaatgtacaattaaattgttgttgactacaggattattt
ttatggtcataataaaacttatatacaaacataagtaaaatccataaaaa
aaa
>NM_006409 3
acttccgggatctgtcagccgctccctctgggcttccgtcctccgcccgc
gcccgacggagcctgttcgcgtcgactgcccagagtccgcgaatcctccg
ctccgagcccgtccggactcccccgatcccagctttctctcctttgaaaa
cactaagaataatgtcactgcatcagtttttactagagccaatcacctgt
catgcctggaacagggatcgtactcagattgccctcagtcccaataatca
cgaagtgcacatctataagaagaacgggagccagtgggtgaaagctcatg
aactcaaggagcacaacggacacatcacaggtattgactgggctcccaag
agcgaccgcattgtcacttgtggggcagaccgcaatgcctatgtctggag
tcagaaagatggtgtttggaagccaaccctggtgatcctgagaattaatc
gcgcagctacttttgtgaagtggtccccccctagagaacaaatttgctgtg
ggaagtggagcacgactcatttctgtttgttactttgagtctgaaaatga
ctggtgggtgagcaagcacattaaaaagccgattcgctccacagtcctca
gcttggattggcatcccaacaacgttttgctggcagcaggatcatgtgac
ttcaaatgcagagtgttttctgcctacattaaagaagtggatgaaaagcc
agccagcacgccctggggcagcaagatgccttttgggcagctgatgtcag
agtttggtggcagtggcactggtggctgggtccacggggtaagcttctct
gccagtgggagccgcctggcctgggtcagccacgacagcaccgtgtctgt
tgctgatgcctcaaaaagtgtgcaggtctcgactctgaagacagagttcc
tgccgctcctaagtgtgtcatttgtctcagagaacagcgtcgtggctgct
ggccatgactgctgcccaatgctctttaactacgatgaccgcggctgcct
gaccttcgtctccaagttagatattccaaaacagagcatccaacgcaaca
tgtctgccatggaacgcttccgcaacatggacaagagagccacaactgag
gaccgcaacacggccttggagacgctgcaccagaatagcatcactcaagt
ctctatttatgaggtggacaagcaagattgtcgcaaattttgcactactg

gcatcgatggagccatgacaatttgggatttcaagaccctcgagtcttcc
atccagggcctccggataatgtgaagctgagtgagcctccgccatccagc
atgacaaactgtggccgaccgcagctgtgccgtggcacgatggcgaggaa
gccagccccaaggaaacactgaaaacacatatcacgccaatgccgtgtgg
ttttgtttgaatataaaattggtgaaagtgttggtttttttaaggcagta
atttttttgtttgttttttttgcgatttcattccattcttgaccaaagctt
ctctttaagtagtttattatggaaaattgtcacactaacttaaaagacag
ggtgagggagatatgtaaattgtccactagaaaattaaataaaagaactg
aatgtggaaaaaaaaaaaaaaaaa
>NM_003846 2
gctagggagtaggggtcgtctgataaggggaagctgtgacgcagacacgc
acagtaatacacagatggaggctcaaaagacacgagtttcgcgtcctgaa
attccgcttccagggccaagctttcttttctgatactgtttgtccctcgc
gaggcaccgttgggtcgcgcagtaggcgtgactaggggcgggaagtgggg
cgggagcagggccgcggagcctgggctgcggctgtcatggacgcctgggt
ccgcttcagtgctcagagccaagcccgggagcggctgtgtagggccgccc
agtatgcttgctctcttcttggccatgcgctgcagaggcatggagccagt
cctgagttacagaaacagattcgacaactggagagccacctgagccttgg
aagaaagcttctacgcctgggtaactcagcagatgcccttgagtcagcca
aaagagctgttcacctatcagatgttgtcctgagattctgcatcactgtt
agtcacctcaatcgagccttgtacttcgcctgtgacaatgtcctgtgggc
tggaaagtctggactggctccccgtgtggatcaggagaagtgggcccagc
gttcattcaggtactatttgttttccctcatcatgaatttgagccgtgat
gcttatgagattcgcctactgatggagcaagagtcttctgcttgtagccg
gcgactgaaaggttctggaggaggagtcccaggaggaagtgaaactgggg
gacttgggggaccagggactccaggaggaggtctgccccaactggctctg
aaacttcggctgcaagtcctgctcctggctcgagtccttagaggtcatcc
cccacttctgctagacgtggtcagaaatgcctgtgatctcttcattcctc
tggacaaactaggcctctggcgctgtggccctgggattgtggggctttgt
ggcctcgtgtcctccatcctgtctattctcaccctaatctatccctggct
acgactcaagccctgaccttccggtacaggataaggaggggacctgaatt
ggtgagatggaatcttagatcgtcccccatgtgccagcctcattcgaatt
ctactctttggttaaagttagaaattcagagatttaggggtggaggaaga
gctttggggaagatgaggtaaggaaagatgactcgtgaagttaataggat
gtctctaatttctagatgtgcctgagcttctgttcttttcctctttcttt
gtgtctctcttgaatatatttactttgtgtctcttaactctgtttaaggt
tctgtgtctatgcatctctctctttctttttcaaccttctcattctcct
atccagggatttaatcagcagaattactttttgataggggaggtataagg
tttggcctgtaaggttctaactgccttctttttctcacagaggtggctt
atggcagatttttcctccttcaaactccaaacataatttttaagactatg
tgccagtggactcttcccttatatctctgcaccacaagttgttggatgtt
tcctcttcctcccttatgtctacctcaccaacctcgctcatcatttggcc
cttatccttccttgtacacctaccttcagatttctgcttacactttgatt
tcagagctttattccccagtctgttcttactcctttgtcgcttatccaga
atgatgctatgtgtagcatcttgctgtaaatcctgtacaatgattctgtg
taaatagctgtggcctatgccaataatgaagagcaagcctttcaggtaag
caaattaaagttcagtttgctcatcgacaaaaaaaa
>NM_001014444 2
tttcaaatggggagtttccctgcacaagctttcttgtctgccactatgtg

agatatacctttcaccttctgccgtgattgtgaggcctcctcagccacgt
ggaactagacggccctggccaacttctccagcggtcccgaaggaggggtc
atgcagcccgtgcgcaccgtggtgccggtgaccaagcacaggggctacct
gggggtcatgcccgcctacagtgctgcagaggatgcactgaccaccaagt
tggtcaccttctacgaggaccgcggcatcacctcggtcgtcccttcccac
caggctactgtgctactctttgagcccagcaatggcaccctgctggcggt
catggatggaaatgtcataactgcaaagagaacagctgcagtttctgcca
ttgccaccaagtttctgaaacctcccagcagtgaagtgctgtgcatcctt
ggggctggggtccaggcctacagccattatgagatcttcacagagcagtt
ctcctttaaggaggtgaggatatggaaccgcaccaaagaaaatgcagaga
agtttgcagacacagtgcaaggagaggtacgggtctgttcttcggtccag
gaggctgtggcaggtgcagatgtgatcatcacagtcaccctggcaacaga
gcccattttgtttggtgaatgggtgaagccaggggctcacatcaatgctg
ttggagccagcagacctgactggagagaactggatgatgagctcatgaaa
gaagctgtgctgtacgtggattcccaggaggctgccctgaaggagtctgg
agatgtcctgctgtcaggggccgagatctttgctgagctgggagaagtga
ttaagggagtgaaaccagcccactgtgagaagaccaccgtgttcaagtct
ttgggaatggcagtggaagacacagttgcagccaaactcatctatgattc
ctggtcatctggtaaataaaacaaaggaacttgatgttgagatggatgct
tgaggaatattgctgctggttctcataatttctagagtaaatgagggagt
ccagtccccagtgaactctccttttgtgcttatcatgttttaccttaaat
gctgagatcctcatttatgtttgtagttggaaagcaaagctaggtagcca
tttcttctgttctaccaagttataatagcattcatttccctttatatttc
cctgaaataaagcacattccaattgtgcagtg
>NM_001888 3
tttcaaatggggagtttccctgcacaagctttcttgtctgccactatgtg
agatatacctttcaccttctgccgtgattgtgaggcctcctcagccacgt
ggaactgtaaaaactcctggaagaaaagatcctgcaatttggctttttgt
gagatggaaaagattacaccttgccctgcaaacttcccccctttaaaggc
gaaggtttgttagagcagcgggcgcgctcataaagggcacagccgagggt
acgtggatcgcggtgcggagactgaggttagaaggcacaggtggcgagat
gagccgggtaccagcgttcctgagcgcggccgaggtggaggaacacctcc
gcagctccagcctcctcatcccgcctctagagacggccctggccaacttc
tccagcggtcccgaaggaggggtcatgcagcccgtgcgcaccgtggtgcc
ggtgaccaagcacaggggctacctgggggtcatgcccgcctacagtgctg
cagaggatgcactgaccaccaagttggtcaccttctacgaggaccgcggc
atcacctcggtcgtcccttcccaccaggctactgtgctactctttgagcc
cagcaatggcaccctgctggcggtcatggatggaaatgtcataactgcaa
agagaacagctgcagtttctgccattgccaccaagtttctgaaacctccc
agcagtgaagtgctgtgcatccttggggctggggtccaggcctacagcca
ttatgagatcttcacagagcagttctcctttaaggaggtgaggatatgga
accgcaccaaagaaaatgcagagaagtttgcagacacagtgcaaggagag
gtacgggtctgttcttcggtccaggaggctgtggcaggtgcagatgtgat
catcacagtcaccctggcaacagagcccattttgtttggtgaatgggtga
agccaggggctcacatcaatgctgttggagccagcagacctgactggaga
gaactggatgatgagctcatgaaagaagctgtgctgtacgtggattccca
ggaggctgccctgaaggagtctggagatgtcctgctgtcaggggccgaga
tctttgctgagctgggagaagtgattaagggagtgaaaccagcccactgt
gagaagaccaccgtgttcaagtctttgggaatggcagtggaagacacagt

tgcagccaaactcatctatgattcctggtcatctggtaaataaaacaaag
gaacttgatgttgagatggatgcttgaggaatattgctgctggttctcat
aatttctagagtaaatgagggagtccagtccccagtgaactctcctttttg
tgcttatcatgttttaccttaaatgctgagatcctcatttatgtttgtag
ttggaaagcaaagctaggtagccatttcttctgttctaccaagttataat
agcattcatttccctttatatttccctgaaataaagcacattccaattgt
gcagtg
>NM_002126 4
actcttgtcagggccgcggcacatgggcggccggatgcgctgagcccggc
gctgcggggccgcggagcgctggggagcagcggccgccggcgcggggagg
ggggtggggtgggacggcgcaccgcctccggtgctggcactaggggctgg
ggtcggcgcggtgtcttctgcccttctgcagccgtcgacatttttttttc
tttctttttttcaattttgaacattttgcaaaacgaggggttcgaggcag
gtgagagcatcctgcacgtcgccggggagcccgcgggcacttggcgcgct
ctcctgggaccgtctgcactggaaacccgaaagtttttttttaatatata
tttttatgcagatgtatttataaagatataagtaattttttttcttccctt
ttctccaccgccttgagagcgagtacttttggcaaaggacggaggaaaag
ctcagcaacattttagggggcggttgtttctttcttatttctttttttaa
ggggaaaaaaatttgagtgcatcgcgatggagaaaatgtcccgaccgctcc
ccctgaatcccacctttatcccgcctccctacggcgtgctcaggtccctg
ctggagaacccgctgaagctccccccttcaccacgaagacgcatttagtaa
agataaagacaaggaaaagaagctggatgatgagagtaacagcccgacgg
tcccccagtcggcattcctggggcctaccttatgggacaaaacccttccc
tatgacggagatactttccagttggaatacatggacctggaggagttttt
gtcagaaaatggcattcccccagcccatctcagcatgaccacagccctc
accctcctgggctgcagccagcttcctcggctgccccctcggtcatggac
ctcagcagccgggcctctgcaccccttcaccctggcatcccatctccgaa
ctgtatgcagagccccatcagaccaggtcagctgttgccagcaaaccgca
atacaccaagtcccattgatcctgacaccatccaggtcccagtgggttat
gagccagacccagcagatcttgccctttccagcatccctggccaggaaat
gtttgaccctcgcaaacgcaagttctctgaggaagaactgaagccacagc
ccatgatcaagaaagctcgcaaagtcttcatccctgatgacctgaaggat
gacaagtactgggcaaggcgcagaaagaacaacatggcagccaagcgctc
ccgcgacgcccggaggctgaaagagaaccagatcgccatccgggcctcgt
tcctggagaaggagaactcggccctccgccaggaggtggctgacttgagg
aaggagctgggcaaatgcaagaacatacttgccaagtatgaggccaggca
cgggcccctgtaggatggcatttttgcaggctggctttggaatagatgga
cagtttgtttcctgtctgatagcaccacacgcaaaccaacctttctgaca
tcagcactttaccagaggcataaacacaactgactcccattttggtgtgc
atctgtgtgtgtgtgcgtgtatatgtgcttgtgctcatgtgtgtggtcag
cggtatgtgcgtgtgcgtgttcctttgctcttgccattttaaggtagccc
tctcatcgtcttttagttccaacaaagaaaggtgccatgtctttactaga
ctgaggagccctctcgcgggtctcccatcccctccctccttcactcctgc
ctcctcagctttgcttcatgttcgagcttacctactcttccaggactctc
tgcttggattcactaaaaagggccctggtaaaatagtggatctcagtttt
taagagtacaagctcttgtttctgtttagtccgtaagttaccatgctaat
gaggtgcacacaataacttagcactactccgcagctctagtcctttataa
gttgctttcctcttactttcagttttggtgataatcgtcttcaaattaaa
gtgctgtttagatttattagatcccatatttacttactgctatctactaa

gtttccttttaattctaccaaccccagataagtaagagtactattaatag
aacacagagtgtgtttttgcactgtctgtacctaaagcaataatcctatt
gtacgctagagcatgctgcctgagtattactagtggacgtaggatatttt
ccctacctaagaatttcactgtcttttaaaaaacaaaaagtaaagtaatg
catttgagcatggccagactattccctaggacaaggaagcagagggaaat
gggaggtctaaggatgaggggttaatttatcagtacatgagccaaaaact
gcgtcttggattagcctttgacattgatgtgttcggttttgttgttcccc
ttccctcacaccctgcctcgcccccacttttctagttaactttttccata
tccctcttgacattcaaaacagttacttaagattcagttttcccactttt
tggtaatatatatattttttgtgaattatactttgttgtttttaaaaagaa
aatcagttgattaagttaataagttgatgttttctaaggcccttttttcct
agtggtgtcatttttgaatgcctcataaattaatgattctgaagcttatg
tttcttattctctgtttgcttttgaacgtatgtgctcttataaagtggac
ttctgaaaaatgaatgtaaaagacactggtgtatctcagaaggggatggt
gttgtcacaaactgtggttaatccaatcaatttaaatgtttactatagac
caaaaggagagattattaaatcgtttaatgtttatacagagtaattatag
gaagttcttttttgtacagtatttttcagatataaatactgacaatgtat
tttggaagacatatattatatatagaaaagaggagaggaaaactattcca
tgttttaaaattatatagcaaagatatatattcaccaatgttgtacagag
aagaagtgcttggggggtttttgaagtctttaatattttaagccctatcac
tgacacatcagcatgttttctgctttaaattaaaattttatgacagtatc
gaggcttgtgatgacgaatcctgctctaaaatacacaaggagctttcttg
tttcttattaggcctcagaaagaagtcagttaacgtcacccaaaagcaca
aaatggattttagtcaaatatttattggatgatacagtgtttttttaggaa
aagcatctgccacaaaaatgttcacttcgaaattctgagttcctggaatg
gcacgttgctgccagtgccccagacagttcttttctaccctgcgggcccg
cacgttttatgaggttgatatcggtgctatgtgtttggtttataatttga
tagatgtttgactttaaagatgattgttcttttgtttcattaagttgtaa
aatgtcaagaaattctgctgttacgacaaagaaacattttacgctagatt
aaaatatcctttcatcaatgggattttctagtttcctgccttcagagtat
ctaatcctttaatgatctggtggtctcctcgtcaatccatcagcaatgct
tctctcatagtgtcatagacttgggaaacccaaccagtaggatatttcta
caaggtgttcattttgtcacaagctgtagataacagcaagagatgggggt
gtattggaattgcaatacattgttcaggtgaataataaaatcaaaaactt
ttgcaatcttaagcagagataaataaaagatagcaatatgagacacaggt
ggacgtagagttggcctttttacaggcaaagaggcgaattgtagaattgt
tagatggcaatagtcattaaaaacatagaaaaatgatgtctttaagtgga
gaattgtggaaggattgtaacatggaccatccaaatttatggccgtatca
aatggtagctgaaaaaactatatttgagcactggtctctcttggaattag
atgtttatatcaaatgagcatctcaaatgttttctgcagaaaaaaaataaa
aagattctaataaaatgtattctcttgtgtgccaggagaggtttcagaaa
cctacctcgtcttacaaatttaaacactttggagtctgtacaggtgcctt
atatgtaggtcattgtcacgatacacacacacgaacactccctctggact
ggctgcctctccatccagggcagttaactagcaaacaaggcagatctgct
tcatggagcgggaggccatggcttgactctgagtgatttgggtcaaccgg
agtcagacgcatgtctgcacgctgcagctattatgagagtccctttgtca
tttttcaccttttcatcctaagcatctttcagagattaattatttggcca
ttaacaatgaatccaaatcatatcatactgacatcatctagacatgattt
ggaaggaacagcttaggacctcctgatgaggtcacattgttgtttctttt

aactagacttggcaaagaaaggcaaaaattgaccagcctatctttctgct
ggtgctgccttaaggaggtagtttgttgaggggagggctgtagatcatta
cttctttctcttcaggaagtggccactttgaaccattcaaataccacatt
aggcaagactgtgataggccttttgtcttcaaatacaacaggcctccact
gacccatccctcaaagcagaaggaccctttgaggagagtacagatgggat
tccacagtggggtgggtggaatggaaacctgtactagaccacccagaggt
tccttctaacccactggtttggtggggaactcacagtaattccaaatgta
caatcagatgtctagggtctgttttcggaagaagcaagaattatcagtgg
caccctccccactgcccccagtgtaaaacaatagacattctgtgaaatgc
aaagctattctttggtttttctagtagtttatctcattttaccctattct
tcctttaaggaaaactcaatctttatcacagtcaattagagcgatcccaa
ggcatgggaccaggcctgcttgcctatgtgtgatggcaattggagatctg
gatttagcactggggtctcagcaccctgcaggtgtctgagactaagtgat
ctgccctccaggtggcgatcaccttctgctcctaggtacccccactggca
aggccaaggtctcctccacgtttttctgcaattaataatgtcatttaaa
aaatgagcaaagccttatccgaatcggatatagcaactaaagtcaataca
ttttgcaggaggctaagtgtaagagtgtgtgtgtgtgtgtgtgcgtgcat
gtgtgtgtgtgtgtatgtgtgtgaataagtcgacataaagtctttaattt
tgagcaccttaccaaacataacaataatccattatccttttggcaacacc
acaaagatcgcatctgttaaacaggtacaagttgacatgaggttagttta
attgtacaccatgatattggtggtatttatgctgttaagtccaaaccttt
atctgtctgttattcttaatgttgaataaactttgaatttttcctttca
aaaaaaa
>NM_002109 3
tcgatagccggaagtcatccttgctgaggctggggcaaccaccgcaggtc
gagacagcaggcggctcaagtggacagccgggatggcagagcgtgcggcg
ctggaggagctggtgaaacttcagggagagcgcgtgcgaggcctcaagca
gcagaaggccagcgccgagctgatcgaggaggaggtggcgaaactcctga
aactgaaggcacagctgggtcctgatgaaagcaaacagaaatttgtgctc
aaaacccccaagggcacaagagactatagtccccggcagatggcagttcg
cgagaaggtgtttgacgtaatcatccgttgcttcaagcgccacggtgcag
aagtcattgatacacctgtatttgaactaaaggaaacactgatgggaaag
tatggggaagactccaagcttatctatgacctgaaggaccagggcgggga
gctcctgtcccttcgctatgacctcactgttccttttgctcggtatttgg
caatgaataaactgaccaacattaaacgctaccacatagcaaaggtatat
cggcgggataacccagccatgacccgtggccgataccgggaattctacca
gtgtgattttgacattgctgggaactttgatcccatgatccctgatgcag
agtgcctgaagatcatgtgcgagatcctgagttcacttcagataggcgac
ttcctggtcaaggtaaacgatcgacgcattctagatgggatgtttgctat
ctgtggtgtttctgacagcaagttccgtaccatctgctcctcagtagaca
agctggacaaggtgtcctgggaagaggtgaagaatgagatggtgggagag
aagggccttgcacctgaggtggctgaccgcattggggactatgtccagca
acatggtggggtatccctggtggaacagctgctccaggatcctaaactat
cccaaaacaagcaggccttggagggcctgggagacctgaagttgctcttt
gagtacctgaccctatttggcattgatgacaaaatctcctttgacctgag
ccttgctcgagggctggattactacactggggtgatctatgaggcagtgc
tgctacagaccccagcccaggcaggggaagagcccctgggtgtgggcagt
gtggctgctggaggacgctatgatgggctagtgggcatgttcgaccccaa
agggcgcaaggtgccatgtgtggggctcagcattggggtggagcggattt

tctccatcgtggaacagagactagaggctttggaggagaagatacggacc
acggagacacaggtgcttgtggcatctgcacagaagaagctgctagagga
aagactaaagcttgtctcagaactgtgggatgctgggatcaaggctgagc
tgctgtacaagaagaacccaaagctactgaaccagttacagtactgtgag
gaggcaggcatcccactggtggctatcatcggcgagcaggaactcaagga
tggggtcatcaagctccgttcagtgacgagcagggaagaggtggatgtcc
gaagagaagaccttgtggaggaaatcaaaaggagaacaggccagcccctc
tgcatctgctgaactgaacaaactatcagaggaaaggaagtgggactggc
actatttgaggttaagacaaactgcatatgtacttcaattgctttgcact
tttccgtttcagcggaagacctgaagagtggtcagaacagagcctttgat
ttttattatggttattttattgattattactggcaaaaacggccaggtac
aacaccttttttcatacaaggcccaggaggcttagtccagtctgtgctcct
gggctacaaggacccagcctgagatggtcccatctgcagggccccgcacc
agttggagcagatgcctccccaccaccaattgccaaaggtccaataaaat
gcctcaaccacggaaaaaaaaaaaaaaaaaa
>NM_022003 3
aaccgaaaagcaacagctgatggcaaatccgctccggcgcttgcacgcct
cctcggcggcagtctcctgttgcttctcggagcttgactgccctttggaa
aaatctctccgcccctcgagaggagagaggagcagaggacgcgcgcgagc
acagagcgggaggacggacagggagaggaggagaggggggtctgcgcgcgg
ccgctacccagaagccagcggacggcagcacggagtgggctgtccccgag
cccagccccgagcgagccccccccccgcccccgcaggacgcgcctcccag
ccagcccgactcctaggaggaggggaggcgggaaagcagctcaagcctca
cccaccgccctgcccccagccccgccactcccaggctcctcgggactcgg
cgggtcctcctgggagtctcggaggggaccggctgtgcagacgccatgga
gttggtgctggtcttcctctgcagcctgctggcccccatggtcctggcca
gtgcagctgaaaaggagaaggaaatggacccttttcattatgattaccag
accctgaggattgggggactggtgttcgctgtggtcctcttctcggttgg
gatcctccttatcctaagtcgcaggtgcaagtgcagtttcaatcagaagc
cccgggccccaggagatgaggaagcccaggtggagaacctcatcaccgcc
aatgcaacagagccccagaaagcagagaactgaagtgcagccatcaggtg
gaagcctctggaacctgaggcggctgcttgaacctttggatgcaaatgtc
gatgcttaagaaaaccggccacttcagcaacagccctttccccaggagaa
gccaagaacttgtgtgtcccccaccctatcccctctaacaccattcctcc
acctgatgatgcaactaacacttgcctccccactgcagcctgcggtcctg
cccacctcccgtgatgtgtgtgtgtgtgtgtgtgtgtgtgactgtgtgtg
tttgctaactgtggtctttgtggctacttgtttgtggatggtattgtgtt
tgttagtgaactgtggactcgctttcccaggcaggggctgagccacatgg
ccatctgctcctccctgcccccgtggccctccatcaccttctgctcctag
gaggctgcttgttgcccgagaccagcccccctcccctgatttagggatgcg
tagggtaagagcacgggcagtggtcttcagtcgtcttgggacctgggaag
gtttgcagcactttgtcatcattcttcatggactcctttcactcctttaa
caaaaaccttgcttccttatcccacctgatcccagtctgaaggtctctta
gcaactggagatacaaagcaaggagctggtgagcccagcgttgacgtcag
gcaggctatgcccttccgtggttaatttcttcccaggggcttccacgagg
agtccccatctgccccgccccttcacagagcgcccggggattccaggccc
agggcttctactctgcccctggggaatgtgtcccctgcatatcttctcag
caataactccatgggctctgggaccctacccttccaaccttccctgctt
ctgagacttcaatctacagcccagctcatccagatgcagactacagtccc

tgcaattgggtctctggcaggcaatagttgaaggactcctgttccgttgg
ggccagcacaccgggatggatggagggagagcagaggcctttgcttctct
gcctacgtccccttagatgggcagcagaggcaactcccgcatcctttgct
ctgcctgtcagtggtcagagcggtgagcgaggtgggttggagactcagca
ggctccgtgcagcccttgggaacagtgagaggttgaaggtcataacgaga
gtgggaactcaacccagatcccgcccctcctgtcctctgtgttcccgcgg
aaaccaaccaaaccgtgcgctgtgacccattgctgttctctgtatcgtga
tctatcctcaacaacaacagaaaaaaggaataaaatatcctttgtttcct
agtggc
>NM_004420 2
ccgaggcgagcgcgagcgaggtccagcaccatgtgctaggtcactcccag
cgcgaggccacacctgggccgtcggagcagcccctcctcacttcaggggt
caccctccccagcacccattgccccaccatggctggggaccggctcccga
ggaaggtgatggatgccaagaagctggccagcctgctgcggggcgggcct
ggggggccgctggtcatcgacagccgctccttcgtggagtacaacagctg
gcatgtgctcagctccgtcaacatctgctgctccaagctggtgaagcggc
ggctgcagcagggcaaggtgaccattgcggagctcatccagccggctgca
cgcagccaggtggaggctacggagccacaggacgtggtggtctatgacca
gagcacgcgggacgccagcgtgctggccgcagacagcttcctctccatcc
tgctgagcaagctggacggctgcttcgacagcgtggccatcctcactggg
ggcttcgccaccttctcctcctgcttccccggcctctgcgagggcaagcc
tgctgccctgctacccatgagcctctcccagccctgcctgcctgtgccca
gcgtgggcctgacccgcatcctgcctcacctctacctgggctcgcagaag
gacgtcctaaacaaggatctgatgacgcaaaatggaataagctacgtcct
caacgccagcaactcctgccccaagcctgacttcatctgcgagagccgct
tcatgcgggtccccatcaacgacaactactgtgaaaaactgctgccctgg
ctggacaagtccatcgagttcatcgataaagccaagctctccagctgcca
agtcatcgtccactgtctggctggcatctcccgctctgccaccatcgcca
tcgcctacatcatgaagaccatgggcatgtcctccgacgacgcctacagg
ttcgtgaaggacaggcgcccgtccatctcgcccaacttcaacttcctggg
ccagctgctggagtacgagcgcagcctgaagctgctggccgccctgcagg
gcgacccgggcacccccctcagggacgccggagcctccgcccagtcctgcc
gccggggccccgctgccacggctgccaccacctacctcagagagcgctgc
cacagggaatgcggctgccagggagggcggcctgagcgcgggcggggagc
cccccgcgcccccccacgcccccggcgaccagcgcactgcagcagggcctg
cgcggcctgcacctctcctcggaccgcctgcaggacactaaccgcctcaa
gcgctccttctccctggacatcaagtctgcctacgcccctagcaggcggc
ccgacggccccgggcccccgaccccggcgaggccccgaagctctgcaag
ctggacagcccgtcgggggccgcgctgggcctgtcctcgcccagcccgga
cagcccggacgccgcgcctgaggcgcgcccacggccccgccggcggcccc
ggccccccgccggctcccccgcgcgctcccccgcgcacagcctcggcctg
aacttcggcgatgcggcccggcagactccgcggcacggcctctcggccct
gtcggcgcccgggctgcccggccctggccagccggccggccccggggcct
gggcaccgccgctcgactccccaggcacgccgtcgcccgacgggccctgg
tgcttcagccccgagggcgcacagggggcgggcggggtgctgtttgcgcc
cttcggccgggcgggcgccccgggaccaggcggcggcagcgacctgcggc
ggcgggaggcagcgagggctgagccccgggacgcgcggaccggctggccc
gaggagccggccccggagacgcagttcaagcgccgcagctgccagatgga
gttcgaggagggcatggtggagggggcgcgcgcgcggcgaggagctggccg

ccctgggcaagcaggcgagcttctcgggcagcgtggaggtcatcgaggtg
tcctgacccctccgctgccctcggccccgccgcccgcagccaggcccgtt
ataaatgtatattatatataatgcaaagaaaggtaaatggttttactggg
atttttatcgagaagtaaatatttcgatttttatttatttaagctgttc
attctggcaatgatttggcaacagtgcgggtggtcctcgagctctatttt
tactgtctggtatttaaactgaaacatacgtttctaagcaatacgaggcc
accttcagtcgcaagctgggtgccaggcctggggcccctcccagttcccc
cgccccaggaaacactgctgacctttgcaaaggctgccgagctttcgtgc
actttttacataacaaaaaggtgaaaaaaaggaaaaaaaaacttctttgc
cacaaactgagccgcagaacccccttctcccccacccacctcccctgc
tccctcccttctctgcgccggcctagggctctgcaccaaagccataggat
ggaggagcaggagctggtgtgccccggagaggtgcggccagccctccatc
agctccaggcaccaaatcttggtggcaaggagggcaccccgctgcccgtt
gccccagagctgttctctggcaggggaggacaggcattgggcttcatggt
gccagggtgttcagaggggctgagaaatagaacagtgtgtgtaggggctt
cgggcagggggttctggaacgtcagatgaggtgcagcccaggggaggaca
gaggtgttagtgcccccaactcctgccagagccccagtccagccacagag
tggctcagaaaggccattcctagagggctgcggccctcccttctcccttg
cccatgcccccagagctgcctgccgggcagggtggcaccattgcaggaga
ggagcttggcctccgggggtcaggcaggaggcgcctggctagccagtgct
ggctccactgggcaggaagccctggacccccaggtatgaggagggggtgg
tcttagggttctgttccaggtctgccccgccccccctcccagccatgcccc
aggcagaacttggaattcaggtgtgcacctgcaggctgaggggctctgtg
agcaggtgctgctcacacagggagttcaggcgccagccaagcccctgtgc
tgctgggataggcctgcttcacttagggagcactgcctcaagacaggtaa
agccccctcgtttgcccccacccccatggggccgctcaggagagaaactc
ccattcacccctttcccagggtgctctctctctaggtggcatgccagccc
ccaaacacaagtggcttttgggcccaggtgggtcagcctgctgcccctgc
cccataccccctcgggccattgggacccctgcccttcagatgtcctaggg
tctaggagtggggccagtcactgtgggaagaggccaggggcttggccgga
gaggcagcccagggcaggacccagtcctgagtcctggagcagggccaggg
aggcgcccatcccgccccagccagccgccctctctgctgtttcttctatt
tgttcttcttttcacccacagctctgtgttcctgtcatccctcctttcag
caaaagtcctgttcccgttccctctgtccccacccactcctgttcccca
agaaaataagctatcgttgtatttgcaatctatggattagaggtttaagt
atttattattattggttaattattattaattatgtaaatttgcctcccat
atgtctgttgcgttgggtttctgaggagaccctgggtgaggaggatgcac
tggcttcccgcttctcgcccccccacccctgtgctgtccgggagacagtgg
tctggggccactggttgggccccccttctcccttccccccttccccttgtcc
cttctgcaggccgttgagggggggctgtctgtctcagtctgtctctgctcc
cactcttgaggcactggttaccgcaaagtgagcagccagcagggggggcga
aggtcctgtgttggccactgcctcctccagtgctgcaggaggcgggctga
ggccccacctggtggctttcacctgacccagccctgagtcctctccaagc
ctctctccggcccctcccacctggccactgcctcctccagtgctgcggga
ggcgggccagggccccacctggtggctttcacctgacccagccctgagtc
ctctccaagcctctctccggcccctcccacctggccactgcctggcattg
ggatcgccccaaaatggacccggcccctcctgttatttgctgggaagtcc
agcggaggagagggtgcaggtcccccgctgagcctccagtctctgtagac
tgggctgccggcccttcagccccccttggagcccctcccgccacagccgc

accttctgctcccggcccctccctttgtatttggagacaatgtgttgtaa
taaagcttaaagtggatgttttcaaaaaaaaaaaaaaaaaa
>NM_014164 5
ccctggccacaccctccgcctggacgcagcagccaccgccgcgtccctct
ctccacgaggctgccggcttaggacccccagctccgacatgtcgccctct
ggtcgcctgtgtcttctcaccatcgttggcctgattctccccaccagagg
acagacgttgaaagataccacgtccagttcttcagcagactcaactatca
tggacattcaggtcccgacacgagccccagatgcagtctacacagaactc
cagcccacctctccaaccccaacctggcctgctgatgaaacaccacaacc
ccagacccagacccagcaactggaaggaacggatgggcctctagtgacag
atccagagacacacaagagcaccaaagcagctcatcccactgatgacacc
acgacgctctctgagagaccatccccaagcacagacgtccagacagaccc
ccagaccctcaagccatctggttttcatgaggatgaccccttcttctatg
atgaacacaccctccggaaacgggggctgttggtcgcagctgtgctgttc
atcacaggcatcatcatcctcaccagtggcaagtgcaggcagctgtcccg
gttatgccggaatcgttgcaggtgagtccatcagaaacaggagctgacaa
cctgctgggcacccgaagaccaagccccctgccagctcaccgtgcccagc
ctcctgcatcccctcgaagagcctggccagagagggaagacacagatgat
gaagctggagccagggctgccggtccgagtctcctacctcccccaaccct
gcccgcccctgaaggctacctggcgccttgggggctgtccctcaagttat
ctcctctgctaagacaaaaagtaaagcactgtggtctttgccccaaaaaa
aaaaaaaaaaaaaa
>NM_144779 2
tctctccacgaggctgccggcttaggacccccagctccgacgtaagtccc
tctcgtgcgccacctccatccgctgcccctctgcccacgggccgggctca
gatgtcgccctctggtcgcctgtgtcttctcaccatcgttggcctgattc
tccccaccagaggacagacgttgaaagataccacgtccagttcttcagca
gactcaactatcatggacattcaggtcccgacacgagccccagatgcagt
ctacacagaactccagcccacctctccaaccccaacctggcctgctgatg
aaacaccacaaccccagacccagacccagcaactggaaggaacggatggg
cctctagtgacagatccagagacacacaagagcaccaaagcagctcatcc
cactgatgacaccacgacgctctctgagagaccatccccaagcacagacg
tccagacagacccccagaccctcaagccatctggttttcatgaggatgac
cccttcttctatgatgaacacaccctccggaaacgggggctgttggtcgc
agctgtgctgttcatcacaggcatcatcatcctcaccagtggcaagtgca
ggcagctgtcccggttatgccggaatcgttgcaggtgagtccatcagaaa
caggagctgacaacctgctgggcacccgaagaccaagccccctgccagct
caccgtgcccagcctcctgcatcccctcgaagagcctggccagagaggga
agacacagatgatgaagctggagccagggctgccggtccgagtctcctac
ctcccccaaccctgcccgcccctgaaggctacctggcgccttgggggctg
tccctcaagttatctcctctgctaagacaaaaagtaaagcactgtggtct
ttgccccaaaaaaaaaaaaaaaaaaaa
>NM_001870 2
agggctgaggcataaaactgccagagggtctcaaggcaggcaaagaagaa
ccatgaggctcatcctgcctgtgggtttgattgctaccactcttgcaatt
gctcctgtccgctttgacagggagaaggtgttccgcgtgaagccccagga
tgaaaaacaagcagacatcataaaggacttggccaaaaccaatgagcttg
acttctggtatccaggtgccacccaccacgtagctgctaatatgatggtg
gatttccgagttagtgagaaggaatcccaagccatccagtctgccttgga

tcaaaataaaatgcactatgaaatcttgattcatgatctacaagaagaga
ttgagaaacagtttgatgttaaagaagatatcccaggcaggcacagctac
gcaaaatacaataattgggaaaagattgtggcttggactgaaaagatgat
ggataagtatcctgaaatggtctctcgtattaaaattggatctactgttg
aagataatccactatatgttctgaagattggggaaaagaatgaaagaaga
aaggctatttttacggattgtggcattcacgcacgagaatgggtctcccc
agcattctgccagtggtttgtctatcaggcaaccaaaacttatgggagaa
acaaaattatgaccaaactcttggaccgaatgaatttttacattcttcct
gtgttcaatgttgatggatatatttggtcatggacaaagaaccgcatgtg
gagaaaaaatcgttccaagaaccaaaactccaaatgcatcggcactgacc
tcaacaggaattttaatgcttcatggaactccattcctaacaccaatgac
ccatgtgcagataactatcggggctctgcaccagagtccgagaaagagac
gaaagctgtcactaatttcattagaagccacctgaatgaaatcaaggttt
acatcaccttccattcctactcccagatgctattgtttccctatggatat
acatcaaaactgccacctaaccatgaggacttggccaaagttgcaaagat
tggcactgatgttctatcaactcgatatgaaacccgctacatctatggcc
caatagaatcaacaatttacccgatatcaggttcttctttagactgggct
tatgacctgggcatcaaacacacatttgcctttgagctccgagataaagg
caaatttggttttctccttccagaatcccggataaagccaacgtgcagag
agaccatgctagctgtcaaatttattgccaagtatatcctcaagcatact
tcctaaagaactgccctctgtttggaataagccaattaatcctttttttgt
gcctttcatcagaaagtcaatcttcagttatccccaaatgcagcttctat
ttcacctgaatccttctcttgctcatttaagtcccatgttactgctgttt
gcttttacttactttcagtagcaccataacgaagtagctttaagtgaaac
cttttaactacctttctttgctccaagtgaagtttggacccagcagaaag
cattattttgaaaggtgatatacagtggggcacagaaaacaaatgaaaac
cttcagtttctcacagattttcaccatgtggcttcatcaatttatgtgct
aatacaataaaataaaatgcacttaatgctttaaaaaaaaaaaaaaaaa
>NM_003120 2
gactatctcccagcggcaggcccttcgataaaatcaggaacttgtgctgg
ccctgcaatgtcaagggagggggctcacccagggctcctgtagctcaggg
ggcaggcctgagccctgcacccgccccacgaccgtccagcccctgacggg
gcaccccatcctgaggggctctgcattggcccccaccgaggcaggggatc
tgaccgactcggagcccggctggatgttacaggcgtgcaaaatggaaggg
tttcccctcgtccccctccatcagaagacctggtgccctatgacacgga
tctataccaacgccaaacgcacgagtattacccctatctcagcagtgatg
gggagagccatagcgaccattactgggacttccacccccaccacgtgcac
agcgagttcgagagcttcgccgagaacaacttcacggagctccagagcgt
gcagcccccgcagctgcagcagctctaccgccacatggagctggagcaga
tgcacgtcctcgataccccatggtgccaccccatcccagtcttggccac
caggtctcctacctgccccggatgtgcctccagtacccatccctgtcccc
agcccagcccagctcagatgaggaggagggcgagcggcagagccccccac
tggaggtgtctgacggcgaggcggatggcctggagcccgggcctgggctc
ctgcctggggagacaggcagcaagaagaagatccgcctgtaccagttcct
gttggacctgctccgcagcggcgacatgaaggacagcatctggtgggtgg
acaaggacaagggcaccttccagttctcgtccaagcacaaggaggcgctg
gcgcaccgctggggcatccagaagggcaaccgcaagaagatgacctacca
gaagatggcgcgcgcgctgcgcaactacggcaagacgggcgaggtcaaga
aggtgaagaagaagctcacctaccagttcagcggcgaagtgctgggccgc

gggggcctggccgagcggcgccacccgccccactgagcccgcagcccccg
ccgggccccgccaggcctccccgctggccatagcattaagccctcgcccg
gcccggacacagggaggacgctcccggggcccagaggcaggactgtggcg
ggccgggcctcgcctcacccgccccctccccccactccaggccccctcca
catcccgcttcgcctccctccaggactccaccccggctcccggacgccag
ctgggcgtcagaccccaccggggcaaccttgcagaggacgacccggggta
ctgccttgggagtctcaagtccgtatgtaaatcagatctcccctctcacc
cctcccacccattaacctcctcccaaaaaacaagtaaagttattctcaat
ccatcaaaaaaaaaaaaaaaaa
>NM_004988 4
agagagaagcgaggtttccattctgagggacggcgtagagttcggccgaa
ggaacctgacccaggctctgtgaggaggcaaggttttcaggggacaggcc
aacccagaggacaggattccctggaggccacagaggagcaccaaggagaa
gatctgcctgtgggtcttcattgcccagctcctgcccacactcctgcctg
ctgccctgacgagagtcatcatgtctcttgagcagaggagtctgcactgc
aagcctgaggaagcccttgaggcccaacaagaggccctgggcctggtgtg
tgtgcaggctgccacctcctcctcctctcctctggtcctgggcaccctgg
aggaggtgcccactgctgggtcaacagatcctccccagagtcctcaggga
gcctccgcctttcccactaccatcaacttcactcgacagaggcaacccag
tgagggttccagcagccgtgaagaggaggggccaagcacctcttgtatcc
tggagtccttgttccgagcagtaatcactaagaaggtggctgatttggtt
ggttttctgctcctcaaatatcgagccagggagccagtcacaaaggcaga
aatgctggagagtgtcatcaaaaattacaagcactgttttcctgagatct
tcggcaaagcctctgagtccttgcagctggtctttggcattgacgtgaag
gaagcagaccccaccggccactcctatgtccttgtcacctgcctaggtct
ctcctatgatggcctgctgggtgataatcagatcatgcccaagacaggct
tcctgataattgtcctggtcatgattgcaatggagggcggccatgctcct
gaggaggaaatctgggaggagctgagtgtgatggaggtgtatgatgggag
ggagcacagtgcctatggggagcccaggaagctgctcacccaagatttgg
tgcaggaaaagtacctggagtaccggcaggtgccggacagtgatcccgca
cgctatgagttcctgtggggtccaagggccctcgctgaaaccagctatgt
gaaagtccttgagtatgtgatcaaggtcagtgcaagagttcgctttttct
tcccatccctgcgtgaagcagctttgagagaggaggaagagggagtctga
gcatgagttgcagccaaggccagtgggagggggactgggccagtgcacct
tccagggccgcgtccagcagcttcccctgcctcgtgtgacatgaggccca
ttcttcactctgaagagagcggtcagtgttctcagtagtaggtttctgtt
ctattgggtgacttggagatttatctttgttctcttttggaattgttcaa
atgtttttttttaagggatggttgaatgaacttcagcatccaagtttatg
aatgacagcagtcacacagttctgtgtatatagtttaagggtaagagtct
tgtgttttattcagattgggaaatccattctattttgtgaattgggataa
taacagcagtggaataagtacttagaaatgtgaaaaatgagcagtaaaat
agatgagataaagaactaaagaaattaagagatagtcaattcttgcctta
tacctcagtctattctgtaaaatttttaaagatatatgcatacctggatt
tccttggcttctttgagaatgtaagagaaattaaatctgaataaagaatt
cttcctgttaaaaaaaaaaaaaaaaaaaaaaaaaaaaaaaaaaaaaaaaa
aaaaa
>NM_002674 2
agcaggctgacagagaaatttttcattttctttcttgtttgactgtatgc
aaacatcaaactaagaatggcaaaaatgaatctctcttcctatatattaa

tactaactttttctttgttttctcaaggtattttactttcagcatccaag
tccataagaaatttagatgatgacatggtatttaatacattcaggttggg
gaaaggctttcagaaggaagacactgcagaaaaatcagttattgctcctt
ccctggaacaatataaaaatgatgagagcagtttcatgaacgaagaggaa
aataaagtttcaaagaacacaggctccaaacataatttcttaaatcatgg
tctgccactgaatctggctataaaaccttatcttgcactaaaaggatctg
tagctttcccagctgagaatggagttcagaatactgaatcaacacaagaa
aagagagaaattggggatgaagaaaactcagctaaatttcctataggaag
gagagattttgacatgctcagatgtatgctgggaagagtctaccgacctt
gttggcaagtctgatacctgttggtccacatcatcttttcagaagaaaat
aaaagcatttaattgccaatgggaggagaagcccatactgctactataac
ttgtgtatgttaaatgtctgttttaaaagaaagtagtgttaagatgtatc
agtaactgaaatgatatgctttctctgtgcattaaactttgtgaaaattc
tgcataa
>NM_021804 2
gttgttgtgatcccatggctacagaggatcaggagttgacatagatactc
tttggatttcataccatgtggaggctttcttacttccacgtgaccttgac
tgagttttgaatagcgcccaacccaagttcaaaggctgataagagagaaa
atctcatgaggaggttttagtctagggaaagtcattcagtggatgtgatc
ttggctcacaggggacgatgtcaagctcttcctggctccttctcagcctt
gttgctgtaactgctgctcagtccaccattgaggaacaggccaagacatt
tttggacaagtttaaccacgaagccgaagacctgttctatcaaagttcac
ttgcttcttggaattataacaccaatattactgaagagaatgtccaaaac
atgaataatgctggggacaaatggtctgcctttttaaaggaacagtccac
acttgcccaaatgtatccactacaagaaattcagaatctcacagtcaagc
ttcagctgcaggctcttcagcaaaatgggtcttcagtgctctcagaagac
aagagcaaacggttgaacacaattctaaatacaatgagcaccatctacag
tactggaaaagtttgtaacccagataatccacaagaatgcttattacttg
aaccaggtttgaatgaaataatggcaaacagtttagactacaatgagagg
ctctgggcttgggaaagctggagatctgaggtcggcaagcagctgaggcc
attatatgaagagtatgtggtcttgaaaaatgagatggcaagagcaaatc
attatgaggactatggggattattggagaggagactatgaagtaaatggg
gtagatggctatgactacagccgcggccagttgattgaagatgtggaaca
tacctttgaagagattaaaccattatatgaacatcttcatgcctatgtga
gggcaaagttgatgaatgcctatccttcctatatcagtccaattggatgc
ctccctgctcatttgcttggtgatatgtggggtagattttggacaaatct
gtactctttgacagttccctttggacagaaaccaaacatagatgttactg
atgcaatggtggaccaggcctgggatgcacagagaatattcaaggaggcc
gagaagttctttgtatctgttggtcttcctaatatgactcaaggattctg
ggaaaattccatgctaacggacccaggaaatgttcagaaagcagtctgcc
atcccacagcttgggacctggggaagggcgacttcaggatccttatgtgc
acaaaggtgacaatggacgacttcctgacagctcatcatgagatggggca
tatccagtatgatatggcatatgctgcacaaccttttctgctaagaaatg
gagctaatgaaggattccatgaagctgttggggaaatcatgtcactttct
gcagccacacctaagcatttaaaatccattggtcttctgtcacccgattt
tcaagaagacaatgaaacagaaataaacttcctgctcaaacaagcactca
cgattgttgggactctgccatttacttacatgttagagaagtggaggtgg
atggtctttaaaggggaaattcccaaagaccagtggatgaaaaagtggtg
ggagatgaagcgagagatagttggggtggtggaacctgtgccccatgatg

aaacatactgtgaccccgcatctctgttccatgtttctaatgattactca
ttcattcgatattacacaaggaccctttaccaattccagtttcaagaagc
actttgtcaagcagctaaacatgaaggccctctgcacaaatgtgacatct
caaactctacagaagctggacagaaactgttcaatatgctgaggcttgga
aaatcagaaccctggaccctagcattggaaaatgttgtaggagcaaagaa
catgaatgtaaggccactgctcaactactttgagcccttatttacctggc
tgaaagaccagaacaagaattcttttgtgggatggagtaccgactggagt
ccatatgcagaccaaagcatcaaagtgaggataagcctaaaatcagctct
tggagataaagcatatgaatggaacgacaatgaaatgtacctgttccgat
catctgttgcatatgctatgaggcagtactttttaaaagtaaaaaatcag
atgattctttttggggaggaggatgtgcgagtggctaatttgaaaccaag
aatctcctttaatttctttgtcactgcacctaaaaatgtgtctgatatca
ttcctagaactgaagttgaaaaggccatcaggatgtcccggagccgtatc
aatgatgctttccgtctgaatgacaacagcctagagtttctggggataca
gccaacacttggacctcctaaccagcccccctgtttccatatggctgattg
tttttggagttgtgatgggagtgatagtggttggcattgtcatcctgatc
ttcactgggatcagagatcggaagaagaaaaataaagcaagaagtggaga
aaatccttatgcctccatcgatattagcaaaggagaaaataatccaggat
tccaaaacactgatgatgttcagacctccttttagaaaaatctatgtttt
tcctcttgaggtgattttgttgtatgtaaatgttaatttcatggtataga
aaatataagatgataaagatatcattaaatgtcaaaactatgactctgtt
cagaaaaaaaaattgtccaaagacaacatggccaaggagagagcatcttca
ttgacattgctttcagtatttatttctgtctctggatttgacttctgttc
tgtttcttaataaggattttgtattagagtatattagggaaagtgtgtat
ttggtctcacaggctgttcagggataatctaaatgtaaatgtctgttgaa
tttctgaagttgaaaacaaggatatatcattggagcaagtgttggatctt
gtatggaatatggatggatcacttgtaaggacagtgcctgggaactggtg
tagctgcaaggattgagaatggcatgcattagctcactttcatttaatcc
attgtcaaggatgacatgctttcttcacagtaactcagttcaagtactat
ggtgatttgcctacagtgatgtttggaatcgatcatgctttcttcaaggt
gacaggtctaaagagagaagaatccagggaacaggtagaggacattgctt
tttcacttccaaggtgcttgatcaacatctccctgacaacacaaaactag
agccaggggcctccgtgaactcccagagcatgcctgatagaaactcattt
ctactgttctctaactgtggagtgaatggaaattccaactgtatgttcac
cctctgaagtgggtacccagtctcttaaatcttttgtatttgctcacagt
gtttgagcagtgctgagcacaaagcagacactcaataaatgctagattta
cacactcaaaaaaaaaaaa
>NM_030765 2
attcccacctcctccagaagccccgcccactcccgagccccgagagctcc
gcgcacctgggcgccatccgccctggctccgctgcacgagctccacgccc
gtaccccggcgtcacgctcagcccgcggtgctcgcacacctgagactcat
ctcgcttcgaccccgccgccgccgccgcccggcatcctgagcacggagac
agtctccagctgccgttcatgcttcctccccagccttccgcagcccacca
gggaaggggcggtaggagtggccttttaccaaagggaccggcgatgctct
gcaggctgtgctggctggtctcgtacagcttggctgtgctgttgctcggc
tgcctgctcttcctgaggaaggcggccaagcccgcaggagaccccacggc
ccaccagcctttctgggctcccccaacaccccgtcacagccggtgtccac
ccaaccacacagtgtctagcgcctctctgtccctgcctagccgtcaccgt
ctcttcttgacctatcgtcactgccgaaatttctctatcttgctggagcc

ttcaggctgttccaaggataccttcttgctcctggccatcaagtcacagc
ctggtcacgtggagcgacgtgcggctatccgcagcacgtggggcagggtg
gggggatgggctaggggccggcagctgaagctggtgttcctcctaggggt
ggcaggatccgctcccccagcccagctgctggcctatgagagtagggagt
ttgatgacatcctccagtgggacttcactgaggacttcttcaacctgacg
ctcaaggagctgcacctgcagcgctgggtggtggctgcctgcccccaggc
ccatttcatgctaaagggagatgacgatgtctttgtccacgtccccaacg
tgttagagttcctggatggctgggacccagcccaggacctcctggtggga
gatgtcatccgccaagccctgcccaacaggaacactaaggtcaaatactt
catcccaccctcaatgtacagggccacccactacccaccctatgctggtg
gggaggatatgtcatgtccagagccacagtgcggcgcctccaggctatc
atggaagatgctgaactcttccccattgatgatgtctttgtgggtatgtg
cctgaggaggctggggctgagccctatgcaccatgctggcttcaagacat
ttggaatccggcggcccctggaccccttagacccctgcctgtataggggg
ctcctgctggttcaccgcctcagcccccctcgagatgtggaccatgtgggc
actggtgacagatgaggggctcaagtgtgcagctggccccataccccagc
gctgaagggtgggttgggcaacagcctgagagtggactcagtgttgattc
tctatcgtgatgcgaaattgatgcctgctgctctacagaaaatgccaact
tggtttttaactcctctcaccctgttagctctgattaaaaacactgcaa
cccaaaaaaaaaaaaaaaaaaaaaaaaaaaaaaaaaaaaaaaaaaaaaaa
aaaa
>NM_002020 4
aggccagccggcgcccgcgcggacactttcagccccgagccgcggccgct
cgggtcggacccacgcgcagcggccggagatgcagcggggcgccgcgctg
tgcctgcgactgtggctctgcctgggactcctggacggcctggtgagtgg
ctactccatgacccccccgaccttgaacatcacggaggagtcacacgtca
tcgacaccggtgacagcctgtccatctcctgcaggggacagcaccccctc
gagtgggcttggccaggagctcaggaggcgccagccaccggagacaagga
cagcgaggacacgggggtggtgcgagactgcgagggcacagacgccaggc
cctactgcaaggtgttgctgctgcacgaggtacatgccaacgacacaggc
agctacgtctgctactacaagtacatcaaggcacgcatcgagggcaccac
ggccgccagctcctacgtgttcgtgagagactttgagcagccattcatca
acaagcctgacacgctcttggtcaacaggaaggacgccatgtgggtgccc
tgtctggtgtccatccccggcctcaatgtcacgctgcgctcgcaaagctc
ggtgctgtggccagacgggcaggaggtggtgtgggatgaccggcggggca
tgctcgtgtccacgccactgctgcacgatgccctgtacctgcagtgcgag
accacctggggagaccaggacttcctttccaaccccttcctggtgcacat
cacaggcaacgagctctatgacatccagctgttgcccaggaagtcgctgg
agctgctggtaggggagaagctggtcctgaactgcaccgtgtgggctgag
tttaactcaggtgtcacctttgactgggactacccagggaagcaggcaga
gcggggtaagtgggtgcccgagcgacgctcccagcagacccacacagaac
tctccagcatcctgaccatccacaacgtcagccagcacgacctgggctcg
tatgtgtgcaaggccaacaacggcatccagcgatttcgggagagcaccga
ggtcattgtgcatgaaaatcccttcatcagcgtcgagtggctcaaaggac
ccatcctggaggccacggcaggagacgagctggtgaagctgcccgtgaag
ctggcagcgtaccccccgcccgagttccagtggtacaaggatggaaaggc
actgtccgggcgccacagtccacatgccctggtgctcaaggaggtgacag
aggccagcacaggcacctacaccctcgccctgtggaactccgctgctggc
ctgaggcgcaacatcagcctggagctggtggtgaatgtgccccccagat

acatgagaaggaggcctcctcccccagcatctactcgcgtcacagccgcc
aggccctcacctgcacggcctacggggtgcccctgcctctcagcatccag
tggcactggcggccctggacaccctgcaagatgtttgcccagcgtagtct
ccggcggcggcagcagcaagacctcatgccacagtgccgtgactggaggg
cggtgaccacgcaggatgccgtgaaccccatcgagagcctggacacctgg
accgagtttgtggagggaaagaataagactgtgagcaagctggtgatcca
gaatgccaacgtgtctgccatgtacaagtgtgtggtctccaacaaggtgg
gccaggatgagcggctcatctacttctatgtgaccaccatccccgacggc
ttcaccatcgaatccaagccatccgaggagctactagagggccagccggt
gctcctgagctgccaagccgacagctacaagtacgagcatctgcgctggt
accgcctcaacctgtccacgctgcacgatgcgcacgggaacccgcttctg
ctcgactgcaagaacgtgcatctgttcgccacccctctggccgccagcct
ggaggaggtggcacctggggcgcgccacgccacgctcagcctgagtatcc
cccgcgtcgcgcccgagcacgagggccactatgtgtgcgaagtgcaagac
cggcgcagccatgacaagcactgccacaagaagtacctgtcggtgcaggc
cctggaagcccctcggctcacgcagaacttgaccgacctcctggtgaacg
tgagcgactcgctggagatgcagtgcttggtggccggagcgcacgcgccc
agcatcgtgtggtacaaagacgagaggctgctggaggaaaagtctggagt
cgacttggcggactccaaccagaagctgagcatccagcgcgtgcgcgagg
aggatgcgggacgctatctgtgcagcgtgtgcaacgccaagggctgcgtc
aactcctccgccagcgtggccgtggaaggctccgaggataagggcagcat
ggagatcgtgatccttgtcggtaccggcgtcatcgctgtcttcttctggg
tcctcctcctcctcatcttctgtaacatgaggaggccggcccacgcagac
atcaagacgggctacctgtccatcatcatggaccccggggaggtgcctct
ggaggagcaatgcgaatacctgtcctacgatgccagccagtgggaattcc
cccgagagcggctgcacctggggagagtgctcggctacggcgccttcggg
aaggtggtggaagcctccgctttcggcatccacaagggcagcagctgtga
caccgtggccgtgaaaatgctgaaagagggcgccacggccagcgagcacc
gcgcgctgatgtcggagctcaagatcctcattcacatcggcaaccacctc
aacgtggtcaacctcctcggggcgtgcaccaagccgcagggcccccctcat
ggtgatcgtggagttctgcaagtacggcaacctctccaacttcctgcgcg
ccaagcgggacgccttcagcccctgcgcggagaagtctcccgagcagcgc
ggacgcttccgcgccatggtggagctcgccaggctggatcggaggcggcc
ggggagcagcgacagggtcctcttcgcgcggttctcgaagaccgagggcg
gagcgaggcgggcttctccagaccaagaagctgaggacctgtggctgagc
ccgctgaccatggaagatcttgtctgctacagcttccaggtggccagagg
gatggagttcctggcttcccgaaagtgcatccacagagacctggctgctc
ggaacattctgctgtcggaaagcgacgtggtgaagatctgtgactttggc
cttgcccgggacatctacaaagaccccgactacgtccgcaagggcagtgc
ccggctgcccctgaagtggatggcccctgaaagcatcttcgacaaggtgt
acaccacgcagagtgacgtgtggtcctttggggtgcttctctgggagatc
ttctctctgggggcctccccgtaccctggggtgcagatcaatgaggagtt
ctgccagcggctgagagacggcacaaggatgagggccccggagctggcca
ctcccgccatacgccgcatcatgctgaactgctggtccggagaccccaag
gcgagacctgcattctcggagctggtggagatcctgggggacctgctcca
gggcaggggcctgcaagaggaagaggaggtctgcatggccccgcgcagct
ctcagagctcagaagagggcagcttctcgcaggtgtccaccatggcccta
cacatcgcccaggctgacgctgaggacagcccgccaagcctgcagcgcca
cagcctggccgccaggtattacaactgggtgtcctttcccgggtgcctgg

ccagaggggctgagacccgtggttcctccaggatgaagacatttgaggaa
ttccccatgaccccaacgacctacaaaggctctgtggacaaccagacaga
cagtgggatggtgctggcctcggaggagtttgagcagatagagagcaggc
atagacaagaaagcggcttcaggtagctgaagcagagagagagaaggcag
catacgtcagcattttcttctctgcacttataagaaagatcaaagacttt
aagactttcgctatttcttctactgctatctactacaaacttcaaagagg
aaccaggaggacaagaggagcatgaaagtggacaaggagtgtgaccactg
aagcaccacagggaggggttaggcctccggatgactgcgggcaggcctgg
ataatatccagcctcccacaagaagctggtggagcagagtgttccctgac
tcctccaaggaaagggagacgccctttcatggtctgctgagtaacaggtg
ccttcccagacactggcgttactgcttgaccaaagagccctcaagcggcc
cttatgccagcgtgacagagggctcacctcttgccttctaggtcacttct
cacaatgtcccttcagcacctgaccctgtgcccaccagttattccttggt
aatatgagtaatacatcaaagagtagtattaaaagctaattaatcatgtt
tataaaaaaaaaaaaaaaaaaaaaaaaaaaaaaa
>NM_182925 4
aggccagccggcgcccgcgcggacactttcagccccgagccgcggccgct
cgggtcggacccacgcgcagcggccggagatgcagcggggcgccgcgctg
tgcctgcgactgtggctctgcctgggactcctggacggcctggtgagtgg
ctactccatgacccccccgaccttgaacatcacggaggagtcacacgtca
tcgacaccggtgacagcctgtccatctcctgcaggggacagcaccccctc
gagtgggcttggccaggagctcaggaggcgccagccaccggagacaagga
cagcgaggacacgggggtggtgcgagactgcgagggcacagacgccaggc
cctactgcaaggtgttgctgctgcacgaggtacatgccaacgacacaggc
agctacgtctgctactacaagtacatcaaggcacgcatcgagggcaccac
ggccgccagctcctacgtgttcgtgagagactttgagcagccattcatca
acaagcctgacacgctcttggtcaacaggaaggacgccatgtgggtgccc
tgtctggtgtccatccccggcctcaatgtcacgctgcgctcgcaaagctc
ggtgctgtggccagacgggcaggaggtggtgtgggatgaccggcggggca
tgctcgtgtccacgccactgctgcacgatgccctgtacctgcagtgcgag
accacctggggagaccaggacttcctttccaacccccttcctggtgcacat
cacaggcaacgagctctatgacatccagctgttgcccaggaagtcgctgg
agctgctggtaggggagaagctggtcctgaactgcaccgtgtgggctgag
tttaactcaggtgtcacctttgactgggactacccagggaagcaggcaga
gcggggtaagtgggtgcccgagcgacgctcccagcagacccacacagaac
tctccagcatcctgaccatccacaacgtcagccagcacgacctgggctcg
tatgtgtgcaaggccaacaacggcatccagcgatttcgggagagcaccga
ggtcattgtgcatgaaaatcccttcatcagcgtcgagtggctcaaaggac
ccatcctggaggccacggcaggagacgagctggtgaagctgcccgtgaag
ctggcagcgtacccccccgcccgagttccagtggtacaaggatggaaaggc
actgtccgggcgccacagtccacatgccctggtgctcaaggaggtgacag
aggccagcacaggcacctacaccctcgccctgtggaactccgctgctggc
ctgaggcgcaacatcagcctggagctggtggtgaatgtgccccccagat
acatgagaaggaggcctcctcccccagcatctactcgcgtcacagccgcc
aggccctcacctgcacggcctacggggtgcccctgcctctcagcatccag
tggcactggcggccctggacaccctgcaagatgtttgcccagcgtagtct
ccggcggcggcagcagcaagacctcatgccacagtgccgtgactggaggg
cggtgaccacgcaggatgccgtgaaccccatcgagagcctggacacctgg
accgagtttgtggagggaaagaataagactgtgagcaagctggtgatcca

gaatgccaacgtgtctgccatgtacaagtgtgtggtctccaacaaggtgg
gccaggatgagcggctcatctacttctatgtgaccaccatccccgacggc
ttcaccatcgaatccaagccatccgaggagctactagagggccagccggt
gctcctgagctgccaagccgacagctacaagtacgagcatctgcgctggt
accgcctcaacctgtccacgctgcacgatgcgcacgggaacccgcttctg
ctcgactgcaagaacgtgcatctgttcgccacccctctggccgccagcct
ggaggaggtggcacctggggcgcgccacgccacgctcagcctgagtatcc
cccgcgtcgcgcccgagcacgagggccactatgtgtgcgaagtgcaagac
cggcgcagccatgacaagcactgccacaagaagtacctgtcggtgcaggc
cctggaagcccctcggctcacgcagaacttgaccgacctcctggtgaacg
tgagcgactcgctggagatgcagtgcttggtggccggagcgcacgcgccc
agcatcgtgtggtacaaagacgagaggctgctggaggaaaagtctggagt
cgacttggcggactccaaccagaagctgagcatccagcgcgtgcgcgagg
aggatgcgggacgctatctgtgcagcgtgtgcaacgccaagggctgcgtc
aactcctccgccagcgtggccgtggaaggctccgaggataagggcagcat
ggagatcgtgatccttgtcggtaccggcgtcatcgctgtcttcttctggg
tcctcctcctcctcatcttctgtaacatgaggaggccggcccacgcagac
atcaagacgggctacctgtccatcatcatggaccccggggaggtgcctct
ggaggagcaatgcgaatacctgtcctacgatgccagccagtgggaattcc
cccgagagcggctgcacctggggagagtgctcggctacggcgccttcggg
aaggtggtggaagcctccgctttcggcatccacaagggcagcagctgtga
caccgtggccgtgaaaatgctgaaagagggcgccacggccagcgagcacc
gcgcgctgatgtcggagctcaagatcctcattcacatcggcaaccacctc
aacgtggtcaacctcctcggggcgtgcaccaagccgcagggcccccctcat
ggtgatcgtggagttctgcaagtacggcaacctctccaacttcctgcgcg
ccaagcgggacgccttcagcccctgcgcggagaagtctcccgagcagcgc
ggacgcttccgcgccatggtggagctcgccaggctggatcggaggcggcc
ggggagcagcgacagggtcctcttcgcgcggttctcgaagaccgagggcg
gagcgaggcgggcttctccagaccaagaagctgaggacctgtggctgagc
ccgctgaccatggaagatcttgtctgctacagcttccaggtggccagagg
gatggagttcctggcttcccgaaagtgcatccacagagacctggctgctc
ggaacattctgctgtcggaaagcgacgtggtgaagatctgtgactttggc
cttgcccgggacatctacaaagaccccgactacgtccgcaagggcagtgc
ccggctgcccctgaagtggatggcccctgaaagcatcttcgacaaggtgt
acaccacgcagagtgacgtgtggtcctttggggtgcttctctgggagatc
ttctctctgggggcctccccgtaccctggggtgcagatcaatgaggagtt
ctgccagcggctgagagacggcacaaggatgagggccccggagctggcca
ctcccgccatacgccgcatcatgctgaactgctggtccggagaccccaag
gcgagacctgcattctcggagctggtggagatcctgggggacctgctcca
gggcaggggcctgcaagaggaagaggaggtctgcatggccccgcgcagct
ctcagagctcagaagagggcagcttctcgcaggtgtccaccatggcccta
cacatcgcccaggctgacgctgaggacagcccgccaagcctgcagcgcca
cagcctggccgccaggtattacaactgggtgtcctttcccgggtgcctgg
ccagaggggctgagacccgtggttcctccaggatgaagacatttgaggaa
ttccccatgaccccaacgacctacaaaggctctgtggacaaccagacaga
cagtgggatggtgctggcctcggaggagtttgagcagatagagagcaggc
atagacaagaaagcggcttcagctgtaaaggacctggccagaatgtggct
gtgaccagggcacaccctgactcccaagggaggcggcggcggcctgagcg
gggggcccgaggaggccaggtgttttacaacagcgagtatggggagctgt

cggagccaagcgaggaggaccactgctccccgtctgcccgcgtgactttc
ttcacagacaacagctactaagcagcatcggacaagacccccagcacttg
ggggttcaggcccggcagggcgggcagagggctggaggcccaggctggga
actcatctggttgaactctggtggcacaggagtgtcctcttccctctctg
cagacttcccagctaggaagagcaggactccaggcccaaggctcccggaa
ttccgtcaccacgactggccagggccacgctccagctgccccggcccctc
cccctgagattcagatgtcatttagttcagcatccgcaggtgctggtccc
ggggccagcacttccatgggaatgtctctttggcgacctcctttcatcac
actgggtggtggcctggtccctgttttcccacgaggaatctgtgggtctg
ggagtcacacagtgttggaggttaaggcatacgagagcagaggtctccca
aacgccctttcctcctcaggcacacagctactctccccacgagggctggc
tggcctcacccacccctgcacagttgaagggaggggctgtgtttccatct
caaagaaggcatttgcagggtcctcttctgggcctgaccaaacagccaac
tagcccctggggtggccaccagtatgacagtattatacgctggcaacaca
gaggcagcccgcacacctgcgcctgggtgttgagagccatcctgcaagtc
tttttcaacagaacttcacagactgttagagctgctgagaagaatttgct
ttccgaattcagcctggaaggcgcccagggacagctgtactgagtctaga
tgactctgacccccgccccaggtcaaggccagcagagcagtcagtgcctc
tggagaaggcccttgctctcccacctggcccagactccgaggagcctggg
tctggagctgccggtctggttcttccctttagagcccggatctgccacct
gcggcccctcccaagccgtgaaccagctcatgagagatgaacactgtggg
atccactcaggaaggctcggggctggcacaaaggaccacccagcattgcc
ctgtgccacccagcactcagtggacattctggggacctgccttcagcctt
ttcctgccctgtgcctgacatcagcaccctggctggtcagaatgccgccc
tcccagaggagcagccgagagatcccctgaaggctggaggcattctgctc
aggacccctatcccagctcacagtgcccaaccatctcaccaggagaaaga
gccacatccccacgttaggaccacggagactgaccaccaccctgaccccc
caaacccacgcaccagacgcttgcaggacaggcgccgcgcagcgggcagg
ggcttgcccggccgaccctcccctccccacctcccccactgcgcgttact
ccaggatatgccgagtgcacgtataaggtcatcttcgtcgtccccgtgga
cctccccccttcctctgcacgtcgtccaacgtgggactggcgtgtcaggct
tccctgggaggatctggaggttgttctctgcagagaaccagcctggctcc
tggcgcgcacctctgctcccttctcctcactacccacccacgcatgtacc
gggaaaaaaactactatgcccttctagaccatgttctgagaaaagatcga
aaatatttaacaagagataataataaatctgatgccggtctttgtgtgtg
ttgcgga
>NM_014384 2
gcgagcgcaacggaggtcgaaggcgttcagactcttagctgaacgcggag
ctgcggcggctatgctgtggagcggctgccggcgtttcggggcgcgcctc
ggctgcctgcccggcggtctccgggtcctcgtccagaccggccaccggag
cttgacctcctgcatcgacccttccatgggacttaatgaagagcagaaag
aatttcaaaaagtggcctttgactttgctgcccgagagatggctccaaat
atggcagagtgggaccagaaggagctgttcccagtggatgtgatgcggaa
ggcagcccagctaggcttcggaggggtctacatacaaacagatgtgggcg
ggtctgggctgtcacgtcttgatacctctgtcatttttgaagccttggct
acaggctgcaccagcaccacagcctatataagcatccacaacatgtgtgc
ctggatgattgatagcttcggaaatgaggaacagaggcacaaattttgcc
caccgctctgtaccatggagaagtttgcttcctactgcctcactgaacca
ggaagtgggagtgatgctgcctctcttctgacctccgctaagaaacaggg

agatcattacatcctcaatggctccaaggccttcatcagtggtgctggtg
agtcagacatctatgtggtcatgtgccgaacaggaggaccaggccccaag
ggcatctcatgcatagttgttgagaaggggacccctggcctcagctttgg
caagaaggagaaaaaggtggggtggaactcccagccaacacgagctgtga
tcttcgaagactgtgctgtccctgtggccaacagaattgggagcgagggg
cagggcttcctcattgccgtgagaggactgaacggagggaggatcaatat
tgcttcctgctccctgggggctgcccacgcctctgtcatcctcacccgag
accacctcaatgtccggaagcagtttggagagcctctggccagtaaccag
tacttgcaattcacactggctgatatggcaacaaggctggtggccgcgcg
gctgatggtccgcaatgcagcagtggctctgcaggaggagaggaaggatg
cagtggccttgtgctccatggccaagctctttgctacagatgaatgcttt
gccatctgcaaccaggccttgcagatgcacgggggctacggctacctgaa
ggattacgctgttcagcagtacgtgcgggactccagggtccaccagattc
tagaaggtagcaatgaagtgatgaggatactgatctctagaagcctgctt
caggagtagaacccacacttgttctggcctggtgttcagtgcgactgcag
tcagtgttgagtggtgccatgtgggccgctctattccaaaggaatcatgg
attagacccaagggctgagctcctctagggcaggacctgcaccctgtgtg
ttggcaccagcatcgggtcttggactggggcagaatccccagtggaaccg
gaagagctggactgatgagaaacatcagaagaacacatactaccttgttt
tcctaatgccagaagggtgaccagtgaagattcaccgtcaaaccatgaaa
gtcctttcttggatccactttatcttgattagtctgcattttactagttc
actggatccctcctctaggggcctggggactttcactgatgctcttcctg
attctagagcaaaggtgtgggaaggggaaatggaggaatgccctcctgtc
tgtgtcgttctctgtgccacagctacagatgcagaaggtttctctggata
gcacacctctgaatgtaaatcatgataaaatggatatttggaaacttact
cctaagctgtgatttagggtgtatttctacttctggactgcctcaatatc
aagggctgagacttttgaattttgaatattcgttgggtttcatgttaaga
agcctgtggtctaggagtgctattcagtgtttcttttcctgataaacact
ttgaatattttttttgtgtttttgtttccttttctgaagctgttcctcct
tttaaatatttttaatcacattgataaaatctatccttcaccacctctgg
ttctactatagttgatttttattttaaatgtttaattgtatttgattaaa
cacttaactggattttggaataataaaactctcgtccaatttggctttta
aaaaaaaaaaaaaaaa
>NM_004170 5
agagcgcggcggcagcaggaggagccgggcgcgcctgccacgcaaaacta
ccgggctggcagggcggcgggcgcggtgcgcgatcccgggtggcggcggc
aacggcggtggtgacggcggcgactgcagcggccggctctcacctctccc
ctgtgcacccgcatctcgccgcgccgccgagcagccagcagtccccgggt
cgcccagcccacgcgcgcacggccgagcccagcgcacaatagcggcgaca
gccatggggaaaccggcgaggaaaggatgcgagtggaagcgcttcctgaa
gaataactgggtgttgctgtccaccgtggccgcggtggtgctaggcatta
ccacaggagtcttggttcgagaacacagcaacctctcaactctagagaaa
ttctactttgcttttcctggagaaattctaatgcggatgctgaaactcat
cattttgccattaattatatccagcatgattacaggtgttgctgcactgg
attccaacgtatccggaaaaattggtctgcgcgctgtcgtgtattatttc
tgtaccactctcattgctgttattctaggtattgtgctggtggtgagcat
caagcctggtgtcacccagaaagtgggtgaaattgcgaggacaggcagca
cccctgaagtcagtacggtggatgccatgttagatctcatcaggaatatg
ttccctgagaatcttgtccaggcctgttttcagcagtacaaaactaagcg

tgaagaagtgaagcctcccagcgatccagagatgaacatgacagaagagt
ccttcacagctgtcatgacaactgcaatttccaagaacaaaacaaaggaa
tacaaaattgttggcatgtattcagatggcataaacgtcctgggcttgat
tgtcttttgccttgtctttggacttgtcattggaaaaatgggagaaaagg
gacaaattctggtggatttcttcaatgctttgagtgatgcaaccatgaaa
atcgttcagatcatcatgtgttatatgccactaggtattttgttcctgat
tgctgggaagatcatagaagttgaagactgggaaatattccgcaagctgg
gcctttacatggccacagtcctgactgggcttgcaatccactccattgta
attctcccgctgatatatttcatagtcgtacgaaagaaccctttccgatt
tgccatgggaatggcccaggctctcctgacagctctcatgatctcttcca
gttcagcaacactgcctgtcaccttccgctgtgctgaagaaaataaccag
gtggacaagaggatcactcgattcgtgttacccgttggtgcaacaatcaa
catggatgggactgcgctctatgaagcagtggcagcggtgtttattgcac
agttgaatgacctggacttgggcattgggcagatcatcaccatcagtatc
acggccacatctgccagcatcggagctgctggcgtgccccaggctggcct
ggtgaccatggtgattgtgctgagtgccgtgggcctgcccgccgaggatg
tcaccctgatcattgctgtcgactggctcctggaccggttcaggaccatg
gtcaacgtccttggtgatgcttttgggacgggcattgtggaaaagctctc
caagaaggagctggagcagatggatgtttcatctgaagtcaacattgtga
atccctttgccttggaatccacaatccttgacaacgaagactcagacacc
aagaagtcttatgtcaatggaggctttgcagtagacaagtctgacaccat
ctcattcacccagacctcacagttctagggcccctggctgcagatgactg
gaaacaaggaaggacatttccgtgagagtcatctcaaacactgcttaagg
aaaagagaaacactaatggccaagtgtacatttgatttgatatacagacc
tccagattattttctatatttggattcacagcctttgcgctctgggtttt
gggatttgggtgtggggtaagttgaagggaaatcaatttaaaggaaagtt
ctattatctgggttttagaaattctataagagacaaagtttggaagtaca
taaagtaataactgttagaattaggtaatggatatgaaagagaaaatgct
ttctcatgcatagacaagtgttttgggtttttaaaaaaaatattctgtca
ttggttacaaatttttactcaggctttctattggcatggatttcctttga
cctctcactttttataaattataatgcatctaaaccacctgtccccagt
taatgtgccaaaatgtcaatttttaacttatctccagccaatttcaaaga
aaacagaccagcatagttctgcaataacagttttaagatgggcatagggt
ttggaagaaagggagaaggattctttttcaatgtactgtattgggacgc
tggtaactgttaacccagtgttcagcatagagctatatatatatatatat
gtatatatttattattttcatataatttgccagacagagatcagaattga
accgtcaatgtgaaataaagagttctccttgtacttgaataataaccacg
attccaacccaggtctgctttggggcttatcagaactcctttctaaggag
cactagaatgagaaatcatgttgttcgatcgtttcacatctgtatatcag
ctctaaagcagagatgtattatggtgatactccaaggtggcatagccatt
catttacaacttccagatttgagctgcctggagggaatccatatcagctc
tgcataagattatatacaaagctgtcactcacaaaaggctggatgtgctt
tcatccaactggaaggctttattcttccaagttcattcatactcaaagag
gccagtactttgccatccttgcacttctgttatcagggcccaaataacag
tggcaagctaccaactaagttgtattttaataaagattccatgggttgaa
caagccacgttgcagaaaaagagcttcccctaacctgggttgttgcagag
taaatcccacgacataagctggtatcagtggttcgggggaaatagttcca
ttctatgactcttgtctcctcctccaggaggactgttctaactagtaatc
ttggccctattcattacatcctctgcttgtcattctgctaatttatgaag

atagtttattatagtctgtacttcagttctcatcttgtaaataatgctta
acataaacttgtacttacactgaaatccaaaatagtcatgtttctgcagt
attctgtagccaacttaaacctgtgctttcatgtttaagaaatgagaaat
tgtgccaaagatagcagaagagtagataagtgctcagtattgacgaccta
catctgaaatctacaacataatgatactgaattgttatgtaaacatcata
aatagtaaataatgattcaatgtgaattttaaaatgcaaatattgctatt
gtttataggaaataaatctaaatataaatgaaattgaatcagtaatttat
cttgggctaaatggttctaccccttactaggttgccccaattagtggcac
tagttggcagagctgttcatgagctgccagttatcattttggagtcagtt
tgaaaccagcctcttaacacactgctgttaactcataaaagagaagagtg
ttccatttcagtctcaataaacacttccctcattctttcaaaaaaaaaaa
aaaaaaa
>NM_004170 5
agagcgcggcggcagcaggaggagccgggcgcgcctgccacgcaaaacta
ccgggctggcagggcggcgggcgcggtgcgcgatcccgggtggcggcggc
aacggcggtggtgacggcggcgactgcagcggccggctctcacctctccc
ctgtgcacccgcatctcgccgcgccgccgagcagccagcagtccccgggt
cgcccagcccacgcgcgcacggccgagcccagcgcacaatagcggcgaca
gccatggggaaaccggcgaggaaaggatgcgagtggaagcgcttcctgaa
gaataactgggtgttgctgtccaccgtggccgcggtggtgctaggcatta
ccacaggagtcttggttcgagaacacagcaacctctcaactctagagaaa
ttctactttgcttttcctggagaaattctaatgcggatgctgaaactcat
cattttgccattaattatatccagcatgattacaggtgttgctgcactgg
attccaacgtatccggaaaaattggtctgcgcgctgtcgtgtattatttc
tgtaccactctcattgctgttattctaggtattgtgctggtggtgagcat
caagcctggtgtcacccagaaagtgggtgaaattgcgaggacaggcagca
cccctgaagtcagtacggtggatgccatgttagatctcatcaggaatatg
ttccctgagaatcttgtccaggcctgttttcagcagtacaaaactaagcg
tgaagaagtgaagcctcccagcgatccagagatgaacatgacagaagagt
ccttcacagctgtcatgacaactgcaatttccaagaacaaaacaaaggaa
tacaaaattgttggcatgtattcagatggcataaacgtcctgggcttgat
tgtcttttgccttgtctttggacttgtcattggaaaaatgggagaaaagg
gacaaattctggtggatttcttcaatgctttgagtgatgcaaccatgaaa
atcgttcagatcatcatgtgttatatgccactaggtattttgttcctgat
tgctgggaagatcatagaagttgaagactgggaaatattccgcaagctgg
gcctttacatggccacagtcctgactgggcttgcaatccactccattgta
attctcccgctgatatatttcatagtcgtacgaaagaaccctttccgatt
tgccatgggaatggcccaggctctcctgacagctctcatgatctcttcca
gttcagcaacactgcctgtcaccttccgctgtgctgaagaaaataaccag
gtggacaagaggatcactcgattcgtgttacccgttggtgcaacaatcaa
catggatgggactgcgctctatgaagcagtggcagcggtgtttattgcac
agttgaatgacctggacttgggcattgggcagatcatcaccatcagtatc
acggccacatctgccagcatcggagctgctggcgtgccccaggctggcct
ggtgaccatggtgattgtgctgagtgccgtgggcctgcccgccgaggatg
tcaccctgatcattgctgtcgactggctcctggaccggttcaggaccatg
gtcaacgtccttggtgatgcttttgggacgggcattgtggaaaagctctc
caagaaggagctggagcagatggatgtttcatctgaagtcaacattgtga
atccctttgccttggaatccacaatccttgacaacgaagactcagacacc
aagaagtcttatgtcaatggaggctttgcagtagacaagtctgacaccat

ctcattcacccagacctcacagttctagggcccctggctgcagatgactg
gaaacaaggaaggacatttccgtgagagtcatctcaaacactgcttaagg
aaaagagaaacactaatggccaagtgtacatttgatttgatatacagacc
tccagattattttctatatttggattcacagcctttgcgctctgggtttt
gggatttgggtgtggggtaagttgaagggaaatcaatttaaaggaaagtt
ctattatctgggttttagaaattctataagagacaaagtttggaagtaca
taaagtaataactgttagaattaggtaatggatatgaaagagaaaatgct
ttctcatgcatagacaagtgttttgggtttttaaaaaaaatattctgtca
ttggttacaaatttttactcaggctttctattggcatggatttcctttga
cctctcactttttataaattataatgcatctaaaccacctgtccccagt
taatgtgccaaaatgtcaatttttaacttatctccagccaatttcaaaga
aaacagaccagcatagttctgcaataacagttttaagatgggcatagggt
ttggaagaaagggagaaggattctttttcaatgtactgtattgggacgc
tggtaactgttaacccagtgttcagcatagagctatatatatatatatat
gtatatatttattattttcatataatttgccagacagagatcagaattga
accgtcaatgtgaaataaagagttctccttgtacttgaataataaccacg
attccaacccaggtctgctttggggcttatcagaactcctttctaaggag
cactagaatgagaaatcatgttgttcgatcgtttcacatctgtatatcag
ctctaaagcagagatgtattatggtgatactccaaggtggcatagccatt
catttacaacttccagatttgagctgcctggagggaatccatatcagctc
tgcataagattatatacaaagctgtcactcacaaaaggctggatgtgctt
tcatccaactggaaggctttattcttccaagttcattcatactcaaagag
gccagtactttgccatccttgcacttctgttatcagggcccaaataacag
tggcaagctaccaactaagttgtattttaataaagattccatgggttgaa
caagccacgttgcagaaaaagagcttcccctaacctgggttgttgcagag
taaatcccacgacataagctggtatcagtggttcgggggaaatagttcca
ttctatgactcttgtctcctcctccaggaggactgttctaactagtaatc
ttggccctattcattacatcctctgcttgtcattctgctaatttatgaag
atagtttattatagtctgtacttcagttctcatcttgtaaataatgctta
acataaacttgtacttacactgaaatccaaaatagtcatgtttctgcagt
attctgtagccaacttaaacctgtgctttcatgtttaagaaatgagaaat
tgtgccaaagatagcagaagagtagataagtgctcagtattgacgaccta
catctgaaatctacaacataatgatactgaattgttatgtaaacatcata
aatagtaaataatgattcaatgtgaattttaaaatgcaaatattgctatt
gtttataggaaataaatctaaatataaatgaaattgaatcagtaatttat
cttgggctaaatggttctaccccttactaggttgccccaattagtggcac
tagttggcagagctgttcatgagctgccagttatcattttggagtcagtt
tgaaaccagcctcttaacacactgctgttaactcataaaagagaagagtg
ttccatttcagtctcaataaacacttccctcattctttcaaaaaaaaaaa
aaaaaaa
>NM_006066 3
aaaaggcgaggctggccccgccccttgcaccgcccacgtggccagcgcca
cctgcctcattgtgcccaggagttctccaaacccgcgctgcggagtgagt
gaccaagttccggccagttcgacctcgaggatccagaggtggagacggta
ctacctcccagctctgttttccatccccttcaggtccttcctcgggaggc
ggcgaaggcggtccaccctgcgcgtgatcctttatgcccggcccctgccc
ctccctccgggtggaacttccccctcaccgccagacttaagctgaggatc
gttggatctctggcggggtgcagaactgagcccaggccacagtaccctat
tcacgctctgtgcttgtgccaaggtttcaagtgatcctcccgcctcagcc

tgcccaggtgctgagattacatgtatgagccactgcacctggaaaggagc
cagaaatgtgaagtgctagctgaaggatgagcagcagctagccaggcaaa
gggggcaatggcggcttcctgtgttctactgcacactgggcagaagatgc
ctctgattggtctgggtacctggaagagtgagcctggtcaggtaaaagca
gctgttaagtatgcccttagcgtaggctaccgccacattgattgtgctgc
tatctacggcaatgagcctgagattggggaggccctgaaggaggacgtgg
gaccaggcaaggcggtgcctcgggaggagctgtttgtgacatccaagctg
tggaacaccaagcaccaccccgaggatgtggagcctgccctccggaagac
tctggctgacctccagctggagtatctggacctgtacctgatgcactggc
cttatgcctttgagcggggagacaaccccttccccaagaatgctgatggg
actatatgctacgactccacccactacaaggagacttggaaggctctgga
ggcactggtggctaaggggctggtgcaggcgctgggcctgtccaacttca
acagtcggcagattgatgacatactcagtgtggcctccgtgcgtccagct
gtcttgcaggtggaatgccacccatacttggctcaaaatgagctaattgc
ccactgccaagcacgtggcctggaggtaactgcttatagccctttgggct
cctctgatcgtgcatggcgtgatcctgatgagcctgtcctgctggaggaa
ccagtagtcctggcattggctgaaaagtatggccgatctccagctcagat
cttgctcaggtggcaggtccagcggaaagtgatctgcatccccaaaagta
tcactccttctcgaatccttcagaacatcaaggtgtttgacttcaccttt
agcccagaagagatgaagcagctaaatgccctgaacaaaaattggagata
tattgtgcctatgcttacggtggatgggaagagagtcccaagggatgcag
ggcatcctctgtaccccttaatgacccgtactgagaccacagcttcttg
gcctcccttccagctctgcagctaatgaggtcctgccacaacggaaagag
ggagttaataaagccattggagcatccataaaaaaaaaaaaaaaaaa
>NM_004505 2
ctaaaaataccattaagtaatagtattagcttttgtattctgagattcaa
cagcagcagtcacttccctccactcctatgtgtatcccaggaccaccctg
ggcggggagggctgaggtcagggaggtctgaagctggtcctgggctccgg
gggtgacagtgatgaggaactgggtgcacacatgagtggggcagccgggc
ctggccagagaagcaacacacacgtgcacagacatgtttatccacataca
catgtgcacgcatgtgcacaaacacattgcaggcaggcatgttgacgcct
caggcagcggaggaccctgactctgggccctgctgacccaggcaaggccc
cattgtgatgcgtgccatgacctcagaatgtcactggtgcttagcaccta
tccgctctccagactgcgtctgtgttctacggcagttacacacacgcagt
ggtattcacaagcggttttgtggactcaaaggttttctccctgagaggca
taacccaggccagctgattcatcagaatcaggtgagtgtgacctgctctc
ttccctccaggctgacttggggacagtggctatggtatgggcggtgttgg
cctctgggcagctacagaggagggtcatccctgagcactcaccgggcgcc
cgttctacactgcccatgtagacgattttctctttcgtcttcatggtggc
ttcgtagagtgggtgctgttcccaaatgtacccattcgacaggtgagccg
tctggggtcagagaggcagtaactggcctgggaatccagacaagaccctg
ggttttgctctcagccctgctgtgtgccatgctagacttcaggcctcaac
cctgagacctccctgctctagatcccaaatctgcccagatttccgatccc
aatggggcagagcctggccctggcagagacactgggatggatccactgtg
ggtggggaggagggaagggtcctcagaacacacctggggcctaagctggg
tcttgatggtcactgtgggacccactggacacacacagtcccttgtctgg
gagtggcatggggagccttctgcccttgggcagttgtggaaagtgaagga
gccctggagagctggctgaggggagactatcttcccttgtgttcaaaggg
gtccaggcactggggctctccccaagtatttcttattctgtctggcctcg

ctttccttttgccctgagtattctcaggagggacggtccatctagatgtc
ctccaggagcaaggacccactgttcttcatcagtgacccaggaaaatgaa
gccccctcctgtggggacagctcagaatggtggagtccacagtccctccc
tgagagacatggtttccatgagcacagtggctgctttggagacagtaatc
attttcatccccaaaaccaaacacactcctgctcaaatggtgttattgct
aaagcagcttcactggttagactgaagggccatggtagcccaagtgatga
gcggggtagaatggagcagtcaggagagatcttgttccccgtaggaaact
gggcatctctgtggccctgaacatcccaggaggccgatcgtacagagacc
tctggtgcctgaccgcagttcacatccacatccctggaatagaccatcac
aggctcttcacccttggcaggtggacaccattcaacctgccggggcagga
tggacatggtagagaatgcagatagtttgcaggcacaggagcggaaggac
atacttatgaagtatgacaagggacaccgagctgggctgccagaggacaa
ggggcctgagcccgttggaatcaacagcagcattgatcgttttggcattt
tgcatgagacggagctgcctcctgtgactgcacgggaggcgaagaaaatt
cggcgggagatgacacgaacgagcaagtggatggaaatgctgggagaatg
ggagacatataagcacagtagcaaactcatagatcgagtgtacaagggaa
ttcccatgaacatccggggcccggtgtggtcagtcctcctgaacattcag
gaaatcaagttgaaaaaccccggaagataccagatcatgaaggagagggg
caagaggtcatctgaacacatccaccacatcgacctggacgtgaggacga
ctctccggaaccatgtcttctttagggatcgatatggagccaagcagagg
gaactattctacatcctcctggcctattcggagtataacccggaggtggg
ctactgcagggacctgagccacatcaccgccttgttcctcctttatctgc
ctgaggaggacgcattctgggcactggtgcagctgctggccagtgagagg
cactccctgccaggattccacagcccaaatggtgggacagtccaggggct
ccaagaccaacaggagcatgtggtacccaagtcacaacccaagaccatgt
ggcatcaggacaaggaaggtctatgcgggcagtgtgcctcgttaggctgc
cttctccggaacctgattgacgggatctctctcgggctcaccctgcgcct
gtgggacgtgtatttggtggaaggagaacaggtgttgatgccaataacca
gcattgctcttaaggttcagcagaagcgcctcatgaagacatccaggtgt
ggcctgtgggcacgtctgcggaaccaattcttcgatacctgggccatgaa
cgatgacaccgtgctcaagcatcttagggcctctacgaagaaactaacaa
ggaagcaaggggacctgccacccccagccaaacgcgagcaagggtccttg
gcacccaggcctgtgccggcttcacgtggtgggaagaccctctgcaaggg
gtataggcaggcccctccaggcccaccagcccagttccagcggcccattt
gctcagcttccccgccatgggcatctcgtttttccacgccctgtcctggt
ggggctgtccgggaagacacgtaccctgtgggcactcagggtgtgcccag
cctggccctggctcagggaggacctcagggttcctggagattcctggagt
ggaagtcaatgccccggctcccaacggacctggatatagggggcccttgg
ttcccccattatgattttgaatggagctgctgggtccgtgccatatccca
ggaggaccagctggccacctgctggcaggctgaacactgcggagaggttc
acaacaaagatatgagttggcctgaggagatgtcttttacagcaaatagt
agtaaaatagatagacaaaaggttcccacagaaaagggagccacaggtct
aagcaacctgggaaacacatgcttcatgaactcaagcatccagtgcgtta
gtaacacacagccactgacacagtattttatctcagggagacatctttat
gaactcaacaggacaaatcccattggtatgaaggggcatatggctaaatg
ctatggtgatttagtgcaggaactctggagtggaactcagaagagtgttg
ccccattaaagcttcggcggaccatagcaaaatatgctcccaagtttgat
gggtttcagcaacaagactcccaagaacttctggcttttctcttggatgg
tcttcatgaagatctcaaccgagtccatgaaaagccatatgtggaactga

aggacagtgatggccgaccagactgggaagtagctgcagaggcctgggac
aaccatctaagaagaaatagatcaattattgtggatttgttccatgggca
gctaagatctcaagtcaaatgcaagacatgtgggcatataagtgtccgat
ttgaccctttcaatttttttgtctttgccactaccaatggacagttacatg
gacttagaaataacagtgattaagttagatggtactacccctgtacggta
tggactaagactgaatatggatgaaaagtacacaggtttaaaaaaacagc
tgagggatctctgtggacttaattcagaacaaatcctactagcagaagta
catgattccaacataaagaactttcctcaggataaccaaaaagtacaact
ctcagtgagcggatttttgtgtgcatttgaaattcctgtcccttcatctc
caatttcagcttctagtccaacacaaatagatttctcctcttcaccatct
acaaatggaatgttcaccctaactaccaatggggacctacccaaaccaat
attcatccccaatggaatgccaaacactgttgtgccatgtggaactgaga
agaacttcacaaatggaatggttaatggtcacatgccatctcttcctgac
agcccctttacaggttacatcattgcagtccaccgaaaaatgatgaggac
agaactgtatttcctgtcacctcaggagaatcgccccagcctctttggaa
tgccattgattgttccatgcactgtgcatacccggaagaaagacctatat
gatgcggtttggattcaagtatcctggttagcaagaccactcccacctca
ggaagctagtattcatgcccaggatcgtgataactgtatgggctatcaat
atccattcactctacgagttgtgcagaaagatgggaactcctgtgcttgg
tgcccacagtatagattttgcagaggctgtaaaattgattgtggggaaga
cagagctttcattggaaatgcctatattgctgtggattggcaccccacag
cccttcaccttcgctatcaaacatcccaggaaagggttgtagataagcat
gagagtgtggagcagagtcggcgagcgcaagccgagcccatcaacctgga
cagctgtctccgtgctttcaccagtgaggaagagctaggggaaagtgaga
tgtactactgttccaagtgtaagacccactgcttagcaacaaagaagctg
gatctctggaggcttccacccttcctgattattcaccttaagcgatttca
atttgtaaatgatcagtggataaaatcacagaaaattgtcagatttcttc
gggaaagttttgatccgagtgctttttttggtaccacgagacccggccctc
tgccagcataaaccactcacaccccaggggggatgagctctccaagcccag
gattctggcaagagaggtgaagaaagtggatgcgcagagttcggctggaa
aagaggacatgctcctaagcaaaagcccatcctcactcagcgctaacatc
agcagcagcccaaaaggttctccttcttcatcaagaaaaagtggaaccag
ctgtccctccagcaaaaacagcagccctaatagcagcccacggactttgg
ggaggagcaaagggaggctccggctgccccagattggcagcaaaaataag
ccgtcaagtagtaagaagaacttggatgccagcaaagagaatggggctgg
gcagatctgtgagctggctgacgccttgagccgagggcatatgcgggggg
gcagccaaccagagctggtcactcctcaggaccatgaggtagctttggcc
aatggattcctttatgagcatgaagcatgtggcaatggctgtggcgatgg
ctacagcaatggtcagcttggaaaccacagtgaagaagacagcactgatg
accaaagagaagacactcatattaagcctatttataatctatatgcaatt
tcatgccattcaggaattctgagtgggggccattacatcacttatgccaa
aaacccaaactgcaagtggtactgttataatgacagcagctgtgaggaac
ttcaccctgatgaaattgacaccgactctgcctacattcttttctatgag
cagcaggggatagactacgcacaatttctgccaaagattgatggcaaaaa
gatggcagacacaagcagtacggatgaagactctgagtctgattacgaaa
agtactctatgttacagtaaagctaccactctggctgctagacagcttgg
tggcgagggagatgactccttgtagctgatacttggcaaaagtgtcactg
aaagacaagctaaatgtagttattttatcctgttagaacaaaaattctaa
ttaaaatagttaacttgaagagtagaaacaattgtattttgaagtctcat

acaagctgtctgatagagaactttcaggcagatcccaccattagcctgta
aacaaaaggtgtggcaccagccacctgggaccaaataagaattgaattgt
gcttgtccagatatgaacaaatatgtagtgagtatagagtttaccaataa
tcataacaaatattaaagatttccttggagtcagaggaaaaaacaaacaa
ttataatgttgtctagggacgacatgatacgctacctcctttttcctgaa
gttttattccattatattgacaagatggagaaagcaagatcatgaaggtg
tgcaaatgattcttacggcatggacaaggatttttcaatttattttttaa
actgtttccataccctttctttttcttgctttttgtttttgccattgtgt
ttacgtttgagacacaaccagtcattggtggcaggggcatagagtggtca
gtctgaaagggaggctctcttaagagctatgtgccttccaaccagaggga
gacccagtagaaagaaaaacatcctgggaaatccagctaccagggccctc
ccagtggaggcatcttacatttaggctacttcaagtatcctcagaaatgt
attctgcacccccggccccgcccatgctgagggaaggggagcagttgcca
atatttgcaccatcttcacatgcacatgttgcaacaagagcttctgggaa
ggtaagcggcatcggagctagatcacgtttcacaattagtggttattctt
ttctgtgtttgttttgcactttaaaaaagagagaacacatgcaaatgaac
ttgcttgtgtgtatttgatggctctaagggctataaattacaaacaaaac
acatcccagacattaggagttcataagtatatttaatgaaattggtggtt
ttaggaagtcaactttagttttgctttgtttgcatgtccactggtttttt
tattttgatatttgtcttttttttaaattttacagtagtcattgaaagtta
tgtttctttgcttacttcatttttccctctaattatttaagattggaac
aaaagtataaatattatttatttgaggtagaattttttttcatgtagtttc
ttaatatatacttgaaggaaatgtttcaccttatttttggtctttgttta
ttcatttagaccctgcaagttgattctcattgccagattccattaccctt
tcttcctcataggtagtaattaccaatgtaactaagcatttgtgttctga
tatctgaggccagtaactattaatatctagttctcagagcatttggaaag
gttatcttaaatggctacctaaattgaaatccttttcagaaaaaatataa
ttgcaagtaggtaggagtggcctaaattgtctaatgtaataaagtcagac
aaaatgcacactttatagtttcaagattttcagtaaataaaatctgtcca
ttcctacctggacatgtcccattaaaaagtggaagattttaaataatttc
tttacagatgttttatttaaacaggtagcacaatctactaatgttgtgtg
atttgtgttatactggttgtaattaattttttttaattcatgaactagcgg
aaaatttattaaattaactattaactacattcaccttgtaaattactgta
taaaacttgttgacaatgcactgactttagaaagatgttaatgtacataa
atagagtgtaaataaaatagtgttgatgtactgaaatatgaactgtatca
aaagtattggtaattgtatatggggtgtacctgtttatctgttaactatt
atccaaacaaattaaatactgtggttgcctctatgtgctgtttttcctca
tacaagtaaacacagaaagtc
>NM_153285 1
cttcctccaggagtctctggtgcagctggggtggaatctggccaggccct
gcttaggcccccatcctggggtcaggaaatttggaggataaggcccttca
gccccaagtgctggagggcacagggaagtcacagatgtatgtgcaacacc
gtgtggccgaggcattccgtgtggctgtggctgctggtgaccccaatctg
ccggtgctgccctatgtccagatcttctatgacacgacaaaccactttct
gcccctggatgagctggagcacagcctgggggagagtgcggcccaggggg
cagctggagtggtgctctgggtgagctgggaaaatacaagaaccaaggaa
tcatgtcaggccatcaaggagtatatggacactacactggggcccttcat
cctgaacgtgaccagtggggcccttctctgcagtcaagccctgtgctccg
gccatggccgctgtgtccgccgcaccagccaccccaaagccctcctcctc

cttaaccctgccagtttctccatccagctcacgcctggtggtgggcccct
gagcctgcggggtgccctctcacttgaagatcaggcacagatggctgtgg
agttcaaatgtcgatgctaccctggctggcaggcaccgtggtgtgagcgg
aagagcatgtggtgattggccacacactgagttgcacatattgagaacct
aatgcactctgggtctggccagggcttcctcaaatacatgcacagtcata
caagtcatggtcacagtaaagagtacactcagccactgtcacaggcatat
tccctgcacacacatgcatacttacagactggaatagtggcataaggagt
tagaaccacagcagacaccattcattccatgtccatatgcatctacttgg
caaggtcatagacaattcctccagagacactgagccagtctttgaactgc
agcaatcacaaaggctgacattcactgagtgcctactctttgccaatccc
cgtgctaagcgttttatgtggacttattcattcctcacaatgaggctatg
aggaaactgagtcactcacattgagagtaagcacgttgcccaaggttgca
cagcaagaaaagggagaagttgagattcaaacccaggctgtctagctccg
gggtacagcccttgcactcctactgagtttgtggtaaccagccctgcac
gacccctgaatctgctgagaggcaccagtccagcaaataaagcagtcatg
atttacttaaaaaaaaaaaaaaaaaaaaaaaaaaaaaaaaaaaaaaaaaa
>NM_153283 1
cttcctccaggagtctctggtgcagctggggtggaatctggccaggccct
gcttaggcccccatcctggggtcaggaaatttggaggataaggcccttca
gccccaaggacatcctggctgccatacctgctcctgacttctcagggctg
gcagtcatcgactgggaggcatggcgcccacgctgggccttcaactggga
caccaaggacatttaccggcagcgctcacgggcactggtacaggcacagc
accctgattggccagctcctcaggtggaggcagtagcccaggaccagttc
cagggagctgcacgggcctggatggcaggcaccctccagctggggcgggc
actgcgtcctcgcggcctctggggcttctatggcttccctgactgctaca
actatgactttctaagccccaactacaccggccagtgcccatcaggcatc
cgtgcccaaaatgaccagctagggtggctgtggggccagagccgtgccct
ctatcccagcatctacatgcccgcagtgctggagggcacagggaagtcac
agatgtatgtgcaacaccgtgtggccgaggcattccgtgtggctgtggct
gctggtgaccccaatctgccggtgctgccctatgtccagatcttctatga
cacgacaaaccactttctgcccctggatgagctggagcacagcctggggg
agagtgcggcccagggggcagctggagtggtgctctgggtgagctgggaa
aatacaagaaccaaggaatcatgtcaggccatcaaggagtatatggacac
tacactggggcccttcatcctgaacgtgaccagtggggcccttctctgca
gtcaagccctgtgctccggccatggccgctgtgtccgccgcaccagccac
cccaaagccctcctcctccttaaccctgccagtttctccatccagctcac
gcctggtggtgggcccctgagcctgcggggtgccctctcacttgaagatc
aggcacagatggctgtggagttcaaatgtcgatgctaccctggctggcag
gcaccgtggtgtgagcggaagagcatgtggtgattggccacacactgagt
tgcacatattgagaacctaatgcactctgggtctggccagggcttcctca
aatacatgcacagtcatacaagtcatggtcacagtaaagagtacactcag
ccactgtcacaggcatattccctgcacacacatgcatacttacagactgg
aatagtggcataaggagttagaaccacagcagacaccattcattccatgt
ccatatgcatctacttggcaaggtcatagacaattcctccagagacactg
agccagtctttgaactgcagcaatcacaaaggctgacattcactgagtgc
ctactctttgccaatccccgtgctaagcgttttatgtggacttattcatt
cctcacaatgaggctatgaggaaactgagtcactcacattgagagtaagc
acgttgcccaaggttgcacagcaagaaaagggagaagttgagattcaaac
ccaggctgtctagctccggggtacagcccttgcactcctactgagtttg

tggtaaccagccctgcacgacccctgaatctgctgagaggcaccagtcca
gcaaataaagcagtcatgatttacttaaaaaaaaaaaaaaaaaaaaaaa
aaaaaaaaaaaaaaaaa
>NM_153281 1
agatgtggggagcgcctctgccctgtcgccccgtccgggatgtgaggagc
gtctctccctggccgccccgtctgagaagtgaggagcccctccgcccagc
agccgccctgtctgagaagctagtcttgaattcctgacctcaggtgatcc
tcccgcgttggcctcccaaagtgctgggattacaggcatgagccaccact
cctggccccagaaagtgacattctttacttaccacaggaggaaaagatcc
acgaaagtaaagtgcctagggcccatcaatgtcagatgcagccacgtgga
caaagaacactccctgagcacaagacacgaaagaacccggtctccagtgt
cacaaacaggcctgcccagcagggtctgcccctgcccacctcctccagcc
taggttgtcctcgaccagtcccgtgccatggcagcccacctgcttcccat
ctgcgccctcttcctgaccttactcgatatggcccaaggctttaggggcc
ccttgctacccaaccggcccttcaccaccgtctggaatgcaaacacccag
tggtgcctggagaggcacggtgtggacgtggatgtcagtgtcttcgatgt
ggtagccaacccagggcagaccttccgcggccctgacatgacaattttct
atagctcccagctgggcacctacccctactacacgcccactggggagcct
gtgtttggtggtctgccccagaatgccagcctgattgcccacctggcccg
cacattccaggacatcctggctgccatacctgctcctgacttctcagggc
tggcagtcatcgactgggaggcatggcgcccacgctgggccttcaactgg
gacaccaaggacatttaccggcagcgctcacgggcactggtacaggcaca
gcaccctgattggccagctcctcaggtggaggcagtagcccaggaccagt
tccagggagctgcacgggcctggatggcaggcaccctccagctggggcgg
gcactgcgtcctcgcggcctctggggcttctatggcttccctgactgcta
caactatgactttctaagccccaactacaccggccagtgcccatcaggca
tccgtgcccaaaatgaccagctagggtggctgtggggccagagccgtgcc
ctctatcccagcatctacatgcccgcagtgctggagggcacagggaagtc
acagatgtatgtgcaacaccgtgtggccgaggcattccgtgtggctgtgg
ctgctggtgaccccaatctgccggtgctgccctatgtccagatcttctat
gacacgacaaaccactttctgcccctggatgagctggagcacagcctggg
ggagagtgcggcccagggggcagctggagtggtgctctgggtgagctggg
aaaatacaagaaccaaggaatcatgtcaggccatcaaggagtatatggac
actacactggggcccttcatcctgaacgtgaccagtggggcccttctctg
cagtcaagccctgtgctccggccatggccgctgtgtccgccgcaccagcc
accccaaagccctcctcctccttaaccctgccagtttctccatccagctc
acgcctggtggtgggcccctgagcctgcggggtgccctctcacttgaaga
tcaggcacagatggctgtggagttcaaatgtcgatgctaccctggctggc
aggcaccgtggtgtgagcggaagagcatgtggtgattggccacacactga
gttgcacatattgagaacctaatgcactctgggtctggccagggcttcct
caaatacatgcacagtcatacaagtcatggtcacagtaaagagtacactc
agccactgtcacaggcatattccctgcacacacatgcatacttacagact
ggaatagtggcataaggagttagaaccacagcagacaccattcattccat
gtccatatgcatctacttggcaaggtcatagacaattcctccagagacac
tgagccagtctttgaactgcagcaatcacaaaggctgacattcactgagt
gcctactctttgccaatccccgtgctaagcgttttatgtggacttattca
ttcctcacaatgaggctatgaggaaactgagtcactcacattgagagtaa
gcacgttgcccaaggttgcacagcaagaaaagggagaagttgagattcaa
acccaggctgtctagctccgggggtacagcccttgcactcctactgagtt

tgtggtaaccagccctgcacgacccctgaatctgctgagaggcaccagtc
cagcaaataaagcagtcatgatttacttaaaaaaaaaaaaaaaaaaaaa
aaaaaaaaaaaaaaaaaaaa
>NM_014236 3
cgcgactgcgcgcatgcgctgcagggccctgcgcggcttccgtcctggct
gagatggcggcgcccgggatcctgtgtagcggctgcagagggtgccgccg
ccctaggcgaagtagggccgtcctgagcgaaagaaccgcccccagcagga
gcaccaccacggcttagcaaagaatcccagaccccgcccgggaaggcagc
cgcaccatggagtcttccagttcatctaactcttatttctccgttggccc
aaccagtcccagcgctgtcgtgctcctctactcgaaggagctcaaaaagt
gggatgagtttgaagatattttagaagagaggaggcatgtcagtgacttg
aaatttgcaatgaaatgctacacacctcttgtctataagggaattactcc
atgtaaaccaattgatattaaatgtagtgttctcaattctgaggagattc
attatgtcattaaacagctttccaaggaatcccttcaatctgtggatgtc
ctccgagaggaagtgagtgagatcttagatgaaatgagtcacaaactgcg
tcttggagccattcggttttgtgccttcaccctgagcaaagtatttaaac
aaattttctcgaaggtgtgtgtaaatgaagaaggtattcagaaactacaa
agagccatccaggagcatcctgttgttctgctgcctagtcatcgaagtta
cattgacttcctcatgttgtcttttcttctatacaattatgatttgcctg
tgccagttatagcagcaggaatggacttcctgggaatgaaaatggttggt
gagctgctacgaatgtcgggtgccttttttcatgcggcgtacctttggtgg
caataaactctactgggctgtattctctgaatatgtaaaaactatgttac
ggaatggttatgctcctgttgaatttttcctcgaagggacaagaagccgc
tctgccaagacattgactcctaaatttggtcttctgaatattgtgatgga
gccatttttaaaagagaagtttttgatacctaccttgtcccaattagta
tcagttatgataagatcttggaagaaactctttatgtgtatgagcttcta
ggggttcctaaaccaaaagagtctacaactgggttgctgaaagccagaaa
gattctctctgaaaattttggaagcatccatgtgtactttggagatcctg
tgtcacttcgatctttggcagctgggaggatgagtcggagctcatataac
ttggttccaagatacattcctcagaaacagtctgaggacatgcatgcctt
tgtcactgaagttgcctacaaaatggagcttctgcaaattgaaaacatgg
ttttgagcccctggaccctaatagttgctgttctgcttcagaaccggcca
tccatggactttgatgctctggtggaaaagactttatggctaaaaggctt
aacccaggcatttggagggtttctcatttggcctgataataaacctgctg
aagaagttgtcccggccagcattcttctgcattccaacattgccagcctt
gtcaaagaccaggtgattctgaaagtggactccggagactcggaagtggt
cgatgggcttatgctccagcacatcactctcctcatgtgctcagcttata
ggaaccagctgctcaacatttttgtgcgcccatccttagtagcagtagca
ttgcagatgacaccagggttcaggaaagaggatgtctacagttgctttcg
cttcctacgtgatgtttttgcagatgagttcatcttccttccaggaaaca
cactaaaggactttgaagaaggctgttacctgctttgtaaaagtgaagcc
atacaagtgactacgaaagacatcctagttacagagaaaggaaatactgt
gttagaatttttagtaggactctttaaaccttttgtggaaagctatcaga
taatttgcaagtaccttttgagtgaagaagaggaccacttcagtgaggaa
cagtacttggctgcagtcagaaaattcacaagtcagcttctcgatcaagg
tacctctcaatgttatgatgtattatcttctgatgtgcagaaaaacgcct
tagcagcctgtgtgaggctcggagtagtggagaagaagaagataaataat
aactgtatatttaatgtgaatgaacctgccacaaccaaattagaagaaat
gcttggttgtaagacaccaataggaaaaccagccactgcaaaactttaat

aatcaacaaatagttatggaaaattcggtcacgtaattactctcatcgaa
ggactcattacaacaaacagggaagtaaaggaagagacacatcctctcat
actccctgagactctgagaacagtggacgcagagggaagagatgatcatt
ggaagcaatcagtttactcttccccaccacagtggttaaaaggcgtttgt
atctgacactatgtgtgtgttttaaaataaacttttggaaacatgtttgg
aaaagcaaagctcagctcatttcactaacacttttcagcttactatatgt
attaaacttttatgttgacttttgaattaaagtatgacaacactgaaagc
tctggatattaaaagaaaatgaaaagggcatatctacgttacttgtagct
tgctttaattaaagttgcctcaaacaagtaaaaaaaa
>NM_003041 3
ggggcagatcctggggagaatggaggagcacacagaggcaggctcggcac
cagagatgggggcccagaaggccctgattgacaatcctgctgacatccta
gtcattgctgcatatttcctgctggtcattggcgttggcttgtggtccat
gtgcagaaccaacagaggcactgtgggcggctacttcctggcaggacgca
gcatggtgtggtggccggttggggcctctctcttcgccagcaacatcggc
agtggccactttgtgggcctggcagggactggcgctgcaagtggcttggc
tgttgctggattcgagtggaatgcgctcttcgtggtgctgctactgggct
ggctgtttgcacccgtgtacctgacagcgggggtcatcacgatgccacag
tacctgcgcaagcgcttcggcggccgccgcatccgcctctacctgtctgt
gctctcccttttcctgtacatcttcaccaagatctcagtggacatgttct
ccggagctgtattcatccagcaggctctgggctggaacatctatgcctcc
gtcatcgcgcttctgggcatcaccatgatttacacggtgacaggagggct
ggccgcgctgatgtacacggacacggtacagaccttcgtcattctggggg
gcgcctgcatcctcatgggttacgccttccacgaggtgggcgggtattcg
ggtctcttcgacaaatacctgggagcagcgacttcgctgacggtgtccga
ggatccagccgtgggaaacatctccagcttctgctatcgaccccggcccg
actcctaccacctgctccggcaccccgtgaccggggatctgccgtggccc
gcgctgctcctcggactcacaatcgtctcgggctggtactggtgcagcga
ccaggtcatcgtgcagcgctgcctggccgggaagagcctgacccacatca
aggcgggctgcatcctgtgtgggtacctgaagctgacgcccatgtttctc
atggtcatgccaggcatgatcagccgcattctgtacccagacgaggtggc
gtgcgtggtgcctgaggtgtgcaggcgcgtgtgcggcacggaggtgggct
gctccaacatcgcctacccgcggctcgtcgtgaagctcatgcccaacggt
ctgcgcggactcatgctggcggtcatgctggccgcgctcatgtcctcgct
ggcctccatcttcaacagcagcagcacgctcttcaccatggacatctaca
cgcgcctgcggccacgcgccggcgaccgcgagctgctgctggtgggacgg
ctctgggtggtgttcatcgtggtagtgtcggtggcctggcttcccgtggt
gcaggcggcacagggcgggcagctcttcgattacatccaggcagtctcta
gctacctggcaccgcccgtgtccgccgtcttcgtgctggcgctcttcgtg
ccgcgcgttaatgagcagggcgccttctggggactcatcgggggcctgct
gatgggcctggcacgcctgattcccgagttctccttcggctcgggcagct
gtgtgcagccctcggcgtgcccagctttcctctgcggcgtgcactacctc
tacttcgccattgtgctgttcttctgctctggcctcctcaccctcacggt
ctccctgtgcaccgcgcccatccccagaaagcacctccaccgcctggtct
tcagtctccggcatagcaaggaggaacgggaggacctggatgctgatgag
cagcaaggctcctcactccctgtacagaatgggtgcccagagagtgccat
ggagatgaatgagccccaggccccggcaccaagcctcttccgccagtgcc
tgctctggttttgtggaatgagcagaggtggggtgggcagtcctccgccc
cttacccaggaggaggcagcggcagcagccaggcggctggaggacatcag

cgaggacccgagctgggcccgtgtggtcaacctcaatgccctgctcatga
tggcagtggccgtgttcctctggggcttctatgcctaagaccaactgcgt
tggacaccataagccacagcctcacaggaagtgggggtgaggagcctgcg
gtgctccccagaaaaggggaaggggcagtggggtgagaaggtcctggctc
cccttctcccggccttcctctgcctggggcccactgcatctgattggcag
tcacttcccatgagggcctggcccacccgctgcagttgccctaaggaaaa
ataaagctgcctttcccctgtccaaaaaa
>NM_182710 2
gcacccggtgggcgtcacgtgacggactcagtagaccgccactggctgtg
cacgttatggggtttccacctagggctcggcctgaggcttgtaacactcc
gttttcccccgagtcacaggggcagtcttgcccctcgcagctgggtcgcg
gtgtctctcaaaggtccccctctacaggggcttcgtgaggcccgggccca
cagggcgctcggtcccggaagtgacgtctcccagaggggccggaagtggc
agtggagggagggaagatggcggaggtggtgagtccggtgcccggggcgg
ggcggagggagccaggggaggtgggtagagcccgaggccccccagtagcc
gaccctggcgtcgcgctgtctccccagggggagataatcgagggctgccg
cctacccgtgctgcggcggaaccaggacaacgaagatgagtggcccctgg
ccgagatcctgagcgtgaaggacatcagtggccggaagcttttctacgtc
cattacattgacttcaacaaacgtctggatgaatgggtgacgcatgagcg
gctggacctaaagaagatccagttccccaagaaagaggccaagacccccca
ctaagaacggacttcctgggtcccgtcctggctctccagagagagaggtg
ccggcctcggcgcaggccagcgggaagaccttgccaatcccggtccagat
cacactccgcttcaacctgcccaaggagcgggaggccattcccggtggcg
agcctgaccagccgctctcctccagctcctgcctgcagcccaaccaccgc
tcaacgaaacggaaggtggaggtggtttcaccagcaactccagtgcccag
cgagacagccccggcctcggtttttccccagaatggagccgcccgtaggg
cagtggcagcccagccaggacggaagcgaaaatcgaattgtttgggcact
gatgaggactcccaggacagctctgatggaataccgtcagcaccacgcat
gactggcagcctggtgtctgatcgaagccacgacgacatcgtcacccgga
tgaagaacattgagtgcattgagctgggccggcaccgcctcaagccgtgg
tacttctccccgtacccacaggaactcaccacattgcctgtcctctacct
gtgcgagttctgcctcaagtacggccgtagtctcaagtgtcttcagcgtc
atttgaccaagtgtgacctacgacatcctccaggcaatgagatttaccgc
aagggcaccatctccttctttgagattgatggacgtaagaacaagagtta
ttcccagaacctgtgtcttttggccaagtgtttccttgaccataagacac
tgtactatgacacagaccctttcctcttctacgtcatgacagagtatgac
tgtaagggcttccacatcgtgggctacttctccaaggagaaagaatcaac
ggaagactacaatgtggcctgcatcctaaccctgcctccctaccagcgcc
ggggctacggcaagctgctgatcgagttcagctatgaactctccaaagtg
gaagggaaaacagggacccctgagaagcccctctcagaccttggcctcct
atcctatcgaagctactggtcccagaccatcctggagatcctgatggggc
tgaagtcggagagcggggagaggccacagatcaccatcaatgagattagt
gaaatcaccagcatcaagaaggaggatgtcatctccactctgcagtacct
caatctcatcaactactacaagggccagtacatcctcacactgtcagagg
acatcgtggatggccatgagcgggccatgctcaagcggctcctgcggatc
gactccaagtgtctgcacttcactcccaaggactggagcaagagggggaa
gtggtgaccagacactgcccactgcagtgccaagacggcagcaggactgg
ggctgatagcccaccccgcccccactgcagctcccacaaagcactctaag
ggagatggggctgaggacagctcaaaaaggagaggacaggcctggcaggg

gcccactggtgcccagcaccaaggcgagctccgggctcagaccaactcca
aggtcagctggccacaggcccaggcctcctctgaagcagggaccagaggg
agccaggcagctgtgtacagtgagaagggatccggatgggggagctctgt
acagagggctggtgattgtaaaaatttcttttgtaaagtagaagttgggg
gtggggtgggtgctggctgcaaaaatttctggcttctcttacccctattg
cccccggcaataaattgtttctatatgccagaaaaaaaaaaaaaaaa
>NM_015971 3
ctgagaggagtgagtgccgtcaccgagggccgcgccagactgcgacggat
acagggagggcaagggtttccttttggcgcttccctttggaccccggagt
gaaaaactctaacgtccagatcagtggagagaaacgcagatttaggaccc
tgaggagtctttttcacccgtttcccgtcactcgctcaggcgcgccgagg
gcagtccttgtggggtcctcgtggccagccaagatggctgcccccgcagt
gaaggttgcccgaggatggtcgggcctggcgttgggcgtgcggcgggctg
tcttgcagcttccagggctaactcaggtgagatggagccgctatagtcct
gaattcaaggatcccttgattgacaaggaatattatcgcaagccagtgga
ggagctaactgaggaggagaaatatgttcgggagctcaagaagactcagc
tcatcaaagctgctccagcagggaaaacaagttctgtgtttgaagaccca
gtcatcagtaaattcaccaacatgatgatgataggaggaaacaaagtact
ggccagatccctcatgattcagactctggaagctgtgaaaaggaagcagt
ttgagaagtaccatgccgcttctgcagaggaacaggcaaccatcgaacgc
aacccctacaccatcttccatcaagcactgaaaaactgtgagcctatgat
tgggctggtacccatcctcaagggaggccgtttctaccaggtccctgtac
ccctacccgaccggcgtcgccgcttcctagccatgaagtggatgatcact
gagtgccgggataaaaagcaccagcggacactgatgccggagaagctgtc
acacaagctgctggaggctttccataaccagggccccgtgatcaagagga
agcatgacttgcacaagatggcagaggccaaccgtgccctggcccactac
cgctggtggtagagtctccaggaggagcccagggccctctgccgcaagaa
acagtgtgagctactgccacgctgaaaactacctgtgggttaaggatgta
gttcctttgtaagggtgggcaggcctcgtaagaaagatgtagcagcatat
tcactatccgttaatccttctttctttgaggctggaacttgctctctctg
cccctatttccttgtaaagagggagcacattgacttgggaatttcctcca
ggaaactcagggctgttttctcttcccttaggttggggcggacctttgga
tatataaaggaagcagttttagtatcagaaaagatttattagaaaattct
cacgctgaactggtgtagcatgtggtgcagcattcagtgaaactggctgg
aggaaataggcttgtttccagagttgtccttatacaaaatgtataaaaag
cagtttctggtgtgaaaaaaaaaaaaaaaaaaa
>NM_000035 3
atctgtcttatttggcagctgctgcctcacccacagcttttgatatctag
gaggactcttctctcccaaactacctgtcaccatggcccaccgatttcca
gccctcacccaggagcagaagaaggagctctcagaaattgcccagagcat
tgttgccaatggaaaggggatcctggctgcagatgaatctgtaggtacca
tggggaaccgcctgcagaggatcaaggtggaaaacactgaagagaaccgc
cggcagttccgagaaatcctcttctctgtggacagttccatcaaccagag
catcgggggtgtgatccttttccacgagaccctctaccagaaggacagcc
agggaaagctgttcagaaacatcctcaaggaaaaggggatcgtggtggga
atcaagttagaccaaggaggtgctcctcttgcaggaacaaacaaagaaac
caccattcaagggcttgatggcctctcagagcgctgtgctcagtacaaga
aagatggtgttgactttgggaagtggcgtgctgtgctgaggattgccgac
>NM_004790 4

aactcattccctccaggccaaggattaaaaactgcccatgcaagggtcag
gtctccagcagaccctgaaagctgagctgccctgacccccaaagtgagga
gaagctgcaagggaaaagggagggacagatcagggagaccggggaagaag
gaggagcagccaaggaggctgctgtcccccacagagcagctcggactca
gctcccggagcaacccagctgcggaggcaacggcagtgctgctcctccag
cgaaggacagcaggcaggcagacagacagaggtcctgggactggaaggcc
tcagcccccagccactgggctgggcctggcccaatggcctttaatgacct
cctgcagcaggtggggggtgtcggccgcttccagcagatccaggtcaccc
tggtggtcctccccctgctcctgatggcttctcacaacaccctgcagaac
ttcactgctgccatccctacccaccactgccgcccgcctgccgatgccaa
cctcagcaagaacgggggctggaggtctggctgccccgggacaggcagg
ggcagcctgagtcctgcctccgcttcacctccccgcagtggggactgccc
tttctcaatggcacagaagccaatggcacaggggccacagagccctgcac
cgatggctggatctatgacaacagcaccttcccatctaccatcgtgactg
agtgggaccttgtgtgctctcacagggccctacgccagctggcccagtcc
ttgtacatggtgggggtgctgctcggagccatggtgttcggctaccttgc
agacaggctaggccgccggaaggtactcatcttgaactacctgcagacag
ctgtgtcagggacctgcgcagccttcgcacccaacttccccatctactgc
gccttccggctcctctcgggcatggctctggctggcatctccctcaactg
catgacactgaatgtggagtggatgcccattcacacacgggcctgcgtgg
gcaccttgattggctatgtctacagcctgggccagttcctcctggctggt
gtggcctacgctgtgccccactggcgccacctgcagctactggtctctgc
gcctttttttgccttcttcatctactcctggttcttcattgagtcggccc
gctggcactcctcctccgggaggctggacctcaccctgagggccctgcag
agagtcgcccggatcaatgggaagcgggaagaaggagccaaattgagtat
ggaggtactccgggccagtctgcagaaggagctgaccatgggcaaaggcc
aggcatcggccatggagctgctgcgctgccccaccctccgccacctcttc
ctctgcctctccatgctgtggtttgccactagctttgcatactatgggct
ggtcatggacctgcagggctttggagtcagcatctacctaatccaggtga
tctttggtgctgtggacctgcctgccaagcttgtgggcttccttgtcatc
aactccctgggtcgccggcctgcccagatggctgcactgctgctggcagg
catctgcatcctgctcaatggggtgataccccaggaccagtccattgtcc
gaacctctcttgctgtgctggggaagggttgtctggctgcctccttcaac
tgcatcttcctgtatactggggaactgtatcccacaatgatccggcagac
aggcatgggaatgggcagcaccatggcccgagtgggcagcatcgtgagcc
cactggtgagcatgactgccgagctctaccccctccatgcctctcttcatc
tacggtgctgttcctgtggccgccagcgctgtcactgtcctcctgccaga
gaccctgggccagccactgccagacacggtgcaggacctggagagcaggt
gggcccccactcagaaagaagcagggatatatcccaggaaagggaaacag
acgcgacagcaacaagagcaccagaagtatatggtcccactgcaggcctc
agcacaagagaagaatggactctgaggactgagaaggggccttacagaac
cctaaagggagggaaggtcctacaggtctccggccacccacacaaggagg
aggaagaggaaatggtgacccaagtgtgggggttgtggttcaggaaagca
tcttcccaggggtccacctccctttataaaccccaccagaaccacatcat
taaaaggtttgactgcgcaccaaaaaaaaaaaaaaaaaaaaaaaaaaaa
cagtgtccatccagcctcgctatccaggaaaacgccaacgccctggctcg
ctacgccagcatctgtcagcagaatggactggtacctattgttgaaccag
aggtaattcctgatggagaccatgacctggaacactgccagtatgttact
gagaaggtcctggctgctgtctacaaggccctgaatgaccatcatgttta

cctggagggcaccctgctaaagcccaacatggtgactgctggacatgcct
gcaccaagaagtatactccagaacaagtagctatggccaccgtaacagct
ctccaccgtactgttcctgcagctgttcctggcatctgctttttgtctgg
tggcatgagtgaagaggatgccactctcaacctcaatgctatcaaccttt
gccctctaccaaagccctggaaactaagtttctcttatggacgggccctg
caggccagtgcactggctgcctggggtggcaaggctgcaaacaaggaggc
aacccaggaggcttttatgaagcgggccatggctaactgccaggcggcca
aaggacagtatgttcacacgggttcttctggggctgcttccacccagtcg
ctcttcacagcctgctatacctactagggtccaatgcccgccagcctagc
tccagtgcttctagtaggagggctgaaagggagcaacttttcctccaatc
ctggaaattcgacacaattagatttgaactgctggaaatacaacacatgt
taaatcttaagtacaaggggggaaaaaataaatcagttattgaaacataa
aatgaataccaaggacctgatcaaatttcacacagcagtttccttgcaac
actttcagctccccatgctccagaatacccacccaagaaaataataggct
ttaaaacaatatcggctcctcatccaaagaacaactgctgattgaaacac
ctcattagctgagtgtagagaagtgcatcttatgaaacagtcttagcagt
ggtaggttgggaaggagatagctgcaaccaaaaaagaaataaatattcta
taaaccttcagctgctatcgggtttcacttttctgctcttgctgtccaaa
gactcagtgtatttcattacttttgactctactagacatgactgggtttc
aacagtaaaggtcttcaactcttgctagtcattggaatcaagccgcaaaa
ttttaaaaactgagatgctcaggccacaccccagctcaattaaatcagaa
accctagacttgggatcctctaactattagatttcttaaagctccctcag
taattccaatgtacagtcaagtttgagaactaccaatctaaatttcaagt
ttgagggtatttgaaaattaaagccattcacaatacgaagccagctaaaa
atgtagaatgattttgagcaacttgtggagtataataagagaattaatgt
gacttcaatgcttggagcattcttgttcaagtggcccaggtttggtgaaa
caggactaccttgtcatctgcacgtccaggcatatttcgtagttttgcag
taaataatattcacataatgatactgtattgactttcaattttcagaatt
aacctatagttacagcacttaagacaaccagagttataaaagagaattta
aatattataactttggacaatataaaagtgatgatttaactgacagaagc
taggaaatataagggggaggagaagtggaagaaagcaaagagagtcagga
atactacttaaaactgatgggttaagaaatagtgctttaattctatttaa
gtaataaaagaaatggatgtaaatcataaaaatatatatctaaaattaaa
atattgatggtagtatgctaaatttc

>NM_005605 4
aaggcggaagggtggggagggcggcgctcggggcgggaggcccggccggg
tccgctaggacagcggggccgctgggaagttgtgagagcggcgctcgggg
gcgcgcttgcgtgcacgagggcccgggccgcgagcagccgcggccgtccc
ggtcgccacccttagcagcggtcgcggtcggtgccgaagcggtgttcccc
gccttagccgctggcgcctcccaagagagcggccggtgggccctcgtcct
gtcagtggcgtcggaggccggcgctgcggtggccgcgcccttctggtgct
cggacaccgctgaggagccggggccgggcacggctggctgacggctccgg
gcagctaaggctgcccgaggagaaggcggcggccgcggcgtaggcgcacg
tccggcgggctcctggagcctggaggaggccgaggggaccatgtccggga
ggcgcttccacctctccaccaccgaccgcgtcatcaaagctgtcccctttt
cctccaacccaacggcttactttcaaggaagtatttgagaatgggaaacc
taaagttgatgttttaaaaaaccatttggtaaaggaaggacgactggaag

aggaagtagccttaaagataatcaatgatggggctgccatcctgaggcaa
gagaagactatgatagaagtagatgctccaatcacagtatgtggtgatat
tcatggacaattctttgacctaatgaagttatttgaagttggaggatcac
ctagtaacacacgctacctctttctgggtgactatgtggacagaggctat
ttcagtatagagtgtgtgctgtatttatggagtttaaagattaatcatcc
caaaacattgtttctgcttcggggaaatcatgaatgcaggcatcttacag
actatttcaccttcaaacaggaatgtcgaatcaaatattcggaacaggtg
tatgatgcctgtatggagacatttgactgtcttcctcttgctgccctctt
aaaccagcagtttctctgtgtacatggaggaatgtcacctgaaattactt
ctttagatgacattaggaaattagacaggtttacggaacctcccgccttt
ggacctgtgtgtgacctgctttggtctgatccctcagaggattatggcaa
tgagaagaccttggagcactatacccacaacactgtccgagggtgctctt
atttctacagttaccctgcagtttgtgaatttttgcagaacaataattta
ctatcaattatcagagcccatgaagcccaagatgctgggtatcgaatgta
caggaagagccaagccacaggctttccatcacttattacaattttctctg
cccccaattacctagatgtctataacaataaagctgctgtgttgaaatat
gaaaacaatgtcatgaatatcaggcagtttaactgttctccacacccccta
ctggcttccaaactttatggatgttttcacatggtctttgccttttgttg
gggaaaaagtcacagagatgctggtaaatgtgctcaacatatgctctgat
gacgaactgatttctgatgatgaagcagaaggaagcactacagttcgtaa
ggagatcatcaggaataagatcagagccattgggaagatggcacgggtct
tttcaattcttcggcaagaaagtgagagtgtgctgactctcaagggcctg
actcccacaggcacactccctctgggcgtcctctcaggaggcaagcagac
tatcgagacagccacagtagaagcggtagaggcccgggaagccatcagag
ggttctcgcttcagcacaagatccggagttttgaagaagcgcgaggtctg
gaccgaattaatgagcgaatgccaccccgaaaggatagcatacacgctgg
tgggccaatgaaatctgtaacctcagcacactcacatgctgcgcacagga
gcgaccaagggaagaaagcccattcatgacttagagtcctgccgtggctc
aggtggatctaaaactcaagaacaaattctatttatttattattggaaaa
tgaaaagcaactcaaaacaacttcaacgtggaggtgcatttataattcag
tctgcatttattctgtaaaaaggtggctgttttataaattcttttaattt
atgttcaatatatataaaaagtgcatctgttttgtttttccctttttct
ccataattttaagaaatgaatctgattgttgtcaacacatttgtgaagtc
ttgtgctataaaggggaacttcccctaataaaagggccttggaaacctca
aacctgggtttctgacttgaaaaaaaaaaaaaaa
>NM_153766 1
gttgcatacagatgagttggcagccggtctgagctggcccacagactcat
aaaatcaacagggcctcgggtaccctcacctagcatatccaaactcttgc
atcaaaggtgcagggacttgctcacatcgagaatctggttgctttcttgg
agaccaagaaaatgagtttttgtttctacatttactccagcaatccatga
ggtttcttctctactgtgccttcatccccatcctgggccctgacaaagac
agtgtgacaagttctgagccccacaggtggaatcccaaaccaagcagctc
ttgctggtcaccccaagatctacttatacatgaagttttgaaaatcatct
cctgaacctcttctgacagagatcagggtgttgacagaaagtatgttcaa
acatcttcggaaatgggtcgtcactcgctttttttgggcattctcggcaaa
gagcaaggctagtctccaaagatggaaggtgcaacatagaatttggcaat
gtggaggcacagtcaaggtttatattctttgtggacatctggacaacggt
acttgacctcaagtggagatacaaaatgaccattttcatcacagccttct
tggggagttggtttttctttggtctcctgtggtatgcagtagcgtacatt

cacaaagacctcccggaattccatccttctgccaatcacactccctgtgt
ggagaatattaatggcttgacctcagcttttctgttttctctggagactc
aagtgaccattggatatggattcaggtgtgtgacagaacagtgtgccact
gccatttttctgcttatctttcagtctatacttggagttataatcaattc
tttcatgtgtggggccatcttagccaagatctccaggcccaaaaaacgtg
ccaagaccattacgttcagcaagaacgcagtgatcagcaaacggggaggg
aagctttgcctcctaatccgagtggctaatctcaggaagagccttcttat
tggcagtcacatttatggaaagcttctgaagaccacagtcactcctgaag
gagagaccattattttggaccagatcaatatcaactttgtagttgacgct
gggaatgaaaatttattcttcatctccccattgacaatttaccatgtcat
tgatcacaacagccctttcttccacatggcagcggagacccttctccagc
aggactttgaattagtggtgtttttagatggcacagtggagtccaccagt
gctacctgccaagtccggacatcctatgtcccagaggaggtgctttgggg
ctaccgttttgctcccatagtatccaagacaaaggaagggaaataccgag
tggatttccataactttagcaagacagtggaagtggagacccctcactgt
gccatgtgcctttataatgagaaagatgttagagccaggatgaagagagg
ctatgacaaccccaacttcatcttgtcagaagtcaatgaaacagatgaca
ccaaaatgtaacagtggcttttcaacgggagtaaagcaaagtctctaaag
ctcctagtacctagaagcattatgaagcagtcaacaatttaggggtacga
aagtaggatgagagccttcaaagtctaccagcacaaagacccctgagccc
cgcaattgtgatcccacaagacatgcatctccacaaggctactgtattag
aacgtgcaatgcatttatatgaaactggtgtatggaagacataggtgctc
tcttgaaatcttaaatatgattatttgagctcatataaggtggattggag
cagataaaattatcaaaagtttcatgaacaggccaaacaaaatatttttt
aaagtttccttaaagaagttatgaactttagaaaggatcaggggacaata
ataatctcattttgattctactgataagaatgactccacttttaatgtgg
acttttactcatggaaaaattgtctcctaatttggggagatgaaccaacc
aatcaatgacaagaaaacgcttacacaaagaacaatttgaggctctaagc
ttctcatgtggtacgtttagacagaggctaaatctgcacactagaatctt
gatgataccttcctgcaagacagaatgctttagttaaaagtggtgatgat
atttctttcaatctgtattggatggcttaaagggctataaatctgtttat
aaagagcatttcctgctcttcgaagacagcaatgaggagttggaaggtgc
aaagtcagtagagaagggaatgtatcattaatgcacctgagaagaaacag
tttcatgtgttcctccacctagagtttgtactggaatgctatttctaaag
aagaagtgggaaagagagaggaatgggatggagccccacagtcagaatgt
tactatgtctttctttccctgacagcccatcttcctaaaagggaccagct
tatggaaggctcgaccttgaggggaaagttttactgtgaaagtcttcttc
agatccccacctgcatcattccgaatgtgtcctggaaaaaaactggtact
caaagctgcttaggaatcaaaatgttttcagtgtgttgattaatatagta
aatttctgaaactgtg
>NM_000220 2
gtctgcatagaaagaccaacaaccagcaccacttccttgctttttccagc
catgaatgcttccagtcggaatgtgtttgacacgttgatcagggtgttga
cagaaagtatgttcaaacatcttcggaaatgggtcgtcactcgcttttttt
gggcattctcggcaaagagcaaggctagtctccaaagatggaaggtgcaa
catagaatttggcaatgtggaggcacagtcaaggtttatattctttgtgg
acatctggacaacggtacttgacctcaagtggagatacaaaatgaccatt
ttcatcacagccttcttggggagttggtttttctttggtctcctgtggta
tgcagtagcgtacattcacaaagacctcccggaattccatccttctgcca

atcacactccctgtgtggagaatattaatggcttgacctcagcttttctg
ttttctctggagactcaagtgaccattggatatggattcaggtgtgtgac
agaacagtgtgccactgccatttttctgcttatctttcagtctatacttg
gagttataatcaattctttcatgtgtggggccatcttagccaagatctcc
aggcccaaaaaacgtgccaagaccattacgttcagcaagaacgcagtgat
cagcaaacggggagggaagctttgcctcctaatccgagtggctaatctca
ggaagagccttcttattggcagtcacatttatggaaagcttctgaagacc
acagtcactcctgaaggagagaccattattttggaccagatcaatatcaa
ctttgtagttgacgctgggaatgaaaatttattcttcatctccccattga
caatttaccatgtcattgatcacaacagccctttcttccacatggcagcg
gagacccttctccagcaggactttgaattagtggtgtttttagatggcac
agtggagtccaccagtgctacctgccaagtccggacatcctatgtcccag
aggaggtgctttggggctaccgttttgctcccatagtatccaagacaaag
gaagggaaataccgagtggatttccataactttagcaagacagtggaagt
ggagacccctcactgtgccatgtgcctttataatgagaaagatgttagag
ccaggatgaagagaggctatgacaaccccaacttcatcttgtcagaagtc
aatgaaacagatgacaccaaaatgtaacagtggcttttcaacgggagtaa
agcaaagtctctaaagctcctagtacctagaagcattatgaagcagtcaa
caatttaggggtacgaaagtaggatgagagccttcaaagtctaccagcac
aaagacccctgagccccgcaattgtgatcccacaagacatgcatctccac
aaggctactgtattagaacgtgcaatgcatttatatgaaactggtgtatg
gaagacataggtgctctcttgaaatcttaaatatgattatttgagctcat
ataaggtggattggagcagataaaattatcaaaagtttcatgaacaggcc
aaacaaaatattttttaaagtttccttaaagaagttatgaactttagaaa
ggatcaggggacaataataatctcattttgattctactgataagaatgac
tccacttttaatgtggacttttactcatggaaaaattgtctcctaatttg
gggagatgaaccaaccaatcaatgacaagaaaacgcttacacaaagaaca
atttgaggctctaagcttctcatgtggtacgtttagacagaggctaaatc
tgcacactagaatcttgatgatacccttcctgcaagacagaatgctttagt
taaaagtggtgatgatatttctttcaatctgtattggatggcttaaaggg
ctataaatctgtttataaagagcatttcctgctcttcgaagacagcaatg
aggagttggaaggtgcaaagtcagtagagaagggaatgtatcattaatgc
acctgagaagaaacagtttcatgtgttcctccacctagagtttgtactgg
aatgctatttctaaagaagaagtgggaaagagagaggaatgggatggagc
cccacagtcagaatgttactatgtctttctttccctgacagcccatcttc
ctaaaagggaccagcttatggaaggctcgaccttgaggggaaagttttac
tgtgaaagtcttcttcagatccccacctgcatcattccgaatgtgtcctg
gaaaaaaactggtactcaaagctgcttaggaatcaaaatgttttcagtgt
gttgattaatatagtaaatttctgaaactgtg
>NM_153765 1
gttgcatacagatgagttggcagccggtctgagctggcccacagactcat
aaaatcaacagggcctcgggtaccctcacctagcatatccaaactcttgc
atcaaaggtgcagggacttgctcacatcgagaatctggttgctttcttgg
agaccaagaaaatgagtttttgtttctacatttactccagcaatccatga
ggactttataggaattttgcaccattctgaatggatacatttggatttct
caacatttgttcagcttcctaatgactgttgtgacaattgctctatacca
gtgaatgccaactgtttatctctgctctgaacagatcagggtgttgacag
aaagtatgttcaaacatcttcggaaatgggtcgtcactcgctttttggg
cattctcggcaaagagcaaggctagtctccaaagatggaaggtgcaacat

agaatttggcaatgtggaggcacagtcaaggtttatattctttgtggaca
tctggacaacggtacttgacctcaagtggagatacaaaatgaccattttc
atcacagccttcttggggagttggtttttctttggtctcctgtggtatgc
agtagcgtacattcacaaagacctcccggaattccatccttctgccaatc
acactccctgtgtggagaatattaatggcttgacctcagcttttctgttt
tctctggagactcaagtgaccattggatatggattcaggtgtgtgacaga
acagtgtgccactgccatttttctgcttatctttcagtctatacttggag
ttataatcaattctttcatgtgtggggccatcttagccaagatctccagg
cccaaaaaacgtgccaagaccattacgttcagcaagaacgcagtgatcag
caaacggggagggaagctttgcctcctaatccgagtggctaatctcagga
agagccttcttattggcagtcacatttatggaaagcttctgaagaccaca
gtcactcctgaaggagagaccattattttggaccagatcaatatcaactt
tgtagttgacgctgggaatgaaaatttattcttcatctccccattgacaa
tttaccatgtcattgatcacaacagccctttcttccacatggcagcggag
acccttctccagcaggactttgaattagtggtgtttttagatggcacagt
ggagtccaccagtgctacctgccaagtccggacatcctatgtcccagagg
aggtgctttggggctaccgttttgctcccatagtatccaagacaaaggaa
gggaaataccgagtggatttccataactttagcaagacagtggaagtgga
gacccctcactgtgccatgtgcctttataatgagaaagatgttagagcca
ggatgaagagaggctatgacaaccccaacttcatcttgtcagaagtcaat
gaaacagatgacaccaaaatgtaacagtggcttttcaacgggagtaaagc
aaagtctctaaagctcctagtacctagaagcattatgaagcagtcaacaa
tttaggggtacgaaagtaggatgagagccttcaaagtctaccagcacaaa
gacccctgagccccgcaattgtgatcccacaagacatgcatctccacaag
gctactgtattagaacgtgcaatgcatttatatgaaactggtgtatggaa
gacataggtgctctcttgaaatcttaaatatgattatttgagctcatata
aggtggattggagcagataaaattatcaaaagtttcatgaacaggccaaa
caaaatatttttaaagtttccttaaagaagttatgaactttagaaagga
tcaggggacaataataatctcattttgattctactgataagaatgactcc
acttttaatgtggacttttactcatggaaaaattgtctcctaatttgggg
agatgaaccaaccaatcaatgacaagaaaacgcttacacaaagaacaatt
tgaggctctaagcttctcatgtggtacgtttagacagaggctaaatctgc
acactagaatcttgatgataccttcctgcaagacagaatgctttagttaa
aagtggtgatgatatttctttcaatctgtattggatggcttaaagggcta
taaatctgtttataaagagcatttcctgctcttcgaagacagcaatgagg
agttggaaggtgcaaagtcagtagagaagggaatgtatcattaatgcacc
tgagaagaaacagtttcatgtgttcctccacctagagtttgtactggaat
gctatttctaaagaagaagtgggaaagagagaggaatgggatggagcccc
acagtcagaatgttactatgtctttctttccctgacagcccatcttccta
aaagggaccagcttatggaaggctcgaccttgaggggaaagttttactgt
gaaagtcttcttcagatccccacctgcatcattccgaatgtgtcctggaa
aaaaactggtactcaaagctgcttaggaatcaaaatgttttcagtgtgtt
gattaatatagtaaatttctgaaactgtg
>NM_153767 2
gttgcatacagatgagttggcagccggtctgagctggcccacagactcat
aaaatcaacagggcctcgggtaccctcacctagcatatccaaactcttgc
atcaaaggtgcagggacttgctcacatcgagaatctggttgctttcttgg
agaccaagaaaatgagtttttgtttctacatttactccagcaatccatga
ggactttataggaattttgcaccattctgaatggatacatttggatttct

caacatttgttcagcttcctaatgactgttgtgacaattgctctatacca
gtgaatgccaactgtttatctctgctctgaacaggtttcttctctactgt
gccttcatccccatcctgggccctgacaaagacagtgtgacaagttctga
gccccacaggtggaatcccaaaccaagcagctcttgctggtcaccccaag
atctacttatacatgaagttttgaaaatcatctcctgaacctcttctgac
agagatcagggtgttgacagaaagtatgttcaaacatcttcggaaatggg
tcgtcactcgctttttgggcattctcggcaaagagcaaggctagtctcc
aaagatggaaggtgcaacatagaatttggcaatgtggaggcacagtcaag
gtttatattctttgtggacatctggacaacggtacttgacctcaagtgga
gatacaaaatgaccattttcatcacagccttcttggggagttggtttttc
tttggtctcctgtggtatgcagtagcgtacattcacaaagacctcccgga
attccatccttctgccaatcacactccctgtgtggagaatattaatggct
tgacctcagcttttctgttttctctggagactcaagtgaccattggatat
ggattcaggtgtgtgacagaacagtgtgccactgccatttttctgcttat
ctttcagtctatacttggagttataatcaattctttcatgtgtggggcca
tcttagccaagatctccaggcccaaaaaacgtgccaagaccattacgttc
agcaagaacgcagtgatcagcaaacggggagggaagctttgcctcctaat
ccgagtggctaatctcaggaagagccttcttattggcagtcacatttatg
gaaagcttctgaagaccacagtcactcctgaaggagagaccattattttg
gaccagatcaatatcaactttgtagttgacgctgggaatgaaaatttatt
cttcatctccccattgacaatttaccatgtcattgatcacaacagccctt
tcttccacatggcagcggagacccttctccagcaggactttgaattagtg
gtgtttttagatggcacagtggagtccaccagtgctacctgccaagtccg
gacatcctatgtcccagaggaggtgctttggggctaccgttttgctccca
tagtatccaagacaaaggaagggaaataccgagtggatttccataacttt
agcaagacagtggaagtggagacccctcactgtgccatgtgcctttataa
tgagaaagatgttagagccaggatgaagagaggctatgacaaccccaact
tcatcttgtcagaagtcaatgaaacagatgacaccaaaatgtaacagtgg
cttttcaacgggagtaaagcaaagtctctaaagctcctagtacctagaag
cattatgaagcagtcaacaatttaggggtacgaaagtaggatgagagcct
tcaaagtctaccagcacaaagacccctgagccccgcaattgtgatcccac
aagacatgcatctccacaaggctactgtattagaacgtgcaatgcattta
tatgaaactggtgtatggaagacataggtgctctcttgaaatcttaaata
tgattatttgagctcatataaggtggattggagcagataaaattatcaaa
agtttcatgaacaggccaaacaaaatattttttaaagtttccttaaagaa
gttatgaactttagaaaggatcaggggacaataataatctcattttgatt
ctactgataagaatgactccacttttaatgtggacttttactcatggaaa
aattgtctcctaatttggggagatgaaccaaccaatcaatgacaagaaaa
cgcttacacaaagaacaatttgaggctctaagcttctcatgtggtacgtt
tagacagaggctaaatctgcacactagaatcttgatgataccttcctgca
agacagaatgctttagttaaaagtggtgatgatatttctttcaatctgta
ttggatggcttaaagggctataaatctgtttataaagagcatttcctgct
cttcgaagacagcaatgaggagttggaaggtgcaaagtcagtagagaagg
gaatgtatcattaatgcacctgagaagaaacagtttcatgtgttcctcca
cctagagtttgtactggaatgctatttctaaagaagaagtgggaaagaga
gaggaatgggatggagccccacagtcagaatgttactatgtctttctttc
cctgacagcccatcttcctaaaagggaccagcttatggaaggctcgacct
tgaggggaaagttttactgtgaaagtcttcttcagatccccacctgcatc
attccgaatgtgtcctggaaaaaaactggtactcaaagctgcttaggaat

caaaatgttttcagtgtgttgattaatatagtaaatttctgaaactgtg
>NM_153764 1
gttgcatacagatgagttggcagccggtctgagctggcccacagactcat
aaaatcaacagggcctcgggtaccctcacctagcatatccaaactcttgc
atcaaaggtgcagggacttgctcacatcgagaatctggttgctttcttgg
agaccaagaaaatgagtttttgtttctacatttactccagcaatccatga
gatcagggtgttgacagaaagtatgttcaaacatcttcggaaatgggtcg
tcactcgctttttgggcattctcggcaaagagcaaggctagtctccaaa
gatggaaggtgcaacatagaatttggcaatgtggaggcacagtcaaggtt
tatattctttgtggacatctggacaacggtacttgacctcaagtggagat
acaaaatgaccattttcatcacagccttcttggggagttggtttttcttt
ggtctcctgtggtatgcagtagcgtacattcacaaagacctcccggaatt
ccatccttctgccaatcacactccctgtgtggagaatattaatggcttga
cctcagcttttctgttttctctggagactcaagtgaccattggatatgga
ttcaggtgtgtgacagaacagtgtgccactgccatttttctgcttatctt
tcagtctatacttggagttataatcaattctttcatgtgtggggccatct
tagccaagatctccaggcccaaaaaacgtgccaagaccattacgttcagc
aagaacgcagtgatcagcaaacggggagggaagctttgcctcctaatccg
agtggctaatctcaggaagagccttcttattggcagtcacatttatggaa
agcttctgaagaccacagtcactcctgaaggagagaccattattttggac
cagatcaatatcaactttgtagttgacgctgggaatgaaaatttattctt
catctccccattgacaatttaccatgtcattgatcacaacagccctttct
tccacatggcagcggagacccttctccagcaggactttgaattagtggtg
tttttagatggcacagtggagtccaccagtgctacctgccaagtccggac
atcctatgtcccagaggaggtgctttggggctaccgttttgctcccatag
tatccaagacaaaggaagggaaataccgagtggatttccataactttagc
aagacagtggaagtggagacccctcactgtgccatgtgcctttataatga
gaaagatgttagagccaggatgaagagaggctatgacaaccccaacttca
tcttgtcagaagtcaatgaaacagatgacaccaaaatgtaacagtggctt
ttcaacgggagtaaagcaaagtctctaaagctcctagtacctagaagcat
tatgaagcagtcaacaatttaggggtacgaaagtaggatgagagccttca
aagtctaccagcacaaagacccctgagccccgcaattgtgatcccacaag
acatgcatctccacaaggctactgtattagaacgtgcaatgcatttatat
gaaactggtgtatggaagacataggtgctctcttgaaatcttaaatatga
ttatttgagctcatataaggtggattggagcagataaaattatcaaaagt
ttcatgaacaggccaaacaaaatatttttaaagtttccttaaagaagtt
atgaactttagaaaggatcaggggacaataataatctcattttgattcta
ctgataagaatgactccacttttaatgtggacttttactcatggaaaaat
tgtctcctaatttggggagatgaaccaaccaatcaatgacaagaaaacgc
ttacacaaagaacaatttgaggctctaagcttctcatgtggtacgtttag
acagaggctaaatctgcacactagaatcttgatgataccttcctgcaaga
cagaatgctttagttaaaagtggtgatgatatttctttcaatctgtattg
gatggcttaaagggctataaatctgtttataaagagcatttcctgctctt
cgaagacagcaatgaggagttggaaggtgcaaagtcagtagagaagggaa
tgtatcattaatgcacctgagaagaaacagtttcatgtgttcctccacct
agagtttgtactggaatgctatttctaaagaagaagtgggaaagagagag
gaatgggatggagccccacagtcagaatgttactatgtctttctttccct
gacagcccatcttcctaaaagggaccagcttatggaaggctcgaccttga
ggggaaagttttactgtgaaagtcttcttcagatccccacctgcatcatt

ccgaatgtgtcctggaaaaaaactggtactcaaagctgcttaggaatcaa
aatgttttcagtgtgttgattaatatagtaaatttctgaaactgtg
>NM_004110 3
gcttgtgggcgggcccgggcaggagcgggcttgccctgcggagcagtagc
taggaacagatccacttgcaggttgctgttcccagccatggcttcgcgct
gctggcgctggtggggctggtcggcgtggcctcggacccggctgcctccc
gccgggagcaccccgagcttctgccaccatttctccacacaggagaagac
cccccagatctgtgtggtgggcagtggcccagctggcttctacacggccc
aacacctgctaaagcacccccaggcccacgtggacatctacgagaaacag
cctgtgccctttggcctggtgcgctttggtgtggcgcctgatcaccccga
ggtgaagaatgtcatcaacacatttacccagacggcccattctggccgct
gtgccttctggggcaacgtggaggtgggcagggacgtgacggtgccggag
ctgcgggaggcctaccacgctgtggtgctgagctacggggcagaggacca
tcgggccctggaaattcctggtgaggagctgccaggtgtgtgctccgccc
gggccttcgtgggctggtacaacgggcttcctgagaaccaggagctggag
ccagacctgagctgtgacacagccgtgattctggggcaggggaacgtggc
tctggacgtggcccgcatcctactgaccccacctgagcacctggaggccc
tcctttttgtgccagagaacggacatcacgaaggcagccctgggtgtactg
aggcagagtcgagtgaagacagtgtggctagtgggccggcgtggacccct
gcaagtggccttcaccattaaggagcttcgggagatgattcagttaccgg
gagcccggcccattttggatcctgtggatttcttgggtctccaggacaag
atcaaggaggtcccccgcccgaggaagcggctgacggaactgctgcttcg
aacggccacagagaagccagggccggcggaagctgcccgccaggcatcgg
cctcccgtgcctggggcctccgctttttccgaagcccccagcaggtgctg
ccctcaccagatgggcggcgggcagcaggtgtccgcctagcagtcactag
actggagggtgtcgatgaggccacccgtgcagtgcccacgggagacatgg
aagacctcccttgtgggctggtgctcagcagcattgggtataagagccgc
cctgtcgacccaagcgtgccctttgactccaagcttggggtcatccccaa
tgtggagggccgggttatggatgtgccaggcctctactgcagcggctggg
tgaagagaggacctacaggtgtcatagccacaaccatgactgacagcttc
ctcaccggccagatgctgctgcaggacctgaaggctgggttgctcccctc
tggccccaggcctggctacgcagccatccaggccctgctcagcagccgag
gggtccggccagtctctttctcagactgggagaagctggatgccgaggag
gtggcccggggccagggcacggggaagcccagggagaagctggtggatcc
tcaggagatgctgcgcctcctgggccactgagcccagccccagccccggc
ccccagcagggaagggatgagtgttgggaggggaagggctgggtccgtct
gagtgggactttgcacctctgctgatcccggccggccctggcttggaggc
ttggctgctcttccagcgtctctcctccctcctggggaaggtcgcccttg
cgcgcaaggttttagctttcagcaactgaggtaaccttagggacaggtgg
aggtgtgggccgatctaaccccttacccatctctctactgctggactgtg
gagggtcaccaggttgggaacatgctggaaataaaacagctgcaaccaag
aaaaaaaaaaaaaaaaaaaaaaaaaaaaaaaaa
>NM_000520 4
agttgccgacgcccggcacaatccgctgcacgtagcaggagcctcaggtc
caggccggaagtgaaagggcagggtgtgggtcctcctggggtcgcaggcg
cagagccgcctctggtcacgtgattcgccgataagtcacgggggcgccgc
tcacctgaccagggtctcacgtggccagccccctccgagaggggagacca
gcgggccatgacaagctccaggctttggttttcgctgctgctggcggcag
cgttcgcaggacgggcgacggccctctggccctggcctcagaacttccaa

acctccgaccagcgctacgtcctttacccgaacaactttcaattccagta
cgatgtcagctcggccgcgcagcccggctgctcagtcctcgacgaggcct
tccagcgctatcgtgacctgcttttcggttccgggtcttggccccgtcct
tacctcacagggaaacggcatacactggagaagaatgtgttggttgtctc
tgtagtcacacctggatgtaaccagcttcctactttggagtcagtggaga
attataccctgaccataaatgatgaccagtgtttactcctctctgagact
gtctggggagctctccgaggtctggagacttttagccagcttgtttggaa
atctgctgagggcacattctttatcaacaagactgagattgaggactttc
cccgctttcctcaccggggcttgctgttggatacatctcgccattacctg
ccactctctagcatcctggacactctggatgtcatggcgtacaataaatt
gaacgtgttccactggcatctggtagatgatccttccttcccatatgaga
gcttcacttttccagagctcatgagaaaggggtcctacaaccctgtcacc
cacatctacacagcacaggatgtgaaggaggtcattgaatacgcacggct
ccggggtatccgtgtgcttgcagagtttgacactcctggccacactttgt
cctggggaccaggtatccctggattactgactccttgctactctgggtct
gagccctctggcacctttggaccagtgaatcccagtctcaataataccta
tgagttcatgagcacattcttcttagaagtcagctctgtcttcccagatt
tttatcttcatcttggaggagatgaggttgatttcacctgctggaagtcc
aacccagagatccaggactttatgaggaagaaaggcttcggtgaggactt
caagcagctggagtccttctacatccagacgctgctggacatcgtctctt
cttatggcaagggctatgtggtgtggcaggaggtgtttgataataaagta
aagattcagccagacacaatcatacaggtgtggcgagaggatattccagt
gaactatatgaaggagctggaactggtcaccaaggccggcttccgggccc
ttctctctgccccctggtacctgaaccgtatatcctatggccctgactgg
aaggatttctacatagtggaacccctggcatttgaaggtacccctgagca
gaaggctctggtgattggtggagaggcttgtatgtggggagaatatgtgg
acaacacaaacctggtccccaggctctggcccagagcaggggctgttgcc
gaaaggctgtggagcaacaagttgacatctgacctgacatttgcctatga
acgtttgtcacacttccgctgtgaattgctgaggcgaggtgtccaggccc
aacccctcaatgtaggcttctgtgagcaggagtttgaacagacctgagcc
ccaggcaccgaggagggtgctggctgtaggtgaatggtagtggagccagg
cttccactgcatcctggccaggggacggagccccttgccttcgtgcccct
tgcctgcgtgcccctgtgcttggagagaaaggggccggtgctggcgctcg
cattcaataaagagtaatgtggcatttttctataataaacatggattacc
tgtgtttaaaaaaaaaagtgtgaatggcgttagggtaagggcacagccag
gctggagtcagtgtctgcccctgaggtcttttaagttgagggctgggaat
gaaacctatagcctttgtgctgttctgccttgcctgtgagctatgtcact
cccctcccactcctgaccatattccagacacctgccctaatcctcagcct
gctcacttcacttctgcattatatctccaaggcgttggtatatggaaaaa
gatgtaggggcttggaggtgttctggacagtggggagggctccagaccca
acctggtcacagaagagcctctcccccatgcatactcatccacctccctc
ccctagagctattctcctttgggtttcttgctgcttcaattttatacaac
cattatttaaatattattaaacacatattgttctcta
>NM_002428 2
acgactctttgatccctcccctaagacctccttcccgtccgcctaggcct
cggtatcccccaccaggtatgagaaggaggagtcttcattcttcccaggc
ggggagaccacgccttccctgcctcctccctccgcggggggtcgcgttgg
aggtcacccccgcccccctaggcgctgggttgggagtgacgcggggtgggc
tggagaggtttcctgccgtctgggaagcgtaaacggaccgcccacctgtc

gggcctcggccgcccgcacctgcttgtgagaagcctgcggctggggcacc
gcccccggtccccgcccgggtccgcgcattgggagcacactggccctta
agagcgcggcggccgcggcgcgcggggaggagggctgggagcgcccggag
ccgcgctgaactcgccggggacgtcgggcgcccgctccttggctggctcg
cttgctctctcgctcgctctctcgggtcggctccctgcgcctcctcccgg
gactgcttggggacccgggcccggtcgggcgcggcggcgagcagaatccc
cgtgcaggaggcgcgcgctccgggcagcgcccggccccgggcatggacag
agggagctgggcgcgcagggagcgccgcgggaccgggcggccggagcgct
gagccagacaaaggctccggagttgcgcttgctctcgggagccgggcccc
aggcgctgggcgccgaggctgcggaacctcgccgggggcagctccggtcg
gccccctttcccgccggctggttccgagctcccggacctgccctgcccgc
ttctcctcgggcttgggaatttgccgaggcgacctaggcggctccggcgg
ggaccgggagcccgaggtccgcggcgcgcctgccgggccaggagccaggg
agcgtcgcaagtttccaaggcgcgtgcgaggatccggcgtgcagtgttcc
gagctgggctgggcgccgagagcatgggcagcgacccgagcgcgcccgga
cggccgggctggacgggcagcctcctcggcgaccgggaggaggcggcgcg
gccgcgactgctgccgctgctcctggtgcttctgggctgcctgggccttg
gcgtagcggccgaagacgcggaggtccatgccgagaactggctgcggctt
tatggctacctgcctcagcccagccgccatatgtccaccatgcgttccgc
ccagatcttggcctcggcccttgcagagatgcagcgcttctacgggatcc
cagtcaccggtgtgctcgacgaagagaccaaggagtggatgaagcggccc
cgctgtggggtgccagaccagttcggggtacgagtgaaagccaacctgcg
gcggcgtcggaagcgctacgccctcaccgggaggaagtggaacaaccacc
atctgacctttagcatccagaactacacggagaagttgggctggtaccac
tcgatggaggcggtgcgcagggccttccgcgtgtgggagcaggccacgcc
cctggtcttccaggaggtgccctatgaggacatccggctgcggcgacaga
aggaggccgacatcatggtactctttgcctctggcttccacggcgacagc
tcgccgtttgatggcaccggtggctttctggcccacgcctatttccctgg
ccccggcctaggcggggacacccattttgacgcagatgagccctggacct
tctccagcactgacctgcatggaaacaacctcttcctggtggcagtgcat
gagctgggccacgcgctggggctggagcactccagcaaccccaatgccat
catggcgccgttctaccagtggaaggacgttgacaacttcaagctgcccg
aggacgatctccgtggcatccagcagctctacggtaccccagacggtcag
ccacagcctacccagcctctccccactgtgacgccacggcggccaggccg
gcctgaccaccggccgccccggcctccccagccaccacccccaggtggga
agccagagcggcccccaaagccgggcccccccagtccagccccgagccaca
gagcggcccgaccagtatggccccaacatctgcgacggggactttgacac
agtggccatgcttcgcggggagatgttcgtgttcaagggccgctggttct
ggcgagtccggcacaaccgcgtcctggacaactatcccatgcccatcggg
cacttctggcgtggtctgcccggtgacatcagtgctgcctacgagcgcca
agacggtcgttttgtctttttcaaaggtgaccgctactggctctttcgag
aagcgaacctggagcccggctacccacagccgctgaccagctatggcctg
ggcatcccctatgaccgcattgacacggccatctggtgggagcccacagg
ccacaccttcttcttccaagaggacaggtactggcgcttcaacgaggaga
cacagcgtggagaccctgggtaccccaagcccatcagtgtctggcagggg
atccctgcctcccctaaaggggccttcctgagcaatgacgcagcctacac
ctacttctacaagggcaccaaatactggaaattcgacaatgagcgcctgc
ggatggagcccggctaccccaagtccatcctgcgggacttcatgggctgc
caggagcacgtggagccaggcccccgatggcccgacgtggcccggccgcc

cttcaacccccacgggggtgcagagcccggggcggacagcgcagagggcg
acgtgggggatggggatggggactttggggccggggtcaacaaggacggg
ggcagccgcgtggtggtgcagatggaggaggtggcacggacggtgaacgt
ggtgatggtgctggtgccactgctgctgctgctctgcgtcctgggcctca
cctacgcgctggtgcagatgcagcgcaagggtgcgccacgtgtcctgctt
tactgcaagcgctcgctgcaggagtgggtctgaccacccagcgctcctgc
taacggtgctcaggggcgcctgtggttctgagatggctcccaggggctc
cctccgcccccaggtaggggcccctctcagccctcacacaccctgtctgc
cccgccctcattatttatgtccaggtgtttgttttgttttgtttttggca
ccttacttgaccatttgtttctgtttccccgactggggcagggtgtttag
aattttctaaatgtagttctgctccagacagggaattaggcccccatcat
cctctggcttggccacagccaggggagcagaggggcagaggcccacattg
gaagagcagcacctcctcagcctgaaccccagggctgtaactgccaggct
ctctttgcccagttggagactgtctggcccccctggtcccctccttccca
agtgagtctctctgggccttaggaagagccttccacccaggggcagcccc
aggccaaaggggacctggaagggaggtgggccgtggcccttgagtcccca
ttgaggcttggttccttcccaatccagtggacttcgcagtccacttctga
cagcctcagtgaccctggctccttgtgccagagaacccagcccacccccg
gcagcagcccccagctcccacctccccttgggcccacacctccttccctc
tctggagaaagggccctgggcctgcctcaccacggaccaaagggagtctg
ccagggcccctctccccagggaagcagcagcctcgcccctggcagagatg
cctccctgagctagaaccctctgttccttccctgtgcctcctccctccct
ctccgactcacaccactagcctcaggggtctgagctccagctcctttggg
cttcagctgccagtgtcctgagccccagggagagggggctggtgggtgcc
taggcctgggcagtggatggccgtgaatgggtgcccacagtgtcaggcac
tgggcatgaggggttcctcccctccagctccctgtgcccccagggtcctg
ggaggagagacactggtggggataggccagccgcgcatcagactgtgaac
cccacgaaggagcccattgtggcctaagaggctgccctcctgtgctcagc
cctgaggacagatgcctccttcctcttttccttcccaaagcaagcaagag
gccgtggctgctgtgggaaatggtactgtacagctggctctacttcccca
tggccctgagcgagtggagtctgccacccaggatccccaaggcacttgag
ggggaaggattctgctggcctctgcgagtggtttcttgtgcactggcacc
aagtgcgggtccggcagcttctgccccctgcagaaccggagagccagcta
aggggtggggctgcgggggttccgtgtccacccccatacatttatttctg
taaataatgtgcactgaataaattgtacagccggcaaaaaaaaaaaaaaa
aaaaaa
>NM_002147 3
gtgaagcacagggttataacgaccacgatccacaaatcaagccctccaaa
atcacccaaatgagctcgtactttgtaaactccttctcggggcgttatcc
aaatggcccggactatcagttgctaaattatggcagtggcagctctctga
gcggctcttacagggatcccgctgccatgcacaccggctcttacggctac
aattacaatgggatggacctcagcgtcaaccgctcctcggcctcctccag
ccactttggggcggtgggcgagagctcgcgcgccttccccgcgcccgccc
aggagccccgcttcaggcaagcggcttcgagctgctccctgtcctcgccc
gagtccctgccctgcaccaacggcgacagccacggcgccaagccctctgc
ttcgtccccctccgaccaggcgacctcagccagctccagcgccaatttca
ccgaaatagacgaggccagcgcgtcctcggagcctgaggaagcggcaagc
cagctaagcagccccagcctagctcgggcgcagccagagcccatggccac
ctccacagccgcgcccgaggggcagactccgcaaatattcccctggatga

ggaagcttcacatcagccatgatatgaccgggccggacgggaaaagggcc
cggaccgcgtatacccgctaccagaccctggagctggaaaaggagttcca
cttcaaccgctacctgacccggcgacggcgcatcgagatcgcccacgcac
tctgcctgtccgagcgccagatcaagatctggttccagaaccggcgcatg
aagtggaagaaggacaacaaattgaaaagtatgagcctggctacagctgg
cagcgccttccagccctgagcccgcccagaggagcccagcggcccaagag
cccgtgccacccccagccctggcccctccaatcctccccgctctgccgcc
gcccgctggggaccggttcccacaagcctgcctcgccttgtgttacgata
tttcgtttggtcttaggtcttcctgtggctccctctctcctggactggtt
atcttgttattattgttaataataattattattattattttccttccatg
ctcccaactcccttctgcttgtcccaaatccgccagtgtttctgaatgtt
tgtgtctgtggttgcagtctttcccccaggaaaaaaaaaaaaaagaaattc
gcatgtttaatgtgaactctcccctccccatctgtgttctaacttattta
taaaaagatgatcgctgtattttgagtttcagctggaaacttctgtaagg
ggcagcagttgaggtggggtagtgccgcagtggggtcaagctgagctggc
ttcggagatggagtcccttttcattctcctcctcctccctcctcactccc
taggcccaagtctcctaggggcttggtcctagggtgggaaggggctaggg
aggaccaaagggatggtattgagaagagagaaagaagatagtgagattta
agttcctgctgcctgggtaggccccacaaggcctggtctgggagtatacg
gaaacaaaaatgatcctcagtgcaaaatgtcttgtgtatttctctgtgaa
tccatgggtctggctagagggcccaaagcttgtaaatatggggatagtct
gggtcagacccatctctcccttacccatcttgcttccaagaccatttgta
gtgagcgagtggatgctgtgctacgtgtgaaatctgtctttgcggggcct
gtctcagtgattcgcttttggtatttgtttgtagctttcctggaagtcaa
ataaatgtttcccccactccaaaaaaaaaa
>NM_002153 2
acccactgggaatatgattatgcttaatctatgctcagttgaaaggggct
ggggctgctttctcccctcccttcttgactctctgttcacagaactcagg
ctgcctccagccagcctttgcccgctagactcactggccctgagcacttg
aaggtgcagcaagtcactgagaatgagcactttcttctcggacacagcat
ggatctgcctggctgtccccacagtactatgtgggacagtattttgcaaa
tacaagaagagctcagggcagctgtggagctggatggtctgcctggcagg
cctctgtgcagtctgcctgctcatcctgtccccttttttggggcttgatcc
tcttctcggtgtcatgcttcctcatgtatacttacttatctggccaagaa
ttgttacctgtggatcagaaggcagtcctggtgacaggtggtgattgcgg
gcttggccatgctttgtgcaagtatctggatgagctgggcttcacggtat
ttgccggagttttgaatgaaaatggcccaggagctgaggaattgcgaaga
acctgctctccgcgcctctcggtgctccaaatggacatcacgaagccagt
gcagataaaagatgcttacagcaaggttgcagcaatgctgcaggacagag
gactgtgggctgtgatcaacaatgctggggtgcttggctttccaactgat
ggggagcttcttcttatgactgactacaaacaatgcatggccgtgaactt
ctttggaactgtggaggtcacaaagacgtttttgcctcttcttagaaaat
ccaaagggaggctggtgaatgtcagcagcatgggaggaggggccccaatg
gaaaggctggcatcttatggctcatcaaaggcggctgtgaccatgttctc
atcagttatgagactggagctttccaagtggggaattaaagttgcttcca
tccaacctggaggcttcctaacaaatatcgcaggcaccagtgacaagtgg
gaaaagctggagaaggacattctggaccacctccccgctgaggtacagga
agactacggccaggactacatcttagcacagcggaatttcctcctattga
tcaactcgttagccagcaaggacttctctccggtgctgcgggacatccag

catgctatcttggcgaagagcccttttgcctattacacgccagggaaagg
cgcttacttgtggatctgccttgctcactatttgcctattggcatatatg
attactttgctaaaagacattttggccaagacaagcccatgcccagagct
ctaagaatgcctaactacaagaaaaaggccacctaggcaatggaagccct
caaagaagtcggaatgtcatagtcttgaaatgaaagggaaactgggaaac
tgggtttctcattaaagttgtttcccactctgtaaaaaaaaaaaaaaaaa
a
>NM_002980 2
aaagccgcgtcctgatcaatggggcgggcggcttagcgcgcgcggccacc
tggtccgaggaggagcagtcccggggcccgccgcaggtcgggtggctcag
ccatggctcctcggggcgcagcggccggccggagcccgggaccctgcgcg
gggcgctgagctcccgagcgggcagagggcacgggcaggcggacgtcggg
gcgccctcggggaacgtgcgggcaccatgcgtccccacctgtcgccgccg
ctgcagcagctactactgccggtgctgctcgcctgcgccgcgcactcgac
tggagcccttccccgactatgtgacgtgctacaagtgctgtgggaagagc
aagaccagtgcctgcaggaactctccagagagcagacaggagacctgggc
acggagcagccagtgccaggttgtgaggggatgtgggacaacataagctg
ctggccctcttctgtgccgggccggatggtggaggtggaatgcccgagat
tcctccggatgctcaccagcagaaatggttccttgttccgaaactgcaca
caggatggctggtcagaaaccttccccaggcctaatctggcctgtggcgt
taatgtgaacgactcttccaacgagaagcggcactcctacctgctgaagc
tgaaagtcatgtacaccgtgggctacagctcctccctggtcatgctcctg
gtcgcccttggcatcctctgtgctttccggaggctccactgcactcgcaa
ctacatccacatgcacctgttcgtgtccttcatccttcgtgccctgtcca
acttcatcaaggacgccgtgctcttctcctcagatgatgtcacctactgc
gatgcccacagggcgggctgcaagctggtcatggtgctgttccagtactg
catcatggccaactactcctggctgctggtggaaggcctctaccttcaca
cactcctcgccatctccttcttctctgaaagaaagtacctccagggattt
gtggcattcggatggggttctccagccatttttgttgctttgtgggctat
tgccagacactttctggaagatgttgggtgctgggacatcaatgccaacg
catccatctggtggatcattcgtggtcctgtgatcctctccatcctgatt
aatttcatccttttcataaacattctaagaatcctgatgagaaaacttag
aacccaagaaacaagaggaaatgaagtcagccattataagcgcctggcca
ggtccactctcctgctgatcccccctctttggcatccactacatcgtcttc
gccttctccccagaggacgctatggagatccagctgtttttgaactagc
ccttggctcattccagggactggtggtggccgtcctctactgcttcctca
atggggaggtgcagctggaggttcagaagaagtggcagcaatggcacctc
cgtgagttcccactgcaccccgtggcctccttcagcaacagcaccaaggc
cagccacttggagcagagccagggcacctgcaggaccagcatcatctgag
aggctggagcagggtcacccacggacagagaccaagagaggtcctgcgaa
ggctgggcactgctgtgggacagccagtcttcccagcagacaccctgtgt
cctccttcagctgaagatgcccctccccaggccttggactcttccgaagg
gatgtgaggcactgtggggcaggacaagggcctgggatttggttcgtttg
ctcttctgggaagagaagttcaggggtcccagaaagggacagggaaataa
atggtgcctgggatgagattcaaaaaaaaaaaa
>NM_005074 3
attgtgtaaagttgagagaaaagtcttcacagcggaagaagaaatcactt
ttttgtcccgaatcagtgggtggaggcaaaacccttcagcaagaagagaa
gcaacttcagccgtgtatgcaaatggataaccggttgcctcccaaaaaag

ttccaggtttctgttcctttcgctatggattgtctttccttgtgcactgt
tgtaatgttataataacagcacagcgtgcgtgcctgaacctcacaatggt
agtcatggtgaatagcacagatccacatggtttgcccaacacctccacaa
agaagctcctggataatataaagaaccctatgtataattggagcccagat
atccagggaatcatcttgagttccacctcctatggtgtcatcatcatcca
agttcctgttggatacttctctggaatatattctacaaagaaaatgattg
gctttgcattatgcctcagctctgtgttaagcctgctcatcccaccagca
gctggaattggagtagcttgggtcgttgtatgtcgagcagttcagggagc
agcccaggggatagttgcaacagcccagtttgaaatatatgtcaaatggg
ctcctcccctggaacgaggccgacttacttctatgagtacatcagggttt
ttgctgggaccctttattgtcctacttgtgactggagttatctgtgaatc
tctgggctggcccatggtcttctatatttttggtgcttgtggctgtgccg
tatgtcttctctggttcgttctgttttatgatgaccccaaagaccaccca
tgtataagcatcagtgaaaaggaatacatcacatcctccctggtccagca
ggtcagttcaagtagacaatctctgcctatcaaggctatacttaagtcgc
ttccagtctgggctatttccactggtagttttacgtttttctggtcacat
aacatcatgacactatacactccaatgtttatcaactccatgcttcatgt
taatataaaagagaatgggttcttgtcttcccttccctatttgtttgcct
ggatctgtggtaacctagcaggtcagttatcagacttcttcctgaccagg
aatattctcagcgtaattgctgtccggaaactcttcacagcagcaggatt
tctccttcctgcaatctttggtgtctgcctgccttacctgagttccacct
tctacagcattgtcattttcctaatacttgctggtgcaacaggcagcttt
tgcttgggtggagtgtttataaatggcttggatattgctcccagatattt
tggatttattaaagcatgttcaactttaactggaatgataggaggactaa
ttgcttccactttgactggattgatccttaagcaggatccggaatccgcc
tggtttaaaaccttcatcctgatggcagccattaatgtgactggcctaat
tttctaccttatagttgctacagcagaaattcaggactgggctaaagaaa
aacaacacacacgtctctgaagtgtgaaacagagcacttgcagagcctgg
gacaacctccttattgaagggaagagggaccagcacatgaggctgaggct
gaggggcagtcaccagcaccaggaagaaggtggtaggaggagtcctaggg
gccagttttaccctcccacatatcctgtgtgcatcgtttttaacatgtg
ttgtttgtaacatgtaatatatctgaattttaaatgggggtggtgattta
tctcagtcttcctctctgcatcagttgctttataaatgattgtcaaacca
ataaatacacacaagataaaattttattctaaggcttcatgaaaaaaaaa
aaaaaaaaa

>NM_000294 2
aattgggcggccggtttccaattggaagggcttctgagcctcagtcttcg
ggcggcgctgaccattggcgggcaggatggaggcggagccttgggttgtc
gcccgccgccgggggttaaggtgagcgactgcaggcaaacccggcgacag
cgcagctcgcgtcgaccctggctcctctgcctgccccctcaggcccccgc
ctccttcaggatgacgctggacgtggggccggaggatgagctgcccgact
gggccgccgccaaagagttttaccagaagtacgaccctaaggacgtcatc
ggcagaggagtgagctctgtggtccgccgttgtgttcatcgagctactgg
ccacgagtttgcggtgaagattatggaagtgacagctgagcggctgagtc
ctgagcagctggaggaggtgcgggaagccacacggcgagagacacacatc
cttcgccaggtcgccggccacccccacatcatcaccctcatcgattccta
cgagtcttctagcttcatgttcctggtgtttgacctgatgcggaagggag
agctgtttgactatctcacagagaaggtggccctctctgaaaaggaaacc

aggtccatcatgcggtctctgctggaagcagtgagctttctccatgccaa
caacattgtgcatcgagatctgaagcccgagaatattctcctagatgaca
atatgcagatccgactttcagatttcgggttctcctgccacttggaacct
ggcgagaagcttcgagagttgtgtgggaccccagggtatctagcgccaga
gatccttaaatgctccatggatgaaacccacccaggctatggcaaggagg
tcgacctctgggcctgtggggtgatcttgttcacactcctggctggctcg
ccacccttctggcaccggcggcagatcctgatgttacgcatgatcatgga
gggccagtaccagttcagttcccccgagtgggatgaccgttccagcactg
tcaaagacctgatctccaggctgctgcaggtggatcctgaggcacgcctg
acagctgagcaggccctacagcaccccttctttgagcgttgtgaaggcag
ccaaccctggaacctcacccccccgccagcggttccgggtggcagtgtgga
cagtgctggctgctggacgagtggccctaagcacccatcgtgtacggcca
ctgaccaagaatgcactgttgagggacccttatgcgctgcggtcagtgcg
gcacctcatcgacaactgtgccttccggctctacgggcactgggtaaaga
aaggggagcagcagaaccgggcggctctctttcagcaccggccccctggg
ccttttcccatcatgggccctgaagaggagggagactctgctgctataac
tgaggatgaggccgtgcttgtgctgggctaggacctcaaccccagggatt
cccaggaagcagaactctccagaagaagggttttgatcattccagctcct
ctgggctctggcctcaggcccactaatgatcctgctaccctcttgaagac
cagcccggtacctctctccccactggccaggactctgagatcagagctgg
ggtggaagggagccattctgaacgccacgcctggcccggtcagtgctgca
tgcactgcatatgaaataaaatctgctacacgccagggagaacaggtgtc
ctgtgtctgtctggcttgggcaggaaagcccagaaggtgctcagcagggt
gcagggatggtgccattctggcccagacctttattggggaaaatgttggg
ggtcacttggtcttgctcttgcctttacccggaggtagctggaagggccg
ctctagtgcagtcaacttgctgctgagcctttcctcgctggccttgagcc
gctcctccaccagcccctggagctgctccagctctttgttcacttgggtc
ttgatgtaggctcggaggacgtggatgtggcctgcaggggcagggaagaa
ggggcagggtgaggagagatgctgtctggcaatggggggatggtccctag
ttgggcaaacagtccccaaatttcccctggtggggatatagaggtagcaa
tgttgtttccctttaggaaatgttagcaagcccttgtgtgagaggtagtt
gggtagggtggctatgctggactttgcagcttcaaattctgatccctact
cagtagctgtgtgaccttaggcaggttatttaacctatctgtgcctcagt
ttccttgtatgaaatgtggatagtgatacctagcacataggattgaggga
ggattaaatgagttaatttatgtaaaatgcttagagcagggcatgcactg
cacacctattgccaagtatgtgctggctgttattgtcattggctttctct
tttttttttttttttgagatggagtcttgctttatcccccaggctggag
tgcagtggcagcgatctcagctcactgcaagctctgcctcccaggttcat
gccattctcctgcctcagcctcccagtagctgggactacaggcacccgcc
accacgcccggctaactttttgtatttttagtagagacggggtttcacag
tgttagccaggatggactcgatctcctgacctcgtgatccgcctgcctca
gcctcccaaagtgctgggattacagttgtgagccactgcacctggcctgt
tctttgtttttttttaactcttaagttctgggatacgagcagaacatgca
ggtttgctacgtaggtatacatgggccatggtggtttgctttacccatca
acccatcatataggttttaagccaccgtcattggctttctaatgatcatt
tctctggagccacttcagctgtgctttttgtttgtttgtttgtttgtttt
gagacagagtcttgctctgttgcccaggctggagtgcagtggccagatct
tggctcactgcagtctccacctcctgggttcatgcagttctcctgcctca
gcctcctgagtagctgggactacaagcgcgcaccaccatgcctggctatt

ttttgtattttagtagatagggggtttcacggtgttgcccaggctggtcg
cgaactcctgagctcaggcaatccgcctgcctcagattgtgctgggatta
caggtgtgagccaccacgcctggcctgcagctgtgcttttatctgtcatc
ttggacttctgcataagggatcatttagagaaaggactctgactaaaaac
aaaaagtctgaagtcctgatgtcatccaaacccttcattctacacctgct
cagagggacagggcagcctgccctctccagatcctcacttggctctggct
ctggtggggccactgtctctagttcctctttctgcggggtaactgcctgc
tcctccatctccttgctttcttctttctctgcaaaccagaagcaggaaga
gactgtggccccaggcctggcccagcccagccctgccctggcactctccc
ttacccgtctcactctctggccacagtttgggtggctgtagccccatgta
agtcaaagacagatccagccgcacctgggaagcaaggcagagggaggctg
aaaggagtgcacctgtgctgaggggaggggcggttgagggtggctcctca
tggtccaccacccccctacctggggtgtgaagacgtatttatagagcttga
agtggcggaagtaagtgttgaccacgtagtcttccaaggccagcagctgt
tcctctttgaagaggtcgatgctgaaaggaggccgctgtggtggtgactc
ggaattagaaccctgactaccttccggagccctggctttgcctccaaaag
cccagcaacaggagcaaggagagcccactggggtcttcacaagaagataa
gggtgatgaatgtgagagagactgggtgagaccttgtctggggatgggta
aagtttccagaatgttccaggggaactgaccctgactccatggcaaaaaa
ggacactggtgaagtagcggtagcactcctccacgttgcccaagggggtt
gctggtagggaaagcaagatgcagcagtgaggccctctctggtatccatt
cattcacttcactcaacagctgtttatgaccatgagcttcagaagcagac
agatctgggtttcagcactggcttgtttccttaactcatgtaacaaatac
tgaatacccactatatgcccactgtatggttttcggcactgaacaagaca
gacaaggtcctgcccttacgaagcttatattctaggggaataaaccaaga
aatagatcatttcagctattaaacattctgaagcaagagctattaaacat
tctaaagcaggtgttattgtgcactatgcatatatttgcatatattattt
ctcagaacagtcctgtaaaatgtgtgctgtatccttaatttctagataag
gaagcagtggattggaaagctgatggcttgtgtatgatgatgctactaat
aatgacatattccaggcagaaatcccctgcttctgcttcccatggtcact
tgtgccaggcccggttcctcttctccctggggctcaaacctggtagctgc
cccctctcaccaatacaagccttgtgaagatcctggagcagggcacaagc
cgctgacgtctgctccagtgagaagccctgctggcggcagaaaatgagcg
cgtggcagaagaggtccagggtgatggcgtccctcaggctctgctcagga
caccctagctccagcagctcagccagcacccttgggagggagagaggagt
gagtgacaactgggcctcctttcatcaccctacacccaccacatgctttg
gagtcagccattcctcatgttttcccaacctgacccctcagcctttgcca
agagctcttcccacgcccccctcagtccctactccctcatctcagcattg
gtggtcttctccagctggtgcatggaatggacgtccaggtacttcctgtt
atagtaaaagaaaaagagtattggtggccgttacctatcatgacaaggct
gtgacagctagtgcgtgagactgggagttggccaccggcgtgaagctggg
ctcttttgccagcttgctgtgtgactatgcaaatcgttaactctctgggc
ctaaatgaactcttatgagcctctccatttctaaagcctagggttaggaa
tcttgaattctttttctgctctgccactaccttgtatgaccttggtcaa
gtactccctgtggccctcagtgtcccctctgtacaatactccttagcgg
ccatgagactccatttacattctgtctttggctcttttttctcacactg
cctcctctttggactggagaggctggaaagccaacttccagtcacctgga
gtaattgcatctccaggcctcagtctcatctgtaaaatggggatgccctg
gccacagacaggactgttggggagacaataaagaacgcaaatattcagtg

taa
>NM_003890 2
ctgcagccatgggtgccctatggagctggtggatactctgggctggagca
accctcctgtggggattgacccaggaggcttcagtggacctcaagaacac
tggcagagaggaattcctcacagccttcctgcagaactatcagctggcct
acagcaaggcctacccccgcctccttatctccagtctgtcagagagcccc
gcttcagtctccatcctcagccaggcagacaacacctcaaagaaggtcac
agtgaggcccggggagtcggtcatggtcaacatcagtgccaaggctgaga
tgataggcagcaagatcttccagcatgcggtggtgatccattctgactat
gccatctctgtgcaggcactaaatgccaagcctgacacagcggagctgac
actgctgcggcccatccaggccctaggcaccgagtattttgtgctcacac
cccccggcacctcagccaggaatgtcaaggagtttgccgtggtggccggt
gccgcaggtgcctcggtcagtgtcacgctgaaggggtcagtgacattcaa
tggcaagttctatccagcaggcgatgtcctaagagtgactctacagccct
acaatgtggcccagctacagagctcagtggatctctcggggtcaaaggtc
acagctagtagccccgtggctgtcctctctggccacagctgtgcgcagaa
acatacgacctgcaaccatgtggttgagcagctgctacccacgtctgcct
ggggcacccactatgtagtacccacgctggcctcccaatctcgctatgat
ttggccttcgttgtggccagccaggccacaaagctgacctacaaccatgg
gggtatcactggctcccgtgggctccaggcaggtgatgtggtagagtttg
aggtccggccatcctggccactctacctgtctgcaaatgtgggcatccag
gtcctgttgtttggcacaggtgccataaggaatgaagtgacttatgaccc
ctacctggtcctgatcccagatgtggcggcctactgcccagcctatgtgg
tcaagagtgtaccaggctgtgagggcgtggccctggtagtggcacagacg
aaggctatcagcgggctgaccatagatgggcatgcagtgggggccaagct
cacctgggaggctgtgccaggcagtgagttctcgtatgctgaagtggagc
tcggcacagctgacatgatccacacggccgaggccaccaccaacttggga
ctgctcaccttcgggctggccaaggctataggctacgcaacagctgctga
ttgcggccggactgtactgtccccagtggagccctcctgcgaaggcatgc
agtgcgcagccgggcagcgctgccaggtggtaggcgggaaggccgggtgt
gtggcggagtccaccgctgtctgccgcgcccagggcgaccccccattacac
caccttcgacggccgtcgctacgacatgatgggcacctgttcgtacacga
tggtggagctgtgcagcgaggacgacaccctgcccgccttcagcgtggag
gccaagaacgagcaccggggcagccgccgcgtctcctacgtgggcctcgt
cactgtgcgcgcctacagccactctgtgtcgctgacccgcggtgaagttg
gcttcgtcctggttgacaaccagcgctcgcgcctgccagtctccctgagt
gagggtcgcctgcgtgtgtaccagagcggaccacgggccgtggtggagct
ggtctttgggctggtggtcacttatgactgggactgccagctggcactca
gcctgcctgcacgcttccaagaccaggtgtgcgggctgtgtggcaactat
aatggtgacccagcagacgacttcctcacgcctgacggggctctggctcc
tgacgctgtggagttcgcaagtagctggaagctggatgatggggactacc
tgtgtgaggatggctgccagaacaactgtcccgcctgcaccccaggccag
gcccaacactatgagggcgaccgactctgtggcatgctgaccaagctcga
tggccccttcgctgtctgccatgacaccctggaccccaggcccttcctgg
agcagtgtgtatatgacctgtgtgtggtcggtggggagcggctcagcctg
tgccgtggcctcagcgcctatgcccaggcctgtctggagcttggcatctc
ggttggggactggagatcaccagccaactgccccctgtcctgccctgcca
acagccgctatgagctctgcggccctgcttgcccgacctcctgcaacggg
gctgcggcgccgtccaactgctccgggcgcccctgcgtggagggctgcgt

gtgcctcccaggcttcgtggccagcggcggcgcctgcgtgccggcctcgt
cgtgtggctgcaccttccagggtctccagctcgctccgggccaggaagtg
tgggcggacgagttgtgccaaaggcgctgcacctgcaacggcgccaccca
tcaggtcacctgccgcgacaagcagagctgcccggcgggtgagcgctgca
gcgtccagaacggcctcctgggctgctaccccgatcgcttcgggacctgc
caggggtccggggacccacactatgtgagcttcgacggccggcgcttcga
cttcatgggcacctgcacgtacctgctggtcggctcatgcggccagaacg
cagcgctgcctgccttccgggtgctggtggaaaacgagcatcggggcagc
cagactgtgagctacacgcgcgccgtgcgggtggaggcccgcggggtgaa
ggtggccgtgcgccgggagtaccccgggcaagtgctggtggatgacgtcc
ttcagtatctgcccttccaagcagcagatgggcaggtgcaggtgttccga
cagggcagggatgccgtcgtgcgcacggactttggcctgactgtcactta
tgactggaatgcacgagtgactgccaaggtgcccagcagctatgctgagg
ccctgtgtggactctgtgggaacttcaacggggacccagctgatgacctg
gctctgcggggtgggggtcaagctgccaatgcactggcctttgggaacag
ctggcaagaagagacgaggcccggctgtggagcaactgaaccgggtgact
gtcccaagctggactccctggtggcccagcagctgcagagcaagaatgag
tgtggaatccttgccgaccccaaggggcccttccgggagtgccatagcaa
gctggacccccagggtgccgtgcgcgactgtgtctatgaccgctgcctgc
tgccaggccagtctgggccactgtgtgacgcactggccacctatgctgct
gcatgccaggctgctggagccacagtgcacccctggaggagtgaagaact
ttgcccactgagctgcccaccccacagccactatgaggcgtgttcctacg
gctgcccgctgtcctgtggagacctcccagtgcccgggggctgtggctca
gaatgccatgagggctgcgtgtgcgatgagggctttgcgctcagtggtga
gtcctgcctgcccctggcctcctgtggctgcgtacaccagggcacctacc
acccaccaggccagaccttctaccctggccccggatgtgattccctttgc
cactgccaggagggcggcctggtgtcctgtgagtcctccagctgcggacc
gcacgaggcctgccagccatccggtggcagcttgggctgtgtggccgtgg
gctctagcacctgccaggcgtcaggagacccccactacaccaccttcgat
ggccgccgcttcgacttcatgggcacctgcgtgtatgtgctggctcagac
ctgcggcacccggcctggcctgcatcggtttgccgtcctgcaggagaacg
tggcctggggtaatgggcgagtcagtgtgaccagggtgatcacggtccag
gtggcaaacttcaccctgcggctggagcagagacagtggaaggtcacggt
gaacggtgtggacatgaagctgcccgtggtgctggccaacggccagatcc
gtgcctcccagcatggttcagatgttgtgattgagaccgacttcggcctg
cgtgtggcctacgaccttgtgtactatgtgcgggtcaccgtccccggaaa
ctactaccagcagatgtgtggcctgtgtgggaactacaacggcgacccca
aggatgacttccagaagcccaatggctcacaggcaggcaacgccaatgag
ttcggcaactcctgggaggaggtggtgcccgactctccctgcctgccgcc
cacccccttgcccgccggggagcgaggactgtatccccagccacaagtgtc
ctcccgagctggagaagaagtatcagaaggaggagttctgtgggctcctc
tccagccccacagggccactgtcctcctgccacaagctggtggatcccca
gggtcccttgaaagattgcatctttgatctctgcctgggtggtgggaacc
tgagcattctctgcagcaacatccatgcctacgtgagtgcttgccaggcg
gctggaggccacgtggagccctggaggactgaaactttctgtcccatgga
gtgccctccgaacagtcactacgagctctgtgcggacacctgctccctgg
gctgctcagctctcagtgcccctccacagtgccaggatgggtgtgctgag
ggctgccagtgtgactccggcttcctctacaatggccaagcctgcgtgcc
catccagcaatgcggctgctaccacaatggtgtctactatgagccggagc

agacagtcctcattgacaactgtcggcagcagtgcacgtgccatgcgggt
aaaggcatggtgtgccaggaacacagctgcaagccggggcaggtgtgcca
gccctccggaggcatcctgagctgcgtcaccaaagacccgtgccacggcg
tgacatgccggccacaggagacatgcaaggagcagggtggccagggcgtg
tgcctgcccaactatgaggccacgtgctggctgtggggcgacccacacta
ccactccttcgatggccggaagtttgacttccagggcacctgtaactatg
tgctggcaacaactggctgcccgggggtcagcacccagggcctgacaccc
ttcaccgtcaccaccaagaaccagaaccggggcaaccctgctgtgtccta
cgtgagagtcgtcaccgtggctgccctcggcaccaacatctccatccaca
aggacgagatcggcaaagtccgggtgaacggtgtgctcacagccttgcct
gtctctgtggccgacgggcggatttcagtgacccagggtgcatcgaaggc
actgctggtggctgactttggactgcaagtcagctatgactggaactggc
gggtagacgtgacgctgcccagcagctatcatggcgcagtgtgcgggctc
tgcggtaacatggaccgcaaccccaacaatgaccaggtcttccctaatgg
cacactggctccctccatacccatctggggcggcagctggcgagccccag
gctgggacccactgtgttgggacgaatgtcgggggtcctgcccaacgtgc
cctgaggaccggttggagcagtacgagggccctggcttctgcggacccct
ggcccccggcacagggggccctttcaccacctgccatgctcatgtgccac
ctgagagcttcttcaagggctgtgttctggacgtctgcatgggtggtggg
gaccgtgacattctttgcaaggctctggcttcctatgtggccgcctgcca
ggctgctggggttgtcatcgaagactggcgggcacaggttggctgtgaga
tcacctgcccagaaaacagccactatgaggtctgtggctcaccctgcccg
gccagctgtccgtcccctgcaccccttacgacgccagccgtatgtgaggg
cccctgtgtggagggctgccagtgcgacgcgggtttcgtgttaagtgctg
accgctgtgttcccctcaacaacggctgcggctgctgggccaatggcacc
taccacgaggcgggcagtgagttttgggctgatggcacctgctcccagtg
gtgtcgctgcgggcctgggggtggctcgctggtctgcacacctgccagct
gtgggctgggtgaagtgtgtggcctcctgccatccggccagcacggctgc
cagcccgtcagcacagctgagtgccaggcgtggggtgacccccattacgt
cactctggatgggcaccgattcaatttccaaggcacctgcgagtacctgc
tgagtgcaccctgccacggaccacccttgggggctgagaacttcactgtc
actgtagccaatgagcaccggggcagccaggctgtcagctacacccgcag
tgtcaccctgcaaatctacaaccacagcctgacactgagtgcccgctggc
cccggaagctacaggtggacggcgtgttcgtcactctgcccttccagctg
gactcgctcctgcacgcacacctgagcggcgccgacgtggtggtgaccac
aacctcagggctctcgctggctttcgacggggacagcttcgtgcgcctgc
gcgtgccggcggcgtacgcgggctctctctgtggcttatgcgggaactac
aaccaggaccccgcagacgacctgaaggcggtgggcgggaagcccgccgg
atggcaggtgggcggcgcccagggctgcggggaatgtgtgtccaagccat
gcccgtcgccgtgcaccccagagcagcaagagtccttcggcggcccggac
gcctgcggcgtgatctccgccaccgacggcccgctggcgccctgccacgg
ccttgtgccgcccgcgcagtacttccagggctgcttgctggacgcctgcc
aagttcagggccatcctggaggcctctgtcctgcagtggccacctacgtg
gcagcctgtcaggccgctggggcccagctccgcgagtggaggcggccgga
cttctgtcccttccagtgccctgcccacagccactacgagctctgcggtg
actcctgtcctgggagctgcccgagcctgtcggcacccgagggctgtgag
tcggcctgccgtgaaggctgtgtctgcgatgctggcttcgtgctcagtgg
tgacacgtgtgtacctgtgggccagtgtggctgcctccacgatgaccgct
actacccactgggccagaccttctaccctggccctgggtgtgattccctt

tgccgctgccgggagggcggtgaggtgtcctgtgagccctccagctgcgg
cccgcatgagacctgccggccatccggtggcagcttgggctgcgtggccg
tgggctctaccacctgccaggcgtcgggagatccccactacaccaccttc
gatggccgccgcttcgacttcatgggcacctgcgtgtatgtgctggctca
gacctgcggcacccggcctggcctacatcggtttgccgtcctgcaggaga
acgtggcctggggtaatgggcgagtcagtgtgaccagggtgatcacggtc
caggtggcaaacttcaccctgcggctggagcagagacagtggaaggtcac
ggtgaacggtgtggacatgaagctgcccgtggtgctggccaacggccaga
tccgtgcctcccagcatggttcagatgttgtgattgagaccgacttcggc
ctgcgtgtggcctacgaccttgtgtactatgtgcgggtcaccgtccctgg
aaactactaccagctgatgtgtggcctgtgtgggaactacaacggcgacc
ccaaggatgacttccagaagcccaatggctcgcaggcaggcaacgccaat
gagttcggcaactcctgggaggaggtggtgcccgactctccctgcctgcc
gccgcccacctgcccgccggggagcgagggctgtatccccagcgaggagt
gtcctcccgagctggagaagaagtatcagaaggaggagttctgtgggctc
ctctccagccccacagggccactgtcctcttgccacaagctggtggatcc
ccagggtcccttgaaagattgcatctttgatctctgcctgggtggtggga
acctgagcattctctgcagcaacatccatgcctacgtgagtgcttgccag
gcagctggaggccaggtggagccctggaggaatgaaactttctgtcccat
ggaatgccctcagaacagtcactacgagctctgtgcggacacctgctccc
tgggctgctcggctctcagtgcccctctgcagtgcccagatgggtgtgct
gagggctgccagtgtgactccggcttcctctacaacggccaagcctgcgt
gcccatccagcaatgtggctgctaccacaatggtgcctactatgagccgg
agcagacagtcctcattgacaactgtcggcagcagtgcacgtgccatgtg
ggtaaagtcgtggtgtgccaggaacacagctgcaagccggggcaggtgtg
ccagccctccggaggcatcctgagctgcgtcaacaaagacccgtgccacg
gcgtgacatgccggccacaggagacatgcaaggagcagggtggccagggc
gtgtgcctgcccaactatgaggccacgtgctggctgtggggcgacccaca
ctaccactccttcgatggccggaagtttgacttccagggcacctgtaact
atgtgctggcaacaactggctgcccgggggtcagcacccagggcctgaca
cccttcaccgtcaccaccaagaaccagaaccggggcaaccctgctgtgtc
ctacgtgagagtcgtcaccgtggctgccctcggcaccaacatctccatcc
acaaggacgagatcggcaaagtccgggtgaacggtgtgctcacagccttg
cctgtctctgtggccgacgggcggatttcagtgacccagggtgcatcgaa
ggcactgctggtggctgactttggactgcaagtcagctatgactggaact
ggcgggtagacgtgacgctgcccagcagctatcatggcgcagtgtgcggg
ctctgcggtaacatggaccgcaaccccaacaatgaccaggtcttccctaa
tggcacactggctccctccatacccatctggggcggcagctggcgagccc
caggctgggacccactgtgttgggacgaatgtcgggggtcctgcccaacg
tgccctgaggaccggttggagcagtacgagggccctggcttctgcggacc
cctggcccccggcacagggggccctttcaccacctgccatgctcatgtgc
cacctgagagcttcttcaagggctgtgttctggacgtctgcatgggtggt
ggggaccgtgacattctttgcaaggctctggcttcctatgtggccgcctg
ccaggctgctggggttgtcatcgaagactggcgggcacaggttggctgtg
agatcacctgcccagaaaacagccactatgaggtctgtggcccaccctgc
ccggccagctgtccgtcccctgcaccccttacgacgccagccgtatgtga
gggcccctgtgtggagggctgccagtgcgacgcgggtttcgtgttaagtg
ctgaccgctgtgttcccctcaacaacggctgcggctgctgggccaatggc
acctaccacgaggcgggcagtgagttttgggctgatggcacctgctccca

gtggtgtcgctgcgggcctgggggtggctcgctggtctgcacacctgcca
gctgtgggctgggtgaagtgtgtggcctcctgccatccggccagcacggc
tgccagcccgtcagcacagctgagtgccaggcgtggggtgacccccatta
cgtcactctggatgggcaccgattcgatttccaaggcacctgcgagtacc
tgctgagtgcaccctgccacggaccacccttgggggctgagaacttcact
gtcactgtagccaatgagcaccggggcagccaggctgtcagctacacccg
cagtgtcaccctgcaaatctacaaccacagcctgacactgagtgcccgct
ggccccggaagctacaggtggacggcgtgttcgtcactctgcccttccag
ctggactcgctcctgcacgcacacctgagcggcgccgacgtggtggtgac
cacaacctcagggctctcgctggctttcgatggggacagcttcgtgcgcc
tgcgcgtgccggcggcgtacgcgggctctctctgtggcttatgcgggaac
tacaaccaggaccccgcagacgacctgaaggcggtgggcgggaagcccgc
cggatggcaggtgggcggcgcccagggctgcggggaatgtgtgtccaagc
catgcccgtcgccgtgcaccccagagcagcaagagtccttcggcggcccg
gacgcctgcggcgtgatctccgccaccgacggcccgctggcgccctgcca
cggccttgtgccgcccgcgcagtacttccagggctgcttgctggacgcct
gccaagttcagggccatcctggaggcctctgtcctgcagtggccacctac
gtggcagcctgtcaggccgctggggcccagctccgcgagtggaggcggcc
ggacttctgtcccttccagtgccctgcccacagccactacgagctctgcg
gtgactcctgtcctgggagctgcccgagcctgtcggcacccgagggctgt
gagtcggcctgccgtgaaggctgtgtctgcgatgctggcttcgtgctcag
tggtgacacgtgtgtacctgtgggccagtgtggctgcctccacgatgacc
gctactacccactgggccagaccttctaccctggccctgggtgtgattcc
ctttgccgctgccgggagggcggtgaggtgtcctgtgagccctccagctg
cggcccgcatgagacctgccggccatccggtggcagcttgggctgcgtgg
ccgtgggctctaccacctgccaggcgtcgggagatccccactacaccacc
ttcgatggccaccgcttcgacttcatgggcacctgcgtgtatgtgctggc
tcagacctgcggcacccggcctggcctgcatcggtttgccgtcctgcagg
agaacgtggcctggggtaatgggcgagtcagtgtgaccagggtgatcacg
gtccaggtggcaaacttcaccctgcggctggagcagagacagtggaaggt
cacggtgaacggtgtggacatgaagctgcccgtggtgctggccaacggcc
agatccgtgcctcccagcatggttcagatgttgtgattgagaccgacttc
ggcctgcgtgtggcctacgaccttgtgtactatgtgcgggtcaccgtccc
tggaaactactaccagctgatgtgtggcctgtgtgggaactacaacggcg
accccaaggatgacttccagaagcccaatggctcgcaggcaggcaacgcc
aatgagttcggcaactcctgggaggaggtggtgcccgactctccctgcct
gccgccgcccacctgcccgccggggagcgcgggctgtatccccagcgaca
agtgtcctcccgagctggagaagaagtatcagaaggaggagttctgtggg
ctcctctccagccccacagggccactgtcctcctgccacaagctggtgga
tccccagggtcccttgaaagattgcatctttgatctctgcctgggtggtg
ggaacctgagcattctctgcagcaacatccatgcctacgtgagtgcttgc
caggcggctggaggccacgtggagccctggaggaatgaaactttctgtcc
catggaatgccctcagaacagtcactacgagctctgtgcggacacctgct
ccctgggctgctcggctctcagtgcccctctgcagtgcccagatgggtgt
gctgagggctgccagtgtgactccggcttcctctacaacggccaagcctg
cgtgcccatccagcaatgtggctgctaccacaatggtgtctactatgagc
cggagcagacagtcctcattgacaactgtcggcagcagtgcacgtgccat
gtgggtaaagtcgtggtgtgccaggaacacagctgcaagccggggcaggt
gtgccagccctccggaggcatcctgagctgcgtcaccaaagacccgtgcc

acggcgtgacatgccggccacaggagacatgcaaggagcagggtggccag
ggcgtgtgcctgcccaactatgaggccacgtgctggctgtggggcgaccc
acactaccactccttcgatggccggaagtttgacttccagggcacctgta
actatgtgctggcaacaactggctgcccgggggtcagcacccagggcctg
acacccttcaccgtcaccaccaagaaccagaaccggggcaaccctgctgt
gtcctacgtgagagtcgtcaccgtggctgccctcggcaccaacatctcca
tccacaaggacgagatcggcaaagtccgggtgaacggtgtgctcacagcc
ttgcctgtctccgtggccgacgggcggatttcagtggcccagggtgcatc
gaaggcactgctggtggctgactttggactgcaagtcagctatgactgga
actggcgggtagacgtgacgctccccagcagctatcatggcgcagtgtgc
gggctctgcggtaacatggaccgcaaccccaacaatgaccaggtcttccc
taatggcacactggctccctccatacccatctggggcggcagctggcgag
ccccaggctgggacccactgtgttgggacgaatgtcgggggtcctgccca
acgtgccctgaggaccggttggagcagtacgagggccctggcttctgcgg
acccctttcatctggcacagggggccccttcaccacctgccatgctcatg
tgccacctgagagcttcttcaagggctgtgttctggacgtctgcatgggt
ggtggggaccgtgacattctttgcaaggctctggcttcctacgtggccgc
ctgccaggccgctggggttgtcatcgaagactggcgggcacaggttggct
gtgagatcacctgcccagaaaacagccactatgaggtctgtggcccaccc
tgcccagccagctgtccgtcccctgcacccccttacgacgccagccgtatg
tgagggcccctgtgtggagggctgccagtgcgacgcgggtttcgtgttaa
gtgctgaccgctgtgttcccctcaacaacggctgcggctgctgggccaat
ggcacctaccacgaggcgggcagtgagttttgggctgatggcacctgctc
ccagtggtgtcgctgcgggcctgggggtggctcgctggtctgcacacctg
ccagctgtgggctgggtgaagtgtgtggcctcctgccatccggccagcac
ggctgccagcccgtcagcacagctgagtgccaggcgtggggtgaccccca
ttacgtcactctggatgggcaccgattcgatttccaaggcacctgcgagt
acctgctgagtgcaccctgccacggaccacccttgggggctgagaacttc
actgtcactgtagccaatgagcaccggggcagccaggctgtcagctacac
ccgcagtgtcaccctgcaaatctacaaccacagcctgacactgagtgccc
gctggccccggaagctacaggtcgacggcgtgttcgtggctctgcctttc
cagctggactcgctcctgcacgcacacctgagcggcgccgacgtggtggt
gaccacaacctcagggctctcgctggctttcgatggggacagcttcgtgc
gcctgcgcgtgccggcggcgtacgcggcctctctctgtggcttatgcggg
aactacaaccaggaccccgcagacgacctcaaggctgtgggcgggaagcc
cgctggatggcaggtgggcggggcccagggctgcggggaatgtgtgtcca
agccatgcccgtcgccgtgcaccccagagcagcaggagtccttcggcggc
ccggacgcctgcggcgtgatctccgccaccgacggcccgctggcaccctg
ccacggccttgtgccgcccgcgcagtacttccagggctgcttgctggacg
cctgccaagttcagggccatcctggaggcctctgtcctgcagtggctacc
tacgtggcagcctgtcaggccgctggggcccagctcggcgagtggaggcg
gccggacttctgtcccttgcagtgccctgcccacagccactatgagctct
gcggtgactcctgccctgtgagctgcccgagcctctcagcacccgagggc
tgtgagtcggcctgccgtgaaggctgtgtctgcgatgctggcttcgtact
cagtggtgacacctgcgtacccgtgggccagtgtggctgcctccatgatg
gccgctactacccactgggcgaggtcttctacccgggccctgagtgtgag
cggcgctgtgagtgtgggccaggtggccatgtcacctgccaggagggcgc
agcctgtgggccccatgaggagtgccggttagaggatggtgtccaggcct
gtcatgccacaggctgtggccgctgcctggccaacgggggcatccactac

atcacccttgatggccgtgtctacgacctgcatggctcctgctcctatgt
cttggcccaagtctgccacccaaagcctggggacgaggacttttccatcg
tgcttgagaagaatgcagctggagatctccaacgcctcctggttactgtg
gctggccaggttgtgagcctagctcaggggcagcaggtcaccgtggacgg
cgaggctgtggccctgcctgtggctgtgggccgcgtgcgggtgaccgccg
agggccgaaacatggttctgcagacgaccaaggggctgcggcttctcttt
gatggcgatgcccacctcctcatgtccatccccagccccttccgtggacg
gctctgtggcctctgtgggaacttcaatggcaactggagtgacgactttg
tcctgcccaatggctcagcagcgtccagtgtggagaccttcggggctgca
tggcgggcgcccggctcctccaagggctgtggcgagggctgcgggcccca
aggctgcccagtgtgcttggcagaggagactgcaccctatgagagcaacg
aggcctgcgggcagctccggaacccccagggccccttcgcgacctgccag
gcggtgctgagtccctctgagtacttccgccaatgcgtatacgacctgtg
cgcgcaaaagggtgacaaagccttcctgtgccgcagcctggcagcctaca
cggcggcctgtcaggcagctggcgtggccgtgaagccctggaggacagac
agcttctgcccgctccattgccccgcccacagccactactccatctgcac
tcgcacctgccagggatcctgtgcggctctctccggcctcacgggctgca
ccacccgctgttttgagggctgtgagtgcgacgaccgcttcctgctttcc
cagggtgtctgcatccctgtccaagattgtggctgcacccataatggccg
atacttgccggtaaactcctccctgctgacctcagactgcagcgagcgct
gttcctgttcctcaagctctggcctgacatgccaggcagctggctgccca
ccaggccgtgtatgtgaggtcaaggctgaagcccggaactgctgggccac
ccgtggtctctgtgtcctgtctgtgggtgccaacctcaccacctttgatg
gggcccgtggtgccaccacctctcctggtgtctatgagctctcttcccgc
tgcccaggactacagaataccatcccctggtaccgtgtagttgccgaagt
ccagatctgccatggcaaaacggaggctgtgggccaggtccacatcttct
tccaggatgggatggtgacgttgactccaaacaagggtgtgtgggtgaat
ggtctccgagtggatctcccagctgagaagttagcatctgtgtccgtgag
tcgtacacctgatggctccctgctagtccgccagaaggcaggggtccagg
tgtggcttggagccaatgggaaggtggctgtgattgtcagcaatgaccat
gctgggaaactgtgtggggcctgtggaaactttgacggggaccagaccaa
tgattggcatgactcccaggagaagccagcgatggagaaatggagagcgc
aggacttctccccatgttatggctgatcagtcatccaccaggaacgaaga
tttcctgaagaagacctggtccctctggaggttgcagtggctgaaggatg
catcatgtgctcctaccctgctctaccgcttttctgggtcacagaggcca
aatgtgagagcattgaataaatatcttaagctaagctgcaaaaaaaaaaa
aaaaaaa
>NM_001886 2
acatgttccctgggcctatctcggaaggggccacaatgaccctgcaatgc
acaaagtcagcgggaccctggaagatggtggtgtgggatgaggacggctt
ccagggccggcggcacgagttcacggccgagtgccccagcgtgctggagc
ttggcttcgagactgtgcgatctttgaaagtgctgagtggagcgtgggtg
ggctttgagcatgctggcttccaagggcagcagtacattctggaacgagg
cgaatatccaagctgggatgcctggggcggcaacacggcctaccccgccg
agaggctcacctccttccggcctgcggcctgtgctaaccaccgtgactcg
aggctgacaatcttcgagcaagagaacttcctgggcaagaaaggagagct
gagcgatgactatccttccctccaggccatgggatgggaaggcaatgaag
tagggtccttccacgtccactctggggcctgggtttgctcccagtttccg
ggctaccgaggatttcagtatgtgctggaatgcgatcaccattccggtga

ctacaaacatttccgggagtggggctctcatgccccgaccttccaggtgc
agagcatccgcaggatccagcagtgaacaggggtgcggcacggaggagcg
catgcgtgcttatctgcaatggaggcgctctggaggctgtggtgtgttct
ctccttctgcctcccctgtaacctgtgtgaacccagcacccatgtgaac
tggtccgtgcacagtcagcacaaaaaactcaaacgaataaaaaagagaaa
gtctggtattaaaaaaaaaaaaaaaaaa
>NM_030960 2
gtcacgggattcggagccgggcggctacgggcggggtgtcgcagctctct
tcgacgtacctgtcctcaggagccgcggcggcgactgcgcctcggacggc
cgtcggggccgagaaccatgagccccaggggcacgggctgctccgccggg
ctgctgatgactgtcggctggctgcttctggcgggcctccagtccgcgcg
cgggaccaacgtcaccgctgccgtccaggatgccggcctggcccacgaag
gcgagggcgaggaggagaccgaaaacaacgacagcgagaccgcggagaac
tacgctccgcctgaaaccgaggatgtttcaaataggaatgtcgtcaaaga
agtagaattcggaatgtgcaccgttacatgtggtattggtgttagagaag
ttatattaacaaatggatgccctggtggtgaatccaagtgtgttgtacgg
gtagaagaatgccgtggaccaacagattgtggctggggtaaaccaatttc
agaaagtcttgaaagtgttagattggcatgtattcacacatctcccttaa
atcgtttcaaatatatgtggaaacttctaagacaagaccaacaatccatt
atacttgtaaatgattcagcaatcctagaagtacgcaaggaaagtcaccc
cttggctttcgagtgtgacacactggataataatgaaatagtagcaacta
ttaaattcacagtctatacgagcagtgaattgcagatgagaagatcaagc
ctaccagccactgatgcagccctaatttttgtgctgaccataggagtcat
tatctgtgtatttataattttcttattgatcttcataatcataaattggg
cagcagtcaaggctttctgggggcaaaagcctctacacctgaggtacaa
tccgagcagagttctgtgagatacaaagattcaacttctcttgaccaatt
accaacagaaatgcctggtgaagatgatgctttaagtgaatggaatgaat
gatgtttgaatgatatataacaaaccaaaggatattacagaatattagat
tcattattacaaaaataaaatacacattgaaatactttaataatgttgcg
atggattgccacagtgtgaaggaaatgcagtgtggggataggactatttt
atcagtgcatttttccagtacagttatcaaatattacttttaatttgttc
tcaacacttatttcaggtaatagcttggggatatttatctaaaggtaccc
ccaacaaatcttctaagtgcatttttgatcactttgataacttcttaggt
gatttgcctgttttgtcttaaataagaacaatgtaatatagaaatgcttt
acatattagactttctctcccctggaagcactgggttgaacttgctaaag
taaatcatactttagaatctcttcagggaatgtgacatacaaagtttgta
agacatgaagtaataacgataatgataacaataaatgcttacttagtgaa
a
>NM_004103 4
gaataggactagcaacttcatagacggttgtgaagacaagctagacggca
gatgaaagttcttggcacagagtgaacacttgataaactatatggcagcc
acagcctccggagccgttgcacacctacctgcccggccgacttacctgta
cttgccgccgtcccggctcacctggcggtgcccgaggagtagtcgctgga
gtccgcgcctccctgggactgcaatgtgccgatcttagctgctgcctgag
aggatgtctggggtgtccgagcccctgagtcgagtaaagttgggcacgtt
acgccggcctgaaggccctgcagagcccatggtggtggtaccagtagatg
tggaaaaggaggacgtgcgtatcctcaaggtctgcttctatagcaacagc
ttcaatcctgggaaaaacttcaaactggtcaaatgcactgtccagacgga
gatccgggagatcatcacctccatcctgctgagcgggcggatcgggccca

acatccggttggctgagtgctatgggctgaggctgaagcacatgaagtcc
gatgagatccactggctgcacccacagatgacggtgggtgaggtgcagga
caagtatgagtgtctgcacgtggaagccgagtggaggtatgaccttcaaa
tccgctacttgccagaagacttcatggagagcctgaaggaggacaggacc
acgctgctctatttttaccaacagctccggaacgactacatgcagcgcta
cgccagcaaggtcagcgagggcatggccctgcagctgggctgcctggagc
tcaggcggttcttcaaggatatgccccacaatgcacttgacaagaagtcc
aacttcgagctcctagaaaaggaagtggggctggacttgtttttcccaaa
gcagatgcaggagaacttaaagcccaaacagttccggaagatgatccagc
agaccttccagcagtacgcctcgctcagggaggaggagtgcgtcatgaag
ttcttcaacactctcgccggcttcgccaacatcgaccaggagacctaccg
ctgtgaactcattcaaggatggaacattactgtggacctggtcattggcc
ctaaagggatccgccagctgactagtcaggacgcaaagcccacctgcctg
gccgagttcaagcagatcaggtccatcaggtgcctcccgctggaggaggg
ccaggcagtacttcagctgggcattgaaggtgcccccaggccttgtcca
tcaaaacctcatccctagcagaggctgagaacatggctgacctcatagac
ggctactgccggctgcagggtgagcaccaaggctctctcatcatccatcc
taggaaagatggtgagaagcggaacagcctgccccagatccccatgctaa
acctggaggcccggcggtcccacctctcagagagctgcagcatagagtca
gacatctacgcagagattcccgacgaaaccctgcgaaggcccggaggtcc
acagtatggcattgcccgtgaagatgtggtcctgaatcgtattcttgggg
aaggctttttggggaggtctatgaaggtgtctacacaaatcacaaaggg
gagaaaatcaatgtagctgtcaagacctgcaagaaagactgcactctgga
caacaaggagaagttcatgagcgaggcagtgatcatgaagaacctcgacc
acccgcacatcgtgaagctgatcggcatcattgaagaggagcccacctgg
atcatcatggaattgtatccctatggggagctgggccactacctggagcg
gaacaagaactccctgaaggtgctcaccctcgtgctgtactcactgcaga
tatgcaaagccatggcctacctggagagcatcaactgcgtgcacagggac
attgctgtccggaacatcctggtggcctcccctgagtgtgtgaagctggg
ggactttggtctttcccggtacattgaggacgaggactattacaaagcct
ctgtgactcgtctccccatcaaatggatgtccccagagtccattaacttc
cgacgcttcacgacagccagtgacgtctggatgttcgccgtgtgcatgtg
ggagatcctgagctttgggaagcagcccttcttctggctggagaacaagg
atgtcatcggggtgctggagaaaggagaccggctgcccaagcctgatctc
tgtccaccggtcctttataccctcatgacccgctgctgggactacgaccc
cagtgaccggccccgcttcaccgagctggtgtgcagcctcagtgacgttt
atcagatggagaaggacattgccatggagcaagagaggaatgctcgctac
cgaacccccaaaatcttggagcccacagccttccaggaacccccacccaa
gcccagccgacctaagtacagacccccctccgcaaaccaacctcctggctc
caaagctgcagttccaggttcctgagggtctgtgtgccagctctcctacg
ctcaccagccctatggagtatccatctcccgttaactcactgcacacccc
acctctccaccggcacaatgtcttcaaacgccacagcatgcgggaggagg
acttcatccaacccagcagccgagaagaggcccagcagctgtgggaggct
gaaaaggtcaaaatgcggcaaatcctggacaaacagcagaagcagatggt
ggaggactaccagtggctcaggcaggaggagaagtccctggaccccatgg
tttatatgaatgataagtccccattgacgccagagaaggaggtcggctac
ctggagttcacagggcccccacagaagcccccgaggctgggcgcacagtc
catccagcccacagctaacctggaccggactgatgacctggtgtacctca
atgtcatggagctggtgcgggccgtgctggagctcaagaatgagctctgt

cagctgccccccgagggctacgtggtggtggtgaagaatgtggggctgac
cctgcggaagctcatcgggagcgtggatgatctcctgccttccttgccgt
catcttcacggacagagatcgagggcacccagaaactgctcaacaaagac
ctggcagagctcatcaacaagatgcggctggcacagcagaacgccgtgac
ctccctaagtgaggagtgcaagaggcagatgctgacggcttcacacaccc
tggctgtggacgccaagaacctgctcgacgctgtggaccaggccaaggtt
ctggccaatctggcccacccacctgcagagtgacggagggtgggggccac
ctgcctgcgtcttccgcccctgcctgccatgtacctcccctgccttgctg
ttggtcatgtgggtcttccagggggaaggccaaggggagtcaccttccct
tgccactttgcacgacgccctctccccacccctacccctggctgtactgc
tcaggctgcagctggacagaggggactctgggctatggacacagggtgac
ggtgacaaagatggctcagagggggactgctgctgcctggccactgctcc
ctaagccagcctggtccatgcagggggctcctgggggtggggaggtgtca
catggtgcccctagctttatatatggacatggcaggccgatttgggaacc
aagctattcctttcccttcctcttcggccctcagatgtcccttgatgcac
agagaagctggggaggagctttgttttgggggtcaggcagccagtgagat
gagggatgggcctggcattcttgtacagtgtatattgaaatttatttaat
gtgagtttggtctggactgacagcatgtgccctcctgagggaggacctgg
ggcacagtccaggaacaagctaattgggagtccaggcacaggatgctgtg
ttgtcaacaaaccaagcatcaggggggaagaagcagagagatgcggccaag
ataggaccttgggccaaatccgctctcttcctgcccctctttctctttct
tcctttactttcccttgcttttccctcttttcttactcctcctctttctc
tccccaacccccattctcatctgcacccttcttttctcatgtgtttgcat
aaacattcttttaacttctttctatttgacttgtggttgaattaaaattg
tcccatttgctttgcaaaaaaaaaaaaaaaaaaaaaaaaaaaa
>NM_173174 2
gcagttcccgcctcctcaggtccgggcgggtccctggccggggtagcacg
gaagggtctcccaggcggcgtagtagggcttccgtgttactggaaaccta
cttccggctgcaaatgggaaaaggagcctctaccttaaccaatccccggg
aacctcaggcccgcggatgggagaaaccagagatgccaacttcctgcttc
cgaagtagtggctgggtcttaaagcaccgatgtttctgcattgaatagcc
ctggaagacagaattcttgtctctccaaagatctggcagacttattccca
attataagagatccttgcaaaggaggtgtcttgcttcaccttcaccttcc
actgtgattgtaagttttctgaggcctcgccagccatgcggaactgaata
ggactagcaacttcatagacggttgtgaagacaagctagacggcagatga
aagttcttggcacagagtgaacacttgataaactatatggcagggagggc
tggaacggggcttgtttgaagagcaatatgagccagggttatagacctga
gtttggggtaagagtggaacatccaaatggaagagtccagcagctgaaag
gacatttgttcaaaggcctttttcattacagttttctccttcttctgatc
cagccacagcctccggagccgttgcacacctacctgcccggccgacttac
ctgtacttgccgccgtcccggctcacctggcggtgcccgaggagtagtcg
ctggagtccgcgcctccctgggactgcaatgtgccgatcttagctgctgc
ctgagaggatgtctggggtgtccgagcccctgagtcgagtaaagttgggc
acgttacgccggcctgaaggccctgcagagcccatggtggtggtaccagt
agatgtggaaaaggaggacgtgcgtatcctcaaggtctgcttctatagca
acagcttcaatcctgggaaaaacttcaaactggtcaaatgcactgtccag
acggagatccgggagatcatcacctccatcctgctgagcgggcggatcgg
gcccaacatccggttggctgagtgctatgggctgaggctgaagcacatga
agtccgatgagatccactggctgcacccacagatgacggtgggtgaggtg

caggacaagtatgagtgtctgcacgtggaagccgagtggaggtatgacct
tcaaatccgctacttgccagaagacttcatggagagcctgaaggaggaca
ggaccacgctgctctatttttaccaacagctccggaacgactacatgcag
cgctacgccagcaaggtcagcgagggcatggccctgcagctgggctgcct
ggagctcaggcggttcttcaaggatatgccccacaatgcacttgacaaga
agtccaacttcgagctcctagaaaaggaagtggggctggacttgtttttc
ccaaagcagatgcaggagaacttaaagcccaaacagttccggaagatgat
ccagcagaccttccagcagtacgcctcgctcagggaggaggagtgcgtca
tgaagttcttcaacactctcgccggcttcgccaacatcgaccaggagacc
taccgctgtgaactcattcaaggatggaacattactgtggacctggtcat
tggccctaaagggatccgccagctgactagtcaggacgcaaagcccacct
gcctggccgagttcaagcagatcaggtccatcaggtgcctcccgctggag
gagggccaggcagtacttcagctgggcattgaaggtgccccccaggcctt
gtccatcaaaacctcatccctagcagaggctgagaacatggctgacctca
tagacggctactgccggctgcagggtgagcaccaaggctctctcatcatc
catcctaggaaagatggtgagaagcggaacagcctgccccagatccccat
gctaaacctggaggcccggcggtcccacctctcagagagctgcagcatag
agtcagacatctacgcagagattcccgacgaaaccctgcgaaggcccgga
ggtccacagtatggcattgcccgtgaagatgtggtcctgaatcgtattct
tggggaaggctttttggggaggtctatgaaggtgtctacacaaatcaca
aaggggagaaaatcaatgtagctgtcaagacctgcaagaaagactgcact
ctggacaacaaggagaagttcatgagcgaggcagtgatcatgaagaacct
cgaccacccgcacatcgtgaagctgatcggcatcattgaagaggagccca
cctggatcatcatggaattgtatccctatggggagctgggccactacctg
gagcggaacaagaactccctgaaggtgctcaccctcgtgctgtactcact
gcagatatgcaaagccatggcctacctggagagcatcaactgcgtgcaca
gggacattgctgtccggaacatcctggtggcctcccctgagtgtgtgaag
ctgggggactttggtctttcccggtacattgaggacgaggactattacaa
agcctctgtgactcgtctccccatcaaatggatgtccccagagtccatta
acttccgacgcttcacgacagccagtgacgtctggatgttcgccgtgtgc
atgtgggagatcctgagctttgggaagcagcccttcttctggctggagaa
caaggatgtcatcggggtgctggagaaaggagaccggctgcccaagcctg
atctctgtccaccggtcctttataccctcatgacccgctgctgggactac
gaccccagtgaccggccccgcttcaccgagctggtgtgcagcctcagtga
cgtttatcagatggagaaggacattgccatggagcaagagaggaatgctc
gctaccgaaccccccaaaatcttggagcccacagccttccaggaacccca
cccaagcccagccgacctaagtacagacccccctccgcaaaccaacctcct
ggctccaaagctgcagttccaggttcctgagggtctgtgtgccagctctc
ctacgctcaccagccctatggagtatccatctcccgttaactcactgcac
accccacctctccaccggcacaatgtcttcaaacgccacagcatgcggga
ggaggacttcatccaacccagcagccgagaagaggcccagcagctgtggg
aggctgaaaaggtcaaaatgcggcaaatcctggacaaacagcagaagcag
atggtggaggactaccagtggctcaggcaggaggagaagtccctggaccc
catggtttatatgaatgataagtccccattgacgccagagaaggaggtcg
gctacctggagttcacagggcccccacagaagcccccgaggctgggcgca
cagtccatccagcccacagctaacctggaccggactgatgacctggtgta
cctcaatgtcatggagctggtgcgggccgtgctggagctcaagaatgagc
tctgtcagctgccccccgagggctacgtggtggtggtgaagaatgtgggg
ctgaccctgcggaagctcatcgggagcgtggatgatctcctgccttcctt

gccgtcatcttcacggacagagatcgagggcacccagaaactgctcaaca
aagacctggcagagctcatcaacaagatgcggctggcacagcagaacgcc
gtgacctccctaagtgaggagtgcaagaggcagatgctgacggcttcaca
caccctggctgtggacgccaagaacctgctcgacgctgtggaccaggcca
aggttctggccaatctggcccacccacctgcagagtgacggagggtgggg
gccacctgcctgcgtcttccgcccctgcctgccatgtacctcccctgcct
tgctgttggtcatgtgggtcttccagggggaaggccaaggggagtcacct
tcccttgccactttgcacgacgccctctccccacccctacccctggctgt
actgctcaggctgcagctggacagaggggactctgggctatggacacagg
gtgacggtgacaaagatggctcagagggggactgctgctgcctggccact
gctccctaagccagcctggtccatgcagggggctcctgggggtggggagg
tgtcacatggtgcccctagctttatatatggacatggcaggccgatttgg
gaaccaagctattcctttcccttcctcttcggccctcagatgtcccttga
tgcacagagaagctggggaggagctttgttttgggggtcaggcagccagt
gagatgagggatgggcctggcattcttgtacagtgtatattgaaatttat
ttaatgtgagtttggtctggactgacagcatgtgccctcctgagggagga
cctggggcacagtccaggaacaagctaattgggagtccaggcacaggatg
ctgtgttgtcaacaaaccaagcatcaggggggaagaagcagagagatgcgg
ccaagataggaccttgggccaaatccgctctcttcctgcccctctttctc
tttcttcctttactttcccttgcttttccctcttttcttactcctcctct
ttctctccccaacccccattctcatctgcacccttcttttctcatgtgtt
tgcataaacattcttttaacttctttctatttgacttgtggttgaattaa
aattgtcccatttgctttgcaaaaaaaaaaaaaaaaaaaaaaaaaaaa
>NM_173176 2
aaggaagtggggaggagagcagcaggggtgtggttaacaactcagaggag
gagggagaatctaacctgtcagcccttttactcagccacagcctccggag
ccgttgcacacctacctgcccggccgacttacctgtacttgccgccgtcc
cggctcacctggcggtgcccgaggagtagtcgctggagtccgcgcctccc
tgggactgcaatgtgccgatcttagctgctgcctgagaggatgtctgggg
tgtccgagcccctgagtcgagtaaagttgggcacgttacgccggcctgaa
ggccctgcagagcccatggtggtggtaccagtagatgtggaaaaggagga
cgtgcgtatcctcaaggtctgcttctatagcaacagcttcaatcctggga
aaaacttcaaactggtcaaatgcactgtccagacggagatccgggagatc
atcacctccatcctgctgagcgggcggatcgggcccaacatccggttggc
tgagtgctatgggctgaggctgaagcacatgaagtccgatgagatccact
ggctgcacccacagatgacggtgggtgaggtgcaggacaagtatgagtgt
ctgcacgtggaagccgagtggaggtatgaccttcaaatccgctacttgcc
agaagacttcatggagagcctgaaggaggacaggaccacgctgctctatt
tttaccaacagctccggaacgactacatgcagcgctacgccagcaaggtc
agcgagggcatggccctgcagctgggctgcctggagctcaggcggttctt
caaggatatgccccacaatgcacttgacaagaagtccaacttcgagctcc
tagaaaaggaagtggggctggacttgtttttcccaaagcagatgcaggag
aacttaaagcccaaacagttccggaagatgatccagcagaccttccagca
gtacgcctcgctcagggaggaggagtgcgtcatgaagttcttcaacactc
tcgccggcttcgccaacatcgaccaggagacctaccgctgtgaactcatt
caaggatggaacattactgtggacctggtcattggccctaaagggatccg
ccagctgactagtcaggacgcaaagcccacctgcctggccgagttcaagc
agatcaggtccatcaggtgcctcccgctggaggagggccaggcagtactt
cagctgggcattgaaggtgccccccaggccttgtccatcaaaacctcatc

cctagcagaggctgagaacatggctgacctcatagacggctactgccggc
tgcagggtgagcaccaaggctctctcatcatccatcctaggaaagatggt
gagaagcggaacagcctgccccagatccccatgctaaacctggaggcccg
gcggtcccacctctcagagagctgcagcatagagtcagacatctacgcag
agattcccgacgaaaccctgcgaaggcccggaggtccacagtatggcatt
gcccgtgaagatgtggtcctgaatcgtattcttggggaaggctttttgg
ggaggtctatgaaggtgtctacacaaatcacaaaggggagaaaatcaatg
tagctgtcaagacctgcaagaaagactgcactctggacaacaaggagaag
ttcatgagcgaggcagtgatcatgaagaacctcgaccacccgcacatcgt
gaagctgatcggcatcattgaagaggagcccacctggatcatcatggaat
tgtatccctatggggagctgggccactacctggagcggaacaagaactcc
ctgaaggtgctcaccctcgtgctgtactcactgcagatatgcaaagccat
ggcctacctggagagcatcaactgcgtgcacagggacattgctgtccgga
acatcctggtggcctcccctgagtgtgtgaagctgggggactttggtctt
tcccggtacattgaggacgaggactattacaaagcctctgtgactcgtct
ccccatcaaatggatgtccccagagtccattaacttccgacgcttcacga
cagccagtgacgtctggatgttcgccgtgtgcatgtgggagatcctgagc
tttgggaagcagcccttcttctggctggagaacaaggatgtcatcggggt
gctggagaaaggagaccggctgcccaagcctgatctctgtccaccggtcc
tttataccctcatgacccgctgctgggactacgaccccagtgaccggccc
cgcttcaccgagctggtgtgcagcctcagtgacgtttatcagatggagaa
ggacattgccatggagcaagagaggaatgctcgctaccgaaccccccaaaa
tcttggagcccacagccttccaggaaccccccacccaagcccagccgacct
aagtacagaccccctccgcaaaccaacctcctggctccaaagctgcagtt
ccaggttcctgagggtctgtgtgccagctctcctacgctcaccagcccta
tggagtatccatctcccgttaactcactgcacaccccacctctccaccgg
cacaatgtcttcaaacgccacagcatgcgggaggaggacttcatccaacc
cagcagccgagaagaggcccagcagctgtgggaggctgaaaaggtcaaaa
tgcggcaaatcctggacaaacagcagaagcagatggtggaggactaccag
tggctcaggcaggaggagaagtccctggaccccatggtttatatgaatga
taagtccccattgacgccagagaaggaggtcggctacctggagttcacag
ggcccccacagaagcccccgaggctgggcgcacagtccatccagcccaca
gctaacctggaccggactgatgacctggtgtacctcaatgtcatggagct
ggtgcgggccgtgctggagctcaagaatgagctctgtcagctgccccccg
agggctacgtggtggtggtgaagaatgtggggctgaccctgcggaagctc
atcgggagcgtggatgatctcctgccttccttgccgtcatcttcacggac
agagatcgagggcacccagaaactgctcaacaaagacctggcagagctca
tcaacaagatgcggctggcacagcagaacgccgtgacctccctaagtgag
gagtgcaagaggcagatgctgacggcttcacacaccctggctgtggacgc
caagaacctgctcgacgctgtggaccaggccaaggttctggccaatctgg
cccacccacctgcagagtgacggagggtgggggccacctgcctgcgtctt
ccgcccctgcctgccatgtacctcccctgccttgctgttggtcatgtggg
tcttccaggggggaaggccaaggggagtcaccttcccttgccactttgcac
gacgccctctccccaccccctacccctggctgtactgctcaggctgcagct
ggacagaggggactctgggctatggacacagggtgacggtgacaaagatg
gctcagagggggactgctgctgcctggccactgctccctaagccagcctg
gtccatgcaggggctcctgggggtggggaggtgtcacatggtgcccta
gctttatatatggacatggcaggccgatttgggaaccaagctattccttt
cccttcctcttcggccctcagatgtcccttgatgcacagagaagctgggg

aggagctttgttttgggggtcaggcagccagtgagatgagggatgggcct
ggcattcttgtacagtgtatattgaaatttatttaatgtgagtttggtct
ggactgacagcatgtgccctcctgagggaggacctggggcacagtccagg
aacaagctaattgggagtccaggcacaggatgctgtgttgtcaacaaacc
aagcatcaggggggaagaagcagagagatgcggccaagataggaccttggg
ccaaatccgctctcttcctgcccctctttctctttcttcctttactttcc
cttgcttttccctcttttcttactcctcctctttctctccccaaccccca
ttctcatctgcacccttcttttctcatgtgtttgcataaacattctttta
acttctttctatttgacttgtggttgaattaaaattgtcccatttgcttt
gcaaaaaaaaaaaaaaaaaaaaaaaaaaaaa
>NM_030770 2
ccagggagacctcatcttccaaccaagcttgctgggctagctttaatcaa
tgctggcctgagaacaggagcggaacattgcctagtagaccctgaggctt
tacaacagtgccactgacccctatgagcctgatgctggatgaccaacccc
ctatggaggcccagtatgcagaggagggcccaggacctgggatcttcaga
gcagagcctggagaccagcagcatcccatttctcaggcagtgtgctggcg
ttccatgcgacgtggctgtgcagtgctgggagccctggggctgctggccg
gtgcaggtgttggctcatggctcctagtgctgtatctgtgtcctgctgcc
tctcagcccatttccgggaccttgcaggatgaggagataactttgagctg
ctcagaggccagcgctgaggaagctctgctccctgcacttcccaaaacag
tatctttcagaataaacagcgaagacttcttgctggaagcgcaagtgagg
gatcagccacgctggctcctggtctgccatgagggctggagccccgccct
ggggctgcagatctgctggagccttgggcatctcagactcactcaccaca
agggagtaaacctcactgacatcaaactcaacagttcccaggagtttgct
cagctctctcctagactgggaggcttcctggaggaggcgtggcagcccag
gaacaactgcacttctggtcaagttgtttccctcagatgctctgagtgtg
gagcgaggcccctggcttcccggatagttggtgggcagtctgtggctcct
gggcgctggccgtggcaggccagcgtggccctgggcttccggcacacgtg
tgggggctctgtgctagcgccacgctgggtggtgactgctgcacattgta
tgcacagtttcaggctggcccgcctgtccagctggcgggttcatgcgggg
ctggtcagccacagtgccgtcaggccccaccaaggggctctggtggagag
gattatcccacaccccctctacagtgcccagaatcatgactacgacgtcg
ccctcctgaggctccagaccgctctcaacttctcagacactgtgggcgct
gtgtgcctgccggccaaggaacagcattttccgaagggctcgcggtgctg
ggtgtctggctggggccacacccaccctagccatacttacagctcggata
tgctccaggacacggtggtgcccttgttcagcactcagctctgcaacagc
tcttgcgtgtacagcggagccctcacccccgcatgctttgcgctggcta
cctggacggaagggctgatgcatgccagggagatagcgggggcccccctag
tgtgcccagatggggacacatggcgcctagtgggggtggtcagctggggg
cgtggctgcgcagagcccaatcacccaggtgtctacgccaaggtagctga
gtttctggactggatccatgacactgctcaggactccctcctctgagtcc
tgctgtttcctccagtctcactgcacaccactgcctcatgcttcctgggg
cctccagcagctccactaatggaggagaggcagtagcctccgacacagaa
cgcatggacctcctactactgtgtgtgaggaacagtcactacccactggc
cagccacccagccaacaggtctctcctcttgggccctgatttcagagtcc
tctttctcactagagactcaatgacagaagagaggctgggacttggttgg
gcatgctgtggttgctgagggatgagggggaggagagaggtaggagctgg
agatgaagaggctgctagaagcagcaggaagcctgcccttctgccctctc
ccctccctgcccctgtgtgagtcttttgggagggtgctgggaggtgcccc

ccgtcccaccttttttcctgtgctctaggtgggctaagtgcctccctagag
gactccatggctgagaggctcctgggcagatggggtcaaggctgggccag
cccagatgaagcctatgggagtcaggaccctctccactctccctctccac
tccccttcctgttctcacctggctgtggctggccctgtgtggggtgggta
cactggaaaacaagaaggttggagttggtctaggacattggttttaaatg
acagttctgtgaactggtccaaggagttctgttattaaagtgatatatgg
tcttggtccaaaaaaaaaaaaaaaaaaaaaaaa
>NM_030775 2
tattcttccaaatggaaactgctaatttttgaagcagaaggttgacagct
tcagtaagatctcaagagagcgagaagactggaatcagggaaccctactc
tggaaactgtcagtcccagggcactggggagggctgaggccgaccatgcc
cagcctgctgctgctgttcacggctgctctgctgtccagctgggctcagc
ttctgacagacgccaactcctggtggtcattagctttgaacccggtgcag
agacccgagatgtttatcatcggtgcccagcccgtgtgcagtcagcttcc
cgggctctcccctggccagaggaagctgtgccaattgtaccaggagcaca
tggcctacataggggagggagccaagactggcatcaaggaatgccagcac
cagttccggcagcggcggtggaattgcagcacagcggacaacgcatctgt
ctttgggagagtcatgcagataggcagccgagagaccgccttcacccacg
cggtgagcgccgcgggcgtggtcaacgccatcagccgggcctgccgcgag
ggcgagctctccacctgcggctgcagccggacggcgcggcccaaggacct
gccccgggactggctgtggggcggctgtggggacaacgtggagtacggct
accgcttcgccaaggagtttgtggatgcccgggagcgagagaagaacttt
gccaaaggatcagaggagcagggccgggtgctcatgaacctgcaaaacaa
cgaggccggtcgcagggctgtgtataagatggcagacgtagcctgcaaat
gccacggcgtctcggggtcctgcagcctcaagacctgctggctgcagctg
gccgagttccgcaaggtcggggaccggctgaaggagaagtacgacagcgc
ggccgccatgcgcgtcacccgcaagggccggctggagctggtcaacagcc
gcttcacccagcccaccccggaggacctggtctatgtggaccccagcccc
gactactgcctgcgcaacgagagcacgggctccctgggcacgcagggccg
cctctgcaacaagacctcggagggcatggatggctgtgagctcatgtgct
gcgggcgtggctacaaccagttcaagagcgtgcaggtggagcgctgccac
tgcaagttccactggtgctgcttcgtcaggtgtaagaagtgcacggagat
cgtggaccagtacatctgtaaatagcccggagggcctgctcccggccccc
ctgcactctgcctcacaaaggtctatattatataaatctatataaatcta
ttttatatttgtataagtaaatgggtgggtgctatacaatggaaagatga
aaatggaaaggaagagcttatttaagagacgctggagatctctgaggagt
ggactttgctggttctctcctcttggtgggtgggagacagggctttttct
ctccctctggcgaggactctcaggatgtagggacttggaaatatttactg
tctgtccaccacggcctggaggagggaggttgtggttggatggaggagat
gatcttgtctggaagtctagagtctttgttggttagaggactgcctgtga
tcctggccactaggccaagaggccctatgaaggtggcgggaactcagctt
caacctcgatgtcttcagggtcttgtccagaatgtagatgggttccgtaa
gaggcctggtgctctcttactctttcatccacgtgcacttgtgcggcatc
tgcagtttacaggaacggctccttccctaaaatgagaagtccaaggtcat
ctctggcccagtgaccacagagagatctgcacctcccggacttcaggcct
gcctttccagcgagaattcttcatcctccacggttcactagctcctacct
gaagaggaaagggggccatttgacctgacatgtcaggaaagccctaaact
gaatgtttgcgcctgggctgcagaagccagggtgcatgaccaggctgcgt
ggacgttatactgtcttcccccacccccggggaggggaagcttgagctgc

tgctgtcactcctccaccgagggaggcctcacaaaccacaggacgctgca
acgggtcaggctggcgggcccggcgtgctcatcatctctgccccaggtgt
acggtttctctctgacattaaatgcccttcatgg
>NM_005855 2
gcggccgccgcctcccaccgctcccgcgcccgctcccgcccccggcgcgt
ctcctccgggcccgcgccgcggcgggctcagtcctcagcggggcgcgtgg
cgagcggactcgactcggcaccgctgtgcaccatggcccgggccctgtgc
cgcctcccgcggcgcggcctctggctgctcctggcccatcacctcttcat
gaccactgcctgccaggaggctaactacggtgccctcctccgggagctct
gcctcacccagttccaggtagacatggaggccgtcggggagacgctgtgg
tgtgactggggcaggaccatcaggagctacagggagctggccgactgcac
ctggcacatggcggagaagctgggctgcttctggcccaatgcagaggtgg
acaggttcttcctggcagtgcatggccgctacttcaggagctgccccatc
tcaggcagggccgtgcgggacccgcccggcagcatcctctaccccttcat
cgtggtccccatcacggtgaccctgctggtgacggcactggtggtctggc
agagcaagcgcactgagggcattgtgtaggcggggcccaggctgcccgcg
ggtgcacccaggctgcagggtgaggccaggcaggcctgggtaggggcagc
ttctggagccttgggacagagcaggcccacaatgccccccttcttccagc
caagaagagctcacaggagtccagagtagccgaggctctggtattaacct
ggaagccccccctggctggaggccaccgccaccctaggaagggggcaggga
cgtgaccttgacttacctctggaaagggtcccagcctagactgcttaccc
catagccacatttgtggatgagtggtttgtgattaaaagggatgttcttg
aacttggaaaaaaaaaaaaaaa
>NM_022449 3
atcagcccaggatctgaccctggactggctcacactcagttgcctctggc
cagtgcagggctcagccagggatggcttctagctgacagtgggaggaatt
aattcatctgaccggaatattcttttctcttctgggctgttggtttttca
agtgcaacaaagattccatacagctccaaggaaggagccaagaaaaacat
tctgtgccaaagtgagatcctggaagtgaaaccccggaataaagctgaaa
aacgggctccagttgggtgccaggaaatgcaggactggaatgtgacttga
cttccggcagcgcgcaggtgctcccgggtcacctgctttgaggtccagcc
tcctgccctgcctcaggtgaccacatgaccactgtggactttgccctgaa
accttctgggaggagaagaggcctgaccttggcgctggggtccagtgggc
attgctctggtccgaggctgctgctcttgacctctgctctgcggctgttt
tccattggagtagaggctcctcctgtcctgtcctgcctgtggagggaagc
aaaccttcccctggaccagagagaggagaaagcggagacaggtagcaacg
ctgtggactggtgatgacaggctcttcagctccctgcaagtgaccgggcc
tggggaacagggcatggcacaggcacacaggacccccagcccagggctg
cccccagccagccccgtgtgttcaagctggttctcctgggaagtggctcc
gtgggtaagtccagcttggctcttcggtacgtgaagaacgacttcaagag
tatcctgcctacggtgggctgtgcgttcttcacaaaggtggtggatgtgg
gtgccacctctctgaagcttgagatctgggacacagctggccaggagaag
taccacagcgtctgccacctctacttcaggggtgccaacgctgcgcttct
ggtgtacgacatcaccaggaaggattccttcctcaaggctcagcagtggc
tgaaggacctggaggaggagctgcacccaggagaagtcctggtgatgctg
gtgggcaacaagacggacctcagccaggagcgggaggtgaccttccagga
agggaaggagtttgccgacagccagaagttgctgttcatggaaacttcgg
ccaaactgaaccaccaggtgtcggaggtgttcaatacagtggcccaagag
ctactgcagagaagcgacgaggagggccaggctctacgggggatgcagc

tgtggctctgaacaaggggcccgcgaggcaggccaaatgctgcgcccact
aggtgcagccactcctgggggctgtggggaagacacccctgcctgggcc
atggccagctctaggtggattctgattcactgtcaatgctgggttgctcc
cgagccctagatgttcctggaagttggccccctttatgaaaaccacttcc
cacagccagtgggaactgccagaggaagatctggcgtcacatggctccca
ggaaagtgctgtgccctatccccactgataccatctgattccccgatgcc
tgtgcctgttccacctggacggtggcccctcagcctggcagcctctgga
cagagaggaaggaaggattggaaaagtccccgcagcacagcgacggtggg
aagatgccttacgtctgatcttgatgggggcactggcctggagcctgggc
ccacctgcttctggggggttggggagcaggccagatggaggtggtggtgc
caggaagaaatggagcgatgactgactgtggggtgggcccaggatttcca
catcttggtgaagttgcccctgggaagggcagctgggggcagtggcgcca
gttcccttccatggtctcccggctggcaatgtggtgaagctgagtttctg
tccaatgagcaggaagattctgagacatttcgcctgagatataagttgta
ctgcgtatgcagtttttcctccaaaaattaaattgcttttgacaatctga
aaaaaaaaaaaaaaaaa
>NM_001013436 2
ggccgcgcgggcctgggctgggcggagcgggcgtaccccacggcccggc
cagtggaaggcgcgggcagcagcggctccgagtggccgcggcggtgggct
gtgccggagtctcctccctttggtccgctgcaggcccgcagcccgagtgt
cgccgccatggcttcgccgcagctctgccgcgcgctggtgtcggcgcaat
gggtggcggaggcgctgcgggccccgcgcgctgggcagcctctgcagctg
ctggacgcctcctggtacctgccgaagctggggcgcgacgcgcgacgcga
gttcgaggagcgccacatcccgggcgccgctttcttcgacatcgaccagt
gcagcgaccgcacctcgccctacgaccacatgctgcccggggccgagcat
ttcgcggagtacgcaggccgcctgggcgtgggcgcggccacccacgtcgt
gatctacgacgccagcgaccagggcctctactccgccccgcgcgtctggt
ggatgttccgcgccttcggccaccacgccgtgtcactgcttgatggcggc
ctccgccactggctgcgccagaacctcccgctcagctccggcaagagcca
acctgctcccgccgagttccgcgctcagctcgaccccgccttcatcaaga
cctacgaggacatcaaggagaacctggaatcccggcgcttccaggtggtg
gactcccgagccactggcaggttccgcggcaccgagcccgagccccgaga
cggcattgaacctggccacatcccaggtaccgtgaacatccccttcacag
acttcctgagccaggaggggctggagaagagccctgaggagatccgccat
ctgttccaggagaagaaagtggacctgtctaagccactggtggccacgtg
tggctctggcgtcacagcctgccacgtggcactaggggcctacctctgcg
gcaagccagacgtgcccatctacgatggctcctgggtggagtggtacatg
cgcgcccggcccgaggatgtcatctcagagggccgggggaagacccactg
aagctgggcaggacacaggcgagctcaggtgatgccggccaccagcaatg
cctggcctggtagctccgcttctgctttcaccaagagagtgtttcttcac
tcaactcaggtggcatttggggtgacatctcaaaggccaggaattccgtt
gacttgttggctgccagtaggggcgggaggaaaggcggaggcgagccctg
gaggagggaggccacaactccgagctgcccacctggtgctgagctggggc
cccgcctcctttctgttttatttttgaggaaataaaataaccaagtgcta
aatcttgtaaaaaaaaaaaaa
>NM_004306 2
ctgttgtaaactttgcctgtaggaggactgatctcttaatgaaatacaga
aaaaccatctcagaaaaaggaaaatgggcaatcgtcatgctaaagcgagc
agtcctcagggttttgatgtggatcgagatgccaaaaagctgaacaaagc

ctgcaaaggaatggggaccaatgaagcagccatcattgaaatcttatcgg
gcaggacatcagatgagaggcaacaaatcaagcaaaagtacaaggcaacg
tacggcaaggagctggaggaagtactcaagagtgagctgagtggaaactt
cgagaagacagcgttggcccttctggaccgtcccagcgagtacgccgccc
ggcagctgcagaaggctatgaagggtctgggcacagatgagtccgtcctc
attgaggtcctgtgcacgaggaccaataaggaaatcatcgccattaaaga
ggcctaccaaaggctatttgataggagcctcgaatcagatgtcaaaggtg
atacaagtggaaacctaaaaaaaatcctggtgtctctgctgcaggctaat
cgcaatgaaggagatgacgtggacaaagatctagctggtcaggatgccaa
agatctgtatgatgcaggggaaggccgctggggcactgatgagcttgcgt
tcaatgaagtcctggccaagaggagctacaagcagttacgagccaccttt
caagcctatcaaattctcattggcaaagacatagaagaagccattgaaga
agaaacatcaggcgacttgcagaaggcctatttaactctcgtgagatgtg
cccaggattgtgaggactattttgctgaacgtctgtacaagtcgatgaag
ggtgcggggaccgatgaggagacgttgattcgcatagtcgtgaccagggc
cgaggtggaccttcaggggatcaaagcaaagttccaagagaagtatcaga
agtctctctctgacatggttcgctcagatacctccggggacttccggaaa
ctgctagtagccctcttgcactgagccaagccagggcaataggaacacag
ggtggaaccgcctttgtcaagagcacattccaaatcaaacttgcaaatga
gactcccgcacgaaaacccttaagagtcccggattactttcttggcagct
taagtggcgcagccaggccaagctgtgtaagttaagggcagtaacgttaa
gatgcgtgggcagggcaccttgaactctggcttagcaagcatctaggctg
cctcttcactttcttttagcatggtaactggatgttttctaaacactaat
gaaatcagcagttgatgaaaaaactatgcatttgtaatggcacatttaga
aggatatgcatcacacaagtaaggtacaggaaagacaaaattaaacaatt
tattaattttccttctgtgtgttcaatttgaaagcctcattgttaattaa
agttgtggattatgcctctaaaaaaaaaaaaaaaaa
>NM_005700 3
aatgaatgggggagccggaagcaggaagtgagtttgcgaacggagcagct
gctgcagggcccatggcggacacccagtacatcctgcccaatgacatcgg
cgtgtctagcctggactgccgtgaggccttccgcctgctgtcacccacag
agcgcctctatgcctaccacctgtcccgtgccgcctggtacggaggcctg
gctgtgctgcttcagacctcccctgaggcccccctacatctatgctctgct
cagccgcctcttccgcgcccaggaccccgaccagctgcgccaacatgccc
tggctgaaggccttaccgaggaggagtatcaggcgttcctggtctatgcc
gcgggtgtttactccaacatgggcaactacaagtcctttggtgacaccaa
gtttgttcccaacttgcccaaggaaaagctggaacgggtgatcctaggga
gtgaggctgctcagcagcacccagaagaagtcaggggcctctggcagacc
tgcggggagcttatgttctctctggagccaaggcttcgacacctcggact
ggggaaggagggaatcaccacctatttctctgggaattgtaccatggaag
atgccaaattggcccaggactttctggactcacagaacctcagtgcctac
aacacccggctcttcaaagaggtcgatggagaagggaagccctactacga
ggtgcggctggcttctgtgcttggctcagagccttccctggactctgagg
tgacttccaagctgaagagctatgaattccggggaagccctttccaggtg
acccggggggactacgcgcccatcctccagaaggtggtggagcagctgga
gaaagccaaggcctatgcagccaacagccaccaggggcagatgctggccc
agtatatagagagcttcacccagggctccatcgaggcccacaagaggggc
tcccgcttctggatccaggacaaaggccccatcgtggagagttacatcgg
gttcatcgagagctaccgcgacccctttggttcccgaggagaatttgaag

gtttcgtagctgtggtgaacaaggccatgagtgccaagtttgagcggctg
gtggcgagcgcagagcagctgctgaaggagctgccctggcccccaacctt
tgagaaggacaagttcctcacccctgacttcacctccctggatgttctca
ccttcgctggctccggcatccctgccggcatcaacatccccaactacgat
gatctgaggcagacggaaggctttaagaacgtgtcgctggggaatgtgct
ggctgtggcctacgccacgcagcgggagaagcttacctttctggaggagg
atgacaaggacctgtacatcctctggaaggggccctccttcgatgtgcag
gtgggcctgcacgagctgctgggccatggcagtggcaagctcttcgtaca
ggacgaaaaaggagcattcaactttgaccaggaaacagtgatcaacccag
agacgggcgagcagattcagagctggtatcggagcggggagacctgggat
agcaagttcagcaccatcgcctccagctacgaagagtgccgggctgagag
cgtgggtctctacctctgtctccacccgcaagtgctggagatctttggct
ttgagggggctgatgcggaggacgtgatctacgtgaactggctcaacatg
gttcgggccgggctgctcgctctggagttctacacacctgaggccttcaa
ctggcgacaggcccatatgcaggcccggtttgtgatcctgagagtcttgc
tggaggctggcgagggactcgttaccatcactcccaccacaggctccgat
gggcgcccagatgcccgggtccgcctcgaccgcagcaagatccggtctgt
gggcaagcctgctctagagcgcttcctgcggagacttcaggtgctgaagt
ccacaggggatgtggccggagggcgggccctgtacgaggggtatgcaaca
gtcactgatgcgcccccgagtgcttcctcaccctcagggacacggtgct
gctgcgtaaggaatctcggaagctcattgttcagcccaacactcgccttg
aaggctcagacgtgcagcttctggaatacgaggcgtcagctgctggcctc
atccgatccttctctgagcgtttcccagaggatggacccgagttggagga
gatcctcacacagctggccacagccgatgcccgattctggaagggcccca
gtgaggccccatctggccaagcttgaggaagatgtgtggccttgcccca
attccatcagaccaaggctgcaagtggccctccattcgtgtgtgtattta
ggggctggggagggggaggggcaggagcttggaccttggtactacctcag
ctgagggtggtgacacaaccccttccatttgtcagcactttccagcctgc
caattgcttcccctctgtgatctcatttcatctgcactgccatacgtgga
gtgagcaagacagggcttaccatcctgtctaccagatgaggaaatggcag
ttctgagaagtcactggtctagatcccgcaggtggcacgtgacagctagg
gttcaaaacgttctcaccaaatccaatgctcctcacatattaattttata
accagacaaataaatattagagacaaccaccatcaaaaaaaaaa
>NM_005029 3
ggagcgcccgagcggagaggcggcccgggagcagggggcggcccccact
ccggccgggtgcccggcccctggcccctgcctgccctctagatcgccgcc
gcagccgccgctactgggagtctgcctgttgcaggacgcactagccctcc
ctccatggagttcggcctgctcagcgaggcagaggcccggagccctgccc
tgtcgctgtcagacgctggcactccgcacccccagctcccagagcacggc
tgcaagggccaggagcacagcgactcagaaaaggcctcggcttcgctgcc
cggcggctccccagaggacggttcgctgaaaaagaagcagcggcggcagc
gcacgcacttcaccagccagcagctacaggagctagaggcgaccttccag
aggaaccgctaccccgacatgagcacgcgcgaggagatcgccgtgtggac
caacctcaccgaggcccgcgtgcgggtgtggttcaagaaccggcgcgcca
aatggcggaagcgcgagcgcagccagcaggccgagctatgcaaaggcagc
ttcgcggcgccgctcgggggggctggtgccgccctacgaggaggtgtaccc
cggctactcgtacggcaactggccgcccaaggctcttgccccgccgctcg
ccgccaagacctttccattcgccttcaactcggtcaacgtggggcctctg
gcttcgcagcccgtcttctcgccacccagctccatcgccgcctccatggt

gccctccgccgcggctgccccgggcaccgtgccagggcctggggccctgc
agggcctgggcgggggcccccccgggctggctccggccgccgtgtcctcc
ggggccgtgtcctgcccttatgcctcggccgccgccgccgccgcggctgc
cgcctcttcccccctacgtctatcgggacccgtgtaactcgagcctggcca
gcctgcggctcaaagccaaacagcacgcctccttcagctaccccgctgtg
cacgggccgcccccggcagccaaccttagtccgtgccagtacgccgtgga
aaggcccgtatgagcggccccgcccgtagatcatccccgagggcgggggc
aacgattcacagcctccgcggactggggtcattttgactggcttgctccc
gccccagggtctgaaaggggtgtttgggcagctgggggggcaccggctcag
gagagggccttcccctcccagccctgaggggtggactaggccctacacac
agaccgcgcccctgggactaaagccaggaacagggaccagctccccgggg
gccaactcacccttggcccatcccgccttctccaggcttcccctccctcg
ttttcaaagataaatgaaataaacgtgcgcggactgtcaaaaaaaaaaaa
aaaaaaa
>NM_000017 2
cggccccgctcccgaggccctacgggcgtggcctctgtcccgggtcccgc
cccccagcactccggaacagcgcgctcgcagcgggaggtcgcgaagcctg
ggactgtgtctgtcgcccatggccgccgcgctgctcgcccgggcctcggg
ccctgcccgcagagctctctgtcctagggcctggcggcagttacacacca
tctaccagtctgtggaactgcccgagacacaccagatgttgctccagaca
tgccgggactttgccgagaaggagttgtttcccattgcagcccaggtgga
taaggaacatctcttcccagcggctcaggtgaagaagatgggcgggcttg
ggcttctggccatggacgtgcccgaggagcttggcggtgctggcctcgat
tacctggcctacgccatcgccatggaggagatcagccgtggctgcgcctc
caccggagtcatcatgagtgtcaacaactctctctacctggggcccatct
tgaagtttggctccaaggagcagaagcaggcgtgggtcacgcctttcacc
agtggtgacaaaattggctgctttgccctcagcgaaccagggaacggcag
tgatgcaggagctgcgtccaccaccgcccgggccgagggcgactcatggg
ttctgaatggaaccaaagcctggatcaccaatgcctgggaggcttcggct
gccgtggtctttgccagcacggacagagccctgcaaaacaagggcatcag
tgccttcctggtccccatgccaacgcctgggctcacgttggggaagaaag
aagacaagctgggcatccggggctcatccacggccaacctcatctttgag
gactgtcgcatccccaaggacagcatcctgggggagccagggatgggctt
caagatagccatgcaaaccctggacatgggccgcatcggcatcgcctccc
aggccctgggcattgcccagaccgccctcgattgtgctgtgaactacgct
gagaatcgcatggccttcggggcgcccctcaccaagctccaggtcatcca
gttcaagttggcagacatggccctggccctggagagtgcccggctgctga
cctggcgcgctgccatgctgaaggataacaagaagcctttcatcaaggag
gcagccatggccaagctggccgcctcggaggccgcgaccgccatcagcca
ccaggccatccagatcctgggcggcatgggctacgtgacagagatgccgg
cagagcggcactaccgcgacgcccgcatcactgagatctacgagggcacc
agcgaaatccagcggctggtgatcgccgggcatctgctcaggagctaccg
gagctgagcccgcggcggactgccccaggactgcgggaaggcgcgggagc
caggggcctccaccccaaccccggctcagagactgggcggcccggcgggg
gctccctggggaccccagatgggctcagtgctgccacccagatcagatca
catgggaatgaggccctccgaccattggcagctccgcctctgggcctttc
cgcctcctcaccactgtgcctcaagttcctcatctaagtggccctggcct
cctgggggcggggttgtgggggggctgagcgacactcagggacacctcag
ttgtcctcccgcgggccctggtgccctggcatgaaggcccagtgcgacag

gcccttggtggggtctgtcttttccttgaggtcagaggtcaggagcaggg
ctggggtcaggatgacgaggcctggggtcctggtgttgggcaggtggtgg
ggctgggccatggagctggcccagaggcccctcagccctttgtaaagtct
gatgaaggcaggggtggtgattcatgctgtgtgactgactgtgggtaata
aacacacctgtcccccaaaaaaaaaaaaaaaaa
>NM_001144 4
cgcgccggcaggggcagcagcgggagcagcgcggggcggagcggcgccag
cagccaggagcggcccggcccggcccggcgcggcggcggcggcggcagcg
gcagcggcagcgacgccagagcccgtgggcgccgttcgcgaggccgccgc
agaggcccggccgcagcgcagggaagcctgggggccagaggtcgccgctg
ccgccatgccgctgctcttcctcgagcgcttcccctggcccagcctccgc
acctacacgggcctcagcggcctggccctgctgggcaccatcatcagcgc
ctaccgcgcgctcagccagcccgaggccggccccggcgagccggaccagc
taacggcctcgctgcagcctgagccgccggcgcccgcccggccgagcgcc
gggggaccccgggcccgcgatgtggcccagtacctgctctcagacagcct
cttcgtgtgggttctagtaaataccgcttgctgtgttttgatgttggtgg
ctaagctcatccagtgtattgtgtttggccctcttcgagtgagtgagaga
cagcatctcaaagacaaattttggaattttattttctacaagttcatttt
catctttggtgtgctgaatgtccagacagtggaagaggtggtcatgtggt
gcctctggtttgccggacttgtctttctgcacctgatggttcagctctgc
aaggatcgatttgaatatctttccttctcgcccaccacgccgatgagcag
ccacggtcgagtcctgtccctgttggttgccatgctgctttcctgctgtg
gactggcggccgtctgctccatcaccggctacacccacggaatgcacacc
ttggctttcatggctgcagagtctcttcttgtgacagtgaggactgctca
tgtgattttacgatacgtaattcacctctgggacctcaaccacgaaggga
cgtgggaaggaaaggggacgtatgtctattacacagactttgtcatggag
ctcactctcctgtccctggacctcatgcaccatattcacatgttgttatt
tggcaacatctggttatccatggccagcctggtcatctttatgcagctgc
gttacctgtttcatgaggtgcaacgtcgaattcgtcggcacaagaactat
ctacgtgtggttggaaacatggaggccaggtttgcagttgcaactccaga
ggagctggctgtcaacaatgacgactgtgccatctgttgggactccatgc
aggctgcgcggaaactgccctgtggacatcttttccacaactcctgtctt
cgttcctggctagaacaagacacctcctgtccaacatgcagaatgtctct
taatattgccgacaataatcgtgtcagggaagaacatcaaggagagaact
tggatgagaatttggttcctgtagcagcagccgaagggagacctcgctta
aaccaacacaatcacttcttccatttcgatgggtctcggattgcgagctg
gctgccgagtttttcggttgaagtgatgcacaccaccaacattcttggca
ttacgcaggccagcaactcccagctcaatgcaatggctcatcagattcaa
gagatgtttccccaggttccataccatctggtactgcaggacctccagct
gacacgctcagttgaaataacaacagacaatattttagaaggacggattc
aagtaccttttcctacacagcggtcagatagcatcagacctgcattgaac
agtcctgtggaaaggccaagcagtgaccaggaagagggagaaacttctgc
tcagaccgagcgtgtgccactggacctcagtcctcgcctggaggagacgc
tggacttcggcgaggtggaagtggagcccagtgaggtggaagacttcgag
gctcgtgggagccgcttctccaagtctgctgatgagagacagcgcatgct
ggtgcagcgtaaggacgaactcctccagcaagctcgcaaacgtttcttga
acaaaagttctgaagatgatgcggcctcagagagcttcctcccctcggaa
ggtgcgtcctctgaccccgtgaccctgcgtcgaaggatgctggctgccgc
cgcggaacggaggcttcagaagcagcagacctcctagcgctcccttgcct

tcctcagctgcctcctgcgccctgtgcccgactgactggaggaggcctgt
cccaattctgcccgctccatggaaaagcgggcttgactgcattgccgctg
tataaagcatgtggtcttatagtgtttggacagctgataaatttaatcct
tctttgtaatactttctatgtgacatttctcttccccttagaaacactgc
aaattttaactgtaggtatgatctcttctggtgttgactggactgcttgg
ggtgggggacgatcaggaggaagtgagcagtcgcctgcctgcagcaggca
gcttctactcctgcctcatgcgtacgtcccacaaatgcaggtgtcctgag
caccacacccagtgggaagagtgtgggggaggcgcacagtgtgagcccgc
ccccacgtcgtggggtaacatctgttatcaaactgctgtcgttgttgtgg
aagcatgtagactgtgccagaggccagacccacgggctcatgcacccctg
agccagcagggcatcttggaaaaggaactcttggttcgatacctggagca
gaggaggggaaagtccagggctatagggtgtgatgaagtcacccctttct
gtcccactacatctgggactgactttccgagcctccagtccaaagccggc
ttgatttccgtgaactctggtgctcctgcatctcatgagtgtgccccatg
ggtcccctcccctctcagcatttccttgtcccgtctggacctggggagtg
gttaggcagcaagctttggtttatggttttcattcattggtgaagtaaat
taggcagtgctaaagcctgtgggtttggtccttgaacaagatgtgggcct
tgcaagatgggagagtaaaccttgaagggctttattaaagaaataaaaaa
gaacttttgtatcttttatcctgggagcactgcgttttcctagctgtgtt
attcctggtttaattcagcagagaaggtaaggtgtgaacctacctgcctt
ggagagggcccaggtcccaaatctcttcaaattcttcacatgtttaactt
taaggatttgaaccatgaagtcataggttacagacctcagttttatgccc
cattggattacttttttttttttttttttttttttttttttactctttg
aaagctttgttttgtggtagtccttttgggaagaatccagtattatccac
aattattggcaaagtttaaatgtattttacataacggaaagtttttagaa
tgttgaaaagtaattgaaaaaggtgataggtaaatttttaggcaaagata
atttatttcaataaatctttcaaaagccttaccttgaaatgctgttagta
aatttctgtgatttttttttttaatttgttttgctgagagcatagctatt
tgtttttattgtaaaacaataataataataaaaagcaaactcta
>NM_005258 2
tcccagctgcgcgtcgcagtcccgacgcgagaagggctggagtcggcgtc
cagcctagagcccccggtgggagccaggccgggacgcgtgcaccatgccc
tacctgctcatcagcacccagatccgcatggaggtgggccccactatggt
gggcgatgaacagtcggatccagagctgatgcagcatctgggggcttcaa
agagaagagccttgggaaacaacttttatgaatactacgtcgatgaccct
ccccgcatagtcctggacaagctggaacgcaggggcttccgtgtgctgag
catgacgggggtgggccagacgctggtgtggtgtctgcacaaggagtgac
cttctcatgctgatttgcagacggggcacccctgtggaggggctgctgtg
ggccctgacctccaagctcctgcctcaccgtctgccttgctcctctcttc
ccaaatcatcaccgccatgggcccagccccaaagggcagtgaatggcctt
ctctgaaaccctgcgtcaagcagtgggagagggcagtgcccggtgccctg
gtgctcccagctgccctcctgcttcgggcctgggccgagggccttgtgta
ggccatgttcctcgggcagctgccccgggccggagctgggcactccagcg
gccctggcgcgtggctcctgcatagctagcccaagccaataaagggctgt
gatgagtggctgc
>NM_024663 3
ggggcggtgccgaggccgggccggagcggggcgaagggggccgagcggcg
ggccgggccgggccgggcagggccggggcgtgggccggcaggaagatggc
gaacgtggggctgcagttccaggcgagcgcgggggactcggacccacaga

gccggcccctgctgctgctcgggcagctgcaccacctgcaccgcgtgccc
tggagccacgtccgcgggaagctgcagccccgggtcaccgaggagctctg
gcaggctgccctgagcacgctcaaccccaaccccacggacagctgtcccc
tctacctgaactacgccaccgtggctgccctgccctgcagggtgagccgg
cacaacagcccctcggccgcccacttcatcacgcggctggtgcggacctg
cctgccgcccggagcgcatcgctgcattgtgatggtctgcgagcagccgg
aggtctttgcttccgcctgtgccctggcccgggccttcccgctgttcacc
caccgctcaggtgcctctcggcgcttggagaagaagacggtcaccgtgga
gtttttcctggtgggacaagacaacgggccggtggaggtgtccacattgc
agtgcttagcgaatgccacagacggcgtgcggctagcagcccgcatcgtg
gacacaccctgcaatgagatgaacaccgacaccttcctcgaggagattaa
caaagttggaaaggagctggggatcatcccaaccatcatccgggatgagg
aactgaagacgagaggatttggaggaatctatggggttggcaaagccgcc
ctgcatcccccagccctggccgtcctcagccacaccccagatggagccac
gcagaccatcgcctgggtgggcaaaggcatcgtctatgacactggaggcc
tcagcatcaaagggaagactaccatgccggggatgaagcgagactgcggg
ggtgctgcggccgtcctgggggccttcagagccgcaatcaagcagggttt
caaagacaacctccacgctgtgttctgcttggctgagaactcggtggggc
ccaatgcgacaaggccagatgacatccacctgctgtactcagggaagacg
gtggaaatcaacaacacggatgccgagggcaggctggtgctggcagatgg
cgtgtcctatgcttgcaaggacctgggggccgacatcatcctggacatgg
ccaccctgaccggggctcagggcattgccacagggaagtaccacgccgcg
gtgctcaccaacagcgctgagtgggaggccgcctgtgtgaaggcgggcag
gaagtgtggggacctggtgcacccgctggtctactgccccgagctgcact
tcagcgagttcacctcagctgtggcggacatgaagaactcagtggcggac
cgagacaacagccccagctcctgtgctggcctcttcatcgcctcacacat
cggcttcgactggcccggagtctgggtccacctggacattgctgcaccgg
tgcatgctggtgagcgagccacaggcttcggtgtggccctcctgctggcg
ctcttcggccgtgcctctgaggaccctctgctgaacctggtgtccccact
gggctgtgaggtggatgtcgaggagggggacctggggagggactccaaga
gacgcaggcttgtgtgagcctcctgcctcggccctgacaaacggggatct
tttacctcactttgcactgattaattttaagcaattgaaagattgccctt
catatgggttttggtttgtctttctggtcgtcagcgtggtggtggaaaca
gctgaagttttaggagacagcttagggtttggtgcgggccacggggaggg
gaccgggaagcgctggggcttgtttctgtttgttacttacaggactgaga
catcttctgtaaactgctacccctggggccttctgcaccccggggtgagg
cctcctgcctgcctggtgccctgtcccagccccaggtcctgtgcagggca
cctgcgtggctgacagccaggctcttactccagccggggctgccagcgca
tccagccagcccagccctgtgaaagatggagctgacttgctgcaggggac
ctgatttatagggcaagagaagtcacactccggcctctcagaattcactt
gaggttcaattaaatacagtcacaccgccccctcaaaaaaaaaaaaaaaa
aaaaaaaaaaaaaaaa
>NM_014303 3
agacagcgtggggtggggagggtcctcggggtccttggcagggcacgtgc
gggaggaagtggagctccctgtacgcgcggccctagtcggctcctcaacg
tggagcgatgggaggccttgagaagaagaagtatgaacgaggctcggcca
ccaactacatcacccggaacaaagcccggaagaagctccagctgagcttg
gctgactttaggcggctgtgcattctgaagggcatttatccccatgaacc
caaacacaagaagaaggttaacaagggttctacagcagcccgaacgtttt

accttatcaaagacatcaggtttctcctccacgaacccattgtcaacaag
ttccgtgaatacaaggtgttcgtccggaagctccggaaggcttatgggaa
gagcgagtggaacactgtagagcgtttaaaggacaataagcccaactaca
aactcgaccacatcatcaaggaacggtatcccacgttcatcgatgccctg
cgggacctggacgatgccctctccatgtgcttcctgttttccaccttccc
gcggactggcaagtgccacgtgcagaccattcagctgtgccgccggctca
ctgtggagttcatgcactacattatcgctgcccgtgccctgcgcaaggtc
ttcctgtccatcaaaggcatttactaccaggccgaggtactggggcagcc
catcgtgtggatcactccctatgccttctcccatgaccacccgacagacg
tggactacagggtcatggccaccttcaccgagttctacaccacgctgctg
ggctttgtcaacttccgcctttaccagttgctcaacctccactatccccc
gaagctcgagggtcaggcccaagcagaggcaaaggccggtgagggcacct
acgcgttggactccgagagttgtatggagaaactggcagccctcagtgcc
agcctggcccgcgtggtggtgcctgccacagaggaggaggccgaggtgga
tgagtttcccaccgatggggagatgtcagcgcaggaggaagaccgcagga
aggagctggaggcgcaggagaagcacaagaagctttttgagggcctgaag
ttcttcctgaaccgagaggtgccccgtgaggccctggccttcatcatcag
gagttttggtggggaagtgtcctgggacaaatctttgtgcattggggcca
cctatgacgtcacagactcccgcatcacccatcagattgtcgaccggcct
gggcagcagacctcagtcattggcaggtgctacgtgcagccccagtgggt
gtttgactcagtgaacgccaggctccttctccccgtggcagagtacttct
ctggggtgcagctgcccccacacctttcacccttttgtgaccgagaaggaa
ggagattacgttccacctgagaagctgaagctgctggctctgcagcgggg
agaggacccaggaaacctgaatgagtcagaagaggaggaggaagaggacg
acaacaacgaaggtgatggtgatgaagagggagaaaatgaggaggaggag
gaagatgcagaggctggttcagaaaaggaggaagaggcccggctggcagc
cctggaagagcagaggatggaggggaagaagcccagggtgatggcaggca
ccttgaagctggaggataagcagcggctggcccaggaggaggagagtgag
gccaagcgcctggccattatgatgatgaagaagcgggagaagtacctgta
ccagaagatcatgtttggcaagaggcgaaaaatccgagaggccaacaagc
tggcggagaagcggaaagcccacgatgaggcggtgaggtctgagaagaag
gccaagaaggcaaggccggagtgagtgcctgcggcccctcacagggctga
ggccagcccctagcagctggatgtggcagaggcaggccagaggacctaag
tgtgatggaccagagtcacttctcctcctcctttctccagccagccctga
cccctcatgctctctggctgggccagtgggcagccctcgcttcccttgga
tggagctgccctgctggtgcctggtcagagaagaggcctctgtgcccagc
ctgattctctgctcccaggagccagtgacatgaggtgcagaggcccaccc
agccccctacctactgcccccattcatcctggctttccacagccccctcc
cacacagttggacccgtgattctcagggtgctgtgatggggtgagggtag
ggggagcatttgttattaaatgactggacttttgtgccaattgcaaaaaa
aaaaaaaaaaaa
>NM_000251 1
ggcgggaaacagcttagtgggtgtggggtcgcgcattttcttcaaccagg
aggtgaggaggtttcgacatggcggtgcagccgaaggagacgctgcagtt
ggagagcgcggccgaggtcggcttcgtgcgcttctttcagggcatgccgg
agaagccgaccaccacagtgcgccttttcgaccggggcgacttctatacg
gcgcacggcgaggacgcgctgctggccgcccgggaggtgttcaagaccca
gggggtgatcaagtacatggggccggcaggagcaaagaatctgcagagtg
ttgtgcttagtaaaatgaattttgaatcttttgtaaaagatcttcttctg

gttcgtcagtatagagttgaagtttataagaatagagctggaaataaggc
atccaaggagaatgattggtatttggcatataaggcttctcctggcaatc
tctctcagtttgaagacattctctttggtaacaatgatatgtcagcttcc
attggtgttgtgggtgttaaaatgtccgcagttgatggccagagacaggt
tggagttgggtatgtggattccatacagaggaaactaggactgtgtgaat
tccctgataatgatcagttctccaatcttgaggctctcctcatccagatt
ggaccaaaggaatgtgttttacccggaggagagactgctggagacatggg
gaaactgagacagataattcaaagaggaggaattctgatcacagaaagaa
aaaaagctgacttttccacaaaagacatttatcaggacctcaaccggttg
ttgaaaggcaaaaagggagagcagatgaatagtgctgtattgccagaaat
ggagaatcaggttgcagtttcatcactgtctgcggtaatcaagtttttag
aactcttatcagatgattccaactttggacagtttgaactgactactttt
gacttcagccagtatatgaaattggatattgcagcagtcagagcccttaa
cctttttcagggttctgttgaagataccactggctctcagtctctggctg
ccttgctgaataagtgtaaaacccctcaaggacaaagacttgttaaccag
tggattaagcagcctctcatggataagaacagaatagaggagagattgaa
tttagtggaagcttttgtagaagatgcagaattgaggcagactttacaag
aagatttacttcgtcgattcccagatcttaaccgacttgccaagaagttt
caaagacaagcagcaaacttacaagattgttaccgactctatcagggtat
aaatcaactacctaatgttatacaggctctggaaaaacatgaaggaaaac
accagaaattattgttggcagtttttgtgactcctcttactgatcttcgt
tctgacttctccaagtttcaggaaatgatagaaacaactttagatatgga
tcaggtggaaaaccatgaattccttgtaaaaccttcatttgatcctaatc
tcagtgaattaagagaaataatgaatgacttggaaaagaagatgcagtca
acattaataagtgcagccagagatcttggcttggaccctggcaaacagat
taaactggattccagtgcacagtttggatattactttcgtgtaacctgta
aggaagaaaaagtccttcgtaacaataaaaactttagtactgtagatatc
cagaagaatggtgttaaatttaccaacagcaaattgacttctttaaatga
agagtataccaaaaataaaacagaatatgaagaagcccaggatgccattg
ttaaagaaattgtcaatatttcttcaggctatgtagaaccaatgcagaca
ctcaatgatgtgttagctcagctagatgctgttgtcagctttgctcacgt
gtcaaatggagcacctgttccatatgtacgaccagccattttggagaaag
gacaaggaagaattatattaaaagcatccaggcatgcttgtgttgaagtt
caagatgaaattgcatttattcctaatgacgtatactttgaaaaagataa
acagatgttccacatcattactggccccaatatgggaggtaaatcaacat
atattcgacaaactggggtgatagtactcatggcccaaattgggtgtttt
gtgccatgtgagtcagcagaagtgtccattgtggactgcatcttagcccg
agtaggggctggtgacagtcaattgaaaggagtctccacgttcatggctg
aaatgttggaaactgcttctatcctcaggtctgcaaccaaagattcatta
ataatcatagatgaattgggaagaggaacttctacctacgatggatttgg
gttagcatgggctatatcagaatacattgcaacaaagattggtgcttttt
gcatgtttgcaacccattttcatgaacttactgccttggccaatcagata
ccaactgttaataatctacatgtcacagcactcaccactgaagagacctt
aactatgctttatcaggtgaagaaaggtgtctgtgatcaaagttttggga
ttcatgttgcagagcttgctaatttccctaagcatgtaatagagtgtgct
aaacagaaagccctggaacttgaggagtttcagtatattggagaatcgca
aggatatgatatcatggaaccagcagcaaagaagtgctatctggaaagag
agcaaggtgaaaaaattattcaggagttcctgtccaaggtgaaacaaatg
ccctttactgaaatgtcagaagaaaacatcacaataaagttaaaacagct

aaaagctgaagtaatagcaaagaataatagctttgtaaatgaaatcattt
cacgaataaaagttactacgtgaaaaatcccagtaatggaatgaaggtaa
tattgataagctattgtctgtaatagttttatattgttttatattaaccc
tttttccatagtgttaactgtcagtgcccatgggctatcaacttaataag
atatttagtaatattttactttgaggacattttcaaagatttttattttg
aaaaatgagagctgtaactgaggactgtttgcaattgacataggcaataa
taagtgatgtgctgaattttataaataaaatcatgtagtttgtgg
>NM_004541 3
agattccgtcgcttcttccggagccgtacgtggcaccgccccgctcgcgg
gcggccgcggggcttgctgggaagagaggcgaagccaggtcacctttcaa
ggacccagaagtagggttttggcctaggtaacggggcagagatgtggttc
gagattctccccggactctccgtcatgggcgtgtgcttgttgattccagg
actggctactgcgtacatccacaggttcactaacgggggcaaggaaaaaa
gggttgctcattttgggtatcactggagtctgatggaaagagataggcgc
atctctggagttgatcgttactatgtgtcaaagggtttggagaacattga
ttaaggaagcattttcctgattgatgaaaaaaataactcagttatggcca
tctacccctgctagaaggttacagtgtattatgtagcatgcaatgtgtta
tgtagtgcttaataaaaataaaatgaaaaaaatgcaaaaaaaaaaaaaaa
aa
>NM_014820 4
cttgagcgcccttagcccgctgtatacgcgcccctcctcggcttcagtag
gcaagagggccatctgcccttctttcctgaaggtagaggggacaacacca
gctacgacggggactccagaagtccatctcccgaacagcagcggggcgaa
aagaaagaaaaagggtttccgaagactcctactcacacccacgctttccc
ttaacccggaagtgatttccgcccctcctctccctcttcggttgatactg
gaggagaaggacggccaggtctggcccggcatgccctgggcttccggtga
cctctggcccttttctgtcgtccgctctctctgcctagcgtgctcgctcg
ctcattgctttccttccctccctcggtcttccttcgcacgctgtttggtg
attgtggcgctcgcgacagacagggaggcggtggcagaggacacttgtca
tggccgcctctaaacctgtggaggcagcggtggtcgcagccgctgtaccg
agctccgggagtggggtgggcggcggcgggactgcgggcccgggcacggg
ggggctgccgcgatggcagctggctctggcggtcggggcacccctgctgc
tgggcgcgggtgccatatacctgtggagtcggcagcaacggcgccgggag
gccagaggccggggcgacgccagcggcctgaagcgcaacagcgaacggaa
gaccccggagggcagggccagtccggccccgggcagcggacaccctgaag
gtcccggtgctcacttggacatgaactctcttgatagagcccaagcagcc
aagaataaaggcaataaatattttaaagcaggaaaatatgaacaagctat
tcagtgctatactgaggctattagcttgtgccctacagagaagaatgttg
acctttctacattttatcaaaacagagctgctgcctttgaacagttgcaa
aaatggaaagaagtggcacaagactgtacaaaagctgttgaacttaatcc
caaatatgtgaaagctctctttagacgtgcaaaagcccatgagaagctag
acaataagaaggaatgtttagaagatgtcactgctgtgtgtatattagaa
gggttccaaaatcaacaaagcatgctgttagccgataaagttcttaaact
ccttggaaaagagaaagccaaagaaaaatataagaatcgtgaacctctga
tgccatctccacagtttatcaaatcttacttcagttctttcacggatgat
atcatttcccagcccatgcttaaaggagagaaatctgatgaagataaaga
caaggaaggggaggctttagaagtgaaagaaaattctggatacttaaagg
ccaaacagtatatggaagaagaaaactacgataaaatcataagtgaatgc
tcaaaagaaatagatgctgaaggcaaatacatggcagaagcattgctact

acgagctaccttctacctgcttattggcaatgccaatgcagccaaaccag
atttagataaagtcatcagtttgaaagaagctaatgtgaagcttcgagca
aatgctctcatcaaaagaggcagcatgtacatgcaacagcagcagccttt
gctgtccactcaagattttaacatggctgctgacatcgatcctcagaatg
cagatgtttatcaccaccgaggacagctgaaaatactccttgatcaagtt
gaagaagcagtggcagattttgatgaatgtattaggttaagacctgagtc
tgctctggcacaagcacagaaatgttttgcattgtaccgccaggcatata
cgggaaacaactcttcacaaatccaagcagctatgaaaggttttgaagag
gtcataaagaaatttccaaggtgtgccgaaggctatgcactatacgccca
ggcattaacagatcaacaacagtttggtaaagctgatgaaatgtatgata
aatgtattgatttggaaccagataatgctacaacatatgttcataaaggt
ttacttcaacttcagtggaagcaagatctggatagaggtttggaacttat
cagcaaggctattgaaattgacaataaatgtgattttgcctatgaaacca
tgggaactattgaagtacaaagaggaaacatggagaaagccattgacatg
ttcaacaaagctattaacctggccaaatcggaaatggagatggcccatct
gtattcactttgcgatgccgcccatgcccagacagaagttgcaaagaaat
acggattaaaaccaccaacattataaaacagggggaaagcagactgaccc
tctttttaaaagtttacccccttcttcaactgaaccctaaagacactgtca
tgaactgtgttgaatggtggaaatcagtatttctgtttgtggtgttgtta
tttgttacatctgtttcatgtctaggtgttgtgggtgtggctgttgaagg
aagtttgcagtcttgcagcttttattccctgtgcaacaaaagattagaac
atgttaaagggatttttaaataaagttgcaaagagtacaaatgataattg
gccatgcaaataaaaactgatttgttgattttttttttaagggggggttgg
cagttgattatgttctggatgattccgtctatatatgtgtgaataatgta
agtattttacagcatgttgatttttaaattaacgtagtaaatgctgtaaa
atagatttatattcagttaaccgctttcagttgatttttttgaaagaaaca
aaggttaaatgggggattaaagtaaaattgagagaccctttaaaccattg
tcagcatgcacaatgcctctgattctgcagttttagaaacttggtggcac
ttattaatcctcttggcccctttccactctaatggatagtgtacattctt
cttaaagtccacaacagcagattttcttgcagtaaattatgcagatgcaa
aatattctaattgatatatgtgttggaagactgagtattgatgggggagt
ggaccagacaaagaggtaagatgaaacagtagtgtgtttataattgtctg
tgactattttctataataattagtactatttaatggtgagcttttaaaaa
tgtaggatagagggtacagtggcactgtatatactatttatagtctcagc
tactagggaggctgaggcaagaggcttgagaccaggagctcgaggctgta
atgtgccatgatgattgcacctgcgaatagccactgcactccagcctggg
caatatagcaagaccccatcctttaaaaaattttagaactttttttaaaat
caaagtgcagattgcttgtatgtaaaacccaaataaaggtagagtaagtg
tgatatatgggagtattaaaatagcttaaaatttctcgtgaaggacatgt
ggctaaagggtcaaaaaggatgtaagacttggagccagagcatagtattt
cctgaaataacaagtttagtgctttaactatggctacatgtgcttaagga
attttgagccacttatttttgaagatgctgaggacatgtagagtgctttt
tgtagtgagctaaccttgatctctaaggactaactacccaggtccaggtc
ttcactaggggtactgacagtgtttaagctttactccacctccttactta
gaaatcactttacgatttatttccattttccacttttatagaccatcctt
ttgcttatatgctagatttttctggtgagggaagggttgtgttcttcagg
ggtcttttgttttgaaatactcaggatggggagaggtttatttaagaacg
aattataattatggtttacactgttgggagtaaaggagcatttttacacc
ccttaagggtgcttaattctgttgaaaccaaaaagatttgtctacaaatg

ctatctttttagaaactattagaaatgactccctttcaaagtcaatctt
tggaaaatattgaggaggtcactaattagttggtgcagttaatataattc
aagatgatttggatgatgggaagtttgagaccgctgcattttgtttttaa
attatgcaccttctgataacccccaaatacagaaatgttctacatctctg
aatgacctctgactttaaaaaagtttttatttgcatggctgtatttacat
taacactgacattttcttctactcttctcccttctttcatcttggggttg
ggtagagaaacacaaaggaaactgaagcatgtgccattctatactgtcat
tccaaattctcatggactattgcctgttgtgaaaatgtttgaaactgcac
tgaaagctgcatctgtctgtatctttcttttgtaaatgacctcacatgta
aattcaccaaataaatattacattcaagctc
>NM_003890 2
ctgcagccatgggtgccctatggagctggtggatactctgggctggagca
accctcctgtggggattgacccaggaggcttcagtggacctcaagaacac
tggcagagaggaattcctcacagccttcctgcagaactatcagctggcct
acagcaaggcctacccccgcctccttatctccagtctgtcagagagcccc
gcttcagtctccatcctcagccaggcagacaacacctcaaagaaggtcac
agtgaggcccggggagtcggtcatggtcaacatcagtgccaaggctgaga
tgataggcagcaagatcttccagcatgcggtggtgatccattctgactat
gccatctctgtgcaggcactaaatgccaagcctgacacagcggagctgac
actgctgcggcccatccaggccctaggcaccgagtattttgtgctcacac
cccccggcacctcagccaggaatgtcaaggagtttgccgtggtggccggt
gccgcaggtgcctcggtcagtgtcacgctgaaggggtcagtgacattcaa
tggcaagttctatccagcaggcgatgtcctaagagtgactctacagccct
acaatgtggcccagctacagagctcagtggatctctcggggtcaaaggtc
acagctagtagccccgtggctgtcctctctggccacagctgtgcgcagaa
acatacgacctgcaaccatgtggttgagcagctgctacccacgtctgcct
ggggcacccactatgtagtacccacgctggcctcccaatctcgctatgat
ttggccttcgttgtggccagccaggccacaaagctgacctacaaccatgg
gggtatcactggctcccgtgggctccaggcaggtgatgtggtagagtttg
aggtccggccatcctggccactctacctgtctgcaaatgtgggcatccag
gtcctgttgtttggcacaggtgccataaggaatgaagtgacttatgaccc
ctacctggtcctgatcccagatgtggcggcctactgcccagcctatgtgg
tcaagagtgtaccaggctgtgagggcgtggccctggtagtggcacagacg
aaggctatcagcgggctgaccatagatgggcatgcagtgggggccaagct
cacctgggaggctgtgccaggcagtgagttctcgtatgctgaagtggagc
tcggcacagctgacatgatccacacggccgaggccaccaccaacttggga
ctgctcaccttcgggctggccaaggctataggctacgcaacagctgctga
ttgcggccggactgtactgtccccagtggagccctcctgcgaaggcatgc
agtgcgcagccgggcagcgctgccaggtggtaggcgggaaggccgggtgt
gtggcggagtccaccgctgtctgccgcgcccagggcgacccccattacac
caccttcgacggccgtcgctacgacatgatgggcacctgttcgtacacga
tggtggagctgtgcagcgaggacgacaccctgcccgccttcagcgtggag
gccaagaacgagcaccggggcagccgccgcgtctcctacgtgggcctcgt
cactgtgcgcgcctacagccactctgtgtcgctgacccgcggtgaagttg
gcttcgtcctggttgacaaccagcgctcgcgcctgccagtctccctgagt
gagggtcgcctgcgtgtgtaccagagcggaccacgggccgtggtggagct
ggtctttgggctggtggtcacttatgactgggactgccagctggcactca
gcctgcctgcacgcttccaagaccaggtgtgcgggctgtgtggcaactat
aatggtgacccagcagacgacttcctcacgcctgacggggctctggctcc

tgacgctgtggagttcgcaagtagctggaagctggatgatggggactacc
tgtgtgaggatggctgccagaacaactgtcccgcctgcaccccaggccag
gcccaacactatgagggcgaccgactctgtggcatgctgaccaagctcga
tggccccttcgctgtctgccatgacaccctggaccccaggcccttcctgg
agcagtgtgtatatgacctgtgtgtggtcggtggggagcggctcagcctg
tgccgtggcctcagcgcctatgcccaggcctgtctggagcttggcatctc
ggttggggactggagatcaccagccaactgccccctgtcctgccctgcca
acagccgctatgagctctgcggccctgcttgcccgacctcctgcaacggg
gctgcggcgccgtccaactgctccgggcgcccctgcgtggagggctgcgt
gtgcctcccaggcttcgtggccagcggcggcgcctgcgtgccggcctcgt
cgtgtggctgcaccttccagggtctccagctcgctccgggccaggaagtg
tgggcggacgagttgtgccaaaggcgctgcacctgcaacggcgccaccca
tcaggtcacctgccgcgacaagcagagctgcccggcgggtgagcgctgca
gcgtccagaacggcctcctgggctgctaccccgatcgcttcgggacctgc
caggggtccggggacccacactatgtgagcttcgacggccggcgcttcga
cttcatgggcacctgcacgtacctgctggtcggctcatgcggccagaacg
cagcgctgcctgccttccgggtgctggtggaaaacgagcatcggggcagc
cagactgtgagctacacgcgcgccgtgcgggtggaggcccgcggggtgaa
ggtggccgtgcgccgggagtaccccggcaagtgctggtggatgacgtcc
ttcagtatctgcccttccaagcagcagatgggcaggtgcaggtgttccga
cagggcagggatgccgtcgtgcgcacggactttggcctgactgtcactta
tgactggaatgcacgagtgactgccaaggtgcccagcagctatgctgagg
ccctgtgtggactctgtgggaacttcaacggggacccagctgatgacctg
gctctgcggggtgggggtcaagctgccaatgcactggcctttgggaacag
ctggcaagaagagacgaggcccggctgtggagcaactgaaccgggtgact
gtcccaagctggactccctggtggcccagcagctgcagagcaagaatgag
tgtggaatccttgccgaccccaaggggcccttccgggagtgccatagcaa
gctggacccccagggtgccgtgcgcgactgtgtctatgaccgctgcctgc
tgccaggccagtctgggccactgtgtgacgcactggccacctatgctgct
gcatgccaggctgctggagccacagtgcacccctggaggagtgaagaact
ttgcccactgagctgcccaccccacagccactatgaggcgtgttcctacg
gctgcccgctgtcctgtggagacctcccagtgcccgggggctgtggctca
gaatgccatgagggctgcgtgtgcgatgagggctttgcgctcagtggtga
gtcctgcctgcccctggcctcctgtggctgcgtacaccagggcacctacc
acccaccaggccagaccttctaccctggccccggatgtgattccctttgc
cactgccaggagggcggcctggtgtcctgtgagtcctccagctgcggacc
gcacgaggcctgccagccatccggtggcagcttgggctgtgtggccgtgg
gctctagcacctgccaggcgtcaggagacccccactacaccaccttcgat
ggccgccgcttcgacttcatgggcacctgcgtgtatgtgctggctcagac
ctgcggcacccggcctggcctgcatcggtttgccgtcctgcaggagaacg
tggcctggggtaatgggcgagtcagtgtgaccagggtgatcacggtccag
gtggcaaacttcaccctgcggctggagcagagacagtggaaggtcacggt
gaacggtgtggacatgaagctgcccgtggtgctggccaacggccagatcc
gtgcctcccagcatggttcagatgttgtgattgagaccgacttcggcctg
cgtgtggcctacgaccttgtgtactatgtgcgggtcaccgtccccggaaa
ctactaccagcagatgtgtggcctgtgtgggaactacaacggcgacccca
aggatgacttccagaagcccaatggctcacaggcaggcaacgccaatgag
ttcggcaactcctgggaggaggtggtgcccgactctccctgcctgccgcc
cacccccttgcccgccggggagcgaggactgtatccccagccacaagtgtc

ctcccgagctggagaagaagtatcagaaggaggagttctgtgggctcctc
tccagccccacagggccactgtcctcctgccacaagctggtggatcccca
gggtcccttgaaagattgcatctttgatctctgcctgggtggtgggaacc
tgagcattctctgcagcaacatccatgcctacgtgagtgcttgccaggcg
gctggaggccacgtggagccctggaggactgaaactttctgtcccatgga
gtgccctccgaacagtcactacgagctctgtgcggacacctgctccctgg
gctgctcagctctcagtgcccctccacagtgccaggatgggtgtgctgag
ggctgccagtgtgactccggcttcctctacaatggccaagcctgcgtgcc
catccagcaatgcggctgctaccacaatggtgtctactatgagccggagc
agacagtcctcattgacaactgtcggcagcagtgcacgtgccatgcgggt
aaaggcatggtgtgccaggaacacagctgcaagccggggcaggtgtgcca
gccctccggaggcatcctgagctgcgtcaccaaagacccgtgccacggcg
tgacatgccggccacaggagacatgcaaggagcagggtggccagggcgtg
tgcctgcccaactatgaggccacgtgctggctgtggggcgacccacacta
ccactccttcgatggccggaagtttgacttccagggcacctgtaactatg
tgctggcaacaactggctgcccgggggtcagcacccagggcctgacaccc
ttcaccgtcaccaccaagaaccagaaccggggcaaccctgctgtgtccta
cgtgagagtcgtcaccgtggctgccctcggcaccaacatctccatccaca
aggacgagatcggcaaagtccgggtgaacggtgtgctcacagccttgcct
gtctctgtggccgacgggcggatttcagtgacccagggtgcatcgaaggc
actgctggtggctgactttggactgcaagtcagctatgactggaactggc
gggtagacgtgacgctgcccagcagctatcatggcgcagtgtgcgggctc
tgcggtaacatggaccgcaaccccaacaatgaccaggtcttccctaatgg
cacactggctccctccatacccatctggggcggcagctggcgagccccag
gctgggacccactgtgttgggacgaatgtcgggggtcctgcccaacgtgc
cctgaggaccggttggagcagtacgagggccctggcttctgcggacccct
ggcccccggcacagggggccctttcaccacctgccatgctcatgtgccac
ctgagagcttcttcaagggctgtgttctggacgtctgcatgggtggtggg
gaccgtgacattctttgcaaggctctggcttcctatgtggccgcctgcca
ggctgctggggttgtcatcgaagactggcgggcacaggttggctgtgaga
tcacctgcccagaaaacagccactatgaggtctgtggctcaccctgcccg
gccagctgtccgtcccctgcaccccttacgacgccagccgtatgtgaggg
cccctgtgtggagggctgccagtgcgacgcgggtttcgtgttaagtgctg
accgctgtgttcccctcaacaacggctgcggctgctgggccaatggcacc
taccacgaggcgggcagtgagttttgggctgatggcacctgctcccagtg
gtgtcgctgcgggcctgggggtggctcgctggtctgcacacctgccagct
gtgggctgggtgaagtgtgtggcctcctgccatccggccagcacggctgc
cagcccgtcagcacagctgagtgccaggcgtggggtgacccccattacgt
cactctggatgggcaccgattcaatttccaaggcacctgcgagtacctgc
tgagtgcaccctgccacggaccacccttgggggctgagaacttcactgtc
actgtagccaatgagcaccggggcagccaggctgtcagctacacccgcag
tgtcaccctgcaaatctacaaccacagcctgacactgagtgcccgctggc
cccggaagctacaggtggacggcgtgttcgtcactctgcccttccagctg
gactcgctcctgcacgcacacctgagcggcgccgacgtggtggtgaccac
aacctcagggctctcgctggctttcgacggggacagcttcgtgcgcctgc
gcgtgccggcggcgtacgcgggctctctctgtggcttatgcgggaactac
aaccaggaccccgcagacgacctgaaggcggtgggcgggaagcccgccgg
atggcaggtgggcggcgcccagggctgcggggaatgtgtgtccaagccat
gcccgtcgccgtgcaccccagagcagcaagagtccttcggcggcccggac

gcctgcggcgtgatctccgccaccgacggcccgctggcgccctgccacgg
ccttgtgccgcccgcgcagtacttccagggctgcttgctggacgcctgcc
aagttcagggccatcctggaggcctctgtcctgcagtggccacctacgtg
gcagcctgtcaggccgctggggcccagctccgcgagtggaggcggccgga
cttctgtcccttccagtgccctgcccacagccactacgagctctgcggtg
actcctgtcctgggagctgcccgagcctgtcggcacccgagggctgtgag
tcggcctgccgtgaaggctgtgtctgcgatgctggcttcgtgctcagtgg
tgacacgtgtgtacctgtgggccagtgtggctgcctccacgatgaccgct
actacccactgggccagaccttctaccctggccctgggtgtgattccctt
tgccgctgccgggagggcggtgaggtgtcctgtgagccctccagctgcgg
cccgcatgagacctgccggccatccggtggcagcttgggctgcgtggccg
tgggctctaccacctgccaggcgtcgggagatccccactacaccaccttc
gatggccgccgcttcgacttcatgggcacctgcgtgtatgtgctggctca
gacctgcggcacccggcctggcctacatcggtttgccgtcctgcaggaga
acgtggcctggggtaatgggcgagtcagtgtgaccagggtgatcacggtc
caggtggcaaacttcaccctgcggctggagcagagacagtggaaggtcac
ggtgaacggtgtggacatgaagctgcccgtggtgctggccaacggccaga
tccgtgcctcccagcatggttcagatgttgtgattgagaccgacttcggc
ctgcgtgtggcctacgaccttgtgtactatgtgcgggtcaccgtccctgg
aaactactaccagctgatgtgtggcctgtgtgggaactacaacggcgacc
ccaaggatgacttccagaagcccaatggctcgcaggcaggcaacgccaat
gagttcggcaactcctgggaggaggtggtgcccgactctccctgcctgcc
gccgcccacctgcccgccggggagcgagggctgtatccccagcgaggagt
gtcctcccgagctggagaagaagtatcagaaggaggagttctgtgggctc
ctctccagccccacagggccactgtcctcttgccacaagctggtggatcc
ccagggtcccttgaaagattgcatctttgatctctgcctgggtggtggga
acctgagcattctctgcagcaacatccatgcctacgtgagtgcttgccag
gcagctggaggccaggtggagccctggaggaatgaaactttctgtcccat
ggaatgccctcagaacagtcactacgagctctgtgcggacacctgctccc
tgggctgctcggctctcagtgcccctctgcagtgcccagatgggtgtgct
gagggctgccagtgtgactccggcttcctctacaacggccaagcctgcgt
gcccatccagcaatgtggctgctaccacaatggtgcctactatgagccgg
agcagacagtcctcattgacaactgtcggcagcagtgcacgtgccatgtg
ggtaaagtcgtggtgtgccaggaacacagctgcaagccggggcaggtgtg
ccagccctccggaggcatcctgagctgcgtcaacaaagacccgtgccacg
gcgtgacatgccggccacaggagacatgcaaggagcagggtggccagggc
gtgtgcctgcccaactatgaggccacgtgctggctgtggggcgacccaca
ctaccactccttcgatggccggaagtttgacttccagggcacctgtaact
atgtgctggcaacaactggctgcccgggggtcagcacccagggcctgaca
cccttcaccgtcaccaccaagaaccagaaccggggcaaccctgctgtgtc
ctacgtgagagtcgtcaccgtggctgccctcggcaccaacatctccatcc
acaaggacgagatcggcaaagtccgggtgaacggtgtgctcacagccttg
cctgtctctgtggccgacgggcggatttcagtgacccagggtgcatcgaa
ggcactgctggtggctgactttggactgcaagtcagctatgactggaact
ggcgggtagacgtgacgctgcccagcagctatcatggcgcagtgtgcggg
ctctgcggtaacatggaccgcaaccccaacaatgaccaggtcttccctaa
tggcacactggctccctccatacccatctggggcggcagctggcgagccc
caggctgggacccactgtgttgggacgaatgtcgggggtcctgcccaacg
tgccctgaggaccggttggagcagtacgagggccctggcttctgcggacc

cctggcccccggcacagggggccctttcaccacctgccatgctcatgtgc
cacctgagagcttcttcaagggctgtgttctggacgtctgcatgggtggt
ggggaccgtgacattctttgcaaggctctggcttcctatgtggccgcctg
ccaggctgctggggttgtcatcgaagactggcgggcacaggttggctgtg
agatcacctgcccagaaaacagccactatgaggtctgtggcccaccctgc
ccggccagctgtccgtcccctgcaccccttacgacgccagccgtatgtga
gggcccctgtgtggagggctgccagtgcgacgcgggtttcgtgttaagtg
ctgaccgctgtgttcccctcaacaacggctgcggctgctgggccaatggc
acctaccacgaggcgggcagtgagttttgggctgatggcacctgctccca
gtggtgtcgctgcgggcctgggggtggctcgctggtctgcacacctgcca
gctgtgggctgggtgaagtgtgtggcctcctgccatccggccagcacggc
tgccagcccgtcagcacagctgagtgccaggcgtggggtgacccccatta
cgtcactctggatgggcaccgattcgatttccaaggcacctgcgagtacc
tgctgagtgcaccctgccacggaccacccttgggggctgagaacttcact
gtcactgtagccaatgagcaccggggcagccaggctgtcagctacacccg
cagtgtcaccctgcaaatctacaaccacagcctgacactgagtgcccgct
ggccccggaagctacaggtggacggcgtgttcgtcactctgcccttccag
ctggactcgctcctgcacgcacacctgagcggcgccgacgtggtggtgac
cacaacctcagggctctcgctggctttcgatggggacagcttcgtgcgcc
tgcgcgtgccggcggcgtacgcgggctctctctgtggcttatgcgggaac
tacaaccaggaccccgcagacgacctgaaggcggtgggcgggaagcccgc
cggatggcaggtgggcggcgcccagggctgcggggaatgtgtgtccaagc
catgcccgtcgccgtgcaccccagagcagcaagagtccttcggcggcccg
gacgcctgcggcgtgatctccgccaccgacggcccgctggcgccctgcca
cggccttgtgccgcccgcgcagtacttccagggctgcttgctggacgcct
gccaagttcagggccatcctggaggcctctgtcctgcagtggccacctac
gtggcagcctgtcaggccgctggggcccagctccgcgagtggaggcggcc
ggacttctgtcccttccagtgccctgcccacagccactacgagctctgcg
gtgactcctgtcctgggagctgcccgagcctgtcggcacccgagggctgt
gagtcggcctgccgtgaaggctgtgtctgcgatgctggcttcgtgctcag
tggtgacacgtgtgtacctgtgggccagtgtggctgcctccacgatgacc
gctactacccactgggccagaccttctaccctggccctgggtgtgattcc
ctttgccgctgccgggagggcggtgaggtgtcctgtgagccctccagctg
cggcccgcatgagacctgccggccatccggtggcagcttgggctgcgtgg
ccgtgggctctaccacctgccaggcgtcgggagatccccactacaccacc
ttcgatggccaccgcttcgacttcatgggcacctgcgtgtatgtgctggc
tcagacctgcggcacccggcctggcctgcatcggtttgccgtcctgcagg
agaacgtggcctggggtaatgggcgagtcagtgtgaccagggtgatcacg
gtccaggtggcaaacttcaccctgcggctggagcagagacagtggaaggt
cacggtgaacggtgtggacatgaagctgcccgtggtgctggccaacggcc
agatccgtgcctcccagcatggttcagatgttgtgattgagaccgacttc
ggcctgcgtgtggcctacgaccttgtgtactatgtgcgggtcaccgtccc
tggaaactactaccagctgatgtgtggcctgtgtgggaactacaacggcg
accccaaggatgacttccagaagcccaatggctcgcaggcaggcaacgcc
aatgagttcggcaactcctgggaggaggtggtgcccgactctccctgcct
gccgccgcccacctgcccgccggggagcgcgggctgtatccccagcgaca
agtgtcctcccgagctggagaagaagtatcagaaggaggagttctgtggg
ctcctctccagccccacagggccactgtcctcctgccacaagctggtgga
tccccagggtcccttgaaagattgcatctttgatctctgcctgggtggtg

ggaacctgagcattctctgcagcaacatccatgcctacgtgagtgcttgc
caggcggctggaggccacgtggagccctggaggaatgaaactttctgtcc
catggaatgccctcagaacagtcactacgagctctgtgcggacacctgct
ccctgggctgctcggctctcagtgcccctctgcagtgcccagatgggtgt
gctgagggctgccagtgtgactccggcttcctctacaacggccaagcctg
cgtgcccatccagcaatgtggctgctaccacaatggtgtctactatgagc
cggagcagacagtcctcattgacaactgtcggcagcagtgcacgtgccat
gtgggtaaagtcgtggtgtgccaggaacacagctgcaagccggggcaggt
gtgccagccctccggaggcatcctgagctgcgtcaccaaagacccgtgcc
acggcgtgacatgccggccacaggagacatgcaaggagcagggtggccag
ggcgtgtgcctgcccaactatgaggccacgtgctggctgtggggcgaccc
acactaccactccttcgatggccggaagtttgacttccagggcacctgta
actatgtgctggcaacaactggctgcccgggggtcagcacccagggcctg
acacccttcaccgtcaccaccaagaaccagaaccggggcaaccctgctgt
gtcctacgtgagagtcgtcaccgtggctgccctcggcaccaacatctcca
tccacaaggacgagatcggcaaagtccgggtgaacggtgtgctcacagcc
ttgcctgtctccgtggccgacgggcggatttcagtggcccagggtgcatc
gaaggcactgctggtggctgactttggactgcaagtcagctatgactgga
actggcgggtagacgtgacgctccccagcagctatcatggcgcagtgtgc
gggctctgcggtaacatggaccgcaaccccaacaatgaccaggtcttccc
taatggcacactggctccctccatacccatctggggcggcagctggcgag
ccccaggctgggacccactgtgttgggacgaatgtcgggggtcctgccca
acgtgccctgaggaccggttggagcagtacgagggccctggcttctgcgg
acccctttcatctggcacagggggcccccttcaccacctgccatgctcatg
tgccacctgagagcttcttcaagggctgtgttctggacgtctgcatgggt
ggtggggaccgtgacattctttgcaaggctctggcttcctacgtggccgc
ctgccaggccgctggggttgtcatcgaagactggcgggcacaggttggct
gtgagatcacctgcccagaaaacagccactatgaggtctgtggcccaccc
tgcccagccagctgtccgtcccctgcacccccttacgacgccagccgtatg
tgagggcccctgtgtggagggctgccagtgcgacgcgggtttcgtgttaa
gtgctgaccgctgtgttccccctcaacaacggctgcggctgctgggccaat
ggcacctaccacgaggcgggcagtgagttttgggctgatggcacctgctc
ccagtggtgtcgctgcgggcctgggggtggctcgctggtctgcacacctg
ccagctgtgggctgggtgaagtgtgtggcctcctgccatccggccagcac
ggctgccagcccgtcagcacagctgagtgccaggcgtggggtgacccccca
ttacgtcactctggatgggcaccgattcgatttccaaggcacctgcgagt
acctgctgagtgcaccctgccacggaccacccttgggggctgagaacttc
actgtcactgtagccaatgagcaccggggcagccaggctgtcagctacac
ccgcagtgtcaccctgcaaatctacaaccacagcctgacactgagtgccc
gctggccccggaagctacaggtcgacggcgtgttcgtggctctgcctttc
cagctggactcgctcctgcacgcacacctgagcggcgccgacgtggtggt
gaccacaacctcagggctctcgctggctttcgatggggacagcttcgtgc
gcctgcgcgtgccggcggcgtacgcggcctctctctgtggcttatgcggg
aactacaaccaggaccccgcagacgacctcaaggctgtgggcgggaagcc
cgctggatggcaggtgggcggggcccagggctgcggggaatgtgtgtcca
agccatgcccgtcgccgtgcaccccagagcagcaggagtccttcggcggc
ccggacgcctgcggcgtgatctccgccaccgacggcccgctggcaccctg
ccacggccttgtgccgcccgcgcagtacttccaggctgcttgctggacg
cctgccaagttcagggccatcctggaggcctctgtcctgcagtggctacc

tacgtggcagcctgtcaggccgctggggcccagctcggcgagtggaggcg
gccggacttctgtcccttgcagtgccctgcccacagccactatgagctct
gcggtgactcctgccctgtgagctgcccgagcctctcagcacccgagggc
tgtgagtcggcctgccgtgaaggctgtgtctgcgatgctggcttcgtact
cagtggtgacacctgcgtacccgtgggccagtgtggctgcctccatgatg
gccgctactacccactgggcgaggtcttctacccgggccctgagtgtgag
cggcgctgtgagtgtgggccaggtggccatgtcacctgccaggagggcgc
agcctgtgggccccatgaggagtgccggttagaggatggtgtccaggcct
gtcatgccacaggctgtggccgctgcctggccaacgggggcatccactac
atcacccttgatggccgtgtctacgacctgcatggctcctgctcctatgt
cttggcccaagtctgccacccaaagcctggggacgaggacttttccatcg
tgcttgagaagaatgcagctggagatctccaacgcctcctggttactgtg
gctggccaggttgtgagcctagctcaggggcagcaggtcaccgtggacgg
cgaggctgtggccctgcctgtggctgtgggccgcgtgcgggtgaccgccg
agggccgaaacatggttctgcagacgaccaaggggctgcggcttctcttt
gatggcgatgcccacctcctcatgtccatccccagccccttccgtggacg
gctctgtggcctctgtgggaacttcaatggcaactggagtgacgactttg
tcctgcccaatggctcagcagcgtccagtgtggagaccttcggggctgca
tggcgggcgcccggctcctccaagggctgtggcgagggctgcgggcccca
aggctgcccagtgtgcttggcagaggagactgcaccctatgagagcaacg
aggcctgcgggcagctccggaacccccagggccccttcgcgacctgccag
gcggtgctgagtccctctgagtacttccgccaatgcgtatacgacctgtg
cgcgcaaaagggtgacaaagccttcctgtgccgcagcctggcagcctaca
cggcggcctgtcaggcagctggcgtggccgtgaagccctggaggacagac
agcttctgcccgctccattgccccgcccacagccactactccatctgcac
tcgcacctgccagggatcctgtgcggctctctccggcctcacgggctgca
ccacccgctgttttgagggctgtgagtgcgacgaccgcttcctgctttcc
cagggtgtctgcatccctgtccaagattgtggctgcacccataatggccg
atacttgccggtaaactcctccctgctgacctcagactgcagcgagcgct
gttcctgttcctcaagctctggcctgacatgccaggcagctggctgccca
ccaggccgtgtatgtgaggtcaaggctgaagcccggaactgctgggccac
ccgtggtctctgtgtcctgtctgtgggtgccaacctcaccacctttgatg
gggcccgtggtgccaccacctctcctggtgtctatgagctctcttcccgc
tgcccaggactacagaataccatcccctggtaccgtgtagttgccgaagt
ccagatctgccatggcaaaacggaggctgtgggccaggtccacatcttct
tccaggatgggatggtgacgttgactccaaacaagggtgtgtgggtgaat
ggtctccgagtggatctcccagctgagaagttagcatctgtgtccgtgag
tcgtacacctgatggctccctgctagtccgccagaaggcaggggtccagg
tgtggcttggagccaatgggaaggtggctgtgattgtcagcaatgaccat
gctgggaaactgtgtggggcctgtggaaactttgacggggaccagaccaa
tgattggcatgactcccaggagaagccagcgatggagaaatggagagcgc
aggacttctccccatgttatggctgatcagtcatccaccaggaacgaaga
tttcctgaagaagacctggtccctctggaggttgcagtggctgaaggatg
catcatgtgctcctaccctgctctaccgcttttctgggtcacagaggcca
aatgtgagagcattgaataaatatcttaagctaagctgcaaaaaaaaaaa
aaaaaaa
>NM_005720 3
ggcaggccccgcccccgcccacgaggaagtggctgctgctccggcgcgg
agcccagagccggttcggcgcgtcgactgcccagagtccgcggccggggc

gcgggaggagccaagccgccatggcctaccacagcttcctggtggagccc
atcagctgccacgcctggaacaaggaccgcacccagattgccatctgccc
caacaaccatgaggtgcatatctatgaaaagagcggtgccaaatggacca
aggtgcacgagctcaaggagcacaacgggcaggtgacaggcatcgactgg
gcccccgagagtaaccgtattgtgacctgcggcacagaccgcaacgccta
cgtgtggacgctgaagggccgcacatggaagcccacgctggtcatcctgc
ggatcaaccgggctgcccgctgcgtgcgctgggcccccaacgagaacaag
tttgctgtgggcagcggctctcgtgtgatctccatctgttatttcgagca
ggagaatgactggtgggtttgcaagcacatcaagaagcccatccgctcca
ccgtcctcagcctggactggcaccccaacaatgtgctgctggctgccggc
tcctgtgacttcaagtgtcggatcttttcagcctacatcaaggaggtgga
ggaacggccggcacccaccccgtggggctccaagatgccctttggggaac
tgatgttcgaatccagcagtagctgcggctgggtacatggcgtctgtttc
tcagccagcgggagccgcgtggcctgggtaagccacgacagcaccgtctg
cctggctgatgccgacaagaagatggccgtcgcgactctggcctctgaaa
cactaccactgctggcgctgaccttcatcacagacaacagcctggtggca
gcgggccacgactgcttcccggtgctgttcacctatgacgccgccgcggg
gatgctgagcttcggcgggcggctggacgttcctaagcagagctcgcagc
gtggcttgacggcccgcgagcgcttccagaacctggacaagaaggcgagc
tccgagggtggcacggctgcgggcgcgggcctagactcgctgcacaagaa
cagcgtcagccagatctcggtgctcagcggcggcaaggccaagtgctcgc
agttctgcaccactggcatggatggcggcatgagtatctgggatgtgaag
agcttggagtcagccttgaaggacctcaagatcaaatgacctgtgaggaa
tatgttgccttcatcctagctgctggggaagcggggagaggggtcaggga
ggctaatggttgctttgctgaatgtttctggggtaccaatacgagttccc
ataggggctgctccctcaaaaagggaggggacagatggggagcttttctt
acctattcaaggaatacgtgcctttttcttaaatgctttcatttattgaa
aaaaaaaaaaaatgcccccaaagcactatgctggtcatgaactgcttcaa
aatgtggaggtaataaaatgcaactgtgtaaaaaaaaaaaaaaaaaaaa
a
>NM_003255 4
cgcagcaaacacatccgtagaaggcagcgcggccgccgagaaccgcagcg
ccgctcgcccgccgccccccaccccgccgccccgcccggcgaattgcgcc
ccgcgcccctcccctcgcgcccccgagacaaagaggagagaaagtttgcg
cggccgagcggggcaggtgaggagggtgagccgcgcgggaggggcccgcc
tcggccccggctcagcccccgcccgcgcccccagcccgccgccgcgagca
gcgcccggacccccccagcggcggcccccgcccgcccagccccccggcccg
ccatgggcgccgcggcccgcaccctgcggctggcgctcggcctcctgctg
ctggcgacgctgcttcgcccggccgacgcctgcagctgctccccggtgca
cccgcaacaggcgttttgcaatgcagatgtagtgatcagggccaaagcgg
tcagtgagaaggaagtggactctggaaacgacatttatggcaaccctatc
aagaggatccagtatgagatcaagcagataaagatgttcaaagggcctga
gaaggatatagagtttatctacacggcccctcctcggcagtgtgtgggg
tctcgctggacgttggaggaaagaaggaatatctcattgcaggaaaggcc
gagggggacggcaagatgcacatcaccctctgtgacttcatcgtgccctg
ggacaccctgagcaccacccagaagaagagcctgaaccacaggtaccaga
tgggctgcgagtgcaagatcacgcgctgccccatgatcccgtgctacatc
tcctccccggacgagtgcctctggatggactgggtcacagagaagaacat
caacgggcaccaggccaagttcttcgcctgcatcaagagaagtgacggct

cctgtgcgtggtaccgcggcgcggcgccccccaagcaggagtttctcgac
atcgaggacccataagcaggcctccaacgcccctgtggccaactgcaaaa
aaagcctccaagggtttcgactggtccagctctgacatcccttcctggaa
acagcatgaataaaacactcatcccatgggtccaaattaatatgattctg
ctcccccccttctccttttagacatggttgtgggtctggagggagacgtgg
gtccaaggtcctcatcccatcctccctctgccaggcactatgtgtctggg
gcttcgatccttgggtgcaggcagggctgggacacgcggcttccctccca
gtccctgccttggcaccgtcacagatgccaagcaggcagcacttagggat
ctcccagctgggttagggcagggcctggaaatgtgcattttgcagaaact
tttgagggtcgttgcaagactgtgtagcaggcctaccaggtccctttcat
cttgagagggacatggcccttgttttctgcagcttccacgcctctgcact
ccctgcccctggcaagtgctcccatcgccccggtgcccaccatgagctcc
cagcacctgactccccccacatccaagggcagcctggaaccagtggctag
ttcttgaaggagccccatcaatcctattaatcctcagaattccagtggga
gcctccctctgagccttgtagaaatgggagcgagaaaccccagctgagct
gcgttccagcctcagctgagtctttttggtctgcacccacccccccaccc
ccccccccccgcccacatgctccccagcttgcaggaggaatcggtgaggt
cctgtcctgaggctgctgtccggggccggtggctgccctcaaggtccctt
ccctagctgctgcggttgccattgcttcttgcctgttctggcatcaggca
cctggattgagttgcacagctttgctttatccgggcttgtgtgcagggcc
cggctgggctccccatctgcacatcctgaggacagaaaaagctgggtctt
gctgtgccctcccaggcttagtgttccctccctcaaagactgacagccat
cgttctgcacggggctttctgcatgtgacgccagctaagcatagtaagaa
gtccagcctaggaagggaaggattttggaggtaggtggctttggtgacac
actcacttctttctcagcctccaggacactatggcctgttttaagagaca
tcttatttttctaaaggtgaattctcagatgataggtgaacctgagttgc
agatataccaacttctgcttgtatttcttaaatgacaaagattacctagc
taagaaacttcctagggaactagggaacctatgtgttccctcagtgtggt
ttcctgaagccagtgatatgggggttaggataggaagaactttctcggta
atgataaggagaatctcttgtttcctcccacctgtgttgtaaagataaac
tgacgatatacaggcacattatgtaaacatacacacgcaatgaaaccgaa
gcttggcggcctgggcgtggtcttgcaaaatgcttccaaagccaccttag
cctgttctattcagcggcaaccccaaagcacctgttaagactcctgaccc
ccaagtggcatgcagcccccatgcccaccgggacctggtcagcacagatc
ttgatgacttccctttctagggcagactgggagggtatccaggaatcggc
ccctgccccacgggcgttttcatgctgtacagtgacctaaagttggtaag
atgtcataatggaccagtccatgtgatttcagtatatacaactccaccag
acccctccaacccatataacaccccacccctgttcgcttcctgtatggtg
atatcatatgtaacatttactcctgtttctgctgattgtttttttaatgt
tttggtttgtttttgacatcagctgtaatcattcctgtgctgtgtttttt
attacccttggtaggtattagacttgcactttttttaaaaaaaggtttctg
catcgtggaagcatttgacccagagtggaacgcgtggcctatgcaggtgg
attccttcaggtctttcctttggttctttgagcatctttgctttcattcg
tctcccgtctttggttctccagttcaaattattgcaaagtaaaggatctt
tgagtaggttcggtctgaaaggtgtggcctttatatttgatccacacacg
ttggtcttttaaccgtgctgagcagaaaacaaaacaggttaagaagagcc
gggtggcagctgacagaggaagccgctcaaataccttcacaataaatagt
ggcaatatatatatagtttaagaaggctctccatttggcatcgtttaatt
tatatgttatgttctaagcacagctctcttctcctattttcatcctgcaa

gcaactcaaaatatttaaaataaagtttacattgtagttattttcaaatc
tttgcttgataagtattaagaaatattggacttgctgccgtaatttaaag
ctctgttgattttgtttccgtttggatttttgggggaggggagcactgtg
tttatgctggaatatgaagtctgagaccttccggtgctgggaacacacaa
gagttgttgaaagttgacaagcagactgcgcatgtctctgatgctttgta
tcattcttgagcaatcgctcggtccgtggacaataaacagtattatcaaa
gagaaaaaaaaaaaaaaaaa
>NM_018961 3
agcctcagccttgcctctccccctcttgctttatctcctcatttctgtgt
gcaggcgagcttcttggcctaagggcaggaagagatggcagcgggggaga
cgcagctctacgccaaggtctccaacaagctcaagagccgcagcagcccc
tcgctcctggagcccctcctggccatgggcttcccggtgcacaccgcgct
gaaagcgttggcagccacggggaggaagacggcggaggaggccttggcct
ggctgcatgatcattgcaatgacccttccctagacgaccccatcccccag
gagtatgcccttttcctctgtccaacggggcccctgctggaaaaacttca
agagttctggagagagagcaagcgccagtgtgcaaagaacagagctcatg
aggtcttcccacacgtgacactctgtgacttcttcacgtgtgaagaccag
aaggtggaatgcctgtacgaggcgctgaagagagctggagacaggctcct
gggctccttccccacggccgtgcctctggctctccactcctccatcagct
acctcggcttcttcgtcagtggcagccccgcagacgtcatccgggaattc
gccatgaccttcgccacggaagcatctctcttagcaggcacttccgtttc
ccgcttctggattttcagccaggtgcctggacatggccctaacctgaggc
tgagcaatttaactagagcctccttcgtgagccactacatccttcaaaaa
tactgctccgtgaagccttgcaccaaacagctgcatctgaccttggccca
caagttctacccccaccaccagaggacgctggagcagctggccagagcca
tccccctgggccacagctgccagtggaccgcagcactctactcccgagac
atgcgctttgtgcactaccagaccctgagagccctattccagtacaaacc
ccagaacgtggatgagctgacgctaagtcctggtgactacatctttgtgg
accccacgcagcaggacgaagccagcgagggctgggtgattgggatctca
cagcggacgggctgccggggcttcctgccggaaaactacacggatcgagc
cagtgagtctgacacgtgggtgaagcacaggatgtacaccttcagtctag
ccacagacctgaactccagaaaggatggtgaagccagcagcagatgcagc
ggggaatttcttccacaaacggcaaggagtcttagcagcttacaggcctt
gcaggctaccgttgcaaggaagagcgtgctggtggttcgccacggggaga
gagtggatcagatcttcgggaaggcatggctgcagcaatgctccactcct
gatgggaaatactacaggccagacctgaatttccccctgcagtctgccaag
acggagtcgtgggatcaaagactttgaaaacgatccccattatcatcgt
gtggcattttccagtccagaattgcaggggacgcgctactggacagtggt
atcagaatcagctctgtgtttgcctccccagccctccgctgtgtgcagac
ggccaaactcatcctggaagaactcaaactggagaaaaaaatcaagatac
gagtggaacctggaatctttgaatggacaaaatgggaagctggcaaaacc
accccaaccctcatgagcctggaagagctgaaagaggcaaatttcaacat
tgacactgattacaggcccgcgtttcccctgtccgccctcatgccggccg
agagctaccaggagtacatggacaggtgcacggcgagcatggtgcaaatc
gtcaacacctgtccacaggacacgggtgtcatcctaattgtgagtcacgg
ctccactctggactcctgcacgcggccactgctcgggctgccgccccggg
aatgtggggattttgcccaactcgtgagaaagatcccttccctgggcatg
tgcttctgtgaagaaaataaagaggaaggaaaatgggagttggtgaaccc
accggtgaagaccctgacccacggggcgaacgcagcatttaactggagga

actggatctcaggcaactgagagccacggtgatgttgtcataacctcaga
gtggagaggcagaaaccatgtgcagaggctgggagatgctgctgtttcca
gaggcgtcttagtctcacccaatgtgatttgtagaagcacgagacgcact
tttatatcccggaatatttccctccggctttcgcctttgtaactcccatc
tgtggacccatcgtccaccagcccagctgcggggagcacagggcaggtgg
ctgggtgaggatgccgccctgcagcatgtacaccgagtgtctgcagctgg
ggacacaactgcccgggactctaacttccaggaattaaagactcaccaca
cacgaaggatctaaccacttcattttccatggtctaatcattaaattccc
aatcgtttttcttttctgggtccatcactctttagccatatccacatg
ggctaaaacaggtgtaatagtcaataaaatggtcccagaaaaccatcgac
caggtc
>NM_000191 2
cacgcggggcccagctggactgccgcgggggattctgggccaagatggca
gcaatgaggaaggcgcttccgcggcgactggtgggcttggcgtccctccg
ggctgtcagcacctcatctatgggcactttaccaaagcgggtgaaaattg
tggaagttggtccccgagatggactacaaaatgaaaagaatatcgtatct
actccagtgaaaatcaagctgatagacatgctttctgaagcaggactctc
tgttatagaaaccaccagctttgtgtctcctaagtgggttccccagatgg
gtgaccacactgaagtcttgaagggcattcagaagtttcctggcatcaac
tacccagtcctgaccccaaatttgaaaggcttcgaggcagcggttgctgc
tggagccaaggaagtagtcatctttggagctgcctcagagctcttcacca
agaagaacatcaattgttccatagaggagagttttcagaggtttgacgca
atcctgaaggcagcgcagtcagccaatatttctgtgcgggggtacgtctc
ctgtgctcttggctgcccttatgaagggaagatctccccagctaaagtag
ctgaggtcaccaagaagttctactcaatgggctgctacgagatctccctg
ggggacaccattggtgtgggcaccccagggatcatgaaagacatgctatc
tgctgtcatgcaggaagtgcctctggctgccctggctgtccactgccatg
acacctatggtcaagccctggccaacaccttgatggccctgcagatggga
gtgagtgtcgtggactcttctgtggcaggacttggaggctgtccctacgc
acagggggcatcaggaaacttggccacagaagacctggtctacatgctag
agggcttgggcattcacacgggtgtgaatctccagaagcttctggaagct
ggaaactttatctgtcaagccctgaacagaaaaactagctccaaagtggc
tcaggctacctgtaaactctgagccccttgcccacctgaagccctgggga
tgatgtggaaataggggcacacacagatgattcatggatggggacatgga
aatgagaataggttaaatggtgcaggtacctcatagccagctctacacag
aggtctctcctggcagaaagcaggcgaagggcaggaggagctgcttggca
gaaggacctcctgcccagacctgaggagtgagaggctttgagggctgaag
tctccctttgttacggaccctggcccaggagttgaatgcctgaggacgtg
tgggaaccccgttccctacttagcatgatccttgagtctcctctctggat
ggaatccgcgagctggccacctggccaccctctacacggctccaccctgc
catggccgtggggcccttgctctctgacttctcaggacacaggtcatgga
ggttcttcccaagctggcagaggccatttgtggaaagtggagagctacgt
ggtggccgtctgccaactccagcatctctggaaaatctccacgctgaatg
tgatttttgaaaacagcttatgtaattaaaggttgaatggcacatcataa
ttccagcaaaaaaaaaa
>NM_002488 4
gcttcctgtttttccctccgaccaaccccgagcgcaaagaaattgacctc
gcagcggtcctacaatacttttatatcattggccaagctttaccccgccc
ctgcctcatgcagcctatgggctaggctttagggtccgcggttggtcaga

ccggagcacttggcctgaagacctggaattggcgacttcgatattaacaa
ggatggcggcggccgcagcaagtcgaggagtcggggcaaagctgggcctg
cgtgagattcgcatccacttatgtcagcgctcgcccggcagccagggcgt
cagggacttcattgagaaacgctacgtggagctgaagaaggcgaatcccg
acctacccatcctaatccgcgaatgctccgatgtgcagcccaagctctgg
gcccgctacgcatttggccaagagacgaatgtccctttgaacaacttcag
tgctgatcaggtaaccagagccctggagaacgttctaagtggtaaagcct
gaagcctccactgaggattaagagcaacagccccagagcctgggctctgc
tggacttagtataatgtgaaaaaaatgtgttctcctattcctcataaagc
ttgtgctgtaaaatactttctcagggtgttcttgtcctcatctaccctct
accccttactgtgcaaccactgaggcaaagtagcttaatataaaaataaa
actttattctgtctcatcaaaagcta
>NM_000435 2
gcggcgcggaggctggcccgggacgcgcccggagcccagggaaggaggga
ggaggggagggtcgcggccggccgccatggggccgggggcccgtggccgc
cgccgccgccgtcgcccgatgtcgccgccaccgccaccgccacccgtgcg
ggcgctgcccctgctgctgctgctagcggggccgggggctgcagcccccc
cttgcctggacggaagcccgtgtgcaaatggaggtcgttgcacccagctg
ccctcccgggaggctgcctgcctgtgcccgcctggctgggtgggtgagcg
gtgtcagctggaggacccctgtcactcaggcccctgtgctggccgtggtg
tctgccagagttcagtggtggctggcaccgcccgattctcatgccggtgc
ccccgtggcttccgaggccctgactgctccctgccagatccctgcctcag
cagcccttgtgcccacggtgcccgctgctcagtggggcccgatggacgct
tcctctgctcctgcccacctggctaccagggccgcagctgccgaagcgac
gtggatgagtgccgggtgggtgagccctgccgccatggtggcacctgcct
caacacacctggctccttccgctgccagtgtccagctggctacacagggc
cactatgtgagaaccccgcggtgccctgtgcaccctcaccatgccgtaac
gggggcacctgcaggcagagtggcgacctcacttacgactgtgcctgtct
tcctgggtttgagggtcagaattgtgaagtgaacgtggacgactgtccag
gacaccgatgtctcaatgggggacatgcgtggatggcgtcaacacctat
aactgccagtgccctcctgagtggacaggccagttctgcacggaggacgt
ggatgagtgtcagctgcagcccaacgcctgccacaatgggggtacctgct
tcaacacgctgggtggccacagctgcgtgtgtgtcaatggctggacaggc
gagagctgcagtcagaatatcgatgactgtgccacagccgtgtgcttcca
tggggccacctgccatgaccgcgtggcttctttctactgtgcctgcccca
tgggcaagactggcctcctgtgtcacctggatgacgcctgtgtcagcaac
ccctgccacgaggatgctatctgtgacacaaatccggtgaacggccgggc
catttgcacctgtcctcccggcttcacgggtggggcatgtgaccaggatg
tggacgagtgctctatcggcgccaaccccctgcgagcacttgggcaggtgc
gtgaacacgcagggctccttcctgtgccagtgcggtcgtggctacactgg
acctcgctgtgagaccgatgtcaacgagtgtctgtcggggccctgccgaa
accaggccacgtgcctcgaccgcataggccagttcacctgtatctgtatg
gcaggcttcacaggaacctattgcgaggtggacattgacgagtgtcagag
tagcccctgtgtcaacggtggggtctgcaaggaccgagtcaatggcttca
gctgcacctgcccctcgggcttcagcggctccacgtgtcagctggacgtg
gacgaatgcgccagcacgccctgcaggaatggcgccaaatgcgtggacca
gcccgatggctacgagtgccgctgtgccgagggctttgagggcacgctgt
gtgatcgcaacgtggacgactgctcccctgacccatgccaccatggtcgc
tgcgtggatggcatcgccagcttctcatgtgcctgtgctcctggctacac

gggcacacgctgcgagagccaggtggacgaatgccgcagccagccctgcc
gccatggcggcaaatgcctagacctggtggacaagtacctctgccgctgc
ccttctgggaccacaggtgtgaactgcgaagtgaacattgacgactgtgc
cagcaacccctgcacctttggagtctgccgtgatggcatcaaccgctacg
actgtgtctgccaacctggcttcacagggcccctttgtaacgtggagatc
aatgagtgtgcttccagcccatgcggcgagggaggttcctgtgtggatgg
ggaaaatggcttccgctgcctctgcccgcctggctccttgcccccactct
gcctcccccgagccatccctgtgcccatgagccctgcagtcacggcatc
tgctatgatgcacctggcgggttccgctgtgtgtgtgagcctggctggag
tggcccccgctgcagccagagcctggcccgagacgcctgtgagtcccagc
cgtgcagggccggtgggacatgcagcagcgatggaatgggtttccactgc
acctgcccgcctggtgtccagggacgtcagtgtgaactcctctccccctg
caccccgaacccctgtgagcatgggggccgctgcgagtctgcccctggcc
agctgcctgtctgctcctgcccccagggctggcaaggcccacgatgccag
caggatgtggacgagtgtgctggccccgcaccctgtggccctcatggtat
ctgcaccaacctggcagggagtttcagctgcacctgccatggagggtaca
ctggcccttcctgcgatcaggacatcaatgactgtgaccccaacccatgc
ctgaacggtggctcgtgccaagacggcgtgggctccttttcctgctcctg
cctccctggtttcgccggcccacgatgcgcccgcgatgtggatgagtgcc
tgagcaacccctgcggcccgggcacctgtaccgaccacgtggcctccttc
acctgcacctgcccgccaggctacggaggcttccactgcgaacaggacct
gcccgactgcagccccagctcctgcttcaatggcgggacctgtgtggacg
gcgtgaactcgttcagctgcctgtgccgtcccggctacacaggagcccac
tgccaacatgaggcagacccctgcctctcgcggccctgcctacacggggg
cgtctgcagcgccgcccaccctggcttccgctgcacctgcctcgagagct
tcacgggcccgcagtgccagacgctggtggattggtgcagccgccagcct
tgtcaaaacgggggtcgctgcgtccagactggggcctattgcctttgtcc
ccctggatggagcggacgcctctgtgacatccgaagcttgccctgcaggg
aggccgcagcccagatcggggtgcggctggagcagctgtgtcaggcgggt
gggcagtgtgtggatgaagacagctcccactactgcgtgtgcccagaggg
ccgtactggtagccactgtgagcaggaggtggacccctgcttggcccagc
cctgccagcatgggggggacctgccgtggctatatggggggctacatgtgt
gagtgtcttcctggctacaatggtgataactgtgaggacgacgtggacga
gtgtgcctcccagccctgccagcacgggggttcatgcattgacctcgtgg
cccgctatctctgctcctgtcccccaggaacgctgggggtgctctgcgag
attaatgaggatgactgcggcccaggcccaccgctggactcagggccccg
gtgcctacacaatggcacctgcgtggacctggtgggtggtttccgctgca
cctgtcccccaggatacactggtttgcgctgcgaggcagacatcaatgag
tgtcgctcaggtgcctgccacgcggcacacacccgggactgcctgcagga
cccaggcggaggtttccgttgcctttgtcatgctggcttctcaggtcctc
gctgtcagactgtcctgtctccctgcgagtcccagccatgccagcatgga
ggccagtgccgtcctagcccgggtcctgggggtgggctgaccttcacctg
tcactgtgcccagccgttctggggtccgcgttgcgagcgggtggcgcgct
cctgccgggagctgcagtgcccggtgggcgtcccatgccagcagacgccc
cgcgggccgcgctgcgcctgcccccccagggttgtcgggaccctcctgccg
cagcttcccggggtcgccgccgggggccagcaacgccagctgcgcggccg
ccccctgtctccacgggggctcctgccgccccgcgccgctcgcgcccttc
ttccgctgcgcttgcgcgcagggctggaccgggccgcgctgcgaggcgcc
cgccgcggcacccgaggtctcggaggagccgcggtgcccgcgcgccgcct

gccaggccaagcgcggggaccagcgctgcgaccgcgagtgcaacagccca
ggctgcggctgggacggcggcgactgctcgctgagcgtgggcgacccctg
gcggcaatgcgaggcgctgcagtgctggcgcctcttcaacaacagccgct
gcgaccccgcctgcagctcgcccgcctgcctctacgacaacttcgactgc
cacgccggtggccgcgagcgcacttgcaacccggtgtacgagaagtactg
cgccgaccactttgccgacggccgctgcgaccagggctgcaacacggagg
agtgcggctgggatgggctggattgtgccagcgaggtgccggccctgctg
gcccgcggcgtgctggtgctcacagtgctgctgccgccagaggagctact
gcgttccagcgccgactttctgcagcggctcagcgccatcctgcgcacct
cgctgcgcttccgcctggacgcgcacggccaggccatggtcttcccttac
caccggcctagtcctggctccgaaccccgggcccgtcgggagctggcccc
cgaggtgatcggctcggtagtaatgctggagattgacaaccggctctgcc
tgcagtcgcctgagaatgatcactgcttccccgatgcccagagcgccgct
gactacctgggagcgttgtcagcggtggagcgcctggacttcccgtaccc
actgcgggacgtgcgggggggagccgctggagcctccagaacccagcgtcc
cgctgctgccactgctagtggcgggcgctgtcttgctgctggtcattctc
gtcctgggtgtcatggtggcccggcgcaagcgcgagcacagcaccctctg
gttccctgagggcttctcactgcacaaggacgtggcctctggtcacaagg
gccggcgggaacccgtgggccaggacgcgctgggcatgaagaacatggcc
aagggtgagagcctgatgggggaggtggccacagactggatggacacaga
gtgcccagaggccaagcggctaaaggtagaggagccaggcatgggggctg
aggaggctgtggattgccgtcagtggactcaacaccatctggttgctgct
gacatccgcgtggcaccagccatggcactgacaccaccacagggcgacgc
agatgctgatggcatggatgtcaatgtgcgtggcccagatggcttcaccc
cgctaatgctggcttccttctgtgggggggctctggagccaatgccaact
gaagaggatgaggcagatgacacatcagctagcatcatctccgacctgat
ctgccagggggctcagcttggggcacggactgaccgtactggcgagactg
ctttgcacctggctgcccgttatgcccgtgctgatgcagccaagcggctg
ctggatgctggggcagacaccaatgcccaggaccactcaggccgcactcc
cctgcacacagctgtcacagccgatgcccagggtgtcttccagattctca
tccgaaaccgctctacagacttggatgcccgcatggcagatggctcaacg
gcactgatcctggcggcccgcctggcagtagagggcatggtggaagagct
catcgccagccatgctgatgtcaatgctgtggatgagcttgggaaatcag
ccttacactgggctgcggctgtgaacaacgtggaagccactttggccctg
ctcaaaaatggagccaataaggacatgcaggatagcaaggaggagacccc
cctattcctggccgcccgcgagggcagctatgaggctgccaagctgctgt
tggaccactttgccaaccgtgagatcaccgaccacctggacaggctgccg
cgggacgtagcccaggagagactgcaccaggacatcgtgcgcttgctgga
tcaacccagtgggccccgcagcccccccggtccccacggcctggggcctc
tgctctgtcctccaggggccttcctccctggcctcaaagcggcacagtcg
gggtccaagaagagcaggaggcccccgggaaggcggggctggggccgca
ggggccccgggggcggggcaagaagctgacgctggcctgcccgggccccc
tggctgacagctcggtcacgctgtcgcccgtggactcgctggactccccg
cggcctttcggtgggcccctgcttcccctggtggcttccccttgaggg
gccctatgcagctgccactgccactgcagtgtctctggcacagcttggtg
gcccaggccgggcgggtctagggcgccagccccctggaggatgtgtactc
agcctgggcctgctgaaccctgtggctgtgcccctcgattgggcccggct
gcccccacctgcccctccaggcccctcgttcctgctgccactggcgccgg
gaccccagctgctcaacccagggacccccgtctccccgcaggagcggccc

ccgccttacctggcagtcccaggacatggcgaggagtacccggcggctgg
ggcacacagcagccccccaaaggcccgcttcctgcgggttcccagtgagc
acccttacctgaccccatcccccgaatcccctgagcactgggccagcccc
tcacctccctccctctcagactggtccgaatccacgcctagcccagccac
tgccactggggccatggccaccaccactggggcactgcctgcccagccac
ttcccttgtctgttcccagctcccttgctcaggcccagacccagctgggg
ccccagccggaagttaccccaagaggcaagtgttggcctgagacgctcg
tcagttcttagatcttgggggcctaaagagaccccgtcctgcctccttt
ctttctctgtctcttccttccttttagtctttttcatcctcttctctttc
caccaaccctcctgcatccttgccttgcagcgtgaccgagataggtcatc
agcccagggcttcagtcttcctttatttataatgggtgggggctaccacc
caccctctcagtcttgtgaagagtctgggacctccttcttccccacttct
ctcttccctcattcctttctctctccttctggcctctcatttccttacac
tctgacatgaatgaattattattatttttatttttcttttttttttaca
ttttgtatagaaacaaattcatttaaacaaacttattattattattttt
acaaaatatatatatggagatgctccctccccctgtgaaccccccagtgc
ccccgtggggctgagtctgtgggcccattcggccaagctggattctgtgt
acctagtacacaggcatgactgggatcccgtgtaccgagtacacgaccca
ggtatgtaccaagtaggcacccttgggcgcacccactggggccaggggtc
gggggagtgttgggagcctcctccccaccccacctccctcacttcactgc
attccagatgggacatgttccatagccttgctggggaagggcccactgcc
aactccctctgccccagccccacccttggccatctccctttgggaactag
ggggctgctggtgggaaatgggagccagggcagatgtatgcattcctttg
tgtccctgtaaatgtgggactacaagaagaggagctgcctgagtggtact
ttctcttcctggtaatcctctggcccagcctcatggcagaatagaggtat
ttttaggctatttttgtaatatggcttctggtcaaaatccctgtgtagct
gaattcccaagccctgcattgtacagccccccactcccctcaccacctaa
taaaggaatagttaacactcaaaaaaaaaaaaaaaaaaa
>NM_001163 3
gggtccgagcagcggctgcggagccggagctgggcgagggcggctgcggt
cccggcggcggcggcggcggcggcagctgcggcgggagaagatggtggcg
ctggaggcagcagcggctggagcggagctttattcccaggcttggcacca
gcgctgtcactagcgccgcctgctcccgcgggcccgcggacccagctgtc
aggcaagcccagtggagcaaaatgagagcgcagtgagccgggcccagctt
ctctcctagccgttccgactcccaccatgaaccacttggaggggtctgcg
gaggtggaggtgaccgacgaggcggcaggtggggaggtgaacgagtcggt
ggaggccgacctggagcaccccgaggtggaagaggaacagcagcagccgc
cgcagcagcagcactatgtgggccgccaccagcgcgggcgagccctcgag
gacctccgcgcccagctcggccaggaggaagaggagcgcggggaatgcct
ggcgcgctcagccagcacggagagcggcttccacaaccacacggacaccg
ccgagggcgacgtgatcgccgcggcccgcgacggctacgatgcggagcgc
gcgcaggaccccgaggacgagagcgcctatgctgtgcagtaccggcccga
ggccgaggagtacacggagcaggcagaggccgagcacgccgaggccacgc
accgccgcgcgctgcccaaccacctgcacttccactcgctggagcacgag
gaagccatgaatgcggcctactcaggctacgtctacacgcaccggctctt
ccaccgcggtgaggacgagccctactccgagccctatgccgactacggcg
gcctccaggagcacgtgtacgaggagataggggacgcgcccgagctggac
gcacgcgacggcctgcggctctacgagcaggagcgcgacgaggcggccgc
gtaccgccaggaggccctgggcgcgcggctgcaccattacgacgagcgct

ccgacggcgagtccgacagccccgagaaggaggccgagttcgcgccctac
ccgcgcatggacagctacgagcaggaggaggacatcgaccagatagtggc
cgaggtgaagcagagcatgagctcgcagagcctcgacaaggcagccgagg
acatgcctgaggccgagcaggacctggagcgtccccctaccccggccggg
ggtcgccccgacagccccgggctgcaggcgccggcggggcagcagcgggc
ggtgggccccgcgggcggcggcgaggcggggcagcggtacagcaaggaga
agcgcgatgccatctcgctggccatcaaggacatcaaggaggccatcgag
gaggtgaaaaccaggaccatccgttcgccttacaccccccgacgagcccaa
agagcccatctgggtcatgcgccaggacattagccccaccagggactgtg
acgaccagaggccgatggacggagattctccgtctcctggcagctcctcc
cccttgggtgcagagtcatcaagcacatctcttcaccccagtgaccctgt
ggaagcgtccactaataaagagtcaagaaaaagcttggcttcattcccaa
cctacgttgaagttccgggaccctgcgaccccgaagacttgatcgatgga
atcatttttgccgccaattaccttggctccactcagctgctctcagacaa
aactccttccaaaaacgtgcgcatgatgcaggcccaggaagccgtaagca
ggatcaagatggcccagaaattagccaaaagcaggaagaaggctcctgaa
ggcgaatctcagccaatgactgaagtggatctcttcatttctacccagag
aatcaaagtgctgaacgccgacacacaggagacaatgatggaccaccctc
tgaggaccatttcctacattgcggacattgggaacatcgttgtgctgatg
gcccgccggcggatgcctcgctccaactcccaggagaacgtggaagcgtc
ccacccatcccaggatgggaaaaggcagtacaagatgatctgccacgtct
tcgagtctgaggatgctcagctgattgcacagtccatcggacaggcattt
agcgtggcataccaggaattcctcagggccaatgggattaaccccgaaga
tctcagccagaaggagtatagtgacctgctcaatacccaggacatgtaca
acgatgacctgatccacttctccaagtcggaaaactgtaaagatgttttc
atagagaagcagaaaggagaaatcctaggtgtggtgattgtggagtctgg
ctggggatccatcctccccaccgtgatcattgccaacatgatgcatggtg
gccctgcggagaaatctgggaagctgaatatcggtgaccagatcatgtcc
attaatggcaccagcctggtgggcctgcctctgtccacctgccagagcat
tattaagggcttaaagaatcagtcccgagtcaagctgaatatcgtgagat
gtcctccggtgaccaccgtgttaatcagaagaccagaccttcgctaccag
ctcggtttcagcgtccagaatggaattatctgcagcctcatgcgaggggg
aatagctgagagaggaggcgtccgtgtggggcaccggatcattgaaatca
atggacagagcgtcgtggccacccccacgagaagatcgtccacattctc
tccaatgctgttggggagattcatatgaagacaatgccagccgcgatgta
caggctgctgacggcccaggagcagcctgtttacatctgaccgcggccac
acgcggtggcatgcatggaggactctcctcttcgtggttgtgtttctcgt
gctgcatccctgtgtccactgagactttcccctctcgcgcccagcatttg
gttttacacaggaagagaagaatccacaaggacctctttactctctccga
tttgcttttttttttttttttttttcaataccagggaagtttcgtatgca
ctcccttgaggatggagagcagccagcacccacctggtactgacccagga
ccatcctggagggctttctgggtgtgtccagggggtgggctgtcactgct
tgagggagaatcctcccttcccaggaggtgcagacttcttaaaaggagct
cgcggggcagcaaagcagctgattcagcagtgcctaaaacccagttgctg
atccctgctctctgagtttatctgtgggaatgtggtagtacccagggcca
gcccacgtcattaaggttatgcactgcctgccatgtagttggggcaccat
acattatttcttcccagaatctctgagccaaccttaaacctcttctattg
ctagttctaatttcaacgtatgtgtgttttctaatacgagccttctcacc
ccaggataaaggggaaataactgccttgggcaaggacaccatagtgtca

cagagagcttggcaattcatggggcatctgaagctttaccaggttgtcct
caagttatcaaccattagataaacacaggcaggtactgtctgcttctctc
tctctcacacacacacacacacacacagtcccatttgcatatcacccttc
cctgctcccaccaccagtgagacaagctgaagattaggcacacagatccc
ctggaaacccacctctctggaaggccccttccctggcaggtggccaggca
ggcgggggctgctcagcctcatcctgagagacctgccctctctgcgctga
ggcccagcccctccagcaccactttggcttaaagcatcctcctcctggac
tcccctccctgccgaacacacatcccaggagtctgggaaggagggactgt
tcccaagaaggcaagctgcaggtgcaattgcgtcttgttttctggcccaa
ggcaagaccaactttcttctctgctcagggccactaggcggatgtgatat
tggaaaggccagaatagtacataaaccacaaaattaaggagacaatagca
acctcaagttatgcctcagaatccccagatgtggtccataaacaccctct
tgctgccttgtgggcctgagagcatttttggctggtgtggaattgttccg
tgttttcatcaggtgcctcatatttatgcttccgtctcagttcagtgtag
atgttttagtaatattataattctgagccagttttcacttggtccaaaga
gcattttgtgttcatgtgaggtgtgatttggctttgcttttgttttgttt
cattcatgacctcagtagagacaggggaagagaaactattatattttgta
attaatatagtccctgttctctgcatccgagatatttatgttgtgtctat
ggaaataggaactcagtgagcaaatggtgggtagcatgtatttatagaga
gaatggagggattctaaggagggagcaaagtgactataacaagattaggg
acaagagacttcaaccagagagttctgtgatcaattccccgagatctctg
catcttctgagttttctgcttagaatcagggaagctcagtgcttgacagg
aacgagcagtggcactctcagtttcactgtgttacaaaggagccctgagg
aagggaggccgctggctgcccaggtgccctgggggggaaagggacttctgc
tgctgcagagagtggcacccggtggggccaggggctctgggatggtccgt
aaagctccccaggacttctttgaaggatgctttacagaatgtttctggga
gaccagcctcaaggtcaacccatcacaaacgtgaggaccaggaaggaacc
atggccagggttctccatttgctgtagagtatctggtgatacagtgagac
actaaagagtcatcagccagttcatctttcctctgccaataagagtggat
agaacaggaatgaagtgttactggtggtatttcaggcatcaggagccttg
gaaaatcatcgtaggggcccatgcctgtctgtccctaggcacctgggcag
tgccacagaggcccgcccacgactggccaggggacagaactccagtagtg
catccctgtacaacctgcacataacggttatgtatgttcacctaaaggag
agaatatactttctattcctattgcacaaaaaccttgtaaactttgagag
gtgctgaaaaagccaattctgcaagctctcctagaggactgacattgtaa
cattagagtgagaagatttctctgccacattcttttgtgttaatccaggt
gtgtcttcacacccagtgagtggaagttgctggagctcggtacaggtcca
cccagctgaagtgggttctttttcccactaccactggggcaagggagtga
gcctaggtatggtcacctcagcagccacagtcttaaaagcaagtcgctag
attttttgaaagtcccaagagaagtggtggtcttcccttctgtcatccca
gtatatatagttatacatgtaaatatctaaataccccatgtaatgggaag
gctatgtacagggcagccttcccaaacgctgtgagggcacagagctgctg
tggctgctccaccagggtgtgctggtggctcagctttggggttggactcc
cacgttcacctccccctcctggtccaggcagaggcaccagaacccatcct
gccttttctccagtgtgagcatttcagggagccaccatcagccttgggtt
catgagaggaagaaacccccaaagaccaatgcctggagcccacttctcca
gcgacacccttccctcgccagagcaggctcctaggagtgcatggcaggggt
gccgggtccctgtgcacgggtttgctttctgaacattgcccatcaacacc
gccttcagggagtcccagagtaacctactacctcccccatcgctcctctg

cccccctcaccctgtgggcaccaacacctcccgcacacagatgaaaacca
tctacagagcccttcccgggtccctcgacccagtggtgtagccgttacca
atgtttacaaccaggggccgcccctccttagatgtgtttactcgcattca
caggccacctgttggtttgagtttcatgtgtctccttccctggcctaggt
tgacttgagcacacaactcgatctttcttcccctccatcaaggcagtgtg
tgaggactgtgtgggaatgacatttcttgtacatacttagcacagcacct
agagtatgaacttgtactattgttctgtaaagagggtgaaatagagctta
ttgtaaagtgtcgggagaagtatagtattgtatttgtaacaatatcgttc
tttgtatacacaaaactcaatgatctatatataaatataaataaatatat
aaataaaatatatctacacgtgagcctggaatgtggacaccgcacaccca
ctgaatgtacttccttgacagtctggtcactggtccctccctccctccct
cctccgtcccgtggccacagtcatccagtttggggttgtgatgtatttac
tgtgctatctatcttcctttatttaactgaacgctaatgtctgtataaga
gttgctgcaacaataaacaagaacttcacctcttaaaaaaaaaaaaaaaa
aaa
>NM_017533 2
atccttcctcaaaattcttgaagtagttgtctgctttgagcctgccacct
tcttcatctgataatacaagaggtatacctagtccagtactgccatcaat
aacctgcagccatgagttctgactctgagatggccatttttggggaggct
gctcctttcctccgaaagtctgaaaaggagcgaattgaagctcagaacaa
gccttttgatgccaagacatcagtctttgtggtggaccctaaggagtcct
acgtgaaagcaatagtgcagagcagggaagggggggaaggtgacagccaag
accgaagctggagctactgtaactgtgaaagaagaccaagtcttctccat
gaaccctcccaaatatgacaagatcgaggacatggccatgatgactcacc
tgcatgagcctgctgtgctgtataacctcaaagagcgttacgcagcctgg
atgatctacacctactcgggcctcttctgtgtcaccgtcaacccctacaa
gtggctgccggtgtacaaccctgaggtggtgacagcctaccgaggcaaaa
agcgccaggaggccccaccccatatcttctccatctctgacaatgcctat
cagttcatgctaactgatcgtgaaaaccagtcaatcttgattactggaga
atctggtgcagggaagactgtgaacacgaagcgtgtcatccagtactttg
caacaattgcagttactggagagaagaaaaagaggaacctgcctctggc
aaaatgcaggggacccttgaagatcaaatcatcagtgctaacccccctact
ggaagccttcggcaatgccaagaccgtgaggaatgacaactcctctcgct
ttggtaaattcatcaggatccattttggtgccacaggcaaactggcttct
gcagatattgaaacatatctgctagagaagtcccgagttacttttcagct
aaaggctgaaagaagctaccacatattttatcaaatcctgtccaataaga
aaccagagctcattgaaatgcttctgatcaccaccaacccatatgacttc
gcatttgtcagccaaggggaaattactgtgcccagcattgatgaccagga
agagctgatggccacagatagtgctgtggacatcctgggtttcactgctg
atgaaaaggtggccatttacaagctcactggagccgtgatgcattatggg
aacatgaaattcaagcaaaagcaaagggaagagcaggcagagccagatgg
cacggaagttgctgacaaagctgcttatctgacaagtctgaactctgctg
acctgctcaaatctctctgctatcccagagtcaaggtcggcaatgagttc
gtaaccaaaggccagactgtgcagcaggtgtacaatgcagtgggtgctct
ggccaaagccatctacgagaagatgttcctgtggatggtcacccgcatca
accagcagctggacaccaagcagcccaggcagtacttcatcggggtcttg
gacattgctggctttgagatctttgatttcaacagcctggagcagctgtg
catcaacttcaccaacgagaaactgcaacagtttttcaaccaccacatgt
tcgtgctggagcaggaagagtacaagaaggaaggcatcgagtgggagttc

attgacttcgggatggacctggctgcctgcatcgagctcatcgagaagcc
tatgggcatcttctccatcctagaagaggagtgcatgttccccaaggcaa
cagacacctccttcaagaacaagctgtatgaacaacatcttggaaaatcc
aacaacttccagaagcccaagcctgccaaaggcaagcctgaggctcactt
ctcactggtgcactatgccggcaccgtggactacaacatcgccggctggc
tggacaaaaacaaggacccccctgaatgagactgtggtggggctgtaccag
aagtctgcaatgaagactctggctttcctcttctctggggcacaaactgc
tgaagcagagggtggtggtggaaagaaaggtggcaaaaagaagggttctt
ctttccagacagtgtcagctcttttcagggagaatttgaataagctgatg
accaacttgaggagcactcacccccactttgtgcggtgcatcatccccaa
tgaaactaaaactcctggtgccatggagcatgagcttgtcctgcatcagc
tgaggtgtaacggtgtgctggaaggcatccgcatctgcaggaaaggcttc
ccaagcagaatcctttatgcagacttcaaacagagatacaaggttctaaa
tgcgagtgctatcccagagggtcagttcattgacagcaagaaggcttctg
agaaacttctagggtctattgaaattgaccacacccagtacaaattcggt
cataccaaggttttcttcaaagctggcctgctgggaactctagaggaaat
gcgagatgaaaagctagctcaactcatcacgcgcactcaagccatatgca
gagggttcctgatgagagtggagttcagaaagatgatggagaggagagag
tccatcttctgcattcagtacaacatccgtgctttcatgaatgtgaagca
ctggccctggatgaagctgtatttcaagatcaagcccctcctcaagagtg
cagagacagagaaggagatggccaacatgaaggaagaatttgagaaaacc
aaagaagagctggctaagacagaggcaaaaaggaaagaactagaagaaaa
gatggtgacgctaatgcaagagaaaaatgacttacaactccaagttcaag
ctgaagcagatgccttggctgatgcagaggaaagatgtgatcagttgatt
aaaaccaaaatccaacttgaggccaaaatcaaagaggtaactgaaagagc
tgaggatgaggaagagatcaatgctgagctgacagccaagaagaggaaac
tggaggatgaatgttcagagctcaagaaagacattgatgaccttgagctg
acactggccaaggttgagaaggagaaacatgccacagagaacaaggtgaa
aaacctcacagaagagatggcaggtctggatgaaaccattgctaagctga
ccaaggagaagaaggctctccaggaggcccaccagcagaccctggatgac
ctgcagatggaggaggacaaagtcaacaccctgaccaaagctaaaaccaa
gctagaacagcaagtggacgatcttgaaggatctctggaacaagaaaaga
aactttgcatggacttagaaagagccaagagaaaactggagggtgaccta
aaattggcccaagaatccacaatggatacagaaaatgacaaacagcaact
taatgagaaactcaaaaagaaagagtttgaaatgagcaatctgcaaggca
agattgaagatgaacaagcccttgcaatacagctacaaaagaagatcaaa
gaattacaggcccgcattgaggagctggaggaggaaatcgaggcagagcg
ggcctcccgggccaaagcagagaagcagcgctctgacctctcccgggagc
tggaggagatcagtgagaggctggaagaagccggtggggccacttcagcc
cagattgagatgaacaagaagcgggaggctgagttccagaaaatgcgcag
ggacctggaagagtccaccctgcagcacgaagccacggcagctgctcttc
ggaagaagcacgcagatagtgtggctgagcttggggagcagattgacagc
cttcagcgggtcaagcagaagctggagaaggaaaagagtgagctgaagat
ggagatcaatgaccttgctagtaacatggagactgtctccaaagccaagg
caaactttgagaaaatgtgccgcaccctagaggaccagcttagtgaaata
aaaacaaaggaagaagagcaacaacgcttaataaatgagttgtcagccca
gaaggcacgtttacacacagaatcaggtgagttttcacgacagctagatg
aaaaagatgctatggtttctcagctatcccgaggcaaacaagcatttaca
caacagattgaagaattaaagaggcagctagaagaggagactaaggccaa

gagcactctggcccatgccctgcagtcagcccgccatgactgtgacctgc
tgcgggaacagtatgaggaggagcaggaagccaaggctgagctgcagagg
ggaatgtccaaggccaacagtgaggttgcccagtggaggaccaagtacga
gacggacgccatccagcgcacagaggagctggaggaggccaagaagaagc
tagcccagcgtctgcaggatgcagaagaacatgtagaagctgtgaattcc
aaatgtgcttctcttgaaaagacaaagcagaggctacagaatgaagtaga
ggacctcatgattgatgtggaacgatctaatgctgcctgcatagctctcg
ataagaagcaaagaaactttgacaaggttctggcagaatggaaacagaag
tatgaggaaactcaggctgaacttgaggcctcccagaaggagtcgcgttc
tctcagcactgagctgttcaaggtgaagaatgcctacgaggaatccctgg
atcatcttgaaactctaaagcgagagaataagaacttacaacaggagatt
tctgacctgacagagcaaattgcagagggtggaaagcatatccatgaact
ggagaaagtaaagaaacaacttgatcatgagaagagtgaactacagactt
ccctagaggaagcagaggcatctcttgagcatgaagaaggcaaaattctt
cgcattcaacttgagctaaatcaggtgaaatctgagattgaccgaaaaat
tgctgaaaaagatgaagaactcgatcagctaaagaggaaccatctcagag
ttgtggagtcaatgcagagtacactggatgctgagatcaggagcagaaat
gatgctctgaggatcaagaagaagatggagggagatcttaatgaaatgga
aatccagctgaaccatgccaaccgccaggctgctgaggcactaaggaatc
ttagaaacacacaaggaatactgaaggacactcagctacatttggatgat
gccatcagaggccaagatgaccttaaggaacaactggcaatggttgagcg
cagagctaacctgatgcaggctgaagttgaagagctcagggcatccctgg
aacggactgagagaggcaggaaaatggcagagcaagagcttctggatgcc
agtgaacgtgtgcaacttctgcacactcagaacaccagcctgatcaacac
caagaagaagctggaaacagacatttcccaaatccagggagagatggagg
acatcgtccaggaagcccgcaatgcagaggagaaggccaagaaggccatc
actgatgctgccatgatggctgaggagctgaagaaggaacaggacaccag
cgcccacctggagcggatgaagaagaacatggagcagaccgtgaaggatc
tgcagctccgtctggatgaggctgagcagctggcgctgaagggtgggaag
aagcagatccagaaactggaggccagggtgagagagcttgaaagtgaggt
ggaaagtgaacagaagcacaatgttgaggctgtcaagggtcttcgcaaac
atgagagaagagtgaaggaactcacttaccagactgaggaggaccgcaag
aatattctcaggctgcaggacttggtggacaaattgcaaaccaaagtcaa
agcttacaagagacaagctgaagaggctgaggaacaatccaatgtcaacc
ttgccaagttccgcaagctccagcacgagctggaggaggccaaggaacgg
gctgacattgctgagtcccaagtcaacaagctgagagtgaagagtcggga
ggttcacacaaaagtcataagtgaagagtaattcattctaatgaaagaaa
atgtgaccaaagaaatgcacgaaatgtgaagttctttgtcactgtcctgt
atatcaaggaaataaa
>NM_005001 3
acgtcaccggctgcgcccttcagtatcgcggacggaagatggcgtccgcc
acccgtctcatccagcggctgcggaactgggcgtccgggcatgacctgca
ggggaagctgcagctacgctaccaggagatctccaagcgaactcagcctc
ctcccaagctccctgtgggtcctagccacaagctctccaacaattactat
tgcactcgcgatggccgccgggaatctgtgccccccttccatcatcatgtc
gtcgcagaaggcgctggtgtcaggcaagccagcagagagctctgctgtag
ctgccactgagaagaaggcggtgactccagctcctcccataaagaggtgg
gagctgtcctcggaccagccttacctgtgacactgcaccctcacggccac
ccgactactttgcctccttggatttcctccagggagaatgtgacctaatt

tatgacaaatacgtagagctcaggtatcacttctagttttactttaaaaa
ataaaaaaatagagacagagtctcaccatgtttcccaggctgatcttgaa
ctcctggcctcaagcgatcctcctgccttgacctc
>NM_004750 4
ggggccgggcggggccagcggcgcattagcgccttgtcaattcggctgct
cagacttgctccggcctccgcgtccgcgcccagcgacgtgcgggcggcct
ggcccgcgccctcccgcgcccggcctgcgtcccgcgccctgcgccaccgc
cgccgagccgcagcccgccgcgcgcccccggcagcgccggccccatgccc
gccggccgccggggccccgccgcccaatccgcgcggcggccgccgccgtt
gctgcccctgctgctgctgctctgcgtcctcggggcgccgcgagccggat
caggagcccacacagctgtgatcagtccccaggatcccacgcttctcatc
ggctcctccctgctggccacctgctcagtgcacggagacccaccaggagc
caccgccgagggcctctactggaccctcaacgggcgccgcctgccccctg
agctctcccgtgtactcaacgcctccaccttggctctggccctggccaac
ctcaatgggtccaggcagcggtcgggggacaacctcgtgtgccacgcccg
tgacggcagcatcctggctggctcctgcctctatgttggcctgcccccag
agaaacccgtcaacatcagctgctggtccaagaacatgaaggacttgacc
tgccgctggacgccaggggcccacggggagaccttcctccacaccaacta
ctccctcaagtacaagcttaggtggtatggccaggacaacacatgtgagg
agtaccacacagtggggccccactcctgccacatccccaaggacctggct
ctctttacgccctatgagatctgggtggaggccaccaaccgcctgggctc
tgcccgctccgatgtactcacgctggatatcctggatgtggtgaccacgg
accccccgcccgacgtgcacgtgagccgcgtcgggggcctggaggaccag
ctgagcgtgcgctgggtgtcgccacccgccctcaaggatttcctctttca
agccaaataccagatccgctaccgagtggaggacagtgtggactggaagg
tggtggacgatgtgagcaaccagacctcctgccgcctggccggcctgaaa
cccggcaccgtgtacttcgtgcaagtgcgctgcaacccctttggcatcta
tggctccaagaaagccgggatctggagtgagtggagccaccccacagccg
cctccactccccgcagtgagcgcccgggcccgggcggcggggcgtgcgaa
ccgcggggcggagagccgagctcggggccggtgcggcgcgagctcaagca
gttcctgggctggctcaagaagcacgcgtactgctccaacctcagcttcc
gcctctacgaccagtggcgagcctggatgcagaagtcgcacaagacccgc
aaccaggacgaggggatcctgccctcgggcagacggggcacggcgagagg
tcctgccagataagctgtaggggctcaggccaccctccctgccacgtgga
gacgcagaggccgaacccaaactggggccacctctgtaccctcacttcag
ggcacctgagccaccctcagcaggagctggggtggcccctgagctccaac
ggccataacagctctgactcccacgtgaggccacctttgggtgcacccca
gtgggtgtgtgtgtgtgtgtgagggttggttgagttgcctagaacccctg
ccagggctgggggtgagaaggggagtcattactccccattacctagggcc
cctccaaaagagtccttttaaataaatgagctatttaggtgctgtgattg
tgaaaaaaaaaaaaaaaaaaaaa
>NM_002019 4
atcgaggtccgcgggaggctcggagcgcgccaggcggacactcctctcgg
ctcctccccggcagcggcggcggctcggagcgggctccggggctcgggtg
cagcggccagcgggcgcctggcggcgaggattacccggggaagtggttgt
ctcctggctggagccgcgagacgggcgctcagggcgcggggccggcggcg
gcgaacgagaggacggactctggcggccgggtcgttggccgcggggagcg
cgggcaccgggcgagcaggccgcgtcgcgctcaccatggtcagctactgg
gacaccggggtcctgctgtgcgcgctgctcagctgtctgcttctcacagg

atctagttcaggttcaaaattaaaagatcctgaactgagtttaaaaggca
cccagcacatcatgcaagcaggccagacactgcatctccaatgcaggggg
gaagcagcccataaatggtctttgcctgaaatggtgagtaaggaaagcga
aaggctgagcataactaaatctgcctgtggaagaaatggcaaacaattct
gcagtactttaaccttgaacacagctcaagcaaaccacactggcttctac
agctgcaaatatctagctgtacctacttcaaagaagaaggaaacagaatc
tgcaatctatatatttattagtgatacaggtagacctttcgtagagatgt
acagtgaaatccccgaaattatacacatgactgaaggaagggagctcgtc
attccctgccgggttacgtcacctaacatcactgttactttaaaaaagtt
tccacttgacactttgatccctgatggaaaacgcataatctgggacagta
gaaagggcttcatcatatcaaatgcaacgtacaaagaaatagggcttctg
acctgtgaagcaacagtcaatgggcatttgtataagacaaactatctcac
acatcgacaaaccaatacaatcatagatgtccaaataagcacaccacgcc
cagtcaaattacttagaggccatactcttgtcctcaattgtactgctacc
actcccttgaacacgagagttcaaatgacctggagttaccctgatgaaaa
aaataagagagcttccgtaaggcgacgaattgaccaaagcaattcccatg
ccaacatattctacagtgttcttactattgacaaaatgcagaacaaagac
aaaggactttatacttgtcgtgtaaggagtggaccatcattcaaatctgt
taacacctcagtgcatatatatgataaagcattcatcactgtgaaacatc
gaaaacagcaggtgcttgaaaccgtagctggcaagcggtcttaccggctc
tctatgaaagtgaaggcatttccctcgccggaagttgtatggttaaaaga
tgggttacctgcgactgagaaatctgctcgctatttgactcgtggctact
cgttaattatcaaggacgtaactgaagaggatgcagggaattatacaatc
ttgctgagcataaaacagtcaaatgtgtttaaaaacctcactgccactct
aattgtcaatgtgaaaccccagatttacgaaaaggccgtgtcatcgtttc
cagacccggctctctacccactgggcagcagacaaatcctgacttgtacc
gcatatggtatccctcaacctacaatcaagtggttctggcacccctgtaa
ccataatcattccgaagcaaggtgtgacttttgttccaataatgaagagt
cctttatcctggatgctgacagcaacatgggaaacagaattgagagcatc
actcagcgcatggcaataatagaaggaaagaataagatggctagcacctt
ggttgtggctgactctagaatttctggaatctacatttgcatagcttcca
ataaagttgggactgtgggaagaaacataagcttttatatcacagatgtg
ccaaatgggtttcatgttaacttggaaaaaaatgccgacggaaggagagga
cctgaaactgtcttgcacagttaacaagttcttatacagagacgttactt
ggattttactgcggacagttaataacagaacaatgcactacagtattagc
aagcaaaaaatggccatcactaaggagcactccatcactcttaatcttac
catcatgaatgtttccctgcaagattcaggcacctatgcctgcagagcca
ggaatgtatacacaggggaagaaatcctccagaagaaagaaattacaatc
agagatcaggaagcaccatacctcctgcgaaacctcagtgatcacacagt
ggccatcagcagttccaccactttagactgtcatgctaatggtgtccccg
agcctcagatcacttggtttaaaaacaaccacaaaatacaacaagagcct
ggaattattttaggaccaggaagcagcacgctgtttattgaaagagtcac
agaagaggatgaaggtgtctatcactgcaaagccaccaaccagaagggct
ctgtggaaagttcagcatacctcactgttcaaggaacctcggacaagtct
aatctggagctgatcactctaacatgcacctgtgtggctgcgactctctt
ctggctcctattaaccctctttatccgaaaaatgaaaaggtcttcttctg
aaataaagactgactacctatcaattataatggacccagatgaagttcct
ttggatgagcagtgtgagcggctcccttatgatgccagcaagtgggagtt
tgcccgggagagacttaaactgggcaaatcacttggaagaggggcttttg

gaaaagtggttcaagcatcagcatttggcattaagaaatcacctacgtgc
cggactgtggctgtgaaaatgctgaaagagggggccacggccagcgagta
caaagctctgatgactgagctaaaaatcttgacccacattggccaccatc
tgaacgtggttaacctgctgggagcctgcaccaagcaaggagggcctctg
atggtgattgttgaatactgcaaatatggaaatctctccaactacctcaa
gagcaaacgtgacttatttttctcaacaaggatgcagcactacacatgg
agcctaagaaagaaaaaatggagccaggcctggaacaaggcaagaaacca
agactagatagcgtcaccagcagcgaaagctttgcgagctccggctttca
ggaagataaaagtctgagtgatgttgaggaagaggaggattctgacggtt
tctacaaggagcccatcactatggaagatctgatttcttacagttttcaa
gtggccagaggcatggagttcctgtcttccagaaagtgcattcatcggga
cctggcagcgagaaacattcttttatctgagaacaacgtggtgaagattt
gtgattttggccttgcccgggatatttataagaaccccgattatgtgaga
aaaggagatactcgacttcctctgaaatggatggctcctgaatctatctt
tgacaaaatctacagcaccaagagcgacgtgtggtcttacggagtattgc
tgtgggaaatcttctccttaggtgggtctccatacccaggagtacaaatg
gatgaggacttttgcagtcgcctgagggaaggcatgaggatgagagctcc
tgagtactctactcctgaaatctatcagatcatgctggactgctggcaca
gagacccaaaagaaaggccaagatttgcagaacttgtggaaaaactaggt
gatttgcttcaagcaaatgtacaacaggatggtaaagactacatcccaat
caatgccatactgacaggaaatagtgggtttacatactcaactcctgcct
tctctgaggacttcttcaaggaaagtatttcagctccgaagtttaattca
ggaagctctgatgatgtcagatacgtaaatgctttcaagttcatgagcct
ggaaagaatcaaaacctttgaagaacttttaccgaatgccacctccatgt
ttgatgactaccagggcgacagcagcactctgttggcctctcccatgctg
aagcgcttcacctggactgacagcaaacccaaggcctcgctcaagattga
cttgagagtaaccagtaaaagtaaggagtcggggctgtctgatgtcagca
ggcccagtttctgccattccagctgtgggcacgtcagcgaaggcaagcgc
aggttcacctacgaccacgctgagctggaaaggaaaatcgcgtgctgctc
cccgcccccagactacaactcggtggtcctgtactccaccccacccatct
agagtttgacacgaagccttatttctagaagcacatgtgtatttataccc
ccaggaaactagcttttgccagtattatgcatatataagtttacaccttt
atctttccatgggagccagctgctttttgtgatttttttaatagtgcttt
tttttttttgactaacaagaatgtaactccagatagagaaatagtgacaa
gtgaagaacactactgctaaatcctcatgttactcagtgttagagaaatc
cttcctaaacccaatgacttccctgctccaacccccgccacctcagggca
cgcaggaccagtttgattgaggagctgcactgatcacccaatgcatcacg
taccccactgggccagccctgcagcccaaaacccagggcaacaagcccgt
tagccccagggatcactggctggcctgagcaacatctcgggagtcctcta
gcaggcctaagacatgtgaggaggaaaaggaaaaaaagcaaaaagcaagg
gagaaaagagaaaccgggagaaggcatgagaaagaatttgagacgcacca
tgtgggcacggagggggacggggctcagcaatgccatttcagtggcttcc
cagctctgacccttctacatttgagggcccagccaggagcagatggacag
cgatgaggggacattttctggattctgggaggcaagaaaaggacaaatat
ctttttttggaactaaagcaaattttagaactttacctatggaagtggttc
tatgtccattctcattcgtggcatgttttgatttgtagcactgagggtgg
cactcaactctgagcccatacttttggctcctctagtaagatgcactgaa
aacttagccagagttaggttgtctccaggccatgatggccttacactgaa
aatgtcacattctattttgggtattaatatatagtccagacacttaactc

aatttcttggtattattctgttttgcacagttagttgtgaaagaaagctg
agaagaatgaaaatgcagtcctgaggagaggagttttctccatatcaaaa
cgagggctgatggaggaaaaaggtcaataaggtcaagggaaaaccccgtc
tctataccaaccaaaccaattcaccaacacagttgggacccaaaacacag
gaagtcagtcacgtttccttttcatttaatggggattccactatctcaca
ctaatctgaaaggatgtggaagagcattagctggcgcatattaagcactt
taagctccttgagtaaaaaggtggtatgtaatttatgcaaggtatttctc
cagttgggactcaggatattagttaatgagccatcactagaagaaaagcc
cattttcaactgctttgaaacttgcctggggtctgagcatgatgggaata
gggagacagggtaggaaagggcgcctactcttcagggtctaaagatcaag
tgggccttggatcgctaagctggctctgtttgatgctatttatgcaagtt
agggtctatgtatttatgatgtctgcaccttctgcagccagtcagaagct
ggagaggcaacagtggattgctgcttcttggggagaagagtatgcttcct
tttatccatgtaatttaactgtagaacctgagctctaagtaaccgaagaa
tgtatgcctctgttcttatgtgccacatccttgtttaaaggctctctgta
tgaagagatgggaccgtcatcagcacattccctagtgagcctactggctc
ctggcagcggcttttgtggaagactcactagccagaagagaggagtggga
cagtcctctccaccaagatctaaatccaaacaaaagcaggctagagccag
aagagaggacaaatctttgttcttcctcttctttacatacgcaaaccacc
tgtgacagctggcaattttataaatcaggtaactggaaggaggttaaaca
cagaaaaaagaagacctcagtcaattctctacttttttttttttttccaa
atcagataatagcccagcaaatagtgataacaaataaaaccttagctatt
catgtcttgatttcaataattaattcttaatcattaagagaccataataa
atactccttttcaagagaaaagcaaaaccattagaattgttactcagctc
cttcaaactcaggtttgtagcatacatgagtccatccatcagtcaaagaa
tggttccatctggagtcttaatgtagaaagaaaaatggagacttgtaata
atgagctagttacaaagtgcttgttcattaaaatagcactgaaaattgaa
acatgaattaactgataatattccaatcatttgccatttatgacaaaaat
ggttggcactaacaaagaacgagcacttcctttcagagtttctgagataa
tgtacgtggaacagtctgggtggaatggggctgaaaccatgtgcaagtct
gtgtcttgtcagtccaagaagtgacaccgagatgttaattttagggaccc
gtgccttgtttcctagcccacaagaatgcaaacatcaaacagatactcgc
tagcctcatttaaattgattaaaggaggagtgcatctttggccgacagtg
gtgtaactgtatgtgtgtgtgtgtgtgtgtgtgtgtgtgtgtgtgtgggt
gtatgtgtgttttgtgcataactatttaaggaaactggaattttaaagtt
acttttatacaaaccaagaatatatgctacagatataagacagacatggt
ttggtcctatatttctagtcatgatgaatgtattttgtataccatcttca
tataataaacttccaaaaacaca
>NM_017459 2
attgcaacttggtctcacagtggcttaggccagggtgggagcagtgaacg
gagtcacaaaagaaatttttcagctgtcctctctgacaccaccccggcct
gcctctttgttgccatgagagctgcctacctcttcctgctattcctgcct
gcaggcttgctggctcagggccagtatgacctggacccgctgccgccgtt
ccctgaccacgtccagtacacccactatagcgaccagatcgacaacccag
actactatgattatcaagaggtgactcctcggccctccgaggaacagttc
cagttccagtcccagcagcaagtccaacaggaagtcatcccagccccaac
cccagaaccaggaaatgcagagctggagcccacagagcctgggcctcttg
actgccgtgaggaacagtacccgtgcacccgcctctactccatacacagg
ccttgcaaacagtgtctcaacgaggtctgcttctacagcctccgccgtgt

gtacgtcattaacaaggagatctgtgttcgtacagtgtgtgcccatgagg
agctcctccgagctgacctctgtcgggacaagttctccaaatgtggcgtg
atggccagcagcggcctgtgccaatccgtggcggcctcctgtgccaggag
ctgtgggagctgctagggtggtgctggcatcctgagtcctggccctcctg
ggatctggggccctcgggccctgcctgacctggtgctttttttccccatcc
ccatgttccttttattctgtaaaaagttagtggactgcagccctgggggt
tgcaggctgcggtgcctcaggcccctccttcagcctgtggccacctctgg
ggcacaatgggggctccccactgcccagtctgcccctcgggttgggggag
tatcccaggcctctctgtgggacctgggcccctgacgggccttctcagcc
cgttttgaggacagacagtcccccgaggtaggctacatcccccaccccca
gctggtctgcttggatttcctacagcccccgtgggcatggaccaccttta
ttttatacaaaattaaaaacaagtttttacaaaaaaaaaaaaaaaaaaaa
aaaaa
>NM_002403 3
gccccgtcgggggcccggagggggactcggagcgggccaagggggcggctc
cggcgggcggactcggagcgggcggcggagtgacccggacagctgtcctc
tctgacaccaccccggcctgcctctttgttgccatgagagctgcctacct
cttcctgctattcctgcctgcaggcttgctggctcagggccagtatgacc
tggacccgctgccgccgttccctgaccacgtccagtacacccactatagc
gaccagatcgacaacccagactactatgattatcaagaggtgactcctcg
gccctccgaggaacagttccagttccagtcccagcagcaagtccaacagg
aagtcatcccagccccaaccccagaaccaggaaatgcagagctggagccc
acagagcctgggcctcttgactgccgtgaggaacagtacccgtgcacccg
cctctactccatacacaggccttgcaaacagtgtctcaacgaggtctgct
tctacagcctccgccgtgtgtacgtcattaacaaggagatctgtgttcgt
acagtgtgtgcccatgaggagctcctccgagctgacctctgtcgggacaa
gttctccaaatgtggcgtgatggccagcagcggcctgtgccaatccgtgg
cggcctcctgtgccaggagctgtgggagctgctagggtggtgctggcatc
ctgagtcctggccctcctgggatctggggccctcgggccctgcctgacct
ggtgctttttttccccatccccatgttccttttattctgtaaaaagttagt
ggactgcagccctgggggttgcaggctgcggtgcctcaggcccctccttc
agcctgtggccacctctggggcacaatgggggctccccactgcccagtct
gcccctcgggttgggggagtatcccaggcctctctgtgggacctgggccc
ctgacgggccttctcagcccgttttgaggacagacagtcccccgaggtag
gctacatcccccaccccagctggtctgcttggatttcctacagcccccg
tgggcatggaccacctttattttatacaaaattaaaaacaagtttttaca
aaaaaaaaaaaaaaaaaaaaaaa
>NM_002084 3
gtcgccccgggacggggaggtggggagctgagggcaagtcgcgcccgccc
ctgaaatcccagccgcctagcgattggctgcaagggtctcggcttggccg
cggattggtcacacccgagggcttgaaaggtggctgggagcgccggacac
ctcagacggacggtggccagggatcaggcagcggctcaggcgaccctgag
tgtgcccccaccccgccatggcccggctgctgcaggcgtcctgcctgctt
tccctgctcctggccggcttcgtctcgcagagccggggacaagagaagtc
gaagatggactgccatggtggcataagtggcaccatttacgagtacggag
ccctcaccattgatggggaggagtacatccccttcaagcagtatgctggc
aaatacgtcctctttgtcaacgtggccagctactgaggcctgacggcca
gtacattgaactgaatgcactacaggaagagcttgcaccattcggtctgg
tcattctgggctttccctgcaaccaatttggaaaacaggaaccaggagag

aactcagagatccttcctaccctcaagtatgtccgaccaggtggaggctt
tgtccctaatttccagctctttgagaaaggggatgtcaatggagagaaag
agcagaaattctacactttcctaaagaactcctgtcctcccacctcggag
ctcctgggtacatctgaccgcctcttctgggaacccatgaaggttcacga
catccgctggaactttgagaagttcctggtggggccagatggtataccca
tcatgcgctggcaccaccggaccacggtcagcaacgtcaagatggacatc
ctgtcctacatgaggcggcaggcagccctgggggtcaagaggaagtaact
gaaggccgtctcatcccatgtccaccatgtaggggagggactttgttcag
gaagaaatccgtgtctccaaccacactatctacccatcacagacccctttt
cctatcactcaaggccccagcctggcacaaatggatgcatacagttctgt
gtactgccaggcatgtgggtgtgggtgcatgtgggtgtttacacacatgc
ctacaggtatgcgtgattgtgtgtgtgtgcatgggtgtacagccacgtgt
ctacctatgtgtctttctgggaatgtgtaccatctgtgtgcctgcagctg
tgtagtgctggacagtgacaaccctttctctccagttctccactccaatg
ataatagttcacttacacctaaacccaaaggaaaaaccagctctaggtcc
aattgttctgctctaactgatacctcaaccttggggccagcatctcccac
tgcctccaaatattagtaactatgactgacgtccccagaagtttctgggt
ctaccacactccccaaccccccactcctacttcctgaagggccctcccaa
ggctacatccccaccccacagttctccctgagagagatcaacctccctga
gatcaaccaaggcagatgtgacagcaagggccacggaccccatggcaggg
gtggcgtcttcatgagggaggggcccaaagcccttgtgggcggacctccc
ctgagcctgtctgaggggccagcccttagtgcattcaggctaaggcccct
gggcagggatgccacccctgctccttcggaggacgtgccctcacccctca
ctggtccactggcttgagactcaccccgtctgcccagtaaaagcctttct
gcagcagctgaaaaaaaaaaaaaaaaaaa
>NM_001124 1
ctggatagaacagctcaagccttgccacttcgggcttctcactgcagctg
ggcttggacttcggagttttgccattgccagtgggacgtctgagactttc
tccttcaagtacttggcagatcactctcttagcagggtctgcgcttcgca
gccgggatgaagctggtttccgtcgccctgatgtacctgggttcgctcgc
cttcctaggcgctgacaccgctcggttggatgtcgcgtcggagtttcgaa
agaagtggaataagtgggctctgagtcgtgggaagagggaactgcggatg
tccagcagctaccccaccgggctcgctgacgtgaaggccgggcctgccca
gacccttattcggccccaggacatgaagggtgcctctcgaagccccgaag
acagcagtccggatgccgcccgcatccgagtcaagcgctaccgccagagc
atgaacaacttccagggcctccggagctttggctgccgcttcgggacgtg
cacggtgcagaagctggcacaccagatctaccagttcacagataaggaca
aggacaacgtcgcccccaggagcaagatcagcccccagggctacggccgc
cggcgccggcgctccctgcccgaggccggcccgggtcggactctggtgtc
ttctaagccacaagcacacggggctccagcccccccgagtggaagtgctc
cccactttctttaggatttaggcgcccatggtacaaggaatagtcgcgca
agcatcccgctggtgcctcccgggacgaaggacttcccgagcggtgtggg
gaccgggctctgacagccctgcggagaccctgagtccgggaggcaccgtc
cggcggcgagctctggctttgcaagggcccctccttctgggggcttcgct
tccttagccttgctcaggtgcaagtgccccagggggcggggtgcagaaga
atccgagtgtttgccaggcttaaggagaggagaaactgagaaatgaatgc
tgagacccccggagcaggggtctgagccacagccgtgctcgcccacaaac
tgatttctcacggcgtgtcaccccaccagggcgcaagcctcactattact
tgaactttccaaaacctaaagaggaaaagtgcaatgcgtgttgtacatac

agaggtaactatcaatatttaagtttgttgctgtcaagattttttttgta
acttcaaatatagagatatttttgtacgttatatattgtattaagggcat
tttaaaagcaattatattgtcctcccctattttaagacgtgaatgtctca
gcgaggtgtaaagttgttcgccgcgtggaatgtgagtgtgtttgtgtgca
tgaaagagaaagactgattacctcctgtgtggaagaaggaaacaccgagt
ctctgtataatctatttacataaaatgggtgatatgcgaacagcaaacc
>NM_004995 2
cagaccccagttcgccgactaagcagaagaaagatcaaaaaccggaaaag
aggagaagagcaaacaggcactttgaggaacaatcccctttaactccaag
ccgacagcggtctaggaattcaagttcagtgcctaccgaagacaaaggcg
ccccgagggagtggcggtgcgaccccagggcgtgggcccggccgcggagc
ccacactgcccggctgacccggtggtctcggaccatgtctcccgccccaa
gacccccccgttgtctcctgctcccccctgctcacgctcggcaccgcgctc
gcctccctcggctcggcccaaagcagcagcttcagccccgaagcctggct
acagcaatatggctacctgcctcccggggacctacgtacccacacacagc
gctcaccccagtcactctcagcggccatcgctgccatgcagaagttttac
ggcttgcaagtaacaggcaaagctgatgcagacaccatgaaggccatgag
gcgcccccgatgtggtgttccagacaagtttggggctgagatcaaggcca
atgttcgaaggaagcgctacgccatccagggtctcaaatggcaacataat
gaaatcactttctgcatccagaattacacccccaaggtgggcgagtatgc
cacatacgaggccattcgcaaggcgttccgcgtgtgggagagtgccacac
cactgcgcttccgcgaggtgccctatgcctacatccgtgagggccatgag
aagcaggccgacatcatgatcttctttgccgagggcttccatggcgacag
cacgcccttcgatggtgagggcggcttcctggcccatgcctacttcccag
gccccaacattggaggagacacccactttgactctgccgagccttggact
gtcaggaatgaggatctgaatggaaatgacatcttcctggtggctgtgca
cgagctgggccatgccctggggctcgagcattccagtgacccctcggcca
tcatggcaccctttttaccagtggatggacacggagaattttgtgctgccc
gatgatgaccgccggggcatccagcaactttatgggggtgagtcagggtt
ccccaccaagatgccccctcaacccaggactacctcccggccttctgttc
ctgataaacccaaaaaccccacctatgggcccaacatctgtgacgggaac
tttgacaccgtggccatgctccgaggggagatgtttgtcttcaaggagcg
ctggttctggcgggtgaggaataaccaagtgatggatggatacccaatgc
ccattggccagttctggcggggcctgcctgcgtccatcaacactgcctac
gagaggaaggatggcaaattcgtcttcttcaaaggagacaagcattgggt
gtttgatgaggcgtccctggaacctggctaccccaagcacattaaggagc
tgggccgagggctgcctaccgacaagattgatgctgctctcttctggatg
cccaatggaaagacctacttcttccgtggaaacaagtactaccgtttcaa
cgaagagctcagggcagtggatagcgagtaccccaagaacatcaaagtct
gggaagggatccctgagtctcccagagggtcattcatgggcagcgatgaa
gtcttcacttacttctacaaggggaacaaatactggaaattcaacaacca
gaagctgaaggtagaaccgggctaccccaagtcagccctgagggactgga
tgggctgcccatcgggaggccggccggatgaggggactgaggaggagacg
gaggtgatcatcattgaggtggacgaggagggcggcggggcggtgagcgc
ggctgccgtggtgctgcccgtgctgctgctgctcctggtgctggcggtgg
gccttgcagtcttcttcttcagacgccatgggacccccaggcgactgctc
tactgccagcgttccctgctggacaaggtctgacgcccaccgccggcccg
cccactcctaccacaaggactttgcctctgaaggccagtggcagcaggtg
gtggtgggtgggctgctcccatcgtcccgagccccctccccgcagcctcc

ttgcttctctctgtcccctggctggcctccttcaccctgaccgcctccct
ccctcctgccccggcattgcatcttccctagataggtcccctgagggctg
agtgggagggcggccctttccagcctctgcccctcaggggaaccctgtag
ctttgtgtctgtccagccccatctgaatgtgttgggggctctgcacttga
aggcaggaccctcagacctcgctggtaaaggtcaaatggggtcatctgct
ccttttccatcccctgacataccttaacctctgaactctgacctcaggag
gctctgggcactccagccctgaaagccccaggtgtacccaattggcagcc
tctcactactctttctggctaaaaggaatctaatcttgttgagggtagag
accctgagacagtgtgagggggtggggactgccaagccaccctaagacct
tgggaggaaaactcagagagggtcttcgttgctcagtcagtcaagttcct
cggagatctgcctctgcctcacctaccccagggaacttccaaggaaggag
cctgagccactggggactaagtgggcagaagaaacccttggcagccctgt
gcctctcgaatgttagccttggatggggctttcacagttagaagagctga
aaccaggggtgcagctgtcaggtagggtggggccggtgggagaggcccgg
gtcagagccctgggggtgagcctgaaggccacagagaaagaaccttgccc
aaactcaggcagctggggctgaggcccaaaggcagaacagccagaggggg
caggaggggaccaaaaaggaaaatgaggacgtgcagcagcattggaaggc
tggggccgggcaggccaggccaagccaagcaggggggccacagggtgggct
gtggagctctcaggaagggccctgaggaaggcacacttgctcctgttggt
ccctgtccttgctgcccaggcagcgtggaggggaagggtagggcagccag
agaaaggagcagagaaggcacacaaacgaggaatgaggggcttcacgaga
ggccacagggcctggctggccacgctgtcccggcctgctcaccatctcag
tgaggggcaggagctggggctcgcttaggctgggtccacgcttccctggt
gccagcacccctcaagcctgtctcaccagtggcctgccctctcgctcccc
cacccagcccacccattgaagtctccttgggccaccaaaggtggtggcca
tggtaccggggacttgggagagtgagacccagtggagggagcaagaggag
agggatgtcgggggggtggggcacggggtaggggaaatggggtgaacggt
gctggcagttcggctagatttctgtcttgtttgttttttttgttttgttta
atgtatatttttattataattattatatatgaattccaaaaaaaaaaaaa
aaaaaaaa
>NM_002964 4
gagaaaccagagactgtagcaactctggcagggagaagctgtctctgatg
gcctgaagctgtgggcagctggccaagcctaaccgctataaaaggagct
gcctctcagccctgcatgtctcttgtcagctgtctttcagaagacctggt
ggggcaagtccgtgggcatcatgttgaccgagctggagaaagccttgaac
tctatcatcgacgtctaccacaagtactccctgataaaggggaatttcca
tgccgtctacagggatgacctgaagaaattgctagagaccgagtgtcctc
agtatatcaggaaaaagggtgcagacgtctggttcaaagagttggatatc
aacactgatggtgcagttaacttccaggagttcctcattctggtgataaa
gatgggcgtggcagcccacaaaaaaagccatgaagaaagccacaaagagt
agctgagttactgggcccagaggctgggcccctggacatgtacctgcaga
ataataaagtcatcaatacctcaaaaaaaaaa
>NM_004429 4
gcacagcggcagcggagggagtctatgcgagctggacagcagtgggaggt
ttgtgaggctcgcactggccgcagaccctcgggctcgatcgcccgggagc
caggactcggcgacgcgaggctgccgggctacccggccgaggcttcgggg
gcgcaaactaatgggactggctcgctcggcagcatctccccgctcttcta
agtacactgagcagggcccgcgctgaagtagaagctgtccgggggcgcgt
agcccggagtcccagtgtggcccggaggaacggagcccgtgccagggcgg

cccagtcgggagcccggggaccgagcttgtgctgtggggaaacccccact
tcttccaagggacagcgatcccgggacggtcgaggcgtcggggcggtcac
cgagacctctgcgggaagaccccgtcggggagagggcgcgcagccccgaa
gcgtctcgggaagtcgagcggaatcgggcgggatcacccgggggcgcaga
gcccccgtcgcgcctcgtgcggcagcggagagcccaggagaacgagccct
cgggggccgaagcccatgcccgggttgggggcggctgcccagtgagtcct
cctggccggccgggcggagaagagcgacaccgaagccggcgggaggggag
cacttcaaggccggcggctgcggaggatgggcgcctgagcggctccgagc
gcagcgcggcagaggaaggcgaggcgagctttggtgaggaggcgccaagg
gatcccgaagtgcagtctgcccccgggaagatggctcggcctgggcagcg
ttggctcggcaagtggcttgtggcgatggtcgtgtgggcgctgtgccggc
tcgccacaccgctggccaagaacctggagcccgtatcctggagctccctc
aaccccaagttcctgagtgggaagggcttggtgatctatccgaaaattgg
agacaagctggacatcatctgcccccgagcagaagcagggcggccctatg
agtactacaagctgtacctggtgcggcctgagcaggcagctgcctgtagc
acagttctcgaccccaacgtgttggtcacctgcaataggccagagcagga
aatacgctttaccatcaagttccaggagttcagccccaactacatgggcc
tggagttcaagaagcaccatgattactacattacctcaacatccaatgga
agcctggaggggctggaaaaccgggagggcggtgtgtgccgcacacgcac
catgaagatcatcatgaaggttgggcaagatcccaatgctgtgacgcctg
agcagctgactaccagcaggcccagcaaggaggcagacaacactgtcaag
atggccacacaggcccctggtagtcggggctccctgggtgactctgatgg
caagcatgagactgtgaaccaggaagagaagagtggcccaggtgcaagtg
gggcagcagcggggaccctgatggcttcttcaactccaaggtggcattg
ttcgcggctgtcggtgccggttgcgtcatcttcctgctcatcatcatctt
cctgacggtcctactactgaagctacgcaagcggcaccgcaagcacacac
agcagcgggcggctgccctctcgctcagtaccctggccagtcccaagggg
ggcagtggcacagcgggcaccgagcccagcgacatcatcattcccttacg
gactacagagaacaactactgcccccactatgagaaggtgagtggggact
acgggcaccctgtctacatcgtccaagagatgccgccccagagcccggcg
aacatctactacaaggtctgagtgcccggcacggcctcaggcccccgagg
gacagtcggcctggaccggacctctcctttcgcccccacacccccctcccc
ttgccagctgtgcccacctttgtatttagttttgtagtttcttggctttt
ataatccccctttttccctgccccctgggcttcggagggggggtgcttgtg
cccctaacccccatgctcttgtgccttccccctctggccaggcctctggg
ctccgtgggggcgccccttcttggaaggcagggctggacactgatggaca
gcaggcagggagacagtcccctggccctgcccctccctcgcccccccttgc
caccttcccaggactgcttgtccgctatcatcactgtttttaatgctttt
gtgttcatttttagctgtcaactcattttcatctgtttttgaagaaaa
atggaaaaatgtaaaaggcagcccctccccaggctttgtgagcctggccc
aagccagtacaagagggcctggggcacgatgtggtcagccaggaagcata
ggatgccatttcttttatagattccttggtatttctggtggggtaagggg
caggccagggctgttcacgcccatgagggaagaggaaagtgccactgggc
aaggtgtcccaccctcccctcctgaccctcctacgaggcttatcctggca
atggggtagtcactgccacccttccacacacacacacacacacacacaca
caaaaaaaaatcccttccttgtgggattcttgggcatctcctgcctccct
cactctcacggtaattaatgtcttaattggctgttgcctggggaacagga
gagctgctgcaggcagatgacctcatgggggggtggagggaggtgaggtgc
ccaggtggctatttgccctgcagagctgggagtttcacccccaccccccca

ccctgttctctccttacctttggcatcctttggcctggtggggaaacaga
ggcccagggtggagacctaagcgggtataagaccaggtggcctgctcctt
ttctgggccctagcacaggtgggtaacccccacccaacccagctcctgct
gctgtcccagtcttgggctggggcctggaaagaggaagaggctgcctggg
gctgggccagcccgctgtgcactttgaccccagttccttgccagcacggc
tgctaacagactgccacttgagtgcgccttgcaggcactcccagagcagc
catggaaggagctggccctcacaccatccacctccacactgcctcctggc
cagctgcccaccccagtgccaggtgggagagggagcagaacagccagccc
cttccaggtggcagtcggaagggtttttgtttttgtttctgttgccattt
gtgtaaatactagtctttttggaaaaaaaaataatgtaaagatgttttgta
taaactctgaattattttcttgttgctttttcttagaaaaaaatgagaa
ctaaaaaaaaaaaattaaccacatggagaaaaaaaaaaaaaaa
>NM_000719 6
ttattttttcaaatggtgtagccgccggaggtgcggtgctcagttcttgg
aaggggcccggatgtactgaggatgcgttacagtttcactcgaggaggca
gtagtggaaaggagcagtttttggggtttgatgccataatgggaatcagg
taatcgtcggcggggaagaagaaacgctgcagaccacggcttcctcgaat
cttgcgcgaaagccgccggcctcggaggagggattaatccagacccgccg
gggggtgttttcacatttcttcctcttcgtggctgctcctcctattaaaa
ccatttttggtccatggtcaatgagaatacgaggatgtacattccagagg
aaaaccaccaaggttccaactatgggagcccacgccccgcccatgccaac
atgaatgccaatgcggcagcggggctggcccctgagcacatccccacccc
gggggctgccctgtcgtggcaggcggccatcgacgcagcccggcaggcta
agctgatgggcagcgctggcaatgcgaccatctccacagtcagctccacg
cagcggaagcggcagcaatatgggaaacccaagaagcagggcagcaccac
ggccacacgcccgccccgagccctgctctgcctgaccctgaagaacccca
tccggagggcctgcatcagcattgtcgaatggaaaccatttgaaataatt
attttactgactatttttgccaattgtgtggccttagcgatctatattcc
ctttccagaagatgattccaacgccaccaattccaacctggaacgagtgg
aatatctctttctcataatttttacggtggaagcgtttttaaaagtaatc
gcctatggactcctctttcaccccaatgcctacctccgcaacggctggaa
cctactagattttataattgtggttgtggggctttttagtgcaattttag
aacaagcaaccaaagcagatggggcaaacgctctcggagggaaaggggcc
ggatttgatgtgaaggcgctgagggccttccgcgtgctgcgcccccctgcg
gctggtgtccggagtcccaagtctccaggtggtcctgaattccatcatca
aggccatggtcccccctgctgcacatcgccctgcttgtgctgtttgtcatc
atcatctacgccatcatcggcttggagctcttcatggggaagatgcacaa
gacctgctacaaccaggagggcatagcagatgttccagcagaagatgacc
cttccccttgtgcgctggaaacgggccacgggcggcagtgccagaacggc
acggtgtgcaagcccggctgggatggtcccaagcacggcatcaccaactt
tgacaactttgccttcgccatgctcacggtgttccagtgcatcaccatgg
agggctggacggacgtgctgtactgggtcaatgatgccgtaggaagggac
tggccctggatctattttgttacactaatcatcatagggtcatttttttgt
acttaacttggttctcggtgtgcttagcggagagttttccaaagagaggg
agaaggccaaggcccggggagatttccagaagctgcgggagaagcagcag
ctagaagaggatctcaaaggctacctggattggatcactcaggccgaaga
catcgatcctgagaatgaggacgaaggcatggatgaggagaagccccgaa
acatgagcatgcccaccagtgagaccgagtccgtcaacaccgaaaacgtg
gctggaggtgacatcgagggagaaaactgcggggccaggctggcccaccg

gatctccaagtcaaagttcagccgctactggcgccggtggaatcggttct
gcagaaggaagtgccgcgccgcagtcaagtctaatgtcttctactggctg
gtgattttcctggtgttcctcaacacgctcaccattgcctctgagcacta
caaccagcccaactggctcacagaagtccaagacacggcaaacaaggccc
tgctggccctgttcacggcagagatgctcctgaagatgtacagcctgggc
ctgcaggcctacttcgtgtccctcttcaaccgctttgactgcttcgtcgt
gtgtggcggcatcctggagaccatcctggtggagaccaagatcatgtccc
cactgggcatctccgtgctcagatgcgtccggctgctgaggattttcaag
atcacgaggtactggaactccttgagcaacctggtggcatccttgctgaa
ctctgtgcgctccatcgcctccctgctccttctcctcttcctcttcatca
tcatcttctccctcctggggatgcagctctttggaggaaagttcaacttt
gatgagatgcagacccggaggagcacattcgataacttcccccagtccct
cctcactgtgtttcagatcctgaccggggaggactggaattcggtgatgt
atgatgggatcatggcttatggcggcccctcttttccagggatgttagtc
tgtatttacttcatcatcctcttcatctgtggaaactatatcctactgaa
tgtgttcttggccattgctgtggacaacctggctgatgctgagagcctca
catctgcccaaaaggaggaggaagaggagaaggagagaaagaagctggcc
aggactgccagcccagagaagaaacaagagttggtggagaagccggcagt
gggggaatccaaggaggagaagattgagctgaaatccatcacggctgacg
gagagtctccacccgccaccaagatcaacatggatgacctccagcccaat
gaaaatgaggataagagcccctaccccaacccagaaactacaggagaaga
ggatgaggaggagccagagatgcctgtcggccctcgcccacgaccactct
ctgagcttcaccttaaggaaaaggcagtgcccatgccagaagccagcgcg
tttttcatcttcagctctaacaacaggtttcgcctccagtgccaccgcat
tgtcaatgacacgatcttcaccaacctgatcctcttcttcattctgctca
gcagcatttccctggctgctgaggacccggtccagcacacctccttcagg
aaccatattctgttttattttgatattgtttttaccaccattttcaccat
tgaaattgctctgaagatgactgcttatggggctttcttgcacaagggtt
ctttctgccggaactacttcaacatcctggacctgctggtggtcagcgtg
tccctcatctcctttggcatccagtccagtgcaatcaatgtcgtgaagat
cttgcgagtcctgcgagtactcaggcccctgagggccatcaacagggcca
aggggctaaagcatgtggttcagtgtgtgtttgtcgccatccggaccatc
gggaacatcgtgattgtcaccaccctgctgcagttcatgtttgcctgcat
cggggtccagctcttcaagggaaagctgtacacctgttcagacagttcca
agcagacagaggcggaatgcaagggcaactacatcacgtacaaagacggg
gaggttgaccaccccatcatccaaccccgcagctgggagaacagcaagtt
tgactttgacaatgttctggcagccatgatggccctcttcaccgtctcca
ccttcgaagggtggccagagctgctgtaccgctccatcgactcccacacg
gaagacaagggccccatctacaactaccgtgtggagatctccatcttctt
catcatctacatcatcatcatcgccttcttcatgatgaacatcttcgtgg
gcttcgtcatcgtcacctttcaggagcagggggagcaggagtacaagaac
tgtgagctggacaagaaccagcgacagtgcgtggaatacgccctcaaggc
ccggcccctgcggaggtacatccccaagaaccagcaccagtacaaagtgt
ggtacgtggtcaactccacctacttcgagtacctgatgttcgtcctcatc
ctgctcaacaccatctgcctggccatgcagcactacggccagagctgcct
gttcaaaatcgccatgaacatcctcaacatgctcttcactggcctcttca
ccgtggagatgatcctgaagctcattgccttcaaacccaagcactatttc
tgtgatgcatggaatacatttgacgccttgattgttgtgggtagcattgt
tgatatagcaatcaccgaggtaaacccagctgaacatacccaatgctctc

cctctatgaacgcagaggaaaactcccgcatctccatcaccttcttccgc
ctgttccgggtcatgcgtctggtgaagctgctgagccgtggggagggcat
ccggacgctgctgtggaccttcatcaagtccttccaggccctgccctatg
tggccctcctgatcgtgatgctgttcttcatctacgcggtgatcgggatg
caggtgtttgggaaaattgccctgaatgataccacagagatcaaccggaa
caacaactttcagaccttcccccaggccgtgctgctcctcttcaggtgtg
ccaccggggaggcctggcaggacatcatgctggcctgcatgccaggcaag
aagtgtgccccagagtccgagcccagcaacagcacggagggtgaaacacc
ctgtggtagcagctttgctgtcttctacttcatcagcttctacatgctct
gtgccttcctgatcatcaacctctttgtagctgtcatcatggacaacttt
gactacctgacaagggactggtccatccttggtccccaccacctggatga
gtttaaaagaatctgggcagagtatgaccctgaagccaagggtcgtatca
aacacctggatgtggtgaccctcctccggcggattcagccgccactaggt
tttgggaagctgtgccctcaccgcgtggcttgcaaacgcctggtctccat
gaacatgcctctgaacagcgacgggacagtcatgttcaatgccaccctgt
ttgccctggtcaggacggccctgaggatcaaaacagaagggaacctagaa
caagccaatgaggagctgcgggcgatcatcaagaagatctggaagcggac
cagcatgaagctgctggaccaggtggtgccccctgcaggtgatgatgagg
tcaccgttggcaagttctacgccacgttcctgatccaggagtacttccgg
aagttcaagaagcgcaaagagcagggccttgtgggcaagccctcccagag
gaacgcgctgtctctgcaggctggcttgcgcacactgcatgacatcgggc
ctgagatccgacgggccatctctggagatctcaccgctgaggaggagctg
gacaaggccatgaaggaggctgtgtccgctgcttctgaagatgacatctt
caggagggccggtggcctgttcggcaaccacgtcagctactaccaaagcg
acggccggagcgccttccccagaccttcaccactcagcgcccgctgcac
atcaacaaggcgggcagcagccagggcgacactgagtcgccatcccacga
gaagctggtggactccaccttcaccccgagcagctactcgtccaccggct
ccaacgccaacatcaacaacgccaacaacaccgccctgggtcgcctccct
cgccccgccggctaccccagcacggtcagcactgtggagggccacgggcc
ccccttgtcccctgccatccgggtgcaggaggtggcgtggaagctcagct
ccaacaggtgccactcccgggagagccaggcagccatggcgggtcaggag
gagacgtctcaggatgagacctatgaagtgaagatgaaccatgacacgga
ggcctgcagtgagcccagcctgctctccacagagatgctctcctaccagg
atgacgaaaatcggcaactgacgctcccagaggaggacaagagggacatc
cggcaatctccgaagaggggtttcctccgctctgcctcactaggtcgaag
ggcctccttccacctggaatgtctgaagcgacagaaggaccgagggggag
acatctctcagaagacagtcctgcccttgcatctggttcatcatcaggca
ttggcagtggcaggcctgagcccctcctccagagaagccattcccctgc
ctcattccctaggccttttgccaccccaccagccacacctggcagccgag
gctggcccccacagcccgtccccaccctgcggcttgagggggtcgagtcc
agtgagaaactcaacagcagcttcccatccatccactgcggctcctgggc
tgagaccacccccggtggcgggggcagcagcgccgcccggagagtccggc
ccgtctccctcatggtgcccagccaggctggggccccagggaggcagttc
cacggcagtgccagcagcctggtggaagcggtcttgatttcagaaggact
ggggcagtttgctcaagatcccaagttcatcgaggtcaccacccaggagc
tggccgacgcctgcgacatgaccatagaggagatggagagcgcggccgac
aacatcctcagcgggggcgccccacagagccccaatggcgccctcttacc
ctttgtgaactgcagggacgcggggcaggaccgagccgggggcgaagagg
acgcgggctgtgtgcgcgcgcggggtcgaccgagtgaggaggagctccag

gacagcagggtctacgtcagcagcctgtagtgggcgctgccagatgcggg
cttttttttatttgtttcaatgttcctaatgggttcgtttcagaagtgcc
tcactgttctcgtgacctggagttaaccggaacagcgtcttcattcattt
ctgttgggaccagacgcggagcctgggtgcgcgagccgccctccgggagg
aaggcgcccggctgcgtctgcagaggcggggagaggaggcggcgagggtc
ccggggcgcgaggaaggcgcctgccctctcccagctcgcaggccccgggc
ccggccgcgcctccgcggggagagcaccccggcttcccgcgcgccctcac
caaaaggaccctacagcaaacgggtgtctttcgactctgcttgtagaaac
catttgcacatattctgtacgagcctcgctgtctccctagagccagggcc
ctgcggatttggagaagggagcggggcaggacttccaggaggaccccaac
ccggcccggagagggaggaggaggcctccaggggcgcggagctctgggga
tgggcgtcgggccggcagtggtgcggctcactccgtccctgcccacctgc
gacgggatcccccgaccggcacgggccacgccgagctcccggccagccgc
cggcccgcaggcagcgcgagggaggagctgcgccgccggctccgcccaac
caggtggtgctgagcttccgctgagcgctcttttgttttgtggtttgaca
cttttcttgacagcatgttgcagtttcttttcggttttggttttttttaa
atgttttattttgctttcccagcgggaggggaggaagaagagtgtttaca
aagtcctgtagccccctcacctttctgttttcacttttgccaatgtacat
cgggtttggttttcttgtattatttaaacggttgtggtttcctttttcca
cggaggttcaatagaagccgctgcaggagagttttaccaaccattgtgta
tgcccaataatttgttatcatttccttaggtagtaacctatttttgttct
ggtttggttcggttatctaatggaaaggtaactggcaatgcacttgatgt
ggtcttgcacatgtgggtgatagagttgggttcctttttatgctgggtgt
acaggtgggtttgggagagaggagcatgcgcgagagagtctccgagtgtg
tgcgacgcgtgtgtgtgtggtgggttgtctgtgtgcatatgtcctgcccg
tgtatatgcacccacaccatgtgcccgtgcacaccagtgactacgcagtc
ccccctttctggtttagctgtgggaagatctgaatctggggccgtttgaa
agcaaaaacaaaccactgtctctgcttctgaaacgggaatcagtaactct
ttgcattttctgtcccacaagatatgcaaaaacaatgcaataatattcat
ttaaaaatacaattgtgagttgtgttggcattaaaactgtattttaaaaa
aagacagaaatttaagggaaaaacacaagaaggcattttgcttcaatata
ttccgtgtaatgttttattgcattgataatgtttctgttgaagaaaccgt
tatacttgaattcaggtcagtttcagtatttttcaaatatttttttaaaa
ctgaattgcaattgtgccaagcgaatataatgaattgaattaagttggtt
ttcggattcacttcttgtatattttgctgcatgtaaagtaaatcattttg
tatttggagtgtgacaagctttacctttgaactcaagtgcttttctatat
gtggttgggggaaagggaacaagttttctttagtttgcacaatgagcaaa
ggtatcaccagtgtagtcattattctgctctccacaaacaggtttggaca
ttactgttttgcatatcttgtgtttgcttacatttccctcaatttttcca
aaatcgtttgctgggtatgtttgtaccgcctcttgctgtgagagaccagg
acctattttattccagtcttcactctgtccactctgctctggtcatctga
tttggtacttctccaagaacagcccttcactgtgaggtgcagggaggcgt
tctgatgagccctcagtcactgggccgtcatccgcatcccccatggaaga
ggtagctggctttcccttcccttccaccacacggaattttctctttggct
tccttaggaaagtgtacactaaccgggaggataaaattaaagtcaggctg
cttggagggaggggcatcctcacttccggattcttgttgctctacccaac
aaggacagcaggggctcgagaaaggaactggtgaaaccctgatccatctg
aaagtcaactctgcgtgctcctttctccatcccttcctcactctggagca
gcctttccttcaggcttgccctaatgtttgggctgccggggagggggcca

ggacaagggaagaggcatccggagctcacagtgggggtgggaacagattt
ttgtgggggcatctctaatgctcacttatatctccctagaacatcactct
tttggtgctgtgtccttcaaatgtatgtcaacagtggtggctgaaaaggg
actgctttggggaaaacaggacccaaccattcacccagaattgacccatt
aaatctcttccagtcctagtgttccctgagcccctcttggcacatatata
agtaagctagaaattacaataagggacagtccattcctctatgacagctt
gctggactgattcatgacaaagtggagaaatgtactcaatactccccggt
taacacagtctagaaacagagtttctttatggatatccacacccaagtca
tccaaactttcttgattccttttcactgccatcaaggtcctctagaaatt
gagtttaggtatcatccctttgaaaagttcccaagatttctaccaggaggt
acacacaggcgttccctgtctagggcaggaggactatcctagcttgacct
tctgatccactagaataagactggcgtatgatgcctgtcatcagaacaga
ctggcacaagtagtgacatcaatgaaccacagcacaatcttccaagtgat
gtctactctccacctaaaatggaattttccccatgaccttgtaaaacata
attgtcacatcttccataccccctcctgacagcccccaagtgtcaggagaa
aacagtcaggggctaagggcccaagggacttgaagaaacaacagtttaag
gtctgcagtttggtcaacttaattcttgtcctccgaccagccctgcctct
ttcatttccagaccttggagaatttttcccagctttgattcagaaggtac
tagttataaccccctttccttcttcttaatccaataggcctcactctcact
gggaaatccactcaaaggaacaaggcaatgtctctcattctatttcccag
ttccaaattccaggtgcttgtctggagtgaagctacccgttactttctcc
cagcttttctccacccagcatgtctcctgcccatgcagctgaagacagtg
gggcaacctcaggagaagcagacctttccatgcccaagttcatctcctga
gcaacagtgacacctagaaaatgaggactttggaagtcacccaaaagatg
gtggctactttatggagtcctgaagatacacagccaccactcctaaaggc
aaagaaagaaaacacgaatgtaggtcagggatagagtggaaccctggtca
tcggggtttttagcctcatcgtgggaaaggtggtaaaggaggatgatggc
atctccatccctagaggccaagaattgaaatatcattgtcaaggattaga
aacaattcagcaaagaggccacaaaaagggcctgctgactcccagaagac
ctctttaaaccccaggggaggcaaatacttgctgatggagtctgggccgt
ttccatattttaaagaagacctgcctctggggcaaatgtcagcacagaga
ggactgggaggagaatggaggcaagaaaagggcatattttgactccctct
gtgcctcttcccagttcatggaaggatgtgttcagcttacccacccacag
tgaccagtgtggtggagccgctgacatctcaaggatctatttgggaaggt
gagaagagtactcattccatctgggggtgttgttccagccacatcagcct
acctggtgggatgtgggggtgtctgccaccctgtccccccctctgctgatg
tccctcccctcaggctgtccaggtgccacctgacacaggctgctgtgcaa
agacaggcggggaagcccaaacctcactcccagggaggccctcagccgcc
agagtccaggttctccagaggctacgatttgaggaggttgagggggaaga
caggagggaaagaaaagtcctacaactgtcaggaatggggcacctttccc
tgtccctaagcaaagctccctcttcccactgccctccccagccccagctc
cctgtcctccccaacacctagtgagaaagacggtgcgtggaagggagtcc
catgggcagatgcttacacgacctctttgtgaagcctcttctgggtttaa
cttcattcatcaatttattcttatgtcaaagcaatgaaacttttctttct
ggagccagataccaatacaacaggtgaacgggtttctgccacatctctac
attgacgggggatgcttgaacaaccccccctcactacacagacacacaccg
ttaaggcacaagggctggggttgagctctagatgagggactttcctgctc
ctgcaagggtgagcactgtatacacagacaaggagggtgcagtagagtga
ctcccttggatggaagtagtaccatcagaacctactattattatgacata

aattctatttacatacattgagagaatactacaatcaacactttttcctg
ggatgactttaagaggtttgagccacagcacctgaagtggcaaagatcca
tggtctttgtagggtattagagaactcttccagtcacctctgaaagcact
ctagatcttgcagctgagtggatgaagtgtaacaaatctgttgcacgctg
agaggagtcagaattagcatttttcatgaaagttccccacgtctctacta
agaatgaggaagaaaagactaagactaggtaattacacagaggcttgaaa
tgttacatcaccagagccaagtcctctcccttcagatcagttactggctg
ctacacagggacacccccaccttttcagggcatcccatgcactccacttc
tcaggatctaaggaatttgactttgtagggatcccagaaagggcactgtg
ccacttcccctggtgtgaatcagacatacattgtacattcatttctaaaa
ttcactcatgcacctcaaaccaaggtcattatccaaaaaaaaaaaaaaaa
agctctgggtggaagagtttgtaagttttaagagagggtcatttctatgt
gaggaaatgcagaaatggacagaatgattcttattcactgtttgggtctg
gagaattcccattgtggaaatcttagagatctcaagtttattaccaaggg
aataaggaaaaaaaggtgagcaggcaccaggccaagcagtggctcccttg
ccaaggaacctgaggctgcaggtttcagggaccccccttgaagaaacctct
cctggccattggccaggagaaaagagaagtctctcctgtagagtcacaag
agaagcaaaagagggtgggtcactgggtcctggacatagcccccaacccc
aagacttcccaatatggagaaactacaccaatgtttaaaaggggaaaagg
aaagaacttgtacatcaagggaagatgatttgtaaacacacagtcctgtg
cagaaagatccccttcaggaggtgtctccagcatcccaaagctgtgcgca
ccttctcttttcctgcctcaggccacctatgcatccagctgcagcccata
cccacacctgaaatccatctcttgaatcccagccaggttatataccaccc
cattgccatgtcctgtcctggccagaatgcatgctgttcccccaagcctc
gtgggagtgaggccatgggaaacagagatgagcatgtctggacaagtctg
tgatggtagtggatgagaataacccatggcaaaacacgcacattcattaa
gaaatagggcgacagattccccgttggtgaagcactgaaaggtctattac
tctcataatattgctgttttattttaatccaccagagctaccatgcaaaa
ctttcctcctgtgaaacgctccagataaagctcctctaatctccccttcc
ctcatgtcctccagctcaaacccaccttcatcccccaaaccaatctgtat
catgcctgttatcagagaggcacagaaagatgggcagtgcctccgttgtc
accattccccacacccctacacacccccacaccctcccctccaggctcca
cgacttcacagtcttactgttgtaaatatcattgtacagtttgtaatcct
caaataatcccattgtcagaggcctcgcctgatgggccttctcaccctcg
agaaaggccagggaatctagaaggggcaacccttcaaggagagcttcagg
gtcatctctgtgtgagacactattgtatattcctgtaagattgcattttt
atctaaggaatgatgttatttaaaaaacaaacaaaaaacacaaaaaataa
gaattgcaaataaatttcttaacaatgtct
>NM_005257 3
ggcacccttcggcgagcgctgtttgtttagggctcggtgagtccaatcag
gagcccaggctgcagttttccggcagagcagtaagaggcgcctcctctct
cctttttattcaccagcagcgcggcgcagaccccggactcgcgctcgccc
gctggcgccctcggcttctctccgcgcctgggagcaccctccgccgcggc
cgttctccatgcgcagcgcccgcccgaggagctagacgtcagcttggagc
ggcgccggaccgtggatggccttgactgacggcggctggtgcttgccgaa
gcgcttcggggccgcgggtgcggacgccagcgactccagagcctttccag
cgcgggagccctccacgccgccttcccccatctcttcctcgtcctcctcc
tgctcccggggcggagagcggggccccggcggcgccagcaactgcgggac
gcctcagctcgacacggaggcggcggccggaccccggcccgctcgctgc

tgctcagttcctacgcttcgcatcccttcggggctccccacggaccttcg
gcgcctggggtcgcgggccccgggggcaacctgtcgagctgggaggactt
gctgctgttcactgacctcgaccaagccgcgaccgccagcaagctgctgt
ggtccagccgcggcgccaagctgagccccttcgcacccgagcagccggag
gagatgtaccagaccctcgccgctctctccagccagggtccggccgccta
cgacggcgcgcccggcggcttcgtgcactctgcggccgcggcggcagcag
ccgcggcggcggccagctccccggtctacgtgcccaccacccgcgtgggt
tccatgctgcccggcctaccgtaccacctgcaggggtcgggcagtgggcc
agccaaccacgcgggcggcgcgggcgcgcaccccggctggcctcaggcct
cggccgacagccctccatacggcagcggaggcggcgcggctggcggcggg
gccgcggggcctggcggcgctggctcagccgcggcgcacgtctcggcgcg
cttcccctactctcccagcccgcccatggccaacggcgccgcgcgggagc
cgggaggctacgcggcggcgggcagtgggggcgcgggaggcgtgagcggc
ggcggcagtagcctggcggccatgggcggccgcgagccccagtacagctc
gctgtcggccgcgcggccgctgaacgggacgtaccaccaccaccaccacc
accaccaccaccatccgagcccctactcgccctacgtgggggcgccactg
acgcctgcctggcccgccggacccttcgagaccccggtgctgcacagcct
gcagagccgcgccggagccccgctcccggtgccccggggtcccagtgcag
acctgctggaggacctgtccgagagccgcgagtgcgtgaactgcggctcc
atccagacgccgctgtggcggcgggacggcaccggccactacctgtgcaa
cgcctgcgggctctacagcaagatgaacggcctcagccggcccctcatca
agccgcagaagcgcgtgccttcatcacggcggcttggattgtcctgtgcc
aactgtcacaccacaactaccaccttatggcgcagaaacgccgagggtga
acccgtgtgcaatgcttgtggactctacatgaaactccatggggtgccca
gaccacttgctatgaaaaaagagggaattcaaaccaggaaacgaaaacct
aagaacataaataaatcaaagacttgctctggtaatagcaataattccat
tcccatgactccaacttccacctcttctaactcagatgattgcagcaaaa
atacttcccccacaacacaacctacagcctcagggggcgggtgccccggtg
atgactggtgcgggagagagcaccaatcccgagaacagcgagctcaagta
ttcgggtcaagatgggctctacataggcgtcagtctcgcctcgccggccg
aagtcacgtcctccgtgcgaccggattcctggtgcgccctggccctggcc
tgagcccacgccgccaggaggcagggagggctccgccgcgggcctcactc
cactcgtgtctgcttttgtgcagcggtccagacagtggcgactgcgctga
cagaacgtgattctcgtgcctttattttgaaagagatgtttttcccaaga
ggcttgctgaaagagtgagagaagatggaagggaagggccagtgcaactg
ggcgcttgggccactccagccagcccgcctccggggcggaccctgctcca
cttccagaagccaggactaggacctgggccttgcctgctatggaatattg
agagagatttttttaaaaaagattttgcattttgtccaaaatcatgtgctt
cttctgatcaattttggttgttccagaatttcttcataccttttccacat
ccagatttcatgtgcgttcatggagaagatcacttgaggccatttggtac
acatctctggaggctgagtcggttcatgaggtctcttatcaaaaatatta
ctcagtttgcaagactgcattgtaactttaacatacactgtgactgacgt
ttctcaaagttcatattgtgtggctgatctgaagtcagtcggaatttgta
aacagggtagcaaacaagatatttttcttccatgtatacaataatttttt
taaaaagtgcaatttgcgttgcagcaatcagtgttaaatcatttgcataa
gatttaacagcatttttttataatgaatgtaaacattttaacttaatggta
cttaaaataatttaaaagaaaaatgttaacttagacattcttatgcttct
tttacaactacatcccattttatatttccaattgttaaagaaaaatattt
caagaacaaatcttctctcaggaaaattgcctttctctatttgttaagaa

tttttatacaagaacaccaatataccccctttattttactgtggaatatg
tgctggaaaaattgcaacaacactttactacctaacggatagcatttgta
aatactctaggtatctgtaaacactctgatgaagtctgtatagtgtgact
aacccacaggcaggttggtttacattaatttttttttttgaatgggatgt
cctatggaaacctatttcaccagagttttaaaaataaaaagggtattgtt
ttgtcttctgtacagtgagttccttccctttcaaagctttctttttatg
ctgtatgtgactatagatattcatataaaacaagtgcacgtgaagtttgc
aaaatgctttaaggccttcctttcaaagcatagtccttttggagccgttt
tgtaccttttataccttggcttatttgaagttgacacatggggttagtta
ctactctccatgtgcattggggacagtttttataagtgggaaggactcag
tattattatatttgagatgataagcattttgtttgggaacaatg
>NM_018222 4
agcgagggagggagcgagggaagggaaaggcgagcgtgagctgcctcaaa
tgcttggaataattccgcttccgtttggaaagccgcagcctcagtcccgc
cgccgcccgctgcgtccgcccagcgccagctccgcgtcccgaccggcccg
cggcagcctgcgccgcgccatggccacctccccgcagaagtcgccttctg
tccccaagtctcccactcccaagtcgcccccgtcccgcaagaaagatgat
tccttcttggggaaactcggagggaccctggcccggaggaagaaagccaa
ggaggtgtccgagctgcaggaggagggaatgaacgccatcaacctgcccc
tcagcccaattccctttgagctggaccccgaggacacgatgctggaggag
aatgaggtgcgaacaatggtggatccaaactcacgcagtgaccccaagct
tcaagaactgatgaaggtattaattgactggattaatgatgtgttggttg
gagaaagaatcattgtgaaagacctagctgaagatttgtatgatggacaa
gtcctgcagaagcttttcgagaaactggagagtgagaagctaaatgtggc
tgaggtcacccagtcagagattgctcagaagcaaaaactgcagactgtcc
tggagaagatcaatgaaaccctgaaacttcctcccaggagcatcaagtgg
aatgtggattctgttcatgccaagagcctggtggccatcttacacctgct
cgttgctctgtctcagtatttccgcgcaccaattcgactcccagaccatg
tttccatccaagtggttgtggtccagaaacgagaaggaatcctccagtct
cggcaaatccaagaggaaataactggtaacacagaggctctttccgggag
gcatgaacgtgatgcctttgacacccttgttcgaccatgccccagacaagc
tgaatgtggtgaaaaagacactcatcactttcgtgaacaagcacctgaat
aaactgaacctggaggtcacagaactggaaacccagtttgcagatggggt
gtacctggtgctgctcatggggctcctggagggctactttgtgcccctgc
acagcttcttcctgaccccggacagctttgaacagaaggtcttgaatgtc
tcctttgcctttgagctcatgcaagatggagggttggaaaagccaaaacc
gcggccagaagacatagtcaactgtgacctgaaatctacactacgagtgt
tgtacaacctcttcaccaagtaccgtaacgtggagtgaggggctgccctg
ggcccaccactgcccaagagttcttgctgttggcgtactggaccctcctc
cgaactgccttaccctgcttattcctgtctcttgcactgtgctctcccac
aagtccagctgcaacccagagatagtggaaactgaaattaggaaggaaat
catcaataactcagtgggctgacccatccctcccaggcgctggggaccaa
cctagcaatgaaggttgggaaggttgttcccttcccggtgccaggtccag
atttccctccatgatttgggaaccagcttaggcaaaagagtccccacaag
atgaaaataaagatcctagttaccattcaaaggatgctaactgtgtgtca
ggccccacactaagtgctctgctctgatatactcaaggccattaatcttc
aggactcccattgacgtaggtgtttcattccccttttacagatgaggaaa
ctaaggcttggaggttaaatgacttgccagaagttggaattttttttcctc
tttgaacataacctctcccttctccctaaaggtaaccactattctgagtc

caatcatcaaggttttgcttttctttttagctaagtatgcattcctcaat
agtagacagtacaacatgtttataacaagccaattacattatgttctttg
catgttctaaagttgtgtatgtgtgtgcacatctgagcacgtgcacatgt
acacctgagccaaaaacacgagaacccactgatctcaccactggggcaag
ctaggtcagagcttagtgattcacactgaaattggcaaattggatttaac
ccaattaatagtgtgtgtgtggcaggagtcatgtccctcacatcctttgt
acaaatgaaaattactcttaattccttcagatttataataactctgtact
ttggtttcagggtgacatttgggaaggattttgtttagaattaatggagt
ggcacattttgcagcctttttgcttgattgcatgtaatggaaatgcccta
tattttcctgcaaaataagtactaaattcattatcgttaagcaaatgtac
aatatgctcaggcaccgcagagagctgggcacgggcccatgtgagcatca
ctttggaagtagggctcttcaacagggacccttgaactttaaagaaagga
acttctttttgccttctaattgatcatttagactattctggctaagtctg
cccacatgtaattaccggctaattcaagcgaggaaaaatgtaagtcattt
agaccaaagccaagcagtttctttgcgtgggttactcaagggcttgtggt
tacttgtatctcctctatgtgaacttgactttgaaagacagagctctagt
gtgccagcctgctaagtcctgtaagaatagggaagggcggagggggggtgg
gcagtgactaggggacgagaagcatggggaaaatatttgcactctaaaca
tacagagatagaggtggggcttgtggtacttaacacttgtagccatcaac
tgactgagaccttgggctaaataatcaattgtgctgatattacatctcgt
tatggaatgttcctaaatatgccaggtagacaccagcccaagtaccctcc
tccagaagtctgtgactaccttgtcactactttaggcccattccacaaag
cccatctctggtttgagaattcattttgatctgtatctacaccacccaaa
gttaggcctcctataatgtccaaaacattcctttcagcctttttatttct
tactgtactgtctcttactgtactgtctatctgcagtaattgaggaccca
taaaatttagataactacatgtctttgctcttagaattgtcactcagcat
aatgagcatttaacatacaaaggcaatgtactgttttgtgttgatctatg
taaaagaatacaattctttttttacataattagtgaaattttattttttat
taggaaacactaaatagtgtaatatttcttttgcttttaaaaaaaattcct
ggtagcaaatcaagataaataattgcttcattttcttgagcaatactgaa
gcaggatgaagtaagaggaatgcattcatttaaacatgctttgctttatg
aattttgtctctttttttggtctctttttcttatattcaagttacaaatgt
acaagtatccttactaagagtgctccttttgtattttacatatatacagt
atgaaaatacattggaacactaggaaagtttttaaataacagttctaatt
tatcagaaaattgtgttttgggattgagttctttgtctcagcccagaatc
ccaggtcctgggcctggttttctaatgctgtcatctcagttcgatatttt
actttagaacctggaatctcctacttaatatatgaccatgactttgaaag
gcaaaagaggaatcaagaataaataaaacaaacttaatcttcatctttaa
aaaaaagaaaaaagaaaccaaaatgagaatcaacacttcataggctcact
gggttttcttttcttttctgttaatttaaactcagttatttttaatgctt
aatacatacatggtgcaaaatttaaaaagcgcaaataggtatctagtgga
aaacctaagcctccctctctcctccggcacccattacctctccctggagg
caactgttttgatccatttcttacacacactgccagagatactctaggca
tgtaaagcacaaacatacatataaaatctgcgggcttcaaaaaatataag
taggatgtcatctatactgtcatacactttgttttttatcacttaatgtt
atatcttggatattgtattaccctgggtattaaaaagaactcctttcaca
ttttaaaataacaatctgagcacttcataaatccaaatgcgtatctccag
tctgctcgagctaggaaaggaaaaaagccctagccgaaggaggaaaggct
gcctagacaatgatttcaaaagtagagtctaaaacctatggggtgcagga

ccagaggctggggcacattatcagaggcatcctcatgtgcctcgaaccga
atgtttttaagtcctcttttggaaccttatggtgaattgtatgcagattg
atgaaggtttcatttttatatattaccaggactacgaatttgtaatccac
taagcatcacaaaggttactacatacgctataatactgtactataaagtc
tagctgccagcaatgcagtgctcgcctgctatattagaaggctccagttt
cacatgtgcaggcacagagatgacatttcccgatctgggagaaggcttct
ttatcagcacatcatcagtgttgcctcgagttggttgcaattttcaattg
ctgctattggtgctttttaattatgaattactgttagcttgcattatcat
cagtttgttttgttgtgggttgcttggttaaccctacagagtatacttga
agcttatttgcatcaacagtattctggagctgtaatttttcaataactag
actctgagacatgtatacattgtggttcaaataggaatcatccatagtcc
agtgctagaacaaatgtatacataatccactccccacaaattatcactca
tttatttttccactggaaaataagactagatggagtcggtaggtgaaaca
gttttgccgtgcctaagtggaggctgatcaaaactaatttgagatccgct
gcctttaatagagctccacctcaggaatcttacctttataatacatccta
gataggcaagagggatatgtcctgaaatcttcactaattccaagattgcg
tgtcatttcccccaaaaatttgcttaactctattggaaaatccaagagaa
ttgctgcagctgctgaaaacaagagagctgcattaagctgcaaggccggg
tagggtaggtagactgactcaccacctatgggacctgcctccacacatca
gcttcccaaatacttgcagatttgggtcttaccaaaagaaaagtcagtat
gagttactcacaatcctaactacaaaggctacatttacaaataccagagt
ttcgactgtgtttttactctctgctccctgaaaactgtaaccccaggcta
gctccaaactctgcttatagcccatgctttactacagtggtggtcgcata
tgtgaacgtgtgcatgcgtgttcacgtgttcatgacaacagcaatgcagc
catgagggtgctgcccccaaaccaagcctgatgaccttgagaacctcagg
cttcccagtaggatcctgcaagaaagcatctcttaaaaataaattctaca
ggatattagtaaatgttagatgaaaaaaaaagtcacgtttaaatatgttt
aggaaacatgaggtgaagaaagttagcttactgacgctttttgttttcta
gttggagcgcacctcagagtcccctacataatgcgtattaggactttcta
agggaagacggggtacgatgggtcccaatcttgtttgatcacaggatgta
tctgtcccctgagaaatttctaggatgagtactctgaactcaggacttca
tttagtcatatattcggcaagtatgtgttgagtgcagcatgtccaagggt
gagtgagagattatagctagcaggggagacagatgaaaaataactatgaa
tcgacgttagttacaaattgtgactgtcgcgatggagaagtactaaaatc
tatgaaagagttctaatgtagatttaaggtcatgagaagtctccggcaaa
gtggcattttaaagtaatccctcagtcgtggagctactccaatgagaagc
ctgccactccagggcgcaccacggaggaggatccccagacaagaagacct
ggctccccagaggagtgcggaaagccagcatggctagaggacacagaatg
agggagaagacggatccgatcgcaggcatcgggagtgctgatttttctcc
tttgaaaaacaggttgccatctacctttttaaatgtcccactgtgtagga
aaactctggggaaagctacgtcagcaatatggagtctggggttgcctttc
tcatgtcataccatggtcctggctggggagcggaggagaccaggagagga
gatcagacctctgcccaccccaacaccaatctggtcttagcaacctctgg
gcatcccatagctcctcctaacctgctgctgctgatggacaggaactcct
ggactacccccagcctctccacaatgtgtcctgagaagagctaggggaac
tggacagagtggacgaggctgcatgtgtggagggaatgaaataaggggcc
taccaggccagaacgctgatccatggagaccagtgcttccagccctctca
cctcggaggaggactcctgttttaccaacggcaggagcaggaatcccacc
attcagcctgtgggcttgacaaactataagcaagcctcctggatgtggaa

taatacattttcaaaattcatactaatcagcatgaactataactgctcca
atgcccaccacattatataaaatctaccaaaagggggtcacttaatgttt
cacaatgtttggtgaaacaactttcatatgaaagtacggttgatccttga
atgatatgggcaccgaccccctgagcagctgaaaatttgcatgtaacttga
ctcccccaaaacgtaactataatactaatagccttactgaacacggtcaa
ttaacacatagctagtatgttatgtgcattatatactatatccttaccat
acggtaagctagagaaaaaatgttactaagaaaaacataagcaagagaaa
atatatttactattaagtgaaagcaggtcatcataaagtcctcatcctca
tggtcatcacattgagtaggcggaggaggaggaggaggaggaggagtagg
ggttggtcttgctgtctgaggggtggcagaggcagaaaaaaaatccatgg
gccgagcacggtggctcacacctgtaatcccagcactttgggaggccgag
acggacagatcacaaggtcaggagattgagactatcctggctaatatggt
gaaaccccgtctgtactaaaaatacaaaaaattagccaggtgtggtggtg
cgtgcctgtaatcccagctactcagggggctgaggcagaagtattgcttg
aacccaggaggcagaggttgcaatgagctgagatcgcatcactgcactca
agcctgggcaacagagcaagactgcgtctcaaaaaaacaaaacaaaacaa
aacaaaaatccatgaacaagtggacctgcccagttcaaatcattgttgtt
caagggtcaattgtatattattttcactggaagttttgaaattccagaaa
tatgcttggaaatccccgcttgaaatctccttggttggaaaccagtccag
tgggtgactggcccagaatgatgcttctcctcggtggacccccctgggctg
agcagcctgtgttcctgcctcaactcccctgaaccctcctgaactttcta
cacacatgtgggcactggctttgatactggcatttggaagggcacttggg
aaattcagagaagtacccattattaagataaatgatgttttatcaatgaa
gcagacttttcatttctttttattgatcttcatgttttcacatcttgaga
tgcaatttgttagcacaggctgtcattccaagacacacaaatgtcattaa
ggcaaccgcttaaaggagtgtgatattttattgaggtagacaggacaata
gataaatatttaatctgttacatgtttgctctgtgtggagccagggttgg
ggctgcacaactctctggctgctatgtgtcttcctggaaaccctgtcaaa
ggccttaccgcctgcctggagaaacacagtgcctgcccttggcaaatata
tgttggtgtatctgaaaaacagctcctggaagctttttctcattcaggct
ttaggggttaccccatctttccttatgtgtgtaatattggagaatgtaca
ctctcactgaactggggatgtttgacttaaaatgatggacaataagatag
tgagcagtaagtgtgctctaggctaggctacgagaggccatgagctcctc
atctcttctctgttctgagctctctgatccactgcacttggggcaggggg
tgcattctctgtgcctctcctgagtctactttctgcatcattggttctcc
cagctcacttccataatgtcctcctaggctgcattggaattgtgtgttgt
ctagacccatggccaagactgtcattgcctgtgagggagaccaagctcac
caccaagggcttttgccagattgctttcatttacagaatttgcccattca
tgtgtctttgtgtttatggattaaatggctttctgaccagcatccctttg
gtgtgctttgtcaataaga
>NM_004684 4
aaaaatgcataaagagccaagtgcttatattctggccaagttatgaggct
ctgagaacaagagcttgaggggaagactgttaaccccatccacgccacca
gaattagctctttccccttttggtttgcaagcactgcctgtaaagccctcg
catgagaggccagcctgctagggaaatccaggaatctgcaacaaaaacga
tgacagtctgaaatactctctggtgccaacctccaaattctcgtctgtca
cttcagacccccactagttgacagagcagcagaatttcaactccagtaga
cttgaatatgcctctgggcaaagaagcagagctaacgaggaaagggattt
aaagagtttttcttgggtgtttgtcaaacttttattccctgtctgtgtgc

agaggggattcaacttcaatttttctgcagtggctctgggtccagcccct
tacttaaagatctggaaagcatgaagactgggctttttttcctatgtctc
ttgggaactgcagctgcaatcccgacaaatgcaagattattatctgatca
ttccaaaccaactgctgaaacggtagcacctgacaacactgcaatcccca
gtttaagggctgaagctgaagaaaatgaaaaagaaacagcagtatccaca
gaagacgattcccaccataaggctgaaaaatcatcagtactaaagtcaaa
agaggaaagccatgaacagtcagcagaacagggcaagagttctagccaag
agctgggattgaaggatcaagaggacagtgatggtcacttaagtgtgaat
ttggagtatgcaccaactgaaggtacattggacataaaagaagatatgag
tgagcctcaggagaaaaaactctcagagaacactgatttttggctcctg
gtgttagttccttcacagattctaaccaacaagaaagtatcacaaagaga
gaggaaaaccaagaacaacctagaaattattcacatcatcagttgaacag
gagcagtaaacatagccaaggcctaagggatcaaggaaaccaagagcagg
atccaaatatttccaatggagaagaggaagaagaaaaagagccaggtgaa
gttggtacccacaatgataaccaagaaagaaagacagaattgcccaggga
gcatgctaacagcaagcaggaggaagacaatacccaatctgatgatattt
tggaagagtctgatcaaccaactcaagtaagcaagatgcaggaggatgaa
tttgatcagggtaaccaagaacaagaagataactccaatgcagaaatgga
agaggaaaatgcatcgaacgtcaataagcacattcaagaaactgaatggc
agagtcaagagggtaaaactggcctagaagctatcagcaaccacaaagag
acagaagaaaagactgtttctgaggctctgctcatggaacctactgatga
tggtaataccacgcccagaaatcatggagttgatgatgatggcgatgatg
atggcgatgatggcggcactgatggccccaggcacagtgcaagtgatgac
tacttcatcccaagccaggcctttctggaggccgagagagctcaatccat
tgcctatcacctcaaaattgaggagcaaagagaaaaagtacatgaaaatg
aaaatataggtaccactgagcctggagagcaccaagaggccaagaaagca
gagaactcatcaaatgaggaggaaacgtcaagtgaaggcaacatgagggt
gcatgctgtggattcttgcatgagcttccagtgtaaaagaggccacatct
gtaaggcagaccaacagggaaaacctcactgtgtctgccaggatccagtg
acttgtcctccaacaaaaccccttgatcaagtttgtggcactgacaatca
gacctatgctagttcctgtcatctattcgctactaaatgcagactggagg
ggaccaaaaaggggcatcaactccagctggattattttggagcctgcaaa
tctattcctacttgtacggactttgaagtgattcagtttcctctacggat
gagagactggctcaagaatatcctcatgcagctttatgaagccaactctg
aacacgctggttatctaaatgagaagcagagaaataaagtcaagaaaatt
tacctggatgaaaagaggcttttggctggggaccatcccattgatcttct
cttaagggactttaagaaaaactaccacatgtatgtgtatcctgtgcact
ggcagtttagtgaacttgaccaacaccctatggatagagtcttgacacat
tctgaacttgctcctctgcgagcatctctggtgcccatggaacactgcat
aacccgtttctttgaggagtgtgaccccaacaaggataagcacatcaccc
tgaaggagtggggccactgctttggaattaaagaagaggacatagatgaa
aatctcttgttttgaacgaagattttaaagaactcaactttccagcatcc
tcctctgttctaaccacttcagaaatatatgcagctgtgatacttgtaga
tttatatttagcaaaatgttagcatgtatgacaagacaatgagagtaatt
gcttgacaacaacctatgcaccaggtatttaacattaactttggaaacaa
aaatgtacaattaagtaaagtcaacatatgcaaaatactgtacattgtga
acagaagtttaattcatagtaatttcactctctgcattgacttatgagat
aattaatgattaaactattaatgataaaaataatgcatttgtattgttca
taatatcatgtgcacttcaagaaaatggaatgctactcttttgtggttta

cgtgtattattttcaatatcttaataccctaataaagagtccataaaaat
ccaaaaaaaaaaaaaaaaaaaaaaaaaaaaaaaaaaaaaaaaaaaaaaaa
aaaaaa
>NM_004684 4
aaaaatgcataaagagccaagtgcttatattctggccaagttatgaggct
ctgagaacaagagcttgaggggaagactgttaaccccatccacgccacca
gaattagctctttccctttggtttgcaagcactgcctgtaaagccctcg
catgagaggccagcctgctagggaaatccaggaatctgcaacaaaaacga
tgacagtctgaaatactctctggtgccaacctccaaattctcgtctgtca
cttcagacccccactagttgacagagcagcagaatttcaactccagtaga
cttgaatatgcctctgggcaaagaagcagagctaacgaggaaagggattt
aaagagtttttcttgggtgtttgtcaaacttttattccctgtctgtgtgc
agaggggattcaacttcaatttttctgcagtggctctgggtccagcccct
tacttaaagatctggaaagcatgaagactgggcttttttcctatgtctc
ttgggaactgcagctgcaatcccgacaaatgcaagattattatctgatca
ttccaaaccaactgctgaaacggtagcacctgacaacactgcaatcccca
gtttaagggctgaagctgaagaaaatgaaaaagaaacagcagtatccaca
gaagacgattcccaccataaggctgaaaaatcatcagtactaaagtcaaa
agaggaaagccatgaacagtcagcagaacagggcaagagttctagccaag
agctgggattgaaggatcaagaggacagtgatggtcacttaagtgtgaat
ttggagtatgcaccaactgaaggtacattggacataaaagaagatatgag
tgagcctcaggagaaaaaactctcagagaacactgatttttggctcctg
gtgttagttccttcacagattctaaccaacaagaaagtatcacaaagaga
gaggaaaaccaagaacaacctagaaattattcacatcatcagttgaacag
gagcagtaaacatagccaaggcctaagggatcaaggaaaccaagagcagg
atccaaatatttccaatggagaagaggaagaagaaaaagagccaggtgaa
gttggtacccacaatgataaccaagaaagaaagacagaattgcccaggga
gcatgctaacagcaagcaggaggaagacaatacccaatctgatgatattt
tggaagagtctgatcaaccaactcaagtaagcaagatgcaggaggatgaa
tttgatcagggtaaccaagaacaagaagataactccaatgcagaaatgga
agaggaaaatgcatcgaacgtcaataagcacattcaagaaactgaatggc
agagtcaagagggtaaaactggcctagaagctatcagcaaccacaaagag
acagaagaaaagactgtttctgaggctctgctcatggaacctactgatga
tggtaataccacgcccagaaatcatggagttgatgatgatggcgatgatg
atggcgatgatggcggcactgatggccccaggcacagtgcaagtgatgac
tacttcatcccaagccaggcctttctggaggccgagagagctcaatccat
tgcctatcacctcaaaattgaggagcaaagagaaaaagtacatgaaaatg
aaaatataggtaccactgagcctggagagcaccaagaggccaagaaagca
gagaactcatcaaatgaggaggaaacgtcaagtgaaggcaacatgagggt
gcatgctgtggattcttgcatgagcttccagtgtaaaagaggccacatct
gtaaggcagaccaacagggaaaacctcactgtgtctgccaggatccagtg
acttgtcctccaacaaaaccccttgatcaagtttgtggcactgacaatca
gacctatgctagttcctgtcatctattcgctactaaatgcagactggagg
ggaccaaaaaggggcatcaactccagctggattattttggagcctgcaaa
tctattcctacttgtacggactttgaagtgattcagtttcctctacggat
gagagactggctcaagaatatcctcatgcagctttatgaagccaactctg
aacacgctggttatctaaatgagaagcagagaaataaagtcaagaaaatt
tacctggatgaaaagaggcttttggctggggaccatcccattgatcttct
cttaagggactttaagaaaaactaccacatgtatgtgtatcctgtgcact

ggcagtttagtgaacttgaccaacaccctatggatagagtcttgacacat
tctgaacttgctcctctgcgagcatctctggtgcccatggaacactgcat
aacccgtttctttgaggagtgtgaccccaacaaggataagcacatcaccc
tgaaggagtggggccactgctttggaattaaagaagaggacatagatgaa
aatctcttgttttgaacgaagattttaaagaactcaactttccagcatcc
tcctctgttctaaccacttcagaaatatatgcagctgtgatacttgtaga
tttatatttagcaaaatgttagcatgtatgacaagacaatgagagtaatt
gcttgacaacaacctatgcaccaggtatttaacattaactttggaaacaa
aaatgtacaattaagtaaagtcaacatatgcaaaatactgtacattgtga
acagaagtttaattcatagtaatttcactctctgcattgacttatgagat
aattaatgattaaactattaatgataaaaataatgcatttgtattgttca
taatatcatgtgcacttcaagaaaatggaatgctactcttttgtggttta
cgtgtattattttcaatatcttaataccctaataaagagtccataaaaat
ccaaaaaaaaaaaaaaaaaaaaaaaaaaaaaaaaaaaaaaaaaaaaaaaa
aaaaaa
>NM_017426 2
ggcctcttaggctaggtcagaaaggaggcggaactcggtgatctgactgg
cggtttcccggccggactgagaaggggagcgcgctgcgcgtcgcaggagt
aacctacttggtctcctgctttcgcgacatggccttcaattttggggctc
cctcgggcacctccggtaccgctgcagccaccgcggcccccgcgggtggg
tttggaggatttgggacaacatctacaactgcaggttctgcattcagctt
ttctgccccaactaacacaggcactactggactctttggtggtactcaga
acaaaggttttggatttggtactggttttggcacaacaacgggaactagt
actggtttaggtactggtttgggaactggactgggatttggaggatttaa
tacacagcagcagcagcaaactacattaggtggtctcttcagtcagccta
cacaagctcctacccagtccaaccagctgataaatactgcgagtgctctt
tctgctccaacgctgttgggagatgagagagatgctattttggcaaaatg
gaatcaactgcaggccttttggggaacaggaaaagggtatttcaacaata
atattccgccagtggaattcacacaagaaaatcccttttgccgatttaag
gcagtaggttatagttgcatgcccagtaataaagatgaagatgggctagt
ggttttagttttcaacaaaaaagaaacagagattcgaagccaacaacaac
agttggtagaatcattgcataaagttttgggaggaaaccagacccttact
gtaaatgtagagggcactaaaacattgccagatgatcagacacagaagttgt
tatttatgttgttgagcgttcgccaaatggtacttcaagaagagttccag
ctacaacgctatatgcccattttgaacaagccaatataaaaacacaattg
cagcaacttggtgtaaccctttctatgactagaacagaactttctcctgc
acagatcaaacagcttttacagaatcctcctgctggtgttgatcctatta
tctgggaacaggccaaggtagataaccctgattctgaaaagttaattcct
gtaccaatggtgggttttaaggaacttctccgaagactgaaggttcaaga
tcagatgactaagcagcatcaaaccagattagatatcatatctgaagata
ttagtgagctacaaaagaatcaaactacatctgtagccaaaattgcacaa
tacaagaggaaactcatggatctttcccatagaactttacaggtcctaat
caaacaggaaattcaaaggaagagtggttatgccattcaggctgatgaag
agcagttgcgagttcagctggatacgattcagggtgaactaaatgcacct
actcagttcaagggccgactaaatgaattgatgtctcaaatcaggatgca
gaatcattttggagcagtcagatctgaagaaaggtattacatagatgcag
atctgttacgagaaatcaagcagcatttgaaacaacaacaggaaggcctt
agccatttgattagcatcattaaagacgatctagaagatataaagctggt
cgaacatggattgaatgaaaccatccacatcagaggtggtgtctttagtt

gacagttcacaaacttgtgtaaaggtttgtgaaatgcatcttcttactgc
atcagaccttccttaagaatgaaaccgaccacatggagggaaaaagaaaa
caattctttcttggattggtttttgagaagtttactgacaaattactgt
tcatcaaatctgaaatagtcacctcacagctcttcaaagaaaacctttga
aagatttatatctaaaagctgtatttactttaaaagaagtgcataattac
caaaattgtatgtactattgtacatttttacaacagcattttcttaaaca
taatctgtgtttaatgattattgtccattgagcctgtactctgctttcca
taccaagtaaatatgaaataatctactttgcacataacagaacaaactat
aattacttggctgttggagatttgtacttgagtataaatgtacaccagtt
tttgtatttgtgaactcatctgtgggaggagtaaagaaaatccaaaagca
tttaatgttttgtttttgttctataaagatatgaaaatgtatttttatat
tattttacttatttggaatttacagagcacacctaagcaattaggatata
acaaaactacttaaccatttttgcaaccattttgttttttaagccttttt
atttctaaaaagatgaaaacttataaataaattcttaatttgtaattact
tttaaaaa
>NM_018222 4
agcgagggagggagcgagggaagggaaaggcgagcgtgagctgcctcaaa
tgcttggaataattccgcttccgtttggaaagccgcagcctcagtcccgc
cgccgcccgctgcgtccgcccagcgccagctccgcgtcccgaccggcccg
cggcagcctgcgccgcgccatggccacctccccgcagaagtcgccttctg
tccccaagtctcccactcccaagtcgcccccgtcccgcaagaaagatgat
tccttcttggggaaactcggagggaccctggcccggaggaagaaagccaa
ggaggtgtccgagctgcaggaggagggaatgaacgccatcaacctgcccc
tcagcccaattccctttgagctggaccccgaggacacgatgctggaggag
aatgaggtgcgaacaatggtggatccaaactcacgcagtgaccccaagct
tcaagaactgatgaaggtattaattgactggattaatgatgtgttggttg
gagaaagaatcattgtgaaagacctagctgaagatttgtatgatggacaa
gtcctgcagaagcttttcgagaaactggagagtgagaagctaaatgtggc
tgaggtcacccagtcagagattgctcagaagcaaaaactgcagactgtcc
tggagaagatcaatgaaaccctgaaacttcctcccaggagcatcaagtgg
aatgtggattctgttcatgccaagagcctggtggccatcttacacctgct
cgttgctctgtctcagtatttccgcgcaccaattcgactcccagaccatg
tttccatccaagtggttgtggtccagaaacgagaaggaatcctccagtct
cggcaaatccaagaggaaataactggtaacacagaggctctttccgggag
gcatgaacgtgatgcctttgacaccttgttcgaccatgccccagacaagc
tgaatgtggtgaaaaagacactcatcactttcgtgaacaagcacctgaat
aaactgaacctggaggtcacagaactggaaacccagtttgcagatggggt
gtacctggtgctgctcatggggctcctggagggctactttgtgcccctgc
acagcttcttcctgaccccggacagctttgaacagaaggtcttgaatgtc
tcctttgcctttgagctcatgcaagatggagggttggaaaagccaaaacc
gcggccagaagacatagtcaactgtgacctgaaatctacactacgagtgt
tgtacaacctcttcaccaagtaccgtaacgtggagtgaggggctgccctg
ggcccaccactgcccaagagttcttgctgttggcgtactggaccctcctc
cgaactgccttaccctgcttattcctgtctcttgcactgtgctctcccac
aagtccagctgcaacccagagatagtggaaactgaaattaggaaggaaat
catcaataactcagtgggctgacccatccctcccaggcgctggggaccaa
cctagcaatgaaggttgggaaggttgttcccttcccggtgccaggtccag
atttccctccatgatttgggaaccagcttaggcaaaagagtccccacaag
atgaaaataaagatcctagttaccattcaaaggatgctaactgtgtgtca

ggccccacactaagtgctctgctctgatatactcaaggccattaatcttc
aggactcccattgacgtaggtgtttcattccccttttacagatgaggaaa
ctaaggcttggaggttaaatgacttgccagaagttggaatttttttcctc
tttgaacataacctctcccttctccctaaaggtaaccactattctgagtc
caatcatcaaggttttgcttttctttttagctaagtatgcattcctcaat
agtagacagtacaacatgtttataacaagccaattacattatgttctttg
catgttctaaagttgtgtatgtgtgtgcacatctgagcacgtgcacatgt
acacctgagccaaaaacacgagaacccactgatctcaccactggggcaag
ctaggtcagagcttagtgattcacactgaaattggcaaattggatttaac
ccaattaatagtgtgtgtgtggcaggagtcatgtccctcacatcctttgt
acaaatgaaaattactcttaattccttcagatttataataactctgtact
ttggtttcagggtgacatttgggaaggattttgtttagaattaatggagt
ggcacattttgcagcctttttgcttgattgcatgtaatggaaatgcccta
tattttcctgcaaaataagtactaaattcattatcgttaagcaaatgtac
aatatgctcaggcaccgcagagagctgggcacgggcccatgtgagcatca
ctttggaagtagggctcttcaacagggacccttgaactttaaagaaagga
acttctttttgccttctaattgatcatttagactattctggctaagtctg
cccacatgtaattaccggctaattcaagcgaggaaaaatgtaagtcattt
agaccaaagccaagcagtttctttgcgtgggttactcaagggcttgtggt
tacttgtatctcctctatgtgaacttgactttgaaagacagagctctagt
gtgccagcctgctaagtcctgtaagaatagggaagggcggagggggggtgg
gcagtgactaggggacgagaagcatggggaaaatatttgcactctaaaca
tacagagatagaggtggggcttgtggtacttaacacttgtagccatcaac
tgactgagaccttgggctaaataatcaattgtgctgatattacatctcgt
tatggaatgttcctaaatatgccaggtagacaccagcccaagtaccctcc
tccagaagtctgtgactaccttgtcactactttaggcccattccacaaag
cccatctctggtttgagaattcattttgatctgtatctacaccacccaaa
gttaggcctcctataatgtccaaaacattcctttcagcctttttatttct
tactgtactgtctcttactgtactgtctatctgcagtaattgaggaccca
taaaatttagataactacatgtctttgctcttagaattgtcactcagcat
aatgagcatttaacatacaaaggcaatgtactgttttgtgttgatctatg
taaaagaatacaattctttttttacataattagtgaaattttattttttat
taggaaacactaaatagtgtaatatttcttttgcttttaaaaaaaattcct
ggtagcaaatcaagataaataattgcttcattttcttgagcaatactgaa
gcaggatgaagtaagaggaatgcattcatttaaacatgctttgctttatg
aattttgtctctttttttggtctctttttcttatattcaagttacaaatgt
acaagtatccttactaagagtgctccttttgtattttacatatatacagt
atgaaaatacattggaacactaggaaagtttttaaataacagttctaatt
tatcagaaaattgtgttttgggattgagttctttgtctcagcccagaatc
ccaggtcctgggcctggttttctaatgctgtcatctcagttcgatatttt
actttagaacctggaatctcctacttaatatatgaccatgactttgaaag
gcaaaagaggaatcaagaataaataaaacaaacttaatcttcatctttaa
aaaaaagaaaaaagaaaccaaaatgagaatcaacacttcataggctcact
gggttttcttttcttttctgttaatttaaactcagttatttttaatgctt
aatacatacatggtgcaaaatttaaaaagcgcaaataggtatctagtgga
aaacctaagcctccctctctcctccggcacccattacctctccctggagg
caactgttttgatccatttcttacacacactgccagagatactctaggca
tgtaaagcacaaacatacatataaaatctgcgggcttcaaaaaatataag
taggatgtcatctatactgtcatacactttgtttttttatcacttaatgtt

atatcttggatattgtattaccctgggtattaaaaagaactcctttcaca
ttttaaaataacaatctgagcacttcataaatccaaatgcgtatctccag
tctgctcgagctaggaaaggaaaaaagccctagccgaaggaggaaaggct
gcctagacaatgatttcaaaagtagagtctaaaacctatggggtgcagga
ccagaggctggggcacattatcagaggcatcctcatgtgcctcgaaccga
atgtttttaagtcctcttttggaaccttatggtgaattgtatgcagattg
atgaaggtttcatttttatatattaccaggactacgaatttgtaatccac
taagcatcacaaaggttactacatacgctataatactgtactataaagtc
tagctgccagcaatgcagtgctcgcctgctatattagaaggctccagttt
cacatgtgcaggcacagagatgacatttcccgatctgggagaaggcttct
ttatcagcacatcatcagtgttgcctcgagttggttgcaattttcaattg
ctgctattggtgctttttaattatgaattactgttagcttgcattatcat
cagtttgttttgttgtgggttgcttggttaaccctacagagtatacttga
agcttatttgcatcaacagtattctggagctgtaatttttcaataactag
actctgagacatgtatacattgtggttcaaataggaatcatccatagtcc
agtgctagaacaaatgtatacataatccactccccacaaattatcactca
tttatttttccactggaaaataagactagatggagtcggtaggtgaaaca
gttttgccgtgcctaagtggaggctgatcaaaactaatttgagatccgct
gcctttaatagagctccacctcaggaatcttacctttataatacatccta
gataggcaagagggatatgtcctgaaatcttcactaattccaagattgcg
tgtcatttcccccaaaaatttgcttaactctattggaaaatccaagagaa
ttgctgcagctgctgaaaacaagagagctgcattaagctgcaaggccggg
tagggtaggtagactgactcaccacctatgggacctgcctccacacatca
gcttcccaaatacttgcagatttgggtcttaccaaaagaaaagtcagtat
gagttactcacaatcctaactacaaaggctacatttacaaataccagagt
ttcgactgtgtttttactctctgctccctgaaaactgtaaccccaggcta
gctccaaactctgcttatagcccatgctttactacagtggtggtcgcata
tgtgaacgtgtgcatgcgtgttcacgtgttcatgacaacagcaatgcagc
catgagggtgctgcccccaaaccaagcctgatgaccttgagaacctcagg
cttcccagtaggatcctgcaagaaagcatctcttaaaaataaattctaca
ggatattagtaaatgttagatgaaaaaaaaagtcacgtttaaatatgttt
aggaaacatgaggtgaagaaagttagcttactgacgctttttgttttcta
gttggagcgcacctcagagtcccctacataatgcgtattaggactttcta
agggaagacggggtacgatgggtcccaatcttgtttgatcacaggatgta
tctgtcccctgagaaatttctaggatgagtactctgaactcaggacttca
tttagtcatatattcggcaagtatgtgttgagtgcagcatgtccaagggt
gagtgagagattatagctagcaggggagacagatgaaaaataactatgaa
tcgacgttagttacaaattgtgactgtcgcgatggagaagtactaaaatc
tatgaaagagttctaatgtagatttaaggtcatgagaagtctccggcaaa
gtggcattttaaagtaatccctcagtcgtggagctactccaatgagaagc
ctgccactccagggcgcaccacggaggaggatccccagacaagaagacct
ggctccccagaggagtgcggaaagccagcatggctagaggacacagaatg
agggagaagacggatccgatcgcaggcatcgggagtgctgatttttctcc
tttgaaaaacaggttgccatctaccttttttaaatgtcccactgtgtagga
aaactctggggaaagctacgtcagcaatatggagtctggggttgcctttc
tcatgtcataccatggtcctggctggggagcggaggagaccaggagagga
gatcagacctctgcccaccccaacaccaatctggtcttagcaacctctgg
gcatcccatagctcctcctaacctgctgctgctgatggacaggaactcct
ggactacccccagcctctccacaatgtgtcctgagaagagctaggggaac

tggacagagtggacgaggctgcatgtgtggagggaatgaaataaggggcc
taccaggccagaacgctgatccatggagaccagtgcttccagccctctca
cctcggaggaggactcctgttttaccaacggcaggagcaggaatcccacc
attcagcctgtgggcttgacaaactataagcaagcctcctggatgtggaa
taatacattttcaaaattcatactaatcagcatgaactataactgctcca
atgcccaccacattatataaaatctaccaaaaggggtcacttaatgttt
cacaatgtttggtgaaacaactttcatatgaaagtacggttgatccttga
atgatatgggcaccgacccctgagcagctgaaaatttgcatgtaacttga
ctcccccaaaacgtaactataatactaatagccttactgaacacggtcaa
ttaacacatagctagtatgttatgtgcattatatactatatccttaccat
acggtaagctagagaaaaaatgttactaagaaaaacataagcaagagaaa
atatatttactattaagtgaaagcaggtcatcataaagtcctcatcctca
tggtcatcacattgagtaggcggaggaggaggaggaggaggaggagtagg
ggttggtcttgctgtctgaggggtggcagaggcagaaaaaaaatccatgg
gccgagcacggtggctcacacctgtaatcccagcactttgggaggccgag
acggacagatcacaaggtcaggagattgagactatcctggctaatatggt
gaaaccccgtctgtactaaaaatacaaaaaattagccaggtgtggtggtg
cgtgcctgtaatcccagctactcagggggctgaggcagaagtattgcttg
aacccaggaggcagaggttgcaatgagctgagatcgcatcactgcactca
agcctgggcaacagagcaagactgcgtctcaaaaaaacaaaacaaaacaa
aacaaaaatccatgaacaagtggacctgcccagttcaaatcattgttgtt
caagggtcaattgtatattattttcactggaagttttgaaattccagaaa
tatgcttggaaatccccgcttgaaatctccttggttggaaaccagtccag
tgggtgactggcccagaatgatgcttctcctcggtggacccccctgggctg
agcagcctgtgttcctgcctcaactcccctgaaccctcctgaactttcta
cacacatgtgggcactggctttgatactggcatttggaagggcacttggg
aaattcagagaagtacccattattaagataaatgatgttttatcaatgaa
gcagacttttcatttctttttattgatcttcatgttttcacatcttgaga
tgcaatttgttagcacaggctgtcattccaagacacacaaatgtcattaa
ggcaaccgcttaaaggagtgtgatattttattgaggtagacaggacaata
gataaatatttaatctgttacatgtttgctctgtgtggagccagggttgg
ggctgcacaactctctggctgctatgtgtcttcctggaaaccctgtcaaa
ggccttaccgcctgcctggagaaacacagtgcctgcccttggcaaatata
tgttggtgtatctgaaaaacagctcctggaagctttttctcattcaggct
ttaggggttaccccatctttccttatgtgtgtaatattggagaatgtaca
ctctcactgaactggggatgtttgacttaaaatgatggacaataagatag
tgagcagtaagtgtgctctaggctaggctacgagaggccatgagctcctc
atctcttctctgttctgagctctctgatccactgcacttggggcaggggg
tgcattctctgtgcctctcctgagtctactttctgcatcattggttctcc
cagctcacttccataatgtcctcctaggctgcattggaattgtgtgttgt
ctagacccatggccaagactgtcattgcctgtgagggagaccaagctcac
caccaagggcttttgccagattgctttcatttacagaatttgcccattca
tgtgtctttgtgtttatggattaaatggctttctgaccagcatccctttg
gtgtgctttgtcaataaga
>NM_000210 2
aacgggctcattcagcggtcgcgagctgcccgcgagggggagcggccgga
cggagagcgcgacccgtcccgggggtggggccgggcgcagcggcgagagg
aggcgaaggtggctgcggtagcagcagcgcggcagcctcggacccagccc
ggagcgcagggcggccgctgcaggtccccgctcccctccccgtgcgtccg

cccatggccgccgccgggcagctgtgcttgctctacctgtcggcggggct
cctgtcccggctcggcgcagccttcaacttggacactcgggaggacaacg
tgatccggaaatatggagaccccgggagcctcttcggcttctcgctggcc
atgcactggcaactgcagcccgaggacaagcggctgttgctcgtgggggc
cccgcgggcagaagcgcttccactgcagagagccaacagaacgggagggc
tgtacagctgcgacatcaccgcccgggggccatgcacgcggatcgagttt
gataacgatgctgaccccacgtcagaaagcaaggaagatcagtggatggg
ggtcaccgtccagagccaaggtccagggggcaaggtcgtgacatgtgctc
accgatatgaaaaaaggcagcatgttaatacgaagcaggaatcccgagac
atctttgggcggtgttatgtcctgagtcagaatctcaggattgaagacga
tatggatgggggagattggagcttttgtgatgggcgattgagaggccatg
agaaatttggctcttgccagcaaggtgtagcagctacttttactaaagac
tttcattacattgtatttggagccccgggtacttataactggaaagggat
tgttcgtgtagagcaaaagaataacactttttttgacatgaacatctttg
aagatgggccttatgaagttggtggagagactgagcatgatgaaagtctc
gttcctgttcctgctaacagttacttaggtttttctttggactcagggaa
aggtattgtttctaaagatgagatcacttttgtatctggtgctcccagag
ccaatcacagtggagccgtggttttgctgaagagagacatgaagtctgca
catctcctccctgagcacatattcgatggagaaggtctggcctcttcatt
tggctatgatgtggcggtggtggacctcaacaaggatgggtggcaagata
tagttattggagccccacagtattttgatagagatggagaagttggaggt
gcagtgtatgtctacatgaaccagcaaggcagatggaataatgtgaagcc
aattcgtcttaatggaaccaaagattctatgtttggcattgcagtaaaaa
atattggagatattaatcaagatggctacccagatattgcagttggagct
ccgtatgatgacttgggaaaggtttttatctatcatggatctgcaaatgg
aataaataccaaaccaacacaggttctcaagggtatatcaccttattttg
gatattcaattgctggaaacatggaccttgatcgaaattcctaccctgat
gttgctgttggttccctctcagattcagtaactattttcagatcccggcc
tgtgattaatattcagaaaaccatcacagtaactcctaacagaattgacc
tccgccagaaaacagcgtgtggggcgcctagtgggatatgcctccaggtt
aaatcctgttttgaatatactgctaaccccgctggttataatccttcaat
atcaattgtgggcacacttgaagctgaaaaagaaagaagaaaatctgggc
tatcctcaagagttcagtttcgaaaccaaggttctgagcccaaatatact
caagaactaactctgaagaggcagaaacagaaagtgtgcatggaggaaac
cctgtggctacaggataatatcagagataaactgcgtcccattcccataa
ctgcctcagtggagatccaagagccaagctctcgtaggcgagtgaattca
cttccagaagttcttccaattctgaattcagatgaacccaagacagctca
tattgatgttcacttcttaaaagagggatgtggagacgacaatgtatgta
acagcaaccttaaactagaatataaattttgcacccgagaaggaaatcaa
gacaaattttcttatttaccaattcaaaaaggtgtaccagaactagttct
aaaagatcagaaggatattgctttagaaataacagtgacaaacagccctt
ccaacccaaggaatcccacaaaagatggcgatgacgcccatgaggctaaa
ctgattgcaacgtttccagacactttaacctattctgcatatagagaact
gagggctttccctgagaaacagttgagttgtgttgccaaccagaatggct
cgcaagctgactgtgagctcggaaatccttttaaaagaaattcaaatgtc
actttttatttggttttaagtacaactgaagtcacctttgacaccccaga
tctggatattaatctgaagttagaaacaacaagcaatcaagataatttgg
ctccaattacagctaaagcaaaagtggttattgaactgcttttatcggtc
tcgggagttgctaaaccttcccaggtgtattttggaggtacagttgttgg

cgagcaagctatgaaatctgaagatgaagtgggaagtttaatagagtatg
aattcagggtaataaacttaggtaaacctcttacaaacctcggcacagca
accttgaacattcagtggccaaaagaaattagcaatgggaaatggttgct
ttatttggtgaaagtagaatccaaaggattggaaaaggtaacttgtgagc
cacaaaaggagataaactccctgaacctaacggagtctcacaactcaaga
aagaaacgggaaattactgaaaaacagatagatgataacagaaaattttc
tttatttgctgaaagaaaataccagactcttaactgtagcgtgaacgtga
actgtgtgaacatcagatgcccgctgcgggggctggacagcaaggcgtct
cttattttgcgctcgaggttatggaacagcacatttctagaggaatattc
caaactgaactacttggacattctcatgcgagccttcattgatgtgactg
ctgctgccgaaaatatcaggctgccaaatgcaggcactcaggttcgagtg
actgtgtttccctcaaagactgtagctcagtattcgggagtaccttggtg
gatcatcctagtggctattctcgctgggatcttgatgcttgctttattag
tgtttatactatggaagtgtggtttcttcaagagaaataagaaagatcat
tatgatgccacatatcacaaggctgagatccatgctcagccatctgataa
agagaggcttacttctgatgcatagtattgatctacttctgtaattgtgt
ggattctttaaacgctctaggtacgatgacagtgttccccgataccatgc
tgtaaggatccggaaagaagagcgagagatcaaagatgaaaagtatattg
ataaccttgaaaaaaaacagtggatcacaaagtggaacgaaaatgaaagc
tactcatagcgggggcctaaaaaaaaaaagcttcacagtacccaaactgc
tttttccaactcagaaattcaatttggatttaaaagcctgctcaatccct
gaggactgatttcagagtgactacacacagtacgaacctacagttttaac
tgtggatattgttacgtagcctaaggctcctgttttgcacagccaaattt
aaaactgttggaatggatttttctttaactgccgtaatttaactttctgg
gttgcctttatttttggcgtggctgacttacatcatgtgttggggaaggg
cctgcccagttgcactcaggtgacatcctccagatagtgtagctgaggag
gcacctacactcacctgcactaacagagtggccgtcctaacctcgggcct
gctgcgcagacgtccatcacgttagctgtcccacatcacaagactatgcc
attggggtagttgtgtttcaacggaaagtgctgtcttaaactaaatgtgc
aatagaaggtgatgttgccatcctaccgtcttttcctgtttcctagctgt
gtgaatacctgctcacgtcaaatgcatacaagtttcattctccctttcac
taaaacacacaggtgcaacagacttgaatgctagttatacttatttgtat
atggtatttattttttcttttctttacaaaccattttgttattgactaac
aggccaaagagtctccagtttacccttcaggttggtttaatcaatcagaa
ttagagcatgggaggtcatcactttgacctaaattatttactgcaaaaag
aaaatctttataaatgtaccagagagagttgttttaataacttatctata
aactataacctctccttcatgacagcctccaccccacaacccaaaaggtt
taagaaatagaattataactgtaaagatgtttatttcaggcattggatat
tttttactttagaagcctgcataatgtttctggatttcatactgtaacat
tcaggaattcttggagaaaatgggtttattcactgaactctagtgcggtt
tactcactgctgcaaatactgtatattcaggacttgaaagaaatggtgaa
tgcctatggtggatccaaactgatccagtataagactactgaatctgcta
ccaaaacagttaatcagtgagtcgatgttctatttttttgttttgtttcct
cccctatctgtattcccaaaaattactttggggctaatttaacaagaact
ttaaattgtgttttaattgtaaaaatggcagggggtggaattattactct
atacattcaacagagactgaatagatatgaaagctgattttttttaatta
ccatgcttcacaatgttaagttatatggggagcaacagcaaacaggtgct
aatttgttttggatatagtataagcagtgtctgtgttttgaaagaataga
acacagtttgtagtgccactgttgttttgggggggctttttcttttcgg

aaatcttaaaccttaagatactaaggacgttgttttggttgtactttgga
attcttagtcacaaaatatattttgtttacaaaaatttctgtaaaacagg
ttataacagtgtttaaagtctcagtttcttgcttggggaacttgtgtccc
taatgtgtttagattgctagattgctaaggagctgatactttgacagtgt
ttttagacctgtgttactaaaaaaaagatgaatgtcctgaaaagggtgtt
gggagggtggttcaacaaagaaacaaagatgttatggtgtttagatttat
ggttgttaaaaatgtcatctcaagtcaagtcactggtctgtttgcatttg
atacatttttgtactaactagcattgtaaaattatttcatgattagaaat
tacctgtggatatttgtataaaagtgtgaaataaatttttataaaagtg
ttcattgtttcgtaacacagcattgtatatgtgaagcaaactctaaaatt
ataaatgacaacctgaattatctatttcatcaaaccaaagttcagtgttt
ttatttttggtgtctcatgtaatctcagatcagccaaagatactagtgcc
aaagcaatgggattcggggtttttttctgttttcgctctatgtaggtgat
cctcaagtctttcattttccttctttatgattaaaagaaacctacaggta
tttaacaacc
>NM_005545 3
aagcagttgttttgctggaaggagggagtgcgcgggctgccccgggctcc
tccctgccgcctcctctcagtggatggttccaggcaccctgtctggggca
gggagggcacaggcctgcacatcgaaggtggggtgggaccaggctgcccc
tcgccccagcatccaagtcctcccttgggcgcccgtggccctgcagactc
tcagggctaaggtcctctgttgctttttggttccaccttagaagaggctc
cgcttgactaagagtagcttgaaggaggcaccatgcaggagctgcatctg
ctctggtgggcgcttctcctgggcctggctcaggcctgccctgagccctg
cgactgtggggaaaagtatggcttccagatcgccgactgtgcctaccgcg
acctagaatccgtgccgcctggcttcccggccaatgtgactacactgagc
ctgtcagccaaccggctgccaggcttgccggagggtgccttcagggaggt
gcccctgctgcagtcgctgtggctggcacacaatgagatccgcacggtgg
ccgccggagccctggcctctctgagccatctcaagagcctggacctcagc
cacaatctcatctctgactttgcctggagcgacctgcacaacctcagtgc
cctccaattgctcaagatggacagcaacgagctgaccttcatcccccgcg
acgccttccgcagcctccgtgctctgcgctcgctgcaactcaaccacaac
cgcttgcacacattggccgagggcaccttcaccccgctcaccgcgctgtc
ccacctgcagatcaacgagaaccccttcgactgcacctgcggcatcgtgt
ggctcaagacatgggccctgaccacggccgtgtccatcccggagcaggac
aacatcgcctgcacctcaccccatgtgctcaagggtacgccgctgagccg
cctgccgccactgccatgctcggcgccctcagtgcagctcagctaccaac
ccagccaggatggtgccgagctgcggcctggttttgtgctggcactgcac
tgtgatgtggacgggcagccggcccctcagcttcactggcacatccagat
acccagtggcattgtggagatcaccagccccaacgtgggcactgatgggc
gtgccctgcctggcaccсctgtggccagctcccagccgcgcttccaggcc
tttgccaatggcagcctgcttatccccgactttggcaagctggaggaagg
cacctacagctgcctggccaccaatgagctgggcagtgctgagagctcag
tggacgtggcactggccacgcccggtgagggtggtgaggacacactgggg
cgcaggttccatggcaaagcggttgagggaaagggctgctatacggttga
caacgaggtgcagccatcagggccggaggacaatgtggtcatcatctacc
tcagccgtgctgggaaccctgaggctgcagtcgcagaaggggtccctggg
cagctgcccccaggcctgctcctgctgggccaaagcctcctcctcttctt
cttcctcacctccttctagccccacccagggcttccctaactcctcccct
tgcccctaccaatgcccctttaagtgctgcaggggtctggggttggcaac

tcctgaggcctgcatgggtgacttcacattttcctacctctccttctaat
ctcttctagagcacctgctatccccaacttctagacctgctccaaactag
tgactaggatagaatttgatcccctaactcactgtctgcggtgctcattg
ctgctaacagcattgcctgtgctctcctctcaggggcagcatgctaacgg
ggcgacgtcctaatccaactgggagaagcctcagtggtggaattccaggc
actgtgactgtcaagctggcaagggccaggattgggggaatggagctggg
gcttagctgggaggtggtctgaagcagacagggaatgggagaggaggatg
ggaagtagacagtggctggtatggctctgaggctccctggggcctgctca
agctcctcctgctccttgctgttttctgatgatttgggggcttgggagtc
cctttgtcctcatctgagactgaaatgtggggatccaggatggccttcct
tcctcttacccttcctccctcagcctgcaacctctatcctggaacctgtc
ctccctttctccccaactatgcatctgttgtctgctcctctgcaaaggcc
agccagcttgggagcagcagagaaataaacagcatttctgatgccaaaaa
aaaaaaaaaaaa
>NM_201526 1
caggccgaggcagggagaactctccactcggaggaggagctggggtcctc
ttccatcccgtcttcatcctgcctggctgcgtgacctcgggaggcaccat
gcaggagctgcatctgctctggtgggcgcttctcctgggcctggctcagg
cctgccctgagccctgcgactgtggggaaaagtatggcttccagatcgcc
gactgtgcctaccgcgacctagaatccgtgccgcctggcttcccggccaa
tgtgactacactgagcctgtcagccaaccggctgccaggcttgccggagg
gtgccttcagggaggtgcccctgctgcagtcgctgtggctggcacacaat
gagatccgcacggtggccgccggagccctggcctctctgagccatctcaa
gagcctggacctcagccacaatctcatctctgactttgcctggagcgacc
tgcacaacctcagtgccctccaattgctcaagatggacagcaacgagctg
accttcatcccccgcgacgccttccgcagcctccgtgctctgcgctcgct
gcaactcaaccacaaccgcttgcacacattggccgagggcaccttcaccc
cgctcaccgcgctgtcccacctgcagatcaacgagaaccccttcgactgc
acctgcggcatcgtgtggctcaagacatgggccctgaccacggccgtgtc
catcccggagcaggacaacatcgcctgcacctcaccccatgtgctcaagg
gtacgccgctgagccgcctgccgccactgccatgctcggcgccctcagtg
cagctcagctaccaacccagccaggatggtgccgagctgcggcctggttt
tgtgctggcactgcactgtgatgtggacgggcagccggcccctcagcttc
actggcacatccagatacccagtggcattgtggagatcaccagccccaac
gtgggcactgatgggcgtgccctgcctggcacccctgtggccagctccca
gccgcgcttccaggcctttgccaatggcagcctgcttatccccgactttg
gcaagctggaggaaggcacctacagctgcctggccaccaatgagctgggc
agtgctgagagctcagtggacgtggcactggccacgcccggtgagggtgg
tgaggacacactggggcgcaggttccatggcaaagcggttgagggaaagg
gctgctatacggttgacaacgaggtgcagccatcagggccggaggacaat
gtggtcatcatctacctcagccgtgctgggaaccctgaggctgcagtcgc
agaaggggtccctgggcagctgcccccaggcctgctcctgctgggccaaa
gcctcctcctcttcttcttcctcacctccttctagccccacccagggctt
ccctaactcctccccttgcccctaccaatgcccctttaagtgctgcaggg
gtctggggttggcaactcctgaggcctgcatgggtgacttcacattttcc
tacctctccttctaatctcttctagagcacctgctatccccaacttctag
acctgctccaaactagtgactaggatagaatttgatcccctaactcactg
tctgcggtgctcattgctgctaacagcattgcctgtgctctcctctcagg
ggcagcatgctaacggggcgacgtcctaatccaactgggagaagcctcag

tggtggaattccaggcactgtgactgtcaagctggcaagggccaggattg
ggggaatggagctggggcttagctgggaggtggtctgaagcagacaggga
atgggagaggaggatgggaagtagacagtggctggtatggctctgaggct
ccctggggcctgctcaagctcctcctgctccttgctgttttctgatgatt
tgggggcttgggagtccctttgtcctcatctgagactgaaatgtggggat
ccaggatggccttccttcctcttacccttcctccctcagcctgcaacctc
tatcctggaacctgtcctccctttctccccaactatgcatctgttgtctg
ctcctctgcaaaggccagccagcttgggagcagcagagaaataaacagca
tttctgatgccaaaaaaaaaaaaaaaaa
>NM_001001392 1
gagaagaaagccagtgcgtctctgggcgcaggggccagtggggctcggag
gcacaggcaccccgcgacactccaggttccccgacccacgtccctggcag
ccccgattatttacagcctcagcagagcacggggcgggggcagaggggcc
cgcccgggagggctgctacttcttaaaacctctgcgggctgcttagtcac
agcccccccttgcttgggtgtgtccttcgctcgctccctccctccgtctta
ggtcactgttttcaacctcgaataaaaactgcagccaacttccgaggcag
cctcattgcccagcggaccccagcctctgccaggttcggtccgccatcct
cgtcccgtcctccgccggcccctgccccgcgcccagggatcctccagctc
ctttcgcccgcgccctccgttcgctccggacaccatggacaagttttggt
ggcacgcagcctggggactctgcctcgtgccgctgagcctggcgcagatc
gatttgaatataacctgccgctttgcaggtgtattccacgtggagaaaaa
tggtcgctacagcatctctcggacggaggccgctgacctctgcaaggctt
tcaatagcaccttgcccacaatggcccagatggagaaagctctgagcatc
ggatttgagacctgcagtttgcattgcagtcaacagtcgaagaaggtgtg
ggcagaagaaaaagctagtgatcaacagtggcaatggagctgtggaggac
agaaagccaagtggactcaacggagaggccagcaagtctcaggaaatggt
gcatttggtgaacaaggagtcgtcagaaactccagaccagtttatgacag
ctgatgagacaaggaacctgcagaatgtggacatgaagattggggtgtaa
cacctacaccattatcttggaaagaaacaaccgttggaaacataaccatt
acagggagctgggacacttaacagatgcaatgtgctactgattgtttcat
tgcgaatctttttagcataaaattttctactctttttgtttttgtgtt
ttgttctttaaagtcaggtccaatttgtaaaaacagcattgctttctgaa
attagggcccaattaataatcagcaagaatttgatcgttccagttcccac
ttggaggcctttcatccctcgggtgtgctatggatggcttctaacaaaaa
ctacacatatgtattcctgatcgccaacctttcccccaccagctaaggac
atttcccagggttaatagggcctggtccctgggaggaaatttgaatgggt
ccattttgcccttccatagcctaatccctgggcattgctttccactgagg
ttgggggttggggtgtactagttacacatcttcaacagacccccctctaga
aatttttcagatgcttctgggagacacccaaagggtgaagctatttatct
gtagtaaactatttatctgtgtttttgaaatattaaaccctggatcagtc
ctttgatcagtataatttttttaaagttactttgtcagaggcacaaaaggg
tttaaactgattcataataaatatctgtacttcttcgatcttcaccttttt
gtgctgtgattcttcagtttctaaaccagcactgtctgggtccctacaat
gtatcaggaagagctgagaatggtaaggagactcttctaagtcttcatct
cagagaccctgagttcccactcagacccactcagccaaatctcatggaag
accaaggagggcagcactgtttttgtttttttgtttttttgttttttttttt
tgacactgtccaaaggttttccatcctgtcctggaatcagagttggaagc
tgaggagcttcagcctcttttatggtttaatggccacctgttctctcctg
tgaaaggctttgcaaagtcacattaagtttgcatgacctgttatccctgg

ggccctatttcatagaggctggccctattagtgatttccaaaaacaatat
ggaagtgccttttgatgtcttacaataagagaagaagccaatggaaatga
aagagattggcaaaggggaaggatgatgccatgtagatcctgtttgacat
ttttatggctgtatttgtaaacttaaacacaccagtgtctgttcttgatg
cagttgctatttaggatgagttaagtgcctggggagtccctcaaaaggtt
aaagggattcccatcattggaatcttatcaccagataggcaagtttatga
ccaaacaagagagtactggctttatcctctaacctcatattttctcccac
ttggcaagtcctttgtggcatttattcatcagtcagggtgtccgattggt
cctagaacttccaaaggctgcttgtcatagaagccattgcatctataaag
caacggctcctgttaaatggtatctcctttctgaggctcctactaaaagt
catttgttacctaaacttatgtgcttaacaggcaatgcttctcagaccac
aaagcagaaagaagaagaaaagctcctgactaaatcagggctgggcttag
acagagttgatctgtagaatatctttaaaggagagatgtcaactttctgc
actattcccagcctctgctcctccctgtctaccctctcccctccctctct
ccctccacttcaccccacaatcttgaaaaacttcctttctcttctgtgaa
catcattggccagatccattttcagtggtctggatttctttttattttct
tttcaacttgaaagaaactggacattaggccactatgtgttgttactgcc
actagtgttcaagtgcctcttgttttcccagagatttcctgggtctgcca
gaggcccagacaggctcactcaagctctttaactgaaaagcaacaagcca
ctccaggacaaggttcaaaatggttacaacagcctctacctgtcgcccca
gggagaaaggggtagtgatacaagtctcatagccagagatggttttccac
tccttctagatattcccaaaaagaggctgagacaggaggttattttcaat
tttattttggaattaaatactttttccctttattactgttgtagtccct
cacttggatatacctctgttttcacgatagaaataagggaggtctagagc
ttctattccttggccattgtcaacggagagctggccaagtcttcacaaac
ccttgcaacattgcctgaagtttatggaataagatgtattctcactccct
tgatctcaagggcgtaactctggaagcacagcttgactacacgtcatttt
taccaatgattttcaggtgacctgggctaagtcatttaaactgggtcttt
ataaaagtaaaaggccaacatttaattattttgcaaagcaacctaagagc
taaagatgtaatttttcttgcaattgtaaatcttttgtgtctcctgaaga
cttcccttaaaattagctctgagtgaaaaatcaaaagagacaaaagacat
cttcgaatccatatttcaagcctggtagaattggcttttctagcagaacc
tttccaaaagttttatattgagattcataacaacaccaagaattgatttt
gtagccaacattcattcaatactgttatatcagaggagtaggagagagga
aacatttgacttatctggaaaagcaaaatgtacttaagaataagaataac
atggtccattcacctttatgttatagatatgtctttgtgtaaatcatttg
ttttgagttttcaaagaatagcccattgttcattcttgtgctgtacaatg
accactgttattgttactttgacttttcagagcacacccttcctctggtt
tttgtatatttattgatggatcaataataatgaggaaagcatgatatgta
tattgctgagttgaaagcacttattggaaaatattaaaaggctaacatta
aaagactaaaggaaacagaaaaaaaaaaaaaaaaa
>NM_001001390 1
gagaagaaagccagtgcgtctctgggcgcaggggccagtggggctcggag
gcacaggcaccccgcgacactccaggttccccgacccacgtccctggcag
ccccgattatttacagcctcagcagagcacggggcgggggcagaggggcc
cgcccgggagggctgctacttcttaaaacctctgcgggctgcttagtcac
agcccccccttgcttgggtgtgtccttcgctcgctccctccctccgtctta
ggtcactgttttcaacctcgaataaaaactgcagccaacttccgaggcag
cctcattgcccagcggaccccagcctctgccaggttcggtccgccatcct

cgtcccgtcctccgccggcccctgccccgcgcccagggatcctccagctc
ctttcgcccgcgccctccgttcgctccggacaccatggacaagttttggt
ggcacgcagcctggggactctgcctcgtgccgctgagcctggcgcagatc
gatttgaatataacctgccgctttgcaggtgtattccacgtggagaaaaa
tggtcgctacagcatctctcggacggaggccgctgacctctgcaaggctt
tcaatagcaccttgcccacaatggcccagatggagaaagctctgagcatc
ggatttgagacctgcaggtatgggttcatagaagggcacgtggtgattcc
ccggatccaccccaactccatctgtgcagcaaacaacacaggggtgtaca
tcctcacatccaacacctcccagtatgacacatattgcttcaatgcttca
gctccacctgaagaagattgtacatcagtcacagacctgcccaatgcctt
tgatggaccaattaccataactattgttaaccgtgatggcacccgctatg
tccagaaaggagaatacagaacgaatcctgaagacatctaccccagcaac
cctactgatgatgacgtgagcagcggctcctccagtgaaaggagcagcac
ttcaggaggttacatcttttacaccttttctactgtacaccccatcccag
acgaagacagtccctggatcaccgacagcacagacagaatccctgctacc
aatatggactccagtcatagtataacgcttcagcctactgcaaatccaaa
cacaggtttggtggaagatttggacaggacaggacctctttcaatgacaa
cgcagcagagtaattctcagagcttctctacatcacatgaaggcttggaa
gaagataaagaccatccaacaacttctactctgacatcaagcaataggaa
tgatgtcacaggtggaagaagagacccaaatcattctgaaggctcaacta
ctttactggaaggttatacctctcattacccacacacgaaggaaagcagg
accttcatcccagtgacctcagctaagactgggtcctttggagttactgc
agttactgttggagattccaactctaatgtcaatcgttccttatcaggag
accaagacacattccaccccagtgggggtcccataccactcatggatct
gaatcagatggacactcacatgggagtcaagaaggtggagcaaacacaac
ctctggtcctataaggacaccccaaattccagaatggctgatcatcttgg
catccctcttggccttggctttgattcttgcagtttgcattgcagtcaac
agtcgaagaaggtgtgggcagaagaaaaagctagtgatcaacagtggcaa
tggagctgtggaggacagaaagccaagtggactcaacggagaggccagca
agtctcaggaaatggtgcatttggtgaacaaggagtcgtcagaaactcca
gaccagtttatgacagctgatgagacaaggaacctgcagaatgtggacat
gaagattggggtgtaacacctacaccattatcttggaaagaaacaaccgt
tggaaacataaccattacagggagctgggacacttaacagatgcaatgtg
ctactgattgtttcattgcgaatctttttagcataaaattttctactct
ttttgtttttgtgttttgttctttaaagtcaggtccaatttgtaaaaac
agcattgctttctgaaattagggcccaattaataatcagcaagaatttga
tcgttccagttcccacttggaggcctttcatccctcgggtgtgctatgga
tggcttctaacaaaaactacacatatgtattcctgatcgccaacctttcc
cccaccagctaaggacatttcccagggttaatagggcctggtccctggga
ggaaatttgaatgggtccattttgcccttccatagcctaatccctgggca
ttgctttccactgaggttgggggttggggtgtactagttacacatcttca
acagacccccctctagaaatttttcagatgcttctgggagacacccaaagg
gtgaagctatttatctgtagtaaactatttatctgtgtttttgaaatatt
aaaccctggatcagtcctttgatcagtataatttttaaagttactttgt
cagaggcacaaaagggtttaaactgattcataataaatatctgtacttct
tcgatcttcaccttttgtgctgtgattcttcagtttctaaaccagcactg
tctgggtccctacaatgtatcaggaagagctgagaatggtaaggagactc
ttctaagtcttcatctcagagaccctgagttcccactcagacccactcag
ccaaatctcatggaagaccaaggagggcagcactgtttttgtttttgtt

ttttgttttttttttttgacactgtccaaaggttttccatcctgtcctgg
aatcagagttggaagctgaggagcttcagcctcttttatggtttaatggc
cacctgttctctcctgtgaaaggctttgcaaagtcacattaagtttgcat
gacctgttatccctggggccctatttcatagaggctggccctattagtga
tttccaaaaacaatatggaagtgccttttgatgtcttacaataagagaag
aagccaatggaaatgaaagagattggcaaaggggaaggatgatgccatgt
agatcctgtttgacatttttatggctgtatttgtaaacttaaacacacca
gtgtctgttcttgatgcagttgctatttaggatgagttaagtgcctgggg
agtccctcaaaaggttaaagggattcccatcattggaatcttatcaccag
ataggcaagtttatgaccaaacaagagagtactggctttatcctctaacc
tcatattttctcccacttggcaagtcctttgtggcatttattcatcagtc
agggtgtccgattggtcctagaacttccaaaggctgcttgtcatagaagc
cattgcatctataaagcaacggctcctgttaaatggtatctcctttctga
ggctcctactaaaagtcatttgttacctaaacttatgtgcttaacaggca
atgcttctcagaccacaaagcagaaagaagaagaaaagctcctgactaaa
tcagggctgggcttagacagagttgatctgtagaatatctttaaaggaga
gatgtcaactttctgcactattcccagcctctgctcctccctgtctaccc
tctcccctccctctctccctccacttcaccccacaatcttgaaaaacttc
ctttctcttctgtgaacatcattggccagatccattttcagtggtctgga
tttctttttattttcttttcaacttgaaagaaactggacattaggccact
atgtgttgttactgccactagtgttcaagtgcctcttgttttcccagaga
tttcctgggtctgccagaggcccagacaggctcactcaagctctttaact
gaaaagcaacaagccactccaggacaaggttcaaaatggttacaacagcc
tctacctgtcgccccagggagaaaggggtagtgatacaagtctcatagcc
agagatggttttccactccttctagatattcccaaaaagaggctgagaca
ggaggttattttcaattttattttggaattaaatactttttttccctttat
tactgttgtagtccctcacttggatatacctctgttttcacgatagaaat
aagggaggtctagagcttctattccttggccattgtcaacggagagctgg
ccaagtcttcacaaacccttgcaacattgcctgaagtttatggaataaga
tgtattctcactcccttgatctcaagggcgtaactctggaagcacagctt
gactacacgtcatttttaccaatgattttcaggtgacctgggctaagtca
tttaaactgggtctttataaaagtaaaaggccaacatttaattattttgc
aaagcaacctaagagctaaagatgtaatttttcttgcaattgtaaatctt
ttgtgtctcctgaagacttcccttaaaattagctctgagtgaaaaatcaa
aagagacaaaagacatcttcgaatccatatttcaagcctggtagaattgg
cttttctagcagaacctttccaaaagttttatattgagattcataacaac
accaagaattgattttgtagccaacattcattcaatactgttatatcaga
ggagtaggagagaggaaacatttgacttatctggaaaagcaaaatgtact
taagaataagaataacatggtccattcacctttatgttatagatatgtct
ttgtgtaaatcatttgttttgagttttcaaagaatagcccattgttcatt
cttgtgctgtacaatgaccactgttattgttactttgacttttcagagca
cacccttcctctggtttttgtatatttattgatggatcaataataatgag
gaaagcatgatatgtatattgctgagttgaaagcacttattggaaaatat
taaaaggctaacattaaaagactaaaggaaacagaaaaaaaaaaaaaaaa
a
>NM_001001391 1
gagaagaaagccagtgcgtctctgggcgcaggggccagtggggctcggag
gcacaggcaccccgcgacactccaggttccccgacccacgtccctggcag
ccccgattatttacagcctcagcagagcacggggcgggggcagaggggcc

cgcccgggagggctgctacttcttaaaacctctgcgggctgcttagtcac
agcccccccttgcttgggtgtgtccttcgctcgctccctccctccgtctta
ggtcactgttttcaacctcgaataaaaactgcagccaacttccgaggcag
cctcattgcccagcggaccccagcctctgccaggttcggtccgccatcct
cgtcccgtcctccgccggcccctgccccgcgcccagggatcctccagctc
ctttcgcccgcgccctccgttcgctccggacaccatggacaagttttggt
ggcacgcagcctggggactctgcctcgtgccgctgagcctggcgcagatc
gatttgaatataacctgccgctttgcaggtgtattccacgtggagaaaaa
tggtcgctacagcatctctcggacggaggccgctgacctctgcaaggctt
tcaatagcaccttgcccacaatggcccagatggagaaagctctgagcatc
ggatttgagacctgcaggtatgggttcatagaagggcacgtggtgattcc
ccggatccaccccaactccatctgtgcagcaaacaacacaggggtgtaca
tcctcacatccaacacctcccagtatgacacatattgcttcaatgcttca
gctccacctgaagaagattgtacatcagtcacagacctgcccaatgcctt
tgatggaccaattaccataactattgttaaccgtgatggcacccgctatg
tccagaaaggagaatacagaacgaatcctgaagacatctaccccagcaac
cctactgatgatgacgtgagcagcggctcctccagtgaaaggagcagcac
ttcaggaggttacatcttttacaccttttctactgtacaccccatcccag
acgaagacagtccctggatcaccgacagcacagacagaatccctgctacc
agagaccaagacacattccaccccagtggggggtcccataccactcatgg
atctgaatcagatggacactcacatgggagtcaagaaggtggagcaaaca
caacctctggtcctataaggacaccccaaattccagaatggctgatcatc
ttggcatccctcttggccttggctttgattcttgcagtttgcattgcagt
caacagtcgaagaaggtgtgggcagaagaaaaagctagtgatcaacagtg
gcaatggagctgtggaggacagaaagccaagtggactcaacggagaggcc
agcaagtctcaggaaatggtgcatttggtgaacaaggagtcgtcagaaac
tccagaccagtttatgacagctgatgagacaaggaacctgcagaatgtgg
acatgaagattggggtgtaacacctacaccattatcttggaaagaaacaa
ccgttggaaacataaccattacagggagctgggacacttaacagatgcaa
tgtgctactgattgtttcattgcgaatctttttagcataaaattttcta
ctctttttgtttttgtgttttgttctttaaagtcaggtccaatttgtaa
aaacagcattgctttctgaaattagggcccaattaataatcagcaagaat
ttgatcgttccagttcccacttggaggcctttcatccctcgggtgtgcta
tggatggcttctaacaaaaactacacatatgtattcctgatcgccaacct
ttcccccaccagctaaggacatttcccagggttaatagggcctggtccct
gggaggaaatttgaatgggtccattttgcccttccatagcctaatccctg
ggcattgctttccactgaggttgggggttggggtgtactagttacacatc
ttcaacagacccccctctagaaatttttcagatgcttctgggagacaccca
aagggtgaagctatttatctgtagtaaactatttatctgtgtttttgaaa
tattaaaccctggatcagtcctttgatcagtataatttttaaagttact
ttgtcagaggcacaaaagggtttaaactgattcataataaatatctgtac
ttcttcgatcttcaccttttgtgctgtgattcttcagtttctaaaccagc
actgtctgggtccctacaatgtatcaggaagagctgagaatggtaaggag
actcttctaagtcttcatctcagagaccctgagttcccactcagacccac
tcagccaaatctcatggaagaccaaggagggcagcactgtttttgttttt
tgtttttgtttttttttttgacactgtccaaaggttttccatcctgtc
ctggaatcagagttggaagctgaggagcttcagcctcttttatggtttaa
tggccacctgttctctcctgtgaaaggctttgcaaagtcacattaagttt
gcatgacctgttatccctggggccctatttcatagaggctggccctatta

gtgatttccaaaaacaatatggaagtgccttttgatgtcttacaataaga
gaagaagccaatggaaatgaaagagattggcaaaggggaaggatgatgcc
atgtagatcctgtttgacatttttatggctgtatttgtaaacttaaacac
accagtgtctgttcttgatgcagttgctatttaggatgagttaagtgcct
ggggagtccctcaaaaggttaaagggattcccatcattggaatcttatca
ccagataggcaagtttatgaccaaacaagagagtactggctttatcctct
aacctcatattttctcccacttggcaagtcctttgtggcatttattcatc
agtcagggtgtccgattggtcctagaacttccaaaggctgcttgtcatag
aagccattgcatctataaagcaacggctcctgttaaatggtatctccttt
ctgaggctcctactaaaagtcatttgttacctaaacttatgtgcttaaca
ggcaatgcttctcagaccacaaagcagaaagaagaagaaaagctcctgac
taaatcagggctgggcttagacagagttgatctgtagaatatctttaaag
gagagatgtcaactttctgcactattcccagcctctgctcctccctgtct
accctctcccctccctctctccctccacttcaccccacaatcttgaaaaa
cttcctttctcttctgtgaacatcattggccagatccattttcagtggtc
tggatttctttttattttcttttcaacttgaaagaaactggacattaggc
cactatgtgttgttactgccactagtgttcaagtgcctcttgttttccca
gagatttcctgggtctgccagaggcccagacaggctcactcaagctcttt
aactgaaaagcaacaagccactccaggacaaggttcaaaatggttacaac
agcctctacctgtcgccccagggagaaaggggtagtgatacaagtctcat
agccagagatggttttccactccttctagatattcccaaaaagaggctga
gacaggaggttattttcaattttattttggaattaaatactttttccct
ttattactgttgtagtccctcacttggatatacctctgttttcacgatag
aaataagggaggtctagagcttctattccttggccattgtcaacggagag
ctggccaagtcttcacaaacccttgcaacattgcctgaagtttatggaat
aagatgtattctcactcccttgatctcaagggcgtaactctggaagcaca
gcttgactacacgtcatttttaccaatgattttcaggtgacctgggctaa
gtcatttaaactgggtctttataaaagtaaaaggccaacatttaattatt
ttgcaaagcaacctaagagctaaagatgtaatttttcttgcaattgtaaa
tcttttgtgtctcctgaagacttcccttaaaattagctctgagtgaaaaa
tcaaaagagacaaaagacatcttcgaatccatatttcaagcctggtagaa
ttggcttttctagcagaacctttccaaaagttttatattgagattcataa
caacaccaagaattgattttgtagccaacattcattcaatactgttatat
cagaggagtaggagagaggaaacatttgacttatctggaaaagcaaaatg
tacttaagaataagaataacatggtccattcacctttatgttatagatat
gtctttgtgtaaatcatttgttttgagttttcaaagaatagcccattgtt
cattcttgtgctgtacaatgaccactgttattgttactttgacttttcag
agcacacccttcctctggtttttgtatatttattgatggatcaataataa
tgaggaaagcatgatatgtatattgctgagttgaaagcacttattggaaa
atattaaaaggctaacattaaaagactaaaggaaacagaaaaaaaaaaaa
aaaaa
>NM_001001389 1
gagaagaaagccagtgcgtctctgggcgcaggggccagtggggctcggag
gcacaggcaccccgcgacactccaggttccccgacccacgtccctggcag
ccccgattatttacagcctcagcagagcacggggcgggggcagaggggcc
cgcccgggagggctgctacttcttaaaacctctgcgggctgcttagtcac
agcccccccttgcttgggtgtgtccttcgctcgctccctccctccgtctta
ggtcactgttttcaacctcgaataaaaactgcagccaacttccgaggcag
cctcattgcccagcggaccccagcctctgccaggttcggtccgccatcct

cgtcccgtcctccgccggcccctgccccgcgcccagggatcctccagctc
ctttcgcccgcgccctccgttcgctccggacaccatggacaagttttggt
ggcacgcagcctggggactctgcctcgtgccgctgagcctggcgcagatc
gatttgaatataacctgccgctttgcaggtgtattccacgtggagaaaaa
tggtcgctacagcatctctcggacggaggccgctgacctctgcaaggctt
tcaatagcaccttgcccacaatggcccagatggagaaagctctgagcatc
ggatttgagacctgcaggtatgggttcatagaagggcacgtggtgattcc
ccggatccaccccaactccatctgtgcagcaaacaacacaggggtgtaca
tcctcacatccaacacctcccagtatgacacatattgcttcaatgcttca
gctccacctgaagaagattgtacatcagtcacagacctgcccaatgcctt
tgatggaccaattaccataactattgttaaccgtgatggcacccgctatg
tccagaaaggagaatacagaacgaatcctgaagacatctaccccagcaac
cctactgatgatgacgtgagcagcggctcctccagtgaaaggagcagcac
ttcaggaggttacatcttttacaccttttctactgtacaccccatcccag
acgaagacagtccctggatcaccgacagcacagacagaatccctgctacc
agtacgtcttcaaataccatctcagcaggctgggagccaaatgaagaaaa
tgaagatgaaagagacagacacctcagtttttctggatcaggcattgatg
atgatgaagattttatctccagcaccatttcaaccacaccacgggctttt
gaccacacaaaacagaaccaggactggacccagtggaacccaagccattc
aaatccggaagtgctacttcagacaaccacaaggatgactgatgtagaca
gaaatggcaccactgcttatgaaggaaactggaacccagaagcacaccct
cccctcattcaccatgagcatcatgaggaagaagagaccccacattctac
aagcacaatccaggcaactcctagtagtacaacggaagaaacagctaccc
agaaggaacagtggtttggcaacagatggcatgagggatatcgccaaaca
cccaaagaagactcccattcgacaacagggacagctgcagcctcagctca
taccagccatccaatgcaaggaaggacaacaccaagcccagaggacagtt
cctggactgatttcttcaacccaatctcacaccccatgggacgaggtcat
caagcaggaagaaggatggatatggactccagtcatagtataacgcttca
gcctactgcaaatccaaacacaggtttggtggaagatttggacaggacag
gacctctttcaatgacaacgcagcagagtaattctcagagcttctctaca
tcacatgaaggcttggaagaagataaagaccatccaacaacttctactct
gacatcaagcaataggaatgatgtcacaggtggaagaagagacccaaatc
attctgaaggctcaactactttactggaaggttatacctctcattaccca
cacacgaaggaaagcaggaccttcatcccagtgacctcagctaagactgg
gtcctttggagttactgcagttactgttggagattccaactctaatgtca
atcgttccttatcaggagaccaagacacattccaccccagtggggggtcc
cataccactcatggatctgaatcagatggacactcacatgggagtcaaga
aggtggagcaaacacaacctctggtcctataaggacaccccaaattccag
aatggctgatcatcttggcatccctcttggccttggctttgattcttgca
gtttgcattgcagtcaacagtcgaagaaggtgtgggcagaagaaaaagct
agtgatcaacagtggcaatggagctgtggaggacagaaagccaagtggac
tcaacggagaggccagcaagtctcaggaaatggtgcatttggtgaacaag
gagtcgtcagaaactccagaccagtttatgacagctgatgagacaaggaa
cctgcagaatgtggacatgaagattggggtgtaacacctacaccattatc
ttggaaagaaacaaccgttggaaacataaccattacagggagctgggaca
cttaacagatgcaatgtgctactgattgtttcattgcgaatctttttag
cataaaattttctactctttttgtttttgtgttttgttctttaaagtca
ggtccaatttgtaaaaacagcattgctttctgaaattagggcccaattaa
taatcagcaagaatttgatcgttccagttcccacttggaggcctttcatc

cctcgggtgtgctatggatggcttctaacaaaaactacacatatgtattc
ctgatcgccaacctttcccccaccagctaaggacatttcccagggttaat
agggcctggtccctgggaggaaatttgaatgggtccattttgcccttcca
tagcctaatccctgggcattgctttccactgaggttgggggttggggtgt
actagttacacatcttcaacagacccccctctagaaatttttcagatgctt
ctgggagacacccaaagggtgaagctatttatctgtagtaaactatttat
ctgtgtttttgaaatattaaaccctggatcagtcctttgatcagtataat
tttttaaagttactttgtcagaggcacaaaagggtttaaactgattcata
ataaatatctgtacttcttcgatcttcaccttttgtgctgtgattcttca
gtttctaaaccagcactgtctgggtccctacaatgtatcaggaagagctg
agaatggtaaggagactcttctaagtcttcatctcagagaccctgagttc
ccactcagacccactcagccaaatctcatggaagaccaaggagggcagca
ctgtttttgtttttttgtttttttgtttttttttttttgacactgtccaaagg
ttttccatcctgtcctggaatcagagttggaagctgaggagcttcagcct
cttttatggtttaatggccacctgttctctcctgtgaaaggctttgcaaa
gtcacattaagtttgcatgacctgttatccctggggccctatttcataga
ggctggccctattagtgatttccaaaaacaatatggaagtgccttttgat
gtcttacaataagagaagaagccaatggaaatgaaagagattggcaaagg
ggaaggatgatgccatgtagatcctgtttgacatttttatggctgtattt
gtaaacttaaacacaccagtgtctgttcttgatgcagttgctatttagga
tgagttaagtgcctggggagtccctcaaaaggttaaagggattcccatca
ttggaatcttatcaccagataggcaagtttatgaccaaacaagagagtac
tggctttatcctctaacctcatattttctcccacttggcaagtcctttgt
ggcatttattcatcagtcagggtgtccgattggtcctagaacttccaaag
gctgcttgtcatagaagccattgcatctataaagcaacggctcctgttaa
atggtatctcctttctgaggctcctactaaaagtcatttgttacctaaac
ttatgtgcttaacaggcaatgcttctcagaccacaaagcagaaagaagaa
gaaaagctcctgactaaatcagggctgggcttagacagagttgatctgta
gaatatctttaaaggagagatgtcaactttctgcactattcccagcctct
gctcctccctgtctaccctctcccctccctctctccctccacttcacccc
acaatcttgaaaaacttcctttctcttctgtgaacatcattggccagatc
cattttcagtggtctggatttctttttattttcttttcaacttgaaagaa
actggacattaggccactatgtgttgttactgccactagtgttcaagtgc
ctcttgttttcccagagatttcctgggtctgccagaggcccagacaggct
cactcaagctctttaactgaaaagcaacaagccactccaggacaaggttc
aaaatggttacaacagcctctacctgtcgccccagggagaaaggggtagt
gatacaagtctcatagccagagatggttttccactccttctagatattcc
caaaaagaggctgagacaggaggttattttcaattttattttggaattaa
atacttttttccctttattactgttgtagtccctcacttggatatacctc
tgttttcacgatagaaataagggaggtctagagcttctattccttggcca
ttgtcaacggagagctggccaagtcttcacaaacccttgcaacattgcct
gaagtttatggaataagatgtattctcactcccttgatctcaagggcgta
actctggaagcacagcttgactacacgtcatttttaccaatgattttcag
gtgacctgggctaagtcatttaaactgggtctttataaaagtaaaaggcc
aacatttaattattttgcaaagcaacctaagagctaaagatgtaattttt
cttgcaattgtaaatcttttgtgtctcctgaagacttcccttaaaattag
ctctgagtgaaaaatcaaaagagacaaaagacatcttcgaatccatattt
caagcctggtagaattggcttttctagcagaacctttccaaaagttttat
attgagattcataacaacaccaagaattgattttgtagccaacattcatt

caatactgttatatcagaggagtaggagagaggaaacatttgacttatct
ggaaaagcaaaatgtacttaagaataagaataacatggtccattcacctt
tatgttatagatatgtctttgtgtaaatcatttgttttgagttttcaaag
aatagcccattgttcattcttgtgctgtacaatgaccactgttattgtta
ctttgacttttcagagcacacccttcctctggtttttgtatatttattga
tggatcaataataatgaggaaagcatgatatgtatattgctgagttgaaa
gcacttattggaaaatattaaaaggctaacattaaaagactaaaggaaac
agaaaaaaaaaaaaaaaaa
>NM_000610 3
gagaagaaagccagtgcgtctctgggcgcaggggccagtggggctcggag
gcacaggcaccccgcgacactccaggttccccgacccacgtccctggcag
ccccgattatttacagcctcagcagagcacggggcgggggcagaggggcc
cgcccgggagggctgctacttcttaaaacctctgcgggctgcttagtcac
agcccccttgcttgggtgtgtccttcgctcgctccctccctccgtctta
ggtcactgttttcaacctcgaataaaaactgcagccaacttccgaggcag
cctcattgcccagcggaccccagcctctgccaggttcggtccgccatcct
cgtcccgtcctccgccggcccctgccccgcgcccagggatcctccagctc
ctttcgcccgcgccctccgttcgctccggacaccatggacaagttttggt
ggcacgcagcctggggactctgcctcgtgccgctgagcctggcgcagatc
gatttgaatataacctgccgctttgcaggtgtattccacgtggagaaaaa
tggtcgctacagcatctctcggacggaggccgctgacctctgcaaggctt
tcaatagcaccttgcccacaatggcccagatggagaaagctctgagcatc
ggatttgagacctgcaggtatgggttcatagaagggcacgtggtgattcc
ccggatccaccccaactccatctgtgcagcaaacaacacaggggtgtaca
tcctcacatccaacacctcccagtatgacacatattgcttcaatgcttca
gctccacctgaagaagattgtacatcagtcacagacctgcccaatgcctt
tgatggaccaattaccataactattgttaaccgtgatggcacccgctatg
tccagaaaggagaatacagaacgaatcctgaagacatctaccccagcaac
cctactgatgatgacgtgagcagcggctcctccagtgaaaggagcagcac
ttcaggaggttacatcttttacaccttttctactgtacaccccatcccag
acgaagacagtccctggatcaccgacagcacagacagaatccctgctacc
actttgatgagcactagtgctacagcaactgagacagcaaccaagaggca
agaaacctgggattggttttcatggttgtttctaccatcagagtcaaaga
atcatcttcacacaacaacacaaatggctggtacgtcttcaaataccatc
tcagcaggctgggagccaaatgaagaaaatgaagatgaaagagacagaca
cctcagtttttctggatcaggcattgatgatgatgaagattttatctcca
gcaccatttcaaccacaccacgggcttttgaccacacaaaacagaaccag
gactggacccagtggaacccaagccattcaaatccggaagtgctacttca
gacaaccacaaggatgactgatgtagacagaaatggcaccactgcttatg
aaggaaactggaacccagaagcacaccctcccctcattcaccatgagcat
catgaggaagaagagaccccacattctacaagcacaatccaggcaactcc
tagtagtacaacggaagaaacagctacccagaaggaacagtggtttggca
acagatggcatgagggatatcgccaaacacccaaagaagactcccattcg
acaacagggacagctgcagcctcagctcataccagccatccaatgcaagg
aaggacaacaccaagcccagaggacagttcctggactgatttcttcaacc
caatctcacaccccatgggacgaggtcatcaagcaggaagaaggatggat
atggactccagtcatagtataacgcttcagcctactgcaaatccaaacac
aggtttggtggaagatttggacaggacaggacctctttcaatgacaacgc
agcagagtaattctcagagcttctctacatcacatgaaggcttggaagaa

gataaagaccatccaacaacttctactctgacatcaagcaataggaatga
tgtcacaggtggaagaagagacccaaatcattctgaaggctcaactactt
tactggaaggttatacctctcattacccacacacgaaggaaagcaggacc
ttcatcccagtgacctcagctaagactgggtcctttggagttactgcagt
tactgttggagattccaactctaatgtcaatcgttccttatcaggagacc
aagacacattccaccccagtgggggtcccataccactcatggatctgaa
tcagatggacactcacatgggagtcaagaaggtggagcaaacacaacctc
tggtcctataaggacaccccaaattccagaatggctgatcatcttggcat
ccctcttggccttggctttgattcttgcagtttgcattgcagtcaacagt
cgaagaaggtgtgggcagaagaaaaagctagtgatcaacagtggcaatgg
agctgtggaggacagaaagccaagtggactcaacggagaggccagcaagt
ctcaggaaatggtgcatttggtgaacaaggagtcgtcagaaactccagac
cagtttatgacagctgatgagacaaggaacctgcagaatgtggacatgaa
gattggggtgtaacacctacaccattatcttggaaagaaacaaccgttgg
aaacataaccattacagggagctgggacacttaacagatgcaatgtgcta
ctgattgtttcattgcgaatctttttagcataaaattttctactctttt
tgtttttgtgttttgttctttaaagtcaggtccaatttgtaaaaacagc
attgctttctgaaattagggcccaattaataatcagcaagaatttgatcg
ttccagttcccacttggaggcctttcatccctcgggtgtgctatggatgg
cttctaacaaaaactacacatatgtattcctgatcgccaacctttccccc
accagctaaggacatttcccagggttaatagggcctggtccctgggagga
aatttgaatgggtccattttgcccttccatagcctaatccctgggcattg
ctttccactgaggttgggggttggggtgtactagttacacatcttcaaca
gaccccctctagaaatttttcagatgcttctgggagacacccaaagggtg
aagctatttatctgtagtaaactatttatctgtgtttttgaaatattaaa
ccctggatcagtcctttgatcagtataatttttaaagttactttgtcag
aggcacaaaagggtttaaactgattcataataaatatctgtacttcttcg
atcttcaccttttgtgctgtgattcttcagtttctaaaccagcactgtct
gggtccctacaatgtatcaggaagagctgagaatggtaaggagactcttc
taagtcttcatctcagagaccctgagttcccactcagacccactcagcca
aatctcatggaagaccaaggagggcagcactgtttttgtttttttgttttt
tgtttttttttttgacactgtccaaaggttttccatcctgtcctggaat
cagagttggaagctgaggagcttcagcctcttttatggtttaatggccac
ctgttctctcctgtgaaaggctttgcaaagtcacattaagtttgcatgac
ctgttatccctggggccctatttcatagaggctggccctattagtgattt
ccaaaaacaatatggaagtgccttttgatgtcttacaataagagaagaag
ccaatggaaatgaaagagattggcaaaggggaaggatgatgccatgtaga
tcctgtttgacatttttatggctgtatttgtaaacttaaacacaccagtg
tctgttcttgatgcagttgctatttaggatgagttaagtgcctggggagt
ccctcaaaaggttaaagggattcccatcattggaatcttatcaccagata
ggcaagtttatgaccaaacaagagagtactggctttatcctctaacctca
tattttctcccacttggcaagtcctttgtggcatttattcatcagtcagg
gtgtccgattggtcctagaacttccaaaggctgcttgtcatagaagccat
tgcatctataaagcaacggctcctgttaaatggtatctcctttctgaggc
tcctactaaaagtcatttgttacctaaacttatgtgcttaacaggcaatg
cttctcagaccacaaagcagaaagaagaagaaaagctcctgactaaatca
gggctgggcttagacagagttgatctgtagaatatctttaaaggagagat
gtcaactttctgcactattcccagcctctgctcctccctgtctaccctct
cccctccctctctccctccacttcaccccacaatcttgaaaaacttcctt

tctcttctgtgaacatcattggccagatccattttcagtggtctggattt
ctttttattttcttttcaacttgaaagaaactggacattaggccactatg
tgttgttactgccactagtgttcaagtgcctcttgttttcccagagattt
cctgggtctgccagaggcccagacaggctcactcaagctctttaactgaa
aagcaacaagccactccaggacaaggttcaaaatggttacaacagcctct
acctgtcgccccagggagaaaggggtagtgatacaagtctcatagccaga
gatggttttccactccttctagatattcccaaaaagaggctgagacagga
ggttattttcaattttattttggaattaaatactttttttccctttattac
tgttgtagtccctcacttggatatacctctgttttcacgatagaaataag
ggaggtctagagcttctattccttggccattgtcaacggagagctggcca
agtcttcacaaacccttgcaacattgcctgaagtttatggaataagatgt
attctcactcccttgatctcaagggcgtaactctggaagcacagcttgac
tacacgtcatttttaccaatgattttcaggtgacctgggctaagtcattt
aaactgggtctttataaaagtaaaaggccaacatttaattattttgcaaa
gcaacctaagagctaaagatgtaatttttcttgcaattgtaaatcttttg
tgtctcctgaagacttcccttaaaattagctctgagtgaaaaatcaaaag
agacaaaagacatcttcgaatccatatttcaagcctggtagaattggctt
ttctagcagaacctttccaaaagttttatattgagattcataacaacacc
aagaattgattttgtagccaacattcattcaatactgttatatcagagga
gtaggagagaggaaacatttgacttatctggaaaagcaaaatgtacttaa
gaataagaataacatggtccattcacctttatgttatagatatgtctttg
tgtaaatcatttgttttgagttttcaaagaatagcccattgttcattctt
gtgctgtacaatgaccactgttattgttactttgacttttcagagcacac
ccttcctctggtttttgtatatttattgatggatcaataataatgaggaa
agcatgatatgtatattgctgagttgaaagcacttattggaaaatattaa
aaggctaacattaaaagactaaaggaaacagaaaaaaaaaaaaaaaaa
>NM_198177 2
agtgccagaactaactttgactttcactcttcgccaagggcttgcagaac
accttaaaggaaaaaagatggaggcgcttagagttcagatgttcatgcca
tgctcctttgaaagcttgtatctcagttccgccgagcatcctggggcctc
caagcctccgataagctcctccagtatgacatcacgcatcttgctacgcc
agcaactcatgcgtgagcagatgcaggagcaggagcgcagggagcagcag
cagaagctgcaggcggcccagttcatgcaacagagagtgcccgtgagtca
gacaccagccataaacgtcagtgtgcccaccacccttccctctgccacgc
aggtgccgatggaagtccttaaggtgcagacccacctcgaaaaccccacc
aagtaccacatacagcaagcccaacggcagcaggtaaagcagtacctttc
taccactttagcaaataaacatgccaaccaagtcctgagcttgccatgtc
caaaccagcctggcgatcatgtcatgccaccggtgccggggagcagcgca
cccaacagccccatggctatgcttacgcttaactccaactgtgaaaaaga
gggattttataagtttgaagagcaaaacagggcagagagcgagtgcccag
gcatgaacacacattcacgagcgtcctgtatgcagatggatgatgtaatc
gatgacatcattagcctagaatcaagttataatgaggaaatcttgggctt
gatggatcctgctttgcaaatggcaaatacgttgcctgtctcgggaaact
tgattgatctttatggaaaccaaggtctgcccccaccaggcctcaccatc
agcaactcctgtccagccaaccttcccaacataaaaagggagctcacaga
gtctgaagcaagagcactggccaaagagaggcagaaaaaggacaatcaca
acctgattgaacgaagaagaagatttaacataaatgaccgcattaaagaa
ctaggtactttgattcccaagtcaaatgatccagacatgcgctggaacaa
gggaaccatcttaaaagcatccgtggactatatccgaaagttgcaacgag

aacagcaacgcgcaaaagaacttgaaaaccgacagaagaaactggagcac
gccaaccggcatttgttgctcagaatacaggaacttgaaatgcaggctcg
agctcatggactttcccttattccatccacgggtctctgctctccagatt
tggtgaatcggatcatcaagcaagaacccgttcttgagaactgcagccaa
gacctccttcagcatcatgcagacctaacctgtacaacaactctcgatct
cacggatggcaccatcaccttcaacaacaacctcggaactgggactgagg
ccaaccaagcctatagtgtccccacaaaaatgggatccaaactggaagac
atcctgatggacgacaccctttctcccgtcggtgtcactgatccactcct
ttcctcagtgtcccccggagcttccaaaacaagcagccggaggagcagta
tgagcatggaagagacggagcacacttgttagcgaatcctccctgcactg
cattcgcacaaactgcttcctttcttgattcgtagatttaataacttacc
tgaaggggttttcttgataattttcctttaatatgaaattttttttcatg
ctttatcaatagcccaggatatattttatttttagaattttgtgaaacag
acttgtatattctattttacaactacaaatgcctccaaagtattgtacaa
ataagtgtgcagtatctgtgaactgaattcaccacagactttagctttct
gagcaagaggattttgcgtcagagaaatgtctgtccatttttattcaggg
gaaacttgatttgagatttttatgcctgtgacttccttggaaatcaaatg
taaagtttaattgaaagaatgtaaagcaaccaaaaagaaaaaaaaaaaga
aagaaagaggaaaagaaatccatactaacccttttccattttataaatgt
attgattcattggtactgccttaaagatacagtacccctctagctttgtt
tagtctttatactgcaaactatttaaagaaatatgtattctgtaaaagaa
aaaaaaaaatgcggccttttcatgaggatcgtctggttagaaaacataact
gataccaaccgaaactgaagggagttagaccaaggctctgaaatataaag
tctaatcttgctctcttttattctgtgctgttacagttttcttcatcaat
gagtgtgatccagtttttcataagatattttattttgaaatggaaattaa
tgtcctctcaaagtaaaatattgaggagcactgaaagtatgttttacttt
tttttattttatttttgcttttgataagaaaaccgaactgggcatattt
ctaattggctttactatttttatttttaaattatgttttactgttcattt
gatttgtacagattctttattatcattgttcttttcaatatatttgtatt
aatttgtaagaatatgcatcttaaaatggcaagttttccatatttttaca
actcactggtggttttccgcattctttgtacacccatgaaagaaaacttt
tatgcaaggtcttgcatttaaaagacagctttgcgaatattttgtaaatt
acagtctcactcagaactgtttttggacacatttaaggtgtagtattaat
aggttaaaaccaggctttctagaaagaataaacttacatatttattttta
ggacatgaaaatagcaatattcttggagattgataaccatagcattaata
cgcccattatggtcatttaaattggggtttatttcagcaaacttgttgaa
tttatttttaagaaagaaatactgtattgggaagttactgttacttgata
acaatgttttaacaagaagcaatgttataaagttagtttcagtgcattat
ctacttgtgtagtcctatgcaataacagtagtgttacatgtatcaagcct
agatgttttatacagatgccatatagtgttatgagccaggctgttgaatg
gaatttctcagtagcagcctacaactgaatagcaagtggcataaagcata
tccattcagaatgaagtgccttaaatatagcagtagtcttttttggacta
gcactgactgaactgtaatgtaggggaaagtttcatgatggtatctatag
tcaagacgaacatgtagcatggtgcctatgtagacaatataagagcttcc
aattttccttcagatatttttaatattaaatatattttagtgacagagtg
ccaacttctttcatcaggaaaccttattcaggagggtttttaaaaagtgt
ttaaatgtcaaatgtgaattggtgatgggtgatggagggttcagagagga
gtgatcgtcagatgtgtgaatggacggtttaggtgaaaataatcaactgc
atagttcccatgcacgctgggcaatgagaatccttggaaacattggtgat

gctatcagttttatagctttatttcttaagggggtagggaaaattagttc
ccattctttcaaccccccttaactgtatagctcttttcctagaatagtgac
gcaaatctgcatgaacagctaattgtaccatagtgttcattgatacaatc
atagcattgtctatttttctcttcatatttatatggggggggagggcgctg
gatgcaaaagttgaagatcgtgatgctatgatgttagttttccttagctg
attttgagggtttttaaaaataaagcaaggttgactaacctacggccacg
ggaacaggaccatggttaagcaaccatatagaaagctttgttgaaagaaa
gtatggcatcttgtaccactgccctgactgtcacaactcctaaccttgcc
attgcctgcctccccctccccttctccttaagagacaatttctgcaggtg
gcaggtgagcaagcccaggagaatgctgcaatcttgggggtggttttatt
tatttctttttgccaaatagagtgtggattcatttcaggggctagctaa
gccaagaggcagtggtttgggcttgttgtttgtaacaagaaaatgatcca
caccactcccccgattcccgggtgcagaattgtaactcggggttgggcct
ctatatggagtgaccaaaatgccaaaattgtccatctgcctctgagtagg
gcaatggaaataccaaaccttctgactttgccaaaaagcatacaagcaac
ctggtcatacataggatgacaaaattctttctggttgtttttaaacaata
aagcaataagaacaaatacaatacataggaagttaaaagcacaaaggaat
gaacttattaatatttttgaaaaatgcactgggaaaaagttgatgtcaat
aacagtataaaacagccctatttcttgataaaaaatgacaaatgactgtc
tcttgcggatgcttggtactgtaatgttaataatagtcacctgctgttgg
atgcagcaataatttctgtatggtccatagcactgtatattatggatcga
tattaatgtatccaatgaaataatcgacttgttcttgatagcctcattaa
agcatttggtttttcacatag
>NM_198159 2
gtaaactccccgcgctggggcgggcggccgcgagccggcgagcgggcaga
gctcggcactgcgccggggcgcacggctcgggggacccaggcccagctac
cttccctccgcccccgggctctgttctcactttccagcagtggaaggacg
ggaagcgggagccatgcagtccgaatcggggatcgtgccggatttcgaag
tcggggaggagtttcatgaagagcccaaaacctattacgaactcaaaagt
caaccgctgaagagcagcagttccgccgagcatcctggggcctccaagcc
tccgataagctcctccagtatgacatcacgcatcttgctacgccagcaac
tcatgcgtgagcagatgcaggagcaggagcgcagggagcagcagcagaag
ctgcaggcggcccagttcatgcaacagagagtgcccgtgagtcagacacc
agccataaacgtcagtgtgcccaccacccttccctctgccacgcaggtgc
cgatggaagtccttaaggtgcagacccacctcgaaaaccccaccaagtac
cacatacagcaagcccaacggcagcaggtaaagcagtacctttctaccac
tttagcaaataaacatgccaaccaagtcctgagcttgccatgtccaaacc
agcctggcgatcatgtcatgccaccggtgccggggagcagcgcacccaac
agccccatggctatgcttacgcttaactccaactgtgaaaaagagggatt
ttataagtttgaagagcaaaacagggcagagagcgagtgcccaggcatga
acacacattcacgagcgtcctgtatgcagatggatgatgtaatcgatgac
atcattagcctagaatcaagttataatgaggaaatcttgggcttgatgga
tcctgctttgcaaatggcaaatacgttgcctgtctcgggaaacttgattg
atctttatggaaaccaaggtctgcccccaccaggcctcaccatcagcaac
tcctgtccagccaaccttcccaacataaaaagggagctcacagagtctga
agcaagagcactggccaaagagaggcagaaaaaggacaatcacaacctga
ttgaacgaagaagaagatttaacataaatgaccgcattaaagaactaggt
actttgattcccaagtcaaatgatccagacatgcgctggaacaagggaac
catcttaaaagcatccgtggactatatccgaaagttgcaacgagaacagc

aacgcgcaaaagaacttgaaaaccgacagaagaaactggagcacgccaac
cggcatttgttgctcagaatacaggaacttgaaatgcaggctcgagctca
tggactttcccttattccatccacgggtctctgctctccagatttggtga
atcggatcatcaagcaagaacccgttcttgagaactgcagccaagacctc
cttcagcatcatgcagacctaacctgtacaacaactctcgatctcacgga
tggcaccatcaccttcaacaacaacctcggaactgggactgaggccaacc
aagcctatagtgtccccacaaaaatgggatccaaactggaagacatcctg
atggacgacaccctttctcccgtcggtgtcactgatccactcctttcctc
agtgtcccccggagcttccaaaacaagcagccggaggagcagtatgagca
tggaagagacggagcacacttgttagcgaatcctccctgcactgcattcg
cacaaactgcttcctttcttgattcgtagatttaataacttacctgaagg
ggttttcttgataattttcctttaatatgaaattttttttcatgctttat
caatagcccaggatatattttatttttagaattttgtgaaacagacttgt
atattctattttacaactacaaatgcctccaaagtattgtacaaataagt
gtgcagtatctgtgaactgaattcaccacagactttagctttctgagcaa
gaggattttgcgtcagagaaatgtctgtccatttttattcaggggaaact
tgatttgagatttttatgcctgtgacttccttggaaatcaaatgtaaagt
ttaattgaaagaatgtaaagcaaccaaaaagaaaaaaaaaaagaaagaaa
gaggaaaagaaatccatactaacccttttccattttataaatgtattgat
tcattggtactgccttaaagatacagtacccctctagctttgtttagtct
ttatactgcaaactatttaaagaaatatgtattctgtaaaagaaaaaaaa
aatgcggccttttcatgaggatcgtctggttagaaaacataactgatacc
aaccgaaactgaagggagttagaccaaggctctgaaatataaagtctaat
cttgctctcttttattctgtgctgttacagttttcttcatcaatgagtgt
gatccagtttttcataagatattttattttgaaatggaaattaatgtcct
ctcaaagtaaaatattgaggagcactgaaagtatgttttactttttttt
attttatttttgcttttgataagaaaaccgaactgggcatatttctaatt
ggctttactatttttatttttaaattatgttttactgttcatttgatttg
tacagattctttattatcattgttcttttcaatatatttgtattaatttg
taagaatatgcatcttaaaatggcaagttttccatatttttacaactcac
tggtggttttccgcattctttgtacacccatgaaagaaaacttttatgca
aggtcttgcatttaaaagacagctttgcgaatattttgtaaattacagtc
tcactcagaactgtttttggacacatttaaggtgtagtattaataggtta
aaaccaggctttctagaaagaataaacttacatatttatttttaggacat
gaaaatagcaatattcttggagattgataaccatagcattaatacgccca
ttatggtcatttaaattggggtttatttcagcaaacttgttgaatttatt
tttaagaaagaaatactgtattgggaagttactgttacttgataacaatg
ttttaacaagaagcaatgttataaagttagtttcagtgcattatctactt
gtgtagtcctatgcaataacagtagtgttacatgtatcaagcctagatgt
tttatacagatgccatatagtgttatgagccaggctgttgaatggaattt
ctcagtagcagcctacaactgaatagcaagtggcataaagcatatccatt
cagaatgaagtgccttaaatatagcagtagtctttttttggactagcactg
actgaactgtaatgtaggggaaagtttcatgatggtatctatagtcaaga
cgaacatgtagcatggtgcctatgtagacaatataagagcttccaatttt
ccttcagatatttttaatattaaatatattttagtgacagagtgccaact
tctttcatcaggaaaccttattcaggagggtttttaaaaaagtgtttaaat
gtcaaatgtgaattggtgatgggtgatggagggttcagagaggagtgatc
gtcagatgtgtgaatggacggtttaggtgaaaataatcaactgcatagtt
cccatgcacgctgggcaatgagaatccttggaaacattggtgatgctatc

agttttatagctttatttcttaagggggtagggaaaattagttcccattc
tttcaacccccttaactgtatagctcttttcctagaatagtgacgcaaat
ctgcatgaacagctaattgtaccatagtgttcattgatacaatcatagca
ttgtctatttttctcttcatatttatatggggggggagggcgctggatgca
aaagttgaagatcgtgatgctatgatgttagttttccttagctgattttg
agggtttttaaaaataaagcaaggttgactaacctacggccacgggaaca
ggaccatggttaagcaaccatatagaaagctttgttgaaagaaagtatgg
catcttgtaccactgccctgactgtcacaactcctaaccttgccattgcc
tgcctccccctccccttctccttaagagacaatttctgcaggtggcaggt
gagcaagcccaggagaatgctgcaatcttgggggtggttttatttatttc
ttttttgccaaatagagtgtggattcatttcaggggctagctaagccaag
aggcagtggtttgggcttgttgtttgtaacaagaaaatgatccacaccac
tcccccgattcccgggtgcagaattgtaactcggggttgggcctctatat
ggagtgaccaaaatgccaaaattgtccatctgcctctgagtagggcaatg
gaaataccaaaccttctgactttgccaaaaagcatacaagcaacctggtc
atacataggatgacaaaattctttctggttgtttttaaacaataaagcaa
taagaacaaatacaatacataggaagttaaaagcacaaaggaatgaactt
attaatatttttgaaaaatgcactgggaaaaagttgatgtcaataacagt
ataaaacagccctatttcttgataaaaaatgacaaatgactgtctcttgc
ggatgcttggtactgtaatgttaataatagtcacctgctgttggatgcag
caataatttctgtatggtccatagcactgtatattatggatcgatattaa
tgtatccaatgaaataatcgacttgttcttgatagcctcattaaagcatt
tggtttttcacatag
>NM_198158 2
ctcgggataccttgtttatagtaccttctctttgccagtccatcttcaaa
ttggaattatagaaagtagagggagggatagtctaccgtctctcactgga
ttggtgccacctaaaacattgttatgctggaaatgctagaatataatcac
tatcaggtgcagacccacctcgaaaaccccaccaagtaccacatacagca
agcccaacggcagcaggtaaagcagtacctttctaccactttagcaaata
aacatgccaaccaagtcctgagcttgccatgtccaaaccagcctggcgat
catgtcatgccaccggtgccggggagcagcgcacccaacagccccatggc
tatgcttacgcttaactccaactgtgaaaaagagggattttataagtttg
aagagcaaaacagggcagagagcgagtgcccaggcatgaacacacattca
cgagcgtcctgtatgcagatggatgatgtaatcgatgacatcattagcct
agaatcaagttataatgaggaaatcttgggcttgatggatcctgctttgc
aaatggcaaatacgttgcctgtctcgggaaacttgattgatctttatgga
aaccaaggtctgcccccaccaggcctcaccatcagcaactcctgtccagc
caaccttcccaacataaaaagggagctcacagagtctgaagcaagagcac
tggccaaagagaggcagaaaaaggacaatcacaacctgattgaacgaaga
agaagatttaacataaatgaccgcattaaagaactaggtactttgattcc
caagtcaaatgatccagacatgcgctggaacaagggaaccatcttaaaag
catccgtggactatatccgaaagttgcaacgagaacagcaacgcgcaaaa
gaacttgaaaaccgacagaagaaactggagcacgccaaccggcatttgtt
gctcagaatacaggaacttgaaatgcaggctcgagctcatggactttccc
ttattccatccacgggtctctgctctccagatttggtgaatcggatcatc
aagcaagaacccgttcttgagaactgcagccaagacctccttcagcatca
tgcagacctaacctgtacaacaactctcgatctcacggatggcaccatca
ccttcaacaacaacctcggaactgggactgaggccaaccaagcctatagt
gtccccacaaaaatgggatccaaactggaagacatcctgatggacgacac

cctttctcccgtcggtgtcactgatccactcctttcctcagtgtcccccg
gagcttccaaaacaagcagccggaggagcagtatgagcatggaagagacg
gagcacacttgttagcgaatcctccctgcactgcattcgcacaaactgct
tcctttcttgattcgtagatttaataacttacctgaaggggttttcttga
taattttcctttaatatgaaatttttttttcatgctttatcaatagcccag
gatatattttatttttagaattttgtgaaacagacttgtatattctattt
tacaactacaaatgcctccaaagtattgtacaaataagtgtgcagtatct
gtgaactgaattcaccacagactttagctttctgagcaagaggattttgc
gtcagagaaatgtctgtccatttttattcaggggaaacttgatttgagat
ttttatgcctgtgacttccttggaaatcaaatgtaaagtttaattgaaag
aatgtaaagcaaccaaaaagaaaaaaaaaaaagaaagaaagaggaaaagaa
atccatactaacccttttccattttataaatgtattgattcattggtact
gccttaaagatacagtacccctctagctttgtttagtctttatactgcaa
actatttaaagaaatatgtattctgtaaaagaaaaaaaaaaatgcggcctt
ttcatgaggatcgtctggttagaaaacataactgataccaaccgaaactg
aagggagttagaccaaggctctgaaatataaagtctaatcttgctctctt
ttattctgtgctgttacagttttcttcatcaatgagtgtgatccagtttt
tcataagatattttattttgaaatggaaattaatgtcctctcaaagtaaa
atattgaggagcactgaaagtatgttttactttttttttattttattttt
gcttttgataagaaaaccgaactgggcatatttctaattggctttactat
ttttatttttaaattatgttttactgttcatttgatttgtacagattctt
tattatcattgttcttttcaatatatttgtattaatttgtaagaatatgc
atcttaaaatggcaagttttccatatttttacaactcactggtggttttc
cgcattctttgtacacccatgaaagaaaacttttatgcaaggtcttgcat
ttaaaagacagctttgcgaatattttgtaaattacagtctcactcagaac
tgtttttggacacatttaaggtgtagtattaataggttaaaaccaggctt
tctagaaagaataaacttacatatttatttttaggacatgaaaatagcaa
tattcttggagattgataaccatagcattaatacgcccattatggtcatt
taaattggggtttatttcagcaaacttgttgaatttatttttaagaaaga
aatactgtattgggaagttactgttacttgataacaatgttttaacaaga
agcaatgttataaagttagtttcagtgcattatctacttgtgtagtccta
tgcaataacagtagtgttacatgtatcaagcctagatgttttatacagat
gccatatagtgttatgagccaggctgttgaatggaatttctcagtagcag
cctacaactgaatagcaagtggcataaagcatatccattcagaatgaagt
gccttaaatatagcagtagtctttttttggactagcactgactgaactgta
atgtaggggaaagtttcatgatggtatctatagtcaagacgaacatgtag
catggtgcctatgtagacaatataagagcttccaattttccttcagatat
ttttaatattaaatatattttagtgacagagtgccaacttctttcatcag
gaaaccttattcaggagggtttttaaaaagtgtttaaatgtcaaatgtga
attggtgatgggtgatggagggttcagagaggagtgatcgtcagatgtgt
gaatggacggtttaggtgaaaataatcaactgcatagttcccatgcacgc
tgggcaatgagaatccttggaaacattggtgatgctatcagttttatagc
tttatttcttaagggggtagggaaaattagttcccattctttcaaccccc
ttaactgtatagctcttttcctagaatagtgacgcaaatctgcatgaaca
gctaattgtaccatagtgttcattgatacaatcatagcattgtctatttt
tctcttcatatttatatgggggggagggcgctggatgcaaaagttgaaga
tcgtgatgctatgatgttagttttccttagctgattttgagggtttttaa
aaataaagcaaggttgactaacctacggccacgggaacaggaccatggtt
aagcaaccatatagaaagctttgttgaaagaaagtatggcatcttgtacc

actgccctgactgtcacaactcctaaccttgccattgcctgcctccccct
ccccttctccttaagagacaatttctgcaggtggcaggtgagcaagccca
ggagaatgctgcaatcttgggggtggttttatttatttctttttgccaa
atagagtgtggattcatttcaggggctagctaagccaagaggcagtggtt
tgggcttgttgtttgtaacaagaaaatgatccacaccactcccccgattc
ccgggtgcagaattgtaactcggggttgggcctctatatggagtgaccaa
aatgccaaaattgtccatctgcctctgagtagggcaatggaaataccaaa
ccttctgactttgccaaaaagcatacaagcaacctggtcatacataggat
gacaaaattctttctggttgtttttaaacaataaagcaataagaacaaat
acaatacataggaagttaaaagcacaaaggaatgaacttattaatatttt
tgaaaaatgcactgggaaaaagttgatgtcaataacagtataaaacagcc
ctatttcttgataaaaaatgacaaatgactgtctcttgcggatgcttggt
actgtaatgttaataatagtcacctgctgttggatgcagcaataatttct
gtatggtccatagcactgtatattatggatcgatattaatgtatccaatg
aaataatcgacttgttcttgatagcctcattaaagcatttggtttttcac
atag
>NM_000248 3
ctcgggataccttgtttatagtaccttctctttgccagtccatcttcaaa
ttggaattatagaaagtagagggagggatagtctaccgtctctcactgga
ttggtgccacctaaaacattgttatgctggaaatgctagaatataatcac
tatcaggtgcagacccacctcgaaaaccccaccaagtaccacatacagca
agcccaacggcagcaggtaaagcagtacctttctaccactttagcaaata
aacatgccaaccaagtcctgagcttgccatgtccaaaccagcctggcgat
catgtcatgccaccggtgccggggagcagcgcacccaacagccccatggc
tatgcttacgcttaactccaactgtgaaaaagagggattttataagtttg
aagagcaaaacagggcagagagcgagtgcccaggcatgaacacacattca
cgagcgtcctgtatgcagatggatgatgtaatcgatgacatcattagcct
agaatcaagttataatgaggaaatcttgggcttgatggatcctgctttgc
aaatggcaaatacgttgcctgtctcgggaaacttgattgatctttatgga
aaccaaggtctgcccccaccaggcctcaccatcagcaactcctgtccagc
caaccttcccaacataaaaagggagctcacagcgtgtatttttcccacag
agtctgaagcaagagcactggccaaagagaggcagaaaaaggacaatcac
aacctgattgaacgaagaagaagatttaacataaatgaccgcattaaaga
actaggtactttgattcccaagtcaaatgatccagacatgcgctggaaca
agggaaccatcttaaaagcatccgtggactatatccgaaagttgcaacga
gaacagcaacgcgcaaaagaacttgaaaaccgacagaagaaactggagca
cgccaaccggcatttgttgctcagaatacaggaacttgaaatgcaggctc
gagctcatggactttcccttattccatccacgggtctctgctctccagat
ttggtgaatcggatcatcaagcaagaacccgttcttgagaactgcagcca
agacctccttcagcatcatgcagacctaacctgtacaacaactctcgatc
tcacggatggcaccatcaccttcaacaacaacctcggaactgggactgag
gccaaccaagcctatagtgtccccacaaaaatgggatccaaactggaaga
catcctgatggacgacaccctttctcccgtcggtgtcactgatccactcc
tttcctcagtgtcccccggagcttccaaaacaagcagccggaggagcagt
atgagcatggaagagacggagcacacttgttagcgaatcctccctgcact
gcattcgcacaaactgcttcctttcttgattcgtagatttaataacttac
ctgaaggggttttcttgataattttcctttaatatgaaattttttttcat
gctttatcaatagcccaggatatattttatttttagaattttgtgaaaca
gacttgtatattctattttacaactacaaatgcctccaaagtattgtaca

aataagtgtgcagtatctgtgaactgaattcaccacagactttagctttc
tgagcaagaggattttgcgtcagagaaatgtctgtccatttttattcagg
ggaaacttgatttgagatttttatgcctgtgacttccttggaaatcaaat
gtaaagtttaattgaaagaatgtaaagcaaccaaaaagaaaaaaaaaaag
aaagaaagaggaaaagaaatccatactaacccttttccattttataaatg
tattgattcattggtactgccttaaagatacagtacccctctagctttgt
ttagtctttatactgcaaactatttaaagaaatatgtattctgtaaaaga
aaaaaaaaatgcggccttttcatgaggatcgtctggttagaaaacataac
tgataccaaccgaaactgaagggagttagaccaaggctctgaaatataaa
gtctaatcttgctctcttttattctgtgctgttacagttttcttcatcaa
tgagtgtgatccagtttttcataagatattttattttgaaatggaaatta
atgtcctctcaaagtaaaatattgaggagcactgaaagtatgttttactt
tttttttattttatttttgcttttgataagaaaaccgaactgggcatatt
tctaattggctttactatttttatttttaaattatgttttactgttcatt
tgatttgtacagattctttattatcattgttcttttcaatatatttgtat
taatttgtaagaatatgcatcttaaaatggcaagttttccatatttttac
aactcactggtggttttccgcattctttgtacacccatgaaagaaaactt
ttatgcaaggtcttgcatttaaaagacagctttgcgaatattttgtaaat
tacagtctcactcagaactgtttttggacacatttaaggtgtagtattaa
taggttaaaaccaggctttctagaaagaataaacttacatatttattttt
aggacatgaaaatagcaatattcttggagattgataaccatagcattaat
acgcccattatggtcatttaaattggggtttatttcagcaaacttgttga
atttatttttaagaaagaaatactgtattgggaagttactgttacttgat
aacaatgttttaacaagaagcaatgttataaagttagtttcagtgcatta
tctacttgtgtagtcctatgcaataacagtagtgttacatgtatcaagcc
tagatgttttatacagatgccatatagtgttatgagccaggctgttgaat
ggaatttctcagtagcagcctacaactgaatagcaagtggcataaagcat
atccattcagaatgaagtgccttaaatatagcagtagtctttttttggact
agcactgactgaactgtaatgtaggggaaagtttcatgatggtatctata
gtcaagacgaacatgtagcatggtgcctatgtagacaatataagagcttc
caattttccttcagatatttttaatattaaatatattttagtgacagagt
gccaacttctttcatcaggaaaccttattcaggagggtttttaaaaagtg
tttaaatgtcaaatgtgaattggtgatgggtgatggagggttcagagagg
agtgatcgtcagatgtgtgaatggacggtttaggtgaaaataatcaactg
catagttcccatgcacgctgggcaatgagaatccttggaaacattggtga
tgctatcagttttatagctttatttcttaagggggtagggaaaattagtt
cccattctttcaacccccttaactgtatagctcttttcctagaatagtga
cgcaaatctgcatgaacagctaattgtaccatagtgttcattgatacaat
catagcattgtctatttttctcttcatatttatatgggggggagggcgct
ggatgcaaaagttgaagatcgtgatgctatgatgttagttttccttagct
gattttgagggtttttaaaaataaagcaaggttgactaacctacggccac
gggaacaggaccatggttaagcaaccatatagaaagctttgttgaaagaa
agtatggcatcttgtaccactgccctgactgtcacaactcctaaccttgc
cattgcctgcctccccctccccttctccttaagagacaatttctgcaggt
ggcaggtgagcaagcccaggagaatgctgcaatcttgggggtggttttat
ttatttctttttttgccaaatagagtgtggattcatttcaggggctagcta
agccaagaggcagtggtttgggcttgttgtttgtaacaagaaaatgatcc
acaccactcccccgattcccgggtgcagaattgtaactcggggttgggcc
tctatatggagtgaccaaaatgccaaaattgtccatctgcctctgagtag

ggcaatggaaataccaaaccttctgactttgccaaaaagcatacaagcaa
cctggtcatacataggatgacaaaattctttctggttgtttttaaacaat
aaagcaataagaacaaatacaatacataggaagttaaaagcacaaaggaa
tgaacttattaatatttttgaaaaatgcactgggaaaaagttgatgtcaa
taacagtataaaacagccctatttcttgataaaaaatgacaaatgactgt
ctcttgcggatgcttggtactgtaatgttaataatagtcacctgctgttg
gatgcagcaataatttctgtatggtccatagcactgtatattatggatcg
atattaatgtatccaatgaaataatcgacttgttcttgatagcctcatta
aagcatttggtttttcacatag
>NM_198178 2
ctcgggataccttgtttatagtaccttctctttgccagtccatcttcaaa
ttggaattatagaaagtagagggagggatagtctaccgtctctcactgga
ttggtgccacctaaaacattgttatgctggaaatgctagaatataatcac
tatcaggtgcagacccacctcgaaaaccccaccaagtaccacatacagca
agcccaacggcagcagggattttataagtttgaagagcaaaacagggcag
agagcgagtgcccaggcatgaacacacattcacgagcgtcctgtatgcag
atggatgatgtaatcgatgacatcattagcctagaatcaagttataatga
ggaaatcttgggcttgatggatcctgctttgcaaatggcaaatacgttgc
ctgtctcgggaaacttgattgatctttatggaaaccaaggtctgcccccca
ccaggcctcaccatcagcaactcctgtccagccaaccttcccaacataaa
aagggagctcacagagtctgaagcaagagcactggccaaagagaggcaga
aaaaggacaatcacaacctgattgaacgaagaagaagatttaacataaat
gaccgcattaaagaactaggtactttgattcccaagtcaaatgatccaga
catgcgctggaacaagggaaccatcttaaaagcatccgtggactatatcc
gaaagttgcaacgagaacagcaacgcgcaaaagaacttgaaaaccgacag
aagaaactggagcacgccaaccggcatttgttgctcagaatacaggaact
tgaaatgcaggctcgagctcatggactttcccttattccatccacgggtc
tctgctctccagatttggtgaatcggatcatcaagcaagaacccgttctt
gagaactgcagccaagacctccttcagcatcatgcagacctaacctgtac
aacaactctcgatctcacggatggcaccatcaccttcaacaacaacctcg
gaactgggactgaggccaaccaagcctatagtgtccccacaaaaatggga
tccaaactggaagacatcctgatggacgacaccctttctcccgtcggtgt
cactgatccactcctttcctcagtgtcccccggagcttccaaaacaagca
gccggaggagcagtatgagcatggaagagacggagcacacttgttagcga
atcctccctgcactgcattcgcacaaactgcttcctttcttgattcgtag
atttaataacttacctgaaggggtttcttgataattttcctttaatatg
aaatttttttcatgctttatcaatagcccaggatatattttatttttag
aattttgtgaaacagacttgtatattctattttacaactacaaatgcctc
caaagtattgtacaaataagtgtgcagtatctgtgaactgaattcaccac
agactttagctttctgagcaagaggattttgcgtcagagaaatgtctgtc
catttttattcaggggaaacttgatttgagatttttatgcctgtgacttc
cttggaaatcaaatgtaaagtttaattgaaagaatgtaaagcaaccaaaa
agaaaaaaaaaaagaaagaaagaggaaaagaaatccatactaaccctttt
ccattttataaatgtattgattcattggtactgccttaaagatacagtac
ccctctagctttgtttagtctttatactgcaaactatttaaagaaatatg
tattctgtaaaagaaaaaaaaaatgcggccttttcatgaggatcgtctgg
ttagaaaacataactgataccaaccgaaactgaagggagttagaccaagg
ctctgaaatataaagtctaatcttgctctcttttattctgtgctgttaca
gttttcttcatcaatgagtgtgatccagtttttcataagatattttattt

tgaaatggaaattaatgtcctctcaaagtaaaatattgaggagcactgaa
agtatgttttacttttttttattttatttttgcttttgataagaaaacc
gaactgggcatatttctaattggctttactatttttatttttaaattatg
ttttactgttcatttgatttgtacagattctttattatcattgttctttt
caatatatttgtattaatttgtaagaatatgcatcttaaaatggcaagtt
ttccatatttttacaactcactggtggttttccgcattctttgtacaccc
atgaaagaaaacttttatgcaaggtcttgcatttaaaagacagctttgcg
aatattttgtaaattacagtctcactcagaactgtttttggacacattta
aggtgtagtattaataggttaaaaccaggctttctagaaagaataaactt
acatatttatttttaggacatgaaaatagcaatattcttggagattgata
accatagcattaatacgcccattatggtcatttaaattggggtttatttc
agcaaacttgttgaatttatttttaagaaagaaatactgtattgggaagt
tactgttacttgataacaatgttttaacaagaagcaatgttataaagtta
gtttcagtgcattatctacttgtgtagtcctatgcaataacagtagtgtt
acatgtatcaagcctagatgttttatacagatgccatatagtgttatgag
ccaggctgttgaatggaatttctcagtagcagcctacaactgaatagcaa
gtggcataaagcatatccattcagaatgaagtgccttaaatatagcagta
gtctttttggactagcactgactgaactgtaatgtaggggaaagtttca
tgatggtatctatagtcaagacgaacatgtagcatggtgcctatgtagac
aatataagagcttccaattttccttcagatatttttaatattaaatatat
tttagtgacagagtgccaacttctttcatcaggaaaccttattcaggagg
gtttttaaaaagtgtttaaatgtcaaatgtgaattggtgatgggtgatgg
agggttcagagaggagtgatcgtcagatgtgtgaatggacggtttaggtg
aaaataatcaactgcatagttcccatgcacgctgggcaatgagaatcctt
ggaaacattggtgatgctatcagttttatagctttatttcttaaggggggt
agggaaaattagttcccattctttcaacccccttaactgtatagctcttt
tcctagaatagtgacgcaaatctgcatgaacagctaattgtaccatagtg
ttcattgatacaatcatagcattgtctatttttctcttcatatttatatg
gggggggagggcgctggatgcaaaagttgaagatcgtgatgctatgatgtt
agttttccttagctgattttgagggtttttaaaaataaagcaaggttgac
taacctacggccacgggaacaggaccatggttaagcaaccatatagaaag
ctttgttgaaagaaagtatggcatcttgtaccactgccctgactgtcaca
actcctaaccttgccattgcctgcctccccctccccttctccttaagaga
caatttctgcaggtggcaggtgagcaagcccaggagaatgctgcaatctt
gggggtggttttatttatttctttttgccaaatagagtgtggattcatt
tcaggggctagctaagccaagaggcagtggtttgggcttgttgtttgtaa
caagaaaatgatccacaccactcccccgattcccgggtgcagaattgtaa
ctcggggttgggcctctatatggagtgaccaaaatgccaaaattgtccat
ctgcctctgagtagggcaatggaaataccaaaccttctgactttgccaaa
aagcatacaagcaacctggtcatacataggatgacaaaattctttctggt
tgtttttaaacaataaagcaataagaacaaatacaatacataggaagtta
aaagcacaaaggaatgaacttattaatatttttgaaaaatgcactgggaa
aaagttgatgtcaataacagtataaaacagccctatttcttgataaaaaa
tgacaaatgactgtctcttgcggatgcttggtactgtaatgttaataata
gtcacctgctgttggatgcagcaataatttctgtatggtccatagcactg
tatattatggatcgatattaatgtatccaatgaaataatcgacttgttct
tgatagcctcattaaagcatttggtttttcacatag
>NM_198256 2
agaggggggcggtgtactgcgcatgcgggaagatggcgggccgggcgact

tgagatccgcgggtctccctcctctttttccgtctgcgtcgggagctccc
gggcacgtgaggccgtgccgcgtttactggcgggcgggacggcctagccg
ggcggcgcctcggaggaagccgcggaccccttaggtgctgggcccttgga
aatcggcgcgtgggggggcggtgctcgagctgagcgcgagagggcgggaga
gctcgtggggtgcgaggggagcaggacgcccggccgggcagcatgagtca
gcagcggccggcgaggaagttacccagtctcctcctggacccgacggagg
agacggttcgccgtcggtgccgagaccccatcaacgtggagggcctgctg
ccatcaaaaataaggattaatttagaagataatgtacaatatgtgtccat
gagaaaagctctaaaagtgaagagacctcgttttgatgtatcgctggttt
atttaactcgaaaatttatggatcttgtcagatctgctcccgggggtatt
cttgacttaaacaaggttgcaacgaaactgggagtccgaaagcggagagt
gtatgacatcaccaatgtcttagatggaatcgacctcgttgaaaagaaat
ccaagaaccatattagatggataggatctgatcttagcaattttggagca
gttccccaacaaaagaagctacaggaggaactttctgacttatcagcaat
ggaagatgctttggatgagttaattaaggattgtgctcagcagctgtttg
agttaacagatgacaaagaaaatgaaagactagcatatgtgacctatcaa
gacattcatagcattcaggccttccatgaacagatcgtcattgcagttaa
agctccagcagaaaccagattggatgttccagctcccagagaagactcta
tcacagtgcacataaggagcaccaacggacctatcgatgtctatttgtgt
gaagtggagcagggtcagaccagtaacaaaaggtctgaaggtgtcgggac
ctcttcatctgagagcactcatccagaaggccctgaggaagaagaaaatc
ctcagcaaagtgaagaattgcttgaagtaagcaactgatggcatttgaga
atttatgtatcactgagtttttttgggaatatcttcgtggagaattacgca
tcaaatttgattctcagagcaataaattatccatgaagtgctctcgttct
cagtagcggcatcatggccagtagtgtctttgaggagttcaccacttaga
ttactgagtaattgtggtttccacatttgaaaacaactccttttataatt
attcactgctttttgtcagtgaaatagacatcttgcctcctgaagtagct
tcatcacagagtgtcatgaagacagacagtcaggctgaaatggacagttc
tttgtggactctacccttcccttcaaggagtatgtcatatatcacaaaag
aaattgccttacactggttcatgtttgcagttactgttgtacattgcata
gatgtacacacgaatttaaatgtgatgtctttgtatatatctgtataatg
ttgagattacttacgaaatatgtctgagtgacacttttcacccttgtaca
gccaaaataatgtatatatggaaagtgacagacaaattctctaatctctt
tggtacctataacttattagaatcctctggatgagggttagaagagactt
tttccaaacttctacatgtagaagtatcataaatgtgctacacatttatg
tttgtggatttaattaaagtattttaatatggttttcagtgctaaaattg
gagtcagatacttcttggttttaagctgtctacctaattgctgtctccca
gcagactggtggcatgcccagtggctttgggggcaaggatagaaatgcca
tcaggaaatagctgaattcattgtgaaacatgaattcagtcatggtgata
attggaaactcctttcaggtttttgcaagtagattttgtaatgtttgtgt
atgcagccttgctgttgagtcagtccaaggggttttacttaggacaagtt
gtaccttgccctctctccagctctgctcccacattttcacatacctagct
gtttctacctcattgggtaagtcatttaccactctgtgcctcagtttact
ctgtagtttaccattagactgtgagctccttgagggactttgtcataatc
actgttacatcccagtgcctcacaccatgcctggcccttaagaagtgctc
aataaatgtctgaacaaataagtgagtggagtgagtgaattgtaagatca
gaataataatatttggtttgtctatcgtacaagattcctgtatcgtttga
atattgcttttaaagaaatatttgaagcgacttcaaattcagactgtgtt
taaaaagatttaaaatcggaatccaaaatcaaagtattaaaaaataatgc

tgagtgtttaggattcacatacctctgagctctggtggcttttgccagtc
ctgtccctctgctgagcaccagcccttcctgcagcagtcctgccggtgtg
tttctgtgcacctggggatgaagtgtgctcctcccccagcggttagcctc
aatgcttttcctccctctgttttattatttgaaattagccaaagaaaagc
tgcagggggcactgctgatcatcactggactggtgggtcgtactgtttga
cagaatagggaatacaggaggaggagcaagcctagggtaaaacttaggtt
tgggacatgcttgatgaaagcaagagttgagcaggttgttgtgtgtatgt
aggtcaggtgctcagcggaaaagtctgagctgtgtagtacgtcaggcaga
tgtcagtttagatgcgatagtttaagccacaagagtaggagaggtcatct
aaagagtacagcaggcctaagggcatctatatttaaggagaaatacagaa
gaagtctcctgagaaggaataatggggcgggtggggagggagaaggcagt
gtcccaaaaaccaacaaaggttttaccaaaggtaataccatcatttctga
gcagcagttccttaacttgtgaatgtggcaaatgttctcctttacaaatt
gttctgaaggtaaaatgagatcatgtaaacgaaaatcctttgtaaattac
agatgtgtgttataaatgaagtatctctcgagtcactcca
>NM_001849 3
gcttactcggcgcccgcgcctcgggccgtcgggagcggagcctcctcggg
accaggacttcagggccacaggtgctgccaagatgctccagggcacctgc
tccgtgctcctgctctggggaatcctgggggccatccaggcccagcagca
ggaggtcatctcgccggacactaccgagagaaacaacaactgcccagaga
agaccgactgccccatccacgtgtacttcgtgctggacacctcggagagc
gtcaccatgcagtcccccacggacatcctgctcttccacatgaagcagtt
cgtgccgcagttcatcagccagctgcagaacgagttctacctggaccagg
tggcgctgagctggcgctacggcggcctgcacttctctgaccaggtggag
gtgttcagcccaccgggcagcgaccgggcctccttcatcaagaacctgca
gggcatcagctccttccgccgcggcaccttcaccgactgcgcgctggcca
acatgacggagcagatccggcaggaccgcagcaagggcaccgtccacttc
gccgtggtcatcaccgacggccacgtcaccggcagcccctgcgggggcat
caagctgcaggccgagcgggcccgcgaggagggcatccggctcttcgccg
tggcccccaaccagaacctgaaggagcagggcctgcgggacatcgccagc
acgccgcacgagctctaccgcaacgactacgccaccatgctgcccgactc
caccgagatcgaccaggacaccatcaaccgcatcatcaaggtcatgaaac
acgaagcctacggagagtgctacaaggtgagctgcctggaaatccctggg
ccctctggccccaagggctaccgtggacagaagggtgccaagggcaacat
gggtgagccgggagagcctggccagaagggaagacagggagacccgggca
tcgaaggccccattggattcccaggacccaagggcgttcctggcttcaaa
ggagagaagggtgaatttggagccgacggtcgcaagggggccccctggcct
ggctggcaagaacgggaccgatggacagaagggcaagctggggcgcatcg
gacctcctggctgcaagggagaccctggaaaccggggccccgacggttac
ccgggggaagcagggagtccaggggagcgaggagaccaaggcggcaaggg
ggaccctggccgcccaggacgcagagggcccccgggagaaatcggggcca
agggaagcaaggggtatcaaggcaacagtggagccccaggaagtcctggt
gtgaaaggagccaagggcgggcctgggccccgcggacccaaaggcgagcc
ggggcgcaggggagaccccggcaccaagggcagcccaggcagcgatggcc
ccaagggggagaagggggaccctggccctgaggggccccgcggcctggct
ggagaggttggcaacaaaggagccaagggagaccgaggcttgcctggacc
cagaggcccccagggagctcttggggagcccggaaagcagggatctcggg
gagaccccggtgatgcaggaccccgtggagactcaggacagccaggcccc
aagggagaccccggcaggcctggattcagctacccaggaccccgaggagc

acccggagaaaaaggcgagcccggcccacgcggccccgagggaggccgag
gcgactttggcttgaaaggagaacctgggaggaaaggagagaaaggagag
cctgcggatcctggtccccctggtgagccaggccctcgggggccaagagg
agtcccaggacccgagggtgagcccggccccccctggagaccccggtctca
cggagtgtgacgtcatgacctacgtgagggagacctgcgggtgctgcgac
tgtgagaagcgctgtggcgccctggacgtggtcttcgtcatcgacagctc
cgagagcattgggtacaccaacttcacactggagaagaacttcgtcatca
acgtggtcaacaggctgggtgccatcgctaaggaccccaagtccgagaca
gggacgcgtgtgggcgtggtgcagtacagccacgagggcacctttgaggc
catccagctggacgacgaacgtatcgactccctgtcgagcttcaaggagg
ctgtcaagaacctcgagtggattgcgggcggcacctggacaccctcagcc
ctcaagtttgcctacgaccgcctcatcaaggagagccggcgccagaagac
acgtgtgtttgcggtggtcatcacggacgggcgccacgaccctcgggacg
atgacctcaacttgcgggcgctgtgcgaccgcgacgtcacagtgacggcc
atcggcatcggggacatgttccacgagaagcacgagagtgaaaacctcta
ctccatcgcctgcgacaagccacagcaggtgcgcaacatgacgctgttct
ccgacctggtcgctgagaagttcatcgatgacatggaggacgtcctctgc
ccggaccctcagatcgtgtgcccagaccttccctgccaaacagagctgtc
cgtggcacagtgcacgcagcggcccgtggacatcgtcttcctgctggacg
gctccgagcggctgggtgagcagaacttccacaaggcccggcgcttcgtg
gagcaggtggcgcggcggctgacgctggcccggagggacgacgaccctct
caacgcacgcgtggcgctgctgcagtttggtggccccggcgagcagcagg
tggccttcccgctgagccacaacctcacggccatccacgaggcgctggag
accacacaatacctgaactccttctcgcacgtgggcgcaggcgtggtgca
cgccatcaatgccatcgtgcgcagcccgcgtggcggggcccggaggcacg
cagagctgtccttcgtgttcctcacggacggcgtcacgggcaacgacagt
ctgcacgagtcggcgcactccatgcgcaagcagaacgtggtacccaccgt
gctggccttgggcagcgacgtggacatggacgtgctcaccacgctcagcc
tgggtgaccgcgccgccgtgttccacgagaaggactatgacagcctggcg
caacccggcttcttcgaccgcttcatccgctggatctgctagcgccgccg
cccgggccccgcagtcgagggtcgtgagcccaccccgtccatggtgctaa
gcgggcccgggtcccacacggccagcaccgctgctcactcggacgacgcc
ctgggcctgcacctctccagctcctcccacggggtccccgtagccccggc
ccccgcccagccccaggtctccccaggccctccgcaggctgcccggcctc
cctccccctgcagccatcccaaggctcctgacctacctggcccctgagct
ctggagcaagccctgacccaataaaggctttgaacccataaaaaaaaaaa
aaaaa
>NM_005157 4
aaaatgttggagatctgcctgaagctggtgggctgcaaatccaagaaggg
gctgtcctcgtcctccagctgttatctggaagaagcccttcagcggccag
tagcatctgactttgagcctcagggtctgagtgaagccgctcgttggaac
tccaaggaaaaccttctcgctggacccagtgaaaatgaccccaacctttt
cgttgcactgtatgattttgtggccagtggagataacactctaagcataa
ctaaaggtgaaaagctccgggtcttaggctataatcacaatggggaatgg
tgtgaagcccaaaccaaaaatggccaaggctgggtcccaagcaactacat
cacgccagtcaacagtctggagaaacactcctggtaccatgggcctgtgt
cccgcaatgccgctgagtatctgctgagcagcgggatcaatggcagcttc
ttggtgcgtgagagtgagagcagtcctggccagaggtccatctcgctgag
atacgaagggagggtgtaccattacaggatcaacactgcttctgatggca

agctctacgtctcctccgagagccgcttcaacaccctggccgagttggtt
catcatcattcaacggtggccgacgggctcatcaccacgctccattatcc
agccccaaagcgcaacaagcccactgtctatggtgtgtcccccaactacg
acaagtgggagatggaacgcacggacatcaccatgaagcacaagctgggc
gggggccagtacggggaggtgtacgagggcgtgtggaagaaatacagcct
gacggtggccgtgaagaccttgaaggaggacaccatggaggtggaagagt
tcttgaaagaagctgcagtcatgaaagagatcaaacaccctaacctggtg
cagctccttggggtctgcacccgggagcccccgttctatatcatcactga
gttcatgacctacgggaacctcctggactacctgagggagtgcaaccggc
aggaggtgaacgccgtggtgctgctgtacatggccactcagatctcgtca
gccatggagtacctggagaagaaaaacttcatccacagagatcttgctgc
ccgaaactgcctggtaggggagaaccacttggtgaaggtagctgattttg
gcctgagcaggttgatgacaggggacacctacacagcccatgctggagcc
aagttccccatcaaatggactgcacccgagagcctggcctacaacaagtt
ctccatcaagtccgacgtctgggcatttggagtattgctttgggaaattg
ctacctatggcatgtccccttacccgggaattgacctgtcccaggtgtat
gagctgctagagaaggactaccgcatggagcgcccagaaggctgcccaga
gaaggtctatgaactcatgcgagcatgttggcagtggaatccctctgacc
ggccctcctttgctgaaatccaccaagcctttgaaacaatgttccaggaa
tccagtatctcagacgaagtggaaaaggagctggggaaacaaggcgtccg
tggggctgtgagtaccttgctgcaggccccagagctgcccaccaagacga
ggacctccaggagagctgcagagcacagagacaccactgacgtgcctgag
atgcctcactccaagggccagggagagagcgatcctctggaccatgagcc
tgccgtgtctccattgctccctcgaaaagagcgaggtcccccggagggcg
gcctgaatgaagatgagcgccttctccccaaagacaaaaagaccaacttg
ttcagcgccttgatcaagaagaagaagaagacagccccaacccctcccaa
acgcagcagctccttccgggagatggacggccagccggagcgcagagggg
ccggcgaggaagagggccgagacatcagcaacggggcactggctttcacc
cccttggacacagctgacccagccaagtccccaaagcccagcaatggggc
tggggtccccaatggagccctccgggagtccgggggctcaggcttccggt
ctccccacctgtggaagaagtccagcacgctgaccagcagccgcctagcc
accggcgaggaggagggcggtggcagctccagcaagcgcttcctgcgctc
ttgctccgcctcctgcgttccccatggggccaaggacacggagtggaggt
cagtcacgctgcctcgggacttgcagtccacgggaagacagtttgactcg
tccacatttggagggcacaaaagtgagaagccggctctgcctcggaagag
ggcaggggagaacaggtctgaccaggtgacccgaggcacagtaacgcctc
cccccaggctggtgaaaaagaatgaggaagctgctgatgaggtcttcaaa
gacatcatggagtccagcccgggctccagcccgcccaacctgactccaaa
acccctccggcggcaggtcaccgtggcccctgcctcgggcctcccccaca
aggaagaagctggaaagggcagtgccttagggacccctgctgcagctgag
ccagtgacccccaccagcaaagcaggctcaggtgcaccagggggcaccag
caagggccccgccgaggagtccagagtgaggaggcacaagcactcctctg
agtcgccagggagggacaaggggaaattgtccaggctcaaacctgccccg
ccgcccccaccagcagcctctgcagggaaggctggaggaaagccctcgca
gagcccgagccaggaggcggccggggaggcagtcctgggcgcaaagacaa
aagccacgagtctggttgatgctgtgaacagtgacgctgccaagcccagc
cagccgggagagggcctcaaaaagcccgtgctcccggccactccaaagcc
acagtccgccaagccgtcggggacccccatcagcccagcccccgttccct
ccacgttgccatcagcatcctcggccctggcaggggaccagccgtcttcc

accgccttcatccctctcatatcaacccgagtgtctcttcggaaaacccg
ccagcctccagagcggatcgccagcggcgccatcaccaagggcgtggtcc
tggacagcaccgaggcgctgtgcctcgccatctctaggaactccgagcag
atggccagccacagcgcagtgctggaggccggcaaaaacctctacacgtt
ctgcgtgagctatgtggattccatccagcaaatgaggaacaagtttgcct
tccgagaggccatcaacaaactggagaataatctccgggagcttcagatc
tgcccggcgacagcaggcagtggtccagcggccactcaggacttcagcaa
gctcctcagttcggtgaaggaaatcagtgacatagtgcagaggtagcagc
agtcaggggtcaggtgtcaggcccgtcggagctgcctgcagcacatgcgg
gctcgcccatacccgtgacagtggctgacaagggactagtgagtcagcac
cttggcccaggagctctgcgccaggcagagctgagggccctgtggagtcc
agctctactacctacgtttgcaccgcctgccctcccgcaccttcctcctc
cccgctccgtctctgtcctcgaattttatctgtggagttcctgctccgtg
gactgcagtcggcatgccaggacccgccagccccgctcccacctagtgcc
ccagactgagctctccaggccaggtgggaacggctgatgtggactgtctt
tttcattttttctctctggagcccctcctcccccggctgggcctccttc
ttccacttctccaagaatggaagcctgaactgaggccttgtgtgtcaggc
cctctgcctgcactccctggccttgcccgtcgtgtgctgaagacatgttt
caagaaccgcatttcgggaagggcatgcacgggcatgcacacggctggtc
actctgccctctgctgctgcccggggtggggtgcactcgccatttcctca
cgtgcaggacagctcttgatttgggtggaaaacagggtgctaaagccaac
cagcctttgggtcctgggcaggtgggagctgaaaaggatcgaggcatggg
gcatgtcctttccatctgtccacatccccagagcccagctcttgctctct
tgtgacgtgcactgtgaatcctggcaagaaagcttgagtctcaagggtgg
caggtcactgtcactgccgacatccctcccccagcagaatggaggcaggg
gacaagggaggcagtggctagtggggtgaacagctggtgccaaatagccc
cagactgggcccaggcaggtctgcaagggcccagagtgaaccgtcctttc
acacatctgggtgccctgaaagggcccttcccctcccccactcctctaag
acaaagtagattcttacaaggccctttcctttggaacaagacagccttca
cttttctgagttcttgaagcatttcaaagccctgcctctgtgtagccgcc
ctgagagagaatagagctgccactgggcacctgcgcacaggtgggaggaa
agggcctggccagtcctggtcctggctgcactcttgaactgggcgaatgt
cttatttaattaccgtgagtgacatagcctcatgttctgtgggggtcatc
agggagggttaggaaaaccacaaacggagcccctgaaagcctcacgtatt
tcacagagcacgcctgccatcttctccccgaggctgccccaggccggagc
ccagatacgggggctgtgactctgggcagggacccggggtctcctggacc
ttgacagagcagctaactccgagagcagtgggcaggtggccgcccctgag
gcttcacgccgggagaagccaccttcccacccccttcataccgcctcgtgc
cagcagcctcgcacaggccctagctttacgctcatcacctaaacttgtac
tttatttttctgatagaaatggtttcctctggatcgttttatgcggttct
tacagcacatcacctctttgcccccgacggctgtgacgcagccggaggga
ggcactagtcaccgacagcggccttgaagacagagcaaagcgcccaccca
ggtcccccgactgcctgtctccatgaggtactggtcccttccttttgtta
acgtgatgtgccactatattttacacgtatctcttggtatgcatctttta
tagacgctcttttctaagtggcgtgtgcatagcgtcctgccctgccccct
cgggggcctgtggtggctccccctctgcttctcggggtccagtgcatttt
gtttctgtatatgattctctgtggttttttttgaatccaaatctgtcctc
tgtagtatttttttaaataaatcagtgtttacattagaa
>NM_001856 3

agtctagccgcctctggtgccgctggagccccctctaggccctcctcctc
ctcctccctggcgggccggtccagctgtgttccattagtgtccctggcag
gcccccagccccctggaaccgcctctcttcccccacccggagtggccggc
agtccccggcaggcgtgcgccgggcagcagacagccgggagagctcgagc
gccaggaagctggggacccgtgaccgtcacggagagatagagaacccccta
gcggacctgaggaacctgggctctgcttcctccgcctgctgcctggactg
ctcagtgaggtgggggggatccccggccaccggtgctgtggctgtagctga
cctctttggaccgggatgtgggtatcctgggctcctggcctgtggctgct
cggtctttgggctaccttcggccatggggcaaatacaggtgcacaatgcc
caccttcacagcaggaaggactcaaattggaacacagtagtagcctgcca
gccaacgtgactggcttcaacctcatccaccgactcagcctcatgaagac
gtctgccatcaagaagatccgcaaccccaaggggcctctcatcctgcgcc
tgggggcggcccccgtgacccagcccacgcgaagagtattccctcggggt
ctcccggaggagtttgccctggtgctgacactactgctgaagaaacacac
ccaccagaagacgtggtatctgtttcaagtgaccgatgcaaatgggtatc
cacagatatccctggaagtcaacagccaagagcggagcctggagctcagg
gcccagggccaggatggcgactttgtgtcctgcatcttcccagtgcccca
gctcttcgacttgcgttggcacaagctgatgctgagtgtggctggacgtg
tggcctctgtgcacgtggactgcagctcagcctcctcccagcctctgggg
ccccgacgacccatgaggcctgtgggccatgtatttctaggcttggatgc
tgagcagggcaagcctgtctcgtttgaccttcagcaggtgcacatctact
gtgacccggagctcgtgctggaggagggctgctgtgagattttaccagca
gggtgcccccccagagacctccaaggcccgccgggacacccagagcaatga
gctcattgagatcaatccacagtctgaaggcaaggtctacacccgctgct
tctgcctggaggagcctcaaaacagcgaggtggatgcccagctgacggga
agaatcagccagaaggcagaaaggggagcaaaggtccatcaggagacagc
agccgatgagtgtccgccctgtgtccatggtgcccgggacagcaatgtca
cacttgctccctctggccccaagggagggaaaggtgagcggggcctgcct
ggtccaccaggctccaagggagagaagggagcacggggcaatgactgtgt
tcgaatctccccggatgccccacttcagtgtgcagaaggcccgaagggag
agaagggggagtcaggagctctgggaccctcaggactcccaggctcaaca
ggcgagaagggccagaaaggcgagaagggcgacggaggcatcaagggcgt
gccgggaaagccaggccgggacggccggccaggagagatctgtgtcattg
ggcccaaagggcagaagggagaccctggctttgttgggcctgaggggctg
gcaggagagcctgggcccccggcctccctggacccccctgggataggact
gcctgggaccccgggggatccaggtggcccaccaggccccaagggagaca
agggcagctcggggatcccaggaaaggaaggccctggtgggaaacctggg
aagccaggtgtgaagggagagaagggtgacccctgtgaagtgtgcccaac
actgcctgaagggttccagaactttgttggacttcctggaaagccagggc
ccaaaggggagcctggtgatcctgtaccagccaggggagaccctggcatc
caaggcatcaaaggagagaagggggagccctgcttgtcctgcagctcggt
tgtaggggcccagcatcttgtgtcctccacaggggccagtggagatgtgg
gttcccctggctttggtctgcctggccttccgggtagagctggggttcca
gggctgaaaggagagaagggtaacttcggggaggcagggccagctggcag
tccagggccaccaggaccagtggggccagcaggcatcaaaggggcgaagg
gggagccctgtgagccgtgcccagccctgtccaaccttcaggatggggat
gtccgtgtggtggccttgcctggcccatccggagagaagggggaacctgg
gcctccaggctttggcttgccaggaaaacagggcaaggctggagagcgtg
gactgaaggggcagaagggtgatgctgggaatcctggagaccctggaacg

ccgggcaccacagggcggccaggactgtcaggagagcctggagttcaggg
ccccgcggggccaaaaggagaaaagggtgatggctgcactgcctgcccca
gcctgcaggggacagtgacagacatggcaggacggcctgggcagcccggc
cccaaaggagagcagggccccgaaggcgtgggccgacctggtaaacccgg
ccaacccggtctaccaggagttcaagggcccccaggactgaagggcgtgc
agggagagccagggcctccaggaaggggagtccagggaccccagggggag
cctggagccccgggtttgcctggcattcagggacttccgggacctcgggg
accacctggccccactggagagaagggtgcccagggatctccaggggtga
aaggagccaccggacctgtgggacctcctggggccagtgtctctgggcct
ccgggccgtgatgggcagcaaggacagacgggactcagaggaacaccagg
tgaaaaaggaccacgaggagagaagggtgagccaggggagtgctcctgcc
cctctcaaggagacctcatcttctctggcatgccgggtgctccgggactt
tggatgggcagctcctggcagccgggcccgcagggtccaccaggtattcc
cggaccaccaggcccccctggagtacctgggctgcagggagtgcctggaa
acaacggtttgccaggacagcctgggctcactgcagaactgggatcctta
ccaattgaacagcacctccttaagagtatctgcggggactgtgtccaggg
gcagagggcccacccagggtacctcgtggagaagggagagaagggagacc
agggcatccctggtgtgccaggcctcgacaactgcgcccagtgctttttg
tcactggagcgcccaagagccgaggaggcccggggtgacaacagtgaggg
agatcctggctgtgttgggagcccaggcctacctggtcctccgggattgc
caggccagagaggagaagagggtccgcctggcatgaggggctccccgggt
cctccaggccctatcggcccccccaggttttcctggtgctgttggctcccc
cggattgcctggccttcaaggagagcgaggtctcacgggcctgactggag
acaaggggggagccgggtcctccagggcaaccaggttacccaggtgccacg
ggcccccccaggactgcctggcatcaagggggagcgtggctacaccgggtc
agcgggagagagaaaggagagccgggcccccaggatctgaaggcctcccag
gccccccaggcccagcgggtcccagaggagagcgaggaccccaaggtaac
tccggtgagaagggcgaccagggatttcaaggccagccaggctttccggg
cccaccgggtcccccctggattcccaggcaaagttggatcacctggcccac
ctggccctcaagcagagaagggcagcgaagggattcgaggcccatcaggc
ctgcctggctcccctgggccaccgggacctcctgggattcagggccccgc
cggtctggatggtttggatgggaaggacggcaagcctggcttgagggggg
accctggtcctgctggccccccctggactcatgggaccaccgggctttaag
gggaaaacaggacatcctggcctcccaggacctaagggtgactgtggcaa
accaggtcccccctggcagcactggccggcctggcgcagagggtgaacctg
gtgccatgggaccccagggaagacccggtcccccgggacacgttgggcca
ccagggcctccaggccagccaggaccagctgggatctctgcagtgggtct
gaaaggagaccgaggagccaccggagaaaggggccttgcaggcctcccag
gccagcccggccccccctggacaccctggccccccaggcgaacctggtacg
gatggtgcagctggcaaagagggacccccctggaaagcagggattctatgg
acctcctggtcccaagggtgatccaggagctgcaggacagaagggccagg
caggagagaaggggagagccggcatgcctggtggacctggcaagagtggt
tccatggggcctgttgggccaccgggccctgcaggagagagaggccaccc
tggagctccggggccttcggggagccctggcttgcctggtgtgcctggct
ccatgggagacatggtgaattatgatgaaatcaagaggttcatcagacaa
gagatcattaaaatgtttgatgagagaatggcttactacacctccaggat
gcagttccccatggagatggcggcagctccgggacgaccagggcctccag
ggaaggatggtgctccgggcaggccaggtgctccagggtcacctgggctc
cctggtcagattggcagagaaggacggcagggcttgccaggagtaagagg

attgcctggtaccaaaggtgaaaaaggggacattggtattggcattgcag
gagaaaatggtcttcccggcccccaggtcctcaaggtcctccaggctat
ggcaagatgggtgcaacaggaccaatgggccagcaaggcatccctggcat
ccctgggcccccgggtcccatgggccagccaggcaaggctggccactgta
atccctctgactgctttggggccatgccgatggagcagcagtacccaccc
atgaaaaccatgaaggggcctttggctgaaattccccacctgcctttgg
atgaaagactccgttgggaataaatggccaaagcttataggactctgtga
caggttgtgaatgttttttttgttgttgttgttgtttttaattgctgtta
atattttttaaataataaagaaacaaaactatctgccctttcccttccag
tgggttcctctggtgctgcagccagagctccctgttgccctccttttccc
gtttagtcccaggaacaaaaaagggcatttgggtacaggggcatatacc
tgtaatcctagctattcaaggggctgaggtgggaggatcgcctgagccca
ggagtttgagaccagtctaagcaacataatgagactcagtctccgaaaaa
taaaaattaaaaaataaagggtatttccctcctt
>NM_002403 3
gccccgtcgggggcccggagggggactcggagcgggccaaggggcggctc
cggcgggcggactcggagcgggcggcggagtgacccggacagctgtcctc
tctgacaccaccccggcctgcctctttgttgccatgagagctgcctacct
cttcctgctattcctgcctgcaggcttgctggctcagggccagtatgacc
tggacccgctgccgccgttccctgaccacgtccagtacacccactatagc
gaccagatcgacaacccagactactatgattatcaagaggtgactcctcg
gccctccgaggaacagttccagttccagtcccagcagcaagtccaacagg
aagtcatcccagccccaaccccagaaccaggaaatgcagagctggagccc
acagagcctgggcctcttgactgccgtgaggaacagtacccgtgcacccg
cctctactccatacacaggccttgcaaacagtgtctcaacgaggtctgct
tctacagcctccgccgtgtgtacgtcattaacaaggagatctgtgttcgt
acagtgtgtgcccatgaggagctcctccgagctgacctctgtcgggacaa
gttctccaaatgtggcgtgatggccagcagcggcctgtgccaatccgtgg
cggcctcctgtgccaggagctgtgggagctgctagggtggtgctggcatc
ctgagtcctggccctcctgggatctggggccctcgggccctgcctgacct
ggtgctttttccccatccccatgttccttttattctgtaaaaagttagt
ggactgcagccctgggggttgcaggctgcggtgcctcaggcccctccttc
agcctgtggccacctctgggggcacaatgggggctccccactgcccagtct
gccccctcgggttgggggagtatcccaggcctctctgtgggacctgggccc
ctgacgggccttctcagcccgttttgaggacagacagtcccccgaggtag
gctacatcccccaccccagctggtctgcttggatttcctacagcccccg
tgggcatggaccacctttattttatacaaaattaaaaacaagtttttaca
aaaaaaaaaaaaaaaaaaaaaaa
>NM_017459 2
attgcaacttggtctcacagtggcttaggccagggtgggagcagtgaacg
gagtcacaaaagaaatttttcagctgtcctctctgacaccaccccggcct
gcctctttgttgccatgagagctgcctacctcttcctgctattcctgcct
gcaggcttgctggctcagggccagtatgacctggacccgctgccgccgtt
ccctgaccacgtccagtacacccactatagcgaccagatcgacaacccag
actactatgattatcaagaggtgactcctcggccctccgaggaacagttc
cagttccagtcccagcagcaagtccaacaggaagtcatcccagccccaac
cccagaaccaggaaatgcagagctggagcccacagagcctgggcctcttg
actgccgtgaggaacagtacccgtgcacccgcctctactccatacacagg
ccttgcaaacagtgtctcaacgaggtctgcttctacagcctccgccgtgt

gtacgtcattaacaaggagatctgtgttcgtacagtgtgtgcccatgagg
agctcctccgagctgacctctgtcgggacaagttctccaaatgtggcgtg
atggccagcagcggcctgtgccaatccgtggcggcctcctgtgccaggag
ctgtgggagctgctagggtggtgctggcatcctgagtcctggccctcctg
ggatctggggccctcgggccctgcctgacctggtgctttttttccccatcc
ccatgttccttttattctgtaaaaagttagtggactgcagccctgggggt
tgcaggctgcggtgcctcaggcccctccttcagcctgtggccacctctgg
ggcacaatgggggctccccactgcccagtctgcccctcgggttgggggag
tatcccaggcctctctgtgggacctgggcccctgacgggccttctcagcc
cgttttgaggacagacagtcccccgaggtaggctacatccccccacccca
gctggtctgcttggatttcctacagcccccgtgggcatggaccaccttta
ttttatacaaaattaaaaacaagtttttacaaaaaaaaaaaaaaaaaaaa
aaaaa
>NM_021738 2
cactgctctgactgcgtcctccccaggctgaagtgtcctcaggtttcatg
tggatttgcccagtaatggatggctccaccgaggccaggttggggaagtt
cagcctcgaggacactaggggacgaaaaacttcaaaaagaagactctttt
tattaatattttggcgttctttttggagaggaacttttgggatgtagcct
ttacctgtttcctgaatttgacatccaaaaagccttttggaaaacaccat
tctaaatttatcctgttctgaatcatcatcgcttctgatagatgcagttg
cttgctgacatcgtgatctttgattttgtacaacgctatttgggctaact
tttgggattttactttgtgcttagaatttgaaagtagtattcattggaaa
gagactccaagaaaatactcgaagacatcaaaagctggattcgagggtat
aacgtgttttgttttgtttttaaattagaaaaataaggaattgcagtgt
gattttctttccccctttaacttagaggaacgaggattatacagaaattt
gaaatttcaacaagaaaaacttaagcttccttggattcccacgtcaaagg
aaagtttcaagctttcagaaggagttctcactcgaagataaagaacagct
cgctaaccacgaaagaggaatcgatgctcagcttttagttgcacttccta
aagttgcagaattaagacaaatctttgaaccaaagaagaaagaattctta
gaaatgaaaagaaaagaaagaattgccaggcgcctggaagggattgaaaa
tgacactcagcccatcctcttgcagagctgcacaggattggtgactcacc
gcctgctggaggaagacacccctcgatacatgagagccagcgaccctgcc
agcccccacatcggccgatcaaatgaagaggaggaaacttctgattcttc
tctagaaaagcaaactcgatccaaatactgcacagaaacctccggtgtcc
acggtgactcaccctatggttcgggtaccatggacacccacagtctggag
tccaaagccgaaagaattgcaaggtacaaagcagaaagaaggcgacagct
ggcagagaagtatgggctgactctggatcccgaggccgactccgagtatt
tatcccgctataccaagtccaggaaggagcctgatgctgtcgagaagcgg
ggaggaaaaagtgacaaacaggaagagtcaagcagagatgctagttctct
gtaccccgggaccgagacgatggggctcaggacctgtgccggtgaatcca
aggactatgccctccatgtgggtgacggctcttccgacccggaggtgctg
ctgaacatagaaaaccaaagacgaggtcaagagctgagtgccacccggca
ggcccatgacctgtccccagcagccgagagttcctcgaccttctctttct
ctgggcgagactcctccttcactgaagtgccacggtcccccaagcacgcc
cacagctcctccctgcagcaggcagcctcccggagcccctcctttggtga
cccacagctatcccctgaggcccgacccagtacagggaaacccaaacatg
agtggtttctccagaaagattccgaaggggacacaccttcacttatcaac
tggccttccagagttaaagttagagaaaaattggtgaaagaggaaagtgc
tcgaaacagccctgaactcgcctcagagtccgtaactcagaggagacacc

agccagcgccagtccattacgtgtcatttcagtctgagcactcagccttt
gatagggtccccagcaaggcagcaggctctacacgacagccaatccgtgg
ctatgtccaacccgcagataccggtcacaccgccaagctagtgacgccag
aaaccccagaaaatgcatctgagtgtagctgggtagcatcagccacccag
aatgtccccaaacctcccagcttgacggttctagaaggtgacggaaggga
tagcccagttctccatgtctgcgagtcaaaagcagaagaagaagaagggg
aaggagaaggagaagaaaaagaagaagatgtgtgcttcactgaagctctc
gagcaaagcaagaaaaccctactggctttggagggtgatgggctagtgag
aagcccagaagatccctctagaaatgaggactttggtaagcctgctgtga
gcacagtcaccttagagcatcagaaggaactggaaaacgtggcacaaccc
cctcaagctccgcaccagcccactgagaggacaggcaggagcgagatggt
tctctacattcaaagtgagcctgtgtcccaagacgccaaaccaactggtc
acaacagggaagcctcgaaaaagcgcaaggtccgtacccgctctctgtca
gatttcacaggccccccctcagctccaggccttgaagtataaggacccagc
ttccaggagagagctggagctgcccagctccaagaccgaagggccttatg
gggagatcagcatgctggacacaaaagtctctgtcgcccagctccgaagt
gcgttcctggcatctgccaacgcctgcaggagacctgaactcaaatcacg
ggtggagaggtcggctgaaggacctggcttgcccaccggtgtggaacggg
agagagggtcccggaaaccaagacgctatttttctcctggtgaaagtaga
aaaacttccgagagatttagaacccaacctataacttcagcagaacgaaa
ggaatcggataggtgcacttcacattcagaaacgccaactgtcgatgatg
aagaaaaggtggatgaacgagccaagctgagcgtcgccgccaagaggttg
cttttcagggagatggaaaaatcttttgatgaacaaaatgttccaaagcg
acgctcaagaaacacagctgtggagcagaggctacgccgtctgcaggaca
ggtccctcacccagcccatcaccactgaagaggtggtcatcgcagccact
gaacctatccccgcttcgtgttctgggggcacccaccctgtaatggcgag
acttcctagccccactgtagctaggagcgctgtgcagcctgccagattgc
aggcctctgctcaccaaaaggccttagccaaggaccagacaaatgagggc
aaagagcttgctgagcaaggagaacctgattcctccactctaagcttggc
cgaaaagttggccttgtttaacaaattgtcccagccagtctcaaaagcga
tttctacccggaacagaatagacacgagacagaggagaatgaacgctcgc
tatcaaactcagccagtcacactgggagaggtggagcaggtgcagagtgg
aaagctcattcctttctcacctgccgtgaacacatcagtgtctaccgtag
catccacggttgctccaatgtatgccggagatcttcgcacaaagccacct
cttgaccacaatgcaagtgccactgactataagttttcttcttcaataga
aaattcggactctccagttagaagcattctgaaatcgcaagcttggcagc
ctttggtagagggtagcgagaacaagggaatgttgagagaatatggagag
acagaaagcaagagagctttgacaggtcgagacagtgggatggagaagta
tgggtcctttgaggaagcagaagcatcctaccccatcctgaaccgagcca
gggaaggagacagccataaggaatctaaatatgctgttcccagaagagga
agcctggaacgggcgaaccctcccatcacccacctcggggatgaaccgaa
ggaattttccatggctaaaatgaatgcacaaggaaacttggacttgaggg
acaggctgccctttgaagagaaggtggaggtggagaatgttatgaaaagg
aagttttcactaagagcggcagagttcggggagcccacttccgagcagac
ggggacagctgctgggaaaactattgctcaaaccacagcccccgtgtcct
ggaagccccaggattcttcggaacagccacaggagaagctctgcaagaat
ccatgtgcgatgtttgctgctggagagatcaaaacgccgacaggggaggg
ccttcttgactcacccagcaaaaccatgtctattaaagaaagattggcac
tgttgaagaaaagcggggaggaagattggagaaacagactcagcaggagg

caggagggcggcaaggcgccggccagcagcctgcacacccaggaagcagg
gcggtccctcatcaagaagcgggtcacagaaagtcgagagagccaaatga
cgattgaggagaggaagcagctcatcactgtgagagaggaggcctggaag
acgagaggcagaggagcggccaacgactcgacccagttcactgtggctgg
caggatggtgaagaaaggtttggcgtcacctactgccataaccccagtag
cctcacccatttgcggtaaaacaagaggcaccacacccgtttccaaaccc
ctggaagatatcgaagccagaccagatatgcagttagaatcggacctgaa
gttggacaggctggaaacctttctaagaaggctgaataacaaagttggcg
ggatgcacgaaacggtgctcactgtcaccggcaaatctgtgaaggaggtg
atgaagccagatgatgatgaaacctttgccaaattttaccgcagcgtgga
ttataatatgccaagaagtcctgtggagatggatgaggacttcgatgtca
ttttcgatccttatgcacccaaattgacgtcttccgtggccgagcacaag
cgggcagttaggcccaagcgccgggttcaggcctccaaaaaccccctgaa
aatgctggcggcaagagaagatctccttcaggaatacactgagcagagat
taaacgttgccttcatggagtcaaagcggatgaaagtagaaaagatgtct
tccaactccaacttctcagaagtcaccctggcgggtttagccagtaaaga
aaacttcagcaacgtcagcctgcggagcgtcaacctgacggaacagaact
ctaacaacagcgccgtgccctacaagaggctgatgctgttgcagattaaa
ggaagaagacatgtgcagaccaggctggtggaacctcgagcttcggcgct
caacagtggggactgcttcctcctgctctctccccactgctgcttcctgt
gggtaggagagtttgcaaacgtcatagaaaaggcgaaggcctcagaactt
gcaactttaattcagacaaagagggaacttggttgtagagctacttatat
ccaaaccattgaagaaggaattaatacacacactcatgcagccaaagact
tctggaagcttctgggtggccaaaccagttaccaatctgctggagaccca
aaagaagatgaactctatgaagcagccataatagaaactaactgcattta
ccgtctcatggatgacaaacttgttcctgatgacgactactgggggaaaa
ttccgaagtgctcccttctgcaacccaaagaggtactggtgtttgatttt
ggtagtgaagtttacgtatggcatgggaaagaagtcacattagcacaacg
aaaaatagcatttcagctggcaaagcacttatggaatggaacctttgact
atgagaactgtgacatcaatcccctggatcctggagaatgcaatccgctt
atccccagaaaaggacaggggcggcccgactgggcgatatttgggagact
tactgaacacaatgagacgattttgttcaaagagaagtttctggattgga
cggaactgaagagatcgaatgagaagaaccccggggaacttgcccagcac
aaggaagaccccaggactgatgtcaaggcatacgatgtgacacggatggt
gtccatgccccagacgacagcaggcaccatcctggacggagtgaacgtcg
gccgtggctatggcctggtggaaggacacgacaggaggcagtttgagatc
accagcgtttccgtggatgtctggcacatcctggaattcgactatagcag
gctccccaaacaaagcatcgggcagttccatgagggggatgcctatgtgg
tcaagtggaagttcatggtgagcacggcagtgggaagtcgccagaaggga
gagcactcggtgagggcagccggcaaagagaagtgcgtctacttcttctg
gcaaggccggcactccaccgtgagtgagaagggcacgtcggcgctgatga
cggtggagctggacgaggaaagggggggcccaggtccaggttctccaggga
aaggagccccccctgtttcctgcagtgtttccaggggggggatggtggtgca
ctcggggaggcgggaagaggaagaagaaaatgtgcaaagtgagtggcggc
tgtactgcgtgcgtggagaggtgcccgtggaagggaatttgctggaagtg
gcctgtcactgtagcagcctgaggtccagaacttccatggtggtgcttaa
cgtcaacaaggccctcatctacctgtggcacggatgcaaagcccaggccc
acacgaaggaggtcggaaggaccgctgcgaacaagatcaaggaacaatgt
cccctggaagcaggactgcatagtagcagcaaagtcacaatacacgagtg

tgatgaaggctccgagccactcggattctgggatgccttaggaaggagag
acaggaaagcctacgattgcatgcttcaagatcctggaagttttaacttc
gcgccccgcctgttcatcctcagcagctcctctggggattttgcagccac
agagtttgtgtaccctgcccgagccccctctgtggtcagttccatgccct
tcctgcaggaagatctgtacagcgcgccccagccagcacttttccttgtt
gacaatcaccacgaggtgtacctctggcaaggctggtggcccatcgagaa
caagatcactggttccgcccgcatccgctgggcctccgaccggaagagtg
cgatggagactgtgctccagtactgcaaaggaaaaaatctcaagaaacca
gcccccaagtcttaccttatccacgctggtctggagcccctgacattcac
caatatgtttcccagctgggagcacagagaggacatcgctgagatcacag
agatggacacggaagtttccaatcagatcaccctcgtggaagacgtctta
gccaagctctgtaaaaccatttacccgctggccgacctcctggccaggcc
actcccggaggggtcgatcctctgaagcttgagatctatctcaccgacg
aagacttcgagtttgcactagacatgacgagggatgaatacaacgccctg
cccgcctggaagcaggtgaacctgaagaaagcaaaaggcctgttctgagt
ggggagacgccagaggagcctcacggtcacgtccaacaacaccactgcac
cagggaaatggatatatatttttggactggtgtttttcacaaagtatttt
tcaatcagagttttcagaacctgacattgttaaagatactgcttgtcccg
gagttgtgtattttgtaaatgttcaagggaactgtttggaaacttctttc
caccattcaggaggttatcagaattaataaaagtatctgttatgtgcact
taagccgcagctgctatagatagcactgccttcttgttccagctaggcaa
tgcctttttttttttttttgaagcagttctctttataaagtgttatttt
gatagtttgtggattctaaaatatatatatatttatataaacaccatata
agtcaaatatgtatttaacaaagcaatatgtattcattcactttcaagat
ttgttttggtgtcaaaataacatgaaaaggtagatggagttgcttctgtt
gaattagctctgccaccaatatgtatcttcatacacgtttggaaatgttt
cctgcagcattaggtatgacttgttctgagtactgcttccggtgctaaaa
tgaacaaagaatttgtacttaatggcatggactctggagaatctatgcga
atcaacctttctaccttaatatctccccaaaaatgtatagtgccttgttt
ttatgtacagtttatatacagaaaagtttgctctgcatttttgatgatgg
tttggaacattatctacaattttactctcaaatagtcaaaataaaaacat
ctcaatttctaataccggttgtaaacagtacacatgtcattttgtgatat
aggactcccaaataaaagtatcagaataaacacaacaattaactggtaaa
aaaaaa
>NM_003174 3
tcggcgggaagcggcgatcctgccaccgggaggtgtggaagagccgggta
gattctggctacattggagattggttgctttctaaaactgaaggagaagc
ccatgaagagatggtggattctcactgagttttgactagcggaagaaaag
agagagttcaagtggatggccttgaggacttgaaaagctgagatatgatg
attttgaagtcatttcacatcgaagccatgatttaaatatcggcgttaag
atttcaacaagaaaaacttaagcttccttggattcccacgtcaaaggaaa
gtttcaagctttcagaaggagttctcactcgaagataaagaacagctcgc
taaccacgaaagaggaatcgatgctcagcttttagttgcacttcctaaag
ttgcagaattaagacaaatctttgaaccaaagaagaaagaattcttagaa
atgaaaagaaaagaaagaattgccaggcgcctggaagggattgaaaatga
cactcagcccatcctcttgcagagctgcacaggattggtgactcaccgcc
tgctggaggaagacacccctcgatacatgagagccagcgaccctgccagc
ccccacatcggccgatcaaatgaagaggaggaaacttctgattcttctct
agaaaagcaaactcgatccaaatactgcacagaaacctccggtgtccacg

gtgactcaccctatggttcgggtaccatggacacccacagtctggagtcc
aaagccgaaagaattgcaaggtacaaagcagaaagaaggcgacagctggc
agagaagtatgggctgactctggatcccgaggccgactccgagtatttat
cccgctataccaagtccaggaaggagcctgatgctgtcgagaagcgggga
ggaaaaagtgacaaacaggaagagtcaagcagagatgctagttctctgta
ccccgggaccgagacgatggggctcaggacctgtgccggtgaatccaagg
actatgccctccatgtgggtgacggctcttccgacccggaggtgctgctg
aacatagaaaaccaaagacgaggtcaagagctgagtgccacccggcaggc
ccatgacctgtccccagcagccgagagttcctcgaccttctctttctctg
ggcgagactcctccttcactgaagtgccacggtcccccaagcacgcccac
agctcctccctgcagcaggcagcctcccggagcccctcctttggtgaccc
acagctatcccctgaggcccgacccaggtgcacttcacattcagaaacgc
caactgtcgatgatgaagaaaaggtggatgaacgagccaagctgagcgtc
gccgccaagaggttgcttttcagggagatggaaaaatcttttgatgaaca
aaatgttccaaagcgacgctcaagaaacacagctgtggagcagaggctac
gccgtctgcaggacaggtccctcacccagcccatcaccactgaagaggtg
gtcatcgcagccacattgcaggcctctgctcaccaaaaggccttagccaa
ggaccagacaaatgagggcaaagagcttgctgagcaaggagaacctgatt
cctccactctaagcttggccgaaaagttggccttgtttaacaaattgtcc
cagccagtctcaaaagcgatttctacccggaacagaatagacacgagaca
gaggagaatgaacgctcgctatcaaactcagccagtcacactgggagagg
tggagcaggtgcagagtggaaagctcattcctttctcacctgccgtgaac
acatcagtgtctaccgtagcatccacggttgctccaatgtatgccggaga
tcttcgcacaaagccacctcttgaccacaatgcaagtgccactgactata
agttttcttcttcaatagaaaattcggactctccagttagaagcattctg
aaatcgcaagcttggcagcctttggtagagggtagcgagaacaagggaat
gttgagagaatatggagagacagaaagcaagagagctttgacaggtcgag
acagtgggatggagaagtatgggtcctttgaggaagcagaagcatcctac
cccatcctgaaccgagccagggaaggagacagccataaggaatctaaata
tgctgttcccagaagaggaagcctggaacgggcgaaccctcccatcaccc
acctcggggatgaaccgaaggaattttccatggctaaaatgaatgcacaa
ggaaacttggacttgagggacaggctgccctttgaagagaaggtggaggt
ggagaatgttatgaaaaggaagttttcactaagagcggcagagttcgggg
agcccacttccgagcagacggggacagctgctgggaaaactattgctcaa
accacagcccccgtgtcctggaagccccaggattcttcggaacagccaca
ggagaagctctgcaagaatccatgtgcgatgtttgctgctggagagatca
aaacgccgacaggggagggccttcttgactcacccagcaaaaccatgtct
attaaagaaagattggcactgttgaagaaaagcggggaggaagattggag
aaacagactcagcaggaggcaggagggcggcaaggcgccggccagcagcc
tgcacacccaggaagcagggcggtccctcatcaagaagcgggtcacagaa
agtcgagagagccaaatgacgattgaggagaggaagcagctcatcactgt
gagagaggaggcctggaagacgagaggcagaggagcggccaacgactcga
cccagttcactgtggctggcaggatggtgaagaaaggtttggcgtcacct
actgccataaccccagtagcctcacccatttgcggtaaaacaagaggcac
cacacccgtttccaaacccctggaagatatcgaagccagaccagatatgc
agttagaatcggacctgaagttggacaggctggaaacctttctaagaagg
ctgaataacaaagttggcgggatgcacgaaacggtgctcactgtcaccgg
caaatctgtgaaggaggtgatgaagccagatgatgatgaaacctttgcca
aattttaccgcagcgtggattataatatgccaagaagtcctgtggagatg

gatgaggacttcgatgtcattttcgatccttatgcacccaaattgacgtc
ttccgtggccgagcacaagcgggcagttaggcccaagcgccgggttcagg
cctccaaaaacccccctgaaaatgctggcggcaagagaagatctccttcag
gaatacactgagcagagattaaacgttgccttcatggagtcaaagcggat
gaaagtagaaaagatgtcttccaactccaacttctcagaagtcaccctgg
cgggtttagccagtaaagaaaacttcagcaacgtcagcctgcggagcgtc
aacctgacggaacagaactctaacaacagcgccgtgccctacaagaggct
gatgctgttgcagattaaaggaagaagacatgtgcagaccaggctggtgg
aacctcgagcttcggcgctcaacagtggggactgcttcctcctgctctct
ccccactgctgcttcctgtgggtaggagagtttgcaaacgtcatagaaaa
ggcgaaggcctcagaacttgcaactttaattcagacaaagagggaacttg
gttgtagagctacttatatccaaaccattgaagaaggaattaatacacac
actcatgcagccaaagacttctggaagcttctgggtggccaaaccagtta
ccaatctgctggagacccaaaagaagatgaactctatgaagcagccataa
tagaaactaactgcatttaccgtctcatggatgacaaacttgttcctgat
gacgactactgggggaaaattccgaagtgctcccttctgcaacccaaaga
ggtactggtgtttgattttggtagtgaagtttacgtatggcatgggaaag
aagtcacattagcacaacgaaaaatagcatttcagctggcaaagcactta
tggaatggaacctttgactatgagaactgtgacatcaatcccctggatcc
tggagaatgcaatccgcttatccccagaaaaggacaggggcggcccgact
gggcgatatttgggagacttactgaacacaatgagacgattttgttcaaa
gagaagtttctggattggacggaactgaagagatcgaatgagaagaaccc
cggggaacttgcccagcacaaggaagaccccaggactgatgtcaaggcat
acgatgtgacacggatggtgtccatgccccagacgacagcaggcaccatc
ctggacggagtgaacgtcggccgtggctatggcctggtggaaggacacga
caggaggcagtttgagatcaccagcgtttccgtggatgtctggcacatcc
tggaattcgactatagcaggctccccaaacaaagcatcgggcagttccat
gagggggatgcctatgtggtcaagtggaagttcatggtgagcacggcagt
gggaagtcgccagaagggagagcactcggtgagggcagccggcaaagaga
agtgcgtctacttcttctggcaaggccggcactccaccgtgagtgagaag
ggcacgtcggcgctgatgacggtggagctggacgaggaaagggggggccca
ggtccaggttctccagggaaaggagccccccctgtttcctgcagtgtttcc
aggggggggatggtggtgcactcggggaggcgggaagaggaagaagaaaat
gtgcaaagtgagtggcggctgtactgcgtgcgtggagaggtgcccgtgga
agggaatttgctggaagtggcctgtcactgtagcagcctgaggtccagaa
cttccatggtggtgcttaacgtcaacaaggccctcatctacctgtggcac
ggatgcaaagcccaggcccacacgaaggaggtcggaaggaccgctgcgaa
caagatcaaggaacaatgtcccctggaagcaggactgcatagtagcagca
aagtcacaatacacgagtgtgatgaaggctccgagccactcggattctgg
gatgccttaggaaggagagacaggaaagcctacgattgcatgcttcaaga
tcctggaagttttaacttcgcgccccgcctgttcatcctcagcagctcct
ctggggattttgcagccacagagtttgtgtaccctgcccgagccccctct
gtggtcagttccatgcccttcctgcaggaagatctgtacagcgcgcccca
gccagcacttttccttgttgacaatcaccacgaggtgtacctctggcaag
gctggtggcccatcgagaacaagatcactggttccgcccgcatccgctgg
gcctccgaccggaagagtgcgatggagactgtgctccagtactgcaaagg
aaaaaatctcaagaaaccagcccccaagtcttaccttatccacgctggtc
tggagcccctgacattcaccaatatgtttcccagctgggagcacagagag
gacatcgctgagatcacagagatggacacggaagtttccaatcagatcac

cctcgtggaagacgtcttagccaagctctgtaaaaccatttacccgctgg
ccgacctcctggccaggccactcccggagggggtcgatcctctgaagctt
gagatctatctcaccgacgaagacttcgagtttgcactagacatgacgag
ggatgaatacaacgccctgcccgcctggaagcaggtgaacctgaagaaag
caaaaggcctgttctgagtggggagacgccagaggagcctcacggtcacg
tccaacaacaccactgcaccagggaaatggatatatatttttggactggt
gtttttcacaaagtatttttcaatcagagttttcagaacctgacattgtt
aaagatactgcttgtcccggagttgtgtattttgtaaatgttcaagggaa
ctgtttggaaacttctttccaccattcaggaggttatcagaattaataaa
agtatctgttatgtgcacttaagccgcagctgctatagatagcactgcct
tcttgttccagctaggcaatgccttttttttttttttgaagcagttct
ctttataaagtgttattttgatagtttgtggattctaaaatatatatata
tttatataaacaccatataagtcaaatatgtatttaacaaagcaatatgt
attcattcactttcaagatttgttttggtgtcaaaataacatgaaaaggt
agatggagttgcttctgttgaattagctctgccaccaatatgtatcttca
tacacgtttggaaatgtttcctgcagcattaggtatgacttgttctgagt
actgcttccggtgctaaaatgaacaaagaatttgtacttaatggcatgga
ctctggagaatctatgcgaatcaacctttctaccttaatatctccccaaa
aatgtatagtgccttgtttttatgtacagtttatatacagaaaagtttgc
tctgcatttttgatgatggtttggaacattatctacaattttactctcaa
atagtcaaaataaaaacatctcaatttctaataccggttgtaaacagtac
acatgtcattttgtgatataggactcccaaataaaagtatcagaataaac
acaacaattaactggtaaaaaaaaa
>NM_014296 2
gggcggctggtgggcggggcgagggccgctggggccgcgaagtggggcgg
ccgggtgggctacaagccgggtctgggctgaggggcgcggcttcgcggtg
gaccccagcccggcaacgggaaggcgagctctcctccaccgtccaaagta
aactttgccgctccttccgcggcgctcccgagtcctcgccgccgccgggc
cgccgcagtccgcgaagagccgtcctgcgtcagggcctccttccctgccc
cggcgcggggccactgcgccatggacgccacagcactggagcgggacgct
gtgcagttcgcccgtctggcggttcagcgcgaccacgaaggccgctactc
cgaggcggtgttttattacaaggaagctgcacaagccttaatttatgctg
agatggcaggatcaagcctagaaaatattcaagaaaaaataactgagtat
ctggaaagagttcaagctctacattcagcagttcagtcaaagagtgctga
tcctttgaagtcaaaacatcagttggacttagagcgtgctcatttccttg
ttacacaagcttttgatgaagatgaaaaagagaatgttgaagatgctata
gaattgtacacagaagctgtggatctctgtctgaaaacatcttatgaaac
tgctgataaagtcctgcaaaataaactgaaacagttggctcgacaggcac
tagacagagcagaagcgctgagtgagcctttgaccaagccagttggcaaa
atcagttcaacaagtgttaagccaaagccacctccagtgagagcacattt
tccactgggcgctaatcccttccttgaaagacctcagtcatttataagtc
ctcagtcatgtgatgcacaaggacagagatacacagcagaagaaatagaa
gtactcaggacaacatcaaaaataaatggtatagaatatgttcctttcat
gaatgttgacctgagagaacgttttgcctatccaatgcctttctgtgata
gatggggcaagctaccattatcacctaaacaaaaaactacattttccaag
tgggtacgaccagaagacctcaccaacaatcctacaatgatatatactgt
gtccagttttagcataaagcagacaatagtatcggattgctcctttgtgg
catcactggccatcagtgcagcttatgaaagacgttttaataagaagtta
attaccggcataatttaccctcaaaacaaggatggtgaaccagaatacaa

tccatgtgggaagtatatggtaaaacttcacctcaatggtgtcccaagaa
aggtgataattgatgaccagttacctgttgatcacaagggagaattgctc
tgttcttattccaacaacaaaagtgaattatgggtttctctcatagaaaa
agcatacatgaaagtcatgggaggatatgattttccaggatccaactcca
atattgatcttcatgcactgactggctggataccagaaagaattgctatg
cattcagatagccaaactttcagtaaggataattctttcagaatgcttta
tcaaagatttcacaaaggagatgtcctcatcactgcgtcaactggaatga
tgacagaagctgaaggagagaagtggggtctggttcccacacacgcatat
gctgttttggatattagagagttcaaggggctgcgatttatccagttgaa
aaatccttggagtcatttacgttggaaaggaagatacagtgaaaatgatg
taaaaaactggactccagagttgcaaaagtatttaaactttgatccccga
acagctcagaaaatagacaacggaatattttggatttcctgggatgatct
ctgccagtattatgatgtgatttatttgagttggaatccaggtcttttta
aagaatcaacatgtattcacagtacttgggatgctaagcaaggacctgtg
aaagatgcctatagcctggccaacaaccccagtacaaactggaggtgca
gtgtccacaggggggtgctgcagtttgggttttgcttagtagacacataa
cagacaaggatgattttgcgaataatcgagaatttatcacaatggttgta
tacaagactgatgggaaaaaagtttattacccagctgacccacctccata
cattgatggaattcgaattaacagccctcattatttgactaagataaagc
tgaccacacctggcacccatacctttacattagtggtttctcaatatgaa
aaacagaacacaatccattacacggttcgggtatattcagcatgcagctt
tactttttcaaagattccttcaccatacaccttatcaaaacggattaatg
gaaagtggagtggtcagagtgctggaggatgtggaaatttccaagagact
cacaaaaataaccccatctaccaattccatatagaaaagactgggccgtt
actgattgagctacgaggaccaaggcaatatagcgttggatttgaggttg
taacagtttctactctaggagatcctggtccccatggctttctgaggaaa
tctagtggtgactataggtgtgggttttgctacctggaattagaaaatat
accttctgggatcttcaatatcattcctagtaccttttttgcctaaacaag
aaggaccttttttcttggactttaatagtattatccccatcaagatcaca
caacttcagtgatggagaaatctcaagttactggcttttatacttaccaa
acatcagttcttcaaataaggacgcaaatcttcaggacagtaagcagaac
aatcagaatggaattaaatctctaaaaacgtgttacagtggaatctggtg
cttgtcagggtgtttggtaagaactgtatatagtcagaattacctaaatc
acctagaggtaccgtttacatggttttgtgtatatagagttggcttgcat
tttaggggccattttgtataaaaagtgcatatgattaaaattagactcag
tcatcactgtgagatgcctttgctaagaggataaaggaactgagaccaga
tgagaaaaagaaaggatatagattccttgagtggaatagtgggctagatt
aatataccgaaatatttccattgtttcccttttttgcagagcatgtggaa
gttaaacctgcttgattctactatacatcttgggcaactagttaccaaat
gaattgtgccaccataactgattttaattttgcattatttatgattttaa
aatatttgttgcccaggtgttatgaaagaataaagcttttaagtatagac
taccttagcatgaagatgctcatgcctaagaatgaaaattgttgaggtta
tctcccattcaatcatgtagcaagaacttaaagaaattcactactgcagt
ttttatttttaaaaaacagtaattgagatattgaagacattacaatttag
tttgtgtggtcttttttttaaattgctgtatcgttcagtctcttgtggcaa
tagcactttgaagaaaatagagaatttaatatatggtgattgggatatgt
agcattcaaaaaaaagtgaattgccaagatactggtgtcatgtaaattccc
actttacataaaaacccatcaggacagaatgatgctcaatattttaaaat
tctaaaaatagggtgggatttttcattgtctctactttataattatcaaa

acttattttgtattgctactaccttaaattgaaataaaatgtttatactt
acggatattgcatagtttaagttagatttattgaaagatttcatctgtcg
tgtttcatgtaaatgagaacagattatttgcatgaaaatatatacttcaa
caaaaatctgttctttaacagagtagtggtagattattacactaatgaga
tttcactttggtaaatacttcatgctttcagttttagcctattaatttta
ggtggacaaatttaacaagttttctgttactttttaaaaagaaaaaatcc
agaacataagaactatattatgaacacatgatttgaacctgttgtggtaa
agatcttgtacaggatgcaaactaaaaacctaatccctgccatcaaattt
attagaagagacctatatatgaacaacttaaaggcactgatttctataat
agagctctaaaaacatgccaccagtgtatgaataagggaaagattaattt
tggctggaccaatataaaaaattgtatttgaagaattgatactttaactt
ggaccttgaaggtaaagcttcaaaagacaggttactgaccattgagtgtt
tactatgtacccaatgtgtatatttttctttttaatcttcccaatagctg
aataaagtatagatactaaaaaaaaaaaaaaaaaa
>NM_014211 2
agagccactccatctgagggtggctgcgtgtccacatacgaggggacagg
gctgaggatgaggagaaccctggggacccagaagaccgtgccttgcctgg
aagtcctgcctgtaggcctgaaggacttgccctaacagagcctcaacaac
tacctggtgattcctacttcagccccttggtgtgagcagcttctcaacat
gaactacagcctccacttggccttcgtgtgtctgagtctcttcactgaga
ggatgtgcatccaggggagtcagttcaacgtcgaggtcggcagaagtgac
aagctttccctgcctggctttgagaacctcacagcaggatataacaaatt
tctcaggcccaattttggtggagaacccgtacagatagcgctgactctgg
acattgcaagtatctctagcatttcagagagtaacatggactacacagcc
accatatacctccgacagcgctggatggaccagcggctggtgtttgaagg
caacaagagcttcactctggatgcccgcctcgtggagttcctctgggtgc
cagatacttacattgtggagtccaagaagtccttcctccatgaagtcact
gtgggaaacaggctcatccgcctcttctccaatggcacggtcctgtatgc
cctcagaatcacgacaactgttgcatgtaacatggatctgtctaaatacc
ccatggacacacagacatgcaagttgcagctggaaagctggggctatgat
ggaaatgatgtggagttcacctggctgagagggaacgactctgtgcgtgg
actggaacacctgcggcttgctcagtacaccatagagcggtatttcacct
tagtcaccagatcgcagcaggagacaggaaattacactagattggtctta
cagtttgagcttcggaggaatgttctgtatttcattttggaaacctacgt
tccttccactttcctggtggtgttgtcctgggtttcattttggatctctc
tcgattcagtccctgcaagaacctgcattggagtgacgaccgtgttatca
atgaccacactgatgatcgggtcccgcacttctcttcccaacaccaactg
cttcatcaaggccatcgatgtgtacctggggatctgctttagctttgtgt
ttggggccttgctagaatatgcagttgctcactacagttccttacagcag
atggcagccaaagatagggggacaacaaaggaagtagaagaagtcagtat
tactaatatcatcaacagctccatctccagctttaaacggaagatcagct
ttgccagcattgaaatttccagcgacaacgttgactacagtgacttgaca
atgaaaaccagcgacaagttcaagtttgtcttccgagaaaagatgggcag
gattgttgattatttcacaattcaaaaccccagtaatgttgatcactatt
ccaaactactgtttcctttgatttttatgctagccaatgtattttactgg
gcatactacatgtatttttgagtcaatgttaaatttcttgcatgccatag
gtcttcaacaggacaagataatgatgtaaatggtattttaggccaagtgt
gcacccacatccaatggtgctacaagtgactgaaataatatttgagtctt
tctgctcaaagaatgaagctccaaccattgttctaagctgtgtagaagtc

ctagcattataggatcttgtaatagaaacatcagtccattcctctttcat
cttaatcaaggacattcccatggagcccaagattacaaatgtactcaggg
ctgtttattcggtggctccctggtttgcatttacctcatataaagaatgg
gaaggagaccattgggtaaccctcaagtgtcagaagttgtttctaaagta
actatacatgttttttactaaatctctgcagtgcttataaaatacattgt
tgcctatttagggagtaacattttctagtttttgtttctggttaaaatga
aatatgggcttatgtcaattcattggaagtcaatgcactaactcaatacc
aagatgagtttttaaataatgaatattatttaataccacaacagaattat
ccccaatttccaataagtcctatcattgaaaattcaaatataagtgaaga
aaaaattagtagatcaacaatctaaacaaatccctcggttctaagataca
atggattccccatactggaaggactctgaggctttattcccccactatgc
atatcttatcattttattattatacacacatccatcctaaactatactaa
agcccttttcccatgcatggatggaaatggaagattttttttaacttgt
tctagaagtcttaatatgggctgttgccatgaaggcttgcagaattgagt
ccattttctagctgcctttattcacatagtgatggggtactaaaagtact
gggttgactcagagagtcgctgtcattctgtcattgctgctactctaaca
ctgagcaacactctcccagtggcagatcccctgtatcattccaagaggag
cattcatccctttgctctaatgatcaggaatgatgcttattagaaaacaa
actgcttgacccaggaacaagtggcttagcttaagtaaacttggctttgc
tcagatccctgatccttccagctggtctgctatgagtggcttatcccgca
tgagcaggagcgtgctggccctgagtactgaactttctgagtaacaatga
gatacgttacagaacctatgttcaggttgcgggtgagctgccctctccaa
atccagccagagatgcacattcctcggccagtctcagccaacagtaccaa
aagtgatttttgagtgtgccagggtaaaggcttccagttcagcctcagtt
attttagacaatctcgccatctttaatttcttagcttcctgttctaataa
atgcacggctttacctttcctgtcagaaataaaccaaggctctaaaagat
gatttcccttctgtaactccctagagccacaggttctcattccttttccc
attatacttctcacaattcagtttctatgagtttgatcacctgatttttt
taacaaaatatttctaacgggaatgggtgggagtgctggtgaaaagaggt
gaaatgtggttgtatgagccaatcatatttgtgatttttttaaaaaaagtt
taaaaggaaatatctgttctgaaaccccacttaagcattgtttttatata
aaaacaatgataaagatgtgaaactgtgaaataaatataccatattagct
acccaccaaa
>NM_016831 1
ggaaaagctcctcggagatgagcgtgacccccctggctcgtggtggccgcc
tgttctcactaacgccatggcggggaccggagtgagaaaccggtgtctgt
cactgactgcaaagtgagcgagaagcaggctgcgggccgtcccagcacga
cgtggagccccgcggagacctcgagatgccccgcggggaagctcctggcc
ccgggagacggggggctaaggacgaggccctgggcgaagaatcgggggag
cggtggagccccgagttccatctgcagaggaaattggcggacagcagcca
cagtgaacagcaagatcgaaacagagtttctgaagaacttatcatggttg
tccaagaaatgaaaaaatacttcccctcggagagacgcaataaaccaagc
actctagatgccctcaactatgctctccgctgtgtccacagcgttcaagc
aaacagtgagtttttccagattctcagtcagaatggagcacctcaggcag
atgtgagcatgtacagtcttgaggagctggccactatcgcttcagaacac
acttccaaaaacacagatacctttgtggcagtattttcatttctgtctgg
aaggttagtgcacatttctgaacaggctgctttgatcctgaatcgtaaga
aagatgtcctggcgtcttctcactttgttgacctgcttgcacctcaagac
atgagggtattctacgcgcacactgccagagctcagcttcctttctggaa

caactggacccaaagagctgcacggtatgaatgtgctccggtgaaacctt
ttttctgcaggatccgtggaggtgaagacagaaagcaagagaagtgtcac
tccccattccggatcatcccctatctgattcatgtacatcaccctgccca
gccagaattggaatcggaaccttgctgtctcactgtggttgaaaagattc
actctggttatgaagctcctcggatcccagtgaataaaagaatcttcacc
accacacacaccccagggtgtgtttttcttgaagtagatgaaaaagcagt
gcctttgctgggttacctacctcaggacctgattggaacatcgatcctaa
gctacctgcaccctgaagatcgttctctgatggttgccatacaccaaaaa
gttttgaagtatgcagggcatcctccctttgaacattctcccattcgatt
ttgtactcaaaacggagactacatcatactggattccagttggtccagct
ttgtgaatccctggagccggaagatttctttcatcattggtcggcataaa
gttcgaacgagcccactaaatgaggatgtttttgctaccaaaattaaaaa
gatgaacgataatgacaaagacataacagaattacaagaacaaatttaca
aacttctcttacagccagttcacgtgagcgtgtccagcggctacgggagc
ctggggagcagcgggtcgcaggagcagcttgtcagcatcgcctcctccag
tgaggccagtgggcaccgtgtggaggagacgaaggcggagcagatgacct
tgcagcaggtctatgccagtgtgaacaaaattaaaaatctgggtcagcag
ctctacattgagtcaatgaccaaatcatcattcaagccagtgacggggac
acgcacagaaccgaatggtggtggtgaatgtaagacctttacttccttcc
accaaacactgaaaaacaatagtgtgtacactgagccctgtgaggatttg
aggaacgatgagcacagcccatcctatcaacagatcaactgtatcgacag
tgtcatcagatacctgaagagctacaacattccagctttgaaaagaaagt
gtatctcctgtacaaatacaacttcttcctcctcagaagaagacaaacag
aaccacaaggcagatgatgtccaagccttacaagctggtttgcaaatccc
agccatacctaaatcagaaatgccaacaaatggacggtccatagacacag
gaggaggagctccacagatcctgtccacggcgatgctgagcttggggtcg
ggcataagccaatgcggttacagcagcaccattgtccatgtcccaccccc
agagacagccagggatgctaccctcttctgtgagccctggaccctgaaca
tgcagccagcccctttgacctcggaagaatttaaacacgtggggctcaca
gcggctgttctgtcagcgcacacccagaaggaagagcagaattatgttga
taaattccgagaaaagatcctgtcatcaccctacagctcctatcttcagc
aagaaagcaggagcaaagctaaatattcatattttcaaggagattctact
tccaagcagacgcggtcggccggctgcaggaaagggaagcacaagcggaa
gaagctgccggagccgccagacagcagcagctcgaacaccggctctggtc
cccgcaggggagcgcatcagaacgcacagccctgctgcccctccgcggcc
tcctctccgcacacctcgagcccgaccttcccacctgccgccatggtgcc
cagccaggccccttacctcgtcccagcttttcccctcccagccgcgacct
cacccggaagagaatacgcagcccccggaactgcaccggaaggcctgcat
gggctgcccttgtccgagggcttgcagccttacccagctttccctttttcc
ttacttggatacttttatgaccgttttcctgcctgacccccctgtctgtc
ctctgttgtcgccatcgtttttgccatgtccattcctgggggcgacagcc
tcttctgcgatatcaccctcaatgtcgtcagcaatgagtccaactctgga
cccacccccttcagtcaccagccaaaggagagaggaggaaaagtgggagg
cacaaagcgaggggcacccgttcattacttcgagaagcagctcacccttg
cagttaaacttacttcaggaagagatgcccagaccctctgaatctccaga
tcagatgagaaggaacacgtgcccacaaactgagtattgtgttacaggca
acaatggcagtgagagcagtcctgctactaccggtgcactgtccacgggg
tcacctcccagggagaatccatcccatcctactgccagcgctctgtccac
aggatcgcctcccatgaagaatccatcccatcctactgccagcgctctgt

ccacaggatcgcctcccatgaagaatccatcccatcctactgccagcaca
ctgtccatgggattgcctcccagcaggactccatcccatcctactgccac
tgttctgtccacggggtcacctcccagcgaatccccatccagaactggtt
cagcagcatcaggaagcagcgacagcagtatataccttactagtagtgtt
tattcttctaaaatctcccaaaatgggcagcaatctcaggacgtacagaa
aaaagaaacatttcctaatgtcgccgaagagcccatctggagaatgatac
ggcagacacctgagcgcattctcatgacataccaggtacctgagagggtt
aaagaagttgtactaaaagaagacctggaaaagctagaaagtatgaggca
gcagcagccccagttttctcatgggcaaaaggaggagctggctaaggtgt
ataattggattcaaagccagactgtcactcaagaaatcgacattcaagcc
tgtgtcacttgtgaaaatgaagattcagctgatggtgcggccacatcctg
tggtcaggttctggtagaagacagctgttgagtgactgtgaggatgaacc
ttcatacccttttccaagacgtgttacacagacagacctttttaagtcctg
gacttttaaatgaccatgaagttatcattgaatgttaagatttttttcttc
ttgatttttttaatacacgtaatctttttgaagcagacattgtatacagaa
tcttacttctctttgttcctgatatattaaaatggccagttaggctcttt
ttgtagttgaattgtcttctaaagagattggatggcctctaaagaggtat
gtgtatctttatttcagatgtcacccagagtaaattataattagaagtat
agctagaatgagccccaaaccttagcctcatttattttgttctgttacat
aagtcattttccccttagagtgcttgaagaaatgccacctacaggttgtg
tacttttcataatggtttccatgaatgtagtacgttcatacaggcttcat
tcaacctggcgttcccctccataattaagatgaaacattccggttttctc
acaacacattagcacatactgtccattagcatatctgggataaccaggtt
ttgggggttgagttttggccttcatccttgtagatccctttcctattgat
ttcccaccttccagtgaaattctgaaagtcttatcttaaaaatcgatccg
cttaccatgggcctattcttgtaagtttcagttagcatttgcatgtgtaa
tattaaaatgaaagagcttcttacccagtgctgttgcccttttgagtatt
tttgtttttaaaataatgattgtaaaatgttttacaagtaatgtaaaagc
tagtatcattcttacatacttctgtgtttaaattttcattcttaccaaaa
cagttaactctttctttccaatcaatttatacaaaagaggtcgctccagc
cctaccacaggtctgactggcactgccttttgtttgcccttgaacagggc
agtgttgtggggactgcaaaagagaaaacgtccaggcgagcccagttgtc
ctcgcccacagggtcctgcaggctccatcagtcaccgctttctatggcgt
ttgtagttgtgtcttttaagaagtgagtgtgattgtttacttgataaatc
agctcactctctggtgctttttagagaagtccctgattccttcttaaact
tggaatgatagatgaaattcacacccctgcagatcagaaaaaacaaatag
aagaaaatgagggttacagtaacctgttgtctttatataacttgcaacaa
actaatttattttttttcctttttttgtttttggtttttatggttttt
taaggaaaatacttttctcctttgaagttttacagctttttgtaaatgcg
tcctgataatgattaggaaaatcgaccttttcatccatgatgaccatcct
catagctcagatctcctttcaaagtagtggctttctggatggtaattcca
tcttaaggtgtcagaactattttcaaatgctgccttttgacagttcttgga
attttctgatattaagcagttccatgcaaatattcgtgttttataaatag
ctctcatagtctgctccatcttgatagttaagtgatttctgaagcgtttg
tgtgtgtgttgatcaggttgtgtgatatttttgcttgatagagaatcaaa
tttgaaacaattaaccagccagtagattgtctgtcagtgaccttctgtag
taataaagtttttgccactgtaaataaaaacagtatccgtagctatcagg
atcattgcgcactcatatatgctaagccttctgttctctaatagaagcct
ttcttttccattgtttctggatatttgtattatccaaatgtgcttatttc

tttgccttagcacacgttttatggagtacttgttatactaggtttgattt
gaaactggtgcttgtcgcagaactgtcagagcatgaggagcgctcctcct
gtgggtggacgcattcacgcactcccaggttgcacctgctgctggcggtg
agcagggggttcagcagcttgaccgatgccccccgagggggctctcccca
gcttaaactttgttgtttaaatttgttaactttttatattaatgactatt
gaaagtggtaataaaaatttatattataggcttcaatgttttcatgaatg
ttacccaaaaagctgtgttttctttggtcagaggtcaaaatttatgaaaa
acaaaatgctgtatgaatggaaatcattttgcaattgagtgacacttcat
tgtaattcacagtgtaaatttaatccaaactgaaattttgtttcaactga
atttgtaattaactctgaatttgtttttaatcattagtaatatttcagtt
gggtatctttttaagtaaaaacaacaaataaactctgtacatgtaaaacg
tga
>NM_201539 1
gggtctgcgggaagcccgagccggggcgggcccgagtcaaaggcaagtga
aggtggaagcggccgcggcggcagcaggccaccatggcggagctgcagga
ggtgcagatcacagaggagaagccactgttgccaggacagacgcctgagg
cggccaaggaggctgagttagctgcccgaatcctcctggaccagggacag
actcactctgtggagacaccatacggctctgtcactttcactgtctatgg
cacccccaaacccaaacgcccagcgatccttacctaccacgatgtgggac
tcaactataaatcttgcttccagccactgtttcagttcgaggacatgcag
gaaatcattcagaactttgtgcgggttcatgtggatgcccctggaatgga
agagggagcccctgtgttccctttgggatatcagtacccatctctggacc
agcttgcagacatgatcccttgcgtcctgcagtacctaaatttctctaca
ataattggagttggtgttggagctggagcctacatcctggcgagatatgc
tcttaaccacccggacactgttgaaggtcttgtcctcatcaacattgatc
ccaatgccaagggttggatggattgggcagcccacaagctaacaggcctc
acctcttccattccggagatgatccttggacatcttttcagccaggaaga
gctctctggaaattctgagttgatacaaaagtacagaaatatcattacac
atgcacccaacctggataacattgaattgtactggaacagctacaacaac
cgccgagacctgaactttgagcgtggaggtgatatcaccctcaggtgtcc
tgtgatgctggtggtaggagaccaagcacctcatgaagatgcagtggtgg
aatgtaactcaaaactggaccccacccagacctcgttcctcaagatggct
gactccggaggtcagccccagctgactcagccaggcaagctgaccgaggc
cttcaagtacttcctgcaaggcatgggctacatggcctcatcctgcatga
ctcgcctgtcccggtctcgtacagcctctctgaccagtgcagcatccgtt
gatggcaaccggtcccgctctcgcaccctgtcccagagcagcgagtctgg
aactctttcttcggggcccccggggcacaccatggaggtctcctgttgaa
tggcccttgttgccctagagtgggacccagccctcacctcccccagagct
aacctgggaggtgctgaaggggcattgggccaccgtaagcaagggaaaaa
gggcagatcatgcggggagatgaccttgatctttgattgctaccctaacc
ttgacctttaacccgtgattccccccagctcctggaagagatgtcctaat
atctcttagggacccagaccccctaaattctcctcctcccccattttgatg
ttaaggtggagagggcatatgcatcctctgtcctgatctaggtgtctata
gctgaggggtaagaggttgttgtagttgtcctggtgcctccatcagactc
tccctacttgtcccatatttgcaaggggaggggatttggggctggggctc
cattcaccaaagctgaggtggcttctcattaaccctttaggactctgaag
ggtatggacctacgtgaatgtgtgtcagggggagacttgctggtgggtta
gtggtcctcaggatgtgatagaaacatccagtgtaaaaaggaagttggaa
tgggagttggcgggcagtgaacgagtgtggggaaggattggtgctggggc

aacaggaaggggcctggggccgtttggctgcactaactttggtagctcag
tgtgcatctagagtgggactggggagggagctaagcttgggctgggctgc
ttggggcttggcatagggtggaaagggctaccctggggctctgaccacac
tgtagtatgtgtggagggtgccctcccgtctcccacaacttctgctataa
caataaactgtagaggaatctgagtaccgttaaaaaaaaaaaaaaaaaa
aa
>NM_201540 1
gggtctgcgggaagcccgagccggggcgggcccgagtcaaaggcaagtga
aggtggaagcggccgcggcggcagcagaccccagagtcagaaggagtgag
aaccctgaccccctaatcccactgcatccagccaataggagcccagccacc
atggcggagctgcaggaggtgcagatcacagaggagaagccactgttgcc
aggacagacgcctgaggcggccaaggaggctgagttagctgcccgaatcc
tcctggaccagggacagactcactctgtggagacaccatacggctctgtc
actttcactgtctatggcacccccaaacccaaacgcccagcgatccttac
ctaccacgatgtgggactcaactataaatcttgcttccagccactgtttc
agttcgaggacatgcaggaaatcattcagaactttgtgcgggttcatgtg
gatgcccctggaatggaagagggagcccctgtgttccctttgggatatca
gtacccatctctggaccagcttgcagacatgatcccttgcgtcctgcagt
acctaaatttctctacaataattggagttggtgttggagctggagcctac
atcctggcgagatatgctcttaaccacccggacactgttgaaggtcttgt
cctcatcaacattgatcccaatgccaagggttggatggattgggcagccc
acaagctaacaggcctcacctcttccattccggagatgatccttggacat
cttttcagccaggaagagctctctggaaattctgagttgatacaaaagta
cagaaatatcattacacatgcacccaacctggataacattgaattgtact
ggaacagctacaacaaccgccgagacctgaactttgagcgtggaggtgat
atcaccctcaggtgtcctgtgatgctggtggtaggagaccaagcacctca
tgaagatgcagtggtggaatgtaactcaaaactggaccccacccagacct
cgttcctcaagatggctgactccggaggtcagccccagctgactcagcca
ggcaagctgaccgaggccttcaagtacttcctgcaaggcatgggctacat
ggcctcatcctgcatgactcgcctgtcccggtctcgtacagcctctctga
ccagtgcagcatccgttgatggcaaccggtcccgctctcgcaccctgtcc
cagagcagcgagtctggaactctttcttcggggcccccggggcacaccat
ggaggtctcctgttgaatggcccttgttgccctagagtgggacccagccc
tcacctcccccagagctaacctgggaggtgctgaaggggcattgggccac
cgtaagcaagggaaaaagggcagatcatgcggggagatgaccttgatctt
tgattgctaccctaaccttgacctttaacccgtgattccccccagctcct
ggaagagatgtcctaatatctcttagggacccagacccctaaattctcct
cctcccccattttgatgttaaggtggagagggcatatgcatcctctgtcc
tgatctaggtgtctatagctgaggggtaagaggttgttgtagttgtcctg
gtgcctccatcagactctccctacttgtcccatatttgcaaggggagggg
atttggggctggggctccattcaccaaagctgaggtggcttctcattaac
cctttaggactctgaagggtatggacctacgtgaatgtgtgtcaggggga
gacttgctggtgggttagtggtcctcaggatgtgatagaaacatccagtg
taaaaaggaagttggaatgggagttggcgggcagtgaacgagtgtgggga
aggattggtgctggggcaacaggaaggggcctggggccgtttggctgcac
taactttggtagctcagtgtgcatctagagtgggactggggagggagcta
agcttgggctgggctgcttggggcttggcatagggtggaaagggctaccc
tggggctctgaccacactgtagtatgtgtggagggtgccctcccgtctcc
cacaacttctgctataacaataaactgtagaggaatctgagtaccgttaa

aaaaaaaaaaaaaaaaaa
>NM_201535 1
cggccccccgcattccccgcccccacctccccgccttgttgtccaacttc
tcccggagcagccggagagcaggcgtcgggacgcagcaaagagaggagag
accccagagtcagaaggagtgagaaccctgaccccctaatcccactgcatc
cagccaataggagcccagccaccatggcggagctgcaggaggtgcagatc
acagaggagaagccactgttgccaggacagacgcctgaggcggccaagga
ggctgagttagctgcccgaatcctcctggaccagggacagactcactctg
tggagacaccatacggctctgtcactttcactgtctatggcacccccaaa
cccaaacgcccagcgatccttacctaccacgatgtgggactcaactataa
atcttgcttccagccactgtttcagttcgaggacatgcaggaaatcattc
agaactttgtgcgggttcatgtggatgcccctggaatggaagagggagcc
cctgtgttccctttgggatatcagtacccatctctggaccagcttgcaga
catgatcccttgcgtcctgcagtacctaaatttctctacaataattggag
ttggtgttggagctggagcctacatcctggcgagatatgctcttaaccac
ccggacactgttgaaggtcttgtcctcatcaacattgatcccaatgccaa
gggttggatggattgggcagcccacaagctaacaggcctcacctcttcca
ttccggagatgatccttggacatcttttcagccaggaagagctctctgga
aattctgagttgatacaaaagtacagaaatatcattacacatgcacccaa
cctggataacattgaattgtactggaacagctacaacaaccgccgagacc
tgaactttgagcgtggaggtgatatcaccctcaggtgtcctgtgatgctg
gtggtaggagaccaagcacctcatgaagatgcagtggtggaatgtaactc
aaaactggaccccacccagacctcgttcctcaagatggctgactccggag
gtcagccccagctgactcagccaggcaagctgaccgaggccttcaagtac
ttcctgcaaggcatgggctacatggcctcatcctgcatgactcgcctgtc
ccggtctcgtacagcctctctgaccagtgcagcatccgttgatggcaacc
ggtcccgctctcgcaccctgtcccagagcagcgagtctggaactctttct
tcggggcccccggggcacaccatggaggtctcctgttgaatggcccttgt
tgccctagagtgggacccagccctcacctcccccagagctaacctgggag
gtgctgaaggggcattgggccaccgtaagcaagggaaaaagggcagatca
tgcggggagatgaccttgatctttgattgctaccctaaccttgaccttta
acccgtgattccccccagctcctggaagagatgtcctaatatctcttagg
gacccagacccctaaattctcctcctcccccatttgatgttaaggtgga
gagggcatatgcatcctctgtcctgatctaggtgtctatagctgaggggt
aagaggttgttgtagttgtcctggtgcctccatcagactctccctacttg
tcccatatttgcaagggggaggggatttggggctggggctccattcaccaa
agctgaggtggcttctcattaaccctttaggactctgaagggtatggacc
tacgtgaatgtgtgtcagggggagacttgctggtgggttagtggtcctca
ggatgtgatagaaacatccagtgtaaaaaggaagttggaatgggagttgg
cgggcagtgaacgagtgtggggaaggattggtgctggggcaacaggaagg
ggcctggggccgtttggctgcactaactttggtagctcagtgtgcatcta
gagtgggactggggagggagctaagcttgggctgggctgcttggggcttg
gcatagggtggaaagggctaccctggggctctgaccacactgtagtatgt
gtggagggtgccctcccgtctcccacaacttctgctataacaataaactg
tagaggaatctgagtaccgttaaaaaaaaaaaaaaaaaaaa
>NM_201537 1
cggccccccgcattccccgcccccacctccccgccttgttgtccaacttc
tcccggagcagccggagagcaggcgtcgggacgcagcaaagagaggagag
gccaccatggcggagctgcaggaggtgcagatcacagaggagaagccact

gttgccaggacagacgcctgaggcggccaaggaggctgagttagctgccc
gaatcctcctggaccagggacagactcactctgtggagacaccatacggc
tctgtcactttcactgtctatggcacccccaaacccaaacgcccagcgat
ccttacctaccacgatgtgggactcaactataaatcttgcttccagccac
tgtttcagttcgaggacatgcaggaaatcattcagaactttgtgcgggtt
catgtggatgcccctggaatggaagagggagcccctgtgttccctttggg
atatcagtacccatctctggaccagcttgcagacatgatcccttgcgtcc
tgcagtacctaaatttctctacaataattggagttggtgttggagctgga
gcctacatcctggcgagatatgctcttaaccacccggacactgttgaagg
tcttgtcctcatcaacattgatcccaatgccaagggttggatggattggg
cagcccacaagctaacaggcctcacctcttccattccggagatgatcctt
ggacatcttttcagccaggaagagctctctggaaattctgagttgataca
aaagtacagaaatatcattacacatgcacccaacctggataacattgaat
tgtactggaacagctacaacaaccgccgagacctgaactttgagcgtgga
ggtgatatcaccctcaggtgtcctgtgatgctggtggtaggagaccaagc
acctcatgaagatgcagtggtggaatgtaactcaaaactggaccccaccc
agacctcgttcctcaagatggctgactccggaggtcagccccagctgact
cagccaggcaagctgaccgaggccttcaagtacttcctgcaaggcatggg
ctacatggcctcatcctgcatgactcgcctgtcccggtctcgtacagcct
ctctgaccagtgcagcatccgttgatggcaaccggtcccgctctcgcacc
ctgtcccagagcagcgagtctggaactctttcttcggggcccccggggca
caccatggaggtctcctgttgaatggcccttgttgccctagagtgggacc
cagccctcacctcccccagagctaacctgggaggtgctgaaggggcattg
ggccaccgtaagcaagggaaaaagggcagatcatgcggggagatgacctt
gatctttgattgctaccctaaccttgacctttaacccgtgattcccccca
gctcctggaagagatgtcctaatatctcttagggacccagacccctaaat
tctcctcctcccccattttgatgttaaggtggagagggcatatgcatcct
ctgtcctgatctaggtgtctatagctgaggggtaagaggttgttgtagtt
gtcctggtgcctccatcagactctccctacttgtcccatatttgcaaggg
gaggggatttggggctggggctccattcaccaaagctgaggtggcttctc
attaaccctttaggactctgaagggtatggacctacgtgaatgtgtgtca
gggggagacttgctggtgggttagtggtcctcaggatgtgatagaaacat
ccagtgtaaaaaggaagttggaatgggagttggcgggcagtgaacgagtg
tggggaaggattggtgctggggcaacaggaagggcctggggccgtttgg
ctgcactaactttggtagctcagtgtgcatctagagtgggactggggagg
gagctaagcttgggctgggctgcttggggcttggcatagggtggaaaggg
ctaccctggggctctgaccacactgtagtatgtgtggagggtgccctccc
gtctcccacaacttctgctataacaataaactgtagaggaatctgagtac
cgttaaaaaaaaaaaaaaaaaaaa
>NM_182764 1
tcccaagaaggcggggcgggcggaggctggaggagccgccgagcggagac
ccgggagcaggagctgggcctaggtctgcgccctgaattagagcccattg
ggaacgatgccaccaccgtcagacattgtcaaagtggccattgagtggcc
aggtgctaacgcccagctccttgaaatcgaccagaaacggcccctggcat
ccattatcaaggaagtttgtgatgggtggtcgttgccaaacccagagtat
tataccctccgttatgcagatggtcctcagctgtacatcaccgaacagac
tcgcagtgacattaagaatgggacaatcttacaactggctatctccccgt
cccgggctgcacgccagctgatggagaggacccagtcatccaacatggag
acccggctggatgccatgaaggagctggccaagctctctgccgacgtgac

tttcgctactgagttcatcaacatggatggcatcattgtgctgacaaggc
tcgtggaaagtggaaccaagctcttgtcccactacagtgagatgctggca
ttcaccctgactgccttcctagagctcatggaccatggcattgtctcctg
ggacatggtttcaatcacctttattaagcagattgcagggtatgtgagcc
agcccatggtggacgtgtcaatccttcagaggtccctggccatcctggag
agcatggtcttgaacagccagagtctgtaccagaagatagccgaggaaat
caccgtgggacagctcatctcacacctccaggtctccaaccaggagattc
agacctacgccattgcactgattaatgcactttttctgaaggctcctgag
gacaaacgacaggatatggcaaatgcatttgcacagaagcatctccggtc
tataatcctgaatcatgtgatccgagggaaccgccccatcaaaactgaga
tggcccatcagctatatgtccttcaagtcctaacctttaaccttctggaa
gaaaggatgatgaccaagatggaccccaatgaccaggctcaaagggacat
catatttgaactgaggaggattgcatttgacgcagagtctgatcctagca
atgcccctgggagtgggaccgaaaaacgcaaagccatgtacacaaaggac
tacaaaatgctgggatttaccaaccacatcaatccagccatggactttac
ccagactcctcctggaatgctggccttggacaacatgctgtacttggcta
aagtccaccaggacacctacatccggattgtcttggagaacagtagccgg
gaagacaaacatgaatgcccctttggccgcagtgccattgagctcaccaa
aatgctctgtgaaatcctgcaggttggggaactaccaaatgaaggacgca
atgactaccacccgatgttctttacccatgaccgagcctttgaagagctc
tttggaatctgcatccagctgttgaacaagacctggaaggagatgagggc
aacagcagaggacttcaacaaggttatgcaagtcgtccgagagcaaatca
ctcgagctttgccctccaaacccaactctttggatcagttcaagagcaaa
ttgcgtagcctgagttactctgagattctacgactgcgccagtctgagag
gatgagtcaggatgacttccagtccccgccaattgtggagctgagggaga
agatccagcccgagatccttgagctgatcaagcagcagcgcctgaaccgg
ctctgtgagggcagcagcttccgaaagattgggaaccgccgaaggcaaga
acggttctggtactgccggttggcactgaaccacaaggtccttcactatg
gtgacttggatgacaacccacaaggggaggtgacatttgaatccctgcag
gagaaaattcctgttgcagacattaaggccattgtcactgggaaagattg
tccccacatgaaagagaaaagtgctctgaaacagaacaaggaggtgttgg
aattggccttctccatcctgtatgaccctgatgagaccttaaacttcatc
gcacctaataaatatgagtactgcatctggattgatggcctcagtgccct
tctggggaaggacatgtccagtgagctgaccaagagtgacctggacaccc
tgctgagcatggagatgaagctgcggctcctggacctggagaacatccag
attcccgaagccccaccccccatccccaaggagcccagcagctatgactt
tgtctatcactatggctgagcctggagccagaaacgacggtacccaggag
aagggattttgggcccaggagaaacacttacattctggtgccttgtcttt
tgcttgtacagaatctgtagtgattttggtggccagtaaatgccagccat
ttctcaaacccacctcggaccacccagagtttcctcttggtccctgtcta
ctaagagtcatgaaggcagggtgctctgcccactccatcaccatgaagcc
tgggattgggccacgaggaacaaacagcagatgcccttgccttccagtcc
aagaaactgcttcttgaaatggatttaacaacagccactcaccttttcct
cctgagcctgctctctgatcagctggatccccacgtgagcaacagctggc
ccaggaaaggctgcctgcagaggacaggtgtgttgggcgtgttgagagcc
ttgaagtgactacctgtatcttagatctgagtacaagcctgaggcttttg
cttttgtctttttgatgagggctcactccagcttcatatggtgccaaga
cgttgctgcttctgaggttggctctaacatctctggtctttagagccacc
agatctctctggcccatacagatatcagagcagacggaaatttctccctg

caagcgctcagtctcatcccagcaagtcaaagacctcctggccaagtcct
gccctcttaagtctccaggaacgctgcagggaaaacccagctgaggcctg
ggcctagactgtggtgaggtcactagattctactgctcttcccccacatt
aatacctttccttcctcagagagaaatctcccctaacctgaattgcagc
cccctccagtttgctttcctttggccttccagaccccaggaagttggcct
tcccttcctagtgctatggtttctgccattggccatgatttcagggagct
ggctgaggccggctgaggccacacctgtgccagtggggcttccctggtgc
tgcagcacttgtaaaccacacacacagcctctctccctggacatacgtta
gcacattggcattcagtattggtggcctggcatggtaggtactacccaat
gaagagtgtactatatattttcattactataggccatacttatacagacg
tgtatatatatttatataagatctacctatcttaggatggaaccttgggg
aaaaataaaattgaggggaagtaaaaagtatgtaacacttccagttgtga
gccaagattgtaaccagagagcagccaggagcttcctgtcagtaaccatg
ttttcaataaatactctttcatgtacaaaaaaaaaaaaaaaaaaaa
>NM_002811 4
gggagcggaagaaggaggccgcgcgagggctgacgaaccggaagaagagg
aactgggcctgaaagggtaccggtgaccgctactgctgccggtgtttgcg
tgtggcagggagccaggcctggcgagcggggtgtgtcgcgatgccggagc
tggcagtgcagaaggtggtggtccaccccctggtgctgctcagtgtggtg
gatcatttcaaccgaatcggcaaggttggaaaccagaagcgtgttgttgg
tgtgcttttggggtcatggcaaaagaaagtacttgatgtatcgaacagtt
ttgcagttccttttgatgaagatgacaaagacgattctgtatggtttta
gaccatgattatttggaaaacatgtatggaatgtttaagaaagtcaatgc
cagggaaagaatagttggctggtaccacacaggccctaaactacacaaga
atgacattgccatcaacgaactcatgaaaagatactgtcctaattccgta
ttggtcatcattgatgtgaagccgaaggacctagggctgcctacagaagc
gtacatttcagtggaagaagtccatgatgatggaactccaacctcgaaaa
catttgaacacgtgaccagtgaaattggagcagaggaagctgaggaagtt
ggagttgaacacttgttacgagatatcaaagacacgacggtgggcactct
gtcccagcggatcacaaaccaggtccatggtttgaagggactgaactcca
agcttctggatatcaggagctacctggaaaaagtcgccacaggcaagctg
cccatcaaccaccagatcatctaccagctgcaggacgtcttcaacctgct
gccagatgtcagcctgcaggagttcgtcaaggccttttacctgaagacca
atgaccagatggtggtagtgtacttggcctcgctgatccgttccgtggtc
gccctgcacaacctcatcaacaacaagattgccaaccgggatgcagagaa
gaaagaagggcaggagaaagaagagagcaaaaaggataggaaagaggaca
aggagaaagataaagataaggaaaagagtgatgtaaagaaagaggagaaa
aaggagaaaaagtaaaacatgtattaaatagctttttttaatttgtaaatt
aaaatcttacaaactaaatcagtgtgctgctagagggttctttttcactt
gacatgcttattagaaagctgacccaacaagagctctctgcctccggtca
ctcttgctgtggtgctacgtggaagtgaatggagactgatctcaaatctg
aactgcagctttcgctgctgtgagttggggatatgatagtcagctcaggc
ttcagattgtatgagaaaaatgaagagaagtcaacaaatattttggtact
cttcattcatttatctctaaaaccaggagttgaattttcctcatcttgaa
agactcttggggtctgtttctggtattttacaaaattgctaagtggaatg
catgaattgcattatgttctctggtaacacgtagagttcagacccttctg
aactctgttgataataccacaccatgttctggacccatagctctggcatc
ctcaggggttgtgatccagctccatatattgtttaccttcaaagatacaa
ttaaatggcttgatttttaaaaaaaaaaaaaaaaaa

>NM_000067 2
cctccccttgtcgcctaggtccacccgagcccctcccccgggccgcccc
cgagcacgaagttggcgggagcctataaaagctggtgccggcgcgacccg
cggacacacagtgcaggcgcccaagccgccgccgccagatcggtgccgat
tcctgccctgccccgaccgccagcgcgaccatgtcccatcactgggggta
cggcaaacacaacggacctgagcactggcataaggacttccccattgcca
agggagagcgccagtcccctgttgacatcgacactcatacagccaagtat
gacccttccctgaagcccctgtctgtttcctatgatcaagcaacttccct
gaggatcctcaacaatggtcatgctttcaacgtggagtttgatgactctc
aggacaaagcagtgctcaagggaggacccctggatggcacttacagattg
attcagtttcactttcactggggttcacttgatggacaaggttcagagca
tactgtggataaaaagaaatatgctgcagaacttcacttggttcactgga
acaccaaatatggggattttgggaaagctgtgcagcaacctgatggactg
gccgttctaggtatttttttgaaggttggcagcgctaaaccgggccttca
gaaagttgttgatgtgctggattccattaaaacaaagggcaagagtgctg
acttcactaacttcgatcctcgtggcctccttcctgaatccttggattac
tggacctacccaggctcactgaccacccctcctcttctggaatgtgtgac
ctggattgtgctcaaggaacccatcagcgtcagcagcgagcaggtgttga
aattccgtaaacttaacttcaatggggagggtgaacccgaagaactgatg
gtggacaactggcgcccagctcagccactgaagaacaggcaaatcaaagc
ttccttcaaataagatggtcccatagtctgtatccaaataatgaatcttc
gggtgtttccctttagctaagcacagatctaccttggtgatttggaccct
ggttgctttgtgtctagttttctagacccttcatctcttacttgatagac
ttactaataaaatgtgaagactagaccaattgtcatgcttgacacaactg
ctgtggctggttggtgctttgtttatggtagtagtttttctgtaacacag
aatataggataagaaataagaataaagtaccttgactttgttcacagcat
gtagggtgatgagcactcacaattgttgactaaaatgctgcttttaaaac
ataggaaagtagaatggttgagtgcaaatccatagcacaagataaattga
gctagttaaggcaaatcaggtaaaatagtcatgattctatgtaatgtaaa
ccagaaaaaataaatgttcatgatttcaagatgttatattaaagaaaaac
tttaaaaattattatatatttatagcaaagttatcttaaatatgaattct
gttgtaatttaatgacttttgaattacagagatataaatgaagtattatc
tgtaaaaattgttataattagagttgtgatacagagtatatttccattca
gacaatatatcataacttaataaatattgtattttagatatattctctaa
taaaattcagaattct
>NM_005175 2
caggacacgtgggtgggggaagctgagcgctgagaccaagggctaaagct
gggaggtgagtctgtcaccttgagccgggcgagcgctgtgggccaagcag
gggttgcagggtagtaggagtgcagactgaaaaaatgcagaccgccgggg
cattattcatttctccagctctgatccgctgttgtaccaggggtctaatc
aggcctgtgtctgcctccttcttgaatagcccagtgaattcatctaaaca
gccttcctacagcaacttcccactccaggtggccagacgggagttccaga
ccagtgttgtctcccgggacattgacacagcagccaagtttattggtgct
ggggcagccacagttggtgtggctggttcaggggctggcattggaaccgt
gtttggcagcttgatcattggctatgccaggaacccgtctctcaagcagc
agctcttctcctatgccattcttggctttgccctgtctgaggccatgggg
cttttctgtttgatggtcgccttcctcatcctcttcgccatgtgaggctc
catgggggtcaccggcctgttgctactgcaactccacaccattcttggt
gctggggtgtgttaagctttaccattaaacacaacgtttctctaaaaaaa

aaaaaaaaaaaaa
>NM_001002027 1
caggacacgtgggtgggggaagctgagcgctgagaccaagggctaaagct
gggagactgaaaaaatgcagaccgccggggcattattcatttctccagct
ctgatccgctgttgtaccaggggtctaatcaggcctgtgtctgcctcctt
cttgaatagcccagtgaattcatctaaacagccttcctacagcaacttcc
cactccaggtggccagacgggagttccagaccagtgttgtctcccgggac
attgacacagcagccaagtttattggtgctggggcagccacagttggtgt
ggctggttcaggggctggcattggaaccgtgtttggcagcttgatcattg
gctatgccaggaacccgtctctcaagcagcagctcttctcctatgccatt
cttggctttgccctgtctgaggccatggggcttttctgtttgatggtcgc
cttcctcatcctcttcgccatgtgaggctccatgggggtcaccggcctg
ttgctactgcaactccacaccattcttggtgctggggtgtgttaagcttt
accattaaacacaacgtttctctaaaaaaaaaaaaaaaaaaa
>NM_002079 2
gcgttccttctcccctgtgccttcgtcgtcagaagctggcgattggttaa
tcgcgttgccaagctttggacgcggctcgaccattggaggccgcgggccc
gcccccgccggctaggtgaaggtgagtgtctcctccagtcgcaacggcca
gacctgacctgccagctccgggcgtggggtgaaatctcttgattcctagt
ctctcgatatggcacctccgtcagtctttgccgaggttccgcaggcccag
cctgtcctggtcttcaagctcactgccgacttcagggaggatccggaccc
ccgcaaggtcaacctgggagtgggagcatatcgcacggatgactgccatc
cctgggttttgccagtagtgaagaaagtggagcagaagattgctaatgac
aatagcctaaatcacgagtatctgccaatcctgggcctggctgagttccg
gagctgtgcttctcgtcttgcccttggggatgacagcccagcactcaagg
agaagcgggtaggaggtgtgcaatctttgggggggaacaggtgcacttcga
attggagctgatttcttagcgcgttggtacaatggaacaaacaacaagaa
cacacctgtctatgtgtcctcaccaacctgggagaatcacaatgctgtgt
tttccgctgctggttttaaagacattcggtcctatcgctactgggatgca
gagaagagaggattggacctccagggcttcctgaatgatctggagaatgc
tcctgagttctccattgttgtcctccacgcctgtgcacacaacccaactg
ggattgacccaactccggagcagtggaagcagattgcttctgtcatgaag
caccggtttctgttccccttctttgactcagcctatcagggcttcgcatc
tggaaacctggagagagatgcctgggccattcgctattttgtgtctgaag
gcttcgagttcttctgtgcccagtccttctccaagaacttcgggctctac
aatgagagagtcgggaatctgactgtggttggaaaagaacctgagagcat
cctgcaagtcctttcccagatggagaagatcgtgcggattacttggtcca
atcccccgcccagggagcacgaattgtggccagcaccctctctaaccct
gagctctttgaggaatggacaggtaatgtgaagacaatggctgaccggat
tctgaccatgagatctgaactcagggcacgactagaagccctcaaaaccc
ctgggacctggaaccacatcactgatcaaattggcatgttcagcttcact
gggttgaaccccaagcaggttgagtatctggtcaatgaaaagcacatcta
cctgctgccaagtggtcgaatcaacgtgagtggcttaaccaccaaaaatc
tagattacgtggccacctccatccatgaagcagtcaccaaaatccagtga
agaaacaccacccgtccagtaccaccaaagtagttctctgtcatgtgtgt
tccctgcctgcacaaacctacatgtacataccatggattagagacacttg
caggactgaaaggctgctctggtgaggcagcctctgtttaaaccggcccc
acatgaagagaacatcccttgagacgaatttggagactgggattagagcc
tttggaggtcaaagcaaattaagatttttatttaagaataaaagagtact

ttgatcatgagacataggtatcttgtccctctcactaaaaaggagtgttg
tgtgtggcggccacgtgcttctatgtggtgtttgactctgtacaaattct
agtcccaaagatcaagttgtctgaaggagccaaagtgtgaatgtgggtgt
cggctgcggcattaaattcatcatctcaacccagagtgtctggtctccct
gctctttctgcatggttgtgtccctagtcctaagctttggttctttaggg
tgactgtggtaagaaggatatttaatcatgacatgcacggacacgtacat
atttaactgaaacaagttttaccaaacagtatttactcgtgatgtgcgta
gtgcattctgatatttttgagccattctattgtgttctacttcacctaaa
aaaataaaataaaaatgttgatcaagaaaaaaaaaaaaaa
>NM_003891 2
ggtgggaatggcaggctgcgtcccactgctccagggcctggtcctggtcc
tcgccctccatcgtgtggagccctcagtatttctcccggcctccaaagca
aacgacgttctggtgaggtggaagcgtgcgggctcctatcttctggaaga
actcttcgagggaaacttggaaaaagaatgttatgaagaaatctgtgtct
atgaagaagcaagagaagtgtttgaaaatgaagtagtcactgatgaattc
tggagacgatataagggcggctccccgtgcatctcccagccctgcctcca
caacggctcttgccaggacagcatctggggctacacctgcacctgctccc
ccggctatgagggcagcaactgcgagctggctaaaaatgaatgtcaccca
gagcggactgatgggtgtcaacacttctgcctcccaggacaggaatccta
cacgtgcagctgtgctcagggctacaggcttggtgaggaccacaaacagt
gtgtgccccacgaccagtgtgcctgcggggtgctgacctctgagaagcgt
gcaccggatctacaggacctcccgtggcaggtaaagttaacaaattccga
aggaaaagacttctgtggtggtgttataatacgggaaaattttgtactga
caacagcaaaatgttcactgttacacaggaatattactgtaaaaacatat
tttaacagaacgagccaagacccgctgatgatcaagataacgcacgtcca
tgtgcacatgcggtatgacgcggacgcgggggagaatgacctgtcactgc
tggagctggagtggcccatccagtgcccaggtgcggggctccccgtgtgc
acccctgagaaagacttcgctgagcacctcctcatcccacgcaccagggg
cctcctcagcggctgggcacgcaatggcactgacctgggcaactcgctga
ccacgcggcctgtcacacttgtggagggggaggagtgcgggcaggtcctg
aatgtgactgtcaccaccaggacctactgtgagagaagcagcgtggcggc
catgcactggatggatggaagtgtggtcaccagagaacacagaggctcct
ggtttctcacgggggtcctgggctcgcagccagtaggagggcaggctcac
atggtccttgtcaccaaggtctccaggtactcactctggtttaaacagat
catgaactaactgaaactcagctagccagaatgaacaacacaaccggaag
cgggattccaagctggcactgccactgtggagggcgctgaaacttcatca
cacactgagaggccgtcacagccccagaccacccgcttggcccacgcagc
agcagagccgccgtttgctgggttgtttaccgagcactgtgacctttctt
tccctggaactctttatctcaatagagaccttaaaagaaaacatgagata
cgttaaataataaaataagataatctgtcagtcataaag
>NM_002084 3
gtcgccccgggacggggaggtggggagctgagggcaagtcgcgcccgccc
ctgaaatcccagccgcctagcgattggctgcaagggtctcggcttggccg
cggattggtcacacccgagggcttgaaaggtggctgggagcgccggacac
ctcagacggacggtggccagggatcaggcagcggctcaggcgaccctgag
tgtgcccccaccccgccatggcccggctgctgcaggcgtcctgcctgctt
tccctgctcctggccggcttcgtctcgcagagccggggacaagagaagtc
gaagatggactgccatggtggcataagtggcaccatttacgagtacggag
ccctcaccattgatggggaggagtacatccccttcaagcagtatgctggc

aaatacgtcctctttgtcaacgtggccagctactgaggcctgacgggcca
gtacattgaactgaatgcactacaggaagagcttgcaccattcggtctgg
tcattctgggctttccctgcaaccaatttggaaaacaggaaccaggagag
aactcagagatccttcctaccctcaagtatgtccgaccaggtggaggctt
tgtccctaatttccagctctttgagaaaggggatgtcaatggagagaaag
agcagaaattctacactttcctaaagaactcctgtcctcccacctcggag
ctcctgggtacatctgaccgcctcttctgggaacccatgaaggttcacga
catccgctggaactttgagaagttcctggtggggccagatggtataccca
tcatgcgctggcaccaccggaccacggtcagcaacgtcaagatggacatc
ctgtcctacatgaggcggcaggcagccctgggggtcaagaggaagtaact
gaaggccgtctcatcccatgtccaccatgtaggggagggactttgttcag
gaagaaatccgtgtctccaaccacactatctacccatcacagacccctt
cctatcactcaaggccccagcctggcacaaatggatgcatacagttctgt
gtactgccaggcatgtgggtgtgggtgcatgtgggtgtttacacacatgc
ctacaggtatgcgtgattgtgtgtgtgtgcatgggtgtacagccacgtgt
ctacctatgtgtctttctgggaatgtgtaccatctgtgtgcctgcagctg
tgtagtgctggacagtgacaaccctttctctccagttctccactccaatg
ataatagttcacttacacctaaacccaaaggaaaaaccagctctaggtcc
aattgttctgctctaactgatacctcaaccttggggccagcatctcccac
tgcctccaaatattagtaactatgactgacgtccccagaagtttctgggt
ctaccacactccccaacccccccactcctacttcctgaagggccctccaa
ggctacatccccaccccacagttctccctgagagagatcaacctccctga
gatcaaccaaggcagatgtgacagcaagggccacggaccccatggcaggg
gtggcgtcttcatgagggagggggcccaaagcccttgtgggcggacctccc
ctgagcctgtctgaggggccagcccttagtgcattcaggctaaggcccct
gggcagggatgccacccctgctccttcggaggacgtgccctcacccctca
ctggtccactggcttgagactcaccccgtctgcccagtaaaagcctttct
gcagcagctgaaaaaaaaaaaaaaaaaa
>NM_000124 2
agcagaagtcggagtcgctgttgggggcggtgtctatggttgagctgagg
gcgcaggcgccacggcccgtcgagctgggttccaaggcggctggcggcgg
tagcgtctctgtttccttgtgggcgctcgcgcggccctgggtagtctgta
gagaatgccaaatgagggaatcccccactcaagtcaaactcaggagcaag
actgtttacagagtcaacctgtcagtaataatgaagaaatggcaatcaag
caagaaagtggtggtgatggggaggtggaggagtacctctcctttcgttc
tgtgggtgacgggctgtccacctctgctgtggggtgcgcatcagcagctc
cgaggagagggccagccctgctgcacatcgaccgacatcagatccaggca
gtagagcctagcgcccaggcccttgagctgcagggtttgggtgtggacgt
ctatgaccaggacgtgctggaacagggagtgcttcagcaggtggacaatg
ccatccatgaggccagccgtgcctcccagctcgttgacgtggagaaggag
tatcggtcggtcctggatgacctcacgtcatgtacgacatccctaaggca
aatcaataaaattattgaacagcttagccctcaagctgccaccagcagag
acatcaacaggaaactagattctgtaaaacgacagaagtataataaggaa
caacagctaaaaaagatcactgcaaaacaaaagcatctccaggccatcct
tggaggagcagaggtgaaaattgaactagatcacgccagtctggaggagg
atgcagagccggggccatccagtcttggcagcatgctcatgcctgtccag
gagactgcctgggaagagctcatccgcactggccagatgacaccttttgg
tacccagatccctcagaaacaggagaaaaagcccagaaaaatcatgctta
atgaagcatcaggcttcgaaaagtatttggcagatcaagcaaaactgtct

tttgaaaggaagaagcaaggttgtaataaaagagcagctagaaaagctcc
agccccagtcacgcctccagccccagtgcaaaataaaaacaaaccaaaca
agaaagccagagttctgtccaaaaaagaggagcgtttgaaaaagcacatc
aagaaactccagaagagggctttgcagttccaggggaaagtgggattgcc
aaaggcaaggagaccttgggagtcagacatgaggccagaggcagagggag
actctgagggtgaagagtctgagtatttccccacagaggaggaggaagag
gaggaagatgacgaggtggagggggcagaggcggacctgtctggagatgg
tactgactatgagctgaagcctctgcccaagggcgggaaacggcagaaga
aagtgccagtgcaggagattgatgatgactttttcccaagttctggggaa
gaagctgaagctgcttctgtaggagaaggaggaggaggaggtcggaaagt
gggaagataccgagatgatggagatgaagattattataagcagcggttaa
ggagatggaataaactgagactgcaggacaaagagaaacgtctgaagctg
gaggacgattctgaggaaagtgatgctgaatttgacgaaggttttaaagt
gccaggttttctgttcaaaaagctttttaagtaccagcagacaggtgtta
ggtggctgtgggaattgcactgccagcaggcaggaggaattctgggagat
gaaatgggattgggcaagaccatccagataattgccttcttggcaggtct
gagctacagcaagatcaggactcgtggttcaaattacaggtttgaggggt
tgggtccaactgtaattgtctgtccaacaacagtgatgcatcagtgggtg
aaggaatttcacacgtggtggcctccgttcagagtggcaattctacatga
aaccggttcctatacccacaaaaaggagaaactaattcgagatgttgctc
attgtcatggaattttgatcacatcttactcctacattcgattgatgcag
gatgacattagcaggtatgactggcactatgtgatcttggacgaaggaca
caaaattcgaaatccaaatgctgctgtcacccttgcttgcaaacagtttc
gcacccctcatcggatcattctgtctggctcaccgatgcaaaataacctc
cgagagctgtggtcgctctttgacttcatcttcccgggaaagttaggcac
gttgcctgtgtttatggagcagttctccgtccccatcaccatggggggat
attcaaatgcttccccagtacaggtcaaaactgcttacaagtgtgcatgt
gtcttacgagataccataaatccatacctactgcggagaatgaagtcaga
tgtcaagatgagcctttctttgccagataaaaatgaacaggtcttatttt
gccgtcttacagatgagcagcataaagtctaccaaaatttcgttgattcc
aaagaagtttacaggattctcaatggagagatgcagattttctccggact
tatagccctaagaaaaatttgcaaccaccctgatctcttttctggaggtc
ccaagaatctcaaaggtcttcctgatgatgaactagaagaagatcagttt
gggtactggaaacgttctgggaaaatgattgttgttgagtctttgttgaa
aatatggcacaagcagggtcagcgagtattgctgttttctcagtcaaggc
agatgctggacatacttgaagtattccttagagcccaaaagtatacctat
ctcaagatggatggtaccactacaatagcttcaagacagccactgattac
gagatacaatgaggacacatccatatttgtgtttcttctgaccacgcggg
tgggcggcttaggtgtcaacctgacgggggcaaacagagttgtcatctat
gacccagactggaacccaagcacggacacgcaggcccgggagcgagcatg
gagaataggccagaagaagcaagtgactgtgtacaggctcctgactgcgg
gcaccattgaagaaaagatctaccaccgacaaatcttcaagcagtttttg
acaaatagagtgctaaaagacccaaaacaaaggcggtttttcaaatccaa
tgatctctatgagctatttactctgactagtcctgatgcatcccagagca
ctgaaacaagtgcaatttttgcaggaactggatcagatgttcagacaccc
aaatgccatctaaaaagaaggattcaaccagcctttggagcagaccatga
tgttccaaaacgcaagaagttccctgcttctaacatatctgtaaatgatg
ccacatcatctgaagagaaatctgaggctaaaggagctgaagtaaatgca
gtaacttctaatcgaagtgatcctttgaaagatgaccctcacatgagtag

taatgtaactagcaatgataggcttggagaagagacaaatgcagtatctg
gaccagaagagttgtcagtgattagtggaaatggggaatgttcaaattct
tcaggaacaggcaaaacttctatgccatctggtgatgaaagcattgatga
aaagttaggtctttcttacaaaagagaaagacccagccaggctcaaacag
aagctttttgggagaataaacaaatggaaaataatttttataagcacaag
tcaaaaacaaaacatcatagtgtggcagaagaagagaccctggagaaaca
tctgagaccaaagcaaaagcctaagaactctaagcattgcagagacgcca
agtttgaaggaactcgaattccacacctggtgaagaaaaggcgttaccag
aagcaagacagtgaaaacaagagtgaggccaaggaacagagcaatgacga
ttatgttttggaaaagcttttcaaaaaatcagttggcgtgcacagtgtca
tgaagcacgatgccatcatggatggagccagcccagattatgtactggtg
gaggcagaagccaaccgagtggcccaggatgccctgaaagcactgaggct
ctctcgtcagcggtgtctgggagcagtgtctggtgttcccacctggactg
gccacagggggatttctggtgcaccagcaggaaaaaagagtagatttggt
aagaaaaggaattctaacttctctgtgcagcatccttcatcaacatctcc
aacagagaagtgccaggatggcatcatgaaaaaggagggaaaagataatg
tccctgagcattttagtggaagagcagaagatgcagactcttcatccggg
cccctcgcttcctcctcactcttggctaaaatgagagctagaaaccacct
gattctgccagagcgtttagaaagtgaaagcgggcacctgcaggaagctt
ctgccctgctgcccaccacagaacacgatgaccttctggtggagatgaga
aacttcatcgctttccaggcccacactgatggccaggccagcaccaggga
gatactgcaggagtttgaatccaagttatctgcatcacagtcttgtgtct
tccgagaactattgagaaatctgtgcactttccatagaacttctggtggt
gaaggaatttggaaactcaagccagaatactgctaaacaacattgcttcc
taaactttcaagtccctttttctaacgggcatttctgattattaatttat
tattaataatcatgtttgtcaatggaagttggctgcacttgatgtttgtt
tgcatgatgtctacctcagaattaaaactttaaggaagaagaaactcttc
tctgaaagttaaaagttttaataatgctagctaaaggagaaaatacttgg
attgatttttttttttttggcaatctaattatattgtaaatcaggtacct
aacagttactccttggagcacatttgttcctttacccaaaagatgctgtc
agggagcacagttagaagtttgcagaacagaaatctcaatattttttttt
attggtgctaaaaacaggtcttacattcagtcagacctgttcaataagtt
catcaatatctgataacagcattattttgatgcttaaactttaaacattt
atatttaccatttgccacccacaaaggtcaggtttgttatttgttgtttg
ataattatattaattttcttggaaagatcctcttttcaaggtactggtaa
attggtgagtatttttattagtaaagcatgaaatagtatggtaataaatg
ataagacatgtatttgtggaaagctgtagggtattcagtttaccctggct
ttcctttaagcagagggcatctttttctctcctacagtcacaaaatgtgt
tatcattaaaaaaaatcaaattaaagccaaaagtaggtacataaaaacca
cacacatgcatgcacacaaacatcactgcagcccacagcagacccagccg
ttgttaccatgaagtgacaccactccaggcctctcttgtctgcaggctgg
caggctgtcttctctccagttgccttcgtcttgcgcctgcctttgcattc
cttgcgacgggctttcttgtttctgcggtttggattccagccaaggctgt
ttgtatctcactactgtttatgtgtttgtggttctgtgatggtgttgctt
tgatcctcagtttattttcttacccatgtttttcttgtttccttctcagg
atgattttatcatctcatctttgaagtgttgttttccgaaattcatcgta
ttcctgaaatttcttcttagctgtcttagtgcagtttgtttcttggattt
gtattctctggcatgctcttttcctctctctcatttttctgtagtatgcc
tgccctcctaccctgctatttctttacatctctctcatgcttaacatgga

tagctgtgtccagatcttctgtctgctcatccatgtgactcagagaggag
ggttctgggcagggggccttgccggactgcatgagaggacatgagtttt
gctttctctgctctaatattttgcttaagccaagaatccttttcttagag
atgttctatatgattcctgtcaggattttctagtttttttggattatag
cttgttcatttcttttgtttttagtttggtttatatataatgagggaaga
agatgattacattatttttgtcactttgccatcattgtttagaagtcata
gaaagaatttttaaataggccaataagtcttaaacttgagtacttggctt
agaagaaagtcaaaactccttcctttttgactaagtggtttgtttctggg
gagctcttaatttctatttttataatcattagcctataaggaaattgtgt
cttccttgttctcagggtgatctgctgaccttgttcactcatgaagcatt
tgggtatcatacttatagtgtctgaaacataaactgtattgagctagaca
aggtatagcctcctcttcaagtagcaaatactatcaaaagctataatgca
gtaggagcaaggtggtccttgttccagtttttgtctcagttctgctgctg
atgtaccatgatcttgggaaggtggtgtctcagtgtggagatctgacaca
ttgttaccgtgcctcctggctggagggacttggagaacaatgcagttaag
tagaatggttttaacaatacagagaaatttattcatttagataaaaatct
gatttttagaactttaaaagctttgtacagtgtaaatagatttaatgtat
ttaacatgctttatcagcacaaataaaggattttaaaattttgtcaaaaa
attaaatgttaatactatcaccattaaaaatgttcaagcaatagtctgcc
tccccacccccacaccatcttgcacctgttccacagctaagtacagccct
aggtttggtgtgtattctccatgcatttagagaatcacatgacacagact
gctgctataatgtcattttcccattcttcctttactaataaaatttttga
gtttta
>NM_003569 2
gagggccgctgtcactcagccccgcgggccaatagaaaaggggtgaaccc
cgccttcttcctgagttgtgctgcgggcatgcgcactgggcgtccccacg
ccaccgcccatcagctgagaattgcagctgagggctccggggtaggtggg
tgacggcggtcggaggtgtaggagggagccgtggaggtccaggtgactgc
ttagaaaactgcacagcatctgatgaaattagcgaataagaacatcaacc
atgtcttacactccaggagttggtggtgaccccgcccagttggcccagag
gatctcttctaacatccagaagatcacacagtgttctgtggaaatacaaa
gaactctgaatcaacttggaacacctcaagattcacctgaattgaggcaa
cagttgcaacagaagcagcagtatactaaccagcttgccaaagaaacaga
taagtacattaaagagtttggatctctgcccaccacccccagtgaacagc
gtcaaaggaaaatacagaaggatcgcttagtggcagagttcacaacatca
ctgacaaacttccagaaggtccagaggcaggctgctgagcgagagaaaga
gtttgttgctcgagtaagagccagttccagagtgtctggcagttttcctg
aggacagctcaaaagaaaggaatcttgtatcctgggaaagccaaactcaa
cctcaagtgcaggtgcaggatgaagaaattacagaggatgacctccgtct
tattcatgagagagagaatcttctatcaggcaacttgaagctgatattatgg
atattaatgaaatatttaaagatttgggaatgatgattcatgaacaagga
gatgtaatagatagcatagaagccaatgtggaaaatgcagaggtgcacgt
tcagcaagcaaatcagcagctgtcaagggcagcagattatcagcgcaaat
ccagaaaaaccctgtgcatcatcattcttatccttgtcattggagttgcg
attatcagtctcatcatatggggattgaaccactgaagttataaaggagc
acactgtcgcactacattgtctaaattatgtaggaagattcctgtaatca
tgttttttaattattattttaaagctattgtataaaggatggttcccat
actttgttatttttattgggggggttggggtggttcctttggattaaatc
tgatattttctaatactgaaagattttctaaatgtcactgctgacataac

tcccttggtcttcaatttaatagttgttaagtttttgcccacattgcata
tgcctttcatttataatttatttaccctgcttgacttagttttgggaatt
cgtaaatttaaaggtgtgtgtattctgtttgcatctccctgtcactgtga
cacacctagatgtgtgttacttcaattaaaattctcaaatttaattttga
tttgcttcagcagggaaaatattctcaataatgtaaaataattaaggtct
atacatgggttgtatttttctggttcacaacagcacaaagtgtctttcat
ttttttgttggttttcttttaagatcttttttaccctgaagtcggtgaat
acttttctagtttatttgatactctttctgtgtatatattaagcttttgc
tgtagattgcctagtaaaattactaaggataggttgtttttacatatggt
ctatttaagtctgatgtttacgggggaaagtgtagttactaaaaatgttt
aacataatttggaagaagagtatgaacaaccaataccaatacctattgcg
tttggattcttaagaccccagtttgttattccactaaactagttatctta
accatatcatctggttttgtgggccattatttaccttcccttatgtctta
tagaataatggttaatatttttaggtcaaaattacttttggaaagtaact
ttcccacaattaactgtttttgagcacctgacaaaatttagtgtttacct
tgcgtgccattttgtgtcatccttcattaaaaaagcaattggaggtttgc
cagttatctcacttccctttttaaatcaatgttgttttaatgcactaatc
tgaattctgtaaagaggattatcttagtttatactttgtattttataatg
ttcttgtatagcagctcggtactgaaggcggtgtttaacttggcaagctc
tgagacttcaaatgggaacaaatagtaagtagctaagtaaaccacatctt
tgcaaccaaaataaagatgagttaaaaggtatctggttaggcctatttca
tgaggactatgctctggtgggaccacaggtcacctgatacttagtgctgt
gctgcctgaaacctcagcatggagacatcaccacatgcactgtggccatt
cagcatctttctggagcaccagttcactccaggtttttattttagggtgt
catcgattttacctactttgtcagactggtagaagttgctttgcatatca
gaaaaactccatttttttccacaaaagggattacagaaaactcttttgtg
agtgagtgattggaacttagagactcctgttgccagaatcagactgccct
agaacagaatggacaatgcagggaggagaattcacacaaacagcacctgt
tctgaggcctgtgccagcccaccaggcctgctcaaatgtggtctttactt
caagtgcacagaggcacatgaggtttctggtgataaaccagcgtcttacc
gctgttttaaagtcccatccccatggctttcacaatcagttccgtttttt
ttgctgtacttgataaaatgtttattctcatacaggtcaagtacatttac
ttctattcacagtgagtacccaataacaacaaaagcgcttacaaatttgg
ggggcgtgattttagtacctttatttgaagtgtaatcattttaaattatt
attattttaactggggcagttatcagtggtttaaacaggaactttagtgg
cttcaatttgtttaagaaacatattaagtttgagggaaaaatttcccatg
aaatatttggaacgtaagagtagtattgattagagaaaattaaataagaa
acatagtatggtagccaaattttttaaaaaatcttgaacttttctgtagg
tcagttttagaactgctgtgaaaagtgaaggttgccctgtggagattaaa
attagagttgttttcataactgacagcatggtgaatccatttgagtcaaa
gtgaagaatttcctcatcaagtgactatacatttgtttttgtgtgctcaa
aagaaatactcaaacacagactgatattaaccagccaggtaaattgaacg
acaatgtggcattaggtatttggctgtttattggtcgttaaatactatgg
ttttgcaatatgattgatggtaaaagagtgatgtcatattgatactagag
tagcttgtttttttagtaggtgtgggaccatctcttttacaagtgcaact
cagtctaggacagccatggatgcagtgtctggagttggaccctctgagcc
cgctggctgccccagtagcatctgcattggtgaccaaggacactgcactt
tgaagaggtcgccactgggttatttagtgtcttcactgcttttgttaaaa
attgtaaaatttgtacacaaaaagttgtgtttttgaatatcaattgttt

agacacacctacaatgataaataagtgcctttaaaggcccctctttccat
gaaatacatctgtggtttagcaaggaaagtacaaaatagtttatgtagtt
ggtataatttttatttgtgtcttcatgtagaaaaaatgaatgtcataata
aaatataaaacttacgtaaagaaaataaagtcattgtccaccttaatagc
taaggtccacaagggtaacttatgcagcatttattttttttgaaagtcaa
aattgaatttatttctttcacatggctggtttgctgcaatatgaagtttc
agaatgggctgaagtaagttgattgagggatttgagttgaatgacatttt
caagttcatttaaatatgataaaaaattcattggtggtaaataacatctgt
ctttcctggaaaaaaaaaaagttgtgtattttcatgattcagttaaaacaa
aaaatgagcctgtgaatcccaggccttttttagtcctccataacatttgaa
cagtttgacttgtcagcaaagaaatacacttatcaaattttaaaccaatg
ggagcctgaaagtgttacag
>NM_005956 3
aattacggccggattccggagtcctttccagctccctcttcggccgggtt
tcccgccgaatacaaaggcgcactgtgaactggctctttctttccgccaa
tcatttccgccagccattcatcaccgattttcttcatcttcccctccctc
ttccgtcccgcagtccccgacctgttagctctcggttagttaagggactc
gggtccttccgaactgcgcatgcgccaccgcgtctgcaggggggagaagcg
ggcaggggcgcaggcgcagtagtgtgatcccctggccagtccctaagcac
gtgggttgggttgtcctgcttggctgcggagggagtggaacctcgatatt
ggtggtgtccatcgtgggcagcggactaataaaggccatggcgccagcag
aaatcctgaacgggaaggagatctccgcgcaaataagggcgagactgaaa
aatcaagtcactcagttgaaggagcaagtacctggtttcacaccacgcct
ggcaatattacaggttggcaacagagatgattccaatctttatataaatg
tgaagctgaaggctgctgaagagattgggatcaaagccactcacattaag
ttaccaagaacaaccacagaatctgaggtgatgaagtacattacatcttt
gaatgaagactctactgtacatgggttcttagtgcagctacctttagatt
cagagaattccattaacactgaagaagtgatcaatgctattgcacccgag
aaggatgtggatggattgactagcatcaatgctgggaaacttgctagagg
tgacctcaatgactgtttcattccttgtacgcctaagggatgcttggaac
tcatcaaagagacaggggtgccgattgccggaaggcatgctgtggtggtt
gggcgcagtaaaatagttggggccccgatgcatgacttgcttctgtggaa
caatgccacagtgaccacctgccactccaagactgcccatctggatgagg
aggtaaataaaggtgacatcctggtggttgcaactggtcagcctgaaatg
gttaaaggggagtggatcaaacctggggcaatagtcatcgactgtggaat
caattatgtcccagatgataaaaaaccaaatgggagaaaagttgtgggtg
atgtggcatacgacgaggccaaagagagggcgagcttcatcactcctgtt
cctggcggcgtagggcccatgacagttgcaatgctcatgcagagcacagt
agagagtgccaagcgtttcctggagaaatttaagccaggaaagtggatga
ttcagtataacaaccttaacctcaagacacctgttccaagtgacattgat
atatcacgatcttgtaaaccgaagcccattggtaagctggctcgagaaat
tggtctgctgtctgaagaggtagaattatatggtgaaacaaaggccaaag
ttctgctgtcagcactagaacgcctgaagcaccggcctgatgggaaatac
gtggtggtgactggaataactccaacacccctgggagaagggaaaagcac
aactacaatcgggctagtgcaagcccttggtgcccatctctaccagaatg
tctttgcgtgtgtgcgacagccttctcagggccccacctttggaataaaa
ggtggcgctgcaggaggcggctactcccaggtcattcctatggaagagtt
taatctccacctcacaggtgacatccatgccatcactgcagctaataacc
tcgttgctgcggccattgatgctcggatatttcatgaactgacccagaca

gacaaggctctctttaatcgtttggtgccatcagtaaatggagtgagaag
gttctctgacatccaaatccgaaggttaaagagactaggcattgaaaaga
ctgaccctaccacactgacagatgaagagataaacagatttgcaagattg
gacattgatccagaaaccataacttggcaaagagtgttggataccaatga
tagattcctgaggaagatcacgattggacaggctccaacggagaagggtc
acacacggacggcccagtttgatatctctgtggccagtgaaattatggct
gtcctggctctcaccacttctctagaagacatgagagagagactgggcaa
aatggtggtggcatccagtaagaaaggagagcccgtcagtgccgaagatc
tgggggtgagtggtgcactgacagtgcttatgaaggacgcaatcaagccc
aatctcatgcagacactggagggcactccagtgtttgtccatgctggccc
gtttgccaacatcgcacatggcaattcctccatcattgcagaccggatcg
cactcaagcttgttggcccagaagggtttgtagtgacggaagcaggattt
ggagcagacattggaatggaaaagtttttttaacatcaaatgccggtattc
cggcctctgcccccacgtggtggtgcttgttgccactgtcagggctctca
agatgcacgggggcggccccacggtcactgctggactgcctcttcccaag
gcttacatacaggagaacctggagctggttgaaaaaggcttcagtaactt
gaagaaacaaattgaaaatgccagaatgtttggaattccagtagtagtgg
ccgtgaatgcattcaagacggatacagagtctgagctggacctcatcagc
cgcctttccagagaacatggggcttttgatgccgtgaagtgcactcactg
ggcagaagggggcaagggtgccttagccctggctcaggccgtccagagag
cagcacaagcacccagcagcttccagctcctttatgacctcaagctccca
gttgaggataaaatcaggatcattgcacagaagatctatggagcagatga
cattgaattacttcccgaagctcaacacaaagctgaagtctacacgaagc
agggctttgggaatctccccatctgcatggctaaaacacacttgtctttg
tctcacaacccagagcaaaaaggtgtccctacaggcttcattctgcccat
tcgcgacatccgcgccagcgttggggctggttttctgtaccccttagtag
gaacgatgagcacaatgcctggactccccacccggccctgtttttatgat
attgatttggaccctgaaacagaacaggtgaatggattattctaaacaga
tcaccatccatcttcaagaagctactttgaaagtctggccagtgtctatt
caggcccactgggagttaggaagtataagtaagccaagagaagtcagccc
ctgcccagaagatctgaaactaatagtaggagtttccccagaagtcattt
tcagccttaattctcatcatgtataaattaacataaatcatgcatgtctg
tttactttagtgacgttccacagaataaaaggaaacaagtttgccatcaa
aaaaaaaaaaaaaaaa
>NM_000192 3
catgccttatgcaagagacctcagtcccccggaacaactcgatttccttc
caatagaggtctgaggtggactcccacctcccttcgtgaagagttccctc
ctctccccccttcctaagaaagtcgatcttggctctatttgtgtcttatgt
tcatcaccctcattcctccggagaaagccgggttggtttatgtctttatt
tattcccggggccaagacgtccggaacctgtggctgcgcagacccggcac
tgataggcgaagacggagagaaatttacctcccgccgctgcccccccagcc
aaacgtgacagcgcgcgggccggttgcgtgactcgtgacgtctccaagtc
ctataggtgcagcggctggtgagatagtcgctatcgcctggttgcctctt
tattttactggggtatgcctggtaataaacagtaatatttaatttgtcgg
agaccacaaaccaaccttgagctgggaggtacgtgctcttcttgacagac
gttggaagaagacctggcctaaagaggtctcttttggtggtccttttcaa
agtcttcacctgagccctgctctccagcgaggcgcactcctggcttttgc
gctccaaagaagaggtgggatagttggagagcagaaccttgcgcgggcac
agggccctgggcgcaccatggccgacgcagacgagggctttggcctggcg

cacacgcctctggagcctgacgcaaaagacctgccctgcgattcgaaacc
cgagagcgcgctcggggcccccagcaagtccccgtcgtccccgcaggccg
ccttcacccagcagggcatggagggaatcaaagtgtttctccatgaaaga
gaactgtggctaaaattccacgaagtgggcacggaaatgatcataaccaa
ggctggaaggcggatgtttcccagttacaaagtgaaggtgacgggcctta
atcccaaaacgaagtacattcttctcatggacattgtacctgccgacgat
cacagatacaaattcgcagataataaatggtctgtgacgggcaaagctga
gcccgccatgcctggccgcctgtacgtgcacccagactcccccgccaccg
gggcgcattggatgaggcagctcgtctccttccagaaactcaagctcacc
aacaaccacctggacccatttgggcatattattctaaattccatgcacaa
ataccagcctagattacacatcgtgaaagcggatgaaaataatggatttg
gctcaaaaaatacagcgttctgcactcacgtctttcctgagactgcgttt
atagcagtgacttcctaccagaaccacaagatcacgcaattaaagattga
gaataatccctttgccaaaggatttcggggcagtgatgacatggagctgc
acagaatgtcaagaatgcaaagtaaagaatatcccgtggtccccaggagc
accgtgaggcaaaaagtggcctccaaccacagtcctttcagcagcgagtc
tcgagctctctccacctcatccaatttggggtcccaataccagtgtgaga
atggtgtttccggcccctcccaggacctcctgcctccacccaacccatac
ccactgccccaggagcatagccaaatttaccattgtaccaagaggaaaga
ggaagaatgttccaccacagaccatccctataagaagccctacatggaga
catcacccagtgaagaagattccttctaccgctctagctatccacagcag
cagggcctgggtgcctcctacaggacagagtcggcacagcggcaagcttg
catgtatgccagctctgcgccccccagcgagcctgtgcccagcctagagg
acatcagctgcaacacgtggccaagcatgccttcctacagcagctgcacc
gtcaccaccgtgcagcccatggacaggctaccctaccagcacttctccgc
tcacttcacctcggggcccctggtccctcggctggctggcatggccaacc
atggctccccacagctgggagagggaatgttccagcaccagacctccgtg
gcccaccagcctgtggtcaggcagtgtgggcctcagactggcctgcagtc
ccctggcacccttcagccccctgagttcctctactctcatggcgtgccaa
ggactctatcccctcatcagtaccactctgtgcacggagttggcatggtg
ccagagtggagcgacaatagctaaagtgaggcctgcttcacaacagacat
ttcctagagaaagagagagagagaggagaaagagagagaaggagagagac
agtagccaagagaaccccacggacaagatttttcatttcacccaatgttc
acatctgcactcaaggtcgctggatgctgatctaatcagtagcttgaaac
cacaattttaaaaatgtgactttcttgttttgtctcaaaacttaaaaaaa
caaacacaaaaagatgagtcccaccccccactaccaccacacccatcaac
cagccacattcacgctactccccagatctcttccccattccttcttttg
ggctctagaaagtcttgcctcattgagtgtttttccctagtgcgtagttg
gagtctgtccctgtcttggtgttaatgttgacattgttatataataaatg
ataatatatttttttctttcaattttcttaatgggacccagtcccttatt
tggggggaggtctgaggcaagtatatttcaaaatatgtacttgcgggatt
cccttcaagtaaaccatccctgaaacctaaattcacgtttccccttgact
aagaaaagcacctacctctgccatgtgatgtttctgaaaagcctctgtat
gtccccatttgctttggttttgtcctgccttctccaatatcacgtgctca
gttttgcctctacttacccatggagtcaggataacactgacgctccctgg
catcctatcttattcagccctaccatcttgccagctctgtctttccagct
gtctgtcgctaaaacgtggcctatagcttcccttccggaaagcttgcttt
gaaaaacttaaaaagcccccgtttacatgtaggcaggactgtgataacag
tgcaagctctgtgttgacaagagttgtggacaaaaagccaaaataaatat

tcttcctgattaaaaaaatttttttgaaaaaaacaaggccagccccaac
cttccaaacctccatcaccaacaacccaaactggatgtcaagcaaaatgc
acaattcctacagaagaggcaagacacagtcaccaatgatatctcgccaa
agaaaccacgcccacaccaatgccaacacaaaactgtgtttactgaaagc
cgaaaacagtattaaaaaaagtgtgtaagtaaagtgttatggtagggttc
ttcagatgtaatattttactggtactatttatttataaataggaattcta
attaagtaataacatgaaatgaaacccagcataggagctggccaagagct
tttaattttattgatactcaaaaccaagtttgtgtttttttgtttttttt
tgtttttttcctctttcgaatgtgctttgctttttttgattaaaaagaat
tttttttttcctttttttataaacagaccctaataaagagaacagggtaag
atgtgaggctgagtgtgtttaagtacgtgagagagtgtgagtgtgtttgt
aagtgagtgtccctatgcgattatgtctctttacgttgctaaggggggag
ggtgaggattaagtactcgtgccttatatttgtgtgccaattaatgccta
ataaataccatgtgcttaaacaagtaaaaaaaaaaaaaaaaaaaaaaaaa
aaaaaaaaaaaaaaaaaaaaaaaaaaaaaaaaaaaaaaaaaaaaaaaaaa
aaaaaaaaaaaaaaaaaaaa
>NM_181486 1
ttcagagagagagagagagggagagagagtgagagagactgactcttacc
tcgaatccgggaactttaatcctgaaagctgcgctcagaaaggacttcga
ccattcactgggcttccaactttccctccctgggggtgtaaaggaggagc
ggggcactgagattatatggttgccggtgctcttggaggctattttgtgt
tctttggcgcttgccaactgggaagtatttagggagagcaagcgcacagc
agaggaggtgtgtgttggaggtgggcagtcgccgcggaggctccagcggt
aggtgcgccctagtaggcagcagtagccgctattctgggtaagcagtaaa
ccccgcataaaccccggagccaccatgcctgctcccccgcctcaccgccg
gcttccctgctaggagcagcagaggatgtggtgaatgcaccggcttcacc
gaacgagagcagaaccttgcgcgggcacagggccctgggcgcaccatggc
cgacgcagacgagggctttggcctggcgcacacgcctctggagcctgacg
caaaagacctgccctgcgattcgaaacccgagagcgcgctcggggccccc
agcaagtccccgtcgtccccgcaggccgccttcacccagcagggcatgga
gggaatcaaagtgtttctccatgaaagagaactgtggctaaaattccacg
aagtgggcacggaaatgatcataaccaaggctggaaggcggatgtttccc
agttacaaagtgaaggtgacgggccttaatcccaaaacgaagtacattct
tctcatggacattgtacctgccgacgatcacagatacaaattcgcagata
ataaatggtctgtgacgggcaaagctgagcccgccatgcctggccgcctg
tacgtgcacccagactcccccgccaccggggcgcattggatgaggcagct
cgtctccttccagaaactcaagctcaccaacaaccacctggacccatttg
ggcatattattctaaattccatgcacaaataccagcctagattacacatc
gtgaaagcggatgaaaataatggatttggctcaaaaaatacagcgttctg
cactcacgtctttcctgagactgcgtttatagcagtgacttcctaccaga
accacaagatcacgcaattaaagattgagaataatccctttgccaaagga
tttcggggcagtgatgacatggagctgcacagaatgtcaagaatgcaaag
taaagaatatcccgtggtccccaggagcaccgtgaggcaaaaagtggcct
ccaaccacagtcctttcagcagcgagtctcgagctctctccacctcatcc
aatttggggtcccaataccagtgtgagaatggtgtttccggcccctccca
ggacctcctgcctccacccaacccatacccactgccccaggagcatagcc
aaatttaccattgtaccaagaggaaagaggaagaatgttccaccacagac
catccctataagaagccctacatggagacatcacccagtgaagaagattc
cttctaccgctctagctatccacagcagcagggcctgggtgcctcctaca

ggacagagtcggcacagcggcaagcttgcatgtatgccagctctgcgccc
cccagcgagcctgtgcccagcctagaggacatcagctgcaacacgtggcc
aagcatgccttcctacagcagctgcaccgtcaccaccgtgcagcccatgg
acaggctaccctaccagcacttctccgctcacttcacctcggggcccctg
gtccctcggctggctggcatggccaaccatggctccccacagctgggaga
gggaatgttccagcaccagacctccgtggcccaccagcctgtggtcaggc
agtgtgggcctcagactggcctgcagtcccctggcacccttcagcccccct
gagttcctctactctcatggcgtgccaaggactctatcccctcatcagta
ccactctgtgcacggagttggcatggtgccagagtggagcgacaatagct
aaagtgaggcctgcttcacaacagacatttcctagagaaagagagagaga
gaggagaaagagagagaaggagagagacagtagccaagagaaccccacgg
acaagatttttcatttcacccaatgttcacatctgcactcaaggtcgctg
gatgctgatctaatcagtagcttgaaaccacaattttaaaaatgtgactt
tcttgttttgtctcaaaacttaaaaaaacaaacacaaaaagatgagtccc
acccccactaccaccacacccatcaaccagccacattcacgctactccc
cagatctcttcccccattccttcttttgggctctagaaagtcttgcctca
ttgagtgtttttccctagtgcgtagttggagtctgtccctgtcttggtgt
taatgttgacattgttatataataaatgataatatattttttttctttcaa
ttttcttaatgggacccagtcccttatttggggggaggtctgaggcaagt
atatttcaaaatatgtacttgcgggattcccttcaagtaaaccatccctg
aaacctaaattcacgtttccccttgactaagaaaagcacctacctctgcc
atgtgatgtttctgaaaagcctctgtatgtccccatttgctttggttttg
tcctgccttctccaatatcacgtgctcagttttgcctctacttacccatg
gagtcaggataacactgacgctccctggcatcctatcttattcagcccta
ccatcttgccagctctgtctttccagctgtctgtcgctaaaacgtggcct
atagcttcccttccggaaagcttgctttgaaaaacttaaaaagcccccgt
ttacatgtaggcaggactgtgataacagtgcaagctctgtgttgacaaga
gttgtggacaaaaagccaaaataaatattcttcctgattaaaaaaatttt
ttttgaaaaaaacaaggccagccccaaccttccaaacctccatcaccaac
aacccaaactggatgtcaagcaaaatgcacaattcctacagaagaggcaa
gacacagtcaccaatgatatctcgccaaagaaaccacgcccacaccaatg
ccaacacaaaactgtgtttactgaaagccgaaaacagtattaaaaaaagt
gtgtaagtaaagtgttatggtagggttcttcagatgtaatattttactgg
tactatttatttataaataggaattctaattaagtaataacatgaaatga
aacccagcataggagctggccaagagcttttaattttattgatactcaaa
accaagtttgtgtttttttgtttttttttgtttttttcctctttcgaatg
tgctttgcttttttttgattaaaaagaattttttttttcctttttttataaa
cagaccctaataaagagaacagggtaagatgtgaggctgagtgtgtttaa
gtacgtgagagagtgtgagtgtgtttgtaagtgagtgtccctatgcgatt
atgtctctttacgttgctaaggggggagggtgaggattaagtactcgtgc
cttatatttgtgtgccaattaatgcctaataaataccatgtgcttaaaca
agtaaaaaaaaaaaaaaaaaaaaaaaaaaaaaaaaaaaaaaaaaaaaaa
aaaaaaaaaaaaaaaaaaaaaaaaaaaaaaaaaaaaaaaaaaaaaaaaaa
>NM_005550 3
gcccgagttaatcatttcctgtggaaagtgtgcgggaggggcgcgagcgg
gctggccgaggaggaggcggcggcgtggagctgcctcctgccggcgggcc
gggccgggccgagccccgggcgctgcggcgacgcctggatcctgcctccg
ccaggccggctgcctggtgccccgaggaggctgctgagccccaggccatg
gtcccctctcgcaggacgtggaacctgggagccacgccctcgctgcgggg

cctgtggagagtgggccgggccccggagcccgagccggggatggctcgcc
ccgccccagccccagccagcccggccgcccgccctttcccacacaccggc
ccggggaggttgagaactgggcgtggaaaagataccccagtctgcggtga
cgaggactccagtgcccgaagtgcagctcgcccagccctagctcagtgcc
gagcccttagcgtggactgggctggccccggaagcccccacgggctctac
ctgaccctgcaggtagaacacctgaaggagaagctcattagccaggccca
ggaagtgagccgactgcgatctgagctgggggcaccgacttggagaagc
accgggacctgctgatggtggagaatgagcgactgaggcaggagatgcgg
cgctgtgaggccgagctgcaagagctgcgcacaaagccagcaggtccctg
cccaggttgtgagcacagccaggagagcgcccagctccgtgacaagctgt
cccagctgcagctggagatggcggaaagcaaaggcatgctgtcagagctg
aacctagaggtgcagcagaagaccgaccggctggctgaggtggagctgcg
actcaaggactgcctggctgagaaggcacaggaggaggagcggcttagtc
ggcgcctgcgtgacagccacgagaccattgccagcctgcgggcccagtcc
ccacctgtcaagtatgtcatcaagacagtggaggtggagtcgtccaagac
caagcaggccctcagcgagtcccaggcccggaaccagcacctgcaggagc
aggtggctatgcagaggcaggtgctgaaggagatggaacagcagctgcag
agctcacaccagctgaccgcgcggctccgggcgcagattgccatgtacga
gtcagagctggagcgggcccatgggcagatgctggaggagatgcagtccc
tggaagaggacaagaaccgggccattgaggaggcctttgccagagcccag
gtggagatgaaggctgtgcacgagaatctagcaggcgtccggaccaactt
gctgaccttgcagccggcactgcggaccctcaccaacgactacaatgggc
tcaagcggcaggtgcgcggcttcccactgctgctgcaggaggccctcagg
agtgtcaaggccgagataggccaggccatcgaggaggtcaacagcaacaa
ccaggagctgctgcgcaagtaccgccgcgagctgcagctgcgtaagaagt
gccacaatgagctcgtgcggctgaaagggaacatccgagtgattgctcgt
gtccggccagtcaccaaagaggatggggaaggacctgaggccaccaatgc
tgtgactttcgatgccgacgacgactccatcatccacctgctgcacaagg
gcaagcctgtgtccttcgagctggacaaggtcttctccccacaggcctcg
cagcaggacgtgttccaggaggtgcaggccctggtcacctcttgcattga
tggcttcaatgtctgcatctttgcgtacggccagacgggcgccggcaaga
cgtacacgatggaggggaccgctgagaacccaggtatcaaccagcgggcc
ctgcagctgctcttctccgaggtgcaggagaaggcgtctgactgggagta
caccatcaccgtcagcgctgcggagatctacaatgaggtcctcagggacc
tgctagggaaagagcctcaggaaaaactggagatccggctgtgcccagac
ggcagtgggcagctgtatgtaccagggctgactgagttccaagtgcagag
cgtggacgacatcaacaaggtgtttgagtttggccacactaatcgcacga
ccgagttcaccaacctgaacgagcacagctcccgctcgcacgcgctgctc
atcgtgacggtgcgaggcgtggactgcagcacaggcctccgcaccacggg
gaagctgaacctggtggacttggctggctcggagcgcgtgggcaagtcgg
gggccgagggcagccgcctgcgggaggcgcagcacatcaacaagtcgctg
tcggctctgggggacgtcattgctgccctgcgctcccgccagggccacgt
gcccttccgcaactccaagctcacctacctgctgcaggattcgcttagtg
gtgacagcaagaccctcatggtggtacaggtgtcccccgtggagaagaac
actagcgagacgctctattccctcaagtttgctgagagggtgcgctctgt
ggagctggggcctgggctacgcagggcagagcttgggtcctggtcaagcc
aggagcatctagagtgggagccggcttgtcagacgccacagccctcggca
cgggcccactcagcccccagctctgggaccagtagccgccctggatccat
ccggaggaagctgcagccctcggggaagtcgcggccactgcctgtgtgac

ggatgtgaccccgctgggcctgaagctgggccctcactggcctgtccctg
ctgcagcgccaggacccccggaggtagaggcgagagtggaggctcttctt
ctgccccgtctcccctcaaagatgagaaacatgttcagaaggaaacggtg
tctctcggctgtggctctgagtgcaaattgcatgggcggaaaggcggggg
tggctgctcttcctggcaggcctgggccatcagcgaactgggccccgtga
ggagggcgggagtgtggaggagggtgggcctctcacccaggctttctcgg
cccctctcctcagcttgcagagctggccagccccctccttagggggtggg
cgaggagcctctgggcagacccaagaaccatggggactggggtgggttgg
tggcaccaatggcagccctccccgcccctctccttcaaggagggttcccg
cagctggggggtgtgcggaggcgcatggcctcccgccacggggccgtgct
gtgtttatggctggcagaggcagccagcgggtgggggattctgctgctcg
ctcacctgcctggctcgctggtctctcgaattttcttccctctgaaatcc
tatttaagaacttttggaagcttagccatttttacttattaaaataaaag
aagcctttttacacaa
>NM_002633 2
cctttcccctcccgccggacctgccaggaggtgggctggcgcggagggag
ggccctgtcccctgtccctttaaggaggagggccaaacgccggcctagag
tgcggcgtagcccccacccgccgtgccctcaccccagagcagctgcagcc
tcagccggccgcccctccgccagccaagtccgccgctctgacccccggca
gcaagtcgccaccatggtgaagatcgtgacagttaagacccaggcgtacc
aggaccagaagccgggcacgagcgggctgcggaagcgggtgaaggtgttc
cagagcagcgccaactacgcggagaacttcatccagagtatcatctccac
cgtggagccggcgcagcggcaggaggccacgctggtggtgggcggggacg
gccggttctacatgaaggaggccatccagctcatcgctcgcatcgctgcc
gccaacgggatcggtcgcttggttatcggacagaatggaatcctctccac
ccctgctgtatcctgcatcattagaaaaatcaaagccattggtgggatca
ttctgacagccagtcacaacccagggggccccaatggagattttggaatc
aaattcaatatttctaatggaggtcctgctccagaagcaataactgataa
aattttccaaatcagcaagacaattgaagaatatgcagtttgccctgacc
tgaaagtagaccttggtgttctgggaaagcagcagtttgacttggaaaat
aagttcaaacccttcacagtggaaattgtggattcggtagaagcttatgc
tacaatgctgagaagcatctttgatttcagtgcactgaaagaactacttt
ctgggccaaaccgactgaagatccgtattgatgctatgcatggagttgtg
ggaccgtatgtaaagaagatcctctgtgaagaactcggtgcccctgcgaa
ctcggcagttaactgcgttcctctggaggactttggaggccaccaccctg
accccaacctcacctatgcagctgacctggtggagaccatgaagtcagga
gagcatgattttggggctgcctttgatggagatggggatcgaaacatgat
tctgggcaagcatgggttctttgtgaacccttcagactctgtggctgtca
ttgctgccaacatcttcagcattccgtatttccagcagactggggtccgc
ggctttgcacggagcatgcccacgagtggtgctctggaccgggtggctag
tgctacaaagattgctttgtatgagaccccaactggctggaagtttttg
ggaatttgatggacgcgagcaaactgtccctttgtggggaggagagcttc
gggaccggttctgaccacatccgtgagaaagatggactgtgggctgtcct
tgcctggctctccatcctagccacccgcaagcagagtgtggaggacattc
tcaaagatcattggcaaaagtatggccggaatttcttcaccaggtatgat
tacgaggaggtggaagctgagggcgcaaacaaaatgatgaaggacttgga
ggccctgatgtttgatcgctcctttgtggggaagcagttctcagcaaatg
acaaagtttacactgtggagaaggccgataactttgaatacagcgaccca
gtggatggaagcatttcaagaaatcagggcttgcgcctcattttcacaga

tggttctcgaatcgtcttccgactgagcggcactgggagtgccggggcca
ccattcggctgtacatcgatagctatgagaaggacgttgccaagattaac
caggacccccaggtcatgttggcccccttatttccattgctctgaaagt
gtcccagctgcaggagaggacgggacgcactgcacccactgtcatcacct
aagaagacaggcctgatgtggtacgtccctccacccccggacccatccaa
gtcatctgattgaagagcatgacagaaacaaaatgtattcaccaagcatt
ttaggatttgactttttcactaaccagttgacgagcagtgcatttacaag
gcactgccaaacaagatgcccttgggagctgtgagggaaagaggacctgc
gggcttagatcaatctcaattccttttcatgccctcctgcattgctgctg
cgtgggtatttgtctccttagccatcaggtacagtttacactacaatgta
agctataggtggagcatcagcagtgagtgaggccattcttcatccttagg
atgtggcaatgaaatgatggtgcaagttcctttctcttttgtgaatcttt
ccccccatttcctgtttacatgtaacccaacaaaatgcaatttctagtgc
cttctgtccaatcagttctttcctctgagtgagacgtacttggctacaga
tttctgccttgttttgcgacattgtcccattcacacagatattttgggat
aataaaggaaaataagctacaaaaaaaaaaaaaaaa
>NM_000727 3
acactaaacttagtggccactcccagctcgacaaccactgccaccccca
agctcggcttgtcacctgccctaggagacgcagccgccggaccctgccca
gggcacccacgcctcggcgaccaccatgtcccagaccaaaatgctgaagg
tccgcgtgaccctcttctgcatcctggcaggcatcgtgctggccatgaca
gccgtggtaaccgaccactgggctgtgctgagcccccacatggagcacca
caacactacctgcgaggcggcccacttcggcctctggcggatttgtacca
agcgcatccccatggacgacagcaagacctgcgggcccatcaccctgccc
ggggagaagaactgttcctacttcaggcattttaaccccggcgagagctc
ggagatcttcgaattcaccactcagaaggagtacagcatctcggcagccg
ccatcgccatcttcagccttggcttcatcatcctgggcagcctctgtgtc
ctcctgtccctcgggaagaagagggactatctgctgcgacccgcgtccat
gttctatgcctttgcaggtctctgcatcctcgtctcggtggaggtcatgc
ggcagtcggtgaagcgcatgattgacagtgaggacaccgtctggatcgag
tactattactcctggtcctttgcctgcgcctgtgccgccttcatcctcct
ctttctcggcggtctcgccctcctgctgttctccctgcctcgaatgcccc
ggaacccatgggagtcctgcatggatgctgagcccgagcactaaccctcc
tgcggccctagcgaccctcaggcttcttccccaggaagcggggtcttggc
ctggaaccttccagagaggaggcgggagcaattttagccccaccctgctc
ccatctgccccctgcaacagtcgcaggctgcttcctctctctgagttcc
tctgggctgccgcaggctcccctgggaatagagcaagacgtgagtcctaa
cctggccacagttgggggaggcagagccagcaggtggacaggtgtttgca
ggggcccaacttcccctggagctcagaggtgtccccactgtaccagcctc
tgataagctgcctccagttgtcctttatgaacattgcagggacaacctgt
gtttgccagctgggtgttccgtgtaaatagccagcctgtctctttctcgg
tgataaaacacaccctctctggtgagcccagcgtcccctccttggcttcc
aggagccctgggaagcatttttaactgggtagaatctgactgtggcttga
aataaaaagctctcagaaaaaaaaaaa
>NM_000696 3
ctgcccctaaaatctcctagaaccgatcccgcggccccgcccctcccgcg
gccccgcccctcccgcggcccgtcagcctctgccgcggagctgcgtccgc
cactcatgtttctccgagcaggcctggccgcgctctccccgcttcttcgc
agtcttcggccctctcctgtcgccgccatgagcactggcaccttcgtcgt

gtcgcagccgctcaattaccgcggcggggcccgcgtggagccggcggacg
cctccggtaccgagaaagctttcgagccagcaaccggccgagtgatagct
actttcacatgttcaggagaaaaggaagtaaatttggctgttcaaaatgc
aaaggctgcttttaaaatatggagtcaaaaatctggcatggagcgttgcc
gaatccttttggaggctgccaggataataagggaacgggaggatgaaatt
gctactatggagtgcatcaacaatggcaagtccatctttgaggcccgctt
ggacattgacatttcctggcagtgcctggagtattatgcgggcttggctg
catccatggctggtgaacacatccagctcccaggtggatcgtttggttat
accagaagagaaccacttggggtatgtgtgggaataggagcatggaacta
ccccttcagattgcctcttggaagtcggctccagcattagcctgtggta
atgccatggtctttaaaccttctccctttacacctgtttctgcattgcta
ctggctgaaatctacagtgaggctggtgtacctcctgggctcttcaatgt
ggtgcagggaggggctgccacaggccagtttctgtgtcagcatcccgatg
tggccaaagtctccttcactggaagtgtgcccactggcatgaagatcatg
gagatgtcagctaaaggaatcaaacctgttaccttggaacttggaggcaa
atctccactcatcatcttctcagactgtgatatgaacaatgctgtaaagg
gggcgctgatggccaacttcctcacacaaggccaggtttgctgtaatggc
acaagagtatttgtgcagaaagaaattcttgataaatttacagaggaagt
ggtgaaacagacccaaaggattaaaattggagatccccttctggaagata
caaggatgggtccactcatcaaccgaccacacctggagcgagtccttggg
tttgtcaaagtggcaaaggagcagggtgctaaagtgttatgtggtggaga
tatatatgtacctgaagatcccaaattaaaggatggatattacatgagac
cttgtgtattaactaattgcagagacgacatgacctgtgtgaaggaagag
atctttgggcctgttatgtccattttatcatttgacactgaagctgaggt
tctagaaagagccaatgataccacttttggactagcagctggcgtcttta
ccagggacatccaacgggctcatagagtggtagctgagcttcaggctggg
acgtgcttcattaacaactataacgtcagcccagtggagttgccctttgg
tggatataagaagtcaggatttggcagagagaacggccgtgtgacaatcg
aatattattcacagctgaagactgtgtgtgtggagatgggtgatgtggaa
tctgctttttgaaaacctgcagtgaaacctattgacatggccacgctgtg
gaatgatgtgaattggccctgtttacagaggcagtacaactgaatgttat
tttacatccagaattttggcgttcagtataagagaatggttcatgttact
ctttctctctccatcagcttcctcactgaaaatgtgcattaagtgccttg
tagatactaatcaagaaagctgtgattctcctcaaagcgtatttttgtga
aatcttttaagagccagtaacatacttctagagaacaggaaaagagacta
ggataatacatcttccacacatttggcccactgataatgttaattctctg
gcgtatttcaaagaacttgttcctggctgatccaagtgcagtggtattta
caactaattgatcacaaccagtttgtagatttctttgttccttctccatt
cccactgcttcacttgcctagtcttgaagaaaaaaaacaaaaaacaaaaa
aaaaccttgttcctttataggttcctggtagaatcagtagagatgatttc
agctcattgacatttttttaagctatatccccttgtcattccattgagaa
agctgacaactgggatagggaggggattagataatagatggggtcaaatt
ctgtgtgaatgtgaacttgcctagtaagcactttgtctctgttcactact
gcgatagaggaaatctatctccctatcttgggtccttgaactacagcctg
ctgtcttacaccagtggagctaccctttaaatgtacaaattatttgtatg
ctaatgtaatatggtgaaattaaaataaatcacactgttaattgttttcc
>NM_002631 2
ggccgcagtttctggagggagccgctgcgggtctttccctcactcgtcct
ccgcgcgtcgccgctcttcggttctgctctgtccgccgccatggcccaag

ctgacatcgcgctgatcggattggccgtcatgggccagaacttaattctg
aacatgaatgaccacggctttgtggtctgtgcttttaataggactgtctc
caaagttgatgatttcttggccaatgaggcaaagggaaccaaagtggtgg
gtgcccagtccctgaaagagatggtctccaagctgaagaagccccggcgg
atcatcctcctggtgaaggctgggcaagctgtggatgatttcatcgagaa
attggtaccattgttggatactggtgacatcatcattgacggaggaaatt
ctgaatatagggacaccacaagacggtgccgagacctcaaggccaaggga
attttatttgtggggagcggagtcagtggtggagaggaaggggcccggta
tggcccatcgctcatgccaggagggaacaaagaagcgtggccccacatca
agaccatcttccaaggcattgctgcaaaagtgggaactggagaaccctgc
tgtgactgggtgggagatgagggagcaggccacttcgtgaagatggtgca
caacgggatagagtatggggacatgcagctgatctgtgaggcataccacc
tgatgaaagacgtgctgggcatggcgcaggacgagatggcccaggccttt
gaggattggaataagacagagctagactcattcctgattgaaatcacagc
caatattctcaagttccaagacaccgatggcaaacacctgctgccaaaga
tcagggacagcgcggggcagaagggcacagggaagtggaccgccatctcc
gccctggaatacggcgtacccgtcaccctcattggagaagctgtctttgc
tcggtgcttatcatctctgaaggatgagagaattcaagctagcaaaaagc
tgaagggtccccagaagttccagtttgatggtgataagaaatcattcctg
gaggacattcggaaggcactctacgcttccaagatcatctcttacgctca
aggctttatgctgctaaggcaggcagccaccgagtttggctggactctca
attatggtggcatcgccctgatgtggagagggggctgcatcattagaagt
gtattcctaggaaagataaaggatgcatttgatcgaaacccggaacttca
gaacctcctactggacgacttctttaagtcagctgttgaaaactgccagg
actcctggcggcgggcagtcagcactggggtccaggctggcattcccatg
ccctgttttaccactgccctctccttctatgacgggtacagacatgagat
gcttccagccagcctcatccaggctcagcgggattacttcggggctcaca
cctatgaactcttggccaaaccagggcagtttatccacaccaactggaca
ggccatggtggcaccgtgtcatcctcgtcatacaatgcctgatcatgctg
ctcctgtcaccctccacgattccacagaccaggacattccatgtgcctca
tggcactgccacctggccctttgccctattttctgttcagtttttttaaaa
gtgttgtaagagactcctgaggaagacacacagtttatttgtaaagtagc
tctgtgagagccaccatgccctctgcccttgcctcttgggactgaccagg
agctgctcatgtgcgtgagagtgggaaccatctccttgcggcagtggctt
ccgcgtgccccgtgtgctggtgcggttcccatcacgcagacaggaagggt
gtttgcgcactctgatcaactggaacctctgtatcatgcggctgaattcc
ctttttcctttactcaataaaagctacatcacactga
>NM_002560 2
aagtgctgggatgacaggtgtgagccaccgcccccggcccctcgcccgcc
ttttgaaggagcctttcgtcctcaagggcgaggccactcccccccgcga
gttccatgccccctagagggtcatcgttcccgacggggaggtggcgccct
cccccgggccccgggccccgaccgcccgtgctgcctccttccgggccctc
ctccgcgatgacggcgccgccagcaggccaggcggactgggcggggctcc
gagcggggactgggacccagaccgactaggggactgggagcgggcggcgc
ggccatggcgggctgctgcgccgcgctggcggccttcctgttcgagtacg
acacgccgcgcatcgtgctcatccgcagccgcaaagtggggctcatgaac
cgcgccgtgcaactgctcatcctggcctacgtcatcgggtgggtgtttgt
gtgggaaaagggctaccaggaaactgactccgtggtcagctccgttacga
ccaaggtcaagggcgtggctgtgaccaacacttctaaacttggattccgg

atctgggatgtggcggattatgtgataccagctcaggaggaaaactccct
cttcgtcatgaccaacgtgatcctcaccatgaaccagacacagggcctgt
gccccgagattccagatgcgaccactgtgtgtaaatcagatgccagctgt
actgccggctctgccggcacccacagcaacggagtctcaacaggcaggtg
cgtagctttcaacgggtctgtcaagacgtgtgaggtggcggcctggtgcc
cggtggaggatgacacacacgtgccacaacctgctttttaaaggctgca
gaaaacttcactcttttggttaagaacaacatctggtatcccaaatttaa
tttcagcaagaggaatatccttcccaacatcaccactacttacctcaagt
cgtgcatttatgatgctaaaacagatcccttctgccccatattccgtctt
ggcaaaatagtggagaacgcaggacacagtttccaggacatggccgtgga
gggaggcatcatgggcatccaggtcaactgggactgcaacctggacagag
ccgcctccctctgcttgcccaggtactccttccgccgcctcgatacacgg
gacgttgagcacaacgtatctcctggctacaatttcaggtttgccaagta
ctacagagacctggctggcaacgagcagcgcacgctcatcaaggcctatg
gcatccgcttcgacatcattgtgtttgggaaggcagggaaatttgacatc
atccccactatgatcaacatcggctctggcctggcactgctaggcatggc
gaccgtgctgtgtgacatcatagtcctctactgcatgaagaaaagactct
actatcgggagaagaaatataaatatgtggaagattacgagcagggtctt
gctagtgagctggaccagtgaggcctaccccacacctgggctctccacag
ccccatcaaagaacagagaggaggaggagggagaaatggccaccacatca
ccccagagaaatttctggaatctgattgagtctccactccacaagcactc
agggttccccagcagctcctgtgtgttgtgtgcaggatctgtttgcccac
tcggcccaggaggtcagcagtctgttcttggctgggtcaactctgctttt
cccgcaacctggggttgtcgggggagcgctggcccgacgcagtggcactg
ctgtggctttcagggctggagctggctttgctcagaagcctcctgtctcc
agctctctccaggacaggcccagtcctctgaggcacggcggctctgttca
agcactttatgcggcaggggaggccgcctggctgcagtcactagacttgt
agcaggcctgggctgcaggcttccccccgaccattccctgcagccatgcg
gcagagctggcatttctcctcagagaagcgctgtgctaaggtgatcgagg
accagacattaaagcgtgattttcttaaaaaaaaaaaaaaaaa
>NM_018962 2
ctccgacccgggcttttccttcgtaccctgcggcccccctccgcacccctc
acggagctcctcgggtcctgccccctcccagcgcttgcccgcgccgcccc
gcccccgtttttaaacctggcgcgcgggtaggtgagcgcgttagcccgag
tggatctaggcgcgctcgtaggccggcgccgcagcaagggggcgcgggctc
cgccggcaccatggagcccgaagcggcggccggagcccggaaggcgcggg
ggcgcggctgtcactgccccggggacgctccctggaggcctccgccaccg
cgcgggccggagagccccgcgccgtggcgaccttggatccagacacctgg
agatgctgagctgaccagaactggaaggccgcttgaacctagggctgacc
aacacacttttggatcaaagggagcctttgggtttcagcatcctgtaaga
gtctatttacccatgtcaaagcgtcaagaatacctgcggagttccgggga
gcaagtactggccagtttcccagtgcaagccacgattgacttctacgacg
atgagtctactgagtctgcttccgaagctgaagagccagaggaaggaccc
ccacccctccatcttctgccccaggaggtgggaggtcggcaggaaaatgg
cccagggggaaagggcagagaccagggcatcaaccaagggcagcgatcct
caggagggggtgaccactgggggggagggtccgctccctcaaggtgtctcc
tcaaggggtggcaagtgctcctcatccaaatgaatcagtctctgtctcct
gggccctgctctgggacctgcccctcacgttctcttggggacacccgagc
caggacactacgcatccctgctgagtgtgcagaggctagaggcttctcgg

gcagcccctggcctgcacactactcatgacagaaagtcagcttttacttt
tctttcccctggcaattcgtttttggtgcactcatgcatgcctttgagaa
aggattctaggagaaagagaaggtctatgtcaacagagttgttatctcat
agagccagttttcaaagctccttctgcattgtcactcactgatcaggtga
tgaattcttcctagatagtcgcccactccacctcctacttaacctgagac
tcattatttagctatttctgcttttgtaaaaataattcagatattaaact
ccaattttaatctatcatccaagggtagatgtagttgcttagtagcattt
tggaaaaaaaagaaaaaaaggtttggtttggttttgtgacggagttttgc
tcttgtctcccaggctggagtgcagtgacacaatctcggctcactgcaac
ctccacttcttgggttcaagtgattctcctgtctcagcctcccaagtagc
tgggattacaggcatgagctaccacgcctggctaatttttttgtattttta
gtagagactcggtttcaccatgttggtcaggctggctttgaactcctgac
ctcaggtgatccacccgccttggcctcccaaagtgctgggattacaggcg
tgagccactgagcccggccaaaaaaaaaatgtatttattaaaaaaaaaattt
ttttaacccgccgaataattcccataagagtaacaaaaaggaccagcctg
accaacgtggagaaactctgtctctactaaaaatacaaaattagccaggc
gtgatggctcatgcctgtaatcccagctactcgggaggctgaggcaggag
aatggctagaacccgggaggcggaggttgccgtgagtcgagatcgcacca
ttgcattccagcctgggcaacaagagcgaaactccgtctcaaaaaaaaaa
aaaaaaaaaaagaaagaaaagagtaacagaaagatagggatttttaggag
acaattagataaaaatgtatgtgatgactgtataaggaggcctgtgtgta
tgaatttatagggagtagaaccgtctctcttcttagttggtgactgtttg
gggcctggtattttaatagatgaacactactttttttaatttttttattttt
ttaaccctacccttaacccatgaacactatttttaattaaagtatctgat
gtaaaattattttgagtttttaatattttgataaacgtgtattcctgaaa
ctttttgacttacttatcttacatgtggtgtcttcctgtatatagtacat
tatatcaatttctacttgaaataaatattttgaaaaaaaaaaaaaaaaaa
aaaaaaaaaa
