# Supplementary material for: A computational method for predicting regulation of human microRNAs on the influenza virus genome
Source: BMC Syst Biol. 2013 Oct 14;7(Suppl 2):S3. doi: 10.1186/1752-0509-7-S2-S3 (PMC3851852; doi:10.1186/1752-0509-7-S2-S3)
Supplement: Additional File 5 — The coding sequence of the gene fragment of NA from 2000 to 2012 recorded in Genbank. [file 1752-0509-7-S2-S3-S5.PDF]

MP

>gi|145278773|gb|CY021694.1| Influenza A virus (A/Memphis/15/2000(H1N1)) segment 7, complete sequence
AAGATGAGTCTTCTAACCGAGGTCGAAACGTACGTTCTCTCTATCATCCCGTCAGGCCCCCTCAAAGCCG
AGATCGCACAGAGACTTGAAGATGTCTTTGCTGGAAAGAATACCGATCTTGAGGCTCTCATGGAATGGCT
AAAGACAAGACCAATCCTGTCACCTCTGACTAAGGGGATTTTAGGATTTGTGTTCACGCTCACCGTGCCC
AGTGAGCGAGGACTGCAGCGTAGACGCTTTGTCCAAAATGCCCTTAATGGGAATGGGGATCCAAATAATA
TGGACAGAGCAGTTAAACTGTATCGAAAGCTTAAGAGGGAGATAACATTCCATGGGGCCAAAGAAATAGC
ACTCAGTTATTCTGCTGGTGCACTTGCCAGTTGTATGGGACTCATATACAACAGGATGGGGGCTGTGACC
ACCGAATCAGCATTTGGCCTTATATGCGCAACCTGTGAACAGATTGCCGACTCCCAGCATAAGTCTCATA
GGCAAATGGTAACAACTACCAATCCATTAATAAGACATGAGAACAGAATGGTTCTGGCCAGCACTACAGC
TAAGGCTATGGAGCAAATGGCTGGATCGAGTGAACAAGCAGCTGAGGCCATGGAGGTTGCTAGTCAGGCC
AGGCAGATGGTGCAGGCAATGAGAGCCATTGGGACTCATCCTAGCTCTAGCACTGGTCTGAAAAATGATC
TCCTTGAAAATTTGCAGGCCTATCAGAAACGAATGGGGGTGCAGATGCAACGATTCAAGTGATCCTCTTG
TTGTTGCCGCAAGTATAATTGGGATTGTGCACCTGATATTGTGGATTATTGATCGCCTTTTTTCCAAAAG
CATTTATCGTATCTTTAAACACGGTTTAAAAAGAGGGCCTTCTACGGAAGGAGTACCAGAGTCTATGAGG
GAAGAATATCGAGAGGAACAGCAGAATGCTGTGGATGCTGACGATGGTCATTTTGTCAGCATAGAGCTAG
AGTAAAAAA
>gi|70907641|gb|CY000450.2| Influenza A virus (A/New York/146/2000(H1N1)) segment 7, complete sequence
AGCAAAAGCAGGTAGATATTGAAAGATGAGTCTTCTAACCGAGGTCGAAACGTACGTTCTCTCTATCATC
CCGTCAGGCCCCCTCAAAGCCGAGATCGCACAGAGACTTGAAGATGTCTTTGCTGGAAAGAACACCGATC
TTGAGGCTCTCATGGAGTGGCTAAAGACAAGACCGATCCTGTCACCTCTGACTAAGGGGATTTTAGGATT
TGTGTTCACGCTCACCGTGCCCAGTGAGCGAGGACTGCAACGTAGACGCTTTGTTCAAAATGCCCTTAAC
GGGAATGGGGATCCAAATAACATGGACAGAGCGGTTAAACTGTATAGAAAGCTTAAGAGGGAGATAACAT
TTCATGGGGCCAAAGAAATAGCACTCAGTTATTCTGCTGGTGCACTTGCCAGTTGTATGGGCCTCATATA
CAACAGGATGGGGGCTGTGACCACCGAATCAGCATTTGGCCTGATATGCGCAACCTGTGAACAGATTGCT
GACTCCCAGCATAAGTCCCATAGGCAAATGGTAACAACAACTAATCCATTAATAAGACATGAGAACAGAA
TGGTTCTGGCCAGCACTACAGCTAAGGCTATGGAGCAAATGGCTGGATCGAGTGAACAAGCAGCTGAGGC
CATGGAGGTTGCTAGTCAGGCCAGGCAAATGGTGCAGGCAATGAGAGCCATTGGGACTCACCCCAGCTCT
AGCACTGGTCTGAAAAATGATCTTCTTGAAAATTTGCAGGCCTATCAGAAACGAATGGGAGTGCAGATGC
AACGGTTCAAGTGATCCTCTTGTTGTTGCCGCAAGTATCATTGGGATCATGCACCTGATATTGTGGATTA
TTGATCGCCTTTTTTTCAAATGCATTTATCGTATCTTTAAACACGGTCTGAAAAGAGGGCCTTCTACGGA
AGGAGTACCAGAGTCTATGAGGGAAGAATATCGAGAGGAGCAGCAGAATGCTGTGGATGCTGACGAAGGT
CATTTTGTCAGCATAGAGCTAGAGTAAAAAACTACCTTGTTTCTACT
>gi|145278906|gb|CY021750.1| Influenza A virus (A/South Australia/44/2000(H1N1)) segment 7, complete sequence
AGATGAGTCTTCTAACCGAGGTCGAAACGTACGTTCTCTCTATCATCCCGTCAGGCCCCCTCAAAGCCGA
GATCGCACAGAGACTTGAAGATGTCTTTGCTGGAAAGAACACCGATCTTGAGGCTCTCATGGAGTGGCTA
AAGACAAGACCGATCCTGTCACCTCTGACTAAGGGGATTTTAGGATTTGTGTTCACGCTCACCGTGCCCA
GTGAGCGAGGACTGCAACGTAGACGCTTTGTTCAAAATGCCCTTAACGGGAATGGGGATCCAAATAACAT
GGACAGAGCGGTTAAACTGTATAGAAAGCTTAAGAGGGAGATAACATTTCATGGGGCCAAAGAAATAGCA
CTCAGTTATTCTGCTGGTGCACTTGCCAGTTGTATGGGCCTCATATACAACAGGATGGGGGCTGTGACCA

CCGAATCAGCATTTGGCCTGATATGCGCAACCTGTGAACAGATTGCTGACTCCCAGCATAAGTCCCATAG
GCAAATGGTAACAACAACTAATCCATTAATAAGACATGAGAACAGAATGGTTCTGGCCAGCACTACAGCT
AAGGCTATGGAGCAAATGGCTGGATCGAGTGAACAAGCAGCTGAGGCCATGGAGGTTGCTAGTCAGGCCA
GGCAAATGGTGCAGGCAATGAGAGCCATTGGGACTCACCCCAGCTCTAGCACTGGTCTGAAAAATGATCT
TCTTGAAAATTTGCAGGCCTATCAGAAACGAATGGGAGTGCAGATGCAACGGTTCAAGTGATCCTCTTGT
TGTTGCCGCAAGTATCATTGGGATCATGCACCTGATATTGTGGATTATTGATCGCCTTTTTTTCAAATGC
ATTTATCGTATCTTTAAACACGGTCTGAAAAGAGGGCCTTCTACGGAAGGAGTACCAGAGTCTATGAGGG
AAGAATATCGAGAGGAGCAGCAGAATGCTGTGGATGCTGACGAAGGTCATTTTGTCAGCATAGAGCTAGA
GTAAAAA

>gi|157367767|gb|CY026156.1| Influenza A virus (A/Auckland/585/2000(H1N1)) segment 7, complete sequence

ATGAGTCTTCTAACCGAGGTCGAAACGTACGTTCTCTCTATCGTCCCGTCAGGCCCCCTCAAAGCCGAGA
TCGCACAGAGACTTGAAGATGTCTTTGCTGGAAAGAATACCGATCTTGAGGCTCTCATGGAATGGCTAAA
GACAAGACCAATCCTGTCACCTCTGACTAAGGGGATTTTAGGATTTGTGTTCACGCTCACCGTGCCCAGT
GAGCGAGGACTGCAGCGTAGACGCTTTGTCCAAAATGCCCTTAATGGGAATGGGGATCCAAATAATATGG
ACAGAGCAGTTAAACTGTATCGAAAGCTTAAGAGGGAGATAACATTCCATGGGGCCAAAGAAATAGCACT
CAGTTATTCTGCTGGTGCACTTGCCAGTTGTATGGGACTCATATACAACAGGATGGGGGCTGTGACCACC
GAATCAGCATTTGGCCTTATATGCGCAACCTGTGAACAGATTGCCGACTCCCAGCATAAGTCTCATAGGC
AAATGGTAACAACAACCAATCCATTAATAAGACATGAGAACAGAATGGTTCTGGCCAGCACTACAGCTAA
GGCTATGGAGCAAATGGCTGGATCGAGTGAACAAGCAGCTGAGGCCATGGAGGTTGCCAGTCAGGCCAG
G
CAGATGGTGCAGGCAATGAGAGCCATTGGGACTCATCCTAGCTCTAGCACTGGTCTGAAAAATGATCTCC
TTGAAAATTTGCAGGCCTATCAGAAACGAATGGGGGTGCAGATGCAACGATTCAAGTGATCCTCTTGTTG
TTGCCGCAAGTATAATTGGGATTGTGCACCTGATATTGTGGATTATTGATCGCCTTTTTTCCAAAAGCAT
TTATCGTATCTTTCAACACGGTTTAAAAAGAGGGCCTTCTACGGAAGGAGTACCAGAGTCTATGAGGGAA
GAATATCGAGAGGAACAGCAGAATGCTGTGGATGCTGACGATGGTCATTTTGTCAGCATAGAGCTAGAGT
AA

>gi|145278619|gb|CY021630.1| Influenza A virus (A/Wellington/4/2000(H1N1)) segment 7, complete sequence

TTGAAAGATGAGTCTTCTAACCGAGGTCGAAACGTACGTTCTCTCTATCGTCCCGTCAGGCCCCCTCAAA
GCCGAGATCGCACAGAGACTTGAAGATGTCTTTGCTGGAAAGAATACCGATCTTGAGGCTCTCATGGAAT
GGCTAAAGACAAGACCAATCCTGTCACCTCTGACTAAGGGGATTTTAGGATTTGTGTTCACGCTCACCGT
GCCCAGTGAGCGAGGACTGCAGCGTAGACGCTTTGTCCAAAATGCCCTTAATGGGAATGGGGATCCAAAT
AATATGGACAGAGCAGTTAAACTGTATCGAAAGCTTAAGAGGGAGATAACATTCCATGGGGCCAAAGAAA
TAGCACTCAGTTATTCTGCTGGTGCACTTGCCAGTTGTATGGGACTCATATACAACAGGATGGGGGCTGT
GACCACCGAATCAGCATTTGGCCTTATATGCGCAACCTGTGAACAGATTGCCGACTCCCAGCATAAGTCT
CATAGGCAAATGGTAACAACAACCAATCCATTAATAAGACATGAGAACAGAATGGTTCTGGCCAGCACTA
CAGCTAAGGCTATGGAGCAAATGGCTGGATCGAGTGAACAAGCAGCTGAGGCCATGGAGGTTGCTAGTCA
GGCCAGGCAGATGGTGCAGGCAATGAGAGCCATTGGGACTCATCCTAGCTCTAGCACTGGTCTGAAAAAT
GATCTCCTTGAAAATTTGCAGGCCTATCAGAAACGAATGGGGGTGCAGATGCAACGATTCAAGTGATCCT
CTTGTTGTTGCCGCAAGTATAATTGGGATTGTGCACCTGATATTGTGGATCATTGATCGCCTTTCCTCCA
AAAGCATTTATCGTATCCTTAAACACGGTTTAAAAAGAGGGCCTTCTACGGAAGGAGTACCAGAGTCTAT
GAGGGAAGAATATCGAGAGGAACAGCAGAATGCTGTGGATGCTGACGATGGTCATTTTGTCAGCATAGAG
CTAGAGTAAAAAA

>gi|131058482|gb|CY020150.1| Influenza A virus (A/Memphis/7/2001(H1N1)) segment 7, complete sequence
ATGAGTCTTCTAACCGAGGTCGAAACGTACGTTCTCTCTATCATCCCGTCAGGCCCCCTCAAAGCCGAGA
TCGCACAGAGACTTGAAGATGTCTTTGCTGGAAAGAATACCGATCTTGAGGCTCTCATGGAATGGCTAAA
GACAAGACCGATCCTGTCACCTCTGACTAAGGGGATTTTAGGATTTGTGTTCACGCTCACCGTGCCCAGT
GAGCGAGGACTGCAGCGTAGACGCTTTGTCCAAAATGCCCTTAATGGGAATGGGGATCCAAATAATATGG
ACAGAGCAGTTAAACTGTATCGAAAGCTTAAGAGGGAGATAACATTCCATGGGGCCAAAGAAATAGCACT
CAGTTATTCTGCTGGTGCACTTGCCAGTTGTATGGGACTCATATACAACAGGATGGGGGCTGTGACCACC
GAATCAGCATTTGGCCTTATATGCGCAACCTGTGAACAGATTGCCGACTCCCAGCATAAGTCTCATAGGC
AAATGGTAACAACAACCAATCCATTAATAAGACATGAGAACAGAATGGTTCTGGCCAGCACTACAGCTAA
GGCTATGGAGCAAATGGCTGGATCGAGTGAACAAGCAGCTGAGGCCATGGAGGTTGCTAGTCAGGCCAGG
CAGATGGTGCAGGCAATGAGAGCCATTGGGACTCATCCTAGCTCTAGCACTGGTCTGAAAAATGATCTCC
TTGAAAATTTGCAGGCCTATCAGAAACGAATGGGGGTGCAGATGCAACGATTCAAGTGATCCTCTTGTTG
TTGCCGCAAGTATAATTGGGATTGTGCACCTGATATTGTGGACTATTGATCGCCTTTTTTCCAAAAGCAT
TTATCGTATCTTTAAACACGGTTTAAAAAGAGGGCCTTCTACGGAAGGAGTACCAGAGTCTATGAGGGAA
GAATATCGAGAGGAACAGCAGAATGCTGTGGATGCTGACGATGGTCATTTTGTCAGCATAGAGCTGGAGT
AA
>gi|73761475|gb|CY002529.1| Influenza A virus (A/New York/220/2002(H1N1)) segment 7, complete sequence
AAAGCAGGTAGATATTGAAAGATGAGTCTTCTAACCGAGGTCGAAACGTACGTTCTCTCTATCGTCCCGT
CAGGCCCCCTCAAAGCCGAGATCGCACAGAGACTTGAAGATGTATTTGCTGGAAAGAATACCGATCTTGA
GGCTCTCATGGAGTGGCTAAAGACAAGACCAATCCTGTCACCTCTGACTAAGGGGATTTTAGGATTTGTG
TTCACGCTCACCGTGCCCAGTGAGCGAGGACTGCAGCGTAGACGCTTTGTCCAAAATGCCCTTAATGGGA
ATGGGGATCCAAATAATATGGACAGAGCAGTCAAACTGTATCGAAAGCTTAAGAGGGAGATAACATTCCA
TGGGGCCAAAGAAATAGCACTCAGTTATTCTGCTGGTGCACTTGCCAGTTGTATGGGACTCATATACAAC
AGGATGGGGGCTGTGACCACCGAATCAGCATTTGGCCTTATATGCGCAACCTGTGAACAGATTGCCGACT
CCCAGCATAAGTCTCATAGGCAAATGGTAACAACAACCAATCCATTAATAAGACATGAGAACAGAATGGT
TCTGGCCAGCACTACAGCTAAGGCTATGGAGCAAATGGCTGGATCGAGTGAACAAGCAGCTGAGGCCATG
GAGGTTGCTAGTCAGGCCAGGCAGATGGTGCAGGCAATGAGAGCCATTGGGACTCATCCTAGCTCTAGCA
CTGGTCTGAAAAATGATCTCCTTGAAAATTTGCAGGCCTATCAGAAACGAATGGGGGTGCAGATGCAACG
ATTCAAGTGATCCTCTTGTTGTTGCCGCAAGTATAATTGGGATTGTGCACCTGATATTGTGGATTATTGA
TCGGCTTTTTTCCAAAAGCATTTATCGTATCTTTAAACACGGTTTAAAAAGAGGGCCTTCTACGGAAGGA
GTACCAGAGTCTATGAGGGAAGAATATCGAGAGGAACAGCAGAATGCTGTGGATGCTGACGATGGTCATT
TTGTCAGCATAGAGCTAGAGTAAAAAACTACCTTGTTTCTACT
>gi|156536319|gb|CY025035.1| Influenza A virus (A/Auckland/597/2000(H1N1)) segment 7, complete sequence
ATGAGTCTTCTAACCGAGGTCGAAACGTACGTTCTCTCTATCGTCCCGTCAGGCCCCCTCAAAGCCGAGA
TCGCACAGAGACTTGAAGATGTCTTTGCTGGAAAGAATACCGATCTTGAGGCTCTCATGGAATGGCTAAA
GACAAGACCAATCCTGTCACCTCTGACTAAGGGGATTTTAGGATTTGTGTTCACGCTCACCGTGCCCAGT
GAGCGAGGACTGCAGCGTAGACGCTTTGTCCAAAATGCCCTTAATGGGAATGGGGATCCAAATAATATGG
ACAGAGCAGTTAAACTGTATCGAAAGCTTAAGAGGGAGATAACATTCCATGGGGCCAAAGAAATAGCACT
CAGTTATTCTGCTGGTGCACTTGCCAGTTGTATGGGACTCATATACAACAGGATGGGGGCTGTGACCACC
GAATCAGCATTTGGCCTTATATGCGCAACCTGTGAACAGATTGCCGACTCCCAGCATAAGTCTCATAGGC
AAATGGTAACAACAACCAATCCATTAATAAGACATGAGAACAGAATGGTTCTGGCCAGCACTACAGCTAA

GGCTATGGAGCAAATGGCTGGATCGAGTGAACAAGCAGCTGAGGCCATGGAGGTTGCTAGTCAGGCCAGG
CAGATGGTGCAGGCAATGAGAGCCATTGGGACTCATCCTAGCTCTAGCACTGGTCTGAAAAATGATCTCC
TTGAAAATTTGCAGGCCTATCAGAAACGAATGGGGGTGCAGATGCAACGATTCAAGTGATCCTCTTGTTG
TTGCCGCAAGTATAATTGGGATTGTGCACCTGATATTGTGGATTATTGATCGCCTTTTTTCCAAAAGCAT
TTATCGTATCTTTAAACACGGTTTAAAAAGAGGGCCTTCTACGGAAGGAGTACCAGAGTCTATGAGGGAA
GAATATCGAGAGGAACAGCAGAATGCTGTGGATGCTGACGATGGTCATTTTGTCAGCATAGAGCTAGAGT
AAAA
>gi|149780528|gb|CY022534.1| Influenza A virus (A/Auckland/605/2001(H1N1)) segment 7, complete sequence
AAAGATGAGTCTTCTAACCGAGGTCGAAACGTACGTTCTCTCTATCATCCCTTCAGGCCCCCTCAAAGCC
GAGATCGCACAGAGACTTGAAGATGTCTTTGCTGGAAAGAATACCGATCTTGAGGCTCTCATGGAATGGC
TAAAGACAAGACCGATCCTGTCACCTCTGACTAAGGGGATTTTAGGATTTGTGTTCACGCTCACCGTGCC
CAGTGAGCGAGGACTGCAGCGTAGACGCTTTGTCCAAAATGCCCTTAATGGGAATGGGGATCCAAATAAT
ATGGACAGAGCAGTTAAACTGTATCGAAAGCTTAAGAGGGAGATAACATTCCATGGGGCCAAAGAAATAG
CACTCAGTTATTCTGCTGGTGCACTTGCCAGTTGTATGGGACTCATATACAACAGGATGGGGGCTGTGAC
CACCGAATCAGCATTTGGCCTTATATGTGCAACCTGTGAACAGATTGCCGACTCCCAGCATAAGTCTCAT
AGGCAAATGGTAACAACAACCAATCCATTAATAAGACATGAGAACAGAATGGTTCTGGCCAGCACTACAG
CTAAGGCTATGGAGCAAATGGCTGGATCGAGTGAACAAGCAGCTGAGGCCATGGAGGTTGCTAGTCAGGC
CAGGCAGATGGTGCAGGCAATGAGAGCCATTGGGACTCATCCTAGCTCTAGCACTGGTCTGAAAAATGAT
CTCCTTGAAAATTTGCAGGCCTATCAGAAACGAATGGGGGTGCAGATGCAACGATTCAAGTGATCCTCTT
GTTGTTGCCGCAAGTATAGTTGGGATTGTGCACCTGATATTGTGGATTATTGATCGCCTTTTTTCCAAAA
GCATTTATCGTATCTTTAAACACGGTTTAAAAAGAGGGCCTTCTACGGAAGGAGTACCAGAGTCTATGAG
GGAAGAATATCGAGAGGAACAGCAGAATGCTGTGGATGCTGACGATGGTCATTTTGTCAGCATAGAGCTG
GAGTAAAA
>gi|237688864|gb|CY040075.1| Influenza A virus (A/Taiwan/567/2002(H1N1)) segment 7, complete sequence
ATGAGTCTTCTAACCGAGGTCGAAACGTACGTTCTCTCTATCATCCCTTCAGGCCCCCTCAAAGCCGAGA
TCGCACAGAGACTTGAAGATGTCTTTGCTGGAAAGAATACCGATCTTGAGGCTCTCATGGAATGGCTAAA
GACAAGACCGATCCTGTCACCTCTGACTAAGGGGATTTTAGGATTTGTGTTCACGCTCACCGTGCCCAGT
GAGCGAGGACTGCAGCGTAGACGCTTTGTCCAAAATGCCCTTAATGGGAATGGGGATCCAAATAATATGG
ACAGAGCAGTTAAACTGTATCGAAAGCTTAAGAGGGAGATAACATTCCATGGGGCCAAAGAAATAGCACT
CAGTTATTCTGCTGGTGCACTTGCCAGTTGTATGGGACTCATATACAACAGGATGGGGGCTGTGACCACC
GAATCAGCATTTGGCCTTATATGTGCAACCTGTGAACAGATTGCCGACTCCCAGCATAAGTCTCATAGGC
AAATGGTAACAACAACCAATCCATTAATAAGACATGAGAACAGAATGGTTCTGGCCAGCACTACAGCTAA
GGCTATGGAGCAAATGGCTGGATCAAGTGAACAAGCAGCTGAGGCCATGGAGGTTGCTAGTCAGGCCAGG
CAGATGGTGCAGGCAATGAGAGCCATTGGGACTCATCCTAGCTCTAGCACTGGTCTGAAAAATGATCTCC
TTGAAAATTTGCAGGCCTATCAGAAACGAATGGGGGTGCAGATGCAACGATTCAAGTGATCCTCTTGTTG
TTGCCGCAAGTATAGTTGGGATTGTGCACCTGATATTGTGGATTATTGATCGCCTTTTTTCCAAAAGCAT
TTATCGTATCTTTAAACACGGTTTAAAAAGAGGGCCTTCTACGGAAGGAGTACCAGAGTCTATGAGGGAA
GAATATCGAGAGGAACAGCAGAATGCTGTGGATGCTGACGATGGTCATTTTGTCAGCATAGAGCTGGAGT
AAAAA
>gi|122855947|gb|CY019342.1| Influenza A virus (A/Memphis/6/2003(H1N1)) segment 7, complete sequence
TTGAAAGATGAGTCTTCTAACCGAGGTCGAAACGTACGTTCTCTCTATCGTCCCGTCAGGCCCCCTCAAA

GCCGAGATCGCACAGAGACTTGAAGATGTATTTGCTGGAAAGAATACCGATCTTGAGGCTCTCATGGAGT
GGCTAAAGACAAGACCAATCCTGTCACCTCTGACTAAGGGGATTTTAGGATTTGTGTTCACGCTCACCGT
GCCCAGTGAGCGAGGACTGCAGCGTAGACGCTTTGTCCAAAATGCCCTTAATGGGAATGGGGATCCAAAT
AATATGGACAGAGCAGTCAAACTGTATCGAAAGCTTAAGAGGGAGATAACATTCCATGGGGCCAAAGAAA
TAGCGCTCAGTTATTCTGCTGGTGCACTTGCCAGTTGTATGGGACTCATATACAACAGGATGGGGGCTGT
GACCACCGAATCAGCATTTGGCCTTATATGCGCAACCTGTGAACAGATTGCCGACTCCCAGCATAAGTCT
CATAGGCAAATGGTAACGACAACCAATCCATTAATAAGACATGAGAACAGAATGGTTCTGGCCAGCACTA
CAGCTAAGGCTATGGAGCAAATGGCTGGATCGAGTGAACAAGCAGCTGAGGCCATGGAGGTTGCCAGTCA
GGCCAGGCAGATGGTGCAGGCAATGAGAGCCATTGGGACTCATCCTAGCTCTAGCACTGGTCTGAAAAAT
GATCTCCTTGAAAATTTGCAGGCCTATCAGAAACGAATGGGGGTGCAGATGCAACGATTCAAGTGATCCT
CTTGTTGTTGCCGCAAGTATAATTGGGATTGTGCACCTGATATTGTGGATTATTGATCGCCTTTTTTCCA
AAAGCATTTATCGTATCTTTAAACACGGTTTAAAAAGAGGGCCTTCTACGGAAGGAGTACCAGAGTCTAT
GAGGGAAGAATATCGAGAGGAACAGCAGAATGCTGTGGATGCTGACGATGGTCATTTTGTCAGCATAGAG
CTAGAGTAA
>gi|82546776|gb|CY006676.1| Influenza A virus (A/New York/494/2002(H1N1)) segment 7, complete sequence
AAAGCAGGTAGATATTGAAAGATGAGTCTTCTAACCGAGGTCGAAACGTACGTTCTCTCTATCGTCCCGT
CAGGCCCCCTCAAAGCCGAGATCGCACAGAGACTTGAAGATGTATTTGCTGGAAAGAATACCGATCTTGA
GGCTCTCATGGAGTGGCTAAAGACAAGACCAATCCTGTCACCTCTGACTAAGGGGATTTTAGGATTTGTG
TTCACGCTCACCGTGCCCAGTGAGCGAGGACTGCAGCGTAGACGCTTTGTCCAAAATGCCCTTAATGGGA
ATGGGGATCCAAATAATATGGACAGAGCAGTCAAACTGTATCGAAAGCTTAAGAGGGAGATAACATTCCA
TGGGGCCAAAGAAATAGCACTCAGTTATTCTGCTGGTGCACTTGCCAGTTGTATGGGACTCATATACAAC
AGGATGGGGGCTGTGACCACCGAATCAGCATTTGGCCTTATATGCGCAACCTGTGAACAGATTGCCGACT
CCCAGCATAAGTCTCATAGGCAAATGGTAACAACAACCAATCCATTAATAAGACATGAGAACAGAATGGT
TCTGGCCAGCACTACAGCTAAGGCTATGGAGCAAATGGCTGGATCGAGTGAACAAGCAGCTGAGGCCATG
GAGGTTGCTAGTCAGGCCAGGCAGATGGTGCAGGCAATGAGAGCCATTGGGACTCATCCTAGCTCTAGCA
CTGGTCTGAAAAATGATCTCCTTGAAAATTTGCAGGCCTATCAGAAACGAATGGGGGTGCAGATGCAACG
ATTCAAGTGATCCTCTTGTTGTTGCCGCAAGTATAATTGGGATTGTGCACCTGATATTGTGGATTATTGA
TCGGCTTTTTTCCAAAAGCATTTATCGTATCTTTAAACACGGTTTAAAAAGAGGGCCTTCTACGGAAGGA
GTACCAGAGTCTATGAGGGAAGAATATCGAGAGGAACAGCAGAATGCTGTGGATGCTGACGATGGTCATT
TTGTCAGCATAGAGCTAGAGTAAAAAACTACCTTGTTTCTACT
>gi|77543343|gb|CY003305.1| Influenza A virus (A/New York/291/2002(H1N1)) segment 7, complete sequence
AAAGCAGGTAGATATTGAAAGATGAGTCTTCTAACCGAGGTCGAAACGTACGTTCTCTCTATCGTCCCGT
CAGGCCCCCTCAAAGCCGAGATCGCACAGAGACTTGAAGATGTATTTGCTGGAAAGAATACCGATCTTGA
GGCTCTCATGGAGTGGCTAAAGACAAGACCAATCCTGTCACCTCTAACTAAGGGGATTTTAGGATTTGTG
TTCACGCTCACCGTGCCCAGTGAGCGAGGACTGCAGCGTAGACGCTTTGTCCAAAATGCCCTTAATGGGA
ATGGGGATCCAAATAATATGGACAGAGCAGTCAAACTGTATCGAAAGCTTAAGAGGGAGATAACATTCCA
TGGGGCCAAAGAAATAGCACTCAGTTATTCTGCCGGTGCACTTGCCAGTTGTATGGGACTCATATACAAC
AGGATGGGGGCTGTGACCACCGAATCAGCATTTGGCCTTATATGCGCAACCTGTGAACAGATTGCCGACT
CCCAGCATAAGTCTCATAGGCAAATGGTAACAACAACCAATCCATTAATAAGACATGAGAACAGAATGGT
TCTGGCCAGCACTACAGCTAAGGCTATGGAGCAAATGGCTGGATCGAGTGAACAAGCAGCTGAGGCCATG
GAGGTTGCTAGTCAGGCCAGGCAGATGGTGCAGGCAATGAGAGCCATTGGGACTCATCCTAGCTCTAGCA
CTGGTCTGAAAAATGATCTCCTTGAAAATTTGCAGGCCTATCAGAAACGAATGGGGGTGCAGATGCAACG

ATTCAAGTGACCCTCTTGTTGTTGCCGCAAGTATAATTGGGATTGTGCACCTGATATTGTGGATTATTGA
TCGCCTTTTTTCCAAAAGCATTTATCGTATCTTTAAACACGGTTTAAAAAGAGGGCCTTCTACGGAAGGA
GTACCAGAGTCTATGAGGGAAGAATATCGAGAGGAACAGCAGAATGCTGTGGATGCTGACGATGGTCATT
TTGTCAGCATAGAGCTAGAGTAAAAAA
>gi|237689037|gb|CY040147.1| Influenza A virus (A/Taiwan/52/2002(H1N1)) segment 7, complete sequence
ATGAGTCTTCTAACCGAGGTCGAAACGTACGTTCTCTCTATCATCCCTTCAGGCCCCCTCAAAGCCGAGA
TCGCACAGAGACTTGAAGATGTCTTTGCTGGAAAGAATACCGATCTTGAGGCTCTCATGGAATGGCTAAA
GACAAGACCGATCCTGTCACCTCTGACTAAGGGGATTTTAGGATTTGTGTTCACGCTCACCGTGCCCAGT
GAGCGAGGACTGCAGCGTAGACGCTTTGTCCAAAATGCCCTTAATGGGAATGGGGATCCAAATAATATGG
ACAGAGCAGTTAAACTGTATCGAAAGCTTAAGAGGGAGATAACATTCCATGGGGCCAAAGAAATAGCACT
CAGTTATTCTGCTGGTGCACTTGCCAGTTGTATGGGACTCATATACAACAGGATGGGGGCTGTGACCACC
GAATCAGCATTTGGCCTTATATGTGCAACCTGTGAACAGATTGCCGACTCCCAGCATAAGTCTCATAGGC
AAATGGTAACAACAACCAATCCATTAATAAGACATGAGAACAGAATGGTTCTGGCCAGCACTACAGCTAA
GGCTATGGAGCAAATGGCTGGATCAAGTGAACAAGCAGCTGAGGCCATGGAGGTTGCTAGTCAGGCCAGG
CAGATGGTGCAGGCAATGAGAGCCATTGGGACTCATCCTAGCTCTAGCACTGGTCTGAAAAATGATCTCC
TTGAAAATTTGCAGGCCTATCAGAAACGAATGGGGGTGCAGATGCAACGATTCAAGTGATCCTCTTGTTG
TTGCCGCAAGTATAGTTGGGATTGTGCACCTGATATTGTGGATTATTGATCGCCTTTTTTCCAAAAGCAT
TTATCGTATCTTTAAACACGGTTTAAAAAGAGGGCCTTCTACGGAAGGAGTACCAGAGTCTATGAGGGAA
GAATATCGAGAGGAACAGCAGAATGCTGTGGATGCTGACGATGGTCATTTTGTCAGCATAGAGCTGGAGT
AAAA
>gi|237689056|gb|CY040155.1| Influenza A virus (A/Taiwan/123/2002(H1N1)) segment 7, complete sequence
AGATGAGTCTTCTAACCGAGGTCGAAACGTACGTTCTCTCTATCATCCCTTCAGGCCCCCTCAAAGCCGA
GATCGCACAGAGACTTGAAGATGTCTTTGCTGGAAAGAATACCGATCTTGAGGCTCTCATGGAATGGCTA
AAGACAAGACCGATCCTGTCACCTCTGACTAAGGGGATTTTAGGATTTGTGTTCACGCTCACCGTGCCCA
GTGAGCGAGGACTGCAGCGTAGACGCTTTGTCCAAAATGCCCTTAATGGGAATGGGGATCCAAATAATAT
GGACAGAGCAGTTAAACTGTATCGAAAGCTTAAGAGGGAGATAACATTCCATGGGGCCAAAGAAATAGCA
CTCAGTTATTCTGCTGGTGCACTTGCCAGTTGTATGGGACTCATATACAACAGGATGGGGGCTGTGACCA
CCGAATCAGCATTTGGCCTTATATGTGCAACCTGTGAACAGATTGCCGACTCCCAGCACAAGTCTCATAG
GCAAATGGTAACAACAACCAATCCATTAATAAGACATGAGAACAGAATGGTTCTGGCCAGCACTACAGCT
AAGGCTATGGAGCAAATGGCTGGATCGAGTGAACAAGCAGCTGAGGCCATGGAGGTTGCTAATCAGGCCA
GGCAGATGGTGCAAGCAATGAGAGCCATTGGGACTCATCCTAGCTCTAGCACTGGTCTGAAAGATGATCT
CCTTGAAAATTTGCAGGCCTATCAGAAACGAATGGGGGTGCAGATGCAACGATTCAAGTGATCCTCTTGT
TGTTGCCGCAAGTATAGTTGGGATTGTGCACCTGATATTGTGGATTATTGATCGCCTTTTTTCCAAAAGC
ATTTATCGTATCTTTAAACACGGTTTAAAAAGAGGGCCTTCTACGGAAGGAGTACCAGAGTCTATGAGGG
AAGAATATCGAGAGGAACAGCAGAATGCTGTGGATGCTGACGATGGTCATTTTGTCAGCATAGAGCTGGA
GTAAAA
>gi|77747423|gb|CY003689.1| Influenza A virus (A/New York/486/2003(H1N1)) segment 7, complete sequence
AAAGCAGGTAGATATTGAAAGATGAGTCTTCTAACCGAGGTCGAAACGTACGTTCTCTCTATCGTCCCGT
CAGGCCCCCTCAAAGCCGAGATCGCACAGAGACTTGAAGATGTATTTGCTGGAAAGAATACCGATCTTGA
GGCTCTCATGGAGTGGCTAAAGACAAGACCAATCCTGTCACCTCTGACTAAGGGGATTTTAGGATTTGTG
TTCACGCTCACCGTGCCCAGTGAGCGAGGACTGCAGCGTAGACGCTTTGTCCAAAATGCCCTTAATGGGA

ATGGGGATCCAAATAATATGGACAGAGCAGTCAAACTGTATCGAAAGCTTAAGAGGGAGATAACATTCCA
TGGGGCCAAAGAAATAGCACTCAGTTATTCTGCTGGTGCACTTGCCAGTTGTATGGGACTCATATACAAC
AGGATGGGGGCTGTGACCACCGAATCAGCATTTGGCCTTATATGCGCAACCTGTGAACAGATTGCCGACT
CCCAGCATAAGTCTCATAGGCAAATGGTAACAACAACCAATCCATTAATAAGACATGAGAACAGAATGGT
TCTGGCCAGCACTACAGCTAAGGCTATGGAGCAAATGGCTGGATCGAGTGAACAAGCAGCTGAGGCCATG
GAGGTTGCTAGTCAGGCCAGGCAGATGGTGCAGGCAATGAGAGCCATTGGGACTCATCCTAGCTCTAGCA
CTGGTCTGAAAAATGATCTCCTTGAAAATTTGCAGGCCTATCAGAAACGAATGGGGGTGCAGATGCAACG
ATTCAAGTGATCCTCTTGTTGTTGCCGCAAGTATAATTGGGATTGTGCACCTGATATTGTGGATTATTGA
TCGCCTTTTTTCCAAAAGCATTTATCGTATCTTTAAACACGGTTTAAAAAGAGGGCCTTCTACGGAAGGA
GTACCAGAGTCTATGAGGGAAGAATATCGAGAGGAACAGCAGAATGCTGTGGATGCTGACGATGGTCATT
TTGTCAGCATAGAGCTAGAGTAAAAAACTACCTTGTTTCTACT
>gi|125664177|gb|CY019884.1| Influenza A virus (A/Memphis/5/2003(H1N1)) segment 7, complete sequence
ATGAGTCTTCTAACCGAGGTCGAAACGTACGTTCTCTCTATCGTCCCGTCAGGCCCCCTCAAAGCCGAGA
TCGCACAGAGACTTGAAGATGTATTTGCTGGAAAGAATACCGATCTTGAGGCTCTCATGGAGTGGCTAAA
GACAAGACCAATCCTGTCACCTCTGACTAAGGGGATTTTAGGATTTGTGTTCACGCTCACCGTGCCCAGT
GAGCGAGGACTGCAGCGTAGACGCTTTGTCCAAAATGCCCTTAATGGGAATGGGGATCCAAATAATATGG
ACAGGGCAGTCAAACTGTATCGAAAGCTTAAGAGGGAGATAACATTCCATGGGGCCAAAGAAATAGCACT
CAGTTATTCTGCTGGTGCACTTGCCAGTTGTATGGGACTCATATACAACAGGATGGGGGCTGTGACCACC
GAATCAGCATTTGGCCTTATATGTGCAACCTGTGAACAGATTGCCGACTCCCAGCATAAGTCTCATAGGC
AAATGGTAACAACAACCAATCCATTAATAAGACATGAGAACAGAATGGTTCTGGCCAGCACTACAGCTAA
GGCTATGGAGCAAATGGCTGGATCGAGTGAACAAGCAGCTGAGGCCATGGAGGTTGCTAGTCAGGCCAGG
CAGATGGTGCAGGCAATGAGAGCCATTGGGACTCATCCTAGCTCTAGCACTGGTCTGAAAAATGATCTCC
TTGAAAATTTGCAGGCCTATCAGAAACGAATGGGGGTGCAGATGCAACGATTCAAGTGATCCTCTTGTTG
TTGCCGCAAGTATAATTGGGATTGTGCACTTGATATTGTGGATTATTGATCGCCTTTTTTCCAAAAGCAT
TTATCGTATCTTTAAACACGGTTTAAAAAGAGGGCCTTCTACGGAAGGAGTACCAGAGTCTATGAGGGAA
GAATATCGAGAGGAACAGCAGAATGCTGTGGATGCTGACGATGGTCATTTTGTCAGCATAGAGCTAGAGT
AA
>gi|73763196|gb|CY002537.1| Influenza A virus (A/New York/227/2003(H1N1)) segment 7, complete sequence
AAAGCAGGTAGATATTGAAAGATGAGTCTTCTAACCGAGGTCGAAACGTACGTTCTATCTATCGTCCCGT
CAGGCCCCCTCAAAGCCGAGATCGCACAGAGACTTGAAGATGTATTTGCTGGAAAGAATACCGATCTTGA
GGCTCTCATGGAGTGGCTAAAGACAAGACCAATCCTGTCACCTCTGACTAAGGGGATTTTAGGATTTGTG
TTCACGCTCACCGTGCCCAGTGAGCGAGGACTGCAGCGTAGACGCTTTGTCCAAAATGCCCTTAATGGGA
ATGGGGATCCAAATAATATGGACAGGGCAGTCAAACTGTATCGAAAGCTTAAGAGGGAGATAACATTCCA
TGGGGCCAAAGAAATAGCACTCAGTTATTCTGCTGGTGCACTTGCCAGTTGTATGGGACTCATATACAAC
AGGATGGGGGCTGTGACCACCGAATCAGCATTTGGCCTTATATGTGCAACCTGTGAACAGATTGCCGACT
CCCAGCATAAGTCTCATAGGCAAATGGTAACAACAACCAATCCATTAATAAGACATGAGAACAGAATGGT
TCTGGCCAGCACTACAGCTAAGGCTATGGAGCAAATGGCTGGATCGAGTGAACAAGCAGCTGAGGCCATG
GAGGTTGCTAGTCAGGCCAGGCAGATGGTGCAGGCAATGAGAGCCATTGGGACTCATCCTAGCTCTAGCA
CTGGTCTGAAAAATGATCTCCTTGAAAATTTGCAGGCCTATCAGAAACGAATGGGGGTGCAGATGCAACG
ATTCAAGTGATCCTCTTGTTGTTGCCGCAAGTATAATTGGGATTGTGCACTTGATATTGTGGATTATTGA
TCGCCTTTTTTCCAAAAGCATTTATCGTATCTTTAAACACGGTTTAAAAAGAGGGCCTTCTACGGAAGGA
GTACCAGAGTCTATGAGGGAAGAATATCGAGAGGAACAGCAGAATGCTGTGGATGCTGACGATGGTCATT

TTGTCAGCATAGAGCTAGAGTAAAAAACTACCTTGTTTCTACT
>gi|89112167|gb|CY008997.1| Influenza A virus (A/New York/484/2003(H1N1)) segment 7, complete sequence
AAAGCAGGTAGATATTGAAAGATGAGTCTTCTAACCGAGGTCGAAACGTACGTTCTCTCTATCGTCCCGT
CAGGCCCCCTCAAAGCCGAGATCGCACAGAGACTTGAAGATGTATTTGCTGGAAAGAATACCGATCTTGA
GGCTCTCATGGAGTGGCTAAAGACAAGACCAATCCTGTCACCTCTGACTAAGGGGATTTTAGGATTTGTG
TTCACGCTCACCGTGCCCAGTGAGCGAGGACTGCAGCGTAGACGCTTTGTCCAAAATGCCCTTAATGGGA
ATGGGGATCCAAATAATATGGACAGAGCAGTCAAACTGTATCGAAAGCTTAAGAGGGAGATAACATTCCA
TGGGGCCAAAGAAATAGCACTCAGTTATTCTGCTGGTGCACTTGCCAGTTGTATGGGACTCATATACAAC
AGGATGGGGGCTGTGACCACCGAATCAGCATTTGGCCTTATATGCGCAACCTGTGAACAGATTGCCGACT
CCCAGCATAAGTCTCATAGGCAAATGGTAACAACAACCAATCCATTAATAAGACATGAGAACAGAATGGT
TCTGGCCAGCACTACAGCTAAGGCTATGGAGCAAATGGCTGGATCGAGTGAACAAGCAGCTGAGGCCATG
GAGGTTGCTAGTCAGGCCAGGCAGATGGTGCAGGCAATGAGAGCCATTGGGACTCATCCTAGCTCTAGCA
CTGGTCTGAAAAATGATCTCCTTGAAAATTTGCAGGCCTATCAGAAACGAATGGGGGTGCAGATGCAACG
ATTCAAGTGATCCTCTTGTTGTTGCCGCAAGTATAATTGGGATTGTGCACCTGATATTGTGGATTATTGA
TCGGCTTTTTTCCAAAAGCATTTATCGTATCTTTAAACACGGTTTAAAAAGAGGGCCTTCTACGGAAGGA
GTACCAGAGTCTATGAGGGAAGAATATCGAGAGGAACAGCAGAATGCTGTGGATGCTGACGATGGTCATT
TTGTCAGCATAGAGCTAGAGTAAAAAACT
>gi|83727843|gb|CY006916.1| Influenza A virus (A/New York/488/2003(H1N1)) segment 7, complete sequence
GTAGATATTGAAAGATGAGTCTTCTAACCGAGGTCGAAACGTACGTTCTCTCTATCGTCCCGTCAGGCCC
CCTCAAAGCCGAGATCGCACAGAGACTTGAAGATGTATTTGCTGGAAAGAATACCGATCTTGAGGCTCTC
ATGGAGTGGCTAAAGACAAGACCAATCCTGTCACCTCTGACTAAGGGGATTTTAGGATTTGTGTTCACGC
TCACCGTGCCCAGTGAGCGAGGACTGCAGCGTAGACGCTTTGTCCAAAATGCCCTTAATGGGAATGGGGA
TCCAAATAATATGGACAGAGCAGTCAAACTGTATCGAAAGCTTAAGAGGGAGATAACATTCCATGGGGCC
AAAGAAATAGCACTCAGTTATTCTGCTGGTGCACTTGCCAGTTGTATGGGACTCATATACAACAGGATGG
GGGCTGTGACCACCGAATCAGCATTTGGCCTTATATGCGCAACCTGTGAACAGATTGCCGACTCCCAGCA
TAAGTCTCATAGGCAAATGGTAACAACAACCAATCCATTAATAAGACATGAGAACAGAATGGTTCTGGCC
AGCACTACAGCTAAGGCTATGGAGCAAATGGCTGGATCGAGTGAACAAGCAGCTGAGGCCATGGAGGTTG
CTAGTCAGGCCAGGCAGATGGTGCAGGCAATGAGAGCCATTGGGACTCATCCTAGCTCTAGCACTGGTCT
GAAAAATGATCTCCTTGAAAATTTGCAGGCCTATCAGAAACGAATGGGGGTGCAGATGCAACGATTCAAG
TGATCCTCTTGTTGTTGCCGCAAGTATAATTGGGATTGTGCACCTGATATTGTGGATTATTGATCGGCTT
TTTTCCAAAAGCATTTATCGTATCTTTAAACACGGTTTAAAAAGAGGGCCTTCTACGGAAGGAGTACCAG
AGTCTATGAGGGAAGAATATCGAGAGGAACAGCAGAATGCTGTGGATGCTGACGATGGTCATTTTGTCAG
CATAGAGCTAGAGTAAAAAA
>gi|157281259|gb|CY025214.1| Influenza A virus (A/Texas/UR06-0012/2006(H1N1)) segment 7, complete sequence
ATGAGTCTTCTAACCGAGGTCGAAACGTACGTTCTCTCTATCGTCCCGTCAGGCCCCCTCAAAGCCGAGA
TCGCACAGAGACTTGAAGATGTATTTGCTGGAAAGAATACCGATCTTGAGGCTCTCATGGAGTGGCTAAA
GACAAGACCAATCCTGTCACCTCTGACTAAGGGGATTTTAGGATTTGTGTTCACGCTCACCGTGCCCAGT
GAGCGAGGACTGCAGCGTAGACGCTTTGTTCAAAATGCCCTTAATGGGAATGGGGATCCAAATAATATGG
ACAGAGCAGTCAAACTGTATCGAAAGCTTAAGAGGGAGATAACATTCCATGGGGCCAAAGAAATAGCACT
CAGTTATTCTGCTGGTGCACTTGCCAGTTGTATGGGACTCATATACAACAGGATGGGGGCTGTGACCACC
GAATCAGCATTTGGCCTTATATGTGCAACCTGTGAACAGATTGCCGACTCCCAGCATAAGTCACATAGGC

AAATGGTAACAACAACCAATCCATTAATAAGGCATGAGAACAGAATGGTTCTGGCCAGCACTACAGCTAA
GGCTATGGAGCAAATGGCTGGATCGAGCGAACAAGCAGCTGAGGCCATGGAGGTTGCCAGTCAGGCCAG
A
CAGATGGTGCAGGCAATGAGAGCCATTGGGACTCATCCTAGCTCTAGCACTGGTCTGAAAAATGATCTCC
TTGAAAATTTACAGGCCTATCAGAAACGAATGGGGGTGCAGATGCAACGATTCAAGTGATCCTCTTGTTG
TTGCCGCAAGTATAATTGGGATTGTGCACTTGATATTGTGGATTATTGATCGCCTTTTCTCCAAAAGCAT
TTATCGTATCTTTAAACACGGTTTAAAAAGAGGGCCTTCTACGGAAGGAGTACCAGAGTCTATGAGGGAA
GAATATCGAGAGGAACAGCAGAATGCTGTGGATGCTGACGATGATCATTTTGTCAGCATAGAGCTAGAGT
AAAAAA
>gi|94959534|gb|CY010765.1| Influenza A virus (A/Canterbury/20/2001(H1N1)) segment 7, complete sequence
GTAGATATTGAAAGATGAGTCTTCTAACCGAGGTCGAAACGTACGTTCTCTCTATCATCCCTTCAGGCCC
CCTCAAAGCCGAGATCGCACAGAGACTTGAAGATGTCTTTGCTGGAAAGAATACCGATCTTGAGGCTCTC
ATGGAATGGCTAAAGACAAGACCGATCCTGTCACCTCTGACTAAGGGGATTTTAGGATTTGTGTTCACGC
TCACCGTGCCCAGTGAGCGAGGACTGCAGCGTAGACGCTTTGTCCAAAATGCCCTTAATGGGAATGGGGA
TCCAAATAATATGGACAGAGCAGTTAAACTGTATCGAAAGCTTAAGAGGGAGATAACATTCCATGGGGCC
AAAGAAATAGCACTCAGTTATTCTGCTGGTGCACTTGCCAGTTGTATGGGACTCATATACAACAGGATGG
GGGCTGTGACCACCGAATCAGCATTTGGCCTTATATGTGCAACCTGTGAACAGATTGCCGACTCCCAGCA
TAAGTCTCATAGGCAAATGGTAACAACAACCAATCCATTAATAAGACATGAGAACAGAATGGTTCTGGCC
AGCACTACAGCTAAGGCTATGGAGCAAATGGCTGGATCGAGTGAACAAGCAGCTGAGGCCATGGAGGTTG
CTAGTCAGGCCAGGCAGATGGTGCAGGCAATGAGAGCCATTGGGACTCATCCTAGCTCTAGCACTGGTCT
GAAAAATGATCTCCTTGAAAATTTGCAGGCCTATCAGAAACGAATGGGGGTGCAGATGCAACGATTCAAG
TGATCCTCTTGTTGTTGCCGCAAGTATAGTTGGGATTGTGCACCTGATATTGTGGATTATTGATCGCCTT
TTTTCCAAAAGCATTTATCGTATCTTTAAACACGGTTTAAAAAGAGGGCCTTCTACGGAAGGAGTACCAG
AGTCTATGAGGGAAGAATATCGAGAGGAACAGCAGAATGCTGTGGATGCTGACGATGGTCATTTTGTCAG
CATAGAGCTGGAGTAAAAAA
>gi|91119028|gb|CY010405.1| Influenza A virus (A/West Coast/33/2001(H1N1)) segment 7, complete sequence
GTAGATATTGAAAGATGAGTCTTCTAACCGAGGTCGAAACGTACGTTCTCTCTATCATCCCTTCAGGCCC
CCTCAAAGCCGAGATCGCACAGAGACTTGAAGATGTCTTTGCTGGAAAGAATACCGATCTTGAGGCTCTC
ATGGAATGGCTAAAGACAAGACCGATCCTGTCACCTCTGACTAAGGGGATTTTAGGATTTGTGTTCACGC
TCACCGTGCCCAGTGAGCGAGGACTGCAGCGTAGACGCTTTGTCCAAAATGCCCTTAATGGGAATGGGGA
TCCAAATAATATGGACAGAGCAGTTAAACTGTATCGAAAGCTTAAGAGGGAGATAACATTCCATGGGGCC
AAAGAAATAGCACTCAGTTATTCTGCTGGTGCACTTGCCAGTTGTATGGGACTCATATACAACAGGATGG
GGGCTGTGACCACCGAATCAGCATTTGGCCTTATATGTGCAACCTGTGAACAGATTGCCGACTCCCAGCA
TAAGTCTCATAGGCAAATGGTGACAACAACCAATCCATTAATAAGACATGAGAACAGAATGGTTCTGGCC
AGCACTACAGCTAAGGCTATGGAGCAAATGGCTGGATCGAGTGAACAAGCAGCTGAGGCCATGGAGGTTG
CTAGTCAGGCCAGGCAGATGGTGCAGGCAATGAGAGCCATTGGGACTCATCCTAGCTCTAGCACTGGTCT
GAAAAATGATCTCCTTGAAAATTTGCAGGCCTATCAGAAACGAATGGGGGTGCAGATGCAACGATTCAAG
TGATCCTCTTGTTGTTGCCGCAAGTATAGTTGGGATTGTGCACCTGATATTGTGGATTATTGATCGCCTT
TTTTCCAAAAGCATTTATCGTATCTTTAAACACGGTTTAAAAAGAGGGCCTTCTACGGAAGGAGTACCAG
AGTCTATGAGGGAAGAATATCGAGAGGAACAGCAGAATGCTGTGGATGCTGACGATGGTCATTTTGTCAG
CATAGAGCTGGAGTAAAAAA
>gi|131052792|gb|CY019998.1| Influenza A virus (A/Waikato/17/2005(H1N1)) segment 7,

complete sequence
ATGAGTCTTCTAACCGAGGTCGAAACGTACGTTCTCTCTATCGTCCCGTCAGGCCCCCTCAAAGCCGAGA
TCGCACAGAGACTTGAAGATGTATTTGCTGGAAAGAATACCGATCTTGAGGCTCTCATGGAGTGGCTAAA
GACAAGACCAATCCTGTCACCTCTGACTAAGGGGATTTTAGGATTTGTGTTCACGCTCACCGTGCCCAGT
GAGCGGGGACTGCAGCGTAGACGCTTTGTCCAAAATGCCCTTAATGGGAATGGGGATCCAAATAATATGG
ACAGAGCAGTCAAACTGTATCGAAAGCTTAAGAGGGAGATAACATTCCATGGGGCCAAAGAAATAGCACT
CAGTTATTCTGCTGGTGCACTTGCCAGTTGTATGGGACTCATATACAACAGGATGGGGGCTGTGACCACC
GAATCAGCATTTGGCCTTATATGTGCAACCTGTGAACAGATTGCCGACTCCCAGCATAAGTCTCATAGGC
AAATGGTAACAACAACCAATCCATTAATAAGACATGAGAACAGAATGGTTCTGGCCAGCACTACAGCTAA
GGCTATGGAGCAAATGGCTGGATCGAGCGAACAAGCAGCTGAGGCCATGGAGGTTGCTAGTCAGGCCAGG
CAGATGGTGCAGGCAATGAGAGCCATTGGGACTCATCCTAGCTCTAGCACTGGTCTGAAAAATGATCTCC
TTGAAAATTTGCAGGCCTATCAGAAACGAATGGGGGTGCAGATGCAACGATTCAAGTGATCCTCTTGTTG
TTGCCGCAAGTATAATTGGGATTGTGCACCTGATATTGTGGATTATTGATCGCCTTTTTTCCAAAAGCAT
TTATCGTATCTTTAAACACGGTTTAAAAAGAGGGCCTTCTACGGAAGGAGTACCAGAGTCTATGAGGGAA
GAATATCGAGAGGAACAGCAGAATGCTGTGGATGCTGACGATGGTCATTTTGTCAGCATAGAGCTAGAGT
AAAAAA
>gi|83744836|gb|CY007468.1| Influenza A virus (A/Canterbury/106/2004(H1N1)) segment 7, complete sequence
GTAGATATTGAAAGATGAGTCTTCTAACCGAGGTCGAAACGTACGTTCTCTCTATCATCCCGTCAGGCCC
CCTCAAAGCCGAGATCGCACAGAGACTTGAAGATGTATTTGCTGGAAAGAATACCGATCTTGAGGCTCTC
ATGGAGTGGCTAAAGACAAGACCAATCCTGTCACCTCTGACTAAGGGGATTTTAGGATTTGTGTTCACGC
TCACCGTGCCCAGTGAGCGAGGACTGCAGCGTAGACGCTTTGTCCAAAATGCCCTTAATGGGAATGGGGA
TCCAAATAATATGGACAAAGCAGTCAAACTGTATCGAAAGCTTAAGAGGGAGATAACATTCCATGGGGCC
AAAGAAATAGCACTCAGTTATTCTGCTGGTGCACTTGCCAGTTGTATGGGACTCATATACAACAGGATGG
GGGCTGTGACCACCGAATCAGCATTTGGCCTTATATGCGCAACCTGTGAACAGATTGCCGACTCCCAGCA
TAAGTCTCATAGGCAAATGGTAACAACAACCAATCCATTAATAAGACATGAGAACAGAATGGTTCTGGCC
AGCACTACAGCTAAGGCTATGGAGCAAATGGCTGGATCGAGTGAACAAGCAGCTGAGGCCATGGAGGTTG
CTAGTCAGGCCAGGCAGATGGTGCAGGCAATGAGAGCCATTGGGACTCATCCTAGCTCTAGCACTGGTCT
GAAAAATGATCTCCTTGATAATTTGCAGGCCTATCAGAAACGAATGGGGGTGCAGATGCAACGATTCAAG
TGATCCTCTTGTTGTTGCCGCAAGTATAATTGGGATTGTGCACCTGATATTGTGGATTATTGATCGGCTT
TTTTCCAAAAGCATTTATCGTATCTTTAAACACGGTTTAAAAGGAGGGCCTGCTACGGAAGGAGTACCAG
AGTCTATGAGGGAAGAATATCGAGAGGAACAGCAGAATGCTGTGGATGCTGACGATGGTCATTTTGTCAG
CATAGAGCTAGAGTAAAAAA
>gi|115607823|gb|CY016700.1| Influenza A virus (A/South Australia/58/2005(H1N1)) segment 7, complete sequence
AGATGAGTCTTCTAACCGAGGTCGAAACGTACGTTCTCTCTATCGTCCCGTCAGGCCCCCTCAAAGCCGA
GATCGCACAGAGACTTGAAGATGTATTTGCTGGAAAGAATACCGATCTTGAGGCTCTCATGGAGTGGCTA
AAGACAAGACCAATCCTGTCACCTCTGACTAAGGGGATTTTAGGATTTGTGTTCACGCTCACCGTGCCCA
GTGAGCGGGGACTGCAGCGTAGACGCTTTGTCCAAAATGCCCTTAATGGGAATGGGGATCCAAATAATAT
GGACAGAGCAGTCAAACTGTATCGAAAGCTTAAGAGGGAGATAACATTCCATGGGGCCAAAGAAATAGCA
CTCAGTTATTCTGCTGGTGCACTTGCCAGTTGTATGGGACTCATATACAACAGGATGGGGGCTGTGACCA
CCGAATCAGCATTTGGCCTTATATGTGCAACCTGTGAACAGATTGCCGACTCCCAGCATAAGTCTCATAG
GCAAATGGTAACAACAACCAATCCATTAATAAGACATGAGAACAGAATGGTTCTGGCCAGCACTACAGCT
AAGGCTATGGAGCAAATGGCTGGATCGAGCGAACAAGCAGCTGAGGCCATGGAGGTTGCTAGTCAGGCCA

GGCAGATGGTGCAGGCAATGAGAGCCATTGGGACTCATCCTAGCTCTAGCACTGGTCTGAAAAATGATCT
CCTTGAAAATTTGCAGGCCTATCAGAAACGAATGGGGGTGCAGATGCAACGATTCAAGTGATCCTCTTGT
TGTTGCCGCAAGTATAATTGGGATTGTGCACCTGATATTGTGGATTATTGATCGCCTTTTTTCCAAAAGC
ATTTATCGTATCTTTAAACACGGTTTAAAAAGAGGGCCTTCTACGGAAGGAGTACCAGAGTCTATGAGGG
AAGAATATCGAGAGGAACAGCAGAATGCTGTGGATGCTGACGATGGTCATTTTGTCAGCATAGAGCTAGA
GTAAAA
>gi|113170883|gb|CY014008.1| Influenza A virus (A/Wellington/11/2005(H1N1)) segment 7, complete sequence
AAGATGAGTCTTCTAACCGAGGTCGAAACGTACGTTCTCTCTATCGTCCCGTCAGGCCCCCTCAAAGCCG
AGATCGCACAGAGACTTGAAGATGTATTTGCTGGAAAGAATACCGATCTTGAGGCTCTCATGGAGTGGCT
AAAGACAAGACCAATCCTGTCACCTCTGACTAAGGGGATTTTAGGATTTGTGTTCACGCTCACCGTGCCC
AGTGAGCGAGGACTGCAGCGTAGACGCTTTGTCCAAAATGCCCTTAATGGGAATGGGGATCCAAATAATA
TGGACAGAGCAGTCAAACTGTATCGAAAGCTTAAGAGGGAGATAACATTCCATGGGGCCAAAGAAATAGC
ACTCAGTTATTCTGCTGGTGCACTTGCCAGTTGTATGGGACTCATATACAACAGGATGGGGGCTGTGACC
ACCGAATCAGCATTTGGCCTTATATGTGCAACCTGTGAACAGATTGCCGACTCCCAGCATAAGTCTCATA
GGCAAATGGTAACAACAACCAATCCATTAATAAGACATGAGAACAGAATGGTTCTGGCCAGCACTACAGC
TAAGGCTATGGAGCAAATGGCTGGATCGAGCGAACAAGCAGCTGAGGCCATGGAGGTTGCTAGTCAGGCC
AGGCAGATGGTGCAGGCAATGAGAGCCATTGGGACTCATCCTAGCTCTAGCACTGGTCTGAAAAATGATC
TCCTTGAAAATTTACAGGCCTATCAGAAACGAATGGGGGTGCAGATGCAACGATTCAAGTGATCCTCTTG
TTGTTGCCGCAAGTATAATTGGGATTGTGCACTTGATATTGTGGATTATTGATCGCCTTTTTTCCAAAAG
CATTTATCGTATCTTTAAACACGGTTTAAAAAGAGGGCCTTCTACGGAAGGAGTACCAGAGTCTATGAGG
GAAGAATATCGAGAGGAACAGCAGAATGCTGTGGATGCTGACGATGATCATTTTGTCAGCATAGAGCTAG
AGTAAAA
>gi|145278925|gb|CY021758.1| Influenza A virus (A/South Australia/51/2005(H1N1)) segment 7, complete sequence
GATGAGTCTTCTAACCGAGGTCGAAACGTACGTTCTCTCTATCGTCCCGTCAGGCCCCCTCAAAGCCGAG
ATCGCACAGAGACTTGAAGATGTATTTGCTGGAAAGAATACCGATCTTGAGGCTCTCATGGAGTGGCTAA
AGACAAGACCAATCCTGTCACCTCTGACTAAGGGGATTTTAGGATTTGTGTTCACGCTCACCGTGCCCAG
TGAGCGAGGACTGCAGCGTAGACGCTTTGTCCAAAATGCCCTTAATGGGAATGGGGATCCAAATAATATG
GACAGAGCAGTCAAACTGTATCGAAAGCTTAAGAGGGAGATAACATTCCATGGGGCCAAAGAAATAGCAC
TCAGTTATTCTGCTGGTGCACTTGCCAGTTGTATGGGACTCATATACAACAGGATGGGGGCTGTGACCAC
CGAATCAGCATTTGGCCTTATATGTGCAACCTGTGAACAGATTGCCGACTCCCAGCATAAGTCTCATAGG
CAAATGGTAACAACAACCAATCCATTAATAAGACATGAGAACAGAATGGTTCTGGCCAGCACTACAGCTA
AGGCTATGGAGCAAATGGCTGGATCGAGCGAACAAGCAGCTGAGGCCATGGAGGTTGCTAGTCAGGCCAG
GCAGATGGTGCAGGCAATGAGAGCCATTGGGACTCATCCTAGCTCTAGCACTGGTCTGAAAAACGATCTC
CTTGAAAATTTACAGGCCTATCAGAAACGAATGGGGGTGCAGATGCAACGATTCAAGTGATCCTCTTGTT
GTTGCCGCAAGTATAATTGGGATTGTGCACTTGATATTGTGGATTATTGATCGCCTTTTTTCCAAAAGCA
TTTATCGTATCTTTAAACACGGTTTAAAAAGAGGGCCTTCTACGGAAGGAGTACCAGAGTCTATGAGGGA
AGAATATCGAGAGGAACAGCAGAATGCTGTGGATGCTGACAATGATCATTTTGTCAGCATAGAGCTAGAG
TAA
>gi|149780695|gb|CY022582.1| Influenza A virus (A/Auckland/619/2005(H1N1)) segment 7, complete sequence
TAGATATTGAAAGATGAGTCTTCTAACCGAGGTCGAAACGTACGTTCTCTCTATCGTCCCGTCAGGCCCC
CTCAAAGCCGAGATCGCACAGAGACTTGAAGATGTATTTGCTGGAAAGAATACCGATCTTGAGGCTCTCA

TGGAGTGGCTAAAGACAAGACCAATCCTGTCACCTCTGACTAAGGGGATTTTAGGATTTGTGTTCACGCT
CACCGTGCCCAGTGAGCGAGGACTGCAGCGTAGACGCTTTGTCCAAAATGCCCTTAATGGGAATGGGGAT
CCAAATAATATGGACAGAGCAGTCAAACTGTATCGAAAGCTTAAGAGGGAGATAACATTCCATGGGGCCA
AAGAAATAGCACTCAGTTATTCTGCTGGTGCACTTGCCAGTTGTATGGGACTCATATACAACAGGATGGG
GGCTGTGACCACCGAATCAGCATTTGGCCTTATATGTGCAACCTGTGAACAGATTGCCGACTCCCAGCAT
AAGTCTCATAGGCAAATGGTAACAACAACCAATCCATTAATAAGACATGAGAACAGAATGGTTCTGGCCA
GCACTACAGCTAAGGCTATGGAGCAAATGGCTGGATCGAGCGAACAAGCAGCTGAGGCCATGGAGGTTGC
TAGTCAGGCCAGGCAGATGGTGCAGGCAATGAGAGCCATTGGGACTCATCCTAGCTCTAGCACTGGTCTG
AAAAATGATCTCCTTGAAAATTTACAGGCCTATCAGAAACGAATGGGGGTGCAGATGCAACGATTCAAGT
GATCCTCTTGTTGTTGCCGCAAGTATAATTGGGATTGTGCACTTGATATTGTGGATCATTGATCGCCTTT
TTTCCAAAAGCATTTATCGTATCTTTAAACACGGTTTAAAAAGAGGGCCTTCTACGGAAGGAGTACCAGA
GTCTATGAGGGAAGAATATCGAGAGGAACAGCAGAATGCTGTGGATGCTGACGATGATCATTTTGTCAGC
ATAGAGCTAGAGTAAAAA
>gi|117572940|gb|CY017316.1| Influenza A virus (A/Waikato/4/2005(H1N1)) segment 7, complete sequence
ATGAGTCTTCTAACCGAGGTCGAAACGTACGTTCTCTCTATCGTCCCGTCAGGCCCCCTCAAAGCCGAGA
TCGCACAGAGACTTGAAGATGTATTTGCTGGAAAGAATACCGATCTTGAGGCTCTCATGGAGTGGCTAAA
GACAAGACCAATCCTGTCACCTCTGACTAAGGGGATTTTAGGATTTGTGTTCACGCTCACCGTGCCCAGT
GAGCGGGGACTGCAGCGTAGACGCTTTGTCCAAAATGCCCTTAATGGGAATGGGGATCCAAATAATATGG
ACAGAGCAGTCAAACTGTATCGAAAGCTTAAGAGGGAGATAACATTCCATGGGGCCAAAGAAATAGCACT
CAGTTATTCTGCTGGTGCACTTGCCAGTTGTATGGGACTCATATACAACAGGATGGGGGCTGTGACCACC
GAATCAGCATTTGGCCTTATATGTGCAACCTGTGAACAGATTGCCGACTCCCAGCATAAGTCTCATAGGC
AAATGGTAACAACAACCAATCCATTAATAAGACATGAGAACAGAATGGTTCTGGCCAGCACTACAGCTAA
GGCTATGGAGCAAATGGCTGGATCGAGCGAACAAGCAGCTGAGGCCATGGAGGTTGCTAGTCAGGCCAGG
CAGATGGTGCAGGCAATGAGAGCCATTGGGACTCATCCTAGCTCTAGCACTGGTCTGAAAAATGATCTCC
TTGAAAATTTGCAGGCCTATCAGAAACGAATGGGGGTGCAGATGCAACGATTCAAGTGATCCTCTTGTTG
TTGCCGCAAGTATAATTGGGATTGTGCACCTGATATTGTGGATTATTGATCGCCTTTTTTTCCAAAAGCAT
TTATCGTATCTTTAAACACGGTTTAAAAAGAGGGCCTTCTACGGAAGGAGTACCAGAGTCTATGAGGGAA
GAATATCGAGAGGAACAGCAGAATGCTGTGGATGCTGACGATGGTCATTTTGTCAGCATAGAGCTAGAGT
AAAAAA
>gi|161139446|gb|CY028196.1| Influenza A virus (A/Kentucky/UR06-0007/2006(H1N1)) segment 7, complete sequence
GTAGATATTGAAAGATGAGTCTTCTAACCGAGGTCGAAACGTACGTTCTCTCTATCGTCCCGTCAGGCCC
CCTCAAAGCCGAGATCGCACAGAGACTTGAAGATGTATTTGCTGGAAAGAATACCGATCTTGAGGCTCTC
ATGGAGTGGCTAAAGACAAGACCAATCCTGTCACCTCTGACTAAGGGGATTTTAGGATTTGTGTTCACGC
TCACCGTGCCCAGTGAGCGAGGACTGCAGCGTAGACGCTTTGTCCAAAATGCCCTTAATGGGAATGGGGA
TCCAAATAATATGGACAGAGCAGTCAAACTGTATCGAAAGCTTAAGAGGGAGATAACATTCCATGGGGCC
AAAGAAATAGCACTCAGTTATTCTGCTGGTGCACTTGCCAGTTGTATGGGACTCATATACAACAGGATGG
GGGCTGTGACCACCGAATCAGCATTTGGCCTTATATGTGCAACCTGTGAACAGATTGCCGACTCCCAGCA
TAAGTCTCATAGGCAAATGGTAACAACAACCAATCCATTAATAAGACATGAGAACAGAATGGTTCTGGCC
AGCACTACAGCTAAAGCTATGGAGCAAATGGCTGGATCGAGCGAACAAGCAGCTGAGGCCATGGAGGTTG
CTAGTCAGGCCAGGCAGATGGTGCAGGCAATGAGAGCCATTGGGACTCATCCTAGCTCTAGCACTGGTCT
GAAAAATGATCTCCTTGAAAATTTACAGGCCTATCAGAAACGAATGGGAGTGCAGATGCAACGATTCAAA
TGATCCTCTTGTTGTTGCCGCAAGTATAATTGGGATTGTGCACTTGATATTGTGGATTGTTGATCGCCTT

TTTTCCAAAAGCATTTATCGTATCTTTAAACACGGTTTAAAAAGAGGGCCTTCTACGGAAGGAGTACCAG
AGTCTATGAGGGAAGAATATCGAGAGGAACAGCAGAATGCTGTGGATGCTGACGATGATCATTTTGTCAG
CATAGAGCTAGAGTAAAAAA
>gi|157281278|gb|CY025222.1| Influenza A virus (A/Michigan/UR06-0015/2006(H1N1)) segment 7, complete sequence
ATGAGTCTTCTAACCGAGGTCGAAACGTACGTTCTCTCTATCGTCCCGTCAGGCCCCCTCAAAGCCGAGA
TCGCACAGAGACTTGAAGATGTATTTGCTGGAAAGAATACCGATCTTGAGGCTCTCATGGAGTGGCTAAA
GACAAGACCAATCCTGTCACCTCTGACTAAGGGGATTTTAGGATTTGTGTTCACGCTCACCGTGCCCAGT
GAGCGAGGACTGCAGCGTAGACGCTTTGTCCAAAATGCCCTTAATGGGAATGGGGATCCAAATAATATGG
ACAGAGCAGTCAAACTGTATCGAAAGCTTAAGAGGGAGATAACATTCCATGGGGCCAAAGAAATAGCACT
CAGTTATTCTGCTGGTGCACTTGCCAGTTGTATGGGACTCATATACAACAGGATGGGGGCTGTGACCACC
GAATCAGCATTTGGCCTTATATGTGCAACCTGTGAACAGATTGCCGACTCCCAGCATAAGTCTCATAGGC
AAATGGTAACAACAACCAATCCATTAATAAGACATGAGAACAGAATGGTTCTGGCCAGCACTACAGCTAA
GGCTATGGAGCAAATGGCTGGATCGAGCGAACAAGCAGCTGAAGCCATGGAGGTTGCTAGTCAGGCCAGG
CAGATGGTGCAGGCAATGAGAGCCATTGGGACTCATCCTAGCTCTAGCACTGGTCTGAAAAATGATCTCC
TTGAAAATTTACAGGCCTATCAGAAACGAATGGGGGTGCAGATGCAACGATTCAAGTGATCCTCTTGTTG
TTGCCGCAAGTATAATTGGGATTGTGCACTTGATATTGTGGATTATTGATCGCCTTTTTTCCAAAAGCAT
TTATCGTATCTTTAAACACGGTTTAAAAAGAGGGCCTTCTACGGAAGGAGTACCAGAGTCTATGAGGGAA
GAATATCGAGAGGAACAGCAGAATGCTGTGGATGCTGACGATGATCATTTTGTCAGCATAGAGCTAGAGT
AAAAAA
>gi|218875170|gb|CY036920.1| Influenza A virus (A/NYMC X-163A(NYMC X-157-St. Petersburg/8/2006)(H1N1)) segment 7, complete sequence
GATGAGTCTTCTAACCGAGGTCGAAACGTACGTACTCTCTATCATCCCGTCAGGCCCCCTCAAAGCCGAG
ATCGCACAGAGACTTGAAGATGTCTTTGCAGGGAAGAACACCGATCTTGAGGTTCTCATGGAATGGCTAA
AGACAAGACCAATCCTGTCACCTCTGACTAAGGGGATTTTAGGATTTGTGTTCACGCTCACCGTGCCCAG
TGAGCGAGGACTGCAGCGTAGACGCTTTGTCCAAAATGCCCTTAATGGGAACGGGGATCCAAATAACATG
GACAAAGCAGTTAAACTGTATAGGAAGCTCAAGAGGGAGATAACATTCCATGGGGCCAAAGAAATCTCAC
TCAGTTATTCTGCTGGTGCACTTGCCAGTTGTATGGGCCTCATATACAACAGGATGGGGGCTGTGACCAC
TGAAGTGGCATTTGGCCTGGTATGTGCAACCTGTGAACAGATTGCTGACTCCCAGCATCGGTCTCATAGG
CAAATGGTGACAACAACCAATCCACTAATCAGACATGAGAACAGAATGGTTTTAGCCAGCACTACAGCTA
AGGCTATGGAGCAAATGGCTGGATCGAGTGAGCAAGCAGCAGAGGCCATGGAGGTTGCTAGTCAGGCTAG
ACAAATGGTGCAAGCGATGAGAACCATTGGGACTCATCCTAGCTCCAGTGCTGGTCTGAAAAATGATCTT
CTTGAAAATTTGCAGGCCTATCAGAAACGAATGGGGGTGCAGATGCAACGGTTCAAGTGATCCTCTCACT
ATTGCCGCAAATATCATTGGGATCTTGCACTTGACATTGTGGATTCTTGATCGTCTTTTTTTCAAATGCA
TTTACCGTCGCTTTAAATACGGACTGAAAGGAGGGCCTTCTACGGAAGGAGTGCCAAAGTCTATGAGGGA
AGAATATCGAAAGGAACAGCAGAGTGCTGTGGATGCTGACGATGGTCATTTTGTCAGCATAGAGCTGGAG
TAAA
>gi|208344085|gb|CY035127.1| Influenza A virus (A/St. Petersburg/8/2006(H1N1)) segment 7, complete sequence
AGATGAGTCTTCTAACCGAGGTCGAAACGTACGTTCTCTCTATCATCCCGTCAGGCCCCCTCAAAGCCGA
GATCGCACAGAGACTTGAAAATGTATTTGCTGGAAAGAATACTGATCTTGAGGCTCTCATGGAGTGGCTA
AAGACAAGACCAATCCTGTCACCTCTGACTAAGGGGATTTTAGGATTTGTGTTCACGCTCACCGTGCCCA
GTGAGCGAGGACTGCAGCGTAGACGCTTTGTCCAAAATGCCCTTAATGGGAATGGGGATCCAAATAATAT
GGACAGAGCAGTCAAACTGTATCGAAAGCTTAAGAGGGAGATAACATTCCATGGGGCCAAAGAAATAGCA

CTCAGTTATTCTGCTGGTGCACTTGCCAGTTGTATGGGACTCATATACAACAGGATGGGAGCTGTGACCA
CCGAATCAGCATTTGGCCTTATATGCGCAACCTGTGAACAGATTGCCGACTCCCAGCATAAGTCTCATAG
GCAAATGGTAACAACAACCAATCCATTAATAAGACATGAGAACAGAATGGTTCTGGCCAGCACTACAGCT
AAGGCTATGGAGCAAATGGCTGGATCGAGTGAACAAGCAGCTGAGGCAATGGAGGTTGCTAGTCAGGCCA
GGCAGATGGTGCAGGCAATGAGAGCCATTGGGACTCATCCTAGTTCTAGCACTGGTCTGAAAAATGATCT
CCTTGATAATTTGCAGGCCTATCAGAAACGAATGGGGGTGCAGATGCAACGATTCAAGTGATCCTCTTGT
TGTTGCCGCAAATATAATTGGGATTGTGCACCTGATATTGTGGATTATTGATCGGCTTTTTTCCAAAAGC
ATTTATCGTATCTTTAAACACGGTTTAAAAAGAGGGCCTTCTACGGAAGGAGTACCAGAGTCTATGAGGG
AAGAATATCGAGAGGAACAGCAGAATGCTGTGGATGCTGACGATGGTCATTTTGTCAGCATAGAGCTAGA
GTAA
>gi|226954748|gb|CY038880.1| Influenza A virus (A/Taiwan/2645/2006(H1N1)) segment 7, complete sequence
ATGAGTCTTCTAACCGAGGTCGAAACGTACGTTCTCTCTATCATCCCGTCAGGCCCCCTCAAAGCCGAGA
TCGCACAGAGACTTGAAGATGTATTTGCTGGAAAGAATACCGATCTTGAGGCTCTCATGGAGTGGCTAAA
GACAAGACCAATCCTGTCACCTCTGACTAAGGGGATTTTAGGATTTGTGTTCACGCTCACCGTGCCCAGT
GAGCGAGGACTGCAGCGTAGACGCTTTGTCCAAAATGCCCTTAATGGGAATGGGGATCCAAATAATATGG
ACAGAGCAGTCAAACTGTATCGAAAGCTTAAGAGGGAGATAACATTCCATGGGGCCAAAGAAATAGCACT
CAGTTATTCTGCTGGTGCACTTGCCAGTTGTATGGGACTCATATACAACAGGATGGGGGCTGTGACCACC
GAATCAGCATTTGGCCTTATATGCGCAACCTGTGAACAGATTGCCGACTCCCAGCATAAGTCTCATAGGC
AAATGGTAACAACAACCAATCCATTAATAAGACATGAGAACAGAATGGTTCTGGCCAGCACTACAGCTAA
GGCTATGGAGCAAATGGCTGGATCGAGTGAACAAGCAGCTGAGGCCATGGAGGTCGCTAATCAGGCCAGG
CAGATGGTGCAGGCAATGAGAGCCATTGGGACTCATCCTAGCTCTAGCACTGGTCTGAAAAATGATCTCC
TTGATAATTTGCAGGCCTATCAGAAACGAATGGGGGTGCAGATGCAACGATTCAAGTGATCCTCTTGTTG
TTGCCGCAAGTATAATTGGGATTGTGCACCTGATATTGTGGATTATTGATCGGCTTTTTTCCAAAAGCAT
TTATCGTATCTTTAAACACGGTTTAAAAGGAGGGCCTTCTACGGAAGGAGTACCGGAGTCTATGAGGGAA
GAATATCGAGAGGAACAGCAGAATGCTGTGGATGCTGACGATGGTCATTTTGTCAGCATAGAGCTAGAGT
AAAA
>gi|256385513|gb|CY044350.1| Influenza A virus (A/South Korea/AF10/2008(H1N1)) segment 7, complete sequence
ATGAGTCTTCTAACCGAGGTCGAAACGTACGTTCTCTCTATCATCCCGTCAGGCCCCCTCAAAGCCGAGA
TCGCACAGAGACTTGAAGATGTATTTGCTGGAAAGAATACCGATCTTGAGGCTCTCATGGAGTGGCTAAA
GACAAGACCAATCCTGTCACCTCTGACTAAGGGGATTTTAGGATTTGTGTTCACGCTCACCGTGCCCAGT
GAGCGAGGACTGCAGCGTAGACGCTTTGTCCAAAATGCCCTTAATGGGAATGGGGATCCAAATAATATGG
ACAGAGCAGTCAAACTGTATCGAAAGCTTAAGAGGGAGATAACATTCCATGGGGCCAAAGAAATAGCACT
CAGTTATTCTGCTGGTGCACTTGCCAGTTGTATGGGACTCATATACAACAGGATGGGGGCTGTGACCACC
GAATCAGCATTTGGCCTTATATGCGCAACCTGTGAACAGATTGCCGATTCCCAGCATAAGTCTCATAGGC
AAATGGTAACAACAACTAATCCATTAATAAGACATGAGAACAGAATGGTTCTGGCCAGCACTACAGCTAA
GGCTATGGAGCAAATGGCTGGATCGAGTGAACAAGCAGCTGAGGCAATGGAGGTTGCTAATCAGGCCAGG
CAGATGGTGCAGGCAATGAGAGCCATTGGGACTCATCCTAGCTCTAGCACTGGTCTGAAAAATGATCTCC
TTGATAATTTGCAGGCCTATCAGAAACGAATGGGGGTGCAGATGCAACGATTCAAGTGATCCTCTTGTTG
TTGCCGCAAATATAATTGGGATTGTGCACCTGATATTGTGGATTATTGATCGGCTTTTTTCCAAAAGCAT
TTATCGTATCTTCAAACACGGTTTAAAAAGAGGGCCTTCTACGGAAGGAGTACCAGAGTCTATGAGGGAA
GAATATCGAGAGGAACAGCAGAATGCTGTGGATGCTGACGATGGTCATTTTGTCAGCATAGAGCTAGAGT
AAAAA

>gi|163964718|gb|CY028460.1| Influenza A virus (A/California/UR06-0442/2007(H1N1)) segment 7, complete sequence
ATATTGAAAGATGAGTCTTCTAACCGAGGTCGAAACGTACGTTCTCTCTATCGTCCCGTCAGGCCCCCTC
AAAGCCGAGATCGCACAGAGACTTGAAGATGTATTTGCTGGAAAGAATACCGATCTTGAGGCTCTCATGG
AGTGGCTAAAGACAAGACCAATCCTGTCACCTCTGACTAAGGGGATTTTAGGATTTGTGTTCACGCTCAC
CGTGCCCAGTGAGCGAGGACTGCAGCGTAGACGCTTTGTCCAAAATGCCCTTAATGGGAATGGGGATCCA
AATAATATGGACAGAGCAGTCAAACTGTATCGAAAGCTTAAGAGGGAGATAACATTCCATGGGGCCAAAG
AAATAGCACTCAGTTATTCTGCTGGTGCACTTGCCAGTTGTATGGGACTCATATACAACAGGATGGGGGC
TGTGACCACCGAATCAGCATTTGGCCTTATATGTGCAACCTGTGAACAGATTGCCGACTCCCAGCATAAG
TCTCATAGGCAAATGGTAACAACAACCAATCCATTAATAAGACATGAGAACAGAATGGTTCTGGCCAGCA
CTACAGCTAAGGCTATGGAGCAAATGGCTGGATCGAGCGAACAAGCAGCTGAAGCCATGGAGGTTGCTAG
TCAGGCCAGGCAGATGGTGCAGGCAATGAGAGCCATTGGGACTCATCCTAGCTCTAGCACTGGTCTGAAA
AATGATCTCCTTGAAAATTTACAGGCCTATCAGAAACGAATGGGGGTGCAGATGCAACGATTCAAGTGAT
CCTCTTGTTGTTGCCGCAAGTATAATTGGGATTGTGCACTTGATATTGTGGATTATTGATCGCCTTTTTT
CCAAAAGCATTTATCGTATCTTTAAACACGGTTTAAAAAGAGGGCCTTCTACGGAAGGAGTACCAGAGTC
TATGAGGGAAGAATATCGAGAGGAACAGCAGAATGCTGTGGATGCTGACGATGATCATTTTGTCAGCATA
GAGCTAGAGTAA
>gi|157281602|gb|CY025358.1| Influenza A virus (A/Kentucky/UR06-0363/2007(H1N1)) segment 7, complete sequence
GTAGATATTGAAAGATGAGCCTTCTAACCGAGGTCGAAACGTACGTTCTCTCTATCGTCCCGTCAGGCCC
CCTCAAAGCCGAGATCGCACAGAGACTTGAAGATGTATTTGCTGGAAAGAATACCGATCTTGAGGCTCTC
ATGGAGTGGCTAAAGACAAGACCAATCCTGTCACCTCTGACTAAGGGGATTTTAGGATTTGTGTTCACGC
TCACCGTGCCCAGTGAGCGAGGACTGCAGCGTAGACGCTTTGTCCAAAATGCCCTTAATGGGAATGGGGA
TCCAAATAATATGGACAGAGCAGTCAAACTGTATCGAAAGCTTAAGAGGGAGATAACATTCCATGGGGCC
AAAGAAATAGCACTCAGTTATTCTGCTGGTGCACTTGCCAGTTGTATGGGACTCATATACAACAGGATGG
GGGCTGTGACCACCGAATCAGCATTTGGCCTTATATGTGCAACCTGTGAACAGATTGCCGACTCCCAGCA
TAAGTCTCATAGGCAAATGGTAACAACAACCAATCCATTAATAAGACATGAGAACAGAATGGTACTGGCC
AGCACTACAGCTAAGGCTATGGAGCAAATGGCTGGATCGAGCGAACAAGCAGCTGAGGCCATGGAGGTTG
CTAGTCAGGCCAGGCAGATGGTGCAGGCAATGAGAGCCATTGGGACTCATCCTAGCTCTAGCACTGGTCT
GAAAAATGATCTCCTTGAAAATTTACAGGCCTATCAGAAACGAATGGGGGTGCAGATGCAACGATTCAAG
TGATCCTCTTGTTGTTGCCGCAAGTATAATTGGGATTGTGCACTTGATATTGTGGATTATTGATCGCCTT
TTTTCCAAAAGCATTTATCGTATCTTTAAACACGGTTTAAAAAGAGGGCCTTCTACGGAAGGAGTACCAG
AGTCTATGAGGGAAGAATATCGAGAGGAACAGCAGAATGCTGTGGATGCTGACGATGATCATTTTGTCAG
CATAGAGCTAGAGTAAA
>gi|158957791|gb|CY027412.1| Influenza A virus (A/Alabama/UR06-0536/2007(H1N1)) segment 7, complete sequence
ATGAGTCTTCTAACCGAGGTCGAAACGTACGTTCTCTCTATCGTCCCGTCAGGCCCCCTCAAAGCCGAGA
TCGCACAGAGACTTGAAGATGTATTTGCTGGAAAGAATACCGATCTTGAGGCTCTCATGGAGTGGCTAAA
GACAAGACCAATCCTGTCACCTCTGACTAAGGGGATTTTAGGATTTGTGTTCACGCTCACCGTGCCCAGT
GAGCGAGGACTGCAGCGTAGACGCTTTGTCCAAAATGCCCTTAATGGGAATGGGGATCCAAATAATATGG
ACAGAGCAGTCAAACTGTATCGAAAGCTTAAGAGGGAGATAACATTCCATGGGGCCAAAGAAATAGCACT
CAGTTATTCTGCTGGTGCACTTGCCAGTTGTATGGGACTCATATACAACAGGATGGGGGCTGTGACCACC
GAATCAGCATTTGGCCTTATATGTGCAACCTGTGAACAGATTGCCGACTCCCAGCATAAGTCTCATAGGC
AAATGGTAACAACAACCAATCCATTAATAAGACATGAGAACAGAATGGTTCTGGCCAGCACTACAGCTAA

GGCTATGGAGCAAATGGCTGGATCGAGCGAACAAGCAGCTGAGGCCATGGAGGTTGCTAGTCAGGCCAGG
CAGATGGTGCAGGCAATGAGAGCCATTGGGACTCATCCTAGCTCTAGCACTGGTCTGAAAAATGATCTCC
TTGAAAATTTACAGGCCTATCAAAAACGAATGGGAGTGCAGATGCAACGATTCAAGTGATCCTCTTGTTG
TTGCCGCAAGTGTAATTGGGATTGTGCACTTGATATTGTGGATTATTGATCGCCTTTTTTCCAAAAGCAT
TTATCGTATCTTTAAACACGGTTTAAAAAGAGGGCCTTCTACGGAAGGAGTACCAGAGTCTATGAGGGAA
GAATATCGAGAGGAACAGCAGAATGCTGTGGATGCTGACGATGATCATTTTGTCAGCATAGAGCTAGAGT
AA
>gi|237688826|gb|CY040059.1| Influenza A virus (A/Taiwan/71720/2007(H1N1)) segment 7, complete sequence
GTAGATATTGAAAGATGAGTCTTCTAACCGAGGTCGAAACGTACGTTCTCTCTATCATCCCGTCAGGCCC
CCTCAAAGCCGAGATCGCACAGAGACTTGAAGATGTGTTTGCTGGAAAGAATACCGATCTTGAGGCTCTC
ATGGAGTGGCTAAAGACAAGACCAATCCTGTCACCTCTGACTAAGGGGATTTTAGGATTTGTGTTCACGC
TCACCGTGCCCAGTGAGCGAGGACTGCAGCGTAGACGCTTTGTCCAAAATGCCCTTAATGGGAATGGGGA
TCCAAATAATATGGACAGAGCAGTCAAACTTTATCGAAAGCTTAAGAGGGAGATAACATTCCATGGGGCC
AAAGAAATAGCACTCAGTTATTCTGCTGGTGCACTTGCCAGTTGTATGGGACTCATATACAACAGGATGG
GGGCTGTGACCACCGAATCAGCATTTGGCCTTATATGTGCAACCTGTGAACAGATTGCCGACTCCCAGCA
TAAGTCTCACAGGCAAATGGTAACAACAACCAATCCATTAATAAGACATGAGAACAGAATGGTTCTGGCC
AGCACTACAGCTAAGGCTATGGAGCAAATGGCTGGATCGAGCGAACAAGCAGCTGAGGCCATGGAGGTTG
CTAGTCAGGCCAGGCAGATGGTGCAGGCAATGAGAGCCATTGGGACTCATCCTAGCTCTAGCACTGGTCT
GAAAAATGATCTCCTTGAAAATTTACAGGCCTATCAGAAACGAATGGGGGTGCAGATGCAACGATTCAAG
TGATCCTCTTGTTGTTGCCGCAAGTATAATTGGGATTGTGCACTTGATATTGTGGATTATTGATCGCCTT
TTTTCCAAAAGCATTTATCGTATCTTTAAACACGGTTTAAAAAGAGGGCCTTCTACGGAAGGAGTACCAG
AGTCTATGAGGGAAGAATATCGAGAGGAACAGCAGAATGCTGTGGATGCTGACGATGATCATTTTGTCAG
CATAGAGCTAGAGTAAAAAA
>gi|237689284|gb|CY040251.1| Influenza A virus (A/Managua/3153.01/2008(H1N1)) segment 7, complete sequence
ATGAGTCTTCTAACCGAGGTCGAAACGTACGTTCTCTCTATCATCCCGTCAGGCCCCCTCAAAGCCGAGA
TCGCACAGAGACTTGAAGATGTATTTGCTGGAAAGAATACCGATCTTGAGGCTCTCATGGAGTGGCTAAA
GACAAGACCAATCCTGTCACCTCTGACTAAGGGGATTTTAGGATTTGTGTTCACGCTCACCGTGCCCAGT
GAGCGAGGACTGCAGCGTAGACGCTTTGTCCAAAATGCCCTTAATGGGAATGGGGATCCAAATAATATGG
ACAGAGCAGTCAAACTTTATCGAAAGCTTAAGAGGGAGATAACATTCCATGGGGCCAAAGAAATAGCACT
CAGTTATTCTGCTGGTGCACTTGCCAGTTGTATGGGACTCATATACAACAGGATGGGGGCTGTGACCACC
GAATCAGCATTTGGCCTTATATGTGCAACCTGTGAACAGATTGCCGACTCCCAGCATAAGTCTCACAGGC
AAATGGTAACAACAACCAATCCATTAATAAGACATGAGAACAGAATGGTTCTGGCCAGCACTACAGCTAA
GGCTATGGAGCAAATGGCTGGATCGAGCGAACAAGCAGCTGAGGCCATGGAGGTTGCTAGTCAGGCCAGG
CAGATGGTGCAGGCAATGAGAGCCATTGGGACTCATCCTAGCTCTAGCACTGGTCTGAAAAATGATCTCC
TTGAAAATTTACAGGCCTATCAGAAACGAATGGGGGTGCAGATGCAACGATTCAAGTGATCCTCTTGTTG
TTGCCGCAAGTATAATTGGGATTGTGCACTTGATATTGTGGATTATTGATCGCCTTTTTTCCAAAAGCAT
TTATCGTATCTTTAAACACGGTTTAAAAAGAGGACCTTCTACGGAAGGAGTACCAGAGTCTATGAGGGAA
GAATATCGAGAGGAACAGCAGAATGCTGTGGATGCTGACGATGATCAGTTTGTCAGCATAGAGCTAGAGT
AAAAAA
>gi|224020931|gb|CY037328.1| Influenza A virus (A/Washington/AF06/2007(H1N1)) segment 7, complete sequence
GTAGATATTGAAAGATGAGTCTTCTAACCGAGGTCGAAACGTACGTTCTCTCTATCATCCCGTCAGGCCC

CCTCAAAGCCGAGATCGCACAGAGACTTGAAGATGTATTTGCTGGAAAGAATACCGATCTTGAGGCTCTC
ATGGAGTGGCTAAAGACAAGACCAATCCTGTCACCTCTGACTAAGGGGATTTTAGGATTTGTGTTCACGC
TCACCGTGCCCAGTGAGCGAGGACTGCAGCGTAGACGCTTTGTCCAAAATGCCCTTAATGGGAATGGGGA
TCCAAATAATATGGACAGAGCAGTCAAACTTTATCGAAAGCTTAAGAGGGAGATAACATTCCATGGGGCC
AAAGAAATAGCACTCAGTTATTCTGCTGGTGCACTTGCCAGTTGTATGGGACTCATATACAACAGGATGG
GGGCTGTGACCACCGAATCAGCATTTGGCCTTATATGTGCAACCTGTGAACAGATTGCCGACTCCCAGCA
TAAGTCTCACAGGCAAATGGTAACAACAACCAATCCATTAATAAGACATGAGAACAGAATGGTTCTGGCC
AGCACTACAGCTAAGGCTATGGAGCAAATGGCTGGATCGAGCGAACAAGCAGCTGAGGCCATGGAGGTTG
CTAGTCAGGCCAGGCAGATGGTGCAGGCAATGAGAGCCATTGGGACTCATCCTAGCTCTAGCACTGGTCT
GAAAAATGATCTCCTTGAAAATTTACAGGCCTATCAGAAACGAATGGGGGTGCAGATGCAACGATTCAAG
TGATCCTCTTGTTGTTGCCGCAAGTATAATTGGGATTGTGCACTTGATATTGTGGATTATTGATCGCCTT
TTTTCCAAAAGCATTTATCGTATCTTTAAACACGGTTTAAAAAGAGGGCCTTCTACGGAAGGAGTACCAG
AGTCTATGAGGGAAGAATATCGAGAGGAACAGCAGAATGCTGTGGATGCTGACGATGATCATTTTGTCAG
CATAGAGCTAGAGTAAAAAA
>gi|224021236|gb|CY037336.1| Influenza A virus (A/Japan/AF07/2008(H1N1)) segment 7, complete sequence
GTAGATATTGAAAGATGAGTCTTCTAACCGAGGTCGAAACGTACGTTCTCTCTATCATCCCGTCAGGCCC
CCTCAAAGCCGAGATCGCACAGAGACTTGAAGATGTATTTGCTGGAAAGAATACCGATCTTGAGGCTCTC
ATGGAGTGGCTAAAGACAAGACCAATCCTGTCACCTCTGACTAAGGGGATTTTAGGATTTGTGTTCACGC
TCACCGTGCCCAGTGAGCGAGGACTGCAGCGTAGACGCTTTGTCCAAAATGCCCTTAATGGGAATGGGGA
TCCAAATAATATGGACAGAGCAGTCAAACTTTATCGAAAGCTTAAGAGGGAGATAACATTCCATGGGGCC
AAAGAAATAGCACTCAGTTATTCTGCTGGTGCACTTGCCAGTTGTATGGGACTCATATACAACAGGATGG
GGGCTGTGACCACCGAATCAGCATTTGGCCTTATATGTGCAACCTGTGAACAGATTGCCGACTCCCAGCA
TAAGTCTCACAGACAAATGGTAACAACAACCAATCCATTAATAAGACATGAGAACAGAATGGTTCTGGCC
AGCACTACAGCTAAGGCTATGGAGCAAATGGCTGGATCGAGCGAACAAGCAGCTGAGGCCATGGAGGTTG
CTAGTCAGGCCAGGCAGATGGTGCAGGCAATGAGAGCCATTGGGACTCACCCTAGCTCTAGCACTGGTCT
AAAAAATGATCTCCTTGAAAATTTACAGGCCTATCAGAAACGAATGGGGGTGCAGATGCAACGATTCAAG
TGATCCTCTTGTTGTTGCCGCAAGTATAATTGGGATTGTGCACTTGATATTGTGGATTATTGATCGCCTT
TTTTCCAAAAGCATTTATCGTATCTTTAAACACGGTTTAAAAAGAGGGCCTTCTACGGAAGGAGTACCAG
AGTCTATGAGGGAAGAATATCGAGAGGAACAGCAGAATGCTGTGGATGCTGACGATGATCATTTTGTCAG
CATAGAGCTAGAGTAAAAAA
>gi|212381605|gb|FJ445070.1| Influenza A virus (A/England/26/2008(H1N1)) segment 7 matrix protein 2 (M2) and matrix protein 1 (M1) genes, complete cds
ATGAGTCTTCTAACCGAGGTCGAAACGTACGTTCTCTCTATCATCCCGTCAGGCCCCCTCAAAGCCGAGA
TCGCACAGAGACTTGAAGATGTATTTGCTGGAAAGAATACCGATCTTGAGGCTCTCATGGAGTGGCTAAA
GACAAGACCAATCCTGTCACCTCTGACTAAGGGGATTTTAGGATTTGTGTTCACGCTCACCGTGCCCAGT
GAGCGAGGACTGCAGCGTAGACGCTTTGTCCAAAATGCCCTTAATGGGAATGGGGATCCAAATAATATGG
ACAGAGCAGTCAAACTTTATCGAAAGCTTAAGAGGGAGATAACATTCCATGGGGCCAAAGAAATAGCACT
CAGTTATTCTGCTGGTGCACTTGCCAGTTGTATGGGACTCATATACAACAGGATGGGGGCTGTGACCACC
GAATCAGCATTTGGCCTTATATGTGCAACCTGTGAACAGATTGCCGACTCCCAGCATAAGTCTCACAGGC
AAATGGTAACAACAACCAATCCATTAATAAGACATGAGAACAGAATGGTTCTGGCCAGCACTACAGCTAA
GGCTATGGAGCAAATGGCTGGATCGAGCGAACAAGCAGCTGAGGCCATGGAGGTTGCTAGTCAGGCCAGG
CAGATGGTGCAGGCAATGAGAGCCATTGGGACTCATCCTAGCTCTAGCACTGGTCTGAAAAATGATCTCC
TTGAAAATTTACAGGCCTATCAGAAACGAATGGGGGTGCAGATGCAACGATTCAAGTGATCCTCTTGTTG

TTGCCGCAAGTATAATTGGGATTGTGCACTTGATATTGTGGATTATTGATCGCCTTTTTTCCAAAAGCAT
TTATCGTATCTTTAAACACGGTTTAAAAAGAGGGCCTTCTACGGAAGGAGTACCAGAGTCTATGAGGGAA
GAATATCGAGAGGAACAGCAGAATGCTGTGGATGCTGACGATGATCATTTTGTCAGCATAGAGCTAGAGT
AAAAA
>gi|224027206|gb|CY037680.1| Influenza A virus (A/Florida/UR07-0022/2008(H1N1)) segment 7, complete sequence
AGATGAGTCTTCTAACCGAGGTCGAAACGTACGTTCTCTCTATCATCCCGTCAGGCCCCCTCAAAGCCGA
GATCGCACAGAGACTTGAAGATGTATTTGCTGGAAAGAATACCGATCTTGAGGCTCTCATGGAGTGGCTA
AAGACAAGACCAATCCTGTCACCTCTGACTAAGGGGATTTTAGGATTTGTGTTCACGCTCACCGTGCCCA
GTGAGCGAGGACTGCAGCGTAGACGCTTTGTCCAAAATGCCCTCAATGGGAATGGGGATCCAAATAATAT
GGACAGAGCAGTCAAACTTTATCGAAAGCTTAAGAGGGAGATAACATTCCATGGGGCCAAAGAAATAGCA
CTCAGTTATTCTGCTGGTGCACTTGCCAGTTGTATGGGACTCATATACAACAGGATGGGGGCTGTGACCA
CCGAATCAGCATTTGGCCTTATATGTGCAACCTGTGAACAGATTGCCGACTCCCAGCATAAGTCTCACAG
GCAAATGGTAACAACAACCAATCCATTAATAAGACATGAGAACAGAATGGTTCTGGCCAGCACTACAGCT
AAGGCTATGGAGCAAATGGCTGGATCGAGCGAACAAGCAGCTGAGGCCATGGAGGTTGCTAGTCAGGCCA
GGCAGATGGTGCAGGCAATGAGAGCCATTGGGACTCATCCTAGTTCTAGCACTGGTCTGAAAAATGATCT
CCTTGAAAATTTACAGGCCTATCAGAAACGAATGGGGGTGCAGATGCAACGATTCAAGTGATCCTCTTGT
TGTTGCCGCAAGTATAATTGGGATTGTGCACTTGATATTGTGGATTATTGATCGCCTTTTTTCCAAAAGC
ATTTATCGTATCTTTAAACACGGTTTAAAAAGAGGGCCTTCTACGGAAGGAGTACCAGAGTCTATGAGGG
AAGAATATCGAGAGGAACAGCAGAATGCTGTGGATGCTGACGATGATCATTTTGTCAGCATAGAGCTAGA
GTAAAAAA
>gi|296240581|gb|CY063607.1| Influenza A virus (A/Aalborg/INS133/2009(H1N1)) segment 7, complete sequence
TAAAGATGAGTCTTCTAACCGAGGTCGAAACGTACGTTCTTTCTATCATCCCGTCAGGCCCCCTCAAAGC
CGAGATCGCGCAGAGACTGGAAAGTGTCTTTGCAGGAAAGAACACAGATCTTGAGGCTCTCATGGAATGG
CTAAAGACAAGACCAATCTTGTCACCTCTGACTAAGGGAATTTTAGGATTTGTGTTCACGCTCACCGTGC
CCAGTGAGCGAGGACTGCAGCGTAGACGCTTTGTCCAAAATGCCCTAAATGGGAATGGGGACCCGAACAA
CATGGATAGAGCAGTTAAACTATACAAGAAGCTCAAAAGAGAAATAACGTTCCATGGGGCCAAGGAGGTG
TCACTAAGCTATTCAACTGGTGCACTTGCCAGTTGCATGGGCCTCATATACAACAGGATGGGAACAGTGA
CCACAGAAGCTGCTTTTGGTCTAGTGTGTGCCACTTGTGAACAGATTGCTGATTCACAGCATCGGTCTCA
CAGACAAATGGCTACTACCACCAATCCACTAATCAGGCATGAAAACAGAATGGTGCTGGCTAGCACTACG
GCAAAGGCTATGGAACAGATGGCTGGATCGAGTGAACAGGCAGCAGAGGCCATGGAGGTTGCTAATCAGA
CTAGGCAGATGGTACATGCAATGAGAACTATTGGGACTCATCCTAGCTCCAGTGCTGGTCTGAAAGATGA
CCTTCTTGAAAATTTGCAGGCCTACCAGAAGCGAATGGGAGTGCAGATGCAGCGATTCAAGTGATCCTCT
CATCATTGCAGCAAATATCATTGGGATCTTGCACCTGATATTGTGGATTACTGATCGTCTTTTTTTCAAA
TGTATTTATCGTCGCTTTAAATACGGTTTGAAAAGAGGGCCTTCTACGGAAGGAGTGCCTGAGTCCATGA
GGGAAGAATATCAACAGGAACAGCAGAGTGCTGTGGATGTTGACGATGGTCATTTTGTCAACATAGAGCT
AGAGTAA
>gi|296240563|gb|CY063599.1| Influenza A virus (A/Bonn/INS128/2009(H1N1)) segment 7, complete sequence
TAAAGATGAGTCTTCTAACCGAGGTTGAAACGTACGTTCTTTCTATCATCCCGTCAGGCCCCCTCAAAGC
CGAGATCGCGCAGAGACTGGAAAGTGTCTTTGCAGGAAAGAACACAGATCTTGAGGCTCTCATGGAATGG
CTAAAGACAAGACCAATCTTGTCACCTCTGACTAAGGGAATTTTAGGATTTGTGTTCACGCTCACCGTGC
CCAGTGAGCGAGGACTGCAGCGTAGACGCTTTGTCCAAAATGCCCTAAATGGGAATGGGGACCCGAACAA

CATGGATAGAGCAGTTAAACTATACAAGAAGCTCAAAAGAGAAATAACGTTCCATGGGGCCAAGGAGGTG
TCACTAAGCTATTCAACTGGTGCACTTGCCAGTTGCATGGGCCTCATATACAACAGGATGGGAACAGTGA
CCACAGAAGCTGCTTTTGGTCTAGTGTGTGCCACTTGTGAACAGATTGCTGATTCACAGCATCGGTCTCA
CAGACAAATGGCTACTACCACCAATCCACTAATCAGGCATGAAAACAGAATGGTGCTGGCTAGCACTACG
GCAAAGGCTATGGAACAGATGGCTGGATCGAGTGAACAGGCAGCAGAGGCCATGGAGGTTGCTAATCAGA
CTAGGCAGATGGTACATGCAATGAGAACTATTGGGACTCATCCTAGCTCCAGTGCTGGTCTGAAAGATGA
CCTTCTTGAAAATTTGCAGGCCTACCAGAAGCGAATGGGAGTGCAGATGCAGCGATTCAAGTGATCCTCT
CGTCATTGCAGCAAATATCATTGGGATCTTGCACCTGATATTGTGGATTACTGATCGTCTTTTTTTCAAA
TGTATTTATCGTCGCTTTAAATACGGTTTGAAAAGAGGGCCTTCTACGGAAGGAGTGCCTGAGTCCATGA
GGGAAGAATATCAACAGGAACAGCAGAGTGCTGTGGATGTTGACGATGGTCATTTTGTCAACATAGAGCT
AGAGTAA
>gi|296240311|gb|CY063487.1| Influenza A virus (A/Boston/110/2009(H1N1)) segment 7, complete sequence
TAAAGATGAGTCTTCTAACCGAGGTCGAAACGTACGTTCTTTCTATCATCCCGTCAGGCCCCCTCAAAGC
CGAGATCGCGCAGAGACTGGAAAGTGTCTTTGCAGGAAAGAACACAGATCTTGAGGCTCTCATGGAATGG
CTAAAGACAAGACCAATCTTGTCACCTCTGACTAAGGGAATTTTAGGATTTGTGTTCACGCTCACCGTGC
CCAGTGAGCGAGGACTGCAGCGTAGACGCTTTGTCCAAAATGCCCTAAATGGGAATGGGGACCCGAACAA
CATGGATAGAGCAGTTAAACTATACAAGAAGCTCAAAAGAGAAATAACGTTCCATGGGGCCAAGGAGGTG
TCACTAAGCTATTCAACTGGTGCACTTGCCAGTTGCATGGGCCTCATATACAACAGGATGGGAACAGTGA
CCACAGAAGCTGCTTTTGGTCTAGTGTGTGCCACTTGTGAACAGATTGCTGATTCACAGCATCGGTCTCA
CAGACAGATGGCTACTACCACCAATCCACTAATCAGGCATGAAAACAGAATGGTGCTGGCTAGCACTACG
GCAAAGGCTATGGAACAGATGGCTGGATCGAGTGAACAGGCAGCGGAGGCCATGGAGGTTGCTAATCAGA
CTAGGCAGATGGTACATGCAATGAGAACTATTGGGACTCATCCTAGCTCCAGTGCTGGTCTGAAAGATGA
CCTTCTTGAAAATTTGCAGGCCTACCAGAAGCGAATGGGAGTGCAGATGCAGCGATTCAAGTGATCCTCT
CGTCATTGCAGCAAATATCATTGGGATCTTGCACCTGATATTGTGGATTACTGATCGTCTTTTTTTCAAA
TGTATTTATCGTCGCTTTAAATACGGTTTGAAAAGAGGGCCTTCTACGGAAGGAGTGCCTGAGTCCATGA
GGGAAGAATATCAACAGGAACAGCAGAGTGCTGTGGATGTTGACGATGGTCATTTTGTCAACATAGAGCT
AGAGTAA
>gi|296240617|gb|CY063623.1| Influenza A virus (A/New York/INS150/2009(H1N1)) segment 7, complete sequence
ATGAGTCTTCTAACCGAGGTCGAAACGTACGTTCTTTCTATCATCCCGTCAGGCCCCCTCAAAGCCGAGA
TCGCGCAGAGACTGGAAAGTGTCTTTGCAGGAAAGAACACAGATCTTGAGGCTCTCATGGAATGGCTAAA
GACAAGACCAATCTTGTCACCTCTGACTAAGGGAATTTTAGGATTTGTGTTCACGCTCACCGTGCCCAGT
GAGCGAGGACTGCAGCGTAGACGCTTTGTCCAAAATGCCCTAAATGGGAATGGGGACCCGAACAACATGG
ATAGAGCAGTTAAACTATACAAGAAGCTCAAAAGAGAAATAACGTTCCATGGGGCCAAGGAGGTGTCACT
AAGCTATTCAACTGGTGCACTTGCCAGTTGCATGGGCCTCATATACAACAGGATGGGAACAGTGACCACA
GAAGCTGCTTTTGGTCTAGTGTGTGCCACTTGTGAACAGATTGCTGATTCACAGCATCGGTCTCACAGAC
AAATGGCTACTACCACCAATCCACTAATCAGGCATGAAAACAGAATGGTGCTGGCTAGCACTACGGCAAA
GGCTATGGAACAGATGGCTGGATCGAGTGAACAGGCAGCAGAGGCCATGGAGGTTGCTAATCAGACTAGG
CAGATGGTACATGCAATGAGAACTATTGGGACTCATCCTAGCTCCAGTGCTGGTCTGAAAGATGACCTTC
TTGAAAATTTGCAGGCCTACCAGAAGCGAATGGGAGTGCAGATGCAACGATTCAAGTGATCCTCTCGTCA
TTGCAGCAAATATCATTGGGATCTTGCACCTGATATTGTGGATTACTGATCGTCTTTTTTTCAAATGTAT
TTATCGTCGCTTTAAATACGGTTTGAAAAGAGGGCCTTCTACGGAAGGAGTGCCTGAGTCCATGAGGGAA
GAATATCAACAGGAACAGCAGAGTGCTGTGGATGTTGACGATGGTCATTTTGTCAACATAGAGCTAGAGT

AA
>gi|296240545|gb|CY063591.1| Influenza A virus (A/Athens/INS122/2009(H1N1)) segment 7, complete sequence
TAAAGATGAGTCTTCTAACCGAGGTCGAAACGTACGTTCTTTCTATCATCCCGTCAGGCCCCCTCAAAGC
CGAGATCGCGCAGAGACTGGAAAGTGTCTTTGCAGGAAAGAACACAGATCTTGAGGCTCTCATGGAATGG
CTAAAGACAAGACCAATCTTGTCACCTCTGACTAAGGGAATTTTAGGATTTGTGTTCACGCTCACCGTGC
CCAGTGAGCGAGGACTGCAGCGTAGACGCTTTGTCCAAAATGCCCTAAATGGGAATGGGGACCCAAACAA
CATGGATAGAGCAGTTAAACTATACAAGAAGCTCAAAAGAGAAATAACGTTCCATGGGGCCAAGGAGGTG
TCACTAAGCTATTCAACTGGTGCACTTGCCAGTTGCATGGGCCTCATATACAACAGAATGGGAACAGTGA
CCACAGAAGCTGCTTTTGGTCTAGTGTGTGCCACTTGTGAACAGATTGCTGATTCACAGCATCGGTCTCA
CAGACAAATGGCTACTACCACCAATCCACTAATCAGGCATGAAAACAGAATGGTGCTGGCTAGCACTACG
GCAAAGGCTATGGAACAGATGGCTGGATCGAGTGAACAGGCAGCAGAGGCCATGGAGGTTGCTAATCAGA
CTAGGCAGATGGTACATGCAATGAGAACTATTGGGACTCATCCTAGCTCCAGTGCTGGTCTGAAAGATGA
CCTTCTTGAAAATTTGCAGGCCTACCAGAAGCGAATGGGAGTGCAGATGCAGCGATTCAAGTGATCCTCT
CGTCATTGCAGCAAATATCATTGGGATCTTGCACCTGATATTGTGGATTACTGATCGTCTTTTTTTCAAA
TGTATTTATCGTCGCTTTAAATACGGTTTGAAAAGAGGGCCTTCTACGGAAGGAGTGCCTGAGTCCATGA
GGGAAGAATATCAACAGGAACAGCAGAGTGCTGTGGATGTTGACGATGGTCATTTTGTCAACATAGAGCT
AGAGTAA>gi|347087891|gb|CY098564.1| Influenza A virus (A/Bangkok/INS520/2010(H1N1)) matrix protein 2 (M2) and matrix protein 1 (M1) genes, complete cds
TAAAGATGAGTCTTCTAACCGAGGTCGAAACGTACGTTCTTTCTATCATCCCGTCAGGCCCCCTCAAAGC
CGAGATCGCGCAGAGACTGGAAAGTGTCTTTGCAGGAAAGAACACAGATCTTGAGGCTCTCATGGAATGG
CTAAAGACAAGACCAATCTTGTCACCTCTGACTAAGGGAATTTTAGGATTTGTGTTCACGCTCACCGTGC
CCAGTGAGCGAGGACTGCAGCGTAGACGCTTTATCCAAAATGCCCTAAATGGGAATGGGGACCCGAACAA
CATGGATAGAGCAGTTAAACTATACAAGAAGCTCAAAAGAGAAATAACGTTCCATGGGGCCAAGGAGGTG
TCACTAAGCTATTCAACTGGTGCACTTGCCAGTTGCATGGGCCTCATATACAACAGGATGGGAACAGTGA
CCACAGAAGCTGCTTTTGGTCTAGTGTGTGCCACTTGTGAACAGATTGCTGATTCACAGCATCGGTCTCA
CAGACAAATGGCTACTACCACCAATCCACTAATCAGGCATGAAAACAGAATGGTGCTGGCTAGCACTACG
GCAAAGGCTATGGAACAGATGGCTGGATCGAGTGAACAGGCAGCAGAGGCCATGGAGGTTGCTAATCAGA
CTAGGCAGATGGTACATGCAATGAGAACTATTGGGACTCATCCTAGCTCCAGTGCTGGTCTGAAAGATGA
CCTTCTTGAAAATTTGCAGGCCTACCAGAAGCGAATGGGAGTGCAGATGCAGCGATTCAAGTGATCCTCT
CGTCATTGCAGCAAATATCATTGGGATCTTGCACCTGATATTGTGGATTACTGATCGTCTTTTTTTCAAA
TGTATTTATCGTCGCTTTAAATACGGTTTGAAAAGAGGGCCTTCTACGGAAGGAGTGCCTGAGTCCATGA
GGGAAGAATATCAACAGGAACAGCAGAGTGCTGTGGATGTTGACGATGGTCATTTTGTCAACATAGAGCT
AGAGTAA
>gi|339855094|gb|CY093206.1| Influenza A virus (A/Chile/115/2010(H1N1)) matrix protein 2 (M2) and matrix protein 1 (M1) genes, complete cds
TAAAGATGAGTCTTCTAACCGAGGTCGAAACGTACGTTCTTTCTATCATCCCGTCAGGCCCCCTCAAAGC
CGAGATCGCGCAGAGACTGGAAAGTGTCTTTGCAGGAAAGAACACAGATCTTGAGGCTCTCATGGAATGG
CTAAAGACAAGACCAATCTTGTCACCTCTGACTAAGGGAATTTTAGGATTTGTGTTCACGCTCACCGTGC
CCAGTGAGCGAGGACTGCAGCGTAGACGCTATGTCCAAAATGCCCTAAATGGGAATGGGGACCCGAACAA
CATGGATAGAGCAGTTAAACTATACAAGAAACTCAAAAGAGAAATAACGTTCCATGGGGCCAAGGAGGTG
TCACTAAGCTATTCAACTGGTGCACTTGCCAGTTGCATGGGCCTCATATACAACAGGATGGGAACAGTGA
CCACAGAAGCTGCTTTTGGTCTAGTGTGTGCCACTTGTGAACAGATTGCTGATTCACAGCATCGGTCTCA
CAGACAAATGGCTACTACCACCAATCCACTAATCAGGCATGAAAACAGAATGGTGCTGGCTAGCACTACG

GCAAAGGCTATGGAACAGATGGCTGGATCGAGTGAACAGGCAGCAGAGGCCATGGAGGTTGCTAATCAGA
CTAGGCAGATGGTGCATGCAATGAGAACTATTGGGACTCATCCTAGCTCCAGTGCTGGTCTGAAAGATGA
CCTTCTTGAAAATTTGCAGGCCTACCAGAAGCGAATGGGAGTGCAGATGCAGCGATTCAAGTGATCCTCT
CTTCATTGCAGCAAATATCATTGGGATCTTGCACCTGATATTGTGGATTACTGATCGTCTTTTTTTCAAA
TGTATTTATCGTCGCTTTAAATACGGTTTGAAAAGAGGGCCTTCTACGGAAGGAGTGCCTGAGTCCATGA
GGGAAGAATATCAACAGGAACAGCAGAGTGCTGTGGATGTTGACGATGGTCATTTTGTCAACATAGAGCT
AGAGTAA
>gi|344995029|gb|CY098099.1| Influenza A virus (A/Khon Kaen/INS461/2010(H1N1)) matrix protein 2 (M2) and matrix protein 1 (M1) genes, complete cds
TAAAGATGAGTCTTCTAACCGAGGTCGAAACGTACGTTCTTTCTATCATCCCGTCAGGCCCCCTCAAAGC
CGAGATCGCGCAGAGACTGGAAAGTGTCTTTGCAGGAAAGAACACAGATCTTGAGGCTCTCATGGAATGG
CTAAAGACAAGACCAATCTTGTCACCTCTGACTAAGGGAATTTTAGGATTTGTGTTCACGCTCACCGTGC
CCAGTGAGCGAGGACTGCAGCGTAGACGCTTTGTCCAAAATGCCCTAAATGGGAATGGGGACCCGAACAA
CATGGATAGAGCAGTTAAACTATACAAGAAGCTCAAAAGAGAAATAACGTTCCATGGGGCCAAGGAGGTG
TCACTAAGCTATTCAACTGGTGCACTTGCCAGTTGCATGGGCCTCATATACAACAGGATGGGAACAGTGA
CCACAGAAGCTGCTTTTGGTCTAGTGTGTGCCACTTGTGAACAGATTGCTGATTCACAGCATCGGTCTCA
CAGACAAATGGCTACTACCACCAATCCACTAATCAGGCATGAAAACAGAATGGTGCTGGCTAGCACTACG
GCAAAGGCTATGGAACAGATGGCTGGATCGAGTGAACAGGCAGCAGAGGCCATGGAGGTTGCTAACCAG
A
CTAGGCAGATGGTACATGCAATGAGAACTATTGGGACTCATCCTAGCTCCAGTGCTGGTCTGAAAGATGA
CCTTCTTGAAAATTTGCAGGCCTACCAGAAGCGAATGGGAGTGCAGATGCAGCGATTCAAGTGATCCTCT
CGTCATTGCAGCAAATATCATTGGGATCTTGCACCTGATATTGTGGATTACTGATCGTCTTTTTTTCAAA
TGTATTTATCGTCGCTTTAAATACGGTTTGAAAAGAGGGCCTTCTACGGAAGGAGTGCCTGAGTCCATGA
GGGAAGAATATCAACAGGAACAGCAGAGTGCTGTGGATGTTGACGATGGTCATTTTGTCAACATAGAGCT
AGAGTAA
>gi|339840968|gb|CY093150.1| Influenza A virus (A/Sydney/DD3-23/2010(H1N1)) matrix protein 2 (M2) and matrix protein 1 (M1) genes, complete cds
TAAAGATGAGTCTTCTAACCGAGGTCGAAACGTACGTTCTTTCTATCATCCCGTCAGGCCCCCTCAAAGC
CGAGATCGCGCAGAGACTGGAAAGTGTCTTTGCAGGAAAGAACACAGATCTTGAGGCTCTCATGGAATGG
CTAAAGACAAGACCAATCTTGTCACCTCTGACTAAGGGAATTTTAGGATTTGTGTTCACGCTCACCGTGC
CCAGTGAGCGAGGACTGCAGCGTAGACGCTTTATCCAAAATGCCCTAAATGGGAATGGGGACCCGAACAA
CATGGATAGAGCAGTTAAACTATACAAGAAGCTCAAAAGAGAAATAACGTTCCATGGGGCCAAGGAGGTG
TCACTAAGCTATTCAACTGGTGCACTTGCCAGTTGCATGGGCCTCATATACAACAGGATGGGAACAGTGA
CCACAGAAGCTGCTTTTGGTCTAGTGTGTGCCACTTGTGAACAGATTGCTGATTCACAGCATCGGTCTCA
CAGACAAATGGCTACTACCACCAATCCACTAATCAGGCATGAAAACAGAATGGTGCTGGCTAGCACTACG
GCAAAGGCTATGGAACAGATGGCTGGATCGAGTGAACAGGCAGCAGAGGCCATGGAGGTTGCTAATCAGA
CTAGGCAGATGGTACATGCAATGAGAACTATTGGGACTCATCCTAGCTCCAGTGCTGGTCTGAAAGATGA
CCTTCTTGAAAATTTGCAGGCCTACCAGAAGCGAATGGGAGTGCAGATGCAGCGATTCAAGTGATCCTCT
CGTCATTGCAGCAAATATCATTGGGATCTTGCACCTGATATTGTGGATTACTGATCGTCTTTTTTTCAAA
TGTATTTATCGTCGCTTTAAATACGGTTTGAAAAGAGGGCCTTCTACGGAAGGAGTGCCTGAGTCCATGA
GGGAAGAATATCAACAGGAACAGCAGAGTGCTGTGGATGTTGACGATGGTCATTTTGTCAACATAGAGCT
AGAGTAA
>gi|334134017|gb|CY090862.1| Influenza A virus (A/Netherlands/2631c/2010(H1N1)) matrix protein 2 (M2) and matrix protein 1 (M1) genes, complete cds

AGATATTTAAAGATGAGTCTTCTAACCGAGGTCGAAACGTACGTTCTTTCTATCATCCCGTCAGGCCCCC
TCAAAGCCGAGATCGCGCAGAGACTGGAAAGTGTCTTTGCGGGAAAGAACACAGATCTTGAGGCTCTCAT
GGAATGGCTAAAGACAAGACCAATCTTGTCACCTCTGACTAAGGGAATTTTAGGATTTGTGTTCACGCTC
ACCGTGCCCAGTGAGCGAGGACTGCAGCGTAGACGCTTTGTCCAAAATGCCCTAAATGGGAATGGGGACC
CGAACAACATGGATAGAGCAGTTAAACTATACAAGAAGCTCAAAAGAGAAATAACGTTCCATGGGGCCAA
GGAGGTGTCACTAAGCTATTCAACTGGTGCACTTGCCAGTTGCATGGGCCTCATATACAACAGGATGGGA
ACAGTGACCACAGAAGCTGCTTTTGGTCTAGTGTGTGCCACTTGTGAACAGATTGCTGATTCACAGCATC
GGTCTCACAGACAAATGGCTACTACCACCAATCCACTAATCAGGCATGAAAACAGAATGGTGCTGGCTAG
CACTACGGCAAAGGCTATGGAACAGATGGCTGGATCGAGTGAACAGGCAGCAGAGGCCATGGAGGTTGCT
AATCAGACTAGGCAGATGGTACATGCAATGAGAACTATTGGGACTCATCCTAGCTCCAGTGCTGGTCTGA
AAGATGACCTTCTTGAAAATTTGCAGGCCTACCAGAAGCGAATGGGAGTGCAGATGCAGCGATTCAAGTG
ATCCTCTCGTCATTGCAGCAAATATCATTGGGATCTTGCACCTGATATTGTGGATTACTGATCGTCTTTT
TTTCAAATGTATTTATCGTCGCTTTAAATACGGTTTGAAAAGAGGGCCTTCTACGGAAGGAGTGCCTGAG
TCCATGAGGGAAGAATATCAACAGGAACAGCAGAGTGCTGTGGATGTTGACGATGGTCATTTTGTCAACA
TAGAGCTAGAGTAAAAAAC
>gi|334127414|gb|CY090838.1| Influenza A virus (A/Managua/0305_10/2010(H1N1)) matrix protein 2 (M2) and matrix protein 1 (M1) genes, complete cds
TAGATATTAAAGATGAGTCTTCTAACCGAGGTCGAAACGTACGTTCTTTCTATCATCCCGTCAGGCCCCC
TCAAAGCCGAGATCGCGCAGAGACTGGAAAGTGTCTTTGCAGGAAAGAACACAGATCTTGAGGCTCTCAT
GGAATGGCTAAAGACAAGACCAATCTTGTCACCTCTGACTAAGGGAATTTTAGGATTTGTGTTCACGCTC
ACCGTGCCCAGTGAGCGAGGACTGCAGCGTAGACGCTTTGTCCAAAATGCCCTAAATGGGAATGGGGACC
CGAATAACATGGATAGAGCAGTTAAACTATACAAGAAGCTCAAAAGAGAAATAACATTCCATGGGGCCAA
GGAGGTGTCACTAAGCTATTCAACTGGTGCACTTGCCAGTTGCATGGGCCTCATATACAACAGGATGGGA
ACAGTGACCACAGAAGCTGCTTTTGGTCTAGTGTGTGCCACTTGTGAACAGATTGCTGATTCACAGCATC
GGTCTCACAGACAGATGGCTACTACCACCAATCCACTAATCAGGCATGAAAACAGAATGGTGCTGGCTAG
TACTACGGCAAAGGCTATGGAACAGATGGCTGGATCGAGTGAACAGGCAGCGGAGGCCATGGAGGTTGCT
AATCAGACTAGGCAGATGGTACATGCAATGAGAACTATTGGGACTAATCCTAGCTCCAGTGCTGGTCTGA
AAGATGACCTTCTTGAAAATTTGCAGGCCTACCAGAAGCGAATGGGAGTGCAGATGCAGCGATTCAAGTG
ATCCTCTCGTCATTGCAGCAAATATCATTGGGATCTTGCACCTGATATTGTGGATTACTGATCGTCTTTT
TTTCAAATGTATTTATCGTCGCTTTAAATACGGTTTGAAAAGAGGGCCTTCTACGGAAGGAGTGCCTGAG
TCCATGAGGGAAGAATATCAACAGGAACAGCAGAGTGCTGTGGATGTTGACGATGGTCATTTTGTCAACA
TAGAGCTAGAGTAAAAAACTAC
>gi|323668719|gb|CY083818.1| Influenza A virus (A/Los Angeles/INS423/2010(H1N1)) matrix protein 2 (M2) and matrix protein 1 (M1) genes, complete cds
TAAAGATGAGTCTTCTAACCGAGGTCGAAACGTACGTTCTTTCTATCATCCCGTCAGGCCCCCTCAAAGC
CGAGATCGCGCAGAGACTGGAAAGTGTCTTTGCAGGAAAGAACACAGATCTTGAGGCTCTCATGGAATGG
CTAAAGACAAGACCAATCTTGTCACCTCTGACTAAGGGAATTTTAGGATTTGTGTTCACGCTCACCGTGC
CCAGTGAGCGAGGACTGCAGCGTAGACGCTTTGTCCAAAATGCCCTAAATGGGAATGGGGACCCGAACAA
CATGGATAGAGCAGTTAAACTATACAAGAAGCTCAAAAGAGAAATAACGTTCCATGGGGCCAAGGAGGTG
TCACTAAGCTATTCAACTGGTGCACTTGCCAGTTGCATGGGCCTCATATACAACAGGATGGGAACAGTGA
CCACAGAAGCTGCTTTTGGTCTAGTGTGTGCCACTTGTGAACAGATTGCTGATTCACAGCATCGGTCTCA
CAGACAAATGGCTACTACCACCAATCCACTAATCAGGCATGAAAACAGAATGGTGCTGGCTAGCACTACG
GCAAAGGCTATGGAACAGATGGCTGGATCGAGTGAACAGGCAGCAGAGGCCATGGAGGTTGCTAATCAGA
CTAGGCAGATGGTACATGCAATGAGAACTATTGGGACTCATCCTAGCTCCAGTGCTGGTCTGAAAGATGA

CCTTCTTGAAAATTTGCAGGCCTACCAGAAGCGAATGGGAGTGCAGATGCAGCGATTCAAGTGATCCTCT
CGTCATTGCAGCAAATATCATTGGGATCTTGCACCTGATATTGTGGATTACTGATCGTCTTTTTTTCAAA
TGTATTTATCGTCGCTTTAAATACGGTTTGAAAAGAGGGCCTTCTACGGAAGGAGTGCCTGAGTCCATGA
GGGAAGAATATCAACAGGAACAGCAGAGTGCTGTGGATGTTGACGATGGTCATTTTGTCAACATAGAGCT
AGAGTAA
>gi|343174016|gb|CY096603.1| Influenza A virus (A/Cambridge/INS528/2010(H1N1)) matrix protein 2 (M2) and matrix protein 1 (M1) genes, complete cds
TAAAGATGAGTCTTCTAACCGAGGTCGAAACGTACGTTCTTTCTATCATCCCGTCAGGCCCCCTCAAAGC
CGAGATCGCGCAGAGACTGGAAAGTGTCTTTGCAGGAAAGAACACAGATCTTGAGGCTCTCATGGAATGG
CTAAAGACAAGACCAATCTTGTCACCTCTGACTAAGGGAATTTTAGGATTTGTGTTCACGCTCACCGTGC
CCAGTGAGCGAGGACTGCAGCGTAGACGCTTTGTCCAAAATGCCCTAAATGGGAATGGGGACCCGAACAA
CATGGATAGAGCAGTTAAACTATACAAGAAGCTCAAAAGAGAAATAACGTTCCATGGGGCCAAGGAGGTG
TCACTAAGCTATTCAACTGGTGCACTTGCCAGTTGCATGGGCCTCATATACAACAGGATGGGAACAGTGA
CCACAGAAGCTGCTTTTGGTCTAGTGTGTGCCACTTGTGAACAGATTGCTGATTCACAGCATCGGTCTCA
CAGACAAATGGCTACTACCACCAACCCACTAATCAGGCATGAAAACAGAATGGTGCTGGCTAGCACCACG
GCAAAGGCTATGGAACAGATGGCTGGATCGAGTGAACAGGCAGCAGAGGCCATGGAGGTTGCTAATCAGA
CTAGGCAGATGGTACATGCAATGAGAACTATTGGGACTCATCCTAGCTCCAGTGCTGGTCTGAAAGATGA
CCTTCTTGAAAATTTGCAGGCCTACCAGAAGCGAATGGGAGTGCAGATGCAGCGATTCAAGTGATCCTCT
CGTCATTGCAGCAAATATCATTGGAATCTTGCACCTGATATTGTGGATTACTGATCGTCTTTTTTTCAAA
TGTATTTATCGTCGCTTTAAATACGGTTTGAAAAGAGGGCCTTCTACGGAAGGAGTGCCTGAGTCCATGA
GGGAAGAATATCAACAGGAACAGCAGAGTGCTGTGGATGTTGACGATGGTCATTTTGTCAACATAGAGCT
AGAGTAA
>gi|343173998|gb|CY096595.1| Influenza A virus (A/District of Columbia/INS527/2010(H1N1)) matrix protein 2 (M2) and matrix protein 1 (M1) genes, complete cds
TAAAGATGAGTCTTCTAACCGAGGTCGAAACGTACGTTCTTTCTATCATCCCGTCAGGCCCCCTCAAAGC
CGAGATCGCGCAGAGACTGGAAAGTGTCTTTGCAGGAAAGAACACAGATCTTGAGGCTCTCATGGAATGG
CTAAAGACAAGACCAATCTTGTCACCTCTGACTAAGGGAATTTTAGGATTTGTGTTCACGCTCACCGTGC
CCAGTGAGCGAGGACTGCAGCGTAGACGCTTTGTCCAAAATGCCCTAAATGGGAATGGGGACCCGAACAA
CATGGATAGAGCAGTTAAACTATACAAGAAGCTCAAAAGAGAAATAACGTTCCATGGGGCCAAGGAGGTG
TCACTAAGCTATTCAACTGGTGCACTTGCCAGTTGCATGGGCCTCATATACAACAGGATGGGAACAGTGA
CCACAGAAGCTGCTTTTGGTCTAGTGTGTGCCACTTGTGAACAGATTGCTGATTCACAGCATCGGTCTCA
CAGACAAATGGCTACTACCACCAATCCACTAATCAGGCATGAAAACAGAATGGTGCTGGCTAGCACTACG
GCAAAGGCTATGGAACAGATGGCTGGATCGAGTGAGCAGGCAGCAGAGGCCATGGAGGTTGCTAATCAGA
CTAGGCAGATGGTACATGCAATGAGAACTATTGGGACTCATCCTAGCTCCAGTGCTGGTCTGAAAGATGA
CCTTCTTGAAAATTTGCAGGCCTACCAGAAGCGAATGGGAGTGCAGATGCAGCGATTCAAGTGATCCTCT
CGTCATTGCAGCAAATATCATTGGGATCTTGCACCTGATATTGTGGATTACTGATCGTCTTTTTTTCAAA
TGTATTTATCGTCGCTTTAAATACGGTTTGAAAAGAGGGCCTTCTACAGAAGGAGTGCCTGAGTCCATGA
GGGAAGAATATCAACAGGAACAGCAGAGTGCTGTGGATGTTGACGATGGTCATTTTGTCAACATAGAGCT
AGAGTAA
>gi|346642608|gb|CY098546.1| Influenza A virus (A/North Fitzroy/INS463/2010(H1N1)) matrix protein 2 (M2) and matrix protein 1 (M1) genes, complete cds
TAAAGATGAGTCTTCTAACCGAGGTCGAAACGTACGTTCTTTCTATCATCCCGTCAGGCCCCCTCAAAGC
CGAGATCGCGCAGAGACTGGAAAGTGTCTTTGCAGGAAAGAACACAGATCTTGAGGCTCTCATGGAATGG
CTAAAGACAAGACCAATCTTGTCACCTCTGACTAAGGGAATTTTAGGATTTGTGTTCACGCTCACCGTGC

CCAGTGAGCGAGGACTGCAGCGTAGACGCTTTGTCCAAAATGCCCTAAATGGGAATGGGGACCCGAACAA
CATGGATAGAGCAGTTAAACTATACAAGAAGCTCAAAAGAGAAATAACGTTCCATGGGGCCAAGGAGGTG
TCACTAAGCTATTCAACTGGTGCACTTGCCAGTTGCATGGGCCTCATATACAACAGGATGGGAACAGTGA
CCACAGAAGCTGCTTTTGGTCTAGTGTGTGCCACTTGTGAACAGATTGCTGATTCACAGCATCGGTCTCA
CAGACAAATGGCTACTACCACCAACCCACTAATCAGGCATGAAAACAGAATGGTGCTGGCTAGCACTACG
GCAAAGGCTATGGAACAGATGGCTGGATCGAGTGAACAGGCAGCAGAGGCCATGGAGGTTGCTAATCAGA
CTAGGCAGATGGTACATGCAATGAGAACTATTGGGACTCATCCTAGCTCCAGTGCTGGTCTGAAAGATGA
CCTTCTTGAAAATTTGCAGGCCTACCAGAAGCGAATGGGAGTGCAGATGCAGCGATTCAAGTGATCCTCT
CGTCATTGCAGCAAATATCATTGGGATCTTGCACCTGATATTGTGGATTACTGATCGTCTTTTTTTCAAA
TGTATTTATCGTCGCTTTAAATACGGTTTGAAAAGAGGGCCTTCTACGGAAGGAGTGCCTGAGTCCATGA
GGGAAGAATATCAACAGGAACAGCAGAGTGCTGTGGATGTTGACGATGGTCATTTTGTCAACATAGAGCT
AGAGTAA
>gi|345722617|gb|JN655534.1| Influenza A virus (A/Pennsylvania/09/2011(H3N2)) segment 7 matrix protein 2 (M2) and matrix protein 1 (M1) genes, complete cds
ATGAGTCTTCTAACCGAGGTCGAAACGTACGTTCTTTCTATCATCCCGTCAGGCCCCCTCAAAGCCGAGA
TCGCGCAGAGACTGGAAAGTGTCTTTGCAGGGAAGAACACAGATCTTGAGGCTCTCATGGAATGGCTAAA
GACAAGACCAATCTTGTCACCTCTGACTAAGGGAATTTTAGGATTTGTGTTCACGCTCACCGTGCCCAGT
GAGCGAGGACTGCAGCGTAGACGCTTTGTCCAAAATGCCCTAAATGGGAATGGGGACCCGAACAACATGG
ATAGAGCAGTTAAACTATACAAGAAGCTCAAAAGAGAAATAACATTCCATGGGGCCAAGGAGGTGTCACT
AAGCTATTCAACTGGTGCACTTGCCAGTTGCATGGGCCTCATATACAACAGGATGGGAACAGTGACCACA
GAAGCTGCTTTTGGTCTAGTGTGTGCCACTTGTGAACAGATTGCTGATTCACAGCATCGGTCTCACAGAC
AAATGGCTACTACCACCAATCCACTAATCAGGCATGAAAACAGAATGGTGCTGGCTAGCACTACGGCAAA
GGCTATGGAACAGATGGCTGGATCGAGTGAACAGGCAGCAGAGGCCATGGAGGTTGCTAATCAGACTAGG
CAGATGGTACATGCAATGAGAACTATTGGGACTCATCCTAGCTCCAGTGCTGGTCTGAAAGATGACCTTC
TTGAAAATTTGCAGGCCTACCAGAAGCGAATGGGAGTGCAGATGCAGCGATTCAAGTGATCCTCTCGTCA
TTGCAGCAAATATCATTGGGATCTTGCACCTGATATTGTGGATTACTGATCGTCTTTTTTTCAAATGTAT
TTATCGTCGCTTTAAATACGGTTTGAAAAGAGGGCCTTCTACGGAAGGAGTGCCTGAGTCCATGAGGGAA
GAATATCAACAGGAACAGCAGAGTGCTGTGGATGTTGACGATGGTCATTTTGTCAACATAGAGCTGGAGT
AA
>gi|358257254|gb|JQ070787.1| Influenza A virus (A/Indiana/10/2011(H3N2)) segment 7 matrix protein 2 (M2) and matrix protein 1 (M1) genes, complete cds
ATGAGTCTTCTAACCGAGGTCGAAACGTACGTTCTTTCTATCATACCGTCAGGCCCCCTCAAAGCCGAGA
TCGCGCAGAGACTGGAAAGTGTCTTTGCAGGAAAGAACACAGATCTTGAGGCTCTCATGGAATGGCTAAA
GACAAGACCAATCTTGTCACCTCTGACTAAGGGAATTTTAGGATTTGTGTTCACGCTCACCGTGCCCAGT
GAGCGAGGACTGCAGCGTAGACGCTTTGTCCAAAATGCCCTAAATGGGAATGGGGACCCGAACAACATGG
ATAGAGCAGTTAAACTATACAAGAAGCTCAAAAGAGAAATAACGTTCCATGGGGCCAAGGAGGTGTCACT
AAGCTATTCAACTGGTGCACTTGCCAGTTGCATGGGCCTCATATACAACAGGATGGGAACAGTGACCACA
GAAGCTGCTTTTGGTCTAGTGTGTGCCACTTGTGAACAGATTGCTGATTCACAGCATCGGTCTCACAGAC
AGATGGCTACTACCACCAATCCACTAATCAGGCATGAGAACAGAATGGTGCTGGCTAGCACTACGGCAAA
GGCTATGGAACAGATGGCTGGATCGAGTGAACAGGCAGCAGAGGCCATGGAGGTTGCTAATCAGACTAGG
CAGATGGTACATGCAATGAGAACTATTGGGACTCATCCTAGCTCCAGTGCTGGTCTGAAAGATGACCTTC
TTGAAAATTTGCAGGCCTACCAGAAGCGAATGGGAGTGCAGATGCAGCGATTCAAGTGATCCTCTCGTCA
TTGCAGCAAATATCATTGGGATATTGCACCTGATATTGTGGATTACTGATCGTCTTTTTTTCAAATGTAT
TTATCGTCGCTTTAAATACGGTTTGAAAAGAGGGCCTTCTACGGAAGGAGTGCCTGAGTCCATGAGGGAA

GAATATCAACAGGAACAGCAGAGTGCTGTGGATGTTGACGATGGTCATTTTGTCAACATAGAGCTAGAGTAA
>gi|338826709|gb|CY092905.1| Influenza A virus (A/Georgia/NHRC0001/2011(H1N1)) matrix protein 2 (M2) and matrix protein 1 (M1) genes, complete cds
TAAAGATGAGTCTTCTAACCGAGGTCGAAACGTACGTTCTTTCTATCATCCCGTCAGGCCCCCTCAAAGC
CGAGATCGCGCAGAGACTGGAAAGTGTCTTTGCAGGAAAGAACACAGATCTTGAGGCTCTCATGGAATGG
CTAAAGACAAGACCAATCTTGTCACCTCTGACTAAGGGAATTTTAGGATTTGTGTTCACGCTCACCGTGC
CCAGTGAGCGAGGACTGCAGCGTAGACGCTTTATCCAAAATGCCCTAAATGGGAATGGGGACCCGAACAA
CATGGATAGAGCAGTTAAACTATACAAGAAGCTCAAAAGAGAAATAACGTTCCATGGGGCCAAGGAGGTG
TCACTAAGCTATTCAACTGGTGCACTTGCCAGTTGCATGGGCCTCATATACAACAGGATGGGAACAGTGA
CCACAGAAGCTGCTTTTGGTCTAGTGTGTGCCACTTGTGAACAGATTGCTGATTCACAGCATCGGTCTCA
CAGACAAATGGCTACTACCACCAATCCACTAATCAGGCATGAAAACAGAATGGTGCTGGCTAGCACTACG
GCAAAGGCTATGGAACAGATGGCTGGATCGAGTGAACAGGCAGCAGAGGCCATGGAGGTTGCTAATCAGA
CTAGGCAGATGGTACATGCAATGAGAACTATTGGGACTCATCCTAGCTCCAGTGCTGGTCTGAAAGATGA
CCTTCTTGAAAATTTGCAGGCCTACCAGAAGCGAATGGGAGTGCAGATGCAGCGATTCAAGTGATCCTCT
CGTCATTGCAGCAAATATCATTGGGATCTTGCACCTGATATTGTGGATTACTGATCGTCTTTTTTTCAAA
TGTATTTATCGTCGCTTTAAATACGGTTTGAAAAGAGGGCCTTCTACGGAAGGAGTGCCTGAGTCCATGA
GGGAAGAATATCAACAGGAACAGCAGAGTGCTGTGGATGTTGACGATGGTCATTTTGTCAACATAGAGCT
AGAGTAA
>gi|338826619|gb|CY092865.1| Influenza A virus (A/Sydney/DD3-59/2011(H1N1)) matrix protein 2 (M2) and matrix protein 1 (M1) genes, complete cds
TAAAGATGAGTCTTCTAACCGAGGTCGAAACGTACGTTCTTTCTATCATCCCGTCAGGCCCCCTCAAAGC
CGAGATCGCGCAGAGACTGGAAAGTGTCTTTGCAGGAAAGAACACAGATCTTGAGGCTCTCATGGAATGG
CTAAAGACAAGACCAATCTTGTCACCTCTGACTAAGGGAATTTTAGGATTTGTGTTCACGCTCACCGTGC
CCAGTGAGCGAGGACTGCAGCGTAGACGCTTTATCCAAAATGCCCTAAATGGGAATGGGGACCCGAACAA
CATGGATAGAGCAGTTAAACTATACAAGAAGCTCAAAAGAGAAATAACGTTCCATGGGGCCAAGGAGGTG
TCACTAAGCTATTCAACTGGTGCACTTGCCAGTTGCATGGGCCTCATATACAACAGGATGGGAACAGTGA
CCACAGAAGCTGCTTTTGGTCTAGTGTGTGCCACTTGTGAACAGATTGCTGATTCACAGCATCGGTCTCA
CAGACAAATGGCTACTACCACCAATCCACTAATCAGGCATGAAAACAGAATGGTGCTGGCTAGCACTACG
GCAAAGGCTATGGAACAGATGGCTGGATCGAGTGAACAGGCAGCAGAGGCCATGGAGGTTGCTAATCAGA
CTAGGCAGATGGTACATGCAATGAGAACTATTGGGACTCATCCTAGCTCCAGTGCTGGTCTGAAAGATGA
CCTTCTTGAAAATTTGCAGGCCTACCAGAAGCGAATGGGAGTGCAGATGCAGCGATTCAAGTGATCCTCT
CGTCATTGCAGCAAATATCATTGGGATCTTGCACCTGATATTGTGGATTACTGATCGTCTTTTTTTCAAA
TGTATTTATCGTCGCTTTAAATACGGTTTGAAAAGAGGGCCTTCTACGGAAGGAGTGCCTGAGTCCATGA
GGGAAGAATATCAACAGGAACAGCAGAGTGCTGTGGATGTTGACGATGGTCATTTTGTCAACATAGAGCT
AGAGTAA
>gi|388770702|gb|CY120748.1| Influenza A virus (A/Brazil/AVS08/2011(H1N1)) matrix protein 2 (M2) and matrix protein 1 (M1) genes, complete cds
AGATATTTAAAGATGAGTCTTCTAACCGAGGTCGAAACGTACGTTCTTTCTATCATCCCGTCAGGCCCCC
TCAAAGCCGAGATCGCGCAGAGACTGGAAAGTGTCTTTGCAGGAAAGAACACAGATCTTGAGGCTCTTAT
GGAATGGCTAAAGACAAGACCAATCTTGTCACCTCTGACTAAGGGAATTTTAGGATTTGTGTTCACGCTC
ACCGTGCCCAGTGAGCGAGGACTGCAGCGTAGACGCTTTGTCCAAAATGCCCTAAATGGGAATGGGGACC
CGAACAACATGGATAGAGCAGTTAAACTATACAAGAAGCTCAAAAGAGAAATAACATTCCATGGGGCCAA
GGAGGTGTCACTAAGCTATTCAACTGGTGCACTTGCCAGTTGCATGGGCCTCATATACAACAGGATGGGA
ACAGTGACCACAGAAGCTGCTTTTGGTCTAGTTTGTGCCACTTGTGAACAGATTGCTGATTCACAGCATC

GGTCTCACAGACAAATGGCTACTACCACCAATCCACTAATCAGGCATGAAAACAGAATGGTGCTGGCTAG
CACTACGGCAAAGGCTATGGAACAGATGGCTGGATCGAGTGAACAGGCAGCAGAGGCCATGGAGGTTGCT
AATCAGACTAGGCAGATGGTACATGCAATGAGAACTATTGGGACTCATCCTAGCTCCAGTGCTGGTCTGA
AAGATGACCTTCTTGAAAATTTGCAGGCCTACCAGAAGCGAATGGGAGTGCAGATGCAGCGATTCAAGTG
ATCCTCTCGTCATTGCAGCAAATATCATTGGGATCTTGCACCTGATATTGTGGATTACTGATCGTCTTTT
TTTCAAATGTATTTATCGTCGCTTTAAATACGGTTTGAAAAGAGGGCCTTCTACGGAAGGAGTGCCTGAG
TCCATGAGGGAAGAATATCAACAGGAACAGCAGAGTGCTGTGGATGTTGACGATGGTCATTTTGTCAACA
TAGAGCTAGAGTAAAAAAC
>gi|338826691|gb|CY092897.1| Influenza A virus (A/South Carolina/NHRC0002/2011(H1N1)) matrix protein 2 (M2) and matrix protein 1 (M1) genes, complete cds
TAAAGATGAGTCTTCTAACCGAGGTCGAAACGTACGTTCTTTCTATCATCCCGTCAGGCCCCCTCAAAGC
CGAGATCGCGCAGAGACTGGAAAGTGTCTTTGCAGGAAAGAACACAGATCTTGAGGCTCTCATGGAATGG
CTAAAGACAAGACCAATCTTGTCACCTCTGACTAAGGGAATTTTAGGATTTGTGTTCACGCTCACCGTGC
CCAGTGAGCGAGGACTGCAGCGTAGACGCTTTATCCAAAATGCCCTAAATGGGAATGGGGACCCGAACAA
CATGGATAGAGCAGTTAAACTATACAAGAAGCTCAAAAGAGAAATAACGTTTCATGGGGCCAAGGAGGTG
TCACTAAGCTATTCAACTGGTGCACTTGCCAGTTGCATGGGCCTCATATACAACAGGATGGGAACAGTGA
CCACAGAAGCTGCTTTTGGTCTAGTGTGTGCCACTTGTGAACAGATTGCTGATTCACAGCATCGGTCTCA
CAGACAAATGGCTACTACCACCAATCCACTAATCAGGCATGAAAACAGAATGGTGCTGGCTAGCACTACG
GCAAAGGCTATGGAACAGATGGCTGGATCGAGTGAACAGGCAGCAGAGGCCATGGAGGTTGCTAATCAGA
CTAGGCAGATGGTACATGCGATGAGAACTATTGGGACTCATCCTAGCTCCAGTGCTGGTCTGAAAGATGA
CCTTCTTGAAAATTTGCAGGCCTACCAGAAGCGAATGGGAGTGCAGATGCAGCGATTCAAGTGATCCTCT
CGTCATTGCAGCAAATATCATTGGGATCTTGCACCTGATATTGTGGATTACTGATCGTCTTTTTTTCAAA
TGTATTTATCGTCGCTTTAAATACGGTTTGAAAAGAGGGCCTTCTACGGAAGGAGTGCCTGAGTCCATGA
GGGAAGAATATCAACAGGAACAGCAGAGTGCTGTGGATGTTGACGATGGTCATTTTGTCAACATAGAGCT
AGAGTAA
>gi|338826655|gb|CY092881.1| Influenza A virus (A/California/NHRC0001/2011(H1N1)) matrix protein 2 (M2) and matrix protein 1 (M1) genes, complete cds
TAAAGATGAGTCTTCTAACCGAGGTCGAAACGTACGTTCTTTCTATCATCCCGTCAGGCCCCCTCAAAGC
CGAGATCGCGCAGAGACTGGAAAGTGTCTTTGCAGGAAAGAACACAGATCTTGAGGCTCTTATGGAATGG
CTAAAGACAAGACCAATCTTGTCACCTCTGACTAAGGGAATTTTAGGATTTGTGTTCACGCTCACCGTGC
CCAGTGAGCGAGGACTGCAGCGTAGACGCTTTGTCCAAAATGCCCTAAATGGGAATGGGGACCCGAACAA
CATGGATAGAGCAGTTAAACTATACAAGAAGCTCAAAAGAGAAATAACATTCCATGGGGCCAAGGAGGTG
TCACTAAGCTATTCAACTGGTGCACTTGCCAGTTGCATGGGCCTCATATACAACAGGATGGGAACAGTGA
CCACAGAAGCTGCTTTTGGTCTAGTTTGTGCCACTTGTGAACAGATTGCTGATTCACAGCATCGGTCTCA
CAGACAAATGGCTACTACCACCAATCCACTAATCAGGCATGAAAACAGAATGGTGCTGGCTAGCACTACG
GCAAAGGCTATGGAACAGATGGCTGGATCGAGTGAACAGGCAGCAGAGGCCATGGAGGTTGCTAATCAGA
CTAGGCAGATGGTACATGCAATGAGAACTATTGGGACTCATCCTAGCTCCAGTGCTGGTCTGAAAGATGA
CCTTCTTGAAAATTTGCAGGCCTACCAGAAGCGAATGGGAGTGCAGATGCAGCGATTCAAGTGATCCTCT
CGTCATTGCAGCAAATATCATTGGGATCTTGCACCTGATATTGTGGATTGCTGATCGTCTTTTTTTCAAA
TGTATTTATCGTCGCTTTAAATACGGTTTGAAAAGAGGGCCTTCTACGGAAGGAGTGCCTGAGTCCATGA
GGGAAGAATATCAACAGGAACAGCAGAGTGCTGTGGATGTTGACGATGGTCATTTTGTCAACATAGAGCT
AGAGTAA
>gi|338826637|gb|CY092873.1| Influenza A virus (A/Illinois/NHRC0001/2011(H1N1)) matrix protein 2 (M2) and matrix protein 1 (M1) genes, complete cds

TAAAGATGAGTCTTCTAACCGAGGTCGAAACGTACGTTCTTTCTATCATCCCGTCAGGCCCCCTCAAAGC
CGAGATCGCGCAGAGACTGGAAAGTGTCTTTGCAGGAAAGAACACAGATCTTGAGGCTCTCATGGAATGG
CTAAAGACAAGACCAATCTTGTCACCTCTGACTAAGGGAATTTTAGGATTTGTGTTCACGCTCACCGTGC
CCAGTGAGCGAGGACTGCAGCGTAGACGCTTTGTCCAAAATGCCCTAAATGGGAATGGGGACCCGAACAA
CATGGATAGAGCAGTTAAACTATACAAGAAGCTCAAAAGAGAAATAACGTTCCATGGGGCCAAGGAGGTG
TCACTAAGCTATTCAACTGGTGCACTTGCCAGTTGCATGGGCCTCATATACAACAGGATGGGAACAGTGA
CCACAGAAGCTGCTTTTGGTCTAGTGTGTGCCACTTGTGAACAGATTGCTGATTCACAGCATCGGTCTCA
CAGACAAATGGCTACTACCACCAATCCACTAATCAGGCATGAAAACAGAATGGTGCTGGCTAGCACTACG
GCAAAGGCTATGGAACAGATGGCTGGATCGAGTGAGCAGGCAGCAGAGGCCATGGAGGTTGCTAATCAGA
CTAGGCAGATGGTACATGCAATGAGAACTATTGGGACTCATCCTAGCTCCAGTGCTGGTCTGAAAGATGA
CCTTCTTGAAAATTTGCAGGCCTACCAGAAGCGAATGGGAGTGCAGATGCAGCGATTCAAGTGATCCTCT
CGTCATTGCAGCAAATATCATTGGGATCTTGCACCTGATATTGTGGATTACTGATCGTCTTTTTTTCAAA
TGTATTTATCGTCGCTTTAAATACGGTTTGAAAAGAGGGCCTTCTACGGAAGGAGTGCCTGAGTCCATGA
GGGAAGAATATCAACAGGAACAGCAGAGTGCTGTGGATGTTGACGATGGTCATTTTGTCAACATAGAGCT
AGAGTAA
>gi|380862309|gb|CY111561.1| Influenza A virus (A/Boston/DOA62/2011(H1N1)) matrix protein 2 (M2) and matrix protein 1 (M1) genes, complete cds
ATATTTAAAGATGAGTCTTCTAACCGAGGTCGAAACGTACGTTCTTTCTATCATCCCGTCGGGCCCCCTC
AAAGCCGAGATCGCGCAGAGACTGGAAAGTGTCTTTGCAGGAAAGAACACAGATCTTGAGGCTCTCATGG
AATGGCTAAAGACAAGACCAATCTTGTCACCTCTGACTAAGGGAATTTTAGGATTTGTGTTCACGCTCAC
CGTGCCCAGTGAGCGAGGACTGCAGCGTAGACGCTTTGTCCAAAATGCCCTAAATGGGAATGGGGACCCA
AACAACATGGATAGAGCAGTTAAACTATACAAGAAGCTCAAAAGAGAAATAACGTTCCATGGGGCCAAGG
AGGTGTCACTAAGCTATTCAACTGGTGCACTTGCCAGTTGCATGGGCCTCATATACAACAGGATGGGAAC
AGTGACCACAGAAGCTGCTTTTGGTCTAGTGTGTGCCACTTGTGAACAGATTGCTGATTCACAGCATCGG
TCTCACAGACAAATGGCTACTACCACCAATCCACTAATCAGGCATGAAAACAGAATGGTGCTGGCTAGCA
CTACGGCAAAGGCTATGGAACAGATGGCTGGATCGAGTGAACAGGCAGCAGAGGCCATGGAGGTTGCTAA
TCAGACTAGGCAGATGGTACATGCAATGAGAACTATTGGGACTCATCCTAGCTCCAGTGCTGGTCTAAAA
GATGACCTTCTTGAAAATTTGCAGGCCTACCAGAAGCGAATGGGAGTGCAGATGCAACGATTCAAGTGAT
CCTCTCGTCATTGCAGCAAATATCATTGGGATCTTGCACCTGATATTGTGGATTACTGATCGTCTTTTTT
TCAAATGTATTTATCGTCGCTTTAAATACGGTTTGAAAAGAGGGCCTTCTACGGAAGGAGTGCCTGAGTC
CATGAGGGAAGAATATCAACAGGAACAGCAGAGTGCTGTGGATGTTGACGATGGTCATTTTGTCAACATA
GAGCTAGAGTAAAAAACTAC
>gi|404425395|gb|CY125776.1| Influenza A virus (A/Boston/DOA90/2012(H1N1)) matrix protein 2 (M2) and matrix protein 1 (M1) genes, complete cds
GTAGATATTTAAAGATGAGTCTTCTAACCGAGGTCGAAACGTACGTTCTTTCTATCATCCCATCAGGCCC
CCTCAAAGCCGAGATCGCGCAGAGACTGGAAAGTGTCTTTGCAGGAAAGAATACAGATCTTGAGGCTCTC
ATGGAATGGCTAAAGACAAGACCAATCTTGTCACCTCTGACTAAGGGAATTTTAGGATTTGTGTTCACGC
TCACCGTGCCCAGTGAGCGAGGACTGCAGCGTAGACGCTTTATCCAAAATGCCCTAAATGGGAATGGGGA
CCCGAACAACATGGATAGAGCAGTTAAACTATACAAGAAGCTCAAAAGAGAAATAACGTTCCATGGGGCC
AAGGAGGTGTCACTAAGCTATTCAACTGGTGCACTTGCCAGTTGCATGGGCCTCATATACAACAGAATGG
GAACAGTGACCACAGAAGCTGCTTTTGGTCTAGTGTGTGCCACTTGTGAACAGATTGCTGATTCACAGCA
TCGGTCTCACAGACAAATGGCTACTACCACCAATCCACTAATCAGGCATGAAAACAGAATGGTGCTGGCT
AGCACTACGGCAAAGGCTATGGAACAGATGGCTGGATCGAGTGAACAGGCAGCAGAGGCCATGGAGGTT
G

CTAATCAGACTAGGCAGATGGTACATGCAATGAGAACTATTGGGACTCATCCTAGCTCCAGTGCTGGTCT
GAAAGATGACCTTCTTGAAAATTTGCAGGCCTACCAGAAACGAATGGGAGTGCAGATGCAGCGATTCAAG
TGATCCTCTCGTCATTGCAGCAAATATCATTGGGATCTTGCACCTGATATTGTGGATTACTGATCGTCTT
TTTTTCAAATGTATTTATCGTCGCTTTAAATACGGTTTGAAAAGAGGGCCTTCTACGGAAGGAGTGCCTG
AGTCCATGAGGGAAGAATATCAACAGGAACAGCAGAGTGCTGTGGATGTTGACGATGGTCATTTTGTCAA
CATAGAGCTAGAGTAAAAAACTA
>gi|404425413|gb|CY125784.1| Influenza A virus (A/Boston/DOA93/2012(H1N1)) matrix protein 2 (M2) and matrix protein 1 (M1) genes, complete cds
GTAGATATTTAAAGATGAGTCTTCTAACCGAGGTCGAAACGTACGTTCTTTCTATCATCCCATCAGGCCC
CCTCAAAGCCGAGATCGCGCAGAGACTGGAAAGTGTCTTTGCAGGAAAGAATACAGATCTTGAGGCTCTC
ATGGAATGGCTAAAGACAAGACCAATCTTGTCACCTCTGACTAAGGGAATTTTAGGATTTGTGTTCACGC
TCACCGTGCCCAGTGAGCGAGGACTGCAGCGTAGACGCTTTATCCAAAATGCCCTAAATGGGAATGGGGA
CCCGAACAACATGGATAGAGCAGTTAAACTATACAAGAAGCTCAAAAGAGAAATAACGTTCCATGGGGCC
AAGGAGGTGTCACTAAGCTATTCAACTGGTGCACTTGCCAGTTGCATGGGCCTCATATACAACAGAATGG
GAACAGTGACCACAGAAGCTGCTTTTGGTCTAGTGTGTGCCACTTGTGAACAGATTGCTGATTCACAGCA
TCGGTCTCACAGACAAATGGCTACTACCACCAATCCACTAATCAGGCATGAAAACAGAATGGTGCTGGCT
AGCACTACGGCAAAGGCTATGGAACAGATGGCTGGATCGAGTGAACAGGCAGCAGAGGCCATGGAGGTT
G
CTAATCAGACTAGGCAGATGGTACATGCAATGAGAACTATTGGGACTCATCCTAGCTCCAGTGCTGGTCT
GAAAGATGACCTTCTTGAAAATTTGCAGGCCTACCAGAAACGAATGGGAGTGCAGATGCAGCGATTCAAG
TGATCCTCTCGTCATTGCAGCAAATATCATTGGGATCTTGCACCTGATATTGTGGATTACTGATCGTCTT
TTTTTCAAATGTATTTATCGTCGCTTTAAATACGGTTTGAAAAGAGGGCCTTCTACGGAAGGAGTGCCTG
AGTCCATGAGGGAAGAATATCAACAGGAACAGCAGAGTGCTGTGGATGTTGACGATGGTCATTTTGTCAA
CATAGAGCTAGAGTAAAAAACTA
